# Inflammatory Transcriptome Profiling of Human Monocytes Exposed Acutely to Cigarette Smoke

**DOI:** 10.1371/journal.pone.0030120

**Published:** 2012-02-17

**Authors:** William R. Wright, Katarzyna Parzych, Damian Crawford, Charles Mein, Jane A. Mitchell, Mark J. Paul-Clark

**Affiliations:** 1 Department of Cardiothoracic Pharmacology, Pharmacology and Toxicology, National Heart and Lung Institute, Imperial College London, London, United Kingdom; 2 Genome Centre, Barts and The London School of Medicine and Dentistry, London, United Kingdom; New York University, United States of America

## Abstract

**Background:**

Cigarette smoking is responsible for 5 million deaths worldwide each year, and is a major risk factor for cardiovascular and lung diseases. Cigarette smoke contains a complex mixture of over 4000 chemicals containing 10^15^ free radicals. Studies show smoke is perceived by cells as an inflammatory and xenobiotic stimulus, which activates an immune response. The specific cellular mechanisms driving cigarette smoke-induced inflammation and disease are not fully understood, although the innate immune system is involved in the pathology of smoking related diseases.

**Methodology/Principle findings:**

To address the impact of smoke as an inflammagen on the innate immune system, THP-1 cells and Human PBMCs were stimulated with 3 and 10% (v/v) cigarette smoke extract (CSE) for 8 and 24 hours. Total RNA was extracted and the transcriptome analysed using Illumina BeadChip arrays. In THP-1 cells, 10% CSE resulted in 80 genes being upregulated and 37 downregulated by ≥1.5 fold after 8 hours. In PBMCs stimulated with 10% CSE for 8 hours, 199 genes were upregulated and 206 genes downregulated by ≥1.5 fold. After 24 hours, the number of genes activated and repressed by ≥1.5 fold had risen to 311 and 306 respectively. The major pathways that were altered are associated with cell survival, such as inducible antioxidants, protein chaperone and folding proteins, and the ubiquitin/proteosome pathway.

**Conclusions:**

Our results suggest that cigarette smoke causes inflammation and has detrimental effects on the metabolism and function of innate immune cells. In addition, THP-1 cells provide a genetically stable alternative to primary cells for the study of the effects of cigarette smoke on human monocytes.

## Introduction

According to the World Health Organisation, approximately 5 million people die each year as a result of smoking cigarettes (http://www.who.int/mediacentre/factsheets/fs339/en/index.html). Smoking represents the second largest global disease burden [1], and is accountable for 90% of lung cancers [2]. It is the leading risk factor for chronic obstructive pulmonary disease (COPD) [3], and is a major risk factor for cardiovascular disease [4], which is the leading cause of death worldwide [1]. Despite the link between cigarette smoking and disease, the biological mechanisms by which cigarette smoke is associated with inflammation and disease are not fully understood.

Cigarette smoke contains approximately 10^15^ free radicals/puff [5] and 4000 different chemicals, including several known carcinogens [6]. Exposure to cigarette smoke causes oxidative stress [7] and is linked to both activation and inhibition of the innate immune system [8]. Hydrocarbons in smoke, for example, bind receptors on endothelial, epithelial and immune cells, causing modifications to metabolic pathways that can result in tumour formation [6]. Similarly, β-aldehydes in smoke are linked to activation of macrophages, which promotes inflammation that is associated with the pathogenesis of COPD [9]. Furthermore, oxidants in cigarette smoke are linked to DNA damage, modification of intracellular signalling, and lipid peroxidation [10]. This highlights the wide-range of effects that the different components of cigarette smoke can have on cells in the body. The cellular processes leading to these effects are currently the subject of intense research.

The acute exposure to cigarette smoke induces an inflammatory response, typified by the recruitment of neutrophils and macrophages in the broncheoalveolar lavage fluid (BALF) in animal models [7], [10] and human smokers [11], [12]. This corresponds with release of inflammatory mediators, such as the neutrophil chemoattractants interleukin 8 (CXCL8) and tumour necrosis factor alpha (TNF-α), which are elevated by smoke *in vivo* and *in vitro* [13], [14], [15]. The intracellular mechanisms by which cells respond to acute smoke have been linked to the transcription factors nuclear factor κβ (NF-κβ) [16], nuclear factor erythroid-derived related factor 2 (Nrf2) [13], [17] and activator protein-1 (AP-1) [13]. Furthermore, recent studies have demonstrated a role for the innate immune receptors Toll-like receptor 2 (TLR2) and TLR4 in the sensing of oxidants in smoke [18], [19].

Monocytes play an important role in the pathogenesis of smoking-related diseases such as COPD [20], and are involved in cardiovascular diseases such as atherosclerosis [21]. Previous research within the group has identified that the acute response of monocytes to cigarette smoke is characteristic of the inflammatory response [13], and that oxidants in smoke may be sensed by TLRs [19]. We hypothesized, therefore, that cigarette smoke would be sensed by monocytes as an injurious stimulus, resulting in the up- and down-regulation of genes associated with immune, inflammatory and oxidative stress pathways. In the current study therefore, a transcriptomics approach was used to assess gene expression changes induced by acute cigarette smoke exposure in the human monocytic cell line, THP-1 cells, and primary human peripheral blood mononuclear cells (PBMCs). THP-1 cells were used as we have previously shown that they respond to a smoke-induced stimulus. We therefore aimed to identify genes and gene networks significantly increased or decreased by cigarette smoke. Furthermore, our aim was to identify a list of genes that can be validated and investigated in future studies, to potentially find and understand the role of novel genes involved in the response to cigarette smoke.

## Results

### Pre-processing and analysis of raw and normalized global gene expression data

Two time points were chosen based on previously published data from our group and others. 8 hours corresponded to maximum RNA levels of our positive control gene, IL-8 [13], and 24 hours was chosen as this is a time point that we have used previously for mediator expression of heme oxygenase 1 and TNFα [13], [22]. RNA from THP-1 monocytes and PBMCs treated with control medium (n = 3–6), 3% CSE-conditioned medium (n = 3) and 10% CSE-conditioned medium (n = 3–6) was hybridized to the Illumina HumanRef-8v3 BeadChip array. Box-whisker plots of raw signal intensity values for each sample showed that the distribution of intensities in each data set was similar for both THP-1 cells and PBMCs. Quantile normalization of the complete dataset made the distribution profiles of all samples identical within each cell type. Very stringent filtering was used to select only probe sets that were measured as “present” or “marginal” in all samples, and resulted in a dataset of 15234 probe sets for THP-1 cells and 18689 probe sets for PBMCs that were suitable for further analysis. All data is MIAME compliant and all results have been deposited on the ArrayExpress database (http://www.ebi.ac.uk/cgi-bin/microarray/magetab.cgi) under the experimental title “inflammatory transcriptome profiling of human monocytes exposed acutely to cigarette smoke”. Raw bead studio export text files for both THP-1 cells and PBMCs are available as Data S1 and Data S2 respectively.

### Gene expression patterns in THP-1 cells treated with 10% CSE or control media

**Figure 1:**
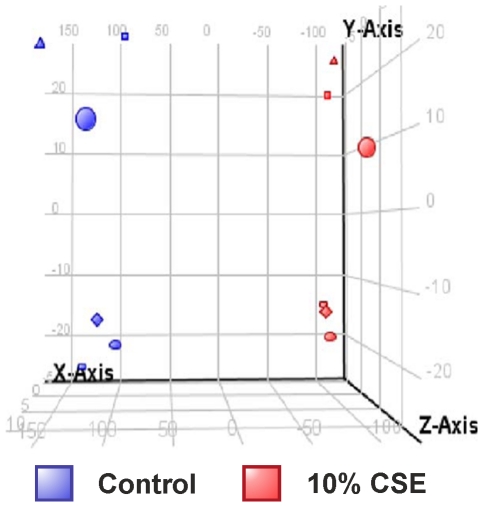
THP-1 samples treated for 8 h with control media or 10% CSE group according to treatment conditions. THP-1 cells were treated for 8 h with control media or 10% CSE, RNA was extracted and gene expression values measured using HumanRef-8v3 Expression BeadChip arrays. Principal component analysis (PCA) by conditions was performed on GeneSpring GX 11.0.2 and represented as a 3D scatterplot of THP-1 monocytes treated with control media or 10% cigarette smoke extract (CSE) for 8 hours from 6 individual experiments.. The PCA plot showed that samples clustered based on their treatment with medium (blue) or 10% CSE (red). Data represent n = 6. Component % variance; PC1 = 81.7%, PC2 = 14.1%, PC3 = 4.2%. RNA was extracted from each sample and gene expression values measured using 2 HumanRef-8v3 Expression BeadChip arrays. This represents stratification according to treatment with 10% CSE (red) and media treated controls (blue) over 6 individual experiments.

Principle component analysis, using 3 principle components (selected by the elbow method) are represented as 3D scatterplot (Figure 1), and show that all samples treated with 10% CSE clearly stratify away from those of control media, indicating detectable differences in the gene transcription patterns of these two groups.

### The effect of 10% CSE on THP-1 monocytes: genes differentially expressed by ≥1.5-fold

**Figure 2:**
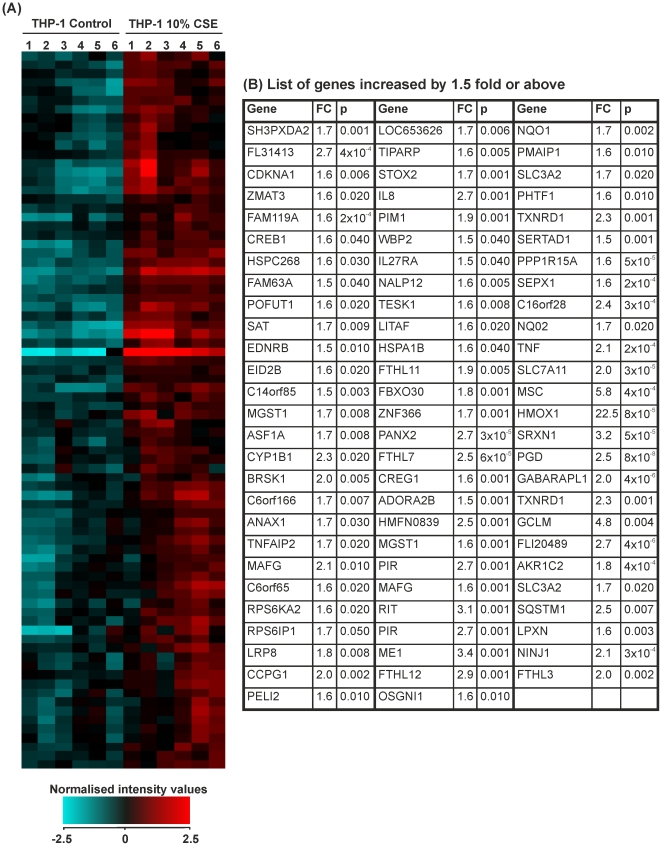
Expression of genes that were increased above a 1.5-fold cut-off in THP-1 monocytes treated with control medium or cigarette smoke extract (CSE) for 8 hours. THP-1 monocytes were treated for 8 hours with RPMI-1640 medium (control 1–6) or CSE (1–6). RNA was extracted from each sample and gene expression values measured using the Illumina HumanRef-8v3 BeadChip array. (**A**) Heat map representation of normalized signal intensity values for genes altered by ≥1.5-fold. Red denotes high expression and turquoise denotes low expression. Order of samples was dictated by hierarchical clustering. (**B**) Table showing gene symbol, fold change and p-value for all genes upregulated by ≥1.5-fold. Statistical significance (p<0.05) was calculated using student's t-test followed by Benjamini-Hochberg false discovery rate correction on GeneSpring GX11.0.2 software. Fold change represents a comparison between mean normalised signal intensity for control (n = 6) versus smoke (n = 6) treated THP-1 monocytes. Refer to Data S1 for full data sets and Entrez Gene IDs.

**Figure 3:**
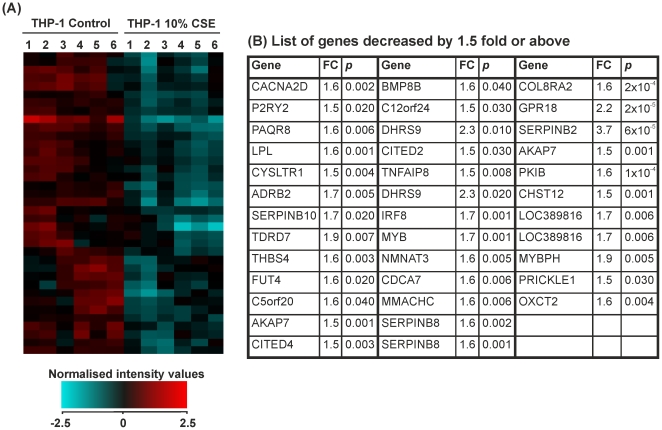
Expression of genes that were decreased below a 1.5-fold cut-off in THP-1 monocytes treated with control medium or cigarette smoke extract for 8 hours. THP-1 monocytes were treated for 8 hours with RPMI-1640 medium (control 1–6) or CSE (1–6). RNA was extracted from each sample and gene expression values measured using the Illumina HumanRef-8v3 BeadChip array. (**A**) Heat map representation of normalized signal intensity values for genes altered by ≥1.5-fold. Red denotes high expression and turquoise denotes low expression. Order of samples was dictated by hierarchical clustering. (**B**) Table showing gene symbol, fold change and p-value for all genes downregulated by ≥1.5-fold. Statistical significance (p<0.05) was calculated using student's t-test followed by Benjamini-Hochberg false discovery rate correction on GeneSpring GX11.0.2 software. Fold change represents a comparison between mean normalised signal intensity for control (n = 6) versus smoke (n = 6) treated THP-1 monocytes. Refer to Data S1 for full data sets and Entrez Gene IDs.

Fold change analysis followed by students *t*-test and Benjamini-Hochberg FDR correction identified 117 genes that were significantly altered by ≥1.5-fold in THP-1 monocytes treated with CSE versus controls. 80 of these genes were upregulated (Figure 2) and 37 downregulated (Figure 3). Hierarchical clustering analysis of conditions using Pearson's centred rank correlation distance metric and Ward's linkage rule on the entire 24526 probe sets distinguished controls from smoke-treated samples. Gene symbols displayed more than once represent transcript variants of the gene.

### The effects of 10% CSE on THP-1 monocytes: genes differentially expressed by ≥2-fold

**Figure 4:**
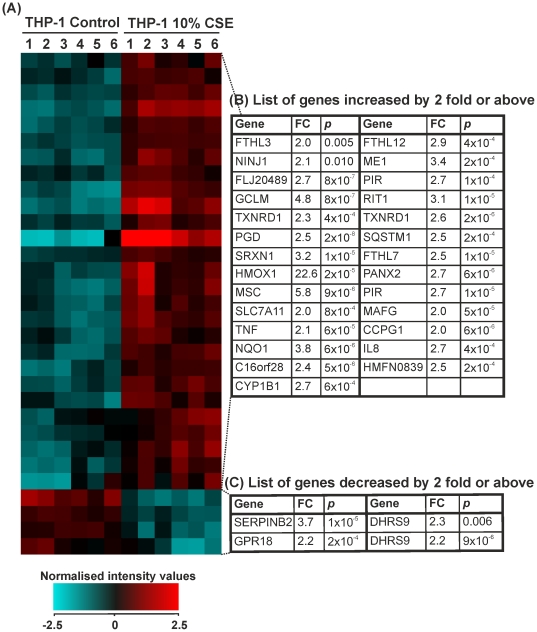
Expression of genes that were changed by 2.0-fold or above in THP-1 monocytes treated with control medium or cigarette smoke extract for 8 hours. THP-1 monocytes were treated for 8 hours with RPMI-1640 medium (control 1–6) or CSE (1–6). RNA was extracted from each sample and gene expression values measured using the Illumina HumanRef-8v3 BeadChip array. (**A**) Heat map representation of normalized signal intensity values for genes altered by ≥2.0-fold. Red denotes high expression and turquoise denotes low expression. Order of samples was dictated by hierarchical clustering. (**B**) Table showing gene name, fold change and p-value for all genes upregulated by ≥2.0-fold. (**C**) Table showing gene name, fold change and p-value for all genes downregulated by ≥2.0-fold. Statistical significance (p<0.05) was calculated using student's t-test followed by Benjamini-Hochberg FDR correction on GeneSpring GX11.0.2 software. Fold change represents a comparison between mean normalised signal intensity for control (n = 6) versus smoke (n = 6) treated THP-1 monocytes. Refer to Data S1 full data sets and Entrez Gene IDs.

Fold change analysis with unpaired *t*-tests and Benjamini-Hochberg FDR correction identified 31 genes that were significantly altered by ≥2-fold in THP-1 monocytes treated for 8 hours with cigarette smoke versus controls. 27 of these genes were upregulated and 4 downregulated (Figure 4).

### Gene expression patterns in PBMCs treated with 3 or 10% CSE or control media

**Figure 5:**
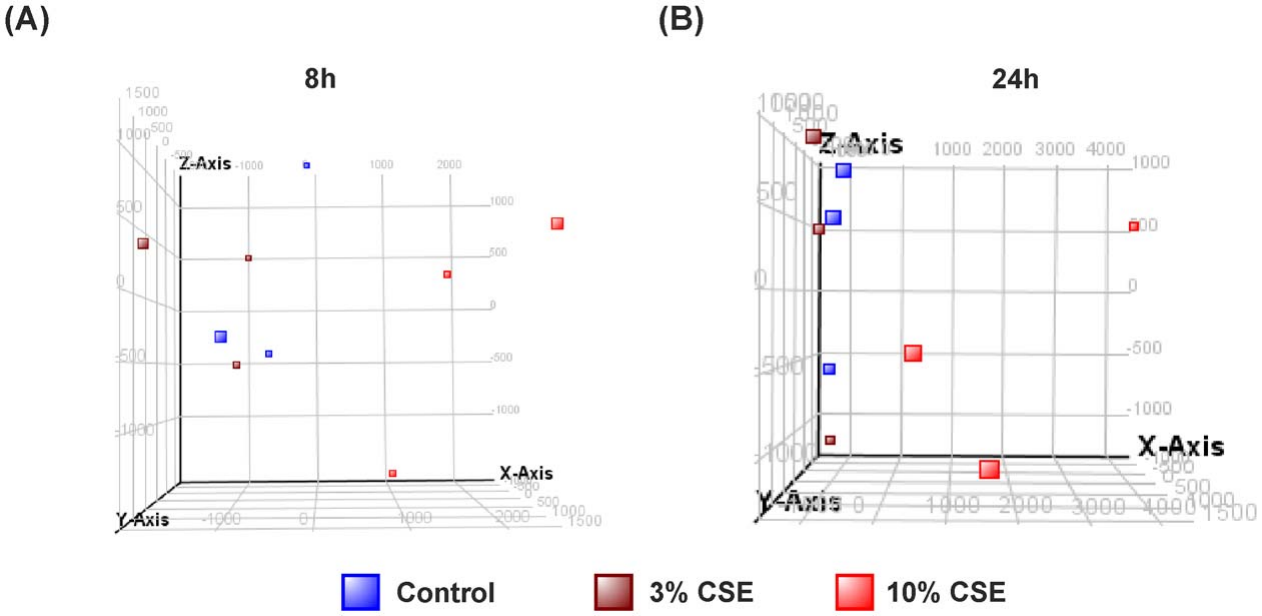
3D Scatterplot from the principle conponent analysis of PBMCs treated with control media or cigarette smoke extract (CSE) for 8 and 24 hours. PBMCs were treated with control media, 3% CSE or 10% CSE for 8 and 24 hours. RNA was extracted from each sample and gene expression values measured using HumanRef-8v3 Expression BeadChip arrays. Samples stratify according to treatment with 3% CSE (maroon), 10% CSE (red) and media treated controls (blue) from 3 individuals for (**A**) 8 hours and (**B**) 24 hours. Component % variance for 8 hours was; PC1 = 35.8%, PC2 = 27.6%, PC3 = 19.2%, PC4 = 17.4%. Component % variance for 24 hours was; PC1 = 44.1%, PC2 = 22.4%, PC3 = 18.8%, PC4 = 14.7%.

Principle component analysis of PBMCs treated with 3 or 10% CSE, using 4 principle components (selected by the elbow method, to determine the true number of components that affect the data) is represented as a 3D scatterplot, where Figure 5A shows 8 hour samples and Figure 5B, 24 hours samples. When looking at both 8 and 24 hours after 10% CSE treatment, there is a clear stratification of samples compared with controls. As predicted by our previous *in vitro* data, there is a less clear difference between cells treated with 3% CSE and controls. This is visually represented by considerable overlap in samples from these two groups. The difference between 10% CSE and the control group indicates detectable differences in the gene transcription patterns of these two groups, and justifies our choice of using a 10% CSE solution to look at the transcriptomic changes in monocytes.

### The effects of 3 or 10% CSE stimulation of PBMCs after 8 hours

**Figure 6:**
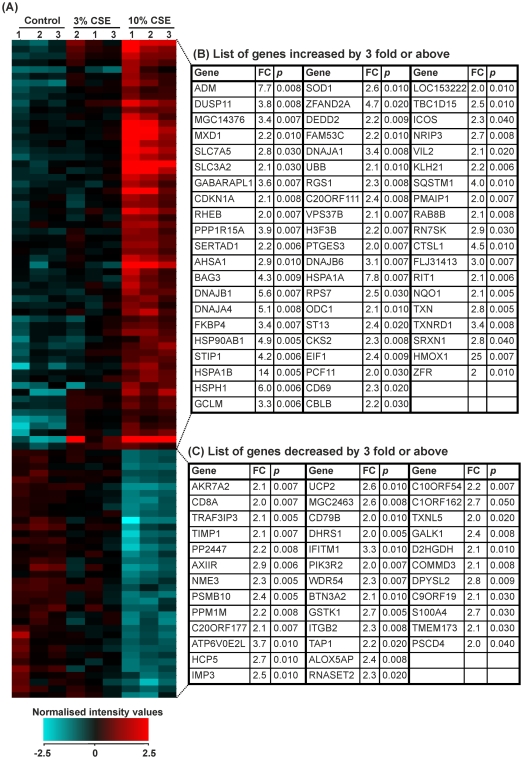
Expression of genes that were changed by 3.0-fold or above in PBMCs treated with control medium or cigarette smoke extract (CSE) for 8 hours. PBMCs were treated for 8 hours with RPMI-1640 medium (control 1–3), 3% CSE (1–3) or 10% CSE (1–3). RNA was extracted from each sample and gene expression values measured using the Illumina HumanRef-8v3 BeadChip array. (**A**) Heat map representation of normalized signal intensity values for genes altered by ≥3.0-fold. Red denotes high expression and turquoise denotes low expression. Order of samples was dictated by hierarchical clustering. (**B**) Table showing gene name, fold change and p-value for all genes upregulated by ≥3.0-fold. (**C**) Table showing gene name, fold change and p-value for all genes downregulated by ≥3.0-fold. Statistical significance (p<0.05) was calculated using student's t-test followed by Benjamini-Hochberg FDR correction on GeneSpring GX11.0.2 software. Fold change represents a comparison between mean normalised signal intensity for control (n = 3) versus smoke (n = 3) treated PBMCs. Refer to Data S2 for full data sets and Entrez Gene IDs.

Using a 1.5-fold cut-off, PBMCs stimulated with 3% CSE for 8 hours resulted in 4 genes significantly changed after analysis with unpaired *t*-tests and Benjamini-Hochberg FDR correction. 4 were upregulated with no genes downregulated. However, when looking at the cells activated with 10% CSE, there were considerably more genes altered, 405. Of these 206 were downregulated and 199 upregulated. To represent the top genes altered after 8 hours of CSE challenge, we chose a 3-fold cut-off. Once again, as could be predicted by the principle component analysis, there were no genes altered after treatment with 3% CSE when analysed using unpaired *t*-tests and Benjamini-Hochberg FDR correction. However, in PBMCs treated with 10% CSE for 8 hours, 91 genes were changed significantly after stimulation with 10% CSE, of these 61 genes was up regulated and 37 down regulated (Figure 6 A–C).

### The effects of 3 or 10% CSE stimulation of PBMCs after 24 hours

**Figure 7:**
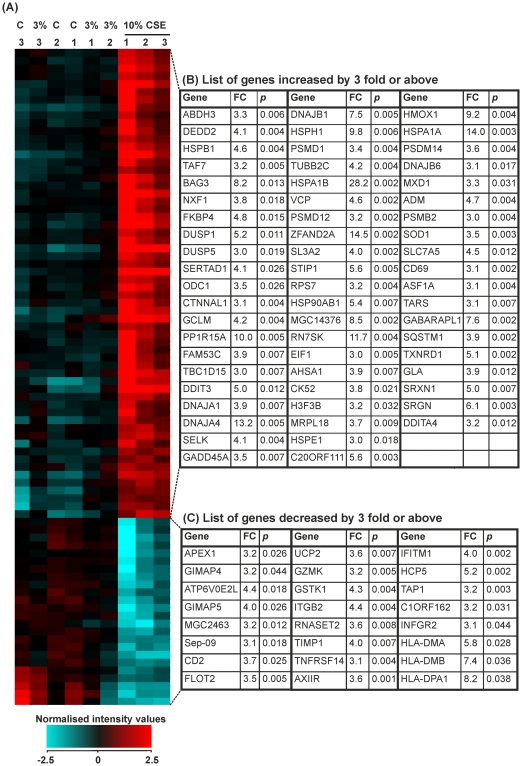
Expression of genes that were changed by 3.0-fold or above in PBMCs treated with control medium or cigarette smoke extract (CSE) for 24 hours. PBMCs were treated for 24 hours with RPMI-1640 medium (control 1–3) or 3% CSE (1–3) or 10% CSE (1–3). RNA was extracted from each sample and gene expression values measured using the Illumina HumanRef-8v3 BeadChip array. (**A**) Heat map representation of normalized signal intensity values for genes altered by ≥3.0-fold. Red denotes high expression and turquoise denotes low expression. Order of samples was dictated by hierarchical clustering. (**B**) Table showing gene name, fold change and p-value for all genes upregulated by ≥3.0-fold. (**C**) Table showing gene name, fold change and p-value for all genes downregulated by ≥3.0-fold. Statistical significance (p<0.05) was calculated using student's t-test followed by Benjamini-Hochberg FDR correction on GeneSpring GX11.0.2 software. Fold change represents a comparison between mean normalised signal intensity for control (n = 3) versus smoke (n = 3) treated PBMCs. Refer to Data S2 for full data sets and Entrez Gene IDs.

Using a 1.5-fold cut-off, PBMCs stimulated with 3% CSE for 24 hours resulted in 3 genes significantly changed after analysis with unpaired *t*-tests and Benjamini-Hochberg FDR correction. 1 was upregulated and 2 were downregulated. However, when looking at the cells activated with 10% CSE, 617 genes were altered. Of these, 306 were downregulated and 311 upregulated. To represent the top genes altered after 24 hours of CSE challenge, we chose a 3-fold cut-off. As predicted by the principle component analysis, no genes were altered by 3-fold after treatment with 3% CSE when analysed using unpaired *t*-tests and Benjamini-Hochberg FDR correction. However, in PBMCs treated with 10% CSE for 24 hours, 85 genes were significantly changed. Of these, 61 genes were upregulated and 24 genes downregulated (Figure 7 A–C).

### Analysis of genes altered in 10% CSE groups in PBMCs after 8 and 24 hours

**Figure 8:**
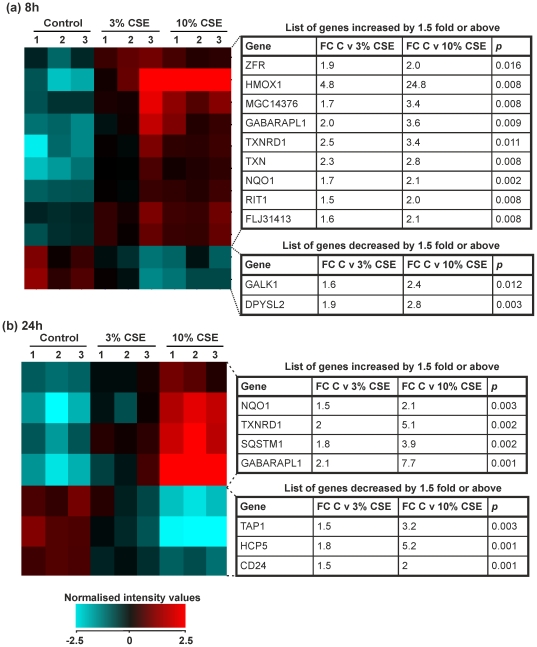
Expression of genes that were changed in both 3 and 10% cigarette smoke extract by 1.5 fold or above compared to control in PBMCs. PBMCs were treated for 8 hours or 24 hours with RPMI-1640 medium (control 1–3), 3% CSE (1–3) or 10% CSE (1–3). RNA was extracted from each sample and gene expression values measured using the Illumina HumanRef-8v3 BeadChip array. (**A**) Heat map representing normalized signal intensity values and list of genes altered by ≥1.5 fold in PBMCs treated with both 3 and 10% CSE after 8 hours. Red denotes high expression and turquoise denotes low expression. Order of samples was dictated by hierarchical clustering. (**B**) Heat map representing normalized signal intensity values and list of genes altered by ≥1.5 fold in PBMCs treated with both 3 and 10% CSE after 24 hours. Red denotes high expression and turquoise denotes low expression. Order of samples was dictated by hierarchical clustering. Statistical significance (p<0.05) was calculated using one-way analysis of variants followed by a Tukey's post-hoc test and Benjamini-Hochberg FDR correction on GeneSpring GX11.0.2 software. Fold change represents a comparison between mean normalised signal intensity for control (n = 3) versus smoke (n = 3) treated PBMCs. Refer to Data S2 for full data sets and Entrez Gene IDs.

There were only a small number of genes elevated in PBMCs treated for both 8 and 24 h after analysis with one-way analysis of variance followed by a Tukeys post-hoc test and Benjamini-Hochberg FDR correction. In the 8 hour group, 9 genes were elevated and 2 genes were repressed (Figure 8A). By 24 hours, 4 genes were upregulated and 3 downregulated (Figure 8B). The majority of genes that were altered over both time points belong to the Nrf2-dependent family of inducible anti-oxidant genes.

### Regulation of target genes; HMOX1, IL8 and TNF in THP-1 cells and PBMCs after stimulation with 3 and 10% CSE after 8 and 24 hours

**Figure 9:**
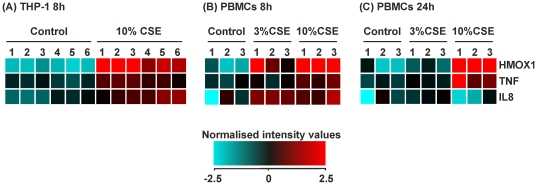
Comparison of target gene expression in THP-1 cells and PBMCs treated with cigarette smoke extract (CSE). We have previously shown that CSE increases the expression of HMOX1, TNF and IL8 in THP-1 cells and PBMCs. RNA was extracted from each sample and gene expression values measured using the Illumina HumanRef-8v3 BeadChip array. (**A**) Heat map representing normalized signal intensities of HMOX1, TNF and IL8 genes from THP-1 monocytes treated for 8 hours with RPMI-1640 (control 1–6) and 10% CSE (1–6). (**B**) Heat map representing normalized signal intensities of HMOX1, TNF and IL8 genes from PBMCs treated for 8 hours with RPMI-1640 (control 1–3), 3% and 10% CSE (1–3). (**C**) Heat map representing normalized signal intensities of HMOX1, TNF and IL8 genes from PBMCs treated for 24 hours with RPMI-1640 (control 1–3), 3% and 10% CSE (1–3). Heat maps were generated using GeneSpring GX11.0.2 software where, red denotes high expression and turquoise denotes low expression.

We have previously shown that monocytes and macrophages stimulated with CSE results in increased levels of heme oxygenase 1, CXCL8 and TNFα [13], [22]. As way of a quality control, here we show that the genes for these products, HMOX1, IL8 and TNF, are elevated in both THP-1 cells (Figure 9A) and PBMCs (Figure 9B) at 8 h after treatment with both 3 and 10% CSE. By 24 h all three genes had returned to control levels in the 3% CSE group. In contrast, in the 10% CSE treated samples HMOX1 and TNF were elevated whereas IL8 gene expression was similar to controls (Figure 9C).

### Ingenuity Pathway Analysis (IPA) of differentially expressed genes in THP-1 cells after 10% CSE exposure for 8 hours

Cigarette smoking is a risk factor for a number of diseases such as COPD, cardiovascular disease, lung cancer and diabetes which have a number of different pathophysiological processes occurring. For this reason, we decided to examine pathways and processes that were affected by smoke in our model using functional pathway analysis tools. We first did a network analysis of genes significantly expressed over a 1.5 fold threshold (Table 1). The pathways shown in Table 1 demonstrate that even a single exposure to 10% CSE results in the activation of genes that are associated with cellular damage and disease (Table 1). Further analysis of genes associated with disease showed that CSE exposure was linked to inflammation, connective tissue disorders and respiratory disease. This fits with a linkage of cigarette smoke to the development of COPD. The molecular and cellular function analysis of these genes (Table S1) also suggests an altered cellular function favouring cell death and abnormal growth.

**Table 1:** Top 5 networks in THP-1 cells after 8 h treatment with 10% CSE.

Associated Network functions	Molecules in Network	Focus Molecules	Score
**Cell Death, Lipid Metabolism, Small Molecule Biochemistry**	AKAP7, CDKN1A, CREB1, CRHR1, CYP1B1, EIF2S1, ERK1/2, FSH, HMOX1, Ifn gamma, IgG, IL5, IL8, IRF8, Jnk, LITAF, LPA, NFkB (complex), NINJ1, P2RY2, PIM1, PMAIP1, PPP1R15A, RNA polymerase II, RND3, SAT1, SERPINB2, SERPINB8, SERPINB10, SOAT1, SQSTM1, TDRD7, TNF, TNFAIP2, TNFAIP3	21	37
**Organismal Injury and Abnormalities, Respiratory Disease, Cell Death**	ADRB2, ANXA1, BIK, CCL18, CTSC, CYSLTR1, DUSP5, DUSP10, G0S2, GBP1 (includes EG:2633), GCLM, GPR109B, IFNGR1, IL5, IL13, IRAK3, KRT15, LGALS3, LPL, LTB, MGST1, MMD, NID1, OSGIN1, RFTN1, RPS6KA2, RRM2, SLC3A2, SP1, SP3, TNF, TP53, TP53I3, TXNRD1, UBQLN2	11	15
**Cellular Assembly and Organisation, Amino Acid Metabolism, Post-Translational modification**	GABARAPL1, HDAC6	1	2
**Molecular Transport, Nucleic Acid Metaolism, Small Molecule Biochemistry**	NME1, SERTAD1	1	2
**Drug Metabolism, Cell Death, Amino Acid Metabolism**	MIR122 (includes EG:406906), SLC7A11	1	2

THP-1 monocytes were treated for 8 hours with RPMI-1640 control medium (n = 6) or 10% CSE-conditioned medium (n = 6). Genes that were significantly modified by ≥1.5-fold, as assessed using student's t-test followed by Benjamini-Hochberg FDR correction, were imported into Ingenuity Pathway Analysis software. The table shows the top 5 networks identified from genes differentially expressed by ≥1.5-fold in THP-1 monocytes treated with 10% CSE.

### Ingenuity Pathway Analysis (IPA) of differentially expressed genes in PBMCs cells after CSE exposure for 8 hours

Network analysis of genes significantly expressed over a 1.5 fold threshold in PBMCs is shown in Table 2. This demonstrates that even a single exposure to 10% CSE results in the activation of genes that are associated with inflammation, cellular damage and growth arrest (Table 2). Further analysis of genes associated with disease showed that CSE exposure was linked to inflammation, infectious diseases and cancer. This fits with a linkage of cigarette smoke to the development of inflammation, increased infection and cancer. The molecular and cellular function analysis of these genes (Table S2) also suggests an altered cellular function favouring cell damage and abnormal growth.

**Table 2:** Top 5 networks in PBMCs after 8 h treatment with 10% CSE.

Associated Network functions	Molecules in Network	Focus Molecules	Score
**Drug Metabolism, Endocrine System Development and Function, Lipid Metabolism**	ACTB, Akt, ARHGEF6, BAG3, CD2, CRTC1, DNAJB1, DNAJB9, FKBP4, FSH, HBP1, HCFC1, Histone h4, Hsp70, Hsp90, HSPA8, IL2RB, IRS2, Lh, MAP1LC3B, MAPK6, MCM5, NQO1, OGT, p85 (pik3r), PGK1, PPP6R3, PRKCB, PSMD1, PTGES3, RAB2A, RBM39, STIP1, VCP, YWHAG	27	35
**Cell Death, Cellular Growth and Proliferation, Hematological System Development and Function**	26s Proteasome, ADRM1, ANXA1, Ap1, Caspase, CD19, CD46, CD81, CDKN1A, COMMD3, Creb,Cyclin E, DPYSL2, DUSP1, EP300, ERK, EWSR1, EZR, HMOX1, IFITM1, Jnk, KLHL21, MAP4K2, NFkB (complex), PDGF BB, PHLDA1, PRNP, PSMA3, PYCARD, RBL2, RELB, RGS1, TXN (includes EG:116484), TXNDC17, UBA7	26	33
**Inflammatory Response, Cellular Movement, Immune Cell Trafficking**	ADAM8, ADM, CD69, CXCR3, DUSP5, Hsp27, HSP90AB1, IFN Beta, Ifn gamma,IgG, IKBKE, IKK (complex), IL12 (complex), Interferon alpha, ITGAL, ITGB2, MYD88, P38 MAPK, PI3K (complex), PMAIP1, PPP1R15A, PSMB9, PSMB10, RGS2, SQSTM1, TAP1, TBK1, TCR, TICAM1, TIMP1, TNF, TNFSF10, TXNRD1, UGCG, ZC3HAV1	25	31
**Cell Cycle, Gene Expression, Connective Tissue Development and Function**	CCNK, CDK9, DDX11/DDX12, DLEU1, E2F1, E2F6, EVL, FEN1, GCLM, GRN, HGF, HIST1H4A (includes others), HSPH1, ID2, ID3, MYC, MYOM2, NRIP1, PHB, PPP2R2A, PRDM5, PRDX1, RAD51, RBL2, SERTAD1, SHMT2, SKP2, SRXN1, SYTL1, TAGLN2, TFDP1, TIAM1, TOP2A, TOPBP1, UXT	15	14
**Cell Death, Cell-To-Cell Signaling and Interaction, Hematological System Development and Function**	ABCB6, AGT, AP1M1, BID, CALR, CANX, CAT, CD8A, CRKL, DOCK2, FXR1, HAGH, HCST, HLA-A,IFIT1, IFITM1, IFNG, IL7, KLRK1, KRT15, MHC Class I (complex), MXD1,Nfat (family), NLRC5, ODC1, PARVG, PELI1, PMAIP1, SAT1, SMARCA4, ST13, TNFRSF1B, TP73, WAS, WIPF1	15	14

PBMCs were treated for 8 hours with RPMI-1640 control medium (n = 3) or 10% CSE-conditioned medium (n = 3). Genes that were significantly modified by ≥1.5-fold, as assessed using student's t-test followed by Benjamini-Hochberg FDR correction, were imported into Ingenuity Pathway Analysis software. The table shows the top 5 networks identified from genes differentially expressed by ≥1.5-fold in PBMCs treated with 10% CSE.

### Ingenuity Pathway Analysis (IPA) of differentially expressed genes in PBMCs cells after CSE exposure for 24 hours

Network analysis of genes significantly expressed over a 1.5 fold threshold in PBMCs is shown in Table 3. By 24 hours, pathways shown in Table 3 demonstrate that there is the activation of protective pathways such as cell death and free radical scavenging. However, there is evidence of considerable intracellular damage, shown through the activation of genes involved in cell survival mechanisms such as cellular assembly and organisation, protein synthesis and cellular maintenance. In addition, clinical pathologies known to be associated with cigarette smoke were upregulated, such as cardiac function and cancer. Again further analysis of the CSE induced inflammation in PBMCs confers cigarette smokes involvement in inflammatory diseases, infection, dermatological conditions and other immune driven disorders (Table S3).

**Table 3:** Top 5 networks in PBMCs after 24 h treatment with 10% CSE.

Associated Network functions	Molecules in Network	Focus Molecules	Score
**Gene Expression, Cell Cycle, Cardiac Damage**	ADA, ATF4, ATP1B3, BTG2, CCNC, CCNL1, CCT3, CCT6A, CDKN1A, DUSP1, ERK, FOXO1, GADD45A, GADD45G, GAPDH, GTF2B, HELZ, Histone h4, HSPB1, IgG, Jnk, KLHL21, MAP1LC3B, MAP4K2, PDGF BB, PPP1R15A, PSMA3, RB1CC1, RGS1, RNA polymerase II, RPL12, SLC38A2, TAF7, TAF10, TXN (includes EG:116484)	29	34
**Cellular Development, Hematological System Development and Function, Hematopoiesis**	ABCC3, BCL11B, CD19, CD37, CD69, CD81, CYBA, CYBB, DNAJB4, EHD1, HLA-A, HLA-F, IFITM1,IFN Beta, Ifn gamma, IFNGR2,IL12 (complex), IL12 (family), IL7R, Immunoglobulin, Interferon alpha, IRF1, IRF8, MHC Class II (complex), MYD88, PMAIP1, PSMB8, PSMB9, SCO2, STAT1, STAT2, TAP1, TCR, TNF, XAF1	27	30
**Cell Death, Free Radical Scavenging, Cellular Growth and Proliferation**	ADM, Akt, DDIT3, DNAJB1, FCRLA, HLA-DMB, HMOX1, Hsp27, Hsp70, Hsp90, HSP90AB1, HSPA8,Igm, IL10,KDM3A, KEAP1, LDL, NAMPT, NCF1, NFE2L2, NFkB (complex), NQO1, P38 MAPK, PI3K (complex), Pkc(s), SQSTM1, STAT5A, STIP1, TAX1BP1, TBK1, TIMP1, TNFRSF25, TNFSF13B, UBA7, VCP	25	27
**Cellular Assembly and Organization, Cellular Function and Maintenance, Protein Synthesis**	ACTB, APP, ARHGDIB, CBLB, CYTH1, CYTIP, DAPP1, DBNL, GABARAPL1, GRB2, HCST, HDAC6, ICAM3, ITGAL, ITGB2, LCP2, LDLRAP1, MAP3K5, MAP4K1, MARS, MFGE8, QARS, RAC2, SMARCA4, SMARCC1, SMARCE1, SNX27, SORL1, SPRY2, SUMO2, SYK, TRIP12, U2AF1, VAV, XPC	18	16
**Cancer, Reproductive System Development and Function, Cellular Development**	ACTN1, Ap1, CD55, CD97, CDC42EP4, CDK14, Creb, DNAJB9, E2f, ERK1/2, FSH, Histone h3, HSD3B2, KIDINS220, Lh, MAL, MAP1LC3B, MAPK6, MFAP1, MT2A, PGRMC1, PPAP2A, PSMD1, PTGER2, PTGS1, RAB4A, RASAL2, RGS12, SH3BP4, SH3GLB2, STK39, TFDP1, TLN1, TUBA1A, YTHDF2	15	14

PBMCs were treated for 24 hours with RPMI-1640 control medium (n = 3) or 10% CSE-conditioned medium (n = 3). Genes that were significantly modified by ≥1.5-fold, as assessed using student's t-test followed by Benjamini-Hochberg FDR correction, were imported into Ingenuity Pathway Analysis software. The table shows the top 5 networks identified from genes differentially expressed by ≥1.5-fold in PBMCs treated with 10% CSE.

### IPA canonical pathway analysis of differentially expressed genes in THP-1 cells after CSE exposure for 8 hours

**Figure 10:**
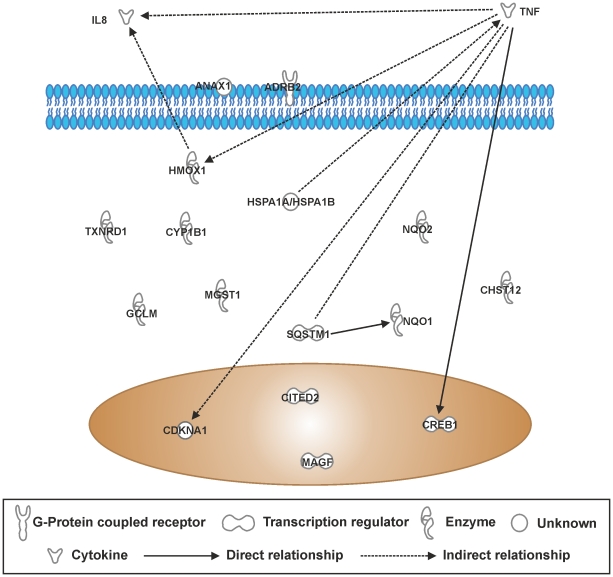
Top 5 canonical pathway gene interactions in THP-1 cells treated cigarettes smoke extract for 8 hours. The top 5 canonical pathways identified from genes differentially expressed by ≥1.5-fold in THP-1 cells treated with 10% CSE. RNA was extracted from each sample and gene expression levels were measured using the Illumina HumanRef8-v3 BeadChip Array.Genes that were significantly modified by ≥1.5-fold according to the student's t-test and Benjamini-Hochberg FDR correction were imported into Ingenuity Pathway Analysis software. Interactions of these genes are represented in this schematic.

A list of genes differentially expressed by ≥1.5-fold in THP-1 monocytes in response to 10% CSE was compiled on GeneSpring GX11.0.2 and uploaded onto IPA to identify canonical pathways represented by the ≥1.5-fold gene list. The “Nrf2-mediated oxidative stress response” pathway was the most significantly represented by genes in the gene list (*p* = 1.39×10^−5^), and was over the threshold for ratio of genes represented in the gene list versus total genes in the pathway (8/188; Table 4). Similarly, the “aryl hydrocarbon receptor signalling” pathway was significantly represented (*p* = 2.21×10^−4^) and was just over the ratio threshold (6/141). “Glucocorticoid receptor signaling” was significantly represented (*p* = 1.01×10^−3^) and passed the ratio threshold (7/278). However, “Airway pathology in chronic obstructive pulmonary disease” was significantly represented (*p* = 1.19×10^−3^) but failed the ratio threshold (2/8). Pathway connections of genes involved in the top 5 canonical pathways, as determined by known experimental association, shows that TNF, IL8 and the oxidant activated enzyme NQO1 are the main molecular hubs in CSE –induced inflammation in these cells, suggesting that CSE is a potent oxidant (Figure 10).

**Table 4:** Top canonical pathways in THP-1 cells after 8 h treatment with 10% CSE.

Canonical Pathways	Molecules in Network	p-value	Ratio
**NRF-2 Mediated Oxidative Stress**	HMOX1, MGST1, NQO2, NQO1, SQSTM1, GCLM, TXNRD1, MAFG	1.39×10−^5^	8/188 (0.043)
**Aryl Hydrocarbon Receptor Signaling**	MGST1, NQO2, NQO1, CDKN1 A, TNF, CYP1B1	2.21×10^−4^	6/141 (0.043)
**Xenobiotic Metabolism Signaling**	HMOX1, MGST1, NQO2, NQO1, CHST12, TNF, CYP1B1, CITED2	2.28×10^−4^	8/265 (0.03)
**Glucocorticoid Receptor Signaling**	IL8, HSPA1B, ANXA1, CREB1, CDKN1A, TNF, ADRB2	1.01×10^−3^	7/278 (0.025)
**Airway Pathology In Chronic Obstructive Pulmonary Disease**	IL8, TNF	1.19×10^−3^	2/8 (0.25)

THP-1 cells were treated for 8 hours with RPMI-1640 control medium (n = 6) or 10% CSE-conditioned medium (n = 6). Genes that were significantly modified by ≥1.5-fold, as assessed using student's t-test followed by Benjamini-Hochberg FDR correction, were imported into Ingenuity Pathway Analysis software. The table shows the top 5 canonical pathways identified from genes differentially expressed by ≥1.5-fold in THP-1 cells treated with 10% CSE. The P-value for association of genes and the described canonical pathways, and the ratio of significantly differentially expressed pathway components compared to the total components in that pathway was generated using Fisher's exact test.

### IPA canonical pathway analysis of differentially expressed genes in PBMCs cells after CSE exposure for 8 hours

**Figure 11:**
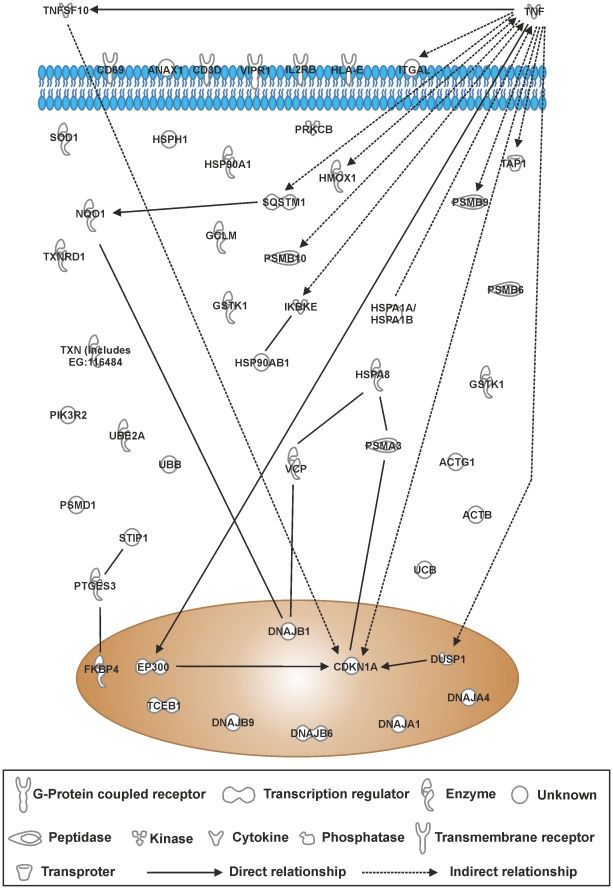
Top 5 canonical pathway gene interactions in PBMCstreated with 10% CSE for 8 hours. The top 5 canonical pathways identified from genes differentially expressed by ≥1.5-fold in PBMCs treated with 10% CSE. RNA was extracted from each sample and gene expression levels were measured using the Illumina HumanRef8-v3 BeadChip Array.Genes that were significantly modified by ≥1.5-fold as assessed using student's t-test followed by Benjamini-Hochberg FDR correction were imported into Ingenuity Pathway Analysis software. Interactions of these genes are represented in the schematic as determined by experimentation in human cells.

A list of genes differentially expressed by ≥1.5-fold in PBMCs in response to 10% CSE was compiled on GeneSpring GX11.0.2 and uploaded onto Ingenuity Pathway Analysis to identify canonical pathways represented by the ≥1.5-fold gene list. As in THP-1 cells, “Nrf2-mediated oxidative stress response” pathway was the most significantly represented by genes in the gene list in PBMCs (*p* = 4.9×10^−12^), and was over the threshold for ratio of genes represented in the gene list versus total genes in the pathway (22/188; Table 5). Similarly, the “protein ubiquitination pathway” was significantly represented (*p* = 8.02×10^−7^) and was over the ratio threshold (19/269). “Aldosterone signaling in epithelial cells” was significantly represented (*p* = 4.17×10^−5^) and passed the ratio threshold (12/157). Similarl to THP-1 cells, “glucocorticoid receptor signaling” in PBMCs was significantly represented (p = 1.70×10^−4^). “crosstalk between dendritic cells and natural killer cells” was also significantly represented (p = 3.33×10^−4^) with a threshold ratio of (8/91), suggesting activation of the adaptive immune system to CSE. Pathway associations of genes involved in the top 5 canonical pathways shows that, similar to THP-1 cells, TNF and the oxidant-activated enzyme NQO1 represent molecular hubs in PBMCs stimulated with CSE (Figure 11). In addition, there appears to be a significant activation of the chaperone/ubiquitination/proteosome pathway, suggesting the presence of irrepairable protein damage/modifications.

**Table 5:** Top canonical pathways in PBMCs after 8 h treatment with 10% CSE.

Canonical Pathways	Molecules in network	p-value	Ratio
**NRF-2 Mediated Oxidative Stress**	AKR7A2, UBB, SOD1, ACTB, NQO1, DNAJA4, DNAJA1, DNAJB9, ACTG1, TXNRD1, EP300, HMOX1, STIP1, VCP, PIK3R2, SQSTM1, DNAJB6, DNAJB1, TXN (includes EG:116484), GCLM, GSTK1, PRKCB	4.90×10−^12^	22/188 (0.117)
**Protein Ubiquitination Pathway**	PSMB9, UBB, PSMA3, UBE2A, HSPA1A/HSPA1B, PSMB10, HSPH1, DNAJB9, DNAJA1, PSMB6, TAP1, TCEB1, HSPA8, HSP90AB1, HSP90AA1, PSMD1, DNAJB6, DNAJB1, UBC	8.02×10^−7^	19/269 (0.071)
**Aldosterone Signaling in Epithelial Cells**	HSPA8, HSP90AB1, HSPA1A/HSPA1B, DUSP1, HSPH1, HSP90AA1, PIK3R2, DNAJB6, DNAJB1, DNAJB9, DNAJA1, PRKCB	4.17×10^−5^	12/157 (0.076)
**Glucocorticoid Receptor Signaling**	HSPA1A/HSPA1B, IKBKE, CD3D, EP300, HSPA8, HSP90AB1, VIPR1, DUSP1, ANXA1, CDKN1A, FKBP4, PTGES3, HSP90AA1, PIK3R2, TNF	1.70×10^−4^	15/278 (0.054)
**Crosstalk between Dendritic Cells and Natural Killer Cells**	HLA-E, CD69, ACTB, TNFSF10, TNF, ACTG1, IL2RB, ITGAL	3.33×10^−4^	8/91 (0.088)

PBMCs were treated for 8 hours with RPMI-1640 control medium (n = 6) or 10% CSE-conditioned medium (n = 6). Genes that were significantly modified by ≥1.5-fold, as assessed, using student's t-test followed by Benjamini-Hochberg FDR correction were imported into Ingenuity Pathway Analysis software. The table shows the top 5 canonical pathways identified from genes differentially expressed by ≥1.5-fold in PBMCs treated with 10% CSE. The P-value for association of genes and the described canonical pathways, and the ratio of significantly differentially expressed pathway components compared to the total components in that pathway was generated using Fisher's exact test.

### IPA canonical pathway analysis of differentially expressed genes in PBMCs after CSE exposure for 24 hours

**Figure 12:**
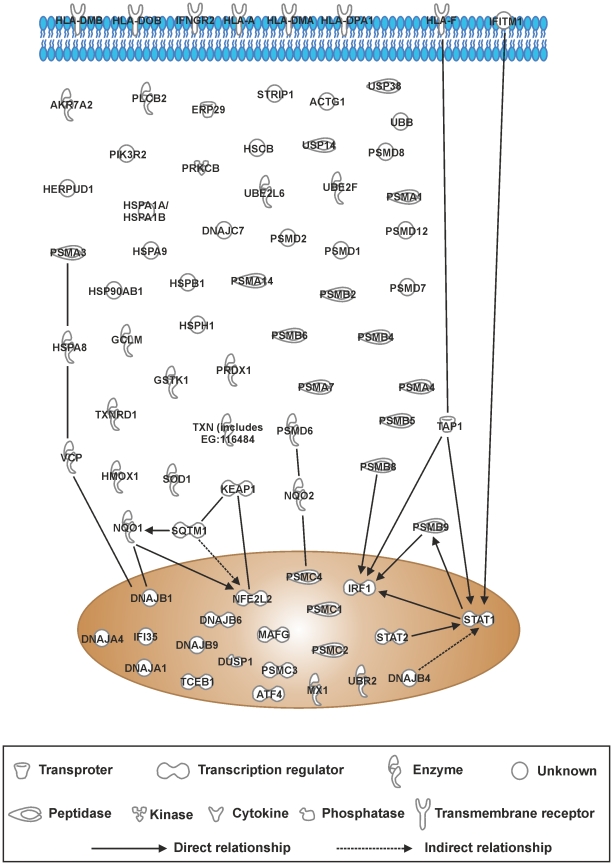
Top 5 canonical pathway gene interactions in PBMCs treated with 10% CSE for 24 hours. The top 5 canonical pathways identified from genes differentially expressed by ≥1.5-fold in PBMCs treated with 10% CSE. RNA was extracted from each sample and gene expression levels were measured using the Illumina HumanRef8-v3 BeadChip Array and genes that were modified by ≥1.5-fold and significantly changed according to student's t-test followed by Benjamini-Hochberg FDR correction were imported into Ingenuity Pathway Analysis software. Interactions of these genes are represented in the figure.

When the ≥1.5-fold gene list of PBMCs treated with 10% CSE was loaded into IPA, only the top two of the top 5 canonical pathways listed passed the significant threshold criteria. These were the same as for the 8 hour samples, however the order was reversed. The “protein ubiquitination pathway” now being the most significant (*p* = 3.13×10^−20^) with a threshold ration of 48/269 and “NRF2-mediated oxidative stress response” pathway had a *p* = 1.6×10^−14^ and a threshold of 31/188 (Table 6). This suggests that survival pathways had been upregulated in these cells. Similarly, to the 8 hour data the major genes upregulated were associated with the chaperone/ubiquitination/proteosome pathway, suggesting extensive cell damage (Figure 12). However, since 98% of these cells were viable, there is the suggestion that these cells were pushed towards a cell survival pathway. To contextualise this finding, it is noteworthy to mention that THP-1 cells and PBMCs treated with 20% CSE results in more than 50% cell death (in house data; data not shown).

**Table 6:** Top canonical pathways in PBMCs after 24 h treatment with 10% CSE.

Canonical Pathways	Molecules in Network	p-value	Ratio
**Protein Ubiquitination Pathway**	USP14, PSMA3, PSMD7, DNAJB4, HSPA1A/HSPA1B, UBR2, PSMB8, DNAJA1, TCEB1, TAP1, PSMB6, UBE2F, HSP90AB1, HLA-A, PSMD14, DNAJB1, PSMC2, PSMB4, PSMB9, UBB, PSMB5, HSPH1, USP38, HSPA9, PSMC4, PSMD6, PSMA1, DNAJB9, UBE2L6, PSMD8, HSPA8, PSMB7, PSMC1, HSCB, PSMB2, PSMD2, PSMD12, PSMA4, PSMD1, DNAJB6, PSMC3, DNAJC7, HSPB1	3.13×10−^20^	43/269 (0.160)
**NRF-2 Mediated Oxidative Stress**	AKR7A2, USP14, PRDX1, DNAJB4, NQO2, DNAJA4, DNAJA1, MAFG, HMOX1, KEAP1, VCP, ATF4, GCLM, TXN (includes EG:116484), DNAJB1, PIK3R2, NFE2L2, GSTK1, UBB, SOD1, NQO1, HERPUD1, DNAJB9, ACTG1, TXNRD1, ERP29, STIP1, DNAJB6, SQSTM1, DNAJC7, PRKCB	1.60×10^−14^	31/188 (0.165)
**Antigen Presentation Pathway**	PSMB9, HLA-DMA, PSMB5, HLA-A, HLA-DOB, HLA-DMB, PSMB8, TAP1, PSMB6, HLA-DPA1, HLA-F	1.62×10^−7^	11/43 (0.256)
**Interferon Signaling**	IFNGR2, IFITM1, MX1, IFI35, STAT2, PSMB8, STAT1, TAP1, IRF1	4.82×10^−8^	9/34(0.265)
**Aldosterone Signaling in Epithelial Cells**	PLCB2, DNAJB4, HSPA1A/HSPA1B, HSPH1, HSPA9, DNAJB9, DNAJA1, HSPA8, HSP90AB1, HSCB, DUSP1, DNAJB6, DNAJB1, PIK3R2, DNAJC7, PRKCB, HSPB1	3.09×10^−6^	17/157 (0.108)

PBMCs were treated for 24 hours with RPMI-1640 control medium (n = 6) or 10% CSE-conditioned medium (n = 6). Genes that were significantly modified by ≥1.5-fold, as assessed, using student's t-test followed by Benjamini-Hochberg FDR correction were imported into Ingenuity Pathway Analysis software. The table shows the top 5 canonical pathways identified from genes differentially expressed by ≥1.5-fold in PBMCs treated with 10% CSE. The P-value for association of genes and the described canonical pathways, and the ratio of significantly differentially expressed pathway components compared to the total components in that pathway was generated using Fisher's exact test.

### Database Searches

PubMed was used to establish which of the ≥2-fold genes identified in THP-1 cells in the current study were also differentially expressed in previous studies involving cigarette smoke (Table 7). Of the genes that were differentially expressed by ≥2-fold in the current study, 17 have previously been shown to be affected by cigarette smoke at a gene- and/or protein- level. In most cases, genes reported in previous studies were regulated in the same direction as the current study, with the exception of TNF (upregulated in the current study and in [15], [23] but downregulated in [24]). In contrast SERPINB2 was downregulated in THP-1 cell, but upregulated in PBMCs in this current study. The data on SERPINB2 in PBMCs is in-line with what has previously been shown [25], [26], [27]. Gene expression changes in previous studies were observed in a range of cells and tissues, including airway epithelial cells, lung tissue, macrophages and monocytes.

**Table 7:** Genes observed to be differentially expressed by ≥2-fold in THP-1 monocytes in response to cigarette smoke were also differentially expressed in previous studies examining the effects of cigarette smoke on various cell types *in vivo* and *in vitro*.

Symbol	Entrez ID	Reg	FC	Description	Reference
**HMOX1** 1 **^,^** 2 **^,^** 3	3162	up	22.6	heme oxygenase 1	[31], [36], [58], [59], [60], [61]
**MSC**	9242	up	5.8	musculin (activated B-cell factor-1)	
**GCLM** 1 **^,^** 2 **^,^** 3 **^,^** 4 **^,^** 5	2730	up	4.8	glutamate-cysteine ligase, modifier subunit	[27], [30], [32], [33], [36], [37], [42]
**NQO1**	1728	up	3.8	NAD(P)H quinone oxioreductase 1	[31], [32], [33], [36], [53], [62]
**RIT1**	6016	up	3.1	Ras-like without CAAX 1	
**SRNX1** 1	140809	up	2.3	sulfiredoxin 1	[36], [63]
**CYP1B1** 1 **^,^** 2 **^,^** 5	1545	up	2.3	cytochrome P450, family 1, subfamily B, polypeptide 1	[27], [32], [35], [36], [43], [52], [64], [65], [66]
**FTHL12**	2504	up	2.9	ferritin, heavy polypeptide-like 12	
**ME1** 1 **^,^** 2	4199	up	3.4	NADP(+)-dependent malic enzyme 1	[32], [40]
**PANX2**	56666	up	2.7	pannexin 2	
**HMFN0839** 1	84803	up	2.5	Glycerol -3-phosphate acetyltransferase 9	[53]
**IL8** 2 **^,^** 3 **^,^** 4 **^,^** 7	3576	up	2.7	interleukin 8	[13], [14], [31], [67]
**FLJ20489**	55652	up	2.7	Solute carrier family 48 (heme transporter), member 1	
**FTHL7**	2500	up	2.5	ferritin, heavy polypeptide-like 7	
**TXNRD1** 1 **^,^** 2 **^,^** 5	7296	up	2.6	thioredoxin reductase 1 (transcript variant 1)	[27], [30], [32], [36], [52], [65]
**SLC7A11** 5	23657	up	2.0	solute carrier family 7	[27]
**PIR** 1	8544	up	2.7	pirin (transcript variant 2)	[32], [35], [68]
**C16orf28**	65259	up	2.4	chromosome 16 open reading frame 28 mRNA	
**FTHL3**	2498	up	2.0	ferritin, heavy polypeptide-like 3	
**SQSTM1**	8878	up	2.5	sequestosome 1	
**TXNRD1** 1 **^,^** 2 **^,^** 5	7296	up	2.3	thioredoxin reductase 1 (transcript variant 4)	[27], [30], [32], [36], [52], [65]
**GABARAPL1** 2	23710	up	2.7	GABA(A) receptor-associated protein like 1	[69]
**PIR** 1	8544	up	2.7	pirin (transcript variant 1)	[32], [35], [68]
**TNF** 2 **^,^** 3 **^,^** 6 **^,^** 7	7124	up	2.1	tumor necrosis factor	[13], [15], [23], [24]
**PGD** 1 **^,^** 4	5226	up	2.5	phosphogluconate dehydrogenas	[29], [30], [32]
**SERPINB2**	5055	down/up	3.8	Plasminogen activator inhibitor	[25], [26], [27]
**GPR18**	2841	down	2.2	N-arachidonyl glycine receptor	
**LOC389816**	389816	down	2.3	Leucine-rich repeat-containing protein 26	
LOC389816*	389816	down	2.2	Leucine-rich repeat-containing protein 26	

1 Observed in airway epithelial cells,

2 Observed in lung tissue,

3 Observed in monocytes,

4 Observed in macrophages,

5 Observed in oral leukoplakia,

6 Observed in peripheral blood mononuclear cells (PBMCs),

7 Observed in bronchioalveolar lavage (BAL), The symbol LOC3898186* is a predicted cDNA sequence similar to LOC3898186.

## Discussion

There are over 10,000 papers on the effects of cigarette smoke in *in vivo* models and on isolated cell systems. Currently, however, there is considerable variation in the responses observed between studies, making it hard to draw useful conclusions [28]. Also, despite the clear link between cigarette smoke and disease we still know little about how smoke causes inflammation in man *in vivo*. In this study, we chose to use a single acute exposure to smoke in a human monocytic cell line, THP-1, and PBMCs.

In THP-1 cells, we identified a total of 80 genes that were significantly altered by more than 1.5-fold, and 33 genes significantly altered by more than 2-fold in response to smoke. Not surprisingly, our study shows that PBMCs are more sensitive to CSE activation than the THP-1 cell line, with a total of 617 genes upregulated in PBMCs compared with 117 genes significantly altered in THP-1 cells by ≥1.5 fold. The increased sensitivity of PBMCs to CSE, when compared with THP-1 cells has previously been seen for individual mediators such as CXCL8 [13]. We also show, through pathway analysis, that the response to cigarette smoke in THP-1 monocytes is driven by genes associated with oxidative stress, TNF and inflammatory pathways, which is similar to observations recorded in other cell types [29], [30]. The major genes pathways in PBMCs stimulated with CSE were similar in both cell types, with the induction of a predominantly anti-oxidant and TNF pathways at 8 hours. In THP-1 cells (8 hours), 10 genes have not previously been associated with the cigarette smoke exposure. These are RIT1, FTHL12, PANX2, FTHL7, C16orf28, FTHL3, SQSTM1, GPR18, LOC389816 and FLJ20489. Interestingly, 2 of these genes were significantly differentially regulated in PBMCs at 8 hours (RIT1 and SQSTM1) and 3 at 24 hours (RIT1, SQSTM1 and FTHL3). RIT1 is associated with tumorgenesis and SQSTM1 oxidative stress and TLR/IL-1 signal activation of NFκB. These results demonstrates that THP-1 cells may be a useful tool and an alternative to primary human monocytes in the dissection of some signalling processes induced by cigarette smoke.

We observed in both THP-1 cells and PBMCs an increase in oxidative stress response genes, like heme oxygenase 1 (HMOX1), NAD(P)H quinone oxioreductase 1 (NQO1), thioredoxin reductase 1 (TXNRD1) and cytochrome P450 genes (CYP1B1), which was consistent with previous studies of cigarette smoke [27], [31], [32], [33]. Furthermore, the observation that our target genes, IL8, HMOX1 and TNF were upregulated confirmed previous research within our group [13], [22] and documented by others [34], indicating that cigarette smoke-induced gene expression changes in this study were characteristic of what is already known to occur.

The up-regulation of genes related to inflammation strengthens the evidence linking this pathway with the response to smoke [25], [29], [30], [33], [35] and also demonstrates that THP-1 cells can be used as a model for studying certain inflammatory profiles of cigarette smoke in primary human monocytes. We observed an up-regulation in the inflammatory mediators, IL8 and TNF, using the microarray. The release of CXCL8 and TNFα in response to smoke has been observed previously in primary monocytes, macrophages and airway smooth muscle cells [13], [14], [15], [23], and may be crucial in the development of COPD, a disease characterized by inflammation. A number of pro-inflammatory and innate immune genes that have previously been associated with the cigarette smoke response, such as IL-1β [27] and IL-6 [36], were not altered in our study. These are known to be early response genes, and may be a result of the time point chosen. In *in vivo* models performed within our group we expect to see the proteins at maximal levels within 2 hours. Furthermore, several studies show that smoke downregulates the expression of monocyte-derived macrophage genes associated with innate immunity and inflammation [33], [37], and potentially has an immunosuppressive effect [38], which may explain the increased susceptibility to bacterial infection in COPD patients. It is possible that the multi-component nature of cigarette smoke has varying effects on different immune pathways and cell types, which together contribute to the pathogenesis of COPD. However, both in THP-1 cells and PBMCs an activation of both innate immune genes and the adaptive immune system was observed using our preparation of CSE. The differences observed in other studies may be due to the use of different cell types and smoke treatment protocols. For example we have previously shown that CSE down regulates the inflammatory response in Type I pheumocytes [39].

It is worth noting that our study differs from many published studies in which macrophages were isolated from lungs of patients who smoked [33], [40], [41]. In many of these studies, macrophages were profiled so that the clues to the pathogenesis of COPD could be identified. In contrast, in this study we have looked at smoke on naive monocytes in an attempt to understand the possible initiating factors that may contribute to the pathogenesis of smoking related disorders such as COPD and cardiovascular disease. The large number of oxidative stress and xenobiotic metabolism genes altered in the current study is consistent with the notion that the oxidative burden caused by repetitive, long-term smoking may contribute to the pathogenesis of COPD. HMOX1, NQO1, CYP1B1 and TXNRD1 in particular have frequently been associated with cigarette smoke in transcriptomic studies in other experimental models [42], [43], [44], [45]. Oxidative stress is associated with the pathogenesis of COPD through mechanisms including the oxidation of arachidonic acid to isoprostanes, which cause bronchoconstriction and release of plasma exudate [20] Furthermore, by modifying carcinogens into active, mutagenic metabolites, NQO1, CYP1B1 and EVL have been implicated in the pathogenesis of human tumours [27], [46].

A possible reason that cigarette smoke is well tolerated by the immune system is alluded to in our data - although cells were 98% viable at both 8 and 24 hours, signs of significant protein modification were observed. Even at 8 hours after a single stimulation with CSE, PBMCs have upregulated significant cell survival mechanisms, including inducible anti-oxidant mechanisms (Nrf2 regulated genes), chaperone/protein re-folding systems (HSPs) and the induction of many components of the ubiquitination/proteosome system (removal of damaged proteins). In addition, TAP1 is induced, which is involved in pumping degraded cytosolic peptides across the endoplasmic reticulum system. It is possible that although PBMCs are pushed down a cell survival route, they may contain residual damaged proteins or miss repaired DNA, which are known to be precursors of inflammation and cancer. Interestingly, by 24 hours the ubiquitination/proteosome system predominates and there are signs of adaptive immune activation as evidenced by increased IFN signaling and induction of a number of HLA cell surface receptors. Our results, therefore, provide a useful insight into possible molecular mechanisms behind cigarette smoke-induced diseases.

A recently developed hypothesis is that monocytes and macrophages change their profile from a pro-inflammatory phenotype (M1) to a tissue remodelling phenotype when in diseased tissue [41], [47]. It has also been shown that in COPD patients there is a polarisation towards an M2/remodelling phenotype [41]. In our study, a number of M1 genes (CD69, TNF, TNFSF10, BBP4) are upregulated after 8 hours CSE exposure. After 24 h the profile is still balanced towards a pro-inflammatory phenotype, with IRF1 and CD69 still upregulated, though there is evidence of anti-inflammatory activity with increased expression of IL-10. The differences between our study and the studies by Morris *et al* [44] and Shaykiev *et al* [45] may be due to the different cell types looked at - macrophages and monocytes - and that in our study, cells were only exposed to one cycle of cigarette smoke. It can be envisaged that repeated exposure to cigarette smoke could push monocytes towards an M2 phenotype.

In the present study it is possible that a change in the phenotype occured between the 8 and 24 hours time point. This was evident by the induction in adaptive immune genes, linked to antigen presentation pathway, IFN signaling and aldosterone signalling (Table 6). Interestingly, the induction of different HLA complexes in our mononuclear cells indicates an activation of monocytes towards an antigen presenting pathway within the peripheral blood. Monocytes in the blood are capable of differentiating into macrophages or dendritic cells [48]. In the current study, this possibility is confirmed by pathway analysis of genes in PBMCs, where there was a significant alteration in genes associated with “crosstalk between dendritic cells and natural killer cells”. This, one can envisage, leads to lymphocyte activation by the presentation of cigarette smoke-derived epitopes to B- and T-cells. Evidence from this study to support this phenomenon is the observed increase in IFN signalling, an event associated with lymphocyte activation. This process can result in immune tolerance (after repeated exposure), which could explain why cigarette smoking is usually well tolerated and can take decades before adverse effects clinically manifest themselves. Alternatively, immune activation can occur which is clearly evident in smoking related diseases such as atherosclerosis and COPD. The likelyhood is that both processes are occurring in people that smoke cigarettes.

In the current study SERPINB2 was downregulated in THP-1 cells but upregulated in PBMCs. This gene codes for the serine peptidase inhibitor plasminogen activator inhibitor 2 (PAI2), which acts by inhibits urokinase plasminogen activator. It is worth noting that tumour metastasis correlates with plasminogen activity due to the degradation of the extracellular matrix by the plasminogen product, plasmin [49]. PAIs are prognostic markers for breast cancer, with PAI2 expression in breast cancer patients being linked with increased survival [49]. Another inhibitor of protease activity, TIMP1, was significantly downregulated in PBMCs. This enzyme inhibits the activity of tissue metalloproteinases, which are known to degrade structural tissue matrix components. These zinc-dependent neutral endopeptidases have been associated with cancer and COPD [50], [51].

In conclusion, we use microarray technology to show that CSE causes significant transcriptomic changes in THP-1 monocytes and PBMCs. A number of studies have also used transcriptomics to study the impact of acute cigarette smoke exposure on other human cells, and observed changes in antioxidant and immune genes that are consistent with our findings [33], [52], [53], whilst other studies report immunosuppressive effects of cigarette smoke in human cells [37]. Such differences may relate to cell type, strength/duration of smoke stimulation, and analysis/interpretation of data, and illustrate the problems in defining meaningful conclusions from analysis of large datasets. Nevertheless, our findings provide insight into the possible mechanisms by which smoke provokes the inflammation that is characteristic of smoking-related diseases, such as COPD. This study provides 10–20 genes to validate and investigate in whole body smoke-induced inflammation, to see if these genes are important in a more complex environment.

## Materials and Methods

### Ethics Statement

Human blood for the isolation of PBMCs was taken after ethical consent from healthy volunteers. This part of the study was covered by Ethics held by Mark Paul-Clark and granted by The Royal Brompton Ethics Committee, project entitled “Role of TLR2 in the sensing of oxidants and ensuing inflammation: implications for Therapeutic Intervention”. Ethics Ref Number: 08/H0708/69.

### Materials

All cell culture plastics and general disposables were obtained from Fisher Scientific (Loughborough, Leicestershire, UK). Unless stated otherwise, all cell culture reagents were supplied by Invitrogen (Paisley, Renfrewshire, UK). General laboratory reagents were purchase from Sigma-Aldrich (Poole, Dorset, UK).

### Cell Culture

THP-1 monocytes were obtained from the European Collection of Cell Cultures (Wiltshire, UK) and cultured under sterile conditions in RPMI 1640 medium supplemented with 10 mM L-glutamine, 10% filtered heat-inactivated foetal calf serum (FCS) and 100 U/ml penicillin/streptomycin. Cells were maintained in a humidified atmosphere at 37°C containing 5% CO_2_. Unless stated otherwise, all cell culture reagents were supplied by Invitrogen (Paisley, Renfrewshire, UK).

### Isolation of Human Peripheral Blood Mononuclear Cells (PBMCs)

Human PBMCs (70% lymphocytes, 30% monocytes) were isolated as described previously [54]. Briefly, fresh citrated blood was layered on a Fircoll gradient (Histopaque gradient, Histopaque 1077 & Histopaque 1119) and centrifuged (400 g; 20°C) for 30 minutes. PBMCs were collected, washed with pre-warmed RPMI 1640 and pelleted by centrifugation (450 g; 24°C). Cells (10^6^/ml) were plated in 6 well culture plates.

### Preparation of Cigarette Smoke Extract (CSE)

To prepare CSE, four full strength Marlboro cigarettes (filters removed) were combusted through a modified 60 ml syringe apparatus and the smoke passed through 100 ml of RPMI 1640. Each cigarette yielded 5 draws of the syringe (to 60 ml mark), with each individual draw taking approximately 10 seconds to complete. CSE was then passed through a 0.25 µm filter to sterilise and remove particulate matter, and was used immediately at a 10% concentration diluted in control media [13]. As described previously [44], smoke extract “strength” was evaluated by measuring nitrite using the Griess reaction [13], [19] to ensure continuity between batches. In all experiments nitrite levels in 100% cigarette smoke extract was between 12 and 16 µM. It is worth noting that CSE (1–100%) made using this method does not contain detectable levels of bacterial antigens for TLR4 (endotoxin; measured by the Eotoxate™, Sigma, Poole, UK) or TLR2 (LTA; measured by in house ELISA).

### Treatment of Cells

THP-1 monocytes were plated at a density of 1×10^6^ cells/ml onto 6-well plates and allowed to equilibrate for 16 h before being treated for 8 h with fresh 10% CSE or RPMI-1640 as described previously [13]. After 8 h, RNA was then extracted using RNeasy™ (Qiagen, Sussex, UK) as described in the manufacturers' handbook. A control and a CSE sample set were prepared on each day for three consecutive days using fresh reagents on each day. The first 3 sample sets (n = 1–3) were prepared and analysed 3 months before the second set (n = 4–6).

### Gene Array

Total RNA was subject to standard microarray procedures. Samples were converted to cDNA, labelled, and hybridized to the Illumina HumanRef-8v3 BeadChip array (Illumina, UK). Quality control and basic interpretation of data was performed at St Bartholomew's and The London Genome Centre (BLGC), Queen Mary, University of London before datasets were received for further analysis.

### Data Analysis

Analysis of datasets was performed using GeneSpring GX 11.3 (Agilent Technologies). Raw data were pre-processed to remove variability across and within array samples. To minimize non-biological variability across arrays, raw data were first log_2_ transformed and then quantile normalized [55], which is the recommended normalization algorithm for Illumina BeadChip array analyses [56]. Normalization at the level of genes was performed on GeneSpring GX 11.3.

Samples were sorted into conditions based on the treatment applied: controls 1–6 and CSE 1–6 for THP-1 cells, and for PBMCs; controls 1–3, 3% CSE 1–3 and 10% CSE 1–3. It is important to note that for the PBMCs samples control 1, 3% CSE 1 and 10% CSE 1 were all from the same donor. Very Stringent filtering of the dataset was performed by selecting only the genes that had detectable signal intensity value in all samples (filter by flags present in all samples). Fold change differences between control- and CSE-treated samples were calculated using cut-offs of 1.5-, 2-fold and 3-fold for statistically significant genes. These genes were identified using unpaired *t*-test (p<0.05) with Benjamini-Hochberg False Discovery Rate (FDR) correction for single group comparisons [57]. Where more than one group were analysed, a one-way analysis of variants followed by a Tukey's post-hoc test and Benjamini-Hochberg FDR correction was used. Significantly differentially expressed genes at each fold-change cut-off were used to generate hierarchical clustering plots using Pearson's centred correlation and Ward's linkage rule, and were displayed as heat maps.

Data sets consisting of genes significantly altered by ≥1.5- fold (compiled on GeneSpring GX11.3) were uploaded onto Ingenuity Pathway Analysis (IPA; Ingenuity ® Systems, www.ingenuity.com) and mapped to Ingenuity's Knowledge Base. The significance of the association between the dataset and the canonical pathways were measured using a ratio of number of genes from the dataset that map to the pathway divided by the total number of genes in that pathway and p-value was generated using the Fisher's exact test. A threshold of 0.10 was used to indicate canonical pathways that are significantly represented by genes in a gene list.

Database searches using PubMed, GeneCards and other sources were performed to identify differentially expressed genes from the current study that were also found to be differentially expressed or important in the response to cigarette smoke in previous studies.

## Supporting Information

Data S1 **Illumina Raw data for THP-1 cells treated with 10%CSE.** THP-1 cells were treated for 8 hours with RPMI-1640 control medium (n = 6) or 10% CSE-conditioned medium (n = 6). Total RNA was extracted using and run on a Human Ref6V2 bead chip for the Illumina platform. Data is presented in the form of raw values.
(PDF)

Data S2 **Illumina Raw data for PBMCs treated with 3 and 10%CSE.** THP-1 cells were treated for 8 and 24 hours with RPMI-1640 control medium (n = 3) or 10% CSE-conditioned medium (n = 3). Total RNA was extracted using and run on a Human Ref6V2 bead chip for the Illumina platform. Data is presented in the form of raw values.
(PDF)

Table S1 **Top bio functions in THP-1 cells after 8 h treatment with 10% CSE.** THP-1 cells were treated for 8 hours with RPMI-1640 control medium (n = 6) or 10% CSE-conditioned medium (n = 6). Genes that were significantly modified by ≥1.5-fold, as assessed using student's t-test followed by Benjamini-Hochberg FDR correction, were imported into Ingenuity Pathway Analysis software. The table shows the top bio functions identified from genes differentially expressed by ≥1.5-fold in THP-1 cells treated with 10% CSE.
(DOC)

Table S2 **Top bio functions in PBMCs after 8 h treatment with 10% CSE.** PBMCs were treated for 8 hours with RPMI-1640 control medium (n = 3) or 10% CSE-conditioned medium (n = 3). Genes that were significantly modified by ≥1.5-fold, as assessed using student's t-test followed by Benjamini-Hochberg FDR correction, were imported into Ingenuity Pathway Analysis software. The table shows the top bio functions identified from genes differentially expressed by ≥1.5-fold in PBMCs treated with 10% CSE. The range of p-values is reflective of the range of molecules that are represented in each network.
(DOCX)

Table S3 **Top bio functions in PBMCs after 24 h treatment with 10% CSE.** PBMCs were treated for 24 hours with RPMI-1640 control medium (n = 3) or 10% CSE-conditioned medium (n = 3). Genes that were significantly modified by ≥1.5-fold, as assessed using student's t-test followed by Benjamini-Hochberg FDR correction, were imported into Ingenuity Pathway Analysis software. The table shows the top bio functions identified from genes differentially expressed by ≥1.5-fold in PBMCs treated with 10% CSE. The range of p-values is reflective of the range of molecules that are represented in each network.
(DOCX)
