# Supplementary material for: Inflammatory Transcriptome Profiling of Human Monocytes Exposed Acutely to Cigarette Smoke
Source: PLoS One. 2012 Feb 17;7(2):e30120. doi: 10.1371/journal.pone.0030120 (PMC3281820; doi:10.1371/journal.pone.0030120)
Supplement: Table S1 — Top bio functions in THP-1 cells after 8 h treatment with 10% CSE. THP-1 cells were treated for 8 hours with RPMI-1640 control medium (n = 6) or 10% CSE-conditioned medium (n = 6). Genes that were significantly modified by ≥1.5-fold, as assessed using student's t-test followed by Benjamini-Hochberg FDR correction, were imported into Ingenuity Pathway Analysis software. The table shows the top bio functions identified from genes differentially expressed by ≥1.5-fold in THP-1 cells treated with 10% CSE. (DOC) [file pone.0030120.s003.doc]

**Supplementary Tables:**

| **Disease and disorders** | **Molecules in Network** | **p-value** |
| --- | --- | --- |
| **Inflammatory Response**  **Connective Tissue Disorders**  **Inflammatory Disease**  **Skeletal and Muscular Disorder**  **Respiratory Disease** | HMOX1, IL8, EDNRB, ANXA1, CDKN1A, CYSLTR1, ADORA2B, TNF, ADRB2  EDNRB, ANXA1, CDKN1A, TNF  HMOX1, IL8, EDNRB, ANXA1, CDKN1A, CYSLTR1, ADORA2B, TNF, ADRB2  ANXA1, CDKN1A, TNF  HMOX1, IL8, EDNRB, CYSLTR1, ADORA2B, IRF8, TNF, CITED2, ADRB2 | 1.79x10^-4^-1.68x10^-2^  1.79x10^-4^-1.37x10^-2^  1.79x10^-4^-1.68x10^-2^  1.79x10^-4^-1.98x10^-2^  2.90x10^-4^-1.98x10^-2^ |
| **Molecular and cellular functions** | **Molecules in Network** | **p-value** |
| **Cellular Development**  **Cellular Growth and Proliferation**  **Cell Death**  **Lipid Metabolism**  **Small Molecule Biochemistry** | IL8, SLC7A11, EDNRB, OSGIN1, SAT1, PPP1R15A, CREG1, SERTAD1, SLC3A2, ZMAT3, PIM1, ANXA1, CREB1, CDKN1A, MYB (includes EG:4602), TNF  TNFAIP8, IL8, SLC7A11, EDNRB, OSGIN1, SAT1, PPP1R15A, CDCA7, CREG1, SERTAD1, CYP1B1, SERPINB2, SLC3A2, HMOX1, ZMAT3, PIM1, ANXA1, CREB1, CDKN1A, MYB (includes EG:4602), CYSLTR1, IRF8, TNF  TNFAIP8, SLC7A11, HSPA1B, OSGIN1, SAT1, AKAP7, SERPINB2, HMOX1, PIM1, ANXA1, CREB1, MYB (includes EG:4602), PMAIP1, IL8, MGST1, EDNRB, NQO1, PPP1R15A, SERPINB10, CYP1B1, TXNRD1, CDKN1A, SRXN1, IRF8, SQSTM1, NMNAT3 (includes EG:349565), TNF  HMOX1, IL8, DHRS9, ANXA1, CDKN1A, LPL, AGPAT9, AKR1C2, TNF, CYP1B1, SLC3A2, ADRB2  IL8, NQO1, SAT1, CHST12, ME1, AKR1C2, TXNRD1, CYP1B1, SLC3A2, P2RY2, HMOX1, PGD, DHRS9, ANXA1, CDKN1A, LPL, ADORA2B, GCLM, AGPAT9, TNF, ADRB2 | 1.28x10^-6^-1.98x10^-2^  1.28x10^-6^-1.98x10^-2^  1.60x10^-6^-1.98x10^-2^  1.13x10^-4^-1.98x10^-2^  1.13x10^-4^-1.98x10^-2^ |
| **Physiological System Development and Function** | **Molecules in Network** | **p-value** |
| **Haematological System Development and Function**  **Haematopoiesis**  **Immune Cell Trafficking**  **Connective Tissue Development and Function**  **Reproductive System Development and Function** | IL8, HMOX1, EDNRB, PIM1, ANXA1, CDKN1A, CYSLTR1, IRF8, TNF, ADRB2  IL8, ANXA1, TNF, ADRB2  HMOX1, IL8, EDNRB, ANXA1, TNF  TNFAIP8, IL8, CDKN1A, PPP1R15A, SERTAD1, TNF, SLC3A2  IL8, TNF | 1.21x10^-4^-1.98x10^-2^  1.21x10^-4^-1.98x10^-2^  1.21x10^-4^-1.98x10^-2^  2.59x10^-4^-1.98x10^-2^  2.59x10^-4^-6.63x10^-3^ |
