# Supplementary material for: Inflammatory Transcriptome Profiling of Human Monocytes Exposed Acutely to Cigarette Smoke
Source: PLoS One. 2012 Feb 17;7(2):e30120. doi: 10.1371/journal.pone.0030120 (PMC3281820; doi:10.1371/journal.pone.0030120)
Supplement: Table S2 — Top bio functions in PBMCs after 8 h treatment with 10% CSE. PBMCs were treated for 8 hours with RPMI-1640 control medium (n = 3) or 10% CSE-conditioned medium (n = 3). Genes that were significantly modified by ≥1.5-fold, as assessed using student's t-test followed by Benjamini-Hochberg FDR correction, were imported into Ingenuity Pathway Analysis software. The table shows the top bio functions identified from genes differentially expressed by ≥1.5-fold in PBMCs treated with 10% CSE. The range of p-values is reflective of the range of molecules that are represented in each network. (DOCX) [file pone.0030120.s004.docx]

| **Disease and disorders** | **Molecules in Network** | **p-value** |
| --- | --- | --- |
| **Cancer** | DPYSL2, CBX7, HLA-DOA, RAB2A, EIF1, RAN, HBP1, ADAM8, ODC1, IFITM2, EZR, ETF1, TXN (includes EG:116484), KAT2A, CLNS1A, KPNA4, CD19, YWHAG, CKS2, NDUFS7, CTNNAL1, TRAPPC1, HSPA8, CTSL1, STIP1, NDUFB7, SQSTM1, SRSF5, TNF, RBM39, ADM, PPP2R2A, PSMB10, TNFSF10, CXCR3, DNAJA1, EP300, DUSP5, CD52, HSP90AB1, VIPR1, ANTXR2, IFITM1, CD24, MAP1LC3B, SH3GLB2, PSMB9, RGS2, PMAIP1, CD79B, CD69, EIF2C2, ITGB2, NUP153, MUM1,PELI1, CDKN1A, DBNL, HAGH, PRKCB, RGS1, AKR1C3, HSPA1A/HSPA1B, S100A4, CD8A, TCEB1, MC1R, RIP1, UBAC1, SLC7A5, PGK1, MCM5, RBL2, EWSR1, TBC1D2B, HIST1H4A (includes others), CCT6A, CD97, CBLB, YPEL5, DUSP1, HIGD1A, HSP90AA1, AKR7A2, CD2, PYCARD, UGCG, H3F3A/H3F3B, IL2RB, GLRX, HMOX1, ADSL, TIMP1, PPP1R2, ANXA1, VCP, ERRFI1, DNAJB1, ACO1, TNFRSF10A, RAB8B, PRNP, SOD1, ETFA, RELB, NQO1, IKBKE, SERTAD1, DNAJB9, CD3D, ACTG1, HLA-E, LPAR5, UBC | 1.58x10^-8^-1.94x10^-2^ |
| **Reproductive System Disease** | RGS1, CBX7, HSPA1A/HSPA1B, EIF1, S100A4, HBP1, TCEB1, ODC1, CRIP1, UBAC1, IFITM2, EZR, CCNK, IRS2, CLNS1A, PGK1, TBC1D2B, NDUFS7, EPHA1, CTNNAL1, CD97, YPEL5, CTSL1, DUSP1, STIP1, HSP90AA1, SRSF5, TNF, RBM39, AKR7A2, H3F3A/H3F3B, CXCR3, UGCG, IL2RB, EP300, HMOX1, HSP90AB1, TIMP1, ANXA1, MXD1, VCP, ANTXR2, CD24, ERRFI1, MAP1LC3B, PIK3R2, SH3GLB2, HSD17B4, TNFRSF10A, PMAIP1, RGS2, SOD1, CD79B, ETFA, ACTB, CD69, RELB, NQO1, IKBKE, ITGAL, MUM1, GALT, CDKN1A, PELI1, LPAR5, DBNL, HAGH, PRKCB | 1.83x10^-5^-1.94x10^-2^ |
| **Dermatological Diseases and Conditions** | CD81, DPYSL2, RGS1, PSMA3, RAN, EIF1, IFI35, ODC1, CRIP1, MC1R, IFITM2, ETF1,TXN (includes EG:116484), MCM5, CD19, HSPA8, CTSL1, DUSP1, HSP90AA1, TNF, ADM, CD2, PSMB10, TNFSF10, H3F3A/H3F3B, CXCR3, PSMB6, IL2RB, ITGB7, HMOX1, CD52, HSP90AB1, SELK, TIMP1, ANXA1, VCP, IFITM1, CD24, TNFRSF10A, CD7, SOD1, CD79B, MAPKAPK3, HSPH1, MAPK6, ALOX5AP, ITGAL, HLA-E, FKBP4, NDUFA3, PRKCB | 1.97x10^-5^-1.94x10^-2^ |
| **Infectious Disease** | CD81, HLA-DOA, PSMA3, RAB2A, CD46, TCEB1, UBA7, IFITM2, CCNK, ETF1, IRS2, TXN (includes EG:116484), KAT2A, CLNS1A, KPNA4, CD19, NDUFS7, ZC3HAV1, TBK1, CD97, TRAPPC1, CBLB, CTSL1, DUSP1, STIP1, NDUFB7, HSP90AA1, FXR1, TNF, TAGLN2, ADM, PPP2R2A, H3F3A/H3F3B, CXCR3, UGCG, TNFSF10, ST13, IL2RB, PSMB6, RRAGD, EP300, HCP5, TIMP1, MXD1, PTGES3, CD24, IFITM1, DNAJB1, RAB8B, PRNP, MYD88, AP1M1, RELB, CD69, EIF2C2, ALOX5AP, IKBKE, NXF1, ITGAL, STX10, ITGB2, NUP153, PELI1 | 2.86x10^-5^-1.94x10^-2^ |
| **Inflammatory Disease** | ADM, DPYSL2, CD81, CD2, HSPA1A/HSPA1B, EIF1, H3F3A/H3F3B, TNFSF10, CXCR3, IL2RB, ODC1, ITGB7, CD52, MC1R, TIMP1, SELK, IFITM2, VCP, IFITM1, TXN (includes EG:116484), PRNP, MCM5, UBB, CD79B, CD69, ITGAL, ITGB2, CTSL1, HLA-E, DUSP1, FKBP4, NDUFA3, TNF, PRKCB | 2.86x10^-5^-1.94x10^-2^ |
| **Molecular and cellular functions** | **Molecules in Network** | **p-value** |
| **Drug Metabolism** | PGK1, HSP90AB1, STIP1, PTGES3, SLC7A5, GCLM, DNAJB1, DNAJA1, TNF, GLRX, HAGH, SLC3A2 | 1.58x10^-8^-1.94x10^-2^ |
| **Lipid Metabolism** | AKR1C3, CD2, UGCG, TNFSF10, DNAJA1, EP300, PTGES2, HSP90AB1, TIMP1, VIPR1, PTGES3, IRS2, DNAJB1, MDH2, HSD17B4, PRNP, RGS2, SOD1, ECH1, ALOX5AP, HSPA8, WAS, STIP1, CDKN1A, PAFAH1B1, TNF | 1.58x10^-8^-1.94x10^-2^ |
| **Small Molecule Biochemistry** | ADM, ABCB6, GNPDA1, GCHFR, AKR1C3, CD2, HSPA1A/HSPA1B, UGCG, TNFSF10, DNAJA1, EP300, OGT, HMOX1, PTGES2, MC1R, HSP90AB1, TIMP1, VIPR1, PTGES3, SLC7A5, IRS2, DNAJB1, ACO1, MDH2, HSD17B4, PRNP, PGK1, RGS2, UCP2, SOD1, UROS, NQO1, ECH1, ALOX5AP, TXNRD1, SLC3A2, HSPA8, PPOX, WAS, STIP1, GALK1, GALT, CDKN1A, PAFAH1B1, TNF | 1.58x10^-8^-1.94x10^-2^ |
| **Cell Death** | ABCB6, CIB1, CD81, PHLDA1, HSPA1A/HSPA1B, S100A4, BAG3, CD46, CD8A, E2F6, UBA7, AHSA1, MC1R, EZR, IRS2, TXN (includes EG:116484), CD19, EWSR1, YWHAG, TBK1, HSPA8, NME3, DUSP1, HSP90AA1, SRXN1, SQSTM1, TNF, TAGLN2, ADM, CD2, PYCARD, UBQLN1, PPP2R2A, CXCR3, TNFSF10, UGCG, IL2RB, GLRX, EP300, HMOX1, DUSP5, HSP90AB1, TIMP1, TICAM1, TNFSF12, ANXA1, VCP, CD24, DNAJB1, PIK3R2, HNRNPC, TNFRSF10A, CD7, PRNP, UBB, PMAIP1, PPM1M, SOD1, ADRM1, UCP2, CD79B, MYD88, CD69, RELB, NQO1, PPP1R15A, IKBKE, ID3, ITGAL, TIAF1, ITGB2, WAS, CDKN1A, UBC, TXNDC17, PRKCB | 1.57x10^-6^-1.94x10^-2^ |
| **Cell Morphology** | ADM, PAPOLA, ULK1, PMAIP1, CHMP1B, UCP2, SOD1, CD2, PYCARD, NQO1, TNFSF10, EP300, TIMP1, WAS, GABARAPL1, CDKN1A, SRXN1, MAP1LC3B, DNAJB1, PARVG, TNFRSF10A, TNF | 1.09x10^-5^-1.94x10^-2^ |
| **Physiological System Development and Function** | **Molecules in Network** | **p-value** |
| **Endocrine System Development and Function** | HSP90AB1, STIP1, PTGES3, DNAJB1, DNAJA1 | 1.58x10^-8^-1.58x10^-8^ |
| **Reproductive System** | RBL2, DUSP1, CDKN1A, TNF, EP300 | 1.83x10^-5^-1.94x10^-2^ |
| **Development and Function**  **Tissue Development** | ADM, CD81, RGS1, GNPDA1, CD2, CXCR3, CD46, ITGB7, EP300, HMOX1, ANXA1, PTGES3, AMICA1, TXN (includes EG:116484), PRNP, CLNS1A, RGS2, RAB21, ITGAL, NXF1, SLC3A2, ITGB2, CDKN1A, PARVG, PAFAH1B1, CHD7, TNF | 2.45x10^-4^-1.94x10^-2^ |
| **Immune Cell Trafficking** | DPYSL2, CD81, RGS1, CD2, MYD88, HSPH1, CXCR3, CD46, ITGAL, ITGB7, ITGB2, HMOX1, TNFSF12, VIPR1, WAS, TIMP1, ANXA1, TXN (includes EG:116484), TNFRSF10A, TNF, CD7, PRNP, PRKCB | 3.69x10^-4^-1.94x10^-2^ |
| **Hematological System Development and Function** | ADM, CD81, RGS1, CD2, TNFSF10, CXCR3, CD46, CD8A, IL2RB, ITGB7, OGT, HMOX1, TIMP1, TNFSF12, VIPR1, ANXA1, CD24, TXN (includes EG:116484), TNFRSF10A, CD7, PRNP, CD19, MYD88, HIST1H4A (includes others), HSPH1, ITGAL, ITGB2, WAS, CDKN1A, TNF, CHD7, PRKCB | 3.76x10^-4^-1.94x10^-2^ |
