# Supplementary material for: Inflammatory Transcriptome Profiling of Human Monocytes Exposed Acutely to Cigarette Smoke
Source: PLoS One. 2012 Feb 17;7(2):e30120. doi: 10.1371/journal.pone.0030120 (PMC3281820; doi:10.1371/journal.pone.0030120)
Supplement: Table S3 — Top bio functions in PBMCs after 24 h treatment with 10% CSE. PBMCs were treated for 24 hours with RPMI-1640 control medium (n = 3) or 10% CSE-conditioned medium (n = 3). Genes that were significantly modified by ≥1.5-fold, as assessed using student's t-test followed by Benjamini-Hochberg FDR correction, were imported into Ingenuity Pathway Analysis software. The table shows the top bio functions identified from genes differentially expressed by ≥1.5-fold in PBMCs treated with 10% CSE. The range of p-values is reflective of the range of molecules that are represented in each network. (DOCX) [file pone.0030120.s005.docx]

| **Disease and disorders** | **Molecules in Network** | **p-value** |
| --- | --- | --- |
| **Genetic Disorder** | MCM6, IDS, DNAJB4, EIF1, GARS, GBAS, SCO2, KEAP1, CYBA, ETF1, ADA, TXN (includes EG:116484), CD19, SPOCK2, GLRX3, BRF2, ARHGDIB, IRF1, HSPA8, NCF1, GALK1, STIP1, SQSTM1, TNF, ADM, HTATIP2, ADAM17, DDIT3, GAPDH, MFGE8, SETX, BCL11B, DNAJA1, YES1, MT1F, GADD45A, GBA, HSP90AB1, HLA-A, VIPR1, LY6E, CD24, IFITM1, PSMD14, LDLRAP1, HGS, IL11RA, GZMM, CD79B, TUBB2C, IL10, MAPK6, IFNGR2, ALOX5AP, EIF2C2, TUBA1A, RNF144A, WAS, GALT, EIF4A1, CDKN1A, FKBP4, CYP4V2, ABCC3, PRKCB, CD81, RGS1, PSMA3, IFI35, ARG2, TAF10, GUSB, IL7R, XPC, CYBB, SLC7A5, TUBA1C, D2HGDH, EIF2C1, GLA, PSMB5, CNTNAP2, ARIH1, FLOT2, DUSP1, HSPB1, HPS6, IL2RB, PSMB6, CD79A, HMOX1, DIAPH1, CD55, UGDH, PPP3CB, SELK, TIMP1, VCP, NAMPT, STAT1, TNFRSF10A, CD7, PSMB4, PSAT1, NDUFV1, UBB, SOD1, ETFA, HSPH1, NQO1, TUBA4A, PLEKHB1, MX1, DNAJB9, UBE2L6, FOXO1, SSTR2, HADH | 2.69x10^-6^-2.99x10^-2^ |
| **Immunological Disease** | CGMDS, AUTS2, UBR2, EIF1, GARS, HLA-DMB, PSMB8, IFITM2, CYBA, ADA, AMICA1, TXN (includes EG:116484), CD19, SORL1, TFDP1, GYPC, SPOCK2, BRF2, IRF1, ARHGDIB, HSPA8, NCF1, PSMB2, ICAM3, STIP1, SYK, ARHGEF18, TNF, BANK1, ADM, HTATIP2, PRDX1, NQO2, BCL11B, PITRM1, TAP1, HLA-DPA1, HCP5, YES1, GADD45A, HLA-A, HSP90AB1, VIPR1, TNFSF12, CD24, IFITM1, PSMD14, ZCCHC6, RSBN1, PSMB9, IL10, TUBB2C, CD69, IFNGR2, HIST1H2AC, EIF2C2, DEDD2, COMMD3, ITGB2, TUBA1A, NUP153, WAS, CDKN1A, FKBP4, TSPO, PRKCB, DERL2, HSPA1A/HSPA1B, NUDC, CD8A, TCEB1, IL7R, H2AFY2, UBAC1, CHD2, CYBB, TMEM156, TUBA1C, EIF2C1, ARL16, TNFSF13B, PSMB5, PGM1, CNTNAP2, CD97, EIF1B, TTC3, ARIH1, ERP29, DUSP1, BTN3A2, TAX1BP1, TMEM173, NFKB1, IL2RB, CD79A, HLA-F, HMOX1, DIAPH1, GTF2B, CD55, PPP3CB, TIMP1, FAM120B, SELK, PPP1R2, VCP, NAMPT, STAT1, TNFRSF10A, CD7, HLA-DMA, UBB, NQO1, CD6, TUBA4A, PLEKHB1, MX1, PHTF1, UBE2L6, SH3PXD2A, PBX3, FOXO1, DYM, HLA-DOB | 2.69x10^-6^-2.99x10^-2^ |
| **Dermatological Diseases and Conditions** | CD81, RGS1, PSMA3, EIF1, GARS, GBAS, IFI35, TAF10, TCEB1, SCO2, MT2A, IFITM2, CYBB, ADA, ETF1, TUBA1C,TXN (includes EG:116484), CD19, PSMB5, RPL12, PSMC4, PGM1, PSMD6, BRF2, IRF1, ARHGDIB, TTC3, ARIH1, HSPA8, DUSP1, BTG2, PSMD12, TNF, TAGLN2, ADM, ADAM17, DDIT3, HPS6, GAPDH, BCL11B, IL2RB, PSMB6, HLA-DPA1, CD79A, HMOX1, YES1, HLA-A, GADD45A, PPP3CB, HSP90AB1, GBA, TIMP1, SELK, VCP, GTF2H5, CD24, IFITM1, NAMPT, STAT1, HGS, TNFRSF10A, CD7, GZMM, IL11RA, PSMB4, SOD1, CD79B, TUBB2C, IL10, HSPH1, TUBA4A, MAPK6, IFNGR2, ALOX5AP, MX1, PSMA1, UBE2L6, NUP153, TUBA1A, FOXO1, TRIM44, FKBP4, TSPO, PSMC3, DOCK10, PRKCB | 3.68x10^-5^-2.99x10^-2^ |
| **Infectious Disease** | CD81, PSMA3, GBAS, TCEB1, IL7R, UBA7, MT2A, IFITM2, CCNK, ZFP161, HIST2H2AA3/HIST2H2AA4, ADA, CYBB, ETF1, XPO1, TUBA1C, TXN (includes EG:116484), EIF2C1, STAT5A, KPNA4, CD19, STK39, NDUFS7, RPL12, GYPC, PGM1, PSMC4, SPOCK2, PSMD6, TBK1, CD97, TRAPPC1, TRMT5, TTC3, CBLB, NCF1, FLOT2, ARAF, DUSP1, STIP1, PSMD12, BTG2, STAT2, IRF8, TNF, TAGLN2, ADM, GAPDH, NFKB1, ST13, PSMB6, IL2RB, CD79A, HCP5, CD55, DIAPH1, PPP3CB, TIMP1, RB1CC1, IFITM1, CD24, PSMD14, DNAJB1, STAT1, HGS, RAB8B, ACTN1, GZMM, PSMB4, APBB1IP, MYD88, IL10, TUBB2C, CD69, IFNGR2, TUBA4A, HIST1H2AC, EIF2C2, ALOX5AP, MX1, PSMA1, UBE2L6, IGF2R, STX10, TNFRSF14, ITGB2, NUP153, TUBA1A, SSTR2, RNF144A, PSMD2, TRIM44, TRPV2, TSPO, PSMC3, DOCK10 | 1.47x10^-4^-2.99x10^-2^ |
| **Inflammatory Disease** | D81, GMDS, HSPA1A/HSPA1B, AUTS2, UBR2,EIF1, HLA-DMB, PSMB8, NUDC, IL7R, IFITM2, UBAC1, TMEM156, TUBA1C, TXN (includes EG:116484), EIF2C1, ARL16, TNFSF13B, PSMB5, SORL1, CNTNAP2, CD97, BRF2, EIF1B, IRF1, ARHGDIB, ARIH1, HSPA8, PSMB2, DUSP1, ICAM3, TNF, ADM, BANK1, DDIT3, PRDX1, TMEM173, GAPDH, BCL11B, NFKB1, TAP1, IL2RB, CD79A, HLA-DPA1, HMOX1, CD55, GADD45A, PPP3CB, HLA-A, SELK, VIPR1, TNFSF12, TIMP1, VCP, IFITM1, CD24, NAMPT, ZCCHC6, STAT1, TNFRSF10A, RSBN1,IL11RA, HLA-DMA, UBB,CD79B, IL10, TUBB2C, CD69, CD6, IFNGR2, HIST1H2AC, TUBA4A, EIF2C2, MX1, PLEKHB1, DEDD2, PHTF1, UBE2L6, SH3PXD2A, ITGB2, NUP153, TUBA1A, SSTR2, PBX3, FOXO1, DYM, FKBP4, HLA-DOB, DERL2, PRKCB | 1.46x10^-4^-2.99x10^-2^ |
| **Molecular and cellular functions** | **Molecules in Network** | **p-value** |
| **Cell Death** | CIB1, AHSA1,MT2A,TXN (includes EG:116484), STAT5A, CD19, YWHAG, TFDP1, TBK1, IRF1, HSPA8, SYK, TNFRSF25, BTG2, SRXN1, SQSTM1, TNF, ADM, ADAM17, HTATIP2, DDIT3, UBQLN1, GAPDH, SETX,YY1, GADD45A, GBA, HSP90AB1, TNFSF12, RB1CC1, DAP3, CD24, PIK3R2, EEF2K, GZMM, PPM1M, PMAIP1, UCP2, ADRM1, GZMK, CD79B, TBC1D9, IL10, MYD88, CD69, OBFC2A, PPP1R15A, CCDC6, IGF2R, ITGB2, TUBA1A, SRGN, WAS, CDKN1A, XAF1, ABCC3, PRKCB, CD81, ABCB6, HSPA1A/HSPA1B, BAG3, CD8A, IL7R, E2F6, UBA7, XPC, XPO1, TNFSF13B, HSPA9, NME3, DUSP1, IRF8, HSPB1, TAGLN2, UTP11L, TAX1BP1, GADD45G, NFKB1, IL2RB, HMOX1, CD55, LAMTOR3, VCP, NAMPT, DNAJB1, STAT1, TNFRSF10A, CD7, UBB, SOD1, CD6, NQO1, MX1, TNFRSF14, SSTR2, FOXO1, ZNF622, FAIM3 | 4.35x10^-7^-2.99x10^-2^ |
| **Cell Morphology** | ADM, ULK1, DDIT3, NFKB1, TIMP1, GABARAPL1, TRIB2, ATF4, MAP1LC3B, EEF2K, DNAJB1, NFE2L2, TNFRSF10A, GZMM, PAPOLA, PMAIP1, RGS19, SOD1, UCP2, GZMK, IL10, NQO1, HERPUD1, SRGN, CDKN1A, SRXN1, PTGER2, PARVG, TNF, HSPB1 | 4.68x10^-7^-2.99x10^-2^ |
| **Cellular function and Maintenance** | ADM, ULK1, DDIT3, NFKB1, TIMP1, GABARAPL1, TRIB2, ATF4, MAP1LC3B, EEF2K, DNAJB1, NFE2L2, TNFRSF10A, GZMM, PAPOLA, PMAIP1, RGS19, SOD1, UCP2, GZMK, IL10, NQO1, HERPUD1, SRGN, CDKN1A, SRXN1, PTGER2, PARVG, TNF, HSPB1 | 4.68x10^-7^-2.99x10^-2^ |
| **Cellular Compromised** | DDIT3, PRDX5, PRDX1, HSPA1A/HSPA1B, DNAJA1, PRDX6, HMOX1, SELK, CYBA, CYBB, ATF4, CD24, AMICA1, NAMPT, TXN (includes EG:116484), GCLM, NQO1, PSMC4, ITGB2, NCF1, DUSP1, SYK, CDKN1A, SRXN1, SQSTM1, TNF | 8.18x10^-7^-2.99x10^-2^ |
| **Cellular Growth and Proliferation** | CD81, AKR1C3, DNAJB4, HSPA1A/HSPA1B, EIF1, CD8A, CD37, IL7R, AHSA1, XPC, ATF4, TXN (includes EG:116484), TNFSF13B, STAT5A, CD19, GLA, YWHAG, TFDP1, CSNK1D, PSMC4, BRF2, IRF1, SLC3A2, FLOT2, ARAF, ICAM3, PSMB2, DUSP1, SYK, BTG2, TNFRSF25, CCT3, PTGER2, IRF8, YTHDF2, TNF, ADM, ADAM17, DDIT3, GADD45G, TAF7, CACNB3, DNAJA1, NFKB1, IL2RB, SURF4, HMOX1, GTF2B, CD55, SEC61A1, GADD45A, TNFSF12, DAP3, NCOA4, CD24, DNAJB1, PIK3R2, SH3GLB2, STAT1, HGS, ACTN1, ATP6V0E2, NDUFV1, SOD1, MYD88, IL10, CD6, EIF2C2, PPP1R15A, MX1, IGF2R, UBE2L6, ACTG1, TNFRSF14, STRAP, PSMC1, SSTR2, FOXO1, WAS, PSMD2, EIF4A1, CDKN1A, FKBP4, VAMP8, PSMC3, PRKCB, DERL2 | 6.59x10^-6^-2.99x10^-2^ |
| **Physiological System Development and Function** | **Molecules in Network** | **p-value** |
| **Endocrine System Development and Function** | YWHAH, HSP90AB1, STIP1, DNAJB1, DNAJA1 | 1.13x10^-5^-2.99x10^-2^ |
| **Hematological System Development and Function** | CD81, ADM, RGS1, ADAM17, CD8A, CD37, IL2RB, IL7R, HMOX1, HLA-A, TNFSF12, ADA, CD24, TNFRSF10A, CD7, TNFSF13B, STAT5A, CD19, MYD88, IL10, HSPH1, CD6, TNFRSF14, ITGB2, WAS, ICAM3, SYK, CDKN1A, IRF8, TNF | 7.33x10^-4^-2.99x10^-2^ |
| **Heamatopoiesis** | IL7R, ADM, STAT5A, ADAM17, HSPA1A/HSPA1B, IL10, TNFSF12, CD6, NFKB1, STAT1, IL2RB, TNF | 8.95x10^-4^-2.99x10^-2^ |
| **Tissue Development** | CD81, RGS1, GNPDA1, RAB21, NFKB1, SLC3A2, HMOX1, ITGB2, CD55, ICAM3, SYK, CDKN1A,  AMICA1, PTGER2, PARVG, TNF | 1.34x10^-3^-2.99x10^-2^ |
| **Tumor Morphology** | ITGB2, IL10, SYK, AMICA1 | 1.34x10^-3^-2.99x10^-2^ |
