# Supplementary material for: Inflammatory Transcriptome Profiling of Human Monocytes Exposed Acutely to Cigarette Smoke
Source: PLoS One. 2012 Feb 17;7(2):e30120. doi: 10.1371/journal.pone.0030120 (PMC3281820; doi:10.1371/journal.pone.0030120)
Supplement: Data S1 — Illumina Raw data for THP-1 cells treated with 10%CSE. THP-1 cells were treated for 8 hours with RPMI-1640 control medium (n = 6) or 10% CSE-conditioned medium (n = 6). Total RNA was extracted using and run on a Human Ref6V2 bead chip for the Illumina platform. Data is presented in the form of raw values. (PDF) [file pone.0030120.s001.pdf]

Illumina Inc. GenomeStudio version 1.5.4
Normalization = none
Array Content = HumanRef-8_V3_0_R0_11282963_A.bgx.xml
Error Model = none
DateTime = 03/03/2010 15:55 (TC1, TC2, TS1, TS2) and 09/07/2010 14:09 (TC3, TC4, TC5, TC6, TS3, TS4,
Local Settings = en-GB

TargetID	AVG_Signa	AVG_Signa	AVG_Signa	AVG_Signa	AVG_Signa	AVG_Signa	AVG_Signa	AVG_Signa
15E1.2	329.5	389.1	194.7	193	157.8	139.8	240.5	342
2'-PDE	428.8	549.5	343.8	318.9	255.4	382.8	351.7	525.9
7A5	88.2	80.8	82.5	91.7	72.4	89.1	89.3	87.3
A1BG	108.4	117.5	113.7	104.4	106.2	116.4	104.2	105.2
A2BP1	86.4	92	97.7	80.3	84.3	90	82.4	90.1
A2BP1	101.2	102.6	103.1	83.8	83.4	94.1	96	97.8
A2BP1	78.8	88.2	105.5	90.3	84.8	90.8	82.3	90.8
A2BP1	101.5	132.3	104.1	96.1	80.8	84	114.2	110.7
A2M	80.3	84.7	86.3	84.5	81.8	83.6	77.6	92
A2ML1	81	80.9	100.4	98.7	86.1	89.1	80.4	76.3
A3GALT2	73	80.3	82.8	80.4	86.3	75.6	71.8	75.8
A3GALT2	83	80.5	98.2	96.7	90.6	90.6	76.9	87
A4GALT	177.7	217.4	150.3	135.5	115.2	126.6	178.4	192.2
A4GNT	120.7	114.5	91.1	95.5	91.5	92.7	109	114.9
AAA1	102.3	111.3	113	92.8	93.3	99.1	93.3	113.8
AAA1	130.9	155.5	110.4	101.5	103.3	103.7	142.6	149.3
AAA1	112.8	111.2	98.4	99.4	85.7	97.3	98.4	112.3
AAA1	68.7	72.3	81.7	76.4	78	70.4	73.9	73.6
AAA1	92.8	103.9	84.4	85.7	78.7	81.1	89.2	98.4
AAAS	165.1	212.7	134.9	125.1	95.3	117.9	150	220.1
AACS	208.5	268.6	210.5	217.4	228.6	264.8	196	195.7
AADAC	101.9	112.1	101.4	93.6	87.6	98.3	91.3	101.1
AADACL1	277.9	333.5	132.1	167.6	129.2	194.6	273.7	389.1
AADACL2	92.6	91	97.9	89.9	79.6	90.1	88	84.4
AADAT	144.5	157.6	103.9	92.2	80	89.1	140	129.7
AADAT	81.7	94.1	91.2	87.6	83.2	86.9	88.2	88.1
AADAT	73.2	82.1	90.3	79.7	88	83.4	93.2	92.1
AAK1	83.3	82.7	98.1	93.9	91	92.6	78.3	79.7
AAMP	329.4	478.3	192.7	171.4	132	185	326.6	418.5
AANAT	78.7	87.7	88.9	83.7	74.2	71.1	82.2	86.2
AARS	5885.8	7945.2	4622.1	3583.6	3033.1	3985.3	5385.3	6295.9
AARSD1	798.4	1088.8	546.3	563	527	838.8	721.4	856.8
AARSL	572.9	644.2	283.9	291.7	296.7	357.2	438.2	485.5
AASDH	469.9	591.6	272.1	277.1	227.3	394.8	424.8	563.9
AASDHPPT	1641.1	1879.5	552.4	658.1	443.9	795.4	1229.9	2143.3
AASS	106.2	127.9	114.7	119.7	96.5	109.4	105.1	116.6
AATF	1479.8	1954.2	1010.7	820.2	626.5	987.9	1271.5	1846.6
AATK	95.9	119.6	106.1	104.9	98.3	102.1	105.4	120.3
AATK	85.8	92.2	85.6	83.4	99.9	85	89.6	92.9
ABAT	99.7	116.5	99.2	91.6	85.4	93.3	104.6	116.8
ABAT	129.7	151.9	94.8	85.5	83.4	89.4	145	176
ABAT	88.5	97.2	96.5	85.1	89.6	89.8	91.2	107

ABC1	247.7	329.9	165	181.8	150.7	162.6	203.3	231.5
ABCA1	451.5	729.9	280.5	724.5	638	1121.6	421.5	559.6
ABCA10	107.8	125	92.5	95.6	102.5	101.9	96	111.5
ABCA11	205.6	228.5	141.1	136	111.1	139.6	141.4	207.2
ABCA12	100.4	119.8	103	77.9	91.8	95.1	100.2	110.5
ABCA12	161.8	177	99.1	85.9	89.4	85.5	150.8	180.4
ABCA13	81.4	86.9	93.7	93.2	87.3	82.6	81	82.6
ABCA2	112	109.6	109.6	103.5	82.5	91.2	106.1	99.5
ABCA2	170.4	185.5	157.5	149.8	130	190.6	131.7	196.4
ABCA3	152.1	169	113.1	92.7	97.9	88.8	121.6	123.4
ABCA4	106	129.6	137.7	140.3	139	137.4	104.4	130
ABCA5	106.5	120.6	116.1	106.8	114.8	104.1	105.2	129.1
ABCA5	87.9	97.4	84.2	89.2	87.1	88.3	84.8	85.1
ABCA5	107.7	120.4	84	86.6	85.5	96.3	95.8	120.6
ABCA6	92.2	85.8	87.3	86.8	87.4	91.7	87.8	87.3
ABCA6	98.6	107.9	115.8	112.5	115.3	110.4	89.7	95
ABCA7	82.6	86.1	79	79.7	86.2	76.9	82.9	85.1
ABCA7	204.6	230.3	226.9	203.3	202.7	312	137.3	161.9
ABCA7	67.3	83	93	91.6	87.5	95.9	78.8	75.5
ABCA8	85.5	87	89.1	80.5	84.5	83.8	83.6	85.7
ABCA9	123.6	167.9	94.9	90.9	84.7	86.5	110.2	127.8
ABCA9	116.1	125.1	98.2	84.4	93.6	94.4	114.1	123.4
ABCB1	79.3	88.7	77.4	81.4	75.9	83.2	84.3	87.1
ABCB10	1638.5	1621.5	831.3	978.1	787.1	1134.5	1478.1	2201.4
ABCB11	84.5	94.2	101.7	97.1	95.9	102	92	82.2
ABCB4	136.5	174.4	122.1	96.6	91.2	114.3	138.7	186.5
ABCB5	89.5	96.3	82.9	74	78.6	78.1	83.1	98.4
ABCB6	147.5	169.1	121.3	121.3	121	129.9	149.9	228.1
ABCB7	967.8	1155.6	296.4	340.3	250.8	446.3	804.5	1444.8
ABCB8	117.7	148.4	94.2	90.2	84.5	98.7	141.1	140.4
ABCB9	84.7	86.9	107.6	101.6	98.5	113.7	81.6	84.3
ABCB9	110	123.6	141.1	137.4	134.6	143.8	103.8	163.2
ABCB9	224.3	276.5	212.8	202.5	176.3	181.1	178.7	196.3
ABCC1	96.9	103.3	105	97	92.6	94.8	91.4	92.6
ABCC1	115.9	156.3	113	97.8	97	96.1	118.3	129.4
ABCC10	139.6	156.1	112.9	103.4	105.1	109.9	113.8	152.7
ABCC11	94.3	95	79.2	79.8	73.9	73.6	95	105.5
ABCC11	135.8	155.9	107.4	99.4	89.9	104.2	131.7	127.5
ABCC11	85.1	93.9	99.2	96.1	97.1	99.6	85.2	89
ABCC12	123.2	141.1	105.8	103.2	95.4	113.8	125.7	135
ABCC13	166.7	191.3	129.7	112.3	103.3	117.9	145.1	155.2
ABCC13	117.7	130.9	107.5	110.7	100.5	99.2	106.5	125.9
ABCC2	112.8	107.5	101.2	99.4	97.3	99.8	107.2	92.6
ABCC3	89.6	93.2	96.6	141.6	131.2	159.8	92.9	107.5
ABCC4	577	730.8	182.5	225.5	165.7	274.9	487	618.2
ABCC5	92.3	101.7	90.3	81.8	85.9	78.4	86.5	97.5
ABCC5	178.8	237	166.4	190.5	201.4	255.5	223.4	215.6
ABCC5	159.3	164.4	140.3	137.3	142.3	166.1	118	159
ABCC6	98.9	113.2	107	95.1	91.7	92.8	105.5	112.8
ABCC6	144.1	193.1	108	92	87.4	101.4	168.3	179.5

ABCC6	75.7	82.3	87.2	81	69.9	77.5	83.4	98.5
ABCC6	100.7	124.5	94.9	98	87.9	87.5	96.2	112
ABCC6	136	173.7	146.9	123.3	118.9	127	131	131.9
ABCC8	97.6	100.8	99	93	83.7	86.9	92.6	90
ABCC9	97.4	95.5	82.9	88.7	77.3	87	86.9	90.7
ABCC9	135.5	131.3	96.8	99.3	83.9	84.1	113.4	136.4
ABCD1	151	170.6	273.4	186.8	193.5	279.1	154.2	290.7
ABCD2	74.9	82.2	89	82	84.8	85.3	79.2	93.9
ABCD4	82.5	99.8	91.2	84.1	87.5	88.9	81.9	89.1
ABCD4	97.1	96.5	111.8	105.1	110.6	107.2	91.6	106.2
ABCE1	2579.6	3057.9	1258.4	871.3	544	1544.5	1725.9	3630.3
ABCE1	2122.2	2881.4	1184.8	813.5	531.9	1401.5	1607.1	3267.8
ABCF1	533.2	711.1	366.7	377.9	317.4	535.1	405.7	745
ABCF1	1380.1	2114.5	1005.1	1000.1	870	1167.3	1035.8	1191.4
ABCF2	114.8	137.4	109.4	91.2	95.9	105.1	112.8	130.6
ABCF2	965.2	1265.6	526.2	372.6	322.3	433	732.8	1122.9
ABCF2	216.8	247.2	192.1	173.2	141.3	224.5	180.6	328.4
ABCF3	150.7	179.1	128.2	146.8	118.7	185.7	147.7	167.6
ABCG1	149.4	171.9	124.4	109	105.4	121.2	136	142.1
ABCG1	96.6	107.6	116.2	94.4	101	97.7	96	100.6
ABCG1	102.6	121.3	91.4	87.6	82	86.1	106.3	110.4
ABCG1	81.2	98.2	82.9	84.2	87.5	91.8	90.1	96.5
ABCG1	111.3	147.4	96.5	97.4	88.5	84.7	131.3	150.8
ABCG1	119.9	122.8	92.4	82.8	76.6	91.3	102.4	137.8
ABCG2	78.8	89.5	86.6	73.7	77.6	75.9	72.6	80.6
ABCG4	119.5	135.4	103.4	99.5	95.9	92.6	121.3	117.8
ABCG5	132.1	157.8	128.9	114.3	97.3	103.7	131.2	152.5
ABCG8	86.2	104.3	99.1	86.1	88.5	90.9	90.7	89.6
ABHD1	115.1	124	108.8	97.2	89.1	91.7	104	123
ABHD10	513.9	648.9	328.9	299.9	232.1	345.6	347.8	532.3
ABHD11	297.9	376.3	178.9	158.4	132.2	144.6	256.1	286.9
ABHD11	107.6	120.2	96.6	79.4	77.5	84.1	87.4	124.4
ABHD11	306.8	390.1	223	197.8	167.5	155.7	282.2	332.2
ABHD12	516.4	670.9	231.8	222.5	174.1	234.4	441.7	730.8
ABHD12	435.2	530.2	376.8	369.3	341.8	508.7	349.8	486.7
ABHD13	91.8	101	95.9	101.4	85.7	94.4	95	113.5
ABHD14A	908.6	1154	560.1	328.5	321.1	364.1	871.5	1141
ABHD14B	155.9	187.7	214.4	280.3	244.4	308.2	135.4	205.4
ABHD2	175.2	227.6	123.4	122.5	104.9	125.7	168.8	217.2
ABHD2	101.9	111.2	100	95.9	81.9	105.8	101.4	119
ABHD2	164.6	234.9	114.2	96.2	84.3	84.4	143.5	198.4
ABHD3	943.6	1130.1	484.3	529	445.7	900.9	843.8	1675.9
ABHD4	110.1	111.6	103.4	97.9	83.7	93.9	117.7	147.9
ABHD5	300.9	348.6	156.5	131.6	112.7	156.6	286.1	497.6
ABHD6	217.7	287.7	155.5	140.2	133.6	150.2	211	302.3
ABHD7	178.8	219.4	117.9	113.2	100	95.8	156.3	207
ABHD8	816.9	976.8	442	351.3	283.6	343.1	779.9	835
ABHD9	128.8	163.3	98	93.1	85.6	90.2	142.9	143.9
ABI1	91.9	114.6	95.7	90.3	83.8	81.5	90	101.7
ABI1	318.1	367.9	169.2	133	139.3	183.4	284.7	500.3

ABI2	226.7	289.5	135.2	114.6	94.2	105.1	189.4	233.8
ABI2	638.5	814.4	413.7	293.2	233.6	346.8	566.2	911
ABI2	228.1	302.6	117.1	101.5	100.8	100.9	193.9	250.5
ABI3	115.7	137.1	111.1	92.1	87.8	104	121	127.9
ABI3BP	99.9	141.2	91.8	81.1	78.8	88.4	101.6	121.3
ABL1	559.8	757.3	600.4	474.3	391.6	405	499.3	593.9
ABL1	116.5	125.3	130.2	143.3	127.6	138	116.8	130.3
ABL1	120	137.5	86.6	86.5	83.1	89.6	118.6	125.8
ABL2	101.3	118.6	107.6	86.2	82.6	111.4	112.1	118.5
ABL2	82.3	82.6	87.4	95.7	87.3	87.5	85.2	88.4
ABL2	123.7	134.8	101.5	94.2	101.7	92.4	117.9	120.2
ABLIM1	100.2	108.9	101.3	95.5	94.6	102.3	98.4	111.9
ABLIM1	90.2	105.3	92.3	82.6	87.8	83.7	88.8	93.5
ABLIM1	87.6	105.5	94.6	87.5	90.4	81.8	94.4	103.9
ABLIM1	90.3	87.7	92.3	86.8	78.2	83	89.8	100.3
ABLIM2	106.7	152.4	100.5	93.8	94.9	104.1	106.6	127.7
ABLIM3	93.5	96.1	113.9	103.2	85.1	104.8	92.7	100.3
ABO	88.6	103.2	99.4	94.9	86.3	87.2	99.2	107.6
ABP1	1187.1	1551.5	720.6	2788.3	2621	2074.2	1039.4	776.1
ABR	1235.5	1564.2	1189.8	1471.7	1114.3	1615.6	1076.1	1030
ABR	131.6	143.3	111.7	100.8	96.8	87.6	116.5	128.2
ABR	107.7	114.8	102.6	95.9	86.4	79.6	97.3	95.4
ABRA	97.5	102.7	99.4	82.9	86	95	96.3	99
ABT1	127.2	188.4	104.5	120.3	99.7	110.8	125.6	186.9
ABTB1	231.8	281.6	154.5	188.8	211.7	198	253	277.7
ABTB1	96.7	133.7	135.2	135.7	168.5	168.9	127.5	183.2
ABTB2	105.8	130.2	114.6	130.4	117.1	172	125.1	194.9
ACAA1	748.8	1016.6	460.7	585.4	504.2	784.3	697.7	930.2
ACAA2	620.8	759.5	196.8	175.9	141.4	228.6	507.3	779.8
ACACA	1111.1	1560.8	820.5	1384.2	1224.3	1736.6	1112.1	1326.3
ACACA	778.3	1204.2	518.2	734.2	722.2	953.1	795.9	906.6
ACACA	90.2	96	98.4	94	89.1	91.1	89.3	99.6
ACACB	273.2	318.1	240.1	426.2	378.2	454.4	211.4	234.2
ACAD10	245.3	262.1	166.7	238	189	285.6	169.1	248.8
ACAD11	338.7	351.8	191.5	239.2	234.7	285.7	227.4	318.5
ACAD8	675.1	902.9	615.9	279.3	235.2	379.4	491.4	706.6
ACAD9	1174.5	1492.7	778.5	930.2	799.7	1028.6	972.9	980.1
ACADL	93.1	99.4	93.3	87.4	77.6	80.9	89.2	88.6
ACADM	3971.4	4368.9	1926.9	2205.7	1815.4	2662.3	2833.7	4107.6
ACADS	233.2	300.6	173	140.7	123.4	159.2	170.7	213.8
ACADSB	120.7	137.5	96.8	99.2	99.4	109.9	116.6	124.7
ACADVL	268.2	338.7	161.2	177.1	152.3	115.5	224.3	269.9
ACADVL	2845.6	3233.3	2335.6	1903.6	1716.9	2552.3	2484.8	4020.9
ACADVL	823.1	911	696.3	625.9	619.3	950.7	682.8	930
ACAT1	5494.5	7064.9	3074.4	2703.5	2102.6	3637.4	4292.1	5637.8
ACAT2	155.1	182.9	115.3	111.7	111.3	119.7	136.6	212.7
ACBD3	1350.4	1668.3	672.6	844.9	661.5	1344.7	1376.5	2512.1
ACBD4	128.3	144	94.6	95.6	95.6	104.8	116.9	145.9
ACBD5	124	149.4	87.8	88.5	80	95.6	115.1	171.7
ACBD5	83.8	92	93	89	84.2	84.6	79.4	87.1

ACBD5	95.5	101.9	93.1	82	97.9	92.4	87.4	103.3
ACBD6	610.2	796.6	439	293.7	282.4	346.5	622.1	821.5
ACBD7	100.3	115.9	96.1	85.6	90.5	90.2	107.7	106.3
ACBD7	79.8	98	91	87.6	84.3	86.8	87.7	95.6
ACCN1	85.9	79.5	92.8	84.7	79.6	87.5	76.3	81.5
ACCN2	330.9	337	153.4	131.4	120.7	155.7	200.5	192.5
ACCN2	92.5	93.4	100.6	92.5	82.4	94.7	82.2	101.3
ACCN3	235.3	228.6	136.6	112	112.9	119.5	188.7	242
ACCN3	102.9	114.2	111	91.5	91.4	102.5	95.9	110.6
ACCN4	105.5	123	108.3	100.4	95.6	101.8	107.8	128
ACCN4	78.4	97.8	100.2	83.5	87.5	86.2	100.6	100
ACCN4	89.9	104.2	97.9	87.3	85.6	81.8	87.3	98.5
ACCN5	92.2	103.9	102.8	78.7	83	84.9	96.2	109.3
ACD	555.8	652.9	535.7	277.4	213.1	264.7	511.1	1007.1
ACE	105.3	119.6	102.5	95.7	87	90.2	103.7	110.3
ACE	94.6	95.2	82.2	78.7	76.4	91.7	92.4	93.7
ACE	103.7	120.2	99.3	83.4	86.3	79.6	115.5	116.9
ACE2	91.5	113	92.3	89	86.4	85.8	101.5	112.2
ACF	94.9	94.9	96.8	84	82	100.5	84.5	101.2
ACF	92.5	94.7	98.1	93.6	85.1	87.5	82.9	94.4
ACF	90.5	98.1	109.9	93.9	98	95.2	86.2	93.5
ACHE	80.6	89.9	83	76.4	79.1	89.1	84.1	89.5
ACHE	86	105.8	96.8	96.4	82.1	95.3	95	91.5
ACIN1	646	890	702.1	556	530.6	1065.2	544.5	914.1
ACLY	3345.2	4455.8	3165.2	4101.9	3216	5100.7	3120.8	3570.7
ACMSD	179.3	174.7	140.6	120.9	129.5	135.8	165.1	174.6
ACN9	1331.5	1373.2	665.5	627.9	403.6	696.7	1033.8	1717.6
ACO1	1316.6	1725.8	1009	1095.5	843	1283.8	1166.4	1676.8
ACO2	592.8	704.4	274.5	209.8	184.9	277.9	489	727
ACOT1	118.9	158.9	96.7	77.8	89.8	86.8	115.9	130.1
ACOT1	426.6	638.1	186.7	123.4	129.1	160.1	339.1	364
ACOT11	555	709.7	398.3	373	280.5	413.8	396.6	420.4
ACOT11	91.7	114.8	117.8	104	112.7	105.7	90.3	94.1
ACOT12	102.3	120.3	100.3	104.5	96.7	95.9	109.8	119.9
ACOT2	93.2	99.5	97.6	97.4	87.1	91.4	90.4	97.8
ACOT4	894.8	1092.5	604.2	253.5	213	303.8	759.2	1208.3
ACOT6	78.7	88.8	85.9	88.7	80.3	83.9	79.8	83.6
ACOT7	93.5	105	84.6	86.1	81.2	93.5	95	114.8
ACOT7	83	95.4	85.6	81.4	87.5	86.1	94.9	92.9
ACOT7	3113.8	4043.5	1424.3	1076	888.6	1022.7	2906	3193.8
ACOT7	99.2	111	102.7	87.6	82.4	85.6	101	114.8
ACOT7	883.3	1330.6	798.3	402.9	327.3	423.1	847.1	1171.1
ACOT7	105.5	108.1	92.6	85.2	71.5	81	99.2	111.6
ACOT8	253.1	310.9	171.5	149	125.8	141.6	194.4	231.1
ACOT9	235.3	308.7	134.6	133.1	127	150.2	239	287.8
ACOT9	799.6	1057.7	353.1	328.7	269	485.9	686.5	1010.9
ACOX1	321.9	436	203.5	171.1	148.4	211.2	290.1	460.1
ACOX1	106.8	120.3	83.6	95	92.2	95.8	115.6	118.4
ACOX1	103.4	117.5	100.5	100	87.5	81.2	87.2	97.8
ACOX2	353.8	478	247.4	262.7	156	258.9	352.1	461.4

ACOX3	645.8	841.1	463.4	361.9	285.6	396.8	526.1	733
ACOXL	81.4	94.2	96.4	92.8	76.9	77.4	86	90.7
ACP1	1983.7	2736.4	1203	1081.6	867.3	1422.2	1895.8	2849.6
ACP1	5925.1	7747	3478.6	2491.1	1818.2	2609.7	5354.4	7777.7
ACP2	256.3	278.9	165.6	128.4	132.3	150.5	251.2	359.2
ACP5	98.4	107	92.4	85.4	91.9	83.1	84	100.5
ACP6	221.8	289.8	173.2	169.8	125.3	171.5	192.3	191.8
ACPL2	124.4	147.9	129.2	98.9	98.7	96.2	113.7	136.8
ACPP	244.4	278.2	188.2	733.6	284.3	963	220.6	366
ACPT	171.6	222.7	200.9	183.8	190.3	155.3	150.7	162.6
ACPT	86	82.8	87.6	89	79.3	89.5	89.8	91.9
ACR	132.1	158.5	102.2	94.4	88.7	98.5	130.4	140.4
ACRBP	106.9	136.1	122.8	112.3	100.5	118.8	103.8	124.8
ACRC	242	345.7	131.1	125.5	113.6	133.4	242.3	315.3
ACRV1	127	149.6	112.3	105.8	98.8	107.9	120.9	149.5
ACRV1	118.6	130.6	102.5	91	83.7	95.7	111.8	117.6
ACSBG1	168.2	186.9	107.6	88.5	85.9	93.1	160	190.9
ACSBG2	136.3	155.9	104.7	98.9	94.9	99.1	125.8	129
ACSL1	536.7	636.2	175.8	137.7	115.9	153.7	400.5	662.3
ACSL3	2506.4	3445.8	1501.4	1719.9	1640.1	2448.6	2262.3	3171.1
ACSL3	267.4	348.6	143.5	153.7	150.4	120.6	203.6	233.7
ACSL3	432.8	558.7	308.6	342.4	313.6	445.4	373.6	438.1
ACSL4	151.8	182	117.3	105.5	94.7	98.6	124.1	133.8
ACSL4	669.6	813.5	285	253.7	164.8	267.7	637.3	839.8
ACSL4	914.4	1131.9	651.5	1121.9	879.3	1444.6	911.9	1545.2
ACSL5	260.2	389.3	182.2	160.7	115.2	193	184.3	280.6
ACSL5	96.7	105.9	91.7	87.6	99	87.1	94.9	94.8
ACSL5	73.6	86.4	91.3	92.4	85	90.7	86.1	85.8
ACSL5	151.5	199.2	101.1	93.3	86.4	84.9	127.1	146
ACSL5	882.8	1157.1	603	538.2	343.6	559.6	645.5	707.2
ACSL6	89.8	100	87.5	83.1	82.3	77.1	89.4	102.9
ACSL6	78.9	87.8	95.8	95.7	85.9	86.5	80.6	89.6
ACSL6	99.2	110.3	102.5	94.6	92.5	92.2	100.3	109.4
ACSM1	70.2	77.6	82.1	86.1	82.6	82	79.7	76.7
ACSM2	108.3	116	93.7	80.6	84.4	79.7	96	106.4
ACSM2	99.3	110.4	104.1	94.5	84.3	92.6	99.7	121.3
ACSM3	97	100.4	104	89.4	95.8	89.6	92.5	112.2
ACSM3	76.6	92.4	103.8	92.6	90.5	97.1	83.8	104.3
ACSM3	103.9	135.8	106.9	107.7	91.8	105.6	109.3	114.1
ACSS1	145.9	197.6	220.9	181.1	180	240	118.3	158.1
ACSS2	131.5	154.2	85.9	78.6	77	87.8	108.8	167.1
ACSS2	99.3	121.5	91.3	82.8	83.2	93.7	98	104.8
ACSS2	1440.1	1840.6	1133.8	1526.7	1396.8	1735.6	1208.7	1342.4
ACSS2	1796.9	2228.1	1362.9	2041.6	2027.3	2370.8	1489.8	1553.5
ACTA1	93.2	102.6	98.6	89.1	92.5	89.6	91.4	103
ACTA2	116.4	146.5	107.4	112.6	91.7	102.3	128.2	141.8
ACTB	31866.2	31406.1	25615	27578.8	23963.8	29893.2	29811.6	32311.5
ACTB	28810.3	31998.4	20140	23234.8	17479.7	30753.7	25435.6	33636.6
ACTBL1	94.4	94.5	86.4	78.4	75	91.1	91.9	96.5
ACTC	88.3	94.1	91.4	91.6	77.9	96.5	82.4	101.6

ACTG1	34708.4	33714.1	41666.1	44609.1	33331.4	41150.9	33335.6	33778.6
ACTG2	84.7	95.4	93.4	99.5	92.1	103.6	93.3	99.3
ACTL6A	4728	6082.4	3745.5	3218.1	2448.7	4058.2	3922.9	5549.3
ACTL6A	3075.3	4312	1100.6	819.1	624.7	1240.8	2271.4	3764
ACTL6B	95.5	87.7	96.7	102.2	95.6	98.6	89.4	104.5
ACTL7A	85.4	91.1	96.2	88.8	83.5	97.3	76.4	95.6
ACTL7B	102.1	134.7	100.4	95	94.6	92	96	111
ACTL8	130.7	131.1	104.8	93	94.1	88.2	110.6	130.1
ACTN1	3648.8	3970.4	3836.6	3928.1	3020.3	4462	2610.7	3626.7
ACTN2	160.7	209.6	114.9	104.5	96.9	112.6	159	195.6
ACTN3	77.9	87.7	97	83.6	84.9	86.8	81.6	85.2
ACTN4	579.3	774.6	808.6	883.4	677.7	980.7	653.6	1416.8
ACTR10	1155.9	1455.1	663.7	603.7	494.1	799	1301.7	2249.5
ACTR1A	917.2	1175.6	570.7	350.7	284.6	315.5	871	952.5
ACTR1B	554.6	615.3	450.6	408.4	384.5	575.6	352.9	560.9
ACTR2	12068	15255.2	8130.7	8205	6004.5	9048.7	10981.4	16770.4
ACTR3	1719.8	1964.9	790.6	943.2	683.8	1411	1626.5	3569.9
ACTR3B	184.8	251.7	147.7	111	101.6	125.6	159	171.4
ACTR3B	98.3	128.8	101.7	89.2	92	89.5	103.4	109.8
ACTR3B	175.7	251.2	115.8	99.9	87.2	95	159.8	176.2
ACTR5	437.5	555.3	247	240.2	217.1	265.7	319.8	433.5
ACTR6	614.4	688.4	342.7	381.7	308.7	484.2	594.9	1034.8
ACTR8	214.5	263.1	167	165.5	162.6	208.3	203.8	266.9
ACTRT1	115	140.7	118.2	114.8	106.3	112.9	113.2	139.2
ACVR1	416.5	578.3	277.6	248.3	207.9	259.9	482	759.3
ACVR1B	115.2	140.5	109.4	106.4	93.5	96.5	117	119.8
ACVR1B	96.5	125.9	98.6	94.6	92.1	107	103.4	117
ACVR1B	469.3	578.6	314.3	331.7	289.1	357.2	473.8	514
ACVR1B	89.5	103.5	93.7	91	86.4	90.3	88.8	113.6
ACVR1C	91.3	104.7	95.7	91.4	83.7	86.9	95.3	110.4
ACVR2A	163.4	186.2	160.6	157.2	154.7	181.5	158.6	230.9
ACVR2B	80.8	118.4	99.4	93.8	79.4	74.6	86.9	122.9
ACVRL1	120.8	151.3	142.4	114.9	106.4	111.1	123.5	136.1
ACY1	911	1203.9	865.4	589.4	453.7	715.3	780.1	1120.3
ACY1L2	441.7	523.3	244.7	199	162.2	297	275.9	781.7
ACY3	102.2	109.6	97.7	86.1	83.4	90	98.4	110.8
ACYP1	276.2	357.9	114.4	111	121.5	129.6	195.3	214.8
ACYP1	1162.3	1216.4	583.2	495.5	473.4	580.8	791.4	1004.6
ACYP1	167.1	191	134.4	117.2	100.9	103.3	140.1	183.8
ACYP2	372.7	463.7	265.1	263	222.6	245.1	380.9	744
AD031	113.5	111.6	84.7	78.6	83.3	77.2	104.2	123.8
ADA	534.5	716.5	505	501.3	469.9	643	440.1	585.6
ADAL	185.9	249.8	137.8	145.9	137	145.7	167.1	248.9
ADAM10	133.7	148	98.7	93.6	93.7	126	124.8	147.4
ADAM11	95.4	102.5	104.9	99.9	93.9	117.8	91.9	115.2
ADAM12	100.3	120.6	83.7	86.1	87.1	91.4	101.3	111.5
ADAM12	106.6	119.1	100.7	80.9	81.7	83.9	106.2	128.3
ADAM12	78.4	101.5	90.5	88.6	80.7	84.8	78.7	85.8
ADAM15	301.1	426.3	261.3	211.3	166.6	204.2	245.8	488.5
ADAM15	5503.9	6870	4062.5	2893.5	2496.5	2461	4716.5	4349.6

ADAM15	200.8	241.7	191.6	174.5	136.3	137.1	157.7	228.4
ADAM17	368.4	518.4	239.4	274.7	244.4	265.9	433.2	543.6
ADAM18	72.4	94.7	98.8	84.2	86.1	88.7	76.3	74.2
ADAM19	107.9	146.6	89.7	89.2	78.2	87.3	113	130.6
ADAM19	89.1	97.4	103.5	95.6	87	91.4	85.5	92.4
ADAM19	117.2	130.6	128.6	156.7	112.2	133.7	105	122.7
ADAM2	100.6	119.7	100.3	96.5	90.6	95.8	97.4	109.6
ADAM20	94.7	83.8	90	78	69.6	86.7	86	86.3
ADAM21	109	109.9	112.7	106.1	98.4	102.8	110.3	106.5
ADAM22	105.1	126.8	102.1	81	92.1	86.8	97.7	124.1
ADAM22	78.6	91	95.4	85.6	79.7	80.4	73.8	89.9
ADAM22	113.7	142	83.2	84.1	72.8	85.3	122.1	139.4
ADAM23	77.2	87.5	93.2	85.7	79.7	83.5	86.1	81.5
ADAM28	84.9	94.2	78.4	88.7	102.3	97.6	90.5	100.7
ADAM28	90.4	102.9	97.5	96.9	85.9	87.7	100.2	116.1
ADAM28	79.3	94.9	92.5	93.7	85.2	101.6	81.5	84.3
ADAM29	91.4	103.9	109.3	89.5	97	92.5	92.3	95.1
ADAM30	90.1	104.4	104	95.7	99.6	83.5	93.1	97.8
ADAM32	126.2	133.6	111.5	107.2	100.1	102.9	121.4	122.2
ADAM32	99.1	132.9	88.3	85.2	73.6	78.2	105.4	111
ADAM33	112.1	130	123	101.9	106	107.8	110.4	112.5
ADAM33	112.3	107.1	97.7	89.7	95.3	101.3	108.8	119.1
ADAM3A	71.5	88	91.8	89.8	80.3	88.9	75	78.5
ADAM6	94.3	100	106.9	97.2	92.7	98.8	84.3	104.5
ADAM7	133.3	146.2	111	94.4	98.2	91.8	132.5	133.5
ADAM8	83.8	94.6	99	92.2	101.2	96.1	85.4	96.8
ADAM9	242.3	291.5	131.2	124.2	127.4	133.2	192	283.7
ADAMDEC1	85.5	97.1	97.1	255.5	249.4	343.4	98.7	107.8
ADAMTS1	208.9	245.4	113.2	117.1	97.1	119.3	154	189.7
ADAMTS10	84.5	96.6	100.2	91.9	91.2	97.5	93.1	116.3
ADAMTS12	89.6	105.6	93.2	83.6	88.7	79.7	95.6	93.7
ADAMTS13	104.7	117.9	90.7	77.2	88.5	84.7	101.7	122.1
ADAMTS13	79.9	81.5	84.7	81.8	82.3	74	79	77.3
ADAMTS13	117.9	147	105.1	95.6	104.2	94.6	128.3	144.5
ADAMTS14	85.2	94.1	93.9	87	80.5	91.9	86.5	89.6
ADAMTS14	97.7	116.2	84.7	83.5	79.4	91.1	103.8	124.7
ADAMTS15	101.9	127.3	93.6	94.8	84.7	90.1	94.6	110.9
ADAMTS16	99.4	112.4	106.3	97.7	107.5	96.2	105.1	113.2
ADAMTS17	107.9	129.5	112.9	100.8	100.2	95.2	122.5	134.1
ADAMTS18	109.3	140.1	107	90.5	90.3	89	128.5	141.3
ADAMTS18	91.7	102.9	98.9	89.4	94.2	85.7	90.9	94.2
ADAMTS19	107.4	111.7	134.4	123.2	118.3	129.7	98.2	112.6
ADAMTS2	113.3	129.9	97.5	111.7	102.1	100	115.1	123.1
ADAMTS2	140.9	169.1	171.2	237.5	194.3	247.9	110.3	176.1
ADAMTS20	92.5	108.3	88.3	84.5	84.8	87.6	94.8	104.2
ADAMTS20	80	90.5	92	82.4	89.4	84.6	75	91.2
ADAMTS3	94.1	94.9	85	78.5	83.3	82.7	91.5	109.7
ADAMTS4	86.7	92.1	115.5	99.9	98.2	91.4	89.2	76.8
ADAMTS5	88.6	96.9	93	77.3	85.1	85	90.9	85.4
ADAMTS6	111.4	95.6	105.6	87	83.8	98.7	99.9	111.7

ADAMTS7	92.3	94.7	85.9	90.3	81.2	84.3	86.7	91
ADAMTS8	131.2	153.4	98.6	91.3	89.1	94.2	132.2	116.6
ADAMTS9	106.4	140.3	109.8	105.7	96.7	93.8	106.8	110.6
ADAMTSL1	91.8	99.3	88.9	97.7	88.5	89.9	83.7	111.1
ADAMTSL1	88.1	93.2	80	84.9	82.5	78.7	85.9	92.2
ADAMTSL1	104.7	127.7	103.8	103.1	93.4	94.2	105.2	110.3
ADAMTSL1	80.6	85.1	82	80.5	79	81.6	85.1	85.9
ADAMTSL2	81.3	89.1	104.3	93.6	90.2	100.6	84.9	81
ADAMTSL3	120.7	136.5	92.7	96.3	81.2	82.4	119.6	139
ADAMTSL5	120.4	143.1	137.9	115.1	117.4	117.4	109.1	151
ADAR	2979.2	3664.5	1319.4	1516.5	1173.7	1676.4	2619.3	3006.1
ADAR	116.1	117.9	85.3	93	81.2	80	103.5	128.2
ADAR	718	1109.6	991.2	787.4	647.8	1360.7	674.1	879.3
ADARB1	110.3	158.4	103	90.6	87.3	89.7	114.5	140
ADARB1	155	194	146.9	140.9	133.3	147.7	137	164
ADARB1	104.8	121.3	93.3	96.3	89.7	89.5	106.2	114.5
ADARB1	378.5	513	269.9	336.9	311.1	410.2	351.7	470.4
ADARB2	103.5	114.4	98.3	95.7	89.6	93	109.7	114.7
ADAT1	413.8	581.3	425.8	373.5	278.2	477.4	326.9	505.2
ADC	104.9	106.9	97.7	89.4	97	103.3	106.7	99.2
ADCK1	213.8	268.2	206.3	200.3	179.2	228.5	173.1	284.8
ADCK2	1423.4	1878.5	1232.6	893.8	647.7	1002	1161.7	999.6
ADCK4	167.3	189.4	119.2	107	88.9	126	153.4	188.5
ADCK5	247.9	305	181.2	149.7	141.3	203.6	198	271
ADCY1	251.8	374.1	281.4	684	429.7	939.4	226.5	456
ADCY2	148.3	161.8	103.7	102.8	90.6	103.6	150	196.2
ADCY3	2339.2	2649.3	2701.2	2306.4	1518.5	2531.7	1608.2	2187.1
ADCY4	166.2	177.8	169.6	149.4	153	173.8	147.7	176.6
ADCY5	80.6	89.7	79.3	72.5	74.6	84.2	87.8	86.9
ADCY6	110.8	139.5	117.4	99.8	100	112.4	117.5	134.1
ADCY6	110.1	140.2	127.5	99.4	117.6	129.1	111.3	124.3
ADCY7	287.3	418.5	204.6	179.7	190.9	278.4	248.5	284.8
ADCY8	104.8	108.9	97	86.5	92.2	90.5	99	105.3
ADCY9	440.7	676.6	391.8	214.5	205.9	259.9	404.9	808.2
ADCYAP1	134.7	130.7	100.5	88.7	85.4	92.7	119	126.6
ADCYAP1R1	101.4	114.7	103.1	92	85.8	96.1	103.9	106.2
ADD1	142.9	166.5	100.3	101.3	87	109.4	146.1	190.7
ADD1	3192.7	3417.8	2043.6	2219.1	1685.7	2410.9	2510.1	2984.5
ADD2	102.2	116.6	92.1	93.5	93	82.8	98.6	117.1
ADD2	77	96.8	91.8	91.6	88	89.7	83.4	98.4
ADD2	123.7	152.8	109.7	86.9	86	84	128.8	129
ADD3	95.6	109.2	98.1	87.8	84.8	90.4	92.3	103.1
ADD3	120.5	142.7	102.9	116.1	99.7	122.4	104	126.7
ADD3	396.5	464.3	193.5	261.2	212.1	381.6	314.7	465.2
ADD3	253.9	305.8	148.2	185.7	165.6	228.9	172.3	248.9
ADFP	1551.1	2097.3	1343.6	1378.7	1184.9	3136.8	1784.5	3147.9
ADH1A	79.4	91.5	82	84.7	86.9	94.2	86.5	92.8
ADH1B	76	77.5	94.1	92.3	87.8	90.3	82.5	83.4
ADH1C	70.7	82.1	89.1	82.5	79.8	86.5	74.8	81.3
ADH4	89	99.3	82.7	77.6	79.3	71.6	85.4	91.7

ADH5	203.9	268.4	117.3	103.1	85.8	110.1	161.1	248.6
ADH6	100.2	118.5	103.8	101.1	93	90.7	100.1	117.9
ADH7	83.1	90.4	82	84.9	80	82.5	79.3	95.5
ADHFE1	98.3	107	98.3	93.1	92.6	92.1	99.3	107.7
ADHFE1	94.3	94.9	90	78	86.9	82.3	96	93.6
ADI1	220.5	270.8	108.1	103.9	92.7	105.5	187.4	296.8
ADIPOQ	164.4	162.8	87.2	91.7	81.6	92.1	144.6	144.2
ADIPOR1	529	693.9	492.4	506.8	397.6	602.8	447.5	1139.7
ADIPOR2	1022.3	1275.9	925.7	962.9	867.4	1111.2	801.3	1279.7
ADK	317.2	391.7	156.6	129.7	129.2	128.5	268.6	262
ADK	84.4	88.9	87.4	84.2	79.4	82.8	85.3	91.8
ADK	422.2	555	279.5	173.3	171.2	229.9	322.3	357
ADM	86.1	102	98.1	162.9	205.6	314.6	130.8	210.7
ADM2	333.7	372.8	190	149.2	129.9	145.2	411.2	561
ADMR	131.5	134.3	96.8	79.8	73.5	86.9	127.4	136.8
ADNP	701.4	939	305.2	303	220.8	368.2	632.4	981.4
ADNP	125.9	178.6	127.6	178.1	155.5	154.2	117.5	151.8
ADORA1	83.3	85.8	89.7	81.4	79.5	83.2	85.8	93
ADORA1	93.9	109.3	91.2	90.4	88.5	89.5	103.3	111.8
ADORA2A	124.6	126.6	110.6	110.7	109.6	108.6	104.7	126
ADORA2B	496.4	661.5	328.3	375.5	261.8	451.3	576.8	1045.7
ADORA3	131.1	147.8	132.3	209.1	151.6	193	138.5	140.2
ADORA3	324.9	381.7	178.5	188.3	169.1	234.6	250.1	315.5
ADPGK	1067.8	1315.8	779.3	970.3	865.3	1220	893.7	1182.3
ADPN	86.9	97	100.4	105.6	111.7	107.9	88.2	104.3
ADPRH	146.2	198.3	100.6	95.4	90.6	97.9	124.7	177.3
ADPRHL1	92	112.5	94.8	96.6	86.1	82	100.6	104.1
ADPRHL1	86.2	100	101	93.3	91.9	87.1	97.8	106.6
ADPRHL1	115.4	128.5	92.7	93.5	88.8	94.9	124.8	123.2
ADPRHL2	459.3	581.8	253.6	192.4	203.2	261.8	391.6	399.1
ADRA1A	77.8	81.8	88.8	83.4	81.1	85.2	84.3	88.4
ADRA1A	80.8	95.9	90.9	82.2	78.6	87.9	84.4	84.1
ADRA1A	126.6	155.2	93.6	87.6	79.4	74.6	121	151.8
ADRA1A	130.9	147.1	110.5	106.8	101.4	101.7	111.2	129.5
ADRA1A	106.7	127.9	99	103.7	95.6	97.4	113.1	113.8
ADRA1B	94.1	111.2	108.9	106.9	99.6	99.7	94.6	100.5
ADRA1D	116.6	151.7	104.1	101.8	95.2	102.3	110.3	126.4
ADRA2A	129.9	152.9	109.6	95.6	101.2	91	152.9	148.8
ADRA2B	190.3	210.2	102.3	115.4	109.8	115.2	202.6	206
ADRA2C	107	131.4	112.4	101	94.2	105.1	109.6	111.6
ADRB1	124.2	142.3	113.5	108.4	112.1	123.4	110.1	129.8
ADRB2	300.9	383	198.5	292.5	296.1	317.3	174.1	209.5
ADRB3	101.6	123	93.4	87.9	95.6	93.1	112.6	106.2
ADRBK1	81.3	84.8	92.7	85.7	83.3	82.8	72.7	80.4
ADRBK2	100	118.3	83.8	87	82.1	87.4	98.7	109.6
ADRM1	684.6	794.3	525.1	356.1	285	395.7	679.7	1279.3
ADRM1	2274.7	2691.5	673	488.6	468	717.3	2530.9	6690.4
ADSL	7255.1	10043.6	5387.7	4898.5	4525.3	6434.1	6291.4	6203.2
ADSS	3123.1	3833.6	2182.2	2356.5	1583.1	2665.4	2580	4140.4
ADSSL1	88.5	105.7	93.8	84.8	81.9	86.8	94.1	102

ADSSL1	238.9	293.8	223.5	207.2	176.2	259.3	195.9	275.3
ADSSL1	175.7	254.4	124.5	94.6	92.5	105	155.4	183.1
AEBP1	120	135.6	77.6	81.6	79	85.5	114.8	130.9
AEBP2	180	183.3	113.4	139	120.1	159.9	142	193.2
AES	864.9	1226.9	927.3	596.5	444.8	781.8	898	1686.9
AES	586.1	800.1	493.3	327.1	262.8	323.7	508.5	1095
AES	110.4	117	83.7	86	92.8	83.8	98	106.2
AFAP	87.7	93.1	91.2	89.7	95.3	96.1	78.3	88.9
AFAP	127.7	159.6	123.3	132.9	112.9	134.8	110	126.7
AFAP	91.2	104.6	116.4	107.3	95.8	101.3	92.8	100.9
AFAP1L2	110.2	115	89.2	85.5	83.7	85.1	112.7	114.9
AFAR3	141.8	165.6	95.7	102.6	88.3	106.7	142.2	171
AFARP1	100.5	126.2	93.8	87.8	84.3	86.8	105.3	127.9
AFARP1	82.1	84.5	97.6	95.3	88.1	96.4	82	86.6
AFF1	482.9	836.4	221.4	306.3	237.6	352.8	338.9	375.8
AFF2	104.5	127.9	101.2	117.6	104.1	121.7	112	124.7
AFF3	784.3	1091.4	256.1	399.6	403.1	462.5	561.1	644.8
AFF3	110.4	122	102	101.7	88.4	98.5	101.1	103.8
AFF4	249.5	349.5	122	145.8	161.9	197.8	218.1	362.4
AFG3L1	329.3	414.5	388.1	393	368.1	639.1	310.2	715.3
AFG3L1	136.6	155.6	128.6	129.2	119.5	112.8	111.3	133.8
AFG3L1	122.8	141.4	88.2	91.9	86.1	81.3	112.2	120
AFG3L1	96.6	105.5	90.3	89.8	88	79.7	101.6	97.8
AFG3L2	1786.6	2452.7	1230.6	997.8	973.9	1488.2	1463.7	1716.7
AFM	86	79.8	86.4	89.4	83.5	78.4	75.4	79.9
AFMID	113.3	136.9	121.1	96.6	94.9	113.1	108.7	110.2
AFP	132.8	136.5	102	92.8	97.4	94.3	111.3	129.6
AFTIPHILIN	145.4	181.5	152.9	120.5	112.4	158	129.5	142
AFTIPHILIN	230.2	273.9	123.7	124.8	100.5	140.1	228.2	382.5
AFTPH	491.4	703.3	258.5	209.5	173.7	416.9	447.7	569.3
AGA	606.7	765.9	307.8	301.6	255.5	309.9	536.9	801.8
AGBL1	91.9	105.4	79.2	81.5	76.3	70.2	88.5	87.6
AGBL2	86.9	92.4	108.6	89.4	83.9	97.2	95.3	88.4
AGBL4	73.9	88.1	79.4	91.5	73.5	83.1	81.3	92.5
AGC1	111.1	119.8	120.3	112.4	90.7	111.3	111.7	107.9
AGC1	74.3	82.8	89.2	83	78.7	78.9	75.2	66.5
AGER	117.2	133	97.1	90.9	76.2	86.6	114.4	121.6
AGER	112.5	127.8	103	102.1	95.6	115.2	93.5	120.2
AGGF1	1176.7	1435.2	782.4	1043.7	854.1	1410.5	1003.8	1318.3
AGL	107.9	113.5	89.5	76.7	78.4	85.2	116.4	123
AGL	90.1	98.1	93.7	88	85.9	90.1	93.3	103.5
AGL	569.6	688.4	365.5	345.9	244.8	461.6	524.3	935.3
AGL	94.4	105.3	95.2	85.8	73.7	85.1	92.1	111.2
AGL	98.4	116.6	101	95.8	92.9	92.3	101	108.9
AGL	215.6	250.7	120.4	123.1	130.1	172.5	170.8	281.1
AGMAT	401	497	384.5	253.8	202	152.2	309.1	472.3
AGPAT1	82.4	88.8	86.8	75.8	73.3	79.1	86.3	89
AGPAT1	81.2	92.4	84.4	88.1	84.8	84.8	84.8	95
AGPAT1	236.8	306.5	251.1	312.3	261.7	395.8	228.6	450.7
AGPAT2	1181.9	1649.8	812.3	583.2	453.2	824.6	1115.2	1550.1

AGPAT2	1486.2	1897.8	591.3	434.2	353.1	287.1	1341.6	1788.5
AGPAT2	1064.5	1507.2	402.2	332.8	276.6	363.9	1029	1504
AGPAT3	93.3	136.2	109.1	107.8	95.5	91.4	105.3	124.5
AGPAT3	211.9	328.3	189.1	215.8	181.9	190.8	176.5	228.1
AGPAT4	136.4	150.3	137.8	158.8	143.4	203.5	120.7	158.4
AGPAT5	968.6	1278.8	496	338.9	282.2	481.7	574.5	704.8
AGPAT6	194.5	241	102.8	95.6	100.8	98.1	171.2	180.3
AGPAT7	136.9	150.8	157.5	99.3	107.9	126.9	125.1	168.7
AGPS	1362.7	1686.9	936.1	1064.7	740.4	1469.6	891.9	1420.2
AGR2	157.1	164.3	99.1	85.7	91	100.4	119.6	154.7
AGR3	88.9	92.3	87.2	86.8	81.9	91.9	83.3	99.6
AGRN	91.9	105.8	90.5	89.9	92.7	91.3	94.1	95.3
AGRP	122.8	119.4	101	90.8	78.4	84.5	106.5	145.3
AGRP	83.8	96.8	94.8	91.5	87.6	95.6	85	101.6
AGT	1166.6	1594.7	825.1	934.8	832	1263.8	1001.9	1466.9
AGTPBP1	91	98.3	85.3	96.4	86.4	97.3	102.8	133.1
AGTR1	75.3	87.7	87.2	85.7	80.7	85.7	71.5	88.5
AGTR1	133.9	151.1	96.6	91.9	84.8	96.8	129.9	125.4
AGTR2	117.5	136.1	103.5	87.7	92.7	101.2	96.8	129.5
AGTRAP	349.7	402.2	201.1	171.4	122.5	176.6	250.9	450.4
AGTRAP	282.3	382.3	207.7	127.8	120.4	198.7	238	319.5
AGTRL1	88.2	104.3	95.6	82.8	83.9	95.3	95.7	99.7
AGXT	87.2	98.3	88.5	84.5	91.2	92	90	80.6
AGXT2	80.4	91.6	85.7	87.5	74.5	84.3	89.6	98.1
AGXT2L1	97.3	107.7	82	82.7	83.8	80.6	94.9	101.6
AHCTF1	829.6	1085.8	548	914.4	591.5	1155.6	698.1	1094.6
AHCTF1	817.2	1236.5	724	699.4	566.3	1291.5	632.5	1123.5
AHCY	5138.3	6658.7	5900.7	5859.9	4379.6	6641.9	4427.1	8680.4
AHCYL1	2569.6	3985.7	1589.3	1187.2	885.4	1717	2428.9	3730
AHDC1	124.8	118.6	112.5	102.1	111.6	117.9	108.9	114.3
AHI1	272.2	320.1	219.5	280.5	256.1	348	200.6	308.9
AHNAK	936.7	1762	494.3	1563.2	1633.8	2026.4	1277.7	1917.1
AHNAK	199.2	256.3	173.4	122.4	120.9	109.7	190.8	184.9
AHNAK	236.5	285.1	111.5	96.7	98.3	101	210.6	318.7
AHR	123.8	136.7	110.1	97.3	92.6	96.9	111.1	137.6
AHRR	111.3	125.3	91.7	116.2	98.1	115.4	120.8	126.4
AHSA1	2558.5	3518.5	1093.3	552.6	506.8	855.2	2529.9	3420.4
AHSA2	537.4	414	167	178.6	178.4	205.7	284.4	532.9
AHSG	137.8	173.4	93.5	92.5	81.1	87.8	131.7	142.4
AICDA	96.1	110.7	100.7	80.9	97.5	94.6	102.9	99.4
AIF1	5851.1	7535.4	3063.3	3861.5	3162.6	5093.8	4665.7	5253.2
AIF1	7394.9	8940.6	6552.3	7859.1	5805	8217	6210.3	11105
AIF1	81.9	107.1	96.5	114.7	106.8	101.5	81.4	98.3
AIFL	141.4	148.9	92.3	89.1	87.4	92.6	121.6	158.1
AIFM1	1899.4	2602.6	1178.5	1377.2	978.3	1602.7	1658.2	2022.6
AIG1	1070.5	1341.5	402.6	720.5	510.8	867.6	895.7	1323.8
AIM1	197.7	265.4	139.1	134.3	130.1	172.5	154.1	210.8
AIM1L	83.2	93.1	95.7	89.6	80.6	88.3	80.6	91.5
AIM2	95.8	104.3	96.5	85.9	78.5	89.1	96.8	114.4
AIP	941.5	1098.8	1016.1	798.8	757.3	1094.5	928.3	1185.7

AIPL1	93.4	116.8	104.8	94.3	90.9	93.8	96.9	107.4
AIPL1	85.3	99.3	106.3	96.7	95.4	90.4	78	93.5
AIRE	116.2	128	96.6	99.2	82.7	93.3	110.7	120.2
AIRE	5940.1	6692.6	2558.7	4188.6	4256.6	3107.8	5095.4	4284.5
AJAP1	74.9	98.6	93.9	81.2	82	77.3	80.8	78.7
AJAP1	106.6	137.2	93.7	95.6	84.6	101.1	106	130.7
AJAP1	140.6	194.9	108.6	107.6	100.5	113.3	145.7	161.6
AK1	553	620.9	300.5	172.1	172.2	172.6	428.7	395
AK2	1683.4	2184.2	403.5	341	284.8	386.5	1536.7	1690.5
AK2	594.5	731.5	456.7	675.6	470.1	811.7	365.9	471.8
AK2	4017.6	5732.9	2984.6	2645.2	2071.9	3278	3393.1	4804.6
AK3	2631.1	3284.3	1718.4	1658.8	1268.4	1838.1	2025.6	2484.8
AK3L1	965.8	1222.1	637.7	712	593.7	805.8	608.2	813.4
AK3L1	88	112.4	89.8	86	79.8	86.7	91.4	102.9
AK5	103.2	111.3	83.6	78.7	83.4	82.2	91.8	100.7
AK5	82	81.4	89.8	81.5	79.9	85.1	78.2	74.5
AK5	79.4	83	103.5	82.8	94.9	82.4	78.4	80.5
AK7	109.6	131.6	90.2	81	86.6	86.4	116.2	119.6
AKAP1	383.6	430.6	174.5	159.8	164.7	210.1	262.5	592.8
AKAP10	139.2	186	111	126.5	128.9	136.3	149.2	141.7
AKAP11	75	83	77.7	83	76.4	78.6	80.2	82.5
AKAP11	427.1	548.8	283.2	449.3	310	693.1	361.1	583.6
AKAP11	121.6	108.2	124.9	105	102.2	101.9	84.7	99.8
AKAP12	87.1	96.1	100.7	97.1	85.3	89.7	99.1	91.9
AKAP12	109.6	142.8	99.8	85.8	72.3	85.5	102.3	121.8
AKAP12	193.2	265	160.1	132.4	127.2	135.7	193.3	237
AKAP13	113.4	113.9	114.7	147.5	149.1	191.2	100	114.9
AKAP13	381.5	548.8	290.7	671.9	527.2	800	304.3	298
AKAP14	70.4	80.9	85.9	83.6	83.2	82	66.6	78.3
AKAP14	79.2	90.1	91.3	92.5	87.5	85.5	84.6	82.6
AKAP14	86.1	102.9	100.3	83.1	87.1	95	104.4	102.9
AKAP2	76.3	88	88	95.9	84.4	87.4	80.2	90
AKAP3	110.9	116.9	108.1	100.3	98.5	109.4	116.1	108.2
AKAP4	115.8	129.9	90.9	94.4	74.3	82.1	132.2	125.9
AKAP4	102	92.1	105.7	108.3	95.9	102.4	87.9	92.8
AKAP4	130.3	152.9	99.7	100.8	99.9	93.2	146.5	133.8
AKAP5	109.5	127.9	86.7	88.1	74.2	87.4	105	109.4
AKAP6	82	87.2	93.1	87.1	77.8	86.2	81.7	88.4
AKAP7	89.6	114.6	84.8	74.7	67.9	73.8	92.9	105.5
AKAP7	109	130.7	99.1	99.1	90.6	100.9	97.3	125.6
AKAP7	1253.9	1728.3	1206.8	1137.2	1095.6	1517.2	700.7	991.7
AKAP7	248.3	325.3	140.9	126.6	136	152.8	116.9	112.3
AKAP8	565.3	734.4	582.2	522.4	451.9	761	508.9	874.7
AKAP8L	246.2	254.6	216.7	188.2	160.3	201.2	242.8	357.7
AKAP9	106.8	121.1	84.9	85.8	85.7	84.5	105.4	113.2
AKAP9	176.9	166	106.8	92.3	86.4	98.8	144.6	141.7
AKNA	245.9	286	278.7	283.5	230.8	398.3	230.9	486.5
AKR1A1	1431.9	1952	1211.7	1372.6	1228	1762	1206	1648.2
AKR1A1	113.1	132.3	94	85.7	84.9	90.1	111.5	133.3
AKR1A1	2122.7	2390.1	815.4	1023.1	911	1212.9	1775	2298.7

AKR1B1	3669.6	4663.3	2501.7	1941.2	1525.4	2142.7	3111.4	4645
AKR1B10	75.3	84.6	97.8	90.1	79.8	81.6	83.3	83.6
AKR1C1	77	79.7	76.6	78.8	81.6	78	76.2	80.6
AKR1C2	431.1	649.3	333.9	262.6	198.1	270	773.7	1037.1
AKR1C3	113.9	135.8	110.2	111.8	93.1	116.5	163.8	215.2
AKR1C4	93.7	101.8	108	92.6	98.7	93.4	88.8	92.3
AKR1CL1	84.7	93.7	101.2	90.6	87.8	89.9	93.6	88.1
AKR1CL2	74.8	95.2	93.2	86.2	75.9	74.7	80.3	88.5
AKR1D1	9416.3	10981.1	3238.9	6768.4	6568.8	4408.4	8082.7	6817.8
AKR7A2	5769.2	7523.2	3381.4	2650.6	2260.3	2898.7	5198	5499.6
AKR7A3	981	1244.1	570.5	504.1	424	556.2	928.3	1831.1
AKT1	3318.4	4010.2	3136.2	2480.6	1985.6	2571.5	2778.8	3999.2
AKT1	1941.1	2612.3	2359.9	1974.3	1439.4	3188.8	1818.3	3748
AKT1	139.1	134.1	95.4	87	71.7	98.4	128.8	150.3
AKT1S1	102.1	125	103.8	104.2	95.8	98	91.4	127.8
AKT2	106.5	126.1	120.8	106.7	92.6	95.8	91	133.2
AKT3	84.8	108.6	102.2	91.1	89.5	102.4	83	111.4
AKT3	119.6	137.8	114.3	89.3	83.5	94.4	124.8	139.2
AKT3	104.2	134.4	98.1	91.1	88.5	90.7	118.1	122.1
AKTIP	362	446.3	288.6	230.1	200.4	298.7	289.9	500.4
ALAD	135	131	99.5	93.1	95	92.1	123.7	130
ALAD	780	1067.2	547.6	533.2	490.5	720.6	575.7	612.5
ALAD	75.4	76.6	90.2	91.6	87.5	89.2	72.8	78.6
ALAS1	124	160.3	118.7	103	95.3	114	114.5	131.9
ALAS1	136.7	193.8	114.7	129.9	119.2	116.7	164.7	206.3
ALAS1	98.3	112.6	107.9	91.4	99	87	90.6	112.5
ALAS2	85.9	101.6	98.9	99.1	93	91.7	88.8	98.5
ALAS2	81.4	93.3	93.5	91.3	82.3	78.2	82.1	83.2
ALAS2	107.5	119.3	104.4	96.4	93.5	83.5	114.9	132.4
ALB	106.1	129.6	91.4	106.6	78.6	96.2	101.6	106
ALCAM	1081	1382.1	496.8	672.7	384.1	1080	844	2258.2
ALDH16A1	273.4	377.4	355.6	255.1	239.9	324.3	233.4	407.4
ALDH18A1	632.2	883.4	405.8	368	266.3	371.6	385.9	421.8
ALDH1A1	101.5	122.1	103.9	92.7	83.9	99.8	87.8	102.7
ALDH1A2	108.6	113.1	101.7	102	85	90.5	99.6	106.7
ALDH1A2	175.8	211.5	111.9	104.4	108.9	112.6	174.5	188.5
ALDH1A3	125.1	137.9	104.8	85.3	81.8	101.9	116.7	129.7
ALDH1B1	642.4	934.8	308.1	217.6	179	258.4	464	713.7
ALDH1L1	74.4	87.4	98.5	87.9	91.3	86.1	81.2	85.8
ALDH1L2	123.1	145.6	90.6	93.5	84.9	90.6	115.7	151.8
ALDH2	1686.9	2111.6	1088.2	1819.5	1517.5	2800.5	1140.9	1569.6
ALDH3A1	89.2	94	88.9	96.8	85.1	97.4	99.3	103
ALDH3A2	144.4	241.5	88.9	104.9	92.9	113.8	146.9	177.2
ALDH3A2	218.6	261.7	166.3	194.2	170.7	210.4	179.5	209.1
ALDH3B1	155.9	190.7	95.9	111.7	84.4	97.8	124.2	168.9
ALDH3B1	603.4	701.5	512.5	677.9	509.3	610.5	521.4	1158.5
ALDH3B2	87.1	114.3	97.1	92.5	91.4	97.4	95.1	117.3
ALDH3B2	97.6	117.7	83.4	89.9	80.9	82.6	102.3	109.1
ALDH3B2	77.9	90.3	94.1	86.7	79.6	91	86	82.8
ALDH4A1	66.8	79.6	95.5	88.4	82.7	101.9	73	92.7

ALDH4A1	187.8	207.7	118.6	135	117.9	131.2	168.2	171.9
ALDH4A1	118.3	129.5	117.9	117.8	96.1	104.4	107.2	129
ALDH5A1	282.2	429	259.8	311.1	240.6	404.4	193.5	336.6
ALDH6A1	143.1	198.4	117.9	108.1	97.6	93.3	129.7	174.1
ALDH7A1	299.3	360.4	190.5	254.7	214.3	299.2	266.8	294.7
ALDH8A1	111.1	136.7	97.3	106.3	106.5	99.7	103.2	129.6
ALDH8A1	75.4	96.9	97.5	90.2	79.3	94.7	84.4	82.5
ALDH9A1	9660.4	11625.2	6893.7	5349.8	4268.9	5689.1	8347.6	9525.6
ALDOA	111.7	114.8	111.2	97.2	104.1	98.8	103.1	107.6
ALDOA	6699.3	9412.6	4753.9	3273.9	3051.4	1910.6	6077.8	8206.6
ALDOA	165.7	175.8	111.2	102.8	89.4	92.6	155	162.9
ALDOA	7122.5	9544.6	5288.4	3963.8	2966	4663.7	6381.9	9412.8
ALDOB	99.9	118.1	107.7	92.6	96.1	108.3	99.8	115.8
ALDOC	706.8	976.8	503.3	447.9	480.1	492.6	648.5	583
ALF	98	92.6	102.3	94.8	87.8	94.5	95.1	99.8
ALF	85.3	96	82.2	86.8	72.4	80.3	84.8	93.9
ALG1	343.8	535.4	234.2	151.2	128	121.9	345	382.5
ALG10	134.7	147.5	109.1	112.9	99.4	116.5	115	115.4
ALG11	215.6	341.4	119.5	175.8	135.2	118.9	257	314.5
ALG12	118.2	134.8	122.3	118.5	130.5	117.6	117.5	125.2
ALG13	129.1	123.4	108.6	102.2	97.9	116.4	122.7	159.5
ALG14	285.4	389.7	149.2	152.4	121.4	142	231.2	285.8
ALG2	101.2	102.7	97.7	94.5	87.9	92.3	103.7	111.3
ALG3	2185	2716.5	1000.2	766.9	585.8	617.2	1879.8	2050.7
ALG5	2281	2610.9	907	770.1	543.5	773	2565.8	4329.7
ALG6	546.8	706.4	380.7	336.7	252.3	452.9	425.9	983
ALG8	2295.4	2899	1061.6	809.4	510	979.4	2086	3231.6
ALG8	83.8	94.1	105.2	92.2	99.5	94.8	88	92.7
ALG8	3238.5	4620.3	2036.6	1594.9	1012.4	1677.9	3538.6	4046.8
ALG9	656.1	871.4	355.4	270.1	194.4	252	530.8	688.7
ALK	101.9	116.7	101.4	99.8	87.4	88.2	104.7	100.2
ALKBH	442.9	650.3	312.5	232.2	196.5	346.8	399	601.9
ALKBH1	168.3	199.7	133.7	114.6	109.3	110.8	139	163.2
ALKBH1	488.7	689.7	338.3	254.8	217.4	354.4	426	644.6
ALKBH2	362.9	438.2	331.1	196.6	175.7	233.9	249.7	266
ALKBH3	824.4	1031.3	680.9	736.1	561.2	1008.1	611.7	958.9
ALKBH4	198.1	262.8	156.7	147.9	148.3	144.5	196.5	204.6
ALKBH5	3124	3746.3	2523.6	2850.1	2327.9	3279.2	2427.7	3676.7
ALKBH6	481.1	615.8	298.6	270.8	243.6	259.4	453.1	564.5
ALKBH7	384.3	492.9	314.5	274.6	230.3	317.7	405	424.5
ALKBH8	346.1	410.7	177	187.7	147.8	151	243.8	334.9
ALLC	100.8	110.7	94.1	92.9	85	92.6	97	110.7
ALLC	91.6	88.6	83.8	82.1	81	83.5	84.2	96.3
ALLC	98.9	107.4	114.1	96.6	102.6	94.3	90.5	92.4
ALMS1	124.8	158.3	143.1	160.9	160	195.2	115.1	133.2
ALOX12	86.3	86.8	88.6	84.8	75.7	86.8	88.1	80.8
ALOX12B	91.5	91.3	94.5	91.4	94.4	88.2	88.7	98.1
ALOX12P2	78.3	76	82.8	87.6	83.1	88.1	76.1	80.4
ALOX15	108.9	126.6	107.9	109.8	104.1	106.6	110.7	114
ALOX15	120	146.3	85.4	86.7	76.8	97.4	127.5	136.3

ALOX15B	105.7	125.9	92.7	90.9	87.4	86	106.8	122.8
ALOX15B	88.6	88.3	98.2	81	86.4	82.7	86.4	90.2
ALOX15B	107.8	131	116.6	102.4	96.6	88.3	104.4	117.6
ALOX5	1291.5	1538	858.7	1522.7	883.3	1631.5	687.3	620.4
ALOX5	105.2	110.5	99.2	106.5	97.6	104.7	105.1	114.6
ALOX5AP	3353.2	4210.4	2347.1	3275.5	2058.1	2258.2	3630.5	4924.4
ALOXE3	87.9	107	98.4	93	92.8	99.8	89.3	97
ALPI	107.1	127.9	103.4	94.7	94.5	104.1	118.5	119.5
ALPK1	182.8	238.2	193.4	207.7	177	245.7	161.6	245.3
ALPK3	83.3	95.4	88.1	88	84.9	93.6	94.5	92.2
ALPL	106.3	109.2	99.8	100	87.7	89.9	100.3	112
ALPP	2146.6	2196.5	1017.3	1840.3	2090	1467.3	1970.8	2267.3
ALPPL2	310	365.9	146.8	167.1	138.6	178.6	220.8	284.8
ALS2	355.4	464.2	288.7	405.4	328.4	459.5	418.4	555.7
ALS2CL	103.9	121.9	99.9	96.9	87.4	104.2	104.3	122.6
ALS2CL	88	88.3	108.8	80.2	90.8	89.2	79.5	97.8
ALS2CL	86.8	102	98.2	78.4	78	84.9	83.8	98.8
ALS2CR11	80.6	86.1	96.7	99.8	89	90.6	76.1	89.3
ALS2CR12	108.8	115.7	95	80.4	79.4	84.3	106.9	100.6
ALS2CR13	1071	1472.7	695	1350.8	923.7	1571.5	814.6	1112.3
ALS2CR15	118	152.4	128.6	111.7	99.3	120	105.5	155.4
ALS2CR16	91.6	91.6	92.4	96.7	94.8	96	90.5	94.3
ALS2CR19	91.1	93.1	99.7	87.4	85.1	83.5	82.3	88.1
ALS2CR2	659.4	813.3	448.6	567.6	432.7	655.6	488.4	706.9
ALS2CR4	531.9	695.6	444.1	393	281.1	467.6	398.2	599.5
ALS2CR7	103.9	109.1	93.3	84.5	82.2	91.1	100.9	112.6
ALS2CR8	112.4	137.5	121.8	93.7	92.2	109.4	97.3	121.2
ALX3	91.7	106.9	81.9	83.5	80.1	87.6	97.9	100.7
ALX4	103.3	112.6	89	86.4	73.5	77.9	85.8	107.5
AMAC1	122.4	159.1	95.7	84	79.3	89.8	106.9	130.5
AMAC1L2	76.5	83.1	106.8	107.1	97.6	88.1	81.9	80.5
AMACR	191.7	230.4	141.8	122.1	132.9	136	154.8	169.7
AMACR	148.3	182.1	136.3	143.1	123.3	159.4	133.9	150.8
AMBN	94.1	114.2	103.4	98.4	95.6	99.6	114.6	122.6
AMBN	86.3	96.8	96.6	81.8	86.2	85	88.8	90.3
AMBP	155.2	194.2	117.3	108.9	100.6	110	157.5	163.3
AMD1	4504.4	5052.8	2769.3	2223.4	1732.2	2420.7	2920.3	4570
AMD1	2098	2798.4	715.4	678.9	498	488.1	1167.2	1608.1
AMDHD1	112.6	125.9	104.2	105.2	96.6	112.1	105.6	116.8
AMDHD2	118.6	147.2	137.9	100	110.8	141.6	92.6	142.2
AMELX	78.7	77	95.3	90.2	96.5	98.5	81.1	82.1
AMELY	92.7	113.4	112.2	102.3	102.4	95	102.8	106.9
AMFR	336.9	433.2	421.3	330.2	262.3	249.7	266.8	563.4
AMFR	156	202.9	119.1	107.1	98.5	109.5	147.3	167.9
AMH	90.1	106.3	104.7	89.1	77.2	88.6	92.4	122.3
AMHR2	77.2	94.7	86.2	92.9	91.9	81	84.7	90
AMICA1	110.9	140.2	116.8	155.5	173.5	183.1	104.8	129.2
AMID	99.5	108.5	111.6	96.2	98.4	115.2	120.6	205.1
AMIGO1	148.6	175.1	93.7	88.8	95.6	94.1	125.4	151.4
AMIGO2	149.2	161.2	103.6	98.9	98.7	98.2	143.4	158.4

AMIGO3	115.6	137	90.6	85.3	98.8	97.9	113.1	125.7
AMMECR1	135.7	194.7	95.3	105.8	107	107	124	165.2
AMMECR1	634.1	683.2	244.1	285.5	181	307.5	578.3	745.3
AMMECR1	265.4	368.7	201.8	194.7	169.8	253.4	236.6	318.9
AMN	96.5	106.7	101.3	99.6	91.5	97.3	92.6	100.2
AMOT	126	148.8	124.5	111.6	108.9	115	118.4	127.3
AMOTL2	115.5	160	87.3	85.9	81.9	98.7	121.5	142.9
AMPD1	87.2	101.9	87.3	82.6	88.4	80.1	92.6	96.1
AMPD2	92.4	113.1	90.4	92	93.6	94.4	90.2	111.1
AMPD2	109.1	110.8	82.3	78.4	81.7	86.1	107.2	120.1
AMPD2	110.4	124.7	85.9	90	93.1	83.5	99.3	123.5
AMPD3	120.4	145.2	111	106.4	97.5	108.2	110.4	118.5
AMPD3	115.4	135.3	87.5	86.1	80.3	79.8	106	106
AMPD3	79.2	87.6	89.7	83	83.5	81.1	87.7	88.8
AMPD3	90.9	100.2	105.1	91.6	96.6	88.7	90.2	86.9
AMPH	99.7	127.1	102.4	99.4	91.6	95	109.7	123.7
AMPH	134.1	149.3	87.4	98.6	81.7	94.7	124.2	152.8
AMT	218.7	196.1	147.4	134.4	131.2	183.8	136.8	144.8
AMY1A	75.3	76.7	91.3	91.6	78.8	82.3	75.7	80.9
AMY1A	883.2	955.9	456.8	591.6	380.8	383.1	655.5	1591.1
AMY1A	71.7	82.5	91	80.5	80.7	89.4	73.8	78.9
AMY1B	82.1	92.3	85.4	86.2	75	75.9	77.4	89.4
AMY2A	90.7	105.8	102.1	89.4	91.1	90.3	83.7	85.1
AMY2B	103.9	110.8	102.6	95.7	103.4	107.6	112.4	124.4
AMZ1	93.6	95.8	90	93.5	85.3	88.2	99.1	106.6
AMZ2	101.8	115.6	106.8	98.6	92.9	101.9	103.5	110.1
AMZ2	1504.6	2189.1	1085.5	1113.2	922.6	1738.2	1128	1741.7
ANAPC1	1262.4	1903.3	1369.3	1608.1	1237.9	1902.6	1093.2	1144.3
ANAPC10	1096.4	1263.2	752.5	658.6	479.8	705.8	1033.3	1721.2
ANAPC11	1173.7	1586.4	1032.7	834.2	639.3	783.9	1074.5	1251.5
ANAPC11	92.4	105.6	92.5	84.3	83.6	93.1	102.3	98.4
ANAPC11	1568.8	1961.8	917.1	927.1	797	938.4	1939.7	1746.7
ANAPC13	5024.8	6476.2	2458.8	2863	2331.5	3021.9	4582.9	5991.7
ANAPC2	117.3	142.3	107.5	103.4	109.4	89.3	111.4	109.4
ANAPC2	134.1	178.2	150.1	117.5	121.5	185.7	109.3	148.8
ANAPC4	1355.9	1651.6	836.1	809.7	668	1139.2	863.4	1083.4
ANAPC5	3908	4186.8	2370.6	2678.8	2401.8	3270.2	2815.8	4440.2
ANAPC7	377	486.7	220.5	147.1	136.2	170.7	248.5	316.6
ANG	151.4	171.3	124.5	126.2	133.8	135.4	160.8	168.5
ANGEL1	174.8	208.5	190.5	189.8	158.2	219.7	147.5	239.2
ANGEL2	850.7	1095.1	749.2	947.6	775.1	1098.9	848.9	1363.4
ANGPT1	99	110	110.8	100.5	95.1	102.7	97.1	107.9
ANGPT2	100.6	111.5	104.8	92	97.1	90.2	102.2	103.5
ANGPT4	79.7	91.1	90.1	89	77.8	86.9	82.4	91.9
ANGPTL1	74.9	92.3	100.4	84.4	79.1	83.8	85.1	86.2
ANGPTL2	131.4	133	107.7	91.9	89.6	98.8	120.1	133.4
ANGPTL3	75.3	79.7	82.9	73.8	82.2	85.6	73.7	93
ANGPTL4	138.7	138.5	96.8	89.8	84.3	97	124.3	146.9
ANGPTL4	133.5	150.4	87	94.2	81.2	86.6	121.5	139.9
ANGPTL4	167.4	210.4	104	100	90.5	95.5	147.2	187.1

ANGPTL5	81.6	88.4	98.5	87.9	84.1	79.3	83.4	90.9
ANGPTL6	96.9	94.9	100.8	126	115.5	149	88.8	101.6
ANGPTL7	87.7	89.3	98.9	100.3	97.1	100.2	95.2	100.8
ANK1	94.4	103	95.6	93.7	95.6	97.3	94.4	104.9
ANK1	119.4	124.4	88.1	85.9	83.1	77.1	112.1	140.5
ANK2	81	93.4	93.2	89.2	84.8	86.1	85.5	110.8
ANK2	99.3	111.2	101.5	89.5	94.9	94.4	98.1	114.4
ANK3	105.2	114.1	110.6	100	94	113.8	97.8	108.4
ANK3	115.8	124.5	106.7	99	94.1	110.4	101.4	97.2
ANK3	124	145	91.6	80.3	83.2	87.3	111.1	131.4
ANKAR	116.6	123.2	96.2	98.1	90.1	99.6	98.7	122.9
ANKDD1A	266	336.3	214.2	138.8	140.9	173.4	268.2	300.3
ANKFN1	93.8	105.5	91.3	81.1	76.8	80	104.4	105
ANKFY1	323.3	485.7	188.8	285.9	215.7	310.3	294.4	318.9
ANKFY1	144.2	187.7	138.5	160.7	160.4	191.7	146.5	181.4
ANKFY1	107	126.6	92.8	100.6	118.1	98	98.6	106.2
ANKH	121.2	122.7	96.5	80	83	80.8	116.9	141.6
ANKHD1	113.1	156.5	118.1	117.4	114.3	95.3	105.8	123.2
ANKHD1	84.5	94.9	80.7	79.9	85.4	91.8	85.6	85
ANKK1	75	91.4	94	81.1	78.4	79.5	79.8	82
ANKMY1	338.8	406.5	276	347.3	360.2	408.4	263.8	254.9
ANKMY1	96.9	126.4	91.6	91.1	87.3	90.8	92.2	102.6
ANKMY1	80.3	89.1	105.3	92.7	90	99.1	80.6	88.6
ANKMY2	785	878.3	381.5	418.1	307	530.9	604	1022.6
ANKRA2	365.2	373.8	243.5	395.8	359.2	560.1	305.2	512.3
ANKRD1	85.9	92.7	101	96.6	84.9	92.2	87.6	93.9
ANKRD10	672	907.2	344.2	294.9	270.3	322.5	648	852.2
ANKRD11	527.2	650.6	371.7	592	525.4	731.4	565.6	676.9
ANKRD12	349.3	473.1	191.9	418.9	435.3	767.4	429.9	574
ANKRD13	828.5	1009.9	504	715.1	609.5	883.3	757.8	1040.5
ANKRD13B	94.6	93.8	97.4	106.5	105.2	106.5	92.4	105.4
ANKRD13C	318.3	350.2	217.9	236.1	184.9	303.7	249.6	414.3
ANKRD13D	126.4	132.2	100.2	97.2	84	97	124.7	155.2
ANKRD15	83.6	89.9	93.1	93.2	91.4	88.6	82	83.3
ANKRD15	78.2	86.9	84.8	78.7	79.2	87	81.9	90.8
ANKRD15	245.8	333.8	356	356.4	230.8	493.2	230.2	514.7
ANKRD16	429.2	568.4	224.2	160.5	136.1	165.9	316.3	326.8
ANKRD16	121	135.3	89.9	95.4	75	89.2	108.9	140.7
ANKRD16	178.8	223.6	138.9	125.5	106.6	138.9	177.8	180.1
ANKRD17	165.1	189.8	121.7	122	112.4	141.4	149.7	189.9
ANKRD17	383.4	543.1	204.8	298.9	212.7	389.5	390.9	596.1
ANKRD17	577.9	934.9	401.4	497.5	388.9	724.2	573.5	1075.9
ANKRD19	95.6	104.3	94.3	98	82.9	88.7	96.9	104.6
ANKRD2	78	74.7	95.4	92.3	93.8	93.5	74.1	80.2
ANKRD20A	193.1	251.2	135.5	178.5	177.7	191.7	159.7	236.7
ANKRD20A	126.1	134.6	105.4	91.1	92.1	95.7	103.9	118.1
ANKRD20A	99.6	111.7	95.1	88.9	90.3	96.5	108.3	114.4
ANKRD20B	75.6	79.7	87.8	90.1	80.8	88.1	73.9	72.5
ANKRD20B	76	83.3	97.8	85.1	76.8	86.4	82.9	86.9
ANKRD21	83.8	94.3	91	87.5	84.6	81.7	87.3	89.8

ANKRD22	115.9	146.7	106.9	82.3	74.5	91.9	103.9	116.6
ANKRD23	114.4	138.3	106	96.1	85.7	109.3	120.8	137
ANKRD24	86.5	103.6	87.8	77.8	78.9	86.1	79.2	94.7
ANKRD25	229.1	348.5	230.3	170.8	169.4	165.4	191	279.5
ANKRD26	148.8	168.7	145	121.5	126.7	190	130.3	153.1
ANKRD27	218.4	307.7	152.7	133.5	118.8	185.8	208.1	281.4
ANKRD28	194.9	206.7	155.8	221.7	156.9	219	210.4	400.2
ANKRD29	118.1	120.1	98	93	87.3	102.7	97.9	116.6
ANKRD30A	88	85.4	91.8	87.4	92.7	88.3	71.9	80.9
ANKRD30A	86.5	101.2	89.5	83	79.1	90.8	83.9	91
ANKRD30B	76.3	91.2	92.9	86.5	83.5	89.2	82.3	87.5
ANKRD32	429.3	494.4	198.2	217.6	177.6	204.1	336.3	484.1
ANKRD33	294.2	337.6	217.5	242.1	209.6	251.8	313.1	284
ANKRD34	120.6	122.2	114.6	82.9	97	98.7	117.6	115.6
ANKRD35	158.3	165.3	100.5	89.1	84.1	92.6	159.8	172.5
ANKRD36	120.4	134	100	99.5	101.8	101.8	99.4	118.9
ANKRD36	94	101.2	88.6	81.1	80	81.3	85.4	97.8
ANKRD36	225.8	233.4	107.5	127.1	130.7	104	114.1	179.9
ANKRD37	131.5	177.1	132.4	190.8	186.5	348.7	119.4	179.6
ANKRD38	143.3	154.4	100.7	111	96.1	112.6	109.7	119.3
ANKRD39	439.9	530.9	377.5	243.4	215.6	252.8	401.9	616.4
ANKRD40	177.5	205.7	108.6	86.4	86	96.7	173.8	195.5
ANKRD41	178.3	198.4	134.3	122.2	112.7	137.7	135.6	159
ANKRD42	83.9	93.9	94.2	87.3	83.2	93.9	87.3	91.1
ANKRD43	89.2	114.2	98.8	93	84	85.9	98.2	105
ANKRD44	149.4	175.7	107.1	106.2	123.5	101.3	140.5	183
ANKRD45	104.9	117.2	99	90.8	97.2	81.2	100.7	102.9
ANKRD46	932	1099.4	609.7	491.5	347	645.3	786.8	1441.3
ANKRD47	113.5	140.8	108.1	101.7	96.5	103.6	125.5	119.7
ANKRD49	791.7	1020.1	500.6	431.7	369.2	764.3	549.2	987.1
ANKRD5	99.9	92.6	90.4	85.4	83	88.4	95	88.4
ANKRD5	73.9	88.4	88	85.1	86.9	81.3	77.7	80.8
ANKRD5	86.9	106.3	89.5	82.6	81.8	92.8	88	96.5
ANKRD50	377.9	456.4	261.4	362	303.2	522.4	349.5	386.6
ANKRD55	201.6	259.7	141.7	160.4	143.3	184.2	132.4	139.7
ANKRD56	85.4	92.9	94.6	82	82.4	81.3	79.1	89.7
ANKRD6	185.9	244.9	189.8	197.6	157.9	227.8	158.3	191.1
ANKRD7	105.8	139.9	111.7	111	102	99.3	107.2	118.5
ANKRD7	96	107.5	93.3	84.3	95.4	90.2	105.7	115.6
ANKRD9	1316.3	1584	838.7	627.7	497.3	609.7	1085.1	1218
ANKS1A	534.5	682.3	485.9	589.7	487.5	683.1	489	628.1
ANKS1B	77.3	95.2	77.6	80	71.8	76.2	86.2	86.2
ANKS1B	80.6	86.5	91.9	81.5	73.6	79.3	81.9	88.7
ANKS1B	95.7	98.1	95.3	89.7	93.8	93.8	85.3	91
ANKS3	113.3	128.5	138.6	124	110.5	132.8	113	137.4
ANKS4B	106.1	137	87.8	86.6	80.7	83	115.2	131.2
ANKS6	99	101	87.7	83.8	87.4	90.7	91.2	100.1
ANKZF1	229.5	299.3	217.1	174.1	178.1	228	188.3	274.3
ANKZF1	98.5	118.3	97.5	98.9	94.6	90.7	101.3	102.4
ANKZF1	125.6	140.5	113	100.9	95.6	113.2	119	144

ANLN	742.7	881.9	351.7	329.6	281.5	518.9	543.8	942.4
ANP32A	313.7	368.1	122.6	110.4	102.2	113	260.9	423.7
ANP32B	19793	24409.3	15059	11773.6	8736.1	14666.3	18818.9	22848.6
ANP32C	91.9	94.4	93	93.9	86.6	109.6	80.2	83.6
ANP32D	85.4	107.3	87.5	78.5	72	80	90.7	110.6
ANP32E	100.2	119.7	94.4	85.2	83.2	95.5	88.9	121.9
ANPEP	366.4	503.6	550.8	768.6	670.4	761.1	362.5	638.4
ANTXR1	346.6	396.9	218.4	322.6	227.1	369.8	267.7	327.4
ANTXR1	179.4	213	130.6	115.7	121.5	120.7	160.5	195
ANTXR1	105.2	115.8	105.8	95.4	91.7	112.4	101.2	119.5
ANTXR2	295.2	423.5	229.4	218.7	183	331.6	289.7	460.4
ANUBL1	83.7	106	93.1	85.9	89.2	92	90.1	93.5
ANXA1	4145.5	4613.4	4042.6	4676	2739.9	5695	4620	10943.7
ANXA10	85.1	109.6	87.6	81.3	83.1	86.4	86.3	99.7
ANXA11	360.6	439.7	195.8	174	180.1	179.1	276.9	405.8
ANXA11	669.7	955.1	476.5	405.1	343.7	363.9	540.1	671
ANXA11	141.1	165.8	104.2	99.4	85.3	91.4	143.9	166.5
ANXA13	83.5	90.6	87.7	80.9	79	88.4	79.4	93
ANXA13	82.9	93.9	97.4	93.2	85.1	92.4	79.3	83.3
ANXA13	98.2	109.5	88.2	92.6	81.2	87.6	98.2	101.4
ANXA2	1207.7	1580.7	629.8	678	506.9	628.6	1169.9	1435.3
ANXA2	231.9	271	211.9	210.7	163.6	202.5	294.3	406
ANXA2	6240.7	7931.1	4712.4	4845.4	4180	6937	7318.3	10177.5
ANXA2P1	942.6	1149	576.4	920.1	575.6	1218.7	1510.4	3091.8
ANXA3	98	112.9	87.8	77.5	71.9	70	83.7	124.2
ANXA4	252.7	305.9	132.7	159.2	133.7	182.6	226.1	510.6
ANXA5	3494.7	4808.3	2769.9	2751.7	2512.3	3830.1	3113.1	3700.3
ANXA6	160.9	197.3	138.3	126.8	130.8	151.6	151.5	209.8
ANXA6	272.5	352.2	221.5	218.8	189.1	259.9	278.6	310.6
ANXA7	83.7	104.4	87.1	88.5	79.6	86.5	79.7	91.2
ANXA7	2025.3	2334.5	1282.4	1112.6	854.6	1380.9	2273.5	3447.3
ANXA8	105.1	110.7	117.3	112.7	104.4	107.5	106.2	107.7
ANXA8L1	182.8	230.5	121.8	113.4	99.6	109.1	129.1	157.1
ANXA9	91.3	99.7	91.4	92.2	83.7	90.4	93.5	100.3
AOAH	118.2	133.4	107.7	111.1	116.9	137.2	107.1	118.9
AOC2	93.4	126.6	114.3	102.6	92.9	105.4	105.6	93.3
AOC2	99.4	106	112.7	92.2	77.5	96.2	99.9	108.8
AOC2	79.1	84.6	102.8	82.7	88.3	86.3	86.7	86.3
AOC3	124.8	152.4	102.4	88.9	78	75	100.9	142.5
AOF1	86.7	105.5	89.5	82.3	89	85.8	91.1	100.3
AOF2	1097.1	1467.6	885.9	1079.3	889.9	1432.6	794.8	1076.7
AOX1	73.6	94.7	87.1	85.8	76.1	83.1	83.4	90.1
AP1B1	100.1	111	120.8	116.4	113.2	167.2	95.2	128
AP1B1	739.2	901.2	581.7	656.1	480.7	565.7	562.5	551.9
AP1G1	462.1	765.1	361.4	408.6	263.3	461.1	464	586.8
AP1G2	100	89.1	89.4	77.3	81.4	75.4	92.3	101.9
AP1G2	255.8	338.9	241	178.1	176.8	239	185	328
AP1GBP1	104.1	121.1	102.7	92.9	94.6	98.4	110.6	115.7
AP1GBP1	100	143.8	89.8	94.2	82.5	94.2	118.6	126.8
AP1M1	2301.5	2442.9	1706.2	1235.5	1031.1	949.5	1544	1534.8

AP1M2	143.3	158.1	102.8	99.8	81.8	85.1	133	152.6
AP1S1	1027.4	1397	580.1	468.1	328.8	555.1	862.9	1232.3
AP1S1	1930.7	2993.3	994.8	572.1	503.5	460.5	1706.7	2284.6
AP1S2	9179.4	11036.2	4967	6947.7	4112.9	6998.1	6843.5	9377
AP1S3	96.2	112.4	92.1	83	77.2	86.8	88.2	125.4
AP2A1	141.4	180.4	142.6	114	106.8	141.4	140.6	184.9
AP2A1	112.5	143	103.7	105.2	91.4	118.4	94.4	144
AP2A1	88.4	113.6	108.6	97.6	103.8	100.9	89.1	101.7
AP2A2	122.2	155.7	139.2	131.1	113.6	154.3	117.7	168.5
AP2B1	137	178.7	151.5	163.2	126.2	196.8	135.6	155.5
AP2B1	157.3	226.2	122.2	149	126.2	179.1	155.6	219.8
AP2M1	1174	1559.7	1111	912.8	806.7	1129.6	1090.9	1716.9
AP2M1	1383.6	1910.3	906.2	1184.8	933.3	1469.5	1272.6	2127.1
AP2S1	8203.6	10041.7	4519.8	2106	1551.9	3047.8	6942.7	10306.5
AP2S1	22533.9	25925.8	22164.1	13906.5	10856.1	12298.8	24034.7	24904.3
AP3B1	2413.4	3402.2	2537.7	2312.6	1981.2	2972.7	2296.4	3688.9
AP3B2	91.4	114.9	99.4	93.2	83.5	103.1	92.6	107.4
AP3D1	89.3	112.3	95.6	88.6	84.1	90.4	99.3	100.5
AP3D1	520.6	637.7	712.8	604.1	465	765.7	511.4	924.3
AP3D1	538.5	701.2	733.3	607.3	483.3	802.4	543.6	1018.6
AP3M1	608.6	837.5	617.9	437.5	347.7	596.8	543.1	1140.7
AP3M1	96.7	98.1	91.1	86.7	86.4	91.1	94.5	92.5
AP3M1	946.8	1214.5	856.5	703.3	531.4	870.7	846.9	1594.1
AP3M2	600.1	750.3	434.3	359.1	288.3	379	376.1	473.1
AP3S1	1361.9	2027.6	672.2	749.5	598.8	875	1117.9	1477.7
AP3S2	161.5	206	155.5	151.8	145.2	166.5	171.8	179.4
AP4B1	1965.8	2224.3	1269.2	808.1	623.3	861	1567.3	1796.1
AP4E1	388.8	546.7	389.7	455.4	380.6	752.3	387.3	618.6
AP4M1	137.2	148.4	107	113.5	99.1	108.1	130.8	148.9
AP4S1	157.9	189.3	128.7	107.2	109.7	104.9	145.7	194.1
APAF1	1193.7	1587.3	619.8	1109.8	885.7	1872.1	1108.2	1434.6
APAF1	124.2	150.5	110	107.9	104.5	94.5	123.8	140.3
APBA1	86.1	93.7	92.1	84.4	84.5	84.5	82.9	92.8
APBA2	111.8	134.8	101.8	98.8	90.3	96.4	100.6	124.8
APBA2BP	94.2	121.3	107	112.1	102.1	135.7	105.4	126
APBA2BP	73.8	88.8	82.6	82.9	77.3	85.6	83.6	76.4
APBA2BP	121.5	145.1	99.3	102.2	87.4	103.5	119.1	159.3
APBA3	396.6	497.1	395.4	219	211.6	256	427.8	458
APBB1	117.2	131.4	87.9	82.7	80.4	76.6	98.4	109.8
APBB1	111.5	114.3	87.7	84.2	79.5	82.4	91.3	119.1
APBB1	79.9	98	92.6	81.8	82.2	82	86.6	88.5
APBB1IP	674.2	929.5	719.2	930.9	682.7	1028.2	467.9	993
APBB2	96.3	122	105.5	87.5	87.3	89.5	108.4	109.2
APBB3	434.7	407.2	361.6	403.2	421.1	564.3	242.5	238.2
APBB3	223.5	247.1	147	165.3	165.2	209.8	148.2	154.8
APC	91.8	111.3	96.7	82.7	84.8	86	96.5	105.4
APC2	85.6	103	96.1	96.3	93.7	93.6	88.9	95.7
APCDD1	117.8	144.4	108.5	118.1	107.9	110.3	120.1	130.3
APCS	118.6	150	126.7	106.4	109.6	116.4	121.4	125.4
APEH	1652.4	2235.2	967.3	931.6	800.7	1109	1392.2	2155.1

APEX1	6374.1	7690.5	4234.3	3231.7	2600.9	4743.8	4417.7	4698.1
APEX1	9087.6	10630.4	8083.5	6897.5	5035.4	9588.6	6826.1	8407.1
APEX2	905.1	1082.1	736.5	499.4	363.8	463.4	762.8	1287.5
APH1A	2419.9	3415.6	2226.4	1675.3	1365.4	1351.1	2070.3	3099.2
APH1A	450	623.7	259.6	194.8	175.4	216.1	421	745.3
APH1B	551.6	755.5	455.7	367.8	316	331.8	581.2	763
API5	1847.9	2330.3	819.8	929.3	618.1	1580.6	1280.6	2683.7
APIN	107.1	117.3	96.3	79.7	86.2	88.8	104.8	113.3
APIP	477.8	575.3	248.6	179.2	191	338.5	386.4	725.8
APITD1	214.2	271.7	136.2	107	93.1	105.7	164.4	171.9
APITD1	1229	1729.5	821	522	422.5	658.4	910.4	1388.4
APLN	109.7	109.5	97.8	91.1	84.2	96.9	120.1	110
APLP1	111.8	109.9	89.1	83.8	86.7	90.3	98.5	124.8
APLP1	102.8	117.1	113.3	83.4	79.7	88.9	108.8	152.4
APLP2	127.5	132.4	101.6	90.9	86.1	104.3	109.7	126.2
APOA1	125.4	142.9	102.9	71.2	76.1	96.7	106.3	125.9
APOA1BP	3315.4	4256.1	2779	2052.4	1514.5	2416.9	2536.7	3640.1
APOA2	98.1	115.5	111.6	105.7	98.7	114.7	110.6	114
APOA4	80.8	93	84.7	78.1	92.3	75.8	88.6	105.4
APOA5	116	149.6	91.5	82.1	88.8	87.8	113.2	131
APOB	123.5	140.3	90	79.5	79.7	83.2	111.2	140.7
APOB48R	240.1	261.3	257.6	274	221.7	337.7	195.2	248.9
APOBEC1	89.7	107.4	102.5	98.6	91.2	95.4	92.4	112.8
APOBEC2	93.9	89.8	81.7	85.1	84.9	84.6	82.7	94.2
APOBEC3A	67.7	84.6	84.6	82.7	82	86.6	74.1	75.5
APOBEC3B	732.6	976	485.5	390.6	337.7	451.3	612.6	651.2
APOBEC3C	167.5	180.1	148.3	160.1	160.4	153.8	154.4	253.3
APOBEC3D	88.8	100.3	93.8	90	78.7	91.6	94.5	98.3
APOBEC3F	321.1	401.6	134.9	146.1	141.1	136.2	241.6	230.4
APOBEC3F	218.1	277.9	173.2	185.4	155	167.5	212	248.4
APOBEC3F	175.5	196.8	124.2	135	125	130.3	163.5	183.8
APOBEC3G	443.2	616.6	233.9	223.4	197.3	254.1	348.8	430.7
APOBEC4	72.4	83.6	96.5	87.2	73.9	84.6	83.3	86.8
APOC1	494.1	597.7	379.6	676.2	484.6	598.3	510	780.3
APOC2	2438.8	2916.5	758	776	616.2	671.9	2465.9	3490.1
APOC3	81.6	85.8	95.4	81.2	84.9	80.8	85.2	87.8
APOC4	92.9	106.3	97.3	86.6	84.8	91.4	94.3	104.1
APOD	71.8	78	100.7	79.2	80.3	79.9	72.9	80.5
APOE	176.5	173.2	141.4	1379.7	1251.4	1764.7	138.3	188.9
APOF	85.6	106.8	95.6	77	84.3	82.6	88.4	106.9
APOH	81.9	97	91.3	88.5	75.2	79.2	80.7	95.2
APOL1	142	226.1	132.1	95.4	98.4	132.2	155	217.4
APOL1	89.7	105.2	87.8	76.8	74.7	80.5	83.5	99.1
APOL2	120	155.3	90.8	90.2	83.7	91.5	120.5	152.3
APOL2	88.4	94.5	118.6	112.4	100.1	106.7	87.2	96.2
APOL2	151.2	211.6	108.5	91.4	83.5	104.8	151.6	164.1
APOL3	71.4	75.9	97	81.5	75.6	75.3	79	81.9
APOL3	113.4	128.3	91.7	99.5	103.4	99.2	111.6	126.4
APOL3	113.9	143.9	90.1	98.8	84.6	93.3	98.9	122.5
APOL4	103.1	126.8	108.5	88.4	77.9	92.4	102.6	126.9

APOL4	100.3	121.5	93.8	98.3	91.8	89.7	100.6	109.1
APOL5	100.1	109.1	94.8	88.2	78.3	86.2	99.9	96.2
APOL6	180	239.6	113.2	126.8	110.5	118.4	152.7	191.3
APOLD1	139.3	150.2	126.9	117.6	111.3	132.3	134.1	210.2
APOM	246.5	324.6	212.3	232.4	183	264.5	248.6	330.9
APP	1362.9	2252.3	1431.1	1483.1	1315.2	2530.9	1269.2	1473.2
APP	139.2	205.3	116.3	122.3	112	117.9	133.1	137.2
APP	1132.8	1683.3	977.5	1196.6	1059.4	1759.8	1041.3	1023.7
APPBP1	231.7	263.3	140.6	128.6	128	146.4	172.7	185
APPBP1	4619.1	5466.6	2082.2	1644.1	1178.3	2321.3	3636.2	4177.9
APPBP1	1470.4	1660.2	766.6	619.5	500.2	849.5	1268.6	2339
APPBP2	402.7	557.3	284.3	575.2	430.8	603.3	421.4	483.5
APPL	324	380.8	174.6	186.7	148.8	225.7	282.2	468.2
APRIN	175.8	217.1	163.3	182.9	141.5	204.3	161.5	190.1
APRIN	781.1	1003.9	410.5	390.3	354.4	537.3	658.3	984.5
APRT	2846.4	3434.5	2645.4	1409.8	1143.4	1597.6	2158	2610.9
APRT	2221.7	2810.5	1200.6	650	530	710.1	1548.8	1908.1
APS	161.3	184.9	161.3	111.9	97.1	112.8	148.6	256
APTX	1100.9	1496.9	691.9	664.7	550	810.6	805.9	1261.5
APTX	73.9	83.6	92.8	89.4	89.8	89.6	76.4	78.6
APTX	456.4	631.8	309.3	247.9	187.5	277.9	376.8	630.5
APXL	93.5	123.8	88.9	98.2	93.6	97	89	100.7
APXL2	95	97.7	112.3	92.8	96.2	106.2	89.7	107.7
AQP1	105.8	138.1	103.9	85.2	98.2	91.7	106.3	135.6
AQP1	110.9	120	96.1	93.3	90	94.5	110.1	130.5
AQP10	81.8	81.3	85.7	93	74.6	76.1	81.2	82.1
AQP11	120.8	128.3	103.2	94.3	99.9	101.8	104.4	108.9
AQP12A	92.4	99.4	98.6	105.8	97.6	91.9	102.7	94.1
AQP2	88.9	94	102	87.9	97.8	102.9	88.6	87.7
AQP3	105.8	141.1	79.2	78.3	70.7	76.9	103.8	153.9
AQP4	94	116.2	97.1	89.3	80.7	95.5	94	97.4
AQP4	89.8	93.4	90.3	85.3	79.8	85.9	74.9	104.8
AQP5	91.2	109.4	96.7	81.9	80.2	82.2	95.2	93.4
AQP6	163.3	187.1	120.7	121.7	111.6	123.4	153.5	180.2
AQP7	82.1	93	90.5	85.8	83.1	80.5	82.1	101.1
AQP7P2	107.1	148	88.7	76.7	73	78	92.9	118.7
AQP8	74.3	89.2	78.8	86.7	84.5	86.3	86.5	86.9
AQP9	99.4	101.3	91	93.7	92.3	106.8	93.7	100.6
AQR	129.8	144.6	103.1	109.9	90.8	114.6	115.6	168
AR	205.6	265.4	151.4	171.2	146.4	195.1	181.4	226.9
AR	109.5	150.7	96.1	105.4	90.5	93.7	103.3	121.3
AR	81.1	98.9	94.5	83.7	87.1	76.8	98.6	105.3
ARAF	1298.2	1525.2	645.2	506.7	482.2	493.8	930	718.7
ARC	140.8	184.2	100	95.8	91.4	99.6	138.6	162.9
ARCN1	2189.2	3216.3	1944.7	1167.5	921	1341.6	1968.6	4289.2
ARD1A	1515.1	1669.8	702.7	640	420.6	639	1442.8	1548.2
AREG	79.4	84.6	101.2	97	84.7	97.5	87.4	81.6
ARF1	889.6	1178.6	425.8	259.3	227.7	296.2	833.1	1152.2
ARF1	6859.4	9583.9	4558.1	3560.2	2788.4	3666.1	6759.9	8493.9
ARF1	140.8	156.7	98.1	90.6	74.8	113.5	126.4	164.6

ARF3	830.3	1249.4	416.5	383.3	280.6	491.6	730.7	1169.2
ARF4	800	1077.8	373.6	344.6	270.5	310	671.5	786.1
ARF4	4233.6	5374	2448.6	2444.8	1645.7	2414.2	3935.1	7450.1
ARF5	1010.1	1405.4	846.3	994.2	776.9	1446.3	894.4	1098.2
ARF6	130.2	149.8	107.8	87.3	84	83.7	131.7	164.4
ARFGAP1	110.5	119.1	102.1	84.1	91.5	91.8	114.7	138.2
ARFGAP1	261.8	256.5	288.9	247.4	257.1	476.6	225.2	343.7
ARFGAP3	102.4	117.5	96.5	82.9	79.6	98.9	109.9	131.4
ARFGEF1	222.7	287.9	148.2	176.4	137.8	240.4	231.9	358.7
ARFGEF2	168	204.5	146.6	131.4	136	145.7	146.7	198.1
ARFIP1	646.3	933.4	329	296.6	241.8	442.7	535.2	851.1
ARFIP1	178.4	221.2	113	97.6	94.3	100.3	137.2	166
ARFIP2	101.2	144.9	91.7	83.6	80.4	88	125.3	160.5
ARFRP1	145.1	163.2	127.4	124.8	113.8	124.6	141.1	156.3
ARG1	79.1	86.9	88.7	82.8	83.6	78.5	83.1	91.4
ARG2	138.2	152.9	107.4	88.2	90.4	91.4	126.3	131.5
ARG99	85.5	101.3	92.1	89.9	88.3	91.2	88	94
ARGFX	86.4	102.1	86.5	83.9	80.6	86.3	84.2	92.9
ARGFXP2	91	102.5	100.8	84.2	77.4	89.6	85	94.9
ARHGAP1	1737.2	2108.7	1661.8	1082.2	785.6	918.4	1612.2	2041.8
ARHGAP10	228	297.1	143.6	112.5	132.5	138.6	216.1	289.8
ARHGAP11	295.7	390.6	163.1	174.7	166.6	201.7	265	291.2
ARHGAP11	249.6	330	170.6	210.5	176.1	307.7	223.3	316.7
ARHGAP11	180	197.1	109.3	107.1	103.4	114.3	157.3	192
ARHGAP12	108.5	114.6	106.5	93.4	89	110.9	117.3	145.8
ARHGAP15	2437	3024.6	1806.1	2073.3	1829.7	3078.5	1931.3	3057.1
ARHGAP17	2131.4	2803.3	1880.8	2203.4	2001.3	2281.8	1700.7	1771.2
ARHGAP17	229.4	326.9	227.9	260.4	242.6	301.6	232.6	412.4
ARHGAP17	102.8	102.3	108	95.1	90.7	89.3	89.7	100.6
ARHGAP18	143.8	167.9	102	108.8	94.4	118.2	135.3	201.8
ARHGAP19	609.8	899	682.4	581	478.4	832	642.8	1043.6
ARHGAP20	86.1	102.1	100.5	85.6	87	90.1	97.1	97.4
ARHGAP21	398	602.7	238.4	229.9	194.9	279.1	528.9	677.9
ARHGAP22	128.5	148.1	102.2	102.4	91	99.5	122.1	117.7
ARHGAP24	80.8	91.1	90.9	92.1	88.1	88.6	86.2	88.1
ARHGAP24	93	102.2	91.4	88.9	90.8	99.1	89.2	90.1
ARHGAP24	134.6	121.9	96.2	85.1	85.4	96	113.7	139.8
ARHGAP24	111.1	115.6	115.9	117.3	115	125.6	111	120
ARHGAP25	103.8	108.2	91.5	85.2	92.8	92.7	96.5	105.9
ARHGAP25	315.1	418.2	181.6	198.8	185.5	304.6	313.8	404.5
ARHGAP25	88.6	103.3	84.1	80	84.7	89.7	87.7	104.2
ARHGAP26	145	196.5	123.1	110.9	113.3	93	141.4	160.9
ARHGAP27	125.2	145.6	96.3	97.6	86.5	99.5	118.8	140.7
ARHGAP28	75.2	96.7	89	80.5	86.5	75.5	82.6	83.9
ARHGAP28	78.1	85.4	103.6	86.6	78.2	89.3	85.6	97.9
ARHGAP28	70	90.1	90.5	88.6	84.3	80.9	86	86
ARHGAP29	72.4	93	107.5	84.2	98.3	94.3	72.9	81.1
ARHGAP30	1055.7	1586.6	736.4	368.6	349.9	409.7	958.5	1310.2
ARHGAP30	1625.9	2130.6	1187.7	710.8	734	734.7	1561.4	1425
ARHGAP30	456.5	657.9	325.9	250.6	236	165.3	364	526.5

ARHGAP4	1318.7	1392.8	1218.2	1247	1127.9	1666.8	926.3	1360.4
ARHGAP5	99.8	118.7	96.9	97.4	82.5	87.3	93.6	111.3
ARHGAP6	99.3	111.4	96.5	89.6	84.4	86.7	106	105.8
ARHGAP6	88.4	99.4	82.8	77.3	82.6	74.2	99.5	98.8
ARHGAP6	76.3	95.3	90.8	74	77.6	89.1	86.5	91.8
ARHGAP6	85.3	101.9	87.9	88.9	79.1	90.6	93	91.8
ARHGAP8	99.5	104.6	85.3	95.1	81.5	85.3	104.8	92.3
ARHGAP8	85.1	83.9	87	81.2	76.1	80.9	80.8	87.6
ARHGAP8	87.3	107.2	86.4	79.2	76.9	74.3	89.2	100.2
ARHGAP9	1757.3	1605.9	917.7	1251.3	1289.1	1676.6	1211.7	1580.5
ARHGAP9	490.2	583.5	315	305.1	297	420.3	371.1	501.1
ARHGDIA	1372.1	1776.4	1087.2	1052.3	659.3	1263.4	1452.6	3916.4
ARHGDIB	22773.2	26617.1	18208.3	19492.3	14216.5	20015.6	22176	23444.3
ARHGDIG	88.9	90.6	95.8	87.7	74	90.4	86.4	91.9
ARHGEF1	184.9	240.1	239.7	173.9	148.5	269.2	170	276.2
ARHGEF1	97.4	109.1	111.8	90.5	96.2	87.2	97.4	112.3
ARHGEF1	133.6	191.2	129.8	108.7	107.6	117.5	118.2	204.1
ARHGEF10	752.1	1087.5	792	472.6	433	639.7	516.3	644.6
ARHGEF10l	120.6	161.7	107.7	93	78.2	90.4	118.8	140.1
ARHGEF10l	112.1	158.5	93	85.5	90.3	98.6	113.8	130.2
ARHGEF10l	103.6	122.4	103	79.4	81	90.7	85.1	130.1
ARHGEF11	82.1	85.6	90.4	118	84.6	83.3	77.9	80.9
ARHGEF11	210.8	231.9	125.8	108.6	112.2	135.3	198.3	235.4
ARHGEF11	118.7	158.3	149.1	129.3	111.3	147	128.2	159.2
ARHGEF12	100.9	125.6	112.6	87.5	82.5	88.1	100.3	126.5
ARHGEF15	172.2	183.4	80.5	85.1	90.6	79.9	131.7	160.5
ARHGEF16	145.5	153.9	89.7	92.5	86.1	90.7	121.5	141.4
ARHGEF17	142.9	159.5	138.9	145.5	135	184.4	129.3	166.3
ARHGEF18	1515.4	2017.8	1784.6	2005.7	1949	2353.5	1124.9	1528.4
ARHGEF19	370.3	481.9	317.5	217	218.2	253.1	414.2	404
ARHGEF2	2405.3	2599.9	2101.6	2402.3	1966.8	2662.7	1901.8	2493.8
ARHGEF3	370.6	511	353.8	472.8	424.1	629.1	437.1	675.9
ARHGEF4	88.8	87.5	83.3	82.3	90.1	79.9	83.1	94
ARHGEF4	100.1	103.6	93.9	86.5	83.1	81.4	97.8	122.4
ARHGEF5	97.4	119.4	105.4	102.6	90.4	122.1	105.6	130.5
ARHGEF6	3791.6	5390.5	3468.4	3391.7	2761.3	4873.2	3360.6	3659.8
ARHGEF7	827.4	1075.3	863.2	1016.3	886.3	1236.9	866.5	1424.8
ARHGEF7	85.2	100.8	86.4	77.9	72.2	84	80.5	99.7
ARHGEF7	129.8	127.3	108.7	99	90.7	97.6	111.1	143.5
ARHGEF9	123.4	123.6	105.3	103.7	96.5	104.3	118.2	128.6
ARID1A	91.9	95.1	91.4	92	97.4	86.6	98.5	101.5
ARID1A	1910.8	2985.7	1193.1	1735.1	1562.7	1769.5	1951.7	1928.5
ARID1B	83.4	94.3	92.7	101	100.3	96.3	86.3	97.2
ARID1B	100.8	107.7	103.5	107	98.2	103.7	104.1	104.4
ARID2	433.1	673.6	186.1	203.3	155.2	270.9	422	580.6
ARID3A	1301.8	1594.7	1315.7	1287.5	1180.6	2242	1197.6	1747
ARID3C	114.4	131.7	90.3	93.7	81.9	90.8	116.4	124.1
ARID4A	213.4	243.4	137.1	141.1	138.8	154.2	173.5	229.7
ARID4A	166.7	230.7	118.9	115.9	118.8	162.2	170.5	233.3
ARID4A	283.9	336.1	188.3	192.9	221.6	388.8	237.5	404.6

ARID4B	215.9	270.9	129.8	112.9	115.2	166	194.4	247.1
ARID4B	690.7	875.9	328.3	403.6	405.6	472.1	575.7	1050
ARID4B	301.1	387.4	167.9	182.7	184.2	160.5	242.7	324.9
ARID5A	91.8	91.1	97.2	86.4	91.2	91.9	99.9	92.7
ARID5A	85.8	85.1	97.7	85	94	86	87.8	93.1
ARID5A	104.6	124	116.7	111.1	101.8	111.1	106.3	141.8
ARID5B	129.1	165.1	133.3	179.5	154.3	213.3	121.3	146.9
ARIH1	439.9	619.4	258.9	249.9	249.7	345	427.4	599.6
ARIH2	1311.5	1452.2	1153.2	1139.7	1034.4	1248.4	1187.4	1130.7
ARL1	722.3	950	456.3	530.6	379.9	677.8	597.5	936.5
ARL10	83.7	109.6	86.6	80.4	87.6	92.9	88.5	92.9
ARL11	101.8	114.6	95.8	96.5	89.5	96.8	100.9	108.7
ARL13A	84.9	92.2	100.8	91	87	95.6	96.3	101.7
ARL13B	167.1	231.8	105	88.3	91.3	101.9	132.9	269.6
ARL13B	132.1	154.3	95.8	107.9	98.8	83.3	143.9	189.9
ARL14	76.1	93.5	90.9	86.4	81.9	95	81.1	97.6
ARL15	135.5	159.3	109.2	104.3	96.2	102.4	122	154.7
ARL16	1442.1	1353.7	610.2	653.1	767.7	743.3	1201.1	1911.3
ARL2	4300	5720	2546.8	1530.2	1341.5	1308.4	3577.9	2674.9
ARL2BP	2015.4	2620.8	1331.9	1222.7	953.3	1386.4	1520.4	2728.4
ARL3	113.2	125.9	135.4	119	98.9	117.9	99.7	143.3
ARL4	283	343	179.5	116	115.7	146.4	206.3	221.1
ARL4	350.4	508	228.1	160.9	163.5	206.5	302.8	393.9
ARL4	259	302.6	166	138.5	122.7	170.2	200.3	282
ARL4A	105.3	107.1	95.3	86.2	79.3	79.8	95.5	119.4
ARL4C	128.5	138.2	104.2	90.7	90.5	84.5	119	149.5
ARL4D	93.2	103.5	94.2	99.1	94.5	87	95.5	113
ARL5	505.4	671.9	185.8	167.4	156.4	171.2	356.8	438.7
ARL5	7775.4	11117.1	3744.9	4749.9	3720	5084.7	6980.1	8454.7
ARL5A	2430.1	2746	1046.3	1273.2	916.4	1564.9	1845.5	2839.4
ARL5B	156.4	184.2	110.8	108.4	91.9	104.5	147.6	193.2
ARL6	99.2	114.3	83.6	81.2	81.9	79.2	103.1	119.2
ARL6	74.3	90.5	102.6	93.7	83.6	91.2	78.6	78.2
ARL6	79.3	89.2	95.2	96	85.1	86.3	74.6	78.1
ARL6IP1	183	189.8	115.9	106.8	99.5	121.4	163	293.6
ARL6IP2	535.4	604.3	295.7	252.4	211.8	391.8	354.1	545.6
ARL6IP4	428.5	641.9	186.9	191.4	204.9	272	434	546.9
ARL6IP4	107.9	121.2	114.1	101.7	101.8	122.7	98.8	116.3
ARL6IP4	505.9	585	316.2	271.8	324.6	411.3	497.2	547.6
ARL6IP5	1823.1	2680.6	669.3	697.4	757.6	903.7	1492.4	1875.9
ARL6IP6	77.8	90.5	96.4	90.5	86.5	88.7	84.4	96.1
ARL8A	128.6	139.2	103.3	89	82.4	100.3	131.2	143.7
ARL8B	2650.1	3238.1	978.9	1599.2	982.7	1929.5	2372.2	3894.6
ARL9	84.6	96.6	89.7	90.7	84.4	86.7	97.1	101.9
ARMC1	1293	1658.8	487.1	407	301.3	511.7	1118.1	1836.6
ARMC10	2477.9	2968.2	1594.5	1606.4	1048.4	1729.6	1850.9	3241.8
ARMC2	109.1	137.7	91.1	84.8	77.6	80	111.2	125.5
ARMC4	93.9	107	115.6	106.9	95.2	116.6	99.2	98.7
ARMC5	165.6	193	106.3	98.5	98	99.2	160.2	333
ARMC6	316.3	339.1	220	176.6	168.8	232.2	272.8	365.7

ARMC7	295.6	396.5	314.4	278.2	274.4	311.3	291.7	467.2
ARMC8	169	201.7	115.2	97.6	95.5	99.9	155.8	214.4
ARMC8	549.5	798.7	282.8	304.1	250.1	359.2	403.8	545.4
ARMC8	111.8	122.2	113.4	105.7	96.4	98.2	104.3	122.2
ARMC8	157.7	229.8	101.8	103.7	86	101.2	137.1	184
ARMC9	125.4	161.8	91.5	91.9	88.3	89.4	123.3	168.5
ARMCX1	177.5	187.7	117.7	163.6	139.3	217.4	147.7	225.8
ARMCX2	77.1	90.7	91.9	80.7	86.4	85.6	94.4	104.2
ARMCX3	313.9	393.9	191	208.5	173.7	249	268.3	468.1
ARMCX3	114.2	151	85.9	89.4	88.2	95	116.2	130.1
ARMCX5	397.2	425.6	187.8	175.1	151.1	226.6	282.5	587.4
ARMCX6	743.6	990.8	373.3	280.8	272.3	363.1	677	916.9
ARMCX6	1250.7	1655	435.4	316.4	281.6	394.1	1067	1382.2
ARMET	7135.9	8388.6	6473	4183.8	3143.4	4919.3	8491.9	11127.7
ARMETL1	101.5	117.5	99.8	116.5	94.6	96.8	110	107
ARNT	216.9	241.7	122	113.6	102.2	123.2	208.2	258.4
ARNT	113.6	134.1	96.1	104.4	95.9	85.7	93	117.2
ARNT2	98.4	98.8	96.3	90	87	84.8	98.3	110.2
ARNTL	142.1	185.1	108.3	98.1	92.3	98.9	138.3	144.6
ARNTL	270.6	357.4	291.3	263.8	269.4	382.2	261.7	473.2
ARNTL2	103.2	126.3	94.2	84.3	82.2	89.6	106.1	113
ARP10	112.1	121.5	106.6	100.1	88.8	98.6	95.7	112.2
ARPC1A	2479	3076.7	1866.4	1888.1	1348.3	1756.6	2145	3508.8
ARPC1B	2651.5	3643.6	1510.3	1131.4	1005	889.1	2426.7	2390.3
ARPC2	107.4	129.7	90.7	77.2	86.9	92.7	121.1	115.9
ARPC2	15160.2	18472.5	10006.9	10358.9	8444.7	11640.8	13858.8	19617.8
ARPC2	105.2	115.6	114.7	90.9	83.8	96.2	106.9	132.7
ARPC3	12512.3	14461.1	9653.2	10371.3	8527.9	12863.3	11016	12418.6
ARPC4	155.9	191.9	104.3	95	85.6	89.7	129.9	150.4
ARPC4	5040	7208.8	4232.9	2879.8	2078.2	1816.4	4625.3	5748.5
ARPC4	3033.1	4256.1	1798.9	1074.3	832.6	946.9	2680.4	2973.1
ARPC5	8662.9	9616.8	2767.2	3517.6	2279	3399.3	8180.8	10777.5
ARPC5L	1643.4	2247.4	1376.1	917.2	659.1	988.9	1494.9	2408.4
ARPM1	145.6	178.6	97.6	90.2	102.3	101.3	137.6	154.3
ARPM2	98.7	123	87.6	90.5	85	89.7	96.4	105.2
ARPP-19	725.7	983.4	317.6	330.6	306.1	356.4	705.9	1049.4
ARPP-21	90	110.3	101.1	89.2	84.9	98.6	77.6	86.9
ARPP-21	130.6	148.5	90.7	79	80.5	77.3	139.5	144.8
ARPP-21	115.9	119.3	89.2	77.4	83.9	81	94.8	106.9
ARPP-21	114.7	132.2	104.2	88	86.7	91	115.8	104.8
ARR3	89.4	104.3	96.7	91.9	84.3	82	92.6	94.3
ARRB1	372.5	445.6	257.3	248.1	203.4	277.6	241.8	365.2
ARRB1	255	318.2	237.9	214.5	195.4	249.9	165	216.7
ARRB2	139.3	165.8	111.8	94.5	89.2	104.6	145.1	149.8
ARRB2	132.1	162	113.9	101.9	95.9	116.9	108.9	151.7
ARRDC1	179.5	227.7	163.9	147	155.9	171.2	137.8	182
ARRDC2	133.2	131.9	103	88.4	85.9	89.5	109.5	143.4
ARRDC2	100.1	110.3	91.6	89.3	86.6	82.5	89.4	107.3
ARRDC2	235.6	255	224.2	174.2	156	200.3	215.2	400.5
ARRDC3	237.6	321.7	169.1	249	250.1	377.6	192.6	259.5

ARRDC4	128.7	150	108.4	160.6	134.4	198.2	148.5	293.6
ARRDC5	91.6	105.7	86.6	79.5	94	87	97.2	100.7
ARS2	6252.7	7360.9	4381.7	3359.4	3133.6	4221.8	4762.5	5264.5
ARS2	94.9	103.2	90.1	81.1	73.8	81.9	92.2	111.9
ARSA	218.4	235.6	111.8	131.9	124.9	121.5	180.4	174.1
ARSB	114.3	126.6	96.7	90.9	88	90.8	111.5	116.5
ARSB	601.3	850.3	377	502.8	360.6	404.7	648.6	810.8
ARSB	218.7	262.8	137	157.2	140.7	161.3	172	221.7
ARSD	190.9	228.5	136.9	202.7	192.5	140.7	144.3	179.1
ARSD	91	113.8	119.6	105.9	93.9	104	104.4	102.7
ARSD	765.7	925.2	792.2	1165.4	875.2	1333.4	704.3	1402.3
ARSE	82.5	87.3	92.9	89.2	80.8	79.9	84.7	89.6
ARSF	77.5	92.1	85.2	87.5	81.6	90.6	79.2	83.8
ARSG	143.5	180.7	113.8	100.6	94.9	124.7	154.1	152.7
ARSH	82	84.7	94	87.6	89.1	107.1	84.3	94.3
ARSI	96.1	112.9	90.5	91	80.7	102.8	94.3	106.4
ARSJ	89.3	98.3	100.9	102.8	93	94.3	94.4	93.2
ARSK	168.1	195.7	140.1	133.8	122.1	142.2	143.5	161.9
ART1	150.6	162.9	105.7	88	94.4	100.3	129.8	161.6
ART3	91.8	107.8	106.9	92.2	87.1	90.2	90.8	97
ART4	101.6	137.2	104.9	103.9	92.4	113.7	117.2	112.2
ART5	105	105.2	90.1	77.5	81.3	74.1	87	96.4
ART5	90.6	107.8	90.2	88.2	90.9	85.1	90.2	94.5
ARTN	85.1	96.3	85.5	74.8	66.3	78.5	83.5	87.8
ARTN	87.4	90.5	94.1	93.4	84.9	83.7	81	92
ARTN	87.9	94.7	94.6	90	92.5	81.9	77.9	98.6
ARTN	102.7	113.4	109.1	100.9	92.7	101.9	91.9	112.9
ARTS-1	151	187.8	113.8	121.9	110.1	111.6	130.5	146.7
ARTS-1	114.6	131.5	89.6	78.4	75	78.9	109.2	122.3
ARV1	2347	2725.5	972.5	1012	722	1115.4	1622.1	2579.6
ARVCF	114.3	117.1	99.7	86.4	73.6	89.5	102.2	128
ARVP6125	96.1	96.3	101.6	103.2	90.8	90.5	89.7	95.3
ARX	106.8	147.4	122.7	103.3	106	104.3	111.3	141.4
AS3MT	116.9	130.9	109.4	94.1	91.1	102.1	122.4	126
ASAH1	290.2	387.8	173.2	198.8	167	248.5	257.5	311.1
ASAH1	141.6	150.7	114.7	101.4	91.4	100.5	137.1	130.6
ASAH2	91.5	97.7	94.8	86.1	81.8	84.7	90.7	94
ASAH3	99.6	119.4	97.2	88	79.2	81.3	102.4	110.5
ASAH3L	103.8	114.1	127.5	105.3	103.4	110.4	103	109
ASAHL	1108.8	1470.1	573.3	426	344.8	519	1048.4	1357
ASAHL	1118	1497.6	650	520.9	471.6	630.3	880.8	1068.6
ASAHL	465.8	606.3	265.5	201.9	184.3	213	399.8	560.8
ASAM	79.6	83.7	85.2	89.8	84.9	90.1	81.4	83.1
ASB1	472	528.8	200.1	209.5	215.7	209	406.7	463.6
ASB10	125.1	118.6	102.7	89.5	87.6	101.9	95.3	135.4
ASB11	80.8	94.2	92.7	83.4	81.4	87.1	90.5	97.4
ASB11	108.3	117.9	82	89	88.2	84.9	104.4	131.1
ASB11	129.1	148.8	108.7	105.6	113.2	125.6	129.9	114.3
ASB12	120.4	134.5	114.1	105.8	101.5	105.1	108.4	124.1
ASB13	357.2	445.7	311.2	245.2	189.8	266.7	290.5	382.7

ASB14	85	107.3	86.2	92	78.3	87.4	84	88.1
ASB15	95.4	99	101	91.3	95.1	93.4	88.5	93
ASB16	94.1	99	97.5	89.3	81.8	89.5	92.4	108.8
ASB17	77.6	80.7	85.7	89.3	82.7	92.5	84.8	88.3
ASB2	111.9	134.3	100.2	89.1	95.6	95.9	103.9	111.1
ASB3	267.7	327.6	153.6	162	170.1	184.3	299.8	465.7
ASB3	315.7	459	210.3	202.4	175.9	202	274.2	353.5
ASB4	105	144.4	90.6	76.5	79.9	80.4	106.9	112.7
ASB4	93.5	102.3	91.4	75.4	81.4	82.6	98.1	103.3
ASB4	83.2	97	92.6	90.4	84	91.8	83	85.3
ASB5	84.6	94.1	88.5	85	92.4	86.4	79.7	89.6
ASB6	85.1	95.6	88.6	81.2	78	79	90	102.5
ASB6	117.7	111	127.3	115.8	112	112.6	123.6	166.9
ASB6	97.1	128.9	129.1	117.7	107.4	128	107.6	166.5
ASB7	124.2	150.4	95.3	87.1	88	94.7	122.1	142.7
ASB7	106.3	136.9	99.5	102.4	88.1	92.3	106.9	114
ASB7	232.6	312.4	144.6	127.9	101.9	144.7	207.9	214.5
ASB8	300.7	391.6	172.3	180.1	133.1	228.6	313.1	428.5
ASB9	78.7	85	90.9	85.9	78.5	87.2	83.9	82
ASB9	74.3	83.2	87	79.1	80.4	77.3	73.8	78.6
ASCC1	931.6	1413.1	901.9	607.4	482.7	805.3	862.6	1075.1
ASCC2	3012.5	3853.8	1818.2	2023.3	1509.6	2076.9	2224.1	1694.7
ASCC3	148.5	218	86	93.6	94	100.7	156.3	189.3
ASCC3	249	305.9	151.2	184.4	120.6	167.9	217.6	369.6
ASCC3	127.8	160.6	100	98	84.5	80.6	123.6	146.7
ASCC3L1	204.8	290.1	125.8	168.9	139.5	178.9	183.2	233.8
ASCIZ	163.7	200.8	112.2	117.9	122.1	129.7	177.1	272.1
ASCL1	99.3	122.1	103.2	103.9	96.4	89.4	102.7	111
ASCL2	317.9	411.5	229.6	233.3	223.9	222	290.9	279
ASCL3	98.1	111.7	93	84.5	75.6	81.6	98.2	103.1
ASCL4	92	109.2	80	86.4	69.3	83.4	91.2	102.2
ASF1A	899.7	1110	630.3	722.1	539.3	1164.6	1162.8	2573.3
ASF1B	937.6	1191.5	615.1	305.6	317.7	364.6	706.7	857.9
ASGR1	142.3	152.1	115.2	95	98.3	80.4	139.3	154.7
ASGR2	90.3	97.6	90	82.4	98.8	87.1	90.2	102
ASGR2	335.3	395.2	237.2	376.7	303.7	394.8	265.1	544
ASGR2	176.8	201.9	150.9	161.8	159.2	201	146.1	218.5
ASH1L	101	129.5	86.7	105.3	92.2	90.4	99.1	102.3
ASH2L	3337.1	4359.1	2362.6	1825.4	1358.7	2343.5	2826.5	3720.6
ASIP	78.9	92	83.2	87.2	86.1	82.9	86.9	75.3
ASL	78.4	93.8	102	88.9	94.4	83.4	90	92
ASL	97.3	121.1	111.4	105	94.5	116.5	107.8	115.3
ASL	220.4	275.4	156.3	157.1	150.7	174.2	216.6	233.3
ASMT	90.1	105	95	96.1	84	93.3	86.4	83.5
ASMTL	173.2	213.5	165.6	194.8	161.3	246.9	181.5	232
ASNA1	109.4	120.9	98.7	74.6	71.4	92.9	104.9	117
ASNS	2574.7	3584.5	1774.4	712.9	493.6	743	2646.6	3092.3
ASNS	353.2	400.7	163.2	127.4	90.2	115.7	325.4	402.6
ASNSD1	2324	2856	1843.7	1760.6	1267.4	2461.4	1500.7	2470.8
ASPA	95.9	101.2	93.7	90.4	82.5	86.8	85.9	104.9

ASPH	289.6	336.2	124.3	128	119.1	128.8	244.6	284.5
ASPH	146.4	194.2	121.4	106	108.3	119	146.6	222.1
ASPH	120.9	147.8	97	100.3	97.9	92.3	116.2	123.3
ASPHD1	480.4	598.3	370.2	235.9	195.6	256.8	408.5	475.2
ASPHD1	185.6	193.7	118.1	107.8	95.1	108.2	169.5	144.6
ASPHD2	179.8	236.2	150.1	118	116.8	120.9	209.5	271.1
ASPM	1825.5	2230.6	827.8	991.7	838.2	1136.4	1789.1	2534
ASPN	82.9	87.2	94.3	98.4	93.2	86.5	85.1	78.8
ASPSCR1	164	198.7	109.5	103.7	104.6	103.4	158.2	198.9
ASPSCR1	2944.1	3217.2	1922.8	1600.1	1503.1	1705.6	2547.7	2484.9
ASRGL1	126.5	167.1	110.9	121.8	115.4	138.2	112	150.1
ASS	2404.3	3197.8	1084.4	741.9	721.6	965.6	2260.8	4466.1
ASS1	1486.3	2020	394.3	257.3	265.5	337.4	1464.5	2881.5
ASTE1	425	534.8	182.2	190.3	139.1	230.3	387.4	459.1
ASTL	99.5	106.3	108.2	91.5	89.6	90.2	109.9	109.7
ASTN	101.9	106	102.8	99.2	89.4	85.9	92	101.2
ASTN	97.7	105.3	94.4	88	86.6	85.1	90.5	120.1
ASTN2	135.7	166	106.8	89.2	88.1	91	108.6	132.4
ASTN2	117	143.4	96.2	92.2	97.2	84.3	100.7	117.1
ASTN2	85.3	96.4	96	85.7	83.9	103.7	91.4	95.8
ASXL1	319.3	431.2	329	372.3	322.4	479.6	290.5	329.9
ASXL2	734.1	1012.7	505.3	1213.3	970.2	1288.3	709.9	740
ASZ1	76.5	83.6	86.8	87.7	75.9	83.8	79.3	85.7
ATAD1	324.1	378.9	125.5	121.8	111.9	127.3	246.6	489
ATAD2	1622.3	2224.1	1285.1	1101.2	921.3	1703.5	1278.6	2175.8
ATAD3A	428.5	528.4	372.7	330.5	271.6	484.1	381.6	690.3
ATAD3B	164.4	214.5	144.5	143.4	131.9	116.7	157	209.1
ATAD3C	123.7	139.5	93.7	99.4	93.8	103.3	112.6	106.7
ATAD4	226.7	278.7	166.2	132.6	121	148.6	242	250.3
ATBF1	106.3	101.1	99.6	127.2	110.9	103.7	102.9	116.9
ATCAY	102.3	125.1	94.5	90.9	90.7	90.3	102.4	112.9
ATE1	109.6	157.4	93	90.6	84.1	92.4	115.5	109.4
ATE1	93.6	92.5	89.5	82.7	79.7	88.1	89.8	109.4
ATE1	118.2	142.3	91.8	85.4	93	81.2	111.8	152.8
ATF1	216.3	253.3	113.9	110.7	94.1	122.8	185.3	321.8
ATF2	320.3	450.1	212.9	213.7	220.1	300.3	275.7	353.4
ATF3	151.5	181.1	121.7	101.8	105.9	114.7	156.1	266.4
ATF3	77.2	98.2	103.5	99.7	86.4	96.6	84	92.7
ATF3	90.8	100.5	105.9	87.9	82	85.7	98.5	95.2
ATF4	12864.5	15313.2	6188.1	5473.2	4269	6403	14738.7	20141.5
ATF4	191.1	165.4	135.6	121.5	115.4	151	141.4	146.2
ATF4	1011.1	1483.6	256.3	230.4	189	292.3	1294.4	1880
ATF5	1424.2	1710.7	725.6	956	1073.4	888.2	1521.5	1639
ATF6	1495.4	1876.6	658.1	823.3	679.5	972.9	1305.1	1661
ATF7	121.4	129.3	95.6	87.7	86	92.6	100.6	124
ATF7IP	103	94.5	96.6	97.6	93.3	90.2	88.7	103.7
ATF7IP2	376.2	488.3	291.8	323.5	248.2	405.2	304.4	501.9
ATG10	229.3	341.5	228.5	246.5	191.5	244.2	209.6	322.8
ATG12	1586.3	1855.3	1145	1094.5	1010.9	977.2	1459.2	2359.5
ATG16L1	447.8	623.7	378.8	277.9	248.6	484.7	350.8	625.3

ATG16L1	471.5	618.6	481.2	388.6	298.8	569.5	335.2	564.5
ATG16L2	219.4	233.2	186.4	189.6	242.8	262.8	195	230.6
ATG3	187.1	207.3	125.1	116.7	122.6	125.2	181.6	209.8
ATG4A	264	400.2	225.9	248.2	216.7	305.5	232.4	334.6
ATG4A	90.4	108.5	78.4	81.3	78	85.9	87.5	102.5
ATG4B	75.3	80.3	93.7	87.7	91.1	92.5	80.7	84.5
ATG4B	1434.4	2089.5	744.2	431.2	467.3	254	1103.4	1576.1
ATG4C	85.4	93.1	94.3	89	71.7	90.2	86.8	88.6
ATG4D	124.9	146.8	91.7	85.5	88.3	89.5	116.7	146.8
ATG5	341.8	412	223.9	257.4	207.8	277	314.2	520.8
ATG7	808.9	1109.4	591.5	721.5	559.1	820.9	673.2	720.6
ATG9A	83.1	89.1	83.9	77.8	83.2	79.2	85.6	85.9
ATG9A	208.8	304	305.3	300.9	261.1	457.2	219.5	417.3
ATG9A	185.9	261	257	201.6	200.5	270.8	205.7	409.5
ATG9B	90.4	99	81.6	87.7	85.7	86.8	91.2	94.1
ATHL1	161.3	154.5	176.4	185.4	181.7	298.3	139.2	171.5
ATIC	5995.7	7402.4	4828.3	3161.1	1911.8	2715.4	4408.9	5224.6
ATM	97.1	119.9	103.4	92.9	92.4	92.9	95.9	99.8
ATM	572.8	878	345.9	664.7	556.7	890.1	548	676.8
ATM	82.5	95.4	71.8	81.9	70.7	77.8	81.3	88.4
ATN1	103.9	118	84.9	87.1	83.2	94	105.3	120.3
ATN1	116.9	137.3	128.4	134.1	135.2	160	120.1	159.9
ATN1	89.3	102.3	86.4	88.5	73.8	72.2	79.6	102.7
ATOH1	96.8	106.6	96.5	90	85.2	83.4	93.7	99.8
ATOH7	90.3	102.8	94	80.3	90.6	87	97.2	110.7
ATOH8	97.1	102.1	91.7	79.4	82.6	90.1	102.9	103.2
ATOX1	3360	3948	2504.6	1895.7	1509.3	1954.9	3688.2	3553
ATP10A	101.6	132.4	102.9	100.5	77.9	85.8	114.3	150.2
ATP10D	154.5	190.5	123.8	120.8	106.7	119.5	134.9	143.9
ATP11A	99.7	122.6	87.8	89.5	74.3	91.2	102.3	111
ATP11A	84.6	90.5	92	77.6	76.4	90.1	82.1	96.8
ATP11B	232	262.1	172	250.9	187.7	297.4	224.6	359.5
ATP11C	158.1	237.9	128.8	130.7	111.1	137.1	148	194.8
ATP11C	330.2	417.3	200.5	270.8	203.3	366.7	306.3	465.9
ATP12A	107.5	132.3	95.6	79.7	100.4	84.8	102	113.7
ATP13A1	738.6	902.2	481.3	391.9	367.9	352.1	540.7	563.4
ATP13A2	116.4	145	140.7	113.8	126.7	126.3	114.6	177.5
ATP13A4	94.2	104	86.4	92.7	77.6	76.7	89.4	98.2
ATP13A5	69.6	91.2	90.4	83.9	84.7	84.5	83.9	96.7
ATP1A1	2112.4	2663.4	2439.2	1563.3	1209.7	1972.6	1842.8	2140.7
ATP1A1	4658.8	5968.4	2648	1947.4	1897.9	2511.2	4054	6375.2
ATP1A1	1474.9	2345.8	857.4	348.5	277.3	323.1	1215.5	1720.3
ATP1A2	80.4	80.5	84.8	88.8	83.1	76.7	84.3	85
ATP1A3	116.1	118.9	105.8	96.7	102	104.3	102.9	104.4
ATP1A4	88.6	94.2	97.1	92.2	84.4	84.3	86.8	93.4
ATP1A4	98.2	116.7	101.6	85.2	86.2	91.1	97.8	110.9
ATP1B1	230.2	312.7	138.6	261.6	195.5	257.2	231.5	421.6
ATP1B1	492.4	632.9	320.5	643.4	431.8	601.4	469.7	794.5
ATP1B1	389.8	525	196.7	300.5	237.3	257.7	337.3	429.6
ATP1B2	309.6	478.6	182.2	225.4	192.2	201.3	329.6	374.6

ATP1B3	4718.9	5793.8	2139.5	1753.7	898.7	1596.5	5710.8	8036.3
ATP1B3	818.8	1148.9	346.2	308.7	244.3	341.2	625.2	760.3
ATP1B3	4234.8	4536.3	926.1	868.4	680.1	1007.4	4182	6679.3
ATP1B4	76	89	104.1	92.1	87.1	95.1	88	90.3
ATP2A1	84.5	93.6	96.8	88.8	83.4	81.9	89.7	94.2
ATP2A2	554.1	915.1	633.1	705.4	620.1	1062.7	620.1	878.9
ATP2A2	934.6	1114	911.7	1264.3	959.6	1682.3	991	2474
ATP2A2	574.6	792.7	585.6	561.9	481.6	911.2	600.1	1012.2
ATP2A3	131.5	134	120.6	111.1	103.9	108.8	129.5	120.5
ATP2A3	102.4	121.2	108.2	110.6	107	141.2	113.8	127.8
ATP2B1	113.2	129.4	105.8	102.6	95.4	107	111	136.8
ATP2B1	92	119.9	106.7	82.8	83.2	93	103	110.9
ATP2B2	94	116.1	93.6	89	89.4	91.7	91.6	96.7
ATP2B2	84.3	94	95.8	86.3	95	87.2	86.4	94.3
ATP2B2	81.3	98.1	85	86.1	87.6	78.5	86.2	91.3
ATP2B3	274	316.7	199.4	253.5	205.4	274.4	252.2	320
ATP2B3	120	131.9	89.6	88.6	83.3	88.5	109.2	123.9
ATP2B3	86.5	86.8	101	96.7	99.1	96.7	81.1	91.2
ATP2B4	115.4	151.3	96	116.9	121.7	110.7	110.9	127.5
ATP2B4	503.7	857.1	498.8	863.3	872.7	1198.9	513.4	631.8
ATP2C1	233.6	287.2	180	232.3	173.6	263.2	208.2	304.1
ATP2C1	157	212.7	111.9	123.5	101.2	107.4	145.6	213.9
ATP2C1	558.1	757.5	364.1	337.6	250.7	449.2	378.6	564.5
ATP4A	104.8	121.7	91.1	84.6	79.2	81.6	86.6	112.1
ATP4B	101.8	100.2	94.3	91.5	84.1	93.3	94.5	93.4
ATP4B	94	99.5	102.4	84.7	85.6	86.1	91.6	99.6
ATP5A1	19414.9	24068.4	17472.1	17314.9	13342.1	19054.8	16875.6	23345.7
ATP5A1	21701.3	25463.5	15829.2	16992.8	13500.7	16899.7	19161.4	23137.8
ATP5B	22424.7	27829.4	17992.7	18643.9	14221.4	21480.2	19262.1	20982.5
ATP5C1	4337.7	5719.2	2664.2	1792.6	1356	2608.3	3821.1	6656.9
ATP5D	476	586.6	538	416	303.1	411.8	496.8	873.5
ATP5D	5261.9	6547.9	5003.8	2915.2	2028.2	2829.7	4502.1	9048
ATP5F1	6644.4	7873.4	2071	968.6	916.7	1231.9	5436.8	6286.9
ATP5F1	19374.6	21801	10115.4	6616.9	5216.9	7994	16840.2	23206.4
ATP5G1	286.2	337.5	178.1	162.8	137.5	170.7	246.9	252.8
ATP5G1	6479.9	7518.3	5466.3	4923.9	3786.1	4182.6	6217.5	6047.6
ATP5G2	84.9	88.6	95.2	100.8	89.4	79.6	86.6	89.2
ATP5G2	228	254.9	197.1	134.4	111.2	105.6	182	219.8
ATP5G2	8637.9	11007.7	5974.8	2900.9	2300.6	2474.6	7966.5	8390.5
ATP5G3	369.3	434.1	220.9	243.4	193.8	240.4	293.6	435.6
ATP5H	10429.4	11138.7	6483.6	6483.7	4753.8	7159.3	9517.2	14305.5
ATP5H	10452.5	12507.6	8102	7825.4	5669.9	8857.6	9815	15488.9
ATP5I	13661.4	14263.5	10820.4	10082.6	8279.5	9646.9	12967.6	15392.1
ATP5I	1053	1294.4	904.9	766.9	642.1	825.8	868.9	999.1
ATP5J	1376.1	1659.8	647.3	755.1	529.9	771	1157.4	1169
ATP5J	13415.2	17353.9	10448	10642.7	8716.1	11707.8	11219.1	13309.9
ATP5J	14941.5	17507.4	11267	11802.5	9243.4	13857.3	12936.1	13956.7
ATP5J2	6942.7	7591.4	4381.9	4110.3	3321.8	4644.5	6577.2	7353.8
ATP5J2	8899	9905.8	6226.1	5808.5	4651	6191	8660.6	10712.5
ATP5L	682.4	836.2	210.2	266	225	268.4	651.6	1050.7

ATP5O	12195.3	14322.2	7062	7740.5	6057.2	8299	10784	14695.9
ATP5S	390.7	418.1	189.1	212.2	169.7	231	315.9	403.8
ATP5S	356.4	467.6	158.2	132.4	143.4	141	266	336.3
ATP6AP1	4743.1	6584	3583.6	4299.7	3819.2	4448.1	4758.9	4303.7
ATP6AP2	4286.4	5545.8	1796.3	2423.9	2143.3	3326.6	4235.5	6089.2
ATP6V0A1	876.8	1095.5	642.2	724.5	629.9	713	992.1	1087.3
ATP6V0A2	421	581.4	338.8	294.8	227.4	307	382.3	500.9
ATP6V0A4	90	93.3	99.5	91	85	79.5	95.7	97.5
ATP6V0A4	110.3	107.8	95.4	98	92.5	92.6	110.8	110.4
ATP6V0A4	84	103.4	84.9	81.9	76	83.6	87.8	85.5
ATP6V0A4	72	83.5	94.2	91.6	97.2	96.2	73.3	72.2
ATP6V0B	6965.2	8956.3	6174.4	5411.2	4314.7	5197.5	7767.4	10379.1
ATP6V0B	709.5	742.6	188.7	170.6	151.6	206.5	669	830.8
ATP6V0C	604.2	951.6	485	280.9	215.3	262.7	562.6	945.2
ATP6V0D1	1302.9	1838.8	1132.1	805.7	654.5	931	1318.1	1802.9
ATP6V0D2	99.3	132.5	90.2	87.1	87.8	86.6	98.2	113.8
ATP6V0E1	6549.8	7655.8	4154	5047.1	3683.6	4375.9	7224.1	8256.1
ATP6V0E2L	1626.9	1970.9	1111.4	712.5	593.2	593.4	1186.5	1218.7
ATP6V1A	3242.2	3889.8	2050.9	3661.8	2744.1	4415.6	4094.5	5961.3
ATP6V1B1	415.3	392	277.1	232.7	266.8	261.9	326	382.6
ATP6V1B2	9456.1	12445.6	8803.7	5931.8	4656.5	6528.6	7926	10968.2
ATP6V1C1	326.7	424.2	258.7	340.9	237.6	441.1	314.4	632.7
ATP6V1C1	139.5	177.4	103.6	81.1	85.8	104.4	136.5	207.9
ATP6V1C2	93.7	116.5	94.4	131.8	131.3	153.7	91.5	98.4
ATP6V1C2	78.9	82	83.1	86.4	80.9	76.6	83.7	79.6
ATP6V1D	1440.1	1985.8	1031	882.7	653.5	1086.6	1311.2	2231.3
ATP6V1E1	5001.7	6418.8	3446.5	3430.8	2891.3	3987.9	4703.8	5129.6
ATP6V1E1	3460.6	4201.4	3029.6	3263.6	2748.6	3219.1	3378.6	3802.3
ATP6V1E2	282	351.4	207.9	246.4	219	308.8	239.4	295.6
ATP6V1F	4047.2	5578.5	2548.8	2464.5	2075	2450.1	4539.8	5565.9
ATP6V1G1	4368.5	5174.6	2101.9	2404.4	1912	3154.7	4122.7	6465.7
ATP6V1G2	188.7	220.9	144.3	113.8	98.9	121.4	158.5	213.5
ATP6V1G2	92.3	111.4	92.7	92.1	88.9	85	98.5	100.3
ATP6V1G3	86	86.2	95.2	92.9	81	87.4	84.4	94.1
ATP6V1H	110.2	102.4	96.2	81.6	83.6	95.8	94.2	120.3
ATP6V1H	627	1002.1	429.3	340.1	241.1	459.8	730.5	1354.5
ATP7A	93.1	110.8	96.4	114.8	106.8	150.9	97.2	121.8
ATP7B	154.4	187.9	103.2	101.8	93.9	99.4	147.1	162.6
ATP7B	113.8	120.6	131.4	180	153.4	205.6	99.7	136.3
ATP8A1	107.3	117.9	90.5	86.4	83.4	95.9	103	103.9
ATP8A2	110.4	126.6	119.4	98.5	101.3	108.3	104	99.3
ATP8B1	137.9	151	90.6	99	84.4	93.8	116.7	138.2
ATP8B2	144.2	129.2	107.9	97.3	92.1	83.6	113.9	169.5
ATP8B2	312	407.5	186.1	143.4	153.5	152.3	268.2	309.9
ATP8B3	226.5	267.8	179.9	205.9	203.7	237.9	185.6	202.9
ATP8B4	3721.5	5316.5	2855.9	5209.3	3949.9	7809.1	3203.6	3665.2
ATP9A	217.8	342	217	373.5	274.8	415.2	235.1	284.7
ATP9B	147.4	160.7	125.4	121.2	109.3	148.4	157	181.2
ATPAF1	946.3	1266.7	555.3	371.4	288.8	452.8	846.1	942.9
ATPAF1	581.3	739.9	468.9	389.5	275.6	442.8	442.9	463.7

ATPAF2	136.3	169.4	124.3	118.4	115.4	137.6	126.9	146.9
ATPBD1B	983.5	1350.9	666.6	606.9	504.1	555.9	792.8	772
ATPBD1C	86.8	91.8	89	86.2	85.8	89	85.1	86.8
ATPBD3	173.1	234.1	147.3	122.8	105.4	120.2	196.7	192.5
ATPBD4	228.7	264.2	119.9	127	86.9	126.9	203.6	266.7
ATPIF1	1229.6	1548.5	571.6	487.7	442	455.6	1089.1	1210.1
ATPIF1	187.5	199.2	120.7	125.9	105.9	145.1	152.6	249.2
ATR	73.5	89.3	90.6	91.9	73.4	90.2	81.5	91
ATR	101.7	134	80.8	90.7	81.1	82.4	106.8	121.9
ATR	91.3	113.7	107.4	95.7	88	92.1	101	102.7
ATRN	101.4	140.2	101.6	142	118.7	148.5	110.5	188.1
ATRN	104	123.6	89.1	94.6	88.4	86.4	102.7	114.6
ATRNL1	84.7	101.2	108.6	89.9	90.4	79.3	84.6	88.7
ATRX	146.5	182.1	101.6	103	96	97.1	121.1	141.5
ATRX	72.2	87.8	81.6	83.8	82.5	83.4	78	82.5
ATRX	83.9	99.3	80	79	82.7	78.3	91.6	114.2
ATXN1	134.3	171.9	127.8	161.7	134.9	165.6	145.4	198.6
ATXN10	178.2	231.2	97	133.2	118.1	124.6	151.6	221.7
ATXN2	1164.4	1491.1	889.6	1342.1	1040.6	1219.2	1124.8	1321.4
ATXN2L	117.3	125.3	176	157.2	146.1	261.1	117.9	135.1
ATXN2L	114.4	110.3	114.9	101.8	100.2	98.9	95.3	122.4
ATXN3	332.2	487.8	254.1	335.2	312.7	289	390.7	432.1
ATXN3	123.4	142.9	100.3	102.9	103.3	101.3	119.1	138.9
ATXN7	96.7	112.8	88.5	81.2	82.6	87	106.6	119.6
ATXN7L2	182.9	218.5	182.8	115.8	116.6	125.4	175.7	301.9
ATXN7L4	101.9	114.8	96.5	97.9	88.3	95.2	86.8	98.1
ATXN8OS	84.7	83	94.1	88.6	84.4	92.3	81.3	80.1
AUH	293	425.9	210.5	155.5	167.1	230.1	235.1	264.9
AUP1	1283.8	1336	374.2	332.2	275	351.6	1223.5	1804.4
AURKA	3392.3	4228	2908	2570.8	2212.6	2917.8	3018.3	4295
AURKA	2961.1	3930.1	2552.3	2180.2	1690.6	2463.2	2882.2	4393.7
AURKAIP1	7196	8390.7	5515.7	4859.3	4314.4	5417.6	6241.6	5932.3
AURKB	1012.3	1121.2	967.9	437.2	379.8	536.4	850.6	1217.4
AURKC	86.3	103.5	88.7	79.3	76	86.8	87.4	90.1
AURKC	75	90.9	90.4	83.3	74.6	89.6	78.1	90.2
AURKC	139	170.8	106.3	87.5	83.7	92.9	139.6	157.9
AUTS2	88.1	103.8	99.8	82.6	81	82.2	100.3	86.5
AVEN	287	345.8	151	139.4	135.9	167.4	279.3	475.6
AVIL	105.5	121.9	113.3	110.4	96.5	102.5	104.3	102.9
AVP	101.6	117.5	109.3	96	84.8	91.7	111	110.4
AVPI1	346.2	475.8	281	231.4	185.6	237.8	363.8	360.4
AVPR1A	93.8	110.6	100.8	89.5	86.3	84.6	97.4	106.7
AVPR1B	106.8	127.4	92.9	87.4	73.5	84.9	93.3	128.8
AVPR2	124.4	145.4	91.1	78.9	80.1	81.7	109.7	128.6
AXIIR	895.1	1025.4	755.4	980.1	823.8	1112.4	667.3	759.5
AXIN1	654.8	697.6	875	710.1	642.4	972.8	611.7	1244.9
AXIN1	103.1	118.5	90.8	81.8	87	111.8	100.6	117.6
AXIN2	89	100.4	102.2	90.8	85.3	91.1	92.2	108.5
AXL	249.9	312.1	191.6	362.2	240.6	232.2	220.9	212.3
AXL	248.9	301.2	170.7	357.6	230.2	294.9	179.1	236.6

AXUD1	309.2	400.6	272.3	269.8	273.6	277.9	381	475.5
AYP1	118.5	127.7	109.8	104.8	106.7	101.4	104.5	117.9
AYTL1	259.7	341.3	140	121.9	108.8	122.2	206.4	250.9
AYTL2	633.9	806.6	377.1	237.7	197.4	227.7	509.1	513.3
AZGP1	116.3	141.8	111.2	91	96.8	95.7	117.6	155.5
AZI1	584.5	734.7	505.6	533.7	495.1	533	392.5	413.6
AZI1	132.8	152.1	89.8	85.6	77.6	86.3	120.9	137.2
AZI2	544.5	720.2	177.4	253.2	178.2	342.4	427.9	678.9
AZIN1	4479.6	5794.1	2982.2	3284	2253.5	4388.5	3941.2	5680.7
AZIN1	563.2	781.5	207.5	217	157.2	163.5	324.3	488.3
AZIN1	297.9	371	162.6	145	113.4	175.6	299.1	535.5
AZU1	13000.7	15809.2	13333.6	9574.1	7633.9	9828.3	10130.6	14989
B2M	11781.2	12771.4	3197.7	3208.6	2560.6	3649.5	10812.4	11565.7
B3GALNT1	416.6	536	289.7	284.1	215.8	360.1	344.3	461.7
B3GALNT2	449.9	633.4	266.6	236.2	178.3	218.2	378.9	498.7
B3GALT1	103.8	117.4	116.2	100.9	103.8	102.1	101.7	100.3
B3GALT2	109	123.7	103.8	98.5	90.6	92.9	113.8	124.8
B3GALT3	167.1	222.1	125.6	131.8	118	153	155.6	174.7
B3GALT3	119.3	142.1	95.8	94.1	84.4	89.8	99.3	114.1
B3GALT3	180.3	206	104.5	96.5	102.2	109.8	147.3	196.7
B3GALT4	118.4	127.7	99.8	97.5	117.4	113.7	111.1	118.5
B3GALT5	88.7	92.2	84.7	79.1	75.2	87.4	84.6	91.9
B3GALT5	103.2	114.9	96.7	89.4	83.2	89.3	105.8	117.6
B3GALT5	74.3	98.1	88	91.5	71.4	77.5	91.1	87.9
B3GALT6	466.3	608.9	477.5	555.2	414.1	654.8	467.5	945.1
B3GALT7	192.8	261.8	137.6	136.6	130.8	166.2	197.4	280.5
B3GAT1	100.7	117.1	100.8	97	104.4	99.1	94	108.3
B3GAT1	107	120.8	83.8	79.8	74.3	85.2	98.6	118.4
B3GAT1	81.7	97.4	97.7	93.9	95.1	99.2	94.3	96
B3GAT2	82.7	83.5	100.8	90.8	86.5	96.7	80.8	87.2
B3GAT3	233.8	254.7	255.4	193.9	167.7	215.7	181.7	221.1
B3GNT1	724.1	1154.1	380.5	307.4	259.8	475.5	453.1	641
B3GNT3	136.6	151.9	93.4	98.2	90.3	96	123.8	142.9
B3GNT4	121.3	155.1	95.9	83.3	92.3	93.7	113	132.1
B3GNT5	122.4	122.6	103.8	101.2	98.1	137.5	106.9	159.7
B3GNT6	280.4	379.9	259	194.5	176.4	204.8	198.2	255.7
B3GN-T6	100.2	111	93	78.9	77.4	76.3	99.6	126.7
B3GNT7	86.7	102.4	99.3	113.2	93	94.5	96.7	90.3
B3GNTL1	216.3	260.2	184.4	188.1	154.3	184.7	172.4	211.1
B3GTL	221.9	259.8	201	159.9	128.5	166.7	163.4	223.7
B4GALNT2	92	90.9	102.3	82.8	82.2	88	89.5	94.2
B4GALNT3	92.6	97.2	93.7	83.5	77.2	87.1	86.2	94.4
B4GALNT4	76.3	87.4	79.7	77.5	83.8	82.3	80.2	89.6
B4GALT1	247.6	301.4	137.1	153.1	142.1	145.9	263	388.3
B4GALT2	93.6	110.6	108.5	97.4	88.2	94.6	94.5	87.5
B4GALT2	111.6	139.1	108.7	97.7	102.7	102.1	109.7	135.7
B4GALT2	144.3	170.3	105	85.5	90.1	90.7	119.4	197.8
B4GALT3	2246.8	2798.7	1484	1043.8	771.2	872	2210.3	2137.1
B4GALT4	290.5	420.4	171.5	147.5	153.2	218.4	299.5	416.5
B4GALT4	135.4	164.1	152.7	128.4	128.3	143.3	155.6	186.1

B4GALT5	2139.6	2514	1220.2	1677.6	989.9	1805	1811.4	2342.2
B4GALT6	112.9	138	110.9	101.4	89.2	111.1	100.2	126
B4GALT7	821.3	1020	504.7	491.8	417.8	445.4	629.2	523
BA16L21.2.	294.9	333.2	150.3	152.2	130.1	183.6	222.7	335.1
BA9F11.1	87.5	111.7	77.5	86.5	76.7	79.7	92.8	96.9
BAALC	125.5	128.8	110	111	97.3	112.5	115	136.6
BAALC	101.3	97.8	102.8	95	85.2	99.5	91.5	104.3
BAAT	81.5	85.7	81.5	75.6	82	81.8	83	102.9
BACE1	215.3	303.5	141.5	136.9	112.9	155.2	208.1	260
BACE1	110.6	124.8	100	95.5	83.2	88.3	108.2	125.5
BACE2	96.7	119.4	93.8	85.9	85	79.3	106.9	125.8
BACE2	117.2	115.6	89.5	94	93.2	86.1	105.2	115.2
BACH1	161	219.5	101	104.8	105.9	94.6	176.3	198.9
BACH1	93.5	99.6	85.5	82.7	80.4	81.7	97.4	105.2
BACH1	84.9	102.5	89.8	86.2	77.6	89.8	93.2	119.7
BACH2	83.6	86.3	88.5	88.8	83.5	82.9	74.1	87.4
BAD	152.8	183.7	115.7	89.9	96.6	94.6	141	145.8
BAD	451.7	594.3	331.3	224.7	213.5	235.2	392.2	460
BAD	100.4	117.2	102.4	93.1	85.6	89.3	96.3	104.2
BAG1	182.5	248.5	148.6	109.6	111.4	119	182.6	261
BAG3	980.2	1253.6	694.2	353.9	322.6	351.4	1199	1868.2
BAG4	333.9	434.5	150.1	133.7	115.8	127.2	289.4	400
BAG5	1600.1	1837.1	1331.1	1359.2	975	1317	1215	1953.9
BAG5	112.9	134.3	99.4	88.9	78.8	86.7	98.6	126.5
BAG5	82.8	105.9	87	91.9	78.7	90.4	88.8	100.9
BAG5	396	546	273.8	249.1	223	374.8	285.7	471
BAGE	138.7	165	100.2	96.9	93.4	106.1	144.4	154.3
BAGE2	94.3	114.9	91.3	88	81	87.8	80.9	105.6
BAGE3	88.6	112.3	94.9	90.2	90.8	87.1	96.6	109.9
BAGE4	78.1	82.9	94.7	88.5	91.8	89	86.3	83.6
BAGE5	123.2	159.7	104	104.6	104.2	113.6	123.8	147.5
BAHD1	120.3	137.4	83.2	100.5	78.6	81.9	112.3	132.4
BAI1	130.9	133.2	97.1	100.3	94.4	104.5	119.2	119.1
BAI1	90.7	96.5	84.9	73.4	93.8	84.7	84.6	93.3
BAI1	81.9	89.9	91	87.7	81.3	90.1	78.8	86.5
BAI2	74.8	81.2	85.6	85	81.6	79.9	81.9	88.6
BAI3	83.7	97.1	91.7	92	86.8	110.9	77.9	94.9
BAIAP2	174.7	245.3	146.5	194	169.1	219.2	174.3	212.1
BAIAP2	149.2	172.5	117.1	103.8	102.1	127.9	134	156.4
BAIAP2	88	106.5	88.5	97.8	95.2	90.1	98.6	115.2
BAIAP2	105.5	117.2	97	79.6	79.3	84.9	95	104.7
BAIAP2L1	104.7	135	108.9	118.6	117.6	135.3	122.6	143.8
BAIAP2L2	108.5	108	109.7	114.7	119.1	123.8	100.9	124.4
BAIAP3	81.9	79.5	82.6	78.3	77.4	81.3	79	77.4
BAK1	367.9	497.9	198.1	146.9	149.7	146	336.8	529.6
BAMBI	157.1	163.4	94.5	112.5	100.9	147.9	191.9	241.3
BANF1	3724.5	4754.9	4330.8	2672.4	2207.9	3193.8	3477.7	5127.3
BANK1	518.6	702	232.2	407.6	333	566.4	358.5	401.5
BANP	148.4	176.6	110.3	109.7	109	132	142.8	180
BAP1	304.5	392.8	285.5	248.8	224.8	330.3	269.2	380.4

BAPX1	1035.6	1209.6	883.8	465.4	457.7	553.4	778.3	819.8
BARD1	1100.6	1592.2	858.5	1047.2	975.3	948.4	1029.1	1165.2
BARHL1	99.5	111.7	97.8	87.7	86.2	86.4	99.7	107.1
BARHL2	113.8	153.2	86.4	86.7	79.4	84.6	136.8	134.6
BARX1	843.8	1129.8	291	202	164	291.9	718.5	1208.4
BARX2	121.3	136	102.8	109.4	104.6	108.3	108.7	128.9
BASP1	3517.7	4452.7	2918.9	5201.6	3490.3	7848	3822.3	7740.9
BAT1	2541.6	3208.7	2077.5	1962.7	1522.1	3158.5	1749.2	3375.9
BAT1	141.7	187	89.6	83.3	80.8	91.9	113.6	139
BAT1	122.9	155.2	98.1	103.4	94.5	107.7	113.2	145.8
BAT2	247.1	352	581.6	559.2	495.3	835.1	254.4	432.4
BAT2	382.2	506.4	881	893.3	707.5	1143.8	368.7	732.7
BAT2D1	1262.4	2016.3	952.3	1508.9	1253	2664.2	1196	1497.2
BAT3	313.2	448.2	348.9	265.5	240.5	238.6	249.2	335.3
BAT3	260.8	335.4	280.2	291.7	245.4	386.6	275.2	415.1
BAT4	239.9	304.6	137.3	108	115.7	121.1	239.8	280.4
BAT5	787.9	1013.7	722.6	795.7	701	1066.7	718.1	1622.4
BATF	675.3	996.9	619.8	386.2	311.1	455.8	718.6	627.8
BATF2	102.5	118.3	100.3	89.3	92	91.6	100.8	106.8
BAX	1312.1	1721.5	1386.2	1282.9	1076.4	1260.2	1417.4	1418.2
BAX	136.9	154.4	84.2	90.6	78.9	87.7	136.9	149.5
BAX	145.6	204.4	162.9	129.2	121.1	157.3	161.9	286.8
BAZ1A	2300.7	2804.7	1774	1744.6	1297.4	2928.4	1918.5	3777.9
BAZ1A	533.5	803.1	343.5	380.5	332.1	419.6	421.4	716.6
BAZ1B	804.7	1078.6	707.7	710.4	673.9	1211.6	775.7	1340.8
BAZ2A	91.3	114.9	103.5	94.3	92.9	97.4	103.6	124.3
BAZ2B	467.3	589.3	317.5	476.4	437	719.1	360.8	512.4
BBC3	108	114.1	88	75.9	81	86.2	106.6	130.9
BBOX1	107.3	139.6	80	82.1	73.2	83	101.3	147.8
BBS1	139.8	163.7	143	141.9	138.6	157.8	109.2	141.9
BBS12	108.9	110.9	124	113.2	110.7	122.9	104.8	97.1
BBS2	309.9	357.4	193.6	300.9	291.7	406.9	245.6	291.9
BBS4	318.8	340.5	226.6	239.3	214.6	305.1	282.2	320
BBS5	123.4	121.8	106.2	93.2	94.6	90.3	115.1	125.1
BBS5	122.2	118	90	89.6	85.9	82.9	102.5	110.5
BBS5	85	95.8	95.2	94.5	82.7	90.3	80.2	90.7
BBS7	114.3	114.5	98.3	95.8	87.4	105.2	111.4	136
BBS7	239.6	255.8	168.4	156.2	128.5	197.2	213.1	271.8
BBS7	92.8	93.1	90.4	85.9	81	85.5	83.5	101.4
BBS9	134.5	135.4	85.1	78.3	77.7	97.1	106.7	122.2
BBX	317.2	420.9	219.1	260.2	265.7	269	266.1	511.2
BC002942	366.1	441.3	484	540.1	417.6	683	309.5	726.3
BC37295_3	85.4	91.4	81.6	84	80.7	80.4	89.3	91.9
BCAM	111.1	112.1	108	93.8	86.2	88.9	108.8	122.9
BCAM	87	98.9	90	81.6	92.4	82.5	84.7	94.5
BCAM	101.8	97.3	120.6	97.9	97.3	106	86.7	87.1
BCAN	149.8	205.9	118.9	111.9	107.6	106.4	143.5	175.5
BCAN	86.6	95	96.3	94.2	84.1	95.9	82.8	96.3
BCAN	93.6	104.1	92.8	87.7	83.4	84.1	89.4	109.2
BCAP29	719.4	848.6	327.1	351.7	258.5	352.5	504.7	828.2

BCAP29	102	106.6	96.6	89.7	87.9	95.2	95.1	96.2
BCAP29	694.5	816.5	260.8	295.4	213.6	313.3	511.8	781
BCAP29	72.3	79.7	103.3	93.9	94.8	94.3	74.4	79.1
BCAP29	331.9	412.5	257.5	299.5	239	337.6	278.9	388.1
BCAP31	4044.3	5541.6	2988.6	2702.8	2071.8	2856	4551	6442.4
BCAR1	104.5	125.2	115	103.6	91.2	107.1	109.3	135.7
BCAR3	123	142.7	124.3	159.7	132.4	157.5	140.5	196.3
BCAS1	108.8	120.7	95	86.2	76.2	86.9	98.4	107.9
BCAS2	132.9	130.7	96.1	85.1	84.4	98	126.9	174.6
BCAS3	77.5	85.3	97.3	95.2	90.7	93.9	80.3	75.3
BCAS4	110.3	139.6	112.9	106.2	109.7	104.4	107.2	117
BCAS4	100	118.2	111.9	90.7	97.5	90.2	101.9	102.3
BCAS4	163.9	208.4	113.2	105.1	108	100.3	134.7	182.9
BCAT1	737.3	1084.5	537.5	264.3	209.7	438.1	673.7	1423.8
BCAT2	546.4	762.9	482.7	306.1	269.5	324.5	514.6	590.9
BCCIP	3319.2	4294.6	2063.9	1644.9	1153	2035.1	2343.7	3231.3
BCCIP	1477.9	1839.1	542.9	378.7	290	539.1	988.3	1458.1
BCCIP	689.1	927	393.1	345.8	257.3	383.8	558.5	795.8
BCCIP	137.5	158.5	99.9	95.5	77.5	87.9	137.1	167.7
BCDIN3	1551.4	1933.2	1247.3	791.8	763.4	683.7	1376.5	1332.1
BCDO2	84.8	91.8	77.5	80.8	84.4	74	82.5	98.4
BCHE	92.4	98.1	102.1	83	86.2	85.3	96.9	97.2
BCKDHA	2117.2	2681.5	1285.8	1175.8	1094.7	1230.1	1740.7	1449.6
BCKDHB	596.6	850.5	236.9	218.2	248.4	245.9	570.4	713.8
BCKDHB	91.1	111.4	112.8	107.7	95.9	96.8	95	94.4
BCKDK	3008.5	3700.3	1750.7	1156.8	1093.3	1103.6	2518.9	2071.7
BCL10	292.4	357.8	124.5	127.1	114.8	103.9	231.1	374.8
BCL11A	806.3	1049.5	225.6	159.5	126.7	166	639.8	681.8
BCL11A	114.3	147	107.2	86.6	86.4	88.1	120	122
BCL11A	177.2	232	147.1	141	119.3	112.7	163.3	188.7
BCL11B	98.2	110.6	98.3	81.8	86.5	90.2	99.9	98.2
BCL11B	83.3	94.5	93.5	90.9	86	87.7	83.9	96.8
BCL2	123.4	150.1	103.5	106.1	89	115.5	115.8	111.1
BCL2	116.8	130.2	81.2	81.4	89.7	78.8	114.9	130.7
BCL2	1921.5	2396.8	1662	1873.3	1247.6	2262.7	1315.1	2075.5
BCL2A1	228.8	263.9	106.7	100.9	94	106	182.7	206.5
BCL2L1	489.9	590.1	633	477.1	377.9	628.4	560.5	1534.5
BCL2L1	171.4	198.2	129	110.9	121.7	108.5	152.1	196.5
BCL2L10	95.3	107.1	89.2	81.1	84.3	87.4	92	105.7
BCL2L11	107.4	113.9	102.2	104.3	89.1	95.2	100.2	111.5
BCL2L11	101	113.8	109.6	104.5	86.9	108.7	100.8	103.3
BCL2L11	122	134	106.5	105.5	99.3	104	110.9	122.8
BCL2L12	265.7	305.8	195.8	150	139.1	127.6	221.8	271.3
BCL2L12	1946.2	2511.7	1388	787.5	688.4	732	1848.9	2150.5
BCL2L12	1716	1929	914.2	542.2	461.4	548	1512.6	1699.5
BCL2L13	2644.2	3335	2675.9	2874.6	2462	3469.7	2030.6	2534.3
BCL2L14	95.9	118.3	92.6	91.1	93.7	85.7	102.2	125.2
BCL2L14	106.4	124	107	104.6	84.6	86.8	108	116
BCL2L14	98.1	121.4	102.6	110.3	94.7	94.2	96.3	102.1
BCL2L14	75.7	79.5	84.9	78.4	85.4	88.4	75.3	78

BCL2L2	538.6	605.7	272.5	269.4	235.2	329.9	424.8	684
BCL3	156.7	166.1	138.5	111	131.4	116.8	183.4	283.8
BCL6	84.1	106.3	92.7	86.3	91.1	86.6	89.6	94.8
BCL6	353.3	412.9	246.9	317.2	226.7	339.4	356.2	571.9
BCL6B	144.5	195.1	108.6	77.3	82.6	81.2	174	167.7
BCL7A	108.6	135.9	101.8	88.9	81.7	93.2	114.9	145.3
BCL7A	114.2	112.6	98	91.6	87.5	94.2	105.3	124.8
BCL7A	91.6	116	108.9	94.2	85.6	92.6	93.1	118.2
BCL7B	434.9	591.2	272.8	222.3	185.2	285.4	401.3	536.9
BCL7B	687.5	841.1	426.3	402.9	363.1	352.7	649.8	729.4
BCL7C	1038.7	1329.2	882.6	477.5	433.4	587.9	876.3	1415.4
BCL9	160.5	193.6	129.6	101.3	109.4	117.5	173.5	237.3
BCL9L	83.6	109.9	95.7	100.2	95.6	88.8	97.6	121.8
BCLAF1	2857.8	3817.5	1701.5	1938.5	1686.8	2883	2371.7	4049.6
BCLAF1	809.9	1121.4	444.3	462.7	380.4	777.6	652.7	1252.9
BCMO1	76.7	88	84.3	80.4	77.9	79.3	83.2	92.4
BCNP1	98.6	109	101.2	91.3	100.3	97.9	103.1	95.2
BCOR	617.8	868.1	659.2	938.2	675.2	1291.5	588.2	1096.9
BCOR	98.9	122.2	113.4	134.1	135	137.8	87.4	115
BCOR	193.4	199.5	134.4	167.6	160.9	148.8	153.6	226
BCORL1	91.8	110.8	96.5	127.9	108.9	131.5	101.3	143.5
BCORL2	85.7	90.8	95.3	85	75.5	78.7	86.5	91.1
BCR	92.5	97.2	92.3	95.1	86.1	108.6	102.2	98.7
BCR	97.3	109.4	94.9	103	104.3	96.8	91.3	104.8
BCR	122.6	144.6	120.5	140.7	131.7	113.3	133.2	131.9
BCS1L	576.6	656.7	417.6	367	284	411.3	426.5	604.8
BDH	105.7	126.5	79	80.8	82.7	79.1	128.7	121.8
BDH	81.3	101.3	83	79.2	77.6	79.9	98	103.1
BDH	189	257.4	170.3	135.2	116.1	156.7	162.3	176.2
BDH2	87.5	107.2	92.8	93.3	79.2	87.3	103.8	119.9
BDKRB1	87.9	92.7	109.5	98.2	92.6	101.1	89.9	90.1
BDKRB2	101.9	103.2	87.9	89.6	81.8	79.1	91.8	104.9
BDNF	84.5	92.4	87.4	86.1	84.9	87.7	92.9	89.9
BDNF	117.2	120.5	98.2	97.4	96	99.7	117.8	109.7
BDNF	111.3	141.1	94.5	82.6	85.9	87.1	131.3	145.8
BDNF	133.8	143.8	110.5	92.5	86.5	102.7	113.7	133.2
BDNF	85	101.6	100.4	85.8	83.9	98.5	95	87.5
BDNF	162.2	194.4	97.1	89.8	82.7	101	159.2	171.4
BDNF	82.6	107	99.7	90.3	93.1	89.4	94.7	98.6
BDP1	102.4	156.6	105.2	122.6	109.6	97.9	124.2	146.9
BECN1	1269.7	1592.7	354.5	456.4	367.4	546.4	971.6	1352.7
BET1	519.1	732.4	216.7	178.2	180.3	270.1	424.5	598.1
BET1L	157	176.9	129.5	105.9	102	134.1	146.4	189.9
BEX1	6260.4	9089	4665.6	839.1	632.1	890.7	6202.2	6397.6
BEX2	81.2	89.9	101.1	91.3	101.7	96.2	93	85.9
BEXL1	208.8	278.2	136.4	107.9	93.4	122.8	176.5	230.8
BEXL1	304.8	449.6	171.1	137.6	131.3	139.6	341	351.7
BFAR	709.4	860.5	420.8	408.4	306.3	450.8	682.5	1422.6
BFSP1	101	120.6	98.6	80	88	84.4	100	114.8
BFSP2	127.7	144.6	99.7	89.8	94.7	90.2	130.2	141.9

BGLAP	164	181.2	125.1	109.7	98.8	115.3	163.3	165.3
BGN	114.6	129.2	107.6	99.4	86.6	110.7	222.6	281.6
BHLHB2	312.4	347.4	345	1502.4	1633.7	2372.4	282.8	498.4
BHLHB3	89.3	95.4	95.4	99.3	83.7	86.3	87.1	91.8
BHLHB4	89.5	97.7	97.4	82.1	85.6	84.7	93.4	97.9
BHLHB5	5964.7	6949.3	4162.7	4499.6	3715.6	5439	5730.3	8443.6
BHLHB8	103.2	115.5	111.2	102	89.8	97.4	96.5	111.1
BHLHB9	164.6	198.8	121.9	129.6	116.4	149.1	152.6	176.2
BHMT	76.6	74.1	79.6	74.3	72.6	76.5	71.3	79.6
BHMT2	77.8	79.4	79	69.4	68.3	76.3	77.7	76.2
BICC1	81.3	100.2	92.7	95.2	80.1	94.7	85.4	87.1
BICD1	97.9	104.3	88.1	84.3	87	92.5	91.2	86.7
BICD1	95.1	101	115.2	113	98.4	102	97.4	108.7
BICD2	73.8	88.7	82.5	89.5	83.9	83.6	88.1	79.5
BICD2	988.8	1115	677.7	815.5	676.1	1085.2	769.7	1181.8
BID	790.2	1020.7	499.2	606.6	564.5	893.4	607.6	945.2
BID	1315.9	1522	430.3	528.5	412	664.8	1124.5	1465.1
BID	104.1	98.4	73.3	76.5	84.5	91.8	87.7	102.4
BIK	390.1	540.3	469.9	522.8	569.6	650.5	327.2	535
BIN1	292.9	365.7	272.6	312	225.2	438.7	296.4	418.6
BIN1	340.9	471.1	426.2	404.2	300.8	589.3	383.7	567.5
BIN2	142.6	163.6	101.1	104.3	91.3	98.9	129.8	165.7
BIN3	187.8	189.9	134.2	142.8	133.7	131	162.1	226.5
BIRC1	90.4	102.5	95.7	94.9	84.8	83.2	96.8	95.9
BIRC1	132.9	148.4	113.2	183.5	167.4	126.7	109.5	124.5
BIRC2	982.5	1009.7	486.9	597.8	428.7	683	808.5	1423.8
BIRC3	573.2	638	342	733.4	684.1	654.1	594.6	743.7
BIRC3	93	98.6	106.1	105.1	120.4	97.3	90.3	92.3
BIRC3	84.6	92.8	105.8	101.3	89.9	91	79.5	85.4
BIRC4	161.8	170.8	100.4	96.9	92.5	93.1	128.8	177.2
BIRC5	1781.9	2271.7	850.2	498.4	406.7	468.7	1463.3	1775.5
BIRC5	279.9	362.5	314.3	171.6	162.1	193.4	250.6	317.8
BIRC6	183.7	307.1	140.9	189.1	163.5	276	203.4	251
BIRC7	83.6	100.3	109.4	91.1	92.3	91.4	83.3	97.7
BIRC7	106.2	129.1	96.8	94.9	91.2	87	103.6	113.1
BIRC8	84.7	83.1	103.2	93.6	91.2	92	92.5	83.8
BIVM	516.2	671.9	458.1	395.6	333.8	648	469.9	1043.8
BLCAP	502.2	638.7	348.1	432.1	333.3	573.9	353.1	540.1
BLES03	126.1	160.2	111.8	99.9	100.3	121.5	120.3	180.4
BLK	83.8	90.9	104.4	88.3	90.4	88.7	92.1	92.3
BLM	681.5	831.4	461.6	665	501.5	915.1	611.1	872.7
BLMH	1000.5	1410	397	317.2	249.7	305.5	917	1129.4
BLNK	81.7	112.4	97.6	85.3	79.4	94.4	93.3	94.3
BLOC1S1	1285.9	1620.4	376.2	323.4	431	514.9	1046.3	1792.8
BLOC1S2	91	119.1	107.5	87.4	86.5	91.9	101	115.9
BLOC1S2	143	172	111.8	90	104.6	104.9	168.7	202.1
BLOC1S3	86.8	93.5	91.2	97.6	92.3	90	81.7	81.5
BLR1	79.9	86.2	78.5	89.8	76.5	87.5	84.7	87.8
BLR1	148.5	218.8	117.7	125.5	121	128.7	158.9	163.7
BLR1	79.2	83.8	96.2	85	84.4	79.4	77.3	76

BLVRA	1000.7	1218.3	287.7	665.9	495.4	739.7	885.8	954.4
BLVRB	436.1	508.1	146.1	162.7	146.9	172.2	485.5	530.2
BLZF1	266.9	300.7	169.3	273	220.9	196.5	299.8	410.4
BM88	116.9	155.2	92.4	92	93.8	93.7	123.4	129.6
BMF	92.2	98.5	82.4	86.5	103	116.3	82.5	107.8
BMF	73.3	92.4	83.4	90.5	84.2	85.7	88.4	88.2
BMF	87.6	88.2	88.8	87.9	85.4	89.2	90.7	90.4
BMF	107.8	117.8	82.6	90.7	95.7	106.2	110.6	120.1
BMP1	89.5	93.8	108.3	92.7	82.2	101.2	92.8	84
BMP1	129.3	144.2	112	105	90.4	100	133.5	142.2
BMP1	203.3	264	275.1	192.8	188.9	219.3	176.2	262.4
BMP10	76	84.7	84.8	88.2	72.3	81	86.6	98.5
BMP15	93	107.3	105.8	103.1	88.9	103.9	90.2	91.8
BMP2	167.9	189.9	156.7	126.7	123	134.6	152.5	198.7
BMP2K	200.6	261.1	145.5	143.3	123.5	170.8	136.8	193.4
BMP2K	146.3	164	112.1	110.8	107.1	110.2	129.5	155
BMP2K	123.2	150	120.4	122.3	123.8	141.9	121.1	158.1
BMP3	113.8	135.7	89	81.5	81	87	111.2	130.4
BMP4	229.6	308.9	193.2	118.3	105.5	131	237.1	363.7
BMP4	99.9	94.7	77.4	76.6	81.3	76.9	96.4	92.9
BMP4	115.1	155.8	96.8	82	81.6	79.7	141	145.3
BMP5	142.9	164.7	94.6	96.4	93.6	89	134.5	158.3
BMP6	101	139	90.2	99.1	78.6	106.9	112.3	165.2
BMP7	107.4	120.6	94.3	102.9	81.8	92.5	112.6	124.9
BMP8A	96.8	121.1	100.4	109.7	106.4	92.9	95	96.2
BMP8B	940.9	1457.7	791.5	301.4	310.8	468.9	520.3	684.6
BMPER	92	106.2	94.7	85	75.1	80	89.5	111.6
BMPR1A	105.3	122.9	90.8	87.6	86.3	90	106.3	108
BMPR1B	89	97.3	89.6	85	77.6	87.6	92.2	92.3
BMPR2	209.4	343.5	142.6	177.8	159.3	205.2	222.9	382.2
BMS1L	1185.9	1469	866.4	672.2	586.4	988.4	924.7	858.3
BMX	94.8	101.9	100.8	90.3	94.7	84.9	94.5	99.4
BMX	84.7	100	96.4	99	88.6	101	90.9	93.3
BNC1	126.6	144	100	102.4	85.8	97.3	125.5	129.1
BNC1	73.4	84.3	83.1	81.9	84.9	83.6	77.7	78.8
BNC2	95.4	100.9	93.7	88.8	85.3	83.8	94.3	113
BNIP1	230.6	304.5	141.5	123	130.9	146.6	274.2	299.3
BNIP1	312.2	428.4	279.2	169.5	199.8	239	318.6	390.5
BNIP2	1618.9	2014.4	1153.6	1582	1081.9	2156.9	1453.3	3072.9
BNIP3	2570.6	3409	1440.4	1431.1	1334	2169.8	2186	3167.5
BNIP3L	113.2	129.4	83.6	98.4	90.7	115.7	121.2	210.8
BNIPL	119.8	141.2	116.2	112.2	102.8	112	109.4	106
BNIPL	137.1	148.8	122.5	115.1	99.8	108.8	140.7	146.7
BNIPL	90.9	108.1	76.5	67.9	76.2	78.8	83.7	106.9
BOC	79.1	85.4	78.1	84.8	77.4	87	81.2	78.2
BOK	134.7	157.8	140.1	115.1	116.5	130	150.1	159.7
BOLA1	209.8	233	111.6	104.7	94.1	119.7	168.2	202.4
BOLA2	4157.1	4780.8	2981.1	2538	1850.6	2809.1	3427.5	3486.8
BOLA2	726.5	743.5	360	317.8	238.6	291.8	650.2	821.6
BOLA3	6540.8	7089	5065.4	4576.2	3268.2	4396.6	6145.4	8085.4

BOLA3	6630.7	7512.1	5662	5145.5	3550.1	4918.7	6198.4	8273.6
BOLA3	113.9	142.2	103.7	95.6	92.5	84.8	117.7	134.1
BOLL	86.2	84.2	93.8	76.7	72.1	87.7	83.5	82.5
BOLL	95.8	100.7	98.4	95.6	98.2	101	89	92.7
BOLL	77.5	78.3	90.1	83.4	81	84.8	75.2	87.8
BOP1	6247.4	7847.1	3325	1896.1	1539.6	1972.1	4871.4	4362.8
BP75	584.3	754.8	276.2	226.4	178.5	307.3	491.2	628.6
BPGM	255.9	332.1	160.1	154	119.6	189.1	204.5	269.3
BPGM	83.4	89.4	83.9	89.5	84.8	82.5	87.6	88.5
BPHL	210.7	275.2	201.7	181.4	158	197.3	191.1	243.4
BPI	96.7	113.2	91.3	91.5	81.4	92.2	99.4	110.3
BPIL1	89.9	101.5	80.7	83.5	78	92.1	84.1	97
BPIL2	83.5	93.9	100	91.8	82.1	89.7	85.3	97.9
BPIL3	80	94.6	80.1	88.5	82.2	82.1	86.9	98.7
BPNT1	338.2	422.2	239.6	191.1	172.1	126.8	251.2	397.3
BPY2	76.2	84.1	95.4	95.1	88.2	80.3	72.9	79.1
BPY2B	135.3	126	105.8	90.6	86.5	92.7	127.4	122.7
BPY2IP1	217.3	254.3	332	240.9	201.6	331.1	213	401.2
BRAF	135.8	158.6	115.6	119.2	106.9	116.3	131.1	196.9
BRAP	112.6	127.5	92.3	85	66.5	84.5	109.5	146.7
BRCA1	1205.7	1420.6	430.5	351	251.1	485.9	816	954.3
BRCA1	1489.5	1978.4	514.3	465.4	395.6	539.1	992.1	1159.6
BRCA1	142.4	152.8	94.9	91.7	87.9	74.9	127	172.6
BRCA2	109.3	119.1	105.9	110.9	87	111.6	128.5	132.5
BRCC2	103.7	115.7	109.3	93.5	93.8	103.2	97.8	107.3
BRD1	177.6	222	198.7	192.6	201.8	277.4	169.5	244.5
BRD2	3161.1	4124.8	2360.1	2207.9	2132.5	2493.7	3088.6	3085.5
BRD3	837.2	1268.1	1077.4	1172.5	931.7	1157.7	838.8	1621.7
BRD4	122.4	143.7	111.2	90.8	92.2	92.2	115.3	135.4
BRD4	84.5	102.2	109.8	102.3	102.1	115.7	90.6	98.8
BRD7	1295.7	1603	639.6	620.7	454.2	803.1	1046.5	1548.5
BRD8	642.5	832.4	363.4	405.5	399.1	657.1	628.6	1142.9
BRD8	79.9	81	98.3	83.4	74.3	81.4	77	93.9
BRD9	2281.4	2547.8	1644.5	1706	1528.4	1993.5	1811.5	2108.3
BRD9	130.9	145.1	100.8	101.7	82.1	89.4	120.8	132.5
BRDG1	98.2	88.2	97.8	100.7	94.1	91.1	93.8	100.4
BRDT	71.3	81	81.7	82.9	81.7	85.9	76.4	81.4
BRDT	69.2	84.3	84.6	83.3	82.4	84.3	76.5	78.7
BRDT	109.5	114.9	88.1	84.5	79.4	91.4	92.8	113.5
BRE	1025.9	1486.2	765	589.9	454.1	543.5	901.3	1166.8
BRE	674.5	963.7	449.4	469.6	343.8	411.6	565.9	968.3
BRE	101.9	111.5	95.2	89.1	81.7	85.7	90.7	88.5
BRF1	106	118.1	89.4	95.5	88.1	94	105.8	114.3
BRF1	124.4	157.9	155.8	120	119.1	165.5	110.4	167.2
BRF1	103	120.3	109.6	101.8	101.8	93	96.5	110.4
BRF2	672.6	819.3	613.2	491.4	397	689.9	723.7	1555.8
BRI3	2557.4	2785.1	2066.8	2378.4	1470	2770	2532.4	5419.2
BRI3BP	217.1	221.9	134.5	121.2	119.2	107.6	179.1	224
BRI3BP	1464.6	1796.1	746.8	804.9	557.6	685.6	1345.5	1415
BRI3BP	746.1	974.4	334	292.9	275.6	275.7	667.2	703.9

BRIP1	118	112.3	97	97.1	90.2	110	106.2	100.9
BRMS1	2577.8	3508.1	2214.1	1722.2	1360.9	1417	2430.8	2463.3
BRMS1	364.7	461.1	327.6	171.9	172.6	162.4	335	436.2
BRMS1L	104.8	114.2	98.9	90.5	93.4	95.1	102.1	105.5
BRP44	2213.9	2413.8	1353.5	1285.1	1026.8	1466	1950.2	3456.5
BRP44L	6416.6	8323	2915.3	2717.7	2428.7	3241.2	6481.8	7931.1
BRPF1	268.7	352.8	272.3	264	231.9	369.5	287.8	501.3
BRPF1	3108.3	3381.6	3223.2	3779.5	3042.1	3914.1	2686.4	4030.4
BRPF3	510.7	647.9	366.5	537.2	443	612.4	504.4	655.6
BRRN1	129	171.2	98	85.1	81.3	98	138.4	146.5
BRS3	98.9	105.6	99.8	89.6	82.8	92.1	88.1	108.1
BRSK1	517.2	558.6	513.6	418	378.5	348.2	621.7	1180.5
BRSK2	117.6	125.7	98.9	84.6	77	89.7	106.4	119.3
BRUNOL4	86.2	97.6	97.3	104.8	99.6	101	83.2	82.3
BRUNOL4	83.3	103.8	92.4	89.7	80.6	79.5	89.7	103.8
BRUNOL5	89.5	98.7	97.7	90.2	93.7	92.6	84.4	89.6
BRUNOL6	91.1	112.5	112.7	97.1	93.2	96.8	99.7	96.1
BRWD1	73.5	88.5	88.9	81.5	81.2	79.8	79.3	77.8
BRWD1	185.5	204.2	89.4	86.4	92.2	96	168.2	201.3
BRWD1	757.7	1075.3	458.8	478.3	396.3	635.7	655.4	854.7
BRWD1	81.8	98	101.1	105	97.2	98.6	83.5	111.8
BRWD2	207	235.7	116.5	103.7	97.6	122.3	191.8	241
BRWD3	109.5	139.1	90.1	96.5	96.7	105.7	124.5	173.3
BSCL2	711	902	404.9	288	288.7	317.8	577.7	571.8
BSDC1	265.4	333	213.6	252.6	214.5	276	285.5	468.6
BSG	2557	3724.6	2960.4	2508.3	1873	3032	2589.5	4466.1
BSG	115	126.4	88.3	101.9	95.3	91.8	120.4	122.9
BSN	111.1	134.7	106	108.3	103.1	105.8	113.8	114.1
BSND	135.6	152.8	107.7	95.4	79.7	86.7	112.6	134.7
BST1	501.7	696.1	361.1	368.7	295	428.6	505.3	554.9
BST2	1586.7	1801.2	799.1	650.2	562.6	712.3	1326.1	1352.5
BTAF1	967.4	1117	717	939.5	827.7	1677.8	671.8	966.1
BTBD1	516.3	697.9	180.9	207.7	168.5	338.6	399.6	664.9
BTBD1	542.7	833.3	218.2	194.1	161.6	231	324.5	533.7
BTBD10	476.2	640.6	217.6	201.8	149.6	358	406.8	694.4
BTBD11	109.7	133.7	98.6	82.3	84.9	79.5	114.4	114
BTBD11	100.1	95.5	94.1	89.2	80	85.1	88.1	97.5
BTBD11	82.2	71.3	90.2	78.8	71.5	90.3	66.5	96.9
BTBD11	88.1	98.5	94	94.6	84.2	87.1	90	99.4
BTBD12	559.2	634.2	298.9	298	244.7	241.7	484.3	405.7
BTBD14A	481	595	333.9	236.4	229.1	253.1	386.7	342.4
BTBD14B	118.3	152.6	106.8	89.2	96.6	104.6	112.8	137.2
BTBD3	126.2	127.5	98.4	93.9	89.3	92.7	121.6	121.4
BTBD3	201	248.2	169.3	164.8	117.9	145.9	184.6	284.5
BTBD4	180.8	212.1	231.6	358.8	317.9	428.2	184.9	287.7
BTBD5	205.3	276.9	202	282.5	223.8	233.7	219.9	344.5
BTBD6	1477.9	1544.9	734.2	517.2	449	538.2	1243.8	1510.7
BTBD7	428.9	656.5	391.5	396.5	328.4	428.5	453.6	826.4
BTBD7	139.4	147.9	88.5	102.1	93.2	89.6	110.9	121.3
BTBD7	301.7	412.2	178.3	192.4	157.6	175.3	232.4	282.1

BTBD8	83	95	90.9	83.2	79.6	78.2	92.1	98.3
BTBD9	145	164.1	99.6	108.2	95	104.8	132.8	142.4
BTC	85.8	85.7	99	87.7	95.1	94.8	84.2	82.2
BTD	192.2	210.2	131.3	128.3	131.2	127.6	160.7	211
BTF3	24906.5	28664.1	18644.5	18243.9	12843.8	17202.1	24961.9	30463.3
BTF3	25574.8	26401.5	25758	23955.5	19414.4	23659.6	21857.8	30464.6
BTF3	377.5	524.8	231.1	210.6	168	211.4	303.4	343.3
BTF3L4	975.5	1289.3	424.9	410.2	315	530.7	780.8	1116.8
BTG1	1440.7	1739.1	635.4	940.7	789.6	1054.5	1199.8	1939.6
BTG2	144.6	186.8	148.7	140.6	162	184.8	185.1	273.3
BTG3	359.5	388.4	212.9	229.8	169.8	216.7	323.3	667.1
BTG4	97.5	124.2	83.6	86.4	82.7	74.2	92.6	102.4
BTK	2602	3244.9	735.5	749	550.4	917.7	2074.2	2273.5
BTLA	103.3	113.4	96	104.5	86	94.8	82.9	96.8
BTN1A1	67.5	75.8	83.4	83.7	77.1	87.8	80.3	78.3
BTN2A1	95.3	103.5	86	96.8	83.6	79.3	85.1	109.5
BTN2A1	480.9	547.9	289.3	276.7	288.1	294.2	390.4	512
BTN2A2	117.3	128.4	127	107	113.1	125.1	114.1	128
BTN2A2	94	113.1	86.7	86.7	85.2	77.3	95	85.2
BTN2A3	78	85.9	96.9	90.5	80.5	84.2	80.6	88.6
BTN3A1	89.2	103.8	96.2	91.1	82.9	84.1	89.5	87.1
BTN3A1	101.8	114	109.9	101.2	99.2	107.8	114.1	114.7
BTN3A2	185.2	224.3	128.2	141.1	98.2	178.7	135.7	224.9
BTN3A3	152.8	170.1	111.2	113.1	93.1	109.8	135.5	158.7
BTNL2	148.1	186.1	109.2	90.9	88.7	94.7	146.2	184.8
BTNL3	92.8	112.3	82	91.2	78.8	79	93.2	101.8
BTNL3	81	95.9	87	83	84.1	84.6	88.5	84.8
BTNL3	87.3	105	107.6	86.5	80.2	99.4	90.3	93.9
BTNL8	77.8	89.2	81.6	84.5	68.5	82.3	85.8	84.1
BTNL9	97.4	105.3	104.7	87.9	82.4	86.8	107.6	130.7
BTRC	116.4	168	107.7	92	83.2	84.4	127.6	160.7
BTRC	86.4	101.5	94.9	103.9	86.9	97.4	93	104.6
BUB1	1842.8	2080.2	1051.4	846.2	720.9	1431.9	1279.2	2330.3
BUB1B	366.2	436.8	139.8	120.1	122.7	131.1	260.2	496.8
BUB3	8195.1	10605.1	6472.6	4217.3	3224.6	4266.1	6828.1	7481.6
BUB3	94.5	100.5	98.6	95.3	92.9	100.4	93.1	114.8
BUB3	1711.6	2469.9	442.4	250.1	171.9	242	1436.1	2358.1
BVES	87.4	86.5	101.4	83.9	81.7	83.3	90.3	96.5
BVES	106	127.6	103.6	90.6	86.8	85	111.4	122
BVES	349.9	551.6	338.9	323.8	263	428.4	333.8	496.3
BXDC1	5502.4	6838.8	3264.4	2839	1904.1	3332.8	5095.8	6389.8
BXDC2	2112.5	2684.6	757.4	589.1	396	718.2	1511.1	2385.3
BXDC5	901	1055.6	352.5	392.2	261.5	462.9	791.8	1407.8
BYSL	1348.8	1706.1	916.3	522.6	327.7	624.1	1085.3	2073.2
BZRAP1	105.6	111.7	120.8	181.5	174.7	229.8	94.9	119.4
BZRPL1	84.7	85.2	80.4	81.7	77.4	80.2	81.4	82.1
BZW1	123.3	131.7	110.8	103.5	97.4	108	123.1	140.1
BZW2	3670.6	4409.7	2153.4	2398.5	1531.6	2693.1	2624.5	4528
C10ORF10	104.1	132.8	109.7	94	88.2	103.6	130.1	145.3
C10ORF104	404.8	511.4	226.7	288.8	212.3	290	407.4	681.7

C10ORF107	86.8	101.4	95.4	91.3	87.2	93.7	94.9	90.8
C10ORF11	492.1	652.1	236.8	187.2	163.6	226.3	505.8	976.7
C10ORF111	83.1	84.3	97.1	88	83.4	90.1	88.9	87.1
C10ORF113	86.1	96.9	84.3	83.6	68.7	82.5	87.3	100.8
C10ORF114	233.7	263.6	161	126.2	128.1	136.1	198.2	242.3
C10ORF116	140.4	169.8	116.7	97	99.4	106.8	150.5	152.7
C10ORF118	107.9	154.2	85.4	90.9	78.8	86.7	108.8	135.2
C10ORF119	196.1	240.8	168.9	141.7	139.1	185	152.6	206.9
C10ORF12	124.8	134.4	109.5	103.6	93.8	104.4	127.4	135.6
C10ORF120	82.9	78.4	82.2	79.3	81.8	84.6	79.2	84.9
C10ORF125	347.6	474.7	264.1	211.4	168.5	217.9	275.3	372
C10ORF129	97.6	107.9	91.6	76.3	78.2	84.8	100.9	108.1
C10ORF13	102.7	142.4	105.2	101.3	93.8	93.5	105.7	123.4
C10ORF130	86.9	92.1	92.4	79	91.7	78.2	87.6	99.5
C10ORF132	80.3	83.9	93.3	92	93.6	76	75.7	91.7
C10ORF137	401.3	465	216.5	184.9	173.7	225.9	343	438.3
C10ORF22	740.2	880.4	412.4	352.5	267.6	450.6	620.6	894.5
C10ORF25	95.3	100.8	78.3	74.3	75.7	86.2	95.4	105.5
C10ORF26	216.8	308	198.8	197.4	187.9	253.3	197.4	276.9
C10ORF27	94.4	99.9	94.7	93.6	89.5	94.8	98.1	94.2
C10ORF28	224.2	296.7	139	128	124	139.5	211.7	307.5
C10ORF30	106.8	119.4	98	93.5	80.9	85.8	100.7	101.3
C10ORF32	483.5	636.8	347	350.1	292.4	363.4	455.4	872.4
C10ORF33	324.6	440.7	260.6	186.7	169.5	188.5	248.9	313.7
C10ORF35	250.9	311.2	205.8	177	177.8	210.7	273.2	264.4
C10ORF37	108.1	115.2	99.4	92.8	98.3	86	121	108.5
C10ORF38	188.7	211.7	156.2	148.9	114.5	156	183	207.5
C10ORF4	96.4	105.1	102.5	102.2	91.5	102.8	92	100.5
C10ORF4	92.3	121.7	81.1	86	82.9	95	92.6	104.2
C10ORF4	134.3	129.1	99.2	89.7	96.5	91.8	119.1	130.3
C10ORF4	98.2	94.5	106.2	96	86.4	86	93.8	110.7
C10ORF4	80.7	92.3	92.4	91.2	92.1	96.8	83.9	89.1
C10ORF42	288.9	402.6	237.8	221.8	163	328.5	215.7	341.4
C10ORF46	139.2	163.7	105.8	100.6	109.3	96.2	108.7	157.7
C10ORF47	275.7	358.3	201.9	139.5	128.8	164.9	249.9	342.3
C10ORF49	95.2	96.8	101	92.8	86.8	87.2	87.3	97.8
C10ORF53	96.3	96.3	92.1	84.8	79.1	90.5	92.2	107.9
C10ORF53	83.3	93.7	91.8	93.5	76.1	84.7	85.9	91.4
C10ORF53	82.1	89.4	110.9	96	99.4	91.1	93.4	90.5
C10ORF54	396.2	460.9	331.5	270.9	226.3	238.6	306.9	322.3
C10ORF55	127.6	152.4	109.1	96.4	93.7	87.5	122.8	143.3
C10ORF56	225.6	316.9	211.6	169.6	157.4	170.2	301.8	535.1
C10ORF57	497.8	633	234.2	219.8	182.9	207.7	379.4	417.7
C10ORF58	4821.9	5573.6	2284.2	1654.2	1993.2	1792.8	4397.7	4391.2
C10ORF59	81.5	91.7	86.3	82.2	81.9	92.9	80.1	96.2
C10ORF59	373.9	467	242.5	230.9	193.4	263.4	345.7	470.3
C10ORF59	209.2	294.9	144.4	128.4	106.7	143.9	196.3	268.1
C10ORF6	341.3	561.8	453.7	547.5	455.2	730.9	334	411.2
C10ORF61	631.6	914.4	374.1	243.9	179.3	269.5	412.4	600
C10ORF61	94	94.5	92.9	87.5	84.8	87.1	88.9	89.3

C10ORF61	257.7	312.4	163.8	140.1	110.8	171.9	208.4	300.1
C10ORF62	80.6	88.1	89.1	79.2	87.3	96.5	71.7	82.7
C10ORF63	86.3	93.5	91.8	87.7	80.8	83.1	80.5	83.2
C10ORF65	83.1	78.5	86.4	79.1	66.2	80.2	75	98
C10ORF67	96.6	101.5	83.6	75.1	75.3	80.9	90.9	98.1
C10ORF68	76.8	81.6	88	92.6	87.2	84.6	80.1	86.3
C10ORF7	2858.8	3934.3	1871.3	1063.3	852.9	1246.3	2323.9	3709.4
C10ORF71	80.8	83.5	79.6	80.7	78.7	82.3	88.6	88.6
C10ORF72	119.6	136	105.9	105.9	93.1	94	112.3	122.8
C10ORF72	121.7	124.4	101.2	98.7	84.5	89.8	114.3	127.5
C10ORF72	120.3	136.4	104.8	93.5	84.2	85.5	111	124.8
C10ORF76	228.3	268.9	290.6	239.3	192.3	291.4	238.2	390
C10ORF77	178.6	216.4	136.2	110.7	94.6	112.8	178.2	192.5
C10ORF78	126.2	161	115	101.8	102.7	99	135.3	155
C10ORF79	81.9	104.5	105.6	89.7	82.6	82.3	85.2	96.1
C10ORF80	93.2	99.4	105.9	102.5	81.4	100.2	91.2	101.2
C10ORF81	93.9	105.8	104.4	96	89.2	94	90.8	98.6
C10ORF82	121.4	149.2	90.7	94.5	70.5	84.5	112.7	119.8
C10ORF83	91.3	110.9	95	90.8	84.6	84.3	91.5	94.8
C10ORF84	152.6	215.9	131.6	121.7	96	120.1	146.5	163.7
C10ORF85	96.1	96.8	104.6	96.3	105.2	90.9	92.6	103.3
C10ORF86	3244.7	3672.6	1605.2	1109.3	749.2	1105.3	2092.5	2604.9
C10ORF87	151.8	198.4	120.9	109	111.6	115.4	145.6	157.9
C10ORF88	170	192.3	96.3	87.8	98	106.5	171.1	258.1
C10ORF89	191	232.9	124.6	111.8	104.3	121.9	186.2	231.9
C10ORF9	258	295.3	143.6	130.9	128.3	153.2	263.6	459.4
C10ORF9	77.2	91.5	85.5	78.1	78.3	76.2	84.4	85.3
C10ORF90	86.9	105.8	89.1	77	75.6	85.8	100.8	103.1
C10ORF91	94.9	103.9	99.5	95.6	98	97	93.2	93.7
C10ORF92	97.1	113.6	109.1	102.5	97.6	97.8	99.9	97.1
C10ORF93	70.8	84.9	97.3	80.7	84.8	93.8	85.5	83.4
C10ORF93	79.9	91.6	79.1	76.1	76.4	67.6	73.6	81.7
C10ORF95	103.6	109.8	111.4	98.2	86	93	104.7	113.7
C10ORF96	90	94	92.7	86.2	79	87	89.5	95.3
C10ORF97	531.5	624.9	235.5	238.1	210	279.5	468.9	656.3
C10ORF99	98	107.7	104.3	94.3	91	90.8	99.3	110.4
C11ORF1	1666.3	1962.7	1306.5	906.9	639.8	1032.5	1532.1	2782.2
C11ORF10	13332.4	15046.3	11198.4	6588	5501.7	7190.5	13125.7	15552.2
C11ORF11	178.6	251.6	179.4	142.7	137.5	142.9	197.5	178.2
C11ORF16	189	221.6	103	102.8	93	97.4	172.7	195.3
C11ORF17	123.3	125	102.6	87.3	85.6	95.8	116.2	123.2
C11ORF17	1151	1659.6	953.5	526.5	492.1	695.2	956	1395.5
C11ORF17	739.3	1082.5	444.2	399.5	330.1	494.7	709	930.3
C11ORF17	108.5	125	88.2	71.8	77.2	86.9	98.3	130.3
C11ORF17	1590.6	2417.5	1217	1101	844.9	1306.1	1727.9	2174.7
C11ORF2	1761.2	2191.5	1584.8	1263.2	1137	1100.2	1663	1261
C11ORF24	400.7	487.2	186.5	157.9	129.9	163	367.2	386.2
C11ORF30	127.2	134.7	108.4	115.7	108	111.8	101.3	123.2
C11ORF31	201.2	262	199.9	185	161.7	213.4	181	233.7
C11ORF35	98.4	126.2	109	144.4	126.2	183.8	103.9	150.9

C11ORF36	87	84.5	85.5	83.3	87.2	84.1	87.1	96.9
C11ORF38	341.3	413.9	184.9	181.8	135.3	151.1	278.1	395
C11ORF40	90.7	100.4	102.8	88.1	85	87.2	91	93.9
C11ORF42	84.6	102.2	89.9	88.4	84.4	95.9	87.1	104.6
C11ORF44	93.8	97.4	99	98.8	86	85.9	98.1	101.1
C11ORF45	155.7	193	168.9	143.6	128.1	166.7	118.6	141.3
C11ORF46	1296.2	1461.5	644.3	523.3	408.2	553	847.2	1535.6
C11ORF47	113.8	142.3	118.2	120.7	124.3	153.7	101.7	127
C11ORF48	1296.5	1504.9	628.1	517.5	401.4	616.9	1060.1	1111.4
C11ORF49	102.1	118.7	99.3	88.2	89.6	96.2	96.1	99.6
C11ORF49	150.8	180.2	143.4	112.3	93.9	102.7	122	156.6
C11ORF49	262.2	361.6	158.3	125	119.1	120	205.7	262.1
C11ORF49	102.7	120.8	100	96.8	88.4	90.3	94.6	115.8
C11ORF51	607.8	863.3	277.2	182.2	178.9	251.9	588.1	747.4
C11ORF52	88.4	92.6	92.3	106.5	100.5	93.7	83.9	105.4
C11ORF53	80.3	106.8	93.8	95.1	98.6	94.2	89.4	91.6
C11ORF54	346.5	463.4	288.9	353.2	319.1	403.8	347.9	631.8
C11ORF55	149.9	185.9	98.4	89.3	91.9	96.7	151.9	170.2
C11ORF56	292.1	349.4	369.7	390.9	364.3	710.7	231.6	365
C11ORF61	120.2	157.9	96.2	102	100.1	96.6	125.1	154.1
C11ORF63	120.1	129.7	99.1	103.8	94.4	97.9	114.4	145.5
C11ORF63	149.4	158.4	110.7	102.4	95.6	100.7	125.3	141.9
C11ORF63	194	217.5	160.8	152.4	171.8	152.6	195.5	292.2
C11ORF65	142.2	171.7	96.6	90.7	88.6	102.6	131.2	168.4
C11ORF70	78.6	85.5	90.1	75.5	81.4	88	84.2	81
C11ORF71	133.8	138.7	117.2	114.3	115.1	141.4	110	137.4
C11ORF76	67.3	78.1	80.4	88.1	79.1	89.6	76	75.2
C11ORF77	181.8	187.5	132.4	131.7	123.6	127.8	138.4	225.5
C11ORF9	94.8	99.8	86.3	89.4	82.7	97.2	93.3	93.2
C12ORF10	1413.4	1804.3	793.6	496.1	393.8	487.8	1297.9	1224.3
C12ORF11	190.1	219.5	100.4	123.2	88	101.7	165.7	294.2
C12ORF12	81.4	99.7	102	88.3	95.9	97.6	91.3	103.8
C12ORF23	138.3	166.9	98.8	93.3	91.8	102.9	130.7	174.1
C12ORF24	1585.5	2186.6	830.7	579.6	496.4	754	1024.8	1113.5
C12ORF25	101.2	109.3	97.1	90.7	81.3	92.3	109.2	92.7
C12ORF26	573.3	707.9	350.3	461.8	348.6	464.7	421	649.5
C12ORF28	95.5	104.5	98.3	98.3	89.5	97.1	98	103.5
C12ORF29	591	684.5	294.1	340.9	211.7	398.2	522.3	890.6
C12ORF30	115.2	109.2	108.9	102	93.2	100.8	90.8	110.5
C12ORF31	1202.9	1713.9	439.2	565.5	421.1	420	1055	1737.9
C12ORF32	612.2	739.9	506	485.8	365.1	501.6	428.4	835
C12ORF34	113.4	137.1	125.5	113	94	110.7	111.9	147.7
C12ORF35	266.1	355.3	116.5	124.1	101.6	189.2	303.9	518.9
C12ORF36	84.1	98.4	95.1	79.7	86.4	89.8	86	91.4
C12ORF38	97.5	107.6	93.3	82.5	79.7	86	95.4	91.4
C12ORF39	82.4	106.6	95.2	102.2	92.3	85.9	81.6	84.2
C12ORF4	236.2	329.5	227.9	229.4	164.7	293.9	216.3	355
C12ORF40	82.7	92.5	92.9	91.9	74.8	90	87.4	89.6
C12ORF40	85	104.9	77.4	74.3	81.1	85.8	86.4	95.4
C12ORF41	1423	1887.4	925.8	790.9	668.5	940.1	1169.4	1395.3

C12ORF42	67.9	75.7	79.2	77.2	75.5	75.7	75.7	79.9
C12ORF43	223.9	320.5	170.8	179.7	128.7	245	228.5	356.9
C12ORF44	443.1	639	458.4	348	313.3	390	510.6	753.7
C12ORF45	409.8	526.2	255.4	190.9	160.8	237.6	392.7	370.6
C12ORF46	263.5	326.5	185.3	190.6	172.6	259.4	227.6	274.6
C12ORF47	1217.2	1463.8	507.4	504	532.9	664.1	966	1081.9
C12ORF48	726.5	783.3	370.5	285.6	264.3	424.2	558.2	858.2
C12ORF49	223	304.6	167.9	138.8	126.4	113.5	189.1	288.2
C12ORF5	258.2	300.1	136.2	116	99.7	130.4	227.6	306.7
C12ORF50	88.5	92.6	104.3	88.3	84.4	94.1	88.7	93.1
C12ORF52	1045.1	1396.7	512.8	411.7	332.1	449.5	894.8	890.5
C12ORF53	81.6	98.5	81.7	87.2	86.5	78.9	87.4	90.3
C12ORF54	102.5	140.6	106.1	85.7	81.5	95	101	123
C12ORF57	2323.1	2683.2	1518.4	977.3	784.2	1093	1876.4	2292.6
C12ORF58	167.2	189.4	94.5	100	99.6	134.5	134.4	151.9
C12ORF59	106.5	114.4	88.2	86.9	80.2	97.1	108.1	103.8
C12ORF60	109.4	124.7	117.2	103	84.5	96.1	102.2	139.3
C12ORF61	95.7	108.3	86.4	85.2	81.5	82.9	88.9	100.1
C12ORF62	429.4	489.8	187.2	168.4	134	164.1	410	497.3
C12ORF64	89.2	104	101.9	82.7	90.1	99.9	98	97.8
C13ORF1	291.9	379.2	154.7	170.4	139	193	294.1	421.8
C13ORF10	478.5	613.1	224.9	253.9	231.4	438.6	420.7	678.5
C13ORF16	82.9	95.1	93.9	89.6	84.2	91.3	88.9	98.5
C13ORF18	1846.5	2553.2	685.3	816.1	713.2	1037.3	1253.8	1718.1
C13ORF21	73	93.4	99	74.6	71	76.2	89.2	84.7
C13ORF23	683	798.4	602.1	808.7	484.9	889.9	684.1	1336.4
C13ORF23	516.7	628.7	462.8	602.9	433.7	675.7	498.8	1026.2
C13ORF23	71.3	78.7	91.7	78.7	76	88.6	78.7	84.2
C13ORF24	141.8	177.9	129.1	143.3	120.6	189.2	143.5	198.2
C13ORF26	78.7	77.5	80.9	76.4	79.6	90.3	72.8	90.6
C13ORF3	689.1	831.3	237.7	207.6	192.8	258.3	492.9	738.6
C13ORF7	741.7	1118.3	349.6	360.6	238.1	527	659.2	1161.7
C13ORF8	2304.4	2898.2	1807.1	2156.4	1367.8	2224.6	1808.4	2166.6
C14ORF1	133.5	145.5	92.9	92	84.9	85.3	106.4	146.8
C14ORF10(	995.6	1255.3	454	323.7	273.3	471.5	958.1	2407.9
C14ORF101	167.4	177.8	104.2	97.6	101.7	105.8	139.2	155.2
C14ORF10:	824.9	880.1	600.2	792.5	730.8	799.8	869.5	1040.6
C14ORF10:	280.4	350	258.6	294.5	291.7	481.2	258.2	379.1
C14ORF10:	143.2	178.8	126.3	114.1	115.5	140.7	153.3	199.2
C14ORF10⁄	459.8	492.7	222.2	145.8	151.2	198	330.5	790.4
C14ORF10!	71.6	83.5	98.9	91.4	89.2	88.3	82	81.9
C14ORF10(	2081.8	2266.8	870.4	1185	879	2014.9	1438.9	2270.5
C14ORF10{	729.9	827.9	263	290.3	211.7	332.4	583.6	881.1
C14ORF11	159	186.5	104.4	91.4	74.1	89.7	158.5	207.2
C14ORF11:	2343.8	2754	1360.5	1218.3	924.7	1374.1	2041.6	2967.6
C14ORF11!	118.9	142.9	95.1	91	87.2	93	105.3	133.5
C14ORF11{	122.1	156.6	113.5	121.3	113.4	112.4	131.1	149.5
C14ORF11{	211	250.2	159.6	171.8	147.8	196.9	172.3	239.7
C14ORF11!	153.9	177.1	107.1	97	87.3	82.8	150.2	190.4
C14ORF12(	1265.4	1782	493	375.9	304.4	545.4	927.5	1207.8

C14ORF121	103.4	130	124.4	112.5	106	109.7	102.8	111.8
C14ORF122	322.7	356.9	196.9	169.9	159.6	163.7	312.4	295.8
C14ORF124	383.6	438.2	153	126.1	121.4	121.1	284.9	295.2
C14ORF126	255.9	314.6	176.6	132.3	119.4	119.1	188.6	238
C14ORF129	206.5	251.6	135.1	121.6	116.2	149.4	209.2	326.6
C14ORF130	647.5	805	401.4	273.6	229.5	327	477.7	796.7
C14ORF130	1336.2	1798.6	670.4	542.6	469.1	839	1023.3	2121.5
C14ORF130	100.3	139.9	83.4	81.2	82.6	87.3	107.2	128
C14ORF131	815.3	950.8	577.2	534.6	506.1	608.4	609.7	612.3
C14ORF133	225.7	352.2	230.2	149.2	153.8	184.1	212.2	261.1
C14ORF135	903.9	1232.1	574	605.2	453.7	809.5	751.9	1128.9
C14ORF138	86.6	82.8	93.4	85.8	89	88.6	83.4	86.8
C14ORF138	384	469.6	243.8	193.3	155.8	236.1	302.5	448.6
C14ORF138	438.9	492	262.6	258.8	206.4	269.9	395.3	642.6
C14ORF139	286.9	403.8	237.7	225.8	197.7	239.5	309	318.7
C14ORF140	89.4	113.7	96.4	96.5	91.9	90.1	91.5	104.8
C14ORF140	100	102.3	98.3	92.2	82.5	86.8	93.9	104.2
C14ORF142	1352.3	1573.9	409.3	366.5	169.2	327.6	1102.9	1669.5
C14ORF143	524.1	585.9	271.9	171.7	137.4	198.9	497.9	718.4
C14ORF145	339.5	453.8	183.5	129.9	152.9	207.6	272.3	319.3
C14ORF147	597.7	776.1	391.5	575	459.9	596.4	527.9	868.2
C14ORF148	84.3	93.9	93.5	91.6	84.1	87.9	82.2	101.1
C14ORF149	511.9	692.7	242.1	243.5	158.6	233.2	488.1	693.9
C14ORF150	88.8	88.5	87.6	79.1	75.8	80.3	77.9	94.1
C14ORF150	142.8	168.9	113.4	112.5	103	106.5	131.7	154.5
C14ORF150	225.6	280.8	115.6	108	102.8	123.9	144.8	183.3
C14ORF151	141	172.2	148.4	127.8	120.2	156	159.1	253.4
C14ORF152	85.7	90.3	100.3	93.5	91.8	99.3	89.9	92.5
C14ORF153	1861.6	1702.1	1415.2	2175.3	2066.1	1736.5	1721.1	3158
C14ORF155	79.3	85.5	88.1	81.4	78.4	96.2	84	84.3
C14ORF156	13714	16295.6	10774.8	8541.1	5670.6	8915.5	10663.9	15011.6
C14ORF159	726.2	921.8	673.1	606.4	566.2	816	513.9	701.2
C14ORF161	80.3	84.8	97.7	95.3	95.1	98.4	83.2	84.1
C14ORF162	111.7	127	87.1	90.2	78.9	92.7	103.1	132.7
C14ORF166	3217.1	3823.2	724.3	540	462.5	704.9	2847.2	4304.7
C14ORF166	80.5	91	94.4	85.5	81.4	95.3	73.2	76
C14ORF169	619	820.7	262.7	204.8	159.7	219.7	594.8	815.3
C14ORF172	148.2	181.1	149	104	102.8	156.5	147.2	194.3
C14ORF173	2272.2	2745.3	2403	1895	1985.9	1614.2	1875	1755.3
C14ORF173	153.6	169.4	245.4	163	167.1	288.3	145.5	207.9
C14ORF19	133.2	156	106.8	94.8	105.1	102.5	150.8	173.2
C14ORF2	1552.9	1997.5	1192.4	1149.8	832.3	1519.1	1377.7	1694.7
C14ORF21	168.5	219.8	123.9	121.7	121.9	132.7	171.5	172
C14ORF24	12467.2	13485.3	4323.6	6300.9	5277	4573.5	11451.5	12701.4
C14ORF24	150.7	170.8	101.4	95.6	87.2	102.5	158.5	233.4
C14ORF24	151.1	205.1	111.7	103.6	87.2	101	154.1	203.2
C14ORF28	152.8	178.9	135.7	137.8	131.5	141.5	132.7	216.1
C14ORF29	103.6	121.8	108.4	103.3	93.6	93.7	106.1	109
C14ORF29	82.7	88.7	93.1	99	87.7	86.2	81.6	92.1
C14ORF32	1159.8	1529	844.2	907.3	640.2	1085.3	1042.1	1634.6

C14ORF37	90.5	108.3	84.6	79.2	75.6	81.4	84.2	111.3
C14ORF39	81.7	95.6	95.7	87.7	79.1	83.9	74.9	90.4
C14ORF4	258.9	287	147.8	139.8	131.3	166	192.4	255.4
C14ORF43	72.9	82.8	76.5	78.7	76.6	64.5	76.9	88.7
C14ORF43	321	434.2	214.6	180.4	159	195.4	309.4	366.6
C14ORF43	556.8	754.9	312.7	294	297.8	364.4	552.1	1089.7
C14ORF44	90.2	113	100.5	88.5	82.5	90.5	90.5	108.5
C14ORF45	76.2	72.9	94.6	94.7	75.3	86.7	75.8	83.1
C14ORF46	402.1	500.2	191.4	167.9	160.1	179.4	372.1	532.3
C14ORF48	127	137.8	108.7	97.7	101.5	105.6	120.9	132.7
C14ORF49	117.6	128.4	124.3	112.4	92.5	102.4	109.6	110.5
C14ORF50	124.6	136.8	90.2	79.9	83.5	94.5	122.6	132.7
C14ORF54	81.5	85.7	95.3	85	87.4	87.4	78.1	89
C14ORF58	285.6	355.1	143.1	161	147.6	177.6	271.1	302.7
C14ORF68	92.3	107.8	96.9	82.1	76.9	78.7	105.2	102.1
C14ORF73	90.8	93.9	86.5	95.3	76.3	83.9	93	96.7
C14ORF73	127.5	133.6	96.2	85.3	88	93.2	114.5	116.5
C14ORF79	170.9	226	163.5	124.6	114.5	138.6	137	177.7
C14ORF8	86.1	87.2	92.2	84.9	78.3	77.4	81.6	84.1
C14ORF80	859.8	1020.8	346.4	241.1	228.4	266.8	691.7	502.1
C14ORF85	3532.2	3485.6	1615.7	1852.9	1899.3	1561	3342.5	5848.8
C14ORF92	187.8	235.2	114.2	116.5	96.8	114.9	191.7	276.9
C14ORF93	534.5	726.7	365.9	306.7	257.8	329.8	452.1	447.4
C14ORF94	850.7	1072.9	519.9	274.5	292.2	385.4	623.4	740.3
C15ORF15	7233.3	8992.6	2846.2	3100.6	2149.5	3399.1	6925	8723.3
C15ORF17	450.1	570.3	304.3	303.2	270.9	379.9	392.5	492.6
C15ORF2	102.8	138.8	92	82.1	96.2	93	104.7	135.4
C15ORF21	131.4	145.5	114	99	99	114.3	128.3	160.1
C15ORF21	108	123.7	108.9	104.9	101	104.3	100.7	93.3
C15ORF21	112	111.5	87.3	92.4	78.3	86.7	108.8	116.6
C15ORF23	908.5	1341.7	303	179.4	181.3	233.2	752.3	951
C15ORF24	5256.9	6605.6	3968.4	3037	2489.6	3993.7	4704.1	6983.9
C15ORF26	107.5	107.3	91.3	90.3	85.8	91.9	89.1	113.9
C15ORF27	119.3	151.2	101.7	102	82.6	99.4	107.3	107.5
C15ORF29	155.1	182.4	103.4	99	95.4	105.6	136.5	175.1
C15ORF32	72.3	94.5	98.3	84.4	88.4	90.8	85.9	93.6
C15ORF33	78.1	93.5	84.2	86.6	78.9	83.3	77.8	88.8
C15ORF37	89.8	107.2	92.5	90.2	88.3	91.4	94.4	104.3
C15ORF38	223.7	273.7	156	130.3	117.2	122.1	179.4	206
C15ORF39	201.4	252	233.3	245.1	200.3	257.1	206.9	342.2
C15ORF40	238	283	187.3	207.2	174	210.2	201.3	294.6
C15ORF41	346.9	508.5	325.8	223.7	178.4	291	231.3	424.6
C15ORF42	194.3	275.4	221.8	214.7	187.8	262.7	178.2	235.2
C15ORF43	80.2	88	96.7	90.9	80.8	83.1	81	83.6
C15ORF44	166.6	218.9	116.5	118.9	104.4	130.2	176.9	217.6
C15ORF44	492	721.9	279.4	184.7	176.4	242.7	410.1	477.4
C15ORF48	120.6	127.4	100.4	99.1	95.6	102.3	119.8	121.5
C15ORF48	71.2	75.8	88.7	80.8	79.5	76.9	75	82.7
C15ORF48	98.2	122.5	86.7	91.5	80.8	89	94.8	126.4
C15ORF5	98.2	106.2	111.7	92.9	81.4	92	96.7	107

C16ORF24	796.3	1131.4	643.5	524.1	442	521.5	689.9	865
C16ORF25	100.7	105.5	113	90.1	91.1	110.4	84	102.6
C16ORF25	82.6	86	98.4	91	77.5	83.1	81.4	96.4
C16ORF28	200.2	208.6	162.7	132.3	123.2	112.9	315.9	813.6
C16ORF3	105	119.8	89	86.4	90.1	83	110.9	120.7
C16ORF30	92.2	98	98.3	83.2	92.5	85.6	80.5	86.8
C16ORF33	4669.4	5422.9	5007.1	3068.1	2637.1	4778.2	3928.5	5798.9
C16ORF34	255.5	293.8	159.8	150	135.5	198.2	234.3	288.2
C16ORF35	90.7	127.1	91.7	90.8	85.3	77.3	89.5	107.3
C16ORF35	1159.1	1459.7	698.2	556	422	476.6	818.4	733.6
C16ORF44	609.4	793.3	430.1	300.2	303.9	335.8	632.6	770.3
C16ORF45	121.1	130.8	105.2	85.5	88.4	90.5	102.5	127.1
C16ORF46	95.6	121.2	92.5	94.9	78	94.5	99.3	105.8
C16ORF48	1023	1219.6	449.1	345.9	300.9	383.2	733.2	715
C16ORF5	119.2	138.8	124.1	99	100.7	112.6	137.2	166.9
C16ORF50	238.4	320.8	112.1	98.1	108.3	110.1	242.1	268.2
C16ORF53	1704.8	2214.6	560.5	347.3	249.4	390.4	1401	1863.5
C16ORF54	124.3	174.9	96.4	83.4	87.7	79.3	131.1	140.4
C16ORF55	97.5	118.5	92.8	86.8	81.5	84.2	90.3	121.6
C16ORF56	360.2	363.2	542.6	411.2	488.3	867.6	251.9	447.8
C16ORF57	850	909	328.5	327.6	259	288.8	749.3	723.3
C16ORF58	2829.3	3433.1	1502	1625.9	1443.7	1688.2	2159.8	1720.6
C16ORF60	1623.5	2233.4	925.9	641.4	542.4	684.4	1392.4	1825.9
C16ORF61	4856.7	6247.1	3057.9	2393.3	1898.7	2424	4407.9	4896.3
C16ORF63	3799	3484.1	2240.3	2225.5	1607.8	2133.1	3314	7070.9
C16ORF69	93.2	110.4	109.5	86.8	93.7	94	88.9	102.9
C16ORF7	143.5	189.7	155.9	172.9	166.8	188.1	141.2	211
C16ORF78	81.7	89.4	91.5	82.9	76.1	74.8	85.3	86.6
C16ORF80	1472.5	1794.9	787.1	624.3	385.4	617.7	1093.2	1640.3
C16ORF9	398.3	508.8	631	422.4	347.7	643.9	400.1	821.9
C17ORF25	1279	1573.7	419.1	346.1	243.6	517.7	962.7	1510.3
C17ORF27	91.2	107.5	100.4	89.4	96.1	89.3	102.2	106.8
C17ORF28	83.2	91.1	98.6	99.7	84.5	86.7	84.6	90.1
C17ORF32	453	539.3	209.1	188.2	159.8	209.6	403.3	836.4
C17ORF37	158	200.5	116.5	112.8	107.1	103.9	139.4	180.1
C17ORF38	89.1	94.5	96.6	100.2	96.2	90.5	99.8	108.1
C17ORF39	212.1	269.4	133.2	107.3	103.7	102.6	214.2	285.5
C17ORF40	3016.8	3414.9	1099	1288.4	919.2	1581.2	2288	2913.8
C17ORF42	79.9	93.7	115	105.2	93.7	110.2	80.4	96.1
C17ORF45	14919.6	21094.6	12171.2	12597.6	9374.3	14663.9	15728.6	17100.1
C17ORF46	130.5	130.4	101	91.6	102.4	91.8	120.9	130.4
C17ORF47	104.2	149.2	102.9	80.7	71	91.7	106.4	140.3
C17ORF47	144.2	179.7	105.3	90.1	90.9	88.7	139.8	181.1
C17ORF48	166.9	204.1	125.8	155	123.2	166.3	156.4	257
C17ORF49	712	933.1	646.5	540.4	452.7	534.6	761.8	1137.9
C17ORF50	86.3	86.9	98.4	106.4	96.7	91.1	84.2	88.6
C17ORF53	851.4	1000.3	615.2	429.3	425.8	424.8	622.2	507.4
C17ORF54	91.9	100.5	110.7	94.2	97.9	87.1	90.4	89.2
C17ORF55	98.5	108.8	140.6	150.1	163.9	197.1	91.5	98.4
C17ORF56	148.6	147.8	121	111.2	113.8	119.5	120.1	170.4

C17ORF57	104.8	112.4	99.3	84.5	79.2	88	96.3	114.6
C17ORF58	1351.7	1698.2	921.9	633.6	491.7	793.2	1024.2	1442.2
C17ORF58	938.9	1101.3	769.1	505.6	388.2	578.2	741.9	1059.1
C17ORF58	118.3	159.3	111.8	104.1	96.8	100.5	117.7	134.8
C17ORF59	98.7	109.7	98.8	99.7	86	98.3	102.1	115.8
C17ORF61	2036.3	2508.5	1787.4	1871.4	1680.3	1867.9	2150.2	1902.8
C17ORF62	772.1	987.8	913.3	689.6	595.6	1173.5	544.6	959.4
C17ORF63	240.8	290.9	240.7	326	270.8	282.5	251.7	479.8
C17ORF64	150	175.3	111.3	102.4	95.9	99.9	145.5	159.3
C17ORF65	127.1	157	222.7	153.1	151.4	173.5	108.7	173
C17ORF66	84.3	82.4	96.1	83.2	89.3	90.2	79.3	83.5
C17ORF68	194.9	231.5	162.9	157.9	147.6	161.5	143.2	142.8
C17ORF68	133.2	156.2	109.9	110.6	111.6	105.6	119.9	113.5
C17ORF69	67.1	76.3	84.5	79.2	72	77.9	72	82.9
C17ORF70	1711.1	2121.4	1490	1434.9	1230.3	1436.8	1059.1	1004.3
C17ORF71	430.9	522.7	194.5	158.5	133.3	169.5	365.6	500.1
C17ORF73	137.9	172.2	104.4	106.7	102.8	114.3	138.2	161.7
C17ORF74	128.2	169.4	116.8	111.3	105	126.8	135.3	151.2
C17ORF75	178.7	180.8	120.8	118.5	109.2	93.4	139.8	177.4
C17ORF76	122.1	137.5	102.9	98.5	90.7	101.7	119	125.5
C17ORF77	70.3	75.5	77.6	80.9	78.6	74.5	76.3	74.2
C17ORF78	92.1	107.1	89.5	91.6	80.1	89.5	89.1	88.9
C17ORF79	780.5	907.4	243.3	229.1	162.1	267.5	681.8	726.2
C17ORF80	282.4	376.3	214	205.2	196.5	295	190.1	244.1
C17ORF81	162.4	169.4	135.4	97.5	100.9	115.5	154.6	190
C17ORF81	322.3	439.3	234.2	202.5	171.1	218.3	270.6	286.2
C17ORF81	161.6	239.7	158.5	149.2	124.5	171.8	151.7	188.4
C17ORF81	106.2	128.7	87	86.1	75.9	82.6	97	111.8
C17ORF83	99.2	99	105.6	92.1	94.2	82.1	92.3	97.3
C17ORF85	262	265.1	215	226.8	220.9	270.8	245.6	302.6
C18ORF1	95.4	118.1	91.7	85.2	85.1	81.1	98.9	110
C18ORF10	948.5	1313.6	365.1	370	261.3	465.6	727.5	1302.2
C18ORF14	84.8	94	85.6	83.9	78.3	85.3	95.2	91.4
C18ORF17	321.7	408.1	246.3	237.3	204.2	321.8	313.6	405.8
C18ORF19	540.1	730.6	222.7	240.7	204.7	241	356.7	473.5
C18ORF21	673.6	977.4	282.1	336.8	275.4	354.6	682.9	1186.8
C18ORF22	502	639	335.1	235.3	218.7	296	352.2	382.7
C18ORF24	379.3	497.9	263.7	210.9	189.2	269.8	322	525.5
C18ORF25	1092.9	1362.5	986.5	1320.3	1010.7	1506.4	981.6	1464.3
C18ORF25	90.8	94.1	78.7	78.6	77.4	83.6	95.4	104
C18ORF26	99.1	95.4	91.6	86.2	88.3	87.4	84.6	108.5
C18ORF34	84.1	91.3	97.1	90.8	91	86.1	89.5	95.2
C18ORF37	815.7	1026.7	429.1	361.9	311.7	471.3	650.9	809.9
C18ORF45	172.8	311.7	122.9	135.2	125.1	108.7	205	202.3
C18ORF51	106.7	122.5	98.3	78.8	85.4	89.1	89.5	98.2
C18ORF51	112.2	119.3	95.4	85.9	82.5	79.6	99.2	130.5
C18ORF54	311.5	410.3	237.4	176.3	175.8	212.2	284.8	390.6
C18ORF55	3425.2	4064.3	2107.4	2284.7	1777.9	2971	2358.2	3634.2
C18ORF56	917.7	1103.4	735	561.4	454.7	384.8	796.7	1231.4
C18ORF8	562	768.4	364.4	353.1	328.2	428.8	513.6	1145.5

C19ORF10	2760.1	3329.6	1808	956	885.8	1159.5	2856.4	3436.2
C19ORF12	93.9	108.8	97.5	90.7	94.2	90.6	83.6	93.7
C19ORF12	280.9	332.2	197.4	136.3	141.7	173.2	245.7	337.4
C19ORF12	747.5	1022.1	440.9	326.5	316.6	406.5	582.6	613.6
C19ORF12	128.3	163.2	93.5	95.8	82.3	76.7	141.1	164.4
C19ORF15	100.6	117.1	99.1	101.6	90	97	94	102.1
C19ORF18	87.5	94.9	89.6	89.4	86.4	90	83.3	90.2
C19ORF19	86.4	93.3	90.5	89.8	90.4	85.3	90.8	101.3
C19ORF2	1233.5	1821.6	499.7	454.9	337.3	589	997.8	1303.4
C19ORF2	4785.2	6254.2	2987.6	2722.5	1922.2	3576	4034.8	4942.5
C19ORF20	116.3	153.1	110.2	102.6	103.7	110.5	126.5	158.8
C19ORF21	111	122.2	124.2	112.4	118.8	107.4	97.2	108.7
C19ORF22	946.2	1305.1	885.5	800.8	595.8	761.9	871.5	1726
C19ORF23	133.6	149.9	115.7	110.3	99	107.3	130.3	152.2
C19ORF24	508.4	612.4	383.6	271.8	212.9	235.9	466	589
C19ORF25	241.3	265.1	296.3	251.5	222.6	260.7	212.8	399.4
C19ORF26	155.4	197	88.3	96.8	74.6	82.5	159.5	190.1
C19ORF28	161.2	176.2	100.4	104.3	84.7	104.8	140.9	191.6
C19ORF28	221.1	257.5	171.6	156	132.8	146.9	208.3	267.7
C19ORF29	164.5	197.3	171.7	160.4	129.3	217.9	156.4	215.9
C19ORF29	144	171.1	118.5	106	100	142	125.6	180.2
C19ORF30	154.7	165.3	102.9	106.2	101.8	104.9	147.9	162.2
C19ORF31	27860.4	28416.7	20720.6	23558.3	25622.1	23808.6	28879.4	27836.1
C19ORF33	78	83.4	83.8	77.5	78.3	85.5	82.4	86.8
C19ORF34	94.2	95.1	84.6	104.3	76.5	87.5	101	114.6
C19ORF35	94.3	105.9	105	109.2	91.4	97	92.2	98.8
C19ORF36	94.7	113.4	93.9	92.3	79.2	104.8	104	108.2
C19ORF36	94.3	94.7	106.7	85.2	89.3	84.3	83.1	90.3
C19ORF37	3129.2	3736.6	1574.4	1384.6	1152.2	1424.7	3164.3	2872.2
C19ORF4	87.2	96.5	97	93.3	99.1	92.9	90.1	101.3
C19ORF40	264.2	360.4	229.7	204.4	203.3	241.7	222	227.1
C19ORF48	1185	1532.5	576.9	276.4	268	297.1	1128.5	1150.9
C19ORF55	107.9	121.4	102.9	95.9	98.2	98	100.4	108.3
C19ORF6	240.1	284.2	198.2	172.4	185.7	134.6	216.9	356.8
C19ORF6	445.2	529	386	407.9	381.2	761.4	377.9	652.7
C19ORF7	1291.8	1501	1148.3	992	711.2	939.5	1189.2	1866.9
C1D	115.1	135.6	95.1	91.5	85.7	89.4	124.6	153.4
C1D	89.6	90.6	80.7	82	84.6	82	93.7	89.4
C1GALT1	199.9	238.2	134.6	128.7	110.5	139.4	168.6	272.8
C1GALT1C1	87.7	88.3	90.3	93.9	91.5	78.5	85.5	97.4
C1GALT1C1	264	309.6	162.3	171.1	150.3	192.3	232.5	296
C1ORF100	93.3	118.8	105.9	85.1	87.5	83.7	96.8	109.7
C1ORF101	141	130.6	141.9	153.2	147.2	172.5	117.3	157.9
C1ORF102	109.7	122.2	99.1	92.9	83.2	85.3	106.6	110.3
C1ORF102	97.8	127.3	107.2	86.2	90	93.4	100.1	134
C1ORF102	99.3	114.2	99.9	94.1	85.9	96.5	91.8	92.5
C1ORF103	87.9	87.5	84.1	80.3	89.7	83.4	82.2	80.7
C1ORF103	333.6	448	154.9	118.8	107.5	119.2	253.7	511.1
C1ORF104	108.3	134.5	103.2	92.1	99.5	103.7	107	118.3
C1ORF105	107.3	137.4	88.3	85.5	80.6	89.3	109.8	134.6

C1ORF106	885.5	995.3	1120.7	399.4	314.4	456.2	524.6	629
C1ORF107	187.2	230.4	176.4	197.7	178.2	233.4	178.9	224.5
C1ORF108	734.9	931.9	448.5	439.2	352.9	359.8	677.8	1122.7
C1ORF108	181.6	227.7	103	102	101.6	83.8	158.3	189.6
C1ORF108	905	1130.8	385.7	329.2	299.4	271.3	665	1009.4
C1ORF109	476	694.7	415.3	322.3	280.4	440.9	377.7	526.9
C1ORF110	102.5	113.5	91.1	94.3	82.4	85.6	93.5	100.6
C1ORF111	85.4	100.7	102.6	101	85.5	101.6	90.7	94.8
C1ORF112	828.1	1026.7	650	459.5	395.1	557	582.2	598.5
C1ORF113	127.7	151.7	97.5	93.6	88.3	89.9	125.1	139.8
C1ORF114	121.1	123.6	90.1	69.6	76.8	84	116	115.4
C1ORF115	105	118.7	114.1	130.6	123.1	177.2	108	122.8
C1ORF116	94.5	93.6	94.2	85.9	85.9	88.3	89.8	90.8
C1ORF117	108.9	135.9	94.3	94	72.3	84	113.4	117.3
C1ORF118	100.2	102.8	90.5	82.4	76.2	93.9	85.6	107
C1ORF119	1024.7	1347	604.1	399.6	311.4	545.8	857.3	1312.2
C1ORF121	1506	2030.7	757	648	399	780.8	1194.7	2153.8
C1ORF122	230.4	291.3	220.3	205.8	177	231.7	215.7	325.5
C1ORF123	1235	1592.3	1031.5	1098.8	872.2	1214.6	899.8	1036
C1ORF124	650.8	893.3	410.1	330.8	253.1	483.6	524.1	733.7
C1ORF124	490.9	604.5	217.7	179.4	163.9	209.5	327.3	491.9
C1ORF124	152.6	204	129.8	100.7	101.3	121.3	145.5	169.6
C1ORF125	97.1	114.8	97.6	91.2	100.8	94.8	101.9	101.3
C1ORF125	80.5	91.3	88.7	95.5	85.6	82.5	91.8	90.7
C1ORF127	106.1	162	96.1	86.5	84	85.2	125.5	149.7
C1ORF128	1293.6	1876.3	1217.3	1155.1	842.8	1740.3	1299.4	1769.8
C1ORF129	121.5	133.7	114.1	101.8	86.9	88.6	115.2	118.3
C1ORF131	3152.5	3707.6	2973.8	2553.6	1975	3311.8	2777.7	4945.1
C1ORF135	459.9	603.9	302.4	226.7	181.5	238.1	381.2	503.2
C1ORF14	74.3	88.6	92.5	86.3	86	86.1	71.5	82.7
C1ORF140	82	101.5	103.9	101.8	97.3	102	79.7	87.7
C1ORF141	72.9	73.8	103.2	101.7	93.6	96.1	81	80.5
C1ORF142	236.6	309	243.6	206.5	148.1	219.4	226.9	349.7
C1ORF144	830.4	1138	495.3	516.9	434.4	604.9	763.7	865
C1ORF146	83.1	82.9	85.1	88.6	91	79.9	80.7	83
C1ORF149	277.1	359.4	244.3	208.6	159.5	256.5	214.3	322.3
C1ORF150	93.4	99.5	89.9	80.8	87.9	83.2	98.4	95.9
C1ORF151	97.7	113.8	91.7	101.3	89	94.7	103.3	102.4
C1ORF152	91.8	103.7	74.1	97.1	74.9	79.5	92.6	99.6
C1ORF155	1104	1430.7	602.2	503.1	395	461.5	890.5	1790.7
C1ORF156	80.2	98	85	79.7	75.4	89.7	90.4	98.2
C1ORF157	82.3	96.1	105.8	83.1	83.5	78.9	93.8	100.2
C1ORF158	91.8	100.8	96.3	99.3	88.1	87.3	86.4	102.3
C1ORF159	194.6	240.6	207.4	191.7	152.3	230.8	163.3	242
C1ORF160	212.3	244	181	155	139.2	183.7	206	390.6
C1ORF162	3557.3	4447.1	2333.4	1903.7	1172.8	1825.3	3209.7	3463.9
C1ORF163	541.1	769.1	301.9	295.8	236.1	248.1	489.5	524.5
C1ORF164	1401.7	1643.2	1160.7	785.2	732.4	913.7	1315.7	1083.7
C1ORF165	112.5	130.9	103.9	94	102	103.7	100	117.9
C1ORF166	203.8	211.5	127.7	146.4	125.6	161.7	174.7	304.8

C1ORF168	78.3	91.2	92.7	82.8	87.6	87.6	76.1	86.4
C1ORF171	623.8	881.1	207.1	225.2	178.4	282.5	502.4	718.3
C1ORF172	98.5	112.4	107.5	93.6	86.2	94	95.6	104.8
C1ORF173	85.2	99.3	108.5	98.4	93.9	93.3	92.3	87.4
C1ORF175	94.1	119.7	93.7	99.8	96.2	97.9	87.2	100.6
C1ORF176	687.1	644.3	377.8	552.9	549.4	404.2	633.5	1118
C1ORF177	98.1	108.7	105.4	99	103.6	101.3	90.2	118.6
C1ORF178	78.3	86.5	85.1	78.3	80.5	80.8	76.9	95.3
C1ORF178	97.7	97.3	98.1	99.3	89.6	96.5	83.8	98.2
C1ORF179	80.5	107.1	89.4	84.9	82.1	76.6	79.7	89.5
C1ORF181	973.6	1301.9	652.6	850.3	605.9	1153.3	845.1	1202.5
C1ORF182	107.2	155.5	120.1	102.6	103.1	108.7	126.9	130.4
C1ORF183	98.3	108.1	92.7	100.7	100.3	90.8	110.7	108.2
C1ORF183	103.7	103.1	89.2	85.5	86.4	99.2	94.9	99.6
C1ORF183	101.2	96.1	97.5	103.6	93.6	111.6	96	103.3
C1ORF186	506.2	519.1	371.8	196.5	152.2	210.9	269.8	372.7
C1ORF187	92.7	114.1	98.8	94.7	90.2	98.2	95.8	102.6
C1ORF188	126.8	148.9	163.6	121.9	131.3	137.8	104	101.8
C1ORF189	83.8	114	91	83	85.4	85.9	84.9	104.7
C1ORF19	2584.8	3620.6	1611.1	1167.8	763.7	1368.7	2150.3	2953.3
C1ORF190	104.3	124	97.9	93.2	90.5	96.8	105.2	113.5
C1ORF192	113.2	133.9	107.6	83.7	84.7	102.1	108.6	121.3
C1ORF198	252.1	323.8	244.5	216.4	186	254.9	256.8	399.9
C1ORF2	2217.1	2988.4	1051	912.2	727.1	742.5	1556.5	1309.5
C1ORF2	78.5	99.4	102.9	95.6	86.1	85.7	84.2	94.4
C1ORF2	122	149.4	142.9	115.9	132.1	162	118.9	143.5
C1ORF201	135.4	157.7	95.1	80.8	87.7	92.3	145.6	155.1
C1ORF21	111.6	124.7	120.6	119.1	122.6	127	116	125.4
C1ORF210	92.2	112.8	100.9	93.4	93.6	86.2	96.5	99
C1ORF24	517.6	676.6	427.9	689.7	553.8	357.1	639.2	941.9
C1ORF25	122	130	95.9	93.8	90.6	82.4	110.2	156.4
C1ORF26	145.6	141.8	102.1	92.8	89.3	93	129.5	159.9
C1ORF27	90.3	108.4	93.3	79.6	84.3	89.4	101.6	103.5
C1ORF31	572.3	635.6	331.6	354.3	303.5	423.9	529.6	888.7
C1ORF32	92.3	116.4	93.8	88.8	90.9	90.7	102.1	112.8
C1ORF33	987.1	1235.2	556.9	509.9	376.7	419.9	733.1	891.7
C1ORF34	97.6	96.1	94.1	89.9	92.8	96	93.9	97.5
C1ORF35	819.8	948	643.1	646.2	556.3	788.7	627.6	939.2
C1ORF36	97.7	124.3	104.9	82.2	82.9	93.2	97.3	130.6
C1ORF37	5143.8	6496.5	3502	3062.3	2284.2	4182.1	5074.4	6388.7
C1ORF38	111.1	122.2	95.6	101.6	87.4	94	121.3	113.5
C1ORF41	3216.9	4079.4	2209.7	2067.1	1692.7	2784.8	2451.3	3104.5
C1ORF42	80.9	89.7	101.7	95.5	93.8	104.1	85.2	90.4
C1ORF43	2467	2912.8	868.8	785.8	607.4	735.7	2071.7	3546.3
C1ORF43	1271	1609.1	396.6	204.4	224.3	332.7	903.7	1522.3
C1ORF45	84.1	94.8	90.2	90.1	84.4	80.7	89.9	88.5
C1ORF48	3129.8	4220.3	2015.1	1714.3	1260.9	1913.8	2955.9	4762
C1ORF49	81.1	97	102.3	81	84.4	86.7	96.3	112.7
C1ORF50	1867.1	2409.8	1650.2	1776.9	1514.7	1961.8	1771.4	1961.5
C1ORF51	130.9	128.6	119.5	92.4	97.7	90.6	101.3	167.1

C1ORF52	922.8	1157.2	738.3	814.7	697.2	860.9	939.9	1328.3
C1ORF53	687.2	819.3	533.2	652.3	503.1	673.1	568.7	1001.6
C1ORF54	5367.3	5727.2	4120.1	3043.9	2326.6	3250.2	5316.3	5929.6
C1ORF55	742.9	861.5	490.9	583.7	487	828.3	637.9	1498.9
C1ORF56	182.6	212.3	114.3	124	106.6	115.2	155	244.7
C1ORF57	1746.3	2203.9	1094.3	1015.5	737.5	1212.2	1597.1	2800.1
C1ORF58	282.4	285.7	114	86.8	87	101.2	186.8	282.6
C1ORF59	3308.4	4258.2	2875.2	1920.5	1417.5	2226.7	2565.8	3644.6
C1ORF60	482.1	483.4	602.4	716	654.1	1151.3	387.4	612
C1ORF61	77.5	79.3	98.5	90.2	83.3	85	78.1	80.4
C1ORF62	98.6	110.6	88.1	94.1	79.1	90.9	104.4	101.7
C1ORF63	488.2	533.6	139.2	173.1	138.8	182.2	405.4	673.8
C1ORF64	91.1	97	89.8	85	90.9	82.9	100.7	94.9
C1ORF65	72.3	80.7	92	79.8	79.6	86	83.5	82.2
C1ORF66	389.6	437.1	512	307.6	322.8	458.5	354.2	445.7
C1ORF69	194.6	239.9	227.6	187.7	177.3	233.7	181.1	255.6
C1ORF71	1468.6	1779	1137.4	1062.6	905.6	863.8	811.1	1515.9
C1ORF73	435.4	595.1	293.2	219.5	202.9	272.6	393	505.6
C1ORF74	359.8	493.1	200.9	149.8	118.3	144.3	391.1	410.8
C1ORF75	98.5	120.4	103.8	95.2	93.3	105.6	86.6	104.6
C1ORF76	154.9	183.3	142.9	154.9	140.8	151.2	133.4	141.5
C1ORF77	1439.4	1800.7	720.2	698	607.4	851.1	1220.3	1184.7
C1ORF78	124.3	134.5	96	97.8	96.5	99.9	122.8	133.6
C1ORF82	449.4	606.7	334.7	535.1	436	574.1	394.2	557.7
C1ORF83	288.6	393	208.7	237.7	205.2	334.8	230.2	303.6
C1ORF84	144.7	136.6	125.3	117.6	121.5	137.5	120.1	163.6
C1ORF84	142	187.6	124.4	130.7	110.7	111.4	121.2	142
C1ORF84	142.2	169	111.5	101.5	114.7	107.3	135	142.1
C1ORF85	1554.6	2119.2	970.5	1020.8	783.1	1001.2	1472	1391.8
C1ORF86	530.8	707	513.4	363.2	325.5	398.3	473.7	670.7
C1ORF87	104.1	118.3	98.2	82.4	80.4	91.4	103.7	110.4
C1ORF88	117.7	141.3	109.9	93.7	104.2	94	123.1	150
C1ORF89	109.7	138.5	100.7	97	90.2	104.7	107.3	112.2
C1ORF9	94.8	102.2	85.5	78.9	82.2	82.2	75.9	100.5
C1ORF9	118.6	115.4	92.4	91.3	86.7	87.5	124.3	129.4
C1ORF9	100.6	121.9	108.7	86.9	91.6	98.5	101.8	115.2
C1ORF90	105.4	134.5	94.8	76.6	81.7	94	107.7	125.6
C1ORF91	107.2	141.9	96.2	84.3	74.3	80.4	100.9	124.3
C1ORF92	105.5	106.1	94.4	86.1	85.2	92	84.8	109.1
C1ORF94	113.9	137.5	99.8	93.5	84.3	84.8	127.1	120.7
C1ORF95	93.5	97.9	84.7	80	78.1	78.4	101.4	111.1
C1ORF96	156.6	199	115.8	109.3	98.6	126.7	140.2	171
C1ORF97	947.9	1085.8	524.9	565.6	396.6	588.6	918	1282.6
C1ORF99	90.7	107.7	87.3	79.4	85.7	81.2	95.3	102.7
C1QA	127.6	152.1	102.5	91.5	87.9	112.4	102.5	129.2
C1QB	99	120.3	95.8	92.9	84.8	95.9	96.2	110.5
C1QBP	5013.6	6262.5	2963.8	2605.3	1632.6	3016.3	4039	7166.7
C1QC	87.7	98.7	97.5	95.2	89.2	94.3	93.1	100.2
C1QDC1	137.3	167.3	106.2	116.2	112.5	165.2	125.3	210.6
C1QDC1	122.7	157.8	120.3	144.2	117.9	183.3	119.6	151

C1QDC2	100.3	90.9	107.8	100.8	99.1	97	85.1	103.1
C1QL1	99.2	111.7	116.3	99.6	95.3	98.3	95.1	110.5
C1QL2	98.5	101.4	109.2	101.4	86.6	98.1	83.8	97
C1QL3	92.8	122.6	103.8	84.3	79.7	86.1	98.9	109.5
C1QL4	93.7	94.7	85	79.5	87.8	103.3	88.4	111.7
C1QR1	3129.7	4044.8	3011	3043.7	2127.5	3525.5	2593.9	4005.1
C1QTNF1	102.5	111.6	112	107.7	110	111.1	99.1	99.4
C1QTNF1	74	80.2	85.5	75.8	88.6	84.4	76.9	81.5
C1QTNF1	82.2	102.5	86.3	76.5	75.2	80.2	83.1	93.7
C1QTNF2	122.2	145.9	100.2	88.8	84.9	93.8	113.9	120.9
C1QTNF3	74.4	82.5	102.7	98.1	92.7	91.8	82.1	88.6
C1QTNF3	85.5	112.4	91.3	90	86.8	83.7	97.9	112.2
C1QTNF4	94	108.3	95.5	78.9	91.2	93.2	81.9	96.6
C1QTNF5	116	163	79.7	77.5	80.3	77.3	124.5	150.8
C1QTNF6	94.8	106.8	99.6	102.5	94.4	92.1	93.3	97.4
C1QTNF6	91.8	107.6	99.1	96.8	97	106.6	86	93.1
C1QTNF6	82.4	93	91.8	94.8	88.1	93.2	75.9	77.8
C1QTNF7	93.5	113.1	89.4	88.2	82.4	88	106.8	103.7
C1QTNF8	126.5	180.2	98.8	84.1	74.2	86.2	145.1	158.8
C1R	95	106.3	92.7	94.7	87.4	83.3	104.3	88.1
C1RL	200.2	194.6	153.8	214.7	181.6	198.7	137.7	156.8
C1S	82.1	79.3	104.5	84.8	102.9	91.8	86.3	81.5
C1S	95.5	114.4	96.9	86.6	92.3	100.2	92.1	104.7
C2	166.9	192.5	112.5	201.4	194.9	233	149.4	184.6
C20ORF10	88.3	99.6	82.1	88.1	79	93.8	97.6	98.4
C20ORF10(	1687.4	2237.2	1444.4	2377.3	1936.4	2016.1	1598.7	1536.3
C20ORF102	85.8	101.9	99.2	91.6	87.1	84.9	87.5	95.5
C20ORF103	2298.2	3066.3	1443.4	1534.2	1253.6	1895.9	1496.7	1868.4
C20ORF106	93.2	105.3	106.9	110.7	116.9	108.7	111.6	136.5
C20ORF107	117.9	121.3	106.3	115.2	108.5	115.5	107.2	144
C20ORF108	170.4	228.2	135.7	187.5	159.4	243.2	174.9	430.9
C20ORF11	1550.2	1862.6	1846.1	2170.3	1654.9	2061.8	1301.5	2103.6
C20ORF111	1459.9	1713	895	713.2	627	1103.5	1360.3	2350.5
C20ORF112	106.9	123.8	80.9	77	69.5	85.4	97.7	123.3
C20ORF114	95.1	105.8	94.4	95.1	87	99.5	91.1	92
C20ORF116	825.7	1001	542.4	420.4	369.7	481.5	868.8	1241.6
C20ORF117	246	382.6	352.2	445.8	382.4	552.8	307.3	492.9
C20ORF117	75.6	105.5	81.6	88.8	81.2	85.2	89.5	98.9
C20ORF118	139.7	163.8	98.6	94.8	95.4	98.8	133.4	142.2
C20ORF12	277	393.4	217.2	221	249.4	273.5	202.1	252.5
C20ORF121	399.5	536.2	478.6	527.5	390.7	610.8	362.5	553
C20ORF121	360.3	483.9	222.6	245.6	194.6	253.7	290.8	411.9
C20ORF128	112	128.6	96	90	91.9	93.9	115.9	134.7
C20ORF129	616.5	919.1	528.4	375.9	284.1	410.8	561.8	1009.3
C20ORF132	95.4	114.2	102	100.7	86.1	96.5	93.9	97.3
C20ORF132	102.9	119.8	120.4	118.2	103.9	113.2	102.7	117.5
C20ORF132	112.6	136.5	96.7	99	90	121.6	114.1	109.2
C20ORF133	90.9	103	84.4	93.6	87.7	84.2	82.1	99.4
C20ORF133	98.7	105.4	86.1	79.3	89.5	84.6	92.6	108.2
C20ORF134	102.6	133.9	113.7	89.1	84.3	91.3	111.5	114.2

C20ORF135	90.5	86.4	87.5	82.7	89.2	81.8	92	94.1
C20ORF14	375.6	534.8	136.8	177.6	132.3	117.1	258.1	339.4
C20ORF141	89.8	96.4	115.6	112.2	108.1	98.5	93.8	91.4
C20ORF142	109.1	110.4	88	90.1	83.6	93.9	108.1	107
C20ORF144	80	96.2	91.7	75.2	85.9	77.1	93.5	94
C20ORF149	284.9	399.4	291.8	216.4	161.6	140.7	246.6	338.7
C20ORF151	99.6	113.6	79	79.8	81.9	74.4	97.2	134.9
C20ORF152	75.8	99.2	89.2	84.8	77.7	81.7	80.8	90.1
C20ORF155	1969.6	2678.1	1109.2	959.4	709.6	1245.8	1656.6	2143.8
C20ORF160	90.5	99.4	105.1	93.6	85.1	90.9	96.6	99.6
C20ORF161	94.1	117.1	96.9	97.7	84.2	86.1	95.6	111.9
C20ORF161	127.3	143.2	108.9	103.9	99.4	109.1	124.9	171.6
C20ORF165	90.3	107.3	91.8	86.2	81	89	92.5	94.5
C20ORF166	86.8	105.2	87.3	85	79.5	84	98.8	106.9
C20ORF172	793.1	1083.5	381.5	350.3	318.8	459.8	692.1	1081.1
C20ORF174	80.5	79.6	87.1	81.1	82.3	78.5	78.5	80.8
C20ORF174	80.6	88.7	96.1	90.1	92	82.3	80.4	80.9
C20ORF177	991.6	1502.7	1175.5	1269	1030.9	1151.4	1179	1986
C20ORF179	89.1	103.2	104.7	108.4	85.9	97.5	95.6	95.9
C20ORF179	72.5	84.1	91.6	81.6	87.6	81.7	80.7	83.3
C20ORF185	87.4	95.1	93.2	84	86.5	82.9	89.7	101
C20ORF186	81.2	87.4	88.2	84.8	77.2	81.9	82.1	78.2
C20ORF19	166.9	191	100.8	96.1	89.2	110.9	157	181.9
C20ORF191	444.2	607.9	157.6	161.9	178.4	213.1	437.4	534.2
C20ORF195	86.7	93	99.1	91.6	87.8	90.6	93.2	102.8
C20ORF20	2603.1	3545.9	1912.9	1726.1	1674.3	2109	2447.6	2754
C20ORF22	398.9	498.5	240.6	161.7	150.5	188.1	444.9	700.9
C20ORF23	87.9	99.3	103.7	100.9	98.3	120.3	91.2	116
C20ORF24	12982.2	15729.9	10954	9709	8220.2	11558.5	16302.6	22994.6
C20ORF24	12806.3	15498	12429.8	12788.7	10038.6	14568	15828.9	22544.9
C20ORF24	6624.3	8980.8	3667	3849.7	2884	4712.6	8334.6	10876
C20ORF26	86.2	94.3	92	96.4	88.4	82.3	86.5	82.3
C20ORF27	1728.9	2284.3	810	630.4	549.5	522.6	1389.6	1135.1
C20ORF28	125.5	164.1	102.8	92.5	78.1	96	128.1	145.4
C20ORF29	152.4	179.1	171.2	142.2	122.4	137.8	138	232.9
C20ORF3	488.6	752.5	381.1	353	242.5	360.4	509	857.6
C20ORF30	2365.7	2873.1	1341.1	1829.9	1188.4	1762.6	1819.5	3474.9
C20ORF30	2467.6	3267.7	2261.6	2834.7	2138.3	3081.1	2145.3	4043.6
C20ORF31	582.7	836.1	333.6	277.6	233.4	281.3	604.3	795.2
C20ORF32	80.7	89.1	85.7	87.2	81.8	81.5	94.2	87
C20ORF35	276.7	388.5	265.5	317	237.5	413.3	236.5	374.2
C20ORF39	86.2	87.2	103.4	101.9	96.7	96.3	85.8	80.9
C20ORF4	690.6	911	828.6	766.9	582.2	946.7	649.8	1442.2
C20ORF42	182.3	201.8	159.6	125.3	105.1	139.8	122.7	170.8
C20ORF43	3850.7	5322.4	3173.2	4141	3279.6	4869.3	3761.2	4668.7
C20ORF44	399.8	622.9	292.1	231.1	173.2	276	357.5	599.2
C20ORF44	232.6	303.5	126.6	125.6	97.7	109.6	213	324.5
C20ORF44	1401.8	1728.4	501.5	523.7	352.3	584.2	1367	1725.6
C20ORF45	1169.6	1613.6	784.5	780.6	621.6	1061.4	828	1009.9
C20ORF46	96.5	95	101.2	86.2	91.5	89.6	84.5	89.2

C20ORF52	7055.2	6800.7	7644.2	6704.9	5244.8	6217.5	7292.3	8780.4
C20ORF54	101.1	97.3	96.5	86.8	83.9	82.8	96.7	101
C20ORF55	177.8	194.9	143.6	105.9	115.3	113.4	167.9	278.4
C20ORF55	91.6	112.4	80.5	79.4	78.2	86.7	82.5	113.9
C20ORF55	265.3	307.6	214.1	177.9	152.5	188	225.5	282.8
C20ORF58	87.1	94.8	96.1	88.6	80.8	93.9	93.7	89.4
C20ORF59	187.6	209.4	190.5	169	163.6	238.1	170.5	238.5
C20ORF6	92.3	106.7	94.6	94.3	81.9	88.3	87.6	96.1
C20ORF67	114.1	147.6	152.9	142.6	141.4	177.5	115.5	157.9
C20ORF7	94.4	95.6	105.3	99	88.5	97.6	87.4	99
C20ORF7	571.8	711.3	327.1	253.2	204.4	341.8	455.3	674.6
C20ORF7	271.6	342	157.4	143.8	120.6	148.3	229.6	381.1
C20ORF70	84.2	104.9	104.4	86	81.3	89.6	89.4	101.5
C20ORF71	93.6	109.2	86.1	87.2	78.8	91.5	94.4	100.9
C20ORF71	84.8	91.9	111.6	105	92.4	100.5	93.8	84.4
C20ORF71	78	87.9	87.7	86.8	83.8	85.2	85.1	86.4
C20ORF72	934.1	1258.8	716	701.2	655.7	908	663.3	907.8
C20ORF74	85.5	102.1	91.5	100.1	86.6	96.9	90.4	95.5
C20ORF74	134.9	146.8	86.1	77.7	75	86.1	117.3	147.1
C20ORF75	112.5	124.1	101.2	94	86	108.8	103.1	111.7
C20ORF77	80.9	93.7	91.7	92.8	93.1	91.6	86.1	103
C20ORF79	89.9	97.5	98.7	99.6	85.2	90.2	90.1	106.8
C20ORF85	89.8	110.4	114.1	93.3	82.2	97.3	87	101.9
C20ORF86	107.6	106.2	93.2	95.1	88.1	95	98.6	121.5
C20ORF94	166.7	196.6	139.4	159.1	144.3	110.7	184	181.9
C20ORF96	97.4	110.4	109.7	104.7	93.8	110.2	113.6	133.8
C20ORF98	283.1	365.8	327.2	297	265.8	292	270.6	416.6
C21ORF10(	83.2	97	103.5	87.1	77.9	96.7	73.7	88.1
C21ORF11(	168.9	214.2	131.7	140.8	111.4	134.6	144.9	185.6
C21ORF12(	78.1	91.3	86	85.2	84.3	80.3	85.5	89.5
C21ORF12(	322.1	429.9	259.1	232.5	184.4	275.8	261.9	324.3
C21ORF12(	77	84.3	93.9	89.6	86.7	91	80.1	83.7
C21ORF13	91.9	106.8	93.2	96.7	90.7	96.5	96.9	97.5
C21ORF2	402.6	465.5	337.9	359.9	348.5	381.9	332.3	345.3
C21ORF24	7767.1	6857	4102.2	6535.8	6472.5	5356.9	7223.2	10556.9
C21ORF25	138.5	238.5	172.6	147.6	132.3	152.9	183	247.2
C21ORF25	397.7	584.9	292.4	324.8	279.1	304.5	375.3	408.6
C21ORF29	87.5	88.2	92.8	80.5	82.8	87.5	85	91.8
C21ORF33	930.1	1083.2	495.6	550.3	420.9	501.3	673.4	761.3
C21ORF33	2919.7	4082	1749.6	1773.6	1347.4	2138.1	1953.1	2447.7
C21ORF33	553.4	692.9	377.7	444.8	336.8	452.1	393.3	472.2
C21ORF34	105.6	140.1	95	89.2	83.3	97.9	119.2	132.8
C21ORF34	88.9	89.3	83.9	78.4	86.6	83.2	98	91.6
C21ORF34	103.5	104.3	80.6	78.3	75.9	74.1	97.7	108.1
C21ORF41	83.8	90.3	87	86.1	82.1	92.9	82.8	89.9
C21ORF45	190.9	270.7	112.1	105.4	84.3	102.4	186.3	210.5
C21ORF49	89.3	101.6	86	80.3	75.9	86.5	90.8	103.5
C21ORF51	618.6	697.2	332.2	472.5	377	637.3	486.9	644.4
C21ORF51	259.3	328.8	187.2	202.6	172.9	329.3	232.6	263.2
C21ORF55	85.6	95	86.8	87	82.2	85.9	83.1	84

C21ORF55	115.4	161.7	94	100.8	99.4	94.6	92.4	105.6
C21ORF55	95.7	98	97.7	89.9	86.6	85.6	83.3	109.2
C21ORF56	356	505.1	207.1	189.6	174.3	160.6	313.5	437.3
C21ORF57	3311.8	4095.9	2156.2	2238.3	2170.8	2930.6	3101.4	4149.2
C21ORF57	396.1	482.8	188.8	266.8	253	346	352.9	512.7
C21ORF57	98.7	108.8	108.3	106.8	95.1	96.6	89.8	94.1
C21ORF58	117.9	116.9	128.8	124.4	122.1	126.1	109.1	123.2
C21ORF58	121.2	135.6	86.3	77.2	79.1	78.7	116.8	120
C21ORF58	94	104.6	115	104.7	102.9	88	92.2	105.1
C21ORF59	88.6	87	93.5	85.1	82.5	85.6	87.9	90.3
C21ORF59	1236.1	1599.5	673.1	647.5	542.4	962.8	978	1152.4
C21ORF6	267.5	363.5	184.7	183.7	147.9	221.4	218.7	238.6
C21ORF62	102.5	128.3	85.4	92.3	76.4	83.6	115.2	123.1
C21ORF63	123.6	136.2	111.2	94.3	91.4	93.8	121.7	146
C21ORF66	254.3	275.8	125.1	146.7	160.3	146.3	159.4	250.9
C21ORF66	179.7	206.8	131.5	135.9	126.5	148.9	133.9	177.2
C21ORF66	217.8	277.3	135.8	137.9	117.6	138.8	154.5	204.4
C21ORF66	1218.3	1459.2	609.8	714	679.5	1222.6	922.5	1226.5
C21ORF67	148.7	152.8	113.4	97	86.7	96.4	116.5	141.9
C21ORF69	140.7	189.5	130.6	119.2	112.1	119.3	121.8	123
C21ORF7	151.2	167.2	108.5	103.2	102	113.8	135.2	161.8
C21ORF70	495.1	619.8	352	321.4	268.1	342.2	476.4	515.6
C21ORF87	144.1	139.2	96.4	84.6	82.3	88.4	126.9	139.2
C21ORF87	115.6	120.5	100	92	76.7	79.2	100	133.6
C21ORF88	118.3	118.9	99.8	89.4	82.6	87.8	110.9	121.4
C21ORF90	118.8	133.2	92.4	87.5	86.6	88.1	116.7	126.5
C21ORF91	165.3	210.3	136.8	132	123.5	151.7	146.8	203.6
C21ORF94	87.2	110	85.6	87	91	98.9	84.4	91.4
C21ORF99	80.7	86.9	82	78.1	74.3	73.1	81.1	91.2
C22ORF13	2781.6	3275.3	2035.9	2285.9	1717.8	2243.4	2257.5	2396.2
C22ORF15	81.3	87.4	114.5	97.3	107.5	97.8	93.2	80.5
C22ORF16	3061.4	3896.9	2842.3	1826.2	1302.7	2183.6	2127.9	3618.2
C22ORF18	1127.3	1544.2	624.3	444.8	361.3	408.1	855.9	943.9
C22ORF23	126.6	156.6	93.9	98.6	103.4	97.3	141.2	144.6
C22ORF25	230.6	251.7	275.1	274.4	261.3	301.3	186.3	292.4
C22ORF5	156.6	196.6	217.1	210.4	187.7	289.9	157.9	250.3
C22ORF8	168.1	225.4	141.9	125.8	123.1	143.3	173.3	196.6
C22ORF9	147.9	179.1	111	109	111.5	86.8	115	158
C22ORF9	988.2	1233.9	769.6	1005.4	886.9	1249.2	758.3	1157
C22ORF9	99.8	92	91.7	94	99.1	84.3	89.7	95.5
C2ORF10	132.8	168.2	125.7	95.3	99.5	137.5	128	163.1
C2ORF11	114	107.3	87	82.3	75.1	87.1	97.9	105.7
C2ORF12	89.9	91.8	93.6	87.1	80.9	96.8	84.4	114.2
C2ORF13	81.6	81.6	92.1	92.7	96.8	95.7	81.7	79.6
C2ORF16	96.1	104.9	98.5	100.4	109.3	88.7	97.6	105.2
C2ORF17	245.9	317.2	279.4	245.7	242.3	342.3	246.1	351
C2ORF18	307.1	499.4	263	214.8	164.4	178.4	284.3	341.8
C2ORF21	131.8	151.1	92	90.9	87.6	79.7	127.1	142.2
C2ORF24	328.4	381	343	266.1	225.5	316.4	285.5	600.2
C2ORF25	4774.3	5111.6	3005.3	3510.1	2131.1	3382.3	4110.7	8986.4

C2ORF26	251	280.5	163	201.9	188.8	213.6	262.1	385.6
C2ORF27	88.9	100.5	84	84.5	82.6	85	83.4	91.8
C2ORF28	6020.4	7454	4363.4	4159.7	2760.6	3993.1	5391.5	6918.1
C2ORF28	3927.9	5290.7	1917.9	1397.9	1331.2	1860	3175.1	4741.5
C2ORF29	3622.4	4393.5	2277.2	2055.8	1535.4	2088.7	2803.2	3403.7
C2ORF3	119.2	145	104.4	104.2	77.2	101.1	111.3	135.4
C2ORF30	1002.7	1215.7	706.5	767.2	579.9	1028.8	897.4	1705.5
C2ORF32	139	188.2	145.6	119.7	110.9	142.3	123.1	153
C2ORF33	823.8	937.5	214.3	246.5	178.2	228.3	782.3	1354.5
C2ORF34	239.8	344.9	193.6	148.3	132.7	184	230.8	292.8
C2ORF4	576.8	896.7	395	340.9	286.9	414.3	452.5	634.5
C2ORF52	88.3	99.9	103.6	93.6	95.9	101	92.1	93.1
C2ORF7	407.7	551.2	223.2	199.8	160.1	248.4	349.1	661.2
C3	96.9	113.2	105.1	102.8	93.3	101.8	100.7	121.6
C3AR1	121.1	135.5	97.3	85.5	85.3	84.4	105.6	110.1
C3ORF1	235.6	293.6	197.1	216.5	184.5	211.1	182.9	226.1
C3ORF10	1662	2069.9	976.2	1357.8	961.5	1289.6	1363.1	1568.8
C3ORF14	2815.4	3592.3	2088.5	1344.7	940.7	1281.3	2434.7	3382.3
C3ORF15	134	161.4	103.2	94.6	85	103.8	129.8	155.1
C3ORF17	2762.3	3244.2	1556	1321.2	1285.1	1750.5	2378.1	3126.6
C3ORF17	166.9	188.9	97	94.4	88.7	93	130.3	162.5
C3ORF17	118.2	171.2	122.3	147.8	104.7	124.7	118.5	149.5
C3ORF18	119.9	128	97.6	89.5	96.5	83.7	117	126.1
C3ORF19	769.3	994.8	616.2	779.2	709	1080.4	574.3	817.2
C3ORF20	85.4	99.5	86.3	85.2	81	82.1	91.8	88.2
C3ORF21	1127.1	1394.7	578	562.6	378.3	421.2	692.5	702.8
C3ORF22	140.1	164.9	102.3	105.8	91.6	102.6	127.2	160
C3ORF23	358.1	494.6	154.2	126.4	102	137.6	256.1	389.3
C3ORF23	98.4	97.6	98.8	92.1	71.5	95.7	97	90.3
C3ORF23	135.7	172	118.2	103.9	102.4	98.4	126.3	140.8
C3ORF23	171.9	240.1	160.5	181.6	145.2	214	154.8	235.3
C3ORF24	75.1	82.4	101.8	86	89.4	82.2	77.9	84
C3ORF25	96.9	111.7	95	102.4	86.7	104.6	99.6	87.6
C3ORF26	2376.8	3238.4	1115.9	1188.9	855.6	1323.7	1826.4	2922.1
C3ORF27	91.3	104.3	95.7	92	84.8	84.5	93.7	98.9
C3ORF28	576	687.2	352.6	286.5	225.1	317.6	532.7	715.7
C3ORF29	2342.8	2844.8	1263.8	1433.8	1378	1468.1	2107.7	1991.6
C3ORF29	520.4	635.4	322.8	364.6	301.7	438.3	487.9	820.2
C3ORF30	91.9	101.3	88.3	77.8	82.3	87.3	90.8	102
C3ORF31	708.1	906.6	428.1	354.5	270.4	367.9	608	788.5
C3ORF32	109.5	132.7	95.8	92.9	86.6	102.1	109.1	124.7
C3ORF33	124.2	157.9	104.1	92.5	87.9	100.6	131	119.4
C3ORF34	270.3	408.5	164.7	180.9	174.4	191	282.6	475
C3ORF34	3365.4	2992.9	2932.3	3340	3097.1	2884.9	3355.5	5722.7
C3ORF35	113.3	131.5	117.8	102.6	101	106.8	117.2	138.1
C3ORF35	85.7	96.3	89.3	80.8	81.5	85.7	88	92.7
C3ORF36	99.3	119.3	96.1	90.1	85.6	91.7	100.3	121.9
C3ORF37	669.5	936.8	501.1	307.2	271.3	323.1	576.7	597.6
C3ORF37	104.6	104.5	96.2	93	85.8	88.8	81.4	108.1
C3ORF38	873.6	1277.6	559.3	470.1	372.8	651.5	602.7	1043.2

C3ORF39	196	238.6	208.4	179.9	139.9	226.3	175.6	272.1
C3ORF40	242.9	311	172.1	135.2	126.6	156.9	254.6	233.6
C3ORF42	92.3	105.7	94.8	91.7	103.7	90.1	92.9	104.9
C3ORF42	84.8	98.5	103.7	99.4	94.8	97.4	94	94.8
C3ORF45	104.7	109.1	93.1	79	80.3	82.9	100.1	110.1
C3ORF48	91	104.4	98.1	90.7	84.7	96.3	89.9	93.2
C3ORF52	87.7	94.4	89.3	92.4	89.5	87.7	94.9	85.5
C3ORF54	499.1	676.1	353.2	281.9	306.2	351.8	484.4	440.5
C3ORF56	89.3	87	87.5	79.7	86.1	79.5	80.2	89.9
C3ORF57	88.1	97.7	93.5	94.8	86.8	94.1	91.8	88.7
C3ORF58	322	385	189.4	228.7	182	243	286.6	610.5
C3ORF59	259.3	297.1	134.4	101.1	89.8	110.7	229.2	243.5
C3ORF60	272.9	326.9	195.9	126.2	117.7	119.6	261.4	262.1
C3ORF60	110	117.2	130.8	114.4	109.5	123.1	98	106.1
C3ORF60	102.6	122.9	84.6	82.8	77.8	84.4	106.5	106
C3ORF60	192.8	249.6	109.7	109.2	106	102.8	173.9	194.8
C3ORF60	11078.1	13395.8	8084.7	7185.6	5514.4	5938.3	11347.7	12406.9
C3ORF62	169.7	194.7	151.2	143.6	158.8	159	168	404.2
C3ORF63	312	499.2	283.7	215.5	221.7	304.3	287.4	307.9
C3ORF64	308.8	358.2	156.9	171.7	159.6	217.1	300	439.2
C3ORF65	85.6	109	92.6	102	90.3	97.6	89.5	98.7
C3ORF9	466.9	664.4	267.3	218.4	192.2	244	382.5	763
C3ORF9	1614.5	2048.6	1010.1	970.5	756.7	1017.5	1181.2	1677.5
C4A	110.2	155	97.1	78.2	73.6	87.3	100.1	130.5
C4BPA	106.8	102.2	94.1	89.7	81.3	86	98.8	107.4
C4BPB	116.6	161.3	109.2	87.7	103.8	97.1	110.6	115.3
C4BPB	88.4	94.5	90.3	81.7	85.3	79.5	96.9	91.4
C4ORF13	167.5	207.7	113.5	101.9	102.5	98.7	137.2	169.2
C4ORF13	354.5	506	242.7	197.5	168.1	260.3	290.7	374.6
C4ORF14	1070.2	1506.7	872.3	693.2	612.3	963	948.6	1466
C4ORF15	95.1	106.6	116.2	102.1	103.2	103.6	92.3	98.8
C4ORF16	99.4	108.4	98.4	87.5	87.5	93.8	96.7	141.6
C4ORF17	81.8	98.6	89.1	81	77.3	85.4	86.2	92.8
C4ORF18	89.5	108.9	96.2	102	99	102.6	100.9	107.4
C4ORF18	90.2	100	92.2	113.4	101.8	115.2	89.2	93.6
C4ORF18	92.7	91.1	96.9	101.1	89.9	86.4	95	102.7
C4ORF28	93.1	102.1	85.6	79.5	80.7	81.7	92.9	86.1
C4ORF32	96.2	101.7	90.1	85.1	93	99.2	90	93.1
C4ORF34	344.7	439.6	188.7	205.7	205.3	215.9	348.2	325.5
C4ORF36	97.3	109.3	114	99.1	80.7	103.7	94.2	101.1
C4ORF6	84	97.4	88	93.9	97.7	93.3	88.3	86.1
C4ORF7	80.3	101.9	97.4	98.6	89.8	98	82.6	89.3
C4ORF8	292.8	324.3	216.8	186.8	173	243.5	259	381.8
C4ORF9	830	1131	711.7	456.1	319.5	495.3	687.8	898.7
C5	963.7	1290.5	770.8	800.5	783.7	1111	706	907.5
C5AR1	89.1	101	91.3	110.8	96.1	107.2	93.8	123.1
C5ORF13	2731.4	3222	1903.5	1790.8	1567.2	2674.1	1704.5	2683.3
C5ORF14	170.6	222.8	116.3	106.4	95.1	98.8	164	175.8
C5ORF15	2066	2824.8	1486.3	1944.5	1657.3	2108.3	1760.3	1979.2
C5ORF16	261.4	305.3	215.4	168.6	157.2	312.8	206.4	350.2

C5ORF20	520.1	662.5	493.6	673.6	624.8	684.9	366.3	402.1
C5ORF21	897	1121	571.6	715.1	556.2	823.6	761.9	1066.3
C5ORF28	2219.7	2269.9	893.3	1537.5	1574	1232.1	1909.1	2403
C5ORF3	173	239.1	138.6	131.3	133.8	156	162.1	225.9
C5ORF34	140.4	145.6	110.3	102.5	100.9	119.9	125.9	169.6
C5ORF36	79.1	86	92.4	80.5	84.2	82.1	78.3	88.7
C5ORF37	592.5	849.3	436.3	436.5	398.7	557.3	551.6	749.3
C5ORF4	92	106.2	88.8	84.4	87.2	90.7	101	95.6
C5ORF4	77.2	89.8	97	85.4	83.2	87.5	77	80.9
C5ORF4	101.5	109.7	99.1	101.5	95.5	96.1	103.5	99.2
C5ORF5	788.3	867.4	392.5	708.3	596.2	966.6	663.7	1278.8
C6	82.7	107.7	102.6	91.5	88.8	94.8	97.6	96.1
C6ORF1	117.4	127.4	108.5	93.1	106.5	101.8	121.1	142.8
C6ORF1	268	317.3	176.2	117.6	129.5	115.9	271.6	381.3
C6ORF1	163.3	216.5	133.6	107.9	110.6	107.6	141.7	197.9
C6ORF10	114.2	125.8	96.7	88.3	90.9	83.3	120.8	124.3
C6ORF105	98.9	109.6	92.6	85.7	82.3	92.6	101.4	109.5
C6ORF106	204.6	267.1	156.6	162.6	147.4	124.1	245.7	234
C6ORF106	204.1	278.5	208.4	216.2	185.5	276.6	209.7	263.8
C6ORF107	124.5	123.1	113.7	91.4	103.4	101.8	116.3	120.4
C6ORF108	119.7	127.5	100	92.7	80.3	101.3	118.5	139.4
C6ORF108	4981.8	6737.6	4777.7	4121.3	3195.5	5280.8	4159.1	4852.3
C6ORF111	1130	931.8	952.6	915.7	842.9	1827.8	565	1156.8
C6ORF113	742.3	949.2	390.8	401.3	360	629.5	662.6	1197
C6ORF114	82.4	95.8	82.5	73.5	69.4	74.7	85.2	92.8
C6ORF117	162.8	206.3	163.9	456.1	401.7	475.8	155.8	188.5
C6ORF118	86.9	99.8	102.7	95.6	89.4	100.9	95	101.3
C6ORF120	217	283.6	159.1	209.8	167	301.4	200.8	358.4
C6ORF122	89.9	92.7	97.4	90.8	88.5	92.5	88.3	87.2
C6ORF125	5968.6	6814.8	4224.9	3830.7	2877.1	4577.6	4442.2	4889.7
C6ORF128	94.6	125.9	96.6	93.6	96.6	104.7	94	107.8
C6ORF129	1431.8	1626.1	1075.5	754.6	609.8	906.8	1443.3	1738.6
C6ORF130	1130	1537.8	893.1	958	803.7	1082.6	1093.4	1630.6
C6ORF134	111.2	136	94.9	81.2	82.4	93.5	100.9	116.1
C6ORF134	94.6	102.3	115.5	93.4	92.5	97.9	83.7	104.2
C6ORF134	93.3	92.5	96.1	91.2	83.2	78.7	85.7	92.4
C6ORF136	509.3	565.8	210.6	179.8	146	144.2	495.1	530.2
C6ORF139	636.5	838.7	313.3	267.8	262.5	422.8	462.7	948.7
C6ORF142	90.3	97.3	90.4	87.8	78.9	86.2	97.3	94.1
C6ORF143	83.3	89.2	104.8	99.7	88.3	100.9	82	83.9
C6ORF145	127.7	128.7	82.4	83.9	73.7	79.5	122.1	166.2
C6ORF146	80.2	91.7	94.6	84	74.4	87	80.1	78.8
C6ORF148	258.9	317.9	217.9	162	139.5	171.3	243.5	356.6
C6ORF149	239.5	296.2	146	165.8	149.7	177.9	220.6	318.9
C6ORF15	98.5	99.4	90	82.1	78.3	81.3	88	97.8
C6ORF150	445.8	603.8	187.7	192.4	166.7	149	339.9	477.1
C6ORF151	114.4	127.1	90.4	107.3	105.3	107.6	115.9	172.4
C6ORF152	124.1	147.1	98.2	104.8	91.5	101.5	118	142
C6ORF153	3266.6	4208	885.9	862.9	704.8	897	2892.6	2975.5
C6ORF154	104.4	113.5	84.4	79.3	82.7	82.2	101.9	121.7

C6ORF157	164.2	181.9	112.3	101	97.7	111.6	142.5	259.1
C6ORF159	96.7	108.9	109.9	99.2	97	92.6	92.7	91
C6ORF162	215.6	293.1	171.8	204.8	167.2	252.6	178.6	243.9
C6ORF162	215.4	287.3	146.5	179	165	212.4	170.5	219.1
C6ORF165	108.2	124.5	96.1	94.3	87.6	101.7	112	122.9
C6ORF165	82.2	97.2	96.6	86.9	84.3	86.5	91.2	90.3
C6ORF165	89.4	107.8	105.6	105.5	99.7	103.6	93.5	100.1
C6ORF166	1221	1497.4	1156.4	1173.8	814.5	1394.8	1534.4	3842.7
C6ORF167	321.8	484.3	159.8	141.7	130.2	163.2	292.9	499.3
C6ORF168	94.9	106.9	104.9	83.4	84.8	90.2	96.4	100.3
C6ORF170	288.4	337.9	215.5	237.9	225.1	245.9	282.8	338.4
C6ORF173	2684.3	2890.6	1229.3	1098	810.7	1169.4	2834.7	4001.1
C6ORF174	83.9	84.1	109.9	104.2	99.7	101.1	79.6	80.9
C6ORF182	135.6	157	118.7	108.9	102	106.4	130.3	170.1
C6ORF188	74.8	77.1	101.4	92.9	92.5	98.1	77.6	87.6
C6ORF190	117.4	121.9	99.4	98.5	124.1	112	117	120.7
C6ORF191	96.9	121.2	87	92.3	80.7	85.4	99.6	111.4
C6ORF192	74.4	90.2	102.3	84.8	84.4	83.9	72	82.1
C6ORF194	82.3	85.4	94.9	76.1	77.3	77.6	82.2	86.9
C6ORF195	84.8	98.3	97.5	100.3	95.3	87.7	87.7	98.6
C6ORF199	89.6	98	91.3	87.7	78.2	93.5	87.8	100.6
C6ORF201	82.2	98.6	99.2	80.1	86.1	94.8	87	97.6
C6ORF203	283.5	337.9	136	119.2	112.8	130	251.6	463.7
C6ORF204	120.2	138.9	96.8	103.9	103	102.8	140.6	128
C6ORF204	150.5	185.6	99.7	114.5	91	94.7	153.9	204.4
C6ORF205	289.1	350.4	132.4	111.9	110.7	129.3	254	430.8
C6ORF205	94.4	97.7	96.5	84.6	94.9	97.6	90.8	93.4
C6ORF206	80	88.1	86.1	85.5	84.1	91.2	69.6	85.7
C6ORF208	121.1	129.5	101.7	89.5	90.5	91.8	107.4	122.6
C6ORF21	123.5	134.3	96.1	89.4	94.5	89.6	113.1	120.3
C6ORF211	187	215.5	121.3	141.9	111.2	150.2	195.2	285.1
C6ORF213	95.1	109.6	107.8	103	89.8	93.8	95.3	96.5
C6ORF25	115.1	144	131	118.6	101.7	109.9	112.2	126.4
C6ORF25	89.6	104.6	93.7	89.7	91.2	96.5	86.4	95.2
C6ORF25	128.3	134.9	102.9	83.5	83.2	87.9	118.9	141.8
C6ORF25	94.7	108.3	89.9	85.9	85.2	87.3	102.8	109.6
C6ORF26	248.6	258	167.5	197.1	205.3	324.7	178.7	218.3
C6ORF27	96.1	113.9	100	101	104.5	93.5	90.6	112.2
C6ORF32	102.3	109.4	94	98	86.3	88.1	89.6	88.8
C6ORF35	119.7	128.4	90.6	100.2	79.3	94.5	121.7	140.9
C6ORF47	556.5	737.6	247.8	287	310.2	305.2	485.6	444.7
C6ORF48	152.1	191.5	123.3	102.4	100.5	110	163.7	155.1
C6ORF48	14402.4	17704.9	11827.8	12646.7	10498.1	12360.7	14481.4	18019.1
C6ORF49	11879.2	14474.7	5476.9	3566.6	3103.7	5279.7	10349.9	11845.6
C6ORF51	92.7	107.4	87.2	86.9	84	82.8	97.4	108.2
C6ORF55	580.6	737.2	505.3	676.8	478.4	870.2	436.5	641
C6ORF57	912.5	1150	514.4	500.8	425	417.7	768.6	1079.3
C6ORF59	117.8	132.2	83.5	97.5	95.4	106.7	121.5	141.6
C6ORF60	171.2	222.2	179.2	175.4	174.3	239.7	177.3	197.1
C6ORF62	84.8	106.1	102	94.7	80.1	91.7	92.1	94.6

C6ORF64	319.3	413.6	224.3	253.3	242.9	245.5	278.5	247.1
C6ORF65	133.4	157.8	158.7	162.2	141.3	194.4	183.9	297.4
C6ORF66	597.9	608.2	209	197	137.7	201.5	548.1	933.7
C6ORF69	926.3	1140.9	912.1	1096.9	835.3	1171.7	928.2	1496.3
C6ORF70	556.4	794.7	423.2	535.9	481.5	774.5	444	721.8
C6ORF71	66.2	82.6	94.3	89.3	79.8	88.1	77.5	73.2
C6ORF72	484	677.2	167.3	126.4	118	133.7	405.2	691.1
C6ORF75	78.2	86.5	89.5	82.5	76	81	70.6	95.3
C6ORF75	85.7	92.2	91.3	86.9	85.8	94.3	87.5	87.6
C6ORF75	953.1	1286.3	544.7	532.4	397.1	853.5	658.3	1013.5
C6ORF78	87.4	93.2	103.9	91.5	87.6	93.7	93.9	100.3
C6ORF79	586	644.1	406.5	369.7	257.7	355.8	514.6	1010.8
C6ORF81	94.6	120.2	92.7	98.6	90.4	91	101.5	112.3
C6ORF84	231.2	247.1	187.8	183.9	183.1	294.7	177.6	243.8
C6ORF85	95.8	99.5	101.2	104.8	99.3	101.8	92.3	103.3
C6ORF89	308.1	434.7	241.8	205.9	177.7	244.4	231.2	395.6
C6ORF96	420.8	497.3	217.2	224.4	171.5	244.8	353.5	590.9
C7	96.5	110.2	79.3	73.6	68.2	83.8	95.1	106.1
C7ORF10	116.9	136.3	112.9	103	94.7	98.5	122.1	139.1
C7ORF11	243.9	249.8	149.5	173.5	120.1	150.4	234	484.7
C7ORF16	87.1	96.4	101.6	89.3	87.9	98.3	88.7	87.3
C7ORF20	546.6	696.9	439.4	369.5	365.5	443.6	484.1	626.5
C7ORF20	261	348.6	166	168.2	154.6	145.1	210.4	247.7
C7ORF21	778.5	993.5	881.8	706.3	556.8	775.9	807.9	1672
C7ORF24	2816.9	3298.4	1201.1	876.3	781.8	1459.3	1849.2	2862.2
C7ORF25	329.4	484.2	211.7	182.3	184.3	227.2	270.4	414.6
C7ORF26	572	635.8	690.7	520.8	426.8	614.9	446.5	1034.4
C7ORF27	1817.8	2033.5	1013.2	810.7	628.8	837.8	1277	1004.7
C7ORF28A	210.8	309.7	151.1	127	118.7	121.2	197.5	205.8
C7ORF28B	1188.1	1468.8	698.4	648.5	538.9	810.2	1118.6	1663.3
C7ORF29	124.2	126.3	111.3	111.9	98.3	120	116.2	120.9
C7ORF30	3814.2	4874	3376.6	3061	2102.8	2638.2	3975.6	5258
C7ORF31	85.6	90.3	97.8	94.9	86.2	88.4	87.2	99.2
C7ORF33	75.1	86.6	102.8	86.4	86.6	94.8	82.5	86.3
C7ORF34	81.1	97.2	85.1	87.3	81	86.3	91.8	93.6
C7ORF36	432.9	494.9	202.7	202.4	146.1	226.8	426.8	739.2
C7ORF44	2066.3	2487.9	1229.3	1484.3	1220.1	1696	1845.6	2153.3
C7ORF9	97.9	108	91.3	84.3	86.5	94.4	95.7	112.8
C8A	90.6	93.1	91.4	83.1	76.3	91.5	103.6	93.9
C8B	83.9	88.5	90.4	76.4	83	91.5	89.2	94.2
C8G	125.1	139.7	105.8	90.1	90.8	100.7	122.2	127.5
C8ORF1	646.3	832.6	242.4	222.1	199.1	323.2	534.7	914.9
C8ORF13	103.6	105.6	94.7	79.1	78.5	101.2	103.2	108.5
C8ORF15	87.8	121	99	89.2	90	94.1	96.9	96.1
C8ORF22	86.7	90.2	92	93.7	87.9	92.1	88.2	86.3
C8ORF30A	93.9	131.3	87.6	92.6	85.2	84.3	108.6	138.1
C8ORF31	93.7	116.9	108.9	92.2	88.6	82.1	103.1	117.2
C8ORF32	664.5	855.9	439.1	286.6	220.4	300.7	572.1	915.7
C8ORF33	1858.6	2413.3	1070.6	880.9	596.5	837.9	1645.1	1802.7
C8ORF34	115.6	125.8	144.4	125.7	125.9	117.7	116.3	103.2

C8ORF35	2411.1	2834.1	1376.7	1097.3	988.4	1785	1800	2647.6
C8ORF36	729.5	949.8	545.7	406.9	432.2	584	702.5	725.3
C8ORF37	3914.2	4581.5	1629.1	2478.5	2855.7	1743.4	3588.3	4595.7
C8ORF38	913.6	1039.9	400.1	298.1	221.4	399.6	793.3	1445
C8ORF4	88.1	100.6	83.9	79.8	73.2	89.9	85.5	113.1
C8ORF40	3694.6	4459.8	1735.2	1362	932.9	1241	3099	3507.5
C8ORF41	522	599.7	247.6	185.5	168.7	203	450.6	592.7
C8ORF42	78	89.7	81.4	86.6	88.8	92.4	86.5	76.6
C8ORF44	145.7	165.4	132.8	130.1	132.8	115.2	127.2	149.5
C8ORF45	1765.3	1776.5	515.1	691.5	838.9	666.5	1617.3	2325.3
C8ORF46	78.6	78.3	102.3	104.7	83.5	88.8	79.9	86.3
C8ORF47	127.7	163.9	116.6	83.6	90.3	92	142.1	171.5
C8ORF48	111.5	134.1	98.9	89.7	91.5	92.7	118.1	121.3
C8ORF49	74.4	92.5	89.4	90.4	81.7	76.9	82.8	83.8
C8ORF52	859.4	988.2	260.6	241.2	195.3	248.2	795	1453.6
C8ORF53	184.3	184.1	136.6	114.8	117	124.7	189.2	308.8
C8ORF55	1182.5	1627.2	643.5	461.4	377.8	418.9	914	608.5
C8ORF58	122.1	147.2	115.8	109.8	101.4	115.5	138.4	171.7
C8ORF61	101.7	106.6	115.2	113.5	103.1	115.9	102.9	148.7
C8ORF70	127.1	157.1	123.6	96	90.8	98.9	144	167.8
C8ORF72	146.6	192.9	124.2	118.7	118	120.8	127.4	151.7
C8ORF74	78.6	84.5	102.4	80.2	88.8	88.7	79.8	82.2
C8ORF76	2483.9	3006.2	1826	1430.5	1069	1579.3	2721	3188.7
C8ORFK32	101.6	136.2	98.8	93.5	94.2	84.4	112.3	116.4
C8ORFK36	111.1	142	94.2	81.4	87.1	82.3	114.9	113.9
C8ORFK36	73.2	88.9	87.8	91	85.8	83.5	80.4	73.9
C9	76.8	89.3	95.8	85.2	78.7	89.3	82.8	90
C9ORF100	269.4	330.6	189.9	106.7	114.5	136.9	197.3	164.8
C9ORF100	562.1	641.7	287.7	238.1	161.7	245.8	421.3	630.4
C9ORF102	256.7	288.4	232.9	263.6	234.9	321.2	226.9	304.8
C9ORF103	605.7	711.8	486.4	406.3	327.5	388.1	485.1	625
C9ORF106	113.3	118.9	113.1	106.4	106.1	118.2	92.6	116.2
C9ORF10O!	493.8	622.5	263.3	211.6	165.5	326.2	374	629.3
C9ORF11	77.9	84.6	105.4	93.2	87.5	84.5	80.8	84.1
C9ORF111	406.3	410.7	392.3	432.5	546.5	440.2	314.4	255.3
C9ORF112	178.8	214.5	161.4	131	118.1	133.1	162	201.2
C9ORF114	1181.5	1642.3	932.6	532.2	414	498.6	949.6	796.6
C9ORF116	396.7	518.5	275.5	156.5	149.7	155.7	321	350.6
C9ORF116	385.4	535.4	252.4	173.4	136.2	173.8	285.9	383.3
C9ORF117	83.5	120.4	75.9	85.9	80.6	74.7	106.9	106.6
C9ORF121	88.2	102.5	90.3	88.9	82.9	85.2	85.9	82.8
C9ORF123	223.4	243.5	182.8	220.4	166.9	218.4	172.1	232.4
C9ORF125	238.8	291.8	193.4	142.9	113	167.5	183.9	269
C9ORF126	102.9	116.4	100.5	86.2	84.1	98.1	95.4	122.5
C9ORF127	206	242.3	178.4	158.1	128.1	166.7	187.9	193.5
C9ORF127	121.7	134.1	89.4	75.3	77.6	85.9	119.7	163.4
C9ORF128	95.7	108.1	94.3	91.4	94	90.6	88.6	104.2
C9ORF132	122.3	121.3	100.5	92.7	85.4	96.9	124	143.9
C9ORF138	89.4	89	90.9	96.4	97.4	86	84.3	93.9
C9ORF140	1056.6	1250.7	1849.3	835.3	734.3	1265.6	979.7	2447

C9ORF142	3833.2	4655.8	4028.1	2075.9	1794.7	2548.8	3145.9	4764.1
C9ORF150	95.3	93.2	105.1	91.6	80.5	83.5	89.3	96.7
C9ORF152	98.5	111.6	81.4	82	78.4	80.9	99	108.3
C9ORF153	83.8	93	88.3	74.9	78.8	80.3	85.6	91.2
C9ORF156	506.3	700.7	440.6	314.7	282.2	357.2	512.6	701.1
C9ORF157	83.7	90	81.5	72	79.5	85.6	81.5	87.8
C9ORF16	205.3	249.9	146.9	128.8	113	117.6	164.5	213.3
C9ORF166	112.5	132.5	87	88	80.7	92.3	110.3	121.1
C9ORF18	87.7	81.1	93	89.6	81.3	83	91.4	85
C9ORF19	2309.7	3095.3	1765.6	1442.7	1057.6	1473.7	1987.6	2073.7
C9ORF21	427.3	522.2	241.8	175.5	165.5	281.5	406.8	608.7
C9ORF23	389.2	513.8	249.3	179.4	147.1	164.8	298.4	395.1
C9ORF23	262.2	298.1	206.5	126.8	126.5	128.6	182.4	211.9
C9ORF23	1013.1	1295.4	791.2	483.7	356.3	511.2	946	1029.4
C9ORF24	100.6	115.8	118.5	103.4	102.6	105.6	98.8	97
C9ORF24	117.9	123.9	118.5	108.8	103.4	110.1	119.2	110.8
C9ORF24	83.9	91.5	91.2	81.8	86.1	109.5	80.3	84.9
C9ORF25	127.8	117.2	114.2	118.3	114.9	119	123.4	141.9
C9ORF28	394.6	452.3	634.3	687.5	532.7	812.6	380.2	782
C9ORF3	102.9	120.6	101.5	101.3	97.4	98.1	109.4	108.8
C9ORF30	507.3	616.4	220.6	178.1	161.4	263.5	488.1	888.4
C9ORF32	535.7	655.4	293.6	194.9	183.2	273.6	461.4	527.5
C9ORF36	72.9	79.2	96.7	89.2	92.3	95.5	74.7	83.5
C9ORF37	278.9	308.8	143.5	141.9	137.7	133.1	176.6	196.9
C9ORF39	257.2	336.3	222.8	231	207	350.2	231.7	274.4
C9ORF4	86.6	105.5	93.1	81.4	82.2	90.8	82.9	94.9
C9ORF40	784.3	952.2	456.1	274.9	215.9	332.5	609.1	733.6
C9ORF41	95.8	105.3	110.1	99.1	90.4	97.6	96.1	94.4
C9ORF43	127.4	152.5	95.8	82.8	74.5	88.6	121.9	144.4
C9ORF45	184.6	254.3	189.3	275.3	271.5	347.9	167.8	172.6
C9ORF46	1909.3	2551.9	1449.2	1631	1157.4	1838	1515.4	2645.3
C9ORF47	102.4	115.9	90.8	81.7	80	81.6	104.9	122.2
C9ORF48	161.5	194.3	166.7	137.8	123.4	205	151.5	211.9
C9ORF5	497.4	661.8	243.9	330.4	208	295.3	595.4	1700.8
C9ORF50	103.6	109.9	98.3	96.5	91.5	82.3	100.6	91
C9ORF52	84.5	88.8	91.7	82.8	77.2	78	85.7	94.9
C9ORF58	111.3	110.7	96	97.6	81.3	85	104.7	122.3
C9ORF58	102.3	102	87.5	81.2	81.1	79.3	99.6	111.2
C9ORF6	634.2	784.9	501.9	440.9	364.8	634.3	484.1	1031.7
C9ORF61	80.8	100.3	108.4	96.8	88.6	79.6	88.3	109
C9ORF64	326.2	471.8	237.4	232.6	201.8	309.8	274.8	412.8
C9ORF66	464.4	590	320.7	327.6	267.9	296.3	339.3	400.5
C9ORF68	91	92.3	88.5	93.4	78.3	85.2	83.6	87.2
C9ORF7	128.7	151.7	125	123.7	123.5	156.6	122.1	146.6
C9ORF71	88.6	78.9	83.5	88.2	80.3	86.3	85.3	77.8
C9ORF72	86.6	93.3	101.3	88.6	92.3	90.1	93.3	100.4
C9ORF72	74.1	88.5	84.8	81.4	82.3	87.9	79.6	87.8
C9ORF72	107.1	130.3	94.8	94	91.3	95.5	113	126.2
C9ORF74	2450.6	2949.1	1800.8	1148.2	898.5	1245.9	2106.3	2070
C9ORF75	210.9	250.2	163.6	121.9	119.4	143.3	195.6	223

C9ORF77	444.2	597.1	215.1	118.9	110.2	133.9	338	464.9
C9ORF77	108.6	138.4	122.3	110.7	109.3	116	110.4	129.5
C9ORF77	424.5	557.1	215.1	174.3	164.7	181.5	398.3	765.4
C9ORF78	3827.6	4772.3	2342.6	2320.4	1638.7	3016.7	3114.8	4547
C9ORF79	107.8	110	108.5	97.8	96.4	92.5	99.8	114.5
C9ORF80	5620.8	6020.8	2255.6	4356.3	4012.5	2749.9	5292.8	5707.7
C9ORF82	288.7	386	195.9	188.8	137	209.5	229.9	312.5
C9ORF84	110.9	97.3	116.1	97.9	98.8	106.6	91.2	85.8
C9ORF84	77	78.5	90.2	85.4	83.6	82.2	72.5	84.3
C9ORF85	225.3	263.8	120.7	117.7	90.5	111.4	232.6	443.8
C9ORF85	127.4	151.4	88.1	93.2	79.9	87.9	146.2	174
C9ORF86	609.7	781.2	765.6	682.1	612.3	1159.8	562.4	952.8
C9ORF88	1642.5	2265.6	1073.1	642.8	443.7	552	1498.3	1444
C9ORF89	553.3	697.8	463.2	300	258.5	362.9	709.1	1033.4
C9ORF9	113.9	142.7	108.8	101.2	102.2	96.3	104.2	106.3
C9ORF90	682.3	831.9	559.2	436.8	381.5	424.2	652.2	599.8
C9ORF91	421.4	591	376.9	254.1	222.2	260.9	405.2	419.4
C9ORF93	118.5	139.4	127.4	115.9	119	156.3	114.5	113.9
C9ORF95	223.8	226.6	112.3	127.7	116.5	130.7	206	280.7
C9ORF96	77.9	87.4	96.1	86	88.9	89.4	86.3	102.9
C9ORF97	147.9	190.2	164.8	139	130.1	135.6	130.9	168.3
C9ORF98	112	117.3	96.1	89	81.8	100.8	104.9	135.6
CA1	84.8	98.8	88.5	101.7	88	82.7	87.7	96.5
CA10	90.6	90.1	84.7	83.8	81.6	80.3	90	102.8
CA11	279.8	330.5	194.5	107	118.6	116.1	237.4	303.2
CA12	92.8	99.4	97.2	81.5	83	92	97.4	96.8
CA12	79.5	85.9	83.5	79.1	64.9	79.2	76.1	89.1
CA13	158	204.1	125.6	95	100.1	98.2	132	157.7
CA14	86.8	97.3	92.3	93.4	87.4	83.2	92.1	99.6
CA2	17262	18585.3	8786.3	6970.2	3838.9	6598.2	15256.1	21638.2
CA3	80	88.6	100.5	95.6	91.3	100.2	75.5	83.3
CA4	102.4	124.9	116.5	107.5	100.4	105.8	108.6	96.5
CA5A	85	89.7	87.5	85.7	86.6	79.5	86.5	82.8
CA5B	142.5	165.5	135.4	118.7	100.6	128	139.8	206.8
CA6	126.3	132.2	113.5	104	107	104	112.5	126.3
CA7	102.3	125.9	99.4	82	83.2	85.2	100.6	116.8
CA7	94	96	99.4	91.6	85.5	88.4	80.2	87.2
CA8	86.3	92.7	87	95.2	88.4	92.1	85.3	101.5
CA9	104	133.8	96.7	101	92.1	90.7	111.4	126.4
CAB39	3791.8	4762	2715.2	3444.5	2476.7	4157.6	3432.7	4492.5
CAB39L	230.2	283.1	176.8	161.7	160.9	185.6	214.7	248.7
CAB39L	73.2	88.7	100.2	84.8	75.4	85	78.5	84.6
CAB39L	143.2	174	111.4	88.4	85.9	102.2	133.4	147.7
CABC1	1635.6	2020.7	1243.8	1073.4	889.8	1294.6	1581.1	2318.7
CABIN1	161.4	184.3	241.2	206.2	197.9	225.8	164.2	260.4
CABLES1	254.5	313	163.2	127.4	108.1	124.6	244.4	252.1
CABLES2	268.1	362.3	247.3	200.8	191	204.8	262.2	344.7
CABP1	93.3	98.1	80.2	87.1	88.1	87.6	108.6	103.3
CABP1	81.2	83.9	92.4	96.3	98.5	99.5	83.3	85.1
CABP1	80.8	88.9	92.4	82.1	77.3	96.4	84.7	86.6

CABP2	87.7	118.3	100.2	89.5	91.1	92.7	96.1	108.1
CABP2	94.8	109.1	96.1	95.3	82.9	97.3	96.4	97.1
CABP2	100.5	136.6	96.2	85.6	91.5	96.2	101.4	115.2
CABP4	78.7	88.3	88.1	87.3	87.2	89	74.8	85.3
CABP5	98.2	94.3	82	81.2	84.3	85	90.6	91.6
CABP7	144.1	154.7	102.2	103.5	97.9	88	126.3	138.9
CABYR	87.7	87.1	75.6	81.4	80.2	78.1	80.6	80.4
CABYR	107.8	116.6	104.3	89.5	87	94.5	98.6	98.1
CACHD1	113.7	127	109.6	94.7	98.3	112.8	103	133
CACNA1A	78.4	93.2	87.8	89.4	76.9	82.1	84.5	92.6
CACNA1A	76.4	94.2	90.4	80.2	82.4	86.7	91.8	95
CACNA1B	240.8	298	141.8	144.7	129.4	159.2	282.7	323.1
CACNA1C	107.4	127.2	91.4	86.4	81.2	83	99.8	111.2
CACNA1D	109.6	121.9	111.6	101.6	131.3	117.1	101.7	95.7
CACNA1E	109.5	118.2	115.2	114.6	105.7	135.1	104.4	95.9
CACNA1F	82.2	102.6	80.2	79.1	82.7	87.2	89.8	100.1
CACNA1G	76.6	94.4	87.5	87.1	86.6	90.3	88.9	90.1
CACNA1G	77.1	87	99.6	91.1	90	91.6	77.8	78.1
CACNA1G	89.1	93.5	97.7	91.7	79.6	86.2	84.2	97.8
CACNA1G	91.6	101.1	85.1	91.1	82.2	83.8	95.5	91.6
CACNA1H	102.4	103.8	109.7	97.7	101.5	104.3	87.6	91.1
CACNA1H	79.2	78.4	97	92.4	83.8	89.2	75	77.3
CACNA1I	81.8	95.7	93.6	85.2	79.5	89.6	81.7	82.5
CACNA1I	86.2	90.8	94.3	82.9	83.8	80.1	87.1	91.1
CACNA1S	90.4	109	84.4	75.9	74.9	85.2	99.5	108.6
CACNA2D1	97.6	100.1	87.9	92.2	88.2	92.8	86.6	87.4
CACNA2D2	138.4	185.7	100	103.2	98.8	96.1	135.5	148.6
CACNA2D2	94.6	130.4	108.2	97.3	91.1	103.3	93.5	101.5
CACNA2D3	1124.1	1436.3	527.7	637.5	506.9	733.5	656.2	667.8
CACNA2D4	124	151.9	111	98.2	93.6	106.2	117.6	117.6
CACNA2D4	411.1	496.8	277.1	204.7	187.6	260.2	347	511.1
CACNB1	96.1	111.1	96.7	100.3	93	83.3	111.9	114.1
CACNB1	96	102.2	96.8	87	83.6	101.9	90.7	93.3
CACNB1	75.9	74.5	86.6	89.2	76.4	85.1	66.8	77.8
CACNB1	79	81.1	100.4	91.7	92.2	82.2	86.8	91
CACNB2	76.6	96.2	100.7	91.3	92.4	85.1	82.9	80.2
CACNB2	98.1	105.7	82.9	95.6	78.4	80.6	93.9	113.4
CACNB2	95	113.1	88.8	87.5	79.6	78.5	97.4	104.7
CACNB2	80	92.2	97	90	83.2	93.9	84.1	96
CACNB2	68.2	87.6	85.9	79.4	75.7	81.8	76.5	81.5
CACNB3	198.4	218.5	192.7	161.4	154.6	178.3	136.5	126.5
CACNB4	122.8	141.1	105.9	93.9	98.8	107.1	123.9	149.1
CACNB4	89	108.9	92.4	71.5	78.9	80.6	80.9	87.9
CACNB4	90.6	102.1	88.6	81.4	87.2	87.4	85.6	93.4
CACNG1	83.9	95.1	92.5	81.1	81	87.4	85	90.6
CACNG2	78	88.4	99.1	83.4	90.3	76.7	87.4	86.7
CACNG3	106.7	111.5	98.5	88.3	91	98.7	119	108
CACNG4	115.8	142.6	94	101	99	98.3	105.7	116.7
CACNG5	101.7	107.6	91.2	82.9	88.1	85.1	100.3	110.2
CACNG5	99.5	130	103.4	92.3	85.6	93.5	103.6	115.8

CACNG5	91.3	113.9	87.8	86.3	74.7	74.5	102.3	95.3
CACNG6	90.3	95.8	91	87	81.3	87.7	95	88.8
CACNG6	83.6	94.3	93.8	81.2	87.4	93.4	83.4	81.8
CACNG6	112.3	133.3	95.7	81.4	82	84	104	127
CACNG7	80.9	91.2	85.1	86.2	77.3	79.2	79.9	77.1
CACNG8	112.7	135	118.2	104.7	97.7	104.4	116.8	116.2
CACYBP	3103.5	3666.7	1857.8	1115.2	1047.5	1556.8	2272.5	2546.6
CACYBP	529.7	713.6	231.5	209.4	184.8	225.5	504	696.1
CACYBP	96.3	102.6	80	84.1	78.7	73.9	101	113.4
CAD	210.1	241.2	278	292.5	196.5	346.2	165.8	219.5
CADPS	78.6	90	89.3	87.6	90.4	83.6	88.8	82.6
CADPS	109.5	115.9	107.1	95.6	93.6	105.4	94.4	124.5
CADPS	78.1	89.2	90.8	70.2	78.2	79.6	93.5	91.6
CADPS2	67.8	88.7	91.2	91.8	82.5	95.4	81.1	86.8
CADPS2	120.1	167.5	133.2	100.2	88.8	106.2	122.3	129.3
CAGE1	103.2	120.5	116.5	118.3	104.5	110.7	111.6	117
CALB1	78.7	98.7	94.9	95.3	89.2	88.1	89.5	102.3
CALB2	106.7	130.5	129.4	106	111.7	97.9	113.4	136
CALB2	83	86.3	92.9	88.8	77	79.9	82.4	87.5
CALCA	96.6	102.2	84.8	87.2	86	81.2	98.4	104.9
CALCA	98	107.3	97.3	102.2	87	91.6	93.4	102.5
CALCA	73.6	93.1	79.5	76.1	86.7	79.6	75.9	85.9
CALCB	82.9	102.5	106.6	95.7	83.3	92.5	93.2	87.4
CALCOCO1	101.9	124.9	104.7	103.1	109.1	126.6	100.4	107.1
CALCOCO2	261.7	342.8	174.1	231.9	186.1	216.6	273.1	307.2
CALCR	101.6	109.7	92.8	95.9	80.6	86.1	98.8	110.7
CALCRL	85.7	81.9	82.1	80.8	75.1	84.3	87.1	94.9
CALD1	122.1	153.1	88.4	92.4	86.3	94.7	124.3	140.7
CALM1	1539.5	2028.2	1543	1340.3	997.8	1756.4	1253.1	2407.3
CALM2	14024.8	16461.9	7145.4	7967.3	5629.2	7235.2	13730.5	17642.2
CALM3	11063.9	13497.1	7042.3	5405.1	4433.7	5071.9	9649.1	9005.2
CALML3	94	98.1	119.4	103.1	96.2	97.5	94.2	94.7
CALML4	93.5	127.1	100.1	87.2	80.3	89.4	91.1	97.6
CALML4	168.6	160.4	118.6	107.9	112.8	127	173.4	179.4
CALML4	1596	1684.8	757.6	700.2	651.2	847	1592.6	1428.8
CALML4	418.5	551.4	293.2	408.7	347.1	560.2	348.9	458.3
CALML5	143	171.5	98.2	89.4	86.1	89.6	118.6	154.6
CALML6	92.9	98.1	92.1	85.3	94.4	91.1	83	92
CALN1	131.1	165	91.6	92.5	89.9	89.5	138.1	138.2
CALN1	265.5	385.2	196.8	182.1	150.8	205.4	279.8	335.8
CALN1	85.5	82.3	110.5	116.8	113.4	110.8	78.7	90.8
CALR	1692.6	2023.6	1002.6	1094.5	1018.4	1387.4	1446.8	2148.4
CALR3	84.9	97.8	105.7	103.7	93.4	99.4	86.3	86.6
CALU	643.4	827.3	469.7	544	359.1	753.8	555.6	932.9
CAMK1	780.7	938.4	789.4	991.4	691.7	1119.2	585.2	879.5
CAMK1D	227	313.9	210.6	142.2	124	131.6	196.8	238.1
CAMK1D	73.6	85.9	76.6	85.1	75.9	82	73.9	88.2
CAMK1D	88.8	98.1	93.1	79.4	88.7	88.1	86.9	87.6
CAMK1G	107.8	136.1	96.1	94.2	87.1	98.5	105.8	131.5
CAMK2A	89.7	107.6	89.3	84.7	82.9	86	88.9	105.9

CAMK2B	103.8	121.6	99.4	90.4	90.7	102.8	102.4	134
CAMK2B	147.2	170.8	194	191.4	171.8	170.2	134.5	213
CAMK2B	133.8	134.3	125.2	123.3	95.8	115.2	120.6	125
CAMK2B	108.1	136.9	98.9	83.1	84.4	80.9	107.8	123.1
CAMK2D	310.8	399.1	181.6	124.2	99.6	139.1	242.8	308.2
CAMK2D	233.7	312.7	131.2	113.4	108.8	124.2	201.1	242.6
CAMK2D	97.8	117.3	101.7	96.6	94.3	96.5	100	114.8
CAMK2D	86.5	98.8	83.8	77.4	75.2	81.1	84.9	95
CAMK2G	300.7	335.3	192	210	207.4	262.3	260.5	326
CAMK2G	159.1	193.4	107.8	107.3	105.5	121.4	167.2	192.5
CAMK2N1	94.2	103	99.1	80.2	78.6	79	102.8	103.2
CAMK2N2	88	97.9	97.1	86.7	85	88.7	83.6	99.3
CAMK4	84.9	92	88.2	90	73.9	73.7	92.3	92.6
CAMKK1	99	107.3	100.2	89.4	91.6	89.4	97.8	112.5
CAMKK1	94.2	89.7	97.6	94.5	95.1	92.9	91.5	98.8
CAMKK2	133.1	135.2	118.3	99.1	95.7	98.2	109	130.7
CAMKK2	133.4	158.1	103.9	96.7	93.4	92	124.6	157.6
CAMKV	178.4	218.6	123.5	209.4	248.9	308.9	152.5	160.9
CAMP	231.7	236.8	198.4	353	231.9	355.2	195.4	236.8
CAMSAP1	517.4	762.6	475.1	574.3	529.8	741	558.3	662.5
CAMSAP1L	385.5	444.7	245.1	151.4	143.8	211	249.1	340
CAMTA1	186	205.8	140.7	132.5	130.2	157.4	156.8	292.4
CAMTA2	77.3	97.4	86.9	83.5	74.3	84	69.6	87.6
CAND1	248.8	300.5	119.4	139	108.6	147.5	226.5	345.6
CAND2	89.2	91.1	100.1	100.1	96.1	87.1	85	85.2
CAND2	267.6	343.1	180.8	163.7	143.5	158	228.4	244.1
CANT1	401.9	515	516.1	601.7	449.2	845.2	358.5	811.3
CANX	4133.5	5473.6	3568.2	5608.7	3838.6	6194.2	4179	5906.8
CAP1	33785.1	33512.5	41694.5	41598	36355.3	50115.7	31902.8	31433.4
CAP2	185.3	206.6	134.3	129.8	119.9	145.5	162.9	205.5
CAPG	738.3	951.2	248.5	171	147	172.7	843.1	1124.1
CAPN1	2262.2	2598.1	1096.2	851.5	700.9	781.8	1707.1	1674.9
CAPN10	372.4	456.5	195.3	161.8	155.1	209.7	361.1	457.8
CAPN10	77.6	86.4	94	89	92.3	88.4	85.5	78.4
CAPN10	88.9	105.1	93.6	91	82.3	94.6	94.3	110.9
CAPN10	80.1	94.6	93.2	86	88	90.8	86.6	106
CAPN11	119	114.3	91.9	96.6	90.5	100.7	103.7	110.3
CAPN12	94.1	100.8	92.8	97.5	111.4	102.9	94.5	101
CAPN13	105	125.9	93	77.9	73.8	87.2	113.9	118
CAPN2	433.7	560.5	214.3	259.6	203.9	277	350.6	421.3
CAPN3	123.5	145.5	91.6	94.6	92.9	90.2	121.6	129.8
CAPN3	89.3	92.2	79.8	83.9	80.4	83.6	83.5	97.9
CAPN3	196.3	211.2	198.2	167.7	169.9	206.1	147.3	223
CAPN3	124.8	132.2	144.9	127.2	141.2	155.6	109.6	132.4
CAPN5	104.6	120.7	98.6	115.7	121.3	120.9	89.5	100.6
CAPN7	201.7	244.3	113.7	120.9	106.8	134.4	180.6	231.6
CAPN9	83.7	93.2	96.3	94.8	84.5	89.7	74.9	95.3
CAPN9	80.2	92.3	89.7	90.5	79.9	89.8	93.9	91.7
CAPNS1	2116.2	2879.4	2458.5	1580	1203.7	1804.3	2226.1	4097.7
CAPNS1	1220.2	1620.7	656.6	433.8	369.7	455.4	1194.5	1778.2

CAPNS2	90.9	109.9	91.5	95.6	92.3	91.7	95.6	99.5
CAPS	100.5	125.1	96.1	82.4	84.9	101.7	114.2	120.8
CAPS	184.7	179.8	108.6	118.2	135.5	125.7	162.7	174.2
CAPS2	65.8	81	85	87.3	81	76.4	74.2	77.7
CAPSL	116.1	123.8	96.5	82.5	97.7	87.8	99.9	134
CAPSL	101.6	107.5	91.6	87.7	90.1	84.5	97.3	98
CAPSL	86.7	96.7	95.1	96.9	88.5	99	91.5	93.8
CAPZA2	2832.6	3537.2	2605.2	2840.3	2010.6	4153.8	2464.6	4785.6
CAPZA3	71.2	80.9	96.6	84.1	82.3	86.3	79.8	76.7
CAPZB	310.1	361	241.3	160.8	164.4	219.4	334.3	470.2
CARD10	149.1	180.4	179.1	160.9	132.9	153.7	153	184.5
CARD11	151.2	193.6	117.3	134.2	129.2	132.3	155.3	154.4
CARD12	139.1	172.4	136.2	153.9	141.6	163.8	116.1	154.3
CARD14	118.6	122.7	101.4	87.5	86.2	92.3	110.3	142
CARD14	77.2	108.4	91.5	76	78	74.3	82.1	101.7
CARD14	87.8	95.1	96.8	90.4	81.6	88.7	78.4	108.2
CARD15	187.6	209.2	208.2	243.8	310.5	275.2	156.1	229.8
CARD6	116.4	135.8	113	107.8	96.2	108.5	114.8	138.8
CARD8	785.9	1064.1	541.4	629.1	572.6	721.8	770.4	1005.1
CARD9	5060.4	5671.5	3676.9	2976.7	2533.8	3561	3163.3	3742.5
CARF	563.1	828.3	363.5	324.3	275.7	486.6	433.9	703.4
CARHSP1	3887.9	4757.9	2358.1	1549.6	1396.9	1401.9	3869.7	3684.9
CARKL	392.4	470.6	405.8	487	449.5	548.3	284.2	323.4
CARM1	2693.6	3504	2089.6	1466.9	1231.1	1842.1	2486.4	2778.5
CARS	171.4	233.4	123.9	107.8	100.2	118.6	177.9	308.1
CARS	824	1235.6	417.4	297.3	290.5	393.1	932.2	1216.2
CART	146.5	161.6	105.6	108.4	97.8	104.4	139.1	144.9
CART1	151.9	194.2	124.8	177.8	169.7	276.5	125.2	185.7
CASC1	71.3	85	96.1	89.3	82.8	91.6	86.7	87.5
CASC2	131	151.5	115.5	111.6	102.8	121.7	140.9	149.9
CASC2	127.5	149.7	109.6	104.1	101.1	102.1	121.4	149.4
CASC3	424.1	596	375.2	381.5	302.7	505.8	384.5	502.7
CASC4	242.1	266	158.6	145.1	146.1	146.1	166.3	210.8
CASC4	824.2	1063.9	551.8	659.3	529.2	1119.3	769.3	1054.4
CASC5	127.2	149.1	97.8	103.3	90.5	99.4	114.3	114.2
CASC5	252.4	378.4	140.2	164.6	141.8	176.6	228.9	302.6
CASD1	219	225.8	140.8	132.1	116.1	174.8	155.8	199.6
CASK	99.6	114.9	86.2	91.3	79.5	83.1	107.5	116.9
CASKIN1	105.5	102.4	94.1	91.9	88.6	88.6	93.8	103.2
CASKIN2	105.3	125.8	87.4	82.2	78.6	78.5	109.3	127.9
CASP1	1864.5	2319.2	1056.5	931.4	611.2	1228.3	1395.6	1772.7
CASP1	79.2	80.5	98.3	74.5	85.5	83.5	80.3	84.4
CASP1	1700.8	2187.7	674.6	675.3	446.9	776.2	1116.2	1469
CASP10	130.3	133.5	119.5	110.7	113.8	116.6	122.1	136
CASP10	97.9	106.7	89.9	90.1	84.3	80.2	98.6	114.7
CASP12	84.1	104.6	91	85.3	76.5	96.1	87.9	95.2
CASP14	83.1	94.5	85.9	88	77.6	93.5	83.3	91.7
CASP2	302.8	379.3	152	148.4	124.6	99.8	198.5	257.7
CASP2	4234.1	5424.3	3426.8	3532.7	2851.3	3206.8	4173.6	5633
CASP2	482.1	616.8	423.1	413.5	338.2	464.9	415.5	696.2

CASP3	867.5	1260.2	630.3	523	401	561.7	899.5	1291.4
CASP3	173.8	219.3	133.7	117.5	100.8	115.1	151.4	182.6
CASP4	85.5	87.9	86	70.4	74.8	80.4	80.5	83.4
CASP4	1287.7	1720.5	454.2	459.4	327.6	433.8	1450.3	1941.2
CASP4	97.6	113.9	91.7	83	86.9	85	104	106.6
CASP4	146.4	177.9	117.6	103.6	98.4	102.5	125.7	156.2
CASP5	85.1	83.9	101.6	95	88.3	93.6	82.3	81.7
CASP6	415.3	494.5	163.2	164.4	134.4	176.4	383	554
CASP6	197.4	233.8	111.5	98.6	106.6	119.8	208.9	230.3
CASP6	182.8	201.2	111	118.8	93.9	88.3	189.4	271.4
CASP7	179.8	253.4	198.5	149.9	141.9	199	185.1	235.1
CASP7	122.4	118.2	92.7	83.5	87.9	89.3	112.5	151.1
CASP7	88.8	108.2	91.1	85.8	79.9	87.7	88.2	102.8
CASP8	152.3	166.6	97.9	98.9	77.1	100.4	125.3	180.7
CASP8	100.3	107.9	90.7	86	82.5	87.2	95.1	102.5
CASP8	297.7	320.7	104.3	106.2	91.1	94.3	191.2	266.7
CASP8	316.7	390.5	137.1	115.6	108.5	96.8	233.6	304.9
CASP8AP2	99.1	101.6	95.5	86.5	80.7	80.5	95	102.4
CASP9	170.1	198.8	110.2	110	102.6	103	165.1	163.8
CASP9	260	319.2	224.7	187.6	184.4	218.4	223.6	309.1
CASQ1	89.5	106	93.6	89.2	84.6	83.9	90.2	92.5
CASQ2	80.4	96.5	95	82.5	80.8	83.6	76.7	90.5
CASR	87.6	90.8	90.6	87	75.3	85.6	79.6	84.9
CAST	376.8	534.2	173.1	195.1	175.9	302.2	341.8	513.6
CAST	104.3	117.1	94.3	103.1	88.8	93.3	106.2	137.8
CAST	88	93.2	91.5	99.9	86.8	83.8	85.6	94.1
CAST	445.4	518.4	235.5	348.9	270.4	577.2	428.8	580.1
CAST1	83.7	93.3	86.3	81.7	84.5	87.1	84.5	97.9
CASZ1	299.2	332.3	222.9	212.4	188.2	208	216.9	267.7
CASZ1	94	105.8	89.8	90.4	83.4	86	89.3	98.7
CASZ1	79.1	91.4	91.5	90.6	84.4	75.6	78.5	84.9
CAT	2388.1	2706.5	656.9	714.7	411.2	687.8	2468.2	4420.1
CATSPER1	536.6	613.9	311.1	219.2	176	249.8	466.1	591.6
CATSPER2	110.3	127.5	103.1	102.8	93.1	120.9	118	118.3
CATSPER3	114.7	132	99.8	76.8	78.2	79.5	95.3	113.7
CATSPER4	98.8	113.3	88.4	85.4	88.5	86.8	105.8	103.9
CAV1	147.3	185.8	135.7	248.9	218.7	329.1	172.8	220.1
CAV2	150.8	189.7	109.5	128.2	111.3	132.7	135.7	169.9
CAV2	109.6	136.8	102.2	94.2	92.7	97.7	113.2	130.6
CAV3	104.7	112.3	103.8	99.2	89.4	94.2	111.9	110.5
CAV3	112.6	115.7	90.1	82.5	72.8	79.7	111.6	132.3
CBARA1	267.9	362.4	279	311.2	209.1	421.2	233.9	419.7
CBFA2T2	168.4	202.9	129.6	147.1	134.1	161.7	158.3	190.8
CBFA2T2	102.4	124.6	96.2	99.8	83.3	98.2	105.5	107.9
CBFA2T2	124.1	114.1	98	97.1	90.3	100.9	107.9	111.7
CBFA2T3	128.1	149.6	94.2	90.5	93.5	90.9	114.4	141.5
CBFA2T3	101	120.2	118.4	115.5	119.2	111.2	106.4	119.6
CBFA2T3	106.1	127.7	91	86.1	89.6	86.7	116.5	110.8
CBFB	2451.8	2857.9	1254.2	1120.7	620.9	1427.4	1903.8	4617.5
CBFB	132.3	149.8	86.7	87.5	77.8	88	111.5	143.2

CBL	396.2	595.6	190	230.2	252.9	378.6	456.1	1213
CBLB	204.9	238.5	154.2	222.6	206.5	296.6	206.2	249.9
CBLC	129	150.4	98.2	96.2	85.1	97.8	136	147.8
CBLL1	1433.2	1763.9	626.3	605.3	480.1	517.5	1191.8	1660.6
CBLN1	76.5	85.3	92.2	95.4	85.6	103.6	85	84.1
CBLN2	92.4	103.3	76.5	70.9	64.8	71.4	83	103.3
CBLN3	158.8	180.8	126	112.5	128.5	118.6	131.1	159
CBLN4	88	85.9	96.3	93.6	87.9	91.9	88.6	88.3
CBR1	300.9	400.4	184.4	152.5	146.2	161.6	300.6	355.5
CBR3	183.3	227.3	148.6	151.4	139.9	170.3	245.3	367
CBR4	378.3	442.5	285.8	353.8	264.7	431.2	240.6	401.7
CBS	2647	3183	1823	883	799.5	1108.6	2165.3	2670.7
CBWD1	118.3	127.7	89.4	90.3	86.7	92.6	109.4	163.9
CBWD2	83.7	94.6	87.9	93.1	86.2	81.9	103.7	89.7
CBWD5	3788	4854.9	1134.2	721.5	604.1	1007.7	2788.8	4015.3
CBX1	488.1	761.5	151.7	128.5	117.8	166.8	499.7	448
CBX2	127.1	163.6	91.6	83.3	91.9	114.2	117.4	135.2
CBX2	80.6	94.8	87.8	88.5	77.5	77	93.5	92.9
CBX2	642.3	860.8	546.4	363	279.8	325.5	554.9	690
CBX3	236.8	277.6	155	152.3	130.1	146	210.6	238.2
CBX3	1809.8	2148.8	961.2	1198.4	957.3	1171.4	1392.9	1687.9
CBX3	79.8	85.5	90.4	81.2	77.7	86.4	75.8	82.3
CBX4	408.2	445.2	294.9	281.7	253	387.6	380.1	792.3
CBX5	1684.2	2207.9	645.7	489.1	379.6	532.9	1285.6	1502.6
CBX6	1364.3	1794.4	801.3	716.8	577	866.9	982.9	1123.2
CBX7	174.8	203.8	199.6	177.6	177.9	225.6	155	215.7
CBX8	156.4	197.1	145.4	132.8	122.7	165.2	161.3	176.5
CBY1	91.6	104.6	97.5	85.7	88.8	96	95.8	101.1
CC2D1A	136.6	145.6	130.5	96.3	104.3	113.9	133.4	183.1
CC2D1B	444.7	441.3	311	448.4	368.4	353.5	372.3	496.7
CCBE1	442.3	412.2	191.8	256.5	248.5	212.3	457.1	657.3
CCBL1	176.2	229.8	129.9	120.1	121.4	128.4	149	161.9
CCBL2	568.6	746.1	207.8	209.6	238.2	296.1	506.5	693.1
CCBL2	229.9	327.2	138.2	158.7	130.6	169.1	173	210.4
CCBP2	422.2	555.1	285.1	237.8	199.5	227.3	286.8	257.8
CCDC104	103.3	110.9	101.8	87	86.2	87	120.5	115.2
CCDC108	76.6	87	89.4	80.7	79.2	86.7	80.4	88.5
CCDC108	97.7	107.9	86.6	79.4	84.1	81.9	90.6	120.8
CCDC11	97.2	107.4	116.2	101.5	93.5	105	93.1	107.1
CCDC110	79.3	86.2	110.8	102.4	87.8	94.5	89.4	82.4
CCDC117	99.3	115.9	96.9	100.8	86.6	111.8	98.7	104.7
CCDC12	1800.9	2390.9	1282.6	1216.7	976	1336.2	1769.3	1682.3
CCDC121	138	170.6	102.9	95.5	91.1	105.7	116.7	141.4
CCDC127	279.7	402.9	166.4	181.2	157.8	217.5	294	457.6
CCDC129	120.3	127.1	95.5	82.2	90.3	86.8	111.4	128.7
CCDC13	90.1	103.5	102.1	96.2	86.8	94.9	95.7	107.2
CCDC14	1790.7	1697	815.4	860.8	743.4	1527.8	1064.7	1416.5
CCDC16	1474.5	1815.6	747.3	738.9	657	1148.4	926.9	1566.4
CCDC17	93.1	92.3	105.1	103.3	97.4	105.3	93.3	99.1
CCDC18	127.4	132.2	107.1	97.8	96.8	100.1	108.4	104.6

CCDC21	465.6	571.9	337.6	335.4	283.3	337.4	446.1	361.8
CCDC22	162.9	204	113	105.8	94.7	123.9	169.1	201.9
CCDC23	2845.9	3667	2682.8	2643.8	2131.2	3008.7	2818.3	4169.4
CCDC24	186	202.5	125	109.3	102	119.5	154.5	169.9
CCDC25	724.8	933.6	258.7	286.9	206.4	308.2	610.4	920.4
CCDC25	1232.3	1505.4	1006.3	1151.5	1040.2	1460	949.2	1104.1
CCDC27	157.4	171.3	111.3	100.9	108.9	104.3	144	165.2
CCDC28A	1534.7	2047.9	939.1	1234	929.9	1283.6	1497.2	2084.5
CCDC28B	204.2	230.5	133.9	126.5	123.3	142.5	187.6	221.9
CCDC3	92.1	115.6	102.8	93.6	95.7	98.6	87.6	107.6
CCDC32	437	594	186.7	169.6	173.6	223.1	414.4	461.8
CCDC32	2222.7	3000	1938.4	2025.9	1604.4	2354.7	2019.2	2749.3
CCDC33	95.4	106.9	102.8	101.9	96.6	98.7	83.9	92.3
CCDC34	78.4	81.1	84.5	91.2	87.1	77.7	77.7	78.6
CCDC34	747	926.9	390.9	352.1	397.5	969.1	469.5	701.1
CCDC34	2842.7	3582.2	2063.3	1962.7	1733.6	2775.1	2159.2	2686.2
CCDC36	122.9	128.6	101.9	100.7	84.9	94.6	122.2	142.3
CCDC37	85	92.4	94.7	83.9	85.5	85.2	82.4	87
CCDC38	85.5	93.6	89.9	92.9	79.9	95.4	87.2	96.1
CCDC4	118.6	163.1	159.2	115.4	115.5	125.8	110.3	157.6
CCDC4	200.8	233.4	126.4	117.1	101.4	114	184.8	194.1
CCDC41	113	126	105.6	96.6	93.7	114.5	101.8	126.6
CCDC41	302.8	352	189.5	256.7	199.1	422.8	228.2	305.9
CCDC42	76.6	74	75	78.6	78.6	71.7	75.3	82.8
CCDC43	431.4	575.5	163.9	187.9	143.7	164.2	369.7	503.1
CCDC44	1392.7	1775.1	753.1	685.4	550.9	774.3	1099.9	1060.9
CCDC45	755.5	852.4	358.9	459.3	381.3	628.9	625.9	677.5
CCDC46	89.8	89.4	90	87	81.1	88.2	81.8	85
CCDC46	73.8	93.1	88.5	74.6	78.9	79.2	81	83.3
CCDC47	327.5	437.9	214.7	249.4	178.8	289.4	289.3	492.3
CCDC48	105	137.1	94.3	76.6	77	88	110.6	137.7
CCDC49	617.4	717.9	386.1	354.5	352.6	427	474.6	650.5
CCDC5	1469.8	1755.1	610.2	603.9	487.4	928.6	1041.5	1095.2
CCDC50	1539.3	1835.3	904.3	932.3	832.5	1259.4	1427.6	1856.6
CCDC50	94.3	108.5	82.5	86.9	80.7	77.8	86	101.4
CCDC51	428.7	531.9	270.5	249.3	194	276.5	352.6	360
CCDC52	79.2	91.8	94.6	105.3	93	90.5	87.4	93.3
CCDC53	1019.1	1303.1	731.2	570.2	470	760.6	976.3	1472.4
CCDC54	84.6	103.7	101.6	90.8	84.6	82.1	95.9	88
CCDC55	491.5	598	168.3	182.1	163.8	288.2	415.3	738
CCDC56	3513.7	4792.7	2297.1	2086.8	1628.9	1966.8	3207.9	3041.1
CCDC57	76.3	82.9	104.8	93.1	85.6	98.2	76.6	83.7
CCDC58	1607.8	1934	732.1	628.1	461.3	1098.1	1276.1	2075.4
CCDC59	3145.3	3375.4	2476.2	2934.2	2443.6	5183.9	2665.8	4864.7
CCDC6	1136	1579.6	1406.6	1276.3	906.6	1470.5	1099.2	1444.6
CCDC60	107.2	102.7	91.3	93.9	93.3	87.4	101.7	103.7
CCDC62	128.9	164.7	95.6	99.3	90.9	107.2	127.8	186.1
CCDC62	109.6	126.2	100.7	87.3	82	89.1	107	121
CCDC63	113	128.2	91.2	89	86.3	87.3	105.5	120.1
CCDC64	87.2	94.8	88.8	89.8	91.4	92.5	91.8	85.7

CCDC65	95	125.2	90	76	82.3	79.9	104	101.1
CCDC66	242	278.9	149.9	146.3	137.6	204.3	191	267.5
CCDC67	102.9	124.3	96.6	91.2	88	90.6	100.7	126.5
CCDC68	108.1	107.6	101.2	88.6	92.5	95.7	95.9	101.8
CCDC69	157.8	163.1	103.2	107.4	80.2	95.5	148.9	204.6
CCDC7	102.1	118.8	114.6	93.9	100.6	93.8	100.7	105.6
CCDC7	83	90.9	92.9	77.3	76.4	90.4	78.8	86.3
CCDC7	92.4	111.4	91.4	87.9	85.1	90.2	101	98.4
CCDC70	136.3	174.3	94.7	79.2	89.1	89.8	139.6	163.5
CCDC71	386.5	666.1	340.7	326.4	253.8	367.3	445.8	522
CCDC72	9415.6	11022.4	4209.4	4223	4283.5	7286.2	7363	8059.5
CCDC74A	125.2	129.7	97.2	87.6	89.4	92.6	111.8	113.7
CCDC74B	94.5	111.8	97.6	101.9	84.7	92	97.5	104.1
CCDC75	130.3	136.5	98.2	108.3	103.4	97.9	112.8	111.7
CCDC76	739.5	864	432.5	294.4	240.9	556.3	480.5	1051.7
CCDC77	496.9	596.6	300.3	337.5	298.5	439.8	587.6	751.3
CCDC8	113.5	127.8	91.2	94.5	98.2	95.5	122.7	121.4
CCDC85A	77.1	85.7	90	88.2	80.9	87.9	78.4	83.9
CCDC9	89	112.5	115.7	95	91.5	106.1	99.2	101.9
CCDC90B	2559.4	3148.1	1899.3	2232.3	1614.8	2488.8	2316.9	3943.9
CCDC91	117.4	145.2	99.9	91.2	86.3	101.7	116.7	156.6
CCHCR1	98.7	129.3	92.4	76.4	79.7	74.1	99	112.8
CCIN	93.3	98.7	100.8	93.1	79.4	94.8	87.3	99.1
CCK	89.8	96.8	99.2	89.4	78.1	87.4	83.6	83.9
CCKAR	90.1	110.2	97.4	98.5	87.7	96.5	98.3	99.3
CCKBR	88.4	103.6	84.3	97.5	83.7	91.8	87.7	99.8
CCL1	92.6	98.9	85.7	79.3	82.6	83.6	85.3	94.6
CCL11	77.4	92.9	93.7	78.3	89.2	90	79.2	88.4
CCL13	133.3	146.9	101.8	106.9	99.3	115.6	131.2	140.3
CCL15	89.7	106.2	84.5	90.6	81.2	86.8	84.9	85.6
CCL15	75.3	85.9	91.2	81.2	81.7	83.7	75.2	83.8
CCL16	137	172.5	101.3	91.9	87.1	94.4	131.1	128.6
CCL17	78.5	92.9	96.9	85	79.2	89.7	80	79.1
CCL18	114	134.8	76.3	84.9	73.9	76	110.3	131.5
CCL19	109.5	124.9	116	102.3	91.7	99.2	104.9	111.1
CCL2	191.4	245.9	141.5	164.8	151.1	178.8	227.1	269.9
CCL20	138.6	174.6	98.8	107.3	102.8	110.5	132.6	167.5
CCL21	92.3	109.3	107.4	90.5	92.7	95.8	97.5	107.6
CCL22	174.2	182.7	134.3	151.7	120.9	143.2	167.2	219
CCL23	236.1	324.8	208.6	226.5	188.9	236.8	239.6	345.6
CCL23	1464.2	1861.9	967	979.1	794.5	881.5	1375.5	1450.7
CCL24	90.3	97.1	84.4	85.3	78.8	81.5	88.9	87
CCL25	91.3	100	85.8	84.8	82.3	96.1	98.4	100.4
CCL25	114.1	123.8	91	83.1	88.7	86.6	97.4	107.2
CCL26	113.3	139.6	103.1	90.2	91.2	92.9	118.4	128.4
CCL27	100.4	94.4	94.8	84.8	87.1	86.3	89.3	92.4
CCL28	172.4	186.3	103.1	95.4	87.6	94.3	152.4	167.1
CCL3	100.8	108.8	102.5	90.9	108.9	94.7	94.2	107.7
CCL3L1	124.2	162.3	100.5	98	99.9	103	139.2	137.2
CCL3L3	170.7	191.6	162.1	151.6	227.8	173.2	177.8	224.7

CCL4L1	89.3	107.7	96.3	96.2	100.3	83.6	101	100.3
CCL5	8668.9	10435	5098.7	3628	2945	2726.7	6921.8	6119.3
CCL7	81.7	88.5	88	83.6	79.6	85	78.6	88
CCL8	90	114.5	99.2	91.9	86.3	89	95.5	104.1
CCM2	207.6	331.7	130	101.7	96.7	89.2	243.4	288.5
CCM2	192	244.5	135.1	142.7	125.9	102.4	221.1	347
CCM2	1442.2	1805.7	1047.4	1429.2	1494.6	1268	1634.1	1635.6
CCNA1	1391.9	1989.9	1171.6	409.1	401.4	668.7	1008.7	1444
CCNA2	3366.7	4764.7	2129.7	1252.4	943.3	1567.2	2456.9	3712.9
CCNB1	870.9	1104.8	310.3	184.8	168.9	174.3	769.1	1442.9
CCNB1IP1	3375.5	4443.1	2044.1	1143.5	986.4	1420.2	3299.2	4157.4
CCNB1IP1	144.1	179.5	112	97.2	88.7	97.8	137.3	160.2
CCNB1IP1	90.9	102.7	96.7	97.8	86	86.5	98	106
CCNB1IP1	1513.4	1903.1	1031.6	596.5	447.2	727.9	1425.9	1841.7
CCNB2	4712.6	6057.8	2763.5	1570.2	1321.7	1684.2	4339.6	5027.5
CCNB3	95.6	99.5	95	92	85.9	90.9	100.2	105.8
CCNB3	90	102.1	90.7	88.5	73.1	94.1	87.6	97.4
CCNB3	81.5	89.7	100.2	90.4	87.7	92.9	85.1	81.4
CCNC	1678.5	2162.7	644.1	691.5	524.2	827.2	1010.4	1370.4
CCNC	2042.9	2659.4	793.8	926.5	578.7	1323.5	1496.7	2262.5
CCNC	1013	1378.1	244.9	269.6	225.8	288.9	906.6	1555.6
CCND1	436.1	555.9	317.7	302.6	205.2	305.8	321.5	339.1
CCND2	98.5	121.5	98.3	99.2	100.5	110.5	87.2	112.1
CCND3	12868.2	15695.4	10279	7658.3	5257	6361.6	12926.7	12894.7
CCNDBP1	186.8	232.4	108.9	95.2	92.6	133.6	146.7	162.8
CCNDBP1	101.3	112.5	96.2	95.2	92.5	98.1	97.9	102.1
CCNDBP1	3770.1	4624.4	2653.5	2687.6	2480.3	3472.7	3006.1	4328.1
CCNE1	968	1322.4	720.9	691.5	565.3	717.1	779.9	999.9
CCNE1	119.1	145.4	107.4	81.8	89.3	85.2	114.7	112.5
CCNE2	86.4	87.9	89.2	82.8	85.6	83.6	88	109.5
CCNE2	1646	2065.4	1072.2	845.6	618.4	1473.3	893.2	1647.1
CCNF	1472.4	2087.2	1434.5	914.1	735.5	816.5	1350.7	1306.1
CCNG1	2649	2891.1	814.3	981.6	826.9	918.9	2774.6	4087.2
CCNG1	84.7	105.8	92.1	87.1	75.4	76.6	93.7	110.6
CCNG2	272.5	334.6	132.2	210.7	220.2	310.3	297.1	417
CCNH	764.6	891.3	276.6	243.8	195.2	267	710.8	1245.3
CCNI	10736.6	14213.7	7900.1	7761.1	5504.3	7401.8	8815.6	10664.3
CCNJ	139.8	154.8	108.6	98.6	87.7	102.3	119.5	148
CCNK	1056.1	1405.2	716.8	1037	667.9	1184.4	976	2389.8
CCNL1	941.6	1125.7	594	693.4	713.1	996.7	694.9	1070
CCNL2	201.8	239.9	119.1	122.2	98.4	112.9	160.6	196.8
CCNT1	157.1	191.8	134.1	120	106.8	127.3	137.5	187.5
CCNT2	142.9	176.2	115	104.4	89.4	105.6	114.7	144.9
CCNT2	325.7	365.2	252.8	261.6	238.7	522.9	245.2	473.5
CCNT2	108.4	123.4	97.7	104	88.9	89.8	102.2	102
CCNY	1271.8	1857.7	913.2	1236.6	1037.6	924.7	1317.1	1367.3
CCNYL1	474.9	478.5	244.2	331.9	239.7	361.2	367.9	641.6
CCPG1	102.1	96.7	95	93.7	96.1	102.4	114.1	155.1
CCPG1	316.7	428.2	279.2	278.4	248.7	434.8	515.1	844.8
CCR1	383.9	454.2	334.4	363.7	302.1	559.8	343	551.6

CCR10	96.1	96.2	90.5	87.3	85.1	93	88.2	95.8
CCR2	77.4	96.5	91.2	85.5	81	88.6	98.3	93
CCR2	394.5	563.6	242.9	244.8	170.7	291.6	265.2	300.4
CCR3	93.7	107.5	101.6	95.7	88.6	92.4	96.3	105.9
CCR3	85.3	86.7	104.7	91	81.1	87.1	100.7	90.9
CCR4	74.5	90.2	87	80.8	73.8	81.6	83.3	79.9
CCR5	85.5	102.6	84.8	82.9	85.6	79.1	87	102.4
CCR6	19499.4	20698	9726.6	14017.2	14050.1	10744.1	18157.4	20824.2
CCR6	82.1	85.6	86.7	94.7	82.8	89.8	80.2	85.4
CCR7	85.3	102.1	94.7	86.1	89.6	83.1	85.2	98.5
CCR8	76.2	89.9	101	89.5	93.5	91	84.3	85.3
CCR9	154	172.4	103.2	90.4	84.7	98.6	129.3	146.9
CCR9	65	73.5	78.7	72.1	79.7	75.5	67.4	78.4
CCRK	701.4	778.5	557.8	357.1	355.1	495.7	434.1	424.5
CCRK	258.3	284.6	215.8	169.5	169.4	254.9	175.5	260.3
CCRL1	91.1	87.3	93.8	82.7	83.5	82.3	92.5	89.1
CCRL1	92.9	90.2	95.2	98.5	92.2	94.9	92.7	95.3
CCRL1	85	84.2	102.6	89.7	90	86.3	84.3	89
CCRL2	85.2	91.4	102.2	89.4	85.5	95.3	84.7	86
CCRN4L	513.3	820	406.2	387.7	296.7	265.8	427.8	473.3
CCS	537.9	682.4	336.9	243.6	220.8	146.1	424.7	486.9
CCS	368.9	438.5	218	217.6	184.2	195.1	310.2	307.1
CCT2	4750.1	6001.7	4111.7	3903.9	3123.3	6056.9	3303.2	5992.3
CCT3	1367.6	1907.2	295.8	277.5	298.7	279.6	1375.3	1355.1
CCT3	4965	6590	3842.8	2335.1	2105.1	3952.1	4173.2	5116.4
CCT4	676	860.8	390.3	441.6	291.1	470.6	552.9	782.3
CCT5	288.8	349.3	144.6	141.8	123.3	187.2	269.3	373.3
CCT6A	3612.9	4790.8	841.3	801.4	543.9	834.7	2453.5	3936.5
CCT6A	2159.3	2612.7	849.9	632.2	477.8	910.8	1550.5	3620.5
CCT6AP1	3002.1	3726.8	1285.8	1248.1	913.7	1590.1	2292.1	5209.7
CCT6B	251.7	338.3	158.1	166.2	128	162.6	222.3	402.3
CCT7	8603.1	11200.8	7257.3	6245.3	5065	8335.2	8166.1	11526.4
CCT7	2464.2	3374.2	496.4	512.7	486.5	854.8	2423	3412
CCT7	4315.1	5288.3	1925.4	1376.1	1076.9	1667.7	3573.6	4674.5
CCT8	13112.4	16248.3	8583	6220.8	4090.8	8113.8	9980.8	16051.3
CD109	120	138.6	117	103.1	106.9	113.8	124.3	138.2
CD14	365.9	459.3	311.2	1626.7	1013.4	1225.5	326.9	318.3
CD14	143.1	152.2	124	231.6	190.2	254.5	118.5	133.2
CD151	653.8	963.8	386.2	374.6	276.2	202.3	601.8	836.5
CD151	6235.5	7969.2	4205.6	3770.1	2789.5	3602.8	5984.5	7544.1
CD151	219	297.4	127.9	124.7	114.5	149	218.3	323.3
CD160	129.6	166.3	105	96	82.3	93.8	133.1	150.9
CD163	93.3	112.8	93.9	96.3	78.3	91.3	98.3	103.9
CD163	87.6	93.1	95.4	95.1	81.3	92.3	91.7	97.4
CD164	262.7	290.1	130.3	160.8	120.2	161.2	276.2	445.7
CD164L2	77.2	83.2	83.8	79.5	74.8	77.8	84.5	80.5
CD177	99.9	119.8	100.3	94.6	92.9	85.9	110.6	126.9
CD180	99.6	115.4	97.5	88.5	89	86.9	98.1	112.4
CD19	186.7	267.7	210.4	135.7	129.1	128	166.5	230.8
CD1A	88.8	106.6	100.5	87.6	81.4	97.6	94.7	98.9

CD1B	76.3	78.7	90.4	94.5	88.3	100.2	78	80.5
CD1C	101.9	126.1	103.9	98.8	93.4	82.8	103.7	104.7
CD1D	881.8	1062.1	436.4	348.6	173.3	473.7	561.3	1152.1
CD1E	103.7	112.4	108.5	100.5	98.5	103.1	106.7	110.5
CD1E	113.5	135.3	89	82.1	77.2	84	127.9	108.5
CD2	76.5	92.6	86.3	79.9	87.6	85.6	79.6	82.3
CD200	162.6	194.8	114.6	112.6	112.6	114.4	144.2	161.5
CD200	71.6	88.1	74.2	74.1	70.8	81.7	78.6	81.1
CD200R1	84.5	84.2	92.1	88.5	81.6	88.4	77.5	82.3
CD200R2	86	106.2	87.3	88.2	93	77.8	89.7	92.1
CD207	80	87.8	98.8	95.4	90.3	89.9	87.8	93.2
CD209	434.1	615.1	446.6	344.9	282.9	402.3	350.2	597.6
CD22	94.9	103.2	90.7	86.6	90.1	88.2	86.1	97.9
CD226	77	89	78.4	79.9	80.3	74.6	84.1	81.7
CD24	110.2	120.2	116.6	195.2	178.8	168.5	109.8	125.6
CD244	219.6	288.1	195	131.1	116.9	177.6	219.3	209.4
CD247	183.1	231.6	129.1	95.2	94.4	114	121.9	133.7
CD247	211.2	214	137.4	116.7	94.5	111.5	148	155.4
CD248	81.1	92.3	85.7	88.3	80.6	81.7	79.9	84.1
CD274	86.5	88.6	91.9	89.5	88	89.9	80.1	82.7
CD276	427.8	588.9	469	652.9	452.6	786.8	400	693.7
CD276	146.2	163.1	123.6	114.8	104.8	87.7	141.7	152.2
CD2AP	261.4	280.8	129.4	129.5	104.7	148.8	183	265.9
CD2BP2	2404	3021.9	1713.9	1252.3	1137.6	1361.2	1966.5	2187.8
CD300A	268.3	356.3	205.7	308.9	250.9	453	247.2	296.7
CD300C	392.6	540.3	203.4	215.5	153.3	177.7	276.4	213.3
CD300E	94.3	109.1	95.8	89.2	84	91.5	108.5	107
CD300LB	94.9	101.7	100.6	92	91.8	83.8	85.6	101.7
CD300LF	687.3	881	514.8	657.9	540.5	775.4	713.3	597.9
CD300LG	207.3	264.9	135.6	338	148.8	306.4	211.8	315.5
CD302	91.6	119.7	92	93.3	77.7	96.9	101.9	108.4
CD320	494	650.8	523.6	332.6	271.6	352.4	442.1	649.6
CD33	2451.9	2752.3	1332.6	1039.9	761.3	1174.3	2018.9	2545.9
CD33L3	96.1	100.4	97.7	88.2	89.3	90.5	92.4	108.2
CD34	118.1	127.9	97.7	93.2	90.2	99	114.1	134.4
CD34	87.2	100.3	95	86.9	79.5	90.1	93.4	97.5
CD34	84	85.2	92.2	95.7	96	96.4	83.7	86.6
CD34	85.9	105.3	95.6	78.5	76.7	80.8	97.9	111.1
CD36	366.7	470.6	220.4	418.4	356.1	624	282.8	443.5
CD36	664.3	885.5	351	613.5	483.9	883.2	583.5	1088.7
CD36	81.1	92.6	82.6	97.5	82.6	87	73.2	87.2
CD37	1480.8	2059	1092.9	640.8	540	605.2	1344.1	2020.2
CD37	2509.6	3264	1465.9	776.5	641.6	811.2	2350.3	4023.9
CD38	356.2	517.7	189.9	132.4	121.3	136.3	330.5	424.5
CD3D	110	126.7	90.4	99.1	82.1	99.2	98.9	117.5
CD3D	82.9	89.5	88.3	91.5	81.8	83.7	83.9	91.7
CD3E	92.3	115.4	103.9	97	88.5	98.7	92.5	102.5
CD3EAP	233.8	280.4	126.7	108.6	107.1	126.3	186.3	189.4
CD3G	108.7	134.1	87.6	88.8	87	92.1	113.8	125.2
CD4	158.5	230.6	203.1	189.6	158.8	213.2	148.6	233.9

CD40	99.5	105.8	89.3	87.3	82.4	87.1	108	115.6
CD40	115.5	146.7	102.7	114.5	122.5	125.6	118.9	157.9
CD40LG	78.6	93.2	98.5	86.5	90.5	89.7	80.7	85.5
CD44	112.4	126.5	121.4	101.3	110.9	122.3	101.9	105.7
CD44	249.8	403.2	221.1	228.7	192.8	385.5	244.5	415.8
CD44	2650.2	3370.6	1839.1	2856.2	1914.4	2930.6	2199.4	2992.3
CD46	1469.2	1826.6	1039.7	1018.5	816.1	1514	1057.5	1967.5
CD47	692.4	777.5	455.4	622.2	462.8	626.9	493.4	657.6
CD47	83.8	98.3	108.4	90.3	92.9	93.7	87.9	97.5
CD48	94.4	109.1	91.3	98.1	90.9	95.1	103.4	107.6
CD5	89.8	96	91.1	99.6	88.4	104.2	81.7	102.9
CD52	780.8	826.5	530.4	5138.9	3828	4470.7	733.5	792.6
CD53	722.1	1099.3	263.6	234.4	172.7	301.4	580.5	769.6
CD53	750.4	966.8	387.2	281.5	233.8	381.3	562.5	843.1
CD55	391.3	538.1	185.4	145	124.2	189.2	420.1	681.9
CD58	1447.4	1559.5	718	401.1	302.1	412.3	1355.8	2837.5
CD59	106	125.7	93.3	102.3	99.3	99.7	107.7	126.9
CD59	111	131.6	85.1	89	82.9	88.8	120.1	134.1
CD5L	106.1	131.3	85	80.2	75.5	89.5	107.4	112.7
CD6	134.1	136	145.5	153.8	122.9	147.3	105.5	101.9
CD63	768.2	902.3	328.8	386.3	326.6	440.2	741.1	602.3
CD68	748.8	927.7	568.2	1260.7	911.4	1705.1	736.4	1296.8
CD68	267.6	367.1	202	443.2	419.1	239.5	280.8	383.6
CD69	77.6	95.8	89.4	87.7	79.9	81.8	86.7	95.1
CD7	101.3	92.9	96.1	98.6	88.9	91.1	87	97.2
CD72	140.4	141.3	103.3	98.6	95.4	99	140.9	150.1
CD74	1176.6	1602.2	1082.8	883.7	762.8	902.8	1127.9	2109.2
CD74	104.1	111.9	107.1	92.1	100.4	91.9	92.9	120.2
CD74	509.6	790.9	412.3	366.9	272.2	353.2	505.9	929
CD79A	103.2	108.2	92.5	96.8	92.3	93.3	109.1	101.1
CD79A	127.3	140.3	126.6	114.2	95.8	113.3	136.9	136.7
CD79A	108.4	115.2	86	76.2	83.4	84.4	99.7	132.5
CD79B	233.7	309.2	169.2	135.1	124.1	137.5	216	234.2
CD79B	125.7	139.8	136.7	124.8	111.4	108.6	114.6	123.3
CD79B	115.4	127.9	102.5	85.9	92.4	94.4	115.2	119.1
CD80	98.8	104.1	90.2	83.3	88.7	92	101.5	114.3
CD81	22401.6	26994.8	17987.8	14865.5	11778.7	12648.4	22086.7	21098.7
CD82	193.7	234.6	217	255.3	254.3	255.4	172.8	290.7
CD82	199.7	285.5	128.4	118.8	129.1	130.9	203.3	231.7
CD83	277.2	342.8	185.5	145.3	178.4	157.6	273.7	441.2
CD83	185.3	280.4	140.7	98.8	101.7	114.6	166.3	250.7
CD84	134.2	167.4	99.7	86.4	92.5	97.1	118.3	166
CD86	148.2	187.5	98.4	136.5	118.4	160.6	134.7	168.2
CD86	130	149.9	132.5	387.8	335.4	420.8	127.9	129.4
CD86	76.7	90.8	101.9	94	90.1	85.2	81.8	75
CD8A	100.1	103.4	91.8	85.3	80.5	82	96.5	95
CD8A	96.3	101.1	96.2	90.1	86.8	97.1	91.1	106.7
CD8A	80.9	95.8	101.3	86.6	80.6	93.7	82.5	96.5
CD8B	83.5	100.4	86.3	82.1	82	77.5	93.1	94.2
CD8B1	94.5	112.4	92	93.3	93.4	83.7	104.4	107.5

CD8B1	76.7	85.9	96	87.9	82.8	88.7	80.7	85.4
CD8B1	78.1	81.6	94.9	88.1	87.3	86.7	74.3	81.3
CD9	117.6	119.2	101.1	99.2	100.1	109.7	120.4	131.1
CD96	88.9	93.2	90.7	76	82.4	91.8	90.9	96.1
CD96	99.4	109	88.3	97.5	83.3	86.3	88.9	107.9
CD97	152.7	163.4	125.7	113.9	105.4	137.3	128.5	152.3
CD97	1671.2	2054.6	1511.4	1013	804.6	833.8	1263.1	1474.5
CD99	801.6	1119.4	424.6	453.2	379.8	498	786.4	1115
CD99L2	330	478.5	264.4	309.5	230.7	350.7	367.4	608.2
CD99L2	2563.3	3195.4	2164.2	2753.7	2192.6	2805.3	2183.7	2643.4
CDA	229.9	295.9	178.1	124.4	109.2	126.4	224	267.3
CDADC1	108.4	115.7	108.1	104.4	93.7	104.4	105.3	140.7
CDAN1	467.5	448.2	365.3	389.7	295.9	411.7	349.1	470.3
CDAN1	6472.3	8058.3	2445.2	4196.7	4271.7	3647.3	6872.2	5805.3
CDC14A	106.8	116.3	105.3	99.3	91.4	99.6	107.6	108
CDC14A	126.3	126.3	99.1	88.4	89.9	80.7	101.1	118.8
CDC14A	80.6	85.3	101.5	76.2	88.6	96.5	85.1	80.6
CDC14A	109.7	119.1	89.4	92	98.1	99.1	89	134.5
CDC14B	175.2	215.5	141.4	116.2	105.6	145.9	176	227.5
CDC14B	338.5	372.8	349.1	360.2	239.3	473.4	326.5	485.8
CDC14B	181.1	238.4	210.6	3277.5	2556.4	262.9	356.4	187.9
CDC16	3658.9	5005.2	1537.8	1199.3	933.4	1565.2	3124.2	3496.8
CDC16	2025.9	2472.1	692.5	536.6	443	833.8	1623	1933.9
CDC16	3271.7	4259.1	2306.2	1778.9	1448.7	2387.9	3020	3256.8
CDC2	2802	3525.9	1106.8	689.7	573.3	955.7	1915.9	2629.2
CDC2	438.9	624.4	227.9	193.5	170.4	233.2	335.7	530.4
CDC20	18354.6	20738.2	9863.5	6687.2	5588.9	6211.7	14822.7	12412.5
CDC23	1616.7	1991.7	881.5	1176.2	775.3	1385.1	1325.5	2197.4
CDC25A	913	1211.9	753.5	475.4	349.6	590.2	731	932.1
CDC25A	247.1	279.2	162	186.3	151.8	147.6	175.4	248.6
CDC25B	448.5	543.7	619.3	384.9	344.2	495.4	424.9	763.4
CDC25C	539	729.7	363.2	287.7	257.9	274.6	408.7	486.5
CDC25C	573	728.2	304.3	221.8	188.9	301.4	466.6	530.8
CDC25C	411.5	598.3	309.3	228.3	216.1	295.5	310.6	386
CDC26	3417.4	4247	1695.9	1128.9	797.7	1027.3	3169.5	4160.1
CDC27	100.9	113.3	103.3	93.4	93.3	93.2	101.4	110.2
CDC2L1	69.9	78.8	85.9	77.3	78.5	84.5	77.3	79.8
CDC2L2	181.8	210.9	96.4	113.7	97.6	85.1	160.5	197.1
CDC2L5	515.4	727.4	312	318.9	313	365.8	445.2	534.1
CDC2L5	593.5	850.3	388.6	479	413.2	534.1	564.9	653.1
CDC2L6	1460.3	2418.1	1118.4	1812.3	1679.1	2045.8	1656.8	1546.4
CDC34	396.3	508.3	256.7	165.1	152.2	164.6	398.3	647.3
CDC37	13230.3	15273.8	12258.4	9090.9	8471.4	8366.4	11101.3	11487.6
CDC37L1	96.8	114.9	93	87.6	83.3	91.9	105.5	114.8
CDC40	1789.7	2423.9	1117.9	1581.4	1137.9	2341.4	1619.8	2175.8
CDC42	109.8	141.5	94.8	91.7	84.4	85.5	119.3	141.2
CDC42	78.8	80.3	92.5	85.2	84.1	80.8	72.6	77
CDC42	536.3	805.7	319.6	234.6	197.4	234.9	434.4	536
CDC42	1385.3	1695.1	467.5	398.9	303.8	350.5	1282.2	2176.6
CDC42BPA	171.3	222.9	135.2	96	90	92.2	199.2	205.2

CDC42BPA	97.4	103.1	84	79.8	77.2	84.2	84.3	101.7
CDC42BPB	93.9	128	122.4	133.3	128.2	185.5	106.4	136.8
CDC42BPG	83.5	98.6	85.8	77.6	78.6	84.8	86.5	92
CDC42EP1	110.3	138	99.7	92.8	83.6	83.1	114.2	124.3
CDC42EP1	172.3	246.8	134.6	99.5	100.7	105.3	171.6	200.6
CDC42EP2	94.1	123.4	120.5	98.6	87.8	88.1	112.8	128.2
CDC42EP3	274.8	406.1	203.8	143.9	126.8	149.5	258.8	330.8
CDC42EP4	457.8	539.3	495.8	709.4	673.3	690.7	567.2	659.9
CDC42EP5	88.5	97.7	106.7	81.3	86.3	84.5	101.5	100
CDC42SE1	606	769.3	457.5	241.3	204.3	242.6	476.3	922.8
CDC42SE1	248.1	315	153.8	139.6	108.1	126	212.2	321.3
CDC42SE2	355.9	435.7	128.7	150.6	118.9	160.3	225	337.8
CDC42SE2	1323.1	1688.4	715.8	1141.2	783.4	1192.4	1189.4	1541.9
CDC45L	3729.2	4619.6	2464.7	2639.5	2064	3163.1	2374	2424.8
CDC5L	1068.9	1317	431.5	500.1	383.4	1009.7	875	1918.4
CDC6	153.2	207.8	147.8	122.2	114.3	135.7	145.3	178.7
CDC7	715.6	735.6	242	318.6	249.7	304.3	495.6	814.3
CDC73	112.1	155.1	94.6	101.4	99.2	102	109.5	126.7
CDC91L1	837.8	933.8	349.6	315.2	302.3	290.6	646.5	708.1
CDCA1	376	529.3	147	142.4	161.1	140.3	250.4	293.9
CDCA2	807.1	1091.5	590.4	340.8	315.1	576	669	838.5
CDCA3	601.2	726.7	300.4	224.9	200.5	242.4	555.8	974.1
CDCA4	157.5	207.9	121.6	108.6	95.2	117.8	144.9	210
CDCA4	863.9	1299.2	628.1	393.6	356.1	520	728.7	983.8
CDCA4	1102.4	1439.3	504.7	284.3	266.9	315.7	950.4	1006.7
CDCA5	3270.7	3660	2708.3	1728.6	1521	1925.8	2386.1	3777
CDCA7	6646	9032.2	4805.2	3416.2	2477.8	3272.4	3576.5	3851.8
CDCA7	125.2	124.5	98.1	89.9	81	83.4	86.1	106.3
CDCA7L	784.4	1053.8	490.2	329.6	278.7	385	441.9	621.8
CDCA8	987.9	1328.8	765.4	585.4	531.1	688.5	822.1	1269.1
CDCP1	99.2	116.4	108.9	109.2	104.9	91.1	95.9	97.8
CDCP1	98.5	102.1	82.9	98.6	91.4	89.7	108.2	102
CDCP1	96.4	99.9	91.9	90.1	79.7	85.1	83.9	93.1
CDCP2	80.4	95.9	97.5	85	88.9	89.7	82.7	84.2
CDGAP	91.3	101.5	92.1	85.1	78.1	79.2	88	112.2
CDH1	82.6	84.5	91.2	88.7	89.6	88.1	80.9	85
CDH10	83.5	90.4	94.3	96.7	86	77	83.4	88.5
CDH11	94.1	100.5	114	100.9	104.4	92.7	101.8	107.6
CDH12	68.5	79.9	80.8	83.6	80.5	80.7	72.1	81.7
CDH12P	103.4	110.1	101.8	93.5	85.2	85.1	103	135.4
CDH13	84.1	83.7	93.9	79.1	83.7	84.4	80.1	84.6
CDH15	109.4	133.5	98.5	112.1	100.3	119.6	131.9	141.6
CDH16	92	94.6	107.5	114.1	88.7	106.9	87.1	80.3
CDH17	80.7	89	94.6	90.6	77.9	82.6	83.3	87.9
CDH18	79.3	82.9	79.9	75	69.7	77.2	82.2	80.1
CDH19	77.9	98.9	90	72.3	84.4	88.1	82.9	90.8
CDH2	956.2	1143.2	595.5	692.7	560.7	573.6	734	954.1
CDH20	147.1	187.9	104.4	96.2	94.6	87.5	152.5	167.6
CDH22	91.4	103.3	84.3	88.2	90.7	81.9	98.3	93.7
CDH23	159.7	190.4	117.2	133	122.6	132.1	152	169.9

CDH23	143.1	164.9	108.1	103.8	98.8	119.4	119.4	140.5
CDH23	100.7	119.4	119	127	117.6	152.2	99.1	103.3
CDH24	99.7	116.7	96.2	90.5	78.2	86.1	104	128.5
CDH24	126.8	124.6	94.1	87.9	95.5	98.8	112.5	138.4
CDH24	82.7	83.7	83.1	81.6	82.6	79.1	82.8	90.7
CDH26	91	108.2	109.5	97.3	103.5	105.2	98.8	95.3
CDH26	103	118.7	92.4	108.8	83	86.9	103.3	125
CDH3	89.4	88.9	94.8	94.9	85.4	86.3	86.4	91.1
CDH4	88.4	109.6	92	80.4	86.9	86.9	93.8	112.2
CDH5	82.5	90.5	91.7	86.5	88.1	84.3	111.7	128
CDH6	81.6	94.4	73.2	78.4	81.5	83.5	77.6	87.1
CDH7	148.1	169.4	95.4	92.5	90.2	102.3	137	141.3
CDH7	87.7	112.7	91.2	94	85	89.4	97.7	103.2
CDH7	86.4	105	90	95.1	90.9	91.8	84.2	96.7
CDH8	84	91	76.8	75.6	77.2	81.8	86.7	97.5
CDH9	99.4	126.8	129.6	113.9	114	119.2	103	104.7
CDIPT	245.9	254.9	128.1	112.6	111.8	154.1	264.8	359.1
CDK10	1674.9	1529.9	1179.2	1113.2	1293.4	1504.7	924.9	890.8
CDK10	130	185.1	115.9	99.1	89.4	122.1	126.8	163.7
CDK2	530.7	652.1	243.4	231.8	217	230.1	385.5	444.6
CDK2	1517.4	2123.3	1015	869.8	816	1251.5	1132.5	1697.6
CDK2AP2	341.3	425.7	202.6	123.2	113.4	133.9	351.9	503.9
CDK3	113.2	124.4	109.7	105	103.2	116.1	104.6	112.8
CDK4	3732.9	4581.1	1779.5	1373.8	1152.3	1438.7	3339.1	4193.3
CDK5	550.1	768.3	285.1	311.2	258.5	330	379.7	502.3
CDK5R1	111.3	110	92.3	96.9	100.2	108.9	98.5	160.2
CDK5R2	92.9	106.2	99.8	88.7	87.7	90.3	97.7	101.9
CDK5RAP1	872.1	1100	491.6	525	477.1	655.8	693.9	875.3
CDK5RAP1	172.2	177	116.6	102.1	92.8	89.1	150.2	154.7
CDK5RAP2	207.7	297.8	163.7	163.8	164.2	245.5	455.3	637.7
CDK5RAP2	1101.3	1626	918	961.5	917.7	1312.3	1452.4	1672.4
CDK5RAP2	99.6	92.2	109.7	107.8	116.7	137.1	95.7	104.9
CDK5RAP3	1401.8	1468.1	1213.9	1154.1	1340.3	1866	841.2	1180.7
CDK5RAP3	496.8	478.3	310	178.6	221.5	191.7	272.2	492.8
CDK5RAP3	1452.8	1556.7	1261.9	1203.2	1365.7	1819	1064.8	1341.7
CDK6	2158.4	3887	1355.2	2066	1812.3	2365.1	2789.6	3242.7
CDK7	780.9	998.7	296.4	267.9	281.4	422.1	642.7	986.4
CDK8	179.8	238.1	137.9	150.2	131	169.9	165	217.7
CDK9	605.6	749.7	398	374.4	353.3	438.6	490.5	626.5
CDKAL1	196.5	276.7	160.3	163.7	125.2	157.5	165.5	188.2
CDKL1	128.1	165.1	119.7	108	99.4	110.4	125.5	136.2
CDKL2	75.2	90.8	79.8	79.8	71.7	78.4	79.7	88.7
CDKL3	348.4	439.7	223.4	206.7	173.3	242.2	354.4	509.1
CDKL4	95.1	102	99.8	105.1	92.7	96.9	88.4	105.7
CDKL5	74.7	84.9	88.5	93.6	83	83.2	88.7	95.8
CDKL5	103.2	100.9	97.4	104	81.8	91.4	102.6	100.1
CDKL5	83.6	91.7	89.8	85.2	89.2	93.9	89.9	87
CDKN1A	362	393.3	214	269.9	244.1	294.5	533.9	605.7
CDKN1A	98.5	134.5	101.8	80.7	92.4	88.5	105.1	114.9
CDKN1B	363.6	438.5	247.6	256.7	238.5	417.8	319.7	510.7

CDKN1C	96	110.3	93.6	110.1	89.8	113.7	104.3	122.8
CDKN2A	119.9	165.2	103.2	95.5	89.6	95.7	125.8	141
CDKN2A	80.4	92.9	105.3	96.2	85.8	94.1	78.4	97.6
CDKN2A	98.9	108.3	97.5	83.1	89.5	94.2	94.9	95.4
CDKN2B	193	235.5	127.6	127.2	118.4	128	161.2	176.6
CDKN2B	86.3	88.3	113.8	85.9	88.4	90.8	81.4	91.5
CDKN2C	1300.4	1644.9	799.7	846.1	811.8	1486.9	858.4	930.1
CDKN2C	117	124.4	92.3	81.9	79.2	92.8	114.6	122.3
CDKN2C	237.5	301.6	130.3	109.8	101.3	95.6	286	305.4
CDKN2D	116.2	119.9	99.8	93.1	85.3	96.1	111.7	131.3
CDKN2D	301.6	392.7	245.7	153.9	183.2	182.9	287.4	425.1
CDKN3	2738.3	3298.3	1005.9	613	521.1	712.3	2186.1	3667.7
CDO1	297.9	333.5	156.8	127.4	110.7	125.1	241.4	377.9
CDON	104.6	106.3	94.9	78.9	87.4	92.8	103.2	100
CDON	92.2	124.1	88.2	94.3	80.3	104.9	113.8	116.7
CDR1	95.8	112.6	108.4	96.9	86	98.9	91.9	95.4
CDR2	590.3	799.4	326	283.2	221	327.6	529.9	698.7
CDR2L	378.3	455.7	284.6	257	224.1	239.5	327.1	359.7
CDRT1	86.1	93.8	94.7	91	88	93.2	87.9	89.9
CDRT15P	138.3	155.6	97.1	94.7	76.2	87.1	132.7	142
CDS1	203.3	278.4	149.5	113	110.1	118.6	192.5	241.2
CDS2	178.9	230.4	185	183.5	159.1	170.2	210.8	330.5
CDSN	95.9	118.1	87.8	86.3	87.8	89.4	90.4	117.8
CDT1	2074.7	2737.4	1144.3	571.3	544.7	486.2	1609.8	1053.7
CDV3	1563.5	2264.9	638.7	751.3	687.8	1467.9	1513.7	2808.5
CDV3	2035.1	2872.5	888.2	1180.9	791.6	1687.7	1875.2	2881.1
CDX1	83.4	91.5	86.1	86	76.9	86	88.7	87.8
CDX2	135.6	146.7	86.3	81.7	76.2	87.2	111	124.2
CDX4	85.8	117	97.9	82.3	88.1	91.3	95.7	108
CDY1	102.9	106.2	94.4	86.2	83.5	88.5	100.4	110.7
CDY1	87	89.5	88.8	77.1	85.1	88.5	79.5	98.2
CDY1B	78	90.3	102.3	93.5	101.2	91.9	82.3	84.2
CDY2A	72.7	81.9	92.9	82.2	80.7	88.7	73.8	79.5
CDY2B	118.4	137	96.4	86	83.2	82.7	108.6	122.2
CDYL	193.6	252.2	133.3	128.3	124.9	154.6	167.3	247
CDYL	78.6	92	96.1	79.5	81	78.6	90	87.6
CDYL2	102	107.6	99.6	92.4	101.9	87.9	97.7	109.3
CEACAM1	140.4	159.2	112.7	119.3	100	109.4	163.3	171.7
CEACAM1	138.2	135.2	103.8	94.9	95	102.3	125.7	143.5
CEACAM1	86.6	95.6	88.1	81	80.1	96	91.9	110.6
CEACAM19	116.6	133.7	99.4	91.2	90.7	102.6	115.1	140
CEACAM20	92.2	103.3	84.1	80.3	88.1	82.7	96	98.4
CEACAM21	119.7	128.7	120.1	109.1	103.6	99.8	118.4	124.7
CEACAM3	89	117.7	96.7	86.8	88.5	92.8	103.6	113.7
CEACAM4	118.8	144.6	109.5	114.1	89.8	102.7	120.8	129.2
CEACAM5	105.5	114.8	90.9	97.4	84.8	99.1	97.2	107.7
CEACAM6	435.5	535.1	353.3	181.5	138.7	189.2	335.3	678.4
CEACAM7	78.8	75.4	102.2	92.4	88.3	99.4	78.9	79.4
CEACAM8	72.3	89.5	98.3	82.8	77.2	83.7	76.6	76.4
CEBPA	4194.9	5271.9	4896	3509.9	2929.3	6341.4	4925.4	10897.5

CEBPB	1846.4	2240.7	1540.8	2040.1	1640.7	2567.6	1774.6	2137.3
CEBPD	1908	2441.5	1577.2	1519.2	1103.7	1892.5	1680.4	3026
CEBPE	168.7	191.7	147.3	118.3	109.7	132.8	166.4	159.9
CEBPG	1251.5	1507.2	829	843.8	692.3	1023.3	1054.4	1956.8
CEBPZ	3311.3	4031.7	1528.1	1681.3	1215	2959.4	2716.2	4071.3
CECR1	140.6	156.3	109.8	130.3	127.4	104.4	117	104.9
CECR1	632	827.5	782.4	1828.1	1459.8	1908.5	577.9	1074.2
CECR1	97.6	133.2	95.7	85.8	79.6	92	98.4	110.3
CECR5	241.8	299	190	186.9	174.7	267.7	202.5	277.8
CECR5	3363.6	4421.8	1793.9	2159.2	1521.2	2353	2458.7	2362.4
CECR6	337.6	393.3	387.4	350.9	286.1	447.5	262.2	547.6
CEECAM1	172	230	180.7	144.4	133	178	161.4	252.6
CEI	105	105.2	104.6	89	89.4	100.5	90.2	97.7
CEL	130.1	118.1	126.3	106.3	95.8	115.7	112.9	202.7
CELSR1	214.2	224.9	141.4	109	110.9	123.3	209.9	211.7
CELSR2	160.9	209.8	123.3	121.8	115.6	131.3	164.5	262.9
CELSR3	137.4	161.5	131.5	102.5	103.3	123.3	127.8	163.4
CEMP1	123	148.6	98.9	85.8	86.2	87.3	127.7	156.5
CENPA	2126.7	2825.1	1141.7	799.6	683.3	902.3	1811.6	2215.9
CENPA	1081.2	1405.8	519.9	315.1	285.6	345	823.7	928.9
CENPB	4574.5	5534.4	2646.5	2065.4	1660.5	2067	3996.2	3792.9
CENPC1	185.4	206.4	105.4	108	93.5	105.9	161.9	226.6
CENPE	441.4	510.4	165.2	177.8	167.2	286.1	330.3	385.8
CENPF	600.9	869.6	560.2	629	618.7	993.4	485.8	472
CENPH	95.8	118.2	102.7	100.2	90.3	86.8	103	79.7
CENPI	83.5	93	97.9	85.7	81.1	80.7	86.6	87.9
CENPJ	575.9	613.7	222.5	229.5	251	333.3	621.6	1018.5
CENPM	1972.6	2600.7	1318.8	1043	861.2	821.2	1608.6	1903.5
CENPM	861.3	1169.8	482.1	327.5	318.9	275.6	657.9	679.4
CENPO	134.3	167	125.6	113.1	109.5	111.1	138.6	151.2
CENTA1	3892.7	4568.4	3149.6	2500.8	2285.7	2936.3	3296.5	3472.8
CENTA2	740.8	1041.6	439.9	363.9	289.6	339.7	495.3	573.1
CENTB1	105	115	103.8	183	175.5	253.1	96.3	121.2
CENTB2	356.5	413.7	161.1	264.1	200.3	308	382	636.8
CENTB5	99.9	115.8	102.6	97.3	90.3	93.7	107.8	109.5
CENTD1	261.2	301.3	149.8	146.2	131.5	141.2	281.8	413.3
CENTD1	86.3	85.6	77.7	85.1	74.4	81.1	82.9	87.4
CENTD2	108.7	119	93.2	85.5	77.7	74.7	100.3	136.4
CENTD2	159.6	192.8	157.1	170.1	143.5	246.2	130.7	189.8
CENTD3	240.9	250.5	197.9	206.5	186.9	202.3	181.9	207.3
CENTG1	92	106.5	90.5	84	87.5	87.6	94.5	104.7
CENTG2	89	110.6	90.1	94.9	88.7	94.3	89.9	105.6
CENTG2	253	299.5	186.2	222.4	186.5	240.1	220.6	309.5
CENTG2	81	101.3	97.3	87.9	85.6	80.1	77.4	93.3
CENTG3	126.9	149.3	101.6	104.6	95.4	95.7	117.6	132.3
CENTG3	188.3	232.1	130.7	110.7	107.4	103.9	181.7	223.8
CENTG3	235.4	300.8	307.5	374.1	383.3	545.2	217.2	530.9
CEP1	641.5	856.7	490.1	534	489.4	963.4	432	608.8
CEP135	667.3	769	528.2	518.3	495.4	755.9	513.6	696.4
CEP152	363.3	379.2	254.8	284	287.5	448.2	248.5	445.9

CEP164	182.2	209.6	305.9	278.1	250.5	374.1	175.1	271.8
CEP170	133.7	191.2	142.5	142.8	134.8	146	120.8	147.1
CEP170	130.6	176.6	101.6	107.3	90.5	87.9	119.2	128.2
CEP192	620.3	928.9	370.3	588.2	530.2	857.3	542.9	542.1
CEP250	126.7	166.4	103.7	105.8	97.8	108.6	116.5	144.9
CEP250	141.7	167.5	133.6	114.9	114.7	146	153.6	155.6
CEP27	632.6	705.7	272.9	343.2	356.6	250.3	510.8	640.7
CEP290	128	138	134.1	154.7	124.8	195.9	119.2	172.3
CEP55	1074.6	1428.1	532.5	347.9	331.5	542.8	684.1	1339.9
CEP57	209.5	228.1	150.9	128.6	132.3	172.4	163.2	384.7
CEP63	1380.1	1649.6	788.8	1026.3	846.1	1519.4	975.8	1608.4
CEP63	94.4	105.5	95.2	89.8	91.2	93.2	90	109.8
CEP63	85.5	101.5	107.3	94.9	92.3	98.6	85.6	87.5
CEP63	122.1	137.1	105.9	100.1	97.3	108.1	111.2	126.4
CEP68	144	183.5	122.2	127.7	100.7	117.9	122.8	160.8
CEP70	313.2	376.4	172.3	152.8	160.1	189.1	226.9	255.3
CEP72	144.4	168.3	163.5	134	144.7	172.5	145.1	197.5
CEP76	137.4	174	134.2	122.8	116.1	123.5	132.9	188.6
CEPT1	153.2	156.5	98.6	98.4	95.7	88.5	127.6	137
CEPT1	974.7	1161.3	280.6	169.6	156.2	173.5	529	781.7
CEPT1	1697.9	2297.9	746.9	419.7	326.2	576.3	1367.8	2232.5
CER1	93.4	105.4	104	85.6	80.3	83.4	98.8	99.2
CERK	892.8	1054.8	689	893.8	687.1	927.2	899.8	1338.8
CERK	758.7	879.9	366.6	444.2	358.6	443.4	636.6	1063.7
CERK	163.4	200.6	130.5	123.4	100.3	127.4	148.3	156.6
CERKL	106.8	143.7	109.5	93	93.9	96.9	93.9	122.8
CERKL	67.5	98.1	91	85.7	79.9	84.7	77.3	85.3
CERKL	85.2	101.6	106.8	89.6	85.2	87.5	83.7	98.4
CERKL	94.6	113.7	94.3	87.7	95.1	85.3	96.8	100.3
CES1	2982.2	3501.9	1536.3	3395.8	2516.2	3652	3638.3	4796
CES2	1813.2	2115.6	1185.3	863.4	748.7	712	1484.4	2355.2
CES2	228.7	234.2	157.8	155.8	141.4	154.1	179.5	296.1
CES3	124.3	119.2	96.6	94.6	89.2	100.6	111.3	128.5
CES4	1129.7	1449.3	522.1	627.8	604.6	417.6	1085.8	1173.1
CES7	98	126.9	93	89.5	86.7	89.1	97.8	107
CESK1	75.2	81	79.6	82.2	82.5	86.1	78.4	87.9
CETN1	81.5	97.5	94.1	86.6	78.2	79.2	86.3	86.3
CETN2	2804.9	3658.8	2114.1	1700.1	1263.8	1778.8	2352.7	3539.6
CETN3	101.8	111.2	100.5	86	80.4	84.5	110.4	117.5
CETP	188.8	231.8	146.9	163.9	145.8	190.4	174.6	192.8
CFB	90	93.2	95.9	105	108.5	116.3	85.8	90.3
CFC1	81.2	96.5	97.5	82.6	83	89.2	82.9	89.1
CFD	11184.2	13777.4	6526.1	9733.4	7985.4	11803.3	10259.6	13854.9
CFDP1	548.5	800.3	161.8	135.8	118.5	153.2	473.9	473.1
CFH	81.7	92.9	93.4	88.7	89.4	79.4	85.7	94.8
CFH	109.6	120.1	118.8	115.9	109.5	113.9	110.2	106.7
CFH	100.6	119.3	82.6	85.9	83.2	86.7	91.9	117.1
CFHR1	105.2	118.5	118.6	98.6	76.5	83.1	106.3	113.7
CFHR2	119.9	136.5	110.9	101.3	102.9	110.3	124.5	127
CFHR3	88.2	82.4	86.7	92.8	81.2	92.6	87.4	85.8

CFHR4	79.6	94.7	88.2	77.7	85.2	82	81	91.6
CFHR5	111.8	126.4	95.8	84.1	82.7	77.2	108	116.4
CFI	120.2	135.9	99.1	75.7	81.3	89.3	111.8	127.9
CFL1	10478.3	14410.5	7139.6	5508.4	4455.6	4674.6	10456.7	13270.5
CFL2	139.3	154.1	99.7	93.8	83.2	80.8	101.2	128.2
CFL2	80.9	85.8	89.3	81.5	87.5	84.9	80.5	84
CFLAR	488.4	647.8	226.4	433.3	453	352.8	454.9	624.8
CFTR	121.8	153.2	103.1	92.7	81.5	90	120.1	131.9
CG018	187.8	246.5	119.5	146	133.9	193	187.4	304.5
CG018	107.1	118.7	87.3	80	74.9	76	115.8	114.8
CG018	170.7	226.2	120.1	186.6	145.5	192.4	199.8	288.7
CGA	110.5	138.9	96.7	83.1	75.8	85.3	116.8	131.1
CGB	122.9	131.1	114.1	96.3	93.7	96.5	116.8	111.8
CGB1	84.4	100.9	107.4	94	91.2	101.1	94.4	94.5
CGB2	75.8	82.7	83.2	88.4	78.5	82.6	78.2	64.9
CGB5	95.2	86.1	90	89.1	89.7	93.4	92.3	88.8
CGB7	124.7	138	91.8	78.6	87.9	81.1	116.1	138.4
CGGBP1	1183.5	1490.9	717.7	580.6	498.3	573.4	1040.5	1260.4
CGGBP1	83.9	95.9	89	89.4	77	79.7	81.1	88.5
CGGBP1	2568.4	3272.6	1671.5	2100.1	1691.6	3123.1	2221.4	3166
CGI-09	138.7	155.6	99.2	78.8	78.7	99.1	123.6	163.1
CGI-115	2209.8	2722.6	1045.3	854.2	586.2	1058.4	1807.8	2920.1
CGI-38	81.8	92	94	89.9	89.5	86.6	83.2	85.8
CGI-38	88.4	102.6	94.9	82.5	78.9	82.9	94.2	99.1
CGI-69	4942.1	5818	3581.6	3251.8	2502.9	2975	3941.4	5116.7
CGI-96	1919.2	2624.3	1267.1	827.6	638.5	884.6	1579.5	2141
CGI-96	629.2	803.5	189	156.5	153.2	169.1	484	615.3
CGN	122.4	137	116.8	106.1	97	94.6	103.5	115.1
CGNL1	161	213	127.7	158	134	192.4	130	156
CGREF1	97.3	113.9	93.1	90.6	95.2	99.3	95.6	104.2
CGRRF1	346.6	411.9	189.8	167.9	144.5	174.7	376.9	536
CH25H	87.9	93.2	94.7	80.8	80.7	88.5	87.6	92.5
CHAC1	149.6	214.1	118.6	98	91	95.7	164.7	190.7
CHAC2	298.2	349.1	158	112.3	102.2	128	207.4	316.2
CHAD	102.3	126.1	102.7	87.8	94.5	96.3	110.8	97.2
CHAF1A	331.9	382.6	180.1	159.2	130	172.8	245.4	250.2
CHAF1B	918.4	1182.6	481.6	400.1	319.1	390.8	602.4	731.2
CHAT	142.8	171.2	117.7	96.7	105.8	101.2	127.5	162.9
CHAT	89.4	99.8	94.5	88.4	91	89	91.9	81.9
CHAT	75.4	88.2	89.8	93.4	77.4	83.3	81	85.7
CHAT	72	92.6	84	77.7	75	78.6	84.2	88.4
CHCHD1	457.8	592.9	246.9	191.8	192.2	210.3	363.4	592.1
CHCHD2	16186.1	18022.1	8073.8	6055	4842.7	6920.4	16798.5	18118.2
CHCHD3	1484.7	1947	510.5	502.8	469.4	819.5	1167.8	1915.1
CHCHD4	2129.1	3005.1	1136.6	936.2	746	1086	1566.6	1399.2
CHCHD5	956.6	1237.5	837.2	626.8	466.5	669.3	911.5	1068.7
CHCHD6	230.3	325.9	181.2	144.7	144.7	228.2	239.2	238.5
CHCHD7	384.1	421.4	132.7	98	98.3	109.1	267.8	363.5
CHCHD7	1000.1	1246.4	580.1	317.7	241.6	362.7	696.8	1091.6
CHCHD8	487	621.8	326.3	240.2	184.6	247.6	400.4	461.5

CHD1	833.1	937.9	289.3	372.1	312.5	567.4	704.4	1054.9
CHD1L	821.9	1160.7	542.7	441.3	420.8	708	616.9	943.9
CHD2	169.8	239.8	111.1	124.6	118.7	141	142.1	214.2
CHD3	137.9	158.7	93.6	82.2	83.6	99.9	127.1	156.6
CHD3	130	150.8	99.9	98.5	92.1	102.5	106.7	110.5
CHD4	854.5	1244.4	1018.4	1132.2	1004.2	1197.2	741.6	1306.8
CHD5	104	120.3	102.5	93.4	90.3	98.9	107.3	100.4
CHD6	137	196.8	110.9	130.7	131.4	166.2	143.2	171.3
CHD7	363.7	603	416	395.7	324	562.6	403.8	479.7
CHD8	495.7	725.2	536.8	762.8	679.5	849.9	558.1	640.8
CHD9	524.2	934.7	536.2	710.6	649.1	1345.4	691.7	879.7
CHDH	78.2	92.8	89.6	77.8	79.4	82.2	73.5	87
CHEK1	1523.4	2037.3	942.6	502	392.1	607.2	1103.8	1563.9
CHEK2	607.5	813.4	282.9	264	315.9	432.5	465.8	780.3
CHEK2	85.1	105.1	92	93.9	81	94.2	82.8	91.6
CHERP	235.1	309.7	197	172.9	153.5	203.3	231.2	286.1
CHES1	702.1	963.7	516.3	677	517.4	750.9	673.4	1215.8
CHFR	1127	1345.4	1309.1	1196.2	1038.8	1568.2	978.2	1424.9
CHGA	89.3	115.7	104.5	99.7	95.5	103.5	98.4	98
CHGB	106.1	108	85.5	93.5	77.9	82.5	97.9	107.5
CHGN	96.7	99	96.1	92.3	81.8	100.5	98.7	98.4
CHI3L1	101.3	111.5	114.7	123.7	116	132.1	94.8	115.1
CHI3L2	86.4	85	104.9	106.9	106.1	98	88.2	86.5
CHI3L2	83.7	96.3	95.1	90.7	82.3	89	85.2	93.6
CHIA	84.8	89.5	90.9	80.4	79.6	79.2	83.3	89.6
CHIA	96.5	91.3	104	91.1	84.3	96.8	84.7	99.9
CHIC1	124.6	146.9	103.9	95.2	88.8	94.8	110.8	123
CHIC1	104.9	117.5	97.4	88.1	90	87.4	98.2	97
CHIC2	1457.1	1732.5	1069.3	961	778	1078.4	1569.4	2901.6
CHID1	97.8	136.7	89.6	84.4	88.1	91.7	88.6	103.5
CHKA	883.6	1069.6	610	628.1	540.6	684.3	817.4	1145.9
CHKA	230.5	310.3	158.8	160	170.7	186.3	218.8	354.1
CHKB	791.6	724.4	700.1	585.7	631.1	1167.8	495.3	962.4
CHKB	93	114.4	80.5	82.4	82.1	84.5	101	141.6
CHL1	98.3	104.5	104.3	99.6	99.8	99.2	99.5	105
CHM	132.2	141.9	94.9	88.6	83.4	86.7	109.3	131.9
CHML	185.5	204.1	132.3	137	120.8	147.1	164.2	177.9
CHMP1B	1072.2	1375.8	602.5	691.6	626.4	966.3	858.4	1149.5
CHMP2A	257.7	342.8	203.2	184.8	138.7	181.4	237.4	222
CHMP2A	471.3	684.8	264.6	182.5	163.8	191.7	491.7	488.8
CHMP2A	2574.3	3442.7	1372	1130.3	929.6	1223.9	2253.8	2503.5
CHMP2B	584.7	775.3	288.4	359.7	257.4	458	557.9	1023.6
CHMP4A	1415.8	1935	665.1	516.1	429.3	513.4	1202.6	1416.6
CHMP4B	904.6	1155.7	800.6	822.6	636.3	892.3	895.9	1294.5
CHMP4C	88.4	95.7	91.6	83.3	88.3	89.5	92.7	110.3
CHMP5	1519.3	2012.1	542	528	436.6	866.7	1078.8	1338
CHMP6	432.9	591.2	297.5	283.9	243	302.5	425.5	452.6
CHMP6	211	266.2	119	117.9	107.7	116.2	181.2	178.6
CHMP7	201.8	239.7	148.1	129.4	125.1	168.6	189.2	259.1
CHN1	123.4	142.3	124.6	101	94.6	107.8	144.5	152.8

CHN1	78.1	79.2	98.7	94.1	84.4	84.3	70.9	82.8
CHN2	108.9	137.2	126	110.7	114.9	124.5	95.3	107.4
CHN2	158.5	225.4	142.1	136.9	154.6	146.7	108.2	115.4
CHN2	98	116	84.7	80.2	74.6	87	89.6	113.5
CHODL	154.1	170.9	103.4	96.8	88.4	109.4	134	151.3
CHORDC1	97.5	103.9	89.4	79.8	76.7	87.7	94.8	119.1
CHP	1007.9	1060.1	321	313	233.2	258.5	993.5	1601.5
CHPF	144.4	173.9	189.2	185.8	165.7	241.5	140.2	178.2
CHPT1	395.6	448.7	168	171.3	148.5	181.4	349.3	659.5
CHR415SYT	67.8	85.7	92.2	82.5	86	84.5	73.7	80.2
CHRAC1	345.8	474.3	265.6	213.7	171.8	206.7	290.2	555.9
CHRD	86.8	103.8	93.3	98.1	87.4	80.4	85.4	92.8
CHRDL1	76	80.6	84.5	80	81.4	81.1	76.7	86.1
CHRDL2	92	105.9	82.8	81.5	70.8	71.8	93.6	105.9
CHRFAM7A	114.2	120.7	95.9	89.3	82.6	94.7	101.9	124.5
CHRFAM7A	80.5	90.4	93.4	94.9	86.3	96.8	87.4	81.9
CHRFAM7A	78.3	86.7	80.4	90.8	76.2	77.5	76	81.3
CHRM1	98.5	102.3	87.6	88.6	82.4	84.9	95.4	105.8
CHRM2	80.8	86.2	84.1	91.1	86.7	88.1	90.4	88.5
CHRM2	86.3	94.6	90.8	88.6	82.3	81.9	90.9	92.5
CHRM4	73.3	79.1	97.4	85	87.9	91.2	84	79.7
CHRM5	95.2	101	86.4	82.3	78	81.1	94.7	98.3
CHRNA1	77.4	97.1	90.6	78.7	84.2	83.6	91.1	86.5
CHRNA1	145.6	156.8	110.7	114.7	105	90.7	119.3	132.2
CHRNA1	76.4	76	84.1	86.2	79.6	78.1	77.3	88.9
CHRNA10	126.3	150.6	102.5	81.8	76.6	87.3	117	156.1
CHRNA2	104.5	114.6	103.3	97	98.6	96.4	94.7	107
CHRNA3	107.9	103.6	121	108.9	96.7	96	99.7	97.6
CHRNA4	101.4	117.5	105.8	89.6	94.6	99	103	114.9
CHRNA5	1563.5	1246	1273.3	1935.2	1752.9	1397.1	1526.9	2902.1
CHRNA6	115.4	137.3	111	105.7	105.2	109.3	112.7	126.1
CHRNA7	107.4	114.4	96.5	95.5	86.1	106	100	107.6
CHRNA9	78.2	103.8	98.4	98.9	90.5	88.7	81.7	92.2
CHRNB1	181.8	294.6	178.9	167	135.8	219.6	203.9	294.7
CHRNB2	89.5	83.7	81.6	84.1	82.8	83.2	84.4	88
CHRNB3	114.6	129.3	99.1	83.9	79.1	81.2	106	127.6
CHRND	96.3	112	96.2	81.2	81	82.6	91.4	111.1
CHRNE	100.4	113.6	100.2	87.9	92.6	96	103.8	117.7
CHRNG	78.9	91.1	91.3	84	83.8	83.4	85.1	91.9
CHST1	107	114.2	93.4	86.3	81.3	94.3	109	114.3
CHST10	257.2	363.4	254	213	174	269.9	248.1	393.9
CHST11	110.7	123.4	94.5	88.5	86.2	85.8	116.5	119.8
CHST12	343.8	414.8	232.6	250.3	229.8	238.2	207.5	208.9
CHST13	3015.2	3738.7	1846.7	1165.1	1007.6	1125.7	2758.4	2012.1
CHST2	134.4	165.5	101.4	86.7	88.5	99.6	124	163.5
CHST3	154.5	191.8	153.9	131.3	128.9	129.2	154	203
CHST4	184.5	206.1	162.8	110.1	100.9	117.1	121.3	121.2
CHST5	81.9	97.4	89.4	81.1	85.9	88.5	75.1	81.3
CHST5	81.1	102.3	102	81.9	86.7	85.4	91.4	91
CHST6	101.3	108.8	115.6	104.3	92.3	98.4	94.1	113.1

CHST7	161.5	224.2	166.2	135.1	122.1	143.3	160.4	188.8
CHST8	88.9	102.7	93	96.9	86.6	96.9	92.5	92.6
CHST9	78.7	86.5	84.7	84.1	87.2	83.5	80.8	89.1
CHSY1	2657.9	3225.6	1420.1	2161.4	1516.2	2827.3	2277.8	3184.1
CHTF18	332.3	324.2	487	345.9	312	579.4	224.2	473.1
CHUK	510.2	627.1	386.1	328.9	246.7	345.3	560.9	878.7
CHURC1	715.7	818.2	423.4	495.6	408.3	722.2	578.2	880
CHX10	112.6	145.5	109.9	93.6	94.8	95.1	116.3	118.1
CIAO1	2008.3	2119.2	1095.5	1128.4	849	1132	1595.1	2321.5
CIAPIN1	580.3	675.3	286.4	202.8	167.9	324.7	520.8	966.8
CIAS1	1744	1994.1	1231.7	1028.3	822.7	1192.8	1452.4	1902.8
CIAS1	296.5	349	176.7	149.2	137	147.8	264.8	334.4
CIB1	4426.8	5679.3	4555.5	4408.8	3723.7	4399.3	3746.9	4111.1
CIB2	104.9	133.4	84.8	85.5	90.2	104.2	119.1	124.9
CIB3	263.8	320.9	177.4	115.7	114.5	129.7	236	224.2
CIB4	79.9	82.5	82.2	82.8	83.3	84.5	73.2	82.1
CIC	210	246.4	150.5	161.4	131	171.8	198.3	293.9
CICE	129.5	141.5	101.4	100.2	87.7	95.5	116.6	140.5
CICK0721Q	121.5	124	100.8	95	95.2	92.2	118.2	124
CIDEA	109	119	88.4	95.7	90	90.6	94.4	110.9
CIDEA	88.5	124.8	88.1	86.5	78.7	81.4	89	145.4
CIDEB	178.3	203.3	130.4	134.3	132.6	139.6	146	192
CIDEC	92.1	106	115.6	96.3	91.2	80.9	98.6	99.7
CIITA	114.3	119.1	87.4	87.5	89.8	86	115.5	111.3
CILP	81.5	89.4	88.5	82.5	84.4	79.9	77.6	75.7
CILP2	101.6	119.9	88.4	91.5	83.7	90.1	101.6	114
CINP	184.9	283.1	146.9	123.7	107.6	120.1	195	235.4
CIP29	5742.2	6517.7	3473.1	2690.8	2365.9	3427.8	4664	5457.1
CIR	408.8	539.2	120.3	134.3	134.1	217.7	424	562.3
CIRBP	3245.8	4505.1	1775	773.7	597.5	793.9	2543.8	3656.3
CIRH1A	2385.4	2953.5	1506.2	1020.8	797.8	1070.2	1960.4	2365.2
CISH	88.8	110.5	106.6	90.1	90.4	107.5	94.4	119.2
CIT	107.1	117.9	105.2	87.9	92	93.4	107.5	113.1
CITED1	104.1	126.2	98.2	91.6	85.5	83	98.3	118.1
CITED2	3825.7	4990.6	1824.6	2113.7	1129.8	2452.4	2813.8	3513.3
CITED4	3259.1	4551.6	3418.4	2768.1	2051.5	2725.2	2261.1	3749.9
CIZ1	114.5	124.7	112.1	100.7	104.7	104.1	104.7	120.8
CKAP1	1526.9	1884.7	784.4	515.5	458.4	666.2	1415	1854.6
CKAP2	682.2	768.5	266.6	273.1	268.6	379.3	581.4	889.7
CKAP4	1116.3	1549.2	1329.7	953.9	706.6	1671	1006.8	1715.2
CKAP5	1665.5	2242.2	2210.5	2552.1	2324.8	3492.1	1597.6	2610.7
CKAP5	269.5	350.4	161	126.4	127.3	132.9	220.2	279.9
CKB	239.7	336.1	219.7	127.5	120.4	145.8	211.9	288.7
CKLF	6015.4	6754.8	4530.7	3872.7	2762.8	4047.2	4425.5	4618.2
CKLF	3838.9	4793.5	2023.2	1712.4	1381	1835.5	2926	3316.6
CKLF	87.7	101.1	83.4	70.4	67.6	74.4	86.4	103.8
CKLFSF4	85	104.6	97.1	94.7	96.9	93.3	99	99
CKM	398.5	482.4	234.9	206.9	164.4	197.3	413.8	639.2
CKMT1A	89.6	105.1	98.8	97.2	82.2	98	90.6	99.3
CKMT1B	78.8	98.8	94.1	79.9	76.4	85.6	83.7	84.6

CKMT2	93.1	113.5	94.6	82.8	83.6	92.9	99.4	113.9
CKS1B	3954.5	4712.2	2233	1358.1	1194.8	1302.6	3764.4	4535.3
CKS2	6675.5	7850.4	4096.4	2676.8	2074.9	3265.3	6321.7	8486.2
CLASP1	370	563.2	332	482	389.9	584.7	358.9	610.5
CLASP2	449.6	756.2	359.5	421.5	375.2	701.1	482.1	619.6
CLC	110.4	142.4	103.1	92.9	76.4	95.6	126.8	117.2
CLCA1	93	104.1	84.9	78.9	78.7	85.5	96.2	97
CLCA2	89.9	98.1	95.7	82	75.9	85.6	94.4	87
CLCA3	69.5	86.5	87.7	90.2	82.4	89.2	80.1	78.3
CLCA4	102.4	101.1	86.7	77.3	82.1	90.1	94.6	104.8
CLCC1	97.1	107.8	93.5	84.1	87.2	88	97.5	91.4
CLCC1	162	212.9	132.8	107.1	100.9	103.4	183.1	228.4
CLCF1	116.4	140	95	95	88.2	112.6	127.9	159.1
CLCN1	99.7	102.1	91.9	89.1	80.1	85.2	93	105.4
CLCN2	144.4	151.8	97.6	92.5	87	93.4	128.9	153.9
CLCN3	87.3	94	89.8	87.5	83.8	86.2	86.5	103
CLCN3	476.8	568.3	290.6	310.9	223.5	311.1	428.3	617.7
CLCN4	94.8	121.4	96.9	102.6	94.3	96	86.2	89.7
CLCN5	103.1	122.1	97.6	102.5	89.5	94.3	101.8	113.4
CLCN6	172.6	180.3	113.9	101.3	88.3	105.8	151.9	180
CLCN6	183	209	161.6	254.4	267.3	278.9	170.5	180.1
CLCN6	168.8	202.9	171	262	248.4	316.8	164.4	233.8
CLCN7	3200.2	3695.8	3382.5	3505.5	2932.1	3390.7	2601.6	2709.8
CLCNKA	104.7	117.5	103.4	105.6	105.1	123.1	98.5	103.5
CLCNKA	99.4	108.9	115.1	118.1	113	147.6	94.2	90.3
CLCNKB	99.8	108.5	105.2	94.1	86.5	99.6	95.7	107.5
CLDN1	85.1	106.9	105.1	106.1	107.7	102.7	104.3	105.6
CLDN10	143.2	185.8	126.7	91.9	87.2	93	145.9	149.4
CLDN10	76.9	79.7	89.8	76.8	77.4	86	78.1	93.8
CLDN10	110.5	130.4	120.3	107.3	88.5	99.5	105.3	111.2
CLDN12	263	301.4	151.8	182.8	153.8	242.9	175.7	242.8
CLDN14	368	479.5	234.9	225.8	207.2	253.3	327.9	338.5
CLDN14	91.6	95.6	81.9	74.8	73.4	84.3	91.5	98.6
CLDN14	103.6	113.2	81.5	82.6	86.1	82.6	97.3	111.4
CLDN15	85.1	90.4	110	94.3	85.3	103.2	89.1	83.3
CLDN15	554.2	533.2	397.8	301.1	399.7	345.5	376.4	527.4
CLDN15	106.5	121.4	119.1	107.2	101.9	108.8	107.3	119.4
CLDN16	90	105.9	96.7	91.6	86.2	89.5	90.5	99.4
CLDN17	97.9	108.3	82.9	86.1	74.8	89.3	90.7	111.5
CLDN18	87.1	99.3	98.2	91.3	81.9	103.4	79.3	87.7
CLDN18	169.8	174.7	132.7	101.1	119.6	118.8	158.6	166.3
CLDN18	81.9	82.5	105.3	96.3	92.9	103.6	85.4	101
CLDN18	106.8	137.6	91	90.2	90	90.7	114.9	125.1
CLDN19	133	131.1	104.8	99.8	93.7	106	122.2	123.2
CLDN2	102.1	102.1	103.9	90.7	93	85	98	100.5
CLDN20	197.4	234	128.5	103.6	98	105.6	183.8	200.5
CLDN23	901.3	1023.3	625.2	624.9	385.1	613.9	995.2	1506.4
CLDN3	104.6	119.6	87	84.7	85.4	76.3	106.8	125.1
CLDN4	129.9	135.6	85.8	95.4	87.3	96.6	114.1	119
CLDN5	99.3	128.3	84.4	81.4	69.5	92.1	94.5	106.1

CLDN6	81.6	103.1	92	83.9	93.7	86.6	88.1	101.1
CLDN7	128.1	126	124.7	116.1	129.9	164.4	108.3	122.2
CLDN8	84.4	90.7	87.4	84.7	79.5	81.5	83.9	85.7
CLDN9	111.2	137	100	93.9	99.5	107.8	109.8	120.6
CLDND1	1051.8	1297.7	458	395.3	399.1	675.4	825.8	1605.9
CLDND1	945.7	1176.6	564.2	591.8	439.2	835.9	835.5	1599.2
CLDND2	241.1	236.2	246	183.2	209.4	186.4	198.2	211.5
CLEC10A	128.1	127.2	106.7	118.6	111.2	121.5	105.9	119.3
CLEC10A	89.9	111.7	99.6	101.9	101.9	105.5	94.9	101.8
CLEC10A	104	112.1	107.7	136.8	114.4	140.2	90.6	104
CLEC11A	5788.5	7794.9	7714.6	2577.7	2220.6	2580	5305	11795.1
CLEC12A	384.5	476.9	193	530.2	409.9	718.1	236.5	275.4
CLEC12A	334.5	410.4	196.5	387.7	337.1	484.1	228.2	255
CLEC12A	188.5	209.6	111.7	141.4	125.1	180.9	146.4	158.7
CLEC12B	113.2	147.8	94.2	94.4	86.6	102.9	127.3	155.8
CLEC14A	107.9	127.5	106.2	79.1	83.9	101.9	123.8	136.8
CLEC1A	88.4	97.2	90.1	91.2	84.7	92.7	87.9	94.6
CLEC1B	87.2	89.8	96.4	86.7	90.4	79.6	82.9	101.7
CLEC2B	123.7	129.8	92.6	85.1	83.1	89.5	111.4	112.5
CLEC2D	75.7	79	91.5	81.4	83.1	99.8	81.8	87.5
CLEC2D	222.1	239.9	190.9	164.2	141.2	191.3	147.4	170.4
CLEC3A	86.1	85.4	93.7	91.1	95.8	87.7	89.5	99
CLEC3B	175.8	210.8	116	133.1	116.2	125.4	140.3	153.4
CLEC4A	94.6	114.9	92.5	110.2	100.8	101.1	96.6	105.6
CLEC4C	87.1	97.6	91.1	84.9	84.4	93.5	93.1	96.3
CLEC4C	99.1	96.3	90.2	91.7	77.1	98.2	90.1	97.3
CLEC4D	135.8	148	92.1	95.6	85.6	84.1	122.3	140.5
CLEC4E	94.9	123.6	92.3	94.7	85.9	81.7	92.8	102.4
CLEC4F	75.1	82	97.8	85.2	82.9	80.9	84.6	76.6
CLEC4G	118.8	139.7	107.3	89.4	84.7	93.4	121.1	136.6
CLEC4M	80.2	77.5	89.4	84.3	74.9	84.4	80.6	70.7
CLEC5A	125.4	134.5	108.8	111.5	88.5	118.3	114.4	142.8
CLEC6A	105.9	121.5	94.3	94.3	82.2	88.5	96.6	110.5
CLEC7A	85.7	91.7	106.6	103.1	96.2	91.1	87.3	88.2
CLEC7A	86.7	100.2	97.7	93.9	86.1	91.2	98.8	94.2
CLEC7A	89.7	89.3	104	101.9	85.7	88.6	86	94
CLEC9A	93.5	97.9	105.6	101.7	101.9	101.8	89.7	96.9
CLGN	170.2	195.9	127.2	136.1	119.8	170.3	146.7	208.2
CLIC1	19662	25449.5	11556.4	10238	7202.6	11182.1	18465.6	23228
CLIC2	182.5	228.3	101.8	104.4	97.6	109.1	179.8	192.7
CLIC3	90.2	107.2	92.1	82.2	82.3	89.7	91.8	102
CLIC4	1605.4	1956.8	568.1	760	639.5	977.9	1662.8	3318.9
CLIC5	97.2	117.3	101.5	93.7	97.7	102.8	95.8	105
CLIC6	271.6	307.9	242.5	230.7	195.9	196.8	269.6	406.5
CLIP1	589.6	679.4	284.9	317.2	308.8	523	770.3	1325.6
CLIP2	101.2	122.5	127.6	119.4	109.1	153.8	91.6	116.3
CLIP2	104.8	125.8	144.4	138.8	137.4	202.4	118.5	140.4
CLIPR-59	117.1	107.1	100.3	103.4	113.3	111.3	99.9	116.1
CLK1	347.3	325.5	175.5	133.4	167.4	184.9	305.3	628.1
CLK1	291.1	307.7	121.1	114.4	103	126.6	241	388.2

CLK2	562	627	284.9	245.2	280	312.8	380.9	540
CLK2	654.7	689.4	483.4	507.5	493.9	721.9	428.8	840.7
CLK2P	79.6	87.2	96.9	94.5	93.2	94	84	92.5
CLK3	393.3	507.6	244.7	179.5	179.1	201.1	351.9	415.8
CLK3	1021.4	1221.2	745.5	838.5	812.9	969.3	934.7	694.8
CLK3	197.6	260.4	106.6	122.1	126.8	135.2	216.4	245.8
CLK4	250	240.1	171.8	194.2	147.1	258.7	197.4	507.3
CLLU1	89.6	102.8	84.6	82	76.5	79.9	93.2	96.1
CLLU1OS	107.1	120.7	92.5	87.1	85.2	92	101.3	114.2
CLMN	118.7	146.5	96.1	92	82.6	96.1	123.7	135.8
CLN3	286.9	388.7	249.5	273.3	232.5	375.9	228	378.5
CLN3	240.6	259.6	215.5	247.6	214.8	304.7	204.7	363.6
CLN5	356.6	414.5	240.2	359.2	269.2	449.2	311.1	558.6
CLN6	292.4	391.1	220.1	230.7	171.5	272.3	229.2	347.8
CLN8	127.1	122	122.6	119.6	85.6	89.6	98.5	134.8
CLNS1A	5511.7	7492	3163	2742.4	2396.1	4332.8	4701.1	5777.3
CLOCK	108.2	133.2	103.6	97.4	96.9	103.2	118.2	106.5
CLPB	106.7	114.9	107.1	110.7	103.4	103.5	107.2	109.4
CLPP	1110.6	1493.3	769.7	408.8	365.6	425.7	914.3	1120.3
CLPS	79.8	83.5	89.4	88.2	80.6	86.9	77.3	75.2
CLPTM1	548	703.9	889.9	572.4	487.7	527.4	520.1	1193.7
CLPX	963.5	1245.4	591.5	575	464.1	817.5	849.3	1815.4
CLRN3	119.2	153.2	87.5	96.6	87.5	88.8	122.8	139.7
CLSPN	157.9	224.1	115.3	94.6	87.1	86.9	139.2	180.5
CLSTN1	968.9	1304.4	1444	1906.2	1371.1	1984.6	1009.1	2052.7
CLSTN1	119.9	150.8	115.9	110.6	102	103.3	107	107
CLSTN2	392.9	471.8	326.9	147.6	122.2	156	358.1	649.6
CLSTN3	91.1	90	98.3	93	92.3	87.6	84.6	95.4
CLTA	3702.3	4643.3	2589.6	1630	1379.7	2167.3	2824.2	4003.1
CLTA	82	93.1	101	89.4	88.1	84.4	74.5	83.5
CLTA	2821.2	3657.8	1782.2	1157.6	1067.2	1696.9	2224.2	2956.1
CLTB	3464.9	4448.9	2333.8	1903.1	1583.8	2505.3	3411.3	4532.5
CLTB	106.4	128	94.3	85.8	78	87.6	102.7	127.2
CLTC	224.1	323.9	152.2	169.1	170.5	205.8	293.8	417.3
CLTCL1	327.2	346.4	289.7	258.4	251.9	288.6	187.4	236.8
CLTCL1	190.3	218.9	128	107.3	121.5	134.1	170.9	184.6
CLU	71.2	81.7	85.1	73.5	67.9	65.5	71.1	69.7
CLU	104	113.8	104.7	85.9	82.3	81.2	111.1	103.1
CLU	91.1	99.3	95.7	87.4	79.4	81.2	90	95.7
CLUAP1	347	475.7	175.4	147.2	133.1	147.8	259.4	292.8
CLUAP1	29932.4	28501.7	18430.5	27535.8	26687.1	21602.5	28920	29730
CLUAP1	481.9	645.1	358.6	269.4	206	356.3	339.9	385.2
CLUL1	80	84.7	91.7	84.8	84.3	83.7	82.3	84.2
CLUL1	108	123.5	101.6	110.7	95.5	106.4	110.9	108.2
CLUL1	99.5	121.1	86.3	84.2	77.8	86.4	97.7	112.9
CLYBL	769.8	1024.9	407.6	510.4	361.6	503.2	766.5	1073.2
CLYBL	613.9	761.7	306.3	324.7	276	346.2	552.1	607.1
CMA1	87.8	101.7	100.5	93.6	89.8	94.6	88.1	93.7
CMAH	100.5	117.1	97.8	87.1	93.9	93.8	89.9	99.1
CMAS	1724.4	2551.6	1252.7	1175.3	897.7	1562.1	1661.4	2446.3

CMIP	1876.8	2272.4	1235.1	948.6	845.3	1504.4	1690	2133.5
CMIP	107.6	119.6	91	96	92.5	88.3	93	96.8
CMIP	109.7	130.6	92.9	83.9	84.3	88.1	111.9	117.9
CMKLR1	94.7	111.5	94.7	90.6	85.6	95.3	93.8	98.2
CMKOR1	150.9	174.8	121	167.1	126.1	165.5	115.8	127.9
CML2	88.9	92.8	101.2	87	90.5	96.7	87.9	84.7
CMPK	987.5	1078.3	804.3	862.9	694.6	1169.8	1103.3	2528.7
CMTM1	92.7	105	106.4	95	104.7	92.3	101.6	99.7
CMTM1	128.3	147.1	93.7	96.8	87.7	90.4	113.5	130.8
CMTM1	95.2	114.9	100.5	88.2	74.3	90.7	103	103.6
CMTM2	81.5	86.3	80.7	79.2	70.7	69.5	78.6	77
CMTM3	113.9	125.3	87.6	83.1	78.2	87.3	111.9	130.1
CMTM3	2284.9	2716.5	1701.6	1566.1	1363	1542.2	2086.6	2755.7
CMTM3	128	141.3	111.2	102.9	102.5	108.3	125.1	135.7
CMTM3	606.6	840.3	476.4	414.5	367.1	467.3	515.4	803.4
CMTM4	646.5	792	390.4	369.9	324.5	381	515.8	502.4
CMTM4	102.9	128.7	105.7	104.4	93.5	105.5	107.9	111.3
CMTM5	103.6	125.9	88.2	93	81.5	90.5	107.5	117.4
CMTM7	6734.4	8086.5	4255.4	3397	2239.1	3027.8	5595.8	5930.8
CMTM7	405.4	506.6	276.5	197.6	153.3	192.8	343.8	381.3
CMTM7	883.2	1145.9	360	311.3	220.4	286	727.4	1574.1
CMTM8	189.7	174.2	140.8	213.1	195.6	202.5	176.6	384.9
CMYA1	70.3	81.7	81.1	72.6	73.6	69.8	81.4	83.8
CMYA3	88	100.4	93.7	88.2	82.7	87.5	88.9	86.7
CMYA3	90.8	100.8	92.5	85.1	82.4	80.8	88	97.2
CMYA5	277.3	335.7	195.4	261.6	194.5	370.1	282.6	329.1
CNAP1	1231.7	1272.1	910.5	762.1	664.9	701.1	804.7	1032.4
CNBD1	73.1	85.8	89.1	84.4	79.2	83	75.6	73.4
CNBP	3366.8	3862.4	1344.4	1365.7	978.6	1832.7	2709.1	6307.1
CNDP1	93.3	104.3	105.3	99.4	90	108.9	96.2	100.5
CNDP2	2821.1	3664	1780.7	1971.6	1365.9	2406.7	2014.8	2401.2
CNFN	518.8	652.1	467.1	279.4	211.7	228	570.8	702.1
CNGA1	83.9	89.4	88.8	77.4	76.6	84.8	87.2	96.3
CNGA2	83.3	98.7	102.9	98.2	94.7	93.6	76.4	80.6
CNGA3	75.4	88	103.9	93.8	89.3	105.9	84.6	87.5
CNGA3	81.1	90.4	87.9	84.4	78	81.5	85.1	100.8
CNGA3	166	185.6	115	102.1	102.5	107.9	154.3	151.5
CNGA4	90.9	104.1	104.2	85.5	83.5	84.6	96.9	96.1
CNGB1	156.3	198.4	118.5	123.7	115.7	121.1	152.7	133.2
CNGB3	85.2	90.3	82.7	80.4	91	81.1	82.7	89
CNIH	3111.2	3469	1485.8	1427.6	911.3	1276.2	2612.3	4525
CNIH	2471.8	2882.9	1213.6	1026.9	701.8	1151.1	1719	2769
CNIH2	88.5	99.1	88.7	84.6	76.5	94.2	91	87.7
CNIH2	85.8	95.4	106.3	96.8	103.1	89.8	86	92.5
CNIH3	122.7	144.6	97	94.1	92.6	123.1	112.8	135.5
CNIH4	8915.9	10275.9	2873.9	2257.3	1517.2	2752	8748.1	10190.1
CNKSR1	97.5	115.7	100.7	95.8	92.1	91.1	95	99.7
CNKSR2	105.9	125.9	95.8	87	100.3	100.2	96.4	103.6
CNKSR3	235.6	320.8	193.9	236.7	203	316.5	266	366.7
CNN1	77	88.3	88.2	75.8	73	79.8	88.2	98.6

CNN2	217.4	317.9	129.3	142.2	120.4	106.2	192	291.9
CNN2	2778	3766.8	1394.4	1298.9	1085.7	1009	2336.4	2412.7
CNN3	678.6	892.6	501.5	339.2	276.5	462.3	506.2	708.3
CNNM1	112.3	123.2	123.7	102.9	120.2	113.9	108.8	108.5
CNNM2	103.1	104.6	89.8	84.6	87.1	90.6	94.4	93.7
CNNM2	127.7	140.6	102.4	110.6	101.1	114.9	125.8	139.8
CNNM2	135.6	160.8	96.5	81.8	81.7	90.8	140.9	186.5
CNNM3	393.9	436.8	201	202.2	181.4	190.3	318.4	394.8
CNNM4	92.5	115.5	137.3	115.8	113.1	124.7	103.3	116.1
CNO	849.4	1207.2	412.9	299.9	305.9	336.6	825.4	962.5
CNOT1	110.6	127.5	110.2	107.5	101.9	104.5	104.3	130
CNOT1	3145	4624	3263.7	4640.9	3502.6	5653.9	3273.4	4005.1
CNOT10	580.8	777	527.6	506.4	419.1	781.6	445.3	552.2
CNOT2	1270.6	1839.1	676.1	903.3	745.3	1296.5	1010.2	1247.3
CNOT3	174.1	252.2	281.7	196	197.4	262.4	152	313.3
CNOT4	644.6	806.2	576.3	583.8	469.5	614.3	715.8	1279.3
CNOT4	123.6	131.5	113.5	118	95.3	107.3	107.9	122.3
CNOT4	116.7	130.8	112.2	106.8	98.7	110.6	122.3	124.3
CNOT6	704.4	1023.2	576.7	782.6	618.1	971.1	650.7	1040.2
CNOT6L	166	221	162.8	148.1	159.2	182.3	149.2	213.4
CNOT7	3081.9	4173	1073.4	709.6	485.4	639.3	2503.1	3338.9
CNOT7	2150.1	2737	1491.9	1354.6	863.6	2218	1750.1	2842.1
CNOT7	128.1	144.9	95.6	107.8	77.8	73.8	109.2	130.5
CNOT8	1243.7	1670.3	410.4	587.7	590.3	798	1075.6	1426.8
CNP	93.3	95.4	90.6	87.8	89.6	89.6	100.4	100.2
CNR1	116.1	153.1	99.3	99.4	93.9	104.6	106.7	132.2
CNR2	80.4	93.5	100.5	98.5	88.8	99.2	88.1	92.5
CNTD	75.6	74.6	102.7	94.1	95.2	102.7	79.7	82
CNTF	95.8	110.2	84.7	90.3	78.7	85	95.2	101.8
CNTFR	93.7	108.3	98.9	87.8	81.5	94.9	85.5	98.6
CNTFR	82.3	100.1	92	93.6	91.6	92.2	90.1	100
CNTFR	82.6	86.6	92.8	86.6	76.3	84.4	85	89.6
CNTN1	82.2	100.2	92.4	90.1	82.8	87.9	87.9	92.2
CNTN1	77.3	90.6	93.8	74.5	75.3	82.7	82.3	84.4
CNTN2	80.3	91.4	96.8	94	84.4	95.4	77.2	82.9
CNTN3	85.3	106	94.3	78.2	78.8	82.8	89.2	88.9
CNTN4	114.9	159.2	95.6	85.6	82.4	97.3	114.2	155.2
CNTN4	80.1	86.3	87.2	79.8	90.7	82.6	80.6	85.3
CNTN4	203.2	264.6	147	124	113.1	170.7	191.9	265.3
CNTN5	77.8	96.8	83.1	80.6	83.4	89.6	80.3	81
CNTN5	93.7	101.8	100.4	84.4	87.6	91.2	92.2	96.2
CNTN6	98.2	118.1	98.2	101.4	86.4	95.6	99.7	110.5
CNTNAP1	108.3	97.4	102.5	92.4	82.1	88.4	111.8	117.1
CNTNAP2	81.1	91.8	96.2	87.6	82	82.3	84.2	78.3
CNTNAP3	104.4	123.5	100.5	96.1	87.2	104.5	116.5	128.6
CNTNAP4	82.3	101.1	100.7	95.7	93.4	88.9	87.7	91.6
CNTNAP4	85.1	104.3	86.4	79.7	83.5	92.6	90.3	98.1
CNTNAP5	135.1	156.9	109.6	92.8	100.3	100.1	137.1	136.9
CNTNAP5	81.1	90.5	113.5	101.5	100.2	106.9	82	78.7
CNTROB	112.3	131.2	119.1	110	109.3	131.8	110.2	124.3

COASY	2477.3	3071.9	1176.9	1149.4	955.6	993.9	1712.9	1645.9
COASY	467.4	542.7	258	218.4	204.6	227.1	337.2	589
COBL	98.9	106.9	92.8	87.1	97.8	89	100.4	125.9
COBLL1	85	83.1	94	89.4	95.8	93.1	95	94.8
COBRA1	440.3	623.5	208.1	170.1	142.3	113.1	323.3	502.5
COBRA1	918.8	1396.4	1331.4	837	773.4	1247	818.3	1895.6
COBRA1	1348.4	1669.8	1843.3	1405.4	1108.6	1800.6	1292	2865.1
COCH	137.2	152.3	101.4	106.4	102	115.2	129.3	157.3
COG1	175.6	223.6	159.4	178.2	150.1	187.2	156.3	196.8
COG2	925.2	1219.3	799.4	757.6	609.4	968.2	781.9	846.7
COG3	173.9	209.1	138.5	161.9	150.1	188.4	183	306.1
COG4	199	226.4	128.6	133.1	126.9	178.3	161.2	220.2
COG5	193.1	239	128	143.2	129.4	154.5	145.9	210.1
COG5	257.3	302.3	139.3	151.1	136.6	204.3	225.6	387.1
COG6	262.3	353.1	187.8	214.2	184	313.2	227.2	293.7
COG7	172.3	213.8	175.5	156.2	124.4	165.2	167	264.7
COG8	227.2	304.1	168.9	120.6	113.2	121.2	193.9	249.6
COIL	1206.8	1730.1	799.9	937.2	734.4	1404.1	1008.5	1735.1
COL10A1	84.5	86.6	97.5	86.1	88	93.8	86.4	82
COL11A1	129	152.6	107.6	97.1	102.7	102.3	127.5	114.9
COL11A1	75.2	98.3	92.1	79.9	70.6	83.8	98.7	88.8
COL11A1	86	94.5	103.8	86.8	85.1	98.3	81.9	102.1
COL11A1	94.4	100	95.9	91.9	82.4	88.2	100.9	127.2
COL11A2	116.8	121.5	97.3	92.4	94.3	96.5	118	123.1
COL11A2	95.8	117.6	102.1	89.8	86.6	97.5	99.5	119.3
COL12A1	159.8	187.8	124.1	106.5	104.9	113.9	150.4	170.9
COL12A1	119.1	139.9	96.6	97.6	85.9	86.4	127.8	150.5
COL13A1	103.7	108.9	113.4	101.5	93.9	102.4	97.5	98.7
COL13A1	120.4	118.5	97.3	94.7	93.1	89.8	107.8	122
COL15A1	124.3	155.2	122.4	141	103.2	171.7	121	167.3
COL16A1	111.7	115.6	97.4	78.4	77.7	90.9	109.5	110
COL17A1	90.2	110.4	82	79.1	84.2	84	102	104.6
COL17A1	107.3	117.6	86	81.4	93.5	80.9	100.3	127.9
COL18A1	107.8	129.1	99.3	187.2	152.4	186	107.4	112.4
COL18A1	114.1	117.6	87.4	86.6	80.8	82.6	104.3	131.2
COL19A1	68.6	80	88.9	89.5	86.8	80.2	86.6	81
COL1A1	101.8	114.6	99.4	114.3	120.1	119.2	103.2	112.6
COL1A2	102.5	108.7	89.1	85	84.4	92.1	95	119.7
COL20A1	106.4	110.1	96.8	93.9	89.2	98.3	106.8	119.7
COL21A1	77.2	77.7	91.3	78.8	76.5	85.5	80.2	77.3
COL22A1	104.1	147.5	103.7	92.7	85.8	95	103	125
COL23A1	210.9	250.9	219.9	268.4	239.2	393.9	184.8	366.5
COL24A1	116.7	144.3	124.3	161.6	156.1	240.1	139.5	195.2
COL25A1	95.7	114.1	93.2	83	86.8	82.5	111.1	128.6
COL25A1	82.6	94.3	87.9	75.6	79.1	85.6	81	94.3
COL25A1	101.5	133.2	91.8	92.3	74.3	82.4	99.1	107.6
COL27A1	127.1	131.2	113.4	102.1	97	117.9	96.8	116.9
COL2A1	143.7	175.6	92.5	90.2	82.8	93.4	131.5	173.7
COL2A1	101.4	114.7	92.5	87.4	88.3	90.7	101.6	95.8
COL3A1	86.4	90.4	94.1	86.2	84.4	76.2	88	90.1

COL4A1	371.5	487.7	239.9	478.4	403.6	547.7	311.7	377
COL4A2	152	172.7	96.5	94.7	104.3	152.7	139.2	161.4
COL4A3	72.2	82.4	82.3	77.8	77.9	74.4	79.3	86.1
COL4A3	110.1	110.8	99.8	93.8	86.2	82.5	104	109.4
COL4A3BP	152.4	161	97.5	99.7	98.4	96.1	131.8	179.2
COL4A3BP	589.7	677.1	284.3	436.1	375.8	409.7	474	728.4
COL4A4	81.9	92.9	90.3	88.2	86.7	81.5	82.2	95.5
COL4A5	173.6	197.7	143.1	106.5	100.8	119.2	155.1	170.9
COL4A5	71.6	69.5	89.2	85.3	83.2	81.4	71.7	79.2
COL4A6	89.8	85.1	89.3	90	88.5	82.9	71.1	77.8
COL4A6	88.7	93.4	91.3	80.3	83.2	85.9	94.1	100.5
COL4A6	111.3	101.1	95.1	80.6	75.2	84.2	94.9	100
COL5A1	103.3	107.1	105.3	96.4	80.9	83.6	107.5	123.7
COL5A2	102	125.3	101.4	118	92.7	100.7	104.4	133.8
COL5A3	79.4	92.9	91.5	83.8	70.5	78.9	88.2	82.3
COL6A1	131	153.7	142.4	196.7	213.1	239.8	121.5	182.5
COL6A2	107.8	109.5	108.5	102.4	88.3	104.1	107.6	107
COL6A2	95.3	106.5	119.1	119	95	111.1	89.4	116.5
COL6A2	184.9	208.4	111.4	97	97.2	107.7	163.9	188.2
COL6A3	70.4	74.2	92	82.6	73.3	82.7	77.4	78.8
COL6A3	104.9	118.5	100.1	107.4	93.9	102.8	109.1	106
COL7A1	136.4	132.2	159.9	154.1	170.9	194.4	117.5	177.6
COL8A1	121	133.8	105.8	100.8	111.6	131.9	140.6	158
COL8A1	79.3	83.9	81	87	76.5	85.4	96.2	96
COL8A1	98.2	98.5	94.8	88.6	89.3	85.1	97.2	99.5
COL8A2	404.5	504.9	234.1	220.4	185.1	282.8	194.7	248.1
COL9A1	112.1	137.8	106.8	106.9	98.9	98.8	116.2	117.6
COL9A1	110.6	124.3	89.3	88.1	78	91.4	104.2	137.8
COL9A1	126.2	197.4	94.5	81.2	78.3	84.6	132	162.5
COL9A1	81.5	83.9	85.6	80.7	81.1	91.1	81.9	92.9
COL9A2	2082.5	2566	3521.2	3667.1	4133.6	7382	1978.6	4095
COL9A3	162.3	177.5	98.5	98.8	91.1	103.8	143.9	151.1
COLEC10	75.1	78.8	97.3	84.5	76.9	81.5	70.4	79.6
COLEC11	85.3	91.6	93.5	85	79.5	85.1	81	98.4
COLEC11	76.9	76.4	90.5	88.9	83	81.3	77.4	78
COLEC11	125.8	172	100.5	94.8	85.3	79.9	127.9	143.8
COLEC12	77.8	91.9	98.5	91.9	102.9	96.6	84.4	93
COLEC12	95.9	91.4	79.1	81.4	76.7	86	98.5	89
COLQ	147.7	157.1	103.6	87.1	83.1	94.6	122.6	156.6
COLQ	91.3	106.2	94.9	89	90.5	81.4	86	94.4
COLQ	89.7	88.6	108.9	92.3	92.3	101.3	87.9	96.6
COLQ	90.1	108.9	110.8	98.5	106.8	98.8	85.4	85.6
COMMD1	1550.6	1619.8	1041.9	1030.1	756.4	894.4	1356.7	1554.9
COMMD10	1239.6	1550.1	739.9	737.9	516.3	1157	955.9	1325.3
COMMD2	1127.2	1329.5	711.8	597.1	457.8	535	1079.9	1493.3
COMMD3	10250.7	12167.3	8388.2	5889.9	5337.6	7111.9	8319.6	10001.4
COMMD4	2480.7	3052.8	1829.6	1356.5	1200.7	1382.1	2455	3144.1
COMMD5	2029.6	2676.5	1153.6	616.1	536.3	746.9	1914.3	2220.3
COMMD5	254.5	330.3	189.1	133.5	117.4	115.1	254.8	250.4
COMMD6	7635.1	9364.6	4527.6	5574.9	4412.6	6271.7	6775.4	7703.8

COMMD6	88.1	89.5	86.9	93.9	79	88.3	72.4	85.7
COMMD7	4457.7	5116.4	3312	2208.8	1759.6	2595.8	4000.4	4756.3
COMMD8	178.3	182	129.2	107.7	100.8	118.2	170.7	278.1
COMMD9	841.9	1114.2	486.3	378.9	364.7	486.4	770.7	997.6
COMP	91.6	103	92.5	72.5	79	92.9	96	101.7
COMT	1014	1310.4	339.9	216.4	212.7	191.1	895.5	975.9
COMT	10564.8	13092.5	11818.5	7448.1	6512.7	7148.7	11619.1	12140.8
COMTD1	224.9	290	209.2	158.7	149.5	156.2	171.9	269.7
COP1	161.8	184.1	105.5	108.2	90.8	104.3	159.7	186.6
COP1	105.6	131.5	105.6	94.5	92.6	99.7	106.1	127.2
COPA	4069.6	4592.8	2770.4	2827.3	2212.6	2804.7	3951.7	4846.5
COPB	1730.5	2162.3	1185.9	1319.4	904.3	2030.9	1396.8	3172.2
COPB2	2116.7	2658.2	1772.4	2299	1878.5	3259.3	1536.4	2247.8
COPE	2169.1	2892.5	1783.4	1110.9	879	985.6	1992.7	2639.5
COPE	3604.6	4603.8	1824.1	1051.3	869.9	1275.4	3351.9	4605.9
COPG	626.6	915.8	552.6	501.7	391.4	529	510.9	813.8
COPG2	226.4	330	190.1	269	245.5	408.4	218.6	245
COPS2	89.2	106.2	90.5	95.6	93.6	87.8	89.2	110
COPS3	2917.1	3717.4	2137.5	1941.3	1493.6	2381.5	2664.7	3454.5
COPS4	1468.2	1932.2	654.7	627.3	407	692.4	1190.4	1851.4
COPS5	6839.5	8697.7	5087.6	3182.8	2519.9	4071.2	5648.9	9319.7
COPS6	930.5	1185.1	726.7	636.6	518.3	706.2	813.3	927.7
COPS7A	1473.2	1812.2	993.5	1164.7	876.1	1531.7	1241.2	1604.8
COPS7B	1134.5	1389.1	938.4	694.6	553.8	739.3	926.8	1174.5
COPS8	105.3	118.6	98.7	85.2	83.7	78.9	89.8	98
COPS8	485.2	698.4	218	177.9	154.4	274.7	438.3	618
COPZ1	1722	2348.1	1306.9	898.4	698.2	1232.9	1513.6	1785.5
COPZ2	225.5	281	163.4	178.7	163.3	201.1	179	267.3
COQ10A	302.5	354.2	224.3	196.3	178.4	221.6	217	278
COQ10B	1239.4	1845.2	990.5	934.7	868.6	1306.6	1087.2	1572.3
COQ2	3162.1	4361.6	2273	1794.4	1198.7	1884.3	2985.7	4458.3
COQ3	1265.7	1616.7	517.1	501	344.9	512.7	1026.5	1137.7
COQ5	2239.8	2968.6	1809.6	1607.6	1284.5	1981	2040.2	2363.1
COQ6	320.4	403.8	193.3	135.8	147.2	172.2	258.2	309.5
COQ6	627.3	783.4	563.2	490.2	385.7	628.6	527.4	736.4
COQ7	215.4	275.8	182.6	179.8	144	160.3	194.8	210.2
COQ9	1221.2	1569.8	962.4	660.3	512.4	935.5	999.6	1769
CORIN	79.4	90.8	92.7	81.7	81.3	79.3	82.5	85.7
CORO1A	3119.2	4513.5	2324.9	1160.4	897.1	1322.9	2563.2	4210.7
CORO1B	214.1	263.8	196.8	140.5	139.4	174.2	201.1	307.7
CORO1B	1191.2	1442.7	1133.6	996	798	1157.3	1194.1	2136.9
CORO1C	572.3	722.6	356.8	428.8	339	613.4	482.7	786.6
CORO2A	645.4	825.5	645.5	721.8	604.9	908.6	478	723.2
CORO2A	180.9	210.1	143.4	150.9	151.1	188.8	139.8	146.6
CORO2A	105.3	125.9	75	74.2	73.3	72.8	102.9	138.5
CORO2B	85.2	88.3	90.1	88.6	94.5	91	86.3	85.1
CORO6	118.8	132.7	124.5	108.3	113.6	132.2	103.7	129.4
CORO7	301.4	375.7	401.9	392.4	316.7	628	264.4	435.2
CORT	94.1	111.6	109.9	98.2	88.1	91.2	101.1	109.8
COTL1	166.9	213	113.8	127.7	117.5	108.9	151.2	292.1

COVA1	420.2	594.4	316.8	360	280.4	431.2	322.2	492.2
COVA1	85.1	95.2	109.9	92.4	89.7	103.6	85.5	94.2
COVA1	124.9	150.4	136.8	140.6	132.3	167.1	122.3	170.3
COX10	785.5	933.6	497.9	552	453.1	688.6	632.6	667.1
COX11	134.6	186.8	113.5	112.2	99	123.5	141.4	168.7
COX11P	76	89.8	84.1	78.2	86.4	84.6	79.4	88.4
COX15	113.2	147.3	99.5	84.5	78.7	85.5	112.9	105.9
COX15	283.6	349.8	273.1	203.9	167.5	205	254.7	257.1
COX17	7569.2	8334.1	6117	4901.5	3732.8	5275.1	6269.3	9058.5
COX4I1	23064.6	26137.6	19719.6	16467.1	14186.9	17369.1	22685.3	24290.8
COX4I2	91.8	97.9	100.9	90.2	89.7	100.2	89.8	99.9
COX4NB	524.5	836.9	372.7	283.8	203.4	359	503.4	821.7
COX5A	15347.9	19242.9	12127.7	11240.9	8386.3	10247.4	18591	22280.4
COX5B	11071	13636.2	7002.2	6464.1	5217.7	7138.3	8792.9	9013.4
COX6A1	2693.9	3486.4	1392.3	1287	1062.7	1782.8	2273.4	2660.1
COX6A2	65.6	84.4	98.7	88.9	86.5	79.4	81.9	81.7
COX6B1	11901	13314.5	11909.1	9604.6	6761.4	10801.1	9809.5	13223.5
COX6B2	92.3	97.3	108.1	96.7	89.4	91.2	88.7	102.8
COX6C	18535.2	20638.5	9394.7	8141.5	7128.6	8207.5	16396	17475.7
COX7A1	103.6	115.2	92.5	79.7	79.3	94.2	116.6	122.3
COX7A2	28119.8	28869.4	19677.1	19190.9	14926.2	21582.9	24397.7	27397.2
COX7B	5090.7	5193.9	1710.4	2332.2	1521.5	2565.4	4540.6	7497.7
COX7B2	85	89.6	84.5	79.1	73.3	75	85.2	87.3
COX7C	24601.8	28685.7	22098.2	19831.7	15180.4	20907.1	22665.4	26627
COX8A	14637.1	17349.7	11430.1	6174	5450.9	6057.1	12180	13337.3
COX8C	118.7	130.5	94.8	93.8	83.2	83.5	105.8	120
CP	86.1	104	99.8	95.7	86.1	90.4	87.9	87.3
CP110	1557.5	1874.1	742.8	623.6	595.4	1065.3	978.1	1461.9
CPA1	90.5	98.9	77.6	79.4	74.7	80.4	87.5	94.9
CPA2	79.8	91.4	95.4	78.4	78.3	85.8	85.5	91.4
CPA3	107	125.4	91.7	93.1	84.7	95.8	117.2	125.5
CPA4	78.6	90.3	93.1	94.1	82.9	85.6	92.7	83.3
CPA5	112.4	118.5	96	83.3	89.6	88.3	112.7	130.8
CPA6	84.3	80.7	86.8	73.6	70.1	75.6	84.2	88.9
CPAMD8	83.4	102	86.4	86.4	82.9	89.5	90.9	111.6
CPB1	129.2	129.4	93.8	87.5	90.9	83.7	120.1	167.9
CPB2	101.2	131.3	84.7	83.9	87.5	86.1	102.1	114.6
CPB2	81.1	81.6	85.8	85.9	77	86.5	76.6	86.2
CPB2	80.9	89	119.2	107.9	94.2	99.1	84.2	87.1
CPD	775.9	1201	458.3	1378	1018.4	1868.7	841.1	977.8
CPE	253.7	277.1	165.5	112	96.6	107.4	247.2	464.4
CPEB1	98.8	128.9	80.1	94.2	80.8	91.2	115.3	121.9
CPEB1	91	96.4	95.8	91.5	81.5	86.3	90.7	106.6
CPEB1	95.1	109.9	99.4	93.9	91.4	99.8	85	100
CPEB2	130.1	166	95.6	80.9	71.7	88.1	114.2	157.4
CPEB2	364.8	492.9	339	446.1	332.4	632.1	366.5	738.2
CPEB3	90.4	91.4	93	97.9	93.5	88.8	99.8	113.6
CPEB4	116.1	140.1	81.9	97	85.4	93.7	126.9	141.8
CPLX1	80.2	89.5	86.4	99.2	81.5	79.2	81.1	88.9
CPLX2	113.6	165.5	106.1	97.5	75.4	76.7	113.3	139.7

CPLX2	91.7	94.1	97.6	89.1	88.5	81	92.8	103.6
CPLX3	83.8	100.8	93.2	86.2	82.7	86	85.9	94.6
CPM	90.2	107.8	102.9	101.7	89.8	108.8	89.4	90.9
CPM	282	381.1	176.1	151.9	123.4	196.1	220.9	284.3
CPN1	84.1	93.1	95.4	84.4	84.9	87.7	83	96.2
CPN2	125.5	158	101	90.8	86.5	98.7	123.9	125.2
CPN2	95.7	90.3	103.3	97.2	90.3	95.7	89.6	91.6
CPNE1	181.2	218.1	100.6	107.3	77.9	88.5	149.9	195.6
CPNE1	1439.5	1945.9	886.4	739.2	614.7	834.9	1281.5	2049.2
CPNE1	1183.7	1490.1	1018.4	632.4	569.8	792.9	966.4	1203
CPNE2	141.1	193.2	145.6	111.5	103.3	126.9	145.3	169.7
CPNE3	1317	1711.9	1226.8	1056.5	808.8	1438.2	1222.8	2523.6
CPNE4	91.4	97.3	110.9	104.6	90.8	100.4	94.8	97.2
CPNE5	89.1	106.5	103.1	92.9	83.3	93.9	93.1	100.3
CPNE6	83.5	90.5	90.5	84.4	86.6	92	83.4	96.9
CPNE7	98.4	127.7	92.3	87.5	82.9	93.5	100.9	124.6
CPNE8	1301.6	1611.9	504.2	637.5	499.9	739.3	910.4	1246.9
CPNE9	124.9	129.6	94.9	97.5	78.5	87.8	115.4	125.3
CPO	117.3	123.8	84.9	81.6	80.8	91.3	107	107.1
CPS1	173	244	181	90.9	89	103	200.5	246.2
CPSF1	1190.2	1237.3	1094.4	666.9	661.3	740.2	787.1	759.7
CPSF2	561.4	899	462.8	381.7	355.3	432.4	498.3	521.5
CPSF3	4174.4	6216.2	1621.6	1783.7	1083.9	2080.4	4070.1	4733.5
CPSF3L	233.2	339.6	193.6	188.8	180.4	210.4	229.8	310.9
CPSF3L	180.5	228.5	142.7	147.7	135.8	160.7	190.9	241.9
CPSF4	3366.1	4108.7	2672.4	2214.2	1971.2	2452.4	2975.4	3234
CPSF4	140.1	177.5	118.7	95.9	102.2	119	143.6	188.3
CPSF6	201.3	226.8	129.8	127.3	117.8	165.9	160	271.1
CPT1A	117.3	151.8	103.6	94.5	85.1	83.6	121.7	126.7
CPT1A	117	122.8	111.3	95.3	97.8	96.8	119.4	141.8
CPT1A	255.8	328.1	220.3	226.1	185.1	189.6	225	362.8
CPT1B	193.2	220.6	190.7	182.6	173.9	285.2	180	276.9
CPT1B	124.3	131.7	112.9	125.8	111.1	107.5	102.4	110.6
CPT1C	88.7	97.3	98.9	91	97.8	90.8	89.6	93.4
CPT2	1479.3	1840.5	1242.7	1337	959.8	1174.1	1293.7	1844.2
CPVL	2095.4	2711.5	1682.5	2278.6	1701.2	2363.9	1710.7	2765.2
CPVL	1104.2	1582.2	581.9	706.8	572.2	901.8	823.8	1228.6
CPXCR1	110	125.1	89.9	83.2	88.6	82.1	99.1	118.6
CPXM	123.2	141.1	100.2	100.4	87.1	82.8	124.4	136.6
CPXM2	84.6	100.3	105.3	99.1	103.1	97.2	89.8	95.6
CPZ	104.5	115.6	92.1	94.2	85.4	92	93.6	99.7
CPZ	91.4	99.8	92.1	87.8	95.4	90.7	85.7	90.4
CPZ	80	84.7	91.3	89.1	90.8	95.5	84.6	84.2
CR1	90.5	97.9	92.8	94.7	81.5	97.8	91.4	97.8
CR1	81.1	95.8	88.8	81	72.7	77.8	83	89.4
CR1	89.7	84.7	73.6	80.6	76.6	76.2	86.9	98.8
CR2	80.4	93.8	86	73.2	71.6	82.1	83.8	93
CR2	86.9	94	101.2	103.2	99.7	104.6	93.9	84.2
CRABP1	934.1	1354.4	705.7	542.6	409.1	670	1016.3	1617.3
CRABP2	133.2	155.7	105.7	92.3	94.9	100.8	130.5	142

CRADD	364.8	469.2	286	288.4	234.1	281.6	376.5	449.3
CRAMP1L	237.2	322.6	263.2	238.7	231.1	305.5	235.5	428.4
CRAT	139.5	136.4	133.2	145	107.6	136.3	134.3	167.2
CRAT	103.3	128.1	100.9	94.6	93.5	99.4	114.6	118.7
CRB1	82.4	101.4	100.7	95.5	88.7	91.6	99.3	90.7
CRB2	102.5	110.7	88.1	84.7	84.6	83.2	97	93.8
CRB3	97.7	95.4	91.8	87	93.7	77.9	82.7	89.7
CRB3	96.5	103	99.6	86.5	96.1	102.7	96.5	98.8
CRB3	81.1	109.8	96.1	101.3	95.1	89.3	90	97.5
CRBN	579.1	738	336.1	291.8	273.3	487.9	551.7	813.4
CREB1	2142.7	1796.6	1330.3	1763.7	1499.3	1284	2112.6	3433.2
CREB3	140.8	178.9	95.9	98.3	83.4	76.8	139.1	165.7
CREB3L1	106.3	124.6	99.5	94.9	91.2	89	96.7	127.6
CREB3L2	678.2	1004.7	698.9	939.6	795.9	1143.8	762.2	1265.7
CREB3L3	80.9	92.5	91	82.7	80	79.7	79.6	82.9
CREB3L4	137.8	167.7	98.5	90.1	87.3	94.6	116.5	140.3
CREB5	101.5	115.7	83.8	72.8	74.4	80.1	97.7	113
CREB5	79.4	84.7	84.7	83	79.1	87.5	74.7	77
CREB5	85.4	99.9	90.9	97.5	95.5	94.9	86.5	89.4
CREB5	94.8	121.4	94.8	87.3	87.3	73.5	103	122
CREB5	104.8	112.1	95.9	92	87.5	102.8	106.3	115.4
CREBBP	92.8	117.5	120.2	113.5	111	110.8	99.4	112.6
CREBBP	796.1	1007.5	614.7	860.9	621.7	987.5	685	1385.1
CREBL1	132.8	196.7	119.8	120.1	101.8	100.5	135.1	154
CREBL2	103.9	119.3	90.6	82.1	80	89.2	102	119.9
CREG1	1966.9	2728.7	1128.5	1191.6	993.2	1590.7	2314.9	4668
CREG2	91.8	106.6	99.1	96.2	91	87.9	92.2	88.3
CRELD1	95.7	116.4	115.9	93.2	93.3	98.2	90.3	98.2
CRELD1	93.7	108.2	101.8	86.8	79.5	87.5	95.4	117.7
CRELD1	497.7	515.9	469.7	377.7	341.4	515.6	395.7	528
CRELD1	79.1	85.6	88.9	90.1	84.6	91	82.3	83.4
CRELD2	2363	2636.4	1783.9	2512.7	2059.6	2923.3	2441.9	2734.3
CREM	86.3	90	104.6	77.4	86.7	88.1	70.5	92.6
CRH	125.4	151.3	116.1	99.6	95.7	114.4	117.5	144.9
CRHBP	87.3	96	86.5	101.7	82.3	84.3	80.3	91.7
CRHR1	170	221.5	143.5	143.7	127.6	145.2	146.7	180.7
CRHR1	70.4	92.3	95.7	90.1	91	89.6	81.7	82.3
CRHR2	68.1	81.8	80.6	78.3	78	81.5	70.7	78.3
CRI1	199.3	237.6	171.7	202.5	192.9	239.2	162.1	225.8
CRI2	452.8	645.1	215.1	198.8	163.8	204.6	379.7	562.3
CRIM1	138.1	150.5	108.6	106.3	98.1	105.6	119.6	130
CRIP1	4467.2	4920	2004.4	1142.8	956.3	1204	4133.5	5089.8
CRIP2	94.8	96	91.6	77.3	72.7	81.1	100.7	138.3
CRIP3	119.3	136.3	92	84.2	89.3	98.4	117.8	129.1
CRIPAK	133.6	130.7	146.6	156.6	177.6	199.6	130.2	166.4
CRIPT	675.5	939.5	556	422.7	360.8	439.9	726.4	820.3
CRISP1	72.2	76.6	85.9	90.6	86.7	90.7	79.6	88.9
CRISP1	93.5	88.8	91.1	85.6	78.6	85	87.8	101.6
CRISP2	107.7	123.3	101.6	103	96.4	92.4	105	111.9
CRISP3	93	99.1	116.8	105.6	109.9	95.6	84.9	91.8

CRISPLD1	185.7	206.2	104.9	103.5	104.8	151.3	144	191.9
CRISPLD2	82.6	84.8	91.8	127.1	97	128.6	81.1	85.8
CRK	554.7	796.3	338	314.6	298.6	368.8	518.7	796.7
CRK	265.7	330.5	146	127.8	111.9	129.9	246.5	341.6
CRKL	2804.3	4065.4	2753.6	2909.9	2099.1	3604.6	2481.9	2941.7
CRKRS	274.5	393.9	269.8	384.8	275.1	413.7	260.7	410.7
CRLF1	102.8	104.6	108.3	96.3	100.6	108.5	95	96.3
CRLF2	102.7	112	91.6	86.1	83.5	99.4	100.4	99.4
CRLF3	316	389.6	219.5	324.5	285.9	419.9	345.1	544.8
CRMP1	130.1	138.6	101.6	103.3	101.3	109.4	96.4	133.6
CRMP1	112.8	143.3	115.6	100.8	87.9	94.8	119.3	129.5
CRMP1	89.5	95.3	103.1	84	94.4	97.4	86.9	94.8
CRNKL1	111	129.2	119.6	128.1	108.9	150.8	105.3	134.6
CRNN	210.2	293	151.7	191	184.3	302.7	197.8	227.1
CROCC	168.6	191.4	121.1	112.2	104.9	110.8	155	178.2
CROP	444	373.5	170.4	193.7	149.1	258	230.8	485.3
CROP	1337.8	1380.3	582.8	677.1	529.9	1088.4	885	1893.8
CROT	155.1	177.1	122.4	144.2	114.4	152.5	138.6	215.2
CRP	88.5	93.8	85.5	90.9	82.7	95.5	84.2	95.1
CRR9	2232.6	3277.1	2317.5	1982.5	1648.3	2414.4	1948.3	3699.7
CRSP2	90.2	92.1	92.5	97.7	92.4	97.4	81.5	88.4
CRSP3	356.2	426.1	229.9	296.2	261.8	423.5	306.6	571.4
CRSP3	129	152.1	116.1	111.1	101.7	120.2	114.1	151.7
CRSP6	103.7	150.1	106.7	101.2	104.4	105	115.4	123.2
CRSP7	190.7	252.8	167.4	146.6	133.3	168.5	186.6	215.1
CRSP9	130.2	143.1	98.5	84.2	82.3	105.7	115.9	173.5
CRTAC1	100.6	105.6	89.2	78.2	78.6	88.5	98.1	106.9
CRTAM	82.6	94	94.9	83.3	80.6	80.7	84.8	98.6
CRTAP	1992.2	3237.1	1074	1212.3	932.9	554.2	1647.3	2105.4
CRTAP	233	285.9	191.7	288.8	271.5	274.2	197.5	266.1
CRTC1	85.2	97.1	124.9	105.8	101.7	115.5	85.8	100.6
CRTC2	148.8	167.4	110	106	91.8	109	156.4	328.6
CRX	129.2	146.8	98.1	87	91.6	95.7	128.8	129.2
CRY1	255.6	291.6	161.9	156.7	125.7	196.9	219.1	366.9
CRY2	209.8	252.8	147.5	135.8	117.4	137.8	185.3	284.1
CRYAA	94.1	109.4	91.7	83.8	78	84.6	107.9	115.4
CRYAB	84	93.2	91.4	83	83.1	85.2	76.7	83.7
CRYBA1	80.7	88.8	92.2	89.9	84.2	85.9	82.7	93.9
CRYBA2	89.9	90.6	95	95.2	85.3	87.9	89.4	90.6
CRYBA2	87.6	98.3	103.4	99.1	98.5	97.9	78.8	93.4
CRYBA4	95.4	99.2	95.2	90.1	88	88.2	96.5	95.1
CRYBB1	131.3	173.3	112.5	105.2	99.6	107	120.9	153.2
CRYBB2	511.2	645.7	395.4	307.7	273.1	386.5	452.5	505.6
CRYBB3	79.8	85	85.3	74.8	80.3	81.5	83.7	86.6
CRYGA	131.7	146.1	119.3	110.1	109.8	116.1	117.3	126.4
CRYGB	116.5	118.5	89.1	83.3	75.5	75.3	101.5	118.4
CRYGC	91	95.1	94.6	80.2	82.9	85.4	91.4	90.6
CRYGD	82.9	84.8	96.2	80	78.8	83.5	81.2	82.5
CRYGN	114.1	126.9	93.2	81.7	84.3	84.5	118.1	136.4
CRYGS	145.5	174.9	114.6	132	122.4	144.3	132.4	179.3

CRYL1	196.2	254	180.6	238.2	236.7	288.8	179.9	252.8
CRYM	138.8	181.7	97.6	86.1	90	95.8	123.9	146
CRYM	87.3	87.6	115.3	106	106.7	102.7	94.4	88
CRYM	79.5	87.3	80.4	72.1	80.5	75.4	82.3	87.3
CRYZ	394.5	521.4	235	256.8	224.7	313.8	400.2	614.8
CRYZL1	1230.8	1475	754.8	750.2	611.7	836.7	954.2	1327.8
CS	110	132	101.8	99.6	96.1	91.3	95.5	107
CS	3412.2	4266.7	4153.4	4407.6	3022.5	4861.2	3137.5	4730.1
CS	462.1	745.7	511.1	489	364.9	642.7	361.4	698.7
CSAD	204.6	196.1	169.2	225.7	212.5	273.2	147.9	325.2
CSAG1	85.3	90.2	102.7	88.6	84.3	90.6	79.5	98.9
CSAG1	101.1	100.4	102.8	84.8	84.8	80.1	83.9	90.9
CSAG2	89.3	106.4	92.3	87.9	81.9	77.9	93.7	97.1
CSAG2	102.9	116	107.7	97.8	87.6	92.7	112.8	109.2
CSAG2	78.2	71.1	92.2	79.3	73.1	86.4	80.7	74.7
CSAG3A	94.9	106.9	108.8	97.7	100.3	90.8	101	106.7
CSDA	7378	8488	3052.9	2332.1	1746.4	1872.7	5815	7368.6
CSDC2	81	86.5	102.1	102.2	92.5	95.6	90.5	85.2
CSDE1	654.3	762.7	244.2	240.5	180.5	184.8	507.1	483.2
CSDE1	362	488.4	144.2	174	170.7	126.5	232.8	401.8
CSE1L	3985	5347.2	2321.4	2012.9	1230.8	2740.6	2964.2	5031.5
CSE1L	5035	6246.4	3511.8	2915.6	2218.6	4123	3707	6608.7
CSEN	173.2	171.4	118.4	97.9	93	105.5	138.9	146.1
CSF1	89	115.8	94.7	94.4	89.8	85.5	102.8	103.4
CSF1	87.9	89	94.7	84.3	89.9	92	86.7	85.2
CSF1	75.3	70	88.5	89.5	78.8	84.2	75.5	77.1
CSF1R	1838.7	2198.4	1244.7	3332.6	2486.6	3168	1503.3	1601.3
CSF2	88.3	97.4	89.1	84.4	92.6	82.6	88.2	107.7
CSF2RA	559.8	701.1	269.8	335	298.7	381.5	580.6	873.9
CSF2RA	2386.1	2455.9	1016.6	1612	1319.8	1359	2322	2741
CSF2RA	622.2	835.7	447.6	615.3	483	699.5	579	981.5
CSF2RB	90.8	100.9	88.8	88.7	77.7	84.8	85.4	105.2
CSF3	95.5	122.8	111.3	101.7	96.3	94.3	96.4	103.5
CSF3	99.7	140.3	83.4	85	85.3	82.7	107.1	130.6
CSF3	96.9	109.8	93.9	92.4	89	100.7	93.2	123
CSF3R	130.5	148.5	147.6	173.6	151.6	201.2	105.9	170.9
CSF3R	1689.5	1848.8	1223.6	2202.5	1997.4	1961	1231.4	1190.1
CSGLCA-T	192.9	201.1	198.6	193	155.9	196.8	187.6	371.5
CSH1	80.1	79.4	95	84.3	83.9	88.9	74.2	79.1
CSH1	96.8	99.4	92.7	100.7	79	89.6	87.6	94
CSH2	93.8	111.3	89.9	89.8	86.4	95.3	95.3	126.6
CSH2	73.1	77.4	89.4	78.8	84.9	88.4	77.1	80.7
CSHL1	102.9	117	96.7	97.1	75.3	92	93.4	119.3
CSHL1	118.6	135.8	96	86.3	82.3	84.8	104.8	134.9
CSK	1814.2	2303.8	2019.2	1569.4	1263.9	1796.5	1432.8	2411.8
CSMD1	65.2	79.1	78	81.4	74.8	79	73.5	78
CSMD2	102.5	129.9	94.5	92.2	78.5	85.7	110.4	128.9
CSMD3	76.5	91.1	77.7	86.4	82.5	92.3	83.5	85.2
CSMD3	91	92.5	85.4	85.8	82.7	87.3	94.3	93.2
CSMD3	73.8	86.3	87.8	82.2	82.2	82.2	80.8	88.2

CSN1S1	92.8	111.2	105.2	84.7	83.3	101.2	93.6	102
CSN1S1	98.2	106.8	88.2	102.6	82.9	88.6	101.8	101.5
CSN2	81	95.3	87.4	81.5	75.4	75.7	84	98.7
CSN3	80.9	86.2	91.4	81.6	80.1	81.4	77.4	86.3
CSNK1A1	544.7	826.5	177	125.6	138.3	205.6	473.9	562.4
CSNK1A1	134.9	141.4	117.8	102.3	103.3	113.3	123.1	159.4
CSNK1A1	831.8	1108.8	492.7	565.3	459.4	654.4	670.6	854.3
CSNK1A1L	82.3	92.2	100	87.6	83.5	80.6	80.9	98.3
CSNK1D	2184.7	2597.7	1243.6	1238	1194.1	1311.7	2028.1	2029.1
CSNK1D	565.5	701	618.5	638.5	519.1	741.3	509.7	1121.6
CSNK1E	2765.9	3379.2	1267.9	1046.2	916.8	1306.1	2513.7	2333.6
CSNK1E	99.1	115.1	113.3	110.7	105.2	114.4	96.4	106.9
CSNK1E	421.1	535.7	378.8	154.5	180.7	209.2	348.5	674.1
CSNK1G1	353.4	486	227.1	250.2	221.5	286.3	371.3	422.6
CSNK1G1	100.5	111.8	101.4	99.7	100.8	87.3	89.1	102.9
CSNK1G2	3042.1	3721.2	556.2	456.8	385.7	648.2	2592.5	3343.7
CSNK1G3	91.5	113.3	96.2	100.1	92.4	93.9	93.9	96.8
CSNK1G3	356.4	429	257	340.9	264.9	568.9	285.3	451.7
CSNK1G3	285	330.2	148.2	151.2	130.1	223.5	252.6	440.6
CSNK2A1	623.2	825	325	259.6	236.5	333.4	439.4	604.7
CSNK2A1	568.2	754.7	314	320.3	217.5	281.7	384.4	525.5
CSNK2A1	152.9	199.6	111.7	101.2	108.8	100.2	129.5	157
CSNK2A1P	410.8	552	174.4	158	126.3	148.2	319.9	421.4
CSNK2A2	502.2	566.1	460.9	329	258.6	300	452.9	831.8
CSNK2B	8966.8	11252.2	8173.8	4798.1	3634.2	5137.1	7562.4	10775.9
CSPG2	216.5	337.1	141.4	557	234.5	679.2	221.4	270.2
CSPG3	127.8	139.7	126.4	111.5	104.3	115.1	115.6	141.9
CSPG4	170.3	210.1	153.9	87.1	88.6	86.4	129	144.5
CSPG4LYP2	87	94	82.4	88.6	80.6	85.4	88.5	88.5
CSPG5	164.5	202.6	167.3	214	187.6	277.3	139.2	245
CSPP1	687.4	715.1	405.5	417.7	380.3	649.7	487.2	704.7
CSPP1	173.2	214.9	131.5	138.8	111.8	154	156.2	206.2
CSRP1	1421	2106.7	1335.8	753.9	554.3	1118.3	1326.1	1864.2
CSRP2	116.3	140.3	88.7	84.4	81.7	88.5	109.6	166.7
CSRP2BP	125.2	152.4	120.8	111.6	103.4	121.2	111.2	149.8
CSRP2BP	759.6	921.7	474	425.2	329.8	427.7	568.7	728.8
CSRP2BP	86.8	99.4	92.5	88.4	88.5	84.3	96.9	88.2
CSRP3	73.1	83.3	89.5	78.4	81.7	88.2	70.7	75.4
CSS3	178.2	223.6	173.6	189.5	144.7	217.7	151.8	198
CST1	89.1	93.2	95.8	87.7	78.4	96.2	91.6	90.7
CST11	74.5	88.1	98.8	90.6	77.8	81	79.3	78.4
CST11	96	107.6	78.8	86	78.9	81.3	93.6	94
CST2	102.6	117.7	78.9	82	81.6	80.4	100.5	118.3
CST3	13279.9	15923.6	12461.3	11759.7	9685.3	13544	11156.6	17292.3
CST4	73.3	90.1	106.1	101.1	97.4	99	86.9	96.2
CST5	100	120	89.9	84.7	80.1	77.6	102.2	112.1
CST6	78	93.5	88.9	85.1	83.5	83.6	77.7	81
CST6	94.9	90.7	92.5	100.2	84.3	84.1	98.3	96.4
CST7	506.4	617.6	357	175.1	174.9	209	484.9	811.5
CST8	82.8	95.8	90.1	83.2	81	87	81.8	93

CST9	198	289.5	126.6	128.2	122.8	144.7	201.8	203.7
CST9L	86.8	100.2	82.1	78.8	80.9	85.8	85.8	88.9
CSTA	232	286.1	170.4	176.6	136.1	157.3	187.4	256.1
CSTB	4946.3	6252.5	3795.6	3725.1	3187.1	4158.2	4378.4	5517.5
CSTF1	191.4	223.3	116.1	110.4	91.3	91.1	160.1	177.6
CSTF1	108.5	129.6	95.7	109.4	92.3	97.4	102.6	117.1
CSTF1	142.4	156	94.8	98.1	91.5	75.9	110.3	153.5
CSTF1	97.4	125.9	109.7	99.9	93.8	89.4	93.6	98.4
CSTF2	1425.5	1642.9	830.4	827.7	560.8	997.1	1220.1	1714.7
CSTF2T	224.5	273.7	130.8	113.8	110.8	133.3	173	217.3
CSTF3	1027.8	1318.8	536.1	585.9	456.5	647	673.3	888.1
CSTF3	236.7	252.2	167.4	191.3	184.2	217.4	152.9	157
CSTF3	3692.1	4510.2	2665.9	2248.7	1990.4	2568.7	2618.9	3690.5
CSTF3	738.3	896.1	224.6	244.9	210.2	245	473	761.8
CSTL1	90.1	97.3	87.8	79.9	82	85.6	91.5	92.5
CT45-1	107.1	120.2	95.6	80.6	75.5	94.4	100	114.7
CT45-4	80.8	97.6	91.3	89.3	81.1	86.7	84.9	88.4
CT45-5	78.2	79.3	86.8	84.7	76.7	84.3	81.9	83.2
CT45-5	86.9	93.1	86.5	84.7	79.3	92	88.5	97.9
CT45-5	90.5	95.8	102.4	93.5	85.1	95.1	88	95.9
CT47.1	82	88.4	104.6	102.1	99.9	109.1	85.1	87.9
CT47.6	92.7	110.9	83.6	86.8	84.8	88.8	92.1	101.6
CT47.7	97.1	100.4	116.9	91.9	94.8	106.4	94.3	101.6
CTAG1A	152.1	172.8	97.6	96	80.2	93.9	128	173.7
CTAG1B	74.4	82.7	99.3	88.8	83.2	86.8	75.3	77.1
CTAG2	96.7	116	99.8	108.8	97.5	95.4	94.5	108.7
CTAG2	94.5	105.5	95.7	87.8	74.1	82.8	90	90.1
CTAGE1	86.2	97.1	91.9	90.4	86	90.4	85.9	86.6
CTAGE5	130.6	138.8	99.7	86.5	73.2	92	115.3	140.3
CTAGE5	85.9	100.2	93.4	86.2	76.5	86.2	87.1	104.9
CTAGE5	79.8	88.3	89.2	84.8	84.6	85.7	79.1	84
CTAGE5	103.8	119.4	98.5	114	96.3	111.2	113.1	99.6
CTBP1	329.3	449.5	182.3	213.9	217.2	125.4	208	381.4
CTBP1	182.9	251.7	191.1	154.9	137.3	210.3	144	224.6
CTBP1	1381.9	1623.3	625.8	480.3	443.1	552	1083.7	1798.1
CTBP2	175.1	230.3	113.9	95	103.1	92.5	150.4	174.7
CTBP2	99.3	126.1	105.6	86.7	89.9	93.8	112.3	112.2
CTBS	688.2	988.1	360.4	507	389.7	402.4	643.9	809
CTCF	561.1	736	633.5	490.9	462.6	684.6	430.8	676.6
CTCFL	70.1	87.7	100.7	95.2	89.2	91.7	84.6	79.1
CTDP1	145	214.7	136.7	128.4	108.5	170.3	147.4	229.4
CTDP1	97.3	128.7	95.8	92.7	93.5	98.7	105.7	101.7
CTDP1	188.3	254.5	151.6	144.4	131.7	152.1	162.8	178
CTDSP1	830.1	1054.4	445.9	433.4	340.8	385.9	698.3	994.7
CTDSP1	134	200.7	149.6	147.9	138.9	115.5	126.5	201.7
CTDSP2	1069.4	1456.2	1017.6	1026.4	1090.1	1009.7	1017.5	1047.8
CTDSPL	610.1	854.2	624.4	480.6	387	525.1	457.3	611.6
CTDSPL2	677.4	973	225.5	184.3	168.5	286.5	481.2	842.3
CTF1	149.9	162	99.3	97.6	94.7	113.8	139.5	159.6
CTF8	170.8	197	107.1	114.4	111.3	86.3	139.8	164

CTGF	153.9	173.5	101.5	101.7	107	111.5	251.6	319.8
CTGLF1	113.2	122.9	102.3	95.2	91.1	113.2	111.8	117.2
CTGLF3	980.2	912.9	638.9	788.3	714.6	1028.3	629.3	880.6
CTGLF4	144.9	174.7	114.5	96.9	106	99.6	121.7	160.5
CTH	131.7	178.4	108.5	117.5	109.5	161.4	166.3	248.2
CTH	103.4	111.6	110.9	109.7	105.8	95.8	109.5	109.8
CTHRC1	79.5	82.9	88.5	89	72.7	84.4	81.6	92.3
CTLA4	78.3	87.2	89.3	75.1	78.2	84.9	75.5	85
CTLA4	120.4	147.2	93.2	93.3	82	91.2	126.8	129.3
CTLA4	114.8	145.5	96.8	81.6	80.2	77.4	123.6	139.2
CTNNA1	1667.2	2317.5	1270.3	1691.8	1200.5	1935.4	1454.4	1742.2
CTNNA2	98.7	108.2	105	87.3	96.7	94.9	98.7	109.6
CTNNA3	87.1	99.4	89.1	87.4	78	82	82.9	83.9
CTNNAL1	576	736.2	290.3	423	242.9	578.3	466.4	1117.5
CTNNB1	1164.9	1539.8	550.2	533.3	444.2	575.8	685.9	1062.9
CTNNB1	86.8	97.8	90.4	84.7	79.7	82.7	77.9	72
CTNNBIP1	139.5	134.7	153.5	189.4	130.2	180.4	144.4	175.5
CTNNBIP1	88.7	104.4	95.8	98.3	89.8	100	89.7	100.7
CTNNBL1	1025.5	1520.7	524.2	470	435.9	757.8	798.2	1027.5
CTNND1	93.8	114.4	99	94	92.2	98	100.6	98.8
CTNND1	124.8	153.6	108.8	93.3	92.9	91.8	107.8	131.7
CTNND2	109	127	89.4	83.6	79.1	86.8	105.3	121.7
CTNS	98	86.1	90.7	74	71.7	89.8	81.5	99.8
CTNS	311.9	358	181.8	172.3	150.5	173.7	254.4	259.2
CTNS	112.5	129.7	112	99	95.2	96.8	92	100.7
CTPS	4226.9	5286.8	2990.8	2637.4	1872.7	3759.1	2946.1	5797.9
CTPS2	95.2	96.9	99.1	86.4	80.7	94.4	78.9	106.1
CTPS2	709.5	905.4	635.2	942.5	814.4	1127.1	567	645
CTPS2	234.6	273.5	107.2	108.4	101.8	133.5	192.7	275.8
CTPS2	177.6	244.5	162.3	184.3	163.8	234.9	151.6	187.7
CTR9	93.5	106.1	100.2	96.4	92.8	118.2	106.8	161.3
CTRB1	95.8	98.7	86.2	84.1	77.2	78.7	87.1	95.2
CTRB1	96.4	101	84.7	85.4	77.8	89.1	90	108.4
CTRB2	117.6	120.8	91.8	93.5	86.8	105.7	113.6	109.6
CTRC	126	135.6	125.2	107.8	110.8	116.9	124.2	128.9
CTRL	152.7	164.9	129.5	119.7	102.6	112.9	133.2	148.8
CTSB	1539.6	1774.1	1103.9	1852.6	1670.6	1283.4	1380	1275.4
CTSC	108.2	134.4	94.1	94	102.1	95	119.7	143.7
CTSC	1254.4	1635	577.4	436.8	376.3	484.8	870.3	875.1
CTSC	2091.1	2752.8	1035	1237.4	817.7	1429.4	1801.3	3203.7
CTSD	383	509.1	412.7	552.6	374.1	665.4	383.7	673.9
CTSE	142.4	168	109	110	101.4	109.5	159.8	166.3
CTSE	82	95.5	93.9	86	92.1	89.2	94.1	93.9
CTSE	149.2	183.1	101.3	91.6	88	112.2	137.3	155.9
CTSF	147.8	184.5	139.3	118.3	120.9	133.5	151.6	176.3
CTSG	27864.3	26131.7	36062.1	35386	32256.9	34549.9	30449.6	28641.7
CTSH	3617.1	4643.7	2550.8	2772.1	2519	3786.6	3069.2	4965.8
CTSH	246.8	320	131.2	133.5	126.1	180.8	210.7	283.5
CTSK	139.7	147.8	118	111.6	108.2	109.8	127.5	177.7
CTSL	310.7	397.7	187.7	200.4	170.4	205.4	286	298.2

CTSL1	2421.6	3180.8	1381.5	2030.9	1641.2	2369	2621.5	2695.9
CTSL1	2375.9	3000.7	1144	1566.2	1408.3	1864.1	2560.9	2896.5
CTSL2	523.2	700.6	186.4	191.9	179.5	252.2	491.6	588.3
CTSO	490.8	575.1	225.8	273.1	230.8	358.5	434.8	746.4
CTSS	218.1	240.4	178.1	176.3	140.6	181.2	194.7	270.6
CTSW	130.4	164.2	101.2	114.3	112.6	117.9	136.3	166.9
CTSZ	1047.4	1830.7	721.3	1468.9	1322.7	1797.9	1746.1	2584
CTTN	157.1	199.4	173.3	88.7	93.7	97.4	158.5	218.5
CTTN	157	203	150.2	103.1	98.7	102.6	138.5	163.8
CTTN	75.1	87.1	85.4	79.9	78.3	92.8	75.9	74.9
CTTNBP2	77.8	89.9	95.2	81.1	91.3	93.7	84.6	98.5
CTTNBP2NI	126.5	183.6	109.4	98.7	93.1	107.9	128.4	157.9
CTXN1	355.6	419.3	250.5	117.8	89.6	117.7	310	443.6
CTXN3	84.9	84	92.3	85.3	86.9	89	79.1	90.2
CUBN	80.6	83.2	97.6	81.9	82	80.6	78.1	83.4
CUEDC1	372.8	427.2	249.3	265.6	234.5	184.4	377	340.7
CUEDC2	1314.9	1775.3	801.7	429.4	354.4	617.7	1346.7	2157
CUGBP1	216.5	336.6	128.3	163.9	142.4	149.4	169.2	234.5
CUGBP1	175	281.8	118.2	139.9	100.6	109.8	139.9	238.9
CUGBP2	152.3	186.4	98.3	93.9	84.6	88.5	138.8	172.9
CUGBP2	397.8	638.7	338.4	435.7	350.5	534.4	348.4	557
CUGBP2	378.6	560.6	215.4	216.2	197.4	249.2	308.9	410.3
CUL1	1002.6	1350	326.8	333.1	244.4	474.3	602.9	857.4
CUL2	741.4	1137.9	593.4	597.2	490.6	746	724.4	1073.6
CUL3	91.9	106.8	97.3	92.8	91.1	94.5	74.9	111.3
CUL4A	119.2	129.2	91.2	81.5	77.8	91.2	93.5	117.7
CUL4A	1221.7	1610	764.5	768.5	671.7	876	1085.8	1861
CUL4B	186.2	211.1	129.1	153.5	148	207.2	156.4	222
CUL4B	202.6	226.1	126.2	130.1	105.1	136.7	142.1	195.8
CUL5	1263	1573.1	619.2	777.1	590	942.7	1178	1871.2
CUL7	114.6	114.4	100.7	95.8	98.5	102.9	109.7	104.7
CUTA	7340.7	8378	6558.9	6272	4609	4947.5	6628.6	7562.4
CUTA	3012.6	4316.2	2226.4	2012.8	1571.9	1703.6	3048	3710.6
CUTA	160.3	198.7	119.3	95.7	98.5	102	157.1	168.1
CUTC	809.2	989.5	502.1	354.9	290.5	479.6	630.5	1258.8
CUTL1	94.8	97.8	91	94.9	81.1	93.3	98.5	95.3
CUTL1	285.2	363.4	290.9	532.1	420.6	875.1	301.7	565
CUTL1	85.8	92	104.8	97.3	98.4	92.2	85	82.5
CUTL2	132.3	175.6	135.2	143	125	162.4	112.4	133.5
CUZD1	127.3	147.7	128.2	98.4	104.6	125.1	123.8	140
CWF19L1	1867.5	2378.8	1323.8	968.8	788.8	1148.4	1999	2948.7
CWF19L2	343.1	467	292	298.9	252	477.5	306.7	481.4
CX36	86.4	105.2	84.8	86.9	87.5	81.5	86.3	90.4
CX3CL1	94.7	101.7	92.2	77.6	70.6	85.3	85.5	94.4
CX3CR1	1459.6	1759	1013.5	1363.3	1276.4	1477	1224.5	1788.5
CX40.1	87.5	108.5	85.1	83.2	76.7	84.7	90.7	94.8
CX62	77.9	87.9	89.4	81.2	84.3	89.5	85.5	87.7
CXADR	79.3	101.6	89.9	83.2	72.8	86.8	88.4	92.1
CXCL1	125.8	136.2	92.2	90.9	86.4	71.7	103.6	147.8
CXCL10	95.6	127.2	99.4	80.4	90.8	90.4	98.7	104.7

CXCL11	83.5	87.1	93.6	89.4	76.5	88.1	87.9	88.1
CXCL12	139.3	175.3	120.2	118.9	109.5	109.6	134.5	159.5
CXCL12	104.8	111.6	102.8	91.6	84.7	96.1	95.2	101.4
CXCL13	99.7	111.7	91.3	87.4	80.3	83.1	102.3	96.1
CXCL14	95.5	103.9	91.7	85.5	83	82.1	92.2	101.6
CXCL16	145	158.5	120.3	124.5	108	113.9	132.9	138.2
CXCL2	82.2	95.4	90.4	99.3	77	88.6	82.5	98.2
CXCL3	114.9	128.2	86.6	90.4	82.5	97.1	115.5	125.7
CXCL5	79.8	103.7	83.8	84.9	80.2	77.6	83.3	103.4
CXCL6	101.9	113.3	94	90.5	83.1	93	98.1	112.2
CXCL9	93	114	98.9	83.2	80.3	82	97.5	109
CXCR3	179.5	212.9	99.9	94.1	91.9	93.7	142.6	149.6
CXCR4	968.1	1501.8	334.1	515.6	395.6	779.5	746.4	922.1
CXCR4	100.3	120.7	88.3	81.8	72.3	78.1	110.4	108.4
CXCR4	3194	4569	1515.6	1749	1767.7	2010.4	2578.5	2372.1
CXCR6	81.7	97.8	91.1	85.3	82.9	91.1	82.2	85
CXCR7	84.9	104.9	91.2	113.2	103.6	100	82.6	91.5
CXORF1	101.4	115.3	93.4	96.1	88.9	87.2	111.7	106.4
CXORF12	257.1	319.2	201.4	189.4	189.8	217.4	231.1	222
CXORF15	138	189.2	121.1	118.8	120.8	109.8	112.4	136.2
CXORF2	109	135.1	98.2	86.1	88.9	85.2	113.1	114
CXORF20	84.1	95.3	91.8	87.2	89.3	85.9	83.8	95.9
CXORF21	262.4	270.6	132.3	150.9	106	130.7	191	393.1
CXORF22	85.6	79.7	83.8	82.6	88	88	77.8	88.6
CXORF23	123.5	142.5	114.6	118.8	110.3	127.5	133.8	181.2
CXORF26	1905.8	2317	604.9	516.2	398.4	684.4	1816.4	2720.4
CXORF33	253.4	336.6	166.9	160.1	136.2	153.3	257.4	303.1
CXORF34	91.2	95.9	102.3	83.8	83	86.9	83	100
CXORF36	116.2	155.2	97.3	84.6	77.9	78	126.5	183.5
CXORF38	632.9	820.9	396.8	424.5	315.1	412	514	713.1
CXORF39	208.7	259.8	171.2	169.7	143.1	193.7	208.1	358
CXORF40A	825.2	1044.2	488.7	501.8	407.3	442.4	690	1163.1
CXORF40B	526.7	713.6	309.4	297.9	266.6	313.9	483.6	931.2
CXORF41	81.8	89.7	86.9	93.3	82.3	93.5	85.1	91.2
CXORF43	103.8	139.9	104.1	95.1	97.1	93.7	113.4	113.8
CXORF44	162.1	176.4	136.7	155.7	136.1	161.5	131.1	158.7
CXORF45	139.1	154.6	157.2	183	193.1	304.1	131.2	188.1
CXORF48	80.7	87.5	93.5	94.7	85.5	95.2	86.2	84.4
CXORF48	78	88.2	86.9	84.1	75.3	84.9	77.9	90.8
CXORF53	581.9	720.9	454.8	533	437.7	957.5	475.5	703.5
CXORF55	93.4	105.3	100	88.6	78.2	88.9	104.4	107.2
CXORF56	299	420.3	145.2	143.6	124.6	113.7	312.3	421.9
CXORF6	119	139.7	94.2	74.9	79.5	85.8	105.3	129.5
CXORF9	247.5	304.2	250.8	325.4	257	542.1	181.8	224
CXX1	1020.3	1290.2	767.9	543.9	394.1	544.6	946	1345.1
CXXC1	2061.5	2461.9	1574.4	1391.3	1277	1418.8	1632.5	1668.7
CXXC4	122	169.3	103.1	93.8	93.9	87.8	118.2	174.9
CXXC5	1906	2524.5	2240.6	2004.6	1640.9	2774.8	1942.9	4442.5
CXXC6	117.5	136.5	109.8	101.9	91	128	91.6	103
CXYORF3	1972.2	2281.8	1774.5	2529.8	2550.1	3559.1	1894.2	2618.9

CYB561	190.4	196.2	142	110.8	115.7	113.5	147.7	175.7
CYB561	182.2	230.2	144.5	116.6	121.2	126	161.5	192.9
CYB561	105	115.6	114.2	106.9	102.2	98.3	102.3	114.2
CYB561D2	612	794.7	542.3	482	387.9	517.3	563.6	685.5
CYB5A	1651.2	2044.1	1013.8	1035.9	847.5	1132.3	1489.3	1993.4
CYB5A	893.8	1175.8	343.7	364	297.1	409.6	775.7	772.9
CYB5D1	235.2	310.5	208	205.6	166.7	245.4	181.8	236.4
CYB5D2	177.7	214	153.3	205.4	192.3	238.1	164	246.2
CYB5-M	7115.9	8516	4410.2	3572.8	2693.5	3741.2	6159.3	8530.1
CYB5R1	475.4	562.9	256.2	277.3	234.6	276.8	529.4	775.3
CYB5R2	446.9	557	222.2	178.2	162.7	201	397.9	374.6
CYB5R3	462.6	711	336.8	320.1	265	293.1	467.4	895.6
CYB5R3	10136.7	12022	6004.4	4919.8	3674.4	3943.5	9275.2	10354.2
CYB5R4	800	1048.7	362.6	452.7	323.8	633.2	586.7	908.3
CYBA	17034	21230	13552.4	10738.9	8390.6	10681.6	14723.7	17927
CYBASC3	561	770.3	590.1	484.1	478.9	578	512.6	733.3
CYBB	118	135.6	104.7	552.3	479.8	704	102.6	124.1
CYBRD1	381.9	481.8	341.3	346.3	343.7	468.8	363.6	686.1
CYC1	9933	11412	8527.3	5268.8	4734.4	6058.8	8909.8	10832.4
CYCS	262.6	346.5	232.6	315.1	243.2	382.3	207.3	362.3
CYCSL1	140.9	165.1	116.1	121.8	115.7	119.9	140.6	164.9
CYFIP1	95.6	122.7	109.5	115.9	102.4	109.3	95	100.2
CYFIP1	535.5	726.5	743	1000.7	839.9	1365.2	426.2	672.8
CYFIP1	117	126	88.2	78.8	88	87.3	116.2	137.8
CYFIP2	102	103.1	101.3	92.1	84.2	85.2	99.9	114.2
CYFIP2	101	117.9	107.4	115.6	108.6	130.9	92	112.8
CYFIP2	222.8	277.8	200.8	168.8	151	180.5	198.3	164.3
CYGB	78.8	99.3	96.2	80.2	69.9	82.5	91.8	91.8
CYHR1	157.9	182.6	122.2	100	103.5	139.8	132.6	152.5
CYLC1	78.7	86.1	84.4	75.4	85.2	78.8	79.9	91.8
CYLC2	160.6	159.6	90.8	98.5	88.5	94.8	146	136.4
CYLD	82.1	88.5	105.7	96.4	88.2	81.6	83.3	90.7
CYLD	126.4	184.8	147	165.5	174.2	167.8	127.1	135
CYLN2	95.6	112.6	86.8	89.5	87.9	89.4	101.2	107.6
CYORF14	178.3	186.3	130.2	151	140	193.1	166.5	175
CYORF15A	590.1	768.4	369.4	481.8	342.1	612.9	402.3	613
CYORF15B	177.2	204	138.3	141.1	133.8	173.5	123.1	161.1
CYP11A1	131.9	147	111.4	133.9	108.3	134.2	122	141.8
CYP11B1	81.9	101.2	78.3	81	81.5	80.7	91.1	104.8
CYP11B2	91.7	120.9	96.2	83.8	94.2	95.5	109.5	128
CYP17A1	101.1	97.5	90.6	87.3	81.9	83.4	90.3	103
CYP19A1	97.7	117.2	98.7	88.2	91.3	89.7	96.8	95.4
CYP19A1	82.8	96.9	99.3	97.4	101.5	83.9	82.6	85.5
CYP19A1	105.9	118.2	98.6	84	94.9	78.5	99.4	130.8
CYP1A1	105.7	111.4	101.2	87.1	94.3	91.3	94.7	110
CYP1A2	82.7	86.9	98	88.9	82.9	86.6	81.8	87.2
CYP1B1	194	248.9	141.4	357.9	225.9	608.5	523.2	1232.3
CYP20A1	555.2	684.4	328	375.7	293.7	437.7	482.7	784.8
CYP20A1	96.6	113.4	98	92.9	81.5	82.9	101.4	100.7
CYP21A2	91	104.9	106.1	90.6	88.4	102.3	94.4	104.6

CYP24A1	87.1	97.1	87.8	85.7	73.6	81.9	76.4	93
CYP26A1	89.4	112.9	90.1	86.6	81.1	79.4	96.4	107.8
CYP26A1	102.5	111.8	91.5	90.6	86.3	87.9	96.3	105
CYP26A1	82.5	84.9	93.2	85.9	82.7	89.2	74.1	85.7
CYP26B1	111.7	133.3	100	90.8	79.8	91.6	120	142.1
CYP26C1	95.3	130.3	85.6	79.9	69.4	81.9	104.3	103.9
CYP27A1	124.1	144.2	132.5	158.4	159.6	159.2	121.1	143.4
CYP27B1	136.7	169.8	155.8	117.1	116.5	120.6	126.7	128.8
CYP2A13	91.2	94	94.1	83.7	79.4	89.1	93.4	97.9
CYP2A6	76.6	79.2	91.4	84.6	80.8	81.5	75.7	79.4
CYP2A7	86.2	99.2	112.6	104	105.9	104.1	91.5	105.6
CYP2B6	75.6	84.6	96.6	85.4	84.5	90.1	82.7	87.7
CYP2B7P1	80.6	87.6	95.5	90.3	81.2	81.1	81.7	93.6
CYP2C18	84	88.6	103.1	94.1	94.3	95.4	90.6	98.8
CYP2C19	95.1	102.6	94.9	85.6	84.1	97.5	90.3	111.8
CYP2C8	114.6	124.2	92.9	86.9	89.4	89.2	109.2	134.1
CYP2C9	69.8	84.8	102.4	105.4	84.3	87.4	75.4	85.7
CYP2D6	129.1	171	88.9	97.6	87	94	122.7	126.9
CYP2D7P1	97.3	111.8	120.7	114.9	110.1	123.5	89.1	96.7
CYP2E1	109	120.2	97.6	92.8	102.9	100.3	107.3	112.1
CYP2F1	85.5	90.1	95.3	88.3	80	86.2	78.9	101
CYP2J2	164.9	188.2	147.8	161.9	126.3	184.1	107.3	143.1
CYP2R1	191.1	221.1	181.6	166.6	146	200.2	182.1	226.8
CYP2S1	158.5	182	144	110.3	103.1	113.9	145	174.8
CYP2S1	389.7	518.9	394.4	332.7	261.1	299.8	365.7	625.6
CYP2U1	130.4	147.4	98	90.3	81.4	100	113.6	168.6
CYP2W1	144.4	162.2	88.8	93.2	74.4	86.9	163.5	188
CYP39A1	75.1	79.5	91.1	88.4	81.2	85.7	72.4	83.3
CYP3A4	91.4	99.3	94.1	86.9	93	83.3	94.7	102.3
CYP3A43	94.9	111.8	96.4	87.4	92	88.1	94.1	97.1
CYP3A43	78.3	81.1	92	94.5	97.4	89	82	78.4
CYP3A5	79.8	85.9	95.7	87.9	91	90.2	89.2	82.6
CYP3A7	96.4	111.8	91.1	90.6	82.2	94	94.3	107.9
CYP46A1	117.5	123.5	108	97.3	94.9	108.4	93.1	111.3
CYP4A11	119.4	137.5	89	82.3	82.3	85.8	112.7	122.1
CYP4A22	87.1	96.1	101.9	83.5	87.1	86	97.7	96.5
CYP4B1	112.1	116.3	97.3	93.8	86.9	91.2	103.2	115.5
CYP4F11	103.1	102.9	101.1	87.4	84	93	102.5	91.7
CYP4F12	82.7	90	87.2	85.8	84	87.8	93	95.9
CYP4F2	77.9	94.5	101.5	89.7	94	95.2	86.7	90.5
CYP4F3	84.9	85.6	82.3	84.5	86.1	84.4	83.9	92.8
CYP4F8	117.1	107.3	95.6	94.7	82.5	86.1	106.3	108.6
CYP4V2	453.3	533.8	360.1	682.6	477.6	755	331.1	400.9
CYP4X1	79.6	88	84.7	70.1	79.2	81.6	82.7	87.8
CYP4Z1	82.2	84.8	101.9	85.3	91.6	90.9	79.8	90.3
CYP4Z1	93.3	96.9	83.5	72.2	74	80.5	84.6	99.2
CYP4Z1	78.7	80	87.3	81.1	75.2	82.9	79.3	83.2
CYP4Z2P	97.1	99.1	92.5	91	83.9	97.5	93.7	101.8
CYP51A1	372.5	487.4	192.8	199.1	177	235.4	460.7	808.3
CYP7A1	106.7	101.1	93.3	90.2	93.9	82.9	92.7	103.4

CYP7B1	112.3	124.3	91.3	91.7	89.4	89.7	112.4	128.3
CYP8B1	94.2	113.3	90.2	88.9	86.7	86.3	97.2	101.3
CYR61	102.6	107.1	86.5	96.1	87.7	100.6	107.4	122.6
CYS1	94.5	98.2	87	90.7	83.9	90.9	95.6	108.8
CYS1	102.4	104.8	83	90.2	88.1	85.5	88.6	108.2
CYSLTR1	954.6	1328.4	636.4	704.1	554	806.4	617.8	749.7
CYSLTR2	113.7	128.6	86.6	72.2	70.2	89.8	123.8	141.1
CYTL1	77.4	83.3	82.9	70.6	74.3	75	83.5	86
CYYR1	85.9	97.4	99.5	91.7	93.8	95.4	94.7	98.1
D15WSU75	971.4	1317.9	809.2	592	459.2	758.8	700.5	969.9
D21S2056E	98.6	123.2	79.5	86.9	68.5	74.8	97.9	115.9
D2HGDH	761	793.8	615.8	941.7	1116.8	1302.4	452.3	419.6
D4S234E	135.9	146.5	104.7	96.1	90.1	93.8	144	140.2
D4ST1	280.4	370.4	380.3	315.5	234.5	299.1	257.4	445.1
DAAM1	107	153.2	100.4	100.8	102	126.6	135.1	166
DAAM2	100	136.6	102.9	91.7	90.3	89.9	107.4	124.4
DAB1	71.5	75.9	83.3	79.4	67.6	77.6	81.3	71.6
DAB2	266.8	305.6	173.6	335.1	313.6	307	259.4	335.6
DAB2IP	85.2	110.6	102.9	82.7	87.1	90	89.8	102.5
DAB2IP	87.1	95.8	92.4	78.8	84.3	88.9	91.7	87.2
DAB2IP	88.9	103.4	87.8	87.6	68.6	89.4	91.8	101.2
DACH1	100.8	111.7	97.3	89.7	84.6	89.6	95	108.8
DACH1	108.2	110.7	96.7	102.3	94.9	99.3	90.2	92.9
DACH1	382.9	487.5	220	380.9	312.7	459.1	212.9	380.7
DACT1	88.2	90.8	100.1	89.7	92.3	91.1	87.9	91.9
DACT1	96.2	101.8	108.4	100.8	95.6	93.2	100.4	97.9
DACT1	105.8	114.7	105.7	103	94.2	86.2	102.4	102.9
DACT2	92.4	95.4	96.2	93.8	84.7	83.6	87.8	104
DAD1	7030.7	8993.3	4727.8	3531.1	2654.2	3867.3	6535	7297.1
DAG1	314.8	396.6	281.2	284	233	343.9	327.6	545.3
DAK	152.8	166.3	145.7	113	113.1	124	132.6	212.3
DALRD3	236.7	290.2	147.5	114.2	110	102	187.6	207.4
DALRD3	88.9	106.3	91.7	77.7	79.8	85.9	87	108.7
DAND5	92.5	90.7	100.5	98.2	87.1	102.9	92.5	104.5
DAO	91.4	106	91.1	97.9	87.6	86.9	96.7	112.3
DAOA	76.2	95.7	88.1	89.2	84.8	85.7	88.9	99
DAP	723.9	884.7	526.6	478.1	365.6	562.5	585.3	1163.2
DAP3	3020.7	4027.8	1894	1782.5	1366.4	2378.8	2765.8	4965.7
DAPK1	152.8	201	119.2	165.6	140.7	235.4	155.3	175.1
DAPK2	124.3	118.6	124	93.4	88.5	96.7	97.5	128.9
DAPK3	228.5	304.1	166.8	113.9	124.3	152.1	204.5	299.9
DAPP1	3429.5	3829.6	1556	1790	2019.4	2160.8	3002.2	2935.8
DARC	109.2	153	92.2	85	69.8	78	109.6	135.4
DARS	10040.7	12852.8	7708.4	7995.2	6496	11106.7	8812.2	10774.3
DARS2	1028.9	1311.3	566	499.3	406.8	583	778.7	903.3
DAXX	395.4	505.5	297.1	241	204.4	268.2	370.2	484.3
DAZ1	86.4	84.6	79.1	79	74.1	82.8	88.1	93.6
DAZ2	86.3	97.4	84.2	90.3	89.6	82.5	102.7	97.5
DAZ2	94.3	95	105.1	94.6	86.7	99	95.4	87.1
DAZ4	92.7	118.6	87.5	86.4	89.1	94.9	92.6	105.9

DAZAP1	91.8	106.8	93.3	84.3	88.9	93.7	101	117.9
DAZAP1	1436.7	1561.2	710.6	437.8	394	324.7	1140.4	1570.3
DAZAP2	13603.2	16805.5	9633.5	11248.7	8091.8	12199.6	11943.1	13899.2
DAZL	83.3	93.1	94	91	85.1	91.9	82.9	95
DBC1	74.7	91.7	90.3	88.1	81.7	86.2	84.9	91.2
DBF4	132.5	150.8	97.5	97.7	94.9	98.3	130.7	157.7
DBF4B	122.4	125.2	109.4	108.5	103.8	98.3	123.7	121.1
DBF4B	78	82.8	96.7	93.9	93.3	102.1	72.2	92.6
DBH	89.3	124.9	90.1	88.7	80	76.6	95.9	113.6
DBI	11657.6	14012.5	7883	7064.5	6419.5	8827.3	9337.3	15379.4
DBI	2171	2677.5	862.9	799.8	724.8	1096.5	1755	2062.3
DBN1	1071.8	1433.3	706	516.5	308.5	492.4	886.6	831.2
DBN1	103.4	135.2	106.5	99.2	89.3	85.4	104.6	134.1
DBNDD1	210.4	284.4	118.4	112.7	99.2	103.5	205.5	245.9
DBNDD2	79.6	92.8	84.3	81.1	89.5	78.1	68.6	83
DBNL	4747.2	6058	3851.4	3448.9	2754.4	3785.1	3674.1	3789.5
DBNL	596.7	779.4	505.4	424.3	368.1	557.4	539.1	674.9
DBP	198.6	242.2	130.1	138.9	114.1	129.5	173.8	210.8
DBR1	368	399.3	179	199.4	138.9	215.2	333.7	445.7
DBT	1649.6	1903.3	1068.6	958.7	823.9	811.2	1459.7	1915.2
DBX1	113	113.8	91.5	96.4	91.9	84.5	106.4	129.2
DBX2	112.4	131.2	107.9	94.3	94.8	92.8	112.4	123.2
DC2	1559.5	1773.1	312.9	290.7	241.9	327.8	1577.5	2864.8
DCAKD	1252.2	1571.8	951.6	1114.4	963.7	844.1	1005.6	1030.7
DCAL1	91.8	88.6	94.7	90.1	78.4	94.2	101.3	98.8
DCAMKL1	163.4	209.6	115.1	101.5	104	110.8	162.5	179.4
DCAMKL2	86.2	98.7	109.5	100.1	93.1	99.5	101.6	94.4
DCAMKL2	86	89	85	86.2	79	83.5	83	88.1
DCBLD1	298.5	357.9	170.4	191.7	157.8	153.2	245.9	423.5
DCBLD2	570.4	716.9	388.1	401.3	368.5	485.6	518.8	840.8
DCC	91.1	105.2	103.1	90.1	90	93.4	97	98.2
DCC1	417.2	492.3	150.1	126.9	130.6	137.1	300.2	382.8
DCD	93.5	108.5	88.2	81.5	83.7	93.2	90.7	100.8
DCDC1	75.8	93.1	90.1	89.7	82.9	90.1	83.3	85.9
DCDC2	84.6	83.6	92.1	90.5	81.9	82.6	78.8	81.8
DCDC5	470	614	289.3	228.5	210.7	235.7	492.4	429.7
DCHS1	172.3	264.1	120.2	112.4	102.4	103	173.5	196.1
DCHS2	83.5	94	79.3	83.7	77.4	73.2	81.9	88.6
DCI	646.3	954.8	266.8	170.3	174	205.7	525.4	564.8
DCK	105.1	128.8	149.7	139.5	125.7	129.8	106.3	149
DCLRE1A	375.8	538.9	242	191.1	209.2	285.5	343.4	413.2
DCLRE1B	201.5	277.8	136.8	114.3	97.8	133.9	173	251.2
DCLRE1C	267.7	301	271.1	268.5	257.6	305.1	269.3	390.9
DCLRE1C	138.1	165.7	106.8	105.5	109.9	117.1	137	158.9
DCLRE1C	366.9	421.6	251.9	384.4	399.6	326.7	344.2	400.5
DCN	108.5	88.9	100.3	93.4	87.2	94.2	88.8	106.3
DCN	94.4	97	86.3	85.8	101.6	91.2	87.3	98.6
DCN	107.6	119.5	111.4	88.1	82.5	83	97.7	100.2
DCP1A	366.7	446.1	236.6	252.6	241.8	331.9	397	642.2
DCP1B	131.1	139	104.2	110.2	95.1	105.8	112.7	134.9

DCP2	1525	2196.2	935.5	2004.8	1316.8	2969.5	1574.7	2186.6
DCPS	977.5	1261.1	839.1	328.1	295.4	449.9	863.6	841.1
DCST1	106.8	111.4	100.7	124.6	94.9	93.5	111.7	129.6
DCST2	77	79.9	90.9	74.9	74.8	79.8	78.8	88.2
DCT	90.7	110.7	99	94	92.4	92.9	93.3	98.5
DCTD	476.7	706.3	186.9	141.8	115	143.4	406.5	556.5
DCTD	1725.6	2434.3	1732.6	1158.6	856	1337.1	1510	2594.3
DCTN1	725.4	877.7	571.6	597.2	488.4	559.7	515.7	927.4
DCTN1	139.6	157.2	90	84.3	89.4	101.7	130.8	167.6
DCTN1	120.7	145.6	168.1	137.2	128.7	171.2	121.6	173.2
DCTN2	2362.4	3019.7	1742.4	1650.5	1257.5	1749.2	2290.6	3122.6
DCTN3	113.5	126.1	95.6	89.8	86.9	99.5	108.4	121.3
DCTN3	3845.4	4529.5	3679.8	2400.1	2137.7	3213.2	3322.2	4406.4
DCTN4	614.2	744.8	306.4	360.6	280.4	450.9	697.6	1080.5
DCTN5	1627.9	2345.6	1538.4	1122.4	786.9	1304.9	1389.2	1998.3
DCTN6	150.6	163.1	133.7	105.8	104.7	98.6	158.3	289.5
DC-UBP	368	506.5	295.6	225.1	215.7	245.2	272.9	343.7
DCUN1D1	86.6	95.4	80.8	88.8	80.5	90.7	97.7	99.3
DCUN1D2	137.5	201.2	127.8	113.1	101.1	137.3	134.2	175.4
DCUN1D3	173.8	243.9	151.1	146.1	143	161.3	197	191.7
DCUN1D4	548.5	673.9	172.1	196.7	151.1	243	417.9	476.1
DCUN1D4	489.7	596.6	400.7	403.7	312.6	545.9	439.2	638.5
DCUN1D5	1719.7	1948.8	1099.3	903.3	575.7	1343.1	1541	3196.1
DCX	98.2	107	112	108.5	97.3	109.4	100.4	115.5
DCX	88.9	103.5	100.6	87.4	82	90.9	103.5	104.6
DCXR	2124.8	2705	1569.8	1099.8	818.9	1068.1	1968.7	2596.8
DDA1	1663.7	1975	1251.5	837.5	726.1	758	1597.1	1960.4
DDAH1	223.8	211.5	133	181.6	120	212.2	170.3	324
DDAH2	192.8	190.7	109.1	112	100.3	119.4	161.8	217.1
DDB1	5888.2	7562.6	5392.4	4963.4	3833	5278.9	5283.5	5821.9
DDB1	224.2	223	120.3	132.6	127.5	110.5	169.4	206.6
DDB2	183.3	202.6	102.8	102.3	89.3	87.7	156.8	211.3
DDC	80.1	97.3	110.3	93	102.7	97.5	88	92.2
DDEF1	231.8	314.9	175.6	222.7	247.2	306.6	200.5	638
DDEF2	205.9	256.4	133.4	111.5	119.6	149.4	203.8	275.3
DDEFL1	133	165.1	110.2	100.6	86.5	90.7	125.3	155.5
DDHD1	93.4	96.2	89.2	88.6	91.5	90.5	89.5	90.3
DDHD2	208	254.1	144.4	118.8	112.5	124.4	180.6	218
DDI1	123.9	156.7	92.4	80.2	89.1	96.3	115.6	158
DDI2	90.1	109.5	101.3	88.6	84.9	85.3	98.4	91.6
DDIT3	485	601.9	319.1	331.6	319.5	584.5	496.1	743.8
DDIT4	541.1	723.3	308.8	637	719.5	1105	697.5	966.6
DDIT4L	120.2	155.4	104.8	121	96.2	132.6	139.4	200.4
DDN	84.5	99.1	93.3	90.9	85.3	91.9	97.1	97.4
DDO	78.9	87.6	102.1	88.8	94.7	94.4	81.8	96.2
DDO	95.3	106.1	108.1	96.7	85.6	94.9	103.7	111.7
DDOST	5808.4	7924	2439.2	2877.4	1916	2648.2	5791.6	7092.7
DDR1	119.2	136.7	104.6	98.7	95.6	96.6	110.7	136.6
DDR1	115.1	157.4	101.8	94.1	81	95.3	126.8	128.4
DDR1	90.4	103.1	103	88.1	95.2	103.3	99.2	97.6

DDR2	105	128.5	93	82.5	90.1	93.7	97.8	111.2
DDR2	87.1	114.6	99.3	103.2	86.1	102.9	94	98.2
DDT	5857.5	7144	3842.1	4060.9	3402.8	4892.9	5584	6045.6
DDX1	3927.3	4591.6	3790.7	3332.5	2619.8	5137.2	2982.5	5183.5
DDX10	920.8	1340.6	770.3	607.9	405.4	978.3	667	1182.8
DDX11	120.6	147	115.4	98.2	93.2	94.5	119.7	130.9
DDX11	166.4	162.5	151.8	127.1	124.3	150.5	139.1	253.1
DDX11	94	116.5	98.7	89.1	86.8	101.9	98.8	127.2
DDX17	1729.2	1860.9	586.2	868.4	548.3	992.5	1683.5	3226.1
DDX17	232.5	360.5	171.2	231.1	245.9	156.2	174.7	223
DDX17	675.2	886.8	352.7	634.6	553.1	279.1	514.2	880.6
DDX18	2829.7	3822.5	2308.3	2515.8	1868.6	3408.5	2165.9	3232.8
DDX19A	531.9	658.7	577.7	543.5	443.9	675.5	410.8	570.5
DDX19B	2190.8	2757.6	1212.2	1121.2	908.6	1364.3	1669.3	1772.5
DDX20	208.4	229.7	136.2	111.8	106.4	125.1	171	237.5
DDX21	3169.4	3698.8	2216.1	1545.4	903.1	3161.3	2424.8	5949.4
DDX23	554.9	700.1	623.9	364.6	321.3	425.7	466.4	907.4
DDX24	1261.9	1616.9	1104.1	789.7	597.5	1281.1	1079.2	1846.5
DDX25	81.1	86.9	85.5	91.2	85.9	94.2	77	90.7
DDX26	1128.2	1706.3	896.2	984.3	906.8	1372.7	1088.6	1510.6
DDX26B	153.4	186.2	109.4	104.3	91.6	106.3	149.4	174.4
DDX27	859.3	1251.6	260.7	265.1	231.2	310.5	840.7	995.5
DDX28	285	394.4	172.7	144	130.8	169.5	257.3	364.7
DDX31	393.4	497	306.3	245.3	182.1	278.3	289.4	334.8
DDX31	84.4	95.9	98.7	91	92.7	82.1	85.7	85.5
DDX31	182.9	210.4	139.2	119	97.5	107.2	138.4	168
DDX39	7382.7	9110.3	4370.2	2831.3	2510.3	3418.8	6670.2	8785.3
DDX3X	93.4	106.9	94.7	91.1	85.3	96.1	94.8	113.2
DDX3X	1609.1	2267.4	1319.9	1516.9	1099.9	2444.4	1287.4	2761.2
DDX3Y	90	108	91.1	89.9	77	87.3	88.9	126.5
DDX4	81.2	85.6	89.3	80.1	82.3	90.4	82.1	77.5
DDX41	734.5	972.1	540.9	475.2	406.2	666.6	647.4	943
DDX42	116.7	125	110.8	129.5	123.9	100.2	116.1	148.4
DDX42	1565.6	1950.3	1160.4	1467.5	1116.9	1875.3	1157.5	1579.2
DDX43	87	114.8	100.2	90	87.6	88.1	97.3	108.8
DDX46	1415.1	1923	741.7	1041	847.6	1341.8	1122.3	1316.4
DDX47	886.4	1165.7	474.3	314.5	237.5	381	709.6	919.8
DDX47	2042.4	2923.8	870.7	679.7	563.7	774.4	1760.1	2190.2
DDX48	5779.1	7648.4	4325.1	3732	2756.9	4892.2	4584.4	6253.8
DDX49	221.3	232.1	120.1	127.6	100.2	117.5	165.3	180
DDX5	8049.7	8871.7	6406.7	6334.9	4932.9	8738	5296.6	9373.9
DDX50	774	1125.1	349.1	259.4	224.5	386.2	617.5	764.5
DDX51	4178.8	4104.1	1971.6	2940	2801.3	1721.8	3569.4	5731.2
DDX52	137.3	159.6	112.4	109.7	104.9	104.7	124.2	190.3
DDX53	114.2	140.9	96.7	90.5	85.8	89.6	105.8	122.4
DDX54	277.2	329.3	221.2	245.7	208.2	252.3	283.3	379.5
DDX55	1398.1	1428.4	852.1	747.2	666.4	953.9	1088.2	1422.9
DDX56	1728.4	1957.5	1524.1	1108.4	817.2	1414	1254	1927.9
DDX58	137.6	180.7	129.7	129.4	124.4	122.6	129.4	146.2
DDX59	759.8	924.8	507.3	516.7	410.6	719.8	716.6	1331.2

DDX59	104.4	108.1	89.7	86.1	76	77.3	108.3	116.2
DDX59	147	165	120.3	110.6	94.1	156	143	240.6
DDX6	126.2	142.3	123.9	101.2	99	102.9	122.7	127.4
DEADC1	119.4	131.4	113.7	118.4	104.9	130.5	106.3	107.9
DEAF1	232.3	291.4	159.1	129.3	118.1	158.4	182.5	236
DEAF1	96.3	115.5	102.1	94.2	100	86.4	107.4	101.3
Dec-01	96.2	98.4	96.3	82.5	73	75.8	96.6	100.3
DECR1	5809.1	7584.7	1404.2	662.2	539	479.7	5450.5	6165.3
DECR2	882.3	999.4	695.4	474.8	398.3	423.1	788	823.8
DEDD	579.3	685.3	542.4	522.4	424	735.4	523.5	1025.1
DEDD	120.1	179	94.8	94.5	84.9	99.9	130.1	163.6
DEDD	267.8	347.6	246.3	189.3	169.5	223.6	242.6	321.3
DEDD2	1150.2	1587.4	436.1	309	320.9	333.5	1260.4	1531
DEF6	1018.9	1325.1	433.7	460.1	430.3	592.6	767.6	741.2
DEFA1	83.8	83	88.3	80.5	85.7	90.4	87.6	99.7
DEFA3	95.3	91.8	89	84.8	76.2	81.8	84.3	96.2
DEFA4	108.3	129.5	110.7	118.5	104.4	117.1	108.7	112.8
DEFA5	97.5	102.8	96.7	95.1	89.6	94.9	99.8	92.4
DEFA6	75.3	90.6	89.3	83.8	80.4	79.5	80.9	85.7
DEFB1	538.3	620.6	447.5	665.3	507	852.9	447.8	549.8
DEFB103A	87.8	95.7	106.1	100	101.7	105.2	84.1	86
DEFB104B	89.1	102.6	82.5	87.7	81.5	83.3	85.2	86.2
DEFB105A	84.7	108.1	101.2	85	81.1	83.6	88.4	95.3
DEFB106A	71.3	82.4	84	83.4	78.6	76.8	71.9	75.6
DEFB106B	95.2	86.9	103.4	95.1	111.4	99.2	94.4	91.9
DEFB107A	100.8	102.5	110.6	108.4	105.7	109.4	100.1	105.6
DEFB108B	119.5	126.8	89.3	88	83.6	83.5	97.3	115.1
DEFB109	86.4	111.2	94.1	88.4	71.7	92.9	95.5	105.8
DEFB110	90	101.1	89.6	84.8	85.2	79.3	92.6	91.2
DEFB111	92.1	107.1	86	84.4	84.3	84.7	85.2	90.9
DEFB112	91.6	104.8	87.8	80.3	75.8	87.9	91.1	100
DEFB114	94.3	110.3	93.1	85.9	82.9	81.8	98.8	108.8
DEFB118	103	102.9	89.5	82.9	93.6	93.3	100.9	102.9
DEFB119	217.2	262.4	135.1	120.4	107.8	129.1	188.4	213.7
DEFB119	72.7	78.8	91	80	82.5	75.8	73.1	83.5
DEFB121	77.6	79.9	82.5	84.3	74.9	71.5	80.3	83.6
DEFB122	99	118.6	101.5	98.4	90.5	104.7	98	107.3
DEFB123	118.1	111.1	97.6	94.3	78.1	101.8	104.1	122.5
DEFB124	83.9	111.1	94.4	93.5	89.4	77.5	93.3	91.1
DEFB125	149.4	163.2	103	93.9	84.6	96.5	129.2	169.9
DEFB126	78	105.5	92.3	81.4	80.5	79.8	83.1	100.7
DEFB127	84.9	90.8	88.4	81.7	80	81.6	78.2	88.2
DEFB128	110	119.9	97.9	93.6	92.3	88.7	97.7	102.1
DEFB129	112.2	123.2	86.7	83.8	72.3	78.8	133.2	116.1
DEFB131	133.2	192.6	110.8	101.8	87.7	102.8	151.7	187.8
DEFB134	81.2	86.9	88.6	80.1	77.2	85.9	81.7	85.8
DEFB136	80.8	86.6	95.4	84.7	81.7	83	89.7	81.6
DEFB137	96.2	113.2	90.8	83.7	80.7	90.8	95	93.2
DEFB32	90.6	92.8	94	79.5	88.6	89.5	80.9	89.3
DEFB4	93.3	102.7	94.4	83.9	82	86.4	87.1	96

DEGS1	7923	8968.8	3039.9	3343.7	2944.8	3814.6	7410.8	10044.5
DEGS1	2958.1	4112.6	1599.2	1327.9	1041.4	1708.8	2790.7	6479.1
DEGS2	92.5	108.4	99.2	96.1	90.4	89.4	93.7	91.6
DEK	2782.5	3461.5	1621	1611.3	1280.1	2530.6	2429.6	4201.3
DENND1A	1088.7	1555.1	632.8	1108.8	919.6	1504.5	958.3	870.3
DENND1A	116.4	126.4	107.5	108.1	103.9	123	120.4	140.6
DENND1A	367	419	208.3	304.5	244.8	368.3	302.6	488.5
DENND1B	89	84.5	97.5	97	91.7	91.9	75.6	89.7
DENND1C	122	139.5	111.7	119.7	105.9	116	118.9	127.5
DENND2A	93.3	104.2	87.8	84.6	87.5	82.4	90.6	95.1
DENND2C	101.4	125.3	89.5	82.4	81.5	89	103.3	132
DENND2D	348.1	424	275.2	306	223.6	306.1	277.5	374.4
DENND3	252.2	298.2	235.2	215.7	222.7	345.6	227.2	420.9
DENND4A	392.7	554.4	187.2	190.2	160.3	252.9	354.4	540.7
DENND4C	263.3	373.5	168.9	250.5	191.6	314.2	296.7	316.4
DENR	255.9	273.5	165.3	239.1	273	238.2	253	329.2
DEPDC1	74.7	79.2	98.5	76	69.1	77.4	79.2	80.2
DEPDC1B	848.6	828.2	260.8	197.8	183.2	276.6	664.4	1036.5
DEPDC2	76.2	84.8	87.8	86.3	81.5	93.7	81.5	88.8
DEPDC2	80.2	83.4	91.5	91.7	88.7	95.9	75.3	84.7
DEPDC4	135.5	142.8	106.7	94.5	98.3	101.8	129.1	149
DEPDC5	172.1	206	185.2	217.6	182.6	240.4	154.4	208.1
DEPDC5	98.4	113.7	101.2	94	90.9	91.9	106.8	103.2
DEPDC5	77.1	83.3	86.2	73	70.8	80.8	78.1	77.2
DEPDC6	220.6	281.3	138.9	157.9	145.4	170.7	200.4	336.3
DEPDC7	210.6	278.4	125.6	99.4	89.5	105	160.4	207.5
DEPDC7	102.5	119.9	100.9	91.6	91.3	99.9	101.3	118.3
DERA	2692.2	3145.4	1673.9	1890.9	1180	1851.5	2094.9	2324.3
DERL1	1385	2026.7	996.2	842.6	627	1142.7	1095.3	2109.3
DERL2	371.8	478.4	200.7	172	186.5	203.5	444.3	526.3
DERL3	91.8	95.5	113.2	93.2	89	95.6	93.1	96.8
DERL3	133.3	139.6	87.5	88.5	88.5	100	116.9	119
DERPC	113.2	127.2	84.4	78.5	85	85.7	117.3	147.4
DERPC	151.1	191.9	138.2	159.9	145.1	108.1	139.8	151.4
DES	86.8	104.2	99.8	97.2	92.7	92.2	89.4	97.2
DET1	348.4	466.4	215.8	225.7	195	287.6	314.3	465
DEXI	2563.3	3421.7	1043.5	858.5	679.5	830.3	2387.7	2335.5
DFFA	479.9	503	314.7	407.1	327.9	226.5	352	415
DFFA	288.4	364.1	140.7	116.9	126.5	128.9	276.9	287.7
DFFB	79.7	92.5	93	82.2	83.6	93.9	88.1	93.8
DFFB	273.1	318.3	243.9	259.2	224.9	293.2	271.7	324
DFNA5	384.4	456.2	213.6	317.9	267.9	425.9	386.4	534.2
DFNB31	94.9	109.4	105.7	92	93.5	103	105.3	103.8
DFNB59	103.3	128.4	99.3	96.3	89.5	93.9	110.3	125.7
DGAT1	562.4	671	472.5	446.8	360.3	469.7	441.7	663.6
DGAT2	172	245.5	113.8	107	85.4	109.2	153	216.7
DGAT2L3	85.1	90.9	106.3	93.3	99.6	115.3	89	79.3
DGAT2L4	77.5	84.2	97.2	99	93.7	97	82.9	80.6
DGAT2L6	81	82.8	88	78	81.9	77.5	79.5	82.1
DGCR13	133.4	145	122.7	110.9	92.7	123.1	119.1	166.5

DGCR14	420.4	552.5	189.3	148.6	136.5	130.8	381	538.1
DGCR2	228.6	292.3	314.2	304.5	262.6	402.5	228.9	351.1
DGCR5	91.7	103.2	98.2	90.6	86	95.8	87.5	92.6
DGCR6	193.9	272.4	167.9	164	166.7	197.7	158.6	201.6
DGCR8	335.1	429.2	468.8	348.5	324.3	477.2	339.9	719.7
DGKA	83.2	90.3	89.9	86.8	80.9	84.5	81.7	79.9
DGKA	88.2	88.5	93.3	94.1	81	93.2	81.9	103.1
DGKA	143.5	167.7	120	110.2	99.4	126.5	127.4	156.2
DGKA	119.7	122.2	81.3	96.7	81.3	81.9	118	129.9
DGKA	135.3	150.2	113.3	82.9	90.6	107.2	115	181.1
DGKB	77.6	104.5	86.7	97.6	80.9	80.7	89.6	100
DGKB	99	129.7	94.4	82.9	85.1	85.7	104.9	121.7
DGKB	74.2	91.1	94.8	85.5	83	87.7	87.1	82.3
DGKD	174.5	195.1	104	102.2	74.9	105.4	163.1	194.5
DGKD	129.3	159.9	114.9	121.2	107.5	110.5	130.6	168.9
DGKD	86.7	87.7	80.4	80.4	73.7	84.2	78.9	88.7
DGKE	114.1	145.7	91.8	83	85.4	99	118.5	142.2
DGKG	108.9	129.6	107.2	104.1	96.2	102.6	127.5	171.6
DGKH	130.9	140.7	95.4	80.7	89.4	87.1	123.6	135.9
DGKH	116.1	155.9	94.3	83	91.6	88.3	123.1	153.7
DGKI	85.1	101.8	93.6	91	83.6	88.2	86.5	94.7
DGKK	78.5	79.3	99.4	85.4	91.4	88	77.3	81.3
DGKQ	444.4	468.7	578.6	519.1	364.5	659.4	371.9	791.5
DGKZ	93.8	103.5	85	78.5	85.2	84.8	86.3	103.5
DGKZ	94.9	116.5	99.6	85.4	90.2	85.8	91.8	101.8
DGKZ	171.7	152.9	126.5	136.6	134.5	97	119.9	163.2
DGUOK	4358.5	5369.1	2836.5	2527.6	1945.6	2246.4	4045	4548
DGUOK	4213.1	5153.9	2996.2	2576.5	1803.1	2486.1	4317.5	5723
DGUOK	510.8	623.1	305.7	337.8	278.7	387.4	422.7	512.4
DHCR24	430.2	559	428	407.4	341.7	365.4	429.3	564
DHCR7	313.8	405.5	296.2	284.3	314.1	359.5	282	380.3
DHDDS	219.1	277.5	238.9	255.8	191.6	329.2	215.9	305.4
DHDDS	208.5	240.9	140.4	154.1	135	178.5	188.5	273.9
DHDH	112.4	130.4	106.4	103.1	98.5	120.8	106.6	108.2
DHFR	481.4	590.3	186.1	184.6	151.7	124.3	337.7	573.6
DHFRL1	112.4	115.8	106.4	99.5	98.6	103.3	109.3	133.8
DHH	90.8	107.9	109	95.1	85.5	89	106.6	113.4
DHODH	134.2	167.8	130.8	104.9	96.7	115.7	105.7	129.9
DHODH	255.5	283.9	139.8	118.6	96.6	105.1	177.2	196.4
DHPS	1187.8	1435.5	922.2	510.1	410.4	667.9	881.8	1217.7
DHPS	938.3	1138.1	808.2	429.1	412.5	618.6	671	908.4
DHPS	1943.3	2164.1	1223.7	969.1	729.3	1289.7	1713.3	2711.2
DHRS1	444.8	633.6	277.5	291.4	274.3	250.8	395.4	408.3
DHRS10	101.7	108.2	104.6	100.4	90.3	94.3	101.7	92.6
DHRS2	134.2	135.2	106.1	94.9	87.7	93.6	120.7	123.1
DHRS2	88.3	97.6	103.7	78.7	75.2	79.2	91.6	94.6
DHRS3	105.2	106.4	99.3	103.5	112.6	118.1	104.1	109.6
DHRS4	3308.8	4448.3	3095.5	2889.1	2320.7	3779.8	2737.3	4548.8
DHRS4L2	538	782.4	321.7	291.3	276.9	380.7	455.4	479.6
DHRS7	1628.9	2112.6	703	414.2	327.5	444.3	1738.8	2592.2

DHRS7B	366.9	481.5	218.9	200.8	178.4	196.4	385.9	479.7
DHRS8	1413.2	2103.3	497.2	356.6	281.5	496	1269.7	1881.9
DHRS9	11074.3	13820.3	9400.6	8376.9	6898.3	7079.7	7069.1	5133.2
DHRS9	489.7	471.8	356.7	647.7	433.6	695.7	432.3	544.5
DHRS9	100.9	106.1	99.8	93.5	92.7	90.7	102	98
DHRS9	5771.8	7873.2	2541	2071.7	1543.3	2722.4	2963.6	3594.4
DHRSX	148	162.4	134.6	138.7	126.6	153.7	143.2	191
DHTKD1	216.6	238.8	117.1	144.4	118.3	155.6	188.4	264.8
DHX15	9623.4	11553.2	5316.5	4361.3	3292.2	5324	7869.2	9947.8
DHX16	535.9	623.1	547.1	629.5	517.5	855.7	463.9	650.9
DHX29	894.2	1300.1	947	1026.5	840.9	1981.2	931.2	2056.9
DHX30	592.7	688	710.9	668.7	503.6	1051.5	506.9	995.6
DHX30	109.4	127	93	89.9	89.7	83.1	104.7	103.6
DHX30	264.9	367.3	279.1	271.1	216.6	354.2	233.2	466.7
DHX32	676.7	825.7	418.4	266	235.9	327	470.2	542.3
DHX33	479.7	623.9	431.5	435.8	354.9	498.4	330.5	562.4
DHX34	221.3	237.4	192.4	169.9	175.1	186.8	181.9	193.8
DHX35	255.5	349.6	236.9	233.4	217.7	319.8	215.2	292
DHX36	363.8	530.8	220.2	220.9	219.3	330.2	342.2	762.3
DHX37	868.4	1190.2	773.6	715	548.7	652.4	569.1	712.7
DHX38	248.1	258.6	218.6	194.1	182.8	272.6	216.9	369.1
DHX40	633	728.9	415.8	478.4	387.9	590.1	543.7	668.7
DHX40	340.1	368.3	185	198.4	174.8	222.2	293.4	373.1
DHX57	98	106.3	90.7	99.1	110.4	95.9	100.7	107.5
DHX57	94.2	94.6	96.6	90.5	91.2	86.9	103.2	96.5
DHX8	210.9	237.4	163	185.7	162.1	209.1	197.4	234.7
DHX9	945.3	1328.4	537.4	528.2	429.1	523.6	577.2	852.2
DIABLO	2344.8	3163.5	2051.2	2271	1631.2	2460.1	2394	3279.7
DIABLO	480.4	650.1	351.4	332.8	265.9	408.7	437.6	468.7
DIABLO	333.7	494.8	207.8	162.9	149.6	151.5	281	323
DIAPH1	291.7	426.1	347.4	257.4	200.2	274.7	260.9	516.8
DIAPH1	914	1218.2	1348.8	1269.6	891.9	1442.6	927.9	1861.9
DIAPH2	499.2	648.6	292.8	464	395	612.3	462.4	635.9
DIAPH3	315.4	437	220.4	151.7	133.5	182.8	247.3	317.3
DIAPH3	277.1	384.6	210.8	156.1	145	169.5	232.8	295.1
DIAPH3	318.9	540.8	225.8	173.7	152.2	218.2	310.8	358.3
DICER1	86.7	100	92.3	83.1	88.5	84.7	80.9	86.2
DICER1	121.1	146.3	98.1	89	81.4	90.2	121.4	136.8
DICER1	125.2	170.5	101.2	108	108.7	137.3	146.1	223.5
DIDO1	151.1	189	140.8	119.4	117.6	126.1	118.9	126.2
DIDO1	181.5	190.4	150.6	170.4	148.7	199.8	141.5	168.4
DIDO1	155.5	186	109	112.2	94.5	96.5	150	168
DIO1	89.8	100.2	91.3	89.6	89.2	84	84	100.9
DIO1	86.9	108.3	98.2	90.8	86.9	90.1	99.4	107.6
DIO1	87.2	96.6	78.1	79.8	73.9	82.2	89.2	92.6
DIO2	169	160.8	98.6	90	85.9	88.3	137.5	144.6
DIO2	89.3	110.6	95.3	78.3	90	77.8	94.8	96.3
DIO3	96.7	99.9	90.5	93.6	84.3	94.7	99.6	92.7
DIP	747.7	928.7	1033.9	1110.8	952.7	1473.4	664.5	988.7
DIP13B	227.2	273.8	245.7	221.9	199.9	323.6	215.9	339.1

DIP2A	90.7	101	91.9	87.7	86.8	80.5	87.8	110.3
DIP2A	88.2	97.4	97.8	95.1	86.7	89.2	86.9	94.9
DIP2A	213.5	334.7	150.2	145.7	134.4	155.1	217.1	253.6
DIP2A	130.8	138	125.4	102	109.1	117.2	113.6	169.7
DIP2A	136	164.5	138	178.6	164.1	204.7	136.3	204.9
DIP2B	403.5	559.3	404	803.8	577.9	994.5	445.9	642.4
DIP2C	84.7	89.8	90.5	93.2	81.8	79.1	92.3	100.1
DIPA	174.8	213.2	107.7	91.7	88.9	92.6	130.2	166.3
DIRAS1	99.4	136.3	100.1	90.7	85.7	102.3	100.1	130
DIRAS2	77.1	86.4	86.9	76.3	76.3	74.8	87.4	86.1
DIRAS3	98.3	114.1	86.1	90.7	98	94.2	94.4	101.7
DIRC1	85.5	93.3	90.4	83.2	79.1	89.2	84.7	88.9
DIRC2	344.2	460	259	234.2	210.6	281.2	292.4	408.2
DISC1	92.3	110.3	108	220.1	222.3	143.1	104.2	90.4
DISC1	80	100	93.3	86.7	77.3	91.7	88.4	96.1
DISC1	112.6	143.5	85.9	89.1	89.1	85.7	111.9	151
DISC1	76.1	90.5	93.7	106.7	89.5	101.1	78.1	83
DISP1	207	253.7	149	137.4	109	140.2	220.3	325.8
DISP2	101.3	112.9	108	104.3	98.6	106.4	104.7	105.9
DIXDC1	126.5	165.8	124.5	120.4	119.4	136.2	106.8	181.7
DIXDC1	83.1	87	88.1	86.4	79.8	82.4	94.3	92.2
DIXDC1	129.4	185.6	124.1	102.1	94.5	134.3	119.2	134.9
DJ122O8.2	2255.2	2861.2	1406.2	1595.7	1331	1902.3	1793.9	1835.2
DJ222E13.2	182.2	200	164.1	145.3	119.3	139.3	137.4	238.4
DKC1	5492.4	6975.9	3996.2	3663.2	2612.8	5228.7	4012.7	6093.4
DKFZP434A	85.6	95.1	89	84	84.1	88.9	87	93.7
DKFZP434A	279.4	318.5	132.2	132.3	136.6	119.7	207	239.1
DKFZP434B	149	181.1	151.1	156.1	138	169.1	137	281.1
DKFZP434B	83.3	87.9	98.5	86.2	89	85.9	74	87
DKFZP434I	92	104.2	102.6	97.9	97.1	111.6	89.6	95.3
DKFZP434K	599.8	844.3	432	313.1	278.1	356.6	565	492.3
DKFZP434C	69.6	88.7	83.3	84.7	77.6	84.6	74	86.9
DKFZP434C	147.3	183.4	102.9	104.3	82.7	101.1	135.2	162.5
DKFZP547C	177.5	208	164.3	163.4	145.5	227.9	185.6	333.1
DKFZP547C	227.1	331.8	215.8	197.4	196.1	274	239.2	319.6
DKFZP547H	86.9	99.9	106	110.6	100.8	100	86.1	87.8
DKFZP564E	496	554.7	341.4	254	223	285.6	367	486.8
DKFZP564J	1379.6	1704.9	875.2	860.6	647.6	1047	1313.7	1377.7
DKFZP564J	84.9	97.9	90	80.1	80.6	80.9	88.7	88.7
DKFZP564J	86.8	103.5	82.8	81.7	88.6	91	108.5	118.8
DKFZP564K	1219	1189.7	809.6	1303.9	1254.7	945.4	1125.1	1923.7
DKFZP564N	100.3	118.2	91.6	92	84.4	86.1	95.9	117.2
DKFZP564C	329.1	423.5	202.2	157.3	152.6	182.8	303.4	387.3
DKFZP564C	103.6	106.9	110.6	98.2	91.8	109	94.3	98.8
DKFZP566N	100.9	128	81.4	87.8	73.6	87.3	100.1	124.3
DKFZP586D	1022	1210.9	785.7	1073.9	770.9	999.5	781.4	1052.9
DKFZP586D	97.6	118	90	102.8	106.1	94.4	89.4	101
DKFZP586H	85	86.8	85.5	84	88.2	83	82.7	84.3
DKFZP586H	71.9	99.2	82.3	75.6	72.7	76.2	83.2	88.1
DKFZP586I	168.5	202.6	117.6	213.7	199.5	267.1	137.8	230.1

DKFZP586F	104.7	119.8	95.7	100.9	97.4	98.6	105.1	112
DKFZP666G	73.3	81.3	91.4	80.9	79.9	74.3	74.2	81.5
DKFZP667G	107.4	128.5	88.9	89.3	86.7	100.5	107.4	112.3
DKFZP686A	77.5	94.1	82.8	81.1	77.7	86.7	82.5	93
DKFZP686A	140.2	154.7	94.8	87.1	81.4	86	133	160.6
DKFZP686D	84.8	102.2	90.2	73.6	74.8	74.9	95.6	88.8
DKFZP686E	131.1	154.5	134.2	124.5	117.2	121.3	115.2	139.7
DKFZP686I:	98.5	105.5	100	87.5	92.3	92.4	92.8	104.5
DKFZP686I:	188.1	270.8	132.8	118.1	122.3	124.7	199	241.5
DKFZP686K	156.1	191.8	130.9	188.3	147.9	140.8	143	284.2
DKFZP686L	540.5	646.5	273.5	323.6	244.5	310.1	564.4	798.2
DKFZP761E	96.1	112	84.7	100.9	92.5	79.2	92.3	104.9
DKFZP761E	146.5	138.4	115.8	114.4	101.3	102.1	127.8	185.5
DKFZP761H	84.4	90.6	93.7	80.8	81.7	88.7	78.3	96.1
DKFZP761I:	242.7	229.2	202.1	228.9	254.3	210.4	204.7	246.6
DKFZP762E	791.4	1121.5	591.5	412.6	459.7	748.7	567	732.4
DKFZP762I:	567.7	581.2	259.3	373	386.1	273.6	508.1	543
DKFZP779E	132.3	162.2	103.6	91.5	90.5	103.9	129.1	141.5
DKFZP779C	103.8	115.5	83.6	88.9	79.7	84.3	106.3	112.7
DKFZP779C	114	124.1	97.5	104.4	98.6	89.5	117.2	116
DKFZP781I:	138	152.6	119.3	105.5	98.7	120	119.3	167.3
DKK1	73.3	77.9	97.5	72.9	73.6	84.4	69.4	78.6
DKK2	130.6	167	114.9	91.8	86.5	105.4	109.8	150.4
DKK3	117.7	135.1	86.7	89.9	83.6	85.5	104.3	121.5
DKK3	81.4	89.6	99	89.2	84.5	91.4	106.3	105
DKK3	88.8	102.2	88.5	90.4	89.3	86.8	97.1	86.4
DKK3	139.4	168.3	87.4	85.8	81	97.9	171.6	186.4
DKK4	84	94.8	105.9	100.5	89.5	78.8	87.7	88.4
DKKL1	122.6	148.1	128.3	107.7	92.1	109.3	125.9	151.2
DLAT	801.7	1061.5	512.5	579.2	425.1	820.3	695	1185.2
DLC1	91.8	107.3	85.9	91.2	83.2	90.6	95.2	103.2
DLC1	120.7	146.9	97.2	82.6	74.3	83.4	132.6	153.7
DLD	1610.7	2189.3	409.4	547.1	397.8	683.8	1651.6	3056.7
DLEC1	116.8	151.2	99.2	97.1	77.9	98.6	137	141.2
DLEC1	78	90.4	94.7	82.9	86.2	98.5	79.2	93.9
DLEU1	164.9	199.9	102.3	101.5	83.7	102.7	149.9	226.1
DLEU1	548.8	722.5	312	377.7	268	449.3	384.4	602.3
DLEU2L	94.1	115.6	98.1	104	91.1	95.8	91.6	122.9
DLEU7	111.2	131	105.6	98.5	98.8	91.7	107.1	118.3
DLG1	129	158.1	86.8	76.6	75.6	96.5	110.4	130.4
DLG2	77.9	96.2	115.5	104.8	98.6	104.1	85.2	88.3
DLG3	120.2	133.6	97.9	88.6	91.1	94.2	126.8	123.1
DLG3	75.2	73.8	83.1	79.1	74	87.7	77.7	79.2
DLG3	82.4	92	101.5	87.2	84.8	91.6	85.7	80.9
DLG4	143.1	166.7	146	126.2	128.9	136.5	122.8	172.6
DLG5	110.3	134.3	92.2	88.4	85.1	86.5	116.8	142.1
DLG7	1522.7	1790.1	659.6	365.6	324	751.5	1053.4	1831.6
DLGAP1	80.6	94.2	88.5	90.6	77.8	88.9	82.3	95.4
DLGAP1	100	107	90.8	90	81.4	81	107.7	114.7
DLGAP2	190.4	217.2	126.8	117.1	113.3	136.9	177.7	207.3

DLGAP3	82.5	96.6	93.3	76.6	83.8	88.1	75.1	93.4
DLGAP4	81.3	94.9	94.5	89.5	95.3	78.4	84.5	84.2
DLGAP4	75	81.2	84	75.7	79.3	80.9	83.1	85.5
DLGAP4	139	161.5	160.9	168.1	141.1	197.1	147.7	282.3
DLK1	94.9	109.2	86.6	89.8	80.5	88.5	97.9	104.6
DLK1	88.1	103.4	99.1	82.5	79.9	89.2	87.6	91.6
DLL1	167.5	229.8	185.3	273.8	255.1	327.8	155.5	214.1
DLL3	81.6	83.7	90.9	80.7	84.1	74.9	81.7	93.4
DLL3	308.1	341.2	269.2	110.4	87.5	105.2	235.6	332.6
DLL4	111.6	120.5	92.4	89.2	82.7	85.4	90.1	109.3
DLNB14	387.2	408.4	285	272.6	284.1	309.4	328.2	501.2
DLST	437.5	640.5	432	425.9	367.6	548.1	450.7	739.1
DLX1	211.1	217	139.9	106.2	103.9	107.5	136	181
DLX1	707.4	764.9	506.6	379.9	302.6	401.2	562.8	788.7
DLX2	104.8	146.6	86.8	86.5	81.7	83.3	101	99.9
DLX3	166.1	187.4	114.1	116.9	95.4	108.2	191.1	232.8
DLX4	105.7	129.1	115.3	114.9	95.3	92.7	91.4	112.1
DLX4	98.9	108.3	98.5	93.3	91.8	88	103.5	97.8
DLX4	86.2	90	103.8	93.4	84.3	103.7	81.2	83.6
DLX5	73.8	82.4	85.6	86.9	86.8	86.6	77.3	77.7
DLX6	81.5	89	100.1	94.7	90.7	84.5	86.4	87.6
DMAP1	644.5	842.6	552.6	552.3	523.6	684	618.6	734.6
DMAP1	416.3	569.6	252.6	241	224.4	287.9	385.1	374.7
DMBT1	77	86.7	88	87.9	82.6	94	82.7	81.4
DMBX1	82.4	101.3	95.2	93.3	94.3	92.6	85.2	94.5
DMC1	2392.4	2254.1	1212.8	1934.1	1746.1	1644.4	2183.1	3020.1
DMD	114.2	150.6	99.5	85	85.9	85.9	115.5	114.9
DMD	78.4	84.2	95	86.9	89.3	87.4	80	89.7
DMD	168.2	199.4	168.5	167.2	165.9	176.9	139.1	173.6
DMD	91.8	100.2	93.5	96.6	86.7	93.2	92.6	106.6
DMD	84.1	80.4	95.3	84.6	79.2	89.9	84.4	82.3
DMD	93.7	108.6	95	88	72.1	87.7	109.7	98.7
DMD	133.5	182.9	117.5	118.3	108.5	118.3	137.6	168.8
DMD	92.2	110.4	86.5	73.2	87.2	76.7	98.3	108.4
DMGDH	92.3	104.8	103.2	98.3	93.5	95.3	86.9	100
DMN	177.2	201	164.4	172.7	137.5	166.8	183.6	235.9
DMN	88.8	114.4	87.3	79.7	78.6	91.6	90.4	94.4
DMP1	73.3	85.4	86.2	91.2	78.2	85	75.8	78.8
DMPK	91.2	108.9	116.6	93.9	94.6	90	94.3	100.3
DMPK	85.2	95.9	95.7	92.2	82.7	91.8	93.4	103.8
DMRT1	148.8	161.7	134.3	101.9	101.7	105.5	111.7	159.6
DMRT2	284.6	332.8	174.6	195	170.7	227.2	172.6	209.6
DMRT2	81	102	102.5	96.1	83.4	98.8	89.9	100.8
DMRT3	154	177.9	126.8	132.6	136.9	169.7	128.4	122.4
DMRTA1	91.6	106.1	91.5	92.8	77.7	87.5	94.1	97.7
DMRTB1	86	98.9	98.2	90.8	97.5	86.8	87.9	83.1
DMRTC2	91.1	102.7	91.8	78.9	79.8	79.4	94	99.4
DMTF1	429.4	551.9	295.5	442.1	387.2	681.7	372.2	520
DMWD	131.9	130	102.3	92.4	93	95.3	121.8	154.3
DMXL1	86.3	98.4	94.8	99.8	97.2	123	101.9	107.5

DMXL2	160.6	182.3	142.9	220.1	222.8	285.3	131.4	146.8
DNA2L	283.7	324.6	182.3	149.2	145.7	212	236.6	265.7
DNA2L	297.1	356.8	186.1	165.8	155.2	168.8	203.2	252.3
DNAH1	150.4	164.7	140.5	150.1	145.7	176	120.5	147.9
DNAH10	91.1	101	103.9	103.6	91.6	95	89.7	92
DNAH11	76.9	87.6	88.1	82.3	79.4	81.9	77.7	73.9
DNAH17	83.4	87.6	91.7	84.6	85.3	92.4	85.6	89.8
DNAH3	99.2	105.6	96.8	81.2	84.4	83.4	97.1	100.3
DNAH5	116.7	137.5	108	95.2	87.2	87.7	118.3	154.3
DNAH7	87.9	93.1	108.7	101.3	97.5	105	91.2	92.4
DNAH8	121.6	142.2	95.6	83.9	87.4	96.6	119	129
DNAH9	117.6	122.5	111.9	82.2	87.9	97.7	116.6	127.3
DNAH9	104.8	112.4	88.1	77.5	75	83.8	100.3	130.7
DNAH9	89.6	99.8	87.2	88.6	79.6	88.7	89.3	105.1
DNAHL1	101.4	98.8	80.9	73.2	65.5	81.5	94.8	114.6
DNAI1	103.3	119.5	100.4	104.6	84.3	94.6	108.6	98.9
DNAI2	212	287.2	136.4	144.2	144.5	181.4	208.2	214
DNAJA1	12715.4	15080.6	8951.6	5887.6	5359.3	9207.5	10739.2	15656
DNAJA2	3272	4096.5	1678.2	1575.1	1071.8	2149.6	2789.7	4889.7
DNAJA3	3332.2	4447	2836.1	1484.6	1136.1	1813.4	2612.1	3337.8
DNAJA4	1470.6	1791.9	1136.8	707.6	558.2	890	1206.3	1983.1
DNAJA5	144.5	192.6	128.5	113.3	104.5	126.2	136.2	142.6
DNAJA5	85.2	84.9	97.1	90.3	87.7	89.2	85.4	95.7
DNAJB1	1236.3	1760.5	953	496.1	411.5	672	1227.1	2508.3
DNAJB11	5680	7354.7	4827.5	5049.2	3770.4	6339.8	6048	8432.7
DNAJB12	200.7	250	109.8	104	111.4	119.7	204	249.5
DNAJB12	212.2	296.1	151.7	128.8	138.2	145	218.4	183
DNAJB12	312.4	385.7	177.4	142.6	110.1	121.8	228.2	282.8
DNAJB13	120.8	144	102.6	82.4	79.5	85.8	113.9	131.6
DNAJB14	167.1	245.9	98	93.2	91.4	87.6	156.2	210.4
DNAJB14	504.4	599.2	187.1	284.4	254.2	241.5	440.8	820.3
DNAJB14	91.3	89	91.9	87.9	86.8	93	88.8	97
DNAJB2	106.7	110.3	96.1	100.3	95.1	104.2	99.2	109.9
DNAJB2	305.4	385.5	275.9	285.4	245.7	373.1	315.5	522.7
DNAJB4	108.3	106.2	98.7	81	80.7	100.1	105.7	171.8
DNAJB5	89.8	99.1	100.9	95.4	97.3	92.6	88.6	102.1
DNAJB5	85.4	94.2	90.3	91.3	85.8	96.9	86.7	95.2
DNAJB6	1812.5	2480.9	898.8	801.6	687.6	928.8	1450	2013
DNAJB6	1415	2006.3	1287.1	1480.3	1310.9	2352.3	1354.7	2621.5
DNAJB7	97	100.1	95.3	92.6	92	92.8	103.6	99.1
DNAJB8	140.1	170	118.5	92.4	97.8	107.1	148.1	166.6
DNAJB9	323.7	407.2	276.2	239.3	241.7	315	339.7	629.6
DNAJC1	170.1	210	158.3	128.4	114.5	145.3	179.6	292.7
DNAJC10	1013.2	1179.7	471.8	599.5	365.6	769.1	867.4	1247
DNAJC11	213.1	280.7	123.7	118.5	111.4	118.7	193	268.4
DNAJC12	102.9	112.9	94.4	84.6	98.4	88.4	96.3	97.5
DNAJC12	78	97	84.4	80	81.6	76.5	75.6	99
DNAJC12	114.5	125.9	79.2	86.2	80.5	80	99.9	142.4
DNAJC13	259	380.6	258.8	238.9	250.9	328.3	269.4	281.2
DNAJC14	270.2	367.9	256.5	367.1	261.7	523.6	251.6	393.4

DNAJC15	817.9	1028.6	431.7	506.1	373.4	652.4	619.7	938.1
DNAJC16	114.9	134.2	99.2	100.7	97.5	103.2	109.1	124.9
DNAJC17	447.7	582.6	259.2	250.6	257.9	340.6	393.2	379.8
DNAJC18	150.2	164.6	124	120.7	114.3	125	124.7	139.4
DNAJC19	304.6	370.2	212.4	206.9	152.1	216.1	242.8	379.9
DNAJC3	295.1	331.3	157.4	152.4	117.3	143.2	344.1	512
DNAJC4	131	144.4	122.8	116.7	104.8	108.5	134.7	132
DNAJC5	270.6	355.6	276.9	240.2	193.7	248	244.7	369.3
DNAJC5B	121.1	151.4	102.3	113.3	114.9	120.6	120.2	156.1
DNAJC5G	121.6	125.6	98	88.6	89.7	90.5	109	118.3
DNAJC6	82.3	93.6	89.3	85.8	74.5	99.2	74.8	86.6
DNAJC7	2145.2	3061.5	734.7	611.6	573.2	788.9	2059.2	2922.1
DNAJC8	8585.9	10701.5	6498.8	7082.4	6886.3	10007	7897.9	8292.3
DNAJC9	4135.1	5349.4	2188.9	1260.8	999.2	1527	3285.7	4048.9
DNAL1	125.6	131.5	96.2	91.5	88.2	90.6	112.3	129.2
DNAL4	537.4	676.3	478.9	529.9	425.2	530.6	481.4	848.4
DNALI1	179.4	212.6	107.6	100.1	89.8	105.4	182.8	198.9
DNAPTP6	590.7	824.6	476.4	478.8	431.2	828.9	543	1334.8
DNASE1	121.2	137.4	101.9	101.3	103	108.6	114.1	127.8
DNASE1L1	355.3	488.7	310.7	383.4	293.6	526.9	334.2	662.5
DNASE1L1	95.9	93.4	93.2	83.8	75.7	88.5	91.2	103.9
DNASE1L1	242.7	264.6	143.4	150.7	130.4	138.5	205.4	240
DNASE1L1	100.2	108.8	96.8	81.6	91	91.7	102.3	107.9
DNASE1L2	98.5	109.3	120.2	96.5	106.1	128.4	92.7	126.5
DNASE1L3	78.5	92.1	108.9	99.7	84.9	78.2	79.3	79.4
DNASE2	1601.8	1718.9	915.7	1469.9	866.9	1463.4	1850.7	2353
DNASE2B	151.1	175.3	101.3	97.7	86.2	98.4	168.9	161.8
DNASE2B	84.2	101.2	99.2	103.1	87	83.7	85.1	100.3
DNASE2B	81.6	86.6	110.4	106.7	100.2	100.7	81	81.6
DNCL2A	1586.3	2177.2	837.5	398.7	348.1	391.4	1622.8	2329.1
DNCL2A	819.7	1023.2	405.4	261.2	229.1	265.9	757.4	1032.6
DND1	93.5	87.6	103.4	99.7	101.3	100.4	80.1	90.3
DNER	79.6	97.7	89.4	88.8	74.2	81.9	86.8	90.1
DNHD1	156.9	162	112.1	111.5	113.3	135.1	145.3	164.2
DNHD2	80.9	85.7	87.7	82.6	81.3	83.4	84.3	96.5
DNHD3	76.7	82.7	93.4	79.6	79.5	88.1	76.6	81.2
DNM1	116.5	133	147.3	118.8	109.2	111.8	126.8	175.1
DNM1L	758.2	1010.6	617.3	837.6	667.6	926.2	646	1265.6
DNM1L	479.4	591.3	228.3	190	141.7	243.7	378.8	726.6
DNM2	130.7	151.2	170.4	149.5	134	165.7	139.2	176.6
DNM2	238.1	297.6	305.7	202.7	230	298.3	226.7	375.2
DNM3	88.9	100.1	101.1	96.6	89.5	102.9	90.6	107.1
DNMBP	90	102.8	99.3	99.7	88.8	95.8	98.1	106.5
DNMT1	7009.2	8969.1	6141.1	5136.9	4751.7	6030.1	5239.8	4862.9
DNMT2	126.7	153.2	111.4	92.1	84.2	106.3	104	133.9
DNMT3A	87.1	97.5	99.7	91	83.3	97.1	88.2	105
DNMT3A	110.4	119.1	95.5	86.2	84.7	86.7	116.9	115.6
DNMT3A	122.9	138.3	93.3	84.3	85.8	97	111.2	116.7
DNMT3B	101.3	130.6	102.5	101.1	103.7	101.9	99.4	107.4
DNMT3B	182.5	213.4	125.1	114.9	98.2	140.4	144.3	204.2

DNMT3B	92	94.9	97.5	91.6	101.9	94.1	92	95.8
DNMT3L	82.9	107.5	82.8	84.5	80.1	82.5	88.3	96.3
DNMT3L	97.4	109.3	103.5	103	97.6	108.6	101.7	93.8
DNPEP	256.5	260.5	159.1	117	102.3	125.7	209.3	268.1
DNTT	93.7	88.7	94.1	88.9	95	84	93.2	98
DNTT	87.7	95.9	95.7	90.7	90.1	88.5	78.3	79.5
DNTTIP1	1570.6	2272.3	726.1	761.2	726.5	1159.2	1898.1	2361.1
DNTTIP2	88.3	104.4	95.8	89.8	86.8	84.9	93.2	125.6
DOC1	304	361.4	237.9	372.7	255.8	653.8	213.1	292.7
DOC1	260.4	353.4	245.7	356.1	268.4	669.7	221.4	303.8
DOC1	93.5	117.6	87.8	91.2	80.4	87.9	98.6	106.5
DOC2A	109.3	125.1	96.5	89.8	88.8	94.6	98.5	116.7
DOC2B	85.1	99.7	100.5	93.3	102.3	93	88.1	94.7
DOCK1	103.9	117.6	106.2	87.7	89.2	99.3	117.4	113
DOCK10	1416.7	1844.1	943.4	1699.5	1066.7	2599.6	1131.6	1654.6
DOCK11	798	1206.5	411.8	666.5	496.8	915.4	814.6	1096.7
DOCK2	3518.5	4696.8	3109.1	6026.9	4992.4	6665.8	3408.2	2884.6
DOCK3	82.9	86.3	92.5	94.1	83.7	82.6	83.4	84.2
DOCK4	87.8	111.2	87.3	83	76	76.2	91.8	112.1
DOCK5	156.4	192	118	113.2	103.5	132.1	153.7	198.8
DOCK6	144.7	160.3	190.6	145.2	140	174.1	125.9	193
DOCK7	85.9	99.7	91.2	80.5	79.9	92.7	92.6	91.7
DOCK8	1565.7	2174.5	1176.8	1570.6	1115.1	2523.6	1614.2	3158.4
DOCK9	109.3	121.6	96.5	96.1	84.1	105.2	102	111.8
DOK1	115.6	133.3	100.6	92.5	80.9	81.1	117.7	141.5
DOK2	178.3	225.6	98	94.3	108.5	86.7	182.9	316.8
DOK2	379.7	495.3	150.6	121.2	112.4	118.2	388.3	628.1
DOK3	114.5	116.5	104	127.3	114.6	152.7	101.6	130.9
DOK4	158.9	194.7	113.9	101.1	88.8	106.7	166.3	197
DOK5	82.3	90.5	95.4	75.9	80.2	74.4	83	90.6
DOK6	81.2	84.8	102.8	100.2	91.7	102.5	83.2	84.3
DOLPP1	376.5	440.9	251.1	177.9	149.7	189.9	378.6	426.9
DOM3Z	455.2	533.3	405.5	372.5	348	522.2	361.2	479.4
DONSON	875.1	1154.4	599.9	522.2	404.8	777.4	617.5	1404.6
DONSON	336	476.9	171.2	144.1	128.3	206.1	225.4	350.8
DOPEY1	144.3	177.8	147.4	168.1	159.3	232.2	130.2	169.8
DOPEY2	583.9	713.5	440.1	699.9	585.9	686.8	522.8	672.7
DOT1L	128.6	149.8	104	103.5	92.1	110.3	123.3	141.9
DP58	81.1	92.8	89	86.9	88.5	79.7	79.2	84.8
DPAGT1	520.1	625.5	244.5	138.2	118.3	115	390.9	479.5
DPAGT1	1292.8	1540.6	737.8	521.2	423.8	535.7	1078	1250.9
DPCR1	93.5	119.6	101.7	97.2	99.8	94.9	115.7	106.8
DPEP1	103.3	108.8	88.2	83.4	87.2	88.2	95.8	108.7
DPEP2	79.1	99.6	103.5	99.1	100.8	109.8	92	86.2
DPEP3	106	116.1	102.2	91.9	95.2	97.6	106.8	119.2
DPF1	107.1	114.4	130.2	100.6	100.5	103.8	104.4	107.3
DPF2	1039.6	1401.6	770.5	473.5	404.9	485.2	1011	1177.5
DPF3	91.7	97.1	104	94.9	96.7	95.5	80.1	105.5
DPH1	118.7	141.7	113.3	97.7	99.4	96.9	115.7	138.2
DPH2	243.4	332	169.4	132.9	115	99.7	182.2	243.4

DPH2	141.8	161.9	131.5	111.3	102.8	105.5	111.7	131.7
DPH2	1827.7	2288.2	940.4	842.2	538	928.1	1266.8	1607.9
DPH3	642	722.9	465.7	423.2	299.1	393.7	564.7	751.6
DPH3	256.4	273.8	162.5	149.2	130.2	172.3	177.1	203.1
DPH5	1686.7	2288	990.2	465.5	356.4	685	1271.2	1273.1
DPH5	1352.7	1778.9	719.3	307.7	231.9	398	1260.2	1456.7
DPM1	4614.3	4629.7	1946	2069.5	1267.6	2038.7	4279	6674.3
DPM2	570.6	752	482.2	215.7	182.1	204.5	537.5	610
DPM3	1659.5	2070.6	1107.9	826.8	851.7	978.1	1754.1	2082.1
DPM3	2009.2	2335.6	1922.1	1607.7	1472.8	1769	2198.1	2443
DPP10	84.6	100.1	102.2	93.9	87.4	87.4	88.8	100.4
DPP10	96.9	115.2	88.7	93.7	83.4	88.4	101.4	112.8
DPP3	271.3	348.1	184	149.1	141.4	209.9	243	340.7
DPP3	242.3	336.6	196.2	150.1	124.4	191.9	190	268.8
DPP3	401.2	561.6	359.1	312	275.1	497.9	357.3	494.4
DPP4	116	116.4	107	94.9	94.5	93.5	99.5	109.3
DPP6	130.3	161	85.7	77.7	78.4	83.5	112.9	161
DPP6	76.8	82.9	87.8	86.1	73.3	87.7	82	86.4
DPP7	1033.9	1417.8	739.2	605.9	563.5	897.8	940	1284.4
DPP7	3277.1	4052.7	1081.8	768.5	813	754.5	3673	1782.7
DPP8	358	491.8	136.2	196.2	170.7	266.6	338	495.9
DPP8	467.8	645.6	308.2	404	297	583.9	437.4	732.2
DPP8	92.1	107.7	90.1	75.1	71.8	89.3	90.7	104.4
DPP9	419.9	529.8	517	315.6	317.3	514.7	406.2	645.9
DPPA2	108.1	123.1	93.1	83.6	85.5	84.5	116.1	118.1
DPPA3	90.1	106.5	97.6	99.4	88.2	94.9	93	99.7
DPPA4	92.3	103.7	91.3	84.4	83.8	84.7	98	100.9
DPPA5	86.4	91.5	94.5	93.5	87.4	85.8	84.8	80.8
DPRX	75.5	78.2	89.3	81.7	76.8	90.8	79.9	78.9
DPRXP4	99.8	105.4	101.6	80.6	82.6	91.9	99.7	110.7
DPT	111.2	130.8	93.3	90.7	92.5	94.5	98.4	121.4
DPY19L2	111.1	115.7	113.1	89.1	87	96.5	91.8	91.4
DPY19L3	175.3	223.4	121.4	103.4	101.8	102.4	133.2	185.7
DPY19L4	443.2	523.9	211.5	200.9	161.1	255.6	303.3	438.3
DPYD	286.3	335.6	185.2	249.4	173.8	330.2	229.3	461.1
DPYS	92.6	97.4	90	91.7	78.1	95.5	89	87.6
DPYSL2	4499.7	6019.2	4934.4	4301.3	3773.1	5338.7	3971.6	5090.3
DPYSL3	103.9	116.2	106.2	99.5	88.9	106.6	117.3	147.9
DPYSL4	133.5	177.2	129.1	90.7	83.5	85.5	140	137.2
DPYSL5	90.7	117.8	98.4	91.7	86.4	86.3	95.1	117.5
DQX1	107.6	134.8	101.2	99.4	95.6	93.2	117.3	125.4
DR1	1293.1	1463.9	485.3	540.3	408.1	644.9	904.1	1878.8
DRAP1	1354.5	1524.3	1699.1	1661.2	1452.1	2869.4	1275.6	3002.3
DRB1	619.3	819.3	289.7	251	182.9	293.1	451.1	680.6
DRD1	90.1	104.9	95.4	89.6	73.7	77.1	91	107.6
DRD1IP	84.7	86.5	90.1	83.4	83.3	79.4	85.9	94.9
DRD2	81.3	83.2	95.2	83.3	83.6	81.8	93.1	91.9
DRD2	97	109.1	92.7	81	80.3	78.7	101.1	121.2
DRD2	126	138.5	93.7	82.7	83.9	78.9	109.8	121
DRD3	115.3	126.9	100.8	91.7	85.3	88.2	109.6	126.8

DRD3	100.6	104.3	101.1	89.4	85	91.8	97.2	105.6
DRD3	97.7	105.8	96.8	87.5	86.8	90.5	96	117.8
DRD4	84.2	87.4	101.1	119.7	124.6	129.1	99.1	103.3
DRD5	81	95.8	106.3	90.7	92	94.9	84.9	89
DREV1	159	209.4	163.1	187.9	151.4	254.2	167.1	402.8
DRG1	3048.1	4264.3	2017.5	1688	1239	2348.8	2317.8	4077
DRG2	232.3	273.1	207.7	185.8	145.1	177	198.4	383.2
DRP2	71	74.7	82.1	78.4	72.8	76.9	74.6	81.1
DSC1	93.7	110	95.5	96	86.9	95.1	100.3	103.8
DSC2	252.5	320.4	174.8	301.8	249.8	358.3	205.5	270.8
DSC2	121	144.5	102.5	111.8	106.5	122.3	112.4	109.8
DSC3	99.5	120.8	96.6	91	82.7	78.5	110.3	104.9
DSCAM	133.2	149.7	146.3	121.9	136.5	130.6	132.5	134.7
DSCAM	135.3	148.2	97.5	94.5	81.4	96.1	126.2	139.2
DSCAM	183.7	167.6	96.2	94.3	88.5	97.8	123.3	166.5
DSCAML1	78.5	84.2	92.5	76.9	84.5	88.2	80.7	78.1
DSCR1	114.1	132.6	89.1	90.9	87.2	78.7	120	121.9
DSCR1	129.7	175.1	103.5	89.2	78	81.6	123.7	132
DSCR1	578.7	853.1	415.6	368.5	273.9	526.3	465.3	778.9
DSCR10	143	166.5	106.7	112.8	101.5	104.1	137.7	148.1
DSCR1L1	105.5	99.2	107.1	107.8	91.8	105.3	88.1	104.6
DSCR1L2	154.4	198.3	115.8	114.2	112.2	123.7	138.7	171.2
DSCR2	2705.2	3390.9	1338.3	1251.3	910.7	1334.2	2143.4	3439
DSCR2	2289.3	3049.2	969.9	755.1	523.6	1164.2	1700.6	2485.2
DSCR3	468.9	582.8	477.8	544.8	498	706.1	462.5	830.2
DSCR4	127.9	143.4	83.8	81.1	71.2	84	112.1	143.5
DSCR5	694.3	918.2	514.5	395.9	285.5	357.3	537.7	608.6
DSCR5	2327.5	2955.9	1611.7	1439	1083.6	1598.8	2002.4	3744.1
DSCR5	128.3	142	94.5	101.7	88.5	93	120.6	131.8
DSCR6	148.9	173.7	119.4	88.3	89.5	104.1	129.8	159.4
DSCR8	91.9	113.5	106.4	95.4	92.4	106.2	91.1	99.4
DSCR8	112.2	129.2	104.8	101.8	93.6	99.8	108.1	111.4
DSCR8	86.7	100.4	88.2	93	85	88.6	84.5	93.1
DSCR9	93.7	110.1	95.9	81.6	81.5	81.5	98.7	89.5
DSEL	86.8	98.7	102.3	98	100.2	118	96.9	91.8
DSG1	103.8	117.7	87.4	85	84.6	85.8	112	120.1
DSG2	97.2	123	111	159.8	143.3	143.9	97.9	115.5
DSG3	93.3	103.7	89.7	82.3	82.9	83.9	89.7	107.9
DSG4	134.4	152.8	105.9	111	89.6	103	131.6	177.3
DSP	100.4	119.1	94.4	83.6	90.4	89.2	99.3	109.2
DSPG3	80.2	76.5	88.7	82.5	75.9	87.2	77.8	87.3
DSPP	102.1	110.7	116.6	93.6	92	92.9	96.8	94.3
DST	91.2	110.7	110.3	99.5	93.7	95.9	83.8	99.5
DST	85	91.4	85.5	85.7	75.1	80.4	84.1	96.7
DST	90.3	101.1	114.1	93	92.1	91.9	99.7	108.3
DST	84.3	86	107.5	95.9	88.8	104	71.6	86.3
DST	78	104.9	92.2	94.4	87.7	97.6	85.8	123.3
DSTN	773.8	969.8	332.4	484.7	265.5	425.4	794.5	1555.1
DSTN	180	221	108	118.6	105.2	108	181.5	189.6
DSTN	766.7	1088.7	369.4	518	347.6	548.3	768	1465.2

DSU	103.4	112.3	98.5	91.4	88.4	87.9	89.2	98.6
DTL	642.4	779.2	435.1	403.9	304.5	481	394.9	757.8
DTNA	268.3	293.4	208.5	160.4	128.4	166.7	200.4	242.3
DTNA	1073.5	1455.3	733.2	400.9	254.9	455.4	878.6	1172
DTNA	265.5	335	207.2	138.8	127.1	152	194.8	245.5
DTNB	134.2	165.1	120.9	116.2	108	110	130.3	126.1
DTNBP1	89.9	101	110.1	106.3	105	110.2	89.2	93.6
DTNBP1	213.5	241.6	165.3	141	143.2	177.4	179.5	266.6
DTNBP1	107.1	129.1	106.6	98.5	101.5	102.9	107.3	120.8
DTWD1	90.3	95.8	91	76.6	96.4	96.2	91.8	102.7
DTWD2	723.4	855.5	373.2	627	594.6	457.9	717.6	953.6
DTX1	101	126.4	86.3	84.2	81	84.5	108.4	121
DTX2	850.1	1274.3	575.8	612.9	624.4	605.7	703.8	627.4
DTX3	88.2	93.9	98.2	87.2	87.9	97.7	91.8	103.4
DTX3L	140.2	200.7	115.7	120.1	118.1	120.6	123.1	106.4
DTYMK	84.6	104.7	96.4	102.9	85.8	92.2	81.6	105.6
DUB3	90	104.7	93.8	86.7	80.6	96.1	88.2	99.8
DULLARD	208.7	271.1	186.4	204.4	159.1	238.5	207	400
DUOX1	84.3	91.2	89	89.4	80.9	82.4	98.1	98.6
DUOX1	97.5	111.9	104.1	96.5	84.8	99.2	105.4	104.5
DUOX1	82.1	101.2	97.3	91.7	84.3	84.9	79.4	85.6
DUOX2	78.8	94.5	89.3	87.4	83.8	79.5	75	100.2
DUPD1	79.1	95	100.4	89.8	88.8	89.8	83.2	97.9
DUS1L	352.1	361.5	246.2	194.6	169.8	283.8	244.8	453.1
DUS2L	520.5	637.6	273.6	226.8	180.1	197.7	409	388.1
DUS3L	1057.3	1249.2	943.2	460.7	386.2	575.6	826.6	1430.2
DUS4L	521.8	653.3	318.5	431.5	313	434.2	418.6	528.8
DUSP1	137.8	140.3	146.3	116.5	114.2	156.3	165.5	212.4
DUSP10	250	307.6	160.3	158.7	161.2	189.2	189.4	248.1
DUSP10	90.8	102.7	84.7	76.3	79.9	84.2	90.3	105.1
DUSP10	362.4	408.1	243.7	499.3	324.8	707.7	303.7	548.9
DUSP11	145	171.9	116.2	99.9	96.3	126	148.5	185.9
DUSP12	2462.9	3175.5	1698	1245.2	1032.3	1597.5	2039	3883
DUSP13	97.8	129	97.3	96.2	82.2	92.1	105.6	118.7
DUSP13	78.3	88.1	101.6	81.9	85.5	86.8	76.5	88.4
DUSP13	123.3	155.7	103.3	101.9	98.7	96.5	119.9	128.9
DUSP13	81.3	98.6	103.4	91.9	89.8	92.8	86.8	95.1
DUSP14	482.9	655.9	286.3	180.5	186.1	218.8	463.7	736
DUSP15	120.4	124.9	91.9	88.9	98	81.6	110.1	128.1
DUSP15	80.7	95	86.4	75.3	83.6	86.9	80.6	86.3
DUSP15	92.6	112.1	87.8	74	83	75.4	97.2	96.9
DUSP16	101	126.4	108.3	93.9	93.9	102.1	104.8	118.6
DUSP18	194.6	232.2	171.7	201.9	196.7	237.7	188	255.2
DUSP19	2596.8	2060.9	1752.9	2742.2	2851.1	2299.3	2519.1	4287
DUSP2	171.3	230.1	150.4	110.2	95.3	127.2	148.9	206.7
DUSP21	83.5	92.1	102.6	91.4	92.1	92.4	90.5	87
DUSP22	129.8	167.4	102.8	133.9	128.4	133.9	114.4	140.9
DUSP22	233.5	282.5	201.1	266	240	288.1	201.6	264.6
DUSP23	5221	6606	3349.1	2502.8	1892.9	2566.4	4498.8	4055.4
DUSP26	193.4	229.1	143.3	150.9	118	141.5	187.5	197.1

DUSP27	106.7	136.5	90.3	100.2	92.6	78	109.8	130
DUSP27	127	160.3	110.1	97.4	91.3	90.9	103.2	134
DUSP3	2184	2783.6	1759.9	2314.4	1918.1	2733.2	1781.3	2933.5
DUSP4	85.3	87.6	91.7	82.7	81.5	88.7	79.7	88.2
DUSP4	95	104.6	100.6	95.5	96	90.4	105.5	160.6
DUSP5	195.1	260.3	166.7	132.6	114.3	136.6	218.5	399.6
DUSP5P	233.2	247.3	168.6	144.5	145	146.4	152.5	193.1
DUSP6	208.3	269.3	117.4	131.6	90.4	109.3	211.6	442.5
DUSP6	128.4	141.9	98.3	100.1	99.4	107.1	116.7	128.1
DUSP7	110.6	135.8	116.4	126.1	123.5	94.5	103.3	120.1
DUSP8	111.1	121.7	116.5	97.7	99.6	91.4	102.6	150.2
DUSP9	146.1	183.6	99.9	87.7	87.5	104.3	127.3	161.6
DUT	321.1	482.7	170.6	178.6	159	145.8	280.5	315.5
DUT	1416.2	2121.4	423.2	372.4	355.1	471.3	1099.2	1375.5
DUT	1294.8	1793.5	727.5	679.1	545.9	649.7	1105.8	1338.3
DUX1	110.7	122.7	101.2	93	90.6	102.3	112.9	116.7
DUX2	117.4	120.6	107.9	83.2	80.7	90.2	102	116.7
DUX3	94.7	96.8	104	106.9	95.6	99	84.1	84.5
DUX4	88.9	101	72	96.2	77.2	78.3	80.5	91.7
DUX4C	94.6	114	83.9	84.8	85.1	88.9	94.1	100.1
DUX5	89.3	113.2	87.6	80.7	87.7	81	96.7	114.3
DUXA	83.6	95.3	84.4	81.2	87.2	81.1	79	83.6
DUXAP3	1862.8	1688.9	602.1	1130.5	838.4	796.2	1727.2	2303.8
DVL1	96.5	105.4	125.3	139.6	136.3	128.8	88.2	99.7
DVL2	198.8	235.5	207	216.3	213.6	268.5	175.9	278.3
DVL3	356.3	473	325.8	291.9	267.5	462.9	345.4	495.5
DXS9879E	2970.7	3654.3	2113.4	1254.5	881.5	1174.1	2814.9	3221.4
DYDC1	87.1	95.6	99.5	89	86.8	83.3	94.4	102.4
DYM	972	1158.4	954.9	844.4	727.2	1281.7	695.6	1483.9
DYNC1H1	425.8	644.5	355.5	604.5	575.9	940.2	421	509.9
DYNC1I1	110.9	107.9	90.7	77.5	82.3	81	102.4	115.3
DYNC1I2	1379.6	2000.4	1068.6	1148.2	992	2221.1	1167.3	1699.3
DYNC1LI1	119.7	140.4	112.8	118.4	107.5	111.7	122.7	196.1
DYNC1LI2	1815	2083.2	1337.3	1714	1546.1	2004.1	1219.5	1747.4
DYNC2H1	202.8	248.9	120.6	106	109	153.9	169.2	228.6
DYNC2LI1	72.6	84.9	91.5	82	82	88.1	78.3	74.8
DYNC2LI1	628.3	761.7	345.4	256.3	234.8	341.9	366.8	473.7
DYNC2LI1	111.2	120.5	104.5	114.4	97.3	125.8	104.9	128.8
DYNLL1	8690.8	10535.3	6540.9	3877.6	2860.5	4702.2	6629.2	8867
DYNLL2	525.4	693.6	385.4	468.5	407.8	410.4	563.1	480.8
DYNLRB1	2842.4	3616.3	1690.6	971	772.8	938.5	2973.7	3691.9
DYNLRB2	139.5	167.5	103.7	92.5	85	96.3	135.2	163.2
DYNLT1	1708.9	1787	1151.1	950.8	780	1203.5	1427.7	2056.1
DYNLT3	84.4	87.2	112.4	93.8	85.1	100	82.4	88.1
DYRK1A	1098.9	983.6	347.8	510	358.5	777.2	586.8	1014.2
DYRK1A	101.2	127.8	92	85	88.9	84	118.7	115
DYRK1A	976.8	863.4	490.7	598.9	425.6	926.2	543.7	945.8
DYRK1A	127.1	152.7	96.1	91.3	74.9	93.5	121	151.8
DYRK1B	80.2	94.9	94.9	90.3	86.6	102.6	92	97.1
DYRK1B	77.9	89.5	106.1	108.7	95.8	84.3	77.3	75.9

DYRK2	434.3	514.1	261.7	475.9	393.8	383	325.8	538.3
DYRK2	100.3	109.1	118.6	110.4	102	105.9	97.4	107.5
DYRK3	101.7	113	102.6	104.5	85.9	85.3	104	114.3
DYRK3	103.4	106.6	92.9	87.1	90.8	86.4	88.7	100.5
DYRK3	77.9	81.1	91.5	93.3	80.8	89.3	76.6	81.6
DYRK4	329	438.6	231.1	188.6	175	224.1	244.5	295.6
DYSF	116.5	160.4	100.9	95.8	87	92.9	131.8	154.2
DYSFIP1	4418.7	5342.8	2091	384.2	341.3	421.7	4255.8	5015
DYX1C1	88.2	92.5	98.3	95.4	83.1	96.2	85.9	85.5
DYX1C1	79.4	84	103.7	89.6	78.9	87.2	86	89.2
DZIP1	124.8	182.5	109.1	93.6	89.9	94.7	121.9	124.1
DZIP1	80.5	96.8	97.3	86.7	88.5	91.9	80.3	80.3
DZIP1L	198.6	259.9	109	115.8	108.6	126.1	191.7	205.4
DZIP3	96.4	115.8	96.6	94.3	92.1	90.8	108.3	98.7
E2F1	121.9	152.2	99.4	91.6	92.1	85.4	112.8	142.2
E2F2	1028.7	1234.2	947.9	1225.5	961	910.6	779.9	818.8
E2F3	1265.7	1535.1	811	936.2	650.6	808.6	1233.9	1925.4
E2F4	1581.8	1863.1	1375.4	860.4	630.8	829.4	1191.6	1843.8
E2F5	421.2	529.6	331	207.5	159.1	250.9	261.2	382.9
E2F6	97.3	117.9	104.1	97.5	101.3	104.5	96.1	112.6
E2F6	702.2	996.5	515.6	386.3	325.9	480.7	809.1	989.7
E2F7	323.4	415.1	273	336.1	253.7	391.2	299.8	422.1
E2F8	199.9	242	117.7	98.6	93.2	112	167.7	167.6
E4F1	1863.2	2108.4	1229.9	1006.6	1069.9	983.3	1515.7	1182.2
EAF1	276.8	340.1	139.6	133.1	101	176.8	278.2	486.5
EAF2	1576.1	1820.6	831.4	1058.4	797.9	1177.5	1084.5	1874.1
EBAG9	2867.3	3688.1	1897	1587.7	1269.8	1979.9	2629.7	4410.1
EBAG9	123.7	158.2	126.5	108.3	106.1	110.6	133.7	124.3
EBAG9	259.3	353.4	139.1	133.2	112.4	112.7	184	219
EBF	118.1	128.2	93.3	100.6	91.7	92.2	132.9	134.6
EBF2	73.5	80.4	81.5	84.7	81.8	77.8	77.6	74.2
EBF3	360.4	524.2	237.3	245	258.1	286.1	423.5	381.6
EBI2	388.4	535.6	188.1	346.6	300.3	443.9	495	672.8
EBI3	98.2	108.4	112.9	119.6	158.4	114.1	97.9	112
EBNA1BP2	5672.9	6785.4	4048.5	4043.9	3022.1	5060.9	4412.9	5672.8
EBP	1699	2279.2	1005.8	665.4	703.7	794.2	1683.1	1764.3
ECAT1	84.8	101.5	90.8	93.1	75.2	82.9	76	92.9
ECAT11	115.6	144.4	88.7	81.7	83.2	94	125.9	138.1
ECD	453	714.8	239.8	201.8	152.4	276.8	402.1	651.7
ECE1	112.3	116.2	104.9	99.2	87.6	110.1	96.4	109.7
ECE2	470.8	667.8	325.2	192.8	154.8	196.4	381	454
ECE2	104.3	108.3	93.5	89	74.1	79.8	98.8	100.9
ECE2	85.1	87.5	95.9	95	77.1	91.1	88.9	93.8
ECEL1	92	102.5	107.5	120.4	109.6	119.8	86.4	91
ECGF1	2884.9	3580	2186.6	2427.5	1879.6	2375.4	2280	3375
ECH1	5462.5	6936.9	5124.1	3667.4	3360.9	4574.4	5416.8	6693.4
ECHDC1	962.6	1081	327.8	412.6	339.4	519	631.3	1059
ECHDC2	2518.3	3222.5	1591.6	1475	1483.8	2121.9	1858.5	2029.5
ECHDC3	359.8	453.8	237.8	171.3	170.2	168.5	379.3	450.3
ECHS1	1955.5	2606.8	1187.9	929.7	759.2	750.1	2008.4	1849.6

ECM1	231.9	286	289.3	222.6	190.9	260.4	225.3	381.6
ECM1	446.2	494.1	158.2	117.2	96.7	112.2	442.5	579.3
ECM2	70.3	94.2	89.9	81.5	79.2	78.7	76.6	91
ECOP	236.6	302.4	224.4	239.5	202.9	203.2	189.6	275.8
ECRG4	83.4	97	80.5	85.4	87.5	83.1	83.1	94.2
ECSIT	187.3	231.2	143.1	127.7	114.5	133	164.7	229.6
EDA	162.7	175.6	94.4	98	83.2	85.2	171.8	169.5
EDA	99.7	102.6	98.5	96.8	92.5	99.7	103.6	108.4
EDA	105.2	119.6	98.6	96.7	88.6	87.3	99.3	110.4
EDA	84.9	96.3	98.3	91.8	83.2	103.6	97	95.8
EDA	88.7	87.2	91.3	84.3	87.2	90.2	85.4	83.2
EDA2R	81.2	97.6	90.6	85.3	80.5	84.8	90	88.3
EDAR	78.5	97.8	102.8	97.3	74.2	91	91.2	98.9
EDARADD	103.2	110.4	92.7	86.7	90.9	87.2	98.7	102.3
EDARADD	114.3	152.3	126.7	119.3	122	125.1	120.5	117.6
EDARADD	99.8	109.5	84.9	74.3	75.8	73.9	87.3	97.4
EDD1	657.1	954.2	305.3	486.6	356.3	562	726.9	1119.2
EDEM1	865.3	1289.2	341.6	402.4	409.9	588	929.5	1214.9
EDEM3	621.7	761.1	323.1	345.4	255.6	394.5	559.6	1232.1
EDF1	3146.3	3764.6	812.4	623	477.9	772.4	2825.2	4476.4
EDF1	11003.8	13192.2	8191.9	7482.3	5690.4	7385.8	10735	14453.6
EDG1	135.4	189.9	90.2	93	89.3	96.5	126.8	163.5
EDG2	464.6	651.2	378.2	214.5	168.1	241.6	477.2	655.3
EDG2	115.1	134.5	98.3	78.7	80.9	85.6	110.3	126.5
EDG3	281.8	368.1	197.8	129.8	112.3	140.8	298.6	471.3
EDG4	685.1	591.8	422.3	455.2	468.9	558.1	502.6	756.7
EDG5	87.3	93.8	100.9	91.2	98.5	91.8	86.1	86.1
EDG6	87.2	91.9	89.9	85.8	86.8	84.2	89	97.6
EDG7	96.3	90.8	106.9	87.2	84	95.9	85.7	97.9
EDG8	211.3	233.3	183.3	190.9	176	201.4	184.2	201.5
EDIL3	101.9	99.8	92.4	98.3	86.5	92.4	98	107.5
EDN1	126.1	147	101.9	93.4	95.1	100.2	159.4	175.6
EDN2	105.5	115	101	96.9	91.2	83.4	85.6	107.7
EDN3	102.3	110.7	98.1	91.2	85.3	86.2	104.1	109.3
EDN3	84.3	99.2	85.7	88.9	77.5	82.3	82.3	92.8
EDNRA	90.9	94.3	82.5	88.4	82.5	94	95.6	98.9
EDNRB	168.7	192.5	116.4	106.5	106.7	110.3	181.6	244.5
EDNRB	82.6	80	93.1	84.8	75.6	82.5	79.2	86.8
EDNRB	71.4	84.3	93.4	83.4	78.3	83.4	71	77.5
EEA1	121.7	146.7	126.4	119.5	124.7	187.1	108.6	129.7
EED	110.1	106.6	107.1	105	96.4	108.9	98.7	91
EED	416.7	555.8	227.9	197.1	142.1	219.9	376.3	813.7
EED	219	272.3	125.6	109.7	93.6	106	206.3	258
EEF1A1	32574.7	34364.2	58560.2	56366.3	55294.1	51504	31077	32683
EEF1A1	30986.7	30960.6	53310.5	46243.3	40456	53855.6	31701.3	30467.3
EEF1A2	2999.4	3795.8	2248.4	464.4	310.6	373.2	2626.2	3828.4
EEF1B2	1643.8	2128.7	793	545.7	410.4	718.8	1191.4	1401.1
EEF1B2	24210	26978.9	13818.7	14394.3	11107	13803.1	27053	27658.5
EEF1B2	1374.8	1719.3	872.5	761.6	612.1	1052.8	1095	1213.4
EEF1B2	20799	22732.3	17758.5	15665	13731	22037.3	16403.3	17598.7

EEF1D	6781.3	9218.5	3023.2	2040.7	1928	2770.7	7031	7924.8
EEF1D	78.8	97.4	84.2	81.1	79.7	86.8	91.3	96.4
EEF1E1	84	108.6	93.6	80.9	74.4	82	84.5	104.3
EEF1G	21166.1	26586	14747	12883.9	11757.1	17245.4	20393.1	24828.6
EEF2	12949.3	17439.1	9720.4	9744.5	7009.7	10966.5	12458.7	13576.8
EEF2K	1028.5	1467.5	738.8	920.3	721.8	886.3	695.2	642.6
EEFSEC	122.6	130	103.5	94.8	81.7	105.6	112.1	139.9
EFCAB1	82.8	98.9	101.8	91.9	87.2	92.1	90.6	97.3
EFCAB2	84.4	115.7	96.7	80.5	76.8	75.3	93.1	141.1
EFCAB3	96.5	121.1	94.5	103.7	95.6	84.3	102.9	113
EFCBP1	91	115.1	96.5	87.3	84.3	87.3	92.8	88.3
EFCBP2	188.2	193.2	172	110.4	112	113.3	171.6	194.4
EFEMP1	88.9	111.2	95.4	96.7	86	90.2	115.6	162.6
EFEMP1	97.5	102.7	100.5	95.1	95.5	91.8	111.3	118.3
EFEMP1	89.6	94.6	91.8	93.6	81.1	92.9	197	261.7
EFEMP2	704.1	736.1	473.6	333.7	306.5	360.7	591	479.4
EFHA1	1724.2	1917.6	620.8	866.7	521.5	1119.8	1612.9	2980.4
EFHA2	77.7	84	90.8	77.7	79.8	80.5	89.7	94.8
EFHB	82.5	95.1	95.3	92.8	91.5	89.2	84.6	96.6
EFHC1	144.2	154.3	108.8	123.2	108.6	138.6	119.7	173.7
EFHC2	93	102.6	93.8	81.5	73.4	86.7	88.7	92.3
EFHD1	359.4	401.6	211.1	277.9	147.7	253.9	221.2	278.3
EFHD2	3311.6	4347.2	3061.1	2520.8	1799.6	2900.6	2493.5	3548.1
EFNA1	88	101.9	89.8	86.2	89.7	87	93.4	99.6
EFNA1	93.8	103	98.2	88	96.4	89.3	96.4	102.6
EFNA2	81.3	92.4	81.3	85.6	81.1	76.9	77.9	80.7
EFNA3	94.6	99.6	87.1	86.7	85.8	82.6	98.5	106.3
EFNA4	160.7	188.1	110.4	95.6	92.4	100.1	143.7	193.6
EFNA4	300.9	396.3	311.8	251.3	236	260.7	334.1	374.6
EFNA5	80.1	85.5	94.9	92.2	78.7	87.8	90.6	91.7
EFNB1	127.3	147.6	110.5	106.4	100.1	114	132.6	135.2
EFNB2	139.8	151.7	115.6	107.8	100.6	105.2	146.6	171.8
EFNB3	68.2	88.3	89.2	90.4	80.8	79.4	79.2	84.1
EFS	124.2	125.2	98.7	91.6	93.9	95.6	108.1	119.5
EFS	100.9	104.2	95	81.5	78.5	96.6	89.1	114
EFTUD1	228.3	321.8	197.6	194.2	167.9	289.5	191.1	281.5
EFTUD1	248.6	372.5	222.2	258.6	231.5	358.5	197.1	287.8
EFTUD1	111.1	122	88.1	100.1	75.3	89.4	98.6	101.6
EFTUD2	1825.3	2513.5	1855.4	1790.8	1309.8	2244.7	1466.3	2572.9
EGF	117	114.6	97.1	86.6	95.9	88.8	106.2	123.9
EGFL5	74.3	88.7	92.9	92.3	87.1	86.3	82.3	86.4
EGFL6	78.4	99.8	97.1	93.7	91.5	94.9	77.7	82.8
EGFL7	174.7	213.9	127.2	119.6	112.6	117.7	162.3	247.6
EGFL7	139.4	182.6	108.3	99.2	84.2	99.6	148.1	159.3
EGFL8	99.3	111.6	79.1	81.8	71.2	67.6	103.3	101
EGFL9	84.7	101.9	91.5	82.4	86.5	90.3	100.3	98.2
EGFL9	108.6	140.1	109.3	103.3	95.9	90.9	107.1	115.8
EGFL9	186	216.5	114.8	88.3	89.1	98.5	183.2	193
EGFLAM	224.1	286.5	157.9	192.8	174.1	192.6	220.3	305.7
EGFR	101	99.5	95.8	94	80.4	92.7	91.7	96

EGFR	78.3	83.6	94.3	91	88.7	90.6	83.6	92.1
EGFR	71.3	77	88.1	88.5	77.7	76.6	83.2	86.9
EGFR	113.1	131.3	98.7	87.9	92.4	89.8	96.3	119
EGLN1	381.4	516.8	214.6	199.3	188.9	253.1	298.6	501.1
EGLN2	103.4	104.7	80.9	88.1	88.3	83.6	108.6	110.7
EGLN2	3501.9	4311.4	2137.4	1912.2	1708	1855.9	3317.2	3094.2
EGLN3	87.2	99.7	86.8	89.4	86.8	96.2	92.5	102.1
EGR1	237.5	285.3	212.6	151.1	124.4	159.2	222.7	354.2
EGR2	111.4	110.7	111.3	107	98.3	111.5	104.6	115.4
EGR3	128.4	146.5	98.3	96.1	93.9	96.1	122	135.4
EGR3	140.9	175	119	117.4	104.7	103.8	152.7	188.7
EGR4	104	109.3	78.8	84.6	82.3	88.8	104.8	111.8
EHBP1	1013.8	1324.9	691.6	758.2	568.9	908.3	843.4	1411.8
EHD1	1521.8	2011.7	1089.7	911.3	707.8	732.3	1301.3	1294.7
EHD2	108.7	129.1	95.3	94.4	90.9	99.5	114.7	134.2
EHD3	95	107.8	101.1	84.1	79.5	78.7	93.7	117.8
EHD4	438.5	516.1	499.1	687.8	512.9	734	297.4	420.9
EHF	80.7	103.9	93.5	87.2	82.7	88.3	84.8	89.2
EHHADH	261.8	418.1	261.5	334.8	251.5	511.8	246.9	291.3
EHMT1	116.8	144.2	126.8	113.4	111.7	119.5	108	129.3
EHMT2	191.6	246.5	172.8	160	168.3	152.9	179.1	255.2
EHMT2	103.4	121.2	84	85.2	72.1	89.7	97.5	122.9
EI24	5110.4	6370.7	2680.8	1727.3	1282.9	1763.9	4404.2	5202.8
EI24	1585.8	2162.9	631.6	400	274.1	416.7	1354.6	2771.8
EID2B	3183.4	2774.8	1237.1	1688.2	1392.3	1277.3	2695.3	4741.3
EID3	94.6	98.6	84.6	86.7	80.7	80.4	101.3	135.7
EIF1	3640.3	4768	2199.5	2035.2	1557.5	2690.4	3655.4	6104.7
EIF1AX	112.7	120.8	105.2	95.5	100.2	98.9	111.8	113.7
EIF1AX	1851.3	2194	863.6	923.7	549.2	755.3	2118.4	3799.5
EIF1AX	172.6	203.2	143.7	151	127.5	174.5	163.8	199.9
EIF1AY	370	459.3	219.5	278.3	207.3	296.8	347.6	542.1
EIF1B	1470.3	1950.3	478.6	398.7	357.2	449.3	2032.9	3085.3
EIF2A	3490.3	4596.3	2175.8	2213.2	1686.9	3073.9	3227.7	5301.1
EIF2AK1	5268.8	6982.8	2705.8	3231.3	2363.4	3384.3	4771.9	5926.7
EIF2AK2	1247	1992.9	674.5	772.6	758.2	784.7	1383.9	1579.9
EIF2AK4	406.6	497.9	227.8	337.5	328.6	354.1	360	366.9
EIF2B1	1147.8	1259.5	809.2	761.9	629	1059.2	802.4	1060.2
EIF2B2	1358	1796.7	1043	948.7	700.7	1116.7	1031.9	1345.7
EIF2B4	2528.7	2966.9	2175.5	1813.9	1560	2391.5	2038.8	2520.7
EIF2B4	2037.2	2643.6	1494.1	1312.7	1162.6	1806	1678.4	2218
EIF2B4	96	106.8	108.2	103.2	95.8	97.4	93.2	101
EIF2B5	1411.2	1950.1	623.9	545.2	495.6	678.3	1196.5	1180.1
EIF2C1	250.2	352.9	219.3	293.9	221	325.2	248.8	326
EIF2C2	757.8	1630	563.8	1184.9	1098	712.2	1095.6	986
EIF2C3	141.8	198.2	115.2	119	107.6	129.8	141.5	181
EIF2C3	118	140.7	120.5	133	118	129	112.9	130.6
EIF2C4	182.2	231.7	125.8	129	115.9	129.7	168.5	199.7
EIF2S1	440.2	605.7	219.5	192.1	176.9	186.2	365.5	542.2
EIF2S2	384.8	485.9	332	385.5	272.6	482.7	294.6	554
EIF2S3	5371.7	6274.7	4700	4989.6	4167.1	5776.2	4396.5	7129.4

EIF3S1	1055	1396.2	546.2	480.6	372.5	977.3	844.2	1676
EIF3S10	1402.8	1657.2	498.3	636.8	542	975	1074.6	1522.1
EIF3S12	7880	9322.3	3556.2	3133.7	2352.3	3606.3	7573.2	11009
EIF3S2	3556.8	4619	2134.5	2278.4	1792.6	2676.5	2966.4	4152.9
EIF3S3	8151	10410	5614.1	4460.1	3718	5653.4	7304.1	9768.1
EIF3S4	3640.2	4610.7	2432.6	1463.3	1290.9	1873.1	3948.1	5113.1
EIF3S5	7496	9062.3	5134.8	4280.3	3109.7	4558.2	6803.4	8890.2
EIF3S6	30314.1	31749.7	20056.5	18102.8	13923	16891.7	27503	30027.2
EIF3S6IP	8205.8	11264.5	5812.3	4772.9	3911.7	6082.8	7095.3	10370.9
EIF3S7	5973.5	7657.5	4887.7	4872.6	3820.3	6575.4	4894.9	10290.7
EIF3S8	177.9	210	106.8	102.9	93.5	115.7	162	184.7
EIF3S9	9427.2	11985	7846.3	8801.7	7549	9265.8	7263.1	7281.1
EIF3S9	441.3	598.3	571.9	468	392.3	639.3	350.4	520.1
EIF3S9	1694.1	2320.5	2146.6	2195.4	1872.8	3173.9	1460.9	2619.1
EIF4A1	20690.7	25346.7	17993.7	16305.2	11766.1	19510.9	19220.3	26974.7
EIF4A2	7004.1	7813.8	4286.6	5005.9	3727.7	7135.6	4709.5	6369.5
EIF4B	3979.6	5962.5	4129.7	4072.4	3017	6721.2	3727.4	5030.9
EIF4E	252.6	283.5	121.1	125.4	106.6	129.6	234.4	545.7
EIF4E2	1718.5	2401.5	758.2	545.9	432.1	628.4	1407.2	1823.1
EIF4E3	174.9	247.8	148	167	137.8	193.1	174.1	258.6
EIF4EBP1	355.1	446	238.4	164.4	135.9	173.4	348.4	497.9
EIF4EBP2	931	1365.1	784.5	566.4	475.3	807.3	707	1043.3
EIF4EBP3	98.9	97.8	97.7	84.6	95.3	87.3	93.1	98.4
EIF4ENIF1	468.7	560.7	413.3	489.2	398.6	576.6	432.8	803.9
EIF4G1	867.2	1077.6	896.3	1036.2	870.6	1153.5	770.6	987.2
EIF4G1	83.4	91.5	82	77.2	77.9	86.6	76.1	92.3
EIF4G1	1616.6	2197	2945.4	3224.3	2588.5	3412.9	1524.5	2093.6
EIF4G1	102.5	116.6	97	93.3	87.3	87.2	106.9	109.7
EIF4G2	7863.8	9167.7	3816.5	3046	2236.7	3077.6	6799.1	12003
EIF4G2	1383.6	1848.1	600.6	430.1	320.4	561	837.6	1188.8
EIF4G2	7012.2	8615.1	6295.4	4776.5	3347.6	8335.6	5918.7	9952.9
EIF4G3	944.2	1505.2	825.5	1133.4	852.9	1611.9	964.1	977
EIF4H	3943.7	5281.3	2721.5	2302.4	1896.5	2914	3188.4	5151.1
EIF5	140.1	168.1	92.5	96.2	100.8	109.1	138	183
EIF5	2426.7	3349.7	1584.6	1512.6	1125.3	1227.7	3017.4	3731.5
EIF5	110	126	103.4	89.8	89.9	91.3	91	108.4
EIF5A	1939.7	2290.5	1178.7	906.1	661.4	570.1	1741.5	2186.9
EIF5A2	102.2	110.1	90.9	86.5	92.3	84.3	114.2	118.8
EIF5B	549.1	857.8	453	568.9	522	1072.9	394.9	722.5
ELA1	93.1	101.1	88.9	128.3	118	128.8	102.4	104.3
ELA2	32115.9	31639.8	27789.5	27450.4	24268.3	23376.7	31004.4	30970.5
ELA2A	98.3	107.4	91.8	79	79.3	92.5	100.1	99.7
ELA2B	101.9	139.1	109.8	89.8	97.5	94.8	120.8	138.5
ELA3A	80.8	86.9	87.3	83.1	77.2	88.6	86.6	87.7
ELA3B	90.9	94.3	100.8	87.7	89.9	91.7	90.6	100
ELAC1	198.4	254.8	203.3	197.5	164.7	196.1	159.8	241.7
ELAC2	225.3	252.3	240.9	272.9	235.8	326.5	226.7	306.9
ELAVL1	200.1	253.9	163	150.8	153.7	168.9	194.4	231.3
ELAVL2	107.2	133.5	105.6	101.4	89.8	96.9	100.2	119.4
ELAVL3	100.7	109.7	93.7	94.4	86.4	91.3	88.1	111.3

ELAVL3	97.2	122.8	99.4	92.3	95.2	99.9	96.8	104.5
ELAVL4	97.1	108.6	102.8	83.8	81.2	94.6	93	101.3
ELF1	5563.1	7124.7	2751.3	3204.8	2198.8	3635.5	4849.8	5477.4
ELF2	207.9	293.7	131.9	135.3	135.2	123.5	207.7	310.2
ELF2	1020.2	1377.1	743.4	748.2	675	1212.1	844.3	1451.5
ELF2	282.1	361.8	170.9	169.3	149.2	129.2	239.1	290.2
ELF3	107.9	137.4	94.5	95.1	91.8	85.2	97.7	109.6
ELF4	885.2	1040.6	774	666.8	552.2	660.6	858	1095.6
ELF5	70.1	88	82.5	86	76.8	75.6	71.5	82.3
ELF5	81	93.1	95.2	84.7	89.5	85	86.4	94.7
ELK1	180.6	180.4	127.9	154.7	153.6	172.4	168.9	222.1
ELK3	113.6	128.9	98.8	103.6	89.4	102.6	103.8	113.8
ELK4	185	233.7	130.6	102.2	105.4	112.7	186.4	247
ELK4	109.2	121.5	92	94	83.1	84.4	99.6	112.4
ELK4	133.2	183.3	114.1	105.4	98.1	96.8	138.9	167.8
ELL	320.3	442.9	317.7	257.1	215.8	255.3	326.6	506.3
ELL3	119.4	123.4	112.1	106	94.2	108	106.8	122
ELLS1	373.3	450.1	229.4	584.4	427.5	583.4	335.7	427.3
ELMO1	105	122.3	113.3	86.3	81.7	90.7	105.1	124.5
ELMO1	255.3	343.9	377	580.7	491.9	870.9	280.8	543.4
ELMO1	101.6	95.5	92	86.6	80.6	88.8	100.8	117.4
ELMO1	141.5	154.5	115.9	115	115.1	107.2	115.6	175.1
ELMO2	120.6	148.1	99.5	118.3	109.6	126.3	113.5	151.4
ELMO2	392.6	494.3	404.8	527	484.2	713.1	335.5	587.2
ELMO2	84.4	97.3	95.1	93.9	90.8	93	83.8	96.4
ELMO3	130.2	146.5	135.4	97	104.3	135.2	113.1	154.3
ELMOD1	85.2	93.7	104.1	97.9	88.3	102	95.1	98.6
ELMOD2	251.9	326.6	167.3	120.2	107.1	141.1	221.4	325.4
ELN	121	138.4	85.2	80.9	74.6	81.7	111.1	135.6
ELN	129.9	154.2	96.2	101.3	79.3	88.7	132.1	135.5
ELOF1	374.1	496.9	158.7	121.4	118.4	139.1	356.4	420.8
ELOVL1	521.7	636.4	276.6	228.3	171.9	230.3	514.7	826.5
ELOVL2	110.5	129.3	107.2	88.2	92.5	105.3	117.5	114.2
ELOVL3	130	135.2	118.1	104	98.3	103.4	115.4	122
ELOVL4	140	141.7	113	121.5	94.8	116.6	137.4	164.8
ELOVL5	2349.9	2732	1386.3	1579.8	1045.8	1880.6	2258.8	4350.4
ELOVL6	122	133.7	111	99.2	83	92.7	113.7	104.9
ELOVL7	83.2	88.6	90.2	83	85.6	90.9	81.6	91.5
ELP3	1395.5	1944.4	953.6	880.1	730	1247.1	1235	1462.8
ELP4	468.7	582.1	297.8	259.4	223.8	318.7	324.9	484
ELSPBP1	84.6	84.3	85.3	88.8	81.1	93.6	71.5	84.2
EMCN	116.9	131.4	101.8	89.7	87.3	91.9	118.1	124.6
EMD	83.5	94.3	100.4	89.2	94.9	91.9	96.7	108.1
EME1	294.7	389.5	166.1	150.3	154	209.7	233.6	278.8
EME2	108.3	137.6	100.1	99	91.4	101.5	114.5	133.4
EMG1	1677.2	2189.3	1149.8	893.3	629.6	1345.6	1524.6	1917.5
EMID1	87	90.7	107.6	104.9	98.9	110.5	92.2	97.5
EMILIN1	85	84.5	90.5	88.2	92	90.5	78	84.4
EMILIN2	2783	3752.1	1742.6	1568.9	1287.1	1406.8	2531.4	2171.2
EMILIN3	103.7	120.7	93.2	99.3	83	97.6	107.9	110

EML1	207.7	316.6	186	123.9	106.5	152.8	167.1	250.6
EML1	84.3	86.7	86.1	84.9	83	84.6	79.6	79.6
EML1	160	201.9	171.1	118.7	100.7	125.4	139.6	181.8
EML3	1550.2	1781.9	1152.5	783.2	712.4	810	977.2	1136.8
EML4	478.2	678.8	270.6	317.6	263.9	339.3	438	636.9
EML5	89.4	96.1	91.3	89	76.7	88.2	96.1	98.3
EMP1	127.8	123.4	114	94.7	83.2	110.1	150.9	251.6
EMP2	132.9	169.2	98.4	93.3	86.8	104.7	137.9	143.5
EMP3	5732.3	7847.8	4421.4	2675.3	1900.4	2646.7	5375.7	6685.9
EMR1	78.8	87.7	91.9	106.5	91	119.1	77.5	94
EMR2	186.8	253.1	156	512.6	412.7	528.4	171.1	201.8
EMR3	72.4	80.2	77.1	74.4	72.7	73.4	78.5	87.7
EMR3	96.8	116.2	96.8	82.9	89.8	85.8	103.3	111.1
EMR4	99.6	117.6	106.5	99	95.1	94.2	91.4	99.5
EMR4	99	99.6	95.4	101	88.7	96.1	85	95.2
EMX1	110.9	122.2	103.1	85.2	91.9	82.7	109.2	117.5
EMX1	87.1	101.1	87.7	81.2	81.1	79.3	90.3	96.4
EMX2	125	139.4	86.8	86	90.3	85.7	116.8	126.9
EN1	99.7	104.7	100.3	91.4	94.1	90.5	94.3	111.5
EN2	107.8	131.2	78.3	82	72.4	95	102.1	135.6
ENAH	87.4	111.6	88.4	73.6	81.2	77.8	76.8	86.1
ENAH	89.8	117.1	92.8	76.9	81.7	86.6	88.3	101.9
ENAH	80.8	89.6	101.7	81.8	74.2	77.9	84.3	85.1
ENAM	79.2	93.1	89.7	91.2	89.2	88.6	82.4	83.2
ENC1	151.9	178.3	144.5	166.3	140.5	174.1	155.1	222.3
ENDOG	805.1	1120.2	474.1	277.4	223.7	286.1	671.9	853.3
ENDOGL1	182.6	212.3	117.4	106.5	97.5	104.6	201.6	239.2
ENG	371.6	516.4	400.6	312.8	199.5	373.1	312.1	565.1
ENO1	20041.8	24067.5	16440.9	13657	10282.4	13413.8	20952.6	23640.2
ENO2	126.7	127.3	95.9	86.4	90.3	102.5	112.2	136.7
ENO3	246	238.2	175.7	173.3	163.6	204.6	171.4	168.4
ENO3	86.8	119.6	108.1	110.7	102.8	109	94.6	98.8
ENOSF1	1121.1	1200.7	427.1	465.7	438.3	657.8	759.7	1222.7
ENPEP	78.4	80.4	83.7	82.3	85.1	87	78.6	84.3
ENPP1	119.7	142.7	111	102.5	109.5	103.1	110.4	114.9
ENPP2	81.8	101.9	106.3	98.7	89	92.4	94.5	97.5
ENPP2	86.1	101.4	81.2	80.9	79.9	78.9	88.7	95.8
ENPP3	91.8	93.7	98	87.4	85.9	94.1	87.1	92
ENPP4	247.7	294.9	188.5	277.5	185.2	440.7	205.4	353.8
ENPP5	97	109.7	98.1	96.9	105.6	94.6	109.4	111.1
ENPP6	81.1	79.2	91.2	83.2	74.8	92.9	74.8	86.9
ENPP7	99.4	121.3	96.9	93	87.8	100.7	96.6	115.8
ENSA	106.7	108.8	93.9	88.6	86.5	91.2	101.3	115.1
ENSA	2040.1	3166.6	667.3	702.8	670.5	651.2	1956.3	2325.6
ENTH	3083.3	3730.9	1988	3226.2	2485.2	5318.7	1823.7	3576.5
ENTPD1	274.1	276.9	139.2	138.9	107.9	148.2	249.2	257.4
ENTPD2	71.6	79.5	85.3	88.5	81.7	78.8	80.5	86.3
ENTPD2	96.9	115.5	90.7	86.8	76.8	79.1	94	108.1
ENTPD3	87.7	107.6	107.5	82.1	66	82.2	101.9	113.8
ENTPD4	232.7	257.5	207.3	136.9	149.4	131	182	251.8

ENTPD5	128.6	157.9	101.8	94.1	88.6	95.5	119.4	123.1
ENTPD6	445.9	496.1	448.9	318.8	307.6	461.5	355.6	563.8
ENTPD7	180.3	216.7	134.3	108.5	100.5	119.1	131.9	196
ENTPD8	84.8	97.3	85.6	86.8	87.8	87.2	88	91.7
ENTPD8	135.5	136.2	96.9	84	82.2	89.9	112.8	161.2
ENTPD8	94.1	106.8	89	83.7	83.7	89.1	90.7	103.3
ENY2	9037.4	10279.4	5292.7	3734.3	2990.7	4655.5	8237.7	12238.4
EOMES	152.7	187.5	111.7	95.1	85	95.2	118.4	167.7
EP300	212.8	270.2	264.7	644.4	562.9	658.3	216.4	520.3
EP400	409.2	589	394.6	912.2	591.3	909.8	436.8	745.6
EP400NL	88.8	94.9	101.3	80.3	84.8	90.4	87.3	91.3
EPAG	90.9	102.7	90.8	89.5	88	99.4	89.5	98.6
EPAS1	270.5	326	295.7	838.8	861.6	868.7	360.8	674.6
EPB41	127.6	153.8	91.6	81.8	77.8	84.5	109.6	160.2
EPB41	76.8	78	96.6	86.1	83.8	81.1	79.6	75.6
EPB41	111.8	128.4	94.1	97.9	91.1	103.8	105.2	129.1
EPB41L1	93.7	92.7	95.4	96.9	99	97	92	99.4
EPB41L1	112.7	131.9	98.5	84.1	76.7	87	116.8	118.5
EPB41L2	124.2	134	87.6	94.1	80.3	102.2	118.4	148.5
EPB41L3	318.5	425.9	383.4	1176	648.7	1971.8	406.5	844.9
EPB41L4B	150.1	183.2	98.6	99.3	96.1	98.9	140.3	172.8
EPB41L4B	124.7	167.2	88.5	90.2	93.9	87.7	138.2	158.5
EPB41L5	89.5	125.6	83.9	84.9	72.1	83.6	91.6	135.4
EPB42	77.7	88	84.1	81.3	82	82.9	75.9	93.3
EPB49	109.5	129	116	101.2	105.8	117.8	103.6	118.9
EPC1	177.1	217.5	149.7	141.4	130.9	147	150.8	165.8
EPC2	122.2	163.7	94.3	96	102.1	127	122.8	144.8
EPDR1	2755.3	3845.4	2290	2292.8	1656.8	2537.3	3139.1	5130.1
EPGN	319.9	439.4	149.7	122.2	109	146.2	277.6	293.3
EPHA1	131.3	158.8	156.4	120.9	99.6	135	118.8	125.1
EPHA10	105.9	115.1	121.1	105.2	104.9	123.8	99.3	109.2
EPHA10	97	97.8	100.9	86.5	90.9	92.9	87.6	98.6
EPHA2	92.8	97.3	90.5	85.2	85.8	87.2	90	104
EPHA3	96.4	108	95.6	91.9	85.8	86.3	86.2	97.5
EPHA3	77.2	85.1	99.9	95.8	98.7	102.4	83.2	79.4
EPHA3	94.8	97.1	84.4	79.6	76.7	87	85	103.1
EPHA4	83.4	93	93.8	90.9	88.4	87.8	94	86.4
EPHA5	110.7	112.4	84.3	80.8	87	85.2	100.4	123.2
EPHA5	81.1	79.8	84.4	76	78.2	72.8	78.2	86.3
EPHA5	122.8	135.5	85.4	87.2	80.8	84	111.2	135.8
EPHA6	84.6	95.5	88	85.4	82.1	80.2	84.4	94
EPHA6	117.3	129.9	120.8	107.9	102.1	108	111.9	117.5
EPHA7	84.1	96.7	100.7	102.1	96.1	90.7	87.8	87.8
EPHA8	124.3	139.1	115.7	125.9	103	101.7	112.8	128
EPHA8	78.1	81	96.8	89.6	90.3	87	76.1	82.5
EPHB1	207.8	254.1	223	495.7	317.3	697.2	191.5	395
EPHB2	107.9	143.5	106	87.8	89.8	88.6	109.2	128.2
EPHB2	100.6	110.6	84	80.1	82.5	86.7	99.9	94.9
EPHB3	112.5	130.6	125.3	121.6	107.8	114.4	94.4	122.9
EPHB4	152.1	180.6	175.8	172.4	150.6	195.5	125.6	181.8

EPHB6	110.1	123.5	138.9	100.8	93.6	123.4	106.6	160.9
EPHX1	196.7	258.9	168.6	132.7	127.2	137	242.7	357.3
EPHX2	159.7	195.6	100.8	107.7	98.5	131.9	149.7	178.9
EPIM	114.9	148.5	91.1	86.3	85.3	95	105.8	132.5
EPM2A	176	195.4	103.4	99.2	106.1	101.1	157.2	211.2
EPM2A	96.2	92.3	101	91.9	91.4	94.4	89	97.5
EPM2AIP1	410.4	531.4	399.8	437.7	347.7	557.6	298.6	702
EPN1	6023	8023.4	4871.5	3119.2	2589.3	3017.8	5668.1	7782.2
EPN2	80.5	98.4	92.2	99.3	85.5	88.2	86.1	99.4
EPN2	84	91.4	97.2	99.4	84.3	99	84.8	83.8
EPN2	91.7	99.6	104.6	111.9	99.4	96.2	84.3	108.1
EPN3	105	123.6	104	87.1	78.8	84	107.3	112.2
EPO	84.1	86.6	91.4	81.7	79.2	85.9	91	91
EPOR	193.6	213.3	131.4	128.3	110	132.1	181	254.8
EPPB9	114.8	128	104.1	92.4	80	93.6	108.7	133.4
EPR1	133.9	175.7	127.1	100.3	109.6	96.4	106.4	109.5
EPRS	8622.3	10916.9	5391	5064.9	4112.9	7097	6963	8737
EPS15	243.7	303.5	191.8	209.1	178.1	277.6	247.3	307.8
EPS15L1	175.3	211.3	167	162.3	148.5	153.1	165.3	198.8
EPS8L1	86	97.3	103.9	98.7	96.1	90.7	88.4	105.7
EPS8L1	124.6	140.1	127.6	107	98.7	106.9	133.2	169.4
EPS8L1	79.7	87.5	98.1	91.8	82.5	81.1	80.3	84.1
EPS8L1	121.2	149.5	97.6	79.6	81.7	76	111.5	143
EPS8L2	109.9	125.8	88.3	79.6	85	78.6	102.6	123.3
EPS8L3	111.9	141.5	93.6	89.1	88.9	92.9	110.8	111.5
EPS8L3	151	140	102.5	83.7	96.4	109.5	139.2	154.1
EPSTI1	214.4	300.2	210.8	188.4	191.8	212.2	190.7	216.3
EPX	120.6	136.9	97.4	83.8	78.5	89.2	105.7	128.7
ERAF	105.4	139.2	92.7	94.8	87.3	90.1	114.8	114.1
ERAL1	1577.2	1794.8	1096.6	843.4	605.4	1049.3	1350.7	2400.6
ERAS	89.8	98.6	87.9	99.4	83.9	88.6	84.7	104.1
ERBB2	96.1	99.8	79	81.2	75	68.7	96.3	111.6
ERBB2	103	128.3	111.4	99.1	97.9	102.2	106.1	111.6
ERBB2	97.7	118.6	117.5	122.2	118.8	145.9	99.7	106.1
ERBB2IP	193	253.8	131.8	124.9	123.4	142.2	156.3	174.3
ERBB2IP	105.2	127.2	107	109.9	111.5	118.6	109.4	121.8
ERBB3	114	133.7	119.3	91.2	98.3	98.7	117.3	120.7
ERBB3	88.5	92.2	94.6	94.5	89.2	91.2	81.6	91
ERBB3	102.5	111.2	94.1	82.5	86.5	96.4	105.1	99.5
ERBB4	118.1	149.3	141.8	134.6	128.7	134.7	136.7	119.2
ERC1	103.8	123.4	93.9	86.1	92	88.2	105.9	123.4
ERCC1	1265.5	1673	1339.4	1077.4	833.2	1245.7	1175.5	1786.8
ERCC1	2104.9	2796.6	1476.6	1062.5	771.1	985.1	2044.9	2111.3
ERCC1	124.5	151.6	101.9	99.1	92.7	107.3	119.6	143.4
ERCC2	530.6	715.6	353.7	261.1	256.7	298.1	501.9	464.3
ERCC3	508.7	659.2	437.9	465.9	454.2	761.2	466.9	697
ERCC4	146.8	162.2	118.8	119.2	99.2	100.3	124.4	138.6
ERCC5	1003.4	1188.6	804.4	1099.2	943.9	1682.8	731.1	1395.9
ERCC6	103.3	108.9	95.9	85.3	91.6	88.1	96.2	125.2
ERCC8	66.6	81.8	89.7	86.3	86.5	85.2	79.2	78.6

ERCC8	326.3	440.1	177.3	180.6	153.7	184.6	262.7	351.5
ERCC8	76.8	87.5	93.5	82.5	80.9	85.2	75.4	85.2
ERCC8	277.7	346.9	155.4	154	121	183.5	207.2	295.1
EREG	103.9	109.8	99.2	96.7	100.4	95.4	103.4	116.3
ERF	252.8	314.8	194	131.9	134.3	158.4	237.3	363.6
ERG	71.7	99.4	93.6	90.1	78.7	82.7	78.3	81.3
ERG	96.3	107.1	94.7	92.4	91.6	85.7	90.9	111.2
ERG	128	157.7	113.6	107.4	96	98.1	138.2	138.4
ERGIC1	406.9	589.3	186.2	211.4	184	221.2	342.4	486.7
ERGIC1	157.3	205.9	103.7	105.8	109.3	96.3	129.7	164.3
ERGIC1	134.9	149.2	97.1	114.2	99.3	100.2	131.2	160.1
ERGIC2	680.1	776	231.7	245.2	181	238.2	611.5	1173.5
ERGIC3	1657.8	2377.5	925.8	665.6	626	662.7	1612.8	1909.4
ERGIC3	4474.2	6189.2	3939.2	2985.3	2494.6	3718.3	4368.4	6024.6
ERH	5636.6	7685.8	2472.3	1925	1652.8	2234.9	4989	5164.2
ERICH1	1603.9	1994.6	931.6	904.4	706.8	1192.4	1271.2	1712.2
ERLIN2	209.7	238.9	139.3	142	135	146.2	171.1	181.8
ERMAP	144.4	169.8	101	158.5	143.9	111.2	112	124.9
ERMAP	747.2	888.5	350.9	458.7	385.9	521	684	977.5
ERN1	221.3	310.5	168.1	175.5	158.3	191	186.3	199
ERN1	79.3	85.4	85.6	85.6	79.9	78.5	86.4	85
ERN2	134.7	145.7	96.6	89.8	90.5	99	130.7	156.9
ERO1L	415.5	540.7	274.8	244.7	199	294.8	333.7	472.5
ERO1LB	160.2	183.2	128.7	114.2	102.2	133.2	160.2	299.6
ERP29	2601.9	3891.3	1729.1	1620.2	1385.7	1050.2	2311.1	2954.7
ERP29	10623.9	12747.9	8717.3	9618.2	8848.4	12274.9	10033.6	12038.7
ERRFI1	217.4	265.8	137.5	154.5	151	202.1	213.6	341
ERV3	95.9	109.6	109.1	149.7	131.5	141	96.1	98.3
ERVWE1	110.7	117.5	98.4	85.6	94.1	89	98.4	108.7
ESAM	121.8	110.1	101.7	93.5	91	95.5	121.1	137.9
ESCO1	150.2	176.8	104.5	120.9	103	139.4	142.3	247.5
ESCO2	184.8	246	106.9	84.6	94.1	92.6	168.1	267.9
ESD	848.2	952.7	188.7	201.8	144.3	205.6	674.6	880.7
ESM1	87.3	100.9	84.6	80	80.5	78.9	107.5	124.5
ESPL1	240.5	308.8	251.3	226.8	192.5	270.3	243.3	325.2
ESPN	160.1	176.3	116.5	102.2	99.9	104.7	159.1	170.6
ESPNL	82.1	78.5	79	82.4	76	81.1	80.3	86.5
ESR1	84.8	91.6	83.7	84.2	86.1	89.1	85.9	86.4
ESR2	98.7	109.3	90.4	90.3	81.1	90.3	101.5	119.9
ESR2	90.2	101	98.7	94.7	88.7	91.8	90.1	94.9
ESRRA	314	412.3	314.1	188.4	153.4	298.7	237.1	371.9
ESRRB	102.3	116.7	102.7	94.3	95.5	90.2	110.1	112.5
ESRRG	142.2	151.6	98.9	101.1	101.3	95.2	140.7	137.8
ESRRG	86.3	103.8	96.5	88.6	87.1	90.1	85.5	99.5
ESRRG	68.4	73.6	73.3	72.2	69.3	76.1	63.5	74.2
ESSPL	74.1	82.8	78.6	84	72.6	77.1	76.7	86.2
ESTRRA	398.1	433.8	206.7	168.8	149.1	181	357.4	361.5
ESX1	90.6	99.4	96.4	84.2	83.2	81.3	92.8	106.8
ET	306.8	367.4	260.1	406.7	391.5	349.7	328.7	468.5
ETAA16	98.2	108.9	101.1	98.2	86.2	93	90.3	122.4

ETF1	4445.7	5827.1	2074.3	2001.1	1413.8	2289	4844	7641.4
ETFA	6319.8	8894.7	4757.4	4905.8	3852.5	6867.5	5109.7	7207.4
ETFB	70.8	77.8	76.6	79.5	72.7	76.1	72.5	69.8
ETFB	3371.3	4303.5	2249.8	1933.6	1785.2	2022.4	3412.6	3734.6
ETFB	2601.3	3388.8	1554.6	1917.1	1605.3	1969.9	2587.6	2651.5
ETFDH	634.4	893.6	528.5	473.6	380.3	705.7	551.2	890.9
ETHE1	127.7	151.9	100.6	101.1	87.3	95.5	111.3	146
ETNK1	99.6	106	96.4	97.1	96.9	101.9	93.5	113.8
ETNK1	242.2	309.3	134.2	138.5	116.9	150	223.5	365.8
ETNK1	305	366.8	170.3	190.2	140.4	203.1	305.6	519.7
ETNK2	85.1	107.7	101.9	85.9	77.3	82.7	90.3	90.9
ETS1	419.6	527.7	230.2	225.2	192.4	288.9	367.6	480.1
ETS2	446.1	587.5	513.1	543.5	567.9	675.6	305.9	529.6
ETV1	79.3	77.9	81.4	77.2	75.8	80	75.6	79.7
ETV2	148.5	149.9	95	88.4	91	87.8	131.8	166.2
ETV3	137.1	141.7	107.1	126.3	119	100.4	123.9	159
ETV4	161.8	172.6	152.6	109.7	102.8	154.2	143.3	188.5
ETV4	111	148.1	113.8	91.9	82.7	109.3	122.1	148
ETV5	468	609.7	450.1	523.3	353.7	488.8	549	755.1
ETV5	139.2	174.4	101.8	112.7	92.7	95.5	148.9	180.9
ETV6	466	606.1	250.8	268.4	234.8	261.3	417	426.3
EVA1	107.5	119.1	101.8	85.9	83.7	86.5	112.4	111.3
EVA1	128.3	149.8	96.8	98.4	94.2	95.7	122	149.5
EVA1	76.9	70.3	92.2	82.7	72.8	78.7	67.6	75
EVC	117.2	149.5	103.5	103	95.1	91	114	111
EVC	121.9	150	103.9	93.4	78.3	100.3	117.1	153.1
EVC2	118.8	160.9	103.7	104.7	93.5	106.7	126	146
EVI1	78.2	84.1	93.9	76.8	89.7	92.2	84.5	88.5
EVI2A	240	295.4	129.9	137.9	121.9	154.4	237.6	327.5
EVI2A	109.1	130.3	102.5	87.3	95.7	103.6	113	125.1
EVI2B	109.8	136.1	93.5	95	84.1	89.6	107	121.3
EVI5L	498.5	703.8	405.1	318.9	295	348.4	497.3	516.4
EVL	3444.2	3978.4	2110.8	1847.4	1874.4	1907.3	2805.1	2652.3
EVPL	88.3	100.8	91.6	104	91.2	85.2	95.4	91
EVX1	135.8	132.1	87.4	92.7	79.7	82	114.7	142.7
EVX2	118.1	119.4	94.9	82.7	82.7	83.8	96.8	122.4
EWSR1	2795	3104.6	1603.1	2190	1679.8	2986.6	2696.8	3086
EWSR1	260.5	268	124.3	98.5	97.5	129.5	201.7	265.3
EWSR1	2689.3	3653.9	2292.3	2690.6	2073	3937.8	2173.8	3536.3
EXDL1	109.2	119.5	94.6	78.1	81.2	80.1	107.1	111.8
EXDL2	279.1	345.2	310.3	282.4	226.2	332.1	246.1	425.4
EXO1	1978.1	2680.2	904.6	884.1	719.8	1185.7	1411.1	1515.8
EXO1	350.3	488.8	232.6	170.1	161.5	255.5	224.4	273.7
EXO1	91.9	129.6	88.2	89.1	88.5	83.8	89.6	103.6
EXOC1	147.2	182.2	140.2	152.4	154.8	121.7	160.5	151.6
EXOC1	387.5	435.1	151.2	157.4	133.7	223.5	267	422.5
EXOC1	505.7	683.4	369.2	426.5	344.4	699.3	419.7	1066
EXOC2	160.7	171.6	132.1	164.2	121.1	207.2	128.2	174.7
EXOC3L	88.6	96.5	103	86.9	86.4	93	89.3	100.8
EXOC4	192.5	271.6	115.9	158	149.8	144.2	155.3	175.2

EXOC6	106.7	129.8	113.2	95.3	94.6	107.7	108.1	110.2
EXOC6	438.1	530.2	273.8	216.7	196.2	311.5	373.3	601.5
EXOC6	116.4	106.5	92.1	85.7	100.1	83.7	105.7	126.2
EXOC7	770	839.3	911.6	661.7	582.4	851.1	715.8	1029.8
EXOC7	139.7	159.9	128.6	146.2	112.4	134.6	121.7	142.8
EXOC8	116.1	127.4	116.2	94.5	92.1	100.2	136.6	135.9
EXOSC1	3016.7	3893.1	2161.2	1505	1127	1753.3	2755.7	3076.5
EXOSC10	975.2	1109.2	555.2	525.4	406.5	646.5	882.8	1325.9
EXOSC10	2634.5	3246.9	896.4	861.6	819.5	1032.2	2131	2390.7
EXOSC10	956.5	1350.4	683.1	571.5	515.8	907.5	661.4	843.3
EXOSC2	1243.2	1641.8	851.6	643.1	502.9	617.3	889.1	1142.3
EXOSC3	4182.3	5411.8	2028.2	1263.7	886.5	1584.7	3277.5	5178.2
EXOSC3	5859.9	7377.5	2395.2	1659.4	1275	1930.3	5241	6455.6
EXOSC4	982.3	1368.2	630.4	405.4	321.9	458.3	909.9	1177
EXOSC5	1154.2	1561	771.7	482.2	380.3	600.7	1006.4	1034
EXOSC6	1074.7	1523.5	971.4	700.5	538	645.6	1195.1	1518.5
EXOSC7	1844.2	2383.8	1245.6	1023.2	744.4	1081.3	1623.1	2020.9
EXOSC8	2753.4	3066.6	963.5	907.7	656.1	987.9	2667.3	3601.2
EXOSC9	826.7	1119.7	232.8	169.2	146.2	213	734.1	1223.4
EXOSC9	1468.5	1973.4	516.4	324.6	269.2	471.8	1194.1	2277.8
EXPH5	108.3	112.4	100.5	94.5	85.7	82.4	110.5	115.9
EXT1	132.1	158.9	138.1	116.2	110.3	110.3	122.5	151.8
EXT2	74	78.3	80.6	80.4	85.1	81.7	77.4	92.7
EXT2	470.7	617.2	332.1	268.4	237.6	358.5	396.6	484.4
EXT2	307.7	398.9	204.4	183.7	142.7	201.3	283.6	364.1
EXTL1	128.6	150.3	107.9	93.3	96.8	103.4	122.4	129.1
EXTL2	302.5	386.9	160	125.4	115.9	111.7	212.8	360
EXTL2	454	530	376.3	251.8	196.9	303.5	336.3	776.9
EXTL3	160.8	213.1	236.9	208.3	180.8	246.4	179.3	220.6
EYA1	109.7	136.3	102.9	81.2	88.1	89.5	93.7	125.4
EYA1	74.3	90.6	78.9	75.1	73	82	78.2	93.5
EYA1	90.9	99	88.6	86.4	89	87.6	79.4	111.8
EYA2	116	150.1	104.8	107.5	91.4	96.8	134.6	135.8
EYA2	84.7	96.9	87.4	94.4	80	95.6	84.3	90.5
EYA2	90.3	98.5	91	97.8	87.5	91.3	98	106.1
EYA2	87.3	84.5	94.4	99.8	92	95.8	78	88.7
EYA2	88.7	102.3	93.2	73	77.5	76.7	89.2	92
EYA3	122	140.6	115.8	114.1	111.3	117.1	104.6	126.8
EYA3	73.3	85	93.4	97.3	84.3	75.3	93.7	94.5
EYA4	94.7	114	88.4	95	84.4	85.2	104.2	115.7
EYA4	85	97.4	100.8	93.3	82	88.3	85.5	93.9
EYA4	94.8	101.4	100.3	89.1	93.7	90.8	102.2	92.1
EZH1	99.4	120.9	115	113	99.1	101.3	103.5	105.6
EZH2	829	1259.2	354.5	341.8	327.2	228.9	670.9	986.9
EZH2	567.8	610.4	317.3	238.1	244.6	355.8	459.3	663
EZH2	778.5	1083.9	577.3	456.9	484.1	687.1	541.4	703.8
F10	78.4	90.5	89.9	84.1	83	93.6	74.3	87.2
F11	95.3	105.2	105.6	96.2	97.4	97.4	90.1	90.4
F11	97.4	100.5	89.7	86	85.5	91.7	86.7	88.6
F11R	97.2	111.1	127.3	99.3	97.5	128.4	101	107.7

F11R	121.3	118.6	95.3	86.1	88	94.5	105.3	106.2
F11R	246.5	300.7	119	157.8	133.2	193.2	260.9	304.7
F12	194.7	226.1	147.3	104.7	105.1	114.7	165.7	163.3
F13A1	97.6	144.6	107.4	233.5	185.5	296.7	97.1	136.8
F13B	84.2	88.9	87.3	90.4	78.4	86.5	78.3	82.8
F25965	150.2	170.3	113	107.5	95.2	105.9	140.3	187.5
F2R	27023.9	28483.7	19804.1	25167.9	27558.7	23013	27042.7	29726.1
F2RL1	83.9	85.3	85.4	88.2	80.3	89.8	86.9	100.4
F2RL2	91.7	105.9	83.6	74.5	77.9	79.8	93	106.4
F2RL3	143	175.7	120.2	109.6	100.1	106.3	159	144
F3	166	157.8	110.9	124.9	135	144.9	184.6	269.6
F5	141.7	142.8	101.1	100.3	84.6	98.4	121.5	139.4
F7	73.7	80.7	85.9	78.4	80.3	77.7	78.2	74.9
F7	126.1	115.9	97.8	92.4	94.6	98.7	108.2	115.2
F7	108.6	120.9	84.1	87.1	80.9	94.1	108.5	125.1
F8	112.1	126.9	98	95.2	97.1	99.1	112.8	106.9
F8	86.2	87	99.5	94.9	94.6	91.3	89.8	81
F8	93.8	124.5	86.8	83.2	85.3	88.6	104.7	114.2
F8A1	513	690.5	465.8	328.8	275.4	376.1	485.4	774
F8A3	123.4	157.9	94.2	83.5	88.3	94.6	123.7	141.5
F9	97.1	102.9	112.4	102.7	98.5	88.1	102.1	98.9
FA2H	72.4	90.6	90.6	84.7	81	77.3	90.8	100
FAAH	101.3	140.4	108	94.2	96.9	125.3	103	140
FABP1	86.7	87.2	96.5	84.7	85	84.6	82.9	89.4
FABP2	93.1	96.1	107.2	107	97.7	97.7	90.8	89
FABP3	126.8	141.9	95.3	87.9	75.4	87.6	119.5	132.7
FABP4	113.2	135.9	92.3	94.6	86.9	98.9	90.6	130.9
FABP5	120.4	134.6	93.1	95.9	93.5	86.4	117.8	143.3
FABP5L3	107	138.1	120.6	99.7	95.1	101.8	118.1	122.4
FABP6	83.8	95.1	84.4	76.1	81.2	88.8	95.3	89.7
FABP6	102.8	106.4	88.3	83.8	83.1	93	110.5	105.6
FABP7	179.8	208.5	119.3	118.2	96	107.6	173	193.8
FADD	1358.4	1990.5	776.3	653	600.1	826.1	1412.4	1488.3
FADS1	990.1	1540.3	1100.6	952.8	1094.7	1254.9	1031.6	2148.2
FADS2	170	228.2	161.8	130.5	140.8	134.2	154.7	218.5
FADS3	152.3	157.5	97	91.7	91.4	84.6	142.1	163.3
FADS6	130.5	145.5	97.1	86.2	81.7	92.5	134.1	157.5
FAF1	1311.6	1691.3	707.5	719.1	664.4	937.6	1249.9	1584.6
FAF1	1477.5	2315	654.5	420.7	340.4	672.6	1014	1565.3
FAH	1060.4	1530.6	583.2	543.5	467.4	776.7	1184.9	1757.4
FAHD1	611.7	723.7	224.1	229	190.2	325.7	420.3	614.7
FAHD1	346.6	515.8	169.6	153.5	121.3	167.8	339.3	507.3
FAHD1	95	95.6	91.6	91.1	87.2	85.6	91.9	110.7
FAHD2A	157.4	192.9	116	109.8	121.5	121.5	141.8	155.7
FAHD2B	391.1	466.4	300.1	306.8	276	433.8	324.1	420.5
FAIM	122	145.3	94.8	89.7	80.8	87.2	119.8	169.9
FAIM	70.7	74.6	96.2	84.2	95	88.8	78.8	78
FAIM	112	142.2	97.8	92.2	79.4	91.4	111.7	126.7
FAIM	108.7	112.2	101.2	91	87.5	97.5	98.4	97.4
FAIM	513.9	601	313.9	179	176.5	233.1	334.3	442.1

FAIM2	83.4	96	105.4	93.5	85.4	96.8	94.6	90.5
FAIM3	89.1	93.6	95.1	87.1	84.2	90	86.1	90.1
FALZ	99.2	109.7	103.5	103.8	93.7	101.5	97.3	96.6
FALZ	162.7	222.2	113.9	127.5	114.2	138.8	173.3	208.3
FALZ	97.8	111.4	93.4	101.9	102.4	127.6	92.4	104.9
FAM100A	140.4	183.3	246.8	176	157.9	234.6	139.1	260.4
FAM100B	154.7	235.3	175.3	149.2	139.5	184.4	203.5	349.3
FAM101A	97.5	130.8	94.3	87	78.9	85	119	124.5
FAM101B	365.4	543.2	288.1	432.2	285.1	275.8	379.2	612.7
FAM102A	179.1	198.6	115.7	116.7	113	110.6	143.6	167.8
FAM102A	85.9	101.9	89	96.3	89.6	88.3	80.8	99.1
FAM102B	674.8	904.2	259.5	280.2	189.7	418.9	714.8	1345.1
FAM103A1	519.5	627.5	240.7	321	227.8	458.1	518.9	963.9
FAM104A	1537	2193.3	953	1005.9	860.6	1603.4	1312.7	1881
FAM105A	499.2	709.3	169.4	147.2	151.9	259.3	342.8	363.1
FAM105B	515.4	702.7	495.5	467.6	377.8	529.3	547.7	743.4
FAM107A	87.8	114.3	93	85.9	88.5	79	103.9	102.3
FAM107B	2260.4	3232.3	1189.1	1213	890.6	1060.3	1640	2069.2
FAM108A1	75.9	88.9	80.1	79.9	73.3	81.2	72.2	78.5
FAM108A2	590.8	923.2	513.5	287	291	203.3	497.9	466.9
FAM108A2	232.7	292.5	152.7	103.1	106.2	112.6	194	270.1
FAM109A	98.4	125.2	111.9	108.7	110	118.1	103.7	136.1
FAM109B	83.1	88.6	115.3	103.1	102.7	99.3	89.5	83.5
FAM10A4	123	164.9	84.2	84.4	73.5	81.2	104.9	132.6
FAM10A6	85.8	90.2	103.1	94.2	90.9	84.9	85.2	85.5
FAM10A7	463.8	596	167.9	156.6	133.1	213.5	346.6	566.4
FAM110C	100.4	101.6	90.4	87.2	83.4	81.6	96.3	94
FAM111A	2618.2	3082.4	1109.8	1038.6	962.2	1036.5	1739.9	2119.6
FAM111A	941.4	1233.3	643.2	636.2	586.6	947.1	649.6	891
FAM111B	163.6	197.2	119.4	118.1	97.4	118.2	155	172.7
FAM112A	112	124	101.8	85.1	90.8	89.3	114.1	115.5
FAM112A	68.8	75.2	97.8	86.9	80.9	83.5	71.7	75.3
FAM112B	5480.8	7115.9	1301	1194.4	906.1	1760.4	4941.3	6575.4
FAM113A	239.6	252.7	134.4	150.3	135.2	204.4	187.6	250
FAM113B	242.5	330.6	170.8	167.6	144.2	167.9	171.2	145.3
FAM119A	2422.4	2288.7	1353.9	1754.3	1334.4	1443.2	2162.9	4156.6
FAM120A	5211.1	5785.9	2016.3	2189.7	1428.4	1882.7	4449.7	7297.1
FAM120C	91.3	98.4	101.9	93.9	80.2	90.2	93.1	109.2
FAM122A	148.4	181.5	93.8	90.5	89.6	99	138.5	155.9
FAM122B	786.1	915.4	646.3	760.3	628.6	1333.8	512.7	931.2
FAM122C	98.4	121.9	109.6	92.3	97.6	99.5	109.7	119.6
FAM123A	87.8	94.4	97.3	91.5	83.8	85	82.3	98.6
FAM125A	1353.5	1802.8	722.1	373.3	318.5	353.6	1315.7	1280
FAM125B	145.9	166.4	110	98.2	94	99	131.9	161.5
FAM125B	70.8	76.9	85.1	85.4	85	91.7	74.2	79.9
FAM126A	73.9	84.9	96.8	90.4	83.6	81.4	75.7	83.9
FAM126B	79.2	85.1	97.5	87.5	82.4	83.7	81.3	86.6
FAM127B	91.5	101.9	94.5	98.7	94	99.9	96.6	97.9
FAM127C	135.7	148	102.3	91.8	83.2	99.4	125.4	154.9
FAM129B	112.2	124	105.2	85.3	83	86.1	96	116.1

FAM129B	135.9	151.8	144.3	103.7	90.9	107.7	143	223.3
FAM12A	102.9	123.2	135.5	115.1	115	118.6	102.7	105.4
FAM12B	94.4	94.1	104.1	96.4	107.8	94	91.8	104.9
FAM130A1	160.3	176.8	152.8	163.6	148.1	180.3	155.2	283.1
FAM130A2	71.7	81.7	98.6	86.8	82.7	96.4	76.4	83
FAM13A1	106.5	113.2	86.3	89.9	73.3	79.6	122.9	119.5
FAM13A1	92.8	110.6	93.3	89.4	83	81.7	104.8	113.7
FAM13A1	95.7	110.1	101.9	88.7	79.9	88.4	98.9	118.7
FAM13C1	91.9	108.9	85.6	85.7	79.3	86.2	90.7	100.7
FAM13C1	72.3	82.7	84.7	80.4	79.9	86.1	77.9	84.2
FAM13C1	86.5	87	86.2	87.1	73.4	78.2	88.3	94.5
FAM14A	798.9	936	253.1	215.5	207.9	221.3	788.8	842.9
FAM14B	164.7	205.7	127.4	105	106.3	112.4	173.8	174
FAM18B	336.1	435.7	148.9	151.7	127.7	203.5	316.6	555.8
FAM18B	546.9	681.4	274.9	332.7	267	489.3	490	1052.2
FAM18B2	104.6	116.5	111.8	89.3	90.5	90.3	97.7	101.7
FAM19A1	76	86.8	90.8	86	82.8	79.6	76.5	85
FAM19A2	81.4	93.3	102.6	90.9	96.7	98.6	75.7	83.6
FAM19A3	81.7	91.6	86.8	80.8	76	83.1	82.8	99.4
FAM19A3	138.5	191.4	91.7	101.8	92.3	93.2	119.6	146.5
FAM19A3	69.4	90.8	80.9	73	70	75	84	89.4
FAM19A4	120.9	148.9	115.5	111.2	104.1	107.1	119.7	136.8
FAM19A4	88	102	101	83.8	86.2	93.9	90.7	107.2
FAM19A4	95	104.7	93.9	93.6	84.2	109.8	99.3	111.7
FAM19A5	103.6	108.6	91.8	86.3	83.7	91.7	99.8	97.3
FAM20A	85.5	89.6	89.6	83.9	82	91.6	79.9	95.6
FAM20B	1271.2	1549.9	690.4	750.9	556.9	864	1006.4	1516.8
FAM20C	95.9	107.5	100.9	85.7	85.9	90.1	89.6	94.4
FAM20C	230.2	317.6	264.3	160.4	135.3	185.8	235.2	402.6
FAM20C	140.2	175.6	125.7	128.1	95.2	107	145.6	163.6
FAM21C	145.2	183.1	125.7	114.1	108.9	114.3	132.3	156
FAM22G	86	103	79.6	83.3	81.5	87.1	88.1	97.4
FAM24A	83.9	104.4	93.2	81.4	86.1	90	79.8	95.1
FAM24B	370	436.1	246.6	221	169	217.1	333.8	357
FAM26B	159	177.7	120.9	109.1	104.7	108	143.3	208.5
FAM26C	185.7	252.9	119.9	107.6	113.9	137.5	199.6	243.7
FAM27L	112.2	122	84	94.3	74.7	91.7	99.8	117.9
FAM29A	88.9	95	92.4	86.6	78.4	85.7	91.1	104.9
FAM29A	115.4	122.3	108.7	116.6	88.1	125.8	105.2	146.8
FAM33A	770.1	950.1	433.7	324.5	267.5	342	705	1031
FAM35A	314.5	423.4	155.3	144.3	128.5	226.2	247.5	411.7
FAM36A	444	521.1	147.7	181.9	130.7	167.6	391.2	580.6
FAM38A	870.2	1020.9	1456.8	1394.8	1316.4	2258	759.9	1210.4
FAM38B	142.3	170.6	141.2	132.9	128.4	131.9	141.8	155.1
FAM3A	873.2	988.4	722.3	679.2	576	703.3	802.6	1162.8
FAM3B	82.2	98.2	86.3	78.6	82.1	83.5	91.9	115.2
FAM3B	86.1	101.7	92.2	89.4	86.2	87.9	98.1	106.1
FAM3C	412.7	560.4	271.5	254.9	190.8	335.3	333.4	555.2
FAM3C	469.9	625.8	236.3	261.2	169	294.2	364.8	607.3
FAM3D	91.1	110.9	99.9	97.5	83.7	93.7	97.1	105.9

FAM40A	363.9	428.1	214.9	166.3	147.6	164.9	342.4	387.8
FAM40B	240.4	270.9	145.9	246.1	247.3	174.2	223.4	260.4
FAM43A	1872.7	2217.6	1466.7	1278.7	1009	1466.5	1773.9	2161.2
FAM43B	84.9	113	99.4	90.2	88	94.1	99.3	96
FAM44A	84.2	92.1	94.9	88.3	75.7	88.9	93.7	87.3
FAM44B	992.3	1249.2	516.8	658.4	516.4	772.5	768.8	940.2
FAM45A	1571.9	1609.3	607	588.2	570.8	731.7	1479.9	2942.5
FAM45B	102.2	140.9	108.4	95.1	90	92.1	110.3	124
FAM46A	12692.1	17940.4	7778.8	11207.5	9785.1	13988.3	12062.9	13583.9
FAM46B	99.7	107.5	103.6	108.1	91.8	88	100.2	113.4
FAM46C	113.8	129.7	112.3	174.4	203	261.3	115.9	131.8
FAM46D	77.1	88.8	89.8	87.6	83.9	95.7	88.3	92.1
FAM47A	89.6	94.9	92.1	85.1	86.2	82.4	92.9	83.8
FAM47B	88.5	100.2	95.3	84.8	75.1	85.3	97	102
FAM47C	84.5	106.9	105	84.2	84.2	95.4	96.3	99.9
FAM48A	401.4	416.1	311.5	381.8	341.8	577.1	291.5	441.5
FAM48A	90.1	95.3	93.5	89.6	87.5	83.5	84.2	98.5
FAM49A	114.6	149.8	105.3	90.9	87.9	96.1	108.2	122.1
FAM49B	1109.3	1186.5	280.8	292.8	197.4	396.9	1075.7	2241.6
FAM50A	3120	3910.6	2557.8	2483.9	2283.7	2742.1	2468.2	2962.9
FAM50B	78.4	91.8	105.3	98.2	93.9	96.7	88.5	90.1
FAM51A1	139.7	176.6	139.4	116.3	109.1	133.2	111.4	149
FAM53A	135.7	145.5	94.4	102.8	91.4	105	135.1	157.8
FAM53B	158.8	175.8	108.6	94.9	82.1	98.5	151.4	171.7
FAM53C	1528.5	2000.3	833.1	839.3	754.6	849.7	1371.6	1072.1
FAM54A	242.3	296.7	118.3	135.6	114.2	128.5	201.9	304.4
FAM55A	81.5	73.4	96.7	83.9	79	86.8	83.4	78.2
FAM55B	75.8	86.9	91	81.8	76.4	86.6	80.1	74.3
FAM55C	277.2	411.8	193.6	199	152.9	151.4	236.3	303.6
FAM55D	96.1	101	87.8	83.4	89	87.2	90.6	94.9
FAM55D	91.1	95.1	92.4	91.7	85.6	91.2	98.2	98.7
FAM55D	80.6	86.2	96.5	80.5	77.9	84	86.8	89.4
FAM57A	269.1	324.6	282.4	155.5	157	171.3	236.3	352.4
FAM58A	946.5	1176	788.1	598.3	509.9	618.3	1014.4	1207
FAM59A	129.8	140.8	112.6	112.1	101.9	116.5	117.7	139.7
FAM5B	106.1	126.6	96.6	95	81.7	89.7	108.3	111.7
FAM5C	107.6	138.2	93	87.9	88.9	88	113.9	129.2
FAM62A	1296.7	1763	991	1195.7	917	1505.7	1058.7	1797.5
FAM62B	2429.8	3629.2	4103.4	5426.7	4416.2	6267.4	2257.9	3321.7
FAM62C	126.4	137	124.1	102.1	99.6	115.6	99.1	139.4
FAM63A	442.7	441.1	341	405.8	314.2	352.8	420	812.6
FAM63B	149.2	196.3	98.7	101.9	99.7	105.4	148.6	176.9
FAM64A	387.2	442.9	343.2	141.5	127.4	151.6	324.7	451.4
FAM65A	990.7	1257.4	715.4	578.3	540.9	612.5	792.9	700.8
FAM69A	206.2	248.3	154.7	147.8	132.4	141.2	171.8	189.5
FAM69B	898.7	1088.9	483.2	424.6	355.8	463	678.6	714.3
FAM69B	123.5	141.1	91	77.2	74.3	84	114.4	160.8
FAM70A	80.1	76.2	94.2	83	82.8	80	85.2	83.2
FAM70B	100.8	116	97.8	104.3	87.9	87.7	100.4	103.9
FAM71A	106.5	116.2	94.4	90.5	84.1	95.5	111.6	92.7

FAM71C	91.4	99.3	108	88.9	95.1	92.2	91.6	99.4
FAM72A	1050.7	1108.7	223.1	228.9	169.7	241	917.8	1696.1
FAM73A	113.2	115.2	94.9	85.7	85.1	87.7	110.1	115.1
FAM73B	155.6	179.6	155.1	139.3	134.4	208.7	139.1	220.2
FAM76A	97.6	128	107	102.6	96.9	108.8	106.3	117.3
FAM76B	292.1	380.3	226.3	232.1	194.3	343.8	210.4	522.2
FAM77C	99.9	115.5	91.3	94.1	88.3	88.1	108.5	104.5
FAM77D	112.1	155.8	106.5	103	99.3	101.9	120.2	130.5
FAM78A	114.4	139.5	156.8	124.1	117.2	141	110.3	156
FAM78B	86.9	103.9	94.9	99.3	94.2	92.8	86.1	95.9
FAM79A	1024.6	1385.7	614.6	664.7	661.5	725.1	952	1012.2
FAM79B	81	94.2	111.4	96.6	96.8	95.2	87.1	88.7
FAM7A2	481	476.9	186.4	292.7	285.5	192.4	305.4	406.5
FAM80A	121.6	142.4	111.2	103.9	107.2	119.6	131.2	146.9
FAM80B	96.9	95.2	102.5	102.5	86.4	122.3	93.4	106.3
FAM81A	163.2	180.6	140.3	99.3	88.9	110.3	112.5	163.3
FAM81B	142	171.4	103.5	96.7	91.8	91.3	136.3	147.3
FAM82A	80.1	88.9	95.8	94.2	93.8	92.4	80	87
FAM82C	750.9	959.7	480	552.8	393.8	713.3	678.5	1313.5
FAM83A	78.6	92.1	96.8	90.3	83.5	86.5	78.5	90.9
FAM83A	84.4	98.2	93.8	86.9	83.3	84.2	95.8	93.4
FAM83A	85.8	91.1	98	96.7	84.1	92.1	80.8	88.7
FAM83E	79	93.8	89.4	81.1	77.1	88	85.6	101.7
FAM83F	108.6	139.9	92.2	90.2	89.9	86.2	112.7	129.5
FAM83G	94.6	101.1	99.7	87.1	87.2	82.3	97.6	111.8
FAM84A	99.9	113	104.5	84.8	86.9	94.5	105.5	110.4
FAM84B	286.8	400.2	283.1	244.2	202.1	293.1	357.2	520.5
FAM86A	710.9	879.5	396.7	241.6	161.8	294.2	512.5	842
FAM86A	412.4	500.3	228.1	141	116.8	155.5	276	285.2
FAM89A	405	539.5	264	233	202.2	271.9	399.8	503
FAM89A	1037.8	1372.6	932.4	816.3	647.1	1172.5	1153.7	1736.6
FAM89B	348.8	455.8	339.9	217.4	213	263	321	502.1
FAM8A1	153.3	167.3	131.5	126.2	110.9	137.2	141.8	188.6
FAM90A1	95.9	104.4	107.9	105.8	110.2	109.8	93.8	108.4
FAM91A1	418.6	513.5	231.4	187.2	162.3	254.1	301.1	463.9
FAM92B	101.2	108.8	83.5	79.5	73.8	81.4	91.6	124.5
FAM96A	8072	8790.9	5473.9	5817.4	4958.7	6151.3	7034.2	10129.7
FAM96A	1903	2363.1	447.6	267.6	287.3	531.1	1802	2521.6
FAM96B	1710.4	2144.1	1238.2	794.5	801.4	735.5	1717.6	1815.7
FAM98A	1206	1304.5	507.8	459.6	273	504.8	1000.5	1552.5
FAM98B	106.3	124.2	84.6	90.6	79.6	86.4	98.9	122.8
FAM98B	103.5	153.3	108.9	186.1	149.8	119.8	125.2	119.9
FAM98C	202.5	229.8	189.6	138.6	148.7	179	172.2	198.9
FAM9A	69.5	70.2	88.2	89.5	81.8	83	83.1	79.3
FAM9B	76.1	81.2	93.5	90.9	79.6	84	79.5	80.5
FAM9C	100.7	119.8	105	89.7	99.7	99.5	106.4	112.3
FANCA	94.1	119.9	114	99.6	98.8	103.8	86.6	108.8
FANCA	100.7	131.3	94.4	91.4	82.9	88.6	93.4	124.6
FANCA	93.3	123	100.6	98.3	97.8	93	92.3	101.5
FANCB	319.2	394.5	133.5	162.3	138.1	150.2	264.7	368.2

FANCB	192.8	257.3	146.2	125.5	138.2	127.3	166.7	252
FANCC	156.6	176.1	95.2	95.1	96.1	95.4	134.3	144.2
FANCD2	1443.6	1560.5	853.7	935.5	1004.8	1105.9	1042.9	1264.7
FANCD2	142.8	143	125.9	116.1	116.3	108.4	121.1	145.6
FANCD2	212.2	287.5	145.3	142.4	113.7	133.4	201.9	228.2
FANCE	946.7	1293.7	512.8	653.9	640	769	684.6	630.3
FANCF	104.3	123.2	103	98.4	82.7	100.6	107	121.1
FANCG	1296.4	1652.7	712.7	398.5	346.4	320.4	888.5	977
FANCL	79	84.3	110.9	98.7	90.5	106.1	84.5	80.8
FANCM	110.6	154.6	94.7	90.4	81.7	89.9	115.7	128.6
FANK1	109.2	129.5	98.6	100.6	91.8	98.8	114.9	110
FAP	80.1	88.1	117.8	101.9	94.4	103.6	72.5	87.7
FARP1	95.4	100.1	100.2	94.4	88.3	86.7	102.2	103
FARP1	113.7	121	88.6	84.1	89.4	87.5	97.4	115.5
FARP1	131.1	143.2	132.8	97.1	101.5	91.8	109.8	113.2
FARP2	99.2	112.1	124	124.8	117.5	114.8	92.8	100.7
FARS2	451.1	689	357.6	321	261.6	371.9	378.4	511.8
FARSB	133.3	126.1	133.5	140.5	129.8	126	119.9	131
FARSLA	734	836.8	559.2	291.6	275.2	332.4	626	790.5
FAS	93.3	104	89.9	89.4	78.2	94.2	94.3	88.9
FAS	73.3	81.3	89.1	86.4	76.4	82.3	80.4	100.3
FASLG	100.9	124.9	101.5	88.5	98.9	88.5	107.9	105.7
FASN	7719.7	9292.7	7584.9	6120.1	5881.6	6645.8	6258.7	7610.4
FASTK	887.4	1003.3	391.5	410.8	321.5	416.9	624.5	752.1
FASTK	327.5	408.4	270	196.1	168	149.6	226.1	349.3
FASTK	272.1	336.2	232.6	208.6	194.2	256.7	234.9	393.8
FASTKD2	253.1	333.7	193.6	159.8	117.9	176.1	201.5	289.3
FASTKD3	458	559.6	286.1	249.7	191.1	317	284.7	465.7
FAT	710.1	1047.6	365.7	601.4	469.9	762.5	761.6	1052.9
FAT2	67.6	70.6	89	85.4	77	83.9	70.4	76.2
FAT4	122.4	145.8	83.6	100.8	89.7	85	102.1	157.6
FATE1	97.1	115.1	104.9	87.7	78.3	84.7	105.7	117.3
FAU	18903.3	23036.5	15885.6	12195.1	10555.3	14268.8	16048	18731.5
FBF1	122.4	119.9	113.9	96.7	94.1	98.6	100.8	111.9
FBL	4767.1	6490.5	2846	1859	1531.1	2002.6	4373.3	4452.7
FBLIM1	110.4	122.1	84.7	87.6	81.5	79.3	102.5	105.5
FBLIM1	97.1	111.1	111.7	97.6	101.6	96.1	86.1	98.1
FBLN1	100.3	113.6	94.1	96	74	85.7	89.6	91.6
FBLN1	100.9	124.8	116.2	101.5	98.9	91.7	102	127.4
FBLN1	427.5	598.3	313	135.7	125	146.4	323.4	353.5
FBLN1	85.3	92.1	85.9	87.4	84.4	81.2	87.8	85.4
FBLN1	119.8	143.5	105.7	90.9	82.3	93.4	119.7	120.6
FBLN2	64.9	79.8	88	91.5	80.1	87	72.3	78.7
FBLN2	107.1	134	96	85.8	81.7	89.4	108.1	123.2
FBLN2	102	115.8	95.5	90.7	85.8	87.1	115.1	123.1
FBLN5	132.9	159.3	108.1	99.7	98.5	104.2	111.6	137.8
FBN1	107.6	116.2	104.3	95.3	92.4	85.8	103.7	124.2
FBN2	529.1	874.1	563.5	1165.8	907.3	1103.7	486.6	604.2
FBN3	88.5	98.6	99.7	99.5	88.8	101.5	87.1	86.2
FBP1	656.3	815.9	508.2	353	252	341.2	502.9	615.8

FBP2	80.4	82.9	99.7	96.5	93	87.1	81.9	82.6
FBS1	123.3	133.5	132.3	123	110.7	134.2	121.1	159.3
FBXL10	125	134.1	91.2	79.3	84.5	82.4	108.3	145.9
FBXL10	3172.3	4123.9	2148.2	2867.4	2685.6	3814.1	2709.3	3537.3
FBXL10	116.4	148.7	94.4	99.1	88.1	92.4	102.3	121.7
FBXL12	284.3	347.9	151.3	140.1	118.5	131.8	243.7	298.9
FBXL13	95.3	118.6	94.3	85.2	92.7	102	101	103.5
FBXL14	84.8	81.1	88	98.5	93.8	90.7	84.2	91.2
FBXL15	304.9	393.1	301	210.1	214.5	264.3	245	304.3
FBXL16	83.3	92.2	103.7	96.5	83.5	85.3	91.6	90.8
FBXL17	160.2	183	115.7	108.5	95.2	145.2	121.9	170.3
FBXL18	106.8	122.6	91.1	87.5	86.3	88.1	107	118.2
FBXL2	115.7	134.8	105.6	92.3	86.8	102.9	104.2	121.9
FBXL20	441.2	598.8	265.4	241.1	243.5	294.6	381.2	366.7
FBXL21	159.9	183.9	95	106.6	80.9	92.6	152.6	160.7
FBXL22	84.7	78.9	82.9	89.8	86	92.7	75.9	81.5
FBXL3	85.7	97.4	83	93.2	84.1	76.7	85.2	108.1
FBXL4	83.5	77.7	100.1	94.3	91.8	88.4	75.5	80.4
FBXL5	98.6	107.6	90.9	84.9	75.1	86.4	99.1	95.2
FBXL5	200.5	237.1	123.1	120.3	109.9	119.6	179.6	269.3
FBXL5	921.5	1200.7	597.3	557	440	851.8	726.6	1086.3
FBXL6	633.9	688.1	598.4	315.5	290.3	498.1	402.2	711.4
FBXL7	123.1	164.1	96.2	94.8	82.7	97.3	111.8	172.9
FBXL8	102.4	107.6	101.1	101.7	84.9	102.3	99	113.7
FBXO10	86.4	93.9	94.6	95.1	93.1	89.6	80.6	102.7
FBXO10	134.1	154.3	172.6	140.6	121.4	148.3	121.6	216
FBXO11	206.1	242.9	155.6	180	135.8	186.5	177.2	204.1
FBXO11	1326.1	1860.7	714.7	956.8	841.6	1577.7	1167.2	1665.2
FBXO11	126.1	130.7	105.7	91.7	95.9	98.9	117.9	143
FBXO11	180	278.8	169.1	168.1	164.2	205	161.9	242.4
FBXO15	448.4	587.8	344.8	176.5	154.7	172.9	428.5	458.5
FBXO16	129.2	135.1	102	103.5	102.8	95.6	110.3	137.6
FBXO17	74.7	85.5	81.5	78.2	76.6	73.8	68.4	81.3
FBXO17	136.6	133.9	96.4	78.4	81.7	88.3	121.3	147.6
FBXO17	84	94.2	97.5	78.3	75.8	86.7	80	90.6
FBXO18	102.8	113.1	100.2	95.7	85.1	96.4	102.2	100.9
FBXO18	598	743	409.1	355.2	308.8	426.3	538.5	524.2
FBXO2	106.2	112.2	95.1	88.6	88.1	91	106.4	105.4
FBXO21	191.3	235.8	131.4	133	131.8	140.2	194.7	230.9
FBXO21	632.7	849.8	404.5	375.2	294.2	393.2	555.7	764.4
FBXO22	215.5	251.7	157.2	144.4	130.6	153	181.3	243
FBXO22	72.8	83.8	83.6	74.8	75.2	81.9	77	75.7
FBXO22	758.1	1034.2	393.6	318.3	255.1	432.2	542.2	655.8
FBXO22OS	91.1	100.9	102.4	96.6	94.2	101	101.1	120.2
FBXO24	108.8	119.9	94.8	99.8	86.5	88.6	101.7	117.6
FBXO24	89.4	113.2	97.8	93.8	85.1	94.8	89.2	102.3
FBXO24	87.4	110.8	97.2	101.2	90.5	93.6	90.5	89.9
FBXO25	102.5	108.6	84.3	77.5	78.1	77.7	108	121.6
FBXO27	89.5	89.7	92.8	91.8	85.9	90	90.1	85.9
FBXO28	1927.8	2755.5	986.1	814	618.3	972.9	1756.9	2634.8

FBXO3	83.4	107	104.2	98.1	86.8	101.2	104.8	105.8
FBXO3	520.4	696.4	247.1	180.8	170.5	262	391.5	553
FBXO3	152.9	179.4	138.3	109.7	107.4	111	122.4	165.3
FBXO30	192.1	230.7	145	159.4	141.7	209.4	301.1	685.1
FBXO31	293.2	371.5	273.1	230.3	181.9	254.3	289.8	444.2
FBXO32	171.1	193.2	113.6	111.1	106.8	110.5	156.9	192.7
FBXO32	93.9	103.2	109	105.1	90.8	96	96.2	106.5
FBXO32	179.5	214.8	149.7	152.8	138.6	201.3	177.7	233.2
FBXO33	1076.5	1362.7	814.9	817.7	599	949.4	1076.1	1540.3
FBXO34	542.3	681.2	435.1	405.8	341.1	642.6	420.5	779.3
FBXO36	107.6	125.4	82.5	80.2	85.2	81.5	111.5	114.6
FBXO38	113.6	133	111.3	98.6	88.7	100.5	109.3	116.3
FBXO38	165	184.7	115.1	122.7	116.1	148.8	173.8	244.5
FBXO39	84.7	90.3	91.6	80.2	90.5	88.3	84.8	89.5
FBXO4	1441.9	1946.1	978.9	707.2	562	1137.7	962.2	1376.1
FBXO4	330.5	493	131.4	143.4	111.3	157.7	274.3	411.4
FBXO40	116.9	139	94	84.9	80.9	86	111.4	128.3
FBXO42	102.3	157.3	90.7	87.1	76.6	91.5	105.4	165.1
FBXO43	172.3	190.9	131.4	102.3	115.6	124.1	136.5	142.5
FBXO43	182.6	204.5	147.3	126.9	129.1	133.4	131.5	144.5
FBXO44	100.9	121.3	109.9	104.2	103.6	100.7	96.9	113.7
FBXO44	125.3	134.3	97.9	86.4	90	93.5	111.4	135.3
FBXO44	78.7	86.7	89.5	76.7	82.9	79.7	76.2	86.5
FBXO46	143.7	199.7	171	158.9	144.1	156	157.9	207.1
FBXO47	72.9	76.4	90.7	87.3	84.8	85.9	76.2	77.8
FBXO5	202.3	239.7	133.3	161.2	144.2	202	178.4	259.1
FBXO6	400.8	546.4	231.4	253.9	222.4	256.3	324.7	341.1
FBXO7	606.9	738.6	295.4	196.1	157.2	185.3	450.7	564.4
FBXO7	171.4	211	117.7	97.1	94.7	106.1	153.2	173.2
FBXO7	944.6	1269.3	651.3	589.2	472.2	830.4	818.9	1124.1
FBXO8	421.7	596.2	208.3	228.6	205.1	267	413.6	646.5
FBXO9	91.5	99.7	89.2	87.1	88.1	90.9	86.8	88.7
FBXO9	380.2	495.2	171.6	136.7	123.2	119.2	308.3	304.6
FBXO9	97.6	124.4	95.7	83.9	84	89.2	105	116.1
FBXO9	108.1	110.2	97.1	82.6	82.2	83.5	90.6	103.8
FBXW10	110.3	123.5	86	83.2	81.9	87	98.6	107.1
FBXW11	82.3	88.1	92.1	93.3	80.2	89.1	79.3	90.5
FBXW11	97.1	106.7	87.2	89.5	86.4	85.7	103.9	109
FBXW11	1146.2	1349.4	832.9	1009.5	773.9	1157.8	902	1621.1
FBXW12	86.9	106	94.2	82.8	78.6	74.7	90.9	88
FBXW2	258.2	365.4	214.9	201.4	188.7	241.4	235.1	315
FBXW4	360.4	449.6	252.2	196	192.7	188.5	299.9	344
FBXW5	496.8	660.1	431.6	352.6	335.6	473.8	445.6	896.2
FBXW7	216.9	235	139.8	145.1	127	125.3	193.2	212.7
FBXW7	115.8	126.4	100	90.7	88.2	97.5	103.8	131
FBXW7	559.5	592.4	271.9	326.1	239.7	403.9	446	868.3
FBXW7	68.4	84.7	91.3	82.1	78.8	82.3	80.7	82.9
FBXW8	81.7	93.9	111.2	99.8	91.1	92	80.4	88.7
FBXW8	97.3	95	105.8	103	95.3	96.5	91.2	90.1
FBXW8	419.6	413.9	214.6	228.6	171.4	215.6	368.2	366.3

FBXW9	409.8	551.3	281.4	209.7	182.2	222.1	279.2	268.9
FCAMR	109.5	131	89.2	91.8	89.5	90.2	104.2	130.2
FCAR	497.2	463.6	440.5	658.8	601.7	481	554.9	974.1
FCAR	109.3	111.2	98.8	83.4	82.3	81	98.1	99.2
FCAR	92.8	97.2	98.7	98.5	103.1	98.1	86.7	90
FCER1G	11910	15217.3	9275.5	8566.4	5941.9	9223.7	10595.1	12807.4
FCER2	112.1	140.8	119.8	86.7	86.9	104.8	125.1	143.1
FCF1	288.6	369	179.8	136.1	124.5	102.9	230.6	348.4
FCGBP	121.6	142.6	86.3	90.8	83.9	92.4	117.7	141
FCGBP	90.7	93.1	101.9	91.3	109.3	97.3	82.2	98.1
FCGR1A	1536.7	2291.2	592.4	316.9	252.7	417.5	1178.8	1424.1
FCGR1B	3545.5	4509.8	1715.2	840.6	625.6	1005.7	2209.9	2482.2
FCGR1B	3239.4	4351.2	1792	931.9	667.9	1216.9	2243	2276.5
FCGR2A	652	928.6	260.1	292.2	205.7	333.3	478.5	704.8
FCGR2A	573.7	829.4	279.8	250.3	211.4	196.8	444.4	508.8
FCGR2B	114.8	123.2	104.7	142.6	116.2	193.8	100.9	120.1
FCGR2C	90	109.3	90.2	79.2	80.2	85.5	93.9	87.4
FCGR3A	81	95.9	84.5	82	85.5	87.7	80.8	88.3
FCGR3B	138.5	180.9	118.4	112	99	117.9	126.9	174.3
FCGRT	2163.8	2755	1861.3	1761.1	1533.2	2623.8	1839.9	2909.8
FCHO1	159.3	204.4	168.2	123.9	108.8	143	145.6	178.5
FCHO2	178.5	184.8	125.8	111.2	106.2	145.5	169.5	233.3
FCHSD1	105.4	114.8	87.8	90.8	83.9	96	111.7	105.8
FCHSD2	396.7	568.6	287	262.9	265	367.4	306.3	373.6
FCMD	8152.3	8812.4	3758.1	3866.1	4423.4	4311.4	7200.3	8720.8
FCMD	129.8	163.5	123.9	100.8	87.3	98	112.3	126.6
FCN1	98.9	102.1	98.8	84.7	88.7	93.4	102.7	106.5
FCN2	86.7	98	86.2	83.1	87.2	88.1	89	107
FCN2	114	115.4	88.4	92.9	79.7	84	112.3	108.6
FCN3	100.7	115	85.3	86.1	72.6	82.4	100.5	117.3
FCN3	110	134	85.3	77.1	70.3	81	110.4	126.1
FCRL1	91.5	95.7	92.2	86.8	77.9	91.5	98.3	92
FCRL2	90.6	98.5	79.7	76	87.6	89.9	83.6	93.8
FCRL2	95	108.9	116	101.2	105.1	102.6	92.3	94.5
FCRL2	82.6	98.2	100.7	82.8	85.5	84	82.2	87.5
FCRL3	94.8	103.4	91.2	87.1	81.9	91.7	87.5	96.9
FCRL3	151.7	134.2	88.7	89.6	89.6	98.1	117.9	124.6
FCRL4	78.4	80.9	81.7	80.6	83.4	65.4	74.4	79.8
FCRL5	123.1	129.1	100.1	104.7	96.5	99.6	110.1	120.3
FCRL6	69.1	79.8	93.6	84.9	82.1	83.3	76.6	75.4
FCRLM1	103.9	114.6	90.2	88.1	81.2	83.8	98.9	111.8
FCRLM2	341.6	397.4	213.1	165.3	122.9	168.2	337.8	470
FDFT1	9215.3	13278.4	6253.9	6395.4	6401.7	7069.7	9755.6	11375.6
FDX1	117	136.6	108.1	96.8	88.4	103.2	121.1	176.1
FDXR	118.2	136.9	92.1	96.6	88.9	94.4	90.6	130.2
FDXR	138.2	134.9	92.9	100.6	79.7	91.7	105.5	135.3
FECH	119.8	120.5	94.4	97.4	99.5	102.1	108.1	144.1
FECH	330.9	452	217.6	322.5	241.1	387.2	361.7	432.8
FEM1A	1114.1	1548.4	1338	1114	842.4	1130.1	871.9	961.1
FEM1B	231.2	299.9	196.1	177.6	178.5	229.6	233.2	278.9

FEM1C	400.3	537.5	306.1	346.8	275.6	577.8	332.3	646.6
FEN1	5865.9	6945.8	4783.7	3482.9	2805.1	3455.8	4218.1	5428.1
FER	130.8	179.9	124.4	125.6	101.8	108.9	131.5	174.3
FER1L3	337	407.1	290.1	199.5	188.1	256.5	320.9	360.7
FER1L3	160.5	222.9	152.3	100.1	110.7	106.6	165.1	156.8
FER1L4	87.5	85.8	81.4	93	88.8	97.8	84.7	84.4
FERD3L	84.4	95	104.2	97.2	89.5	89	83.2	88.3
FES	2469.6	2870.5	2962.7	1837.6	1699	2228.3	2191.5	2576.8
FETUB	112.3	124.8	95.9	91.2	90.1	96.9	104	114.7
FEV	94.4	105.9	103.6	80.6	80.4	85.3	101.9	118
FEZ1	230.3	320	137.3	113	105.1	115.1	192.4	219.4
FEZ1	173.5	211	193.8	167.2	187.6	171.8	155.4	178.5
FEZ1	560.2	673.1	434.5	283.5	392.3	313	475.5	626.3
FEZ2	1387.5	1862.3	1187.1	1634.1	1074	2086.5	1118.9	3055.8
FEZ2	2778.3	3335.1	2367	3249.6	2085.2	3815.1	2554.3	5369.4
FFAR1	117.9	139.8	105.8	96.7	109.2	102	108.4	110.4
FFAR2	118.8	128.6	94.5	98.3	91.7	103.2	121.8	121
FFAR3	97.6	105.6	99.4	98.3	94.1	101.2	91.6	91.2
FGA	80.5	91.3	89.8	79.4	72.9	88	86.3	89.4
FGA	289.6	284.7	165.3	157.7	158	155.1	257.8	313.5
FGA	88.9	112.3	81.4	82.6	82.9	81.4	99.6	103.1
FGB	101.4	125.9	88.3	93	88.5	85.1	96.4	105.2
FGD1	98.6	128.4	122.6	99.9	95.8	107.3	97.8	111.5
FGD2	107.3	105.6	88.5	92.9	85.1	77.6	97.4	96.1
FGD3	997	1254.4	641.2	556.5	583.8	654	979.3	979.1
FGD4	109.8	125.2	107.8	92.1	80.4	84.7	101.5	117.4
FGD5	93.2	97.6	106.5	94.2	88.6	93.6	94.9	102
FGD6	82.2	82.9	92.7	94.7	85.6	89.4	82.7	77.5
FGF1	111.8	115.5	107.4	96.5	90.3	96.4	107.9	102.7
FGF1	115.5	118.9	99	79.1	79.1	82.3	103.1	129.1
FGF10	85.7	116.2	87.8	75.7	82.8	81.2	91.6	89
FGF11	99.5	115.2	99.9	94.1	81.4	85.7	106.5	110.2
FGF12	131.3	118.1	89.6	83.1	82.2	88	114.5	117.1
FGF12	90	89.9	94.8	86.7	87.1	87.8	83.1	93
FGF12	101.7	118.1	99.6	81.3	86	88.6	92.4	97.8
FGF13	89.9	113.9	102.9	86.9	83.2	93.4	98.1	106.8
FGF13	76.8	91.9	83.4	76.7	76	75.2	86.7	91.4
FGF13	87.1	91	92.8	83.2	79.2	86.5	86.3	105.7
FGF14	89.6	95.2	94.8	82.7	82.3	93.8	97.9	103.2
FGF14	84.1	100.7	93.4	78.6	80.2	77.9	83.5	90.9
FGF16	97.9	87.9	83.5	77.8	74.8	85	82	94.5
FGF17	110.8	128.2	110.9	91.7	101.5	87.5	107.6	124.2
FGF18	138.3	172.7	145.9	127.9	117.1	117.1	135	165.6
FGF18	70.7	93.5	99.1	93.8	74.8	91.9	83.8	85.1
FGF19	104.9	127.2	103.8	88.2	90.5	83.7	93.8	122.2
FGF2	129.4	145.4	112.8	92.7	80.9	102.5	116.8	126.3
FGF20	151.9	176.8	109.9	100.4	94	117.5	140.9	153.6
FGF21	129.3	134.1	96.6	97.2	100.1	96.1	115.4	118.3
FGF22	81.9	105	96	83.3	81.8	84.1	98.6	99.3
FGF23	82.8	96	96.9	93.9	88.3	93	78	93

FGF3	96.1	105.9	91.8	99.5	85.7	90.1	103.1	107.3
FGF4	92.2	109.1	83.9	73.4	67.4	76.6	94.6	122
FGF5	91.8	115.5	105.3	100.1	89.2	90.5	97.7	104.9
FGF5	92.1	101.4	97.9	91.2	87.7	84.8	89.6	112.6
FGF6	85.5	114.3	86.9	79.6	69.7	81.3	97.3	100.5
FGF7	74.1	81.3	96.9	75.7	78.8	80.7	80.6	81.4
FGF8	98.8	106.6	102.1	92.2	91.9	89.9	105.6	94.8
FGF8	82.6	87.5	92.5	82.1	80.3	82.4	80.3	81.3
FGF9	80.4	97.3	83.8	85.5	77.6	87.9	77.4	97
FGFBP1	81.7	93.1	90.1	81.8	78.9	85.4	88.4	92.7
FGFR1	92.7	111.8	94	97.7	92.9	93.6	87.9	95.9
FGFR1	96.3	127.5	89.1	82.2	71.1	87.1	94.5	114.3
FGFR1OP	529	692.3	345.7	374.3	332.2	478.9	412.9	599
FGFR1OP2	201.5	261.5	126.8	108	110.2	143.7	184.2	272.1
FGFR2	78.2	96.2	92.5	82.1	81.1	77.5	95.4	88.2
FGFR3	177.5	203.1	93	97.4	78.3	89	155.6	155.8
FGFR3	260.6	305.8	250.2	263.4	195.3	306.6	239.7	360.7
FGFR4	114.6	142.4	117.2	108	105.1	125.5	106.9	138.7
FGFR4	79.6	106.2	86.6	88.6	84.9	92.9	75.8	82.3
FGFR4	93	109.7	95.5	98.1	92.9	103	89.9	101.2
FGFRL1	292.4	301.6	395.7	338	278.3	465.1	348	1010.7
FGFRL1	120.8	138.9	137.4	119.4	104.8	177.1	124.2	220.5
FGG	77.3	89.9	89.4	83.6	83.4	93.8	79.5	95.3
FGG	92.8	116.6	88.3	82	92.6	90.6	97.6	107.3
FGG	92	102.5	98.4	86.2	76.8	79.7	90.1	101.1
FGL1	104.4	111.9	91	85.4	80.8	85.9	101	98.5
FGL1	88.9	103.1	98	95.3	94.9	96.6	85.1	93.1
FGL2	177.6	235.3	118	460.5	346.1	526.5	135.6	164.3
FGR	241.2	309.1	180.8	147.5	119.9	150.3	197.4	300.3
FGR	166.6	157	102.4	92.9	91.7	91.8	139	175.5
FGR	675.1	772	427.1	328	238.8	309.1	407.1	562.5
FH	6437	7979.9	2660.3	2173.2	1288.5	2275	6012.3	8629.6
FHIT	98.5	100.7	100	83.4	88.3	93.8	100.6	92.7
FHL1	138.8	151.9	122	134.2	129.9	126	144.6	180.1
FHL2	509	699.4	289.4	205.3	188	286	562.8	746.8
FHL2	299.3	387.8	230.4	193.8	190.5	234.9	274.4	384.7
FHL2	92.7	85.6	102.7	99.4	101.1	96.5	90	89
FHL2	85.7	99.4	91.6	82.7	88.2	90.7	82.3	96.1
FHL3	363.3	467.8	258.9	259.1	211.2	234	349	445.7
FHL5	90.8	98.9	97.8	86.6	85.9	82.2	87.5	97.9
FHOD1	497.7	636.9	701.1	493.3	388.5	703.7	439.9	819.9
FHOD3	82.3	87.9	99.7	96.5	88.7	101.9	89.2	85.5
FIBCD1	269.1	319.7	249.8	139.9	126.2	134.2	187.3	258.7
FIBP	2147.1	2594.8	1407.8	1040.3	883.6	810.9	2096	1735.1
FIBP	321.9	399.9	211.6	159.3	162.7	163.3	283.7	282.8
FIGF	103	130.5	101.6	81.9	91.3	91.5	105.1	128.6
FIGLA	112.2	142.9	101.8	84.9	85	80	121.2	129.6
FIGN	96	97.9	98	81.7	86.9	92.4	92.8	89
FIGNL1	453.4	571.4	294.9	250.1	182.3	307.8	338.2	432.3
FIGNL1	552.3	724.3	277.7	269.8	230.2	337.3	384.2	526.2

FILIP1	94.2	118.7	116.1	120.7	105.4	122.2	103.2	105.6
FILIP1L	201.9	251.9	158.1	229.8	166.2	370.8	187.4	189.4
FIP1L1	86	87.2	97.9	78.5	85.2	80.3	80.5	92.2
FIS1	1157.4	1515.7	647.6	490.1	438.1	639.4	1137.5	1323.4
FJX1	99.4	115.2	109.6	99.4	87.3	105.6	97.6	102.3
FKBP10	115.3	128.4	102.4	89.8	83.5	101.7	100.7	117.4
FKBP11	245.9	253	177.3	252.6	326.7	311.7	160.6	193.9
FKBP14	1062.7	1029.1	720.3	1241.6	1313.4	901.7	987.7	2239
FKBP1A	389.7	502.2	388.3	269.7	245.3	292.4	380.4	545.5
FKBP1A	683.9	897.4	528.2	864.8	669.9	1042.1	548.6	850.1
FKBP1A	1457.3	2160.9	1445.4	1269.5	999.3	1839.5	1082.6	2370.7
FKBP1B	114.5	129.8	92.6	84.5	92.3	93.5	114.5	125
FKBP2	672	790.8	308.7	266.8	232.9	255.9	667.2	745.9
FKBP2	166.8	188.5	98	96.1	83.6	103.5	148.8	198.1
FKBP2	209.6	238.6	162.6	152.1	137.6	134.4	183.9	207.5
FKBP3	1340.8	1618	301.7	290.7	204.9	336.5	1132.2	2038.8
FKBP4	540.3	712.9	325.8	162.5	138.7	157.8	431.5	550.6
FKBP5	982.3	1194.2	581.3	660	650	736.5	848.3	896.8
FKBP6	82.6	88	85.5	87.2	83.7	91	75.7	87.2
FKBP7	119.1	134.2	91.8	79.9	90.3	87.5	88.1	117
FKBP8	170.6	217.2	111.5	117.8	104.5	114.2	177.6	229.2
FKBP9	84.9	89.6	107.8	101.1	98.4	104.4	84.2	87.3
FKBP9L	606.3	771.8	484.7	619.3	470.1	775.3	516.4	878.2
FKBPL	161.4	220.8	163.5	126	126.2	143.9	177.5	169
FKHL18	74.2	88.4	94.2	82.4	84.9	90	89.3	86.6
FKRP	131.7	134.5	140.1	130.3	113.5	112.8	128.3	202.3
FKRP	123.7	155.5	110.7	98.2	94	97	126.5	154.6
FKSG14	1001.8	1221.7	438.1	565.8	415.8	991.1	651.5	945.9
FKSG2	93.9	93.9	90.2	94.6	83.8	88.8	85.4	104.8
FKSG24	213	222	132.4	104.7	102.6	112.4	210.3	220.3
FKSG30	88.3	84.5	91.8	91.1	86.9	91.6	84.3	96.1
FKSG44	80.2	81.2	92.7	88.7	89.5	92.3	80.1	78.4
FLAD1	202.1	244.1	110.7	98.7	94.7	89.3	175.5	201.4
FLAD1	1155.9	1297.1	798.9	525.4	413.2	529.4	891.9	930.1
FLCN	90.5	103.1	103	111.8	93.2	102.8	99	113.7
FLCN	88	105.1	96	91	83.5	89.3	91.3	106.3
FLCN	113.2	134.3	113.1	101	87.6	93.4	128.4	131.2
FLG	77.3	76.3	85.9	77.6	80.9	86.7	77.8	77.5
FLG2	81.2	90.6	100.1	92.4	83.8	82.7	77.5	90.4
FLI1	217.7	247.5	99.7	98.9	90.5	90.9	205.3	249.5
FLII	239.2	281.4	316.6	306.2	279.6	479.6	246.5	329.8
FLJ00060	83	89.7	97.6	92	92.9	83.7	85.8	99.2
FLJ10006	844.2	1344.4	593.7	601.2	517.9	1108.2	670.9	903.9
FLJ10081	446.7	531	437	406.1	369.1	556.7	404.1	819.5
FLJ10099	949.9	1328	590.7	593.7	429.4	787.5	975.2	1557.6
FLJ10120	133.8	159.5	141.1	173.4	174.4	219.3	117.8	134.2
FLJ10120	117	124.9	107.7	105.8	98.3	96.3	105	136.6
FLJ10154	3369.6	3766.6	1890.6	1845.7	1755.2	3301.4	2320.1	3619.9
FLJ10159	196.9	240.3	128.4	120.3	122.5	125.8	164.2	149.5
FLJ10178	247.1	285.6	252.7	102	98.5	123.8	201.5	298.5

FLJ10213	260.2	248.2	150.5	234.8	214.9	247.3	189	249.9
FLJ10241	1575.9	2202.5	1674.5	992.8	712.8	1153.3	1470.1	2815.2
FLJ10260	141.1	171.7	107	109.8	89	105.6	134.5	142.1
FLJ10292	151.2	183.6	120.6	117.8	110.6	115.5	127.7	206.7
FLJ10324	84.2	90.2	116.6	91.9	97.9	94.3	85.4	107.7
FLJ10357	106.4	142.8	107.7	103.9	113.2	112.3	108.3	126.7
FLJ10374	237.7	325	125.2	121.9	113.9	120	265.4	204.6
FLJ10379	458.9	594.1	409.1	517	381.8	708.1	377.9	612.7
FLJ10490	102.5	107.3	111.9	102.7	91.2	99.6	98.3	116.7
FLJ10661	250.7	283.7	198.5	138.5	122.8	153	192.4	268.9
FLJ10726	362.3	458.8	227.5	226.6	194.4	315.4	272.3	451.7
FLJ10769	639.9	931.5	557.5	587.2	552	802.2	597.5	931.9
FLJ10781	114.1	107.2	91.9	90.2	88.1	79.8	92.5	123.3
FLJ10786	78.5	88.1	80.4	82	74.2	82	70	84.4
FLJ10815	101.9	135.1	95.5	98.9	77.6	99.5	107.1	125.2
FLJ10847	144.9	182.1	133.2	177.2	163	193.3	164.9	190.5
FLJ10916	205.1	232.5	163.6	198.2	191.8	198.9	179.3	176.7
FLJ10922	160.2	177.2	161.3	156.6	116.7	140.9	138.9	184.5
FLJ10925	161	163.4	155.6	150.1	142	180.1	130.1	195
FLJ10945	125.5	150.9	98.5	97.7	79.5	96	134.9	145.1
FLJ10986	450	619.7	407.2	501.8	434.3	639.4	422.1	622.7
FLJ10996	109.8	123.1	99.2	98.7	79.8	120.3	101.3	115.4
FLJ11000	153	186.7	114.5	119	116.6	127.9	144.7	147.9
FLJ11017	118.1	146.2	106.5	103.9	97.2	107.4	119.6	153.5
FLJ11151	160.4	176.4	107.8	121.4	113.8	122.1	156.6	205.3
FLJ11155	358.1	412.7	181.6	224.9	163.9	233	318.3	427.5
FLJ11171	328.5	369.5	239.4	191.7	156.8	262.5	290.1	590.2
FLJ11184	146.5	155.8	105.5	98.6	95.4	105.7	124.6	180.6
FLJ11193	247.3	356.5	135.7	125.4	120.2	145.4	236.4	318.8
FLJ11200	434.1	537	210.1	182.7	156.4	185.5	365.2	576.8
FLJ11259	179.4	234.9	179.4	134.2	155.7	159.7	149.5	232.1
FLJ11286	222.3	249.8	150.5	131.7	132.2	144.9	190.3	185.4
FLJ11305	4282.2	5710.9	3407.5	2818.4	2276.6	3241.3	3562.9	4061
FLJ11331	83.7	97.8	91.5	90.6	85.9	89.4	85.7	96.1
FLJ11712	1150.6	1002.2	370.9	406.1	291.8	502.4	1080	1865.2
FLJ11783	106.8	116.7	85.8	90.9	92.4	96.9	105.4	112
FLJ11795	187.1	230.8	161.4	201.8	169.1	240.6	112.7	122.6
FLJ11806	113.7	127.7	96.3	75.1	78.8	91	111.5	124.4
FLJ11806	175.4	205.8	157.8	121.4	101.3	141.7	144.5	160
FLJ11806	78	75.3	94.5	87.4	85.5	96.1	91.8	85.3
FLJ11806	177.1	205.8	159.1	143.9	116.2	166.4	147.4	333
FLJ12056	91.3	111.7	90.1	86.7	91	91.6	85.9	102.6
FLJ12118	439.3	486.6	151.5	171	132.5	174.2	358.2	364.4
FLJ12331	71.3	92.3	92.8	89.5	85.8	81.5	77.3	90.1
FLJ12505	115	145.3	97.8	97.4	98.9	97.1	97.6	102.6
FLJ12529	94.6	121.4	108.7	91.1	86.8	100.5	101.3	118.1
FLJ12571	223.9	280.7	180.4	137.7	106.7	144.8	170.3	196.8
FLJ12681	101.9	106.3	93.5	94.8	86	90.4	110.3	102.8
FLJ12684	277.7	337.7	203.6	122.2	98.1	137.9	166.9	191
FLJ12716	405.4	547.8	271.6	286.3	222.6	404.2	300.7	339.6

FLJ12716	620.1	839.9	242.8	289.1	283.8	358.9	568	773.2
FLJ12788	576.7	584.6	547.6	527.3	508.4	727.9	391.6	519.9
FLJ12886	268.2	290.8	227.1	175.5	137.9	175.5	223.5	374.6
FLJ12949	219	262.1	154.7	134	126.1	145	183	222.1
FLJ12949	389.9	457.7	479.6	301	252	325.4	300.7	424.6
FLJ12998	155	179.3	157	130.5	139.6	173.8	137.9	201.2
FLJ13096	167.5	205.2	124.3	109.3	99.7	129.1	143.4	193.4
FLJ13149	565.3	719.9	412	406.1	342.6	460.8	496.3	587.2
FLJ13231	105.9	115	99	87.7	85.7	93.3	96.5	111.3
FLJ13236	124	163.2	110.7	99.9	89.2	92	123.7	126.9
FLJ13265	94.3	124.2	104.8	95.9	91	100.5	94.7	111.2
FLJ13273	175.7	211.9	122.9	125.6	117.2	105.3	130.3	171.2
FLJ13273	933.4	1143.5	331.3	272.2	197.9	244.3	935.3	1146.4
FLJ13305	169	209.3	119.2	117	108.3	112	119.7	135.8
FLJ13391	88.3	91.4	89.5	74.2	85.5	84.3	98.5	103
FLJ13576	338.1	357.4	192.8	203.8	168.1	241.5	212.5	274.5
FLJ13611	109.7	118.6	92.8	83.6	89.1	83.2	109	124.5
FLJ13614	130.4	166.5	104.2	93.7	92.1	90.9	125.9	139.1
FLJ13639	193	207.7	144.5	138.2	124.9	152.2	173.4	200.1
FLJ13639	109.1	120.3	131.4	114.4	99.5	110.4	100.3	117.5
FLJ13639	161.4	222.8	144.9	143.9	119	161.8	166.7	162.6
FLJ13841	87.4	95.5	95	85.2	90.4	83.8	93.1	105.6
FLJ13909	769.5	838.2	520.1	325.3	265.3	295.1	537.8	533.2
FLJ13910	126.9	159.8	122	110.9	98.4	110.2	117.6	158.9
FLJ13912	1235.3	1660.3	791	506.8	408.4	576.1	935.7	1110.2
FLJ13946	125.1	130.8	98.6	89.6	85.3	97.4	102.2	124.7
FLJ13984	123.2	148	114.4	99.8	89.5	111.7	130.5	138.1
FLJ14054	87.8	104.7	104.5	90.4	78.9	90.5	86.2	90.1
FLJ14107	93.8	111.7	103.9	83.6	101.5	106.7	94.3	119
FLJ14154	190.1	257	161.4	122.9	110.9	125.7	187.5	320.9
FLJ14166	100.1	123.2	90.8	96.2	78.5	89.3	104.2	132.1
FLJ14213	108.9	123.3	104.8	98.8	83.5	93.5	94.1	110
FLJ14346	128.6	119.1	93.1	82.6	80.8	81.7	124.6	174.8
FLJ14397	91.3	102.5	99.6	87.9	82.2	87.1	100.1	121.6
FLJ14466	220.9	263.3	224.1	184.4	140.6	181.7	221.8	291.8
FLJ14640	152.7	196.3	128	117.6	103.8	141.9	150	184.2
FLJ14668	2113.8	2551.9	1179	942.4	782.4	1152.5	1516.9	1665.6
FLJ14768	198.9	240.7	167.4	141.6	134.2	153.2	195.6	239.3
FLJ14803	1079	1380.6	743.2	832.3	554.7	1195.4	960.1	2134.3
FLJ14816	74	88.1	94	86.1	91.3	75	79.8	86.6
FLJ14834	76.6	80.1	96	80.7	79	84	79.1	79.7
FLJ16008	137.9	157.3	94.9	99.1	84.1	92.6	120.8	146.2
FLJ16124	75	70.1	90.9	81.9	84.8	88.9	80.2	81.2
FLJ16165	112.6	108.7	100.7	88	102	100.4	106.2	136.6
FLJ16231	178.5	227.5	152.1	118.5	101.3	146.1	155	192.3
FLJ16237	129.4	140.9	96.8	86.6	89.2	93.3	122.8	127.3
FLJ16331	104.1	133.8	109.7	103.1	100.9	86.7	105.1	119.4
FLJ16478	86.1	84	88.4	90	85.7	82.4	87	87.6
FLJ16542	122.1	146.6	102.1	101.5	86	96.2	120.4	175.4
FLJ16636	110.1	128	99.4	93.9	84.8	101.8	107.8	125.7

FLJ16641	107.2	128.9	97.5	86.3	92.6	92	109.3	123.4
FLJ16734	134.2	157	110.5	99.1	98.4	100.2	130.1	144.5
FLJ16793	112.9	135	114.6	100	94.3	106.5	99.9	107.5
FLJ20032	126.5	128	97	88.6	86.8	82.9	97.4	120.2
FLJ20035	197.5	254.1	188.9	197.7	168.6	290.9	160.7	210.9
FLJ20054	489.5	665.2	465.7	873.5	699.1	1394.8	365.9	516
FLJ20097	175.4	191.6	117.1	141.3	118.2	160.5	168	225.2
FLJ20097	131.6	127.4	125.5	107.8	114.2	104.5	105.7	127.5
FLJ20097	69	79.7	84.4	80.7	74.7	83.6	73.5	80.9
FLJ20105	154	204.2	117.8	105.5	99.4	116.1	145	203.5
FLJ20105	211.1	288.3	190.3	145.3	123.1	193	179.1	203.4
FLJ20125	251.5	339.5	181.7	164.2	124.7	243.4	202.3	320.3
FLJ20130	180.2	239.8	151.7	88.6	79.2	83	137.3	157.6
FLJ20152	105.5	133.3	99	89.6	80.2	100.9	95.2	119.7
FLJ20152	161.6	221.9	125.7	102.4	89.8	112.4	162.8	168.8
FLJ20152	272.4	324	183.9	157.4	125.3	199.1	226.1	369.5
FLJ20160	158.8	210.5	147.6	168.1	151.1	188.1	167	265.3
FLJ20184	86.9	108.2	93.5	80.9	84.5	75.8	104.9	109.7
FLJ20186	568	777.7	275.1	195.2	189.5	226	611.4	684.7
FLJ20186	541.6	712.3	194.6	162.6	138.2	158.8	593.5	930.2
FLJ20186	775	759.9	804.3	608.1	540.5	747.7	574.6	782.3
FLJ20232	1416.2	2104.9	833.4	702.8	533.2	693.1	1185.8	1385.3
FLJ20245	316.4	395	494.3	361.1	295.5	630.8	291.7	645.2
FLJ20254	249.6	293.1	170.1	124.3	115.2	135.1	217.5	339.7
FLJ20272	1207	1674.6	457.2	393.1	287.8	439.5	825	1095.7
FLJ20273	171.9	224.4	135.5	170.5	167.6	267.9	209.9	371.6
FLJ20280	402.9	535.9	291.4	280.8	216.7	252.9	299	408.6
FLJ20294	118.2	135.4	113.9	106.5	94.6	91.9	108.9	126.9
FLJ20297	156.5	168.9	130.4	131	117.6	119.2	140.5	158.8
FLJ20298	103.1	109.3	90.1	76.2	76.2	79	104.3	116
FLJ20298	94.4	116.5	106.1	95.3	80.3	93.7	98.7	99.6
FLJ20309	78.6	85.1	98.8	107.4	96	93.9	102.5	100.6
FLJ20323	485.8	607.5	316.1	455.7	328.4	718.8	365.5	594.1
FLJ20364	1598.4	1781.5	840.4	816.1	658.4	1177	1128.7	1641
FLJ20366	88.2	98.5	81	96.1	71	77.6	90	95.6
FLJ20397	88.8	108.5	97.1	77.7	82.3	90.3	100.7	106
FLJ20422	189.6	241.2	138.1	123.6	111.6	148.8	172.5	215.2
FLJ20433	88	91.9	88.5	85	84	94.3	80.3	87.5
FLJ20489	188.8	199	168.2	143.8	126.4	155.8	403.2	712.6
FLJ20512	2180.3	2930.1	2060	1335	1032.3	1290.3	2129.7	2107.8
FLJ20516	1103.3	1551.8	487.6	286.3	203.2	373.4	802.4	1361.4
FLJ20534	948	1398.7	479.4	384.3	290.1	503.9	760.5	1595
FLJ20551	259	309.3	188.8	181.3	173.3	230.2	227.9	306.7
FLJ20558	416.6	602.2	204.4	195.2	148.3	219.6	341.7	474.4
FLJ20581	112.2	127.8	119.4	110.1	107.6	101.7	111.6	109.2
FLJ20582	610.9	864.9	331.9	333.8	257.5	367.9	584.5	818.3
FLJ20625	1997.6	2315	1987	1577.7	1266.2	1404.9	1876.7	2461.9
FLJ20628	403.3	457.1	203.8	186.9	162.7	220	345.5	508.6
FLJ20643	1083	1451.1	727.1	412.5	372.3	520.7	1035	1151.2
FLJ20647	835.5	1069	408.7	339.9	284.3	377.4	751	1091.3

FLJ20674	104.6	138.5	90.7	80.6	79.3	90.6	99.4	118.7
FLJ20674	404.2	559.1	253.4	248.4	220.4	195.8	324.5	388.3
FLJ20699	367.7	505.1	223.5	217.3	173.7	256.7	301.4	499.8
FLJ20701	103.4	130.6	105	119.6	104.8	119.9	104.3	110.2
FLJ20718	685.2	968.9	537.6	383.7	298.1	232.2	426.5	746.6
FLJ20718	102.3	127.9	116.3	97.4	89.9	90.8	98.2	120.3
FLJ20758	113.2	135	107.9	92.3	99.4	117	104.7	133.3
FLJ20850	124.3	145	119.5	101	82.5	103.8	131	136.8
FLJ20920	128.2	155.5	103.1	98.6	99.6	111.5	120	158
FLJ21062	98.4	99.3	101.4	104.1	82.6	106	93.8	95
FLJ21075	88.5	105.8	93.2	86.5	81.5	90	85.8	95.1
FLJ21103	205.2	276	146.2	104	110.3	113.3	210.8	223.1
FLJ21106	250.1	295.3	238.5	249.4	232.4	242.3	239.4	335.1
FLJ21125	246.7	318.5	275.3	267.7	220.2	343.1	189.3	325.4
FLJ21127	531.6	759.5	313.9	312	267.2	320.3	492.5	496.6
FLJ21159	113.4	132.9	102.8	79.8	76.3	104.4	104.8	130.9
FLJ21511	83.9	87.3	106.8	108.8	104.8	108.3	81	75.3
FLJ21687	113.6	132.5	101.9	88.2	81.6	90.2	118.2	148.5
FLJ21742	183.1	209.5	136.9	103	111.4	109.5	144	133.2
FLJ21749	242.8	326.1	201.4	177.9	158.5	210.4	226.6	284.7
FLJ21816	309.6	419.4	302.2	226	196.5	338.4	252.2	319.5
FLJ21820	414.2	572.9	373.3	338.2	280.1	447.8	327.8	583.3
FLJ21827	258.8	336	189.2	109.7	109.7	129.9	199.1	299.2
FLJ21839	304.4	387.8	323.9	264.2	250.5	444.4	244.6	379.9
FLJ21839	120.7	148.1	158.2	184.7	152.4	243.2	115.8	156.8
FLJ21865	166.9	177.7	287.9	281.2	276.5	443.8	137.6	233.3
FLJ21901	597.9	603.6	262.7	279.8	225.8	395	423	688.5
FLJ21908	90.2	114	87.8	95.4	91.2	82.9	98.5	106.9
FLJ21945	424.1	538.4	326	302.2	239.8	293.4	337.3	394.6
FLJ21963	94.9	116.9	87.2	81.1	84.9	98.1	92	119.5
FLJ21986	90.7	94.6	98.2	106.9	100.9	130.2	84	97.3
FLJ22104	317.2	362.4	185.5	239.3	177.8	240.9	302.5	563.8
FLJ22167	132	157	142.8	112.6	103.7	122.7	99.2	117
FLJ22184	88.5	102	98.8	89.4	89.9	97.1	86	95.2
FLJ22184	110.9	123.8	96.2	107.2	111.4	99.8	117.5	121.7
FLJ22222	132.5	152.9	133.7	118.3	114.6	133	137.7	179.4
FLJ22222	504.4	692	492.1	511.4	445.4	650.5	495.3	689.4
FLJ22222	174.3	218.1	125.7	120.2	100.6	143.9	166.7	209.3
FLJ22318	217	267	194.7	171.5	164.5	212.6	190.6	231.6
FLJ22349	154.6	190.9	123.8	111.1	103.8	117.9	154.4	145.4
FLJ22374	137.3	171.8	122.6	114.5	87.5	103.8	130.1	133.1
FLJ22386	237	314	231.1	192.2	188	282.9	206.8	312
FLJ22471	965.1	1210.2	508.1	520.8	400.4	432.5	993.1	778.2
FLJ22531	179.1	239.4	135.9	136.9	140.2	129.1	171.3	172
FLJ22555	1528	2128.9	959.9	986.3	669.8	1080.9	1375.3	2493.6
FLJ22624	1303.8	1590.1	709	563.2	435.1	730	958.5	1746.2
FLJ22639	110.9	124.5	109	112.1	95.9	127.7	101.8	118.1
FLJ22662	127.6	141.6	133.8	330	254.4	443.2	123.1	157.7
FLJ22671	81.9	90.8	96.7	87.8	85.1	84.3	77.9	89.9
FLJ22688	103.5	116.8	120	102.7	92.1	86.1	96.2	109.7

FLJ22709	568.1	584.5	463.1	347.3	308.8	339	505	515.4
FLJ22746	265.9	322.2	207.3	198.3	154.1	216	248.1	258.8
FLJ22795	106.4	110.1	120.1	104.6	97.9	97.6	107.7	110.6
FLJ23049	159.7	189.4	115	118.3	113.1	108.7	165.7	155.4
FLJ23191	91.6	97.2	84	71.7	74.5	80.7	97.2	96.2
FLJ23356	96.8	121.5	84.6	103.5	80.1	80.2	105.8	125.9
FLJ23436	157.4	190.5	95.4	100.6	97.6	125.4	125.9	189
FLJ23447	101.7	106.2	96.4	88.4	99.1	95.8	101.9	105.2
FLJ23514	106.3	116.3	98.4	93.1	86.2	94.9	93.6	106.1
FLJ23518	92.1	94.8	93.2	81.5	78.9	90.1	90.8	96.2
FLJ23577	86.7	94.6	108.5	104.6	94.2	93.9	90.1	96.1
FLJ23577	166.3	184	107.1	100.2	91	99.7	137.4	172.3
FLJ23577	97.6	116.5	86.4	85.2	86.3	90.1	96.8	114.6
FLJ23584	307.4	375.8	302.2	298.9	282	401.1	266.1	371.6
FLJ23588	86.5	94.7	93	88.8	84.9	88	88.3	94.6
FLJ23588	105.6	91.6	87.1	80.6	87.7	82.7	88.4	100.1
FLJ23588	115.1	143.8	98	90.1	94.4	91.3	115	136.9
FLJ23657	86.3	96.7	87	80.9	84.3	84.2	93.8	103.6
FLJ23834	80.3	89.5	99.6	99.8	85.9	105.3	87.3	88.9
FLJ23861	98.3	121.8	96.9	90.4	89.8	97.3	104.9	112.6
FLJ23878	87.4	99.2	91.8	87.4	79.7	98.5	95.3	100.1
FLJ25006	217.7	215.8	149.2	229.9	219.7	314.8	135.9	181.3
FLJ25067	175	244.3	165.6	161.6	137.4	188.4	180.6	241.7
FLJ25084	87.9	91.4	104.6	95	96.4	106.1	95.5	95.5
FLJ25102	136.2	185.6	113.8	108	114.6	103.7	140.8	153.2
FLJ25143	165.9	176.8	100	81.4	87.7	95.7	162.5	191.3
FLJ25222	750.1	775.4	442.1	423.6	471.3	619.6	601.6	1235
FLJ25328	83.5	88.3	80	79.7	86.5	92.9	83.2	90.8
FLJ25369	75.7	83.1	88.7	83.7	80.2	82.2	70.2	80.8
FLJ25371	88.6	96.4	101.5	90.6	84.7	87.5	80.9	88.6
FLJ25410	128.6	150.4	103	106.7	98.8	113.7	129.8	165.4
FLJ25416	695.6	800.9	468.8	382.1	267.6	451.8	497.8	940.7
FLJ25421	123.9	136.4	97.9	83.3	80.9	89.3	124.2	154.5
FLJ25439	89	105.2	100.2	97.7	93.1	93.7	95.2	99.8
FLJ25444	79.7	88.3	91.7	85.8	75.1	86	90.1	90.9
FLJ25476	577.3	794.5	434.8	403.1	346.1	425.1	507.5	648
FLJ25477	104.1	124.7	106.5	90	98	106.4	108.3	116.1
FLJ25660	113.9	117.1	92.2	84.6	80.4	81.1	117.2	115.6
FLJ25680	84.8	108.9	101.2	98.4	98	96.3	95.6	99.1
FLJ25770	82	95.1	83.4	81.4	78.4	79.5	74.9	90.9
FLJ25773	99.8	120.1	84.9	83.1	75.1	78.2	101.8	107.6
FLJ25791	95.4	98.5	118.4	105.6	90.2	101.2	98.6	109.1
FLJ25801	84.5	96	95.5	93.7	85.7	91.9	89.5	90
FLJ25976	86.1	102.4	82.1	86.6	79.1	75.3	87.2	90.1
FLJ25996	87.4	92.9	90.9	76.1	76.2	92.3	87.8	98.7
FLJ26175	125.2	135.6	111.9	95.4	106.1	110.5	108.5	142.4
FLJ26443	95.4	104.3	94.2	89.8	90.8	90.9	99.3	102.5
FLJ26850	170.7	235.3	105	95	91.1	100.8	166.1	199.7
FLJ27465	91.7	101.4	130.2	123.9	124.7	113.9	91.9	96.8
FLJ27465	99.6	103.8	86.1	95	84.2	90.9	102.8	99.2

FLJ27505	80.5	83	89.2	81.9	84	88.9	81.9	89.7
FLJ27523	105.6	129.9	99.6	98.8	89.9	95.1	110.5	110.6
FLJ30046	1650.4	2017.1	694	424.5	295.7	450.5	890	1294.5
FLJ30046	533.7	768.9	243.5	163.7	135.5	209.4	288	372.1
FLJ30058	152.2	162.7	123.9	93.9	90.7	114.7	146.9	158.6
FLJ30277	110.9	130.4	91.8	90.4	80.1	82.3	107.6	110.3
FLJ30294	97.4	104.6	89.2	89.6	84.4	89.5	92.4	129.9
FLJ30313	82.4	88.5	109.3	90.6	86.8	97.3	96.1	87.9
FLJ30596	266	354.8	140.2	140.4	108	148.5	188.9	321.3
FLJ30655	275.9	354	140.6	126.9	120.2	188.4	218.7	303.6
FLJ30707	80.4	96.9	86	79.2	77.7	82.2	90.5	97.6
FLJ30834	132.2	154.1	104.2	94.5	95.8	94.7	121.6	136.5
FLJ30851	74.2	80.4	98.4	89.3	83.7	93.4	79.5	78.4
FLJ30934	152.1	196.2	106.1	98.1	94.5	95.3	146.2	151.7
FLJ30990	142.8	163.2	113.1	111.5	90.1	98.7	114.5	145.1
FLJ31196	95.8	116	86.7	83.5	67.9	79.1	98.3	111.1
FLJ31204	113.6	148.7	111.3	96.8	85.2	91.4	110.9	130.7
FLJ31401	94.5	97.7	85	93.9	83.6	82.2	86.8	91.9
FLJ31413	778.8	976.6	572.7	539.6	506.8	557.7	982.6	1253.8
FLJ31438	155.8	167.9	102.2	112.8	98.6	137.5	126.5	179.5
FLJ31568	99	104.5	96	97.8	91.4	88.8	88.2	101.4
FLJ31659	86.6	98.7	113.2	104.4	98.3	96.7	82.2	86.7
FLJ31846	89.5	96.7	113.6	110.8	99.8	100.6	90.6	91.9
FLJ32011	111.7	154	110.8	109.2	93.1	105.7	121.5	129.7
FLJ32063	88.8	89.7	99.1	91.6	87	86.4	86.6	92.1
FLJ32110	95.7	105.2	88.2	91.4	82.9	82.6	95.3	107.7
FLJ32130	101.7	113.3	106.1	106.1	92.9	95	89	105.2
FLJ32206	120.4	146.9	91.4	94.9	98.5	92.5	122.1	118.3
FLJ32312	139.3	168.6	109	106.4	96.7	122.7	134.3	144.1
FLJ32447	172.4	183.2	135.5	133.4	141	135.7	164.6	153.7
FLJ32549	176.2	252.2	104.4	96.3	88.8	92.2	171.3	188.8
FLJ32569	107.5	111.6	87.5	85.4	81.1	79.1	95	98.6
FLJ32569	77.2	79.6	91.2	91.1	84.1	89.5	84.2	82.7
FLJ32658	95.6	119.1	100.7	94.4	86.7	99.5	97	100.5
FLJ32679	98.3	103.5	94.6	93.7	81.5	99	80.6	93.3
FLJ32745	365.1	459.6	261.3	226.4	190.7	297	264	386.4
FLJ32771	118.9	137.7	99.1	93.4	89.5	93.4	110	127
FLJ32784	83.8	95.9	96.2	88.7	95	96.2	84.4	84.8
FLJ32786	97.3	110.8	103.1	95.3	89.7	94.2	99.6	105.1
FLJ32867	78.6	94	99.4	101.2	90.8	95.2	82.6	87.1
FLJ32871	76.4	89.5	87.9	79.6	76.5	83	80	89.1
FLJ32894	125.1	125.2	118.6	99.2	96	99.4	118.1	115.8
FLJ32926	83.1	85.3	90.7	84.2	82.2	84.3	78.1	85.6
FLJ33360	87.4	94.1	85.2	76.8	74.9	89.3	84.6	89.1
FLJ33590	86.8	92.5	105.8	89.9	100.2	89.3	88	101.4
FLJ33641	1534.6	2041.6	884.9	2787.1	2083.3	3682.6	975.1	1357.2
FLJ33718	146.5	214.7	108.8	99.8	88.7	104	143.8	165.4
FLJ33790	507.4	573.9	310	248.1	195.3	247.8	361.2	303.8
FLJ33860	140.8	153.6	133.6	438.7	319.3	670	114.9	156.5
FLJ34306	71.5	86	89.5	91.9	82	88.2	81.5	86.7

FLJ34503	112.1	106.4	90.6	88.3	82.2	91.3	99	104.4
FLJ34931	129.9	145.7	95.6	111.4	87.1	97.8	113.3	128.8
FLJ34969	592.1	656.8	356.7	521.9	391.5	593.5	552.7	1592.6
FLJ35119	157.6	154.4	147.2	147.8	135.6	131.5	121.3	189.3
FLJ35220	108.3	127.2	104.4	108.5	116.5	121.8	110.9	112.3
FLJ35409	88.5	86.4	77	81.5	83.5	86.3	88.1	93.4
FLJ35695	79.1	77.4	90.6	81.4	81.3	83.8	79	83
FLJ35725	103.3	133.2	120	95.4	102.3	106.4	113.4	134
FLJ35725	113.6	167.7	105.3	86.6	82.7	77.2	125.1	143.4
FLJ35767	212.4	264.3	140.9	101.9	99.2	106.6	273.9	302.7
FLJ35773	97	123.4	85.1	86.2	83.4	89.7	99.5	110.3
FLJ35776	81.8	87	102.2	90.8	83.6	85.2	85.4	85.6
FLJ35784	82	85.5	92.2	101.8	92.7	95.5	74	84
FLJ35816	84.4	89.8	96.4	102.2	90.3	95.8	94.4	103.8
FLJ35834	95.1	111.2	107.2	93.4	98.7	95	98.1	101.3
FLJ35848	86	87.6	82.1	81	79.3	83.1	86.1	91.6
FLJ35880	98	110.2	96.7	91.6	101.6	90.7	100.5	107.8
FLJ35894	80	87.5	84.1	83.8	79.6	82	81.7	92.5
FLJ36004	76.2	81.3	83.8	83.6	85.5	71.4	75	84.7
FLJ36031	87.8	101.7	84.9	80.4	89.4	84.4	100.8	104.5
FLJ36046	113.3	116.2	118	96.6	98.4	103.2	107.5	105.3
FLJ36070	106.9	121.4	114	94.2	92.3	92.3	112.4	112.9
FLJ36090	169.6	232.3	110.8	119.1	111.3	158.4	179.8	254.8
FLJ36166	121.6	100.8	110.9	94.6	99.6	113.1	81.2	109.4
FLJ36180	118.1	127.4	113.5	99	88.4	97.8	114.5	115.9
FLJ36208	149.2	178.7	124.6	105.7	102.9	114.1	139	155.5
FLJ36268	82.2	95.7	88	90.7	75.9	95	87.3	98.3
FLJ36445	93.7	114.2	84.5	85.7	86.5	84.9	100.2	104.4
FLJ36492	92.6	101.4	82	84.2	83.1	86.6	91.4	94
FLJ36492	87.1	91.6	83.4	80.8	87.2	79.7	88.9	86
FLJ36748	106.2	109.3	115	112.1	100.3	99.2	97.4	95.5
FLJ36779	90.8	113.9	92	88.8	86.5	105	90.1	99.2
FLJ36874	1601.2	2232.8	1178.6	1103.5	903.4	1213.3	1514.5	2221.7
FLJ36888	110.8	152.9	106.8	103.9	94.2	111.9	112.9	150.8
FLJ36980	119.3	130.9	93.6	84.5	88.5	82.4	118.4	136.8
FLJ37078	97	109	97.3	97.8	101	94.8	91.9	119.8
FLJ37266	86.4	92.7	87.7	78.5	82.9	90.9	93.3	98.5
FLJ37357	87.8	97.4	96.5	93.1	89	98	84.7	88.7
FLJ37440	106.2	111.6	116.7	90.4	83.8	88.2	107.6	119.3
FLJ37464	150.2	139.4	103.9	103.6	96.7	98.5	141.4	133.2
FLJ37538	107.8	138.4	103.3	88.7	82.4	101.7	115.9	122.6
FLJ37562	190.8	234.6	148.2	171.3	135.7	199.4	181.6	277.2
FLJ37927	99.7	105.6	109.6	108.6	100.8	112.5	101.5	95.5
FLJ37953	89	96.3	99.1	97.2	89.7	88.3	88.5	116.8
FLJ37970	137.4	158.4	162.4	167.9	195.1	240.9	134.1	192.3
FLJ38020	96.9	104.1	90.1	86.7	88.3	87.7	104.9	112.3
FLJ38159	230.3	274.8	130.2	139.6	111.1	131.4	210.9	252.7
FLJ38288	153.5	228.3	124.9	101.5	102.4	111.8	180.2	234.8
FLJ38377	97.4	104.9	92.2	86.2	88.2	94	95.7	100
FLJ38451	246.4	269.6	188.4	179.3	149.2	174.9	226.9	253.6

FLJ38482	1185.5	1287.1	804.9	868.6	531.6	690.4	1209.8	1861.8
FLJ38596	77	103.9	78.8	83.2	75.2	73.3	88	88.9
FLJ38663	507.1	576.2	249.2	225.9	156.8	240.5	421.4	428.3
FLJ38723	88.7	91.2	101.8	95.1	92	87.4	82.2	86.3
FLJ38725	138.3	162	85	87	78.8	79.2	137.3	161.7
FLJ38964	85.3	89.7	95.8	83.4	80.9	83.2	86.2	96.3
FLJ38973	143.4	156.8	96.7	101.1	87	86.7	116.3	200.2
FLJ38984	229.9	271.6	174.7	161.4	149.3	187.5	253	322.3
FLJ38991	106	127.2	107.5	108	103.7	100.9	92	115.4
FLJ38991	111.5	135.1	108.3	100.7	94.8	104.5	117.3	128.5
FLJ39061	118.8	114.2	92.9	88.8	87.6	85.8	111.5	113.7
FLJ39155	96.6	110.6	88	81.7	87.3	87.8	96.1	110.6
FLJ39155	84.6	90.3	102.5	96.7	82.3	92.9	88.4	104.6
FLJ39155	74.8	83.3	88.5	87.9	79.7	85.7	77.2	76.2
FLJ39237	96.6	99.5	90.8	91	93.1	99.6	98.4	104.6
FLJ39378	93.9	106.2	111.5	98.4	86.8	89.7	97	116.4
FLJ39501	106.1	120.6	91.7	97.9	91.7	103.6	97.3	111.5
FLJ39502	100.5	89.5	94.7	81.6	84.4	90.6	84.9	96.5
FLJ39531	99.8	129.5	94.2	78.7	81.2	88.6	95.5	123.2
FLJ39575	124.9	138.4	109.6	103.9	122.7	118.4	116.6	164.7
FLJ39599	171.2	238.6	121.4	117.1	108.6	115.9	175	213.4
FLJ39660	124.3	142	108.4	97	99.9	96.9	119.6	130.3
FLJ39743	92.1	107.5	86.2	86	84.4	83.5	94.7	99.6
FLJ39779	113.8	137	90.2	99	88.3	98.4	104.7	140.1
FLJ39822	144.6	167.9	91	90.1	74.4	93.5	142.4	173.6
FLJ39827	438.3	548	424	463.8	378.8	506.1	329.4	357.5
FLJ40113	79.3	97.8	86.8	78.8	76.8	82.8	82.9	85.2
FLJ40125	124.2	140.8	111.3	97.8	103.6	120.2	97.4	110.7
FLJ40142	225.2	295.5	207.7	273.2	296.8	300.2	184.8	241.8
FLJ40172	101.7	128.3	85.7	82	77.9	88	109.1	116
FLJ40235	90	102.6	101.1	92.6	84.6	89.5	86.3	105.6
FLJ40243	79.3	86.5	77.3	73.7	75.5	79	73.4	90.7
FLJ40244	71.8	79	90.1	79.4	73.1	80.2	68.5	75.2
FLJ40288	103.4	129.1	104	90.4	80.8	86.8	111.9	116.9
FLJ40296	81.3	82.4	99	90.7	88	100	68.4	84.1
FLJ40298	75.9	83.5	91.6	76.8	75.2	78	78.5	90.2
FLJ40365	88.6	91.7	97.9	84.7	80.2	94.2	82.3	94.9
FLJ40411	161	205.5	126.4	105.5	84.5	102.3	164.5	179.1
FLJ40448	119.6	146.4	120.5	98.8	95.2	117.9	125.7	139.2
FLJ40453	94.5	103.5	97	89.5	81.2	79	101.2	118
FLJ40504	100.2	100.9	94.7	99.7	93.3	91.7	108.9	105.3
FLJ40629	957.3	1197.8	718.8	653.9	552.9	757	833.6	1143.2
FLJ40722	111.8	136.5	96.6	83.9	85.4	94.6	98.6	121
FLJ40852	106	141.4	113.2	98.5	100.7	104.4	107	114.1
FLJ40869	97	117.9	90.4	80.4	76.5	97.3	93.1	111.9
FLJ40919	88.8	93.9	95.6	82.3	78.3	78.4	88	104.5
FLJ41046	86.1	106.8	92.4	92.6	86.9	85.5	95.6	104.3
FLJ41131	508	563.2	273.5	284.4	251	269.7	349.5	356.2
FLJ41170	80.8	96.2	103.8	88.8	83.9	87.4	83.5	87.7
FLJ41423	485.9	554.5	251.3	276.9	326.5	288.7	473.7	430.9

FLJ41603	75.5	83.1	92	97.6	80	94.4	94.6	83.3
FLJ41649	114	145.4	93.1	97.2	84.7	87.2	108.6	131.5
FLJ41733	104.1	117.8	94.4	86.3	89.4	89.5	90	103.6
FLJ41821	88.1	96.4	79.6	80.5	83.7	84.9	85.5	90.2
FLJ41841	79.8	86.9	100	101.1	98.2	85.5	75.6	83.9
FLJ41993	97	112.3	97.3	93.2	79.9	87.5	103.5	105.5
FLJ42102	82.9	90.5	79.6	73.3	84.8	74.6	80.8	91.2
FLJ42117	120.8	143.7	107.2	98.2	86.9	89	116.7	129.9
FLJ42133	80.5	87.4	102.3	106.2	98.6	109	87.9	93.5
FLJ42177	84.5	91.2	89.8	82.4	87.4	97.4	81.6	91.2
FLJ42258	96.7	97	99.5	96.5	95.5	91.1	97.3	94.5
FLJ42280	77.4	100.8	96.1	93.3	90.9	90.6	92.2	95.7
FLJ42289	94.8	108	91.9	87.8	80.4	87.3	106	120.8
FLJ42461	72.8	81.8	75	78.6	79.8	73.2	78.4	76.5
FLJ42486	126.9	136.4	101	88.9	82.5	101.9	114.3	124.5
FLJ42953	96.1	95.6	95.4	83.4	87.8	90.1	93.7	95.4
FLJ42957	76	89.4	87.4	88.9	84.4	95.7	71.2	89.7
FLJ42986	275.3	259.1	220.1	153.5	187.7	207.9	204.1	371.4
FLJ43093	1337	1352.7	1398.6	1706.5	1657.7	2336.2	741.3	710.1
FLJ43276	99.7	116.6	99	95.1	94.9	109.9	102.8	97.8
FLJ43339	263.5	281.3	177.9	187.4	193.7	207.6	165.1	181.4
FLJ43374	160.8	174.3	113.4	159.5	139.3	166.2	110.2	122.1
FLJ43505	78.6	88.4	96.2	82.9	83	73.9	78.2	95.1
FLJ43692	383.5	526.5	271	282.5	230.4	329.4	340.4	438.3
FLJ43752	114	119.6	118.8	98.2	97.4	108.9	108.3	118
FLJ43763	91.7	93.6	91.7	83.7	79.7	85.1	89.2	76.6
FLJ43806	102	118	98.5	93.2	84.8	87	94.5	108.1
FLJ43855	80	86.6	82.6	83.5	88.2	85.7	85.9	89.1
FLJ43855	124.9	144.1	160.1	174.3	200	253.9	134.8	189.3
FLJ43860	88.6	107.9	100.5	87.1	84.5	93.4	100.8	102.6
FLJ43860	114.6	139.7	94.3	89.6	88.1	91.9	107.3	111.2
FLJ43980	93.8	108.2	90.6	80.5	78	85.6	97	97.2
FLJ43987	97.4	109	87.9	84.2	79.5	95.9	86.4	105.5
FLJ43987	92.6	96.4	106	101.1	94.2	106.1	93.5	97.4
FLJ44048	93.6	103.9	90.3	94.1	84.7	92.1	98.5	98.4
FLJ44060	82.1	79	87.4	86.4	81.5	82.6	86.3	83
FLJ44076	89.8	103.3	101.9	81.6	92.5	92.6	86	92.2
FLJ44112	93.1	105.3	85.4	94.1	82.5	84.4	90.8	106.1
FLJ44124	2332	2355.5	829.5	1134.1	1222.7	1010.9	2162.4	2453.4
FLJ44216	788	1043.7	838.7	801.5	647.7	1023.2	561	926.8
FLJ44216	94.9	103.2	85.4	80.9	92	78.9	88	104
FLJ44290	88.7	99.9	95.2	86.1	84.4	82.7	88.4	91.5
FLJ44379	114	137.7	90.2	81.9	84.5	89.3	116.7	135.8
FLJ44635	111.6	114.7	87.8	88.2	78.1	89.1	120.2	120.3
FLJ44635	86.8	85.6	95.7	84.4	88.8	83.4	82	91.8
FLJ44653	95.3	116.2	101.1	89.9	89.8	92.2	90.7	103.5
FLJ44691	86.4	98.9	94.3	87.6	71.9	89.5	86.8	95.4
FLJ44815	91.8	114.3	105.8	92.8	87.4	91.8	90.4	94.9
FLJ44894	100.1	112.2	85.4	87.4	76.8	80.6	105.2	106.8
FLJ44955	118	143.6	89.1	79.4	80.2	79.9	108.9	126

FLJ44968	133.1	147.4	103.1	105.6	102.5	129.7	110.2	149.1
FLJ45032	146.1	183.1	148.9	155	130.1	143.2	145.7	174.2
FLJ45079	104.7	124.5	90.1	108	95.2	82.4	101.8	111.3
FLJ45121	89.1	88.1	88.6	81.5	81.2	87.6	88.4	95.5
FLJ45139	83.2	92.7	88.4	86.2	78.2	84.9	89.4	90.7
FLJ45224	98.1	108.9	100.8	93.3	86	94	93.7	99.2
FLJ45256	109.6	113.1	116.7	118.8	102.1	109.9	101.2	98.8
FLJ45337	121.8	155.7	118.3	123.3	118.2	124.4	120.9	141.4
FLJ45422	107.7	130.8	93.1	87.2	85.9	80.2	107.2	136.9
FLJ45445	90.9	110.8	100.1	93.7	97.7	91.4	88.3	102.9
FLJ45455	105.9	119.8	100.6	95.9	95.6	92.2	101.9	106.1
FLJ45530	73.6	87.4	93.8	92.2	86.5	89	81	87.5
FLJ45537	71.9	79	95.9	96.6	87.3	89.5	77.3	76.5
FLJ45557	165.7	233.8	141.6	127.1	115.1	147.2	177	249.1
FLJ45645	83.7	107.3	99.2	97.7	89.7	95	81.5	93.6
FLJ45717	88.4	113.3	109.7	94.8	86.2	93.7	97.4	101
FLJ45721	76.8	92.1	94.6	83.3	89.7	79.2	83.6	104
FLJ45803	77.3	87.9	98.7	83.4	82.3	89.6	85.3	91
FLJ45831	84.9	89.2	103.9	100.3	79.8	85.9	87.8	92.9
FLJ45850	162.4	205.8	117.8	91.9	86	95.6	140.4	158.2
FLJ45909	284.7	318.2	229.5	212.2	197.2	171.5	273.6	343.1
FLJ45910	91.6	108.6	102.1	91.7	85	98.4	93.7	95.4
FLJ45964	99.4	104.8	95.5	89.9	81.5	98.5	90.3	86.6
FLJ45966	107.5	127.3	109.2	90.1	89.4	100	108.9	118.3
FLJ45974	109.6	129.6	102.4	92.9	95.6	96.9	107.5	118.1
FLJ45983	109.8	115.8	91.5	85.6	85.9	84.3	110.4	111.4
FLJ46010	80.2	83.3	89.9	90.6	91.2	101.1	86.5	87.3
FLJ46072	253	279.6	291.7	253.9	192.2	292.4	235	412.4
FLJ46082	79.7	91.3	95.9	80.4	84.3	83.9	83.8	83
FLJ46109	76.7	90.1	95.9	102	95.7	91.6	78.3	90.3
FLJ46109	186.5	225	124.1	150.1	156.2	134	201.9	226.8
FLJ46210	94	112.7	89.4	85.1	74.6	83.6	93.3	100.3
FLJ46247	111.3	121.2	86.6	82.5	82.5	91	104.5	118.2
FLJ46247	87.6	118.4	100.4	80.5	74.4	89.5	91.2	108.3
FLJ46247	71.5	90.3	106.3	108.4	88	92.3	79.9	93.9
FLJ46257	80.6	89.1	91.1	88.3	79.6	94.6	77.8	86.1
FLJ46266	85.2	105.2	112.3	92.7	89.1	101.2	89.3	92.6
FLJ46309	2887.4	2947.9	785.3	1368.5	1241.7	899.3	2577.1	4200.3
FLJ46321	79.1	81.4	86.7	84.1	82.3	87.3	81.4	87.7
FLJ46347	101.2	107.4	90.4	89.4	78.3	81.8	98.2	99.6
FLJ46385	72.8	80.6	85.2	83.9	80.2	84.4	84.3	90.3
FLJ46481	128.8	159.1	119.8	120.2	110.3	104.5	127.3	127.6
FLJ46536	87.8	103.6	92.6	100.8	87.2	103.8	89.1	113.3
FLJ46688	92.8	116.1	112.1	100.5	96.9	97.2	96.8	99.3
FLJ46831	91.1	107.4	100.7	94.1	90.8	83.5	93.8	97.9
FLJ46836	138.2	161.3	103.9	93.1	78.8	97.3	113.2	163.1
FLJ46838	113.9	135.9	95.9	93.7	89.9	85.9	111.3	120.7
FLJ90013	236.8	321.6	147	139.7	125.9	161.1	231	420
FLJ90166	96.4	101.8	93.9	85.6	82.2	87.2	90	97.3
FLJ90396	460.5	510.7	179.8	211.9	162.5	270.2	384.9	789.2

FLJ90575	125.8	136.3	113.3	89.4	94.4	109.8	103.9	136.3
FLJ90586	83.1	96.2	84.5	75.3	83.3	83.5	88.5	94
FLJ90650	104.1	121.6	79	83.4	84.8	85.5	96.4	112.6
FLJ90652	465.4	592.8	486.1	254.9	235.9	373.3	369.3	747.8
FLJ90709	374.6	441.9	208.2	321.7	235.7	348.8	329.9	481.5
FLJ90757	195.1	230.7	191.7	240.5	186.3	203.8	156.5	211.9
FLJ90805	531.1	556.9	329.9	245.9	243.8	245.7	310	372.3
FLJ90834	87.1	88.3	91.7	92.6	90.3	90.9	84.9	89.6
FLNA	151.5	187	84.8	89	89.3	88	131.1	169.2
FLNB	260.4	441.6	461.2	693.7	743.2	1090.2	315.6	527.8
FLNC	101.1	106.8	95.8	92.3	78	85.8	95	119.4
FLOT1	202.1	254.7	186.2	123.6	125.4	190.4	202.4	232.6
FLOT2	6908.5	8693.4	5099.7	7080.9	5390.1	7116.1	5088.2	5454.9
FLRT1	164.5	178.1	126.7	116.9	103.8	129.6	180.9	213.7
FLRT2	113.7	127.7	103.9	98.1	94.6	94.1	115.8	114.2
FLRT3	93.2	103.6	82.5	86.9	86.5	86.6	70.7	87.2
FLRT3	77.3	99.8	96	87.3	86	87.5	80.7	98
FLT1	103.5	110.5	88	76.4	87	93	99.8	116.2
FLT3	444.7	622.2	338.9	250.8	219	291.7	367.4	635
FLT3LG	116	132.5	127.9	105.6	96.8	113.4	115	135.3
FLT4	92.3	101.5	96.3	87.7	93	87.9	101.6	97.1
FLT4	75.5	91.7	82.8	79.6	78.5	88.1	87.8	84.6
FLVCR	321.9	410.2	158.6	130.3	138.6	156.9	286.5	396.9
FLYWCH1	152.5	160.6	111	97.8	97.8	101.4	123.4	128.4
FLYWCH1	97.3	105.5	104.3	92.6	88.1	95.7	105.5	97.7
FMN2	87.4	100.3	96.2	81.7	88.2	92.2	92.6	86.1
FMNL1	110.5	123	107	108.5	109.4	124.2	111.7	119.3
FMNL2	109.4	121.8	107.1	90.6	85.5	96.6	101.1	114.6
FMNL3	85.4	101.1	91.5	101	94.5	97.6	87.8	92
FMNL3	92.2	109.2	100.2	97.4	84.1	95.5	88.4	103.2
FMNL3	88.4	85.5	96	85.7	82.1	86.5	85.8	100.2
FMO1	94.1	102.4	99.9	91.9	90.5	92.6	96.2	110.7
FMO2	113.6	102	104.8	103.2	108.9	101.1	98.4	112.7
FMO3	73.7	87.1	82.7	74.5	77.7	79.2	72.7	80.5
FMO3	92.1	97	87.7	84.9	82.9	95.2	88.2	102.3
FMO4	164.8	187.4	153.4	155.1	142.1	175.1	168.7	262.2
FMO5	177.3	210.9	122.7	130.8	133.6	176.7	151.8	200.6
FMO6	166.8	183.5	164.5	132.3	146	155.7	154.3	172.2
FMO6P	112.8	138.1	91	91.1	83	85.5	101.7	119.9
FMOD	99.7	115.4	101.5	87.1	91.5	94.8	97.9	109.2
FMR1	83	91.3	93.2	87.3	91.1	86.8	86	88.7
FMR1NB	87.9	92.4	107.8	90.1	79.4	95.8	88.4	91.2
FN1	135	132.1	90.2	80.3	90.7	83.8	112	136.6
FN1	73.9	89.6	96.4	84.9	87	87.1	81.2	86.1
FN1	106.7	113.5	92.8	82.3	75.2	91.8	115.1	121.5
FN3K	103.8	109	109.7	98.2	96.8	87.1	100.2	101
FN3KRP	2234.1	2739	756.2	678.4	694.5	886.5	1600.8	1537.8
FN5	992.6	1254	361.1	364.9	296.9	352.8	1042.2	1111.3
FNBP1	1727.3	2173.8	1162.6	1913.7	1696.5	1937.9	1557.8	1571.4
FNBP1L	84.8	89.7	92.1	91.6	85.7	83.3	81.3	85.3

FNBP1L	172.2	227.4	107.6	117.8	121.9	129.8	150.5	173.9
FNBP4	1463.3	1597.8	1446.8	1679.8	1731	2373.4	1005.7	1774.7
FNDC1	77.3	90.5	88.3	75.9	79.6	85.2	81.4	84.3
FNDC3A	238.1	255.1	142.4	159.6	142.7	234.4	228.9	421.6
FNDC3A	99.9	102.3	105	89.7	94.4	85.9	101.3	106.1
FNDC3A	230.2	260.8	156.9	214.7	170.6	305.4	225.8	415.9
FNDC4	98.9	111.8	111.7	90.1	82.4	90.2	90.7	98.2
FNDC5	83.2	89.2	97.6	92.5	88.9	90.3	82.2	86.5
FNDC6	129.6	138.9	120	111.4	105.5	108.2	127	158.1
FNDC7	86.3	83.1	101.3	94	94	96.6	88.6	83
FNDC8	78	109.4	95	95.2	81.4	95.3	89.2	94.9
FNTA	1399.3	1914.2	740	561.2	485.3	766.7	929	1140.1
FNTA	106.9	132.5	93.6	78.2	77.2	77.7	109.8	136.5
FNTA	3260	4288.7	2668.3	2012.9	1416.8	2983.8	2566.7	4669.1
FNTB	474	595.9	364.2	318.6	265.1	297.7	362.5	409.3
FOLH1	76.4	93.6	97.3	95.1	94	102.2	90.1	96
FOLH1	121.2	127.8	129.3	132.4	131.7	113.8	104.5	110.8
FOLR1	85.2	97.7	92.6	88.2	77	87.9	87.5	85.9
FOLR1	107.3	127	95.8	90.5	88.3	85.6	105.5	119.4
FOLR1	87.4	93.2	92.5	93.6	84.6	92.3	91.4	94.6
FOLR1	86.3	91.2	111.4	110.7	113	108.4	88.8	83.9
FOLR2	145.9	169.1	117.1	105.5	99.7	88.5	137.9	154.7
FOLR3	98.8	110.2	95.2	91	85.9	94.5	96.8	99.2
FOS	591.4	727.5	323.6	312.1	249.5	316.3	547.2	987.9
FOSB	118.6	139.5	123.6	110.4	112.5	113.4	127.6	141
FOSL1	145.3	191.6	143.6	111.6	100.9	107.8	163.1	374.3
FOSL2	152.4	194.7	103.7	86.3	82.1	94.7	149.6	162.8
FOXA1	87.4	94.8	92.6	81.9	87.5	81.6	86.2	117
FOXA2	91.5	94.7	96.3	91.5	95.1	93.5	83.1	86.5
FOXA2	79.6	89	83.3	77	85	78	73.3	84.2
FOXA2	79.9	87.2	91	84.8	86.4	83.6	80.5	89
FOXA3	80.5	93.2	104.7	111	99.7	108.1	89	85.5
FOXB1	92.5	127.4	103.1	88.4	85.5	99.5	99.4	109.1
FOXC1	459.9	626.1	331.3	133.2	122.7	105.3	391	354.6
FOXC2	128.3	163.6	104	105.9	99.7	100.9	136.4	139.3
FOXD1	1710.2	2103.9	1278.6	1299.5	1183.7	1324.9	1552.5	1927.3
FOXD2	379.1	421.7	273.7	276.3	230.2	212.5	310.8	509.1
FOXD3	107.6	124.1	87.8	88.2	88.1	91	108.7	112.4
FOXD4	519.5	655	611.1	446.2	408.8	534.5	434.6	897.8
FOXD4L1	512.7	605.8	187.3	170.2	158.7	179	390.8	340
FOXD4L2	82.8	93.5	87.5	80.3	86.2	81.8	87.8	94.1
FOXD4L3	128	141.2	93.3	91.4	86.8	93.4	123.9	159.3
FOXE1	127.1	156.8	109	111.1	91.6	92.7	120.9	134.2
FOXE3	98.3	101.7	100	94.2	87	81.9	99.4	105.8
FOXE3	104.6	143.6	101	83.5	77.5	92.9	104.9	111.2
FOXF1	102.7	118.7	103.8	87.4	85.2	89.6	110.1	118
FOXF2	167.2	203.1	132.5	95.8	94.1	100.8	142.8	279.4
FOXG1B	105	108.5	91.4	83.1	83.4	80.3	97.4	116
FOXH1	99.3	118.7	105.6	87.5	91.3	98.6	101.5	106.2
FOXI1	83	99.3	84.8	81.4	86.7	82.2	83.5	104.4

FOXI1	97.8	111	116	103	88.4	100.6	95.1	111.4
FOXJ1	138.3	159.5	98.2	90.8	82.3	78.1	122.8	157.4
FOXJ2	578.3	750.9	586.7	627.9	585.4	561.8	561.3	595.1
FOXJ3	2471.6	2911.5	2157.1	3017.5	2213.9	3526.4	2267.2	3445.3
FOXK1	741.7	1143.4	835.9	980.2	739	1203.8	917.1	1644.8
FOXK2	122.4	134.4	87.4	90.4	84.4	93.7	110.5	133.8
FOXK2	127.6	152.2	119.9	110.8	87.1	111.2	119.8	155.1
FOXL1	98.3	108.3	89.5	90.2	86.7	88.5	97.6	108.1
FOXL2	95.9	126.2	109.9	91.4	83.5	96.8	103	142
FOXL2	192.6	221.8	220.6	107.4	84.5	98.3	192	479.6
FOXM1	222.2	304.7	309.8	254.5	221.7	320.4	229.2	400.5
FOXM1	177.1	217.3	205.7	200.1	173.3	219.1	152.6	242
FOXN1	107.5	119.1	93.8	91.9	96.3	89.1	115.9	110.3
FOXN4	81.6	96.5	96.3	86.7	85.2	85.6	92.3	96.5
FOXO1A	114.9	171.2	118.2	147.5	132.7	156.9	122.8	148.6
FOXO3A	80.1	90.8	89.3	82.8	74	85.7	85.3	91.1
FOXO3A	1052	1784.3	945.3	2013.9	1975.3	1898.8	1124.4	1005
FOXP1	116.8	148.7	104.4	86.8	92.9	103.4	110.1	156
FOXP1	142.7	174.4	105.9	95.3	90.7	92.5	129	151.2
FOXP1	128.7	150.3	101	86.1	97.8	89.6	125.1	144.5
FOXP2	201.7	299.5	131.4	167.7	131.6	196.6	159.4	164.3
FOXP2	188	281.9	128.6	173.2	141.9	226.9	159.3	171.2
FOXP3	106.2	132.8	88.8	91.2	84.2	89.2	110.9	133.6
FOXP4	119.8	159.8	130.9	120.8	127.8	135.9	133.2	189.4
FOXP4	97.8	108.1	100.6	88.2	98.3	91	97.8	109.1
FOXQ1	94.7	110.1	118.2	96.7	96.6	83.9	93.2	103.1
FOXR1	73.3	97.6	102.6	94.3	92.5	89.4	90.6	91.1
FOXR1	84.2	87.3	96.3	91.1	92.1	97.1	83.6	97.6
FOXR2	84.1	89.6	90.1	83.2	82.2	84	83.4	89.1
FOXRED1	1313.5	1438.1	722.6	585.9	461.1	552.6	996.1	1074
FOXRED2	585.1	673.1	428.1	549.8	429.7	397	501	400.3
FPGS	127.9	125.7	103.2	98.2	99.8	111	112.5	132.1
FPGS	140.4	162.8	91.6	79.1	79.3	90.7	116.7	164.3
FPGS	91.7	103.9	102	103.9	98.9	87.9	82	104.9
FPGT	78.5	97	89.9	86.3	89.2	90.7	76.1	95.8
FPR1	106.6	113.5	95.3	134.2	100.6	153.7	104.4	110.6
FPRL1	96.4	104.9	106.1	108	91.2	105.6	91	87
FPRL1	99.7	101	94.5	110.7	91.9	123.8	93.4	98.2
FPRL2	90.5	98.2	101.5	85.8	92	91.4	89.1	87.1
FRAG1	818.2	1054.7	300.3	271.3	216.2	289.2	580.6	639.1
FRAP1	454.1	640.8	551.3	641.6	550.3	894.1	469.5	721.2
FRAS1	77.7	78.4	92	81.3	84.8	87	76	72
FRAT1	172.8	219.8	135.3	129.2	123.3	122.7	144.9	156.3
FRAT1	119	146.4	124.4	112	99	107.6	95.5	116.4
FRAT2	1828.9	2524.8	1530.5	1185.7	919.3	1243.3	1675.8	2056.4
FREM1	101.1	120.7	88.7	72.8	80	81.7	104.8	114.3
FREM2	122.6	144.9	110.8	92.5	95.3	98.9	135.4	131.1
FREQ	276.5	314.9	323.7	205.2	165.2	285.2	210.6	256.7
FRG1	2144.3	2630.9	381.3	394.4	327.3	463.8	2147.8	3589.4
FRG2	72.5	76	87.5	80.5	73.7	79.6	77.5	66.3

FRK	87.5	95.6	86.2	79	82.5	89.4	91.9	93
FRMD1	85.1	100	89.6	95.9	79.2	95.4	84	97.3
FRMD4A	114.5	140.8	104	112.2	103	142.9	109.9	120.7
FRMD5	92.5	100.3	96.5	91.9	83.5	91.1	93.9	99.8
FRMD6	103.2	130.8	89.8	83	89.2	84.3	113.6	123.5
FRMD6	102.7	108.4	104.4	96.8	102.3	91.3	91.9	112.1
FRMD6	144.9	167.8	111.8	97.4	89.9	98.9	122.5	155.4
FRMPD1	116.8	147.6	98.9	92.6	83.9	102.4	102.6	144.6
FRMPD2	90.3	89.2	101.7	93	85.3	78.8	86.8	85.3
FRMPD2	82.9	101.2	93.8	87.7	84.6	84.1	90.7	96.4
FRMPD2	90.4	102.6	97.9	93.3	84.6	88.6	86.9	89.7
FRMPD2	85.6	97.6	101.8	99.3	89.4	96.5	93.7	89.7
FRMPD2	77.3	80.7	102.8	88.5	91.1	78.8	89.7	83.1
FRMPD2	79.4	86.7	92.1	88.2	80.1	93.3	85.5	88.3
FRMPD4	117.6	134.8	107.4	104.4	92.9	111.5	125.6	120
FRRS1	131.7	158.6	113.8	92.2	100.7	104.9	134.8	153.8
FRS2	99.9	104.9	96.8	102.1	95.8	98.6	98.1	119.4
FRS2	96.8	124.1	97.8	95.1	86.4	79.6	92.5	113.9
FRS2	77.5	71.5	88.3	84.4	79	83.5	73.6	83.6
FRS3	338.4	361.9	210.5	220.5	205.7	244.4	239.9	239.2
FRY	169.4	201.8	124	130.5	124.9	177.1	154.6	195.1
FRYL	104.3	137.1	108.6	102.9	91.4	99	103.9	119.8
FRZB	118.5	136.8	98.7	95.9	94.6	100.8	114.1	138.1
FSCN1	5962.4	8190.8	4918.4	4302.6	3069.1	4132.7	5381.3	7676.7
FSCN2	87.1	95.2	76.7	80.8	75.6	84.2	84.9	99
FSCN2	79.1	81	100.1	83.7	79.5	93.6	89	83.4
FSCN3	93.8	108.3	106.7	89.1	92.4	92.3	92.8	102.7
FSD1	107.1	106.8	92.9	85.4	92.2	96.6	90.9	118.2
FSD1	80.5	99.3	98.4	74.2	75.1	78.8	100.8	113.5
FSD1	98.1	103.6	105.9	105.9	102.9	107.8	96.3	83.1
FSD1CL	101.6	92.4	95.7	90.4	90.4	91.6	95.3	97.7
FSD1NL	134.3	149.9	104.1	119.9	109.6	107.1	119.8	143.2
FSD2	84	84.2	103.9	87.5	86.8	87.2	83.1	84
FSHB	74.1	87.5	98.2	92.5	87	87	74.2	81.1
FSHB	122.3	146.3	105.4	80.7	80.2	94.4	109.3	157.3
FSHR	79.7	91.1	90.7	82	77.9	87.9	80.1	86.8
FSHR	83.7	86.5	100.5	108.4	101.9	96.2	78.3	80.3
FSIP1	87.8	126.9	98.2	90.9	93.8	94.2	90.6	95.5
FSIP2	100.4	116.9	96.2	93.8	84.8	89.8	111.7	110.4
FST	126.4	123.9	83.3	89.9	80.2	88.6	112.2	133
FST	290.6	353.4	177.9	211.9	154.4	242.2	258.5	318.4
FSTL1	177.2	198.6	119.6	99.4	91.8	103.5	160.8	250.9
FSTL3	163.6	168.2	122.8	97.3	113.4	127.3	133.5	195.6
FSTL4	69	80.5	94.6	81.9	79.6	85.1	68.1	87.4
FSTL5	68.8	75.1	81.5	83.6	72.8	81.6	71.8	78.3
FTCD	88	84.3	96.3	84.8	77.9	89.6	79.1	92
FTCD	81.5	102.8	93.5	78.5	77.8	79.9	99.6	108.7
FTHL11	204.7	271.3	123.8	136.8	155.3	167	446	804.6
FTHL12	1167.7	1526.5	484.6	593.9	441.7	649.4	3154.3	7256.4
FTHL17	90.1	123.5	85.8	87.6	79.8	80.4	96.5	116.6

FTHL2	104.4	135.8	91.3	90.4	96.9	97	134.6	212.8
FTHL3	266.1	387.4	170.7	204.9	240.5	228.7	613.6	1230.5
FTHL7	6895.3	9212.8	4401.8	6447.4	6004.6	6613.9	16568	24031.7
FTHL8	108.1	127.7	84.9	78.2	93	87.4	116.7	121.5
FTL	27813.9	29051.4	47906	48185.5	42681.3	49295	29530.8	30029.7
FTMT	75	81.2	92.6	79.1	81.8	88.3	79.3	83.2
FTO	171.6	247.9	163.5	149.4	116.2	146.2	172.5	256.9
FTS	142.7	204.1	102.9	87.1	81.6	107.9	126.7	184.3
FTSJ1	994.6	1146	458.4	458.7	392.4	631.4	728.9	1226.3
FTSJ1	108.8	113.2	112	98.3	95.4	91.3	94.6	109.8
FTSJ2	469	586.5	223.7	141.4	136.5	165.7	314	485.5
FTSJ3	222.2	316.5	129.6	140.9	122.7	156.7	202	227.2
FUBP1	350.3	475	244.1	246.2	203.1	324.5	291.5	430.3
FUBP3	2436.8	3256.1	1603.8	1933.2	1440.5	2613.2	1875.7	3652.6
FUCA1	410.7	479	291.3	371.9	262.7	390.3	349.9	475.5
FUCA2	190.4	219.6	132.7	123.2	107.5	145.3	141.1	217
FUK	252.4	299.9	189.7	178.3	164.3	216.2	184.1	213.2
FUNDC1	97.9	114.4	96.5	90.2	81.4	88.2	100.7	104
FUNDC2	124.1	149.8	99.7	87	90.7	105.7	115.3	130.1
FURIN	138.6	154.3	137.4	130.9	120.9	151.4	134.4	174.3
FUS	418.8	496.4	221.1	196.1	171.6	169.9	337.3	414.3
FUSIP1	78	83.3	84.6	76.1	77	81	81.1	80.5
FUSIP1	859.8	1053	439.7	287.6	246.5	253.9	489.1	473.4
FUT1	109.2	124.1	88.6	82.8	74	79.6	113.3	136.4
FUT10	89.5	121.4	116.8	111.7	98	116.2	95.6	110.5
FUT11	130.3	137.5	81.1	81.2	82.2	81.9	105	150.9
FUT2	72.9	81.7	75.2	71.1	72.1	70.3	78.9	71.3
FUT3	89.4	111.1	95.8	93.9	82	91.7	99.3	109.1
FUT4	3483.4	4498	3072.9	3794.3	2867.9	4327.9	2462	2714.9
FUT5	108.7	112.9	94.9	92.4	87.9	89	110.3	104
FUT6	148.5	148.4	121.8	123.2	98	139.6	134.2	201
FUT6	91.1	95.6	110.8	99.5	99.7	91.9	86.5	89.3
FUT7	220	270.1	161.1	135.5	146.5	162.8	187.4	275.2
FUT8	390.7	521.5	188.1	152.1	118.5	173.3	279.8	509
FUT8	74.2	83	89.8	89.9	93.9	98.4	71.1	81.6
FUT9	77.6	83.4	84.4	80.9	84.5	72.2	87.2	89.5
FVT1	562.1	769.1	329.5	345.9	266	401	573.3	1148.2
FXC1	393.3	487.6	256.1	222.1	175.9	222.8	299.4	413.4
FXN	185.8	242	121.8	132.2	102.7	132.9	173.4	194.3
FXN	119.3	145.7	100.8	89.7	87.6	107.6	104.7	121.7
FXR1	1394.2	1709.9	973.9	771.2	716.6	1320.8	1229.5	2142.6
FXR1	100.5	124.3	92.9	94.8	84.3	90	111.1	122.5
FXR1	1513.6	2027.7	735.9	801.3	705.9	1162.2	1279	1657.7
FXR2	145	148.2	140.8	162.8	159.5	220.3	128.7	207
FXYD1	115.1	129.8	108	94.9	87.8	98.3	113.1	114.3
FXYD1	118.3	144.4	101.6	85.5	85.2	92.1	112.3	133.2
FXYD1	145.2	171.1	101.5	99.2	88.3	92.3	127.4	147.8
FXYD2	167.2	194.8	110.3	104.6	103.5	115.4	167.1	169
FXYD2	80	78.8	80.4	81.4	79.4	79.9	85.8	76.9
FXYD2	118.5	139.3	91	92.2	79.9	88.3	117.6	124.6

FXYD2	94.8	103.7	93.6	85.3	91.3	92.9	90.7	96.3
FXYD3	91.9	113.8	97.5	90.6	91.7	89.6	96.2	96.4
FXYD3	85.8	107.4	87.7	82.9	82.7	86.9	90.6	101.8
FXYD3	91.7	90.6	95.1	87.9	97.1	85.8	89.2	95.1
FXYD4	83.7	104.3	91.2	85.9	86.8	79.9	81.1	94
FXYD5	2004	2250	1123.9	607.5	455.8	456.5	2431.1	3199.3
FXYD5	119.4	132.3	133.8	115.8	114.4	122.9	97.1	111.8
FXYD5	4565.5	5965.8	2736.6	1601.1	1483	1286.6	4838.7	5921
FXYD6	105.5	115.7	107	95.3	93.7	96.9	98.4	99.5
FXYD7	92.5	112	100	92.1	79.1	95.9	91.6	95
FYB	94.2	100.6	105.8	104.9	98.7	107.3	94.1	118.6
FYB	306.6	323.3	221.1	671.7	543.4	1140.8	217	440.3
FYCO1	242.4	318.7	183.3	234.8	201.8	283	261.9	294
FYN	218.3	283.5	170.9	179.2	147.2	236.2	248.5	449.9
FYN	129.4	199.8	126.1	129	113.8	142.2	140.7	182.9
FYN	265	348	157.8	129.5	116.5	143.6	231.6	300.5
FYN	110.6	124.5	117.1	138.8	115.6	161.4	101.7	111.9
FYTTD1	153.7	146.8	105	111.8	110.7	94.5	137.8	189.3
FYTTD1	584.4	716.2	188.5	171.2	162.4	245.3	564.8	997
FYTTD1	238.1	278.1	116.3	106.6	113.1	113.9	168.6	284.5
FZD1	130.6	188.6	108.7	129.1	124.4	167.1	151.6	163.3
FZD10	119.8	128	95.6	86.7	84.9	94.2	114.7	118.8
FZD2	416	626	322.2	377.8	303.8	404	392.8	513.4
FZD3	141.7	207.2	143.7	113.5	106.5	122.9	136.7	170.9
FZD4	129.1	148.5	126.7	126.1	113.6	133.8	137.5	147
FZD5	87.4	107.5	101.9	89.3	87.5	92.5	107.3	133.4
FZD6	255.9	268.8	125.6	121.9	99.8	126.4	211	351
FZD7	248.9	291.1	241	209.1	201.7	183.4	228	247.4
FZD8	87.9	99.8	94.1	88.5	81.6	86.9	86.9	101.3
FZD9	530.8	651.1	376.6	275.8	213.4	254.6	561.6	625.1
FZR1	160.1	182.7	114.7	95.2	95.7	97	155.7	170.8
G0S2	120.1	153.6	97.4	97.8	99.3	99.6	130.7	128.7
G10	3642.7	4055.1	2207.4	1551.9	1158.8	1635.7	3916.8	6419.5
G1P3	2082.6	2570.6	1647.6	1204.9	1089.8	756.5	2028.6	2077.3
G3BP1	443.9	554.8	363.8	361.2	354.7	454.3	382.1	920.2
G3BP1	530.9	743.5	233.3	226.3	172	251.1	408.9	526
G3BP2	136.6	158.2	101.3	84.7	100.4	99.4	115.9	147.5
G3BP2	2396.4	3033.7	1457.1	1556.7	956.6	2280.6	1795.5	3511.6
G6PC	93.1	97.6	88.9	88.2	84.4	89.7	85.2	92.7
G6PC2	126.1	132.1	103.3	97	95.7	96.9	115.3	123.3
G6PC2	87	100.2	90.9	91.6	82.9	86.5	93.6	100
G6PC2	78.3	94.7	82	83.9	75.8	77.6	83.9	88.6
G6PC3	482.7	632.9	436.3	337.4	272.7	366.3	429.6	709.5
G6PD	282.6	323.9	350.3	373.6	269.2	433.8	354.2	715.6
G6PD	1523.4	2121	1021.9	1158.7	926.2	1005.3	2018	2900.5
G6PD	103.6	108.7	94.1	92.4	82.6	87.2	99.7	112.8
GAA	261.2	299.7	386.4	309.6	278	591.8	248.6	401.9
GAA	173.6	204.5	181.3	173.1	167.9	285.1	145.7	271.2
GAB1	70.4	79.3	87.7	84.6	79.1	90.1	74.9	81.4
GAB1	111.1	136.6	94.6	106.4	87.8	91.4	110	123.7

GAB1	95	126.4	89.2	81.8	78.4	90	96.5	119.3
GAB2	251.9	334.5	192	290.1	274.9	465.2	268.6	342
GAB2	116.3	118.3	96.4	106.4	103.4	90.1	108.8	122
GAB2	96.4	115.2	86.3	84.2	78.1	88.4	100.9	107.7
GAB3	121.8	143.7	121.6	139.3	124.6	137.5	108	166.1
GABARAP	181	198.2	121.6	127.7	104.2	104.6	164.8	175.3
GABARAPL	124.7	131.4	119.2	119.2	122.9	132.1	246.8	443.3
GABARAPL	272	284.4	121.6	112.1	102.6	118	283.7	434.2
GABBR1	109.9	120.1	117.2	99	104.9	118	91.3	91
GABBR1	99.1	111.1	85.6	92.9	78.4	81.8	95.7	104.2
GABBR1	83.6	99.5	99	91.6	97.5	96.9	92.6	92.1
GABBR2	242.8	329.6	273.8	797.6	788.3	1030.4	220.8	435.1
GABPA	78.1	77.8	84.5	76.9	84.3	89.3	72.1	92.3
GABPB2	68.7	83.6	82.2	86.1	80.8	80.9	83.3	78.2
GABPB2	736.9	953.8	308.3	227.5	184.6	281.3	594.4	1014.9
GABRA1	71.5	82	84	82.7	76.3	85.9	77.8	73.4
GABRA2	89.3	128.2	97.1	88.1	87	101.5	93.6	107.2
GABRA3	94.6	115.3	93.3	91.5	74.9	81.4	99.2	99.4
GABRA4	86.9	88.9	98.4	92.6	89.9	81.7	83.3	77.8
GABRA5	93.8	105.6	84.2	83.1	89.3	84.5	92.5	99
GABRA6	107.6	127.2	96.2	84.6	84.2	89	106.8	118.5
GABRB1	71.7	78.7	93.2	84.1	85.8	83.9	70.3	86.2
GABRB2	92.5	99.2	91.9	92.2	86.6	75.2	94.8	99
GABRB2	80.4	92.7	101.9	86.5	88.2	86.7	83.2	95.7
GABRB3	78.7	82.8	98.5	96.1	77	84.6	94.6	90.3
GABRB3	99.3	100.4	103.7	90.5	90.3	94.5	96.7	103.6
GABRB3	104.6	111.5	104.7	89	84.7	97.9	107	120
GABRB3	107.5	109.2	98.2	92.7	85.8	80.7	103.4	106
GABRD	114	128.6	93.6	89	84.6	96.5	115.2	135
GABRE	97.5	113	104.5	94.5	103	104.1	94.7	104.8
GABRE	104.6	131.1	86.1	83.9	79.7	91	113.1	124.3
GABRE	121.6	117	122.1	108.7	106.4	102.8	102.7	118.1
GABRG1	93	95	100.6	82	78.7	90.5	103.4	95.7
GABRG2	120.7	131.6	109	118.8	97.8	118.1	118.4	131.9
GABRG2	169.5	195.2	157.6	145.6	152.4	155	174.7	221.3
GABRG3	88.2	104.5	96.9	86.1	85.5	97.8	102.1	115.8
GABRQ	77	95.1	80.9	76.5	75.3	75.2	91.3	91.2
GABRR1	77.9	82.6	94	86	84.7	85.6	67.7	86.4
GABRR2	89.5	97.7	81.8	79.4	84.4	87.4	94	107.4
GAD1	71.8	90.2	82.5	79.3	78.7	80.3	73	76.3
GAD1	97	103.8	100.3	91.2	89	95.8	92.5	109.3
GAD1	92.6	93.3	85.7	86.4	66.8	87.5	83.4	88.2
GAD2	87.4	101	100	96.9	97.8	92	82.1	90.6
GADD45A	595.7	714.4	321.9	329.9	294.9	405.8	821.6	1191
GADD45B	240.5	281.1	135.5	128.8	127.4	132.7	213.6	279.7
GADD45G	83.6	87.4	85.5	93.5	93.6	94.8	85.7	83.2
GADD45GI	138.4	149.9	98.4	95	88.9	90.1	141.6	214.3
GADL1	70.7	79.9	102.2	95.7	96.1	90.6	77.3	82.9
GAGE1	100.8	127.4	91.8	91.3	94.7	84.8	113.6	122.2
GAGE1	94.2	106.8	98.6	98.9	85.6	93	95.7	111.4

GAGE1	86.1	91.1	116.3	105.3	104.6	115.3	83.3	82.6
GAGE2	81.8	80.5	112.6	105.4	94.9	103.8	83.3	86.9
GAGE4	118.6	122.9	101.4	84.9	98	87.9	106.3	119.2
GAGE5	77.3	96.3	85	86.9	84	82.6	83.1	87.6
GAGE6	104.1	121.5	92.1	91.7	79.8	86.7	110.1	113.4
GAGE7	91.3	108.3	106.4	90.4	88.9	100.7	96.2	107
GAGE7B	81.3	89	95.2	83.1	86.3	78.9	83.5	85.7
GAGE8	88.7	99.8	84.6	78.3	79.2	79.8	89.1	95.6
GAK	2048.1	2340.9	2192.4	1815.6	1696.1	2028.9	1546.8	1620.7
GAL	1211.8	1414.7	1046	665.4	427.6	670.2	1182.8	2134.2
GAL3ST1	94.8	101.8	98.4	85.7	90.4	81.3	92.2	98.6
GAL3ST2	163	167.6	138.3	111.7	107.2	126.7	138.9	160.3
GAL3ST3	89.8	93.7	95.3	95.1	89.7	85.5	89	104.6
GAL3ST4	124.2	133.1	91.8	81.3	78.7	91	112.6	136.9
GALC	292.6	344.3	164	140.2	114.4	139	226.6	291.2
GALC	826.7	944.7	694.8	960.3	620	1055.4	611.9	948.1
GALE	84.9	95.4	93.4	91.6	84.4	89.6	87.2	90.9
GALE	274.6	285.7	207.7	230.8	171.9	206.9	234.5	190.6
GALE	102.6	99.5	95.6	97.8	97	101.8	94.6	95.7
GALGT	134.5	174.6	100.2	89.4	87.2	98.1	162.5	191.9
GALIG	91.2	109.3	92.2	91.3	92.2	85.9	95.1	106.2
GALK1	5118.5	6576.4	3880.3	3386.9	2688.1	2916.4	4594.4	4650.8
GALK2	566.1	773.9	298.9	225.2	204.6	252.5	468.2	691.4
GALK2	194.8	303.9	159.6	227.1	197.7	236.3	205.8	273.1
GALK2	179.3	216	105.6	107.6	96.7	105.3	149.6	161.9
GALM	366.8	494.5	335	411.7	361.4	604.5	366.5	500.7
GALNAC4S-	74.9	93.5	76	83.8	71.5	76.3	89.9	109.3
GALNACT-2	134.4	169.1	92.5	88.1	76.8	92.2	116.8	166
GALNS	155.1	188.9	103.5	102.4	96.8	108.7	149.3	187.4
GALNT1	76.1	95.1	108.4	93.2	87.4	84.9	75.5	81.1
GALNT10	104.8	150.3	89.5	88.5	86.9	87.3	131.2	126
GALNT10	125	172.2	128.9	133.9	126.2	166.5	110.1	163.1
GALNT10	115.7	132	117.9	109.2	103.4	121.5	100.3	145.1
GALNT11	422.3	548.5	248.3	254.2	207.3	422.1	407.6	619
GALNT12	512.4	677.9	316.5	377.4	310.7	494.7	386	524.1
GALNT13	83.5	84.7	102.5	97.5	94	82.7	79.7	75.4
GALNT14	149.5	198	106.1	86.7	76	97	154	196
GALNT17	100.3	124.2	97.3	99.3	88.2	98.1	115	122.6
GALNT2	116.7	136	108.2	107	99.3	110.8	103.8	124.4
GALNT3	222.4	217.7	158.6	176.7	175.6	153.8	193.9	262.4
GALNT4	157.9	194	99.7	105	102.1	101.6	152.8	193.8
GALNT5	75.8	83.6	89.7	79.4	79.9	80	81.6	76.3
GALNT6	174.7	195.5	132.1	131.2	119.7	129.5	153.1	206.7
GALNT7	87.7	105.3	91.1	86.3	80.8	85.2	79.9	99
GALNT8	104.4	136	101.7	90.3	84.4	83.8	112.4	125
GALNT9	131.9	149	97.8	74.1	84.7	88.1	116.4	123.8
GALNT9	79.6	102.6	79.6	70.4	70.4	67.5	87.9	98
GALNTL1	79.1	92.9	108.7	99.1	93.3	90.7	81.6	84.8
GALNTL2	99.6	113.7	94.4	90.3	76.8	85.9	100.3	111
GALNTL4	99.9	120.5	96.6	80.4	83.5	79.2	98.6	106.3

GALNTL5	72.7	87.5	87.6	84.2	80	83.5	75.9	78.3
GALP	81	88.1	89.8	88.4	76	89.4	79.9	92.5
GALR1	90.4	91.5	96.6	80.8	84.6	92.8	89	84.2
GALR2	1143.6	1567.7	696	282.4	253.2	261.8	890.6	547.6
GALR3	102.6	110.1	101.8	84.6	83.3	84.4	99.6	99
GALT	1267.6	1427.1	626.3	506.4	489.6	547.6	1073.9	887.2
GALT	373.7	420.7	310.9	206.8	214.5	235.4	268.9	311.3
GAMT	2256.7	2850.2	1862.1	1378.3	1182.6	1337.6	2206.7	2174.7
GAMT	118.7	146.2	84.3	84.3	73.2	87.7	98.7	124.6
GAMT	1358.5	1880.6	1114	695.7	550.1	674.9	1182.9	1727.5
GAN	126.6	131	96.8	96.8	88.7	96.5	122.1	134.1
GANAB	448.3	513.3	414.9	398	358.9	383.3	443.7	579.5
GANC	95.6	112.7	104.8	90.3	90.2	103.1	90.1	124.4
GAP43	90.8	120.3	90.4	86.2	84.1	81.7	92.3	100.6
GAPDH	5031	6904.7	2742.7	2418.3	1472.3	2170.7	4072.8	6066
GAPDH	2544.7	3316.8	728	449.2	404.2	715.7	2368.9	3421.8
GAPDH	12859.3	16555.4	9670.7	5166.5	3760.7	5378.3	11280.8	13497.7
GAPDHS	91.6	93.4	97.3	84.6	80.9	84.3	85.8	93.9
GAPVD1	1272.1	1917.2	774.1	971.5	782.6	1238.9	1402.8	1789.8
GARNL1	262.7	333.9	169.1	167.4	159.9	224.3	286.5	376.3
GARNL3	94.5	108.1	94.5	95.7	99.4	97	83.8	92.6
GARNL4	138.4	187.2	146.6	130.6	158.1	169	138.5	165.8
GARS	4218.1	5981.4	3056.1	1760	1196.9	2356.3	4978.1	9277.3
GART	1759.8	2379.3	1748.5	1659	1122.3	1938.7	1371.4	2049.2
GART	1100.5	1617.5	432.9	315.3	238.4	330.4	777.4	907.6
GART	108.1	124.7	101.5	100.5	89.4	94.8	103.8	107.3
GAS1	134	158.1	120.7	100.2	96.7	100.3	130.5	164.6
GAS2	82.5	78.5	96	91.7	86	78.4	75.6	80.6
GAS2	91.2	97	85.7	88.1	80.8	78	76.3	99.8
GAS2L1	106	121	111.4	100	92.1	105.6	112.7	124.7
GAS2L1	89.7	103.5	99.8	90.2	88.9	102.6	79.9	104.8
GAS2L1	92.5	115.5	95.8	85.1	74.8	88.6	83.3	108
GAS2L2	133.9	177.2	92.4	84.3	84.3	96.8	144.1	159.9
GAS2L3	198.8	239.9	152.6	217.3	188.6	194.3	186.7	198.9
GAS6	852.2	1043.3	891.6	1450.7	1183	1983	708.9	1365.4
GAS6	826.2	1067	887.5	1498.8	1168.6	1948.4	699.7	1406.5
GAS6	165	208.6	138	162.3	142.2	112.7	145.6	170.5
GAS7	94.5	101.7	98.1	87.2	86.5	85.9	88.6	102.4
GAS7	153.9	208.4	157.2	224.8	229	316.2	163.1	193.5
GAS7	86.9	96.5	93.6	104.6	93.6	92	82.1	90.9
GAS7	85.4	85.3	117.5	107	104.3	106.7	76.9	91.5
GAS8	168.8	193.4	214.9	165.2	169.8	193.2	150.5	239.3
GAST	121.4	143	122.3	110.7	111.8	120	113.8	135.2
GATA1	87.4	95.5	97.5	92.5	91	90.9	85.3	91.6
GATA2	212.6	286.6	228.7	132.8	113.8	136.4	217.5	324
GATA3	92.7	89.9	94.6	98.7	85	97.4	100.5	87.1
GATA4	84.3	95.4	94.8	86.7	87.1	85.6	87.1	93
GATA5	87.7	93.9	99.8	94.2	92.9	83.1	87.4	87.6
GATA6	96	107.7	85.5	89.6	82.1	87.2	97.4	99.8
GATAD1	237.4	369.9	143.9	152.7	152.7	223.9	233.5	383.3

GATAD2A	1763.3	2271.4	1636.3	1504.5	1111.8	1592.7	1667.5	1842.7
GATAD2B	240.2	352.7	208.8	250.4	234.8	353.7	223.2	427.4
GATM	390.3	601.8	269.8	259.1	255.5	409.4	257.9	370
GATS	166.6	229.8	147.7	177.5	177.8	161	154.1	158.8
GBA	938.3	1291	950.6	828	673.9	856	807.9	1174.6
GBA2	201.6	247.4	225.1	155.8	142.2	203.7	184.5	253.4
GBA3	77.2	84	99.9	93.5	90	95.9	78.7	82.9
GBAS	1022.6	1181.4	640.5	775.4	516.7	1046.3	862.5	1584.3
GBE1	947.4	1357.6	500.1	763	566	1243.7	741.6	1200.1
GBF1	181.7	235.5	181.8	196.6	172.1	253.2	169.6	201.4
GBGT1	470.6	536	269.3	120.7	110.3	131.7	295.3	292
GBL	467.6	550.1	373.7	282.8	224.9	339.9	433.3	830.4
GBP1	94.6	108.3	89.4	90.6	96	96.5	100.5	101.9
GBP2	220.1	277.6	163.7	243.8	235.5	312.1	212.2	220.1
GBP3	195	229.2	133.7	124.3	113.2	145.6	168.3	187.5
GBP4	146.4	173.5	112.9	117.7	115.3	122.6	130.6	153.7
GBP5	126.1	147.2	105.5	141.2	125.8	162.8	119.8	133.1
GBP6	121.8	135.5	90.3	76.9	77.6	87.1	106.8	124.7
GBP7	83.4	90.5	92.7	84.5	78.1	81.4	82.4	88.1
GBX2	138.2	175	104.8	101.3	96.8	110.7	126.5	155.3
GC	88.1	104.6	93.6	78	78.5	81	89.3	89.3
GCA	917.6	1090.5	477.1	551.4	427.9	629.2	633.1	1103.9
GCAT	340.6	443	209.2	221.3	214.4	275.6	320.7	488.1
GCC1	453.2	588.8	266.9	250.4	246.8	363.9	464.1	615.9
GCC2	109.6	127.3	101.1	94.2	91.3	98.1	104.6	116.2
GCC2	73.9	80.6	83.4	86.3	86	82	72.4	78.2
GCC2	111.9	129.4	94.8	88.3	82	110.3	105.2	121.9
GCDH	206.1	233.3	169.6	146.4	131.9	196.3	173.3	218.2
GCDH	544.5	674.8	253.1	219.6	174.2	257.3	425.2	527.7
GCET2	112.4	118	91.7	80.6	82.2	80.4	109.5	104.4
GCET2	136.4	137	90	81.7	87.1	88.9	110.4	131.3
GCG	95.4	112.8	97	97.8	89.2	99.9	97.8	102.3
GCGR	90.6	97.3	89.9	93.8	90	88.1	92.7	85.2
GCH1	136.7	162.8	102	104	96.2	96	124.4	148
GCH1	115.5	134.8	104.9	103	89.6	93.3	95.9	116
GCH1	147.9	149.8	89.4	83.9	84.5	87.3	122.6	183
GCHFR	631.2	760.8	505.1	365.4	355	464.1	539.4	557.3
GCK	97.3	100.7	84.7	77.5	80.9	87.7	97.8	105.2
GCK	79.6	91.5	89	80.5	86.5	91.6	78.9	84.9
GCK	103.6	112.8	88.3	84.7	79.3	83.8	104.6	114
GCKR	90	110.9	105.9	89.3	91.4	98.5	92.5	97.1
GCLC	87.5	87.7	89.8	82.5	80.3	88.5	81.3	99.3
GCLM	163.8	188.7	154.9	157.3	133.1	134	561.9	1602.3
GCM1	105.3	114.7	95.6	91.4	92.7	92.7	99.5	117
GCM2	74.9	86.5	108.3	94	87	87.5	86.8	85.8
GCN1L1	697.8	914	296.1	316.9	266.3	471.1	675.3	713.2
GCN5L2	1778.4	2115.8	931.4	1017.8	932.3	1160.2	970.9	998.4
GCNT1	88	114.2	86.7	85.2	87.2	93.2	92.5	100.5
GCNT2	83.3	86.6	90	72.9	97.9	81.3	85.7	87.3
GCNT2	88.2	94.6	97.9	87.6	90.1	90.6	87.6	95.8

GCNT2	119.1	144	105.7	111.8	111.6	115.9	131.1	164.1
GCNT2	90.2	107.2	98.7	91.7	87.7	93.1	90.6	97.3
GCNT3	71.5	85.6	98	78.6	80.8	86.3	73.9	87.9
GCOM1	96.7	100.5	98.6	88.4	97	88.1	94.7	97.9
GCOM1	106.7	118.4	93.6	93.5	88.8	96.1	101.5	125.6
GCOM1	98.9	101.8	94.2	94.5	86.8	94.3	87.9	95.2
GCOM1	89.7	110.8	107.8	111.1	95.6	102.3	85.1	101
GCS1	232.3	296.2	231.1	191	143.6	202.3	168.7	271.4
GCSH	598.2	759.2	280.7	271.4	213.9	251.4	449.5	513.8
GDA	91.9	92.2	94.4	78.2	80	80.2	101.5	98.5
GDAP1	125.7	167.9	102.9	89.3	80	89.8	108.2	119.6
GDAP1	168.8	214.2	115.8	104.1	95.3	91.7	126.3	127.6
GDAP1L1	111.1	127.3	104.7	94.1	81.6	86	105.5	124.7
GDAP2	239.7	336.6	203	172.9	144.1	217.7	202.6	264
GDDR	106.1	120.4	106.4	97.5	95.2	92.5	94.8	122.1
GDF1	107.4	115.2	95	92.2	84.2	91	101	136.3
GDF10	90.5	93.1	83.8	83.9	86.4	81.7	85.9	91.9
GDF11	130.7	180.8	94.2	81.7	79.7	88.5	127	153.9
GDF15	91.3	103.6	116.3	100.5	92.8	101.1	98.3	101.3
GDF2	78.8	77.9	98.1	78.1	84.2	87.9	85.1	80.3
GDF3	89.8	92.6	105.3	112.6	90	97.2	89	91.7
GDF5	99.1	113	97.1	97.8	89.8	89.1	97.5	90.8
GDF6	94.2	96.6	91.6	79.3	75.3	73.2	86.4	97.2
GDF7	147.1	143	101.1	89.3	92.6	87.5	136.4	144.1
GDF8	89.7	98.3	92.9	86.7	90.8	90.4	86.6	102.6
GDF9	96.2	108	104.5	94.8	92.7	99.2	88.5	86.5
GDI1	265.8	285	207.1	173.9	198.9	282.7	228.8	606.4
GDI2	3342.9	4710.9	1116.7	952.1	622.9	1347	2917.3	5812.2
GDNF	92	104.7	99.7	101.9	89.8	89.8	94.8	100.8
GDNF	95	112.6	85.5	83.2	81.5	73.5	84.8	98.3
GDNF	136.6	156.9	93.6	82.9	85.3	85	134	144
GDPD1	223.1	245.8	137.5	153.6	149.2	169.2	206.1	307.2
GDPD2	102.6	137.5	99.9	82.9	88.5	96.7	99.6	104.2
GDPD3	121.1	149.4	112.4	101.5	112.9	114.1	125.9	149.6
GDPD3	82.8	104.3	96.8	81.9	78.8	91.1	93.2	112.1
GDPD4	113.2	139.5	99.7	85.5	79.1	84.7	108.8	132.2
GDPD5	156.1	190.3	177.7	189.4	164.5	254.6	140.2	253.4
GEFT	105.7	114.8	92.2	95.4	89	84	102.6	131.5
GEFT	107.2	122.8	100.5	87.3	80	94	105.4	111.4
GEFT	105.8	125.7	112.8	103	96.1	109.8	101.8	106.8
GEM	98	117.9	90.8	90.8	83.2	78.6	95.5	115.7
GEM	105.1	116.9	97.1	101.5	94.3	99.9	108.7	129.1
GEMIN4	1401.3	1837.5	1063.4	895.3	620.5	785.2	940.6	918.2
GEMIN5	305.4	413.5	287.4	183.1	146.9	268.8	223	278
GEMIN6	1743.2	2135.1	1152.7	672.4	496.3	813.7	1288.3	1465.8
GEMIN7	122	147.8	92.6	87.2	79.1	81.6	121.2	144.8
GEMIN7	84.9	93.3	98.8	95.6	90.4	92.6	95.7	97.7
GEMIN8	145.7	185.5	134	113.9	95.8	123.3	129.4	156.4
GENX-3414	185.2	240.2	131	106.3	106.1	125.7	188.1	243.9
GFAP	100.7	113.9	97.3	91.4	82.5	91.5	99.2	103.9

GFER	138.9	144.6	86.3	88.7	79.9	89.4	111	173.4
GFI1	2555.8	3003.9	2099.2	1492.1	1028.3	1856.9	1611.3	2441.4
GFI1B	90.6	96.2	89.6	89	91.3	102.9	85.1	91.1
GFM1	1534.1	1906	771.4	881.1	641.4	1198.5	1236.7	1815.5
GFM2	155.5	199.4	103.4	84.5	85.1	142.4	136.2	187.5
GFOD1	227.3	273.3	140	146	142.9	175.2	186.1	228.5
GFPT1	389.9	521	215.1	264.7	243.1	330	397.8	741.2
GFPT2	85.5	92.9	104	99.5	104.2	109	78.2	90.5
GFRA1	130.7	136.9	90.1	86.6	81.5	94.4	104.5	122.9
GFRA1	96.6	121.5	93.3	92.9	101.4	91.6	106.3	100.9
GFRA1	117.3	124.7	105	101.8	94	92.7	102.3	111.3
GFRA2	183.2	207.2	167.9	201.3	190.6	243.8	154.6	190.5
GFRA3	83.4	88.9	106.3	105.4	94.5	110	81.5	110.3
GFRA4	80	86	93.1	93.5	80.9	88.5	82.2	86.1
GFRA4	109.7	121.6	133.2	103.3	105.1	125	114.2	123.7
GGA1	115	112	108.2	116	103.1	97.1	102	113.4
GGA1	24053.9	23155.8	13798.9	18055.8	16391.7	14077.1	21515.5	28720.8
GGA1	124.9	131.4	108.8	105.5	95.5	92.3	115.4	137
GGA1	132.8	134.8	90.4	92.9	112.8	88.7	120.7	135.2
GGA2	621.3	810.3	467.2	810.6	696.8	928.1	489	533
GGA3	243.6	268.2	221.3	176	180.8	228.5	209.6	330.6
GGA3	280.3	357	234.5	239.7	198.2	250.5	263.3	454.1
GGA3	109.9	131.3	95.2	81.6	71.6	86.1	113.5	168.5
GGCX	151.4	187.7	152.7	200.4	158.3	215.5	139.2	167.3
GGH	529	602.4	290.6	197.2	173.2	240.9	493.8	1009.8
GGN	75.4	84.3	90.7	82.5	80.6	85.3	75.9	80.2
GGPS1	141.5	178.9	132.3	131.4	122.9	139.2	134.2	165.4
GGPS1	2043.7	2590.9	1002.4	984.3	658.6	1289.1	1739	3447.5
GGT1	78.2	91.1	93.7	105.6	102.6	99.1	81.8	99.2
GGT1	189.3	261.3	214.8	209.4	202.5	291.4	171.5	226.5
GGT3	128	163.1	106.2	106.8	101.7	109.4	134.4	153.3
GGT6	87.8	94.8	103.6	101.5	90	98.8	97.2	98.4
GGTA1	88.1	97.9	86.1	89.5	83.8	88.8	81.3	85.6
GGTL3	122.4	132.4	93.5	88.7	92.3	102.9	120.1	121.6
GGTL4	155.3	182.8	154.8	147.4	124.5	189.9	140.5	180.9
GGTLA1	118.6	111.9	93.2	88.3	85.8	94.4	115.7	116.1
GGTLA4	93.9	100.8	100.7	95	101.5	101.8	85.5	104.9
GGTLA4	141	171.4	107.5	103.2	107.9	106	129.4	133.3
GGTLA4	121.9	133.2	103.7	101.1	100.2	89.7	119.6	112.2
GH1	86.7	81	90.6	92.4	93	91.5	82.5	89.4
GH1	77.9	89	81.5	78.8	73.4	83.3	81.2	88.2
GH2	75.7	85.8	92.1	89	80.9	84.8	85.4	94
GH2	80.5	97.3	93.2	89.8	79.7	88.5	86.4	94.4
GH2	88.4	106.8	96.1	91.5	85.8	88.1	89.8	93.3
GHITM	7486.3	10109.9	5256.1	5136.5	3625.9	5678.4	7799.3	8920.7
GHR	110.1	129.5	90.8	93	76.7	92.9	93	90.8
GHRH	90	93.1	91	93.1	88.9	80.6	89.1	95.1
GHRHR	75.8	87.3	90.6	82.4	78.4	85.5	82.6	91.9
GHRHR	96	139.9	93.2	84.5	86.2	91.8	115.4	122
GHRHR	89.4	103.4	82.3	77.8	74.9	73.3	91.1	105.2

GHRL	150	179.3	125.5	151.3	254	139.7	131.1	132.8
GHSR	82.5	96.8	89.3	84.3	77.7	86	103.8	112.8
GHSR	94.7	111.8	93	94	92.4	95.1	106.5	95.2
GIF	94.9	98.8	90.6	88.9	89.1	80.8	94.2	103.6
GIMAP1	137.5	180	91.1	89.8	78.2	101	159.9	177.7
GIMAP2	354.5	382.7	164.1	169.9	117.1	187	256	418.9
GIMAP4	81.7	97.9	97.1	143.5	117.5	140.9	75.1	91.9
GIMAP5	204.4	261.9	179.7	143.5	136.4	199.2	177.9	262.2
GIMAP6	132.3	154.7	88.2	93.2	76.8	88.5	121.9	154.5
GIMAP6	457.4	682.3	366.6	332.5	237.3	401	422.3	480.3
GIMAP7	103.8	136.5	96.7	80.7	78.8	87.6	113.1	126.9
GIMAP8	189.3	241.6	134.1	148.4	141.7	182	179.4	208.8
GIOT-1	97.9	104.1	91.4	81	79.6	85.8	87.3	88.8
GIP	96.2	99.3	90.7	90.7	80.6	85	95.6	100.6
GIPC1	106.9	121.8	85	76.8	89.6	91.6	125.4	122.4
GIPC1	569.1	774.6	658.9	519.9	389	591.1	508.4	1119
GIPC2	98.1	111.9	95.3	87.7	82.3	84	102.5	103.5
GIPC3	72.8	79.9	82.6	79.1	74.1	79.7	78	81.8
GIPR	95	104.3	90.5	83	79	83.5	95.5	104.3
GIT1	143	179	154.3	146	140.8	176	141	180.9
GIT2	560.2	806.5	263	329.7	290.8	424	407.4	463.7
GIT2	268	359.8	178.8	169.8	165.9	176.2	221.9	263.3
GIYD1	522.1	601.9	216.1	155.4	136.6	150.8	348.3	498.8
GIYD1	254.3	355.5	183.2	116.8	120.4	103.1	197.8	226.1
GIYD2	77	86.6	86	76.3	75.8	82.7	77.3	80.4
GJA1	85	97.1	82.7	79.1	81.7	80	102	111.6
GJA10	77.4	89.2	92.9	94.3	97	78.9	76.6	92.3
GJA12	126.1	158.5	119.3	99.2	101.2	109.9	131.7	114.2
GJA3	88.3	89.1	100.3	91.4	90.8	87	71.8	89.9
GJA4	87	97	88.4	88.2	80.8	92.2	89.2	95.8
GJA5	165.2	192.1	108.1	98.5	87.2	87.9	154.2	151.9
GJA5	103.7	111.9	100.8	101.8	102.5	96.7	93.1	105.7
GJA7	154.3	179.2	94	87.4	85.8	97.2	117.5	173.6
GJA7	8942.4	9033	3738.8	7031.9	6975.8	4497.7	7583.8	8977.9
GJA8	111.3	117.2	109	99.8	86.1	99.9	115	113.2
GJB1	116.8	151	102.7	95.8	82.3	87.7	123.6	131.8
GJB2	87.6	92.3	99.7	91.8	81.6	90.3	87.6	97.2
GJB3	135.5	190.8	85.3	90.9	82.4	93.6	142.9	170.5
GJB3	76.9	87.2	98.6	84.2	84.9	78.7	83.7	89
GJB3	82.1	90.2	102.8	92	87.6	89.4	90.2	84.4
GJB4	107	148	84.9	80.4	82.7	93.6	106.7	119.9
GJB5	85.5	117.1	90.9	84.8	75.3	81.8	85.8	112.2
GJB6	82.2	96.7	93.1	78.8	83.2	82.4	75.1	80
GJB7	89.3	94.6	89.2	90.7	93.2	88.9	87.1	93.8
GJC1	157.9	195.5	155.2	180.7	135.3	206.5	166.3	198.5
GJE1	106	122.8	88	93	76.1	88.2	101.5	122.5
GK	390.1	545.7	232.7	220.9	238.4	283.3	324.4	426.6
GK2	335.8	424.9	172.4	162.9	148.6	155	351.2	466.2
GKAP1	94.2	116.3	93.3	87.3	91	94.4	99.6	122.5
GKN1	110.3	115	105.1	93.8	88.1	95.2	99.1	116.3

GLA	5038.6	6595.1	3564	2868.8	2400.8	3007.2	5575.1	6696.8
GLB1	2653	3311.3	2384.3	3067.6	2417.3	3071.2	2407.9	3109.7
GLB1	105.7	96	91.3	89.5	88	86.6	100.6	91.2
GLB1	1728.1	2261.8	2009.4	2945.3	2170.6	2744.7	1635.6	2575.6
GLB1L	135.7	160	107.3	100.8	100.4	105.6	121.6	127.8
GLCCI1	209.7	219.7	157.9	186.6	174.2	214.4	175.7	243.1
GLCE	278.2	391.5	183.9	223.3	168.7	257.5	248.1	383.2
GLDC	579.4	737.9	560.3	135.7	117.3	156.9	365.7	438.2
GLDN	77.5	85.7	88.5	84	86.9	82.4	79.9	91.3
GLE1L	2014.9	2405.8	1395.2	1056	878.9	1267.3	1507.2	1854.6
GLE1L	384.7	498	243.4	159.5	140.1	179.9	289.5	313.7
GLG1	2054.3	2630.8	995.9	1002.9	854.7	1142.6	1745.1	1821.9
GLI1	141.6	172.4	139.3	110.3	117	117.7	136.5	149
GLI2	90.2	94.5	81.1	91.4	88.6	81.1	105.3	105.9
GLI3	113.5	121.8	105.5	104.5	96.8	99.5	115.9	112.3
GLI4	112.9	125.8	135	112.2	104	113.5	100.5	124
GLIPR1	531	676.3	256.6	511.4	457.4	482.4	447.7	486.6
GLIS1	74	91	94.9	99	84.9	89.8	81.3	83.8
GLIS2	78.7	85.1	96.3	73.6	86	85.8	85.2	82
GLIS3	97.3	95.7	98.9	88.6	88.6	87.5	99.9	101.2
GLIS3	94.4	106.4	92.9	82	84.1	88.6	102.2	90.6
GLIS3	83.5	97.8	87.7	97.3	86.9	88.9	84.4	91.8
GLMN	90.8	96.1	95.8	94	93.4	96.8	90.9	88.8
GLMN	1216	1474.4	592.1	750.9	589.7	1038.7	934.1	1317.2
GLO1	16875.4	19985.5	17599.4	17196.1	11837.8	25030.3	13422	20951.3
GLOXD1	274.2	331.7	247.4	145.9	125.9	140.8	194.5	217.5
GLP1R	84	95.1	99.1	94	85.5	86.2	88.2	88.6
GLP2R	79.9	92.9	78.2	85.4	78.9	78.3	89.8	92.2
GLRA1	95.7	102.3	89.3	87.9	92.4	86.1	112	107.2
GLRA2	102.4	95.7	83.1	78.6	82.6	87.2	104.9	117.6
GLRA3	79.3	96.8	89.8	93.8	83.1	89	91.9	95.9
GLRA3	81.2	98.2	86.1	78.4	81.1	87.6	91.8	92.9
GLRB	218.9	275.2	172.7	169.3	154.4	204.4	180.1	324.3
GLRX	961.2	1070.9	243.9	446.4	321.6	548.2	926.4	1114.1
GLRX2	433.7	580.9	440.7	337.7	267.1	411.1	412	401.2
GLRX2	127.6	157.9	102	100	92.6	98.9	133.7	136.8
GLRX2	991.3	1189.9	323.9	260.8	226.3	310.3	1077.9	1810.7
GLRX5	8591.9	11601.8	5045.8	4421.3	3423.5	4631.9	7929.7	8658.9
GLS	318.9	406.4	187.3	185.9	176.3	235.6	278.8	372.7
GLS2	92.4	104.7	92.3	78	85.2	81.1	88.4	94
GLS2	76	86.8	91.5	88.5	86.8	79.9	74.4	84.2
GLT1D1	122.7	135.1	78.7	82.6	76.3	80.1	103.6	143.2
GLT25D1	2827.5	3604.5	3777.2	2855.2	2510.5	3180.9	2384	3356.6
GLT25D2	120	146.7	136	113.2	110	124.3	112.9	127.8
GLT6D1	93.3	93.5	87.7	87.8	88.4	93.5	99.1	93.6
GLT8D1	380.5	458.6	135.9	144.1	130.2	141.5	276.3	364.8
GLT8D1	91.9	100.2	101	89	84.4	89.5	80.9	87.2
GLT8D1	163.2	199.5	116.8	93	91.1	112	141.9	227.8
GLT8D1	128.1	150.5	114.2	102.2	84.3	96	119.8	114.4
GLT8D2	91.1	102	101.5	87.7	93.8	94	97.3	102.7

GLTP	1172.2	1537.3	938.9	799.8	600.8	869.1	1126	1472.2
GLTSCR1	245.5	314.2	227.8	183.4	197.4	196.5	238	256.7
GLTSCR2	10992.3	14523.8	9153.3	8099	7818.6	10174.4	11775.6	12934.6
GLUD1	2304	3224.4	1580.9	1114.3	979.9	1713.1	1997.2	2969.9
GLUD2	238.9	268.6	132.7	139.3	104.1	126.6	175.8	245.3
GLUL	212.7	276.5	136.8	107.1	104.9	98.2	188.3	227.8
GLUL	81.5	95.1	104.5	96.9	94.8	96.7	77.8	80.2
GLUL	85.3	106.2	101	93.3	93.1	91.7	90.8	90.6
GLULD1	87.7	88.2	84.4	86.9	79.2	85	85.2	88.9
GLYAT	95.2	110.5	98.5	82.1	86.2	84.2	106.3	100.7
GLYAT	97.7	109.4	96.6	92.8	80.8	98.8	93.5	113.7
GLYAT	121	154.2	84.4	80.9	79.3	83.1	120.4	142
GLYATL1	96.9	103.8	101.9	88.4	96.6	95	92.2	99.4
GLYATL2	77.9	92.6	87.7	82.1	86.7	83.4	82.1	85.6
GLYCAM1	81.8	88.3	85.8	88.1	81	89.2	83.9	94.9
GLYCTK	170.1	202.5	144.8	114.5	116.4	136.6	147.1	165.7
GM2A	191	269.1	151.7	184.2	141.3	147.2	173	234.6
GM632	323.4	358.8	230.8	238.5	199.7	225.5	222.4	203.7
GMCL1	931.6	1190.2	363.4	490.5	338.1	435.5	686.2	1190.4
GMCL1L	79.5	76	100.7	89.3	82.5	99.8	74.7	77.2
GMDS	1093.3	1382.7	764.2	747.3	618.9	675.4	896.2	912.9
GMEB1	286.8	376.8	179.9	205.9	182.9	212.5	236.4	375
GMEB1	270.9	389.5	207.2	208.6	161.7	192.2	202.5	335.7
GMEB2	547.2	598.8	456.8	527.1	400.5	541.3	459	582.8
GMFB	2294.9	3068.5	1845.2	1773.7	1472	2138.8	1957.7	3022.3
GMFG	5293.7	6462.4	1875.9	1425.3	1204	1798.6	5177.9	6408.8
GMIP	142.9	181	192.3	230.3	186	270	159.3	291.2
GML	114.2	108.5	80.4	81	80.4	91.4	97.7	137.4
GMNN	768.5	929.8	305.7	312.5	257.3	402.5	670	1268.9
GMPPA	578.5	733.2	552.1	398.8	334.1	493.9	492.9	763.6
GMPPA	210.1	257.2	110.4	112.6	97.4	110.2	204.8	248.2
GMPPB	514.3	626.9	413.6	245.4	219.6	290.5	431.1	444.3
GMPPB	256.7	291.9	153.6	137.4	122.5	143.2	222.8	231.9
GMPPB	95.2	96.9	98.7	100.3	85.3	87.4	89.4	91.7
GMPR	114.5	125.1	98.7	97.4	91.9	99.7	108.9	113.6
GMPR2	1013.8	1260.2	395.5	235	263	328.6	857.9	1307.2
GMPR2	151.6	191.1	110.4	93.9	89	85.6	144.8	158.1
GMPR2	1923.7	2373.2	728.9	667	546.9	894.8	1844.7	2437.1
GNA11	139.6	195.5	187.1	182.4	182.5	209.6	133.9	176.9
GNA12	553.3	656.6	437.2	512.7	379.8	486.8	460	579.1
GNA13	430	512.6	190.5	201.7	193	273.7	383.1	780.4
GNA14	129.6	169.6	126.8	117.9	117.1	135.1	123.7	139.6
GNA15	996.2	1205.9	922	867.1	909	871	1128.8	1264.4
GNAI1	97.5	105.2	96.6	82	89.9	91	93.5	110.9
GNAI2	2162.4	3025.8	2061.7	2118.3	1798.7	2603.1	2049.3	5185.4
GNAI3	532.6	719.2	245.8	234.8	182.1	259.2	500.1	819.6
GNAL	98.5	124.9	93.7	87.3	90.3	90.4	91.1	107.8
GNAL	81.6	91.5	98.7	96.8	100	93.3	91.9	97.1
GNAO1	87.5	102.9	101.3	87.2	74.1	89.4	91.4	88.3
GNAO1	98.6	105.8	108.8	94.4	83.6	93.9	99.6	95.6

GNAO1	97.7	106.4	97.5	89.3	81.9	91.7	102.4	100.6
GNAQ	404	590.6	245.2	268.2	255.8	434.6	258.5	407.2
GNAS	170.1	211.2	122.9	110.1	104.4	107.8	156.7	188.7
GNAS	84.8	80.8	87.3	80.9	77.4	88.7	78	86.6
GNAS	12308.9	17489	11285.1	10667.2	9130.4	14240.3	10876.6	18444.2
GNAT1	106	132.2	102.1	95.6	97.4	100	110.8	130.4
GNAT1	106.3	125.2	102.6	102.5	92.6	96.8	107.9	113.2
GNAT2	112.9	126.4	90.1	86.7	87.9	93.7	106.9	153.4
GNAZ	113.7	121.5	97.9	88.6	85	84.3	104.3	126.2
GNB1	6125.8	7617.9	4051.6	4277.2	3407.9	4875.8	5711.4	6138.8
GNB1L	213.6	294.4	142.4	95.9	91.5	100.3	234.9	240.1
GNB2	203.5	270.7	206.6	150.6	129	174.5	182	402.1
GNB2L1	26198.1	25237.2	19745	18548.9	15417	18606.8	27742.7	24569.4
GNB3	101.4	120	96	91.8	86.9	86	103.9	115.3
GNB4	210.5	328.5	155.9	111.6	107.8	89.4	271.3	308.4
GNB5	737	1082.4	338.6	381.3	357.8	277.5	680.3	999
GNB5	166.2	229.2	160.2	165.3	146.5	146.8	192.3	273.9
GNB5	105.1	105	92	88.3	71.2	89.2	100.8	112.3
GNE	925.9	1230.7	259.7	333.5	282.6	394.8	860.5	1237.5
GNG10	2519	3560.5	1028.8	729.2	589.9	819.2	1776.3	2099.5
GNG11	122.7	165.1	119.1	87.9	81.4	93.8	179	221.8
GNG12	116.6	130.8	110.7	95.9	97.5	108.7	111.3	137.6
GNG13	80.7	87.7	90.9	84.8	88.7	85.7	78	82.4
GNG2	85	98.2	96.7	92.7	92.1	96.6	87.4	96.4
GNG3	110.6	140.4	98	80.2	90.3	92.2	103.7	136.9
GNG4	407.3	545.6	387.3	161.5	128.1	169.2	361	554.9
GNG5	832.4	961.5	417.1	436.4	295.8	404.6	742.8	1628.3
GNG7	171.7	215	136.2	151.5	151.6	189.2	167.9	187.7
GNG8	84.2	88.7	88	86.4	81	83.3	95.3	88.5
GNGT1	109.6	135.9	97.3	87.4	92.1	99.5	108	120.1
GNGT2	101.7	126.7	87.1	82.5	85.5	89.1	106.8	119.2
GNL1	541.3	789.1	336.2	218.6	194	232.8	407.6	360.3
GNL2	4315.4	5430.6	1724.3	1734.4	1429.9	2984.6	3458.1	4272.6
GNL3	2699.9	3312.7	800.8	690.6	625.2	1223.6	1866.3	2934.1
GNL3	4325.9	5607.8	2439.6	1774.4	1326.3	3071.1	2708.2	3540.1
GNL3	155.9	175.8	115	102	109.3	93.8	145	157.3
GNL3L	2056.1	2495.3	1066.9	1071.7	790.7	316.2	1737.6	2442.3
GNLY	108.6	124.5	87.6	90.1	77.2	88	126.3	115.4
GNLY	96.7	102.8	97	101.8	105.4	111.7	95.9	114.5
GNLY	87.1	105.7	81.6	81.2	82.3	95	88.1	93.4
GNMT	97.1	104.2	109.5	104.5	116.4	119.3	98.9	102.9
GNPAT	2612.1	3288.3	1447.1	1486.9	1086	2449.5	2073	3239.5
GNPDA1	1959.2	2460.4	1551.2	1673.2	1166.7	1712.2	1896.2	2098.4
GNPDA2	98.8	109	90	80.1	84.8	79.9	99.8	118.7
GNPNAT1	141.3	166	104.4	95.1	78	95.3	120.5	177.5
GNPTAB	113.5	126.1	89.1	95.2	84.2	94	108.8	121.8
GNPTG	828.6	929.8	869.2	1038	995.3	1164.1	749.5	803.6
GNRH1	184.3	183.6	134.7	132.4	122.1	146.3	171.3	214.3
GNRH2	83	97.3	89.7	85.9	84.1	86.5	74.6	94
GNRH2	107.8	131.1	93.4	94.2	84.1	87.5	122	168.9

GNRHR	76.5	75.7	87.8	81.6	92.7	87.5	77.4	70.9
GNRHR2	104.5	113.7	95.1	84.3	71.7	81.6	94	123.1
GNS	2167.4	2850.2	1875	4323.3	3574	4418.7	1742.9	2072.8
GOLGA	116.6	135	108.3	101.7	103.6	104.3	108.1	118.1
GOLGA1	163	187.8	130.7	172.8	173.4	245	143.8	175.4
GOLGA2	263.2	384.2	180.1	218.9	196.3	244.8	294.9	392.9
GOLGA2L1	97.5	134.3	83.7	93	87.2	92.6	109.8	131.6
GOLGA2LY:	108.8	119.2	112.7	83.5	85.3	89.5	105.9	123.1
GOLGA3	916.7	1268.3	595.9	955	770.4	1330.7	916.4	1752.6
GOLGA4	136.3	197.7	126.1	129.3	115.7	162.2	137.5	213.4
GOLGA6	83.3	97.2	103.5	94.9	93.8	93.9	86.2	94.6
GOLGA7	3519.7	5095.4	2505.8	2376.5	2076.7	3625.7	2991.2	5206.2
GOLGA7	139.6	143	90.3	91.3	88.1	87.7	116.6	125.8
GOLGA7	206.6	244.1	108.3	96.8	91.3	95.7	205.3	225.7
GOLGA8A	267.3	150.7	142.2	128.2	147.5	121.3	128.2	176.8
GOLGA8B	903.7	787.7	698.3	1182.8	1131.5	2241.9	476.5	939
GOLGA8E	82.1	100.6	96.7	93.2	82.6	94.1	82.4	85.4
GOLGA8F	86.7	86.7	101.9	83	90.9	89.8	85.4	84
GOLGA8G	95.6	94.8	99.8	97.3	94.1	88.4	87.4	97.1
GOLGB1	106.8	111.2	86	88.3	84.3	115.8	101	107.6
GOLPH2	489.9	666.6	278.8	230.9	190.1	270.5	406.8	741.9
GOLPH2	150.2	216.4	94.8	96.4	84.9	88.9	141.7	166.8
GOLPH3	2077.9	2769.3	871.5	1136.3	812.7	1816.5	1628.1	3091.8
GOLPH3L	421.4	489.9	338.2	363.4	264.9	425	408.6	756
GOLPH4	343.5	515.5	349.6	411.3	312.2	424.4	298.4	523.6
GOLT1A	80.5	92.4	89.8	81.9	82.1	91.6	87.9	85.7
GOLT1B	328.4	407.8	141.7	120.7	113.8	145.2	259.8	486.6
GON4	98.8	134.6	90.4	84.1	82	82.3	104.7	112.9
GOPC	502.2	558.8	209.1	182.6	182.6	203.7	375.3	786
GORASP1	467	507.9	461.3	492.5	396.1	644.4	394.7	953
GORASP2	1191.9	1511.8	632.1	700.9	508.3	685.1	1070.9	1423.6
GOSR1	201.1	226.6	166	122.3	117.6	123.6	193.6	259.2
GOSR1	154.6	218	113.1	106.3	97	110.5	152.1	209.6
GOSR2	164.9	198	129	119.6	123.6	132.8	162.6	216.1
GOSR2	600	751.1	285.2	255.8	208.2	280	461.2	559.2
GOSR2	104.3	156.3	117.1	99.8	90.4	98	104.7	120.2
GOT1	660.2	788.6	412.4	401.3	258.9	420.9	525.6	910.2
GOT1L1	96.9	112.6	105.7	94.6	85.8	88.9	94.4	94.9
GOT2	1417.3	1886.5	1422.2	1108.9	737.3	1308.9	1047.4	1804
GP1BA	188	206.9	143.3	136.7	132.5	141.3	191.1	203.8
GP2	128.8	164.9	92	81.2	86.1	92	115.4	155.7
GP6	114.8	142.2	108.5	122.2	96.5	128.8	122.4	128.2
GP9	287.5	353	147.4	146.4	134.1	133.2	260.3	212.2
GPA33	147.3	165.7	134.2	153.4	165.9	191.8	125.2	142.6
GPAA1	2249.9	3345.5	2491.8	1071.3	768.5	977	1954.7	3068.1
GPAM	184.2	204.3	120.8	115.3	86.5	121.6	163.1	272.7
GPATC1	340.5	445.8	250.3	245.9	210.7	322.7	328.5	461.3
GPATC2	380.7	464.4	301.1	342.4	309.6	569.9	314.5	598.5
GPATC3	188.1	238.7	145	148.6	132.3	181.5	200.9	266.9
GPATCH4	285.5	316.3	125.6	110	97.6	88.6	201.8	279.6

GPATCH4	2032.1	2630.6	1288.1	1050.2	882.1	1484.5	1475.7	1846.6
GPATCH4	1030.9	1331.6	583	477.7	349.5	731.7	834.5	1110.9
GPBAR1	109.2	119	102.4	98.1	101.8	102.4	98.4	119
GPBAR1	113.1	126.7	99.9	128.5	103.7	114.8	117.2	103.4
GPBP1	344	441	139.5	153.9	127.3	193.7	323.9	786.9
GPBP1L1	226.6	230.9	127	149.2	132.2	178.8	187.5	296.2
GPC1	109.5	134.8	115.5	101.9	96.1	112.7	115	141.2
GPC2	160.2	200	116.8	116.1	107.8	121.5	157.6	177.9
GPC3	551.7	764.2	360.6	392.8	319.2	525.6	468.9	661.1
GPC4	322.8	408.7	263.9	305.2	242.3	353.7	251.7	390.4
GPC5	105.9	102.6	107	109.7	106.1	126.7	103.8	114.3
GPC6	205.9	225.9	149.9	147.5	131.2	151.9	174	244.1
GPD1	84.5	81.8	89.7	79	82.2	85.7	89.8	88.1
GPD1L	1242.4	1756.2	764.2	910.7	633.2	952.9	861.8	899
GPD2	97.1	101.4	95.3	88.6	78.3	86.5	107.8	114.1
GPHA2	112.9	156.5	82.2	87	85.3	99.2	138	163
GPHB5	110.3	118.2	116	117.2	114.3	115.1	104.6	102.2
GPHN	367.7	482.3	268.8	271.7	216	292.5	302.1	311.5
GPHN	137.3	171.1	117	105.3	94.2	102	113.5	148
GPI	2132.5	2600.4	1943.1	1035.3	824.3	1285.1	1762.2	2744.6
GPIAP1	149	162.2	108.2	110.2	87.4	115.8	125.2	152.6
GPIAP1	1434.9	1927.8	909.5	732.4	556.1	943.9	1159.6	1791.8
GPIAP1	1453.5	2115.4	604.5	538.4	482.5	957.7	892.4	1234.1
GPKOW	438.8	587.8	134.7	141.2	132.9	143.6	415.4	494.3
GPLD1	95.2	95	90.4	77.6	82.9	86.4	87.7	92.7
GPLD1	81.1	94.3	94	95.6	88.6	80.4	88.1	85.2
GPM6A	95.3	102.1	99.9	93.7	96.2	94.6	88.1	103.4
GPM6A	89.2	97.6	93.3	91.6	84.3	89.6	98.1	103.3
GPM6A	149.4	180.1	123.2	97.8	125.8	107.8	160.3	167.9
GPM6B	182.7	216.7	137.7	192.1	171.1	190.4	129.5	175.6
GPM6B	96.4	94.1	97	95.6	84.1	93.7	93.8	94.3
GPM6B	152	191.9	109.8	152.5	131.4	166.1	121.4	142.4
GPNMB	109.1	137.4	107.6	224.8	230.2	403.4	115.5	125.4
GPNMB	108.5	138.2	113.5	522.4	434.6	930.4	105.7	163.8
GPR1	683.5	678.6	273.9	400.5	490.9	328.4	672.6	904.8
GPR101	105.7	117.7	95.4	93	92	89.1	106.1	105.2
GPR103	87.4	87.7	100.3	90.6	102.7	89.4	80	86.9
GPR107	110.5	117.1	109.9	99.5	90.3	95.9	103.6	133.1
GPR108	356.9	451.4	239.9	181.7	199.3	248.6	344	452.7
GPR109A	78	82.1	112	82.8	81.5	79.6	89.9	91
GPR109B	86.7	92.3	99.2	94.3	92.5	98	85.5	92.3
GPR110	86.6	83.2	87.1	87.7	83.1	94.4	86.5	92.6
GPR110	83.8	84.4	81.6	79.3	77.4	85.4	74.7	86.9
GPR110	89.8	95.4	96.1	94.7	90.5	91.9	97.3	92.9
GPR111	101	112.8	99.4	85.3	83.4	85.5	95.9	107.5
GPR112	125.3	132.7	89.4	92.3	86.7	80.8	115.7	123.4
GPR113	110.7	121.6	125.9	105.7	109.7	105.1	113.8	117.5
GPR114	714.5	862.1	662.2	287.6	245.7	274.8	383.4	419.3
GPR115	80.6	85.8	98.6	80.1	79.2	75.6	76.7	83
GPR116	131	126.3	92.2	84.8	90.3	87.2	120.7	134.7

GPR119	175.5	214.3	104.6	107.4	93	108.1	143.6	186.7
GPR12	81.9	85.4	94.1	82.1	76.8	72.9	91.3	82.1
GPR120	90	97.3	90.5	80.9	68.9	84.4	85.9	102.3
GPR123	77.2	95	75	90.6	88	92	82.6	84.9
GPR124	443.1	556.2	415.3	524	396.8	689.8	394.3	711.3
GPR125	140.4	153.3	101.2	85.4	87.4	93.1	135	148.2
GPR126	107.7	139.6	84.8	80.4	77.3	84.1	100.8	118.3
GPR128	94.4	106.9	93.1	93.4	90.1	86	97.2	105.1
GPR132	123.2	145.1	178.8	170.9	170.8	202.6	123.8	155.8
GPR133	104	117.7	97.9	92	90.5	89.5	99.5	111.2
GPR135	100.8	113.1	91.8	91.3	87	92.7	105.2	114.4
GPR137	755.4	946.6	610.5	492.8	444.9	466.4	735.9	797.1
GPR137B	222.7	279.2	190.9	182.6	160.4	197.1	207.6	331.2
GPR139	93.8	96.5	93.2	101.6	96.6	100.4	112.9	103.8
GPR141	99.7	125.4	99.2	104	97.7	96.3	102	106.8
GPR142	101.8	102.5	94.5	96.7	79	85.4	91.5	106.7
GPR143	118.8	138.2	113.5	97.8	86.9	98.7	115.4	174.4
GPR146	123.7	143	108.9	114.9	112.2	125.9	117.3	138
GPR148	125.5	149.9	98.8	84.8	85.3	89.3	121.3	135.6
GPR149	68.6	86.2	77.6	79.7	81.2	83.9	81.4	79.1
GPR149	144.3	192.8	96.8	89.5	86.8	89.9	153.3	165.1
GPR15	92	90.2	101.3	99.6	82.7	93.4	85	93.2
GPR150	86.7	89.7	92.5	92	88.8	85.7	102.5	106.1
GPR151	85.4	97.5	92.7	90.9	74.8	84.4	83.3	99.9
GPR152	85.6	84.3	79.1	85.4	80.7	87.3	78.8	88.2
GPR153	151.4	167.3	105.5	99.8	94.6	106.7	128.6	133.2
GPR154	102.3	120.9	97.4	83.9	70.4	90.1	103.1	111.2
GPR154	84.2	92.6	101.7	87.7	85.7	86	82.8	87.4
GPR154	252	366.3	150.3	107.5	113.8	123.4	252.9	223.2
GPR155	95	105.9	88.8	73.5	75.7	76.6	89	97.9
GPR155	80	91.9	87.1	92.9	84.1	88.7	81.4	91.3
GPR156	128	158.8	102.2	101.3	101.4	87	121.4	133.5
GPR157	76.7	82.7	84.9	79.5	76.3	83.9	82.9	80.9
GPR158	103.8	111	96.2	82.8	76.5	82.1	116.7	133.9
GPR160	1747.8	2223.3	841	2221.7	1585.4	2499.8	1795.8	2755.6
GPR161	93.5	106.2	115	101.4	93.2	96.4	98.9	90.7
GPR161	87.9	104.3	101	96.6	72.8	81.4	83.5	113
GPR162	284.3	363.1	224.8	320.9	414.4	315.6	327.6	305.2
GPR162	79.4	82.9	91.4	97.5	91	87.6	82.5	90.3
GPR17	102.3	115.6	100.8	87.1	75	90.4	104.2	116.7
GPR171	103.1	114.3	95.2	92.8	83.2	89.7	98.6	119.1
GPR172A	1256.8	1589.5	1428.3	833	698.6	883.1	1517.4	2958.2
GPR172B	77.1	89	79.9	91.8	82.1	93.6	80.9	81.4
GPR173	96	111.9	101.9	92.9	92.7	87.1	100.3	112.4
GPR173	94.9	103.5	100.2	92.1	93	93.6	92.5	97.1
GPR174	84.2	95.3	88.4	82.8	82.6	91.7	81.4	88.1
GPR175	407.4	460.5	352.1	258.2	214.4	290.2	420.6	864.8
GPR176	97	120.2	96.1	100.3	92.8	96.4	95.3	106.9
GPR177	126.7	158.7	121	104.6	95.6	109	128.6	165
GPR177	315.3	425.5	252.7	247.5	217.9	350.5	294.9	379.8

GPR177	181.8	235.3	124.1	112.5	106.3	113.1	165.7	223.8
GPR179	104.8	114.9	119.8	104.1	108.6	103.3	104.6	106.1
GPR18	874.2	1269	609	508.6	369.8	631.3	394.2	445.7
GPR19	85.2	83	85.3	91.8	87.8	78.7	85.4	97.9
GPR20	117.1	126.1	92.6	93.4	86.4	89.1	111	128
GPR21	104.1	116.1	106.1	93.9	89.4	97.4	99.7	102.1
GPR22	91.2	105.9	102.8	101.1	94.3	94.2	85.7	98.3
GPR23	432.8	565.6	236.4	228.1	196.3	171.4	360.5	470.1
GPR24	92.3	99.8	104.6	85.3	81.4	93.5	97.8	101.4
GPR25	84.5	88.3	87.9	82.3	89	78.1	76.8	93.5
GPR26	112.4	135.4	98.7	94.2	81.8	82.6	109.3	126.9
GPR27	252.3	290.2	169.9	125.6	120.9	143.5	229.4	267.7
GPR3	119.6	137.4	115.8	108	92.5	110	112.8	126.9
GPR30	85.4	103.3	90.8	131	133.2	142.4	90.2	89.7
GPR30	138.4	157.7	98.2	128.4	123.4	117.8	121	149.1
GPR30	102.1	136.6	92.4	91.9	82.4	88.3	103.2	115.9
GPR31	88.1	102.7	85.7	89.7	81.9	91.3	106.2	105.6
GPR32	80.6	94.8	85.4	86.3	81.9	89.1	86.3	93.9
GPR34	97.9	99.7	84.2	81.3	81.4	84.3	103.5	102.7
GPR34	83.7	85.5	88.3	95.7	86.5	87.6	82.2	86.7
GPR34	95.1	116.5	87.1	84.9	84.9	86.3	98.7	113.7
GPR35	143.7	165.2	99.3	94.9	96.4	105.8	166.4	259.9
GPR37	108	114.2	116.6	106.7	107.3	113.4	92.8	109.7
GPR37L1	75.8	78.8	90.5	83.5	86	85.8	73.9	87.4
GPR39	90.7	96	94.8	93.6	93.2	87.9	91.4	87
GPR4	91.2	110.4	82.7	93	79.6	90.6	88.9	95.6
GPR42	80.1	78.7	87.7	85.7	82.5	86.9	80.8	72.8
GPR44	76.3	94	81.6	78.7	88.1	78.1	75.2	89.3
GPR45	74.2	90.1	90.6	87.1	89.5	84.1	81.3	79.2
GPR50	98.1	97.3	89.7	84.2	84.9	83.3	87.7	97.1
GPR52	122.5	136	95.8	88	79.3	86.8	120.3	133.4
GPR55	136.3	151.3	101.3	98.1	103.3	108.7	113	130.7
GPR56	142.7	180.9	99.9	93.8	98.8	95.8	143.6	165.1
GPR56	79	83.8	110.3	107.1	106.2	109.1	91.9	97.2
GPR56	101.3	118.3	99.4	92.5	87.3	94.9	108	112
GPR6	118.9	132.3	101.4	90.3	97.4	93.1	118.4	116.5
GPR61	98.8	116.4	86.6	79	77.2	83.5	104.9	114.5
GPR62	77.4	89.4	91.8	79.8	75.5	87	93.5	90.8
GPR63	330.9	454.6	243.4	193.6	173.2	196.1	278.8	260.8
GPR64	85.8	92.3	104.2	106.1	101.7	93.8	97.3	87.2
GPR64	83.3	96.2	86.9	84	77.8	74.1	89.9	80.6
GPR65	238.3	271	136.6	127.4	112.3	133.4	221.5	350.1
GPR68	106.3	103.9	90	94.8	83	89.5	103	96.3
GPR75	83.7	90	86.5	96	84.4	93.8	80.8	93.7
GPR77	78.5	83.8	86.2	81.4	82.4	71.9	72.3	83.3
GPR78	121.3	135.6	105.8	90.9	95.1	98.4	118.9	127.5
GPR81	79.9	98.7	102.1	105.1	87.8	90.1	89	92.6
GPR82	83.8	96.5	103.1	92.4	90.2	85.7	85.3	81.7
GPR83	85.9	103.5	93.7	91.7	91.8	93.2	84.3	97.1
GPR84	346	427.9	245	108.7	92.9	114.9	272.7	411.5

GPR85	100.5	116	95.6	102.5	112.4	107.1	97.5	105.6
GPR87	103.7	116.4	107.5	105.7	95.7	111.2	100.6	114
GPR88	83.9	97.1	98	106.8	91.8	93.3	84.6	96.9
GPR89A	548.1	723.2	250.2	204.1	171.2	207.2	362.4	492.7
GPR92	247.4	336.9	241.3	361.7	258.3	383.1	174.9	196.7
GPR97	94.6	99.1	88.1	80.9	80.1	84.8	84.1	96
GPR97	81.8	110.7	90.2	92.2	75.6	87.9	95.6	105.1
GPR98	88.5	105.7	98	86.6	79.4	94.7	99.8	99.6
GPRASP1	113.8	131.7	96.4	89.7	84	84	112.6	126.4
GPRASP2	287	341.7	251.3	244.5	193.7	324.8	225.2	430.2
GPRASP2	120.6	143.3	100.2	90	88.6	95.5	119.7	145.9
GPRC5A	99.1	106.7	94.7	78.4	82.5	86	97.8	103.2
GPRC5B	130.4	146.7	102.9	99.8	88.9	94.4	122.9	132.5
GPRC5C	136.3	160.9	109.6	104.9	116.9	112.7	133.6	150.5
GPRC5C	139.7	157.6	103	93.2	86.6	108.9	124.1	156.4
GPRC5C	348.2	405.2	164.6	157.7	165.7	222.3	274.3	256.3
GPRC5D	85.7	99.8	84.2	84.6	86.5	83.3	84.6	92.4
GPRC6A	103.4	107.9	111.6	94.4	92.8	101.4	98.8	97.9
GPS1	124.5	142.1	124.7	121.2	119.1	101.4	124.7	125.4
GPS1	2430.3	2935.5	1561.5	1362.7	1154	1500.5	2010.5	2030
GPS1	331.5	487.8	234.6	179.4	178.7	209	297	384.4
GPS2	315.5	402.8	272.6	203.1	206.8	252.2	326.5	478.9
GPSM1	91.7	117.7	106.4	83.4	87.5	76.9	88.5	121.9
GPSM2	1144.1	1580.8	840.5	393.9	334.3	506.2	823	1171.9
GPSM3	787.6	915.4	649.9	502	383.6	437.9	704.5	1505.3
GPSN2	9762.1	13204	8018.3	4221.6	3446.5	3907.1	8109.7	11837.7
GPT	116.3	129.3	120.1	99.8	99.1	102.6	116.3	117.6
GPT2	849.4	1179.4	842.2	436.3	402.1	480.1	779.2	1030.4
GPX1	15038.8	16767.4	11341.8	8434.6	6860.7	10736.2	10910.6	15125.6
GPX1	206.8	176.4	122.4	93.9	89.5	108.7	149.6	174.9
GPX2	86.6	101.2	101.1	97.3	93.3	105.9	87.4	76.7
GPX3	90	93	87.3	78.5	75.5	87.3	94.2	107.4
GPX4	5952	7741.3	2305.1	2058.5	1539.3	2714.4	4774	5937.8
GPX4	12118	13576.4	10023.3	9089.7	6909.7	9859.6	9101.5	13344.5
GPX5	93.3	99.1	85.7	84.8	85.3	80.2	87.6	100.9
GPX5	79.3	89.4	100.8	89.9	81.9	82.7	73.7	78.5
GPX6	84.7	95.8	108	98.7	97.2	94.5	83.9	92.6
GPX7	2200.7	2662	1262.9	1738.1	1093.7	2060.1	1692.7	2727.6
GRAMD1A	497.9	612.6	305	208.6	205.5	253.8	395.6	307.2
GRAMD1C	75.6	81.3	89.5	86	96.7	84	82.9	78.4
GRAMD2	83	93.9	90	87.7	84	78.4	88.9	88.5
GRAMD3	299.7	348.1	220.4	361.4	326.3	437.8	241	373.1
GRAP	117.1	122.7	94.4	93.8	86.4	84.7	107.6	116.9
GRAP	706.1	898.1	466.7	423	357.1	400.3	678.5	761.5
GRAP2	75	80.7	89.6	84.1	72.7	73.9	78.4	85.3
GRASP	92.1	110.5	117.6	104.3	96.4	110.6	87.9	96.5
GRB10	84.9	83.4	105.3	90.3	86.2	95.3	85	85.4
GRB10	226.3	295.1	172.7	134.4	132	128.9	260	324.3
GRB10	116.1	111.9	97.5	91.2	90.4	87.8	114	128
GRB10	124.9	154	152	117.9	111.9	105.5	136.2	215.2

GRB14	92	98.1	100.3	93.5	83.4	91.6	93.9	87.9
GRB14	96.6	111.5	85.4	80.5	80	81.4	120.9	121.3
GRB2	868.3	1152.1	834.6	796.3	673.8	1245.1	763.2	1679.9
GRB2	2436.8	3441.3	1152.2	1129.8	912.5	625.8	2181.3	2951.4
GRB7	94.3	107.6	107	87.3	93.7	94.1	105.3	120.1
GRB7	91.9	95	89.2	85.4	73.7	79.3	89.7	91.4
GRB7	91.5	127.9	97.1	94.6	85.9	86.2	84.9	84.8
GREB1	87	106.7	88.5	79.7	79.3	88.7	98.6	108.9
GREB1	95.9	105.7	106	99.3	103.3	98.8	96.8	93.9
GREB1	103	107	106.2	83.7	83	87.5	110.9	114.7
GREB1	124.8	144.1	103.3	88	90.2	93.7	114.5	167
GREM1	88	96.6	96.6	99.5	92.5	94.3	93.6	105.8
GREM2	78.9	92.3	96.5	86.8	90	78.2	88.8	82
GRHL1	166.4	196.7	104.5	95.3	88.3	95.7	167.3	165
GRHL1	106.3	132.3	100.5	82.3	90.4	92.1	110.6	124.5
GRHL1	84.6	82	94.7	93.1	81.7	95.6	85.9	84
GRHL2	94.7	100.6	93.5	87.8	89	82.9	95.5	94.5
GRHL3	82.1	96.2	98.2	90.9	86.5	89.5	84.6	86.1
GRHL3	71.1	80.6	100.3	95.3	86.4	86	79.1	83.2
GRHL3	88.9	113.1	105.8	104.8	94.5	90.9	93	93.9
GRHPR	800.5	959.2	360.7	340.5	255.8	377.6	750.1	1105.7
GRIA1	113.9	150.8	91.2	89.9	79.8	85.7	108.6	113.3
GRIA2	108.6	111.6	94.5	93.4	88.5	82.2	100.2	104.8
GRIA3	117.2	157.3	92.8	85.2	86.2	99	118.4	119.4
GRIA3	96.9	112.1	98.6	95.3	79.5	96.9	93.9	102.6
GRIA3	106.9	113.6	121.7	105.8	111.8	108	102.9	102.7
GRIA4	120.6	127.2	100.7	96.9	95.5	88	113.3	125.9
GRIA4	80.8	83.5	88	81	71	77.6	77.9	81.4
GRID1	90.5	87.9	82.8	93.5	83.5	92.4	89.9	94.6
GRID2	71.9	88.1	87.9	78.3	76.9	78.9	78.3	85.5
GRIK1	105.6	110.7	82.2	83.2	79.8	82.4	108.3	130.4
GRIK1	83.4	89.9	97.4	89	82	96.3	95.7	104
GRIK1	81.7	77.3	87.5	86.6	79.9	81.4	83.8	82.1
GRIK2	102.6	104.9	84.6	88.1	83.6	86.2	99.5	98.6
GRIK2	78.7	89.1	99.1	93	90.9	82.6	74.4	88.5
GRIK3	111.7	138.3	110.7	107.5	98.1	97.5	117.4	114.3
GRIK4	102.7	116.9	84.8	82.5	73	83.5	106.2	116.7
GRIK5	92.2	105.4	108.3	91.4	89.3	90.8	95.1	108.7
GRIN1	82.9	89.3	95.9	98.7	86.9	85.8	83.3	86.1
GRIN1	81.2	95.2	89.5	88	91	89.6	85.9	90.7
GRIN1	409.8	421.6	212.5	181	179.5	194.7	395.4	380.3
GRIN2A	106.1	126.1	109	95.7	87.7	94.1	117.5	143.6
GRIN2B	120.4	127	78.9	84	78.5	82.9	118.4	161.2
GRIN2C	123	161	101.5	96.2	96.6	88.2	128.5	151.1
GRIN2D	84.2	85.5	98.6	85.4	84.9	94.1	87.8	86.1
GRIN3A	85.7	100.3	85.4	85.3	76.3	90.6	96.7	112.9
GRIN3B	202	211.4	188.8	216.3	189.6	206.7	164.4	161.2
GRINA	285	367.1	430.6	332.3	308.7	327.7	270.5	512.6
GRINA	868.2	1066.4	755	751.8	463	468.4	942.1	1886
GRINL1A	75.1	88.7	82.1	71.5	77	77	73.6	80.2

GRINL1A	252.6	311.8	152.3	130.3	139.7	145.1	275.1	353.9
GRINL1A	346.4	468.4	129.3	137	126	164.5	258.1	329
GRIP1	84.5	97.6	105.8	84.9	86.8	94.6	75.8	77.4
GRIP2	75.8	82.7	86.4	91	80.4	87.8	80.7	77.6
GRIPAP1	276.1	347	249.5	252.9	237.6	338.3	243.1	306.4
GRIPAP1	170.7	255.8	148.7	135.6	135.6	156	172.6	195.4
GRIPAP1	1740.1	1464.3	1259.8	1840.9	1743.4	1369.5	1514.4	2756.1
GRK1	122.3	131.8	101.4	115.6	100.6	111.7	112.6	118.1
GRK4	98.5	108.1	88.6	88.3	78.9	88	95.8	93.1
GRK4	93	96.7	95.9	98.9	91	107.4	95.9	94.3
GRK4	97	124.4	92.7	86	93.6	98.2	97.8	111
GRK5	154.2	189.4	107	90.6	87.2	98.7	138.8	161.2
GRK6	187.2	241.5	202.3	166.2	158.6	172.7	143.3	201.6
GRK6	204.9	283.3	186	150.6	148.1	200.6	193.6	260.3
GRK7	81.8	83.3	88.9	75.1	79.4	78	78.8	90.3
GRLF1	136.8	146.6	98.3	92.2	101.2	96.1	125.6	152.8
GRM1	86.2	96	85.4	78.5	84.7	94.8	84.9	95.7
GRM2	133.2	156	135.3	98.3	99	97.1	128.7	167
GRM3	83.2	107.7	86.1	85.1	78.2	84.7	93.7	98.6
GRM4	116.7	127.8	111.4	95.9	90	96.7	110.5	118.8
GRM5	124.3	152.8	89.4	76.7	77.7	79.5	112.7	144.1
GRM6	92.1	99.3	86.8	88.9	69.9	74.5	86.5	96.9
GRM7	76.4	78.3	99	102.6	95.6	97.4	82.5	83.3
GRM7	83.7	93.9	102.7	88.4	93.4	94.6	84.2	83
GRM7	97.6	109	88.9	91	93.8	94.8	102.8	100.6
GRM8	95.8	114.5	93.2	99.8	99.2	105	93.1	122.8
GRN	1776.8	2267.1	1458.4	1856.5	1347.4	1985	1491.1	2671.5
GRN	2055.8	2828.6	825.3	1419.6	1112	1035.7	1740.5	2080.4
GRP	83.7	92	80.8	86.2	79.9	82.1	90.4	93.1
GRP	105.4	114.2	95.3	91.5	85.7	93.5	93.7	108.5
GRPEL1	1483.1	1934.7	821.7	666.9	440.1	762	1309.1	2822.6
GRPEL2	1121.1	1356.2	727	790.1	533.7	970	1052.6	1575.5
GRPR	95.1	116.4	90.8	99.4	97.4	103.4	97	121
GRRP1	92	100.1	97.5	80.9	77.8	84.4	86.6	92.6
GRSF1	1643.8	2207	1145.9	980.1	687.7	1103.5	1318.9	2115.8
GRTP1	99.2	105.7	112.7	112.9	105.3	97.9	98.5	105.9
GRWD1	985.2	1419.8	853.6	361.4	293	456.3	967.6	1712.3
GSC	112.4	148.2	100.4	95.1	86	74.2	114.2	145.9
GSCL	81.3	93.4	98.8	76.4	79.8	88.7	90.4	96.5
GSDM1	150.5	175.4	123.5	106.7	96	122.5	138.4	126.2
GSDMDC1	2012.7	2200	1043.5	707.6	692.6	799.7	1356.1	947
GSDML	128.2	136.7	113.3	116.2	126.2	115.1	114.7	138.2
GSDML	131.1	166.4	102.4	111.7	111.3	95.5	135.9	177.1
GSDML	175.5	184.1	130.5	138.6	150.1	151.4	154.3	190
GSG1	92.1	93.5	83	79.8	70.7	86.8	87.5	99.1
GSG1	103.1	118.5	103.3	99.8	96.3	99.2	104.5	109.9
GSG1	102.7	108.5	112.3	107	98.3	107	93.3	112.2
GSG2	211.7	281.9	173.9	135.8	132.5	148	185.5	206.2
GSH1	109.4	141.3	86.6	77.5	86.9	82.4	114.7	121.9
GSH2	75.7	89.9	79.4	73.5	71.2	82.3	77.6	89.6

GSK3A	151.6	203.5	115.1	105.6	92.8	98.5	129.6	166.1
GSK3B	711.2	897.6	423.6	546.5	435.3	441	645.7	688.8
GSN	499.2	836	439.7	275.4	233.6	391.4	448.6	657.6
GSN	102.1	98.8	83.3	85.3	78.4	79.3	89.3	99.1
GSPT1	7458.8	8110.4	5027	4256.3	2975.5	4312.4	6764	7449
GSPT2	512.5	751.4	346.4	315.3	241.3	402.4	398.7	477.1
GSR	185.7	214.2	127.6	117.2	96.4	103.6	186.3	185.9
GSS	597.7	779.3	549.1	420.3	414	703.8	509.9	664.6
GSTA1	78.1	91.7	91.9	76.8	79.5	85.7	84.3	85.7
GSTA2	73.2	79.6	89	92.2	91.3	91.6	82.7	85.7
GSTA3	92.5	89.5	102.1	96.5	92.9	97.8	81.8	89.5
GSTA4	180.1	224	119.2	135.2	99.9	104.7	159.1	226.3
GSTA5	77.5	118.7	91.6	88.9	82.7	83.7	92.6	106.2
GSTCD	106.8	102.4	113.4	127.1	120.8	109.8	99.2	110.6
GSTK1	3411.2	4206.1	2363.7	2758.5	1996.4	3665.4	2406.6	3453.6
GSTM1	1834.9	2258.4	1766.4	653.8	545.7	589.8	1579.8	2969.3
GSTM1	10295.1	12590.3	7494.2	3989.2	3019.9	2911.6	9686.1	11480
GSTM1L	76.7	85.4	92.7	88.5	88.5	87.2	71.1	76.4
GSTM2	10681.6	13817.6	7511.5	3600.5	2866.8	2874.6	10189	12547.8
GSTM3	346.1	430.1	223.1	287.3	277.6	364.9	317.1	392.2
GSTM4	265.5	367.7	213.9	135.4	118.3	120.9	244	343.7
GSTM4	350.5	404.8	214.8	133.2	131.2	187.9	311.7	441.9
GSTM5	96.1	114	93.3	84.5	90	93	104.5	107.4
GSTO1	5018.1	5280.6	2326	1915.8	1320.2	1900.6	4799.7	5953
GSTO2	117.1	120.9	99.7	96.8	81.1	87	105	107.4
GSTP1	9421.1	12084.6	8001.1	6042.5	5435.5	5179	11451.5	11385
GSTT1	77.3	75.9	81.4	68.8	73	73.2	78.1	71.2
GSTT2	120.5	137.3	97.5	91.3	88	95.6	132.1	129.3
GSTT2	93.9	103.6	104.2	96.1	95.5	97.1	94.6	95.3
GSTZ1	116.3	140.6	95.9	78.6	81.8	86.9	102.7	159.4
GSTZ1	396.1	555.4	262.7	325.9	265.6	303.5	356	339.7
GSTZ1	99.3	96.4	97.4	94.8	87.1	89.9	87.7	97.2
GTDC1	210.7	254.6	119.4	107.7	97.6	115.2	157.4	210.2
GTDC1	261.7	305.9	153.5	125.5	127	176.5	182.6	317.4
GTDC1	95.8	118.8	107.6	103.7	95.8	105	101.8	108.4
GTF2A1	104.9	118.8	87	89.1	84.4	78.6	102.9	108.4
GTF2A1	99.5	113.6	100.9	111.7	99.3	88.1	97.2	93.9
GTF2A1	163.7	197.9	101.8	104.1	112.9	104.1	174.1	168.5
GTF2A2	3305.1	4302.7	1248.3	1502.6	1006.5	1830.9	2501.1	3689.8
GTF2B	1285.6	1704.2	788.1	700.3	597.7	787.2	1008.5	1544.5
GTF2E1	562.6	756.9	455.8	446.8	320.2	662.4	483.6	1016.9
GTF2E2	3044.6	4072.5	2416.6	1363.3	1168.7	1577.2	2926	3180.2
GTF2F1	705	896.7	711.9	775.6	711.4	1136.2	655.9	1268.8
GTF2F2	2615.2	3123.8	1492.7	1514.3	1069.3	1959.2	2307.6	3485.3
GTF2H1	163.2	204.6	110.6	106.7	100.1	136.6	155	241.1
GTF2H2	252	274.8	130.6	102.4	95.6	131.9	209.9	330.1
GTF2H3	911.4	1170.4	341.3	321.7	246.7	286.6	684.6	892.8
GTF2H4	323.8	375	296.5	276	251.8	336.5	232.5	284.5
GTF2H5	3764.7	4970.7	2173.7	2259.5	1643.5	3055.6	3405.2	4304.6
GTF2I	703.4	808.6	263.2	238.1	193.2	148.2	327.4	476.5

GTF2I	187.2	186.3	116.9	92.4	94.4	94.9	142.2	206.4
GTF2IRD1	147.1	176.4	158.8	164.1	151.1	196.9	156.7	251
GTF2IRD1	98	113.5	114.3	107.2	95.4	95.5	99.1	99.9
GTF2IRD2B	88.3	102.5	114.8	101.6	89.3	102.4	95.5	93.3
GTF2IRD2P	89.9	95.4	107.6	90.9	89.9	95	93.2	97.4
GTF3C1	191.1	223.8	257.2	220.4	184.8	315.3	171.9	280.9
GTF3C2	1705.8	2063.4	1201.2	1453.4	1124.8	1980.3	1456.8	2401.2
GTF3C2	409.4	499.9	216.3	233.8	209.2	248	291.3	405.3
GTF3C3	1289.7	1556.3	640.1	749.6	435.1	820.2	1018.8	1676.2
GTF3C4	111.8	140.2	88	90.2	84	97.4	101	138.7
GTF3C5	529.9	708	547.9	324.2	269	465.2	404.1	617.9
GTPBP1	113.6	136.5	116.6	96.4	105.9	111.3	111.4	126.1
GTPBP10	131.6	150.4	115.1	124.9	105.8	115	119.6	137.1
GTPBP2	136.6	153.7	131	137.2	122.8	152.3	131.7	180.7
GTPBP3	209	282	154.8	126	116.1	138.1	159.5	165.7
GTPBP3	73.7	90.7	87.7	90.3	83	90.3	86	90.1
GTPBP3	1661.9	2143.7	1468.4	881.7	791.3	1071.9	1147.3	1222.5
GTPBP4	3288.3	4582.6	1903.9	1511.4	1106.7	1989.3	2663.8	4176
GTPBP5	115	130.1	100.3	95.3	86.3	86.4	105.2	138.2
GTPBP6	2803.9	3606.1	1608.3	1947.5	1763.2	1968.7	2068.2	1805.5
GTPBP8	838.2	1133.1	501.4	557.6	477.7	554.7	694.2	1001.3
GTPBP8	451.8	480.4	235.2	272.4	229.3	225.4	377.3	789.3
GTPBP8	458.2	593.4	211.2	165.7	168.6	173.6	376.6	493.1
GTPBP9	85.6	96.8	91.4	85.2	84.6	86.4	90	97.3
GTSE1	543.9	611	463.4	287	282.8	321.1	529.8	687
GUCA1A	112.7	127.5	113.3	112.6	101	101.7	100.8	119.8
GUCA1B	139.7	152.6	124.1	125.8	114.5	132.1	133.4	210.9
GUCA1C	111.3	127.8	121.5	110.6	100	112.1	111.6	108.4
GUCA2A	120.5	143.8	98.2	91.9	87.2	90.8	102	128.5
GUCA2B	106.8	112.9	98.4	85.2	82.2	89.2	94.8	119.9
GUCY1A2	86.1	108.9	101.3	94.6	92.8	98.4	99.2	107.5
GUCY1A3	92	102.4	91.8	89.1	81.5	83.5	114.3	97.4
GUCY1B3	129.6	132	112.6	97.7	99.1	100.6	116.1	115.3
GUCY2C	88.5	98.8	90.3	84.6	68.3	75.8	90.8	95.6
GUCY2D	114.8	122.5	101	91.5	84.4	91.6	98.8	115.7
GUCY2F	124.6	117.8	93.9	90.1	87.5	87.7	113.1	125
GUF1	565	764	311	218.1	198.8	255.4	415.6	563.8
GUK1	3054.4	3816.5	2147.6	1790.5	1590.1	2590.7	2767.8	4614.9
GULP1	88.6	100.5	96.2	94.4	83.2	90.1	85.9	84.2
GUP1	205.5	264.7	99.6	93	93.2	94.4	195.6	201.1
GUSB	2567.9	2987.1	2131.7	2306.9	1902.9	2736.6	2260.3	2820.6
GUSBL2	363.4	472.1	206.4	341.8	290.5	240.8	262	370.8
GYG1	2524.7	3442.6	1362.1	824.3	610.2	1250.7	1930.1	4204.6
GYG2	586.9	826.6	491.1	596.9	422.3	567.8	373.2	348.9
GYG2	82.6	90.9	86.2	84	83	84.5	87	86.4
GYG2	139.5	182.2	138	141.8	122.6	131.1	121.4	129.8
GYLTL1B	90.6	100.2	94.5	90.9	81.3	82.8	86.4	99.8
GYPA	73.2	86.8	80.6	80	76.7	74.3	80.5	92.1
GYPB	80.7	88.5	88	88.1	83.1	85.1	75.3	81.6
GYPC	1710.2	2198.7	914.2	1107.2	949.4	1303.5	1492.6	1845.5

GYPC	1753.2	2128	904.8	1207.7	852.7	1372.7	1641.3	2126
GYPE	94.1	122.7	88.9	86.3	94.2	90.1	110.6	106.3
GYPE	105.5	131.5	88.1	87.7	82.1	80.1	96.9	123.3
GYPE	69.4	81.8	91.5	94.9	88.2	88.8	71.1	77.5
GYS1	211.6	286.4	216	236.6	179.7	321.4	190.7	278.3
GYS2	100.8	107	101.8	111	96.5	107.3	93.6	99.6
GZMA	125.9	133.9	93.5	91.8	81.8	86	120	128.3
GZMB	77.8	88.5	92	81.4	87.3	88.5	81.8	87.1
GZMH	86.2	93.2	99.4	115.7	111.4	101.7	82.6	86.5
GZMK	86.3	94.8	78.2	90.5	83.4	84.8	90.7	98.3
GZMM	85.6	94.3	100.2	86.9	83.3	90.5	90.4	85.3
H19	101.2	116.8	94.3	83.1	82.8	93.6	97.4	100.4
H1F0	3574	4368.5	2597.8	1596.6	1454.5	2622.5	2511.6	4552.5
H1FOO	80.9	81.2	90.1	83.3	97.2	78.6	81.5	81.7
H1FX	346.9	487.8	420	282.6	273.7	390.5	245.9	448.2
H1T2	110	128.9	109.2	97.1	98.4	100.6	121.6	116.7
H2AFB1	83.7	102.6	83.8	90.9	93.3	90	85.2	91.6
H2AFB2	104.9	137	98	99.2	86.3	84.7	97.4	116.7
H2AFB3	73.2	81	90.2	74.1	80.6	87.4	84.3	86.5
H2AFJ	100.7	92.8	89.3	85.3	89.6	84.7	81.1	99.2
H2AFJ	774.2	1052.8	409	320.6	255.8	327.4	718.3	784.2
H2AFV	218.1	319.4	129.7	125.1	112.4	122	196.7	253.2
H2AFX	311.9	423.6	325.6	144.4	117.6	144.5	245.5	498.5
H2AFY	622.5	805	227.3	147.5	147.3	121.4	468.3	598.1
H2AFY	3671	4902.1	1734	1662	1432.1	1795.9	2791.8	3970.9
H2AFY	27047.1	29976.7	21523.6	20153.2	18810.5	23330.8	23777.7	27104.6
H2AFY2	639.9	832.6	594.8	485.8	357.9	572.2	477.4	578.3
H2AFZ	26880.7	30074.5	20969.1	16695.4	12729.9	15215.1	24824.3	28271.9
H2BFS	270	386.6	185.6	244.5	216.4	195.3	225.7	234.6
H2BFWT	81.4	101.5	95.4	83.4	78.3	86.6	87.9	81
H2BFXP	83.8	103.9	87	86.9	86.7	91.6	85.5	82.9
H3F3A	30987.2	32776.1	36142.8	28586.7	23956	24334.1	31007.9	31500.6
H3F3B	1108.8	1399.8	772.8	1000.4	753.6	1222.2	1082.4	2229.3
H6PD	100.8	115.8	95.8	88.9	90.3	91	95.4	107.3
HAAO	127.4	146.4	106.9	98.9	101.5	105.9	136.4	152.7
HABP2	85.8	91.1	88.7	82.2	78.8	85.8	87.1	86
HABP4	255.5	338.5	143	150.4	124.2	173.2	263.5	441.6
HACE1	170	198	111	102.9	91.2	114.6	165.9	202
HADH2	617.3	822.1	541.2	430.6	363.1	512	636.1	759.8
HADH2	966.8	1248.1	701.6	578.4	469.5	710.4	870	1179
HADHA	875.1	1212.3	700.9	555.5	525.5	637.5	728.7	728.2
HADHB	900	1169	213.2	229.9	214.3	311.1	732.7	1094.3
HADHSC	3982.3	5046.8	2428.7	2264.9	1602.5	2459.6	3060.6	3772
HAGH	300.6	391.5	225.5	151.3	161.6	183.4	331.1	369.5
HAGH	1348.1	1559.8	968.1	776.4	683.5	748.1	1350.1	1318.5
HAGHL	201.3	221.7	208.8	185.3	185.1	261.2	138.4	222.2
HAGHL	191.3	253.2	107.7	94.7	94.5	99.2	192.6	229.7
HAGHL	390.1	425.6	335.1	315.8	292.8	440.4	303.1	451.9
HAK	108.8	141.5	100.4	91.9	84.9	78.5	116.6	127
HAL	186.1	220.5	128.6	228	176.9	301	140.1	139.9

HAMP	96.2	94.7	89.9	86.7	89.1	87.6	92.4	97.8
HAND1	100.4	95.5	93	80	79.8	91.6	85.3	116.4
HAND2	113	109.8	99.8	98.7	87.5	96.9	101.6	104.8
HAO1	115.2	142.7	101.2	88	84	95.1	127	133.4
HAO2	106.3	127.6	89.3	89.1	78.5	82.9	111.6	117.2
HAO2	105.6	131.2	93.1	89.4	90.2	81.6	101.7	109.5
HAP1	94.7	93.6	93.5	91	80.9	93.1	104.1	100.3
HAP1	127.1	151.7	107.1	96.4	99.5	101.3	142	121.5
HAP1	96.3	96.5	89.1	96.9	90	86.1	81.4	91.8
HAPLN1	74.7	79.9	87.3	81.1	73.6	84.9	82.4	83.8
HAPLN2	96.5	113.9	113.1	104.3	101.8	96.1	97.5	103.1
HAPLN3	116.3	141.2	114.1	98	85.5	98.2	101.5	136
HAPLN4	91.3	92.9	108.5	105.3	95.5	103.8	88	83.3
HARS	1477.2	1817.1	944.3	818.3	675	903.2	1353.3	1330.7
HARS2	390.7	625.6	348.6	291.6	255.4	430	328.5	457.8
HARSL	1717.9	2011.7	1052.2	1257	851.8	1325.7	1471.2	2473.2
HAS1	86.7	99.8	80.7	83.1	85.5	86.7	86.2	105.3
HAS2	82.9	91.1	94.9	91	89.1	92	87.1	77
HAS2	77.4	96.9	87.2	84	79.6	78.6	88.2	85.9
HAS3	103.7	117.2	98.3	85	85.5	96.4	107.9	124.4
HAS3	92.3	104.7	109.9	112.3	93	105.7	99.5	109.3
HAT1	2443.7	2579	768.3	647.3	450.8	933.6	1984.7	4552.2
HAT1	1070.7	1343.8	210.8	193.6	176.6	283.3	848.7	1445.4
HAVCR1	85.1	110.7	87.3	79.5	80	91.1	93.7	101.4
HAVCR2	127.3	142.9	130.3	127.5	113.3	146.1	147.4	207.4
HAX1	1497.2	1701.1	624.1	373.1	367.9	552.4	1566.6	1956
HAX1	451.7	658.6	341.6	248.7	227	298	437	485.8
HAX1	431.3	529.7	234.1	177.4	168.6	230.1	449.8	671.4
HB-1	104.7	126	86.6	77.8	81.9	88.5	109.8	119.7
HBA2	119	115.1	106.6	90	85.3	92.6	117.3	110.6
HBB	98.6	108.8	88.5	100.5	97.9	100.2	97.4	92.7
HBD	93	93.6	98.4	89.9	91.7	84.3	87.2	90.2
HBE1	86.4	92.9	90.8	83.5	81.4	81.6	89.1	98.3
HBEGF	245.1	275.3	172.6	148.5	111.1	157.6	229.1	454.4
HBG1	114.3	136.3	90.9	87.1	89.3	91.3	113.7	127
HBG2	76.5	93.4	100.4	98.5	85.4	94.6	76.3	85.3
HBM	92.5	94.8	86.8	88.3	87.5	91.7	90.1	95.5
HBP1	291.4	391.5	220.7	279.6	258.2	340.9	348.7	422.5
HBQ1	152.6	177.9	126.4	88.2	90.5	89.3	144	144.1
HBS1L	99.9	108.9	92	85.3	74.6	97	106.5	102.2
HBXAP	132.7	151.1	94.4	85	92.7	87.7	119.3	163.5
HBXIP	9676.9	11482	7424.8	4444.8	3331	5146.4	10136.4	12353.5
HBZ	99.3	117.4	97.3	85.8	84.2	80.5	98.6	108.5
HCA112	90.2	112.7	91.9	94.6	103.1	96.3	94	101.3
HCAP-D3	561.4	803	618	540.3	520.5	783.7	531.3	845.2
HCAP-G	2290	2332.1	811.6	715.1	583.2	947.4	1742.5	2648.8
HCAP-H2	345.5	406.4	205.1	186.3	143.2	230.7	293.5	375.6
HCAP-H2	112.3	128.4	104.3	90.7	92.2	90	108.9	141.4
HCAP-H2	293.4	382.5	172.1	129.3	131.9	125.2	259.7	318.4
HCCA2	204.1	235	171.5	141.7	137.4	160	214.7	301.4

HCCS	157.1	157.7	101.2	100.4	91.1	102	138	188.5
HCFC1	3129.3	4154.3	2001.2	2376.9	1775	2104.2	3020.2	3793
HCFC1R1	371.5	400.1	163.9	141	121.6	116.9	301.9	138.7
HCFC1R1	1152.7	1317.6	839.3	755.2	518.8	607.5	1108	1980.4
HCFC2	148.1	189.3	131.2	140.6	121.1	191.7	132	217.8
HCG27	128.5	128.6	106.7	102.6	93.1	110.3	113	110.5
HCG2P7	2746.7	2793	1676.6	2904	2659.5	2059	3276.5	4875.3
HCG3	108.6	116.2	94.7	95.9	94.6	93.8	95	140.3
HCG4	98.4	109.3	92.7	83.2	80.5	85.3	93.4	102.4
HCG9	82.9	89.1	88.2	81.9	79.7	83.3	87	89.9
HCK	414.6	525.9	383.5	557.7	574.9	743.4	419.9	714.8
HCLS1	7042.3	8611.2	5309.4	5197.3	4194.8	6947.8	6909.5	9371.6
HCN1	103.9	106.2	101	86.5	85.1	91.4	107	102.7
HCN2	96.7	104.3	93.8	73.8	79.1	84.8	87	93.9
HCN3	138.2	142.5	151.6	114.4	136.8	136.8	118.5	126.9
HCN4	97	122.9	110.7	106.2	107.8	105.6	93.6	111
HCP1	98.2	123.3	124.9	119.4	114	106.9	95.8	110.6
HCP5	252.6	326.5	220.4	142.3	137.1	161	165.5	166.8
HCRT	98.1	102.7	129.5	112.5	120.5	117.7	102	101.5
HCRTR1	92.7	102.8	97.3	94.8	81.2	89.5	93.1	97
HCRTR2	104.9	110.1	113.6	108.7	99.2	102.1	97.1	116.2
HCST	2940.6	3665.4	2695.4	1212.9	1018	983.1	3382.1	4677.6
HCST	314.4	389	225.9	146.4	143	135.2	402.4	417.9
HCTSL-S	109	129.2	101.2	91.6	86.7	84.1	96.8	120.4
HDAC1	7949.3	9426	4756.1	4461.8	3803.2	5131	6978.3	6750.4
HDAC10	127.4	136	139.7	124.1	108.7	137.1	104.3	126.9
HDAC11	121.2	126.4	88.7	91.2	80.5	92.1	109.1	143.7
HDAC2	5830.6	7138.4	2322	2547.4	1548.9	3323.2	3873	9008.9
HDAC3	1150.2	1406.2	874.7	845.4	681.8	990.1	883.9	1735.9
HDAC4	396.1	519.5	240.4	287.1	232.1	272.1	317.6	433.2
HDAC5	144.1	163.8	112.5	84.2	77.2	86.8	157.1	149.3
HDAC5	98.9	103.3	110.1	105.7	97	104.5	90.1	114.7
HDAC6	229	273.3	245.7	288.5	252.2	368.1	203.1	287.7
HDAC7A	80.1	93.4	89.3	85.7	88.6	93.3	86.6	88.7
HDAC7A	100.1	120	98.8	82.1	85.1	90.5	100	120.5
HDAC8	276.5	388.3	185.4	166	140.9	202.2	244.1	268.5
HDAC9	140	172	107.6	98.7	89.1	100.4	131.3	156.9
HDAC9	106.5	138.7	88.5	103.1	99.8	172.5	98	107.5
HDAC9	94	91.4	100.9	98.5	92.3	109.1	89.5	103
HDAC9	84.3	77	74.9	82.2	78	94	89.1	83.8
HDAC9	108.7	119.6	87	106.6	97.2	131.5	84.7	97.5
HDC	90.8	111.7	81.9	81.9	77.6	82.9	89.9	98.9
HDCMA18I	1421.8	1697.7	552.7	518.4	441.7	995	1219.9	2106.4
HDDC2	11039.3	12048.7	4451.2	4798.7	3993.7	5479.6	10270.9	12080.8
HDDC3	1040.5	1312.4	776	562.5	427.6	655.6	776	1075
HDGF	2015.1	2549.2	2084.7	866	757.2	1115.4	1582.2	3749.6
HDGF2	686.9	802.1	322.1	246.2	220.1	287.4	634.7	889.5
HDGF2	395.2	532.3	379.7	367.2	354.7	467.1	339.1	394.8
HDGFL1	108	94.2	100.3	93.3	84.9	95.8	84.8	102.2
HDGFRP3	93.3	120.8	89.5	93.5	87.3	75.8	106	127.4

HDHD1A	632.9	903.8	397.4	341.9	250.3	313.6	520.3	565.8
HDHD2	592.8	714.7	166.9	189.5	194.2	216.6	479.6	527.5
HDHD3	213.4	277.8	167.8	121.5	128.7	127.9	200.4	294.3
HDLBP	165.8	221	104.7	151.9	137.4	122.6	154.5	178.6
HDLBP	97.1	124.4	106.3	101.3	93.9	87.7	98.7	108.1
HDLBP	98.2	96.9	108.9	116.3	110.3	94.4	97.9	101.8
HEAB	543.4	739	353.6	326.2	266.1	338	466.2	704.1
HEATR1	2098.8	2927.3	1638.7	1343.2	945.5	1593	1517.4	1484
HEATR5B	360.9	612.8	317.6	412.8	342.8	582.3	304.5	493.1
HEBP1	784	1064.5	276.2	411.2	252.2	443.3	1116.5	1544.3
HEBP2	3553.6	4055.1	1284.9	1395.7	1042.6	1815.8	3585.2	5148.1
HECA	202.8	267.1	131.6	180.8	158.1	264.7	246.6	391.9
HECTD1	958.6	1560.7	1246.9	2125.2	1798.1	3647.9	1051.2	1821.7
HECTD2	104.8	123.9	86.5	91.8	83.7	86.8	100.8	123
HECTD2	109.8	105.9	95	83.2	78.2	87.6	84.5	102.9
HECTD2	157.3	209.1	101.4	111.4	102	109.5	146.4	166.9
HECTD3	252.9	308	221.2	216.3	201.6	203.4	209.2	261.1
HECW1	97.4	106.8	84.7	81.5	74.3	71	94.2	100.1
HECW2	98.2	117.2	106	99	104.4	98.4	101.6	105.7
HEL308	324.5	395	269.9	263	210.4	376.9	273.8	510.4
HELB	135.7	169.6	109	109.3	111.3	116.8	122.1	146.6
HELLS	549.5	730.6	245.5	178.7	168.7	233.2	402.5	610.7
HELT	79.2	86.9	87.3	91.9	86.2	88.7	87.5	92.4
HELZ	230.6	378.6	252.5	568.8	508.9	782.8	254.1	466.1
HEM1	661.3	819.7	313.7	532.3	469.2	475.9	662.9	654.4
HEMGN	99.7	113.6	96.1	92.6	89.2	83.2	99.9	108.8
HEMGN	77.2	88.5	101.5	89.7	90.3	86.1	78.1	91.7
HEMGN	76	87.5	87.2	84.5	84.9	86.8	76.6	73.5
HEMK1	161.5	172.9	151.9	194.5	174.4	235.8	142	162.1
HEPACAM	87.6	85.9	89.2	81.8	81.2	84.9	90.4	88.3
HEPH	65.7	71	87.7	79.3	83.2	81.4	85.3	85.2
HEPH	80.9	100.4	80.5	76.3	79.9	83.2	76.5	86.7
HERC1	257.6	409.2	318	669	630.7	1121.6	258.1	327.3
HERC2	438.1	586.9	361	584.6	557	760.5	453.2	499.5
HERC2P2	118.2	159	112.4	102.7	115.1	103.8	115.3	120.5
HERC2P4	89.7	102.4	91.4	82.3	83.9	80.4	90	94.9
HERC3	225.7	325.3	175.9	167.1	148	258	194.5	266.1
HERC4	168.3	191.8	126	127.8	106	122.5	163	230.5
HERC4	131.5	151.3	105.2	90.3	81.3	99.5	129.8	189.9
HERC4	612.9	816.3	467	343.5	289.6	587.4	498.2	952.7
HERC5	573	700.8	384	314.5	277	369.6	445.5	477.9
HERC6	174.7	208.3	187.8	173	149.4	204.9	158.2	230.5
HERPUD1	1153.1	1448.6	595	367.7	266.1	427.4	1553.2	2881.1
HERPUD2	119.7	121.4	105.7	102.9	102.5	112.3	128	179.1
HERV-FRD	124.4	124.2	100.4	109.6	98.1	95.8	98.6	114.9
HES1	84.5	97.5	93.5	85.7	77	81.9	89.5	106.5
HES2	91.2	109.2	79	86.5	86.2	82.2	91.7	119.1
HES4	154.6	181.7	123.2	94.9	102.9	101.3	139.8	152.6
HES6	1980.9	2173.1	974.2	912.5	893.9	1065.6	1480.8	1674.7
HES7	71.9	77.1	81.5	62.5	71.7	82.4	76.8	85.7

HESX1	161.3	216.9	147.5	137.3	152.2	178.3	174.3	218.5
HEXA	117.6	152.2	103.8	98.3	94.8	112.8	122.5	136.8
HEXB	9393.1	11805.3	8756.9	14033.1	11485.9	17228.2	8208.5	12713.4
HEXDC	151	181.8	114.1	104.4	102.5	104.4	134.4	136.9
HEXIM1	132.3	138	107.1	100.1	93.7	100.8	106	140.1
HEXIM2	172.7	228.7	140.2	141.1	126.8	149.6	149.3	154.6
HEY1	109	113.2	84.3	77.5	83.8	87.7	105.5	106.4
HEY1	89.4	106.1	103.4	92.4	94.5	92.3	93.7	109.8
HEY2	95.9	98.8	97.2	88.1	79.4	83.5	96.7	126.6
HEYL	606.7	802.1	564.3	524.8	426.8	562.3	488.2	582.2
HFE	82.7	93.2	101.8	85.8	83.5	78.4	81.9	92.9
HFE	90.4	102.2	94.2	86	89.4	91.1	100	101.5
HFE	125.5	134.7	106.2	84.1	81.2	94.9	105.2	127.7
HFE2	80.5	101.5	89.3	82.4	81.9	84.1	90.7	104.2
HFE2	81.6	98	89	92.2	86.6	83.1	81.4	86
HFE2	82.9	87.2	91.9	92	76.4	83.4	79.6	84.6
HGD	118.6	131.5	108.4	91.4	94.1	102.2	143.7	179
HGF	106.4	113.6	78.9	74.5	72.9	89.2	100.2	124
HGF	167.6	174.2	108.3	106	95.1	112	145.7	179.9
HGF	97.7	102.4	88.5	81	80.9	94	94	108.6
HGFAC	86.2	101.4	94.3	76	75	82.6	89.8	105.3
HGS	3171.4	3667.4	2873.8	2035.9	2002.3	2214.2	2816.4	3126.1
HHAT	114.1	132.9	92.6	90.2	84.5	84.2	114.6	133.7
HHEX	605.7	813.7	161.1	132.3	125.9	155.8	677.7	1082.7
HHIP	78.7	91.7	88.7	85.8	80	89.2	85.4	93.9
HHLA1	97.1	95.1	94.3	95	92.9	95.9	89.1	95
HHLA2	84.2	100.9	99.3	86	79.7	79.1	77.7	86.9
HHLA3	135.5	170.5	140.6	106.4	112.1	117.5	123.1	160.3
HHLA3	85.7	104.6	98.1	79.5	84.8	82.9	91	105.4
HHLA3	109.5	117.1	98.5	80.2	81.7	82.5	91.1	116.8
HIAT1	181.5	200.7	118.7	91.1	98.5	100.3	182.7	303.7
HIATL1	1332.7	1607.7	1193.7	1040.2	684.6	1798.9	1055.9	1676.2
HIBADH	1022	1414.5	718.1	920.6	750.9	1367.8	756.5	1025.2
HIBCH	766.8	992.3	557.6	538.2	380.8	664.9	585.3	936.4
HIC1	88.5	98.2	123.4	99.6	102.4	107.4	84.6	90.6
HIC2	736.4	935.7	582.7	497.3	466	497.1	649.4	804
HIF1A	209.5	213.8	118.8	146.1	123.4	172.4	212.7	309.6
HIF1A	318	396.7	167.4	163.2	122.9	204	255.8	395.5
HIF1AN	221.9	261.9	183.7	143	126	153.9	217.3	254.9
HIF3A	97.8	102.2	105.2	85.4	89.3	85.8	110.6	96.8
HIF3A	100.7	136.7	99.6	88.1	87	82.7	115.1	129.3
HIF3A	97.5	94.4	93.4	90	85.4	83.8	94.5	102.3
HIF3A	97	101.9	103.5	90.7	93	92.1	98.2	111.3
HIF3A	79.5	86.5	93.8	84.6	81.1	82.5	84.8	99.4
HIG2	349.5	429.5	150.3	161.4	158.8	216.4	272.5	278.2
HIGD1A	1612.5	1657.9	789.7	783.9	488	795.7	1640.4	3449.4
HIGD1B	86.6	101.8	90.3	81.8	86.8	88.6	102.2	115.6
HIGD2A	3577.1	4834.4	2086.8	1978.9	1496.4	1934.1	3360.3	4895.6
HIGD2BP	79.1	88.4	92.2	84.1	95.5	88.5	83.3	89.2
HIGD2BP	100.6	104.5	85.3	83.8	81.6	84.5	96.3	100

HILS1	92.1	108.3	98.2	86.5	87.4	92.4	93.1	88.8
HINT1	13339.9	15559.5	11174.4	12263.4	10279.1	13228.4	11203.9	16302.2
HINT2	1482.2	1675.2	1136.7	1085.3	861	1021.1	1407.4	1872.6
HINT3	91.6	102.3	91.3	91.6	93.3	89.4	92.4	89
HIP1	197.4	354.9	218.9	133.6	133.2	167.9	238.5	297.2
HIP1R	88.9	123.9	104.2	101.7	92.8	97.1	93.5	116
HIP1R	102	119.5	98	88.5	80.7	95.4	97.7	121.5
HIP1R	108.2	106.7	103.6	88.8	99	98.9	96.1	114.3
HIP2	101.9	110.9	86.7	85	84.8	91.7	99.2	118
HIPK1	70.5	88.5	94.9	83.3	81.5	80.1	82	82.4
HIPK1	79.5	92.8	94.2	87.4	80	95.1	88	83.3
HIPK1	100.1	104.4	105	102.5	93.6	90.2	98.5	112.4
HIPK1	93	120.7	92.1	81.7	84.2	89	96.3	108.3
HIPK2	168	226.8	115	224.4	146.9	158.6	224.4	297.5
HIPK3	155.9	214	131.9	96.6	110.7	134.1	117.8	124.5
HIPK3	195.2	256.8	116.2	112.2	99.9	120.2	153.8	213.6
HIPK4	86.7	103.8	96.8	82.8	83.9	91.8	87.8	99.2
HIRA	131.7	162.6	98	92.4	94.2	102.6	115.2	145.1
HIRIP3	1464.1	1959.2	888.5	580.7	508.7	598.4	1019.5	694.1
HISPPD1	90.3	91.4	94.5	91.3	85.5	90.3	87.2	102.6
HISPPD2A	220.7	286.1	359.2	406.7	329.6	555.3	212.3	300.6
HISPPD2A	72.4	79.5	88.8	87.5	94.3	82.9	86.9	77
HISPPD2A	89	114.1	103.5	98.9	97.6	89.7	92.3	96.2
HIST1H1A	103.6	117.9	112.2	104.2	106.6	110.3	102.4	95
HIST1H1B	86.2	97.4	110.1	103.1	92.8	98.3	88.4	89.8
HIST1H1C	263.7	313.1	120.5	138	118.1	117.8	267	314.1
HIST1H1D	92.4	111	101.2	99.1	96.3	93.3	92.7	99.1
HIST1H1E	75	82.6	88	86.7	80	76.3	83.6	76.1
HIST1H1T	90.1	105.6	93.8	98.9	88.4	87.6	91.6	100
HIST1H2AA	96.5	101.8	110.1	96.9	97.8	96	93.7	99.2
HIST1H2AB	112.1	112.7	81	86.9	86.1	76.9	94.1	113.9
HIST1H2AC	91.7	116.9	83.2	98.7	96.1	96.2	101	103.8
HIST1H2AD	80.8	83.1	87.5	79.4	78.7	80	77.2	79.1
HIST1H2AE	83.3	91.1	89.3	91.8	90.8	87.7	88	85.9
HIST1H2AG	75.3	75.8	90.4	78.1	77.5	82	81.9	76.8
HIST1H2AH	91.9	94.1	95	98.5	90.3	92.3	88.5	89.3
HIST1H2AI	106.8	118.8	94.5	96.3	90.6	96.7	107.1	112.1
HIST1H2AJ	94.7	96.9	87.8	92.7	84.6	91.7	92.9	95.7
HIST1H2AK	94.3	115.7	93.5	81.5	80.4	81.3	85.6	106.5
HIST1H2AM	106.7	104	101.5	106.6	109.8	94.4	101.7	102.5
HIST1H2BA	77.9	80.9	93.7	78.3	80.6	92.5	80.1	78.6
HIST1H2BB	89	101.2	91	89.9	84.6	85.8	88.6	96
HIST1H2BC	122.9	138.5	102.1	98	88.8	99.9	135.5	147.4
HIST1H2BD	143.8	187	138.4	130.6	133.5	135.6	152.1	175.5
HIST1H2BD	133.5	146.2	114.2	140.5	117.2	110.5	116.8	128.6
HIST1H2BD	78	77.1	95.1	84.8	75.9	86	72	81.7
HIST1H2BE	119.6	139.2	108.2	100.8	103.1	96.9	117.8	133.7
HIST1H2BF	111.8	129	112.5	157.7	161	113.3	112.2	125.3
HIST1H2BG	129.7	160.7	100.1	107.7	105	109.8	129.6	133.3
HIST1H2BH	763.5	960.2	269.3	281.7	221.5	238.3	674.9	782.4

HIST1H2BI	72.3	84.2	89.6	80.5	77.6	77.7	79.5	81.6
HIST1H2BJ	840.6	833	415.3	339.4	335.5	378	749.3	790.2
HIST1H2BK	1486.7	2117.4	944.3	1848	1268.5	1826.5	1483.6	1868.2
HIST1H2BL	85.6	92.9	86.4	81.4	87.1	96.2	89.3	94.1
HIST1H2BN	128.7	144.9	104.3	93.6	90.1	96.1	108.2	126.5
HIST1H2BO	79.7	88.5	93.8	90.3	83.2	82.3	81.8	83.2
HIST1H3A	131.9	152	105.4	98.9	96.4	108.8	126.6	143.3
HIST1H3B	97.1	103.5	91.4	93.8	80.9	90.5	91.7	86.8
HIST1H3C	155.1	193.1	120.1	128.4	102.8	135	167.3	174.5
HIST1H3D	131	154.5	117.6	102.4	110	109.7	135.3	143.6
HIST1H3F	119.5	151.1	100	106.3	91.7	97.2	116.9	150.4
HIST1H3G	99.7	134.6	105.5	98.4	94.8	102.2	115.5	135.2
HIST1H3H	104.4	94.2	100.8	109.2	95.1	111.9	105.4	106.1
HIST1H3I	101.4	107.9	114.6	117.9	99.1	110.6	108.6	105.9
HIST1H3J	113.1	133.9	103.1	120.6	121.1	134.3	124	118.1
HIST1H4A	94.7	125.4	96.6	92.3	80.1	92	102.4	127.1
HIST1H4B	90.9	101.3	115.7	127	119	103.7	99.8	101.2
HIST1H4C	23877.8	23647.7	17831.1	14033.3	13444.7	6926.8	19749.8	19253
HIST1H4D	72	85.2	95.2	72.2	77.1	89.8	73	80
HIST1H4E	86.5	102.6	96	106.5	94.9	103.3	107.2	107.9
HIST1H4F	79.4	98.5	92.8	99.7	91.7	104.1	87	85.8
HIST1H4G	84.8	88.5	96.8	86.7	79.6	89.5	84.6	88.6
HIST1H4H	110.1	122.2	90.6	91.9	95.7	77.2	109.9	114.7
HIST1H4I	90.8	96.7	98.6	90.2	85.8	94.7	94.4	103.1
HIST1H4J	100.6	106.7	94.5	79.6	88.1	96.9	102.4	111.8
HIST1H4L	81.8	92.8	89.6	83.9	79.4	82.1	89	91
HIST2H2AA	390.3	521.2	272.9	368.3	261.2	316.1	393.3	518.9
HIST2H2AB	134.4	148.7	106.4	111	94	109.9	133	136.8
HIST2H2AC	1100.4	1297.1	973.2	898.7	717.8	843.5	1126	1269.4
HIST2H2BE	132.8	145.2	98	101.7	93.1	104.7	128.2	166.5
HIST2H2BF	189.2	270.5	140.5	142.4	132.9	112.8	157	171.3
HIST2H3C	122.9	135.3	99.8	126.1	119.5	109.2	114.3	124.7
HIST2H4A	140.4	167.7	118.4	124.9	117	112.1	153.7	163.9
HIST3H2A	218.4	301.6	159.2	116.5	119	143.6	198.4	243.8
HIST3H2BB	96.3	101.1	101.8	99.8	88.4	96.7	98.1	97.9
HIST3H3	98.1	122.4	98.2	92.2	86.5	85.6	93.7	106.2
HIST4H4	90.4	91.1	91	87.2	86.1	89.1	76.9	85.8
HIVEP1	104.1	124.2	102.1	99.7	97.2	111.6	112.5	120.5
HIVEP2	247.4	293.1	175.5	144.6	136.8	157.6	262.6	288
HIVEP3	104.1	119.1	111.2	106.5	99.4	109.2	97.9	121.4
HK1	1486	1829.2	1872.3	2137.3	1463.1	2298.2	1138	1801.7
HK1	102.6	124.5	97.4	110.8	90.2	86.5	100.4	104.7
HK1	86.6	104.1	89.6	88.8	79.4	78.5	95.4	96.5
HK1	77.6	88	94.7	96	84.6	87	87.6	78.8
HK2	380.4	489.4	153.1	182.1	158.4	251.9	311.8	504.2
HK3	159.9	186.9	228.2	201.4	184.7	332.9	130.4	197.3
HKDC1	116.3	136.1	81.3	77.3	78.2	80.9	109.7	121.4
HKR1	134.8	201.2	114.7	106.4	110	127.9	141.2	132.9
HKR2	107.3	119.1	112	99	91.4	106.3	94.5	125.8
HKR3	702.5	768.6	590	478.3	493.1	586.7	602.8	837.4

HLA-A	10010.9	13684.9	9065.6	7121.3	5332.4	9270.9	8049.4	13220.6
HLA-C	69.9	70.8	87.5	77.5	74	75.7	71.2	74.7
HLA-DMA	579.9	708.7	390.9	505.1	502.4	441.7	535.4	492.8
HLA-DMB	150.3	145.9	124.3	199.3	169.1	222.7	119.4	137.4
HLA-DOA	81.5	88.1	81	78.7	86.1	78.1	76.2	92.6
HLA-DOB	102.2	115	108.6	100.1	90.1	94.4	96.5	110.7
HLA-DPA1	179.2	210.7	138	147.4	127.6	150.2	160.8	179
HLA-DPB1	76.1	86.9	85.2	82.6	78.2	86.1	81.4	83.4
HLA-DPB2	80.9	91.5	92.4	77.6	84	87.8	83.2	82.3
HLA-DQA1	81	84.2	89.2	92.9	83.2	90.6	79.2	83.4
HLA-DQA2	109.4	118.1	93.5	84.6	95.5	92.1	101	123.8
HLA-DQB1	92.3	98.8	102.4	90.6	80.6	97.1	93.8	97.3
HLA-DQB2	86	93.8	108.3	103.2	95.2	85.7	100	88.7
HLA-DRA	655.8	841.3	421.4	362.5	344.8	458.4	561.3	1112.6
HLA-DRB1	1887.5	2580	579.7	813	623.6	746	1699.1	2150.9
HLA-DRB3	454.6	565.2	220.2	206.8	186.6	292.5	402.4	522.1
HLA-DRB4	85.4	100.4	105.9	98	94.8	82.8	91.9	104
HLA-DRB5	1012.4	1333.2	663.2	395	320.9	449.2	837.4	1165.2
HLA-DRB6	571.3	839.3	241.4	233.9	192.9	224.2	635.8	698.5
HLA-E	366.4	473.4	177.1	137.4	129.9	187.3	335.5	450.5
HLA-F	781	1060.1	401.8	394.1	347.7	466.5	604.4	803.8
HLA-G	186.4	225.5	130.9	109	106.4	115.7	185.5	263.4
HLA-H	2670.2	3737.7	1637.9	1288.6	1144.3	1786.3	2225.3	2687.6
HLCS	92.6	107	81.7	90.1	82.9	86.2	84.5	108
HLF	108.1	113	100.3	92	89.2	90	95.9	107.7
HLRC1	190.4	234.4	103.5	92.6	86.7	94.1	179.9	315
HLX1	523.2	744.9	474.8	371.9	345.7	478	420.3	750.1
HLXB9	107.3	117.2	100.2	108.4	105.4	107.9	115.8	139.9
HM13	78.4	100	91.4	92.4	90.1	87.3	85.2	86.1
HM13	876.6	1142.2	574.9	408.6	348.2	336.2	799.2	1029.4
HM13	130.6	137.7	110.9	118	113.4	106.2	115.9	134.6
HMBOX1	278.3	383.5	143.3	161.5	140.6	188.1	309.4	488.1
HMBS	117.9	135.5	89	93.5	93.8	90.4	115.5	151
HMBS	1783.1	2649	811.8	488.4	412.3	531.7	1324.2	1511.8
HMBS	2485.1	3534.3	1489.1	962.2	731.8	1140.5	1951.9	1641.4
HMCN1	80.2	80.7	85.3	88.1	89.9	93.9	83.2	85
HMFN0839	215	294.3	157.8	297.6	156.3	264.3	509.1	1082.7
HMG1L1	1108.6	1483.9	271.6	233.6	185.4	317.9	996.6	1660.5
HMG20A	194.7	298	180.2	201.2	162.9	307.2	174.9	233.8
HMG20B	2223.2	2588.2	1466.6	1287.1	1390.2	1604.4	1902.1	1948.2
HMG2L1	201.6	274.4	135.5	103.4	114	145.8	165.8	204.5
HMG2L1	125.4	134.3	94.9	95.9	90.7	105.5	127.4	164
HMG4L	92.4	95.9	88.4	92.6	84.4	79.9	89.8	93.9
HMGA1	131.5	167.7	99.6	100	87.9	90.5	127.2	160.8
HMGA1	80.7	95.7	96.8	86.5	87.7	92.4	84.6	92.7
HMGA1	108.1	129.8	97.5	85	94.5	82.9	106.7	114.7
HMGA1	2388	3564.7	3826.3	3296	2595.5	4527.3	2082.5	5094.7
HMGA2	106.1	126	85.8	85.9	79.9	88.9	107.9	131.6
HMGA2	119.9	125.2	105.2	97.3	84.4	92.4	106.3	112.8
HMGB1	478.8	515.8	179	217	173	209.4	400.5	814.8

HMGB2	6584.9	6214.5	2560.5	1938.3	1923.9	3172.8	4879.7	8225.2
HMGB3	659.8	965.2	462.1	425.7	316.5	508.1	627.4	820.5
HMGB4	94.5	107.3	102.5	104.5	97.4	93.2	88.9	98
HMGB4	90.5	102.6	96.1	87.6	85.8	84.7	87.3	83.5
HMGCL	225.7	298.6	183.3	208.2	192	216.1	227.1	270.5
HMGCLL1	83.2	88.7	95.6	93.4	88.8	93.2	71.1	88
HMGCLL1	99.7	114.9	93.3	88.7	78	98.5	102.9	109.4
HMGCLL1	92.3	110.2	91.9	91.1	87.6	88	84.5	85.9
HMGCR	1329.3	2087.4	1082.8	1935.8	1662.1	2269.7	1162	1545.7
HMGCS1	1313.4	1725.4	754.4	1980.5	1729.6	2133.3	1265.7	1913.9
HMGCS2	104.9	130.5	105.2	87.3	76.1	84.4	105.9	135.3
HMGN1	22238.1	27720.2	18888.1	22561.6	19496.6	26935	19703.6	25793.6
HMGN2	10364.9	13797.1	3482.2	1828.9	1237.1	2010.5	10171.3	11088.5
HMGN3	142.7	174.8	85.4	104.4	93.8	96.4	162.4	199.3
HMGN4	1179.7	1524.1	563.4	565.3	426.6	710.4	1133.4	1525.5
HMHA1	352.8	370.8	250.9	184.3	201	210.1	293.7	221.9
HMMR	2005.6	2204.9	755.4	547.8	465.5	899.6	1674.1	2487.1
HMMR	2584.9	2895.6	1302.9	940.5	946.5	2044.2	1810.4	2538.6
HMOX1	108.6	119.4	126.3	99	90.4	85.6	4005.1	17776.5
HMOX2	116.7	138.8	90.5	84.6	78.2	97.6	109.2	154.5
HMP19	108.7	138.3	99.2	80	79.1	90.4	106.3	137.7
HMX1	83.1	93.6	105.6	86.9	93.2	96.3	87.8	87.7
HMX2	164.6	207.5	228.4	108.4	111.9	117	154.1	272.4
HN1	681.4	941.3	256.2	159.3	108.3	178.3	543.5	808.1
HNF4A	108.2	128.7	98.2	98	80	90.9	117.3	121.8
HNF4A	88.3	110.2	103.3	92.2	92	85.6	95.5	97.7
HNF4G	261.7	346.1	241	168.4	139.3	267.4	208.5	287.3
HNMT	2690.8	3518.4	863.6	818.3	505.6	986.8	2053.9	2957.9
HNMT	526.2	655.7	419.5	321.5	257.4	355.4	581.4	764.8
HNMT	511.5	615.5	276.3	286.3	200.2	379.4	322.4	464.9
HNMT	103.1	132	103.7	112.1	100.5	116.4	95	108.7
HNRNPG-T	76.8	89	84.7	85.2	74.6	85.2	83.6	94.1
HNRPA0	1901.6	2532.9	1135.4	1074.6	660.9	1418.9	1222.9	2142.7
HNRPA1	737	1194.4	358	274.4	210.8	268.2	484.7	534.6
HNRPA1	440.2	522	181.5	139.1	128.2	153.1	369.8	365.8
HNRPA1L-2	3068.7	4701.8	666.2	482	461	706.4	2728	3000.7
HNRPA2B1	18268.1	20637.9	10452.2	9257.5	7070	11786.3	17253.2	22817.5
HNRPA3	1764.2	3015.8	555.1	962.6	802.8	618.2	1594.1	1541.2
HNRPA3P1	262.2	351.1	112.2	159.4	125.5	125.4	260.2	312.1
HNRPAB	2015.4	2696	1422.2	989.6	806.6	1375.2	1489.5	1817.3
HNRPAB	5277.6	7233.2	2829.7	2215.6	1503.3	2563.9	4346.3	6171.2
HNRPAB	14130	18255.7	13586	9281.8	7474.8	10403.1	12462	17974.1
HNRPC	953.2	1281.8	283.3	252.2	209.9	395	768.8	1060.7
HNRPC	1173.2	1565.7	789.3	642.7	503.1	765	900.8	1102
HNRPCL1	109.7	123.8	96.2	88.5	81.1	87.2	105.7	117.7
HNRPD	19824.4	25063.8	9978.7	8956.8	6429.9	9660.6	19233.2	22441.3
HNRPDL	891.7	1158.6	333	382.1	256.3	420.3	656.3	956.4
HNRPDL	1582.8	1615.2	957.5	642.9	768.3	920.5	740.6	774.7
HNRPF	295.7	344.7	177.3	172.4	137.1	204.4	263	361.8
HNRPH1	95.9	114.5	83.4	92.1	95.9	99.5	80.6	99.3

HNRPH2	638	1027.4	238.6	272.4	190.6	296.2	550.6	692.3
HNRPH3	949.7	990.5	313	285.7	212.5	426.9	736.9	1214.4
HNRPH3	86.4	92	92.9	85.6	80.8	90.7	85.6	94.3
HNRPK	6096.7	7699.1	4835.8	4005.1	3058.6	6511.2	4494.1	8175.8
HNRPL	1593.5	2294.4	1812	1302.4	1312	1344.7	1441.8	3670.1
HNRPLL	355.6	392.2	132.7	139.6	126.1	192.9	316.8	547.8
HNRPM	308.5	466.1	132.1	116.5	116.4	103.8	248.5	342.7
HNRPM	9850.6	12718.6	6407.4	5699.2	4444.8	6839.1	8281.4	10906.6
HNRPR	3148	4987	2572.3	2671.4	2279.7	3195.2	3269.2	3430.4
HNRPU	620.1	684.2	422.8	713.7	563.1	506	639.9	925.9
HNRPU	687.8	969.5	595.4	906.8	756.9	946.6	523.8	728.1
HNRPUL1	2460.6	2984.5	2009.2	1697.5	993.9	1940.6	2286.2	5325.6
HNRPUL1	5201.1	5915.1	5309.7	3034.8	2710.1	2774.9	5101.6	5595.7
HNRPUL2	905.8	1018.2	374.9	396.6	366.9	356	740.8	932.3
HNT	68.9	78.5	99.1	90.7	91.3	85.3	75.5	75.1
HNT	102.7	103.2	101.9	98.4	90.1	95.3	98.5	103.4
HNT	109.8	120.5	96.2	87.4	78.3	79.3	110.4	122.1
HOMER1	134	151.1	127.2	108.1	85.6	126.8	110.2	173.1
HOMER2	104.4	117.4	113.7	118.9	113.3	129.5	101.5	116.8
HOMER2	168.5	197.6	137.7	168.2	136.4	192.8	151.1	190.2
HOMER3	525.1	726.6	457.4	315.9	240.2	307.8	459.8	634.8
HOM-TES-1	100.4	105.9	96.4	96.4	84	86.2	88.7	122.6
HOM-TES-1	137.4	151.1	108.9	104.9	100.9	113	133.2	148.8
HOM-TES-1	937.3	1007.5	494.2	411.4	444.1	502.1	635.4	716.3
HOOK1	94.7	116.5	102.3	89.4	91.6	90.2	102.9	100.3
HOOK2	265.2	268.1	209.2	152.8	155.3	208.3	204.5	308.2
HOOK3	203.5	333.7	124.4	116.2	115.7	114.5	183.3	227.3
HOP	123.2	122.3	105.7	90.1	84.7	89.6	119.8	118.3
HOP	112.2	136.3	80.8	88.8	89.4	77	108.9	128.6
HOP	69.8	77.5	81.7	81.8	81	88	80.7	86.6
HORMAD1	89.9	89.3	99.6	82.4	88.4	83	90	91.8
HORMAD2	79.4	99.2	89.3	87.7	100.2	87.8	85.1	86.4
HOXA1	79.5	92.2	103.7	89.7	88.2	93.2	83.2	85.4
HOXA1	107	111.1	108.6	96.7	100.3	96.5	96.2	105
HOXA10	935.6	1161.8	425.6	463.4	356.1	433	954.8	1800.1
HOXA10	180.2	197.2	137.6	95.1	92.6	93.4	174.2	183.5
HOXA10	250.4	285.3	122.6	107.3	105.6	92.1	240.6	331.7
HOXA11	163.2	217.6	139	148.1	138.3	151.8	141.4	212.1
HOXA13	558.5	673.7	373.6	439.4	373.5	387.8	420.6	906.3
HOXA2	97.5	104.4	89.7	87.2	80.8	82.1	101.3	105.8
HOXA3	112.5	125.1	90.7	89.8	83.4	92.8	125	116.7
HOXA3	91.9	99.4	100.7	93.3	91.2	95.5	95.9	104.1
HOXA3	81.5	94	91.7	79.6	90.8	93.9	77.9	85.6
HOXA3	75	87.1	84.5	83.8	81.6	85.9	78.2	92.6
HOXA4	92.5	105.8	107.5	103.6	106.8	114	99	139.7
HOXA5	537.5	760.1	402.4	284.2	276.7	355.5	575.1	617.1
HOXA6	132.9	142.7	112.3	101.6	102.8	101	124.3	156.9
HOXA7	105.4	113.4	98.3	96.2	87.9	86.8	112.3	113.1
HOXA9	2572.4	2967.3	1145.7	1228.9	939.1	1516.1	2837.3	3946.1
HOXA9	161.9	178.1	130.8	100.5	102.6	123.6	130.9	161.2

HOXB1	275.5	342.8	163.2	155.6	137.3	195.1	225	264.8
HOXB13	76.2	92.5	93.6	99.2	77.4	80.3	76.2	86.5
HOXB2	86.7	84.1	88.1	87.4	84.4	82.1	82.4	85.2
HOXB3	88.7	88.1	106.4	86.7	95.2	90.8	83	86.1
HOXB4	112.4	124.2	114.9	104	90.6	104.5	101.3	103.9
HOXB5	124.1	150.5	103.8	78.3	87.9	86.7	116.2	138
HOXB6	136.9	160.9	142.2	133.7	133.1	129.6	113	137
HOXB6	195.2	217.6	141.2	113.6	118.1	96.2	188.1	247
HOXB7	195	283.3	222.3	109.8	104.7	118.3	210.7	248.8
HOXB8	99.4	94.2	78.2	88.5	79.7	79.3	85.8	103.5
HOXB9	105.3	118.3	92.9	79.8	77.3	85.3	96.5	112.1
HOXC10	102.7	103.2	93.9	98.1	86.9	94.3	88.2	100.1
HOXC11	230.4	242.3	97.8	118.3	114.8	136.8	208	251
HOXC12	88.8	98.5	90.3	79.4	76.7	84.1	87	98.9
HOXC13	101	118.6	86.5	90.5	80.5	85.1	94.6	117.1
HOXC4	87.4	92.4	113.1	95.3	91.1	95.5	85.8	88.9
HOXC4	85	109.7	79.5	84.7	88.9	95.7	96	94.6
HOXC5	94.3	96.7	99.5	86.1	94.3	84.9	93.8	97.7
HOXC6	86.2	99	90.9	99.8	96.3	101.9	83.9	89.9
HOXC6	98.2	110.2	104.7	101.6	94.9	89.6	90.3	99.9
HOXC6	157.6	199.7	125.5	108.6	109.1	104.2	141.3	196.1
HOXC8	112.7	116.7	96.1	101.6	95.8	90.5	101.9	111.4
HOXC9	95.2	117.9	92.8	81.2	85.8	82.8	89.8	112
HOXD1	79.2	85.5	102.9	87.2	86.8	87.6	88.2	87
HOXD10	87	98.7	89.6	86.1	76.6	84.9	93.7	96.9
HOXD11	124.9	152.1	96.3	79	75.6	91.4	98.8	137.4
HOXD12	175.4	242.1	140.5	116.6	103.4	117.8	181.6	202.4
HOXD13	289.8	324.6	151	136.1	125.5	141.6	275.6	292
HOXD3	117.4	147.5	124.8	113.6	103.6	121.7	122.9	129.6
HOXD4	124	139.9	100.1	93.5	86.2	98.4	127.3	130
HOXD8	119.3	119	103.5	96.6	92.9	91.4	119	116.8
HOXD9	85.2	93.3	89.8	87.4	85	90.1	78.7	81.3
HP	125.3	148.9	108	150.2	114.5	189.1	116.8	120.8
HP1BP3	304.4	400.3	168.1	151.6	136.9	139.9	232.6	283.3
HPCA	84.2	95.4	98.7	89.4	96.5	89	79.7	88.9
HPCAL1	105.9	121.7	106.4	86.4	90.6	87.5	119.9	149
HPCAL1	3243.9	4600.6	2113.2	1730.3	1648.6	2226.7	5324.2	5378.6
HPCAL1	156.6	185.1	91.8	89.6	83.6	80.7	142.8	212.1
HPCAL4	112.1	124.6	91.4	78.3	82.4	81.2	94.8	129.7
HPD	119.8	148.2	91.2	88.3	84.5	85.2	127.3	122.3
HPGD	117.3	123.8	87.1	90.1	93.8	88.8	116.4	120.8
H-PLK	88	97.3	90.8	92.5	87.5	92.8	81.7	90.8
HPN	79.9	96.2	97.8	89	92.3	89.7	89.3	86.7
HPN	104.7	114.7	102	89	89.2	97.7	102.4	112.4
HPN	84.2	91.7	92.5	90.6	88.9	78.4	74.3	78.8
HPR	125.7	151.3	97.7	97.9	86.1	89.5	129.3	132.1
HPRT1	5340.1	6129.5	3468.3	3434.5	2526.9	3651.8	5041.1	8023.6
HPS1	153.9	194.6	183.2	184.3	154.8	160.5	158.1	198.9
HPS1	328.7	370.1	308.1	216.5	183.9	253.7	334.6	409.1
HPS1	129.9	166.8	127.4	123.4	113.1	124.2	133.5	138.8

HPS3	359.4	539.9	247.3	181.9	147.9	279.8	280.9	369.9
HPS4	95.1	121.3	93	84.2	77.3	85.1	97.2	118.4
HPS4	123.8	131.2	115	122.1	117.2	135.6	112.5	116.9
HPS4	209.2	218.4	186.7	215.9	182.4	214.2	159.5	160.6
HPS4	111.8	135.2	101.3	87.4	100.7	81.4	104.5	127.3
HPS5	567.5	641.8	474.1	460.9	371.1	559.5	439.7	775.6
HPS5	87	97.7	98.1	91.8	90.2	87.3	87	91.9
HPS6	1963.9	2346.7	1530	952.3	811.2	855.4	1732.9	1705.3
HPSE	293.2	392.8	155.9	153.9	126.5	173.5	259.9	413.2
HPSE2	81.8	83.6	87	86.1	85.6	82.8	82.4	86.4
HPX	112	126.4	93.3	79.4	80.9	92.4	113.6	123.9
HR	65.6	78.3	82.5	79.3	75.9	66.7	71.5	66.5
HR	85.3	104.7	85.6	79.3	74.7	79.3	104.6	106.7
HRAS	669.1	755.3	370.1	248.3	221.7	228.6	648.2	1049.3
HRAS	105.7	121.1	93.1	94.7	82.5	94.5	108.4	120.8
HRASLS	84.3	106.6	96.6	91	87.7	93.2	87.4	88.4
HRASLS2	116.4	130.3	97.7	83.9	86	93.8	111.4	135.9
HRASLS3	73.5	82.3	99.2	97.8	84.3	103.6	82.6	83.9
HRASLS5	74.9	80.6	102.9	91.3	96.9	85.3	76.9	85.1
HRB	89.9	109.4	97	86.5	94.5	99.5	91.8	93.4
HRB2	217.5	262.3	175.2	170.4	151.4	209.9	165	245.7
HRBL	146.7	176.4	113.5	108.9	102.4	108.9	151.3	167.3
HRC	106.2	119.9	124.5	113.6	113.4	116.4	108.1	135.6
HRG	77.7	96.2	92.2	82.3	89.6	79.9	96.5	77
HRH1	120.4	159.8	100.2	99	80.3	93.5	105.9	145.2
HRH2	102.7	112.7	87.3	94.5	91.6	81.6	106.9	109.6
HRH3	97.1	132.5	98.8	95.9	84.5	82	91.3	108.7
HRH4	101.8	118.2	90.9	88.5	79.4	90.1	101.6	116.9
HRIHFB212	232.8	262.7	177.5	166.2	161	174.5	175.6	179.7
HRIHFB212	125.1	149.7	116.1	117.9	131.6	136.8	125.9	128.8
HRIHFB212	767.2	861.1	660.4	535.7	460.1	533	688.9	951.8
HRK	71.1	79.3	80.3	77.4	75.2	76.4	80.4	79
HRNR	98	104.5	84.8	94.3	79.1	86.5	101.5	110.3
HRSP12	291.9	351.5	180.1	152.7	132.3	137.5	277.4	483.1
HS1BP3	206.3	295.8	215.5	168.2	147.6	208.2	215.1	351.9
HS2ST1	173.1	175.2	108	108.4	98.8	131	138.4	214.8
HS3ST1	108.9	113.7	96.3	88.3	88.4	94.9	98.7	99.9
HS3ST2	93.8	100.7	98.7	98.1	89.1	99.1	86.2	94.1
HS3ST3A1	222.9	248.4	161.8	126.9	110.6	129.5	178.4	259.6
HS3ST3B1	102.1	118.3	95.9	93	90.3	93.9	115.6	113.2
HS3ST4	110.6	138.3	89.3	98.4	92.3	80.6	120.3	114.9
HS3ST5	95.8	103.3	85.5	84.3	78.3	80.8	92.8	114.2
HS3ST6	85.5	89.3	96.5	85.2	82.2	87.8	88.3	103.3
HS6ST1	127.9	142.5	112.4	106.7	110	101.9	116.4	126.3
HS6ST2	77.3	87	92.1	91.4	86	92.4	76	87.8
HS6ST2	109	121.8	90.2	84.4	78.1	85.8	103.4	121.8
HS6ST2	98.7	108.7	92.9	84.1	81.2	82.2	104	116.3
HS6ST3	84.7	109	93.8	92.6	95.3	91.3	93.5	94.3
HS747E2A	74.3	83.8	83.4	80.4	73.6	72.2	88.6	87.6
HSA9761	2246.5	2993.4	889.6	820.1	618.2	1026.1	1685	2150.7

HSBP1	2308.9	3095.2	1907.7	1772.4	1463.7	1833	2096.3	2517.9
HSCARG	1948.6	2637.4	818.4	753.2	594.4	765	1721.5	1407.1
HSCB	601.2	753	350.3	290.2	325.3	348.1	676.8	756.9
HSD11B1	1772.2	2342.1	1002.4	526.6	359.1	713.1	1075.9	1192.1
HSD11B1	1766.1	2366.8	1341.7	637.5	466.7	858.2	1118.9	1468
HSD11B1	1598.9	1993.8	586.7	307.8	239.8	389.2	1075.5	1024.4
HSD11B1L	121.6	143	118.1	110.1	97.8	102	118.2	123.8
HSD11B2	85.6	104.5	99.5	83.4	82.3	79.2	90.3	82.3
HSD17B1	92.4	104.9	101.8	91.3	80.3	94.1	93.9	97.1
HSD17B12	6833.4	9195.1	3273.2	4240.5	2641.1	3875.6	7010	10246.5
HSD17B13	92.9	114.1	96.6	96.9	82.6	87.2	99.2	111.2
HSD17B2	80.6	91	86.4	83.1	92.2	89.5	84.5	89.3
HSD17B3	117.5	122	94.8	91	89.5	107	109.9	114.2
HSD17B4	1791.7	2339.8	1092.3	1780.5	1239.4	2655.1	1385.3	2309.3
HSD17B6	181.2	238.5	160.8	123.6	110.2	125.7	187.6	245
HSD17B8	301.3	392.8	228.8	228.3	214.6	274.4	277.5	298.9
HSD3B1	113.4	108.7	90.4	90.8	74.5	92.6	108.5	121
HSD3B2	87.6	112.5	99.4	93.5	90.8	88	88.5	99.9
HSD3B7	97.9	130.3	116.3	95.5	102.1	105	109.6	135.8
HSDL1	154.9	178.7	153.3	140.9	139.8	171.7	137.8	188.8
HSDL2	556	680.5	293	358.8	250.4	470.7	498.9	1047.3
HSF1	151.1	212.5	143.2	135.1	127.2	92.1	138.6	167.2
HSF2	691.6	863.9	427.3	461.7	354.9	732	459.8	874.4
HSF2BP	140.9	150.2	135.2	177.7	156.2	192.6	121.2	150.7
HSF4	78.5	95.9	92.6	90.7	98.9	121.2	80.8	92.3
HSF4	100.8	126.3	96.1	69.7	70.3	100.5	102.1	125.3
HSF5	79.6	93.5	83.1	88.3	77.3	81.3	90.5	92.4
HSFX1	88.8	93.2	97.8	88.4	84.6	93.7	99.5	86.1
HSFY1	101.9	117.1	96.1	90.6	94.5	88.4	98.3	116.5
HSFY1	76.1	85.8	90.2	86.4	80.2	84.5	72.4	82.1
HSFY2	73.3	89.2	99.4	85.3	95.6	96.6	74.2	85
HSFY2	81.6	105.1	86.8	74	67.7	83.3	93.9	107.4
HSH2D	302.5	381.9	282.6	184.2	229.6	273.7	181.4	223.6
HSMPP8	449.8	572.4	398.5	461.9	498.6	644.4	360.6	491.9
HSN2	93.5	107.9	92.8	113.5	111.5	107.6	88.5	93.2
HSP90AA1	105.4	119.5	99.8	95.1	93.6	88.9	109.4	125.4
HSP90AA1	22620.8	25999.7	14055.7	10703.6	10639.8	17785.9	24382	28577.8
HSP90AB1	8537.4	11904.8	8066	6135.5	4935.1	8968.4	6834.9	12789
HSP90AB2F	136.4	166.3	112.2	95.8	87.3	104	131	157.3
HSP90AB4F	80.1	103.1	88.2	87.4	79.6	80.7	93.2	82.6
HSP90B1	9768.1	12067.2	8413.1	12301.3	11464.7	15709.4	8349.6	8740.8
HSP90B3P	84.9	105	107.3	87.6	79.8	81.8	100.4	97.8
HSPA12A	76.1	87.9	97.9	77.6	85.8	94.2	86.3	89.3
HSPA12B	91	100.9	94	95	95.7	101	94	111.9
HSPA14	1991.1	2670.8	1046.4	513.4	448.4	666.4	1657.1	2617.6
HSPA1A	117.7	145.1	113.4	125.7	122.5	138.4	141.5	269.7
HSPA1B	2273.9	3118.6	2489.9	1646.1	1193.4	1554.6	3079	6950
HSPA1L	217.8	241.1	171.5	174.6	158.4	206.1	214.4	297.6
HSPA2	108.1	123.1	108.4	115.4	94.4	107.4	104	144.1
HSPA4	640	877.8	208.5	171.6	184.1	263.8	619.5	1416

HSPA4	260.6	348.8	149.9	140.3	142.6	171.2	210.7	284.8
HSPA4	4317.3	5648.3	1903.1	2112.6	1498.1	2928	4292.7	5376.3
HSPA4L	494.4	646.1	260.8	197.5	167.3	305.6	350.1	627.5
HSPA5	1318.3	1744.5	810.4	1315.9	783.4	1345.4	1360.9	1947.3
HSPA8	19480.6	21992.2	14986.5	9871.1	7221.3	13691.2	17390.1	26770.3
HSPA8	18126.5	22556.1	11694.6	5996.8	4879.7	8730.4	16991.9	28266.6
HSPA9B	3844.3	5477.7	2777.3	2078.2	1753.7	4027.4	3097.9	5385.2
HSPB1	10535.5	11969.5	3861.7	2672.9	2166.9	2908.6	10956.6	13352.7
HSPB2	133.4	143.7	105.2	94.8	94.8	95.7	114.8	119.5
HSPB3	86.4	107.8	107.7	90.7	83.6	95	91.2	88
HSPB6	121.2	159	112.4	116.2	99.7	100	122	134.4
HSPB7	109.1	141	135.1	142.5	135	198.6	107.6	113.5
HSPB8	82.2	90.2	82.5	78.5	83.2	83.2	84.3	93.1
HSPB9	91.1	109.9	90.8	90	84.7	80.6	93.4	100.4
HSPBAP1	374.6	483.7	212.3	193.6	173.2	228.9	276.4	323.4
HSPBP1	209	294.6	132.7	118.3	113.6	111.1	207.2	223.5
HSPBP1	268.4	338.6	152.4	110.9	107.5	114.2	215.9	297.1
HSPC023	2004.2	2348.3	1570.1	1292.8	1115.5	1441.5	2118.2	2371.6
HSPC049	728.6	954.5	809.4	556.4	418.9	725.5	338.9	710.9
HSPC065	99.3	114.1	97.6	96.2	85.6	89.1	89.4	112.1
HSPC072	93.8	103.5	81.1	79	78.3	85.4	90.7	102.3
HSPC105	97.7	106.2	89.9	88.1	80.8	88.1	89.8	104.9
HSPC111	1272.2	1439.4	558.5	447.3	316.4	570.9	893.2	1176.5
HSPC117	1682.9	2230.9	1126.6	1050	886.7	1469.5	1379.6	1198.7
HSPC142	775.8	1151.5	441.6	352.3	369.9	349.7	818.4	903
HSPC142	1957.4	2517.9	1738.5	1413.6	1141.5	1586.6	1928	2660.7
HSPC148	2738.4	3157.2	1256.6	1113.7	933.3	1399	2218.9	2662.6
HSPC152	4672.4	6256	1720.2	1090.9	814	1199.5	4238.9	5273.9
HSPC159	197.4	246.8	128.3	93.6	86	101.2	161	192.8
HSPC171	779.8	994.1	592	531.5	379.2	549.2	772.9	1320.6
HSPC176	3051.1	3814.2	2668.3	1980.8	1801.7	2159.8	3093.1	2957.5
HSPC196	768.5	891.2	473.4	428.8	393.4	491.6	668.3	741.1
HSPC268	707.8	734.1	442.4	564.6	586.5	450.6	763.4	1315.1
HSPD1	17128.7	18243.2	7533.1	6648.2	4664.6	7871	14451.3	18930.9
HSPD1	106.8	107.4	103.4	97	91.8	98.3	96.6	103.3
HSPD1	451.6	533.2	182.4	145	120.1	141.8	293.6	393.7
HSPE1	4784.6	5478.7	1153.2	1130.8	719.4	1447.6	4258.7	6511.8
HSPG2	111.4	118.6	103.5	93.8	83.4	95.1	112.4	126.7
HSPH1	6044.6	7574.2	3340	2382.2	1752.5	3506.6	6031.3	9368.7
HSU79303	1883.7	2304.9	1095.7	413	367.5	319	1414.3	1164.8
HSZFP36	642.9	740.8	457.6	395	316.4	492.8	539.8	852.4
HSZFP36	446	590.9	236.6	206.5	216.6	308.5	376.3	481.9
HTATIP	113.6	159.3	112.2	111.1	100.1	107	119.7	131.8
HTATIP	169.8	192.5	108.5	83.9	82.1	90.7	174.6	193.7
HTATIP2	1930.1	2589.9	1243.1	763.3	631.2	928.8	2637.3	3485.7
HTATSF1	92.4	119.6	91.2	97.3	79.5	91.8	87.3	110.8
HTF9C	146.2	198.5	111.9	105.5	100.4	109.4	156.3	173.7
HTF9C	131.1	156.6	113.8	111.5	107	98.1	117.7	146.2
HTF9C	108.6	128.9	112.3	118	99.6	127	105.3	114.5
HTLF	1250.1	1627.2	722.9	878	647.6	1324.5	1199.4	1980.2

HTN1	74.6	85.5	85.5	83.9	79.8	76.9	77.4	84.2
HTN3	121.3	128.6	114.1	100.1	93.7	96	121.6	128.4
HTR1A	83.9	93.1	89.6	96.1	75.1	83.7	83.6	91.9
HTR1B	78.8	85.5	87.1	91.6	88.4	75.9	74.9	84
HTR1D	112.3	121.1	99.1	92.7	84.5	98.9	104.1	111.5
HTR1E	152.6	176.5	122.8	100.3	100.1	99.2	143.2	177.8
HTR1F	75.7	73.6	84.9	85.7	86.5	94.5	82.1	79.1
HTR2A	1632	2019.4	522.4	493.7	524.3	630.1	1602.5	2234
HTR2B	88.7	106.2	103.5	93.3	90	93.8	83.5	97.2
HTR2C	79.7	77.5	109.4	103.2	105.2	102.7	78.8	82.2
HTR3A	99	94.7	108.3	96.2	87.5	91.1	95.9	105.3
HTR3A	96.6	118.2	84.9	85.7	91.4	89.2	96.7	109.3
HTR3A	91.8	106.7	91.2	89.3	93	88.7	94.1	86.8
HTR3B	103.4	122.4	93.2	96.2	86.3	93.1	91.5	98.6
HTR3C	94.3	103	98.4	104.6	90.7	85.6	86.9	91.4
HTR3D	85.9	92	90.9	91.2	84.4	90.9	82.2	89.7
HTR3E	90.2	83.2	92.5	84.8	84.6	87.5	88.6	82.9
HTR4	78.2	86.3	76.2	69.2	70	78.2	77.9	96.3
HTR4	83	96	95.5	85.3	93.2	88.7	84.4	91.6
HTR4	125.4	144.2	103.3	101.1	106.2	93.3	112.8	112.6
HTR4	96.2	125	88.6	86.3	73.7	85.7	103	123.2
HTR5A	84.2	87.9	82.1	72.3	74.8	85.3	85.2	90.3
HTR6	134.6	160.6	95.3	96.7	90.2	94.2	133.7	160.3
HTR7	100.8	115.8	92.5	85.1	93.5	96	111.6	125.5
HTR7	141.9	180.8	107.9	107.2	99.3	98.1	138.2	170.9
HTR7P	124.8	132	99.2	85.3	90.1	94.6	124.5	141.3
HTRA1	87.9	91	87.8	78.5	94.4	91.7	99.9	106.6
HTRA2	671.1	902.7	338.6	209.3	199.9	277.5	464.5	816.6
HTRA2	353.3	437.6	207.1	185.2	174.7	257.9	300.5	380.7
HTRA3	96.3	111.7	94.4	98.6	90.6	84.1	90.7	99.2
HTRA4	95.8	101.3	96.7	97.8	92.8	105.8	97.4	100.5
HUNK	84.4	93.7	102.5	97.6	92.5	93.6	85.9	96.8
HUS1	76.3	99	96.9	84.1	83	84.8	84.4	78.2
HUS1B	259.9	303.4	106.7	117.3	108.7	134.3	195.6	259.4
HUWE1	153.6	228.1	137.1	176.9	165.6	277.4	155.9	195.8
HYAL1	112.2	116.5	93.5	89	90.3	92.9	101.7	112.6
HYAL1	82.8	89.6	86.3	84.6	81.2	88.2	86.4	93.5
HYAL1	104.4	128.4	98.5	90.7	89	88.8	101.2	102.3
HYAL1	93.6	108.8	87.9	83.5	83.6	84.5	86	105.9
HYAL2	120.9	135.9	86.2	82.4	79.8	94	131.1	144.9
HYAL2	233.4	264.8	184.6	176.2	151.4	250	168.9	231.4
HYAL2	96.1	105	82.5	80	77.1	75	84.6	123.8
HYAL3	1480.5	1962.7	1112.8	1108.5	991.4	1397.6	1318.4	1982.8
HYAL4	104.5	120.8	87.7	84.9	84.2	81.8	102.8	122.4
HYDIN	86.4	90.1	97.9	91.4	91.9	93	83.8	94
HYDIN	104	106.4	94.9	83.4	83.7	86.2	115.2	105
HYDIN	79	90.8	88.7	85.1	80.6	75.8	88.4	85.6
HYDIN	97.4	109.3	97.4	91.1	87.3	95.9	100.6	103.7
HYI	272.2	337.4	210	190	164.4	213.5	224.8	298.7
HYLS1	1242.9	1593.3	736.4	396.6	328.1	526	957.9	1461.6

HYOU1	332.2	415.7	453.2	355.9	268.2	488.8	332.1	608.1
HYPB	705.5	1062.4	636.3	1011.9	902.8	1477.9	772.2	923.2
HYPE	212.9	290	136.6	164	130.8	119.4	226.9	327.3
HYPK	2242.3	2365.1	1598.5	2817.4	2587.9	2020.6	2273.5	3309.9
IAH1	2429.1	3217.6	1870	1788.7	1284.9	2159.9	1848.7	4084.4
IAPP	108.8	113.4	99.8	90.8	93.1	100	93.7	101.5
IARS	2024.2	2881.7	1580.8	1210.6	828	1931.8	1647.1	2695.7
IARS	116.9	124.8	97.7	84.4	77.4	84.6	95.9	110.3
IARS2	3721	4946.9	2142	2159	1665.6	3474.4	2796.8	4400.5
IBRDC1	129.3	120.6	119.9	106.1	96.2	126.6	110.7	143.4
IBRDC2	106.4	117.4	100.3	147.3	151.6	156.2	98.4	103.1
IBRDC3	99.4	132.9	131.5	123.5	128.3	193.2	114.4	182.1
IBSP	101.4	120.5	100.8	102.8	96.5	104.8	107	122.8
IBTK	1114.6	1256.5	471.7	742.7	455.4	920.6	957.5	1732.7
ICA1	314.3	386.6	221.1	271.7	240.9	328.9	314.1	436.9
ICA1	429.2	606.7	337	455.4	402.1	502	428.7	498.2
ICA1	124.9	130.1	104.6	98.9	86.2	98.1	114.9	150.2
ICA1L	96.5	95.6	98.3	97.3	95.2	100.1	104.3	101.7
ICA1L	133.5	153.6	115.2	99.2	91.3	105.3	110.6	141.7
ICAM1	123.9	145.1	98.7	80.4	81	93.2	120.9	163.8
ICAM2	239.4	330.4	231	267.2	233.8	291.2	233.4	247.6
ICAM3	914.8	1082.9	1083.5	1421.2	1195.3	1782.4	777.6	875.3
ICAM4	92.8	114.9	110.2	100.5	106.3	119.3	100.7	100.5
ICAM4	113.4	121.6	112	110.3	107.9	104.5	113.3	124.2
ICAM4	86.2	98.8	87.2	95	95.8	100.5	82.9	92.7
ICAM5	90.5	102.7	93.9	79.3	82	91.1	83.9	108.7
ICEBERG	92.7	90.7	92	78.5	81.9	83.1	80.2	98.3
ICK	139.1	169.1	105.2	95	82	90.2	140	171.3
ICK	73.7	73.2	83.8	82.5	84	82.7	78.9	85.3
ICMT	670.7	972	491.4	474.7	391.7	606.2	555.2	918.2
ICOS	131.8	140.1	114	98.9	93.4	109.7	121.3	132.7
ICOSLG	167.1	206.3	144.9	155.2	122.2	194.1	163.6	204.6
ICT1	1259.5	1666.1	565	415.8	373.4	668.9	1119.7	1604.6
ID1	156.4	123.6	123.7	94.5	81.9	89	115.5	128.8
ID1	667.9	711.9	321.8	249	140.9	260.6	586.9	1087
ID2	1546.5	1724.3	717.4	697.8	650.6	833.7	1619.8	2961.8
ID3	492.3	633.3	340.3	297.8	176.7	313.4	671.4	987.3
ID4	80.9	89	85.4	75.7	77.6	80	83.2	94.5
IDE	409.1	563.2	272.1	204.3	171.8	241.1	440.9	622.2
IDH1	2651.3	3314.8	1779.4	1459.1	1164.4	1956.5	2150.2	4106.8
IDH2	2211.6	3185.8	1934.4	1331.6	1194.2	2021	1948.8	2755.3
IDH3A	546.5	699.8	521.2	562.6	432.6	853.6	460.8	572.2
IDH3B	2228.5	2931.2	1434.3	1034	860.6	1282	1925.5	2381.2
IDH3B	95.5	92.5	103	103.5	90	92.2	84.9	105.6
IDH3B	1214.4	1865	989.2	659.7	651.5	868.4	1111.1	1414.9
IDH3G	113.1	140.7	103.5	98	92.5	97.4	105.8	171.8
IDH3G	1083.7	1312	998.6	717	695.7	773.2	973	1100.8
IDI1	611.3	720.4	205.4	237.8	201.9	278.8	555.7	1122.4
IDI2	73.9	81.6	86.3	84.5	85.4	80.7	80.4	78.7
IDS	349	419.4	305	351.1	305	438.6	330.8	504

IDS	152.9	161.7	107.3	122.5	94.3	116.3	148.9	204.1
IDS	338	483	259.2	237.1	200.2	277.5	359.9	688.6
IDUA	165.6	162.8	191.3	187.6	154.1	354.6	120.5	202.1
IER2	135.7	182.6	112.6	93.7	95.1	93	146.3	166.1
IER3	115.5	116.5	90.4	89.5	87.1	86.2	105	119.7
IER3IP1	1096.8	1261.9	432.4	288.4	287.3	287.2	1048.2	2022.6
IER5	336.8	456.1	306.6	212.9	210.7	257.6	386	758.8
IER5L	139.3	165.5	145	107.7	112.3	124.6	153.8	230.9
IFI16	374.9	465.8	177.6	166.5	152.2	284.8	263.6	345.4
IFI27	121.6	145.6	119.1	90.2	89.5	87	164	188
IFI30	127.4	164.9	105.7	197.1	182	233.2	120.6	133.2
IFI35	978.3	1230.8	680.7	638.2	568.6	734.6	921.4	1136.7
IFI44	181.4	241.5	153.9	133.6	114.1	138.3	191.1	294.4
IFI44L	94.9	108.2	96.1	87.4	88.6	88.6	90	91.9
IFI6	356	418.6	218.8	140.5	144	113.7	289.1	368.9
IFIH1	352.7	339.2	162.1	154.8	150.5	150.7	280.1	367.5
IFIT1	160.7	192	128.2	101.1	100.6	98	156.7	174.1
IFIT1L	82	90.7	74.9	81.6	78.2	80.6	91.6	80.7
IFIT2	213.1	248.1	185	114.2	108.2	125	215.2	256.4
IFIT3	160.7	186.8	121.9	99.8	88.8	95.9	163.8	165.9
IFIT3	125.2	153.1	121.3	99	116	111.7	128.6	133.8
IFIT3	86.3	102.1	93.6	92.6	86.3	85	97.5	95.9
IFIT5	104.6	132.8	97.8	98.2	91.7	84	115.6	127.1
IFITM1	721.5	1014.4	693.9	246.4	247.2	205.8	677.7	824.9
IFITM2	4643.4	5454.6	3438.7	1910.9	1723.8	1566.1	5493.3	6410.4
IFITM3	2201.8	2581	1413.2	839	617.6	649	2325.1	3787.7
IFITM4P	80.9	93.4	100	85.7	86.5	84.1	89.5	90.3
IFITM5	82.1	97.1	90.3	90.4	92.1	89.5	92.9	83.3
IFNA1	89.6	104.2	87.7	88	86.8	88.7	88.8	100.1
IFNA10	165.9	174.4	117.9	110.2	98.6	108.9	157.8	161.4
IFNA13	90.7	108	101.1	83.9	82.1	77	102.9	106.7
IFNA14	93.4	108	104.4	102.7	85.5	102.6	90.8	100.3
IFNA16	88.4	96.9	104.9	98.7	93.4	100.4	82.1	83.5
IFNA17	82.5	89.1	88.5	75.7	88.8	85.1	75.7	77.2
IFNA2	83.1	97.9	91.6	79.3	83.2	89.5	99.1	99.4
IFNA21	101.8	114	111.5	102.5	100.4	109.5	96.4	112.5
IFNA4	81.8	89.9	86.3	84	82	79.2	82.1	89.2
IFNA5	90.8	108.4	101.9	79.1	85.7	85	96.6	100.1
IFNA6	79.4	83.8	84.5	73.1	74.7	84.9	80.9	84.7
IFNA7	113.5	121.2	92.2	96.8	86.5	90.5	111.4	129.4
IFNA8	322	436.9	192.9	158.6	149.2	177.5	342.5	375.7
IFNAR1	306.8	424.6	306.6	397.3	354.9	452.3	305.5	381.1
IFNAR2	353.5	432.1	245.4	406.8	345.3	233	397.6	666.3
IFNAR2	1236	1748.3	867	908.2	663.1	1183.2	969.6	1541.7
IFNAR2	107.2	140.7	91.5	82.2	76.2	86.3	104	101.9
IFNB1	82.2	110.9	87.6	72.1	75.2	71.7	91.3	89.2
IFNE1	78.6	82.5	101	101	91.8	98.6	88.3	89.7
IFNG	85.3	91.8	99.6	90.6	95	98.2	84.8	84.2
IFNGR1	2192.5	2831.2	1499.9	2091.9	1360.8	2672.1	2033	2668
IFNGR2	4217	4798.3	2952.4	3327.8	2862.7	4601.3	3230.3	4176.3

IFNK	101.9	118.1	92.8	83.4	76.5	81.8	113.6	115.7
IFNW1	71	85.6	87.2	90	90.6	94.1	84.3	93.6
IFRD1	561.6	677.1	189.5	207.7	150.3	246.8	624.3	994.3
IFRD1	107.5	104.2	83.6	81.7	92.9	90.6	132.4	196.2
IFRD2	628.9	860.7	527.8	364.7	277.8	389	510	619.7
IFRG15	119.4	136.7	112.5	95.6	85.4	90.1	102.2	129.6
IFT122	85.8	117.3	89.2	74.7	84.5	82.7	88.2	109.4
IFT122	221.2	295.6	204.4	231.4	196.5	259	227.2	330.6
IFT122	421.6	587.4	357.4	351.9	344.3	350.2	376.2	373.6
IFT140	102	111.2	102.5	86.7	94.4	99.2	96.7	114.5
IFT172	151.6	167.9	125.8	125.4	118.6	135	140.4	151
IFT52	326.8	464.2	186.6	204.7	150.9	289.3	342.3	613.8
IFT57	186.2	210.2	145.9	139.4	113.7	154.6	146.9	169.6
IFT74	656.2	866.6	192.2	172.8	204.4	267.2	543.4	707.6
IFT80	94.9	112.7	92.7	79.2	79.3	79.4	93.3	96.9
IFT81	92	93.2	123.1	108.9	108.6	109.7	89.2	90
IFT81	131.7	134.7	98.3	97.7	97.6	100.9	111.5	133.5
IFT81	183.2	207.6	127.4	102.3	100.1	111.1	159.5	179.7
IFT88	183.9	236.8	132.6	121.7	111.1	150.1	161.5	233.7
IGBP1	1044.9	1351.9	439.8	468.5	378.7	597.9	1025.4	1757.9
IGF1	89.1	99.4	89.6	88.9	76.7	80.1	93.3	96
IGF1R	103.4	109.9	93.3	94.8	87.5	91.2	93.1	95.6
IGF2	77.3	83.6	87.3	83.3	83.1	82.1	70.5	76.4
IGF2	80.9	89.7	98.7	84.3	84.3	94.2	81	94.2
IGF2AS	93	117.5	86.1	78.1	86.3	81.1	93.5	106
IGF2BP1	115.1	126.1	97.9	96.1	84.2	87	106.4	151.5
IGF2BP2	2606.8	2721.5	1341.9	1855.1	1488.8	2084.8	2389.6	5550.2
IGF2BP3	118.4	140.2	113	127.1	120.7	140.5	131.6	180.8
IGF2R	2017.5	3254.4	1701.5	4537	3587.5	4465.7	2589.5	2769.8
IGFALS	110.4	100.5	97	96.8	100.1	90.2	94.8	110.6
IGFBP1	135.8	151.2	103.2	81.3	80.5	91.9	135.8	142.2
IGFBP2	312.6	472	371.4	637.7	549.2	897.1	292	454.6
IGFBP3	86.2	99.2	95	81.9	80.5	87.3	88.9	103.3
IGFBP3	98.4	124.7	102.8	93.2	88.8	91.9	102.1	109.3
IGFBP4	301.7	403	437.9	224.8	197	412.6	248	430.2
IGFBP5	116.9	100.3	88.9	89.2	80.8	92	93.7	111.8
IGFBP6	108.9	107.7	93.5	103	100.7	91.8	90.9	100.9
IGFBP7	3249.7	4141.6	2575.6	1992.5	1661	2165.5	2850.6	5382.6
IGFBPL1	115.6	145	83.8	89.8	86.3	87.7	112.3	140.9
IGFL1	79.7	81.7	95.9	87.8	87.8	92.1	84	83.1
IGFL2	78	84.5	102.9	90.7	90.5	88.3	78.7	81.3
IGFL3	125.8	138.1	97.4	82.7	84.2	84.1	100.8	125.1
IGFL4	84.2	95.1	91.3	79.9	88.8	81.1	86.8	88.6
IGHMBP2	130.1	149.9	146.1	123.5	130.6	143.1	113.4	145.4
IGJ	97.7	101.6	93.7	86.4	79.3	78.2	98.8	96.5
IGLL1	90.7	99.8	96.6	90	81.1	92	88.3	107.9
IGLL1	93	109.9	102.4	98.3	94.5	100.6	88.4	91
IGSF1	105.4	130.8	127.6	96.9	83.7	106.1	109.6	148.3
IGSF1	170.4	205.1	110.9	83.5	91.5	96.4	157	218.6
IGSF1	98.3	102.6	116.6	102.5	101.3	94.2	99.5	97.8

IGSF10	99	131.8	81.5	78.9	75.5	82.7	97.8	119.3
IGSF11	84.3	99.5	91.8	85.4	77.5	84.6	87.7	95.4
IGSF11	100.9	102.4	103.3	93.5	87.9	89.8	94.1	112.2
IGSF11	114	140.5	105.5	104.5	102.4	105.1	109.9	113.3
IGSF2	103.3	114.9	100.8	98.8	96.5	102.6	98.2	105.8
IGSF21	76.4	76.6	93.1	77.9	83.9	88.8	78.9	91.2
IGSF22	75.9	84.1	79.4	82.1	74.4	72.9	82.5	82.5
IGSF3	293.9	474.7	288.8	245.8	185.3	236	309.7	442.5
IGSF3	145.4	181.2	90.4	98.2	86.4	85.8	126.8	157.1
IGSF4	96	114.4	102.3	110.2	92.4	110.2	86.8	111.3
IGSF4B	81	92.4	86	84.4	86.3	86.3	81.3	93.5
IGSF4C	74.8	94.8	83	86.3	74.5	71.4	84.8	86.9
IGSF4D	75.1	86.9	85.1	93.6	83	83.5	76.1	86
IGSF6	345.2	493.7	158.3	199.9	158.3	164.3	212.4	227.1
IGSF8	108.8	137.9	97.1	94.4	94.8	98.9	110.2	117.5
IGSF9	96.8	113.3	90.5	76.3	79.1	79.6	99.5	107.4
IHH	101.6	115.4	99.4	79.6	74.9	88.9	96.7	101.8
IHPK1	143.6	160.7	92.4	84.9	88.8	88.8	140.1	150.8
IHPK1	855.7	1189.1	594.9	475	453.9	520.1	802.4	750.8
IHPK1	523.1	696.5	823.2	655.5	544.7	726.9	584.9	1126.4
IHPK2	382.4	519.7	215.6	197.7	187.2	211.6	315.1	422.7
IHPK2	288.5	366.7	150.7	133.9	120.5	155.1	223	279.1
IHPK3	113.5	133.7	106	105.4	100.3	108.4	113.5	133
IIP45	272.3	351	297.8	189.4	172.9	249.5	274.5	369.2
IIP45	100.4	118.5	120.4	110.4	93.2	105.3	84.2	119.9
IK	1673.5	2676.1	853	902	787	1492.3	1406.5	2012.6
IKBKAP	115.4	136.1	116.6	120.3	112.6	118.2	105.4	110.8
IKBKB	113	130.1	101.9	108.7	90.8	103.9	118.3	151.5
IKBKE	259.3	292.7	250.2	166.2	182.2	195.2	189.2	259.1
IKBKG	1586.5	2000.1	927.6	894.7	746.9	932.5	1825.4	1925.2
IKIP	211.5	306.9	148.6	172	153.3	192.3	196.2	196
IKIP	168.3	218.3	122.4	128.4	111.3	143.2	144.1	166.7
IKIP	201	226.3	124.5	135.3	135.1	156	168.3	191.7
IKZF3	83.9	95.5	85	83.4	74.6	77.5	90.6	95
IL10	1832.3	2043.9	513.1	929.1	976.8	560.7	1534.4	1479.3
IL10RA	155.3	167.8	134	142.2	139.4	137.7	158.4	201.7
IL10RB	8046.4	10162.9	5328.4	4755.7	3745.8	3745.1	8375.8	9387.8
IL11	111.5	139.3	81.7	80.6	79.3	76.5	115.9	114
IL11RA	154.4	181.2	153.4	137.6	137.2	145.9	143.5	173.3
IL11RA	704.4	843.9	651.8	547.6	595.4	634	538.2	571.2
IL11RA	101.7	105.3	89.2	98.6	86.1	93.7	98.1	106.8
IL12A	132.1	132.2	109	86.9	87.4	92.3	121.4	127.6
IL12B	94	108.5	94	97.5	92.9	87.4	94.7	91.1
IL12RB1	120.8	150.7	103.7	93.9	92.3	99.3	127.3	120.4
IL12RB1	176.6	232.3	148.3	123.4	113	107.6	178.6	195.1
IL12RB1	293.8	391.6	165.4	128.7	110.5	113.3	255.2	263.2
IL12RB2	214.1	292.6	128.2	134.2	126.6	171.1	199.8	247.7
IL13	85	110	76.2	83.1	71.4	69.1	87.8	102.6
IL13RA1	1418.8	1598.9	1074	1976	1588.6	2202	1097.9	1672.1
IL13RA2	76.4	81.6	87.5	79.1	74.2	83.3	74.6	85.5

IL15	71.5	101.8	93.2	83.2	83.5	81	90.7	91.7
IL15	82.1	85.6	93.8	79.1	80.1	82.1	82.5	94
IL15	162.8	192.5	121.6	111.4	104.1	120.9	147.3	209
IL15RA	90.7	94.6	85.6	85.5	81.3	86.3	86.2	91.4
IL15RA	136.7	160.5	98.5	89.5	90.7	95.4	115.1	134.4
IL15RA	89	101.9	82.4	105.9	88.9	90.6	91.5	92.4
IL16	84.6	89.5	88.1	100.7	91.3	97.2	97	93
IL16	101.9	129.4	111.9	126.1	116.2	147.1	111.8	133.2
IL17	80	82.7	86.5	81.3	77	81.1	86.9	78.6
IL17B	104.6	98.6	91.4	82.7	88.2	84.7	89.2	97.7
IL17C	101.5	122	106.1	87.9	92.1	95.9	103.4	121.4
IL17D	190.6	250.9	163	163.8	139.7	165.9	177.2	185.4
IL17E	130.7	139.7	112.7	95.8	89.6	95.2	107.6	120.6
IL17F	88	97.3	90.8	84.9	83.1	80	82.8	81
IL17R	330.2	473.1	348.6	381	281.5	452.5	321.2	598.1
IL17RB	107.6	115	103.7	84.8	85.4	90.7	88.6	97.1
IL17RC	86.6	101.4	85.1	78.5	72.6	83.8	87.9	98.4
IL17RC	99.9	105.4	96.7	90.7	86.4	94.1	92.6	109.7
IL17RC	116.3	129.5	89.1	84.4	87.2	82.6	110.8	126.4
IL17RD	112.9	114.7	87.6	91.9	82	82.5	119.7	111.4
IL17RD	772.9	762.7	403.8	665.5	666.1	534	722.8	892.1
IL17RD	73.1	78	91.6	79.6	81.5	80.8	73.5	78.9
IL17RE	114	137.9	100	85.3	78.1	89.7	111.6	135.2
IL17RE	99.3	100.7	96	87.6	87.2	91.9	94.8	104.2
IL17RE	81	96.5	85.7	80	79.7	83.9	88.6	104.6
IL18	8058.5	9217.5	3462.3	5350.5	5519.4	4659.6	7311.1	7604.4
IL18BP	89.1	90.6	93.5	87.9	88.5	88.7	83.2	88.6
IL18BP	494.5	599	351.4	770.8	491.9	640.8	485.6	654
IL18R1	91.2	95.6	98.7	96.4	85.9	105.5	90.5	94.6
IL18RAP	112.2	142.8	108.3	97.9	99.8	107.1	97	103.2
IL19	81.4	96.7	81.6	79.6	78.8	74.5	89.8	89.2
IL19	133.8	156.2	117.8	110.2	103.3	105.4	125.2	139.8
IL1A	90.2	100.9	98.7	88.6	79	88.5	88.1	108.1
IL1B	157.3	191.9	144.1	128.7	138.2	143.5	201.2	282.6
IL1F10	75.2	80.7	89.1	94.5	83.8	87.9	81.8	72.4
IL1F10	92.1	88.8	88.5	92.3	84.1	86.1	91.9	93.2
IL1F5	103.2	117.3	91.2	85	88.4	86	91.6	116.3
IL1F5	91.3	84.8	114.6	90.2	98.1	92.9	80.4	93.5
IL1F6	90.2	107.6	104.4	84.4	92.8	86.1	88.6	89.7
IL1F7	82.4	102.5	100.7	102.8	93.3	98.8	95.3	93.3
IL1F7	340.8	405.1	138.7	160.4	134	155.3	283.2	336.3
IL1F7	74.4	93.1	95.3	85.1	75.8	95.3	86.5	88.5
IL1F8	75.7	81.8	85.7	83.6	84.2	79	85	103
IL1F8	110.1	121.2	95.3	88.2	96.3	100.7	103.7	122.3
IL1F8	84.7	96.9	99.8	98.9	88.4	86.1	82.4	89.6
IL1F9	83.9	97.8	94.4	83.2	82.3	89.5	87.2	85.7
IL1R1	102.9	140	85	89.6	73.9	82.8	114.5	165.2
IL1R2	85.8	86	83.9	84.3	78.1	93.2	85.4	95.8
IL1R2	135.9	163.1	93.3	87.1	76.4	90.6	126.7	137.7
IL1R2	89.4	86.5	97.1	90.1	88	96.7	87.2	88.1

IL1RAP	130.2	128.7	103.1	112.3	100.6	112.7	121.2	131.3
IL1RAP	83.7	95.6	85.8	84.5	84.3	94.3	83.7	101.5
IL1RAP	176	209.9	121.4	134.2	107.3	152.5	167	306.4
IL1RAPL1	78.9	89.6	93.5	88.1	89	87	80.3	82.7
IL1RAPL2	80.1	100.7	94.2	90.1	83.1	84.4	86.4	96.7
IL1RL1	80.2	82.1	99.4	96.1	89.9	97.4	83.4	89.6
IL1RL1	136.7	188.7	93.9	92.3	87.6	94.1	146.7	180.1
IL1RL1	77.7	82	95	88.9	89.2	96.8	110.6	132.8
IL1RL2	95.8	101.7	101.2	94.7	84.1	97.6	102.1	120.4
IL1RN	112	118.2	111.3	79.7	84.1	92.3	112.9	131.9
IL1RN	543.6	719.6	376.2	176	146.4	143.2	446.5	559.8
IL1RN	830	1068.5	796.8	431.3	290.9	456.9	971.4	1773.9
IL2	83.5	93.9	87.9	85.6	84.4	84.1	80.2	85
IL20RA	133.6	156.4	99.4	94.1	84.2	95.3	139.9	155
IL21	97.7	106.2	88.9	93.1	84.4	88.4	107.7	99.3
IL21R	138.6	163.4	117.6	100.6	97.4	107	132.7	154.7
IL21R	128.3	143.9	113.1	101.8	105.3	116.2	130.4	126.3
IL21R	109.2	129.4	97.4	95.9	94.6	87.8	109.9	123
IL21R	103.4	114.5	78.9	69.9	68.9	70.5	85.2	113.6
IL22	82.1	76	90.1	92	89.6	91.9	79.2	81.3
IL22RA1	109.9	118	95.2	79.7	81.3	86.6	99.7	124.9
IL22RA2	139	145.8	96.3	93.1	89.5	86.8	122.9	147.7
IL22RA2	81.8	97.6	85.6	79.5	78.4	95.4	87.2	86.8
IL23A	123.4	140.9	131.5	121.5	116.5	141.5	110.8	141.5
IL23R	90	94.1	94.4	90.5	83.4	86	99.9	88.5
IL24	77.1	101.4	79.7	83.1	80.2	79.8	88.1	92.8
IL24	75.4	75.8	87.1	83.4	87.6	92.2	81	87.2
IL24	97.4	118.9	88.3	86.2	80.8	87.9	92.8	90.9
IL25	90.8	104.9	96.4	91.9	94.3	87.7	87.3	102.3
IL25	110.7	123.5	79.2	91.4	81.2	84.6	109.6	120.9
IL26	85.1	86	93.3	85.4	94.6	90.9	80.5	83.9
IL27	85	90.2	86	87.7	78.7	77.9	90.2	88.5
IL27RA	754.6	801.3	736.1	430.3	319.5	368	846.3	1895
IL28A	84	85.8	94	86.5	84.2	86.2	91.6	100.7
IL28B	102.6	109.8	96.5	85.2	90.4	89.8	97.4	103.5
IL28RA	81.9	105.3	92.6	83.7	81.7	92.9	95.6	97.3
IL29	103.4	117.5	86.1	84.9	78.9	86.4	97.5	104.1
IL2RA	112.1	125.6	85.8	89.4	87.9	85.5	96	136.1
IL2RB	212.3	250.1	178.2	155.1	123.2	151.9	163.4	217.3
IL2RG	79.9	88.1	94.1	90.9	81.2	78.7	88.7	84.9
IL3	80.7	84.8	86.6	87.8	93.3	87.6	79	86.4
IL31	93.7	86.3	82.4	74.4	81.9	85.3	88.3	98.3
IL31RA	90	99.5	102.5	93	91.3	95.7	85	92.5
IL32	129.6	145.9	91	92.6	78.4	86.3	116.5	146.2
IL32	86.4	101.1	97.5	106.5	86.9	103.8	85.8	90.7
IL33	78.1	104.8	94.1	96.1	84.5	85.8	80.2	87.8
IL3RA	155.5	204.7	138.1	133.1	108.3	116.5	156.4	181
IL4	80.4	83.8	94.9	84.7	82.2	78.9	77.9	83.4
IL4	93.5	103.3	99.7	95.4	95	96.4	86.8	105.5
IL4I1	111.9	124.7	103.7	112.9	139.5	108.4	114.9	131.4

IL4I1	79.2	89.9	91.6	80.9	83.9	89.4	86.9	93.3
IL4R	87.3	99.1	100	101	93.2	97.3	83.4	88.3
IL4R	270	343.8	177.4	164.5	121.6	142.6	240.7	329.4
IL5	81.9	89.5	95.6	96.2	80.7	84.3	83.1	92.9
IL5RA	132.9	156.8	123	115.4	119.7	108.8	113.9	144.4
IL5RA	118.1	141.9	92	88.9	88.8	91.9	114.6	99
IL6	103.3	123.9	93.1	89.8	88	88.9	106.2	127.7
IL6R	289.3	387.5	214.4	263.8	266.5	234	217.7	268.7
IL6R	634.1	965.6	405.5	366.3	339.8	343.7	540.2	649.9
IL6ST	77.8	94	84.7	83.8	102.6	90.2	84.1	82.3
IL6ST	102.5	116.6	97.1	96.2	88	90.8	102.3	138.1
IL7	73.7	83.4	99.9	81.8	78.8	87.9	72.2	81.1
IL7R	99.1	118.8	108.1	80.3	99.1	83.7	119.6	129
IL8	556.5	644.7	369.4	579.4	519.4	625.8	1063	2361.4
IL8RA	101.3	134.1	97.5	81.8	75.2	76.8	100	132.2
IL8RB	385.6	531.3	256.5	280	245.9	275.7	355	386.6
IL9	94.2	109	93.3	87.7	90.1	86.7	103	100.7
IL9R	74.4	78.2	93.6	80.6	85.4	89.5	83.3	91
ILDR1	98	114	96.6	92.9	81.3	91.7	108.1	103.4
ILF2	7862.4	11219.3	5416.2	3770.5	3064.7	5965.2	5910.9	8725.7
ILF3	667.7	784.8	745.1	638.4	531.6	958.3	564.6	1054.2
ILF3	207.3	295.7	124.4	117	90.6	99.9	176.3	242
ILF3	1170.1	1742.6	972.2	649.7	542.8	1021.1	767.4	1215
ILK	79.5	93.1	96.5	89.1	82.4	92.9	85.8	96.4
ILK	2969.4	3926.6	1890.1	1534.4	1447.9	1831.5	2434.9	3508.9
ILK	278.6	330.4	141.3	121.1	127.5	156.5	275.6	397.5
ILKAP	825.3	983	446.2	427.6	331.9	491	691.1	748.2
ILKAP	91.6	97.2	95.2	87.9	92.7	96.7	83.1	100.5
ILVBL	834.4	1057.9	543.4	356.9	321	418.8	688.2	1027.3
ILVBL	1330.4	1733.4	958.6	772.9	660.7	821.8	1131.7	943.3
IMAA	121.2	127.8	100.5	106.6	105.5	87.7	105.3	108.8
IMAA	28995.5	27083.3	26964.2	34250.2	33390.9	28596.1	28551.4	29995.1
IMMP2L	453.5	566.9	296.2	368.9	311	433.3	394.1	571
IMMT	2053	2394.8	670.5	725.7	545.9	968.2	1647.2	3091.6
IMP3	5543.9	6832.1	3874.6	3302.5	2275.2	3534.8	4770.4	5443.8
IMP4	4514.1	6071.8	3335.9	2276.7	1525.4	2231.4	3480.3	3057.6
IMP5	118.9	145.4	101.5	88.3	83.1	89.7	109.8	138.8
IMPA1	1563.2	1691.9	453.2	372	332.8	544.7	1318.9	2218.5
IMPA2	1260.1	1582.4	1353.7	1570.4	1387.1	1772.6	1294.7	1983.6
IMPACT	119.9	182.3	126.1	112.9	115.4	136.3	107.6	131.2
IMPAD1	889.1	1210.8	379.2	307.5	243.7	341.9	787.5	1049.4
IMPDH1	2403.3	2873.6	2104.9	1664.9	1423	1674.9	2073.1	2163.2
IMPDH1	118.8	127.4	102	86.3	90.2	91.2	108	129.4
IMPDH1	98.6	119	81.6	80.7	79.6	81.5	106.2	128.8
IMPDH2	5507.7	6900.3	3516.2	2465.8	1691.4	2503.4	4292.1	6490.5
IMPG1	79.4	89.3	84.1	87	78.9	102.1	84.2	90.2
IMPG2	133.5	148.2	128.5	122.4	113.6	117.9	134.2	143.6
INA	97.7	115.3	111.1	96.9	98.3	96.5	110.1	110.6
INADL	156.3	205.8	96.4	83.3	82	82.4	132	151.4
INADL	90.7	94.6	108.1	79.4	91.6	89.8	90.8	109.4

INCA	85.8	102.7	99.6	93.5	87.1	96	93	93.5
INCA1	95.3	90.5	83.9	78	76.4	78.4	82.9	100.4
INCENP	483.9	518.2	339.5	369.1	317.9	299.5	352.3	403.6
INDO	66.1	88.7	95.1	89.8	97.7	79	76.2	80.5
ING1	82.9	93.8	88.9	91.3	93.8	85.1	86.6	95.3
ING1	313	447.2	297.8	286.6	218.2	365.3	254.1	460.7
ING1	135.6	145.7	96.9	81.4	77.9	90	114.9	122
ING1	88.7	114.8	78.7	80.7	74.9	85.8	102.5	114.4
ING2	735.6	970.6	353.9	332.6	220.8	348.1	743.3	1397.4
ING3	358.5	482	219.4	219.9	192.5	203.9	272.7	304.9
ING3	113.3	116.3	106.3	85.6	80.8	95.3	103.1	125.1
ING3	993.9	1223.6	924.7	1021.5	829.6	1280.7	839.1	1438.4
ING4	149.7	174.9	118.9	118.6	102.5	112.8	145.4	179.3
ING5	137.7	202.6	102.9	103.4	93.3	103.5	125.5	163
ING5	81.1	94.3	106.6	93.3	82.9	81.3	90.4	99.9
INGX	112.1	113	110.8	91.6	87.3	83.6	107	107.2
INHA	85.5	93	84.7	81.4	75	81.4	90	98.5
INHBA	94.6	108.3	87.8	90.8	78.2	96.6	98.5	105.3
INHBB	126.7	146.7	112.7	102.3	90.4	104.3	131.7	138.8
INHBC	82.2	100.4	93.1	77.1	80.4	83.6	84.7	92
INHBE	126.6	164.6	99.5	104.3	90.8	93.6	134.4	153.3
INMT	84.6	98.6	78.9	85.5	89.6	71.1	77.4	93.8
INOC1	126.7	167	90.3	80.2	80.5	95.9	133.5	144.9
INOC1	238	325.6	242.6	256.1	224.2	317.4	241.1	357.1
INPP1	1447.9	1810.1	804.7	510.5	470.1	575.8	1304.1	1211.4
INPP4A	69	77.3	83.9	73.6	76.5	77.9	63.7	87.1
INPP4A	127.3	153.6	113.5	116.8	96	139.5	112.8	129.5
INPP4A	109.1	137.8	81.4	85.3	85.1	87.5	117.4	119.8
INPP4B	495.7	606.7	306.1	303	218.3	418.2	371.7	687.9
INPP5A	298.2	387.2	223.2	192.8	172.7	233.5	308.7	679.6
INPP5B	265.2	334.5	218.6	228.2	192.5	222.4	246.5	306.6
INPP5D	664.5	814.8	499.2	407.5	364.4	399.8	557	765.3
INPP5E	428.3	390.3	317.9	285.7	269.8	315	323.7	759.3
INPP5F	96.8	95.7	73	76.6	75	78.7	96.6	95.6
INPP5F	104.7	121.1	107.3	103.2	92.8	96.5	107.3	101.3
INPP5F	168.5	170.8	164.7	148.6	161.4	151.7	150.8	181.5
INPPL1	1421.8	1760	1729.5	1351.4	1352.1	1947	1277.4	1075.8
INS	135.7	149.3	96	89.2	91.3	92.3	125.8	123.4
INSC	115.6	132.5	97.6	94.8	74.3	86.1	122.3	132.8
INSIG1	322.2	422.4	202.3	280.8	263.7	228.7	285.9	715.3
INSIG2	327.5	495.5	298.4	345.2	271.7	431.3	318.2	541
INS-IGF2	82.8	87.7	85.3	87.5	85.2	90.6	87	92.9
INS-IGF2	73.3	87.4	78.5	82.8	83.8	77.3	83.2	85
INSL3	72	75.8	87.7	78.2	75.5	83.1	80.8	75.5
INSL4	86.2	99.9	91.6	84.1	85.5	98.4	88.5	88.3
INSL5	116.8	126.8	102.9	92	85	79.8	100.2	117.7
INSL6	76.3	87.5	90.3	85.8	75.7	88.6	87.2	83
INSM1	145.2	160.7	98.7	97.9	105.4	94.9	131.4	153.2
INSM2	81.9	86	83.4	86.7	82.5	84.2	85.2	86
INSR	141	170.4	109.5	93.6	94.3	88.7	122.2	163.5

INSRR	121.4	151.2	87.8	87.2	81.8	83.5	130	134.4
INT1	157.8	214.9	215.4	214.4	198.2	280.8	158.6	234.7
INT4	447.5	601.7	321.2	331.2	288.6	400.6	379.3	520.1
INTS1	653.2	757.9	452.2	484.7	429.5	508.2	473.9	367.7
INTS6	132.7	141.6	102.2	92.5	95.7	96.3	105	130
INTU	146.3	140.5	113.1	102.2	82.7	147.6	113	142.7
INVS	110.5	154.6	109	103.5	84	133.9	99.7	132.8
INVS	353.4	476.4	428.3	326.7	274.7	501.6	331.7	498.3
INVS	124.6	153.4	108.9	108.7	99.2	102.8	113.4	129
IPF1	108.7	122.3	106.3	94.2	85.4	86	105.1	112
IPMK	108.3	132.9	90.5	92.7	81	87.8	140.4	140.7
IPO11	976.4	1101.7	891.3	1036.4	731.6	1133	651.6	760.4
IPO13	174.2	218.4	171.2	185.1	135.8	205.1	149.8	247.6
IPO4	243.9	319.1	311.2	215.3	173.3	295.6	196.4	265.3
IPO7	428.4	544.8	242.8	304.5	231	268.2	313.4	408.9
IPO8	194.4	260.3	139.3	168.7	131.6	177.7	169.2	215.4
IPO9	554.5	670.2	364.8	376.8	290.9	455.7	418.2	680.8
IPP	170.8	201.6	105.3	109.2	108.4	100.9	144.9	198.7
IPPK	115.1	159.8	109.6	98.3	90.9	92.3	121.6	147.1
IQCA	75.8	83.5	95	86.5	84.6	78.8	73.4	79.3
IQCB1	114.6	128.4	100.5	85.3	77.2	106.9	115.8	132
IQCB1	885.6	1123.4	427.6	408.9	418.1	848	575.6	825.3
IQCC	284.2	359	143.9	116.4	115	120.2	197.8	212.3
IQCD	117.7	136.7	127.8	128.9	117.5	157.2	99.6	104.7
IQCE	98.3	114	122.2	109	111.4	127.6	103.5	120.4
IQCF1	70.4	90.8	88.2	76.6	74.1	85.9	83	92.7
IQCF2	76.3	88.4	95	88.4	98.1	95.1	90.1	92.2
IQCG	141.9	150.8	101.7	86.6	91.7	94.6	120.1	150.9
IQCH	74.3	79.4	86.2	82.3	82.2	88	76.8	78.4
IQCH	104.9	111.6	105.5	86.4	87.8	93.2	108.4	113.5
IQCK	329.3	412	200.1	158.5	123.7	147.1	263.9	361.4
IQGAP1	685.9	1033.2	353.5	477.5	350.7	826.9	750.8	1987.2
IQGAP2	116.7	139.1	119.3	105	113.1	129.8	109.5	131.2
IQGAP3	135.1	153.9	128.3	132.4	118.4	128.3	130.7	121.9
IQSEC1	168.5	209.6	154.7	242.2	187	266.4	154.6	195.1
IQSEC2	118.7	123	112.1	98.3	102.3	108.1	112.3	126.8
IQSEC3	110.7	121.9	85.7	88.7	78.3	85.2	114.9	122.9
IQWD1	1183.8	1564.1	896.9	894.9	879.9	1355.7	1069.3	1990.2
IQWD1	147.4	182.4	122.9	102.9	108.6	111.6	113	152.1
IQWD1	141.6	181.9	100.5	103.2	112.2	127.4	142.8	261.2
IRAK1	1792	1987.9	1346.2	1153.3	1145.1	2010.2	1599.2	3732.1
IRAK1BP1	298.1	358.1	187.6	185.5	131.2	181.6	230.6	336.6
IRAK2	186.7	245.4	153.8	132.2	133.5	143.4	201.2	237.5
IRAK3	1031.1	1364.5	687.4	855.7	697.6	1440.4	1094.9	1592.5
IRAK4	271.3	357.7	207.6	161.9	158.2	186.2	216.1	308.1
IREB2	851.7	1214.1	532.7	612	473.8	621.7	752.4	946.9
IRF1	515.5	636.4	385.9	320.5	329	251.7	588.8	559.7
IRF2	82.6	92.5	89.4	89.7	78.5	92.7	92.5	108.4
IRF2BP1	119.9	158	157.6	128.3	134	178.4	127.9	160.3
IRF2BP2	1209.2	1871.9	937.4	1351.6	939.8	1573.1	1096.6	1643.5

IRF2BP2	772.4	912.9	751.5	941	820	1038.1	793.2	1168.1
IRF3	384.4	441.5	399	268	244	360.9	343.7	521
IRF4	157.9	164.5	95.5	79.1	78.3	85	139.9	190
IRF5	1207.4	1382.8	709.7	629	662.6	722.2	960.4	644.9
IRF6	89.4	103	84.7	82.6	82.3	78	95.3	96.9
IRF7	125.3	135.7	136.5	93.9	91.7	112.7	122.4	161.5
IRF7	90.4	112.9	98.1	97.2	88.2	93.5	98	99.7
IRF7	380.6	440.5	280.9	204.6	220.2	222	317.7	292.4
IRF8	17805.8	20287.3	13073.2	11602.3	9073.9	14317.7	12753.9	11117.3
IRGC	87.3	103.3	88.9	80.2	85.3	90.7	91.4	106
IRGQ	68.8	74.4	89.4	87.2	81.1	84.2	75.9	81.6
IRS1	94.5	113.4	89.6	92.1	83.5	101.8	101.9	105.6
IRS2	540.3	753.3	475.2	518.3	441.2	772.2	395.4	797.4
IRS4	114	142.2	101.1	104.6	98.1	100	112.7	117.7
IRX1	149.2	165.2	111.7	103.1	97.3	92.4	146.3	161.5
IRX2	91.8	105.8	90.6	89.2	79.6	87.1	91.6	94.7
IRX3	13741.4	16575.9	13311.1	8813.2	8109.7	6536.4	10147.3	10551.9
IRX4	100.7	118.5	111.5	109.2	93.9	109.5	104.7	112.3
IRX5	532.5	700.1	210.5	210.9	143.1	168.1	432	495.5
IRX6	127.6	149.6	109.6	96.8	86.8	91.1	108.2	138
IRXL1	74.5	95.7	79.6	82	75.9	71.7	72.6	90.3
ISCA1	411.9	548.1	140.1	138.8	115.6	197.5	404.1	935
ISCA2	397.1	508.8	300.5	247.5	196	249	337.4	440
ISCU	1676.1	2097.5	1313.6	1044.6	918	1042.7	1470	1704.6
ISG15	370.3	385.3	233.2	193.6	169.1	203.1	334.8	318.6
ISG20	184.8	223.6	132.2	112	111.8	124.3	195.1	213.2
ISG20L1	1027.6	1133.4	565.9	492.5	323.2	468.8	735.1	800.6
ISG20L2	1547.5	1908.3	1127.7	761.7	513.5	626.3	1309.6	2205.2
ISGF3G	717.4	868	498.6	327.4	307.9	402	732.2	1216.2
ISL2	191.9	239.2	122.9	100	95.1	107.4	184.6	243.4
ISLR	88.9	108.6	87.3	79.3	78.3	76.1	86	92.5
ISLR	81.1	84.7	92.9	92.1	78.2	85	90.5	84.1
ISLR	99.3	110.3	93.9	88.9	88.6	86.4	97.3	97.8
ISLR2	91.9	102	103.9	90.4	86.5	85.8	94.1	103.9
ISOC1	648.7	833.4	299.8	252.4	248.8	320.2	510.1	912.6
ISOC2	1040.2	1287.7	317.7	360.9	213.8	350.8	827.6	1350.7
ISYNA1	113	128.7	114.6	117.9	98.8	119.1	110.1	134.6
ITCH	313.1	391.1	231.5	316.6	258.8	331.1	286.1	434.4
ITFG1	101.2	117.6	90.6	94.1	81.9	81.5	96	109.9
ITGA1	88.2	94	85	77.7	82.2	81.5	85.4	91.6
ITGA10	92	101.1	100.3	108.7	109.1	104.9	94.6	100.2
ITGA11	81.6	91.3	108.7	107.9	102.2	112.5	92.9	99.5
ITGA11	101	116.7	112.4	103.7	90.2	111.2	101.5	119
ITGA2	169.3	216.7	106.4	98.3	94.3	95.7	144.1	186.1
ITGA2B	106.3	120.5	86.4	71.4	79	89.3	101.1	117.7
ITGA3	111.8	153.9	96.9	100.8	88.8	99.2	112.4	116.1
ITGA3	333.9	376.4	196.9	207.7	156.1	204.9	361.4	359.8
ITGA4	384.8	530.7	249.1	309.6	267.4	486.1	287.1	473.4
ITGA5	567.4	737.3	434.5	446.5	400.7	481.4	536.6	691.4
ITGA6	107.4	140.3	98.8	90.7	85	94.4	105.5	136.1

ITGA6	81	79.1	93	78.3	82.5	87	73.4	86.3
ITGA7	108.8	129.4	103.7	114.9	101.6	110.7	104	105.5
ITGA8	70.8	81.9	93.2	79.7	84	77.7	69.7	74.2
ITGAD	105.5	126.2	93.7	86.1	81.7	83	98.8	124
ITGAE	964.6	1123	366.5	345.7	281.7	414.2	1089.8	1598.4
ITGAL	633.4	771.8	688.6	386	310.9	493.1	566.6	1218.4
ITGAM	290	297.7	211	486.9	390.7	607.7	209.5	434.4
ITGAV	255.9	314.5	168.7	186.3	166.8	304	265	546.4
ITGAX	195	198.9	239.6	301.5	293.5	385.2	180.3	267.4
ITGAX	82.9	101.8	100.7	100.1	95.8	95.1	89.1	99.9
ITGB1	731.7	1178.2	431.4	347.7	297.6	552.8	596	752.6
ITGB1	2905.9	3870.4	1296.7	1487.3	1319.2	2363.7	2985.2	4001.9
ITGB1	1417.4	2139.7	882.2	824.4	668.5	1432.2	1219.5	1659.5
ITGB1	98.9	115.6	100.6	99	89.9	89.8	97.5	100.9
ITGB1	110.1	113.1	98.9	72.5	81.6	85.8	95.6	111.5
ITGB1	77.7	81.5	89.1	96	87.6	89.7	89.4	79.6
ITGB1BP1	125.1	144.7	118.3	98.4	84.9	101.8	96.9	136.2
ITGB1BP1	2687.2	3035.6	922.6	701.1	562.9	703.6	2370.2	3625.5
ITGB1BP2	120.6	155.8	105.3	106.1	90.4	101.4	121.7	135.3
ITGB1BP3	141.6	169.9	99	102.9	94.8	90.9	145.6	130.2
ITGB1BP3	83.6	99	97.1	97.1	80.3	90.5	90.4	96.2
ITGB2	17389.2	20645	17616	25831.6	23029.2	25459.5	15292.6	18598
ITGB3	101.1	106.6	90.3	94.3	89.6	85.4	102.7	94.5
ITGB3BP	691.7	814.3	320	328.4	217.1	359.7	679.4	934.9
ITGB4	90	88.9	107	96.9	100.8	96.4	88.4	93.5
ITGB4	86.8	94.3	80.8	80.9	88	84.1	83.8	84.5
ITGB4	95	120.7	97.1	102.9	86	82	91.3	103.4
ITGB4	109.6	125.4	93.6	85.6	80.2	83.2	106.5	124.3
ITGB4BP	2319.6	2724.6	1620.3	1205.6	935.2	1340.9	2104.5	2812.7
ITGB4BP	133.5	150.9	90.9	81.6	79.1	88.4	112.1	137.7
ITGB4BP	3967	5398.9	3086.3	1782.3	1223.1	1993.6	3858.9	4424.4
ITGB5	438.9	593	218.1	143.8	128	142.4	399.9	489
ITGB5	655.9	915.8	604.7	291.1	257.2	335.4	578	701.5
ITGB6	101.5	113.2	84.3	86	83	80.4	98.4	106.6
ITGB7	415.1	564.5	528.1	809.4	585.7	1307.5	344.3	562.1
ITGB8	109.1	107.4	91.5	92.3	89.6	87.1	102.1	101.3
ITGBL1	123.3	146.2	93.1	77.4	83.6	100.7	128.4	126.4
ITIH1	110	122.3	96.2	96.3	80	92.5	104.8	127.8
ITIH2	92	114	94	76.5	74.9	87	99.5	96.5
ITIH3	126.9	146.7	97.5	84	79.4	79.3	115.2	145.1
ITIH4	87.7	101.9	84.8	89	96.7	105.4	90	106.6
ITIH5	97.2	99.9	87.6	81.3	86.6	87.1	81	89
ITIH5	25800.1	25432.9	12767.1	19777.5	17199	14843.8	23210.1	26492.1
ITIH5	111.8	125.3	96.2	81.5	77.9	96.2	109.7	113.7
ITIH5	83.3	90.6	100.2	94.7	91.9	95.3	79.2	85.2
ITIH5L	75.4	87.4	88.5	86.9	77	82.2	84	83
ITK	100.6	126.7	103.3	103.9	105.9	102.8	90.5	103.9
ITLN1	85.3	93.4	120.5	103.1	102.5	109.8	86.8	87.9
ITLN2	103.6	110.5	83.3	89.9	89.3	84.9	99.4	96.6
ITM2A	97.1	107.1	103.9	89.8	87	96.7	96.9	102

ITM2B	79.9	84.7	92.2	85.9	78.8	85.7	84.6	101.7
ITM2C	1803.7	2293.2	1453.1	2229.5	2016.9	3039.6	1725.7	3087.6
ITM2C	2321.6	3494.6	2084.5	3736.5	3270	4949.9	2200.8	2936.6
ITPA	585.6	760.9	311.7	247.2	197.2	268.2	463.5	579.2
ITPA	590.3	773.3	361.2	389.8	294.2	386.1	536.4	547.7
ITPK1	194.5	224.6	178	210.6	169.5	302.2	172.6	279.7
ITPKA	371.2	489.4	275.3	326	395	376.5	386.5	323.9
ITPKB	261.2	334.3	242.4	202.5	177.7	238.6	235.9	338.5
ITPKC	106.9	127.4	111.5	88.8	102.1	91.6	119.9	152.6
ITPR1	777.8	1381.4	686.2	1218.5	892.6	2009.1	765.2	1672.4
ITPR2	117.3	150.9	117.8	135.4	115.4	147.5	125.9	131.2
ITPR3	997.1	1164.4	1184.1	896.2	893	851.1	537.1	528.9
ITR	437.4	547.1	281.3	225.2	167.1	241.6	343.2	734.9
ITSN1	90.5	94	106.8	93.7	91.7	98.3	82	88
ITSN1	96.1	115.1	84.2	85.4	90.1	100.8	94.9	103.2
ITSN1	297.7	401.5	287.9	267.2	275.3	284.2	251.8	235.8
ITSN2	144.6	152.3	101.4	89.5	74.8	79.2	153.7	151.2
ITSN2	141.2	182.3	95.9	86.7	77.7	100.9	144.3	170.6
IVD	467.3	664	297.8	319.9	238.4	341.1	332	466.7
IVL	114	117	88	91.6	80.8	77.4	105.5	118.1
IVNS1ABP	518.7	665.8	334.3	347.4	245.4	552.1	324.6	519.8
IVNS1ABP	522	575.5	244.5	330.3	232.3	460.9	391	569.6
IVNS1ABP	144.2	163.7	110.8	95.2	99.2	98.3	126.3	139.3
IXL	685.3	791.3	383.1	385.1	315.9	356.6	586.7	576.9
IZUMO1	90	104.8	101.6	98.4	95.5	90.7	99.5	96.1
JAG1	104.2	121.7	103	92.6	95.9	102.7	103.3	128.1
JAG2	331.5	359.3	365.8	263.3	196.8	334.4	276	347.2
JAG2	109.8	127.4	77.6	81.1	83.9	94.5	107.9	112.8
JAG2	178.4	226.2	233.3	135.8	126.9	172.9	149.3	185.3
JAK1	356.4	536.9	264	233.4	200.2	325.9	307.7	451.6
JAK2	282.8	366.2	197.3	207.8	202.6	391.2	231.2	388.1
JAK3	101.1	104.5	104.9	97.7	80	75.8	93.1	111.4
JAKMIP1	88.7	120.5	95.7	79	75.2	83.6	88.7	105.7
JAKMIP2	82.1	112.8	95	93.3	82.2	90.2	94.4	103.5
JAM2	91.2	111.8	100.4	94.7	93.1	100.5	96.3	103
JAM3	129.7	157.6	99.8	95	91.3	101.5	113.5	147.2
JARID1A	68.6	74.7	78.3	82.3	69.5	75.4	66.1	78.7
JARID1B	564.8	747.8	559.4	760.4	618.7	1127.9	458.7	685.3
JARID2	711.2	830.6	535.8	758.7	616.7	1013.6	601.1	852.8
JAZF1	218.8	263.4	122.3	120.2	96.2	140	201.1	240
JDP2	147	202.9	118.8	96.1	80.1	105.8	146.9	189.9
JM11	136.4	165	120.2	130.6	113.3	121.3	148.7	150.5
JMJD1A	209.2	221.7	131.2	149	145.1	237.4	203.3	434.2
JMJD1B	373.1	534.5	328.5	457.9	414.1	691	345.4	495.6
JMJD1C	1669.6	2158.7	787.6	1422.1	1098.1	2154.6	1407.8	2460.5
JMJD1C	861.3	1431.8	584.8	764.8	628.3	1636.2	710.8	1203.5
JMJD2A	307.8	389	241.1	269.2	223.4	327.7	265.2	291.9
JMJD2B	91.3	108.8	94.8	78.7	82.7	89.4	94	112.7
JMJD2C	139.6	173.6	118.5	114.7	102.2	128.7	123.9	156.7
JMJD2D	111.9	110.8	88.1	82.2	85.8	84.7	103.8	120.5

JMJD3	82.6	87	85.5	82.6	79.8	88.1	76.5	82.1
JMJD4	276.4	366.4	267.8	229	178.1	247.3	218.3	347.6
JMJD5	111.3	135	95.7	91.8	85.9	96.1	100.7	121.4
JMY	118.4	132.2	107	94.4	91.6	116	110.1	136.9
JOSD1	395.8	526.6	185.8	179.2	163.9	195.7	347.5	452.7
JOSD2	227.7	302.3	181.7	144.5	122.3	151.8	231	359.8
JOSD3	175	190.8	127	97.7	103.5	129.6	173.1	265.1
JOSD3	340.9	308.3	184.5	127.1	117.3	199.8	197.6	316.4
JPH1	176.6	194.4	158.1	105.7	102.3	121.4	131.3	179.7
JPH2	140.3	175.5	115.2	105.1	87.8	103.4	148.1	179.3
JPH2	115	132.4	102	96.8	93.9	98.8	132.3	122.5
JPH2	86.4	87.8	99.6	99.3	83	93.6	84.9	90.8
JPH3	122.5	157.6	100.6	93.5	89.3	94.7	125.2	136
JPH4	97.4	102.6	93.2	88.3	86.2	89.4	102.4	106.8
JRK	143.2	189.1	144.3	128	130.2	125.3	132.7	144.7
JRKL	169	243.1	118.3	113.8	105.6	119.5	148.5	183.3
JSRP1	77.7	77.6	91.1	87.7	84.2	98.5	79.4	85.5
JTB	12717	14727.3	15041.1	11254.7	7986.6	10991.3	11500.4	17409.1
JTV1	4230.3	6020.1	2591.6	1755.9	1375.3	1857.9	3834.2	3801.8
JUB	107.2	135.1	96.6	91.5	86.8	95.1	107.1	147.2
JUB	88.4	111.4	86.2	82.4	75.1	84.5	88.1	95.5
JUB	101	105.5	92.7	97.1	84.8	87.5	91	107.7
JUN	218.9	225.1	186.3	236.7	242.3	372.9	246.1	426.4
JUNB	142.5	180.8	140.1	132.8	123.7	143.7	142.9	185.7
JUND	5073.9	6370.9	5228.4	2849.7	2433.9	3465.2	5359.6	10095.8
JUP	116.3	116.8	97.2	98.6	80.3	77.4	119.6	120.1
JUP	89.1	105.8	100.2	88.6	107.7	97.6	96.2	95.6
JUP	101.7	119.2	130.5	180.8	196.9	188.3	113.9	134.9
K5B	101.6	105.9	106.8	90.7	82	90.8	95.1	108.2
K6HF	98.7	106.2	96.6	98.2	86.5	91.3	109.9	130.8
K6IRS2	96.7	110.9	106.2	100.9	94.9	94.4	102.7	109.5
K6IRS3	100.9	100.6	93.1	85.3	93.9	86.9	90.4	100.9
K6IRS4	96.9	116	88.3	86.6	75.2	78.2	109.5	115.1
KA21	72.4	94.7	90.2	82.3	86	78.7	80.6	86.6
KAAG1	84.6	92.6	90.1	86.4	94.9	93.3	83	95
KAL1	200.8	238.3	142.1	207.5	174.8	264.4	160.9	181.4
K-ALPHA-1	31032.9	29480.8	22526.2	19323.3	17266	17744.1	29774	29132.7
KALRN	126.9	126.4	96.3	90	81.6	78.7	98.2	136.4
KALRN	86.9	90.9	94.4	93.6	91.9	87.7	81.7	86.1
KALRN	81.6	102.4	89.4	85.9	83.8	95.1	92.5	84.4
KARCA1	85.6	96.9	82.6	84.4	78.6	87.9	80.2	97.3
KARCA1	72.3	76.8	86.2	78.6	74.3	85	70.7	82.7
KARS	1039.6	1383.2	649.9	744.3	518.2	978.3	807.1	1432.1
KATNA1	2148.9	2458.7	1582.4	1483.6	1208	2218.6	1909.1	2869
KATNAL1	142.3	179.8	109.7	88.7	97.9	107.8	144.1	150.4
KATNAL1	134.4	187.2	116.4	138	120.2	134.2	197.3	266.9
KATNAL1	99.1	109.7	106.2	92.3	89.4	94.7	93.8	102.2
KATNAL2	196.3	232	153.9	143.4	118.4	185.8	170.2	202.7
KATNAL2	130.2	144.3	106.8	87.8	80.1	89.1	111	150
KATNB1	609.9	712.8	441.5	333	303.2	408.4	540.5	546.3

KAZALD1	101.8	114.2	106.1	87.4	93.6	97.7	103.6	109.4
KBTBD1	111.9	117.9	97.9	84.9	78.9	89.1	95.5	134.4
KBTBD10	86.8	106.4	87.2	84.8	82.6	83.5	82	95.5
KBTBD11	4261.3	4790.7	2700	2336.3	1607.7	2524.7	2812.9	3031.3
KBTBD2	967.9	1255.2	627.1	626.8	529.5	848.9	919.7	1331.7
KBTBD3	100.8	108	98.6	96.6	100.7	106.8	95.6	108.1
KBTBD3	191.7	215	160.3	168.3	133.9	160.9	181.7	256.4
KBTBD4	89.3	111	82	84.1	75.5	82.9	89.4	112.1
KBTBD4	127.6	168.2	122.6	119	96.3	135.5	114.6	130
KBTBD5	65.7	80	92.9	92.4	81.8	90	75.2	79.1
KBTBD6	118.3	131.6	95.6	95	80.8	82.8	115.1	153.7
KBTBD7	251.5	282.2	154.6	205.8	167.5	206.8	217.2	356.3
KBTBD8	247.6	289.7	172	133.7	122.8	172.2	228.1	390.9
KCMF1	191.7	202.8	102.2	88.2	91.1	94.5	158.4	176.2
KCMF1	153.6	197.7	103.6	96.8	85.5	96.7	136.5	198.7
KCMF1	232.2	319.4	173.1	153.9	124.9	166.7	213.8	290.2
KCNA1	98.4	113.3	97.9	101.1	83.1	92.5	94.9	109.3
KCNA10	83	95	89.5	89.1	85	82.3	77.5	87.2
KCNA2	74.6	79.2	82.1	71.7	75.1	78	77.5	73.3
KCNA3	81.1	88.5	87.7	84	83.9	82.1	74.2	78.8
KCNA4	84.2	88.3	94.9	82.8	72	77.4	78.9	81.4
KCNA5	85.8	101.2	84.7	74.3	74	77.9	90.4	84.7
KCNA6	97.1	122.2	88.9	73.2	79.2	87.6	100.4	116
KCNA7	87.6	120	94.3	84.1	83.5	81.1	91.7	116.5
KCNAB1	103	135.5	78.3	92.7	85.4	103.2	97.7	104.6
KCNAB1	87.3	91.9	84.5	82.5	83.2	97.7	99.5	101.5
KCNAB1	85.3	96	96.2	90.5	84.9	100.8	85.1	82.3
KCNAB1	98	116.4	95.1	91.5	85.6	95.7	96.7	105.6
KCNAB2	118.1	144.6	133.4	123.5	127.1	141.7	112	130.1
KCNAB2	99.3	122.5	125.1	106.9	99.1	111.8	100.1	109.8
KCNAB3	85.7	84.3	103.4	97.1	89.3	97.2	78.2	89.9
KCNB1	84.8	110	100.7	79.5	81.6	87.4	94.5	99
KCNB2	94.2	115.2	93.4	80.6	82.7	92.7	100.6	106.2
KCNC1	72.2	88	93	87.4	82.3	87.8	85.2	95
KCNC2	73.5	91.8	74.3	81.6	83.5	83.2	80.7	83.4
KCNC2	84.3	91.9	91.8	85.7	82.2	81.6	82.3	86.4
KCNC3	88	107.9	86.2	88.3	85	96.3	86.8	98.5
KCNC4	93	101.5	94.5	91.2	82	96.4	93.2	95.1
KCNC4	97.2	121	97.6	86.8	86.3	96.2	94.2	102.1
KCNC4	86.3	93.9	100	91.7	83.3	91.6	89.3	91.8
KCNC4	82.9	100.7	100.4	91.8	90.2	94.4	89.6	94.2
KCND1	120.1	119.4	102.5	87.4	91.9	103.7	107.9	131.3
KCND2	78.7	95.5	96.8	105.4	92.7	96.6	78.8	86.5
KCND3	83.8	108.5	86.9	95.9	73.3	79.1	87.7	94
KCND3	146.6	158.3	123	113.6	92.6	114.6	118.5	161.3
KCND3	90.7	113.3	98.3	92.8	87.3	91.7	95	99.2
KCNE1	98.1	100.6	82.8	78.8	84.8	84.7	85.6	114.3
KCNE1L	116.6	149.2	101.8	96.4	105.2	95.3	110.9	124.6
KCNE2	86.4	96.8	93.4	87.8	79.5	80.8	96.5	94.3
KCNE3	90.1	104.4	87.5	93.4	87.7	92.7	100.6	108.1

KCNE4	83	94.1	94	80.6	77.4	75.7	79.6	90.9
KCNF1	113.5	120.1	111.3	106.5	107.1	102.4	101.2	106.2
KCNG1	562.8	728.5	347.1	201.8	173.7	202.8	495.3	501.8
KCNG1	130.3	155	95.3	90	87	88.5	121.2	139.3
KCNG1	97.8	105.8	112.3	96.1	92.7	96.7	93.3	97.3
KCNG2	101.9	110.8	117.4	114.4	100.5	112.5	117.1	110.6
KCNG3	87.9	93.6	93.3	80.3	71.9	86.6	82.4	94.8
KCNG3	97.2	104.8	113.8	86.6	100.2	97.2	90.6	98.9
KCNG4	75.6	76.4	77.1	78.3	71	76.8	76.5	78
KCNG4	80.3	100.5	82.8	76.4	79.8	78.4	88.4	84.7
KCNH1	89.3	97.8	90.2	83.2	87.4	92.1	82.9	114.5
KCNH1	83.1	87.5	98.7	85.6	91.6	96.7	74.4	94.4
KCNH2	101.1	101.4	108.2	89.9	99.9	97.6	105.8	98.4
KCNH2	112.3	127.6	90.4	85.7	88	83	117.2	124.3
KCNH2	132	153.1	115.3	108.7	102.1	128.7	118.2	130.8
KCNH2	90.1	102.2	95.1	85.5	80	87.1	95.1	101.4
KCNH3	80.8	93.2	95.4	88.2	98.8	94.9	86.1	85.6
KCNH4	117.1	112.5	119.9	112.7	111	106.8	100.8	99
KCNH5	92.9	110.8	91.6	88	83.7	91.5	100.7	105.2
KCNH5	86.6	89.7	92	83	86.3	88.7	86.4	93.1
KCNH6	86.3	99.1	91	82.8	88.8	84.1	86.9	89.5
KCNH6	5745.1	5300.4	2264.3	4057.1	3945.1	2792	4520.3	7337.4
KCNH7	86	93.7	87.5	82.8	81.5	84.3	90.3	103
KCNH7	86.6	103.6	91.5	77.5	81.9	90.5	86.3	111.7
KCNH7	103.8	113.8	88.4	76.4	75.8	84.4	95.6	114.1
KCNH8	112.8	140.8	110.2	99.7	91.9	110	109.9	123.2
KCNIP1	100.3	112.3	100.2	95.4	88.5	95.5	106.2	105.8
KCNIP1	76	88.6	84.3	93	80.3	92.1	80.3	80.7
KCNIP1	80.9	89.5	96.3	88.3	82.7	80.9	86.3	91.9
KCNIP2	100.4	109.9	87.8	88.1	82.6	82.8	106.1	105.5
KCNIP2	99.5	128.5	98.1	90.1	85.7	92.2	101	125.5
KCNIP3	101.6	127.4	101.9	94.9	93.4	94.1	94.5	107.7
KCNIP3	276.7	342.2	192.3	159.9	148.6	161.4	300.5	307.6
KCNIP4	88	96.5	101.2	79	81.9	85.7	81.8	82
KCNIP4	86.9	90.1	84.2	96.5	86.1	89.7	82.6	106.4
KCNIP4	84	96.5	78.9	81.4	77.3	81.3	81.4	95.1
KCNIP4	75.6	83.4	94.7	81.4	82.4	79.3	81	86
KCNIP4	87.1	113	95.8	86.8	75.2	87	92.9	95.7
KCNIP4	111	128.4	92.6	84.1	87.5	83.3	104.1	111.6
KCNJ1	92.6	108.3	102.1	86.5	79.7	87.1	106.9	100.6
KCNJ1	82.3	105.2	102.6	102.7	92.7	97.9	88.2	87.7
KCNJ1	86.2	97	101.9	105.2	92.9	92.4	91.7	86.8
KCNJ10	105.5	126	91.3	95.3	88.1	92.1	100	118
KCNJ11	76.1	88.7	91.7	85.4	86.2	81.9	82.9	85.4
KCNJ12	71.3	83.3	86.7	81.9	76.3	73.7	75.6	80.8
KCNJ13	115	138.9	95.3	97.7	91.1	103	105	129.5
KCNJ14	155.3	195.7	95.3	93	95.8	97.4	153.9	160.2
KCNJ14	87	97.7	99	84	88.2	88.1	95.5	98.5
KCNJ14	112.8	116	107.8	107.3	95.1	95	111.3	105.5
KCNJ15	107.7	139.1	91.8	88.4	88	98.1	109.4	106

KCNJ15	82.7	95.6	95.8	96.9	96.1	91.1	96.3	96.3
KCNJ15	83.6	88.7	104.4	89.5	89.7	91.4	90	99.9
KCNJ16	91.7	107.1	104.2	99.5	104.8	100.3	99.7	98.7
KCNJ2	107	121.2	104	138	127.9	130	103.3	140.7
KCNJ3	83	90.2	101.3	88.5	83.1	91.1	80.8	92.5
KCNJ4	104.7	118.8	90.6	80	74	79.3	113.1	123.6
KCNJ4	74.9	102.8	91.3	81.1	80.8	89	92.1	100
KCNJ4	80.3	83.7	96.7	93.3	85.7	92.8	83.1	89.1
KCNJ5	123.6	134.2	111.8	87.2	106	111.8	111.1	108.9
KCNJ6	101.5	109.6	96.1	83.6	78.5	80.3	101.3	109.8
KCNJ8	117.2	138.1	101.2	93.3	85.3	97	100.8	124.5
KCNJ9	93.4	103.8	96.2	92.7	82.9	86.4	88.6	88.5
KCNK1	119.7	144.9	113.7	87.5	90.6	92.9	130.8	143.9
KCNK10	102.2	101.6	100.9	92.7	82.8	85.5	98.2	101.9
KCNK10	153.8	182.8	122.9	103.5	116.8	119.6	130.2	170.9
KCNK10	86.8	98.5	94.4	77.4	86.5	93.2	91.9	92.6
KCNK10	146.6	174.8	101.1	98.4	88.2	91.5	127	149.3
KCNK12	310.4	343	213.6	197.5	165.2	170.8	299.2	322.8
KCNK13	196	235	137.5	95.8	88.2	121.4	183.5	271.6
KCNK15	130.9	128.3	96.3	75.2	79.4	86.8	115.9	149.9
KCNK16	104.4	116.3	114.5	94.3	87.9	93.4	108	112
KCNK17	106.3	125	98.7	93.2	97	84.2	108.6	116.1
KCNK18	122.8	146.1	109.5	102.4	91.6	101.5	127.9	127.1
KCNK2	91.3	93.3	99.3	93.1	82.6	95.3	88	88.6
KCNK2	87	110.4	95.3	92.1	85	94	94.7	96.3
KCNK2	74.6	81.5	88.4	82.3	81.4	80.6	75.4	75.2
KCNK2	134.6	174.6	101.7	95.8	92.7	100.1	148.8	147.5
KCNK3	111.1	163.8	117.3	101.5	95.5	91.6	107.7	142.9
KCNK4	83.4	88.8	98	99.4	95.1	93.8	88.1	90.1
KCNK4	91.5	92.5	99	88.7	86	91.7	83.6	96.8
KCNK5	146.5	182.7	118.6	103.7	90.8	122.7	138.5	187.8
KCNK6	140.1	137.7	124.8	119.2	123.3	150	136.8	192.3
KCNK7	112	101.7	116.2	97.9	100	108.4	118.3	102
KCNK7	97.6	101	95.5	86.5	89.4	87	87.9	103.7
KCNK9	107.7	132.1	102.4	87.5	84.3	94.6	123.8	136.1
KCNMA1	91.9	95.7	91.7	94.2	84.6	85.2	92.7	99.8
KCNMA1	118.9	123.2	96.8	91.8	92.8	90.7	107.8	138.2
KCNMA1	80.7	90	89.6	91.1	84.7	85.2	88.5	86.3
KCNMB1	790.6	1036.3	545.5	828	610.3	878.6	593.8	692.3
KCNMB2	154	162.4	101.3	91.2	87	97.2	143.4	155.2
KCNMB2	76.3	87.1	91.5	84.8	92.2	87.3	81.7	78.6
KCNMB2	94.9	113	105.7	90.8	86.2	87.7	97.1	109
KCNMB3	77.3	77.4	80.8	88.3	80.7	84.8	73.1	76.6
KCNMB3	96.5	103.3	98.3	94.7	85	97.9	92.5	110.7
KCNMB3	251.4	324.4	224	257.5	242	328.6	218.9	353.6
KCNMB4	92.7	120.3	85	85.2	86.7	89.2	91	118.6
KCNN1	122.5	125	95.1	105.5	93.9	96.5	122.4	132
KCNN2	340.2	500.3	241	207.3	202.5	229.6	251.3	310.6
KCNN2	1619.3	2011.8	1214.3	1476.6	1330.5	1792.6	1429.8	1465.3
KCNN3	108.1	107.8	100.6	94.9	89.4	89.7	107.1	109.7

KCNN3	155.6	179.2	110.6	102.1	91.6	100.5	154.4	138.6
KCNN4	321.8	421.8	205.5	164.6	156.1	168.4	344.3	409.6
KCNQ1	94	104.6	86.2	92.9	84.2	91.9	95	92.2
KCNQ1	257.3	309	168.4	160.9	141.7	149.6	246.6	275.3
KCNQ1	98.1	112.6	99.2	87.9	83.6	90.7	111.4	105
KCNQ1DN	85.1	87.2	88.5	81	75.4	87.4	88.7	89.2
KCNQ2	77.3	82.6	89.5	83.3	82.4	84	79	81.7
KCNQ2	147.1	173.9	99.9	107.8	89.2	103.3	153.1	154.9
KCNQ2	109.8	125.5	100.1	94.2	89	104.5	111.3	123.4
KCNQ3	139.6	172.4	129.9	122.1	106.1	138	148.2	144.6
KCNQ4	107.4	128.2	95.5	91.3	81.7	94.2	106.4	122.7
KCNQ4	79	84.1	78.4	82.2	79.7	80.8	85.7	86.9
KCNQ4	131.1	139.5	112.2	93.1	83	83.2	128.4	143.7
KCNQ5	90.5	101.8	96.8	90.2	90.1	86.4	80.9	84.4
KCNRG	87.5	92	113.3	116.6	102	113.6	85.9	86.5
KCNRG	93.3	110.5	98.9	111.7	108.6	114.5	92.9	105
KCNS1	171.1	224.5	169	103.6	95.2	99.2	144.3	142.8
KCNS2	87.8	105.1	89.5	99.1	91.7	95.9	93	86
KCNS3	92.6	112	93.5	84.5	81.6	87.6	106.1	108
KCNT1	75.3	77.7	87.3	78.7	83.2	87	75	77.1
KCNT2	77.7	82	84.3	75.7	77.7	87.2	78.1	80
KCNV1	78	78.1	100.3	99.5	85.4	93.9	81.7	87.5
KCR1	153	197.4	120.1	101.6	97.6	108.4	152.5	200.7
KCTD1	128.2	157.6	103.9	88.9	91.1	89.4	140.6	152.4
KCTD10	239.8	325.7	154.3	217.5	173.8	210.6	289.8	316.8
KCTD11	141.7	159.4	117.1	110.5	117.5	109.6	131	147.7
KCTD12	208.8	214.7	121.5	137.8	115.5	159.9	171.5	258.8
KCTD13	251.3	283.2	248.3	191.4	182.6	247.4	264.9	556.5
KCTD14	85.4	108.7	102.4	97.6	82.8	85.1	90	98.2
KCTD15	264.4	340.5	226.4	153.5	144	165.2	204.3	234.1
KCTD16	79.7	104.9	93.9	84	76.6	81.7	84.5	91.2
KCTD17	131.4	170.6	89.3	102.6	88.1	100.1	113.4	136.6
KCTD18	296.2	341.4	189.7	141.4	132.3	201.5	220.4	363.9
KCTD2	90.4	102.6	103.5	106.6	102.3	89.3	90.9	107.5
KCTD2	155.1	170.5	155.9	131.9	122.1	145.1	148	186.3
KCTD3	1353.3	1716.3	708.1	789	568.6	1155.9	1173.7	1863.3
KCTD4	78	89.8	91.7	97	82.6	91.2	80.8	80
KCTD5	1569.4	1933.8	1391.2	1209.1	1060.4	1343.6	1515.3	1976.3
KCTD6	463.9	539	306	261.5	202.7	256.2	498.3	877.8
KCTD7	89.8	96.3	88	86.5	84.8	87.7	93.7	104.2
KCTD8	140.2	192.6	103.9	107.3	92.1	108.3	139.4	166.7
KCTD9	492.8	550.3	346.5	322.7	219.2	397	559.9	1194.4
KDELC1	125.8	169.9	98.2	104.5	92.9	104.6	143.8	190.1
KDELC2	619.7	890.9	337.9	379.4	300.6	581.2	515.3	1137.4
KDELR1	2016.1	2358.8	1096.3	1008.1	756	774.5	2233.4	2301
KDELR2	2550.8	3069.2	1585.5	1247.6	869.2	1241.7	1901.3	3526.4
KDELR3	448.2	648.7	287.8	257.7	189.5	306.2	343.7	412
KDELR3	327.2	423.2	178.4	210.6	135.8	195.3	247.3	370.6
KDELR3	136.1	177.4	124.9	120.4	89.9	93.8	126	160.8
KDR	99.7	121	100.9	96.7	84.5	88.5	106.2	122.2

KEAP1	816.5	1314.6	618.5	432.2	358	636.3	1128	1844.8
KEAP1	87.2	101.3	98	86.7	84.6	91.1	84.4	105.3
KEAP1	1098.1	1494.9	715.2	461.3	404.8	810.2	1310.3	2268.3
KEL	80.2	91.5	90.9	87.4	82.3	90.1	72.7	81.8
KENAE	232.9	310.2	149.1	170.8	136.6	151.1	194.4	209.9
KERA	138.1	139.9	97.8	86.1	83.7	90.6	131.7	138.6
KGFLP1	74.1	88.5	88.1	81.7	84.4	79.1	77.6	76.2
KHDRBS1	6789.8	7677.5	4224.4	4810.5	3278.7	4744.2	7100	11032.2
KHDRBS2	109.4	124	90.8	87.9	80.1	90.5	105.3	121
KHDRBS3	102	110.4	84.4	97.4	82.6	80	92.9	125.2
KHK	147.6	175.6	132.2	108.6	98.9	125.7	123.8	155.6
KHK	138.4	150.5	109.8	98.4	99.1	102.4	149.4	168.4
KHK	138.9	191.2	125	101	104.4	99.6	116.1	118.6
KHSRP	1303.8	1681	1145.5	721	732.2	631.8	909.1	895.4
KIAA0020	2899	3488.3	2537.9	2180.2	1630.6	3696.5	1974.7	3416.7
KIAA0082	108.9	155.2	98.9	110.9	99.5	108.2	111.6	121.2
KIAA0090	453.8	574.6	324.2	521.5	361.6	508	402.1	584.6
KIAA0100	149	197.3	106.3	97.3	91.3	101.5	159.7	168.9
KIAA0101	10111.8	12217.6	7596.7	5701.1	4758.2	6412.1	9116.2	9505.5
KIAA0101	936.3	1104.3	663.5	697.3	537.2	726.1	993.4	1355.7
KIAA0101	24148	23601.2	10398	12935	14399.6	13034.5	23489.4	25326.9
KIAA0103	208.5	274	138.9	109.6	108	127	212.1	282
KIAA0133	336.2	477.1	314.8	147.8	120.9	157.9	290.1	346.4
KIAA0141	319.6	413.9	334.4	456.8	404.1	640.4	250.9	445.4
KIAA0143	1507.5	2082	1217.3	1046.4	761.4	1211.6	1311.2	2140.4
KIAA0152	1585.9	2368.5	1953.3	2012.6	1351.9	3385.6	1355.2	2432.3
KIAA0157	1364	1882	804.4	658	545.9	682.2	1225	1669.2
KIAA0174	3312.2	4233.1	1991.8	1637.1	1335.2	1615.3	2434.6	3002.4
KIAA0179	291.8	387	285	327.7	219.3	424.6	222.5	462.3
KIAA0182	658.8	915.7	587.1	921.3	796.3	1013.4	657.6	901.6
KIAA0195	138.2	172.4	269.2	251.9	235.6	332.7	120.6	194.1
KIAA0196	1712.3	2467.4	1693.8	1416.8	1137.5	1892.4	1359.9	2304.3
KIAA0232	212.3	243.7	148	175.9	147.7	159.6	214.9	322
KIAA0240	582.8	760.7	405.3	681.5	565.5	775.3	675.4	912.6
KIAA0241	157.3	228.4	134.7	141.3	130.8	168.3	147.2	196.3
KIAA0247	270.3	392.8	204.2	248.5	215.7	288	290.7	490.6
KIAA0251	240	358.4	146.2	142.2	147.6	135.9	189.1	217.1
KIAA0256	173.9	204.5	136.6	193.8	175.8	228.4	190.7	244.8
KIAA0258	97	109.6	95.2	93	93.7	88.4	89.8	100.5
KIAA0261	430.8	628.8	258.8	248.3	207.7	417.4	324.9	472.1
KIAA0265	121.3	154.5	117.2	106.2	106.5	134.4	117.9	147.8
KIAA0274	458	545.8	380.8	481.8	408.6	676.6	378.7	481.1
KIAA0280	107.6	119.2	98.9	82.7	80.4	96.9	101.9	108.4
KIAA0284	92.2	103.2	100.8	102.1	87.8	108.2	91.1	105.9
KIAA0286	466	660.8	400.1	428.4	325.8	593.9	532.9	1010.7
KIAA0310	1043.7	1169.3	1338.5	1657.7	1308.6	1862.3	1208.5	1877.6
KIAA0319	74.6	90.1	81.3	83.4	81.7	83.8	80.7	87.4
KIAA0319L	190.7	236.2	155.5	173.1	159.6	168.2	169.7	196.2
KIAA0319L	172	210.3	199.8	217.5	188.4	230.3	168.2	404.4
KIAA0319L	119.4	126.4	96.3	96.2	101.9	104.3	112.3	115.1

KIAA0323	208.6	199.3	250.8	171.9	207.4	232.3	209.7	353.9
KIAA0329	88.4	93.3	104	91.8	87.6	82.5	77.7	92.1
KIAA0350	546.2	742.2	629.4	664.5	607.2	645.2	458.8	555.2
KIAA0355	202.6	237.2	174.8	182.9	202.5	236.7	269.6	322.1
KIAA0367	97.5	112.8	95.9	93.6	91.5	99.1	95.5	106.2
KIAA0368	120.3	144.8	131.7	132.5	116.4	138.5	123.3	148.5
KIAA0372	601.3	723.5	294	572.1	413	778.3	492.8	711
KIAA0376	1208.7	1651.8	1294.6	1744.2	1337.9	2125.2	1112.5	1341.2
KIAA0391	1005.2	1295.5	516.8	449.7	323.7	500.2	810.7	1004.7
KIAA0404	170.7	206.3	209.8	230.7	204.3	226.3	180.9	234.9
KIAA0406	228.8	284.1	157.2	179.2	147	247.6	206.8	261.1
KIAA0408	184.2	189.9	120.8	120.1	111.7	116	155.6	215.1
KIAA0409	561.9	718.3	286.5	196.5	161.8	252.3	478.7	519.8
KIAA0415	136.6	163.9	130.6	134.3	122.5	146.4	149.6	208.1
KIAA0423	201.8	242.8	146.9	132.7	130.2	162.7	168.9	232.7
KIAA0427	116.5	128.9	166.3	185.3	156.3	216.1	115.5	160.6
KIAA0446	1159.2	1394.2	456.1	372.4	302.4	404.7	1005.3	1193.4
KIAA0460	125	158.3	131.4	137.9	130.4	241.9	102.2	154.7
KIAA0467	112	116.4	77.8	94.1	80.5	78.5	107	123.9
KIAA0494	742.1	1088.7	568.7	880.7	685.5	1242.4	647.3	1026.9
KIAA0495	138.2	161.8	114.3	111.8	95.5	117.2	136.1	147.3
KIAA0513	89	102.4	102.7	95.7	96.3	108.3	96.2	135.4
KIAA0514	76.3	80.1	87.7	78	63.8	81	71.9	69.8
KIAA0523	115.9	144.9	84.1	70.5	89.1	78.6	146.3	141.1
KIAA0528	1139.7	1492.1	455.9	434.8	323.9	539.1	821.1	1111.6
KIAA0553	92.8	122.2	105.1	94.7	97.6	118.3	109.6	125.6
KIAA0556	222.6	270.1	162.8	183.8	153.3	196.3	200.6	235.8
KIAA0562	122.4	138	159.2	144.4	145	163.5	117.5	164.4
KIAA0564	199	220.3	167.9	177.1	149.5	212.3	172.5	274.3
KIAA0564	143.2	189.7	109.9	121	112.3	142.7	143.9	189.8
KIAA0586	405.9	545.7	240.4	214.3	216	302.5	325.5	324.5
KIAA0644	100.6	117.5	94.8	91.9	89.1	95.6	96.6	106.5
KIAA0649	141.6	167.1	129.8	112.5	111.8	154.9	148.4	142.7
KIAA0652	112.6	126.2	96.1	107.4	92	111.7	104.2	125.7
KIAA0664	188	204.5	264.5	236.4	185.3	404.6	169.4	191.8
KIAA0672	137.9	168.2	178.2	145.5	144.8	152.9	111.8	104.4
KIAA0683	445.4	481.1	337.8	300	264.4	327.1	367.9	297.3
KIAA0690	425.2	485.1	412.4	277.2	183.2	326.9	305.5	527.8
KIAA0701	114.5	141.8	130.9	110.5	110.2	124.3	113.8	111.4
KIAA0703	77.5	92.3	88.8	86.6	89	93	85.7	88.3
KIAA0738	4883.7	5356.1	1829.5	3071.5	2562.5	2636.9	4546.2	5356.7
KIAA0746	643.1	898.5	405.8	335.7	341.3	465.5	712.6	923.8
KIAA0753	139.6	160.3	100.9	115.5	91.8	121.8	126	167.6
KIAA0773	225.2	278	129.7	123.4	99.5	115.9	205.1	250.2
KIAA0773	105.6	134	117.8	112.9	110.7	107.3	121.4	113.6
KIAA0773	81.2	85.9	101.1	91.4	87.8	90	82	86.1
KIAA0774	98.8	118.7	101.3	92.2	91	99.2	95.3	96.7
KIAA0774	88.8	79.8	79.4	83.3	77.2	80.3	82.3	87.8
KIAA0774	84.8	97.7	103.6	82.8	76.6	80.7	91.8	123.5
KIAA0776	295.8	338.8	154.8	155.7	141.4	153.4	219.7	354.8

KIAA0789	112	141.1	116.9	88.3	78.8	103.7	119.7	121.9
KIAA0802	82.1	103	98.8	103.2	88.3	106.7	86.1	87.3
KIAA0828	141.3	171.8	144.2	159.5	135.7	186	127.8	179
KIAA0831	326.5	401.5	173.7	182.5	166.7	208.7	334.6	594.8
KIAA0853	92.9	120.1	90.6	91	82.5	85.6	87.9	100.5
KIAA0859	578.9	678.6	472.8	432.4	334.7	534.2	385.1	540.6
KIAA0859	2350.1	2913.1	1960.8	1579.3	1243.8	1961.5	1538.1	1980.9
KIAA0892	443.6	465.7	368.9	275.8	252.7	282	436.8	682.3
KIAA0895	94.1	106.7	88.4	74.9	75.9	77.9	87.8	106.5
KIAA0907	1880.8	2058.9	631.6	655.6	575.1	1169.3	1456.3	2365
KIAA0913	198.4	239.2	255	268.4	298.9	384.3	188.7	310.9
KIAA0922	417.2	552.6	420.5	367.4	325.7	466.6	300.6	410
KIAA0961	132.8	138.1	129.9	91.6	88.9	103.1	107	224.3
KIAA0963	130.9	146.9	158.9	114.3	112.8	150	123.2	223.3
KIAA0980	117.5	140.6	110.1	107.9	103.8	114.8	107.8	149.2
KIAA0999	457.7	648.2	220	247.9	260.4	319.9	450.9	511.4
KIAA1005	110.8	116.4	109.3	97.9	86	101.8	108.5	117.2
KIAA1008	189	243.8	143.4	140.5	143	172.3	151.1	196.4
KIAA1012	1322.6	1787.8	1036.4	1599.3	1273.2	2100.3	1495.5	2110.9
KIAA1024	126.1	151.7	113.3	116.3	113.7	120.3	137.1	163.2
KIAA1026	123.1	121.6	117.9	113.8	99.1	108.6	112.4	120.5
KIAA1026	127.1	172.2	101.2	96.9	95.9	104.6	131.6	140.2
KIAA1026	95.3	109.4	92.9	88.5	89.6	85.7	88.9	105
KIAA1026	137.5	162.7	143.4	108.3	105.2	113	119.3	155.3
KIAA1026	94.3	103.4	92.9	90.9	80.4	82.2	96.7	101.3
KIAA1033	110.7	141.6	88.3	74.9	85	108.8	130.3	156.3
KIAA1128	145	178.4	164.8	150.6	145.3	237.9	158.4	223.9
KIAA1143	78.5	81.6	94.5	90.1	82.7	84.9	84.8	82.1
KIAA1147	291.8	393.3	301.9	525.8	409.8	571.5	293	417
KIAA1160	1249	1652.9	419.4	312.9	297.5	329.4	862.5	1150.8
KIAA1161	128.4	158.5	89.4	92.9	83.1	93.7	126.9	138
KIAA1166	112.3	150.7	115.8	95.6	100.8	103.4	122	135.2
KIAA1171	83.5	90.6	80.6	85.6	72.8	78.9	95.4	103
KIAA1189	101.7	131.4	110.8	99.7	86.3	100.4	95.7	106.2
KIAA1189	80.8	92.4	85.8	78.4	80.8	84.8	81.2	85.8
KIAA1189	88.4	100.2	89.1	88.4	82.4	90.4	99	105.1
KIAA1191	899	1084.3	695.1	840.9	653	828.5	777.5	1266.1
KIAA1191	913	1342.2	459.2	510.7	441.4	635.2	824.1	1198.1
KIAA1193	111.4	138	101.8	90.6	84.2	91.1	117.4	158.7
KIAA1199	82.5	96.3	91.2	89.9	85.9	93.8	85.2	96.7
KIAA1212	226.2	296	145.7	174.7	160.7	267.5	227.9	331.7
KIAA1217	83.5	98.7	82.9	80.5	73.8	89.1	82.7	103.9
KIAA1219	254.2	353.6	260.3	234.8	178.5	324.9	229.3	304
KIAA1244	109.6	117.2	105.2	98.5	89.5	81.8	100.2	119.3
KIAA1267	788	872.4	548.1	703.1	594.4	961.4	728.2	1182.6
KIAA1274	131.8	168.6	100.3	96.4	80.7	96.5	116.7	164.4
KIAA1279	106.4	131.2	99.9	95.6	100.5	92.6	120.2	143.7
KIAA1285	150.4	216.1	108.7	85.6	88.6	105	140.8	184.9
KIAA1287	477.8	478.3	231.8	316.8	236.6	339.4	399.1	526.2
KIAA1324	90.1	107.8	96.9	91.5	85.7	88.4	92.7	102.9

KIAA1324L	267.5	350.7	227.9	239.8	210	346.5	222	301.9
KIAA1328	179.5	187.8	122.9	124.1	108.1	137.7	153.1	171.7
KIAA1333	207	242.6	116.7	114.2	116.5	146.6	179.3	270.8
KIAA1344	152.3	186.5	119.6	114.2	93.9	130.7	121.6	182.2
KIAA1345	71.9	73.4	94.1	90.3	93.8	91.1	76.5	82.4
KIAA1370	139	144.5	112	125.6	127.6	152.3	142.8	230.5
KIAA1377	85.2	106.1	90.4	87	74.2	97.4	87.7	97.5
KIAA1407	102.5	113.6	110.7	104.9	104.4	122	96.6	113.9
KIAA1409	108.4	110.2	94.6	104.6	99.5	103.7	98.4	103.3
KIAA1411	302.9	335.4	195.5	203.2	186	254	269.2	410.7
KIAA1429	352.8	518.5	209.7	236	205.9	286.4	243.2	304.7
KIAA1432	89.4	118.8	91.2	94.4	86.9	97.2	100.4	97.1
KIAA1434	318.5	326	157.6	336.7	250.5	492.8	267.1	471.1
KIAA1443	121.2	136.4	92.3	90.4	88.1	87.5	104.8	134.8
KIAA1446	165.8	188.8	213.1	162.8	141.6	185.4	159.2	202.9
KIAA1456	92.8	101.5	95.7	97.8	85	94.4	93.1	98.1
KIAA1467	150.1	191.5	148.9	107.7	103.7	123.1	167.8	236.3
KIAA1468	339.5	406	185.4	221.4	159.3	325.3	300.6	478
KIAA1505	88.7	100.8	115.6	104.4	99.7	127.1	89.5	96.3
KIAA1522	107.5	140.2	111.1	108.1	108	134.7	139.9	213.2
KIAA1524	77.2	93.1	93.5	83.9	77.9	88.9	88.7	87
KIAA1529	76.7	90.9	75.1	83.7	78.6	87.8	77.9	86.5
KIAA1530	200.7	214.2	176.9	214.8	200	231.3	171.9	241.2
KIAA1530	122.2	152.4	101	97.1	90.8	94.7	143	139.2
KIAA1539	152.6	167.8	153.5	206.6	179.8	233.3	139.6	315.6
KIAA1542	94.6	99.9	91.2	92.9	80.8	83.4	84.8	110
KIAA1543	96.1	104.6	97.2	99.9	88.5	99.9	97.1	122
KIAA1543	83.2	87	96.8	98.2	92.8	85.1	96.9	100.9
KIAA1553	123	134.8	94.1	82.3	90.8	94	108.9	153.1
KIAA1553	147.2	240.8	191.9	176.9	142.7	180.7	137.4	186
KIAA1559	119.9	141.7	83.6	98.9	98.4	89.5	126.6	158.3
KIAA1576	467.6	543.4	169.3	126.7	111.3	139.8	407.4	549.5
KIAA1586	194.2	247.2	115.5	145.2	127.7	187.5	170.8	233.2
KIAA1598	428.4	548	330.5	416	322.3	658.4	369.1	640.6
KIAA1600	220	259.7	212.2	168.1	140.4	211.4	228.9	477.6
KIAA1604	168.2	163.8	98.8	103	88.8	132.5	145.3	298.1
KIAA1609	100.7	129.4	110.4	106.6	101.6	94.7	102.3	118.4
KIAA1618	224	286.6	191.6	186.7	169.2	214.7	213.3	254.6
KIAA1622	83	90	94	83.3	78.5	94.9	80.7	85.3
KIAA1622	84	82.6	90.5	83.2	88.7	88.2	86.9	72.1
KIAA1622	76.4	70.9	85.3	84.9	74.2	78.1	74.7	77.4
KIAA1627	262.3	311.8	110.1	121.4	120.8	135.1	223.3	234.6
KIAA1632	110.6	140.4	97.4	109.3	109.5	114.2	100.6	110.7
KIAA1641	129.6	149.3	122.6	106.6	93.4	116.6	120.6	129.4
KIAA1683	109.2	167	85.8	90.6	82.9	89.9	120	106.8
KIAA1688	107	99	105.5	90.3	81.8	95.6	91.5	110.4
KIAA1698	248.1	340.3	320.6	224.1	183.7	233	234.8	436.1
KIAA1704	87.2	112	83.1	82.7	78.8	81.9	89.1	91.9
KIAA1706	104.2	134.4	130.6	135.3	151.5	143.9	128.8	151.9
KIAA1712	952.7	1113.5	723.4	742.6	705.9	1181.2	715.6	898.2

KIAA1715	340.7	541.7	372.7	590.7	430	615.4	342.8	497.3
KIAA1727	115.6	126.9	98.7	97.4	89.7	111.4	116.1	121.7
KIAA1729	494.2	594.9	437.2	204.5	173.7	242.1	436.8	818.6
KIAA1737	448.4	533.9	304.4	329.7	252.7	387.9	375.9	656.2
KIAA1751	962.4	980.5	436.1	524.4	548.4	443.2	858.4	1542.6
KIAA1754	292.7	338.3	239.4	188.2	181.4	224.4	294.6	435.5
KIAA1754L	91	104.3	93.4	87.5	79.3	87.5	90.5	93.3
KIAA1754L	106.7	122.5	92.4	94.6	81.8	85.3	100.4	123.6
KIAA1754L	86.9	97.6	87.7	88.7	86.8	88.5	88	85.2
KIAA1755	122.5	144.1	93.1	85.4	88.2	85.9	115.9	145.5
KIAA1772	76	89.5	97.2	88.2	92.9	96.7	80.2	87.2
KIAA1787	135	132.2	188.4	184	166.3	247.6	129.8	140.3
KIAA1794	761.8	931.8	215	196.5	161.9	333.2	626.5	847.9
KIAA1797	280.6	365.1	227.4	220.4	181.5	263.4	243.6	379.3
KIAA1799	80.2	98.3	110	93.6	93.8	91.1	88.8	106.3
KIAA1804	177.8	208.4	119.3	123.7	110.3	145.4	147.4	297.9
KIAA1815	255.8	340.4	169.2	189.2	184.8	247.7	227.5	273
KIAA1822	92.3	103.5	99.8	102.4	93.9	89.9	91.1	105.9
KIAA1822L	110.8	142.9	94.7	80.6	79.8	89.8	122.9	129
KIAA1826	824.6	1022	619.5	523.7	392.9	791.3	619.6	938.8
KIAA1838	1380.3	1953.8	1299.9	1921.3	1556.7	2095.9	1104.6	1297.5
KIAA1840	488.8	641.8	386.2	841.5	694	1243.6	461.7	799.3
KIAA1841	67.9	72.8	74.1	74.2	68.4	72.9	72.4	77
KIAA1853	97.4	107.5	90.2	77.8	74.8	91.9	94.7	120.7
KIAA1862	112.1	139.5	140.8	126.9	122.4	135.6	106.8	128
KIAA1875	111.6	151.2	125.7	96.2	116.3	130	99.4	121
KIAA1893	122.3	132.3	109.3	112.6	105.1	129.8	106.1	131.9
KIAA1900	88.2	92.5	92.2	92.4	86.7	93.7	88	102.4
KIAA1904	120	143.8	97.6	89.7	80.5	87.7	108	144.2
KIAA1909	76.5	86.1	77.4	80.4	73.5	74.5	74.2	87.2
KIAA1912	89.8	94.1	103	83.2	76.7	83	86.5	106.5
KIAA1913	81.1	87.1	108.2	118.5	104.9	116.7	84.1	86.6
KIAA1914	91.6	107.7	108.7	114.7	102.6	106.3	94.4	99.3
KIAA1919	91	110	122	108	98.1	98.4	97.2	99.1
KIAA1920	99.9	115.9	108.8	96.9	96.1	101.1	98.8	109.1
KIAA1924	108.2	130.1	84	76.4	68.6	86.7	107.7	126.5
KIAA1944	81.7	88.6	93	82.5	79.5	90.8	78.1	85.5
KIAA1946	124.6	149.7	94	86.4	84.8	95	121.1	147.2
KIAA1958	175.9	249.4	128.1	104.6	110.9	106.1	163.9	161.6
KIAA1961	82.8	93.7	87.5	79.5	75.9	81.8	83.3	98.2
KIAA1961	402.8	667.4	390.4	497.6	483.3	778.4	434.8	745.3
KIAA1967	461.2	648.4	401.7	296.8	247.9	497.2	377.2	607
KIAA1967	146.2	159.7	121.7	143.3	120.2	88.3	104	135.6
KIAA1967	131.7	138.5	105.6	97.7	81.6	88.5	124.8	166.6
KIAA1970	451.5	590.7	432.6	356.9	225.2	505.9	308.4	542.4
KIAA1984	95.6	105.3	106.7	97.3	93.2	97.2	100	103
KIAA2010	100.7	119.9	99.4	89.7	81.6	81	104.4	115.9
KIAA2010	938.1	1271.6	691.3	500.2	411.7	843.8	776.5	1134.6
KIAA2013	614.4	822.4	475.5	443.8	409.9	593.9	582.9	897.2
KIAA2018	81.7	95.3	84.8	84.6	79.4	79.4	92.7	98.8

KIAA2022	83.2	100.3	88.9	102.3	95.5	100.2	92.9	111.3
KIAA2026	269.8	383.8	334.8	441.2	384	716.1	300.1	539.6
KIDINS220	783.4	1132.6	663.8	1101.9	847.9	1922.5	809.4	2011.2
KIF11	758.9	911.2	441.5	319.2	288.9	499	726.8	1377.2
KIF12	86.9	92.9	102.5	100.6	87.9	98.2	90.6	92.9
KIF13A	87.5	95	105.6	93.2	85.8	95.5	93.3	90.1
KIF13B	176.8	236.9	231.5	229.8	201.6	299.9	187.2	344.2
KIF14	843.3	986.9	466.1	426.2	417.3	618.1	797.9	1195.5
KIF15	992.4	1169.3	612	715.6	611	1078.7	667.8	1091.2
KIF17	306.7	410.9	329.3	212.1	174.3	220.1	283.7	484.3
KIF18A	289.7	326.4	153.3	131	116.9	155.4	235.2	333
KIF19	83.7	92	98.8	97.8	89.9	96.1	93.7	106.1
KIF1A	84.9	89.8	85.2	78.9	76.2	81.2	80.1	93.2
KIF1B	113.6	128.8	102.8	92.7	96.8	97.1	111.2	113.7
KIF1B	116.5	160.5	96.9	93.8	99.9	120.2	160.3	148.7
KIF1B	192.5	266.5	169.4	310.2	229.3	351	197.7	227.6
KIF1C	129.6	127.3	97.5	101.1	89.7	129.9	109	161.2
KIF2	740	1030.5	686.7	687.5	520.9	1012.2	624	1115.4
KIF20A	1078	1338.7	977.2	913	884.2	1225	729.3	1016
KIF21A	150.3	175.9	105.9	98.8	87.9	132.9	153.2	204
KIF21B	78.3	72.4	94.6	88.4	80.7	77.7	74.5	88.4
KIF21B	155.6	210.3	129.6	108.1	95.3	98	193.3	271.7
KIF23	140	180.2	102.4	93.8	92.5	99	121.8	145.2
KIF23	683.7	813.4	249.9	242	218.4	295.7	441.1	674.2
KIF25	69.6	82.9	91.3	90.8	83.8	85.9	76.4	78.3
KIF25	134.6	166.7	112.7	96.8	93.2	116.2	132.5	148.8
KIF26B	78.4	83.4	100.6	85	75.2	90.4	80	80.4
KIF27	120.5	131	115.8	100.9	111.2	114.3	115.6	134.5
KIF27	107.3	113.1	93.2	93.5	79.8	89.8	87.5	102
KIF2B	87.6	89	96.1	94	91.1	84.5	84.9	93.5
KIF2C	1094.9	1328.5	762.2	608.5	571.5	722.3	866.6	1055.6
KIF3A	81.1	91.3	89.2	79.2	80.8	86.9	83.4	81.9
KIF3B	293.9	395.4	289.1	318.2	272.5	406.5	251.1	525.5
KIF3C	102.3	116.4	108.5	98.2	96.1	109.6	112.9	138.9
KIF4A	356.7	501.6	308.3	371.4	355.5	626.1	311.9	445.1
KIF5A	87.8	101.9	103.4	84.5	91	100.3	94.6	93.9
KIF5B	657.6	960.2	414.2	352.4	282.7	621.6	549.4	701
KIF5C	100.9	115.8	99.8	108.9	84.1	92.6	85.6	117.4
KIF6	190.7	202.2	115.5	117.3	101.5	119.2	164.8	183.7
KIF7	121.3	130.4	127.3	200.8	110.6	245.4	107	139
KIF9	97.4	111.4	98.8	90.8	83.6	86.7	110.3	106.9
KIFAP3	384.5	460.7	238.9	245.7	189.2	262.6	325.5	375.4
KIFC1	2097.7	2732	2445.8	2532	2419	3338.3	1880.1	2528.6
KIFC2	315.1	261.5	243.9	179	196.1	279.2	251.3	451.3
KIFC3	120.9	135.9	100.4	95	87.3	91.1	113.4	130.9
KIN	93.1	116.7	112.7	106.9	103.3	106	93.6	99
KIR2DL1	98.6	102.4	87.3	97.4	90.4	91.9	95.2	97.8
KIR2DL3	81.6	92	101.9	100.1	92	99.2	82.6	89.2
KIR2DL3	131.3	171.5	118.1	92.9	86.1	104.9	130	153.4
KIR2DL4	89.9	107.9	99.1	89.1	86.4	91.6	84.1	97.1

KIR2DL5A	95.1	106.5	91.2	90.6	88.2	95.8	97.9	104.2
KIR2DL5A	90.4	89.7	101	94.6	86.1	91.2	80.3	95.2
KIR2DL5A	89.1	87.6	96.2	84.9	86.5	84.2	81.2	88.4
KIR2DL5B	85.7	105.5	88.5	79.4	76.3	81.5	83.2	113.8
KIR2DS1	84.2	80.8	91.3	81.3	91.1	87.4	75.4	81.6
KIR2DS3	75.5	81.5	103.2	91.1	82.6	87.2	80.4	80.4
KIR2DS4	127	144	90.6	89.2	79.2	99.8	133.6	143.2
KIR2DS5	94.5	102.1	96.5	92.3	89.4	82.7	92	96.6
KIR3DL1	103.4	107	96.8	89	85.7	97.2	105.6	114.3
KIR3DL2	93.7	98.9	98.3	94.1	86.1	94.3	100.1	98.4
KIR3DL3	98	105.2	97.9	86.3	86.9	88.3	95.4	106.9
KIR3DP1	118.4	134.7	104.5	97.7	88	102.5	121.3	138.1
KIRREL	96	96.6	95.3	88.3	86.1	88.6	100.4	115.5
KIRREL2	113.3	119.8	114.8	88.6	91	88.5	110.4	156
KIRREL2	107.9	131.1	110.8	98.3	86.9	98.2	105	114.7
KIRREL2	86.3	82.4	122.5	103	114	96.4	78.8	93.5
KIRREL3	91.7	92.2	88.4	76.5	83.8	86.1	81.9	97
KISS1	93.4	98.8	100.3	102.4	95.8	102.3	86.3	89
KISS1R	108.5	132.2	99.1	95	101.3	94.9	101.4	102.6
KIT	157	169	128.9	138.6	126.8	163.4	138.9	169.4
KITLG	140	171.6	111.8	139.7	96.6	157.1	192.7	372.2
KITLG	84.6	97.5	95.1	85.5	87.2	92.6	85.3	93.7
KL	115.7	141	118.7	125.8	111.5	128.5	120.1	150.7
KLB	108.5	134.5	104.6	109.1	88.3	100.1	112.1	123.3
KLC2	86.1	96.3	97.3	82.7	81.6	87	93.3	94.3
KLC2	116.4	110.7	115.5	100.6	99.6	103.1	106.7	109.7
KLC3	103.4	114.9	85.2	84.5	80.6	88.4	95.5	109
KLC3	83.7	99.4	90.5	87.8	85.7	85.5	80.7	102.4
KLC4	118.3	155.8	97.7	103.5	92.8	91.3	122.3	130.9
KLC4	92.1	108.5	80.5	80.7	80.4	83	97	103.7
KLC4	101.4	130.9	89.5	84.3	74.8	80.8	111.5	135.8
KLF1	104.8	116	100.7	100.7	89.9	97.8	108.4	127.2
KLF10	142.8	165.3	115.1	140.3	147.8	163.9	132.2	166.4
KLF10	154.7	199.5	109.7	106	100.6	114.1	160	217.4
KLF10	194	236.3	116.4	129	100.4	104.5	171.5	230.7
KLF11	239.4	311.3	141.4	141	128.2	156	255.2	315.8
KLF11	926.8	1244.7	622	786.8	706.9	626	843.7	911.2
KLF12	121.6	135.7	92.4	117.2	102.4	136.1	106.5	136.8
KLF12	77.6	92.2	98.2	96.5	93.4	97.5	79.5	83.3
KLF13	500	679.8	538.4	827.8	658.6	1147.3	486.9	603
KLF14	84	90.4	86.1	93.8	85.9	81.3	90.3	91.4
KLF15	146.6	173.2	140.1	107.2	104.7	119.3	133.4	124.7
KLF16	91.1	96.3	86.5	82.5	83.6	83.6	80.8	91.8
KLF17	95.2	102.9	91.8	91.6	88.5	86	101.7	113.6
KLF2	452.6	557.6	254.9	146.3	162.1	143.1	441.6	662.5
KLF3	96.5	111.9	97.7	88.2	82.6	101.9	101.2	111.9
KLF4	360.2	401.8	306.8	280.5	215.5	372.7	244.8	711.7
KLF5	87.4	94.7	84.9	87	96.7	91.6	93.8	102.6
KLF6	318.1	490.4	137.4	151.4	156.6	183.7	404.9	487.5
KLF6	526.2	799.8	351.2	376.5	359.3	385.7	749	1125.2

KLF7	104.3	126.1	94.5	84.8	80.9	96.3	106	122.4
KLF8	200.2	228.6	146.2	128	117.6	136	158.4	187.4
KLF9	782.1	1006.2	580.1	643.5	532.7	692.5	852	1095.2
KLHDC1	78.6	79.2	99.5	85.9	83	76.1	81.9	82
KLHDC2	668.3	889.8	288.5	264.2	232.3	315.5	583.8	684.4
KLHDC3	5370.7	6447.1	2652.1	1994.9	2093.8	2228.9	4806.9	6216.2
KLHDC4	446.6	636.7	298.5	184.6	167.5	217.9	428.8	607
KLHDC5	822.4	953.1	454.6	528	400.8	671.7	702.1	1215.7
KLHDC6	91.9	94.3	89.2	75.7	79.9	79.1	87.1	82.1
KLHDC7A	105.9	122.7	103.7	101	108.5	98.1	82.5	98.1
KLHDC7B	79.1	88.5	92	89.4	83.4	77.1	86.5	97
KLHDC8A	109.9	132.4	81.8	76.1	72.9	83.4	103.2	122.3
KLHDC8B	133	154.3	161.7	239	269.3	366.6	157.5	179.4
KLHL1	89.5	106.4	105.1	94.9	91.4	96	91.2	94.7
KLHL10	85.8	101.6	96.3	91.4	89.3	92	91.1	95.9
KLHL11	90.2	101.9	111.3	93	95.6	98.3	89.3	94.8
KLHL12	1781.7	2276.7	1139	1086.7	849.3	1499.1	1592.6	2162.1
KLHL13	127.5	142.1	103.5	92.1	94.6	112	110.3	118.2
KLHL14	83.6	88.2	91.4	78.1	84.7	89.2	87.9	84.9
KLHL15	99.6	106.7	101.1	96.6	90.7	104.8	98	104.9
KLHL17	89.4	103.7	103.6	95.4	81.8	88.7	93.9	105.9
KLHL17	137.9	181.3	153.9	132.6	128.3	204.2	107.1	169.4
KLHL18	198	189.7	131	137.2	108.4	133	192.1	271.2
KLHL2	311.6	366.8	177.4	169.1	129.3	182.4	320.6	606.2
KLHL20	410.3	509.8	249.7	322.7	287.8	443.4	357.8	709.1
KLHL21	149.6	155.8	155.3	133.6	101.8	166.2	155.2	243.7
KLHL22	717	952.3	508.7	524.6	473.5	483	563.7	408.6
KLHL23	146.8	228.5	118.9	106.1	100.9	92	132.2	155
KLHL25	133.2	136.1	98.7	93.2	88.7	91.5	120.3	136.9
KLHL26	139	147.3	136.5	133.1	99.7	123.8	127.7	187.2
KLHL3	183.2	225.7	178.8	189.1	174.1	235.9	164.5	173.3
KLHL4	75.9	88.1	86	77.1	74.9	85.6	77.2	93.3
KLHL4	95.3	94.8	108.1	92.2	92.5	90.4	91.6	95.9
KLHL5	93.5	113.6	97.8	91.9	93.5	87.3	106.7	105.1
KLHL5	497.7	666.6	544.1	520.6	523.5	722.4	522.8	937.1
KLHL5	329.8	464.6	227.4	197.2	212.5	270.5	283.1	419.7
KLHL7	189.9	240.7	166.7	175	167.2	253.3	181.5	245.9
KLHL7	111	136.9	109.1	114.1	98.6	114.1	106.4	132.4
KLHL8	873.9	1246	673.3	707.8	581.6	797.8	1052.5	1112.2
KLHL9	94.9	94.6	90.4	86.5	75.8	86.5	97	84.4
KLK1	201.1	253.9	177.1	127.5	113.1	134.6	178.4	228.4
KLK10	87.7	87.1	82.5	80.7	74.5	84.7	83.5	91.5
KLK10	124.3	106.6	111.1	99.3	103.1	89.3	91	108.6
KLK11	107.8	120.7	99.6	98.6	94	98.8	99	113.6
KLK11	96.6	96.7	97.2	97.9	87.6	85.1	105	96.6
KLK12	128.9	167.7	94.5	80.7	89.8	90.1	132	137.5
KLK12	91.4	108.1	85.3	75.3	81.4	76	96	120.9
KLK13	83.9	76.8	88.5	82.6	94.8	74	73.8	84.7
KLK14	89.9	97.5	102	89.1	86.4	97.6	96.9	104.2
KLK15	99.8	96.8	89.2	97.1	86.3	87.7	97.6	104.6

KLK15	81.6	89.9	99	83.8	81.3	88.9	84.9	93.7
KLK15	103.8	120.8	91.4	87.9	91.7	87	98	115.6
KLK2	117.2	129.5	91.2	99.1	91.8	93.4	117.9	122.1
KLK2	104.8	138.3	101	84.6	92	94.3	106.1	138.5
KLK3	78.6	90.9	86.3	71.9	75.3	77.2	85.1	97.5
KLK3	87.7	99.8	80.3	88.6	76.1	77.4	91.9	105.7
KLK3	104.8	127.5	92.8	81.4	86.3	81.8	96.7	135.9
KLK4	86.7	99.3	88.9	83.8	84.5	84.4	83.1	85.8
KLK5	94	101.8	81	83.3	87	83.1	84.4	102.1
KLK5	101.6	128.7	84.5	77.2	84.8	80.9	90.6	134.3
KLK5	89.1	98	100.3	89.6	91.2	80.8	88.7	106.7
KLK6	90.2	90.9	96.9	86.7	81.3	83.3	90.7	79.3
KLK6	129.5	140.7	93.5	86.7	79	87.2	115.5	149.4
KLK7	103.4	109	104.1	96	96.7	89.4	106	97.4
KLK7	117.8	129.2	125.9	113.1	112.6	125.7	113.3	125.6
KLK8	94.4	120.2	98.5	84.4	88.4	96.3	101	98.9
KLK8	96.7	118.5	95.8	92.7	83.8	92.4	105.2	113.1
KLK9	85.6	93.1	86.7	88.3	74	80.7	80.7	85.3
KLKB1	130.1	154.9	86.8	85.4	91.5	92.3	116.6	120.1
KLKBL4	82.7	81.5	82.3	85.5	78.2	83.1	88.8	95.1
KLKP1	89.3	100.3	88.9	91.1	85	80.8	87.5	100.9
KLP1	295.6	412.4	170.3	112.9	127.6	156.8	252.6	312.5
KLRA1	99.4	131.6	91.5	105.5	90.2	103	106.7	125.9
KLRB1	75.4	87.5	93.6	78.9	91.6	89.4	75.3	84.1
KLRC1	78.6	86.6	103.9	85.2	78.9	90.6	76.1	95.1
KLRC2	115.6	154.3	93.5	85.3	77.4	81.6	117	129.6
KLRC3	78.8	87.4	95.6	80.8	92.4	87.9	86.7	88.8
KLRC3	82.4	96.4	103.1	85.5	91.1	95.6	87.6	87.5
KLRC3	76.4	89.6	85.7	87.8	77.1	93	85.8	88
KLRC4	122.1	129.6	114.9	94.1	94	96.4	125.6	123.8
KLRD1	83.8	90.3	85.1	83.2	79	83.9	81.8	88.5
KLRD1	86	86.6	93.2	84.2	89.5	91.1	89.2	95.4
KLRF1	118.9	124.4	105	117.4	102.5	123.8	108.4	114.4
KLRG1	83.8	103.2	94.4	91.6	78.9	85	100.6	88.2
KLRG2	159.9	209.3	139.9	115.9	110.8	122.6	131.5	167.8
KLRK1	130.2	139.7	100.5	99.7	106.5	105.3	119.4	117.3
KMO	127	175.9	99.5	113.6	103.5	110	137.4	163.1
KNDC1	102.7	121	91	93.3	94.3	83.9	107.7	115.8
KNDC1	100	114.7	103.3	92.9	84.1	88.7	90.4	100.8
KNG1	93.8	103.5	98.9	98.7	90.4	96.1	99	106.8
KNS2	235.9	322.1	183.3	162.9	134.1	175.1	224.5	225.3
KNS2	392.7	552	316.3	247.5	219.8	309.1	402.2	550
KNTC1	987.1	1117.6	770.2	1189.3	1125.1	1529.7	779.3	870.6
KNTC2	968	1132.5	423.6	395.6	296.6	550.4	866.7	1409.2
KPNA2	306.6	390.8	163.9	143.6	147.2	140.3	258.6	520.9
KPNA3	663.3	894.1	316.8	393.5	269	534.1	568	1682.1
KPNA4	1568	1867.3	1097.3	1138.2	850.7	2029.2	1438.5	2011.3
KPNA5	157.8	172.9	117.1	122.5	129.4	123.5	133.8	173.1
KPNA6	400.1	538.4	617.9	986.8	822.1	1601.3	368.2	846.6
KPNB1	11805.9	13311.6	6479.3	7015.9	5211.1	9771.3	11353.9	14420.8

KPTN	110.8	124.3	97.7	88.2	84.1	86.9	96.3	115.1
KRAS	322.7	415.5	275.3	237	208.2	293.8	312.4	531.2
KRAS	92.6	107.3	100.2	85.6	94.7	91.1	92	110
KRCC1	549.8	720.6	223.1	204.2	171.3	336.8	503.9	987.1
KREMEN1	120.1	129.8	92.5	85.8	77.6	79.2	120	132.8
KREMEN1	207.9	265.2	140.3	119.1	109.9	126.6	209.4	256.3
KREMEN2	107.5	129	93.2	85.9	79.2	81.8	92.5	133.4
KREMEN2	233.6	282.8	245	156.2	144.1	183.5	161.1	196.3
KRIT1	84.8	104.5	96	103.1	86.5	103.4	89.9	96.4
KRT1	80	93.1	82.3	84.3	83.2	85.6	88.6	84.1
KRT10	1767	2324.4	1339.7	1223.9	1004.6	1408.6	1755.9	2070.3
KRT12	110.5	138.1	113	89.5	85.2	89.6	109.2	134.4
KRT13	89.5	106	91.3	93.5	91	92.1	100.8	98.1
KRT14	77.8	83.1	107.6	88.7	85.2	84.6	76.7	81
KRT15	104	106.6	97.5	94.2	95.6	79	94.9	105.6
KRT16	110	113.3	96.8	102.5	89.4	89.8	116.2	142.9
KRT17	94.4	97.6	93.1	136	142.5	163.8	94.4	101.3
KRT18	74.3	88.4	81.2	76	89.7	77.7	78.4	82.1
KRT18	92.3	90.3	98.4	82.4	91.7	97.8	90	100.7
KRT19	90.9	83.8	97.2	77.9	79.5	88.3	87.5	93.3
KRT1B	73.1	94	90.6	84.2	90.1	87.9	92.3	86.8
KRT20	82.3	95.2	87.4	81.3	81.6	80.4	81.9	85.8
KRT23	205.6	238.2	118.4	121.7	103.4	123.4	179.5	184.3
KRT24	148.7	192.9	111.1	91.4	91.2	99.9	134.1	163.1
KRT25A	100.8	118.7	83.5	75.8	77.8	84.8	98.7	104.4
KRT25C	83.1	98	88.4	83.6	76.8	88.9	82.5	91.5
KRT25D	79.9	89.7	99.7	90.2	82.8	89.7	76.2	80.7
KRT26	108.2	112.5	91.8	87.7	85.7	86.1	92	104.5
KRT2A	79.1	95.1	88.7	78.9	79.2	78.9	91.3	98.7
KRT2B	78.8	85.2	98.2	96.8	98.9	89.5	84.3	85.7
KRT3	92.5	112.5	105.2	92.7	90.7	94.4	92.4	96.5
KRT33A	97.4	101.4	112.1	94.7	99.3	95.6	97.1	103.9
KRT39	80.8	79	96	85.9	75	87.7	80.9	74.3
KRT4	102.9	113.6	89.4	94	78.7	82.7	87.3	111.1
KRT40	79.7	87.9	81.2	79.2	76.1	82	91.7	88.7
KRT5	77.2	81.7	95.3	84.2	88.7	86.6	79.6	88.6
KRT6A	103.5	105.6	100.5	94.9	94.3	98.7	98.3	96.8
KRT6B	75.8	83.2	98.1	90.6	85.5	80.9	81.6	96.7
KRT6E	78.9	83.2	96.2	92.3	81.7	82.1	71.3	75
KRT6IRS	91.2	108.9	88.5	87.3	85.4	82.2	91.5	101.7
KRT6L	79.7	82.3	96.6	94.4	99.6	87.4	74.8	88.1
KRT7	86.4	97.8	93.1	87.3	86.5	87.4	102.9	101.1
KRT8	80.2	104.9	96.9	90.4	80.5	103.3	94	92
KRT86	78.2	84.8	91.1	100.6	90	95.1	83	89.3
KRT9	111.2	122.8	121.7	116.3	102.8	106.7	101.4	124.2
KRTAP10-1	80.2	101.2	87.4	87.1	81.6	91.6	79.9	96.6
KRTAP10-1	99.2	104.4	121.2	100.4	102.2	104	105.1	114.4
KRTAP10-1	128.9	140.4	99.6	97.6	101.9	116.8	122.9	152.1
KRTAP10-1	79.5	89.5	88.5	76.1	76.9	79.5	74.2	92.1
KRTAP10-2	129.7	131.9	104.2	107.7	89.3	96.3	124.3	125.4

KRTAP10-2	68.5	83.5	97.3	81.5	78.6	85.5	78.4	84.7
KRTAP10-3	80.9	81.4	95.5	93.2	83.1	96	87.8	90.1
KRTAP10-4	114.4	124.4	95.6	85.4	88.5	89.4	105.9	124.4
KRTAP10-5	133.6	162	78.4	87.9	88.2	79.9	122	152.7
KRTAP10-7	87.5	89.9	92.4	77.5	78.1	80.9	83.2	84.5
KRTAP10-8	98.5	96.5	87	88	81.2	87.6	90.6	84.5
KRTAP10-9	100.9	117.6	112.8	111.3	104.2	105.2	96.2	102
KRTAP1-1	76.7	85.6	83.8	83.6	80.3	73.1	90.7	87.6
KRTAP11-1	95.8	104.3	90.1	77.4	84.6	80.5	91.7	111.1
KRTAP12-1	82.6	93	108.7	97.2	86.8	93.4	82.6	85.2
KRTAP12-2	93.9	110	94.6	88.2	80	94.6	96	104.3
KRTAP12-3	82.9	94.4	98.6	88.5	83.1	79.2	85.7	93.7
KRTAP12-4	108	109.2	106.4	89.9	92.9	91.3	108.9	107.2
KRTAP1-3	95.2	99.9	102.3	95.7	85.7	92.4	94.3	105.5
KRTAP13-1	75.9	95.8	99.8	94.6	84.1	94.3	77.9	85.9
KRTAP13-2	86.6	94.6	89.4	83.6	86.1	78.2	90.4	100.2
KRTAP13-3	80.7	98.4	89.2	98.9	81	91.1	88.4	88.6
KRTAP13-4	81.2	95.5	100.3	89.4	81.6	91.7	92.9	95
KRTAP15-1	96.8	98.1	96	75.9	86.4	87.2	93	94.4
KRTAP17-1	97	106.2	95.3	83.5	93.7	87.6	99.1	105.1
KRTAP19-1	97.3	106.7	90.9	84	83.6	79.6	94.8	99.6
KRTAP19-2	92.2	106.4	90.4	88.4	85.5	85	97.1	87.5
KRTAP19-3	80.9	99.3	101.5	87	77.5	83.8	89.9	97
KRTAP19-4	94.5	105.2	99.6	87.5	97	87.6	91.9	100.4
KRTAP19-5	93.9	135.4	83.9	81.9	75.6	85.1	107.4	131.2
KRTAP19-6	208.6	234	134.1	120.1	107.6	122.8	186.3	225.5
KRTAP19-7	117	133.2	90.6	101.1	81	93.3	119.7	123.8
KRTAP20-1	78.6	90	91	83.2	76.9	82.2	83.1	82.3
KRTAP20-2	116.7	140.4	95.8	93.5	90.9	96.9	105.5	112.3
KRTAP21-1	95.6	94.4	100.2	104.1	92.1	92.7	83.1	90.7
KRTAP21-2	87.2	87.8	98.1	98.3	96.7	91.2	84.8	84.3
KRTAP22-1	94	110	92.9	86.7	98.8	86.1	96.6	113.8
KRTAP23-1	88.6	106.8	98.8	89.3	91	83.6	92.1	99
KRTAP26-1	83.4	99.9	96.6	94.8	87.9	92.5	79.6	96.1
KRTAP27-1	89.1	96	97.2	87.6	93	87.8	96.5	93.3
KRTAP3-1	78	78.4	88.5	75.9	80.6	79.8	77.5	80
KRTAP3-2	88.4	104.6	86.9	89.6	80.1	80.3	88.5	97.8
KRTAP4-10	88.4	110	94	90.7	101.1	89.7	86.4	86.8
KRTAP4-12	99.3	117.4	97.1	104	82.5	96.6	104.9	112.6
KRTAP4-14	81.4	84.4	93.5	91.3	84.7	92.3	91.9	89.4
KRTAP4-2	78.8	79.4	89	84.6	75.1	87.8	76.8	88
KRTAP4-4	84.6	100.9	91.1	90.6	82.4	81.7	83.2	102.5
KRTAP4-5	78.2	82.1	87.9	88.2	71	80.5	82.4	90.8
KRTAP4-7	110.3	115	90.8	89	84.2	86.3	109.8	119.7
KRTAP5-1	99.4	124.4	82.6	79	85.2	92.4	105.9	106.8
KRTAP5-10	83.7	97.1	87.6	87	81	79	93.5	99.2
KRTAP5-11	121.4	122.2	93.7	89.5	86.4	91	99.6	116.4
KRTAP5-2	78.5	87.1	91.1	82.1	83.2	86.2	89.6	96.5
KRTAP5-3	88.2	88.9	88.5	80.5	70.4	81.6	84.7	107.3
KRTAP5-4	88.4	94.5	85.2	79.7	88.3	83.4	87.4	101.5

KRTAP5-5	77.7	86.8	78.9	78.6	84.6	82.1	82.1	83.3
KRTAP5-6	77.3	98.7	83.9	81.2	72.9	87.8	86.6	85.3
KRTAP5-7	81.4	96.6	96.3	99.3	82.8	92.3	75.7	95.3
KRTAP5-8	78.1	87.1	87	91	75.9	79.2	79.2	86.2
KRTAP5-9	95.8	113.7	91.4	86.8	90.8	88.5	97	130.7
KRTAP6-1	95	117.5	90.6	94	86.1	93.3	101.5	107.1
KRTAP6-2	97.5	103.8	95.9	89.4	89.1	97.2	96.2	105.4
KRTAP6-3	94.6	103.3	90.7	82.4	84.4	81.5	94.9	99.3
KRTAP8-1	91.7	97.5	87.7	83.9	80.1	78.4	86.5	97.6
KRTAP9-3	73.8	79.7	99	82.9	84.4	93.4	77.8	83.4
KRTAP9-4	92.5	113.7	98.7	96.3	94.7	95.7	93.8	105.5
KRTCAP2	9013	9874.3	5885.7	3963.1	3518.9	3440.2	9703.6	7431.4
KRTCAP3	139.7	176.9	104.5	93.5	81.3	91.7	145.1	164.5
KRTHA2	99.6	118.5	83	82	84	92	116.4	106.5
KRTHA3B	83.9	92.1	93.7	90.7	79.1	81.8	90.4	97.1
KRTHA4	93.8	93.1	105.3	92.2	88.6	84.1	87.8	97.8
KRTHA5	80.8	81.6	91.2	95.2	86.4	91.7	65.9	81.6
KRTHA6	108.3	123.9	96.3	95.8	87.4	95.2	112.5	124.1
KRTHA7	136.7	144	91.5	82.8	82.2	77.3	130	144.3
KRTHA8	79.5	102.3	101	92.5	96.9	87.6	81	87.1
KRTHB1	134	152	97.4	94.9	88	86.1	124.7	133.6
KRTHB2	85.7	90.8	89.4	87.2	81.1	85.8	90.3	96.6
KRTHB3	95.7	100.7	124	106.7	112.1	114.6	85.6	91.1
KRTHB4	89.8	98.4	89.8	81.3	86.9	81.5	87.9	101.2
KRTHB5	83.7	95.9	99.6	85	89.3	99.5	93.6	88.4
KSP37	97.3	102.7	96.6	90.3	85	92.9	94.6	110.9
KSR1	104.8	119.8	101.1	95.4	85.4	95.6	108.7	114.9
KSR2	91.6	111.7	100.5	89.3	82.6	79.8	85.1	95.9
KTI12	647.2	844.9	322.4	262.9	220.5	412.3	550.5	874.4
KTN1	1030.8	1470.2	461.7	564.2	338.9	1170.3	912.4	1833.6
KTN1	175.5	218.2	175	207.9	174.2	351.3	162.8	243.9
KUA	822.8	982	444.9	415.5	365.1	431.9	732.1	840.7
KUA-UEV	637.8	864.8	122.1	123.9	91.6	123.4	515.4	667.7
KY	204.9	252.7	124.5	117.9	98.8	119.8	198.5	184.7
KYNU	1222.1	1603.1	566.9	1164	1216.7	1716.6	1035.6	2040.2
KYNU	643.3	982.2	343.8	539.1	552.4	836.3	462.3	640.6
L1CAM	131.5	151.3	106.7	109.2	96.5	101.7	138	159.7
L1CAM	248.1	271.4	141.1	131.6	110.2	119.1	214.5	234.5
L3MBTL	101.6	115.1	98.5	86.7	95.8	96.3	98.5	99.6
L3MBTL	87.7	93.9	89.6	85.7	88.4	85.7	86.3	97.5
L3MBTL	83.5	99.6	112.3	81.7	87.1	91.1	91.8	99.5
L3MBTL	127.8	141.3	102	96	86.2	109	111.6	121.8
L3MBTL2	83	91.5	107	94.1	82.9	87	88.6	86.3
L3MBTL2	843.8	1048.4	1356	1055.4	799.4	1255.7	691.3	1260.1
L3MBTL3	248.1	284.1	188.7	193.9	154.5	234.7	208.9	293.4
L3MBTL3	123.6	149.2	102.5	91.7	95.6	79.4	119	119.1
L3MBTL3	92.3	87.4	94.1	85.9	78.2	77.3	87.4	86.4
L3MBTL4	85.8	92.3	93.1	85.9	87	78.1	87.7	88.3
LACE1	147.2	173.9	129.6	128.7	116.8	141.7	144.9	170.3
LACRT	93.3	104.9	100.7	89.4	92	91.9	91.9	92.4

LACTB	282.2	330.1	130.5	133.2	110.4	142.4	260.3	418.7
LACTB	934.1	1291.8	776.5	1010.8	891.2	1202.3	998.6	1334.5
LACTB	135.9	164.7	116.8	131.3	115.3	142.7	124.9	177.3
LACTB2	1355.6	1810.6	672.7	525.7	361.5	515.1	1190.6	1307.1
LAD1	110.1	125.3	93.1	75.8	85.9	91.8	108.8	106.6
LAD1	80	86.6	99.6	88	89.5	95.1	86.4	86.7
LAG3	109.3	119.9	105	102.8	95.5	94.9	111.2	109
LAIR1	760.7	920.1	422.5	264.9	221.8	214.5	527.9	590.2
LAIR1	27318.6	28776.3	30366.3	31560.6	33932.1	30124.6	32913.4	30387.7
LAIR2	180.3	220.4	126.3	128.7	102.9	120.4	151.2	217.7
LAIR2	226.4	275.4	166.4	175.2	164.3	195.8	178	234.9
LALBA	83.7	99.3	92.1	88.2	88.9	86.9	97.5	99.5
LAMA1	92.7	100.5	95.2	83.4	80.2	85.5	84.3	105
LAMA1	73.5	85.5	86.6	76.7	74.9	70.9	70.4	80.8
LAMA2	116	138.8	113	114.4	103.5	106.9	111.7	111.9
LAMA2	105.7	148.8	101.3	104.2	90.1	93	103	113.6
LAMA3	94.7	107.9	93	94.9	91.3	100.5	97.1	114.4
LAMA3	81.8	79.4	96.4	88.6	84.5	85	76.7	87.3
LAMA3	105.4	128	105.3	95.6	95.8	110.8	108	108.8
LAMA3	104.3	127.9	104.7	101.8	102.5	124	100	121.4
LAMA4	94.3	102.2	85.9	79.4	87.3	86.5	107.5	114.6
LAMA5	477.3	520.4	457.3	356.8	404.1	457.1	367.5	555.3
LAMB1	91.4	105	96.3	97.9	81	96.8	99.7	112.5
LAMB2	121.5	126.8	85.4	88.9	83.1	82.4	113.3	128.3
LAMB3	129.9	148.7	130.9	144.9	121.3	154.7	126.1	142.5
LAMB3	94.8	111.4	94.4	94.8	86.8	88	101.6	110.2
LAMB3	86.9	96.2	82.3	90.3	83.9	87.6	86.6	92.4
LAMB4	96.5	107.4	92.4	97.6	91.9	88.7	96.6	85.2
LAMC1	1083.9	1811.7	904.6	1664.3	1095.2	1744.9	1501.2	1750.4
LAMC2	120.6	141.5	105.8	93.5	83.6	103.8	124.9	123
LAMC2	82.3	91.8	93.5	76.3	80.5	90.7	84.9	96.5
LAMC2	108.2	110.2	98.7	85.8	85.4	95.8	102.5	101.5
LAMC3	102.8	108.3	90	89.2	83.8	91.3	92.9	100.5
LAMP1	4229.2	6487.7	3408.7	4074.9	3177.9	4378.1	3991.8	5885.6
LAMP2	4920.4	5866.2	3726.1	6180.9	4683.4	7099	4720.5	7039.5
LAMP2	1535.1	1917.1	666	943.8	629.7	1051.7	1282.9	2264.2
LAMP2	1366.8	1803.9	583.3	650.7	612.7	832.3	1413.6	1872.1
LAMP3	69.2	86.6	83.1	75.7	83.7	85	76.6	78.9
LANCL1	1396.5	1930.4	750.2	621.8	440.3	842	1313.9	1494.3
LANCL2	334.3	432.3	215.9	291.9	238	316.1	275.5	354.9
LANCL3	82.3	86.6	88.1	85.9	77.3	89.2	83.1	87.9
LAP3	1844.3	2048.6	669.5	645.6	470.1	730.8	1393.5	2538.8
LAPTM4A	266.6	318.5	124.5	125.9	113.4	134.2	281.1	373.7
LAPTM4B	3788.5	5187.9	2677.8	1591.9	1071.1	1748.6	3250.1	5818.4
LAPTM5	1865.6	2928	1434.8	1576.2	1142.3	1511.8	1808.5	3502.6
LARGE	93.4	103	103.9	103.6	88.7	95.9	89.9	94.6
LARGE	81.2	96.1	80.7	85.1	80.6	80.7	86.2	95.6
LARGE	84.3	94.5	89.7	78.4	77.6	79.1	87.6	88
LARP1	103.1	99.2	94.9	85.4	86.5	80.4	96.9	106.7
LARP1	606.8	886.6	649.5	723.5	519.5	1175.7	551.4	1463.1

LARP2	169	200.8	104.9	96.5	92.2	99	152	224.6
LARP2	219	265	181.8	233.7	190.9	331.8	204.1	307.5
LARP2	101.8	120.1	103.2	99	96.1	102.5	101.6	107
LARP2	675.5	768.9	315.3	317.3	277.9	386.7	637.8	993.2
LARP4	132.8	143.6	142.2	118.4	123.9	132.2	117.8	131.5
LARP4	347.6	445.3	197.7	209.8	150.9	245	362.7	606.4
LARP5	894.6	1245.8	858.1	884.2	725.9	1278.5	913.5	1208.3
LARP6	131.6	147.8	138.6	126.5	111.2	130.5	170	173.7
LARP6	176	173.3	146	141.9	126	129.6	171.5	233.2
LARP7	2136.6	2749	1482.7	1452.6	1325.2	3030.7	1673.3	2588.1
LARS	1884.2	2580.6	1113.9	1184.6	903.8	1670.1	1386.8	1742.6
LARS2	260.4	338.1	288.1	298.4	223.4	413.2	260.2	306.1
LAS1L	811.3	1029.4	470.6	508.6	379.4	585.7	663.5	759.9
LASP1	5667.9	7633.1	4438.1	4809.7	3917.8	4441.8	4786.1	4981
LASS1	92.3	105.5	94.8	81.2	88.5	96.5	87.4	94.5
LASS1	170.2	197.2	138	110.6	103.7	112.2	170.4	195.2
LASS1	79.6	97.2	93.5	80.8	90.6	80.4	85.1	92.1
LASS2	819.1	1152.7	464.5	379.2	258.7	423.1	664.5	1075.4
LASS2	1512.7	2007	822.3	588.7	499.1	770.6	1157	1704.6
LASS3	85.1	86.8	95.3	77	84.2	75.4	75.5	88.3
LASS4	106.3	135.7	95.4	94.7	98	106.7	113.6	122.5
LASS5	213.6	301.9	165.3	218.6	204.7	251.9	203.3	246.7
LASS6	2449.2	3477.9	2747.6	4505.3	3646.6	5011.3	2131.3	4578.5
LAT	184.4	204.9	159.8	158.6	138.9	182.1	165.4	220.3
LAT	136.2	132.1	118.5	121.1	103.9	131.4	114.8	132.9
LAT	90.6	106.6	99.3	93.8	77.6	85.5	84.7	85.7
LAT	84.4	104	99.8	98.8	94.7	91	94.1	92
LAT1-3TM	839.7	885.9	338.2	296	257.1	396.1	692.7	1236.4
LAT1-3TM	109.8	120.5	114.4	92.9	96.3	98	109.4	113.6
LAT1-3TM	96	110.9	84.9	91	90.8	80.3	90.9	114.6
LAT2	2171.4	2671.6	1565.8	1089.9	860.9	1439.4	2135.7	2832.6
LAT2	2274.9	3020.6	1197.1	823.7	695.6	1163.3	2200.2	3658.1
LAT2	481.8	658.6	195.8	173.1	168.8	137.7	458.3	586.3
LATS1	93.2	110.1	97.4	82.1	89.6	85.9	95.4	103.5
LATS2	110.7	112.4	120.3	117.5	124.3	122.3	103.3	128.8
LAX1	165.7	232.4	116.5	111.6	120.7	120.6	154.7	146.8
LAYN	100.5	115.5	102	93.8	85.1	95.1	100.6	99.8
LBH	104.5	114.9	93.8	85.5	76.6	73.4	98	102.9
LBH	143.1	194.5	126.3	103.2	95.6	109.2	121.6	168.6
LBP	119.1	136.7	93.5	78.7	81.2	87	112.2	148.5
LBR	639.8	750.7	230.5	141.7	138.4	110.6	413	565.4
LBR	1669.9	2195.4	869.1	663.5	577.6	616	1566.2	2004.5
LBR	109.3	121.8	92.3	86.3	85	79	116.1	136.5
LBX1	80.3	99.8	86.9	75.5	74.9	81.8	80.7	96
LBX2	109.1	126.5	136.8	95.6	98.6	110.2	114.1	126.5
LBXCOR1	110.6	142.1	92.2	94.4	88.3	91.4	116.3	135.2
LCAP	100.2	108.6	108.9	101.4	99.2	93.5	98.7	103
LCAT	125.6	141.9	108.6	92	86.3	96.3	105.6	139.2
LCE1A	81.8	93.2	91.5	88.1	88.7	83	92.3	99.5
LCE1B	91	106.3	100.2	95.8	94.7	92.1	94.1	100.5

LCE1D	85.7	83	104.7	95.2	91.7	94.7	92	88.6
LCE1E	103.5	123.7	96.7	87.3	83.7	93.7	101.7	130.7
LCE1E	80	85	89.6	84.6	78.1	91.3	84.8	87.5
LCE1F	96.5	116.3	92.3	88.9	79.1	94.7	98.5	112.7
LCE2A	81.7	98.7	81.2	86.2	83.1	94	96.3	83.6
LCE2B	81.9	98.3	92.9	82	82.6	88	81.8	95.2
LCE2C	83.7	98.5	104.6	86.7	84.8	92.5	81.1	89.7
LCE2D	76.3	79.8	94.2	86.3	84	91.7	86.2	82.1
LCE3A	93	108.3	84.8	74.8	82.4	89.2	92	108.6
LCE3B	87.6	95.5	98	83.9	84.2	80.9	84.2	98.8
LCE3C	80.9	87.3	93.9	88.2	86.6	84.4	77.6	90.3
LCE3D	89.3	105.7	90.8	79.7	77.3	88.6	91.7	101.2
LCE3E	87	88.4	99	85	91.1	89	77.7	76.6
LCE5A	110.2	139.5	109.8	86.3	89.6	93.8	113.6	130.4
LCK	160.7	173.7	113.1	100.8	101.5	104.9	157.2	150.3
LCK	100.5	90.7	93.2	89.5	87.8	90.9	91.7	106.7
LCK	85.1	96.8	93.9	93.8	90.6	92.8	93	97.1
LCMT1	1140.5	1559	643.6	506	380.4	450.5	1003.3	859.7
LCMT1	642.5	831.2	393.3	280.3	256.9	273.2	478.3	588.9
LCMT1	416.4	549.7	321.6	239.4	186.8	211.8	341.6	401.8
LCMT2	732.7	946.9	682.7	705.7	507.1	956.9	539.1	1046.5
LCN1	119.2	138	89.3	92.2	94.9	100.6	116.8	130.4
LCN10	93.5	113	92.6	85.7	74.2	79.8	109.7	102.4
LCN12	83	90.2	83.8	78.8	83.4	84.4	82.4	97.8
LCN2	108.8	101.8	85.1	79.6	85.9	78.5	103	105.1
LCN6	79.4	80.8	91.2	79.2	78.6	83.2	81.8	88.3
LCN8	81	86.9	91.7	91	77.7	76.5	86.3	95.5
LCN9	90	92.5	88.4	80.6	85.8	87.3	86.4	97.2
LCOR	142.2	171	124	147.8	145.9	180.8	156.1	221
LCORL	70.7	82.5	87.1	75	71.6	74.6	68.3	81.8
LCP1	15942.4	19579	11088.5	17119.6	12481.1	21326	14405.7	16587
LCP2	168.1	201.9	142.2	169.4	136.4	182.7	149.9	142.5
LCT	91.6	86	101.7	94.5	92.3	91.3	92	91.9
LCTL	155.2	188.9	101.6	99.6	83.6	109.1	133.9	177.1
LDB1	111.2	131.6	91.8	92.5	85.7	87.5	112.6	136.2
LDB2	105.8	134.4	95.3	83.7	73.5	85.8	105.7	121.1
LDB3	108.6	122.5	111.7	91	100.3	102.1	96.3	110.6
LDB3	80.6	89.7	74.4	73.9	81.9	76.9	80.4	94.9
LDB3	76.5	78.6	90.3	90.7	81.3	92.1	73.8	79.8
LDHA	15279	17540.2	11517.1	10629.1	7051.6	13786.1	13479	23395.3
LDHAL6A	97.8	107.5	100.3	90.8	98.2	98.1	92.5	124.5
LDHAL6B	73.9	78.3	94.1	82.4	86.8	79.7	83.5	78.3
LDHB	8741.6	10129	2143.4	1425.8	1302.2	2597	7395.4	12131.3
LDHC	115.9	129.4	97.2	94.1	84.3	100.7	103.8	117.9
LDHC	79.7	82.1	88.1	87.5	78.9	85.9	88.5	99
LDHD	103.2	107.1	92.3	92.7	79.2	84.3	92.1	103.2
LDHD	97	142.7	114.8	112	127	138.6	100	116
LDLR	1987.6	2642.7	1068	1535.3	1477.3	1511.9	1804.8	1439.5
LDLRAD1	92.2	98.9	100.7	88.7	88.1	96.4	86.2	96.6
LDLRAD2	76.9	93.6	97.4	91.9	89.3	92.3	81	92

LDLRAD3	132.1	126.7	95.4	114.2	84.9	104.4	102.1	107.9
LDLRAP1	99.3	135.1	125.8	123.8	134	146.7	121.8	135.8
LDOC1	106	114.2	101.2	87.8	96.9	92.8	105.1	141.7
LDOC1L	485.7	564.7	396.7	476.4	276.6	566.8	432.6	1089
LEAP2	205.8	235	144.1	149.3	136.2	148	170.3	222.5
LECT1	94.8	112.9	117.8	117	109	117.6	99.9	94.8
LECT2	74.8	83.8	102.7	80.9	80.1	85.7	79.7	81.8
LEF1	192.7	230.2	160.5	97.9	83.4	108.8	146.1	254.2
LEFTY1	77.1	89.8	87.2	90.6	84.6	89	88.4	98
LEFTY2	88.7	98.9	92.7	85.8	88.7	86.1	85.1	96.8
LELP1	92.8	114.9	91.4	97.1	87.7	81.6	100.1	102.4
LEMD1	82.4	107.1	90.6	85.3	76.8	90.2	95.5	100.5
LEMD2	697.1	835.3	395.8	385.7	366.5	415	599.4	661
LEMD3	1047.6	1492.1	645	831.1	555.7	995.5	1123.1	1821.3
LENEP	74.8	86.9	85	86.5	76.6	75.2	84.1	89.3
LENG1	187	222.9	113.2	100.3	94.5	101.7	170.1	207.5
LENG4	277.2	355.2	297.5	290.5	255	355.6	255.2	627.2
LENG8	132.1	155.3	121.7	99.2	105.2	111.8	121.2	167
LENG9	98.5	120.9	104.7	97.1	98.9	108.9	99.8	114.4
LEO1	360	498.3	120.3	117	97.3	122.4	282.6	429.6
LEP	300.7	330.9	136.3	202.4	150.9	130.1	285	333.4
LEPR	107.2	139.8	84.2	77	86.3	89.6	109.7	122.9
LEPR	167.3	214.7	139.1	142.7	138.1	166.2	175.4	236.9
LEPR	92.7	100.2	102.9	95.3	86.2	97.2	85.8	98.2
LEPRE1	900.1	1143.4	848	1218.9	1072.1	1297.7	829.8	1243.4
LEPREL1	122.5	144	106.6	100.9	92.5	106.9	116.5	113.6
LEPREL2	92.4	101	94.9	88.6	99.3	94.7	92	93.6
LEPROT	1596	2143.7	810.5	1066.8	858.8	873.1	1653.1	1882.1
LEPROTL1	1309.1	2014.1	714.3	459	417.1	650.6	1418.6	1851.4
LETM1	200.3	242.3	168.2	143	121.9	141	162.5	219.5
LETM2	127.2	140.2	100	103.9	86.8	86	118.7	130.2
LETMD1	200.1	215.6	141.6	127.9	139.9	120.5	149.6	181.3
LETMD1	436.2	511.6	387.8	559.4	461.4	603.2	325.4	461.4
LFNG	102.8	120.1	95	81	85.8	84.2	89.9	104
LFNG	98.9	111.9	104.9	106	93.3	108.7	86.8	92.5
LFNG	300.8	382.6	275	245	214.6	227.8	308.1	451.1
LGALS1	25073.1	29319.5	17224.8	15764.9	12627.4	13705.8	25374.4	25663.4
LGALS12	145.5	162	128.2	108.8	109.7	106.6	122.2	147.9
LGALS13	99.7	111.2	96.4	86.2	76	92	99.5	119.2
LGALS14	154.4	165.4	112.4	97.5	93.9	105	116.3	150.9
LGALS14	84.1	104.9	78.4	88.5	82.3	82	86.4	107.5
LGALS2	152.1	173.4	96.6	91.6	87.4	105.2	144.9	192.6
LGALS3	160.4	192.8	117.2	195.6	150.7	229.3	158.5	205.6
LGALS3BP	110.6	136.3	102.9	104.1	99.5	104.1	113	127.1
LGALS4	83.1	97.5	97.9	89	103.8	98.5	84.3	98.2
LGALS7	107.5	116.4	95.3	77.5	89.4	85.2	104.9	134.1
LGALS8	92.5	113.5	97.5	96.4	86.9	86	100.1	99.8
LGALS8	967.3	1192.7	271.5	272.3	256	386.9	917.9	1432.4
LGALS8	121.1	135.3	84.8	85.2	75.9	83.7	110.7	143.4
LGALS8	1431.1	1654.2	525.9	575.1	433.6	596.7	1305	1833.7

LGALS9	554	859.9	609	569.3	391.8	504.4	416.5	984
LGALS9	230.7	310.8	287.7	223.4	191.7	253.1	217.3	365.4
LGI1	93	90.9	87.9	76.6	79.6	76.1	85.3	90.5
LGI2	131.4	150.8	111.6	97.8	92.4	97.7	147.3	153.2
LGI3	88.4	98.5	86.3	82.1	73.8	83.2	96.8	98.4
LGI4	119.2	152.5	89.4	95.4	78	84.9	114.2	146.9
LGICZ1	111.2	120.4	88.6	85.7	85.1	95.2	112	134.4
LGMN	78.7	90.8	97.8	86.6	85.1	92.6	88.8	89.3
LGMN	119.7	135.2	105.8	100.3	101	91.5	120.3	144.6
LGMN	120.8	126.2	114.9	87.3	94	97	113.4	132.7
LGP1	363.6	463.4	417.6	407	331.7	611.1	281.1	575.8
LGP2	120.9	126.4	107.5	131.5	136	151.3	101.8	124.9
LGR4	199.6	250.6	140.9	132.4	136.1	157.3	142.4	147.4
LGR5	88.1	103.4	92.9	84.5	89.8	96.5	92.3	95
LGR6	109.5	111.4	95.1	77.9	78.1	85.6	94.2	108.8
LGR6	128.3	140.3	95.9	86.8	95.1	98.1	105.8	129.4
LGR6	117.3	117.1	85.3	75.5	73.9	79.2	93.9	130.9
LGR6	80.9	84.1	81.2	75.8	65.7	79.6	85.4	79.2
LGR7	220	261.9	134.6	120.2	118.7	149.7	137.1	197.7
LGR8	76	79.4	89.4	78	80.8	83.1	80.9	77
LGTN	607.1	766.8	583.6	692.6	634.7	932.3	505	775.9
LHB	100.5	102.5	91.4	86.2	87.8	83.6	93	94
LHCGR	98.9	97.4	97.7	88.1	77.3	82.8	94.9	100.4
LHFP	141	159.2	107.4	94	91.9	97	129.5	162.8
LHFPL1	94.2	129.7	102.9	96.2	81.1	83.1	106.4	148.5
LHFPL2	145.9	198.2	144	147.5	139.3	163.6	142.6	155.1
LHFPL3	118.3	122.4	109.9	102	89.3	111.2	104.6	128.5
LHFPL5	78.1	80.8	81.1	83.7	72.6	82.1	78.6	86
LHPP	93.1	100.6	90.6	83	84.4	91.3	83.7	98.7
LHX1	103.8	123.8	107	92.9	100.3	96.1	110.2	108.2
LHX2	1347.8	1684	1085.3	441.7	405.2	431.8	1269.6	2012.3
LHX3	78.4	89.3	97.8	90.9	85	94.4	79.2	80.1
LHX3	133.4	137.1	99.3	92.3	81.5	88.7	124.1	145.6
LHX3	88.4	110.7	85.3	92.3	78.6	89.8	99.3	118.6
LHX3	107.1	116.7	81.3	82.1	76	78.3	127.8	105.2
LHX3	84	91.9	95.2	87.2	86.4	88	76.4	84.6
LHX4	122	154.4	110	104.8	92.6	110.6	118.9	148.9
LHX5	100.2	116.4	92.1	95.5	89.3	89.3	112	102.3
LHX6	191.6	256.5	119.6	111.1	110.2	131.3	197.1	255.8
LHX6	101.6	98	95.6	84.4	74.2	91	91.7	85.8
LHX8	85.6	88.3	91.9	86.7	75.4	82.6	92.6	102.6
LHX9	68.6	71.4	84.2	75.1	71.4	81.8	75.6	82.7
LHX9	77.9	94.6	94	86.1	83	80.6	78.4	82.8
LIAS	201.2	264.2	154.4	124.7	110.3	122.1	184.3	225.7
LIAS	486.5	677	285.6	280.6	223.6	321.5	460.6	759.1
LIF	92.8	113.2	93.7	93	86.1	87	82.9	89.2
LIFR	86.8	100.4	98.4	82.2	88.6	94.3	95.5	90.2
LIG1	548.5	712.4	585	541.3	507.8	851.3	434.3	630.4
LIG3	99.2	123.3	86.1	85.5	80.5	89.3	92.9	103.8
LIG3	148.2	220.2	124.4	115.6	105.5	120.2	133.3	151.5

LIG3	353.1	476.6	276.1	270.7	222.7	344.4	246.2	251.7
LIG4	88.5	110	89.7	94.7	82.7	96.7	93.5	132.8
LIG4	103	123.2	98.6	87.5	77.7	82.5	112.4	118.8
LILRA1	106.1	98.1	92.3	82.2	88.4	87.5	92.6	104
LILRA2	1265.7	1680.9	453.9	474.1	422	538.7	1108.2	853.1
LILRA3	177.4	207	159.1	106.5	121.3	130	162.9	185.1
LILRA3	75.1	79.2	95.1	80.3	86.2	84.4	85.3	89
LILRA4	127	151	85.9	87.5	85.5	101	131.6	154.1
LILRA5	81	105.4	81.7	79.5	74.7	82.7	90.8	97.1
LILRA5	112.8	138.2	103.3	168.2	152.6	124.9	106.3	101.5
LILRA5	77.4	87.3	94.2	89.5	93.3	83.3	77.9	82
LILRA6	92.3	104.6	85.3	87.5	86.9	90.2	90.2	98
LILRB1	76.2	88.8	109.5	87.5	90.2	91.4	83	90.3
LILRB1	4814.1	5443.4	2104.3	3466	3641.7	2585.7	4291.2	3876.6
LILRB2	158.7	172.7	158	218.4	194.6	259.7	131.5	169
LILRB3	96.7	116.6	88.6	97.8	93	86.8	95.2	108.3
LILRB3	13669.8	14875.5	9802.1	14269.1	14807.6	10060.7	11263	11715.2
LILRB4	1185.3	1602.3	502.3	281.7	210.8	273	1130.5	1351.8
LILRB5	74.3	80.5	89.4	80.5	76.3	87.2	80	82.1
LILRB5	101.5	122.1	101.2	98	93.9	96.5	106.9	120.3
LIM2	71.4	90.2	90	78.1	77.1	89.2	70.3	85.9
LIMA1	315.7	402.2	249.9	323.8	266.7	672.1	202.5	401.6
LIMD1	76.8	93.8	88	84.1	86.7	93.1	80.4	85.8
LIMD2	87.5	120.1	90.4	86.1	80	90.5	103.6	108.8
LIME1	367.6	348.3	294.9	258	276.3	348.3	269.6	414.9
LIMK1	116.2	120.1	106	104.2	89.7	124.7	110.7	142.6
LIMK1	86.7	92.1	101.1	99	85.7	97.4	92	92.6
LIMK2	201	260.1	157.3	120.6	115.4	120.1	154.2	182
LIMK2	158	205.2	123.1	87.3	89.9	100.6	117.2	162.2
LIMK2	211.2	215.8	128.9	100.9	100.5	128.2	178.3	284.3
LIMK2	111.8	128.8	96.9	102.8	99.8	90.5	100.8	103.9
LIMS1	529.4	753.5	194.2	282.8	224.2	160.7	454.2	515.4
LIMS1	93.7	116.1	86.7	83.4	80.1	89.4	97.2	110.5
LIMS2	116	132.5	98.2	88.1	84.3	90.3	116.8	127.3
LIMS3	87.9	102.7	93	99.7	85.9	92.4	83	106.4
LIN10	155.9	195.6	160.4	143.7	144.4	159.2	148.5	207.9
LIN28	79.7	89.5	79.4	82.2	79.2	79.8	79.8	96.9
LIN28B	113	120.8	95.5	86.5	81.9	84	106.3	117.3
LIN7A	245.4	310.5	202.3	231.9	213.1	326.7	248.4	322.8
LIN7B	222.9	235.2	159	128.7	124.8	153.7	185.5	261.7
LIN7C	132.7	134.1	114.1	133	107	139.6	126.9	188
LIN9	590.8	736.3	357.5	340.7	274.7	596.8	466	1173.1
LINCR	80.2	81.1	101.3	89.8	87.2	92.7	81.8	88.3
LINGO4	84.9	89.5	110.1	108.6	114.4	107.2	82	95.7
LINS1	200.3	257.9	118	119.9	102.2	140.2	174.4	273.8
LINS1	135.8	177.9	101.8	92.3	96.7	95.8	108.8	122.6
LIPA	1641	2131.5	1330.3	1587.2	1031	1535.4	1704.6	2991.5
LIPC	77.2	84.4	88.4	74.5	83.4	80.7	87.3	88.4
LIPE	109.7	116.9	108.4	106	92.4	103.4	106.7	106.8
LIPF	98	103.9	122.6	110.7	108.4	107.8	86.6	91.6

LIPG	90	83.7	98.8	95.4	94.6	93.9	86.9	87.5
LIPH	115	139.7	88.5	92.8	93.8	92.6	107.8	111.1
LIPI	89.8	94.2	88.5	80.7	83.9	80.7	84.8	94.6
LIPK	109.8	124.4	108.2	94.7	91.3	98.7	99.2	120.1
LIPL1	97.3	120.3	108.4	109.5	96.3	106.4	97.3	103.3
LIPT1	449.2	582.9	305	302.2	213.7	434.5	330.2	493.9
LIPT1	210.8	266.4	122.5	124.2	119.4	137.1	190.5	207.1
LITAF	2515.6	2946.3	2832	2244.1	1462.3	2319.3	2831.3	7781
LIX1	105.5	122	99.8	106	92.7	93.9	106.3	124.3
LIX1L	384.8	534.7	220.7	185.3	176.3	205.3	295.1	309.5
LKAP	684.7	994.9	808.9	1526.4	1262.5	1691	783.2	1082.8
LLGL1	184.8	223.2	196.4	250	210.1	291.8	201.7	209.4
LLGL2	101.2	119.8	93.4	94.5	91.8	94	108.7	113.6
LMAN1	246.2	308.5	101.4	102.2	98.4	116.8	180.2	261.5
LMAN1L	143.2	164.3	98.8	94.3	94.1	103.1	137.1	153.1
LMAN2	269.5	292.1	166.5	173.1	147.1	164.4	262.5	309
LMAN2L	605.8	778.4	286.7	289.7	201.7	248	501	712.4
LMBR1	941.4	1353.8	629.9	637.2	458.2	801.4	776.7	1133.8
LMBR1L	124.6	172	109.6	111.6	104.5	109.1	124.5	141.3
LMBRD1	2138.6	2584.7	1012.8	1187.6	1106.8	1623.7	1907.9	2742.5
LMBRD2	129.3	135	99.8	95.4	98.4	107.6	112.8	138.9
LMCD1	105.7	120.2	90.8	77.9	76	89.7	101.4	98
LMLN	135.1	158.3	103.7	99	87.6	95.8	112.9	133.4
LMNA	750.6	1008.5	767.6	805.6	516.4	833	845.7	1402.4
LMNA	3202.6	4053.4	1912.7	1632.4	1126.7	1701.6	3109.2	2452.4
LMNB1	2476.8	4709.8	1392.1	1542.1	1218.1	2194.7	2676.7	2641.3
LMNB2	1956.8	2533.4	2741.3	2461.3	1998.1	2745.8	1761	2608.4
LMO1	96.9	101.6	86.4	78.3	75.8	72.3	87.9	114.5
LMO2	3263.8	4395.3	910.6	762.8	900.3	1001.8	2660.3	4267.9
LMO3	93.7	111.4	88.8	89.6	83.9	93.6	83	93.5
LMO3	86.6	95.5	116.1	97.3	101.1	100.8	86.2	97
LMO3	75.3	81.8	86.6	80.3	81.5	78.5	81.1	83
LMO4	2350.4	2518.8	1419	2068.7	1682.7	2169.9	2555.4	4727.9
LMO6	98.8	126.3	112.5	96	99.7	100	107.2	143.6
LMO7	92.9	113.2	107.7	103.1	94.9	103.4	92.8	111.5
LMOD1	108.8	126.5	90	94.9	85	90.7	110.6	105.9
LMOD3	585.5	518.3	388.8	605.2	559.8	375.4	548.3	924.4
LMTK2	108.6	137	99.5	97.2	84.8	82.6	117.2	133.5
LMTK3	202.4	246.7	138.6	102	109.6	110.4	180.8	271.9
LMX1A	73.8	90.8	89.9	78.9	80.3	77.1	71.1	85.5
LMX1A	85	94.6	89.8	85	74.1	77.7	94.8	98
LMX1A	83.4	91.9	100.5	100.7	86.2	93.3	72.7	84
LMX1A	90.3	105	94.1	85.8	85.3	81.8	86.9	103.6
LMX1B	112.3	113.8	102.7	90.2	88.4	91.2	103.8	123.1
LNK	629.2	905.4	832.8	850.7	735.5	1348.5	698.2	1300
LNPEP	90	110.6	120.7	102.4	104.3	107.2	82.4	98.7
LNPEP	315.7	464.9	204.5	226.5	211.5	246.6	272.2	336.2
LNX1	81.2	92.9	86.7	89.5	87.2	92	84.4	87.3
LNX2	151.4	186.5	132.7	143.8	117.6	163.6	131.8	237.4
LOC11270З	142.5	165.1	131.2	101.7	95.5	121.7	137.7	178.4

LOC112714	153.4	176.1	98.8	109.5	98.8	108.7	138.4	194.9
LOC112869	179.8	220	133.3	124.1	102.4	130.5	167.2	205.1
LOC112869	309	392.3	231.8	194.7	166.9	199.1	312.5	309.3
LOC113179	181.7	252.1	211.4	127.9	135	134.7	180.3	278.5
LOC113386	916.9	1224.8	724.8	433.5	414.6	542.5	768.3	822.1
LOC113444	313.8	461.3	206.9	245.5	212.2	283.1	315.1	395
LOC114984	261	317.9	216.8	192.9	187.5	253.8	257.3	394
LOC115098	251.3	300.7	196.9	159.7	149	182.1	255.1	320.7
LOC116143	676.3	726.2	343.5	345.7	276.5	284.3	494.6	551.9
LOC116236	404.6	509.4	189.4	208.8	176.9	303.2	356.2	559.1
LOC119710	827.2	1020.3	644.5	571.1	463.4	676.1	858.5	1146.3
LOC120379	86.5	93.5	89	81.3	73.3	73.7	75.5	99.5
LOC122258	130.9	139.7	94.6	75.5	72.9	79.8	120.5	111.8
LOC123688	132.1	168.3	150.6	185.4	164.2	160.2	165.4	218.2
LOC123876	80.1	85.1	73	70.3	74.6	85.7	85.9	88.1
LOC124220	85.8	90.1	92.4	83.5	76.9	81.9	91.9	93.2
LOC124446	1160.1	1483.7	1044.4	826.5	703.5	909.4	1006.5	1864.5
LOC124512	319.7	439.1	237.9	249.2	191.1	243.3	243.1	292.2
LOC124512	642.7	826.3	183.1	191.9	166.6	216.3	491.8	690.7
LOC124512	365.1	447.4	233.1	210.6	166.4	203.4	250.6	302.5
LOC124751	95.1	124.3	87.2	91.4	87.6	87.4	104.3	121.3
LOC124842	103.1	120	90.1	79.9	89.1	83.9	95.4	108
LOC125893	621.9	803.1	499.7	394.2	298.6	478.5	444	676.8
LOC126075	100.2	102.7	88.7	84	78.9	88.9	87.6	85.2
LOC126147	72.3	86	103.4	77.7	82.1	86.9	76.6	94.7
LOC126208	1848.9	2072.4	1324.9	1114.9	1023.1	1132.5	1715.6	1759.1
LOC126248	107.6	129.9	83.8	74.4	67.5	79.5	105	131.3
LOC126295	191.6	218.4	118.1	114.7	99.4	117.7	151.7	186.3
LOC126536	115.1	125.2	119.3	110.6	102.2	117.7	108.6	116.2
LOC128153	100.9	118.4	89.8	87.5	85.4	98.5	101.3	113.7
LOC128439	274	336.9	207.2	149.5	154.4	157.7	248.6	210.5
LOC128977	270.5	331.3	244.8	247.8	210.4	276.7	229.3	259.6
LOC129138	543.6	721	424.7	354.1	298.9	499.9	485	653
LOC129285	414.8	549.8	228.6	275.9	201.5	344	340.9	368.6
LOC129530	115.7	122.9	120.1	99.7	101.7	111.9	99.7	122.9
LOC129531	1511.2	1848.7	837	963.5	935.6	1332.2	1348	1757.3
LOC129607	170.7	208.2	132.5	116.3	93.6	137.8	145.2	190.2
LOC130074	229.2	356.1	204.6	273.2	230.7	303.4	210.2	356.1
LOC130355	494.3	607.3	243.8	171.2	163.3	244.8	594.2	1101.8
LOC130576	100.6	125.7	110.8	93.8	96.1	94.1	108.7	118
LOC130940	81.1	85.2	89.9	95.8	91.1	83.3	80.4	83.5
LOC130951	123.8	142.4	104	100.4	89	97.5	126.6	160
LOC131149	102.6	102.3	85.8	88	80.9	83.3	87.9	118.8
LOC132203	78.6	92.3	94	84.8	91	90.6	86	85.6
LOC132241	129.6	130.1	100.2	90.8	102.7	91.1	109.7	133.7
LOC132321	267.9	332.8	123	122.8	98	169.7	243.5	403.9
LOC133308	225.3	267.4	138.1	106.3	98	113.5	170.3	250
LOC133619	826.2	1154.3	379.8	607.9	432.7	633.6	924.5	1451.2
LOC134145	87.8	91.7	94.9	92.4	95	86.6	74	91.3
LOC134147	107.7	115.6	119	163.2	130.5	152.5	105.9	152.1

LOC134997	22056.5	24809.7	9566.6	5620.6	4031.8	7847	19180	23462.1
LOC136242	98	114	92.2	94.2	82.7	79.2	108.5	105.8
LOC136263	130.1	156.1	98.7	84	93.2	95.3	115	130.3
LOC136306	78.1	88.8	83.2	89.4	85.4	94.1	77.5	90.7
LOC138046	74.3	83.1	70.4	68.9	75.7	70.7	66.3	73.4
LOC138255	95.8	105.5	93.4	97.9	89.6	92.4	89.8	100.1
LOC139886	706	775.7	469.3	610.8	398.7	707.2	512.8	804.6
LOC143188	113.1	119.8	82.4	71.4	80.1	78.6	95.1	110.2
LOC143543	371.9	400.5	189.2	167.7	146.6	188.8	270.3	443.9
LOC143941	81	104.1	118.1	111.7	97.8	113.2	89.6	89.1
LOC144097	130	146	94.5	95.3	82.1	89	128.9	182
LOC144233	537.2	686.3	380	356.5	264.1	394.7	501.2	629.4
LOC144363	706.6	855.6	498.9	619.4	432.5	618	619.2	1050.4
LOC144404	94.4	112.8	100.6	80.6	83.2	91.4	81.3	92.6
LOC144404	100.9	127.2	111.7	127.2	107.8	108.2	98.8	104.8
LOC144501	115.2	144.8	77.6	81.9	80.7	84.4	105	125.2
LOC144501	87	107	98.5	75.1	78.4	85.7	98.4	102.6
LOC144983	139.5	201.4	102.3	93.2	84.6	102.9	147.1	150.1
LOC144983	71.7	83.1	76.2	82.6	83.7	76	75.5	85
LOC146167	97.3	127.2	94	92.3	91.4	86.3	111	107.2
LOC146325	97.9	93.7	77.8	79.4	79.8	78.6	88.8	96.4
LOC146562	92.2	104	94.1	89.5	90.3	88.2	92.6	105.7
LOC146713	94.1	113.1	83.7	85.8	95.1	87.4	104	116.4
LOC146909	99.4	114.9	90.8	105.4	98.3	87	90.7	109.8
LOC146909	97.1	109.6	105.5	106.2	95.6	108.5	103	106.9
LOC147808	167.9	198.3	143.2	130.6	132.4	122.3	184.3	183.3
LOC148898	100.4	114.9	111.3	98	97.3	98.9	96.9	91.9
LOC149643	90.7	99.8	104.5	96.3	91.8	104.6	89.5	97
LOC149950	106.1	123.7	97	89.7	82.9	82.8	104.7	115.2
LOC150084	98.4	97.4	113	102.5	96.5	97.9	95.2	102.2
LOC150223	521.7	640.3	300	223	166.9	232.3	341.6	483.3
LOC150297	94	93.5	87.2	86.7	86.1	86.2	92.2	102
LOC150383	92.3	94.9	84.1	94.4	79.7	79.1	91.2	100.8
LOC150383	213.3	290.3	208.6	160.1	160.4	194.2	188	236.9
LOC150383	122.7	143.5	103.3	87.5	84.4	108.6	111.6	121.4
LOC150763	152	172.9	102.6	95.6	91.4	95.9	137.2	187.5
LOC150786	87.1	96.8	85.8	86.8	79	86.2	88.7	86.8
LOC151121	87.1	100.5	96.1	80.6	85.2	74.9	84.3	86.5
LOC152195	111.5	122.7	108.4	145	132.7	154.6	108.3	98.8
LOC152485	124	137.4	116.1	99	98.3	97.6	125.1	119.7
LOC152573	118	142.5	134.4	129.6	107.5	143.6	93	120.2
LOC152667	77	91.4	98.9	80.7	89.2	92.7	78	93.5
LOC153222	170.4	180	166.6	266.4	228.6	257.8	198.8	406.8
LOC153328	166.6	206.8	135.1	113.9	111.6	107.3	167.3	176
LOC153364	166	209	147.7	164	138.2	194.6	147.8	209
LOC154907	80.6	88.4	84.7	84.5	82.8	79.2	76.8	80.2
LOC155100	90.1	100.8	90.6	84.3	82.5	82.3	88.8	83.2
LOC158160	633.4	731.3	210.6	198.7	180.5	228.2	533.9	670.7
LOC158160	95.2	104.2	80	89.1	85.4	99.7	94.1	102.1
LOC158160	195.9	236.5	122.5	111.2	123.4	120.8	181.6	219.8

LOC158381	108.9	128.3	108.5	99.3	107.6	100.4	116.7	124.5
LOC158830	106.1	120.6	99.3	90.8	87.4	85.6	100.4	115.9
LOC161247	108.2	116.2	101.6	94.8	86.2	91.7	94	109.5
LOC161931	96.5	122.7	86.9	85.5	81.9	84.4	109.2	121.8
LOC162073	104.7	116.3	91.3	84.9	81.4	82.7	121.9	110.6
LOC162427	129	130.5	94	83.8	94.1	92.1	122.2	141.7
LOC163131	105.8	123.7	98.8	84	88.1	90.2	102.7	115
LOC164380	75.8	85.3	86.1	88.1	88.2	84.1	77.4	82.4
LOC164714	97.7	115.8	107.5	90.8	96.8	92.9	111.1	109.5
LOC165186	116.1	142.7	94.7	96.8	83.5	92.8	92.8	129.1
LOC168474	144.6	165.4	110.3	118.7	95	112.9	114.6	147.1
LOC168850	644.4	862.3	513.2	626.7	453.2	690.4	582.3	1026
LOC196264	162.4	188.6	104.3	91.3	84.3	88.2	150.8	165.2
LOC196394	112.7	133.9	100	87.8	83	112.6	112.1	136.7
LOC196463	118.5	151.8	154.6	226.5	162	223.2	132.3	252.2
LOC196541	87.3	102.6	85.2	80.6	80.9	87.7	94.5	104.8
LOC196913	88.5	99.1	93.9	105.7	81	86.8	87.3	102.3
LOC197322	314.4	360.8	235.3	216.6	201.1	228.4	266.1	287.6
LOC197336	350.5	339.9	348.2	266.6	220	296.1	228	283.3
LOC198437	116	128.8	111.3	89.2	84.5	88.8	100.9	126.1
LOC200312	97.5	122.9	101.5	103.5	99.6	113.3	94.1	138.3
LOC200420	117.9	132.4	110.3	90.3	91.6	97.2	116.7	137.7
LOC200810	110.8	121.6	103.8	101.2	90.5	94.9	98.5	105.9
LOC201164	232	344.9	231.3	171.5	136.1	204.4	135.8	148
LOC201175	212.9	282.2	150.5	126.8	107.4	119.1	202.7	178.6
LOC201181	110.6	135.1	100.8	92.7	78.4	88.6	98.8	109.3
LOC201229	107.1	116.7	92.2	92.8	88.4	104.5	95	116.3
LOC201229	95.3	108.1	107.4	106.9	96.1	111.5	97.2	95.2
LOC201725	424.3	486.7	188.9	192	126.8	189.3	352.9	687.2
LOC202134	188.2	239	120.6	141	140.2	131.1	177.6	182.9
LOC202459	110.6	151	96	94.8	80.9	102.1	127.5	144.2
LOC203076	99	114.4	102.8	97.9	97	90.3	99.6	112.7
LOC203413	88.4	105.9	91.4	80	90.2	86.5	80.9	92.9
LOC203547	2869.4	3745	1733.8	2175.6	1523.8	2463.4	2361.6	2666.1
LOC205251	495.9	612.4	176.3	152.6	131.2	179.3	318.9	292.3
LOC219854	1976.9	2340.5	1057.3	798.2	637.7	873.2	1496.3	1949.4
LOC220115	79.8	91.2	80.6	79.3	74	82.4	79.7	77.3
LOC220115	81.2	103.9	99.1	92.6	82.9	76.8	77.4	100.9
LOC220429	86	106.8	95.9	83.5	87.5	90.5	96.3	103.9
LOC221091	84.7	109.5	83.8	82.2	82.8	86.1	92.9	84.8
LOC221091	80.9	85.4	96.8	86.4	87.7	86.3	84.4	83
LOC221143	994	1087.5	481.9	475.7	335	510.9	809.1	1089.4
LOC221711	106.5	110.5	105.2	100.2	108.9	106.2	96.2	99.9
LOC221955	857.4	1075.9	517.5	656.6	530.6	595.5	662.1	753.2
LOC222967	94	91.5	94.7	88.2	86.2	94.7	98.9	109.1
LOC222967	116.8	138.8	94.3	89.3	83.3	87.5	122.2	121.9
LOC253012	95.3	119.3	92.7	86.3	83.8	82.5	101.1	114.5
LOC253012	94.2	105.9	119.4	100.7	102.2	104.9	96.9	94.2
LOC253012	73.2	83.5	71.6	88.3	79.7	80.2	76.9	74.9
LOC255374	97	123.4	99.4	82	79.8	90.4	102.6	107.9

LOC257358	94	99	89.6	89.3	87.9	85.1	100.1	101.1
LOC260339	85.3	105.5	99.9	90.4	93.7	93.1	99.2	103
LOC260341	88.9	101.7	91.3	85.9	86.3	96.5	82.8	94.1
LOC283129	88.5	92.2	87.4	84.1	89.2	85.6	84.6	93.2
LOC283129	87.2	99.3	92.8	82.7	79.6	85.8	96.1	96.2
LOC283130	91.9	113.9	98.9	88.5	79	83.8	100.1	122.1
LOC283152	102.9	112.4	95.8	86.3	89.3	87.4	89.4	106.9
LOC283152	84.5	92.4	96.2	86.9	90.4	99.3	75.7	93.7
LOC283174	91.1	99.1	106.4	104.7	100.3	97.1	99.7	91
LOC283219	663.2	724.6	429.1	316.4	283.1	371.3	695.9	703.9
LOC283345	823	1066.3	475.7	359.6	291	411.8	630.9	1190.5
LOC283377	141.8	190.9	121.7	108.9	96.8	110.9	142.7	151.7
LOC283392	96.7	91.6	100.2	93.9	100	93.6	86.9	82.8
LOC283514	109.6	140.1	92	90.4	91.6	89.4	95.5	117.4
LOC283537	301	431.6	309.2	339.6	255.7	433.5	265.5	532.3
LOC283551	83.7	97.9	95	88.9	80	89.5	94.7	87.2
LOC283677	90.5	97.1	94.7	85.1	86.5	79.4	96	111.5
LOC283755	82.8	91.2	87	77.8	83.7	91.4	78.2	84.7
LOC283871	198.8	225.6	211.3	175.6	157.3	193.8	163.2	306.3
LOC283874	193	210.2	132.9	114.9	122.8	138.1	170.6	211
LOC283951	1353.1	1849.2	955.4	733.1	562.8	921.9	1158.6	1803.5
LOC284009	75.8	89.6	93.7	87.3	83.8	83.1	95.6	94.6
LOC284194	173.5	197.6	162	127.9	108.7	124.6	159.5	207.4
LOC284274	94.5	126.6	89.6	91.6	80	79.3	100.1	101.6
LOC284323	106.7	116.4	90	84.5	83.6	86.4	95.1	110.6
LOC284361	84.3	81.3	91	83.9	76.9	91.2	79.1	92.6
LOC284361	136.4	172.2	127.5	99	96.2	110.4	144.2	241.9
LOC284379	81.8	88.8	116.8	102.8	102.9	102.9	79.1	81.2
LOC284402	94.3	114.5	101.1	85.9	81.8	93.7	95.1	100.5
LOC284434	80.1	84.3	86.4	80.2	80.2	80.1	76.8	78
LOC284441	87.1	91.9	101.3	87.5	90.5	86.1	89	91.2
LOC284739	91.5	106	86	83.1	83	86.5	89.9	94.4
LOC284751	89.5	106.3	86.9	86.9	69.2	82.9	87.1	110.7
LOC284757	100.6	119.2	86.5	140.2	155.2	122.7	112.4	108.6
LOC284912	120.2	138	95.8	82.8	84.2	95	126.1	135.2
LOC284948	88.5	87.3	100.2	84	82	93.3	83.6	92.5
LOC285016	120.7	138.7	102.7	102.4	98.3	100.9	119.1	119.6
LOC285033	105.7	112.9	91.5	87.9	97.3	106.4	104.4	127.9
LOC285074	672.9	1035.7	433.5	484.2	413.6	602.3	704.8	983.8
LOC285193	471.7	629.4	431.1	450.2	375	473.8	430.1	558.1
LOC285359	167.2	212.5	103.8	100.9	79.2	89.8	170	221.8
LOC285382	188.7	239.5	128.9	102.5	108.2	111	157.7	179.1
LOC285429	128.2	138.6	106.1	110.9	98.1	128.3	129.8	146.1
LOC285453	156.9	218.3	129.4	102.9	92.6	113.8	155.9	159.4
LOC285498	176.3	217.4	127.1	154.4	141	162.9	147.7	172.3
LOC285513	94.7	96.3	98.5	80.3	85.5	86	93.7	90.8
LOC285527	90.5	109.8	87.8	97.4	95	92.3	90.3	102
LOC285636	686.5	887.8	517.1	603.6	488	1124.8	489.5	961.2
LOC285735	88.2	99.1	92.5	85.2	77.6	81.2	94.1	96.1
LOC285908	134.9	162.7	122.9	159.4	165.4	168.1	129.6	141.2

LOC285989	605.7	698.5	347.3	572.2	539.8	741.7	497.9	599.9
LOC285989	125.3	137	102.7	96.9	101.9	104.5	104.8	107.1
LOC285989	352.7	427	225	437.3	383	448.8	282.3	360.9
LOC286076	97.5	105.3	84.7	88.6	84.6	79.1	89.6	112
LOC286187	92.7	98.4	87.4	79.2	83.2	92.8	83	112.3
LOC286526	94.3	93.5	101.8	86.4	85	86.7	91.7	95.7
LOC338328	91.4	114.8	91.2	76.7	80.1	79.6	100.8	116
LOC338328	86	85.9	98.7	91.8	91.2	90.2	86.7	94.9
LOC338799	104.2	109.4	140.9	105.1	110.1	132.7	84.4	100.1
LOC339123	4930.9	6164.1	3252.2	2439.5	2093	2208.3	4267.5	3396.6
LOC339229	2296.1	3065.4	1901.3	1594.7	1412.3	1720.2	1867.8	1714.1
LOC339229	269.6	312	229.4	169.5	159.5	200.1	194.4	226.2
LOC339240	92.3	108.2	88	84.7	73.2	81.4	101.1	101.8
LOC339344	1448.1	1633.2	1095.5	770.2	642	748	1198.4	1707.2
LOC339457	105.6	114.2	101.8	94.4	92.2	98.3	108.4	108.1
LOC339745	120.8	131.6	119.1	103.5	91.7	118.3	120.2	158.3
LOC339977	96.1	109.2	90.9	79.7	87.6	82.4	93	126.7
LOC340069	95.9	103.7	91.6	87.3	88	90.5	96	88.6
LOC340156	96.3	121.1	95.2	92.2	86.1	95	109.9	111
LOC340260	89	111.6	86.9	80.7	83	85.6	105.3	97.6
LOC340527	160.4	187.2	90.2	90	94.5	83.3	146.2	170.5
LOC340527	142.3	154	114	102.6	93.3	108.3	113.5	137.6
LOC340527	83	97	97.4	93.6	86.2	83	85	94.5
LOC340529	106.4	113.5	95	79.8	85.9	89.6	110.4	120
LOC340578	114.3	166.6	107.1	113.9	105.3	106.4	113	142.7
LOC340602	87.7	94.6	88.5	98	87.7	93.5	89.5	82
LOC340843	96.4	107.1	100.4	92.9	82	97.4	99.7	105.3
LOC342897	114.2	117.5	95.4	95.4	88.3	94	104.5	108.2
LOC343066	105.4	124.5	90.7	84.6	81.2	89.8	88.7	112.1
LOC343521	135.3	156.7	97.2	93.5	96.3	97.6	122.2	133.8
LOC344405	114.4	131.5	106.8	101.3	92	102.5	109.2	115
LOC344405	127.5	145	103.2	98.2	103.1	103.1	117.4	136.3
LOC344657	101.4	106.2	91.2	82.2	81	78	88.7	97.5
LOC345222	122.9	141.7	92.9	85.9	79.7	81	138.3	123.3
LOC345222	102.9	105.5	97.6	96.4	88.4	87.2	95.4	98.9
LOC346157	82.7	101.6	93.1	78.9	79.6	87.1	86	99.5
LOC346653	92.4	99.6	98.8	84.8	82.2	89.2	95	100.5
LOC346673	99.1	118.7	101.4	86.9	93	100.5	88.4	99.5
LOC347273	119.1	138.1	104.6	101.3	100	104.4	112.3	128.6
LOC347487	92.4	103.5	81.5	74.1	73.6	81.5	89.3	110.9
LOC348174	99.6	113	98.6	81.8	86.1	87.2	102.2	124.4
LOC348180	125.1	171.1	117.4	100.4	100.4	106	126.2	136
LOC348180	173.1	241.7	162.1	124.2	127.2	136.6	143.6	217.7
LOC348180	140.9	163.2	92.5	68.7	73.8	83.9	130	142.4
LOC348262	327.6	456	272.9	190.5	161.3	183.7	316.3	573.3
LOC348645	103.2	126.2	104.6	186.3	171.7	116.2	104.4	108.3
LOC348840	193.3	203.7	112.5	95.7	96	90.2	173.2	187.5
LOC349114	190.6	181.4	154	153.7	146.8	213.7	183.5	259.7
LOC349136	98.7	121.8	99.2	96.6	85.2	91.9	109	110.1
LOC349236	79.3	100.5	88.4	90.5	99.5	97.8	92.1	85.7

LOC349338	267.2	293.2	195.5	182.2	199.4	281.8	232.9	334.2
LOC352909	86.6	113	94.5	88.3	80.8	88.9	88.7	106
LOC374395	5276.7	6135.5	3793.7	3018.3	2234.8	2482.1	5224	6937
LOC374491	76.3	86.7	97.2	90.3	78.2	96.6	78.4	79.2
LOC374491	91.7	105	123.5	104.4	106.7	101.6	90.7	98.9
LOC374920	192.6	212	114.2	114.4	106.2	118.8	186.4	192.2
LOC375323	88.7	103.3	113.4	102.4	100.8	101.7	93.8	92.2
LOC375449	113.3	147.4	91.4	89.6	92.6	86.2	112.3	120.6
LOC375748	97.5	114.6	92.9	93.5	91	91.5	91.3	106.9
LOC387601	82.2	97.5	90.1	89.1	82.9	83.4	81.9	91.2
LOC387646	81.6	102.9	99.9	99.1	84	99.2	93.1	92.7
LOC387680	109.6	154.9	104.2	99.7	98.7	102.4	111.3	130.7
LOC387755	101.4	100.6	93.5	90.2	87.3	88.3	102.6	97.7
LOC387758	90.3	102.8	90.5	86.2	85	90	92.7	102.5
LOC387775	92.2	110.4	90.2	83.8	84.2	88	90.3	92.4
LOC387775	103.9	120.5	91.3	77.3	85.5	92.8	107.4	116.1
LOC387778	104.5	116.7	103	98.4	102.4	101.6	109.7	105.4
LOC387856	99.5	107.1	91	89.7	79	78.9	104.6	106.4
LOC387856	88.7	103.2	81.5	77.7	80.4	81.9	91.2	99.6
LOC387856	118.4	116.6	105.5	113.8	93.3	97	106.9	127.9
LOC387911	92.6	110.2	92	82.8	79	81.4	118.7	100.9
LOC387921	136.2	168.1	109.7	99.5	98.2	109.5	117.3	132.1
LOC387921	400.4	459.4	320.2	359.1	311.6	548.6	255.2	437.9
LOC388135	111.8	113.1	102.5	87.2	88.4	100.1	101.9	103.2
LOC388199	80	86	95.5	89.3	77.8	80.3	78.5	88.3
LOC388284	154.7	198.8	127.4	107.9	95.6	110.1	121.1	152.7
LOC388323	79.9	94.7	84	73.8	79.6	81.6	90.3	94.2
LOC388335	80.2	97.2	108.9	97.5	106	101.6	82.2	86.3
LOC388389	138.1	161.7	138.4	113.1	104.3	117.4	160	180.5
LOC388407	96.7	122.1	105.1	99	92.9	93.7	104.6	108.5
LOC388419	88.1	104.5	86.3	85.3	83.2	79.8	88.4	101.9
LOC388503	124.3	146.4	106.5	96.4	103.3	109.7	104.2	121.6
LOC388524	32434.7	32864.7	33410.8	22590.5	19594.3	22152.2	34308.2	32545.1
LOC388595	84.3	86.7	92.2	91.6	84.8	89.8	81.9	89.1
LOC388610	133.3	159.5	132.8	113.3	104.3	127.8	128.9	142.5
LOC388692	137.8	134.4	133.7	165.9	165.8	232.8	118	140.9
LOC388882	107.5	106.6	89.7	93.9	90.6	85.4	90.9	111.5
LOC388886	133.5	162.8	110.5	115.7	103.2	157.8	123.4	186.2
LOC388910	75.8	86.7	92.3	83.6	84	86	77.8	80.4
LOC388915	71.9	69.6	91	78.4	81.2	82	70.5	70.3
LOC388931	88.1	90.8	97.9	93.1	83.4	90.3	86.4	93.2
LOC388955	109.6	113.7	107.8	97.5	94.8	100	101.1	122.4
LOC388965	105	97.1	103.9	88	84.4	88.7	96.3	114.8
LOC389031	168	195.6	111.7	108.3	95.9	105.4	187.1	163.3
LOC389072	85.5	107.7	95.1	102.7	76.3	92.3	91.3	93.3
LOC389118	132	142.2	104.1	87.5	73.6	93.4	112.9	149.6
LOC389151	82.2	92.1	84.1	83.1	88.7	80.8	85.8	91
LOC389257	77	85.1	100.5	107.9	95	89	83	87
LOC389257	79.4	95.3	94.7	88.3	82.1	90.4	91.7	85.5
LOC389286	388.3	570.9	225.2	204	240.1	144.8	292.7	185.2

LOC389293	74.3	92.6	95.4	80.9	79.2	87.9	85.6	83.7
LOC389429	86.4	97.3	88.5	86.6	87	84.5	86.1	97.3
LOC389432	84	98	95.1	90.1	79.9	92.2	87.4	90.8
LOC389458	100.5	135.3	94.4	90.5	78.4	96	94.5	110.6
LOC389517	70.8	75.4	82.3	82.9	69.1	79.9	78.9	86.6
LOC389541	1501.2	1599.1	590.7	677.4	656.6	895.5	1208.7	1302.8
LOC389549	78.9	81.1	102.3	93.4	88	94.1	76.8	77.6
LOC389607	86.7	104.4	95.5	98.3	83.9	91.7	95.7	110.3
LOC389813	78.4	85.8	98.5	94.1	84.7	86.2	73.3	91.1
LOC389816	1904.6	2552.4	1425	1035	899.4	715.1	966	938.8
LOC389816	1610.2	2001.4	1268.6	1010.7	855.1	640.9	763.3	787.4
LOC389827	82.2	89.8	94.4	94.4	88	93.8	77.9	90.5
LOC389827	93.5	104.8	100.2	91.8	92.2	100.8	97	116.5
LOC389832	113.6	128.6	106.5	81.2	85	91	107.3	121.6
LOC389834	87.9	90	95.4	89.3	79.8	95.4	87.8	97
LOC389936	92.5	101	84.7	75.6	77.1	83.6	85	89
LOC390110	109.5	128.2	106	100.4	88.5	97	108.9	119.5
LOC390243	97.2	95.5	90.6	80.8	70.9	76	88.6	100.6
LOC390243	82.7	84.9	86.8	96.4	85.5	86.7	74.3	78.9
LOC390637	129.8	153.8	113.9	99	100.2	101.7	124.6	106
LOC390667	122.8	139.2	94.1	92.6	80.7	101.7	114.4	135.6
LOC390688	112.2	124.1	92.8	89.6	95.4	93	104.5	135.5
LOC390748	83.3	103	89.8	84.2	88	90.6	88.2	96.7
LOC390748	103.9	120.3	108.9	98.5	92.2	104.3	116.1	105.9
LOC390927	107.1	104.5	99	99.8	89.9	100.2	102.9	118
LOC390999	96.6	101.4	95.9	95.2	78.1	90.3	88.8	98.8
LOC391356	531.4	690.7	356.4	378.7	287.9	331.3	567.4	607.8
LOC392196	76	79.1	93.7	88.1	83.9	94.4	81.2	75.4
LOC392395	81.7	97.4	86.7	84.4	76.1	89.2	84.3	92.1
LOC392465	102.9	136.4	96.6	87.2	79	87.3	101.6	137.5
LOC399706	85.4	100.6	85.4	79.8	75.2	80	81	92.1
LOC399744	125	159.5	97.7	112.8	104.2	118.2	143.4	167.5
LOC399818	96.5	112.6	109.4	110.4	103.3	102.2	92.9	102.8
LOC399898	85.1	110.9	108	92.6	94.4	96.4	84.7	91.7
LOC399900	7410.1	7648.5	6271.1	10404	10102.5	7359	8496	12156.9
LOC399947	83.4	85.8	105	91.9	86.1	99.2	77.9	89.3
LOC400120	114.6	133.9	127.3	118.7	110.9	123.6	105.2	108.2
LOC400258	136.2	141	101.8	95.1	87.1	93.6	132.2	128.1
LOC400451	339.4	461.2	241.7	193.7	172.6	178.7	341.4	313.4
LOC400464	186.9	223.1	159.9	234.5	225.2	276.1	161.9	178.9
LOC400464	125.6	140.2	117	212	238.1	214	124.2	133
LOC400499	86.7	88.7	102.1	99.5	99.6	95.4	89	94.9
LOC400506	273.5	384.1	184.1	148.7	137.4	192.9	246.1	250.8
LOC400566	438.8	522.7	289.3	252.7	187.2	239.9	289.9	439.3
LOC400566	898.9	1145.9	504.5	465.9	340.4	431.9	562.9	568.3
LOC400657	637.2	749.1	427.9	364.4	295.6	499.6	455.5	891.1
LOC400707	119.3	123.6	96.8	86.9	79.2	84.8	94.9	108.2
LOC400891	90.6	112.2	97	93	91.1	91.6	87	80.7
LOC400924	125.6	156.3	98.7	112.3	100.8	101.8	139.7	164.3
LOC400965	84.1	86.3	95.7	89.9	83.5	93.5	81.1	90.3

LOC40098€	79	100	86.7	86.1	84.3	90.9	83.8	85.3
LOC40098€	256.6	264.4	107.7	157.5	143.8	158.6	129.3	378.7
LOC40101C	177.7	230.5	123.9	102.6	92.6	104	155.1	199.1
LOC40101S	28822.9	27757.2	14698.9	8843.2	6344.3	9041.8	28683.3	28664.5
LOC40105Z	245.5	283.1	181.7	205.7	184.2	246.3	212.8	248.4
LOC40107Z	96.9	98.8	105.6	102.3	91.2	93.5	83.7	98.7
LOC401137	92.9	120	101.9	89.5	92.6	90.6	111.7	121.1
LOC40115Z	400.6	498.1	268.4	285.6	243.1	278.3	392.5	601.9
LOC40123E	196.3	259.5	126.5	217.3	167	302.3	226.4	375
LOC40125Z	99.3	103.7	93.2	87.4	76.9	88.1	91.9	107.9
LOC40128€	88.9	100.1	98.6	89.6	77.5	96.2	90	97.6
LOC40129€	102.2	110.9	117.3	109.3	112.9	110.4	91.7	99.1
LOC401357	424.8	481	312.4	298.7	313.2	362	409.4	393
LOC401387	71	104.8	94.3	88.7	79	87.1	82.9	100.1
LOC40139E	100.7	131.6	82	90.1	79.9	89.8	94.4	106.7
LOC40141C	91.6	84.7	92.5	84.9	83.6	80.3	80.3	91.6
LOC401431	108.2	137.5	134.8	107.7	105.6	115.7	99.4	109.6
LOC40149E	77.3	96.7	88	80.8	78.5	78.9	79.7	86.2
LOC401551	164.1	198.5	127.3	98.2	93.8	104.5	155.1	177.4
LOC40156E	91.6	112.2	102	96.4	92.3	90.8	101.4	108.2
LOC40158S	136.2	170.1	99.1	102.8	95.9	92.9	135.1	169.7
LOC40189E	101	119.8	102.9	96.8	85.1	82.1	100.5	107.6
LOC40205E	595.1	727.9	198.4	155	143.6	200.5	405.8	628.9
LOC402057	227.9	278.6	131.6	128.3	120.8	132	241.2	812.9
LOC40211C	97.8	113.1	97.9	74.9	91	81.6	101.9	103.6
LOC402117	90.7	101.6	92.2	81.4	79.2	88.9	87.9	92.7
LOC40217€	86.2	100.7	106.9	102.1	99	97.3	94.2	109.3
LOC40257E	106.2	109.3	94.1	89.8	83.9	87.4	106.6	120.5
LOC40268Z	137.5	166.1	94.2	86.8	90.8	92.3	135.6	159.7
LOC40575E	85.2	104.7	95.3	88.5	85.5	89.8	89.5	96.6
LOC40783E	603.9	860.1	625.4	505.2	383.9	591.4	520.6	1021.6
LOC43236S	16838.9	18997.9	13317	11007.2	9198.4	12355.7	13466.6	15664
LOC43998E	80.9	89.3	98.8	93.3	89.7	86.3	80.7	85.2
LOC44005E	75.8	84.3	78.7	80	78	76.7	78.4	98.9
LOC440087	98.4	113.7	97.1	88	87.5	87	94.7	113
LOC44009E	91.7	105.8	97.2	89.1	85.5	85.2	93.6	117.3
LOC44014E	508	613.2	223.3	213.1	170.2	237.6	419.6	813.8
LOC44014E	496	639.5	213.3	226.3	167.2	257	436.9	884.4
LOC440157	254.3	259.3	178.3	199.4	189.9	220.9	183.7	218.9
LOC44024E	100.6	102.1	105.9	100.9	98.2	96.2	97	101.2
LOC44029E	93.1	102.1	92.2	88	76.6	79.3	88.4	93.2
LOC44031E	87.3	109.5	91.8	88.8	88.9	87.5	98.6	100.3
LOC44034E	325.4	379.7	262.7	229	244.8	317.1	275.3	463
LOC44035C	130	110.9	110.2	122.6	106.2	144.1	92.4	115.6
LOC44035Z	145	169.4	112.4	123.7	126.7	108	127.5	157
LOC44039€	140.7	179.8	111.9	100.6	94.9	103.8	129.5	156.2
LOC44050E	90.7	89.5	97.2	100.1	87.9	84.8	84.9	95.2
LOC44050E	78.3	86.7	85.8	81.6	84.6	85.7	83.4	82.4
LOC44055E	76.9	73.6	93	92	90.3	88.5	75.8	88.3
LOC44057C	88.3	92.9	93.9	78.4	86.1	89.5	87	81.9

LOC440686	223.1	226.9	140.4	207.1	159.1	161.7	198.9	545.1
LOC440742	93.4	104.2	89.5	92.4	78.2	78.7	102.9	95.3
LOC440799	95	124.2	86.7	88.3	102.7	93	104.4	106.4
LOC440836	88.5	108.1	96.4	94.5	87.9	91.1	91.7	92.8
LOC440925	108.7	101.1	97.1	96.6	86.2	86	92.5	101.4
LOC440926	842.8	881.5	409.3	420	297.9	357.7	848.5	1816.2
LOC440944	95.3	109.8	96.2	93.1	82.3	83.2	94.8	105.2
LOC441046	101.4	98	97	98	94.2	84.8	97.7	102.7
LOC441070	97.5	134.8	81.7	76	69	76.9	102.9	138.3
LOC441108	92.3	99.9	96.2	82.8	83	92.4	94	93.9
LOC441120	83.9	109.7	99.8	83.5	79	86	95.1	114.9
LOC441150	199	260.5	175.4	147.8	161.3	151.3	192.7	208.6
LOC441161	83.8	90.9	95.4	85.7	83.9	89.1	74.3	89.8
LOC441168	109.8	126.2	117.2	112.5	105.4	117.1	107.8	102.6
LOC441177	81.6	83.6	87.1	84	77.6	86	90.6	78.2
LOC441178	98	96.2	95	96.2	88.2	83.1	92.4	104.9
LOC441208	103.7	122.4	91.7	96.9	85.8	88.7	103.6	110.1
LOC441233	81.3	88	96.8	75.2	84.4	89.7	70.9	86.6
LOC441242	293.3	388.2	238.8	232.1	179.7	287.2	251.2	500.1
LOC441244	205.6	266.4	115.2	98.9	105.1	133.3	167.6	264.1
LOC441251	96.9	111.6	95.1	80.7	80.7	86.4	112.4	116.6
LOC441257	82	80.7	89.9	85.4	78.4	88.1	83.7	81.6
LOC441268	136.7	155.1	114.2	118.3	135.6	119.5	132.4	147.7
LOC441282	89.5	102.7	99.6	87.3	90.1	91.9	92.4	105.8
LOC441294	97.8	127.9	113.7	116.8	107.2	125.4	104.9	126.2
LOC441376	97.5	94.3	88.1	83	92.4	89.4	89.9	98
LOC441426	81.4	88.5	88.9	80	81.8	88.8	82.4	93.4
LOC441476	83.7	95.3	87.9	91.4	82.1	92.9	84.7	94.3
LOC441581	88.6	94	95.6	96.7	80.1	94	81	81.4
LOC441581	91.7	103.2	93.7	81.8	81.3	95.2	94.7	100.7
LOC441601	83.3	88.5	92.4	84.1	78.9	82.3	81	89.8
LOC441734	83.4	85.9	91.3	92.7	95.4	83.4	82.2	93
LOC441743	242.7	261.4	156.3	155.2	130.4	152.3	219.1	255.6
LOC441931	78.5	101.6	101.1	101.4	87.3	96.5	86.1	92.4
LOC441956	84.7	89.2	91.8	84.4	88.3	86.5	84.4	91.7
LOC441956	86.7	106.3	96.5	93.4	96.6	96.2	87.8	84.4
LOC442213	128.9	161.7	116.3	98.5	106.2	115.1	129	138.8
LOC442245	94.6	105.3	113.1	101.5	97.6	101.5	91.6	101.3
LOC442424	80	79.4	87.6	83.5	77.8	86.1	83	81.1
LOC442425	115.5	140.4	92.7	86.9	79	86.4	101.6	132.3
LOC442578	1518	1546.4	1026.7	1394.4	1385.8	1318.1	1228.9	1723.1
LOC442582	168.2	151	95.7	96.3	89.9	96.1	122.4	189.2
LOC442715	93.9	105.7	97.9	88.2	80.2	91.1	90.2	95
LOC492303	81.2	95.8	86.7	85.3	79.2	87	84.1	90.1
LOC492311	214.5	299.2	151.6	221.6	208.2	272.1	210	302.9
LOC493829	103.3	144.6	94.9	93.7	83.2	81.8	104.9	121.1
LOC493856	480.1	586	231.8	193.2	181.2	241.8	447.5	793.7
LOC493860	86.8	86.7	95.9	84.5	79.4	87.9	80.9	99.1
LOC493869	102.2	107.3	100.8	103.9	86.7	91.4	90.1	110.6
LOC497190	132.7	140.6	100.3	96.5	96	105.5	126.2	120.4

LOC497661	153.5	180.1	103.3	113.4	97.5	115.2	147.9	242
LOC51035	3104.1	3848.2	1976.8	1597.2	1396.8	1666.7	3140	3992
LOC51057	102.3	107.3	91.7	94.1	83.6	94.4	98.1	108.7
LOC51057	104.4	131.8	108.8	82.4	83.6	82.3	104.3	144
LOC51136	744.6	941.2	356.4	377.6	352.1	557.5	465.7	757
LOC51149	192.8	239.1	119.9	117.2	105.9	127.7	176.7	194.3
LOC51149	79	101.6	92.1	84	76.3	78	83.8	95.8
LOC51233	85.7	92.3	89.1	85	83.8	85.2	82.8	90.6
LOC51252	107.4	113.4	94.9	92.2	87.8	99.9	118.8	111.6
LOC51255	2666.1	3372	1619.7	1848.3	1552.8	2290.9	2871.6	4210.8
LOC51333	119	131.1	96.5	97.3	97.3	100.1	119.1	132.4
LOC51334	167.2	199.8	137.1	127.3	118.2	131.7	161.2	187.2
LOC54103	153.4	162.5	101.5	97.5	95.3	96.8	139.2	209.2
LOC541469	119.7	125.7	87.4	87.7	88.7	89.9	110.5	146.2
LOC541473	85.6	101.6	81.6	88.9	89.5	92.4	101.9	100.1
LOC550631	91.7	91.6	104.7	99.1	86.1	90	87.2	95.3
LOC552891	74.5	85.1	107.5	90	100.7	86.3	68.9	83.5
LOC554226	84.2	96.7	85.5	76.7	73.6	83.4	93.1	88.8
LOC554235	89.7	103	98.2	94.7	85.4	90.1	87.1	94.9
LOC554251	99.4	101.8	87.3	86.7	78.5	82.5	86.1	94.4
LOC55565	234.4	273.8	197.5	161.1	166.5	186.5	205.5	175.5
LOC55908	146	184.4	109.9	97.6	101.8	108.2	145.1	148.7
LOC56901	122.2	151.1	123.1	100.4	90.8	101.9	129.2	180.2
LOC56902	1069.5	1202.6	397.6	345.2	209.4	444.8	802.4	1176.4
LOC56964	81.9	91.4	88.2	86.8	80.3	88.2	85.1	88.9
LOC57149	980.1	1251.2	610.2	672.1	482.5	812.7	905.6	1111.6
LOC57228	88.9	93.7	95.8	87.6	93.7	99.7	85.2	101
LOC57228	121.4	137.7	111	124.8	114.1	144.5	117.6	136.5
LOC57228	95.8	97.9	80.2	83.1	90	86.2	96.4	101.5
LOC57228	93.2	89.9	104.1	98.2	96.6	95.2	86.9	89.8
LOC594834	72.1	82	84.8	76.5	69.6	79.2	72	79.4
LOC594834	101.3	114.3	83.7	90.6	74.7	80.1	102.9	116.2
LOC594834	87.6	93.7	85.8	70	79.2	75.6	90.4	95.5
LOC595101	131.5	153.5	105.9	102.2	96.9	105.4	104.9	141.2
LOC606495	159.7	194.1	109.9	101.7	109.4	129.2	140.1	166.9
LOC606724	1514.2	1951	1033.4	630.2	520.2	823.7	1228.4	1786.1
LOC613037	728.5	917.6	464.7	685.8	706.1	1195.1	741.2	1009.9
LOC613212	81.1	89.3	93.1	98	89.4	86.3	80.4	75.7
LOC619207	91.9	99.9	80.8	85.9	85.1	82.3	95.7	94.9
LOC619208	102.9	105.8	86.4	84.4	86.3	85	98.5	103.4
LOC63920	232.7	247.5	204.4	196.4	156.9	216.3	179.2	228.9
LOC63928	110.1	122	94.4	78.1	78.8	85.6	100.6	111.7
LOC641522	109.5	132.7	112.8	104.2	94.8	102.4	103	120.7
LOC641847	83	89	91.7	87.3	84.3	94.7	87.2	86.2
LOC641949	134.2	147.2	113.4	113.3	101.6	121.8	107.1	126.2
LOC642090	411.6	566.7	352.7	546	438.1	728.4	429.8	777.2
LOC642181	97.6	99	86.8	79.7	82.8	82.1	80.3	93.6
LOC642201	126.9	133.1	85.8	85.9	80.1	86.2	112.3	118
LOC642265	73.4	80.2	88.9	79.7	82	81.1	74.6	86.9
LOC642293	72.4	76.6	81	76.6	86.7	82.8	68.7	78.1

LOC642316	92.1	92.6	92.6	98.3	91.4	88.8	102.3	88.2
LOC642323	373.8	476.3	197.3	240.7	179.8	210.2	216.4	309.7
LOC642782	100.5	102.3	94.2	88.6	87.6	83.5	87.7	93.6
LOC642944	89.7	96.8	90.2	87.8	93.2	100.5	96.4	82.7
LOC642947	8623.3	9226	3949.4	7518.6	7689.1	5385.6	7399.5	9082.8
LOC642968	131	145.8	93.6	86.4	88.4	90.5	132.9	153.6
LOC642980	97	122.8	92	97.5	83.9	102.9	92.4	114.7
LOC642980	95.8	112.6	95.6	93.3	82.2	97.6	100.4	117.1
LOC643057	28616.1	26888.2	36978.8	37493.4	30824.1	35396	28119.6	28790.7
LOC643165	85.6	109.6	90.8	96	81.9	90.4	101	99.5
LOC643196	91	105.8	99.3	90.1	82.6	80.3	93.8	102.1
LOC643226	79.4	85.3	103.5	103.8	93.2	108.1	77.4	76.3
LOC643226	73.9	81.2	87	77	78.6	73	81	77.2
LOC643253	90.7	86.8	94.7	95.6	85.9	91.9	87.5	95.3
LOC643298	114.6	128.5	107.6	95.7	98.9	107.8	108.8	137.8
LOC643366	103.6	114	95.9	95.6	88.5	102.9	99.2	114
LOC643394	77.5	89.4	84.9	86.5	93.3	88.1	78	96.1
LOC643414	87.7	91.3	88.4	86.5	81.3	79.3	83.1	85.7
LOC643425	78.9	93.5	87	101.1	85.7	88.3	88.2	82.7
LOC643516	8167.1	10078.8	3182.6	2481.6	1884.4	2537.1	7227	8481.1
LOC643542	83.8	84.1	92.8	83.3	75.8	76.1	88.2	101.1
LOC643591	303	348.1	137.8	180.6	167.6	145.1	194	290.7
LOC643668	7361.4	9690.1	3496	3218.5	2571.7	3696.9	6829	8895.3
LOC643736	94.7	106.7	104.4	98.6	88.4	92.4	105.2	108.1
LOC643812	90.6	95.3	107.5	100.4	99.8	99.7	89.4	97.6
LOC643842	107.2	133.1	103.5	93.9	91.7	94.3	104.3	118.6
LOC643853	126.6	192.9	100	96.7	93.7	95.2	128.6	141
LOC643853	124.6	163.5	149.2	149.5	126.2	162.8	122.1	184.3
LOC643866	96	105.9	91	82.3	82.3	88.2	99.3	103.7
LOC643905	128.9	130.1	99.7	80.8	81.2	105.3	103.1	107.7
LOC643923	105.5	108.3	102.6	97.5	91.2	90.9	106.7	111
LOC643923	95.3	94.3	81.1	91.2	87.5	84.8	93.9	96.7
LOC643940	2594.7	3105.4	1315.6	1180.8	722.5	1241.2	2336.7	3437
LOC643995	2559.9	3588.1	658.6	355.5	244.9	528.2	2104.4	2558.5
LOC644076	76	84.4	83.7	75.2	82.2	87.5	78.7	82.4
LOC644096	641.8	825.9	483.3	339.3	282.1	396.6	614.2	961.4
LOC644168	114.4	133.4	111.2	92.3	95.9	97.3	120.2	113.2
LOC644376	91.2	94.2	97.6	95.4	90	99.2	91.1	95.6
LOC644398	130.5	152.4	100.8	94.5	80.4	93.9	136.4	158.6
LOC644407	98.6	112.1	103.9	96.9	88.6	92.5	98.3	106
LOC644529	93.2	103.7	106.9	99.7	87.6	102.1	90.5	91.7
LOC644613	90.6	105.3	92.6	87.2	76.7	84.8	97.7	107.8
LOC644615	562.3	832.6	354.9	252.8	234.6	319.1	521.5	717.3
LOC644685	128.9	166.1	95.4	85.5	84	85.1	107.6	174.2
LOC644784	101	108.8	91.4	86	82.4	86.1	99.5	101.3
LOC644792	98.7	104.3	92.6	85.8	87.9	86.4	103.5	112.5
LOC644890	113.5	123.8	105.9	95.2	99.7	95.2	112.5	131
LOC644943	87.4	95.4	92.5	98.7	84.1	91.5	96.5	91.2
LOC644997	102.3	121.3	96.1	93.9	92.5	86.6	105.8	104.7
LOC644997	165.6	203.3	113.2	102.3	96.1	97.9	152.8	160.1

LOC645039	90.1	112.8	101.9	87.9	89.9	94.1	98.5	97.7
LOC645437	95.9	96	95.9	85.9	78.8	88.9	94.9	112.7
LOC645562	129.3	149.7	106.9	97.4	77.5	85.3	98.8	120.1
LOC645849	83.3	89.1	90.1	81.4	85	87.7	77.1	90.1
LOC645922	70.9	82.2	96.6	90.1	75.1	81.1	80.4	82
LOC645974	86.9	89.8	98.1	87.8	84.7	96.4	81.2	90.2
LOC645974	110.3	125.4	96.3	96.3	82	82.8	97.9	130.2
LOC646182	94.6	94.1	94.7	87.6	86.4	99.6	86	96.6
LOC646200	21347.9	25021.7	11621.4	13387.3	10084.8	13926	20719	24838.8
LOC646480	75.6	86.2	90.7	91.8	79	85.2	75.4	83
LOC646600	77.7	81.2	87.6	100.2	90.6	88.2	73.4	82.6
LOC646962	77.9	90.4	88.1	77.4	78.4	83.7	80.1	101.3
LOC647043	101	142.7	94.3	83.8	89.5	81.8	115.7	127.8
LOC647141	88	105.1	90	86.3	85.8	79.2	85.7	95.6
LOC647649	80.1	86.2	100.2	87.1	88	86.3	82	81.6
LOC647649	76.2	80.8	100.2	89.3	94.4	93.9	75.8	81
LOC647760	154.4	173.7	115.4	108.6	94.9	115	143.3	156.8
LOC647881	89.3	92	78.4	77.2	66.5	80.1	85.5	95.4
LOC648148	137.9	148.3	89.9	80.9	80.5	94.4	116.9	150.2
LOC648293	837.1	1171	752.4	884.9	687.9	1230.1	788.8	1301.8
LOC648303	93.4	102.9	84	89.4	82.8	90.3	93.5	100.7
LOC648367	82.4	107.8	98.5	83.8	86.4	85.5	102.4	99.5
LOC648832	110.1	126.4	111.6	104.2	87.5	96.8	106.9	122.6
LOC649019	107.2	111.7	93.8	103	89.9	101.3	108.2	108.7
LOC649159	76.2	86.6	91.7	79.7	82.8	83.4	82.1	80.8
LOC649159	150.5	179.4	111.5	95.2	76.2	90	143.4	184.2
LOC649230	99	97.1	89.3	82.3	80.6	83.2	95.9	97.6
LOC649377	122.9	151.8	91.4	80.7	78.9	89.6	119.6	143.6
LOC649387	122.6	147.3	83.4	88.2	77.4	86.3	113.3	139.3
LOC649387	78.6	83	94.4	80.6	81.4	81.4	80	83.4
LOC649431	78.3	84	97.2	95.6	92.9	101.8	80.8	85.1
LOC649446	88	88.7	94.9	89.3	68.7	80.7	85.3	93.3
LOC649516	98.3	120.8	94.1	102	104.1	97.9	96.8	111.9
LOC649555	101.8	118.2	95.6	79.8	77.1	91.8	96.6	118.8
LOC649588	85	96.8	83.8	67.7	69.2	84.3	99	101.6
LOC649708	303.6	366.5	147.8	125.4	122.5	124.6	267.2	370.9
LOC649853	2374.8	2978.8	1544.2	1004.3	864.1	1000.2	1949.3	2758.3
LOC649946	2434.1	3181.1	1206	984.6	808.9	1072.9	2278.3	5106.1
LOC650000	78.2	88.4	95	82.9	81.5	92.1	77.4	88.6
LOC650040	142	169.4	100.9	98.8	99.2	101.1	140	164.1
LOC650137	90.2	99.2	92.7	90.7	81.3	90.5	92.2	97
LOC650137	71.3	85.8	88.4	84.7	76.9	89.4	76.1	77.7
LOC650239	101.5	118	100.2	96.1	94	106.1	95.5	99.5
LOC650254	95	95	94.3	81.4	78	84.2	86.9	102.4
LOC650293	81.2	89.1	96.7	101.2	84.1	90	75.2	77
LOC650586	127	154.5	100.8	93	90.2	97.6	119.9	131.3
LOC650645	102.9	113.1	97.6	80.2	78.1	84.8	85.7	117.4
LOC650695	80.8	82.5	93.7	93.1	89.9	87.9	74.3	77.5
LOC650798	138.1	153.2	89.1	97.2	77.5	79.8	140.5	155.9
LOC651076	249.4	287.2	161.4	120.9	104.6	155.4	209.6	261

LOC651137	85.3	97.3	82.6	83	81.1	85.2	78.2	88.6
LOC651321	107.3	117.8	114.9	110.1	107.9	107.1	95.9	107.6
LOC651423	325.9	446.4	192.8	194.5	161.9	171.7	255.5	280.5
LOC651503	93.2	104	112	100.9	103.3	103.4	89.1	99.6
LOC652020	85.6	86.9	97	85.5	89.1	92.7	89.8	86.7
LOC652236	97.2	108.6	112.4	90.6	89.1	91.9	100.3	117.9
LOC652461	104.5	114.9	108.6	88.1	81.9	90.6	104.5	122.9
LOC652480	77.4	71.5	96.6	90.5	84	87.4	76.4	86.3
LOC652705	82.7	110.7	80.6	85	68.5	79.3	92.5	101.5
LOC652744	91.2	103.1	98.6	77.3	77.6	76.2	91.4	96.9
LOC652870	91.9	97.9	99.6	96.2	93.6	90.9	97.6	88.3
LOC652888	89.6	98.6	91.5	88.5	87.5	86.8	88.3	85.7
LOC652924	329.4	422.2	192.6	260.9	198.4	235.9	280.8	366.4
LOC652968	165.6	180.8	133.1	114.6	104.8	117.3	153.9	199.3
LOC653033	116.5	124.3	103	89.7	94.6	94.8	104.8	114.7
LOC653056	93.1	95.5	96.7	86.8	88	96.5	90.1	99.7
LOC653062	89.2	95.9	89.4	88.2	74.2	90.4	88.6	97.6
LOC653084	138.3	154.6	113.3	90	86.4	90.3	117.1	123.3
LOC653098	142	165.1	127.9	109.7	98.3	112	141.7	160.6
LOC653101	119.8	149.1	96.7	91.4	87.6	96.4	120.8	180
LOC653122	86.4	78.6	94.9	84.5	90.1	98.2	79.1	85.9
LOC653131	100.1	112.5	88.3	85.7	86.3	96.8	103.9	122.8
LOC653133	199.9	247.6	141.9	221.8	197.4	143.5	170.2	219
LOC653142	71.9	86.9	80.3	75.4	77.9	87.7	78	77.3
LOC653150	144.2	145.3	99.6	98.1	89	90.2	112.5	169.3
LOC653152	129.7	183.4	107.8	94.7	88	91.2	130.9	149.8
LOC653170	105.7	117.4	97.4	84.2	88.2	93.3	98.6	124.1
LOC653179	110.8	127	106	121.9	97	101.3	119.2	117.5
LOC653216	88.8	95.7	95.4	96.7	100.5	95	87.1	94.4
LOC653216	102	105.6	121.9	121.7	123.5	125.7	92.6	90.5
LOC653216	72.2	85.6	100.5	97.3	93.9	88.2	77.4	84.5
LOC653281	97	116.1	99.1	85.1	85.8	90.8	100.9	106.8
LOC653282	166.9	195.3	115.8	111.8	107.9	121.4	150.6	182.9
LOC653285	101	118.5	100.1	102.4	89.7	99.5	99.2	100.3
LOC653314	12223.5	12792.3	3933.5	3062.2	2837.3	3051	11714	12224.5
LOC653316	79.7	91.3	91.5	87.5	78.5	80.8	76.5	92.2
LOC653319	108.8	125.3	115.4	97.6	90.3	94.3	96.3	121.2
LOC653328	9670.5	10994.4	4627.5	3491.7	2699.9	3648.9	10710.3	13358.1
LOC653375	256.9	382.2	236.1	189.3	185.5	248.4	241.7	354.6
LOC653386	113.1	122.2	102.1	88.8	88.1	99	108.7	149.5
LOC653417	137.3	148.7	114	96.9	87.5	95.7	119.2	136.2
LOC653419	95	109.6	95.6	92.5	90	80.9	99.8	128
LOC653423	120.9	130.3	96.9	82.3	90	101.4	116.6	134.6
LOC653466	98.7	102.8	86.9	85.9	84.1	95.5	98.5	101.8
LOC653484	95.8	97.5	91.7	91.1	88	102.2	106.9	104.4
LOC653490	101.3	111.8	107.3	108.6	93	107.2	98.3	92.3
LOC653499	92.5	93	101.4	93.5	89	83.4	100.3	103.1
LOC653502	97.3	117.3	83.9	81.7	83.8	85.6	94.4	111.5
LOC653517	87.7	81.9	94.1	88.6	81.6	87.6	89.9	84.2
LOC653542	724.1	438.2	215.7	227.7	178.3	270.8	239.8	530.4

LOC65354(	92.9	97.2	83.9	92.7	83.2	84.9	94	88.7
LOC65355‹	140.8	125.3	101.9	101.8	96.6	102.2	108.1	135.4
LOC65356(	2081.7	2710.2	577.5	540.8	413	559.2	2196.3	2983.7
LOC65356‹	84.6	95.2	81.3	82.8	76.2	79.2	91.9	98.2
LOC65360(	83.5	92.1	88.6	94	85	88.5	82.4	76.3
LOC65360ž	103.2	122.7	104.6	96.6	92.7	91.7	110.4	106.5
LOC65361ŝ	70.6	81.5	80.8	86.7	77.5	75.5	82.5	78.4
LOC65361⁄	87.2	101.5	95	101.1	88.8	85.6	89.1	99
LOC65362(	101.6	121.6	85.4	86.8	79.7	90	94.3	104.2
LOC65362ŝ	91.5	101.6	106.6	97.9	91.8	92.6	81.9	91.2
LOC65362(	166	201.3	148.1	183.1	142.3	273.6	219.6	410
LOC65365‡	96.3	137.4	90.2	86.9	79.4	79.8	104.9	127.4
LOC65368‡	71.5	91.3	88	87.3	83.7	78.7	79.2	89
LOC65368(	76.5	82.4	89.5	85.5	90.2	84	73.5	80.1
LOC65369ŝ	105.3	134.9	109.8	95.2	97	108.4	115.6	131.8
LOC65369⁷	83.3	93.1	88.9	89.2	90	87.9	85.4	91.6
LOC65370ŝ	95.6	109.6	101.4	94.3	89.4	98.7	99.1	111.6
LOC65370‡	183.5	209	144.8	114.8	105.7	125.1	177.4	217.2
LOC65370ƍ	258.8	284.7	127.2	114.6	114.8	134.9	237.7	446.3
LOC65371(	84.5	97	73.1	76.9	76	74	92.8	96.3
LOC65372ŝ	106.8	119.5	98.7	98.6	83.7	82.1	92.4	117.5
LOC65374ž	97.4	110.7	110.1	103.5	95.7	99.7	102.8	105.6
LOC65374⁄	158	207.8	103	93.8	80.6	78.1	144.7	162
LOC65374‡	103	127.5	98.7	104	105.3	98.8	95.3	106.7
LOC65374(	92.5	106.4	88.2	81.6	79.8	88.8	79.8	97.4
LOC65374ƍ	80	92.6	85.9	75.2	79.9	76.8	82.8	90.8
LOC65375⁷	87.6	109	102.5	85.7	90.7	97.9	93.8	102.8
LOC65375ƍ	82.8	83.6	82	78.4	77.1	83.4	84	89.8
LOC65376ž	103	97.3	102.9	97.1	82.4	98.2	98.3	105.9
LOC65376⁷	86.4	95.7	104.5	94.2	85.3	97.9	93.5	91.9
LOC653771	80.5	84.1	86.1	85.9	85.2	82.3	79.2	91.7
LOC65379ž	79.1	86	94.1	79.7	93.2	85.8	79.3	82.6
LOC65379‡	86.2	89.7	86.6	89.1	75.9	79.5	84.7	95.5
LOC65379⁷	92.6	111.3	92.5	87.4	86.8	88.8	94.7	95.1
LOC65380ŝ	185.5	210.6	126.8	137	117	114.8	125.9	140
LOC65381ŝ	79.9	90.6	79.5	80.9	86	81.5	79.1	90.3
LOC65381⁄	70.5	85.2	85.1	85.1	72.1	81.3	68.4	76
LOC65382⁷	72.7	89.5	85.9	76.9	88.9	82.1	70.3	83.2
LOC65386(	72.1	95.3	96.3	91	93.6	97.9	86.7	93.8
LOC65390(	109	112.4	96.6	76.6	80.6	84.9	109.3	118.4
LOC65395(	129	151.5	111.3	200.4	191.8	194.1	120.5	134.4
LOC65398(	78.1	73.5	82.4	76.9	78.3	79.5	75.8	76.8
LOC65398(	91.1	106.2	100.5	87.5	91	91.5	100.3	103.8
LOC65401⁷	308.5	339.5	218.4	242.9	202.3	305.9	264.1	488.3
LOC65405ž	109.9	123	102.8	108.9	108.6	103.8	124.6	139.4
LOC65408⁄	93.9	112.8	93.4	109.8	92.7	116.8	95.6	102.8
LOC65411ƍ	83.2	91.5	95.9	85.2	82.7	101.8	76.6	92.4
LOC65412ŝ	160.1	176.5	143.8	138.4	133.1	143.7	148.9	161.3
LOC65412ŝ	87.7	95.6	100	103	96.5	96.5	92.8	92
LOC65412ƍ	88	110.9	99.3	99.3	92.2	96.8	90.8	88.5

LOC654146	104.8	107.5	109.8	98.9	94.5	93.1	93.1	103.9
LOC654151	88.5	91.5	91.3	92.9	91.9	91.3	91.7	102.4
LOC654182	93.1	98.6	88.1	77.9	79.9	78.5	88	97
LOC654229	135.4	141.7	96.1	108.9	78.3	101.1	128.3	142.7
LOC654346	94.4	104.8	73.1	69.8	74.2	70.2	90.9	118.2
LOC723972	1228.6	1815.5	292.6	271.5	253.9	290.6	1000.8	1156.7
LOC727936	99.8	99.6	92.1	87.3	86.3	90.1	98.9	102.6
LOC728137	114.9	113.9	91.6	85.9	81.4	77.3	98.3	130.5
LOC728215	81.5	84.9	85.4	74	80.1	77.3	85.6	84
LOC728269	94.7	115.4	93.5	92.8	79.7	88.2	95.4	111.3
LOC728489	1228	1543	941.5	679.3	593.2	694.8	1142.3	1220.6
LOC728621	81.1	82.9	90	78.6	80.2	74	80.6	81.9
LOC728635	149.5	183.3	108.3	103.4	95.1	101.3	116.7	128.9
LOC728643	2641.8	4147.4	743.4	642.5	477.4	793.2	2514.4	4006.8
LOC728758	140.4	176.5	138.7	203.8	195.8	192	133.7	168.6
LOC729164	89.9	104.6	95.2	95.3	96.3	89.3	97.5	95.6
LOC729440	127.2	152.7	92.6	77.1	81.6	97.6	109.5	131.4
LOC729603	9398.9	10336.4	3666.9	7699.9	7297.2	4731.4	8594	8983.4
LOC81558	128.6	135.2	96.5	104.6	99.8	105.6	98.7	146.5
LOC81691	844.7	1023.3	763.6	611.9	574.6	692.5	583.3	701.6
LOC84661	3311.2	3989.9	2325.8	2574.2	1775	2730	3122	4973.8
LOC89944	90.6	89.3	98.5	85.5	84	88.3	83	86.6
LOC90167	81.1	97.4	81.7	79	72.7	82.5	83.4	94.5
LOC90321	131.7	175.1	94.5	92	78.2	92.2	134.4	166.1
LOC90355	231.3	287.8	194.5	202.7	165.3	245.6	215.4	267.1
LOC90379	128.9	159.1	120.1	103.9	99	98.1	125.5	157.7
LOC90379	272.2	320	284.1	219.2	209.8	292.1	227	431.7
LOC90580	715.9	960	643.9	403.9	305.6	442.3	663	1102.7
LOC90586	1776.9	1768.5	1079.3	1607	1896.3	1393.2	1468.2	2173
LOC90639	545.7	539.7	320.6	380.6	375.1	458.5	410.9	431.8
LOC90693	170.6	181.8	109.1	101.2	88.9	109.4	151.2	243.8
LOC90826	352.4	463.9	207.2	196.5	169.6	228.8	287.2	374.8
LOC90835	1924.8	2303.2	1462.3	757.5	721.8	793.7	1436.4	1352.9
LOC90925	97.3	122.2	93.6	87.6	99.1	97.3	108.7	119.3
LOC91137	1390.2	1721.5	545.7	636.1	430.7	933.2	1146.3	1740.5
LOC91353	103.6	124	112.6	92.9	89.6	90.9	113.5	119.9
LOC91431	204.4	251.1	115	117.6	104.5	157.8	166.8	285.4
LOC91461	114.5	126.1	130.3	108.7	110.4	115.6	102.2	167.7
LOC91561	31902.9	31382.2	51947.2	45655	44710.4	48656.4	29830.2	29939.5
LOC91614	181.5	244.6	125.8	119.4	104.2	126	150.4	222.2
LOC91689	316.9	396.1	215.5	213	183.3	214.7	249.6	327.1
LOC92017	84.6	123	96.8	132.2	127.5	124.4	94.7	90.7
LOC92196	93.2	109.2	87.8	91.2	97.5	96.5	94.7	105.9
LOC92270	151.4	176	125.7	123.9	119.6	150	141.3	166.2
LOC92345	259.3	302.2	216.7	173	144	202.4	212.5	290.7
LOC92689	111	134.2	103.5	90.4	95.3	88.3	108.8	130.2
LOC92691	128	144.9	117.4	94.4	93.5	101.9	124.8	135.7
LOC93081	3120.2	3739.6	1257	1365.6	1166.2	1430.8	2977.8	3963.7
LOC93349	119.8	138.3	104.6	88.8	92.2	93.1	103.8	127.4
LOC93622	637.2	862.9	408.5	328.7	267.8	367.6	564	779.9

LOH11CR2/	108.3	137.1	104.3	93	101.6	108.4	111.3	122.1
LOH11CR2/	138.5	181.6	99.7	93.4	94.4	92.8	134.5	175.8
LOH12CR1	182.3	218.7	103.8	111	99.5	112.9	180.6	212.9
LOH3CR2A	87	91.2	87.4	80.9	78	82.7	79.8	84.9
LONP2	159.4	172.6	107.4	118.2	110.9	129.1	155.5	141.5
LONRF1	258.1	345.4	231.3	192.4	178	244.8	274	478.1
LONRF2	91.6	100.5	89.5	82.2	84.2	88	84.1	104.6
LONRF3	161.5	203.5	151.2	131.3	119.4	167.2	163.1	275.4
LONRF3	98.7	105.3	86	86.3	90.6	77.2	89.8	102
LOR	76.6	83.9	88.5	84.8	78.5	76.1	71.8	75.3
LOX	93.1	90.9	77	84.7	88.2	81.8	91.7	92.8
LOXHD1	129.4	138.7	102.9	97.9	84.5	91.7	114.7	147.4
LOXL1	68	81.6	83.2	80.1	77.2	73.3	69.2	83.7
LOXL1	89.5	95.9	103.4	96.7	91	80.6	94.3	96
LOXL2	78	87.3	84	76.5	74.2	79.1	83.5	92.1
LOXL3	139.3	146.1	154.1	138.4	125.2	151.8	133.4	142.6
LOXL4	101.8	105.8	96.6	97.6	95.8	97.1	89.2	107.7
LPA	95.7	116.4	105.3	102.2	84.1	83.7	104	110.4
LPA	83.5	86.3	100.6	93.5	104.5	95.7	87.5	82.1
LPAL2	78.4	95.7	98.3	89.5	86.9	97.1	80.3	106.9
LPAL2	93	94.7	91.2	93.3	80.8	88.2	83.4	89.8
LPAL2	78.2	84.9	96.1	95.8	85.9	97.3	77.4	92.8
LPGAT1	134.7	166.9	110.3	104.7	92.5	101.3	124.5	130
LPHN1	250.9	360	381.3	284	237.8	428.2	269.2	497.7
LPHN1	145.9	157.3	99.7	83.6	96.3	98.2	156.3	154.1
LPHN2	685.9	1011.5	432.6	573.3	474.3	748.3	624.4	1129
LPHN3	268.3	362	143.9	300.5	223.7	408	241.9	608
LPIN1	929.1	1127.5	580.7	890.6	781.7	1039.4	933.2	1430.2
LPIN2	183.7	236.4	144.8	117.6	97.1	119.6	169.4	185.4
LPIN3	139.5	131.6	110.4	97.4	106.5	105.6	120.9	141.2
LPL	622.4	700.8	277.6	418.6	265.1	548.5	333.9	428.8
LPO	85.4	101.7	93.8	93.3	95.9	100.9	88.8	103.7
LPP	724.8	1352.8	374.1	1074.1	967.1	1548.3	859.8	833.4
LPPR2	146.8	223.7	163.2	202.6	139.6	225.9	150.9	242.3
LPPR4	91	97.2	94.6	94	81.1	87.1	85	109.6
LPXN	1936.4	2374.7	976.9	1026.2	820.9	966.5	2621.6	3809.7
LRAP	5193.5	4597.2	3634.9	4437.3	4467.2	3387.6	4544.2	7925.5
LRAT	89.6	95.1	87.5	88.1	80.8	97.3	96.2	98.1
LRBA	250.1	314.6	281.5	420.7	330.1	479.5	245.8	304.9
LRCH1	92.9	99.8	86.3	86.8	87.5	78.8	97.3	99.3
LRCH2	104.7	119.7	94	90	86.4	89.9	104.1	116.9
LRCH3	235	291.1	166.8	186.9	168.1	178.3	189.5	248.5
LRCH4	1263.4	1608	676.6	642.4	680.8	620.6	970.9	651
LRDD	244.9	249.7	373.1	240.6	244.6	424	170	290.1
LRDD	204.1	210.6	218.5	177	176.3	263	194.5	213.5
LRDD	83.4	100.2	89.4	76.3	79.7	79.5	76.5	89.1
LRFN2	73.1	83.1	93.2	80.5	84.1	81.3	83.2	75.3
LRFN3	561.4	727.7	456.1	330	282.4	325.4	485	493.9
LRFN4	170.8	204.5	210.5	169.5	143.1	240.7	168.1	320.7
LRFN5	83.6	96.7	92.9	92	88.6	96.2	95	98

LRG1	1230.7	1583.6	493.2	336.7	354.3	374	942.9	702.5
LRIG1	428	630.1	366.8	611.8	587.5	875.3	428.7	902.7
LRIG2	230.1	259.4	174.4	133.4	120.8	163.1	178.6	246
LRIG3	90.4	99.1	94.1	84.9	88	92.4	91.2	93.9
LRMP	496	611	402.9	494.8	529.3	670.6	354.1	516.4
LRP1	109.7	124.7	92.7	87.5	84.3	92.8	128.2	145.1
LRP10	521.6	553.3	215.7	162.9	167.7	165.2	586.8	939.2
LRP11	504.1	671.6	239.5	386.4	288	543.2	403.7	696.7
LRP12	125.4	127.8	99.5	98.5	113.4	95	118.3	134.8
LRP16	101.9	123.8	98.4	80.5	86.6	87.7	99	111.7
LRP1B	85.1	96.6	92.2	96.9	82.2	86.6	92.7	88.1
LRP2	80.5	92.5	92.5	84	85.1	93	78.3	82.4
LRP2BP	124.2	147.4	104.7	90.2	87.4	99.4	132.5	140.4
LRP3	2350	2880.3	1798	1551.7	1131.2	1350.5	1806.7	1992.4
LRP4	89	91.6	102.5	103.1	84.8	102.7	78.3	91.4
LRP5	97.3	107.5	132.2	130.2	115.2	133.2	94.4	105
LRP5L	255.2	249.7	162.9	245.8	262.6	295.4	189.2	192.1
LRP6	121.5	140.6	117.2	117.4	111.1	122.1	132	125.6
LRP8	166.9	165.3	112	110.5	93.1	115.3	197.1	411.9
LRP8	782.6	896.7	586.6	811.2	609.3	1179.2	1141.7	2225.5
LRPAP1	521.9	745.3	360.6	307.7	270.9	396.9	542.5	634.3
LRPPRC	735.7	1127.6	704.5	788	588.1	979.3	589.2	929
LRRC1	150.6	178.5	99.2	90	85.3	86.9	118.9	175.2
LRRC1	435.1	490.7	263.7	312.2	218	325.4	386.3	557.5
LRRC10	83.7	87.3	93.8	95.2	87.5	81.3	90.7	82.7
LRRC14	723.4	750.9	445.8	252.4	237.6	262.2	453.2	518.9
LRRC15	153.1	241.1	118.3	108.9	102.8	108.9	182.2	184.5
LRRC16	118.4	132.1	106.6	104.3	94.8	93.8	99.4	107.6
LRRC17	236.6	259.6	176.7	211.4	186.8	246.4	152.6	181.5
LRRC17	90.1	111.9	96.4	97.5	91.4	101.9	96.2	103.4
LRRC17	121.7	157.7	104.4	108.7	98.8	129.9	101.3	128.6
LRRC18	97.8	115.1	92.6	82.1	86	87.8	96.9	100.1
LRRC19	116.5	140.6	100.6	95.4	92.3	78.8	112.6	131.4
LRRC2	89.6	101.6	82.5	82.9	77.7	85.7	93.4	97.2
LRRC20	69.2	76.4	88	83.2	81.9	80	75.9	82.1
LRRC20	731	950	492.3	368.4	294.1	333	500	400.8
LRRC20	133	188	182.6	163.5	135.5	174.7	125.2	152.9
LRRC21	85.9	103.5	93.4	86.3	80.6	87	91.3	119.2
LRRC22	79.2	89	99.4	93.9	93.5	81.8	78.5	78.1
LRRC23	104.4	112.9	105.6	100.9	99.3	109	106.4	113.9
LRRC23	90	99	107	90.7	96.2	86.9	91.6	97.3
LRRC24	92.3	103.3	96.5	92.6	86.3	79.6	87.4	107.7
LRRC25	84.5	93.6	104.4	101.4	91.6	94.6	82.9	86.1
LRRC27	98.6	111.8	91.8	89.7	82.3	83	101.8	99
LRRC28	343.3	459.1	209.5	236.2	204.5	229.3	342.7	351.9
LRRC29	91	103.2	98.8	96.6	91	95.7	84.5	86.1
LRRC29	144	188	131.4	126.7	114.1	118.8	146.8	195.4
LRRC3	193.5	234.7	121.7	159.1	117.4	141	180.2	213
LRRC31	112.7	123.4	104.3	110.1	110.7	113.4	105.5	128.1
LRRC32	90.4	97.6	84.4	94.5	87	85.9	93	85.5

LRRC33	767.4	1017.6	992.9	880	711.3	1421.6	721.9	1587.6
LRRC34	220.6	283.3	105.8	98.3	83.6	94.3	160.6	233.8
LRRC35	108.7	149.5	99.4	84.6	82.4	86	112	128.2
LRRC36	94.7	110.1	93.5	87.5	80.8	90.4	89.4	106.9
LRRC37A2	91.4	115.5	94	91.8	99.1	88.3	89.6	104.8
LRRC37B	579.2	724.9	353.3	608.6	520	645.2	535.9	851.8
LRRC39	112.6	129.8	95.9	90.3	86.5	91.6	105	139.8
LRRC3B	102.1	115.2	115.5	100.3	98.6	110.6	128.1	182.4
LRRC4	88.5	110.1	86.2	83.2	82.6	84	79.6	110.2
LRRC40	131.8	140.7	124.6	117.4	103.6	120.7	122.9	192.3
LRRC41	670.9	859.5	251.9	200.1	171.2	210.4	536.3	487.8
LRRC42	1003.9	1232.2	625.7	845	648.7	730	1036.7	1396.7
LRRC43	97.1	101.3	106.7	105.8	107.2	115.7	87.1	94.3
LRRC45	202.7	227.5	365.2	337.7	338.6	579.2	137.5	178.8
LRRC45	153.2	169.1	142.2	144.5	120.2	177	110.8	126.7
LRRC46	108.5	151.4	93.8	101.5	79.9	97.8	122.1	154.3
LRRC47	539.8	649.4	319.5	400.5	279	365.9	445.4	609.4
LRRC48	105	110.9	100.8	102	89.7	84.6	98.7	149.1
LRRC48	80.7	88.7	104.1	98.6	91.1	90.4	78.5	80.6
LRRC49	95.8	104.8	90.7	101.2	89.4	96.5	94.9	111.4
LRRC4B	94.3	111.6	92.6	91.9	87.9	83.5	94.4	110.6
LRRC4C	97.5	92	90.9	89.4	98.4	81.5	87.5	92.9
LRRC50	106.5	121.9	98.8	96.1	82	86.5	98.1	114.2
LRRC51	99.7	108.9	90.7	104	96.8	100.4	93.6	85.9
LRRC52	91.3	93.3	90.6	83.6	85.1	90.1	94.5	108.2
LRRC54	217.6	264.6	199.4	117.7	103.3	116	210.8	346.7
LRRC55	86.8	95.7	93.8	93.4	89.5	92.8	99.7	92.2
LRRC56	169.6	205.8	128.8	106.6	108.6	115.3	144	132.2
LRRC57	435.5	507.1	207.1	174.8	157.7	215.2	447.3	606.6
LRRC59	159.7	224.3	112.3	86.1	79.9	99.6	147.4	200.8
LRRC6	110.4	124.5	101.5	85.6	85.7	87.3	103	111.3
LRRC61	88.9	101	92.2	82.7	81.6	80.5	86.4	112.7
LRRC7	74.6	82.7	89.2	81.7	86.6	79.2	71.8	74.6
LRRC8A	286.9	314.3	192.1	156.3	150.5	145.5	322.3	386.1
LRRC8B	98.7	107.2	91.6	84.4	88.1	85.8	98.8	110
LRRC8C	375.1	559.8	245.5	235.2	214.5	333.7	329.4	608.3
LRRC8D	480.2	662.8	299.6	320.5	232.2	360.3	421.9	580.6
LRRC8E	122.3	147.9	92	82	86.8	97.6	108.9	133.7
LRRCC1	130.7	115.4	104.1	89.2	82.3	92.3	128.8	138.7
LRRCC1	2095.8	2700.4	676.3	591.8	525.3	823.1	1381.6	2450.4
LRRCC1	116.4	118.4	104.4	104.4	96.6	91.9	95.3	121.8
LRRFIP1	1437.6	1564.3	1753.4	2697.3	2033	3003.2	1849.8	4019.7
LRRFIP2	77.7	92.6	84	81.4	80.6	78.1	71.5	90
LRRFIP2	453.5	681.6	447.1	562.7	497.4	802	587.7	1092.5
LRRFIP2	507.1	644.4	270.3	288.3	287.6	415.8	515.4	667.8
LRRIQ1	81.3	84.3	84.4	88.3	80	77.9	80.9	77.1
LRRIQ2	78.6	87.6	93.8	85.8	87.7	85.4	87	90.7
LRRK1	151.9	157.5	101	118.9	93.7	116.9	147.8	202.6
LRRK2	87.6	109.2	95.5	87.9	78.5	83.7	92.1	90.9
LRRK2	94.9	128.6	94.3	91.5	87.1	97.4	95.5	122.3

LRRN1	92	112.1	98.8	83.6	87.3	92.6	88.9	106.1
LRRN3	100.8	112.4	96.3	89.5	85.1	83.7	106.7	118.2
LRRN5	99.3	113.7	95.5	100.3	96	104.8	95.2	103.2
LRRN5	97.6	112.7	92.6	98.7	83.9	78.2	98.4	97.4
LRRN5	118.6	127.3	90.6	90.3	81.5	83.9	104.8	123.3
LRRN6A	108.3	115	97.9	90.2	85.4	87.9	105	124.8
LRRN6C	80.8	95.9	93.1	86.2	89.7	92.1	91.4	79.9
LRRTM1	120	126.5	93.5	83.2	85	84.6	121.9	119.7
LRRTM2	95.3	101.9	99.2	93.1	97.6	86.9	90.8	96.6
LRRTM3	83.3	97.5	99.4	85.1	76.4	87.4	85.7	94.4
LRRTM4	165	194.1	106.6	102	102.8	93.9	158	173.5
LRSAM1	80.5	84.8	91.5	85.1	86.5	78.8	81.6	84.1
LRSAM1	471.7	542.9	373.9	236	233.3	263.6	373.1	398.4
LRSAM1	94.7	113.8	94.8	84	85.4	90.8	109.5	119.7
LRTM1	94.4	112.4	97.9	93.5	88	84.2	93.5	121.2
LRTM2	133.5	149.1	107.1	91.7	91	94	102.6	141.9
LSAMP	116.3	130.4	112.2	106.7	106.8	115.8	102	131.2
LSDP5	183.4	209.4	119.5	124.4	134	110	158.2	212.4
LSG1	383.3	378	247.2	235.6	167	259.5	299	575.3
LSM1	8780.3	10885.9	8180.7	6172.5	4479.2	6418	7948.2	9325.7
LSM10	654.5	802.3	368.7	278.9	236.1	345.6	583.1	661
LSM11	123.7	129.9	120	98.1	95.2	88.3	99.7	110.8
LSM12	1014.1	1438	499.5	416	308.1	419.6	798.9	1344.2
LSM14A	2826.9	3575.8	2200.7	2168	1761.6	2180.4	2793.2	2669.3
LSM14B	230.5	314.9	120.6	115.8	116.6	122.2	230.8	267
LSM16	177.6	206.9	209.9	203.4	175.6	256.4	173.7	285.6
LSM2	7100.9	8186.4	3623	3281.4	2623.6	3394.7	6710	6877.2
LSM3	4968.3	4953.9	3075.4	3556	2279	3410.7	5310.7	9075.6
LSM4	3310.2	4785.6	2758.1	1228.9	1067.5	1705.3	2794.7	3222.9
LSM5	575.6	660.2	342	332.7	225	279.3	541.1	977.9
LSM6	466.8	530.4	183.5	142.3	106.3	123.5	459.5	852.8
LSM7	7076.8	8379.7	4293.7	3049.5	2853.8	4236	6434.6	6821
LSM8	680.6	855.7	416.7	386.1	316.3	408.7	495.3	665.5
LSMD1	767.2	1040.6	361.5	392	273	464	710.5	932.2
LSP1	129.9	131.3	117.5	169.8	160.4	222	113.7	168.9
LSP1	85.7	94.1	96	93.7	94	91.8	86.2	102.9
LSP1	91.2	100.7	98.7	210.2	190.7	173.3	96	93
LSP1	108.5	131.4	96.7	90	76	78.2	95.1	128.6
LSR	86.8	90	105.5	91.4	91.8	101.5	93.4	93
LSR	109.9	140.4	94.7	101.9	85.9	99.2	101.6	119.7
LSR	126.5	145.7	89.6	79.1	89.7	100.8	108.4	179.2
LSS	101.2	127.7	98	106.5	113.9	122.2	105.4	121.7
LSS	135.9	152.8	113.4	123	115.6	117.1	123	137.4
LSS	291.6	383	386.5	529	702.3	773.6	226.5	377.1
LST1	4088	4779.5	3766.7	1740.5	1479.4	2045.5	3381.1	3416.7
LST1	253.5	293.6	245	154.9	137.8	143.6	190.2	274.2
LST1	3544.2	4178	1752.4	1095.1	911	1385.5	2991.1	3506.4
LST1	3412.9	4359.3	1645.7	1320.1	1026.4	1189.7	3137.5	3405.9
LST-3TM12	102.5	105.8	93.7	87.9	91.3	84.7	99.9	117.2
LTA	188.5	163.7	112.9	109.1	99.7	105.6	167.1	185.9

LTA4H	2506	3268.2	1111.9	1775.2	1299.8	2121.7	2055.7	2687.5
LTB	533.3	715.1	337.5	279.7	221.5	219.4	717.9	800.9
LTB	165.8	231.3	139.4	129.2	123.4	118.7	220.8	265.9
LTB4DH	985.9	1484.7	672.5	534.1	413.3	583.4	978.4	1246.5
LTB4R	159.9	168.8	167	177.4	153.6	254.2	144.9	158.3
LTB4R2	94	102	102.5	92.5	92.1	97.7	89.1	99.5
LTBP1	103.4	126.6	93.5	80.7	74.8	82.6	112.3	124.5
LTBP1	102.2	120.7	93.8	92	87.3	91	117.2	108.4
LTBP3	101.8	110.6	90.4	96.8	92.7	87.8	99.3	97.8
LTBP3	97.2	95	88.5	79.7	80.7	81.5	93.8	99.3
LTBP3	217.9	281.9	178.9	119.8	116.5	141.5	233.4	285.4
LTBP4	98.7	101.8	101.4	97.8	90.9	99.2	98.9	102
LTBP4	195.3	232.9	183.6	181.7	162.3	193.1	211.5	332.4
LTBR	309.6	430.8	455.2	434.9	355.4	549.8	366.1	1224.1
LTC4S	793.2	1062.9	489.8	598.5	487.3	397.4	659.3	677.7
LTF	105.8	113.3	99.1	81.6	86.7	85.4	90.2	120.3
LTK	137.2	136.5	89.8	97.7	90.4	97.7	122.9	161.7
LTK	87.9	110.6	97.8	98.1	86	85.7	100.6	101.6
LTV1	3029.3	4055.1	981.2	717.7	642.9	1197.9	2508.8	3741.5
LUC7L	124	126.6	99.9	89.9	97	100.1	117.1	138.6
LUC7L	447.5	582.1	297.3	310.1	280.8	399.1	349.9	443.2
LUC7L2	184.1	196.6	145.1	172.1	157.3	181.2	156.2	176
LUM	85.3	91.5	102.6	94.9	89	97.8	82.2	89.4
LUZP1	567	843.1	567.1	725.8	621.9	914.3	533.2	607.9
LUZP2	102.4	129.3	111.1	106.9	107.1	101.1	94.6	99.9
LUZP4	73.8	82.1	74.8	81.5	80.5	75.4	80.2	82.2
LXN	86.4	97.8	99.5	86.6	87.7	84.1	97.1	101.6
LY6D	85.5	108	92.1	78	92.1	82.6	99.8	102
LY6E	889.3	1234.9	876.7	193	197.8	249.2	725.4	1439.8
LY6G5B	104.8	119.9	104.6	101.4	98.7	96.9	92.2	116.9
LY6G5C	221.7	257.2	140.8	133.9	115.5	104.9	199.6	240.3
LY6G5C	109.9	135.7	94.6	88.3	77.5	83.3	102.4	114.1
LY6G5C	79.1	79.8	98.4	86.6	86.3	87.4	81.2	75
LY6G6C	119.4	148.7	91.9	79.9	76.3	85.5	107.6	151.8
LY6G6D	87.2	95.6	103.8	84.2	88.6	82.8	84.1	103
LY6H	276.3	321.3	195.4	126.7	138.1	159.5	257.2	266.3
LY6H	97.4	128.9	84.3	88.4	88.5	88.2	98.6	115.4
LY6K	85.6	100.6	88.4	84.5	82.7	87.5	77.5	95.2
LY75	82.9	89.8	95.8	82.7	92.3	84.1	84.8	100.4
LY86	1600.7	2115.5	1276.7	1467.9	1205.6	1457.2	1611.5	1871.2
LY9	107.5	147.2	106.6	85.8	79.3	82.7	108.5	115.8
LY9	84.5	97.9	113.9	101.6	102.4	102.1	82.6	89.4
LY9	104.9	130.4	95.5	91.4	82	92.8	103.5	130.5
LY96	188.8	230	176.2	277.1	246.5	356.6	253.6	311.5
LYAR	2379	2880.5	609.6	590.9	539.6	949.4	1626.4	2542.4
LYCAT	103.3	123.4	89.6	91.3	82.7	92.2	100.8	114.2
LYCAT	927	1178.3	615.4	578.5	413.8	700.1	730.1	1250.5
LYG2	100.6	116.1	95.5	88.8	83.1	100.4	100.5	126.8
LYK5	82.4	90.2	89.2	78.5	76.9	88	82.6	108.4
LYK5	1380.1	1817.1	711.3	725.8	676.8	705.4	1134.1	1146.9

LYK5	171.4	208.9	151.6	138.6	127.6	172	164.2	254.9
LYL1	2771.8	3467.8	1448.1	1621.5	1539	1838.3	2016	2010.8
LYN	3651	4842.6	3235.6	3193.4	2302.9	2945.9	3160.7	5917.9
LYNX1	86.4	81.1	91	80	79	82.4	87.9	94.7
LYNX1	87.3	111.9	86.1	75.8	76.4	83.9	99.5	106.6
LYNX1	92.7	103.6	81.2	77	74.6	69.2	100.6	103.9
LYPD1	95.6	117.1	84.7	87.5	75.4	95	100.6	100.1
LYPD1	140.2	179.9	131.9	113.1	107.4	115.2	121.1	153.5
LYPD2	120.1	140.2	101.1	106.1	99.5	110.5	119	134.8
LYPD3	95	108.2	94.5	89.1	88.1	85.1	92.1	103.4
LYPD4	136.3	148.4	96.2	77	78.7	93	114.1	133.4
LYPD5	75.4	83.8	70.7	75.1	75.8	70.2	85	77.4
LYPD5	137.5	202.3	126.1	102	106.2	105.7	130	143.9
LYPD5	105.9	123.5	107.2	92.6	89.4	90.2	108.4	111
LYPLA1	103.4	113.1	91.9	89.4	80.2	88.9	111.3	163.5
LYPLA2	245.5	358.4	205.3	163.6	130.5	148.9	221.1	288.6
LYPLA2P1	133.5	168.8	106.7	94.6	87.8	104.1	139.6	145.6
LYPLA3	197.4	260.5	167	144	141.9	160.8	211.3	372.8
LYPLAL1	2534.1	2799.6	1378.3	1244.6	975.6	1170.8	2258.6	3495.6
LYSMD1	103.4	114.7	107.2	103	93.5	105.8	102.8	118.3
LYSMD2	1269.8	1593.4	751	854.8	672.3	891.8	1247.8	1765.8
LYSMD3	153.7	193	90.9	91.4	79.1	93.3	171.3	206
LYSMD3	102.1	109.2	92.8	84.8	83	83.3	92.4	109.7
LYSMD4	373.5	435.7	276.1	303.1	332	379.6	285.8	328.7
LYST	1135.6	1559.1	755.8	1341	954.6	2449.8	954.8	1844.5
LYST	114.5	134	92.5	96.4	87.3	94.2	103.6	114.9
LYST	92.7	109.6	97.5	86	81.7	79.1	102.3	102.2
LYZ	419.2	631.5	334.8	416.9	370.3	529	368.5	350.8
LYZL1	81.9	92.6	87.4	89.1	79.7	88.3	89.4	95
LYZL2	86.8	83.9	107.3	106	99	110.5	87.1	80.1
LYZL4	74.6	83.3	103.8	96.6	86.5	89.1	86.5	90.2
LYZL6	97.5	114.9	118.4	104.9	101.1	101	98.1	101.4
LZIC	297.1	369.9	174.3	162.2	130.5	183.7	244.9	316.9
LZTFL1	280	319.6	193	209.6	170.6	238.8	215.3	424.5
LZTR1	729.5	799.8	861.1	686	628.3	936.8	515.4	774.1
LZTR2	106.3	116.4	90.6	89.7	82.7	73	100.1	128.4
LZTS1	136.5	140.7	158.3	99.8	96.5	112.9	120.2	167.9
LZTS2	158.2	168.6	139.5	129.1	115.5	145.6	142.6	228.1
M160	1090.7	1164.1	989.4	1179	729.4	1168.5	969.8	1080.8
M6PR	2093.8	3047.9	1051.2	934.8	737.2	1071.3	1566.3	1950.5
M6PRBP1	8192	11078.5	2993.1	2533.3	2063.4	2459.8	7848.1	8632.7
MAB21L1	100.5	123	100.2	93.4	83.1	92.9	106.3	112
MAB21L2	82.3	82.5	92.9	88.9	78.8	92.4	93	84.2
MAB21L2	95.1	137.2	95.4	85.7	83.6	84.6	98.5	107.2
MACF1	119.6	137.8	109.1	116.7	111.4	102.4	126.5	134.6
MACF1	91.1	93.3	93.8	88.5	96.8	88.4	94	103.8
MACF1	292.5	435	266.4	611.4	503.2	783.5	316.9	400.4
MAD1L1	569.5	774.7	435.5	287.2	267.2	400.1	545.5	667.6
MAD2L1	262.4	256.6	146	126.7	106.8	121.3	209.6	367.6
MAD2L1BP	92.8	94.6	112.1	95	95.5	100.7	84.5	92

MAD2L1BP	877	1043.1	359	287.6	226.5	239.7	665.2	710.3
MAD2L1BP	1530.1	2022.9	909.3	640.6	532.1	788.7	1501.2	2491
MAD2L2	3166.3	4087	2534.3	2045.9	1597.2	2558.1	2786	3885.1
MADCAM1	79.1	97.9	93.8	88.9	85.1	94.9	90.7	91.1
MADCAM1	89.7	112	114.1	90.9	93.7	86.9	84	88.1
MADD	80.7	91.7	96.6	99.6	96.7	91.6	81.8	82.9
MADD	167.7	176.5	197.1	202.8	183	287.2	152.7	199.6
MADD	277.4	328	180.3	186.5	196.1	189.2	260.5	273.1
MAEA	1229.6	1545.9	739.8	271.9	247.4	255.7	931.8	1567.8
MAEA	604.3	748.8	243.9	185.5	161.3	119.6	532.5	682.1
MAEL	74	83.5	92.7	86.9	93.2	84.2	81.6	89.2
MAF	90	125.9	86.6	92.9	81.1	86.2	97.4	114.4
MAF	84.8	84.5	97.9	103.6	103.6	104.9	84.9	84.4
MAF1	306.9	411.3	259.7	181	164.7	222.1	287.7	472
MAFA	212.9	241.1	146.1	121.8	111.4	126.8	214.8	207.7
MAFB	222.6	275.6	145.4	282.7	221.7	322.5	270	431
MAFF	95.2	112.5	98.1	103.8	87.7	93.4	97.5	112.1
MAFF	691.4	944.6	230.7	189.8	165	178.2	630.3	935.6
MAFF	113.1	129.5	94.8	87.8	94.7	100.2	107.5	146.2
MAFG	238.3	258	258.4	255.1	250.2	309.6	357.2	653.1
MAFG	213.8	244.7	129.7	121.6	113.4	108.7	250.7	428.5
MAFK	112.4	137.1	95.2	94.8	86.1	90.1	100.7	131.9
MAG	82.6	91	97.1	91.7	91.6	93.6	83	103.3
MAG	81	85.1	94.1	91.8	79.9	88.1	93.7	82.6
MAGEA1	127.3	124.8	89.5	87.4	73.3	85.7	119.1	133.2
MAGEA10	82.4	82.8	78.4	87.4	87.2	80.4	84.9	79.8
MAGEA10	93	119	102	89.1	77	78.2	94.1	109.7
MAGEA11	107.3	109.9	83	86	79.6	81.2	102.4	134
MAGEA11	74.6	79.4	90	87.4	75.3	77.7	78.6	88.6
MAGEA11	175.9	199.7	111	98	100.2	102.4	179.2	174.9
MAGEA11	86.4	118.3	93	77.2	72.7	74.1	102.2	114.3
MAGEA12	90.5	111.4	95.9	93	89.4	100.4	103.3	94.2
MAGEA2	107.6	110.6	90.2	80.6	88.9	80.3	111.4	103.8
MAGEA2	79.3	71.5	90.3	92.3	84.4	87	71.7	87.3
MAGEA2B	80.1	93.1	86.5	89.3	77.8	77.3	84.9	84.2
MAGEA3	87.8	94.5	93.8	90.8	86.9	85.9	80.5	90.1
MAGEA4	81.3	91.8	96.6	88	103.3	100.2	82.7	93.5
MAGEA5	96.3	107.9	93.2	95.9	95.9	104.4	90.8	110.3
MAGEA6	112.1	123.1	101.8	103.2	86.1	101.7	111.3	120.3
MAGEA8	72.8	81.2	92.6	85.1	87.2	86.8	76.7	78.9
MAGEB1	77.8	85.2	83.9	82.7	78.8	85	80.5	77.9
MAGEB1	85.6	93.9	85.7	84.8	77.7	84	99.4	96.1
MAGEB10	96.4	103.3	82.2	82.6	77.3	79.6	94.4	111.5
MAGEB18	94.3	104	93.9	88	77.8	82.9	100.3	104.8
MAGEB2	97.2	126.8	81.6	78.1	72.1	85.1	92.1	125.3
MAGEB3	140	178.2	91.3	86.1	89	87.8	134.2	149.4
MAGEB4	77.9	83.7	86	76.2	78.4	88.1	79	90.2
MAGEB6	106.6	122.2	101.5	82.6	90.6	86.9	100.6	99.4
MAGEC1	88.4	88.2	93.9	79.4	82.5	84.4	89.2	84.1
MAGEC1	82.4	83.7	84.2	72	76.5	80.6	78.9	90.9

MAGEC2	84.3	103.9	84	73.3	74	83.1	90.7	95.9
MAGEC3	81.3	88.4	90.8	93.7	76.8	99.2	80.7	90.1
MAGEC3	84.4	99.4	88.1	77	82.3	80.2	93.9	89.9
MAGEC3	83.9	92.5	78.9	85	80.5	82.4	92.8	89.5
MAGED1	389.3	465.6	289.2	1271.3	864.4	1204	329.3	433.3
MAGED1	174.7	196.2	140.7	297.8	234.9	330.4	169.5	200.6
MAGED1	102.3	105.4	96.2	101.2	101.6	84.3	101.5	115.8
MAGED1	94	102	90.3	91.3	82.9	92.8	88.1	97.4
MAGED2	224.8	284.6	121.7	131.7	120.8	107.5	186.5	230.4
MAGED2	143.5	155.9	97.1	98.6	98.4	102.5	125.4	147.7
MAGED2	197.5	259.6	129.4	122	104	138.8	176.3	223.7
MAGED2	163.9	189.6	96.8	85.3	87.9	91.5	142.9	192.5
MAGED4	103.1	125.9	109	106.5	102.9	99	110.4	121.5
MAGEE1	170.6	213.5	164.3	169.7	146.9	207.4	144.3	184.3
MAGEE2	103.7	111.7	98	87.7	88.2	93.1	104.9	102.7
MAGEF1	234.6	325.1	131.1	125	126.5	155.3	171.1	169
MAGEH1	145.7	162.8	131.2	138.9	130.8	123.8	141.3	161.1
MAGI1	80.6	89.8	89.3	92.4	79.7	79.3	85.6	86.4
MAGI1	73.5	86.3	78.1	76.6	78.8	75.4	78.6	86.9
MAGI1	109.8	130.8	83.3	82.2	84.2	87.8	119.8	139.5
MAGI2	87.3	103.6	88.2	83.2	75.5	83.1	100.3	100.3
MAGI3	83.3	92.6	98.9	86.1	86.6	92.2	88.3	81.2
MAGI3	82.5	89.7	95.1	90.3	97.8	92.5	84.6	97
MAGMAS	1830.6	2039.6	1598	933.2	729.2	1550.6	1453.7	1782.1
MAGOH	1573.3	2251.9	852.8	820.4	738.1	851.3	1369.7	1495.7
MAK	87.3	90.2	93.2	89.4	87	82.2	84.9	88.3
MAK10	203.5	228.4	120.2	97.1	80.2	110.9	185.7	304
MAL	113.4	122.1	96.3	92.5	89.9	93.5	113.5	126.6
MAL	102.9	110.8	116.7	94.5	89.3	103.9	102.7	101.2
MAL2	78	104.6	83.5	82.1	79.4	73.8	91.8	103.2
MALAT1	113	109.9	81.3	84.5	83	83	101.5	96.6
MALL	89.9	96.8	105.3	102.9	87.2	95.7	88.6	94.2
MALT1	267.8	372.5	143.8	159.9	147.3	175.9	308.4	406.9
MALT1	85.9	103	86.2	84.1	83.2	85.6	86.4	100.7
MAMDC1	84	99.3	93.6	98.4	85.9	95.9	86.4	88.6
MAMDC2	71.5	87.5	91	78.5	73.8	93.9	81.7	98.4
MAMDC4	102.6	110.5	121.3	116.3	122.2	145.9	88.8	112.7
MAML1	82.6	87.6	94.9	89.3	90.6	89.8	84.4	89.3
MAML1	145.3	194.7	166.3	178	161.9	224	149.1	189.7
MAML2	81.1	100.9	84.4	89.8	79.4	86.1	88.1	103.1
MAML3	90.6	100.7	82.8	82.6	67.6	73.4	94.3	103.3
MAN1A1	308.5	378.4	181.8	151.8	154.3	147.8	270.6	352.2
MAN1A2	143.7	182	109.5	97	96	123.1	121.9	189
MAN1B1	1022.8	1404.1	834.2	938.2	789.3	1002.3	876.1	846.2
MAN1C1	193.4	243.2	190.6	159.9	145	164.2	183.5	307.1
MAN2A1	88.1	115.5	90.5	90.5	82.6	93.1	99	104.4
MAN2A2	147	155.4	136.4	188.8	181	230.3	140.6	216.3
MAN2B1	358.3	479.2	395.3	398.2	334	487.9	310.1	484.6
MAN2B2	1257.4	1790.9	292.9	316.6	254.6	349.4	1179.9	1164.5
MAN2C1	251.5	282.2	424	427	436.6	674.4	165.7	276.6

MANBA	331	452.6	220.3	324.2	302.3	488.8	275.7	449
MANBAL	846.6	1088.5	715.2	658.4	541.3	776.4	798.5	1089.1
MANBAL	103.4	112	113.5	86.2	94.1	101.5	96.4	106.2
MANBAL	337.9	389.9	153.4	135.7	121.5	137.2	340.9	333.2
MANEA	78.4	91.7	82.3	80.8	79.3	87	88.6	88.3
MANEA	460.1	606.2	217.6	227.4	195.1	223.4	308.9	453.1
MANEAL	78.4	84.8	92.4	99.3	99.9	90.2	87.4	103.1
MANEAL	106.2	113.3	95.7	82.1	82.5	80.3	95.7	89.5
MANEAL	97	110.8	94.9	85.3	84.8	75.7	95.8	110.8
MANSC1	109.2	133.6	101.7	101.5	90.1	107	113.3	123.7
MAOA	81.9	100.9	87.3	92.6	84.5	91.2	86.4	94.3
MAOB	112.3	117.2	95.9	85.7	87.4	90.7	94.9	106
MAP1A	109.1	117.3	137.1	139.8	136	133.2	103.2	112.3
MAP1B	88.1	102.6	94.5	88.3	82	83.2	85.9	87.3
MAP1B	119.1	151.1	135	155	165.7	171.5	141.3	211.4
MAP1B	92.8	126.7	88.3	85.4	91.2	88.4	115.7	151
MAP1D	134.4	153.9	92.7	94.3	85.8	85	112.2	150.2
MAP1LC3A	87.1	93.8	91.2	91.6	79.5	78.5	82.8	84.8
MAP1LC3A	92.9	100.3	89.3	91.2	93.4	97	96.9	95.9
MAP1LC3A	933.9	1264.8	1004.5	968.8	843	803.5	1015.6	1813.8
MAP1LC3B	603.6	739.4	391.6	334.6	252.2	394.5	593.5	1279.5
MAP1LC3C	119.3	155.7	113.7	101.9	112.9	97.8	117.8	115.7
MAP2	80.8	82.1	90.9	82.2	79	85.9	80.9	95.8
MAP2	102.2	104.5	88.7	80.4	81.3	80.3	97.7	116.2
MAP2	95	101.2	98.9	87	84.2	94.8	99	105.8
MAP2	104.7	125.1	96.9	92.6	86.3	107	101	106.3
MAP2K1	865	1076.3	529.2	695.8	510.7	754.9	863.5	1554.4
MAP2K1IP1	1568.3	1894.4	755.8	902.6	592.8	1224.8	1400.3	2591.5
MAP2K3	89.2	95.8	88.2	84.3	81.6	79.5	75.6	103.9
MAP2K3	102.7	130.7	109.1	94	90.9	95.7	118.2	124.2
MAP2K3	85.6	97.6	94.7	90.1	85.3	96.5	88.7	91.7
MAP2K3	122.2	161.1	87.9	86.7	92.1	86.9	117.3	135.1
MAP2K4	228.2	298.2	197.6	233.3	177.2	269.7	220	486.2
MAP2K5	258.7	305.7	232.2	275.1	216.2	289.7	261.7	379.9
MAP2K5	155.7	172	117.9	128.1	114.9	150.4	152.4	220.9
MAP2K6	153.4	184	95.1	117.8	102.9	126.7	94.3	89.8
MAP2K7	133.6	162.3	137.1	120.5	118.5	146.2	133.6	176
MAP3K10	115.3	111.1	91.8	78.8	73.4	80.9	100.6	127.1
MAP3K11	241.5	297.3	374.7	299	251	317.4	227.2	514.9
MAP3K12	140.6	144.9	97.9	90.6	75.7	86.7	118.3	174.5
MAP3K13	85.3	90.8	91	94.9	93.4	91.2	80.3	99.3
MAP3K14	118.7	126.9	108.7	105	105.1	115.3	110.5	120.4
MAP3K15	108.3	109.4	96.9	90.8	93.1	102.6	105.9	108
MAP3K2	187.9	235.8	119.9	141.4	133.2	149.9	205.2	280
MAP3K3	118.8	125.1	88.2	108.2	85.3	94.3	102.4	116.3
MAP3K4	101.5	107.9	101.2	97.1	85	91.2	104.6	108.1
MAP3K4	814.1	1117.3	1156.4	1021	998.8	1591.3	740.6	978.2
MAP3K6	499.5	572.6	330.6	254.5	233.6	320.6	334.7	342.5
MAP3K7	647.4	968	251.7	220.5	204.7	316	507.1	670.7
MAP3K7	2092.5	2715.8	1275.1	1556.3	1088.9	2341.6	1716.9	3216.4

MAP3K7IP1	114.5	120	93.7	101.1	85.9	95.8	117.6	116.5
MAP3K7IP1	244.3	270.7	285.6	242.4	194.5	267	264.7	534.5
MAP3K7IP1	92.3	98	92.6	82.5	88.4	85.2	108.4	112.2
MAP3K7IP2	158.3	198.1	105.5	102.3	109	125.1	144.3	229.8
MAP3K8	159.7	182.5	128.6	128.4	141.7	155.4	140.3	250.8
MAP3K9	92.9	106.4	90.3	84.8	81.2	88.2	95.1	108
MAP4	135.4	193.7	110.4	147.2	124.7	114.5	135.4	171.5
MAP4	92.5	91.2	95.1	90.1	86.1	88.9	93.5	99.8
MAP4	97.5	111.8	94.1	92	78.4	92.1	95.3	111.3
MAP4K1	2017.3	2431.1	1381	670.8	522.8	726.5	1563.6	2154.6
MAP4K1	491.7	636.2	744	388.4	325.5	459.5	430.8	896.3
MAP4K2	2457.8	3103.4	1803.5	1501.4	1350.1	1462.5	1892.9	1773.5
MAP4K3	224.8	263.8	122.5	119.1	116.3	171.4	178	232
MAP4K4	105	104.5	100.2	97.4	82.2	97	99	110.7
MAP4K4	236.2	295.7	178.4	254.9	215.2	351.5	198.6	379.8
MAP4K5	205.1	250.4	113.6	85.7	88.2	99.4	184.9	253.6
MAP4K5	293	339.6	165.3	162.2	139.2	217.2	255.6	386.5
MAP6	84.9	99.4	94.6	83.5	92	88.1	83.6	94.8
MAP6	86.5	83.8	90	84.4	81.9	87.2	79.4	85.5
MAP6	101.1	123.7	89.6	85.4	76	83.8	107.2	112.1
MAP6D1	511.7	748.1	355.1	345	275.3	356.1	450.2	435.8
MAP7	138.8	187.4	119.5	128.8	123	170.5	132.7	154.4
MAP7D2	75.5	87.8	87.5	81.9	82.4	95.9	81.9	88
MAPBPIP	4205.1	5255.9	2341.9	1896.2	1469.1	1872.4	3727.6	4126.8
MAPK1	129.4	175.1	107.8	136	115.3	141.8	118.6	140.5
MAPK1	1283.8	1696.1	843.3	797.7	632.2	866.6	1277.4	1727.8
MAPK1	587.3	757.7	393.6	335.4	284.7	396.9	487.1	833.8
MAPK10	69.5	85.5	86.5	80.5	74.8	76.2	84.5	83.1
MAPK10	103.7	118.7	111.5	94.1	86.5	93	91.3	101.1
MAPK10	99.4	114.8	105.9	94.6	97.9	100.9	103.6	103.4
MAPK10	75.3	91.8	95.9	86.7	80.8	81.6	81.9	91.3
MAPK10	100.3	100.5	95	81.2	83.1	87.1	96.5	99.6
MAPK11	145.8	181.3	139.5	137.8	113.5	162.8	137.5	162.8
MAPK11	96.2	103.1	97.3	93.3	93.6	97.8	93.1	103.3
MAPK12	342.5	464.7	224.2	170	153.5	233	270.2	425.2
MAPK13	156.2	195.4	131.5	158.8	141.4	161.4	133.7	139.6
MAPK14	107.5	126.6	98.3	96.4	85.2	89.9	96.2	113.3
MAPK14	266.8	297.2	167.7	203.9	158	151.3	191.4	288.2
MAPK14	192.7	261.4	142.5	176.2	154.3	142.4	132.2	179.7
MAPK15	104.6	138.2	90.4	87.2	87.8	91.9	104.2	112
MAPK3	1200.9	1495.4	752.2	474.9	423	434.9	1096	1032.5
MAPK3	441.2	486.6	327.5	253.8	230.5	288.7	452.1	845.9
MAPK4	84	95.3	100	89.9	91.6	81.6	79.8	101.5
MAPK6	2051.2	2540.5	1499.2	1864.9	1447.4	2539.6	1753.1	3240.7
MAPK7	126.7	143.6	102.2	114.5	82.4	97.9	123.4	143.3
MAPK7	145.3	161.4	186.7	227.4	189	239.9	148.7	272.3
MAPK7	105.2	109	97.3	99.6	94.7	102.3	102	96.4
MAPK8	112.8	136.8	106.6	92.3	80.3	98.6	99.1	104.3
MAPK8	107.7	136	90.6	82.8	85.4	85.4	103	109.8
MAPK8IP1	81.2	94.7	85.9	88.9	85.2	80.8	78.4	84.1

MAPK8IP2	106.3	145	86.7	89.5	86.5	82.9	105.5	125.9
MAPK8IP2	79.3	79.8	88.4	87	88.2	79.5	76.8	86.7
MAPK8IP2	91.4	105.6	93.2	95.4	84.8	93.6	95.7	94.7
MAPK8IP2	96.8	100.8	97.1	94.4	94.4	82.8	94.4	95.2
MAPK8IP3	101	104.9	93.7	78.5	90.2	90.7	103.8	120.7
MAPK8IP3	192.7	202.1	259	239.3	272.4	370.4	171.8	380.3
MAPK9	1478.7	2370.8	1204.9	1114.1	885.5	1709.2	1340.4	2112.4
MAPKAP1	237.7	291.8	182.7	254.2	213.6	290.8	199.2	242.6
MAPKAP1	789.3	1057.4	478.5	406.1	306.4	425.8	624.2	807.3
MAPKAPK2	104.1	124.4	99.3	85.3	77.7	97.8	122	124.2
MAPKAPK2	215.3	299.2	153.6	124.9	120.2	151.6	225.7	414.2
MAPKAPK3	4723.7	6022.4	3336.9	3059.1	2373.1	2367.7	3927.3	3197
MAPKAPK5	1050.6	1269.3	342	288.7	281.8	451.3	936.3	1589.8
MAPKBP1	96.4	99.2	99.7	119.8	111.4	132.2	86.6	106
MAPRE1	4500.7	6726.9	2839.2	3341.6	2305.3	3633.6	4355.1	6249.5
MAPRE2	1007.4	1667.4	767.7	735.8	611.3	955.4	813.7	1232.5
MAPRE3	120.7	139.5	121	110.1	97.9	111.3	132.5	171.2
MAPT	98.4	126.5	109.9	86.5	76.7	82.9	104.9	119.4
MAPT	75.2	88.7	95.8	85.4	80.4	87	75.8	79.4
MAPT	74.9	92.6	100.5	100.7	92.8	106.7	81.3	85.2
Mar-01	87.2	98.9	95.4	101.2	84.8	103.9	88.3	88.6
Mar-02	245.4	276.4	192.6	165.3	157.8	214.9	209.7	302.8
Mar-02	125.1	151.3	135.8	138	120.9	128.1	123.9	164.2
Mar-02	181.4	190.9	97.8	102.7	94.3	111	150.3	189.9
Mar-02	109.3	111.5	100.9	84.3	85.1	94	99.9	108.1
Mar-03	197.3	278.1	144.4	144.9	145.3	134.2	153.8	173.6
Mar-03	471.1	622.2	360.9	525.7	459.6	610	344.2	472.3
Mar-03	482.9	658.1	356.4	480.4	433.2	589.8	345	421.4
Mar-04	93.6	98.7	92.1	80.1	82.3	81.3	84.7	88.5
Mar-05	262.8	377.9	119.8	129.7	113.1	134.4	330.3	511.8
Mar-06	250.7	303.6	268.9	598.1	586.8	414.3	242.4	299.2
Mar-07	3079.8	3087.5	2504.9	2933.2	2438.4	4420.3	2183.1	3618.2
Mar-08	88.7	93.6	85.6	85.9	80.4	83.4	82.2	88.9
Mar-08	92.5	111	94.1	78.9	85	83.7	106.2	106
Mar-08	79.9	83.2	77.4	79.4	75.1	81.1	75.8	76.8
Mar-09	106.9	110.6	113.2	99.1	95.7	103.1	97.1	93.9
MARCKS	1629.7	1788.8	812.4	2707.3	2232.7	3024.8	907.3	1188.2
MARCKSL1	702.6	973.3	611.8	770.5	448.7	742.5	569.5	1156.5
MARCO	111.1	132	115.2	102	108.2	118.4	119.6	133.9
MARK1	90.4	99.9	104.7	98.9	104.7	105.1	93.5	104.4
MARK2	99.4	116.3	96.4	102	102.1	91.7	102.2	118.6
MARK2	86.8	92.3	88.9	90	80.2	94.3	83.7	95.9
MARK2	142.3	165.9	131	134.5	117.4	130.5	120.8	197.6
MARK3	263.7	361.4	256.9	213.7	207	265	248.2	421
MARK4	119.5	142.4	99.6	88.6	74.6	86.2	118	150.4
MARS	401	521.1	195.5	188.6	177.5	278.2	398.4	703.9
MARS2	144.1	165.2	110.6	103.8	102.5	103.1	132.2	180.5
MARVELD2	81.5	93.2	87.5	82.5	80.2	75.6	92.7	98.9
MARVELD2	78.7	91.7	97.9	81.6	85	85.7	84.7	89.3
MARVELD2	75.5	94.6	85.1	79.5	78.7	74.4	78.5	77

MARVELD3	89.2	92.6	83.7	88	87	84.4	93.1	91.1
MARVELD3	101.7	112.1	95.8	95.2	83.8	95.2	104.2	111.8
MARVELD3	74.8	94.2	107.9	105	82	97.2	82.3	79
MAS1	90	99.8	102.3	92.3	92	87.4	84.6	93
MAS1L	104.8	120.5	100	97.4	93.8	88.1	103.8	119.7
MASA	3538	4358.4	1607.9	1562.5	1042.2	1750.1	2537.7	3351.7
MASK	1474.1	1884.5	545	653.7	526.8	775.6	1228.6	2165.5
MASK-BP3	137.3	166.9	136.6	120.3	126.2	157.7	131.1	164
MASP1	80.2	92.7	88.4	84.4	84.2	93.9	78.4	86.2
MASP1	91.4	117.8	93	89.9	79.6	83.6	101	105.6
MASP1	111.5	132.1	79.3	72.2	78.8	80.3	109.5	131.6
MASP1	86.6	98.4	99.8	96.2	97.1	95.7	83.8	91.6
MASP2	94.1	100.4	96	77.5	91.9	87.3	93	95.7
MASP2	79.4	84	89.3	82	89.1	82.8	74.7	75.4
MASP2	91.7	100.4	101.4	94.7	93.7	86.7	88.3	105.8
MASS1	92	113.6	82.6	85.6	75.2	91.4	94.3	93.9
MAST1	74.8	88.4	100.3	88.1	85.7	89.8	81.1	102.9
MAST2	131.3	165.3	100	99.5	89.5	99.3	120.2	161.4
MASTL	222.1	249.5	124.9	132.9	142.6	156.9	147.3	311
MAT1A	75.1	89.8	85.9	87.1	72.3	73.7	80.3	87.6
MAT2A	6136.3	7305.8	6285.8	4516.4	3952.9	5382.4	3878.6	5354.2
MAT2B	3280.9	3927.4	1690.9	2073.7	1515.6	2403.7	3232.8	5390.9
MAT2B	277.8	340.1	131.7	110.3	115.7	160.9	217.6	250.6
MAT2B	452.3	536.9	182.9	152.7	155.2	182.8	291.6	310.6
MATK	122.7	133.7	94.2	97.5	92	88.4	129.2	136.3
MATK	2382.3	2919	1185.7	556.7	406.4	437.5	1481.7	982.6
MATK	203.2	293.9	142.8	101.7	92.8	89.9	153.6	180
MATN1	107.7	118.3	92.9	83.4	84.9	93.6	106.1	108.3
MATN2	94.8	115.2	97.5	93.5	87.6	91.1	91.1	105.1
MATN2	293.2	351.6	229.5	261.7	208.3	324.6	235.7	360.9
MATN3	92	103	98.9	92.5	80.8	97.7	90.3	105.5
MATN4	66.3	80.8	90.7	77.2	78.3	80.5	75.8	71.5
MATN4	84	87.6	86.3	82.5	87.8	85.3	84.9	85.4
MATN4	79.1	96.6	73.6	74.1	80.6	81	81.7	84.9
MATR3	338.4	426	170.6	231.2	183.7	136.1	212.8	252.5
MATR3	265.8	307.2	99.6	103.1	100.3	95	160.1	220.7
MATR3	6388	7029	1959.6	2188.2	1593.4	3201.8	5654	7743.4
MATR3	1326.6	1915.3	477.8	414.3	328.8	424.9	785.6	1277.9
MAWBP	85.3	107.7	79.2	81.2	82.4	86.6	88.2	110.5
MAWBP	102.6	117.6	98.3	81.9	79.5	86.2	100.2	108.2
MAWBP	123.3	135.1	114.5	101.2	100.5	93	100.6	109.7
MAX	252	335.2	193.1	173.8	156.4	172.3	245.1	251
MAX	338.4	443.5	136.9	102.5	97.1	108.6	310	442.3
MAX	270.3	363.1	173.8	135	123.4	121.8	253.1	284
MAX	102.7	108.9	107.3	82.2	78.1	93.2	98.1	97.2
MAX	76.7	91.9	92	88.8	84.1	84.7	76.3	84.4
MAZ	116.7	130.9	126.9	102.6	97.4	100.5	106.7	131.3
MAZ	107.7	115.6	122.9	101.3	99.3	104.6	105.1	116.7
MAZ	254.6	294.7	198	271.8	233.2	443.8	176.8	355.7
MB	101.9	126.4	103.7	97.8	90.1	96	96.3	120.2

MB	91.2	92.5	89.9	83	85.6	81.7	73.5	90.1
MB	99.5	114.7	91.8	96.1	89.4	100.8	107.8	115.3
MB	85.1	92.6	90.2	80.8	82.4	80.7	83.1	80.5
MBD1	268.5	352.7	235	205.7	187.1	294.2	252.4	336.4
MBD1	293.6	334.9	259.2	248.1	255.6	355.5	266.2	366.6
MBD2	83.3	92.3	97	81.2	86.8	85.2	83.4	93.3
MBD2	95	99.4	88.6	88	77	88.5	96.3	90.2
MBD2	88.6	99.5	84.5	87.8	85.6	78.6	96.8	92.5
MBD3	282.4	358.7	265.4	166.4	182.6	253.4	267.7	600.9
MBD3L1	83.5	89.1	84.7	74.5	76.4	74.9	85.7	98.8
MBD3L2	96.9	115.3	88.4	87.8	81.5	83.8	94.3	102
MBD4	1224.6	1322.6	1111.8	1857.5	1681.3	1494.8	1206.4	1990.5
MBD5	79.4	101.7	96.8	84.2	87.9	99.5	83.6	90.9
MBD6	644.7	577.3	605.9	625.7	718.1	693.7	522.9	548.2
MBIP	360.8	449.6	217	248.8	189.6	335.5	320.5	634.7
MBL2	82.7	89	101.6	88.7	86.6	80.2	78.2	91.9
MBNL1	1448.5	1561.6	535.1	808.6	523.1	939	1099.4	2532.9
MBNL1	4158.7	4428.3	1533.8	2497.6	1237.8	1655.7	3027.4	4482.4
MBNL1	119.1	137.7	94.3	85.4	90.1	91.4	92.4	104.9
MBNL2	87.6	88.5	94.9	93.3	86.1	88.5	80.5	80.4
MBNL2	82.4	86.7	96.3	81.6	87.8	91.6	82.7	89.1
MBNL2	80.9	97.5	90.4	89.6	94.8	91.9	87.9	90.3
MBNL3	112.3	132.6	93.4	102	87.3	88.9	110.6	133.9
MBNL3	92.8	111.7	83.4	85.3	78.4	82.4	87.3	87.2
MBOAT1	121.1	143.6	89.4	77.9	77.3	87.5	121.7	130.6
MBOAT2	149.1	162.5	127.4	130.7	117.8	128.2	146.7	219.4
MBP	521.4	697.6	307.8	506.4	460.4	329.8	506.7	588.6
MBP	2350.9	3406.5	1959.3	3741	2946.4	4537.5	2250.6	2416.3
MBP	405.4	517	278.8	430.1	372.5	264.7	386.2	438.8
MBTD1	149.1	160.8	114	155.9	127.4	113.6	135.3	153
MBTPS1	621.3	846.6	826.9	939.9	806.2	1495.8	531.1	1070.1
MBTPS1	179.8	206.7	156.3	138.7	114.3	201.6	136.2	182.1
MBTPS2	225.8	277	158.3	152.6	139.2	188.2	164.5	217.6
MC1R	313.3	430	196.1	202.4	173.5	170.1	275.2	208.2
MC2R	75.1	87.8	90	86.6	76.6	85.4	79.6	79.5
MC3R	80.8	91.9	89.8	74.7	82.3	75.9	89.1	84.9
MC4R	98.2	104.1	85.1	81.2	80.2	89.2	100.7	96.9
MC5R	102.1	111.1	89.2	86.3	79.5	80.6	96	98.6
MCAM	101.1	118.8	100.3	84.2	76.7	86.9	104.4	110.8
MCART1	206	257.8	115.7	115.8	99.7	108.9	207.1	272.6
MCART2	80.2	88.3	89.9	84.8	74.9	89.5	82	85.6
MCART6	86.5	82.6	101	103.8	94.4	90.8	86.7	86.4
MCC	119.1	133.3	98.2	85.3	86.6	90.6	113.6	117.8
MCCC1	1076.9	1426.8	730.3	857.7	740.3	1295.7	844.7	1340.9
MCCC2	321.5	426.7	231.1	116.9	129.7	137.3	266.1	353.9
MCCD1	127.8	168.5	108.9	108.1	104.1	111.9	134.9	139.5
MCEE	852.9	1108.6	650.6	860.6	695	852.4	735.1	826
MCEMP1	4837	6198.8	4775.3	2579.9	1814.8	3390.3	4218	4938.1
MCF2	109.2	112.6	93.5	85.1	80.9	85.3	111.2	125.5
MCF2L	86	82.7	105	96.5	84.7	91.5	87.1	85

MCF2L2	94.8	115.1	90.5	81.4	90.4	86.7	95.4	90.5
MCFD2	129.6	157.3	118	131.4	111.4	139.8	146.2	184.8
MCHR2	94	105.6	94.7	93.1	80.3	97.6	95.9	104.1
MCHR2	113.2	162.8	108.2	110.4	94.1	107.5	141.3	141.4
MCL1	570.3	756.8	235.7	254.9	216.8	281.3	511.3	998.5
MCL1	556.7	747.7	225.5	198.7	182.2	180.7	428.4	642
MCM10	364	456.3	301.9	267.3	205	286.3	258.5	402.6
MCM10	1671.4	1841.6	1476.6	960.6	724.8	1068.2	1165.1	1826.5
MCM2	1583.6	2108.6	2807.8	1366.1	909.7	1726.4	1092.1	2127.1
MCM3	14251.1	18500.1	14692	15942.6	11129.4	20027.1	9330	12200
MCM3AP	775.9	956.1	501.5	584.5	529.2	593.8	697.7	761.9
MCM3APA	172.3	217.9	173.5	247.2	208.3	267.6	145.4	183
MCM4	2126.3	2712.9	1172.7	873.5	546.8	789.4	1548.2	2497.8
MCM4	6369.2	8166.9	5604.3	3314.4	2981.3	3193.6	4069.5	5033.3
MCM5	2062.7	3221.3	2231.5	980.2	818	1195.4	2423.5	4198
MCM6	9585.7	12544	10192.6	8252	6887.9	9409.1	6528.5	8516.8
MCM7	1622.2	2219.5	707.2	450	511.1	177.3	912.4	1019.1
MCM7	12610.2	15011.7	6369.2	5396	4549.3	5837.2	8903	8903.1
MCM7	280.1	306.3	160.2	131.3	136.8	109.6	172.5	183.1
MCM8	210	271.7	147.7	127.1	135.1	147.3	160.1	251
MCM8	79.7	93.1	93.6	97.2	76.8	80.9	88.4	90.3
MCM8	2820	3317.6	902.6	1491.6	1440.2	932.9	2369.1	2716.7
MCMDC1	150.6	171.5	109.3	137.4	120.3	127.1	125.3	166.3
MCOLN1	234.8	274.4	242	193.1	170.4	215.7	229.3	408.1
MCOLN2	2687.1	3106.7	2296.4	1808.9	1713.6	2114.6	2272.8	3681.1
MCOLN3	142.7	135.6	148.3	214.8	193.8	267.1	122	166.6
MCPH1	230.7	334.9	194.6	149.1	143.3	152	213.1	254.2
MCRS1	1462.5	2197.2	1290.5	916.4	777.2	934.4	1292.3	1733.3
MCRS1	348.5	463.7	231.9	155.4	144.6	98.6	282.7	345.5
MCTP1	81.5	104	77.2	74.2	75.1	82.3	85.4	94.6
MCTP1	312.3	391.9	236.5	246.6	195.6	257.3	242.5	274.5
MCTP1	80.8	90.6	92.8	88.5	85.5	76.6	81.5	96.8
MCTP2	75.2	90.9	87.4	76.7	72.9	78.4	83.7	81.5
MCTS1	812.5	987.9	292.9	300.6	196	305.1	697.6	1049.4
MDAC1	81.7	93.6	84.3	84.7	75.2	82	77.9	87.4
MDC1	578.2	721.3	254.2	291.3	287.5	380.3	493	806.5
MDFI	141.8	167.5	88.5	84.4	87.1	91.3	116.5	173.5
MDFIC	396.4	510.7	195.7	214	156.1	296.3	264.3	383.1
MDGA1	151.5	178.7	113.6	96.9	102.5	107.6	153.3	174.1
MDH1	12876.3	16466.4	9841.5	10421.7	8545.1	13301.5	10796.4	15368.2
MDH1B	99.5	109.2	93.8	78.1	81.8	86.7	101	106.6
MDH2	18216.5	25839.1	15625.8	15110.7	11640.4	13961.2	18449.4	22268
MDK	91.7	115.9	109.7	87.3	95.3	90.6	90.2	104.9
MDK	87.9	107.2	91.6	89.3	90.4	82.6	86.9	102.2
MDK	246.3	207.4	268.6	214.9	197.4	276.5	164	247.5
MDK	98.5	130.3	84.6	81.1	89.4	78.1	101.8	129.4
MDK	85.9	102.8	90.9	80.3	76.2	77.6	83.4	90.2
MDM1	93.2	106.1	95.1	96.7	81.5	88.8	88.7	99.1
MDM1	144.9	187.3	112.8	95.6	92.5	103.2	128	171
MDM1	181.6	253.2	140.8	120.2	109.5	113.4	140.5	166.1

MDM2	163.2	222.1	130.4	140.2	117.4	160.6	144.2	162.6
MDM2	88	99.5	105.1	97.2	88.6	99.9	89.6	95.3
MDM4	219.6	281.1	121.2	127.7	150.6	115.9	173.7	243
MDN1	130	187.4	122.2	132.8	127.9	129.6	125.6	123.7
MDP-1	403.2	457.3	187	147.9	124.7	155.7	340.3	394.5
MDS028	243.8	336.7	227.7	266.5	222.8	352	246.7	320.9
MDS032	297.3	340.4	156.9	138.3	126.7	143.7	264.7	275
MDS1	120	131	90.5	79.2	78.4	84.7	109.1	118.4
ME1	642.6	759.5	269.6	235.2	193.8	309.7	1495.9	3875.6
ME2	1661.9	2037.3	648.4	667.1	524.4	1003.7	1389.2	1930.6
ME3	118.7	153.3	108.4	104.1	97.3	110	113.8	124.9
ME3	256.6	257.5	180.2	181.8	183.6	286.8	173.2	234.5
ME3	78.2	92.4	86.2	84.4	79.5	93.2	71.4	81.3
MEA1	790.2	1004.7	491.7	427.2	331.7	549.6	676.3	881.6
MECP2	92.7	125.3	102	101.6	107.9	127.9	93.4	112.7
MECR	142.2	177.1	105.9	106.4	96.3	85.8	127.7	130.1
MECR	171.3	206	166.3	139.8	127.4	166.4	182.3	305.7
MED12	131.6	162.6	169.4	289.4	245.7	386.3	131.5	147.8
MED12L	91	82	89.5	82.9	76.1	77.4	89.4	101.2
MED18	123.5	133	89.9	87	89.4	80.6	113.6	138.5
MED19	617.1	752.3	518.4	422.5	410.2	474.4	581.5	552
MED25	379.3	431	382.4	237.5	216	222	378.1	599.6
MED28	1927.5	2762.3	1315.3	794.6	611.7	772.9	1740.8	2528.5
MED31	100.7	128.8	88.9	86.7	77.1	78	102.1	129.4
MED4	967.3	1385.9	786.1	819.3	666	1066.2	865	1325.9
MED6	3346.3	4178.9	2116.8	1878.8	1359.5	1683.7	3644.2	3981.1
MED8	873.2	1024.2	462.2	405.4	409.8	397.7	780	874.7
MED8	1160.1	1568.1	445.3	352.6	314	393.3	1100.2	1334
MED8	515.6	879.4	430.7	321.9	266.5	339.4	488.2	686
MED9	191.9	222.6	122.8	111.3	96.3	116.4	180.4	225.2
MEF2A	154.2	194.9	108	123.9	110.2	129.1	134.6	163.3
MEF2B	101.5	98.9	92.5	98	113.6	97.3	93.1	99.7
MEF2C	845.5	1246.4	481.8	682	518.5	493.3	690.7	775.3
MEF2D	2418.2	2855.3	1869.5	1804.9	1665.5	1375.3	1926	1648.1
MEFV	123.9	165.7	109.9	110.7	119.9	139	124	140.7
MEG3	99.4	113.5	87.8	97.2	82.9	87.6	95.8	110.1
MEG3	99.8	111.1	94.2	87.4	86.5	79.7	96.5	104.1
MEGF10	77.9	92.5	89.1	85.4	82.4	88.7	77.9	91.7
MEGF11	97.8	100.2	89.5	80.1	75.6	83.1	91.3	98.6
MEGF8	137.3	168.1	132.6	130.7	103.7	98	141.3	174.3
MEGF9	147.6	168.2	95.2	86.3	74.4	95.7	123.4	194.1
MEI1	141.4	150.5	122	140.3	115.1	130.8	145.5	157.2
MEIG1	74.4	85.4	77.9	66.3	65.5	69.7	73.7	84.9
MEIS2	447.6	633.5	404.7	281.1	242.6	366.7	318.5	482.9
MEIS2	104.3	127.2	82.2	88	80.4	88.3	87	103.6
MEIS2	97.3	114.7	96.2	88	74.5	86.7	93	114.2
MEIS2	121.6	137.6	82.9	83.1	88.7	100.5	122.9	147.3
MEIS3	125.6	128.7	98.6	83.2	79.7	84.2	116.8	132.8
MEIS3P1	558.6	628	270.7	229.5	217.7	290	501.7	778.6
MELK	1306.9	1602.6	1170.2	846.9	810.1	1241.4	1237.5	1716

MEN1	118.8	127.4	123.5	115.5	99.4	125.2	113	150.4
MEN1	112.3	124	96.2	96.2	92.3	87.7	113.8	123.1
MEN1	121.9	136.8	102	88.7	80.9	94.5	118.6	130.8
MEN1	227.4	267	266	201.2	178.7	258.4	184.5	301.9
MEOX1	105.5	130.3	99.6	87.7	86.1	89.7	118.1	117.3
MEOX1	83.4	91.3	85.5	81.9	83	82.1	90.5	79.4
MEOX1	92.7	115.3	100.8	91.4	89.2	88.5	91.3	110.2
MEOX2	82.5	78.8	78.8	75	82.6	86.9	76.6	84.1
MEP1A	96.8	114.4	103.9	110.1	98	105.7	95.2	96.6
MEP1B	148.6	190.8	89.7	94.7	94.3	92.8	164.6	196.1
MEPE	98.6	121.2	94.9	85.6	81	84.7	97.7	101.8
MERTK	110.4	126.3	116	211.8	196.3	302.1	117.5	120.2
MESDC1	101.7	116.2	120.9	114.4	95.6	145	101.6	172
MESDC2	97.8	111.1	99.1	93.9	79	84.3	97.8	95.9
MESP1	257.3	322.4	143.9	140.2	125.8	125.9	225.6	232.1
MEST	96.8	119	95.1	81.6	82.7	92.4	94	116.2
MEST	356	436.4	155.1	120.4	111.8	116.4	201.3	278
MEST	74.9	82.7	101.8	94.8	88.8	84	82.4	85.8
MET	101	123.5	92.3	85.1	79.2	75.1	97.5	119.5
METAP1	2663.9	3444.3	2282	1690.1	1081.9	2160.8	2204.2	4012.4
METAP2	4089.5	4598.9	1797	2074.5	1532.6	2790.8	3113.9	5901.4
METRN	169.5	204.1	157.3	136.4	100.8	130	155.7	211.3
METRNL	168.5	211.6	147.1	125.9	115.3	116.1	205.5	295
METRNL	102.5	102.2	91.9	84.6	82.6	95.1	115.9	120.4
METRNL	129.5	144.3	109.6	104.4	92.4	93.5	134.1	199.6
METT10D	269.5	336.9	147.1	173.7	172.9	181.6	275.8	238
METT11D1	100.8	111.4	102.2	93.9	94.6	94.6	95.8	101.6
METT11D1	284.4	302.2	138.2	127.3	106.7	124.5	194.3	351.7
METT5D1	220.8	243	115.4	103.9	96.4	117.8	192.9	216.5
METTL1	490.8	623.1	254	228.6	159.3	200.7	413	408
METTL1	102.1	117.1	93.4	81.2	78.4	85.6	93.8	122.1
METTL2A	282.5	291.3	166.3	199.6	142.6	134.5	251.9	429.9
METTL2A	115.3	138.5	118.8	105.1	103.7	109	103.3	124.3
METTL2B	133.5	154.8	107.2	99.1	106.8	103.5	126.7	142.3
METTL3	1078.5	1324.7	738.8	607.3	490.3	998.1	696.4	1287.1
METTL4	210.8	204.9	196.1	164	145.5	196.9	173.1	264
METTL5	3593	4048.3	2084.7	1871.3	1218.6	1951.9	3158.3	6129.4
METTL6	293.4	366.4	115.1	115.5	96.6	125.6	243.5	361.5
METTL7A	135.1	151.9	126.6	106	106.5	124.7	122.7	138.4
METTL7B	824.7	1062.3	827.4	761.4	663.3	1184.7	575.6	826.9
METTL9	158.1	177	114	109.8	100	121.7	156.5	219.2
MFAP1	868.8	1108.4	319.8	361	288.6	432.5	725.1	1181
MFAP2	97.9	105.3	83.7	80.6	79.7	81.9	103.3	109.1
MFAP2	106.1	128.6	83.4	89.7	84.5	81	92.2	112.3
MFAP2	86.6	88.1	92.1	85.7	81.2	92.9	86.8	88.4
MFAP3	276.3	357	215.4	143.1	143.6	153.4	220.8	306.6
MFAP3L	101.7	106.6	84.7	84.6	87.9	80.8	99.9	113.3
MFAP3L	102	122.9	95.2	79.9	77.2	81	118	120.3
MFAP3L	74.3	80.3	95.7	85.4	83.7	82.9	86.2	80.8
MFAP4	136.8	151.6	115.7	205.8	205.1	194.8	152.8	145.6

MFAP5	95.9	108.8	102.8	102.5	108.3	106.5	105	103.8
MFGE8	595.1	748.1	366.6	471.4	439.9	418.2	517.3	446.3
MFHAS1	146.7	244	161.9	160.9	164.9	223.1	143.1	192.6
MFI2	108.4	126.2	99.3	107.4	99.8	100.8	111.4	98
MFI2	160.6	200.6	137.2	129.1	131.5	132.6	132.3	141.6
MFI2	104.9	124.3	106.9	91.9	83.3	86	109.9	118.8
MFN1	73.6	101.6	94.8	84.2	78	91.5	77.9	89.9
MFN1	123.5	171.8	123.2	109.7	107.3	110.8	117.9	145.4
MFN2	317.2	455.7	312.9	304.2	266.3	455.8	279.3	481.2
MFNG	1770.6	2217	940.3	367.7	259.6	370.5	1308.2	1588.4
MFRP	71.6	85.3	92.7	88.9	82.8	76	76.3	81.3
MFSD1	6073.3	6575.1	2927.2	4456.4	3073.9	5342.7	6267.7	9376.2
MFSD2	400.6	547.9	277.4	224	199.6	287.7	311.8	455.1
MFSD3	2240.1	3415.3	1604.5	701.3	543.4	658.1	1752	1197.7
MFSD4	80.5	104.7	85.5	82	78.6	87.5	91.6	100.1
MFSD5	681.7	821.5	476	420.4	294.8	378.2	674	899.7
MFSD7	124	131	104.9	96.1	90.2	101.5	105.8	114.9
MFSD8	287.7	362.6	282.3	268.6	235.8	288.1	262.2	410.8
MGA	288.4	408.5	170.6	201.9	175.8	282.6	334.5	471.1
MGAM	110.7	144.5	90.3	100.3	83.8	102.4	96.8	128.1
MGAM	105.8	120.5	75.6	79.9	70.9	88	103.4	123.1
MGAT1	922.6	1151.2	828.8	975.6	872.9	1527.7	945.4	2076.5
MGAT2	2753.3	3112.2	1186.8	1103.1	926.4	1146.6	2295.8	3576.5
MGAT2	162.7	192.5	97.4	99.5	81.9	86.7	156.7	196.2
MGAT3	83.7	84.4	94.1	84.7	79.4	81.7	70.6	84.9
MGAT4A	386.6	474.7	262	356.6	266.1	372.7	343.9	553.5
MGAT4B	143.8	204.7	161	145.8	142	107.7	144.2	199.2
MGAT4B	84	104.2	86.8	80.3	83.5	79.1	85.4	95.4
MGAT4B	288.8	420.1	300.7	213.3	214.2	302.4	220.9	462.8
MGAT4C	84.7	88.4	90.9	87.6	83.4	83.2	82.4	83.1
MGAT5	99	122	102.6	102.5	98.2	103	119.3	121.1
MGAT5B	142.5	173.7	109.4	100.7	99.9	98.4	138.4	165.7
MGAT5B	106.3	113.7	84.8	83.8	77.6	83.7	104.3	111
MGC10334	154.6	190.7	163.6	147.6	118.1	168	143.5	215.7
MGC10433	1073.2	1364.2	1013	704.9	486	964.3	1034.2	2322.4
MGC10471	2507.7	2513.2	1915	1257.7	1379.8	1626.8	1976.2	2274
MGC10911	406.6	553.9	252.6	222.7	212.2	279.9	317.1	376.2
MGC10992	418.3	486.9	302.3	153.9	177.5	159.1	303.8	321.1
MGC10993	925.9	1293	781.8	396.5	315.8	436.3	743.8	1051.4
MGC10997	2316.1	3353.3	1568.2	1730.7	1502.4	1843.1	2120.6	2365
MGC11102	146.7	152.4	100.1	86.7	89.3	99.3	130.1	176.8
MGC11257	4376.6	5505.1	2919.6	2597.8	2178	2628.1	4015.2	4459.7
MGC11271	91.5	100.8	99.5	86.4	87.9	90.1	95	104.8
MGC11332	101.6	102.5	101.5	87.6	87.8	89.2	101.9	129.2
MGC11335	329.4	429.1	188.9	130.3	134.8	138.3	329.1	274.4
MGC11929	102.3	138.1	97	77.3	91.1	82.8	102.9	130.6
MGC12965	106.6	95.1	95.6	90.7	88.7	99	100.4	125.7
MGC12966	98.6	99.3	89.7	88.9	91	91.8	96.1	121.5
MGC12966	1286.3	1675.8	980.6	972.3	774	1301.6	962.3	1691.8
MGC12981	793.3	1145.1	569.1	510.2	354.7	659.4	702.9	1130.8

MGC13017	9431.8	9086	5601	7321.8	7626.3	4870.3	8292.7	13508.7
MGC13024	100.8	123.9	113.4	114.8	120.9	124.1	99.1	134.3
MGC13034	138.4	156.2	107.1	85.7	77.1	82.9	123.4	149.2
MGC13057	86.8	84.7	86.2	92.9	79.2	87.2	84.2	87.8
MGC13057	90.3	109.7	100.3	99.8	103.6	99.9	96.8	102.9
MGC13096	445.5	606	359.4	208.9	169.8	230.1	289.9	347.8
MGC13114	1150.4	1397.3	868	498.9	368.1	525.5	1005.2	1647.7
MGC13114	237	312.5	140.8	112.5	112.3	103.3	233.8	229.8
MGC13125	377.9	521.7	236	180.9	181.3	204.4	311	422
MGC13138	125	137	104.1	95.8	91.9	104.3	115.3	152.8
MGC13170	135.7	192.3	135.4	98.1	94.6	95.9	141.1	142.7
MGC13170	2917.7	3794.8	1668.9	1059	911.8	1096.1	2567.9	3529.7
MGC13379	1226.5	1644.2	700.3	471.2	377.3	494.5	1127.2	1164.2
MGC14141	1006.3	1338.5	403.8	369	324.3	375.9	854.1	767.8
MGC14327	2774.9	3446.9	2241.5	1864.9	1392.1	2293.9	1860.2	2709.3
MGC14376	97.7	94.9	101.9	108.3	98.4	102.8	101.5	164.4
MGC15416	747.8	1042.9	380.4	241.2	181.5	329.8	551.4	687.5
MGC15476	140.4	158.8	135.2	144.1	144.4	137.7	124	156.7
MGC15523	188.1	204.2	222.2	209.8	209.4	352	151.4	189.8
MGC15523	92.9	99.7	95.6	93.3	91.9	83	96.9	113.9
MGC15523	573.8	757.8	539.3	415.2	358.3	517.8	521.3	859
MGC15619	220.8	318.3	161	149.3	161.9	185	190.4	218.6
MGC15619	303.3	465.1	204.8	232.6	222.5	247.2	266.9	315.8
MGC15763	312.4	492.9	214.6	198.9	179	282.7	221.9	308.3
MGC15875	232.2	292.3	236.8	284	275.5	380	196.4	264.9
MGC15875	139.7	165.1	106.1	97.3	99.1	108.6	116.4	156
MGC15912	387.9	516.3	141.3	251.7	226	218.9	326.9	394.8
MGC16028	291.8	337.2	184.4	191	167.6	223.4	244.4	303.6
MGC16169	325.2	404.6	203.1	238.6	193.5	366.6	269.1	338.6
MGC16186	108	123.2	105.3	104	103	95.2	105.2	101
MGC16291	84.7	86.2	84.6	92.5	79.5	82.7	80	82
MGC16372	80.2	95.1	90.9	85.5	77.2	78.5	78.6	85.8
MGC16703	18933.2	19867.6	9667.6	12755.3	12637.7	10637.7	17910.6	20914.8
MGC16824	139.3	186.4	105.3	117.1	109.5	130.9	131.6	228.8
MGC16943	132.1	156.3	100	84.8	85.2	97	139.7	156
MGC17330	123.9	158.6	127.4	107.1	118.1	114.3	144.9	146.6
MGC17624	170.4	178	115.1	127.7	126.3	149.9	135.1	123.1
MGC17839	321.1	393.1	236	131.1	103.4	126.9	282.2	368.9
MGC18079	224.8	390.8	150.5	132.4	122.2	146.4	226.5	382.9
MGC18216	101.4	128	86	81.4	76.4	83.4	101.5	106.8
MGC19604	314.8	350.9	256.2	178.2	164.3	179.9	266.8	349.8
MGC19764	88.7	101.4	101.7	84.6	88.6	97	89.7	108.5
MGC20255	352.1	463.1	342.6	286.9	216.7	348.8	343.5	876.2
MGC20470	84.2	100.4	91.6	81.9	79.7	86.9	84	82.1
MGC20983	132.6	162.9	136.9	124.4	107.2	108.5	128.7	139
MGC21644	81.8	96.6	93.1	87.6	84.3	88.8	93.6	92.7
MGC21675	126.8	124.7	104.2	97.3	89.3	85.1	115.7	126.9
MGC21830	171	180.1	148	142.1	149.3	175.9	149.1	186.8
MGC22001	92.8	97.6	94.4	93.6	95.4	95.5	96.2	101.4
MGC22793	1125.5	1367.6	724.4	721.4	522	813.4	920.3	1257.4

MGC23280	169.6	221.8	103.1	102.8	85.9	100	148.6	173.3
MGC23909	828.6	1056.5	306.2	323.3	197.3	351	785.2	1502.8
MGC23985	86	87.3	101.9	93.3	85.5	82	87	98.8
MGC24039	125.7	137.1	110.8	109.1	114	115.7	109	191.2
MGC2408	603.5	702.5	324.2	272.4	186.4	233.2	526.9	706.9
MGC24381	224.7	232.3	133.8	116.2	105.9	140.9	207.1	323.7
MGC2463	77.4	78	101.2	93	83.2	88.1	73.6	72.5
MGC24665	1148.7	1709.1	659	458	442.2	540.9	807	994.2
MGC2474	121.5	151.6	110.8	92.8	96.7	101.1	126.5	144.8
MGC24975	130.6	146.3	115.4	111.1	107.6	121	117.5	141.7
MGC2654	572.9	713.4	400.4	366.4	295.5	472.9	520.1	806.1
MGC26647	89	103.6	92.2	88.7	86.2	83.9	93.6	107.8
MGC26718	84	91.5	106.5	106.3	87.2	97.2	79.1	100.1
MGC26733	98.2	119.5	112.5	107.5	98.1	98.6	98.6	101.7
MGC26816	74.1	81.1	88.7	76.7	82.3	85.8	79	81.6
MGC26856	103.6	103.8	108	100.6	93.4	92.8	96.5	104.6
MGC26885	407.4	526.8	184.8	132.1	134.8	161	415.9	534.9
MGC26963	89.7	94.7	91.2	86.2	80.8	84.3	92	90.6
MGC27016	69.4	75.4	87.5	72.1	83.1	71.1	72.8	73.3
MGC27121	81.9	89.2	111.9	102.8	102.9	109.5	91.4	91.2
MGC2747	980.1	1386.9	502.3	338.9	299.8	348.8	860.8	1229.6
MGC2749	1027.8	1062.9	512.8	277.1	229.2	283.9	957.9	1636.1
MGC2752	166.3	221.3	140	106	107.4	119.2	147.3	226.9
MGC2803	2118.8	3084.9	433.7	341.4	430.9	530.8	2031.9	3089.6
MGC29506	98.3	100.2	89	98.4	85.6	89.6	93.8	95.6
MGC29671	304.3	314.5	208.8	151.5	161.1	217.4	248.7	356.5
MGC29891	10832.5	11365.3	3631.3	4786.2	5153.6	3960.4	9747.8	9834.4
MGC3101	84.4	104	97.2	90.3	83.3	84.2	97.1	111
MGC3121	2165.7	2475.3	1047.2	621.5	585.1	669.9	1736.2	1477.2
MGC3123	161.9	188.3	107.3	105.3	96	102.6	134.3	165.3
MGC3196	392.7	495.1	304.4	246.2	197.3	268.9	338.6	601.6
MGC3196	207.6	271.6	174.7	150.3	128.2	155.8	191.4	324.1
MGC31967	187.2	238.2	141.8	109.4	100.4	122.3	184.4	226.2
MGC3207	899.3	1097	876.9	733.1	620.1	828.1	713.5	1341.5
MGC3207	125.6	130.2	86.6	96.6	81.3	79.3	111.2	142.6
MGC3207	295.5	329	229.6	174.3	143.4	157.6	215.6	282.8
MGC3207	2040.2	2295	1237.6	1010.4	746	1070.3	1472.7	1897.7
MGC3265	209.6	236.3	117.3	109.5	89.5	120.2	186	291.5
MGC33212	359.2	457	305.8	213	195.5	226.7	382.2	483.6
MGC33212	1374.3	1581.8	1168.2	943.9	728.3	873.4	1371	1938.6
MGC33214	158.3	180	123	93	85.4	109.3	134.6	169.8
MGC33407	99.7	100.4	89.2	91.9	83.1	85.1	102.1	119.3
MGC33486	86.8	102.3	94.2	78.2	83.7	80.1	94.4	110.2
MGC33530	84.7	88.3	103.1	102	95.5	93.9	97.8	92.2
MGC33556	1978	2264.4	1251.5	413.7	396.5	677.1	1376.2	2527.1
MGC33584	173.6	210.9	139.1	151.4	136.2	215.9	180.8	260.7
MGC33600	85.7	96.2	78.7	90.1	83.9	76.2	77.9	93.1
MGC33648	331.7	419.5	254.9	292.4	210.8	362.3	279.3	444.4
MGC33657	94.3	121.1	99.5	95	96	92.3	101.5	91.7
MGC33692	688.9	959.6	495.6	614.5	491.1	484.4	642.2	803

MGC33846	134.2	154.7	123.9	117	100.8	105.7	149.3	139.9
MGC33887	130.4	169.3	113.1	103	93.7	107.5	125.1	157.8
MGC33894	73.7	82.5	86.8	87.3	80.2	72.4	76.6	89.7
MGC34646	87.6	89	97.6	80	87.2	80.6	82.5	88
MGC34647	77.7	80.1	101.8	93.7	88.1	93.9	81.1	83.4
MGC34725	95.4	101.1	81.4	95.3	80.7	83.8	85.4	103.3
MGC34732	80.7	90.4	86.6	78.4	78.4	83.8	81.8	85.5
MGC34761	155.7	162.1	95.5	88.8	86.8	86.5	138.2	161.9
MGC34774	81.2	99.4	95.7	88.4	81.2	89.2	88.8	95.8
MGC34821	88.2	98.5	97.5	90.6	90.5	97.2	88.6	89.5
MGC34824	113.6	132.9	92.7	87.1	90.9	94.5	124.5	119.5
MGC34830	81.3	87.9	102.7	87.6	93.5	96.1	79.3	82
MGC35043	88	109.8	95.3	80.8	87	94	96.7	97.7
MGC35154	82	92.4	106.2	94	93.9	88.7	87.7	88.2
MGC35206	167.9	181.4	92.5	96.8	83.1	96.9	146	188.3
MGC35212	3523.4	4240.6	2472.3	1594.6	1189	2207.1	2211	2760
MGC35295	94.7	105.1	96.5	88.7	86.7	85.2	95.9	104
MGC35308	120.6	155.2	115.9	100.1	100.5	100.9	113.7	124.5
MGC35361	172.6	245.1	138.8	135.9	121.9	165.4	136.7	177
MGC35402	88.9	90.4	99.4	102.6	103.5	91.9	83	88.3
MGC35440	132.4	167.1	97.5	90.8	84.9	90.7	127.8	157
MGC39497	90.2	118.1	92	91.7	85.5	96.5	88.7	114.7
MGC39581	110.1	116.7	96	91.5	86.2	92.1	100.8	111.5
MGC39606	94.5	95.9	89.7	85.4	71.1	89.2	89.7	95.8
MGC39633	325.4	389.5	186.3	244.4	226.8	509.1	233.5	327.6
MGC39662	81.2	82.5	92.4	95.1	86.8	78.6	81.6	85.1
MGC39681	110	120.8	98.8	96.1	84.1	91.1	124.6	112.3
MGC39715	102.1	101.4	101.2	97.5	87.7	94.3	100.5	104.7
MGC39900	975.4	1127.9	497.3	347.4	291.8	350.4	698	732.3
MGC39900	119.7	137.5	104.6	100	89.3	101.1	108.8	142.1
MGC39900	900.5	1146.7	509.8	360.4	293.8	424.3	740.5	796.4
MGC40069	99.2	95	97.4	87.5	86.5	95.4	86.4	95.6
MGC40222	87.5	121.4	86.6	96.6	84.3	90.4	94.9	100.9
MGC40405	1079.5	1080.2	288.7	354.5	368.5	469.3	969.1	1384
MGC40405	579.4	727.2	376	367	351.8	517.3	455.4	736.7
MGC40499	135.7	175.1	105.7	99.3	90.3	93.4	123.8	134.4
MGC40499	318.7	364	146.7	125.5	116.8	141.9	235.1	363
MGC40579	232.6	313.2	176.7	247.4	198.7	184.4	201.2	227.4
MGC4093	352.3	434.9	193	156.2	157.3	160.9	306.6	336
MGC4172	290	370.1	211.3	240	177.2	217.6	207.4	266.8
MGC41945	119.9	136.7	110.3	96.6	87.6	92.4	99.1	125.4
MGC41945	103	115.8	89.3	85.3	88.2	90	99.3	119.7
MGC42090	81	85.2	97.2	93.8	96	88.7	83.1	84.8
MGC42105	121.2	132.3	103.3	109.5	92	111.8	123.5	130.6
MGC42174	168.7	209.5	214	192	177.3	219.6	143.4	170.9
MGC42367	151	161.4	149.5	256.6	218.8	284.5	123.4	163
MGC4266	95.7	105.2	89.6	78.1	81.4	84.6	80.8	100.3
MGC4268	353.5	421.1	165.5	164.2	152.2	195.7	300.7	477.8
MGC4399	88.8	100	96.1	92.9	82	96	96.1	110.5
MGC44328	88.6	96	100.2	85.7	80.7	95.8	82.9	98.6

MGC44505	94.3	100.2	106.2	100.5	101	100.4	84.2	87
MGC45438	125.6	136.9	96	95.6	87.9	92.5	120.3	137.6
MGC45491	86	121	96.5	89.4	75.5	84.4	106.5	105.2
MGC4562	1552.4	1813.5	997.7	811.2	610.2	768	1180.5	1452.8
MGC45800	92.8	100.2	106.7	90.5	94.4	95.8	103.8	101.1
MGC45840	298.7	308.4	224.5	344.8	393	499.4	241.2	283.3
MGC4618	864.5	872.4	488.8	371.8	419.6	497.2	645.8	624.4
MGC46496	84.7	95.9	103.9	100.5	98.6	96.7	95.1	90.8
MGC4655	91.8	99.1	94.1	85.4	77	85.2	80.6	92.6
MGC4677	83.2	89.5	81.6	78.9	77.2	84.3	79.7	92.5
MGC4677	3153.5	3397.3	2719.9	2662.5	1974.8	2841.6	3777.8	3737.4
MGC4728	409.4	592.4	298.2	246.9	219.3	234.4	440.9	647.6
MGC4825	425.2	589	199.6	170.2	139.3	188	352.2	532.5
MGC48595	82.1	96.4	86.2	86.3	80.1	81.8	87.9	99.1
MGC48628	87.2	106	88	83.2	82.9	93.9	91.6	109.1
MGC48915	79	83.5	85.3	79.1	87.7	78.5	81.3	71.2
MGC50273	71.8	79.1	84.2	83.5	80.5	75.3	79	77
MGC50372	85.1	89	93.8	91.5	82.1	83.5	81.9	88.2
MGC50559	112.8	111.4	90.2	85.3	80.7	100.5	113.7	127.8
MGC50721	103.4	113.2	83.1	85.6	84	86.6	106.2	103.7
MGC50722	88	107.3	109.7	91.9	103.4	102.3	92.9	94
MGC50811	99.3	104.9	84.3	92.5	79.3	94.2	92.5	94.6
MGC51025	76.4	91.8	86.7	87.8	82.8	90	86.4	81.7
MGC5139	186.7	206.7	107.9	98.2	90.4	97.3	147.6	177.2
MGC52057	142.8	176.4	125.1	108.2	89.2	108.5	138.3	149.9
MGC52110	746.2	843.8	355.2	478.3	454.5	661.1	470.7	669.8
MGC5242	935.9	1139.2	350.5	312.6	283	390	715	1046.4
MGC52498	114.6	112.7	110.4	110.8	95.3	108.4	85.6	99.8
MGC5509	542.6	710.8	296.8	290.4	245	391.6	469.6	543.4
MGC57359	93.8	101.3	110.7	101.8	90.4	100.7	87.3	79.1
MGC57359	114.1	118.6	82.1	80.5	76.5	81	95.6	124.2
MGC59937	110.1	131.4	110.5	100.3	84.3	91.8	107.5	114.7
MGC61571	1182.5	1344	726.8	556.2	481.6	701.7	790.6	948.3
MGC61598	87.4	83.9	87.1	84.6	90.2	90	74.8	93.8
MGC62100	86.5	95.7	105	93	96	92.8	84.2	93.2
MGC7036	2032	2847	1198.9	830.9	781.4	991.5	1809.3	2684.1
MGC70857	314.8	386.5	294.7	271.8	244.2	298	352.4	646.2
MGC70863	86.9	93.5	94.8	96	85.6	91.3	92.5	86.9
MGC70863	154.9	202.1	130	131.2	120.1	132.2	126.9	131.4
MGC70870	94.9	125.9	86.7	100.8	94	87	105.3	106.6
MGC70924	137.8	168.7	110.5	94.8	89.3	92.2	116.5	143.8
MGC71993	1699.2	2201.7	1251.8	760.5	620.1	660	1601.1	2371.7
MGC72075	91.5	104.4	113.4	106	93.7	120	83.4	91.8
MGC72080	258.5	362.3	135	127.3	114.8	143.5	248.4	312.3
MGC75360	78.2	105.9	89.4	79.1	78.5	87.4	82	94.5
MGC87631	131	139	114.4	100	94.9	100.2	104.4	109.2
MGC88374	74.7	85	86.5	85.5	75.5	81.9	86.5	90.7
MGC88387	162.8	175.7	119.7	101.9	99.3	100.9	160.6	157.8
MGC9712	114.4	141.4	91.1	82.7	81	87.6	122.9	120.8
MGC9850	857.1	1287.7	631.3	694.1	505.5	876.1	890	1550.5

MGC99813	96.6	115	104.6	103.5	107.8	135	97.4	109.1
MGEA5	1014.8	1387	759.9	919.9	987.7	1551.8	1032.7	1209.8
MGLL	96.5	101.4	91.2	124.5	115.4	124.6	81	99.8
MGLL	89.8	107.8	93.5	99	88.4	88.3	87.8	85.8
MGLL	100.5	98.1	112.1	139	120.3	147.2	89.9	101.3
MGMT	1146.7	1460.5	593.4	471.6	460.4	497.4	1245.7	1357
MGP	92.2	97	102.8	86.1	80.5	88.1	128.9	152.3
MGRN1	186	271.8	329.5	199.4	167.1	290	173	348.1
MGST1	76.9	88.8	83	80.8	80.7	74.1	80.3	90.2
MGST1	454.9	546.7	288.6	350.7	223.6	388.8	588.6	598.3
MGST1	287.1	366.3	159.8	235.2	175	291.2	296.7	351
MGST1	1666.1	2491.4	1160.3	1332.3	945.2	1588.5	2308.3	3221.7
MGST2	5455.9	6595	4146.6	3088.5	2190.6	3257.2	4475	6520.2
MGST3	4825.1	6290.6	4221	3220.1	2364.1	3090.4	4436.9	6543.1
MIA	106	122.4	90.1	80.3	79.8	77.7	105.6	138.5
MIA2	77.5	75.9	88.8	85.6	76	74.8	72.1	73.1
MIA3	158.7	175	145	119.9	117.5	135.9	149.4	163.3
MIB1	158.7	218.1	133.2	165.5	124.2	225.7	144.7	267.2
MIB2	207.1	206	254.9	202	214.9	343.5	168.6	228
MIB2	170.7	170.5	196.8	152.4	157.6	241.1	147	211.2
MICA	138.6	153.8	101.4	97.4	96.1	107.2	126.3	133.1
MICAL1	824.5	961.2	1273.1	920.3	940.5	1673.8	748.5	1375.9
MICAL2	124.6	160.7	93.7	103.6	87.9	89.4	125.7	137.8
MICALCL	80.5	90.7	98.5	132.9	112.1	133.9	82.5	104.7
MICAL-L1	121	186.2	161.8	135.6	133.5	159.8	135.6	166.5
MICAL-L2	93.4	99.5	106.5	108.3	110.3	102.2	88.7	98
MICAL-L2	121.5	142.4	103.4	91.1	103.5	107.6	142	155.1
MICB	387.3	552.1	295.3	247.2	231.4	251.7	412.5	399
MID1	243	297.7	182.1	176	152	200.2	196.8	226.6
MID1	75.3	77.7	80.4	81.1	82.5	93.9	74.2	83.3
MID1	92.6	88.3	94.4	84.5	85.8	93.9	87.6	97.8
MID1IP1	690	858.4	389.7	494.6	462.9	737.9	516.6	819.5
MID2	103.9	143	104.5	117.7	103.4	108.1	101.4	116.3
MID2	86	99.8	116.2	109.1	109.8	101.9	90.9	86.1
MID2	122	155.1	112.7	136.9	122	164.6	124.6	146
MIDN	387.7	423.1	360	321.8	317.8	255.2	397.5	663.7
MIER1	78.8	92.8	96.3	95.1	88	81	93.2	87
MIER1	770.8	1044.3	421.2	414.5	355.7	535	710.3	905.9
MIF	14456.4	17211.8	6968.5	5012	3817.1	6340.7	13956.7	17742
MIF4GD	796.7	974.4	233.3	278.9	241.2	245.7	530.4	538.7
MIMITIN	2078.3	2529.4	1452.4	1630.5	1272.4	2533.9	1992.1	2502.2
MINA	232.6	303.1	132.6	125	112.8	124.9	179	191.1
MINA	131.3	147.7	85.7	86.6	78.8	89.8	110.4	135.5
MINK1	104.3	99.3	101.8	86.7	88.4	95.1	78	115.8
MINK1	104.7	142.6	93	73.1	73.5	86	124.5	130.1
MINPP1	214.4	267.7	130.9	136.1	104.7	144.2	225.9	355
MIOX	80	96.6	90.9	95.7	89.7	89.6	77.6	90
MIP	93.2	99.5	85.2	83.9	85.8	85.9	88.1	96.9
MIPEP	238.1	295.3	170.7	172	128	187.8	169.9	255
MIPOL1	95.4	103.5	93.1	91.7	81.2	86.2	93	98.4

MIR16	484.6	606.5	499.3	366.7	292.3	354.6	414.1	1046.5
MIS12	523.7	721.9	303.8	259.7	240.2	281.6	506.9	806.8
MIST	83.2	87	98.1	94.6	85.6	92.1	91.1	92.6
MITF	130	151.2	107.5	112.9	106.6	119.6	123	155.9
MITF	85.4	87.5	89.2	81.2	72.6	87	77.6	84.1
MITF	270.6	318.9	144.6	124.3	111.1	134.3	262	375
MIXL1	78.9	79.1	89.4	74.3	76.7	77.7	82.8	85.4
MIZF	160.3	166.3	109.9	98.4	95.6	109.6	133.4	141.7
MIZF	376.6	472	336.2	286.4	231.8	332.6	371.8	556.7
MIZF	509.2	681.9	322.2	235.5	203.5	267.3	431.8	499.1
MKI67	136.6	159	129.4	106.9	118.4	149.1	131.6	149.2
MKI67IP	3159.7	3701.2	1490.2	1375.5	968	1822	2226.6	3387
MKKS	287.9	332	168.6	184.9	159.3	224.3	223.8	270.8
MKKS	917.4	1059.8	480.6	466.8	405.5	504.9	742	870.9
MKKS	110.1	120.7	91.5	84.2	84.4	82.9	114.6	131.5
MKL1	275.1	373	490.1	463.9	358.3	660.6	226.5	378.8
MKL2	115.4	133.8	99.5	102.2	95.5	109.4	107.4	157.5
MKLN1	801	1207.9	657.4	1048.3	908.7	1436.7	743.6	1116.2
MKNK1	104.3	113.7	105.4	100.7	90	98	90.9	104.9
MKNK1	583.5	746.1	537	530	513.5	947.4	472	1112.1
MKNK2	1246.6	1328.4	727.1	631	600.4	532.6	1235.6	1726.2
MKNK2	153.2	181.8	175.2	166.8	149.9	193.4	147.2	218.9
MKRN1	2611.9	3248.7	2031	2644.6	2169	3159.8	2207.9	3337.6
MKRN2	1062.4	1498	988.8	1055.7	866.4	1750.5	929.1	1365.8
MKRN3	136.7	150.3	99.2	99.9	116.8	106.3	136.3	146.7
MKS1	154.5	171.2	137.9	133.3	126.1	164.1	106.2	125
MLANA	72.4	73.4	78.4	77.4	71.9	79.4	79	77
MLC1	268.6	274.7	153.2	152.3	141	105.4	213.7	275.3
MLC1	354.7	513.3	253.9	150.3	143.4	199	236	407.9
MLC1	432.6	523.5	235.5	259.2	221.5	132.8	303.1	436.9
MLC1SA	2014.5	2456.5	1380	1153.1	1052.3	1167.8	1941.3	1698.4
MLCK	92.5	105	89.5	83.7	83.8	81.3	93.5	101.9
MLF1	109.3	146.8	98.1	95.3	89.1	101.9	126.9	165.3
MLF1IP	283.3	300.4	138.1	135.1	142.7	172.5	243.4	357.9
MLF2	471.9	673.6	421.7	299.7	278	408.5	433.4	845.9
MLH1	971.1	1240.2	455.7	525.1	411.1	612.1	702	1138.8
MLH3	191.5	236.6	132.8	116.7	121.4	110.2	161.5	204.2
MLH3	109.7	146.1	140.7	112.4	109.1	126.2	108.1	113.1
MLKL	1384.4	1848.6	446.4	267.8	231.5	340.6	928.2	1054.8
MLKL	322.8	386.5	187.1	132.8	125.4	144.1	229.9	291.2
MLL	116.6	139.2	122.8	118.9	110.4	147.3	111.3	126
MLL2	181.5	187.2	100.2	103.2	80.1	87.5	159.3	170.8
MLL3	144.2	215.5	96.5	122.9	108.6	121.5	132.2	176.3
MLL3	134.3	186.1	95.9	125.6	110.5	115.3	141.6	178
MLL3	102.9	121.6	109.3	88.8	98.9	100.8	93.4	117.5
MLL3	267.2	368	186.1	228.7	195.7	236.9	249.4	329.9
MLL4	243.6	285	224.8	181	189.6	258.1	203	334.1
MLL5	117.4	147.8	113.9	145.7	149.5	263.3	120.6	137.7
MLL5	123.6	142.5	109.8	114.4	114.9	163.4	134.7	125.7
MLL5	365.4	563.7	154.4	282.6	327.4	259.8	377	407.4

MLLT1	86.3	104.7	101.3	88.2	88.1	90.7	95.8	100.2
MLLT10	114	134.3	89.1	82.8	78.7	80.3	101.9	105.2
MLLT10	686.2	919.1	397.8	509.9	436.1	710.1	570.3	680.5
MLLT11	118.5	134.1	105.2	105.5	100.6	136.5	151.1	229.3
MLLT3	104.6	111.3	84.5	89.4	84.8	91.2	104.1	127.4
MLLT6	534.3	564	520.5	375.8	419.7	418.3	365.2	474.4
MLLT7	181.5	218.3	107.5	98.2	112.1	118.3	237.5	361.2
MLN	84.5	106.6	91.8	86.8	77.8	84.7	102.1	104.1
MLN	112.4	122.5	117.7	85.7	84.3	78.6	111.1	128.3
MLNR	90	103.8	93.7	99.3	85.4	97.8	89.7	95.4
MLPH	119.4	137.9	108.1	84.5	88.9	87.7	115	117.8
MLSTD1	705	849.6	313	283.3	233.4	328.4	440.3	648.5
MLSTD2	560.2	734.6	496.4	700.3	523.5	831.9	467.3	1044.9
MLX	118.2	125.7	105.9	103.6	97.4	106.3	96.2	116
MLX	534.6	660.1	255.2	215.7	177.5	256.3	380.7	517.2
MLXIP	87.5	94.7	105.4	98.8	100.8	92.1	91.8	90.7
MLXIPL	134.6	135.7	123.7	103.2	99.5	100.3	101.7	108.8
MLYCD	225.6	301.3	148.9	124.4	122	146.8	192.1	215.5
MLZE	98	101.9	96	84.9	84.1	85.4	95.7	99.9
MMAA	131.6	177.9	96.3	90	80	90.8	146.3	159.9
MMAB	134.9	179.9	153.8	191.7	195.5	201.4	156.8	166.6
MMACHC	874.1	1119.1	524.8	446.9	286.7	384.9	492.8	539
MMD	1026.9	1210.3	563	547.7	436	660.2	899.6	1813.7
MMD2	68.9	78.9	82.4	81.7	83.7	80.7	74.1	78.3
MME	93.2	103.4	97.6	95.6	81.5	89.4	88	88.3
MME	91.8	112.3	85.5	84.8	87.3	77.3	91.7	101.1
MME	89.9	93.1	79.2	85.2	76.3	78.4	80.6	103.1
MME	82.2	85.1	89.4	74.2	75.1	82.4	80.3	91.7
MMEL1	95.4	120.1	92.3	79.2	72.1	87	105.3	111.4
MMP1	113.5	120.3	90	94.3	85	81.9	107.7	112.2
MMP10	82.2	104.8	87.4	86.5	75.6	85.4	91.3	104.8
MMP11	82.5	103	108.7	115.5	101.5	115.6	93.2	97.4
MMP12	80.6	82.3	96.5	88.5	76.3	86.8	76.7	78.2
MMP13	82	96.8	94.7	87.8	87.9	88.9	87.7	92.4
MMP14	128	157.8	112.7	97.9	95.4	109.5	131.3	145.7
MMP15	121.7	146.3	144.1	133.1	133.1	144	121.4	118.7
MMP16	75	84.4	102.4	90.9	85.4	88.9	72.7	67.9
MMP16	71.6	85.7	85.5	79.8	84.6	82.7	84	83.2
MMP16	119.3	149.4	93	95.3	80.8	96.1	120.9	136.1
MMP17	87.3	95	98.7	83	86.2	90.8	92.1	110.6
MMP19	91.9	106.7	102.4	96.4	96.5	93.8	94.9	104.1
MMP19	121.1	147.6	92.5	94.7	91.9	91.7	120.5	118
MMP2	138.7	163	118.1	122.1	118.3	118.9	147.1	141.6
MMP20	87.7	92.6	90.2	88.7	89.8	105.4	84.3	95.7
MMP21	83.6	87.9	84.1	81.6	80.3	98.4	81.9	88.5
MMP23A	94.7	91.9	82.3	89.1	75.8	84.5	104.3	107.6
MMP23B	123.2	131.5	119.4	118.2	116.7	119.1	96.8	109.9
MMP24	115.4	132.9	95	86.9	83	102.8	105	123.6
MMP25	79.3	85.9	101.9	98.2	86.6	89.3	87.5	82
MMP25	180.1	191.3	144.7	124.1	131.5	123.1	174.3	170.6

MMP26	84.7	88.4	95.2	88.9	76.6	87.7	89.4	89.3
MMP27	102.6	125.2	96.4	96.4	91	90.7	94.2	106.3
MMP28	99.7	108.7	86.5	83.9	85.3	90.7	98.5	109.6
MMP28	79.6	106.5	93.1	79.9	79.6	90.6	87.7	91.3
MMP3	172	199.7	113.1	92.6	97.4	99.5	145.8	183.7
MMP7	79.9	80.3	85.7	83.3	78.1	75.1	79.9	90.2
MMP8	76.1	85.9	87	70.4	78.8	78.7	78.4	86.7
MMP9	107.3	130.1	121.4	444.8	481.5	473.1	112.7	161.4
MMRN1	93.6	105.6	85.9	91.7	84.7	85.6	98.9	114
MMRN2	125.1	130.2	94.2	89.1	81.4	87.9	117.5	113.2
MMS19L	720.2	965.4	821.8	729	588.3	907.8	537	788
MN1	97.2	110.7	102.8	93.4	93.4	89.6	100.5	116.9
MNAB	193.6	254.5	149.3	139.9	119.7	169.1	194	296.8
MNAT1	437.5	505.1	147.9	132.9	106	178	291.6	634.2
MND1	1207.9	1505.9	544.2	378.7	286.2	344.8	1043.5	1216.1
MNDA	1969.2	2683.2	834.8	808.7	608.5	1366.4	1249.3	1507.9
MNS1	611.7	717.8	260.9	239.8	230.9	427.5	384.8	455.4
MNT	232.1	262.5	215.4	253.5	218.2	265.4	195.7	302.1
MOAP1	329.3	391.6	246.7	279.4	241.9	419.5	330.7	703.9
MOBK1B	122.8	184.6	94.7	86.7	92.6	104.8	135.9	169.4
MOBKL1A	152.1	163.4	82	89.2	85.5	88.5	158.9	204.9
MOBKL2A	519.9	697.5	298.7	292.2	251.6	268.3	571.1	457.6
MOBKL2B	77.9	83.2	88.8	84.2	74.2	87.2	86.6	85.6
MOBKL2C	107.6	116.9	98.2	100.4	88.8	88.2	106	107.4
MOBKL2C	327.3	426.3	233.2	291.2	303.3	333.6	326.5	329.4
MOBKL2C	90.2	103	93.9	89.7	80	80.9	81.1	88.3
MOBP	74.9	84.2	87.7	79.7	75.2	80.4	83.3	85.3
MOBP	84.3	102.2	95.6	88	78.4	89.5	93.6	97.3
MOBP	83.4	103.3	98.1	87.1	89.3	87.5	91	111.9
MOBP	83.2	100.1	95.9	89.2	87.7	84	91	97.5
MOCOS	192.4	249	222.6	204	157.3	210.4	188.7	252.1
MOCS1	291.1	295.5	162.6	180.4	142.8	180.9	253.1	248.8
MOCS1	90.9	142.3	107.2	88.3	84.8	94.5	100.7	118.2
MOCS1	423	529.5	207.4	116.6	101.9	119.5	375.9	521.8
MOCS2	88.4	96	92.2	83.6	82.3	85.6	103.3	94.4
MOCS2	142.8	165.6	97.6	106.9	91.9	100.1	145.2	161
MOCS2	472.7	612.2	271.4	276.6	213.4	449	337.1	480.9
MOCS3	126	164.2	117.4	130	110	143.8	114.6	121
MOG	81.8	91.1	88.1	84	88.3	85.7	82.9	88.4
MOGAT1	87.7	103.6	107.8	89.6	89.5	94.3	86.3	88.7
MOGAT2	99.6	107.1	103.2	91.6	86.6	90.2	102.6	106.6
MOGAT3	85.9	88.5	96.9	92.7	90	87.4	85	89.1
MON1A	175.6	238.6	118	111.9	92.2	98.8	163.3	238.9
MON1B	223	286.2	254.5	222.4	192.6	251.2	195.8	307
MON2	384.5	458.8	291.8	414.6	371	567.5	366	515.2
MORC1	101.3	112.2	87.5	98.2	92	93.6	96.7	105.5
MORC2	386.9	479.8	421.3	386.3	296.2	443.7	304.1	568.7
MORC3	294.9	337.3	167.9	171.8	157.5	293.2	244.4	438.9
MORC4	127.8	144.1	108.6	108.2	92.2	86.2	118	168.8
MORF4	96.5	104.5	99.9	85.4	81.1	90.6	88.7	105.4

MORF4L1	4991.1	6260.4	1498.4	1514.7	1095.9	2087.6	3779.8	6805.7
MORF4L1	98.4	113.9	91.4	87.4	87.6	90.2	101	111.1
MORG1	323.9	395.5	196.5	169.8	156.6	176	311.8	270.2
MORN1	94.9	115.6	86.5	85.1	84.4	89	94.2	122.3
MORN3	111.7	131.3	91.1	91.2	82	92.5	108.7	113.3
MOS	96.7	118.5	85	82.1	76.7	76.5	94.2	108.7
MOSC1	960	1182.8	715.1	1376.8	909.8	1819.6	532.5	697.5
MOSC2	154.5	166.2	102.6	205.4	127.9	193.5	135.1	187
MOSPD2	191.9	207.1	98.7	114.3	98.8	122	192.5	303.4
MOSPD3	354.2	434.8	337.4	302.7	265.4	333.3	360.9	474.6
MOSPD3	285.3	364.6	296.8	222.8	179.3	246.8	259.8	412.4
MOV10	285.6	310.7	273.6	197.9	219.8	283	222.5	319.8
MOV10L1	97.5	120.4	95.8	105.1	82.7	88.6	101.3	118.4
MOXD1	182.1	224.4	163.7	189.4	177	229.1	162.7	248.3
MOXD1	118.9	142.6	112.9	130.6	135.2	144.9	96.1	128
MPDU1	372.8	463.4	196	165.7	133.3	145.2	320.7	418.9
MPDZ	89.7	112.7	92	94.8	72.1	88.1	89.1	115.1
MPFL	145.7	186.2	111.6	102.4	102.2	94.4	151.9	163.5
MPG	82.8	82.2	93.3	91.2	81.4	94.4	80.5	89.1
MPG	131.3	149.5	119.7	106.3	97.3	103.8	121.9	135.6
MPG	92.7	106.1	106.8	95.6	97.1	108.1	99.2	89.1
MPHOSPH1	1699.4	2312.7	923.9	696.4	694.1	1407.1	1258.9	1740.9
MPHOSPH1	98	129.9	92.3	84.2	86.1	94.6	109.5	161.9
MPHOSPH6	549.7	685.3	167.2	145	127.1	171.4	403.8	704.2
MPI	174.5	219.6	144.9	158.5	139.4	169.9	127.7	144.2
MPL	100.3	94.8	97.6	90.5	85.9	92.7	89.3	104.1
MPN2	86.7	84	109.6	94.4	83.4	92.2	83	84.7
MPND	165.2	171	143	133.9	113.2	139.8	121.6	156.3
MPO	142.4	170.9	179.8	252.3	211.2	257	129	167.4
MPP1	650.4	928.2	397.9	418.7	334.1	534.2	582.9	831.7
MPP2	129.5	152	131.7	103.3	91.5	109.1	115.1	128.8
MPP3	122	120.9	130.3	129.4	127.6	146.7	108.8	136.6
MPP4	74.4	75.7	81.7	82.6	83.2	77.4	73.8	78.2
MPP5	229.8	285	207.7	239.2	170	318.5	197.7	325.4
MPP6	416.8	587	344.7	203.5	165.3	242.9	339.3	521.3
MPP7	98.9	110.1	82.9	86.4	84.7	82.9	98	95.7
MPPE1	1583	1972.5	1039.9	945.4	771.2	1225.5	1298.6	1743.4
MPPED1	113.5	115.8	108.1	91.3	83.7	85.9	114.1	115.3
MPPED2	98.1	107.5	94.2	77.3	82.2	77.4	103.3	102.6
MPST	141.4	173.7	110.3	107.2	101.2	101.4	129.1	154.8
MPST	545.6	696.2	331.7	272.4	230.2	292.6	510.6	703.5
MPST	166.7	207.4	110.8	110.1	96.1	133.8	157.9	201.9
MPV17	990.2	1286.6	768.4	499.7	413.1	593	833.5	958.6
MPZ	101.2	125	100.8	86	90.5	103.3	99.3	125.3
MPZL1	85.1	111.6	92.3	81.7	86.7	83.8	89.1	102.3
MPZL1	150.5	199.4	149.9	167.5	149.9	170.3	152	187
MR1	768.7	1076.6	501.6	320	318.5	451.4	606.5	878.8
MRAP	93.4	101.3	79	83.6	72	81.4	83.1	98.9
MRAP	102.6	98.5	95.1	107.2	94.9	97.9	91.8	95.5
MRAS	170.2	216.9	199.9	134.2	98.4	124.8	159.1	227.3

MRC1	79.3	86.1	84.1	82.6	75	85.1	74.9	74.6
MRC1L1	81.2	93.1	82.1	75.8	84	89.3	95.7	101.1
MRC2	141.9	162.9	142.4	182.9	145.4	159	119.5	146.6
MRCL3	18385.5	18884.5	13379.2	17134.4	9383.9	15876.9	19818.5	26397.1
MRE11A	151.8	171.1	137.1	116.4	115.2	103.8	130.5	163
MRE11A	319.1	416.5	151.3	172.6	160.5	162.4	250	261.2
MRE11A	532.7	686.2	251.4	223.6	192.1	368.1	357.8	518.9
MRFAP1	4446.8	6096.8	3077.3	2627.6	1995.8	3283.7	3749	5330.8
MRFAP1L1	2489.4	3293.2	1519.7	1506.9	1330.3	2232.7	2273.2	2832.1
MRFAP1L1	171.3	209.8	144.2	125.4	130.9	127.6	153.8	192.3
MRFAP1L1	207.1	219.7	114.7	95	102.8	104.3	152.8	173.9
MRGPRD	93.6	109.9	99.1	93.5	90.6	96.3	100.8	91.8
MRGPRE	86.2	99.8	116.2	91.7	90.1	89	96.3	105.9
MRGPRF	77.7	100.6	112.1	104.3	95.3	92.5	83.3	84.7
MRGPRX1	79.9	85.1	75.3	89.1	77.1	81.9	73.9	81
MRGPRX2	91.9	104.1	97.1	89.3	84	86.9	93.8	103.6
MRGPRX3	156.2	168	117.7	97.8	87.8	102.5	146	150.1
MRGPRX4	87.8	101.9	97.8	84.3	80.8	94.4	88.1	94.2
M-RIP	103.1	127.5	113.2	98.3	92.5	102.5	105.3	109.1
M-RIP	317.2	398.8	336	420.1	336	509.8	293.8	461.2
MRLC2	9336	12085.5	6621	5446.9	3766.3	6164.7	7780.8	10573.3
MRM1	141	171.3	105.8	88.2	84.9	96.5	114	127.9
MRO	76.4	87.5	84.3	78.1	78.9	82.6	81.6	81.5
MRP63	340.4	376.9	211.5	219.1	200.9	244.4	286.9	467.7
MRPL1	990.1	1120.4	257.2	226	200.6	299.6	718.5	1322.6
MRPL10	445.8	544.7	140.8	143	138.1	157.4	446.7	642.4
MRPL10	98	129.9	105.4	106.5	94.3	103.4	91.7	117.9
MRPL11	1852.5	2182.7	660.9	710	589	750.5	1831.8	1604.6
MRPL11	157.4	190.8	105.4	103.4	91.8	104	145.8	168.5
MRPL11	2237.2	2895.1	1963.1	1613.1	1506.7	2204.6	1805.2	2300.1
MRPL12	1830.1	2119.4	1558.2	1171.4	943	1587.3	1294.2	1731.4
MRPL13	2844.7	3266.4	1010.7	941.2	531	638.9	2832.5	3470.8
MRPL14	2637.7	3141.6	2582.9	2297.9	1873.3	2446.4	2439.2	2518.4
MRPL15	3645	4177.6	923	872.2	591.1	920.2	3207.2	4050.9
MRPL16	732.2	1070	242.1	224.5	198.2	185.1	617.5	814.5
MRPL17	2412.2	3107.6	1286.9	814	813.1	809.1	2054.5	1816.9
MRPL18	8278.9	9268.4	2068	2491.3	1764.5	3633.5	8519	14156
MRPL19	433.5	553.9	145.7	111.6	124.8	110.9	371	540
MRPL2	520.9	692.5	582.6	394.8	301.1	506.2	503.8	834.1
MRPL20	1489.9	1938.7	803.6	710.2	507.5	695.1	1122.2	1281.8
MRPL21	2560.8	3282.6	1143.1	998.8	782.1	1530.8	1728.5	1722.7
MRPL21	2550.2	2782.9	1533.4	1535.9	998.1	1878.3	1947.1	3106
MRPL22	2486.6	3312.8	1278.7	943.6	835.9	1484.1	2245.6	2425.2
MRPL22	4129.7	4968.9	2258.1	1793	1655.6	2196.7	3811.5	3962.5
MRPL23	5260.3	6284	3772	2517.7	2024.2	2469.1	4232	5376.6
MRPL24	997.3	1208.2	450.9	367.2	279.4	573	807.9	917
MRPL24	4100.2	5152.4	2755.9	2430.7	1672.2	3523.2	3095.8	3638.2
MRPL27	1459.5	1840.9	860.8	815.6	642.6	1003.2	1331.2	1509.8
MRPL27	120.1	114.3	94.3	89.4	84.9	90.3	98.1	109.2
MRPL27	219.2	235.3	123	118.4	123.5	127.1	177.7	250.5

MRPL28	428	496.7	271.7	179.9	167.3	168	360.7	430.2
MRPL3	156.6	165.1	112.7	105.8	99.3	112	148.6	221.9
MRPL30	250.5	325.2	111.8	101.3	104.9	116.5	202.6	298.5
MRPL30	904.6	1197.5	363.8	256.4	244	299.5	805.3	1194.9
MRPL32	1442	1839.7	507.6	520.9	394.9	560.6	1184.4	1654
MRPL33	4247.5	4995.9	1951.3	2018.8	1156.6	2512.2	2714.3	4323.7
MRPL33	8477.7	10029.8	7418.6	6313.4	5356.7	8723.7	5940.8	6270.8
MRPL34	1323.3	1526.7	729.4	559.7	422.1	635.8	945.4	910
MRPL35	3530.3	4368.1	2580.7	2061.3	1369.8	2440.4	2830	4106.9
MRPL35	577.3	782	353.8	330.6	288	423	468.1	517.3
MRPL35	514.2	590.3	239.4	236	175.2	325.7	393.1	599.1
MRPL36	2807.6	3419.8	1971.8	1600.8	1231.5	1681.9	2318.8	2480.7
MRPL37	7852.1	10363.5	5948.2	4861.1	4141.4	4692.8	7146.1	6719.7
MRPL38	877.6	1022	263.8	215.9	191.7	285.8	796.4	1373.2
MRPL39	88.5	98.5	108.1	102.9	98.2	104.3	89.1	102
MRPL39	2286.6	2827.4	1136.5	1081.5	758.7	1327.8	1792.8	2488.4
MRPL4	87.2	92.3	104.5	84.2	89.3	82.4	86.4	99.9
MRPL4	371.4	468.9	240.2	120.2	119.9	183.1	307.2	430.3
MRPL4	221.1	322.9	220.5	149.8	133.4	157.6	181.4	263.9
MRPL40	1348.9	1854.7	791.7	855.8	612.3	1080.4	1157.1	1802.3
MRPL41	2711	3157.7	2221	1368.9	1067	1596.9	1740.8	1829.8
MRPL42	559.8	788.5	192.5	175.6	151.1	209.2	411.1	775.2
MRPL42P5	95	115.3	101.3	98.3	96.1	89.1	92.5	128.5
MRPL43	994.2	1379.3	849.3	600.6	449	755.5	749.2	1112
MRPL43	118.7	140.2	124.4	103	93.1	101.3	98.3	101.9
MRPL43	428.3	534.3	254.1	175.5	132	173.3	324.8	364.5
MRPL44	97.6	104.5	97	87.5	85.5	84.5	105.7	114.2
MRPL45	1880.4	2694.8	608	543.6	455.8	748.2	1778.7	2918.7
MRPL45	1369.4	1986.3	520.7	443.5	333.8	511.1	1246.9	1754.4
MRPL46	1393	1966.4	935.3	775.4	670.5	1151.8	1261.4	1868.3
MRPL47	433.8	587.8	196.7	168.5	139.4	202.7	389.9	661.7
MRPL47	584.5	684.4	279	258.1	232.4	319.2	523.1	673.3
MRPL48	779.9	984.4	381.7	248	220.9	313.5	667.3	817.1
MRPL49	338.3	458.4	331.1	210.6	195	230.1	343.5	532.8
MRPL50	2859.6	3140.1	1140.9	589.5	358.1	675.6	2567.1	3913.9
MRPL51	2664.5	3499.2	1758.5	1489.1	1178.6	1595.5	2608.7	3677.4
MRPL52	1143.6	1285.5	688.1	450.1	337.4	407.5	909.5	1101.2
MRPL52	760.1	887.6	421.6	295.8	201.9	280.5	681.3	839.7
MRPL53	893.5	1025.3	459	389.1	296.3	438.4	821.1	1039.7
MRPL54	1972.8	2501.8	1194.1	798.9	714.9	1012.2	1564.3	2106.7
MRPL55	2391.6	2655.9	805	777.5	519.7	743.5	2243.8	2786.8
MRPL55	913.6	1249.1	852.3	596.7	503.9	686.4	747.8	906
MRPL55	1347.6	1626.2	1139.1	938.5	677.7	991.1	1082.3	2282.3
MRPL9	757.5	1113.5	536.8	415.2	356.6	593.5	692.8	1167.4
MRPS10	4600.1	5710.8	2753.5	2781.6	1925.5	2983.8	4160	5509.2
MRPS11	1513.4	1790.6	1275.1	1402.4	1103.4	1667.4	1151.2	1503.9
MRPS11	848.8	1039.5	635.8	674.5	519.4	796.6	701.6	661
MRPS12	4444.4	5868.4	2134.3	1393.8	997.4	1158.8	4071	3778.7
MRPS12	90.7	118.5	87.2	91.1	84.3	93.2	98.7	103.6
MRPS12	958.9	1266.7	796.6	508.3	357.8	510.2	798.9	1154.3

MRPS12	243.2	342.3	196.9	115	127.8	125.6	186.6	251.3
MRPS14	233.5	277.3	165.9	133.5	109.6	139.2	173.1	221.6
MRPS15	1621.9	2043.7	1341.9	1452.6	1083.9	1982.2	1338.1	2527.6
MRPS16	974.7	1254.1	521.7	430.4	367.5	526	787.7	937.7
MRPS17	2390.2	2856.8	1048.2	963.2	593.3	758.2	1843.6	2316.7
MRPS18A	550.4	702.7	340.9	326.9	302.3	398.2	595.1	555.6
MRPS18B	1087.6	1328.7	521.2	546.6	383.4	449.1	985.1	1239.2
MRPS18C	113.4	118.7	97.6	85.6	92.6	84.9	104.1	119.4
MRPS2	772	1060.8	725.9	389.6	315	462.1	646.9	1054.2
MRPS21	6725.1	7999.9	2119.6	1804.6	1509.3	1907.2	5379.1	6884.9
MRPS21	1420.9	1516.3	889.2	605	551	845	889.4	1117.4
MRPS22	3267	4122.3	2472.9	2188.4	1940.1	2848.7	2940.1	3996.8
MRPS23	1203.1	1467.3	389.1	419	291.1	491.7	947.2	1157.4
MRPS24	3321.3	4423.6	2768.5	2121.4	1753.8	2727	3247.8	4151.5
MRPS26	835.1	904.3	380.1	302	268.9	354.9	660.3	626
MRPS27	1635.9	2187.2	984.8	776.1	607.1	1201.9	1376.8	2299.9
MRPS28	1549.9	2010.8	634.6	548	406	673	1410.6	1943
MRPS30	2608.4	3315.3	1946	1639.9	1198.9	2068.4	2027.2	3570.2
MRPS31	1370	1712.8	846.4	860.9	744.5	1054.7	1098.1	1363.1
MRPS33	2856.1	3484.8	1889.8	1984.1	1238.2	2298.7	2176.5	3073.4
MRPS33	280.7	388.1	157	168.2	139.2	212.5	231.5	294.8
MRPS34	674.2	951.3	331.4	225.1	179.5	188.5	515.2	600.4
MRPS35	1313	2003.7	446.5	378.4	304.7	530.3	1065.6	1872.8
MRPS36	171.8	227.8	144.9	183.3	146.5	182.1	171.7	237.2
MRPS5	936.4	1128.4	606.4	613	606.8	958	732.3	1069.1
MRPS6	5644.5	7833.2	3896.3	4478.5	3965.9	5344.1	5397.4	6795.8
MRPS7	1763.2	2149.5	698.7	603.1	552.1	844.3	1403.5	1488.4
MRPS9	1265.1	1900.4	1378.1	1091.8	830	1401.9	1099.9	2042.3
MRRF	775.3	936.2	626.4	383.9	329.6	479.9	611.2	729
MRRF	1598.5	2254	940	561.8	462.6	709.3	1347.9	1629.7
MRRF	196.4	217.1	126	111.7	91	103.5	168.1	178.4
MRS2L	342.6	435.8	174.5	175.5	134.3	211.2	278.8	433.4
MRVI1	86	98.3	87.4	83.5	82.4	81	85.5	94.8
MRVI1	92.4	102.9	114.1	116	107.3	104.2	84.3	86.9
MS4A1	127.1	153.4	100.1	95	95.8	95.2	119.6	138.1
MS4A1	89	90.3	88.1	78.7	74.3	84.7	88.5	91.1
MS4A1	82.1	91	87.9	84.3	77.7	87.7	80.3	82.7
MS4A10	67.2	81.5	76.8	74.5	81.6	82.6	72.1	80.2
MS4A12	83.7	96.1	90.7	82.7	88.1	81.6	91.6	93.6
MS4A13	114.9	131.2	99.3	86.5	87.9	85.7	115.4	118.9
MS4A2	104.2	112.8	92.2	94.8	89.2	105.8	100.2	113.1
MS4A3	1428.2	1853.6	786.6	3619.7	2311.1	4550.4	802.4	1070.6
MS4A3	149.9	175.1	103.3	162.7	117.6	174.2	112.8	158.8
MS4A3	2903.2	3378.4	2196.3	7444.2	5206.3	8646.9	2263.1	2997.1
MS4A4A	91	101.4	93	89.2	81.2	83.5	93.1	93.9
MS4A4A	77.6	97	83.5	76.9	80.2	83.2	76.4	86.3
MS4A4A	171.2	190.4	121.3	160.9	121.1	186.7	145.9	176.3
MS4A5	85.4	88.1	78.8	85.3	86.3	95	87.2	104.4
MS4A6A	3196.4	4174.3	2215.2	6547.6	5116.6	8774.9	2137.7	2750
MS4A6A	2267.7	2830.6	794.1	2789.7	1830.9	3151.9	1423.4	1912.8

MS4A6A	978.9	1360.8	655.4	1880.5	1478.5	2465.8	576.3	621.8
MS4A6E	85.5	86.1	95	85.3	76.6	80.3	75	88.3
MS4A7	3620.6	4191.9	2196.6	3945.6	2741.7	4695.6	2370.1	3443.9
MS4A8B	80.9	93.7	93.8	88.5	89.1	94.6	81.3	89
MSC	126.3	186.1	134.2	98.9	101.9	101.7	999.9	2067.8
MSH2	130.8	144.8	103.9	96	95.7	99	113.6	164.2
MSH3	19345.7	19094.5	9055.2	11214.8	11412.6	9928.4	19118.7	21375.5
MSH4	88.4	114.1	94.8	96.9	87.7	89.5	90.8	94.2
MSH5	251.2	245.8	137.7	133.9	119.8	106.9	165.7	205.6
MSH5	398.7	495.3	284.5	183.6	226.5	329.7	346.4	425.9
MSH5	344.1	374.8	292.9	212.8	260.5	319.4	262.9	345.2
MSH5	163.3	189.2	122	113.8	109.5	114.6	144.8	163.6
MSH5	263.1	332.6	267.2	193.3	203	272.3	226.4	243.4
MSH6	3097.3	3851.1	2035.6	2549.1	1884.5	3155.6	2446.8	2825.5
MSI1	86.1	95.7	106.6	103.1	105.2	96.9	85.4	91
MSI2	105.5	128.8	95.4	88	85.2	82.4	113.1	120.6
MSI2	182.9	244.9	146	112	123.2	122.5	177	214.6
MSI2	192.8	245.1	180.9	176.1	169.3	203.7	173.6	203.9
MSL2L1	131.3	150.6	97	93.3	81.2	98.8	121.9	151.8
MSL3L1	529.6	471.6	201.5	436.8	321.6	210.8	381.1	672.6
MSL3L1	79.2	91.5	79.8	73.3	74.6	86	83.6	85.1
MSL3L1	192.8	276	133	159.7	186.1	242.5	162	198.5
MSL3L1	208.2	256.6	167.9	213.2	201.1	237.2	178.5	187
MSLN	85.1	103.8	105.1	99.5	97	91.1	87.3	88.9
MSMB	99.3	114	86.1	90	72	84.1	93.5	104.3
MSMB	89.2	104.6	96	91.7	79.2	85.3	83.7	98.8
MSMB	74.5	84.7	79.4	84.8	83.8	85.9	83.5	76.9
MSN	3171.7	4824.9	3931.3	7622.3	6292.8	9344	2907.7	4692.4
MSR1	91.1	107.3	93.4	81.2	84	84.1	78.3	101.9
MSR1	133.5	141.6	92.2	87	89.1	82.7	131.8	152.5
MSR1	118.1	138.4	103.1	89.7	81.7	82.9	119.4	110.6
MSRA	1616.4	1931.7	1294.9	789.3	603	716.3	1412.9	1803.1
MSRB2	4173.7	5126.6	2686.4	1838.2	1595.4	1643.1	3477	3133.3
MSRB3	86.7	94.9	102.6	122.7	110.3	142.6	86.9	97
MSRB3	92	88.1	101.6	186.4	177	229.9	89.9	95
MSRB3	83	90.8	95.6	86.8	88.2	88.8	79.4	82
MST150	238.4	332.2	169.3	144.8	130.1	147	236.2	286.9
MST1R	78.2	94.1	98.4	77.2	76.1	82	91.9	95.1
MST4	930.6	1163.1	419.5	559	378.9	814.6	671.7	1781.2
MSTP9	133.9	149	90.5	102.1	86.2	94.5	121.8	139.3
MSX1	708.5	810	495.1	254	222.1	320	741.7	1329.1
MSX2	99.3	134.9	97	83	83.1	104.1	99.7	135.4
MSX2P	87.4	104.8	98.8	90	76.2	96	86.1	92.7
MT	559.9	805.5	464.3	342.4	250	278.5	377.8	466
MT	133.6	167.1	98.9	88.5	89.9	86	114.8	157.8
MT1A	4285.9	5364.6	3097.1	1248.6	966.2	1306.7	3271.8	3678.4
MT1B	98	104	101.2	107.4	90.4	102.5	102.5	106.8
MT1E	5929.1	6262.8	3187.9	992.2	1008.7	949.5	4229	4242.8
MT1F	7404.9	8649.2	5295.1	2586.9	2029.3	1949.7	6649	8414.7
MT1G	1662.1	2053.3	1278.7	250.1	184.5	204.9	1272.5	1707.2

MT1H	551	593	235.1	123.4	120.4	118.2	392.9	383.7
MT1M	98	120.5	97.2	85.8	79.6	79.5	116.6	146
MT1P2	110.4	114	95.3	85.2	78.2	82.8	100.8	118.4
MT1X	3733.2	4685.8	3282.2	1182.3	753.2	1020.4	2391.8	2382.2
MT2A	919.5	997.5	818.3	494.6	415.1	422.8	781.6	1036.3
MT3	92.4	104.9	113.9	103.8	102.2	94.3	91.3	94.5
MT4	74.4	87.6	88.1	80.6	83.6	74.5	74.2	80.2
MTA1	532.3	706.4	568.1	502	402.3	687.9	439	1029.4
MTA2	1019.6	1162.8	833.5	497.2	459.6	377.6	752.3	1365.7
MTA3	271.1	388.5	209.4	172.4	143.3	193.4	263.5	324.9
MTAC2D1	69.2	81.6	82	78.2	73.9	89.1	73.5	73.3
MTAP	74.6	91	103.7	101.5	82.7	98.2	82.8	92.5
MTBP	272.1	343.4	222.4	176.8	149.1	252	242.2	419.1
MTCH1	9024.9	10985.3	6859.9	7082.3	5371.9	7817.8	8476.3	11118.3
MTCH2	165.7	178.7	113.1	100.6	101.9	117	136.9	169.7
MTCP1	764	889.4	545.9	733.8	530.3	763.5	608.4	1209.5
MTDH	2466.1	2846.7	464.7	502.8	377.5	817.4	2144.9	5397.3
MTE	561.7	680.1	393.5	189.3	166.9	205	436.3	584.5
MTERF	151.4	157.6	93.2	88.5	88	82.7	148.2	148.8
MTERFD1	2322.1	3079.3	1065.6	589.1	452	720.4	1951.5	2828.3
MTERFD2	110.9	132.9	103.8	100.6	103.5	110.8	125.2	128.4
MTERFD3	111.5	118.9	90.3	91.5	89	93.1	96.4	89.5
MTERFD3	88.6	101.7	108.4	98	93.8	101.8	80.3	105.4
MTF1	273.1	402.4	348.2	492	404.6	550.4	254.2	656.4
MTF2	673.9	660.8	293.8	213.5	242.7	279.6	598	817.9
MTFMT	80.2	101.4	97.2	91.1	92.3	96.6	87.9	89.8
MTFR1	721.5	897.5	551.9	554.4	378.9	702	701.4	1632.6
MTG1	183.4	214.2	165	130.2	121.4	130.8	126.6	152.7
MTHFD1	345.3	464.5	287.6	201.8	196.8	373.1	229.7	341.7
MTHFD1L	1029.5	1663.9	1018.8	771.3	536.6	1097.6	900.1	1747.4
MTHFD2	2126.5	2880.8	780.7	534.1	423.3	501.9	2005.1	2872
MTHFD2	81.3	97.7	90.6	81.4	75.4	83.4	86.8	91.1
MTHFD2	3540.3	4899.8	2180.6	1804.4	1478.1	3097.1	3128.5	5289.2
MTHFD2L	297.9	337.1	196.7	206.8	175.5	239.9	284.3	479.3
MTHFR	136.4	148	134.5	164.7	170.3	185	133.9	151.7
MTHFS	516	622.4	418.3	474.7	350.6	514.8	516.9	972
MTIF2	2400.9	2719.7	896.3	1076.9	870.6	1687	1560.3	2292.8
MTIF2	96.3	102.7	91.6	77.7	86.5	83.9	88.4	99.2
MTIF3	1143.8	1544.6	359.7	392.2	304.8	481	1017.7	1292.7
MTL5	573.6	791.5	344	294.3	255.6	277	485.1	452.8
MTL5	133.9	144.8	107.6	106.3	102.6	112.5	118.3	161.6
MTM1	578.7	797.7	475.5	398.8	306.1	643.1	396.1	624.3
MTMR1	76.9	83.5	94.9	89.1	84.8	88.4	81.2	83.6
MTMR11	1114.7	1588.2	597.7	290.9	232.1	306.7	924.2	989.3
MTMR12	189.7	253.6	175.8	191.2	154.9	303.4	174.3	194
MTMR14	310.4	363	204.4	224.6	173.8	223.7	263	354.7
MTMR15	267.8	301.2	249.3	215.3	207.2	280.4	221.5	379
MTMR2	96.5	136.5	106.6	88.4	77.5	99.6	94.5	140.5
MTMR2	218.3	287.2	182.4	197.5	151.8	193.5	208.8	193.8
MTMR3	403.5	468.6	376.9	606.5	465.5	900.7	379.8	759.6

MTMR3	86	97.4	87.4	91.6	89.1	87.4	92.5	88.5
MTMR3	117.8	166.7	109.5	119.4	94.6	122.5	120.5	150.5
MTMR4	470	554.8	315.6	341.9	274	506.4	444.3	782.6
MTMR6	578.1	785	251.8	249.3	202.5	365.3	590.9	1080.5
MTMR7	93.6	116.8	91.4	77.7	84.5	84.3	93.2	110
MTMR8	113	128.2	101.6	92.4	89.4	94.3	108.5	109.7
MTMR9	682.3	939.4	511.6	470.4	372.1	515.8	671.6	919.4
MTNR1A	80.6	88.2	95.9	95.1	85.7	80.1	79.5	85.8
MTNR1B	76.4	108	91.3	93.2	90.7	89.5	82.6	85.2
MTO1	143.3	167.5	117.6	103.3	100.1	108.6	130.9	175.1
MTO1	2570	3266.9	1836.5	1636.3	1251.7	2216.6	2110.2	3472.2
MTP18	355.9	514.1	233.6	152.3	168.4	195.2	290.6	375.7
MTP18	219.6	285.5	183.9	134.1	129.2	148.8	173.4	192.1
MTP18	2000.3	2381.3	943.1	647.5	531.6	700	1635.6	1475.5
MTPN	6030.4	7145.5	3309.5	4123.1	2970.9	5012.8	5386.8	9830.4
MTR	670.4	814.9	192.5	279.1	140.1	289.3	559.8	837.5
MTRF1	251.4	262.7	126.7	104.5	110.5	131.4	189.8	263.5
MTRF1L	1168.7	1765.6	519.5	530.2	415.1	671.9	1128.5	1436.5
MTRR	111.6	152.2	89.1	93.2	72	89.3	127.9	136.1
MTRR	307.8	360.4	133.5	128.9	116.7	157.7	199.3	252.7
MTRR	1838.2	2515.8	1077.4	1282.2	790.5	1427.8	1411.5	2024.1
MTSS1	188.3	217.6	107.1	113.7	133.5	144.9	177.3	221.4
MTTP	77.4	87.5	91.2	89.1	87.4	81.3	81.3	93.3
MTUS1	74	83.4	85.1	79.9	79.2	81.2	81.3	84.6
MTUS1	128.5	141.9	82.6	69.4	82.3	84.6	126.1	134.4
MTUS1	96	104.5	87.9	79.9	71.9	97.2	94.8	117.8
MTUS1	74.9	76	91.8	76	69.3	78.6	78.9	76.1
MTUS1	80.9	92.4	87	87.7	86.2	80.6	89.3	90.7
MTX1	755.8	998.1	452.2	277.5	247.3	244	635.7	662.7
MTX1	1031	1270.3	755.4	575.3	457.4	575.7	967.6	1134.1
MTX2	1551.2	1962.5	834.5	912.1	636.3	987.9	1205.9	2216
MTX2	82	96.4	91.8	95.4	83	89.2	81.3	88.1
MTX3	453.2	565.5	367.3	438.6	358.8	628.3	366	660.2
MUC1	95.2	107.8	111	94.8	95	101	97	109.2
MUC1	128.1	161	138.3	150.1	167.5	179.8	129	145.1
MUC1	320.2	341.4	198.6	224.8	220.7	224.9	231.6	237.9
MUC13	104.8	140.8	85.2	78.4	67.7	79.2	106.7	121
MUC15	92.6	108.2	87.5	87.5	80.6	83.5	99.7	101.3
MUC16	136.7	166	101.6	87.8	82.4	90	124.9	152.4
MUC17	95.8	107.9	93.5	92.2	87	99.1	94.1	101.9
MUC2	115.3	127	99.8	91.6	96.1	100.4	92.8	110.8
MUC20	78.6	83.3	86.7	86.2	88.1	88.6	81.2	74.7
MUC4	115.1	137.7	91	78	79.1	85.5	115.5	129.1
MUC4	107.7	113.8	107.7	102	95.2	105.8	100.8	101.7
MUC4	72.8	82.2	82.1	93.1	78.9	82.6	69.3	78
MUC4	129.5	179.9	94.4	92	80.9	91.2	122.6	151.9
MUC7	83	87.2	85.1	82.3	78.8	88.2	79.4	89.4
MUCDHL	84.2	93.2	89.7	85.8	83.5	87.3	76.4	87
MUCDHL	103.4	111.8	106.5	84.8	92.9	92.6	92.1	116
MULK	649.6	850.4	432.1	466.3	369.3	651.8	517.8	736.5

MUM1	1198.3	1402.8	1299.9	1167.2	980.6	1374.8	878.6	1271.2
MUM1L1	75.8	82.3	89.4	82.2	86.3	81.4	90.5	88.3
MUS81	432.9	524.3	292.3	299.2	291.6	443.9	305.3	612.6
MUSK	223.2	291.5	146	136.2	134.9	164.2	222.7	292.3
MUSTN1	93.6	126.7	103.9	102.5	91.9	104.3	98.5	105.5
MUT	86.8	89.6	95.5	97.7	90	89.6	89	93.1
MUTED	634	851.5	346.5	298.8	227.7	372.8	514.5	797.1
MUTYH	127	166.4	125.3	111.5	96.1	111.7	122.9	154.1
MUTYH	994.5	1088.6	671.6	390.3	364.8	483.5	700.7	718.6
MUTYH	137.8	148.6	102.4	101.5	90.4	99.9	130	158.7
MVD	188.1	280.2	183.4	145.3	164.6	215.7	228.3	346.7
MVK	136.9	157	116.2	130.9	120.5	126.7	125.1	150.6
MVP	150	219.7	158.5	131.2	109	190.2	166.8	241.2
MVP	296.4	380.7	335.5	255.7	251.8	380.4	272.2	427
MX1	417.9	562.8	410.4	338.4	352.8	316.2	323.3	314.1
MX2	246.3	329.4	334.4	529.4	396	609.5	239.9	279.7
MXD1	169.6	210.6	164	221.5	221.3	281.5	225.6	366.8
MXD3	328.7	391.8	222.9	235.3	210.4	283.7	253.8	265.3
MXD4	997.6	1223.9	913.5	1117.8	1167.5	1024.5	1018.3	1059.7
MXI1	105.4	133.4	92.2	84.9	83.2	82.6	105.3	100.8
MXI1	77.6	83.1	92.9	74	84.8	80.7	76.8	79.5
MXI1	130.6	193.4	112.3	109	97.5	127	129.6	132.7
MXI1	157.7	203.3	114.7	112.5	109.3	113.5	139.7	140.9
MXRA5	143.2	171.5	122.1	95.7	106.3	113.8	129.6	155.3
MXRA7	160	181.8	107.9	94.2	86.1	97.2	147.7	181.2
MXRA7	211.3	292.9	153.3	128.3	122.4	146.7	165.2	204.5
MXRA7	108.8	122.2	101.4	96.1	81	95	101.1	131.7
MXRA8	75.5	87.7	89.3	82.8	81.5	85	80.6	84.6
MYADM	81.6	88.7	102.7	86.8	86.9	93.3	79.2	78
MYADM	238.5	346.7	129.9	131.6	114.5	115.9	199.5	384.9
MYADM	2815.2	3756.9	2517	2293.2	1992.3	2524.7	1950.3	2730.8
MYADML	118.9	122.9	98.6	89.4	88.3	90.5	98.8	111.5
MYB	11286.9	13381.3	9184.3	7672.6	5933	9639.4	6697.5	7202.9
MYBBP1A	136.9	179.6	133.6	119.3	86.8	113.6	120.4	150.7
MYBL2	114.6	140.8	102.5	88.2	82.6	92.6	109.2	133.7
MYBPC1	81.7	89.4	80	74.4	65.1	82.1	78.9	92
MYBPC1	96.6	107	89.7	87.2	90.2	94.9	94.5	132.1
MYBPC1	116.1	120.7	98.7	89.1	96.6	99.4	102.9	108.8
MYBPC1	88	102.6	93.1	84.9	88.1	97.5	92.1	102.8
MYBPC2	81.2	91.1	100.6	89.9	78.7	90.4	83.4	82.2
MYBPC3	104.7	108.4	123.4	116.9	130.9	134.4	97.4	103.8
MYBPH	895.4	1216.1	537.8	404.2	406.6	373.1	410.8	311
MYBPHL	77	98.9	83.6	109.4	116.2	110.1	88.8	96.8
MYC	2203.5	2887.4	630.4	773.7	487.8	725	1631.1	2355.4
MYCBP	190.5	238.2	132	130.5	106.7	159	156.2	176.1
MYCBP2	556.5	917.7	544.1	1118.1	847.6	1344.7	637	1178.3
MYCBPAP	115.8	125.2	98.4	102.6	101.9	116.5	100.6	124.3
MYCL1	96.6	120.9	82.2	77.7	90.5	98.5	94.1	103.9
MYCL1	141.7	162.4	107.7	100.6	97.2	105.1	131.4	161.4
MYCL1	108.7	135.3	97.8	86	80.1	82	116.7	138.2

MYCN	107	113.2	93	73.7	80.8	93	105.2	122.9
MYCT1	120.7	123.5	86.2	93.3	95	93.6	113.9	119.4
MYD88	391.5	534.9	365.4	370.9	280.9	484.7	298.1	541.4
MYEF2	118.4	141.3	91.3	90.2	72.2	81.8	93.6	131
MYEOV	108.5	109.8	88.5	93.2	92.8	90.7	105.4	107.4
MYEOV2	137.1	161.7	95.6	96.8	87.2	90.8	112	137.9
MYF5	91.6	92.4	96.3	95.3	95.1	88.4	83.1	92.2
MYF6	96.7	104.4	97.3	89.8	81	86.4	99.1	106.3
MYH1	99.3	109.1	89.3	92.2	92.4	85.6	101.4	104.1
MYH10	232.3	313.6	200	509.3	442.7	648.3	190.3	243.3
MYH11	89	97.7	85.1	84.7	76	80.2	90.7	95.5
MYH11	85.5	81.2	100.4	92.3	83.1	96.1	84.4	75.3
MYH13	78.2	88.2	82.5	88.6	73.2	80.5	87.3	85
MYH14	98.6	120.8	103.2	85.9	87.3	103.2	98	117
MYH14	102	101.9	95.4	99.2	95.6	85.7	89.5	101.2
MYH16	161.1	179.3	106.1	82.6	87.6	92.5	168.3	168.2
MYH2	82.3	84.4	88.3	81.9	73	88	79	78.4
MYH3	84.6	86.4	89.9	89	95.4	90.7	83.1	88.2
MYH4	227.1	279	143.2	149.9	121.7	153	224.2	213.9
MYH6	95.3	114.5	114.2	96.4	102.6	93.9	99.6	94.3
MYH7	105.3	130.3	110.8	101.3	96.9	97.5	107.2	113.1
MYH7B	103.1	112.3	87.6	88.9	86.4	84.3	100.1	108.3
MYH8	81.4	85.5	88.7	78.9	81.9	95.2	79.8	94.8
MYH9	3329.5	4621.7	5077.8	8189.8	6160.5	10539.1	3155.4	6721.3
MYL1	97.3	118.8	101.9	100.2	87.3	103.2	99.6	117.5
MYL1	80.2	87.2	87.8	90.9	86	81.3	80.7	89.6
MYL2	100.5	102.4	103.5	99.9	87.2	92.4	97.9	97.1
MYL4	83.1	94.1	84.3	82	76.7	73.9	76.2	90
MYL4	90.8	98.2	87.7	88.7	87.1	86.1	91.3	91.1
MYL5	396.2	502.9	351.1	297.8	294.2	366.2	319.8	555.7
MYL6	21063.2	22029.7	17678.2	24265.7	21386	27961.4	20469	21099.6
MYL6	18703.4	19723.4	17854	24623.3	21415.4	30714	18813.5	21850.3
MYL7	115.7	142	102	89.3	94.1	102.7	110.9	116.3
MYL9	122.5	138.8	94.4	89.1	97.8	77.8	117.1	131.5
MYL9	105.6	125.1	124.8	161.7	131.3	192.4	102.8	140.9
MYLC2PL	97.7	93.4	91.7	83.8	84.9	76.8	85.7	88.9
MYLIP	772.3	967.5	414.1	720.8	672.6	1169.8	542.3	747.3
MYLK	84.7	89.8	124.6	126.4	117.2	117.1	92	94.3
MYLK	93.3	118.5	89	87.9	84.3	91.8	83.7	110.6
MYLK2	132	143.3	113.4	91.6	99.7	91.7	115.3	117.6
MYLPF	96.8	108.7	93.2	81.4	80.7	84.7	97.4	105.7
MYNN	92.7	89.7	90.8	90.5	90.4	88.5	83.3	94.3
MYO10	171.7	219.3	132.9	136.4	135.8	196.7	171.9	196.5
MYO15A	106.2	111	85.9	85	78.3	79.2	100.3	118.6
MYO18A	187.9	208.2	157	172.8	191.2	205.2	164.2	194.5
MYO18A	112.8	144.5	94.3	94.2	88.4	99.4	130.2	151.2
MYO18B	75.6	85.7	86.8	84.1	75.8	89	79	82.7
MYO1A	108.5	123	113	117.4	109.8	103.2	116.1	113.5
MYO1B	105	131.9	95	90.9	102.7	108.1	104	125
MYO1C	104.7	111.1	136	110.7	103.3	101.4	104.4	154.4

MYO1C	70.9	78.1	81.3	85.2	80.4	78.2	75.8	87.6
MYO1C	104.2	132.2	130	120.9	123.3	151.4	106.2	125.5
MYO1D	78.7	90.3	83.7	92.5	76.1	90.4	76.9	86
MYO1E	136.1	163	103.4	93.3	84.9	94.3	135.3	136.9
MYO1F	386.1	477	484.4	368.4	288.3	708.1	308.8	509
MYO1G	725.1	871.3	757.8	834.7	773.3	1363.5	663.2	1169.7
MYO3A	81.6	93.1	87	89.3	86	82.8	81.6	88.4
MYO3B	168.7	172.6	152.6	158.8	165.8	151	167.9	260.2
MYO5A	258	328.9	159.1	212.8	166.8	211.1	296.6	403.5
MYO5B	83.1	100.3	100.6	87.3	87.3	95.1	89.5	94.8
MYO5C	108.6	118.9	104.9	169	125.8	227.8	111.3	104.4
MYO6	265	342.6	164.8	210.8	187.3	222.2	262.5	299.8
MYO7A	89.4	99	99	130.3	109.4	152	89	97.5
MYO7B	87.1	99.4	94.1	85.5	90.7	90.6	94	95.9
MYO9A	188.3	247.7	225.9	409.3	381	631.6	199.4	220.2
MYO9B	225.3	246.1	265.7	235.6	243.4	258.2	242.7	449
MYOC	81	94.5	96.3	75.3	81.8	74.6	95.1	88.1
MYOCD	95.7	112	111.9	97.8	83	98.3	90.1	101.1
MYOD1	80.6	89.9	109.2	98.4	88.7	98.5	91	88.6
MYOG	100.1	123.8	91.9	87.5	81.7	84.6	100.3	121.3
MYOHD1	128.4	148.9	93.7	91	95.2	90.4	117	123.5
MYOHD1	99.5	106.8	105.4	101	94.5	97.3	96.1	99.3
MYOHD1	208	255.4	211.2	185.2	159.8	199.5	176.9	253.3
MYOM1	83.2	85.6	101.3	97.3	88.7	94.8	81.7	85.6
MYOM2	118.9	143.9	113.5	104.6	94.7	106.9	128.6	142
MYOM3	86.9	106.3	101.6	99.6	89.7	85	94.7	92.9
MYOT	94.4	103.8	95.6	100.6	92.6	93.4	89.4	94.3
MYOZ1	204.2	274.8	177.1	153.2	122.2	146.4	175.7	187.8
MYOZ2	77.6	84.4	93.3	85.5	86.1	95.2	83.8	74.5
MYOZ3	107.4	128	112.8	103.1	105	100.9	101.4	97.6
MYPN	79.5	81.8	98.8	84.2	88	90.3	89.2	85.5
MYR8	99.2	98.8	88.5	84.8	77.5	85	92	98.7
MYRIP	78.7	88.7	91	80.3	73.3	72.9	80.3	82.7
MYST1	651	874.9	431.2	324.3	279.8	388.1	582.8	552.9
MYST2	131.6	163.9	110.9	105.2	95.2	120.2	122.4	177.7
MYST3	556.6	881.2	671.8	949.3	686.1	1745.4	644.2	1627.2
MYST4	91.8	100.1	100.2	108.7	95.9	115.3	89.5	103.9
MYT1	88.8	102.3	92.8	80.7	80	82.4	98.2	89.9
MYT1L	76.2	92.6	90.3	77.8	76.8	94.1	87	96.8
N4BP1	180.8	184.7	135.5	148.4	129.5	150.5	165.8	190.2
N4BP2	929.1	1022.3	417.2	640.4	635.9	422.4	747.3	834.9
N4BP3	78.7	89.8	88.2	78.5	84.8	86.1	86.1	90
N6AMT1	144	167.9	149.1	169.7	151.7	208.8	121.6	134.8
NAALAD2	129.9	130.2	93.5	90.5	75.1	88.1	117.2	159.2
NAALADL1	85.2	84.1	90.9	88.1	83.9	92.5	88.2	95
NAALADL2	125.6	146	82.3	92	83.1	83	116.2	147.7
NAB1	384.8	410.2	246.9	280	233.1	420.9	310.2	646.9
NAB2	144.6	144.3	109	98	105	100.8	129.1	235
NACA	17721.2	19124.9	5577.7	6626.7	5294.3	8353.4	15198.8	20216.2
NACAL	102	116.8	79.6	75.6	78.4	75.7	96.1	114.2

NACAP1	545.6	705.7	215.9	143.4	152	227.1	502.1	734.3
NADSYN1	409.2	418.7	253.9	258.6	215.4	297.8	340.6	586.1
NAG	154.7	182	135.4	154.9	130.4	165.9	156.3	162.8
NAG18	22709.4	23369.2	10716.8	15659.9	13054.3	10952.8	23618.5	23809.7
NAG6	211.3	220.7	166.6	165.6	176.9	203.9	165.7	157.2
NAG8	165.8	219.6	116.9	363.7	300.7	316.9	219.3	213.6
NAGA	305	375.6	278.1	314.7	244.3	386.8	213.3	327
NAGK	448.8	596.8	319	359.6	361.8	476.6	351.7	497.3
NAGLU	209	319.4	155.9	150.6	127.1	186.9	195.3	302.2
NAGPA	314.8	447.7	320.5	229.4	205.7	273	318.6	506.7
NAGS	125.8	157.6	98.1	87.2	87.4	96	119.6	146.2
NAIP	118.3	124.7	87.5	89.8	89	94.2	120.6	147.6
NAIP	126.7	165.8	102.7	162.7	166.4	122.8	118.8	131.1
NALP1	99.2	119.4	96.7	92.2	86.7	96.1	108.1	116.2
NALP10	161.4	182	111.8	107.8	107.7	108.9	142	148.2
NALP11	87.2	105	84.8	86.5	81.5	82.7	89.5	104.5
NALP12	121.5	134.1	101.6	87.7	83.6	93.4	102.5	128.4
NALP12	240.9	242	246.3	172.6	120.8	217.6	324.3	697.3
NALP12	95	120.2	114.7	96.6	86.6	96	111.4	116.5
NALP13	84.6	88.6	92.1	90.9	96.5	87.4	88.7	88.4
NALP14	83.1	80.4	90.1	87.4	76.9	89	76	78.8
NALP2	90.3	129.1	92.2	176.3	166.4	202.3	93.3	96.7
NALP4	101	109.6	86.2	74.9	78	86.7	85.2	92.6
NALP5	95.3	119.9	94.1	95.5	90.7	94.5	103.4	98
NALP6	87.1	97.1	87.8	81.6	81.4	79.4	86.5	95.5
NALP7	116.7	142.4	89	104.6	92.4	95.7	129.7	148.5
NALP7	89.7	97.2	83.4	82.4	82.8	94.9	92	108.3
NALP7	90.1	88.9	91.9	98.5	85.2	85.3	88.4	101.7
NALP9	87.4	95.7	83.3	83.8	72.7	75.2	91.7	96
NANOG	87	92.5	93.9	84.7	88.6	85.8	85.7	97.4
NANOS1	118	150.7	111.4	108.8	98.5	102.6	116.1	135.6
NANOS1	86.6	92.5	97.7	95	83.4	93.8	94.5	100.5
NANOS1	97.1	107.4	87.2	85.9	80.8	84.1	94.8	100.1
NANOS2	96.7	109.5	93.9	79.6	79.4	84.6	89.4	96.2
NANP	210.1	291.4	110.9	105	104.1	129.1	187.6	264.7
NANS	934.2	1169.3	299.3	163.7	139.8	203.9	800.9	1079.3
NAP1L1	552.1	758.2	269.9	260.4	258.3	455.9	415.9	813.6
NAP1L1	98.6	108.5	83.3	81.9	75.3	88.1	100.1	128.9
NAP1L2	87	110.4	103.7	97.7	94.7	88.3	87.9	90
NAP1L3	139.7	170.8	117.9	103.7	102.1	129.6	132.7	160.1
NAP1L4	2889.2	4140.3	2442.1	2050.9	1757.5	3110.9	2451.4	3670.5
NAP1L5	430.6	487.2	322.9	273.2	204.3	335.3	354.7	516.7
NAP5	75.3	81.9	107.3	84.7	82.9	97.1	70.4	83
NAP5	121.4	121.3	83.1	80.4	76.3	79.1	101.5	138.6
NAP5	70.2	88.4	84	73.8	68.3	69.1	79.1	85.1
NAPA	159.5	198.1	142.8	107.9	108.6	106.8	152.9	234.8
NAPB	246.1	251.1	199.5	315.5	263	320.8	214	287.5
NAPE-PLD	178.4	244.1	126.2	83.5	97.9	112.4	170	216.9
NAPG	235.2	292.8	121.7	110.1	112.7	129.6	204.1	326.8
NAPRT1	4263.1	5204.5	2276.6	1827.7	1708.9	1991.8	3361.6	3022.8

NAPSA	240.5	299.7	202.4	157.6	162.3	202.7	208.5	228.3
NAPSB	1656.3	2090	1444	1260.8	1067.3	1582.5	1080.7	1644.7
NARF	508.3	683.4	436.4	505.2	524.6	668.7	638.9	848.1
NARFL	266.1	307.5	180.1	146.3	125.3	211.1	265.6	315.1
NARG1	375.8	454	269.3	271.9	212	383.8	307.1	459.3
NARG1L	414.9	400.2	212.3	243.4	214.8	267.7	311.8	546.5
NARG1L	246.8	320.9	161.5	139.6	130.9	158.4	186.4	222.5
NARG1L	97.1	114.8	104.4	95	95.9	97	96.9	115
NARG2	176.2	210.8	109.1	100.4	91.9	108	177.6	227.4
NARG2	238.6	273.6	124.3	151.9	110.5	129.7	167.3	264.2
NARG2	108.1	143.7	115.9	129.1	111.8	133.7	123.3	159.4
NARS	4799.3	6514.6	1738.7	2436.5	1478	3200.7	4278.2	5956.5
NARS2	443.6	545.6	210.4	210.8	183.5	301	312.4	529.6
NASP	114.6	126.3	95.3	88.8	98.7	94.6	111	127.1
NASP	829.6	1105.6	518	638.5	620.1	839.5	648.3	751.4
NAT1	200.1	213.2	119.1	95.8	88.3	113.5	142.3	186
NAT1	763.5	1078.1	430.2	296.7	253.3	420.8	571.4	780.4
NAT10	2109.5	2744.6	1668.5	1496.4	1162	1600.4	1729.1	1570.5
NAT11	132.6	145.3	137.4	114	103.7	129.6	122.5	154.9
NAT12	237.1	287.5	122.6	119	95.4	139.5	207.1	299.2
NAT13	413.2	525.1	175.9	152.1	133.1	184.6	367.8	791.8
NAT2	85.5	89.3	92.2	90.3	84.2	90.3	88.2	93.4
NAT5	4799.4	6584.9	3754	4230.6	3124.9	4801.6	3968	4296
NAT5	79.5	97.7	101.1	88	92.4	88.7	88.4	79.5
NAT5	3022	4132.4	2139.2	2175.2	1547.9	2761.4	2300.2	2856.3
NAT6	239.1	280	257.4	352.5	282.4	378.3	209.4	383.4
NAT8	86.2	90.1	90.7	95	96	92.7	82	79.3
NAT9	576.7	590.1	452.3	381.9	347.3	498.6	456	628.8
NAV1	114.6	150.3	110.1	103.1	90	115.7	109.1	124.3
NAV2	101.2	120.6	95.4	99.1	91.5	87	105.3	99.4
NAV2	123.8	158.6	113.1	92.7	90.9	94.2	109.1	131.5
NAV3	210.8	257.7	157.2	258.4	216.9	412.1	145.4	164.2
NBEA	80.6	68.4	113.7	93.3	67.7	76.8	92.7	93
NBEAL1	83.6	85.3	86.8	91.8	78	92	74.7	89.5
NBL1	362.1	455.3	542.1	318.7	236.4	331.8	409.5	710.5
NBL1	214.6	304.5	197.6	145.7	135	183.9	230.8	381
NBLA1038:	86.6	107.9	88.3	102.8	101.5	116.6	99.5	123.3
NBN	96.3	120.1	88.9	83.3	82.8	85.6	92.2	88.3
NBN	163.9	226.8	126.8	113.1	96.6	139.8	149.2	188.2
NBPF1	188.9	216.3	128.7	133.3	147.2	124.3	146.7	202.8
NBPF1	120.8	143.1	108.6	89.5	88	97.7	113.2	138.7
NBPF10	2090.9	4045.3	798.3	1590	1819.7	1646.3	2368.8	2981.7
NBPF11	691.9	874.2	491.6	615	592.1	1100.8	534.7	973.7
NBPF14	171.5	212.3	141.4	134.1	156.7	207.4	139.1	172.3
NBPF15	79.8	99.1	83.7	84.5	73.6	77	87.1	84
NBPF20	1809.4	4017.6	1066.4	2865.5	2979.9	2239.8	2229	2215.9
NBPF3	257.7	324.7	212.3	236.3	234.6	353.3	201.1	396.4
NBPF3	108.3	123.4	108.9	100.8	88.5	92.3	104.1	120.5
NBPF7	89.5	105.7	94	93	83.6	87	98.1	95.4
NBPF9	93	103.1	97.5	111.2	107.6	92.9	94.5	93

NBR1	115.8	140.5	106.7	123.2	120.2	92.7	105.5	113.9
NBR1	84	100.2	100.2	95.1	95	90.3	78.5	92.2
NBR2	225.6	286	105.9	102	92.9	105.1	196.8	232.7
NCALD	492.7	630.2	412.6	655.9	558.3	773.3	328.3	351.2
NCALD	322.6	400.7	287.7	487.4	431.7	541.5	273.3	255.2
NCALD	83.5	79.9	88.3	94.4	83	91.7	82.2	82.9
NCAM1	111.2	114.5	84.9	78.3	82.8	88.4	102.1	112.3
NCAM1	139.1	171.5	103.2	101	95.7	101	141.2	140.9
NCAM1	256.3	298.1	217.7	108.5	100.9	118.3	249.1	266.5
NCAM2	599.2	800.4	403.5	1293.7	1154.3	2408	509.6	722.6
NCAPG2	1233.1	2050.9	1221.7	1168.7	948.9	1744.9	1014.1	1368.8
NCBP1	1367	1681	566.4	520.2	324.6	566.9	1087.7	1440.9
NCBP2	1964.4	2451.5	753.3	779.4	459.5	1011.8	1395.1	2366.8
NCBP2	142.9	167	120.9	126.3	104.7	111.9	118	162.6
NCBP2	759.9	938.5	353.5	401	350.8	444.5	545.8	742.9
NCDN	109.6	140.3	96.7	99.2	73.8	94.5	107.7	145.1
NCDN	107.3	118.2	129.3	116.7	112.4	116.9	104.6	110
NCDN	117.5	131.9	160.4	132.6	120.4	164.4	116.4	130.9
NCF1	909.6	1255.3	546.9	368.3	371.2	259.4	779.7	1031.2
NCF1B	105.3	109.7	100.7	94.5	94	92.3	97.8	107.3
NCF1C	412.3	583.4	258.3	200.6	219.7	224.5	385.7	513.8
NCF2	159.3	205.9	95.4	101	89.5	105.5	159.2	209.7
NCF4	934.9	1113.3	295.4	247.8	304.5	303.1	774	691.5
NCF4	267.8	293.5	210.9	198.4	230.2	291.2	210.6	362.7
NCF4	1762.6	2289.4	1444.6	1024.2	962.6	1342.3	1396.1	1575.2
NCK1	989.4	1317.1	616.7	776.1	627.2	1212.2	724	1569.5
NCK2	145.4	170.3	96.1	96.4	85	98.2	113.2	164
NCKAP1	83.4	90.4	92.5	88.2	80.9	94.2	77.1	96.4
NCKAP1	85.8	93.2	102.2	92.1	93.2	94.9	81.9	99.3
NCKIPSD	125.4	170.5	94.7	96.2	91.1	91.8	127.4	150.9
NCKIPSD	731.8	906.9	313.5	257.4	233.2	284.5	453.7	429.1
NCL	9705.7	11703.1	4192.6	4488	3152.8	6878.8	8741.1	11271.6
NCOA1	524.8	694.9	282.1	398	358.7	475.7	465.7	465.6
NCOA1	99.7	124.2	107	122.8	107.8	122.5	101	115.3
NCOA1	103.3	105.7	102.3	96.5	95.1	98.3	105	123.7
NCOA2	133.5	166.9	102.4	111.9	88.9	103.7	119.9	147.7
NCOA3	104.3	121.8	101.6	109	97.2	106.8	103.5	120.9
NCOA3	415.9	557.1	270.9	451.8	321.1	533.2	471.5	799
NCOA4	5969.1	7457.8	4586.2	6284.3	5380.3	8045.9	4696.5	5558.3
NCOA5	520.4	685.6	788.8	599.1	554.1	738.1	489.1	1103.4
NCOA6	739.1	909.9	516.7	858.4	672.1	843.3	660.2	890.8
NCOA6IP	527.7	572.6	297.4	202.7	191.8	227.6	373.7	344.8
NCOA7	529.4	713.8	413.4	553.3	430.7	724.8	663.2	964.1
NCOR1	190.5	270.4	160.7	167.2	176	237.3	202.2	266.3
NCOR2	365.1	521	363.9	421.5	319.8	694	367.7	617.5
NCOR2	784.3	1058.8	1263.2	1276.5	912.9	1795.5	763.1	1593.6
NCOR2	86.3	91.2	114.4	97.1	102	108	88.9	92.8
NCR1	122.7	159.4	91.6	80.6	80.8	72.4	128	142
NCR2	89.6	107.7	97.5	85.6	86	83.2	91.3	97.6
NCR3	136.8	163.7	149.2	120.8	98.8	119.6	148.8	157.3

NCSTN	4815.4	5731.4	4040.8	4703.8	3820.7	4111.5	4002	4699.8
NDE1	486.1	641.8	246	171.2	196.1	181.7	505	598.1
NDEL1	705.3	1013.2	665.5	748.1	584.9	867.1	890.6	1133.2
NDEL1	107.6	132.3	122.1	127.4	101.2	129.6	117.4	147
NDFIP1	254.4	314.4	170.3	214.9	168.8	262.2	207.7	322.2
NDFIP2	1833	2279.3	1369	1845.4	1273.8	2346	1607.8	2421.2
NDN	77.8	86	94.7	86.4	85.4	91.2	81.1	83.6
NDNL2	167.6	212	112.7	95.9	95.6	97.9	157.3	199.6
NDOR1	110.1	121.5	84	81.5	77.3	83	116.6	136.6
NDP	120.8	134.3	96.7	88.2	92.1	105.8	120.7	128.3
NDRG1	333.7	387.6	158.4	209.5	219.6	234.3	242.7	300.9
NDRG2	147	169.5	106.3	85.3	86.5	88.5	126.7	181.2
NDRG2	545.1	751.1	427.3	308.7	217.3	281.2	503.7	620.8
NDRG3	342.9	437.2	183.2	226.2	178	248.3	290	472.2
NDRG3	182.7	280.4	160	125.7	106.4	130.8	151.6	195.3
NDRG4	100	127.3	91.1	83.2	80.3	84.8	99.1	139.7
NDRG4	89.4	96.6	109.6	100.1	99.6	106.8	92.2	93.5
NDRG4	102.2	105.7	111.1	92.3	96.7	101	94.2	98.5
NDST1	129.2	177	152.6	124.4	109.1	183.6	144.2	175.8
NDST2	316.9	356.9	325.3	229.4	217.7	232.2	234.6	244.5
NDST3	89.8	89.7	92.9	93.1	91.6	86.2	92.6	90.8
NDST4	84.1	72.8	102.6	97.6	99.5	107.8	73.9	75.2
NDUFA1	17621.2	21291.1	15453.1	14270.6	12193.4	15645.6	14346.2	18259.6
NDUFA10	750.9	928	326.7	183.1	193.1	214.8	528.8	642.6
NDUFA11	4830.8	5363.1	4025.8	2437	2129.9	2919.6	3729.6	3619.3
NDUFA12	4499.8	5149	3216.4	2679.6	2277.9	2963	3727.3	3991.6
NDUFA13	3345.1	3793.1	2545.3	2216.5	1870.1	2479.4	3157.5	3695.9
NDUFA3	6834.1	7236.6	6173.1	3987.5	2857.4	3258.7	5788	7186.5
NDUFA4	14974.9	16206.7	7919.9	9984.9	6011.9	8765.2	16890	20907.9
NDUFA5	96.6	103.6	98.9	84.8	81.2	99.7	96.1	116
NDUFA7	1461.7	1793.4	1170.7	736.3	526.5	622.6	1449.4	1680.1
NDUFA8	4823.4	6444.6	3815.3	2965.9	2244.7	2709.4	4174.7	4896.1
NDUFA9	931.2	1399.7	575.4	469.2	373.8	508.6	808.8	1126.4
NDUFAB1	7759.7	8362.7	3978	3318.3	1999.2	3027.8	7449.4	10240.7
NDUFAF1	646.3	974.3	397.7	357.2	286.3	443.7	604.5	818.1
NDUFB1	141	172.6	153.7	122.4	119.2	135.8	120.4	146.9
NDUFB10	5210.8	5559.4	1114.4	946.4	939.7	888.6	5090.6	5628.7
NDUFB11	5786.2	7591.4	4297.6	3653.8	2799.8	3637	5786.2	8339.3
NDUFB2	6099	7422.9	4210.5	5093.5	4153	5336.7	5199.7	6032.7
NDUFB3	7733.8	8643.9	4600.5	4537.1	3274.2	5557.2	7474.6	10581.8
NDUFB4	75.3	84.2	84.7	83.5	77	84.9	81.5	83.9
NDUFB5	7169.3	9398.9	7050.3	8801.3	6792.8	8957.7	7601.7	9136.9
NDUFB6	2243.7	2571.6	1334	1242.1	945.9	1052.4	2216.8	2090.6
NDUFB7	3573.8	4213.2	2663.6	2062.9	2044.7	2655.2	3265.7	3517
NDUFB8	12029.3	15230.3	11548.2	8017.2	6944.1	9216.1	12017.3	13603.6
NDUFC1	1876.5	2122	1273.6	832.4	656.1	799.3	2131.9	3797.1
NDUFC2	111.9	119.9	109.7	108	109.2	92.5	106.1	102.4
NDUFS1	223.3	261.7	154.2	196.4	163.6	162.9	187	259
NDUFS2	324.9	385.2	156	145.1	154.8	136.9	300	315.9
NDUFS3	6268.3	7106.8	2986.2	2617.7	2453.3	2295.8	5339.9	5238.4

NDUFS4	5268.7	6178.6	2634.2	2728.1	1897.1	2539.5	4994.3	6294.2
NDUFS5	9042.4	11335.7	6015.2	4908.3	3632.9	5104.1	8521.2	10673.8
NDUFS6	97.5	105.7	89.9	92.9	81.1	81.1	99.4	104.2
NDUFS7	3625.3	4764.2	3018.9	1975.7	1690.5	1721.3	3576.5	3232.8
NDUFS8	6082.9	7865.3	3650.4	2788.1	2087.2	2676.8	6536.6	6792.4
NDUFV1	2293.1	3025.4	1960.8	1862.6	1562.2	2536.3	2292.2	2757.4
NDUFV2	4416.5	4845.5	3397.7	3862.9	2217.1	3292.1	4246.3	7609.9
NDUFV3	136.5	183.7	118.6	108.8	99.3	108.8	148.2	133.7
NDUFV3	514.6	610.7	256.4	192.1	146.3	222.5	379.9	561.6
NEB	119.1	153.3	98.7	92	88	100.1	111.1	138.8
NEBL	106.5	137.2	98.6	93.3	88.1	90.7	121.6	125.9
NEBL	81.2	87.9	81.2	74	75.3	78.6	89.2	93.9
NEBL	108.3	141.3	95.8	86.9	81.8	95	117.5	123.4
NECAP1	344.3	395.9	256.2	254.5	240.8	352	290.8	432.6
NECAP2	863	1191.1	802.1	702.8	719.8	881.7	770.3	1121.3
NEDD1	94.6	113.5	99.4	98	93.3	98.2	94.4	125
NEDD4	177.5	215.8	103.4	116	100.5	109.4	180.2	190
NEDD4	81.5	93	90.1	83.1	84.3	85.5	77.8	93.6
NEDD4	214.1	307.7	194.9	212.2	184.8	273.7	203.4	216.7
NEDD4L	115	149.9	138.3	126.6	126	145.1	118.1	152.4
NEDD8	14633.3	18714.6	13038.3	10139.6	8543	10700.5	15604	17933.4
NEDD9	105.2	123.3	84.5	89.8	96.6	88.5	101.4	91.5
NEDD9	144.8	186.5	106.7	114.1	96.9	125.4	142.4	189
NEDD9	122	158	86.5	72.5	90.5	87.6	136	152.8
NEFH	726.7	1112	531.8	261.3	214.1	302.2	581	655.7
NEFL	108.1	111.7	97.7	83.3	89.7	75.8	109.3	131.7
NEFM	110.2	111.4	104.5	89.3	96.2	93.1	96.4	102
NEGR1	81.2	87.1	91.5	97	82.1	85.5	90.9	91.3
NEIL1	157.9	178.1	108.8	110.8	105.9	106.8	142	142.5
NEIL2	286.3	379.1	189	133	119.6	139.4	244.3	392.5
NEIL3	87.7	104.8	86.4	76.4	76.8	74.9	82.5	104.2
NEK1	173.9	213.5	178.5	225.5	197.4	339.4	167.7	229
NEK10	101.5	113.5	105.9	98.3	106.4	109.3	84.7	93.9
NEK10	123.6	138.7	96.6	84.1	79.5	90.2	112.5	131.5
NEK11	95	118.9	97.8	93.9	85.1	98.5	98.8	102.2
NEK11	147.8	144.2	102.9	94.9	84.5	106.1	121.6	129.4
NEK2	946	1171.7	525.2	412.8	356.8	494.3	757.8	1339
NEK3	97.8	107.7	94	87.9	78.7	77	101	112.6
NEK3	403.9	518	180.1	174.2	158.3	209.7	343.3	499
NEK3	97.5	105.5	89.8	94.1	89.5	87.5	101.9	109.2
NEK4	131.9	170.2	104.6	109.9	110	123	122.3	124.4
NEK5	78.8	96	84.2	78.6	75.2	81.7	85.7	89.3
NEK6	497.8	655.5	432.2	360.5	291.3	395.3	502.2	714.4
NEK7	237.7	311.5	109.5	92.4	92.5	98.5	168.4	216.5
NEK8	174.4	188.1	146.3	193.4	198.9	179	130.3	143.5
NEK9	102.5	112.9	104.3	96.7	87.3	98.6	100.9	104.3
NELF	1517.3	1600.5	539.1	478.8	376.6	556.8	1151.4	878.9
NELL1	196.6	207.9	120.1	124.6	108.3	124	174.6	200.3
NELL2	127.4	148.8	126.1	130.4	102.7	138.5	101.3	127
NENF	4663.7	5974.8	2902.3	3510.3	2842.8	2886.8	4484.8	3881

NEO1	200.3	243.3	125.6	152.6	156.7	161.6	192.4	229.9
NES	102.5	120.6	98.2	91.8	87.9	99.2	111.7	121.2
NET1	1195.7	1553.3	792.4	561.6	411.2	725.7	1306.2	2466.3
NET1	134.5	150.8	90.5	86.9	89.2	94	126.1	129.5
NET1	377.8	491.3	218.7	142.1	163.5	170	316.5	355.9
NETO1	79.2	94.4	94.1	91.1	90.4	84.7	82.2	85.7
NETO1	74.8	85.2	104	98.2	87.4	94.5	81	87.2
NETO1	83	88.8	90.2	77.5	80.6	81.3	84.4	91.5
NETO1	75.1	71.1	90.9	83.7	84.9	92.3	77.8	66.4
NETO2	775.1	902	552.4	344.8	290.9	505.9	594.5	999.8
NEU1	764.1	1043.3	608.7	785.8	695.4	1057.2	823.8	1158.6
NEU2	80.5	92.4	95.1	94.1	92	89	76.6	88.5
NEU3	131.2	140.4	111.3	103.9	95	92.3	113.8	131.1
NEU4	111.9	142.7	92.5	86.4	82.2	85.7	103.6	125.4
NEURL	104.3	120.9	108.5	104.8	90.3	99.6	101.7	109.7
NEURL2	126.1	127	119.6	94.3	104.5	107.8	106.7	116.1
NEUROD1	94.5	112.9	101.8	87.9	90.8	98.2	100.2	99.9
NEUROD2	90	100	95.3	93.2	98.1	94.1	95.5	86.6
NEUROD4	95	124.9	91.9	89.7	83.1	86.4	104.4	114.1
NEUROD6	99	119.3	98.3	101.8	87.1	89.9	109.1	114.5
NEUROG1	95.1	112	98.4	98.5	102.2	96	102.9	114.3
NEUROG2	130.9	134.2	96.6	94.1	90.1	104.3	129.1	136.9
NEUROG3	125.6	141.8	94.9	95.4	92.4	94.8	111.7	133.4
NEXN	287.6	373.2	148.5	105.5	95.6	117.6	160.9	207.9
NF1	111.9	148.2	101.1	113.3	108.4	137.3	118.8	129.1
NF2	127.7	187.6	110.3	129.3	114.2	102.5	122.1	150.8
NF2	105.3	137	92.4	96	95.3	83.6	105.3	104.6
NF2	112.5	131.2	111.2	101.8	103.2	108.5	109.9	111.6
NFAM1	126.9	143.6	93	95.4	86	91.2	114.8	175.9
NFASC	112.3	126.4	90.8	85.7	80.9	88.4	106.3	131.9
NFAT5	123.8	144.8	96.5	104.7	101.6	93.7	122	129.1
NFAT5	129.5	153	96.4	104.8	109.8	91.8	124.9	141.8
NFAT5	77.5	106.1	89.6	89.3	81	87.2	81.9	86
NFATC1	354.9	493.5	154.2	142.3	128	148.6	306	367.8
NFATC1	117.1	127.2	85.9	81.5	75.1	90.3	104.6	150.2
NFATC1	81.6	94.2	93.6	83	78.6	92.2	75.8	87.4
NFATC1	118.3	152.4	109.3	79.7	70.4	81.3	106.9	155
NFATC2	74.9	80.8	79.4	86.8	79	72.8	73.2	74.1
NFATC2	84.3	93.7	95.9	92.7	88.9	86.9	82.8	90.4
NFATC2IP	308.5	325.7	148.8	150.7	157.2	150.3	256.7	356.3
NFATC2IP	145.9	169.1	108.8	88	93.2	95.9	122.5	162.3
NFATC3	243.5	346	199.7	227.9	207.1	246.3	216.9	271.3
NFATC3	75	89.1	94.3	91	88.5	95	83	85.6
NFATC3	198.8	282.3	157.9	159.5	172.2	203.5	183	197
NFATC3	134.7	184.8	141.2	156.1	131.1	179.2	141.9	162.9
NFATC3	99	121.7	112	112.6	97.9	109.8	99.9	109
NFATC4	79	102.1	90.7	85.9	70.6	86.1	82	86.1
NFE2	494.2	682.3	297	586.4	597.5	570.2	338.2	287
NFE2L1	333.9	449.9	365.8	393.2	364.7	642.4	375.8	544.6
NFE2L2	1567.4	2051	935.5	1035.4	809.8	1437.1	1516.5	4306.2

NFE2L3	307.3	411	183.2	197.1	238.4	240.7	240	286.6
NFIA	92.7	96.1	90.9	91.5	74.7	84.4	96.6	107.2
NFIB	161.2	145.8	91	92	84.4	87.9	110.6	115.5
NFIC	114.1	117.8	102.4	90.4	80.4	96.1	110.1	139.6
NFIC	93.5	93.7	104.4	100.5	94.6	98.2	93.3	106.8
NFIC	105.4	116.2	105.5	100.1	91.4	103.5	103.2	116.9
NFIL3	321.5	383.9	134.9	139.3	104.3	141.6	325.7	522.9
NFIX	361.4	415.9	358.5	222.1	146.3	203.2	338.6	975.7
NFKB1	2961.2	3825.7	2449.9	1879.7	1640.8	1815.7	2336.8	3114.3
NFKB2	115.9	138.9	113.3	96.9	97.3	105.4	116.1	129
NFKB2	99.3	124.2	114.9	107.3	103.3	106.2	116.7	120.5
NFKB2	92.5	100	90.4	86.3	78	81.7	79.1	92.6
NFKBIB	226.4	278.3	146.2	116.3	102.2	113.7	221.3	290.5
NFKBIB	142.1	183.6	102	92.5	91.5	84.3	147.9	193.9
NFKBIB	183.9	240.6	172.9	137.8	127.7	137.9	198.5	286.1
NFKBIE	659.7	780.3	299.4	230.5	245.3	246.4	511.6	686.5
NFKBIL1	158.4	185.9	101	109.8	89.8	104.2	143	195.5
NFKBIL2	150.7	152	176.2	124.1	122.3	138.1	111.7	141.8
NFKBIZ	130.7	163.5	110.6	111.1	108.5	108.3	122.6	136.3
NFKBIZ	391.1	396	301.6	450.3	518.9	525.7	317.6	481.4
NFRKB	156.2	185.4	174.2	111.5	126.5	148	141	208.3
NFS1	200.4	234.6	118.7	104.9	101.4	107.1	175.5	190.6
NFS1	1594.4	1962.8	576.6	382.5	341.6	298.8	1277.4	1037.8
NFU1	1635.4	1793.4	1211.2	1084.7	834.4	1602.9	1395.5	2408.5
NFX1	355.7	477.8	174.1	160.6	163.9	158.8	296.6	288.1
NFX1	636.8	950	325.8	236.7	211.8	275.6	431.8	590.9
NFX1	157.9	138.8	131.9	122.6	131.2	115.9	131.5	180.2
NFX1	2503.2	2359.3	726.6	735.7	558.2	818.1	2014.6	2994.5
NFXL1	374	414.7	216.2	244.9	224.9	374.8	370.4	560.3
NFYA	457.1	678.3	310.8	260.4	199.9	399.6	278.3	320.4
NFYA	89.9	113.9	100.8	100.2	89.7	91.5	89.8	112.5
NFYB	140.9	151.3	116.1	129.6	121.7	133.2	114.3	125
NFYC	2893.3	4112.4	2369.4	1972.9	1456	2679.8	2068.8	3487.1
NGB	165.6	193.9	115.5	110.5	113.1	124.4	156.5	172.2
NGDN	140.8	166.1	111.4	130.2	122.7	133.4	141.4	166.8
NGDN	2128.6	2700	897.6	818.2	555.2	1020	1831.7	2700.2
NGEF	91.8	105	97.4	81.5	76.3	88	97.6	97.8
NGFB	131.3	147.3	122.3	112.4	116	119.7	118.6	115.8
NGFR	117.6	124.8	87.6	76.6	78.9	96.5	108.9	154.4
NGFRAP1	270.7	235.9	163.1	124.5	118.7	128.3	163.6	215
NGFRAP1	1174.7	1737.2	555.3	329.6	264.4	360.4	1042.1	1404.6
NGFRAP1	6623.4	7943.8	3659.4	2092.2	1558.3	1758.4	6312	7958.5
NGFRAP1L1	333.9	457.2	232.5	91.8	85.6	95.5	295.4	426.2
NGLY1	836.6	924.4	316.5	381.4	333.2	457.2	632.9	1105.4
NGRN	3797.3	5289.4	2836.7	2661	1859.1	3261.8	3590.7	4412.1
NGRN	717.8	975.9	422.9	247.6	210.5	213.4	520	639.1
NHLH1	78.8	91.1	96	87.2	89.4	86.5	83.3	93.9
NHLH2	95.1	110.1	90.5	89.2	83.5	89.2	101.5	113.1
NHLRC1	85.4	89.2	94.1	84.1	79	82.8	76.7	91.2
NHLRC2	230.5	317	203.9	150.9	159.1	202.2	208.4	239.1

NHN1	234.3	249.1	194.2	149.5	157.9	178.1	200.1	281.3
NHP2L1	3999.1	4938.3	2018.2	1748.3	1320.6	1562	3206.4	3246.1
NHP2L1	1463.5	2136.8	1113.2	819	621.6	732.7	1248.7	1508.7
NHP2L1	1074.7	1508.9	569.5	401.1	270.1	447.1	852.5	932.9
NHS	88.5	93.8	94.7	82.5	77.4	83.7	82.5	98
NIBP	121.8	142.8	170.2	127.4	113.4	178.4	119.1	196.2
NICN1	933.4	1061.4	649.6	639.1	539.4	600.6	609.6	652.5
NID1	177.4	226.1	157.3	138.7	139.8	161.8	153.4	189.1
NID2	83.7	94.6	107.4	99	89.6	81	85.3	88.6
NIF3L1	2691.6	3465.6	1861.1	1692	1196.5	1941.9	2409.3	2881.3
NIF3L1BP1	9770.9	11232.2	6060.9	5643.2	4460.7	6129.9	8335.6	10336.6
NIFIE14	3394.8	4158.6	2779.9	1612.1	1258.3	2005.6	3020.9	4527.5
NIFUN	373.5	525.1	228.1	177.6	162.8	194.9	401.4	459.4
NIN	308.8	490.1	228	191.8	222.8	288.7	318.2	375.1
NIN	216.1	325.8	215.8	226	242.3	377.1	234.7	295.4
NIN	72	75.7	91.4	85.4	80	88.5	72.3	79.1
NIN	562.1	790.4	476.7	902.4	789.2	1475.2	572	761.6
NINJ1	1215.6	1475.2	856.8	701.6	502.7	541.8	2132.9	3136.8
NINJ2	1664.9	1950	1063.1	1271.3	946.3	1021.4	1559.4	1268
NIP	85.2	86	85.2	78.1	81.8	87.5	94.2	92.7
NIP30	351	480.3	338.4	354.9	304.7	482.4	281.9	438
NIP7	3180.3	3622.7	1622.8	1183.9	709.9	1243.5	2254.7	2782.3
NIPA1	179.4	233.1	130.9	146.8	138.3	152.2	200.4	291.7
NIPA2	237.8	269.3	101.8	97.9	91.5	97.7	172.3	202.7
NIPA2	4410.6	5720.8	3494.9	2595.8	1800.3	2645.8	3448.6	4987.1
NIPBL	77.1	76.4	91.2	75.3	76.1	84.3	72.4	70.9
NIPBL	208.7	302.5	147.9	175.3	166.8	243.5	226	270.6
NIPBL	434	615.6	369.9	721.9	681.6	1221.6	433.7	528.6
NIPSNAP1	712.4	897.1	383.7	347.1	278.6	395.7	681	1069.2
NIPSNAP3A	77.6	86.6	93.3	88.5	89.1	89.4	80	84.9
NIPSNAP3E	114.6	126.7	82.1	90.8	93.9	93.2	90.6	137.1
NISCH	531.2	623.2	493.4	587	490.4	788.8	483.4	619.6
NIT1	137.6	162.4	108.4	105.9	100.1	114.2	119.3	139.4
NIT2	1401.6	1779.5	679.6	662.7	529.1	699.3	1143.5	1313.9
NKAP	181.9	227.4	116.9	108.2	91.7	146.6	148.8	238.2
NKD1	101	111.8	125.7	117.5	101.4	130.6	110.9	120.2
NKD2	1200.9	1390.4	855.8	593.8	651.9	620.6	1342.7	1662.3
NKG7	1092.9	1484.9	1075.3	638.8	503.2	621.5	1120.6	1483.1
NKIRAS1	268.5	340.3	174.9	190.6	174.6	224.8	228.2	335.6
NKIRAS2	120.8	125.4	98.8	100.8	98.7	90.6	112.8	123.5
NKIRAS2	85.7	100.9	90.1	88.2	90.2	86.8	93.8	111.5
NKIRAS2	292.9	329.3	187.3	201.7	152.2	172.1	297.1	605.1
NKPD1	96.6	95.1	89	90.3	78.5	89.3	82.9	106.4
NKRF	732.9	849.7	428.8	455.3	328.4	507.8	516.5	771.2
NKTR	231.8	306.8	164.2	206.9	221.3	294.5	205.1	246.5
NKTR	1039.5	1168.3	731.5	1491.9	1447.2	2378.8	725.8	930.4
NKX2-2	115.1	151.8	125.6	194.6	186.3	195.7	111.8	138.6
NKX2-3	89.5	102	101.2	90.9	90.6	81.9	92	86.6
NKX2-5	73.3	78.3	94.5	80.7	84	87.7	79.1	82.2
NKX2-8	122.1	142.6	101.1	102.4	83.1	97.8	118.4	141.3

NKX3-1	138.2	158.6	110.4	95.1	91.6	97.2	136.4	212
NKX6-1	112.5	103.9	90.9	80	83.7	83.4	111.1	108.6
NKX6-2	92.5	106.5	95.1	85.2	82.4	87.5	87.5	99.1
NKX6-3	139.1	180.3	116.8	102.7	93.1	112	149.5	160.5
NLE1	137.9	166.4	117.2	100.4	91.6	104.6	129.9	170.8
NLE1	107.7	145.3	129.1	112.4	110.4	109.8	113.9	126.2
NLF1	77.6	84.3	87.4	79.9	80.8	78.6	77.6	76
NLF2	91.3	102	83.8	83.9	78.8	80.9	87.9	91.5
NLF2	96.9	113	100.6	84	83.4	92.2	79.2	94.1
NLGN1	97.9	108.4	95.7	97	94.4	107.4	99.4	98.2
NLGN2	111.4	129.9	120	107.7	110.3	98.4	109.1	136.8
NLGN3	79.9	90.7	85.7	81.6	79.1	87.2	86.9	92.2
NLGN4X	78.2	75	85.4	92.7	82.7	93	80.7	74.3
NLGN4X	85.7	95.8	101.9	88.2	86.9	91.8	85.4	87.2
NLGN4Y	197.9	261.9	151.2	146.5	121.5	152.9	182.9	260.2
NLK	220	244.7	137.2	143.9	117.1	165.4	203.6	300.9
NLN	319.9	448.6	254.1	160.2	130.6	225.5	360.7	471.7
NLRP1	78.4	86.6	81.2	82.5	87.3	74	81.2	91.8
NLRP3	981.2	1289.6	871.6	777	679.6	984.1	879.6	1205.2
NLRP8	2301	2284.6	1059.3	2194.4	2102.3	1368.8	2358.8	3055.5
NMB	640.8	816.2	539.9	250.3	210.3	273.4	532.3	894.7
NMBR	85.1	93.9	93.2	83.3	83.1	85.6	82	90.6
NMD3	1401.9	1836.5	484.3	349.5	338.9	763.2	957.4	1724.2
NME1	18991.4	22163.1	11396.7	9470.2	6925.4	12190.8	15403.2	17875.2
NME1	403.2	494.5	234.7	184.6	159.2	194.2	326.1	354.7
NME1	878.6	1174.7	503.4	415.4	386.4	468.1	728.2	818.9
NME1-NMI	416.2	507.8	264.7	154.9	163.3	169.7	369.1	402.3
NME2	85.3	86	89.7	80.5	94.2	80.6	86.7	88
NME2	411.5	510	315.7	212.2	180.1	221.5	319.5	329.5
NME2	110	129	102.8	101	78.3	95.9	130	208.6
NME2	442.9	491.7	190.6	174.5	159.3	180.9	403.3	349.1
NME2P1	95.8	103	104.2	96.2	90.5	101.1	102.5	98.9
NME3	1400.1	1588.4	1080.6	886.8	820.1	1172.2	1071.1	1534.3
NME4	3366.3	4400.7	3262.9	2410.3	1809.1	2552.1	3013	5438.7
NME5	75.4	91.6	80	76.7	75.4	77.9	85.2	85.1
NME6	444.3	569.5	322.7	264.4	217	269	455	507.1
NME7	221.5	292.7	131.3	103	105.1	95.2	189.5	304.8
NME7	638.9	767.7	303.2	205.2	173.4	223.5	466.9	648.5
NMI	2846.9	3305	862	698.3	598.1	993.2	2413.1	4256.5
NMNAT1	231	312.4	153	154.8	146	136.1	210.2	275.1
NMNAT2	97.2	123.6	99.1	96.5	97.8	92.9	99	121.6
NMNAT2	105.2	111.8	92.4	82.8	81.8	83.8	100.1	103.4
NMNAT2	75.8	90.4	88.6	93.4	85.3	99.3	80.7	86.9
NMNAT3	638.3	859.3	320.5	261.6	257	311.1	362.4	337.7
NMS	95.6	105.8	85	82.7	91.6	88	97.1	101.2
NMT1	260	292.4	218.2	225.4	233.4	244.7	228.1	212.3
NMT2	109.2	127.3	105.5	117.8	87.2	114.8	111.2	169
NMU	81.4	102.8	96.7	81.9	82	76.9	82.5	99
NMUR1	83.3	89.4	114.3	105.9	102.2	108.4	88.7	90.2
NMUR2	85.1	90.4	93.4	76.9	86.4	87.6	82.9	94.1

NNAT	88.2	85.1	80.6	87.6	88.5	86.9	79.9	85.3
NNAT	102.3	111	111.3	94.6	103.9	94.5	95.3	108.2
NNMT	218.3	256.4	143	132.6	121.2	135.2	242.7	310.8
NNT	85.3	102.1	86.4	86.8	91.6	86.9	76.7	86.6
NNT	975.1	1402.6	558.2	760.2	415	1031.9	904.6	1757.6
NNT	696.2	854.5	344.8	367.1	296.8	556.7	661.4	929.1
NOB1P	414.1	481.4	235.9	154	151.1	222.6	332.6	512.3
NOBOX	86	94.1	92.7	83.4	85.7	84.1	86.6	96.2
NOBOX	100.4	118.2	95.4	93	104.3	82.7	108.6	115.4
NOC2L	166.5	204	139.7	134.1	97.6	171.7	130.5	171.7
NOC3L	1185.5	1375.8	765.7	685	480.1	1057.8	689.8	1113.2
NOC4L	278.7	397.9	210.5	130.6	109.5	106.5	208.4	329.7
NOC4L	294.8	375.3	247.5	194.5	159.2	246.6	237.5	275.7
NOD1	151	183.2	215.4	197.9	181.8	265.7	154.6	167.2
NOD27	174.2	188.7	158.1	117.6	118.3	124.7	126.7	142.9
NOD3	94.2	100.6	105.7	107.9	103.8	102	95.7	96.4
NOD9	1352.2	1775.2	917	528.7	425.7	528.5	987.5	936.3
NOD9	182.1	207.8	197.2	137.7	126.5	140.5	165.2	271.4
NODAL	143.3	155.5	106.6	103.5	97.4	104.5	130.6	137.1
NOG	90.3	93.5	97.4	108.6	94.3	93.5	82.2	97.4
NOL1	1781.9	2191.2	1127.1	808.9	626.3	1017.1	1190.7	1317.7
NOL1	117.8	134.9	92.6	82.3	86	78.9	111.6	119.2
NOL1	273.6	293	110	111.5	84.9	136.6	190.5	280.5
NOL10	177.7	206.9	130.2	144.8	148.1	167.3	140.1	165.1
NOL11	2773.3	3674.2	1082.4	932.7	663.4	1246	1942.8	2667.8
NOL12	633.7	604	380.9	341.3	334.1	427.3	421	470.2
NOL3	115.6	122.7	106.1	100.1	107.9	118.5	107.1	124.9
NOL4	105.6	106.1	116.1	101.5	95.3	92.9	94.4	100.4
NOL5A	4017.6	4508.9	1039.5	700.4	625.2	1177.5	3050.4	3243.9
NOL6	2553	2944.4	1844	1288.9	905.2	1347.5	1519	1802.6
NOL6	142.4	168.9	162.1	143.4	123.8	114.5	107.5	136
NOL7	5764.8	7564.5	3183.2	2844.6	2398.9	3232.7	4895.8	6373.2
NOL8	400	429.2	276	273.7	236.3	452.9	317	744.7
NOL9	135.2	167.9	132.6	127.6	111.6	99.8	126.9	140.7
NOLA1	679.1	1033.9	358.1	274.4	242.8	189.2	563.8	639.8
NOLA1	4987.1	5544.8	4144.3	3035.4	2076.2	3308.7	3817	4891.3
NOLA1	887.6	1230.2	619.9	427.2	326.9	513.5	724.2	803.7
NOLA2	2575.9	3518.1	980.6	893.2	752	1242.2	2569.9	3158.9
NOLA2	2077.1	2591.3	828.1	665.5	518	737.2	1861.8	2605.7
NOLA3	11806.7	14210.8	8874	8256.1	5611	7515	11500.1	13221.6
NOLC1	694	1019.8	587.7	307.9	262.4	421.9	507.9	797.3
NOM1	86.2	95.1	103.5	90.9	91.6	95.4	85.4	88.7
NOMO1	408.5	485.5	277.6	253	222.3	355.7	337.6	476.6
NOMO2	84.3	99.4	91.7	85.6	83.7	97.3	85.4	93.3
NOMO3	373.5	424.3	180.9	165.8	162.2	106.9	181	284.5
NONO	9610.8	10330.4	5253.2	5741	5332.4	6927	8063.6	10187.7
NOP5/NOP	2555.3	2699.3	350.9	278.9	248.1	412.5	2030.8	3077.9
NOPE	135.8	165.7	97	98.2	104.4	101.1	135.6	144
NOS1	87.4	75.4	88.9	82.1	85.1	86.5	80.2	83.1
NOS1AP	82	96.6	97.6	85.8	90.3	91.8	87.8	98.1

NOS2A	91.2	99.1	95.8	93.4	85.2	92.7	85.9	101.6
NOS2A	86.9	99.4	86.7	88.7	77.6	84.7	87.7	100
NOS3	289.1	348.9	205.2	170.8	129.6	142.5	220.6	168.3
NOSIP	2226.8	3157.7	1200	673.8	610.5	946.7	2114.7	2904.7
NOSTRIN	77.1	86.4	91.9	91.5	96.6	88.4	87.9	91.2
NOTCH1	959.1	1344.1	750.6	1039.3	848.5	932.5	919.4	752.5
NOTCH2	169.6	222.4	160.6	211.9	179.3	206.3	164.5	198.9
NOTCH2NL	124.8	127.9	97.7	89.9	83	94.1	123.9	118.2
NOTCH3	83.7	88.1	99.1	97.5	94.9	98.5	90.3	82.6
NOTCH4	99.2	108.4	104.1	97	97	90.2	92.8	101.2
NOTUM	117.5	136.3	113.6	106.4	87.1	100.8	101.4	112.5
NOV	86.5	91.6	114	95.3	88.4	93.5	92.1	102.4
NOVA1	97.7	104.1	108.8	93.1	87.8	91.5	109.6	102.9
NOVA1	87.3	104.3	86.4	75.9	74.5	77	87.1	102.1
NOVA2	147.5	181.5	98	99.3	89.4	98.9	153	150.3
NOX1	85.3	88.5	94.5	83.5	77.5	84.2	92.6	92.7
NOX1	102.9	101.6	92	86.5	88.6	93.7	90.9	99.2
NOX1	98.7	106.8	101.2	93.9	96.4	93.4	98	107.3
NOX3	77.5	83.7	102.6	86.7	90.2	88.4	90.8	75.9
NOX4	107.8	108.3	101.1	95.9	92.6	99.5	99.2	111.6
NOX5	105.7	129.5	90.6	82.8	77.1	93.8	95	107.2
NOXA1	195.1	255.6	248.3	207.7	267.4	350.4	178.7	256.2
NOXO1	114.8	134.5	89.1	83.4	83.7	80.9	103.1	139.9
NOXO1	78.1	90.2	85.5	83.5	76.6	85.3	84.5	90
NOXO1	109.4	130	110.7	103.7	98	96.4	102.3	117.3
NP	3024.9	4533.8	2728	1446.8	1055.5	1754.6	2635.7	2996.9
N-PAC	668.9	840	477.2	415.6	346.6	554.4	652	1436.5
NPAL1	86.7	97.6	88	86.6	79.1	77.5	86.3	84.7
NPAL2	111.7	121.3	102.6	95.6	93.1	104.3	92.1	120
NPAL3	304.2	380.9	289.7	360.4	281.2	336.3	266.7	437.2
NPAS1	133.6	165.6	126	115.2	116.7	111.2	116.6	131.8
NPAS2	125.3	140.5	112.5	100.8	101.7	103.4	117.1	136.3
NPAS3	110.9	130.8	84.2	84.6	83.8	84.2	130.9	138.5
NPAS3	83.6	98.7	84.5	85.3	85.6	85	87.2	90.7
NPAS3	74.6	88.4	90.2	83.3	83.5	81.2	83.3	85
NPAS4	82.3	96	119.3	96.1	97.4	102	89.9	88.2
NPAT	152.6	202.2	124	131	123	133.9	135	179.8
NPB	97.4	122.6	98.1	80	76.5	83.3	90.9	105.1
NPBWR1	78	85.9	85.6	80.6	78.9	79.4	76	83.2
NPBWR2	99.6	109.4	121.9	97	107.4	103.3	101.7	102.6
NPC1	346.8	465.3	389.2	448.3	392.3	650.6	504.1	866
NPC1L1	107.9	134.2	94.1	97.8	87.2	93.4	117.4	122.8
NPC2	8401.7	10901.4	7477.5	7686	6213	7056.2	8461.2	10614.2
NPDC1	198.6	228.2	183.6	160.9	130.5	172.1	153.7	245.7
NPEPL1	450.9	536.8	323.2	405.3	387.6	419.5	325.7	319.2
NPEPPS	572	778.9	387.5	554.2	431.8	803.4	567.4	1351.1
NPFF	115.5	114.4	92.3	91.7	83.8	97.4	110.3	133.8
NPFFR1	106	113.4	92.3	80.3	79.9	84.9	104.2	110.6
NPFFR2	88.3	105	93.8	90.7	83.3	89.2	88.3	108.1
NPFFR2	92.4	107	109.7	90.5	99	107.8	98.3	116.8

NPHP1	92.4	98.9	90.4	84.1	83.5	94.9	102.2	88.4
NPHP1	107	121.8	107.6	85.6	85.1	98.5	110.5	124.8
NPHP1	87.5	102.5	83.3	79.7	78.5	85.5	91.8	101.9
NPHP3	175.9	204.4	186.3	252.1	225.4	340.7	155.1	231.5
NPHP4	173.8	191.2	175.4	176.5	145.2	213.1	137.2	211.2
NPHS1	88.1	90.5	87.2	89.4	82.6	98.2	84.2	87.7
NPHS2	115.8	149.4	88	80.1	82.7	88.1	117.1	123.8
NPIP	249.9	298.1	228	206.5	192.3	315.1	259.6	494.9
NPL	273.9	332.8	195.4	172	148.4	207.9	241.2	391.5
NPLOC4	253.7	314.6	301.7	224.6	232.5	372.8	264.9	450.5
NPM1	106.3	120.7	105.3	95.2	91.9	81.8	97.2	103.2
NPM2	156.2	204.4	119.3	131.8	123.7	169.2	141.4	153.3
NPM3	1616.1	1879.2	1515.7	748	475.8	799	1350.5	1628.8
NPNT	102.8	110.9	92.6	89.9	79.4	89	90.4	110
NPPA	84.3	89.7	96.5	84.5	92.9	94.9	86.7	89.1
NPPB	90.6	111.7	86.3	89.2	83.7	84.8	89.5	95
NPPC	99.2	121.7	100.2	85.7	91.7	93.1	93.3	109
NPR1	159.6	193.9	94.6	106.7	93.6	93.8	152	168.6
NPR2	124.8	126.2	107.7	107.7	97.3	118.4	104.8	136.4
NPR3	117	125.2	91.9	89.9	80.8	87.2	120.5	127.4
NPTN	3086.6	3986.5	2582.9	3208.7	2332.3	3835.8	2889.4	5453.6
NPTX1	83	87.4	83.9	84.2	84.8	92.3	83.7	96.7
NPTX1	165.1	170.1	146.8	286.4	120.4	267.9	197.7	376.4
NPTX2	573.9	736.9	623.4	249	183.1	305.9	363.6	822.7
NPTXR	106	114.7	97.7	98.9	93.3	96.9	94.3	112.2
NPY	201.5	249.1	165.8	95.1	89.2	94.3	213.2	280.8
NPY2R	85.7	90	93.2	88.9	86.1	89	84.3	92.1
NPY5R	105.1	107.4	101.3	77.4	82.7	79.4	91.5	111.1
NPY6R	117.5	137.1	103.3	96.1	94.1	98.8	124.8	112.9
NQO1	92.6	110.6	99.5	92.5	85.3	93	133.7	202.2
NQO1	1318.1	1978.7	886.5	590.9	445	628.8	4420.4	6381.8
NQO2	253.1	314.5	117.7	135.7	93.4	136.4	407.8	721.5
NR0B1	95.4	110.1	94.6	94	79.5	89.5	95.4	98.7
NR0B2	81.3	91.5	88.9	88.8	83.1	90.8	91.2	82.2
NR1D1	94	108.4	98.6	85.4	88.1	86.6	104.4	101.6
NR1D2	123.3	134.9	109.9	108.3	103.3	114.9	127.3	145.7
NR1H2	205.9	235.1	224.9	183.9	170.6	216.4	261.1	295.1
NR1H3	655.2	785.7	243.8	247.6	209.2	252.9	486.1	410.9
NR1H4	95.8	122.4	96.1	86.4	95	97.1	108.2	108.9
NR1I2	98.9	123.7	91	93.1	84.2	91.8	93.9	101.7
NR1I2	119.9	142.1	96.5	96.7	89.1	94.7	119	126.8
NR1I3	85.3	106.7	97.8	99.1	99.4	92.8	94.8	91.1
NR1I3	79.3	84.9	99.8	75.8	76.7	85.8	82.9	89.4
NR2C1	245.7	269	122.2	149	128.1	188.1	196.5	289.7
NR2C1	148.6	176.3	115.3	133.8	107.8	141	144.6	164.5
NR2C1	295.8	383.3	192.6	226.4	217.1	290.6	205	260.1
NR2C2	198.8	252.6	160.3	168.7	160.4	178.2	187.7	236.5
NR2E1	86.9	99	98.9	93.9	86.8	100.1	82.6	86.1
NR2E3	96.7	99.4	93.2	87	81.8	87.4	94.6	106.7
NR2E3	75.8	94.1	92.9	78.9	76	81.6	76.9	85.1

NR2E3	80.8	90.8	95.6	88.2	97.8	88.8	93.6	89.1
NR2F1	109.6	117.4	107.7	103.8	98.6	101.1	101.7	122.8
NR2F2	159.6	173.6	113.9	93.7	76.5	98.5	147.8	226.9
NR2F6	383.4	418	360.2	304.8	252.2	340.3	380.7	412.8
NR3C1	166.7	208.9	123.8	156.1	139.3	180.2	148.5	169.1
NR3C1	96.1	101.2	104.3	105.2	95.9	85.4	96.5	96.5
NR3C1	115.6	140.1	92	100.1	83.5	83.2	123.6	139.9
NR3C2	103.1	136.2	105.2	94.5	84	96.6	105.1	112.1
NR4A1	118.5	128.4	75.7	85.6	82.7	88.4	105.2	122
NR4A1	89.4	89.8	93.6	88.3	82.4	85.6	84.6	91.2
NR4A1	114.8	110	87.5	89.8	72.2	87.7	104.6	106.5
NR4A2	91.3	95.9	90.5	87.3	83.4	85.8	94.8	106.2
NR4A3	94.1	100.1	104.7	93.3	94.1	90.9	85	94.8
NR4A3	88.1	100.3	79.8	84	74.9	84.2	91	103.8
NR4A3	78.6	93.7	86.9	87.9	78	81.1	84.2	97.5
NR4A3	97.1	101.3	107.7	82.5	86.7	100.2	87.8	100.3
NR4A3	81.7	89.8	96	91.1	91.1	90.5	88.7	89.3
NR5A1	83.5	88.9	97.4	83.2	91	85.3	88.1	86.3
NR5A2	99.9	107.2	91.8	88.8	89.4	84.5	91.5	109.2
NR5A2	91.3	100.7	87.2	87.5	86.8	86.5	91.5	101.9
NR5A2	96.5	112.1	86.1	81.2	92.1	75	93.7	116.3
NR6A1	83.2	121.7	97.9	104.4	106.5	108.8	91.6	99.8
NR6A1	124.9	118.7	92.5	89.9	85.9	85.5	114.8	119.7
NRAP	72.4	83.2	99.7	90.1	89	94.4	78	80.7
NRAP	69.3	82.3	91.4	79.9	84.6	86.1	80.9	76.2
NRAS	1186.3	1337.7	665	479.7	323.4	567.1	874.6	1444.2
NRBP1	184.6	226.8	124	119.2	102.8	142.7	156.1	211.9
NRBP2	487.4	418.4	384.1	484.4	439.9	587.6	280.3	362.8
NRCAM	106.5	107.7	94.3	91	85.3	92.4	108	117.4
NRCAM	117.4	108.2	85.6	72.9	75.6	88.2	128.9	135.7
NRD1	2695.6	3911.1	2901.4	3282.9	2399	5581.3	2334	4190.4
NRF1	93.7	116.5	96.4	101.1	97.3	86.6	91.7	110.3
NRF1	107.9	120	104	103.4	90.5	97.7	90.7	111.3
NRF1	99.3	106.6	105.1	95.5	83.5	89.7	89.2	99.3
NRG1	82	91.3	92.7	84.1	78.3	82.7	91.1	88.2
NRG1	79.1	97.3	91.2	94	92.8	89.3	90.7	88.5
NRG1	103.9	111.7	92.3	99.9	90.9	98	99.7	116.9
NRG1	105.5	104.4	99	92.1	75.4	91.6	89.4	115.8
NRG2	100.4	125.2	98.4	96	76.2	98.2	97.6	127.7
NRG2	83.1	84.3	95.8	100.8	85.6	94.2	81.8	85.5
NRG3	97.8	100.3	99.1	86.6	90.5	86.9	90.1	105.6
NRG4	253.2	295.4	186.1	121.4	110.9	119.5	137.4	150.9
NRGN	329.2	467	275.7	183.1	160.7	191	243.4	538.9
NRIP1	383.1	476.7	379.9	386.1	327.9	620.9	329.9	641.5
NRIP2	121.7	154.1	97.7	96.5	101.5	99.4	121.7	137.5
NRIP3	1120.7	1824.9	598.2	367.3	284.1	207.2	1385.9	1804.3
NRK	120.6	143	118.6	121.1	103.5	114.6	108	125.7
NRL	80	89	108	87.9	87.1	89	82.5	88.9
NRM	220.7	251.9	175.4	178.2	135.8	140.5	216.9	353.8
NRN1	167.7	263.5	105.1	97.7	84.6	87.9	147.9	162.6

NRP1	122	143	96.7	88.4	82.7	93.7	117.5	138.1
NRP1	184.3	287.6	166.1	139.4	128	177	175.8	281.5
NRP2	98.4	115.2	99.5	86.5	76.5	86.5	107.2	108.9
NRP2	161.8	194.4	91.6	98.1	86.9	92.9	142.5	159.6
NRP2	87.3	101.9	90.7	81.7	80.4	84.3	96.1	104.2
NRTN	91.6	103.5	107.8	86.5	89.4	94.2	91.6	113
NRXN1	87.6	106.8	84.3	75.7	71.7	68.3	87.6	107.6
NRXN1	101	113	84.8	71.5	73.3	88.2	107.9	108
NRXN1	87.9	104.5	87.4	84.8	81.1	78.8	96.6	96.6
NRXN2	87.7	95.3	100.7	91.5	89.8	89.8	89.2	91.4
NRXN2	97.3	114.3	113.5	106.9	97.4	106.8	89.8	103.7
NRXN3	84.2	90.9	92	82.1	77.9	83.3	90.3	85.4
NRXN3	72.9	77.4	100.3	92.2	89.8	92.4	78.5	83.5
NRXN3	107.1	124.4	90.1	88.9	81.5	83	116.2	130.5
NS4ATP2	1071	1283	313.3	257.3	203	364.6	1074.9	1339
NSBP1	68.8	75.7	95.2	83	78.7	83.1	72.6	71.2
NSD1	121.8	159.6	100.1	105.3	100.3	109	123.2	147.2
NSD1	133.6	162.1	118.8	139.6	135.3	118.3	129.1	142.1
NSDHL	882.1	1239.8	562.7	624.5	543.8	764.1	790.7	850.9
NSF	299.2	402.9	129.7	176.7	146.6	120.4	186.7	279.1
NSF	735.1	1099.9	515.2	581.4	443.3	930.4	595.6	968.5
NSF	4924.3	6624.8	3778	5060.4	3771.4	6079.4	4056.4	5554.1
NSFL1C	327.9	382.3	313.8	301.7	248.8	346.6	327.4	610.5
NSFL1C	396.1	500.6	334.3	355.6	276	356.5	386.6	471.6
NSL1	162.9	196.6	100.2	95.6	88.8	98.1	143.8	178.6
NSL1	124.7	145.9	98.9	94.1	88.8	81.1	127.4	136.6
NSMAF	4008.4	4722.2	3093.1	2424.1	1828.9	3059.5	3295.6	4640.8
NSMCE1	1211.6	1501.1	615.2	492.4	407	493.5	1375.4	1403.8
NSUN2	2724.1	3731.4	1252.5	1599.3	1162.9	2028.1	2419.8	3644.4
NSUN3	244.8	308.3	176.8	137	118.1	156.1	237.9	336.4
NSUN4	884.1	1122.6	643.4	814	537	832.1	770.1	978.4
NSUN5	593.4	620.4	298.7	232	238.7	324.8	449.3	607
NSUN5	156.1	134.4	141.2	117.6	127.4	145.8	140.5	153.2
NSUN5	366.1	496.7	317.9	179.4	167.4	214.9	299.9	417.7
NSUN5B	685	861.7	411.3	285.7	264.6	334	483.1	623.2
NSUN5C	127.5	142.5	95.1	91.2	77.6	79.7	108	157.9
NSUN5C	161.1	201.5	117	111.9	101.5	103.8	173.3	207.7
NSUN6	159.7	184.1	97.6	89.5	85	102.6	138.7	157.4
NSUN7	113.5	131	97	90.1	76.8	89	108.6	116.2
NT5C	2723.9	3113.3	1347.8	975.6	829.7	979.6	1986.2	1737.4
NT5C1A	83.4	97.1	103.4	93.5	83.8	92.9	85.4	95.4
NT5C1B	77.4	80.5	102	91.2	90.3	90.5	84	82.6
NT5C1B	76.6	91.4	87.3	85.4	84.3	90	77.2	86.2
NT5C2	999.6	1298.6	392.4	431.5	252.1	503.2	1042.9	1371.2
NT5C3	2030.7	2630.4	1201	1039	716.5	1207.1	1821.3	3522.9
NT5C3L	674.2	904.8	439	320.8	277.8	348.1	637.5	700.4
NT5DC1	127.7	121.8	102.5	102.5	100.1	108.5	112.3	142.6
NT5DC2	430.6	526.2	458.3	244.6	188.8	263.2	357.4	628.1
NT5DC3	91	105.6	98.5	107.1	112.4	99.2	91.4	100.5
NT5DC3	812.8	980.9	522.8	687.4	578.5	818.9	577.7	785.2

NT5E	159.4	204.1	117.9	116.8	113.8	130.4	136.2	192.9
NT5M	95.2	120.8	102.4	99.1	102.7	103.3	89.9	104.3
NTAN1	961.2	1150.1	564.9	465.9	367.3	579.1	883.4	1618.4
NTE	506.8	662.8	609.7	407.7	317.6	533	403.1	737.6
NTF3	85.4	109.9	101.3	88.5	90.8	92.9	102.7	95.4
NTF5	94.2	107.6	92.4	80.5	93.2	94.3	100.7	98.2
NTHL1	671.7	739.3	460	268.8	225.6	339.2	451.9	564
NTN1	90.8	111.1	102.6	84.4	90.5	85.3	88.4	93.8
NTN2L	85.7	95.4	89	81	71.6	77.6	84.6	91.9
NTN4	76.1	87.7	89.1	87	80.7	85.7	86.6	91.7
NTNG1	72.2	74.5	85.4	79.6	70.6	79.8	78.6	84
NTNG2	112.9	124	93.4	87.4	87.3	87	100.1	107.4
NTRK1	125.3	140.6	96.8	88	88.8	89.8	117.6	129.6
NTRK1	92.5	115.5	104.4	103.6	95.6	102	91.3	94.4
NTRK1	91	92.8	89.4	87.8	85.5	82.6	94.1	96.4
NTRK2	91.5	97.2	88.4	93.1	86.6	85	94	105.9
NTRK2	100.1	107.3	95.4	89.2	91.3	89.2	94.8	95.4
NTRK2	88.5	92	88.8	81.8	77.4	86.2	87	87.7
NTRK3	96.1	97.3	103.1	92.5	88.2	101.2	89.7	101.4
NTRK3	89.8	114.7	93.1	84.5	80.4	87.7	87.7	105.1
NTS	74.9	85.5	80.8	82.3	77.5	84.9	71.1	81.6
NTSR1	271.6	325.2	190	158.3	138.5	155	209.7	223.8
NTSR2	94.1	99.7	90.8	96.3	83.7	84	85	95.9
NTSR2	96.7	117.2	103.9	101.5	85	93	98.8	110.1
NUAK1	170.5	179.5	123.9	109.8	98.5	103.5	148.3	198.7
NUAK2	82.1	94.3	89.5	83.7	83.4	84.5	96.2	85.2
NUB1	89.3	101.5	101.2	79	89.6	102.2	88.3	87.3
NUBP1	382.5	467.6	350.9	279.8	270.8	337.4	345.1	440.2
NUBP2	310.1	383.2	245.2	161.5	137.1	191.5	272.1	345.8
NUBPL	696	799.7	478.6	829.5	825.1	612.4	602.4	695
NUCB1	6281.4	8418.7	6733.7	6980.3	5767.3	7560.7	6772.2	10082.4
NUCB2	5053.3	5943.5	2407.3	1770.9	1394.8	2456.4	4868.5	10249.2
NUCKS1	704.8	750.5	169	199.2	178.3	197.8	536.9	739
NUDC	4803.1	6141.8	4141.6	3935.3	2793.5	3863.2	4198.4	4390.4
NUDCD1	179.3	223.1	135.3	114	110.3	141	152.4	214.7
NUDCD2	350.4	380.2	153.6	147.7	112.7	171.3	324.6	458.6
NUDCD3	279.6	383.3	350.6	281.5	249.7	351	235.5	356.7
NUDT1	384.3	473.8	172.2	141.9	129.4	139.7	375.9	359.2
NUDT1	3719.1	4228.5	3118.4	2400.4	2066	2356.2	3179.3	3084.4
NUDT10	93.7	100.4	71.6	73	67.9	79.6	97	110.5
NUDT11	165.3	186.3	140.9	105.7	96.1	127.1	131.8	203
NUDT12	75.6	104.4	91.5	78.3	78.2	81.5	84	106
NUDT13	145.7	181.4	114.1	100.4	104.1	122.2	151.2	162.4
NUDT14	1965.1	2286.7	1928.9	1587.1	1194.3	1646.1	1916	3068.5
NUDT15	327.2	353.4	140.1	170	126.2	136.7	271.3	412.3
NUDT16	231	318.9	185.5	167.7	157.8	238.5	179.3	212.8
NUDT16L1	896.3	1204.5	753.4	336.4	326.9	370.9	793	1193.4
NUDT17	109.4	135.7	96.3	83.1	89.6	90.8	95	109.3
NUDT18	1025.7	1343.9	799.3	519.3	382.9	643.8	740.6	956
NUDT2	685.7	826.2	417.9	441.8	360.5	545.6	599.1	680.2

NUDT2	1429.1	1748.3	975	1020.3	807.9	1396.2	1233.2	1774.6
NUDT21	102.1	115.9	102.7	101	92.8	106.6	106.7	117.5
NUDT22	442.1	504.4	278.9	197.9	167	216.2	352.1	579.3
NUDT3	2674.6	3615.2	1935.5	1522.9	1352	2303.3	2280.6	2616.5
NUDT4	83	95.5	85.5	84.6	87.8	73.2	80.7	85.7
NUDT4P1	117.3	132.9	83.9	77.4	74.4	83.7	103.6	112.6
NUDT5	2802.1	3682.3	1221.2	564.3	399.8	520.4	2645.9	3530.9
NUDT6	174.4	218.6	113.1	92.1	88.7	114.9	143.1	123.1
NUDT6	314.9	379.2	203.7	150.1	145.1	215.5	215.6	257.5
NUDT6	190.5	210.3	138.6	110.9	105.8	115.7	137.6	149.1
NUDT8	92.8	120.9	100	100.4	94.8	97.1	94	93.1
NUDT9	209.7	285.7	115.2	100.4	103.7	103.7	214.1	210.2
NUDT9	1036.7	1368	640.7	497.6	438.1	558.4	957	1445.4
NUDT9	137.9	154.8	111.8	97.6	86.8	98.8	132.5	131
NUDT9P1	78.2	88.2	87.9	84.6	79.8	92.1	70.8	86.7
NUF2	190.9	237.1	130.3	124.4	106.7	135.8	171.3	350.8
NUF2	87.5	91.8	104.5	109.1	90.4	106.6	87.4	93.5
NUFIP1	132.9	153.3	111.1	97.6	88.2	95.4	119.6	151
NUFIP2	1137.4	1347.2	382.6	488.8	373.1	564.9	1001.7	1396.5
NULP1	2442.5	3271.7	1264.6	1151.4	1064.7	1346.1	2140.6	1953.4
NUMA1	202.1	278.5	252.2	305	254.7	408.2	228.5	366.8
NUMB	1499.9	1777.4	995.1	993.1	754	1019	1329.6	1873.3
NUMBL	130.6	141.9	100	86.6	87.4	89.8	122.8	170
NUP107	1585.1	1805.8	514.7	531.6	571.8	913.9	1081.8	1356.2
NUP133	1331.2	1677.3	1298.8	837.8	644.4	1171.2	979.5	1829
NUP153	349.6	461.5	287.5	388.2	303.9	684.4	380.6	632.7
NUP155	159	203.5	109.2	130	112	87.1	122.8	126.3
NUP155	1305.6	1647.5	1411.7	1538.8	1066	2036.1	948.8	1693.1
NUP160	1351.1	1751.5	1638.5	1968.2	1268	2851.5	1326.9	2320.6
NUP188	275	320.5	403.3	377.8	289	412	254.4	534.7
NUP205	1841.3	2472.8	1701	1950	1421.6	2362.7	1811.1	2704.2
NUP210	425.3	642.4	418.8	432.2	379	576	364.5	567
NUP210L	79.5	80.2	108.3	94.9	92.7	93.4	79.7	90.1
NUP214	1290	1714.7	1001	1033.8	822.1	1392.9	1282.1	1828.5
NUP35	969.1	1244.2	337.7	228.9	186.8	298.3	721.4	991.5
NUP35	680.7	892.1	182.7	129.7	104.5	172	497.4	732.7
NUP37	4404.1	6093.9	3107.4	2443.2	1790.4	2546.3	3509.4	4129.1
NUP43	230.3	281.2	140.7	174.6	137.5	117.6	207.6	333.5
NUP50	1602.9	1944.9	819.6	703.1	651.1	919.1	1313.2	1686.7
NUP50	139.2	165.5	92.1	94.4	92.6	89.2	120.2	124.2
NUP50	162.3	218.5	137.1	125.2	140.7	122.2	137.4	150.7
NUP54	1719.3	2202.8	1095.9	1040.9	814.8	1442.3	1441.8	2913.6
NUP62	623.2	793	208.4	156	148	167.2	476.9	995.6
NUP62	8521	9788.5	5794.6	4216.3	3665.3	3642.6	5717.9	6631.3
NUP85	2941.5	3966.3	2172.1	1588.9	1336.1	2225.7	2218.5	3122.2
NUP88	2618.9	3124.4	1257.2	1265.8	988	1739.1	2242.9	2716.8
NUP93	1469.9	2130.9	1470.9	1188.6	828.8	1598.2	1044.2	1563.8
NUP98	190.7	229.1	123.5	105.1	108.2	114.2	148.7	153.4
NUP98	221	263.4	197.7	146	133.6	158.2	173.5	251.5
NUP98	143.6	158.9	92.4	94.8	99.7	104.5	135.6	162.1

NUPL1	84.1	97.3	90.1	82.8	84.3	97.4	79	84.6
NUPL1	223.5	242.1	112.9	109.1	102.6	98.4	172.9	240.7
NUPL1	278.6	387.1	157.8	127.9	147.2	134.9	234.4	297
NUPL2	1296.1	1684.1	711.5	870.1	724.8	1131.5	868.1	1210
NUPR1	189.7	240.3	146.7	173.5	196.1	318.7	270.5	312.2
NUS1	169.9	199.8	111.2	129.6	120.1	132.9	156.3	226.4
NUSAP1	7527.3	7922.3	4558.7	3771.1	3087	4765.4	5945.2	7203.2
NUSAP1	2456.8	2955.8	812	515.4	417.2	863.3	1807.4	2494
NUT	90.8	112	97.4	109.2	95	104.4	103.7	103.4
NUTF2	428.8	539.4	156.2	118.3	110.9	116.5	348.3	434.8
NVL	105.2	101	97.1	87.8	89.4	82.4	90.3	105.2
NVL	684.4	925.7	427.5	390.1	383.2	615.6	494	547.4
NXF1	1036.9	1288.5	987.4	623	641.4	878.4	925.6	1952.2
NXF1	895.2	1057	853.9	669.4	613.2	716.1	787.3	1261
NXF2	106.9	108.9	99.8	94.1	93.3	89.6	96.6	102.3
NXF2	73.8	78.2	90.9	95.4	84.9	80.3	77.2	83.6
NXF3	500.4	533.9	231.8	228.5	268.9	291.6	392.9	391.8
NXF4	114.4	133.5	100.5	91.8	82.9	99.5	106	115.4
NXF5	92.8	103.9	93.8	91.4	83.6	88.1	99	100.7
NXN	242.1	281.5	199.7	303.5	265	325.6	226.1	270.9
NXPH1	92.6	102.9	105.4	100.1	93.3	97.7	87.5	104.2
NXPH2	91.1	92.3	82.3	79.1	85.1	78.8	95.3	90.2
NXPH3	101.8	115.6	104	93	89	93.3	104.1	111.7
NXPH4	86.6	102.1	109.4	89.2	83.5	82.2	85.4	107
NXPH4	99.7	93.7	101.5	101.5	86	97.4	91.6	103.2
NXT1	2915.3	3899.8	2161.5	1504.7	1101.8	1627.1	2548.1	2728
NXT2	1054.9	1145.9	341.2	252.7	230	302.4	711	1403.4
NYD-SP14	78.2	97.3	107.7	105.2	89.4	99.3	93.4	95.4
NYD-SP18	96.1	103.4	90.4	80.5	77.3	75	89.4	88.8
NYD-SP21	368.8	390.7	197.4	171.2	164.9	271.3	269.9	467
NYD-SP21	250.1	304.3	151.7	137.5	138.8	152.4	201.4	295.2
NYD-SP26	91.5	100.8	97	93	87.8	93.5	93.9	92.5
NY-REN-7	91.1	94.3	95.6	88.9	93.2	106.4	97.2	96
NY-SAR-48	272.3	350.2	139.8	118.4	105.3	131.4	271.8	314.2
NY-SAR-48	341.2	432.1	254	173.7	163.9	204.4	346.7	471.1
NYX	94.4	115.1	90.8	80.1	79.9	87.3	104	123.1
OACT2	133.3	179.2	103.8	99.7	88.5	103.8	142.2	148.9
OACT5	363	493.5	231.4	232.8	172.7	273.5	326.6	465.8
OAF	1566.5	1905.8	1961.6	720.7	520.8	872.8	1132	2761.3
OAS1	76.5	83.8	97.1	78.8	76.3	77.7	73.7	87.3
OAS1	172.6	220.3	126	104.9	112.5	122.8	174.1	160.5
OAS1	182.6	229.3	140.5	112.4	128.1	126.9	173.4	168.8
OAS1	171	193.6	106.8	88.1	93	90.2	158.6	183
OAS2	100.9	126.9	113.9	96.3	92.7	105.8	102.2	102.5
OAS2	101.2	103.8	101.3	90.8	88.8	94.3	95.6	115.9
OAS2	104	108.6	83.7	84.4	75.4	79.9	97.3	104.4
OAS2	324.1	437.3	306.7	275.7	275.8	247	263.2	271.4
OAS3	86.7	98.7	87.4	77.4	87.9	87.4	80.7	98.3
OASL	81.5	87.2	91.1	81.7	79.7	85.6	85.2	83.1
OASL	130.5	164.5	104.3	79.3	71.6	88.3	127.7	178.1

OAT	1468.8	2240.2	1201.4	1024.5	858.2	1489.2	998.4	1431.6
OATL1	114.8	132.1	103.5	106.3	103.9	93.2	103.1	109.7
OATL1	87.5	108.2	97.5	104.8	95.5	109.5	99.2	113.1
OAZ1	33343.2	31823.3	48060.3	35773.8	32694.3	36220.9	32546.9	35268.7
OAZ3	163.5	175.4	102.2	115.5	109.3	122.7	150.8	171.3
OBFC1	373.5	466.8	301.6	279	253	390.7	340.8	403.6
OBFC2A	404.2	463.7	239.7	271.8	253.2	338.3	330.8	465.3
OBFC2B	402.2	515.5	520.9	426.6	334	528.3	349.3	647.8
OBP2A	85.4	98.3	102.2	92	90.7	88	90.6	112
OBSCN	103.2	106	98	88.5	93.2	102.1	101.3	119.4
OC90	86.5	98.1	94.5	88.2	91.1	81.5	85	101.4
OCA2	84.2	91.7	105.2	89.5	80.8	84.7	82.3	98
OCIAD1	2983.8	3061.2	1250.4	2530.4	2637.4	1658.3	2860.3	3625.8
OCIAD1	3897.5	4775.1	2375.2	2275.9	1509.4	3109.8	3000.6	6290.8
OCIAD2	711.1	921.9	438.6	265.9	233.9	265.3	701.4	872
OCIAD2	2041.3	2531.6	1825	1443.1	1155.6	1372.4	2300.1	2854.9
OCLM	117.6	128.8	94.9	90.4	89.5	85.2	111.4	126.2
OCLN	81.3	89.8	100.6	86.9	87.7	77.6	77.1	85.1
OCLN	82.9	95.5	108	106.5	84.9	84.8	82.7	84.5
OCM	79.2	71.8	90.2	87.1	86.4	89.7	72.2	83.2
OCRL	320.5	451.9	314.9	472	397.4	717.4	251.3	452.3
OCRL	223.6	299.4	168.6	221.6	156.7	287	180.9	232.9
ODC1	4203.9	5386.7	3250.5	2275.3	1701.6	3566.5	2885.7	5256.4
ODF1	82.8	98.9	87.2	92.4	85.1	90.4	85.7	94.2
ODF2	342.8	459.3	270.8	247.9	182.1	243.9	323.8	457.5
ODF2	164.5	208.5	141.2	122.5	101.8	117.7	153.6	175.9
ODF2	855.4	1187.6	810	655.3	545.4	1063.1	732.7	1427
ODF2L	96.3	110.5	101.6	102.1	97.5	99.7	117.4	97.8
ODF2L	106.3	107.9	90.8	79.5	93.6	85.2	95.7	97
ODF3L1	109	110.1	110.6	90.4	88.6	100.5	103.1	102.9
ODF4	112.1	130.7	94	92.6	84.9	84.7	117.8	112.3
ODZ1	79.7	79.7	89.1	86.9	83	80	74.7	73.6
ODZ2	104	109.9	106.3	101	101.4	104.8	104.4	101.7
ODZ3	116.8	160.7	134.9	95.5	93.8	108.7	145.1	146.4
OFCC1	81.1	96.5	86.8	79.3	84.7	82.8	81	95.3
OFD1	299	311.8	164.9	205.6	180.8	292	239.9	408.5
OGDH	112.6	130.3	111.5	107.6	101.3	105.3	102	105.1
OGDH	89.7	96.1	84.7	93.2	88	90.1	84.7	101.8
OGDH	196.3	247.8	234.1	301.6	231.4	461.5	152.8	215.7
OGDHL	116.9	121.1	129.8	93.9	85.4	92.7	89.2	114.4
OGFOD1	171.2	219.4	99.7	87.5	74.4	88.8	135.4	183.9
OGFOD1	756.9	904.4	499.6	334.7	256.8	513	674.4	1109
OGFOD1	1434.7	1747.6	1043.1	719	507.8	683.6	1107.8	1563.2
OGFOD2	115.3	120.9	107.6	106.5	92.1	94.6	109.5	119.8
OGFR	402.5	505.9	642	409.1	369.4	467.1	365.4	637.3
OGFRL1	903.1	1214.8	314.4	290.8	216	368.9	801.4	1391.8
OGG1	222	294.7	120	121.7	131.9	116	163	185.6
OGG1	455.6	567.4	269.8	242.3	202	208.7	267.8	320.3
OGN	107.5	109.4	94.1	87	90.9	82.3	106.9	109.7
OGN	77.9	84.4	93.1	99	91.9	85	79.2	80.5

OGN	74.1	82.9	91.1	83.2	83.3	81.1	70.3	80.7
OGT	1588.6	2197.5	1271.2	1649.2	1397.7	2116	1121	1213.6
OGT	631.1	919.8	422.3	457.2	391.8	458.5	437.4	498.7
OIP5	2518.3	2886.5	1898.8	1463.7	1123.3	1312.7	2251.2	3330.6
OIT3	112.1	132.3	83.4	86.5	77.8	94.6	105.4	133
OLFM1	107.5	119	91.9	87.8	83.9	85.8	117.2	121.8
OLFM1	78.6	94.1	93	81.6	82.8	78.4	77.8	85.2
OLFM1	123.1	149.8	101.3	93.8	83.2	101.3	116.9	155
OLFM2	94.3	107.9	86.7	69.7	96.9	79.7	92	105.3
OLFM3	81.7	78.1	84.5	84.1	70.5	73.3	82.4	82.3
OLFM4	89.2	89.3	89.1	84.2	85	81.9	82.7	89.3
OLFML1	78.9	90.2	103.9	93.3	86.2	86.8	84.4	92.5
OLFML2A	81.8	93.4	86.9	80.9	84	91.4	86.8	94.6
OLFML2B	100.8	98.7	96.1	89.5	86.2	95.6	96.8	98.6
OLFML3	211.7	275.7	181.3	237	190.5	361.1	203.1	293.3
OLIG2	415.5	603.7	423.3	397.7	396.5	459.5	388.8	481.3
OLIG3	104.7	104.2	90.4	101.8	93.6	99.3	94.8	107.2
OLR1	86.2	106.2	85.4	88.3	90.9	101.3	84.6	95.1
OMA1	149.5	161.7	97.7	101.5	82.8	105.6	142.8	173.2
OMD	113.5	139.4	97	99.5	86.7	94.1	118.8	129
OMG	98.4	103.3	85.4	81.3	87.8	82.8	87.4	104
OMP	82.9	80	87.9	89.8	78	81.1	76.4	85
ONECUT1	116	119.3	97.2	78.5	76.3	86.9	110.5	125.5
ONECUT2	125.6	140.8	113.7	147.9	131.9	114.6	100.5	115.1
ONECUT3	72.6	86.2	99.3	88.1	82	92.5	73	81.8
OPA1	682	888.9	495.5	708.5	451	866.9	562.9	1067.3
OPA1	94.1	91.2	95.8	91.1	81.8	90	88.8	100.1
OPA3	136.4	160.1	94.6	96.3	94.8	86.6	133.3	143.8
OPA3	595.9	741.2	269.6	397.3	295.3	221	523.4	492.5
OPCML	90.4	99.1	87.5	78.2	76.9	82.2	86.2	93.7
OPCML	119.5	130.8	101.9	112.9	97.6	111.2	113.5	122.9
OPHN1	83.3	99.4	98.1	88.5	88.3	88.1	92.4	98.2
OPLAH	137.2	158.6	116.9	118.2	100.3	114.6	122.4	158.8
OPLAH	563.8	648	501.7	576.5	480.7	622.9	426.9	575.5
OPN1LW	99.3	101.2	89.9	84.6	80.9	83.6	106.9	105.4
OPN1MW	77.6	80.8	91.7	87.7	77.1	87.9	78.2	83.3
OPN1SW	99.3	113.5	110.2	104.9	103.8	92.5	103.7	103.7
OPN3	535.3	796.7	387	294.5	237.8	353.2	426.2	644.5
OPN3	179	226.5	134.3	98.9	98.8	122.1	144.5	170.9
OPN3	242.4	307.5	152.2	119.7	106.2	121.6	186.4	236.7
OPN4	83.7	98	88.1	78.3	86.4	86.9	84.6	95.2
OPN4	83.9	97.4	93.9	82.9	84.8	94.1	88.8	98.7
OPN5	87.7	98.1	98.5	93.1	92.8	90.5	87.4	92.1
OPN5	117.1	127.6	104.7	93.3	90	94	116	124.9
OPRD1	141.6	176.9	107.4	90.2	91.7	102	143.7	140.1
OPRK1	131.1	149.6	99.2	103.6	98.4	93.8	127.5	124.1
OPRL1	345.9	366.7	263.4	312.7	264.4	351.3	251.5	273.4
OPRL1	105.3	141.4	89	89.3	92.4	88.8	123.2	139.6
OPRL1	81.8	94.6	81.9	75.5	79	74.5	87.2	94.5
OPRM1	166.5	194	115.5	115.6	99.2	115.2	144.8	166.1

OPRM1	66.1	79	86.5	81.5	87.3	84.1	78.8	74
OPRM1	155.2	159	118.4	101.5	103.1	112.6	148.4	158.8
OPRM1	78.5	91	85.6	79.9	80.6	81.8	83.1	85.5
OPRM1	71.9	78.6	79.1	79	72.7	78.8	75.9	76.8
OPRM1	78.3	96.4	89.7	89	84.6	93.4	89	97.7
OPRS1	233.5	345.3	217.3	198.8	185.6	300.5	212.2	564.1
OPRS1	1213.8	1457	686.9	443.2	345	650.9	936	1884.6
OPTC	93.7	102.9	98.9	96.5	94.2	96.2	101.8	96
OPTN	140.3	156.5	147.2	165.6	202.4	180.6	130.8	165.9
OR10A2	83.2	96.9	88	83.7	83.3	87.3	92	96.4
OR10A3	82.6	90.4	94.1	80.8	78.6	84.3	84.7	88
OR10A4	79.9	87.5	86.4	87.5	81.9	76.9	87.3	88.2
OR10A5	73.2	83.2	83	74.8	73.6	74.8	71.7	73.6
OR10A6	97.4	119.5	90.3	88.7	93.1	89.9	100.5	95
OR10A7	101.3	110	96.1	93	97.1	92.1	94.1	116.8
OR10AD1	97.5	101	105.6	99.5	93	98.2	101.8	111.2
OR10AG1	102.9	114.1	81.9	79.9	80.2	87.1	103.5	95.8
OR10C1	75	77	94.4	91.5	87.1	90.8	81.1	77.8
OR10G2	90.4	116.6	95.3	86.5	83.1	81.6	97	97.9
OR10G3	81.2	98.7	98.1	94.1	91.8	97.6	82.4	94
OR10G4	83.7	99.2	92.2	83.7	89.5	91.4	91.9	97.9
OR10G7	100.3	111	97.2	94.6	86.1	89	97.9	101.3
OR10G8	79.8	93.5	103.7	89.3	82.5	90.7	82.9	87.3
OR10G9	77.9	81.8	93.7	82.6	81.3	77.7	78.9	84.3
OR10H1	70.9	84	83.4	76.9	76.9	79	73.8	82.1
OR10H2	88.5	95	87.3	79.5	79	83.9	88.2	93
OR10H3	82	89.4	95.9	90.1	88.4	94.5	90.6	87.7
OR10H4	77.5	73.8	93.9	84.2	76.3	81.8	77.3	77.9
OR10H5	96.3	95.8	90.4	85.4	87.4	78.7	84.3	103
OR10J1	117.5	128.1	116.6	111.4	96.1	101.8	124.3	120.3
OR10J3	75.6	89	97.5	88.5	79.8	86.5	78.8	80
OR10J5	74.3	88.2	99.9	95.6	83.5	88.7	86.6	88.4
OR10K1	82.1	90	99.5	93.6	87.9	85.3	82.7	78.2
OR10K2	72.9	80.7	98.6	82.4	88.1	87.8	80.4	93.3
OR10P1	79	87.7	98.9	84.7	84.3	88.5	87.7	80.3
OR10Q1	68.6	81.6	91.8	82.2	76.5	83.5	72.6	81.5
OR10R2	93.7	90.7	104.8	88.5	87.9	96.2	87.1	89.6
OR10S1	86.7	98.2	107.2	97.8	97.7	100.5	83.3	95.6
OR10T2	81.1	95.5	89.6	83.8	86.3	84.5	94.6	101.8
OR10V1	82.8	103.5	79.4	91.9	79.6	91.4	85.2	108.9
OR10W1	89.9	93.1	94.4	96.2	87.8	103.4	83	87.9
OR10X1	82.7	96.2	94.8	82.6	80.7	80.4	86.6	99.1
OR10Z1	81.7	92.5	86.6	86.3	74	86.8	92.6	95.4
OR11A1	89	105	93.1	89.2	79.6	74	85.4	88.5
OR11G2	104.2	124.7	89.7	80.8	89.6	78.6	98.4	100.7
OR11H1	89.6	109.9	90.5	83.7	76.4	77.4	89	106
OR11H12	92.8	108.3	98.2	96.8	88.3	90.3	89.8	95.3
OR11H12	141.2	156	102.5	95.5	89.5	93.3	140.8	146.1
OR11H4	104.2	106.1	107.2	87.5	96.5	103.9	97.4	107.3
OR11H6	85.2	82.4	86.7	81.5	79.2	88.7	89.5	86.6

OR11L1	176.1	201.5	137.1	114.3	92.4	116.6	143.3	179.6
OR12D2	100.1	113.8	104.9	98.2	89.2	97.9	101.2	102.9
OR12D3	78.4	93.8	85.3	82.4	80.4	87.4	91.7	95.3
OR13A1	94.3	103.2	108.5	107.8	80.7	97.2	99.1	112.8
OR13C2	97.8	90.8	104.4	86.7	88.4	92.6	91.4	94.7
OR13C3	96.4	93.4	92	87.3	85.4	76	81	87
OR13C4	74.8	98.4	82.1	84.5	88.5	81.6	78.8	97.6
OR13C5	78.4	82.9	86.7	84.1	87.3	81.9	87.9	82.4
OR13C8	82.1	87.7	96.7	94.3	77.8	85	89.7	86.9
OR13C9	98.6	122.3	103.9	88.5	89.6	93.9	101.1	98.9
OR13D1	76	92.4	101.9	80.1	87.6	94.4	78.3	84.2
OR13F1	84	88.9	91.7	77.9	77.8	85	79.6	80.5
OR13G1	82.4	92.2	101.6	98	88.5	97.3	85.4	85.8
OR13H1	98.6	111.6	88.7	88.4	77.6	93	100.1	109.2
OR13J1	81.3	99.9	92.8	94.8	89.5	89.6	84	70.8
OR1A1	81.7	99.4	95.1	91.6	80.7	89.8	86.9	102.3
OR1A2	91	111.4	107.2	93.1	93.5	91.1	93.4	112.7
OR1B1	85.9	93.7	93.2	89.6	93	86.9	91	101.6
OR1C1	92.1	101.8	93.4	90.4	84	96.3	95	94.3
OR1D2	135	176.6	100.7	93.7	92.3	88.8	145.6	159.9
OR1D4	102.3	115.3	103.1	96.6	89.6	89.2	112.2	102.7
OR1E1	93.9	123.4	91.4	88	88.6	88.7	106.1	113.7
OR1E2	77.5	85	82.6	78.1	75	78.8	79.9	79.5
OR1F1	84.8	97.4	90.7	90.5	83.8	81.1	92.2	90.9
OR1F2	81.6	80.2	90.8	97.3	88.3	84.1	89.3	91.7
OR1G1	109	141.3	94.6	92.6	89.2	89.3	110.1	102
OR1I1	95.9	121.1	93.1	86.4	79.4	86.3	91.8	114.9
OR1J1	92.5	98.8	99.1	98.4	92.6	93.2	94	106.8
OR1J2	103.2	127.4	102.9	91.3	88	105.2	100.2	106.1
OR1J4	99.4	108.9	99.4	95.8	84.4	89.6	92.6	107.2
OR1K1	100.6	130.4	78.4	91.7	83.5	78.8	93.4	119
OR1L1	81.4	93.3	100.4	98.4	87.3	87.9	91.9	101.2
OR1L3	71	80.5	92.5	78.5	76.2	72.9	76.4	83.7
OR1L4	88.7	94.2	98.6	94.4	84.1	87.3	93.3	106.4
OR1L6	106.9	127.6	84.5	92.4	78.9	93.6	99.4	118.2
OR1L8	108.5	127.6	93.5	89.8	82.3	92.3	111.5	112.5
OR1M1	81.2	88	86.8	90.1	83.5	89.1	86.7	78.3
OR1N1	97.9	110.6	99.8	102	96.2	90.2	95.4	96
OR1N2	110.9	113.1	98.5	93.8	86.4	96.5	112.2	114.5
OR1Q1	85.7	85.1	82	77.2	77.5	78.9	80.2	83.1
OR1S1	80.7	83.9	87.4	85.1	79.8	79.5	80.8	77.3
OR1S2	68	83.7	90.4	83.3	85.6	83.8	74.5	85.4
OR2A1	128.4	172.5	124.4	118.6	114.9	112.4	130.4	145.9
OR2A12	87.5	93.5	91.3	85.7	75.9	87.5	86.9	88.2
OR2A14	106.4	128.4	95.3	87.3	85.3	78.2	110.4	130.7
OR2A2	103.2	114.3	88.3	93.1	79.5	88.8	100	110
OR2A20P	115.2	150.3	98.7	86.4	77.8	88.2	112.8	153.7
OR2A25	93.5	116.2	94.8	79.9	83.7	95.3	96.9	112.3
OR2A42	100.1	101.5	82	80.8	76.8	80	100.1	133.8
OR2A5	140.3	167.5	103.3	105.5	94.4	107.8	136	156.1

OR2A7	85.5	107.2	98.6	89.6	74.6	86.2	86	96.4
OR2A9P	226.8	338.3	220.1	194.5	170.6	208.1	230.4	388.1
OR2AE1	81.4	92.9	87.8	81.2	79.8	86	80	88.8
OR2AG1	88.2	101.2	107.9	99.5	109.5	110	85.1	97.1
OR2AG2	88.6	109.1	99.3	82.5	95	90.4	98.6	98.9
OR2AK2	130.5	135.2	91.5	93.2	79.8	93.7	130.9	135.6
OR2AT4	88.9	97.2	77.6	80.1	73.7	77.7	99.9	91.3
OR2B11	87.6	99.4	92.2	91	93.3	92.3	88.5	89.4
OR2B2	96.6	131.4	99.5	92	92.3	93.2	99.2	116.7
OR2B3	85.6	99.7	83.8	91.3	81	78.4	95.7	108
OR2B6	85.1	85.9	102.7	97.7	98.8	89.8	90.1	87.8
OR2C1	107.4	113.3	93.6	86.3	81.4	92.5	88.2	112
OR2C3	95.2	107.7	86.1	97.9	84.6	92.3	89.8	104.1
OR2D2	91.6	93.4	86.2	88.3	85.2	84	84.6	94.7
OR2D3	87.6	106.8	98.8	85.8	90.4	79.9	87.9	83.1
OR2F1	82.1	99.7	84.8	78	82.9	85.7	82.4	91.7
OR2F2	88.6	109.7	92.9	88.2	84.8	83.5	92.8	96.7
OR2G2	90	100.8	90.2	89.9	83.3	92	93.5	99.8
OR2G3	99.9	102.8	102	94.9	103.4	98.5	98.1	87.8
OR2G6	82.4	91.5	80.7	86.1	81.6	82.7	78.8	92
OR2H1	79.3	81.1	101.1	87.5	80	82.1	75	82.5
OR2H2	179.2	226.3	118.4	106.2	88.6	106.6	170.1	195.2
OR2J2	79.2	79.5	89.2	83.5	87.6	82.6	79.6	83.7
OR2J3	95.5	90.2	101.4	87.6	85.6	82.1	93.2	99
OR2K2	73.1	80.7	84.4	83.5	74.7	76.6	71.8	74
OR2L13	83.7	102.7	93.8	87.2	86.1	86.8	86.5	89.2
OR2L1P	75.7	82.3	97.2	95.5	75.7	82.5	76.6	82.1
OR2L2	81.1	82.6	92.6	83.9	76.6	83.6	73.6	76.9
OR2L3	135.2	146.1	105.8	102.2	89.3	94.9	117.6	158.4
OR2L8	94.5	101.1	90.3	84.6	72.9	87	99.7	100.5
OR2M1P	87.5	100.7	80.4	78.7	87.9	90.6	80.5	95.3
OR2M2	109.6	110.5	95.5	78.7	80.6	87.2	110.7	107.3
OR2M3	78.2	94.4	93.5	84.7	81.9	91.8	84	89
OR2M4	90.2	88.5	89.8	88.3	88.2	95.9	79.9	88.1
OR2M5	88.4	112.4	97.9	88.2	84.4	89.4	103	110.6
OR2M7	81.6	91.3	99.1	83.4	84.9	91.7	85.7	89
OR2S2	83.5	91.3	101.2	89.5	86.9	86	84.2	97.3
OR2T1	116	139.1	90.3	82.2	83.3	92.4	113.4	137.9
OR2T10	80.3	95.7	86.9	88.4	90.8	69.9	72.9	80.2
OR2T11	112.4	125.5	95.5	92.3	94.2	106.3	109	123.3
OR2T12	76.6	85.2	87.9	68.1	82	79	79.3	78.9
OR2T27	71.9	94.8	87.1	81.7	83.9	79.6	81.2	82.6
OR2T3	84.7	79.9	86.7	82.8	87.8	74.6	75.3	78.7
OR2T33	106.6	120.1	94.4	98.3	97.4	89.8	116.7	101.4
OR2T35	95.2	109.6	94.2	82.6	78.5	92.1	106.4	102.1
OR2T4	82.5	90.9	86.1	80.3	77.5	78.7	78.6	95.5
OR2T5	85.5	85.7	89.3	84.2	91.3	87.5	82.9	85.7
OR2T6	88.4	104.1	104.2	97.8	90.5	98.6	95.1	108.9
OR2T8	105.8	134.4	90.7	78.1	72.7	79.1	114.8	125.2
OR2V2	89.9	96.7	99.5	96.6	94.5	90.8	96.5	101.8

OR2W1	81.4	100	78.2	70.9	70	78.4	85	95.1
OR2W3	129.9	153	97.1	107.7	114.2	94.9	126.4	116.7
OR2W5	102.3	121.9	125.2	112.3	98.2	122.3	89.4	103.3
OR2Y1	75.8	81.6	99.3	96.8	86.8	94.5	68.7	86.2
OR2Z1	87.3	103.9	86.3	93.3	92	82.2	89.8	98.9
OR3A1	111.8	132.1	93.6	88.4	86.6	88.1	101.3	140
OR3A2	104.1	107.4	102.7	101.1	89.8	99.6	100.1	107
OR3A3	76.4	93.2	92.7	78.7	79.4	79.3	81.2	88.1
OR3A4	199.9	290.3	146.2	180	152.6	191.9	203.9	252.7
OR4A15	148.4	160.7	106	85.2	77.1	91.1	148.7	165.6
OR4A16	98.9	104.4	85.9	88.6	75	82.2	88	110.7
OR4A47	80.2	97.2	96.1	79.6	82.4	81.5	75.9	94.4
OR4A5	67	84.7	93	87	81.5	90.7	76.8	87.1
OR4B1	87.2	101.3	105.6	89	93.8	87.7	89	89.2
OR4C11	105.4	114.1	99.7	91.6	91.6	98.2	100.5	109.5
OR4C12	81.9	96.4	83.7	80	88.4	86.6	78.4	85.4
OR4C13	100.4	113.4	78.5	88.2	84	93.3	98.5	116.7
OR4C15	115.8	142.6	92.1	89.8	95.8	92.7	119.2	117.7
OR4C16	153.6	176.2	92.3	92.8	87.8	96.4	153.6	183.7
OR4C3	79.6	81.8	92.9	83.5	87.7	94.6	79.1	84.1
OR4C45	101.2	115.6	101	99.8	100.8	99.9	96.8	88.9
OR4C46	134	158.5	90.1	88.8	77.9	94.1	120.4	135.3
OR4C6	91.3	109.6	87.6	91.4	83.7	85.5	86.2	96
OR4D1	179.7	218.5	120.7	95.8	89.8	101.7	160.8	204.7
OR4D10	81.7	111.2	90.6	81.1	85.4	86.4	102.5	89.7
OR4D11	103.8	118.5	108.3	100.4	92.7	103.9	98.9	117.5
OR4D2	127.2	147.8	88.6	79.4	74.8	84.1	106.3	119.9
OR4D5	103.7	115.5	88.9	84	83.1	85.9	107.3	107.8
OR4D6	91.6	118.2	104.3	98	89.4	98.5	104.5	99.1
OR4D9	75.4	84.7	93.1	89.6	80.4	78.5	86.1	91
OR4E2	90.8	92.6	97.8	89.1	89.7	87.3	93.8	98.6
OR4F15	81.9	92.5	85.2	89.4	76.9	87.6	85.2	91.6
OR4F21	98.8	113.4	99.2	97.2	86.8	102	96.4	105.5
OR4F3	84.4	99.3	102.7	90.4	93	88.8	90.5	84.3
OR4F4	104	126.9	107.4	103.8	91.6	102.6	101.5	111.7
OR4F5	86.4	96.8	101.5	98.1	99.3	98.5	87.4	93.8
OR4F6	82.8	85.1	101.9	85.1	90.3	83.6	85	88.3
OR4K1	102.6	105.2	85.5	84.2	79.3	87.5	99	95.3
OR4K13	97.3	114.1	99.4	92.2	93.9	85.6	92.9	100.1
OR4K14	94.1	106.1	107.5	94.1	88.1	88.2	89.4	102.3
OR4K15	76.9	81.3	92.6	82.9	79.7	91.6	79	80.8
OR4K17	77.4	89.1	94.9	89.7	82	85.3	90.3	100.4
OR4K2	76.3	82.4	89.4	97.8	97.5	93.8	78	83.5
OR4K5	83.6	91.5	85.9	89.6	90.2	89	78.7	86.2
OR4L1	121.1	135.9	101.9	77.5	83.6	85	112.5	126.9
OR4M1	88	90.3	83.1	86.4	81.5	85.8	85.7	86.9
OR4M2	89	95.5	90.3	74.4	78.8	86	84.4	102.8
OR4N2	103.9	104.2	94.8	86.6	81	83.9	90.2	103.9
OR4N4	74.4	81.2	94.6	81.7	79.5	88	84.1	78.9
OR4N5	83.6	84.2	101.3	89.4	86.3	91.7	87.3	81.1

OR4P4	119.5	167.2	102.6	101.9	91.3	110.5	120.1	132.1
OR4Q3	68.3	75.1	96.9	83.9	88.1	94.9	73.1	83.7
OR4S1	115.4	160.5	88.6	88.3	88	91.6	116	133.6
OR4S2	82.2	89	87.1	88.6	94	92.3	85.1	89.8
OR4X1	89.8	109.7	99.7	89.2	94.4	81.1	99.8	98.1
OR4X2	83.9	85.2	94.3	74.8	80	87.2	83.1	91.1
OR51A2	85.4	90.1	94.3	79.2	78	83.8	80.3	92
OR51A4	71.6	79.9	87.8	84.9	78.6	76.8	76.7	73.8
OR51A7	96.3	103	95.2	96.6	88.4	95.5	100.6	99.5
OR51B2	96.2	115.1	94.1	87.7	76.8	91.1	89.7	103.4
OR51B4	85.5	100.4	88.2	79.4	75.9	87.1	86.1	95.1
OR51B5	79.8	107	96.3	81.4	76	81.8	82.2	83.9
OR51B6	89.8	117.5	91.1	82	77.8	82.4	87.3	95.7
OR51D1	86.7	93.4	106.9	97.1	93.8	99.4	81.3	86
OR51E1	73.2	80.7	93.3	85.8	91.5	83.8	79.8	88.5
OR51E2	83.8	90.8	99.6	102.3	98.9	86.3	82.2	86.3
OR51F1	81.5	86.7	96.7	84.9	85.8	81.4	75	83.8
OR51F2	73.7	80.9	90.9	82.4	83.2	75.1	75.5	74.6
OR51G1	89.2	90.9	90.8	80.8	80.6	76.3	85.9	83.4
OR51G2	93.1	101.8	105.6	91.6	85.9	92	82.5	109.5
OR51I1	85	100.2	102.6	97.1	103.2	103.2	86	92.1
OR51I2	90.4	101.7	92.1	82.5	85.1	82.5	89.5	96.2
OR51L1	82.9	94.3	89	85.5	79.1	81	87.2	83
OR51M1	93.5	107.3	96.8	95.9	97.8	95.9	95.3	104.9
OR51Q1	122	131.2	96.6	97.5	95.6	90.7	122.3	134.7
OR51S1	83.1	94.3	93.2	89.6	85.4	78.9	81.5	89.4
OR51T1	262.6	287.9	111.2	124.8	101.2	123.1	271.4	332.5
OR51V1	71.4	86.9	87.6	81	75.1	84	74.8	80.9
OR52A1	73.5	85.5	86.9	76.3	78.2	83.6	95.7	97.6
OR52A4	76	89.2	86.1	91.7	86.6	88.1	81.6	86.7
OR52A5	142.7	174.2	123.3	119.5	103	115.9	133.6	149.7
OR52B2	94	106.9	90.2	80.8	87.8	89.8	93.7	103.5
OR52B4	82.4	86.3	83.8	85.7	76.6	78.6	78.3	83.6
OR52B6	118.9	130.4	108.9	92.5	89.9	99.4	125.2	118.5
OR52D1	133.7	159.8	100.5	97.2	95	83.9	148.1	158.6
OR52E2	96.6	89.4	87.1	85.8	79.5	87.1	90.4	93.2
OR52E4	77.8	76.9	102.9	104	94.1	91.7	83.7	87.5
OR52E6	86.5	106.5	111	92.7	94.3	93.8	93.3	97.8
OR52E8	79	82	94.3	82.8	80.2	81.4	74.8	84.8
OR52H1	87.4	92.5	99.7	93.2	93.6	91	91.7	85.7
OR52I1	89.9	87.5	88	87.3	89.7	97.7	82.4	87.7
OR52I2	82.2	84.9	91	84.1	79.4	79.3	77.1	87.2
OR52J3	88.3	88.5	95.9	87.7	82	82.4	84.3	80.9
OR52K1	115.5	136.6	118.1	96.4	104	108.2	124.3	119.2
OR52K2	106	100.6	96.4	85.8	76.2	95.5	95.9	96.6
OR52L1	80.6	90.5	100.2	94.7	92.9	92.9	84.8	87.7
OR52M1	86.8	93.8	94	97.2	90	83.9	82.4	90
OR52N1	102.8	106.9	99.4	79.2	83.1	105.7	99.3	105.6
OR52N2	132.8	159.1	114.7	100.4	105.5	103.5	116.2	135.3
OR52N4	76.9	88.6	105.7	80.9	84.3	81.9	84.4	89.9

OR52N5	101.8	119.5	102	97.6	88.8	100.6	107.9	126.1
OR52R1	95.9	113.2	93.5	88.6	82	78.6	95.8	117.1
OR52W1	113.7	119.5	89.7	84.4	81.1	78.3	113.8	120.6
OR56A1	82.7	86.5	113	103.9	101.8	101.3	88.9	81.2
OR56A3	74.2	84.1	79.6	84.2	86.3	88.1	80.7	79.7
OR56A4	102	109.2	96.7	84.5	86.8	93.1	102	99.9
OR56B1	116.7	137.2	86.2	88.9	89.4	90.4	121.6	136.3
OR56B4	101.7	121.2	99.1	100.2	97	103.6	102.4	113.4
OR5A1	91.3	94	80	82.4	82.6	78.4	84.1	99
OR5A2	91.6	99.5	84.1	78.9	68	78.5	79.8	88
OR5AC2	84.5	101.3	106.5	92	87.5	86.2	83.5	88.2
OR5AK2	82.9	89.5	81.2	73.7	79.6	86.8	82.2	87.2
OR5AN1	100.7	102	99.5	91.6	88	92	92.3	101.2
OR5AP2	98.8	103.1	91.4	77.2	79.5	82.4	94.2	101.5
OR5AR1	89.1	100.8	98.2	85	85	87	94.3	108.6
OR5AS1	93.1	107.3	83.5	87.5	82.9	85	93	105.5
OR5AT1	81.9	104.9	91.8	85.2	92.2	94.5	80	94.9
OR5AU1	94.2	96.2	89.5	92.8	85.3	84.4	88.9	107.4
OR5AY1	87.6	109	91.4	86.1	88.7	89.8	87.6	103.5
OR5B12	119.9	138.3	100.5	103.3	89.6	89.1	110.5	114.8
OR5B17	83.1	85.2	87.5	83.9	87.9	92.6	82.2	87.3
OR5B2	108.3	116.6	94.3	91.5	86.1	94.4	105.9	109.2
OR5B21	88	108.1	93.6	85.7	87.3	93.4	90.8	102.3
OR5B3	86	89.9	103.3	91.9	88.6	84.3	90.3	98.9
OR5BF1	93.3	95.4	85.7	90.2	88.7	88.7	94.8	88.8
OR5BU1	80.6	112.1	91.7	80.5	87.7	81.3	85.5	81.7
OR5C1	91.8	113.2	90.1	100.7	84	93	90.7	102.2
OR5D13	81.9	93.5	97.5	95.8	79.6	93.5	89.2	102.2
OR5D14	98.6	101.8	99.6	88.1	82.3	93.8	90.6	106.9
OR5D16	90.5	104.8	105.3	94	95.8	107.3	91.9	97.7
OR5D18	74.5	81	87.9	73.6	71.2	73.6	76.4	78
OR5F1	87.3	98.6	97.7	89.6	87.9	75.6	92.2	87.5
OR5H1	88.5	88.7	95.1	87.7	85.2	90.2	89.6	92.1
OR5H14	75.7	80.7	83.4	65.3	71.9	80.3	78.1	81.8
OR5H15	82.3	83.9	88	83.5	79.6	84.2	84.7	82.8
OR5H2	88	86.3	90.2	81.7	81.8	83.9	84	94.3
OR5H6	82	88.5	94.9	83.5	83.4	84.6	82.8	87.2
OR5I1	74.7	88.3	92.3	84.7	90.1	89.1	78.1	86.4
OR5J2	85.9	94.4	82.3	83.7	70.9	73.5	81.6	96.4
OR5K1	76.6	93.3	94.8	87.1	76.4	86.2	83.6	73.3
OR5K2	81.7	103.6	105.2	89.7	86.2	106.6	89.8	112.9
OR5K3	87.2	91.3	82.6	83.6	79	88	83.6	93.7
OR5K4	84.6	95	97.2	82.8	78.7	92.4	85	90.4
OR5L1	106.3	121.9	93.2	88.9	83.8	93.3	102.8	107.4
OR5L2	77.1	80.1	84.3	86.7	73.7	89.6	72.3	81.6
OR5M10	94.2	97.9	94.8	91.3	83.9	92	93.6	98.3
OR5M11	76.6	87.1	90	86.9	88	82.7	78.4	81.3
OR5M3	124	164.8	104.7	97.9	92.5	84.5	122.2	145.4
OR5M8	96.7	114.8	99.9	91.2	83.9	92.7	92.3	101.6
OR5M9	116.8	145.2	94.3	84.1	80.9	98.6	107.8	135.2

OR5P2	76.8	80.6	92.8	80.9	67.1	79.4	74.7	80.3
OR5P3	93	117.7	105	95.6	97.3	92.8	94.3	96.3
OR5R1	91.3	119.8	95.2	80.9	77.4	112.1	93.8	108
OR5T1	85	91.9	82.6	73.1	75.2	78.8	85.7	85.1
OR5T2	78.1	85.1	80.7	92.2	83.3	91.9	83.2	80.6
OR5T3	94.2	106.1	92.4	95.1	87.6	93.1	95.5	102
OR5U1	83.7	98.9	105.4	95	87.6	89.7	91.2	99.3
OR5V1	85.1	96	107.4	92.5	94.5	96.5	85.3	100.2
OR5W2	94.4	110.9	85.7	78.1	75.4	86.9	89.4	106.3
OR6A2	92.4	95	95.8	92.6	83	98.6	87.6	99
OR6B1	98	108	94.3	84.5	80.1	73.1	99.1	97.6
OR6B2	94	108.9	89.6	91.4	89.1	91.5	84.7	96.8
OR6B3	83.7	105.5	98.7	93.5	82.4	81	88.5	100.2
OR6C1	98.2	105.2	86.2	75.1	80.6	88.6	89	96.2
OR6C2	80.4	85.5	87.1	95.3	89.3	82	85.2	87.1
OR6C3	134.2	139.3	96.3	87.4	81.7	87.7	119.8	121
OR6C4	98.7	111.2	89.7	85.6	87	85.8	92.9	113.3
OR6C6	84.9	92.2	92.7	84.7	82.8	85.6	85	89.4
OR6C65	92.7	103.3	101.6	89	83.8	98.9	89.9	89.4
OR6C68	87.6	91.7	85.5	73.3	75.5	77.1	82.1	90.2
OR6C70	82	78.2	91.7	92.4	83.7	94.9	77.2	75.6
OR6C74	75.1	90.2	95.9	93.8	89.3	74.6	86.6	89.3
OR6C75	88.9	90.5	91.9	83.3	87	78.4	88.1	98.6
OR6C76	91.9	109.5	86.8	83.3	77.8	86.6	79	110
OR6F1	96	108.1	97.2	86.4	78.3	89.3	110.5	106.4
OR6K2	91	91.9	93.4	88.4	89.1	96.6	80	82.3
OR6K3	93.1	96.5	88.7	83.7	84.6	82.8	85.6	101.6
OR6K6	81.3	80.2	94.2	79.8	85	81.3	83.2	92.1
OR6M1	84.9	104.3	87.5	88.4	91.5	75.4	87.2	105.6
OR6N1	90.1	94	88.7	91.7	87	86.7	95.7	100.3
OR6N2	94.3	118.7	94.6	94	87.2	78	95.4	100.8
OR6Q1	85.6	99	95.5	82	88.9	96	88.5	86.2
OR6S1	104.7	113	93.3	85.4	76.4	80	105.7	117.4
OR6T1	72.5	82.8	87.3	86.6	82.7	79.7	78.9	78.5
OR6V1	137.8	151.1	92.9	94.3	100.2	107.4	131.3	138
OR6W1P	88.9	89.1	101.1	81.6	85	93.8	91.5	91.2
OR6X1	73.6	77	86.2	90.6	87.8	102.6	82.6	83
OR6Y1	81.6	94	99.7	89.7	91.6	98.1	89.3	86.9
OR7A10	87.7	96.7	90.1	91	81.9	92	90.8	96.9
OR7A17	117	129.2	105.6	85.8	86.9	84.3	110.2	132.9
OR7A5	80	80.9	88.4	84.3	81.8	89.3	84	83.9
OR7C1	72.1	82.5	96.9	84.6	85.6	84.4	75.1	89.7
OR7C2	111.1	119.7	99.6	95.1	82.3	96.1	104	117.3
OR7D2	84.1	96	88.6	82.8	78.5	83	86.9	92.9
OR7D4	80.6	101.2	106.8	85.3	81.9	87.3	88.4	94.5
OR7E156P	134.8	169.7	121	119.8	103.8	121.4	152.6	149.8
OR7E24	139.2	164	96.1	100.8	90.1	95	124.2	130
OR7E37P	79.9	90.3	85.8	80.2	77.6	78.5	81.9	92.2
OR7E91P	86	90.5	85.6	85	78.6	80.8	80.5	86.2
OR7G1	115.6	120	90.2	88.3	81.1	80.4	93	110

OR7G2	119.2	119.3	87.1	75.9	82.3	78.1	113.3	122.4
OR7G3	101	121.4	102.6	96.1	92.1	109.2	109.6	113.2
OR8A1	95.7	97.1	101	85.9	91.5	81.2	89.9	95
OR8B12	132.4	160.1	91.9	95.2	96.5	94.4	133.6	136.8
OR8B2	84.5	94.9	96.5	82.7	87.3	96.5	83.3	91.7
OR8B3	85.1	79.6	80	79	82	86.3	80.7	84.4
OR8B4	90.9	102.9	92.6	85.3	79.3	88.3	97.6	99.3
OR8B8	80.5	85.4	80.7	77.8	80.5	80.4	80.6	91.6
OR8D1	80.5	109.8	97.1	89.5	75.3	90.2	87.6	101.1
OR8D2	99.4	95.6	112.7	98.2	89.5	94.2	101.8	87.1
OR8D4	83.3	91.2	103.2	95.1	84.3	83.9	87.4	93.4
OR8G1	96.3	114.2	94.9	88.9	89.7	91.2	93.4	97
OR8G2	99.4	119.2	98.8	96.5	86.9	99.8	105.1	114.7
OR8G5	89.8	86.3	87.3	92.5	83.6	88.3	89.8	92.1
OR8H1	103.5	102.4	97	96.7	85.9	85.6	95.6	113
OR8H2	77.4	82.8	95.4	80.4	81.4	88.5	82.4	82.1
OR8H3	165.3	189.6	109.2	105.7	92.1	99.5	154.7	189.6
OR8I2	83.5	102.2	77.4	84.2	63.1	76	94.4	89.7
OR8J1	84.6	76.4	94.5	84.5	81.8	87.9	80.1	77.7
OR8J3	90.9	101.2	101.8	83.8	91.1	88.5	94.9	94.2
OR8K1	77.2	91.7	97.4	93.7	83.8	85.1	77.5	81.6
OR8K3	83.8	94.3	90.7	93.2	91.2	82.2	96.3	103.2
OR8K5	101.2	130.4	94	84.6	83.9	93.1	117.8	132.8
OR8S1	95.4	98.5	86.8	82.6	89	82.9	88.3	102.4
OR8U1	106.5	114.5	93.5	91.2	80.1	96.4	105.1	106.4
OR8U8	86	73.5	85.7	85	80.2	76.1	69.8	74.4
OR8U9	78.6	81.6	85.5	85.8	79.3	76.7	72.5	79.8
OR9A2	105.2	110.4	100.4	93.3	87.9	102.1	103.3	108.4
OR9A4	127.5	140.2	80.6	84.6	75.9	69.1	106.3	130.3
OR9G1	90.6	98	86.4	93.8	82.9	79.6	88.8	94
OR9G4	79.8	80.3	88.5	87.4	82	87.6	83.5	87.9
OR9G9	87	91.2	102.9	94.2	94.4	96.3	89.4	92
OR9I1	95.8	92.8	117.8	96.5	107.8	107.4	86.1	90.1
OR9K2	85	90.2	89.6	82.1	81.3	80.9	86.2	90.3
OR9Q1	92.6	82.9	82.9	75.9	69.5	67.3	81	87.3
OR9Q2	83.9	88.2	92.3	83.9	82.5	86.4	83.6	95.6
ORAOV1	423.2	492.1	322.9	279	277.9	321.4	365.4	321.7
ORC1L	400.2	581.2	457.3	293	241.1	264.5	287.3	477.2
ORC2L	744.4	835	515.9	516.2	337	706.1	570.6	926.4
ORC3L	1268.4	1588.7	523.8	674.3	455.3	1075.5	1019	2239.1
ORC4L	118.4	133.2	106.8	113.2	104.7	117.2	106.9	140.2
ORC5L	1284.9	1759.7	969.4	1056.6	771.3	1110.2	1178.8	1900
ORC5L	209.8	290.4	108.2	93.7	87.1	117.1	192.9	283.4
ORC5L	79.1	85.8	97.7	86.9	86.8	95.7	75	81.2
ORC6L	21583.6	23486.3	11879.2	15510.4	14600.1	11623.6	20971.6	22980.4
ORF1-FL49	316.9	339.8	213.4	175.6	150.7	166.9	238.5	341.3
ORM1	527.9	671.8	366.7	416.8	366.3	573.2	460.6	693.6
ORM2	96.7	114.8	101.3	117.7	116.3	124.7	94.7	89.4
ORMDL1	251.9	283.9	140.4	132.2	131.2	156.8	191.7	264.5
ORMDL2	125.8	144.6	104.3	91.9	102.4	93.5	110.1	137.3

ORMDL3	137.5	155.6	110.5	100.2	107	102.1	121.3	188.4
OS9	198.9	246.1	154.8	160	140.9	192.9	177.8	290.5
OSAP	88.9	97	100.8	90.3	86.2	86.3	81.4	115.6
OSBP	1500.8	2144.2	1076.7	1255.4	1056.5	1708.2	1659.9	1910.1
OSBP2	97.4	104.9	99.5	90.6	85.6	101.3	89.9	108
OSBPL10	91.4	99.5	92.8	85.8	87.9	87.9	84.8	108.1
OSBPL11	131.7	150	112.1	117.9	106.9	125.8	148.3	208.7
OSBPL1A	357.6	447.4	147.7	199.6	170.6	218.8	279.2	338.7
OSBPL1A	91.3	111.7	101.6	102.6	93.8	92.6	88.7	85.2
OSBPL1A	1112.7	1286.9	443	788.5	628.8	967.4	778.2	780.2
OSBPL2	108.3	134.7	93.3	105.6	92.9	120.8	108.3	153.1
OSBPL2	344.1	440.2	157.7	178.7	162.7	220.1	313.5	412.8
OSBPL3	113.9	160.4	109.9	97.2	85.8	104.6	132.5	138.3
OSBPL3	134.5	167	139.6	165.5	122.3	195.9	119	171.2
OSBPL5	430.1	524.7	540.4	597.8	624	716.3	387.9	596.3
OSBPL5	457.3	586.2	447.1	529.1	538	595.1	426.6	425.1
OSBPL6	246.3	246.1	144.5	137.9	126.6	117	240.9	236.4
OSBPL6	107.5	111.7	104.5	89.1	85.1	88.1	124	141.4
OSBPL7	357.1	351.8	295.5	273.9	281.1	343.7	264.2	415.4
OSBPL7	122.8	142.9	124.7	102	97.5	110.7	117.7	147
OSBPL8	379	491.8	195.2	199	172.7	218.3	312.6	399.6
OSBPL8	439.1	487.8	192.5	237.9	197.4	400.1	414.4	805
OSBPL9	88.3	97.1	91.4	86.2	90.5	90.7	82.3	96.2
OSBPL9	1644.8	2217	1227.6	1875.6	1626.6	2778.4	1445.5	1973.4
OSCAR	102.8	117.5	118	122.5	118.5	132.3	91.7	104.8
OSCAR	248.6	280.6	159.5	231.9	160	193.3	197.9	231.5
OSGEP	870.5	1096.5	405.7	360.7	320.4	454.8	604.8	657.9
OSGEPL1	412.1	506	253.2	258.7	208.9	315.6	304	578.5
OSGIN1	144.4	178.1	118.1	108.2	92.5	92.5	237.4	530.5
OSGIN1	81.7	81.5	94.9	82.8	85.5	87.4	81.1	79.4
OSM	126.1	140.5	110.5	96	96	102.1	118.8	126.5
OSMR	76.4	84	98.6	96.2	100	82.9	82.8	79.1
OSR1	81.1	88	77.7	77.9	80.9	82	78.8	86.1
OSR2	125.3	139.8	107.9	93.3	94.8	89.9	114.9	131.6
OSR2	192	233.7	151.4	152.1	133.7	168.5	154.2	255
OSRF	180.2	185.7	123.4	119.3	112.3	154.5	138.8	254.4
OSTALPHA	154.8	167	103.6	127.4	130.8	151.3	129.8	143.7
OSTBETA	75.9	83.2	90.5	79.6	85	87.2	79.9	88.5
OSTF1	3771.4	4769.3	2855.8	2603.3	2146.6	3413.3	2965.7	4491.2
OSTM1	298	334	219.9	329.9	264.2	498.7	254.9	532.3
OSTN	81.1	82.1	97.9	90.5	83	91.5	89.2	83.4
OTC	79.8	89	94.1	90.8	76.6	85.7	95.7	86.9
OTEX	87.9	94.1	93.9	89.1	81.8	90.1	89.4	93.5
OTOA	71.3	88	85.5	87.4	82.6	78.6	80	82.3
OTOA	77.6	92.5	107.7	109.1	95.5	103.3	83.7	91.4
OTOA	96.3	113.3	106.5	98.8	97	99	82.9	92.1
OTOF	84.4	85.7	90.9	94.7	74.9	78.2	79.3	83.3
OTOF	89.8	83.1	93.9	88.9	100.9	92.5	86.3	95.5
OTOF	101.5	105.2	87.4	86.8	79.9	77.8	90.3	109
OTOP1	114.1	133.7	99.7	97.3	90.5	93.3	126.2	140

OTOP2	86.7	108.4	91.9	84.5	82.3	85.2	88.3	104.6
OTOP3	101	109.8	98.5	88.7	81.8	77.2	93.5	99.2
OTOR	74.7	79.5	83.5	75.3	67.7	80.2	81.4	83.4
OTOS	79.5	79.7	85.4	79.8	79.7	84.4	83.1	78.7
OTP	190.3	235.5	127.8	104.2	107.3	99	169.9	176.4
OTUB1	90	93.6	96.2	101.3	86.6	90.1	95.6	96.9
OTUB1	187.3	220	157.2	112.6	114.3	182.4	199.4	295.2
OTUB1	280.8	341.6	190.7	170.2	150.8	168.8	244.3	284.7
OTUB2	151.3	184	142.8	119.4	112	136.1	150.8	139.4
OTUD4	384.5	547.9	382.4	320.4	257.7	508.1	332.1	418.8
OTUD4	88.8	97.7	105.1	95	87.7	87	98.5	106.4
OTUD4	96.3	100.3	108.5	100.1	98.3	93.4	82.1	98.7
OTUD5	134.1	168.4	100.4	82.5	91.9	94	126	138.4
OTUD6A	97.9	108.5	107.9	101.9	96.9	104.1	97.7	102.3
OTUD6B	508.9	625.2	274.9	255.3	185.3	282.7	328.8	605.3
OTUD7	80.5	86.7	91.8	85.4	73.1	85.8	78.6	80.9
OTUD7A	108	102.7	102.4	91.9	82.2	90.2	107.6	105.6
OTX1	1382.7	1600.2	412.3	498.4	336.7	288.6	867.3	1298.3
OTX2	90.7	91.6	95.6	89.2	82	82.9	86.2	88.3
OTX2	98.3	109.7	112.8	99.3	88.1	101.4	100.6	97.5
OTX2	112.1	134.5	100	91.6	98.9	95.1	116.3	128.6
OVCA2	138.3	163.3	100.9	81.4	102.1	101.1	120	142.2
OVCH1	102.6	115.2	86.9	72.8	74.7	82.1	102.8	108
OVCH2	84.7	100.1	83.4	75.3	86.3	77.7	95.5	94.2
OVGP1	317.7	397.8	177.8	194.9	181.5	261.8	293.3	410.9
OVOL1	84.9	99.2	88	86.8	75.2	76.4	85.7	96.8
OVOL2	565.9	734.3	499.1	314.7	268.3	305.6	454.2	549
OVOS2	76.6	75.2	81.8	84.4	80.4	74	85.6	78
OVOS2	144	154.5	118.6	103.9	114.4	110.3	135.7	149.8
OXA1L	2003.7	2804.8	1139.8	692.2	598.5	1039.8	1792.5	2406.4
OXCT1	335.3	464.3	169.7	161.9	159.3	228.6	216.3	341.8
OXCT2	585.5	658.7	508.7	382.9	356.1	438.7	304.8	282
OXER1	109.3	124.1	97.8	90.2	92.9	102.7	105.7	124.6
OXGR1	82.6	88	84.8	79.9	79.1	81.3	87.8	89.1
OXR1	1388.1	1733.7	1529.7	1573.8	1229.5	2247.4	1355.8	2710.9
OXSM	250.6	346.6	134.5	141.2	122	178.2	252.9	326.7
OXSR1	910.7	1179.3	851.6	1092.5	944.8	1741	913.8	1334
OXT	89.8	106.7	94.5	79.4	80.1	84.1	87.4	100.6
OXTR	125.1	145	97	89	86	82.1	128.3	147.6
P11	94.6	103.1	113.5	109.2	101.1	96.2	96.7	103.8
P15RS	2145.2	2827.5	1749.6	2501.6	1715.7	2931.5	1745.2	2431.8
P18SRP	265.7	366.3	168.8	170.1	147	195	189.1	264.7
P2RX1	227.4	238.6	182.6	219.2	236.7	283.2	169.7	233.5
P2RX2	78.7	89.3	98.1	86.1	84.8	97.5	86.6	90.1
P2RX2	80.3	86.7	91.2	96.2	82.4	88.3	85.8	81.2
P2RX2	95	98.9	102.1	103.5	89.5	95.4	93.3	103.9
P2RX2	104	116.5	105.7	97	97.7	102	101.7	128.4
P2RX3	130.9	140.4	90.2	84.3	92.5	89.4	124.6	130.2
P2RX3	83.5	96.3	85.1	82.2	74.1	89.4	78.9	84.8
P2RX4	375.7	484	426.2	306.2	282.3	326.9	314.1	520.6

P2RX5	89.9	98.3	104.6	92.7	86.8	88.3	90.5	86.2
P2RX5	118.1	141.5	85	90.9	88.6	86.7	110.6	123.1
P2RX7	253	268.8	178.4	331	424.7	467.5	209.3	314.6
P2RXL1	92.8	110.4	94.9	79.5	74.9	81.6	101.6	105
P2RY1	104.8	125.2	99.2	92.8	84.5	97.3	104	123.6
P2RY10	89.7	102.3	92.7	78.5	65	82.8	97	99.2
P2RY10	153.7	191.8	90.5	87	79.3	95.4	153.7	185.4
P2RY11	529.2	587.6	436	259.8	267.4	242.4	431.3	394.9
P2RY13	107.7	102.7	95.8	93	81.5	97.2	95.5	108
P2RY13	92.6	110.4	109.4	101.1	92.6	98.4	92.2	94.8
P2RY13	73.1	74.3	82.2	79	82.3	75.8	78.7	76.3
P2RY14	77	87	95.9	78.7	81.6	82.5	87.9	81.9
P2RY14	91.9	91.8	85.4	85.2	86	87.4	85.3	101.5
P2RY14	86	102.2	81.7	78.7	73.6	84.1	83.7	81.8
P2RY2	82	98.9	99.7	97.9	84.5	96.6	83.9	100.5
P2RY2	380.5	470.6	307.9	397.8	294.5	597.4	292.5	371.2
P2RY2	1385.6	1871.8	674.2	893.6	755.2	1200.3	964.3	879.8
P2RY2	152.5	191.1	116.6	128.2	111.2	100.8	125.2	150.7
P2RY4	137.9	131	93.1	94.7	87.1	96.9	113.9	135.8
P2RY5	176.3	190.1	129.3	350.2	265.9	561	134.3	190.5
P2RY6	115.1	145.2	109.3	101	94.8	104.9	94.5	126.3
P2RY6	320.8	380.2	196.1	136.8	129.7	154	347.7	541.3
P2RY6	152.1	144.2	106.4	84	85.5	89	130.3	164.5
P2RY8	1716.5	2293.1	2375.3	2620.6	2152.8	3158	1353.4	2483.7
P4HA1	127.7	158.3	110.8	100.5	99.1	109.7	124.1	173.8
P4HA1	483.3	663.7	193.4	166.7	174.5	316.9	395.6	723.9
P4HA2	207.4	256.5	155	157.1	128.5	169.6	208.4	286.7
P4HA2	148.5	169.9	93.8	86.2	87.5	86	138.8	147
P4HA3	93.6	103.5	86.7	82.8	91.4	91.8	92.4	102.8
P518	99.5	100.6	95.9	81.4	80	86.6	90.4	113.5
P53AIP1	87.3	95.9	93.3	74.2	79.3	84.6	85.2	89
P8	198.1	222	112.5	119.2	141.2	223.9	234.7	282.1
PA2G4	1984.4	2496.3	1187.9	984.6	826.4	1701.3	1648	3485.6
PA2G4P4	113.6	153.9	96	81.1	81.9	92.2	116.4	150.6
PABPC1	5624.3	5121.1	1407.5	1164.8	650.2	1161.7	5797.4	13281.6
PABPC3	72.8	78.8	89.6	79.6	71.6	74	68.3	78.1
PABPC4	5790.3	6729	8405.6	9871	7984.5	11670.3	4792.3	7629.6
PABPC5	87.6	88.4	80.1	84.1	77.3	75.9	84	89.4
PABPC5	93.9	91.9	83.2	88.1	86.3	80.2	89	102.3
PABPC5	81.4	86.4	96	89.2	87	95.8	79.4	90.3
PABPN1	1812.1	2316.8	1520.6	1699.1	1469.2	2309	1299.7	1678.1
PACRG	96.7	100.7	88.8	83.7	79.2	80.3	96.3	104.9
PACRG	114	137.8	104.7	93.6	86	77.9	111	138.3
PACRG	105.5	124.2	80.9	81.7	66.2	85.5	105.1	114.7
PACRG	86.1	90.9	106.1	111.8	104	104.1	77.4	86.3
PACS1	96.3	110.2	137.8	153.9	125	159.5	105.1	111.4
PACS2	183.4	196.3	155.9	132.7	129.4	160	145.4	188.6
PACSIN1	94.7	99.6	107.9	126.8	121.2	129.5	92.7	88.9
PACSIN2	2618.4	3262.9	1968.8	2399.6	1935	2607.1	3409.8	3281.4
PACSIN3	212.4	212.9	120.1	134.3	102.7	134.5	176.2	210.8

PADI1	95.6	120.9	109.9	89.7	90.9	97.4	94.7	111.1
PADI2	105.5	147.3	90.4	84.4	77.2	94.7	106.5	161.3
PADI3	101.2	125.6	89.9	91.5	81.3	93.2	103.8	111.4
PADI4	88.7	100.1	94	92.3	85.9	88.4	94.1	102.8
PADI6	152.9	167	100	98.3	88.9	98.4	151.1	173.2
PAEP	82.3	83.2	88.4	94.2	81.6	83.4	80.7	81.8
PAEP	92.6	123.7	88	83.2	77.3	77.9	103.8	114.9
PAF1	902.3	1000.2	438.7	340.4	286.9	409.3	675.6	741.1
PAFAH1B1	461.1	596.7	310.5	315.1	311.2	334.3	455.5	559.2
PAFAH1B2	181.3	252.8	121.9	106.8	104.5	103.8	166.5	221.6
PAFAH1B3	3060.5	3823.8	2260.1	652.7	659.6	1125.2	2427.5	2408.8
PAFAH2	251.8	356.4	208.1	197.2	152	257.9	231.7	379.5
PAG1	718.1	972.7	288.9	361.1	278.3	385.9	606	891.9
PAGE1	81.5	84.2	98.9	97	87.4	95.2	78.6	94.7
PAGE2	82.9	84.4	86.8	86.4	80.7	84.4	86.1	90.5
PAGE2B	78.6	107.3	87.7	84.5	83.9	86.3	103.6	93.7
PAGE3	93.5	106.7	96.3	90.2	86.4	88.7	100	102
PAGE4	95.2	119.5	91.8	83.1	79.7	81.4	101.3	113.2
PAGE5	80.6	90.4	96.6	89.1	86.1	84.5	95	94.1
PAGE5	83.1	88.7	84.9	79.7	86.6	75.8	87.8	94
PAH	79.1	95.1	91.2	88.3	81.7	82.2	83.4	88.5
PAICS	5469.6	7753	3776.3	3040.7	2211.4	3697.2	4455.7	7395.4
PAICS	205.1	217.7	125.1	113.3	101.7	105.1	131.7	158.5
PAICS	6156.1	8438.9	4779.5	4002.3	2816.9	4446.4	4746	7446.2
PAIP1	149.1	183.7	124.2	134.5	118.5	122.1	114.1	168.5
PAIP1	125.2	141.1	101.1	93.8	88.6	92.7	107.3	135.9
PAIP2	4703.9	5786.4	3132.9	3413.6	2694	4132.8	4077.5	7981.1
PAIP2	92.4	105.8	82.1	90.5	87.7	90.1	90.6	91.5
PAIP2	177.4	218.9	107.7	106	103.6	99.4	186.7	225.2
PAK1	569.3	669.8	355.7	396.3	318.4	465.1	454.3	732.6
PAK1IP1	246.9	267.6	108	116.4	106.4	127.9	195.8	274.3
PAK2	1050.7	1591.9	566.4	1088.6	684.3	1195.1	932.5	1691.2
PAK2	330.2	648.8	206.4	328	231.5	176.6	484.4	426.5
PAK3	80.4	86.3	108.5	102.6	99.6	94.6	81.6	79.3
PAK4	84.7	96.6	92.9	89.7	82.8	99.9	83.7	109.4
PAK4	108.1	115	94.6	89.5	85.4	100	91	120.1
PAK4	289.9	313.1	198.6	183.2	168.6	181.2	247.5	347.5
PAK6	103.2	110.3	94.6	85.7	82.8	89.3	96.9	102.5
PAK7	94.5	102.5	98.6	92.3	92	93.3	91.8	106.4
PAK7	123.2	145.7	102.5	95.5	90.9	107.5	112	127.3
PALLD	108.3	120.6	81.9	88.4	88.4	86.4	120.2	134.9
PALM	110.6	141.1	92.8	96.3	82.3	83.5	115.7	131.2
PALM	100.3	126.5	134.2	116.4	109.8	146.7	118	149.4
PALM	465.2	619.3	376.9	254.2	271.2	218.5	394.3	453.9
PALM2	84.1	105.7	89.2	75.7	73.5	83.4	83.2	97.2
PALM2	80.3	92	93.6	81.4	79.2	81.8	89.5	105.9
PALM2-AK/	81	78.7	93.1	90.7	101.6	88.6	85.4	89.9
PALM2-AK/	84.6	91.7	96.6	86.5	84.3	85.7	81.7	78.8
PALMD	119	119.4	100.4	100.7	91.7	97.4	108.7	110
PAM	976.9	1320.9	790	724.3	624.6	996.1	801.2	1054.7

PAMCI	87.7	93	94.2	91	84.4	98.9	90.6	99.2
PAN3	1133.9	1305.8	790.9	1052	894.7	1530.5	996.2	2029.1
PANK1	90.9	101.8	107.5	98.6	88.8	86.8	85.8	88.6
PANK1	374.2	530.2	245.1	194.9	159.5	237	324.1	443
PANK2	850.5	1037.1	607.7	737.6	557.6	868.3	723.7	1183.2
PANK2	545.2	744.9	319	347.4	269.9	441.1	452.7	824.9
PANK2	86.5	81.4	91.4	80.4	84.2	83.6	79	77.2
PANK3	159.3	202.6	132.7	134.6	125	154.1	145.9	162.6
PANK4	145	170.5	151.2	149.4	133.5	191.4	133.2	173.9
PANX1	156.7	232.1	142.9	133.7	106.9	140.2	165.4	260
PANX2	185	221.4	120.5	143	152	149.2	538.1	812.3
PANX3	103.9	127.5	112.9	116	110.5	102.8	96.7	103
PAOX	122.5	141	117.9	106.5	106.6	119.8	108.8	135.5
PAOX	358.6	469.7	203	171.7	166	184.4	259.6	258.1
PAOX	109.7	150.7	88.7	78.1	71.5	88	109.2	133.7
PAP2	89.3	93.5	99.3	95.3	90	89.2	101	102
PAP2D	83.3	83.3	97	76.8	97.3	78	79.2	85.6
PAPD1	1104	1409.1	693.3	547.3	376.2	497	853.7	1233.5
PAPD4	1527.9	1888.9	950.2	1158.8	1010.9	1784.6	1241.4	2785
PAPD5	464.5	635.8	174.7	188.2	147.9	135.8	406.3	496.9
PAPLN	183.2	204.9	119.7	180.7	172.1	202.4	137.9	184.5
PAPOLA	3204.9	3915.6	1682.1	1807.6	1223.5	2721.7	2329.1	3705.1
PAPOLB	84.3	92.8	94.9	77	72.8	83.6	86.4	91.2
PAPOLG	123.9	138.3	121.9	115	113.6	111.3	119.2	120.6
PAPPA	78.4	78.5	88.3	80.2	85	87.9	79.6	79.5
PAPPA2	71	78.9	94.4	89.5	91	78.1	84.6	80.6
PAPPA2	84.1	105.4	94.5	92.9	88.3	85.6	88.8	80.8
PAPSS1	122.6	129.3	120	130.5	120.1	160	109.2	145
PAPSS2	207.5	268.1	178.2	228.8	201.9	212.9	171.9	241.2
PAQR3	475.8	578.9	294.9	285.3	200.9	373.5	387.3	731.7
PAQR4	2358.8	3145.9	1521.2	865.6	708.4	819.7	1648.9	1637.9
PAQR5	80.6	84.6	86.8	103.3	84.1	78.8	77.1	81.2
PAQR6	122.8	136.1	91.4	93.1	83.9	90	109.9	145.8
PAQR6	110.3	109.4	109.3	105.9	100.4	118.6	104.1	95.5
PAQR6	100.4	106.2	111.1	106.6	107.9	146.4	84	103.4
PAQR7	156.3	207.4	130.5	94.8	101.2	117.5	157.1	186.1
PAQR8	2372.6	3226.3	1032.2	1999.9	922.8	2142.7	1245.6	1340.5
PAQR9	88.8	113.1	97.9	96.7	92.7	94.5	91.9	112.4
PARC	180.1	184.2	130.2	123.1	120.7	144.2	129.9	201.9
PARD3	114.3	129.7	93.3	98.3	90.6	85.8	113.4	119.5
PARD3B	102.5	108.2	97.8	85.1	88.8	90.5	94.3	101.8
PARD6A	153.3	164.8	109.8	95.4	86.4	96.8	132.4	157.5
PARD6A	112.9	126.7	100.3	101.3	87.4	88.4	117.8	138.5
PARD6B	120.8	119.7	92.5	100.5	87.2	94	118	124.7
PARD6G	130.8	168.2	103.2	110.7	100.5	114.8	120.7	163.5
PARD6G	100.1	128.3	106.5	101.6	93.2	103.5	107.3	119.7
PARG	133	169	114.3	91.2	90.5	104.5	115.1	168
PARK2	89.8	91.5	86.4	90.1	95	90.5	87.3	92
PARK2	85.8	97.7	96.4	84.3	90.8	94.9	88.3	92
PARK7	7728.3	9210.9	4400.1	4230	3403.1	4098.4	7242.1	8483.4

PARL	4275.9	5251.9	3489	4068.9	3531.3	5139.6	3675.7	5016.9
PARL	2215.4	3759.8	924.3	1149.5	1008.5	1462.3	2219.1	2689.2
PARN	790.5	1090.1	310.7	244.3	216.8	288.3	765.7	706
PARP1	9381.7	10265.8	6258.2	5547.2	4443.6	7475.8	7598.7	10102.7
PARP10	152.4	169.3	112.2	115.1	91.2	130.2	129.8	152.8
PARP10	361.8	383.7	200.3	166	176.1	192.9	204.8	224.8
PARP11	107	139.3	106.9	132.2	120.7	162.2	107.9	137.6
PARP12	186.2	211.1	179.8	151.5	157.2	174.6	152.7	221.1
PARP14	282.1	369.1	183.1	139.4	143	164.4	274.2	292.3
PARP15	121.2	136.7	97.1	90.5	84.2	85.2	105.1	135.1
PARP16	145.9	173.6	133.3	176.1	125.2	174.3	129.9	186.3
PARP2	833.8	1042.1	458	370.3	290.7	475	721.3	1398.6
PARP2	426.1	543.4	275.3	166.2	171.5	214	307.2	466.7
PARP3	131.6	139.4	93.6	101.3	91.8	90.8	111.9	127.4
PARP3	576	640.1	393.3	386	326.7	459.5	417.4	528
PARP3	157.5	175.1	148.5	157	147.7	181.6	112.9	175.2
PARP4	1515.5	1954.5	1493.8	1960.3	1667.5	2205.8	1559.2	1472.7
PARP6	134.7	168.4	115.4	124.9	125.3	105.5	120.6	152.4
PARP6	232.6	274.5	138.1	146.8	130.9	188.3	191.8	297.1
PARP6	105.4	106.5	106.2	104.8	97.4	106.5	82.6	95.3
PARP8	1772.1	2019.7	605.9	659.2	516.4	608.4	1150.5	1318.9
PARP9	140	162.1	106.8	87.4	96.2	103.8	135.2	175.8
PARS2	117	166.3	106.2	88.4	97.2	98.5	119.6	151.5
PART1	81.7	97.8	89.8	88.7	92.2	94	94.7	99.5
PARVA	125.3	142.9	130.2	127	109.3	130.8	124.9	161.5
PARVB	689.6	872.9	856.3	865.1	634	1354.7	660.7	1792
PARVB	105.3	145.3	101.6	118.4	109.5	117.7	108.3	129.3
PARVB	183.9	185.6	116.2	114.9	105.2	88.3	144.1	174.2
PARVG	3121.2	4272.8	3715.6	2950.8	2702.9	4558.1	2809.2	4371.1
PASD1	82.4	87.9	87.5	92.8	82.3	86.2	78	78.9
PASK	194.2	215.5	192.5	142.7	145.7	178.8	161.1	186.9
PATE	116.3	136.2	107.1	98.8	96.6	99	108.8	114
PATZ1	135.2	165	98.9	88.7	83.5	82.1	107.7	145.1
PAX1	100.5	110.3	98.2	88.7	87.3	92.3	102.9	102.4
PAX2	97.5	101.3	121.7	97.3	110.3	97.2	91.7	90.7
PAX2	80.1	86.8	86.1	83.1	82.1	89.3	82.8	88.3
PAX2	103.4	126.8	97.9	103.8	94	95.6	107.3	117.6
PAX3	89.7	116.3	90.6	94.1	84.5	90.8	104.2	98.3
PAX3	132.3	153.5	88.6	82.8	75.7	83.6	118	153
PAX3	91.9	100.3	84.8	78.9	73.6	101.2	100.2	103.9
PAX3	110.6	97.8	93.6	92.2	92.6	92.4	100.1	112.3
PAX4	100.6	123.5	116.4	90.8	98.6	90.8	107.6	122.1
PAX5	82.9	90.6	89.2	74.6	74.6	80.4	80	97.4
PAX6	78.6	79.6	93.4	77.7	76	86.9	79.1	82.3
PAX6	124.5	163.7	94.7	73.5	77.7	78.3	113.5	152.5
PAX7	457.8	484.9	268.2	243.3	203.8	266.3	394.8	497
PAX8	136.1	159.2	98.4	96.8	86.7	101.8	138.4	172.5
PAX8	98	102.2	111.5	86.9	89	91.8	93.9	95.2
PAX8	83.3	87.1	95.6	88.5	74.9	80.1	76.4	88.3
PAX9	106.6	155	107	84.4	83.8	92.8	121.8	127

PAXIP1	1018.8	1321.3	791.5	837.6	729.2	889.3	710.4	734.5
PB1	87.7	111.8	118.7	102.6	100.2	116.8	92.9	102
PB1	231.1	300.2	183.6	205.7	175	203.9	199.5	210.9
PB1	199.2	198.8	157.8	159.2	157.6	207.9	165.8	321.6
PBEF1	340.4	481.2	135.4	97	102.6	144.8	363.8	532.8
PBEF1	255.6	327.8	143.8	118.2	115.6	138.2	219.2	289.5
PBK	2200.2	2952.8	1003.3	549	532.8	645.8	1441.3	1925.2
PBOV1	90	115.2	93	85.2	83	98.2	93.4	108.7
PBX1	89.7	102.8	95	95.3	77.7	89.5	98.6	90.4
PBX2	483.1	576.8	276.3	432	434.1	432.9	433.3	385.8
PBX3	8971.6	10367.6	6778	6249.7	5030.2	6213.2	7991.1	8750.5
PBX4	115.6	126.3	123.3	112.7	99.1	112.7	113.8	155.8
PBXIP1	105	138.2	111.4	96.6	108.1	111.5	101.2	125.7
PC	87.4	98.3	91.3	84.5	81.3	87.3	81.4	95.9
PC	78.3	99.1	100.2	85.5	92.8	89.2	90.2	106.2
PC	81.9	100.6	135.1	109.1	104.1	125.5	85.3	102.7
PC-3	96.8	102.6	89.7	83.9	87	85.6	101	107.4
PCAF	98.7	107.6	90.7	94.5	91.5	103.2	107.7	116.2
PCBD1	529.6	694.8	359.8	271.1	232.5	303.4	452.6	655.7
PCBD1	527.6	665.4	432.8	310	277.3	318.2	496.9	583.6
PCBD2	231.3	272.6	156.7	175.1	170.5	190	204.8	252.1
PCBP1	10573.4	13058.9	5369	5207.7	3403.7	5251.5	8631.6	10444
PCBP2	8839.5	11180.2	5106	5712.6	4830.2	6922.9	7816.5	13307.8
PCBP2	3105.4	4449.7	2405.7	2971.9	2554.6	3716.4	2855.8	4165.8
PCBP3	90.7	94.5	93.9	95.6	90.8	84.3	78.8	105.1
PCBP4	677.2	945.9	212.6	203.6	184.5	288.8	581.6	772.9
PCBP4	104.9	123.8	103.1	101.9	92.4	84.7	101.8	107.8
PCBP4	105.9	134.8	95.4	102.7	84.9	92.8	110.4	138.9
PCBP4	422.7	551.1	252.1	233.9	225.5	349.2	433.7	495.4
PCCA	1104.8	1346.9	525.6	515.4	452.1	657.6	932.2	1588.4
PCCB	4605.6	5757.8	3606.4	2778.3	2241.2	3311.3	3808.6	4894.7
PCDH1	87	99	91.3	87.9	76.9	83.5	87.4	96.5
PCDH1	95.4	112.5	101.7	94.4	92.4	80.2	106.4	111.6
PCDH10	80.9	97.7	80.7	79.6	80.8	83.5	90.8	91.6
PCDH10	126.9	158	91.7	91.7	80.2	85.3	110.2	132.1
PCDH10	85.9	97.7	90.8	92.9	85.9	90.7	113.2	90.6
PCDH11X	98.4	115.1	99.8	90.2	100.7	104.9	113.2	106.3
PCDH11X	87.2	91.5	97.8	87.6	96.9	84.7	94	89.2
PCDH11X	78	90.8	100.5	92.4	94.6	97.8	83.9	89.2
PCDH11Y	68.6	80.6	90.2	81.5	84.5	88.2	71.2	84.5
PCDH11Y	87.1	78.5	88.4	78.6	83	89.7	78.7	83
PCDH11Y	87.8	110.3	89.5	93.1	84.7	88.9	101.9	102.5
PCDH12	134.8	165.7	93.7	93.8	85	88	149.1	160.4
PCDH15	79.4	94.3	98.2	98.8	89	91.7	87.1	92.2
PCDH17	91.6	99	110	93.3	87.3	88.8	85.4	92.6
PCDH18	95.3	113.3	114.3	106.6	86.5	93.5	99.6	104.1
PCDH19	89.4	92.2	101.4	99.7	92	95	82	84
PCDH20	227.3	218.5	157.9	160.6	133.3	164.1	200.5	288.2
PCDH21	132.2	170.9	92.6	85.7	77.8	97.7	144.8	172.7
PCDH7	86.2	109.8	92.2	119.5	101.3	109.3	93.2	92.8

PCDH7	87.2	95.2	84.9	77.7	81.3	83.4	85.7	91
PCDH7	91.6	88.5	96.8	96	90	103.5	82.5	102.8
PCDH7	84.4	87.6	88.3	90.2	92.4	93.1	81.1	92.5
PCDH8	90.4	102.4	114.6	91.1	97.1	95.3	91.1	101.9
PCDH8	106.5	108.6	95.1	81.1	80.4	86.9	92.8	117.6
PCDH9	82.3	96	87.1	87.7	82.9	88.7	79.8	88.5
PCDH9	87.1	93.4	90.6	95.8	85.9	78.7	82.3	92.8
PCDH9	127.8	156.8	93.7	91.6	76.9	106.8	119.8	164.8
PCDHA1	94.8	103.6	97.9	83.1	85.2	92.3	90.3	96
PCDHA1	75.4	84.2	93	77.9	86.5	101.6	84.3	80
PCDHA1	76.3	77	105.6	93.9	88.7	101.8	77	81.9
PCDHA10	92.8	89.5	99.8	85.5	82.2	91.7	81.9	97.4
PCDHA10	75.5	91.7	90.3	77.9	79.8	86.2	81.2	85.8
PCDHA11	141.4	160.2	106.4	105.1	91.2	96.7	133.4	140.6
PCDHA12	98.2	107.5	87.7	80.5	75	95.1	90.6	108.2
PCDHA12	81.3	83.4	92.6	98.8	89.6	95.1	87.1	84
PCDHA13	90.4	99.4	84.2	81.8	83.4	81.5	93.4	102.9
PCDHA2	77.2	86.5	88.5	71.8	74.1	84.3	88.8	88.1
PCDHA3	97.7	122.9	85.8	80.5	80	87.5	93.3	109.8
PCDHA3	80.7	97.4	88.3	84.7	81.4	86.6	82.8	83.9
PCDHA3	89.4	98.7	95.9	88.3	82.3	89.2	87.4	88.5
PCDHA4	93.3	98.7	102.4	106.2	83.7	117.4	91.9	100.9
PCDHA5	144	193	115.9	101.8	103.9	112.3	144.7	187.1
PCDHA6	77.2	80.5	98.8	90.5	90	91.6	79.4	85.8
PCDHA6	108	139	95.2	91.1	87.5	90.9	113.2	127
PCDHA7	89.7	92.5	96.2	80.6	86.5	90.1	82.5	92.7
PCDHA7	123.2	141.3	122.8	111.2	107.1	106.4	116.3	121.9
PCDHA7	79.1	86.8	90	83.5	82.5	85.6	73.5	85.8
PCDHA8	99	118.7	95.7	87.2	82.8	82.4	93.5	118.5
PCDHA9	85.5	89.4	101.8	93.8	94.4	84.3	84.1	100.6
PCDHA9	78	86.8	86	81.2	81.6	87.6	79.7	83.5
PCDHAC1	90	95	95.3	87.2	82.3	82.2	85.6	84.3
PCDHAC1	75.3	82.6	83.6	83.9	84.9	87.3	72.7	76.1
PCDHAC2	117.2	134.5	86.8	79.8	90.4	108.8	111.4	137.6
PCDHAC2	89.2	97	93.6	90.9	78.2	87.7	90.8	105
PCDHAC2	105.4	136.3	99.4	121.5	101.1	118	110.5	127.6
PCDHB1	77.8	73.9	95.9	79.5	79.4	78.5	72.8	78.5
PCDHB10	93.9	89.2	103.3	103	89.6	102.4	88.1	115.4
PCDHB11	71.2	78	93.7	83.5	72.6	74.6	70.3	83
PCDHB12	108.1	118.4	98.9	83.8	89.6	97.3	101.1	110.6
PCDHB13	92.8	100.8	90	96.3	90.8	93.8	98	105.8
PCDHB14	105.3	124.5	107.5	100	96.9	104.3	98.4	113.7
PCDHB15	73.6	83.3	91	79.8	74.2	85.7	71.6	80.1
PCDHB16	91.7	100.6	92	95.7	81.5	91.2	87.8	98.2
PCDHB17	209.7	249.6	171.5	131	126.5	147.9	192.7	287.5
PCDHB18	88.3	96.7	94.3	83.4	85.4	82.3	86.5	97.5
PCDHB2	1133.7	1396.4	841.8	563.2	420	579.8	992.7	1284.7
PCDHB3	94.8	108.2	94.5	80.6	81.2	86.1	109.4	97.4
PCDHB4	97.8	104	92	93.1	95.9	102	89.5	119.7
PCDHB5	303	384.9	239	201.6	165.7	221.8	255	421.7

PCDHB6	71.2	82.4	85.2	79.5	77.6	79.8	65.9	77.1
PCDHB7	71.5	88	88.6	86.9	79.1	84.2	71.8	77.8
PCDHB8	89.7	104.4	97.2	94.4	81.2	85.1	85.9	103.2
PCDHB9	248.9	303.1	152.5	213.8	228.8	160.7	252.1	255.1
PCDHGA1	109.3	134.5	94.3	90.2	83.1	93.7	104.3	120.2
PCDHGA1	96	104.8	95.7	95.5	90.9	95.8	94.5	100.2
PCDHGA1	80.8	83.8	90.2	93.8	86.8	83.5	76.5	87.6
PCDHGA10	97.9	123.5	98.8	88.2	100.1	88.2	103.4	105.4
PCDHGA11	84.8	106.2	92.9	84.9	83.7	84	100	103.9
PCDHGA11	81.7	88.5	90.9	82.4	82.9	84	84.2	84.3
PCDHGA12	85.5	94.3	89.4	84.9	93.8	88.4	83.2	94.5
PCDHGA2	81.2	85.7	90.6	84.8	78	90.4	84.2	79.4
PCDHGA3	82.9	90	88	97.6	80.3	80.7	83.7	97.2
PCDHGA3	84.3	90.2	87.1	90.3	90.6	99.4	89	86
PCDHGA4	72.1	85.6	78.4	75.3	74	70.3	74.7	74.6
PCDHGA4	90.8	94.5	76.6	72.4	76.6	73.5	76.9	86.8
PCDHGA5	93.2	110.5	95.5	93	87	102	93.1	107.7
PCDHGA5	87.5	100.2	88.5	102.5	74.7	84.3	89.5	110.4
PCDHGA6	85.7	86.1	105.7	98.7	92.5	106.2	80.8	74.8
PCDHGA7	79.8	86.6	93.8	89.7	85.6	87.3	79.7	86
PCDHGA7	72.1	74.4	84.8	76.5	72.8	76.8	70.4	76.2
PCDHGA8	82.9	91.7	93.7	81.1	82.1	79.7	81.6	97.5
PCDHGA9	134.6	158.3	91.8	77.2	76.5	82.6	123.1	165
PCDHGA9	139.3	180.4	96.7	88	98.7	97	141.7	155.2
PCDHGB1	106.4	122	99.3	90.7	94.5	90.7	107.1	112.4
PCDHGB2	89.7	99.2	100.6	86.2	91.3	102.9	86.6	94.8
PCDHGB3	84.8	99.2	101.1	95.9	94.8	92.1	92.3	102.6
PCDHGB4	140.7	168.3	106	96.8	96.3	95.7	116.2	143.3
PCDHGB5	79.5	85.4	96.3	98.9	92.6	94.8	82.5	81.3
PCDHGB6	87.4	91.5	91.4	96.7	93.6	87.4	85.1	95.1
PCDHGB6	192.2	204.6	122.8	110.1	112.2	122.9	176.7	212.8
PCDHGB7	79.3	100.6	95.3	85.9	89.6	93.5	86.4	93.7
PCDHGB7	73.3	78	90	88.8	87.3	78.9	69.6	88.4
PCDHGC3	97.3	120	95.3	86.4	92.7	88.4	96.2	115.5
PCDHGC3	104.9	110	109	101.2	104.6	102.5	100.3	101
PCDHGC3	146.9	169.7	106.1	110.6	105.2	105.2	133.7	150.5
PCDHGC4	108.3	110.9	105.1	94.5	84.4	89.9	98.3	114.6
PCDHGC4	82.5	81.2	78.2	78.6	77.2	75	70.4	85.4
PCDHGC5	77.6	88	85.7	89.3	90.8	91.8	77.8	92.1
PCDHGC5	76.4	84.8	101.5	90.8	95.4	109.4	84	82.4
PCF11	378.9	439.6	262.1	288.1	231.9	360.6	300.3	681.3
PCGF1	391.7	485.6	334.1	307.8	240.9	476.6	361.6	596.1
PCGF2	166.1	215.6	172.4	110.5	89.8	105.1	166.2	232.8
PCGF3	109	129.4	110.1	90.1	90.3	98.4	110.5	114.7
PCGF4	5136	5831.1	2398.5	1823.5	1333.5	2257.5	3935.7	5733.3
PCGF5	127.1	132.8	113.5	103.6	80.4	104.2	129.4	211.5
PCGF6	383.9	475	201.8	164.7	154.8	172.5	358.7	629.4
PCGF6	427.6	554.4	223.3	180.4	144.1	175.7	301.9	471.5
PCID1	2464	3383.9	898.4	687.6	543.4	762.4	2456	4263.9
PCK1	75.5	81.3	80.3	87.8	80.4	90.9	79.5	78.5

PCK2	190.6	276.3	182.3	113.8	116.2	112.3	167	247.9
PCK2	1180.6	1415	973.1	499.2	426.9	634.1	1037.9	1769.7
PCK2	146.9	174.5	114.7	89.2	85.7	87.3	133.1	203.9
PCLKC	176.6	199.8	135.8	111.8	114	113.8	154.7	158.6
PCM1	2468.7	2926.9	1085.8	1447.3	1134.6	1982.4	2057.3	3082.8
PCMT1	5402	6485.7	3689.2	3872.6	3132.5	4468	5028.7	6625.8
PCMTD1	406.7	524.9	173.4	204	167.3	255.5	434.9	770.5
PCMTD2	427.1	515.5	399.7	519.1	499.4	883.8	359.1	604.4
PCNA	150.5	172.8	107.5	97.5	90.4	98.7	131.3	158.8
PCNA	172.6	226.6	129.2	132.5	125.5	133.6	149	167.5
PCNA	3786.3	4737.8	1280.7	1098.2	741.7	1118.8	2853.6	5640.9
PCNP	4006.6	4469.6	3051.4	3750.9	2866.8	4574.4	3714.8	6016.3
PCNT2	504.7	658.4	506.5	850.6	902.3	1271.3	447.2	500.4
PCNX	402.9	531.6	324.2	355	348.6	468.8	411	347.9
PCNXL2	123.6	133.5	103.3	103.3	90.2	116.2	119.9	131.8
PCNXL2	102.1	119.9	110.3	117.7	103.8	117.3	98.8	107.3
PCNXL2	97.1	101.1	117.2	118.8	120.1	120.9	93.6	90.6
PCOLCE	108.1	125.8	113.2	97.5	98.2	114.5	100.7	145.6
PCOLCE2	186.8	211.2	120.5	239.5	146.5	244.6	157.6	195
PCOLN3	396.9	551.2	401.5	297.3	236.1	402	383.4	837
PCP2	111.4	124.8	116.3	96.8	96.2	101	105.6	124.5
PCP4	79.2	77.6	101.6	92	80.6	88.3	86.9	82.4
PCQAP	115.8	148	136.3	150.6	126.3	184.8	116.6	164.7
PCQAP	119	125.6	142.3	126.4	122.1	127	98.9	110.9
PCSK1	85.7	107.4	84.6	75.9	76.4	86.6	96.1	104
PCSK1N	108.6	134.8	92.6	75.3	75.7	79.5	95.6	125.8
PCSK2	79.3	90	84.5	81.5	80.7	85.2	93.6	79.9
PCSK4	120.3	129.1	101.8	94.4	98.8	98.7	113.1	111.6
PCSK6	105.9	113.1	85.3	89.3	81.1	88.7	95	103.6
PCSK6	93.2	113.1	90.9	92.4	78.9	91.4	93.5	103
PCSK6	123	166.9	115.3	108.4	104.3	106.5	127.2	133.5
PCSK6	86.4	99.3	104.7	106	91.7	97.5	90.3	94.1
PCSK6	92	117.7	86.2	97.8	94.3	92.6	96.7	107.1
PCSK7	341.6	382.1	292.8	292.8	236.1	340	292.6	414.2
PCSK9	107.1	113.2	103.1	91.6	82.9	101.2	108.6	119
PCTK1	107.4	126.9	115.3	100.9	91.3	83.6	91.5	113
PCTK1	72.5	85.4	91.3	83.2	85	83.2	76.1	77.6
PCTK2	132.9	147.7	102.9	98	78.6	95.5	130.6	155.4
PCTK3	113.8	136	117.4	113.6	120	123.7	108.9	135.2
PCTK3	124	135.7	79.7	86.1	78.3	86.4	115.9	121.8
PCTK3	132.3	120.3	93.6	95.2	77.9	90.2	112	127.3
PCTK3	81.5	87	87.7	78.2	82.6	92	79.1	86.2
PCTP	375.8	484	167.3	154.3	115.8	188.7	318.7	520.1
PCYOX1	425.6	491.4	307	541.1	506.7	465	366.9	611.9
PCYT1A	108.2	129	98.3	91.7	97.4	84.1	112.4	120.8
PCYT1B	96.2	99.4	96.3	100	79	91.7	93.4	91.7
PCYT2	166	193.3	93.6	96.5	96.3	107.2	153.9	178
PDAP1	131.3	174.1	80.9	79.7	70.6	84.1	110.3	148.4
PDC	88.6	87.4	101.5	99.8	85.6	103.4	85.4	89.9
PDC	79.2	86.7	93	79.2	82.9	86.2	84.6	90.1

PDCD1	75.9	92.2	99.1	89.8	83.9	81.5	86.7	80.1
PDCD10	362.7	448.7	141.6	168.6	140.5	213.4	269.4	357.4
PDCD10	190.4	233.2	108	102.2	104.5	105.6	189.5	162.4
PDCD10	2308.9	2724.9	889.4	960.6	749.8	1357.3	2028.2	3070.2
PDCD10	212.6	237.6	132.1	117.5	103.8	159.4	196.9	334.8
PDCD1LG2	65.1	84.7	81.7	72.9	75.4	73.3	74.7	79.4
PDCD2	1222.6	1584.1	1163.3	1073.7	870.6	1281.4	810.4	1489.8
PDCD2	2669.8	3282.2	2175.6	2397.5	1946.8	2838.7	1787.1	2612.6
PDCD4	1338.4	1690.2	946.5	1627.5	1596.9	1682.8	1345.6	1739.9
PDCD5	2135.7	2483.7	1189.5	1069.2	802.7	1065.8	1816.6	2851.9
PDCD6	5777.5	7168.1	3347.6	4023.9	3191	4813.6	4379	6444.1
PDCD6IP	79.2	84.8	92.1	86.7	80.4	81	76	83.5
PDCD7	13614.1	14439.6	4694.7	6330.3	6700.1	5043.3	12793.3	14632.8
PDCD8	483.2	667.3	205.5	134.6	133.1	141.1	439.9	558.4
PDCD8	530.6	799.3	334.4	284.7	249.8	375.2	473.2	588.3
PDCD8	99.3	134	97.4	91.4	79.1	78.8	100.7	128
PDCL	335.2	446.3	161.8	137.3	112.8	125.2	302.1	390.1
PDCL2	82.7	102.9	94.5	84.8	89.1	83.9	91.2	95.6
PDCL3	1286.8	1855.1	559.4	596.5	424.5	662.1	1069.3	1149.9
PDDC1	145.4	183	144.5	130.3	114.5	120	126.6	154
PDDC1	411.3	379.6	276.5	230.8	209.2	263.1	222.7	325.2
PDE10A	79.3	83.2	98.3	79.9	78.1	85	84.4	95.4
PDE11A	78.6	89.9	88.8	86.2	76.7	80.7	85.1	85.5
PDE11A	79.6	92.9	103.2	102.6	93.4	104.1	83.5	77.8
PDE11A	88.9	97.6	114.1	101	98.6	98	85.4	93.3
PDE1A	89	92.3	97.3	93	86.1	90.9	90.8	88
PDE1A	80.4	83.1	100.6	94.5	96	93.4	79.5	88.6
PDE1A	97.7	102.2	99.5	87.8	97.4	103.6	87.6	105.3
PDE1B	124.6	134.2	125.2	160.9	150.3	224.4	112.3	164.5
PDE1C	82.2	88.6	91.4	91.3	84.3	88.1	84.4	91
PDE2A	84.7	101.2	88.7	97	84.5	92.9	90.3	85.6
PDE3A	105.5	120.6	89.2	84.3	90	85	108.2	111.7
PDE3B	466	597	385.1	542.9	440.4	896.9	522.2	808
PDE4A	127.3	145.4	86.4	92	85.3	90.9	120.7	160.1
PDE4B	106.7	113.9	114.9	216.6	134.6	281.2	97.2	110
PDE4B	84	98.2	86.9	90.7	91.8	104.6	89.5	87.6
PDE4B	77.4	94.3	92.6	93.2	86.9	88.9	77.4	93.8
PDE4B	93.5	137.7	86.9	83.3	80.8	89.5	93	101.4
PDE4C	79.6	89.1	93.6	87.5	87.1	96.8	83.5	97.2
PDE4D	151.5	196.4	130.8	129.2	109.3	177.4	159.3	215.3
PDE4DIP	92.5	94.9	104.8	95.9	94.6	87.9	79.2	97.4
PDE4DIP	160	169.3	78.7	85.4	83.6	83.4	150.4	159.2
PDE4DIP	79.1	85.8	86.7	82.6	81.1	79.2	76.8	82.6
PDE5A	85.8	84.7	103.8	87.8	93.9	88.7	80.3	90.3
PDE5A	91.1	122.6	107.3	99.8	91.8	93.3	97	106.1
PDE5A	80.9	102.6	84.7	90.1	94.4	81.9	87.3	93
PDE5A	72.3	83	87.8	76.8	75.8	78.7	82.2	91.1
PDE5A	77.4	93.2	96.1	90	82.7	82.8	84.7	85.5
PDE6A	107.4	127.2	99.6	86	77.8	83.9	111.3	118.8
PDE6B	86.4	85.6	81.6	92.5	70.4	78.1	86.4	89.3

PDE6C	84.5	99.4	91.6	82.5	67.6	78.5	86.6	105.5
PDE6D	613.1	968	267.4	198.6	173.2	227.7	627.1	766.6
PDE6G	124.2	141.9	108.6	106.7	98.4	105.7	117.5	125.6
PDE6H	174.1	183.2	104	100.7	92.9	90.9	171.5	174.9
PDE7A	622.9	884.5	310.3	253.8	246	384.9	486.6	683.9
PDE7A	425.4	497.7	287.2	590.7	700.8	706.8	363.9	439.9
PDE7A	100.5	109	108.2	104.5	89	104.9	95.5	117.6
PDE7B	409.4	588.4	267.5	207.7	172.4	177.8	301	249.8
PDE8A	306	372.8	167.6	153.8	135	163.4	312	380.7
PDE8A	107.6	116.3	102.4	95.2	95.3	108.3	101.8	110.8
PDE8A	97.9	112.4	91.3	90.2	83.7	92.7	100.6	106.8
PDE8B	120.5	133.1	110.5	95	91.9	103.6	101.5	113.5
PDE8B	82.5	92.6	93.9	92.5	92.3	78.6	78.4	82.1
PDE9A	91	98	107	99.2	95.5	91.5	91.9	105.8
PDE9A	135.1	164.3	115.2	116.1	131.1	120.1	122.7	138.3
PDE9A	128.2	148.3	90	105.7	85.6	92.3	113.8	134.2
PDE9A	91.1	105.7	89	91.8	88	87.9	84.4	101.1
PDF	82.4	83.4	99.8	94.6	93.4	86.4	87.9	101.1
PDGFA	155.8	142.4	90.5	78.6	80.3	81.9	120	137.2
PDGFA	112	106.4	93.9	91.9	82.9	90.5	109	114.8
PDGFB	99.6	115.9	94.3	89	87.3	87.9	94.3	106.7
PDGFB	95.9	103.7	93.5	94	89.2	90.5	97.6	109.2
PDGFB	117.4	128	101.8	108.9	102.2	107	108.6	119.5
PDGFC	183.3	237.1	117.5	114.3	90.9	125.3	164.8	236.6
PDGFD	130.9	158.6	106.8	134.9	121.5	189.2	111.3	129.5
PDGFD	166.5	210.9	125.2	138.5	111.1	164.5	120.9	163.3
PDGFRA	235.2	286.9	160.9	161.7	151.5	159.9	245.4	289.3
PDGFRB	120.8	147.2	103.4	115.7	111	137.2	113.2	129.3
PDGFRL	132.4	159.6	127.9	108.6	113.6	108.3	144	133.8
PDHA1	1284.2	1700.8	465.7	516.1	435.6	514	1145.1	1390.8
PDHA2	81.9	82.5	88.6	83.3	81	87	90	88.3
PDHB	5371.5	6648.3	3850.7	4134.1	3308.4	4984.4	4283.9	5095.8
PDHX	991.9	1141.6	454.6	549.2	358.1	656.8	888.1	1746.6
PDIA2	100.3	104.1	90.5	90.9	87.9	90.5	93	98.1
PDIA3	112.8	129.5	99.7	85.8	85.8	90.3	101.8	126.1
PDIA3P	632.9	818.2	226.6	211	215.3	260.9	565.6	998.1
PDIA4	188.2	204.7	127.2	132.9	108.4	158.6	161.8	282.5
PDIA5	571.6	757.3	434	626	530.7	873.7	503.3	735.1
PDIA6	2029.2	2857	1049.5	933.4	775.9	943.6	1940.2	2387.9
PDIK1L	242.8	301.5	201.1	204.2	158.1	265.7	228.7	371.1
PDILT	78.4	88.5	92	90.5	82.1	82.9	85.4	86.5
PDK1	114.9	138	109.1	97.2	95.7	102	107.9	137.6
PDK2	121.7	153.7	115.5	100.1	102.4	95.5	124.7	136.7
PDK3	311.2	458.4	194	140.4	127.4	176	285.4	351.5
PDK4	99	120.2	101.6	101.3	104.5	125.7	107	160
PDLIM1	807.3	1215.3	685.1	266.2	212.3	248.4	829.4	1201
PDLIM2	93.2	97.9	94.9	90.9	80.3	85	95.7	100.7
PDLIM2	80.5	104.2	80	70.6	79	72.8	83.9	94.3
PDLIM3	102	116.3	90.2	89.2	98.1	92.7	98	115.6
PDLIM4	73.7	81.4	95.4	88	87.2	79.7	81.6	80

PDLIM5	194.8	237.2	133.5	110.5	113.1	113.5	142.6	172
PDLIM5	128.2	150	110.3	88.4	94	97.5	98.1	131.9
PDLIM5	149.9	182.1	103.5	94.1	87.6	88.1	121.8	139.8
PDLIM7	151.9	201.5	159.2	130.7	113.8	136.6	123.5	163.7
PDLIM7	517.3	576.3	253.5	245.5	208	230.4	447	584.9
PDLIM7	313.4	405.1	265.1	190.2	151	166.2	321.2	411
PDP2	152.9	157.7	128.6	107.4	105.6	95.4	160.3	200.8
PDPK1	91.6	110.7	95.6	106	98.1	89.1	97.7	110.9
PDPK1	694.4	853.8	275.6	293.6	301.9	234.5	536.8	961.9
PDPK1	698.8	879.8	659.6	939.2	716.1	1219.5	772.9	1553.4
PDPN	160.2	205.7	154.2	89.7	94.9	98	140.3	146
PDPN	108.9	94.2	89.4	95	83.4	93.2	105.5	103.8
PDPR	219.2	315.6	189	221.4	222	247.8	213.4	285
PDPR	365.3	504.8	335.7	409.2	377.2	412.2	385.7	488
PDPR	121.9	152	98.5	86.5	82.3	86.2	108.6	124.6
PDRG1	603.9	834.3	475	360.8	303	494.8	574.2	755.2
PDSS1	1078	1411.9	533.9	400.4	297.2	454.5	763.6	1301.3
PDSS2	1593.6	1872.5	723	875	824.4	935.6	1418.5	1622.5
PDXK	1171.5	1584.1	1328	2061.3	1665.8	2184	1207.5	1138.6
PDXP	2433.8	3370.3	1470.4	1164.2	980.7	1183.6	2194	1879.9
PDYN	151.5	172.6	98.7	95.7	86.9	85.2	155.1	160.9
PDZD11	93.5	111.1	83.5	89	82.2	96.2	94	97
PDZD3	115.4	140.8	87.2	84.1	84.8	89.9	107.1	124.4
PDZD4	111.4	126.1	100.3	105.5	93.3	98.3	108.6	122.7
PDZD7	129.5	146.3	163.3	112.6	109	125.5	126.3	179.2
PDZD8	219.2	295.4	143	132.6	132.1	165.4	209.2	279
PDZK1	90.3	99	98.3	86.1	90.5	80.4	89.1	92.5
PDZK1IP1	81.7	99.1	84.2	81.8	81.6	81.8	84.4	90.2
PDZK3	80.9	85.6	101.5	92.2	82.9	89.8	82.2	94.4
PDZK3	90.5	92.3	85.4	83.3	80.9	88.1	92	96.1
PDZK5B	91.7	100.6	90.6	87.5	95.2	92.1	97.4	96.6
PDZK5B	105.2	119.5	89.8	87.5	87	84.6	94.6	106.2
PDZK5B	77.2	80.7	87.7	79.1	84.1	75.6	79.8	84.5
PDZRN3	91.6	106.1	86.1	91	85.8	88.6	93	105.3
PDZRN3	79	86.5	92.7	83.8	91.6	86.8	80.7	84.9
PDZRN3	90.1	94.7	82.1	81.5	75.7	82.6	83.1	92.8
PDZRN4	109.6	107.2	92.9	93.6	91.5	88.5	99.7	103.9
PEA15	2717.1	3571.3	1983.8	1346.7	1195.6	1668.4	2553.2	2888.5
PEAR1	95.4	105.8	108.5	102.3	88.5	103.4	93.2	104.8
PEBP1	1902.9	2065.5	674.4	612.5	441.6	723.7	1733	4309.7
PEBP4	114.5	120.9	90.4	86.8	82.6	91.5	110.2	116.9
PECAM1	1074.8	1552.7	688.9	710.8	569.2	1223.5	754.8	1165.2
PECI	553.1	762.2	179.6	237.5	154.8	234.5	535.3	790.2
PECI	376.1	495.1	223.4	223.7	174.7	242.4	349.3	502.9
PECR	415.1	550.5	359.7	289.2	243.8	334.3	392.2	413.2
PEF1	369.5	511.8	270.4	302.9	220.6	346.5	353.9	475.4
PEG10	114.9	123	94.1	83	79.5	75	108.2	122.7
PEG3	103.1	90	88.3	88.4	86.6	90.1	95.9	92.8
PELI1	187.8	221.4	144.2	151.2	140.3	181.6	192	276.9
PELI2	403.6	525.9	313.9	361.2	280.4	560	497.1	934.2

PELI3	98.1	105.9	99.2	111.9	98.9	126.1	105.1	117.2
PELO	1286.7	1641.2	980.8	777.3	633.3	936.1	1470.2	2243.9
PELP1	307.3	344.9	256.1	179.4	157.1	219.7	276.8	474.2
PEMT	394.2	503.9	342.2	272.7	218.1	234.9	393	591.9
PEMT	100.9	112.4	90.1	85.4	89.3	84.6	115	108.4
PEMT	87	102.9	93.8	88.3	79.9	86.4	98.4	85.3
PEMT	165.9	180.2	110.4	98.5	97.3	88.5	137.2	157.2
PENK	1348.7	2101.1	786.1	436.1	393.2	490.7	1236.2	1153.4
PEO1	263.8	343.6	235.5	181.7	133	197.1	206.5	293.9
PEPD	984.4	1332.3	718.5	653.9	493.6	857.8	867.4	1333.8
PER1	107.5	133.1	86.4	75.4	81	89.3	97.6	113.9
PER2	87.8	87.8	90.1	67.9	80	80.3	79	87.6
PER2	1581.9	1937.2	1077.4	1136.4	740.4	956.3	1312.8	1682.5
PERF15	87.4	88.8	101.4	91.4	96.8	93.3	86.6	93
PERLD1	559.2	620.4	404.7	428.6	341.6	435.7	457.2	403
PERP	276.4	286.1	136.7	122.2	93.6	123.1	183.4	483.9
PERQ1	108.2	136.1	128.2	132.8	125	118.2	111.3	131
PES1	279.8	355.2	145.7	134.6	110.4	143.8	242.4	207.9
PET112L	531.2	700.6	285	194.6	212.7	249.9	450.3	425.2
PEX1	162	210	145	156.1	125.1	221.6	127.2	210.4
PEX10	114.1	152.4	90.1	78.8	81.8	87	102.2	140.7
PEX10	86.2	101	88.4	90.3	97.4	82.8	85.5	87.6
PEX11A	188.2	248.6	104.5	96.4	95.8	95.2	173.7	232.3
PEX11B	1498.7	1905.9	975.5	635.6	646.3	884.9	1206.8	1705
PEX11G	143.6	173	106.9	78.8	87.5	99.9	123.5	134.2
PEX12	101.5	122.7	98.4	84.6	79	87.8	102	114.1
PEX13	90.2	96.1	90.5	79.5	73.7	83.6	91.4	100.7
PEX14	179.1	202.6	181.9	167.4	128.9	181.6	201.7	468.4
PEX16	1042.3	1409.7	511.4	496.3	434.7	491.8	998.4	976.4
PEX16	146.3	156	125.1	109.4	99.5	115.7	135.7	174.9
PEX16	884.1	1324.8	851.7	600.4	455.5	551.5	904	1207.7
PEX19	680.8	873.4	498.9	646.9	475.8	758.8	625.1	881.8
PEX26	110.6	126.2	100.8	107.8	102.5	110.7	106.2	120.1
PEX3	87	97.4	94.5	103.2	92	113.6	90.4	105.2
PEX5	670.6	890.8	493.7	589.8	459.1	636.5	560.3	717.9
PEX5L	88.5	120.4	93.2	92.9	92.3	86.9	92.1	121.8
PEX6	192.8	257.8	160.2	144.6	141.4	202.6	197.7	244.1
PEX7	547.5	768.3	353	425.4	317.9	424.8	519.5	767.7
PF4	84	95.3	103.3	92.3	89.1	91.7	80.7	86.7
PF4V1	104.3	122.3	99.8	99	89.1	96.7	109.1	110.1
PFAAP5	345.3	435.5	260.6	294.4	289.5	374	265	303.2
PFAS	733.2	844.3	411.9	420.5	337.9	502.3	533.5	682.6
PFC	166.3	236.9	168.4	282.7	204.7	263.7	139.5	210.4
PFDN1	1868.4	2470.1	1612.7	1352.6	1056.8	1666.4	1698.5	1908.3
PFDN2	135.3	170.7	116.3	88.8	92.9	89.5	138.8	167.8
PFDN4	131.6	162.9	114.3	112.2	111.6	130.1	142.8	170.1
PFDN5	9171.6	10502	3185.3	3882.8	3578.9	4540	7785.4	10287
PFDN5	7943	8933.9	2057.9	2509.2	1785.3	3395.3	6636.1	9266
PFDN6	1204.1	1480	508	529.4	505.7	657.4	1372.1	1055.2
PFKFB1	98.3	104.8	98	89.4	86.8	95.4	88.3	108.6

PFKFB2	99.6	121.6	91.2	97.7	94.5	94.6	101.5	113.4
PFKFB2	83.4	93.8	103.4	89.9	94.3	101	98.4	105.1
PFKFB2	99.5	107.9	102.8	92	98.5	102.9	90.5	102.4
PFKFB3	695.8	883.3	701	505.4	475.1	722.8	716.5	1213.2
PFKFB4	399.5	472.8	437.3	586.2	689	696.4	321.7	349.3
PFKL	103.6	127.3	94.4	85	86.2	98.1	95.8	111.5
PFKL	535.3	749.4	622.4	700	623.6	846.7	483.5	1148.1
PFKM	154.8	174.1	109.2	126.4	128.5	140.3	139	138.8
PFKP	1379.5	1595.7	1198.8	518.4	420.2	766.9	1226.8	2062.5
PFN1	24770.6	26397.8	21373	20190.1	15690.6	20187.2	23437.6	25363
PFN2	82.6	85.9	95.9	92.8	85.1	99	87.9	87.4
PFN2	102.8	118.3	100.7	105.2	93.8	105.2	91.2	121.3
PFN3	122.6	137.3	82.3	73.2	86.3	93.7	119.2	137.3
PFN4	144	148.2	115.4	121.3	117.9	113.1	124.1	147.3
PFS2	3764.1	5473.1	1958.6	1100.5	929.5	1132	2815.4	2580.5
PFTK1	141.9	177.1	151.2	160.1	124.9	198.1	132.6	203.7
PGA3	82.8	90.5	93.5	99	93.1	92.6	89	98.6
PGA4	91.5	105.1	88.8	94.1	87.7	92.5	113.1	103.6
PGA5	111.5	124.3	105.7	125.9	109.3	116.6	107.7	114.3
PGAM1	4333.4	6005.8	2738.2	1359.5	1122.5	1381.7	3380.3	4274.8
PGAM2	100.8	119.5	102.8	92.3	81.3	88.9	93.5	116
PGAM4	107.4	132	99.4	89.2	78.2	87.8	96.9	132.5
PGAM5	82	94.1	102	94.4	96.5	84.4	77.2	93
PGAP1	101.2	107.9	100.5	82.9	86.4	103.4	123.5	117.9
PGBD1	148.4	180.2	130.3	117	120.5	137.3	147.3	167.6
PGBD2	265.3	299	191.1	186.9	191.9	225.7	195	311.3
PGBD2	142.6	149.3	101.5	103.2	106.6	102.4	109.8	133.9
PGBD3	128.8	157	102.9	100	82.2	111	121	143.4
PGBD4	144.5	166.1	100.9	108.8	100.1	124.1	119.5	125.5
PGBD5	183.8	200.3	209.5	180.6	160.9	251.7	185.5	359.3
PGC	81.3	78.1	93.7	100.6	85.5	81.1	79.4	84.3
PGCP	1148.5	1539	713.1	629.1	488.9	867.7	1175.6	1408.2
PGD	2543.2	3597.7	1717.3	1405.7	1178.3	1800.2	4915	7517.4
PGDS	90.6	94.1	84.9	94.9	81.3	91.1	84	94.1
PGEA1	395.7	568	329	303.2	263.9	358.8	359.4	450.8
PGEA1	113.1	139.6	101.3	98.1	102.7	103.4	107.5	114.5
PGF	98.5	99.8	78.2	74.2	70.8	80.2	106.5	112
PGGT1B	396.2	455.3	250.5	260.6	240.5	297.7	293.6	394.5
PGK1	2512.2	3513.8	1304.9	1483.3	972.8	1819.4	2238.5	3488.8
PGK2	87.3	91.5	77.5	77.1	73.9	75.6	81.1	96.7
PGLS	2147.5	2710.8	1818.4	1354.6	1073.8	1536	1567.6	2873.1
PGLYRP1	102.3	116.5	107.1	89.7	97	92.4	107.7	111.7
PGLYRP2	93.9	100.7	89.9	88.9	82	88.4	91.5	99.6
PGLYRP3	85	94.9	91.7	91.7	87.3	86.8	87.6	85.4
PGLYRP4	137.9	166.6	106.3	107.3	99.5	116.8	133.1	151.2
PGM1	1165.4	1595.7	1091.6	1362.6	1019.7	1584.8	888.9	1288.7
PGM2	944.2	1035.2	620.6	498.9	384.7	696.9	655.6	962.4
PGM2L1	101.6	114.8	97.5	98.4	91.6	111.1	115.2	137.2
PGM3	305.3	386.6	193.5	179.4	135.8	201.5	251.7	398.7
PGM5	74.1	73.9	99.2	94.3	82.2	89.7	76.4	83.5

PGM5	90.9	104.3	115.4	97.9	93.3	94.7	86.4	103
PGM5	84.6	89.6	106.1	96.9	89.4	102.5	85.1	96
PGM5P2	84.4	86.5	91.7	79.7	82.9	83.5	82.5	77.6
PGM5P2	137.7	151.1	87.8	83.8	80	83.6	121.7	147.1
PGPEP1	107.4	131.9	102.8	95.8	91.5	106.3	131.2	128.9
PGR	79.9	94.6	92.5	90.1	89.5	95.1	78.2	93.4
PGRMC1	3714	4630.2	2177.5	1881.9	1392.7	2268.2	3171.1	4182.5
PGRMC2	2744	3965.2	1855	1515.7	1205.3	1640.2	2483.2	3103.1
PGS1	501.3	577.5	416.5	434.6	396.9	548.8	439.3	831.3
PH-4	113.8	124.2	105.8	100.4	102.9	95.8	97	124
PH-4	202.5	231.3	144.8	128.2	112.4	134.2	187.6	237.5
PH-4	465	648.6	426	316.7	326.5	370.5	404.7	325.1
PHACS	225.9	227.8	234.4	254.8	273	379.5	162.8	170.3
PHACTR1	209.7	282.1	147	258.9	257.4	248.4	187.8	195.6
PHACTR2	760.1	1034.9	514.7	884.5	654.5	718.9	716	1051.5
PHACTR3	94	100.4	78.6	83.7	84	79.6	91.3	96.5
PHACTR3	4160.3	5194.8	2621.1	2513.5	1976	2902.1	3014.4	3271.9
PHACTR3	879.8	1392.3	377.2	334.8	244.9	479.2	583.3	698.1
PHACTR4	1348.8	1862.3	979.3	1189.3	1155.7	1396.6	1291.2	1522.3
PHACTR4	103.5	125.4	90.1	79.2	86.8	94.7	118.2	117.9
PHACTR4	74.4	95.6	83.2	88	85.2	84.3	76.1	94.2
PHB	1027.9	1471.8	675.9	735.1	512.5	861.4	800.2	838
PHB2	1314.4	1595.5	472.4	326	307.8	438.4	1058.8	1636.3
PHC1	110.5	132.8	123	107.7	108.8	125.7	117.8	118.4
PHC2	88.8	114.1	86.8	87.1	87.8	88.9	100.6	113.1
PHC2	149.9	195.9	142.7	140.4	128.8	132.9	143	176.6
PHC2	77.1	94	93.5	105.5	88.4	101	77.3	91.2
PHC3	187.8	235.4	133.6	163	149.4	198.5	162.7	230.9
PHCA	383.2	560	302.5	316.9	281.6	276.6	438.7	497.4
PHEX	106.3	122.3	102.9	91.5	82.8	75.8	108.8	111.9
PHF1	97.3	118.2	96.8	91.5	85.1	120.7	102.3	125.3
PHF1	134	168.8	130.4	128.2	134.7	159.7	137	227.7
PHF1	192.6	248.6	199.2	170.6	171.4	276.3	231.1	378.2
PHF10	2269.6	2802.7	1156.5	826.6	741.1	1115	1501.5	2711.6
PHF11	1564.5	1984.1	866.9	647.3	498	769.9	1429	1643.7
PHF11	1920.7	2484.6	1387.9	1011.1	886.2	1229.6	1807.8	2070.5
PHF11	131.8	165.9	98.9	87.6	84.9	85.9	130.5	134.3
PHF12	81.2	101.1	103.6	103.8	104.9	109.1	92.7	93.7
PHF12	163.8	202.9	179.6	192.7	178.8	253.7	164.6	260.8
PHF12	81.3	89.9	91.6	77	79.1	80.8	78.4	96.3
PHF13	503.7	698.7	433	360.2	286.8	346.9	545.9	847
PHF14	96.9	127.3	92.6	85.2	91.8	92.4	106.2	113.9
PHF14	169.4	191	141.7	130.4	121	160.9	145.3	167.1
PHF14	816.6	1096.3	295.8	257.3	223.6	435.4	595.8	862.5
PHF15	318.7	418.5	468.4	347.8	354.7	373.9	345.6	377.9
PHF16	371.1	492.2	262	298.8	258.1	390.1	362.7	463.4
PHF16	108	123.1	93.2	80.8	80.7	89.9	106.1	140.3
PHF17	130.3	192.6	126.8	121.7	106.2	125.9	127.9	148.5
PHF17	508.5	667.9	526.1	374.6	307.7	447.8	433	479.2
PHF17	232.8	263.3	140.4	126.6	113.3	140.1	181.1	238.5

PHF19	520	560.9	327.3	233.1	226.8	313	389.5	465.4
PHF19	380.3	421.9	293.6	223.8	198.5	209.5	385.6	347.4
PHF19	302.2	405.8	265.5	171.6	147.4	164.8	288	522.2
PHF2	109.5	133.8	97.4	84.7	91.1	83.6	104.1	128.3
PHF2	318.1	446.1	328.7	299.3	259	441.1	322.5	501.4
PHF20	220.2	327.8	170.8	195.8	214.2	294.3	220.2	348.2
PHF20L1	341.1	472.6	127.4	117.2	120.3	168.6	301.4	463.4
PHF20L1	869	1169.6	769.3	808.6	688.5	1678.1	693.7	1485.3
PHF20L1	119.8	129.5	108.2	120	98.3	107.3	100	128.3
PHF20L1	99.5	117.1	94.8	103.9	91.7	104.2	108.4	120.4
PHF20L1	344.9	494.3	206.6	217.2	185.1	245.3	346.4	495.7
PHF21A	1095.8	1563.3	856	1013.4	962.4	1216.8	990.6	1192.4
PHF21B	106.5	119.4	92.8	93.4	102.3	92.1	100	105.7
PHF22	1410.3	1643	977	816.1	684.1	1134.5	1105.7	1619.3
PHF23	276.1	343.7	133.3	155.9	111.1	131.3	250.8	330.1
PHF3	153.3	206.6	117.6	116.7	117.1	160.2	159.6	208.8
PHF5A	3654.2	4859.2	2174.2	1516.1	1244.7	1590.2	3250	3597.2
PHF6	124.2	136	107.1	87.2	87.5	91.5	112.6	119.9
PHF6	66.5	86	93.6	82.6	83.5	80.7	68.6	79.3
PHF7	193	217.2	137.3	141.1	173.4	240.5	144	149.8
PHF7	83.2	85.2	98.6	94.4	91.6	108.9	83.7	89
PHF7	113.2	126.7	113.6	110.1	104.6	115.6	102.9	109.9
PHF8	102.1	101.9	94.8	80.7	79.8	91.1	94.2	97.9
PHGDH	12489.7	17242.2	11269.3	3597	2950.8	3439.4	14014.2	13524.2
PHGDHL1	1366.3	1836.5	538.2	625.9	568.8	568.2	1309.7	1216.7
PHIP	2384.6	3039.5	883.3	1169.5	1080.1	2522	2011.8	2548.5
PHKA1	140.5	169.1	145.4	150.1	129.2	155.8	124.4	126.8
PHKA2	324.3	359.2	339.5	470.6	521.5	585.8	237.4	359.6
PHKB	247.4	315	230.7	264	207.2	331	272.6	485.4
PHKB	169.2	236.3	102.7	92	91.8	107.1	142.4	184.1
PHKB	663.4	885.2	585.4	623.9	472.7	889.1	626.9	910.4
PHKG1	99	115.9	106.9	109.5	93.3	111.1	99.4	122.7
PHKG2	270.4	349.7	144.5	117.2	123.3	142.2	220.6	265.5
PHLDA1	102.4	117.5	128.1	105.3	96.3	114.9	128.3	179.2
PHLDA2	133.2	159.3	112	94.1	95.8	98.3	136.1	154.8
PHLDA3	107.5	133.4	91.2	92.5	84	85.7	124.2	144.1
PHLDB1	311.4	385.9	300.7	191.2	209	195.4	311.6	288
PHLDB2	80.5	98.8	90.8	76.4	78.5	86.1	84.3	90.8
PHLDB3	113.7	129.8	85.7	87.8	80.5	82.3	111.1	142.6
PHLPP	206.4	290.8	188.1	229.8	197.6	327.8	185.4	240.8
PHLPPL	114.8	138.5	124.8	108.9	98.2	119.4	115.6	193.1
PHOSPHO1	86.7	100.1	92	85.7	81.8	89.8	85.1	83.6
PHOSPHO2	74.5	73.5	88.2	84.1	76.7	85.6	71.7	79.1
PHOX2A	77.2	84.7	90.1	95.7	91.5	92.2	87.2	89.2
PHOX2B	97.6	114.7	93.3	80.8	73.7	82.6	99.3	103
PHPT1	4194.8	4963	1911.6	1747.2	1420.3	1928.7	3819.7	4828.6
PHTF1	665.9	787.7	427	292	233.1	402.9	753.2	1595.4
PHTF2	141.8	131.1	103.2	96	90.6	102.5	120	142.7
PHYH	194.2	279.9	143.7	118.8	128.1	155.6	194.3	231.4
PHYH	709.6	1007.1	381.1	261.2	278.1	315.1	611.3	688.2

PHYH	1736	2404.7	743.9	605	481.5	650.6	1416.5	2045.4
PHYH2	1734.6	2238.4	1055.4	1362.4	1005.9	1438	1463	1674
PHYHD1	82.5	84.2	84.8	80	77.6	84.1	77.8	97.2
PHYHIP	123.4	150.1	119.2	121.3	96.7	109.2	106.7	120.9
PHYHIPL	89.5	104.6	97.9	82.8	73.6	83.8	95.2	92.3
PI15	227.1	235.8	136.9	117.3	91.5	143.6	201.4	224.1
PI16	95.8	110.2	99.5	95.6	80.6	89.1	89	94.2
PI3	94.6	112.9	94.4	85.5	86.1	86.8	99	94.4
PI4K2B	123.7	118.3	121.7	112.1	98.1	119.6	114.4	185.7
PI4KII	140.9	154.7	130.2	111.6	99.8	128.9	125.1	152.9
PIAS1	1169.6	1643.5	540	693.7	557.2	913.8	938.4	1351.7
PIAS2	77.1	74.1	80.3	79.8	76.3	77.7	75.9	80.3
PIAS2	572.6	776.2	372.2	317.4	239.8	359.3	468.3	752.8
PIAS2	206.2	252.1	144.6	106.8	104.7	116.1	168.8	273.2
PIAS3	138.2	161.4	129	115.2	125.4	122.7	123.5	211
PIAS4	1017.9	1368.4	685.5	661.1	615.6	637.7	1106.6	965.4
PIB5PA	193.4	181.3	133.7	96.3	97.2	99.8	173.9	171.8
PIB5PA	114.6	152.2	90.1	79.5	78.8	82.2	105.4	114.1
PICALM	144.3	154.6	93.6	96.2	81.3	91	117.6	168
PICALM	833	1110.4	366.6	375.9	318.6	671.9	712.7	1223.5
PICK1	230.2	257.6	159.3	156.3	158.9	178.3	211.6	249.6
PIF1	185.4	185.8	145.9	122.2	134.9	148.7	142.8	162
PIG38	122.6	147.5	89.1	74.1	74.7	82.7	116.6	126.2
PIGA	867.9	1036.7	546.4	751.5	536.4	890.6	798.9	1321.1
PIGA	122.3	159.9	87.3	86.3	77.2	84.4	112.4	153.2
PIGA	197.2	226.4	125.7	113	113.1	130.7	161.5	221.6
PIGB	1333.9	1802.8	746.4	852.2	641.4	1131.8	900.7	1380.9
PIGC	1332.5	1789.9	948.3	808.7	657.9	1061.3	964.2	1625.7
PIGC	459.1	650.4	293.7	231.7	206.9	282.2	360	410.1
PIGF	1045.8	1473.5	649.3	690.5	471.3	711.5	812.3	1529.6
PIGF	158.7	192.9	101.5	115.7	100.7	137.4	139.6	173.1
PIGG	281.3	390.2	194.2	141.7	132.3	197.4	256.8	352.8
PIGH	385.2	507.4	188	154.9	133.6	207.1	396.9	641.6
PIGK	832.8	1166.4	392.9	372.2	270.8	445.8	675.9	1130.7
PIGL	114.5	127.3	109.2	99.3	94	95.1	111.9	126.1
PIGM	639.3	839.3	196.7	206	204.6	304.4	447.3	652.4
PIGN	139.1	139	92.7	93	85	77.5	106.7	119.3
PIGN	490.5	690.7	397.5	446.3	378	570.4	438.1	490.6
PIGN	478.2	707.7	485.7	700.2	539.3	980.5	422.4	644
PIGO	182.8	221	142.9	108.3	93.9	113.3	158	253.1
PIGO	287	350.9	249.3	183.1	163.5	200.9	252	369.9
PIGO	98.7	115.9	127.5	112	98.4	106.5	103.3	111.8
PIGQ	637.1	740.4	369	239.9	215.3	268.5	372.3	274.2
PIGR	82	89.8	99.1	96.3	88.2	89	77.1	82.3
PIGS	610.3	801.4	526	714	572.4	818.8	619.6	1068
PIGT	1247.4	1718.4	886.4	801.9	688.6	866.8	1038.1	2363.5
PIGV	382.2	464.4	199.1	255.1	207.4	276.7	263.9	412.6
PIGW	106.7	105.3	81.9	90.5	81.2	85.2	106.7	107.6
PIGX	225.6	271.2	109.8	128.8	109.8	117.5	212.3	285
PIGY	4806.1	6246.5	2675.1	2518.6	1511.4	2693	3839.5	6604.5

PIGZ	132.6	148.1	135.3	189.9	183	236.4	119.2	149.1
PIK3AP1	362.8	412.9	157.2	130.5	146.3	152.6	427.5	648.7
PIK3C2A	158	214	116.1	137.9	118.2	158	145.9	201.9
PIK3C2B	110.5	150	106.6	103.5	100.2	114	124.7	131.2
PIK3C2G	75.7	89.1	85.6	89.5	85.9	91.5	84.6	69.9
PIK3C3	254.8	275.3	122.9	110.1	102.2	142.6	229	324.5
PIK3CA	146.6	160.8	103.8	104.3	92.7	111.8	127.6	199.7
PIK3CB	527.2	685.1	242.8	201.5	178.2	367.2	423	609.3
PIK3CD	492.6	689	488.5	404.8	394.1	523.4	355.8	443
PIK3CG	496.4	629.8	263.2	328.4	236.1	322.5	377.3	606.9
PIK3R1	846.4	1063.6	348.8	670.4	558.3	674.4	694.8	1090
PIK3R1	73	82.8	83.1	81.5	78.4	74.2	75.5	76
PIK3R1	129.2	150.8	97	91.4	83.9	95.6	119.3	158.9
PIK3R1	101.7	112.1	103	83.9	93.4	92.2	106.5	116.9
PIK3R1	114.9	147.3	111.6	102.1	109.5	126.4	116.4	161.8
PIK3R2	1640.3	2114.6	1306.6	1625.3	1273.8	1372.5	1080.2	923.9
PIK3R3	124.1	146.4	94.1	89.6	91.2	98.4	126.6	145.2
PIK3R4	201.5	254.2	144.6	187.5	153.8	270.4	200.9	255.6
PIK3R5	116.2	133.7	103.3	99.6	87.2	94.8	118.1	137.2
PIK4CA	1872.7	2113.6	1151.5	1541.9	1327.9	1640.8	1427.5	1560.6
PIK4CB	477.2	550.7	613.8	477	391.9	498.5	396.2	619
PILRA	143.9	152.9	121.6	168.4	156.1	153.6	153.4	203.3
PILRA	123.9	152.7	101.5	98.7	84.6	95	115.5	128.8
PILRA	115.7	118.1	122.4	119.6	114.7	129.7	114.3	115.8
PILRB	107.1	93.8	107.5	104.6	103.2	98	92.3	105.1
PILRB	434.7	391.5	403.5	462.8	437.7	609.2	313.5	541.3
PILRB	82.7	97.7	84.5	79.6	81.5	80	86.7	94.5
PILRB	105	117.3	95	122	128.7	91.4	102.2	118.2
PIM1	213.7	289.7	202	197.8	188	190.6	286.1	633.4
PIM2	624.2	936.4	506.2	490.2	371.4	402.1	492.5	677
PIM3	199.6	271	198.1	167.1	150.4	187.3	212.1	429.6
PIM3	117.6	115.1	93.7	92.2	87	84.3	105.9	132.8
PIN1	3934.5	4614.2	2559.3	1993.4	1623.5	2218	3401.4	3590.8
PINK1	233.9	295.5	144.4	106.1	114.6	126.4	245.6	357.3
PINX1	272.7	352.7	224.9	183.2	146.9	193.4	252.7	319.7
PIP	82.9	98.1	90	101.6	88.8	78.6	86.2	89.7
PIP3-E	471.4	671.5	247.5	275.4	211.7	311.6	601.2	821.3
PIP5K1A	135.7	176.6	110	100.9	97.8	93.9	133	151.9
PIP5K1B	110.6	110.9	92.2	95.6	87.1	95.6	101.9	96.6
PIP5K1C	265.3	369.9	428	350.3	298	439.1	286.3	581.7
PIP5K2A	139.4	163.1	140.1	132.6	117.2	133.1	135.5	218.1
PIP5K2B	204.4	252.3	166.5	285	245.6	148.8	167.6	221.6
PIP5K2B	172.8	244.5	112.4	96.6	96.8	101.3	160.2	207.3
PIP5K2C	177.1	225.7	120.9	113.1	99	117.9	169	212.5
PIP5K3	91.7	101.7	103.8	102.5	106.1	97.2	105.1	100.8
PIP5K3	81.6	107.6	95.6	91.7	87.9	102.2	82.4	98.5
PIP5K3	103.8	112.7	97.5	110.9	99.4	95.7	110.8	104.4
PIP5KL1	101.4	114.8	128.9	121.2	118.1	124.1	104.1	109.9
PIPOX	145.1	185.5	130.7	121.9	112.8	126.8	139.8	196.1
PIR	350.9	493.5	161.6	153.6	142.9	251.8	748.6	976.6

PIR	473.7	536.2	201.8	187.2	152	266.7	1019	1881.8
PISD	489.3	640.6	351.2	340.9	299.5	519	363.2	621.7
PITPNA	201.6	260.5	136.6	126.9	125.9	148.9	223.1	230.8
PITPNB	451.7	639.7	412.7	357.1	313.1	515	401.9	651.3
PITPNC1	203.9	282.5	216.7	198.5	152.2	267.1	225.8	383.9
PITPNC1	118.3	140.8	87.8	88.9	75.2	91.4	115.1	146.6
PITPNM1	285.7	346.7	561.7	524.8	440.2	835.6	258.3	481.9
PITPNM2	163	211.6	155.6	140.6	138.9	148.5	129.6	147.4
PITPNM3	79.8	79.4	85.1	81.5	79.6	82.5	77.5	84.4
PITRM1	327	415.7	262.1	243.3	201	355.3	266.9	352.9
PITX1	1539.7	1965.3	1290	543.2	419.1	467.2	1188.8	1496.1
PITX2	109.1	112.2	105.1	106.5	92.9	100.4	106.4	116.6
PITX2	171.7	229.4	125.4	97	94.9	115.5	172.9	187.9
PITX2	71.3	84	97.2	96.2	88	91.2	86.4	86.3
PITX3	96.1	117.2	87.5	86.7	76.8	81.9	99.3	102.5
PIWIL1	96.6	109.3	102.9	86.7	92.6	82.6	88	107.3
PIWIL2	74.5	89.4	94.1	85	81.1	81.4	83.1	96.9
PIWIL2	86	89	109.1	96.4	91.8	85.9	83.2	83.4
PIWIL3	96.3	108.3	85.3	89.3	87.2	81	109	111.2
PIWIL4	499.9	604.1	240.8	214.7	194.2	257.1	368.9	556.9
PJA1	92.7	124.8	106.6	98.8	102.7	98	91.8	112.2
PJA1	242.1	406.9	216.9	217.2	188.6	266	209.4	295.1
PJA1	95.4	111.5	93.2	82.7	73.8	84.8	105.6	107.8
PJA2	2511.5	3204.9	1216.3	2008.8	1519.7	3224	2420.9	3877.3
PJCG6	97.1	110.8	110.1	108.3	96	98.5	89.5	98.5
PKD1	111.9	147.1	157.4	173.8	201.8	307.4	130.2	164.1
PKD1	105.7	121.5	135.1	138.2	114.6	176.8	116.1	131.6
PKD1L1	107.1	126.5	97.2	92	92.9	99.9	103.7	116.7
PKD1L2	80.7	76.3	89.7	83.9	85.1	84.7	75.7	81.9
PKD1L2	81.7	94.6	85.7	79.8	84.2	96	75.4	88.7
PKD1L2	92.9	97.6	97.3	92.3	82.1	92.1	83.9	87
PKD1L2	78.5	87.8	95.9	95.9	96.9	91.4	78.2	84.4
PKD1L3	127.7	134.8	74.6	88.5	80.6	84.9	116.2	128.2
PKD2	275.7	330.1	170.6	204.6	169.2	300.9	278.8	639.3
PKD2L1	81.8	91.9	96.6	82.8	86.5	91.8	88.7	108.4
PKD2L2	95.6	102.6	98	96.1	88.1	101	96.2	90.6
PKDREJ	104	104.3	94.1	86.4	81.4	86.2	100.5	132.7
PKHD1	87.8	98.3	84	93.5	91.2	87.6	87.5	96.2
PKHD1	79.1	91.4	95.6	71.7	78.1	88.4	94.2	94.3
PKHD1	136.3	129.8	103.2	93.8	95.9	103.4	109.8	142.2
PKHD1L1	126.5	147.7	95.9	90.4	89	90.2	119.2	143.7
PKIA	156.6	194.7	150.8	111.8	93.8	92	159.2	176.3
PKIA	87.3	95.9	86.9	88.4	87.6	84.6	83.7	99
PKIA	112.9	118.4	105	92.3	105	100.6	116.1	133.7
PKIB	128.2	142.4	90.6	115.6	101.1	116.1	101	125.2
PKIB	2591.7	3223.6	1403.3	1527.1	1100.5	1726.8	1403.5	1443.1
PKIG	106.2	118.2	93.5	96.6	88.7	94.1	96.4	125.6
PKIG	145.6	181.2	95.2	88.1	86.8	96.7	142.9	163.4
PKIG	123.2	135	93.9	100	85.3	84.4	123.9	143.1
PKLR	78.4	85.3	99.8	87.8	84.2	91	77.4	80.7

PKLR	91.3	98.1	98.1	83.5	79.6	76.6	88.5	85.9
PKLR	147.8	174.1	118.2	94.2	94.4	105.6	153.3	144.7
PKLR	93.1	102.4	84.2	93.5	92.7	92.9	94.9	105.4
PKM2	937.3	1203.2	1475.7	1422.4	1167.6	2158.4	1044.5	2384.5
PKM2	395.1	488.4	417.6	328.2	352	580.4	373.2	865.4
PKM2	290.3	416	434	448.9	391	345.3	282.9	591.6
PKMYT1	197.8	220.2	201.5	110.7	107.4	142	187.4	271.7
PKN1	81.4	95.6	92.8	86.3	74.6	72.3	88.2	89.9
PKN1	157.4	187.2	127.8	106.4	102	139.2	154.1	192.5
PKN1	162.3	238.3	150.9	118.4	104.9	154	142.8	252
PKN1	131.4	152.3	98.7	79.6	84.3	98.8	128.7	189
PKN2	137.9	159	109	111	111.8	99.2	131.9	186.1
PKN3	228.5	269.9	272.3	138.3	144	200.5	181.3	369.8
PKNOX1	227.4	280.2	138.4	160.2	124.3	144.5	185.9	226.4
PKNOX1	115.5	148.1	108.3	107.9	97.3	98.9	120.8	122.7
PKNOX2	108.7	119.3	103.3	82.8	92	90.4	89.3	110.9
PKP1	101.7	122.2	112.2	91.2	92.6	102	110.2	104.4
PKP1	105.3	121.3	85.1	84.2	81.5	94.2	99.8	116.6
PKP2	92.5	96.1	107.3	112.3	105.4	122	85.3	86.6
PKP3	115.8	160.7	86.2	84.7	87.4	84.9	114.4	139.2
PKP4	165.3	197.6	141.6	133.7	131.1	181.2	133.8	132
PKP4	1676	1917.8	1151.4	871	691.2	1169	1165.8	1312.3
PLA1A	81.5	90.3	95.5	104	84.4	105.5	85.1	91.1
PLA2G10	99.4	97	96.3	99.3	82.8	92.8	85.2	107.3
PLA2G12A	110.1	118.6	97.5	91	85.9	94.3	97.2	113
PLA2G12B	114.8	141.8	108.2	94.8	87.8	95.4	118.2	122.9
PLA2G1B	82.7	88.4	86.4	81.3	80.1	84	82	88.4
PLA2G2A	98.1	108.4	102.5	87.2	86.9	91.7	88.7	105.6
PLA2G2D	277.2	314.4	160	234.5	222.7	175	240.3	223.3
PLA2G2E	90.7	94.6	99.4	95.2	88.6	86.3	97.8	103
PLA2G2F	108.2	108	81.8	90.9	76.9	79.9	98.9	107.4
PLA2G3	74.3	92.2	96.9	91.6	88.6	83.9	82.9	88.6
PLA2G4A	141.3	142	137.8	130.8	123.3	166.7	133.8	183.4
PLA2G4B	437.3	462.3	481.3	391	439	499.2	341.5	419.2
PLA2G4C	240.7	295.9	174.2	126.8	131.9	138.9	197.5	215.8
PLA2G4D	94.8	106.1	85.2	92.7	90.5	90.3	102.5	100.8
PLA2G4E	83	96	88	85.2	93.4	88.8	80.3	87.9
PLA2G4F	82.2	95	85.1	85.5	80.5	81.8	78.3	88.9
PLA2G5	92.2	94.6	87.4	84.6	78.4	90.3	88.7	89.6
PLA2G6	102.8	125.4	104.8	94.1	98.8	91.7	98.7	111.8
PLA2G6	102.4	117	107.2	88.8	86.6	86.6	112.4	113.8
PLA2G7	87.4	95.7	96.9	515.7	402	873.9	94.1	102.7
PLA2R1	90.8	92.6	99.8	86.7	83.1	94.4	83	96.7
PLA2R1	78.2	88.3	95.7	80.4	80.9	96.1	76.4	82.5
PLAA	91.1	110.6	116.8	103.8	105.7	111.9	101.5	103.1
PLAA	177.4	226.7	113.4	89.8	93.2	98.6	165.1	173.2
PLAA	242.2	277.4	112.3	107.7	110.6	104	256	278
PLAC1	94.6	105.8	92.6	96.5	92.4	88.7	97.8	108.5
PLAC2	147.4	188.1	107.5	111.6	80.8	120.4	152	185.1
PLAC4	126.4	143.1	96.5	93.8	83.7	91.2	111.5	124.6

PLAC8	13095.5	15767.6	6839.7	8871.5	4869.6	8051.6	9709.7	13594.6
PLAC8L1	104.5	124.3	114.6	100.6	94.1	102.1	102.1	137.2
PLAC9	91.1	112.7	108.9	92.6	89.4	82.9	96.2	102.8
PLAG1	101.5	111.2	91.5	89.4	75.3	85.3	101.6	107
PLAG1	105.2	109.8	95	92.5	90.7	94.2	101.2	121.8
PLAGL1	212.3	252.8	206.8	320.5	265.1	445	166.4	286.5
PLAGL1	167.7	164	130.6	132.3	121.6	141.3	161.4	220.1
PLAGL2	551.1	678.6	633	596.4	512.6	649.9	499.2	747.9
PLAT	85	97.8	84	81.4	79.2	81.5	92.9	87.2
PLAU	716.5	902.7	479	287.4	259.7	453.2	727.5	1052
PLAUR	471.8	660.7	250.6	222.6	167	225.6	509.1	495.9
PLAUR	238.2	313.8	160.2	135.8	130.4	132.3	283.7	353.6
PLAUR	563.6	776.2	429.8	375.5	310.8	410.5	648.8	795.9
PLB1	227.1	276.5	135.2	123.7	114.3	150.7	166.5	190.3
PLCB1	130.3	143.2	96.5	101.4	88.4	122	128.1	159.3
PLCB1	105.7	108.1	98.3	99.3	84	86.3	97.5	110.1
PLCB2	323.4	303.4	372.2	344.5	319.4	401.5	194.9	325.5
PLCB3	146.3	165.6	96.5	98.3	89.9	104.5	140.4	145.2
PLCB4	97.3	114.2	107.3	96.2	100.9	105.4	88.5	104
PLCB4	83.2	87	87.1	75.9	77.6	73.9	83.7	84.5
PLCD1	112.8	133.5	106.1	94.2	92.4	115.6	114.8	118.6
PLCD3	103	111.8	98.9	79	88.2	71.3	99.3	119.1
PLCD4	91.1	118.7	101.2	83.9	81	86.9	99.7	96.3
PLCE1	102	105.5	78.7	82.7	84.5	93.1	86	101.2
PLCG1	152.3	121.8	117.3	105.5	103.8	116.2	119.3	220.7
PLCG1	323.3	324	167.7	170	160.8	195.6	206.3	396.5
PLCG2	2953.9	3430.3	2100.8	2595.7	2186.5	3034.3	2234.6	2678.8
PLCH1	77.6	80.1	95.4	90.3	85.3	87.4	75	81.8
PLCH2	146.2	167.3	111.4	100.6	97.8	98.8	134.8	153.5
PLCL1	149.4	187.7	102.2	84.8	74.8	93.8	123.3	175.4
PLCL2	396.6	549.6	275.6	341.2	236.4	338	353.5	452
PLCXD1	837.1	783.1	637	826	686	960.4	611.9	1246.9
PLCXD2	115.7	120.3	79.3	76.8	75.4	79.8	104.2	119.9
PLCXD3	83	85.2	102.5	81.8	90.7	88.6	79.1	90.9
PLCZ1	92.8	117	97.2	85.4	80.1	89	91.5	98.7
PLD1	190.7	208.9	132.8	142.3	134.9	181.8	163.1	170.5
PLD2	93.6	107	100.5	97.6	97.8	103.1	99.2	110.3
PLD3	112.9	136.9	94.7	81.8	84.5	78.2	115.6	133.3
PLD3	1261.5	1555.1	763.9	2765.8	2408.1	1800.9	1180.9	1395.8
PLD3	100	141.2	113.4	101.4	92.9	103.4	100.1	116.2
PLD4	145.8	173	141.1	133.5	134.8	133.4	129.3	170.1
PLD5	76.1	78.8	82.4	81.3	85.7	78.8	73.4	74.5
PLDN	1279	1504.7	728.5	788.9	718.7	918	1227.7	2588.1
PLEC1	126.4	168	195	194.3	146.6	243	137.5	185.9
PLEC1	81.1	99.2	86.1	89.2	83.5	87.1	96.2	94.8
PLEC1	109.3	110.9	106.1	92.6	84.6	89.7	110	120.7
PLEC1	95	98.3	101.1	94.5	85.4	94.6	92.1	96.1
PLEC1	87.5	99.8	94.6	89.1	88	94.1	87.7	94.9
PLEC1	82.4	105.6	94.7	94.1	86.5	87.3	85.3	100.8
PLEC1	93.9	100.5	88	90.6	85.1	86.4	90.3	107.4

PLEC1	108.7	115.1	99.6	95.5	98.5	91.5	98.6	106.7
PLEK	3105.1	4045.1	2340	2855.7	3330.6	3521.2	2648.4	2519.5
PLEK2	102.4	123.2	95.3	85.2	88	91.7	92.7	125.7
PLEKHA1	81.1	96.5	101	88.2	82.9	94	93.6	118.3
PLEKHA1	82.1	103.5	99.3	89.1	83.6	87.4	88.9	115.2
PLEKHA3	287.4	405.3	187.9	186.6	163.1	249.8	265.6	343.8
PLEKHA4	123.7	154.5	117.1	103.8	103.3	100.5	118.4	170
PLEKHA5	120.1	146.6	108.7	112.5	101.1	97.1	113.6	139.1
PLEKHA6	91.3	104	105.6	94.8	88.9	95.8	94.3	100.2
PLEKHA7	96.1	114.1	106.9	109.2	98	112.9	101.8	96.7
PLEKHA8	119.7	129.5	106.1	85.4	92.1	96.2	101.4	108.3
PLEKHA9	597.3	837.6	362.9	402.8	324.1	437.9	450.7	524.1
PLEKHB1	118.7	119	115.2	95.3	105.2	119	103.2	116.3
PLEKHB2	689.3	827.7	351.6	366.6	282.7	263.2	651.6	1041.5
PLEKHB2	1995.6	2521.5	865.9	671	546.9	395.9	1659.5	2166.2
PLEKHB2	96.1	97.6	81.7	91.5	91.7	95.2	85.6	93.6
PLEKHC1	111.3	126.6	101.6	89	88.8	95.7	119.1	155.7
PLEKHF1	307.1	399.6	244.9	138.4	131.1	136.7	389.8	322.1
PLEKHF2	437.2	584.4	178.2	125.5	116.3	166.6	322.5	360.3
PLEKHG1	80.6	80.7	97.3	84.2	89.3	79.9	84.7	82.6
PLEKHG2	131.4	146.3	132.3	105	98.2	148.3	128.1	193.5
PLEKHG3	257.7	309.9	268.3	249.6	231.3	280.2	222.6	268.5
PLEKHG4	182.2	202.8	212.6	153.2	141.2	174.9	134.6	153.2
PLEKHG5	75.4	89.8	100.2	88.3	88.9	93.1	74.3	77.2
PLEKHG5	123.6	135.8	95	92.3	84.4	84.7	102.6	129.6
PLEKHG5	126.2	145.2	82	77.6	80.7	88.4	132.3	125.8
PLEKHG5	92.5	90.6	102.5	84.3	96	81.6	89.5	98.5
PLEKHG5	107.5	107.9	101.9	93.6	88.2	86.8	101.3	101.6
PLEKHG6	87.5	90.7	90.2	85	85.8	87.7	92.4	104.3
PLEKHH1	117.9	143.5	104.9	116.9	109.1	142.6	104	144
PLEKHH2	78.4	92.9	87.5	86.5	95.2	86.6	86.5	88.5
PLEKHH3	225.6	261.9	136	121.8	125	126	233.4	273.1
PLEKHJ1	233	303.6	180	163.4	147.4	184	197.2	220.7
PLEKHM1	165.8	196.2	181.7	160.7	144.9	172.3	170.8	285.6
PLEKHN1	185.3	220.2	125.2	121.6	105.7	122.4	180.9	209
PLEKHO1	221.2	265.6	131.9	143.1	198.3	157.1	265	317.7
PLEKHQ1	146.2	175	126.9	120.3	121.1	157.5	162.4	356.7
PLG	92	96.4	89.1	92.7	77.5	86.9	87.1	103.6
PLGLB1	118.1	151.4	125.5	126.9	132.9	138.9	117	153
PLIN	85.2	88.2	89.6	76.9	82.5	75.8	88.1	84
PLK1	583	636.7	496.1	262	233.6	275.6	513.7	939
PLK2	102.5	117.8	93.4	88.3	79.6	87.8	97.5	120.7
PLK3	120.5	133.1	100	79.9	78.1	89.9	125.4	155.7
PLK4	1552.9	1906.9	1225.3	864.2	781.9	1588.4	1032.6	1848.1
PLN	119	123	96.9	87.9	87.3	86.5	112.4	109.7
PLOD1	1249.9	1568	875.1	1081.1	905.4	1087.1	1028.8	1044.1
PLOD2	129.3	144.7	90.1	87.2	81.4	97.7	111.5	140.1
PLOD2	134.6	131.5	98.1	104.4	84.6	92.7	121.1	147.3
PLOD2	389.4	485.6	352.5	249	225.9	339.9	310.9	612.5
PLOD3	2676.9	2965.5	2906.8	2303.1	1636.3	2119.9	2402.7	4049.2

PLP1	117.3	119.9	101.1	94.8	86.5	100.1	112.8	128.5
PLP1	96.8	114.5	85.4	76.4	80.5	97.2	100.6	105.4
PLP1	116.9	141.2	93.3	82.4	85.1	88.8	109.8	129.5
PLP2	268.7	383.7	170	135.3	140.9	164.4	264.5	334.6
PLRG1	2090.5	2718.2	960.9	793.4	579.4	1166.8	1730.1	2439.7
PLS1	113.9	129.7	122	96.5	98.5	112.6	108.6	145.9
PLS3	826.6	1033	377.8	297.5	255.3	368.5	950	1732.1
PLSCR1	292.8	355.6	141.1	149.9	125.1	146.7	228.1	264.8
PLSCR2	89.7	109.4	100.8	98.4	98.6	97.4	93.4	95.5
PLSCR3	928.2	1120.6	630.7	560.1	472.1	445.8	780.4	846.7
PLSCR4	130.5	174.5	105.5	101.2	100.8	119.4	130.5	167.4
PLTP	238.1	341.8	178.2	150.5	142.6	118.9	204.1	287.3
PLTP	829.2	907.6	502.1	590.8	457.7	837.6	622.8	938
PLUNC	84.9	88.5	97.6	85.1	78.4	87.7	84.9	85.1
PLVAP	95.4	95.6	95.6	96.4	86.3	84.6	98.6	93
PLXDC1	124.4	141.6	112.7	101	104.8	103.7	117.7	125
PLXDC2	228	276	130.8	128.3	111.1	145.5	168.8	241.1
PLXNA1	157.9	168.3	176.4	134.4	123	135.1	137.8	180.5
PLXNA2	133.4	185.7	95.8	87.1	81.8	94.8	123.5	135.4
PLXNA3	110.7	115.2	136.2	139.4	124	181.7	104.2	148
PLXNA4B	88.3	99.7	82.6	86.3	77.6	76.2	88.7	93.7
PLXNB1	111.6	125.6	135.4	116.4	107.3	127	110.8	130.2
PLXNB3	87.2	92	86.5	88.1	84.3	87.6	88.1	91.9
PLXNC1	79.5	91.1	106.4	115.3	100.6	110.2	86.1	85.2
PLXND1	110.7	134.6	84.4	80.5	97.3	88.5	94.9	151
PMAIP1	395.7	451.4	188.9	160.6	136.6	191.2	499.9	1060.7
PMCH	74.3	78.1	94	95.3	92.8	87.4	83.2	82.8
PMCHL1	99.1	110.3	104	90.9	93.2	93.5	107.2	117.2
PMFBP1	117.3	141.2	95.2	101.1	85.1	95.7	114.3	123.2
PML	85.7	147	97.4	90.9	97.3	86.4	111.4	131
PML	91.7	96.1	105.8	90.3	93.2	99.3	87	99.7
PML	156.4	181.8	120.1	117.6	97.4	106.3	149	172.6
PML	104.7	164.7	106.9	104.3	87.1	86.9	124.3	143.5
PML	108.2	125.1	102.7	104.5	92.9	105.1	106	104.7
PMM1	1463.4	1932.5	1055.5	1003.4	823.3	1098	1356.9	1647.2
PMM2	586.6	689	418.7	317.7	223.1	336.8	588.4	595.4
PMP2	76	91.6	85.6	88.2	73.5	86	81.9	84.8
PMP22	84.2	82.1	100.8	92.1	88.8	88.1	85.8	90.2
PMP22	100.4	112.2	91.2	80	92.3	93.8	97	115.9
PMP22	160.9	206.1	120.9	127.7	123.8	126.7	172.7	178.9
PMP22CD	96.1	104.4	102	95.7	87.2	90.7	91.9	113.6
PMPCA	1440.7	1715.8	1211.1	1049.9	835.1	1287.7	1256.1	1388.6
PMPCB	2438	3278.1	1394.7	1398.6	1130.7	1753.6	2055.6	2518.1
PMS1	182.6	192.1	126.6	143.7	106.2	179	141.3	243.8
PMS2	486	600.9	221.2	181.8	158	208.5	352	401
PMS2	174.2	232.4	135.4	113.5	120	101.3	148.4	151.3
PMS2CL	171.4	206.5	134.2	125.5	130.9	130.4	124.1	118.2
PMS2CL	99.2	104.9	75.2	90.3	80.8	92.2	93.9	105.1
PMS2L2	112.6	144.9	103.2	101.9	86	94.3	121.8	132.2
PMS2L3	291.6	348.5	171.1	169	176.3	189.6	242.2	314.5

PMS2L5	458.6	623.5	205.3	167.5	147.2	178.8	393.5	601.9
PMVK	434.8	653.9	204.3	181	170.8	205.3	457.6	442
PNCK	130.7	140.4	110.9	234.4	302.3	455.7	114.3	132.1
PNKD	231.3	256.3	344.5	303.7	326.8	406	192.8	342.3
PNKD	144	180.4	97.2	94.6	104.2	111.2	151.4	162.2
PNKD	302.3	338.6	196	171.1	173.2	249.5	244.3	240.1
PNKP	847.6	998.1	694.9	493.2	420.5	586.2	725.2	1173.8
PNLDC1	86.9	105.1	86.8	80.3	80.2	80.2	85.8	103.5
PNLIP	106.3	121.6	91.5	93.7	89.1	90.6	117.8	123.1
PNLIPRP1	87.1	107.5	95.2	84.7	89.5	85.5	87.8	94.4
PNLIPRP2	80.8	82.6	81.5	79.2	74.8	84	73.2	86.4
PNLIPRP3	75.1	80.2	86.8	77.9	87.6	84.2	81.6	84.6
PNMA1	222.3	263.8	161.2	154	131.1	160.2	160.2	271.7
PNMA2	141.3	163	135.8	437.3	419	524.9	132.6	120.7
PNMA3	407.9	483.5	253.1	144.2	131.6	143.5	230.9	247.1
PNMA5	83.9	104.6	82.5	77.5	83.3	84.9	74.6	84.2
PNMA6A	147.6	183.9	110.4	89.7	99	99.8	121.9	175.6
PNMT	78.9	95.1	90.2	92.3	97.7	99	83.9	81.9
PNN	2655.6	2638.7	1140.8	1134.3	1032.3	1974.2	2063.2	3944.3
PNOC	159.6	175.3	129.7	87.1	86.7	93.3	144	178.2
PNPLA1	162.4	178.8	123.2	101.2	92	110.9	188.3	217.6
PNPLA1	116.7	131.5	102	91.5	87.9	106.8	127.5	182.9
PNPLA2	190.8	252.4	167.4	153.1	151.1	212.9	178.2	318.5
PNPLA4	95.7	114.9	103.8	92.8	93.3	105.5	88.6	113.2
PNPLA5	90.6	106	91.9	81.4	80.2	79.4	84.4	90.2
PNPLA8	108.5	137	92.5	102.3	92.1	116	113.5	164.7
PNPO	2445.6	3107.9	1180.3	1073	812.8	1237.6	1885.4	2031
PNPT1	8251.1	8696.1	3214.2	6019.3	5554.3	4159.3	6632.6	7613.5
PNRC1	111.4	113.8	106.4	99.9	97	100.6	96.9	121.6
PNRC2	3659.5	4463.8	1423.4	1802.1	1376.5	2096.4	2724.2	3901.1
PODN	89.2	97.4	99.7	98.1	99	88.9	88.7	109.7
PODXL	175.5	217.8	173.7	162.7	132	179.4	158.1	187.6
PODXL	77.2	86.8	100.8	94.2	91.1	87.2	81	83.2
PODXL	119.8	124.2	100.9	95.4	92.8	107.6	114.2	131.6
PODXL2	290.2	320.7	373.7	382.3	303.3	488.7	220.1	343.1
POF1B	129.5	139.5	93.6	79.8	87.7	97.2	103.3	147.7
POFUT1	2066	2369.7	935.6	1560.7	1631.5	1280.6	2379	3775.7
POFUT1	104.8	136.1	105	109.2	94.2	93.9	108.7	121.8
POFUT1	99.7	122	92.3	96.6	95	85.6	89.1	106.7
POFUT2	300.5	323.9	284.8	308.2	282.5	368.2	267.6	461.7
POFUT2	221.5	284.7	144.8	139.1	108.5	143.3	194.1	272.9
POFUT2	262	266.5	274	306.3	249.6	396.3	251.6	467
POGK	2266.1	3422.6	2445	1476.2	1125.2	1973.8	1880.5	2609
POGZ	228	297.3	179.2	207.4	222.1	190.1	218	223.1
POGZ	146.5	174.7	126.1	108.6	109.8	105.7	119.3	162.3
POGZ	115.4	149.3	114.2	123.4	108.8	109.7	110.6	120.7
POL3S	125.4	120.2	114.8	110	111.5	125.3	98.3	120.9
POLA1	490.7	671.4	305.2	327.7	270.6	367	408	458
POLA2	1765.9	2368.4	1245.7	1183	854.8	1104.1	1226.7	1311.4
POLB	1038.5	1363	637	588.2	495.2	670.3	1007.1	1133.1

POLD1	416.1	498	460.5	360.1	316.9	513.8	304.6	569.8
POLD3	93.3	115.8	101.8	96.2	102.6	107.5	110.9	97.5
POLD4	286.7	327.7	157.4	175.6	158.6	211.2	199.6	286.7
POLDIP2	379.1	500.3	344.8	304.7	242.5	379.4	328.5	456
POLDIP3	935.3	1117.3	816.9	646.3	494.5	714.2	863.7	1591.5
POLDIP3	174.1	250.3	153.9	232	197.1	111.2	156.3	189.7
POLE	260.5	343	419.1	328.3	328.7	592.5	229.6	356.4
POLE2	1783.6	2296.3	908.8	500.5	396.2	550.2	1443.4	1700.8
POLE3	8712.9	11767.1	6097.8	4629.2	3333.3	4481.2	7052.8	8474.2
POLE4	5693	7235.5	5125.9	4903.5	4166	5511.5	5309.6	7514.2
POLG	420.8	535.5	432.6	371.4	331.3	459.9	360.2	573.5
POLG2	380.1	371	139.8	130.8	101.2	143.1	285.7	365.7
POLH	98.7	118.7	104	104.5	86.9	83.3	101.3	131.4
POLI	88.2	90.8	95.2	95.7	78.7	81.7	91.1	97.4
POLK	77.9	87.1	85.1	83.5	85.7	81.1	77.6	90.6
POLL	128.4	137.6	104.9	100.3	88	104.6	107.9	146
POLM	253.6	304.3	281.1	230.8	183	266.3	215.9	438.1
POLN	113.4	108.3	102.5	92.7	79.2	88	97.8	115.1
POLQ	1270.3	1887.1	988.4	1220.5	1096.1	1751.7	1011.8	1233.3
POLR1A	126.8	144.1	103.5	106.2	83	88.5	127.7	128.3
POLR1B	135	150.8	111.3	106.2	98.5	110.1	113.4	144.8
POLR1C	2601.7	3161.6	1398.4	1055.7	802.2	1297	2094.7	2313.3
POLR1C	1241.8	1662.9	854.9	668.9	460.4	858.5	1204.8	1643.1
POLR1D	5628.6	6473.8	4020.4	4094.6	3194.7	4053.6	4781.9	6627.1
POLR1E	3136.5	3802.2	1146.8	826.4	775.4	1020.2	2655	3226.2
POLR2A	778.7	930.6	953.6	1347	1076.8	1517.7	808.5	1276.7
POLR2B	1256.3	1766.8	562.9	558.6	354.4	731.3	904.8	1506
POLR2C	346.3	506.7	178.7	138.3	129.5	163.1	317	430.2
POLR2C	1809.2	2432.9	1407	955.3	727.6	1411.8	1476.8	2260.1
POLR2D	1477.9	2218.3	1238.5	822.1	656.2	1076.6	1121	2179.4
POLR2F	5343.1	6444.6	4668.9	4166.8	3346.1	5329	4962.5	6085.1
POLR2G	3032	3634.6	1497.1	1199.7	905.3	1242.7	2900.3	3829.6
POLR2H	2975.6	3499.7	1329.7	997.8	806.8	1123.4	2244.3	2847.2
POLR2I	2230.1	2712.9	1098.5	673.1	520.3	709.4	1594.2	2254.5
POLR2J	322	443.3	224.5	194.9	171.3	212.3	301	460.9
POLR2J2	95.8	109	103.1	101.2	93.5	85.3	93.8	97.8
POLR2J2	125.1	125.9	102	96.4	99.3	101.7	119.3	135.2
POLR2J3	2467.1	3148.1	523.2	418.5	374.7	480.2	2283.8	2899.8
POLR2K	236.8	265.4	147.3	120.2	116.3	130	237.2	369.3
POLR2L	217.9	256.4	198.3	167.4	136.6	147.9	219.1	292.9
POLR3A	278.4	349.3	353.3	300	259.2	354.6	263.7	463.1
POLR3B	433.1	578.6	342.1	370.4	250.1	436.1	309.5	476.9
POLR3C	2388.1	2954.7	1677.6	1444.7	987.7	1734.4	2167.1	2736.3
POLR3D	178.3	242.6	184	124	112.2	132.3	166.4	229.3
POLR3F	191.1	223.1	112.8	101.7	100.4	119.9	165.3	186.8
POLR3G	161.6	193.2	132	100.1	86.3	96.5	144.1	127.2
POLR3GL	2686.3	3096.5	1325.7	1343.1	964.3	1554.6	2181.8	3121.6
POLR3H	701	943.9	641.8	408.6	363	401	601.2	567.5
POLR3H	114.9	117.6	108.5	86.4	78.3	88.3	100.3	117
POLR3H	109.5	144.9	98.5	90.6	96.2	92.9	103.6	128.1

POLR3K	1551.7	1686.6	713.6	486.7	365.3	486.8	1346.8	1503.6
POLRMT	397.4	441	568.9	346.1	277	427.8	296.7	525.9
POLS	427	503.8	278.4	302.3	299.2	355.4	443.9	586
POM121	92.4	97.9	97.8	173.5	133.7	97.9	91.6	99.6
POMC	118.7	152.8	115.6	114.5	98.1	127.7	119.1	123.1
POMC	102	124.7	93.6	90.4	89.8	96.6	111.4	117.3
POMGNT1	684.3	829	312.8	334.3	295.4	361.9	501.8	527.9
POMP	83.1	84	91.5	88.6	81.8	84.6	73.2	86.4
POMT1	110.4	134.7	96.6	92.1	97.2	89.2	105.6	135.3
POMT1	338.9	411.8	266.2	212.7	211.3	300.2	270.1	482.9
POMT2	142.3	156.4	132.8	143.6	123.7	181.1	138.9	201.3
POMZP3	98.9	107.6	112	95.9	100.2	103.8	95	149.1
PON1	93.8	108	82.6	79.1	78.9	81.2	97.6	120.5
PON2	1273.5	1392.4	584.4	767.2	480.8	679.5	1022.1	1660.8
PON3	123.2	112.5	101.3	92.3	91.5	95.7	93.4	108.3
POP1	541.8	742.2	392.9	234.8	202.6	285.5	470	773.1
POP4	513.5	642.1	157.9	138.5	146.4	163.8	466.2	733.2
POP5	878.4	977.3	495	569.7	481.2	654.4	569.7	674.4
POP7	705.7	838.4	571.9	328.8	328.1	390.8	622.7	642.6
POPDC2	100.8	125.2	99.1	95.7	87.6	92	110.6	112.2
POPDC3	96.2	110.8	90.1	92.5	90.3	96.7	100.7	126.3
POR	249.3	346.5	229.9	226.5	176.2	288	242.7	325.1
PORCN	87.9	111.3	89.1	86.8	86.7	106.6	84.8	101.7
PORCN	146.6	171.4	91.3	97.6	87.1	91.4	153.6	165.1
POSTN	91	94	93.8	84.9	81.7	91	94.1	100
POT1	100.8	114	93.3	87.8	89.8	95.8	107.2	135.8
POT1	342	460	178.6	157.8	126.1	243.2	234	319.7
POT1	87.3	87	90.4	95.9	90.7	93.7	80.9	95
POTE15	106	119.3	92.6	95.1	85.6	93.6	104.3	131.4
POTE2	82.5	96.2	88.3	81.3	85.2	75.7	78.1	94.2
POTE8	88.1	101.3	104.4	92.8	89.7	95.4	97.5	121.1
POU1F1	77.3	83.5	92.5	85.2	79.9	81.7	74	80.1
POU2AF1	109.8	139.3	89.6	81.2	86	92.9	103.1	117.4
POU2F1	161.9	232.1	122.7	143.7	147.2	168.2	158.5	195
POU2F2	90.7	101.5	86.9	79	84.4	89	92.9	99.1
POU2F3	91.1	114.7	100.4	92.3	89.5	98.2	100.9	100.8
POU3F1	77.9	87.9	92.4	80.3	82.8	89.7	87.3	93.3
POU3F2	132.4	142.4	91	87	82.5	96.9	126.8	122
POU3F3	89.8	86.1	84	82.9	73.2	81.5	94.3	98.6
POU3F4	98.4	96.8	93	92.6	99.4	85.5	94.7	93.2
POU4F1	130.9	154.8	140.9	112.1	104.8	113.4	105.3	139.1
POU4F2	113.3	141.9	122.1	341.6	249	354.5	91.5	91.2
POU4F3	90.9	93.8	109.6	98.1	82.2	95.3	84.7	88.6
POU5F1	81.5	87.5	94.3	84.1	91.6	82.5	70.2	85.5
POU5F1P1	85.4	110.9	84.3	88.2	84.3	84.6	89.4	96.2
POU6F1	107.9	135.5	103.2	100.9	100.7	100.5	106.7	120.8
POU6F2	72.4	87.7	85.4	80.1	84.4	77.4	74.7	77.8
PP2447	5864	6158.3	4072.2	3651.8	3734.1	4445.5	3558.2	3412.7
PP8961	83	110.5	84.8	89.7	89.2	88.2	82.2	84.4
PPA1	11244.7	13186.7	7360.3	6769.2	5151.3	7985.4	10213.8	12810.7

PPA2	5172	6306.4	1880.6	1275.1	892.5	1612.8	4546.8	5703.7
PPA2	2471.1	2625.5	1353.6	2723.3	2898.3	1725	2119	2437.8
PPA2	76.9	97.2	88.5	84.1	80.6	78.1	83.2	93.7
PPAN	456.8	636.8	406.6	276.8	238.2	332.4	397.8	458.3
PPAN-P2RY	164.9	180.6	114.9	100.8	104.2	98.7	144.9	169.4
PPAP2A	84.5	98.5	90.9	84.5	85	86.9	82.1	94.1
PPAP2A	105.1	124.2	114.8	106.2	101.8	94.4	104.9	100.9
PPAP2A	101	101.9	108.2	109.5	100.1	109.1	101.1	128.1
PPAP2B	114.3	119.5	105.3	91.5	93.1	92.1	107.5	137.8
PPAP2B	77.5	90.6	104.2	85.8	81	84.8	76.5	83.1
PPAP2C	132.2	137	91.1	90.7	93.9	76.2	107.8	146.7
PPAP2C	100.3	138.5	101.5	88.4	91.5	98.2	107.1	126.1
PPAP2C	81.2	94.2	105.6	103	90.9	96.8	82.9	83.8
PPAPDC1A	108.6	131.4	81	73.5	82.8	93.6	106.8	118.4
PPAPDC1B	470.5	554.2	176.2	121.5	127	139.9	370.5	530.8
PPAPDC2	447.9	590.3	292.2	290.8	230.1	369.3	344.9	661.1
PPAPDC3	187	220.2	169.6	126.3	107.4	130.2	199	267.7
PPARA	111.3	139.5	105.5	99.1	105.5	110.4	132.7	138
PPARA	120.7	134.7	92.3	95	84.5	88.7	125	142.9
PPARBP	581.9	753.4	158.8	238.5	186.3	228.1	632.1	956.7
PPARD	101.6	109.5	98.4	94.2	96.9	105.8	91	115.7
PPARD	107.3	139.2	151.6	163.9	147.8	223.7	124.5	149.2
PPARG	100.9	105.3	100.7	91.8	93	91.9	89.7	99.5
PPARG	886.6	1123	561.8	1062.7	706.1	1299.6	706	829.6
PPARG	192.9	226.5	105.8	124.8	115.6	144.8	174.9	263
PPARG	111.6	133.5	90.3	82.5	76.9	86.9	106.2	137.7
PPARGC1A	79.2	91.8	96.8	95.5	85.1	96.8	84.2	92.6
PPARGC1B	182.2	206.6	142.6	169.1	125.3	131.9	152	261.5
PPAT	688.7	891.3	423.7	335.1	229.5	361.6	534.4	1018.8
PPBP	91.7	99.1	91	91.6	82.6	106	102	116.5
PPCDC	96.7	116.9	104.1	94.7	83.6	98.3	104	118.9
PPCS	2913.1	3079.4	1307.5	1906.2	1415.7	2505.3	2119.7	4636.5
PPCS	5477.4	5805.8	2878.7	4668.5	3135.4	5323.7	4389.3	6425.9
PPEF1	92.5	103	84.7	89.8	83.9	87.3	97.5	111.2
PPEF1	89.1	100.1	85.9	97.6	87.2	97.7	87.3	99.5
PPEF2	87.2	96.9	87.3	87.5	76.8	82.7	87.6	89.9
PPFIA1	1016	1289.3	594.9	651.1	597	1026	981.5	1383.7
PPFIA1	197.4	224.4	165.2	146.3	141	162.7	168.4	182.6
PPFIA1	290.3	377.6	181.8	157.4	135.2	209.2	257.5	335.4
PPFIA2	85.7	100.5	90.1	88.4	84.5	103.4	92	97.9
PPFIA3	121.2	118.2	92.3	97.4	87.2	95.3	103.3	121.8
PPFIA4	137.6	145.6	155.3	129.3	146.2	166.9	110.6	116.6
PPFIBP1	92.6	105.7	93.2	96.1	87.7	84.5	88.9	91.7
PPFIBP1	147.6	158.5	104.2	103.8	99	88.1	120.2	145.4
PPFIBP1	93.6	101.7	94.2	79.7	85.3	85.3	103.2	92.4
PPFIBP2	459.7	568.1	327.4	363.5	402.3	471.3	347.5	350.3
PPGB	557.1	669.4	414.7	424.1	329.8	479.8	516	792
PPHLN1	1682.5	2159.5	870	753.7	609.3	811.7	1664.9	2320.4
PPHLN1	107.3	121.7	113.3	110.8	108.9	113.4	101.7	114.1
PPHLN1	124.8	136	119.7	101	121.4	124.7	105.7	121.1

PPHLN1	1748.1	2564.2	1280	1099.7	812.2	1105.4	1674.5	2084.7
PPIA	471.2	718.7	152.1	132.9	99.2	143.5	354.3	481
PPIB	197.6	229.9	119.9	116.2	123	142.5	199.1	266.3
PPIC	2134.6	2683.5	1254	1923.1	1416	2461.2	1927	3105.4
PPID	91	106.6	89.4	78	86.7	87	104.3	97.7
PPIE	3208.7	4259.8	1705.4	1367.3	1155.4	1977.2	2640.1	3506.2
PPIE	129.9	133	121.3	102.5	100.9	106.5	117.3	136.5
PPIF	176.3	227.4	145.5	146.9	112.3	126.4	206.6	294.7
PPIG	104.8	115.1	102	86.3	89.2	105.4	95.5	149.9
PPIH	8457.8	10157.2	4553.1	2630	2256.2	3344.2	7410	9776.5
PPIL1	1579	2074.8	1050.4	844.6	479.7	1049.6	1174.4	2752.3
PPIL2	95.3	111.4	91.7	86.9	81.6	88.2	86.2	95.8
PPIL2	89.2	110.7	107.5	101.1	107.6	96.9	88.8	98.6
PPIL2	81.6	98.1	88.6	96.5	79.7	80.2	95.9	100.6
PPIL3	148	162.9	104.8	91.3	88.8	85.6	124.2	145.1
PPIL3	94.4	126.9	93.3	78.9	77.1	84.5	104.7	108.9
PPIL3	3542.3	4686.8	2174.8	1904.8	1500.6	2216.2	3184	3879.6
PPIL3	149.9	190.9	117.6	116	113.6	122.4	157.8	195.2
PPIL4	82.9	98.9	92.9	90	83.7	92.8	87.3	95.7
PPIL5	2928.3	3679	1799.4	1085.4	802.2	1183.7	2332.5	3858.4
PPIL5	94.9	107.2	89.9	92.3	90.2	78.8	91.3	88.9
PPIL5	175.5	214.3	108.9	92.9	95.3	101.8	136.6	242.7
PPIL6	111.2	130.2	106.3	86.5	85.5	85.1	112	135.9
PPL	96.6	120.2	96.8	84.2	77.8	82.8	103	105.6
PPM1A	113.8	103.6	90.9	90	84.3	85.6	96.2	124.4
PPM1A	316.3	480.2	172.8	189.9	178.7	181.1	271.1	341.6
PPM1A	138.7	165.3	118	96.8	86.8	113.5	122.2	150
PPM1A	214.1	231	127.2	124.6	115.2	115.9	152.5	206.3
PPM1B	149.5	197.9	91.7	98.4	82.7	73.3	138.7	162.3
PPM1B	1083.2	1378.7	690.2	929.6	795.7	1236.8	1005.3	1550.6
PPM1B	121.7	123	99	156.5	162.1	103.7	122.6	115.4
PPM1B	150.6	164.3	104.8	109.1	110.6	108.5	130	223
PPM1B	80.5	81.9	84.1	81.5	85.3	90.4	75.7	80.3
PPM1D	475.8	651	233	214.8	193.3	275.7	462	648.4
PPM1E	166	235.3	161.8	143.9	134.7	170.2	152.6	184.3
PPM1F	1090.6	1390.7	1153.8	940.8	805.6	867.1	1029.5	636.6
PPM1G	257.9	376.2	154.2	102.4	103.3	96.4	165.1	211.7
PPM1G	8312.5	10434.2	5645.2	4643.8	4141.8	4748	6387.6	6497.1
PPM1J	90.3	98.9	102.6	89.2	79.4	81.4	85.4	94.8
PPM1K	222.3	250.4	150.9	203.1	212.6	185	249.3	327.4
PPM1L	92.8	107.5	84.7	89.4	98.3	85	95.6	100.9
PPM1M	261.8	287.1	251.2	190.8	171.6	214.4	208.2	261.4
PPM2C	99.7	127.6	105.8	97.5	86.4	106.3	109.4	150.1
PPME1	352.7	425.7	275.3	244	197.9	301.6	336.3	535.3
PPOX	415.6	450.7	310.4	217.9	218.4	278	277.2	287.1
PPP1CA	451.7	718.6	374.3	270.9	231.8	305.4	480.8	663.6
PPP1CB	1126.5	1355.6	581.8	728.8	544.5	862	784.4	1608.2
PPP1CC	7764.5	9916.2	3559.7	3817.4	3553	5714.6	6152.5	6938
PPP1R10	246.2	323.6	182.7	198.6	184.3	257.5	205.5	365.4
PPP1R11	1449.9	1714.6	874.6	773.3	583	775.2	1306.7	1422.1

PPP1R12A	244.5	262.6	130.6	134.7	145.1	192.4	191.9	364.7
PPP1R12B	288	358.1	196.8	185.7	162.7	176.4	265.7	329.7
PPP1R12C	193.7	214.9	121.5	109.4	131	154.4	174.4	213.9
PPP1R13B	182.4	219.7	166.1	145.1	152.1	164.7	180.7	194.5
PPP1R13L	122.2	133.9	108.2	94.1	99.9	106.5	120.3	138.4
PPP1R14A	167	183.8	147.2	114.9	122	107.7	146	170.1
PPP1R14B	2852.9	3311.1	3294.1	2403.7	1983	2589	2202.7	3579.3
PPP1R14C	159.8	209.1	146.9	136.8	124.1	157.4	200.2	321.5
PPP1R14D	150.2	182.1	110.2	115.9	101.8	104.8	138.7	162.9
PPP1R15A	870.4	1060.3	539.2	452.9	489.5	577.8	1189	1717.6
PPP1R15B	114.8	115.6	115.3	91.4	84.6	100	110.5	159.1
PPP1R16A	496.5	610.2	231.1	152.5	116	146.8	364.8	360.1
PPP1R16B	108	119.7	129.5	115.2	105.7	136.1	109.7	137.5
PPP1R1A	95.6	96.1	97.6	94.4	84.6	88.7	88.4	96
PPP1R1B	100.4	103.7	90.6	84.1	88.1	85.9	98	112.2
PPP1R1B	79	91.4	92.6	100.8	96.5	104.9	92.1	88.1
PPP1R1B	127.4	149.4	113.9	117.9	87.7	103.1	134.6	150.9
PPP1R1C	96.4	109.9	88.2	94	78	90.1	98.6	104.9
PPP1R2	305.3	393.3	262.5	245.7	197.2	277	268	422.8
PPP1R2P3	82.4	97.9	100.4	95.1	80.7	91.1	89.4	102.3
PPP1R2P9	86.8	96.8	95.3	88.7	77.5	86.4	92.8	105.8
PPP1R3A	87.4	87.8	94.8	86.5	94.5	91.1	85.4	92
PPP1R3B	82.2	97.4	95	82.6	77.9	79.5	92.2	93.1
PPP1R3C	120.8	144.2	116.1	108.8	95.8	129.5	117.2	132.9
PPP1R3D	257.7	326.3	194.6	201.8	147.8	211.7	212.5	355.9
PPP1R3F	119.2	139.8	102.9	106.4	95.5	107.5	118.4	148.4
PPP1R7	1038	1317.3	558.8	549.7	460	674.9	963.4	1256.4
PPP1R8	540.2	686.4	304.5	250.9	217.5	288.1	450.5	565.4
PPP1R8	179.8	236.6	114.7	108	89.8	123.6	162.9	218.6
PPP1R8	88.3	95.6	104.2	81.6	82	89.1	97.3	105.9
PPP1R9A	96.6	90.6	112.4	102.6	100.6	99.3	85.9	84
PPP1R9B	106.6	120	104.8	115.2	98.5	111.2	107.6	133.4
PPP2CA	2293.9	3145.3	1578.2	1980.6	1412.5	2156.6	1847.1	2868.5
PPP2CB	228.2	234.2	136.5	114.5	129.6	103.5	199.6	286.6
PPP2CB	288.4	353.8	173	148.2	123.9	213.8	329.8	720.9
PPP2R1A	2951.7	3807.3	2491.3	1915.4	1419.5	3031.3	2329.1	3669.7
PPP2R1B	117	148.8	113.7	104.9	104.7	103.1	112.2	132.1
PPP2R1B	197.8	266.3	113.1	104.4	95.9	105.9	162.2	225.4
PPP2R1B	488.7	660.4	209.8	177	142.7	223	408.9	536.5
PPP2R2A	1841.8	2362.8	1797.6	1209.9	885.8	1415	1854.9	2814.3
PPP2R2B	422.2	484.6	208.4	214	204.1	224	406.6	443.9
PPP2R2B	81.4	88.7	85	85.8	81.4	76.8	73.8	82.2
PPP2R2B	99.2	110.5	93.5	85.5	91.2	85	99.3	106.4
PPP2R2B	117.3	130.6	98.5	111.1	92.1	89.3	133.4	130.1
PPP2R2B	120.6	149.7	122.7	129.3	106.1	127.2	129.2	149.3
PPP2R2B	78.2	87.7	95.6	82.5	77.7	82.6	87	106
PPP2R2C	87.9	103.1	92.5	93.6	80.5	83.4	108.7	101.5
PPP2R2C	88.7	98.4	110.7	91.8	90.3	98.4	87.6	94.3
PPP2R2C	102.9	112	80.8	91	71.1	78.4	102.6	116.8
PPP2R2C	96.2	111.1	90.8	94.6	85.4	91.2	101.6	106

PPP2R2D	304.1	394.1	149.2	100.5	105.1	112	247.2	334.1
PPP2R2D	94.9	113.4	108.9	94.6	89.8	95.6	91.3	104.1
PPP2R2D	588	774.3	394.5	277.3	256.4	325.9	531.2	797.2
PPP2R3A	89.7	100.6	95.9	93.1	94.2	97.7	89.4	97.3
PPP2R3A	97	111.1	107.9	94.9	79.6	84.9	101.2	109.4
PPP2R3A	103.4	124	114.7	105.2	112.6	109.2	89.4	142.9
PPP2R3B	132.3	166.8	122.4	112.3	110.2	120	125.6	175
PPP2R3B	120.6	135.1	127.1	125.4	119.4	162.1	118.4	133
PPP2R3B	86.9	91	108.2	101.9	114.3	183.4	83.8	99.1
PPP2R3C	2057.5	2408.3	817.8	628.1	455.9	708.3	1801.5	2515.7
PPP2R4	96.3	101.5	107.1	102.2	86.7	90.1	90.4	105.6
PPP2R4	110.7	141.6	93.4	85.9	86	80.4	104.1	134.2
PPP2R4	124.4	134.9	107.1	107.2	95.4	112.9	124.4	130.3
PPP2R4	1167.5	1344.4	852.2	505.3	403.6	449.3	1024	1401.9
PPP2R5A	710.4	1037.5	687.3	506.7	423.5	725.8	678.5	1021.3
PPP2R5B	105.8	129	109.5	115.9	100.7	121.8	104.5	144.5
PPP2R5C	2086.6	2921.1	1460.3	967.7	797.3	1364.8	1815.6	2353.1
PPP2R5C	193.5	217.9	116.3	123.5	100.5	100.7	148.3	186.8
PPP2R5C	128.8	131.7	119.8	105.7	95.6	99.8	118.5	173.8
PPP2R5D	248	341.2	131.9	110	115.3	93.7	218	285.5
PPP2R5D	166	230	195.7	193.8	159.6	221.8	172.4	294.3
PPP2R5D	533.4	659.6	436.5	291	236.9	398.8	521.4	1184.2
PPP2R5E	1915.7	2552.7	1304.1	1300.9	935	1409.9	1517.3	2064.9
PPP3CA	353.3	441.5	156.7	151	128.7	169.6	367.9	559.5
PPP3CB	903.6	1089.6	965.5	822.9	597	919.2	773.3	1246
PPP3CC	465.4	593.5	285.3	188.4	203.3	262.8	420.5	651.8
PPP3R1	1243.4	2196.6	1156.4	1081.5	731.4	1061	1280.7	1766.3
PPP3R2	96.2	111.7	101.5	92.3	86.9	94.5	90.8	85.7
PPP4R1	768	940.6	414.7	449.7	359.1	600.1	715.6	1380.6
PPP4R1	415.6	539.3	315.7	268.5	265.6	436.3	369.2	721.2
PPP4R1L	100.1	115.8	94	90.6	80.8	83.6	109.7	133.8
PPP4R2	126.6	127.3	95.1	91.6	88.8	103.3	103.4	148.3
PPP5C	121.3	133.7	93.2	88.6	87.1	89.5	104.2	154
PPP6C	4557.3	5990.4	2286.4	2111.5	1460.5	2646	4606.5	6489.3
PPRC1	675.1	793.7	616.8	437.8	308.2	465.8	529.6	569.7
PPT1	24887.1	26364.9	14382.4	14403.6	11392.9	13626	24558.4	27677.1
PPT2	86.1	98.6	90.2	91.5	85.1	83.9	86.2	91.2
PPT2	90.9	105.7	95.5	95.9	101.9	113.1	90.7	98.3
PPWD1	849	1015.3	484.1	452.4	404.4	783.3	563	881.8
PPY	93.8	104.5	97.3	88.8	81.1	84.3	91.8	98.8
PPY2	99.1	129.3	85.3	81	72.7	90	107.5	137.1
PPYR1	83.3	90.4	91	85.7	86.8	80.2	79.6	90.6
PQBP1	361.1	497.8	148.3	143.5	123.6	180.6	425.6	446.6
PQBP1	1248	1458.8	716.9	586.9	517	726.1	1219.5	1449
PQBP1	139.3	159.4	107.7	104.7	104.6	101	113.1	135.5
PQLC1	2828.5	3576.8	2431.3	2478.4	2060.9	2561.7	2448.7	3271.5
PQLC2	156.9	172.6	116.9	106.1	101.6	101.4	139.1	172.7
PQLC2	100.9	118	92.2	86.5	75.5	83	100.7	123.4
PQLC3	1326.7	1877.8	826.2	1212.1	844.3	1674.4	1035.6	1896.2
PRAC	97.2	110	98.4	89.5	85.5	85.1	93.8	103.1

PRAF2	98.1	110	95.7	78.9	86.8	84.3	107.4	112.9
PRAM1	219.7	236.4	258.9	259.5	193.5	255.4	127.6	183.1
PRAME	136.9	145.3	108.2	89.6	88.6	90.4	101.1	131.1
PRAME	594.3	760.1	477.9	310.4	286.3	368	452.9	533.8
PRAME	735.7	1026.3	574.6	440.3	409	697.4	609.8	905.3
PRAMEF1	72	78.3	104.6	98.6	87.4	91.8	74	77
PRAMEF10	124	136.3	112.2	102.7	101.8	101.4	115.5	135.3
PRAMEF10	86.7	87.8	82.6	77.4	78.4	75.9	80.9	88
PRAMEF10	84.9	83.2	86.5	79.4	76.7	83.4	86.2	79.4
PRAMEF16	79.1	88	104	86.7	85.4	92.3	83.4	78.1
PRAMEF4	110.5	127.5	89.8	83.3	79.9	86.6	103.6	124.9
PRAMEF5	188.1	204.3	145.1	124.1	130.3	135.4	184.7	199.9
PRAMEF6	81.6	83.3	104.9	93.6	91.8	90.5	85.6	81.9
PRAMEF7	89.5	92.4	94	92.8	89.5	92.3	87.7	101.6
PRAMEF8	92.3	99	102	96.4	93.7	93.9	97	103.5
PRAMEF9	115.3	140.1	84.2	84.6	80.5	83.9	100.3	128.3
PRAP1	88.8	90.7	87.8	93.6	87.7	99.2	96.8	97.7
PRB1	113.1	130.3	95.6	87.3	94.7	90.3	116.2	127.9
PRB1	117	113.9	122.3	103.6	104.8	104.5	101.3	113.6
PRB2	109.2	121.8	105.7	94	99.9	92.8	115.8	121.6
PRB3	105.5	125.7	104.9	107.3	98.2	98.9	99.5	97.8
PRB4	90.6	103.2	91.2	86.2	79.4	92.3	94.1	94.4
PRC1	3032.2	3566.9	2805.2	3026.9	2403.5	4065.3	2610.7	4848.5
PRCC	227	241.2	167.6	138.6	129.7	184	174.5	294.3
PRCC	431.7	477.2	374.8	339.7	273.3	348.1	360.3	478.2
PRCP	2447.4	3231.2	1556.4	2237.3	1600.1	2026.3	2414.8	3262.5
PRCP	724.7	1020	406.7	543.8	385.6	607.4	691.5	1145.6
PRDM1	167.1	203.3	98.5	90.8	93	94.3	149.2	164.7
PRDM1	87.2	93.5	86	92.4	87.1	97.9	91.5	112.6
PRDM1	120	117.7	91.5	103.1	107.2	84.9	101.4	122.4
PRDM1	249.3	281.7	162.2	478.1	419.1	642.6	336.6	516.3
PRDM10	353.6	512.6	286.9	372.6	295.8	489.8	393.1	518.5
PRDM11	88.2	88.7	96.3	81.8	81.9	88.8	87.4	84.2
PRDM12	87.5	105.2	79.6	78.2	81.5	82.9	90.4	99.8
PRDM13	111	137.5	92.5	91.1	90.3	79.3	101.7	124.5
PRDM14	82.5	93.3	82.5	81.1	92	90.3	78.6	87.9
PRDM15	90.4	102.2	100.5	89.8	88.6	90.4	92	97.1
PRDM16	84.7	99.7	98.1	88.3	84.7	76.8	97	98.1
PRDM16	75.7	85.4	84.3	87.5	84.4	86.6	72.7	71.9
PRDM16	87.6	85.4	95.7	90.5	86.3	85.3	80.2	92.3
PRDM2	106.3	123.6	119.2	120.1	103.2	105.7	106.1	122.1
PRDM2	81.3	91.5	87.6	90.7	79.4	79.1	82.6	87.9
PRDM4	383.9	493.4	195.5	218.2	181.9	242.1	300.4	370
PRDM5	86.2	100.6	96.6	87.9	94.2	94.6	88.9	100.4
PRDM7	88.7	100.2	95.2	98.2	94.7	88.3	85.3	86.8
PRDM8	227.7	296.6	190.3	187.5	151	237.1	215.3	304.2
PRDM9	127.6	133.6	131	104.6	97.9	121.4	110.3	129.3
PRDX1	9957.1	12541.5	8034.1	7651.4	5982	7064.6	11176.8	14699.2
PRDX1	6400.8	8554.7	2857.6	2824.1	2034.7	3085.8	6424.4	9552.7
PRDX2	82	89.3	85.5	89.9	91.6	89.5	87.4	83.9

PRDX2	3122.1	3943.4	1518.8	694.2	732.9	707.8	2869.2	2952.3
PRDX3	7860.7	10730.3	5060.5	3418.1	2542.2	4190.8	6102.4	7820.1
PRDX4	6718.9	9069.3	3248.8	2488.3	2169.9	2720.8	5899.3	7778.8
PRDX5	2789.9	3578.2	2060.9	2179.5	2231.8	3084.8	2981.1	3036.2
PRDX5	7014.7	8389.8	5583.9	5828.2	4788.4	9281.6	7139.6	9157
PRDX5	6595.1	7985.2	5540	5967.5	5285.3	7507.8	7114.7	9294.6
PRDX6	6739.8	8712.9	4680.4	4788.6	3373.7	4915.5	7039.8	8120.6
PREB	671.7	788.2	460.3	459.5	354.1	473.6	539.8	1147.3
PRED57	109.7	140.4	98.1	96.1	84.7	94.8	116.1	140
PREI3	790.4	1102.5	552.3	507.2	435.1	628.7	689.2	1075.6
PREI3	339.1	434.7	156.4	166.4	186.3	220.1	300.7	353.4
PREI3	75.9	84.8	91.2	80.2	78.8	77.6	83.2	79.6
PRELID1	1921.3	2695.3	2083	1974.2	1595.4	2156.8	1836.9	2656
PRELID2	140.4	150.5	104.7	99.6	91.4	91.5	126.9	168.7
PRELP	92.2	112.9	92.8	96.8	86.1	97.6	92.3	108.1
PREP	1198	1753	610.6	534.2	400.4	573.7	811	1127.5
PREPL	306.3	409.5	267.1	324.2	267	575	212	327.8
PREX1	167	247.3	152	240	231.8	350.1	181.3	221.6
PRF1	81.6	92.7	109.2	101	98.4	90.2	90.1	97.1
PRG2	621.4	871.6	929.5	683.6	509.3	592.1	524.4	811.3
PRG2	297.6	385.2	214.9	158.4	131.5	152.5	253.4	272.2
PRG3	82.5	92.1	95.6	96.1	84.4	88.5	84.9	95
PRG-3	117.5	132.1	122.2	112	105.6	121.7	131.6	143.3
PRG-3	79.3	92	84.2	78	82	89	74.1	93.2
PRG-3	111.7	116.7	88.2	84.8	83.9	88.1	99.7	107
PRH1	98.9	122.5	103.3	78.9	85.8	92.5	98.7	123.4
PRH2	106.4	119	90	97.6	84	85.8	111.2	109.6
PRIC285	638.1	801.2	438.3	397.7	386.3	369.5	624.8	760.2
PRICKLE1	952.1	1150.5	755.6	706.1	594.3	521.2	565.8	464.4
PRICKLE2	90.2	96.5	105.1	104.7	88.3	92	90.8	102.2
PRIM1	2546.8	3626.9	1457.4	1142.4	896.4	1173.8	1778.5	2681.1
PRIM2A	142.1	203.5	130.9	107.7	100.2	136.9	126.9	118.2
PRIMA1	96	110.4	97.9	85.9	84.9	85	88.4	112.5
PRKAA1	192.5	214.3	160.8	238.8	191.8	286.8	173.5	219
PRKAA1	617.4	736.3	256	374.1	351.4	542.2	454.3	743.9
PRKAA2	80.6	85.6	96.6	83	77.2	84.2	82.4	87.8
PRKAB1	259.7	379.9	202.4	191.8	180.2	230.8	242.7	359.3
PRKAB2	177.8	211.7	156.2	149.7	148.5	213.2	179	302
PRKACA	108.1	142.1	98.5	90.4	87.1	98	110.6	112.4
PRKACA	109.6	143.9	103.9	92	87.1	95.1	104.4	143.3
PRKACB	103	108	92.1	89.2	82.3	90.1	109.4	120.9
PRKACB	223.1	267.8	135.5	139.5	115.7	143.8	167.3	201.1
PRKACB	103.3	132.2	79.4	83.8	74.1	75	104.3	131
PRKACB	83.8	101	103.3	91.3	95.5	102.5	88.9	102.2
PRKACG	93.3	104	108.3	99.9	89.8	96.2	91.7	94.1
PRKAG1	373.9	480.8	191.2	169.6	155.9	215.4	317.3	394.8
PRKAG1	302	441.8	307.8	322.9	268.4	460.9	289.1	381.3
PRKAG2	378	505.4	266.4	321.5	275.5	380.7	376.9	592.5
PRKAG2	240.6	296	126.6	136.6	121.2	162.8	227.9	318
PRKAG2	113.7	134.7	105.8	94	93.4	92.5	111.8	150.9

PRKAG3	75.2	92.7	105.6	95.9	85.1	98	82.4	89.2
PRKAR1A	113	127.4	96.2	102.5	85.3	93.4	100.1	112
PRKAR1A	1325.6	1661.8	578.1	479.7	436.1	548.7	1323.3	2113
PRKAR1B	252.8	312.4	202.9	246.6	222	254.3	192.5	244.6
PRKAR2A	254.5	386.6	210.2	305.5	259.9	167.9	237.4	274.1
PRKAR2B	95.9	108.6	99.4	100.6	85.7	92.5	96.2	98.7
PRKCA	322.1	475.1	292.5	769.6	521.2	1001	306.7	593.9
PRKCABP	112.6	128.2	117.1	100.4	103.1	117	111.9	130.1
PRKCB1	341.1	363.1	116.5	173	102.4	150.8	275.2	447.7
PRKCB1	370.6	560.2	195.2	322.6	195.1	411.9	341.7	494.3
PRKCB1	3457.2	4102.9	2240.7	2356.1	2318.7	2628.4	3121.8	4060.6
PRKCBP1	287.1	392.4	203.4	213	185.6	273.4	193.6	267.1
PRKCBP1	99.9	108.2	90.9	98.8	84.4	97.4	95.5	101.4
PRKCD	3297.8	4357	2943.2	2967.5	2924.6	3285.8	3166.5	4259.9
PRKCD	117.5	161.5	101.9	100.7	99.3	93.9	107.6	144.1
PRKCDBP	193	253.4	156.6	284.9	216.9	282.4	183.9	163.9
PRKCE	111.7	145	107.7	100.6	95.7	109.2	100.3	116.7
PRKCG	80.7	102.4	100.4	96.1	84.9	92.6	89.5	104.1
PRKCH	281.3	386.5	229.6	143.1	121.2	170.2	245.1	333.7
PRKCI	191.3	206.8	112.5	110.9	100.6	140.1	181.2	313.3
PRKCQ	1065.7	1363.3	493.1	405.1	327.3	553.9	871.6	1503.8
PRKCSH	1412.8	1888.6	884.1	700.6	566	592.6	1196.3	1756.3
PRKCSH	202.8	282.9	133.1	116.3	126.5	121.7	207.7	337.7
PRKCZ	196.9	253.2	111.1	92.2	93.7	103	197.5	209.9
PRKCZ	89	96.3	108.7	93.5	89.5	93.4	100.3	97.6
PRKCZ	314.3	406.2	171.4	209.2	179.4	199.8	284.7	284.9
PRKCZ	87.8	101.2	97.4	90.5	95.5	91.2	86.8	91.6
PRKD1	68.7	89.9	77.2	76.5	76.8	66.7	78.3	79.6
PRKD2	148.1	185.4	129.2	111	115.6	111.7	162.2	233.8
PRKD2	118.6	121.4	120.5	92.9	91.4	104.7	107.5	122.7
PRKD3	123.4	168	94.1	90.9	92.6	93.6	123.1	143.4
PRKDC	1262	2412.1	1052.1	1538.7	1311.6	1644.6	1043	784.2
PRKDC	188.5	286.6	131.2	161	132.5	255.6	187.1	247.5
PRKDC	236.8	434.7	132.8	151.2	150.6	170.3	221.1	256.1
PRKG1	72.2	77.9	90.2	79	81.2	76.2	77.8	78
PRKG2	83.7	100.7	90.9	79.8	81.6	77.5	86.4	80.4
PRKRIP1	481.6	537.6	332.1	369.8	344.7	413.7	364.9	388
PRKRIR	2796.3	3196.2	1786.4	1903.4	1346.8	2432.8	2011.4	3228.2
PRKX	177.1	248.9	175.9	177.8	151.6	195.2	160.6	211.3
PRKY	125.6	134.6	78.7	82.2	91.5	109.3	113.8	126.6
PRL	74.4	84.5	89	92.3	85	88.6	88.2	88.1
PRLH	78.5	85.1	96.4	91.7	87.1	84.4	80.4	84.9
PRLHR	93.8	90.1	95.4	87.1	94	85.8	107.1	101.2
PRLR	98.9	131.2	88.9	82.7	82.5	83.7	99.2	101.7
PRM1	104.6	117.2	97	86.8	84.3	87.9	99	100.4
PRM2	84.5	91.4	89.3	78	78.6	82.7	87.1	83.4
PRM3	90.4	110.2	97	90.1	79.3	79.2	87	85.9
PRM3	97.3	127.3	98.3	97.1	95	93.6	105.2	111.4
PRMT1	178.6	227.6	114.8	100.5	99.9	92.9	169.1	161.2
PRMT1	2591.6	3978.9	1801.2	1070.6	961.2	1260.4	2310.5	2675.9

PRMT1	5874.4	7433.6	4141.9	2457.7	2213.1	3266.6	5670.6	7002.4
PRMT2	340.8	378.2	211.9	233.6	226.5	303.5	315.9	413.6
PRMT2	163	185.3	102.7	112.7	113.5	106.9	119.6	160.9
PRMT2	338.6	400.2	255	280.9	294.4	394.7	263.6	469.1
PRMT5	1452.6	1918.6	606.4	386.8	316.3	635.3	966.8	1366.8
PRMT5	1321	1606	685.2	525.3	366.3	585.1	897.2	1366.2
PRMT6	3151.2	4020.1	1654.5	721.2	611.7	1121.1	2516.3	3964.4
PRMT7	355.8	453.9	210.2	158.3	130.9	161.7	281.8	300.1
PRMT8	84.1	97.4	97.6	92.4	86.8	83.1	90.3	86.1
PRND	78	80.1	94.3	90.6	92.2	89.9	76	91.3
PRNP	560.4	813.8	322	396	294.9	607	525.7	841.8
PRNP	1743.4	2058.7	1132	1504.1	1094.3	1832.1	1634.4	3143.7
PRNPIP	124	205.4	97.1	93.1	76.4	89.2	124	153.2
PRNT	89.9	87.7	83	83.9	83.3	91.2	84	93.2
PRO0149	479.7	650.6	369.5	335.8	283.6	376.5	505.4	879.7
PRO0611	92.1	113.8	85.9	81.5	78.1	88.3	95.2	112.7
PRO0628	114.4	138.8	120.6	154.4	152.3	117.7	123.5	148.2
PRO1853	203.2	251	116.4	122.5	108.8	114.7	197.3	278.1
PRO1853	190.2	193.7	123.9	118.7	117.9	139.2	143.9	196.3
PROC	77.5	81	90.1	98	94.8	108.2	78.4	99.9
PROCA1	125	132.2	95.4	93.7	86.4	95.6	109.6	121.1
PROCR	292.5	387.9	189.2	141.8	134.8	164.2	251.7	347.3
PRODH	77.4	88.4	84.1	84	79.5	85.9	82.1	89.5
PRODH2	98.6	112.6	93.4	81	79.2	72.5	85.1	125.8
PROK1	106	135.2	95.5	79.9	82.2	80.7	107.3	127.4
PROK2	128.3	160.6	117.2	105.2	95.7	114.2	144	214.6
PROKR1	77.9	80.9	84.4	82.4	83	75.8	74.1	74.3
PROKR2	86.8	98	101.1	94.8	88.9	90	88.3	96.7
PROL1	89.7	100.8	87.2	86.2	87	88.6	95.5	95.8
PROM1	77.7	78.7	80.4	76.4	79.1	77.7	77.9	79.4
PROM2	91.1	112.5	82.1	93.4	72.1	83.3	95.7	112.2
PROP1	124.5	151.1	89.2	84	91.2	100.6	132.8	147.6
PROS1	1706.5	2168.5	1229.9	1621.2	1254.4	2105.8	1472.2	2014.6
PROSAPIP1	76.7	80	91.5	88	93.4	84.8	79.2	94.3
PROSC	2701.5	3720.5	2807.1	2168.4	1651.8	2894.7	2420.2	5133.2
PROX1	77.8	84	85.1	82.7	81.8	80.7	83.9	84.3
PROZ	78.4	75.4	87.1	76	71	88.2	78.9	79.9
PRP2	86.1	102.1	91.9	85.7	84.9	87.6	91.2	99
PRPF18	436.3	524.5	229.7	204.4	148.5	221	394.4	648.7
PRPF19	992.5	1335.7	446.4	470.4	300.9	545.8	867.4	1394.6
PRPF3	2393.5	2727.7	1370.9	948.8	912.3	1582.3	1736.6	3069.8
PRPF31	1514.7	1905.7	862.4	688.3	523.4	806.1	1252.5	1991.5
PRPF38A	908.6	1095.2	471.9	514.9	415.3	715.7	707.7	1059.4
PRPF38A	385.4	525.5	141.8	160.6	118.1	179.1	335.8	472.8
PRPF38B	437.3	545.5	310.4	229.3	177.9	352	363.7	765.9
PRPF39	132	136.3	107.7	101.2	88	102.2	119.3	152
PRPF4	2792.8	3260.5	2488.7	1730.1	1183.5	1784.5	2521.3	4368.6
PRPF40B	85.5	102.1	99.4	91	92.8	93.4	94.1	92.2
PRPF40B	74.4	73.9	95	86	84.6	83	69.2	80.7
PRPF4B	124.3	120.5	106.1	114.6	87.4	119.9	106.3	141.5

PRPF4B	68.2	79.9	86	83.3	73.8	74.5	73.6	84.3
PRPF4B	183.6	189.2	172.6	205.8	200	366.4	141.8	235.5
PRPF8	1405	1852.3	1498.2	1465.4	1151.7	1916.8	1535.3	2073
PRPH	152.2	185.2	128.8	110.9	107.6	117.1	135.2	172.2
PRPS1	1758.3	2525.2	1022.7	776.5	566.6	1124.2	1573.1	2207.7
PRPS1L1	101.7	133.1	90.6	92.4	86.1	86.9	98.6	117.8
PRPS2	899.8	1318.5	252.7	287.6	304.7	370.9	706.3	849.9
PRPS2	1696.4	2016.1	685.8	738.9	524.5	791.3	1299.6	1536.7
PRPSAP1	1384.6	1748.4	933.8	994.4	889.7	1143.6	1286.7	1960.6
PRPSAP2	470.9	661.5	336.5	343.1	295	390.4	468.7	666.9
PRR11	724.6	1011.4	358.6	339.4	333.5	258.8	680.1	855.2
PRR13	17848.9	22696.1	13332.5	13730.6	9639	13586.8	16414.6	17569
PRR15	132.5	147.4	85.2	89.4	84	88.1	123.4	166.3
PRR3	210.8	261.3	117.1	88.8	91.7	99.2	197	173.3
PRR3	180.4	293.1	103.9	110.8	87.2	105.6	179.2	168.8
PRR4	115.7	119.2	120.4	105.9	94.8	118	111.4	170.3
PRR5	100	114.8	94.8	100.9	86.3	85.5	93.4	95.9
PRR5	343.8	466.9	353.1	239.9	242.1	334.1	229.6	429.4
PRR5	177.9	201.1	171.1	147.6	121.9	167.4	164.2	213
PRR6	1567.7	1946.6	1294.9	1004.8	897.6	1018.9	1622.4	1084.9
PRR7	123.8	162.5	110.2	101.3	85.3	99.3	111.8	169
PRR8	124.6	138.9	82.6	96.2	93.1	104.2	116.6	151.4
PRRG1	316.9	391.3	271.3	252.6	224.9	320.2	303.6	435.6
PRRG2	109.8	112.9	96.8	87.7	86.1	85.8	105.8	99.7
PRRG3	84.5	101.9	88.7	87.8	86.2	85.3	88.2	101
PRRG4	205.9	184.2	135.2	142.8	128.1	110.9	195.1	307.2
PRRT1	128.6	136.6	105.2	101.8	95.3	102.8	126.7	155.6
PRRT2	107.5	128.3	95.6	81.1	87	86.7	103	126
PRRT3	172.9	188.4	117.8	100.3	96.9	105.1	209	298.5
PRRX1	80	89.7	106	86.1	87.3	93.1	92	83.9
PRRX1	98.4	112.9	101.9	95.9	80.6	84.9	92.1	107.5
PRRX2	123.5	137.5	89.3	82.6	87.8	85	109.4	130.9
PRSS1	102	116.7	99	86.6	80	80.8	87.6	110.4
PRSS12	247.4	328.2	217.6	210.9	178.8	267.2	189.9	237.6
PRSS15	1339.4	1588	1436.9	1119.3	958.4	1911.2	1184.9	2695
PRSS16	101.1	126.8	101.3	93.7	94.3	101.6	96.9	116
PRSS2	104.3	123.4	92	87.5	73	74.7	107.7	117.9
PRSS21	129.5	156.3	91.5	92.3	87	93.2	117.3	148.1
PRSS21	92.9	88.6	85.9	82.5	82.1	81.9	84.9	90.1
PRSS22	98.5	118.6	97.8	94.2	81.1	87.1	105.4	120
PRSS23	90.6	104.6	95.3	82	93.4	85.8	110.5	131.8
PRSS27	92.4	117.3	105.4	108.2	106.8	115.7	100	104.4
PRSS3	88.7	103.3	103.2	102.3	95.6	98.3	93.2	105.5
PRSS33	78.4	92.1	89.3	90.2	77.5	81.9	86.4	84
PRSS35	109.5	133.3	83.7	91.2	79.3	95.7	125.5	136.1
PRSS7	179.7	217.3	136.8	110.6	113.6	122.9	151.8	191.6
PRSS8	102.4	121.5	79.6	80.9	80.8	82.3	113.9	130.3
PRSSL1	126.4	153.7	110.7	95.4	101.2	100.3	121.7	152.1
PRTFDC1	121.4	151.6	99.8	72.7	82.9	79.3	120.6	145.5
PRTG	84.9	113.6	95	95.1	78.6	85.9	87.8	108.8

PRTG	76.3	80.1	101.9	86	84.7	80	71	83.9
PRTN3	10640.9	14488.1	8668.7	7386.6	5570.3	8987.4	7950.2	13062.6
PRUNE	297.3	409.4	216.2	230.3	200.4	294.2	287.6	471.4
PRUNE2	107.2	110.2	99	95	83.6	83.4	105.8	114.6
PRX	106.2	96	101.9	90.1	89.4	95.9	93.4	105.9
PRX	120.2	122.6	109.3	97.3	87.5	90	115.8	118.1
PRX	93.2	104.6	108.2	100.7	107.6	108.4	89.2	99.2
PRY	96.8	102.9	87	83.8	85.4	78.1	96.8	105.1
PSAP	3654.8	4925.7	1774.5	3618.9	3107	3769.3	3365.5	3154.1
PSAP	3446.1	4377.7	1770.4	3412	3135.3	3794.9	3095.6	3149.3
PSARL	4334.4	5867.3	3564.8	4504.1	3720.8	5724.7	4345.9	5256
PSAT1	3253	3763.6	2614	969.9	680.5	1062.6	3856.2	5798.4
PSAT1	78.7	82.8	88.9	86.4	80.7	76.5	78.2	77.1
PSCA	116.7	130	98.8	96.3	92.9	90.5	122	113.6
PSCD1	170.2	200.9	127.8	112	124.8	147.9	165.4	248.9
PSCD1	551.9	757.5	734.4	844.7	850.4	1224.5	499.8	907.4
PSCD2	204.2	279.5	136.4	121.9	117.3	130.2	228	289.1
PSCD2	129.5	141	117.7	105.5	87.1	107.8	134.1	151.4
PSCD3	125.1	149.7	140.8	133.1	138.2	173.5	136.6	196.7
PSCD4	886	1002.3	892.5	1027.1	1021.2	1065.3	765.5	615.2
PSCDBP	71.1	86	78.3	78.3	71.7	78.9	86.1	88.2
PSD	93.8	102.4	94.3	84.9	83.6	88.3	99.4	101.8
PSD2	87.8	102.5	89.2	94.3	85.1	81	87.5	89.3
PSD3	120.2	133.5	106	104.5	94.6	98.9	106.1	117.2
PSD3	177.3	189.8	108.1	97.3	92.5	111	159.7	172.6
PSD3	160	220.4	162	167.8	148.2	231.6	132.7	155.9
PSD4	340.6	367.4	499.1	442.1	352.5	502.7	301.8	346.6
PSEN1	233.3	252.9	132.3	140	131.5	110.8	206.7	239.9
PSEN1	254.3	338.9	108.7	97.1	95.3	105.9	236.8	327.9
PSEN2	330.7	451.8	265.6	241.1	167.5	258.5	297.2	346.3
PSEN2	489	588.8	361.3	313.4	240.7	331.9	433.9	549.1
PSENEN	315.5	389.6	189	142.6	117.8	122.9	271.4	400.8
PSF1	118.6	162.4	96	84.3	79	94.9	102.2	133.6
PSG1	87.3	93.5	87.5	79.6	77.9	78.6	86.3	94.9
PSG11	83.8	82.1	94.3	79.6	79.5	87.4	88.1	89.1
PSG2	75.5	76.7	96.1	96.8	92.7	95.8	85.8	84.3
PSG3	81.6	99.4	81.6	75.6	68.5	74.1	93.3	103.9
PSG4	82.5	82.3	96.4	86.3	91.8	92.6	82.9	85.8
PSG4	107.2	138.6	100.1	83.2	81.4	90.9	114.7	108.5
PSG4	67.3	87.1	95.8	80.8	83.3	89.4	76.4	78.2
PSG5	71.1	81.5	96.4	90.8	80.9	87.6	74.8	79.7
PSG6	81	82.9	101.4	76.8	78.8	87	81.7	86.2
PSG6	83.6	97.6	100.5	98.5	98.8	102.7	81	97.7
PSG6	84.2	89.2	92.8	91	84.4	84.5	81.7	89.5
PSG7	99.1	106.2	91.6	85	79.7	91.4	89.8	98.3
PSG8	80.6	82	100.7	93.3	90.3	87.7	79.2	79.3
PSG9	82.2	105.4	81.2	89.5	77.5	83.5	88.8	92.3
PSIP1	101.5	114.9	93.3	91.5	87.9	81.4	95.2	105.3
PSIP1	1068.8	1225.7	557.3	555.3	554.8	912.8	653.5	1223.5
PSITPTE22	85.5	91.7	83.5	87.4	72.6	81.1	87.1	92.8

PSKH1	92	107.7	89.2	92	86.8	82.3	92.1	113.3
PSKH2	82.7	87.9	76.2	69.7	70.9	76.9	82.2	100.9
PSMA1	145.8	165.7	95.1	98.5	84.4	89.6	125.6	146.1
PSMA1	986	1249.5	447.6	371.2	236.8	334.5	933.6	1049.2
PSMA1	8527.7	9706.9	4720.9	3416	2471.1	4005.9	8172	12064
PSMA2	709.5	864.9	408.5	410.3	307.8	416.1	657.9	1152.7
PSMA3	4583	5719.8	1407.9	881	783.2	1171.9	4763.3	6641.9
PSMA3	1880.5	2739.3	427.5	280.2	241	383.7	2248.7	3566.4
PSMA4	6191.2	6741.6	1755.7	1295.1	1200.3	1807	5261.2	6957.8
PSMA5	12532.4	15627.2	8517.7	4276.8	3105.5	4305.7	12127.5	17370.5
PSMA6	14777.5	15794.6	12192	11068.8	8695.9	12823.5	13468.6	17822.2
PSMA8	80.1	89.8	94.2	91.6	82.9	79.9	75.2	78
PSMAL	87.7	100.3	87.9	95.2	83.9	81.5	85	93.2
PSMB1	17851.5	21391.3	9520	8541.3	7328	9771.9	18266.6	21205.2
PSMB10	12459.4	14881.1	6535.7	4996.3	4643.7	5411.8	12011.5	11142.8
PSMB2	3361.8	4835.2	2521.9	1998.6	1568.8	1964.4	3042.1	3413.8
PSMB3	9383.6	10799	8467.9	7044.8	5296.5	7643.6	8265	10998.1
PSMB4	4929.5	5820.4	4165.9	3141.1	2423.5	3427.2	5139.8	8149.7
PSMB5	3188	4529.7	1525.6	1023.6	807.2	1242.6	2880.8	3605.8
PSMB6	5573.5	6664.3	3812.5	3208.3	2737.4	2788	5896.7	4851.1
PSMB7	6176.8	9526.8	5013.5	4008.4	3381.6	4886.6	7500.1	8388.3
PSMB8	3016.7	4200.2	968.5	998	861.4	1293.4	2176.9	2294.3
PSMB8	1205.6	1526	252.6	308	239.9	388.1	944.7	1084.8
PSMB8	95.6	113.3	106.7	94	86.2	94.7	100	105.3
PSMB8	4146	5782.8	3392.6	3212.2	2812.5	3942.5	3267	3569.2
PSMB9	1049.3	1321	678.4	525.6	450	598.2	794.3	770.9
PSMB9	158.2	193.8	100.6	91.8	89.9	102.5	147.3	152
PSMC1	9804.3	12863.8	7770.7	6874.2	5706.9	9065.5	9912.6	12409.6
PSMC2	4723.5	6466.4	2840.8	3292.9	2602.6	4080.3	4830.1	7781.6
PSMC3	3424.3	4501.1	1650.5	1627.9	1215.4	1583.5	3596.9	3717.1
PSMC3IP	356.3	407.9	162.9	109.4	105.3	109.6	288	375.6
PSMC3IP	365.8	482.1	283	234	203.7	219.2	277.8	396.1
PSMC4	1435.5	1822.5	674.6	351.2	310.7	314.1	1086.9	1352.1
PSMC4	1200.9	1568.3	488.7	274.5	258.1	278.4	1015.4	1061.7
PSMC5	3972.1	5126.7	976.6	818.1	786.3	1288.9	3759	4848.4
PSMC6	124.5	129.7	104.4	104.2	86.1	109.2	111.4	220.4
PSMD1	1401.5	1565.4	1341.5	1666.4	1243.1	2204.8	1629.7	3186.7
PSMD10	1997.2	2772.2	1234.8	1373.3	920.8	1889.8	1645	2541
PSMD10	268.7	348.3	106	102	97.7	126.5	231.9	317.6
PSMD10	790.2	1034.3	333.9	424.8	328.7	565.9	643.1	831.3
PSMD11	244.2	306.1	152.9	141.8	102.7	146.5	252.7	385.6
PSMD12	2082.8	2354.3	1299.4	1533.8	898.8	1760.9	1917.3	3495.7
PSMD13	188.6	257.5	120	113.9	95.1	104.2	204.2	200.4
PSMD13	273.1	348.2	174.7	165.5	150	193.3	226.8	285.4
PSMD14	3086.3	3887.6	1214.5	995.9	580.8	1348.6	3174.8	5566.6
PSMD2	1365.1	1608.9	991.2	953.2	859.6	1194.3	1206	1636.8
PSMD3	234.8	336.2	179.7	172.4	168.6	201.2	263.9	366.1
PSMD4	163.9	192	179.4	146.9	150.6	125.7	160.4	276.3
PSMD4	2999.9	4410.1	1851.2	1520.2	1166.4	2060.8	3248.8	3109.2
PSMD4	4166.1	5420	3413.6	2729.7	2234.5	3514.5	4306.3	4233.3

PSMD5	390.6	467.9	251.6	141.9	132	166.5	319.7	346.8
PSMD6	4831.1	6108.3	2371.8	2125.8	1593.8	3040.7	4468.9	8132.1
PSMD7	2780.7	3611.6	2393.6	1787.1	1493.6	2506.3	2632.9	5467.8
PSMD8	1502	1963.4	779.7	513	482.4	576.3	1636.6	1428.4
PSMD9	204	250.8	175.6	180.1	139.5	180.5	188.1	262.6
PSME1	7223.4	9185.7	4726.4	3057.8	2266.4	3178.6	5918.6	9385.8
PSME1	143.9	163.5	98	98.6	98.2	91.4	132	182.5
PSME2	5620.2	6928.6	1988.4	1382.1	1128.2	1574.2	5198.9	5434.6
PSME3	1207.6	1454.3	639.7	886.9	546.8	783.9	850.7	1004.1
PSME3	851.9	1034.3	699.4	928.3	603.5	1029.3	664.7	1202.6
PSME4	1488	2033.2	889.2	1072.1	756.5	1340.9	1639.3	1872.1
PSMF1	115.5	126.7	90.9	87.4	86.3	88.3	117.8	144.1
PSMF1	1497.8	2004.3	1000.6	1164.6	896	1187.5	1188.7	1308.5
PSORS1C1	209.9	266	121.5	154.4	118	143	177.8	201
PSORS1C2	97.7	106.6	113.2	94.7	95.8	101.3	100.7	104.6
PSPC1	964.9	1116.5	303.8	224.8	170	290.8	728.7	1146.7
PSPH	219.1	337.6	148.3	122.7	104.8	135.3	234.5	188.9
PSPN	77.1	100.1	87.9	75.5	75.6	78.6	90.1	111.6
PSRC1	1757.8	1914.5	1071.3	359.8	346.6	431.9	1281	1339.1
PSRC1	239.7	357.1	122.9	83.1	96.7	116.5	228.8	329.2
PSRC1	111	116.9	118.4	104.4	101.9	104.6	94.8	110.2
PSRC2	148.7	165.6	124.6	131.3	120.8	172.5	114	145
PSTPIP1	324.6	340.5	242.4	247.4	223.6	277.1	243.5	285.7
PSTPIP2	357	427.1	154.7	161.5	159.6	230.3	248.3	312
PTAFR	131.2	141.7	80	87.8	89.6	99.4	130.4	143.2
PTBP1	10043.9	12257.8	7389.3	6925.4	4647.6	6384.2	9195.2	10049.9
PTBP1	15256.4	18095.3	14117.1	12594.3	10041.7	13843.1	13175.1	14724.3
PTBP2	317.3	349	187.4	217.8	217.9	307.7	240.7	460.4
PTCD1	1104.9	1382.9	783	513.5	400.5	545.4	871.2	848.6
PTCD2	547.4	758.3	372.4	315.6	263.2	375.3	442.5	622.9
PTCH	110	119	124.1	112.8	110.2	116.9	113.3	121.8
PTCH2	114.8	143.2	96.8	95	89.8	90.2	123.5	139.5
PTCHD1	81.1	89.7	86	83.7	80.6	80.9	80.5	83
PTCHD3	103.8	114.7	103.4	102.2	103.6	102.3	102.4	110.6
PTCRA	112.8	128	103.9	102.5	91.5	91	118.4	117.1
PTD004	87.7	95.2	93.6	93.7	84.6	87.7	101.3	93.7
PTD004	107.4	121.4	97	110.3	113.1	138	93.3	132.7
PTD008	3196.5	3775.3	1713.9	1269.4	953.4	1033.1	3413.5	3669.4
PTD015	737.8	949.1	337.7	319.9	277.6	289.6	690.6	719.8
PTDSR	97.9	123.2	96.7	91.1	88	78.6	99	103.7
PTDSR	230.3	246.4	151.2	122.2	121	187	234.7	304.2
PTDSS1	4877	6329.2	4779	2705.8	1771.1	3324.3	3713.5	6226.6
PTDSS2	160.2	182.2	152.4	113.8	99.1	148.7	177.1	219.3
PTEN	82.6	85.5	97.7	170.7	149.1	190.2	85.4	91.6
PTER	76.5	83.6	81.5	82.5	78.4	78.7	85.9	91
PTER	93.7	98.1	99.3	89.2	95.3	91	100.9	113.5
PTER	124.1	159.4	108.6	109.7	97.3	105.8	114.5	123.5
PTF1A	102.1	108.5	93.2	103.9	81.3	84.9	94.4	109.7
PTGDR	70.2	82.4	96	91.3	93.6	95.1	75.4	87.3
PTGDS	93.2	105.2	87.4	202.9	171.1	174.6	93.3	108.1

PTGER1	148.4	178.2	115.2	111.7	96	101.2	152.2	146.9
PTGER2	97.7	102.2	105.2	103.9	101.5	103.6	84.7	93.5
PTGER3	108.9	124.3	94	88	89.3	86.8	89.7	96.6
PTGER3	96.6	105.5	92	87.1	81.5	91.1	95.2	95.5
PTGER3	92.1	95.6	112.3	101	90.4	99.3	87.2	93.7
PTGER3	77.1	73.3	90.9	75.3	82.9	75.2	73.3	80.4
PTGER3	88.1	98	82.9	88.9	74.9	82.1	93.8	109.4
PTGER3	90.5	93.4	89.5	91.2	86	90.6	79.6	96.1
PTGER4	1386.2	1844.7	528.8	968.9	864.5	1104	992.4	1309.7
PTGES	77.8	85.1	85.7	83.7	80	93.5	77.1	84.6
PTGES2	746.6	1078.5	648.5	316.5	268.4	381.1	652.3	830.4
PTGES2	1483.8	2118.4	993.6	538	430.6	582.9	1276.1	1134.3
PTGES3	2506.7	2577.7	1642.3	1418.8	943.6	1723.7	2174.2	4366.7
PTGFR	100.7	131.1	88.4	80.7	80.6	82.1	110.5	121.4
PTGFR	103.1	125.5	95.4	87.6	85.5	84.3	106.6	123.4
PTGFRN	139.8	179.7	92.3	85.4	78.7	93.2	146.7	253
PTGIR	96.2	105.8	104.2	97.7	100.9	115.4	85.9	99.7
PTGIS	76.5	89.2	90.5	80.9	74.6	78.4	77.2	77.7
PTGS1	303.6	317	202.7	224.5	180.2	246.9	247	318.8
PTGS1	136.9	184.4	119.3	94.2	93.2	93.2	126.1	158.2
PTGS2	188.3	210.3	160.1	154.3	135.1	152.6	196.9	292.9
PTH	95.2	95.4	76.3	74.7	66.7	76	93.2	102.2
PTHLH	95.9	102.8	76.3	93.6	88.5	82.2	100.3	118.3
PTHLH	95.7	107.6	95.1	87.2	82.1	92.1	90.9	99.3
PTHR1	113.1	128.1	112.1	109.9	112.6	98.7	97.5	111.6
PTHR2	93.7	88.6	93.7	82.4	83.2	87.8	88.6	99.2
PTK2	1167.7	1575.6	917.9	219.1	157.2	253.8	1076.1	1311.7
PTK2	94.3	107	89.4	88.1	78.1	83.2	95.5	95.2
PTK2B	174.8	222	116.2	104.2	102.3	114.3	156.6	179.8
PTK2B	307.3	346.1	182.1	168.7	148.5	212.9	234.2	362.5
PTK2B	97.8	111.8	101.1	85.6	94.2	104.4	98.5	98.5
PTK6	143.1	142	145.8	135	138	135.4	112.2	124.5
PTK7	92.4	108.7	115.7	107.3	105.3	103.5	85	98.3
PTK7	141.6	153.4	106.4	107.9	80.2	102.9	132.1	166.3
PTK9	111.9	130.6	97	88.4	91	103.9	108.2	127.9
PTK9	194.5	233.2	114.8	103.8	92.1	104.4	160.9	223.3
PTK9L	830.8	1144.1	949.3	639.7	553	785.6	903.4	1273.2
PTMA	1889.9	2581.8	907.4	776.3	696.4	1231.8	2437	5538.4
PTMS	149.4	182.5	182.4	182.7	141.8	193.8	155.3	302.1
PTN	520.9	752.1	226.9	232.6	191	261.1	503.3	598.4
PTOV1	1809.9	2012.7	1655.1	1197.3	933.4	1205.5	1813.4	4222.1
PTP4A1	134.9	151.7	86.5	93.1	93.2	100.8	127.7	169.4
PTP4A2	4278.6	5363.8	2785.4	4196.2	3175.8	4095.6	4156.8	5142.4
PTP4A3	221.3	288.7	169.3	133.9	123	139.6	191	239
PTP4A3	218.1	296.4	178.3	163.9	144.2	122.5	181.6	229.8
PTPDC1	89.8	95.7	98	89.5	84.6	82.4	87.3	87.9
PTPDC1	143.6	174.1	149	153.3	126.2	200.3	148	201.3
PTPDC1	85.2	78.9	84.8	81.9	90.8	81.5	80.6	89.5
PTPLA	627.6	815.3	576.8	384.4	282.9	442.6	591.6	1139
PTPLAD1	1982.3	2316.7	1241.4	1192.6	886.8	1017.1	1396.9	2743.3

PTPLAD2	560.3	609.6	277.4	366.3	399.5	334.9	520.1	608.3
PTPLB	1264.5	1484.5	811.4	973.6	666.2	1046.6	1091.5	1778.7
PTPN1	836.4	1094.5	741.2	1204.3	973.9	1311	747.4	1066.3
PTPN11	2224.2	2743.4	1044.9	1560.3	1009.7	1989.6	2109.3	2947.5
PTPN12	1647	2115.6	737.6	1166.7	958.7	1918.8	1474.6	1964.4
PTPN13	71	80.4	87.9	93	83.5	97.5	74.2	80.6
PTPN14	82.5	88.4	101.2	97.5	89.5	99.7	80.9	92.5
PTPN18	155	192.6	107	98.3	93.1	100	151.2	190.3
PTPN2	95.2	90.7	83.5	73.8	74.5	85.2	82.2	98
PTPN2	802	979.3	429.2	448.1	408.6	621.1	429.3	589.3
PTPN20A	70.7	75.5	74.2	79.4	77.5	71.2	70.8	73.2
PTPN20B	73.1	76.8	85.7	82.5	82.7	82.9	74.6	75.3
PTPN21	83.7	87.6	88.4	81.1	78	82.3	79.2	92.3
PTPN22	181.3	216.7	139.5	113.6	105.1	127	102.8	113.6
PTPN22	80.7	93.8	85.4	79.4	77.4	86.7	90.4	90.2
PTPN22	291.4	338	172.4	154.4	124.5	200.3	164.7	205.7
PTPN23	104	128.6	146	146	134.8	166.3	103.4	126
PTPN3	157	191	111.4	113.7	105.8	116.2	138.3	170.4
PTPN4	192.5	191.8	146.6	179.3	133.9	196.6	157.6	215.6
PTPN5	119.2	158.5	98.5	94.2	82.4	84.2	131.7	150.1
PTPN5	114.2	143.2	99.4	91.5	91.7	82.2	119.2	152.3
PTPN6	108.6	126.8	78.5	90.7	83	83.4	108.4	136.4
PTPN6	1284.1	1671.2	1446.6	865.1	708.3	1607.2	1147.1	1786.6
PTPN7	122.3	133.1	117.1	117.1	106.4	122.1	127.7	135.1
PTPN7	1224.7	1438.9	1330.6	722	572.2	937.1	1051.9	2105.1
PTPN9	137.1	176	178.7	150.7	145.1	185.7	144.6	259.9
PTPN9	121.3	141.4	104.3	93.5	97.7	95.6	103.1	121.7
PTPNS1	1635.6	1975.4	735.5	857.4	897.3	969.2	1433.8	1995.8
PTPNS1	165.6	248.3	193.6	241.2	201.5	337.9	161.4	237
PTPRA	92.3	106.6	104.4	91.4	87.4	93.6	88.7	110.3
PTPRA	289.7	384.7	211.7	251.4	228.2	314.9	260.8	317.9
PTPRA	276.4	321.6	159.1	184.6	157.4	217.9	235.8	431.4
PTPRA	182.1	204.8	124.6	109.7	109.9	119.5	168.4	176.2
PTPRB	98.1	116.7	98	96	86.9	101.6	95.8	94.2
PTPRC	75.6	82.9	90.3	75.7	79.3	75.8	80.9	74.9
PTPRC	294.9	392.2	119.9	115.1	106.8	93.6	226.5	255.7
PTPRC	377.4	517.3	109.7	119.6	113.7	85.6	268.9	347.9
PTPRC	147.2	174.6	111.7	93	89.3	80.6	136.9	149.5
PTPRCAP	118.5	138.9	112.7	102.7	96.3	106	119.4	138.1
PTPRD	91.7	96.9	97.3	84.4	90.3	91.1	99.4	109.6
PTPRD	83.1	79.8	101.9	87.9	91	86.9	77.7	85.3
PTPRE	437.6	558	499.1	771.1	595.7	907.9	546.5	1219.1
PTPRE	129.7	131.6	99.7	107.5	97.8	93.8	123	144.7
PTPRE	147.3	148	112.9	97.6	85.5	97.2	128.6	159.2
PTPRF	2120	3050.2	839.8	1054.3	1079.6	1261.6	1943.7	1887.2
PTPRF	448.5	624.5	620	983.1	812.5	1120.1	434.7	793.9
PTPRG	155.2	160	104.2	93.3	96.3	104.8	136.2	169.3
PTPRH	133.5	151	101.3	90	90.4	98.2	124.7	138.8
PTPRK	303.5	330.1	277.4	316.1	232.1	408.1	257.2	357.9
PTPRM	75.1	83.9	93.6	80.1	77.8	87.2	72.3	82.6

PTPRN	78.4	87.6	95.8	81.8	82.5	88.7	81.1	94
PTPRN2	319.7	387.4	208.1	210.8	161.5	269.8	266.4	385.4
PTPRO	162.2	208.7	146.1	156.5	120.9	154.7	166.6	268.7
PTPRO	204	250.6	168.4	179.9	131	243.9	200.3	316.4
PTPRR	87.6	102.4	100.3	84.6	92.9	85.1	94.3	107.3
PTPRR	118.1	138.8	125.4	115	117.9	121.1	119.7	116
PTPRS	99	109.6	91.4	85.4	83	97.6	111.1	113.8
PTPRS	76.4	96	92.1	86.7	97.2	91.3	87.1	107.1
PTPRT	91.4	102.7	87.1	89.2	77.9	80.5	103.8	106.9
PTPRT	143.3	176.3	89.1	90.7	90	86.4	128.1	155.1
PTPRT	90.1	97.5	71.8	83.6	84.6	81.2	82.2	93.1
PTPRU	123.1	122.2	103.5	100.9	79.4	101.8	95.6	120.8
PTPRU	86.3	134.5	84.1	90	79.9	76.4	103.3	114.5
PTPRZ1	127.8	137	87.8	90.8	95.1	89.6	122.4	127.5
PTRF	117.6	147.1	80.8	81.8	75.8	87.6	153.6	187.2
PTRH1	380.2	492.9	265	186.9	173.2	182.6	310.7	304.5
PTRH2	1818.6	2104.8	1284.5	1326.1	911.8	1376.6	1546.7	2213.3
PTRH2	220.2	269.3	168.5	139.2	133.1	171.5	177.4	233.3
PTS	1151	1361.8	531.8	534.2	421.6	649.7	1241.1	2035.9
PTTG1	7755.5	10025.4	5527.5	2401.2	1668.8	1808.9	6876.2	7355.2
PTTG1IP	3952.1	5116.5	1986.2	3123.8	2442.4	3329.7	3544.9	5063.1
PTTG2	380.2	465	209	199.6	171.4	186.6	321.6	381.8
PTTG3	3691.9	4790.8	1818	649.3	543.4	648.7	3604.2	4870.8
PTX3	95.4	101.9	93.6	90.8	76.3	81.4	84.6	95.4
PUM1	1423.8	1903.1	1389.5	2247.5	1634.4	2412.6	1277.2	1682.1
PUM1	2145	2551.1	1442.7	1810.8	1339.4	1632.3	2136.5	2946.1
PUM2	1087.8	1542	1085.5	1230.1	896.9	1865	1038.4	2072.9
PUNC	76	86.9	83.6	81.4	73.8	82.5	77.7	97.3
PURA	495.3	636.5	372.9	423.4	288	669.3	454.5	1062.1
PURB	1429	1889.6	1124.4	2282.4	1777.7	2468.3	1212.9	2388.1
PURG	109.6	115.6	155.9	152	140	154.3	107.1	126.5
PURG	105.3	107.2	78.7	81.5	79.8	89.5	88.4	115.6
PUS1	3772	4490.1	2569.9	1355	1194.5	1457.7	2795	2670.6
PUS1	124.6	163.4	109.6	95.7	91.2	101.6	130.8	137.6
PUS1	569.3	661.6	405.9	204.2	203.9	209.5	473.7	697.9
PUS3	407.4	500.6	162.1	121.9	113.4	172.7	382.4	710.6
PUS7	1187	1604.8	684.7	676.6	453.2	968.5	920.8	1096.5
PUS7L	283.9	316.5	275.9	241.5	156.7	299.7	231.4	397.9
PUSL1	300.9	383.1	274.9	227.2	183.3	240.3	283.2	483.9
PVALB	77.3	80.1	80.8	80.2	82.2	90.9	74.2	78.9
PVR	241.3	280.6	149.4	137.9	116.8	164.3	228.9	282.9
PVRL1	105.2	148.3	97.8	106.6	90.2	99.1	96.9	118.3
PVRL1	132.6	154.8	106.8	95	94.5	102.4	130.5	205.8
PVRL1	94.6	93.7	103.6	79.4	77.9	92.3	108.1	103
PVRL1	79.4	78.5	81.3	87.6	82.9	78.3	79.2	87
PVRL2	314	402	241.6	122.9	127.3	113.6	342.8	530
PVRL2	344.5	483.4	387.2	171.7	169	210.6	383.2	624
PVRL2	120.1	145.1	121.8	95.1	97.8	99.4	112.4	151.9
PVRL3	144.2	133.6	101.7	97	83.4	95.9	123.7	163.3
PVRL4	102.2	119.1	90.5	77.3	85.9	96	97	104.3

PWP1	2065.9	2473.8	1210.1	1319.4	859.1	1618.8	1589.7	2565.5
PWP2H	139	155.2	104.7	91.4	88.9	94.6	148.4	150.2
PWWP2	208.4	285.5	270.4	189.9	201.9	262.7	224.8	416.5
PWWP2	109.7	142.4	117.6	89.1	93.6	103.2	116.8	165.1
PXDN	113.4	136.9	83.6	88.8	83.8	84.9	108.5	155.7
PXDNL	83.5	96.5	93.4	82.6	87.9	89.3	84.5	85.3
PXK	335.1	497.9	228.1	287.5	294.3	448.8	485.5	648.3
PXMP2	1853.1	2437.1	1653.3	1461.8	1142.3	1554.5	1408.1	2078.2
PXMP2	794.1	987.6	550.1	467.6	389	484.4	584.6	668.2
PXMP3	1494.6	1872.1	877.3	683.9	477.8	1013.2	1223.2	1890.2
PXMP4	222.9	263.6	158.7	150.4	130.7	129.7	177.3	222
PXMP4	158.1	170.8	144.9	168.4	132.8	118.8	154.6	183.7
PXN	98.1	105.4	109.9	106.2	100.7	107.4	100	110.8
PXT1	76.2	84.4	89.4	84.7	75.9	78.8	75.2	77.1
PYCARD	852	1006.4	444.5	336.2	311	361.1	674.7	670.1
PYCARD	8221.2	9958.5	7944.8	6685.1	5248	7214.8	6498.5	7475.6
PYCR1	89.9	114.4	99.2	84	77	87.2	97.8	110.3
PYCR2	2209.1	2762.1	1336	1004.5	819.2	1130.7	1700.7	2678.3
PYCRL	143.9	173.8	98.8	103.4	81.2	94.3	121.2	188.6
PYDC1	88.6	94.2	96.5	87.1	87.8	85.3	96.5	100
PYGB	372.9	486.8	394.6	245.6	204.7	358.6	344.7	592.1
PYGL	2096.6	2504.2	594.1	434.4	380.9	693.4	1563.9	2857.1
PYGM	111.4	133.8	96.9	90.2	92	98.9	109.4	128.5
PYGO1	93.9	107.8	109.2	104	107.4	101	96	104.7
PYGO2	121.4	162.1	146	129.4	112.3	134.4	124.9	178.7
PYHIN1	111.5	127	103.3	95.2	92.8	104.1	118.5	119
PYHIN1	105.4	124.2	101.8	110.1	105.6	123.2	107	104
PYHIN1	120.6	163.7	131.3	132.4	130.1	157.4	116.1	121.7
PYY	81	91.5	87.6	88.4	88	84	86.6	94.6
PYY2	67.7	80.1	70.4	76.7	72	80.8	80.6	73.6
PZP	103.7	122.1	92.9	91	90.9	91.3	97.1	107.2
QARS	8919.2	11544.4	5075.3	5000.4	3673.4	5575.7	7576.9	7489.8
QDPR	2124.1	2841.3	1391.6	998.9	747.2	1059.3	1839.5	2397.9
QIL1	3544.5	4453.4	2147.1	1542.7	1220.9	1960.6	2822.5	3279.2
QKI	290.2	409.9	96.6	103.8	90.1	93.3	245.7	318
QKI	297.5	435	144.7	136.2	144.6	171.9	217.2	263.1
QKI	291.7	414.1	141.3	162.3	148.5	169	223.2	262.8
QP-C	13735.8	15677.8	11830.8	10985	8794.4	12597.4	11023.8	14170.4
QPCT	263.8	304.4	165.1	156.9	127	234.6	229.4	370.9
QPCTL	151.7	178	144.9	147.5	134.5	99.4	138.8	179.3
QPRT	4186.9	4905.7	2835.7	2805.9	2696	2996	3678.1	3623
QRICH1	124	152.2	106.8	88.6	84.6	98.9	109	156.5
QRICH1	71.8	90.6	93.5	88.7	82.8	81.4	82.6	86.7
QRICH1	1652.3	2381.1	1807.1	2147.5	1855.8	2587.8	1382.8	1710
QRICH2	75.9	84.5	94.5	84.5	78.3	77.8	76.5	90.1
QRSL1	214.1	231.2	136.6	155.1	122.3	157.5	164.7	242.5
QSCN6	1348.4	1449.9	711.5	542.7	405	710.9	1263.8	1786
QSCN6	219	305.7	242.2	221.9	170.2	346.6	184	301.5
QSCN6L1	407.7	480.6	350.8	265.6	254.2	320.3	294.6	347
QSER1	224.6	275	115.9	117.5	91.3	107.4	217.2	268.5

QTRT1	347.5	398.8	163.2	119.7	121.4	159.2	277.9	337.2
QTRTD1	520.4	642.2	193	178	143.2	279.9	419.1	580.9
R3HDM1	191	234.8	133.3	124.3	114	156.6	184.2	276.3
R3HDM2	134.3	170.8	122.3	126.9	118.7	127.4	139.2	152.3
R3HDML	89.4	93.3	88.9	91.7	82.4	78.4	91.6	109.8
RAB10	4399.5	5465.6	2718.5	3722.1	2395.5	4200.5	4050.4	5490.1
RAB11A	3555.9	5122.9	2879.5	3004.3	2102.3	3198.5	3041.9	3978.5
RAB11B	116.5	155.1	116.7	93.1	107.1	85.9	111	150.8
RAB11FIP1	100.4	113.3	100.9	81.3	86.6	85.3	99.1	125.1
RAB11FIP1	144.2	198	115.1	119.5	104	107.9	147.6	284.8
RAB11FIP1	2327	2846.1	1455.7	2342.5	1792.6	2599.5	2174.3	2847
RAB11FIP2	460.7	536.4	299.3	295.8	250.7	376.8	329.1	633.1
RAB11FIP3	420.8	515.8	399.6	349.4	358	468.9	346.4	386.7
RAB11FIP4	82.6	93.8	89	80.9	90.9	87.6	84.9	97.3
RAB11FIP5	373	426.2	338.2	242.7	241.9	251.6	371.2	385.5
RAB12	109.5	130	101.6	85.2	87.8	92.7	104.8	128
RAB13	329.4	390.5	159.3	131.7	124.7	116.8	357	360
RAB14	110.2	120.3	115.8	106	89.4	100.3	110.9	134.1
RAB15	140	198.4	104.1	97	92.7	95.8	147	204.4
RAB17	292.2	358	260.8	184.8	178.6	221.9	237.3	174.9
RAB18	155	168.2	121.4	113.1	103.1	151.4	137.3	294.5
RAB19B	83.4	97	87.7	86.4	84.7	88.5	89.1	96.8
RAB1A	428.1	588.9	268.9	375.2	259	383.8	392.6	552.1
RAB1B	246.5	322.3	203.4	187.7	190.4	161.3	245.1	430.2
RAB2	870.5	1217.9	462.4	428.1	301.9	403.5	796	1280.7
RAB20	132.6	169.3	116.7	118.1	114.7	118.8	139.7	161.3
RAB21	782.7	1003.9	327.8	365.8	267.8	463.2	730.2	1223.2
RAB22A	605.9	942	423.1	996.2	705.8	1034	559.1	644.4
RAB23	84.2	84.3	87.7	81.6	81.7	85.9	83	86.5
RAB23	101.9	125.5	88.5	86.2	80.9	87.4	108.1	125.3
RAB23	1119.4	1471.2	784.5	698.6	548.7	818	922.8	1457.5
RAB24	246	268.8	175.1	181.1	177.7	218.6	189	191.8
RAB24	820.4	1009.7	669.9	728.8	707.5	1221	644.2	857.2
RAB24	596.3	661.7	240	346.1	331.9	444.7	497.7	604.5
RAB24	171.7	242.5	113.9	120.4	110.5	113.6	170.1	178.5
RAB25	134.1	122.7	89.1	93.9	94	91	107.6	121.2
RAB26	149.1	168	114.8	110.5	96.7	105	130.5	133.8
RAB27A	335.7	317.3	282.2	301.3	223.5	327.9	342.6	429.2
RAB27A	143.7	182.4	116.4	110.5	105	117.5	141	128.2
RAB27A	122.4	156.1	94.4	109.4	91.6	109.4	93.9	114
RAB27B	106	120.3	90.1	90.7	87.3	93.6	108.6	116
RAB28	393.4	636.2	251.2	190.4	162.2	236.2	349.7	505.5
RAB28	341.2	461.5	275.8	232.4	193.7	269.1	263.2	349.6
RAB2B	1217.7	1524.8	820.3	647.6	640.6	789.4	1248	1853.5
RAB30	80.7	111.2	90.8	75.8	86.5	86.3	85.7	99.8
RAB31	4002.5	4722.9	3484.8	6705	4221.8	7524.6	3625.2	5231.1
RAB32	2558.1	3298.5	2561.3	2301.9	1935.5	3077.5	1854.1	3262.8
RAB33A	401.9	543.3	319.3	258.1	246.8	352.7	348.7	370.4
RAB33B	146.3	148.5	102.5	109.3	101.8	118.5	139.5	192.5
RAB34	79.2	87.9	83.4	85.8	80.3	84.3	82.2	88.3

RAB35	852.8	1089.1	436.1	402.1	363.9	425	820.5	680.1
RAB36	84.9	99.5	80.5	85	75.2	82.6	84.9	104.8
RAB37	130	111	118.9	208	219.4	271.3	116.5	144
RAB37	103.6	107.9	105.6	151.5	157.9	155.5	100.5	110.8
RAB37	117.4	132.7	96.5	97.2	88	85.7	117	139
RAB38	150.6	201.1	126.5	126.2	110.6	131.8	167	232.3
RAB39	92.1	117.3	101.3	88.8	85.2	92.6	95.2	104.1
RAB39B	74.9	79	85.1	77.6	73.2	80.4	71.5	77.5
RAB3A	105.6	111	92.6	74	81.1	83.8	103.5	123.9
RAB3B	102.9	132.7	106.6	97.7	94.5	87.2	103.3	109.7
RAB3C	123.6	155.4	113.5	256.8	223.3	367.2	130.7	128.8
RAB3D	110.7	137.4	102.5	115.3	86.2	105.1	123.1	115.9
RAB3GAP1	1078.7	1521.9	1018	1714.9	1358.6	1809.6	962.2	1581.8
RAB3IL1	834.6	1152.3	667.3	393	311.5	430.2	752.7	836.7
RAB3IP	236.2	268.8	162.1	216.3	224.3	232.5	185.3	196.7
RAB3IP	244.5	324.9	235.9	267.8	217.6	252.8	242.4	331.5
RAB40A	68.2	79.8	94.1	99.2	91.6	91	81.4	82.1
RAB40B	552.8	676.4	345.3	532.7	453.4	598.7	508	622.8
RAB40C	2632.5	3392.6	2010.4	1508.2	1450.4	1593.7	2046.5	2074.5
RAB41	100.7	117.9	101.3	86.5	86.3	89	103.5	116.3
RAB42	100.6	110.9	116.4	111.4	102	101.4	92.7	92.6
RAB43	153.4	173.4	138.6	168.1	159.1	207.2	146.5	208.1
RAB4A	912.6	1257.8	623.4	523.8	398.6	633.8	666.4	1253.1
RAB4B	229.6	281.3	202	165.5	171.7	185.9	219	286.1
RAB5A	772.6	1029	380.1	387.6	290.3	537.8	687.8	1180.5
RAB5B	1027.6	1418.1	1021.2	1259.8	1240	1329.7	890.4	1266.3
RAB5C	1089	1428.3	547.3	556.6	492.5	893.5	859.9	1868
RAB5C	804.1	1180.5	430	379	339.3	521	749.4	1170.8
RAB6A	132.9	162.5	89.2	91.4	90.3	108.3	128.7	217.2
RAB6B	173.3	203.2	115.3	121.5	121.7	128.7	154.2	186
RAB6C	83.3	86.3	86.3	87.3	81.6	82.7	84	92.6
RAB6IP1	432.6	555.6	520.8	542	539	839.9	621.9	1350.2
RAB6IP2	96.3	110	78.7	81.3	77.3	80.6	94.9	104
RAB7	5158.6	6251.2	3032.6	4258.5	3239.1	5765.5	4539.5	5286
RAB7B	503.6	589.3	385.9	469.2	485.1	517.5	560.8	781.5
RAB7L1	592.9	847.3	591.5	393.3	325.1	563.7	471.1	892
RAB8A	2359.2	3260.6	1693.1	1480	1086.9	1604.8	2153.3	3018.7
RAB8B	1008.7	1367.3	346.2	577.4	581.9	884.2	1048.3	1748.3
RAB9A	1109.7	1345.3	650.7	946.3	729.9	1114.4	924.5	1508.7
RAB9B	193.6	257.8	142.9	169.8	149.7	151.9	173.2	185.5
RABAC1	414.4	529.6	336.3	307.3	237.8	303.5	430.7	588.5
RABEP1	368.9	543.9	437.4	512.4	379.8	946.9	277	505.1
RABEP2	93.6	115.5	91.1	79	87.7	83.9	93.5	113.7
RABEP2	103.9	125	89.4	92.1	84.8	85.8	107.6	130.2
RABEPK	1507	2016	644.7	542.8	338.7	550.4	1199.1	1500.4
RABGAP1	324.5	420.2	362.7	468.5	468.6	507.5	376.2	516
RABGEF1	124.7	131.6	100	102.1	94.7	98.6	122.3	165.7
RABGGTA	123.6	132.7	118.8	98.2	103.3	112.6	115.4	115
RABGGTA	138.8	188.6	134.9	111.1	100.7	124	140.1	171.6
RABGGTB	613.8	759.6	295.2	242	210.9	336.8	477.3	781.3

RABIF	129.6	148.5	102.9	92.5	93.8	115.6	116.4	153.6
RABL2A	117.7	137.5	115.4	113.7	104.6	105.5	120.3	147.8
RABL2A	158	195.4	122.3	126.9	102.4	130.3	129.4	148.8
RABL2B	585.1	674.5	318.8	295.6	275.8	352.9	346.8	372.6
RABL3	912.4	1189.6	417	381.6	313.9	543.1	694	925.2
RABL4	369.3	495	256.4	240.1	188.3	260.5	349.7	487.4
RABL5	98	117.2	92.6	79.7	82.4	79	98.5	112.9
RAC1	8535.4	10709.4	6588	5254.6	4030.6	5255.8	7570.3	12274
RAC1	121.1	137.4	88.9	96.7	85.7	91.4	109.1	142.5
RAC1	75.2	84.5	85.8	82.2	77.4	82.3	77.7	76.2
RAC2	10545.6	12764.2	6463	4591.2	4553.6	4828.8	10480.6	12373.2
RAC3	251.1	282.2	256.3	201.9	208.9	293.1	203.9	307.6
RACGAP1	866.9	1051.7	638	636.1	498.5	725.8	739	1130.7
RAD1	270.1	320	170.5	161.4	148.1	146.9	226	334.8
RAD1	76.8	105.1	92.2	87.5	80.6	82.7	86.7	91.2
RAD17	90.4	102.1	92.8	89	77.6	86.7	89.5	104.1
RAD17	201.9	251.3	181.9	175	157.7	209.4	174.7	244.4
RAD17	112.2	130.2	95	94.1	89.9	76.5	102.3	134.7
RAD18	99.6	108.5	111.8	111.7	90	105.9	96	108.9
RAD21	3109.2	3293.6	978.7	892.9	539.5	1136.5	2613.8	4527.1
RAD23A	1468.5	1993.1	1116.6	671.9	533.7	865.6	1473.5	3123.3
RAD23B	730.5	909.8	675.6	827.9	489.4	902	776.7	1560.7
RAD50	267.6	413.8	249.1	367.5	328.7	589.3	246.5	314.4
RAD50	94.9	92	93.7	87.1	78.5	90.9	94.9	99.3
RAD51	448.9	602.7	295.7	302.9	236.5	335.3	378.8	485.3
RAD51	310.6	374	188.3	125.7	143.7	154.4	245.4	348.5
RAD51	600.1	783.9	210.2	150.2	152.1	178.5	393.9	476.9
RAD51AP1	1302.7	1490.2	692	793.7	673	1043.2	1085.7	1651.3
RAD51C	1815.2	2253.6	1094.7	791	626.5	843.1	1536	2618.9
RAD51C	77.6	77.9	96.9	89.8	78.2	90.2	74.8	85.9
RAD51C	2478.7	3072.6	1273.2	864.7	766.9	1013	1979.2	2421.3
RAD51L1	105.7	121.1	91	83.8	80.4	88.2	101	101.5
RAD51L1	132.5	171.7	97.1	81.2	85.5	90.5	127	150.4
RAD51L1	103.7	145.9	96.1	85.4	78.3	97.5	129.8	137.2
RAD51L3	217.5	266.5	185	138.8	127.6	140.4	188.3	189
RAD51L3	106.5	120	98.3	94.7	86.1	83.9	97.4	96.9
RAD51L3	187.8	239.5	145.8	122.5	118	127.1	160.5	162.9
RAD52	97.9	105.7	99.1	94.2	84.5	98.3	98.9	113.8
RAD52B	108.4	130.9	84.6	88.4	83.5	82.7	114.9	130.4
RAD54B	80.2	82.3	85.8	86.2	77	79.8	79.5	86.1
RAD54B	537	696.9	334	287.3	214.5	425.4	332.6	534
RAD54L	485.5	520.9	230.9	189.1	158.2	233.7	342	494.3
RAD9A	321.2	334.1	306.3	235.9	209.4	231.2	310.5	670.4
RAD9B	94.6	108.5	93.1	89.3	89.3	98	89.4	96.9
RAE1	358.6	409.7	180.7	120.6	114.5	94	303.9	315.8
RAE1	76.4	76.4	78.3	79.9	83.6	72.5	77.7	75.8
RAE1	1441.5	2174.6	1632.9	1111.5	916.3	1201.2	1285.9	2267.2
RAET1E	100.4	119.3	110.3	85.4	80.8	95	104.9	96
RAET1G	90.3	95	96.6	93.8	76.5	88.7	85.7	99.5
RAET1L	79.4	86.1	86.3	82.4	91	94.6	89.3	95.2

RAF1	692.5	877.6	556.5	666.5	577.6	930.1	599.4	1069.6
RAFTLIN	799.9	1092.8	303.2	338.3	231.7	421.1	682.4	759
RAG1	88.9	96.6	83.7	90.7	78.2	81.3	91.8	101.4
RAG1AP1	208.8	250.7	116.2	107.8	112.5	118.4	194.1	268.4
RAG2	80.1	94	87	82.1	80.5	74.9	76.4	83.5
RAGE	163.4	197.6	142.4	112.5	110.4	131.9	142.1	172.6
RAI1	241	310.2	255.6	283.2	227.8	294	228.6	350.4
RAI14	580.7	624	494	356.5	276.1	398	381.8	519.9
RAI16	196.3	207.3	116.3	106.6	98.6	114.9	178.9	237
RAI17	824.4	1178.1	518.3	541	461.5	613.2	910.7	1265.7
RALA	2196.8	2637.4	1343.5	1892.1	1558.9	2399.2	1921.9	2376.4
RALB	2647	3568.2	2124.9	1661.3	1232.8	1784.9	2056.4	2969.8
RALBP1	470.8	604.5	239.8	374.8	315.4	481.1	447.3	617.7
RALGDS	77.1	83.9	86.3	78.5	88.1	90.1	85.3	78.1
RALGDS	1926.2	2302.7	1346.8	939.8	892.3	1164.4	1553.1	1485.2
RALGPS1	96.8	101.1	98.8	107.1	104.3	114.7	87.9	101.6
RALGPS2	103.7	111.9	106.9	107.6	94	103	95.2	111
RALGPS2	90.7	89.3	98	96.3	80.3	86.3	87.5	97.1
RALGPS2	72	88	88.4	83.8	80.3	97.9	87.2	87.8
RALY	1015.5	1383	870.5	944.4	835.2	1007.8	820.4	1235.9
RALY	1425.6	1831	1662.6	1974	1584.4	2507.2	1462	2405.9
RAMP1	76.3	94.4	98.4	95	78.5	93.3	83.6	90
RAMP2	114	133.1	132.5	111	101	113.7	113.8	123.2
RAMP3	83.5	101.9	103.2	89.3	86.4	92.9	84.9	103.4
RAN	1576.3	1931.4	662.7	723.6	490.7	743.3	1045.5	1583.8
RANBP1	8156.7	9198	2906.4	2357.5	1464.6	2103.6	7159.4	9077.4
RANBP10	211.8	255.2	210.4	235.3	202.4	376.1	195.2	346
RANBP17	88.7	93.7	88.9	85.3	91.7	102.9	84.7	95.1
RANBP2	115.1	131.3	89.9	108.2	99.6	97	115.2	146.9
RANBP3	717.8	984.3	482.4	409.9	342.2	438.9	662.9	973.3
RANBP3	229.6	276.9	226.1	165.5	124.1	178.3	274.7	403.3
RANBP3L	85.9	96.4	91.1	90.2	84.8	94.5	87.4	90.4
RANBP5	294.8	429.8	239.5	205.1	156.7	289.6	233.2	287.3
RANBP6	134.9	146	105.5	104.6	102	96.2	122	167.4
RANBP9	175.5	196.5	110.7	111.6	105.1	95.8	156	176
RANGAP1	3217.4	3839.6	2356.3	1321.4	1165.6	1487.5	2710.4	1899.9
RANGNRF	103.3	102.7	96.5	91.2	76.4	83.3	92.3	115.4
RAP1A	249.8	290.1	169	138.9	111.1	160.1	187.8	287.4
RAP1A	419.1	610.3	170.5	172.5	137.9	155.2	280.1	420.4
RAP1B	100.5	113.1	81.7	92.5	85.7	104.9	106.1	131.7
RAP1B	447.3	492.3	255.1	245.7	186.9	289.4	374	749.2
RAP1GA1	223	250.6	214.3	203.3	190.8	230.2	161.6	186.4
RAP1GDS1	530.9	692.3	323.4	270.3	189.7	316.5	437.8	880.1
RAP2A	255.1	323.1	250.7	86.5	92	95.2	248.2	450.3
RAP2B	90.7	107.5	84.6	92.2	90.7	112.1	88.2	113.7
RAP2C	419.4	495.1	192.8	216.3	156.8	242.6	371.6	732.9
RAP80	2077.7	2612.7	1248.6	1468.7	1475.6	1923	1623.2	1508.2
RAPGEF1	94.7	99	107.7	101.4	96.5	99.5	96.8	99.6
RAPGEF1	783.8	1002.5	756	817.7	771.5	811.4	1091.3	1023.5
RAPGEF1	76.3	87.2	78	106.3	109.8	87.4	83.6	82.2

RAPGEF3	115.5	120.6	95.7	88.1	94	92.2	97.1	122.8
RAPGEF4	138.1	186.5	95.5	87.2	85.3	88.3	130.6	155.5
RAPGEF6	302	391.7	296.6	513.7	380.9	790.8	283.6	481
RAPGEFL1	148.6	155.8	126.2	122	130.9	123.5	134.2	158.5
RAPH1	89	100.5	93.4	82	75	86.4	87.4	93.6
RAPH1	97.5	104.3	95.6	92.2	89.3	97	94.5	85.6
RAPH1	80.2	90.1	96.4	86.2	89.3	84.5	88.6	89.3
RAPH1	84.4	81.9	87.7	95.2	89.8	96.8	81.6	85.9
RAPSN	155.9	219.5	107.2	113	113.9	110.4	144.1	172.4
RAPSN	101.8	122.4	99	96.1	84.7	93.6	109.3	115.7
RAPTOR	93.4	99.8	102.3	109.7	87	94.2	102.5	93.1
RARA	104.7	119.4	125.1	114.1	101.1	110.7	107.7	111.9
RARA	759.8	844.7	663.6	987	766.2	778.6	756.7	1525.6
RARA	85.1	104	95.3	93.1	79.1	88.5	89.4	99.2
RARB	106.9	126.4	111.6	109.4	104.2	101.2	94.1	105.6
RARB	77.8	96.2	84	69.9	84.4	84.7	83.5	91.4
RARB	81.1	96.8	98.6	91.6	88.9	86.3	85.6	85.3
RARG	106.4	106.3	100.8	101.9	93.2	93.7	97	98.8
RARRES1	119.4	156.9	90.9	81.6	75.6	92.4	103.3	106.8
RARRES1	126.9	140.8	116.5	104.7	100.4	118.2	111.2	118.7
RARRES1	83.2	102.4	102.5	91.3	96.5	104.9	86.1	88.2
RARRES2	86.5	97.2	96	101.1	89.4	99.2	82.4	87.2
RARRES3	138	176.7	148.4	145.4	146.2	177.2	137	168.1
RARS	3625.5	4435.6	2146.2	2218.9	1443.8	2638.5	2919.6	4179.8
RARSL	1281.7	1656.6	976.1	1110.9	1020.2	1578.1	1094	1583.9
RASA1	87.6	111.2	89.3	77	85.6	83.9	93.1	101.4
RASA1	659.9	980.3	501.3	781.7	700.1	1090.7	527.1	710.7
RASA1	102.7	124.1	89.6	86	93.9	94.2	110.5	114.8
RASA2	175.5	216.6	129.5	105.4	105.2	106.5	146.8	175.6
RASA3	172.9	211	154	149.8	134.8	210.3	178.7	228.8
RASA4	73.3	91	92.2	102.8	103	95.5	84.3	89.1
RASAL1	100.8	109.7	104.5	93	94.6	100.7	94.6	104.6
RASAL2	237	322.8	137.7	157	148	131.6	202.9	244.6
RASAL2	107.8	133	86.4	90.8	99.9	104.8	98.7	108.5
RASAL2	79.1	91	82.7	78.9	79.7	75.5	81.6	84.5
RASD1	103.7	119.8	103.6	92.6	84.2	99.1	108	120.3
RASD2	160.4	181.2	131.3	112.3	102.3	117.5	149.8	171.6
RASEF	85	100.2	97.6	86.9	87.2	86.7	82	98.2
RASGEF1A	91.8	101.8	86.7	86.9	80.6	90.8	80.3	80.2
RASGEF1B	91.7	119.2	91.2	83.3	84.4	84.8	104.7	117.5
RASGEF1C	103.2	103.6	95.6	101.4	85.6	94.8	90.6	100.4
RASGEF1C	73.2	83.3	90.1	89	82.3	81.9	81.8	81.3
RASGEF1C	99	106	97.1	94.2	87.4	95.5	104	97.1
RASGRF1	99.5	112.9	105.7	101.3	101.4	102.6	103.1	109.4
RASGRF1	104.2	117.4	89.6	91	81.4	92.2	89.2	101.6
RASGRF2	87.5	90.5	91.7	90.7	76.1	86.6	86.4	86.7
RASGRP1	135.6	143.6	97.8	94.5	88.4	91	126.6	140.3
RASGRP2	1041.8	1107.2	868.1	660.9	685.7	953.8	690.2	1105.3
RASGRP2	1169.7	1402.4	521.7	455.9	386.8	563.4	761.3	998.4
RASGRP2	276.4	354	181.5	119.1	114.8	100.5	226.5	242.4

RASGRP3	88.1	96.3	91.9	89	97.4	100.2	91.6	91.9
RASGRP4	112.5	109.1	102.6	98.2	91.2	103.6	99.7	106.7
RASGRP4	102.9	130.2	111.6	95.1	93	111.3	98.6	116.8
RASGRP4	73.8	97.7	80.7	84.7	75.8	77	80.1	95.6
RASIP1	102.7	109.4	110.8	100.8	90.6	102.3	99.9	112.5
RASL10B	84.2	89.8	87.5	93.4	75.6	88	81.5	78.9
RASL11A	134.9	141.3	124.2	103	97	104.4	113.2	125.3
RASL11B	195.2	233.5	202.5	111.3	98.8	117.5	154.7	181.8
RASL12	107.3	101.8	92.1	92.7	86.8	89.6	101.5	112.7
RASSF1	500.1	647.4	490.6	322.4	315.5	405.3	471	677.5
RASSF1	862.8	996.4	655.4	433.9	398.1	552.9	658.2	1156.3
RASSF1	88.6	90.3	85	86.9	75.5	81.5	87.6	85.5
RASSF1	290	329.4	179.5	151.8	145.5	134.9	278.1	334.9
RASSF2	413.1	532	387.2	662.5	489	947.3	491.1	1256.3
RASSF2	138.2	158.4	90.3	102.7	95.6	94.6	132.6	148.4
RASSF2	1326.8	1583.9	885.3	1847.1	1356.8	2045	1557.8	2210
RASSF3	118.2	139.4	83.4	96	82.8	93.6	114.9	124.7
RASSF4	497.9	647.1	255.7	179.8	188.2	224.4	345.6	314.7
RASSF4	179.5	239	108.5	94.7	94.8	95.4	156.5	181.3
RASSF5	171.9	255.6	98.9	100.4	99.4	83.5	143.3	181.2
RASSF5	175.5	216	91.1	103.4	94.9	79.7	151.4	174.6
RASSF5	1086.5	1459.3	968.5	832.1	534.2	990.5	965.8	2410.1
RASSF6	229.7	257.9	133.5	129	151	141.2	204.9	273.2
RASSF6	101	95.5	82	85.6	85.4	81.3	93.7	99.2
RASSF6	86.2	97.6	87.9	90.1	79.5	82.2	91.3	100
RASSF7	1329.8	1441.5	997.2	910.9	939.3	1187.2	1154.4	1355.1
RASSF8	129.2	163.8	105.8	98.5	86.1	91.6	128.2	144.5
RAVER1	318.2	460.7	583	457.1	365.9	644.3	372.8	719.9
RAVER2	172	193.3	157.9	146.7	131.2	192.4	151.4	165.4
RAX	90.5	108.9	101	93.4	82.9	95.3	84.3	90.9
RAXL1	348.7	495.4	195.1	380.3	349	265.9	457.8	364
RAXLX	93.1	81.1	85.4	94.3	89.1	91.8	80.5	89.3
RB1	456.3	592.5	263.5	277.1	227.9	494.1	337.7	477.9
RB1CC1	851.2	1004.1	442.8	597.8	520.6	929.3	840.4	1590.2
RBAF600	1108.3	1529.3	800.8	752.6	606.3	805.8	1021.1	1247.8
RBAK	143.5	168.7	115.4	117.2	100.5	129.2	119.8	135.3
RBBP4	175.3	204.3	139.6	136.6	123.7	178.2	147.1	215
RBBP5	793	926.5	461.7	459.2	311.4	445.6	645.6	999.6
RBBP6	73.9	85.3	87.8	82.2	80.2	79.2	77.3	81.8
RBBP6	103.1	107.7	96	86.7	83.4	83.5	97.5	121.8
RBBP6	79.9	92.1	93.7	89.9	89.9	88.1	81.6	95.5
RBBP7	2156.7	2211.6	429.9	409.9	401.4	613.3	1730.3	3655.6
RBBP8	142.1	164.5	109.2	94.6	93.8	97.2	118.4	151
RBBP8	100.6	111.2	76.2	87.5	78.9	84.7	102.2	94.3
RBBP8	1650.9	1896	1059.3	1129.5	890.2	1669.1	1219.5	2071.4
RBBP9	147	205.4	100.2	102.3	87.8	91.8	135.9	194.1
RBBP9	264.1	281.8	160.8	198.8	163.4	196.4	182.8	215.9
RBCK1	741.6	880.7	426	275	258.9	310.9	601.9	765.7
RBCK1	338.6	380.2	336.4	258.4	221.8	373.4	301	553.9
RBED1	126.3	135.4	125.7	113.7	111.2	146.4	130.1	208.8

RBJ	221.7	277.3	165.1	167.1	132.2	182.9	196.1	235.1
RBKS	459.6	527.4	227	271.3	253.4	335.4	355.7	421.2
RBL1	98.8	107.8	105.7	98.6	99.6	96.5	100.8	105.9
RBL1	403.4	635.3	309.4	248.1	225.4	373.6	336.2	361.4
RBL2	866.9	1233.1	769	906	881.4	1634.5	700.6	773.9
RBM10	253.6	327.4	235.2	216.4	203.2	284.2	243.7	387.4
RBM10	3005	3348.1	2317.1	2481	1998.5	2566	2357	3232
RBM10	99.2	95.9	89.4	98.2	95.3	87.6	90.7	96.3
RBM11	104.1	126.7	96.1	98.8	84.7	90.6	102.3	118
RBM12	384.9	550.7	212.6	272.8	191.8	319.4	278.6	314.7
RBM12	946.4	1090.6	765.8	701.2	461.3	841.5	655.7	1051.3
RBM12B	564.1	617.8	219.5	233.9	195.5	203.1	414.1	705.2
RBM13	1873.9	2270.7	813.9	536.4	344.6	706.5	1271.8	1890.2
RBM14	3761.1	4599	3408.3	2385.5	1701.8	2284	2317.7	2174.9
RBM15	1499	2043.5	786.6	826.9	643.6	1027.6	1397.9	1926.4
RBM15B	153.3	198.6	135.6	121	91.5	119.2	148.6	196.3
RBM17	712	913.3	626.3	527.3	444.1	687.8	642.9	1331
RBM18	436.4	522	293.3	256.8	204.9	290.9	342.8	598
RBM19	111.1	137	109	98.2	93.4	99.9	131.3	127.1
RBM21	249.1	276.3	226.2	178.6	159.8	205.2	258.4	408.7
RBM22	5299.7	6979	4263.5	3673.2	2866.2	4575.7	4572	5830.1
RBM23	1522.8	1935	813.7	681.7	463.1	682.7	1340.6	1974
RBM23	1895.3	2596.7	1568.4	1152.5	922.8	1600.5	1536.1	2647.1
RBM24	95.7	111.9	98	76.9	80.7	78.3	104.9	99.4
RBM25	1942.8	2307.5	523.7	580.8	716.2	1102.1	1438.8	2187.6
RBM28	396	475.7	362.7	313	332.6	556.5	281.5	463.7
RBM3	180.2	163	107.1	95.2	77.4	93.8	119.9	152.4
RBM3	203.8	223.6	114.1	114.5	102.4	87.3	167.9	222
RBM33	543.3	670.7	402.1	660.4	740.9	826.3	395.5	514.7
RBM34	1739.3	2069.8	363	388.2	299.8	655.3	1642.8	2752
RBM35B	87.2	104.4	97.3	95.3	87.8	86.1	88.2	89
RBM38	280.6	364	297.1	245.2	173.5	318.6	311.4	679.9
RBM39	1199.6	1498.6	747.5	1016.7	951.4	738	1044.6	1603.5
RBM4	989.3	1228.8	366.7	347	299.9	346.5	809.5	943.3
RBM4	2218.8	3149	1044.9	911.2	761.3	957.3	1792.8	2441.9
RBM4	551.8	706.5	287.3	174.1	185	201	417	434.8
RBM41	100.2	106.2	93.3	91.4	77.5	93.3	94.1	118.1
RBM4B	949.8	1267.8	638.1	481.4	469.4	566.9	790	1085.2
RBM5	1941.4	2004.1	1341.9	1366.7	1460.3	1863.3	1420.5	2128.7
RBM6	510.6	618.5	320.1	409.6	355.1	738.4	370.8	594.8
RBM7	417.1	501.2	182.1	198.9	138.2	177.6	376.1	680
RBM8A	125	152.2	104.7	94.4	97.4	98.2	122.8	138.7
RBM9	358.7	456	279.3	260.7	233.4	228.9	325.4	493.4
RBM9	132	149.9	91.7	91.7	92.5	91.9	123.1	138.4
RBMS1	911.3	1252.1	542.4	640.6	487.9	542.1	837.4	1094.6
RBMS1	85.9	94.3	95.3	87.6	83.5	88.4	84.3	74.4
RBMS2	116.4	158.7	105.5	89.5	87.7	100.6	126.9	128.2
RBMS3	85.5	84.8	92.5	94.4	78.8	89	86.6	86.2
RBMS3	91.7	105.7	89.1	88.1	88.4	89.7	91.9	103.7
RBMS3	86.8	93.4	89.6	73.6	75.8	88.7	90.2	106.9

RBMX	3649.7	4989.1	2051.4	1344.4	991.7	1961.6	2368.1	3442.2
RBMX2	488.5	653.4	211.4	208.2	164.5	284.6	419.1	934.5
RBMXL1	75.8	107.6	91.4	89.7	80.2	101.9	91.4	106.2
RBMY1A1	73.9	87	78.9	70.7	77.9	81.2	76.6	80.7
RBMY1A1	76.2	78.5	87.1	94.2	86.4	86.1	73.6	77.3
RBMY1A3P	84.3	88.3	104.3	90.1	79.6	92.9	85	98.7
RBMY1B	79.2	86.8	99.8	85.1	74.9	83.5	78.4	89
RBMY1E	78.5	88.8	89.5	94.6	82.6	85.9	82.9	86.9
RBMY1F	79.7	87	101.9	83	87.6	90.5	81.1	71.7
RBMY1J	80.1	90.1	95.7	89.8	86.6	96.8	87.6	93.9
RBMY2EP	79.5	88.3	87.1	95	87.4	95.1	82.4	84.5
RBMY2FP	89.8	85.5	88.3	91.2	97.3	87.4	86	89.1
RBP1	103.1	113	92.7	87.7	86.8	91	103.1	95.3
RBP2	99.5	117.9	107.1	94.2	93.4	94.8	93.4	92.5
RBP3	82	99	81.6	71.4	85	84.1	101	96.1
RBP4	94.9	111.8	99.7	88.9	83.6	89	101.5	104.1
RBP5	172.6	205.9	109.1	104.5	90.7	96.5	160.8	171.5
RBP7	120.9	148.8	104.4	111.4	90.4	126.9	119.8	163.2
RBPJ	144.1	167.6	96.2	87.8	87.1	98.8	115.4	130.7
RBPMS	104.6	111.7	102.2	88.8	86.5	96.3	103.1	104.9
RBPMS	110	110.3	96.7	92.4	89	87.1	98.4	116.7
RBPMS	76.7	93.6	94	88.2	83.8	89.9	72.6	78.2
RBPMS2	221.8	307.3	168.9	181	163.4	198.8	209.7	214
RBPSUH	157.5	210.2	98.5	96.5	89.5	92.1	127	168.1
RBPSUH	1965.4	2355.7	837.5	795.5	611.9	468.6	1394	2064.3
RBPSUHL	109.8	103.3	132.6	113.4	108.2	119.8	93.6	121.3
RBX1	5360.3	6200.2	2003.9	1775.8	1438.8	2279.4	5326.3	6787.3
RC3H1	104.8	123	92.3	65.8	82.7	87	86.5	129
RC74	1506	1957.7	1074.5	807	563.2	712.7	1353.1	1598.9
RCBTB1	167.9	190	129	123.3	111.8	145.6	159.3	219.9
RCBTB2	406.2	449.2	275.1	476.3	343.1	749.4	322.1	705.8
RCC1	374.4	426.8	229.4	184.9	165	145.9	241.5	272.3
RCC2	5643.7	6516.7	4311.5	5306.1	3919	4990.6	4141.8	3904.8
RCCD1	143.8	188.8	118.8	112.6	100.8	118.5	122	137.1
RCCD1	101.5	113.4	106.5	86.5	89.7	86.3	94.5	105.8
RCCD1	513.9	453.1	291	292.2	273.2	371.7	270.4	348.8
RCD-8	1129.1	1488.8	1476	1177	1004.7	1132.9	886.6	997.7
RCE1	508.8	614.5	463	316.3	247.2	362.1	512.7	893.7
RCE1	158.6	196.2	183.6	139.1	128.4	155.5	154.2	224.3
RCHY1	244.9	288.4	195.2	145.4	133.8	193.4	149.9	223.6
RCHY1	122.7	139.2	92.8	85.2	80.8	92.2	121.3	152.9
RCL1	598	841	445.5	394	299.5	472.5	412.6	655.7
RCN1	80.9	84.8	120.2	100.8	104.7	116.8	79.7	92.6
RCN2	83.6	90.3	95.8	89.6	79.4	90.7	88	94.8
RCN3	123.6	133.9	123.6	143.1	147	167.9	124.1	126.7
RCOR1	82.9	101.5	98.9	110.4	103.1	94.4	91.3	91.6
RCOR2	198.9	223.2	196.5	147.2	127.8	139.6	151.1	122.9
RCOR3	533.9	708.6	481.1	617.6	572.4	1006.6	486.4	838.3
RCP9	1579.4	1636.7	590.6	682.7	861.4	519.6	1458.9	1671.5
RCP9	148.1	160.6	105.7	101	93.4	85.7	109	127.3

RCP9	77.3	76.2	83.5	78.3	74.6	74.5	84.9	95.1
RCSD1	695	946.5	409.7	319.4	280.4	473.6	516	585.5
RCV1	92.4	94.8	97.9	91.9	89.6	79.3	85.4	89.3
RDBP	580.7	730.2	366.6	329.7	310.6	325.7	493.8	392.6
RDH10	522.8	576.6	435.9	344.1	241.2	422.4	432.9	902.7
RDH11	571.6	622.7	240.9	194.3	190.8	216.9	571.1	920.7
RDH12	93	93.8	94.2	85.1	81	84.8	90.3	88.9
RDH13	365.7	447.1	332.9	261.6	199.7	247.3	360.3	472.2
RDH14	2159.5	2434	1621.5	1407.8	1172.5	1609.7	1804.2	3038
RDH16	86.2	91.2	88	79.8	78.8	84.4	89.3	89.1
RDH5	219.5	247.6	182.9	168	187.4	159.7	179.8	179
RDH8	125	164.5	88.5	91.2	88.4	86	130.1	134.9
RDHE2	113	138	97.3	93.7	85	87.6	96.4	108.5
RDM1	101.2	111	119.3	119.9	98.9	123.9	103.5	94.2
RDS	91.6	108.9	115.2	101.6	90.3	106.4	93.5	96.8
REC8L1	240.9	234.1	214	157.3	186.3	208.3	241.1	332.9
REC8L1	113.8	119.1	109.3	98.2	114.4	107.3	103.4	123.7
RECK	260.5	329.9	184.3	194.9	132.4	208.6	244.2	381.2
RECQL	352.1	453.7	250.1	233.6	203.9	300.9	274.3	375.9
RECQL	722	832.3	278.2	309.7	233.5	350.8	545	898.8
RECQL4	349.3	393	445.1	299.5	294.3	561.6	280.4	419.2
RECQL5	118.8	134.7	118.6	104.4	94.8	105.6	112.5	117
RECQL5	155.6	177.7	125.3	152.8	139	165.8	126.5	158.6
RECQL5	110.6	132.6	116.2	97.9	94.3	104.8	104.4	113.9
REEP1	107.1	116.4	110.4	102.2	86.6	96.6	91.7	130.2
REEP2	96.2	107.5	86.4	83.5	74.2	82.9	93.3	103.5
REEP3	176.7	219.2	126.4	99.4	106.3	108.6	134	186.5
REEP4	298.4	405.5	294.5	163.8	157.7	205	264.9	426.8
REEP5	1456.9	1779.6	635.6	674.8	564.9	812.4	1253.3	1679.6
REEP6	503.7	616	375.4	469.7	345.1	351.4	468.6	410.5
REG1A	89.3	97	90.1	93.9	85.1	87.5	91.1	104.4
REG1B	87.4	97.9	96.7	85.3	86	84.2	86.4	96.1
REG3A	111.7	121.6	95.1	91.5	90.3	80	105.8	134
REG3A	88.8	94.6	101.5	93.7	83.4	86.2	85.3	102.1
REG3G	79.3	83.8	97.1	93.1	86.8	95.6	81.9	86.1
REG3G	91.9	96.4	105.5	91.5	85.9	91.4	92.7	92.1
REG4	98	122.1	95.1	97.3	84.7	95.8	106.9	115.8
REL	145.6	186.2	116.7	101.7	97.8	121.5	146.5	208.9
RELA	151.3	228.1	171.8	150.5	114.9	134.2	160.8	246.4
RELB	119.1	161.2	145.5	128.2	156.9	147.6	130.1	176.3
RELN	96.8	110.9	90.9	78.6	71.7	81.9	102.6	111.3
RELN	140.4	131.8	99.9	98	97.9	105.2	132.4	144.2
REM1	88.2	99.3	80.8	88.3	83.6	86.1	89.5	104
REN	108.2	120.6	99.8	90.3	81.3	90.7	102.3	131
RENBP	366.5	500	379.2	557.9	487.3	730.4	373	520.6
RENT1	256.2	300.5	317.8	195.9	176.3	296.7	240.1	430.2
REP15	80.6	103.7	96.1	95.1	88.2	98.7	85.4	96
REPIN1	171.8	217.8	106.7	94.6	86.2	96.7	158.4	192.7
REPIN1	101.1	136.5	109.3	114	116.4	108.3	105	107.5
REPIN1	2284.4	2846.2	1445.1	1739.8	1726.9	2258.7	1317.4	1091.5

REPS1	156.6	175.5	169	161.7	145.2	202.3	140.6	194.2
REPS2	90	98.4	110.8	108.1	103.9	97.2	104	94.5
REPS2	89.1	101.9	106.6	107.9	99.8	105.9	93.2	103.6
REPS2	84.8	104.5	99.7	93.8	90.4	94.9	90.8	96
REPS2	91	110.4	98.5	131.9	139.7	144.8	98.3	104.6
REPS2	98.1	110.8	101.9	86	76.7	91.7	87.8	94.7
RER1	261.6	325.8	130.9	109.4	106	131.7	223.3	254
RERE	168.2	236.2	182.7	300.6	275.2	354.5	164.3	243.4
RERE	100.4	110.2	94.1	86.3	79.6	80.7	95.5	105.8
RERE	100.7	136.6	112.6	132.6	133.9	145.9	114.6	131.7
RERG	78.9	91.3	87.2	84.7	73.6	87.7	91.1	84.5
RESP18	93.3	87.2	92.1	81.4	84	92.2	89.2	96.7
REST	128.1	147.4	108.3	114.9	98.6	100.2	121.5	160.3
RET	1794.5	2549.4	1403.6	877.3	746.3	770.3	1480.1	1335.4
RET	306.5	468.6	233.3	234.1	196.2	290.5	214.7	217.9
RET	803.7	1142.5	857.6	397.9	350.8	613.1	638.4	1221.8
RETN	81	83.3	96.3	81.9	78	83.1	86	83.3
RETNLB	101.9	108.9	100.7	93.5	97.3	93	95.8	102.5
RETSAT	176	250.9	123	123.9	114.3	144.4	164.8	195.8
REV1	686.9	756	301.2	359.1	265.2	352.9	536.7	823.2
REV1L	738.4	810.3	353.2	411.8	376	625.1	601.1	891.8
REV3L	140.2	203.7	156.8	170.7	154.3	226.4	128.2	151.4
REXO1	229.3	241.7	228.8	177.6	183.7	166.4	237.1	308.8
REXO1L1	87.5	116.7	81.2	78.7	72.9	77.7	96.8	110
REXO2	1707.6	1965	1070.3	964.1	648.9	1036.9	1421.6	2212.7
REXO4	1003.3	1364	443.7	367	286	341	854.2	631
RFC1	911.4	1251.6	511.2	620.5	460.4	1039.9	809.3	975.1
RFC2	255.8	333.7	173.7	163.5	151.3	153.7	239.7	267.7
RFC2	1051.4	1214.7	349.3	241.9	189.8	297	817.9	1133.2
RFC3	1597.1	2173.7	593.5	462.4	311.4	483	904.1	1501.8
RFC3	778.6	1042.3	465.8	368	285.7	376.2	475.3	672.5
RFC3	323.9	429.8	217.2	154.6	146.4	169.5	297.6	457.6
RFC4	3333.9	3940.3	1096.7	1044.7	854.1	1072.9	2496.9	3645.7
RFC4	3506.2	4682.6	2161.1	1786.9	1556.9	2934.1	2613.9	3766.6
RFC5	2182.8	3040.9	1176.2	1080.9	851.4	1191.2	1749.9	2223
RFC5	98.7	101	102.5	94	84.5	91.7	102.8	100.2
RFESD	245.8	317.3	191.9	184.5	147	205.6	214.3	252.7
RFFL	103.9	123.2	78.2	86.5	74	85.5	99.6	121.1
RFFL	94.4	105.2	81.1	84.6	75.7	85.9	83.4	98.7
RFK	143.4	157.5	99.3	88.1	80.3	94	120.8	181.1
RFNG	117.9	133.5	128.8	105.9	100.7	105.8	114.8	136.2
RFNG	503.4	610.9	552.2	439.8	417.1	538.8	405.5	885.7
RFNG	98.4	115.3	88.3	84.1	82.6	93.2	95.9	113.1
RFP	238.9	334.7	186.9	181.5	154.9	211.9	180.4	289.5
RFP2	124.3	135.8	101.6	89.1	103.4	97.3	94.8	109.7
RFPL1S	101.1	136.9	112.6	106.9	102.1	97.7	113.5	129.9
RFPL3S	116.4	105.6	93.8	82	80.2	91.3	105.2	126.3
RFPL3S	91.5	103.8	91.3	88.7	79.8	96.7	93.7	99.3
RFPL4B	79.2	90.3	87.8	85.4	83.7	84.4	83.1	97.9
RFT1	122.6	132.9	98.9	91.1	87	88.7	116	132

RFWD2	3198.9	3909.8	2051.9	1952.8	1642.8	2611	2688.9	3537.8
RFWD2	94.9	99.3	93.2	86	92.9	92.6	93.3	89.1
RFWD2	2670	3592.7	1562.8	1434.7	1084.3	2056.1	2439.6	2967.4
RFWD3	1993.5	2758.8	1206.6	696.7	603.1	766.4	1544.6	1711.2
RFX1	239.7	270.4	232.2	138.2	149.5	169.4	204.8	302.6
RFX2	112.8	130.9	149.5	114.8	120.4	148.8	98.2	121.7
RFX2	107.2	112.5	129.7	115.3	118.3	141.4	93.4	112.2
RFX3	79.3	96.1	92.3	92.9	89	83.8	87.9	96.9
RFX3	102.8	114.2	119.4	96.1	93.9	110.5	121.5	119.1
RFX3	89.2	120.7	105	90.7	90	102	90.9	110.2
RFX4	75.8	80.3	95.6	87.8	78.5	95	82.9	80.8
RFX4	97.8	140.4	89.6	85.7	78.5	78.4	106.2	135.9
RFX4	110.2	105.4	85.9	90.4	86.5	98.9	108.2	117.3
RFX4	81.5	92.8	106.5	83.4	85	80.9	96.1	101.8
RFX5	90.6	116.6	82.8	73.9	83.2	83.3	96.1	115.9
RFX5	1983.9	2222.9	1905.4	1850	1678.1	2574.8	1351.5	2312.2
RFXANK	996.6	1258.5	509.8	334.8	284.7	365.3	903.6	958.4
RFXANK	431.9	521.5	213.6	146.3	136.6	164.3	334.7	365.4
RFXANK	1975.9	2885.4	1749.8	1356.7	1061.7	1463.1	2374.5	2798.3
RFXAP	158.1	188.6	95.8	87	97.5	91.8	136.4	171
RFXDC1	100	112.1	113.2	89.5	101.8	98.7	98.3	100.1
RFXDC2	113.9	121.2	103.9	116.3	96.2	114.2	116.2	136.3
RG9MTD1	602	691	277.5	247.6	220.7	313.9	540.2	1075.5
RG9MTD2	221.4	272.7	123	130.7	124.7	147	175.1	271
RG9MTD3	216.7	243	178.9	157.9	157.2	145.2	188.8	250.4
RGAG1	106.3	115.2	94.5	80	78.1	85.4	104.1	104.5
RGAG4	89.3	108.5	105.4	102.7	84.7	101.6	92.5	100.9
RGAG4	224.6	260.8	125.9	93.2	91.2	106.3	199.2	232.8
RGC32	537	676.1	452.3	359.8	288.8	417.9	482.7	737.7
RGL1	123.3	151.5	117.4	173.6	199.1	211.1	153	186.9
RGL2	233.3	265	180.2	159.9	192.8	241.4	201.5	421.1
RGL3	133.2	143.4	89.4	81.4	82.6	80.7	123.2	154.6
RGMA	192.1	207.3	189.3	190.9	180.2	195.4	130.2	147.9
RGMB	98.3	105.3	104.3	91.9	90.8	92.3	91.9	100.5
RGMB	82.2	88.7	85.6	81.9	86	93.6	83.2	90.4
RGMB	101.4	121.3	100	95	100.2	84.6	113	124.8
RGN	106.5	119	116.8	92.6	97	101.3	113	129.2
RGN	120.3	120.2	90.3	88.9	77.1	84.3	108	134.7
RGNEF	107.8	128.5	97.5	84.1	83.8	87.2	104.8	131.4
RGNEF	75	85.1	95.1	89.1	84.1	87.3	70.5	78.5
RGPD1	179.4	227	175.6	240.6	273.8	296.3	148.9	165.3
RGPD2	182.5	225.1	114.1	112	104.4	119.1	157.6	190.9
RGPD5	88.6	92.2	97.4	86.8	79.6	82.5	92.5	86.9
RGPD5	104	130.8	105.4	103.1	92	102.1	114.3	124.5
RGPD5	80.1	89.9	87.5	92.4	86.3	80.1	79.5	89.4
RGPD7	100.1	115.9	99.7	88.3	98	86.2	97.7	118.9
RGR	79	88.1	91.9	87.1	80.6	86.9	84.1	88.9
RGR	130.9	146.9	117.9	108.4	113.4	113	126	135.2
RGR	115.7	121.4	114.2	103.1	111.1	100.1	116.3	121.7
RGS1	97	101.3	85.1	77.7	83.2	89.1	90.9	104.9

RGS10	771.9	1014.4	529.6	293	208	329.7	478.5	504.6
RGS10	3111.5	3955.1	1026	753	542.9	772.1	2891.1	3450.8
RGS10	117.7	131.8	97.3	73	77.2	82.8	108.1	121
RGS11	106.4	121.5	94.2	95.8	86.7	94.8	96.1	119.4
RGS11	115.9	133	100.2	85.4	84.9	89.6	112.5	124.5
RGS12	101.9	114.6	106.1	94.4	87.2	88.5	103.5	101.1
RGS12	339	427.3	365	329	352.1	393	299.9	439.5
RGS12	117.1	130	81.7	89.7	85.2	81.2	110.7	109.3
RGS13	88.7	93.2	94.6	85.4	92.2	94.5	93.4	94
RGS14	147.6	176.9	136.1	142.5	131.5	186.6	127.3	164.2
RGS16	120.5	126.2	144.1	128	142.8	164	112.8	150.5
RGS17	96.9	112.3	123.1	108.8	109.4	110.8	101.4	109.4
RGS18	1438.6	1754.2	826.4	684.2	469.4	990.9	700	1251.5
RGS19	1476.3	1857.1	1285.4	1380.2	1086.8	1833.3	1211	2470
RGS2	220.2	274.8	181.4	170.7	174.4	214	265	489.9
RGS20	72.7	88.5	90.6	84.7	85.2	85.9	82.2	83.1
RGS20	101.4	103.5	90.1	115.5	117.6	122.1	107.8	137.2
RGS22	77.6	90.3	92.6	80.1	80.5	84.4	88.7	86.7
RGS3	92.5	106.2	96.3	89.6	83.5	90.7	85.5	88
RGS3	124.5	136.2	93.5	91.5	88.8	91.2	105.7	130.7
RGS3	106.6	109.8	93.4	84.9	81	81.5	96.5	101.2
RGS3	72.3	85.7	80.9	77.9	80.1	75.8	79.5	80.6
RGS3	90.4	104.7	85.2	87.1	74.6	85.9	98.8	115.9
RGS3	105.4	106.2	109.7	103.5	114.4	106.7	103	112.6
RGS4	103.6	113.6	94	88.9	91.4	97	109.1	113.4
RGS5	91.7	93	101.4	104.5	88.8	99.5	90.2	103.7
RGS6	112.5	123.4	111.9	93.1	101.9	107.2	118.6	127
RGS7	87.7	103.5	87	84.1	77.5	80.4	90.3	90.3
RGS8	91.2	124.8	87.2	84.9	81.1	81.6	96.3	104.6
RGS9	89.7	98.9	88.8	77.1	87	78.3	84.2	103.3
RGS9	79.7	89.5	86.9	77.2	79.4	83	88	84.9
RGS9BP	91.4	100.9	111.5	93.3	95.4	87.6	94.3	99.3
RHAG	81.1	87.7	96.6	88.5	83.1	89.4	82.2	89.3
RHBDD1	300.4	385.2	226.8	253.7	217.2	278.3	296	338.7
RHBDD2	743.5	923.6	983.3	494	349.9	446.7	829.3	1609.7
RHBDD2	865	1074.1	1074.8	632.3	435.7	506.9	906.4	1874.2
RHBDD3	231.6	317.7	200.6	157.1	145	208.3	214.1	408.7
RHBDF1	685.9	758.7	447.1	521.9	515.9	536	449.4	494.3
RHBDF2	311.6	367.3	342	392.5	369.9	373	194.7	200.3
RHBDF2	110.2	131	96.6	90.7	90.8	97	111	131.8
RHBDF2	363.2	440	281.9	319.1	247.4	301.4	253.5	292.2
RHBDF2	77.4	85.2	93.4	82	80.6	79.5	80.6	82.1
RHBDL1	206.4	240.1	188.6	172.1	187.5	193.1	147	238.2
RHBDL2	162.7	172.5	104.1	99.8	93.3	103.8	162.5	195.9
RHBDL3	121.6	130.1	115.7	105.3	98.6	113.9	117.1	123.3
RHBG	70.1	84	87.2	82.5	70.5	81.4	76.2	85.6
RHCE	139.8	163.9	101	94.7	86.8	94.7	127.2	146.6
RHCE	81.4	97.1	91.1	89.7	84.9	83.5	85.1	84.6
RHCG	82	116.6	79.4	77.6	69.3	82.8	103.4	108.7
RHD	81.6	96.3	84.7	97.3	77.8	85.9	76.3	104.7

RHEB	5090.2	6662.8	2516.8	2437.1	1603.4	2917.8	4617.2	6867.1
RHEBL1	126.9	143.8	102.2	88.1	94.5	89.1	104.4	118
RHO	75.5	89.9	87.4	94.5	80	89.1	93.8	89.1
RHOA	12931.6	14487.6	4475.3	5408.2	2906.5	3674.1	13494.4	18977.1
RHOB	116.4	132.2	94.6	96.2	94	90.7	125.9	189.3
RHOBTB1	106.9	94.2	100.4	89.9	85.7	91.6	84.1	141
RHOBTB1	92.4	91.5	93.9	92.3	91	88.4	85.3	77.6
RHOBTB1	98	113.9	94	81.7	80.8	83.2	95.7	109.9
RHOBTB1	103.4	116.2	106.2	87.9	83.1	89.2	95	106.8
RHOBTB2	152.6	177.2	137.8	153.9	136.5	157.9	143.2	247.9
RHOBTB3	195.2	232.1	207.6	294	244.2	362.9	239.6	314.9
RHOC	2423.3	3051.7	1008.5	592.4	425.5	736.6	2792.7	4319.9
RHOC	1060.7	1303.8	357.3	223.4	212.1	214.8	1181.7	959
RHOD	78.7	82	95.4	85.1	82.7	88.3	82.2	81.5
RHOF	97	111.8	105.6	84.4	94	99.4	103	126.1
RHOG	2266.1	3166.8	2265.2	1613.6	1128.9	1485.4	2569.8	4315
RHOH	96.8	116.8	90.6	94	76.2	72.9	99.2	117.1
RHOJ	79.7	85.8	101.1	82.2	93.6	81	89.2	90.9
RHOQ	1479.6	2004.4	946.6	1256.6	985.8	1019.4	1746.6	2015.2
RHOT1	694.4	928.3	389.8	608.6	447	982	475.9	696.8
RHOT1	1402.8	1887.3	734	1103.9	946.9	1543.8	1145.8	1670.8
RHOT1	201.4	269.1	113	102.7	108.6	141.2	172.9	235.8
RHOT2	580.2	635.7	634.2	468	431.3	743.9	387.4	794.2
RHOV	100.8	117.5	84.4	80.1	77.1	80.6	97.5	123.8
RHPN1	143	172.8	128.7	96.1	93.4	118.5	143.7	172.5
RHPN2	101.6	120.3	107.1	104.9	100.1	107.6	99.3	116.8
RIBC1	149.8	159.1	111.3	89.9	88	99.7	118	152.7
RIBC1	101.3	125.4	82	85.1	80.2	82.3	99.8	103.8
RIBC2	86.7	97.5	96.1	91.1	81.6	88	86.5	94.3
RIC3	80.4	92.3	86.2	80.7	86.9	82.8	80.2	85.9
RIC8A	867	1116.4	815.8	679.6	570	837.2	816.2	1495.6
RIC8B	169.1	203.4	99.9	99.1	85.1	97.3	162.2	221.9
RICS	192.1	297.9	236.8	289	260.9	444.8	189.8	345.7
RICTOR	126.3	142.6	100.3	91.2	88.9	93.4	112	134.2
RIF1	399.5	592.6	253.8	296.3	246.5	520.6	371.8	506.2
RILP	287.3	321.5	155.4	180.2	135.8	189.1	241.4	354.3
RIMBP2	96	99.9	106.4	99.9	97.9	88.8	95.7	96.2
RIMS1	83.3	85.2	94	91.1	89.2	88.1	86.7	88.3
RIMS2	132.7	138.1	86.2	77.5	79.1	82.2	126.2	134.5
RIMS3	1462.5	1679.5	1694	2126.8	2084.6	1993.2	1360.6	1339.9
RIMS4	135.7	155.4	100.4	89.9	81.3	92.1	136.8	156.3
RIN1	209.5	271.6	145.7	107.8	92.4	135	196.8	259.3
RIN2	76.6	85.7	99.8	94.8	84.2	90.6	75.3	90.2
RIN3	109.2	119	107.4	100.6	85.4	98	93.1	124.7
RING1	5437.3	6929.7	3270.5	2759.9	2371.7	3395.8	4545.1	5999.3
RINT-1	262	343.2	223.4	223.8	214	267.8	215.1	355
RIOK1	479.4	635.3	321.8	301.9	228.9	373	384.5	494.5
RIOK1	88.8	87.9	89.9	77.3	80.2	83.4	87.6	98.2
RIOK1	331.4	457	152.8	119.1	108.6	121.7	264	374.5
RIOK2	1462.7	1972.5	816.2	645.3	469.4	1140.6	1050.3	1679.4

RIOK3	923	1073.1	451.9	492.4	428.5	491.5	679	926
RIOK3	1637.4	1851	958.5	1562.8	1271	2041.2	1478	2604.7
RIOK3	78.1	95.5	100.6	90.3	81.3	88.5	82.7	94.5
RIP	970.7	1285.9	827.7	913.2	801.8	1231	751.7	892.7
RIPK1	137.4	159.7	205.4	210.3	213.8	245.2	160.4	197.5
RIPK2	992.6	1337.3	687.5	457.1	339.2	549.4	794.8	1321.4
RIPK3	344.5	431.8	215.8	180.6	166.3	187.4	285.1	317.4
RIPK4	150.4	166.4	124.1	110.5	115	115.6	150.1	149
RIPK5	135.1	166.4	134.7	123.3	122.2	146	128.6	149
RIPK5	569.3	751.6	381.3	418.7	294.3	401.6	562.9	736.3
RIS1	2018.1	2611.9	1310.9	1083.9	707.7	1033.9	1580.7	2227.9
RIT1	287	376.8	159.1	128.6	134.5	172.8	823.7	1486.4
RIT2	80.2	91.3	86.7	83.2	73.9	78.1	80.1	91.1
RKHD1	89.2	95.4	85.6	81.8	78.4	80	90.6	100.3
RKHD2	537.5	664.9	276.7	352.6	260.3	369.6	506.7	934.6
RKHD3	98.1	93.6	94.7	80.6	83.7	93	97.7	126.4
RLBP1	87.7	90.1	101	97.4	88.2	89	95.2	91
RLF	758.2	1027.9	276.7	478.2	363.3	767.4	856.8	1322.9
RLN1	87	92.4	100.8	86.2	83.4	81.8	88.3	90.8
RLN2	81.7	93.9	83.5	80.9	79.7	75.9	88.1	97.5
RLN2	95.6	102.7	92	90	90.1	89.6	93.8	101.1
RLN3	85.2	98.9	86.5	85.5	86.5	85.6	97.2	99.2
RLN3R1	111.5	124.7	100.6	95.1	88.5	97.5	108	112.5
RLN3R2	124.4	136.7	90.7	83.3	86.8	82.4	117.6	118.1
RLTPR	87.1	102.2	94.4	84.4	80.7	79	89.1	100.4
RMI1	1941.6	2336.6	977.3	936.5	581	1038.1	1502.1	2347.7
RN7SK	645.4	736.2	407.1	1005.4	899.5	369.5	740.6	766.7
RN7SL1	26300.9	25051.8	12468	18143.7	18257.6	14028.5	24924.1	26195.5
RNASE1	131.8	145	106.3	101.6	99.7	107.5	114.5	134.1
RNASE1	109.2	110.9	91	105	101.1	105.8	110.5	108.4
RNASE10	116.8	137.4	117	111.2	100.2	113.5	106.8	117.4
RNASE11	87.6	113.3	93.3	83.9	85.4	91.9	101	107
RNASE12	72.3	87.3	96.8	86.3	80.6	80.9	78	80.1
RNASE13	90.2	98.3	98	86.4	84.6	85.3	93	92.7
RNASE2	26554.4	27065.9	14332.3	11237.8	8614	12275.8	24729.7	32036.4
RNASE3	1368	1771.8	1058.6	1125.4	909.1	1240	1149.3	2012.6
RNASE4	76.7	86.7	96.3	96.3	89.5	91.7	79.5	87.2
RNASE4	90.3	110.8	94.5	107.1	102	96.3	94.3	100.4
RNASE4	99.9	113.1	122.6	129.7	112.2	127.5	101.6	132.1
RNASE4	138.1	144.4	105.1	92.2	97.3	105	143.8	148.8
RNASE6	259.2	390	245	209.3	213.2	254.6	212.2	254.1
RNASE7	85	93.5	73.5	76.6	80.3	88.2	84.9	94.3
RNASE8	83.8	84.3	83.7	73.7	78.2	87.1	88	85.3
RNASE9	74.7	81	88.7	80.8	74.8	76.6	75.4	80.3
RNASEH1	1609.9	1911.4	1238.5	1339.9	990.3	1036.4	1289.9	2272.5
RNASEH2A	1019.8	1304.4	667.4	390.4	332.4	401.6	875.2	1030
RNASEL	721.6	845.4	335	393	312.2	527.3	661.3	1069.6
RNASEN	114.5	126.7	91.4	84	89.8	98.6	112.7	120.5
RNASET2	21168.6	26141.3	14469.3	15846.3	13014.4	15950.5	19279.1	19466
RND1	92.5	92.7	90.9	92.3	86.4	104	90.4	94.5

RND2	115	124.6	120.2	106.8	96.3	105.2	106.4	124.8
RND3	108	127.5	90.4	92	86.4	98.5	106.3	137.5
RNF10	200.7	263	197.9	200.3	154	227.2	200.6	342.8
RNF11	84.2	101	103.1	95.4	103.5	95.8	89.2	90.1
RNF111	112.1	125.7	98.4	89.5	83.6	94.8	109.2	141.4
RNF113A	765.3	961.6	373.6	386.9	336.5	440.2	684.9	693.3
RNF113B	127.3	155.9	103.5	91.7	98.7	97.1	119.3	128.8
RNF12	73.8	81.1	86.6	97.9	80.7	81.6	79.3	97.6
RNF12	86.7	91.9	89.9	88.7	83.8	87.7	99.4	100.7
RNF12	104.2	114.3	106.1	107.9	93.9	107.5	92.1	111.7
RNF121	157.9	198.8	132	138.7	114	137.3	158.5	185.3
RNF121	457.8	635.3	335.5	355.2	294.9	311.3	462.9	621.4
RNF122	129.6	153.1	103.3	85.6	88.2	92.7	122.6	151.2
RNF123	238.5	272.3	223	196.1	165.3	200.1	205.5	292.8
RNF125	157.5	203.2	127.6	115.2	105.3	100.4	145.8	172.7
RNF126	1042.7	1297.9	441.9	393.4	333.4	366.6	971.5	779.2
RNF126	308.4	449.6	301	225.4	188.4	296.3	307.3	466.9
RNF126P1	158.2	171.4	119.9	115.2	113.8	116.1	135.3	166.2
RNF126P1	108	128.1	95.7	86.2	84.5	96.9	106.6	108.4
RNF128	111.9	132.3	97.3	87.1	94.4	90.8	112.8	126.5
RNF128	89.1	116.1	110.2	109	103	105.8	107.8	97.4
RNF128	123.3	164.3	119.1	108.8	104.7	105.3	123	131.1
RNF13	498.6	669.2	282.6	423.9	338.4	613.9	515.9	1625.9
RNF13	110.3	124.3	90.1	105.8	100.1	93.7	103.6	106.4
RNF130	1146.6	1603.9	475.3	696	580.9	940.7	1137.9	2167.9
RNF133	98	93.4	107.6	99.5	92.2	100.7	91.1	94.4
RNF135	472.8	643.5	333.6	336.4	224.1	411.4	521.2	1327.8
RNF135	231.3	331	191.5	180.9	166	151.4	213.5	275.4
RNF135	124.3	153.3	109.7	100.3	90.1	97.1	144.9	190.8
RNF138	436.4	513.8	255.3	323.8	233.2	408.2	350.5	586.9
RNF139	97.3	130.5	91.6	88.9	86.4	80.8	103.1	120.8
RNF14	120.6	120.5	93.4	96.9	87.6	95.7	109.4	110.2
RNF14	1021.2	1334.2	590.4	516.8	363.1	848.2	1013.2	1826.2
RNF14	571.2	808.1	368.2	327.3	249.7	496.6	517.6	828.5
RNF141	205	220.3	114	116.7	112.3	118.6	177.1	226.8
RNF144	454.3	625.3	357.1	357.5	305	429.7	353.9	326.6
RNF146	785.5	1253.5	441.8	432.6	384.1	633.2	699.3	838.9
RNF148	125.6	136.4	129.2	109.6	106.4	116.7	117	130.7
RNF149	129.1	165.6	100.7	115.1	108.7	99.6	133.8	184
RNF150	104.3	120.2	108.8	114.6	102.8	140.2	99.8	105.3
RNF151	85.6	84.5	101.6	93.4	82.2	86.7	93.1	96.6
RNF151	82.5	96.7	89.6	84.1	76	84.5	79.8	89.2
RNF152	84.9	86.8	102.8	97.8	92.2	88.2	91.7	97.9
RNF157	113.5	141.6	107.3	99.2	89.6	99.9	122.3	136.8
RNF165	152.8	191.1	166.3	121.1	120	125.1	112.6	144.5
RNF166	178.4	195.6	149.4	118.4	116.2	141.4	159.8	281.2
RNF167	580.3	765.1	223.6	211	177.1	212.2	461	688.3
RNF168	127.4	128.3	93	96.2	92	88.9	123.7	131.8
RNF17	75.6	78.9	90.5	82.4	82	81.4	79.9	91.3
RNF17	91.3	98.3	92	79.3	86.8	89.9	91.5	93.8

RNF170	336.9	468.5	212.6	186.1	157.4	219	333.8	560.2
RNF175	854.7	1013.6	263.4	217.4	206.4	215.3	676.8	860.9
RNF180	106	112.2	96.3	85.1	81.1	95.2	101	101.6
RNF183	120	166.4	101	101.5	84.3	93.8	129.8	141.6
RNF185	106.1	117	127.2	125.4	101.8	146.5	119.7	123.7
RNF186	99.9	123.8	93.6	86.5	78.7	91.9	101.6	137.4
RNF19	176.7	183.2	131	143.4	140	109.4	146.1	203
RNF19	98.1	109.7	96.6	93.5	80.6	82.9	105.9	119.9
RNF19	654.6	860	464.5	444.9	347.5	821.1	471.5	709.3
RNF190	75.8	86.1	90.9	79.1	83.4	91	85.9	86.9
RNF2	116.6	111.8	94.7	89.3	93.1	89.7	94.2	113.4
RNF20	1014.9	1303.8	569.7	543.4	456.9	759.5	829.3	1349.9
RNF24	421.5	514.4	406.7	838.5	603.1	1089.8	418.9	794.6
RNF25	164.3	165.2	138.4	110.9	126.2	126.9	130.8	165.6
RNF26	549.2	728.4	379.1	285.9	214.2	267.8	507.2	994.5
RNF31	268.4	283.7	282.9	219.2	179.7	268.9	242	386.6
RNF32	127.6	166.1	102.8	103.9	97.7	102.9	121.6	155.1
RNF34	2340.6	3297.4	1674.3	1578.2	1351.2	1900.2	2170.3	3092.9
RNF34	151.8	199.4	124.6	87.6	103.1	99.1	142.3	140.1
RNF34	92.4	96.4	81.9	94.6	77.1	85	91.3	109.6
RNF36	81.8	86.5	81.3	81.9	73.7	80.3	86.7	93.6
RNF36	125.8	154.9	101.4	95.6	96.8	92.8	112.7	130.1
RNF38	121	152.2	85.6	85	73.6	92.7	116.3	168.4
RNF38	211.5	332.7	172.5	156.4	135.1	145.7	205.3	221.8
RNF38	579.7	886.9	666.3	829.6	749.8	997.5	720.2	1068.6
RNF39	89.5	102.9	87.1	85.2	78.8	89.2	87.6	101.4
RNF39	112.6	104.8	92.1	89.5	83.9	94.5	95.7	106.9
RNF4	361.5	484.2	275.8	223.7	196.1	322.7	285.5	544.1
RNF40	281.5	405.9	405.9	331.8	277.1	562.2	292.4	537
RNF41	210.5	282.5	153.6	135.3	152.3	192	225.2	260.5
RNF41	104.1	113	101.1	104	95.8	101	86.5	104.6
RNF43	95.9	99.7	107	101.6	93.4	93.6	101.3	118.9
RNF44	245.4	245.5	215.4	256.8	258.5	433	168.6	239.3
RNF5	705.3	885.8	423.7	371.4	342.3	475.3	660.1	756.6
RNF5P1	2336.5	3100.3	1329.8	1278.1	1098.1	1613.5	2203.6	2536.1
RNF5P1	658.3	828.7	359.1	319.6	293.6	374.7	603.7	771.5
RNF6	115.2	129.2	95.1	91.7	94	97.8	107.6	117.7
RNF6	103.2	115.3	98.5	85.8	94.4	96.2	90.3	104.1
RNF6	262.7	285.9	119.6	126.5	107.1	111.3	160.2	241.7
RNF7	111.5	129.8	109.5	99.6	95.4	102.6	115.3	122.9
RNF8	116	138.7	111.2	98.2	93.3	90.7	105.8	146.7
RNF8	725.2	1001.4	344.1	290.6	252.4	406.3	574.9	780.6
RNGTT	1406.4	1780.8	825.1	1269.3	915.1	1291.1	1232.8	1737.2
RNH1	2201.9	3035.6	2304.3	1376.3	1191.8	1602.9	1849.9	2567.1
RNH1	736	944.5	379.9	300.7	259.1	427	768.4	1677.5
RNMT	530.1	640.6	536.2	841.3	707.7	949.8	497.6	683.7
RNMTL1	920.7	1308.8	504.8	401.7	378.3	433.3	817.4	893.6
RNPC1	178.4	234.6	202	174.5	148.5	200.7	174	277.7
RNPC2	100.1	112.1	93.4	84.3	85	95.1	101.2	112.8
RNPC3	386	424	185.5	149.7	136.3	160.6	261.7	426.2

RNPEP	1556.3	1952.1	631.2	545.5	519.2	559.8	1251.8	950.1
RNPEPL1	125.7	163.8	109.6	116.4	89.9	113.5	114.4	191.8
RNPS1	2621.5	3815.6	2192	1055.8	905.6	1345.4	2193.7	3488
RNPS1	247	380.7	208.9	126.3	116.4	128.1	227.8	333.8
RNU2	156.3	134.8	102.9	100.9	101.8	97.1	100.8	123.7
RNU3IP2	356.8	420	170.8	107.8	106.2	127.3	250.7	333.7
RNUT1	1241.6	1682.6	1027.9	839.2	808.1	1073.2	1195.7	2153.1
RNUXA	227.4	252.4	171.7	220.1	223.6	185.3	237.5	379.8
ROBO1	94.6	106	94.2	94.6	86.5	95.9	91.3	98.2
ROBO1	137.6	143.1	101	107.6	101.3	135.2	124.5	158.4
ROBO1	112.4	108.1	88.9	75.6	80.5	83.2	102	121.7
ROBO2	78.9	92.9	90.2	83.5	82.9	86.1	89.8	97.9
ROBO3	360.2	335.4	238.5	150.1	148.6	174.8	273.8	553.5
ROBO4	94	128	88.4	83.3	87.5	88.7	105.7	107.1
ROCK1	310.5	469.1	152.2	185.6	187.5	199.5	321.6	449.2
ROCK2	19785.8	21470.5	9638.2	14897.9	14645.1	10837.4	17637.5	19091.8
ROD1	2879.6	3832.5	1298.3	1593.7	1046.1	1827.2	2839.7	4503.4
ROM1	174	203.3	126.3	127.9	116.7	135.7	156	177.3
ROPN1	98.5	101.3	96.5	90.8	85.6	90.4	93.6	107.1
ROPN1B	104.2	103.8	96.9	88.6	97.3	85.7	99.5	108.1
ROPN1B	105.6	133.1	105.5	98.2	91.9	102.9	104.1	118.3
ROPN1L	149.3	183.6	141.5	88.8	94.1	90.7	151.8	179.7
ROR1	86.8	88.5	101.2	85.7	79.7	94.4	83.8	100.2
ROR2	94.8	102.1	94.6	93.3	87.1	86.1	98.9	102.2
RORA	89.1	95.9	95.1	84.8	85.4	91.5	88.8	104.2
RORA	69	86.2	88.4	79.2	85.1	77.4	78.8	77.6
RORA	93.9	88.1	92.1	83.3	87.6	89.5	82.8	95.2
RORA	108.2	130.2	118	106.1	95.6	114.2	125.8	133.2
RORA	82.1	91.2	95	86.7	79.5	75.6	77.8	88.8
RORA	87.1	96	98.2	85.5	77.9	81.2	85.6	87.5
RORB	92.6	96.5	82.9	79.6	80.6	80.5	80.8	94.7
RORC	80.8	94.7	91.2	81.9	77	88.9	84.8	91
RORC	96.4	113.9	94.8	87.7	72.9	93.4	94.4	116
RORC	102.6	116.3	103	92.6	98	102.6	99.9	112.9
RORC	84.2	94.2	91.3	84.1	86	90	87.8	96
ROS1	96.6	118.4	91.1	87	90.4	96.9	103.3	97.3
RP1	80.1	106.2	98.3	97.2	96.2	98.5	82.7	81.9
RP1-112K5	788.8	1014.8	402.9	312.7	272.4	345.9	766.1	913
RP11-19J3.	276.9	347.9	245.4	198.8	178.3	224.3	217.7	307.5
RP11-450P	87.2	102.7	106.3	86.2	79.5	90.7	84.3	97.3
RP11-529I1	269.7	285.1	174.3	140.9	128.1	169.7	208.8	244.9
RP11-564C	109.1	135.8	101.7	97.8	91.5	100.3	109.9	128
RP13-11B7	117	120.3	96.2	69.2	75.7	95.2	99.4	147.1
RP13-15M1	101.5	114	99.4	93.5	87	90	109.9	103.6
RP13-15M1	112.2	134.7	141.3	126.5	114.1	130.4	105.9	131.7
RP1-32F7.2	104.5	119.8	89.9	86.4	90.7	89.4	108.7	119.3
RP1L1	83.3	85.8	94.4	91.9	82.5	92.4	85.9	88.5
RP2	607.2	860.6	428.8	497.9	348	536.8	449.2	717.8
RP9	831.2	1111.2	524.4	609.6	483.3	751.3	728.4	846.2
RPA1	1984.5	2669.2	775.9	938.6	574.1	978.5	1543.1	2077.1

RPA2	2994.6	3429.3	2137.1	1753.8	1409.1	2002	2781.3	3348.5
RPA3	6403.1	6558.7	3892.6	3546.1	2660.4	3194.9	6115.3	8141.3
RPA4	110.2	123.6	95.9	122.6	120.3	134.2	108.8	113.2
RPAIN	1586.8	1749.1	1389.9	1378.2	1162.2	1721	1308.5	1757.2
RPAP1	186.8	210.7	171.8	192.8	173.6	176.5	175.7	234.2
RPE	121.2	122.3	94.7	81.4	75.7	78.6	121.3	107.3
RPE	1880.1	2527.2	951.3	701.8	552.3	1265.2	1241.6	1872.4
RPE	86.7	102.8	100.4	90.1	99.8	88.3	88.1	95.6
RPE65	87.7	87.4	102.4	85.4	92.4	86	87.1	82.4
RPESP	98.4	101	98.3	99.6	93.8	93.3	98.6	102.6
RPGR	91.6	102.7	74.9	87.7	82.5	83.4	90.2	107.3
RPGR	274.1	327.6	139.6	128	121.4	173.5	242	467.9
RPGR	243.8	315.8	105.6	105.5	91.2	96.2	192.4	251.2
RPGRIP1	79.1	92.6	78	91.2	83	88.3	77.8	93.1
RPH3A	106.5	117.3	101.8	98.7	99.3	100.3	94.2	102.4
RPH3AL	92.9	112.5	109	93.6	88	94.3	104.3	102.1
RPIA	1662.8	2237.4	589.4	512.7	375.8	568.8	1292.2	1723.2
RPIB9	100	112.9	77.4	82	90.4	83.1	94	127.8
RPIP8	83.6	92.7	92.6	85.6	77.2	94.6	83.6	94.9
RPL10	105.2	114.6	90.1	78.5	76.7	83.5	97.3	140.7
RPL10A	30648	30786.2	25267.2	23645.5	19272	22396.6	30897.2	30565.7
RPL10L	105.3	126.4	88.2	90.1	87	89.3	106.6	133.4
RPL11	24792.1	26495.5	13341.9	13653.4	13828.9	15378.7	25019.4	27665.1
RPL12	31313.3	30145.6	37481.2	27130	23619.9	30080.6	29252	29572.2
RPL13	347.8	417.1	216.2	140.1	118.3	112.6	279.1	305.9
RPL13	1219.9	1529.9	399.5	321.1	265.4	340.5	1102.9	1343
RPL13A	5424.5	6952.5	2034.9	1080.9	986.1	1390.8	4858.8	5929.5
RPL14	1372.7	1617	391.9	286.4	273.7	442.9	2045	2421.7
RPL14	2893.7	3570.1	1509.1	1639.3	1152.6	1623.7	2870.5	3274
RPL17	25241.3	27088.6	19805.1	18397.1	16267.1	21807.2	23925.7	25691
RPL17	3398.9	4331	2323.9	2825.4	2170.7	3430.8	2811.6	3131.6
RPL18	30935.2	28463.7	23895.2	17718.8	14726.9	13940.7	29914.5	31592.6
RPL18A	32081.7	27742	32118	22272.2	18694.2	19608.9	28699	28905.3
RPL19	31960.8	31668	24855.9	20004.1	15769.6	25843.2	28918.8	33417.4
RPL21	7680.8	8440.9	5417.3	4518.9	2646.6	5581	5394.9	6761
RPL22	4577.1	5963.6	4022.6	5127.4	3919.1	5902.1	4631	6785.3
RPL23	7285.7	8192.7	2095.6	1345.9	1730.2	1792.6	7172.9	9503.6
RPL23A	1517.1	1813.7	464.5	545.9	324.9	552.5	1391.4	2568.6
RPL23AP13	190.4	248.7	131.5	123.7	120.8	132.1	196.8	295.7
RPL23AP7	486.1	656.7	261.7	274.8	210.3	292.3	441.2	523.2
RPL24	24927.5	26878	20165.8	20274.6	14940	21911	22248.8	23292.7
RPL26	7059.5	7367.1	4245.5	4973.4	3322.2	6391.9	5598.7	6496.9
RPL26L1	2131.8	2390.5	1938.7	2236.1	1433.7	2399.9	1850	3348
RPL27	32218	32611.6	30375.3	33117.3	27787.3	32030.7	29479.5	29138.2
RPL27A	26638.5	28355	14528.1	12487.2	10720	16134.4	26022.7	25864.8
RPL28	748.9	929.3	484.3	331.8	274.7	321.4	807.6	1136.1
RPL29	2299.6	2923.1	1880.9	841.2	535.3	942	1328.7	1450.1
RPL29P2	121.9	140.4	132	111.4	120.6	116.5	118.4	113.6
RPL3	25009.7	28682.3	11175.7	8341.9	6468.3	12961.9	24309.9	27272.8
RPL3	24375.4	26092.6	11645.7	10855.4	11223.8	10011.3	23682.3	25828.1

RPL30	30428.5	31670.5	25841.2	19055.2	14699.1	20377.9	26254	29012.6
RPL31	10142.3	12297	4063.7	5030.9	3738	5186.7	8992.7	8668.5
RPL32	463	603.2	420.5	343.8	258.2	318.6	444.6	731.6
RPL32P3	119.6	153.6	120.6	93.1	105.5	104.1	121	140.3
RPL34	968	1333.8	848.4	1008.3	638.7	1205	754.3	1100.1
RPL34	2343	2914.4	1319.1	1110.3	796.6	1416.3	1622.1	2122.5
RPL35	21482.2	23405	9673.5	9419.8	7893.6	11987.8	21050.4	21877.3
RPL35A	28054.8	28787.5	28371.3	24664.2	18667.1	28587.2	20457.7	20233.9
RPL36	13204.7	15154.2	9283.3	5913.8	4550.5	6746	11359.4	13753.8
RPL36	320.3	350.6	285.8	174	151.6	165.9	333.5	298.9
RPL36A	631.3	805.6	435.4	306.2	209.2	367.3	526.3	599.8
RPL36AL	12416.9	15117.1	4516.3	3281.8	3120.6	4319.2	10948	14387.5
RPL37	195.1	230.4	122.1	130.9	116.5	120.5	160.4	207.6
RPL37A	18717.1	21602.5	7178.8	5139.1	4983.6	5080.4	19511.4	23841.6
RPL38	30320.6	29192.7	43566.2	43735.7	35327.9	44653.5	32022.8	31257.5
RPL38	31970.3	30757.8	30029.4	30503.7	24863.3	28324.4	33370.8	32536.1
RPL39	25328.4	25888.4	24999.6	23468.8	17335.8	27270.6	22434	23314
RPL39L	2770.2	3261.5	2210.1	1751.3	1361.6	2109.8	2093.7	3847.6
RPL3L	95.6	96.7	98.6	94.8	82.9	88.9	85.8	96.4
RPL4	15670.9	20860	10115.4	11366.2	10161.2	14101.9	14528.8	17026.2
RPL41	168.3	193.9	144.4	104.6	102.5	106.2	133.2	161.7
RPL41	33697.2	29331.9	34910.9	34792.1	30763.1	35197.7	34262	32962.2
RPL5	16046.2	18152.1	8166.2	8348.5	6044.5	8508.7	14374.5	17004.8
RPL6	1452.9	1809.2	819	794.3	628.9	1075.9	963.3	1157.2
RPL7	4036.5	5111.1	1102.3	953.2	532.6	1302.2	4514.6	6173.9
RPL7A	1342.3	1763.7	327.6	300.9	254.1	376.8	1655.4	1815.3
RPL7L1	11643.2	12704.6	6258.6	7443.2	6714.9	5957.1	10479.6	10632.4
RPL8	1261.7	1832.1	842.8	429.5	380.6	286.7	1007.2	1190.6
RPL8	31319.4	29852.1	33976.9	27360.3	22934.4	27687.6	32545.3	28617.4
RPL9	1079	1520.3	568.2	551.1	422.5	686.2	776	873.8
RPL9	7078.1	7042.6	2670	2221.4	1844.4	2724.8	7171.3	13426.9
RPLP0	117.6	115.8	87	74	77.9	83.2	108.4	119.3
RPLP0	4236.4	5173.2	2590.8	1869.1	1540.7	1902	3048	3520.2
RPLP0P2	98.9	113.9	100.1	95.2	94.6	93.7	89.1	99.3
RPLP1	27832.9	28106.5	17175	13865.2	10042.8	11605.7	27737.1	28735.9
RPLP1	18952.7	21343	5820.2	6986.2	4915.6	5607.9	17716.9	22634.8
RPLP2	28011.5	27488.5	22914	18106.6	15582.2	17323.9	26511.3	28050.7
RPN1	6854.4	8684.2	6000	5843.5	5220.5	7969.6	6075.9	7431.9
RPN2	1285.6	1740.1	464.9	399.3	288.6	698.1	1198.5	2435.9
RPP14	132.3	135.2	85.5	73.7	76.5	94.7	129.9	144.8
RPP21	911.4	1111.7	729.6	656.7	534.1	580.7	895.3	872.6
RPP25	419.1	615.2	281.6	168	157.1	226.3	367.6	653
RPP30	88.2	94.3	98.5	88.2	88.3	86.3	83.9	96.7
RPP38	230.4	284	203.2	130.2	122.4	144.2	186.8	242.4
RPP38	128.5	157.8	96.5	90.8	86	95.1	144	149.3
RPP40	1530.8	1979.4	1113.9	1073.5	699	987.9	1355.8	1607.5
RPRC1	2214.6	2691.1	1354	1477.8	1215.8	1137.1	1939.7	2122.1
RPRM	89.9	106	103.4	93.9	75.2	83.8	89.4	96.4
RPRML	161.7	200.1	117.7	112.5	97.5	102.1	137.2	148.8
RPS10	27516.7	27322.5	38454.3	33903.5	31389.6	38436.4	27100	28153.1

RPS11	33058.6	31020.8	25078.4	20985.9	19893.6	26000.9	27934.4	31086.1
RPS12	30787.3	27462.7	39228.2	38451.4	33609.1	38997.4	33152.4	30334.7
RPS13	25304.7	28068	12084	10293.6	7094.5	12253.9	21848.3	24871.9
RPS14	28154.6	28175.5	19483.6	18869.2	16902.3	18422.7	27811.8	29578.2
RPS14	17416.1	20994.4	10521.3	10401.5	8234.7	10415	17192	17884.1
RPS15	23238.5	25989.9	14241.2	9449.1	7527.3	10246.7	20729.3	22660.2
RPS15A	15827.2	15333	4487.1	4901.3	3322.6	5211.2	15662.3	16727.3
RPS15A	33275	29346.2	29686.5	28841.1	24431.4	31531.5	31511.7	25975.7
RPS15A	11072.1	13752	8165.3	6349.3	4820.8	7624.7	9974.9	9514.9
RPS16	26077.9	25241.7	24526.7	20962.1	21498.9	22222.1	27789.2	27311.8
RPS17	26494.9	25276.2	25602.6	26860.1	22126.6	24613.4	27210.5	24210.9
RPS19	26978	26850.3	10745.6	8345.7	6573.6	6288.6	25853.3	25764.3
RPS19BP1	909.6	1014.6	547	450.6	403.8	708.7	739.7	1022.2
RPS2	26075.7	30173.6	16874.3	9273.4	9014.3	10322.2	25960.5	27449.5
RPS20	26964.7	30128	13326.2	11435.4	8886.4	11843.3	27622.8	27945
RPS21	6469.8	7473.6	5486.8	3477.9	2571.7	4139.3	5005.6	4854.6
RPS23	2404.3	2993	1734.9	2094.7	1512.5	2045	1721.4	2397.6
RPS24	4195.2	5319.9	2918.2	1705.6	1270.4	3022.2	3194.4	3359.3
RPS24	10292.4	12560.5	4359.8	3599.1	3131.7	3669.2	10871.1	12516.8
RPS25	31015.3	30888	55355.2	45035.8	39169.1	47765.9	32488.8	32265.5
RPS26	7235.3	8005.4	3547.8	3139	2104.5	3173.5	6309.8	9867.9
RPS26L	4332.8	4986.5	1848.1	1448.7	909.8	1239.6	3943.9	6771.8
RPS26L1	84.6	97.5	80.3	82.5	92.4	84.2	80.1	100.9
RPS27	29338.6	27373.1	47753.2	47582.3	43591.2	47875.8	27496.6	27941
RPS27A	29802.8	27052.8	39948.9	37512.1	33862.5	34561.7	28057.6	30952.3
RPS27L	84.5	91.6	102.9	88	76.2	92.4	80.4	97.1
RPS28	9445.7	11795.9	4195.5	2440.6	1957.7	2410.4	9827.7	19967.2
RPS29	20842.6	20283.6	13795.7	10783.2	7389.9	11753.9	15506.2	16204.8
RPS29	748.8	988.8	573.2	381.8	333.9	390.1	894.7	899.7
RPS29	25871.8	29176.2	43229.1	40472.9	35846.3	39108.1	27148.3	28533.7
RPS29	28727	30052.5	33541.7	30088.1	27425	30747.5	30811.4	29613.9
RPS3	32242.9	32183.6	34871.4	23603	21149.1	23723.7	33242.7	34100.7
RPS3A	22943.8	24082.3	9391.3	8286.9	5874.2	6067.9	24091	26491.4
RPS4X	10312.4	14084.8	7766.1	6660.3	5451.1	7682.9	8604.6	8669.6
RPS4Y1	10350.8	13359.2	5819.9	6320.2	4827.5	6708.4	8548.1	12121.4
RPS4Y2	845.3	1129.2	330.3	357.7	222.5	426.4	680.9	1087
RPS5	28506.7	30225.9	17958.9	13332.5	10455.3	17032.9	24225.1	26613.1
RPS6	25015.8	25591.8	9944.7	9452.7	7768.3	10477.3	22256	25257.9
RPS6KA1	114	105.1	87	92	81.4	87.1	106.3	101
RPS6KA1	639.3	723.7	540.8	627.5	541	668.2	539	668.8
RPS6KA2	85.6	84.7	96.2	93.5	92.7	97.4	83	104.8
RPS6KA2	144.2	196.6	164.7	203.1	201.2	228	234.3	430.4
RPS6KA2	93.8	102	107.2	96.4	91	109.1	88.2	99.7
RPS6KA3	141.4	220.3	168.1	235	198.5	220.9	188.5	192.5
RPS6KA3	170.8	211.2	111.5	95.8	97.6	109.6	134.7	180.8
RPS6KA3	291.2	428.8	128.1	140.1	118	133.8	231.7	286
RPS6KA4	381.4	426	314.3	311	235.9	265.4	277.1	725.2
RPS6KA5	165.5	190.4	147.8	195.1	192.9	256.1	165.7	296.2
RPS6KA5	80.6	82.3	101.6	95.5	93.4	99.7	90.7	86.3
RPS6KA6	109.7	144.8	114.7	101.4	104.8	112.6	104.3	133

RPS6KB1	843.8	1074.9	492.3	679.6	510.8	969.9	723.7	968.6
RPS6KB2	3173.7	3940.6	1645.2	1259.1	1196.6	1167.6	2823.2	2330.3
RPS6KC1	138.6	165	114.8	95.2	97.4	117.1	165.8	335.9
RPS6KL1	128.2	136.9	141.1	107.7	103.6	117.8	109.6	130.4
RPS7	2189.6	2799.7	1578.2	1186.2	837.4	1709.8	1538.4	1672.3
RPS9	10622.4	13587.7	7613.2	5985.4	5274.6	7437.1	10595.3	10885
RPS9	13205.8	16659.8	10191.8	8377.1	7506.4	10521.2	13133.9	13801.4
RPSA	33204.2	33357.8	43080.3	23205.6	20513.5	27492.9	33473.8	33377
RPSA	194	216	122.8	104.2	113.2	117.5	194.9	270.7
RPUSD1	768.5	871.1	712.1	359.4	245.8	371.7	701.2	1210.1
RPUSD2	1902.1	2683.4	1474.3	1208.5	857.1	1104.1	1446.2	1547.8
RPUSD3	2078.3	2211.7	1289.7	738.9	626.3	690.7	1598.2	1808.4
RPUSD4	3505.3	4009.6	2370.2	1293.4	903.5	1522.9	2659.2	3182.5
RQCD1	1338.8	1817.1	1122.4	987.7	760	977.1	1130.7	1582.6
RRAD	87.6	90.2	95.3	87.1	80.8	73.6	89.1	93.1
RRAGA	2781.5	3710.2	2481.4	1620.5	1323.1	2203.2	2207.1	3403.8
RRAGB	238.9	340.7	127.8	108.8	104.8	122.9	199.1	281.2
RRAGB	77.9	95.6	88.4	87.9	87	84.9	83.8	94.6
RRAGB	365.5	460.6	263.1	296.4	282.1	426.6	322.4	410.9
RRAGC	409.5	573.3	269.4	413.4	396.3	469.2	466.6	598.5
RRAGD	834.1	1189.6	512.4	837.9	796.9	878.4	774.8	1144.1
RRAS	508.2	654.8	357.1	251.5	241.6	287.7	549.5	567.7
RRAS2	401.8	506.5	218.8	247.8	182.3	281.7	351.6	537.2
RRBP1	155.4	143.3	117	129.6	120.2	143.7	133.2	139.1
RRBP1	603.5	659.4	496.4	616.4	600.3	750.4	507.6	796.9
RRBP1	159.2	157.9	139.8	161.7	159.6	153.7	123.4	165.5
RREB1	86.1	100.1	108.5	92.2	93.9	88.6	82.1	91
RREB1	165.8	262.1	146.6	166.7	144.4	195.9	149.2	165.2
RREB1	113	117.5	111.3	95.4	106.4	98.8	102.5	98.9
RREB1	94.7	116.5	121.7	106.4	110.2	123.5	92.1	100.7
RRH	81.9	90.2	90.3	89.2	77.7	81.8	79.3	90.5
RRM1	7206.2	8545.1	7369.8	6607.1	4933.9	6751.3	5887	10864.1
RRM2	793.1	1254.7	506.8	488.4	429.7	607.9	571.4	847.2
RRM2B	150.8	179.8	122.1	125.9	112.5	131.6	190.6	293.8
RRN3	274.8	339.8	104.7	131.5	96.9	129.2	217.5	339.8
RRP22	243.8	256.4	151.1	118.2	108	118.9	215.9	180.4
RRP22	117.6	122.4	93.1	100.4	82.9	100.5	117.2	132.8
RRS1	1814.4	2619.8	1270.9	729.7	500.1	954.1	1276	1890.3
RS1	95.6	98.8	93.2	91.6	80.8	84.1	86.5	101.8
RSAD1	238.2	258.7	129.5	117.2	115.9	133.7	166.6	238.3
RSAD2	145.4	190	109.3	112.2	94.9	118	138.9	158.1
RSAFD1	405.6	548.6	267	317	288.3	439.7	355.3	449.7
RSBN1	1295.6	1765.7	974.5	779.5	636.8	1071.1	1141.5	1529.5
RSBN1L	560.8	689.5	250.6	245.3	276.3	395.5	479.9	763.2
RSC1A1	147.7	184.1	136.5	119.9	111.3	148.1	135.2	179.3
RSHL1	82.8	104.3	93.3	97.8	81.3	98.8	85.4	98.1
RSHL2	355.2	471.4	233.3	356.6	270.4	391.9	349.2	413.7
RSHL3	77.5	82.9	114.2	102.3	99.3	105.1	75.4	79.8
RSL1D1	4001.8	5840.2	2828	2274.4	2107.8	4094.9	3112.8	4372.3
RSN	81.9	97.8	85.1	93.1	86.4	84.5	88.2	116.6

RSNL2	116.6	141.4	127.4	132.5	124.9	141.8	120.9	153.5
RSPO1	99	135.6	79.8	75.1	72.4	79.2	111.6	107.9
RSPO2	126.3	135.3	164.7	143.5	152.5	158.5	110.3	137.5
RSPO3	130.4	158.3	122	111.6	99.2	124	134.6	166.9
RSPO4	101.2	108.8	96.6	90.9	81.6	90.6	91.4	109.5
RSPO4	89.3	96.8	106.4	94.9	90.6	90.7	91.9	87.6
RSPRY1	1692.2	2164.5	1315.4	1287.7	1076.5	781	1459.9	1774.4
RSRC1	1654.3	2234.8	1060.5	1053.9	849.3	1398.3	1417.4	2149.1
RSRC2	1432.2	1754.1	350.6	457.3	275.2	539.4	1230.7	1920.4
RSU1	857.6	1027.1	405.7	829.1	637.1	982.6	677.3	1071.6
RSU1	142.1	149.5	109	113.7	99.8	148.4	119.9	155
RTBDN	88.3	92.7	90.3	81.8	73.7	84.1	95.7	92.8
RTBDN	105.7	118.7	101.7	83	88.2	89.1	96.9	102.6
RTBDN	109.5	117.5	100.1	92.1	86.7	97.8	100.7	106.3
RTCD1	2540.3	3370.3	587.8	422.2	402.5	510	2357.7	3591.9
RTDR1	72	84.5	90.8	83.5	83.3	79.9	82.9	79.4
RTEL1	183.4	193	108.7	94.6	89.1	93.9	152.4	181.6
RTEL1	97.2	118.7	92.2	92	84.7	82.4	101.1	117.6
RTF1	141.4	146.9	165.5	159.9	156.7	193.4	130.5	160.3
RTKN	103.2	120.7	115.6	107.3	108.3	111.9	104.3	108.3
RTKN	502.3	634.9	266.5	221	212.9	233.2	391.6	278.1
RTKN	129.5	155.1	103.5	96.7	79.9	86.2	125.8	144.8
RTKN	86.4	105	92.6	86.1	85.2	86.1	90.1	106.8
RTN1	85.2	92	90.2	84.3	82.7	85.6	92.6	95.9
RTN1	102.3	119.1	100.7	94.4	82	98.1	101.7	111.5
RTN1	170	209.5	198.3	97.8	93.4	110.8	147.2	225.7
RTN2	177.4	243.1	160.6	202.9	189.4	217.3	188.1	325.5
RTN2	552.9	678.2	349.8	360.4	310.2	377.7	559.1	567.1
RTN2	119.8	181.4	87.6	84	87.6	105.8	116.9	169.2
RTN3	678.9	933.5	281.9	293.1	267	424.6	624.1	1173.9
RTN3	102.7	122.6	108.4	105.6	105	101.5	97.8	114
RTN3	94.9	105.8	90	92.3	83.4	94.3	86.9	104.7
RTN3	1635.6	2277.4	1302.6	1458.9	1224.1	1829.1	1356.9	1974.4
RTN4	785.5	895.4	409.3	560.2	385.4	695.5	955.6	2268.4
RTN4	87.9	82.9	90.1	81.1	77.9	81.7	88.5	87.6
RTN4	3995.5	4627.3	3318	3989.9	2739.5	6035.6	4528.2	9338.9
RTN4	102.4	111.5	101.8	94	92.3	104.2	92.2	100.1
RTN4IP1	726.9	1043.1	342.4	320	238.2	297.5	579.2	846.7
RTN4R	148.9	197.3	104.3	105.7	97.6	97.1	142.3	139.9
RTN4RL1	126.7	147.8	112.9	87.7	89.4	95.2	120	135
RTN4RL2	88.8	97.3	87	89.9	90.8	91.8	92.3	97.7
RTP1	104.4	114.4	104.8	91.4	100.3	89.7	106.6	111.3
RTP2	127.7	141.4	101.6	92.5	94.6	94.7	135.8	150.3
RTP3	94.1	88.4	88.3	85.1	82.3	86.6	97.6	98.3
RTP4	230.3	294.2	171.9	150.5	147.4	196.4	203.3	278.4
RUFY1	106.5	119.2	95.7	103.9	93.2	99.4	99.7	107.6
RUFY1	422.4	556.5	304.3	308.4	314.9	476.7	335.2	609.1
RUFY2	121.7	136.9	103.3	97.3	89.3	92.9	109.4	146.1
RUFY2	141.1	172.4	123	107.6	108	106.6	134.1	150.2
RUFY2	134.5	150.6	98.7	97.1	83.6	92.4	146.7	153.1

RUFY3	131.5	149.8	90.1	91.7	91.3	92.8	126.6	150.3
RUFY3	100.7	142.2	104.2	103.8	104.7	138.6	111.6	167.8
RUFY3	115.4	135.6	103.9	95.9	100.5	92.3	128.8	118.3
RUNDC1	562.8	725.6	460.1	642.4	507.5	954.4	440.7	744.1
RUNDC2A	111	116.1	105.4	98.2	98.8	102.7	113.6	119.1
RUNX1	136.2	171.3	95.6	94.9	86.9	95.8	134.8	147.9
RUNX1	96.6	98.9	103.3	111.2	102.6	118.2	98.9	99.2
RUNX1	599.1	862.8	480.2	719.6	608.6	781	798	932.3
RUNX1T1	81.4	95.5	85.7	85.2	76.1	84.5	89.9	92.1
RUNX1T1	105.1	124.1	86.7	85.7	79.5	88	100.9	117.5
RUNX1T1	89.4	89.9	88.2	84.2	85.1	87.2	83.7	96.4
RUNX1T1	96.1	104	110.9	96.8	94.1	100.9	93.8	96
RUNX1T1	99.2	108.3	86.8	83	80.4	89.6	109.1	116.2
RUNX2	130.1	148.4	84	87.4	89.3	94.9	122	141.8
RUNX2	663.2	878.5	397.8	444.1	355.1	681.2	339.4	494.8
RUNX2	88	116.5	104	95.1	78.3	92	90.6	89.2
RUNX3	1958.4	2583.5	1219.6	1030.7	803.5	868.5	1791.2	2114.6
RUNX3	115.7	117.3	104.4	107.7	102.6	95.9	112.7	122.5
RUSC1	2811.2	3643.7	2015.6	1393.4	1187.6	1267.4	2448.8	2673.8
RUSC2	133.6	145.3	110.3	116.6	89.2	108	131.7	159.8
RUTBC1	860.5	1012	900	1070.8	1058.6	1206.2	583.8	645.4
RUTBC2	88.2	89	98.8	90.4	90.1	95.6	85.5	104.2
RUTBC3	156.5	184	146.1	135.5	133.6	168.9	147.5	194.6
RUVBL1	1050.8	1471.5	491	303.1	251.5	345.3	650.2	779.1
RUVBL2	2089.6	2832.7	1340.1	561	471.3	686.3	1878.5	2188.7
RWDD1	192	241.7	162.8	153.2	133.4	199.8	143.8	182.8
RWDD1	6552.3	7696.5	5303.5	5895.1	4554.8	6639.4	5628.9	9096.6
RWDD2	365.3	493.8	305.2	322.4	258.4	384.8	296.6	393.3
RWDD4A	806.6	1075.5	464.8	416.2	356.9	520	666.2	965.6
RXRA	2060.9	2404.5	2829.3	2695.2	2024.6	3074.6	2788.7	5384.2
RXRB	782.1	920.2	289.2	316.7	330.3	275.7	521.5	411.1
RXRG	83.6	90.5	93.6	89.1	75.2	77.6	79.9	93.1
RXRG	76.9	85.3	105.7	95.3	93.6	93.8	77.2	78.5
RXRG	102.9	132.2	92.4	90.3	85.5	92.1	110.1	113.7
RY1	2005.8	2436.7	832	897.3	667.1	931.6	1803.6	2545.3
RYBP	869.6	1145	411.9	783.1	559.3	569.2	790.6	1219.2
RYK	319.2	395.8	207.2	205.9	190.6	289.6	231.6	291.6
RYK	447.5	604.3	331.6	328	278.8	494.3	373.9	464.6
RYK	1318.6	1733.4	710.6	896	695	1121.6	1122.8	1522.1
RYR1	146.2	218.8	115.6	116.6	111.8	130.7	145.4	157
RYR1	220.9	331.2	217	241.2	210.7	305.5	210	326.2
RYR2	112.3	123.4	108.3	92.9	80.1	99.7	103.7	125.2
RYR3	76.9	88.1	114.5	104.5	95.8	105.9	87.7	87.3
S100A1	94.5	127.8	97.2	104.7	100.6	99.7	102.9	128.5
S100A10	4400	5366.1	3666	3125.9	2008.1	2604.1	4546.6	6960.7
S100A11	1591.9	1993	744.7	608.9	436.7	624.9	1370.1	2027
S100A12	103.1	116.6	106.5	142.8	121.2	182.2	94.4	140.1
S100A13	89.1	99.8	110.9	93.1	89.6	109.4	95.4	104.3
S100A13	91.8	99	87.6	99.3	103.9	87.1	92.7	102.3
S100A13	233.4	264.5	154.8	125.1	131.4	177.4	194.1	302.3

S100A13	225.8	276.3	124.5	134.9	132.5	156.8	217.9	232.4
S100A13	120.8	145	121.1	98.2	103.7	110	117.3	138.9
S100A13	185.4	237.4	155.9	155.5	163.4	143.9	197.2	216.6
S100A14	123.7	147.1	92.8	82.9	83.4	84.5	113.2	130.8
S100A16	101	114.6	88.8	98.1	83.6	87.6	125	120.6
S100A2	187.8	235.8	109.7	124.6	98	114.3	184.6	226.5
S100A3	81.7	101.3	96.3	86.1	83.4	87.2	93.6	96.4
S100A4	17843.5	21563	13676	9960	9621.5	11364.2	14589.1	16305.1
S100A4	20282.2	23844.9	19905.8	16638.8	15105.3	15487.4	16341.2	18131.7
S100A5	132.3	180.8	96.3	86.2	95.3	83.8	142.7	152.2
S100A6	4171.5	4793.9	2236.5	2885.9	2523.3	2328.4	4591.6	3867.1
S100A7	74.3	83.1	77.8	79	75.9	79.4	85.1	85.5
S100A7L1	81	80.3	85	94.7	91.7	82.7	74.6	82.5
S100A7L2	116.9	127.9	89	107	90.7	94.7	111.9	105.9
S100A8	1632	1711.3	635.6	2845.8	2457.1	3782.8	1597.2	1653.9
S100A9	2503.4	2239.2	1514.6	3481.5	2331.8	4018.4	2401.2	3516.1
S100B	74.6	86.4	84.3	77.5	77.9	76.1	81	89.4
S100G	79	90.2	89.6	90.8	73	85.2	81	90.5
S100P	325.2	358	255.8	186.6	188.2	207.5	458.5	619.6
S100PBP	166	224.7	107.4	118.6	107	103.7	158.6	210.2
S100PBP	276.4	402.9	143.4	142.8	125.1	165.8	247.6	348.7
S100Z	74.1	82.4	90.7	73.5	77	79.9	83.8	81.1
SAA1	111.2	113	89.9	86.8	72.6	98.4	124.7	130.1
SAA1	112.6	138	93.4	95.2	82.5	89	110.4	134.1
SAA1	85.5	104.1	100.4	93.4	98.1	106.2	89.4	88.6
SAA2	108.2	119.5	98.3	93.1	94.5	91.4	109.5	118
SAA4	104.2	116.1	86	77.6	79.6	88.7	101.9	135.4
SAAL1	2135.1	2400.4	1081.3	861.6	665	987.6	1707.3	2404.6
SAC	86.3	115.8	88.7	82	86.4	86.8	95.4	108
SACM1L	1013.9	1112.1	391.8	533.2	298.5	648.9	792.5	1725.4
SACS	246.9	344.6	151.3	195.4	155.2	237.8	237.8	285.4
SAE1	9560.4	11412.6	7876.5	5784.7	4482.4	6233.2	8132.3	9942.3
SAFB	1913.2	2644.8	1153.1	997.3	994.8	1659.6	1480.3	1239
SAFB2	767.1	870.3	680.4	619.1	485.3	714.5	604.6	1304.5
SAG	93.6	95.9	95.1	89.5	86.7	95.2	86.1	94.1
SAGE1	80.1	105.5	96	83.3	73.1	83.1	76.6	82.2
SALF	83.1	95.5	91	85.4	75.8	78.8	90.9	98.6
SALL1	82.7	91.6	90	87.7	83.1	104	84.1	86.7
SALL2	99.6	108.8	102.7	95.2	90.8	101.5	92.8	107.6
SALL3	234	243.1	130.3	134.2	115.9	134.7	241.4	247.9
SALL4	102	128.3	118.5	135.1	145.4	145.4	94.1	109.5
SAMD1	168.9	196.9	170	135	115.7	142.7	141.8	248.9
SAMD10	112.9	121.7	109.2	93.2	85.8	99	99	112.5
SAMD11	114	123.3	118.6	118.2	102.9	122.9	111.4	117.6
SAMD12	87.2	88.1	104.4	92.4	85.9	85.9	89.8	92
SAMD13	158.5	201.7	134.7	110.5	100.4	118.6	130.7	195.9
SAMD14	111.4	129	108.8	103.1	96.8	102.2	115.9	108.3
SAMD3	73.8	85.4	98.9	88	88.8	84.6	80.1	74.6
SAMD3	130.7	155.6	102	101.9	92	99.9	131.7	148.7
SAMD3	92.9	93.6	92.1	77.3	77.7	80.1	82.3	87.7

SAMD4A	90.6	88.2	95.7	93	86.1	71.8	89.6	107.3
SAMD4B	116.5	162.3	150.1	165.6	129.6	188.5	126.9	208.7
SAMD7	177.8	189.8	105.8	91.2	88.8	97.8	166.4	172.7
SAMD8	101.3	117.2	100.7	93.2	85.5	103.6	111.9	106.3
SAMD9	553.5	736.7	299	354.5	321.8	584.7	427.5	759.9
SAMD9L	113.7	129.4	117.4	154.4	164.3	235.5	114.7	131
SAMHD1	254.9	359.6	119.4	166.8	155.7	149.7	213.1	252.9
SAMM50	3133.1	4075.9	2797.6	2656.1	1832.7	2884.8	2712.1	2834
SAMSN1	75.9	101.4	86.8	96.3	96.2	96.8	90.2	96
SAP130	528.8	709.1	491.6	522.3	466.9	570.9	496	721.6
SAP18	387.5	505.1	277.3	329.3	258.4	354.5	381.8	585.2
SAP30	1446.5	1538.9	706.2	485.8	355.7	576.6	1518.7	2865.7
SAP30BP	148.5	170.5	155.4	143.6	129.4	151	128.9	187.2
SAPS1	353.6	401.7	386.7	262	239.4	318.4	270.3	453.5
SAPS2	89.6	104.1	91.8	89.8	88.2	96.7	92.5	118.4
SAPS3	452.1	717.5	483	520.9	461.1	697	460.3	613.7
SAR1A	809.7	936.2	245.6	164.8	138.9	174.6	685.4	1146.1
SAR1B	133.3	155.7	117.7	95.6	93.8	98.1	132.3	130.2
SAR1B	1843.7	2057.7	974.9	863.9	534.5	1032.9	1772.8	3171.9
SAR1B	753.6	902.2	304.5	248.5	165.2	319.8	630.2	939.4
SARDH	74.4	76.8	91.5	84.2	83.8	87.3	75	87.7
SARM1	78	89.7	80.7	81.9	75	87.5	77.3	79.6
SARS	1022.8	1472.1	223.5	179.3	157.4	202.6	1113.1	1412.4
SARS2	639.3	702.4	364.4	250.2	230.6	277	526.3	657.6
SART1	106.1	116.5	90.8	89.1	101.5	88.4	110.9	119.8
SART2	90.5	94.3	90.3	83.6	81.5	86.7	86.7	96.2
SART2	86.7	99.9	95.2	86	93	84.4	88.1	91.1
SART2	184.7	211.5	165.2	202.7	202.5	240.4	189.2	302.8
SART3	136.8	186.8	129.9	122.6	132.7	144.9	128.3	205
SAS10	79.9	93.7	90.1	76.8	83	82.7	87.9	90.8
SASH1	1019.8	1353.8	436.5	994.2	783.1	1320.5	974.8	1391.6
SASS6	556.4	616.2	300.4	187.4	200.5	367.7	439.6	860.7
SAS-ZFAT	80.2	97.3	84.4	89	80.7	77.9	84.4	95.2
SAT	809.9	983.4	389.6	489	489.6	711.3	999.6	1427.2
SAT2	235	279.9	130.7	131.2	128.6	154.7	265.5	414.1
SATB1	346.8	436.4	169.8	178.5	136.7	260.4	249.1	307.8
SATB2	1466.4	2007.6	1166.9	1754.6	1324.7	2282.4	1359.7	2147.7
SATL1	100.3	120.6	101.5	92.9	90.4	89.4	88.3	99.7
SAV1	245.1	307.3	219.6	181	167.5	220.5	232.5	483.4
SBDS	670.2	946.9	327.7	298.4	266	467.6	644.3	1109.8
SBDSP	442.8	628.1	198.7	191.1	161.3	229.1	339.1	504.8
SBEM	122	139.2	99.4	91.7	83.1	94.4	117	129.1
SBF1	496.1	571.6	560.7	576.2	466.1	657.2	389.6	447.9
SBF2	96.9	117.9	103	101.3	100.6	118	98.8	131.2
SBK1	80.3	113.2	79.2	75	76.4	73.6	89.2	92.4
SBK1	167.8	193.7	130.9	107	100.1	106.7	155.4	181.5
SBNO1	188.5	257.1	102	101.2	91.4	118.6	166.4	235.3
SBP1	156.7	140.4	100.9	111.3	93.7	101.7	132.5	142.4
SBSN	90.8	97.9	106	89.3	82.9	86.4	97.2	98.8
SC4MOL	441.2	658.9	194.6	233.8	170.3	221.4	317.1	384

SC4MOL	1106.2	1426.4	466.2	566.8	597.8	678.3	842.5	1152.8
SC4MOL	90.9	109.6	106.3	86.9	86.8	85.4	104.4	101.2
SC5DL	682.1	851.6	453.5	443.7	417.3	496.1	705.6	831.9
SC65	301.4	353.8	213.2	206.6	169.3	190.3	266.9	464.7
SCAMP1	1480.7	1960.3	1059.9	1437.9	1021.3	1618.2	1131.1	1740.5
SCAMP1	353.8	408.2	123.5	146.3	124.2	125.1	215	350.7
SCAMP2	306.7	429	268.6	262.5	201.3	231.5	289.4	352.8
SCAMP3	214.9	279.7	151.4	124.6	106.8	133.6	207.5	300.9
SCAMP3	2857.9	3457.4	2071.9	1125.4	848.8	1058.8	2717.1	3253.3
SCAMP4	138.6	154.9	125.6	127.2	112.2	128.7	133.3	175.6
SCAMP5	195.9	257.5	282.3	341.3	317	381.6	174.3	235.9
SCAND1	966.1	1290.2	611.1	459.3	412.8	465.8	760.6	936.3
SCAND1	376.4	467.3	214.8	164.4	144.3	121.2	335.6	408.8
SCAND1	1539.4	1962.5	1088.3	774	680.6	846.7	1209.6	1459.3
SCAP	1288.3	1547.9	671.2	711.6	600.3	740.9	1050.3	924.7
SCAP1	110.2	125	86.3	80.9	74.7	82.2	105.2	118.3
SCARA3	110.8	110.3	96.6	87.5	85.4	93.9	102.5	108.5
SCARA3	83.1	78.1	89.3	90.6	81.5	90.7	80.9	85.7
SCARA3	85.3	98.1	103.1	77.9	81.4	79.1	89.1	93.7
SCARA5	84.5	106	88.7	87.5	93.4	94	83.4	90.3
SCARB1	613.4	767.8	888.3	874	708.5	1124	480.6	894.5
SCARB2	2012.9	2794.7	1478.5	2425.7	1669.4	2880	1923	2441.1
SCARF1	102	105.5	99	83.8	88.9	89.1	96.5	106.5
SCARF1	122.2	148.8	94.6	93.2	83.3	89.1	123.7	138.8
SCARF2	98	112.3	105.7	94.1	75.7	79.5	96.4	119.7
SCARF2	78.4	93.9	80	76.1	89.2	84.1	98.6	85.5
SCARNA15	84.4	87.2	93.8	83.3	79.2	85.1	80.7	81.2
SCC-112	655.6	1032.7	423.8	413.1	309.6	722.6	506.6	1043.9
SCCPDH	259.5	295.7	170.6	183.7	152.1	185	205.6	319.5
SCD	5534.6	6952.5	4422.4	6068.1	5337.9	5463.7	5247	7330.1
SCD5	161.1	200.9	121.3	109	96.4	102.8	168.9	211.1
SCD5	109	129.6	82.8	89.2	92.1	93.9	109.5	111.5
SCD5	300.3	408.9	255.3	133	121.3	156.6	216.4	298
SCDR10	155.6	189.9	118.8	106.6	99.1	125.1	128.2	161.7
SCEL	142.8	168.3	108.7	91.4	93.2	97.2	132	145.5
SCFD1	1407.7	1783.7	866.1	915	678.3	1115.3	1158.6	2003.3
SCFD1	87	85.5	88.7	88	83.1	93.1	87.2	84.4
SCFD2	179.6	243.4	101.1	88.5	91	110	162.3	206.3
SCG2	86.8	97.7	86.6	84.8	74.8	89.2	81.8	109.1
SCG3	196.7	199.7	147.9	146	137.8	183.6	178.4	236.7
SCG5	110.7	122.9	111.5	99.7	100.1	112.8	96.4	129.9
SCGB1A1	99.1	115.3	93.3	88.8	83.2	94.2	99	113.3
SCGB1C1	88.5	125	105.9	87.3	89.7	90.5	93.2	103.1
SCGB1D1	159.1	209.8	132.8	108.5	91.7	108.3	155	164.4
SCGB1D2	87.3	101.9	95.5	84.3	81.8	84.7	90.1	91.3
SCGB1D4	76.6	98	94.6	81.2	79	80.8	93.1	101.6
SCGB2A1	83.9	86	102.1	96.9	92.8	90.6	71.7	88.2
SCGB2A2	85.1	102.6	95.9	87.7	86.2	95.9	93.6	101.2
SCGB3A1	91.9	105.7	99.9	102.3	89.7	93.7	109.6	119
SCGB3A2	96.5	109.6	84.9	78	79.8	82	94.7	115.3

SCGN	89.4	103.1	99.2	95.1	94.5	91.7	89.7	92.8
SCHIP1	147	163.7	133.9	107.5	109.7	123.4	151.7	156.5
SCIN	146.2	170.5	153.1	123.1	113.5	156.1	132.7	147.7
SCLY	194	239.2	232.3	194.2	164.6	199.2	176.5	303.1
SCMH1	148.4	180.6	127.8	212.8	215.1	116.8	117.9	140.7
SCMH1	1900.1	2268.9	843.7	862	918.4	988.6	1432.3	1551.7
SCMH1	344.5	384.3	138.6	229	216.1	110.8	249.2	355.5
SCMH1	365.4	394.2	336.1	309.6	257.9	541.8	249.4	420.3
SCML1	98.6	117.1	91.8	86	89.4	91.9	110.3	110.8
SCML1	121.3	133.6	109.4	106.7	96.4	93.6	117.4	136.8
SCML1	82.3	81.5	89.2	80.9	77.4	83.2	75.9	88
SCML2	241.1	320.2	223.5	143.8	140.5	177.6	224.4	370.7
SCML4	85.9	84.5	88.8	95.4	87.1	84.2	78.3	75.6
SCN10A	90.8	105	94	81.7	87.1	95.4	84.9	92
SCN11A	79.5	100.5	90.4	95.8	67.5	80.2	84.2	93.8
SCN1A	93.8	107.1	89.4	81.5	72.7	86.5	96.6	102.1
SCN1B	93.3	89.5	103.7	102.6	99	98.4	88.8	96.3
SCN1B	81.1	93.3	94.9	89.6	85	91.1	77.2	83.7
SCN2A2	72.7	88.6	93.6	91.8	86.9	94.7	71.3	78.2
SCN2A2	90.3	107	97.9	85.9	88	85.2	94.5	104.1
SCN2A2	85.8	86.2	105.9	90.1	82.5	93.4	86.6	95.9
SCN2B	81.2	95	98.5	94.6	80.6	85.4	72.7	86.4
SCN3A	85.9	97.2	84	83.5	76.1	78.2	81.7	96.5
SCN3A	580.3	726.4	328.1	308.3	208.6	362.5	466.2	538.5
SCN3A	111	119.8	104	93	92	100.8	93.7	123
SCN3B	79.7	97.3	93	83.5	80	80.7	89	86.9
SCN3B	92.2	101.9	96.3	86.4	90.9	88.2	86.3	99.2
SCN4A	88.7	102	105.5	95	92.2	88.6	86.4	106.5
SCN4B	129.4	154.7	95.2	88.2	84.9	90.5	144	150.4
SCN5A	78.1	83.2	89.4	92.2	96.4	86.5	78.4	81.6
SCN5A	92.1	98	81.2	78.7	79	85.3	83.4	100.2
SCN7A	116.6	122.2	109.9	99.1	101.4	96.9	110.6	113.7
SCN8A	75.5	83	93.3	88.8	88.4	84.2	78.1	89.3
SCN9A	96.9	96.7	109.5	97.6	98.8	97.6	99.3	103.6
SCNM1	1380.1	1821.9	801.9	526.6	467.4	568.5	1238.1	1519
SCNM1	2301.7	2874.1	1478.7	959.6	834.8	1108.6	2178.2	1961.8
SCNN1A	96.9	103.4	101.3	94.5	94.1	100.1	89.3	94.5
SCNN1B	119.3	128.2	95.6	104	86.2	95.7	116.6	123.9
SCNN1D	206	191.7	153.3	129.1	161.4	172.8	141.5	160.1
SCNN1G	88.8	106.9	91.2	84.3	91.5	85	92.9	109.6
SCO1	1061.8	1470.7	613.7	556.2	417.5	705.4	789.4	1016.6
SCO2	1193.9	1574.9	1266.7	787.1	752.7	993.5	933.7	1147
SCOC	1265.6	1529.2	414.9	430	335.5	764	914.9	2293.9
SCOTIN	1967.6	2811.8	1222	830.9	602.2	862.2	1723.2	3408.7
SCP2	111.4	132.9	93.9	89.5	81	93.4	96.8	130.2
SCP2	434.6	581.5	197.2	217	226.7	189.2	258.1	363
SCPEP1	2108.7	2690.1	1254.9	2336	2191.6	3208.2	1654.3	2357.3
SCRG1	102.7	109.3	90.5	87.8	96.2	93.8	109.4	112.5
SCRIB	153.9	173	121.9	106.2	101.5	104.3	155.9	187.9
SCRIB	1343.5	1592.5	1002	660.2	535.7	635.1	1020.6	1005.2

SCRIB	100.2	122.9	128.5	110.1	113.7	136.8	90.4	106.5
SCRL	83.2	94	87.6	89.7	95.8	87.1	85.5	91.7
SCRN1	121	146.1	120.2	145.8	143.2	163.6	113.2	128
SCRN2	129.6	152	120	113.9	112.5	120	125.7	117.1
SCRN3	140.3	169.1	114.5	117.9	107.2	117.4	117.1	143.5
SCRT1	81.6	92.6	99.8	99.4	91.7	94.2	87.6	87.3
SCRT2	95.8	93.5	99.6	89.9	82.2	96	81.9	102.1
SCT	116.5	151.9	102.9	90.1	80.2	94.4	120.1	139.8
SCTR	108	135	100.2	92.8	89.9	90.2	104.5	127.4
SCUBE1	102.3	111.4	118.2	103.6	98.4	123.6	98.3	123.9
SCUBE2	82	88.3	104	98	96.1	105.2	86.2	73.3
SCUBE3	100	117	87.1	93.8	87.3	94.7	93.8	90.6
SCYE1	95.3	112	115.5	94.6	94.7	100.7	100.4	120.6
SCYL1	450.5	575.4	284.1	247.9	217.9	270.1	443.4	643.7
SCYL1	2310.7	2982.8	862.7	632.1	580.4	657.7	1999	1952.5
SCYL1BP1	167.7	216.8	136.1	142.7	141.8	183.1	146.2	288
SCYL2	757.5	843.8	405.7	387.7	358	562.7	736.2	1408.7
SCYL3	273.9	322.4	135.8	127.5	132.9	158.7	246.2	385.1
SCYL3	161.6	186	122.5	119.8	107.6	141.9	162.4	234.7
SCYL3	89.4	99.3	93.2	80.8	86.6	88.3	91.7	91.7
SDAD1	2823.6	3269.6	2011.3	1416.2	1058	2550.9	2455.4	4231
SDC1	116.1	141.5	102.6	82.4	83.3	86.3	140.8	154.7
SDC1	191.9	237.8	126.8	127.8	115.1	123.9	148.7	158.5
SDC1	72.2	80.7	105.3	90	91.2	94.5	80.4	85.6
SDC2	362.5	492.3	149.1	108.5	100.4	120	239.4	319.5
SDC3	121.1	134	97.6	94.7	86	97.9	111.3	112.9
SDC3	79.1	86.8	100.6	104	103.8	98	85.8	75.7
SDC4	155.5	178.9	123.1	116.6	94.4	145.5	138	204.4
SDCBP	2327	2965.1	1051.5	1241.4	808.8	1763.3	2541.5	5586.9
SDCBP2	107.4	110.4	97.9	92.3	86.6	84.8	112.4	112
SDCBP2	93.2	94	92.1	80	82.4	82.8	83.6	94.5
SDCBP2	96.6	123.2	93.9	78.5	80.1	89.2	95.5	105.5
SDCBP2	97.6	111.5	87.4	83.7	73.6	77.7	100.7	99.1
SDCCAG1	696.2	996.7	385.8	445.7	367.9	484.5	559.4	794.5
SDCCAG10	2246.3	2837.2	859	993.2	885.1	1605.3	1521.4	2221.1
SDCCAG3	1051.7	1296.9	778.8	401.2	337.9	541.4	679.2	1090.2
SDCCAG3	135.4	174.6	103.6	101.4	99.8	91	115	158
SDCCAG3	2647.1	2923.8	1890.8	1202.3	930.5	1500.4	1727.2	2010.6
SDCCAG33	339	469.9	286.3	258.9	208.1	318.2	336.4	593.6
SDCCAG8	299.6	481.9	215.8	226.3	226.7	326	260.4	313.2
SDF2	1268.7	1538.7	854.4	893.6	740.1	1002.6	1150.1	2065.2
SDF2L1	7081.9	8541.2	5297.7	4851.4	3736.8	4942.6	8567.7	9641.6
SDF4	1242.6	1512.7	1037.6	958.3	754.4	954.9	1178.3	2373.5
SDF4	100.9	114.4	105.8	91.8	84.4	90.6	93.6	117.4
SDF4	1011.4	1578.6	700.7	554.2	442.7	369.7	995	1768
SDFR1	1336.9	1434.3	382.8	370.3	221.9	531.1	1105	1934
SDFR1	104.7	115.3	103.2	84.1	88	89.1	104.6	114.1
SDHA	1438.6	2095.1	1088.6	1000.1	952.3	1431.2	1395.2	1909
SDHAP3	190.2	191.4	133.8	145	140.5	141.2	149.4	173.5
SDHB	4403	5313.4	2956.5	2904.2	2387.1	3609.5	3492.9	4872.7

SDHC	478.4	563.8	278.8	333.9	268.8	416.1	420.6	635.5
SDHD	2609.3	2913.3	680.1	913	555.2	973.1	2439.4	3940.8
SDK1	109.9	107.2	96.3	93.7	94	94.6	98.4	101
SDK1	103.8	118	109.7	99.9	105.4	102.8	113.3	122.9
SDK2	102.8	110.3	89.4	92.3	83.6	88.7	99.6	101.9
SDPR	137.9	156.7	109.4	97.3	100.8	107.1	132.8	193.6
SDR-O	88.7	98.4	86	88.9	83.6	83.8	90	115
SDS	105.1	119.6	99.6	108.3	113.1	144.8	111.9	107.6
SDSL	665.6	905.8	448.4	588.5	458.2	799.3	612.6	627.2
SEBOX	75.6	84.9	94.2	87.7	81.2	88.4	81.4	92.4
SEC10L1	137.2	164.5	118.7	120.9	110.5	140.8	126.3	159
SEC11L1	6750.7	8974.1	4694.7	5728.3	4968.8	6282.9	6236.3	8058.8
SEC11L3	1241.3	1287.6	260.8	356.1	257.8	329.6	1188.3	1908.4
SEC14L1	101.7	136.2	111.8	116.5	102.9	116.8	111.1	159.1
SEC14L1	192.7	233.2	161.4	138.6	132.6	146.5	185.7	215
SEC14L2	79.3	101.2	97.9	91.2	90.4	92.2	87.8	95.5
SEC14L3	92.4	129.8	87	85	77.6	87.5	92.4	90.9
SEC14L4	131.4	159.2	95.2	76.9	74.4	86.9	112.5	139.3
SEC22C	630.9	901.3	725.2	869.4	772.3	1000.5	660.5	1201.2
SEC22C	427.5	572.3	307.3	249.1	244.6	280.5	380.8	418.3
SEC22L1	1310.3	1458.3	735.7	583	458.1	583.9	1101.7	1579.6
SEC22L2	786.6	1057.4	467.7	461.1	366.7	496.9	823.1	1070.5
SEC22L3	307.2	451.2	176.7	156.1	134.9	138	297.1	365.9
SEC23A	85.4	97.5	87.5	77.2	81.5	82.6	79.7	88.4
SEC23B	1234.3	1642.9	618.7	683.1	523.5	750.4	767.3	1102.4
SEC23B	1409.2	1748.8	892.1	889	658.3	1210.9	823.1	1186
SEC23IP	562.9	695.9	412.2	379.3	272.6	536.2	479.8	919.3
SEC24A	156.3	202.8	111.8	129	101.4	142.9	196.9	351
SEC24B	410.6	478.4	180.5	206.9	240.1	214.9	414.2	650.3
SEC24B	76	81.5	83.8	76.4	73.3	81.6	69.6	78.1
SEC24B	213.4	273	149.3	109.4	119.9	143.9	184.3	246.5
SEC24B	225.2	257	119	98.2	90.7	104.2	166	239
SEC24C	1030.6	1149.6	1616.3	1063.7	917.4	1424.8	969.1	1294.6
SEC24C	157.9	174.5	141.2	111.6	113.5	115.8	145.2	163.7
SEC24D	408	470.3	191.7	185.2	148.3	284.8	426.6	1135.2
SEC31L1	117.8	143.7	115.8	110	92.5	100.1	107.8	110.3
SEC31L1	1243.1	1844.7	542.3	601	551.3	935.6	1119.1	1847.7
SEC31L2	74.3	84.9	82.8	81.6	79.4	86.1	82.1	87.8
SEC31L2	110.3	121	90.6	91	83.6	98.6	114.2	132.6
SEC61A1	3540.5	5120	3130.1	3445.9	2780.8	3614.5	3365.7	3416.6
SEC61A2	95.8	121.4	108.9	98.6	105.1	118.5	97.9	118.2
SEC61B	8609.2	10155.9	3248.8	3220.7	1943.6	2732.5	9296.8	12424
SEC61G	8011.4	10617.7	7320.7	5944.5	3689.8	6039.7	7077.3	9059.7
SEC61G	9932.8	11110.9	5715.7	4638.2	3521.8	5780.9	8311.1	12211.5
SEC63	223	298.2	129.6	167.5	112.9	180.5	219	305.3
SEC6L1	488.4	608.3	586.6	451.6	460.3	726.6	450.1	717.6
SEC8L1	234.5	267.6	118.1	106.7	99.4	121.7	247.9	236
SECISBP2	408.7	528.3	307.7	401	366.7	552	342.5	446.9
SECTM1	143.9	166.1	92.5	76	78.9	89.5	141.4	156.1
SEDLP	703.8	866.3	286.7	255.1	242.2	354.2	628.4	826.5

SEH1L	628	763.2	465.6	427.9	361.1	621.8	374	548.3
SEH1L	468.6	600.6	264.1	297.5	189.4	360.6	308.8	554.9
SEH1L	1274.1	1674.6	526.3	560.8	412.7	701	797.2	1021.8
SEL1L	129.7	157.7	117.6	137.1	117.2	164.2	125.8	161
SELE	110.5	125.5	96.5	89.5	95	89.6	115.2	132.7
SELENBP1	98.7	130.5	95.4	92.8	86.9	94.8	108.3	114.5
SELI	211.3	313.1	212.7	226.1	195.7	241.7	200.7	293.4
SELK	1254.1	1527.7	629.1	502.9	442	581.1	1444.9	2084.9
SELL	87.4	93.5	97.4	87.6	85	86.4	88.9	88.5
SELM	93.6	103.4	103.5	96.3	94.3	95.3	97.7	115.7
SELO	273.1	327.4	228.5	188.9	195.1	288.1	234.8	360.6
SELP	103.4	109	95	90.5	89.9	86.4	97.4	102.2
SELPLG	93	108.3	97.2	104	110.5	91.9	89.4	96.2
SELS	131.3	152.6	96	97	91.8	97.7	119.9	150.5
SELS	3250.4	4169	2120.1	1786.7	1489.6	2266.7	3811.9	4717.3
SELS	1005.1	1293.5	734.2	637.2	612.4	943.4	1030.6	1188.4
SELT	97.6	118.6	93.9	92.5	81	88.9	92.6	106
SELV	90.8	93.8	89.9	79.3	79.4	87	91.5	91.2
SEMA3A	124.9	162.4	93.7	98.2	102.2	107.9	110.6	123.6
SEMA3B	188.7	215.9	113.9	93.9	82.2	97.8	160.2	209.5
SEMA3B	98.6	130.1	91.7	88.1	82	93.6	103.1	124.7
SEMA3C	79.4	86.1	78.2	82.9	82.3	87.1	83.3	80.5
SEMA3D	85.2	95.4	91	79.8	73.2	81.8	91.1	86.3
SEMA3E	990.4	843.4	800.7	1167	896.7	817.3	980.7	1722
SEMA3F	132.1	147.2	118.8	116.8	103.7	122.7	110.2	153.1
SEMA3G	129.6	134.5	110.1	89.7	84.5	103.6	125.9	138.8
SEMA4A	588.2	752.4	585.9	505.7	434.3	564.8	509.3	919.4
SEMA4B	121.5	133.3	164.3	147.9	150.5	199.5	118.7	171
SEMA4B	106.5	88.5	89	84.4	82.5	93.1	99.3	93.6
SEMA4C	117.4	141.2	109.9	106	102.5	106.5	113.2	131.9
SEMA4D	356.2	507.5	514.3	659.9	535.7	693.7	352.5	513.6
SEMA4F	179.8	206.3	152.4	148.2	123.7	146.4	181.6	217.5
SEMA4G	93.6	106.7	90.6	88.8	84.5	83.7	88	90.4
SEMA5A	81.6	99.9	111.6	96.1	95.9	100.9	88.6	93.1
SEMA5B	70.4	87.4	88.3	95.2	91.9	86.8	78.6	90.2
SEMA6B	118	145.6	113.8	96.3	95.1	93.9	128	153.8
SEMA6B	85.4	101.9	98.4	91.1	80.5	88.2	85.5	99
SEMA6B	84.8	89.6	104.6	89.1	84	85	91.2	87.9
SEMA6C	96.2	107.6	100.3	89.2	77	84.6	96.2	124.3
SEMA6D	86	88.6	93	90.4	87.6	96.2	82	86.1
SEMA6D	82.7	90	89.1	85	79.2	82.1	84.2	92.2
SEMA6D	88.5	113.5	106.9	91.9	98.2	91.7	87.6	99.2
SEMA7A	95.9	110.3	96.3	86	89.2	94.8	92.1	100.9
SEMG1	283.7	363.5	185.5	229.5	194.8	294.9	214.4	248.6
SEMG1	231.9	300.3	147.1	162.3	145.8	235.1	180	236.8
SEMG2	75.1	84.1	96.7	93.2	83.8	82.5	81.9	85.3
SENP2	351.3	446.9	212.8	273.1	191.6	330.5	301.4	471.3
SENP3	128.2	153.5	100.7	94	77.1	81.2	136.2	173.9
SENP5	210.5	242.1	133.7	124.6	115.2	149.2	179.7	213
SENP6	90.7	106.1	90.3	98.2	90.4	98.2	99.9	107.2

SENP7	123.2	146.5	88.6	75.4	80.1	91	114.8	134.2
SENP7	254.2	318.1	171.6	157.9	161.9	179.8	215.1	289.8
SENP8	101.6	94.1	95.1	81.8	89.6	84.5	91.4	121
Sep-15	5174.3	5407.1	2332.7	2850.5	2065.7	2959.9	5078.8	7751.7
SEPHS1	1643.2	2113.8	818.6	644.8	454.1	790.3	1090.7	1608.6
SEPHS2	2045.2	2589.2	447.3	342.2	297.4	402.3	1887.9	2155
SEPN1	82.4	89.9	95.1	91.7	91.2	98.3	85.1	84.8
SEPN1	196	245.6	179.6	178.8	146.9	162.5	184.1	255.2
SEPN1	417.3	565.8	847.9	895.7	724.2	1200.3	396.8	875.2
SEPP1	90.2	90.9	91	84	89.6	90.9	85.4	92.7
Sep-01	99.2	111	87.1	88.1	91.9	85	106.4	119.6
Sep-10	91.2	93.6	91.8	86.7	92.2	87.5	85.1	84.5
Sep-10	79.8	93.9	85	83.8	77	79.9	83.9	78.4
Sep-11	883.5	1096.8	494.8	554.5	403.4	605.4	738.4	1536.9
Sep-02	8934.4	11624.7	5781.8	7496.2	5870	7854.2	7666.2	8352.1
Sep-02	5899.8	7719.1	5356.5	7516.5	4737.5	8449.6	5262.3	9652.3
Sep-02	135.9	146.3	108.8	98.2	94.3	108	113.9	139.2
Sep-02	329	432.3	112.4	113.1	102.5	109.2	245.7	358.3
Sep-03	823.2	937.8	467.1	553.2	499.9	614.6	587.3	760.4
Sep-03	262.6	327.8	206.4	248.3	174.1	266.7	229.8	415.1
Sep-04	117.9	132.5	117	120.8	108.2	114.3	113.9	121.3
Sep-04	156.7	168.2	147.4	122.8	115.1	118.1	131.1	142.4
Sep-04	112.7	123	96.8	78	79.5	89.1	103.7	124.6
Sep-04	135.3	154.9	96.9	85.4	76.9	88.8	116	140.2
Sep-05	84.1	98.2	99.2	98.9	101	97	91.3	92.9
Sep-06	94.5	114.9	91.8	93.6	89.7	94.3	105.6	125.2
Sep-06	123.2	163.5	159.8	196.1	219.2	306	108.5	129.6
Sep-06	406.8	557.1	247.1	320	326.2	301.4	311.9	402.4
Sep-07	313.3	380.2	106.2	135.5	102.2	157.5	266.9	550.4
Sep-09	3558.9	4799.5	3052.1	4047.7	2929.8	4542.6	2652	2877.1
SEPW1	601.7	710.2	407.7	335.4	262.4	284	570.4	971.5
SEPX1	3364.6	4254.1	2967.2	2687	1887	2261.2	4754.8	7352.6
SERAC1	96.9	106.9	94.8	92.9	88.5	90.9	98.6	117.2
SERF1A	114.4	124.2	89.2	85.3	93.5	99.7	89.7	109.9
SERF1A	105.1	119	93	86	89.3	92.1	96.5	120.9
SERF1B	80.1	87.8	96	77.2	78.2	79.5	75.6	77.3
SERF2	11311.5	15595.9	7705.9	7345	6730.5	9639.9	10973.9	11282.9
SERGEF	430.5	617.1	377.7	271.4	236.6	352.2	372.5	532.1
SERHL	112.8	125.9	105.6	96.5	98.9	103.4	95.8	110.4
SERHL2	107.1	112.1	98.8	98.3	95.5	98.1	104.2	107.9
SERINC1	659.7	745.8	270.7	457.1	335.9	513.1	710.4	1744.2
SERINC2	105.6	117.9	119.4	119.9	104.9	124.5	100.6	141.7
SERINC3	149.1	170	111	129.9	113.8	126.5	143.6	177.1
SERINC3	157.4	172.1	91.3	109.6	97.5	95.4	153.9	251.1
SERINC3	1379.7	2091.3	1183	1196.3	1115.2	1109.5	1471	2097.8
SERINC4	123.8	166.3	84.6	83	87.1	85.9	107.4	125.4
SERINC5	72.1	84.8	80.3	86	78.5	83.9	76.9	73.4
SERP1	905	1211.8	268.1	254.1	184.7	406.6	668.9	1324.7
SERPINA1	159.1	208.6	119.2	136.1	132.7	171.2	151	161.8
SERPINA1	73.1	76.6	85.4	79.4	82.3	84.9	71	76.6

SERPINA1	89.1	111.3	93.5	122.5	102.1	135.8	88.1	106
SERPINA1	77.2	88.7	94.9	85.1	99.3	96.6	80	89.7
SERPINA10	99.9	104.3	87	77.2	78.1	89	118.6	102.2
SERPINA11	111.5	146.4	97.5	89.8	89.8	92.3	107	125.9
SERPINA11	75.5	88	86.5	88.2	74.4	69.2	87.5	96.8
SERPINA12	81.1	89	90.7	94.1	85.3	86.1	77.3	80.9
SERPINA13	83.3	94.7	93.3	86	78	83.6	84.7	87.1
SERPINA3	85.4	94.8	91.8	96.9	87	85.2	81.2	84
SERPINA4	130.7	158.8	100.5	91.6	81.8	82.1	118.5	137
SERPINA5	96.8	117.7	88	82	71.4	82.7	109.5	109.8
SERPINA6	85.3	92	81.7	82.5	77.9	83.3	96.7	94.7
SERPINA7	91.9	100.3	94.4	93.3	88.3	91.2	100.6	104.9
SERPINA9	97.6	98.2	105.9	106.8	95.1	100.4	88.7	94.6
SERPINA9	83.2	80.6	86.3	84.2	86.8	80.5	84.1	74.2
SERPINB1	763.5	1092.2	408.5	509	417.1	614.1	671.1	1114.9
SERPINB10	447.1	603.9	226	413.7	351.4	620.3	232	283.1
SERPINB11	89.3	102.7	108.7	92.5	96	94.4	90.1	103.2
SERPINB12	67.7	76.2	85.1	80.9	78	78.1	66.6	66.8
SERPINB13	99	111.3	73.9	69.9	72.1	71.6	104.8	110.4
SERPINB2	2772.5	2914.1	1007.3	1049.3	589.7	1657.5	591.8	569.5
SERPINB3	91.3	93.9	83.1	92.6	75.8	79.8	89.3	87.5
SERPINB4	69.4	84.5	90.7	85.2	75.1	83.4	75.9	79.3
SERPINB5	103.3	114.1	102.6	102.9	90.7	93.6	104.4	110.2
SERPINB6	2157.3	2599.6	1356.5	893.4	747.6	846.8	1968.2	2114.1
SERPINB7	83.9	112.2	84.4	81.4	79.5	88.8	91.1	102.6
SERPINB7	90.6	94.7	98.3	88.2	91.3	84.8	92.8	100.4
SERPINB8	1636.4	2274.7	1032.3	727.8	560.9	924.5	862.2	1090.9
SERPINB8	1436.6	2203	883.8	708	523	888	839.4	992.8
SERPINB9	87.1	90.6	108.7	81.9	88.5	94	82.2	91.8
SERPINC1	105.5	120.1	103.1	98.9	92.5	95.8	96.5	114.1
SERPIND1	88.5	89.6	107.2	93.4	84.5	86.3	92	96.1
SERPINE1	90.7	89	86.7	87.5	88.1	89.5	153.3	217.3
SERPINE2	807	1038.7	583.2	585.5	473.1	666.6	726.3	1020.3
SERPINF1	704.7	837.1	290.8	632.7	410.5	578.6	592	731.1
SERPINF2	99.3	109.8	85.5	83.2	80.8	90.9	92	98.7
SERPING1	128.2	138.7	95	94.3	93.2	99.1	117.1	136
SERPING1	126.4	139	108.8	94.4	102.7	95.6	122.4	129.3
SERPINH1	696.2	954	491.7	371.8	272.8	498.7	669	992.7
SERPINI1	86.2	104.1	86.1	85	85.7	90.5	99.6	116.5
SERPINI2	164.6	168.3	120.4	137.4	149.8	174.1	133.3	214.8
SERTAD1	262.9	312.1	232.8	184.9	153.2	201	385.8	614.3
SERTAD2	2207.5	2739.3	1554.1	1490.5	1291.1	1442	1863.4	2156
SERTAD3	470.6	657	384.1	294.5	231.9	292	446.2	566.8
SERTAD3	126.6	149.4	103.5	86.7	79.5	84.2	123.4	125.9
SERTAD3	215.8	261.6	149.6	140.7	124	109.2	194.3	210.9
SERTAD4	96.4	111.4	101.1	94.3	95	92.9	99.9	106.2
SESN1	369	510	302.5	521.3	452.4	692.4	365.9	620.9
SESN2	89.1	95.2	101.3	84	85	93.9	91.4	113.4
SESN3	291.7	352.5	173.9	198.6	148.1	208.3	180.5	204
SESTD1	472.9	585.8	324.8	310.8	275.3	403.3	342.3	589.4

SET	3826.6	4163.8	1906.6	1412	864	1597.4	2892.9	5159.5
SET7	100.2	110.2	103.7	83.2	84	88	112.5	108
SETBP1	114.2	125.1	115.1	124.7	95.3	131.8	103.1	113.5
SETD1A	312.9	338.7	490.8	436.1	379.8	564.9	277.3	538.3
SETD3	307.5	481.4	162.9	139	125	168.3	244.8	314.4
SETD3	101.4	104.3	104.8	101.7	99.4	101.4	101.4	115.2
SETD3	3791.8	4860.9	2937.2	2730.1	2457	3970.1	3244.8	3933.5
SETD4	287.2	372.5	260.2	202.7	165.8	214.9	251.7	278
SETD6	100.4	119.7	79.8	78.2	65.9	78.6	101.4	116.4
SETD8	459.9	627.6	342.9	309.4	289.6	367	415.1	688.1
SETDB1	600.2	703	621.4	554.1	442.1	748.1	445.1	898.7
SETDB2	864.9	1103	282.8	392.2	387.2	830	692.8	912.8
SETMAR	1097	1372.6	795.8	772.7	529.7	845.3	770.7	1066.2
SETX	257	357.4	141.3	149.8	138.2	186.9	274.9	332.6
SEZ6	113.5	117.1	81.3	89.2	84.9	74.8	108.4	138.6
SEZ6L	67.3	83.5	81.8	75.5	78.3	70.7	71.9	78.2
SEZ6L2	119.5	166.5	107	96.6	104.7	100.8	133.8	149.6
SEZ6L2	92.1	102.3	92.6	80	81.1	91.1	88.2	94.6
SEZ6L2	1695.2	2109.2	1356	2056.2	1539.5	2016	1499.8	1634
SF1	136.2	132.5	112.5	121.6	107.9	90.8	126.5	161
SF1	236	212.3	113.5	163	159.8	85.7	153.8	233.6
SF1	138.9	203	180.1	128.3	142.1	154.9	147.7	205.3
SF3A1	107.1	123	105.1	109.7	106.9	96.7	114.6	116.5
SF3A1	421.8	575.2	434.9	528.1	438.9	653.5	385.5	795.6
SF3A2	2446.4	2920.5	2128.2	1485.8	1127.4	1434.5	2441.6	3760.8
SF3A3	3890	4628.2	1825.4	2037.5	1679.8	2218.2	2905	2161.6
SF3B1	107.4	117.1	103.3	97.1	94.7	102.1	97	111.5
SF3B1	2025.5	3119.7	1943.9	2254.8	1896.9	4056.3	1857	3285.9
SF3B1	744.6	801.6	235.5	344.3	276.8	211.3	522.9	928.7
SF3B14	2746.8	3191.8	845.1	594	523.1	720	2656.3	4077.4
SF3B2	1653.5	2213.1	1351.6	1158.3	983.7	1734.1	1351.9	1891.7
SF3B3	1219.5	1342.7	889.4	850.6	647.3	898.2	1141.2	965.3
SF3B4	4540.9	5611.1	3059.4	2270.8	1445.7	1997.1	4350.2	7070.9
SF3B5	4238.3	4494.3	3434.3	2946.8	2037.9	3456.8	3527.3	4277.1
SF4	783.7	1011.4	523.3	418.9	420.8	518.3	704.2	953.6
SFI1	95.5	119.4	98.3	89.3	84	92.8	101.9	114.3
SFI1	128	134.4	106	106.9	100.4	104.5	104.9	126.6
SFI1	108.8	118.7	102.9	104.3	84.9	101.6	93.3	114
SFMBT1	241.1	338.6	184.4	151.6	145.7	201.4	205.2	346.1
SFMBT1	285.5	379.7	258	191.6	189.6	244.7	246	338.9
SFMBT1	75.1	85.1	84.5	91.4	82.6	88.2	79.1	83.4
SFMBT2	134.4	218.3	184.1	387.2	333.7	151	155.5	135
SFN	85.9	94.8	90.6	91.5	84.4	91.2	87.6	102.4
SFPQ	320.5	372.2	150.4	124.2	106.9	156.1	333.4	440.1
SFRP1	262	350.8	321.7	694.7	534.9	991.6	196.5	319
SFRP2	79.7	86.4	88.4	92.9	85.8	82.6	77.9	79.4
SFRP4	85.5	97.7	86.4	82	82.8	90.6	88.8	81.7
SFRP5	74.7	79	92.7	82.2	79.2	80.8	75.4	80.6
SFRS1	8225.9	9547.1	2070.5	2185.4	1193.2	2053.4	6910.5	8473.2
SFRS10	5181.8	7451.9	2945.4	2199.5	1732.1	2471.5	4172.1	5718

SFRS11	341.9	380.5	159.4	167.3	147.5	250.5	302.7	487.4
SFRS12	239.9	285.2	156.3	161.5	143.1	252.5	196	348.3
SFRS12	633.6	740	259.1	322.4	283.4	474.3	461.2	852.7
SFRS12	173.3	234.5	127.8	131.6	117.8	96.4	133.5	124.2
SFRS14	1918.6	1870.7	1167.7	1025.8	1075.7	1406.9	1286.7	1438.3
SFRS14	804.6	814.2	545	476.7	507	672.9	545	981.4
SFRS14	196	186.5	236.4	172.2	195.4	150.8	141.6	235.4
SFRS15	363.8	541.5	355.8	531.5	382.4	567.5	394.8	599.8
SFRS16	194.5	223.1	167.5	133.9	138.4	210.3	153.5	237.5
SFRS2	14297.3	16381.9	10494.6	7544.3	6476.6	9358.4	10723.2	18107.8
SFRS2IP	831.4	1280.7	481.3	747.3	659.8	1134.3	675.9	888.4
SFRS3	134.4	156.8	88	84.6	91	96.1	113.6	158.1
SFRS4	2320.1	3084.4	1910.5	2116	1772.7	2602.8	2269.6	3521.7
SFRS5	4592.1	5533.6	3303.4	2841.3	2874.9	3113.7	3655.6	3973.9
SFRS5	5910.4	6575.3	3499.7	2939.5	2720.6	3180.3	4635.5	5084.1
SFRS6	6566.5	8389.4	4934.1	5165.8	3581.4	4926.1	5168.7	6797.5
SFRS7	1735	2073	1180.3	1070.7	740.3	1339.9	1257.5	1958.6
SFRS8	240.8	344.1	168.3	163.2	145.9	222.2	244.7	513.3
SFRS9	8763.4	10922.9	6872.9	6277	5185.9	8159.6	6816.9	10186.3
SFT2D1	10210.1	12977.1	9583.4	11123.4	9000	12525.6	9512.4	13756
SFT2D2	133.1	157.8	114	95.6	99.8	97.9	118	152.1
SFT2D3	149.1	180.5	126.2	132.4	119.1	135.8	118.2	178.6
SFTPA1	100	96.9	95.1	74.8	82.7	77.5	93.1	104.4
SFTPA2	87.4	104.7	91.6	88.9	84.2	89.4	107.5	96.6
SFTPB	87.3	101.2	90.3	92.5	91.9	93.9	87.5	91
SFTPB	80.5	99.6	111	103.5	91.5	99.1	94.2	89.4
SFTPC	83.8	84.9	98.2	89.3	90.3	89	76.8	80.2
SFTPD	277	339.1	256.9	347	291.5	396.9	241.2	294.9
SFTPG	112.4	135.5	105	104	92.1	109.4	123.2	118.1
SFXN1	556.3	817	485.9	439.9	292.3	366.6	442	799.6
SFXN2	165.1	187.6	154.3	125.2	111.2	136.2	139.7	151
SFXN3	150.3	174.6	119.4	112.5	100	97.2	137.3	201.8
SFXN4	1146	1685	932.3	594.1	471.4	800.2	810.2	1103.8
SFXN4	2004.5	2586.2	1268.9	1054.2	825.2	1006.3	1699.2	2135
SFXN4	186.9	245.7	126	129.5	111.2	147.6	197.8	239.5
SFXN5	133.6	143.8	165.2	178	183.3	237.8	133	230.8
SGCA	101.1	108	107.2	93.6	104.5	104.5	102.8	106.8
SGCB	129.4	163.4	99.5	104.6	98.6	103.4	133	164.1
SGCD	86.9	101.8	86.7	80.7	81.1	91.5	87.9	101.2
SGCD	109	112.1	105.4	94.2	90.7	89.7	98.1	104
SGCD	98.5	123.6	105.5	92.6	83.5	88.6	98.3	108.4
SGCE	73.9	87.8	87.7	83.9	91.3	84.7	85.4	100.8
SGCG	91.8	95.5	90.3	92.3	82.8	90.9	88.7	104.3
SGCZ	121.9	119.7	102.1	93	92.3	95.8	102.7	112.1
SGEF	102.2	113.8	88	88.6	82.5	85.6	101.3	93.9
SGIP1	622.2	741.8	462.7	278.4	213.3	327.9	383.6	556.4
SGK	180.7	197	136.4	221.7	240.4	377.1	159.4	284.3
SGK2	87.8	103.7	84.5	85.2	86.3	80.2	85.9	95.2
SGK2	73.5	86.3	86.3	93.1	83	87.8	70.6	76.3
SGK2	107	138.2	96.3	84.1	83.3	83.1	107.1	121.9

SGK3	93.2	120.7	104.6	86.8	87.9	84.8	96.8	104.9
SGK3	1490.6	1777.7	693.2	561.1	522.2	694.5	1298.5	1832.2
SGK3	1144.8	1491.7	464.6	385.2	368.3	368.4	867.7	1069.6
SGOL1	417.6	561.4	278.8	235.6	229.8	272.9	385.6	500.4
SGOL2	93.8	92.2	85.6	84	83.8	85	87.3	99.3
SGPL1	137.4	181	128.1	119.7	112.5	122.2	137.8	164.9
SGPP1	119.6	146.4	94.5	88.3	91.2	92.3	106.4	124
SGPP2	96.1	105.6	119.1	91.2	95.2	89.1	101.6	98.5
SGPP2	83.4	83.3	98.4	85	84.6	88.8	76.6	80.6
SGSH	126.9	154.1	163.9	138.6	123.4	164.6	132.2	211.2
SGTA	236.4	337	151.6	115.4	113.9	168.5	218.2	395.5
SGTB	164.3	202.7	101	104.9	97.1	120.7	194.6	248.3
SH2B1	197.9	200.9	261.3	192.4	157.6	224.6	161.6	381.7
SH2D1A	183.3	221.8	112.7	102.2	102.6	112.7	180.3	224.5
SH2D1B	101.4	106.6	99.1	81.3	85.6	91.3	89.6	104.2
SH2D2A	138.5	180.2	96.3	92.9	95.1	100.6	149.3	142.1
SH2D3A	94.8	122.2	100.9	86.9	98.6	97.6	105.5	116.9
SH2D3C	136.4	168.3	208.6	180	157.6	183.7	136.2	231.3
SH2D3C	101.4	104.6	112.3	105.8	109.1	106.2	92.3	113.4
SH2D3C	120.8	156.8	100.9	90.8	90.9	96.4	119.7	154.6
SH2D4A	222.3	284.4	148.8	176.6	143.2	228.3	200.8	189.4
SH2D4B	88.8	98.6	86.3	81.1	82.8	80.4	89.1	96.3
SH3BGR	148.6	174.7	107.5	94.4	91.9	86.1	144.2	167.4
SH3BGR	174.8	227.1	112.7	123.5	106.3	126.1	170.4	183.2
SH3BGRL	78.1	80	88.3	77.6	77.1	72.8	79.8	80.7
SH3BGRL2	166.6	214.7	157	109.9	100.7	143.2	159.7	152.9
SH3BGRL3	1980.3	2574.6	1819.9	1741.4	1232.3	1611.4	1965.4	3366.6
SH3BP1	186.7	227.2	247	255.2	227.7	266	171.2	320.6
SH3BP2	111	134.1	100.4	96.2	96.1	95.9	110.7	119.9
SH3BP4	99.9	122	101.2	87.7	71.9	76.4	113.7	140.4
SH3BP5	82.8	94.9	96.3	84	86.8	86.8	95.5	103.7
SH3BP5	97.1	107.3	97.9	90.2	81.7	89.6	89.4	98.9
SH3BP5L	251.8	310.2	325.9	367.5	352.9	468.7	264	535.8
SH3D19	89.2	98.4	97.3	90.9	83	89.2	86.3	112.5
SH3GL1	365.2	475.3	187.4	138	123.1	133.1	385.5	265.2
SH3GL2	104.2	111.1	117.2	106.1	106	98.2	102.7	103.8
SH3GL3	83.3	88.8	103	92.5	87	91.8	89.5	86.2
SH3GLB1	1204.2	1641.1	981	872.7	680.3	1281.9	1077.1	1705.3
SH3GLB2	1491.1	1926.7	1542.2	1026.6	928.8	1315	1240.5	1566.4
SH3KBP1	7384	7780.5	5454	4931.6	4196.7	5349.6	6520.7	8508.6
SH3KBP1	113.9	123.6	93.5	98.9	84.4	92	109.6	113.5
SH3KBP1	3377.6	4986.7	3956.2	4749.3	4284.5	5589.5	2965.5	5235.7
SH3MD2	149.9	188.7	134.9	114	105	131.5	135.1	147.9
SH3PX3	112.7	150.2	148.2	146.4	118.4	182.2	125	166.8
SH3PXD2A	227.3	331.6	217.3	158.8	147.2	188.1	343.1	372.4
SH3PXD2B	108.9	126.8	90.1	100.8	83.9	107.4	100.9	129.6
SH3RF2	110.8	126.9	99.1	103.4	91	86.1	124.3	117.5
SH3TC1	88	111.5	98.5	112.6	123.2	161.7	95.7	118
SH3TC2	127.2	164.7	102.4	106.1	95.5	97.6	132.9	133.5
SH3YL1	220.8	295.5	142.8	136.6	138.9	136.9	188.3	290.9

SHANK1	85.8	88.4	103.9	94.3	85.2	93.5	81.7	90.1
SHANK2	104.9	125	103.6	92.9	82.3	83	104.1	108.3
SHANK2	94.1	119.5	93.4	91.9	78.8	85.3	101	111.6
SHANK3	140	162.5	139	176.8	156.5	196.1	134.6	150.5
SHARPIN	330	423.5	200.4	145.2	139	145.4	265.8	359.7
SHB	114.1	130.4	135.3	115	107.5	133.7	117.1	166
SHBG	113.9	138.7	83.6	88.4	86.4	95.8	110.8	113.9
SHC1	136.4	158.9	124.1	111.7	106.8	114.7	141.5	173.7
SHC1	87.7	93.8	86.5	83.3	87.6	88.5	80.7	95
SHC1	1275.6	1641.8	1388.4	1048.7	793.7	1279.5	1373.2	3399.1
SHC3	116.1	130.4	83.9	89.4	79.2	97.4	111	134.9
SHC4	109	117.1	122.2	98.1	94.2	104.7	105.4	129
SHCBP1	10492	11221.6	4942.3	7094.3	5673.5	5861.9	9468.8	8653.5
SHD	84.6	90.4	90.1	87.4	87.9	90.9	89.6	82.3
SHE	135.1	153.4	118.1	99	96.5	113.1	122.5	134.1
SHF	110.1	118.1	103.6	90.7	95.9	97.2	105.1	122.7
SHFM1	12512.2	15125.2	11674.2	9180.5	7876.7	11266.1	11999	16140
SHH	107.5	129.9	101.6	91	90	102.3	109.4	125.3
SHKBP1	235.1	316	253.7	188.9	151.9	246.2	244	413.6
SHMT1	706.8	843	328.8	426.2	348.6	469.3	438.6	391.8
SHMT1	201.8	241.2	119.6	116.5	98.4	113	202.9	262.5
SHMT1	150.8	208.7	119.7	121.1	113.6	119.8	156.4	183.1
SHMT2	5261.3	7104.5	4671.9	3148.3	2349.3	3305	4607.7	6411.4
SHOC2	403	488.7	135.7	138.3	132.6	188.5	296.1	490
SHOX	89	104.3	98	90.3	80.8	95.1	99	99.9
SHOX	94.5	97.7	101.9	96.8	83.9	92.8	84.4	99.8
SHOX2	315.7	407.8	188.4	272.2	283.3	353.3	299.5	463.6
SHOX2	120.3	149.5	89.9	95.8	97.7	89.1	112.8	126.1
SHPRH	145.2	168.9	117.4	148.6	147	185.5	113.7	126
SHPRH	193.9	249.8	162.2	214.3	174.9	271.2	179.3	241.1
SHQ1	497.5	671	262.5	220.8	174.5	291.4	385.6	550.4
SHRM	92.9	86.3	102.8	89.7	96.5	91.5	91.5	85.4
SHROOM4	979.3	911.7	811.5	1174.3	997.9	780.9	913.4	1895.7
SI	79.6	90	95.4	88.4	77.9	82.9	89.6	93.8
SIAE	120.2	131.4	91.8	88	78.9	95.7	111.2	132
SIAH1	124.2	136	91	88.8	92.1	102	120.8	112.5
SIAH1	105.4	103.8	96.7	85.1	94.1	89.5	94.9	103.5
SIAH1	588.5	748.8	381.5	423.3	364.6	512.1	524.1	709
SIAH2	286.4	401.9	229.2	169.2	160.8	211	246.5	399.2
SIAHBP1	8810	10689	6548.8	3316.3	2993.2	3486.7	8123.5	6605.7
SIDT1	97	101.7	95.9	90	84.3	92.8	78.9	82
SIDT2	678.1	824.3	561.3	485.7	398.2	530	379.3	464.6
SIGIRR	235.3	246.8	125.5	99.8	122.4	134.5	210.6	268.2
SIGIRR	186.1	264.8	147.8	161.8	161.8	141.2	172	160.2
SIGLEC10	169.4	207.3	127.8	120	117.4	127.8	141.7	135.1
SIGLEC11	131.6	126.5	94	93.8	85.6	88.3	132.1	132.2
SIGLEC12	118.1	144.6	108.1	118.2	97.1	98	126.7	119.6
SIGLEC12	154	180.2	115.9	104.8	90.5	95.7	122.8	174.8
SIGLEC12	93.1	117.3	83.7	77.9	85.9	83.9	99.5	115.3
SIGLEC5	104.3	110.9	105.6	104.8	83.8	97.8	100.3	116.6

SIGLEC6	165.7	229.9	167.7	107.1	92.4	103.6	154.5	161.4
SIGLEC7	108.2	124.8	111.2	107.7	86.2	130.6	105	135.6
SIGLEC7	83.3	92.4	89.6	88.9	75.6	97.2	79.6	92.7
SIGLEC7	108.2	127.4	98.1	88.3	83.7	111.1	99.3	124.7
SIGLEC8	134.6	142	88.8	81.5	83.9	90	126	140.6
SIGLEC9	97.6	101.2	91.4	87.6	87.1	86.7	81.9	112
SIGLECP16	206.5	243.3	175.3	163.5	137.8	163.3	147.4	164.7
SIGLECP3	172.9	237.2	102.8	100.6	83.3	105.6	151.7	162.3
SIGLECP3	151.1	166.3	135	140.1	128.2	149.1	134.5	154.3
SIKE	240.3	337.2	138.8	109.1	107.8	112.8	187.3	267.4
SIL1	390	534.4	245.5	341	299.1	425.2	421.4	379.5
SIL1	140.9	170.5	108.8	108.8	94.9	96.4	129.2	134.3
SIL1	1069.9	1515.2	584.7	1001.7	774.3	1035.4	1146.8	963
SILV	92	116.3	92.6	83.8	100	101.5	98.9	107.1
SIM1	89	99.7	95	88.1	86.2	89.7	79.7	87.2
SIM2	98.6	110.4	100.8	93.9	82.3	90.4	101.7	101.1
SIM2	88	131.5	102	87.4	92	100.8	92.5	111.2
SIN3A	957	1504	1030.3	1382	1264.7	1872.2	957.7	1046.5
SIN3B	108.8	117.5	134	127.4	105.2	133.9	102.3	128.9
SIP1	1024.7	1363.3	834.6	525.5	417.1	714.5	821	1381.7
SIPA1	188.5	219.5	168.4	150.3	153.8	200	134.9	208.1
SIPA1	1322.1	1624.6	1115.2	948.4	851.5	916	878.7	648
SIPA1L1	187.3	231.4	114.6	89.1	93.4	106.1	164.3	195.9
SIPA1L2	176.1	224.7	118.4	154.9	129.4	191.3	179.4	209.8
SIPA1L3	155.5	204.1	127.4	130	104.2	125.8	145.8	190.3
SIRPB1	217.5	234.2	193.8	190.6	139.7	200.8	190.7	244.2
SIRPB2	89.2	110.4	83.3	79.6	78.2	79.9	93.2	95.6
SIRPD	159.4	237.1	111.4	105.7	109.2	106.8	148.4	163.6
SIRPG	994.4	1132.2	383.1	392.8	308	308.8	916.2	1172.9
SIRPG	77.4	84.8	89.1	96.3	86.8	95.7	77.8	80.7
SIRT1	311	395.8	205.1	213.9	214.5	342.7	306.8	664.3
SIRT2	162.5	220.3	101.6	95.7	94	101.6	148.7	237.4
SIRT2	177	238.7	119.1	108.5	89.2	112.3	175.2	282.5
SIRT3	107.9	126.1	99.6	82.6	83.2	94.9	105.1	120.3
SIRT3	131.5	142	81.8	77.2	83	82.9	123.9	142.6
SIRT4	131.9	152.4	125.1	126	106.3	113.7	144.6	159
SIRT5	100.1	131.1	100.2	102.2	102.6	125.3	105.6	120.9
SIRT5	122.4	144.2	106.6	117.1	101.5	108.3	127.6	117.4
SIRT5	219.2	323.1	191	215.3	187.4	271.7	219.7	301
SIRT6	104.2	126.7	112.5	92.9	91.4	112.2	101	136.7
SIT1	90.5	89.1	96.5	97.4	90.8	94.7	91.7	100.4
SIVA	3496.8	4067.7	2155.1	1920.5	1862.1	1826.5	3313.7	3006.4
SIVA	2296.6	2892.7	1701.6	1174.7	1011	1477.3	1740.5	2102.1
SIX1	100.7	109.5	89.2	96.3	84.4	96.8	102.8	113.9
SIX2	100.4	125.7	102.4	90	88.8	91.2	108.7	127.4
SIX3	133.3	149.6	101	93.8	98.5	99.9	136	162.8
SIX4	102.1	127.5	115.6	110.5	111.8	108.8	100.6	98.6
SIX5	513.2	598.9	360.4	259.5	257.8	264.5	424.6	407.8
SIX6	88.1	91.5	96.3	76.6	76.4	89.3	90.4	89.9
SKAP1	80.6	86.2	91.1	88.4	83.8	84.1	83.9	95.2

SKAP2	394.4	502.9	186.1	199.7	141.9	247.1	365.6	843.8
SKI	203.3	226	180.1	185	165	169.5	194.7	207.6
SKIL	71.6	86.2	84.9	80.2	76.8	81.3	76.9	82.8
SKIP	110.5	128.6	100.1	108.8	106.5	94.5	96.7	121.2
SKIP	69.3	92.8	87.9	86.2	81.7	93.1	76.4	87.9
SKIV2L	895.2	885.4	1023.1	1106.4	958.1	1290.5	687.7	713.5
SKIV2L2	1359.8	2076.1	682.5	555	420.3	1054.6	1137	2066.6
SKP1A	1602	2057.4	1100.6	1080.5	760.5	1161	1428.2	2284.3
SKP1A	80.4	96.6	95.6	98.8	87.6	75.2	87.9	89.3
SKP2	3268.6	4069.1	2588.7	3414.5	2064.6	3425.3	2184	3335.4
SKP2	1290.7	1657.1	674.7	504.4	365.8	516.5	697.5	955
SKP2	339.5	366.4	119.8	98.5	102.1	104.2	188.6	239.9
SLA	147.5	183.4	120.8	126.4	109.8	100.6	127.6	139.2
SLA	377.1	423.3	135.8	128.8	134.2	126.8	326.2	363.8
SLA/LP	255.9	343.6	175.5	165.9	135.3	180.8	205.6	282.9
SLA/LP	141.4	174.8	120.4	101.6	93.9	104.3	126.3	180.3
SLA2	79.3	88.9	92	86.9	85.2	83.1	90.4	87.1
SLA2	109.5	115.7	106.6	101.5	91	101.8	107.8	108.7
SLAMF1	121.9	138.7	122.7	78.3	90.7	79.4	95.1	98.1
SLAMF6	170.7	222.5	137.2	147.5	144.8	156.7	161.1	149.6
SLAMF7	90.9	133.2	89.5	88	81.5	86.6	93.8	121.4
SLAMF8	146.1	157.3	106.4	80.3	93.4	94.1	134.6	148.5
SLAMF9	147.2	161.1	102	101.4	85	99.2	124.7	122.5
SLBP	1939.6	2452.9	553.7	454.1	348.4	455.2	1502.7	2560.6
SLC10A1	75	83.3	88.3	85	85.1	90.9	83.1	88.6
SLC10A2	85.9	94.9	92.7	86.2	90.5	84.6	85.9	83.2
SLC10A3	645.7	815.2	426.2	396.3	330.2	446.7	645.9	992.4
SLC10A4	878.3	1024.2	362.5	282.4	197	315.5	820.9	1652
SLC10A5	85.4	100.2	87	80.1	74.7	87.8	82.9	95.8
SLC10A6	67.2	92.3	80.5	80.1	75.9	83.1	82.5	86.1
SLC10A7	78.9	85.7	105.7	84.3	84.6	82.4	87.7	87.1
SLC11A1	380.2	472	312.7	251.3	177.6	220	263.2	264.5
SLC11A1	83.7	90.5	98.4	91.8	87.8	93.9	83.7	86.7
SLC11A2	264.2	330.1	221.2	346.2	338.4	506.6	231.2	326.1
SLC12A1	89.4	101.1	99.3	101	92.5	90.8	89.5	92.6
SLC12A2	145	162.8	114.5	125.3	123.6	131.5	142.3	185.1
SLC12A3	89.7	94.4	82.5	78.6	72.2	98.1	75.6	96.6
SLC12A4	149.1	189.9	109.7	94.4	95.3	103.5	127.9	171.2
SLC12A5	81.6	99.4	92.6	94.7	88.8	89.9	90.6	100.5
SLC12A6	117.2	145.3	96.8	91.2	92.6	100.8	121.4	149.6
SLC12A6	94.7	129.6	92.2	76.4	78.8	85.2	95.6	96.2
SLC12A7	101.1	100.1	94.8	92.3	85.9	98.9	103.5	96.4
SLC12A8	115.7	128.2	106	102.2	91.8	99.7	131.8	163.2
SLC12A9	2478.7	2593.9	1772.6	1410.7	1164.8	1485.1	1651.5	1329.2
SLC13A1	93.6	100.3	92.9	90	84.4	84.5	85.8	95.3
SLC13A2	100	121.2	93.5	87.4	81.4	95.1	104	119.5
SLC13A3	168.3	185.9	145	140.1	148.1	137.2	154	196.2
SLC13A3	88.3	91.2	89.8	82.9	77.6	83.8	86.5	92.5
SLC13A3	81.4	93.3	101.7	97.5	98.1	85.2	85.3	86.2
SLC13A4	93.1	100	106.7	113.7	91.4	88.6	89.7	97.7

SLC13A5	98.2	133	93.9	76	81.3	97.3	105.1	123.9
SLC14A1	100.8	100.3	81.2	89.2	91.1	80.9	91.2	101.7
SLC14A2	95.9	104.1	109.9	102.8	103.1	102.7	88.3	94.9
SLC15A1	118.2	119	81.6	87.1	81.1	83.4	101.9	112.9
SLC15A2	123.8	126.2	132.9	155.5	144.3	199.6	104.9	115.3
SLC15A3	836.1	1080.9	562.4	1267.1	833	1153.3	781.7	686.1
SLC15A4	671.2	806.4	359.2	552.1	462.9	745.5	568.7	862.8
SLC16A1	113.2	139.5	97.2	94.6	80.2	86.8	112.7	120.1
SLC16A10	550.8	739.1	314.8	207.2	152	233.3	352.1	533.3
SLC16A11	95.1	96.6	98	103.5	84.4	97.5	97.9	99.5
SLC16A12	7699.6	9463.8	3156.3	5318.6	5586.3	3704	7200	8306.9
SLC16A13	91	122.3	105.8	96.5	96.5	89.2	106.4	113.5
SLC16A14	90.8	93.9	87.3	92.5	88.4	97.5	81.7	86
SLC16A2	108.5	153.4	117.3	117.7	108.1	134	117.6	144.7
SLC16A3	4706.7	5910.5	2290.5	2235.9	1799.3	2402.6	5370.1	6537.9
SLC16A4	108.3	109.1	78.7	76.3	80.9	77.7	82.2	115.8
SLC16A5	315.9	438.7	272.2	263.7	270.4	278.2	243.6	262.3
SLC16A6	91	99.7	96.8	102.3	95.8	102.5	90.8	89.3
SLC16A7	105.4	124.8	92.2	97.8	89.4	96.3	104.9	114.1
SLC16A8	81.6	94.5	90	79.5	74.8	90.4	83.1	98.5
SLC16A9	290.4	297	213.8	168.8	147	179	231.1	348.3
SLC17A1	109.2	136.9	83	88.2	76.6	77.2	106.1	136.4
SLC17A2	96.2	110.2	91.5	88.4	83.1	78.1	91.7	98.7
SLC17A3	107.7	116.8	112.3	108.5	105.9	108.2	111.1	111.7
SLC17A4	76.5	90.2	91.1	80.6	92.1	86.7	82.4	95.6
SLC17A5	121.6	126.7	102.8	89.7	92.4	89.5	114	112.9
SLC17A6	83.9	85.3	94	86.4	81.9	85.5	84.7	97.2
SLC17A7	115.4	127.2	104.9	96.6	100.3	95.2	114.1	119.6
SLC17A8	72.8	91.5	91.1	84.4	83.4	80.9	83.6	85.6
SLC18A1	100.3	115.7	114	109.4	107.5	100	95.3	104.4
SLC18A2	78	81.4	84.7	78.2	80.5	90.8	70.3	78.1
SLC18A3	80.2	98.3	85.9	86.4	80.1	81	82.6	91.5
SLC19A1	371.2	465.1	263.9	259	191.1	195.4	274.6	562.6
SLC19A2	102.7	124.8	117.7	112.2	102.4	107.9	103.5	121.5
SLC19A3	89.5	105.5	86.5	84.1	86.6	95.4	88.4	90.2
SLC1A1	99.5	124.5	122.1	113.1	102.1	106.4	96.8	98.1
SLC1A2	98.7	130.7	94.4	84	81.9	82.6	101	118.2
SLC1A3	374.1	463.8	207.4	367.1	285.3	450.2	213.3	229.5
SLC1A4	201.3	270	230.1	195.9	186.6	204.1	222.7	353.3
SLC1A5	737.8	883.4	580.3	444.7	304.3	452.1	686.2	1530.4
SLC1A6	84.9	102.2	89.3	78.5	83.6	76.1	85.7	85.6
SLC1A7	129.9	139.2	93.2	94	100.8	108.8	123.4	141.6
SLC20A1	1941	2382.6	1967	1538.9	1062.8	2016.9	1485.7	2760.2
SLC20A2	451	570.8	422.3	344	239.1	387.8	448.6	498.1
SLC22A1	135.7	136.2	99.1	107.7	87	93.2	114.7	121.1
SLC22A1	96.4	111.5	85.5	80.3	90	80	109.4	120.4
SLC22A10	105.5	129.8	83.7	89.4	82.7	88.4	111.9	108.4
SLC22A11	94.5	103	91.2	92.4	85.4	83.2	102	97.7
SLC22A12	106.2	117.8	94.7	88.2	90.6	91.1	119.8	126
SLC22A12	79	96.3	99.2	99	83.3	87.5	89.3	81.3

SLC22A12	84.6	99.6	105.5	84.4	78.6	86.3	85.2	94.1
SLC22A13	94.8	98.3	96.8	96.6	93.3	93.5	93.5	92
SLC22A14	84.2	89	103.1	91	87.7	101.5	88.8	85
SLC22A15	1272	1442.8	972.1	530.8	422.7	909.8	957.1	1572
SLC22A16	2837	3498.3	1484.3	1517.6	1239.2	1674.1	2497.2	3102.4
SLC22A17	84.8	93.3	102.3	85.2	88.1	88.9	86	92.4
SLC22A17	170.6	208.1	132.2	132.9	104.4	138.6	166.6	289.5
SLC22A18	162.4	218.9	143.2	108.5	100	101.3	173.3	171.2
SLC22A18	332.9	441.7	475.6	462.2	385.8	466.4	370.7	617.2
SLC22A18A	169.9	202.8	167	147.9	126.9	187	157.2	200.2
SLC22A2	85.4	97.8	104.7	84.3	97.4	93.6	87.3	95.7
SLC22A2	86.9	95.2	88.6	75.4	83	82.8	91.5	97.9
SLC22A3	87.4	111.6	103.7	89	86.6	87.9	87.6	88.4
SLC22A4	360	387.8	210.8	324.9	236.1	333.9	294.6	419.3
SLC22A5	353.4	451.2	276	295.2	193.4	327.9	271.6	379.7
SLC22A6	111.6	143.1	119.9	99.4	93	106.9	102.3	114.8
SLC22A6	80.3	79	92.5	76.2	76	77.3	82.9	94.1
SLC22A7	79.9	92.3	92	79.8	78.8	83.8	78.8	87.9
SLC22A7	91.5	106.6	107.4	95.7	95.9	100.6	97.5	96.3
SLC22A8	79.6	89.3	83.8	86.6	84	83.4	77.7	97
SLC22A9	81.1	89.7	91.7	84.8	79.7	82.4	81.4	89
SLC23A1	114.9	144.7	103.5	90.1	100.4	90.1	113.5	125.8
SLC23A1	145.8	172.7	112.8	94.8	90.2	104.1	146.2	153.1
SLC23A2	106.2	127.5	92	77.2	84.4	85.1	94.7	111.1
SLC23A2	155.1	183.1	161.4	182.8	195.7	290.8	157.1	206.3
SLC23A2	94.9	110.3	88.6	84.7	88.7	83.5	87.2	102.3
SLC23A3	96.2	116.5	102.5	94.8	97.6	99.1	96.4	107.1
SLC24A1	169.5	197	113.4	143.8	121.9	147.5	147.6	157
SLC24A2	95.4	107.1	102.5	92.9	82.1	89.7	96.8	95
SLC24A3	126.9	136.3	92.4	93.1	78.1	85.6	108.3	127.7
SLC24A4	107.2	107.2	106.7	95.2	104.6	104.8	104.3	129.3
SLC24A4	116	141.9	112.5	119.3	112.4	121.7	105.8	121.9
SLC24A4	116.3	139.6	119.1	119.3	125.4	123.7	117.6	116.5
SLC24A5	88.8	92.5	87.8	87.3	85.7	91	92.9	103.3
SLC24A6	467.1	608.5	344.8	403.5	290.1	345.4	448.3	447.5
SLC25A1	1634.7	2238.4	2132.8	1282.5	1056.3	1471.6	1425.5	2384.3
SLC25A10	906.8	1141	412.1	348.4	324.6	475.4	709.4	385.6
SLC25A11	280.5	368	141.2	133.5	104.9	136.2	251.6	292.9
SLC25A12	243.3	283.7	191.8	172.1	129.5	205	176.2	284.9
SLC25A13	517.3	604.4	230	301.2	239	417	428.2	729
SLC25A14	399.4	468.4	164.5	161.5	158.3	198.5	339.4	344.5
SLC25A14	261.4	292.8	186.5	143.9	128.5	144.8	207.8	230.8
SLC25A15	375.3	463.2	237	246.1	192	204.6	266.4	354.8
SLC25A16	99.9	107.1	91.8	91.4	86	105.2	91.1	107.6
SLC25A17	264.2	376.4	208.7	216.7	165.4	228	251.7	315.3
SLC25A18	84.3	100.5	98	101.6	100.8	94.8	87.5	91.6
SLC25A19	1785.3	2308.7	876.7	934.8	823.4	873.9	1509.1	849.9
SLC25A2	102	115.4	97.3	87.4	79.4	79.6	88.5	102.9
SLC25A20	279.5	403.8	134.7	107	100.2	104.9	263.8	338.3
SLC25A21	74.1	85.1	89.3	88.2	85.4	81.7	76.1	76.4

SLC25A22	682.9	838.2	558.6	329.3	269.7	297.8	422.2	321
SLC25A23	2410.5	2962	2215.9	1602.5	1225.1	1405	2202.1	2985.9
SLC25A24	100.8	129.9	86.4	83.9	76.6	85.7	111.4	118.1
SLC25A24	130.9	166.7	117.9	92.8	88.3	92.1	135.1	141.4
SLC25A24	637.5	881.9	284.8	188	179	254.2	455.5	674
SLC25A25	244.6	306.8	219.5	221.3	208.4	230.3	261.1	398.6
SLC25A25	78.4	99.8	89.9	91.8	78.9	88.2	88.7	99.9
SLC25A25	96.9	112.9	93.2	86.6	80.6	89.3	102.3	110.2
SLC25A25	132.2	127.5	107.9	102	101.1	98.9	129	154.3
SLC25A26	384.2	536.5	244.4	210.3	193.1	241.3	361.3	423.8
SLC25A26	178.3	235.6	122.1	119.6	119.2	145.2	149.9	186.6
SLC25A26	389	492.3	230.8	172.9	131.6	150.3	327.2	371
SLC25A27	148.5	188.8	105	95.3	96.1	112.8	128.7	173.2
SLC25A28	1682.1	2005.4	748.5	578.8	546.6	734.1	1288.5	1272.4
SLC25A29	122.6	135.9	122.3	117.8	101.5	134.6	125.6	185.5
SLC25A29	140.9	164.9	142.9	124.1	124.8	173.4	138.2	184.4
SLC25A3	10281.4	11792.9	5848	4637.9	2887.5	4288.8	9663.1	15588.7
SLC25A3	229.1	284.1	116.6	101.9	88.2	95.4	188.3	236
SLC25A30	130	152.7	105.7	100.2	97.1	93.4	113.9	116.1
SLC25A31	102.9	104.9	100.3	86.1	86.8	75.8	100.1	125.7
SLC25A32	95.6	108.4	99.6	81.9	82.3	94.8	93.8	117.5
SLC25A34	135.2	127.3	105.1	101.5	97.4	98	105.9	110.7
SLC25A35	99.7	107.8	116.6	108.5	121.1	124.3	100.7	112.2
SLC25A36	432.6	614.8	282.1	381.8	287.4	281.6	385.5	554.3
SLC25A37	551.4	663.5	513.7	426.6	412.8	625.1	482.1	930.8
SLC25A4	904.3	1143.7	763.4	536.8	413.6	667.2	623.2	1056.7
SLC25A40	781.8	1004.9	448.5	486.2	367.3	607.6	591.3	898.7
SLC25A42	577.7	740.6	511.3	423	383.3	415.9	490.4	449.1
SLC25A43	1640.2	2165.5	948.6	1011.9	811.3	1317.9	1429	1599.7
SLC25A45	83.8	90.3	89.3	84.7	83.5	88.2	82.6	81.3
SLC25A5	20192	24572.4	15949.3	12964.1	10101.9	14900.5	20225.7	23670.6
SLC26A1	133.1	148.8	105.9	102.3	84.5	90.7	126.5	156.6
SLC26A1	103.4	103.4	89.3	90.4	92.1	88.3	98.5	95.3
SLC26A1	85.7	92.4	97.9	90	84.9	90.2	85.6	84.2
SLC26A10	95.5	103.5	98.3	93.1	83.1	93.1	104.5	116.5
SLC26A11	85.2	90.8	93.9	76.9	84.2	92.9	82.5	88.6
SLC26A11	85.5	116	95.4	88.1	93	89.3	89.3	105.7
SLC26A2	200.3	241.2	108.7	105.3	101.6	111.5	172.4	210.5
SLC26A3	81	86.7	85.1	90.3	75.7	95.7	78.3	90.3
SLC26A4	99.9	117.7	87.2	84.6	76.2	93.2	102.9	105.7
SLC26A5	73.7	81.1	85.8	75.3	83.5	82.7	81.6	78.3
SLC26A5	93.7	112.5	99.7	90.3	94.1	96.3	98	106.4
SLC26A5	90.1	109.5	113	98.6	100.8	89.5	95.3	100
SLC26A6	916.4	1071.8	514	455.7	485.5	539.4	758.5	607.3
SLC26A6	116.3	149	93.4	88.1	74.7	85.9	117.3	140.5
SLC26A6	179.7	192.9	117.6	100.6	101.5	119	153.9	195.4
SLC26A7	84.8	93.1	76.2	82.4	77.7	81.5	81.3	99.4
SLC26A7	82.1	89.2	103.4	94.8	91.5	89.7	79.8	91.2
SLC26A8	86.1	117	98.3	92.8	86.7	86.3	91.6	97.6
SLC26A8	82.6	89.9	81.2	87.1	89.6	78.5	80.4	84.1

SLC26A9	109.6	118.8	85.2	69.9	69.6	81.3	104.7	117.7
SLC26A9	137.6	132.1	92.8	90.9	83.6	101.8	129.8	173.9
SLC26A9	98.7	104.1	95.3	89.9	95.2	92.4	98	101.8
SLC27A1	189.8	206.5	202.7	231.4	208.1	262.7	165.2	203.4
SLC27A2	718.1	928.1	416.1	362	277.7	373.4	524.9	663.3
SLC27A3	442.4	498.8	244.3	252.9	261.1	261.9	327.9	283.7
SLC27A4	119.5	145.4	83.2	82.4	79.3	72.2	98.7	154.7
SLC27A5	305.3	353.2	217.6	112.9	103.2	119.2	231.8	279.9
SLC27A6	112.4	134	101.3	97.6	97.8	111.6	117.1	142
SLC27A6	99.8	103	96.5	83.7	73	76.3	93.7	116.9
SLC28A1	78.2	89	83.3	85.7	79	74.2	76.2	79.5
SLC28A1	121	143.3	94.2	75.9	82.1	87.5	103.5	147.1
SLC28A1	115.4	131.4	100.6	90.3	83.4	93	117.8	123.4
SLC28A3	80.8	81.5	97.3	87.5	85.2	89.6	84.5	89.7
SLC29A1	104.4	114.2	109.3	95.4	88.1	82.1	111.6	116.3
SLC29A1	775.5	1090.4	905.7	685.5	578.3	1090.9	575.8	795.2
SLC29A1	565.3	774.3	531.1	484.1	366.7	653.9	379.9	706.2
SLC29A2	173.9	189.9	131.8	113.5	120.8	133.9	145.9	192.8
SLC29A3	188.2	213	170.2	146.8	109.8	134.4	210.8	306.6
SLC29A4	92.2	106.4	110.1	120	102.9	106.7	90.9	104.5
SLC2A1	1515.6	2042.7	854.3	791	709.6	1110.5	1432.9	1426.1
SLC2A10	193.5	226.5	183.4	234.5	189.4	283.6	190	270.9
SLC2A11	83.4	88.2	99.5	96.8	95.3	99.7	78.2	90.3
SLC2A11	130.9	121.4	117.4	119	109.7	124.8	107.2	113.6
SLC2A11	154.9	214.9	127.6	115.7	123.5	129.2	146.7	172.1
SLC2A12	93.3	88.9	79	85.8	85.1	84.4	93.9	108.4
SLC2A13	83.8	96	91.1	99.6	90.1	100	81.6	94.3
SLC2A14	110	125.5	99.9	103	97	98	104	105.8
SLC2A2	83	91.9	100.6	87.5	86.3	90.5	78.8	86.4
SLC2A3	485.3	566.6	313.8	309	295.5	402.8	465.8	729.7
SLC2A4	101.8	133.9	96.4	87.1	87.5	85.6	104.7	117
SLC2A4RG	964.5	1167.1	459.6	391.3	357.9	403.5	811.1	678.8
SLC2A5	2336	2872.5	2087.6	1845.6	2047.6	2402	1660	2247.1
SLC2A6	969.1	1261.5	587.9	628.3	772.1	435	833.9	807
SLC2A7	75.6	79	95.1	95.8	92	95	76.3	81.5
SLC2A8	757.4	990	479.4	492.8	429.9	482.7	757.1	634.3
SLC2A9	96.9	134.4	91.6	84.5	80.6	90.4	105.6	155.6
SLC2A9	144.6	193	113.1	102.8	92.6	83.4	147.2	148.4
SLC2A9	809.9	1012	637.1	524.3	545.2	732.8	577.9	815.9
SLC30A1	206.6	244.4	135.3	118.8	111.7	152	265.9	471.3
SLC30A10	85.6	107.1	95.3	94.7	85.4	88.6	90.8	85.1
SLC30A2	87.8	89.4	90.8	81.7	77.6	86.8	86.8	97.5
SLC30A2	76.3	76.4	103	104.1	88.1	92.8	74.4	82.8
SLC30A3	424.8	558	297.1	124.8	115.2	119.4	397.3	417
SLC30A4	71.2	71.6	107.5	91.9	89.7	80.1	79.7	78.7
SLC30A5	1444.8	1933.3	942.5	973.9	653.1	840.5	1141.7	1781.7
SLC30A5	139.2	146.9	116.5	127.2	99.1	148	139.5	236.7
SLC30A5	104.8	116.3	98.2	101.3	86.8	102.5	110.9	109.3
SLC30A6	95.3	105.8	88.6	92.1	82.3	94.5	112.3	94.7
SLC30A7	1345.1	1709.6	693.6	617.5	506.2	742.3	1226.9	1704.4

SLC30A8	95.6	100.1	99.6	96.4	91.2	94.5	88.3	92.8
SLC30A9	1436.1	1720.6	763.8	764.2	509.1	1132.2	1362.8	2172.1
SLC31A2	324.5	397.7	263	332.8	286.1	383.5	314.2	448.1
SLC32A1	78	75.2	94.4	90.2	81.5	86.5	71.6	78.6
SLC33A1	314.4	362.3	134.1	113.3	102.7	207.5	280	531.9
SLC34A1	126.2	158.6	85	84.8	82.4	88.6	133.2	139.5
SLC34A2	109.6	123.7	100.8	97.4	84	94.2	113.4	120.3
SLC34A3	91.5	93.8	101.6	84.1	89.2	82.4	85.9	86.8
SLC35A1	2483.9	2804.1	1354.5	1790.6	1496.5	2621.8	2059.1	2966.7
SLC35A2	897.6	1116.1	761.5	592	484.5	708.7	890.6	1248.8
SLC35A2	138.3	152.2	130.1	120.9	110.5	121.2	135.5	142.1
SLC35A2	237.2	272.1	180.9	191.7	161.1	172.2	250.9	348.3
SLC35A2	142	178.9	158.5	141.4	129.7	166.4	146.4	193.6
SLC35A3	1243.4	1558	585.5	442.8	377.2	569.1	821.3	1534.8
SLC35A4	423.6	640	633.2	509.6	397.3	649.8	463	703.6
SLC35A5	1014.8	1201.3	533.3	564.7	415.4	605.5	948	1272.6
SLC35B1	3789.1	4889	3391.1	2870.7	2033.1	3183.9	3487.9	6141.7
SLC35B2	948.4	1279.2	897.7	862.4	735.8	1269.1	780.4	1135.8
SLC35B3	688.9	880.8	467.6	518.7	401.5	604.9	673.4	1088.2
SLC35B4	195.9	226.1	169.1	200	149.4	217.5	185.5	285.3
SLC35C1	172.2	232.1	169.8	145.7	110.9	158.2	161	250.7
SLC35C2	233.1	300.6	157.8	174.9	144.3	215.9	209	273.6
SLC35C2	424.9	521.8	249.6	236.5	196.9	232.2	356.2	462.2
SLC35C2	92.7	104.3	93.3	88.8	77.6	80.7	96.2	96.6
SLC35D1	124.2	122.6	121.1	110.6	100.4	99.5	102.3	118.3
SLC35D2	181.5	232.3	167.4	162.6	156.7	209.5	172.6	271.1
SLC35D3	125	143.6	96.8	92.8	78.4	84.6	114.3	123.2
SLC35E1	1030.8	944.9	385.5	646.2	580.5	447.1	858.9	1296.3
SLC35E2	96.9	121.2	107	100.6	112.8	107.4	100.4	102.5
SLC35E3	448	489.1	296.3	379.7	310.9	379.4	358.2	552.4
SLC35E4	90.6	92.9	102.8	86.7	89.6	85.1	93.6	98.9
SLC35F1	104	123.1	116.3	115.1	92.5	123.6	109.7	118.6
SLC35F2	1814.1	2091.6	939	727	514.5	793.9	1391.8	1773.5
SLC35F3	80.8	92.5	89	80	80.8	85.8	86.9	93.2
SLC35F3	116.9	123.1	83.8	83.3	79.8	76.8	102.6	117.5
SLC35F4	81.9	94.7	94.3	94.5	88.7	93.7	83.8	83.2
SLC35F5	255.7	327.1	144	165.4	131	190.2	228.1	463.5
SLC36A1	275.6	337.2	184.6	195.2	140.6	244.3	278.5	477.5
SLC36A2	87.3	87	86.9	73.2	81.3	80.7	84.3	83.7
SLC36A3	98.7	88.8	86.6	83.5	76.5	80.9	86.7	81.1
SLC36A4	918.9	1089.8	455.4	462.8	351.3	351.2	891.6	1764.9
SLC37A1	181.1	233.8	136	117.6	125.7	116.9	180.4	214.6
SLC37A2	77.1	90.7	89.6	72.7	76.1	77.4	89.3	89.1
SLC37A3	163.9	210.1	138	110.9	112.5	140.1	157.4	207.1
SLC37A3	85.4	94.8	96.8	90.6	97.3	99.8	81.8	97.4
SLC37A4	1472.9	1900.6	622.2	421.5	350.9	441.8	974.4	801.9
SLC38A1	344.8	414.8	113.9	91.3	96.2	87.4	209.3	302.2
SLC38A1	1455.2	1923.9	690.3	960.6	664.3	746.4	1234.3	2176.5
SLC38A2	754.7	867.3	348.9	433.9	409.8	606.2	758.9	1431
SLC38A3	73	79.5	94.2	102.9	92.5	88.5	79.4	88.5

SLC38A5	223.3	278.1	151.4	114.6	105.1	138.3	200.4	237.4
SLC38A6	276	373.2	153.8	157.3	139.3	179.9	403.5	504.5
SLC39A1	1551.9	1829.6	1411.1	1345.5	1096.8	1483.9	1507.8	2121
SLC39A10	262.1	355.7	169.3	246.6	181.2	369.3	216.4	476.7
SLC39A11	667.6	836.4	424.9	517.2	364.2	662.7	548.2	920.5
SLC39A12	93	107.8	102.7	91.1	93.8	90.9	88.7	99.2
SLC39A13	145.1	182.1	110.5	102.3	98.2	105.3	132.4	169.9
SLC39A14	735.1	1031.5	545.6	241.4	190.9	273.2	665.8	1066.7
SLC39A2	93.4	110.7	104.7	87	94.1	93.6	96	108.7
SLC39A3	815	1163.5	423.2	295.7	241.2	178	825.1	922
SLC39A3	3870.3	4967.9	2978.5	1972.2	1705.3	1962	3356.8	3498.9
SLC39A3	178.7	262.8	275.5	165.7	138.2	233.9	161.8	325.5
SLC39A4	71.1	82.2	90.7	86.4	82.5	86.3	78.8	71.4
SLC39A4	110.3	119	101.1	82.5	88.8	93.1	109.5	114.4
SLC39A5	115.3	131.8	90.1	86.8	82.3	82.4	110.3	131.4
SLC39A6	381.4	499.6	163.5	203.6	155.2	312.7	288.6	397.2
SLC39A7	188.5	201.2	110.6	103.2	89.7	113.8	158.9	211
SLC39A7	163.2	208.3	118	126.5	112	141.6	181.2	185
SLC39A8	959.9	1090	413.9	306.5	231.7	307.9	786.8	1604
SLC39A9	238.7	346.7	175.6	120	95.2	124.5	215.2	335.7
SLC3A1	84.1	89.7	87.8	81.8	75	96.5	84.4	88.7
SLC3A2	565.9	774.4	356.6	224.1	194.2	188.4	866.2	1242.3
SLC3A2	287.7	384.3	120.7	91.2	95.4	106.6	287.1	540
SLC3A2	928.7	1279.8	643.2	414.6	322	572.7	1407.2	3046.7
SLC40A1	166.6	192.3	156.9	275.6	192.1	408.3	136	230
SLC41A1	199.4	213.9	158	137.1	132.5	154.3	203.5	302.6
SLC41A2	87.6	113.3	88.3	84.3	82.6	91.9	97.1	115.2
SLC41A3	1112.6	1579.5	796	564.9	496.5	479.3	1070.2	915.9
SLC41A3	78.3	86.3	85.3	75.6	71.6	73.7	82.8	88
SLC41A3	1230.8	1680.1	436.6	366.4	284.5	322.9	1114.5	1107
SLC43A1	199.1	181.4	119.4	95.6	98.2	103	134.3	154.9
SLC43A2	258.6	343.9	192.7	255.5	216.8	290.1	204.9	177.8
SLC43A3	1132.4	1857.4	913.3	743.3	607.1	1196.1	931.2	1327.5
SLC43A3	96.3	97.3	75.2	83.5	78	92.8	88.5	115.7
SLC43A3	671.2	858.9	230.1	262.8	257.8	321.2	524.4	886.9
SLC43A3	249.1	296	134.7	124.1	117.7	110.6	185.6	283.3
SLC44A1	4192.2	5077.5	2989.3	4633.6	3393.2	4799.4	3580.6	3615.1
SLC44A1	1728.9	2187	1122	1585.6	1162.1	2150.5	1244.5	1818
SLC44A2	102.9	121.9	93.4	106.4	114.2	102.9	109.4	121.7
SLC44A3	120.7	127.5	105.3	105.9	100.7	112.6	116.3	120.8
SLC44A4	121.1	137.8	97.5	97.1	95.2	100.3	125	127.3
SLC44A4	7056	8211.3	3186.2	3831.7	4668	3454.3	6200.6	6071.2
SLC44A4	92.2	116.9	84.3	99.1	91.6	97.2	97.6	108.6
SLC44A5	79.2	88.8	100.3	92.1	93	91.7	87.3	79
SLC45A1	115.3	128.5	113.6	95.1	100.6	93.4	123.2	125.5
SLC45A2	107.4	148.2	93.3	92.9	74.9	84.7	126	134.4
SLC45A2	84.4	99	100.9	95.4	77.6	85	86	86.4
SLC45A2	108.8	149.6	95.6	85.9	89.7	90.4	123.7	129.1
SLC45A3	210.5	260.1	210.3	205	172.1	232.1	214.7	316.7
SLC45A4	165.5	248.9	243.1	205.6	179.1	274.7	186.4	245.1

SLC4A1	100.9	112.2	93.1	77.8	78.6	95.9	81.8	107.1
SLC4A10	90.7	99.3	86.9	87.3	81.8	82.1	94.6	96.2
SLC4A11	177.6	207	236.7	180.5	156.4	205.7	156.5	259.6
SLC4A1AP	305.8	353.9	130.5	134.9	116.1	152.5	294.5	475.2
SLC4A2	191.5	231.1	290	179.4	173.8	279.4	185.9	323.5
SLC4A3	117.6	135.4	93.3	98.4	105.4	126.8	116.1	142.2
SLC4A3	120.8	144.8	96.4	92.2	79.2	81.9	119.6	129.7
SLC4A4	92.5	99.5	89.6	82.7	87.6	89.6	90.5	91
SLC4A5	472	662.5	272.2	456.5	551.2	337.8	445.6	457.3
SLC4A5	91.5	103.7	90.8	85.3	80.1	88.5	92.8	94.6
SLC4A5	88.8	96.7	107.3	97.4	96.9	89.1	96.2	90.7
SLC4A7	574.3	733.7	343.4	442.5	289.7	583.5	497.7	680.7
SLC4A8	99.6	143.7	100.5	91.8	110.1	109.2	122.8	119.3
SLC4A8	97.3	108	91.7	90.1	84.6	97.5	93.4	100.1
SLC4A9	102.6	124.1	102.2	83.1	76.7	82	95	104.9
SLC5A1	83.1	88.3	91.6	85.8	93.3	84.1	74	93.1
SLC5A10	125.1	141	98.1	95.4	83.4	90.2	120.7	127.4
SLC5A10	87.8	93.2	104.9	96.4	88.2	99.9	86.2	85.6
SLC5A10	78.8	92.9	82.9	80.7	79.2	75.3	73.8	78.5
SLC5A10	110.8	126.3	91.2	91	100.5	97.1	126.4	119
SLC5A11	91.2	84.8	87.6	90.6	85.7	80.6	82.9	89.5
SLC5A12	85.2	87.3	95.6	93.2	84.5	84.1	85	88.7
SLC5A12	96.6	97.8	82.8	88.5	73.9	79.5	102	115.7
SLC5A12	93.5	106.3	84.3	82.3	74.8	83.8	93	100.3
SLC5A2	93.5	90.1	95.5	89.7	84.2	93.6	87.2	92.9
SLC5A3	113.2	129.4	80.8	92.2	84.3	85	108.7	124
SLC5A4	105	89.9	94.6	89.6	81.5	82.7	94.1	97.8
SLC5A5	91.5	101.1	92.1	80.2	88.5	94.5	103.4	97.1
SLC5A6	805.9	964.1	937.5	521.6	384.1	695.9	542.6	1011.2
SLC5A7	82.3	90	90.8	86.4	84.3	88.3	77.6	84.5
SLC5A8	166.9	187.3	109.9	129.4	133.1	122.6	166.1	197.6
SLC5A9	99.7	101.5	96.5	98.4	95.8	94.1	97.5	103.5
SLC6A1	86.3	90.1	86.3	81	78.3	78.6	83.2	97
SLC6A11	100.5	114	87.8	92.8	83.8	90.2	109.6	99.5
SLC6A12	104	117.7	112.6	96.1	95.8	99.9	110.6	110.2
SLC6A13	158.6	197.2	129.8	111.3	103	121.8	158.3	166.4
SLC6A14	81.6	84.2	99.2	84.8	91.4	88.3	80.6	83.8
SLC6A15	98.6	126.3	108.2	99.1	102.1	102.1	115.4	116.1
SLC6A15	95.2	102.7	95.5	87.5	82.4	93.7	99.3	93.6
SLC6A15	78.6	81.5	88.6	90.6	88.8	91.4	85.3	74.4
SLC6A16	123	142.9	96.8	93.7	88.5	99.8	109.6	132.7
SLC6A17	87.7	108.5	94.6	82.9	80.9	79.9	100.7	93.1
SLC6A18	129	167.8	113.5	92.3	97.5	110.1	121.4	129.4
SLC6A19	96.1	103.8	83.6	84.5	72.3	76	101.2	100.4
SLC6A2	119	136.2	102.3	111.3	88.6	104.7	119.7	135.6
SLC6A20	87.8	100.2	99.7	100.1	91.4	94	93.2	91.4
SLC6A20	97.3	89.3	80.1	80.9	76.5	81.4	85.7	94.3
SLC6A20	98.2	114.2	98.2	92	91.3	95.1	94.5	108.2
SLC6A3	110.8	125.8	109.2	93.4	91	101.8	104.7	106
SLC6A4	73.6	84.3	98.8	91.9	79.1	87.3	83.8	87.7

SLC6A5	86.7	104.1	83.5	80.1	75.9	79.7	80.8	99.8
SLC6A6	80.4	113.2	104	94.5	90.8	92.7	104.9	110.1
SLC6A7	115.2	160.1	95	79.2	77.6	82.8	114.9	128.6
SLC6A8	102.5	126.7	133.2	143.8	130.9	201.8	114.3	127.7
SLC6A9	85.4	94	110.2	93.1	91.7	90.6	85.1	94.3
SLC6A9	111.4	137.8	103.3	87.2	84.8	93	127.7	134
SLC6A9	87.5	105.1	100.7	84.5	91.6	81.9	94.8	109.7
SLC7A1	1771.6	2617.9	1137.1	1288.4	856.2	1291.2	1786.6	2084.2
SLC7A10	108.6	129.1	122.3	125.9	108.6	125.8	110.7	112.6
SLC7A11	106.9	135.2	111.2	105.1	103.5	116.8	290.2	464.2
SLC7A13	74.1	92.2	94.7	77.5	81.8	82.2	81.3	82.1
SLC7A14	89.8	84.6	97.8	85.6	75.7	85.2	80.1	89.7
SLC7A2	92.6	101.5	95	85.6	77.9	84.2	95	99.9
SLC7A2	82	103.8	85.5	89.7	78.6	77.7	91	92.7
SLC7A2	290.2	437.1	260.4	147	108.4	138.1	242.1	384
SLC7A3	79.2	96.7	96.9	100.7	89.7	92.8	90.6	93.5
SLC7A3	92.8	110.5	92.6	89	68.9	82.8	88.5	106.5
SLC7A4	109.4	115.6	88.1	88.9	84.8	91.2	111.8	110.9
SLC7A5	2416.9	2880.3	2245.1	1078.3	652.4	907.7	2796.9	3378
SLC7A6	321.8	403.3	254.7	185.5	184.5	230.8	287.5	422.7
SLC7A6	324	442.4	229.3	147.1	124.9	174	292.6	378.1
SLC7A6OS	242.1	362.9	157.4	138.6	142.9	154.6	236.8	275.5
SLC7A7	1001	1472.6	805.3	720.2	595.5	935.1	826.8	1314.4
SLC7A8	81.4	96.7	86.4	85.8	76.7	80.9	86.3	109.2
SLC7A8	98.3	97.1	102.7	96.3	98.6	100.3	96.4	109.2
SLC7A8	135.8	161	104	100.3	87.5	100.4	135.1	154.3
SLC7A9	173.7	174.1	126.4	118.3	134.1	131.6	146	169.6
SLC8A1	118.4	134.6	96.9	89.5	84	98.6	104	117.3
SLC8A2	87.9	118.9	94.5	92.5	94.3	102.2	94.6	98.5
SLC8A3	89.2	98.5	95.3	85.3	83	92.5	90.5	94.9
SLC8A3	94.4	103.7	99.5	90.5	88.1	96.3	97.5	97.5
SLC8A3	73.4	81	89.3	72	81.2	79.5	76.1	87.1
SLC9A1	473.8	613.2	477.2	391.9	373.6	407.7	475.3	477.7
SLC9A10	81.6	89.9	106.8	93.6	84.8	88.3	90.8	83.2
SLC9A11	76.1	87.1	92.2	88.2	90.7	83.7	77.5	80.6
SLC9A2	110.2	127.1	85.9	91.6	81.6	90.4	103.8	132.6
SLC9A3	108.5	128.8	94.6	90.5	80.6	99.8	101.2	148
SLC9A3R1	1055.7	1395.9	608.8	513.5	430.9	542.8	839	698.1
SLC9A3R2	99.8	123.8	87.7	80.6	84.8	86.1	109.7	128.8
SLC9A4	556.1	389.4	738.2	195.1	149	342.5	365.7	957.3
SLC9A5	103	113.2	101.3	95.1	89.9	95.6	108	122.9
SLC9A6	82.8	96.8	88	79.1	81.9	79.1	91.3	109.3
SLC9A6	84.8	96	97.1	93	95.7	93.9	86.1	96.7
SLC9A6	449.3	626.6	380.6	376.2	323.6	509.5	434.7	831.2
SLC9A7	127.9	154.3	97.6	100.3	85.2	99.9	110.7	149.1
SLC9A8	146.2	166.4	171.1	148.6	129.8	162.9	147.5	233.6
SLC9A9	83.6	97.4	91.3	104.5	90.1	86	84.2	100.4
SLCO1A2	77.5	85.4	81.3	75	84.4	80.8	78.5	82.9
SLCO1A2	80.8	93.9	99	80.8	86.2	79.7	94.5	89.9
SLCO1A2	79.1	95.7	86.7	81.9	77.8	78.1	72.7	88.3

SLCO1A2	84.9	88.7	103.8	94.3	85.7	92.4	82.6	83.4
SLCO1A2	122.1	136.1	86.5	81.6	76	92.8	117.8	135.7
SLCO1B1	69.6	81.1	89.3	77	76.7	79.5	77.3	70.3
SLCO1B3	91.5	91.7	83.1	82.7	73.1	74.1	77.6	89.5
SLCO1C1	81.6	95.2	87.9	78.4	70.9	86.8	78.8	95
SLCO2A1	89	98	91.1	86.5	85.5	91.7	89	96.4
SLCO2B1	82.8	101.5	95.8	112.3	100.7	102.6	93.7	94
SLCO3A1	110.1	129	105.2	98.6	88	88.5	113.2	134.8
SLCO3A1	161.5	223.2	140.3	136.4	128.9	141.5	179.2	203.1
SLCO4A1	219.5	284.6	302.7	183.1	171.5	202.4	176.7	264.6
SLCO4C1	97.9	99.7	96.4	99.8	104.3	125.9	104.3	109.7
SLCO5A1	100.2	97	107.5	98	84.1	81	89.9	94.9
SLCO6A1	78	96.2	87.8	80.1	78.6	81.2	93.1	100.3
SLD5	962.5	1059.3	527.1	276.6	227.9	176.7	806.6	1085.7
SLFN11	679.4	859.9	328	297.3	267.4	467.6	605.7	1122.8
SLFN13	96.9	104.7	105.9	97.1	104.4	102.9	86.6	94.4
SLIC1	161.9	205.2	104.2	101.2	104.9	100.5	128.4	138.2
SLIC1	121.3	154.4	122	113.4	108.5	104.9	109.8	103
SLIT1	119.4	142.8	111.9	104	88.3	101.5	115	133.6
SLIT2	110.8	135.7	111.1	88.4	83.6	97.1	98.7	115.7
SLIT3	72.8	77.8	100.1	94.3	86	89.2	71.7	85.4
SLITL2	107.3	123.2	104	115	108.2	108.5	112.4	138.9
SLITRK1	89.5	93.2	94	81.4	85.7	77.4	88.1	94.2
SLITRK2	98.7	114.7	84	74.5	82.4	77.4	97.2	108
SLITRK3	97.4	112	105.9	93.8	92.9	90.7	97.4	98.4
SLITRK4	519.9	693.6	340.8	386.5	329.2	568.7	463.4	604.6
SLITRK5	188.3	218	113.5	116.2	113.3	123	125.6	128.7
SLITRK6	95.3	102.9	95.3	95.7	100.1	93.4	98.8	99
SLK	316.4	374.2	158.6	210.2	175.8	293	296.6	464.9
SLMAP	962.9	1341.8	590.8	689.9	531.2	983.1	983.1	1441.7
SLMO1	422.3	433.7	284.2	183.2	152.2	208.7	343.1	484.2
SLN	85.4	103.4	94.1	95.8	88.5	91.8	87.8	109.3
SLPI	581.9	737.8	400.6	530.1	396.2	590.6	366.3	505.4
SLTM	1428.7	1675	974.2	1126.2	1089	1668.4	1140.1	1637
SLTM	1255.6	1573	540.6	524.1	510.8	726.6	902.5	1206.7
SLTM	429.8	685.5	176.7	158.8	129.6	149.8	375.8	604.7
SLU7	664.6	810.3	527.3	613.7	492.4	1087.3	657.4	1342.2
SLURP1	97.2	116.5	96.3	91.3	81.3	88.7	92.1	103.4
SMA4	301.2	331.7	135.9	219.9	191.8	176.3	242.1	403.5
SMA5	89.6	101.6	95.3	84.5	84.8	83.8	96.4	107.9
SMAD1	127.4	160.1	103.9	91.3	86.8	88.2	119.4	138.3
SMAD1	87.1	94.8	80	78.2	76.7	76	88.1	89.9
SMAD2	166.1	226.2	188.3	180.3	151.6	184.2	154.7	209.8
SMAD2	99.2	125.1	94.1	88.4	86.2	89.2	110.2	135.9
SMAD3	271	347.4	240.8	394.9	355.6	549.1	214.1	314.4
SMAD4	1091.2	1439.5	888.9	899.8	752.5	1271.3	989.2	1667.3
SMAD5	97.1	99.7	93.6	92.2	89.9	90.1	95.9	100
SMAD5	101.1	102.9	99.5	85.5	83.6	95.4	99.2	117.8
SMAD5	1062.7	1358.8	588.7	862	593.1	934.7	725.1	1236.7
SMAD6	513.7	651.1	290.2	317.3	255.2	349.9	492.7	518

SMAD7	127.8	134.4	96.5	117.9	93	109.2	121.1	177.5
SMAD9	100.8	112.2	88.8	86.8	82.8	87.1	93.9	124.5
SMAF1	119.8	134.9	102.4	92.6	95.6	93.2	111.7	125.1
SMAP	246	298.7	126	123.2	107.4	133.1	241.1	445.6
SMAP1	519.3	675.6	556.7	703	504.2	937	452.5	819.6
SMAP1	276.8	329.9	137	140.1	128.6	168.8	209.8	267.2
SMAP1L	16273	20886.3	11637.8	12717.8	11424.9	13460	13551	13113.8
SMARCA1	90.5	86.9	96.8	87.5	84.8	79.8	81.5	90.5
SMARCA2	87.7	103.8	99.5	89.8	83.4	88	86.2	93.8
SMARCA2	1951.7	2898.9	1255.6	1679.3	1601	2900.9	1893	2407.3
SMARCA3	270.1	310.7	135.7	137.5	110.4	195.2	163.7	240
SMARCA3	607.9	697.3	437.8	619.7	478.9	1075.6	507.7	908.2
SMARCA4	3320.4	4233.4	1840.4	1443.6	1099.6	1280	2534.6	2489.1
SMARCA5	627.8	718.8	209.2	183.7	138.6	306.7	534.3	1141.1
SMARCAD1	299.1	368.5	230.9	224	150.9	331.8	254.1	405.4
SMARCAL1	480.3	629.7	381.3	396.9	336.3	465	404.9	502.8
SMARCB1	320.1	460.6	423.4	279.9	215.1	341.6	302.2	517
SMARCB1	679.5	742	491.7	381.7	341	582.4	526.3	1388
SMARCC1	1476.5	2244.6	1140.3	1696.5	1218.6	2671	1302.8	2575.1
SMARCC2	142.7	170.6	99.5	96.1	90	115.3	146.4	159.3
SMARCC2	335.7	333.4	449.9	818.9	644.8	682.6	291.8	498.7
SMARCC2	112	121.8	155.5	197.9	189	239	100.1	133.7
SMARCD1	90.2	112.5	94.3	81.5	90.6	88	96.7	108.8
SMARCD1	769.6	932.9	597.4	581.3	483.3	734.2	623	1011.7
SMARCD2	625.9	844.4	491.7	317.5	275.6	334.7	470.7	699.1
SMARCD3	121.5	158.6	99.5	115.2	102.9	134.5	116.4	171.2
SMARCD3	93	99.9	94.7	85.2	87.8	82.9	87.9	100
SMARCD3	295.3	361.5	219.4	260.8	232	317.8	319.6	402.9
SMARCE1	865.1	1119.5	307.2	418.3	272	491	750.2	1160.5
SMBP	662.6	768.6	239.9	204.5	154.2	251.8	551.5	890.2
SMC1L1	98	122.6	113.8	120.8	117.9	148	108.2	112.5
SMC1L2	109.1	121.2	112.4	110.2	99.3	109.1	112.8	124.7
SMC2	266.7	322.3	155	138.4	130.3	122	195.8	246.1
SMC2	712.9	836.8	458.6	484.5	392	837.7	547.8	725.4
SMC2L1	728.3	887.7	380.7	301.8	267	601	527.5	868.8
SMC3	144.3	166	112.2	93.9	91.3	113	142.2	290.8
SMC4	3986.1	4789.2	1980.6	2379.5	2043.3	3416.2	3518.3	6633.8
SMC4	406.8	577	136.3	111.3	129.8	113.1	432.4	524.8
SMC4L1	140.8	189	126.3	103.1	120.3	101.3	133	168.5
SMC5L1	89.3	93.1	86.8	84.2	82.8	76.4	85.3	98.4
SMC6L1	116	123.9	90.1	94.2	85.8	85.2	106.1	132
SMCP	116.6	163	86.8	86.1	93.6	86.4	112.2	131.2
SMCR7	87.1	90.7	91.8	85.6	75.7	85.1	82.3	88.2
SMCR7	96.9	107.2	93.5	93.1	89.3	93.9	93.9	101.2
SMCR8	95.7	97.2	92.8	77.9	80	79.3	83.2	102
SMCX	114.7	128.3	114.6	109.8	104.7	120.2	105	139.2
SMCY	274.8	295.3	268.8	261.1	305.4	417.7	202.4	276.2
SMEK2	1381.4	1829.7	756.4	1064.7	589.8	1207.3	1215.4	2023.5
SMEK3P	88.3	95.5	99.9	87.2	84.5	86.4	91.2	92.3
SMG1	439.3	833.5	366.3	641.3	537	1183	573.7	832.7

SMG5	812	915.2	559.9	443.7	368.4	572.8	806.9	1045.3
SMG6	118.4	120.7	106.5	95.6	90.5	94.7	101.8	117.9
SMG7	212.2	284.6	192.7	190	181.8	203.7	219.6	252.1
SMG7	106.6	123.9	111.5	99.3	90.6	98	101.7	92.8
SMG7	389.6	536.9	512	773.1	565.3	1216.2	394.7	1111.2
SMN1	78.2	91.5	85.4	74.3	74.3	79.5	82.3	85.6
SMN2	188.2	206.9	146.1	131.2	118.8	131.2	164	221.1
SMNDC1	1195.9	1318.5	646.5	492.5	406.3	553.8	1090.8	1926.7
SMO	115.1	140.6	113.5	109.3	107.5	122.3	118.6	129.7
SMOC1	68.2	77.7	75	72.3	77.3	73.8	72.1	78.9
SMOC1	94.2	103	97.6	91.9	86	91.2	85.7	95.5
SMOC2	101.5	105.3	95.3	88.9	82.6	91.9	94.8	96.5
SMOX	171.1	201.7	131	123.4	121.4	129.8	160.8	191.5
SMOX	108.3	115.6	109.3	90.4	87.9	110.1	125.8	161.2
SMOX	76.4	82.6	95.1	86.8	84.6	92.7	75.8	83
SMPD1	153.4	226.8	152.3	133.3	122.5	130	160.9	161.1
SMPD1	125.8	145.6	105.1	85.8	92.1	90.4	127.5	195.3
SMPD2	219	281.9	208.3	160.2	169.5	189.3	186.9	257.3
SMPD3	96.4	112.6	103.4	102.9	114.1	92	102.8	99.5
SMPDL3A	94.5	105.7	110.6	105	97.7	108.4	109.5	159.7
SMPDL3B	93.7	96.3	103	108.2	104.6	101.9	87.4	80.5
SMPDL3B	93.4	104.5	85.6	100.9	92.6	101.2	91.4	95.6
SMPDL3B	108.8	124.3	100.9	88.7	93.7	90.3	107.8	114.7
SMPX	72.9	79.8	81.1	89.4	86.6	73.8	83.6	84.6
SMR3A	84.3	84.7	82.9	85.4	81.7	82.6	83.4	83.3
SMR3B	80.4	94.1	91.7	80	84.4	91.8	83.7	90.8
SMS	1863.8	2485.4	924.5	997.7	746.6	1486.1	1283.7	1794.7
SMTN	126.3	132.8	102.5	97.3	81.2	84.7	119	136.2
SMTN	78.6	89.1	90.9	88.6	91.3	94.9	82.4	103.5
SMTN	208.7	223.2	261.8	211.8	199.6	289.8	176.8	298
SMU1	371.1	507.5	189.8	137.4	135.6	154.8	290.4	375.4
SMUG1	808.4	1037.7	418.3	528.7	427.9	544.5	683.1	906.2
SMURF1	116	125.7	115.2	104.8	104.5	121.5	96.1	104.1
SMURF1	94.8	93.9	105.4	93.7	81.3	93.9	96.7	104.7
SMURF2	107.9	129.3	111.2	109.5	85.8	102.2	102.2	122.7
SMYD1	75.8	82.5	72.7	65.8	74.1	78.1	73.8	79
SMYD2	504.9	668.6	236.5	279.1	241.6	341.2	344.7	614.1
SMYD3	6614.9	8255.6	2780.4	2279.4	1709.7	2571.3	6717.7	9427.7
SMYD4	269.3	315.2	223.7	220.3	196.5	261.8	246.2	426
SMYD5	106.7	116.1	111.2	97.2	99.9	97.9	95.8	125.3
SN	90.8	103.8	86.5	87.3	89.4	90.3	96.2	99.5
SNAG1	110.2	120.4	103.7	89.4	83.4	98.2	105	132.3
SNAI1	85.1	81	87.4	88.7	81.1	86.5	75.7	87.3
SNAI2	103.3	114.4	104.2	82.4	85.2	89.2	108	186.3
SNAI3	104.1	126.5	117.9	107.4	101.8	109.2	100.9	171.9
SNAP23	852.3	1104.9	260	287.6	222	268.8	552	781.9
SNAP23	323.8	373.8	165.4	210.3	171.2	244	316.7	509.7
SNAP25	157.5	187.4	107	106.3	94.3	113.8	147.9	174.7
SNAP25	145.5	174.2	130.9	116.7	102.4	122.5	141.1	175.1
SNAP29	404.5	629	259.6	327.1	242.8	352.6	400.7	580.3

SNAP91	115.4	120.1	96.2	152.7	167.2	180.5	102.2	108.4
SNAPAP	246.4	264.9	135.7	104.6	98.9	117.1	198.3	286.3
SNAPC1	217.2	225.5	152.8	194.6	207.5	183.9	213.7	217.2
SNAPC2	534.6	649.4	359.5	270.7	253.4	234.1	592.2	656.7
SNAPC3	81.9	109.1	78.6	72.7	67.4	81.5	88.4	104.4
SNAPC4	1289.9	1547.1	1197.6	923.4	901.6	938.7	956.3	891.5
SNAPC5	449.1	567.3	193.4	183.6	149.4	210.6	375.3	472.4
SNCA	103	115	122.2	96.5	84.2	91.7	109.8	148.4
SNCA	85.2	87.1	92.4	87.9	80.4	83.5	90.6	98.1
SNCAIP	96.5	105.1	87.5	82.4	79.7	78.3	105.4	108.5
SNCB	92.4	108.4	93.9	70.6	87.3	82.3	89	97.8
SNCB	92.2	98.9	104.5	96.9	87.4	84.5	90.7	93.1
SNCG	92.3	104.1	92.9	83.3	74.8	88.1	90.5	101
SND1	898.7	1251.2	1149.3	1428.7	1154	1911.8	759.7	1281.7
SNF1LK	116.2	144.3	113.3	114.4	108.7	112.5	113.7	147.7
SNF1LK2	106.6	122.7	103.1	105.9	89.2	104.9	108.8	124.8
SNF8	1068	1134.5	329.7	361.6	272.7	346.2	1024.4	771
SNFT	527.7	643.9	366.5	194.4	181.6	216.9	467.8	419.9
SNHG10	99.6	117.5	100.3	89.9	86.6	86.2	109.2	97.2
SNHG3	82.3	102.2	94.5	82.4	84.3	77	93.9	100.2
SNHG3-RC(	175.2	233.1	86	78.8	89.2	84.7	153.5	185.9
SNHG5	11639.1	12238.7	5947.7	6672.9	4651.7	8341.8	8553.7	9269.2
SNHG8	107.5	125.8	100.2	101	83.9	91	96	113.5
SNIP	86.5	102	92.2	80.3	72.9	92.4	91.4	94.6
SNIP1	461.1	557.5	203.1	245.5	196.2	248.4	381.8	517.3
SNN	174	217.8	105.2	121.2	93.7	105.8	162	199.1
SNORA32	169.7	151	131.9	122.8	106.8	169.3	114	155.7
SNORD107	78.5	88.3	88.3	80	82.2	83.7	79.7	81.9
SNORD116	90.5	81.6	96.5	87.4	91.8	83.8	80.5	92.4
SNORD116	82.5	89.7	97.2	84.9	92.6	90.6	87.9	86.1
SNORD36A	333.8	332.3	143.2	125.6	110.8	141.8	232.1	338.6
SNORD36C	296.3	268.3	142	123.2	104.8	151.2	181.6	337.2
SNORD48	496.6	409.3	266.1	180.4	203.9	174.5	258.1	331.5
SNORD56	250.5	266.3	224.5	177.2	160	213.9	169	257.4
SNPH	110.3	129.3	101.4	84.7	91.1	99.9	111	133.3
SNRK	1334.7	2008.7	846	1149.1	1057.5	1761.8	1159.1	1724.5
SNRP70	543.5	704.1	335.5	262.5	238.6	344.6	458.8	718.7
SNRP70	4568.7	5768.7	3288.8	2845.3	2501.8	3350.4	4228.1	6694.6
SNRP70	93.6	94.8	95.1	97.7	98.8	92.5	96.7	103.5
SNRPA	1261.2	1606	822.2	602.7	493	713.4	1025.1	1436.8
SNRPA1	7092.9	9181.6	5084.1	3730.4	2760.7	4158.7	5907.6	8254
SNRPB	5632.5	7008.5	3511.7	1887.4	1345.9	1473.7	6184.9	5784.9
SNRPB	5210.2	6368.1	4608.6	2402.5	1883.9	2854.4	4671.4	6067.6
SNRPB2	4534.9	5507	2885.1	2765.9	2040.2	3263.9	3895.2	5325.6
SNRPB2	1466.4	1727.3	1033.9	899.5	695.1	997.2	1038.3	1245.2
SNRPC	3725.4	4467.2	1890.9	2001.1	1467.1	2275.9	3024.4	5256.4
SNRPD1	769	990.1	271.1	223.3	192.2	235.8	573.7	845.6
SNRPD2	202.1	240.6	111	97.1	89.5	91	223.7	191
SNRPD2	25875.3	27420.5	23885.8	17904.1	16834.9	21302.8	23077.8	23862.7
SNRPD3	716.4	998	356.5	273.7	241.8	164.3	629	1029.3

SNRPE	107.9	106.7	97.9	94.4	81.7	94.3	103.6	105.3
SNRPF	12040	14130.9	9120	8155	6418.1	8594.5	9097.8	12691.8
SNRPG	22486.1	22693.1	20637.7	19053.5	11950.8	18569.9	22128.2	25970.1
SNRPN	3436.2	4679.6	2088.2	1561	1383.1	1511.4	3267.4	3416.4
SNRPN	82.2	90.2	92.2	90.2	80.7	85.2	84.2	87.9
SNTA1	190	231.7	172.4	177.9	161.8	203.9	194.8	248.6
SNTB1	926	1267.7	648.4	464.5	433	611.5	712.9	847
SNTB2	94.3	110.1	87.1	86.7	80	83.3	97.9	113.8
SNTB2	194.1	269	153.5	130.2	116.4	127.4	232.6	299.2
SNTG1	70.9	82.8	84.4	77.6	76.1	85.1	76.4	74.3
SNTG2	79.6	95.9	98	93.9	87.4	95.2	75.4	88.4
SNUPN	1086.8	1352	754.7	561	491.5	786.6	1064.8	1804.2
SNURF	3512.8	5106.3	2026.8	1299.2	1222.7	1537.7	2971.6	3380.9
SNURF	137.4	165	118.6	102.9	97.4	98.8	123.3	168.8
SNW1	429.6	539.7	149.9	128.4	112.7	217.5	374.2	590
SNX1	199	241.2	140.6	179.9	188.7	248.9	163.9	216.8
SNX10	609.7	716.4	230.7	377.9	255.1	571.4	396.2	631.6
SNX11	820.5	1084.8	530.1	496.7	406.8	584.5	814.3	1158.6
SNX11	115.9	162.6	103.6	97.2	102.2	89.4	117.6	134.3
SNX12	222.4	297.5	107.4	122.7	119.4	91.1	218.2	252.8
SNX13	252.9	346.2	171.8	179.4	160.3	292.5	259.9	462.5
SNX14	157.1	165.6	87.8	80.8	79.5	90	119.2	172.2
SNX14	1720.7	2092.3	486.8	542.4	382.5	989.1	1375.9	1967.2
SNX15	108.2	120.7	107.4	108.7	94.3	102.4	107.4	97.1
SNX15	107.9	120.8	102.4	96.9	101.5	97.7	107.3	110.8
SNX16	315.8	316.7	196.8	199.1	181	290.9	255	418.1
SNX16	83.2	92.1	76.7	87.4	88.7	83.6	81	90.6
SNX17	1921.6	2430.4	1584.2	1442.9	1074	1855.6	1346.2	2159.4
SNX19	277.1	355.4	294.4	247.4	216.3	347	251.4	476.3
SNX2	3012.1	3701.1	1634.7	1808.7	1609.1	2473.3	2811.1	3244.2
SNX21	80.9	89.2	84.9	80.8	80.9	83.8	80.2	79.9
SNX22	114.2	130.8	97.6	89.6	86.4	84.1	107.2	128.3
SNX24	137.9	164	91.9	114.3	102.5	130.7	122.9	179.4
SNX25	123.3	169.4	110.7	113.9	96.1	123.4	150.2	189.7
SNX26	786	613.2	610.9	538.2	672.8	565.3	380.6	442.4
SNX27	1941.6	2798.8	1520.4	1428.2	1222.1	1694	1663.7	1488.9
SNX3	16474.9	17375.9	8128.4	10677	7745.5	12046.3	14034	18517.2
SNX4	1403.8	1793.5	785.2	878.4	677.5	1102.6	1165.3	1717.9
SNX5	939.5	1308.2	480.5	347.8	285.7	328.4	588.7	688.8
SNX5	510.1	623.8	318.8	343	288.6	423.8	298	454.1
SNX6	444.2	616.9	329.9	225.8	194.5	242.4	546.8	463.8
SNX6	109.9	137	96.6	87.1	95.8	87.3	100.9	122.9
SNX7	93.2	107.4	85.4	80.7	83.2	87.8	86.2	91.6
SNX7	261.4	322.8	124.4	109.4	112.3	107.6	223.5	286.5
SNX7	97.7	109.1	114.9	113.1	110.7	104.1	113.7	114
SNX8	441.9	679.8	373.4	328.6	280.2	414.3	369.7	648
SNX9	88.3	104.1	86.9	78.2	84.1	81.3	87.7	95.9
SOAT1	275.1	346.9	141.1	134.5	127.3	159.2	253.3	577.3
SOAT2	107.2	117	88.3	89.7	69.4	83.3	104.6	126.6
SOCS1	80.4	94.7	99.5	96.2	100	91.5	87.2	105.2

SOCS2	150.8	164.2	130.6	120	107.9	132.8	109.2	138
SOCS3	108.2	111.5	94.2	84.5	88	92.6	105.2	99.3
SOCS4	106.4	128.5	120.6	110.2	95.6	105	101.8	121.2
SOCS4	482.3	671.4	403.2	370.1	288.1	474.6	361.7	906.1
SOCS5	81	96.4	76.6	75.7	74.2	73.4	77.1	88.9
SOCS5	91.2	95.6	86.5	81.2	77.4	91.6	94.5	95.5
SOCS5	147	187.5	115.5	119.3	111.3	160.3	139.4	184.5
SOCS6	133.2	142.1	102.8	97.2	93.8	107.7	119.9	147.6
SOCS7	118.6	146.1	107.8	108.9	97.8	106.5	119.7	146.7
SOCS7	125.9	154.1	109	91.9	86.4	91.6	128.4	146.4
SOD1	14411.6	15722.1	5357.9	4359.4	3693.5	5303.7	13189.7	19103.4
SOD2	413.5	556.8	240.1	197.7	228.4	212.9	375.1	397.3
SOD2	1572.4	2202.7	1570.2	1199.9	1061.9	1347.8	1466.1	2368.9
SOD2	85.3	118	103.1	87.1	90.7	85	88.3	96.7
SOD3	87.4	97.4	88.6	81.2	82.1	82.3	90.7	92
SOHLH2	80.2	98.7	85.2	84.8	83.6	91.7	73.5	79.2
SOLH	143.4	156.9	189.5	154.5	132.7	237.9	134.2	208.2
SON	257.7	348.4	248	382.7	313.1	550.3	230.7	360.2
SON	379.8	516.6	170.2	128.1	132	117.3	253.3	378.9
SON	484.6	750.6	268.3	303.6	297.5	304.6	365.4	443.8
SON	3420.5	4699.8	1928.6	2996.5	2555.5	4040.7	3462	5241.1
SORBS1	75.8	78.8	108.4	94.7	94.9	90.6	77.4	82
SORBS1	93.1	115.3	98.6	89.5	94.6	87.2	100	113.1
SORBS1	83.1	94.9	102	93.3	88.7	87.8	87.5	89.8
SORBS1	79.5	83.3	89	83.2	73.4	80.4	84.6	93.9
SORBS1	85.9	89.1	102.8	95.2	86.7	92.9	78.4	83.9
SORBS2	90.4	112.7	103.4	96.7	92.4	96.4	95.3	112.1
SORBS2	80.2	84.8	88.5	86.5	84.4	75.5	83	90.1
SORBS2	86.9	101.2	106.4	96.9	87.4	98.8	89.3	99.6
SORBS3	94.4	103.2	106.5	93	87.6	82.9	94.6	98.9
SORBS3	107.6	120.1	99.8	115.8	97.4	97.2	98.5	109.8
SORBS3	958.2	1220.7	512.1	263.6	197.6	242.7	816	1232.8
SORCS1	81.6	89.1	84.7	77.6	81	80.7	78.9	86.9
SORCS1	107.5	127.4	91.2	92.5	89.7	93	124.6	124.8
SORCS1	84.8	105	92.1	86.6	88	97.5	93.4	89.9
SORCS2	100.9	110.5	101.1	81.9	80	98.4	87.5	111.3
SORCS3	98.5	108.7	82.7	79.7	74.8	86.3	117.3	122.5
SORD	361.5	457.6	138.8	132.7	111.3	115.5	215.1	296.6
SORD	321.2	327.2	176.5	148	142.4	219.7	246.1	495.1
SORL1	144.9	182.7	131.4	191.1	168.1	185.2	131.1	148.5
SORT1	140.5	187.3	171.9	142.1	150	183.4	118.4	160.1
SOS1	134.6	148.8	126.8	135.9	121.8	126.9	135.5	150.8
SOS2	129.3	157.1	105.7	103.6	113.3	114.6	131.4	151.5
SOST	101.4	92.9	84.8	77.5	82	87.8	89.2	96.4
SOSTDC1	139.8	149.7	116.2	101.7	104.6	112.8	141.4	140.2
SOX1	77.3	90.6	92.6	84.7	82.1	85.3	88.6	79.7
SOX10	88.2	107.1	98.5	74.6	78.9	90.4	88.4	96.1
SOX11	96.9	96.8	85.4	89.8	80	77.9	88.8	95.1
SOX12	110.7	127.9	130	111.5	102	114.7	108.3	117.9
SOX13	136.6	159.5	133.1	186.9	170.7	223.7	124.1	146.9

SOX14	108.6	122	105.2	91.9	91.5	89.5	102.6	128.1
SOX15	104.5	124.2	105.8	99	100.7	99.1	100	107.7
SOX17	78.4	82.9	88.4	84	86.4	83.7	79.3	91.1
SOX18	286.5	386.2	184.2	130.4	123.2	109.2	310.5	346.9
SOX2	73.2	83.4	84.1	83.1	78.8	83.4	68.7	81.4
SOX21	87.6	110.6	107	104.6	105.7	109.5	87	98.9
SOX3	139.1	173.2	151.6	108.8	83.9	99.6	138.8	164.5
SOX30	95.2	95.9	87.4	87.8	75.3	87.8	91.9	108.2
SOX30	99.2	105.4	115.2	101.1	96.6	94	94	102.2
SOX4	248.1	308.2	142.9	115.5	119.4	151.3	201.6	261.2
SOX5	91.1	90.1	86.7	80.8	83.5	91.4	84.2	86.9
SOX5	100.7	104	85.9	86.2	75.4	90.5	105.1	107.6
SOX5	86.3	97	97.7	94.6	92.9	91.8	82.4	94.5
SOX6	75.1	101.1	86.5	88.3	83.5	85	91.6	89.1
SOX6	118	102	104.7	99.3	89.4	87.6	94.8	97.5
SOX6	84.4	91	83.3	84.3	84.5	89.5	77	90.7
SOX6	77.4	89	85.4	84.4	76.1	89.3	76.5	82.6
SOX7	98.1	111.3	97.7	90.2	87.1	96	101.7	104.1
SOX8	99.7	126	105.2	96.6	90.3	101.3	86	100.7
SOX9	76.6	85.1	101.6	95.6	100.2	103.6	77.4	73.6
SP1	519.7	748.6	341.9	390.2	354.4	357.3	481.2	744.8
SP100	185.4	248.5	173.1	168.9	167.7	231.6	192.2	259.4
SP100	132.2	152.3	120.7	128.6	120.9	178.9	127.9	168.4
SP100	263.2	358.3	150.2	159.8	128.9	204.9	255.7	418.2
SP110	1084.4	1409.4	712.3	613	508.7	833.2	757.6	961.5
SP110	696.5	926	547.9	481.3	397.5	588.5	573.2	825
SP110	108.2	115.6	102.6	110.3	113.1	111.9	99.6	104.7
SP140	95.9	109	96.4	90.7	74.8	91.1	89.6	97.6
SP140	100.6	118.2	110.8	96.7	89.3	108.1	91.5	112.6
SP140	83.5	93.7	99.6	85.8	88.7	94	84.8	99.3
SP2	211.4	232.6	258.3	296.8	259.1	359.9	199.2	490.6
SP3	290.2	339.9	161.2	143.2	134.1	227.7	305.1	543.6
SP3	92.2	110.8	103.1	91	86.7	89.2	109.9	104.5
SP3	122.3	142.8	109.2	103.4	91.6	93.8	111.1	123.7
SP4	198.6	262.5	109.4	102.9	106.5	117.1	179.2	279.8
SP5	130.1	158.1	102.5	101.2	94.3	104.8	124.9	134.1
SP6	139.4	155.8	95.1	105.8	97.7	99.8	122.8	143.8
SP7	82.3	105.9	94.1	84.3	88.3	81.2	100	100.5
SP8	74.9	79.2	90.1	79.9	86.9	79.9	79.6	78.5
SP8	99.9	106.7	93.8	92.6	95.1	97.5	89.2	94.3
SPA17	1010.9	1261.6	569.7	326.8	256.1	378.2	783	975.7
SPACA1	104.2	133.8	103.9	100.3	92.4	100	105.3	103.9
SPACA3	128.2	129.3	104.6	94.4	93.4	102.4	127	131.3
SPACA4	93.9	111.6	87.4	86.1	88.6	87.3	92.4	95.2
SPACA5	85.3	81.6	94.5	91.3	82.5	89.2	84.1	83.2
SPACA5B	103	116.9	94.9	91	82.5	76.8	98.5	108.3
SPAG1	287.3	302.5	147.3	137.7	117.1	145.1	241.1	397.8
SPAG1	123.7	120.9	116.2	109.2	117.5	101.9	111.8	119.5
SPAG1	122.7	130	96.7	82	77.1	83	104.9	105.3
SPAG11	80.7	92.7	89.2	83.4	79.2	76.2	86.9	85.1

SPAG11A	94.1	130.4	107.9	98.8	92.2	101	97.4	105.6
SPAG16	88.2	108.4	84.4	85.9	74.8	80.6	89.9	99.3
SPAG16	141.4	165.7	108.3	86.7	88.4	88	135.4	191
SPAG16	99.2	97	105.1	94.2	90	92.7	101.9	96.5
SPAG17	85.3	111.3	94.2	91.6	98.8	79	81.4	92.5
SPAG4	112.6	144.8	102.7	97.3	104.4	115.4	119.6	120.8
SPAG4L	97.8	105.2	97.7	99.4	92.3	103.9	87.8	103
SPAG5	392.2	478.8	199.1	158.2	157.2	206.7	313.2	499.2
SPAG6	148.1	181.5	101.4	92.9	90.1	102.4	119.8	176.6
SPAG6	144.7	173.4	133.6	107.7	102.8	128.6	122.9	138.4
SPAG7	360.4	414.8	253.4	260.1	259.8	327.9	324.8	411.3
SPAG8	83.7	96	99.6	75.7	87.8	102.9	92.7	84.6
SPAG8	92	104.8	89	89.4	86.4	85.3	93.4	87.6
SPAG9	244.4	486.3	282.1	258.6	261	331	327.5	390.1
SPAG9	191.9	254.9	107.4	119.1	116.9	168.6	176.4	281.4
SPAG9	1082.6	1654.8	714.7	839.8	689	1164	1203.2	1906.1
SPAM1	95.5	113.8	105.7	90.9	95.1	90.8	81.7	88.2
SPAM1	84	89.4	82.3	90	100.8	79.8	81.8	87.9
SPANXA1	78.9	93.5	88	89.4	83.2	81	85	92.6
SPANXB1	79.3	82.4	92.4	79.7	80.1	89.5	74.6	80.5
SPANXC	79.6	90.4	92.4	90.8	71.3	86.7	84.8	77.4
SPANXE	88.7	83.9	93.5	94.4	83.9	97.5	87.9	83
SPANX-N1	75.6	83.6	94.7	88.3	77.5	84	79.9	84
SPANX-N2	81	79.8	95.9	84.2	86.2	87.1	73.9	75.7
SPANX-N3	82.3	89.2	82.9	79	75.7	85.7	87.1	90.9
SPANX-N4	74.2	81.2	84.3	79.4	75.3	81	74.1	89.5
SPANX-N5	79.6	88.9	91.4	88.8	79.9	75.2	80.4	84.4
SPARC	80.7	87.4	84.6	79.8	85.8	84.7	106.6	110.2
SPARCL1	85.2	92.7	83.5	82.1	83.6	81.4	82.7	89.5
SPAST	117	145.1	112.1	139	110	144.7	143.1	186.5
SPAST	105.7	122.6	108.5	97.6	86.9	98	100.5	105.4
SPATA1	96.4	108	98.2	100.6	81.7	85.2	95.6	101.9
SPATA12	83.5	101.2	101.1	87.3	79.5	91.2	92.1	93.4
SPATA13	123.6	130.9	99.6	92.6	90.6	90.2	116.8	125.5
SPATA16	93.4	101.8	90.5	95.3	83.6	74.7	96.5	91.2
SPATA18	81.8	79.7	94.8	95.1	100	102.5	86.5	85.4
SPATA19	93.1	112.4	93.1	92.3	79.9	92.7	97.2	106.4
SPATA2	156	219.6	193.4	199.6	167.3	244.2	162.9	271
SPATA20	693.8	895.4	555.6	548.5	527.1	705.1	469.6	509
SPATA21	95.4	83.5	90.8	86.9	83.4	90.8	83.8	82.5
SPATA22	81.2	76.6	102	92.2	94.9	91.5	79.7	83.4
SPATA3	95.2	104.2	93.8	92.2	85.6	93.7	101.2	100.3
SPATA4	85.9	94.8	103.7	100.6	91.4	99.6	89.6	82.7
SPATA5	116.6	124.6	92.3	81	87.2	79.6	103.7	118.9
SPATA5L1	676.1	872.4	622.7	610	442.4	866.7	574.1	1354.9
SPATA6	115.8	137.4	94.3	86.4	75.5	95.8	112.3	118.8
SPATA7	371.8	456.1	313.4	262.5	258	307.3	232.9	333.4
SPATA7	100.1	133.1	84.5	95.4	85.9	87.2	103	112.7
SPATA7	144.1	172.2	112.5	106.2	98.4	124.6	116.3	135.4
SPATA8	89.9	90.3	110.4	101.9	103.6	106.6	84.7	85.9

SPATA9	106.6	124.3	95.1	94.3	91.1	95.7	96.6	117.7
SPATC1	100.6	115.3	82.6	85.6	82.1	87.4	105.6	114
SPATC1	96.9	105.1	101	92.4	95.2	100.6	102.8	105.4
SPATS1	72.2	84.3	80.4	78.7	67.6	78	71.2	87.2
SPATS2	130.8	157	118.3	112.4	103.3	110.1	128	166.3
SPC24	1767.3	2070.4	954	676	560.6	467.3	1277	1203
SPC25	332.4	325.7	268.9	107	91	131.8	242.3	392.4
SPCS1	8818.9	11697	8739	8277	5467	7830.5	7724.1	16137.1
SPCS2	96.7	109.1	100.5	92.4	80.8	82.5	93.3	99.8
SPCS3	536.9	600.5	271.8	206.5	162.3	222.8	324.3	670.9
SPDEF	83.7	93.2	88.9	80.8	86.7	89.2	90.1	82.1
SPDY1	112.5	129.4	110	105.1	106.5	101.6	113.4	110.7
SPDY1	86.6	87.3	98.3	85.8	91.6	89.3	86.6	89.3
SPDY1	69.3	78.5	88.4	88.8	84.6	82.8	71.9	78.8
SPECC1	121.8	161.4	108.9	94.8	94.3	94.1	130.7	142.2
SPECC1	109.2	128.6	107	86.9	91.4	102.3	109.4	139.3
SPEN	628.4	893.4	652.5	1213.2	1020.7	1403	737.9	907.7
SPERT	98.8	113.5	102.1	105.9	91.5	93.5	91.5	104.4
SPESP1	81.3	100.4	94	84.1	80.8	95	89.5	97.5
SPFH1	269.2	334.6	170.8	139.2	127.4	164.8	248.1	314.5
SPFH2	189.6	206.5	106.6	105.4	104	106.3	146.4	163
SPFH2	866	1093.9	579.9	745.5	481.7	887.8	673.1	897.2
SPG20	238.9	282.8	142.8	161.6	127.4	172.6	233.9	347.7
SPG21	468.1	647.7	220.8	132.9	131.3	127.1	337.1	414.4
SPG21	1720.9	2375.6	979	809.1	701.7	773.9	1116.9	1339.5
SPG3A	214.5	237.4	131.2	132.1	104	141.3	178.6	257.1
SPG7	178	195.8	215	173.4	163.7	228.3	144.3	179
SPG7	114.1	137.8	100.1	100.3	109.6	101.2	101.4	143.5
SPG7	1048.6	1211.2	849.5	847.7	1008	1012.8	795.9	679.2
SPHAR	214.8	269.2	131.4	138.2	117.1	116.4	196.5	245.2
SPHK1	103	131.6	116	129.6	105.2	129.3	115.3	149
SPHK1	87.4	98.1	83.5	82.5	79.7	93.8	95.8	99.7
SPHK2	1777.3	2170.5	1292.8	764.9	580.4	627.9	1454.7	1425.8
SPI1	596.1	799.2	413.3	276.2	226.3	363.2	592.2	1451.8
SPI1	1547.9	1824.9	947.1	682.7	458.5	708.7	1387.2	2857.7
SPIB	155.6	182.1	174.6	112.1	109.5	114.5	127	203.3
SPIC	83.8	99.4	95.5	93.6	91	91.2	81.4	83.7
SPIN	320.9	462.1	188.1	239.3	197.4	125.5	316.3	458.5
SPIN1	1153.4	1357.1	531.6	619.4	532.7	550.8	914.5	876.3
SPIN2	82.8	97.8	102.6	88.3	84.4	88.5	83.8	80.9
SPIN-2	108.3	151.2	87.8	79.8	81.2	98.5	112.6	169.3
SPIN2B	104.7	122.7	101.8	99.6	82.1	93.2	94	93.6
SPINK1	103.7	103	101.5	80.5	82.9	84.4	96.4	99.6
SPINK2	93.3	103.5	87	87.4	90	87.1	92.4	97.5
SPINK4	84.4	88	85.5	77.5	89.6	81.3	81.4	80.4
SPINK5	111	125.1	109.7	98.7	86.6	87.3	103.3	120.9
SPINK5L2	82.2	90.2	94.8	90.8	95.2	89.2	82.8	89.1
SPINK6	87.4	102.1	93.8	94.2	87.6	98.9	89.6	94
SPINK7	82.7	84	82.5	88.2	82.3	86.9	77.2	88
SPINK9	96.8	114.3	114.8	95.2	97.3	96.9	112.8	105.6

SPINLW1	78.3	91.9	84.8	93.5	76.6	88.4	79.1	85
SPINLW1	119.9	125.1	145.5	144.8	130.7	133	121.7	116.9
SPINLW1	95.2	109.4	90.7	87.7	84.3	81	93.2	104.1
SPINT1	98.7	113.1	90.2	88.1	84.3	81.7	92.2	118.6
SPINT1	113	117.4	125.2	106	101.7	123.8	101.8	126.1
SPINT2	213	265.3	186.3	145.5	137.4	156.5	202.6	247.1
SPINT4	80.6	93.4	90.8	82.6	88.8	87.4	86.4	93.9
SPIRE1	621.6	790.5	521	457	378	659.6	543.3	856.8
SPIRE2	101.9	111.4	123.5	90	97.6	107.5	101.9	123.2
SPN	2127	2638.8	1708.3	1009.4	932.8	599.2	1754.1	2724.9
SPN	140.5	169.3	98.5	95.2	86.4	94.7	118.1	168.4
SPN	179.3	227.9	113.3	120.9	115.3	88.1	150.1	212.2
SPO11	98.5	108.2	93.1	86.1	83.3	87.8	98.7	102.6
SPO11	109.3	128.4	118	105.1	106	102.9	116.8	121.4
SPOCD1	183	223	87.2	85.9	91.6	91.3	196.4	209.5
SPOCK	204.6	234.5	176.6	627.8	542.7	660.7	198.7	261.6
SPOCK2	105.2	123.1	105.7	104.9	101	106.8	107	115.7
SPOCK3	77.7	83.3	82.6	75.8	74.2	78	79.2	87.8
SPON1	81.1	96.9	94.3	89.9	86.1	100.1	85.6	103.6
SPON2	91.4	113	84.1	88.3	79.6	83.7	93.9	109.7
SPOP	2451.3	3260.3	1693.5	2112.8	1954.3	2247.1	2253.5	2400.6
SPOP	87	102.3	86.1	93.5	89.7	97.4	87.4	102.7
SPP1	121.8	157.3	106.9	114.1	127.5	193.1	110	126
SPP1	129.2	161.3	110.2	105.5	119.4	144.7	110.4	119.4
SPP2	84.7	89.2	96.7	85.1	76.8	79.8	88.9	89.8
SPPL2A	1696.2	2191.6	1143.8	1181	1004.9	1496.3	1355.1	2676.2
SPPL2B	99.3	110	111.5	105.5	105.3	99.3	94.4	113.3
SPPL2B	86.8	89.8	99.8	92.8	82.8	92.9	93.5	89.6
SPPL2B	124.8	139.1	105.6	126.6	119.5	133.4	113.6	149.3
SPPL3	149.4	162	88.7	100.6	92.8	92	139.3	168.1
SPR	563.1	763.6	313.1	172	146.5	253.7	540.2	727.4
SPRED1	316	416	230.2	101.1	103.3	113.8	329.8	451.9
SPRED2	308	402.3	220.6	233.1	195.2	239.1	286.9	289
SPRED3	102.3	113.8	96.7	79.4	89	95.3	112.1	124.7
SPRED3	92.3	100.5	95.7	90.8	83	90.5	85.8	96.2
SPRED3	88.7	95	99.1	97	85.7	84.6	80.5	90
SPRN	103.5	111	101.3	94.9	98.2	99.1	96.1	113.9
SPRR1A	106.3	129.3	83.7	87.7	73	75.7	113.5	133.4
SPRR1B	147.2	160.4	117.1	117.4	108.8	111.5	137.8	136.6
SPRR2A	82.6	90.8	93.1	77.5	83.6	86.4	77.1	80.3
SPRR2B	86.3	102.5	97	96.9	87.9	96.7	85	90.2
SPRR2C	84.6	96.1	92.5	79.3	88.2	88.5	74.9	81.1
SPRR2D	78.2	87.2	88	79.5	76.7	78.5	76.4	105.1
SPRR2E	77.8	82.6	84.8	77.3	82.1	86.1	74.3	68.3
SPRR2F	124.3	134.3	90.6	80	83	90.9	116.6	143.9
SPRR2G	82.8	112.7	102.6	98.5	91.5	90.5	87.8	109.3
SPRR3	112.4	130.2	108.9	84.5	95.7	106.3	101.5	110.8
SPRR4	100.4	120.4	94.3	89.8	87.2	83.5	103.3	115.1
SPRY1	87	104	91.2	91.5	88.5	85.3	85.2	96.9
SPRY1	108.3	119.2	106.6	98	94.7	105.1	98.4	95.1

SPRY2	862.7	896.5	328.5	272.2	168.7	265.9	730.5	1166.6
SPRY3	85.3	97.6	87.2	93.3	97.8	92.9	97.7	91.1
SPRY4	134	150.8	157	167.8	112.9	207.3	153.1	226
SPRYD3	512.8	689.1	292.3	228	192.8	227.7	373	232.6
SPSB1	108.5	133.6	100	100.1	96.8	112.9	124.2	179.7
SPSB2	133.9	135.5	160.6	146.4	153.3	191	112.2	224.8
SPSB3	972.7	1211.9	633.4	579.7	766.3	729.8	1005.4	649.6
SPSB4	113.9	141.9	101.1	90.7	97	86.3	111.4	124.5
SPTA1	139.5	183.1	94.2	93.8	78.3	92.8	150.6	202
SPTAN1	182.9	233.9	266.3	300.1	283.8	407.8	171.9	284
SPTB	103.1	110.3	102.1	88.6	87.2	88.9	105.2	123
SPTB	103	117	103.3	91	86.2	83.8	111.8	109.4
SPTBN1	115.6	136.2	95.4	94.5	85.1	95.8	112.8	117.3
SPTBN1	113.8	150.6	100.1	100.2	90.2	82.6	104.5	113.6
SPTBN1	166.3	261.3	191.7	149.8	150.7	206	170.1	195.4
SPTBN2	89.7	111.6	92.9	102.5	94.3	94.6	100.2	109.6
SPTBN4	83.1	107.2	88.9	87.2	91.2	94.1	90.3	99
SPTBN4	87.4	93.6	85.6	87.1	81.4	88.1	92.1	102.5
SPTBN5	91.3	115.6	95.8	85.5	85	85.7	90.4	109.7
SPTLC1	2999.4	3354.5	1214.2	1491.9	1903.2	1716.5	3006.2	4700.7
SPTLC1	386.5	497.4	194.1	167.4	153.4	247.8	271.3	367.9
SPTLC1	675.9	843.5	301.6	222	168.5	220.3	467	772.6
SPTLC2	159.2	216	156.3	170.9	142.6	183.9	147.4	163.1
SPTLC3	96.6	110.1	87.6	86.7	81	85	102.5	103.2
SPTY2D1	190.7	341.6	177.7	171.8	137.6	148.8	273.7	350.6
SPZ1	85.2	91.5	90	96.6	83.1	93	91.2	94.9
SQLE	2932.9	4622.8	1842.3	2413.1	2216.7	3670	2801.9	5105.7
SQRDL	769.5	1012.2	379.9	379	272.3	420.1	991.8	1500
SQSTM1	1353.3	1714	596.1	545.7	469.4	455.6	2694.5	3643.5
SR140	1496.1	1907.2	1044.3	1148.9	979.7	2184.6	1147.5	1622.9
SRA1	296.9	365.5	188.7	191.5	159.7	226.1	289.1	352.5
SR-A1	98.7	112.4	113.7	106.6	104.7	105.3	89.6	121.7
SRC	83.8	97.1	93.6	88	73.5	84.6	74.2	90.3
SRC	127.6	148.5	103.4	99	94	95.3	120.7	118.7
SRC	170.4	238.6	178.5	153.1	154.5	203.4	171.5	270.8
SRCAP	107.8	136.7	94.9	89.1	91.6	97.4	111.6	145.1
SRCRB4D	107.3	116.9	87.3	87.8	84.8	83.6	108.8	114.9
SRD5A1	102.5	131	101.7	99.3	98.2	106	104.7	133.7
SRD5A2	110.1	130.4	92.4	90.1	84.4	89.5	106.7	111.9
SRD5A2L	265.8	326.6	153.6	141.5	127.6	139.2	256.4	356.2
SRD5A2L2	86.8	93	88.1	79.9	80.5	86.6	79.4	84.9
SREBF1	296.8	337.4	284.2	262.1	275.3	325.7	258.8	479.8
SREBF1	120.4	108	87.3	75	90.7	79.5	92.6	118.1
SREBF1	130.1	144.7	150.6	126	136.9	178.6	111.5	144.5
SREBF2	160.1	215.2	105.1	111.1	100.6	102.8	168.7	180.5
SRF	2609.5	3153.2	1964	1703.6	1585.7	2206.5	2251.5	2183
SRFBP1	571.3	837.6	338.4	496.5	363.4	358.9	453.8	681.2
SRGAP1	85.7	96.5	92.7	97.5	84	86.8	90.6	94.1
SRGAP2	362.5	452.9	249.1	253.7	265.5	359	351.2	447.8
SRGAP2	83.1	99.4	90.7	80.4	86.5	78.3	92.6	95.7

SRGAP2	148.5	188	100.7	103.1	103.9	125.7	142.8	188.7
SRGAP2	84.1	108.5	102.5	85.5	83.6	91.4	106.9	111.5
SRGAP3	127.7	159.2	120.2	136.7	140.5	120.5	117.3	110.5
SRGAP3	100	115.1	114.8	112.1	91.2	107.2	106.5	113.7
SRGN	30551.5	29009.4	15288	13348.4	11452	13405.7	30575.4	30312.7
SRI	73.2	80.9	88.1	86.2	80.8	81.6	77.1	86.1
SRI	393.7	519	243.8	200.7	170.6	238.4	342.8	341.5
SRI	348.4	455.2	282.6	398.9	296.2	485.3	271.6	443.2
SRM	1111.2	1501.7	1355.5	793	560.2	738.7	1044.8	2972.7
SRMS	116.2	139	98.8	89.8	92.1	91.8	105.1	119.4
SRP14	9874.8	13040.5	6177.7	6647	5778.3	8241.6	9565.1	10507
SRP14P1	5062.3	6288.8	1440.9	1675.3	1389.2	2016.2	4778.4	5574.2
SRP19	1937.2	2025.2	929.2	852.2	684.4	1028.8	1796.2	3006.7
SRP46	1081.6	1565.2	902.8	743.7	550.7	941.2	859.9	1360.3
SRP54	2531.7	3645.1	1697.4	1491.4	1151.4	2134.9	1996.7	2958.1
SRP68	1626.1	2171.5	1327	1246.4	933.2	1737.5	1491.4	1953.4
SRP72	929.6	1164.6	311.9	295.1	264.3	474.4	662.2	1114.3
SRP9	15146.7	17213.2	7749	8468.6	6182	9583.8	13702.9	19713.8
SRPK1	2745.7	3653.6	1443.2	1752.1	1192.5	2089.4	2154.4	3018.3
SRPK2	209.9	274.3	121.7	120.2	123.2	126.3	158.8	194
SRPK2	183.2	256.1	116.2	104.6	107.7	99.5	183.1	195.2
SRPK2	130.2	152.4	112.4	115.1	95.3	127.8	127.4	225
SRPR	804.7	1072.9	766.9	361.5	288.3	459.3	696.3	1144
SRPRB	3823.9	5224.8	2872	1701.5	1101.9	1811.4	3696.6	3948
SRPX	117.9	141.9	127.5	94.7	89.2	111.8	168.6	216.7
SRPX2	108.1	120.8	95.1	89	75	91.9	91.7	109.4
SRR	170.4	180.3	125.4	118.6	113.6	137.2	132.1	214.8
SRRM1	3610.8	4288.2	2149.5	2561.8	1823.6	2956	2776.3	4566.2
SRRM2	2124.3	1867.6	2541.9	3198.1	2719.3	3889	1194	2306.5
SRRP35	136.9	172.7	103.4	101.4	95.7	131.3	133	149.3
SRXN1	366.6	484.2	344.9	219.1	182	315	864.4	2396.1
SRY	107.1	120.1	92	80.9	82.5	89	101.2	104.6
SS18	137.9	170.4	127.7	98	101.5	108.8	147.6	109.6
SS18	148.3	153.6	114.3	111.1	108.7	122.5	114.6	148.6
SS18L1	339.1	364.4	163.7	224.6	165.3	262.8	270.4	458.6
SS18L1	122	144.6	90.1	72.2	80.5	84.5	105.1	132.8
SS18L2	7943.4	9240.7	6852.3	6345.1	4414.1	6012.6	7404.1	9129.2
SSB	101.7	90.6	95.7	80.5	80	89.8	92.8	114.1
SSBP1	6376.1	7067.6	2613.1	2753	1547.5	3116.6	6043.6	10039.2
SSBP2	477.6	609.9	329.4	371.6	379	470.4	416.2	505.1
SSBP3	137.1	173	148.8	173.7	174.3	209.3	136.1	190.9
SSBP3	93.9	103.2	124.3	150.1	136.8	193.8	100.8	164.2
SSBP4	483.6	614.8	457.4	299.6	244.7	363.3	324.1	761.7
SSFA2	261.4	394.2	137	159	151.5	207.8	202.8	274
SSH1	100.1	109.9	106.2	128.6	121.4	114.7	109.3	139.5
SSH2	341.1	531	316.9	514.7	486.3	640	376	452.5
SSH3	917	1047.2	479.6	528	381.5	534.2	704.5	807.2
SSH3	78.9	86.3	87.6	77.3	83.8	78.4	77.5	88.8
SSNA1	1114.4	1488.2	931.6	588.5	572.6	773.2	1115.3	1496
SSPN	94.7	109.2	97.2	88.9	85.2	89.3	101.4	108.9

SSR1	2029.9	2312.1	1465.3	1746.4	1164	2285.4	1926	3434.2
SSR2	3497.1	4833.7	1710.1	1117.8	1030.7	1334.2	3230.1	3609.5
SSR3	263.8	371.9	153.1	124.1	123.7	132.6	230.6	354
SSR4	7769.1	9975.9	5608.6	5638.6	4294.4	5227	7637.3	10895
SSRP1	448.2	522	289.3	241.5	206.5	285.9	363.8	402.1
SSSCA1	294.5	324.9	207.2	130.7	111.7	137.1	269.1	304.7
SST	76.5	83.5	85.3	82.4	83.9	77.2	81.9	80.5
SSTR1	88.2	111.2	89.3	88.2	78.9	74	89.4	101
SSTR2	256.8	269.4	174.6	225.6	199.7	189.4	258.7	329.4
SSTR3	100.6	117.7	104.5	93.1	85.6	98.5	101.3	109.2
SSTR4	81.5	85.1	87.5	92	89	90.2	90	93.6
SSTR5	78	84.8	89.9	93.6	85.9	83.5	80.7	83.7
SSU72	3573.7	5376.6	3074	2453.8	2071.3	2273	3886.8	4392.4
SSX1	79.4	79.5	98.1	86.6	75.7	86.8	84.2	88.1
SSX2	105.7	122.3	107.5	82.1	83.4	91.2	105	101.4
SSX2	126.2	150.1	86.1	85.8	77.2	84.7	136.9	150.4
SSX2IP	95.3	108	105.7	106	103.9	128.8	90.7	103.8
SSX3	86.8	92.5	92.8	87.5	85.5	97.7	85.3	85.4
SSX3	98.9	104.2	105.5	101.8	93.3	102	101.9	111.8
SSX3	97.5	115.2	96.4	80.6	81.2	84	112.9	117.4
SSX4	86	83.3	97.1	92.1	72.2	87.1	80.6	86.4
SSX4	81.6	87.3	99	94.1	86.9	81.8	87.2	93.9
SSX4B	100.5	115	99.2	84.9	83	89.9	104.2	105
SSX4B	116	129.8	103.3	84.5	78.8	81	111	128.7
SSX5	109.7	120.1	86.3	90.4	82.9	84.2	107.6	117.4
SSX5	86.4	93.5	95.1	94.5	91.2	91.8	90.3	76
SSX6	92.8	95.5	86.9	81.7	86.8	91.2	92.5	102.9
SSX7	84.6	90.2	94.4	84.5	94.8	89.5	82.7	88.2
SSX9	170	215.3	112	105	100	100.2	178.5	167.7
SSX9	75.9	76.1	89.2	78.7	77.6	78	72.8	90
ST13	1377.3	1754.6	670	709.9	563.3	1099.8	1136.2	1942.2
ST14	445.1	585.1	524.5	369	313.4	457.2	421	510.9
ST18	79.3	83.7	84	81.1	82	80.2	76.6	89.4
ST3GAL1	275	375.4	306.6	446.4	393.6	661.9	289.2	525.9
ST3GAL1	120.7	147.9	110.1	112.6	115.6	107.6	111.4	115.2
ST3GAL2	114.6	143.5	111.3	115.8	114.1	129.3	113.4	103.6
ST3GAL3	360.9	439.7	334	238.6	234.9	260.3	298.8	337.9
ST3GAL3	1313.7	1811.4	681.7	556.2	561.3	637.9	1092	1068.4
ST3GAL4	383.3	495.4	332.8	210.2	178.7	272.5	342.8	668.5
ST3GAL5	135.1	184.4	111.9	125.3	107.1	127.3	142.9	169.4
ST3GAL5	214.4	266.3	176.8	212.4	212.2	264.6	196.5	308.7
ST3GAL6	739.6	874.6	381.4	459.5	312.7	602.9	597.8	889
ST5	100.8	99.5	90.5	88.4	78.8	85.8	89.6	89.1
ST5	161.5	221.2	114.2	111.8	98.1	113.7	149.8	176.9
ST5	97.7	112.8	84.9	90.2	82.5	97.6	96.6	98
ST5	109	119.5	108.2	96.3	99.8	97	116.5	122.3
ST6GAL1	101.3	123	95.2	89.7	93.1	85.7	109	126.6
ST6GAL1	524.7	671.7	445.6	503.6	307.9	622.3	408.8	712.4
ST6GAL1	77.6	91	90.1	100.7	91.9	87.4	88.1	89.4
ST6GAL2	83.4	99.5	98	84.1	84.6	92.9	81	90.8

ST6GALNA(	83.9	91.5	90.7	79.8	75.5	80.2	81.7	88
ST6GALNA(	125.4	151.3	87.3	96.2	84.6	87.3	122.1	142.6
ST6GALNA(	105.4	124.3	96.7	85.8	83.7	91.8	113.1	143.7
ST6GALNA(	399.2	530.5	361.4	204	166.3	257.7	354.7	659
ST6GALNA(	126.1	160.2	123.9	106.3	95.8	95	108.8	135.9
ST6GALNA(	294.7	403.5	208	164.7	141.6	171.7	258.3	435.6
ST6GALNA(	96.1	118.2	99.1	88.1	79.6	92.6	114.5	119.7
ST6GALNA(	211.2	288.6	230.5	159	158.7	237.8	192.1	229.1
ST7	301.8	357.9	195.9	172.2	143.3	218	268.9	388.1
ST7	266.7	314.4	141.3	125.6	110.8	143.1	242.2	288.2
ST7	385.2	517	309.3	285.9	211.9	328.5	314.9	477.9
ST7L	283.2	346.8	132.4	99.9	93.9	129.8	244.1	388.7
ST7L	287.7	332.9	185	119.3	112.8	145.2	245.4	327.8
ST7L	106.9	117.1	106.8	96.1	88.9	95.3	105.6	114.1
ST8SIA1	83.7	96	91.3	83.9	79.3	83.8	79.1	91.4
ST8SIA2	90.8	97.8	94	91.6	86.2	92.6	89.7	99.8
ST8SIA3	108.6	118.3	106	108.2	97.9	98.5	107.1	110.4
ST8SIA4	106.7	129.9	118.8	121.9	107.7	107.5	103.6	111.2
ST8SIA4	69.6	86.2	92.4	83.4	86	84.7	79.9	77.9
ST8SIA4	162.9	222.2	135.9	113.4	110.4	144.9	143.8	172.8
ST8SIA5	188.9	256.7	131.8	110.7	104.6	108.7	192.4	196.3
ST8SIA6	98	96.9	97.3	97.5	99.7	90.1	88.4	92.9
STAB1	81.4	93.2	92.2	82.1	81.9	82	82.9	83.8
STAB2	69.9	82.7	99.3	84.2	84.8	90.6	74.6	72.9
STAC	107.7	137.4	93	91.4	84.5	101.4	121.2	136.3
STAC2	113.2	141	103.3	113.4	105.5	112.1	113.6	126.8
STAC3	176.1	235.2	126.3	114.5	104.9	115.5	162.8	186.3
STAG1	554.8	740.8	356	328.1	276.3	414.8	440.4	594.1
STAG2	163.1	199.5	128.1	101.5	108.1	117	153.1	179.3
STAG2	618	858.6	378.9	561.2	455.8	1212.7	586.1	1720.3
STAG2	111	114.8	94.5	87.4	96.3	95.7	108.9	126.2
STAG3	140.2	181.5	112.2	108.7	97.9	119.4	148.4	164.5
STAM	375.3	416.3	288.8	236.3	193.7	283	290.7	464.7
STAM2	209.8	235.6	132.9	127	133	174	151.9	234.1
STAMBP	1887.6	2635.7	870.2	867	738.7	1149.6	1700.2	2094.4
STAMBP	100.4	95.8	93.9	89.6	77.7	84.9	85.4	98.5
STAMBP	848.6	1301.4	474.6	436.8	367.2	710.3	704	1010.8
STAMBPL1	198	226.2	163.4	124	109	146.2	167.6	257.8
STAP2	99.1	107.8	99.6	89.9	92.4	94.6	99.5	116.7
STAP2	98.3	109.3	87.3	88.7	81.9	87.4	96.1	114.5
STAP2	146.5	147.7	121.8	105.7	112.9	132.6	121.6	139
STAP2	89.4	97.3	106.3	96.2	90.2	91.7	97.8	100.9
STAR	135.6	143.4	151	121.8	121.3	124.3	114.5	166.7
STAR	244	326.7	172.2	110.6	102.4	125.8	209.3	224.3
STAR	122.5	134.1	159.6	132.7	134.3	128.1	117.5	129.1
STARD10	143.8	188.8	142.7	115.8	147.2	154.2	150	186.2
STARD13	86.2	88.8	87.6	84.3	83.9	84.5	83.8	82.8
STARD13	93.7	99.7	113.2	109	98.4	111.2	98.1	99.7
STARD13	167.5	241.9	142.4	165.5	139	169.3	162.4	239
STARD13	94.3	107	116.5	98.3	110.8	107.4	95.8	91.7

STARD3	172.1	187.4	128.2	118	103.5	142.6	175.7	234.7
STARD3NL	599.6	862.1	455.3	487.5	404.9	670.7	599.6	1073.8
STARD4	96.5	108.6	85	84.4	78.5	90.3	93.3	114.5
STARD5	76.4	83	92.6	81.2	75.7	83.8	70.2	92.3
STARD5	160.7	212	149.7	140.2	128	136.2	159.2	199.5
STARD6	84	82.4	86.2	83.9	81.4	80.8	83.3	87.8
STARD7	4874.9	6359.2	5386.8	6077.3	5044.1	7543.3	3597.4	6664.9
STARD7	1742.6	2469.1	1020.6	1282	934.3	2075.5	1549.5	2642.1
STARD7	297.6	326.5	134.5	113.9	110.4	81.8	162	224.5
STARD8	229.5	291.6	128.5	128.3	109.4	121.2	186.6	207.1
STAT1	474.4	606.9	247.2	191.1	181.7	278.3	485.6	788.7
STAT1	1107.9	1510.1	668.9	528.9	446.7	735.3	1069.5	1520.4
STAT1	1782.4	2192.9	1345.4	1705.9	1336.3	2135.1	1773.2	2128.5
STAT2	544.3	684.1	527	1655.2	1507.7	1994.5	414.4	494.6
STAT3	515.5	718.3	618.5	803.1	674.6	1129.8	443.6	779
STAT4	116.9	130.7	121.5	89.3	86.8	101.9	118.3	151.3
STAT5A	250.5	299.3	273.5	287.5	244.4	391	252.5	496.1
STAT5B	178	213.2	211.7	223.7	211	321.3	157.3	209.6
STAT6	415.9	498.9	396.7	432.4	422.3	544.1	386.4	512.3
STATH	87.2	93.5	96.3	90.4	90.5	97.4	78.1	82
STATIP1	895	1285.3	701.8	728.4	644.4	1064.5	841.8	1194.6
STAU	95.6	108.8	87.6	91.3	95.2	90.6	90.6	105.2
STAU	785.6	1028.2	633.9	642	446	872.7	718.1	1621.8
STAU1	3721.5	4732	3631.9	4591.7	3179.7	4237.5	3335.1	4709.6
STAU2	475.6	560.1	354.4	275.1	207.4	381.3	347.3	572.2
STC1	94.9	106.9	104	89.4	90.7	98.1	83.9	102
STC2	107	134.1	113.3	76.6	84.9	85.5	127.7	173.7
STCH	151.8	179.8	128.1	144.9	126.3	167.6	170.5	239.2
STEAP1	71.3	78.9	87.9	83.2	68.8	79.3	72.4	86.4
STEAP2	75.6	99.7	100.7	87.5	98.2	99.3	80.5	81.1
STEAP2	80.9	80.5	90.5	82.3	80.5	75.6	71.9	79.7
STEAP2	81	91.8	87.1	80.9	78.5	81.5	89.7	103.6
STEAP3	86	110.1	85.9	77.9	81.1	79.3	86.1	98.1
STEAP3	479.6	570.3	475.1	628.2	397.9	697.4	341.4	432.8
STEAP3	82.1	82.7	85.1	73	74.2	85.2	74.2	90.6
STEAP4	84.5	99.4	83.7	87.7	77.9	80.2	83.9	90.6
STGC3	85.5	101.7	90.5	90.8	78.3	79	89.7	90.2
STH	120.3	151.4	93.2	95.5	92.2	94.8	126.9	138.5
STIL	1581.7	1964	1443.9	1681.7	1503.9	2250.4	1318.5	1979
STIM1	295.2	398	189.4	222.4	191.2	276.2	274.6	388.5
STIM2	210.2	237.9	113.1	117.9	100.6	143.5	182.6	217.7
STIP1	1923	2649.7	1869.7	745.4	640.6	923.4	1698.8	2319.2
STK10	191	270.8	267.8	253.1	217.1	291.7	196.7	290.4
STK11	134.7	186.7	109.3	109.8	96	149.5	123.4	176.7
STK11IP	174.8	202.7	285.7	259.7	244.3	351	158.3	250
STK16	253.4	328.3	129.7	106.7	111.3	120	233.7	310.1
STK16	158.8	186.1	128.9	106	89.7	94.1	151.5	177.4
STK16	125.4	158.6	98.4	86.4	92.9	90.2	111.7	149.6
STK17A	78.7	95.4	92.5	83.3	84.2	85.3	84	86.9
STK17B	75.5	94.9	114.5	96.4	91.9	94.4	87.8	98.5

STK19	758.2	906.9	549.8	568.2	516	665.9	734.7	827.1
STK19	419.8	636.9	423.7	418.2	398.1	506.7	497.8	629.2
STK23	98.9	124.3	119.8	101.8	96.8	101.3	103.1	107.9
STK24	88.7	110	86.4	87.8	68	89.9	85.9	111.1
STK24	151.6	151.8	100.7	89.1	93.8	91.2	114	132.1
STK24	5622.2	7265.2	4061.9	3214.4	2337.3	3007.9	4893.6	6061.1
STK25	559.7	655.6	445.3	473	469.9	644.2	420	558
STK3	907.1	1100.7	541.6	538.5	414.2	711.5	796.6	1616.1
STK31	104.5	110.8	82.2	87.9	79.9	86.6	100.2	105.9
STK31	117.1	127.7	104	108.4	98.6	94.8	121.9	124.5
STK31	84.6	83.3	86.5	82.9	82.9	79.6	77.4	80.9
STK32A	82.6	104.7	83.4	86.7	68.2	84.9	88.7	110.3
STK32B	101.7	113.7	97.4	92	96.7	94.6	98.8	96.9
STK32C	173	176	120.2	97	90.1	99.1	141	161.6
STK33	120.2	168.4	94.8	94.8	82.4	94.7	121.9	149.5
STK35	134.8	162.5	131.6	122.5	112.7	128.6	140.3	177.9
STK36	359.4	388.3	448	610.8	530.6	722	234.7	285.8
STK38	571.3	840.1	641.8	742.7	651.5	1164	454.7	881.4
STK38L	114.9	122.8	106.9	99.7	97	105.2	105.6	134.4
STK39	1103.6	1258.9	813.4	1059	782.2	1419.8	772.4	1127
STK4	3244.3	3794.8	1971.2	2069.5	1574.2	3023.7	2844.6	3792
STK40	627.3	631.7	683.3	984.6	803.2	1180.1	796.2	1567.6
STK6P	100.5	124.8	109.2	98	98.3	103.9	95.7	99.8
STMN1	103.7	124.5	116.8	108	95.8	98	101.3	106.5
STMN1	4434.7	6069.9	3311.6	2811.1	2190.5	3233.4	3731	5325.3
STMN1	527.7	681.3	371.4	265.1	245.8	289.9	420.9	499.8
STMN1	108.1	122	95.2	76.9	67.3	75.3	110.8	102.7
STMN2	117.9	136.6	95.1	88.5	77	83.5	119.7	136
STMN4	88.8	103.5	87.5	80.7	83.1	82	85.5	97.7
STOM	2128.9	2865.3	551.4	456.3	337.3	416.4	1941.9	2434
STOM	1101.3	1560.3	422.3	189.9	181.4	224.9	684.9	918.4
STOML1	108.3	147.8	111.2	108.9	104	111.2	112.3	142.4
STOML2	5326.3	5895.3	4373.9	2294.2	1682.6	2774	3993.8	6327.3
STOML3	83.7	87.9	101.5	85.3	84	88.9	81.7	79.2
STON1	82.5	85.9	99.5	87.4	75.4	86.1	70.6	81.6
STON2	89.9	95.3	87	82.3	77.2	90.8	83.9	81.6
STOX1	133.9	150.4	108.2	93.1	80.3	90.4	119.8	142.9
STOX2	184.8	215.8	150.1	161.1	132.2	160.4	238.5	374.7
STRA13	3445	4576.1	2896.6	2300.6	2164.5	2564.6	2970.2	3454
STRA6	76.7	78.4	88.3	92.2	91.8	85.3	78.6	76.3
STRAP	1396	1652.5	411.7	372.9	339.1	504.7	1192.9	2112.9
STRBP	496.3	579.3	423.3	477.8	417.5	818.2	390.4	722.2
STRC	99.9	105.1	100.9	108.8	103.2	106	97.1	100.9
STRN	155.6	202.9	115.8	116.3	109.7	126.4	140.4	207.1
STRN3	936.2	1206.8	570	555.7	517.1	770.6	686.6	1075.9
STRN4	152.7	182.2	174.7	124.5	122.5	181.9	161.5	335.5
STRN4	155	170.5	108.6	102.4	99.2	112.7	155.9	184.7
STS	96.3	115.3	97.8	92.1	98.2	93.1	95.9	112.6
STS-1	379.9	475	257.2	375.1	289.6	432.7	289.6	300.4
STT3A	3064.5	4573	1851.8	1461.3	1117	1953.5	2436.9	3302

STT3B	769.8	1097.1	275.9	335.4	256.1	466.4	554.2	922.5
STUB1	1528	1771	1183.1	842.9	658.6	1013.5	1222.4	1679
STX10	680.1	869.3	737.7	727.2	701.9	999.8	641.1	715.3
STX11	280.1	386.3	245.1	283.3	251.3	373.5	342.2	770.4
STX12	448.3	536.6	234.1	234.9	204.2	268.3	400	536.2
STX16	125.4	122.6	89.4	87.4	93	79.1	98.5	128.6
STX16	2779.3	2462.6	1712.5	2620.7	2849.7	2908	1874.4	1965.7
STX17	121.2	153.6	112.9	102.9	89.6	97.1	118.1	165.2
STX18	124.8	168.8	106.1	101.4	95	118.5	110.6	137.4
STX19	117.6	142.6	116.5	113.1	104.7	114.2	120.4	138
STX1A	112.3	131.3	118.2	110.2	112.6	136.8	131.3	159.3
STX1B2	79	91.5	93.6	87.8	87.1	88.8	86.7	91.1
STX2	406.5	427.2	326.2	338.3	348.6	449.9	295.3	362
STX2	317.7	360.9	219.1	273.5	254.5	359	286	399.9
STX3A	516.1	621.5	252.7	291.7	240.6	401.3	631.3	950
STX4A	1353.1	1602.8	834.2	628.1	553.2	651	1300.7	1437.3
STX5A	1394.2	1827.7	1076	951.3	934.3	1004.3	1702.9	1696.2
STX5A	131.7	178.3	101.9	102	88.4	113.4	165.3	250.2
STX5A	335.6	490.2	403.3	310.2	320.5	447.5	416.7	783.4
STX6	687.6	904.3	457.6	640.5	518.3	871.8	629.2	968.9
STX7	89.4	94.1	97.6	80.3	85	90	89.9	101.8
STX8	594.7	837.3	246.9	214.9	190.3	292.6	544.5	626
STXBP1	98	124.6	95.9	85.7	85	93.4	95.5	123
STXBP1	114.7	145.8	133.9	116	126	124.3	116.4	150.2
STXBP1	111.4	121.4	99.2	91.7	82.6	93.1	106.4	100.4
STXBP2	1193.7	1493.9	1677.6	981.4	812.2	1558.8	1217.5	2696.9
STXBP3	1477.3	1879.6	638.9	400.8	367.8	655.2	1277.3	2818.6
STXBP5	492.2	680.3	379.6	759.6	556.3	973.1	619.4	998.6
STXBP6	219.1	276	144.1	123	115.7	118.1	160.4	185.1
STYK1	83.7	87.2	89.1	86.1	75.5	83.7	81.4	94.4
STYX	75.3	86.3	94.7	83.5	86.5	79.7	76.9	87.5
STYXL1	1054.2	1464.7	1104.4	1089	821.2	1330.8	938.7	1062.9
SUB1	1146.3	1407	574.2	557.3	393.2	698.6	1069.7	1621.7
SUCLA2	1037	1420	688.5	664.6	498.1	1305	700.7	1292
SUCLG1	1984	2687.1	1402.2	1209.8	960.9	1592.5	1619.6	2563.7
SUCLG2	1686.6	2170.5	501.5	462.4	393.8	578	1368.5	1714
SUDS3	114.8	138.9	94.4	86.2	80.3	90.8	119.4	135.5
SUFU	123.3	137.6	95.2	75.6	81.6	85.6	103.9	136.4
SUGT1	694.3	866.5	417.9	302.2	246.4	348.6	554.7	927.1
SUHW1	133.5	164.4	106.9	106.1	84.7	104.1	119.6	147
SUHW2	148.2	191	137.3	98.7	88.6	104.4	132.8	171.7
SUHW3	385	422.5	278.3	324.1	250.8	395.7	321	510.5
SUHW4	200.5	242.1	165.7	172.6	141	192.8	188.8	259.4
SUHW4	351.8	415	153.8	145.6	137.1	156.8	294.7	407
SUHW4	174.4	185	129.7	123.4	116.8	142.3	136.8	164.1
SULF1	77	102.7	100.1	90.1	93	86.8	87.1	95.4
SULF2	422.1	565.8	214.6	272.8	253.2	346.2	383.6	340.7
SULF2	83.6	93.5	89.8	104.1	109.3	132.1	81.8	94.1
SULT1A1	96.2	100.3	109.5	111.8	101	103	89.4	97.9
SULT1A1	93.9	97.7	87.2	106.3	104	96.4	88.2	99.8

SULT1A1	3503.6	3731.3	1235.2	2231.1	2237.6	1958.3	3344.8	3692.8
SULT1A1	103.9	109.8	89.5	78.9	77.5	98.2	93.3	112.6
SULT1A2	87.8	96.9	94.7	91.1	85.6	101.4	83.4	99.8
SULT1A2	352	396.2	166.1	164.6	184.4	203.5	385.2	546.7
SULT1A3	198.1	224.5	139.8	118.2	110.3	114.2	156	186.8
SULT1A4	363.3	392	318	264.3	274.1	330.4	362	518.7
SULT1C1	101.3	109.9	86.4	83.1	86.6	81	98	106.3
SULT1C1	73.6	80.8	76.1	76.4	79.3	75.3	72.9	68.9
SULT1C2	96.6	118.3	93	88.4	72.5	85.9	104.6	115.8
SULT1C3	84.1	94.9	93.5	97.8	88.9	77.6	85.2	99.5
SULT1E1	103	127.7	87.9	80.3	81.6	80	103.5	124.6
SULT2A1	82.5	94.4	94.2	99.2	90.7	92	78.9	88.1
SULT2B1	139	169.1	128.6	95.1	87.7	101.7	156.2	139.9
SULT2B1	376.3	436.9	178.8	155	150.3	198.4	381.1	464.4
SULT2B1	112.3	142	98.1	96	94.3	102.6	110.7	134
SULT4A1	133.9	134.4	94.6	84.4	88.3	78.3	120.6	121.8
SULT6B1	101.4	116.1	116.8	103.8	114.2	113.9	99.1	100
SUMF1	495.9	632.1	244.7	274.5	222.3	256.4	394.7	475.8
SUMF2	588	832.1	215.9	146.3	153.1	149.4	502.8	782.4
SUMF2	4603.7	5899.2	3691.3	3275.1	2681.7	2651.5	4171.7	5182
SUMO1	139.4	141.6	99.4	92.3	89.8	100.7	141.7	255.2
SUMO1	85.2	79.8	94.2	80.4	86.9	102.4	77	81.2
SUMO1P1	107	115.7	88.5	79.7	71.4	91.2	98	113.6
SUMO1P3	92.3	117.9	79.8	80.7	75.7	95.1	109.5	122.3
SUMO2	1607	1906	553.9	464.5	366.9	414.5	1394.8	2792
SUMO2	7538.9	9333.6	3910.1	3547.6	3116.1	4117.1	5837.3	6073
SUMO3	12186.5	15548.1	9138.2	9798.9	6476.5	10289.7	10930.2	13593.6
SUMO4	98.3	111	101.1	92.5	89	92.2	96.6	105.1
SUNC1	167.3	221.2	110.4	132.1	100.4	108.7	158.3	206.1
SUNC1	88.8	89.8	96.3	89.5	90.2	84.5	87	94.1
SUOX	559.5	695.1	428.7	466.6	424.3	521.6	405.9	470.1
SUOX	101.1	108.8	86.2	90	93.2	87	83.8	97.3
SUOX	172.2	225.8	150.7	153.3	141.8	219.1	138	223.4
SUPT16H	2333.5	3547.8	2998.1	2739.9	2132.1	4549.5	1877.8	3221.3
SUPT3H	195.8	226.4	144.3	132.6	121.2	152.3	160.5	175.7
SUPT3H	226.6	323.2	155.9	123.3	104.4	130.5	211.5	263.4
SUPT4H1	876.1	1131.8	658	695	598.9	875.6	895.2	1530.5
SUPT5H	377.6	526.5	561.7	615.3	452.3	840.2	413.2	732.4
SUPT6H	162.2	243.5	147.3	180.9	143.6	215	157.9	244.1
SUPT7L	103.9	114.6	121.2	100.1	97.4	101.9	97.6	112.4
SUPV3L1	340.9	415.6	313.5	286.7	210.4	362.8	273.7	333.7
SURB7	117.5	128.9	111.5	95.9	110.2	97.3	113.3	144.7
SURF1	766.3	966.6	452.2	451.3	354.6	556.8	613.9	865.9
SURF2	180.6	197.5	153.3	122	109.9	129.9	141.6	172.7
SURF4	1419.7	2133.6	1172.2	686.1	593.2	1252	1357.2	2640.9
SURF5	187.7	235	138.3	100.9	110.7	121.5	150	199
SURF5	839.3	1028.6	600.5	416.4	356.4	456.8	621.6	600.9
SURF6	1665.9	1874.1	1142	1072.3	764.9	1241.8	1187.9	1950.7
SUSD1	677.9	851.9	598.1	708.6	591.4	1013.3	631.2	956.4
SUSD2	83	93.7	91.3	97.8	84.8	93.7	87.4	89.9

SUSD3	712.6	897	423.3	358.9	341.1	381.2	615.9	682.1
SUSD4	75.7	78.9	89.2	83.6	84.2	80.4	89	89
SUSD4	93.9	92.6	82.3	84.4	74	87.5	93.7	98.5
SUV39H1	2585.2	3000.4	1912.5	1284.4	938.8	1082.4	2027.5	2509.8
SUV39H2	137.8	175.4	136.6	127	142.1	130.3	128	154.2
SUV420H1	130.5	145.7	116.9	103.3	95	103	113.7	138.6
SUV420H1	285	467.1	185.4	168.4	155.2	208.3	208.4	265.7
SUV420H1	272.9	335.2	147	150	126.6	200.6	229.2	394.3
SUV420H2	110.7	123.3	97.1	84.6	85	89.4	112.9	124.8
SUZ12	7260.1	8653.9	5222.5	6746.3	5848.1	8705.1	6765.9	9318.6
SV2A	128	154.5	129.7	112.1	102	106.5	123.8	154.8
SV2B	119.7	133.6	97	82	86.7	99.5	94.7	119.9
SV2C	80.5	90.6	92.5	90.7	86.5	91.2	83.9	89.7
SVEP1	147.9	193.8	169.7	103.2	98.9	110.3	143.2	168.9
SVIL	96.6	104.5	102.9	93.1	88.9	85.5	97	94.6
SVIL	131	163.8	113.4	108.6	102.1	91.2	130.7	165.9
SVIL	270.5	328.5	233.9	267.4	218.4	341.4	267.9	456.5
SVOP	93.6	109.9	97	78.2	86.2	94.4	87.6	95.2
SWAP70	921.1	1169	579.1	737.3	626	1060.4	1118.6	1586.7
SYAP1	338.3	428.3	169.8	180.3	212.4	156.1	308.5	383.1
SYBL1	2446	3115.5	1343.7	2330.4	1724.9	2548.9	2275	4234.8
SYCE1	102.3	103.5	96.6	95.9	100.2	106.5	106.8	95.4
SYCE1	82.4	84.3	80.6	84.7	76.8	78.4	83.2	83.5
SYCN	91.7	95.9	92.4	86.6	87.9	86.7	90.4	94.9
SYCP1	70.8	88.1	88.6	85.5	80.5	91.2	87.2	83.9
SYCP2	95.3	96.3	94.6	104.7	93.9	96	101.7	101.8
SYCP3	86.3	88.3	102.7	96.1	88.7	89.2	83.4	85.6
SYDE1	108.8	131.3	118.8	90.5	86.9	89.8	109.2	117.5
SYF2	758.4	999.7	454.8	592.9	574	671.2	801.7	965.2
SYF2	95.2	99	91.6	92.2	82.3	88.4	92.2	99.9
SYF2	2083.3	2987.2	1383.3	1579.2	1568.6	2271.5	2184.6	2672
SYK	2749.5	3330.6	2432.9	2390.2	1974.1	2516.4	2076.4	3676.6
SYMPK	207.7	280	223.5	149	132.6	198.4	206.4	293
SYN1	81.3	98	87.8	90.5	90.6	80.4	89.4	91.5
SYN1	80.5	93.1	99.6	94.3	90.3	93.6	92.5	98.8
SYN1	85.7	91.2	84.9	82.9	82	76.7	93.1	89.9
SYN2	188.3	227.8	109.5	100.1	76.1	102.5	175.9	227.5
SYN2	132.8	128.9	120.1	96.2	83	95.3	126.2	173.2
SYN3	96.1	123.6	94.7	84.5	90.8	97.8	95.8	111.1
SYN3	90.4	96.6	96.8	98.5	80	89	82.7	97.3
SYNC1	191.9	240.6	109.3	114.4	105.4	112.8	170.4	262.4
SYNCRIP	5958.6	7455.5	2949.2	2881.3	1861.4	2696.8	5332.7	7837.4
SYNE1	81.1	109.3	93.3	82.3	82.5	93.4	98.3	93.4
SYNE1	92.2	86.8	84.1	90.4	77.8	80.1	84.1	94.6
SYNE1	139.5	179.2	110.1	102.8	88.8	105.3	138.5	166.2
SYNE1	82.6	76.8	87.1	79.3	74.1	84.2	73.4	75.9
SYNE2	92.3	89.4	84.9	77.5	76.5	86.2	85.8	93.4
SYNE2	80.5	87.6	100.7	81.6	78.8	84.1	78.3	89.8
SYNE2	118.1	128.5	109.6	95.5	93.3	100.5	112.2	128.6
SYNE2	75	81.2	101.8	93.1	81.2	76.8	83.5	80

SYNGAP1	81.1	99	86	78	81.3	79	85.5	96
SYNGR1	409.1	482.1	207.2	144.9	162.2	162.2	349.7	396.3
SYNGR1	104.1	103	89.7	87.5	91.7	95.5	107.2	110.8
SYNGR1	99.2	97.8	109.9	103.2	106.1	93.1	84.3	104.5
SYNGR1	220.2	272.6	233.5	189.8	172.7	218.3	177.3	249.9
SYNGR2	102.2	109.2	88.6	95.8	93.5	109	106.3	115.9
SYNGR3	78.5	94.2	105.4	90.7	98.1	100.6	75.4	92.5
SYNGR4	107.4	126	120.2	108.4	101.9	103.4	104.6	114.4
SYNJ1	210.8	267.6	145.6	163.4	149.1	211.6	206.5	378.5
SYNJ1	146.2	145.9	94.6	95.4	93	95.9	129.2	153.6
SYNJ1	81	102.2	88.2	98.7	81.3	84.5	86.9	90.2
SYNJ2	281.4	359.5	231.4	264.1	218.6	246	205	325.2
SYNJ2BP	445.7	559.6	249.9	321.5	254.8	265.9	345.1	416.3
SYNPO	136.2	148.5	104.2	87.9	87.3	89.8	127.3	136.9
SYNPO2	114.8	123.1	116.8	90.6	91	106.1	111.5	119.3
SYNPO2	106.6	114.7	92.1	87.2	89.2	96	100.9	114.5
SYNPO2L	96.5	121.2	96.5	93.7	75.1	90.7	97.9	114.7
SYNPR	94.2	100.5	89.1	88.6	89.4	78.2	86.6	97.9
SYP	91	87.2	97.2	87.9	81.6	96.8	91.7	101.3
SYPL1	3648.2	4697.2	2926	3134.8	2201.6	3738.2	3232.1	5175.3
SYPL1	541.7	552.7	271.9	216.4	178.6	196	321.3	404.4
SYPL1	89.2	98.5	99.9	96.9	80.5	83.9	89.8	95.3
SYPL2	84.9	101.9	89.7	78.8	70.1	78.9	87.7	100.3
SYT1	95.3	103.7	97.8	94	89.4	94.8	93.3	96.2
SYT10	74.1	86.9	86.6	72.2	72.5	80.3	74.7	81
SYT11	177.3	197.8	150	105.8	113.6	112	159.8	152.9
SYT12	103.8	112.2	101.1	88	87.8	91.7	96.9	105.2
SYT13	85.2	89.6	102.8	90.4	87.7	85.2	87.8	90.8
SYT14	89	109.6	98.3	86	89.4	93.4	106.7	99.6
SYT15	216	270.4	193.4	119.5	114.2	117.1	218	253.1
SYT15	108.1	133	107.3	97.6	87.2	98.6	107.4	120.2
SYT16	122.8	156.5	93.9	98.5	93.6	92.2	117.3	165.2
SYT17	120.1	119.3	108.5	100.1	108.7	111.8	106.3	104.3
SYT2	86.5	106.3	88.3	73.8	72.7	73.7	79.4	91.9
SYT3	132	164.6	136.2	93.9	99.8	111.1	126.7	178.4
SYT4	118	118.3	92.8	84	78.5	86.7	97.5	110.6
SYT5	104.5	104.2	94.7	89.1	75.4	82.3	74.7	100.7
SYT6	97.2	103.2	93.2	94.5	94.1	90.3	93.7	94.4
SYT7	105.2	128.9	93.2	80.6	86.8	97.3	106.2	125.5
SYT7	151.2	196.7	104.2	94.7	87.9	99.6	148.7	177.6
SYT8	76.8	86	100.9	86.4	100.8	82.5	78.1	84.5
SYT9	93.7	97.3	105.8	94.6	93.4	98.4	93.2	94.6
SYTL1	167.1	214.6	139.5	121	92.2	147.8	140.3	196.3
SYTL2	94	114.5	88.2	87.3	76.5	80.9	110.3	103.4
SYTL2	130.2	159.3	106.7	85.9	80.8	97	129	154.2
SYTL2	82.8	86.1	105	92	78.5	91.9	82.5	86.2
SYTL2	104	132.1	110	108.7	102.5	110.6	104.7	128.4
SYTL3	161.8	171.6	118	101.4	92.2	104.3	151.2	175.8
SYTL4	196.7	240.4	172.4	154.8	150.4	198.1	166.7	186.3
SYTL5	103.1	120.6	98.9	95.9	85.9	93.5	105.3	111.1

SYVN1	774.7	891.7	366.6	326.2	310.9	380.2	709.9	950.2
SYVN1	99.1	114.2	101.9	93.7	89.4	102.4	90.4	116.3
T	92.9	90.4	83.7	89.1	77.8	83.8	85.3	89.8
T2R55	88.4	97.9	92	87.5	79.3	80.8	93	87.1
TAAR1	136.9	157.9	109.3	95.8	90.7	103.6	125.6	138.1
TAAR2	107.2	113.5	94.2	89.8	82.2	90.2	117.5	108
TAAR5	88.1	108.5	96.7	95.4	97.1	84.9	100.3	97.4
TAAR8	94.9	124.5	102.6	98.7	96.6	94.9	96	109
TAAR9	98.8	108.2	97.8	89	93.9	95.1	99.5	108.9
TAB3	101.5	101.8	102.9	94.6	98.7	97.8	96.4	98
TAB3	78.5	106.3	102.7	103.3	98.1	99.3	87.7	97.6
TAC1	81.3	105.2	84.4	79.8	85.6	81.3	90.9	91.2
TAC3	97.1	126.9	86.1	80.1	79.1	91.2	100.9	124.1
TAC3	131.1	145.4	116.6	99.9	102.1	97.1	134.4	162.1
TAC3	71.9	82.4	85.6	79.5	77.7	90.8	78.3	80.8
TAC4	77.9	93	86.8	86	79.3	93.7	86.1	94.6
TAC4	137.1	170.5	98.3	100.6	91.4	91.6	154.8	139.7
TACC1	4559.6	6705	3650.5	2938.4	2192.6	3512.2	4793.3	5581.8
TACC2	159.5	189.8	187	127.4	106.6	135.2	193.4	337.3
TACC2	79.6	92	89.1	83.8	79.8	91.7	83.8	89.3
TACC2	110.5	121.6	106.8	92.7	80.7	90.6	123.3	164.1
TACC3	614.6	770.1	382.6	215.1	192	320.6	523.2	1164.4
TACR1	81.6	84.6	86.1	82.4	80.3	82.6	79.6	85.1
TACR1	88.4	94.2	99.4	92.9	95.4	79	81.2	89.6
TACR1	89.9	94	101.8	96.1	90.9	94.4	91.2	93.4
TACR2	90.4	102.8	94.9	77.1	83.4	91.7	90.5	104.6
TACR3	123.9	144.6	110	106.4	101.3	102.6	113.9	123
TACSTD1	168.4	204.2	99.5	89.3	85.1	93.1	154.1	171
TACSTD2	117.8	132.2	102.3	93.3	87.5	87.8	112.1	119.3
TADA1L	622.6	778.5	285	268.2	229.9	290.2	527.9	905.6
TADA2L	181.3	261.5	98.9	107.5	102.7	99.5	160.8	197.9
TADA2L	296.2	366.2	135.8	113.5	105.2	136.5	238.1	340.5
TADA2L	112.4	116	107.9	95.1	93.8	97.7	102.2	109.3
TADA3L	377.9	466.3	266.1	243.4	201.2	292.9	327.5	536
TADA3L	451.7	481.5	323.9	325.8	276.5	412	347.3	498.5
TADA3L	100.1	92.4	95.9	83.2	79.6	85.5	89	107.6
TAF1	209.9	318.7	140.7	115.3	138	117.4	201.3	239.9
TAF1	113.8	153.9	99.4	103.4	104.6	117	118.3	125.6
TAF10	1674.3	2195.9	1538.9	1117.3	930.3	1307.1	1473.4	2508.9
TAF11	144.7	161.7	104.4	91.9	94.8	96.6	139.2	136
TAF13	216.8	255	143.4	118.5	106.4	99	197.6	228.6
TAF15	4022.5	6490.5	3449	4944.1	4093	4363.6	4622.4	5530.3
TAF15	642.4	1052.6	283.1	280.4	268.5	277	784.5	707.9
TAF1A	233.5	281.3	121.5	120.3	116.3	123	179.9	243.6
TAF1A	101.1	103.2	88.4	76.5	79.4	92.5	91.3	106.6
TAF1B	777.9	825.2	243.1	256.5	226.3	266	541.3	846.6
TAF1C	91.4	106.5	89.4	84.9	83.5	87.1	95.7	116.9
TAF1C	192.5	209.9	192	157.9	160.1	177	159.8	207.7
TAF1C	1776.6	1687.1	1555	1337.4	1040.5	1512.8	1148.5	1584.6
TAF1L	271.7	431.1	188.4	233.2	224.9	321.8	231.7	243

TAF2	928.8	1285.3	763.4	630.3	500.8	1072.2	810.1	1244.4
TAF4	1070.1	1381	830.2	1096	910.8	1300.3	1018	1216.6
TAF5	539.7	686.9	366.2	312.2	231.9	397.1	474.6	977.3
TAF5L	144.4	144.5	129.9	128.4	107.1	135.7	117.6	219.9
TAF5L	217.5	311.8	184.4	381.3	324.3	556	237.5	300.4
TAF6	140.8	159	170.2	127.1	118.3	126.5	115.5	162.2
TAF6	113.9	144.9	106	99.4	92.7	96.8	110.5	157.7
TAF6L	464.8	629.2	398.1	331.8	319.4	368.6	401	598.2
TAF7	1078.4	1330.1	289	284.1	237.2	456.5	766.8	1308.5
TAF7L	84.6	96.2	87.1	72.4	70.6	79.2	94.9	96.1
TAF9	632	739.7	170.3	189.2	148.8	227.6	424.5	695.8
TAF9	503.7	695.3	152	169.9	157.3	174.2	388.9	620.6
TAF9B	77.1	89.9	86.8	81.9	80.9	87.7	79.9	88.7
TAGAP	115.7	128.4	120.5	123.6	112.5	119.1	110.3	109.3
TAGAP	147.6	171.8	116.4	124.1	122.6	109.4	125	146.4
TAGAP	175.6	170.7	140.2	118.1	113.4	125	130.7	155.3
TAGLN	187.2	218.4	168.1	171.8	146.7	169.2	190.2	227.9
TAGLN	111.6	139.1	102.1	93.7	85.3	96.7	123.2	156.3
TAGLN	77.6	91.5	89.4	88.4	85.3	86.8	86.9	90.4
TAGLN2	5225.9	7653.5	6023.9	3626.2	2768.2	4972.8	5156.5	9006.9
TAGLN3	97	103.6	96.9	95.1	87.5	93.3	95.4	100.3
TAGLN3	89.8	88.6	91.1	79	75.2	88.5	87.9	98.3
TAL1	111.4	133.4	102.8	92.5	96.8	92.9	108.7	130.6
TAL2	157.3	177.6	77.7	89.5	81.1	88	135.8	144.6
TALDO1	10972.2	13860.5	8224	8146.2	6281.4	9167.4	12235.5	17860
TANC1	197.8	254.6	173.1	165.7	166.9	290.1	172.4	343
TA-NFKBH	113.6	137.6	119	102.5	98.7	104.1	130.7	167.6
TANK	172.3	194.2	102.4	105.8	82.9	104.2	155.8	241.5
TANK	98.5	107.3	88.5	80.3	77.2	93.7	94.6	112.7
TAOK1	155.4	229.5	209	328.6	260	157.6	241.9	238
TAOK2	144	196.4	219.9	161.5	168.3	165	141.1	203.3
TAOK2	106.6	138.2	90.5	81	93.3	91.1	112.8	150
TAOK2	97.5	141.3	91.5	83.1	86.4	85.9	109.6	118.2
TAOK3	95.8	96	86.5	79.8	73.3	83.6	99.5	95.8
TAP1	1544.2	1791	1479.5	1532.7	1410.9	1724.8	1056.3	1246.3
TAP2	433.7	574.8	261.8	228	214.6	218.9	378.6	492.8
TAP2	498.1	615.5	479.4	486.9	372.7	641.2	399.8	824.9
TAPBP	1185.8	1757.4	1724.7	2554.5	2117	2940	1114.4	1907.2
TAPBP	171.6	173.1	139.6	136.7	132.8	140.2	152.8	215.6
TAPBP	305	438.8	189.6	188.5	175	134.8	231.2	417.9
TAPBPL	197.8	261.8	136.7	128.5	130.8	142.4	186.2	168.6
TA-PP2C	338.2	427.2	227.3	212.9	175.4	184.6	324.5	457.5
TARBP1	568.5	752.2	404.4	640.3	630.3	900	404.9	576.6
TARBP2	267.5	352.2	254.7	180.1	167.8	193.4	227.3	345.8
TARBP2	652.5	823.5	281	229.2	219.2	227.9	567.4	430
TARDBP	211.5	263.2	123.9	125.3	127.4	150.9	179.6	264.1
TARP	992.4	1283	281.5	249.2	165	316.8	649.6	824.8
TARS	2174.4	2749.2	908.1	955.7	676.4	1542.8	2013.3	4302.7
TARSL1	284.7	335.4	155.9	124.4	108.5	158.4	207.9	269.3
TAS1R1	83.6	87.9	89.8	78.8	77	86.2	90	91.1

TAS1R1	85.7	100.8	88	90.3	81.3	90.9	89.9	98.8
TAS1R2	74.3	88.8	106	94.5	90.9	96.8	83.4	88.3
TAS1R3	82.2	87.2	85.1	75.7	76.8	71.1	77.3	86.7
TAS2R1	98.2	112.8	103.4	96.9	89.2	94.7	99.3	110.5
TAS2R10	93.4	97.9	97.3	96.9	93.5	93.6	95.4	99.7
TAS2R13	80.4	95.3	80.8	81	77.6	79.8	90.4	97.5
TAS2R14	98.1	110.1	97.6	85.4	91.5	84.3	99.7	106.4
TAS2R16	100.7	108.2	98.9	84.9	88.6	92.7	107.3	102.8
TAS2R3	137.2	159.1	98.4	94.7	87.2	94.3	139.3	154.4
TAS2R38	106.6	129.6	89.7	92.3	86.5	89.1	104	116.5
TAS2R39	90.8	101.7	91.4	96	97.9	93.7	87.1	90.7
TAS2R4	87.1	98.9	87.8	93.2	81.1	78.7	88.4	97.6
TAS2R40	77.5	87.3	81.1	83.4	77.4	83.6	92.3	85
TAS2R43	94.9	108	112.2	96.9	100.7	101.4	87.5	95.3
TAS2R44	82	94.1	84.6	89.2	85.8	84.7	78.9	92.8
TAS2R45	80.2	98.4	99.6	93.6	84.4	93.9	79	82.8
TAS2R46	79.3	87.7	101.1	90.6	83.5	87.6	91	86.4
TAS2R48	91.2	93.6	96.3	90.2	87.1	92.3	97.9	98
TAS2R49	97.1	114.8	104.8	92.9	88.2	96.8	103.3	111.5
TAS2R5	93.6	101.9	106.4	84.8	79	82.2	88.5	102.9
TAS2R50	82.8	105.7	99.2	85.3	71	82.4	81.7	104.7
TAS2R60	92.9	112.1	95.5	84.3	78.6	96.3	87	96.4
TAS2R7	84.2	88.5	92.9	82.8	82.7	82.7	81.8	93.5
TAS2R8	106.5	118	88.1	77.2	78.4	87.4	110.6	121.6
TAS2R9	97.1	122.2	94.3	88.8	94.9	97.5	96.8	103.2
TASP1	201.5	262.3	163.6	131	133.3	179.9	157.6	195.9
TAT	93.9	104.9	91.7	90.1	94	94.7	93.2	100.5
TATDN1	731.8	962.3	298.8	212.8	165	243.3	825.1	1371.4
TATDN2	450.2	587.7	302.6	254	225.6	365.7	436.3	732.3
TATDN3	543.8	704.9	362.1	331.1	245.6	365.9	572.6	800.1
TATDN3	632.3	818.3	374	326.1	255.4	419.3	555.9	725.9
TAX1BP1	2198.6	3128.7	1552.1	2650.6	2797.5	5400.7	1971.1	3276.6
TAX1BP1	3671.9	4756.5	1968.4	3586.7	3973	6122.3	3094.1	4427.5
TAX1BP3	872.3	1177	608.8	421.2	345	425.3	826.8	867.5
TAZ	293.1	292.4	205.7	181.4	172	224.1	214.2	361.6
TAZ	137.6	129.7	107.6	99	95.7	101.2	120.5	127.9
TAZ	191.4	210.6	183.5	162.4	189.7	241.2	153.1	194.8
TBC1D1	123.3	144.5	101.8	95.7	92.3	98.1	115.8	121
TBC1D10A	128.2	135.4	108.9	98.3	95	85	122.8	175.9
TBC1D10B	174.6	215.7	165.8	117.3	119.2	133.9	162	256
TBC1D10C	125.6	146.1	114.1	154.3	147	177.1	123	153.5
TBC1D13	323.1	409.7	428.1	366.2	297.3	364.1	327.1	551.4
TBC1D14	624	939.5	903.6	821.7	760.6	1178.6	712.9	1147.7
TBC1D15	865.1	1090	475.8	559	486.2	823.8	701	1274.6
TBC1D16	312.2	390.4	270.5	251.7	232.9	282.8	277.3	267.5
TBC1D17	96.1	98.7	111	106.1	103.7	112.4	92.3	105.1
TBC1D19	157.8	190.6	115.2	99.8	93.9	106.3	132.3	184.5
TBC1D2	128.5	160.6	129.3	112.7	114.5	119.2	156.3	172.9
TBC1D20	311.4	354.3	258.6	313.6	299	405	277	504.4
TBC1D21	122.6	156.5	85.4	88	76.9	85.4	106.4	152.4

TBC1D22A	118.5	139.9	98.2	92.4	90.9	99.1	108.3	129.7
TBC1D22B	167.4	203	234.6	244.7	233	322.3	163.9	232
TBC1D23	376.3	467.2	162.2	182.3	140.9	218.5	307.2	493
TBC1D24	127.9	137.7	144.4	123.1	121.2	122.1	137.8	176.4
TBC1D2B	359	496.6	426	414.3	323.2	521.9	331.2	584.3
TBC1D3	91.7	103.6	96.4	85.1	79.7	79.1	92.7	97.6
TBC1D3B	121.4	126.5	114.1	113.3	123.9	157.5	115.1	143.1
TBC1D3C	83.6	99.2	82.6	76.1	78	74.8	86.2	99.2
TBC1D3G	125	138.1	110.2	113.6	121.7	127.5	140.4	139.1
TBC1D4	106	132.3	92.6	87.8	91	105.4	91.8	131.2
TBC1D5	150.8	188.7	132.1	122.5	113.2	139.3	146.9	155.6
TBC1D7	422.2	537.4	234.8	178.2	170.1	216	390	594.1
TBC1D8B	76.5	84.8	97	91.7	86.8	85.1	82.9	76.9
TBC1D9	150.9	187.8	130.6	116	114.4	127.7	154	214.3
TBC1D9B	251.3	265.7	270.1	286.6	254.7	384.2	207.3	457.5
TBC1D9B	433	490.7	215.8	222.5	165.1	313.5	400.5	601.9
TBCA	16398.8	19625.1	12016.1	13166.5	11211.8	15085	15175.6	20176.5
TBCC	687.6	869.5	501.1	361.7	304	441.5	628.3	822
TBCCD1	98.2	108.6	122.9	102.5	100.3	98	102	88.8
TBCD	120.1	148.2	103.3	101.5	102.9	104.3	116.2	146.7
TBCD	354.2	447.1	449.7	508.3	399.5	728.1	333.2	465.6
TBCE	3368.3	4413.8	1716.3	1593.3	1337.7	2355.4	3564.4	4181.6
TBCE	1088	1465.9	269.1	303	215.6	432.1	936.5	1352.7
TBK1	673.2	832.6	496.1	630.3	529.2	960.5	696.5	1324.7
TBKBP1	104.6	117.5	101.7	97.7	93.2	90.9	103.1	126.5
TBL1X	415.3	533	279.5	400.4	318.6	350.5	568	588
TBL1XR1	283.8	385.4	158.9	127.3	150.3	160.9	252.3	394.1
TBL1Y	102.6	113.8	96.9	94.9	83.4	90.7	87.8	101.5
TBL1Y	92.9	106	100.6	88.6	105.3	90.9	85.8	102.6
TBL2	479.3	675.9	341.1	221.3	198	295.6	378.8	537.1
TBL3	189.1	199	185.9	134.1	133.4	140.8	143.7	181.6
TBN	113.2	138.4	100.3	92.1	82.3	82.2	119.4	138.4
TBP	1157.3	1653.9	961.5	927.2	806.2	1358.2	1021.6	1340.9
TBPIP	83.7	87.2	98	89.3	82.5	90.3	88.5	109.4
TBPL1	1011.8	1290.7	674.8	661	479.8	709.7	974.4	1726.6
TBPL2	123.9	137.9	98.7	100.9	95.6	94.1	115	137.2
TBR1	82.1	96.4	86.5	82.9	79	85.9	88.4	94.7
TBRG1	126.8	151.4	93.8	102.5	93.3	90.7	113.8	112.1
TBRG4	559.1	676.7	480.1	426.2	348.1	529.1	437.7	599.6
TBRG4	1128.6	1319.5	834.7	633	507.5	1040.9	994.1	1462.8
TBRG4	221.3	288.5	148	138.5	107.8	111.1	198.7	196.8
TBX1	138.2	153.3	102.1	90.9	82.8	94.1	120.3	201.7
TBX1	80.9	98.6	91	86.6	76.8	83.1	91.3	90.9
TBX1	128.7	139.8	130.2	101.4	102.3	87.8	114	136.8
TBX1	139.6	172.9	111.2	105.9	111.1	117.4	141.3	148.4
TBX10	93.3	111.6	102	104.6	99.6	101.1	97.4	98.4
TBX15	90.7	119.5	102.3	113.5	104.8	140.2	95.8	112.5
TBX18	324.5	405.4	199.9	164.5	139.7	157	248.6	321.1
TBX18	386.6	461.9	205.9	198.2	209.1	288.6	299.2	633.6
TBX19	188	228	146.5	130.6	132.6	143.7	188.4	228.7

TBX2	132.2	145	116.5	103	87.5	116.9	146.4	241.6
TBX20	129.2	136.8	91.2	82.9	82.8	100	128.6	148.1
TBX20	77.8	90.1	99.4	92.6	92.5	93.8	83.4	87.2
TBX21	120.1	143.5	94.7	83.6	78.4	86.3	113.6	170.2
TBX22	75.8	81.7	88.7	90.1	86.4	87.3	80.7	72.6
TBX3	90.9	114.5	104.1	81.3	93.4	92.2	99.9	99.8
TBX3	146.9	171.9	130.6	107.8	98.9	109.5	129.2	139.5
TBX4	91.8	91.9	94	93.3	88.8	101.2	94.3	94.3
TBX5	88.5	96.2	83.5	88.7	85	93.6	84.9	91.5
TBX5	77.3	91.5	86.2	84.1	82.6	84.7	86.6	86.9
TBX5	88.5	83.6	92.4	78.2	79.5	87.2	73.6	82.8
TBX6	103.5	112.4	100.2	93.9	94.3	100.5	100.9	107.9
TBX6	93.3	106.3	93.9	88.6	82.7	88.6	99.8	103.6
TBX6	83	89.3	99.6	92.2	90.7	85.8	85.9	90.2
TBXA2R	91.2	100.5	97.8	99.3	85.1	90.5	97.7	118.5
TBXA2R	82.4	111.5	91.4	87.5	72	68.8	100	129.8
TBXA2R	96.5	131.1	93.1	87.6	86.4	84.6	105.5	132.6
TBXAS1	152.2	184.3	112.3	214.8	192	309.7	217.7	217.5
TCAP	102.5	120.3	89.1	93.9	83.9	86.6	100.6	129.4
TCBA1	83.8	92	93	83.8	83.1	91.9	85.5	77.4
TCEA1	3598.3	5657.9	1525.7	1017.5	791.1	1603.6	2770.9	3806
TCEA1	600.7	706.6	159.9	145.9	131.6	137.1	355.1	681
TCEA1	626.5	742.8	153.4	162.6	139.5	192.9	379.9	717.5
TCEA2	394.6	591.5	361.6	336.9	330.2	370.5	387.7	557.9
TCEA2	129.5	141.9	126.6	125.4	121.9	115.9	127.1	121.1
TCEA2	111.6	123.1	90.8	80.2	83.3	92.3	100.5	121.3
TCEA3	94.8	122.6	86	164.9	117.5	148.8	93.9	118
TCEAL1	428.2	493.2	203.2	201.8	147.6	230.3	329.1	561.1
TCEAL1	178.8	216.3	124	137.2	110.9	139.8	156.3	188.2
TCEAL2	220.8	282.9	183.8	123.6	111.7	125.4	194.7	264.1
TCEAL3	2475.1	3443.5	1612.6	1250.5	1070.8	1341.2	2349.2	2287.3
TCEAL3	4854.8	6587.8	3740.9	3017	2426.8	3100.1	4675.9	4902.6
TCEAL4	122.2	144.9	109.5	89.1	89.8	97	125.9	131.7
TCEAL4	538.4	718.5	286.4	277.6	251.1	315.1	453	576.5
TCEAL4	4556.2	6065.7	3194.8	3358.2	2707.5	3724.7	4010.6	4468.7
TCEAL5	125.3	123.8	90.7	83.6	89.3	86.5	116.9	133.6
TCEAL6	77.4	90.6	84.3	92	92.3	88.9	81.2	84.2
TCEAL7	73	82.8	98.2	89.7	88.8	87.4	74.4	73.4
TCEAL8	1571.8	1925.4	643.8	559	439.1	616.6	1102.2	1364.4
TCEAL8	1152	1441.1	406.1	316.3	255.7	317.7	821.2	1239.9
TCEAL8	1606	2070.1	596.2	458.6	405.7	593.7	1439.6	1954
TCEB1	9298.7	10202.3	4372.4	3114.5	2087.6	3153.2	9046.3	11636.4
TCEB2	323.3	391.6	255.9	180.3	178.8	222.4	374.2	492.5
TCEB2	8275.9	9957	7268.8	4928.3	4033.3	4991.8	8142.1	9895.5
TCEB3	459.2	578	384	363.9	331.7	398.6	391.5	370.2
TCEB3B	75	90.3	80.2	87.4	76.1	80.6	78.4	84.9
TCEB3C	82.7	84.8	85.7	90.2	84.1	91.2	84.3	89.1
TCERG1	774.5	1051.2	462.8	454.5	443.3	733.9	676.5	738.9
TCERG1	145.1	195	108.4	109.1	107.9	96	136.4	182
TCERG1	821.8	1085.1	299.3	304.6	315.8	441.7	653.9	1212.8

TCERG1L	82.5	90.6	89.9	73.2	74.9	78.8	80	85.3
TCF1	81.8	78	86.8	91.9	82.1	78.7	90.3	88.2
TCF12	994.8	1342.3	698.3	1220.4	910.4	2031.3	873.3	1278.8
TCF12	116.3	144.9	101.2	121.5	111.7	103.6	105.7	106
TCF15	116.7	148.6	125.7	106.2	96.6	103.9	121.4	114.8
TCF19	129.6	140.1	90.5	87.4	77.5	88.9	110.6	141.9
TCF19	130.9	158.7	105.5	97.5	91.5	96.4	107.8	145
TCF2	84	104.4	84.6	87	81.9	84.5	86.6	90.7
TCF2	81.9	90.4	100.3	94.5	93.5	90.7	83.6	98.6
TCF2	81	84.5	87.8	80.8	79.3	79.2	91.8	89.6
TCF20	246.9	356.8	152.9	189.9	152.6	183.6	244.1	306.8
TCF20	102.2	114.8	101	91.8	96	89.7	111.1	114.7
TCF20	500.4	674.5	375.2	629.8	587.9	774.8	515.1	658.5
TCF21	99	102	89	82.6	84.2	79	82.4	94.3
TCF21	72.3	83.3	100.9	91.6	80.6	94.6	79.3	84.3
TCF21	81.5	94.8	96.5	96.8	80.2	87.9	97.6	91.7
TCF23	118	139.7	101.7	86.4	84.8	96.4	113.5	130.1
TCF3	290.3	430.2	358.3	295.4	262.9	386.4	276.1	578.3
TCF4	136.3	178.2	126.1	116.8	107.1	113.2	124.6	153.3
TCF7	100.2	106.4	93.2	94.4	96.8	95.2	98.7	106
TCF7	104.9	131	103.8	92.4	93.6	97.3	111.2	115.7
TCF7	98.7	107.2	88.1	87.2	76.9	81.7	83.1	112
TCF7	78.2	83.6	85.8	85.1	78.9	80.1	75.2	80
TCF7	80.7	84	90.1	86.5	85.3	98.8	83.1	84
TCF7	71.8	92.3	87.2	83.4	79.1	87.2	80	100
TCF7L1	90.8	93.4	98.8	84.9	95.5	94.2	87.2	100.8
TCF7L2	95	106.7	92	94.2	83.9	90.8	93	105.9
TCF8	94.2	99	102.1	98.2	85.6	104.8	83.6	94.8
TCFL5	790.8	927.6	762.9	1042.5	763.6	1424.2	570.3	1339.5
TCHHL1	70.5	76.6	92.9	78.5	83.8	70.8	83.1	83.4
TCHP	264.2	375.4	168.5	137.1	131.7	165	205.4	270.1
TCIRG1	619.1	727.2	773.6	673.3	651	1127.8	437.7	858.7
TCIRG1	88.3	107.3	104.6	96.6	101.7	93.4	97	94.7
TCIRG1	201.8	223.8	127.8	170.6	157	103.9	141.5	215
TCL1A	89	92.4	97.4	82.3	87.4	94.1	89.6	94.4
TCL1B	109.4	144.7	91.4	78.6	86.7	79.1	99.5	120.8
TCL1B	75.7	83.2	84.9	84.9	85.1	85.1	82.5	86.5
TCL6	70.8	77.4	75	68.9	82.2	80.5	77.6	82.4
TCL6	150.5	178.9	101.5	89.9	91	100.3	146.8	127.7
TCL6	98.6	107.9	94.2	99.6	88.8	97.8	100.4	98.3
TCN1	156.2	188.9	125.6	194.9	147.5	201.3	141.5	161.4
TCN2	77.3	88.6	101.9	102.9	84.5	98	89.8	91.7
TCOF1	76.2	100.2	101.8	104.2	83.3	109.6	87.7	79
TCOF1	118	124.2	98.2	95.6	94.8	100.3	101.6	127.6
TCOF1	107.8	124	89.2	90.2	76.9	87.3	108.3	119.6
TCOF1	123.2	159.1	107.1	99.7	93.4	113.8	125.1	139.9
TCP1	3090.5	4010.4	582.9	493.1	378.1	520.6	1977.7	3382.3
TCP1	321.9	376.2	140	119	123.2	158.8	226.5	338.7
TCP10	90.4	92.5	94.7	84.9	86.5	79.5	88.3	84.5
TCP10L	121.3	171	109.2	88.2	95.1	97.6	151.8	167.6

TCP11	90	93.2	95.9	91.5	92.2	89.4	89.5	91.6
TCP11L1	73.6	84	88.4	83.4	79.3	82.4	70	74.7
TCP11L2	85.6	92.2	78.1	79	83.3	77.9	88.6	97.6
TCTA	242.7	315.8	197.5	181.7	171.3	233.6	223.5	297.6
TCTEX1D1	1189	1360.7	882	673	549.9	809.3	982	1628
TDGF1	89.2	96	107.7	101.5	92	88.5	96.4	102.4
TDGF3	91.9	100.6	82.1	82.3	76.4	86.2	89.7	101.4
TDH	80.5	90.4	86.5	84.3	85.9	92.4	83.5	83.3
TDO2	78.1	92.4	92.8	85.7	89.3	84.8	78.8	88.3
TDP1	800.7	708.8	514.3	474.8	428.4	361.5	703.4	1111.4
TDP1	216.4	254.1	165.8	148.5	111.1	144	171.5	178.8
TDP1	360.7	478.4	272.7	224.5	203.3	221.2	339.7	305.8
TDRD1	328.1	288.4	300.9	412.6	345.1	304.4	315.5	567.8
TDRD10	89.8	89.2	91.2	100.3	84.8	99.3	93.3	89.4
TDRD3	230.3	254.6	141.5	137.6	123.7	143	180.6	284.2
TDRD4	83.5	87.5	99	85.4	92	89.8	85.1	94.2
TDRD5	73.7	75.8	93.8	86.3	83.6	79.6	76.2	84
TDRD6	121.1	141.8	104.6	94.1	91.2	87	114.9	128.8
TDRD7	3883.6	4556.6	2671.8	3567.8	3069.4	4692.3	1760.8	1995.9
TDRD9	179.3	225.4	97.7	97.8	91.4	94.8	172.8	171.3
TDRKH	111.1	124.9	109.8	102	91.1	106	97.1	121.1
TEAD1	129.4	137.3	103.3	83.3	86.3	96.7	120.8	130.4
TEAD2	340.2	370.7	266.3	171	165.3	168.2	273	317.9
TEAD3	98.4	114.4	95.7	85.7	89.2	91.9	104.9	122
TEAD4	96.7	124.2	85.2	80.5	72.4	83.9	96.6	128.4
TEAD4	123.2	135.7	117.9	107.1	99.9	115.8	113.6	119.5
TEAD4	618.1	825.1	266.9	174.4	161.2	156.8	486.1	375
TEC	183.8	190.1	108.2	101	94.1	96.4	139.3	160.1
TECTA	199	216.6	111.7	103.8	87.8	110	200	190.9
TECTB	77.6	116.7	95.3	83.6	85.9	84.7	94.8	94.8
TEDDM1	110.9	124.7	97.6	99.7	91	97.4	100.8	109.4
TEF	272.4	349.9	201.4	202.9	161.4	197.3	266.8	435.8
TEGT	3821.4	4965.8	2851.8	2593.3	2064.1	4015.4	3493.5	5319.3
TEK	88.3	103.3	106.6	103.5	91.9	93.5	97.2	111
TEKT1	109.7	124.1	100.8	104.9	93	98.6	104.1	132.4
TEKT2	101.2	115.5	122.1	113.3	115.7	104.9	88.3	96
TEKT3	97.2	100.3	90.3	82.2	82.4	76.5	84.3	98.4
TEKT4	87.7	89.6	90.5	77.2	75.7	76.1	87.8	94.5
TENC1	90.9	103.8	87.4	94.8	90.1	87.9	87.6	97.2
TENC1	100.8	114.1	88.9	89.5	81.8	84.1	93	96.3
TENC1	101.5	114.9	100.9	95.2	91.7	90.1	99.3	112.6
TENC1	88.3	108.7	82	90.7	82	83.7	84.5	97
TENR	87.2	90	92.5	82.3	79.5	89.7	86	89
TEP1	89.4	98.7	95.9	93.6	97.4	91.6	80.6	88.6
TEPP	98.1	124.7	98.1	92.8	92.9	83.2	102	119.4
TEPP	100.7	118	94.5	90	88.5	92.2	98.5	120.8
TEPP	103.6	122.4	87.3	87.9	85.6	87	115.3	115.9
TERF1	647.8	892	238.5	250.7	222.2	251.5	731.6	1122.3
TERF1	77	91.4	80	85.1	81.3	79.7	80.3	81.6
TERF1	150.6	181.6	89.1	85.4	88.7	91.4	144.6	222.7

TERF2	479.1	714.5	380.3	311.5	231.9	459.9	428.5	842
TERF2IP	796.5	1204	563.3	333	348.4	523.7	710.5	1177.3
TERT	90.1	100.1	104.5	90.3	89.1	93	88.3	104.2
TERT	112.4	130.1	92.1	87.7	86.7	98.4	114	109
TES	131.2	170.3	105.9	96	92	124.4	128.7	147.8
TES	247.5	300	125.8	108.1	114.4	104.4	214.1	277.2
TESC	793.1	1014.8	419.9	198.9	181.2	218.3	577	860.2
TESK1	174.5	204.9	221.7	178.3	167.1	176	243.4	507.8
TESK2	131.6	171.3	118.9	135.8	122.3	155.4	129.2	196
TESSP2	152	166.3	93	90.7	88.7	77	141.5	170
TESSP5	130.7	139.5	108.4	92.4	96.7	95.7	112.3	116.4
TETRAN	13843.1	16728.7	9946.3	7119	6180.4	6629.6	11463.6	12861.4
TEX10	674.4	946.7	551.6	359	299	500.1	501.6	714.8
TEX101	91.9	106.2	96.2	81.6	78.5	80.1	103.1	100.5
TEX11	112.9	111.4	100	93.6	86.6	88.5	112.3	103.5
TEX11	86	81.5	96	76.3	75.7	82.2	80.6	89.1
TEX11	74.3	82.3	82.8	80.2	83.2	77.8	73.4	79.7
TEX12	96.1	104.2	91.8	83	77.7	84.5	95.3	91.4
TEX13A	128.4	146.4	107.7	95.7	100.1	102.4	123.9	136.2
TEX13B	91.3	91.6	91.4	85.7	94.5	92	83.1	97.9
TEX14	100.5	109.8	90.4	90.2	82.2	88.4	93.5	97.8
TEX14	83.1	91.9	90	86.5	79.6	80	85	88.3
TEX15	201.1	258.5	132.9	102.1	97.5	132.3	132	206.4
TEX2	760.1	965.9	669.2	965.3	689.7	1217.3	551.3	717.7
TEX261	258.7	339.5	230.2	173.8	149.9	179.4	229	242.2
TEX264	747.6	938.4	431.6	283.9	258.9	263	714.8	874.6
TEX9	84.3	99.7	93.2	91.4	83.7	91.6	92	95.2
TF	77.5	81.9	105.4	98	87	100.7	78.6	86.7
TFAM	1606.1	1991.3	1149.1	941.1	638.6	1075.2	1275.9	1914.8
TFAP2A	228.6	224.2	133.9	147.6	141.8	145.1	212.7	264.9
TFAP2A	220.9	285.6	126.9	141.2	129.9	143.1	168.2	213.6
TFAP2A	113.8	127.3	90.2	86.9	85.4	82.9	118.6	120
TFAP2A	111.2	102.9	95.5	93.1	90.1	91.5	101.6	104.4
TFAP2B	86.9	96.2	91.4	88.6	79.1	79	80.7	87.4
TFAP2C	138.7	162.5	104	244.8	209.7	257.1	168.5	217
TFAP2D	81.4	91	104	91.9	88.1	92.6	86.5	100.4
TFAP2E	94	96	105.2	91.2	100.1	96	92.9	125.2
TFAP4	145.7	192.5	142.9	120.5	99	136	131.4	146.8
TFB1M	932.8	1251.3	720.7	771.2	678.8	754.3	753	852.9
TFB2M	2603.7	3534.9	1552.5	1299.6	739.6	1016.7	2100.5	3866.6
TFCP2	708.8	939.7	547.9	533.5	467.2	658.3	592.2	880.4
TFCP2L1	75.9	89.9	88.9	89.4	81.2	85.6	88	77.4
TFDP1	6856.4	7973.9	4759.2	4276.4	3165.8	4963.2	5754.8	8939.7
TFDP2	199	256	157.9	127.8	125.6	144.5	203	237.7
TFDP3	81.1	90.4	101.6	99.4	97	100.9	78.9	82.3
TFE3	135	155.3	129.2	136.2	127.8	146.7	168.8	259.8
TFEB	92.6	98.1	114.4	100.2	92.4	109.2	88.7	109.5
TFEC	96	122.8	109	99.9	94.9	91	104.5	130.3
TFEC	83.1	103.2	82.3	106.7	89.2	79.6	84.7	92.9
TFF1	84.1	105.7	99.9	89.8	79.4	101.1	93.6	108.7

TFF2	85.9	98.9	98	85.5	80.7	87.6	75	95.1
TFF3	82.1	97	97.4	83.4	83.2	88.1	94	91.9
TFG	358.8	456	167.2	107.6	97.9	91.7	223.1	244.9
TFG	4489.5	5442.8	2092.6	2702.6	1896.4	2731.2	4668.9	7826.6
TFIP11	339.8	440.5	319.5	285.9	243.6	362.2	285.1	410.5
TFIP11	408.9	483.8	330.3	289.4	247.1	382.4	375.8	463
TFIP11	249.9	317.3	190.5	212.7	174.4	198.7	239.1	284.7
TFPI	81.5	110.7	92.6	85.3	92.5	91.3	108	116
TFPI	79.5	87.5	91.2	93.9	87.9	91	78.7	78.5
TFPI	98	114.5	81.5	85.9	81.4	84.8	108.8	149.2
TFPI2	80.3	79.7	89.7	87.1	86.1	83.6	90.5	83.8
TFPT	747.7	854	556.9	449.8	396.8	559.1	754	875.2
TFR2	107	118	91.3	95.5	87.6	100	103.9	117.8
TFRC	5945.6	7733.5	4376.6	4343	3190.7	5151.2	5630.5	6765.5
TG	79.7	100.4	97.9	93.2	97.4	94.8	94.4	82.9
TGDS	910.8	1243.5	483.6	492.4	342.2	552.3	761.7	1364.9
TGFA	87	105.6	99.3	96	94.9	97.2	85.2	112.8
TGFB1	102.3	105.4	108.9	106.1	97.5	98	91.2	114.5
TGFB1I1	107.9	115.9	94.5	89.1	87.4	87.3	104.4	115.2
TGFB1I1	121.2	160.4	108.7	99.2	89.3	94.8	124.9	151.1
TGFB2	89.7	95.5	95.7	80.5	78.6	86.1	80	85.1
TGFB3	106.4	133.4	106.3	97.7	102	112.9	104	131.9
TGFBI	2471.3	2906.7	2324.1	7438.3	5399.5	7900.6	2247.9	3651
TGFBR1	116.1	138.4	101.6	106.3	100.4	105.7	104.7	137.8
TGFBR2	148.6	161	124.7	109.9	114.2	108.9	142.3	157.6
TGFBR2	465.8	604.6	361.9	549.9	420.7	617.4	506.3	855.6
TGFBR3	269	390.5	220.1	250.1	243.3	365.8	221.6	266.4
TGFBRAP1	255.5	330.4	216.4	199	161.3	134.5	240.8	263
TGIF	99.6	103.9	98.8	97.9	85.9	93.9	86.1	94.5
TGIF	99	105.1	88.2	87.7	76.7	79.2	95.7	99.4
TGIF	98.3	102.8	105.3	100.5	95.4	97.6	99.9	105.2
TGIF1	98.5	125.5	90.7	75.2	79.8	81.3	105.8	107.6
TGIF1	433	568.7	314.6	336.6	268.5	520.7	466.7	881.8
TGIF1	100.1	122.2	111	103	92.6	94.8	108.7	122.2
TGIF2	488.5	616.6	251.2	252.1	214.1	274	396.7	467.7
TGM1	85.2	94	89.1	94.5	91.7	85	93.5	102.7
TGM2	131.9	155.4	91.7	85.2	80.9	117.6	151.2	154.5
TGM2	113.7	127.6	117.6	98.5	94.4	94.2	104.4	101
TGM2	151.7	152.6	95	93.7	84.5	91.3	140.7	173.1
TGM3	124.5	153.8	92.5	87	81.4	79.3	130.9	135.7
TGM4	111	140.8	98.3	110.2	105.8	111.6	103.3	107.1
TGM5	666.7	849.2	489.6	514.6	398.1	689.4	415.6	559.7
TGM5	364.6	487	263.9	268.4	206.9	312.8	239.5	294.9
TGM5	100.9	131.8	96	86.4	82.5	80.8	98.9	104.1
TGM6	88.8	98.5	94.1	86	76.8	80	86.3	101.5
TGM7	173.2	189.8	102.1	92.9	100	87.6	145.6	167.6
TGOLN2	1898.6	2403.5	1055.3	1275.6	1083.2	1271.2	2001.8	1826
TH	101.2	105.7	101.4	85.4	86.4	84.1	96.2	100.8
TH	87.1	103.4	90.5	78.5	77	80.2	84.1	106.6
TH1L	1743.6	2465.7	1133.6	896	804	1027.8	1712.2	2135.6

THADA	310.5	376.2	314.8	358.3	265.8	444.1	246.7	272.3
THADA	91.5	119.1	86.6	85	73.8	91.4	85.1	90
THAP1	659.3	996.4	282.4	197.3	161.9	191.8	531.2	650.3
THAP1	193	208	111.6	123.3	107.4	132.8	162.3	322
THAP10	486	597.7	263.5	223.9	224.9	291.4	496.2	759.3
THAP11	2645.4	3452.6	1788.3	1188.5	920.1	1274.9	2231.3	2723.9
THAP2	75.8	76.9	101.2	92.6	95.2	98.9	71.4	89
THAP3	97.5	135.8	108	94.1	90.9	93.9	112.4	128.8
THAP4	197.4	234.4	133.1	116.1	108.5	88.7	161.4	175.2
THAP5	97.1	94.4	96.3	85.6	76.1	83.5	83.6	100.4
THAP6	237.3	303.5	256.4	215.4	212.5	269	203.7	291.3
THAP7	211.4	226.8	137	111.1	116.4	118.9	153.2	202.4
THAP7	399.8	506.3	302.4	222.2	202.1	286.2	308	378.8
THAP7	107.2	117.4	103.2	98.3	90.2	88.4	105.3	122.9
THAP8	78.6	97.3	84.7	78.8	73	81.4	80.6	103.8
THAP9	86.6	96.7	96.7	91.7	84.5	100.9	91.6	109.1
THBD	169.7	225.6	149	157.2	127.2	180.6	183	216
THBS1	75.2	81.6	93.7	87.7	78.6	87	126.3	175.9
THBS2	93.9	121.1	106.2	84.5	85.4	102.5	94.1	113.3
THBS3	159.8	166.3	100	93.8	82.7	98.5	145.3	146.9
THBS4	690.1	628.8	591.6	567	486.5	819.4	354.7	427.5
THEDC1	106.8	124.9	98.2	86.9	93.3	96.3	99.9	123.5
THEG	97.5	115.4	122	107.5	103.2	114.4	95.7	109.2
THEG	99	108.6	104.6	98.2	95.1	87.8	91.6	95.1
THEM2	665.7	788.2	474.2	514.1	388	692.7	587.1	790.3
THEM4	112.3	127.4	95	99.6	81	92.8	115.7	124.8
THEM5	93.7	105.2	107.6	88.6	84.1	91.8	96.9	105.1
THEX1	109.8	129.5	75.4	76.7	73.4	85.4	96.5	139.2
THG1L	217.1	265.6	180.2	170.9	153.7	181.3	198.6	252.3
THNSL1	162.8	229.7	129	121.3	114.9	153.7	134.5	190.6
THOC1	1024.5	1180.1	411.3	460.4	333.1	572	785.7	1316.4
THOC2	898.8	1262.2	282.9	480.8	428.6	463	756.1	977.1
THOC2	220.5	301.4	164.2	138.7	121.4	136.8	204.8	283.3
THOC3	2283.3	3246	623.9	563.2	428.4	560.8	1723.8	2480.1
THOC3	1554.1	2560.6	1097.3	965.2	773.1	1219.7	1313.3	1289.2
THOC4	2833.5	3775.8	832.5	397.5	321.2	250.7	2877.4	3751
THOC5	850.1	1146.7	641.9	406.1	396.9	538.9	741	663.6
THOC5	670.8	865.1	663.7	412.2	390.6	578.6	541.2	739.8
THOP1	818.8	980	1050.9	617	500.2	693.3	699.3	1261
THPO	91.2	83.9	83.3	81.4	85.4	82	83.9	101.3
THRA	114	128.5	114.1	125.3	100.4	85.7	124.9	113.6
THRA	108.7	123.4	91.5	91.3	90.9	87.4	117	137.7
THRA	106.4	120.8	110.9	100.5	103.4	92.6	103.3	110.3
THRAP1	103.3	141.7	104.5	85.3	81.1	95.5	117.8	140.3
THRAP2	133.8	170.5	118.5	130.7	133.4	168	160.5	220.2
THRAP3	199.7	218.9	108.2	92.5	91.7	99.8	161.7	192.7
THRAP4	317.8	407.7	331	251	213.3	331.7	317	517.6
THRAP4	362.1	445.2	517.8	353.6	325.1	581.8	263.6	470.1
THRAP5	938.2	1218	417.7	401.8	264.4	276	741.7	629.7
THRAP5	887.4	1123.1	675	619.9	457.8	505.8	696.2	822.3

THRAP5	241.7	296.8	233.4	197.8	181.9	179.4	194.5	279.1
THRAP6	3944.6	4392.8	3033.8	1919.6	1600.1	2019	3994	4724
THRB	87.1	102.3	80.3	80.1	80.8	91.1	84.2	104.6
THRSP	74.2	82	87.9	89.9	85.6	86	71.9	84.4
THSD1	80.2	96.7	85.9	80.9	76.5	79.1	85	88.8
THSD1	79.5	93.6	86.9	82.6	74.8	87.1	86.6	96.3
THSD1	94.8	116.8	93.1	91.6	82.6	91	100.3	108.6
THSD1P	128.9	158.2	108.7	79.6	81.8	102.4	127.4	155.8
THSD1P	93.7	119.4	93.5	81.5	83.2	80.1	86.9	110.3
THSD3	118.8	139.4	85.1	79.8	74.3	77	102.8	136.4
THSD3	101.8	121.2	97	81.2	77.5	85.1	122.2	116.1
THSD3	83.3	103.5	93.7	80.8	89	91.8	89.4	94.2
THSD4	97.7	100.6	99	99.1	98.6	102.1	101.9	99.6
THSD7B	82.4	94.8	97.2	91.1	79	86.6	79.6	84.1
THTPA	100	115.8	101.5	99.4	84.2	93.7	100.8	123.4
THUMPD1	777.6	969.2	412.6	366.4	343.2	647.9	652.3	1246.4
THUMPD2	744.4	936.4	330.9	329.8	259.5	378.6	600	897.4
THUMPD3	303.5	357.9	174.1	172.1	143.7	220.2	269.2	383.6
THY1	89	93.9	94.7	76.6	79.4	81.2	96.6	97.8
THYN1	1493.8	1875.3	1051.9	613.1	460	881.6	942	1285.6
THYN1	207.1	247.2	132.2	92	88.4	99.9	154.1	174.1
THYN1	1192.9	1525	768.2	427.2	321.6	704.8	713.6	785.5
TIA1	1540.1	1828.8	982.9	1001.3	856.2	1398	1162.6	1892.8
TIAF1	1025.3	1288.1	885.8	1205.3	1228.6	1323.5	830.9	1076.1
TIAL1	502.7	705.4	292.3	279.2	212.1	394.9	473.5	1043.5
TIAL1	1099.7	1423.4	867.8	715.6	485.3	986.2	1125.8	2605.5
TIAL1	448.4	448.9	183.4	174	136	169.3	375.2	664.7
TIAM1	86.3	103.4	102.3	90.4	93.3	81.9	98	96
TIAM2	95	98.5	99.3	97.6	74.9	81.8	89.4	102.4
TIAM2	91.9	104.9	93	107.8	107	103.9	83.9	94.7
TICAM1	103.1	98.1	123.8	112	91.7	109.9	95.2	134.9
TICAM1	737.1	983.9	436	380.1	356.9	381	693.9	748.2
TICAM2	495.5	623	369.5	366.6	265.4	368.6	521.4	869.6
TIE1	136.8	158.6	93.7	98.5	86.7	93.9	134.1	183.7
TIFA	182.8	248.7	137.9	132.7	109.6	136.9	143.9	150.7
TIGD1	102.6	127.8	98	100.9	91.8	104.9	102.7	117.5
TIGD2	437.8	505.7	375.5	303.1	214	364.5	351.4	723.2
TIGD3	113.4	133.8	91.2	94.9	85.7	91.6	94.9	123.1
TIGD4	84.9	93.4	83.6	79.4	75.7	78.9	84.3	84.6
TIGD5	1380	1722.4	1142.7	608.2	505.5	718.3	1041	987.3
TIGD6	126.9	163.3	118.5	117	121.9	123.8	113.6	130.8
TIGD7	252.5	282.8	159.5	157	152.6	209.3	192.1	265
TIMD4	77.4	92.4	81.8	88.4	79.9	88	82.7	82.4
TIMELESS	1104.7	1339.5	980.5	783.1	710.8	813.9	898.2	925.6
TIMM10	2771.1	3429.5	2735.5	1735.8	1207.1	1899.7	2525.1	3058
TIMM13	115.8	147.4	102.3	87	78.8	89.2	135.9	164.7
TIMM17A	202.3	223	124.2	110.6	102.3	113	178.3	218.6
TIMM17B	616.9	747.6	390.1	299.7	277.2	280.9	576.9	824.2
TIMM22	1039.2	1473.3	680.6	572	453.8	491	890.8	908.8
TIMM23	1372.2	1751.4	730.9	487.8	385.5	574.3	1203.9	2279.8

TIMM23	5798	6806.4	2581.3	1734.8	1249.7	2248.5	4518.5	6837.8
TIMM23	2001.2	2681.6	1300.5	889.4	642.1	1131.9	1466.8	1961.3
TIMM44	408.5	573.6	301.2	229.8	200	316.8	363.9	531.8
TIMM50	88.1	87.9	80.2	87.8	82.1	82.7	86.2	88.8
TIMM8A	426.8	545.5	165.1	146.1	129.1	144.1	344.3	408.1
TIMM8B	340	391.1	157.8	133.3	121.1	130.8	315.6	347.6
TIMM9	1008.4	1194.7	343.7	234.3	195.8	275.1	772.9	1611.2
TIMP1	5067.7	6419.4	4583	5097.1	4205.7	4194.4	5399.4	6821.7
TIMP2	920.2	1375.4	1030.1	2060.1	1716.6	2586.5	917.6	1346.1
TIMP2	275.9	353.1	189.8	236.9	220.9	208.3	257.6	396.8
TIMP3	321.4	481.6	263.6	155.6	154.9	248.7	255.3	260.4
TIMP4	108.1	110.2	88.9	85.3	84.6	83	95.1	102.5
TINAG	116.1	137.5	94.5	89.1	86.5	89.6	106.3	122.3
TINAGL1	79.5	96.3	90	76.6	82.1	81.7	84.7	79.8
TINF2	819.8	1177.6	621.6	521.2	480.1	672	857.2	762.1
TINP1	10277.1	12800.1	7877.8	9192.8	7668.9	12913.1	10067.2	13278.9
TIP39	99.3	111.8	103	102.8	95.4	88.5	95.9	98.9
TIPARP	651.2	810.8	383.1	519.8	431.7	739.4	819.7	1344.7
TIPRL	1834.4	2349.4	789.2	662.9	510	790.7	1230	2160.2
TIPRL	409.1	530	326.3	276.1	213.3	493.4	364.2	776.9
TIRAP	110.3	150.8	88.1	95.3	84.4	82.5	116.7	166.1
TIRAP	140	170.4	103	98.1	95.2	82.8	120.2	169.7
TIRAP	172.8	224.8	108.2	101.9	95.1	96.9	156.5	208.9
TITF1	76.7	82.9	82.1	83.4	73.5	78.4	81.2	79.4
TITF1	95.7	94.8	94.7	81.1	81.2	91.7	92.5	89.1
TJAP1	2444.7	2992.3	1719.3	1521.4	1556.6	1641.2	2121	2033.2
TJP1	81.1	104.6	85.3	76.2	82.6	86.8	90.7	89.2
TJP1	115.4	142.1	97.2	90.7	80.2	97.4	122.5	122.3
TJP2	128.8	147.6	116.7	108.9	92.8	113.3	121.1	133.9
TJP2	106.2	152	118.2	92.9	89.6	115.5	102.8	97.1
TJP3	101.9	117.3	130.5	113.8	114.3	124.1	105.7	117
TK1	3106.1	4019.6	3174.9	1716.8	1509.3	1627.6	2932.3	3958.7
TK2	205.4	258.5	166.8	138.3	135.9	166	168.8	227.4
TKT	5411.7	7158.1	5107.5	4888.9	4242.8	5728.1	6812.4	9658.4
TKTL1	99.4	135.7	85	82.2	78.5	89.1	100.2	109.5
TKTL2	69	93.5	88.6	88.2	75.3	91.6	80.7	77.7
TLCD1	123.2	151.7	120.6	115.6	92.6	106.8	123.1	136.1
TLE1	314.8	366.9	174.2	157.4	146	157.7	253.4	274.7
TLE2	82.4	82.9	94.1	95.4	87.7	92.1	85	91.2
TLE3	112.6	126.8	132.1	127.9	122.6	148.8	122.4	131.6
TLE4	695.3	920.9	825.5	851.7	581.6	1152.3	687.2	1427.5
TLE6	165.7	186.1	112.8	83.5	84.2	85.4	128	165.1
TLK1	382.4	437.7	184.4	251.6	187.7	247.2	351.3	736.4
TLK2	576.3	802.9	419.3	449.1	421.1	773	492	718.9
TLL1	74.8	91.9	76.6	75.1	75.3	74.9	78.2	81.5
TLL2	73.8	88.7	87	72.8	81	80.6	72.7	79.8
TLN1	572.3	694	473.2	603.4	467.5	516.3	537.2	717.8
TLN2	99.9	115.7	114.4	158.8	123	175.8	103.9	117.3
TLOC1	314.4	477.9	240.9	561.6	398.5	573.2	301.3	411.6
TLR1	110.5	122.7	98.8	83.6	91.1	106	121.6	152.9

TLR10	94	110.1	104	88.9	99.4	108.4	100.5	105.8
TLR10	96.4	113.7	90.3	107.7	86.7	82.3	88.9	109.8
TLR2	109.6	136.2	98.9	124.2	108.3	114.5	96.8	106.2
TLR3	71.1	78.8	88.9	76.6	79.2	83.1	77.1	76.5
TLR4	204.2	246.5	154.7	209.1	136	227	171.8	207.1
TLR5	91.3	102.2	103.6	95.1	89.7	103.6	102.1	105.7
TLR6	91.7	112.7	95.5	84	88.4	90.9	97.2	109.4
TLR7	95.4	97.4	94.9	97	102.5	98.6	93.8	96.1
TLR8	79.5	79.9	86.7	86.9	79.1	92	75.4	79.6
TLR8	87.2	93.6	87.7	93.7	87.1	90.4	84.1	93.1
TLR8	74.6	87.3	85.7	93.1	94.8	92	76.7	82.9
TLR9	97.2	120.7	101.6	101.6	93	108.4	99.6	120
TLX1	103.5	120.7	94.4	84.1	87.5	92.7	102	134.9
TLX2	98.9	132.9	96.5	96	96	99.4	93.4	108.2
TLX2	89.4	108.2	100.3	81	84.7	83.8	91.3	81.3
TLX2	83.9	94.8	92.2	76.5	82.8	94.1	88.6	91.4
TLX3	103.4	130.7	91.7	92.4	79.8	82.5	105.4	128.6
TM2D1	1649.6	1936.4	875.2	889.7	726.4	1034.3	1687.2	2705.9
TM2D2	326.2	424.7	251.3	214.2	167.1	273.5	310.4	637.6
TM2D3	232.3	292.3	152	130.9	101.1	117.1	231.7	255
TM2D3	1267	1698.4	643.3	695.6	500.1	745	1271.6	2379.4
TM4SF1	93.4	98.5	93.6	89.9	82.8	89.6	162.6	231.9
TM4SF11	99.6	115.6	99.1	94.3	78.2	93.1	95.7	108.8
TM4SF18	81.5	84.8	95.1	91.9	83.1	87.7	84.3	88.2
TM4SF19	88	102.4	103.3	91.3	88.6	94.8	93.6	92
TM4SF19	89.2	85.2	90.4	94.4	82.5	79.7	91	97.3
TM4SF20	83	75	86	86.2	80.4	88.1	76.3	76.8
TM4SF4	81	91.2	93.6	75.8	79.8	89.6	71.8	83
TM4SF5	77	81.9	76.2	73.9	73.4	76.2	81.4	89.7
TM6SF1	146.1	152.6	128.9	174.6	132.2	225	126.8	171.8
TM6SF2	78.2	108.1	76.9	86.9	78.5	91.2	88.3	95.1
TM6SF2	80.6	87.9	94.8	100.6	96	91.4	86.4	92
TM6SF2	81.4	94.2	91.9	76.2	69.7	76.4	86.3	90.8
TM7SF2	516.6	721.3	456.9	231.1	269.2	325.5	515.7	660.9
TM7SF3	602.1	785.3	285.6	283.9	195.4	249.9	406.4	718.7
TM7SF4	83.5	91.4	92.8	83.1	84.3	85.6	96.3	98.3
TM9SF1	390.8	554.8	332.8	268.7	212.8	248.2	330	602.2
TM9SF1	204.4	249.7	157.1	112.9	104.3	126	160.3	205.4
TM9SF1	134.7	143.4	143.7	128.6	114.7	134.4	114.6	130.5
TM9SF2	4077.9	5057	2227.9	2193.5	1601.6	3132.2	3415.8	5537.1
TM9SF4	712.9	790	440.9	637.5	449.6	760	583.4	1300.4
TMBIM1	196.5	205.8	153.4	139.1	122.5	131.3	249.6	267.2
TMBIM4	8012.4	10053.3	6012.3	8860.3	6483.9	7590.5	7793.2	8624.2
TMC2	96.7	119.1	84.9	87.1	85.9	91.1	100.2	141.9
TMC4	98.3	111.5	93	88.6	85.1	98.3	93.7	108.2
TMC5	106	124.8	93.1	79.8	93.5	89	106.3	137.8
TMC6	613.1	742.3	519	433.6	300.9	442.3	508	822.6
TMC7	99	126.8	95.5	87.8	91.2	96.2	97.8	100.8
TMC8	144.7	156.9	107.6	108.7	91.1	102.6	138.7	144.9
TMCC1	92.1	103.8	86.4	82.2	82.5	78.7	95.9	95

TMCC1	103.3	125.1	98.5	99.6	93.6	102.6	129.7	141.3
TMCC2	92.7	107.4	92.5	81.7	71.8	104.1	100.8	104.6
TMCC3	115.5	130	92.7	88.5	78.7	82	116.7	130.1
TMCO1	6867.3	8580.6	4583.4	3745.5	2768.1	3913.4	6708.2	8701.1
TMCO2	103.9	103.1	94.2	77	74.8	83.7	95.1	142.3
TMCO3	430.9	602.3	480.6	420.2	351.9	555.3	376.8	640.4
TMCO4	172.9	185.1	140.5	127.8	120.6	142.5	165.2	190.7
TMCO5	133.8	169.3	92.5	87.6	83.5	87.5	123.1	168.2
TMCO6	191.1	233.5	157.1	124.3	116.6	143.7	163.7	167.5
TMDCII	81.5	86.9	85.5	82	88.2	88.1	92.5	93.9
TMED1	716	879.3	291.2	184.5	234.3	257.8	691.7	738.9
TMED10	2137.7	3550.3	1578.2	1787	1357.2	2677.3	2185.9	3029.3
TMED10P	6085.4	7958.9	3632.9	2294.7	2010.5	2734.1	5895.2	7371.2
TMED2	2389.1	3733.1	800.8	798.6	555	1117.4	1855.4	2736.8
TMED3	8156.8	10370.3	6181.1	6514.2	4570.8	5576.4	7909.2	7832.4
TMED4	1641.5	2012.5	1232.3	1236.2	1036.1	1011.3	1215.4	1605.9
TMED5	1290.6	1571.6	769.7	823	754	1186.5	1239.3	2405.8
TMED6	85.7	88.2	97.2	82.9	82.2	89.1	78.1	106
TMED7	953.2	1130.4	480.4	738.1	449.4	903.5	979.1	1695.4
TMED8	131.3	158.3	112.6	117.2	100	91.3	113.1	141.2
TMED9	4318.5	6075.4	2483.6	2371.6	1854.7	2914.8	4486.3	7748.7
TMEFF1	107.8	132.8	89.2	90.2	94.6	99.3	104.5	133.7
TMEFF2	78.1	81.4	81.9	83.6	79	83.8	82.9	90.1
TMEM1	228.9	298.6	131.9	159.9	136.4	161.7	270	344.6
TMEM1	100.8	119.9	93.3	91.2	85.4	84.7	97.8	97.6
TMEM1	158.4	183.3	101.6	93.3	110.6	97.2	174.9	185.6
TMEM10	91.4	99.2	97.5	94.9	76.1	85	88.4	96.1
TMEM10	71.6	86.7	98.4	87.7	88.4	84.4	76.5	78.6
TMEM100	110.7	111.2	107.8	95.9	95.8	108.9	96.7	113.6
TMEM101	1416	1938.8	1199	853.5	740.1	1126.2	1357.8	1574.2
TMEM102	86.5	111.5	100.2	92.7	91.2	95.9	88.1	114.4
TMEM103	887.5	1106.2	517.8	960.7	806.6	1105.5	648.2	691.7
TMEM103	241.3	300.3	190.1	173.1	191.5	261.3	177	222.4
TMEM104	116.4	155.8	174.9	154.9	134.1	191.8	113.4	162.7
TMEM105	193	226.9	151.7	103.6	122.2	122.2	175.8	151
TMEM106B	113.1	130.5	106.7	109	104	121	98.4	170.9
TMEM106C	1563.2	1902.4	596	462.4	389	635.8	1256.1	1420.1
TMEM107	102.5	112.3	96.5	97.4	80.7	93.6	93.5	112.6
TMEM108	169.7	211.5	133.6	134.9	118.9	98.4	184.7	238.2
TMEM109	1100.4	1484.4	875.1	780.4	556.7	862.8	905.8	1419.5
TMEM11	258.1	358.5	173.1	150.1	146.9	175	291.2	378.4
TMEM110	143.5	170.5	117.2	114.5	105.8	121.8	122.2	131.1
TMEM113	1079.2	1413.3	934.4	909.8	660.6	767.3	1081.3	2163.1
TMEM115	740	981.3	442.8	376.2	323	396.6	718.3	722.9
TMEM116	564.8	736.5	461.9	481.4	464.2	625.1	589	851.1
TMEM117	168.3	231.4	115.6	119.1	98	102.3	164.2	246.9
TMEM118	125.3	181.7	137.4	103.3	100.8	108.8	148.9	142.2
TMEM119	81.5	87.6	88.8	93.8	88.6	90.8	78.2	92
TMEM121	75.9	90.2	99.9	88.9	88.3	88.5	84	82.3
TMEM122	80.6	97.1	90.6	95.5	83	81.2	88.2	95.7

TMEM123	4012	4935.4	2486.8	2773.4	2033.1	4509.8	2917.5	7088.5
TMEM125	96.5	114.9	105.3	92	86.8	88.4	85.8	109.2
TMEM126/	3601	4059.2	3349.2	3110.2	2168.4	3032.4	3479	5614.6
TMEM126E	3271.8	3811.1	1873.2	2072.2	1625.7	2567	2535.9	2702.3
TMEM127	169.9	194.8	130	163.1	150.9	171	156.5	164.7
TMEM128	223.2	255.9	133.6	135	132.2	138	228	395.3
TMEM129	103.8	120.2	109.3	98.4	85	93	87.9	128.9
TMEM130	98.1	109.8	90.1	90.7	85.2	91.8	97.9	100.1
TMEM132/	126.8	151.6	94.1	81.2	91.1	92.3	118.7	155.9
TMEM134	299.6	353	195	149.8	134.8	204.7	243.7	267.8
TMEM14A	1743.2	2145.1	981	1057.4	771.9	1162.2	1219	1882.4
TMEM14B	3929.7	4647.3	1510	1202.2	1006.9	1289.2	4007.5	4913.4
TMEM14C	3614.2	4678.7	2357.8	2599.2	2039.4	2790.4	3573.4	4020.7
TMEM15	441.5	569.3	364.1	284.1	218.1	345.8	428.9	724.4
TMEM150	287.2	345.5	190.2	235.9	193.1	257	210.6	222.9
TMEM151	79.4	84.2	95.5	92.8	88.9	89.5	83.7	81.3
TMEM151	81.4	94.5	100.6	90.5	81.5	91.7	85.2	81.4
TMEM154	892.7	1060.6	347.2	423.7	374.7	399.9	768.1	1274.2
TMEM156	137	183.2	122.8	120	120.4	127.9	134.8	140.6
TMEM157	126.6	146.5	101.6	96.6	78.6	100.4	137.1	173.6
TMEM159	119.8	164.5	120	108.8	100.9	115.5	108	130.5
TMEM164	154.9	203.1	112	99.2	94.7	103.5	138.3	166.5
TMEM16A	80.2	89.5	86.1	88.8	82.2	84.2	81.5	82.1
TMEM16B	87.3	99.5	95.6	96.2	82.2	91	86.4	82.4
TMEM16C	78.9	87.5	94.1	94.9	88.1	93.8	84.4	75.3
TMEM16D	116.9	125.4	105.6	98.6	104.3	95.5	116.4	107.9
TMEM16E	91.1	101	92.4	82.3	96.7	90.8	95.9	92.9
TMEM16F	658.9	849.6	449.6	625	593.1	767.5	600.9	886.6
TMEM16G	80.7	90.9	86.2	83.4	81.7	81.1	79.8	86
TMEM16G	127.4	181.1	111.5	113	106.3	113.3	130.2	173.7
TMEM16G	93.3	96.5	79.7	76.4	74.7	77.4	92.4	104.8
TMEM16H	122.4	125.3	109.9	110.4	109.3	130.6	109.7	128.2
TMEM16J	189.2	239	113.4	99.5	93.2	97.5	178.9	199.5
TMEM16J	82.4	77.4	84.1	88.2	76.1	82	78.3	91.8
TMEM16K	212.4	225.6	181.7	152.1	124.7	153	172.1	285.9
TMEM17	3110.4	2757.5	3179.5	4592.6	4820.5	3399	2784.5	6612.9
TMEM170	488.3	597.9	256.4	208.4	155.3	160.5	444	584.7
TMEM173	4522.8	6171.1	3671	3558.7	3126.9	3761.5	3958.8	4999.6
TMEM176E	123.1	139.3	102.3	95.7	99.5	90.8	118	143.6
TMEM178	82.4	94.1	93.3	87.2	79.3	85.9	82	96.5
TMEM18	252.1	290.9	193.1	211.3	168.5	229	192.4	388
TMEM183E	5408	6234.2	3442.5	3031.2	2460	4225.7	4878.6	6723.4
TMEM185/	280.8	379.2	280.2	264.8	226	285.9	262.4	516.7
TMEM185E	701	1040	424.4	343.7	233.7	394.5	559.4	792.3
TMEM186	197.2	250.1	118.8	115.2	109.5	114.5	189.6	202.1
TMEM19	113.9	146.7	94.4	84.5	76.1	88.3	109.3	144.6
TMEM2	112.8	135	120.6	109.5	106.1	113.8	152.4	205
TMEM20	194.8	209.8	142.3	108.9	99.1	117.5	160.5	238.5
TMEM22	109.5	129.5	114.3	111.6	94.8	105.8	132.5	198.7
TMEM23	83.3	102.7	93.1	97.4	90.2	100.6	93.7	97.4

TMEM24	108.3	136	134.4	124	108.6	126	115	162
TMEM25	93	102.5	93.7	88.7	80.9	94.5	87.5	90
TMEM26	100.6	119.5	100.1	86.4	81.9	86.8	104.1	107.5
TMEM27	106.2	119.5	90.2	103.4	96.4	96.8	94.4	109.6
TMEM28	106	144.8	102.9	92.2	87.2	91.6	105.3	118.5
TMEM29	320.2	302.6	397.3	357.3	295.2	490.9	219.8	360.7
TMEM30A	764.9	1058.6	502.2	1075.6	830.7	1337.8	727.3	923.5
TMEM30B	94.1	105.5	88	89.7	80.1	81.1	87.7	84.1
TMEM31	181.1	237.2	160.4	119.3	107.8	117.2	170.4	190.5
TMEM32	1694	2175.4	1596.5	2150.7	1360.3	2129	1486.5	2993.9
TMEM34	146.3	178.8	117.5	102.1	102	106.1	147	187.2
TMEM35	96	106.9	125.1	105.8	107	118.2	99.4	105.8
TMEM37	110	123.9	120.1	104.9	106.1	110.7	122.1	137.7
TMEM38A	103.2	142.7	94.5	81.7	86.4	86.8	95.5	128.5
TMEM38B	89.5	107.6	100.1	88.9	87.4	95.3	105.8	117
TMEM39A	272.6	337	208.6	191.8	164.2	267.2	246.8	382.1
TMEM39B	424.3	519.7	475.7	522.4	416.4	598.1	385	976.1
TMEM4	1674	2115.5	712.4	647.5	576.1	845	1440.6	1793.2
TMEM40	84.9	83.1	90	97.2	96	95.3	85	84.9
TMEM41A	424.1	593.2	238.1	189.1	187.1	219.4	400.3	458.9
TMEM41B	1057.9	1364.9	516.6	577.2	503.9	661	968.8	1505.1
TMEM42	659.6	793.9	542.1	469.8	419.4	558.8	479.8	467.6
TMEM43	1578.9	1979.8	1289.1	1872.1	1287.8	2324.1	1296.7	1617.8
TMEM44	273.6	325.7	141.3	113.6	118.4	130.7	272.6	198.6
TMEM44	197.6	245.5	108.9	98.7	92.5	102.3	177.7	169.1
TMEM45A	290.8	337.5	159.6	294.9	270.6	420.2	217.2	314.3
TMEM45B	133.3	158.1	86.6	95.3	88.7	94.8	129	143.4
TMEM46	80.6	86.6	99.6	87.5	79.5	84.1	81.5	84.5
TMEM47	108.8	117.3	116.3	122.5	108.4	111.4	110	117
TMEM48	1132.6	1454.9	685.4	559.3	427.6	689.2	698.2	1351.1
TMEM49	1627.2	2059.2	1227.4	1511.5	1217.5	1705.2	1455.6	1762.4
TMEM5	2145.8	2317.4	1408.3	944.6	642.1	961.5	1748	2326.8
TMEM50A	188.1	245.6	135	152.9	128.3	180.9	193.1	314.2
TMEM50B	1706	2129.6	1084.8	1017.3	691.7	1196.6	1288.7	1726.4
TMEM51	499.7	706.8	415.2	498.5	498.7	633.5	586.5	866.9
TMEM52	102.2	101.5	110.4	99.7	95.6	92.5	92.2	106.1
TMEM53	145.9	185.9	109.8	113.2	103.5	105.9	143.1	123.8
TMEM54	265.1	309.9	293.4	247.6	186.8	275.8	242.8	388
TMEM55A	470.4	553.1	320	363.1	288.5	423	480.2	1060.5
TMEM55B	556.6	688.1	466.4	322.1	341.4	425.9	474.3	707.7
TMEM56	90.6	112.1	85.4	85.6	87.1	78.8	94.6	104
TMEM57	198.9	267.8	176.2	168.7	152.9	201.2	210.5	302.2
TMEM58	140.4	166.9	108.6	92.4	87.2	89.2	141.8	143.8
TMEM59	5686.7	6697.2	4428.7	4748.9	4325.1	6271.5	5471.7	7594.8
TMEM60	2345.4	3147.6	1630.1	2261.1	2010.7	2315.4	2120.7	2529.3
TMEM61	85.9	99.5	96.9	85.2	86.4	92.4	101.7	100.1
TMEM62	357.2	431	282.2	291	241.6	415.5	274.5	417.4
TMEM63A	127.7	140.6	150.3	170.3	154.7	200.2	115.8	142.4
TMEM63B	148.9	173.9	96.6	85.1	84.2	102.4	142.1	172.7
TMEM63C	126.8	123.6	87.8	93.7	89.5	98.7	116.9	123.6

TMEM64	227.6	288.3	154	145.7	122.1	148.9	210.1	374.6
TMEM65	83.8	97.2	114.6	100.6	105.8	107.9	84.9	87.5
TMEM66	8356.1	10080.8	4067.9	3232.7	2808.3	4750.7	9045.6	12914.7
TMEM67	99.9	113.6	101	87.6	84.8	107.5	90.2	108.9
TMEM68	149.6	181.4	118.2	94.4	94.2	104.7	149.4	141.4
TMEM69	1517.5	2203.8	1077.5	1078.1	909.6	1094.5	1122.3	1197.3
TMEM70	282.7	342	139	108.8	99.1	130	246.5	470.1
TMEM70	526.2	722.1	356.8	254.2	178.8	245.7	471	719.4
TMEM71	153.8	182.4	125.5	125.9	119.1	150.9	134.4	156
TMEM74	86.1	93.1	99.3	100.6	92.9	93.8	81.1	92.4
TMEM75	84.2	89	88	85	79.7	91.5	76.2	88.6
TMEM77	1175.8	1442	767.7	381.4	287.3	527.1	828.2	1690.2
TMEM79	562.5	654.9	430.9	285	279.8	326.2	474.6	527.8
TMEM8	234	366.8	309.5	335	258.3	450.3	251.4	482.6
TMEM80	255.5	301.5	167.1	221.8	173.5	215.3	204	281.3
TMEM80	325.7	379.3	167.4	149.4	152.3	192.9	281.7	332.1
TMEM80	98.1	113.3	94.3	94.6	79.9	79.3	107.4	115.1
TMEM81	118.6	144	90.3	81.5	82.1	79.2	114.8	133.2
TMEM83	121.2	135	94.2	83.2	79.4	78.5	117.5	138.6
TMEM85	4245.4	5413.7	3234.5	3397.9	2642.9	4172.8	3926	5738.5
TMEM86A	100.8	123.9	99.5	96.9	91.3	107.2	125.5	124.9
TMEM86B	255.9	233.9	370	284.2	273	345.8	183.6	286.2
TMEM87A	4570.6	5645.8	3249.2	3652.8	2723.4	3562.1	4189.5	4755.9
TMEM87B	143.7	161.6	105.7	112.2	105.9	114	130.8	194.2
TMEM88	100.2	107.4	90	93.2	83.3	90.8	88.4	100.5
TMEM89	91.7	97.1	120.1	95.8	99.8	93.7	85.9	95.4
TMEM9	468.7	572.6	364	300.7	293	405.2	428	606.4
TMEM91	347.5	394.4	183.7	222.2	238.1	192	265.2	267.7
TMEM92	84.4	91.8	101.1	100.1	89.1	96.8	92.4	81
TMEM93	605.8	776.9	357.7	364.6	278.6	378.4	580.5	646
TMEM93	143.4	195.5	119.5	115.5	105.2	109.7	147.3	154.8
TMEM93	1902.3	2491.5	755.8	616.1	588.2	690.2	1931.1	1799.8
TMEM95	103.8	102.2	88.4	86.8	75.9	81	97.4	107
TMEM97	3728.3	4865.1	2017.6	2095.8	1452.1	2073.2	2805	2994.2
TMEM98	95.2	120.3	98	90.7	78.7	85.5	102.9	124.5
TMEM98	87.8	89.8	101	91.8	90	92.6	84.9	105.4
TMEM99	1212.3	1549.4	841.6	628.7	493.8	666.2	1246.2	1745.1
TMEM9B	1435.6	1820.7	987.8	1033.4	659.2	1133.7	1194.4	1645.8
TMEPAI	74.9	95.4	89.6	81.8	89.1	85.3	77.1	82
TMEPAI	88.2	110.4	83	89.9	73.7	80.6	98.3	103
TMEPAI	109.7	120.6	111.3	102.2	96.2	103.6	107.4	122.1
TMEPAI	94	100.3	96.8	90	84.5	88	91.6	104.7
TMF1	409.5	517.4	266.7	306.2	281.1	600.3	353.3	467.4
TMIE	94.7	100.7	94.8	88.7	95.5	97.5	89.1	110.6
TMIGD	91.7	104.8	108.2	99.3	96.8	102.1	89.1	90.9
TMIGD2	73.3	91.2	93.9	85.7	89.3	78.3	87.8	83.2
TMLHE	203.1	268.9	138.2	130.2	139.8	154.4	187.6	195.8
TMLHE	94.9	131.2	112.5	108.5	108.5	114.8	93.1	110
TMOD1	402.8	537.9	274.5	327.8	305.2	370.1	312.8	314
TMOD2	76.2	83	84.5	79.9	76.8	79.8	78.1	75.7

TMOD3	222.4	305.6	145	157.3	122.5	158.9	198.6	227.9
TMOD4	118.4	125.6	120.3	116.7	104.8	119	102.7	113.7
TMPIT	316.8	438.9	146.5	147.7	162.9	183.3	334.2	400.7
TMPO	263.4	324	120.6	108.6	102.8	95	246.6	291
TMPO	545.6	802.4	327.4	220.8	174.3	313.6	335	499.7
TMPO	91.4	91	89.1	89.5	82.8	87.4	91.1	102.6
TMPRSS11/	78.7	84.3	98.4	87.3	88.9	92	83	89.6
TMPRSS11I	98.6	100	96.5	88.4	91.2	96.2	90.4	100
TMPRSS11I	97.8	100.1	93.8	86.5	81.6	101.3	96.3	85.4
TMPRSS12	74.4	85.5	103.2	83.6	84.1	92.8	78.1	80.2
TMPRSS13	105.8	135.5	107	101.1	107.4	91.5	111.8	113.1
TMPRSS2	80.8	87.3	83.7	87.4	84	90.2	84.8	95.6
TMPRSS3	88.2	93.2	85.1	80.5	78.4	85.9	88.6	91.2
TMPRSS3	84.4	112.8	83.2	86.6	75.6	81.3	98.6	117.7
TMPRSS3	102.7	118.7	105.1	98.6	95.6	95.2	97.7	106.3
TMPRSS3	112.6	124.8	88.7	89.3	91.4	95.9	109.7	130.8
TMPRSS3	101.9	118.1	78.5	70.1	75.8	73.2	90.1	103.4
TMPRSS4	132.8	175.5	110	100.4	88	103.4	134.3	149.2
TMPRSS4	100.3	113.3	77.4	85.2	78.4	83.4	100.2	103
TMPRSS5	78.5	86.3	103.5	93.5	92	92.1	82.8	88.4
TMPRSS6	83.6	95.3	92.8	82.3	82.6	80	92.2	96.8
TMPRSS7	130.4	157.8	106.5	93.9	89.7	100	103.8	114.1
TMPRSS7	83.9	98.2	96.5	90.2	86.5	86.7	78.1	86.9
TMPRSS9	86.7	100.6	88.4	85.3	86.3	77.2	89.8	87.7
TMSB10	16066.8	18282.5	8406.5	9050.3	6531.1	9237.5	13426.1	16998.6
TMSB4X	147.8	195	105.8	105.4	91.5	104.4	151.9	174.1
TMSB4Y	116.6	127.2	129.6	138.7	114.6	148.9	97.7	120.4
TMSL8	1448.9	1788.1	1166.9	618.2	487.2	587.1	1377.9	1670.6
TMTC2	115.1	130.8	109.9	94.3	98.8	104.1	107	110
TMTC3	223.3	253.8	132.9	138.7	116.7	124.5	169.8	253.3
TMTC4	362.9	524.8	235.4	254	192.3	236.6	280.2	373.1
TMTC4	164.4	185	107.4	112.6	95.2	107.6	121.9	163
TMTC4	494.2	595.9	315.2	343.2	231.7	331.9	433.6	816.4
TMUB2	274.5	371	227.8	228.2	229.3	305.5	284.1	474.9
TMUB2	587.3	671.3	244.1	337.3	345.3	329.6	498.7	273.4
TNC	301.9	327.2	159.5	124.7	135	136.1	266.6	299.4
TNF	491.5	580.8	358.5	263.4	258.5	262.8	949.7	1465.8
TNFAIP1	203	278.7	173.9	161	151.3	235.2	178.8	348.6
TNFAIP2	147.1	168.7	209.8	165.9	176.7	174.9	202	296.8
TNFAIP3	190.5	263.7	211.8	160.1	257.7	209.9	180	299.5
TNFAIP6	95.4	109.7	105.7	106.1	99	96.7	97.5	100.1
TNFAIP8	1346.7	1877.3	526.6	532.7	413	635.1	683.8	743.6
TNFAIP8	105.5	136	96	91.2	79.9	94	111.2	139.1
TNFAIP8L1	322	415	174.6	166.7	138.8	102.1	353.8	382.2
TNFAIP8L2	534.9	698.2	325	262.6	203.1	332.4	430.6	553.1
TNFAIP8L3	160.6	187.2	120.2	106.9	92.9	102.5	142.7	171.7
TNFRSF10A	290.2	414.3	257.4	226.8	195.3	219.3	298.7	493.3
TNFRSF10E	111	123.7	117.9	84.4	94.8	89.3	105	109.1
TNFRSF10E	97.3	108.5	102.9	90.7	86.1	99.1	98.3	104.6
TNFRSF10E	785.6	1016	443.5	293.1	232	305.3	784.5	1130.5

TNFRSF10C	123.6	135.5	128.5	136.3	119.3	116.3	126.8	133.9
TNFRSF10D	111	133.1	97.1	70.7	81.7	85.7	102.6	110.9
TNFRSF11A	85.8	96.3	95.3	83	80.2	76.1	87.2	100
TNFRSF11B	90.5	110.5	90.2	90.2	77.2	82.1	89.2	101.7
TNFRSF12A	107	122.2	98.1	91.8	84.1	92.3	152.4	204.8
TNFRSF13B	81	96.2	93.8	89.8	84.2	89.1	88.8	87.3
TNFRSF13C	148.6	186.3	114.3	99.4	99.7	124.7	148.2	185.8
TNFRSF14	355.1	391.8	325.2	781	729.8	869.2	190.2	166.4
TNFRSF17	98	98.3	99.6	81.9	80.6	90.6	87.3	94.8
TNFRSF18	94.9	105.6	96.1	90.9	82.6	86.2	96.9	113.7
TNFRSF18	91.2	83.3	75.6	83.9	77.8	78.7	85.4	98.3
TNFRSF19	83.7	93.9	97.4	88.9	76.6	88.8	85.3	98.6
TNFRSF19	133.6	154.1	106.5	97.5	112.1	109.5	130.4	131.6
TNFRSF19	77	88.9	73.9	76.2	79.9	76.9	89.8	84.1
TNFRSF19L	75.4	79.8	83.5	89.8	81	84.7	75.3	78.8
TNFRSF19L	114.8	145.3	91.8	81.1	87.6	97.2	128.1	140.7
TNFRSF19L	102.9	102	89.6	87.9	72.6	82.7	89.9	102.8
TNFRSF1A	617.7	744.9	636.8	597.8	528.8	729.3	627.6	1214.7
TNFRSF1B	333.9	391.5	132.6	161	147.3	154.6	257.5	214
TNFRSF21	789.5	1117.5	943.7	1348.5	767.7	1649.5	687.7	1032.4
TNFRSF25	105.9	128.1	90	87.6	81.9	90.6	109	110.3
TNFRSF25	101.7	96.3	104.2	112.3	108.9	109.2	89.3	117
TNFRSF4	82.2	87.9	98.7	102.1	86.6	89.4	79.3	91.3
TNFRSF6B	89.3	101.6	103.2	92.7	97.2	105	96.8	98.1
TNFRSF6B	122.9	123.8	152.7	151.7	154.9	197.9	94.7	123.5
TNFRSF7	128.4	155.5	91.2	80.8	82.1	87.5	113.9	140.2
TNFRSF8	81.9	84.2	93.7	89.8	88.8	87.7	85.8	84.9
TNFRSF8	107.9	134	97.4	88.9	77.9	99.3	109	131.6
TNFRSF9	111.6	158.7	86.5	100.3	103.8	104.7	101.5	125.3
TNFSF10	439.5	603.3	326.4	261.4	224.4	369.8	346.9	422.7
TNFSF11	110	134.7	92.9	94.4	91.6	96.4	113.6	124.6
TNFSF11	146.5	151.6	101.1	89.7	94.3	87.3	117.2	148
TNFSF11	76.2	79.1	91.3	82.6	79.9	83.9	76.1	73.8
TNFSF12	94.6	98.4	92	80.9	89.1	86.7	87.4	98.7
TNFSF13	93.4	113	119.1	102	91.1	99.2	87	96.9
TNFSF13	84.6	99.9	101	103.7	88.3	96.4	86.6	98.2
TNFSF13B	1012.2	1296.3	286.3	412.4	431.6	526	906.7	1073.1
TNFSF14	396.2	505	249.2	361.9	326.2	385.3	514.9	681.7
TNFSF14	192.7	272.7	159.1	151.8	140.8	136.7	219.7	239.4
TNFSF14	723.8	890.9	248.6	374.1	423.7	337.9	754.2	786
TNFSF15	271.9	250	199.3	272.1	272.3	231.5	270.5	419.2
TNFSF18	100.8	112.7	99.5	88.6	81.2	93.4	86.7	119.9
TNFSF4	103.4	111.4	104.4	103.2	98.7	99.1	95	123.3
TNFSF5IP1	5405.5	6976.1	4840.6	3678.4	2707.4	4494.3	3617.3	8347.5
TNFSF7	2450.4	3143.8	1766.7	1515.1	1346.7	2431	1967.3	1937.4
TNFSF8	111.4	121.4	98.4	95.4	92.7	90.5	106.1	125.2
TNFSF9	205.8	278.7	168.2	140.1	120.9	174.5	253.2	390.5
TNIK	87.1	111	99.5	104	96.9	100.7	82.4	94.6
TNIP1	431.1	543	343.5	240.1	206.6	303.2	442.8	572.4
TNIP2	189.5	228.9	140.5	107.2	107.3	131.3	165	200

TNIP3	87.6	94.3	94	98.3	97.6	92.9	88.9	96.7
TNK1	82.4	84.3	80.5	77.3	85.2	76.7	72.2	86.3
TNK2	412.5	466.3	387.5	476	516.9	542	389.3	392.7
TNK2	102.1	124.4	99.5	107.5	87.9	91.6	102.7	126.9
TNK2	100.3	112.7	97.8	95.3	84.4	100	100.8	101.4
TNKS	114.9	128	116.4	94.1	105.3	109.7	118.3	118
TNKS1BP1	133.7	174.3	136.3	129	117.7	159.8	123.4	166.9
TNKS2	116.7	128.4	78.6	86	79	91.4	102.6	138.2
TNMD	112	115	101.2	92.9	96.3	99.3	105.8	104.1
TNN	102.9	124.1	107.2	102.1	84.8	111.9	102.1	114.3
TNNC1	102.8	127.6	92.6	96	76.5	94.3	103.8	118.3
TNNC2	95.1	103.9	95.3	81.3	88.8	80.8	86.9	101.9
TNNI1	90	95.5	80.8	79.3	75.3	74.9	92.3	95.5
TNNI2	90.8	102.9	96	94.6	102.2	118.6	94.5	87.4
TNNI3	118	151.7	94.7	92.1	97.3	107.3	113.4	136.8
TNNI3K	94.6	122.1	91	96.9	90.8	96.4	85.5	103.7
TNNT1	354.8	421.7	156.5	132.7	143.2	190.9	303.9	403.4
TNNT2	171.3	215	109.5	110.4	100.2	112.8	157.2	194.1
TNNT2	118.8	140.6	101.7	91.4	90.5	103.1	130.2	140.6
TNNT3	112.1	154.1	87.4	77	86.9	81.8	100.3	129
TNNT3	97	102.5	91.9	76.1	88.5	82.6	92.5	93.2
TNP1	131.8	161.7	115.2	103.3	94.9	101.6	129.3	124.1
TNP2	92.5	107.8	93.3	88.6	82.7	88.9	85	93.4
TNPO1	567.7	740.6	187.8	207.5	185.2	135.4	352.8	518.7
TNPO1	184.4	231.5	101.7	114.6	99.9	97.1	164.6	224.9
TNPO1	1649.6	2308.4	788	1421.5	1108.8	952.4	1862.9	2567.5
TNPO2	1962	2249.6	1422	847.7	815.6	869.9	1720.3	2329
TNPO3	751.3	1035.5	796	1150.9	993.7	1565.8	644.7	949.3
TNR	105.1	108.8	103.6	92	88.9	92	112.5	103.2
TNRC15	353.3	569.6	387	461.8	420.9	708.8	286.6	547.9
TNRC4	76.9	94.8	83.2	88.1	92.9	85.8	85.6	86.5
TNRC5	1650.4	2172.4	1103.1	1058.9	904.9	745	1531.3	2996.4
TNRC5	917.9	1294.7	728.2	634.5	513.6	737.6	944.6	1373.7
TNRC6A	149.8	182.6	105.6	102	95.8	100.2	148	163.7
TNRC6A	196.7	239.2	219.7	272	270.4	424.5	214.7	359.5
TNRC6A	107.5	118.2	99.9	98.7	95.3	90	110.6	117.4
TNRC6B	495.2	800.4	396.9	726.1	686.9	428.9	524	636.5
TNRC6B	124	122.8	95	92.1	84.7	84.3	103.8	139.3
TNRC6B	94.2	95.4	92.1	92.4	91.1	92.3	92.8	101.1
TNRC6C	73.4	77.4	94.6	88.3	81.5	79.5	70.8	73.7
TNRC9	79.8	87.8	75.2	87.6	75.6	87.2	84.5	93.3
TNS1	124.1	147.3	140.1	125.3	101.3	110.3	127.7	194.3
TNS3	1396.1	1929.7	1518.8	2715.4	2026.9	2958.5	1106.2	1288.2
TNS4	88.9	95.9	95	89.3	89.3	92.2	90.3	93
TNT	93.1	98.4	92.8	86.8	75.9	83.7	87	90
TNXA	109.7	135.1	94.1	86.6	79.3	84.9	110.7	118
TNXB	78.8	96.5	116.8	90.7	89.3	98.3	82.5	84.5
TNXB	95	104.2	101.3	95.5	81.7	81.7	99.3	105.6
TOB1	541.7	764	348.9	462.7	415.6	438.3	558.8	774.4
TOB2	154.6	217.6	149.6	126.4	118.2	128.4	157.5	222.7

TOE1	298.3	345.8	173.4	126.1	115.5	141.1	226.2	298.6
TOLLIP	176.8	220.6	225.6	201	191.6	240	217.6	347.7
TOM1	441.4	544.6	302.9	325.1	270.4	323.6	518.8	621.6
TOM1L1	155.4	166.4	131.4	115.7	104.5	132.7	123.8	154.9
TOM1L2	86.5	91.7	93.5	93.3	96.9	88.1	87.9	97.9
TOM1L2	104	130.6	100	96.8	96.9	101.6	102.4	115.1
TOM1L2	110.8	122.9	97.8	99.9	90.4	92.9	104.6	107.7
TOMM20	6189.7	8267.9	4090.1	5038.2	3349	6366.5	4431.6	9140.3
TOMM22	2457.4	3083.7	1046.6	1008.4	818.1	1091.9	2034.7	2282.1
TOMM34	1834.8	2277.3	1118	770	660.3	890.9	1605.7	1609.9
TOMM40	6812.5	8583.8	5331.3	2950.6	2228.1	2768.8	5625.2	6427.5
TOMM40L	593	822.7	314.8	241.1	186.8	361.5	539.4	986.3
TOMM7	14234.7	15506.5	10284.4	12064.5	8925.6	12121.4	15030.1	18413.5
TOMM70A	2050.5	2553.6	1216.2	1473.4	1142.3	2239.2	1568.4	2404.3
TOP1	178.4	211.6	126.6	132	127	163.3	161.5	229.8
TOP1MT	2241.4	2872.9	1727.6	1567	1361.6	2213.6	1713.2	2370.5
TOP1MT	782.9	965.2	599.9	485.5	412.6	694	583.7	731.4
TOP1MT	629.1	881.7	312.6	244.5	190	239.6	522.8	542.4
TOP1P1	85.6	95.3	96.1	89.5	86.9	96.3	89.3	103.8
TOP1P2	311.6	359.2	209.3	260.8	235.2	339.5	254.6	455.7
TOP2A	5259.9	5751.4	4764.4	3893.7	3547.3	6313.1	3823.3	6944.2
TOP2B	2706.5	3993.4	3049.1	3636.8	3453.6	6481.7	2464.2	3651.4
TOP3A	314.5	348.7	217.7	273.9	244	201.6	220.5	272.6
TOP3B	482.3	558.4	518.5	453.5	407.6	422.9	463.7	605.5
TOPBP1	1558.2	1654.5	368.9	496.2	424.1	565.2	1298.5	1566.3
TOPORS	84.3	99.9	87.5	89.4	79.2	84.6	80.5	108.4
TOR1A	687.3	882.8	331.7	225.5	199.2	293.1	504.3	767.3
TOR1AIP1	1899.2	2392.1	1115.1	1413.2	945.3	1892.1	1385.9	3720.8
TOR1AIP2	214.2	251.8	149.6	157.1	136.3	183.9	186.6	290.1
TOR1B	351.2	478.6	264.8	249.5	232.5	312	355	555
TOR2A	258.3	343.9	202.5	161.4	121.9	130.2	249.9	357.3
TOR3A	1269.1	1947	1007.8	787.1	584.5	1025.2	1268	1839.5
TOX	90.6	110.8	88	72.1	78	78.2	97.1	98.9
TP53	85.9	94	88.3	83.1	87.3	91.5	82.1	104.6
TP53BP1	306.3	420	334.1	274.9	243	428	256.1	362.2
TP53BP2	846.4	1145.7	696.3	898.8	836.4	1025.5	894.9	953.5
TP53BP2	109.1	147	122	104.6	95.2	113.4	121.1	114.5
TP53I11	104.5	129.3	128.7	117.3	110.3	137.8	101.8	112.7
TP53I11	107.8	125.4	94.5	90.8	74.7	93.7	105.4	111
TP53I13	805.3	1140.7	395.2	419.1	449.6	494.5	729.3	737.7
TP53I3	155.1	192	153	109.3	110.4	125.6	148.7	192.2
TP53I3	130.6	153.1	118.3	106.9	105.9	116.6	145.1	158.1
TP53INP1	93.7	105.3	102.9	96.6	86.1	88.1	87.3	125.9
TP53INP2	297.2	359.6	221.6	235.1	195.6	231.8	287.9	410.3
TP53RK	357	443.3	318.4	268.5	248.7	451.6	283.8	451
TP53TG3	90.1	99.7	102.7	94	88.6	96.4	86	87.1
TP53TG3	91.4	88.9	104.4	87.1	85.8	99.2	80.3	90.9
TP73	84.7	96.9	92.2	79.4	87.3	106.6	88.6	100.3
TP73L	81.4	97.2	98	87.3	84.9	84.8	77.5	91.6
TPARL	372.7	417.5	172.5	148.3	158	188.4	305.3	426.5

TPCN1	164.8	198.4	182	205.3	186.1	195.1	136.9	195.9
TPCN2	418	557.4	250.1	233.7	236	234	351.7	296.9
TPD52	67.7	85.8	76	74	78.9	85.6	80	93.8
TPD52L1	106.9	101.4	119	100.1	98.8	111.8	111.8	120.6
TPD52L1	95.9	108.2	100.1	101	88.5	76	90.8	106.7
TPD52L1	87.5	104.3	95	84.4	85	94.6	101	113.5
TPD52L2	437.1	565.5	277	301.6	256.1	370	408.4	580.9
TPD52L2	2945.7	3879.9	1740.6	2122.3	1760.6	2243.8	2913.4	2449
TPD52L3	97.9	123.3	106	90.7	87.1	94	106.5	126.7
TPD52L3	78.8	92.2	87.8	89	83.6	84	78.4	79.1
TPD52L3	89.7	90.4	113.5	110.9	106.6	102	93.8	94
TPH1	129.9	142.3	97.1	96.9	95	100.5	110.2	122.2
TPH2	89.2	104.5	102.3	92.9	90.2	98.5	90.5	99.7
TPI1	7014.9	8813.9	5121	4571.5	3367.2	5646.3	6664.6	11438.7
TPK1	963.2	1232.1	778.6	692.3	457.6	768.8	714.6	1462
TPK1	231.4	257.3	121.9	89.1	91.8	120.6	190.1	252
TPM1	276.8	327.8	166.8	299.8	252.3	431.8	252.5	326
TPM1	86.3	108.4	95.6	126.5	113.2	121.4	102.8	99.7
TPM1	339.1	414.9	220.1	510	420.9	842.9	300.3	395.2
TPM2	1390.9	1131.3	826.5	993.4	1350.2	1684.9	686	857.8
TPM2	102.4	121.1	100.2	90.1	95.9	97	97	120.3
TPM2	507.4	475.9	406.2	480.6	549.7	942.5	323.5	616.3
TPM3	3971.6	5491.2	1608.8	1038.7	950.1	738.2	3071	3590.9
TPM3	102.4	113.6	90.6	86.5	80.7	89.8	95.5	100.5
TPM3	131.1	127	83.8	77.8	80.3	87.6	102.8	146.4
TPM4	173.6	243.4	132.9	131.1	108.4	161.2	207.3	342.1
TPMT	308.9	368.3	304.9	568.5	435.8	507.7	277.9	335.5
TPO	79.4	89.4	82.4	83.5	82	87.9	80.7	100
TPO	108.1	125.6	100	108.2	95.3	87.8	109	122.6
TPP1	1515.5	1722	1546.3	2232.5	1732.3	2166.5	1686.3	2847.3
TPP2	169.7	198.1	132.1	127.2	120.6	152.4	151.3	215.6
TPPP	112	130.8	91.7	83.5	80.9	86.4	120.1	125.8
TPR	987.7	1487.6	1168.3	1612.4	1537.7	3423.1	948.9	1235.1
TPRKB	173.3	204.2	101.9	91.9	93.9	103.9	183.4	282
TPRX1	116.3	109.1	86.5	88.9	86.4	84	121.7	105.5
TPRXL	77.8	86.7	80	81.6	75.1	77.1	83.5	86.6
TPSAB1	85	85.1	83	81.6	77.4	85.1	84.2	83.2
TPSD1	78.6	87.9	79.6	81.4	77.1	81.8	82.5	85
TPSG1	101.7	106.8	98.9	90.5	87.1	93.7	98.8	100.8
TPST1	158.7	185.6	132.2	175.8	154.5	229	185.7	258.9
TPST2	1460.2	2138.7	1163	1103.2	827.9	1078.1	2296.5	2838.1
TPT1	30990.4	30666.6	57260.5	53274.2	53226.3	51629.1	31479	31554
TPTE	81.3	83.6	86.8	84.2	78.2	77	77.1	77
TPTE2	77.6	89.3	98.2	93.6	83.3	88.5	79.6	83.2
TPTE2	119.1	151.3	113.1	146	154.6	153.8	105.2	110.4
TPTE2	81.4	97.5	87.8	93.5	88.6	97.9	85.2	93.8
TPX2	1122.3	1440.2	725	605.4	578.7	1080.2	746.1	1021.2
TR2IT1	86.8	92.7	94	82.8	79.7	80.7	89.6	88.4
TRA16	596.9	713.5	317.4	194.2	158.9	191.1	448.7	541
TRA1P2	270.3	356.9	207.1	310.8	219.3	498.5	259.4	364.3

TRA2A	1081.6	1620.7	643.4	610.5	538.2	714.8	777.3	1043.1
TRADD	198.9	263.1	162.7	146.6	150.2	117.6	195.9	222.4
TRADD	259.5	331.9	203.8	164.8	150.3	187.8	221.4	319.1
TRAF1	100.9	116.5	105.9	96.4	105	98.5	104.4	107
TRAF2	191.2	221.9	218.5	180.6	146.9	236.2	182.6	301
TRAF3	201	241.3	196.7	171	163.3	222.3	181.7	223.1
TRAF3IP1	203.3	242	167.1	173.1	150	172.4	178.3	262.8
TRAF3IP2	85.5	97.7	89.4	85.8	74	73.4	89.9	99.2
TRAF3IP2	132.9	148.4	108.2	105.6	103.9	96.9	110	140.1
TRAF3IP2	898.7	1160	541.5	500.7	380	561.2	825.1	731.8
TRAF3IP3	505.1	695.4	397.4	350.1	396.4	555.2	316.6	372.5
TRAF4	109.6	122.2	107.5	99.4	88	100.6	113.4	133.6
TRAF4	103.6	130.6	75.8	79	75.5	74.8	105	135.4
TRAF5	71.4	87.5	87.8	89.8	90.6	87.3	72.9	82.1
TRAF5	93.6	89.4	81.8	83.9	74.5	82.6	84.9	84
TRAF5	100.5	135.9	92.5	82.6	85.6	87.1	99.2	144.9
TRAF5	77.1	89.3	87.5	83.4	77.7	91.2	75.4	91.1
TRAF5	103.1	121.4	95.2	86	86	86.9	94.1	116.8
TRAF6	87.5	84.9	97	94	92.7	93.7	88.6	93.4
TRAF6	163.5	219.9	146.3	144.9	136.3	154.2	163.8	248.3
TRAF6	219.1	264.2	202	165.1	138.9	183.2	191.2	302.3
TRAF7	224.6	292.5	245.3	231.2	166.5	215.7	203	284.1
TRAF7	88.9	102.5	92.5	88.8	87.6	93.7	93.3	110.3
TRAFD1	214.4	295	215.5	201	221.5	305.7	242.4	396.7
TRAIP	275.9	341.4	147.8	125.3	117.8	135.5	233.2	313.7
TRAK1	486.6	612.5	360.7	410.3	329.4	452	405	548.3
TRAK1	325.9	425.9	381.1	447.7	417.3	694.9	329.9	631.6
TRAK1	142.8	192.3	179.1	233.7	241.5	275.7	131.7	192.6
TRAK2	385.3	493.8	258.6	330.8	245.3	373.4	379.7	449.9
TRAM1	6293.9	7778.6	4270.8	4956.4	3407.2	5749.6	6222.1	9754.3
TRAM1L1	106.7	118.4	83.2	87	80.1	89.8	108.7	123.2
TRAM2	497.3	644.3	379.6	468.6	349.7	443.3	516.7	754.8
TRAP1	1083.9	1513.6	748.8	464.8	403.8	776.3	820.3	1062.7
TRAPPC1	1167.5	1437.7	630.3	548.1	441.6	529.1	1226.8	1672
TRAPPC2	156.3	175	116.9	131.2	110.9	97.4	118.9	144.9
TRAPPC2	730.3	963.6	487.1	633.4	546.2	851.3	645.1	987.8
TRAPPC2	88.3	108.7	92.1	91.4	97	96.1	90.7	88.2
TRAPPC3	1604.4	2254.9	1005.7	1177.4	927.2	1249.2	1673.9	1753.6
TRAPPC4	2877.8	3935.8	1820.5	847.1	663	980.2	2482	3024.3
TRAPPC5	1643.1	2013.9	873.2	736.4	574.1	976.3	1368.8	1433.6
TRAPPC6A	421.8	452.5	234.7	255.5	241.7	281	426.5	391.8
TRAPPC6B	129.9	171.7	110.3	103.9	93.6	108.8	126.5	213
TRAPPC6B	155.5	173.7	103.7	93.9	92.4	95	132.3	189.4
TRAPPC6B	195.9	226.2	114.6	100.1	101.5	139.8	205.6	277.2
TRAT1	91	99.4	95.6	97.6	95.8	97.7	85.2	94.9
TRDMT1	156.3	200.5	135.7	118.2	114.2	126.5	134.8	141.6
TRDN	103.5	123.9	102.5	84.1	83.5	99.1	102.6	138.6
TREH	113.2	117.5	91.4	86.5	84.7	83.9	111.3	108.3
TREM1	164	197.5	88.1	87.3	87.2	98.7	139.8	164
TREM2	295.9	341.5	153.3	170.4	142.4	169	208.4	259.5

TREML1	111.8	134.7	103.6	94.6	81.4	91.9	99.3	113.6
TREML2	91.7	119.1	95.1	94.1	94.7	103	95.1	110.3
TREML2P	107.7	138.2	85.1	93.4	80.8	89.3	113.8	122.9
TREML4	87.2	89.6	118	109.4	104.6	118	86.3	93.8
TRERF1	202.1	253.1	177.2	193.5	194.8	277.3	192	237.5
TRERF1	80.6	84.3	83.1	81.5	71.9	84.2	72.6	76.1
TREX1	98.6	135.8	97.3	91.9	96.6	87.8	105.7	116.6
TREX1	451.3	563.2	479.9	402.6	346.1	500.9	374.3	541.1
TREX1	496.8	563.5	402.3	300.1	282.5	382.5	378.4	514.4
TREX1	122.4	126.2	87.7	94.7	85.5	91.8	110.3	127.1
TREX2	83.1	104.3	90.7	90.9	83.3	90	91.8	96
TRFP	2884.8	3784.9	1955.2	1838.2	1487.4	2285.2	2805.2	3399.9
TRG20	940.1	1247.7	792.2	853	652.6	832.4	920.2	1483.4
TRH	89.3	90.5	87.7	78.7	84.4	80.1	89.3	82
TRHDE	89.1	97.2	102	102.2	97.2	109.3	81.2	79.5
TRHR	91.5	108	100.7	93.4	87.3	81.7	93	97.4
TRIAD3	114	127.9	92.5	91.3	88.1	87.7	97.4	124.7
TRIAD3	879	1042.8	675.1	657.8	599.5	655.7	763.4	1117.9
TRIAP1	747.9	921.1	398.9	418	297.8	411	694.8	760
TRIB1	184.1	222	249.3	190.9	179.7	206.6	184.9	232.6
TRIB2	120.9	119.3	125.9	131.4	111.7	121.7	112.4	157.5
TRIB3	1376.2	1884.3	663.7	426.1	346.7	474.7	1550.6	1972.6
TRIM10	80.7	92.5	80.7	79.8	83.5	76.7	83.2	84
TRIM10	75.1	79.3	93.7	85.6	81.8	86.4	86.9	92.1
TRIM10	93.6	108	102.8	95.4	91.6	98.9	95.4	102.4
TRIM11	324.1	376.2	190.7	162.8	146	167.7	293	403
TRIM13	621.3	549.4	367.8	465.7	478.4	337.6	577.7	931.4
TRIM13	292.7	447	221.5	239.1	222	381	225.3	369.8
TRIM13	523.4	671.4	302.5	654.8	592.7	492.9	475.6	549.4
TRIM14	151.2	150.3	113.1	101.8	86.1	84	128.6	125.4
TRIM14	84.1	92.9	100.3	87.5	89.4	88.5	88.7	96.6
TRIM14	134.9	161.5	112.5	105	98.2	95	135.3	152.1
TRIM14	190.5	249.1	118.3	111.5	96.3	92.2	163.9	187.3
TRIM15	102	110	95.6	83.9	81.3	85.2	100.8	107.4
TRIM15	68.9	69.7	80.3	85.5	74.4	78.5	72.4	71.3
TRIM15	91.4	98.6	85.7	91.8	81.4	85.6	91.8	99.9
TRIM16	88	96	79.3	83.5	77.6	77.7	121.9	134.4
TRIM16L	88.3	106.1	87.7	84.2	79.1	85	92.3	90.6
TRIM17	97.2	120	107.4	96.4	92	91.1	99.2	122.7
TRIM17	78.2	81.9	84.2	86.3	83	83.1	76.5	95.4
TRIM2	84.5	96.4	88.8	103.5	99.3	124.7	88.7	115
TRIM21	485.3	660.4	403.4	389.5	299.6	452.3	396.8	690.4
TRIM22	132.5	147.9	130	154.6	140.5	221.3	106.9	109.6
TRIM23	298.2	389.2	177.1	216.7	174.7	195.2	221.5	375.6
TRIM23	118.8	150.3	93.8	86.8	89.6	91.3	132.6	134
TRIM24	300.6	400.6	142.3	214.3	162.2	249.5	264.9	332
TRIM24	96.6	101.2	92.9	100.4	94.7	96.3	93.7	91.9
TRIM24	748.1	772.6	610.1	1038.8	793.3	1352.7	535	1177.2
TRIM25	487.9	655.1	735.6	644.3	582.4	739.8	537.4	1177.8
TRIM26	161.3	206.1	134	139.8	116	161.3	190.4	273.4

TRIM28	790.3	1020.4	941.6	520.7	400.4	817.7	587.8	1161.7
TRIM29	83.1	92.9	92.8	88.1	77.8	81.2	83.3	88.1
TRIM29	179.9	223.2	127.8	114.3	114.9	121.1	153.4	172
TRIM3	105.5	110.6	112.4	92	97.7	97.9	99.1	109.7
TRIM3	80.8	96.5	87.7	81.2	75.9	82.1	81.2	93
TRIM31	114.8	132.4	99.9	88.3	81.9	86.4	116.7	147.8
TRIM31	123.3	146.3	91.8	92	94.3	85	110.4	124.3
TRIM32	313.2	418.7	312.8	257.1	214	372.1	268.1	449.5
TRIM33	714.9	913.6	507.8	354.9	318.5	508.6	730.9	998.4
TRIM33	1015	1562.1	865.6	968.6	748.7	1274.9	1108.2	1857.3
TRIM33	76.7	85.9	85.4	79.7	80.6	75.5	76.2	87.8
TRIM34	81.4	79.4	96	103.5	90.3	91.7	89.5	83.3
TRIM34	96.3	109.4	95.5	83.6	80.4	82.2	93.8	94.4
TRIM34	89.6	83.1	93.2	86.2	86.7	83.4	81.8	87.5
TRIM34	90.5	107.1	89.6	86.8	88.6	81.3	81.9	110.5
TRIM35	196.8	285.3	140	135.5	108.4	120.4	170.9	276.6
TRIM36	120.7	146	110.1	90.3	83.8	96.6	124.6	159
TRIM36	133.6	160.8	104.8	110.5	101.7	134.1	138.6	240.2
TRIM36	97.7	103.7	104.2	98	100.5	103.8	99.1	105.1
TRIM36	89.9	104.5	102.8	92.5	90.6	94.8	96	116.3
TRIM37	146.5	130.7	112.4	113	120.7	120.6	130	138.7
TRIM37	186.8	246	191.7	149	136.4	203.1	156.9	224.8
TRIM38	153.5	181.4	121.3	186.2	163.8	185.1	132.6	200.9
TRIM39	80.5	100.1	89.3	82.9	84.6	86.9	84.8	91.2
TRIM39	236.6	273.7	173.5	205.7	197.6	230.6	195	241.6
TRIM39	99.5	123.9	114.9	101.8	100.9	131.4	102.1	107.3
TRIM4	1177.8	1419.4	791.6	781.1	663.3	837.4	1100.8	1535.5
TRIM4	127	143.4	118.1	97.4	88	100.1	127.1	149.3
TRIM40	95.1	104.7	98.5	74.9	80	95.9	99.5	124.1
TRIM41	448.1	508.2	323.1	311.7	307.3	407.6	422.7	743.9
TRIM41	132.9	162.6	116.1	104.8	112.8	119.4	124.1	169.2
TRIM42	90.5	102.2	95.2	83.4	81.4	85.6	93.1	104.7
TRIM43	98.1	125.3	87.9	88.1	74.9	86.8	93.5	123.2
TRIM44	1083	1497	1107.1	1736.3	1383.9	2956.4	888.2	1282.6
TRIM45	272.2	357	261.8	198.7	170.5	225.6	220.3	321.3
TRIM46	149.2	164.6	155.8	127.8	110	115.9	134.2	195
TRIM47	93.3	95.5	79.5	78.6	86.6	96.5	90.8	99.3
TRIM48	95	96.5	90.6	82.2	88.6	87.8	82.6	93.5
TRIM49	225	298.6	134.4	130.8	122.2	136.5	242.7	295.4
TRIM5	338.2	424.1	214.7	240.1	194.5	243.7	279.6	365.7
TRIM5	125.6	146.4	99.2	91.8	87.2	100.4	106.3	132.8
TRIM5	230.6	307.5	129.4	125.5	125.9	126.3	206.2	215.5
TRIM50A	78.1	88.7	101.2	89.7	86.2	93.3	83.3	83.3
TRIM51	103.7	124.9	110.9	103.9	106.6	99.4	108.5	132.7
TRIM52	190.1	199	129.5	141.2	129.4	127.9	153.9	213.9
TRIM54	108.8	129.5	96.5	97.4	88.7	91.2	103.5	115.7
TRIM54	87.8	93.2	98.4	76.4	82.2	84.3	87.3	94.9
TRIM54	81.7	86.4	96.7	85.7	89.6	85.8	77.8	89.6
TRIM55	90	99.5	81.7	87.2	78.4	85.9	93	91.9
TRIM55	92.1	104.9	88.3	83.6	80	88.5	90.1	88.7

TRIM55	84.6	99.7	94.3	70.9	92.8	87.2	88	85.2
TRIM56	359.5	404.5	174.9	254.8	253.9	307.2	371.7	576
TRIM58	174.8	195.3	129.2	135.4	135.3	158.2	156	198.6
TRIM59	143.7	165.8	113.5	92.5	90.2	88.4	125.3	157.1
TRIM6	84.8	86.7	85.9	82.5	83.2	87.6	82.6	86
TRIM6	107.2	118.6	113	134.1	107.7	157.6	100.1	106.8
TRIM60	77.8	74.2	90	82.5	82	83.9	74.4	82.9
TRIM62	113.2	154	92.1	77.5	76.7	87.6	111.8	153
TRIM63	118.4	139.9	83.6	72.5	82.6	88	112.8	144.2
TRIM67	125.5	144.3	83.7	97.4	90.5	97.6	120.4	136.8
TRIM68	501.4	592.7	326.2	260.1	237.3	295.1	494.8	584.7
TRIM69	91	117	100.6	86	86.9	97.4	99.6	111
TRIM6-TRII	88.2	96.3	93.8	86	87.4	92.6	85.5	80.5
TRIM7	103.1	126.8	96.8	92.8	91.2	88.5	111.7	133
TRIM7	102.4	128.1	104.1	97	90.6	95	108	107
TRIM7	84.2	100.2	106	96.9	97.8	94	88.3	99
TRIM71	89.7	113.9	97	95.5	91.2	95.3	101	108.7
TRIM74	87.6	98.7	87.3	91.1	86	90.3	92.4	94.3
TRIM8	563.6	736.4	584.7	529.6	452.8	515.8	590.3	634.5
TRIM9	134	151.5	110.3	91.7	85.5	104.6	123	149.6
TRIM9	219.8	284.9	194.9	128.2	116.2	146.9	180.1	197.9
TRIM9	160	177.9	117.7	101.4	85.3	100.2	160.2	175.6
TRIO	102.7	110.7	114.1	100.4	102.4	101.5	107.6	106.3
TRIOBP	165.5	207.5	90.1	88.1	76.4	94.2	152.8	179.9
TRIP10	191.4	213.4	178.9	136.2	131.5	172.3	192.9	312.2
TRIP11	348.4	528.3	237.9	297.9	307.3	432.8	319.5	419.1
TRIP12	801.1	1235.2	518.4	667.5	549.5	1153.7	771	1584.4
TRIP13	1637.7	2268.3	1451.2	1299.2	924.9	1471.2	1140.8	1749
TRIP4	502.6	765.6	388.9	496.4	444.5	693.7	429.2	646.1
TRIP6	1892.5	2267.7	1663.5	808.2	677.5	966.2	1982.3	2884.4
TRIT1	2034.9	2182.7	727.5	811.8	616.1	1230.3	1339.6	2243.2
TRMT1	710.9	812.3	472.1	349.7	323	519.3	513.9	567.8
TRMT12	661.2	779.3	219.8	204.1	181.4	253.5	528.4	664.4
TRMT5	15421	20204.6	6129.5	5688	3645.1	6577.2	14024.7	17055.7
TRMU	187.4	242.8	128.4	121.1	116.8	151.7	173.2	237.9
TRNT1	108.1	142	89.3	88.9	75.4	92.4	108.6	128.3
TRNT1	580.1	713.1	348.5	282.5	211.6	356	515.6	763.5
TRNT1	141.4	153.3	95.1	98.7	82	94.8	118.9	178.7
TRO	146.4	204.7	166.2	212	201.9	255.5	152.7	156.9
TRO	96.3	102.6	113.8	103.8	110.6	121.8	97.1	105.3
TRO	220.9	264.8	151.6	173.7	182	224.4	178	217.4
TROAP	493.6	445.6	524.2	325	311.1	398.9	400.3	728
TROVE2	1370.1	1788	660.9	769	621.5	1112.2	1114.5	1845.3
TROVE2	157.1	186.3	100	102.1	84.2	119.9	145.2	285.5
TROVE2	834.9	1170.2	359.1	330.5	265.1	242.6	596.1	748.5
TROVE2	2388.6	3483.3	1512.3	1848.6	1312.4	1825.3	1752.9	3282.5
TRPA1	92.4	94.5	95.9	95	84.1	92.7	83.2	101.2
TRPC1	90.9	106.2	93.4	88	83.3	92.1	92.9	102.5
TRPC2	248.6	311.1	187.2	121.7	114.7	116.2	248.1	279.9
TRPC3	99.3	119	95	100.9	95.9	95.1	96.7	97.7

TRPC4	139.7	166.1	111.1	99	86.6	98.1	130	147.6
TRPC4AP	782.8	962.6	390	349.4	354	458.7	669	831.4
TRPC4AP	868.1	1006.6	918.6	814.6	648.5	1225.4	647.7	1011.5
TRPC5	123.5	159.7	103.7	84.6	76.3	85.5	118.3	176.5
TRPC6	97.5	99.5	103.6	97.2	96.8	98.3	92.2	96.8
TRPC7	82.1	99.9	98.2	86.5	94.4	90.7	93.7	93.7
TRPM1	82.4	81.2	91.4	80.8	80.3	81.3	78.5	80.6
TRPM2	236.1	289.8	126.5	124.1	109.3	178.6	204.6	218.8
TRPM2	85.4	88.1	116.6	101.9	101.6	103.6	81.6	80.2
TRPM3	98.2	107	94.1	90.8	82.9	86.7	91.1	101.7
TRPM3	82	95	82.3	78.9	83.2	77.5	81.4	91.5
TRPM4	1476.2	1986.8	514.1	290.3	282.1	384.1	1552.8	1342.8
TRPM5	71.5	80.2	77.3	79.6	81.8	85	71.7	83.4
TRPM6	86.6	90.7	101.3	91.6	86	85.2	82.8	86.1
TRPM8	81.4	100.5	85.8	96.7	95.5	104.7	85.4	100.1
TRPS1	189.9	220.2	101.9	98.8	96	98	173	233.7
TRPT1	70.2	76.6	81.1	77.7	78.1	80.3	70.2	71.6
TRPT1	2275.2	2340.5	1611.4	1038	871.6	985.9	1681.7	1522.4
TRPV1	94.8	107.9	106.1	90.6	94.4	96.8	90	93.4
TRPV1	126.3	133.6	87.5	81	76	83.4	117.4	128.9
TRPV1	85.7	98.9	100.5	89.8	80.9	95.2	99.6	87.7
TRPV1	79.2	88.6	88.4	82.9	82.7	85.1	83.4	89.9
TRPV1	74	91	98.6	89	84.7	77.5	85	106.3
TRPV2	350.1	408.6	444.4	405.6	340.1	543.6	313.8	477.8
TRPV3	118.5	151.7	111.3	100.7	95.4	98.4	112.2	130.2
TRPV4	85.1	93.9	87.4	89	83.3	92.5	88.9	103.3
TRPV4	86.1	84.6	100.6	99.1	99.2	99	75.8	88.4
TRPV5	95.3	111.8	111.8	84.4	88.6	82.4	94.8	107.9
TRPV6	113.6	136.8	88.3	89.3	88.4	88.8	119.7	123.6
TRPV6	75.9	81.2	85.1	91.2	85.9	87.6	85.9	83.5
TRPV6	79.5	93.3	86.7	78	84.8	80.2	80.2	94.6
TRRAP	749.4	1186.6	1084.2	1769.4	1551.7	2132.5	777.2	1122.8
TRSPAP1	143.6	171.8	112	118.9	106.2	130.2	140	208.5
TRSPAP1	123.5	142.5	92.5	83.4	82.5	99.6	116.3	169
TRUB1	196.4	221.2	123.3	112.6	91.6	112.5	142.4	173.7
TRUB2	193.4	300.2	133.3	113	120.7	131.6	190.5	227.6
TRY1	119	143.7	98.9	86.3	84.3	96.9	122.3	126.5
TRY6	103.2	110.1	90.8	84.3	77.6	86.6	107.5	111.1
TSC1	82.9	98.4	94.6	85.7	93	87.9	88.8	112.2
TSC1	493.2	709.1	222.3	200.4	219.9	236.8	543.9	757.7
TSC1	97.4	126.5	102.1	104	89.5	91.2	102	118.7
TSC2	154.2	177.9	151.2	130.7	119.3	159.6	152.6	192.7
TSC2	77	84.3	103.8	93.1	86.5	95.3	77.9	88.6
TSC22D1	165	203.4	106.9	165.1	125.8	170.7	148.7	217.7
TSC22D1	326.7	392.6	154.8	165.6	185.1	193.8	235.2	265.2
TSC22D1	83.6	108.2	107.9	102.7	99.1	98.6	84.9	93.7
TSC22D2	333	400.1	212.1	232.4	198	275.7	299.1	593.8
TSC22D3	108.9	128.7	95.7	90.1	86	97.1	94.5	110.3
TSC22D3	235.5	406.4	192.4	180.1	207.7	263.2	279.3	377.7
TSC22D3	377.9	465	180.7	190.7	156.4	178.4	394.6	748

TSC22D3	80	95.2	94.3	85.4	81.4	77.1	83.4	88.3
TSC22D3	85.7	87.4	82.9	87.3	86.1	82.4	84.4	87.5
TSC22D4	153.3	212.1	172.8	164	163.6	257.9	161.8	262.8
TSCOT	178.3	198.2	93.3	105.2	79.9	94.4	208.7	259.2
TSEN2	993.8	1240.7	649.9	651.4	543.6	877.3	656.7	760.9
TSEN34	1541.7	2078.2	745.6	798.9	628.9	784.9	1444.1	1600
TSEN34	603.7	740	408.6	503.2	349.3	528.3	590.8	966.8
TSEN54	278.1	349.6	325.9	194.5	200.5	264	235.1	356
TSFM	352.5	465.3	255.7	215.6	217.8	241.4	301.8	411.8
TSG101	2326	2858.7	1591.5	1250.2	932.8	1652.2	1780.6	3420.9
TSGA10	100.3	122.7	116.6	121.3	113.6	142.1	93.9	123.5
TSGA10	108.7	131.7	108.1	107.7	90.3	108.8	106.5	104.8
TSGA10	100.7	103.3	96.9	107.2	96.7	98.4	106.8	114.6
TSGA10IP	124.4	130.2	101.1	86.8	85.8	93	127.2	135.2
TSGA13	120	116.9	96.5	87.2	93.8	87.2	108.4	125
TSGA14	301.9	366	304	207.9	169.6	213.6	269.8	511.3
TSGA2	106.8	117.3	86.7	93.7	84.4	91.3	103.9	105.2
TSHB	84.5	93.9	81.9	89.7	80.7	93.9	86.8	88.5
TSHR	97.5	98.5	98.2	95.1	85.7	87	101.4	109.7
TSHR	108.5	114.4	93	76.4	88.3	84.1	106.9	104.3
TSHR	86.4	97.5	90.1	82.1	77.8	84.5	86.4	93.4
TSKS	95.5	104.1	96.2	87.2	84.8	93.2	94.2	95.9
TSLP	128.7	156.2	115.1	93	89.8	100.5	126.2	144
TSLP	110.1	133.6	102	91.5	77.3	97.2	116.6	138.5
TSN	124.6	153.9	97.4	87.3	83.7	102.3	111.9	160.9
TSNARE1	108.1	129.2	96	87.6	84.2	87.8	116.8	121.3
TSNAX	162.4	184.7	130.4	121.4	110.6	224.9	188.7	468.4
TSNAXIP1	107.1	147.3	81.1	82.9	73.3	87.4	116.3	142.7
TSP50	94.2	100.1	86.8	83.7	80.2	84.4	103.9	112.1
TSPAN1	108.9	109.1	129.9	124	100.2	112.6	109.3	105.8
TSPAN10	166.6	204.3	120.8	129.4	127.2	142.5	134.2	139.9
TSPAN11	87.1	96.3	97.3	82	79.8	85.2	85.6	93.2
TSPAN11	84.1	98.3	96.6	79.2	83.7	74.5	87.6	90.8
TSPAN12	85.9	95.9	87.8	86.7	83.9	85	80.9	94.5
TSPAN13	98.6	111.9	87.6	88.7	84.3	89.2	101	130.2
TSPAN14	140.7	162.7	121.1	108.9	108.5	125.1	142.6	227.2
TSPAN15	110.4	120	111.3	108	87.3	110.9	110.8	118.2
TSPAN16	89.6	108.2	110.3	90.6	93.7	98.6	93.1	93.7
TSPAN17	219.5	277	144.9	129.5	106.6	117.3	207	248.1
TSPAN17	1545.6	1782.5	1185.6	1003.1	993.2	880.5	1282	1182
TSPAN18	104.2	113.9	105.9	97	97.3	114.4	98.9	108.7
TSPAN18	96.4	116.1	114.9	102.2	93.7	95.5	89	117.7
TSPAN18	125.2	156.8	93.2	85.2	74	96.5	121.6	148.2
TSPAN2	80.3	104.1	93.3	92.5	82.1	86.9	79.3	104.7
TSPAN3	2220.7	2898.7	892.5	547.8	494.4	867.1	1475.1	2320.1
TSPAN3	3082.2	4545	1542.8	857.6	690.9	992.4	2053.5	2956.4
TSPAN31	603.7	781.8	427	470.6	400.2	431.7	556.1	565.6
TSPAN32	1256	1351.8	998.1	1533.1	1752.6	1858.4	616.6	514.6
TSPAN32	292.5	303.2	244.2	334.6	356.2	442.3	193.5	235.4
TSPAN32	298.7	299.3	237.7	291.1	333.5	224.6	182.5	205.2

TSPAN33	352.1	440.1	183.9	157.5	140	146.2	237.2	206.2
TSPAN4	281.8	364.7	179.9	141.4	156.6	150.7	256.1	276.5
TSPAN4	107.1	124.2	87.5	92.2	81.7	86.7	108.2	112.3
TSPAN4	172	231.3	167.4	115.2	118	141.1	161.9	190.6
TSPAN4	93.1	110.1	90.6	76.7	71.4	81.3	91.9	106.1
TSPAN5	367.9	485.9	195.6	180.8	153.3	119.4	301.8	503.6
TSPAN6	101	118	106.7	109	115.3	107.2	106.9	107.5
TSPAN7	81	94.3	84.6	77.8	78.5	87.3	82.6	104.8
TSPAN8	123.4	126	83.4	89.3	79.4	85.3	111.3	139.7
TSPAN9	147	182.9	134.7	112.1	105.1	115.9	154.9	174.8
TSPO	4607.8	6874.9	3701.2	2116.1	1728.2	1761.9	4254.9	5756.6
TSPO	2110.5	2777.8	817.3	552.1	384	687.9	1888.9	2836.5
TSPO	21658	26639.2	22294.1	16434.1	13111.7	14032	21132.5	21676.8
TSPY1	65.9	75.6	84.3	73.4	80.6	67.8	73.8	78.5
TSPY2	76.4	81.4	93.9	88.7	80	88.9	79.9	88.2
TSPYL1	1209.9	1536.9	761.2	1065.9	712	1508.6	1019.4	1895.7
TSPYL2	89.6	100.5	97.9	79.9	82.3	84.2	97.3	109.4
TSPYL2	109.6	120.3	109.9	92.5	85.9	91.4	94.3	161.8
TSPYL2	99.8	100.4	94.5	85.5	79.5	88.8	91.7	109
TSPYL2	122.7	128.3	126.8	106.4	101.4	115.7	108.4	170
TSPYL3	79.2	88.6	89.6	86.6	88.5	83	84.2	81
TSPYL4	149	175.6	126.8	116.1	107.1	116.6	138.8	170.4
TSPYL5	616.6	792.7	532.2	339.9	254.1	396.5	506.6	699.3
TSPYL6	91.5	120.9	85.5	92.1	84.8	87.5	98.4	116.7
TSR1	96.7	111.6	88	80.8	86.9	83.5	90.3	112.8
TSRC1	162.9	198.2	148	190.7	161.5	188.8	159.3	143.8
TSRC1	84.8	98.7	96.2	99.9	91.3	124.9	85.1	92.7
TSRC1	161.1	188.2	131.6	151.6	127.3	158.8	134.9	127.6
TSSC1	973.3	1356.5	927.2	950.7	733.8	1293.8	826.7	1210.1
TSSC4	841.1	1099.7	499.8	384.2	365.2	456.4	867.2	793.1
TSSK1	104.4	123	107.6	93.3	90.1	97	110.3	113.1
TSSK2	82.8	100.2	96.8	91.7	84.3	101.2	95.8	85.4
TSSK3	132.2	149.2	98.6	101.8	90.6	102.9	112.7	134
TSSK4	94.9	98.9	98.2	94.8	78.1	87	98.6	91.9
TST	4907	6312.9	3308	2561.2	2314.9	2177	3822.5	3041.1
TSTA3	607.2	722.6	283.6	199	171	267.6	456.1	731.3
TTBK1	84.6	108.3	97.3	91.5	84.6	90.2	86	95.4
TTBK2	109.3	128.6	110.5	96.9	97.4	107.9	103.7	107.7
TTC1	1186.9	1500.4	711.1	733.2	602.8	1120.5	934.5	1319.2
TTC10	121.1	130.2	111	90.9	99.5	98.3	101.7	112.2
TTC10	245.7	338.2	178.8	161.7	156.9	212.8	198.8	288.1
TTC12	174.1	226.7	154.9	136.6	104.5	142.9	142	209
TTC13	1087.7	1387.3	931.2	974.5	746.6	1388.5	801	1213.3
TTC13	129.5	137.3	119.1	102.5	97.2	113.9	103.1	114.9
TTC13	524.2	689.2	374.1	323.9	288.8	433.3	421.2	508.7
TTC14	508.2	599.3	337.1	287.5	278.5	565.8	314.7	577.1
TTC14	441.9	478.8	280.5	306.7	269.9	420.6	318.7	605.4
TTC15	638.9	866	756.1	650.1	593.4	806	579.2	1074.6
TTC16	187.2	233.3	138.7	129.7	126.9	125.1	183.7	222.7
TTC17	352.1	470.4	270.9	362.1	389.6	523.2	309.2	446.8

TTC18	111.3	114.2	94.5	90.9	80.7	93.2	93	122.8
TTC19	616.9	691	527.6	605.8	411.4	895.7	469	789.8
TTC21A	92.9	100.6	93.8	76.8	81.6	95.4	90.5	125.6
TTC21B	79.5	86.7	94.2	82.2	79.3	86.2	83.6	84.3
TTC22	83.4	81.5	90.8	97.8	90.2	93.8	77.3	90.1
TTC23	164.1	207.6	102.4	109.9	101.1	103	167.8	224.4
TTC23	379.1	470.1	343.4	281	267.3	350.7	364.2	480.3
TTC25	141.6	175.9	107	111.6	87.2	108.7	133.3	160.9
TTC3	843.9	1100	776.3	1612.1	1100	2102.8	766.5	1661.9
TTC3	107.1	125.1	88.1	87.6	84.7	94.5	109.8	115.7
TTC32	335.7	366.4	176.2	178.4	185.3	202.7	301.6	434.8
TTC4	2144.1	2676	1334.1	1317.8	943.4	1559.9	1829	2494
TTC5	404.5	593	312.1	246.4	209.8	280.4	364.4	402.2
TTC6	73.5	81.2	93.1	87.8	69.8	74.4	72.1	79.6
TTC7A	228.8	275.2	250.7	311.8	275.7	393.9	172.5	223.8
TTC7B	109.7	123.7	109.5	108	98.2	96.6	110.3	121.1
TTC8	164.8	205.1	101.6	103.4	86.4	100.7	146.1	151.9
TTC8	235.7	305.9	154.8	160.7	135.1	193.9	193.8	228.7
TTC9B	75.8	88.4	92.7	93.6	87.2	92.5	84.9	89.5
TTC9C	362.4	522.6	230.2	173.4	190.4	213.7	335.4	392.8
TTF1	114.9	120.8	82.6	79.1	79.1	80.8	106.9	126.5
TTF2	2076.9	2731.3	1453	1237.2	1038.6	1467	1655.7	1816
TTK	2045.2	2248.7	1136.7	1146.1	900.1	1568.1	1535.6	2776.1
TTL	429.2	557.8	226.5	233	219.3	249.3	398.1	490.2
TTLL1	98.3	111.8	99.1	80.7	91	90.3	89.8	107.7
TTLL1	154	177.1	115.3	105.3	102.4	134.5	137.7	172.5
TTLL1	210.2	283.7	150.4	131.4	127.2	123.2	180.2	201.1
TTLL10	76.3	82.9	94.9	88.4	74.9	82.5	76.6	90.2
TTLL12	785	1040.4	762.8	661.5	507.9	808.8	651.4	1159.3
TTLL13	102.9	110.9	92	89.9	79.7	85.3	93.6	98.4
TTLL2	74.4	80.5	89.9	82.1	78.7	84.2	75.5	78.8
TTLL3	214.1	220.6	243.7	257.4	251.4	338.4	163.7	300.1
TTLL3	93.1	102.3	120.9	104.1	97.6	98.9	93	114
TTLL4	152.9	172.7	134.2	134.5	120.4	144.6	154	231.7
TTLL5	308.2	407.5	231.5	216.4	192.4	231.6	254.5	266.5
TTLL6	100.8	100.6	101.3	86.1	92.2	92.6	100.6	96
TTLL7	99	108.7	117.9	100.7	105.1	109.9	92.7	111.6
TTLL9	95.2	119.4	101.5	79.8	91.5	87.5	109.8	117.9
TTMA	102.9	94.2	107.8	99.2	91.6	101.6	91.9	89
TTMB	96.5	112.1	85.3	77.8	77.4	76.2	93.9	117.2
TTN	98.6	107.9	96.3	87	87.1	95.7	90.7	114.6
TTN	87.1	91	92.2	81.7	80.8	89.1	77	94.5
TTN	113.1	117.9	99.5	90.9	90.6	98.7	109.4	100
TTN	96.8	107.8	91.6	86.3	81.2	87.4	97	105.9
TTPA	71.8	77.5	94.3	79.6	81.9	90.1	76	78.7
TTR	91.2	102.8	96	96.2	101.4	108.3	93	92.2
TTRAP	117.6	138.4	93	91.6	87.9	95.1	117.8	189.2
TTTY1	119.3	119	91.2	83.3	84.9	82.2	108.7	116.5
TTTY10	146	159.3	98.4	105.9	86.5	87.6	140.4	182.2
TTTY11	98.1	109.9	121.6	108.6	94	100.2	88.8	99.1

TTTY12	96.3	101.4	95.9	82.4	71.1	73.5	91.6	99.2
TTTY13	84.7	106.6	83.5	82.7	82.1	95.1	80.9	102.8
TTTY14	147.7	199.1	97.7	90.7	83.2	91.7	138.1	165.8
TTTY17A	101.3	107.4	81.1	78.1	92.2	85.5	92.1	100.4
TTTY19	101.4	103.3	112.4	99.2	97.1	99.2	95.8	97.4
TTTY2	88.8	102.9	85	83.4	79.1	82.3	92.1	102.2
TTTY21	83.5	88.7	91.7	88.8	88.5	87.5	86.3	94.9
TTTY5	96.5	103	92.9	94.7	93.6	91.5	87.5	90.8
TTTY6	109.5	118.2	95.4	94.5	89.4	83.8	101.9	113.3
TTTY6B	83.9	100	91.3	80.1	80.2	83.6	89.7	84.7
TTTY7	87	88.1	92.7	84.8	80.8	88.8	92.1	92.8
TTTY8	89.5	102.4	91.2	85.3	93.4	92.4	98.5	100.5
TTTY9B	87.5	104.5	99.8	89.3	86.2	91	100.3	100.2
TTYH1	97.2	119.4	93.4	84.1	71.6	80.1	90.9	104.2
TTYH1	94.7	104.1	90.2	82.2	74.7	83.8	92.1	109.6
TTYH2	97.1	112	106.4	121.6	113.9	126.7	101	100
TTYH2	97.5	108.4	119	108.7	101.2	112.3	107.3	96.9
TTYH2	107.4	116.2	91.3	84.8	87.8	94.2	91.5	110
TTYH3	304.3	401.7	458.6	528	424.6	668.4	251.2	448.8
TUB	95.2	113.4	97.9	97	82.4	95.3	93.4	96.2
TUB	112	110.7	89.8	84.9	88.9	89.3	111.7	113.8
TUB	138.2	141.8	97.3	96.1	84.5	85	116.3	143.5
TUBA1	1126.7	1273.3	452.8	363.6	296.4	415.5	1148.4	1853.9
TUBA1A	16560.4	17020.4	7758.8	6407.7	5355.1	5446.9	14944.4	17858.5
TUBA2	77.9	93.7	94.3	90.8	91	90.8	76.2	87.6
TUBA3D	83.9	97	83.7	81.8	77.6	79.3	88	90.1
TUBA4	72.9	81.8	84.1	87.1	78.4	82.5	83.4	77.3
TUBA6	22654.3	27416.9	17397.7	8018.5	7274.7	8899.2	16548.3	21969.8
TUBA8	74	87.1	89.3	86.3	89.8	81	82	93.1
TUBAL3	80.5	89.3	101.3	97.4	90.5	88.2	75	87.8
TUBB	123.6	131.3	88.3	87.5	82	84.3	128.5	159.8
TUBB	7963.1	9576.7	6559.8	6542	5243.3	7780.1	6104.4	7265
TUBB2A	124.3	155.7	103.7	82.6	80.7	88.5	129.1	173.2
TUBB2B	141.1	122.6	91.5	84.9	89.1	84.6	114.9	142.8
TUBB2C	6518.6	7628.6	3514.7	1705.3	1187.5	1367.2	5155.6	6641.4
TUBB3	251.8	281.8	181.8	124.2	118	112.8	257.4	259.1
TUBB4	123.2	141.3	127.6	104.7	91.2	107.7	114.7	171
TUBB4Q	454	598	195.8	158.2	118.1	141.6	432	442.9
TUBB6	1841.2	2352.1	629	406.2	330.3	252.9	1382.1	1508.6
TUBB6	1795.4	2597.5	1356.9	1025	733.4	1300.3	1422.8	2485.6
TUBB8	90.7	102.4	82.8	88.3	87.5	79.5	96.9	92.8
TUBD1	192.2	247.9	110	101.5	97	109.5	213.2	386.1
TUBE1	130.8	136.1	95.2	97	92.2	104.7	122.3	203.2
TUBG1	214.6	258.8	103.6	87.7	89.8	96.3	208.7	274.2
TUBG1	4091.8	5090.8	2604.9	1950.8	1681.3	1703.2	4038.4	2970.2
TUBG1	1973.8	2463.2	1264.1	1025.2	766.2	1158.1	1554.5	2792
TUBG2	340.1	381.2	229.5	230.2	209	234.8	299.4	313.2
TUBGCP2	495.4	728.2	542.1	439.3	417.1	579.2	428.2	602.5
TUBGCP3	147.3	187.8	125.7	111.8	122.2	136.6	129	148.8
TUBGCP5	399	425.2	218.1	209	163.9	228.5	301.7	320.4

TUBGCP6	115.2	152.5	120.7	112.3	102.8	133	109.6	123.9
TUBGCP6	243.9	249.1	156.2	157.5	180	196.6	189.6	287.3
TUBGCP6	87.9	87.8	98.8	100.2	86.5	97.3	90	82.6
TUFM	2306.1	3173.4	1592.4	1339.3	829.4	1638.6	1723.9	3708.3
TUFT1	135.1	169.7	117.3	110.3	97.3	139.4	160.4	200.4
TULP1	109.8	122.9	79.1	81	73.9	94	104.9	122.5
TULP2	80.5	88.1	97.9	86.8	82.1	85.6	77.3	83.6
TULP3	125.2	136.6	119.6	125.5	99.7	121.7	136.4	148
TULP4	191.5	291.2	149.8	174.2	146.9	196.9	167.9	221.9
TULP4	298.3	451.4	254.4	329	290.4	395.3	290.5	451.1
TULP4	99.3	121.1	92.4	95.9	81.3	87.1	109.8	116.7
TUSC1	325.5	452.3	302.1	246	232	334.8	296.6	506.8
TUSC2	285	404.2	195.7	155.6	149.2	192.8	241.3	361.7
TUSC3	82.3	79.1	93.3	85.4	84.6	79.5	69.8	86.3
TUSC3	85.9	97	90.9	82.5	86.2	82.9	89.4	94.4
TUSC4	1116.7	1529.6	716.1	666.5	615.3	882	839.7	922.1
TUSC5	96.7	109.2	95.3	78.7	85.9	85	104.7	115.7
TWIST1	887.2	1361.6	654.3	432.6	433.4	693.3	629.1	814.5
TWISTNB	474.4	602.3	231	211.4	151.4	269.9	320.8	439.2
TWSG1	1026.9	1251	658.9	574.6	506.2	757.1	937.4	1557.8
TXK	81	104.7	89.6	79.2	83.1	96.6	93	106.2
TXLNA	1802.2	2147.8	1244.7	966.1	864.6	1231.5	1458.8	1730.7
TXLNB	98.6	112.3	98	90.6	85.6	93.9	96.3	101.9
TXN	14642.3	17851.4	6585.2	4944.7	3190.1	4989.2	15099.4	16445.6
TXN	24276.7	28077.9	18949.1	13510.6	10374.2	14749.8	22610.5	23544
TXN2	133.1	177.7	100.2	97.1	92.3	95.8	127.2	166.7
TXNDC	4026	4883	2244.5	1879.3	1320.4	2043.4	3575.1	4350.3
TXNDC10	406.7	525	270.9	402.2	318.7	713.5	357.3	924.1
TXNDC11	269.4	352.7	205.2	179	165.2	257.8	244.2	398.3
TXNDC12	5261.6	6533.9	4567.5	4576.3	3602.8	4741.3	5058.5	6605.4
TXNDC13	447.5	599.5	221.9	290	217.5	248.3	389.4	664.6
TXNDC14	3283.6	4368.4	1815	1418.6	1003.8	2011.9	2240.7	3662.3
TXNDC3	80.2	95.3	97.6	84.8	87.3	98.8	81.4	83.4
TXNDC4	101.7	119.1	98.4	85.1	82.7	88.3	89.9	123.9
TXNDC5	69.9	74.1	78.2	75.3	77.6	75	69.2	83.4
TXNDC5	343.4	468.5	236.6	160.6	133.3	164.6	272.3	410.8
TXNDC5	164.8	213	111.1	121.8	99.2	102.2	128.4	165
TXNDC6	108.2	113.1	108.1	85.4	65.6	84.6	113.4	122.8
TXNDC8	83	90.8	101.5	96.8	96	102.5	81	90
TXNDC9	806.1	1085.8	370.4	293.4	253.2	304.5	724	1340.4
TXNIP	2025.7	2528.1	583.4	580.7	530.4	685.3	1206	1229.8
TXNL1	436.9	599.5	152.8	159.5	127.2	184.5	419.2	1101.9
TXNL2	2708.2	3404.4	989.7	641.1	446.8	1007.5	2176.8	3835.3
TXNL4A	171.1	194	130.3	147.6	130.9	128.2	148.4	175
TXNL4B	141.9	162.6	133	135.3	111.7	132.2	147.2	188.8
TXNL5	5508.2	5893.9	2984.1	3337.1	2260.6	3131.3	5109.4	6652.5
TXNL6	87.3	103.4	105.5	98.2	90.6	86.7	97	107.1
TXNRD1	851.5	1300.3	758.5	640.5	556.5	1004.2	2067.8	3736.3
TXNRD1	705.9	1034	386.8	273.5	221.1	362.2	1346.5	2290.7
TXNRD2	557.5	722.2	392.4	296.8	274.4	322	473.4	540.5

TYK2	2680.1	2606	1953.7	1646.3	1667.4	2068.3	1584.1	1464.5
TYMS	9396.9	11012.2	6464	5938	4414.3	6186.4	7827.5	12804.6
TYR	80.9	87.6	101.9	90.2	86.1	90	82.1	91.1
TYRO3	175.4	219.8	198.6	202.3	171.5	216.3	171	325.2
TYRO3P	86.3	95.8	95.1	81.2	80.5	86.3	98.9	90.3
TYROBP	5897.1	6533.2	5041.5	7585.4	6099.1	8275.1	4639.3	5652.4
TYRP1	81.9	91.5	80.1	89.9	79.5	77.4	86.5	76.8
TYSND1	1093.1	1352.1	698.8	338.5	299.1	328.5	710.2	612.8
TYSND1	181.3	217.7	145.7	102.4	90.3	90.3	135.3	160.1
TYSND1	121.7	140	121.4	111.2	98.7	130.1	102.8	137.2
U1SNRNPB	533.6	609.9	364.3	321	304.7	292.4	459.1	450.8
U1SNRNPB	239.5	285.4	130	118.9	114.6	123.8	228.4	241.8
U2AF1	1812.5	2541.4	818.4	807.7	570.4	831.8	1825.2	2986.1
U2AF1	584.9	767.9	193	193.9	155.2	201.3	522.4	715.1
U2AF1L2	569.2	630.2	338.9	363.7	366	389.5	466	547.7
U2AF1L3	243.9	260.5	174.8	135.5	120.4	137	215.9	306.9
U2AF1L3	126.7	147.2	109.7	112.9	95.4	126.4	126.2	148.4
U2AF1L4	524.8	693	548	499.8	405.5	455.3	471.9	597.8
U2AF2	4365.6	5435.9	2693	2182.1	1840.5	1398.8	3691.7	5669.9
U2AF2	212.2	302.4	141.7	143.2	128.2	99	179.7	247
UACA	76.6	87.9	87.4	92.1	82.7	88.3	80.4	79.7
UACA	104.5	115.8	108.5	86.5	98.7	98.3	90.1	106.4
UACA	108.5	121.6	101.1	86.3	90.1	87.2	94.3	115.8
UAP1	763.3	1028.2	192.9	188	152.5	203	667.7	1004.5
UAP1L1	264.4	382.2	441.1	432.2	438	778.9	313.4	622.4
UBA2	299	400.9	104.8	101.5	97.4	109.9	205.5	297
UBA52	32984.4	30541.9	50086.7	36895	31483.7	32332.3	33008.6	32564.6
UBA52	517.7	640.2	325	286.9	221.6	283.7	503.6	526.8
UBADC1	3396.8	4473.8	3442.3	2747.8	2253.4	3253.2	2558.2	4257.1
UBAP1	641.9	824.2	574.3	501.6	380.1	653.2	614.5	1121.1
UBAP2	327.9	384	212.8	184.7	164.6	234.4	251.5	409
UBAP2L	332.5	414.5	338	550.2	375.6	630.6	258.3	513.7
UBASH3A	119.2	149.4	99.3	79.1	76.1	78.4	122.3	126.4
UBASH3A	84.5	81.6	91.1	89.7	83.1	85.9	73.9	87.1
UBB	24053.4	27831.6	22875.6	18796.5	15911.6	18055.9	25208.2	25342.5
UBC	24496.4	28207.6	17519.3	19244	16556.7	20068.1	27852.6	31421.5
UBC	23699	26699.2	18239	17643.4	15096.5	19111.8	27178.6	32266.3
UBC	107.2	127.4	93.7	108	106.9	103.4	121.6	128.5
UBD	82	91.9	90.6	88.7	88	86.1	84.7	89.4
UBE1	1163.1	1449.5	1489	1334.1	1049.6	1672.2	869.8	1407.5
UBE1	283.3	291.5	146.9	140.2	126.4	85.6	160.6	197.2
UBE1C	926.5	1199.1	402.3	410.6	295	574.6	677.2	1212.7
UBE1C	272.5	286.2	120.4	124.1	109.9	131.5	243.9	356.2
UBE1DC1	726.1	872.4	329.3	284.3	189.2	360.4	503.5	872.6
UBE1DC1	101.6	113.7	95.6	83.1	80	80.5	96.3	111.3
UBE1DC1	254.1	281.7	133.6	136.4	122.5	162.3	206.6	376.5
UBE1L	809	956.1	635.2	548.7	560.2	656	585.5	630.1
UBE1L2	520.9	718.6	276.4	201.4	192.2	259.1	563	844
UBE2A	124.3	141.4	100.6	87.7	84.4	85.6	105	150.5
UBE2A	2308.7	3086.8	2004.4	1345.5	1042	2089.5	1909.6	3033.8

UBE2B	116.1	135.5	94.8	103.9	112	106.6	126.5	148.4
UBE2C	4020.8	4313.4	1955.8	996.4	760	921.8	2920.3	4283.6
UBE2C	2905.4	3319.1	1749.1	906.1	620.5	926.6	2109.2	3279.7
UBE2C	206.4	214.2	178.6	136.9	136.1	139	136.3	180.6
UBE2C	86.3	91.2	91.2	81.7	85.5	80.6	79.5	82.4
UBE2D1	114.7	136.2	98.5	96.6	85.4	91.8	127.6	118.7
UBE2D2	265.1	336.7	165.4	200.3	172.9	208.6	226	344.6
UBE2D2	210.3	278.4	127.4	181.2	166.3	176.8	189.7	246.4
UBE2D2	170.3	187.2	122.1	113.7	103.4	99.6	165	208.5
UBE2D3	93.1	107.6	90.3	88.1	85.5	88.5	86.6	97.4
UBE2D3	3217	4043	1940.1	1606.4	1446.5	2138.4	2444.3	3757.2
UBE2D3	9302.2	12584.3	5867.3	4145	3328.8	5674.7	7922.1	10606.7
UBE2D3	105.6	133.8	100.7	96.2	89.8	87.1	112.9	138.6
UBE2D4	648.2	742.9	419.2	357.7	259.5	370.1	554.5	797.2
UBE2E1	6751.1	7852.5	3157.7	3605	2303.9	3560.2	5773.9	8656.8
UBE2E1	1245.1	1729.1	288.5	188.5	165.7	262	910.7	1004.7
UBE2E2	1666.3	2218	1010.7	925.6	684.3	1045.6	1458.4	1735.4
UBE2E3	708.3	860.8	312.6	246.3	225.3	353.8	504.9	581.6
UBE2E3	1830.5	2386.3	1833.8	1508.7	1175.9	1815.2	1434.1	3759.3
UBE2F	1876.4	2478.1	1653.1	1699.8	1608.4	2317	1798.5	1868.6
UBE2G1	456.9	726	320.1	343.4	261.5	296.5	460.2	553.3
UBE2G1	709.3	979.5	314.8	282.4	219.5	363.6	552.8	868.7
UBE2G2	1677.3	1710.8	1001.6	1446.5	1087.1	1437.7	1169.1	1790
UBE2G2	104.9	118.2	103.4	110.9	101.8	108.5	92.6	99.4
UBE2H	235.1	280.2	131	121.7	131.7	119.9	226.5	423.4
UBE2H	328	369.1	128	139.1	122.4	128	307.9	486.4
UBE2I	7440	9770	5597.6	3292.9	2807.6	3448.7	6916.6	8647.7
UBE2I	106.3	153.7	100.7	88.9	88.6	89	105.4	112.7
UBE2I	99.3	117.2	95.8	94.4	86.7	91.7	99.3	98.6
UBE2I	180.2	230.9	134.8	111.8	104.2	102.1	148.6	171.7
UBE2J1	939.5	1187.5	784	892.7	628.1	968.4	770.9	1003.2
UBE2J2	151.7	177.2	148.2	137.6	125.4	160.5	123.2	152.6
UBE2J2	396.5	502.2	196.1	152.7	154	187.1	343.6	307.1
UBE2J2	76.7	72.4	102.6	85.9	79.6	81.1	76.9	80.6
UBE2L3	665.5	807.8	484.7	574.1	437	421.1	604.6	659.2
UBE2L3	229.2	297.2	173.5	211	153.4	175.9	202.7	288.5
UBE2L6	87.9	92	100.9	96.2	97.1	88.7	82.7	83.5
UBE2L6	1126.3	1413.9	611.5	449	432.3	436	1128.2	1081
UBE2L6	276	351.8	182.4	150.9	141.2	138.1	252.2	277.3
UBE2M	5620.3	7021.8	3412.4	2400.7	2290.5	2814.6	5053.5	5250
UBE2MP1	101.9	133.3	97.7	90.9	85.4	79.3	90.2	109.9
UBE2N	1902	2589.4	986.6	1302.6	872	1293.8	1702.8	2077.9
UBE2NL	78.9	94.5	87	81.2	74.5	87.8	81.3	86.4
UBE2O	191.6	236.1	260.5	257.6	221.8	271.7	180.1	265.1
UBE2Q1	734.3	850.1	533.5	594.7	460.9	673.3	547.5	771
UBE2Q2	1159.2	1216.3	509.2	542.2	367.9	570.3	956	1529
UBE2R2	151.7	183.2	108.6	95.7	96.6	107.9	143.4	153.5
UBE2S	94.7	116.6	87.2	77.1	86.1	80.6	99.4	102.4
UBE2T	2712	3046.7	1063.3	618.4	448.3	751.4	1943.5	2584.9
UBE2U	105.8	113.9	104	102.4	89.1	96	104.5	103.7

UBE2V1	223.5	273.7	130	123.9	125.6	117.7	194.7	240.7
UBE2V1	101.6	108.2	96.4	77.6	84.2	84.3	104.2	100.7
UBE2V1	96.9	113.8	89.4	88.4	90.9	86.8	97.9	107.1
UBE2V2	75.6	88.2	84.1	80.4	81.8	80.8	82.9	80.4
UBE2W	103.4	118.7	93.3	87.9	84.7	93.1	117.4	112.4
UBE2Z	230	334.1	173.8	141	130	129.2	210.5	333.6
UBE2Z	490.3	645.6	362.8	398.2	351.5	510.9	486.8	1058.5
UBE2Z	155.2	165	105.5	104.8	103.2	98.3	122.3	171.7
UBE3A	1589.8	2025.4	820.6	849.2	664.5	1289.2	1237.9	1598.2
UBE3A	1115.7	1423.1	560.9	559.9	403.4	722.3	959.9	1336.2
UBE3A	105.4	121.1	109.9	103.7	94	96.9	102	121.5
UBE3B	137.3	173.8	112	111.3	95	122.7	163.4	221.9
UBE3C	1763.1	2252.8	1272	1676.8	1137.1	1953.1	1917.2	2318.3
UBE4A	614.8	767.6	259.8	316.6	235.1	379	574.2	892.2
UBE4B	311.9	426	179.1	222.5	198.4	241.4	369.5	544.3
UBIAD1	472.7	644.7	204.2	158.8	126	139.5	452.9	565.4
UBL3	669.4	830.6	348.4	521	362.9	624.5	537	802
UBL4A	644.6	832.3	616.9	407.4	345.7	466.5	611	630.4
UBL4B	98.8	107.3	86.4	78.1	83.3	86.8	97.9	113
UBL5	384.4	421	206.5	172	149.1	186.7	259.5	281.4
UBL5	4530.3	5095.7	1464.2	1025	745.2	1196.5	3956.1	4619.6
UBL7	325.2	420.9	201.1	208.8	229.2	168.1	311.1	330.7
UBL7	486.8	601.5	337.2	358.9	308.7	381.8	476.6	705.2
UBLCP1	1082.2	1152.7	434.5	594.6	372.2	527.7	992.3	1478.3
UBN1	116.6	143	98.3	85	78.9	87.8	112.2	142.6
UBN1	1260.2	1787.4	1483.2	1666.6	1404.2	1717.8	1354.1	1433.9
UBOX5	116.7	141	107.9	103.1	113.3	104.2	124.3	124.5
UBOX5	129.8	153.4	108.5	89.7	90.9	98.8	123.7	149.9
UBP1	1925.9	2446.1	1818.2	2088.2	1707.4	3755.4	1616.1	3291.7
UBPH	251.8	319.4	150.6	136.1	108.7	152	243.5	325.1
UBQLN1	923	1237.6	474.4	366.9	288.6	467.6	819	1458.7
UBQLN1	2770.9	3082.4	1829.8	1654.4	1114.2	1966.1	2971.6	4813.5
UBQLN2	1312.2	1498.3	654.3	707.9	525.7	871.2	1069	1819.6
UBQLN3	168.2	197.1	136.2	115.6	114.4	126.3	161.8	191.8
UBQLN4	1377.7	1704.9	973.3	944.1	687.6	935.4	1071.2	1289.7
UBR1	158.1	193.3	161.9	149.5	123.5	187.7	134.2	173.2
UBR2	750.6	897.7	465.3	621.7	482.5	885.3	663.2	838.8
UBTD1	196.4	233.6	229.8	200.2	167.7	236.1	182.1	318.7
UBTF	149.4	170.3	160.4	149	111.8	172.4	147.1	247.9
UBTF	146.1	173.8	98	94.7	84.9	90.5	125.7	151
UBTF	205.2	240.6	197.2	174.4	152.2	248.7	145.8	224.9
UBTF	107.1	133.8	94.7	89.3	80.7	90	113.1	123.8
UBXD1	418.8	532.6	402.7	323.6	298.7	333.3	426.3	601
UBXD2	1933.5	2275	1189	1531.3	1202.9	2455.2	1985.8	2676.2
UBXD3	98.9	105.2	147.7	130.2	135.3	140.3	96	105.7
UBXD4	570.6	712.2	474.8	453.2	393.1	546.6	523.1	738.3
UBXD5	88	121.4	101	101.3	79.8	91.4	114.8	116.8
UBXD5	93.7	104.5	91.2	91.6	78.9	92.8	85.9	100.6
UBXD5	118.8	136.5	96.2	96.7	94.8	101.2	117.6	128.6
UBXD5	304.4	394.5	302.7	217	205.6	376.7	234.5	336.6

UBXD5	97.4	118.8	96	91.3	79.8	75.7	99.5	124.5
UBXD6	447.8	568.8	127.9	93.9	93.5	141.8	400.6	645
UBXD8	2670.2	3539.5	1909.4	2543.7	1970.6	2947.4	2545.7	3263.8
UCHL1	100.3	124.8	98.4	83.2	68.6	81.5	101	107.9
UCHL3	2461	3158.3	1477.4	1045.7	757.5	1094.6	1686.9	3112.4
UCHL5	2557.8	3469.9	1399.4	1587	1074.6	1893.1	2030.5	3378.6
UCHL5IP	1544.8	1710.2	1127.9	1018.1	897.5	1165.1	1219.3	1202.4
UCK1	147.5	162.3	133.2	120.6	112.6	118.1	144.1	169.4
UCK2	1551.4	1831.9	1139.1	821.6	639	1091.3	1212.9	1405.9
UCKL1	844.6	975.6	888.1	1034.4	854.5	1409.3	627.3	1405
UCN	234	229.1	157.3	134.3	129.8	135.5	155.4	193.3
UCN2	88.1	121.5	95.4	84	86.5	86.8	94.3	117.6
UCN3	131.7	153.1	96.6	84.8	83.3	90.5	121.3	147.2
UCP2	609.7	956	970	744.8	583.8	660.1	607.7	1338.5
UCP3	92.4	108.6	89.4	86	72.5	83.7	102.6	109.3
UCP3	118.4	130.6	101.7	100.5	103.5	110.9	114.3	125.4
UCP3	117.8	143.4	94.7	94.1	88	108.4	123.2	157.5
UCRC	1027.1	1258.9	717.5	513.8	377.4	511.5	907.1	1139.1
UCRC	514.9	645.8	339.9	452.6	333.1	483.6	433.9	759
UCRC	446.7	629.5	400.7	576.2	436.3	502.6	448.5	393.5
UEV3	285.6	343.9	114.7	111.3	103.2	130.7	245.1	349.5
UEVLD	154.3	187.3	101.1	96.8	90.7	95	141.7	192.6
UFC1	5825.4	7997.4	5117.6	3063.2	2639.2	3677.7	5689.5	5995.5
UFD1L	187.9	244.4	155	135.6	136.7	142.3	184.8	245.5
UFD1L	150.6	202.4	135.4	102.2	105.9	109.7	143.7	196.1
UFM1	209.6	238.1	147.1	112.9	123.9	160	209	357.8
UGCG	518	526.7	238.3	196.9	169.2	159.9	514.2	686.7
UGCGL1	79.2	87.3	98.6	84.8	74.9	95.2	82.6	93.5
UGCGL1	161.5	242.7	145.7	222.9	212.5	189	150.8	182.8
UGCGL1	269.6	442.6	243.5	523.8	346.8	326.2	326.1	418.4
UGCGL2	266.5	346.8	205.6	178.4	181.1	244.7	231.9	259.6
UGDH	643.8	810.2	282.3	336.6	273.7	389.8	630.6	947.2
UGP2	1170.3	1237.6	557.1	679.8	388.5	845.3	1211.8	2146.9
UGP2	101.9	114.6	104.2	96.8	89.9	97.9	96.3	98.5
UGP2	3899.1	5137.1	2064.7	2035	1578.5	2912.9	2791.2	4799.9
UGT1A1	99.4	99.6	91.2	89	85.4	97.9	90.7	100.9
UGT1A10	87.1	96.3	82.5	78.7	70.2	82.2	77.9	80.4
UGT1A3	121.3	125.1	93	82.2	83.2	79.2	116.8	131.9
UGT1A4	89.6	107.1	88.7	80.2	74.9	84.9	85.3	104
UGT1A5	95.8	109.8	88.6	90.7	78.4	84.4	102.2	108.1
UGT1A6	82.9	96.1	88.8	93.6	84.9	81.9	83.3	95.7
UGT1A6	79.2	101.3	100.3	87.5	88.8	81.7	83.7	85.9
UGT1A6	75.6	81.1	90.9	88.9	88.2	79.1	76.9	86.7
UGT1A7	86.2	90.1	79.2	82.9	76.1	89.6	87.9	82.4
UGT1A8	80	90	84.6	90.9	83.2	85.5	86.2	88.5
UGT1A9	88.3	95.2	102.9	98.4	90.3	94	86	91.2
UGT2A1	77.2	80.3	86.6	77.6	85.5	78.5	76.4	78.9
UGT2A3	85.7	98.1	92.5	92.6	82.8	100.2	82.9	95.5
UGT2B10	88.3	103.2	94.4	84.7	80.1	80.4	90.7	85
UGT2B11	93.2	102.5	102.8	101.1	90.4	95.8	98.6	105.2

UGT2B15	73.5	85.4	92.8	84	79	86.1	74.7	81.3
UGT2B17	83.4	96	92.6	83.2	94	86.7	87.2	79
UGT2B17	93.4	88.4	88.6	93.8	85.8	99.1	89.2	98
UGT2B28	77.1	91.5	97.7	93.6	87.8	94.2	81.2	89.3
UGT2B4	90.4	97.8	97.1	77.5	92.9	94.8	98.7	99.8
UGT2B7	86.7	83.7	90.3	85.6	85	87.5	89.9	85.8
UGT2B7	83.7	102.6	98.5	90.3	90.5	90.8	91.6	86.4
UGT3A1	90.3	93.2	110.8	111.5	100.9	111.8	82.7	85.2
UGT3A2	343.3	437.8	246.5	215.7	194.9	230.6	272	330.4
UGT8	83.4	86.8	94.7	77.1	77.4	89.9	87.6	79.8
UHMK1	339	370.2	265.7	287.7	232.6	126	271.5	383.4
UHRF1	279.8	410.2	232.3	143.2	142.8	114.7	167.5	166.8
UHRF2	478.8	622.7	217.6	199.6	222.4	356.2	323.6	454.3
UIP1	199	222.3	148.1	121.5	114.1	156.4	148.5	174.4
ULBP1	133.1	160.5	120.8	102	92.6	116.6	165.3	247.3
ULBP3	128	146.1	94.5	96	93.4	95.7	118.4	144.3
ULK1	960.1	1191.7	958	707.3	789.4	678.1	912.1	958.1
ULK1	1011	1258.2	874.6	608.3	693.9	627.1	926.1	913.1
ULK1	85.6	89.5	94.3	89.5	83.5	88.8	85.3	86.3
ULK2	127.7	183.8	97.7	81.7	87.5	90.6	124	154.3
ULK3	136.4	171.8	131	117.7	123	164.6	129.3	163.9
UMOD	78.7	91.3	100.9	84.7	77.3	81.9	70.9	86.7
UMOD	130.7	156.5	99.5	87.4	94.6	98.2	132.6	149.7
UMODL1	82	86.3	99.7	78.2	79.6	86.9	81.2	90.2
UMODL1	84.8	94.8	93.3	89.9	89.3	79.6	88.3	94.8
UMPS	150.1	174.6	125.7	99.9	105.7	126.5	121.6	140.6
UNC119	157.8	151.6	113.9	93.3	98.1	103.6	124.3	177.9
UNC119	379.1	461.4	220.6	179.6	151.3	204.1	309.9	320.6
UNC13A	82.8	91.1	92.3	91.3	81.1	85.8	82.8	82.9
UNC13A	136.6	165.8	106.4	111.2	95.9	95.5	147.6	194.7
UNC13A	103.7	133.1	93.4	84.4	84.1	85.6	115.6	113.9
UNC13B	116	143.8	105.3	100.2	95.7	114.2	106.6	141.3
UNC13C	85	87.4	92.2	84.9	84.4	83.5	82	90.1
UNC13D	114.6	121.7	137	131.6	126.6	184.4	100.6	112.6
UNC45A	794.2	943.2	428	420	413.1	502.2	813.8	1339.4
UNC45A	193.6	215.9	116.9	106.1	107.2	101.8	186	192.7
UNC45A	458.7	527.1	598	498.6	411.8	711.1	506.5	736.2
UNC45A	88.7	94.3	102.7	97.8	84.8	95	87	100.5
UNC45B	93.6	102.3	95.8	89.4	89.7	89.1	89.6	100.6
UNC50	4158.4	5155.4	3123.3	2809.5	2040.1	2956.3	3922.3	5298.9
UNC5A	102.7	109.2	93.5	83.5	84.8	87.4	93.2	102.6
UNC5B	105.3	117.4	106.6	90.8	95.1	96.1	94.8	112.7
UNC5C	157.1	207.4	129.8	106.8	110.1	118.5	163.8	154.5
UNC5CL	84.5	117.6	79.2	86.4	78.1	86.3	92.9	92.2
UNC5D	74	78.4	85.7	79	77.4	84.1	82.3	83.7
UNC84A	489.1	690.6	387.7	335.8	320.7	580.8	391.7	616.6
UNC84B	4607.4	6448.6	2959.4	3039.8	2750.7	3361	4205.5	4108
UNC93A	93.3	109.2	99.8	84	82.6	89.8	99.8	106.2
UNC93B1	1038.3	1320.8	1027.1	886.4	704.2	822	804.4	1645.5
UNCX4.1	88	96.6	100.6	91.5	90.9	86.1	87.1	83.1

UNG	185.7	218.7	101.3	88.9	82.9	87.9	136.8	160.4
UNG	2400.5	3085	1198.7	1029.1	871.2	1351.2	1406.4	2209.5
UNG	122.2	132.2	87.7	93.6	78.6	87.5	106.2	128.5
UNG2	255	283.4	150.7	103.2	106.9	112.4	196.9	248.4
UNK	76.6	93.8	91.4	97.9	84.8	85	84.4	91.9
UNKL	89.3	95.6	98.6	100.5	100.8	101.6	80.9	94.4
UNQ1940	135.3	146	93.4	86.7	83.8	94.8	131.9	132.9
UNQ2446	85.4	97.3	93	84	91.8	88.2	88.8	93.4
UNQ2541	76.7	89.6	93.8	84.8	73.2	78.7	75.4	90.1
UNQ3033	87.4	101	98.5	95.2	83.8	95.1	86.8	99.4
UNQ3045	102.2	111.9	117.4	106.9	100.7	98.8	94.5	97.7
UNQ467	107.7	136.1	112.5	106	99.8	108	127.7	112.8
UNQ470	117.1	133.9	98.3	149.5	166.1	160	116.8	128.9
UNQ473	98.1	92.9	97.6	98	88.5	88.5	95.4	108.1
UNQ501	3293.4	4232	2487.2	2453.4	2176.1	2107.9	3551.1	2965.8
UNQ5783	108.4	157.2	113.7	108.9	102.3	116.8	95.4	104.6
UNQ5810	94.9	99.6	87.4	90.7	89.5	84.8	92.7	103
UNQ6411	107.4	110	97.3	98	84.3	93.4	109.1	117.7
UNQ689	80.2	79.3	97.3	83.2	82.2	83.9	76.1	87.5
UNQ739	144.6	161.3	100.8	94.9	93.1	95.1	132.4	158
UNQ830	119.7	129.1	116.9	114.1	105.4	114.5	115.7	120.2
UNQ846	154.8	181.3	123.1	98.7	101.8	100.7	134.7	164
UNQ9217	123.5	146.1	101.8	105.2	84.5	118.2	117.7	137
UNQ9356	109.4	129.8	96	92.4	87.6	92.1	107.6	142.5
UNQ9391	117.2	134.1	89.5	92.2	83.3	85.2	105.6	127
UNQ9433	144.5	176.9	90.7	108.7	87.9	114.7	166.4	221.3
UNQ9438	99.7	105.7	85.7	91.7	84.8	96.6	97.3	105.4
UPB1	97.1	117.6	99.7	101.3	95.2	100.8	103.3	94.7
UPF2	2036.5	2596.8	1292.4	1446	1322.7	2383.1	1876.9	2391.4
UPF2	115.9	129.7	98.4	84.2	98.5	99	101.4	121.5
UPF2	946.2	1148.7	570.4	804.6	724.1	772.6	939.4	1056.5
UPF3A	528	652.3	311	410.2	355.4	382.5	435.5	826.8
UPF3A	845.3	1072.5	550.8	549.3	506	835.2	630.1	1246
UPF3B	806.3	1033.3	615.3	668	632.4	1085.2	739.6	1438.8
UPF3B	1318.2	1752.4	687.5	708.5	653.2	1358.1	1139.9	1689.7
UPK1A	97.4	111.7	88.1	97.1	92.7	88.8	87.3	107.7
UPK1B	110.8	119.2	92.7	82	90.4	94.4	102.3	132.2
UPK2	92.4	103.1	103.2	92.9	89.1	102.6	87.3	90.3
UPK3A	129.2	167.4	101.6	86.3	92.7	100.2	134	152.1
UPK3B	97.1	112.8	88.7	88.4	78.6	83.5	106.8	115
UPK3B	83.4	88	98.9	75.2	88	90.5	80.1	85.5
UPK3B	123.6	164.8	99.7	94.2	94.8	100.8	123.4	139.8
UPP1	102.6	119.5	89.2	93	83.9	96.6	96.8	118
UPP1	105.6	124.6	90.4	83.5	81.7	77.4	92.8	110.2
UPP1	225.6	279.6	164.2	155.3	146.5	162.9	234.1	247.8
UPP2	93.9	98.7	90.9	82.4	81.4	84.6	94.4	97.7
UPRT	809	1071.8	409.7	462.9	376	565.1	695.5	1049
UQCR	263	304.2	178.2	129.1	107	141.7	248.1	273.6
UQCRB	231.1	308.1	132.7	111	103.7	112.7	194.9	264
UQCRC1	1749.6	2519.7	1255.5	1125.8	861.8	1259.9	1742.7	2698.3

UQCRC2	1444.7	1957.5	394.1	362.8	223	471.1	1281.6	2093.5
UQCRFS1	8711.8	11327.8	7397.9	5326	4060.6	5663.8	6932.3	10816.5
UQCRH	21129.6	22003.5	16326.3	15549.3	14318.3	19278.4	16413.6	17159
URB	136.7	156.2	101.5	109	101	97.7	139.5	145.9
URB	72.1	97	97.5	81.8	80.6	82.1	92.4	92.8
URG4	292.9	366.3	307.4	241.2	261.3	329.3	311.4	401.8
URG4	292.5	331.7	331.8	292.4	259.2	355.2	299.1	551.1
UROC1	103.1	134	99	91.1	82	90.6	107.4	116.3
UROD	1796.9	2570.9	1051.9	1072.4	848.5	1219.3	1789.7	1922.6
UROS	3775	4588	2379	1638.5	1285.9	1638.6	3804.2	3167.9
URP2	850.1	944.5	1003.5	1296.3	1191.3	1508.5	994.8	1791.2
URP2	278.6	362.9	265.9	235.7	215.7	321.8	289.8	516.2
USF1	440.4	506.9	211	193.4	173.6	171.2	269.9	347.6
USF1	650.5	868.3	346.3	309.5	286.1	312.7	516.3	613.1
USF2	143.8	153.1	95.7	89.9	93	82.4	119.1	136.3
USF2	281.1	397.1	358.6	274.1	283.7	355.1	243.4	416.8
USF2	228.8	301.9	176.2	136.8	122.4	165.9	212.4	370
USH1C	89	104.4	95.4	92.6	88.1	88.7	96.6	100.7
USH1C	87.6	96.6	96	82.2	88.8	78.1	90.8	108.6
USH1G	143.6	166.8	107.2	105.7	102	106.4	140.1	155.6
USH2A	93.4	113.6	101.8	104.3	96.9	102	95.4	95.7
USH2A	115.9	121.1	80.3	96.6	82.2	88.4	109.2	116.3
USH2A	81.5	97.3	95	79.2	86.8	84.3	86.8	94.8
USH3A	116.4	152	105.8	109.2	98.7	105.2	117.2	131.7
USH3A	84.8	78.5	98.4	97.5	93.5	91	82.2	86.6
USHBP1	122.2	167.1	98.3	86.9	90.5	91.2	123.1	136.8
USMG5	237	293.8	169.6	158.7	135.7	161.6	218.3	280.7
USP1	91.4	97.8	91	97.5	83.8	82.7	87.2	98.5
USP1	1479.5	1766	603.6	577.3	445.1	740.9	1123.8	1783.1
USP1	568.3	650.4	269.2	271.1	259.9	195.8	447.8	443.2
USP10	757.8	1097.7	297.6	264.2	216	327	616.5	1074.7
USP11	262.7	293.2	202	148.3	143.4	169.8	229.8	328.2
USP12	183	186.5	99.8	90.4	89	99.7	149.2	222.4
USP13	506.2	689.7	292.3	427.5	330.9	500	482.3	673.2
USP14	443.9	572.2	348.4	442.7	362.5	450.9	453.5	1108.7
USP14	314.8	480.4	233.3	247.6	214	354.4	282.9	367.7
USP15	179.9	201.9	125.1	125.4	112.6	175.9	151.1	179.5
USP16	1010.1	1220	636.4	1054.8	784.6	2144	745.4	1522.2
USP16	84.2	97	97.3	96.9	89.5	89.5	95.8	99.9
USP19	93.2	95.6	110.9	101.6	99.4	107.1	96	105.6
USP2	100	98.3	87.4	81	80.4	87.2	83.9	93.1
USP2	157.4	176.9	122.6	107.5	101.8	114.1	148.7	140.1
USP2	93.1	100	104.5	90.6	95.9	94.8	99	105.1
USP20	90.8	105.2	90.1	96.9	82.3	88.5	95	99.6
USP20	108.6	163.9	97.4	97.9	84.3	94.7	121.6	136.4
USP21	580	792.4	507.5	249.7	210	288.8	461.8	811.3
USP21	149.1	196.5	114.3	89	89	80.9	130.9	162.1
USP21	389.5	502.8	331.9	197.6	181.3	223.7	321.2	531.3
USP25	108.6	110.5	107	98.8	94.6	96.1	112.2	119.3
USP26	208.2	261.2	138.2	139.6	106.9	130.3	228.1	258.7

USP28	90.7	106.3	99.5	86.2	91	89.8	93	96.8
USP29	101.1	102.7	92.8	94.9	86	93.8	95.7	96.8
USP3	1608.6	1904.8	1179.1	1081	763.7	1156.3	1447.1	2390.2
USP30	119.8	138.2	103.9	94.5	88.2	93.4	101.4	130.8
USP31	91.2	93.9	98.5	81.1	82.6	81	91	97.4
USP32	102.7	138	100.8	87.9	82.4	105.8	99.6	109.8
USP32	138.8	161.3	101.3	144.9	151.5	184.6	117.3	150.1
USP33	236.2	249.9	132.1	129.9	125.7	122.2	164.4	202.1
USP33	273.3	388.4	168.9	266.5	218.8	265.4	223.1	305
USP34	265	432.5	245.8	338.3	284.1	576.2	271.5	314
USP37	287.3	379.1	203.3	222.6	176.9	269.8	254.8	387.4
USP38	752.1	976.2	499.1	482.3	332.7	525.5	746.1	1083.9
USP39	1636.9	2313.5	1356.5	1021.8	956.3	1694.4	1436.1	1986
USP4	234.4	301.8	132.8	162.1	132.7	130.8	202.9	236.7
USP4	941.5	1208.1	619.1	685.4	508.4	690.1	901.5	1204.9
USP40	133.6	118	104	103.4	93.4	90.2	117.6	137.2
USP42	112.8	131.2	111.7	104.4	105.4	93.5	103.1	126.1
USP42	575.7	715.1	529.6	585.5	509.6	657.7	509.7	829.8
USP43	133.7	146.7	98.3	102	89	91.5	130.9	140.7
USP44	72.6	90.2	93.8	90.1	84.1	91.5	79.4	87.4
USP44	82.2	97.7	98.2	95.7	94.3	99.5	86	94.6
USP45	148.3	183.3	115.1	102.7	97.9	120.9	130.7	170.4
USP46	163.6	215.9	117.8	104.2	97.6	100.4	152.5	201.6
USP47	165.7	208.6	119.7	159.2	117.3	161.6	153.1	238.9
USP48	291.6	309.4	177.3	265.7	249.7	268.7	233.1	373.5
USP48	355.6	489.6	156.1	154.5	148.8	182.6	276.5	432.4
USP48	781.9	1059.8	282.3	399.7	274.7	442.1	741.7	1146.7
USP49	1177.5	1329.1	553	1072.5	965.5	661.9	1132.4	1662
USP5	707.1	845.1	920.9	779.1	633.1	1318.3	639	1414.7
USP50	75.3	92.8	85.2	79.5	80.4	85	80	81.7
USP51	96.1	94.6	93.6	101.2	88.5	90.7	92	98.2
USP52	303.1	339.7	228.9	262.5	232.9	391.8	206.2	317.4
USP53	128.7	127.5	102.5	95.2	89.2	96	101.9	121.9
USP54	91.8	94.6	93.6	92.3	90.4	92.2	86.4	95.3
USP6	96.3	122.5	85.4	78.1	97.4	114.7	84.4	116
USP6NL	221.7	226	141.6	130.2	123.5	160.2	184.8	266
USP6NL	83.5	81.1	90.5	85.3	81.1	81.1	73.7	83.7
USP7	264.6	391.1	256.4	180.3	171.6	189.9	289.7	350.6
USP8	347.9	497.8	180.4	200.5	180	303.5	366.9	568.3
USP9X	109.2	117.6	100.5	98.6	95.7	91.9	105.8	120.1
USP9X	581.1	906.1	350.4	541.6	434.3	702.3	642.1	801.8
USP9Y	90.6	100.9	123.6	103.4	100.1	102.9	85.7	96.5
USPL1	469.8	646	215	187.6	192.6	253.6	442.6	724.1
UST	115.1	146.3	128.7	96.7	107.5	123.2	118.1	161
UTF1	91.4	112.2	99	82.9	84	92.2	95.6	112.7
UTP11L	2189	2791.8	1145.2	893.1	637.5	1004.6	1803.5	2656.6
UTP14A	1454.5	1954.6	1056.7	896.8	701.6	1435.7	1049.2	1746.3
UTP14C	707.4	925.9	466	612.2	503.8	788.6	668.8	1000
UTP15	271.1	328	176.8	169.8	126.6	209.5	229.9	293.5
UTP20	106.5	121.6	100.6	96.6	76.5	99.4	96.5	100.8

UTRN	273.1	314.7	127.6	117	115.8	138.3	261.3	238
UTS2	172.9	201.9	126.5	112	107.5	122.1	150.8	195
UTS2	518.3	706.9	244.8	265.9	199.6	304.6	448.9	581.5
UTS2	355.5	499.8	219.3	200	158.3	206.1	344	462.9
UTS2D	85.7	98.3	91.7	95.7	84	88	89	93.5
UTS2R	101.8	120.9	99.7	94.3	84.5	79.1	90.3	123.1
UTX	74.2	94.5	92.1	89.6	79.6	78.7	78.2	88.8
UTY	100.9	107	104.1	92.8	88.9	91.9	95.9	112.5
UTY	171.3	171.7	97.4	120.5	114.4	143.9	143.9	180
UTY	94.1	100.4	90.3	80.1	78.2	92.7	95.1	93.4
UVRAG	342.5	511.1	228.4	227.1	209.8	269.3	303.9	485.2
UXS1	133.8	142.8	97.4	86.1	90.1	103	139.4	158.7
UXT	4389.5	5379.7	3466	2898.7	2274.3	3287.4	3262.2	5864.8
UXT	106.8	121.4	92.8	88.7	81.3	96.5	105.5	116.8
VAC14	102.6	127.3	111.1	89.8	95.6	92.6	116	126.9
VAC14	146.3	176.6	162.8	133	114.8	184.3	130.5	154.3
VAMP1	127.2	124	100.3	90.4	90.7	95.4	116.7	118.5
VAMP1	107.1	117.7	89.3	71.7	76.1	76.4	103.1	99.3
VAMP1	218.4	226.7	179.1	172.5	162.8	173.5	154.7	225.9
VAMP2	147.8	205.8	115.4	129.9	120.6	148.8	153.3	172.2
VAMP3	691.8	953.1	441.7	459.3	358.9	515.9	643.8	1266.1
VAMP4	238	309	146.2	110.7	106.4	125.6	220.7	277.2
VAMP4	298.5	348.5	156.7	124.6	114.1	162.4	284.8	498.3
VAMP5	200	250.8	191.7	208.5	183	273.7	198	271.4
VAMP8	14953.3	18463.1	12755	12815.3	12330.8	14674.1	15431.2	14097.9
VANGL1	127.9	147.4	119.5	101.1	102.7	98	123	160.3
VANGL2	74.6	95.1	104.1	92.9	81.2	92.2	91.6	100.1
VAPA	115.7	128.3	97	98	100	135.2	120.6	163.8
VAPA	95.7	100.9	97	97.4	82.3	99	100.2	124.1
VAPA	410.1	660.2	285.6	268.6	216.5	317.3	325.9	420.9
VAPB	93.1	116.2	86.3	88.6	84.6	84.8	77.4	97
VARS	132.6	156.1	123.9	142.9	126.2	127	134.1	161
VARS	2355.4	2526.8	2007.8	1353.6	891.3	1545.9	1881.1	3132.5
VARSL	304.4	351.4	282.6	185.3	158.9	227.2	238.8	197.1
VASH1	346.6	464.9	335.2	264.5	193.5	283.3	243.2	267.2
VASP	185.9	280.6	131.6	122.5	98.6	112.4	164.9	277.3
VASP	91.5	117.8	113.5	117.1	99.5	93	98.8	129.6
VAT1	290.8	355.1	214.3	207.6	170.6	256.4	291.7	410.2
VAV1	499	588.5	304.8	245.8	248.3	318.2	461.9	580.4
VAV2	163.1	178.5	116.6	107.1	104.9	118.7	164.7	161.7
VAV3	150.9	180.9	125.5	130.2	112.6	124.9	130.5	158.8
VAV3	701.2	966.4	405.3	375.7	338.6	484.2	606.8	729.4
VAV3	2113.4	2894.7	1618.6	1336.1	1235.1	2192.3	1706.7	2365.3
VAX1	93.2	102.1	86.7	84.2	75.2	86.3	91.4	99.2
VAX2	98.5	114.6	105	102.8	95.5	96.6	98.1	114.4
VBP1	4406.1	4869.6	2605.4	2727.4	2020.6	3436.2	4127	7698.8
VCAM1	85.9	86.9	90.9	75.1	85	82.3	87.4	92.2
VCL	1907.9	2343.9	1281.5	1114	824.2	1426	1467.1	2086.7
VCL	181.9	227.2	144.1	135.4	114.1	145.6	143	180.2
VCL	71.7	80.3	87.1	88.9	70.3	79.6	74.2	75

VCP	1595.1	2022.8	956.4	838.2	675.6	824.3	1279.6	1599.7
VCPIP1	606.8	754.2	360.6	356.3	297.9	586	569.8	925.2
VCX	97.1	113.5	93.6	111.6	111.8	127.4	94	109.6
VCX2	81.4	73	82.2	87.2	81.3	92.2	80.6	70.6
VCX2	87.9	104.9	102.9	88.2	83.5	100.1	93.3	85.8
VCX3A	107.3	108.8	95.6	97.1	80.8	88.5	113	97.4
VCX3A	139.3	158.8	93.5	113.7	107.7	135.3	125.9	141.3
VCX-C	93.8	109.9	116.5	140	107.7	146.7	91.2	97.7
VCY	103.2	96.7	81.9	96.7	83.5	99.8	90.7	100.9
VCY1B	77.6	92.1	91.1	78.3	71.9	83.3	77.3	90
VDAC1	9182.2	10460.2	8252.6	5756.7	3760	6329.3	6598.3	14680.1
VDAC2	110.9	111.6	78.3	82.8	78.4	81.1	97.3	107.4
VDAC3	13446.7	17073.3	10706	8633.8	6403.3	8757	12720.2	16541.4
VDP	1860.5	2378.5	767.6	651.1	591.2	1133.4	1526.8	2006.1
VDR	85.4	106.9	94	89	79.9	83.6	96.6	104.4
VDR	121.3	184.2	143.2	110.9	103.9	123.9	136.7	191.6
VEGF	109.4	153.9	104.1	96.6	105	103.3	119.5	116.4
VEGF	86.7	99.2	98.3	80.8	84.7	79.5	90.4	81.4
VEGFA	196.8	231.3	174.4	150.8	191.2	197	191	155.9
VEGFB	2171.3	2916.1	1431.2	1665.2	1442	1372.4	1870.1	1525.9
VEGFB	90.7	99.9	100	89.3	86.2	90.7	91.2	94.6
VEGFB	234.6	291.3	129.2	122.5	108.9	153.2	216.7	337.6
VEGFC	72.1	84.7	90.1	79.1	85.4	78.2	76.1	88
VENTX	127.9	139.3	94.3	89.9	79.8	84.4	113.5	122.5
VENTXP7	92.3	106.5	91.7	86.7	80.3	84.1	111.9	109.2
VEPH1	85.7	102.1	97	85.3	78.3	89.7	86.8	84
VEZT	82.1	87.7	101.7	91.7	99.3	87.3	73.8	97.4
VGCNL1	90.9	99.9	97.9	90	99.3	91.3	96.9	92.6
VGF	199.8	226.9	146.4	102.2	97.4	106.7	177.9	212.1
VGLL1	88.1	100.9	107.4	102.9	98	102	98.4	85.6
VGLL2	95.1	106.4	102.2	96.4	80.4	92.5	94.3	114.6
VGLL2	92.7	112	102.3	87.9	91.9	89	101.2	111.6
VGLL3	82.3	91.9	98.5	83.2	78.5	82.7	79.2	87.8
VGLL4	1015.3	1314.8	752.2	882.5	837.4	966.8	833.6	905.6
VHL	908.3	1082.5	286.1	323.4	241.4	242.1	829.5	1165.8
VHL	2298.2	2578.2	1801.4	1806.3	1465.7	1335.2	2101.9	3204
VHL	606.1	720	269.4	220.4	182.3	167.3	416.6	548.7
VHLL	90.5	103.1	95.6	84.6	85	87.1	86.9	96.2
VIL1	104.5	122.7	81.1	75.9	75.6	92.1	103.2	126.8
VIL2	4761.3	6591.5	4439.7	4385.6	4032	5949.8	4445.2	4496.8
VILL	112.4	128.3	122.6	130.1	121.8	137.8	96.9	111.1
VIM	6109.9	8096.7	2029.8	974.8	580.6	903.8	4671.4	8859.8
VIP	88.5	83.8	90.4	88	77.2	100.6	91.2	88.2
VIP	89.2	105.6	103.6	88	87.5	93.2	91.6	105.1
VIPR1	202.6	238.8	207.9	234.9	233.5	215.5	153.7	183.5
VIPR2	94.6	117.7	94.4	81.3	84.3	91.2	109.6	114.3
VISA	114.2	148.3	99.9	94	92.9	103	113	154.6
VIT	118.8	148.2	93.6	96.9	108.3	112.4	116.4	121.5
VKORC1	6083	7194	4799	4195.9	3345.8	3760.4	6042.2	7437.8
VKORC1	780.8	1039.5	293.5	280.8	262.9	384.8	813.5	1072.4

VKORC1L1	1072.6	1307.2	832.2	634.1	474.4	617.7	1118.6	1345.1
VLDLR	801.7	1017.5	459.4	477.4	419.1	783.8	687.4	1075.9
VLDLR	75.8	73.2	96.4	92.3	86.5	79.1	72	79.2
VMAC	140.3	126.8	112.8	113.1	100.5	109.9	121.7	117.2
VMD2	289.5	284.7	223.4	174.4	151.2	222.2	193.5	267.8
VMD2L1	104.9	117.9	88.9	74.7	75.4	91.3	104.9	118.5
VMD2L2	89	95.5	95.2	91.4	88.3	84.2	86.2	89.4
VMD2L3	91.1	95.1	96.6	90.7	85.4	99.5	90.8	104.8
VMD2L3	102.7	97.4	94.2	89	78.5	92.2	87.9	99.2
VMO1	117.9	126.1	104.1	93.5	88.6	91.1	119	115.9
VMP	90.3	92.1	96.1	88.3	83.2	85.8	79	86.1
VN1R1	90.1	94.7	85.5	78.8	86.8	80.4	82	93.4
VN1R2	96.8	103.5	113.4	99.8	83.1	96	106.3	105.6
VN1R4	94	94.5	98.8	92.4	88.5	100	92.7	107.1
VN1R5	114	121.9	94.9	107.1	85.4	87.4	111.3	128.5
VNN1	85.2	100.9	97	94.3	98.6	97.1	85	97.7
VNN2	117.3	125.7	107.7	108.3	89	98.5	110.1	118.3
VNN2	77.8	90.6	94.2	98.4	80.3	92.5	77.5	83.7
VNN2	76.3	85.8	91.3	73.3	85.7	86.7	85.1	85.4
VNN3	92.9	98.3	95.4	84.5	89.5	98.2	84.3	94.6
VNN3	91.9	89.9	96.6	90.9	89.2	90.9	84.9	93.2
VNN3	96.8	106.5	101.8	80	91	84.5	98.9	104.7
VPRBP	198.2	273.1	163.9	159.1	143.2	169.1	175.5	204.2
VPREB1	84.8	92.7	94.9	91.4	89.3	88.2	86.9	80.8
VPREB3	95.6	88.6	110.1	87.2	91.6	97.7	93.3	117.3
VPS11	224.9	298	145.6	139.1	132.5	140.9	201	211.3
VPS13A	78.5	88.3	93.2	88.7	76.2	88.9	80.9	77.4
VPS13A	184.3	228.9	141.3	136	141.5	162	141.3	168.2
VPS13A	101.4	123.4	99.6	92.4	102.4	107.6	115	119.7
VPS13A	164.7	214.5	113.6	148.1	111.8	156.5	154.4	182.5
VPS13B	85.8	88.9	82.8	82	81.5	78.2	88.4	93.9
VPS13B	150.3	145	118.1	125.6	126.8	101.9	122.8	158.1
VPS13B	93.8	103.8	99.7	87.9	87.5	82.2	102.5	94.5
VPS13B	119.7	124	99.2	95.6	97.4	84.7	98.4	123.1
VPS13B	113.5	119.2	101.3	95.3	93.5	99.8	96.7	119.2
VPS13B	156.2	159.3	117.5	132	112.8	102.5	129.4	173.6
VPS13C	112.3	162.4	105.6	140.2	133	175.9	111.2	127.7
VPS13C	161.7	216.2	110.2	169.2	168	120.4	140.8	166.8
VPS13D	83.1	90.8	88.5	103.8	85.9	92	88.3	83.2
VPS13D	89.3	96.6	87.6	86.2	78	84.7	96.4	117.7
VPS13D	100.1	115.6	95.1	101.8	95	100	99.3	106.5
VPS16	139.6	158.4	105.3	101.5	92.8	110.1	125	163.3
VPS16	762.3	913.4	406.2	423.5	443.4	490.8	573.5	441.7
VPS16	114.9	142.2	98.4	83.8	89.4	93.7	115.5	121.1
VPS18	225.3	293	279.9	274.5	209.5	324.2	185.6	255.8
VPS24	1608.7	2001.4	996.3	661.5	588.3	856.7	1482.9	1384
VPS24	100.5	131.2	98.7	87.5	93	95.5	94.2	112.1
VPS25	1044.8	1372.9	593.2	535.2	476.4	785.4	784.3	1021.7
VPS26	288.6	323.8	140.7	156.2	139	220.8	248.2	438.1
VPS26A	2633.9	3341.8	1401.3	1411.4	1042.6	1799.2	2225.1	3268.4

VPS26B	569.2	627.3	514.6	350.9	320	464.1	450.3	760.5
VPS28	1495.7	1978	1256.1	820.1	689.3	1111	1392.4	2255.8
VPS28	1195.2	1510.3	845.9	497	408.5	595.8	1064.1	1341.6
VPS29	5304.8	6172	3022.1	3349	2435.5	4073.2	4573.8	5344.1
VPS33A	241.8	318.5	227	221.7	180.3	207.3	218.2	287.7
VPS33B	470.4	607.7	374.1	374.6	305	484	377.9	571.2
VPS35	2887.5	3431.8	1403.3	1440.8	1143.4	1928.6	2334.1	3211.7
VPS36	900.4	1067.8	444.2	583.6	475.5	966.3	683.8	1014.6
VPS37A	697.7	812.4	306.6	267.9	244.4	347.4	640.6	1100.3
VPS37B	252.1	337.9	307.1	267.7	215.2	319.4	254.5	620.5
VPS37C	3417.6	3907.1	2493.4	2337.8	1781.6	2087.4	2549.1	3181.6
VPS37D	296.2	370.5	157.3	141.3	127.8	151.4	286	331.1
VPS39	158	209.2	179.6	206	177.1	266.3	163.9	197.3
VPS41	273.3	334.2	117.6	175.4	156.2	116.5	204.9	273.2
VPS41	368.9	416.2	221.5	293.6	250.7	217.2	299.9	301.5
VPS41	1417.2	1744	1204.3	1551.1	1083.4	1688.9	1085.6	1619
VPS41	101	114.3	103.6	95	108.3	107.2	110.3	154.6
VPS45A	649	870.5	539	429	395.9	693.5	463.8	736.1
VPS4A	260.6	345.9	245.5	185.4	157.4	233.4	237.7	481.2
VPS4B	1386.1	1797.6	1198.5	1367.3	1280.2	2499.6	1231.5	2655.5
VPS52	544.2	654.2	584.5	566.6	488.3	745.1	425.6	863.1
VPS53	97.3	110.1	97.9	88	91.9	89.9	101.2	105.9
VPS54	183.7	211.4	131.7	115.1	102.3	155.3	154.7	241.9
VPS54	237.7	293.6	203.1	188	156.4	246.9	237.7	392
VPS72	678.7	808.8	464.2	298.7	247.9	343.3	564	737
VPS8	189.1	262.1	224.7	227.4	251.8	294.5	185.1	280.1
VPS8	92.4	109.3	81.5	83.8	83.6	94.9	99.5	111
VRK1	1454.5	1721.9	521.2	368.5	314.8	487.1	1320.3	2568.4
VRK2	460.6	528.3	183.8	169.1	125.9	212.3	352.7	632.4
VRK3	295.4	321.9	208.7	195.1	182.4	212.2	315.2	376
VRK3	260	405.3	166.1	172.7	152.1	141.8	222.2	307.9
VRK3	827.3	1092.3	595	442.3	407	406.5	816.8	907.6
VSIG1	86.4	103.7	88.6	81.5	80.3	86.3	87.1	101.1
VSIG2	90.1	114.4	96.5	91.8	92.3	83.1	102.4	102.7
VSIG4	102.1	120.5	103.2	83.2	84.8	96.7	99.2	107.4
VSIG8	92.2	99.5	104.7	97.7	92	86.3	94.4	98.4
VSIG9	107.1	122.9	86.1	91.5	93.8	90.7	93.7	114.4
VSNL1	83	105.2	93.5	81.8	78	89.9	92.7	116.9
VSX1	77.4	90	93.9	100.3	81.7	85.2	80	83.6
VSX1	107.2	106.5	101.9	85.4	85	101.4	104.9	107.2
VSX1	80.1	75.8	77.7	84.4	71.5	83.3	77.9	83.1
VTCN1	82.5	89.8	85.2	80.7	81.4	75	83.5	96.7
VTI1B	489.9	696	166.4	160.2	131.9	149.6	430.2	667.1
VTN	81.8	93.9	83	81.7	78.3	76.2	81.5	76.2
VWA1	97.5	99.1	94.4	78.6	83.8	74.6	99.1	103.8
VWA1	115.1	138.1	113.6	108.4	104.3	107.8	112.7	113.2
VWA2	81	90.2	99.3	82.9	81.7	84.4	78.4	90.5
VWCE	98.4	108.2	102.8	95.3	81.1	89.2	102	116.9
VWF	83.1	93.6	89.1	95	89.8	92.7	98.7	132
WAC	1226.2	1692.9	705.1	632.6	543.8	1071.1	1030.8	1809.1

WAC	173.4	211.5	100.8	96.3	93.5	85.8	122.4	157.1
WAC	2175.1	2654.2	1505.4	1346.4	1149.2	2206.5	2281.8	4550.2
WARS	1099.8	1458.2	1000.1	678	468.1	930.7	1118.8	1822
WARS	1816.3	2264.5	1287.3	1079.6	668.1	1146.3	1929.8	2598.2
WARS2	113.3	130.8	107.3	102.1	92.5	97.9	98	142.1
WAS	5709	6644.7	3946.3	3412.5	2601.5	3626.8	4622.7	6581.2
WASF1	84.1	99.1	98.1	93.4	88.5	94.8	102.4	121.4
WASF1	121.7	139.9	115.3	95.2	104.5	117.9	128.8	136
WASF2	197.4	277.3	244.3	284.5	299.8	475.5	211.8	332
WASF3	89.9	86.5	110.9	99.1	88.9	91.4	86	102.8
WASL	383.3	420.7	324.3	441.4	352.2	573.9	351.7	530.3
WASPIP	566.6	665.3	385.1	658.6	507.7	875.9	468.6	911.4
WBP1	273.4	322.3	150.7	142.8	141.2	122.7	251.4	324.9
WBP11	1289.9	1634	1356.9	1498.8	1164.7	1886	984.8	2841.4
WBP2	3211.4	3636.7	3233.9	3727	2944.4	3606.7	3973.6	9506
WBP4	412.2	482.8	306.6	312.1	287.5	562.9	369.9	778.8
WBP5	150.7	167.2	86.7	90.9	83.9	98.1	152.6	218.8
WBSCR14	105.4	117.7	118.3	95.5	100.6	107.7	108.4	124.4
WBSCR16	267.7	370.2	161.9	115.3	112.8	96.6	255.1	327.3
WBSCR17	84.7	94	101.2	98.3	94.1	91.5	85.3	79.8
WBSCR18	255.8	339.7	199	275.2	307.6	273	231.4	393.4
WBSCR19	83.2	99.7	100.8	89.9	85.8	88.6	84.1	93.3
WBSCR19	73.5	86.8	88.2	83.7	96.4	89.1	80	87.4
WBSCR22	1976.7	2634.1	1520.7	1274	1067.3	1678.3	2049.2	2129.4
WBSCR23	90	99.1	90.6	98.3	80.9	79.7	97.8	104.3
WBSCR27	105.7	132.9	105	109.2	97.2	103.6	103.9	114.1
WBSCR28	89.7	103.6	99.9	84.5	88	81.9	90.9	97
WDFY1	3518.4	4599.3	2199.2	2364.9	1769.5	3858.4	2626.2	3105.4
WDFY2	581.8	774.2	494.1	660.3	585.2	849.8	387	457.9
WDFY3	134.7	166.2	104.8	101.3	102.1	98.8	136.1	154.3
WDFY3	143.2	176.2	102.8	103.7	91.5	97.5	135.5	182
WDFY3	78.5	83.5	83.8	96.2	90.2	87.8	85.4	86.9
WDHD1	121.3	132.6	110.4	103.7	85.9	92.9	117.4	142.7
WDHD1	290	358.2	177.5	180.1	135.1	250.6	234.4	329
WDHD1	252.7	300.5	136.1	127.9	121.5	111.3	177.8	226.7
WDR1	656.2	934.5	347.5	341.8	304.1	212.7	524.3	767
WDR1	7676	8781.1	6088.5	5759.7	4088.4	7262.5	6080	9357.3
WDR12	2015	2488.1	972.1	947	527	1174.1	1563	2594.5
WDR13	451.4	557.1	350.6	276.5	261.8	340.1	445.5	543.4
WDR16	101.3	126	84.8	85.2	82.7	83.4	95.6	110.8
WDR16	109.5	122.4	91.3	89.5	83.6	92.9	116.7	136.7
WDR17	109.6	112.4	98.3	93.3	84.7	91.8	125.9	118.7
WDR17	96.4	118.2	104.5	101.5	80.7	83.3	95.2	104.8
WDR18	3051.8	3784.6	2014.6	1108.3	861	895.8	2585.5	2541.8
WDR19	223.7	275.4	194.2	194.4	198.4	266.4	181.7	238.7
WDR20	361	447.7	271.8	249.1	220.6	273.9	389.5	541.9
WDR20	273.6	382.6	182.5	152.1	159.9	202.3	261	323.9
WDR20	191.8	248.6	136.5	116.1	110.5	124.4	188.4	217.5
WDR21A	400.4	473.2	339.1	296.8	218.1	404.5	288.6	460.5
WDR21C	142.3	139	101.4	97.3	104.6	97.3	119.5	148.3

WDR22	306.9	375.1	231.5	246.3	182.9	261.5	226.2	287.6
WDR23	172	196.4	126.5	112.3	101.1	84.5	150.3	177.2
WDR23	321.8	327.2	226.8	309.4	267.2	276.4	241.6	281.3
WDR24	109.9	131.1	143.4	130.7	119.2	169.6	99.4	120.7
WDR25	167.7	223.5	130.6	101	88.5	94.7	156.1	178.4
WDR26	286.1	353.6	185	228.2	229.6	342.6	267.8	496.5
WDR27	194.5	230.1	227.8	253.2	255.1	352.5	139.8	193
WDR3	135.2	185.8	136.5	108.8	101.8	111.5	130	128.8
WDR31	100.4	125.9	112.1	95.2	92.4	94.4	102.6	106.8
WDR33	134.7	151.5	121.8	202.8	186.8	276.4	113.8	117.1
WDR33	1532.7	2086.1	1056.8	1096.1	840.4	1467.6	1172.4	1488.1
WDR33	364.4	426.6	132.7	120.9	110.7	133.5	290.8	433
WDR33	110.4	131.9	127.1	134.8	111.2	155.2	108.4	161.7
WDR34	436.8	578.1	342.4	229.1	179	263.8	342.8	622.1
WDR35	101.1	116.8	106.7	95.2	99.5	96.6	100.4	110.4
WDR36	512.7	768.4	511.5	652.4	437.1	726.8	432.4	673.8
WDR37	536.9	655.6	546.2	550.3	479.6	621.6	468	978.3
WDR4	2534.3	3049.5	1827.5	1660.8	1111.4	1532	1809.2	2216.3
WDR4	198.5	272.4	130.6	114.4	118.3	132.9	176.1	205
WDR4	148.3	177	126	124.7	112.7	123.3	129.7	175.4
WDR40A	3308.4	4017	1674.6	1542.4	1161.4	1747.1	2454.9	3820.6
WDR40B	383.1	491	261.4	249.2	195.7	275.6	253.7	342.8
WDR41	1327.7	1717.4	615.7	459.6	376.9	485.7	1166.4	1101.6
WDR42B	74.9	85.4	91.2	83.6	84.3	88.2	78.6	75.8
WDR44	106.2	110.7	89.8	98.5	91.6	102.4	109.9	114.2
WDR45	159.5	191.6	171.9	177.1	205.6	243.7	162.4	261.1
WDR45	120.9	153.9	105.7	96.4	88.7	94.9	128.3	138.1
WDR45	178	193.7	104.8	104.2	95.2	99.7	161.6	168.6
WDR45L	467.8	535.3	356	255.7	264.1	299.8	390.4	527.3
WDR46	1062	1249.5	946.3	1064.2	858.5	1453.6	734	990.1
WDR47	90.3	84.5	92.8	84	81.5	91.5	89.2	97.4
WDR48	388.1	482.5	315.1	354.2	310.3	431.3	326.6	389.4
WDR49	319.1	377.2	213.6	222	227.3	302.6	255	334.1
WDR5	255.4	308.5	145.9	112.2	99.9	100	198	254.2
WDR5	785.7	900.4	1061.8	605.9	485.9	935.3	692.2	1441.4
WDR50	1001.5	1305.3	392.5	260.2	286.3	546.8	699.9	1196.6
WDR51A	2412.7	3451.1	1853.6	1309.6	1140.6	1239.8	1932	1693.1
WDR51B	299.5	354.5	107.2	120.3	108.5	155.3	262.8	374.8
WDR52	217.7	274	121.8	105.5	107.9	118.1	195.1	240.3
WDR53	432.5	529.5	228.7	237.7	208.7	280.3	353.4	448.2
WDR54	2231.2	2858.3	1749.4	1086	943.8	1193.4	2027.8	2108.3
WDR55	491.3	629.5	273.8	328.1	255.4	413.2	360.2	595.5
WDR57	2179.5	2850.3	1859.1	1629.5	1365.9	2477.8	1734.2	2016.1
WDR58	711.4	982.8	635.2	403.1	346.9	536.5	559.4	688.6
WDR59	701.8	816.6	544.4	652.2	541.8	751.2	601.1	879.8
WDR5B	145.9	142.4	132.1	154.4	134.9	158.7	102.2	144.8
WDR6	1295.2	1275.5	1815.9	1440.2	1517.3	2355.8	898	1118
WDR60	304.8	359.1	202.6	287.5	249.1	385.6	256.1	334.1
WDR61	3557.6	4329.2	3100.4	3291.5	2478.3	3971.8	3033.9	4718.9
WDR62	110.2	136.9	134.6	126.4	137.8	168.9	117.7	163.3

WDR63	106.2	146.2	92.1	86.4	78.3	94	113.2	119.4
WDR64	103.1	118.6	98.1	88.7	91.6	95.7	106.3	108.3
WDR65	119.7	143.8	84.1	88.3	81	88.2	109.5	136.2
WDR66	88.8	104.2	100.9	94	88.6	88.5	85.7	92.2
WDR67	1041.1	1321.7	479.2	323.6	296	398	832.6	1019
WDR68	1971.8	2728.1	1693.8	2362.1	1598.9	3170.9	1849.7	4040.5
WDR69	75.7	83.4	81.4	85	74.2	76.7	79	87.2
WDR7	92	101.5	104.4	92.3	91.8	82.7	99.1	92.6
WDR7	234.8	333	209.9	237.2	209.2	354.7	206	282
WDR70	1015.1	1118.8	730	1072.1	906.3	1773.3	760.2	1266.6
WDR71	1316.8	1858	786.8	483.1	356.5	514.7	1073.9	1574.6
WDR72	82.7	87.2	92.6	79	90.4	88.9	79.3	87.3
WDR73	447.8	492.8	343.3	318.1	297.1	238.4	393.7	511.3
WDR74	1154.3	1521.4	758.7	468.8	368.4	469.5	1047.1	795.4
WDR74	322.1	332.7	184.2	292.8	292.3	188.9	287.2	323.1
WDR74	560.1	755.5	277.9	185.5	131.1	192	445.2	571.9
WDR75	2311.3	2598.8	1461.4	1323.5	1025.5	2075	1908.3	2764.8
WDR77	831.7	1007.8	657.2	366.2	281.4	435.6	619.8	843.8
WDR78	82.7	97.5	99.1	90.7	86.7	82.8	83.5	96.7
WDR78	106.2	122.4	87.2	83.4	84.3	81.5	108.9	126.2
WDR78	90	100.5	92	90.7	86.2	84.2	91.5	98.8
WDR79	331.1	340	183.7	168.4	153.1	136.6	270.3	247.6
WDR8	809.9	975.9	838.6	619.4	559.2	816.4	718.6	1301.3
WDR81	110.3	139.5	96.1	109.5	105.1	120.1	101.8	138.3
WDRPUH	96.3	99.5	96.5	93.9	93	89.6	95.9	106.3
WDSOF1	211.6	258.8	120.2	102.5	93.7	103.2	183.1	357.5
WDSUB1	823.8	984.5	374.5	532.8	396.2	637.3	805.2	1217.8
WDTC1	108	130.2	87.4	78.1	86.3	81.8	99.7	134.3
WEE1	666.7	879.6	438.4	353.9	303.6	461.1	494.1	967.9
WFDC1	86.3	89.4	98.8	98.8	87.6	94.6	89.5	107.2
WFDC10A	90.6	90.7	116.5	110.4	103.3	101.2	86.6	83.2
WFDC10B	76.7	99	89.5	78.8	77.1	75.5	84.1	83.7
WFDC10B	200.7	218.9	93.5	90.7	95.2	93.8	194	189.2
WFDC11	74.2	100	89.7	89.8	95.5	92.3	87.3	88.6
WFDC12	71.8	84.5	91	98.9	87.1	91.3	82.3	86.5
WFDC13	90.9	115.5	90.3	69.7	75.1	74.6	99.6	116.7
WFDC2	84.8	100.9	90.5	81.1	79.9	82	91.7	95.9
WFDC2	143.3	141.9	105.2	97.4	94.2	104.4	119.6	152.1
WFDC3	112.4	123.5	99.6	97.9	88.2	86.1	109.2	119.3
WFDC3	145.9	175.5	111.3	112.8	116.5	115.9	142.4	168.4
WFDC3	99.4	111.2	87.3	93.9	85.3	88.6	99.8	109
WFDC5	81.4	95.5	92.5	90.8	79.5	83	76.8	86.8
WFDC6	87.3	97.7	101.6	77.7	88.8	92.5	84.2	87.2
WFDC8	96.9	104.7	94.1	91.9	90.3	88.8	105.6	100.7
WFDC8	108.6	127	91.5	90.4	81.6	88.9	101.4	125.5
WFDC9	74.5	77.5	88	88.3	81.2	91.3	77.5	84.2
WFIKKN1	87.2	108	100.6	81.8	82.3	82.1	85.4	102.5
WFIKKN2	78.4	95.5	74.8	69	73.8	86.3	79.8	91.3
WFS1	110.5	117	112.9	100.6	98.6	102.5	109.8	129.7
WHDC1	172	220.7	117.7	145	151.6	137.5	154	192.1

WHDC1	159.6	208	112	104.9	90.8	127.5	160.7	203.4
WHSC1	98.8	128.1	92	102.7	91.3	84.8	97	126.6
WHSC1	140.1	157.9	110.2	93.8	95.9	99.5	120.8	153.3
WHSC1	114.2	130.8	103.8	103.4	86.1	103.4	108.8	148.5
WHSC1	102.5	112.1	96.8	77.5	92.7	86.8	96.5	105.7
WHSC1	118.5	161.7	88.8	89.7	87.4	87.2	102.9	147.8
WHSC1L1	127.8	177.2	88.8	93.8	78.2	94.7	140	168
WHSC1L1	161.1	173.9	107.8	99.3	93.8	99.7	136.4	170.4
WHSC1L1	216.4	287	171.8	178.2	143.1	205.1	189	251.3
WHSC2	149.3	202	129.6	108.5	108.4	104.6	145	241.9
WIBG	612.7	789.6	340.3	446.7	407.1	695.4	593.5	857.4
WIF1	127.4	164.5	97.6	85.2	88.4	82.4	123.6	151.4
WIPF1	83.4	93.6	92.3	99.2	100.2	118.1	85.9	86.2
WIPF1	304.9	414.1	157.4	179.7	151.9	201.5	256.2	414.6
WIPF3	82.4	88.2	96.2	89.3	82.5	94.5	78.7	83.8
WIPI1	105.8	114.9	115.4	123.9	124.6	148.5	121.2	160.3
WIPI2	352	522.9	248.5	262.4	231	288.4	378.5	501.1
WIPI2	99.1	111.8	91.5	87.8	84.1	93.7	105.9	103.5
WIRE	152.3	190.6	119.4	109.1	119.4	108.8	150.7	156
WISP1	78.8	92.9	88.9	78.6	79	85.9	76.9	83.7
WISP1	106.4	95.8	95.1	86.4	90.3	91.6	91.7	95.8
WISP2	93.8	101.5	94	88	87.9	85.2	92.2	118.9
WISP3	77.1	101.4	85	89.2	95.1	86.9	85.9	90.7
WISP3	80	84.1	98.5	79.7	83.6	86.8	74.8	93.6
WISP3	138.9	167.4	97.9	85.8	88	92.1	119.2	154.1
WIT-1	183.4	175.9	140.9	108.6	89.9	109.1	130.3	161
WNK1	170.9	212.9	204.3	244.5	223.4	220.3	163.3	216.6
WNK2	91.8	99.3	97.1	91.2	87.3	92.6	92.1	92.5
WNK3	125.7	136.3	96.1	94.6	80.1	93.1	103.3	125.9
WNK3	126.8	144.2	95.5	92.6	94.3	89.1	119	134.9
WNK4	93.3	104.3	111.9	93.5	87.2	98.4	91.8	115.4
WNT1	70.5	86	93.5	90.7	82.1	88	73.2	81.4
WNT10A	112.7	119.1	101.1	88.5	85.7	93.4	109.9	127.4
WNT10B	109.6	131.6	103	75.1	78.1	92	118.8	142.6
WNT11	93	97.5	94.3	89.2	81.8	96.7	99	95.9
WNT16	148.4	155.2	108.9	104.3	97.5	116.8	151.6	165.2
WNT16	99	91.3	108.3	87.7	92.6	89.5	92.9	92
WNT2	121.9	138.3	103.9	91.2	95.2	90.2	118.5	124.5
WNT2B	129.4	158.9	102.9	94.5	98.1	91.8	125.9	138.1
WNT2B	106.2	122.6	99	91.3	82.8	79.9	96.3	111.9
WNT3	298.4	317.2	195.6	206.2	218.5	173.4	196.5	179.5
WNT3A	91.7	88.8	89	86.3	86.3	88.5	78.7	94.5
WNT4	76.4	89.8	83.3	83	81.4	93.1	76.9	86
WNT5A	81.1	90.9	88.1	85.5	75.3	71.2	78.4	82.1
WNT5B	102.7	128.6	100.3	87	79.4	85.3	104.8	119.8
WNT5B	79.2	93.5	96	84.3	77	81.4	81.8	90.6
WNT6	125.7	166.2	104	101.9	90.2	110.1	131.3	147.2
WNT7A	101.8	111.5	89	91.6	81.5	77.3	97.6	106.7
WNT7B	106.9	125.8	139.3	119.8	122.3	136	105.3	112.3
WNT8A	82.6	97.7	100.9	90.9	92.1	92.4	83.7	96.6

WNT8A	137.6	178.5	96	92.3	85.3	80.4	147	162.6
WNT8B	78.9	78.7	88.6	81.7	79.9	83.5	67	88.9
WNT9A	111.6	129.9	103.4	91.3	87.6	95.3	100.7	117.4
WNT9B	117.1	131	86	82.8	77.5	77.9	104.8	135.7
WRB	595.8	734.6	283.5	251.8	267.3	242.5	494.1	762.2
WRN	701.1	923.2	593.9	553.5	480.9	838.1	614.1	939.2
WRNIP1	205.4	329.6	161.8	124.1	121.5	110	186.4	241.3
WRNIP1	1014.6	1211.4	884.5	1261.5	886.8	1595.3	907.1	1826.6
WSB1	915.2	972.9	368.4	365.2	349	398.6	1060	1738.6
WSB1	893.2	837.2	440.2	520.8	471.3	664.1	742.6	1509.2
WSB2	231.6	285.1	127.5	111.7	103.9	142.4	234.2	371.6
WT1	273.7	367.2	248.4	137.9	127.8	199.4	203.2	245.2
WT1	352.1	517	275.1	161.9	125.6	195.7	266.6	389.7
WTAP	1730.4	2156.1	1089.3	940.1	689.4	995.8	1377.9	2386.2
WTAP	164.8	176.1	121	110.8	108.3	123.3	116.2	129.2
WTAP	165.1	194.1	128.8	111.2	111.5	135.7	152.8	174.9
WTIP	95.3	104.1	84.8	81.5	84.7	85.8	94.3	105.2
WTIP	198.2	204.1	109.4	105.6	104.1	115.3	165.7	170.2
WWC1	463.6	581.1	277	212.1	186.4	255.4	311.1	329.3
WWC2	123.9	140.2	112.5	89.5	95.8	105.8	114.7	134
WWC3	130.6	142.2	156.3	159.8	136.1	179.2	110.8	136.3
WWOX	100.6	107.8	91.1	86.3	84.4	87.5	101.2	109
WWOX	172.4	217.2	138.7	109.5	98.2	99.3	158.8	181.3
WWOX	102.1	104.8	80.7	77.8	72.8	79.3	87.9	113.6
WWOX	200.7	285.5	129.3	103.9	108.5	94.8	195.2	209.7
WWP1	95.9	97.6	96.9	96.8	84.3	92.2	89	109
WWP2	82.8	102.1	81.8	84.6	88.9	89.3	75.7	80.2
WWP2	140	192	123.1	102.1	105.5	109.2	167.6	172.6
WWP2	420	559.6	269.6	263.9	233.2	314.5	390.5	411.7
WWTR1	93.3	95.7	85.6	86	80.8	94.4	88.5	109.7
XAB1	2005.7	2716.1	1714.7	1287.9	1016.2	1717.7	1549	2222.3
XAB2	773	930.6	761.9	551.4	402.6	819.2	715.3	1401.8
XAF1	722.6	815.8	365.6	318.4	324.7	398.1	501.2	499.6
XAF1	180.6	182.8	108	124.6	116.6	123.3	135	157.7
XAGE1	87.6	95.1	91.4	88.1	81.4	93.2	91.2	96.6
XAGE1	476.2	594.8	338.9	259.1	230	218.6	396.9	400.7
XAGE2	81.4	85.9	94.3	90	82.5	87.7	76.3	96.4
XAGE3	95.2	94.9	99.5	96	94.6	100.2	98	103.4
XAGE3	97.2	105.6	87.1	84.9	86.6	80.8	91.7	95.2
XAGE5	71.3	90.8	88.9	72.4	80.9	76.8	78.2	84.4
XBP1	4412.5	6212.9	2700.7	1602.6	1131.3	2316.1	4510.8	8702.4
XBP1	5511.1	7312.3	4597	3025.6	2286	3696.5	6129.5	8984.1
XCL1	94.7	95.2	91.6	89.1	81.6	87.1	83.3	93.3
XCR1	87.6	92.1	92	90.2	82.1	85.8	86.9	95.4
XCR1	81.8	93.2	87	84.5	82.1	86.7	92.5	105.7
XCR1	115	135.8	102.4	109.3	102.4	109	121	124.8
XDH	84.3	109.6	91.5	76.4	68.9	81.6	92.9	114.6
XG	69.5	69.9	85.5	90.9	79.7	69.8	69	77.3
XK	137.3	185.6	128.2	207.6	156.9	256.2	124.5	181.4
XKR3	86.8	102.3	93.4	88.2	71.8	89.4	89.7	100.3

XKR4	72.5	78.5	86.7	87.2	81.2	82.8	77.4	85.5
XKR5	98.1	110.4	88.7	83.6	84.7	88.2	93.9	103.4
XKR6	73.2	81.2	90.6	88.9	83.9	86.8	76	81.7
XKR6	131.5	152.6	91.5	82.8	83.8	95.4	118	152
XKR6	227.4	281.6	166.3	126.8	121.6	134.8	200.2	247.9
XKR7	92	96.9	91.5	85.8	83.1	83.1	95.5	109.9
XKR8	225.2	279.4	191.7	164.3	145.8	176.3	213.5	405.9
XKR9	77.5	88.2	86.2	87.3	80.9	87.2	83.5	87.1
XKRX	102.2	125.1	96.9	87.7	85.2	88.2	97.4	108.3
XKRY	85.7	89	87	82.6	77.5	80.5	80.8	86.6
XLF	94.4	110.8	103.2	93.5	81.6	91.9	95.9	103.7
XLKD1	78.6	81.2	90	82.7	84.9	87.1	82.4	87.3
XPA	125.1	153.3	92.8	84.7	77.4	90.6	119	161.9
XPC	536.2	738.5	608	769	606.4	1117.2	498.4	1045.4
XPNPEP1	2379.5	3098.6	1647.4	1127.5	852.3	1341.5	2141.4	3200.3
XPNPEP2	128.1	150.6	107.8	98.7	90.5	95.6	124.2	148.4
XPNPEP3	10721.8	10983.3	6359.8	6168.9	5981.4	4883.4	11235.3	16905.7
XPO1	2757	3093.1	2558.1	3480.7	2622.9	5669.9	2800.1	4387.9
XPO4	516.4	718.6	253.1	281.7	192.2	301.5	489.4	674.1
XPO5	850.2	1048.3	548	436.2	337.9	400.6	718.1	845.2
XPO6	646.5	844.9	543.7	491.5	405.8	590.7	513.1	765.3
XPO7	773.5	1098.9	576.6	476.7	369.8	596.2	700.4	691
XPOT	717.8	851.8	300.2	214.1	189.4	220	459.3	588.4
XPR1	1632.6	2144.5	1057.5	1228.3	809.7	1581.7	1595.8	2377.3
XRCC1	424.9	585.2	369.9	290.3	234	330.6	424.2	482.1
XRCC2	918.5	721.2	631.1	1131.6	923.8	653.9	850.3	1601.1
XRCC3	286.2	344.2	307.9	209.8	160	276.4	242	441
XRCC4	98.5	117.8	90.8	85.3	73.7	86.6	104.1	134.8
XRCC5	1609.3	2340.8	557.8	551.1	341.8	754.1	1157.2	1983
XRCC6	5243.3	6454.9	4700	3608	2981.6	4811.3	4635.4	6362.3
XRN1	113.3	133.7	102.2	106.9	96.8	101.8	102.4	112.4
XRN1	310.6	472.8	252.1	486.1	388.1	628.9	365.3	511.1
XRN2	86	89	84.9	78.5	80.6	82.3	84.2	94.1
XTP3TPA	3400.6	4536.7	2653.1	1352.5	952.4	1394.5	2842.8	3280.2
XTP7	82.6	83.2	78.8	78.3	79.6	76.7	80	94
XYLT1	88	94.6	121.2	116.5	113	110.1	86.7	93.3
XYLT2	727	828.4	457.6	523.7	445.1	546.8	562.6	720.5
YAF2	212.3	267.5	105.9	112.7	99.8	110	256.1	332.5
YAF2	97.2	91.2	100.1	94.3	90	86.9	100.2	101.8
YAF2	161	196.2	133.1	131.8	122.4	151.7	159.4	163.7
YAP1	99.1	100.8	93.9	105	90.3	96.7	82.9	116.1
YARS	3793.1	5261.1	1761.1	1897.5	1294.4	2175.8	3710.1	4356.3
YARS2	1130.2	1560	577.4	399.1	348.6	457	777.9	1295.6
YBX1	15747.9	19670.9	5314.8	5193.5	5677.5	6699.4	16082.3	20846.2
YBX2	94.2	115.5	100.6	91.6	88.7	96.3	102.2	119.3
YEATS2	357.4	480.2	366	536.8	443.2	803.7	389.4	633.2
YEATS4	1190.8	1486	604	623.7	496	852.9	887	2086.6
YES1	475.4	575.4	204.5	200.1	225.3	411	453.3	867.8
YIF1A	1196.5	1645.1	528.3	405.5	317.8	346.8	989	951.8
YIF1B	307.2	381.5	167.2	122.4	96.8	103	227.6	351.8

YIF1B	643.5	728.4	540.2	360.2	339.4	454.8	557.5	826.2
YIF1B	1667.5	1988.9	547	491	349.2	511.5	1515.6	2345.5
YIPF1	784.5	1038.2	496.5	566	430.5	621.1	706.7	1242.8
YIPF2	201.7	289.1	130.1	112.9	112.5	110.7	222.6	256.5
YIPF3	1718.3	2181.6	704.7	584	492.9	494.8	1680.9	2171.9
YIPF4	106.8	123.8	86.7	87.3	78.7	83.7	97.7	117.5
YIPF5	166	220.5	112.7	149.1	124.1	149.9	198.1	338.8
YIPF5	111.3	134	98.3	88.1	89.3	92.8	113.5	113.9
YIPF6	947.1	1178.7	580.9	791	685.8	868.4	907.7	1281.1
YKT6	123.7	139.4	88.4	80.9	87.3	78.9	120	152.7
YME1L1	1522.4	2099.7	841.4	656.5	538.4	843.7	1194.4	1747.7
YOD1	90.9	84	89.9	87.6	72.3	84.6	81.9	94.8
YPEL1	99.4	114.6	115.5	114.1	132.4	128.7	106.8	102.6
YPEL2	124.1	175.9	167.5	207.2	187.9	277.1	133.6	152.6
YPEL3	139	176.4	129.2	102.3	108.7	108.4	149.1	217.3
YPEL4	83.6	88.8	97.6	87.7	82.6	84.2	84.2	84.4
YPEL5	299.6	403.7	247.5	516.4	432.1	623.7	394.5	843.2
YRDC	679.8	890.5	421.3	406.6	278.5	396.6	590.4	889.1
YSK4	90.5	89.7	98.8	94.3	79.3	83.7	84.4	92.4
YSK4	80.3	87	92.5	87.7	84.5	83.3	77.3	81.7
YSK4	83	93.4	102.7	91.1	90	87.3	90	94.4
YTHDC1	139.6	174	98.6	82.8	89.6	93.1	132.1	187.7
YTHDC1	164.5	195.2	108.6	110.2	119.7	109.3	143.8	213.7
YTHDC1	2942.2	3516.9	2135.9	2630	2083.3	3707.3	2674.9	4217
YTHDC2	191	230.3	141.2	143.3	132.7	198.7	157.5	218.4
YTHDF1	1246.7	1476.2	809.2	642	493.2	672.7	1386	1890.5
YTHDF2	1336.6	1689.7	731.9	798.6	547.1	1014.9	1095.9	2756
YTHDF3	485.5	620.6	205.6	200.1	155	219.2	374.1	585.3
YWHAB	14554.5	16402.2	9037.3	12310.8	7876.5	10314	14374.6	19632.8
YWHAB	687.6	989.2	443.5	443	323	418.2	572.8	660.1
YWHAE	368.6	494.6	321.2	199.1	166.5	299.6	308.7	635.1
YWHAG	3424.8	3973.2	791.5	984.6	909.2	1299	3478.3	5620.2
YWHAH	10400.2	13131.2	10002.2	9574.5	6642.9	10042.8	9382.7	12536.2
YWHAQ	13902.1	18515.6	8363.3	7474.7	5667	8827.4	12564.4	16587.5
YWHAZ	10023.6	14046.2	7936	6638.2	4050.1	8349	7667.2	11520.4
YWHAZ	811.4	1057	217.8	167.6	132.7	179.2	461.2	676.4
YY1	1083.6	1284.2	424	382.1	294.2	533.9	1120.1	1897.9
YY1AP1	2081.4	2808.5	1210.6	1148.2	1053	1168.5	1713.7	1997.5
YY2	89.2	108.7	102.3	87	82.5	102.4	93.4	99.6
ZA20D2	3492.7	3645.9	2529.9	2200.6	1720.5	3293.9	3200.4	6313.3
ZADH1	300.4	285.3	196.1	176	173.8	164.8	238.3	377.9
ZADH2	387.9	501.9	236	380.8	355	286.1	327.2	392
ZAK	472.7	592.2	163.4	127.9	117.4	116.3	330.4	440.9
ZAK	1294.8	1434.4	969.5	848.8	588.7	956.9	1177.7	1916.1
ZAN	84.9	96.7	91.1	83.2	82.3	85.5	88	100
ZAN	115.6	135.3	97	105.9	91.8	107.9	127	143.6
ZAN	91.8	120.9	90.8	87.9	88.4	84.6	99.7	109.4
ZAP70	96.2	101.9	90.7	85.3	97.7	88.8	82.3	90.9
ZAP70	94	81.8	95.3	87.4	86.4	92.3	78.5	88.5
ZAP70	101.7	114.9	89.6	87.3	88.1	83.5	98.3	110.3

ZAR1	79.8	86.7	88.6	90	82.5	71.6	84.3	88
ZBED1	1118.3	1565.2	1236.2	1825.3	1873	2107.1	1042.6	1958.7
ZBED2	73.1	84.9	88	87.6	84.4	86.2	80.2	84.8
ZBED3	115.3	117.6	96	84.7	79.6	79.9	104.5	125.4
ZBED4	471.2	628	484.5	525.2	458.9	697.8	406.5	890.1
ZBED5	936.5	1138.5	276.7	220.2	241.7	271.4	678.8	809.6
ZBP1	362.9	481.9	195.9	160.4	127.8	175.5	370.1	452.9
ZBTB1	87.7	93.4	99.7	94.3	91.2	103.8	89.2	89
ZBTB10	126.8	187.9	96.6	90.5	94.5	83.9	117.7	137.9
ZBTB11	106	151.7	109.7	109.9	97.7	139.9	139.7	181
ZBTB12	95	105.5	102.8	103.3	97.9	98.7	97.2	89.1
ZBTB16	81.1	99.4	83.3	87.6	76.2	89.9	88.4	88.4
ZBTB16	79.4	87.1	96.9	92.7	97.3	95.8	77.2	77.6
ZBTB16	96.7	94.9	89.4	88.3	85.8	86.6	94.3	85.9
ZBTB17	267	316.5	293.3	213.1	194	278	219.9	410.2
ZBTB2	153	182.7	112.3	112.1	97.2	117.7	161.3	313
ZBTB20	110.8	123.9	104.7	153.7	166.8	167.2	105.1	110.4
ZBTB24	628	798	370.9	399.6	339.7	496	490.6	590.6
ZBTB25	213.4	260.3	165.9	123.2	116	159	169	213
ZBTB26	102.8	135	114.5	106.5	92.9	116.4	98.3	104.3
ZBTB3	228.3	282.3	111.9	113.6	101.2	108.6	199.2	237.1
ZBTB32	117.7	124.7	103.1	111	101.5	102.3	113.2	110.5
ZBTB33	2896.3	3615.4	2104.4	3111.3	2283.7	4409.7	2004.1	2687.9
ZBTB37	88.6	96.4	81.9	87.2	78.2	85.1	82.6	88.1
ZBTB38	130.3	161.3	85.9	80.1	87.9	100.3	110.4	150.3
ZBTB39	140.4	164.7	142.9	119.7	117.4	133.7	122.3	178.3
ZBTB4	382.9	433.7	348.3	441.7	351.1	542.9	367.9	597.7
ZBTB40	271.4	296.3	186.9	259.4	320.2	247.2	234.9	233.4
ZBTB41	110.6	146.1	108.4	95.4	88.4	103	107.2	124.5
ZBTB44	411.3	557.5	163.7	119.9	120.1	126.8	296.5	510.8
ZBTB5	197.7	219.5	210.3	185.6	165.1	212.3	198.6	330.4
ZBTB7A	93.7	118	99.9	84.1	79.7	88.1	112.7	133.4
ZBTB7B	113.4	126.8	126.2	113.7	106.2	124.9	114.5	134.8
ZBTB8	95.9	105	93.2	86	85.9	92.8	93.7	98.4
ZBTB8	151.3	186.5	87.2	92.8	106.3	109.2	164.8	206.3
ZBTB8OS	326	348.4	139.8	117.3	102	118.5	262.6	448.5
ZBTB9	479	638.9	364.6	328.5	240	390.4	368.6	626.9
ZC3H10	204.9	228.1	149.5	145.2	140.4	165.7	169.7	247.9
ZC3H11A	90.3	108.6	87.8	91.3	77.4	78.8	95.9	110.8
ZC3H12A	143.4	154.4	157.1	118	120.7	134.1	145.2	232.6
ZC3H12B	125.4	151.2	126.4	124.2	100.8	132.8	126.2	135.9
ZC3H14	519.1	684.3	329.6	324.8	243.8	404.7	351.3	547.9
ZC3H15	1837.9	2246.4	520.7	460.5	339.3	798.7	1185.5	2620
ZC3H3	904.7	1081.2	943.5	748	592	730.6	829.5	1180.2
ZC3H6	207.3	240.5	139.3	137.4	117.2	159.9	169.6	241.4
ZC3H7A	136.8	161.3	105.1	112.7	99.9	104.5	138.3	183.5
ZC3H7B	120.6	126.5	95	93.5	82	102.3	121.5	142.3
ZC3H8	351.5	411.1	113.2	132.9	101.1	149.9	356.8	326.7
ZC3HAV1	175.9	234.9	147.8	142.5	105.8	176.4	160.8	257.6
ZC3HAV1	296.1	403.7	152.1	158.5	121.1	153.6	238.9	371.8

ZC3HAV1	2220.1	3006.7	1176.5	1288.6	976.9	1247.1	1964.2	2230.5
ZC3HAV1L	108.8	120	95.9	94.4	80.4	92	113.7	109.2
ZC3HC1	2587.1	3266.1	1877.9	1377.4	1233.5	1568.5	2304.4	2543.4
ZCCHC10	93.9	91	106.3	97.3	88.7	90.1	81.1	100.3
ZCCHC11	306	374.3	238	255.6	238.3	409	237.8	411
ZCCHC11	192.1	232.9	150.8	155.5	156.6	260.7	167.5	254.2
ZCCHC12	94.2	111.4	99.7	87.6	81.3	92.5	91.3	109
ZCCHC13	87.9	94.2	81.1	80.8	75.7	82.4	90.8	81.4
ZCCHC14	323.9	461.3	299.6	397.9	347.9	459.7	318.7	492.8
ZCCHC16	77.2	77.6	100.9	80.1	78.4	79.8	75.3	83.2
ZCCHC17	991.8	1302.4	889.7	906.8	768.9	1331	860.5	1441
ZCCHC2	93.4	105	97.4	89	91.6	91.7	101.5	99.3
ZCCHC3	379.3	519.1	373.7	335.5	266.2	467.4	317.6	548.6
ZCCHC5	189.7	237.4	119.1	111	97.9	99.1	152.8	147.3
ZCCHC6	163	212.1	105.3	105.2	99.6	113.1	156.9	180.6
ZCCHC7	1449.9	1987.9	963	1036.4	833.5	1727.1	1163.4	1723.7
ZCCHC8	510.1	637.8	367.8	371.4	360.4	609.6	378.3	544.9
ZCCHC9	2167.6	2859.9	1551.2	1769.2	1326.4	2474.9	1761.4	3116.3
ZCD1	3288.2	3942.8	1965.1	1710.2	1081.5	1458.1	3275.6	4751.7
ZCRB1	206.5	248.9	160.9	177.5	132.1	198	229.7	321.1
ZCSL2	571	649.7	389.2	323.9	242.9	294.3	456.8	653.6
ZCSL3	447.8	508.5	332.4	243.6	205.1	343.9	340.4	1014.5
ZCWPW1	333.5	392.1	181.2	207.6	244.8	279.8	270.9	312.1
ZCWPW2	165.8	211.7	121.5	131.2	115.7	141.8	166	224.7
ZD52F10	95	110.9	88.4	85.9	79.3	75.1	88.8	105.3
ZD52F10	95.2	112.9	90.2	86.1	82.8	88.7	87.5	97
ZD52F10	87.8	94.5	102.8	91.2	94	98.1	85.5	97.5
ZDHHC1	246.3	263	192.6	117.6	103.7	118.3	169.2	193.6
ZDHHC11	144.7	165.3	107.5	128.3	156.8	189.7	139.3	148.4
ZDHHC11	94.7	105	115.8	93.2	88.7	99.7	96	102.3
ZDHHC12	481.3	631.8	501.1	374.8	313.5	470.8	403.4	755.4
ZDHHC13	489.9	715.5	431.9	326.2	279	474.8	436.9	606.3
ZDHHC13	373.5	525.2	166	158.4	140	182.7	342.9	518
ZDHHC13	194.9	232.1	110.5	95.7	98.2	115.7	155.1	214.8
ZDHHC14	308	394.2	194.5	195.9	170.5	185.6	251.1	334.7
ZDHHC15	85.1	91.5	84.7	76	75.7	85.1	85.6	90.8
ZDHHC16	547.4	666.3	444	321.2	265.9	380.4	474.9	683
ZDHHC16	102.5	117.9	87	89.6	84.1	83.3	94.5	105.9
ZDHHC16	2410.2	3302.9	1706.5	1166	987.9	1234.1	1936.3	1828.3
ZDHHC17	307.4	382.5	234.7	252.9	232.7	425.4	276.5	492.5
ZDHHC18	147.3	180.2	166.8	138.1	115.8	146.9	147.7	233
ZDHHC19	109.8	123.1	108.3	103.8	102.1	99.5	112	114.2
ZDHHC2	164.6	193.6	118.9	107.7	104.7	126.7	139.2	213.2
ZDHHC20	165.9	189.1	103.2	110.8	106.8	103.6	137.4	183.3
ZDHHC21	114.7	127.1	99	96.1	83.1	95	103.8	137.9
ZDHHC22	123.9	147.3	90	93.1	90.7	94.2	127.1	166.6
ZDHHC23	144.6	209.3	108.8	103.1	98.2	123.3	143.9	156.1
ZDHHC24	167.6	224.3	151.3	118.2	108.8	123.1	161.5	254.4
ZDHHC3	429.5	628.7	369.4	349.7	236.6	461.3	309.3	501.4
ZDHHC4	104.8	124.1	89.7	87	85.6	83.3	100.4	113.4

ZDHHC5	584.4	736.3	464	542.2	455.7	520.5	493.8	456.6
ZDHHC6	1809.2	2239.7	1285.4	770.4	579.8	1171.6	1424.4	2851.9
ZDHHC7	969.3	1289.5	663	818.2	646.1	841.6	1013.2	1463.9
ZDHHC8	1891.5	2269.3	1977.9	2121.7	1790.1	2372.2	1230.1	1383
ZDHHC9	1337.7	1674.7	686.7	362.2	336.8	358.1	1047.5	769
ZDHHC9	85.8	99.2	100.9	95.4	87.9	99.3	89.8	82
ZDHHC9	125.8	148.9	99.9	106.7	89.6	87.3	106	126.4
ZF	705	849.3	325.9	314.8	202.5	345	518.3	819.4
ZFAND1	590.9	782.1	279.9	257.5	199	285.1	546.1	825.8
ZFAND2A	1193.4	1451.9	631.3	478.9	485.7	430.5	1236.7	1684.7
ZFAND2B	623	717.4	537.9	484.6	428.6	415.9	686	921.4
ZFAND3	196	255	215.9	277.9	247.5	331.9	198.6	315.7
ZFAND6	303.7	359.2	148.6	166.1	118.3	172.1	314.4	636.3
ZFHX1B	794.6	1102	606.2	789.9	679.2	1788.6	796.3	1167.4
ZFHX4	75.2	83.8	88.4	78.4	78.2	82.3	83.9	84.6
ZFP1	131.5	179.6	94.5	108.5	93.6	109	129	181.8
ZFP106	713.1	1331.9	610.6	1010.3	870.3	1924	705	1021.5
ZFP161	519.3	730	342.6	341	301.7	491.9	435	534.2
ZFP2	143.7	159.5	131.8	141.7	142.4	171.7	128.1	157.9
ZFP260	353.1	442.6	294	355.4	306	379.3	311.6	456.8
ZFP28	115.3	120.8	106.4	110.2	101.1	114	99.9	116.1
ZFP3	167.2	189.4	177.3	173.7	175.4	233.6	169	184.3
ZFP36	1807.5	2103.9	735	685.8	594.8	607.8	1748.6	2011.1
ZFP36L1	95.5	111.5	83.7	88.4	98.7	106.2	110.5	138
ZFP36L2	2161.2	2572.6	887.9	711.4	752.2	995.4	1446.5	1405.2
ZFP37	90.3	111.5	84.8	100.5	75.5	90.7	93.5	101.5
ZFP41	96	134	99.8	98.1	96.2	100.8	114.3	105.4
ZFP41	94.2	119.7	99.4	89.9	82.2	78.1	89	103.5
ZFP42	92.4	108.5	94.5	99.1	89.9	91.3	97.9	118
ZFP64	119.4	132.7	122.9	123.3	118.8	129.3	111.7	130.8
ZFP64	131.4	171.6	123.6	110	115.5	108.6	120.9	137.3
ZFP90	1243.6	1360.7	538.4	643.1	459.9	626.1	869.4	1189.9
ZFP91	3698.7	4674.2	2347.8	2917.8	2370.7	4480.7	3424.1	4723.3
ZFP91	328	443.2	149.7	175.9	178.7	118.1	346.1	482.8
ZFP95	105.7	118.4	109.5	90	82.4	90.7	99.8	117
ZFP95	107.3	134.3	89.5	88.4	82.8	88.9	105.8	117.2
ZFP95	150.4	183.8	148.2	146.3	138	163.5	137	163.4
ZFPL1	195.6	230.3	169.9	146.9	123.4	155.4	175.2	285.6
ZFPM1	247.1	301.8	321.1	242.8	184.8	199.9	229.6	421.7
ZFPM2	108.6	112.7	89.7	83.1	80.2	79.9	104.3	126.8
ZFR	3637.7	4508.5	1154.3	703.5	650.1	1248.4	3093.9	5358.2
ZFX	164	221.3	127.1	139.1	133.9	171.6	155	201.3
ZFY	173.5	244.8	153.5	155.5	154.3	247.7	150.6	334.7
ZFYVE1	171.2	188.3	107.2	111.6	103.1	104.2	164.1	145.7
ZFYVE1	120.1	139.1	96.7	116.7	100.1	110.3	105.1	137.2
ZFYVE16	143.3	151.7	156.4	156.3	130.3	160.2	142	176.6
ZFYVE19	435.7	536	395.5	389.5	399	631	354.6	635.7
ZFYVE20	899.4	1232.7	575.4	882	635.7	984.3	812.9	997.6
ZFYVE21	784	1351.6	715.9	541.8	492.4	680	781.5	1481.6
ZFYVE26	286.7	396.9	298.5	437.3	397.2	583.8	265.6	331

ZFYVE27	111.2	133.6	90.5	98.5	76.9	87.7	120.4	134.6
ZFYVE27	120.2	155.6	128	112.2	111.4	110.9	131.9	186.8
ZFYVE28	90.4	100.2	93.3	81.3	81.6	79	95.2	93.7
ZFYVE9	97.9	119.5	100.7	104.8	92.5	94.1	92.3	114.3
ZFYVE9	88	83.5	94.4	93.5	80.4	83.4	85.7	83.1
ZFYVE9	80.8	97.8	97.1	93.6	82.4	91.5	89.6	92
ZFYVE9	93.5	103.7	94.8	98.1	89.9	89.1	86.5	91.4
ZGPAT	145.3	180.1	106.7	110.5	100.4	119.3	139	164.9
ZGPAT	110.9	141.9	94.8	93.9	82.3	86.1	111.4	123.7
ZGPAT	751.5	951.1	581.9	479.3	467	499.5	739.6	736
ZGPAT	94.5	101	103.5	97.5	100.2	102.7	99.6	101.2
ZHX1	359	448.7	161.1	133.7	128.7	230.8	281	429.1
ZHX1	129.4	136.2	104.9	96.7	88.9	91.1	111.8	130.9
ZHX1	218.1	334.3	141.2	113.9	115.3	158.1	197.6	231
ZHX2	226.5	276.1	154.2	178.2	184.7	201.8	181.4	166.5
ZHX3	230.9	354.9	128.9	167	135.9	181	208.4	211.1
ZIC1	92.1	105.3	98.6	85.8	70.4	95.1	98.7	106.9
ZIC2	1762	1981.9	1344.4	1317.8	977.5	1445.4	1883	2894.3
ZIC3	103.1	108.8	76.3	75.2	73.5	78.6	92.1	103.2
ZIC4	86.1	89.8	84.1	91.3	81.5	94.1	91.6	93.3
ZIC5	159.1	188	105.5	107.4	101.5	104	144.7	167.7
ZIK1	92.2	92.4	82.9	87.1	81	75.9	90.5	93
ZIM2	87.5	82.9	93.1	81	83.5	84.7	86.3	98.3
ZIM3	109.5	137.2	93.7	82.3	81.4	82.8	103.8	124.4
ZKSCAN1	111	114	103.1	113.1	89.2	102.8	116.4	208.8
ZMAT1	112.5	132.2	105.1	101.4	94.6	86.9	112	118.3
ZMAT1	86.8	82.1	99.9	86.8	94.9	91.7	89.3	90.5
ZMAT1	94.1	107.6	87	81.2	87.5	90.6	98.2	94.3
ZMAT2	1638.2	2094.8	1104.4	1058.3	885.1	1270.6	1494.8	1838.2
ZMAT3	3628.9	2868.9	1795.9	2310.6	1893.4	1646.1	3062.2	4867.9
ZMAT5	91.9	100.8	93.4	78.6	80.8	80.2	88	96.6
ZMAT5	297.8	377.3	208.6	172.8	126.1	162.2	280.1	344.3
ZMIZ2	199.3	218	137.8	129	125.7	130.2	166.4	195.8
ZMPSTE24	16363.7	21376	10223.6	9635.8	5746	11071.4	15551.9	20291
ZMYM1	410.7	491.5	316.6	331.9	314.8	436.6	371.3	457.5
ZMYM3	108.7	129.2	86.9	84.5	72.3	77.5	103.3	138.6
ZMYM3	219.9	291.9	162.8	195.2	200.5	252.9	192.8	249.1
ZMYM3	100.6	113.5	94.8	104.4	95.1	91.9	99.4	108.9
ZMYM4	556.2	784.5	424.3	837.2	676.7	1464.8	484.3	707.2
ZMYM5	211.6	256.7	126.5	150.3	129.9	147.9	176.4	227.5
ZMYM5	212.4	270	124.8	135.2	130.3	134.4	179.2	236.8
ZMYM6	80.7	102.6	109	103.3	92	82.3	90.6	84.8
ZMYND10	87.6	85.5	102.7	92.3	93	94.2	85.7	87
ZMYND11	141.6	139.2	120.5	102.5	98	99.7	120.5	164
ZMYND11	157	163.6	97	94.8	89.1	83.8	158.2	144
ZMYND12	131.7	169.7	117.3	146.1	125.6	162	122.1	150.7
ZMYND15	79.6	86	93.6	84.1	82.6	87.1	78.4	76.8
ZMYND17	113.2	111.7	108.5	104.2	89.3	102.8	105	108.2
ZMYND19	1933.5	2600.1	1531.1	1149.7	1023.1	1400.7	1622.8	1952
ZMYND8	286.2	406.1	185.7	178.6	151.2	233.8	229.9	261.7

ZNF10	127.3	135.5	126.2	128.6	102.1	162.7	104.3	139.8
ZNF100	95.4	88.7	81.9	87.3	83.6	84.9	88.4	93.3
ZNF101	236.3	283.7	170.1	151.7	136.5	162	200.6	279.7
ZNF114	107.6	116.9	115.8	91.4	89.9	111	105	121.5
ZNF117	83.7	87.8	90.9	91.9	86.3	83	79.4	89.6
ZNF11B	268.5	312.3	176.7	216.6	186.6	339.6	198.9	317.7
ZNF12	193.2	216.1	117.2	128.3	131.2	166.6	167.6	309.3
ZNF121	103.3	104.5	91.6	115.8	94.7	78.9	95.1	109.1
ZNF124	260.6	319.8	133.1	122.5	101.4	120.4	225.5	334.3
ZNF131	126.7	169.4	80.9	93.1	80.6	88.5	132	142.5
ZNF132	147.1	153.5	109.5	91.6	82.6	101.8	129	149
ZNF133	176	224.9	173.1	207.2	234.8	311.9	157.7	183
ZNF134	207.5	238.3	203	146.9	132.6	175.9	140.5	212.9
ZNF135	79.9	78.8	88.9	96.5	79.4	87.5	80.5	82.5
ZNF136	176.2	228.5	118.5	115.3	95	116.5	154.9	219.8
ZNF136	237	265.7	168.4	138.1	109.4	129.7	195	275.4
ZNF137	105.1	128.9	106.3	108.1	117.9	115.1	112.8	127.8
ZNF14	105.6	119.7	93.5	84.4	78.4	86.8	99.4	132
ZNF140	175.1	207.5	115.9	91	89.6	120.5	165.6	271.8
ZNF141	92.9	106.3	92.7	90.2	83.6	99.6	91.6	94.9
ZNF142	268.3	301.2	199	182.4	153.8	179	259.9	302
ZNF143	423.6	520.5	294.5	368.2	238.2	492.7	397.4	691.5
ZNF146	1536.2	1705.5	779	828.5	604.9	1128.5	1309.3	2323.1
ZNF148	1430	2096.5	1177.8	1572.4	1258.8	1223.4	1207.1	2043.6
ZNF155	113.1	135.9	95.3	86.8	94.4	84.9	110	124.3
ZNF155	153.6	183.5	114.5	86.3	84.6	87.4	134.1	161.8
ZNF155	127.4	169.2	100.4	98	92.7	94.1	113.1	120.5
ZNF157	94.7	114.4	86.4	85.1	79	84.1	102.1	118.6
ZNF16	311.2	396.7	217.7	191.2	161.6	195.5	316.9	370.6
ZNF16	92.1	100.5	96	91.7	97.8	89.8	93.4	94.9
ZNF16	187	210.6	142.4	117	114.4	141.1	166.5	212.6
ZNF160	192.4	263	182.6	184.9	201	214.2	168	255
ZNF160	86.6	107.9	101.7	117.8	107	95	97.1	95.8
ZNF160	274.7	391.3	189.5	304	313.6	376.7	266.5	379.8
ZNF161	994.9	1357.2	771.7	1096.7	832.8	935.6	969.3	1072.2
ZNF165	122.1	144.9	121.4	104.7	105.8	110.5	118.1	135.9
ZNF167	100.9	120.3	101.7	96.4	100.7	118.9	98	103.9
ZNF167	92.1	100.3	97	92.7	91	97.2	93.1	100.5
ZNF167	102.9	110.3	101.5	92	88	97.4	102.8	96.8
ZNF169	138.4	173.1	141.1	118.9	113	134.1	126.8	145.8
ZNF17	442.8	578.6	380.7	292.6	239.1	442.9	298.5	404.7
ZNF174	172.9	237.9	116.5	110.5	92.3	109.9	172.6	192
ZNF174	138.7	176	140.3	127.9	115.8	153.3	153.1	199.6
ZNF174	126.9	152.1	108.6	99.5	103.6	92.6	119.6	137.7
ZNF175	351.4	377.6	206.1	167.1	151.5	207.3	273.8	358.1
ZNF177	182.7	235.9	220	190.5	181.7	299.2	174.4	289.8
ZNF179	139.6	150.6	114.5	116.3	102.7	103.5	141.3	146.3
ZNF18	190.6	216.4	172.1	195.7	160.7	235	157.8	243.3
ZNF181	138.8	148.3	116.3	92.5	104.5	115.8	134.6	181.5
ZNF182	260.3	321	286.9	344.8	295.3	666.5	219.3	286

ZNF184	153.2	194.1	100.6	107.9	83.7	122.9	148.8	203.6
ZNF187	92.8	119.2	98.4	98.8	79.8	80.3	105.2	105.7
ZNF187	193.9	225.3	111.3	128.9	96.1	129.9	176	207.1
ZNF189	806.4	919.3	568.5	478.1	343.1	589	630.8	1048.1
ZNF189	79.4	93.9	101.3	86.2	83.2	88.6	85.7	91
ZNF19	95.7	114.3	86.7	81.6	78.6	87.9	80.2	89.1
ZNF192	121.6	149.6	104.9	91.5	88.1	91.1	117.1	136.7
ZNF193	235.7	259	175	171.8	171.2	224.3	188.2	259.7
ZNF195	820	1083.7	434.6	428.1	275.2	719.6	551.9	1066.6
ZNF197	209.7	272.6	152.5	141.2	136.3	176	215.7	276.7
ZNF197	121.9	131.2	103.7	88.7	88.2	95.9	107.8	111
ZNF197	183.3	224.7	145.5	139.5	126.6	190.6	189.1	305.7
ZNF2	304.9	367.4	184.5	185	166.7	162.3	246.4	283.7
ZNF2	95	111.4	85.1	89.7	79.1	94.5	90.7	99
ZNF20	297.2	417.4	223.6	234.7	201.4	267.2	248	311
ZNF200	108.1	104.1	90.2	88.3	83.1	81.2	98.6	109.5
ZNF200	345.6	490	167.9	156.4	132.8	178.2	261.1	404.7
ZNF200	752.8	1100.1	511.3	523.4	393.4	503.4	679.5	747.1
ZNF202	196	256.2	198.6	114	116	148.6	170	242.9
ZNF204	70.4	76.4	85.5	80.4	83.2	90.7	77.3	77.8
ZNF205	88	105.6	104.3	86.4	84	91.3	94	102
ZNF205	111.2	145.4	120.2	94.5	100.7	122.1	117.5	133.1
ZNF206	88.3	99.3	85.3	90.2	77.7	77.6	83.2	89.2
ZNF207	4706.8	5499	2752.6	2449.3	1515.2	2486.7	3675	5284.2
ZNF207	4915.8	6115.8	3189.5	2618	1902.4	2857.9	4513.3	6282.4
ZNF208	81.1	100.5	80.1	79.6	83.4	91.5	89.1	94.8
ZNF21	123.8	142.9	116.6	100.1	93.3	95.3	139.3	139.1
ZNF211	307.8	324.2	220	205.5	185.8	268.2	240	345
ZNF212	305.3	418.3	200.5	259.2	210.9	293.9	334.5	429.3
ZNF213	313.1	384.6	318.8	217.5	184.7	188.5	303.5	351.2
ZNF214	77.6	90.1	95.7	92.2	86.1	85.5	78.1	86.7
ZNF215	222.6	238.7	141.9	117.6	105.3	131	151.6	224.1
ZNF217	2024.6	3037.1	725.6	1059.9	697.6	1782.5	1682.6	2577.7
ZNF218	93.1	94.6	106.7	87.5	95.5	96.5	98	96.1
ZNF219	133.4	156.7	145.8	123.4	131.2	151.7	133.8	197.5
ZNF22	2270.4	2814.9	953.1	805.9	756.6	1189.6	1686.6	2146.9
ZNF221	90.8	109.1	83.7	83.3	76.7	85	91.9	101.2
ZNF222	129.2	130.6	109.8	109.2	90.9	103.6	119.6	178.7
ZNF223	424.4	458	228.1	349.9	361.2	260.6	362.6	382.8
ZNF224	219.9	254.8	163.3	142	117.9	177.2	168.8	243.4
ZNF225	170.5	209.3	154.7	144.1	141.1	200.7	164.1	193.5
ZNF226	157.1	160.3	103.6	102.4	85.1	121	149.4	194.3
ZNF226	162	174.6	119	124.6	111.3	128.2	158.2	220
ZNF227	222	252.9	133.4	127.1	108.5	136.4	229.4	343.1
ZNF228	121.5	121.9	98.7	96.6	91.2	94.6	110.7	113.2
ZNF229	80.2	93.2	98	77.9	83.4	79.1	80.7	92.8
ZNF23	145.1	186.4	90.4	94.6	80.1	109.7	151.6	181.9
ZNF230	208.7	275.6	188.5	174.6	154.3	272.4	157.9	204.3
ZNF232	170.6	179.5	133.5	82.6	89	83.5	164.2	229
ZNF233	80.7	86.9	87.5	88.5	75	84.1	82.4	88.2

ZNF234	333.1	466.2	184.2	182	178.8	155.4	304.7	386
ZNF235	158.1	186.8	172.8	146	137.1	186.1	156.1	254.2
ZNF236	136.9	177.2	125.5	137.5	128.9	158.3	127.7	142.3
ZNF238	93.6	103.2	106.6	105.6	103	106.8	88.4	98.3
ZNF238	80.1	94.1	83.5	82.2	78.9	76.4	83.4	81.9
ZNF238	121.1	133.8	120.5	107.8	97.4	108.1	113.9	140.4
ZNF239	366.5	406.3	249.3	205.9	150.4	262.6	241.2	287.7
ZNF24	239.2	338.2	185.2	148.3	136.7	225.7	187.1	265.4
ZNF248	156.2	174.6	107.8	90.1	100.4	121.7	139.7	162.7
ZNF25	682	818.4	361.5	327.9	293.5	379.6	471.5	637.5
ZNF250	298.5	414.2	237.9	188.6	174.5	195.8	299.2	331.8
ZNF253	81.7	96.6	91.5	74.9	91.5	81.5	82.5	97.6
ZNF254	208.3	247.6	123.8	137.8	121.2	195.9	178.1	231.2
ZNF256	245.8	337.1	171.3	128.6	104.7	144.8	201.1	249.1
ZNF257	77.8	79.7	96.9	97.3	81.8	91.4	81.5	80.1
ZNF259	581.3	721.6	228.3	200.1	151.6	303.9	466.4	732.4
ZNF26	372.6	475	326.8	334	310.9	539.2	282.8	418
ZNF263	1057.1	1140.7	484.1	349.2	323	431.2	960	1401.7
ZNF264	329	453.9	156.4	152.4	147.1	132.8	358.2	472.2
ZNF265	424.5	565.3	236.7	278.2	273.2	423.2	279.3	329.8
ZNF265	1930.7	2202.7	759.8	941.9	686.7	1354.8	1042.3	1724.5
ZNF266	359.5	456.6	178.7	194.7	172.5	191	327.8	495.5
ZNF266	171.7	188.2	129.7	116.4	115.4	146.6	167.8	293.3
ZNF266	96.3	94.6	111.9	107.8	98	98.7	97.9	109.6
ZNF267	89.7	115	93.1	73.1	78.5	83.5	88.2	103.6
ZNF268	88.7	95	98.7	89.5	79.3	85	88.8	97.5
ZNF268	82.1	92.7	90.2	88.2	81.9	96.5	82	87.9
ZNF271	495.8	580.3	276.1	247.5	227.5	402.1	429.1	523.7
ZNF273	181.9	202.1	119.1	135.6	109.7	181.2	162	220.2
ZNF273	88.2	84.3	111.7	99.6	82	101.2	80.5	77
ZNF274	1144.7	1391.2	966.3	667.5	624.1	891.3	976.2	1539.9
ZNF274	250.5	317.7	94.5	93.6	96.6	98	205.2	267.2
ZNF274	378.4	527.1	246.9	175.5	162.5	211.4	311.9	424.4
ZNF275	482.5	665.7	418.7	584.7	467.5	573.4	439.7	587.8
ZNF275	105.4	95.1	88.3	84.4	84.4	82.2	94.5	88.5
ZNF276	112.5	135.2	106.3	91.3	83.2	95.7	114.6	130.2
ZNF277	1101.5	1510	629.7	794.7	653.8	959	1062.9	1568.7
ZNF278	136.2	179.1	118.4	136.6	110	114.2	127.1	146
ZNF278	78.9	94.1	83.7	77.7	76	74.2	79.8	68.1
ZNF28	112.8	148.6	91.2	91.9	85.1	82.1	115.9	134.3
ZNF284	85.9	104.2	87.9	80.1	79.5	87.1	84.3	88.8
ZNF285A	156.4	177	148	125.8	108.7	122.8	129.2	150.3
ZNF286	140.9	167	129.4	118.6	101.8	127.6	127.6	175.7
ZNF287	71.6	82.9	82.8	84.6	78.1	88.4	78.5	83.9
ZNF289	474.5	631.6	280.4	279.7	269.2	284.7	375.5	386.5
ZNF291	181.3	241.7	136.4	162.6	181.7	215.8	183.9	207
ZNF294	95.5	106.7	111.6	102.2	92.6	105.2	98.7	97
ZNF295	231.1	284.9	223.6	215.7	186.3	288.1	215	414.3
ZNF297B	392.5	474.5	175.8	289.9	246.1	280.5	329.8	595.6
ZNF3	189.2	234.3	137.2	128.7	139.3	147.3	169.1	216.8

ZNF3	269.8	332.5	144.2	120.3	130.2	136	263	309.4
ZNF3	161.9	237.4	111.8	102	115.6	132.1	154.5	188.8
ZNF3	84	88.2	91	93.3	91.4	90.5	85.7	87.9
ZNF30	164.9	198.8	132.9	163.1	134.9	213.8	154	191.1
ZNF300	74.6	80.4	87.5	83.2	80.5	86.4	79.9	88.9
ZNF302	86.7	95	90.2	85	83.5	84.9	83.7	95.1
ZNF304	141.4	147	111.6	115.3	99.6	106.2	126.9	151.5
ZNF306	156.6	185.8	130.5	110.8	110.7	150.7	138.2	162.2
ZNF307	224.6	329.6	178.5	201.7	194.6	239.1	197.9	208.1
ZNF31	98.9	118.3	97.8	82.9	85.7	94.8	92	107.9
ZNF311	104.5	116	97.5	110.3	108.5	141.6	87.3	107.7
ZNF312	87.7	116.8	81.3	81.6	80	76.7	105.8	120.3
ZNF313	2229.9	2668.8	924.5	1097.3	799.8	1133.7	2130.8	3367.4
ZNF317	571.9	717.7	315.5	234.3	205.6	278.8	497.2	481.2
ZNF318	249.6	381.3	173.4	235.9	189.6	337.7	268.8	524.4
ZNF319	446.6	549.9	354.4	302.4	248	316.1	432.4	635.4
ZNF32	117.7	142.4	92.1	85	91.6	100.7	123.9	151.8
ZNF32	407.4	526.4	236	186.9	136.8	183	318.7	487.6
ZNF32	290.9	317.7	184.3	141.6	130	142.8	209.9	274.5
ZNF320	125.3	155.2	101	91.2	87.2	96.5	133.2	141.1
ZNF322A	159.5	193.1	93.5	91.7	83.9	129.4	139.9	152.1
ZNF322B	120.6	136.5	95.8	100.2	76.2	99.1	117.8	140.2
ZNF323	153.5	194.8	129.3	148.4	121.4	140.7	140.6	181.3
ZNF323	105.9	113.1	98.3	92.6	84.3	103.7	102.8	104.4
ZNF324	220.9	265.5	244.9	230.9	201.8	350.5	181.7	255.2
ZNF326	182.9	234.7	106	102.2	104.9	92.1	158.2	215.4
ZNF326	87.8	89.5	92.4	95.9	93.5	88.8	82.2	87.2
ZNF326	280	357.1	133.1	129	117	104.3	216.8	275.1
ZNF329	230.8	259.4	197.1	156	141.4	180.1	243.3	435.4
ZNF330	594.1	656	234.1	208.3	177.6	269.6	444.3	719.8
ZNF331	173.9	166.6	111	100.7	95.7	105.6	135.4	183.7
ZNF331	139.2	180.6	104.8	82.1	85.3	92.8	133.1	163.5
ZNF331	112.1	117.2	88.6	91.3	82.8	101.1	91.3	119.6
ZNF333	164.1	194.1	127.4	120.3	119.2	158	150.4	247.5
ZNF334	75.5	81.3	77.8	81.4	86.9	77.6	86.2	92.3
ZNF334	103.5	110.4	107.4	87.7	86.2	89.2	96.8	114.6
ZNF335	119.1	129.2	115.9	99.7	91.6	120.9	113.6	135.8
ZNF336	131	178.6	109.1	130.5	105.2	131.3	141	158.2
ZNF337	288.6	325	348.3	290.2	283.9	433.1	211.9	319.3
ZNF33A	278.4	305.3	129.9	131.5	120.9	185.5	223.7	293.3
ZNF33A	116.1	132	94.4	106.8	95.6	107.7	114	124.7
ZNF34	205.4	247.8	197.5	158.3	119.6	170	187.8	217.8
ZNF341	174.9	172.8	181.4	184.7	152.9	190	174.3	251
ZNF342	832.7	1106.5	569.8	448.2	350.9	351.4	731.5	811.1
ZNF343	138.6	145.1	100.3	78.3	75.9	104.2	114.9	144.8
ZNF345	94.2	99.6	104.8	87.6	93.1	94.9	90.7	94.1
ZNF346	129.2	137.5	136.3	136.5	137.7	162.8	122.4	137.9
ZNF347	97.6	107.4	93.6	91	80.8	94.7	93.9	104.1
ZNF35	361.6	467.5	304.7	277.2	227.3	395.1	339.9	397.1
ZNF350	187	209	187.2	138.7	143.5	174.8	159.6	196.5

ZNF354A	215.8	196.8	120	128.8	107.9	141	177.3	224.6
ZNF354B	79.1	91.9	84.8	88.6	95	95.4	84.2	96.4
ZNF354C	81.5	96.5	87.8	72.7	85.2	76.9	98.8	89.4
ZNF358	507.2	657.9	233.6	168.5	163.4	155.8	461.5	422.1
ZNF364	1265.6	1575	987.4	880.6	727.1	707.5	1296.3	1582.8
ZNF365	80.9	102.2	84.8	87.2	85.5	90.5	83.6	90
ZNF365	88.4	88.5	95.7	84.5	83.9	82.9	85.4	102
ZNF365	81.7	98.8	100.2	92.4	92.8	91	85.5	91.3
ZNF365	84.7	86.6	93.3	88.5	87.6	76	80.6	90.5
ZNF366	171.2	224.2	111.9	115.2	97.1	105.3	190.9	337.6
ZNF367	152.6	196.6	109.5	96	94	102.5	122.6	137.9
ZNF37A	95.3	112.4	104.8	91.2	89.5	102.4	95.6	94.4
ZNF37A	102.5	107.2	85.9	86.7	75.4	81.4	98.5	103.7
ZNF37A	125.5	133	100.8	93.2	83.7	88.3	129.7	115.2
ZNF382	84.3	84.5	92.5	79.9	76.1	84.5	82.8	97.6
ZNF383	179.1	199.4	117	113.9	102.6	121.6	156.9	249.4
ZNF384	131.6	158.5	99.1	97.4	90.2	98.9	128.5	136
ZNF384	162	207	173.3	160.5	136.6	218.8	144.9	238.8
ZNF385	137.9	189.6	187.4	227	205	313.2	137.8	217.9
ZNF394	1553.9	1492.4	1037.8	1317.8	1319.8	1109.3	1420.9	2655.2
ZNF395	338.9	446.6	307.1	399	385.8	504.1	280.6	426.9
ZNF396	117	138	108.6	106.4	97.1	122.6	122.6	132.2
ZNF397	150	179.7	115.9	119.1	99	135.6	127.9	150.4
ZNF398	216	255.5	110.4	97.2	92.8	102.4	190.2	256.2
ZNF398	227.3	244.7	211.2	177.5	163.9	229.6	217.7	359.8
ZNF403	1097.4	1406	1009.1	1245.6	1107	1873.2	1022.6	1777.9
ZNF404	89.9	103.9	92.1	87.1	96	88.3	94.3	100.6
ZNF406	110.5	125.8	89.8	85.1	90.4	93.6	117	108.9
ZNF406	120.8	135.8	92.6	83	90.7	91.9	123.3	145.4
ZNF407	76.1	89	90.6	92.5	88.4	85	76.7	82.3
ZNF408	141.7	166.4	152.8	138.6	109.1	124.2	142.1	233.6
ZNF41	97.9	111	90.2	89.4	83.3	78.6	94.8	90.1
ZNF41	92.2	113.3	93.7	76.7	81.8	74.4	108	108.8
ZNF41	97.4	121.2	104.9	105.8	99.8	121.7	105.5	119.6
ZNF410	979.5	1258	608.3	366.9	285.5	524.2	796.5	1146.3
ZNF414	115.2	142.8	137.2	111.1	119.3	113	118.9	166.3
ZNF415	93.5	109.4	113.5	102.2	104.2	100.6	96	112.6
ZNF416	196.8	291.8	207.8	161.2	140.3	197.6	194.8	251.8
ZNF417	145.8	188.7	152.4	127.8	125.6	106.3	126.1	154.2
ZNF418	96.6	103.1	86.4	83.8	76	85.3	97.6	93.5
ZNF419	317.3	424.7	192.2	183.2	147.8	213.8	268.4	374.4
ZNF42	528.9	454.6	452.7	412.1	440.1	502.7	313.6	338.3
ZNF42	141.4	133.9	103.1	94	88.1	81.8	103.6	127.6
ZNF420	108.8	116.2	98.4	92.4	87.6	91.1	92.5	138.2
ZNF423	80.6	84.1	99.7	91.1	83.6	88.7	82.5	93
ZNF425	126.3	143.6	99.4	126.5	114	128.5	156.4	197.8
ZNF426	180	209.3	144.6	152.3	121.7	151.8	158.2	211.7
ZNF429	174.3	212.2	108.9	98.1	96.9	96.2	138	183.7
ZNF43	91	110.2	95.2	87.6	79	82.5	90.4	105.4
ZNF430	4974.3	5489.1	1573.5	2824	2400.4	1855.5	4882.3	4861.1

ZNF431	293.9	338.4	129.4	131.7	145.4	170.6	245.4	341.5
ZNF432	137	166.8	114.3	104.8	111.8	115.2	130.3	160.2
ZNF433	208.6	223.8	135.1	138.8	148.4	199.3	186.3	271.1
ZNF434	568.1	762	383.7	343.7	280.6	468.8	576.9	846.4
ZNF435	154.5	179.5	121.9	114.5	112.8	131.3	129.2	215.4
ZNF436	136.7	172.3	94.1	90.9	84.2	95	125.3	148.4
ZNF436	86.8	88.5	85	86.2	80.8	80.6	82.7	82.7
ZNF436	126.1	130.1	114.4	93.9	77.9	98.5	115.6	167.7
ZNF438	834.4	1164.5	499.1	372.7	283	493.7	616.4	752
ZNF439	167.5	208.7	139.5	154	145.7	202.8	143.2	190.1
ZNF44	83.4	84	96.5	78.5	83.2	80.4	83.5	92.9
ZNF440L	117.4	152.2	120.3	140.7	137	147.3	112.9	133
ZNF441	114.1	126.2	110.6	93.7	82.6	104.1	105.3	136.8
ZNF442	176.3	208.1	138.3	140	154.3	153.1	155.4	183
ZNF443	346.5	381.8	278.8	255.9	200.3	365.5	281.4	452
ZNF444	196.5	227.6	152.7	133.5	126.6	138.1	184.2	483.2
ZNF445	91.7	89.8	88.1	87.5	83.9	87.5	87.2	97.6
ZNF446	157.7	196.6	207.3	149	145.7	169.3	145.6	221.8
ZNF447	92.9	117	96.2	85.6	85.9	90.7	102.2	105.6
ZNF449	92.1	110	108.2	96.7	91.1	102.4	111	117.4
ZNF45	379.7	413.2	245.8	239.1	223.4	292	330.9	516.7
ZNF451	86.7	96.7	85	81.4	79.7	80.9	85.4	102.6
ZNF451	516.7	724.3	313	468.3	453.3	728.3	483.4	932.2
ZNF451	167.6	209.2	105.8	105.1	125.6	144.8	154.7	316.2
ZNF452	83.4	95	88.4	81.8	82.1	83.4	78.1	89.6
ZNF454	127.2	149.4	83.8	86.2	79.4	99	105.6	145.8
ZNF460	149.9	167.7	109.8	103.1	88.7	106.1	144.6	196.7
ZNF462	133.8	157.4	145.7	90.7	100.5	99	120.3	147.9
ZNF467	251	283.4	213.6	271.4	280.8	256.2	191.3	144.6
ZNF468	91.6	108.1	91.3	81.1	86.6	100	95.8	112.8
ZNF471	76.1	94.2	96.3	83.3	85.1	90.3	77.4	96.9
ZNF473	395.2	525.9	401.4	310	226.4	352.1	382.4	545
ZNF473	113.7	148.4	94.7	97.1	91.3	113.8	96.3	112.9
ZNF474	82.4	95.3	96.2	90.6	87.2	97.6	85.8	92.4
ZNF480	453.2	583.8	318.5	248.2	193.2	413.2	362.7	500.8
ZNF482	136.7	168.3	111.2	94.9	93.2	103.8	132.9	169.1
ZNF483	98.8	101.4	80.3	81.1	73.8	84.9	84.1	96.6
ZNF483	356.9	344.5	251.8	351.8	381.1	261.8	320.6	504
ZNF483	125.3	144.8	106.2	93.2	87.7	96.8	109.4	139.2
ZNF484	139.6	171.5	137	121.6	115.2	163.5	140	178.8
ZNF485	166.7	190.3	90.1	90.6	87.3	89.7	148.5	185.9
ZNF490	101.9	110.3	89	84.1	85.9	90.4	106	102.3
ZNF491	102.8	99.2	97.6	87.4	88.9	91.8	90.4	96.1
ZNF493	107.5	107.3	98	101.9	83.5	102.6	105.4	127.1
ZNF493	87	92.7	97.9	93	95.1	98.5	82.9	108.6
ZNF496	102.7	126.4	95.8	89.1	91.9	89.8	103	114.5
ZNF497	115.6	128.5	97.8	95.1	84.7	86.1	112.6	117.3
ZNF498	119.9	152.8	90.7	91.4	84.6	79.7	124.4	172.1
ZNF499	342.5	454.7	183.9	111	111.6	113.6	307.3	290.8
ZNF500	218.8	244.8	216.9	221.7	205.5	345.8	176.3	279.1

ZNF501	76.8	95.9	84.4	75	85.9	77.2	90.7	100.5
ZNF502	96.1	98.2	92.8	85.9	83.7	83.5	84.1	90.4
ZNF503	283.2	331.3	243	178.2	163.2	218.3	317.3	505.7
ZNF507	151.8	162.7	117.6	139.5	116.3	133	123.8	131.5
ZNF508	1709.7	2091.4	1167	1069.9	740.3	1111.1	1587.2	2169.7
ZNF509	124.4	129	127.8	124.7	138.2	167.8	113.6	129.3
ZNF510	114.1	139	108.1	101.6	96.6	116.9	98.1	107.2
ZNF511	1618.3	2072.9	723.3	681.5	488.5	614.7	1669.1	1831.6
ZNF512	874.1	1006.6	951.2	1120.9	825.4	1646.9	620.2	1193.4
ZNF513	156.3	188.7	117	110.6	105.4	119.6	168.5	196.4
ZNF514	195.9	236.2	196.3	148.7	169	184.3	150	205.5
ZNF517	94.6	94.7	99.4	81.4	91.2	99	90.1	97.3
ZNF518	337.9	446	194.9	237.3	207.5	318.3	323.7	449.2
ZNF519	122.4	138.5	98.7	89.6	84.4	92.5	107.3	129
ZNF519	390.6	474.7	251.9	264.7	277.9	240.8	282.7	329.2
ZNF521	1181.4	1372.1	470.9	400.1	426.5	587.3	954.2	1497.9
ZNF524	321	434.1	344.8	285.1	251	322.4	354.3	491.1
ZNF526	300.7	315.2	125.3	118.9	111.4	135	258.7	308.5
ZNF528	106.8	112.1	100.9	94.5	90	87.8	106.5	104.8
ZNF529	438.9	559.8	307.4	382.3	293	414.9	376.6	546.6
ZNF530	114.6	126	119	112.6	93	107.3	120.3	124.3
ZNF532	191	249.8	127.5	171.9	139.2	179.9	166.5	251.2
ZNF533	82.9	84.7	94.7	85.7	74.6	88.1	79.5	81.1
ZNF536	152.2	141.3	107.6	86.5	85.7	95.5	105.3	138.6
ZNF537	649	747	404.8	367.8	293.8	431.8	526.6	644.2
ZNF539	142	160.9	98.6	110.2	99.9	122.7	128.7	184.5
ZNF540	85.2	91.2	87.1	87	80.2	72.9	78.2	84.4
ZNF542	88.5	113.1	89.6	85.3	81.7	86.6	89	99
ZNF543	169.1	184.4	144.1	164.8	124	161.9	150.6	216
ZNF544	102.4	110.3	90.3	88.1	78.8	81.8	96.6	112.3
ZNF545	223.9	271.2	189	136.6	113.5	146.9	196.7	226.6
ZNF546	75.5	80.5	86.2	81.5	83.5	87	83.3	84.3
ZNF547	86.7	95.4	87.3	84.5	76.9	89	80.2	85.3
ZNF548	288.7	323	270.1	330.9	282.4	428.4	231	343.7
ZNF549	5723.3	5068.3	3896	5974.5	5710.4	4118.3	4609.4	9564.7
ZNF550	117.8	142.2	115.6	115.4	100.7	107.7	119.5	137.3
ZNF551	142.3	211.3	130.6	117.9	114.7	127.2	128.3	193.3
ZNF552	112.2	144.3	103.1	91.8	88.1	90.4	113.7	125.1
ZNF553	106.4	136.7	96.8	91.6	79.1	94.2	102.6	132.9
ZNF554	96.6	106.8	102.3	97.3	91.9	86.8	93.4	97.2
ZNF555	108.7	130.2	111.6	95.5	92.7	99.7	103.3	150.4
ZNF556	195.7	245.1	100.1	95.5	105.4	109.3	167.4	210.6
ZNF557	101.1	121.4	86.8	86.2	84.7	81.3	107.1	132.4
ZNF557	436.5	439.8	196.3	159.6	193.9	170.3	347	524.2
ZNF558	146.4	171.3	113.8	150.1	105.4	175.3	115.8	207.9
ZNF559	448.2	600.8	217.3	203.6	155.4	244.7	369.9	552.3
ZNF560	102	104.3	87.8	87.3	92.4	90.7	109.1	106.1
ZNF561	129	159.6	99.5	101.2	89.4	104.3	131.8	149.3
ZNF562	568.6	679	317.7	373.4	270	334.3	504.6	777.1
ZNF563	112.4	135.3	101	100.5	109.1	105.2	103.9	119.5

ZNF564	337.5	449.8	419.6	358.8	283.5	506.4	307	435
ZNF565	262.9	314.6	148.2	136.4	122.6	136.6	229.9	292
ZNF566	150.6	177.1	141.2	125.6	120.7	125.6	114.7	158.3
ZNF567	78.2	83.9	86.5	85.5	71.8	77.4	81.4	86.1
ZNF568	97.7	104.7	102.6	88.2	94.9	96.4	91.5	103.4
ZNF569	99.4	113.5	96.8	91.6	86.8	92.5	107.2	109.5
ZNF570	113.4	123.9	102.8	93.4	86.9	100.1	105	128.4
ZNF571	107.3	114.4	90.2	89	81.4	86.3	92.7	124.9
ZNF572	83.7	81.8	97.8	84.6	82.3	92.5	84.6	89.8
ZNF573	506.7	705	281.8	386.7	337.6	369.3	414.5	494.6
ZNF574	106.9	119.6	137.7	109.9	97.8	114.9	115.1	150.2
ZNF575	96.1	100.9	94.9	85.4	86.8	95.8	97.7	127
ZNF576	491	567.7	303.4	203.6	188.3	206.6	420.7	412.9
ZNF577	421.3	461	272.6	407.8	417.4	360.9	453.9	804.2
ZNF579	131.8	166.2	94.2	83.7	98.6	99.9	123.9	208.5
ZNF580	129.8	119.9	103.1	79.1	93.4	81.1	113.5	139.8
ZNF580	182.9	188.7	193.1	138.9	134	154.6	167.3	348.1
ZNF580	143	153.9	93.6	84.1	83.7	87.6	134.9	183.8
ZNF581	759	1022.3	719.4	391.7	346.3	489.3	686.4	1030.7
ZNF582	104.5	118	92.6	75.7	91.1	86.3	105.7	115.6
ZNF584	135	178.7	119	109.8	98.3	112.5	131.1	155.5
ZNF585A	285.7	354.7	204.2	251.2	189.3	292.1	224.7	269.1
ZNF585A	122.1	138	113.4	110.2	118.2	113.9	107.7	128.6
ZNF585B	96	110.5	97.8	90.1	89.1	101.6	96.5	113.6
ZNF586	343.9	440.7	300.2	226.8	217.3	308.4	278.6	371.1
ZNF586	742	1032.3	535.4	427.5	373.3	589.2	560.3	621.8
ZNF586	151.6	194.6	117.2	94.2	86.7	90.2	121.9	138.9
ZNF587	98.4	126.5	94.4	107.7	99.9	95.9	100.2	115
ZNF588	82.8	82	83.4	87	79.8	92.9	86.7	83.5
ZNF589	259.4	271.3	284.1	316.5	236.6	476.9	196.6	297.7
ZNF592	117.6	127.3	98.5	84.9	95	96	122.8	129.5
ZNF593	1195	1331.6	701.8	507.3	353.8	507.1	1139.6	1053.9
ZNF595	76.8	73.6	85.2	71	70.5	69.2	78.3	90.2
ZNF596	102.2	118.2	93.6	88.1	77.4	88.5	92.9	139.6
ZNF596	94.8	106.5	116	90.6	90.3	105.2	99.7	113.9
ZNF597	163.9	210.5	139.9	129.5	118.4	130.7	127.4	180.7
ZNF598	1772.8	2097.5	1464.3	1091.8	949.2	1283.6	1365.7	1875.8
ZNF599	83.6	84	88.5	79.7	71.7	77.4	74.6	89
ZNF599	86.5	93.9	85.6	82.8	77	86.8	82.5	95.5
ZNF6	101.9	122.6	95.5	89.6	85.4	104.1	98	136.9
ZNF600	140.9	147	96.2	98.5	99.2	103.4	130.9	142
ZNF606	145.3	187.6	102.9	101.2	90.9	115.5	133.4	180.5
ZNF607	74.9	85.7	84	85.3	72.5	86.7	78.1	89.9
ZNF608	86	99.5	111.9	108.5	93.4	96.2	89.3	93.7
ZNF609	118.5	158.6	142.4	131	129	156.6	116.7	194.4
ZNF610	75.1	89.3	87.5	81.3	71.6	79.9	79.8	82.7
ZNF611	140.6	157.3	103.8	103.2	89.1	106.1	118.6	156.4
ZNF613	364.1	425.5	266.6	243.4	197.2	268.5	287.6	379.3
ZNF613	84.8	105	90.8	90.8	88	87.6	96.8	84.3
ZNF614	402.1	519	307.5	273.5	221.2	440.7	349.3	586.7

ZNF615	302.2	401.9	249	205.5	165.3	281.7	292.7	390.9
ZNF616	175.4	213.1	205.7	156.5	130.4	172.8	160.3	207.1
ZNF618	258.6	340.2	203.4	197.1	193.4	174.7	250.6	303.2
ZNF619	82.3	90.6	96.2	84.2	79.4	88.4	87.1	91.7
ZNF620	104	115.4	89.7	85.9	81	93.3	103.7	115.2
ZNF621	742	882.6	387.3	406.6	358.9	496	585.7	818.7
ZNF622	1851.9	2710.7	734.1	834.7	606	1083.9	1726.1	2009
ZNF623	86.4	108.4	86.2	81.7	86.3	85.8	91.6	96.1
ZNF624	98.8	122.4	107.1	99.5	87.9	109.5	103.1	135.3
ZNF625	92	105.3	114.2	99.4	106.7	103.6	94.6	89.5
ZNF626	82.1	84.2	101.2	97.7	90.8	97	79.9	88.1
ZNF626	81.6	92.4	91.4	85.8	76.9	85.7	84	85.9
ZNF626	156.1	211.5	106.5	106	95.1	99.4	173.4	204.6
ZNF627	1229.3	1516.5	719	669.4	468.6	825.7	1011.7	1458.9
ZNF628	94.5	103.6	90.4	84.6	90.6	94.5	98	108.4
ZNF629	134.8	172.1	166	146	130.3	160.4	149.2	172.6
ZNF629	206.4	318.7	160	130.9	94.2	128.4	233.6	313
ZNF630	95.3	125	106.9	96	91.5	106.7	104	120.2
ZNF630	87.5	98.2	121.1	133.5	123.8	147	90.9	98.4
ZNF639	111	110.8	108.2	92.3	92.7	88.2	112.9	107.1
ZNF641	154.8	165.3	129.9	180.1	144.8	161.8	112.5	163.1
ZNF642	599	698.6	269.4	358.3	241.1	381.5	486.6	954.8
ZNF643	245.3	325.5	155.8	167.9	153.1	179.5	197.9	259.6
ZNF644	87.5	105	95.9	90.2	89.5	83.4	99.8	101.8
ZNF644	586.3	673.7	326.3	396	291.4	624.7	522.1	1104.8
ZNF644	328.3	378.7	151.4	177.2	157.1	158.2	229.1	367.2
ZNF645	84.8	101.8	89.4	83.2	87.6	88.3	105.8	100.8
ZNF646	111.4	148.5	120.8	106.2	103.9	127.8	114.9	137.7
ZNF648	98.3	113.1	102.3	80.7	85.9	80	91.4	107
ZNF649	130.1	156.2	116	99.1	94.8	97.3	122.3	145.5
ZNF650	330.4	473	338.1	393.7	330.5	471.3	321.1	411.9
ZNF651	176.5	213.4	149.7	150.6	148.5	168.4	165.3	160.2
ZNF652	1744	1979.1	715	1198.3	1378.8	794.5	1461	1671.1
ZNF653	314.6	354.8	262.3	184.5	199.9	180.9	299.8	265.6
ZNF654	157.8	184.2	112.7	130.6	123	133.6	145.7	184.1
ZNF654	149.9	194.8	129.2	174	179.5	178.9	155	246
ZNF654	193.2	218.3	127.7	152.6	143.2	176.8	170.8	253.2
ZNF654	132	142.5	85.1	97.9	100.8	97	133.2	192.3
ZNF655	173.5	182.7	97	85.6	87.2	94.5	140.2	166.6
ZNF655	130.8	164.9	118	99.4	81.9	96.4	135.2	148.7
ZNF655	104.2	114.8	93.7	89.6	103	97.4	93.4	97.2
ZNF655	330.4	349.4	185.2	200.5	212.6	172.8	299.3	268.2
ZNF658	260.9	314	141.1	118.8	108.8	147.3	204.3	304
ZNF658B	66.3	78.6	94.2	87.9	86.5	77.6	72.8	75.7
ZNF659	103.4	108.8	115.5	100.2	93.6	108.3	96.9	103.2
ZNF660	127	140.5	140.7	127.8	120.3	113.3	131.9	169
ZNF662	82.2	95	99	86.6	85	94.9	90.5	80.1
ZNF664	240.1	297.1	131.8	145	132	156.1	198.8	247.7
ZNF665	73.9	91.9	87.6	82.6	67.7	86.1	91.1	95.7
ZNF667	114.9	132.8	92.5	80.3	83.1	89.2	112.8	135.5

ZNF668	677.7	866.4	482.8	368.3	303.6	360.4	562.2	673.3
ZNF669	392	427.7	256.6	263.9	224.3	185.1	339	439.1
ZNF670	623.7	733.5	329.6	236.1	153	212.3	421.4	673.1
ZNF671	380.1	476.4	316.8	253.2	222.2	284.5	323.7	354.3
ZNF672	1321.5	1585.8	1029.9	976	959.6	1079.6	1199.8	827.4
ZNF673	152.1	174.8	104.6	97.2	89.9	110.1	156.1	166.4
ZNF675	117.2	104.2	101.2	94.1	89	102.1	97.4	123.8
ZNF677	98.5	100.2	94.3	84.4	86.8	85.3	95.6	109.2
ZNF678	75.7	84.2	84.6	69.2	68.4	77.2	82	85.1
ZNF680	288.6	326.3	111.2	151.3	107.9	173.7	209.5	340.6
ZNF681	179.4	189.8	122.3	124.4	103.2	162.2	165.3	327.1
ZNF682	102.2	103.5	102.9	92.7	79.3	91.7	102.8	121.2
ZNF682	184	263.7	150	190.2	168.7	181.1	166.3	167.4
ZNF682	1978.3	1766.8	718.5	955.6	994.8	577.2	1626.7	2573.2
ZNF683	107.3	125.6	98.7	85	88.3	88.3	99	118.4
ZNF684	1424.1	1690.6	715	834.5	507.8	1175.7	1346.2	2472.3
ZNF687	107.3	117	95.6	106.8	86.3	104.5	105.1	131.6
ZNF688	173	203.4	123.5	109.5	102.4	111.2	137	203.4
ZNF688	83.2	101.5	102	104.9	95.3	87.5	83.3	90
ZNF689	1129.3	1445.3	765.1	656	575.5	637	761.9	654.7
ZNF69	1247	1203.1	645.2	1207.6	1042.7	770.8	1101.8	2098.2
ZNF690	167.4	199.9	119.6	106.4	111.9	110.5	155.4	163.9
ZNF691	203.1	249.3	189.6	180.3	146.1	242.9	173.9	246.8
ZNF692	490	493	499	471.5	510.8	791	268.3	386.3
ZNF694	237.6	298	155.8	156.9	147.7	177.9	200.1	206.9
ZNF695	276.2	358.3	239.1	168.2	164.6	168.7	281.3	290.7
ZNF696	534.1	675.2	368.3	276.2	285.7	304.2	458.1	531
ZNF699	165.7	216.3	106.4	99.4	99.5	113.4	159.7	170.5
ZNF7	647	723.8	484.1	429.8	381.6	724.7	478.9	868.8
ZNF70	81	98.7	92.6	75.1	81.4	80.7	86.7	98.7
ZNF700	990.6	1315.5	926.9	1079.3	953.9	1644.5	789.1	1157.1
ZNF701	131.6	151.1	103.6	107.5	94.2	111.2	118.3	152.3
ZNF702	81.6	100.2	97.4	87.3	73.1	84.8	90.7	97.3
ZNF703	99.4	107.1	92.8	80.4	70.1	85.5	98.8	100.6
ZNF703	89.4	80.4	89.1	87.3	77.1	79.6	93.9	104.4
ZNF704	103.4	112.8	102.6	104.8	98.2	103.9	92.5	115.2
ZNF706	233.2	288.3	177.2	165	122.6	175.7	193.8	282.3
ZNF706	86	93.4	121.5	109	98.1	108.9	84.4	92.7
ZNF706	289.1	339.1	223.6	187	146.4	205.9	226.5	290.9
ZNF707	208.4	255.4	186.7	143.1	146.3	171	215.5	337.7
ZNF708	89.4	87.8	102.3	90.2	92.4	96.8	82.8	107.5
ZNF709	97.2	94	89.9	88.1	89.4	88	94	92.8
ZNF71	113.7	126.8	94.7	105.5	95.8	99	104.3	121
ZNF710	83.9	91.4	80.5	86.5	80.5	78.9	83.1	88
ZNF713	121	111.8	106.9	101.8	98.6	100	107.7	115.4
ZNF714	111.2	124.1	97.5	90.9	88.9	98.4	106.8	113.7
ZNF718	102.8	122.4	111.1	105.2	102.9	98.1	103.1	105.8
ZNF721	1059	1359	394	298.2	279.9	431.1	905.8	1527.7
ZNF74	94.7	109	95.9	92.5	79	89.3	91	97.5
ZNF74	146.4	165.7	125.4	93.2	103.5	101.5	133.7	141.2

ZNF74	234.4	282.6	147.5	173.9	140.5	169.6	179.3	213.9
ZNF740	98.5	120.7	105.5	119.2	114.9	115.5	96.8	99.5
ZNF749	115.4	146	99	87.3	77.6	85.2	111.5	119.4
ZNF75	198.2	216.7	152	229.3	164.2	247.8	209.4	311.3
ZNF75A	339.7	388.7	165.3	131.1	109.4	154.2	317.1	445.1
ZNF76	179.2	222.2	213.9	182	200.9	322.8	154.1	212.2
ZNF765	68.8	83.1	77.8	78.7	79.5	72.9	77.5	80.4
ZNF767	197.5	198.9	168.4	159.1	169	169.7	155.2	175.4
ZNF77	231.6	304.8	210.5	179	158.6	239.4	216.5	325.8
ZNF781	95.8	114.1	82.8	87	91.9	97.1	98.9	101.1
ZNF79	143.8	161.7	100.2	103.2	98.8	109.8	139.4	184.1
ZNF8	135.1	131.5	113.7	94.4	90.7	100.7	113.8	124.5
ZNF80	92.6	102.7	113.6	104.8	104.3	98.6	91.5	91.2
ZNF805	142.7	205	113.7	129.8	113.2	157.4	164.8	194.1
ZNF808	84.2	94.5	100.5	89.7	82.1	95.4	82.8	95.8
ZNF81	87.5	97.8	86.3	81.7	83.7	84.6	93.3	93.5
ZNF83	318.4	364	170.8	167.3	171.9	244.7	218.1	390.5
ZNF85	149.9	145.8	106.9	83.6	90.4	132.2	110	173.8
ZNF91	444.6	550.3	219.6	225.2	146.7	306.4	405.7	734.9
ZNF92	155.5	162.6	98.6	86.6	92.1	88.9	109.9	133.2
ZNF92	81.4	96.9	106.9	90.4	93.1	96.2	88.5	97.5
ZNF93	388.8	524.3	166.8	174.4	144.4	205.3	349.6	458.5
ZNF93	266.3	364.8	152.2	139.8	124.1	202	207.2	339.3
ZNF96	119	143.6	108.2	96.5	82.4	105.2	109.1	136.9
ZNF99	110.3	109	100.9	90.1	84.4	90.6	92.1	110.1
ZNFN1A1	1043.4	1203.9	438.2	496.5	437.2	431.4	516.8	554.8
ZNFN1A2	165.6	166.8	111.3	107.2	93.7	107.3	145.1	141.4
ZNFN1A2	108.4	143.6	104.4	88.7	82.8	89	114.2	129.6
ZNFN1A3	127	138	102.1	95.2	95.1	86.7	126.5	147.1
ZNFN1A4	155.9	194	132.6	124.3	107.8	124.2	152.6	223
ZNFN1A5	253.7	371.6	201.6	167.4	137.7	223.5	235.2	389.6
ZNFX1	310.1	414.2	339.4	250.8	184.4	248.2	260.8	517.3
ZNHIT1	1747.4	2024.1	1242.5	1273.5	1008.4	1288.3	1738	1835.3
ZNHIT2	249.9	347.1	140.5	91.8	93.9	124.6	193.1	237.3
ZNHIT3	3513.6	4701.6	3037.2	3653.3	3023.2	3581.7	3516.3	4630.5
ZNHIT3	103.8	110.3	90.9	86.4	80.9	81.8	102.4	100
ZNHIT3	919.8	1216.3	300.3	278.5	269.2	440.7	820.5	1075.9
ZNHIT4	160.7	215	131.8	129.1	115.3	110.8	157.8	179.7
ZNRD1	348.8	504.6	183	122.1	134.9	157.6	314.5	336.4
ZNRD1	498	662.3	359.2	247.7	188.8	309.6	377.9	498
ZNRD1	457.9	556.5	264.5	203.7	163.4	231.9	356.9	423.6
ZNRF1	103.5	119.6	106.5	89.6	90.1	87.5	104.6	129.8
ZNRF2	87.9	105.3	80.5	85.9	80.9	88.7	86.7	102.8
ZNRF4	78.2	91.8	91.4	82.6	83.2	87.3	74.1	82.4
ZP1	79.3	88.6	93.9	101.3	86.1	91.4	81.6	97.1
ZP2	73.8	76.6	88.3	87.3	71.4	87.1	81.9	74.7
ZP3	158.4	187	105.1	93.3	93.8	88.1	145.9	179.9
ZP4	79.6	87.2	90	84.7	79.8	82.6	75.1	81.1
ZPBP	78.9	83.3	90.5	78.5	85.5	90.1	82.1	84.2
ZPBP2	81.5	86.7	99.8	96.6	90.5	92	80.9	83.6

ZPBP2	75.2	87.2	96.8	92.2	82.5	87.1	82.7	86.7
ZPBP2	82.5	96.9	97.1	79.8	86.2	90.6	82.6	95.1
ZPLD1	74.1	77.1	79.5	71.3	73.4	72.8	81.8	79.2
ZRANB1	174.2	259.3	146.4	124.7	120.2	138.3	178	252.5
ZRANB3	85	106.4	93.3	88.8	84.4	86.9	99.2	96.9
ZRF1	137.6	145.4	103.4	88.9	83.4	99.3	148.5	263.1
ZSCAN1	95.2	119.6	88.8	73.4	75.2	80.8	104.8	118.1
ZSCAN2	140	169.7	113.3	114.5	123.7	133.3	140.1	164.5
ZSCAN2	298	354.1	239.9	293.1	251.6	296.6	288	292.3
ZSCAN2	114.4	145.4	110.3	119	121.6	120.8	104.4	125.3
ZSCAN2	90.8	105.7	88.8	79.3	91	96.3	87	93.5
ZSCAN4	81.4	95.2	103.6	93.4	86.4	97	93	95.5
ZSCAN5	302.4	399.7	183.1	169.3	134.3	156.1	251.4	310.1
ZSWIM1	657.7	809	381.6	442.4	389.2	380.6	609.9	677
ZSWIM2	78.2	84.1	97.2	82.6	84.5	91.1	78.5	79
ZSWIM3	113.1	137.5	96	99.1	93.1	82.1	112.3	121.8
ZSWIM3	197	246.8	203.1	187.5	161.3	202.8	205.5	326.4
ZSWIM3	112.4	130.7	102.6	108.2	87.8	97	110.9	116.6
ZSWIM3	136.2	148	95.7	94	88.7	86.2	115	118.6
ZSWIM4	207.1	281.5	176.5	178.3	174.1	181.9	223.3	256.1
ZW10	1605	2211.8	841.8	793.1	713.6	1117.2	1370.5	1414.6
ZWILCH	250.5	322.4	118.7	109.8	94.8	110.1	182.6	274.3
ZWILCH	256.1	292.7	115	105.6	92.1	112.6	198.9	395.3
ZWILCH	1945.8	2478.6	1254.9	1005.3	891.2	1412.1	1440.3	2419.8
ZWINT	167.8	194.4	145.1	102.4	93.4	102.9	130.4	169.3
ZWINT	924	1034.6	259.9	188.7	179.7	238.1	730.2	1003.4
ZWINT	156.6	181.8	100.8	101.5	78.3	90.7	142.2	160.9
ZXDA	120.5	133.6	120.2	106.8	104.2	113.2	121.9	126.4
ZXDA	108.9	117	97.2	87.1	87.3	92.8	97.1	105.9
ZXDB	152	205.6	127.5	115.6	105.6	145.8	128.7	149
ZXDC	125.2	157.8	111.8	123	106.2	108.7	125.9	136.8
ZXDC	225.5	284.1	203.8	263	239.8	250.3	176.2	164.4
ZYG11A	99.3	105.8	99.3	85.9	95	92.8	98.2	102.1
ZYG11B	1147.1	1816.9	913.6	1524.5	1215.1	1943.8	1076.3	1504.5
ZYG11BL	189.3	215.6	134.3	115.3	104.3	119.6	185.4	226.3
ZYX	536.7	765.8	657	643	523.2	935.8	530.3	1280.2
ZYX	2901.8	3598.2	2188.4	2607.9	2038.3	2100	2712	4414.2
ZZEF1	250.9	370.9	189.9	267.9	243.6	284.1	294.2	339.1
ZZZ3	800.9	908.1	501.3	459.1	320.5	671.3	581.1	1116.8

TS5, TS6)

AVG_Signa	AVG_Signa	AVG_Signa	AVG_Signa	ACCESSION
188.9	169.8	156.5	139.2	NM_176818.1
361.3	287.7	224.7	185	NM_177966.4
80.7	89.9	85.1	92.5	NM_182762.2
109.7	105.1	107.7	90.9	NM_130786.2
89.4	85.6	84.1	80.2	NM_018723.2
100.1	95.5	83.7	94.3	NM_145891.1
92.5	85.4	85.1	87.5	NM_145893.1
100.4	93.2	75.1	85.5	NM_018723.2
77.1	86	80.3	89.6	NM_000014.4
109.3	89	86.2	92.2	NM_144670.2
84.9	83.3	78.8	78.6	XM_940724.1
91	88.7	92.9	90.7	NM_001080438.1
146.1	113	130.8	109.6	NM_017436.4
95.8	92.8	92.5	92.5	NM_016161.1
114.9	104.4	91.4	94.7	NM_207284.1
103.8	103.1	99.8	95.5	NM_207290.1
101	86	88.6	89.5	NM_207285.1
76.8	66.1	69.7	71.9	NM_207289.1
83.5	83.7	78.7	80.7	NM_207283.1
125.2	110.7	102.8	99.8	NM_015665.3
169	233.5	221	161.5	NM_023928.2
98.3	94.7	90.7	88.1	NM_001086.2
159.7	151	121.2	151.7	NM_020792.3
89.9	88.3	84.1	85.9	NM_207365.1
97.2	81.9	91.7	92.4	NM_016228.3
88.5	81.3	89.9	84.8	NM_182662.1
84.4	82.3	84	94.9	NM_182662.1
87.6	92.6	96.3	89.6	NM_014911.2
164.4	197.3	171.4	144.8	NM_001087.2
90.1	75.6	75.4	82	NM_001088.1
4730.4	3964.3	3757.5	2302	NM_001605.1
493.5	548.7	525.1	436.5	NM_025267.2
256.7	330.2	275.3	229.6	NM_020745.1
302.3	308.9	263.5	255.8	NM_181806.2
767.1	491.9	385.1	338.1	NM_015423.2
109.1	106	97.7	98.8	NM_005763.2
1032.7	722.1	640.3	526.6	NM_012138.3
103.4	102.3	94.9	101.3	XM_927215.1
76	79.3	80.6	81.5	NM_001080395.1
91.2	82	87	86.9	NM_020686.3
87.8	86.6	87.4	90.7	NM_020686.3
98.6	89.9	88.9	84.1	NM_000663.3

138.1	156.7	153.2	113.9	NM_022070.3
297.6	870.1	838.7	608.4	NM_005502.2
98.4	90	99.8	95.1	NM_080282.2
122.6	139.9	105.4	116.1	NR_002451.1
91	80.6	81.3	86.7	NM_173076.2
86.8	90.4	92.4	81.1	NM_015657.3
95.4	91.9	85.5	90.2	NM_152701.2
89	93.9	90.1	89	NM_212533.1
131.9	146.1	124	121.4	NM_001606.3
97.1	94.1	93.8	93.7	NM_001089.1
143.4	131.5	136	139.4	NM_000350.1
93.8	101.2	112.3	110.4	NM_172232.1
83.9	90.7	82.4	89.9	NM_018672.2
91.7	85	91.4	99	NM_018672.2
84.7	88.8	88	92.1	NM_172346.1
106.5	120.7	106.1	96.6	NM_080284.2
83.9	83.9	69.6	80	NM_019112.2
203.7	265.4	272.7	222.5	NM_019112.2
96.5	90.2	89.7	96.5	NM_033308.1
80.8	83.9	73.3	86.8	NM_007168.2
98.2	97.3	85.6	87.8	NM_080283.2
96	75.9	86.6	85.4	NM_080283.2
87.3	85.3	78.1	80	NM_000927.3
810.8	779.3	702.2	684.2	NM_012089.1
94.6	100.2	95	100.9	NM_003742.2
119.6	75.9	91.4	86.7	NM_000443.2
82.5	84.5	79.3	74.7	NM_178559.3
146.3	177	168.7	162.8	NM_005689.1
381.5	352.6	341.6	393.2	NM_004299.3
92.8	82.5	86.8	84.7	NM_007188.2
92.8	96.3	98.2	102.8	NM_203444.1
136.5	129.4	119.1	134.1	NM_019624.2
183.2	191.1	194.5	142.6	NM_019624.2
90.9	94.4	90.3	97.4	NM_019901.1
126.2	124.4	112.5	119.7	NM_019900.1
114.2	104	97.6	109.2	NM_033450.2
78	73.7	72.1	80	NM_145186.2
113.3	102.4	99.6	86.8	NM_033151.2
96.3	86.7	96	101.5	NM_145186.1
115.1	105.2	99.9	109.4	NM_145188.1
118.4	89.7	100.3	102.2	NM_172025.1
101.3	105.8	104.4	100.9	NM_172024.1
114.7	98.2	93.2	94.4	NM_000392.1
115.9	766.7	487.8	438.4	NM_020037.1
207.4	208	156.3	173.3	NM_005845.2
94.6	85.6	79.6	89.5	NM_001023587.1
194	320.6	287.3	197.4	NM_005688.2
158.8	147.5	132.8	138.5	NM_001023587.1
83.1	94.6	92	96.5	NM_001171.2
77.9	81.3	83.3	95.3	XM_936351.1

80.9	82.9	73.7	82.3	NM_001079528.1
88.7	87.1	96.6	96.8	NM_001171.2
126.4	119.1	138.3	109.9	NM_001079528.1
91.4	92.6	88.5	89.2	NM_000352.2
89.3	87.7	79.5	88.5	NM_020297.1
98.6	92.3	92.3	94.2	NM_020298.2
222.9	212.5	183.9	212.7	NM_000033.2
89.5	80.4	83.6	81.4	NM_005164.2
85.7	91.9	83.5	95.2	NR_003256.1
116.9	111	104.5	121.1	NM_020323.1
1314.8	635.8	444	606.8	NM_001040876.1
1286.7	610.6	434.7	572.4	NM_002940.1
438.2	373.6	327.4	293.4	NM_001025091.1
846.6	896.8	825.2	510	NM_001090.2
103	90.8	87.8	92.8	NM_005692.3
507.6	434.1	329.8	287.9	NM_005692.3
185.3	168.6	143.9	146.9	NM_007189.1
141.1	119.8	127	121.8	NM_018358.1
98.6	96.5	105.5	108	NM_207629.1
107	102.2	92.7	95.2	NM_207627.1
101.3	97.3	85.9	83.1	NM_207630.1
89.5	97.1	96.3	91.7	NM_016818.2
96.5	92.6	91.1	97.3	NM_004915.3
87.2	82.2	86.4	97.9	NM_207174.1
78	75.6	74.8	76.9	NM_004827.2
101.3	90.2	90.4	91.7	NM_022169.3
106.2	101.4	99.4	102.4	NM_022436.2
93.9	91.7	83.5	93.7	NM_022437.2
95.6	85.1	85.2	87.5	NM_152870.1
295.3	277.8	231.6	179	NM_018394.1
189	158.1	150.3	139.4	NM_148916.2
92.7	87.3	81.6	80	NM_031295.3
223.5	195.5	170.6	145.8	NM_148914.2
232.5	239.1	197.5	203.6	NM_001042472.1
319.8	367.9	332.9	291.6	NM_001042472.1
92.2	90.6	83	88.1	NM_032859.2
467.1	308.1	321.7	210.9	NM_015407.3
173.5	219.5	206	188.6	NM_032750.1
120.3	118.7	113.5	107.6	NM_152924.3
95	92.1	93.9	97.6	NM_007011.4
93.9	92.9	92.2	93.8	NM_007011.4
614.6	687.3	558.4	616.4	NM_138340.3
107.7	98.7	101.2	98.2	NM_022060.2
168.8	134.6	121.1	129.8	NM_016006.3
150.8	165.2	147.6	153.8	NM_020676.4
104.7	94.4	98	83.7	NM_173567.2
418.5	359.4	312	194.2	NM_024527.3
103.4	93.8	86	94.5	NM_024794.1
84.3	93.8	80.4	85.1	NM_005470.2
201.4	138.3	109.5	131.1	NM_001012751.1

123.8	115	96.2	101.8	XM_938458.1
414.1	307.8	213.7	207.9	NM_005759.3
110.9	112.1	99.6	109.1	NM_005759.3
86	102.9	97.6	102.5	NM_016428.2
81.2	92.5	84.5	83.8	NM_015429.2
512.2	413.8	428.6	233.3	NM_007313.2
120.5	120.6	124.9	125.6	NM_007313.2
89.9	92.6	90.8	82.3	NM_005157.3
99.2	93.8	91.9	92.7	NM_005158.2
92.3	81.2	85.8	86.5	NM_005158.2
97.5	113.6	94	100.3	NM_007314.1
101.3	92.8	95.3	100.6	NM_006720.3
87.9	83.8	83.7	90.6	NM_006720.3
92.7	90.3	86.4	87.6	NM_002313.5
84.1	82.9	75.6	79.9	NM_001003407.1
105.6	85.7	91.1	92.1	NM_032432.2
96.9	98	94.2	98.1	NM_014945.1
84	93.1	83.8	93.1	NM_020469.2
634.3	2090.4	2510	807.8	NM_001091.2
1108.3	1380.6	1341.7	522.6	NM_021962.2
96.1	105.3	97.9	98.6	NM_001092.3
94.4	93.2	91.7	90.5	NM_021962.2
84.2	82.5	90	91.7	NM_139166.2
114.1	99.9	97.2	92.9	NM_013375.2
178.2	221.3	272	156.5	NM_172027.1
143.1	152.8	172.9	213.3	NM_172028.1
154.1	169.5	141.8	134.3	NM_145804.1
482.9	571	583.7	452.7	NM_001607.2
189.9	174.6	161.6	119.3	NM_006111.1
781.9	1017.1	1158.1	767.4	NM_198836.1
546.1	546.9	711.6	426.9	NM_000664.3
98.8	96.2	90.4	86.7	NM_198839.1
226.5	368.6	455	226.7	NM_001093.2
170.5	217.7	217.6	131.9	NM_025247.3
236.3	271	310.4	253.1	NM_032169.3
534.6	285.8	256.9	221.5	NM_014384.2
680.2	660.2	688.4	413.5	NM_014049.3
90.3	83.7	79.2	84.5	NM_001608.2
2002	1434.8	1525.1	1348.8	NM_000016.2
160.1	145.6	167.2	128.1	NM_000017.1
104.1	112	102.2	110.5	NM_001609.2
173.8	185.5	178	168.3	NM_000018.2
2399.5	2384.6	2175.5	1516.1	NM_000018.2
683.1	840.6	672.3	594	NM_000018.2
2721.7	2258.3	2105.7	1509.5	NM_000019.2
111.6	106	96.6	106	NM_005891.1
987.9	866	745.6	863.2	NM_022735.3
94.6	92.2	86.6	84.5	NM_024722.1
99.6	94.9	85.6	85.8	NM_145698.1
100.5	92.2	86.5	91.8	NM_145698.1

100.1	96.9	87.9	92.8	NM_145698.1
451.6	295.1	300.6	206.5	NM_032360.2
104.6	88.7	78.2	86.4	XM_928013.1
77.1	89	79	92.4	NM_001039844.2
84.9	86.3	77.5	87.5	NM_183377.1
141.2	113	128.4	106.4	NM_020039.2
92	79.6	97	86.9	NM_020039.2
133.7	119.4	114.3	106.4	NM_020322.1
99.1	93	93.6	90.8	NM_020321.1
97.3	94.9	84.1	85.7	NM_018674.3
93	86.1	85.6	82.8	NM_018674.3
82	92.2	80.5	83.5	NM_018674.3
83.4	83.8	87	83.8	NM_017419.1
469	255.5	257.1	200.2	NM_022914.1
97.9	93.7	87.3	95	NM_152831.1
89.2	81.6	73.2	79.9	NM_152830.1
85.5	87.1	96.6	89.9	NM_000789.2
87.6	89.9	91.3	87.5	NM_021804.1
81.5	87.9	84.9	96.1	NM_138932.1
91.2	85.6	84.3	83.1	NM_138933.1
93.2	89.3	96	95.7	NM_138933.1
80.7	90.6	82.8	87.1	NM_000665.3
98.8	88.1	85.5	94.3	NM_015831.1
634.8	563	542.4	477.2	NM_014977.1
3227.8	2911.6	2573.9	2197.3	NM_198830.1
135.2	135.6	127.5	112.6	NM_138326.1
703.5	366.9	355.5	357.6	NM_020186.1
1007.5	889.4	762.6	581.4	NM_002197.1
190.5	219.2	232.9	203.8	NM_001098.2
98.3	87	89.1	90.5	NM_001037161.1
155.4	132	136.7	112.5	XM_929560.1
347.5	292.2	240.2	165.4	NM_147161.2
113.2	100.6	103.1	113.7	NM_015547.2
105.4	95.3	92.2	98.7	NM_130767.1
88.4	97.5	88.8	91.3	NM_006821.3
492.3	248.8	207	190.8	NM_152331.2
77.9	82.5	81.7	78.3	NM_001037162.1
81.4	86.1	86.5	82.8	NM_181865.2
76.8	83	79.5	79.8	NM_181866.2
1272.3	938.1	868.8	558.3	NM_181866.2
87.6	91.2	84.6	82.3	NM_007274.3
776.9	442.8	394.5	306.1	NM_007274.3
91.1	77.3	78.4	76.2	NM_181864.2
138.7	140.8	132.6	119.3	NM_183386.1
130.4	135.9	143	117.3	NM_001037171.1
451.5	328.2	313.4	286.5	NM_001033583.1
210.7	169.6	146.6	163.1	NM_004035.4
87.7	94.6	90.3	89.9	NM_007292.3
97.6	92.7	86.6	89.6	NM_004035.4
261.4	246.6	214.4	158.4	NM_003500.2

411.8	316.4	330.5	229.1	NM_003501.1
91.5	82.6	77.8	76.8	NM_018308.1
1410.6	984.3	1012.9	807.9	NM_007099.2
3565.8	2494.7	2426.9	1700.3	NM_001040649.1
152	150.5	136.1	112.3	NM_001610.1
92.5	93.8	86.9	81	NM_001611.2
134.8	131.5	131.5	124.6	NM_016361.2
119.1	103.7	92.4	99.5	NM_152282.3
199.9	359.7	230.5	236.3	NM_001099.2
170.6	190.4	184.9	149.3	NM_033068.2
96.1	79.4	86.6	91	NM_033068.2
94.8	85.6	97.4	90.2	NM_001097.1
112.4	109.8	97.6	102	NM_032489.2
130.3	123.9	109.1	97.8	NM_052957.2
124.9	98.2	95.4	95.1	NM_001612.4
94.6	87	80.1	92.3	NM_020109.3
88.4	86	90.6	89.5	NM_015162.3
93.8	96.7	85.2	105.3	NM_030924.2
173.6	143.1	128.4	121.1	NM_001995.2
1718	1782	1608.7	1197.6	NM_203372.1
164.5	151.2	154.8	160.4	NM_004457.3
313.7	309.5	286.8	289.7	NM_004457.3
98.2	91	87.1	88.6	NM_022977.1
255.8	173.6	183.2	160.3	NM_022977.1
783.7	845.9	806.2	613.7	NM_004458.1
162.5	120.3	113.6	122.5	NM_203379.1
96.7	88.4	90.2	90.4	NM_203379.1
95	91.6	87.7	93.1	NM_203380.1
96.6	96.4	87.8	96.1	NM_016234.3
563.5	366.1	359.7	215.9	NM_203379.1
87.7	89	69.6	92.4	NM_015256.2
93.9	86.7	82.4	89.3	NM_001009185.1
100.1	93.7	94	89.5	NM_015256.2
86.7	90.4	84.4	84.4	NM_052956.1
82.9	91.6	84.9	86.7	XM_936876.1
94.7	91.5	85.3	88	NM_182617.2
102.9	90.6	100.2	90.3	NM_202000.2
105.1	94.4	87.2	98.1	NM_202000.2
105.5	99.4	109.4	101.5	NM_005622.3
152.6	158	187.9	171.3	NM_032501.2
76.1	85.6	79.4	89.5	NM_018677.2
96.9	88.7	79.6	94.2	NM_001076552.1
1018.2	1215	1469	828.9	NM_001076552.1
1371.9	1621.2	1822.3	1122.8	NM_139274.1
99.5	96.1	102.6	89.1	NM_001100.3
98.9	98.5	97.6	96.2	NM_001613.1
22856.6	22728	19355.3	16778.5	NM_001101.2
20773.4	17653.2	15463.9	17648.2	NM_001101.2
77.3	75.6	82.1	83.3	NM_001004053.1
93.6	87.5	76.4	87.9	NM_005159.3

38970.5	35004.4	31842.7	25771.9	NM_001614.2
87.3	88.9	90.3	93.8	NM_001615.3
3630.6	2427.5	2094.2	1554.3	NM_177989.1
956.9	639.3	541.8	495.7	NM_004301.3
96.5	84.3	86.5	98	NM_016188.3
87.8	82.2	74.8	86	NM_006687.2
99.9	93.2	95.6	99	NM_006686.2
96.9	89	94.6	90.7	NM_030812.1
3204.2	3070.2	2502.4	1799.8	NM_001102.2
107.3	109.1	103.1	93.1	NM_001103.1
88.1	79.1	81.9	80.4	NM_001104.1
815.6	882.2	677.1	587.7	NM_004924.3
754.5	677.4	577.9	418.2	NM_018477.1
630.6	320.9	314.3	213.6	NM_005736.2
297.2	387.7	365.8	295.6	NM_005735.2
9327.2	6468.4	5028.5	4967.3	NM_001005386.1
999.7	733.9	607.9	694	NM_005721.3
133.2	116.1	98	110.9	NM_020445.4
87.3	86.7	81.7	97	NM_020445.4
111.5	94.1	87.6	97.1	NM_020445.4
216.9	219.8	240.1	166.4	NM_024855.2
472.5	360.8	334.1	342.1	NM_022496.2
180.5	167	156.4	153.9	NM_022899.3
113.5	112.3	112.1	101	NM_138289.1
330	282.1	272.9	221.4	NM_001105.2
97.9	99.9	97.1	92.2	NM_020327.2
103.7	94.9	91.5	97.2	NM_020328.2
363.5	327.5	301.7	216.6	NM_004302.3
83.4	91.9	78.3	92.4	NM_020327.2
87	85.4	95.3	95.4	NM_145259.1
160.7	174.4	163.4	145	NM_001616.3
89.2	90.9	72.8	86.3	NM_001106.2
106.9	110.3	108.5	105.2	NM_000020.1
836.9	538.7	454.9	403.5	NM_000666.1
234.5	173.4	162	207	NM_001010853.1
91.7	82.3	86.6	82	NM_080658.1
122.3	106	93.6	93.9	NM_001107.3
506.5	432.9	478.5	367.2	NM_203488.1
118.3	100.8	104.7	108.1	NM_203488.1
260.6	279.6	217.8	255.4	NM_138448.2
84.7	80.1	78.1	81.5	NM_032021.2
380.9	479.8	472.7	331	NM_000022.2
127.1	146.4	134.5	138.8	NM_001012969.1
99.8	90.7	90.3	91.3	NM_001110.2
99.1	104	106.1	102.6	NM_002390.3
92.1	77.7	82.2	85.8	NM_021641.2
84.5	82.8	76.8	81.7	NM_003474.3
81.3	79.3	84.2	79.5	NM_021641.2
218.7	176.6	175.2	178.1	NM_003815.3
3485.3	2506	2611.1	913.7	NM_207195.1

163.3	148.9	142	125.5	XM_937888.1
299.9	414.9	345.7	285.2	NM_003183.4
92.6	84	88.4	101.6	NM_014237.1
97.6	88.8	84	89.4	NM_023038.3
108.7	89.1	100.3	87.4	NM_023038.3
103.7	123.5	101.8	106.7	NM_033274.2
100.9	93.5	88.1	86.1	NM_001464.3
84.3	86.2	84.5	85.6	NM_003814.4
108	98.1	97.6	99.4	NM_003813.2
99.6	85.4	82.1	89.7	NM_004194.2
83.1	79.7	86.4	89.8	NM_021722.2
84.1	73.6	83.3	76.5	NM_021721.2
82.8	86.7	83.7	95.2	NM_003812.2
79.3	95.8	84.4	101.2	NM_021777.2
97.7	92.3	95.9	102.8	NM_021777.2
84.8	93.6	89.4	86.5	NM_014265.3
92.3	99.4	94.5	85.6	NM_014269.3
85.2	91.6	88.3	93.5	NM_021794.2
107.9	102.2	109.5	99.6	XM_940500.1
94.6	86.6	83.2	80.4	NM_145004.4
112.4	127.3	107.5	103.6	NM_025220.2
104.4	102.7	94	85.7	NM_153202.1
88.7	85.7	86	84.7	NR_001569.1
103.9	100.9	96.9	94.2	NR_002224.1
94.3	104.4	102.2	87.1	NM_003817.1
98.5	85.6	108.2	104.4	NM_001109.2
182.7	142.9	129.1	140.5	NM_003816.2
102.8	267.8	232.1	249.1	NM_014479.2
104.8	121.3	92.9	101.2	NM_006988.3
83.4	89.4	84.4	83.4	NM_030957.2
86.1	94.6	77.9	89	NM_030955.2
72.3	83.8	69.9	79.9	NM_139028.2
84.1	83.1	80.2	81.4	NM_139025.2
101.1	89.9	96.8	96.7	NM_139027.2
100.8	92.5	83.7	94.1	NM_139155.1
86.2	81.4	73.7	81.6	NM_139155.2
98.9	100.2	87.3	83.5	NM_139055.1
104.9	96.3	100.6	95.3	NM_139056.1
104.8	100.5	93.1	96.1	NM_139057.1
84.1	90.2	83.2	95.8	NM_139054.2
91.7	86.4	87.5	92.5	NM_199355.1
120.1	117.8	114.6	113.7	NM_133638.2
96.4	108.6	102.8	106.3	NM_014244.1
150.8	207.1	192.2	184.4	NM_021599.1
91.2	83.7	72.1	90	NM_175851.1
80.5	86.5	78	76.8	NM_025003.2
76.9	88.5	83.4	93.2	NM_014243.1
94.8	100.9	93.9	96.6	NM_005099.3
89.6	79.8	82.8	81.4	NM_007038.2
98.2	99	90.9	85.1	NM_197941.2

82.4	79.8	81.7	85.6	NM_014272.2
98.5	93.8	84.6	84.4	NM_007037.3
102.4	106.2	92.5	86.5	NM_182920.1
97.9	94.6	94.3	86.5	NM_139264.1
78.9	77.5	70.4	78.9	NM_139238.1
103.2	89.9	92.8	93.2	NM_052866.2
83.1	80.9	73.7	76	NM_052866.2
91.1	93.3	80.4	86.4	NM_014694.2
92.6	84.5	89.2	83.3	NM_207517.1
135.7	121.8	120.7	106.2	NM_213604.1
1118.2	1009.5	902.1	611.9	NM_015840.2
87.1	89.1	101.7	92.3	NM_001025107.1
694.5	759.5	611.3	521.3	NM_015840.2
115.3	95.4	95.7	91.4	NM_001112.2
142.4	172.2	164.5	142.1	NM_015833.2
91.6	88.9	77.5	86.7	NM_001033049.1
294.8	361.7	409.6	309.8	NM_001033049.1
90.7	84.8	84.1	81.1	NM_018702.1
348.2	360.4	295	288.7	NM_012091.2
101.3	99.7	95	90.6	NM_052998.2
191.6	194.4	189.5	168	NM_020421.2
833.9	692.5	675.2	372.8	NM_052853.3
104.8	105.1	93.1	100.1	NM_024876.2
173.9	166.2	150	144.1	NM_174922.3
251	756.1	577	531.5	NM_021116.1
116.7	104	93.4	95.3	NM_020546.1
2258.3	1565.2	1351.4	935.8	NM_004036.2
186	186.1	233.1	176.4	NM_139247.2
79.7	75.7	80.2	83.7	NM_183357.1
106.7	99.5	91.9	101.1	NM_020983.2
123.3	131.3	120	119.6	NM_015270.2
179.9	250.8	231.1	169.9	NM_001114.2
94.3	97.8	84	85.2	NM_001115.1
368.1	189.4	194	181.8	NM_001116.2
106.7	84.4	91.7	92.2	NM_001117.2
97.7	93.8	92.1	96.3	NM_001118.3
100.3	101.1	94.7	91	NM_001119.3
2272.2	1746.1	1805.6	1302.5	NM_001119.3
97.3	92	87.2	90.4	NM_017483.2
91.6	89.3	92	83	NM_017488.2
89.1	82.8	81.7	91.1	NM_001617.1
87.1	86.8	85.5	85.5	NM_019903.3
91.5	104.9	96.6	104.9	NM_016824.3
200.7	205.4	173.1	214.3	NM_016824.3
158.6	165.3	138.9	180.4	NM_001121.2
1763	3494.4	2156.9	1943.7	NM_001122.2
89	88.9	85.3	87.1	NM_000667.2
89.1	92.7	85.4	92.5	NM_000668.3
79	77.1	82	83.7	NM_000669.3
79.2	79.8	82.2	81.4	NM_000670.3

113.7	107.9	104.2	92.7	NM_000671.3
104.4	93.1	101.1	93.3	NM_000672.2
86.7	83.1	84.1	91.2	NM_000673.3
103.8	87.1	95.4	88.2	NM_144650.1
88.3	83.8	81.8	85.1	NM_001077593.1
102.9	91.1	91.2	106.7	NM_018269.1
98.5	90.9	86.4	85.5	NM_004797.2
460	518.5	409.6	373.3	NM_015999.2
1020.2	872.2	782.6	539	NM_024551.2
147.4	117	124.3	120.4	NM_006721.2
92.1	80.9	83.9	92	NM_001123.2
226.1	182	174.2	164.3	NM_001123.2
124.5	176.1	166.2	149.1	NM_001124.1
261.6	199.3	188.8	154.5	NM_024866.3
98.7	90.7	87	89.1	NM_007264.3
371.3	306.4	278.8	258.5	NM_181442.1
118.6	167.6	159.5	179.4	NM_015339.2
88.2	76.3	79.3	78.6	NM_000674.2
98.1	87	82.1	95	NM_000674.2
127	115.5	108.7	117.7	NM_000675.3
475.6	589.6	467.2	344.6	NM_000676.2
113.6	155.8	152.5	131.7	NM_020683.5
183.3	174.1	158.9	117.7	NM_000677.2
773.2	1073	846.7	657.4	NM_031284.3
98.3	107.8	112.9	113.9	NM_025225.2
98.2	102.6	86.1	95.4	NM_001125.2
89.1	81.7	85.6	89.6	NM_138430.3
94.5	95.6	89.8	95.4	NM_199162.1
91.7	93.1	92.7	87.9	NM_138430.3
192.8	191.5	212.7	139	NM_017825.1
90.3	85.4	82.5	84.4	NM_000680.1
84.8	79.2	81.9	86.5	NM_033302.1
84.4	79.9	77.5	79.5	NM_000680.1
97.9	107.2	91.2	87.4	NM_033304.1
106.3	90.1	84.2	92.3	NM_033303.2
105.5	99.8	107.9	90.4	NM_000679.3
98.5	84.1	84.6	93.8	NM_000678.2
96.6	97.6	93.5	94	NM_000681.2
117.3	112.7	109.7	92.5	NM_000682.4
103.7	110.4	100	92.5	NM_000683.3
104.7	95.9	98.1	107.3	NM_000684.1
147.8	136.9	170	141.4	NM_000024.3
91.7	92.5	89.9	93.1	NM_000025.1
86.2	86.5	89.5	93.2	NM_001619.2
83.9	99.5	89.7	77.5	NM_005160.2
548.5	403.9	299.3	315.4	NM_175573.1
1041.3	729.9	548.6	547.4	NM_007002.2
4595	4013.6	3579.3	2541.6	NM_000026.1
2597.4	1729.4	1376.6	1374	NM_001126.2
89.3	87	84.9	90.9	NM_199165.1

191.5	190.2	189.3	159.7	NM_199165.1
108.8	107.2	101.9	96.6	NM_152328.3
87.8	79.9	72.5	83.1	NM_001129.3
125.1	107.6	115.3	112.3	NM_153207.3
874.5	581.1	497.1	463.9	NM_198969.1
493.8	316.3	261.9	310.6	NM_198970.1
93.9	93.8	83.3	90	NM_198969.1
82.5	95.8	99.3	89.8	NM_021638.3
123.9	124	125.8	94	NM_198595.1
108.7	105.1	98.2	106	NM_198595.1
90.6	92.6	79.9	86.9	NM_032550.2
95.1	96.1	95.8	87.8	NM_201252.1
83	86.1	84	95.5	NR_002796.1
96	96.8	88.2	90.3	XR_000989.1
182.6	142	218.7	186.6	NM_005935.1
105.2	110.6	108.9	111.3	NM_002025.1
230.2	289.6	340.5	278.4	NM_002285.2
90.2	101.1	97.5	91.3	NM_001025108.1
194.3	190.1	159.4	140.9	NM_014423.2
535	499.3	407.6	446.8	NM_001031805.1
133.9	128	150.4	123.8	NM_001031805.1
94.4	98.4	84.1	82.2	NR_003228.1
85.7	87.5	94.7	93.9	NR_003228.1
1147.2	935.9	1168.3	635.8	NM_006796.1
74.4	84.6	85.5	82.7	NM_001133.2
93.4	96.7	88	90.8	NM_001010982.1
94.4	90.6	73.6	79.9	NM_001134.1
134.4	102.6	130.8	112	NM_203437.2
111.4	111.6	116.3	104.4	NM_203437.2
204	258.2	168.4	176.8	NM_017657.3
334.1	229.3	234.8	225.4	NM_000027.2
83.7	91	88.8	88.7	NM_152336.2
99.5	99.1	88	84.7	NM_024783.2
85.8	82.6	82.2	82.5	NM_032785.1
106.8	110.9	98.9	104.7	NM_001135.1
81.2	77.4	73.2	90.4	NM_013227.1
89.4	92.7	81.6	86.2	NM_001136.3
100.3	120.9	103.4	112.6	NM_001136.3
802.6	945.4	774.1	616	NM_018046.3
86	90	83.3	89.9	NM_000642.1
90.6	86.5	99.5	102.8	NM_000646.2
357.4	298.3	284.6	312.1	NM_000645.2
94.5	88.7	81	88.5	NM_000028.1
91.8	101.4	97.1	86.3	NM_000645.2
150.9	120.3	122.7	144.9	NM_000645.2
322.2	209.2	192.6	155.7	NM_024758.3
88.8	82.3	83.1	82.5	NM_032741.3
92.7	96.1	83.7	88.4	NM_006411.2
229	313.8	236.8	293.2	NM_006411.2
732.6	545.3	527.2	480.2	NM_006412.3

652.4	442.3	383.8	345.4	NM_006412.3
442.6	342.3	299.9	231.4	NM_001012727.1
92.3	98.4	93.5	103.8	NM_020132.4
166.5	200.1	192.1	168.7	NM_020132.3
149.9	166	164.1	170.6	NM_001012734.1
455.7	288	271	206.9	NM_018361.2
89.3	89.9	80.2	102.6	NM_178819.2
116.2	114.8	110.3	110.4	NM_153613.1
1044.1	887.1	683.4	646	NM_003659.1
88.4	78.2	70.2	82.7	NM_006408.2
88.5	89.4	81.3	86.3	NM_176813.3
100.8	79.1	84.7	89.2	NM_198576.2
86.7	82.8	76.7	89.1	NM_001138.1
101.1	87.2	85.5	89.1	NM_007316.1
701.9	874.9	777.5	576.2	NM_000029.2
96.4	100.3	81.2	105.1	NM_015239.1
74.6	89.2	79.2	86.4	NM_032049.1
82.9	90.7	83.8	99.7	NM_004835.3
88.7	109.3	92.9	79.8	NM_000686.3
187.4	152.4	134.4	139.1	NM_020350.3
147.1	128.9	137.8	113.1	NM_001040194.1
94.3	91.4	79.1	86.1	NM_005161.2
86.5	86.8	97.2	82	NM_000030.1
86.9	94.9	77.6	85.7	NM_031900.1
80.6	82.6	87	81.1	NM_031279.2
577.8	637.6	586.3	547.3	NM_015446.3
719.9	748.9	554.6	522.8	XM_942402.1
5220.2	4680	3407.4	3345.1	NM_000687.1
1653.7	1069.4	1023.1	833.6	NM_006621.3
111.3	109.2	113	125.9	NM_001029882.1
219.4	272.6	246.3	227.4	NM_017651.3
765.5	1641.8	1809.9	1371.7	NM_001620.1
140.4	111.3	121.9	113.8	NM_024060.2
112.4	85.1	92.4	94.8	NM_024060.2
112.6	94.8	93.8	99.1	NM_001621.3
105	106	106.6	106.7	NM_020731.2
1039.4	653.4	585.9	432.1	NM_012111.1
164.4	142.8	197.5	197.3	NM_152392.1
101.9	89.3	88.9	89.2	NM_001622.1
87.3	86.3	79.3	85.4	NM_020661.1
2692.1	4010.6	2929.7	2272.3	NM_001623.3
7030.7	6045.4	4991.4	7242.5	NM_001623.3
105.6	102.3	96.2	111.4	NM_004847.2
93.5	92.3	92.5	86.5	NM_144704.1
1039.4	1018	1044.6	674.2	NM_145813.1
418.3	649.2	478.8	481.2	NM_016108.2
114.9	152	122.2	117.9	NM_001624.1
94.2	88.3	86.4	90.4	NM_017977.1
93.1	86.7	86.6	92.6	NM_004833.1
896.9	752.3	702.6	561.8	NM_003977.1

98.9	88.6	108.5	89.6	NM_014336.3
97.3	101.5	89.4	86.7	NM_001033054.1
96	99.7	90.9	88.2	NM_000659.1
2512.8	4101.8	5157.3	2879.1	NM_000383.1
81.1	88.4	75.8	87.5	NM_018836.2
84.8	91.5	71.4	87.3	NM_001042478.1
105	103.2	100.7	98.6	NM_001042478.1
260.5	199.8	180.6	139.7	NM_000476.1
344.9	229.4	272.1	221.9	NM_001625.2
391.6	375.8	335.4	252.6	NM_013411.3
2658.2	1875.9	1918.1	1269.3	NM_001625.2
1705.8	1252.2	1232	947.3	NM_016282.2
515.5	464.2	394.1	407	NM_013410.2
83.7	86.1	85	82.2	NM_203464.1
90	81.1	79.2	78.6	NM_174858.1
85.8	94.5	80.3	83.2	NM_012093.2
83.2	90.8	89.6	92.1	NM_012093.2
94.9	86.9	92	87.5	NM_152327.1
154.7	142.7	131.5	189.9	NM_003488.2
120.1	135.3	129.2	118.4	NM_007202.2
80.4	80.3	71.7	79.7	NM_144490.1
280.3	346.8	328.2	341.7	NM_016248.2
119	127.1	123.6	120.8	NM_144490.1
92.5	90.8	91.3	99.3	NM_005100.2
77.5	92.8	77.2	82.6	NM_144497.1
151.6	139.2	102.9	112.8	NM_005100.2
117.5	169.2	142.3	131.9	NM_006738.4
246.7	554.9	545.4	292.9	NM_007200.3
85.7	70.4	89.5	77.4	NM_178813.5
90	79.5	82.3	91.4	NM_001008535.1
101.3	93.5	89.3	93	NM_001008535.1
86.5	91	79.6	96.6	NM_001004065.2
96	92.8	102.3	91.8	NM_006422.2
90.6	93.6	78.4	89	NM_003886.2
109.2	105.8	91.5	96.6	NM_139289.1
101.6	106.5	95.8	92.7	NM_139289.1
76.2	77.4	75.7	79.7	NM_004857.2
84.6	90.6	85.9	91.4	NM_004274.3
81.6	81.5	73.8	77	NM_138633.1
100.8	99.8	86.7	99.2	NM_016377.2
796.7	664.8	676.6	601.7	NM_016377.2
102.8	102.6	112.2	95.5	NM_004842.2
613.3	540.7	462.5	384.5	NM_005858.2
227	217.9	199.9	186	NM_014371.2
87.3	87.5	83.5	92.9	NM_147185.1
89.9	88.5	86.2	90.6	NM_147166.1
270.3	341.3	323.7	298.3	NM_030767.2
1071.8	1197.9	1236.6	939.3	NM_006066.2
101.4	84.2	100	100.9	NM_006066.2
824	817.4	825.4	697.3	NM_153326.1

2421.6	1898.3	1520.7	1336.8	NM_001628.2
88.9	84.2	79.8	83.7	NM_020299.3
82.9	70.5	73.3	86.3	NM_001353.5
542.3	551.2	378.5	272.1	NM_001354.4
123.4	139.5	114.7	106.1	NM_003739.4
101.3	97.2	93.1	99.1	NM_001818.2
107.5	105.7	96.6	92.5	NM_001007536.1
94.2	85.7	88.1	81.9	NM_031436.2
4109.8	7120.3	8995	5059.1	NM_005989.2
2519.7	1984.3	2145.4	1086.6	NM_003689.2
545.4	417.5	358.1	287.3	NM_012067.2
2932.6	2541	2126.8	1256.8	NM_001014432.1
2216.6	1540.2	1539.9	1145.9	NM_001014431.1
85.3	93.5	91.3	78.9	NM_005163.2
104.4	111.5	102.6	93.8	NM_032375.2
113.1	114.8	107.8	100.3	NM_001626.2
99.6	90.8	81.4	93	NM_181690.1
94.6	91.8	84.6	84.4	NM_005465.3
98.1	89	99.1	82.3	NM_181690.1
312.3	217.6	199.2	209.7	NM_001012398.1
87.9	87.4	77.6	86.5	NM_000031.4
412.2	491.3	414.6	284.6	NM_001003945.1
91.4	81.6	78.7	85.9	NM_001003945.1
106.3	103.6	94.9	112	NM_000688.4
122.6	110	111	92.5	NM_199166.1
94.5	93.6	86.6	94.7	NM_000688.4
87.7	91.2	86.8	85.9	NM_000032.1
87.7	86.9	88.9	85.1	NM_001037968.1
98.1	88.8	88	98.1	NM_001037968.1
95.3	104.9	91.1	90.6	NM_000477.3
427	600	372.7	485.3	NM_001627.2
375.7	262.2	231.6	224.4	NM_153329.2
312	281.2	234.6	163.2	NM_002860.3
86.1	88.6	83.3	95.3	NM_000689.3
87.8	104	88.9	95.6	NM_170696.1
114.9	104.1	108	101.1	NM_170696.1
92.4	86	84.8	96.7	NM_000693.1
265.1	163.8	171	146.3	NM_000692.3
98.2	88.7	83.8	83.8	NM_012190.2
89.3	92.8	83.3	88.3	NM_001034173.1
831.2	1424.5	1112.2	909.5	NM_000690.2
94.8	98.2	85.2	98.2	NM_000691.3
102.9	114.3	100.3	96.7	NM_001031806.1
171.8	234.8	214	168	NM_001031806.1
97.5	112.8	91.6	107	NM_000694.2
473.2	547.8	509.9	404.3	NM_001030010.1
91	93.8	96.8	89.2	NM_001031615.1
73.8	82.9	72	88.8	NM_000695.3
87.8	81	85	87.4	NM_000695.3
93.4	84.8	75.9	92.7	NM_003748.2

111.3	121.3	111.8	98.3	NM_170726.1
106.7	111.5	100.3	94.7	NM_170726.1
210.3	253.3	208.8	220.4	NM_001080.3
103.3	94.2	96.2	96.4	NM_005589.2
182.9	200.8	208.3	171.9	NM_001182.2
112.1	116.6	122.1	116.4	NM_022568.2
83.1	81.8	90.5	89.4	NM_022568.2
6701.8	4413.3	4361	2614.5	NM_000696.2
114.9	109.5	113.7	98.5	NM_000034.2
5466.1	4371	4071	3483.3	NM_184041.1
107.3	108.9	99.3	90.6	NM_184043.1
5443.2	3654.3	3123	2319.4	NM_184041.1
90.3	105.7	99.2	96.6	NM_000035.2
502.2	407.2	402.1	232.4	NM_005165.2
100	94.2	89.8	90.7	NM_172196.1
83.7	76.9	83	85.4	NM_006872.2
259.4	143.9	150.4	124.1	NM_019109.3
109.7	99.1	105.3	98	NM_032834.2
113.6	143.6	133.1	155.4	NM_001004127.1
120.5	111	118.8	100.9	NM_024105.3
115	94.1	93.1	102.5	NM_018466.2
167.3	125.7	124.2	133.7	NM_144988.2
95.4	94.7	88.8	93.2	NM_033087.2
915.3	615	566.2	333.4	NM_001006940.1
1107.6	663.8	561.5	699.9	NM_013338.3
365.4	324	263.2	283.6	NM_013339.2
1022	595.7	499.9	551.9	NM_001007027.2
89.5	96	94.9	96.5	NM_001007027.2
2198.2	1222.8	995	842.9	NM_001007027.1
382.1	243.8	231.9	177.4	NM_024740.1
95.1	87.3	92.7	82	NM_004304.3
339.7	200.1	193	199	NM_006020.1
140.7	122.1	105.4	123.9	NM_006020.2
350.8	228.9	193.8	209.8	NM_006020.2
227.7	164.2	162.1	134.2	NM_001001655.1
582.7	590.1	540.9	490.7	NM_139178.1
152.3	163.6	120.6	121.2	NM_017621.2
2714.1	2720.3	2561.5	1327.2	NM_017758.2
316.5	289.9	313.3	199.3	NM_198867.1
308.9	290.5	244.3	204	NM_032306.2
194.8	164.5	158.9	129.4	NM_138775.1
88.2	91	88.7	93.8	NM_199232.1
87.9	74.1	73.3	80.7	NM_018436.2
83	95.8	95.2	89.2	NM_018436.2
154.9	175.1	164.2	151.2	NM_015120.2
85.1	87.6	85.4	87.4	NM_000697.1
94	86.3	85.9	84.8	NM_001139.1
89.7	82.3	78.5	95	NR_002710.2
107.9	92.1	99.7	99.3	XM_937556.1
90.1	91.7	74.4	78.7	NM_001140.3

96.8	90.1	77.8	82.2	NM_001141.2
94.8	85.6	88.3	90	NM_001141.1
102.6	97.1	112.7	86.4	NM_001039131.1
558.1	1147	971.5	476.2	NM_000698.2
105.1	104.4	95.2	102	NM_000698.2
2431.8	2480	2173.7	1535.8	NM_001629.2
99.9	104.7	98.7	90.8	NM_021628.1
95.6	91.5	87.6	90.8	NM_001631.2
213.1	198.4	178.8	184.6	NM_025144.2
87.3	90.5	87.2	90.8	NM_020778.1
99.7	94.6	88.6	92.4	NM_000478.2
1239	1989	2867.8	1895.5	NM_001632.2
136.9	134.3	139.6	122.4	NM_031313.1
354	491.9	413.3	352.8	NM_020919.2
97.4	90.5	81.1	90	NM_147129.2
100.5	93.8	82.3	93.6	NM_147129.2
83.9	87.3	81.8	85.6	NM_182775.1
100.7	103.2	94	90.7	NM_152525.3
93.4	73.7	84.6	83.2	NM_139163.1
537.2	888	772.4	723.8	NM_173511.2
114.6	114.4	109.2	111.1	NM_138468.3
87.8	99	94.7	99.5	NM_205543.1
90.7	87.5	88.3	90.7	NM_152526.3
424.9	483.5	430.3	369.5	NM_018571.4
387.6	275	235.2	204.2	NM_152388.1
83.9	85.9	86.3	94.4	NM_139158.1
118.8	100.6	101.9	99.8	NM_024744.12
81.1	84.4	80	81.9	NM_006492.2
88.7	87	83.7	81.7	NM_021926.2
91.4	100.1	77.7	83	NM_152462.1
100.7	104.5	98.2	106.9	NM_054028.1
124.3	144.8	117.7	111.9	NM_014324.4
132.6	141.2	121	123.6	NM_203382.1
97.5	96.5	86.8	100.7	NM_016519.4
89.6	81.9	84.4	91.2	NM_016519.4
106.9	105.4	97.2	92.5	NM_001633.2
2575.7	1521.5	1559.3	1048.5	NM_001033059.1
746.9	546.2	533.6	377.6	NM_001634.4
99.4	105.5	101.4	90.1	NM_152435.1
121.5	109.3	99.1	109.5	NM_015944.2
94.2	86.6	96.4	97.6	NM_001142.2
109.8	100.7	105	95	NM_001143.1
393.1	331.5	236.2	240.3	NM_138958.1
121.2	120.1	98	112.1	NM_001144.3
93.8	79.9	78.5	92.1	NM_000479.2
92.1	85.9	87.4	87.2	NM_020547.1
114.5	142.1	148.9	141.5	NM_153206.1
148	137.6	118.3	131	NM_032797.4
93.7	109.6	89	89.2	NM_020703.1
106.4	86.9	96.1	97.1	NM_181847.2

87.2	83.3	99.4	92.9	NM_198722.1
105	102.4	92.6	98.4	NM_015365.2
211.4	198.9	175	190.4	NM_001025580.1
199.9	174.9	149.7	152.4	NM_001025580.1
106.6	97.3	98.3	96.7	NM_030943.1
106.4	121.9	96.9	101.9	NM_133265.2
88.1	87.6	85.2	81.1	NM_016201.2
90.2	75	84.6	85.3	NM_000036.1
91.1	89.2	83.2	92.7	NM_004037.6
74	85.2	79.2	85.6	NM_203404.1
94.2	84.4	86.8	84.8	NM_139156.2
107.9	101.3	101.2	106.2	NM_001025389.1
86.5	93.8	92	93.1	NM_000480.2
110.6	96.3	84	90.6	NM_001025389.1
96.3	87.4	77.3	98.8	NM_001025390.1
100.9	93.1	97.2	88.6	NM_001635.2
88.1	94.8	87.4	85.5	NM_001635.2
148.7	141.4	168.4	102.5	NM_000481.2
93.2	79.6	84.3	85.6	NM_001008221.1
457.3	350.7	345.2	352.1	NM_004038.3
91.4	79.3	80.3	76.6	NM_004038.3
86.6	92.4	80.8	83.1	NM_001008218.1
102	95.3	87.4	84.7	NM_000699.2
101.9	105.9	101.2	110.8	NM_020978.3
89.7	91.8	86.2	101.5	NM_133463.1
96	88.2	96.3	85.4	NM_001033574.1
1081.1	1222.2	974.2	853.1	NM_016627.4
1119.6	1208.8	1106.6	718.6	NM_022662.2
750.6	492.7	424.9	387.9	NM_014885.1
926.7	670.8	676.5	397.9	NM_001002246.1
91.5	95.3	88.7	93.3	NM_001002244.1
819.1	807.6	810.5	464	NM_001002247.1
2832.6	2259.8	2158.8	1483.2	NM_015391.1
98.5	117.6	106.7	101	NM_013366.3
130	140.9	114.2	112.4	NM_013366.3
741.1	705.4	712.3	448.3	NM_013367.2
2044.3	2175.2	1800.3	1465.1	NM_016237.3
199.6	147.2	141.7	152.4	NM_016238.1
121.9	138.6	150.5	115.2	NM_001145.2
179.4	171	148.5	159	NM_015305.2
901.5	830.7	1070.6	778.8	NM_144567.3
104.5	107.2	100.3	88.2	NM_001146.3
103.2	98.3	92.1	99.4	NM_001147.1
78.8	80.6	81.6	85.8	NM_015985.2
94.7	93.4	82.7	78.3	NM_004673.3
96	92.6	79.8	75.3	NM_012098.2
87.8	88	83.2	86.1	NM_014495.2
91.5	88.4	78.4	86.3	NM_139314.1
85.6	85.9	90	81.2	NM_001039667.1
94.3	93.5	85.5	87.3	NM_139314.1

87.7	89.5	87.7	97	NM_178127.2
95.3	112.8	123.6	129.9	NM_031917.2
91.8	94.9	86.9	100.5	NM_021146.2
100.6	98.5	88.3	90.3	NM_020475.2
85.6	93.7	77.4	76.6	NM_020480.2
91.2	90.3	77.9	78.6	NM_020977.2
98.2	85.6	90.3	91.7	NM_001148.3
100.4	102.6	95	94.7	NM_001149.2
105.3	103.5	98.8	82	NM_001149.2
107.2	92.6	79.9	84.4	NM_020987.2
93.9	88.8	86.9	94.8	NM_144708.2
202.3	176.2	171.9	162.7	NM_182703.2
76.9	82.5	76.1	86.5	NM_153228.1
165.6	277.6	249.5	169.7	NM_016376.2
134.9	159.2	172.1	166.7	NM_020740.1
89.4	103.4	105	101.7	NM_020740.1
81.3	80.3	83.6	93	NM_054027.3
115.1	117.3	129.8	112.1	NM_024668.2
79.5	79.1	81.4	75.1	NM_017978.1
88.5	77.8	82.2	89.9	NM_178510.1
240	329.1	394.2	218	NM_016552.2
86.9	94.4	80	84.9	NM_017844.2
89.2	94.6	93.1	93.5	NM_016552.2
433.5	356.2	341.2	288.3	NM_020319.1
310.3	449.6	429.8	338.7	NM_023039.2
92.8	91.7	98.7	94.2	NM_014391.2
412.6	321.1	384	345.7	NM_017664.2
439.3	634.4	610.6	426.9	NM_013275.4
275.6	494.9	479.6	405.7	NM_015208.2
536.2	551.6	530	472.3	NM_033121.1
111	95.8	97.4	103.1	NM_152345.3
276.7	220.7	210	221.1	NM_030816.2
97.5	102.3	91.3	93.5	NM_207354.1
96.3	92.7	89.3	93.7	NM_015158.2
89.3	88.6	73.9	94.4	NM_153186.3
321.9	276.7	230.4	266.6	NM_153186.3
182.6	128.4	126.5	111.4	NM_001009941.1
83.1	91.3	83.2	86.4	NM_019046.1
150.9	102.6	104.3	114.1	NM_001009941.1
120.9	117.3	110	109.4	NM_032217.3
231.8	249.9	246.1	206.2	NM_198889.1
439.2	401.2	323.1	398	NM_198889.1
90.8	97.2	82.2	81	NM_001010925.1
90.2	100	92.5	94.8	NM_020349.2
144.6	182.3	180.4	192.6	NM_032250.1
90.3	96.5	90.2	90.7	NM_001012419.1
89.2	85.2	75.8	85.7	NM_001012419.1
88.8	83.5	86.7	90.2	NR_003366.1
85.6	88.3	76.4	99.3	XM_934275.1
87	86.7	90.9	94	NM_174981.2

85.6	76.9	76.8	80.9	NM_144590.1
110	99.5	91.3	88.4	NM_144994.7
79.5	80.5	89.9	83.4	NM_133475.1
184.9	180.8	170.5	163.5	NM_015493.4
127.8	125.7	119.1	123.3	NM_014915.1
143.7	113.3	104.5	127.2	NM_032139.1
194.8	180.9	178.8	177.6	NM_015199.2
100.4	101.1	90.7	98.6	NM_173505.2
103.5	83.9	84.1	84.5	NM_052997.1
89.7	78.3	87.3	85.7	XM_940707.1
88.5	82.5	82.4	92.5	NM_001029862.1
202.1	191.6	168	170.9	NM_032290.2
225.4	307.5	249.6	185.6	NM_182608.2
105.9	95.6	99.8	87.9	NM_001039888.2
87.6	82.9	81.9	79	NM_144698.3
104.2	100	105	100	NM_198555.3
88.6	80.2	87.3	94.8	NM_198555.3
127.5	127.8	151.8	130	NM_198555.3
119.7	202.3	171.7	174.7	NM_181726.1
89.7	103.9	96.5	94	NM_181712.2
334.6	231.5	209.9	192.5	NM_016466.4
87.5	104.2	90.4	89.4	NM_052855.2
124.4	108.4	106.1	120.5	NM_152363.2
88.8	88.4	95.4	85.3	NM_182603.1
88.8	89.4	89.8	90.7	NM_175873.3
109.2	130.5	134.3	124.2	NM_153697.1
88.9	82.3	83.1	95.4	NM_198493.1
645.8	436.1	368	337	NM_198401.2
104.7	100.2	96.9	108.9	NM_198471.1
575	405.8	343.8	429.3	NM_017704.2
89.2	91	84.4	90.4	NM_022096.4
81.5	80.5	76.2	79.3	NM_022096.4
83	86.6	84.1	88.8	NM_198798.1
280.8	309.2	288	270.4	NM_020337.1
113.8	124	110.5	106.2	NM_001039935.1
86.8	89.3	80.7	93.7	NM_001029870.1
196.1	187	200.3	184.8	NM_014942.2
100.1	98.5	98.9	85.7	NM_001077708.1
100.1	96.8	89.6	85.6	NM_019644.1
687.1	575.6	516.8	280.7	NM_152326.2
440.1	542.6	508.2	357.4	NM_015245.1
80.8	77	78.1	80.7	NM_181670.2
83.7	83.8	77	79.8	NM_152788.3
93	93.8	98.5	79.5	NM_020140.2
129.7	118.3	134.3	125.2	NM_133450.1
81.6	94	77.1	93.5	NM_145865.1
90.3	85.1	85	87.7	NM_173551.3
245.2	226.2	225.2	182.9	NM_018089.1
101.8	103.7	107.4	93.1	NM_018089.1
100.9	97.5	109.7	114.8	NM_018089.1

375.7	252.8	248.7	312.7	NM_018685.2
106.6	113.8	102.5	99.2	NM_006305.2
16167.2	9892.3	8760.2	8050.7	NM_006401.1
99.7	100.3	80.2	78.5	NM_012403.1
78.6	75.6	75.8	83.3	NM_012404.2
84.1	88.9	74.4	85.9	NM_030920.2
573.2	774.5	740.1	588.9	NM_001150.1
214.9	212.2	215	188.9	NM_032208.1
119	124.3	100.6	105.5	NM_018153.2
102.2	92.9	99	99.8	NM_053034.1
231.3	223.5	200.6	210.5	NM_058172.3
88.7	86.8	81	83.7	NM_174890.1
5847.5	6432.9	4329	5753.2	NM_000700.1
79.9	77.3	76.3	81.6	NM_007193.3
209.9	154.6	151.7	155.1	NM_001157.2
432.2	343.7	375	253.4	NM_145868.1
97.1	97.9	94	105.8	NM_145869.1
81.7	78.9	76	88.4	NM_004306.2
95.5	89.5	80.2	100.1	NM_001003954.1
103.6	92.3	93.3	92.5	NM_001003954.1
864.1	736.8	613.6	468.1	NM_001002857.1
272.5	225.9	202.5	172.6	NM_001002857.1
6137.6	6219.9	4896.9	3439.5	NM_001002858.1
833.9	804.4	604.7	792.6	NR_001562.1
87.5	74.2	76.4	76.9	NM_005139.1
141.9	139	146.6	152.6	NM_001153.2
3108.6	2971.7	2550.9	2072.8	NM_001154.2
123.7	140	125.8	115.6	NM_004033.2
244.8	193.7	196.4	174.7	NM_001155.3
87.9	83.7	86	88.1	NM_004034.1
1843.1	1021.9	956.2	934.9	NM_004034.1
112.9	109.8	113.5	93.1	NM_001630.2
116.4	107.8	86.6	92.5	NM_001040084.1
85	89.3	86.5	95.5	NM_003568.1
106	109	117.8	103.9	NM_001637.1
102.6	108.9	106.2	89.9	NM_009590.2
106.5	88.6	87.9	93.3	NM_001158.3
91.9	93.8	81.4	86.3	NM_001158.3
90.1	83.2	82.9	80.1	NM_003734.2
81.3	90.8	85.8	85.9	NM_153042.2
683.8	874.8	758.5	515.6	NM_015013.2
87.2	86.4	84.8	84.5	NM_001159.3
116	114.8	95.7	101.8	NM_145730.1
513.6	515.6	502.5	245.5	NM_001127.2
380.7	339	240.6	268.9	NM_001128.5
87.8	82.8	79.4	74	NM_080545.1
224.7	190.4	201.3	167	NM_003917.2
94.8	102	98.2	93.5	NM_007247.3
91.4	88.1	91.1	94.1	NM_007247.3
1437.6	1028.2	974.2	409.9	NM_032493.2

87.2	98.6	76.8	79.6	NM_005498.3
428.9	389.1	406.4	278.5	NM_057089.1
780.5	560.7	491.8	421	NM_001283.2
4701.3	4384.5	4048.2	3297.1	NM_003916.3
85	81.1	78.6	85.3	NM_178814.2
111.7	113.4	99	108.1	NM_130787.2
110.7	95.3	110.2	94.7	NM_130787.2
107.6	100.6	94.7	103.1	NM_014203.2
136.2	128.6	112.3	121.9	NM_012305.2
137.5	148.1	132.1	125.1	NM_001282.2
132.9	122.8	110.6	122.4	NM_001282.2
919.9	943.6	762.5	618.9	NM_004068.3
868.8	927.1	896.2	753.2	NM_004068.3
4468.2	2088.9	2208.1	1720.4	NM_004069.3
20100.6	12127.9	11993.7	7744.1	NM_004069.3
2650.7	2108.2	1710.9	1543.9	NM_003664.3
94.3	95.6	86.1	103.9	NM_004644.3
79.9	90.4	95.9	92.7	NM_001077523.1
688.2	610.3	552.6	368.3	NM_003938.4
755.8	651.4	502.1	413.1	NM_001077523.1
637.4	407.5	316.7	350	NM_012095.4
98.5	94.1	80.7	101.7	NM_207012.1
884.5	640.1	450	398.7	NM_207012.1
413.8	280.3	286	180.3	NM_006803.2
653.9	598	615.1	503.6	NM_001002924.1
143.3	147.7	150.4	123.7	NM_005829.3
1236.2	703.6	673.9	523.2	NM_006594.1
388.9	393.9	366.6	359.9	NM_007347.3
97.3	112.4	115	100.6	NM_004722.2
97.9	101.8	100.3	104.8	NM_007077.2
680.2	920.5	839.5	849.5	NM_181869.1
108.6	93.9	103.4	109.8	NM_181869.1
96	88.6	96.7	98.8	NM_001163.2
84.1	88.6	79.1	96.4	NM_005503.2
112.6	127.8	119	110	NM_031232.2
79.3	73.9	74.3	84.2	NM_031232.2
95	93.7	90.3	91.2	NM_031232.2
336.5	241.5	219.6	174.4	NM_004886.3
76.5	89.5	78.4	74	NM_001164.2
91.1	85	71	83.9	NM_145689.1
97.1	81	78.6	81.9	NM_001164.2
550.7	595	428.6	411.8	NM_019043.3
96.1	86.9	90.9	89	NM_173075.2
271.9	526.8	517	295.9	NM_006051.2
125.2	204.1	212.7	156.5	NM_133173.2
87	79.7	76.1	85	NM_000038.3
91	98.1	94.5	98.6	NM_005883.2
123.5	125.4	102.4	117.5	NM_153000.3
129.6	114.8	118.7	98.5	NM_001639.2
819	798.3	658	531	NM_001640.3

3279.6	2467.6	2254.3	1577.8	NM_001641.2
6394.4	5136	4115.3	3888.2	NM_080649.1
572.2	392.7	344.1	286.9	NM_014481.2
1765.8	1363	1142.3	1073	NM_016022.1
173.6	172.7	129.2	168.3	NM_001077628.1
488.4	343.8	368.1	256.7	NM_031301.2
835.1	841	543.8	686.7	NM_006595.2
94.3	87.9	84.5	81.8	NM_017855.2
205.7	237.1	208.2	205.2	NM_015957.1
117.8	105.6	103.2	106.4	NM_199295.1
631.1	469.3	449.7	402.9	NM_198544.2
90.3	92.3	86.1	89.1	NM_017413.3
92.7	83.2	81	74.1	NM_005166.3
107.2	88.9	80.8	95	NM_005166.3
93.2	95.5	96.3	84.2	NM_001642.1
88.6	86.2	77.9	86.5	NM_000039.1
2394.4	1508.3	1443.6	1070.9	NM_144772.1
119.7	104.5	112.9	98.8	NM_001643.1
84.1	71.4	76.1	78.8	NM_000482.3
90	87.8	81.6	88.9	NM_052968.3
83.6	78.4	84	86.7	NM_000384.1
255.3	300.4	292	231.5	NM_018690.1
99.8	89.9	98.7	94.8	NM_005889.2
85.6	88	83.4	92.9	NM_006789.2
87.5	85	84.7	89.8	NM_145699.2
403.1	281.1	294.4	216.4	NM_004900.3
146.1	161.6	135.3	145.7	NM_014508.2
94.9	85.8	85.9	86.1	NM_152426.2
138.6	159.2	119.4	122.5	NM_001006666.1
172.8	153.5	160.9	130.4	NM_001006666.1
114.6	107.6	109.6	111.1	NM_145298.4
199.4	199.2	170.9	159.2	NM_021822.1
79.6	90.4	93.6	90.2	NM_203454.1
346.6	517.9	544	352.5	NM_001645.3
706.4	621.5	526.6	584.6	NM_000483.3
95.3	90.1	86.6	85.5	NM_000040.1
86.2	92.3	91.2	93.1	NM_001646.1
84.8	83.9	74.2	88.5	NM_001647.2
171.8	1366.8	1325.7	1130.7	NM_000041.2
88.3	85.7	84.8	79.1	NM_001638.2
88.1	79.4	82.9	80.8	NM_000042.1
99.7	105.3	89.1	115	NM_145344.1
88.8	82.6	83.7	81.4	NM_145343.1
93	83.1	80	84.3	NM_030882.2
94	115.5	114.9	99.8	NM_145637.1
100.2	99.5	97.7	84.7	NM_145637.1
81.8	83.1	81.4	80	NM_014349.2
85.4	87.7	87.4	94.1	NM_030644.1
89.9	76.5	78	85.4	NM_145641.1
90	85.3	94.1	81.5	NM_030643.3

92.7	92.2	90.7	95.2	NM_030643.3
87.8	82.3	88	85	NM_030642.1
127.9	119.6	114	112.8	NM_030641.2
155.8	109.9	117.8	131.8	NM_030817.1
183.8	186.3	163.8	154.6	NM_019101.2
937.1	1411.2	1019.2	1151.4	NM_201414.1
103.4	113.4	107.6	119.4	NM_000484.2
580.3	1110.8	825.9	718.9	NM_201414.1
130.1	124.5	130.6	109.4	NM_001018159.1
2109.1	1421.6	1157.9	816.3	NM_003905.3
825	548.6	518.9	557.3	NM_001018160.1
349.5	527.9	511.3	376.3	NM_006380.2
170.4	165.6	163	175.7	NM_012096.1
155.9	138.3	132.1	142	NM_015032.1
346.1	390.2	354.6	358.7	NM_015928.1
2322.9	1457.3	1328.2	1133.6	NM_000485.2
1091.9	671	570.6	480.4	NM_001030018.1
169.3	138.5	133.7	114.7	NM_020979.1
638	502.8	465.5	337.5	NM_175069.1
82.4	89.9	84.1	88	NM_017692.2
244.9	230	224.1	192.7	NM_175073.1
95.8	89.1	85.7	83.8	NM_001649.2
94.7	89.5	93.4	89	NM_133456.1
97.8	94.1	94.4	95.7	NM_000385.3
96.7	86.1	81.4	87.9	NM_198098.1
89.7	84.8	75.8	83.9	NM_080429.2
95.2	92.5	99.7	99.2	NM_173039.1
101.5	94.9	99.9	97.4	NM_198998.1
97.5	91	89.6	83.6	NM_000486.3
81.1	83.2	80.9	79.6	NM_004925.3
110.5	95.1	86.7	89.8	NM_001650.4
86.6	82.6	76.5	79.5	NM_004028.3
86.7	87.2	89.1	74.5	NM_001651.1
126.9	108.3	113.2	94	NM_001652.2
88.3	82.6	82.5	95.8	NM_001170.1
79.4	74.8	70.3	75.4	NR_002823.1
89.2	84.5	83.3	83.2	NM_001169.2
99.8	99.3	89.2	89.4	NM_020980.2
95.2	103	98.9	101.2	NM_014691.2
141.2	167.3	166.9	163.6	NM_000044.2
94.2	103.8	88.9	91.9	NM_000044.2
86.3	94.8	82.9	86.9	NM_001011645.1
588.5	491.9	552	229	NM_001654.1
108.1	86.5	95.7	95.1	NM_015193.3
2170.5	1188.6	933.7	983.2	NM_001655.3
603	444.9	459.5	304.3	NM_003491.2
98.9	97.2	86	97.5	NM_001657.2
390.6	263	256.1	210.8	NM_001024228.1
4745.6	3545.5	3250.1	1840.2	NM_001658.3
92.3	94.9	85.6	87.1	NM_001024226.1

403.3	279.3	249.2	224.5	NM_001659.1
390.7	402.5	259.4	242.2	NM_001660.2
2534.7	2487.7	1714.9	1777.4	NM_001660.2
801.1	868.2	857.7	725.1	NM_001662.2
87.2	83.9	75.2	87.8	NM_001663.2
99.7	82.3	89.5	92	NM_175609.1
260.4	285.6	307.4	177.1	NM_175609.1
94.8	80.8	83.8	81.9	NM_014570.3
159	116.2	124.5	140.8	NM_006421.3
163.2	135	129.4	120.7	NM_006420.1
332.2	240.8	226.3	228	NM_001025593.1
118.2	110.6	95.6	103.9	NM_001025595.1
85	85.8	75.2	87.8	NM_012402.2
113.2	123.9	100.6	102	NM_003224.2
80.6	83.5	85.2	86.2	NM_000045.2
111.2	93.6	95	92.9	NM_001172.3
91.7	91.6	92.3	86.4	NM_175861.1
89.6	88.2	82.7	86.4	NM_001012659.1
94.9	84.9	78.6	92.1	NR_002222.1
1452.6	807.7	771.9	492.1	NM_004308.2
133.6	120.1	112.8	120.9	NM_024605.3
170.2	164.3	159	150.3	NM_199357.1
180.2	155.2	155.6	158.6	NM_014783.2
105.8	106.4	103.4	105.7	NM_199357.1
93.5	92.2	96.2	99.3	NM_018287.4
1833.3	1795.2	1704.7	1575.5	NM_018460.2
1564.4	1210.1	1441.6	672.4	NM_018054.4
234.6	204.6	195.9	190.7	NM_018054.4
95.5	92	88.3	98	NM_001006634.1
104.6	98.7	87.4	100.8	NM_033515.2
638.7	539.7	396.8	369.8	NM_032900.4
100.6	97	78.5	97.5	NM_020809.2
270.7	268.4	225.9	213.2	NM_020824.2
86.4	91.8	90.8	94	NM_021226.2
87.6	90	87.9	90.6	NM_031305.1
89.3	87.8	94.4	97.7	NM_001042669.1
96.5	101.3	95	98.9	NM_001025616.1
113.7	121.2	122.4	108	NM_001025616.1
96.1	88.4	92	96.3	NM_001007231.1
207	311.4	250.8	227.3	NM_014882.2
89.6	92.7	77.3	83.2	NM_014882.2
112.3	109.4	121.5	106.6	NM_015071.2
92.2	94.7	93.7	92.5	NM_199282.1
93	74.2	70.1	80.1	NM_030672.2
87.9	89.7	86	99.8	NM_001010000.1
86.1	85.2	83.1	86.3	NM_030672.2
93.3	87.6	87.7	92.8	NM_004815.2
748.6	419.7	398	333.6	NM_181720.2
1089.6	820.9	788.8	498.8	NM_181720.2
319.4	241	247.4	205.3	NM_001025598.1

1088.7	1033.3	1067.5	624.2	NM_001666.2
92.8	98.8	97.7	73.7	NM_001173.2
99.6	93.4	101.7	83.5	NM_013422.2
82.1	85.3	75.6	84	NM_013423.1
94.4	81.7	84.2	80.6	NM_001174.2
95.9	92.6	88.3	89	NM_006125.1
86	85.4	79.3	89.9	NM_181335.2
82.3	86.6	72.1	83.8	NM_001017526.1
86.8	73.2	79.5	80.4	NM_001017526.1
1021.2	1372.3	1409.8	1092	NM_032496.1
245.1	337.8	364.5	361.6	NM_001080156.1
1044.8	723.4	651.9	629.3	NM_004309.3
17960	15568.3	15052.7	11472	NM_001175.4
89.3	90	81.2	84.3	NM_001176.2
183.8	222.1	177.1	174.2	NM_199002.1
110.6	102.3	95.6	88.6	NM_199002.1
108.3	116.8	117	119	NM_199002.1
629.8	375.1	357	274.9	NM_014629.2
88.1	91.7	98.1	96.5	NM_001011722.2
89.4	100.3	94.2	80.4	NM_018125.3
94.6	87.2	90.3	86.7	NM_001011722.2
84.5	90.3	89	97.6	NM_198236.1
120.9	113	121.9	117.3	NM_014784.2
123.7	140.5	127.7	128.4	NM_014784.2
113.8	90	88.4	87.6	NM_015313.1
92.2	80.7	85.1	80	NM_173728.2
94.6	78.6	78.5	80.5	NM_014448.2
135.2	152.2	140.3	140.6	NM_014786.2
1519.3	1467.2	1599.6	984.7	NM_015318.2
340.4	268.5	255.7	159.5	NM_153213.2
1795.2	1946.1	1660.9	1130.6	NM_004723.2
467.5	410.6	347.2	354.1	NM_019555.1
97.4	88	80.3	83.6	NM_032995.1
76.9	95.3	80.3	85.8	NM_032995.1
108.5	116	101.6	102.8	NM_001002861.1
3346	2786.7	2824.8	1955.5	NM_004840.2
966.3	923.4	967.7	722.4	NM_003899.2
90.2	79.2	79.2	81.6	NM_145735.1
105.2	94.3	98.1	102.9	NM_003899.2
94.5	101.6	101.6	104.6	NM_015185.1
90.7	88.2	85.4	98.6	NM_006015.3
1182.9	1461.8	1615	830.9	NM_139135.1
94.8	107	90.6	104.5	NM_175863.2
100.6	102.2	102.4	98.2	NM_020732.2
191.1	149.1	175.4	158.4	NM_152641.2
1182	1313.9	1144.6	1171.9	NM_005224.1
89.2	94.9	81.2	90.6	NM_001017363.1
136.8	152	132.5	150.7	NM_023000.2
123	136.9	119.3	131.6	NM_002892.2
220.2	215.7	193.2	223.1	NM_002892.2

134	131.4	130.8	132.7	NM_016374.4
365.8	459.2	432.2	443.3	NM_016374.5
186.7	187.7	213	178.3	NM_016374.5
93.6	93	94.6	92.3	NM_212481.1
94.4	91.8	94.5	102.9	NM_212481.1
114.3	111.2	107.4	108.2	NM_212481.1
110.5	138.9	121.7	123.2	NM_032199.1
254.7	302.9	303	249.6	NM_005744.2
1173.6	1264.6	1339.4	697.8	NM_006321.1
476.2	411.6	402.7	340.6	NM_001177.3
88.7	88.5	89.9	87	NM_173664.3
99.7	93.7	91.3	95.7	NM_138450.3
91	85.6	91.7	93.3	NM_001012990.1
118.5	102.4	92	106.7	NM_144996.2
105	95.6	81.3	88.8	NM_182896.1
79.6	87.7	78.8	91.6	NM_025047.1
104	100.9	97.4	100	NM_019087.1
736.2	780.9	1092	837.5	NM_001040025.1
2084.3	1230	1396.7	640.2	NM_001667.1
1299.9	1059.5	925.6	668.4	NM_012106.3
126.3	108.5	103.2	94.4	NM_004311.2
157.6	118.1	105.4	111.2	NM_212460.1
229.3	169.5	146	163.7	NM_005738.2
156.3	125.8	113.6	129.3	NM_005738.2
79.7	87.3	78	85.7	NM_001037164.1
88.2	89.7	89.8	90.2	NM_005737.3
102.9	97.1	98.4	95.7	NM_001661.2
194.9	171.5	166.8	125.1	NM_012097.2
3744.1	3502.7	3726.8	2680.2	NM_012097.2
1016.8	896.7	794.2	673.7	NM_001037174.1
125.4	106.2	96.2	95.6	NM_178815.3
71	73.6	76.8	87.5	NM_032146.3
94.4	86.9	80.1	80.6	NM_032146.3
90.1	92.1	89.6	87.3	NM_177976.1
127.6	98.4	93.3	99.3	NM_015161.1
343.9	236.2	215.9	242.7	NM_022374.1
188.6	206.3	206	174.3	NM_018694.2
118.4	109.7	100.5	105.8	NM_001002252.1
276.6	314.2	303.7	256.4	NM_001002251.1
659.9	530.6	483.6	517.6	NM_006407.3
86	84.4	86.5	86.8	NM_152522.2
84.1	100.6	88	91.3	NM_138795.2
1113.7	1273.4	1027.1	1111.1	NM_018184.2
96.9	94.5	83.4	85.3	NM_206919.1
455.1	297.3	292.5	227	NM_018120.3
1559.1	1274.8	1176.8	1104.5	NM_031905.2
79.8	88.1	85.3	84.1	NM_032131.3
99.1	109.8	110.5	95.4	NM_018076.2
117	99.1	94.3	103.9	NM_024742.1
189.8	160	145.6	137.5	NM_033415.2

322.7	317.3	261.5	266.7	NM_024585.2
110.3	107.6	102.3	94.1	NM_015396.3
340	278.6	238	196.4	NM_014154.2
102.7	96.8	102	96.2	NM_213654.1
96.8	96.2	80.2	86.2	NM_014154.2
89.8	87.6	89.5	85.5	NM_025139.2
138.8	142.4	150	146.2	NM_016608.1
81.9	80.2	93.3	86.5	NM_177949.1
264.7	276.6	217.6	243.1	NM_177947.2
87.5	79.1	84.3	83	NM_177948.2
209.9	156.1	131.2	178.3	NM_022838.2
377	329.1	253.7	256.1	NM_019007.3
388.7	348	257.9	251.6	NM_019007.3
7937.1	5858	4691.3	3277.1	NM_006010.2
104.1	100	90.2	102.9	NM_001029954.1
112	106.4	102.9	110.6	NM_178426.1
88.6	95.7	92.4	89.7	NM_001668.2
89.4	90.7	90.1	85.6	NM_014862.3
91.3	96.8	92.2	93.1	NM_001030273.1
331.2	312.6	270.1	297	NM_001030272.1
84.9	83	74.7	79.4	NM_020183.3
106.5	100.7	91.8	96	NM_181773.2
1893.8	1458.4	1322.6	951.8	NM_006409.2
1309.3	1438.1	1252.1	909.6	NM_005720.2
91.9	86.6	80.1	93.7	NM_152862.1
9396.2	7925.9	7305.9	7422.2	NM_005731.2
88	100.5	93.8	95.3	NM_005731.2
9217.3	8815.8	9354	7710.1	NM_005719.2
98.9	108.4	93.7	95.3	NM_005718.3
3944	2478.4	2203.6	1435	NM_001024960.1
1481.1	766.6	750.4	492.6	NM_001024959.1
3266	2130.8	1893.5	2189.2	NM_005717.2
1243.6	726.7	666.9	527.2	NM_030978.1
101.8	101.5	90.7	92.8	NM_032487.2
92	81.3	84.9	84.7	NM_080431.3
265.2	284.1	317.3	222.2	NM_006628.4
92	82.3	78.8	89	NM_016300.4
86.5	85.5	83.3	81.2	NM_198399.1
78	74	80.7	81.6	NM_001025068.1
91.4	92.4	90.9	90.9	NM_001025068.1
94.7	81	83.5	90.7	NM_004312.1
196.9	174.8	152.3	173.6	NM_020251.2
183.9	162.9	159	154.1	NM_004041.3
93.7	101.7	98.3	91.4	NM_004313.3
110.5	114.6	109.2	109	NM_199004.1
120.4	135.4	154.8	143.6	NM_152285.2
93.9	87.3	79.7	83.5	NM_001025604.1
92.4	87	92.8	89.2	NM_015683.1
245.1	182.7	176.2	183.7	NM_015683.1
209.5	274.9	227.6	207.8	NM_020801.1

134.8	172.7	159.6	181.8	NM_183376.1
82.7	87.3	81.8	85.6	NM_001080523.1
4219.2	3381.3	3371.4	1936.9	NM_015908.4
86.9	80.9	77	80.2	NM_182800.2
125.6	128.7	146.8	94.2	NM_000487.3
88.6	86	85.3	83.3	NM_198709.1
335.9	354.3	351.7	275	NM_000046.2
139.7	153.8	136.2	130	NM_198709.1
127.2	184.6	175.8	165.4	NM_009589.2
118.1	108.4	102.1	95.1	NM_009589.2
707.2	972.5	719.2	733	NM_001669.2
80.5	79.1	82.2	87.6	NM_000047.1
89.7	86.4	80.5	90.5	NM_004042.3
98.7	115.2	104.9	102.5	NM_014960.2
89	91	89.6	98.4	NM_001011719.1
88.5	84.6	83.2	95.3	NM_001012301.1
96.7	90.8	94.4	100.5	NM_024590.2
147	165.7	138.9	139.5	NM_198150.1
102	100.3	90.9	93.2	NM_004314.1
93.5	102.9	94.5	96.7	NM_001179.2
101.4	100	100.1	102.8	NM_021071.2
83.4	85.6	81.8	89.2	NM_053017.2
89.4	84.4	88.1	92.4	NM_053017.3
78.8	74.4	78.3	77.9	NM_057091.1
84	80.6	86.8	87	NM_003976.2
88.6	93.8	81.3	92.9	NM_057160.1
104.4	94.2	92.9	93.7	NM_003976.2
124.6	114.4	121.6	103.6	NM_001040458.1
71.9	90.7	76.4	76.9	NM_016442.2
870.1	680.4	650.2	512.8	NM_022786.1
88	79.7	83	89.3	NM_001670.1
95.3	97.8	88.3	87.3	NM_001030078.1
107.8	114.3	104.2	102.6	NM_139058.1
109.5	98.8	94	90	NM_020682.2
165.6	177.9	169.4	147.4	NM_177924.1
92.5	101.4	92.8	94.6	NM_004315.2
89.1	76.3	73.9	99	NM_019893.1
90.5	90.8	87.5	91.4	NM_133492.1
109.6	94	101.9	106.2	NM_001010887.2
524.4	415.1	344.4	359.9	NM_014435.2
609	447.5	439.7	351.6	NM_014435.2
208.4	176.8	169.6	182.7	NM_014435.2
95.9	98.3	84.6	95.5	NM_024769.2
244.4	219.1	210.5	144.4	NM_016114.3
95.8	97.7	84.7	90.6	NM_080871.2
81	77.5	78.2	82.9	NM_001012428.1
87.7	81.7	82.2	87	NM_001012428.1
110.6	109.1	103.5	89.9	NM_080873.1
122.1	105.8	99.4	105.7	NM_130388.2
318.2	215.3	199	167.9	NM_024701.2

84.3	88.1	89	78.2	NM_130387.4
89.4	90.5	92.8	93.3	NM_080928.2
85.5	89	88	86.3	NM_080863.4
91.4	95.1	89.7	80.2	NM_080868.1
95.9	101.4	96.1	95.1	NM_016150.3
175.6	168	172.7	159.4	NM_145863.1
219.1	170.4	170.5	157.9	NM_016115.3
92.1	81	78.1	81.7	NM_145872.1
86.4	90.5	86.4	92.6	NM_145872.1
88.4	99	93	87.3	NM_016116.1
88.7	74.8	85.5	93.7	NM_080874.2
85.3	79.6	70.2	85.4	NM_017873.2
142.2	115.4	105.8	119	NM_177999.1
155.4	117.4	116.4	103.4	NM_177999.1
94.4	85.5	86.5	98.9	NM_024708.2
99.1	107.9	87.5	95.1	NM_198243.1
126.4	122.1	103.8	99.3	NM_024708.2
171.1	166.4	168.6	155	NM_024095.3
86.6	84.5	85.8	87.8	NM_024087.1
88.5	77	76	85.6	NM_024087.1
849.2	524.6	456.2	338	NM_015947.2
1653.2	1822	1866.4	748.5	NM_032204.3
102.6	90.6	91.5	91.4	NM_022091.3
135.2	153.6	154.3	139.7	NM_006828.2
94.8	86.1	93.3	98.1	NM_022091.3
127.4	132.3	137.5	103.5	NM_014014.2
118.7	124.3	119.4	126.9	NM_015251.2
99.6	90.5	83.6	108.1	NM_004316.2
210.5	209.2	207.5	157.8	NM_005170.2
96.7	82.3	79.1	77.9	NM_020646.1
80.3	81.2	88	80.1	NM_203436.1
1480.3	911.8	696	878.3	NM_014034.1
379.5	286.3	279.7	161.1	NM_018154.2
103.2	86.1	98	103.3	NM_001671.2
90.2	82.2	78.1	84.6	NM_080912.1
214	266.5	248.7	258.1	NM_080914.1
143.5	157.3	146.3	129.9	NM_080914.1
83.3	84.6	91	101.6	NM_018489.1
2209.1	1437.4	1262	1036	NM_004674.1
79	90.5	83.7	87.2	NM_001672.2
91.6	80.6	88.9	91.2	NM_000048.3
99.2	106.1	118.8	102.3	NM_001024943.1
155	142.9	159.7	131.4	NM_001024943.1
91.7	90.7	93.6	90.1	NM_004043.1
156.6	193.7	189.2	175	NM_004192.1
90.5	89.6	88.3	79.2	NM_004317.2
1820.1	828.7	841.9	512.9	NM_133436.1
178	116.5	95.5	107.9	NM_133436.1
1782.3	1479.2	1075.2	1103.3	NM_019048.1
76.3	87	84.3	89.2	NM_000049.2

113.6	114.2	103.4	114.3	NM_032468.2
118.7	109.8	92.7	103.6	NM_020164.2
91.6	89.1	89.3	88	NM_004318.2
303.7	208.5	174.2	164.9	NM_181718.3
110.6	93.9	95	81.8	NM_181718.3
133	125.2	115.8	114.3	NM_020437.3
1033.7	644.6	701.7	621.3	NM_018136.2
95.5	94.6	95	99.8	NM_017680.3
111.3	114.7	104.7	103.8	NM_024083.2
1774.8	1650.8	1981.8	794.7	NM_024083.2
103.4	95.3	116.8	114.7	NM_025080.2
1146.2	676.5	600	730	NM_000050.3
309	269.1	229	297.1	NM_054012.3
183.1	166	162	131.1	NM_014065.2
104.4	102.6	99.8	93.2	NM_001002036.2
99.6	87.3	87.9	89.2	NM_004319.1
90.6	92.4	95.2	81.5	NM_207108.1
90.1	90.9	86.6	87.8	NM_014010.3
99.3	91.9	87.5	103.9	NM_198188.1
94.3	85.5	92.8	92.4	NM_198187.2
261.8	324.4	285.7	221.7	NM_015338.3
568.9	1040.8	935.7	581.3	NM_018263.3
84.1	76.6	73.6	81.5	NM_130768.1
128.7	119.5	98.5	109.4	NM_032810.2
1401	1026.4	800.3	786.7	NM_014109.2
295.7	271.4	224.4	211	NM_018188.2
138.4	143.8	133.3	137.6	NM_031921.4
96.4	96.3	96.7	102.5	NM_001039211.1
144.2	133.8	146.1	125.6	NM_024320.2
87	101.9	120	94.1	NM_006885.2
89.7	88.9	92.3	93.9	NM_033064.2
99.4	82.1	85.8	90.1	NM_001001976.1
86.4	86	77.3	85.3	NM_007041.2
95.7	93.1	87.4	84.5	NM_007041.2
102.3	98.4	103	103.9	NM_005171.2
224.7	229.2	210.7	187.6	NM_001880.2
147.9	119.5	100.9	118.1	NM_001040619.1
99.1	86.7	92	94.5	NM_001030287.1
93.4	85.6	85.2	82.6	NM_004024.3
6623.3	5449.4	6156.8	4470.6	NM_001675.2
128.6	136.8	128.6	109.3	NM_001675.2
384.9	220.1	258.3	193.7	NM_182810.1
874.4	1078.2	954	575.8	NM_012068.2
790.2	648.1	597.7	533.8	NM_007348.2
102.7	88.7	98	88.2	NM_006856.1
104.6	81.2	98.2	93.6	NM_018179.3
337.6	280.8	271.5	221.6	NM_024997.2
206.3	215.2	206.7	223.4	NM_031482.3
1331.6	1145.1	1167.8	908.6	NM_004707.2
411.3	285.8	274.6	229.8	NM_030803.5

474.9	311.5	336.5	257.8	NM_017974.3
208.1	230.5	232.2	221.4	NM_033388.1
123	120.2	106.4	105	NM_022488.3
221.6	236.4	225.1	157.4	NM_178270.1
84.3	84.6	73.8	83.4	NM_178271.1
92.3	84.6	85.4	93.5	NM_013325.4
771.8	521.9	500.9	377.7	NM_178326.2
100.2	87.2	90.8	88.6	NM_032852.2
92.6	82.1	80.9	97	NM_032885.4
222.7	268.6	221.6	204.6	NM_004849.1
484.6	582.8	604	374.3	NM_006395.1
80.7	84.7	78.5	89.9	NM_001077198.1
349.9	403.5	315.8	325.7	NM_001077198.1
257.4	274.2	206.1	236.2	NM_024085.2
88.1	89.2	96.3	91.6	NM_173681.3
152.5	242.6	223.4	229.8	NM_025092.1
3942.5	2127.7	1847.3	1329.1	NM_004044.4
91.4	101.3	87.6	88.1	NM_000051.3
408.2	597.1	737.6	528.7	NM_000051.3
79.6	76.3	72.4	79.5	NM_138292.3
100	95.6	92.3	104.1	NM_001940.3
154.3	140	125.7	126	NM_001007026.1
91.9	84.5	91.8	83.9	NM_001007026.1
89.4	88.3	88	88.3	NM_005172.1
89.9	92	90	77	NM_145178.2
88.5	85.3	79.7	83.7	NM_032827.3
2367.7	1565.3	1532.8	1021.2	NM_004045.3
89.8	96.6	78.1	79.6	NM_024490.2
105.5	116.6	114.6	110.4	NM_020453.2
91	87.4	89.2	84	NM_015205.1
79.2	84.6	76	71.4	NM_032189.2
191	195.8	187.3	216.3	NM_014616.1
132.7	133.6	112.8	112.5	NM_173694.3
210.5	227.2	222.9	206.6	NM_173694.3
92.2	94.8	98.1	84.5	NM_001676.3
435.3	363.4	360	231.5	NM_020410.1
130.7	135.8	118.9	116.5	NM_022089.1
77.2	80	72.8	76.9	NM_032279.2
89.5	77	78.9	85.4	NM_198505.2
1951.9	1495.7	1253.4	971.4	NM_000701.6
2432.2	2168.9	1706.6	1656.1	NM_001001586.1
616.1	281.6	301.6	237.2	NM_000701.6
84.7	79.8	76.3	94.8	NM_000702.2
105.9	108.9	101.1	85.3	NM_152296.3
91.5	93.3	86.6	86.3	NM_144699.2
93.3	86.1	84.2	88.9	NM_001001734.1
181.4	255	207	254.1	NM_001001787.1
331.9	670.8	467.2	582.8	NM_001001787.1
204.4	349.5	233.8	243.2	NM_001677.3
171.3	245.1	222.5	153.6	NM_001678.3

1638.5	1126.9	982.6	1194.4	XM_945505.1
366.9	291.1	259.6	238	NM_001679.2
1080	751.9	466.2	708.7	NM_001679.2
99.7	97.1	92.2	87.3	NM_012069.2
93.4	81.8	90.1	85.3	XM_937782.1
679.4	812.2	634.7	493.4	NM_001681.2
1276.2	1322.8	944.6	1149.9	NM_001681.2
626.7	702.7	499.6	372.8	NM_170665.2
102.5	107.6	106.8	86.1	NM_174958.1
95.8	110.1	112	111.8	NM_174955.1
101.5	103.5	107.1	108.2	NM_001001323.1
82	83.7	81.2	85.9	NM_001001323.1
88.1	82.5	85.6	85.7	NM_001683.2
89.4	84.2	83.3	79.9	NM_001001331.1
85.5	82.6	85.1	82.6	NM_001683.2
192.7	199.5	201	164.6	NM_021949.2
90.4	108	86.4	88.4	NM_021949.2
94.8	99	93.1	99.2	NM_001001344.1
95	122.4	138.7	114.2	NM_001001396.1
405	836.8	707.7	639.2	NM_001684.3
186.6	196.3	169.4	176.4	NM_014382.2
101.2	116.4	99.7	99	NM_001001487.1
345	359.4	272.3	290.5	NM_001001486.1
89.3	85.8	76.6	91.8	NM_000704.2
93.4	92.4	85.2	94	NM_000705.2
86.5	87.1	91	90.6	NM_000705.2
18649.4	13361.2	10935	10059	NM_004046.4
15731.1	12508.8	12552.1	8930.1	NM_001001937.1
17414	14593	13195.1	8911.1	NM_001686.3
2252.2	1496.8	1218	1628.4	NM_005174.2
474.6	337.1	302.3	252.8	NM_001687.4
4415.5	2272.7	1960.5	2109.1	NM_001001975.1
1773.7	804.9	988.6	647.4	NM_001688.3
9570.7	5445.1	4747.3	4112.7	NM_001688.3
162.7	148.9	152.1	108.9	NM_005175.2
4253.7	3635.8	3336.9	2220.9	NM_001002027.1
87.8	92.4	81.1	89.1	NM_001002031.1
155.5	109.4	100.6	107	NM_005176.4
5497.3	2552.4	2390.6	1928.8	NM_005176.4
228.2	212	190.2	180.6	NM_001002256.1
6513.3	5855.2	4842.4	4784.8	NM_001003785.1
8446.8	8144.8	5858.8	6823.5	NM_006356.2
11051.9	8532.7	8201.6	5778.8	NM_007100.2
906.8	740.9	750	526	NM_007100.2
579.7	636.4	502.5	452.9	NM_001003703.1
9666.1	9958	7775	6000	NM_001685.4
10913.2	10231.8	8400.4	6890.2	NM_001003696.1
4183.3	3054.5	3234.2	1956.5	NM_001003714.1
5929.3	4642.8	4593.9	2851.5	NM_004889.2
295.2	231.2	181.8	185.1	NM_006476.4

7183.8	6417.6	5688.3	5486.6	NM_001697.2
183.6	202.4	171.4	152.6	NM_001003803.1
127.9	132.4	123.3	107.6	NM_015684.2
3794.7	3891.2	4298.6	1632.4	NM_001183.3
1890.6	2428.8	2256.8	1941.6	NM_005765.2
875	838	869	518.1	NM_005177.2
329.8	273.9	256.1	190	NM_012463.2
83	93.5	74.8	83.9	NM_020632.1
96	93.5	94.7	91.7	NM_130841.1
85	86.2	80.9	82.7	NM_130840.1
92.7	96	89.4	99.5	NM_130840.2
6352.3	4829.4	4742.1	3004.8	NM_004047.2
162.9	194.8	164.1	163.2	NM_001039457.1
425.9	294.6	274.6	227.6	NM_001694.2
1105.7	751.2	680.9	450.9	NM_004691.3
90.7	90.7	90	88	NM_152565.1
4256.6	3555.9	3688.3	2616.1	NM_003945.3
834.4	664.6	660.6	309.2	NM_145230.1
3751.4	4451.2	3833.9	3368.9	NM_001690.2
280.7	297.1	374.4	214.1	NM_001692.2
8358.6	4509.1	3615.4	2769.8	NM_001693.2
248.8	283.3	228.7	264.9	NM_001695.3
95.3	100.2	83.5	100.2	NM_001695.3
94.3	126.4	137.5	114.9	NM_144583.2
85.6	83.8	80.7	79.8	NM_144583.3
1124.1	860.3	661	640.4	NM_015994.2
3525.5	2949.7	2829.2	1747.1	NM_001039367.1
3233.7	2634.6	2413.1	1475.2	NM_001696.2
197.3	223	215.7	181.8	NM_080653.3
2918.9	2123.3	2152.1	1329.4	NM_004231.2
2315.5	2120.2	1793	1584.7	NM_004888.2
128.9	103.5	101.6	97	NM_130463.2
89.2	99.3	84.8	94	NM_130463.2
98.8	80.4	81.4	84.2	NM_133262.2
105.4	93.6	82	82.5	NM_213620.1
452.5	356.8	327.6	262.6	NM_213619.1
96.9	117.7	112.3	118.6	NM_000052.2
104.9	89.4	96.7	97	NM_000053.2
115.3	188	182.1	162.2	NM_000053.2
90.1	85.6	80.1	83.6	NM_006095.1
117.4	114.2	104.4	97.9	NM_016529.3
90.2	88.3	91.2	93.8	NM_005603.2
100	96.9	84.1	98.4	NM_001005855.1
164.5	135.7	150.3	120.9	NM_020452.2
176	193.8	191.9	145	NM_138813.2
3096.9	3938.1	4227.9	3232.6	NM_024837.2
206.7	338.6	325.3	241.2	NM_006045.1
134.4	129.5	116.8	111	NM_198531.3
482.1	361.2	304.8	244.9	NM_022745.2
357.4	326.4	288	201.9	NM_001042546.1

120.5	115.3	115.5	93.2	NM_145691.3
557.9	429.3	476.7	285.7	NM_018066.3
91.3	81.9	83	85.1	NM_016301.2
141.9	120	121.4	106.1	NM_145232.2
131.6	97.5	106.1	101.1	NM_080650.2
470	442.2	369.5	272.6	NM_016311.3
108.9	105.2	106	116.6	NM_178191.1
90.5	84	79.8	97.1	NM_001184.2
86.3	71	85.7	78.8	NM_001184.2
94	85	96.3	89.1	NM_001184.2
98.1	128.7	112.6	129.1	NM_139322.1
89.5	95.8	87.9	77.6	NM_139321.1
94.7	91	83.1	86.3	NM_207303.1
89.5	109.7	94.3	101.2	NM_138270.1
82	76.6	77.3	80.6	NM_000489.2
83.4	85.2	75.4	74.7	NM_138271.1
127.2	144.3	142.4	143.2	NM_000332.2
103.8	108.8	101.7	103.6	NM_013236.2
932.6	1143.4	1200	676.4	NM_002973.2
163.9	162.2	146.6	169.7	NM_007245.2
97.8	114.7	115.5	123.8	NM_017492.2
279.8	335.2	416	275.7	NM_030660.2
115.8	115.5	116.4	122.7	NM_030660.2
89	78.8	80	80.1	NM_000333.2
185.2	114.3	112.7	118.3	NM_153340.2
96.9	93.6	92	85.1	NM_152749.2
81.6	90.7	88.8	98.7	NR_002717.1
176	187.5	175.1	150.3	NM_001698.1
410.6	303	355.5	215.9	NM_012103.2
2845.1	1967.9	1668	1254.5	NM_198434.1
2434.3	1654.2	1417.1	1035.6	NM_198434.1
4058.3	3925.1	3824.1	2070.6	NM_017900.1
756.5	355.7	331	292.5	NM_004217.1
86.3	89.3	80.6	83.8	NM_001015878.1
83.2	84.4	80.5	84	NM_001015878.1
119.4	96.2	96	83.9	NM_001015879.1
86.6	85.9	81.4	80.5	NM_015570.1
144.7	119.1	123.2	145.4	NM_020371.2
106.9	113.2	111.9	95.7	NM_006576.2
100.4	94.3	91.5	91.6	NM_000490.3
254.1	327.3	245.3	183.4	NM_021732.1
94.9	90.3	94.7	94	NM_000706.3
82.3	81.4	78.4	77.4	NM_000707.2
84.2	88.9	76.2	80.5	NM_000054.2
604.8	839.5	814.6	613.2	NM_001014279.1
959.9	724.6	685.8	451.2	NM_181050.1
91.4	94.1	84.4	88.4	NM_003502.2
106.8	85.1	84.7	90.3	NM_004655.2
141.5	252.6	204.8	154	NM_001699.3
153	264	201.4	213.5	NM_021913.2

348.3	396	349.4	208.9	NM_033027.2
109	110.9	104.4	92.7	NM_032193.2
159.2	112.6	112.8	111.4	NM_017839.3
298.1	212.4	227.3	147.5	NM_024830.3
94.5	89.5	88.9	87.6	NM_001185.2
460	515.6	526.9	279	NM_014984.1
77.3	90.5	78.4	87.2	NM_014984.1
201.5	245.2	189.9	209.3	NM_022461.2
3227.7	2309.2	1996.8	1654.8	NM_015878.4
263.4	173.8	174.2	164.5	NM_015878.4
159.8	108	111.9	110.2	NM_148174.2
11310.1	8861.1	7839.3	7459.3	NM_001700.3
3114.4	2683.2	2615.6	2000	NM_004048.2
277.9	251.5	223.6	205.9	NM_033168.2
277	195	178.9	162.1	NM_152490.1
97	113.2	111.3	96.8	NM_020981.2
92.2	99.2	96.7	90	NM_003783.2
120.1	115.3	124.1	120.3	NM_003781.2
97.1	85.7	75.7	85.4	NM_033169.1
98.4	97.4	100.3	93.3	NM_033169.1
97.2	111.8	98.7	108.7	NM_003782.3
87	88.4	79.1	80	NM_033172.1
92.1	90	81.7	93.3	NM_006057.1
90.4	86.5	87.6	87.3	NM_033173.1
456.5	404.9	368.5	414.9	NM_080605.2
131.9	126.5	124	116.7	NM_198540.2
93.7	98.6	108.4	96.6	NM_054025.1
89	88.9	86.5	91.4	NM_018644.3
100	87.6	89.9	89.9	NM_054025.1
95.2	95.9	85.6	100.2	NM_080742.1
225.7	196.7	163.3	163.5	NM_012200.2
373	264.6	217.7	176.1	NM_006577.3
100.6	88.6	89.1	98	NM_014256.2
85	82.3	77.9	83	NM_030765.2
112	99.6	100.9	110.3	NM_032047.3
208.2	173.7	152.5	164.7	NM_006876.1
87.8	94.9	80.8	89.6	NM_138706.2
88.5	87.4	92.3	100.3	NM_145236.1
173.5	170.5	180.3	160.2	NM_001009905.1
186.8	154.5	107.5	153.8	NM_194318.2
92.7	87	84.5	85.5	NM_153446.1
77.2	79.9	75.7	94.6	NM_173593.3
75.8	77.8	78.6	80.8	NM_178537.3
146.9	148	122.9	143	NM_001497.2
99	97.9	88.9	99.9	NM_001005417.1
102.2	107.3	92.1	103.1	NM_003780.3
107.5	82.1	85.8	93.2	NM_001005417.1
1570.9	756.6	784.7	385.5	NM_003779.2
195.7	153.1	151.4	148.8	NM_003778.3
141.3	129.5	133.8	131.1	NM_003778.3

1078.5	1044.7	900.1	608.7	NM_004776.2
99	100.3	84.8	91.5	NM_004775.2
428.4	411	401	235.5	NM_007255.1
198.6	158.4	131.5	134.6	NM_001015882.1
78.1	88.3	73	76.6	NM_001001663.1
107.7	104.3	95	99.9	NM_001024372.1
89.7	95.1	90	89.1	NM_024812.2
79.1	75.2	72.1	82.9	NM_001701.2
134.7	139.6	115.3	149.7	NM_138972.2
101.2	102.1	86.4	81.2	NM_012104.3
87	81.6	74.4	89	NM_138991.1
89.4	90.5	97.6	96	NM_012105.3
106.2	106.8	111.5	103.9	NM_001011545.1
79.9	80.4	83.5	82.7	NM_206866.1
80.9	85.9	82.4	91.1	NM_001011545.1
86.7	89.1	84.6	87.1	NM_021813.1
98.5	97.3	94	89.5	NM_032989.1
293.5	214.9	231.4	192.9	NM_004322.2
98.6	94.2	91.8	103.6	NM_004322.2
128.2	121.2	108	116.4	NM_004323.3
874.8	396.4	443.8	245.2	NM_004281.3
168.8	113.1	109.7	112.4	NM_004874.2
1416.4	985.9	963.5	757.8	NM_004873.2
91	85.2	75.5	84.4	NM_001015048.1
85.5	91.7	83.7	79.6	NM_001015049.1
241.9	235.2	206	166.8	NM_001015048.1
98.9	92.4	92.8	95.6	NM_001187.1
90.2	93.3	85.4	88.2	NM_182482.1
82.7	83.8	82.5	93.4	NM_182481.1
88.8	89.5	82.3	87.2	NM_181704.1
95.4	121.5	102.9	109.6	NM_182484.1
90.5	86.3	86.8	90.4	NM_014952.2
109.9	88.1	91.5	85.9	XM_940726.1
88.9	80.9	82.5	85.1	NM_001702.1
87.4	80.4	82.9	82.1	NM_001702.1
77.8	80.1	79.1	87	NM_001703.1
89.1	109.4	97.6	94.5	NM_001704.1
144	181.6	184.9	184.8	NM_006340.1
109.6	113.7	118.7	116.7	NM_017450.1
92.5	98.3	89.5	105.9	NM_006340.1
87.8	78.5	83.7	90	NM_017451.1
117.9	101.9	112.4	121.9	NM_018842.3
111.3	135.1	143	119.1	NM_025045.3
79.6	78.9	77.1	85.1	NM_003933.3
153.3	165	153.6	118	NM_001188.2
148.8	127	109.9	107	NM_012342.2
3622.5	2468.5	2151.9	1886.3	NM_003860.2
251.2	291.6	264.4	222	NM_017935.2
124.1	116.5	99.7	113.9	NM_017869.3
286.6	269	217.3	182.5	NM_004656.2

765.3	529.6	577.6	341.8	NM_001189.2
689.4	785.2	822.4	627.5	NM_000465.1
97.5	88	83.8	91.3	NM_020064.2
89.9	86.3	86.1	81.4	NM_020063.1
291.8	179.2	175.2	166.1	NM_021570.2
100.9	107	108.7	94.4	NM_003658.3
3450.9	5204.9	3910.8	4784	NM_006317.3
1868.7	1680.4	1395.5	1815.5	NM_004640.4
96.1	97.1	86	85.1	NM_004640.4
107	115.6	86.7	93.6	NM_080598.4
491.3	598.5	520.1	572.4	NM_080686.1
804.9	900.9	754	750.5	NM_080686.1
1044	1617.2	1230.2	1340.3	NM_015172.2
245.9	234	217	130.9	NM_080703.1
285.8	262.6	256.9	197.4	NM_080703.1
144.1	126.6	117.2	109.6	NM_033177.2
742.2	935.1	771	734.3	NM_021160.1
463.1	353.5	306.5	202.5	NM_006399.2
98.3	90.1	96.9	95.2	NM_138456.3
1509.8	1277	1132.7	1045	NM_138765.2
91.4	83.7	83.5	91.9	NM_138764.2
169.6	180.1	138.6	159.8	NM_004324.3
2263.6	1490.4	1155	1398.8	NM_182648.1
337.4	343	303.5	277.2	NM_013448.2
659.1	840.1	577.3	680.9	NM_023005.2
93.4	108.3	87.7	92.7	NM_013449.2
331.5	437.2	471	435.5	NM_013450.1
83.3	80.7	75.5	86.6	NM_014417.2
99.1	88.8	78.9	88.5	NM_003986.1
137.3	128.9	139.7	120.1	NM_024649.4
105.6	108.4	114.8	117	NM_152618.2
196.9	265.8	266.5	228.6	NM_031885.2
248.2	280.7	247.5	202.1	NM_033028.2
88.2	92.9	85	101.2	NM_152384.2
94.4	79.1	84.6	85.2	XM_939889.1
85.8	93.6	86.9	89.2	NM_152384.2
96.4	98.6	89.6	95.1	NM_018190.2
172.5	164.7	166.6	161.1	NM_176824.1
86.4	83.7	82.6	83.1	NM_018190.2
91.5	91.9	89.6	82.7	NM_198428.2
254.1	279.3	267	269.1	NM_020235.2
472.6	495.3	424.4	452.7	NM_033200.1
85.2	88.5	90.9	86.5	NM_001005850.1
94.3	85.6	88.8	94.6	NM_005581.3
86.7	85.6	78.7	81.3	NM_001013257.1
110.7	104.7	101.6	95.2	NM_005581.3
106.7	109.4	112.3	101.1	NM_198427.1
92.3	86.4	85	91.4	NM_198427.1
110.4	81.6	82.8	81.7	NM_021948.3
361.6	319.2	292.3	298.8	NM_001008406.1

90.2	99.1	84.8	93.4	NM_001008407.1
262.4	224.4	235.5	270.8	NM_018844.2
105	98.4	103.8	100.1	NM_001008405.1
255.3	247.2	221.7	202	NM_001008406.1
3079.5	2461.6	2456.1	1867.3	NM_005745.6
122.7	103.2	107	102.1	NM_014567.2
146.9	231.3	193.2	164.8	NM_003567.2
85.9	85.5	86.3	85.2	NM_003657.1
90	85	84.8	81.9	NM_005872.2
97.4	88	92.9	86	NM_017679.2
115	103.5	112.4	115.2	NM_017843.3
98.9	93.4	100.1	81.2	NM_017843.3
111.2	102.1	102.2	107.7	NM_017843.3
551	287.1	202.7	194.7	NM_005504.4
442.6	257.2	255.3	199.7	NM_001190.2
1871.6	1188	1026.8	792.5	NM_078468.1
487.4	380.8	275.4	317.9	NM_078468.1
366.2	276	235.4	219.6	NM_016567.2
92.1	87.3	84.6	77.8	NM_078469.1
949.2	838.1	771.5	432.8	NM_019606.4
82.6	81.5	77.1	77.2	NM_001037290.1
93.7	78.4	82.7	94.6	NM_000055.1
927.5	915.2	903.9	528.4	NM_000709.2
201.7	202.2	207.7	180.1	NM_000056.2
102.5	91.8	95	86.3	NM_183050.1
1532.7	1008.4	1057.2	489.5	NM_005881.1
139.6	119.4	124.6	114.5	NM_003921.2
183	161.7	130.9	148.1	NM_022893.2
94.9	92.7	94.3	93.8	NM_138559.1
138.1	125.4	115.4	111.3	NM_138553.1
88.8	82.3	84	89.4	NM_022898.1
91.3	90.9	87.3	87	NM_138576.2
93.2	86.4	98	95.1	NM_000657.2
87.4	78.6	80.2	82.4	NM_000657.2
1422.4	946	744.6	879.6	NM_000633.2
105.5	87.5	90	85.2	NM_004049.2
697.9	532.3	434.5	457.7	NM_138578.1
120.8	118.4	119	120.4	NM_138578.1
90.2	82.5	76.5	82.9	NM_020396.2
92.3	94.3	90.4	101.8	NM_138625.2
101.7	91	97.7	87.4	NM_207002.1
99.3	97	91.5	88.4	NM_006538.3
183.7	149.6	131.3	135.6	NM_138639.1
1195.7	658.1	665.8	415	NM_001040668.1
937.3	450	480.8	305.1	NM_052842.2
2409.4	2621.6	2384.1	1444.4	NM_015367.2
94.8	95.9	86.5	90.3	NM_030766.1
83.9	94.1	91	85	NM_138724.1
103.3	104.6	100.7	102.4	NM_138723.1
76.7	75.8	76.3	81	NM_138724.1

259.8	247.5	219.6	199.2	NM_004050.2
166.4	145.9	125.6	137.3	NM_005178.2
81.2	81.2	80.6	82.8	NM_138931.1
276	315.6	288	229.1	NM_001706.2
95.4	81.3	85.9	88	NM_181844.2
88.7	85	84.9	89.8	NM_020993.3
91.1	86.6	90.3	87.4	NM_020993.3
98.9	92	85.8	92.6	NM_001024808.1
262.7	227.6	232	182.3	NM_001707.2
471	355.1	377.1	190	NM_138707.1
831.6	455.8	395.7	436.6	NM_004765.2
149.8	141.8	123.6	118.6	NM_004326.2
99.7	90.5	87.3	97.5	NM_182557.1
2057.9	1536.3	1504	1477.5	NM_014739.1
549.3	383.1	356.6	424.3	NM_014739.2
84.4	77.9	76.5	75.5	NM_017429.2
96.9	94.8	91	97.8	NM_173544.2
631.5	769.1	585.6	678.1	NM_017745.4
114.4	122.5	129.2	135.1	NM_020926.2
145.7	152.6	168.6	158.1	NM_020926.2
113.2	137.1	126.4	126.4	NM_021946.2
84.4	82.2	80.1	88.3	NM_173700.1
87.9	101.8	82.7	85.1	NM_004327.2
90.8	103	95.1	96.1	NM_004327.2
123.4	139.3	123.5	120.2	NM_021574.2
389.7	349.5	301.7	217.1	NM_004328.3
86.9	79.4	76.7	85.5	NM_203314.1
75.7	81.6	83.4	83.3	NM_203315.1
152.3	119	142.8	112.3	NM_004051.3
86	81	85.5	83.9	NM_020139.3
97.1	100.7	102.2	94.1	NM_000710.2
80.4	79.4	84.1	96.4	NM_000623.2
100	79.9	85.8	86.9	NM_170732.3
103	101.5	95.3	88.2	NM_170731.3
107.5	87.8	80.3	84.1	NM_170734.2
107.5	103.8	101.7	78.2	NM_001709.3
83.9	89.1	94.6	84.6	NM_170735.4
90	92.5	87.1	85	NM_170733.2
83.5	95.8	79.2	96.2	NM_001709.3
104.1	121.1	134.8	125.8	NM_018429.1
355.9	432.8	323.7	343.1	NM_003766.2
258.9	174.5	199	177.5	NM_005868.3
116.3	119.5	101.5	109.9	NM_016526.3
3890.2	596.4	635	441.8	NM_018476.3
91.1	97.4	88.2	94.5	NM_032621.2
129	106.7	110	107.5	NM_001080425.1
187.9	148.4	154.4	129.7	XM_936467.1
450.7	333.2	291.4	294.4	NM_016561.1
101.2	84.5	88.5	86.3	NM_001195.2
91.4	102.3	91	89.1	NM_003571.2

116.4	119.8	110.2	107.9	NM_199173.2
109.6	102	99.6	100.2	NM_001711.3
356.8	1509.2	1388.7	1042.2	NM_003670.1
91	85.4	95.1	91	NM_030762.1
88.9	90.1	84.1	85.1	NM_080606.2
5264.3	4158.2	3814.3	3177	NM_152414.3
94.8	93	97.3	97.6	NM_177455.2
130.6	128	130.9	121.5	NM_030639.1
73.8	72.1	72.8	72.6	NM_001713.1
77.2	76.8	79.5	80.7	NM_017614.3
94.9	95.1	86.8	92.7	XM_498431.2
92.9	91.2	86.1	86.9	NM_001714.2
117.6	109.9	105.3	106	NM_001003398.1
85.6	88	79.7	91.5	NM_001003800.1
756.7	815.6	742.6	608	NM_001003800.1
515.5	613.7	463.5	479.7	NM_197967.1
344.6	429.7	378.8	326	NM_001196.2
83.6	84.8	77.7	83	NM_197966.1
340	343.6	364.6	246.5	NM_001197.3
292.9	360.8	329	306.1	NM_139343.1
415	581.2	457.3	443	NM_139348.1
96.2	96.6	86.2	105.6	NM_016293.2
148.5	133	147.1	120.4	NM_018688.3
89.5	94	85.3	84.8	NM_004536.1
110	182.7	202.1	175.4	XM_936944.1
584.6	466.7	420.2	555.1	NM_001166.3
436.5	735.9	912	806.2	NM_182962.1
100.7	104.8	103	123.8	NM_001165.3
99	92.5	86.3	92.2	NM_001165.3
89.4	91.7	86	87.2	NM_001167.2
616.9	373.6	378.2	272.7	NM_001168.2
306.9	159.2	163.8	130.1	NM_001012271.1
145.3	168.2	159	143.3	NM_016252.2
87.8	88.3	96	90.4	NM_022161.2
89	86.9	86.7	95.3	NM_139317.1
95.3	93.7	102.9	93.4	NM_033341.3
471	376.7	340.1	495.8	NM_017693.2
284	359	339	297.6	NM_006698.2
103.5	94.4	101.2	105.8	NM_031450.2
92	98.8	92.8	94.5	NM_001715.2
459.7	447.3	342.5	422	NM_000057.1
333.5	290	241.4	237.4	NM_000386.2
98.3	85.4	74.5	85.7	NM_013314.2
343.1	335.2	314.5	344.1	NM_001487.1
103.5	94.7	95.6	93.4	NM_001001342.1
128.3	115.1	104.6	108	NM_001001342.1
86.8	82.4	96	96.6	NM_212550.1
82.2	79.7	80.7	84.9	NM_032966.1
122.3	119.3	121.7	116.7	NM_032966.1
91.6	93	86.9	87.4	NM_001716.2

228.5	501.1	482.1	322.1	NM_000712.3
146.5	198.4	207	141.5	NM_000713.1
230.4	243.2	336.1	326.2	NM_003666.2
100.8	93.4	90.7	93.8	NM_016564.2
86.9	95.4	91.5	98.4	NM_001003942.1
92.4	89.9	91.2	91.9	NM_033503.3
87.3	86.2	87.7	91.5	NM_001003940.1
83.9	100.1	90.8	109.3	NM_001003943.1
88.4	76.6	89.9	91.8	NM_006128.1
109.5	89.4	92.4	92.8	NM_006131.1
225.4	186.1	178.1	153.9	NM_006129.2
89.2	83.6	82.9	96.9	NM_014482.1
103.6	95.5	93.4	90.4	NM_005448.1
161.8	101.4	103.7	116.7	NM_001200.1
162.3	127.7	120.2	114.5	NM_017593.3
114.5	109.2	102	106.2	NM_017593.3
109.6	129.4	111.3	122.1	NM_198892.1
85.6	83.3	77.8	81	NM_001201.1
195.8	100	96.6	105.6	NM_001202.2
83.2	87.1	78.6	86.6	NM_001202.2
98.9	93.1	84.3	82.1	NM_130851.1
90.8	107.8	86.2	87.8	NM_021073.2
92	81.2	78.7	89	NM_001718.2
96.2	86.7	83.5	81.6	NM_001719.1
87.7	100.9	92.8	92.2	NM_181809.1
405.5	220.5	223.6	210.3	NM_001720.3
89.9	75.2	78.8	80.8	NM_133468.2
96.6	89.9	82	88.3	NM_004329.2
77.7	82.9	81.5	80.6	NM_001203.1
164.7	228.2	159.1	141.9	NM_001204.5
828.3	690.4	566.6	380.4	NM_014753.2
101.7	100.9	89.5	102	NM_001721.4
93.5	94.1	94.2	88.6	NM_203281.1
105.7	88	96.3	104.7	NM_001717.2
85.4	83	79.5	88.2	NM_001717.2
88.9	96.7	89.1	82.4	NM_017637.4
177.9	123.1	138.5	117.2	NM_013978.1
265.4	209	199.5	167.9	NM_013979.1
1596.6	1330.9	1162.5	1150.1	NM_004330.1
1513.5	1199.1	1071.6	868.5	NM_004052.2
94.8	103.8	104.4	123.7	NM_004331.2
107	105.3	116	94.9	XM_936962.1
116	110.3	117.5	108.9	NM_138278.1
83.5	84.6	80.5	79.1	NM_138279.1
85.1	81.8	75.6	90	NM_033254.2
125	120	129.8	114.5	NM_032515.3
105.9	99.1	96.3	95.6	NM_016074.1
2486.3	2046.1	1744.5	1285.6	NM_001031827.1
313.7	231.5	204.4	194.9	NM_001031827.1
4821.8	3376.9	2894.9	2806	NM_212552.1

5500.5	3712.9	3740.2	3033.7	NM_212552.2
83.6	88.9	87.5	91.5	NM_212552.2
83.1	82	77.1	80.9	NM_033030.3
103.1	102.2	94.8	95.8	NM_197970.1
85.1	81.1	81.8	76	NM_033030.3
2437.6	1385	1337.1	569	NM_015201.3
213.5	207.3	169.5	208.2	NR_002730.1
160.7	145.1	152.9	152.4	NM_001724.3
90.9	90.4	87.3	87.4	NM_199186.1
174	171.5	175.9	143.4	NM_004332.1
85.4	89.6	79	89.8	NM_001725.1
85	81.3	82.5	81	NM_025227.1
95.2	93.5	83.4	90.6	NM_174932.1
88.9	81.1	76.2	82.2	NM_174897.1
253.5	186.1	184.1	172.6	NM_006085.3
85.9	89.3	91.3	94.2	NM_004678.1
95.3	82.5	87.5	93.3	NM_001002760.1
268.8	212.5	178.1	182.9	NM_018174.4
116	122.1	103.9	102.8	NM_004333.2
86.8	71.2	85.8	98.8	NM_006768.2
336.2	344.5	316.8	231.4	NM_007304.2
407.5	347.9	367.4	314.1	NM_007301.2
97.5	87.9	91.9	91.3	NM_007295.2
104.5	103.3	91.4	113.2	NM_000059.1
105.4	96.1	95.6	100.7	NM_001001786.1
185.2	212.9	229.5	176.8	NM_014577.1
2516.5	2689	2497.2	1330.9	NM_005104.2
887.6	1032	957.7	883.7	NM_007371.2
92.3	92.8	102.9	100.9	NM_014299.1
103.9	100.7	104.7	101.3	NM_058243.1
660.1	517.4	396	382.2	NM_013263.2
380.6	445.4	371.3	483.4	NM_183359.1
85.6	87.5	81	86.8	NM_139199.1
1822.7	1358	1616.2	672	NM_023924.2
99.1	97.8	88.9	98.8	NM_023924.2
97.3	90.1	89.1	99.9	NM_012108.1
78.3	81.3	77.2	85.6	NM_207189.1
74.9	76.5	80.1	73.8	NM_001726.2
88.3	85.2	74.8	92.2	NM_001726.2
785	541.5	487.4	416.1	NM_199191.1
523.6	354	352.8	263.5	NM_199191.1
80.8	82.5	80.7	83.5	NM_004899.3
86.2	89.1	86.7	94	NM_145696.1
150.6	136.9	122.8	125.4	NM_145696.1
110	108.5	101.1	105.1	NM_001519.2
806.4	542.4	472.8	456.6	NM_018310.2
2438.8	2347.2	2142.3	2394.2	NM_015379.3
128.9	118.5	115	124	NM_080626.5
676	526	480.3	285.2	NM_080626.5
318.6	288.1	293.4	188.6	XM_941876.1

102.7	95.6	94.8	99.5	NM_032043.1
1965.9	1554.6	1334	895.4	NM_001024958.1
272.4	207.2	167.8	146.3	NM_015399.3
112.6	101.8	95.4	93.5	NM_032352.3
1559.5	1093	952.5	810.9	NM_015415.1
2667.3	1944.9	2285.4	1834.2	NM_016098.1
226.5	268	249	240.5	NM_001003694.1
3683	3437.3	3491.9	2429	NM_004634.2
371.4	540.1	544.1	350.8	NM_015695.1
92.3	87.3	83.9	87.1	NM_015341.2
94.8	90.1	97.8	92.3	NM_001727.1
859.5	721.1	625.8	739.6	NM_032430.1
87.3	87.2	89.4	81.8	NM_003957.1
112	109.1	108.3	99.7	NM_020180.2
80.9	85.2	85.6	88.1	XM_943559.1
110.7	104.4	92.3	99.8	NM_021938.3
93.4	100.6	86.4	80.2	NM_052840.3
75.9	73.3	73.6	83.8	NM_018963.3
90.1	103	90.1	94.3	NM_001007246.1
440.7	408.3	402.3	263	NM_001007246.1
89.6	99.8	86.3	91.6	NM_033656.2
116.2	97.4	109.9	106	NM_018117.10
100.7	80.3	82.4	98.1	NM_153252.2
434.8	357.7	359.2	206.4	NM_032667.4
240.1	292.1	290	259.7	NM_018045.4
2451.9	2063.4	1710.3	1758.9	NM_198589.1
96.6	90.7	90.7	94.8	NM_001728.2
101.7	92.7	98.6	87.1	NM_003458.1
91.4	88.6	86.4	91.4	NM_057176.2
336.7	291.4	270.2	222.6	NM_004334.1
783.1	581.2	555.1	353.3	NM_004335.2
818	892.2	885.9	670.4	NM_003972.2
210.1	208.5	143	187.1	NM_001011885.1
243	186.4	143.9	152.4	NM_025238.3
270.4	210.1	168.9	191.2	NM_032320.4
98.8	87.3	77.6	84.5	NM_152322.2
90.2	88.2	82	85.4	NM_001017523.1
98.9	81.2	81.8	82.8	NM_001018072.1
88.5	89	80.7	87	NM_152322.2
285.1	235.6	262.8	154.6	NM_032444.2
275.9	202.8	207.8	138.1	NM_144653.3
101.3	92.9	96.9	98.2	NM_052876.2
88.1	96.4	84.8	88.5	NM_181443.1
128	147.2	149.2	117.4	NM_014962.2
302.1	387.3	351.4	280.1	NM_025224.2
182.7	295.6	257.5	233.2	NM_017658.2
634.3	509.9	403.3	305	NM_033271.1
355.8	377.7	357.4	329.4	NM_001002860.2
99.1	113.9	100.9	104.7	NM_018167.3
163	158.2	142	121.3	NM_018167.3

81.8	85.6	88.7	88.3	NM_183242.1
99.6	109.5	102.3	96.4	NM_152733.1
101.2	97.1	95.4	91.9	NM_001729.1
119.9	118.9	122.4	107.8	NM_000060.2
19753.1	14893.6	13231	13206.7	NM_001037637.1
26470.7	19683.2	18145	17477.5	NM_001207.3
199.3	182.5	155.1	127.7	NM_001037637.1
365.7	344.2	279	224	NM_152265.1
574.8	1145.1	820.3	627.4	NM_001731.1
165.7	197.3	149.2	193	NM_006763.2
210	160.8	149.2	197.6	NM_006806.3
85.4	87.1	73.9	79.9	NM_017589.2
578.7	624.9	511.6	346.1	NM_000061.1
100.5	96.1	88	96.7	NM_181780.2
82.8	78.5	83.5	76.8	NM_001732.1
97.2	83.7	81.4	91.6	NM_078476.1
302.8	275.7	249.6	191.4	NM_007049.2
110.8	108.8	109	112.3	NM_181531.1
85.1	90.4	79.2	89.1	NM_006995.3
87.6	86.4	81.7	88.4	NM_024018.1
86.6	92.5	83.8	79.4	NM_194441.1
93.2	100.7	95.6	106.5	NM_007048.3
118.6	120.5	106.1	103.5	NM_007047.3
99.5	94.2	90.2	88.8	NM_197974.1
125.2	90.2	84.9	86.1	NM_019602.1
88.6	83.9	90.5	84.4	NM_197975.1
102.8	88.6	79.9	80.5	NM_197975.1
96.2	99.8	79.6	85.2	NM_006707.2
82.6	81.5	71.1	77.5	NM_024850.1
95.7	91.3	85.2	87.9	NM_152547.2
87	95.2	92	81.2	NM_003939.2
101.2	95.5	84.3	92.1	NM_033637.2
1213.4	703.8	565.9	540.1	NM_004336.2
140.5	112.6	100.4	116.6	NM_001211.4
5885.2	2871.7	2625.9	1742.1	NM_004725.2
100.5	98.5	94.4	87.9	NM_004725.2
308.2	182.3	139.1	154.9	NM_001007793.1
90	85.2	87.9	85.5	NM_147147.2
99.9	86.2	87.7	78.2	NM_007073.3
333.4	338.8	287.8	278.4	NM_007073.3
3021.4	2180.6	1890.5	1593.8	NM_032194.1
549.5	418.7	343.9	258.4	NM_018321.3
352.5	295.2	265.4	285	NM_025065.5
613.3	392.7	319.5	282.9	NM_004053.3
122.3	169.3	170.9	155.9	NM_004758.1
80.4	77.7	78.5	80.3	NM_001010873.1
105.2	100.5	98.5	94.3	NM_014670.2
2071.4	1588.3	1268	1259.5	NM_014038.1
104.4	99.1	86.6	96.2	NM_007021.2
260.6	205.4	212.2	277.4	NM_173473.2

91.5	90.1	82.5	87.6	NM_173554.1
222.3	195.3	185.8	168.7	NM_032024.2
84.7	96.6	85.4	85.2	NM_153244.1
89	82.1	83.9	79	NM_001010896.1
113.9	119.1	110	93.4	NM_001010911.1
111.6	103.4	103.2	105.6	NM_006829.2
87.3	84.9	83.4	79.6	NM_018017.2
149.6	148.1	122.1	129.6	NM_024834.1
113.9	93.3	90.1	97.2	NM_015652.2
80.9	84.1	84.1	86.9	NM_001010912.1
275.7	159.8	145.9	134.9	NM_198472.1
85.5	80.5	75	73.3	NM_207321.1
108.1	107.7	95.9	103.6	NM_152429.2
80.7	86.7	90.5	95.1	NM_001001732.1
92.3	104.9	91.6	90.8	NM_001010917.1
194.1	172.9	195.2	173.5	NM_015608.2
448.9	294.8	259.1	260.6	NM_032804.5
87	79.4	80.2	84.2	NM_001039380.1
194.7	203.5	185.5	162.5	NM_017787.3
101	98.2	94.4	84	NM_152710.1
130.6	130.3	124.2	112.9	NM_014472.3
88.2	84	78.5	88.8	NM_152751.1
382.2	339.8	274.3	290.5	NM_144591.1
214.9	150.7	160.3	141.4	NM_032709.1
204	210.2	250.7	159.4	NM_145306.1
92.2	83.5	87.7	83.1	NM_001080827.1
129.7	134.6	122.9	115	NM_001010924.1
94.5	89.5	87	91.5	NM_203441.2
84.8	84.9	85.5	88.4	NM_145246.3
105.1	107.1	90.5	90.8	NM_203440.2
102.7	98.5	89.1	98.9	NM_203439.2
95.6	98.4	94.5	94.4	NM_203438.2
198.2	211.1	158.2	184.8	NM_138357.1
125.9	95.1	98.8	105.3	NM_153810.3
184.9	122.3	125.7	118.3	NM_153256.2
99.6	93.2	85.1	86.3	NM_145314.1
98.4	90.6	89.8	94.8	NM_182554.2
85.4	91.3	87.6	92.2	NM_001042427.1
98.1	96	97.3	105.7	NM_001042427.1
288.5	293.2	252.1	167.6	NM_022153.1
99.5	97.9	89.6	76.5	NM_001001791.1
257	213.2	200	191.7	NM_153367.1
182.7	176.6	146.1	135	NM_025125.2
2010.4	1941.1	2644.4	1308.5	NM_032333.2
89.3	93.7	87.2	86.6	NM_018363.1
246	187.3	178	170.8	NM_001031709.1
129.4	122.7	110.7	109	NM_001031709.1
457.6	500.2	553.1	369.1	NM_018121.2
345.1	200.5	171.9	178.8	NM_001013840.1
76.4	88.4	87.7	90.9	NM_015631.2

161	130.4	116.8	121.7	NM_001013840.1
91	87.8	89.9	71.9	NM_001009997.1
91.7	89	73	88.6	NM_145010.2
86.4	82	78	79	NM_138413.2
80.4	76.7	75.1	84.3	NM_153714.1
85.9	84.5	84.1	75.8	NM_024688.1
1926.3	903.4	854.4	659.2	NM_006023.1
81.2	75.7	81	76.6	NM_199459.2
100.8	94.2	87.4	91.3	NM_001031746.1
93.1	89.8	73.7	80.9	NM_001031746.1
92.8	90.9	82.8	81.7	NM_144984.1
311.5	240.2	189.5	193.2	NM_024541.1
123.6	110.4	100	100.3	NM_024789.3
105.6	96.1	100.8	95.9	NM_145247.4
96.9	82.9	78.7	95.7	NM_025145.4
98.2	102.1	98.1	108	NM_001008723.1
96.8	104.9	90.1	103.9	NM_024889.3
88.2	90.6	84.8	82.7	NM_144661.2
93.3	88.8	77.7	81.3	NM_178832.2
109.4	102.1	103.1	97.9	NM_022063.1
100.6	97.4	96.4	95.3	NM_001012711.1
1029.5	685.1	667.3	464.7	NM_017615.1
113.1	98.5	91.5	104.3	NM_144587.2
107.1	102.1	97.6	95.2	NM_024942.1
120.4	111.1	102.3	101.1	NM_153336.1
155.6	143	133.9	139	NM_181698.1
88.1	83.8	82	82.4	NM_181698.1
81.6	84.3	79.7	80.5	NM_001004298.2
101	84.9	94.8	90.8	NM_173541.2
105.7	98.3	90.7	105	NM_017609.2
93.9	88.5	84.5	86.8	NM_173572.1
72.8	72.9	71.1	73.5	XM_945168.1
98.4	97.4	98.2	100	NM_024886.1
78.8	84.3	71.3	84.2	NM_198515.1
261.2	193.6	193.9	194.7	NM_024948.2
102.9	91.1	105.9	97.1	NM_207373.1
1164.3	736.5	547.8	719.3	NM_022761.2
10893.2	6273.8	5062.5	4215.2	NM_014206.1
157.7	164.7	149.5	109.5	NM_006133.1
101.8	84.8	92.7	88.3	NM_020643.1
102.7	93.6	89.6	103.9	NM_020642.3
973.8	574.7	587.3	437.8	NM_020642.3
457.1	294.5	315.3	232.5	NM_020642.3
86.2	75.9	79.7	80.7	NM_182901.1
1393.8	951.9	971	600.6	NM_020642.3
1375.2	1048.3	1160.8	490.7	NM_013265.2
170.6	153.2	153.2	121.3	NM_022338.2
109.7	103.6	110.3	94.4	NM_020193.3
196.4	152.6	156.7	159.6	NM_170746.2
110.4	139	133.4	156.4	NM_173573.1

80.4	87.8	82.9	85.3	NM_173590.1
128.2	138	197.2	144.7	NM_212555.1
97.2	85.8	87.4	85.2	NM_144663.1
88.9	85	83.1	96.4	NM_173525.1
91.7	88.4	87.9	94.2	NM_173580.1
141.9	130.3	112.3	110.8	NM_145013.1
600.3	492.7	366.4	399.6	NM_152316.1
108.9	127	131.4	127.9	NM_173589.2
495.3	410	330.2	271.9	NM_024099.2
94.2	98.7	87.2	91.3	NM_001003678.1
113.2	109.2	106.4	104.7	NM_001003678.1
151.9	132.1	130.1	121	NM_001003677.1
96.1	91	91.7	98.1	NM_001003676.1
247.2	199.1	183.5	156.8	NM_014042.1
96.2	102.4	93.3	88.6	NM_080659.1
84.8	102.7	87.7	94.9	NM_198498.1
345	376.4	360.5	389.7	NM_014039.2
101.9	98.6	91.5	91.6	NM_207428.1
300.4	436.2	389.3	313.8	NM_032127.1
120.5	98.2	88	100	NM_024631.1
97.8	94.8	90.5	89.4	NM_024806.2
108.9	100.3	94.3	88.3	NM_199124.1
179.3	172.2	203.1	180.4	NM_199124.1
89.6	95.5	88.3	94.3	NM_152587.3
85.6	86.6	84.7	84.4	NM_032930.1
106.4	113.7	112.5	110.6	NM_019021.1
89.1	82.8	70.5	82.4	NM_145308.2
137.3	119.8	122.8	126	NM_173811.2
92.4	87.1	77.5	87.8	NM_013279.1
715	457.5	386.1	240.5	NM_021640.2
96.9	100.9	93.2	104.9	NM_018164.1
98.6	84.3	80	87	NM_152638.2
95.7	93.5	92.5	94.2	NM_152261.1
607.8	362.3	329.5	293	NM_013300.1
93.5	78.9	83	93.6	NM_032130.1
354.2	359.6	360.1	316.3	NM_032230.1
95.8	101.9	95.9	100.9	NM_182530.1
304.5	223.6	239.6	204.7	NM_001009894.2
91.3	89.1	92.8	102.2	NM_024953.2
440.9	318.8	251.9	250.4	NM_032338.2
387.8	357.6	303.3	272.1	NM_031465.2
108.8	109.6	100	106.1	NM_032829.1
125.5	141.3	137.1	157.2	NM_018169.2
89	89.3	73.1	91.7	NM_182558.1
80.2	79.1	78.5	79.2	NM_024809.2
93.6	92.3	96.4	86.8	NM_030572.1
188.8	187	176.8	190.1	NM_020374.2
89.5	87.3	74.5	84.3	NM_001031748.1
84.3	76.9	81.9	80.1	NM_001031748.1
823	682.4	615.6	438	NM_017822.2

75.1	82.1	72.6	78.5	NM_198521.1
177.7	122	136.5	143.9	NM_022895.1
442.9	354.7	319.1	284	NM_021934.3
212.8	175.5	174	140.9	NM_152318.1
165.8	180	189.6	158.7	NM_152321.1
404.7	430.1	405.3	305.3	NM_016534.2
347.9	251.8	248.8	220.9	NM_017915.1
161.3	151.7	108	135.5	NM_024738.1
123.9	119.8	100.2	109.1	NM_020375.1
100.8	96.2	90.7	82.9	NM_152589.1
405.1	390.2	360.2	249.2	NM_032848.1
92.9	87.6	78.8	93	NM_153685.2
99.5	88.8	74.2	86.1	NM_152319.2
1496.3	723.7	827.2	616.2	NM_138425.2
85.2	98.6	87.4	92.8	NM_024685.2
91.8	88.4	84.9	86.9	NM_153022.1
114.5	105	90	89	NM_175874.2
82.1	84.9	78.6	92.1	NM_175895.2
139	162.3	159.5	125.6	NM_032901.2
90.3	98.9	99.4	83.4	NM_173591.1
154.6	149.4	151.4	140.5	NM_020456.1
257.3	238.1	203.2	242.8	NM_022118.3
108.6	98.3	92.4	85.7	NM_152324.1
656.8	716.3	703	461.9	NM_025113.1
89.8	82.5	77.3	92.5	NM_001010897.1
663.3	598.5	529.7	461.4	NM_025138.3
533.9	478.5	397.6	409.7	NM_025138.3
86.9	87	83.4	84.7	NM_025138.3
115.3	131.1	121.4	137.6	NM_006346.2
80.7	75	72.4	76.7	NM_152325.1
215.6	175.4	174.4	139	NM_145061.3
454.5	347.4	258.4	263.2	NM_024546.3
1661.4	1271.7	1251.8	770.6	NM_032436.1
94.4	87	80.9	80.7	NM_007176.1
549.4	318.7	265	361.1	NM_016475.2
114.2	117.6	96.1	96.2	NM_017799.2
682.5	914.2	887.8	679.9	NM_017970.2
258.7	296.7	287	284.4	NM_199043.1
122.3	119.8	115.3	112.6	NM_018036.4
184.8	141.7	133.8	127.2	NM_018139.1
102.9	91.5	104	91.8	NM_018168.1
1009.9	711.3	770.4	934.5	NM_018353.3
322.6	234.5	227.8	236.9	NM_018229.2
93.4	93.3	90	90.3	NM_018453.2
1395.8	948.6	731.8	698.2	NM_016468.3
89.8	80.4	92	87.7	NM_018228.1
106	116.8	102.1	102.7	NM_017926.2
164.9	181.8	156.3	171.2	NM_017926.2
94.5	98.8	90.2	97.2	NM_017924.2
496.8	322.8	260.7	234.2	XM_945152.1

113.9	113.7	116.2	96.9	NM_138360.2
186.7	139.7	134.4	116.7	NM_016049.3
160.4	120.1	140.1	107.5	NM_020195.1
162.6	129.4	116.6	110.9	NM_080664.1
164.2	121.7	102.7	121.8	NM_016472.3
382.3	258.4	211	218.9	NM_175748.1
605.5	484	364.7	358.3	NM_018108.2
89.1	87.9	74.4	86.7	NM_175748.1
533.1	508.9	557.1	335.4	NM_018335.2
224.2	170.8	145.3	123.7	NM_022067.2
652.7	514.3	524	443.5	NM_022495.3
83.8	87.9	85.8	93.9	NM_024558.1
281	190.9	185.5	149.8	NM_024558.1
281.6	204.4	211.5	187.6	NM_024558.1
231	213.8	213.8	168	NM_024633.2
102.3	93.2	85.3	94	NM_001042430.1
87	83	87.3	89.5	NM_024643.1
357.2	310.6	260.8	219	NM_032490.3
230.9	181.1	148.1	161.2	NM_145231.2
183.9	145.2	133.8	132.8	NM_152446.2
440.4	497.7	493.9	384	NM_138288.2
88.9	87.3	86.8	82.7	NM_138791.1
237.2	162.4	161.7	182.3	NM_144581.1
84.6	79.1	78.3	78.8	NM_001008726.1
112.1	104.3	103.3	90.6	NM_001008726.1
124.9	109.7	101.2	105	NM_080666.2
162.9	145.8	130.8	136.1	NM_032714.1
88.4	95.2	91.1	89.3	NM_138344.2
1926.1	2586.2	3066.7	3209.3	NM_032374.2
95.4	87.3	79.6	88.4	NM_032135.2
10111.1	6985.9	5480.9	5432.7	NM_031210.3
559.3	589	605.8	433.1	NM_024952.4
109.4	100.6	95.1	95.4	NM_024764.2
90.5	81.3	83.7	85.7	NM_020181.1
722.5	582.4	442.3	432	NM_016039.1
91.4	92.8	91.5	79.2	NM_001081002.1
312.4	183	173	175.6	NM_024644.1
135.1	105.7	97.8	89.5	NM_152307.1
2334.9	2212.9	2284.3	777.9	NM_022489.2
205.7	205.2	188.1	174.7	NM_022489.1
106.5	101.7	95.5	87.1	NR_002937.1
1150.8	974.3	764.7	732.3	NM_004894.1
119.8	107	111	103.8	NM_174913.1
4864.8	5427.4	8599.9	5338.8	NM_173607.2
125.7	111.6	106.2	95.9	NM_173607.2
117.4	115.3	98.9	103.3	NM_173607.2
160.4	149.3	143.9	132.3	NM_001017923.1
96.1	98.7	102.6	98.9	NM_181533.3
91.7	85.7	81.7	89.8	NM_181533.3
968.8	721.6	653.1	544.4	NM_144578.2

88.1	87.7	94.1	77.7	NM_001001872.2
91.9	85.9	83.6	86.7	NM_174978.1
131.5	131.3	128.8	129.5	NM_024496.2
90.3	80.9	83.2	83.3	NM_194278.3
222.7	190.6	192.5	145.4	NM_194278.3
296.9	282.6	268	274.8	NM_194278.2
93.9	85.4	87.4	85.7	NM_152445.1
84.3	90.9	80.3	88.5	NM_025057.1
198.4	167	160.8	157.4	NM_001024674.1
94.7	107.4	99.8	85.2	NM_152777.2
105.4	101.6	92	94.1	NM_152592.2
84.2	86.3	81.8	85.5	NM_172365.1
87.6	86.8	80.5	83.5	NM_173526.1
157	164.6	151.7	144.5	NM_017791.1
87.8	87.7	79.4	83.7	NM_207117.2
93.3	92	85	89.4	NM_001077594.1
92.9	95.9	84.7	87.2	XM_941093.1
158.4	123.5	119.8	103.9	NM_174891.2
81.8	81.6	91.2	86.4	NM_173846.3
326	219.4	223.6	137.2	NM_173608.1
1667.9	2381.8	2984.3	2301.7	NR_002808.1
108.1	111.6	96.1	105.8	NM_014828.1
320.3	261.3	283.5	171.9	NM_021944.1
421	335.8	292.6	258.3	NM_017815.1
2995.6	2610.8	2187.4	2092.6	NM_016304.2
323.6	317.9	287.7	245.7	NM_020447.2
82.5	83.7	78.7	83.4	NM_018958.1
109	96.1	97.6	108.3	NM_001005269.1
102.2	105.3	106.5	98.7	NM_001005267.1
80.4	93.3	71.4	82.5	NM_173609.2
293.4	198.1	155.5	146.9	NM_033286.2
3837	2961.1	2339.4	2019.7	NM_020154.2
83.8	88.6	83.2	77	NM_173528.1
94.8	77.5	83.6	82.3	NM_152335.1
105.8	110.2	91.4	90.7	NM_024713.1
87.9	87.4	76.6	77.9	NM_153040.1
80.4	81.6	80.1	84.6	NM_152647.1
90.7	95.1	80.2	85.6	NM_175898.2
139.8	109.6	115.9	108	NM_182616.1
203.4	249.7	207.5	146.9	NM_015492.3
187.8	173.7	176.1	163.5	NM_144597.1
265.2	195	159	174.1	NM_032499.3
178.2	194.9	166.5	136.6	NM_152259.2
92.8	83.4	80.9	88.4	NM_152448.1
105.3	120	98.7	92.3	NM_030800.1
302.1	204.2	190.3	165.4	NM_030800.1
99.7	94.4	84.2	87.6	NM_032413.2
82.6	82.3	87.2	84.4	NM_032413.2
94	81.9	77.8	87.9	NM_197955.1
89.6	91.8	89.5	93.1	NM_030944.1

464.7	470.1	418	358.6	NM_023933.1
93.9	99.3	92.8	89.5	NM_173476.2
88	80.3	79.2	84.6	NM_173476.2
440.9	229.3	236.2	235.8	NM_023076.2
94.5	93.9	84.9	88.8	NM_001214.2
88.6	87.8	83.4	91	NM_024600.1
3748.2	2437.7	2160.9	1542.1	NM_024571.2
179.1	145.5	145.6	126.5	NM_144570.2
78	80.7	87.4	81.4	NM_001077350.1
630.4	483.8	503.2	266.5	NM_001039476.1
472	343.6	335	222.5	NM_024731.2
81.5	84.5	94.3	85.8	NM_033201.1
90.4	90.6	86.4	97.2	NM_152337.1
384.7	302.6	369.1	185.6	NM_032140.1
123.7	109.2	96.5	94.7	NM_013399.1
110.4	103.9	103.8	97.6	NM_032269.3
335.4	297.2	269.5	167.2	NM_024516.2
98.9	95.7	85.2	89.8	NM_175900.2
91	87.6	89.4	86.9	NM_153025.1
475.4	493.7	442	476.5	NM_025082.1
361.3	345.4	253.7	161.9	NM_024598.2
1405	1466.8	1466.7	612	NM_022744.1
932.4	561.4	490.5	418.4	NM_018455.3
2913.2	1833.7	1640.7	1206.1	NM_020188.2
3613.6	1890.7	1744.9	1919.5	NM_144600.1
97.5	98.6	91.9	95.6	NM_153261.3
162.2	203.9	219.5	147.5	NM_004913.1
93.7	81.2	75.9	81.4	NM_144602.2
715.5	421.8	463.4	351.1	NM_013242.2
561.5	475.4	411.3	407	NM_032039.1
354.9	364.9	304.6	268.8	NM_016080.2
91.2	91.1	76.5	89.2	NM_020914.3
101.9	92.8	86.5	88.2	NM_030630.1
212.6	211.3	169.2	175.7	NM_152464.1
108.4	109.3	105.6	97.6	NM_032339.3
96.2	100.6	95.6	91.5	NM_001010855.1
119.1	110.5	108	93.6	NM_024052.4
705.7	873.2	807.9	584.6	NM_018428.2
112.3	107.5	97.5	106	NM_024683.2
11954	10113.9	10415	6936	NM_152350.1
106	94.4	91.8	89.1	NM_152343.1
82.2	89.1	82.5	91.1	XR_000576.1
105.7	93.3	91	90.9	NM_001038704.1
154.3	142.2	122.7	121.6	NM_020233.3
657.5	472.4	423.2	373.8	NM_174893.1
90.8	93.4	85.6	88.3	NM_145272.2
590.1	416.7	440.5	217.5	NM_024032.2
89.3	84	97.4	87	NM_182564.1
117.8	136.8	150.4	132.3	NM_178519.2
120.7	108.8	121.1	117.3	NM_144679.1

84.2	94.5	92	90.3	NM_152347.3
847.3	475.2	437.9	401.4	NM_181656.1
603.8	410.4	361.8	335.8	XM_942508.1
109.2	106.8	95.5	81.7	NM_181656.1
95	93.9	91.5	93.8	NM_017622.1
1354.2	1423.7	1540.2	762.7	NM_152766.2
780.2	639.5	528.1	505.4	NM_001033046.1
277.8	331.2	274.6	252.6	NM_018182.1
94.4	108.4	99	105.8	NM_181707.1
167.1	157.7	142.1	150.6	NM_178542.3
91.2	92.4	95.7	84.1	NM_152781.2
151.1	157.7	169.1	125.7	XM_938283.1
115.1	117	114.7	90.3	NM_025099.2
79.4	78.3	79.5	78.3	NM_152466.1
1084.3	1046.1	1137.4	542.3	NM_025161.3
165.6	141.7	124.4	115.3	NM_018149.5
118.3	107.3	108.3	99.6	NM_017928.1
109.6	118.1	103.2	99	NM_175734.3
121.9	102.1	104	109	NM_022344.2
103.3	93.7	100.8	103.6	NM_207387.1
89.9	81.8	77.4	80.2	NM_152460.2
84.7	87	83.1	86.7	NM_173625.3
251.2	214.9	190.6	169.5	NM_018405.2
208.4	236.9	172.1	185.4	NM_017941.3
123	108.9	100.6	107.8	NM_203413.1
205	206.2	160.4	154.4	NM_015362.3
138.5	133.1	126.1	110.9	NM_203413.1
90.2	78.9	79.8	80.3	NM_203414.1
89.5	95.2	91	87.4	NM_199339.2
205.3	230.8	216.9	164.2	NM_018553.1
88.4	87.1	76.1	89	NM_181483.2
364.7	329.2	314.9	285.2	NM_015476.2
80.7	91.2	82.5	93	NM_024781.1
223.6	213	220	184	NM_153211.1
210.7	187.7	183.8	154.1	NM_152352.1
306.4	301.4	315.1	261.4	NM_031446.3
248	206.2	195.8	190.8	NM_024805.1
250.3	215.2	174.6	158.1	NM_145060.1
1190.9	1122.6	1067.7	871.6	NM_001008239.1
80.1	74.7	81	78.1	NM_145055.2
79.3	70.2	90.7	80.6	NM_173629.1
95.3	81.4	86.9	86.7	NM_198995.1
353.7	348.6	351.6	255.5	NM_194281.2
112.5	113.9	120.5	104.5	NM_032933.3
83.3	85.1	85.1	83.7	XM_058931.9
94.2	85.6	80.8	90.2	NM_001044369.1
217.2	172.8	170.5	138	NM_173529.3
2108.3	1805.7	1526.4	1291.3	NM_014177.1
666.3	392.1	375.5	376.1	NM_001012716.1
478	438.5	368.3	324.8	NM_013326.2

1720	1069.7	996.5	686.1	NM_019107.3
95.6	84	96.6	98.5	NM_001031726.1
202	145.5	148.5	120.7	NM_001031726.1
460.7	341.7	334.1	216.4	NM_031448.2
110.9	89.9	77.4	82.9	NM_001031726.1
103.6	84.9	98.6	98.6	NM_021185.3
88.5	94.9	87.7	81.6	NM_152474.2
92.7	88.1	78.9	88.7	NM_182577.1
490.3	415.2	309.7	323.4	NM_134447.1
2768	2212.8	1802.4	1526.4	NM_134447.1
102.6	117.3	94.6	105	NM_033513.2
124.2	111.2	107.2	103	NM_173481.2
904.2	660	589.7	475.6	NM_138774.2
125.4	116.1	109.3	99.6	NM_152480.1
370.4	257.9	216.4	180.6	NM_017914.2
306.9	241.5	216.9	199	NM_152482.1
85.5	91.5	89.2	82.6	NM_152769.1
95.4	100.4	88	88	NM_001042680.1
144.6	149.7	133.9	129.3	NM_174983.2
163	139.7	129.1	147.2	NM_001080543.1
117.8	117.7	100.2	101.7	NM_001080543.1
111.6	103	97.7	93.8	NM_174947.2
21246.2	26244.8	27067	24882.9	NM_001014373.1
88.8	85.3	72.2	80.3	NM_033520.1
76.8	79.1	78.2	86.4	NM_152771.1
86.9	97.8	104.7	97.2	NM_198532.1
89.2	92.4	91.5	86.4	NM_001031735.1
102.7	92.1	87.5	95.6	NM_001039846.1
1286.8	1277.8	1236.3	683.6	NM_182498.2
94.4	88	85.7	91.9	NM_012109.1
188.1	218.2	177	182.1	NM_152266.1
505.8	280.7	228.1	209.4	NM_199250.1
94.4	92.4	94.3	91.1	NM_001039887.1
206.8	235.2	210.2	209.1	NM_001033026.1
351	470.5	380.5	376.9	NM_001033026.1
1201.8	737.9	665.4	676.2	NM_015168.1
101.6	90.1	84.8	94.6	NM_006333.2
84.9	79.2	78.7	84.3	NM_173177.1
131.1	116.6	121.2	125.8	NM_020156.1
90.3	81.1	75.7	84.8	NM_152692.3
170.6	137.4	153.5	145.6	NM_001011551.1
93	90.8	94.5	85.6	NM_001012970.1
142.3	179.8	167.5	170.5	NM_173807.2
92.7	91.5	86.4	82.6	NM_206837.1
97	91.1	88.8	94.4	NM_206837.1
90.1	98.2	87.3	88.8	NM_145047.3
79	87.7	87.6	83.8	NM_001006945.1
153	112.9	108.7	121	NM_018372.3
102	97.6	92.5	80.2	NM_001039517.1
90.9	91.5	82.9	91.7	NM_139240.2

789.7	307.2	242.7	168.3	NM_018265.1
195.5	207.7	174.1	147.9	NM_014388.5
502.7	503.4	487.7	372.8	NM_024595.1
105.9	98	87.9	97.8	NM_024595.1
438	466.8	364.9	322.4	XM_941120.1
423.3	320.6	267.1	256.2	NM_017850.1
87.2	91.1	79.8	90.2	NM_178550.3
100.5	96.5	93.7	95.5	NM_182581.1
560	390.4	370.6	222.6	NM_018186.2
102	95.4	85.6	85.4	NM_024676.3
80.9	75	79.9	69.2	NM_021179.1
119.8	159.1	130.9	131.1	NM_024709.2
94.6	91.9	91.4	98.2	NM_023938.4
83.8	79.6	82.6	77.3	NM_182623.1
79.9	80.6	82.4	74.9	NM_199343.1
676.6	393.5	347.1	272.8	NM_020141.3
789	551.6	450.6	390.5	NM_016076.2
195.6	207.3	173.7	168.4	NM_198446.1
822.4	988.5	932.6	583	NM_017887.1
440.9	290.8	246.4	264.3	NM_032018.4
218.2	159.5	154	159.7	NM_032018.4
140.8	112.3	102.6	102.9	NM_001010984.1
85.5	83.1	88.3	82.5	NM_182766.2
88.9	85.1	83.6	80.2	NM_144696.3
95.5	82.8	84.5	89.8	NM_173507.1
1176.4	1185.1	926.3	758.4	NM_020362.2
91.7	96.4	84.2	87.5	NM_025063.1
3072.6	2151.3	1725	1739.9	NM_152379.2
297.1	198	205.3	164.3	NM_024037.1
92.1	78.5	81.1	88.3	NM_030933.1
96.6	98.8	89.5	91.1	NM_001010913.2
98	94.1	83.3	95.5	NM_001013674.1
190.8	183.1	156.3	153.8	NM_053052.2
492.3	461.7	411.3	296.4	NM_015609.1
93.7	90.6	82.4	83.5	NM_001012425.1
200.4	191	160.2	179.3	NM_022756.3
93.9	95.2	77.9	79.8	NM_145278.1
93.3	92.6	85.9	95.3	NM_001032363.1
92.9	90	76.2	78.2	NR_003242.1
722.4	390.4	321.4	322.6	NM_033319.1
86.2	78.2	86.5	83.6	NM_033418.1
98.9	83	77.8	80.2	NM_182579.1
82.2	87	88	88	NM_152290.1
185.9	179.6	189.1	156.6	NM_017891.2
155.5	143.2	147.6	116.2	NM_032125.1
2185.3	1749.8	1453.4	803.6	NM_174896.2
281.6	269.3	249.9	157.1	NM_023077.1
1018.9	740.6	752.1	321.8	NM_018150.2
100.3	97.5	97.4	112.9	NM_024603.1
135	126.3	116.6	125.6	NM_024544.1

95.3	91.1	83.9	91.8	NM_001004303.2
259.7	244.8	163.3	194	NM_138467.1
85.6	92.2	84.2	87.8	NM_152365.1
95.9	90.2	88.3	104	NM_001002912.2
93.9	106.1	98.9	94	NM_198547.1
497	638.1	704.5	739.1	NM_022774.1
98.8	97.9	95.3	101.2	NM_152607.1
90.1	90.3	80.4	86.8	NM_001010922.1
102.6	91.8	93.1	94.6	NM_001029946.1
90	88	80.8	77.4	NM_176782.1
644.4	701	588	600	NM_017953.2
115	106	95.4	109.4	NM_144627.1
96.3	95.5	103.2	95.1	NM_019099.3
88.5	82.4	88.4	84.7	NM_198926.1
110	107.1	106.4	110.5	NM_198926.1
253.5	214	171.5	166.1	NM_001007544.1
102.2	89	89	91.3	NM_198545.3
124.3	120	123.7	120.5	NM_173795.2
91.7	80.8	95.4	72.1	NM_001010979.1
1587.6	953	855.2	598.2	NM_052965.1
106.8	91.6	86.9	80.2	NM_001013615.1
98.1	82	89.5	84.8	NM_001013625.2
218	231	203.3	165.6	NM_032800.1
855.9	661	724.3	333.3	NM_006589.2
104.8	92.4	89.5	93.4	NM_006589.2
126.4	125.1	125.8	107.7	NM_198264.1
105.6	89.4	85.7	97.7	NM_178122.2
112.2	121.5	125	112.3	NM_030806.3
95.5	87.2	90	97.2	NM_182517.1
672.3	662.8	642.6	430.3	NM_022083.1
91.4	87.2	82	95.9	NM_030934.3
85.4	97.6	83.9	90.8	NM_017673.4
101.9	77.8	83	86.2	NM_017847.3
296.8	354	275	271.1	NM_001012985.1
98	79.7	79.6	82.9	NM_199351.1
501.5	360.8	311.5	214.5	NM_016183.2
83.1	90.2	82.8	89.7	NM_001080494.1
758	589.7	564.5	491.3	NM_024319.2
77.9	81.6	81	76.9	NM_183059.1
3511.9	2595.8	2285.1	1912.9	NM_138391.3
85.8	98.2	84.5	91.7	NM_004848.1
2040.8	1941.9	1745.8	1578.8	NM_016126.1
103.6	104.9	91.6	104.3	NM_019060.1
799	690.1	542.8	682.9	NM_015449.1
414.7	266.8	212	251.7	NM_015449.1
86.7	89.5	86.5	86.3	NM_001025231.1
2384.5	1530	1370.9	1148.9	NM_015471.2
97.4	81.6	88.6	95.9	NM_032126.1
1513.3	1710	1625.2	1057.2	NM_024097.1
127.2	108.5	99.4	101.4	NM_144697.2

831.1	706.9	727.7	556	NM_198077.1
439.2	502	462.7	379.9	NM_001024594.1
4224.5	2629.3	2403.8	1844.5	NM_024579.1
612	561.8	542.4	460.9	NM_152608.2
153.6	117.3	113.3	141.3	NM_017860.3
1163.6	864.3	691.8	723.1	NM_032324.1
125.1	105.4	98.1	101.7	NM_144695.1
2476.5	1706.4	1486.6	1143.8	NM_144584.1
628.1	786.8	690.1	626.4	NM_023015.3
92.8	86.8	86.7	90.7	NM_006365.1
99.8	88	81.1	89.4	NM_152763.3
155.9	158.9	195.7	193	NM_020317.3
84.8	79.8	84	87.9	NM_178840.2
89.1	82.9	69.2	84.1	NM_152610.1
473.5	404.4	420.9	366	NM_015997.2
205.5	209.1	189.6	170.6	NM_001010867.1
1315.9	1026.4	872.8	760.6	NM_152609.1
242.3	181.6	212.8	168.1	NM_015434.1
210.8	147.1	123	108.6	NM_152485.2
85.7	88.6	91.4	85.8	NM_018252.1
121.2	136.3	120.6	111.9	NM_173509.2
737.3	580.2	588.9	390.1	NM_015607.2
108.7	107.1	103.1	101.4	NM_018166.1
382.4	501.1	465.6	375.3	NM_024813.1
189.3	239.8	199.3	177.5	NM_153035.1
129.9	123.2	135.1	125.7	NM_001012960.1
109.5	122.6	112.8	131.7	NM_001012961.1
101.2	109.3	97.8	97.2	NM_182518.2
926.7	695.6	850.3	406.8	NM_144580.1
397.9	336.6	322.4	307.4	NM_182533.2
88.9	87	83.5	88.4	NM_152377.1
89.7	102.2	93.6	95.3	NM_181643.2
94.8	83.2	89.2	84.6	NM_030907.2
83.6	70.9	72.8	78.1	NM_014283.2
84.2	93.7	99.4	88	NM_016227.1
93.7	93.9	80.6	94.2	NM_014283.2
85.1	78.2	82.2	83.2	NM_032648.1
93.4	84.3	78.8	87.8	NM_019118.3
98.3	93.2	87.6	102.1	NM_144702.1
99.8	94.4	93.3	94.3	NM_032884.2
84	78.2	88.9	87	NM_001003665.1
110.9	102.8	87.3	104.7	NM_145257.2
550.8	453.7	424.7	485.4	NM_032705.3
86.3	85.7	82.6	89.4	NM_001012274.1
91.5	111.8	80.5	95.5	NM_015991.1
97.2	84.7	84.2	92	NM_000491.2
3043.1	2007.1	1605.3	1491.6	NM_001212.3
100.2	99.8	82.4	90.6	NM_172369.2
136.1	143.5	134.2	131.8	NM_023925.3
124.1	170.7	144.2	163.8	NM_023925.3

107.2	103.2	115.6	107.2	XM_371208.3
110.3	101.9	98.2	100	NM_006688.3
91.5	97.6	100	99.1	NM_182528.1
92.4	86.1	85.9	90.2	NM_001010908.1
88.4	95.9	86.8	83.9	NM_001008223.1
2985.9	1909.7	1744.2	1237.7	NM_012072.2
101.1	103.1	100.1	94	NM_198593.1
83.1	77.3	78.4	84.8	NM_198594.1
80.8	80.2	76	80.6	NM_030968.2
98	97.4	86.6	97.3	NM_031908.3
97.1	88.1	89.3	98.4	NM_181435.4
77.8	84.3	78.2	78.7	NM_181435.4
86.1	95.1	90.4	89.1	NM_031909.1
75.1	86.9	75.2	85.5	NM_015645.2
86.9	88.6	94.5	90.1	NM_031910.3
86.5	98.9	99.5	89.8	NM_031910.3
98.4	90.7	86.6	85.9	NM_182486.1
94	86.7	88.6	88	NM_031911.3
86.9	93	83.1	88.5	NM_207419.1
93.2	88.5	87.1	91.3	NM_001733.4
121.3	177.1	176.4	149.2	NM_016546.1
93.2	95	89.9	81.2	NM_201442.1
90	89.2	88.7	90.9	NM_001734.2
114.9	182.2	165.9	155.4	NM_000063.3
88.1	94.1	79.8	89.2	NM_014477.2
1557.5	2360.6	2461.6	1240.8	NM_032883.1
97.1	91.3	90	89.6	NM_080607.1
968.6	837.5	834.3	798.8	NM_012261.2
120.9	109	129.1	134.4	NM_001012971.2
113.9	132.9	135.4	128.9	NM_001013646.2
139.4	203.9	168	226.9	NM_080821.1
1829.4	1619.7	1568.2	1172.1	NM_017896.2
1124.5	832.3	596.7	621.5	NM_016470.6
80.4	77	81.8	79.9	NM_080616.1
103.7	90.3	87.7	94.7	NM_033197.2
524.9	514.7	438.2	289.3	NM_023935.1
333.2	393.9	360.4	360	NM_199181.2
83.7	84	80.9	87.2	NM_199181.2
94.8	88.5	92	94.8	NM_080628.1
159.3	149.8	158.3	126.4	NM_018152.2
421.4	400.8	351	317.7	NM_024331.3
216	225.9	203	164	NM_024331.2
89.3	85.2	93.2	88.3	NM_178468.2
497	251.8	190.1	176.4	NM_030919.1
92.4	90.5	96.5	91.8	NM_213631.1
109.4	117.3	96.4	110.9	NM_213631.1
96.6	81.6	87.5	95.7	NM_152503.2
91	87.6	90.6	85.5	NM_001033086.1
93.7	81.5	77.5	92.1	NM_001033087.1
98.5	85.9	85.4	85.3	NM_001024675.1

95	88.8	81.9	83.4	NM_080622.2
171	169.6	132.9	147	NM_012469.2
103.9	105.2	99.1	106	NM_080739.2
100.1	84.4	82.9	82.1	NM_001080472.1
89	89	79.4	88.5	NM_080825.2
224.2	176.4	200.6	189.4	NM_024299.2
91.3	84.3	71.4	76.9	NM_080833.1
90.6	92.8	84.8	87.4	NM_080834.1
1086.8	837.1	718.8	562.4	NM_019095.3
101.1	100.7	91.4	95.4	NM_080625.2
94.5	81.8	86.5	90.7	NM_033421.2
89.8	99.8	102	100.1	NM_033421.2
84.5	81.6	78.5	83.8	NM_080608.3
99.1	91.7	79.7	85.2	NM_178463.2
435.3	340.1	297.8	281.9	NM_024918.2
79.8	80.7	71.6	87.8	XM_940057.1
99.5	90.1	89.8	91.5	NM_178457.1
1443	1209.9	1159.6	977.1	NM_022106.1
93.3	99.3	83.5	88.2	NM_178477.2
92.6	81.6	79.8	89.6	NM_178477.2
90	88.9	96.3	88.6	NM_182658.1
78.3	87.3	75.6	87.5	NM_182519.1
90.9	95.7	97.6	97.6	NM_018474.2
171.5	224.1	185.8	194.5	NM_001039379.1
103.4	97.1	84.5	99.2	NM_024059.2
1685.1	1646.2	1645.9	965.8	NM_018270.3
263.3	183.4	187.3	161.4	NM_015600.2
101.3	104.4	95.1	96.1	NM_024704.3
13487.7	13339.1	11111.9	9276.6	NM_018840.2
16240.6	15974.1	14022.3	11652.2	NM_199485.1
4876.6	4456.7	4056.4	3105.9	NM_018840.2
87.7	86.7	91	92.7	NM_015585.2
683	426.5	462.4	203.1	NM_017874.2
96.3	98	88.4	88.2	NM_015417.2
157.6	144	130.4	139.1	NM_018347.1
373	307.1	278.1	236.5	NM_020531.2
1211.9	1505.7	1288	909.8	NM_001009923.1
2045.1	2443.1	1974.2	1506.3	NM_001009924.1
339.7	245.6	286.9	210.4	NM_018217.1
90.3	93.8	80.5	93.6	NM_020356.2
267.5	274.4	224.6	208.9	NM_033542.2
107.1	102	97.9	99.7	NM_024893.1
753.5	632.3	527.6	509.5	NM_015511.2
146.8	102.6	89.6	98.5	NM_017671.3
3005.4	3338.7	3059	1907.7	NM_016407.1
271.7	225.5	169	185.1	NM_199487.1
135.8	119.9	109.6	117.4	NM_018244.3
598.3	563.6	503.9	403.3	NM_199513.1
811.9	799.9	601.9	490.3	NM_016045.1
99.1	89.3	96.8	95.2	NM_018354.1

6798.4	5279.6	5367.5	3400.7	NM_080748.1
86.4	94.8	74.4	85.8	NM_033409.2
150.3	112.9	104.1	107.6	NM_031424.3
80	77.6	77.1	80.3	NM_001042353.1
199.4	182.3	149.7	137.8	NM_001042353.1
81	84.9	80.3	87.7	NM_152864.2
167.8	202.2	144.5	178.9	NM_022082.2
98	83.5	81.3	96.3	NM_016649.3
139.2	141.7	124.4	126.9	NM_022104.3
95.1	97.9	97.4	86.6	NM_024120.3
308.6	198.2	197.6	208.1	NM_024120.2
151.3	117	125.6	121.4	NM_024120.3
93.9	97.5	95.4	90.4	NM_080574.2
93.4	83.5	82.8	85.6	NM_178466.3
108.9	94.8	104	95	NM_178466.2
76.9	84.8	81.9	77.9	NM_178466.3
630.1	638.1	569.6	388.5	NM_052865.2
97.2	91.7	92	89.4	XM_945389.1
75.9	87.3	79.8	76.2	NM_020343.2
98.9	92.2	89.3	94	NM_152611.2
94.9	95.3	88.6	90.8	NM_021215.2
94.2	94.2	89.3	85.7	NM_178483.2
98.2	93.2	95.3	93.1	NM_178456.2
96.4	88.8	88.7	88.8	NM_080674.1
133.4	162.3	166.4	142.4	NM_001009608.1
95.2	105.9	101.6	108.3	NM_153269.1
267.4	247.4	244.9	161	NM_024958.1
102.5	86.5	85.8	91.3	NM_145033.1
120.9	115.1	121.9	109.9	NM_032910.2
92.6	82	73	77.8	NM_199175.1
224.8	194.5	212.5	200.9	NM_013240.2
96.2	85.7	80.4	87.9	NM_152506.1
94.9	96.9	88.8	94.5	NM_152505.3
319.9	422.3	435.3	211.9	NM_004928.1
5047.1	7119.7	8790.9	7260.4	NM_001001789.1
154.9	147.7	159.3	149.4	NM_199050.1
276.2	311.4	327.2	230.7	NM_199050.1
81.6	84.5	81.3	84.1	NM_144991.2
449.5	456.5	443.2	379.3	NM_004649.4
1342.4	1290.2	1174.7	784.3	NM_004649.4
296.5	325.5	297.2	263.8	NM_198155.1
92.1	87.6	93.9	95.3	NM_001005732.1
90	83.6	80.8	90.9	NM_001005732.1
89.9	76	70.2	80.6	NM_001005734.1
83.4	89.6	84.6	85.3	NR_002215.1
112.5	93.1	101.8	91.5	NM_018944.2
92.2	90.6	77.5	91.5	NM_001006116.1
357.1	433.4	383.1	370.7	NM_001042401.1
177.9	189.7	174.5	167.5	NM_001042401.1
91.7	94.6	90	82.6	NM_017833.3

102.7	87.3	93.1	92.7	NM_001040192.1
96.2	89.2	89.8	86.9	NM_017833.3
221.6	159.9	152.8	146.8	NM_032261.3
2148.5	2492	1766.9	1734.7	NM_058181.1
184.2	240.6	245.7	238.5	NM_058181.1
102.8	99.4	106.7	92.3	NM_001006114.1
120.1	110.2	132.3	114.7	NM_199071.2
86.9	76.1	81.5	89.8	NM_058180.3
107.7	92.8	91.1	99.8	NM_199071.2
86.3	82.7	86.1	84.5	NM_021254.1
524.8	518.6	436.4	457	NM_017835.1
141.5	163.9	149.9	129.8	NM_016940.1
90.5	88.5	89.5	79.5	NM_019596.2
106.5	88.1	98.1	96.9	NM_058187.3
142.1	145.7	143	165.2	NM_016631.3
117.6	123.9	108.7	111.9	NM_013329.2
124.3	130.4	124.3	137.1	NM_016631.3
634.7	693.5	688.3	656.8	NM_016631.3
92.4	91.2	88.2	82.3	NM_058188.1
116.6	99.6	95.7	97.8	NM_058189.1
118	109.2	104.2	103.7	NM_020152.2
262.7	233.5	234.6	152.1	NM_058190.2
91.6	83.1	83.3	79.1	XR_001009.1
87.7	76	80.5	83.1	NM_001040140.1
87.2	79.1	90	76.7	NM_153754.1
84.7	82.3	84	82.5	NM_153204.1
130	135.8	129	111.1	NM_017447.2
86.6	94.1	93.7	86.9	NM_145180.2
74	73.6	77.3	82.7	NM_153773.1
1972.5	1989.7	2128.4	990.7	NM_031444.2
125.1	111.2	107.4	107.8	NM_182520.1
2279.8	1531.9	1293	1241.2	NM_213720.1
496.2	372.4	373.6	246	NM_024053.3
94.1	100.6	92.6	90.8	NM_032561.2
227.7	224.5	209.4	232.6	NM_152906.2
203	206.8	200.3	189.4	NM_012264.3
148.8	134.6	130.3	129.9	NM_017911.1
99.5	114.6	110.7	114.9	NM_001009880.1
656.5	817.5	823.3	497.6	NM_015264.1
91.9	89.9	90.1	101	NM_015264.1
102.7	127.4	110.3	97.9	NM_194250.1
100.1	82	85.7	90.7	NM_144629.1
91.4	89.6	86.7	89.1	XM_944033.1
95.2	94.4	88	94.9	NM_173545.1
91.4	101.1	95.1	104.2	NM_032266.2
242.6	284.7	257.3	243.4	NM_024293.2
262.8	216.1	189.2	181.6	NM_017877.2
100.4	99.5	93.9	83.5	NM_182587.1
313	273.8	228.7	238.8	NM_015680.3
3797.1	2638.9	2277.9	2545.4	NM_015702.1

182.3	287.9	271.9	235.1	NM_023016.2
89.8	86	85.7	85.6	NM_013310.3
4533.3	2874	2913.6	2080.2	NM_080592.2
1834.4	1281.7	1341.1	927.9	NM_080592.2
2363.4	1735.4	1531.8	1023.3	NM_017546.3
96	91.4	85.5	94.2	NM_003203.3
841	729	572.1	591.3	NM_015701.2
128	132.6	114.2	114.4	NM_015463.1
265.5	185.5	225.4	209.9	NM_020194.4
196	168.9	168.4	120	NM_024766.1
377.9	309.1	266.1	220.8	NM_015955.2
98.2	87	88.4	92.6	NM_173513.2
203.7	179.3	151.1	185.6	NM_032319.1
86.8	96	94	88.6	NM_000064.1
87.8	84.2	88.6	81.3	NM_004054.2
172.6	195.4	171.7	174	NM_016589.3
923.5	949.6	951.1	633.9	NM_018462.4
2052.4	1059.7	921.3	673.7	NM_020685.3
95.1	91.3	84.8	87.6	NM_033364.2
1359.1	1528.8	1170.7	1216	NM_015412.3
93.9	100.4	89.1	100.6	NM_015412.3
105.5	103.7	112.3	107.2	NM_001025072.2
89.4	83.4	73.8	84.5	NM_016210.2
680.9	771.4	780.8	579.9	NM_016474.3
83.8	86	80	86.1	NM_032137.3
407.8	395.9	346.1	214.5	NM_152531.3
105.2	105.2	90.4	98.3	NM_152533.1
184.5	142.6	118.6	108.4	NM_001029839.1
97.2	92.9	79	81.6	NM_001029839.1
116.9	100.6	98.2	94	NM_001029840.1
178.6	179.1	139.4	149.6	NM_173826.1
89.7	95.3	85.2	92.9	NM_173472.1
99.1	97.5	111.7	82.5	NM_207307.1
1131.9	947.4	850.9	577.1	NM_032359.2
87.5	93.8	91.9	92	NM_007354.1
304	224.8	222.4	205.9	NM_014367.3
1276.9	1374.9	1535.8	855.6	NM_022485.2
320	350.4	340.1	273.1	NM_022485.2
88	85.4	88.2	89.5	NM_152539.1
479.7	273	264.5	236.6	NM_138807.2
102	96.5	93.1	93	NM_015931.1
104.2	88.1	86.4	95.1	NM_173657.1
176.1	180.9	183.2	172.7	NM_032898.2
3469.6	4276.5	4746.8	5048.5	NM_032898.2
98.7	103	93.8	97.7	NM_178342.1
93.3	90.3	84.1	87.8	NM_178343.1
94.4	94.4	91	86.6	NM_025041.2
416.4	314.7	358.8	229.1	NM_020187.2
93.4	86.8	89.6	97.7	NM_001006109.1
663.3	481	378	346.1	NM_173824.2

190.6	165	145.6	145.8	NM_032806.4
164.2	151.6	157.1	110.6	NM_144635.3
93.1	87	97.6	93.6	XR_001028.1
98.5	91.1	86.9	98.2	NM_001039102.1
82.2	82.8	82.5	87.7	NM_153215.1
90.1	89.3	89.7	93.1	NM_144714.2
95.5	90.3	87.2	88.3	NM_024616.1
355	277.1	273.1	178.7	NM_203370.1
89.8	85.5	77.6	86.6	NM_001007534.1
94.3	87.3	83.3	89.1	NM_145035.2
185.2	219	169.2	213.8	NM_173552.2
110.7	102.9	100.7	101.6	NM_178496.2
195.2	120.4	118.3	109.2	NM_199074.1
125.4	119.7	112.7	102.1	NM_199073.1
91	87.1	88.4	87.7	NM_199417.1
103	116.2	102	91.6	NM_199073.1
7145	4932.2	4915.6	2842.8	NM_199069.1
165.4	168	157.1	145.1	NM_198562.1
221.5	238	237.1	201.9	NM_015224.1
202.2	154	157.2	162.6	NM_173654.1
92.1	89.9	96.2	101.2	NM_001039788.1
289.3	249.2	224.3	200.2	NM_152305.1
1057.6	925.9	838.4	660.6	NM_020231.3
87.6	81.3	81.4	89.4	NM_007293.2
97.1	94.6	85.7	89.3	NM_000715.3
88.4	93.2	94.8	81.8	NM_001017367.1
86.6	90.3	84	92.6	NM_000716.3
108.6	110	103.7	103.5	NM_001030316.1
263.6	230.7	196	175.3	NM_001029998.1
857.1	637.6	565.7	473.5	NM_032313.2
108.4	101	107.7	97	NM_024511.4
94.9	89.5	84.1	90.3	NM_018569.2
87.7	82.4	78.4	82	NM_032149.1
86	90.2	83.3	92.5	NM_016613.4
97.7	90.3	92.9	96.8	NM_016613.4
87.5	88.4	89.3	85.3	NM_001031700.1
87.1	77.9	78.3	78.2	NM_145048.2
89.7	82.9	80.9	83.4	NM_152400.1
174.1	242.9	270.6	183.5	NM_174921.1
106.7	99.4	99.7	100.6	NM_144645.1
90.5	90.2	84.8	90	NM_005750.2
89.9	91.9	85.3	91.9	NM_152997.2
200.5	200.7	208.1	190	NM_003704.3
616	414.6	366.9	275.3	NM_003703.1
749.2	726.4	730.6	600	NM_001735.2
96.7	114.4	103.1	101.7	NM_001736.2
1647.4	987.6	932.2	827.6	NM_004772.1
105.4	100.5	101.9	101.5	NM_024715.2
1486.6	1487.8	1447.6	903	NM_020199.1
220.4	184.9	166.7	182.9	NM_173828.3

310.6	342.7	334.1	265.8	NM_130848.1
694.5	600.4	547.9	436.4	NM_032042.3
960.9	1660.7	2376.6	1710.2	NM_022483.3
156	153.4	123.8	128.8	NM_018691.1
97.1	108	102.7	105.8	NM_198566.1
89.3	77.1	79.5	84.4	NM_173665.1
440.3	361.3	367.3	308.4	NM_152408.1
88.3	86.2	82.9	85.4	NM_016348.1
89.4	83.1	72.2	85.1	NM_016348.1
97.3	92.1	99.9	97.4	NM_032385.1
485	516	607.4	534.1	NM_016603.1
103.7	92.4	89.7	87.5	NM_000065.1
113.8	100.8	101.9	103	NM_178508.3
147.2	135.8	151.1	144.6	NM_001008704.1
142.6	117.2	118	110.9	NM_001008703.1
87.4	84.2	84.6	90.1	NM_006781.2
98.7	96.4	87.2	94.3	NM_032744.1
146.9	182.6	166.8	155.5	NM_024294.2
203.4	225	208.4	168.2	NM_022758.4
108.5	94.2	96.9	101.8	NM_017754.2
84.6	75.3	86.4	85.7	NM_199184.1
3636.1	3332.6	3036.3	2590.9	NM_199184.1
946.7	1094	907	1153.7	NM_032870.1
484.5	392.8	348.5	375	NM_145062.1
75.3	70.5	80.6	76.4	NM_033069.1
161.2	385.8	393.7	296.9	NM_138409.1
108.2	85.7	92.7	94.9	NM_144980.1
187.8	171.4	167.6	168.6	NM_001029863.1
92	95.4	91.2	92.3	NM_207502.1
3577.9	3029.7	2708.9	2094.2	NM_032340.1
101.9	97.2	94.7	103.6	NM_145316.2
994.9	780.1	735.8	551.9	NM_138493.1
957.9	914.8	844.5	644.8	NM_145063.2
96.6	86.4	88.2	88.6	NM_001031722.1
108.2	96.8	97	95	NM_024909.1
93.7	81.8	84.1	85.9	NM_001031722.1
221	150.7	171.5	124.8	NM_145029.1
274.7	274.1	214	251.9	NM_018132.3
86.4	89.8	83.4	88.9	NM_138569.1
96.9	93.7	93.6	98.3	NM_001010872.1
81	74.9	80.4	81.7	NM_183373.2
94	83.7	82.5	92.5	NM_173563.1
201.3	183.2	143.9	141.3	NM_030568.3
169.5	143.6	142.3	126.3	NM_020408.3
76.2	76.2	76	91.4	NM_014070.1
198.2	174.7	169.3	161.1	NM_138441.1
96.9	111.5	100.5	105.7	NM_152551.3
104.7	93.7	95.7	90.5	NM_181714.1
870.7	745.5	739	461.3	NM_033112.2
82.2	79.8	75	79.9	NM_001012974.1

109.5	88.7	94.7	107.3	NM_198920.1
86.8	96.1	100.9	87.2	NM_001009994.1
151.9	177.7	176.4	155.8	NM_020425.3
135.2	159.8	161.5	133.3	NM_020425.3
94.7	103.2	95.3	105.4	NM_001031743.1
75.9	91.9	89.4	92.4	NM_178823.2
91.4	101.1	98	96.4	NM_178823.2
1637.4	1566.7	1235.8	1203.4	NM_018064.2
157.9	157.3	120.3	125.5	NM_198468.1
98.6	89.9	81.8	89.6	NM_032511.1
216.5	262.1	321.9	234.2	NM_152730.4
1027.3	649.5	773.3	645.5	NM_001012507.1
94.6	93.2	99.9	104.5	NM_001012279.1
106.3	109	93.6	103.6	NM_173830.3
93.2	85.5	90.3	97.8	NM_153711.1
104.2	111.3	128.6	95.1	NM_001010923.1
86.7	80.8	80.6	80.6	NM_001010876.1
81.6	86.3	91.6	80.7	NM_052831.2
85.6	91.7	83	86.2	NM_001007531.1
91.9	95.2	90.4	93	NM_152554.1
83.2	88.5	85.4	93.2	NM_145025.2
86.1	96	81.6	88.2	NM_206834.1
114.2	117.8	109.6	118.9	NM_016487.2
87.8	116.6	93.6	103.1	NM_001042475.1
101.5	96.4	101.6	107.6	NM_206921.1
121.4	113.9	111.4	111.4	NM_001010909.1
99.9	97.5	86.5	97.4	NM_001010909.1
81.7	87.6	84.8	92.8	NM_152732.2
90.8	99.8	88.4	92.9	NM_025002.1
90.3	92.5	88.6	95	NM_001003693.1
128.8	132.3	117.2	129.9	NM_024573.1
96.4	92.1	95.2	94.4	NM_001010852.2
110.4	112.8	114.2	110.4	NM_138276.1
95.4	97.4	93.2	92.6	NM_138275.1
81.7	88.3	84.9	85.3	NM_138277.1
88.5	78.1	72.3	81.8	NM_138274.1
154.8	223.9	210.2	187.9	NM_001039651.1
98.3	91.6	88.3	90.5	NM_025258.1
85.1	86.3	82.1	83.3	NM_015864.2
87.7	91.5	92.7	91.6	NM_018452.2
276.2	306.2	318.2	158.3	NM_021184.3
109.5	95.1	89.5	102.8	NM_001040437.1
11626.9	11244.7	11838.9	8690.6	NM_001040437.1
5709.6	3445.9	2586.1	2374.3	NM_013397.3
87.3	80.5	77.7	85.7	NM_138408.2
474.6	535.5	449.6	367.8	NM_016485.3
446	444.8	354.1	330.4	NM_145267.2
96	96.1	98.6	99.2	NM_024929.1
152.5	201.6	197.9	163.2	NM_024581.3
91.1	89.4	84.1	91.1	NM_030939.3

227.9	236	276	153.9	NM_018322.1
213.1	237.9	211.9	233.7	NM_152731.1
188.7	146.4	138.5	162.5	NM_014165.1
1033.7	1117.6	939.8	1009.9	NM_173562.3
406.1	563.3	525.2	490.1	NM_018341.1
85.1	82.3	78.7	89.2	NM_203395.1
160.7	134.5	139.9	119.9	NM_138785.1
85.4	81.5	78.2	85.8	NM_021820.1
88.4	83.6	86.6	92.7	NM_001031712.1
468.4	492.2	395.9	343.4	NM_001031712.1
83.3	95.6	80.7	93.3	NM_153036.2
379.7	327.9	313.1	282.6	NM_022102.1
90.2	88.9	88	100.7	NM_145028.3
193.5	191.7	156.4	137.3	NM_014895.1
107	104.9	102.9	95	NM_021945.4
245.7	212.7	178.6	148.4	NM_152734.2
229.6	186.8	172.6	205	NM_017909.1
74.7	85.5	74.7	84.5	NM_000587.2
97.7	93.6	100.6	104	NM_024728.1
146	129.4	127.8	129.7	NM_138701.1
83.7	93.6	93.1	99.5	NM_006658.2
449.8	465.6	387.1	385.5	XM_946079.1
158.9	198.6	160.2	155.8	NM_015949.2
757.6	683.6	607.5	582.9	NM_031434.2
995.5	886.7	869.6	778.2	NM_024051.2
200.8	206.8	182.1	173	NM_024054.1
630.8	488.3	467.3	393.2	NM_024067.2
710.6	808.6	849.2	271.8	NM_152743.2
131.2	127	138.9	117.8	NM_015622.5
698.3	532.1	515.2	466.8	NM_198097.1
109.9	106.4	104.4	110.6	NM_138434.2
3165.9	1894.1	2007.7	1432.8	NM_138446.1
95.5	93.2	82.1	95.6	NM_138811.3
96	88.3	91.8	93.5	NM_145304.2
83.3	88.9	74.8	76.6	NM_178829.2
186.7	172	158.2	177.2	NM_020192.1
1045.5	1162.2	1136.5	750.8	NM_018224.2
91.3	98.5	92.9	76.3	NM_022150.2
85.7	78.8	82.9	73	NM_000562.1
90.6	74.3	94.8	86.4	NM_000066.2
102.3	96.9	89.2	106.9	NM_000606.1
259.7	236.3	229.2	189.2	NM_004337.1
89.9	88.1	86.3	92.7	NM_053279.1
91.7	100.1	97.8	89.9	NM_001033662.1
97.5	91.7	86.7	106	NM_001007176.1
101.3	86.1	83.5	78.2	NM_016458.2
95.1	90.2	87.1	95.9	NM_173687.1
433.3	218.8	194	193.4	NM_018024.1
1144.7	618.1	607.5	348.6	NM_023080.1
128	131.5	137.3	131.6	NM_052958.1

1385	1088	916.7	725.3	NM_018142.1
486	478.3	393	293.7	NM_173685.1
1772.1	2716.9	3449.2	2609.2	NM_177965.2
375.7	200.3	231.9	247.2	NM_152416.1
92.2	76.5	80.9	85.8	NM_020130.2
1716	945.9	1077.1	773.1	NM_138436.2
255.8	197.4	169.5	164	NM_025115.1
84.7	92	91	89.3	NM_175075.2
132	150.8	165.9	142	NM_019607.1
556.1	1480	1400.9	1081.1	NM_173518.2
99	96.1	88	93.3	NM_152765.2
103.4	94.3	81.5	88.8	NM_173549.1
90.5	88.6	81.8	86.2	NM_001007090.1
85.6	88	83.5	84.6	NM_001031839.1
245.4	189.7	169.1	213.3	NM_017864.2
139.4	126.7	116.5	129.8	NM_032334.1
505.1	401.4	404.2	179	NM_016647.1
114.1	116.8	97.2	100.3	NM_001013842.1
117.2	125.3	113.9	115.9	NM_001034061.1
121.6	92.5	101.6	89.9	NM_016010.1
127.7	137.9	133.6	106.7	NM_147189.1
82	87.2	80.6	88.5	NM_001040032.1
2125.5	1335.5	1322.2	1018.2	NM_032847.1
95.9	89.1	84.7	99.2	NM_015912.2
95.4	82.7	78.3	85.5	NM_001081675.1
98.2	83.8	85.6	86.1	XR_001454.1
79	83.5	78.5	87.7	NM_001737.2
144.5	107.6	93.1	91.6	NM_001031728.1
231.7	157.4	129	135.4	NM_001031728.1
243.1	251.7	222.2	165.1	NM_020207.1
426.3	314.9	299.3	242	NM_001001551.1
111.3	101.8	102.5	108	NM_001012715.1
273.4	214.9	181.5	194.2	NM_198841.1
96.7	88.2	82.9	93.2	NM_020641.1
350.3	551.8	781.3	337.9	NM_152286.2
158.1	125.7	105.2	125.4	NM_138778.1
734.5	488.9	462.6	311.3	NM_016390.2
177.8	140.6	143.6	121.3	NM_144654.1
214.3	141.3	124.9	124.7	NM_144654.1
82.4	88.7	79.9	70.9	NM_001012502.2
89.6	90.4	78.9	93.2	NM_145283.1
163.3	188.8	163.2	155.3	NM_033428.1
167.5	123.8	118	118.7	NM_032342.1
105.9	99.3	90	101.4	NM_173690.1
176.1	156.8	173.8	120	NM_016446.2
90.8	79.6	72.3	76.2	NM_001042590.1
104	93.1	97.8	91.9	NM_001012446.1
95.8	81.2	94.6	97.2	NM_203305.1
86.3	85.4	84.4	78.1	NM_153707.1
1744.6	829	636	557.3	NM_178448.2

3147.3	2147.6	1900.7	1605.5	NM_183241.1
81	82	88.6	93.9	NM_203403.1
88.1	84.7	87.7	87	NM_001012993.1
91.2	76.6	78.8	86.6	NM_001010907.1
386.2	293.1	295.1	223.4	NM_016481.2
78.8	81.7	76.9	80.6	NM_001012415.1
144.7	118.6	110.9	97.2	NM_024112.2
80.8	82.7	83.1	91.2	NM_001080515.1
89.3	77.1	82.4	86.8	NM_198469.1
1501.7	1280.2	1314.4	716.4	NM_022343.2
233.6	184.3	198.1	139.4	NM_153698.1
212.3	130.9	144.5	112.9	NM_148178.1
178.6	117.7	123.3	125.1	NM_148179.1
749.8	358.4	371.5	275.9	NM_148178.1
107.2	107.7	106.1	100.2	NM_147168.1
100.7	107.5	101	102.8	NM_147168.1
87	92.1	83.4	94.8	NM_032596.3
119.4	121.2	112.8	100.7	NM_147202.1
582.8	565.9	526.1	409.6	NM_033446.1
101.2	93.7	90.3	104.4	NM_032823.3
193	169.8	171.8	187.7	NM_080655.1
210.1	201.1	178.4	155.2	NM_014064.2
98	84.5	86	96.4	NM_015667.1
141	142.4	140.3	102.8	NM_032937.3
177	236.8	179.7	193.5	NM_017738.1
93.4	79.7	89	88.7	NM_014334.1
443.1	239.1	226.4	200.1	NM_017998.1
110.9	95.3	100	109.2	NM_152420.1
85	89.7	87.9	75.4	NM_152786.1
168.6	247	306.4	186	NM_030814.4
1403	1443.7	1029.5	1181.7	NM_018465.1
93.3	89.6	81.5	82	NM_001001938.1
140.6	138.7	120.9	128	NM_194313.1
350.4	316.7	227.2	277.8	NM_032012.1
100.3	89.3	82.9	91.3	NM_199350.1
85.4	74.2	76.9	82.6	NM_152574.1
100	87.2	95.1	91.7	NM_031426.2
87.7	80.4	77.6	89.9	NM_001002260.1
467.2	458.9	349.4	304.3	NM_017832.2
97.2	94.5	97.5	99	NM_004816.2
205.6	223.5	198.2	145.3	NM_032307.2
240.4	189.3	232.4	169.2	NM_152569.1
89.9	84.7	71.5	85.2	NM_001039395.1
117.4	127.9	128.6	127.5	NM_017586.1
89.5	84.6	75.9	89.4	NM_153237.1
104.2	89.8	84.3	90	NM_018325.1
90.9	80.9	77.4	88.4	NM_145005.3
92.4	91.3	82.1	81.7	NM_145005.3
1341.3	938.7	945	487.2	NM_030914.1
139.9	138.8	146.2	99.5	NM_173691.1

205.7	143.7	129	122	NM_016014.2
102.6	104.6	94.3	107.1	NM_016014.2
251.9	164.2	146	141	NM_001025780.1
2309.9	1947.2	1716.4	1317.2	NM_016482.2
100.1	86.3	105.4	98.3	NM_178828.3
2705.7	4555.2	5504.9	3958.5	NM_021218.1
168.7	148.7	137.6	143.4	NM_024828.2
108	108.1	106.7	101.3	NM_173521.3
89.9	93.6	84.3	82.5	NM_173521.3
135.7	101.1	101.8	98.4	NM_182505.3
85.4	86.7	82.2	103.2	NM_182505.3
630.7	728.5	620	639.8	NM_024718.2
1008.4	718.1	669.9	295.4	NM_022833.2
554.6	441.3	404.1	344.9	NM_032310.2
95.5	96.6	98.3	98.9	NM_018956.3
541.6	410.8	440.6	256.3	NM_197956.1
374.4	258.3	255.5	190.6	NM_153045.2
111.6	121	128.1	122.4	NM_173550.2
121.9	134.2	148.7	130.4	NM_017881.1
90.6	102.3	95.6	100.5	NM_153710.3
137.6	143.5	122.9	120.3	NM_139246.3
94.3	76.9	81.9	96.4	NM_152572.2
84.9	96.1	88.7	90.4	NM_001738.1
87.6	90.5	80.9	88.9	NM_020178.3
178.2	106.7	112	117.9	NM_001217.3
92.8	95.9	83.7	85.2	NM_001218.3
78.2	80.1	75.8	76	NM_001218.3
100.1	110.9	106.4	86.9	NM_198584.1
89.3	87.8	80.4	82	NM_012113.1
8784.9	3891.1	3396	3261.7	NM_000067.1
84.3	97.4	88.9	92.2	NM_005181.2
110.3	102.7	96.4	103.1	NM_000717.2
79.9	82.7	66.8	70.9	NM_001739.1
124.5	109.2	96.3	121.5	NM_007220.2
102.7	99.4	102.4	94	NM_001215.2
87.3	81.8	74.4	92	NM_005182.2
94.6	88.9	83.7	87.4	NM_001014435.1
82.2	96.8	96.7	99.5	NM_004056.4
98.1	88.6	95	98.9	NM_001216.1
2734.9	3014.3	2542.3	1752.8	NM_016289.2
179.9	181.6	161.9	150.3	NM_001031724.1
84.1	76.7	78.4	80.5	NM_001031724.1
99.9	98.6	100.9	89	NM_030925.1
1309.9	1116.6	1221.3	895.9	NM_020247.3
210.7	185.5	159	150.6	NM_012295.2
141.2	115	110.4	109.3	NM_138375.1
231.5	173	183.5	162.3	NM_031215.1
97.6	90.8	92	80.9	NM_031205.2
94.7	94.4	87.4	85.7	NM_001033677.1
96.2	85.3	88.8	87.7	NM_001033677.1

102.6	89.5	85.5	85.8	NM_031204.2
90.2	93.5	94.6	91	NM_031204.2
85.8	97.5	89.4	78.8	NM_016366.1
88.5	76.2	81	88.5	NM_145200.2
90.1	81.1	79.7	86.2	NM_019855.3
101.9	104.8	100	94.5	NM_182527.1
71	79	70.1	71.9	NM_153770.1
108.4	99.9	100	82.6	NM_153768.1
99.4	94.2	94.4	97.7	NM_020925.1
86.3	82.9	80.9	77.9	NM_023035.1
89.2	87.1	79.5	83.3	NM_000068.2
144.4	116.6	111	131.7	NM_000718.1
86.6	85.3	76.9	89.2	NM_000719.4
111	108.9	89	102.1	NM_000720.1
96.5	88.6	98.3	89.6	NM_000721.2
86.1	86.6	76.5	85.6	NM_005183.2
89	81.4	82.9	78.7	NM_198397.1
85.5	97.6	85.9	98.1	NM_198396.1
95.3	88.8	80.5	76.5	NM_198376.1
95	86.6	82.1	77.4	NM_198397.1
107.4	100	103	97.7	NM_021098.2
96.7	87.4	82.3	97.8	NM_001005407.1
82	77.3	78.9	81.8	NM_021096.3
92.5	78.7	85.9	89.7	NM_001003406.1
78.9	79	79.3	80	NM_000069.1
98.8	87.2	81.6	93.1	NM_000722.2
98.8	93.9	88	85.2	NM_001005505.1
99.9	99.3	92	96.5	NM_001005505.1
289.2	357.9	393.5	320.8	NM_018398.2
99.9	96.9	100.3	104.8	NM_001005737.1
222.3	204.5	212	143.7	NM_172364.3
91	91	88.9	81.6	NM_000723.3
92.9	96.6	89.4	95.9	NM_199247.1
89.3	77.6	80.7	79.9	NM_199247.1
82.9	79.6	87	92.5	NM_199247.1
93.4	85.3	87.6	100.5	NM_201572.1
80.9	78.8	80.6	82.5	NM_201570.1
84.4	76.3	77.9	76.9	NM_201590.1
99.3	88.7	96.1	90.6	NM_201572.1
84.3	78.9	76.2	82.6	NM_000724.2
168.1	152.3	169.1	140.7	NM_000725.2
109	98.2	93.6	95.4	NM_001005747.1
79.8	75.3	72.1	76.9	NM_000726.2
82.7	83.6	83.3	82.8	NM_001005746.1
88.8	92.4	79.7	87.5	NM_000727.2
96	85.8	86.8	89.6	NM_006078.2
90.8	87.2	83.4	92.1	NM_006539.2
92.9	98.3	91.7	88.9	NM_014405.2
93.8	97.5	82.9	90.7	NM_145811.1
87.2	92.6	78.8	87.5	NM_014404.1

90.7	84.6	77.2	89.8	NM_014404.1
96.3	89.6	90.1	80.8	NM_031897.2
99.7	84.1	88.9	89.6	NM_145814.1
82.8	84.3	80.2	78.2	NM_031897.2
76	81.7	77.1	74.3	NM_031896.3
110.3	111	102.9	109.4	NM_031895.4
1756.8	1344.2	1207	874.3	NM_014412.2
238.3	198.8	171.4	170.2	NM_001007214.1
77.2	81.8	77.6	76	NM_001007214.1
219	209.4	203.6	173.8	NM_004341.3
83.3	77.9	74.5	83.5	NM_183394.1
115.2	112.4	104	89.5	NM_183394.1
86.4	80.4	75.5	84.8	NM_003716.2
86.7	74.1	78.1	77	NM_017954.8
111.6	97.2	90.2	96.8	NM_017954.8
115.8	133.6	108	112.5	NM_205864.1
92.6	91.2	86.4	93.9	NM_004929.2
121.2	93.9	102.4	98.6	NM_007088.1
83.6	83.7	78.7	85.8	NM_001740.2
87.5	74.3	74.3	83	NM_001033952.1
101.1	88.2	94.7	93.4	NM_001033953.1
77.4	79.6	76.8	73.6	NM_001741.2
93.7	97.4	88.2	86.6	NM_000728.3
106.4	124.7	107	107.9	NM_020898.1
178.5	189	186.8	196.3	NM_005831.3
84.5	82.7	88.2	86.7	NM_001742.2
79.6	77.9	73.5	73.3	NM_005795.2
90.4	82.4	89.3	85.9	NM_033138.2
1565.5	1282.5	999.6	936.5	NM_006888.2
8604.4	5019.8	4535.2	4446.5	NM_001743.3
6290.7	4472.3	4224.6	2483.9	NM_005184.2
106.1	109.9	99.8	105.8	NM_005185.2
92.6	84.9	86.1	92.1	NM_001031733.1
118.1	127.9	107.8	99.3	NM_001031733.1
741.9	694.2	603.6	500.5	NM_033429.1
339.9	379.6	403.1	350.2	NM_001031733.1
87.8	83.6	82.9	84.2	NM_017422.3
87.8	89.6	91.4	98.5	NM_138705.2
103.6	96.2	84.6	90.2	NM_001017440.1
188.3	176	159.7	142.7	NM_031468.2
111.8	110.5	109.4	102.2	NM_031468.2
902.6	1066.4	806.3	820.8	NM_004343.2
101.5	96	97.8	106	NM_145046.2
423.5	597.2	413	376.1	NM_001219.2
673.1	719.7	674.5	579.8	NM_003656.3
165.7	152.1	124.3	119.8	NM_020397.2
80.8	73.8	77.6	78.9	NM_153498.2
90.6	85.3	81.5	93.1	NM_020397.2
95.9	91.8	97.4	98.9	NM_020439.2
93.4	82.6	66.5	76	NM_171825.1

96	98.2	94.1	87.1	NM_172078.1
157.8	171.5	147.5	137	NM_172080.1
116.9	101.7	108.4	106.2	XM_936314.1
87.1	80.1	80.7	80	NM_001220.3
156.5	126.6	117.5	112.7	NM_172115.1
145.8	119.3	104.7	119.9	NM_172128.1
102.9	90.6	92.9	95.6	NM_001221.2
91.2	74.6	80.5	88.2	NM_172115.1
203.1	209.9	227.9	155.6	NM_172169.1
107	132.7	108.4	112.5	NM_172172.1
94.6	80.2	80.3	91.4	NM_018584.4
91.6	86.1	83.3	89.9	NM_033259.2
84.8	80.5	75.8	81	NM_001744.3
91.1	93.7	85.5	97.4	NM_172207.1
101.8	93.1	94.3	88.1	NM_032294.2
97.1	112	97.5	98.9	NM_172215.1
86.4	91.1	82.4	89.7	NM_172215.1
103.4	178.7	214.1	125.8	NM_024046.2
190.4	334	247.5	189.9	NM_004345.3
491.6	553.5	538.2	380.8	NM_015447.1
220.2	128.3	138.4	142.4	NM_203459.1
170.3	152.8	142.7	137.3	NM_015215.1
75.3	78.2	87	76.7	NM_015099.2
104.1	100.3	98.7	113.9	NM_018448.2
99.7	97.7	92.7	90.6	XM_932503.1
159.8	131	132.8	118.1	NM_012298.1
495.5	557.2	427	506.6	NM_138793.2
4609.1	4750.2	4190.5	3572.5	NM_001024649.1
38084	39426.3	32247.2	32819.7	NM_006367.2
127	136.3	119.8	119.5	NM_006366.2
258.5	172.7	158.7	157.4	NM_001747.2
984.7	629.6	730.7	344.7	NM_005186.2
237	167.8	166.3	163.1	NM_023083.2
89.6	89.3	97.1	93.6	NM_023089.1
93.1	97.6	89.2	90	NM_021251.2
85.1	93	90.1	101.1	NM_023083.2
96.2	101.6	101.3	85.7	NM_007058.2
112.9	100	115.8	111.8	NM_144691.3
74.4	91.6	71	80.8	NM_144575.2
209.1	196.2	173.5	152.1	NM_001748.3
97.2	88.2	80.6	83.8	NM_212464.1
88.7	85.4	79.7	87.1	NM_212467.1
200.4	184.7	207.6	174.9	NM_000070.2
155.8	150.6	143.9	131	NM_173087.1
86.4	106	121.8	100.2	NM_004055.3
122.8	117.1	118.8	124.8	NM_014296.2
82.5	82.5	86.4	94	NM_016452.1
83.7	81.2	81.5	82	NM_016452.1
2482.8	1272	1144.8	978	NM_001003962.1
754.3	453	384.4	383.1	NM_001749.2

94.6	103.9	95.2	93.2	NM_032330.1
92.5	95	100	96.8	NM_004058.2
116.9	133.3	146.2	95.5	NM_080590.1
86.8	78.5	87	82.6	NM_032606.2
88.4	78.6	86.8	86.2	NM_144647.2
85.1	86.6	83.2	91.5	NM_144647.2
104.4	84.2	85.4	87.1	NM_144647.2
2796	2185.1	1838.9	2117.9	NM_006136.2
86.2	88.2	81.9	96.4	NM_033328.2
199.8	168.6	162.6	179.2	NM_004930.2
167.4	157.1	145.8	132.1	NM_014550.3
120.2	138	143	113	NM_032415.2
139.4	157.7	157.7	146.3	NM_021209.3
99.1	91.7	92.7	91.9	NM_052819.1
78.7	76.5	74.8	75	NM_052819.1
90.6	78.8	80.8	93.5	NM_024110.2
185.1	288.8	251.3	251.3	NM_022162.1
97.2	94.9	91.4	97.5	NM_032587.2
689.8	670.2	731	644	NM_014959.1
2792.9	2655.2	2870.8	1340.5	NM_052813.2
433	332.6	286	308.6	NM_017632.1
2032.8	1217	1258.3	724.8	NM_014316.1
320.2	349.7	354.7	206.8	NM_013276.2
1879.7	1350.5	1153.4	837.8	NM_199141.1
101.9	121.1	110.5	108.6	NM_001751.4
421.1	350.3	368.8	261.5	NM_001014438.1
104.9	95.5	102.1	96.4	NM_004291.2
113.3	182.3	177.5	214.8	NM_006982.1
87.1	89.9	89.8	86.4	NM_018272.2
114.3	115.5	110.3	118.6	NM_178816.2
101.4	112.1	85.8	95	NM_201377.1
340.3	429.3	332.9	307.4	NM_007359.3
156.9	147.9	137.7	153.2	NM_138423.2
731.6	767.2	540.7	676.3	NM_177974.1
101.2	106.1	94.5	97.7	NM_144508.2
194	132.8	135.6	134.8	NM_170589.2
140.1	138.7	115	122	NM_022900.3
91.2	88.3	82.6	86.6	NM_003688.1
91.4	85.5	85.8	96.7	NM_020764.2
79.5	83.3	78.1	83.1	NM_020753.2
1015.6	686.5	602.6	484.9	NM_033294.2
80.3	72.9	73.8	81.5	NM_033292.2
601.7	482.3	430	353.3	NM_033294.2
101.6	114.1	113.2	107.2	NM_032974.2
87.3	93.5	89.6	93.1	NM_001230.4
93.3	85.1	85.7	88.1	NR_000035.2
94.9	84.5	84.6	86.6	NM_012114.1
146.8	171	155.8	133.2	NM_032982.2
3817.5	2918	2986.8	2572.3	NM_032982.2
417.1	399.9	301.5	324.5	NM_001224.3

650.5	450.1	468.9	320.3	NM_032991.2
125.2	114	121.8	118.3	NM_004346.3
89.2	75.3	75.2	79.7	NM_033307.2
391.3	479.1	316.3	332.1	NM_033306.2
101.9	95.3	84.4	84.7	NM_001225.3
117.5	106	106	97.5	NM_033306.2
90.2	95.8	94.6	94.1	NM_004347.1
184.4	162.1	138.6	138.7	NM_032992.2
113.9	100	102.1	107.2	NM_001226.3
114	93.1	100.7	102.6	NM_032992.2
197.4	161.5	154.8	144.5	NM_033339.3
91.8	83.3	80.2	89	NM_033338.4
85.1	79.8	77.9	80.3	NM_033340.2
122.5	111.8	110	109.2	NM_033356.2
89.4	89.7	95.5	92.9	NM_001228.3
110.1	104.4	101.5	94.8	NM_033355.3
122.4	123.3	123.9	108.5	NM_033358.2
93.7	81.3	84.1	85.9	NM_012115.2
116.9	103.5	106.9	100.4	NM_001229.2
222.8	237.7	206.6	169.9	NM_001229.2
91.8	84.3	83.2	88.5	NM_001231.2
92	88.2	77.1	87.8	NM_001232.1
89.6	86.3	83.3	89.8	NM_000388.2
183.3	209.9	204.2	240.3	NM_001750.4
96.1	96.2	91.6	89.2	NM_001750.4
87.2	91.4	84.7	77.5	NM_173061.1
222.6	307.6	263.7	293.9	NM_001042442.1
88.1	78.1	87.8	81.6	NM_015576.1
194	197.8	190.3	127.8	NM_017766.2
85.1	78.7	78.1	84.6	NM_001079843.1
87.7	81.6	80.7	79.2	NM_001079843.1
692.1	700	585.7	629.7	NM_001752.2
301.3	176.1	192.9	161.5	NM_053054.2
93.4	113.6	96.8	96.5	NM_054020.2
79.2	86.6	74.4	79.2	NM_178019.1
92.5	90	84.8	89.6	NM_198137.1
111.4	165	161.3	166.2	NM_001753.3
111	129.2	114.1	107.8	NM_001233.3
90.1	99.3	104.9	82.2	NM_001233.3
103.3	85.9	79.3	102.6	NM_001234.3
83.2	85.2	83.2	77.3	NM_033337.1
239.4	266.6	210.7	203.9	NM_006077.1
117.8	142.3	149.7	125.2	NM_005093.3
91.9	90.9	90.7	90	NM_005093.3
104	99.3	99.1	108.4	NM_001032999.1
94.3	93.7	83.5	94.1	NM_005187.4
110.5	105.9	106.3	116.8	NM_005187.4
87.7	84	71.8	80.1	NM_175931.1
1653	870.4	475.6	723	NM_001755.2
100.2	79.4	74.2	89	NM_001755.2

243.1	173.5	209.6	212.5	NM_005188.2
182.2	299.1	320.1	198.7	NM_170662.3
92.2	95.4	94	87.9	NM_012116.2
708.6	474.3	519.6	352.6	NM_024814.1
96.1	83.9	87.8	87	NM_004352.1
69.9	80.8	74	69.2	NM_182511.2
122.1	115	114.1	107.1	NM_001039771.2
95.2	90	85.8	94.3	NM_080617.4
164.2	154.3	153.7	141.3	NM_001757.2
210.3	161.8	159.8	138.5	NM_001236.3
276	244	257.6	215.2	NM_032783.3
1709.2	915.7	935.3	524.6	NM_000071.1
97.1	82.2	79.5	87.8	NM_018491.3
99.1	89.5	84.5	78.7	NM_172003.2
1103.9	661.4	546.8	506.5	NM_001024916.2
143.2	130.6	109.5	146.3	NM_006807.3
88.4	91.5	90.2	94.2	NM_032647.2
82.7	79.7	82.6	90.6	NM_032647.2
583.2	315.1	282.7	217.8	NM_005189.1
155.8	146	132.9	119.5	NM_007276.3
866.8	972.6	1034	726.5	NM_016587.2
87.5	83.9	80.5	90.2	NM_007276.3
310.6	363.3	313.5	236.7	NM_003655.2
565.4	433.3	322.2	302.2	NM_012117.1
771.9	593.4	516.8	322.1	NM_014292.3
179.8	196.2	188.8	169.5	NM_175709.2
134.5	132.9	123.3	119.2	NM_020649.1
92	92.3	90.4	86.2	NM_015373.3
111.1	111.6	104.2	102.7	NM_017721.2
360.4	442.3	439	373.3	NM_032449.1
228.3	265	343.1	282.3	NM_133459.1
117.3	143.8	145	115	NM_004059.3
232.7	215	197.1	188.8	NM_001008661.1
147.3	134.6	139.3	145.7	NM_001008661.1
196.2	164.7	159.4	130.9	NM_001296.3
99.8	97.3	85.5	95	NM_080667.3
88.9	85.6	75.3	88.8	NM_152389.2
83.2	80.7	68.1	77.9	NM_152389.2
107.5	91.5	93.9	93.4	NM_145020.2
107.7	100.8	91.2	103.8	NM_152775.1
97.7	97.1	96.9	91.2	NM_173510.1
1087.6	950.7	952.7	544.8	NM_144716.1
87.1	90.8	89.2	90.8	NM_024584.2
231.9	188.1	178.1	166.1	NM_145265.2
84.6	86.3	81.6	82.2	NM_194300.1
89.7	89.7	88	92.8	NM_144719.2
897	833.9	877.9	800.7	NM_022757.3
746.9	790.3	613.7	718	NM_052857.2
108.2	106.5	100.1	104.4	NM_152500.1
97.4	95.3	100.1	95.2	NM_206886.2

369.1	350.7	348	161.6	NM_022778.2
100.6	111.9	104.2	93.5	NM_014008.1
2412.3	2375.6	2213.6	1902.9	NM_199342.1
115.5	110.9	126.4	94.9	NM_152499.1
281.4	242.7	239.9	254.7	NM_001031708.1
892.5	851.3	857.5	514.9	NM_018246.1
96.2	103	107.2	102.3	NM_152492.1
978.7	1078.3	1230.2	844	NM_015439.2
124.2	115.3	108.5	103.2	NM_024296.3
98.7	94.2	94.1	92.7	NM_031455.2
214.2	177.6	157.3	149.7	NM_001080791.1
1953.9	1498.2	1427.6	934.3	NM_052849.2
96.7	95.8	103.5	90.3	NM_182791.1
88.6	89.3	82.6	87.3	NM_080654.2
338.9	344.9	356.7	291.5	NM_030771.1
1554.8	1658.2	1387.8	1047.1	NM_030771.1
92.4	81.3	79.3	87.6	NM_178173.2
82.1	83.7	83.3	90.2	NM_182628.1
89.8	83.3	83.9	90.2	NM_182496.1
142.9	102	99.7	105.6	NM_207406.1
114	114.7	100.7	101.5	NM_207406.1
93.2	100.2	88.8	94.1	NM_016122.1
178.4	226.7	218.1	224.2	NM_016122.1
74	80.7	81.7	85.3	NM_144681.1
155.7	137.3	137.2	111.8	NM_144609.1
725.6	622.1	535.9	360.9	NM_016360.2
384.3	444.4	424.8	426.3	NM_138363.1
89.2	79.6	81.3	90.5	NM_145036.2
82.2	81.9	76.6	78.8	NM_145036.2
243.2	228.1	202.9	209.4	NM_020198.1
88.7	86.8	85.5	92.1	NM_024768.1
403.1	413.8	354.8	305.2	NM_017748.3
637.4	492.2	407.6	376.9	NM_138443.2
958.5	751.7	822.3	613.4	NM_174908.2
79.3	80.3	78.5	81.6	NM_178335.1
217.2	216.6	198.6	136.5	NM_024661.2
90.7	85.6	87.6	98.1	NM_144718.2
717.3	543.7	429.9	577	NM_016053.1
82.7	84.8	99.5	79.7	NM_032600.2
184	196.7	182.8	150.5	NM_032141.2
1839.6	1564.3	1566.1	941.9	NM_001040431.1
86.8	95	99.7	99.7	NM_198082.2
785.2	525.7	409.9	464.5	NM_001017928.2
2986.5	3022.7	2429.6	2565.9	NM_014167.1
1462.6	935	864.9	636.9	NM_005436.2
92.3	92.4	89.9	98.5	NM_178499.2
101.6	92.7	95.4	103.4	NM_201435.2
91.4	87.9	81.5	85.3	NM_032573.3
103.5	97.5	81.6	91.8	NM_152591.1
94.4	83.7	88.9	94.7	NM_207311.1

74.6	80.5	79.5	74.6	NM_033124.2
135.6	149.7	152.4	145.8	NM_001012506.1
93.2	100	86.3	92.2	NM_181645.2
96.8	97.6	91.4	91.4	NM_025214.1
91.3	90.9	90.5	101.1	NM_015621.2
93	92	89.3	90.5	NM_001026383.1
77.4	81.9	82.8	76.4	NM_001026383.1
92.4	89	85.7	91.4	NM_145023.4
89.1	81.7	87	83	NM_031290.1
212	250	216.7	191.8	NM_022903.2
3649	3795.3	4223.2	2787	NM_015933.2
101.1	84.1	77.8	90.1	NM_138770.1
112.1	88.1	88.4	99.5	NM_207310.1
95.4	103	105.9	82.3	NM_174931.1
552.5	329.4	271.6	356.7	NM_019083.1
388.2	472.2	423.6	369.7	NM_032358.2
92.1	101.8	88.5	96.6	NM_032040.1
91.2	84	78.7	82	NM_001080433.1
98	91.6	97.4	92.9	NM_015603.1
2224.1	1801.6	1732.3	1489.7	NM_021825.3
93.2	93.8	95.3	102	NM_018318.3
86	78.5	73.3	81.7	NM_019052.2
95.3	91.9	84.5	88.2	NM_005893.1
88.1	82.2	86.9	80.8	NM_000729.3
98.1	94.2	87.2	93.1	NM_000730.2
84.2	97.5	89.5	92	NM_176875.2
93	79.9	83.1	78.6	NM_002981.1
81.3	81.3	78.4	80.5	NM_002986.2
83	106.9	90.7	85.6	NM_005408.2
86.4	86.5	87.6	87.4	NM_004167.3
85.8	86	85.4	83.9	NM_032965.2
89.1	93.1	89.8	89.5	NM_004590.2
92.2	81.8	79.3	89.5	NM_002987.2
80.9	82.7	71.8	79.8	NM_002988.2
104.2	102.7	91.9	93.2	NM_006274.2
149.7	182.1	134.8	139.4	NM_002982.3
98.6	113.2	89.4	100.5	NM_004591.1
94.4	90.1	88.3	95.1	NM_002989.2
136.1	125.3	138.9	110.7	NM_002990.3
177.2	185.3	186.4	148.6	NM_005064.3
841.8	664.6	723.3	419.4	NM_005064.3
81.5	81.4	83.7	79.2	NM_002991.2
91.6	86.8	81.1	87.5	NM_148888.1
87.8	72.3	83.7	83.7	NM_005624.2
92.2	106.7	93.9	84	NM_006072.4
90.3	87	97.1	100.1	NM_006664.2
91.2	89.2	81.4	85.1	NM_148672.2
100.5	99.3	91.5	100.3	NM_002983.1
110.9	95	88.3	104	NM_021006.4
169.3	161.2	159	189.1	NM_001001437.3

86.9	81.8	86.6	97.5	NM_001001435.2
4471.3	2558.1	2883.9	1263	NM_002985.2
81.9	83.7	85.8	86.8	NM_006273.2
99.2	94.6	85.1	91.5	NM_005623.2
116.9	111	101	103.8	NM_031443.3
153.1	164.1	171.8	162.8	NM_001029835.1
1148.1	1443.7	1642	750.8	NM_031443.3
993.2	328	314.8	300.2	NM_003914.2
1883.5	949.9	617.9	613.3	NM_001237.2
245.7	187.8	131.5	139.7	NM_031966.2
1998	1002.5	957.4	734.3	NM_182851.1
98.2	86.1	91.4	101.3	NM_182852.1
92.4	88.6	85.5	89.2	NM_182851.1
928.3	537.9	455.5	350.6	NM_021178.2
2388.3	1017.3	961.7	727.9	NM_004701.2
90.7	84.4	79.2	85	NM_033031.1
84.7	89.2	82	94.4	NM_033670.2
97.4	102.6	86.8	95.8	NM_033031.1
772.6	665.4	615.1	476.2	NM_005190.3
963.5	858.6	626.8	808.6	NM_001013399.1
345.9	208.4	192.2	229.7	NM_005190.3
278.9	186.7	165	122.6	NM_053056.1
93.2	83.3	97.7	93.2	NM_001759.2
10569.5	8586.1	7850	3518.9	NM_001760.2
98.1	104.2	93.6	89.4	NM_012142.2
102.6	101.4	107.9	96.3	NM_037370.1
3038	2418.8	2419.1	1861	NM_037370.1
731.1	628.6	615.2	425.3	NM_001238.1
107	81.7	90.1	89	NM_001238.1
97.3	83.9	86.6	87.7	NM_057749.1
1234.2	909	656.4	829.2	NM_057735.1
1219	643.5	593.6	330.4	NM_001761.1
1084	819.2	978.5	851.2	NM_199246.1
79.7	82.3	82.7	89.9	NM_199246.1
173.4	173.5	186.1	175.1	NM_004354.1
258.9	194.6	164.7	145.3	NM_001239.2
7880.7	6879.3	5667.5	3188.9	NM_006835.2
106.4	83.2	78.1	80.9	NM_019084.2
996.9	773.1	674	686.8	NM_003858.2
599.1	751.1	838	588.8	NM_020307.2
114.9	119.2	128.3	110.8	NM_030937.3
115.8	118.1	106.1	99	NM_001240.2
110.2	100.5	89.5	106.6	NM_001241.2
271.4	310.2	240.9	267.7	NM_001241.2
97.3	96.8	83.3	94.1	NM_058241.1
1104.4	1083.9	1117.9	571.7	NM_145012.3
343.7	389.1	344.6	315.8	NM_152523.1
116.3	106.6	101.4	120.1	NM_020739.2
538.9	593.6	493.6	525.5	NM_004748.3
312.5	313	252.7	265.9	NM_001295.2

94.4	88.7	98	82.4	NM_016602.1
93.1	94.3	83	92.2	NM_000647.4
233.3	147.7	129.1	133.7	NM_000647.4
93.4	96.9	95.7	94	NM_178329.1
84.2	88.3	78.4	89.7	NM_001837.2
82.2	79.8	76.6	80.9	NM_005508.4
85.3	87.2	93	89	NM_000579.2
10945.3	14244.1	18412.4	15212	NM_031409.2
83.2	84.1	82.1	87	NM_031409.2
82.1	90.2	90.7	94.8	NM_001838.2
88.5	92.5	80.1	87.7	NM_005201.2
95.3	81.8	84.8	89.1	NM_031200.1
77	72	80.7	76.1	NM_031200.1
412.1	340.1	402	201.6	NM_012119.3
187.9	159.9	152.5	155.5	NM_178432.1
95	93.6	79.3	85.3	NM_016557.2
103.4	90.3	99.3	90.9	NM_178445.1
93.4	87.8	82.4	100	NM_178445.1
88.1	86.6	92.8	84.3	NM_003965.4
309.8	250.2	221.6	182.1	NM_012118.2
364.3	252.1	263.3	194.8	NM_005125.1
203.1	192.9	227.4	150.4	NM_005125.1
3709	3264.5	2101.4	2026.5	NM_006431.2
281.9	235.2	287.8	177.8	NM_001008800.1
3390.1	2329.7	1880.8	1398	NM_005998.3
412.7	311.2	280.6	238.9	NM_006430.2
157.7	134.7	130.6	116.7	NM_012073.3
997.2	628.3	436.9	440.8	NM_001762.3
1082.7	628.3	483.3	533.6	NM_001009186.1
1371.5	997.1	706.6	818.5	NR_003110.2
166.2	150.8	140.1	141.6	NM_006584.2
6622.2	5771.5	5063.6	3462.4	NM_001009570.1
572.6	565	561.6	453.6	NM_006429.2
1927.6	1526.6	1166.6	1093.8	NM_006429.2
7975.9	4649.3	3718.8	4116.6	NM_006585.2
103.6	108.5	98.9	109.3	NM_133493.1
277.9	1853.9	1555.3	726.4	NM_001040021.1
118.7	270.8	228.3	201.5	NM_000591.1
340.1	345.5	290.1	259.3	NM_004357.3
3827.6	3132.7	2805.6	1752.5	NM_139030.3
141.6	126.1	122.6	116.5	NM_139030.2
95	82.3	81.1	92.3	NM_007053.2
88.9	99.1	90.4	83.1	NM_203416.1
96.1	97.1	82.3	93.2	NM_004244.3
152.1	131.5	120.4	131.2	NM_006016.3
89.8	82.2	74.6	83.2	NM_207397.1
87.7	86.1	86.1	86.9	NM_020406.1
94.9	86.1	81.4	97.8	NM_005582.1
176.6	127.3	121.7	123.3	NM_001770.3
87.1	80.6	81.2	78.8	NM_001763.1

92.5	86.7	92.8	90	NM_001764.1
105.5	101.8	94.3	94.2	NM_001765.1
324.3	394.9	204.5	208.2	NM_001766.2
92.7	102.5	85.5	96.2	NM_030893.1
87.8	91.9	80.7	83	NM_001042586.1
90.7	85.9	81	84.3	NM_001767.2
110.9	107.9	109.6	85.4	NM_001004196.1
76.5	74.5	74.2	75.8	NM_001004196.1
103.8	79.3	80.1	85.2	NM_138940.2
91.6	88.7	94.5	81.8	NM_001008784.1
89.9	91.4	85.7	91.6	NM_015717.1
375.3	259.5	238.7	214.4	NM_021155.2
86.5	87.9	83.2	87.9	NM_001771.1
85.9	78.6	71.2	83.3	NM_006566.1
96.1	160.2	224.4	170	NM_013230.2
183.4	153.1	135.5	117.9	NM_016382.2
120.2	96.2	99.7	100.8	NM_198053.1
110	97.5	101.3	105.4	NM_198053.1
92.4	89.8	87	81	NM_020404.2
102	96.4	90.8	91.8	NM_014143.2
422.7	475.7	392.2	383.9	NM_001024736.1
97.2	113.2	108	103.8	XM_945867.1
123.4	110	110.3	114.5	NM_012120.1
1466.3	1053	1026.6	615.9	NM_006110.1
181.6	299.2	242.3	249.7	NM_007261.2
161.6	146	159.5	106.4	NM_006678.2
87	94.5	93	93.3	NM_181449.1
89	100.7	89.7	87.5	NM_174892.1
484.7	687.6	612.2	257.6	NM_139018.2
130.9	234.8	174.9	147	NM_145273.2
83.4	88.8	82.1	93.3	NM_014880.3
424.5	281.1	240.1	187.6	NM_016579.2
1098	1016.4	789.7	607.9	NM_001772.2
92.8	98.1	84.3	87.5	NM_213602.1
96.3	94.8	92.4	86.4	NM_001773.2
85	83.7	79.5	84.2	NM_001773.1
88.6	91.1	90.7	87.4	NM_001773.2
76.8	81.3	81.3	75.1	NM_001025109.1
250.9	463.7	307.9	347.2	NM_001001548.1
476.5	556.5	471.6	539.3	NM_001001548.1
84	80.4	91.2	83.3	NM_001001547.1
1068.9	596.9	635.8	431.1	NM_001774.1
1353.4	733.6	722.2	784	NM_001774.1
166	122.7	122.7	116.5	NM_001775.2
86.7	82.5	84.6	88.5	NM_000732.4
92.1	89.7	86.2	89.7	NM_001040651.1
96.2	101.7	92.1	99.5	NM_000733.2
131.6	107.7	99.3	93.4	NM_012099.1
87.3	79.4	81.4	88.4	NM_000073.1
176.2	169.6	134	140.8	NM_000616.2

87.2	92.6	82.7	88.6	NM_152854.2
107.2	125.8	109.6	119.7	NM_001250.3
90.2	86.1	85.3	85.6	NM_000074.2
103.3	107.3	112.2	115.4	NM_000610.3
205	252.2	200.9	226.1	NM_001001392.1
2135.1	2028.8	1937.9	1438.1	NM_001001390.1
1064	928.2	794.8	784.4	NM_172358.1
471.6	489.9	440.1	426.9	NM_001025080.1
99.6	98.1	92	91.4	NM_001777.3
93.3	91.1	87.6	79.1	NM_001778.2
91.9	96	89.2	83.5	NM_014207.2
475.9	3959.8	2995.3	2247.5	NM_001803.2
238.3	193.7	181.7	168.9	NM_000560.2
287.6	250.1	181.7	204.1	NM_000560.2
220.5	186.4	146.3	160.6	NM_000574.2
717.3	397.2	307.3	341.3	NM_001779.1
96.7	102.6	97.6	90.3	NM_203329.1
98.1	95.1	85.2	85.6	NM_203331.1
87.9	88.6	76	77.3	NM_005894.1
116.3	128.8	124.2	110.1	NM_006725.2
351.2	364.9	248.4	277.9	NM_001780.3
580.1	1287.1	1152.5	1035.6	NM_001251.2
217	549.7	498	443.9	NM_001251.2
88.2	90.6	86.3	86.8	NM_001781.1
98.6	96	88.7	92.6	NM_006137.6
100.6	98.2	96.2	102.6	NM_001782.1
816.3	719.3	646.5	648.9	NM_001025158.1
98	92.3	91.9	84.2	NM_001025159.1
319.1	227.2	225.5	248.3	NM_004355.2
95.3	92.7	95.1	95.9	NM_001783.2
109.6	113.5	106.1	106.2	NM_001783.2
80.9	87.4	86	80.1	NM_021601.3
158.4	143.3	128.8	107.3	NM_001039933.1
131	114.9	104.2	108.7	NM_021602.1
108.5	92	87.4	88	NM_000626.1
88.6	95.7	90.6	85.8	NM_005191.2
16319.6	11597.9	11100.7	5857.1	NM_004356.3
178.9	242	226.3	201	NM_001024844.1
120.9	120.5	121.9	116.4	NM_002231.3
200.4	150.6	131.6	142.7	NM_004233.2
132.8	98.4	97.9	107.5	NM_004233.3
85.1	98.9	96.9	99.2	NM_003874.1
101.8	139.1	126.7	121.9	NM_175862.2
110.5	359.7	294	204	NM_175862.2
102.3	93.1	93.3	99.9	NM_006889.2
87.1	86.4	80.3	85.3	NM_001768.4
84.6	89.9	81.2	90.1	NM_001768.4
80.5	81.9	94.9	79.9	NM_171827.2
80.7	80.8	85.1	80.3	NM_004931.3
87.8	84.7	91	97.2	NM_172213.1

94	92.1	97.7	90.8	NM_172101.1
95	88.3	82.2	83.4	NM_172099.1
106.4	99	111.5	109.2	NM_001769.2
86.3	86.1	76.6	86.3	NM_198196.1
105.7	85.5	83.9	84.5	NM_005816.4
123.2	129.3	95	109.9	NM_001784.3
1141	619.3	709.3	363.1	NM_078481.2
384	359	363.2	305.4	NM_002414.3
308.7	315.2	276.7	213.2	NM_134446.2
2387.1	2549.5	2380.3	1186.3	NM_134445.2
151.3	114.9	122.9	109.2	NM_001785.1
105.9	109.1	98	107.2	NM_030911.1
374.9	327.3	338.9	272.3	NM_138477.2
2915.5	3888.1	5741.1	3157.6	NM_138477.2
91.8	90.2	94.9	99.5	NM_003672.2
101.5	91.9	92	97.7	NM_033313.1
91.1	81.5	85.4	84.8	NM_033312.1
93.7	98.5	97	115.9	NM_033313.1
133.6	135.4	134.6	130.9	NM_033331.1
332	353.7	289.2	238.3	NM_033331.1
155.6	1541.5	1533	978.5	NM_003671.2
1402.6	1369.1	977.8	799.4	NM_001078645.1
711	561	549.6	387.1	NM_003903.2
2309	1665.7	1675.8	1098	NM_001078645.1
999.2	483.1	524.4	323.8	NM_033379.2
198.1	138.6	132.9	115.9	NM_001786.2
8821.8	4493.8	4229.4	1939.4	NM_001255.1
1117.6	899	744.5	709.7	NM_004661.2
687	454.1	342.4	320.7	NM_001789.2
145.5	182.6	155	139.5	NM_001789.2
612.5	333.7	287.6	254.2	NM_004358.3
354.7	217.8	211.6	188.6	NM_001790.3
265.1	200.6	221.1	167.3	NM_001790.3
314.5	204	199.2	167.8	NM_001790.3
1708.6	1059.6	1012.9	814.4	NM_139286.3
105.7	84.9	87	83.7	NM_001256.2
82	82	74.7	81.4	NM_033487.1
103.9	89.6	94	90.4	NM_033531.1
269.7	343.2	340.5	285.5	NM_031267.1
401.5	388.9	390.3	381.1	NM_003718.2
1176.2	1670.6	1880.8	1034	NM_015076.3
297.3	194.7	161.3	157.7	NM_004359.1
10147.2	8948.1	9193.7	5049.2	NM_007065.3
91	96.4	83.6	87.4	NM_017913.2
1330.9	1181.6	1088.6	930.6	NM_015891.2
79.8	90.1	86	81.5	NM_044472.1
94.5	88.8	83.8	88	NM_001039802.1
285.7	217	180.8	158.3	NM_044472.1
423.1	329.9	269.8	336.5	NM_001791.2
135.9	94.8	84.1	90.2	NM_014826.3

88.3	77.5	72	85.1	NM_003607.2
116.1	136	140.2	155.9	NM_006035.2
78.4	77.5	80.8	82.7	NM_017525.1
98.4	97	81.3	95.8	NM_007061.3
124.7	101	100.1	105.9	NM_007061.3
112.3	97.7	99.2	97.5	NM_006779.2
197.1	119.2	124.7	112.5	NM_006449.3
615.8	881	936.1	493.4	NM_012121.4
92	98.5	85.1	86.2	NM_145057.2
444	270.8	211.4	241.6	NM_001038707.1
157.7	113.8	126.2	108.7	NM_020239.2
148	126.6	126.1	126.9	NM_020240.1
675.3	711.1	650.3	692.9	NM_001038702.1
1778.1	1837.2	1736.4	1216.9	NM_003504.3
440.8	431.6	412.2	440.4	NM_001253.2
140.6	125.1	116.8	124.9	NM_001254.3
263.4	302	245.9	233.9	NM_003503.2
97.9	91.3	100.2	94.7	NM_024529.3
264.2	285.4	293.5	195.7	NM_080476.4
175.7	163.2	150.3	123	NM_145697.1
566.4	323.2	233	239.7	NM_152562.2
242.4	153.3	147.6	177.5	NM_031299.3
122.6	105.4	102	101.4	NM_145701.1
577.8	326	302.9	221.2	NM_017955.2
423.5	238.2	226.9	162.5	NM_017955.2
2655.9	1497.6	1411.1	868.4	NM_080668.2
3325.2	1935.2	2032.2	1223.2	NM_145810.1
87.1	81.3	88.4	87.9	NM_031942.3
419.5	241.9	222.8	203.4	NM_018719.2
745.8	430.6	370	337.2	NM_018101.2
108.4	106.8	103.1	102.1	NM_178181.1
94.8	85	87.5	91.6	NM_022842.3
86.7	90	82.9	78.4	NM_178181.1
86.8	85.1	69.9	82.4	NM_201546.2
80.6	86.2	79.2	96.8	NM_020754.1
84.1	84	83.2	77.2	NM_004360.2
97.5	86.8	90.4	88.6	NM_006727.2
104.6	101.6	109	97.2	NM_001797.2
91.6	84.8	75	81.7	NM_004061.2
107.5	101.3	94.7	91.5	NR_002209.1
88.2	82.6	77.9	88.5	NM_001257.3
107.9	111.3	104.7	102.3	NM_004933.2
95.9	98.4	101.4	83	NM_004062.2
87.5	81.4	80.9	87.4	NM_004063.2
79.9	75.2	72.4	80.2	NM_004934.2
75.7	86.6	80.4	83.1	NM_021153.2
600.1	526.2	507.8	385.2	NM_001792.2
100.6	100	91	87	NM_031891.2
89.6	82.6	76.3	84.4	NM_021248.1
109.3	128.1	111.5	111	NM_052836.1

106.4	97.3	95.1	101.4	NM_052836.1
97.9	131.9	133.1	122.9	NM_022124.2
103	89.9	84.3	93.6	NM_144985.2
96.2	83.3	95.6	80.4	NM_022478.2
79.7	81.9	70.9	85.3	NM_144985.2
97.3	101.8	96.9	98.3	NM_177980.1
80.4	100.8	90.6	91.9	NM_021810.3
99.8	80.2	86	87.7	NM_001793.3
95.3	85	80.6	81.7	NM_001794.2
83	81.2	84.2	88.8	NM_001795.2
81.2	90.3	76.4	79.6	NM_004932.2
99.4	96.2	91.2	87.1	NM_004361.2
91.6	88.8	89.1	95.5	NM_033646.1
98	86.3	81.3	88.8	NM_033646.1
87	73.5	85.2	82.3	NM_001796.2
123.6	111.2	114.6	102.3	NM_016279.2
129	116.3	114.7	110.1	NM_006319.2
1061.4	1250.2	1539.7	711.5	NM_052988.2
107.7	100.9	96.9	91.1	NM_003674.2
252.9	220.7	209.3	170.1	NM_001798.2
1004.7	774.6	662.1	579.3	NM_001798.2
219.6	179.3	165.8	145	NM_005851.3
105.9	113.5	115.3	111.8	NM_001258.1
1572.3	980.7	1090.5	699.2	NM_000075.2
188.8	262.6	238.1	168.2	NM_004935.2
95.3	97.8	79.7	94	NM_003885.2
86.5	93.7	85.2	81.1	NM_003936.3
492.3	518.6	527.2	336.2	NM_016408.2
115.7	110.7	107.3	98	NM_016408.2
206.2	234.5	204.3	172.4	NM_001011649.1
1201.1	1181	1134.4	579.1	NM_001011649.1
112.1	101.6	101.3	105.5	NM_018249.4
1141.9	1475.9	1407.5	1219.5	NM_025197.2
280.6	301.1	287.2	275.7	NM_176095.1
1207.7	1513.4	1392.9	1212.2	NM_176096.1
1445.9	1650.5	1766.5	974.6	NM_001259.5
347.1	357.3	258.6	236.9	NM_001799.2
146	156.3	123.2	126.3	NM_001260.1
363.9	366.5	352.8	262.1	NM_001261.2
155	155.2	127.6	115.5	NM_017774.1
130	99.8	98	97.7	NM_004196.3
79.1	70.3	80.2	78.6	NM_003948.2
260.1	246.4	224.1	214.3	NM_016508.2
85.9	94.2	88.7	87	NM_001009565.1
88.2	95.8	81.5	86	NM_003159.1
90.5	90	92.2	83.4	NM_003159.2
96.6	94.7	82.2	82.4	NM_003159.2
294.1	508	391.1	264.7	NM_078467.1
94.1	91.4	88.8	92.5	NM_078467.1
234.8	243.7	220.7	227.5	NM_004064.2

94.7	122.2	102.3	110.3	NM_000076.1
94.8	89.8	81.4	77.1	NM_058197.2
89.8	84.4	81.9	90.8	NM_058195.2
86	90.4	86.4	87.5	NM_058197.2
124.1	116.6	108.3	98.1	NM_078487.2
92.5	85.9	81.1	89.2	NM_078487.2
856.2	804.7	815.4	697.1	NM_078626.1
83.9	89.1	88.7	90.9	NM_001262.2
126.4	111.7	94.9	93.8	NM_001262.2
87.9	79.9	87	82.9	NM_001800.3
219.6	173.9	159.7	143	NM_001800.3
1069.7	507.7	442.9	420	NM_005192.2
121.8	110.2	103.2	120.9	NM_001801.2
99.9	84.1	68.6	77.9	NM_016952.3
102.1	91.9	90.6	94.6	NM_016952.3
107.1	87.4	88.4	82.7	NM_004065.2
292.7	278.5	258.6	186	NM_001802.1
305.2	257.7	272.1	160.6	NM_014603.1
87.1	93.4	91.2	96.2	NM_006382.1
88.2	87	76.7	90.2	NR_003261.1
144.7	117.1	133	104.5	NM_001263.2
224.7	198.2	171.6	172.9	NM_003818.2
90.3	82.9	83.8	80.2	NM_001264.3
951.4	525.2	666.8	228.3	NM_030928.2
781.6	828.2	673.4	668.1	XM_945271.1
819.5	993.6	670.7	765.8	NM_017548.3
91.5	78.1	89.7	81.5	NM_001804.1
88.3	88.4	81	85.7	NM_001265.2
98.8	86.4	84	87.2	NM_005193.1
85.6	88.4	82.9	85	NM_004680.2
85.4	90.8	82.2	78.8	NM_004680.2
87.1	93.7	90.7	102.3	NM_001003894.1
88.4	82.4	86.8	90.9	NM_004825.2
85.4	80.1	83.8	80.4	NM_001001722.1
145.5	135.9	130.8	115	NM_004824.2
88.2	91.4	76.7	85.4	NM_004824.2
106.6	94.7	92	98.9	NM_152342.1
104.9	112.6	110.3	114.2	NM_001024912.1
103.8	95	84.7	99.8	NM_001712.3
83.3	93.1	83.1	87.4	NM_001024912.1
96.5	94.3	86.1	93.5	NM_020219.2
80	76.5	72.4	79.5	NM_198444.1
108.2	105.9	101	94.9	NM_033543.2
102	86.1	88.7	91.9	NM_001815.1
100.2	107.2	96.7	104.3	NM_001817.2
101	90.2	89.2	96.6	NM_004363.1
287.7	141.5	144.2	126.4	NM_002483.3
88.8	84.4	84.9	85.3	NM_006890.1
84.5	78.8	81.8	82.7	NM_001816.2
6172.2	3942.8	3409.4	3648	NM_004364.2

1591.6	2546.5	2326.5	1249.3	NM_005194.2
1236.5	1482	1181.7	1121.3	NM_005195.2
110.9	111	115.4	99.4	NM_001805.2
951.4	805	693.6	557.7	NM_001806.2
1600	1601.5	1226.5	1235	NM_005760.2
100.4	127.8	125.7	131.7	NM_017424.2
806.9	1377.7	1466.5	926.7	NM_017424.2
108.8	93.5	85.1	91.1	NM_177405.1
179.6	187.4	151.9	144.8	NM_017829.5
1523.8	1444.5	1428	860.9	NM_017829.5
439.7	278.9	253.8	218.9	NM_031890.2
169.9	133.7	141.5	136.4	NM_016174.3
87	93.8	90.6	88.4	NM_178569.2
147.8	127.6	115.8	117.9	NM_001807.2
127.3	113	116.6	104.7	NM_014246.1
125.5	115.1	113.6	104.4	NM_001408.1
126.2	121.4	136.4	116.8	NM_001407.1
94.4	89.8	91.1	84.1	NM_001048216.1
1035.8	513.3	614.8	377.7	NM_001809.2
424.8	241.1	244.3	189.2	NM_001042426.1
2131	1798.3	1746	775.9	NM_001810.5
117.7	96.3	87.4	94.9	NM_001812.2
149.3	157.1	180.4	119.8	NM_001813.2
514	446.4	376.6	292.1	NM_016343.3
93.1	84	93.5	101.2	NM_022909.3
90.9	84.9	73.5	83.7	NM_006733.2
266.1	209.6	283.2	267.7	NM_018451.2
974.5	720.8	833.7	441.5	NM_001002876.1
369.8	290.8	295.4	238.1	NM_001002876.1
112.3	100.3	100.2	91	NM_024322.1
2900.4	2344.3	2557.3	1196.5	NM_006869.1
376.9	334	298.6	218.2	NM_018404.1
108.6	181	184.7	162.9	NM_014716.2
244.2	310.2	280.8	232.9	NM_012287.3
92.1	103.3	84.8	99.3	NM_030649.1
171.8	136.6	120.7	140	NM_015230.2
82.2	81.9	74	75.5	NM_139182.1
82.2	87.7	85.9	94.2	NM_015242.2
143	181.6	175.9	154.8	NM_015242.2
174.8	218.8	239.8	157.8	NM_022481.5
96.6	83.2	90.7	87.2	NM_014770.2
92.9	98.7	86	98.9	NM_001037131.1
194.2	179.5	170.7	166.6	NM_014914.2
90	87.3	86.1	89.7	NM_001037131.1
93.2	101.9	93.3	106.9	NM_001042535.1
123.4	104	100.1	94.9	NM_001042535.1
337.2	388	307.2	348.6	NM_031946.3
453.1	445.2	392.6	369.6	NM_007018.3
552.2	481.9	465.6	349.1	NM_025009.3
239.5	272.9	235.4	253.6	NM_014985.2

294.7	253.7	221.5	230.1	NM_014956.3
137.2	141.3	142.3	134.4	NM_014812.2
98.4	101.5	99.2	104.7	NM_001042404.1
345.9	533.6	511.8	369.1	NM_032142.2
110.7	99.2	110	101.9	NM_001035518.1
120.7	134.3	112.2	107.6	NM_007186.2
303.2	322.4	469	273.4	NM_018097.1
124.4	152.2	125.3	181.9	NM_025114.2
572.4	374.5	263	269	NM_018131.3
147.9	128.2	121.7	164.8	NM_014679.3
750.1	924.2	890.7	766.1	NM_025180.2
88.9	91.1	88.4	93	NM_001042384.1
92.3	89.9	85.7	86	NM_001042384.1
90.1	106.7	99.6	91.4	NM_001042400.1
124.8	110.3	98.7	112.4	NM_015147.1
160.7	129.9	142.9	122.9	NM_024491.2
152.1	161.2	149	128.6	NM_018140.2
126.9	121.2	111.1	117.2	NM_024899.2
96.4	86.4	90.6	93.6	NM_001007794.1
352.7	210.2	172.8	166.6	NM_006090.3
795.3	445.3	330.2	328.3	NM_006090.3
90.5	80	80.7	86.4	NM_005454.2
760.2	739.8	696.7	368	NM_182661.1
353.8	395.3	445	284	NM_182661.1
113.9	115.3	106.7	120.2	NM_022766.4
87.1	91.4	86.4	80.7	NM_201548.3
81.9	79.5	78	89.2	NM_001030311.1
89.6	94.5	90.1	85	NM_001030313.1
92.6	86.3	91.4	85.9	NM_001030311.1
2040.8	4194	3143.2	2911.1	NM_001025195.1
1106.2	813	755.2	628.2	NM_198061.1
134.9	168.1	150.1	143.1	NM_003869.4
94.2	103.9	91.3	96.3	NM_024922.3
665	1106.6	936.6	661.3	NR_003276.1
97	103.3	83.7	94.6	NM_145024.1
81.1	98.2	77.9	88.7	NM_014406.4
92	85.7	79.5	89.4	NM_004066.1
2347	1327.3	1137.7	909.6	NM_004344.1
92.5	88.8	89.5	97	NM_004365.2
139.7	147.4	152.4	130.4	NM_000078.1
97.5	111.7	112.3	119.1	NM_001710.4
82.6	77.2	74.2	88.4	NM_032545.2
4921.5	8998.6	9082.6	6178.3	NM_001928.2
147.2	119	125	119.8	NM_006324.2
83.6	75.2	86.5	77	NM_000186.2
105.5	118	106.2	103	NM_001014975.1
105.6	80.3	80.6	85.7	NM_001014975.1
90.6	88.1	86.1	87.1	NM_002113.1
104.9	118.9	103.4	96.5	NM_005666.2
93	78.3	84.7	89.2	NM_021023.4

84.9	83.9	83.3	85.2	NM_006684.1
79.1	76.5	82.9	76.2	NM_030787.1
91.4	80.2	83.1	81.2	NM_000204.1
6419.4	4642.5	4120.8	2119.7	NM_005507.2
94.6	80.8	86.2	92.6	NM_138638.1
83.4	91.6	84.7	86.5	NM_021914.5
270.7	544.6	613.8	372.1	NM_003879.3
103.9	83.1	80.5	79.6	NM_000492.2
126.5	175.3	140.9	140.5	NM_052818.1
92.9	85	75.2	80.9	NM_001079691.1
131.1	166	164.7	152.4	NM_001079691.1
90.9	93.3	87	82.6	NM_000735.2
99.9	103	106.3	96.7	NM_000737.2
96.3	79.5	94.3	89	NM_033377.1
78.9	81.7	73.4	89.2	NM_033378.1
87.3	89	82.4	89.2	NM_033043.1
87.2	86.9	76.8	85.6	NM_033142.1
794	533.3	546.4	427.2	NM_003663.3
90.8	92.3	81.7	82	NM_001008390.1
2077.6	1752.8	1939.6	1538.7	NM_003663.3
89.4	88.2	79.5	90.1	NM_015939.3
1212.6	655.5	543.6	449.7	NM_016052.2
97.1	81.8	89.5	91.4	NM_016140.2
89.7	87.3	81.5	85.3	NM_015964.2
3272.9	2702.2	2500.7	1463.6	NM_016016.1
834.5	611.7	571.9	351.5	NM_015703.3
159.4	156.2	132	116.8	NM_015703.3
107.3	93.3	93	83.7	NM_020770.1
134.3	134	123.6	105.1	NM_032866.3
109.5	92.3	87.9	98.1	NM_006569.2
194.4	177.7	166.3	140.5	NM_006568.2
86.9	92.5	78.9	80.9	NM_003956.2
100.8	94.9	86.6	94.6	NM_024111.2
142.2	96.4	112.4	110.5	NM_001008708.1
90.5	95.7	95	85.5	NM_001267.1
168.9	151.1	140.2	131.7	NM_005483.2
381.5	352.7	334.9	203.4	NM_005441.2
113.2	98.4	85.2	96.4	NM_020549.2
94.7	85.8	88.4	86.1	NM_020984.1
87.1	83.3	76.8	96.8	NM_020986.2
87.4	78.8	74.9	79.9	NM_020984.1
233.5	176	158.4	144.7	NM_203298.1
8074.9	4939.8	4655.3	4334.1	NM_016139.2
465.1	524.5	393.3	485.3	NM_017812.1
745.7	704.6	621.1	437.9	NM_144636.1
746.1	542	530.3	353	NM_032309.2
157.8	162	142.1	123.2	NM_032343.1
136.3	100.7	100	101	NM_001011670.1
538.2	274.2	251.6	248.2	NM_001011668.1
243	236.5	202.6	160.8	NM_016565.2

331.2	373	363.8	405.6	NM_001270.2
445	444.9	350.6	302.9	NM_004284.2
137.7	131.7	125.7	129	NM_001042572.2
82.5	82.2	75.5	87.3	NM_005852.2
102	92.8	95.7	94.3	NM_001005273.1
973.2	1077.7	998.8	783.2	NM_001273.2
104.6	83.3	89.8	87.6	NM_015557.1
109.1	127.3	147.5	102.1	NM_032221.3
406.3	419.9	318.8	321.2	NM_017780.2
491.8	704.8	641	426.1	NM_020920.1
502.3	785.7	724.9	668.6	NM_025134.3
83.4	80	69.3	76.8	NM_018397.1
870.4	426.1	358.2	306.6	NM_001274.2
338.1	279.9	257.6	247.5	NM_007194.3
82.9	83.4	89.6	90.7	NM_001005735.1
176.5	160.9	147.3	129.7	NM_006387.4
468.6	642	462.9	487.8	NM_005197.2
1323.5	1165.6	1104	620.4	NM_018223.1
97.3	94.9	95.5	101.7	NM_001275.2
88.8	84.6	71	80.8	NM_001819.1
85.4	86.6	78.4	87.8	NM_018371.3
108	114.5	108.5	116.8	NM_001276.1
100.7	101.4	89.5	106.3	NM_004000.2
94.9	88.9	87.1	91	NM_001025199.1
86.3	81.5	76.2	80.2	NM_201653.1
96.3	91.8	81.4	97.8	NM_021797.2
87.2	95.8	89.4	95.9	XM_942851.1
90.9	92.4	95.2	93.5	NM_001039840.1
1450.6	864.7	885.8	757.5	NM_012110.2
95.4	95.8	88.1	94	NM_023947.2
705.4	549.9	587.6	338.1	NM_001277.2
173.4	151.6	137.3	134.7	XM_942105.1
692.9	748.2	723.4	663.4	NM_005198.3
92.5	87.8	81.4	90.5	NM_152253.1
99.4	102.1	93.8	90.2	NM_006614.2
93.3	92.9	79.4	88.2	NM_000390.1
131.8	127.3	128.2	112.8	NM_001821.2
656.5	688.9	686.3	505.2	NM_020412.3
188.1	173	167.7	133	NM_198426.1
235.3	203.8	188.8	148.3	NM_014453.2
1331.5	911.5	949	575.9	NM_014453.2
400.7	345.9	347.9	305.3	NM_014043.2
621.1	488.5	343.3	317.2	NM_014169.2
817.6	667.5	566.1	437.4	NM_176812.3
91.2	89.7	97.3	79.2	NM_152284.3
594.2	520.7	526.9	398.4	NM_016410.2
246.6	224.2	218.1	146.5	NM_024591.3
127.4	119.9	115.5	101.4	NM_024591.3
153.8	132.5	122	116.8	NM_152272.2
124.4	97.2	100	102.2	NM_001822.3

83.9	89.2	85.5	91.1	NM_001822.3
104.9	98.3	115.1	108.6	NM_004067.1
119.9	148.6	139.2	122.7	NM_004067.1
83.1	86.9	78.2	75	NM_004067.1
111.8	91.9	82.9	98.2	NM_024944.2
88.6	85.9	79.1	81.7	NM_012124.1
344.1	246.2	219.7	181.5	NM_007236.3
138	176.8	161.7	173.7	NM_024536.4
190.9	186.3	135.4	148.2	NM_020244.2
89	90.8	94.6	83.9	NM_001014372.2
238.9	184.8	173.2	160.8	NM_017444.3
84.1	94.4	84.3	92	NM_177978.1
86.3	83.8	75.9	87.6	NM_145234.2
76.3	76.6	82.9	78.9	NM_015424.3
95.8	86.9	89.2	87.8	NM_139320.1
93.1	86.8	84.5	92.1	NM_139320.1
76.3	78.2	81	81.3	NM_139320.1
76.7	90.9	78.1	83	NM_000738.2
87.5	88.4	80	81.3	NM_001006630.1
90.8	76.8	86	93.8	NM_001006626.1
87.1	87.2	91.3	88.9	NM_000741.2
87.2	81.1	83.6	85.4	NM_012125.2
79.7	83.4	81.1	81.8	NM_001039523.1
100.5	113.6	125.9	120.7	NM_000079.1
86.6	86.5	80.8	81.4	NM_001039523.1
98.6	90.8	83.4	81.7	NM_020402.2
97.7	99.7	100	92.6	NM_000742.1
95.8	103.1	100.3	94.3	NM_000743.2
105.4	93.5	94	93.1	NM_000744.2
1864.5	2144.5	2668.3	2904.4	NM_000745.2
111.2	107.9	115.5	104.7	NM_004198.2
89.8	96.7	86.8	95.2	NM_000746.2
96.1	93.6	87.1	93.3	NM_017581.2
191.6	172.4	158.8	159.7	NM_000747.2
88.8	85.5	80.8	74.6	NM_000748.1
89.3	92.6	74.7	78.3	NM_000749.2
82.8	80.1	71.7	83.4	NM_000751.1
101.4	88.7	86.8	90.5	NM_000080.2
84	91.2	85.1	98.4	NM_005199.4
93.8	86.9	81.7	83.4	NM_003654.2
255.7	199.1	175.6	182.6	NM_004854.3
84.5	90	82.6	87.2	NM_018413.2
144	147	181.9	126.8	NM_018641.2
1560.7	1176.2	1167.7	570.4	NM_152889.1
96.3	92.3	92.3	103.2	NM_004267.3
150	123.7	116.9	113.1	NM_004273.2
130.3	95.5	102.4	96.8	NM_005769.1
91.7	88.7	85.2	78.6	NM_012126.1
106.3	91.6	90.5	94.5	NM_024533.2
102.6	101.3	94.8	104.4	NM_021615.3

155	120.8	130.8	114.5	NM_019886.2
94.9	95.5	87.8	93.2	NM_022467.3
91.7	78.4	77.9	89.8	NM_031422.1
1441	1410.8	1544.6	1351.7	NM_014918.3
426.6	401.4	335.2	375.2	NM_022092.1
479.5	334.5	273.3	232.5	NM_001278.3
374.4	454.7	396.7	381.4	NM_145165.2
91.8	97.5	95.3	101	NM_182894.1
1164.9	942.4	1069	625.1	NM_004804.2
297	205.3	167	186.7	NM_020313.1
1206.4	925.5	870.2	605.5	NM_004895.3
194.5	176.2	154.2	125.8	NM_004895.3
3943.4	3526.7	3347.9	2171	NM_006384.2
94	85.2	90.9	86.3	NM_006383.2
167.4	110.7	107.4	100.5	NM_054113.2
92.6	79.3	71	78.6	NM_001029881.1
171.2	194.4	176.2	163.6	NM_015125.2
108.5	93.1	92.8	102.6	NR_002786.1
92.2	97.7	92.9	100.2	NM_001080858.1
92.3	89.1	91	85	NM_198289.1
89.1	88.3	81.6	85.5	NM_198289.1
134.8	121.5	120	105.7	NM_014430.1
100.7	86.9	91.7	92.6	NM_022094.2
85.8	83	89.2	89.2	NM_000246.2
84.4	80.8	81.5	85.1	NM_003613.2
79.4	84.7	78.3	82.1	NM_153221.1
134.3	107.4	111.2	108.9	NM_032630.2
3559.9	2364.3	2126.6	1716	NM_033082.1
153.6	158.9	161.8	141.9	NM_004882.3
1604.3	611.3	604.1	417.2	NM_001280.1
1595.7	755.1	651.9	459.3	NM_032830.1
97.5	97.9	90.8	95.2	NM_145071.1
83.7	86.7	80.7	84.8	NM_007174.1
99.8	90.2	89.3	91.6	NM_004143.2
1281.4	796.7	794.7	705.2	NM_006079.3
1964.3	1107.9	1141.6	964.5	NM_133467.2
114.4	108.1	98.4	109.1	NM_012127.2
754.2	521.1	459.6	385.2	NM_001281.2
310.9	213.2	225.7	213	NM_018204.2
1344.8	996.8	677.3	652	NM_006825.2
2152.1	1950.8	1581.9	1158.8	NM_001008938.1
167	123.8	128.4	111.3	NM_001008938.1
166.7	105.7	108	105.8	NM_001823.3
3574.5	2983.8	2693.8	2000.1	NM_016326.2
1443	1271.7	1032.5	823.1	NM_001040139.1
74.5	80.9	75.7	84	NM_001040139.1
100.9	85.1	93.9	89.2	NM_181521.1
230	196	159.1	171.8	NM_001824.2
89.7	91.6	85.8	94.2	NM_001015001.1
84.5	88.2	81.1	92.8	NM_020990.3

90.9	85.9	75.8	81.5	NM_001825.1
1902.5	1132.2	1008.2	750.4	NM_001826.1
3896.5	1536.2	1491.8	1424.2	NM_001827.1
328.7	442.1	393.4	359.3	NM_015282.1
431.8	487.3	442.2	432.8	NM_015097.1
99.5	94.7	91.7	87.9	NM_001828.4
95.1	78	73	92	NM_001285.1
87.9	77.6	80.5	87.7	NM_006536.4
87.6	95	94.5	87.3	NM_004921.1
86.5	88.5	83.8	82.5	NM_012128.2
80.9	89.3	84.7	85.8	NM_015127.2
150.5	113.6	101.4	98.9	NM_015127.2
108.7	110.9	110.6	106.3	NM_013246.2
87	83.5	87.2	76.7	NM_000083.1
90.9	100.9	84.5	86.2	NM_004366.2
84.9	83.9	80.8	88.7	NM_173872.2
312.2	293.9	252.4	220.2	NM_001829.2
97.7	91.2	82.6	97.1	NM_001830.2
92	90.9	85.8	97.2	NM_000084.1
114.4	100.3	107	101.5	NM_001286.1
178.4	237.1	315.3	175.1	NM_021737.1
191.7	268.7	297.6	221	NM_021737.1
3452.7	3700.5	4110.3	1596.6	NM_001287.3
99.6	112.6	120.9	97.4	NM_004070.2
109.8	110.2	118.8	107.9	NM_001042704.1
105.1	94.5	92.9	92.5	NM_000085.1
90.6	95.4	93.3	91.6	NM_021101.3
109.3	104.2	105.2	78.1	NM_006984.3
90.3	90.6	89.2	82.7	NM_182848.2
112.6	90.9	96.3	94.1	NM_006984.3
122.1	145.9	140.1	126.1	NM_012129.2
233.5	220.4	239.6	177.6	NM_012130.2
90.9	80.6	76.2	89.1	NM_144492.1
86.7	83.8	85.7	81.4	NM_012130.2
88.6	104.1	113.4	101.2	NM_138429.1
449	440.7	502.7	283	NM_014343.1
115.9	111.5	112.9	114.6	NM_138429.1
91.3	96.1	86.6	86.8	NM_006580.2
81.3	89	83.9	85.4	NM_012131.1
98.8	89.2	83.6	88	NM_016369.3
106.4	115.3	111.6	103	NM_001002026.2
99.1	93.6	102.6	90.6	NM_016369.3
91.7	82.4	79.6	88.1	NM_016369.3
105.3	86	93.1	97.8	NM_148960.1
101.6	89.3	88.7	99.1	NM_020384.2
107.5	113.2	106.9	109.9	NM_001001346.1
727.6	543.4	445.9	407.9	NM_194284.1
89.3	93.3	89	86.7	NM_001306.2
97.1	94.2	91.4	89.2	NM_001305.3
81.7	76.2	78.4	81.3	NM_003277.2

91.4	86	86.5	88.6	NM_021195.3
142	145	141.7	136.5	NM_001307.3
84.3	80.2	80.1	84.3	NM_199328.1
103	104.3	115.4	101.1	NM_020982.2
546.4	569.6	493.8	507.2	NM_001040181.1
669.2	697	504.4	593.7	NM_019895.1
253.6	187.2	204.1	172.5	NM_152353.1
98.9	108.9	104.4	96.3	NM_182906.2
98.6	96	93.1	96.1	NM_006344.2
96.4	99	118	112.1	NM_006344.2
6977.4	2726.3	2599.4	2274	NM_002975.2
183.5	459.6	326.8	313.7	NM_138337.4
163.4	351	311.6	216.3	NM_201625.1
101.3	138.7	134.9	96	NM_138337.4
93.2	93.3	91	95.8	NM_205852.1
85.7	78.2	82.9	83.4	NM_175060.1
87.9	89.1	87	85.3	NM_016511.2
88.8	80.5	87.9	91.2	NM_016509.2
89.3	85.8	83.9	78.4	NM_005127.2
92.9	91.5	79.6	88.1	NM_001004419.1
171.1	162.4	150.5	179.6	NM_013269.2
86.7	91.5	87.6	90.1	NM_005752.2
106.3	108	117.3	106.9	NM_003278.1
92.7	109.9	112.1	99.1	NM_194448.1
93.9	95	87.6	87.8	NM_203503.1
83.1	90.7	84.1	93.6	NM_130441.2
93.7	87.8	87.8	78.2	NM_080387.4
89.7	83.6	86	92.9	NM_014358.1
90.9	95.7	99.9	96.1	NM_173535.2
97	97.2	94.9	90.9	NM_198492.1
88.6	81.3	78.3	86.4	NM_214679.1
101.4	113	106.3	94.5	NM_013252.2
79.1	85.4	87.7	78.1	NM_001007033.1
104	108.3	97.7	101.6	NM_197950.2
93.8	89.2	88.5	86.8	NM_197954.1
101.3	99.7	99.3	97.1	NM_197953.1
114.1	100.7	92.8	100	NM_207345.1
128.5	120.2	118.3	137.5	NM_004362.1
11992.4	9445.4	7538.7	6364.9	NM_001288.4
100.6	95.2	99.5	98.5	NM_001289.4
91.6	83.8	80.3	93.7	NM_004669.2
890.5	646.6	570.5	670.8	NM_013943.1
94	91.8	93.2	101.7	NM_016929.1
241.2	237.4	228.1	186.6	NM_053277.1
352.2	489.3	363.4	434.8	NM_002956.2
113.4	130	107.3	108.5	NM_032421.2
135.4	155.2	139	132.9	NM_032421.2
104.3	125.1	156.1	114.7	NM_015526.1
192.6	135.7	155.5	208.2	NM_001024646.1
129.3	117.8	111.2	119.2	NM_004071.2

253	275.3	269.7	171.9	NM_001291.2
529.8	558.3	510.4	473.6	XM_941392.1
83.2	82.5	89.9	88.7	NR_002711.1
217.9	205.2	205.2	182	NM_003992.1
704.5	873.9	930.1	437.1	NM_003992.1
117.5	108	107.5	104.8	NM_001292.1
200.3	198.6	167.8	232.3	NM_020666.2
79	85.4	83.6	85.6	NM_001025233.1
88.2	86.4	80.9	90.4	NM_001025232.1
97.6	82.3	85.1	90.8	NM_024734.2
237.4	341.8	300.8	264.5	NM_000086.1
206.9	257.3	256.4	238.3	NM_000086.1
347.4	343.2	309.6	249.1	NM_006493.1
218.5	173.1	182.4	160.5	NM_017882.1
120.4	126.7	102.6	114	NM_018941.2
3520.6	2613.8	2251.7	2099.2	NM_001293.1
102.9	111.9	90.2	97.3	NM_004898.2
90.6	97.9	97.3	93.5	NM_030813.3
602.3	334	350	241.7	NM_006012.2
80.3	75.4	79	82.4	NM_001832.2
784.3	554.5	432.6	433.3	NM_001294.1
660.5	540	397.6	428.3	NM_006660.3
85.7	85.3	95	82	NM_152311.1
89.2	96.4	83.9	87	NM_022111.2
1371.4	1361.9	1181	987.7	NM_001009566.1
106.6	104.7	107.5	104.8	XM_937951.1
304.7	125.6	124.6	128.1	NM_022131.1
86.3	88.2	89.2	91.6	NM_014718.2
2418.7	1634.8	1432.4	1109.9	NM_001076677.1
96.8	89.3	81.1	100.3	NM_007096.1
1605.3	1166.2	1088	787.6	NM_007096.1
2047.4	1687.4	1370.2	968.4	NM_001834.2
89.4	80.5	83.5	85.5	NM_007097.2
170.5	157.4	148.4	187.2	NM_004859.2
223.1	220.4	248.1	214.2	NM_007098.1
112.1	115.5	98.5	114.1	NM_007098.1
75.4	72.1	77.6	76.6	NM_001831.2
88.5	87	75.9	82.8	NM_203339.1
85.9	85.4	87.8	87.8	NM_001831.2
174.4	140.1	128.7	125.8	NM_015041.1
21274.3	27388	29163.3	24252.9	NM_024793.1
304.2	249.6	221.4	170.5	NM_015041.1
89.3	89.2	95.3	88.2	NM_199167.1
105.2	103.3	97.2	96.1	NM_014410.4
82.1	84.1	74.3	79.7	NM_199167.1
476.5	348.6	381.1	316.4	NM_206808.1
280.2	257.7	232.9	201.8	NM_206808.1
96	84.9	99.6	89.3	NM_001836.2
95.8	98.6	83.1	86.9	NR_002174.1
1317.2	1099.6	963.7	781.9	NM_018686.3

1298.4	1010.2	895.8	653.1	NM_030629.1
86.1	94.6	86.9	96.3	NM_198390.1
84	85.8	84.2	81.3	NM_030629.1
89.4	90	90	89.7	NM_004072.1
106.2	116.6	113.1	103.2	NM_020311.1
98.3	100.4	93.1	89.9	NM_016347.1
800.8	844.3	615.6	812.8	NM_016308.1
106.1	97	100.1	105.3	NM_181270.1
84.9	89.8	91.6	87.8	NM_181289.1
104.3	99.9	102.4	89.8	NM_181301.1
86.3	78.1	75.6	77.7	NM_144673.2
82	82.6	84.6	89.4	NM_144601.2
1794.3	1346.2	1340.4	783.2	NM_181555.1
106.9	114.8	110.4	95.6	NM_181553.1
482.4	377.3	348	323.1	NM_001048251.1
393.3	315.6	395.6	184.2	NM_178818.2
101.7	87	92.2	85.2	NM_178818.2
87.8	79	79.4	75	NM_138460.2
4004.5	2678.2	2661.7	1456.5	NM_138410.2
267	199.9	207.5	187.8	NM_138410.2
317.7	273.9	267.1	172.5	NM_181472.1
178.2	276.7	283.9	279.9	NM_178868.3
79.5	78.4	79.1	77.2	NM_194293.2
91.3	76.5	81.6	85.4	NM_152381.3
79.5	88.6	84.8	93.7	NM_001079810.1
211.9	170.2	168.8	147.6	NM_153610.3
817.6	559.2	509.1	365.8	NM_014865.2
86	90.7	79	78.5	NM_173538.1
1660.3	1337	1047.4	1322.8	NM_003418.1
100	94.3	96.7	100.9	NM_032649.5
1751.5	1705.6	1557.7	1053	NM_018235.1
428	206.5	209.4	151.2	NM_032488.2
88.2	82.8	86.6	84.5	NM_000087.2
97.6	87.9	102.2	95.3	NM_005140.1
103.5	89.5	96.9	96.8	NM_001298.1
85.1	79.5	84.3	76.4	NM_001298.1
100.5	107.2	107.9	101.3	NM_001298.1
101	89.8	85.9	100.5	NM_001037329.1
102.7	104	117.9	104.9	NM_001297.1
86.1	88.2	77.7	90.9	NM_019098.2
1908.6	1017.2	1117.2	739.9	NM_001009551.1
1471.5	851	709.9	603	NM_005776.2
93.6	83.5	85.8	89	NM_182553.1
101.9	95.9	97.2	102	NM_182553.1
90.7	85.8	87.3	90.9	NM_152495.1
2758.4	1279.1	1935	1478.7	NM_014184.1
92.5	99.6	95.2	93.4	NM_006314.1
94.3	93.9	87.8	91.5	NM_014927.2
229.4	232.2	200.5	209.2	NM_173515.2
77.4	78.2	75.4	83.7	NM_001299.4

118.2	104	140	126.1	NM_004368.2
1319.3	1149.8	1162	540	NM_201277.1
475.4	334.2	320.8	261.1	NM_001839.2
110.4	121.2	110.4	101.3	NM_020348.1
87.5	82.9	76.7	90.7	NM_017649.3
104.9	117.4	102	116.4	NM_199077.1
82.4	87.6	79.1	82.8	NM_199077.1
232.2	223.2	235.1	154	NM_017623.3
128.5	116.6	110.4	113.1	NM_020184.2
389.4	299.6	302.1	207.8	NM_018366.2
106.5	101.9	98.3	98.2	NM_206999.1
3195.5	3509.7	3144.4	1845.1	NM_016284.3
458.7	460.9	410.8	338.2	NM_015442.1
676.7	822.9	713.6	590.7	NM_014515.3
215.2	220.3	189.4	190.3	NM_014516.2
652.1	615.2	515.7	472.8	NM_013316.2
95.1	111.3	104.8	100	NM_013316.2
104.6	98	103.4	102.9	NM_001008225.1
687.9	771.7	582.1	578.7	NM_015455.3
158	149.3	139.9	123.6	NM_144571.2
836.3	435.8	451	424.3	NM_054026.1
1451.2	1139.4	724.1	1023.5	NM_013354.4
81.5	84.3	76	79.1	NM_054026.1
506.1	450.1	407.2	358.7	NM_004779.4
91	83.1	82	93.8	NM_033133.3
103.7	96.5	105.7	105.9	NM_016083.3
105.7	95.7	87.4	99.7	NM_001841.1
101	94.1	99.9	106.1	NM_173478.1
81.9	80.8	88.1	78	NM_000614.2
93	85.1	90.4	88.2	NM_147164.1
94.7	87.4	88.1	92.1	NM_147164.1
92.3	78.4	79.8	85.7	NM_001842.3
90	93.5	83	89.8	NM_001843.2
90.1	78	83.1	84.4	NM_001843.2
103.9	98.1	92.4	103.1	NM_005076.2
89.7	86.2	83.5	88.6	NM_020872.1
100.5	83.5	85.3	83.9	NM_175612.1
88.8	81.7	80.5	85.6	NM_175607.1
164.1	115.9	124.5	108.9	NM_175613.1
87.8	75.7	83.3	87.3	NM_014361.2
88.9	80	77.2	89.3	NM_014361.2
96.6	91.6	94.9	99	NM_014461.2
93.2	86	87	77.1	NM_003632.1
91.3	80.6	76	92.1	NM_014141.3
104	91.5	86.5	95.9	NM_033655.2
102.5	101.5	97.8	96.7	NM_138994.1
105.3	82.4	87.7	83.6	NM_033401.2
96.6	97.9	88.1	92	NM_130773.2
102	97.1	100.6	100	NM_138996.1
114.2	123.8	125.2	112.7	NM_053051.1

799.6	925.6	987.9	582.4	NM_025233.4
241.2	224.6	192	183.2	NM_025233.4
103.5	97.5	84.3	95.8	NM_015198.2
97.3	96.6	89.3	85.9	NM_014900.3
171.5	155.6	135.6	133.9	NM_015456.2
1031.5	984.8	776.3	881.2	NM_015456.2
1685.1	1296.6	1147.3	1132.9	NM_015456.2
110.2	95	85.8	97.6	NM_004086.1
172.4	161.7	174.6	137.8	NM_018714.1
730.5	621.8	606.9	366.5	NM_007357.1
152.9	167.7	176.6	160	NM_031431.2
146.8	155.2	127.2	125.9	NM_015386.1
147.2	147.9	121.8	119.9	NM_006348.2
171.2	142.7	123.2	139.6	NM_006348.2
173.8	237	221.6	193.5	NM_020751.1
165.6	158.6	130.3	131	NM_153603.1
149.2	117.1	104.9	118.4	NM_032382.2
753.6	803.6	655.6	630.4	NM_004645.2
88	84.9	93.6	83.6	NM_000493.2
105.4	106	91.2	99.5	NM_001854.2
92	83.9	81.9	92.5	NM_080629.1
90.6	93.1	90.1	87.2	NM_080629.2
91.3	93.5	85.5	83.3	NM_080629.2
99	97.9	95.1	94.8	NM_080681.2
96.7	89.7	91.6	90.3	NM_080680.1
112.1	109.9	93.9	109.4	NM_080645.1
85.4	83.5	89.5	85.9	NM_004370.4
97.9	97.7	97.2	90.8	NM_080805.2
91.3	92.7	81.2	84.9	NM_080815.2
114	119.9	107	109.5	NM_001855.2
82.8	96.6	84.3	95.1	NM_001856.2
86.1	82.3	73.2	73.6	NM_130778.1
90.8	87.2	84.5	75.7	NM_000494.2
109.9	160	144.5	108.8	NM_030582.2
95.4	88.1	89.6	79.7	NM_130445.1
84.5	86.9	86.2	80.2	NM_001858.4
90.5	116.7	111	110.4	NM_000088.2
86.7	91.4	77.7	90.6	NM_000089.3
86.6	97.6	92	99	NM_020882.1
90.8	83.3	89.4	88.9	NM_030820.2
101.8	83.9	90.6	92.5	NM_152888.1
211.4	302.5	256.6	244.4	NM_173465.2
187.3	268.1	235.1	257	NM_152890.4
93.1	91.7	79.2	89.6	NM_198721.1
86.8	78.3	76.6	81.7	NM_032518.2
89.4	83.2	87.1	82.2	NM_198721.1
108.9	104.5	92.8	104.9	NM_032888.2
92.5	84.1	85.2	90	NM_001844.3
91.5	91.9	82.3	92.7	NM_001844.3
92	83.6	91	84.9	NM_000090.2

189	394	334.9	281.1	NM_001845.3
92.7	127.6	107.3	100.4	NM_001846.1
78.8	73.3	73.6	81.6	NM_000091.3
94.5	84.4	93	87.6	NM_000091.2
126.2	122	98	107.7	NM_005713.1
354.1	503.2	446.4	347.9	NM_005713.1
87.9	87.2	87.1	89.5	NM_000092.3
139	108.2	104	105.6	NM_033381.1
88.5	80.4	77.5	90.7	NM_033380.1
91.8	79.4	84.6	88.7	NM_033641.1
90.5	81	74.4	85.2	NM_001847.1
89.9	90.6	82.8	79.8	NM_001847.1
96.9	89.7	76.6	80.1	NM_000093.2
103.5	90.4	95.9	91.2	NM_000393.2
80.6	86.2	85	66.3	NM_015719.2
141	213.4	204.7	195.9	NM_001848.1
93.8	96.7	102.1	105.8	NM_058174.1
115	112.2	105.2	104.6	NM_001849.2
104	104.6	101	93.9	NM_058175.1
83.8	80.9	80	81	NM_004369.2
105.1	102	93.1	97.3	NM_057164.2
191.5	209.3	201.5	167.8	NM_000094.2
93.7	101.3	104.2	106.1	NM_020351.2
90.4	86	77	81.8	NM_020351.2
86.7	90.7	80.3	87.3	NM_001850.3
169.9	160.7	116.2	118.4	NM_005202.1
100	94.4	95.4	98.6	NM_001851.3
80.4	92.6	85.3	79.8	NM_078485.2
83.7	83.5	80.2	89.1	NM_001851.3
92	84.1	72.2	74.1	NM_001851.3
2779.7	4116.9	3248.7	3505.3	NM_001852.3
90	102.3	91.4	96.1	NM_001853.2
93.5	77.2	91.2	78.8	NM_006438.2
81.2	83.1	82.3	87.1	NM_199235.1
[truncated: 400,754 more chars]
