# Supplementary material for: Inflammatory Transcriptome Profiling of Human Monocytes Exposed Acutely to Cigarette Smoke
Source: PLoS One. 2012 Feb 17;7(2):e30120. doi: 10.1371/journal.pone.0030120 (PMC3281820; doi:10.1371/journal.pone.0030120)
Supplement: Data S2 — Illumina Raw data for PBMCs treated with 3 and 10%CSE. THP-1 cells were treated for 8 and 24 hours with RPMI-1640 control medium (n = 3) or 10% CSE-conditioned medium (n = 3). Total RNA was extracted using and run on a Human Ref6V2 bead chip for the Illumina platform. Data is presented in the form of raw values. (PDF) [file pone.0030120.s002.pdf]

TargetID	AVG_Signa	AVG_Signa	AVG_Signa	AVG_Signa	AVG_Signa	AVG_Signa	AVG_Signa	AVG_Signa
15E1.2	128.9	128.2	128.8	118.8	112.4	100.6	128.8	131.9
2'-PDE	207.8	238.4	180	203.8	212.8	157.4	249.3	260.1
7A5	110	113.3	116.3	115.5	108.7	95.4	113.1	108.3
A1BG	123.1	124.1	126.8	122.8	115	100.3	137	113.1
A2BP1	103.9	115.6	121.3	106.3	101.4	99.1	105.3	107.2
A2BP1	114.4	110.6	117.3	118.7	109.2	100.9	122.2	119.5
A2BP1	120.6	119.7	118.4	126	107.6	98.4	106.7	118.6
A2BP1	112.6	126.6	124.2	125.2	105.3	95.7	115.3	116.6
A2M	118.4	111.3	133.1	122.5	104.4	105.8	120.2	114.3
A2ML1	113.4	124.9	116.5	115.8	107.6	94.6	118	115.8
A3GALT2	104.7	112.3	120.7	112.7	103.6	99.4	120.5	110.8
A3GALT2	108.2	112.9	111.5	120.7	103.2	101	120.4	119.3
A4GALT	111.2	112.6	122.1	115.5	106.8	109.2	117.4	112.6
A4GNT	128	119.4	118.6	128.7	116.1	99.1	125.1	121
AAA1	118.2	110.9	119.1	128.3	109.4	100.7	115.4	105.4
AAA1	108.8	122	120.9	118.1	100.1	106.3	115	116.6
AAA1	118.2	110.4	116.5	107.8	106.8	101.5	120.1	108.7
AAA1	113.3	114.7	108.7	108.6	106.8	98.6	117	111.7
AAA1	116	116.2	116.6	115.8	111.7	98.1	110.4	110.7
AAAS	137.3	125.5	133.8	130.1	119.6	100.6	127.9	127.8
AACS	177.3	171.6	144.5	184.9	144.7	124.1	170.8	172.7
AADAC	109.2	110.5	116.9	117.5	100	98	119	109.4
AADACL1	155.4	170.2	189.9	140.7	126.4	119.7	153.1	126.7
AADACL2	115.9	115.7	112.4	118.4	103.1	95.9	116.5	111.4
AADAT	105.3	120.7	120.3	114.1	113.2	89.6	116.6	115.1
AADAT	111.9	118.2	118.3	112.5	108	97.2	118.4	126.2
AADAT	106.6	114.4	116.9	114.5	103.5	100.1	120.3	121.7
AAK1	111.1	115.3	118.1	119.7	107.2	99.8	118	116
AAMP	176.8	171.1	169.5	192.2	133	146.8	182.1	194.5
AANAT	116.5	107.3	111.1	117.9	101.9	98.6	119.2	119.1
AARS	1143.2	1281.2	1073.8	1321.8	1051.6	996.2	950.3	1052.5
AARSD1	190.7	208	241.5	229	162.8	171.5	168.4	172.6
AARSL	136.8	132.6	159.6	175.7	131.9	129.9	153	148.9
AASDH	203.2	197.4	208.3	206.7	173.3	155.9	173.6	182.1
AASDHPPT	304.5	372.2	354	295	238.2	297.5	335.2	304.2
AASS	115.5	120.9	119.4	116	111	100	123.8	114.6
AATF	393.8	359.1	410.3	409.4	319.9	403.6	391.9	420.8
AATK	119.8	117	123.9	119.5	117.3	120.8	126	126.8
AATK	121.5	114.9	121.6	117.7	121.6	96.8	118.2	112.6
ABAT	118.8	127.1	122.4	115.9	117.9	110.3	112.9	110.7
ABAT	125.7	120.4	133.9	138.7	119.2	111.6	123.6	134.1
ABAT	114	116.6	116.1	124.7	112.2	104.2	132.1	116.8
ABC1	148.8	143	154.8	155.2	132.7	116.9	138.3	135.3
ABCA1	280.2	223.7	270.4	562.3	329	470.5	649.8	251.9
ABCA10	121	112.5	126.2	125	121	104.4	120.9	124.4
ABCA11	117.5	123.6	129.6	122	117.1	100	123.1	125.5
ABCA12	115.9	122.9	115.5	117.2	120.9	98.7	114	114.8
ABCA12	115.3	112.8	117	115	112.2	101.2	116.2	115.3
ABCA13	115.1	110.4	122.3	118.2	104.5	97.1	110.2	111
ABCA2	110	115	113.5	121.4	112.9	99.7	109.5	115.8

ABCA2	144.7	140.4	139.5	144.8	110	110.5	126.5	131
ABCA3	121.2	126.2	127.1	131	124.1	101.4	126.7	122.7
ABCA4	130.5	140.8	134.5	151.5	145.2	118.6	144	137
ABCA5	133.8	136.6	144.9	139.6	133.6	109.2	125.9	135.3
ABCA5	116.2	123.3	122.8	127.2	118.5	106.5	113.7	108.9
ABCA5	114.8	123.8	127.1	129.8	118.7	110	119.6	116.6
ABCA6	105.6	107.4	122.3	119.8	101.7	105.8	108.2	108.8
ABCA6	126.8	135.6	114.7	123.9	116.4	100.3	129	119.8
ABCA7	114.2	107.5	106	110.4	108.9	96.4	122.5	111.5
ABCA7	401.9	334.9	309.7	320	220	219.7	257.2	225.4
ABCA7	109.1	118.3	105.6	113.1	113	97.7	104.8	108.7
ABCA8	109.9	114.2	119	124.9	105.3	96.1	116.5	109.1
ABCA9	117.1	117.6	128.3	125.6	127.9	106.3	120.5	122.9
ABCA9	115.4	121.1	130.4	121.5	112.6	92.2	127.2	116.9
ABCB1	203.3	245.8	400.1	266.3	271.1	277.7	318.6	362
ABCB10	173	206.7	220.4	226.4	186.4	192.4	191.6	185.1
ABCB11	114.1	118.8	117.3	116.9	106.7	102.9	115.3	112.1
ABCB4	107.3	115.2	137.6	124.1	106.8	101.2	115.5	119.2
ABCB5	106.9	109.7	109.7	120.7	109.4	97.2	115.6	111.2
ABCB6	131.4	154.9	141.3	195.6	174.3	136.6	228.6	220.9
ABCB7	204.3	224.3	258.3	267.3	234.1	153.7	203.7	186.9
ABCB8	112.2	135.8	111.6	113.6	108.6	106.8	112	117.1
ABCB9	113	120.2	112.5	116.7	104.9	110.9	125.9	105.2
ABCB9	121.8	128.8	121.7	129.8	111.5	105.4	144.6	122.1
ABCB9	124.1	136.4	123.8	136.5	124.2	100	130.9	125.2
ABCC1	120.1	108	117.6	120.3	110.9	97.9	119.8	122.5
ABCC1	138.9	128.4	128.3	158.2	121.7	115.2	152.6	120.8
ABCC10	148.8	141.4	153.1	155.4	131.1	129.6	139.4	148.8
ABCC11	112.7	113.9	117.3	110.2	105.7	95	109.7	112.7
ABCC11	118.2	119	123.3	119.2	110	104.3	131.7	117.8
ABCC11	113.5	116.6	119.4	122.3	114.4	98.9	117.5	129.7
ABCC12	122.9	116.8	135.5	128.7	118	106	122.7	126
ABCC13	123.6	132.6	127.3	130.3	107.8	105.2	126.9	118.5
ABCC13	123.2	139.2	116.7	127	117	105.7	122.4	112.9
ABCC2	129.7	116	120	132.1	114	98	140.3	132.6
ABCC3	236.7	237.6	381.5	203.4	168.4	234.7	167.2	141.6
ABCC4	132.2	118.7	170.9	132	119.4	114.9	130.8	107
ABCC5	110.4	109.4	112.5	104.7	99.4	98.2	109.9	114.5
ABCC5	274.5	243.7	295.7	263.2	227.6	175.6	243.6	204.1
ABCC5	198.6	182.9	191.7	186.2	146.2	145.3	162	137.6
ABCC6	111.6	111.4	119.3	115.9	109.2	96	121.3	117.4
ABCC6	122.5	118.3	154.6	131.9	122.6	106.9	143.9	129.1
ABCC6	109.6	110.9	112.9	108.5	98.1	92.7	107.6	117.3
ABCC6	108.3	116.6	119.1	122.1	113.3	98.3	114.3	119.4
ABCC6	127.2	129.1	123.7	131.3	120	98.9	125.8	110.9
ABCC8	110.9	113	117.4	111.6	106	96	114	117.6
ABCC9	114.4	114.8	105.6	113.6	101.8	94.3	116.4	107.2
ABCC9	131.2	121.5	146	117.4	115.1	119.6	134.2	118.9
ABCD1	120.9	120	125.5	154.5	107	114.9	160.6	124.9
ABCD2	119.8	111.6	113.2	123.5	99.9	96.9	119.1	102.5
ABCD4	125.3	123.2	111.3	118.3	117.7	99.8	124	115.6

ABCD4	129.9	121.6	129	123.6	115.6	99.8	123.3	121.2
ABCE1	336.8	318.7	342.8	356.7	352	265.1	333.4	341
ABCE1	324.8	276.6	285.4	322.8	362.4	204.1	302	339
ABCF1	397.1	570.6	274.7	533.5	363.6	276.8	459.3	471.3
ABCF1	992.4	1270.5	721.7	1044.2	817.2	582.2	1187.4	1209.3
ABCF2	130.6	154.7	144.5	134.1	119.6	120.3	147.2	133.2
ABCF2	164.3	185.5	150.5	184.2	148.9	127.6	180.9	149.7
ABCF2	147.4	162.8	130.1	159.5	135.7	120.8	155.2	148.1
ABCF3	145.7	145.7	146.5	151.6	126.8	116.9	148.6	138.1
ABCG1	135.9	143.5	129.7	146	137.5	107.5	146.5	124.5
ABCG1	117.1	111.4	109.4	125.5	116.1	102.9	131.2	109
ABCG1	118.1	117.2	122.5	113.9	106.4	100.6	116.1	117.1
ABCG1	124.8	134.6	136.3	144.7	137.5	114.2	144	132.3
ABCG1	122.4	127.3	133.5	142.5	114.6	111.8	125.2	134.2
ABCG1	116.2	122.8	125.9	122.7	112.9	116.8	118.5	124.2
ABCG2	111.2	121.8	115.5	108.8	107.2	94.9	109	112.9
ABCG4	111.4	117.1	119.7	115.8	108.6	101.9	119.3	113.8
ABCG5	130.6	122.4	134.1	132.2	111.2	103.9	124.1	118.4
ABCG8	111.2	110.6	119.4	128.8	107.2	102.4	115.7	124.3
ABHD1	124	117.7	118.5	124.2	116.6	102.6	127.6	111.6
ABHD10	133.6	134.2	143.3	116.6	129.3	119.9	132.5	133.2
ABHD11	121.9	114	137.2	131.8	115.9	104.4	129.9	129.4
ABHD11	119.4	110.3	120.3	116.1	121.5	100.9	123.1	119.7
ABHD11	130.7	128.3	140	136	126.8	121	124.9	127.6
ABHD12	129.9	123.9	147	147.5	133.1	134.6	139.9	132.5
ABHD12	174.7	156.4	297.8	186.2	133.5	151.6	172.9	148.2
ABHD13	111.7	116	122.2	131.8	113.4	104.5	125.1	119.8
ABHD14A	191.4	205.1	213.6	177.4	142.8	159.5	134.3	129.5
ABHD14B	189.9	218.5	217.7	196.8	143.1	160.6	171.6	182.5
ABHD2	142.3	125.3	145.2	141.3	130.7	127.5	137.3	124.5
ABHD2	112.1	122.3	113.7	126.4	103.4	94.4	109.5	115
ABHD2	127	123.2	130.2	145.8	118.4	122.9	123.9	145.6
ABHD3	293.1	214.3	333.6	329.6	243.3	292.1	277	388.6
ABHD4	138.2	143.7	148.1	179.4	131.5	139.1	184.5	194.5
ABHD5	130.2	133.9	139.9	149.4	123	117.4	147.2	137.8
ABHD6	176.8	161.1	140.6	161.8	145.3	151.5	161.5	146.7
ABHD7	122.2	145.8	143	135.5	145.4	120.6	124.8	132.6
ABHD8	288.4	304.1	261.6	259.7	199.3	229.3	247.3	232.3
ABHD9	113.7	120.4	130.2	116.5	113.6	96.8	121.2	117.9
ABI1	108.8	118	114.6	119.7	112.1	111	120.6	108.5
ABI1	197.5	243.2	178.7	224.6	193.4	255.9	211.4	274.9
ABI2	121.6	121.6	122.7	127.1	111.5	108.1	126.4	118.1
ABI2	174.9	146.6	148.4	157.9	135.9	133.4	144.4	157.1
ABI2	114	116	118.5	122.9	114.2	99.6	115.8	123
ABI3	165.4	176.3	305.9	183.8	154.4	206.2	140.7	137.5
ABI3BP	124	130.1	125	130.4	118.9	105.7	125.4	118.8
ABL1	226.2	264.5	240.4	247.8	190.3	192.9	235.4	240.8
ABL1	114	113.3	115.5	121.1	119.3	111.8	127.1	128.4
ABL1	110.9	104.8	110.3	113	102.8	105.3	112.9	108.6
ABL2	129.3	116.7	118	137.6	111.9	105.9	133.5	124
ABL2	120.2	126.5	126	119	108.8	105.7	118.6	122.8

ABL2	107.7	111.7	110.8	114.1	109.3	97.3	113.7	118.5
ABLIM1	2354.2	3093.8	2731.3	3134.7	2851	2624.2	3408.1	4150.2
ABLIM1	195.8	212	237.9	208.1	250.1	146.7	185.2	233.4
ABLIM1	113.9	113.3	123.7	126.1	110.5	101.3	126.9	113.5
ABLIM1	433.8	602.1	605.2	635	715.3	556.3	605.4	605.7
ABLIM2	130.7	137.3	129.4	137	136.2	117	133	125.2
ABLIM3	119.9	126.8	128.4	122.4	106.6	100.8	125.4	123.9
ABO	115.2	117.4	131.1	119.4	108	100.7	117.6	113.8
ABP1	132.5	111.3	119.3	132.2	109.2	107.3	117.2	118
ABR	667.6	580.5	619.6	772.7	393.8	482.6	819.2	605.3
ABR	125.1	118.4	138	124.7	123.9	107.1	132.3	124.3
ABR	123.4	123.3	114.9	118.4	102.3	98.8	122.4	116
ABRA	121.4	112.5	112.5	123.6	102.2	97.9	114.4	117.7
ABT1	139.2	121	133.5	139.3	104.1	110.5	141	142.2
ABTB1	422.2	424.5	450.4	576	394.1	405.3	462.4	482.1
ABTB1	205.3	267.2	226.2	268	202.6	196	266.9	213
ABTB2	105.5	106.9	113.8	112.8	102.9	90.8	108.3	121.1
ACAA1	317.5	400.3	358.5	365	272.8	224.7	301.4	267.2
ACAA2	140.3	143.5	145	154.6	130.1	149.9	146.1	143.3
ACACA	177.9	179.3	164.2	212.1	156.6	141.3	209.4	258.9
ACACA	132	144.1	137	140.7	143.3	108.6	155.7	183.7
ACACA	109.1	115.3	112.3	122	108.4	101.2	122.9	124.7
ACACB	191.8	208.8	191.3	172.8	195	159.1	159.6	159.5
ACAD10	131.3	123.6	127.9	129.1	124.1	126.6	141.5	132
ACAD11	274.1	244.7	395.5	285.1	238.6	285.2	257.6	219
ACAD8	328.5	346.2	348.1	310.2	253.7	236.6	235.4	276.1
ACAD9	242.7	388.5	285.8	243.5	302.3	185.9	207.7	326.8
ACADL	115.8	119.4	111.2	118.4	106.8	105	115.2	117.8
ACADM	717.1	800.8	707	936.1	758.3	512.6	861.6	1006
ACADS	130.9	128.4	145.4	131.5	112.6	120	121.7	123.8
ACADSB	122.8	121.7	115.5	122.4	113.3	108.1	118.3	135
ACADVL	119.6	124.2	141.2	114.1	115.7	109.8	117.4	115.2
ACADVL	1384.2	1172.8	1725.3	1116.7	900.2	1202	1110	894.8
ACADVL	378.6	411.7	511.3	401.9	295	338.8	306.6	254.9
ACAT1	284.5	275.9	438.7	364.6	269.6	264.1	285.5	295.5
ACAT2	122.1	122	124.9	121.8	111.8	98.3	119.5	126.4
ACBD3	342.1	402.1	306.8	394.8	274.8	259.6	365.4	412.3
ACBD4	123	134.1	137.7	138.1	112.8	104.7	127.2	126
ACBD5	115.8	129	137.4	133.7	121.3	119	130.7	117.6
ACBD5	111	111.8	113.9	113.4	112	100.8	121.4	108.5
ACBD5	112.4	110.1	116	117.9	104.2	103.2	109.7	117.9
ACBD6	142.4	159.7	158	151	128.4	121.4	144	141
ACBD7	111.1	109.7	110.4	116.7	109.2	90.3	124.2	116.7
ACBD7	113.1	127	113.7	124	112.5	98.4	121	114.2
ACCN1	106	119.2	105.6	109.7	111.5	98.9	113.1	110.3
ACCN2	116.7	122.9	113.5	117.2	103.5	96.7	116.3	116.5
ACCN2	113	110	121.2	123.2	101.6	96.1	117.9	114.8
ACCN3	131.3	129.6	125.6	127.4	118.3	108.5	135	127.1
ACCN3	105.2	112.7	116.7	132.4	108.8	109.9	122.1	111.4
ACCN4	119	120.1	138.1	118.4	117.9	107.1	133.3	126.5
ACCN4	108.4	105.2	108	115.5	103.5	95.9	110	119.1

ACCN4	116.5	115	114.6	123.3	108	100.6	112.9	112.1
ACCN5	115.5	115.2	123.9	114.7	112.8	95.6	118.7	115.5
ACD	226.6	250.6	216.5	233.6	216.3	220.2	255.6	242.7
ACE	117.7	119.2	112.3	121.3	106.5	97.4	120.3	115
ACE	113.6	109.1	127.2	106.9	105.7	96.2	115.9	111.3
ACE	123.6	114.2	112.1	121.8	113.7	103.3	112.2	112.8
ACE2	120.4	117.1	120	129.8	106.9	100.9	120.4	113
ACF	103.9	116.2	117	116.1	111.6	105	119.9	125.6
ACF	119.2	117.7	117.9	121.7	114.9	106.2	118	117.5
ACF	108.7	129.2	116.8	127.1	117.8	98.2	120.2	120.1
ACHE	122.5	110.9	120.1	115.3	102	100.1	111.8	109.3
ACHE	113.9	108.2	111.5	115.7	100	96.8	116.3	114.1
ACIN1	460.5	421.6	418.1	441.7	343.7	366.9	586.3	598.9
ACLY	633.7	570.6	503	659.3	553.2	547.8	587.4	627.4
ACMSD	132	141.5	137.9	149.3	136.5	119	141.5	143.5
ACN9	392.4	389	444.8	421	340.8	261.8	254.6	248.1
ACO1	315.6	316	290	317.5	230.2	209.3	205.9	167.8
ACO2	148.1	181.2	165.3	142.6	152.6	150.2	146.7	153.3
ACOT1	118.7	123.2	119.6	118.7	110.9	101	120.8	116.7
ACOT1	119.2	133.4	132.4	120.8	111.8	97.7	114.2	126.5
ACOT11	121.3	113.1	142.1	124.3	102.6	96.3	124.5	108.6
ACOT11	112.3	121.4	117.2	119.7	119.9	99.7	118.9	114.6
ACOT12	112.7	118	131.2	118.8	112.7	113	124.2	119.9
ACOT2	117.3	117.5	119.1	121.3	115	98.5	121.6	110.1
ACOT4	127.9	122.7	134.7	120.3	113.1	97	124.9	122
ACOT6	115.2	112.4	113	117.2	101.3	93.1	109.8	111.9
ACOT7	114.4	109.1	123.7	122	110.3	98.4	120.2	119.3
ACOT7	112.5	104.1	113.3	111.2	108.5	100.8	118.1	113.5
ACOT7	147.2	186	165.9	164	157.4	138.9	212.7	334.4
ACOT7	123.6	113.9	132.7	125.8	120.1	108.4	129.8	112.1
ACOT7	121.4	119.4	125.1	137.3	118.8	118.7	150.4	198.4
ACOT7	119.8	120.7	113.9	113.2	106.8	99	119.7	126.5
ACOT8	127.7	136.6	127.9	125.3	117.6	106.7	130.5	123.7
ACOT9	141.6	128.4	140.5	153.2	120.6	141.5	165.8	128.7
ACOT9	215.3	164.8	229	244.6	181.7	238.7	221.6	166.9
ACOX1	143.3	150.5	142.4	147.6	121.9	112.2	147.7	133
ACOX1	114.2	105.5	113.3	118.7	105.6	90.7	109.2	106.8
ACOX1	115	110.2	116.1	110.3	107.9	101.5	115.6	106
ACOX2	110.1	103.9	109.5	114.5	103.8	102.2	111.4	103.8
ACOX3	148	154.4	181	167	136.2	141.3	179	154.2
ACOXL	116.5	115.8	107.8	107.3	99.4	96.8	111.3	108.9
ACP1	418.8	382	472.4	416	398	478.8	405.6	503.9
ACP1	663.9	794.7	1119.5	894.9	842.1	779.1	696.6	689.4
ACP2	163	153.4	151.4	154.1	138.3	125.1	134.8	146.3
ACP5	543.7	306.2	510.2	473.5	229.6	254.9	285.6	175.5
ACP6	130.4	139	133.1	139.8	128.7	116.2	122.1	112.5
ACPL2	156.9	210.6	166.4	156.6	176.1	129.6	145.8	139.2
ACPP	108.4	118.4	130.8	115.6	110.1	100.3	124	121.5
ACPT	155	163.3	168.3	158.8	146.2	132.2	161	155
ACPT	123.6	112.7	106.5	123.2	110.7	103.4	122	107.8
ACR	116.5	122.5	125.2	121.8	106	98.4	117.5	111.1

ACRBP	116.3	127.1	129.7	120.4	115.2	108.8	129.2	123.9
ACRC	145.4	149	164.1	145.5	131.7	121.2	148.7	154.1
ACRV1	118.1	131	120.3	115.8	114.9	97.4	118.8	126.1
ACRV1	116	115.3	117.8	117.1	115.4	102.8	123	115.4
ACSBG1	115.2	107.6	134.2	119	112.7	95.4	116.4	110.3
ACSBG2	115.5	117.4	117.2	125.7	117	89.9	116	117.6
ACSL1	184.7	152.8	127	178.7	142.1	128.6	166	132.2
ACSL3	317.6	340.3	295.6	315.7	329.1	243.4	446.2	360.1
ACSL3	109.8	122.2	124.9	124.2	123.4	100.2	122.4	129.3
ACSL3	132.2	140.2	143.4	158.9	136.7	123.8	146.6	143.6
ACSL4	119.3	122.9	119.6	126.5	118.7	100.4	113.2	115.7
ACSL4	158.8	156.5	146.9	146.3	141.9	120.3	157.9	146.7
ACSL4	392.7	353.4	359.8	426.7	326.7	323	422.2	340.7
ACSL5	160.9	159.4	197.5	192.2	165	142.1	156.6	147.1
ACSL5	112.1	113.5	115.6	117.3	112.3	106.5	120.9	109.1
ACSL5	116	120.2	115.1	115.7	110.7	96.1	121.3	112.8
ACSL5	116	112.7	112.2	125.3	119.8	95	124.5	110.7
ACSL5	632.3	609.6	571.6	591.7	449.7	427.1	648.2	484.7
ACSL6	105.6	110.5	124.5	117	118.1	99.3	113.8	128.5
ACSL6	109.8	124.8	118.4	126.3	114.1	99.6	126.1	107.4
ACSL6	118.7	115	120.1	117.4	115.1	96.4	118.1	115.9
ACSM1	106.6	106.9	113	112.1	102.5	90.2	103.7	103.4
ACSM2	109	109.6	110	119.6	106.3	95.3	115.5	107.1
ACSM2	118	111.7	113.2	115.7	113.6	97.5	115.9	115
ACSM3	119.2	126.1	131.3	127.1	117.4	105.1	118.1	118.1
ACSM3	121.2	128.4	111.8	119.5	110.5	96.2	118.1	111.2
ACSM3	155.8	136.7	155.2	165.4	145.8	132.2	147.4	151.2
ACSS1	329.8	428.9	215.7	307.7	239.1	170.5	306.9	273.1
ACSS2	130.3	116.2	126.7	113.5	120.3	119.2	127.2	112.5
ACSS2	117.3	113.3	109.2	136.2	121.2	102.2	132.3	111.1
ACSS2	280.3	328.1	508.6	351.5	252.4	400.6	266.9	277.6
ACSS2	453.3	423.1	675.8	481.6	329.3	496.3	334.7	347
ACTA1	115.6	116.8	111.4	123.8	111.8	101.8	113.9	123.7
ACTA2	277.7	183.9	326.9	286	146.4	215.8	273.4	175.6
ACTB	3948.5	3579.6	6996.9	3873.8	2768.6	4938.8	2941.9	2139.7
ACTB	3314.5	2950.7	5638.2	2900.8	1917.6	4457.6	2450.3	1812.6
ACTBL1	105.3	113.7	120.4	116.5	109.4	105.6	117.9	99.7
ACTC	115.1	112.6	120.3	116.2	101.4	105.3	115.9	113.5
ACTG1	9457.2	7900.6	10806.5	8007.7	5664.1	9237.1	6334.4	5294.3
ACTG2	115.9	117.6	122.9	119.3	116.5	97.9	118.6	121.8
ACTL6A	564.4	559.6	576.4	602.4	519.4	510.4	490.7	444.2
ACTL6A	268.2	232.7	260.2	235.5	190.4	194	230.6	204.3
ACTL6B	110.9	114.4	117.1	117.5	105.6	101.3	119.8	117.1
ACTL7A	111.5	113.6	112.5	114.3	102.8	92.4	112.8	114.2
ACTL7B	127.4	125	124.5	121.5	109.3	98.9	123.3	111.9
ACTL8	113.9	123.3	139.7	125.2	112.3	107.2	121.3	121.6
ACTN1	1075.7	1347.3	1010.8	1079.5	1075.1	648	975.6	1083.7
ACTN2	119	118.1	128.3	124.3	120.2	102.1	139.2	119.6
ACTN3	111.2	109	119.6	122.2	106.7	89	118.4	123
ACTN4	347.8	286.2	298.2	370.1	243.4	324.6	437	322.8
ACTR10	300.1	326.3	388.7	343.9	327.8	435.9	473.8	406.5

ACTR1A	481	649.1	501	614.3	391.3	436.9	776.5	610.9
ACTR1B	330.3	329.7	327.3	330.1	246.6	259.9	229.1	226.2
ACTR2	2340.9	2553	3130.5	2908.6	2075.5	2270.3	2667.1	2413.6
ACTR3	506.5	495.2	615.3	669.7	480	632.7	778	668.6
ACTR3B	118.9	123.3	117.8	118.7	115	105	120.9	126.9
ACTR3B	113.7	113.1	113.3	118.9	104.8	96.6	124	125.2
ACTR3B	122.2	125.9	123.2	135	119.1	105.6	129.7	128.4
ACTR5	224.3	197.4	187.4	205.9	180.9	156	218.6	202.2
ACTR6	475.9	536.9	543.6	568.4	423.3	401.9	400.3	382.9
ACTR8	155.9	182.7	165	180.4	149.9	128.2	160	149.6
ACTRT1	136	135	124.9	130.8	124.8	108.1	127.8	123.5
ACVR1	477.2	442.1	298.1	678.3	535.6	537.1	571.1	443.1
ACVR1B	108.5	124.8	129.2	118	112.8	105.2	127.6	119.3
ACVR1B	123.5	129.4	115.8	129.1	123	106.3	137.9	140.2
ACVR1B	190.6	239.7	207.2	222	203.3	190	189.9	213.3
ACVR1B	111.6	115.1	112	123.3	102.8	104.3	112.6	125
ACVR1C	114.2	108.5	122.1	119.7	106	100.9	110	117.8
ACVR2A	186.3	180.7	214	227.3	169.8	123.4	200.8	170.7
ACVR2B	134.1	111	117	118.6	106.9	105.4	115.5	114.1
ACVRL1	108.1	109.9	118.9	117.8	103.7	98	114	108.3
ACY1	147.8	146	140.9	143.9	133.2	124.2	136.8	129.3
ACY1L2	134.6	140.8	137.2	141.1	135	115.9	141.6	131.4
ACY3	114.3	108	115.6	117.7	106.4	94.7	109.9	112.9
ACYP1	121.6	142	139.5	141.8	121.9	111.9	125.6	116
ACYP1	256	222.6	286.5	212.1	197.5	186.4	206.4	176.4
ACYP1	129	129.7	125.9	127.2	115.3	103.1	140.8	125
ACYP2	189.7	207.2	171.5	197.4	186.4	192.1	208.5	203.8
AD031	122.6	118.9	132.4	120.7	107.6	106.7	112.8	111.4
ADA	445.9	420.4	852.5	358.9	280.7	598.4	244.1	227.9
ADAL	127.8	132.9	132.6	133.2	124.8	105.9	134.5	135.2
ADAM10	114.7	126.3	129.6	124.8	123.7	115.1	119.3	130.4
ADAM11	115.5	120.9	116.1	118	114.9	108.7	124.8	123.8
ADAM12	119.7	108.6	123	125.2	111	99.1	122.1	117.9
ADAM12	121.8	133.6	116	119.3	111.4	111.7	137.8	116.1
ADAM12	113.3	120.5	115	122	114.1	99	116.8	114.8
ADAM15	117.7	114.5	135.1	133.7	112.9	112.4	123	118.9
ADAM15	283.7	256.8	496.5	291.9	204.1	227.1	275.8	287.8
ADAM15	118.1	117.9	116.5	121	113.6	97.3	117.4	114.4
ADAM17	187.6	157.3	151	179.4	143.2	152.5	237.3	192.3
ADAM18	108.3	111.9	106.7	106.3	107	92.5	114.4	111.5
ADAM19	121.7	124	132.1	127.1	135.3	106.2	124.6	121.6
ADAM19	110.2	120.3	117.1	126	105.9	96.6	128.1	115.1
ADAM19	288.8	272.5	310.1	279.5	212	287.5	204.5	180.3
ADAM2	115.5	135.7	133.1	120.5	111.8	102.3	127.3	125.3
ADAM20	112.3	115.6	111.6	117.8	109	105.5	125.9	109
ADAM21	109	120.9	127.5	131.6	116.8	106.8	127.7	119.4
ADAM22	120.4	109.6	114.3	121.9	106.6	100.4	111	111.5
ADAM22	112.3	120.1	118.1	123.3	107.6	96	126.1	119.3
ADAM22	107	108.6	126.3	120.7	108.9	120.3	119.4	121
ADAM23	114.2	125.7	121.9	124.8	108.9	101.5	121.7	118.8
ADAM28	121.5	132.3	123.4	126.2	123.3	99.3	123.9	112.9

ADAM28	146.5	123.4	133.3	121.7	123.5	102.1	120.1	120.8
ADAM28	115.2	116.4	135.4	117.1	110.7	104.9	118.8	117.7
ADAM29	102.6	119.5	126.4	121.4	121.8	109.8	125.2	121.2
ADAM30	116	110.5	109.8	105.4	101.9	101.8	123.8	113.4
ADAM32	115.6	114.6	117.2	121	111.2	98.4	114.9	116.6
ADAM32	121.1	108.4	121.1	120.8	110.7	96.1	116.9	124.2
ADAM33	118.2	118.2	123.9	121.2	122.2	104.7	122.1	126
ADAM33	118.3	119.7	130.2	132.6	115.9	112.1	120	114.8
ADAM3A	102.8	105.8	107	114.5	106	105	119.1	111.3
ADAM6	115.3	115.7	120.8	133.9	108.2	102.3	124.9	123.6
ADAM7	162.3	174.1	159.8	153.8	149.5	129.1	164.2	149
ADAM8	333.7	283.4	289.6	287.1	167.1	178.7	218.6	183.7
ADAM9	122	118.6	145.8	133.9	116	148.6	131.3	132.4
ADAMDEC1	246.7	150.6	219.9	263.5	148.9	140.7	189.9	149.8
ADAMTS1	115.2	112.5	124	135.1	125.9	101.1	124.8	121.2
ADAMTS10	108.9	133.6	127.9	123.6	108	105.1	115.1	129.5
ADAMTS12	109.3	119.6	113.9	116.2	106.6	104.3	122.8	119.4
ADAMTS13	118.2	110.8	136.3	135.9	115	101.3	114.3	120.6
ADAMTS13	111	111.8	110.8	105.9	104.6	95	114.5	115.9
ADAMTS13	118.6	120.3	131.5	127	119.8	106.3	127.3	122.2
ADAMTS14	115.1	115.7	113	117.5	111.8	102	121.2	120.1
ADAMTS14	110	115.2	113.8	123.7	112.1	96.4	124.3	112
ADAMTS15	119.5	123.1	115.8	115.2	114	98.2	123.4	119.2
ADAMTS16	119.6	123.9	120.2	116.2	99.7	98.7	116.5	117.1
ADAMTS17	118.5	112.1	113	118.1	106.8	102.4	109.1	121
ADAMTS18	119.9	114.7	131.1	121	118	102.2	124.3	117.6
ADAMTS18	116.9	114.6	117.9	110.5	106.1	95.8	125.9	114.9
ADAMTS19	136.3	132.5	136.3	140	139.5	115.3	132.7	140.5
ADAMTS2	112.6	112.4	120.7	116.3	99.5	97.9	114.3	104.2
ADAMTS2	112.8	112.6	111.2	118.9	108.3	98	114.1	112.4
ADAMTS20	121.9	115.6	127.2	123.7	116.6	104.2	133.8	128.7
ADAMTS20	115.3	112.8	123.8	116.1	111.5	98	117.8	112.8
ADAMTS3	111.8	110.2	123.4	130.3	118.6	96.5	114.2	104.5
ADAMTS4	119.4	117.1	119.4	124.7	115.3	96.7	110.6	121.4
ADAMTS5	110.6	111.4	104.7	119.3	103.7	92.2	109.2	109.7
ADAMTS6	118.8	125	128.4	122.4	109.8	112.2	125	125.3
ADAMTS7	117.4	121.4	117.9	119.6	113.2	108.7	120.9	114.7
ADAMTS8	116.5	111.8	112.1	110.9	104.9	93.1	115.9	110.6
ADAMTS9	117.4	116.2	113.9	118.6	108	102	114.2	113
ADAMTSL1	121.5	111.6	119.4	129.5	112.7	99.1	119.1	113.8
ADAMTSL1	109.4	114.2	116.6	115.6	107.8	103.1	108.7	119.6
ADAMTSL1	106.5	118.1	107.8	116.1	110.8	100.3	115.1	120.5
ADAMTSL1	117.2	111.1	114.9	114.6	99.5	97.5	123.2	105.7
ADAMTSL2	113.9	126.1	128.5	113.1	110.8	103.5	113.3	121.3
ADAMTSL3	124.5	112.7	128.1	136.2	122.8	110.7	124.9	120.6
ADAMTSL5	119.9	128.9	129.9	129	114.6	103.4	131	131.2
ADAR	920.9	877.9	875.8	809.9	507.7	653.5	672	590.1
ADAR	115.7	108.2	112.6	147.3	110.6	92.1	127.3	118.2
ADAR	451	598	515.9	572.6	406.8	394.6	458.4	439.3
ADARB1	124.3	117.9	120.5	115.5	111.5	106.5	122.9	117.4
ADARB1	179.1	170.5	179.9	171.9	160.1	156.4	166.4	140.2

ADARB1	118	113.2	113	118.7	105.4	91.2	121.7	117.1
ADARB1	402.6	441.5	495	443.4	371.6	354.9	346.7	297.7
ADARB2	114.5	130.4	131.5	123.9	127.3	112.1	117.9	135.1
ADAT1	258.1	343.4	272.3	271.7	221.7	187.7	285.9	244
ADC	113.2	144.9	128.9	130.2	120.3	108.3	129.5	128.8
ADCK1	169.1	166.7	154.4	157.8	136.7	119.8	149.6	137.8
ADCK2	317.1	361.5	364.5	324.5	279.6	255.4	352.4	376.3
ADCK4	127.9	136.7	129.5	130.9	109.9	107.2	133	122.5
ADCK5	152.7	150.4	169.6	150.3	132.5	128.9	154	138.1
ADCY1	116.3	109.3	120.8	134.2	111.6	100.3	123.9	116.1
ADCY2	119.5	110.9	124.9	124.7	103.3	101	134.5	124.3
ADCY3	429.9	464	402.6	411.4	295.3	245.9	364	280.6
ADCY4	139.3	123.1	128.9	116.3	120	111	124	124.7
ADCY5	110.5	104.2	108	114.9	103.5	101.4	120.1	117.5
ADCY6	112.7	115.2	115.9	120.4	115.4	95.4	115.6	115.2
ADCY6	121.1	117	116.5	120.4	106.1	100	124.9	114.6
ADCY7	197.4	205.9	239.4	198.3	183.6	164.6	181.3	167.3
ADCY8	113.9	110.4	116.6	110.2	103.6	105.4	110.4	109.9
ADCY9	134	144.4	144.2	151.2	118.1	123.6	144.4	132.8
ADCYAP1	118.3	109.6	114.8	115.7	107.8	99.1	107.7	126.1
ADCYAP1R	116.2	120.4	123.7	125	111.1	99.4	114.7	118.4
ADD1	127	123.8	137.4	127	118.7	113.1	129.2	119.4
ADD1	1819.3	1971.5	1837.2	1901.1	1596.4	1463.2	1977.4	2106.3
ADD2	113	110	109.2	122.3	107.7	101.5	108.7	119.6
ADD2	120.1	121.2	125.5	110.2	112.1	106.9	128.1	124.1
ADD2	117.2	123.2	128.6	117.4	112.2	105.9	125.2	120.1
ADD3	110.9	108.9	111.3	112.3	106.6	97.3	110.4	111.7
ADD3	725	645.2	640.6	517.2	702.7	356.4	657.1	705.6
ADD3	1052	1033.9	816.2	888.4	895.3	587.8	1067.3	1395.6
ADD3	498.1	593.4	579.6	552.6	612.3	440.5	516.8	517.9
ADFP	682.3	759.9	672.1	799.2	599	861	885.7	986.2
ADH1A	107.4	114.6	111.6	113.6	108.7	95.9	115.5	117.5
ADH1B	114.3	119.2	121.7	114.9	101.4	115.7	117.8	109.4
ADH1C	113.9	112.2	117	126.9	104.7	98.3	118	109.8
ADH4	110.6	111.7	114.1	107.2	103.9	100.8	112	109.7
ADH5	127.6	133.4	125.1	122.9	128.4	126.1	123.8	127.5
ADH6	114.8	116.7	132.1	122.5	114.4	105.9	115.3	120.9
ADH7	120.7	120.2	116.4	121.4	112.6	95.2	121.3	108.9
ADHFE1	185.6	165.8	241.7	152.8	146.9	164.7	148.1	142
ADHFE1	115.8	112.8	131.7	112.3	121.2	108.6	119.1	109.4
ADI1	123.3	117.6	127.6	124.8	120.7	103.5	131.1	124.4
ADIPOQ	110.8	111	108	108	110.8	97.5	121	106.8
ADIPOR1	371.7	314.4	272.6	412.1	238.3	317.7	437.9	409.3
ADIPOR2	414.3	453.4	387.2	433.8	464.3	320.7	547	527
ADK	122.5	116.5	134.6	124.3	123.1	106.3	122.7	127.3
ADK	122.2	111.8	118.8	113.2	106.5	99.3	123.2	121.4
ADK	142.1	140	153.9	156.5	137.5	116	124.8	139.2
ADM	275.6	181.9	225.4	489.5	265.7	597.6	1264.8	1304.6
ADM2	138.7	136.8	138.1	146.7	143.6	130	158.6	144.6
ADMR	117.5	113.1	129.4	121.8	94.3	104.8	119.4	116.2
ADNP	173.3	195.1	172	179.7	209.3	133.2	191.8	177

ADNP	147.2	146.3	144.4	153.9	150.3	109.4	140.4	139.9
ADORA1	121.7	118.5	120.9	118.9	110.5	101.3	124.5	115.9
ADORA1	123.9	116.9	125.5	126.2	117.6	105.7	114.7	128.8
ADORA2A	329.4	356.6	216.4	298.2	241.3	158.9	226.9	194.4
ADORA2B	121.8	130.4	144.3	125.6	116	108.8	126	115.7
ADORA3	105.7	109.7	230.6	109.2	100.9	114.2	110.4	111
ADORA3	132.2	145.3	211.6	157.3	103.5	120.4	138.2	121.6
ADPGK	299.8	326.5	416.6	402.6	245.5	316	294.8	278.1
ADPN	109.1	109.7	111.3	117.3	106.3	95.7	109.5	111
ADPRH	148.1	131.4	143.1	147.5	128	137.4	135.2	129.4
ADPRHL1	117	121	114.7	115.7	109.8	102.6	117.5	109.3
ADPRHL1	116.8	117.2	116.9	126.2	115.5	97.6	114.8	129.5
ADPRHL1	111.6	122.2	127.9	124.7	111.2	97.7	120.6	115.9
ADPRHL2	243.4	236.7	225.9	226.4	187.9	178.8	251.2	251.6
ADRA1A	98.3	108.8	113.8	125.6	107.1	97.5	112.5	103.6
ADRA1A	103.7	113.8	109	112.8	104.1	95.9	107.9	111.9
ADRA1A	112.7	118.2	127.9	123.2	108.5	102.5	120.5	120.4
ADRA1A	119.9	124	131.6	126.3	122.6	107.2	127.7	124.7
ADRA1A	118.4	119.3	118.9	119.9	109.7	104.7	123.4	118.6
ADRA1B	116.1	126	117.7	128.4	123.2	102.6	118.5	119.3
ADRA1D	127.1	124	124.3	119.4	120.7	104.7	124.7	113.7
ADRA2A	122	115.4	103.9	121.2	110.8	106	127.9	125.3
ADRA2B	119.8	118.1	131.3	120.6	120.9	110.6	132.6	126.2
ADRA2C	128.6	123.3	128.9	126.1	111.8	101.3	121.4	127.5
ADRB1	111.4	113.9	126.6	124.2	108.1	101.5	117.7	114.4
ADRB2	369.8	372	311.2	395	341.1	219.1	353.3	282.8
ADRB3	116.7	117	117.9	129.8	114.4	101.9	127	115.2
ADRBK1	107.2	102.2	118.2	110.1	111.2	95	115.5	108.5
ADRBK2	122.6	110	118.8	115.7	114.7	99	134.3	117
ADRM1	231.2	223.9	196.6	224.4	167.3	186.1	321.6	295.1
ADRM1	268.4	308.7	263.2	363.3	275.5	285.6	493.1	449.7
ADSL	905.3	1233.3	926.5	904.3	708.5	534.5	724.8	638.4
ADSS	893.6	1007.4	963.6	1168.2	757.3	990.7	1150.7	992.5
ADSSL1	113.2	125.9	115.9	122.3	112.4	99.4	122.6	119.5
ADSSL1	117.9	117.4	136.7	133	118.2	107.8	120	129.7
ADSSL1	117.9	116.9	121.7	130	115.4	104.7	132.9	118.3
AEBP1	170.4	153.6	144.7	169.2	117.9	120.6	161.1	153.5
AEBP2	140.5	152.6	150.6	143.1	137	124.4	158.2	164.2
AES	2289.4	2323.8	1425.1	2282.3	1507.4	1546.4	2865.1	2351.3
AES	654.9	710	479.6	699	459.3	562.3	671.2	476.5
AES	116.7	111.9	120.3	138.2	116.7	105	120.4	115.2
AFAP	110.7	113.4	117.6	117.8	107	86.9	117.2	108.2
AFAP	125.3	120.9	132	137.1	114.9	118.9	141.7	126.4
AFAP	123.3	124.7	125.1	116.5	122.3	105.5	120.1	108.1
AFAP1L2	126.9	128.4	127.3	134.6	117.9	107.9	130.1	121
AFAR3	124.8	124.2	123.4	122.9	123.4	106.4	126.8	125.5
AFARP1	124	122.2	124.8	118.5	110.4	108.1	118.6	120
AFARP1	118.1	117.9	115.2	117.3	108.3	96.3	117.5	112.1
AFF1	139	135.9	118.9	135.9	133.3	111.9	134.3	129.8
AFF2	119.3	115.2	110.9	121.1	100.2	99.6	114.2	121.7
AFF3	163.9	166.6	157.9	175.6	133.9	124.7	156.2	128.6

AFF3	112.9	114	116.4	128.7	109.5	102.1	109.7	108
AFF4	166.4	170.1	212.3	190.5	174.8	165.4	224	199.7
AFG3L1	306.3	324.2	268.7	284.5	265.3	266.5	326.2	320.9
AFG3L1	125.2	122	136.2	135.6	123.7	106.5	132.1	134.6
AFG3L1	116.1	106.6	120.6	120.5	109.6	96.9	118	132.7
AFG3L1	116.3	119.5	121.2	127.8	101.5	103.2	116	109.6
AFG3L2	284	347.1	251.1	401	289.1	244.4	345.7	326.1
AFM	109.5	107.7	108.3	105.9	101.8	103.8	113.3	110.3
AFMID	124.5	131.5	141.8	129.4	115.2	108.8	128	128.4
AFP	119.4	117.9	117.9	117	116.6	101.2	122.4	118.4
AFTIPHILIN	161	158.3	140.7	169.6	147	132.6	164.9	165.4
AFTIPHILIN	144.2	145.1	135.5	142.4	127	109.7	150.3	142.1
AFTPH	206.7	214.4	199.8	252.5	184.3	202.7	258.5	288.4
AGA	196.7	193	248.5	222.1	146.9	164.8	169.9	149
AGBL1	106.1	110.4	105.4	113.8	103.1	93.9	111	118.8
AGBL2	113.8	116.6	112.8	117.1	108.9	96.6	112.3	116.8
AGBL4	115.5	111.7	106.7	113.5	111.9	93.8	119.4	107.4
AGC1	113.5	112.6	117.9	116.2	116.8	101.2	118.3	118.2
AGC1	120.6	118.3	110.9	116.7	108.7	102.2	113.3	114.4
AGER	116.3	122.2	133.4	127	117	107.6	130.9	136.8
AGER	121.9	130.2	128.6	122.5	116.2	108.9	143.7	124
AGGF1	327.2	345.7	409.3	301.9	253.3	294.9	261.3	322.1
AGL	112.8	120.4	120.5	130	105.2	102.2	116.7	119.6
AGL	109.4	120.1	117.7	115.4	103.1	104.6	117.1	110.6
AGL	354.9	413	386.8	378.9	314.5	243.6	324.5	302.3
AGL	117.5	111.9	119.4	127.8	112.8	101.4	109.4	116.2
AGL	121.7	119.9	121.1	124.3	108.3	95.1	120.1	113.4
AGL	154.2	162.6	145.3	144.4	134.5	114	131.2	132.3
AGMAT	158.8	162.4	208.9	157.8	149.5	137.6	139.4	130.1
AGPAT1	116.1	116	108.4	116.1	108.4	96.5	114.9	115.7
AGPAT1	113.6	114	112.2	114.9	101.6	102.6	116.2	119.5
AGPAT1	147.5	153.9	150	151.1	126.5	136.5	166.2	149.6
AGPAT2	129.1	129.3	146.7	136.1	119.7	112.9	143.7	119
AGPAT2	123.9	118.3	142	142.3	119.5	110.6	132.9	119.6
AGPAT2	154.9	139.3	142.3	135.3	117.1	110	123.7	141.8
AGPAT3	119	109.3	131.3	114.6	110.2	100.2	128.3	117
AGPAT3	142	129.1	137.7	131.6	116	116.6	137.2	119.8
AGPAT4	206	211.2	218.2	211	178.2	150.3	174.4	166.3
AGPAT5	170.8	213.1	163	223.4	163.3	139	192.8	181.2
AGPAT6	126.1	116.8	119.7	113	116.6	104.1	129.2	124.8
AGPAT7	144.4	152.5	129.3	147.6	123.7	118.3	135.2	130.5
AGPS	212.6	212.2	230.2	210.4	192.9	179	205.4	195.9
AGR2	116.2	116.3	117.7	127	119.2	111.1	120.5	120
AGR3	115.9	116	122.8	117.7	103.5	100.8	124	119.2
AGRN	113.1	116.3	112.6	126.3	117.5	102.3	119.5	121.7
AGRP	110.7	113.5	123.7	126.4	108.5	104.8	138.1	114
AGRP	118.2	114.2	115	117.1	100.5	105.6	112	114.8
AGT	112.8	113.3	111.8	110.7	106.3	95.5	106.7	117
AGTPBP1	129.5	128.5	147.7	128.9	134	109	143.3	133.3
AGTR1	110.4	116.3	107.3	130.1	103	103.8	108.2	106
AGTR1	108.3	120.8	106	114.2	101.2	91.9	102.9	105.7

AGTR2	127.3	124.2	126.6	133.5	116.8	114.8	127.2	134.7
AGTRAP	129.3	122.7	151.5	129	124.9	116.1	129.3	134.6
AGTRAP	134.4	119	133.7	123.3	113.4	99.3	121.1	114.4
AGTRL1	114	114.7	122.6	118.6	110.5	103.2	123.7	117.9
AGXT	119.3	128.8	118.1	125.8	111.9	103.7	115.2	119.4
AGXT2	106	117.7	111.1	117.4	103.6	93.6	117.9	110.4
AGXT2L1	118.7	112.1	114.5	115.4	117	96.6	113.9	122.5
AHCTF1	223.8	245.9	267.5	261	240.3	198.6	247.6	275.2
AHCTF1	300.2	314.3	256	274.3	238.7	196.8	288.9	352
AHCY	681.6	659.1	477.5	707	445.3	326.2	798	539.2
AHCYL1	445.7	602.7	501.1	641.8	505.1	415.1	490.3	403.5
AHDC1	131	145.3	127.1	127.7	124.1	102.1	154.1	143.6
AHI1	142.8	132.9	132.5	162.8	139.6	119.8	176.5	139
AHNAK	828	516	531.2	672	667.1	565.3	591.2	438
AHNAK	134.7	114.7	131.6	133.1	115.9	109.7	135.8	119.9
AHNAK	115.6	115.3	127.1	121.6	116.5	101.5	117.6	127.9
AHR	124.6	119.9	139.1	136.2	118.2	111.3	124.9	122.5
AHRR	117.1	127.4	127.1	130.8	114.1	109.7	125.9	122.6
AHSA1	281.1	298.2	258	347.1	251.1	302.7	491.4	816.7
AHSA2	169.9	152.3	192.3	149.5	154.2	150.8	167	154.4
AHSG	116	116.9	124.3	110.8	110.1	92.5	123.9	110.6
AICDA	109.4	114.6	109.1	114.3	108.5	97.2	113	110.1
AIF1	265	125.3	1332	234.7	115.6	654.9	161.8	105.5
AIF1	664.9	402.7	3403.1	532.8	361.2	1903.5	389.9	322.3
AIF1	109.4	115.2	113.3	115.4	106.9	98.2	116.9	125.7
AIFL	120.2	124.6	124	128.7	124.6	111.4	134	128
AIFM1	257.8	319.7	286.5	311.7	253.7	227.2	268.7	275.8
AIG1	124.1	117.9	123.5	128.1	122	102.4	126.9	117.5
AIM1	140.8	139.2	150.9	148.9	130	124.3	140.2	145.1
AIM1L	112.2	117.2	116.5	113.8	110.2	103.9	122.3	113.4
AIM2	117.4	121.2	128.1	113.5	106.2	100.5	118.2	120.9
AIP	1018.1	1127.4	826.6	1001.2	745.9	688.8	863.7	915.6
AIPL1	118.7	117.7	115.4	117.8	124.9	95.1	121.1	121.5
AIPL1	114.3	105.1	117.8	111.1	108.2	102.7	116	110.3
AIRE	115	109.3	122.8	117.8	94.8	97.1	121	119.8
AIRE	3911.9	3661.8	3742	3608.1	4094.6	2060.9	3994.5	3544.3
AJAP1	121.5	113.5	117.4	117.9	106.7	94.5	121.6	114.3
AJAP1	114.4	112	122.9	128.4	106.3	103.4	117.6	114.9
AJAP1	125.6	131.3	120.8	131.7	114.1	109.9	121.1	114.3
AK1	278.9	266.5	382.7	243.6	247.8	215	203.5	229.3
AK2	149.4	140.5	144.8	150.2	121.7	121.4	140.1	126
AK2	134.3	163.7	165.6	167	140.2	124.3	147.5	139.8
AK2	334.5	310.6	308.8	304.6	248.8	222.4	258.6	209
AK3	401.4	589.9	387.6	435.2	485.6	318.7	337.8	361.6
AK3L1	127.8	146.9	121	141.1	127.9	100.2	118.4	121.9
AK3L1	106.2	115	114.4	107.5	110.8	101.8	115.7	113.1
AK5	116.6	120.9	120.1	121.3	114.8	99.1	113.8	114.3
AK5	121.6	115.6	115.5	120	101.5	94.3	118.7	123.1
AK5	133	160.9	122.3	150.6	128.2	105.5	130.8	135.4
AK7	114.1	107.7	114.5	116.7	113.4	105.8	117.5	108.7
AKAP1	144.6	132.6	129.3	143.2	137.3	113.8	138	145.4

AKAP10	127.5	129.1	142.1	149	131.5	116.3	140.5	139.6
AKAP11	105.6	116.9	111.4	109.6	102.4	96.6	111.9	108.8
AKAP11	330.6	334.9	313.5	393.7	303	284.9	282.2	315.3
AKAP11	131.9	130.7	164.5	144	135	110.2	125.5	113.8
AKAP12	119	113.9	115.8	121.4	106.7	99.2	111.2	116.1
AKAP12	127.7	119.9	113.9	133.2	110.3	114.7	115.9	123
AKAP12	115	124.4	119.9	118.9	112.1	104.1	127.3	115.8
AKAP13	214.6	208.8	240.4	258.2	201.4	170.8	249.6	218
AKAP13	1098	1191.8	1019.8	1156.4	910.5	727	1286.2	1171
AKAP14	101.7	109.9	104.5	114.8	102.9	94.8	120.3	113.2
AKAP14	105.7	109.7	114.3	118.3	111.8	90.7	114.3	116.8
AKAP14	127.3	109.6	116.8	110.8	101	105.1	116	116
AKAP2	112.7	112.7	113.8	123.6	118	94.7	121.4	121.2
AKAP3	120.5	122.9	123.1	132.5	116.7	102.3	122.9	128.4
AKAP4	109.3	105.3	117.3	111.4	108.3	101.4	116.5	106
AKAP4	111	113	116.1	112.2	109	96.5	120.9	115.6
AKAP4	107.4	121.6	139.2	133.7	121	102	116	122.6
AKAP5	102.2	118.3	120.7	116.1	106.8	98.9	115.6	102.6
AKAP6	105.2	110.1	109.4	123.7	103.9	88.7	114.5	101.3
AKAP7	109.5	97.2	109.7	104.7	97.2	96.3	113.3	104.1
AKAP7	150.5	130.5	131.5	141.9	134	108.1	137	145.7
AKAP7	323.4	392.1	490.3	320.5	279.2	313.3	214.4	197.1
AKAP7	106.8	119.2	113.2	124.3	115	100	124.6	116.2
AKAP8	409.8	422.8	361.8	381	346.9	309.3	452.9	472.5
AKAP8L	191.1	202	179.8	219.9	209.2	165.7	265	245.4
AKAP9	111.4	107.9	117	122.8	107.7	98.2	119	127.5
AKAP9	118.8	118.1	122.7	121	113	100.1	125	120.3
AKNA	903.1	1203.6	669.2	966.5	749.2	738.3	908.4	803.3
AKR1A1	535	520	1024.8	581.3	360.4	580.9	405	327.7
AKR1A1	109.9	114.1	117	112.9	107	93.9	113	102.9
AKR1A1	450.5	380.2	793.7	454.7	314.3	441.1	425.6	256.1
AKR1B1	1188.6	1889.2	664.1	1225.5	1145.8	466.5	1222.9	1008.9
AKR1B10	113.5	112.8	105.6	112	106	97.5	113	117.7
AKR1C1	107.6	102.2	108.6	114.8	99.1	96.9	117.1	112.7
AKR1C2	115.7	112.9	113.6	122.5	109.5	133.8	132.4	115.6
AKR1C3	234.7	277.2	225.9	374.3	266.9	507	372.5	494.6
AKR1C4	117.3	121.6	118.3	114.5	106.6	105.2	121	122.1
AKR1CL1	112.2	123.5	116.9	134.4	112.1	109.4	122	125.9
AKR1CL2	106.5	119.4	118.7	115.2	100.6	98	117.3	113.9
AKR1D1	4138.9	4113.6	4711.6	5696.9	3811	1753.1	3490.9	3944.4
AKR7A2	459.5	579.4	609.7	453.9	365.6	461.3	280.4	254.5
AKR7A3	158.7	181.5	181.2	195.4	137.2	149	148.5	137.3
AKT1	729.7	694.5	737.7	825	582.1	721.8	865.1	663.3
AKT1	571.2	553.3	501.8	572.2	402.9	545	657.4	437.4
AKT1	119.4	131.6	137.3	127.6	125	112.8	119.7	119.8
AKT1S1	108.8	114.8	112.3	114.9	100.3	102.8	126.1	118.1
AKT2	113.4	117.9	119.7	117.3	118.1	104.2	116.1	126.2
AKT3	108	116.9	126	120.2	109.3	103.5	118.8	124.7
AKT3	123	126.1	128.5	143.6	121.6	109.7	125	125.4
AKT3	112.8	132.3	127.3	129	115.4	114	132.3	124.3
AKTIP	620.9	897.7	700	762.6	846.9	501.3	632	756.5

ALAD	120.7	135.4	123.2	145.5	121.1	111.9	121.3	130.8
ALAD	219	224.3	240.8	224	185.3	173	160.6	158.3
ALAD	115.9	110	122.3	120.3	104.5	94.9	122.1	121.9
ALAS1	127.2	116.1	117.9	124.9	107.7	99.6	124.6	114.5
ALAS1	125.7	135.4	133.7	130.6	117.5	105.3	133	122.3
ALAS1	126	116.5	131.4	122.6	115.7	113.6	131.4	124.7
ALAS2	113.8	112	131.1	120.9	111.9	102.6	120.8	119.1
ALAS2	113.4	115.8	138	118.3	105.1	116.7	124.7	107.8
ALAS2	116.1	124.4	127	119.3	107.3	99	126.8	122.1
ALB	109.2	108.6	112.1	103.3	99.9	87.4	111.5	111.6
ALCAM	158.4	143.9	209.1	165	136.8	204.5	163.7	142.2
ALDH16A1	194.6	191.4	202.3	184.8	140.3	148	175.5	151.1
ALDH18A1	147.4	166	141	165.2	126.4	114.3	158.7	165.2
ALDH1A1	234.8	200.9	132.1	236.8	137.1	124.7	161.8	133.4
ALDH1A2	137.6	138.3	124.3	146.2	136.9	117.3	150.7	122.4
ALDH1A2	113.1	123.2	120.5	122.6	112.3	113.7	129.6	115.5
ALDH1A3	112.5	118.1	123.5	113.6	112.8	99	119.7	114.5
ALDH1B1	119.5	126.4	118.5	132.3	109.7	105.1	117.7	112.9
ALDH1L1	123.7	111.3	124.8	123.7	109.6	107.4	119.3	111.9
ALDH1L2	123.8	118.1	132.6	124.4	114.6	110.4	123.2	125
ALDH2	141.3	125.5	301.5	132.8	107	130.8	130.3	119.7
ALDH3A1	107.2	116	114.3	111.8	112.6	86.2	125.7	120.7
ALDH3A2	124.6	120.4	119.3	117.1	105.8	105.3	122.3	113
ALDH3A2	237.7	169.6	217	239.2	161.5	189.9	193.4	138.1
ALDH3B1	115.6	119.3	118.7	112.5	117.7	106.5	119.4	116.4
ALDH3B1	118.8	123.3	150.4	121.3	109.7	108.4	116.8	117.1
ALDH3B2	124.5	117.8	122.6	126.6	118.2	99	122.1	108.9
ALDH3B2	109.2	118.7	120.5	116.4	109.3	98.8	120	123.4
ALDH3B2	113.6	116.2	109.1	117.6	111.6	98.9	107.8	112.6
ALDH4A1	121.2	110.8	119.6	116.5	109.1	99	116.9	110.3
ALDH4A1	116.2	130.7	134.3	127.1	116.8	95.5	127.7	127.9
ALDH4A1	112.3	127	113.9	122.6	113.7	100.2	118.9	125.9
ALDH5A1	184.2	182.9	235.3	178.4	160.3	149.9	138.3	143
ALDH6A1	135.1	132.4	124	124.9	111.4	100.2	120.3	127.1
ALDH7A1	127.6	138.3	128.9	129.2	118	99.1	129.7	126.9
ALDH8A1	113.4	116.3	109.7	109.8	112.7	101.3	119.7	133.6
ALDH8A1	109.1	119.7	123.6	116.3	110.6	109.4	117.1	117
ALDH9A1	2135.2	2438.6	2544.1	2276.3	1787.9	1707.5	1865.4	1448.6
ALDOA	116.8	119.9	126.6	133.4	120.3	103.2	127.1	120.9
ALDOA	1121.2	994.7	1300.9	1642.4	1054.4	1311.1	1040	1057.5
ALDOA	124.1	126.5	125.4	128.4	117.7	111.9	125.3	127.4
ALDOA	1077.6	1050.7	996.2	1100.3	777.2	1096.2	1193.8	961.3
ALDOB	125.3	123	122.6	131.1	128.4	103.4	137.6	120.7
ALDOC	395.5	475.9	503	424.3	339.5	302.2	375.6	370.7
ALF	114.8	123	115.8	111.1	102.6	100.2	116.1	121.6
ALF	111.3	113.9	107.6	113.5	105	91.8	111.4	114.4
ALG1	140.3	141.9	143.6	131.4	129.9	112.2	123	127.8
ALG10	114.6	117.4	120.2	126.5	110.4	102	123.7	123.3
ALG11	125	109.7	122.1	130.8	113.7	92.6	115.8	119.9
ALG12	123.3	115.9	122.5	139.6	116.5	118.8	118.6	125.2
ALG13	145.1	150.1	159.3	161.8	122.7	120.3	138.1	133.7

ALG14	118.2	118.8	123.2	122.8	114.5	103.7	111.1	129
ALG2	113.9	122.7	113.5	119.7	106.1	100.9	116.5	109.4
ALG3	260.7	247	220.2	280.3	213.2	159.7	230.8	203.4
ALG5	280.6	317.9	333.3	312.2	246.1	250	333	268
ALG6	331.2	324.6	300	289.7	266.7	227.8	259.9	239.9
ALG8	241.1	229.8	235	225.2	188.2	196.1	181.2	207.6
ALG8	115.8	116.7	117.8	117.5	101	93.2	119.5	117.4
ALG8	312.6	305.8	414.7	361	289.6	251.2	306.8	283.2
ALG9	192.2	174.1	221.6	202.9	160.4	182.5	172.5	170.6
ALK	121.4	116	123.8	124.9	119.6	97.6	116.9	116.8
ALKBH	189.9	193.6	186.7	197.8	192.9	184.5	181.4	174.1
ALKBH1	119.1	110.1	125.2	123.4	112.2	99.9	121.9	116.4
ALKBH1	219.7	220.8	211.2	216.6	202.5	184.1	189.1	210.1
ALKBH2	154.6	186.7	166.6	175.3	165	124.7	152.2	142.5
ALKBH3	214.1	245.3	262.2	213.2	204.3	206.3	179.6	188
ALKBH4	132.6	144.6	131.2	142.4	132.1	108.1	130.9	137.4
ALKBH5	2346	2782.1	1950.2	2948.9	2282.7	1773.3	2611.7	2465.9
ALKBH6	213.8	214.9	261.2	237.2	208.7	205.5	213.7	231.3
ALKBH7	166.6	203.3	203.7	193.9	199.1	164.1	221.2	229.2
ALKBH8	190.3	183.7	177.3	182.9	187.8	131.2	170	178
ALLC	112.4	107.7	121.5	116.1	109	98.8	113.5	122.9
ALLC	112.5	121.3	125.8	112.1	106.4	94.6	111.4	118.3
ALLC	112.2	125.6	126.6	115.8	106.6	95.3	117.8	119.9
ALMS1	156.8	162.6	136.5	174.4	151.9	133.7	157.3	141.9
ALOX12	118.3	115.6	116.5	121.8	106.8	101.8	117.1	122.6
ALOX12B	117.7	116.9	115.3	127	111.1	95.4	127.1	108
ALOX12P2	110.8	120.2	123.5	123.6	110.1	95.9	114.3	116.6
ALOX15	125.1	121.4	124.9	107.6	125.2	102.3	129.1	127.9
ALOX15	106.6	113.8	136.7	128	110.2	111.1	128.1	113.2
ALOX15B	113.8	118.7	120.6	119.3	114.6	102.6	114.9	119.5
ALOX15B	133.9	120.1	121	122.6	109.3	104.4	117.8	112.9
ALOX15B	119.2	120.1	134	131.8	114.5	107.1	117.3	120.2
ALOX5	506.1	369.5	1656.3	424	223.1	459.2	199.3	169.4
ALOX5	123.1	116.5	112.2	118.3	122.2	104.4	118.8	130.8
ALOX5AP	1394.9	1956.2	1582.4	1305.6	1290.7	915.3	636.2	840.5
ALOXE3	111.4	118.1	123.3	123.9	114	102.9	120.4	121.9
ALPI	121.7	126.1	122.6	120.3	106.2	99.6	125.2	119.6
ALPK1	142.2	142	172.7	163.7	129.3	133.7	149.4	132.3
ALPK3	113	104.8	107.2	116.3	107.5	100.4	118.8	111.7
ALPL	115.5	112.1	117.8	127.5	107	104.8	126.7	115.1
ALPP	1633.4	1136.7	1581.2	1194.7	2186.9	906.6	1855	1642.2
ALPPL2	121.7	121.6	118.2	124	110.4	109.7	124.1	118.5
ALS2	188	219.9	185.1	198	191.4	163.4	181.4	231.8
ALS2CL	112.2	113.5	112.6	114	115.7	102.7	115	110.2
ALS2CL	119.2	123.3	133.7	124	104.5	107.9	120.7	103.3
ALS2CL	111.7	106	122.1	121.6	101.3	97.9	122.4	117.4
ALS2CR11	108.5	116.5	107.8	110.1	105.6	101.9	119.4	112.8
ALS2CR12	114.1	114.7	129.8	117.5	114.3	102.3	127	112.5
ALS2CR13	1110.9	1395.8	743.8	1287.2	1020.6	496.3	1306.6	1473.9
ALS2CR15	117.6	118.8	121.2	128	108.1	101.9	131.5	114
ALS2CR16	118.3	122.7	131.2	125	115	98.4	127.8	123.9

ALS2CR19	107.5	111.4	119.7	127.2	106.2	98.4	115.9	106.7
ALS2CR2	174.7	175.8	196	176.2	161.4	164.4	173.2	168.9
ALS2CR4	134	161.8	134	153.7	136.8	110.9	128.5	131.9
ALS2CR7	110.1	115.9	127.7	115.9	104.7	99	123.6	117.7
ALS2CR8	110	117.9	120.3	125.6	120.6	105.6	118.9	115
ALX3	110	113.2	119.3	118.1	110.6	96.3	112	115.8
ALX4	113.1	118.4	120.6	127.2	122.3	102.2	116.1	115.4
AMAC1	121.8	115.2	133.3	125.9	110.2	101.5	135.2	124.1
AMAC1L2	115.7	110.8	118.8	125.7	115.1	94.4	120.8	114
AMACR	111.6	127.3	124.1	130	119.7	108.7	129.2	131.5
AMACR	122.6	142.6	131.4	142.9	130.9	98.3	119.2	130.2
AMBN	114.8	120.1	113.8	119.4	112.3	106	118.7	108.2
AMBN	108.6	119.7	115.2	118.6	109.8	93	114.7	108.9
AMBP	121.6	127.9	122.2	122.5	112.3	102.2	131.5	119.3
AMD1	804.6	820.3	835.7	923	749.6	544.4	872.1	1129.8
AMD1	291.9	286.8	361.9	337.7	269.5	202.6	333.5	337
AMDHD1	116.1	115.2	130.1	119.9	117.5	112.5	114.1	111.8
AMDHD2	161.4	169.3	146.2	172.1	135.1	122.4	126.6	142.5
AMELX	108.9	111.3	106.8	114.3	105.1	100.3	114.1	111.2
AMELY	124.8	119.3	127.7	122.5	109.2	99.4	116.5	127.5
AMFR	311.9	410.1	238.5	370.3	381.9	294.8	496.1	510.3
AMFR	122	126.7	124.6	143.3	120.2	108	135	135.8
AMH	106.7	116	121.4	117.7	105.5	100.3	113.7	114.7
AMHR2	120.7	112.9	123.7	119.8	112.8	105.3	112.9	110.7
AMICA1	603.3	918.5	874.1	654	578.7	499.7	519.6	437.1
AMID	113.5	124.4	113.9	133.2	132.7	112.4	130.2	138.9
AMIGO1	123.8	116.9	127.2	127.7	111.8	103.6	120.6	120.4
AMIGO2	118.9	128.9	130.8	123.8	118.4	108.7	126.1	130.3
AMIGO3	113.7	123.6	119.2	118.6	105	95.7	122.7	119.9
AMMECR1	111.9	113.1	121.5	120.8	115.3	95.6	120	119.4
AMMECR1	141.6	152.7	152.4	155.4	154.8	134.2	153	144.6
AMMECR1	138.1	129.7	145.2	137.4	136.4	119.5	130.5	127.2
AMN	112.8	112.4	115.2	115.8	112.2	95.9	112.8	117.9
AMOT	123.5	133.6	130.2	144.8	116.4	122.3	150.6	141.5
AMOTL2	135.5	117.3	123.7	130.3	108.7	117.3	128	124.7
AMPD1	111.9	113.1	111	119.8	104.1	104.1	112.3	119.2
AMPD2	113.5	117.7	118.4	119.8	113.7	112.3	116.7	118.9
AMPD2	117	123	131.2	129	108.4	108.9	122.5	119.7
AMPD2	121.8	118.3	120.2	128.3	113.8	104.8	125	114.3
AMPD3	126.7	136.7	136.4	132.9	120.9	104.5	123.9	128.2
AMPD3	116.6	116.7	123	117.4	108.4	100.2	125	122.2
AMPD3	117.2	116	121.3	127.7	106.2	101.2	119.5	119
AMPD3	119.6	116.9	114.8	118.8	105.4	96.6	121.5	116.2
AMPH	124.4	128.1	125.8	128.7	116.9	108	124.3	116.5
AMPH	127.3	102.5	125.5	116.4	109.5	104.4	122.2	127.4
AMT	194.5	166	202.7	191.2	138.7	144.9	158.4	163.5
AMY1A	109.1	120.9	110.2	115	113.9	94.1	123.8	110.7
AMY1A	186.9	163.9	230.7	194.3	160	142	150.6	170.2
AMY1A	111.4	117.9	104.9	118.4	102.9	95.7	111.2	108.5
AMY1B	118	120.6	147.4	116.5	116.2	119.3	118	122.1
AMY2A	113.1	117	132.5	115.1	101.6	98.5	122.1	118.2

AMY2B	154.7	123	194.8	141	140.2	160.7	157.7	141.1
AMZ1	120.4	114.3	124.6	122.9	110	98.6	124.9	110
AMZ2	120.9	121.3	120.7	122.5	108.2	102.3	115.2	124.4
AMZ2	804.9	909.3	942.6	990.5	853.6	769.4	808.3	1157.6
ANAPC1	406.9	445	327.5	469.1	403.6	276	419.5	420.2
ANAPC10	218.9	238.2	273.6	237.3	210.8	218.4	175.4	227.3
ANAPC11	229.4	236.6	291.3	243.8	196.2	217.7	240.9	208.7
ANAPC11	118.5	110.4	113.1	119.9	107.5	100.4	116.6	122.9
ANAPC11	205.2	204.9	268.5	224	203.1	181.3	189.8	212
ANAPC13	833.1	718.2	909.9	825.6	492.3	576	667.3	578.2
ANAPC2	114.7	111.1	111.1	112.5	104.8	98.5	109.9	105.4
ANAPC2	127.1	129.4	117.4	138.4	111.7	116.8	124.1	109.2
ANAPC4	276.7	360.7	379.1	354.4	321.3	255.7	278.7	276.1
ANAPC5	761	745.4	749.2	745.1	613	561.7	514.6	587.5
ANAPC7	131.8	133.4	145.9	125.7	126.2	122.2	129	140.4
ANG	123.2	128.6	203.5	134.4	116.8	162.8	134.2	121.8
ANGEL1	178.5	168.5	142.5	181.4	169.9	145.5	183.9	159.1
ANGEL2	696.6	666.6	660	659.5	618.2	595.7	800.7	946.8
ANGPT1	123.8	119.1	135.4	124.9	116.7	108.1	120	119.1
ANGPT2	116.4	119.3	114.5	124.6	110.5	101.3	118.2	114.5
ANGPT4	109	123.4	116.7	109.3	107.7	100.4	114.4	106.8
ANGPTL1	121.1	116.5	116	119.4	111	98.8	114.7	112.4
ANGPTL2	118.6	116.1	122.9	121.2	118.5	107.4	119.9	121.6
ANGPTL3	112	115.7	112.5	113.7	111.1	90	107.1	111.3
ANGPTL4	127.8	114.6	134.2	129.4	109.1	98.6	121.3	118
ANGPTL4	135.7	115.6	141.4	123.7	118.7	105.7	124.6	115.9
ANGPTL4	120.1	126.1	129.4	118.8	121.6	109.8	118.9	129
ANGPTL5	110.8	110.3	109	106.8	95.1	91.2	117.6	101.6
ANGPTL6	126.5	123.9	130.1	125.2	114	107.8	114.9	114
ANGPTL7	112.8	116.3	111.6	116.2	106.5	97.6	116.4	118.8
ANK1	118.9	124.6	114.2	126.4	110.1	108.3	129.8	113.1
ANK1	130.9	117.7	141.1	131.7	127.1	107.8	129.7	122
ANK2	109.7	112.9	116.2	115.2	105.3	101.2	110.4	106.6
ANK2	117.9	112.9	120.8	112.2	109.3	103.1	118.2	111.2
ANK3	121.6	141.3	129.3	133.2	114.2	108.8	143.4	135.1
ANK3	141.8	137.1	157.3	155.1	138.8	104.5	138	142.1
ANK3	124.5	108.8	128	123.5	115.3	102.2	130	127
ANKAR	124.8	122.3	119.7	122.6	121.7	111.7	121.3	123.8
ANKDD1A	184.7	157.1	174.7	215.8	195.1	133.9	238.1	199.1
ANKFN1	120.7	117.2	115.7	114.6	103.5	100.8	113.5	124.9
ANKFY1	228	225	226.1	227.2	173.3	177.3	230.1	193.4
ANKFY1	162.8	157.4	146.2	202	156.3	114.6	161.4	155.3
ANKFY1	121.1	118.5	119.1	125.1	122.2	96.8	134.8	119.5
ANKH	115.6	124.7	145	129	113.6	119.3	133.7	126.2
ANKHD1	126.8	116.1	119.6	128.4	117	104.8	120.1	118
ANKHD1	117.8	113.5	115.3	117.5	107	94.4	118.3	115.8
ANKK1	111.5	111.4	126.2	116.7	101.4	106.4	119.5	120
ANKMY1	249.3	330.3	270.7	237.8	227.8	189.8	187.5	207.9
ANKMY1	121.7	115.6	119.2	125.8	111.9	105.5	125.1	119.5
ANKMY1	109.8	110.8	112.3	113.4	105.5	102.1	111.7	122.5
ANKMY2	163.2	158.2	172.5	176.3	156.5	141.3	153.3	170.1

ANKRA2	495.1	507.6	655.8	538	478.3	418.1	381.9	508.2
ANKRD1	125.4	123.3	118.9	119.3	113.2	96.4	117.9	111.1
ANKRD10	232.8	266.9	238.6	245.3	260.5	163.3	225.4	248.9
ANKRD11	709.1	673.5	433.4	660.8	566.6	378.7	655.7	807.5
ANKRD12	617.9	423	503.9	582.3	516.5	472.6	491.7	630.5
ANKRD13	670.5	587	666.9	567.4	492.1	428.8	486.5	482.9
ANKRD13B	117.5	127.9	124.3	120.2	105.6	101.6	123.8	118.5
ANKRD13C	176.2	193.4	169.8	191.9	203.4	135.2	189.9	172.1
ANKRD13D	100.9	119	116.6	116.5	110.4	93.9	116.5	113.6
ANKRD15	114.4	117.8	116	119	112.6	97.3	115.9	117.9
ANKRD15	114.6	123.5	114.7	111.2	111.8	96.4	119.1	125.9
ANKRD15	250.1	212.5	197.4	269	173.4	208.8	208.2	166.3
ANKRD16	121.3	138.4	129.6	139.3	120.7	113.7	136.8	123.1
ANKRD16	120.9	135.8	128.4	136.8	116.6	103.2	124.5	125.4
ANKRD16	132.3	128.9	128.3	133.3	120.2	105.2	119.5	124
ANKRD17	133.1	133.4	127.3	137	124.6	105	128.7	133.4
ANKRD17	197.1	175.9	160.2	223.3	229.7	168.6	200.8	207.6
ANKRD17	250.6	250.8	257.9	327.6	246.8	225	257.8	316.7
ANKRD19	117.4	139.9	136.9	128.2	125.9	105.4	125.9	117.1
ANKRD2	118.6	111.3	116.6	119.6	110.7	105	117.2	108.3
ANKRD20A	136.3	181	141.5	160	182	107.9	141.6	146.5
ANKRD20A	109.8	117.2	108.7	108.7	103	103.3	118.7	109.9
ANKRD20A	112.6	137	120.5	117.6	114.3	106	115.5	126.8
ANKRD20B	111	112.4	121.2	111.2	107	94.1	113.3	110
ANKRD20B	118.6	118.6	118	113.3	101.9	97.1	111.9	120.5
ANKRD21	124.1	112.4	118.7	117.7	106	104	110.4	108.8
ANKRD22	149.5	117.3	183.9	126.4	108	102.3	115.1	112.8
ANKRD23	121.2	127.8	123.7	130.8	120.4	118.4	128.6	132.5
ANKRD24	105.8	121.3	118.8	114.5	98.9	91.9	120.8	113.9
ANKRD25	112.8	111.7	106.5	116.5	105.4	101.7	110.6	110.3
ANKRD26	141.1	154.8	137	140.6	152.5	108.7	144	148.5
ANKRD27	128.3	141.6	122.4	132.4	123.4	122.5	142.1	134.4
ANKRD28	172.1	172.1	151.2	167.3	166.8	142.4	198.5	178.9
ANKRD29	113.1	104.8	101.7	114.6	102.1	94.8	113.7	107.5
ANKRD30A	112.3	115.5	128.4	110	102.4	101.8	111.9	104.3
ANKRD30A	109.2	113.8	121.7	120.8	111.7	97.6	117.1	113.1
ANKRD30B	112.9	108.8	118.2	120.5	111.4	95.3	124.1	117.3
ANKRD32	132.1	130.6	142.3	130.7	111.1	127.6	127.1	127.1
ANKRD33	248.2	230.7	248.7	274.9	163.3	211.7	236.6	192
ANKRD34	131.7	127.6	133.2	141.2	126.3	109.4	129.1	132.6
ANKRD35	125.5	123.6	138.9	129.6	110.6	117.8	130.4	123.5
ANKRD36	130.4	131.1	125.2	126.8	133.3	101.3	117.6	118.9
ANKRD36	121.7	112.9	121.1	123.1	103.5	99	119.2	108.2
ANKRD36	162	162.4	150.4	158.2	175.1	111.8	131.7	150
ANKRD37	149.5	173.7	150.8	185.8	161.6	123	199.3	247.6
ANKRD38	115.2	115.5	134	116.5	118.1	103.7	121.5	125
ANKRD39	173.8	203.8	169.7	204	184.4	148.2	181.3	161.4
ANKRD40	115.1	116.9	119.1	120.8	108.8	104.4	121.2	118
ANKRD41	118.7	129.8	127.5	131.2	121.5	98.8	132.6	118.4
ANKRD42	113.4	102.9	113	118.7	119.6	101.7	113	117
ANKRD43	113.6	112.4	117	117.6	108.9	93.2	118.3	121.8

ANKRD44	181.7	155.8	163.3	151	141.3	135.4	148	144.2
ANKRD45	111.8	121	118.7	112.5	107.5	99.2	121.6	124.8
ANKRD46	280	275.4	307.8	275.5	249.4	209.2	217.8	220.7
ANKRD47	123.3	130.5	118.8	125.6	108.1	104.7	118.7	122.3
ANKRD49	386.7	467.3	591	400.5	402.8	356.5	322	344.8
ANKRD5	114.4	113.2	117.9	106.6	107.1	96.4	116.2	105.3
ANKRD5	117.4	106.1	109.5	108	111.6	100.5	108.2	105.8
ANKRD5	120.6	120.4	119.2	119.2	113.1	104.9	115.2	112.7
ANKRD50	118.5	120.9	144.2	137.5	118	103.4	124.6	130.5
ANKRD55	136.5	141.6	141.8	151.1	136.1	116.7	137.7	126.2
ANKRD56	112.3	119.8	115.5	121	107.7	97.5	116.8	119.5
ANKRD6	156.6	157.5	149	150.5	134.3	116.8	162.6	169.7
ANKRD7	127.2	133.9	126.3	122.7	116.6	103.1	128.9	116.6
ANKRD7	116	114.1	125.7	125.7	109.2	101.2	115.3	121.9
ANKRD9	163.5	153.8	143.6	179	136.3	130.5	159.3	154.9
ANKS1A	231.2	208.4	201.9	240.7	189.2	171	200.5	221.6
ANKS1B	118.8	124	126.7	125.3	107	99.8	108.8	115.7
ANKS1B	106.7	112.3	105.2	112.2	100.1	96.8	113	111
ANKS1B	114.9	119	115.3	115.9	108.9	95.5	110.4	103
ANKS3	138.2	157.8	134.6	159.9	122.8	103.7	149.5	145.3
ANKS4B	109.1	111.3	116.9	108.3	106.5	92.2	115.3	104.6
ANKS6	118.8	105.2	123.7	110.6	107.7	100.2	124.1	114.3
ANKZF1	289.9	345	324.3	319.8	197.6	222	235.2	197.5
ANKZF1	124.2	112	125.3	122.8	112.2	98.8	109.9	113.1
ANKZF1	123	122.4	125.8	112.5	110.7	107.8	115.9	119.1
ANLN	114.4	120.4	117.2	113	119.2	105	118	112.3
ANP32A	110	125.1	121.8	127.4	118.8	105.9	123.6	108.6
ANP32B	4012.9	4436.9	4654.6	4025.5	3513.9	4455.1	3578.6	3896.2
ANP32C	129.8	117.9	117.7	123.8	110.4	102.2	130.7	116.3
ANP32D	115.8	116.5	116.5	114.3	100	105.2	113.6	109.1
ANP32E	110.3	115.8	125	130.7	112.5	101.4	112.6	121.5
ANPEP	248.3	194.9	639.4	246.4	165.7	487.8	183.5	131.4
ANTXR1	124	130.8	123.5	127.2	116.9	98.7	131.4	121.5
ANTXR1	120.7	143.2	142.6	149.6	126.6	126.5	143.3	129.7
ANTXR1	118.2	117.2	126.5	118.7	107.9	97.7	117.2	121.7
ANTXR2	322.3	296.8	375.8	310.8	206.4	261	201	169.1
ANUBL1	127.7	130.4	126.8	121.6	106.6	99.7	114	109
ANXA1	3202.5	3028.4	2971.1	4205.8	2899.5	2655.1	6207.8	4603.9
ANXA10	107.3	106.1	123.9	105.5	109.2	95.3	110.1	112.9
ANXA11	174.7	212.3	228.3	220.1	191.9	164.7	169	184.8
ANXA11	464.2	534.5	595.8	560.8	422.6	307.4	404.4	444.6
ANXA11	132	126.7	126.1	141.7	127.3	104.6	124.7	121.4
ANXA13	105.2	106.4	107.5	108.7	105.2	97.2	111.3	108.5
ANXA13	113.2	103.6	108.5	119.3	105	96.7	115.4	115.6
ANXA13	108	117.4	116.5	115.2	114.5	93.8	118.8	115.9
ANXA2	317.3	233.9	598.3	378.9	236.7	387.8	287.5	240.9
ANXA2	191.8	166.4	204.2	214.6	154.6	166.6	211.9	164.5
ANXA2	2674.8	1773.9	4207.6	3248.5	1401.1	2988.5	3232.8	2132.8
ANXA2P1	1003.2	520.1	1127	871.5	461.5	916.4	874.6	572.4
ANXA3	111.6	118	125.2	125.6	112.3	106.2	122.2	111.9
ANXA4	116.7	119.5	132	130.7	116.3	106.3	118.7	115.4

ANXA5	1917	1852.1	1669.3	3148.8	1651.6	1653.1	3520	1852.9
ANXA6	126.5	141.8	137.3	137	141.6	112.3	138.9	134.2
ANXA6	175.1	203.5	140.6	148.5	151.7	118.7	147	170.7
ANXA7	120	110	117.2	123.9	112	92.1	111.3	112.3
ANXA7	765.9	1003.2	917	1157.6	886.6	923.7	1053.2	1143.1
ANXA8	124.2	119.3	115.3	127.3	115.1	106.8	134.1	114.7
ANXA8L1	109.3	112.3	128.3	124.1	113	96.3	122.2	115.6
ANXA9	123.4	118.1	120.4	124.9	102.6	101.8	124.8	115.6
AOAH	214.7	212.9	201.1	235.6	181.2	140.7	187.6	148.7
AOC2	123.9	126.9	131.6	137.6	109	100.2	127.4	133.8
AOC2	117.6	118.2	118.3	116.3	108.4	102.1	118.7	113.5
AOC2	115.1	114.7	113.8	123	108.7	93.9	121.1	109.6
AOC3	128.4	138.6	142	133.4	128.3	99.9	126.6	129.1
AOF1	110.2	109.5	124.5	112.2	107.8	94	118.9	116.9
AOF2	281.4	331.4	259	290.2	269.6	248.1	286.4	247.4
AOX1	121.9	127.7	113.4	115.6	108	102.3	119.9	113.1
AP1B1	121	121.7	141.1	117.8	113.3	109	134.6	129.5
AP1B1	276.8	268.1	354.6	264.2	213	195.3	246.9	296.3
AP1G1	271.9	311	246.3	309.5	253.3	247.6	307.3	366.8
AP1G2	101.4	116.5	109.8	109.3	106.2	92.5	100.2	111.2
AP1G2	628.3	568.9	468.5	577.7	484.7	344.7	551.7	539.2
AP1GBP1	139.3	135.2	126.4	132.5	130.1	105.9	128.4	133.5
AP1GBP1	137.1	138.3	142	131.1	135.7	111.8	139.5	143
AP1M1	753.2	700.8	804.6	825.4	529.3	513.5	536.3	468.6
AP1M2	108.7	134.3	125.8	128.9	120.6	99.2	119.6	122.6
AP1S1	151.7	174	172.6	152.4	162	140.9	138.5	161.6
AP1S1	171.9	156.9	206.3	179.6	138.5	142	144.3	151
AP1S2	859.3	679.4	1407.9	753.6	556.7	729.5	687.2	728.8
AP1S3	114.5	110.8	110.1	108.7	109.7	99.9	109.6	113
AP2A1	121.6	114.8	140.6	125.5	114.6	111.6	137.8	122.6
AP2A1	115	109	114	118.7	107.6	104.4	123.7	113.2
AP2A1	114.2	108.6	117.9	116.7	113.2	98.7	120.1	119
AP2A2	159.2	188.2	184.3	167	136.3	147.1	184	163.1
AP2B1	168.6	190.2	156.3	167.3	141.9	127.7	185.5	167.9
AP2B1	158.3	161.9	158.1	150.4	148.3	140.7	171	164.4
AP2M1	370.2	313.9	432.4	388	255.7	302.5	397.3	348.5
AP2M1	419.7	327.4	370.5	397.8	256	324	334.1	319.7
AP2S1	606.1	474.2	1063.6	448.4	424.2	598.3	444.9	314.4
AP2S1	3326	3252.5	5578.9	3547	2066.1	3413	3012.3	2060
AP3B1	645.6	644.3	979.4	747.1	417.8	649.4	616.5	547.6
AP3B2	112.6	110.5	115.1	117.2	108.7	101.5	116.9	115.5
AP3D1	117.8	110.3	115.8	114.6	107.5	98.8	116.6	114.3
AP3D1	370.5	348.8	313.8	376.9	244.8	303.9	388.7	343.1
AP3D1	326.4	296.2	276.4	390.2	265.6	283.7	355.8	313.7
AP3M1	253.2	275.5	342.6	290	264.1	331.6	281.1	274.1
AP3M1	120.5	109	115.9	128.5	103.4	97.3	111.6	114.3
AP3M1	306.7	315.5	391.8	361.3	295.7	407	329.7	297.1
AP3M2	339.6	385.3	383.3	369.6	263.1	298.8	292.1	291.4
AP3S1	199.9	224.3	269.8	226.8	187.5	210.7	209.2	232.7
AP3S2	139.9	119.6	152.5	134	120.4	122.6	134.9	129.5
AP4B1	415.2	405.7	508.8	475.3	442.3	315.7	474.1	597.9

AP4E1	204	232.8	229.9	245	231.2	186.6	229.8	221.6
AP4M1	120.2	119.1	135	121.4	106.8	105.8	128.1	115.6
AP4S1	132.7	129.1	133.4	140.8	120.6	107.2	122.3	125.4
APAF1	298.5	244.8	372.5	310.4	266.3	247.2	301.3	329.1
APAF1	129.7	127.9	140.7	124.5	131.7	110.5	130.1	125.9
APBA1	113.5	124.3	128.3	122.8	105.9	110.6	116.6	117.6
APBA2	140.9	161.5	120.9	116.3	136.9	107.8	125.7	126
APBA2BP	110.7	127.1	117	120.5	105.9	102	118	108.5
APBA2BP	109	114.7	111.1	119.5	112.5	100.5	113.3	113.3
APBA2BP	137.4	128.7	146.1	125.8	125.4	124.5	126.1	133.2
APBA3	336.8	501.3	366.9	370.7	301.5	242.8	282.3	299.3
APBB1	128.7	119.2	114.8	124.5	118.3	113.3	126.3	121.2
APBB1	108.1	121	118.5	127	113.6	100.3	120.5	117.3
APBB1	111.3	116.3	111.3	113.4	112.2	104.6	124.3	126.7
APBB1IP	633.4	791.1	639.5	655.5	483.5	493.6	568.8	448.4
APBB2	103.3	107.6	112.8	112.6	109	99	115	115.3
APBB3	557.2	441.5	764.2	588.6	346.8	590.3	361.1	229.4
APBB3	312.9	233.2	382.1	292.5	218.8	240.6	211	160.7
APC	112.8	113.7	129.6	120.5	112.1	109.3	111.7	118.3
APC2	108.2	117	115.1	118.8	104.5	101.4	113.7	107.1
APCDD1	122	120.8	118.4	117.8	108.9	107.6	119.3	114.4
APCS	140.8	136.6	142.6	143.3	123.1	109.2	140.5	136
APEH	274.6	367.3	289.8	342.1	181	284.3	228.9	206
APEX1	738.1	929.7	625.2	746.3	669.4	469.2	519.2	500.8
APEX1	1297.3	1662.7	1410.5	1502.1	1201.7	919.7	1010.5	895.2
APEX2	231.1	190.5	258.5	231.6	175.2	225.5	195.3	197
APH1A	660.7	609.4	652.5	660.9	428.6	497	619.7	519.6
APH1A	147.1	165.2	168.5	163	145.2	134.4	166.1	169.8
APH1B	245.5	194.4	340	238.7	161	235.1	180.9	158.2
API5	369.6	466	473.2	492.8	360	363.2	471.5	360.1
APIN	112.1	111.4	115.7	113.6	115.5	97.6	112.2	111.9
APIP	147.4	159	141.4	170	150.3	131.2	170.8	156.6
APITD1	114.3	117.1	121.9	124.5	104.4	103	123.1	115.3
APITD1	175.9	181.6	179.7	177.8	150.7	139.1	167.9	150.6
APLN	124.4	117.3	125.5	121.6	112.4	103.9	112.2	121
APLP1	112.9	134.8	121.9	123.3	109.7	97.6	127.2	111.2
APLP1	108.7	108.2	110	114.7	108.6	95.1	120.8	107.6
APLP2	117.4	111.5	123.5	116	115.4	102.9	106.6	122.5
APOA1	110.1	128	123.7	118.5	113.8	100.2	123.3	112.7
APOA1BP	548.7	555.5	526.9	602	447.4	353.9	386.6	349.2
APOA2	120.1	113.9	117.8	121.5	102.9	92.6	114.2	119
APOA4	104.9	105.1	105.3	114.5	113.7	99.5	115	113.7
APOA5	105.8	110.2	120.8	103.9	109.1	102	117.7	109.8
APOB	116.1	116.2	123.4	117.5	106	100.5	112.9	120.6
APOB48R	183.1	168.6	218	190.8	127.6	172.7	170.6	163.3
APOBEC1	114.1	107.5	116.3	120.1	107.9	92.7	112.9	120.2
APOBEC2	118.6	117.3	116.7	117.3	102.8	94.4	118.2	118.5
APOBEC3A	140.7	116.9	125.7	139.8	112.6	104.2	129.8	115.4
APOBEC3B	114.3	113.5	117.2	116	103.7	98.8	114.5	116.8
APOBEC3C	158.5	148.9	185.3	161.3	127.3	130.6	132.9	134.5
APOBEC3D	118.5	123.9	123.4	134.8	108.3	105.9	116.5	119

APOBEC3F	186.1	173.7	251.2	162.8	149.1	177.7	146.5	141
APOBEC3F	207.8	165	236	201.9	160.8	170.9	171.4	166.6
APOBEC3F	141.6	147.1	175.9	148.6	124.1	133.1	145.6	148.4
APOBEC3G	348.6	367.6	606.1	328.7	334.8	471.6	251.9	248.6
APOBEC4	110.5	128.9	117.6	124.5	104.9	103.4	121.6	111.1
APOC1	121.2	116	130.9	119	104.6	98.3	120.9	124.7
APOC2	121.4	95.8	130.1	143.2	102.9	104.4	115.5	110.3
APOC3	116.6	113.9	121	116.9	104.5	95.3	110.2	116.5
APOC4	107.9	112.7	114.4	114.2	104.7	102.1	112.2	115.4
APOD	109.7	113.8	120.8	119.9	113.6	102.7	116.4	109.1
APOE	140.8	224	118.8	125.9	168.8	106.6	129	197.4
APOF	116.6	122.9	113.9	123.4	118.1	98.9	123.1	117.8
APOH	105.4	115.8	115.7	113.3	108.6	100	114.5	118.7
APOL1	131.8	120.9	124.2	121.7	120.4	100.5	120.7	121.7
APOL1	107.9	108	108.4	124.5	104.7	96.1	109.1	111.2
APOL2	129.2	114	139.4	127.8	111.5	103.9	129.5	128.8
APOL2	114.1	119.3	119.2	115.9	103.2	103.5	129.1	121.4
APOL2	158.6	131.6	160.5	154	133.8	119.3	146.7	135.2
APOL3	110.1	112.7	106.3	105.4	104.4	102.2	105.4	114.9
APOL3	1183.9	1196.8	940.4	1102.4	962.8	834.8	1161.1	1106.5
APOL3	129.4	119.7	125.7	122.4	113.1	101.7	120	111.2
APOL4	119.8	125	138	130.5	118.4	103.9	118.4	116.7
APOL4	110.8	108.2	120.6	117.2	109.5	94.6	119.8	115.3
APOL5	125.7	116	121.7	117.6	111.4	99.9	121.4	120.3
APOL6	126.8	132.2	133.1	133.8	120.4	103.8	120.9	119.1
APOLD1	125.8	116.5	120.3	131.3	119.4	104	129.4	119.6
APOM	120.5	131.3	143.7	115.1	113.4	117	124	124.9
APP	191.6	191.2	258.3	202.4	174.2	224.7	192.6	176.1
APP	116.7	121	122.1	113.3	119.7	107.2	121.4	127.3
APP	155.5	147.9	191.4	167.1	140.9	160.6	166.2	157.8
APPBP1	116.8	117.8	127.6	121.6	108.6	102.9	120	107
APPBP1	422.3	643.8	538.4	438.8	427.7	384.1	477.6	423.5
APPBP1	358.5	423.3	458.9	394.4	333.7	352.8	340.8	343.2
APPBP2	508.5	513.3	453.5	471.2	391.3	341.5	596.5	556.1
APPL	175	183	159.8	171.5	147.5	139.8	159.4	165.1
APRIN	159.8	174.1	176	171.8	151.1	140.9	180.7	202
APRIN	189.7	238.7	174.6	215.1	207.5	156.6	256.9	243.6
APRT	722.5	888.3	914.3	755.5	643.8	730.4	660.4	559.1
APRT	299.8	381	363.4	307.8	296.9	260.2	262.4	278.8
APS	131.1	124.2	112.2	129.6	112.3	102.6	125.7	112.3
APTX	207.5	221.5	220.6	230.6	190.8	181.3	249.8	222.4
APTX	109.5	113.8	107.9	103.6	107	97.6	107.9	108.5
APTX	141.4	157.6	173.5	161.3	141.8	131.7	149.1	163.8
APXL	114.8	120.7	117.1	122.4	112	100.6	122.1	120.6
APXL2	113.7	123.2	116.8	120.8	114.5	100	119.3	110
AQP1	129.8	111.2	134.1	121.9	115.1	112.6	124	127.8
AQP1	117.7	116.3	119.7	115.9	113.9	98.6	117.2	118.1
AQP10	108.8	114.4	114.3	110.5	102.1	95.6	114.7	105.6
AQP11	122.6	122	116.8	135.3	121.8	102	117.5	108.8
AQP12A	112.5	118	119.3	122.6	107.6	100.7	119.1	113.9
AQP2	112	105.6	121.1	108.7	108.4	98.1	110.5	108.4

AQP3	127.6	133.7	142.1	125.8	126.5	113.3	117.5	126.6
AQP4	116.8	120	111.4	127.6	111.6	112.3	118.2	119.4
AQP4	104	96.7	113.3	114.6	99.6	94.6	113.1	113.7
AQP5	118.6	117.3	112.5	122.6	108.8	95.8	134	115.4
AQP6	120.7	116.6	118.9	116.7	115	103.4	128.9	131.9
AQP7	110.8	114.7	110.6	115.9	110.3	96.1	115.7	105
AQP7P2	120	119.5	134.9	136.6	119.5	112.2	122.3	117.5
AQP8	111.2	117.1	115.5	116	102.9	96.8	114.3	125.9
AQP9	292.1	326.4	121.4	304	236.5	120.1	329.3	235.9
AQR	120.3	115.7	137.1	143.1	114.3	110.5	129.7	133.4
AR	123.4	130.4	128.3	128.5	111.5	111.2	116.7	123.2
AR	110.2	108.3	110.3	118.8	105.1	94.5	115.4	107.8
AR	111.6	117	121.9	120	109.7	98.8	124.5	112.5
ARAF	588.2	735.9	511.1	604.1	444.7	289.3	467.5	466.9
ARC	120	128.8	125.7	123.6	107.9	102.9	132.8	119.8
ARCN1	510.9	621.5	540	690.6	496.5	571.7	759.8	761.3
ARD1A	220.9	239.8	212	199.3	198	188.5	190.8	185.5
AREG	107.8	111.6	109.8	114.5	116.5	97	109.3	111.9
ARF1	141.7	146.4	135.9	167.2	138.6	146.8	167.3	171.5
ARF1	1159.4	1285.8	1018.4	1319.1	807.4	1062.7	1246.7	1585
ARF1	128.8	132.5	135.4	133.1	119.3	117.2	123.8	133.3
ARF3	213.6	314.9	300.5	224.7	194.3	242.3	201.1	174.6
ARF4	151.6	172.9	166.2	163.8	142.7	165	161.6	194.8
ARF4	540.9	540.7	486.8	573.7	513.1	625.6	990.5	1076.5
ARF5	447.1	477.5	744.3	567.5	391.4	578.7	526	497.9
ARF6	123.4	119.4	130.7	114.6	116.9	106.8	124.8	122.8
ARFGAP1	119.6	117.9	129.8	122.8	111.6	98	124.4	119
ARFGAP1	340.2	357	246.1	292.8	201.9	182.3	321.5	297.1
ARFGAP3	118.5	119	128.2	119.3	121.5	114.9	113.3	123.8
ARFGEF1	161.3	176.9	184.9	171.8	151.3	154.9	178.9	177.8
ARFGEF2	123	124.8	121.9	140.2	130	111.7	139.8	143.1
ARFIP1	195	205.4	222.8	220	164.1	161.3	194.7	167.3
ARFIP1	118.2	121.2	120	124.6	108.6	98.2	120.1	122.9
ARFIP2	118	136.6	130.9	131.5	117.4	128.6	106.7	115.5
ARFRP1	136.4	147.9	143.5	145.9	132.7	123.9	137.2	136.2
ARG1	113.4	109	124.1	117.9	106.6	84.3	122.7	112.7
ARG2	157.8	139.7	120.3	188.5	132.2	116.5	245	195.9
ARG99	114.4	113.7	119.7	118.9	109.8	98.6	116.6	111.2
ARGFX	106.6	115.9	111.4	118.4	109.4	97.4	111	113.7
ARGFXP2	112.4	120.1	117.8	121.6	116.8	97.6	116	123
ARHGAP1	362.3	353.6	381.8	420.7	410.7	380.3	380	350.2
ARHGAP10	132.3	137.2	149.9	163.4	120.6	147.8	144.1	132.8
ARHGAP11	144.9	141	134.1	126.5	120.2	119.2	140.9	138.6
ARHGAP11	110.1	117.1	110.9	115.8	110.2	104.9	115.9	111.4
ARHGAP11	125.6	124.9	126.7	124.2	113.3	114.2	126.6	125.7
ARHGAP12	117.1	132.2	136.6	143.5	131.1	113.6	127.1	134.6
ARHGAP15	1005.9	1629.1	1112.2	1145.5	1669.8	993	1242.3	1275.9
ARHGAP17	874.1	738.1	593.3	974.7	629.7	444.3	1054.1	979.4
ARHGAP17	167.5	148.1	154.6	167.4	144.4	135.6	184.4	167
ARHGAP17	122.6	116.8	114.5	124.9	105.5	100.3	119.9	121
ARHGAP18	124.2	122.1	139	126.6	100.5	115.6	119.2	119.8

ARHGAP19	218.5	232.8	277.8	262	184.3	228.8	198.8	191.9
ARHGAP20	110.7	125.7	119.4	129.1	112.1	106.9	117.6	113.1
ARHGAP21	246.9	236.2	278	251.6	228.8	236.6	338.9	430.4
ARHGAP22	131	114.9	144.7	126.2	114	105	123.1	108.7
ARHGAP24	129.1	120.1	126.2	137.2	117.5	102	114.6	118.7
ARHGAP24	126.5	122.5	134.4	131.1	122.9	104	120.9	122.2
ARHGAP24	120.9	122.2	125.2	141.5	117.2	111.3	124.2	124.5
ARHGAP24	147.4	185.4	171.7	180.1	160.1	145.4	165.9	136.7
ARHGAP25	115.5	115.5	120.2	123.5	111.6	102.4	125.1	117.5
ARHGAP25	907	792.2	422.9	743.1	713.9	453.2	538.9	601.7
ARHGAP25	110.9	119.5	119.5	113.5	110.3	102.1	125	119.6
ARHGAP26	119.9	136.6	133	120.8	110.7	114.2	134	126.1
ARHGAP27	117.5	116.3	116.9	118.8	106.4	106.2	124.2	112.6
ARHGAP28	112.3	113.5	116.2	120.3	109.6	95.9	119.4	109.3
ARHGAP28	115.5	119.9	119.8	120.7	112.7	93.3	115	103.3
ARHGAP28	109.4	122.2	116.7	118	108.6	94.9	123.6	110.6
ARHGAP29	119.2	123.1	116.1	116.5	105.5	97.3	116	126.6
ARHGAP30	462.6	644.1	547	569.9	499.9	526.1	495	505.7
ARHGAP30	683.5	1050.3	898.7	923.3	841.6	821.6	932.3	756.7
ARHGAP30	249.5	233	335.2	253.9	206.4	229.8	195.2	208.2
ARHGAP4	781.5	813.3	999.9	679.2	491.4	466.8	430.3	438.2
ARHGAP5	108.8	117.5	119.9	132.5	112.9	97.7	134.6	109.7
ARHGAP6	112.8	114.3	124.6	120.2	116.7	101.5	116.2	128.6
ARHGAP6	108	117.4	117.8	119.5	106.5	104.5	119.1	121.5
ARHGAP6	118.4	112	109.6	118.4	110.2	94.5	111.5	109.8
ARHGAP6	117.9	114.3	124.3	109.8	112.3	100.3	115.9	111.4
ARHGAP8	108.3	110.8	109.1	113.9	102.4	97.2	109	101.3
ARHGAP8	119.8	114.7	116.8	120.6	103.3	98.2	114.1	123.3
ARHGAP8	116	113.8	122.1	115.7	104	107.7	124	118.7
ARHGAP9	1984.5	1790.2	1781	1981.8	1099.4	1012	1349.2	1038.7
ARHGAP9	496.7	402.5	334	418.2	375.1	279	386.5	318.3
ARHGDIA	463.4	479.8	382.8	357.5	322.4	469.6	635.7	640.1
ARHGDIB	8988	10827.8	13771	9306.5	8599.1	9500.2	8960.6	8589.8
ARHGDIG	103.4	113	106.8	113.6	104.1	98.5	111.9	108.2
ARHGEF1	183.5	187.8	206.7	195.9	200.7	159.8	204.6	176.3
ARHGEF1	126.1	123.1	137.1	131.7	109.1	107.2	107.8	125.2
ARHGEF1	166.9	133.6	138.5	146.4	138.2	126.6	129.9	140.6
ARHGEF10	146.7	222.8	137.7	159.3	163.7	119.5	150.1	199.6
ARHGEF10	126.4	132.7	145.2	139.7	128.3	120.8	132.7	127.9
ARHGEF10	117.7	124.5	137.6	131.9	113.8	103.7	124	116.6
ARHGEF10	116.5	115.5	132.6	116.4	105.1	104.9	111.5	116.2
ARHGEF11	109.4	112.5	115.1	111.1	109.1	104	107.9	110.9
ARHGEF11	133.4	124.6	138.7	119.6	123.8	106.2	150.1	123.4
ARHGEF11	133.5	128.8	124.3	130.9	110.9	109	126.6	124.9
ARHGEF12	112.4	119.5	111.1	120.5	106.6	107.4	125.3	116.9
ARHGEF15	115.1	122	125.6	121.8	110.5	100.5	109.5	118.9
ARHGEF16	116.8	131.8	129	129.7	115.3	118.8	122.7	115.9
ARHGEF17	103.1	116.3	117.9	120	108.1	99.7	116.6	108.7
ARHGEF18	2814.9	3203	2444.6	2943	2128.7	1777.6	2346.8	1855.1
ARHGEF19	191.1	176.9	196.8	173.1	155.1	143.4	205.8	176.6
ARHGEF2	1335.6	1282.4	1230	1472.9	995.4	1074.8	1607.3	1289.6

ARHGEF3	1996.7	2065	1659.9	1956.4	1531.5	1558.3	2071.3	1771.7
ARHGEF4	108.6	113	120	124.5	115.4	93.5	115.6	111.9
ARHGEF4	107.5	118.4	131.8	130.7	110.9	109.2	122.4	113.4
ARHGEF5	118.6	148.3	114.2	147	112.3	103.7	155.5	145.5
ARHGEF6	1574.2	1905.7	2146.4	1362.1	1350.9	1469	873	1039.9
ARHGEF7	606.6	635.1	621.2	658	626.2	542.8	896.5	769.8
ARHGEF7	109.9	107.4	106	121.6	108.3	97.1	116.3	118.1
ARHGEF7	120.8	120.6	121.4	118.5	106.8	108.5	124.5	127
ARHGEF9	110.2	131.1	127.4	134.2	112.2	103.4	120.4	111
ARID1A	111.9	125.4	123.1	120.3	108.6	97.8	121.6	109.2
ARID1A	710.1	837.3	665.7	849.7	834.4	521.2	684.9	812.7
ARID1B	103	112.5	106.6	113.1	108	98.1	109.7	112.3
ARID1B	122.5	117.8	118.5	119.9	111.6	101.7	118.3	123.7
ARID2	161.1	148.3	161.1	151.6	155.9	145.3	180.5	156.7
ARID3A	266.6	226.1	272.2	363.9	235.8	280.4	407.3	269.1
ARID3C	126.1	121.6	115.4	135	112.6	99.3	120	125.6
ARID4A	155	176.3	144.2	181	141.5	130.8	162.4	188.5
ARID4A	152.5	144.2	147.8	177.9	142	122.1	155.9	165.2
ARID4A	185.5	209.9	212.2	187.6	195.8	170.4	217.3	218.9
ARID4B	134.8	143.8	134.3	126.7	136.9	115	137.2	126.9
ARID4B	557.8	642.7	485.4	747.7	498.8	313.8	712.5	845.6
ARID4B	202.1	217.4	198.4	271.8	197.7	137.9	267.7	289.7
ARID5A	127	127	122.4	119.5	115	111.7	133.3	133.4
ARID5A	129.2	115	111.6	110.2	105.1	98.5	117.9	117.9
ARID5A	134.9	155.7	160.8	166.5	129	139.2	186.1	157.1
ARID5B	682.8	733.6	387.1	804.4	642.1	472.6	849.7	769.8
ARIH1	205	214.1	171.9	227.2	189.1	170.2	258.3	226
ARIH2	872.2	824.8	778.2	1008.5	910.6	567.9	1023.7	1335.8
ARL1	286.9	301.7	304.5	331.1	234.9	231.1	249.4	273.4
ARL10	106.9	124.1	110.8	121.3	107.8	96.3	118.5	114.4
ARL11	106.4	116.3	119.5	116.4	98.2	93.5	112	106.9
ARL13A	109.6	116.1	117.2	124.8	105.9	97.4	114.6	121.2
ARL13B	119	122.4	113.4	137	127.5	103.5	132.3	116.4
ARL13B	119.7	111.1	120.9	122	110.9	104.2	114.6	119.5
ARL14	110.3	114.3	118.2	113.9	105.2	101.8	112.5	111.9
ARL15	128.7	127.7	122.8	142.1	110.7	105.6	135.1	115.8
ARL16	717.6	726.6	692.6	591.5	629.8	379.6	664.6	673.1
ARL2	589.3	794.9	633.1	634.1	523.1	311	434.5	520.5
ARL2BP	648.5	587.4	727.6	524.9	467.4	486.6	455.7	502.1
ARL3	120.8	119.4	124	129.6	112.5	106.3	137.3	127.3
ARL4	127	128.7	137.1	138.8	124.2	115.3	138.2	128.6
ARL4	127	156.6	120	124.4	118	102.9	131.6	134.6
ARL4	128.9	127.1	123.9	139.2	112.9	108.5	129.3	129.7
ARL4A	119.1	115.5	120.1	121.6	109.9	105.1	119.2	125.9
ARL4C	150.8	148.2	152.6	159.1	136.9	133.9	175.4	138.2
ARL4D	109.9	125.7	118.2	118.9	112.8	105	115.2	121.1
ARL5	129.3	141.9	138.3	138.6	127.5	117.9	135.1	133.1
ARL5	1085.7	1424.7	1279.1	1459	931.5	766.8	1052.7	1066
ARL5A	427.7	312.9	399.9	389.7	265.7	341.5	367.6	368.9
ARL5B	128.3	142.2	122.6	131	121.1	114	136.8	151.8
ARL6	121	114.8	117.5	124	110.5	93.2	113.2	111.3

ARL6	119.8	116.6	118.5	114.2	108	98.1	126	122.1
ARL6	107.7	103.8	109.3	125.1	108.5	98.5	115.9	113.5
ARL6IP1	127.3	137.8	126.4	138	113.7	131.2	138.7	128.8
ARL6IP2	149.4	193.5	179.9	172.3	168	149.8	153.7	160.9
ARL6IP4	134.8	126.6	132.9	156.7	111.7	119.9	130.9	129.6
ARL6IP4	136.5	145.3	131.5	142.1	121.1	116.5	134.5	132.7
ARL6IP4	179.8	171.7	217.7	201.8	154.4	167	191.2	149.6
ARL6IP5	1277.6	1282	1633.3	1032.7	751.7	1111.5	1246.8	1270.8
ARL6IP6	111.3	108.8	125.3	110.9	105.1	102.3	115.9	109.3
ARL8A	122	117.9	131.6	138.2	109.6	103.9	140.5	124.9
ARL8B	381.4	353	358	415.6	410.8	334.7	441.5	431.1
ARL9	114.2	103.6	122.3	118.1	114.6	102.1	123.3	127
ARMC1	250.4	230.7	234	257.1	222.4	159.3	210.7	209
ARMC10	339.3	425.9	468.2	465.2	370.9	316.1	375	354
ARMC2	105.6	112.1	127.9	111.5	106.1	109.1	116	118.7
ARMC4	109.9	115.4	115.2	119.7	98.4	99.7	116.5	109.2
ARMC5	131.3	118.8	141.2	135	116.2	99.8	144.9	138
ARMC6	134.9	144.5	135.3	145.5	121	109.3	128.5	138.8
ARMC7	308.9	309.1	257.8	274	240.7	209	246.7	222.4
ARMC8	118.2	132.5	120.1	121.1	115.6	109.4	123.8	118.2
ARMC8	148.6	190.7	173.9	178.3	181.7	165.2	159.9	174.4
ARMC8	122.4	118.4	129.7	128.1	110.9	102.5	128.4	110.7
ARMC8	108.2	124.7	117.3	132.4	118.7	101.9	118.3	115.6
ARMC9	125.4	117.9	121.4	126.1	114.7	109.8	119.6	118.3
ARMCX1	123.6	111.1	126.7	114.3	111.7	106.1	115.4	113.7
ARMCX2	245.6	248.8	190.6	219.3	189.4	142.4	208.5	229.2
ARMCX3	175.2	204.5	181.1	200.7	183.9	174.2	177.6	222.8
ARMCX3	108.6	116.2	123	121.9	113.6	103.9	113.3	110.3
ARMCX5	138.8	133.1	149.6	138.3	131.4	127.5	138	146.9
ARMCX6	197.8	208	190.9	194.6	169.8	179.4	198.5	181.3
ARMCX6	175.9	199.3	159.5	161.9	179.8	147.3	165.9	184.2
ARMET	709	848.1	1329.1	1020.1	943.8	977.8	1058.6	1205.2
ARMETL1	109.4	120.2	116.7	119.7	110.6	100.3	119	120.3
ARNT	138.1	130.9	131	131.3	121	114.8	151	136.9
ARNT	107.4	114.9	117.4	125.7	105.1	105.8	112.9	121.3
ARNT2	114	110.2	110.2	125.3	113.9	101.6	114.9	121.8
ARNTL	114.5	132.6	132.8	135.1	116.4	107.9	128.5	145.5
ARNTL	476.8	519.7	585.8	565.1	513.1	423.1	558.3	547
ARNTL2	114.7	115.5	115.9	123.6	110.7	99.3	114.5	126.5
ARP10	136.4	134.4	174.3	136.8	130	137	124.1	131.2
ARPC1A	336.4	337.4	359	421.6	305	329.5	317.4	287
ARPC1B	310.6	366.1	516.1	360.9	279.7	346.4	240.3	220.4
ARPC2	109.3	113.9	106.6	110.6	110.1	88.3	99.8	121.7
ARPC2	3385	2943.4	3857.5	3383.6	2174	3836.6	4125.3	3309.1
ARPC2	129.3	121.3	118.5	126.8	119.5	106.4	142.6	136.1
ARPC3	2836.9	2641.7	3674.3	2783.4	2671.4	3208.3	3122.2	2424.3
ARPC4	115.2	114.5	122.6	110.6	107.3	104.3	116.9	115
ARPC4	503.3	443.1	900.1	592.1	532.6	763.6	602.6	496.3
ARPC4	351.7	337.7	486.6	388.2	278.4	403.5	442.9	456.4
ARPC5	1463	882.8	1371.9	1297.5	1054.2	1363.2	1399.4	1199.8
ARPC5L	376.7	422	374.5	347.8	312.9	345	466	435.1

ARPM1	121.3	112.9	121.7	129.8	111.8	107.2	125.8	118.2
ARPM2	119.2	115.5	108.6	123.4	111.1	103.1	119.2	112.7
ARPP-19	198.3	177.8	194.9	246.3	181.2	183.2	213.9	231.6
ARPP-21	114.1	115	115	114.4	112.1	95.7	125.8	115
ARPP-21	104.2	103.6	102.5	119.2	104.5	93.1	116.2	115.8
ARPP-21	111.2	111.7	113.3	120	109.1	102.5	130.3	114.8
ARPP-21	110.3	118.3	113.1	115.3	106.3	99.9	108.7	114.2
ARR3	117.7	116.8	114.7	121.2	111.1	98.6	114	111.3
ARRB1	163.5	162.5	201.3	176	130.6	138.4	175.4	143
ARRB1	151.1	146.6	181.4	153.7	121.3	129.4	133.8	118
ARRB2	133.7	120.3	125.4	134.6	119.8	104.6	129.9	128
ARRB2	157.1	152.7	136.1	157.8	149	161.6	153.3	148.4
ARRDC1	133.2	133.5	123.1	138.6	119.1	102.5	132.8	118.3
ARRDC2	124	124.8	128.7	123	100.2	108.3	123.7	127.3
ARRDC2	132	130.7	147.7	138.8	148.6	117	144.8	141.1
ARRDC2	291.6	343.2	322.8	370.9	321.6	252	383.6	500.4
ARRDC3	139.7	144.2	156.1	161.9	141.3	131.6	148	180.6
ARRDC4	136.2	120	134	149.7	125.7	130.5	154.2	145.7
ARRDC5	171.5	167.8	154	178.3	162.8	112.8	138.1	146.8
ARS2	1518.2	1743.9	1373.3	1627.5	1499.6	871.5	1735.3	1768.4
ARS2	114.6	119.9	117.5	115.9	112.8	95.4	115.2	112.5
ARSA	149.1	142.9	146.4	150.8	162	129.1	136	143.7
ARSB	119.4	118.7	116.5	122.4	103.9	107.1	113	128
ARSB	160.4	135.1	177	156.5	130.3	144	172.4	131.7
ARSB	122.9	124.9	127.5	128.7	120.4	105	125.4	112.2
ARSD	126.2	113.5	122.7	120.1	113	103.6	116.3	119.7
ARSD	119.5	115.4	125	116.9	119.9	107.3	139.4	126.1
ARSD	163.6	172.3	212.3	158.1	133.9	162.8	158.1	156.3
ARSE	118.8	114.5	114.2	108.8	105.7	98.7	123	111.1
ARSF	115.8	106.6	111.6	113.5	109.7	96	120	109.9
ARSG	118	128.1	141.5	130.3	122.7	113.6	123.9	102.7
ARSH	114.5	111.8	119.7	119.7	115.1	98.4	113.7	122.8
ARSI	123.2	112.6	120.1	110.1	107.6	100.7	124.7	120.3
ARSJ	120.8	117.1	126.8	128.4	104.8	102.6	129.1	107.3
ARSK	143.3	135.2	140.7	153.1	141.8	114.8	133.7	139.9
ART1	116.8	115.1	121.3	126.3	116.8	101.7	129.7	118.7
ART3	124.7	116	119.7	117.3	111.2	100.8	121.9	116.7
ART4	117.3	131.4	119.7	114.7	111.7	99.5	125.3	117.6
ART5	112.1	115.3	113.2	116.3	101.9	96.7	113.7	120.5
ART5	112.3	108.9	119.3	113.8	102	93.5	117.7	110.8
ARTN	112.5	117.3	107	108.4	109.8	94.4	115.9	113.4
ARTN	112.8	113.5	113.3	120.1	106.5	96.6	118.6	115.3
ARTN	114.2	105.5	116.4	108.4	106.8	101.8	123.7	110.9
ARTN	111.8	116.1	114.6	122.8	109.3	96.7	120.6	120
ARTS-1	122.8	130.3	125.6	134.9	132.7	105.8	123.1	130.5
ARTS-1	114.8	130.5	138.1	126.2	107.8	107.7	121.4	118.2
ARV1	157.5	171.1	201.8	182	187.4	152.1	175.4	166
ARVCF	119.9	120.3	125.5	118.8	113.4	99.8	123.6	110.8
ARVP6125	108.6	118	114.6	128.7	113.8	104.6	116.9	114.7
ARX	117.9	119.5	115.4	114.3	110.3	95.5	119.5	115.8
AS3MT	113.5	119.7	123	117.6	109.4	100.8	114.6	118.4

ASAH1	145.2	125.7	169.8	143.7	136.8	127.1	144.6	137.2
ASAH1	117.4	116.8	120.7	127.1	110.4	110.2	133.6	131.8
ASAH2	108	113.4	115.5	114.5	105	98.3	118.5	106
ASAH3	110.8	114.6	112.4	113.5	101.8	92.2	107.8	113
ASAH3L	114.2	110.1	111.6	114.7	108.9	99.3	117.3	110.1
ASAHL	174.2	221.1	239.6	179.1	169.1	168.3	152	163.1
ASAHL	183	218.5	284.2	189	190	192	160.2	170.4
ASAHL	137	164.2	220.2	154.6	154.3	159.5	146.4	141.3
ASAM	112.1	126.5	99.8	117.7	106.9	105.3	115.5	113.1
ASB1	188.6	203.5	180.2	214.4	195.5	169.1	248.3	243.8
ASB10	117.9	113.7	124.9	110.9	109.8	100.6	122.1	112.7
ASB11	120.2	132.5	122.4	127.3	101.9	101.8	124.7	114.7
ASB11	124	123.8	133.6	118.7	107.2	107.7	123.4	118
ASB11	114.3	126.3	122.1	139.1	122.1	102.3	116.9	116.3
ASB12	112.6	115.9	110.3	122.7	109	101.8	114.2	111.9
ASB13	195.3	169	192.5	187.3	134.8	129.3	135	115.6
ASB14	105.7	110.1	113.4	118.6	104.1	100.5	115	101.7
ASB15	119.6	105.6	107.9	114.6	108.5	94.2	116.1	105.5
ASB16	113.8	116.7	120.2	119.2	110.3	101.3	106.8	118.4
ASB17	104.7	107.4	107	117	101.2	93.5	111.9	111.3
ASB2	118	119.9	132.3	129.2	117	110.9	120.4	122.3
ASB3	154.4	154.8	174.5	207.2	148.4	136.5	159.8	160.3
ASB3	145.2	135.2	173.8	149.1	127.6	129.1	142.1	131
ASB4	114.8	117.7	119.3	115.7	102.5	101.1	110.2	116.9
ASB4	116.6	107.2	110.6	117.9	115.3	111	116.5	115.5
ASB4	127.1	123.4	129.4	118.9	108.9	95.2	120.8	119.1
ASB5	115.5	118.8	119.2	126.1	112.5	105.5	113.3	113.7
ASB6	107.4	112.8	106.2	114.5	101.5	101.5	113.2	109.5
ASB6	239.1	196.8	174.4	194.7	162.4	141.4	202.5	255.4
ASB6	186.8	186.4	168.7	205.9	161	155.2	219.6	212.3
ASB7	118.5	114.4	116.3	112.8	104.9	102.1	126.3	124.5
ASB7	126.7	123	130.7	127.4	108.7	108.5	120.2	116.9
ASB7	131	124.7	129.4	135.8	112.4	99.4	123.2	116
ASB8	223.5	253.8	213.9	234.2	163.8	171.2	249.4	235.1
ASB9	112	118.7	116.1	122.6	113.2	91.3	113.8	116.5
ASB9	109.9	114.1	113.7	108.6	111.3	97.7	115.4	113.9
ASCC1	370	394.9	356.9	385.9	309.8	285	364.3	321.5
ASCC2	587.9	601.3	718.9	585.2	422.2	407.6	512.3	466.2
ASCC3	116.2	123.2	135.4	132.6	118	112.6	136.7	126.4
ASCC3	151.2	135.3	155.5	136.3	127.9	111	152.6	149.5
ASCC3	122.1	110.1	123.4	105.6	108.7	102.7	112.5	121.3
ASCC3L1	148.1	140	139.3	133.7	126.5	128.8	138.6	134
ASCIZ	169.4	151	170.6	188.6	157.6	159.8	178.9	177.9
ASCL1	118	113.1	116	122.1	113.3	100.9	119.4	112.5
ASCL2	322.8	222.5	300.9	288.4	157.3	151.4	215.3	152.9
ASCL3	111.2	116	116.1	113.5	103.3	95.8	117.8	111.8
ASCL4	110.7	106.9	133.9	120.4	105.6	98.4	122.5	126.5
ASF1A	403.1	411.7	603.3	454	451.6	543.3	616.3	729.4
ASF1B	119.7	126.1	122.6	124.1	104.3	102.3	121.7	111.6
ASGR1	135.3	127.5	195.2	149	120.5	110.3	136.1	129.6
ASGR2	114.2	110.2	114.9	112.3	104.6	94.4	111.5	113.3

ASGR2	124.9	120.5	180.5	118.6	108.2	100.8	116.2	123.1
ASGR2	113.5	112.3	146.4	116.4	103.2	109.1	121.8	116.9
ASH1L	118.5	131.2	129.1	127.3	110.5	101.3	131.5	130.8
ASH2L	321.5	369.2	322.1	335.5	341	289.8	306.7	241.1
ASIP	107.1	108.9	103.8	114.3	108.4	93.3	125.6	116.2
ASL	120.8	112.2	115.4	112.7	108	102.5	117.4	114.1
ASL	123.7	123.7	120.5	123.4	107.4	98.7	122.7	116
ASL	148.1	137.4	147.6	141.6	129.6	116.7	143.1	127
ASMT	126.2	112	121.7	120.8	108	102.5	118.8	113.6
ASMTL	141.8	162.1	151.7	167.3	143.8	160	179.9	153.4
ASNA1	111.1	116.9	113.3	124.2	113.3	106.8	124.4	117.1
ASNS	469.7	540.2	475.1	509.9	437.8	489.7	390	446.2
ASNS	143.2	118.8	129.8	147.1	133.6	118.4	139.2	126.7
ASNSD1	1055.8	1226.5	1028.8	1024	1201.2	942.7	1038.7	1276.7
ASPA	105	108.4	121.5	116.1	109.2	103.5	121.5	115.1
ASPH	119.4	121.3	132.7	137.9	124.7	135.6	144.7	128.7
ASPH	123.2	129.6	122.7	156	137.7	125.2	187.9	131.6
ASPH	113.6	116.5	118.9	118.2	108.7	105	123.2	111.8
ASPHD1	119	123.8	114.8	115.7	112.7	102.9	122.2	111.2
ASPHD1	120.9	114.5	119.3	122.4	107.4	102.5	124.5	120.2
ASPHD2	132.3	138.4	135.9	141.5	121.3	102.7	129	137
ASPM	118.9	121.1	130.9	123.6	107.9	99.8	119.9	109.2
ASPN	115.8	115.1	108.9	117.5	101.9	97.2	106.6	120.8
ASPSCR1	115	121.6	110.3	125	109.7	98.8	125.8	115.5
ASPSCR1	544.3	498.2	452.1	487.4	402.7	338.4	495.4	495.9
ASRGL1	133.4	107.8	124.8	141.3	105.3	103.3	112	114.4
ASS	118.1	119.6	125.1	116	102.9	102.4	116.7	112.4
ASS1	121.1	114.7	117.5	127	112	103.5	129.8	123.1
ASTE1	162.5	191.3	202.8	185.9	194.6	141.4	160	161.1
ASTL	124.7	127.5	123.5	129.7	116.2	97.8	118.2	126.5
ASTN	114.3	112.7	114.6	118.9	106	102	113.2	116.4
ASTN	109.2	119.4	125.7	117.3	116.8	99.5	120.2	114.3
ASTN2	124.7	125.6	132.6	124.7	113.5	109.3	126.2	124.4
ASTN2	123	113.4	123	126.6	120.6	97.8	119.1	117.3
ASTN2	108.9	109.2	127.4	121.5	118.2	102.6	131.9	108.5
ASXL1	238.1	307.1	219.5	264.5	238.1	179.9	343.2	273.5
ASXL2	449.3	513	408.4	611.9	601.4	285.1	678.8	944.4
ASZ1	113.4	113.4	117.8	111.9	112.5	95.7	107.5	110.9
ATAD1	152.2	151.1	162.3	160.2	128.3	130.9	145.2	151.9
ATAD2	143.6	141.7	167.6	151.3	140.1	145.4	137.5	141.5
ATAD3A	165.4	143.4	139.8	140.2	128.8	107.3	164.5	158.3
ATAD3B	117.6	115.2	117.3	123.9	114.5	99.4	127.2	120.5
ATAD3C	110.4	120.3	109.3	111.5	103.3	96.5	107.9	123.9
ATAD4	132.5	134.8	134.1	136	125.1	113.6	137.8	133.1
ATBF1	146.1	123.6	140.2	130.4	119.4	125.3	126.1	125
ATCAY	120.2	118.5	142	135.8	116.1	107.7	122.1	116.3
ATE1	108.9	120.7	126.2	114.5	117.2	101.7	124.8	114.4
ATE1	107.6	115.3	113.4	122.8	94.1	102.1	114.4	114
ATE1	136.7	118.8	132	132.5	124.7	109.1	138.4	120.6
ATF1	136.2	139.5	146.2	161.2	142.8	151.4	141.4	158.4
ATF2	143.1	136.9	143.6	154.3	136.5	117.4	146.2	145.4

ATF3	236.7	223	152.4	337.9	294.9	168.8	524	393.2
ATF3	111.6	116.9	102.5	111	115	97.1	122.6	113.5
ATF3	110.4	109.1	124.3	121.9	108.4	100.9	114.9	103.5
ATF4	3736.6	4214.5	2983.9	3948.9	3005.6	3416.2	5889	6409.8
ATF4	121.6	123.6	140.1	120.4	118	115.7	139.6	133.9
ATF4	186	188.4	174.4	201	172	164.1	239.5	292.8
ATF5	318.3	184.6	323.1	349.6	171.4	285.6	307.3	240.6
ATF6	267.7	351.8	288.7	295	313.8	225.2	237.7	278.9
ATF7	120.4	118.6	110.2	123.6	115.3	104.6	121.4	118.9
ATF7IP	113.6	119.3	127.4	128.2	112.9	92	116.5	130.5
ATF7IP2	160.7	251.3	179.9	174.3	230.5	157.4	176.9	208.1
ATG10	129.7	117.9	139.2	134.8	123.8	116.1	130.3	118.3
ATG12	632.3	820.1	863.9	851.2	748.4	586.7	968.3	840.7
ATG16L1	386.1	381.6	347.7	466.7	327.6	339.2	398.4	423.8
ATG16L1	495.4	505.5	381.5	513.3	424.8	351	525.6	511
ATG16L2	175.4	154.1	189.1	167.4	144.6	153.3	156.8	147.2
ATG3	126.3	127.2	135.7	128.3	114.9	109.6	124.9	148.2
ATG4A	179.8	180.9	166.5	183.2	161.4	130	169.8	191.6
ATG4A	114.8	119.5	120.8	116.9	111.5	100.9	113.2	108.7
ATG4B	113.7	113.9	111.8	116.5	109	95.8	117.4	117.2
ATG4B	304.4	274.1	280.2	289.4	224.6	234.7	289.8	240.3
ATG4C	109.5	113.4	110.6	127.7	103.5	101.7	108.1	113.4
ATG4D	118.9	128.6	123.8	125.5	120.3	106.4	117.1	116.8
ATG5	137.3	160.6	129.2	143	159.7	139.8	150.2	146.3
ATG7	231	180.8	297.3	255.8	157.9	175	204.4	160.1
ATG9A	106	109.6	110.6	114	103.5	98.3	105.7	110.5
ATG9A	278.4	271	267.6	286.4	215.7	223	303.2	257.5
ATG9A	247.5	256.3	246.4	286.3	173.6	213.7	259.6	239.6
ATG9B	114.3	122.7	119.8	128	106.8	97.4	121.7	122.7
ATHL1	727.5	1048.3	494.6	676.8	563.2	386.9	878.3	940.6
ATIC	650.8	778.1	663.7	684	542	490.1	758.3	547.9
ATM	117.1	114.7	119.4	119.1	109	96.6	122.9	120.9
ATM	1425.1	1289.4	1126.3	1652.9	1515.1	814.5	1406.1	2120
ATM	104.5	117	113	109.5	102.6	90.1	110.2	109
ATN1	128.2	112.1	114.9	114.8	111.8	102.6	118.4	121.7
ATN1	252.7	266.5	180.1	276.7	168.7	175.1	314	290
ATN1	118.5	107.4	113.9	115.6	112.3	92.9	112.4	110.9
ATOH1	105.2	112.1	118.7	116.3	114.4	100.7	115.1	112.5
ATOH7	120.1	122.1	113.5	118.2	119	91.8	113	117.5
ATOH8	110.8	116.5	127.4	115.2	112	105.9	125.2	122
ATOX1	394	291.5	481.8	384.2	209	319.1	309.2	244.8
ATP10A	130.3	128.6	127.1	125.1	117.4	109.3	130.1	129.4
ATP10D	128.1	119.5	121.1	128.8	120.8	108.4	129.5	117.5
ATP11A	108.4	112.1	118.2	110.6	103.2	92.7	117.5	117.4
ATP11A	118.4	112.1	111.9	124.5	102.8	95.9	117.8	109
ATP11B	343.4	311	273.2	390	311.8	214.6	424	503.3
ATP11C	120.1	117.6	118.1	126.1	115.7	105.1	123.8	117.2
ATP11C	208.3	232.1	229.3	250.3	205.3	207.7	234.8	254.1
ATP12A	119.6	131.3	137	126.9	118	110.2	118.8	125.7
ATP13A1	272.9	271.6	225.1	310.4	241.2	187.7	264.4	266.1
ATP13A2	147.7	167.6	156.1	160.7	134.8	126.4	150.8	163.9

ATP13A4	108.6	115.5	124.1	121.3	104.1	91.2	116.8	119.4
ATP13A5	110.6	114.8	115.2	108.1	113.3	100.3	120.4	113.7
ATP1A1	859.7	1302.8	674.8	989.7	857.6	649.4	1076.8	1243.7
ATP1A1	1371.7	1168.9	1108.1	1336.9	933.4	1234.5	1484.5	1834.5
ATP1A1	315.7	344.4	262.5	297.8	273	249	305.9	312.8
ATP1A2	114.4	106.2	107.9	106.7	102	92.9	118.9	114.4
ATP1A3	115.1	130.7	124	124.2	120	113.9	126.9	119.5
ATP1A4	112.7	111.2	115.8	118.8	110.1	96.3	115.5	118.7
ATP1A4	111.6	111.1	115.9	119.1	109.6	106.1	118.9	114.2
ATP1B1	125.6	132.9	168.3	137.1	120.9	122.5	118.1	124.3
ATP1B1	178.7	181.5	258	181.2	149.9	161.1	168.2	171.7
ATP1B1	157	134.1	169.1	138.5	133.9	124.8	131.3	147
ATP1B2	157.1	183.4	157.4	173.6	150.2	138.4	162.3	182.4
ATP1B3	881.3	726.3	620.7	1075.2	657.1	656	1409.2	1035.4
ATP1B3	203.7	177.3	166.5	244.2	191	169.9	288.4	227.7
ATP1B3	574.1	460.4	342.7	402.9	417.1	333.8	630.4	567.4
ATP1B4	108.4	110.3	118.8	122.8	116	102	118.7	123.4
ATP2A1	112.3	113.1	111.8	115.5	106	92.9	113.9	116.8
ATP2A2	202.1	175.1	215	216.7	185.4	227.1	238.9	208.8
ATP2A2	300.2	326.5	406.5	307	282.7	295.9	342.1	412.9
ATP2A2	280.3	244	292.7	307.1	219.8	274.1	431.3	325.5
ATP2A3	147.6	137.1	147.1	155.2	125.1	115.2	135	131.2
ATP2A3	148.6	150.3	145.6	157.6	126.8	124.5	156.6	139.2
ATP2B1	149	143.4	143	134.2	128.7	115.5	143.5	134
ATP2B1	122.6	126.5	125.7	122.8	108.8	109.4	121.3	116.4
ATP2B2	117.2	116	113.9	119	112.5	105	136.7	119.3
ATP2B2	119.2	114.4	116.8	116.8	109.6	101.1	118.3	116.5
ATP2B2	114.1	118.3	113.7	123.1	108.2	101.1	118.3	120.8
ATP2B3	113.8	111.3	120	119.3	107.7	96.7	121.9	122
ATP2B3	124	130	124.9	124.8	117.6	105	124.5	117.1
ATP2B3	104.7	113.8	112.2	124.6	111.2	102.5	109.5	118.3
ATP2B4	113.2	135.9	129.9	131.8	124.3	102	127.9	135.5
ATP2B4	377.1	426.8	395.8	415.7	346.7	259.8	262	305.1
ATP2C1	162.4	155.6	197.5	188.8	172.5	151.9	173.1	202.4
ATP2C1	128.3	135.5	121.3	137.4	142.3	103.6	133.1	140.4
ATP2C1	173.6	177.8	240.4	199.7	201.3	176.3	184.1	203.8
ATP4A	113.9	107	116.2	113.1	103.6	97.4	109.7	107
ATP4B	113	117.3	102.9	123.6	97.4	100.2	115.5	109.3
ATP4B	116.2	122.6	119.7	115.4	109.5	95	116.5	112.8
ATP5A1	4047.2	4192.3	4482.2	4770.7	3182.9	3526	3274.8	3921.9
ATP5A1	3956.7	4280.2	4244.8	4312	3286.2	2697.2	3232.3	3313.5
ATP5B	4212	5727.6	5011.4	5117.3	3580.2	4160.8	4577.5	5763.5
ATP5C1	477.5	641.7	540	512.2	538.7	519.8	430.7	426.4
ATP5D	211	233.5	164.4	235.7	178.8	162.7	249.4	183.1
ATP5D	1124	1356.7	1161	978.1	696.3	933.9	797.6	722.6
ATP5F1	345.8	287.6	322.6	270.4	174.7	244	221.7	219.1
ATP5F1	1430.5	1366.9	1611.4	1506.7	1149.2	1692.4	1139.4	1084.7
ATP5G1	127.1	123.4	124.5	119.4	119.2	103.2	120.8	125.6
ATP5G1	639.7	712.8	877.8	671.6	482.3	485.6	405.5	445.1
ATP5G2	113	110.7	115	125.2	115.5	104.3	119.8	115.8
ATP5G2	124.4	116.9	128	126.8	106.6	104.8	125	126.4

ATP5G2	931.7	1050.9	2015.3	1341.5	967	1625.7	906.6	1007.3
ATP5G3	130.2	128.4	139.5	129.9	113.8	110.9	125.9	124.9
ATP5H	1317.9	1420.3	1994.4	1662	1199.4	1561.6	1264.9	1313.8
ATP5H	1666.4	2172.8	2729.8	2030.5	1734.6	2274.8	1406.6	1438.6
ATP5I	2206.7	2747.9	3409	2529.4	2157.2	1992.3	2155.3	2140.3
ATP5I	280	271.3	358.3	325.3	290.3	263.9	310.8	316.9
ATP5J	215.9	223.6	285.2	251.9	212.5	201.9	206.6	206
ATP5J	1461	1817.8	2525.1	1874.4	1438.3	1520.5	1544.4	1563.6
ATP5J	1786.6	1914.2	2442.2	2019.8	1775.9	1838.2	1612.9	1772.9
ATP5J2	566.7	539.6	747.6	609.6	458.3	518.3	584.5	500.6
ATP5J2	774.4	678.8	1192.2	969.5	603.3	774.1	685.4	641.4
ATP5L	198.1	207.5	286.8	203.1	190.7	179.2	199.9	197.8
ATP5O	1696.9	1699.3	2165.9	1939.6	1855.7	1744.9	1533.1	2018.1
ATP5S	167.5	154	165	151.6	142.7	132.4	170.1	143
ATP5S	120.7	126.2	136.5	134.1	120	106.1	137.1	132.5
ATP6AP1	2996.3	2452.7	2479.5	2631	1548.4	1723.6	2089	1895.6
ATP6AP2	1400.2	909.8	1830.7	1033.8	591.7	1109.2	1122.5	975.6
ATP6V0A1	402.3	233.1	685.2	458.3	224.5	440.8	574.3	397.9
ATP6V0A2	212.8	236.9	198.2	226.9	189	174.3	235.4	224.6
ATP6V0A4	110.5	110	127	127	118.4	92.1	122.6	110
ATP6V0A4	113.1	114.3	126.6	120.5	109.1	99.1	132.8	123
ATP6V0A4	111.1	112.5	104.7	117.7	108.1	104.2	129.8	117.3
ATP6V0A4	113.6	113.1	113.8	110.2	110.7	99.7	125.4	117.7
ATP6V0B	1988.7	1753.6	2270.4	2755.8	1374.7	2033.8	2415.6	1758.5
ATP6V0B	158.1	139.3	158.4	171.7	145.6	158.5	144.6	159.2
ATP6V0C	277.7	260.7	299.7	345.3	199.9	273.4	383.5	334.9
ATP6V0D1	505	453.4	657.5	599.5	402.9	463.2	630.9	600.1
ATP6V0D2	116.7	111.7	110.8	116.5	108.9	102.4	116.6	114.2
ATP6V0E1	2318.8	2212.7	2600.4	2975.2	2192	1938.2	2766	2562.9
ATP6V0E2L	1295.9	1664.7	1228.3	1172	848.3	701.5	510.3	485.2
ATP6V1A	1521.5	1149.1	1007.3	1832.2	893.5	1101.8	2095.4	1406.6
ATP6V1B1	124.6	118.1	116.2	122.4	114.2	102.4	114.5	122.3
ATP6V1B2	2295.5	1704.9	2432.2	2826.4	1342.9	2472.1	3996.8	2469.1
ATP6V1C1	194.7	173.3	200.8	193.8	147.2	176.6	274.4	208.7
ATP6V1C1	117	115.7	125.2	127.4	111	96.2	120.5	117.2
ATP6V1C2	110.9	113	111	124.4	115	102.6	123.6	113.2
ATP6V1C2	110.9	113.4	111	114.2	111.2	94.2	116.5	107.7
ATP6V1D	642.6	571.2	629.1	765.2	486.2	564.7	705.4	542
ATP6V1E1	1502.1	1597.2	1501	1755.3	1114	1342.7	1655.7	1308.9
ATP6V1E1	1001.4	1114.4	1063.6	1304.4	840.6	1004.9	1231.1	958.4
ATP6V1E2	145.4	171.9	197.1	150.9	153.3	152.4	188	198.3
ATP6V1F	1207.7	1135.9	1303	1331.8	982.9	1028.7	1395	1035.1
ATP6V1G1	1257.8	1574.6	1657.7	1580.3	1392	1456.5	1824.1	1886.6
ATP6V1G2	124.3	128.3	150.1	138.4	117.3	109.2	125	125.2
ATP6V1G2	117.4	118.8	123	119.9	114.2	102.1	112.7	118
ATP6V1G3	113.1	119.3	120.2	110	111.8	103.3	119.7	115.6
ATP6V1H	121.9	118.3	123.5	118	113.2	101.1	121.2	119.5
ATP6V1H	220.5	214.5	195.7	217.7	175.8	167	222.4	224.3
ATP7A	143.7	129.5	137.2	150.9	141.9	118.6	141.6	149.7
ATP7B	114.3	126.9	117.3	120.6	110.7	110.2	124.9	124.7
ATP7B	124.9	126.7	118.6	116.9	119.4	103.4	127.5	122

ATP8A1	124	122.7	121.9	124.8	125.6	106.2	125.2	125.5
ATP8A2	132.8	123.9	128.5	137.4	117.5	107	122.9	111.8
ATP8B1	108.1	109.8	121.6	124.1	112.5	104.6	124.3	118.9
ATP8B2	127.5	135.6	124.8	130.2	135.8	105.7	124.3	124.4
ATP8B2	235.4	341.3	228.3	212.6	211.6	199.4	174.3	261.4
ATP8B3	117.5	113.9	114.9	122.4	110.6	101.5	128.3	114.4
ATP8B4	186	219.3	226.3	182.1	196.6	131.2	180.2	177.5
ATP9A	261.2	215	238.2	268.2	153.7	162.3	243.5	188
ATP9B	131.2	136.8	136.3	152.8	120.7	108	144.7	129.4
ATPAF1	181.1	194.6	256.6	195	178.7	204.8	193.9	166.5
ATPAF1	179.5	157.4	209.4	198.3	169.1	153.8	176.7	162.2
ATPAF2	137	126.2	138.9	132.9	118.9	113.3	131.2	127.1
ATPBD1B	330.4	325.9	317.5	330.5	253.3	213.4	296.4	317.8
ATPBD1C	109.7	109.8	111	122.9	105.5	103.4	118.7	113.9
ATPBD3	144.5	146.9	165.2	155	136.1	115.7	155.1	151.1
ATPBD4	124.1	127.5	140.8	119.3	115.2	112.6	118	111.8
ATPIF1	235.2	271.2	249.3	220.5	193.3	170.1	187	186
ATPIF1	192.2	154.1	178.5	181	122.4	158.2	145.9	137.6
ATR	118.7	121.8	114.5	117.1	106.4	100.9	120.2	107.4
ATR	115.7	115.5	121	116.2	103.8	96.1	108.4	109.5
ATR	135.7	171.6	151.2	145.7	136.9	117.1	131.8	156.9
ATRN	125	122.6	117.4	137.7	119.9	109.5	126.4	123
ATRN	112.5	115.9	117.5	118.1	110.2	106.1	119.7	117.8
ATRNL1	116.9	119.6	130	116.1	111.2	94.6	118.2	111.8
ATRX	123.9	120.4	122	140.4	122.3	101.4	134.7	129.7
ATRX	111.1	118.7	112.7	119.7	103.6	92.8	114.7	113.7
ATRX	120.9	115	124.4	125.4	116.8	99.6	125.5	121.4
ATXN1	274.4	320.3	287.5	362.3	289	219.8	338.4	312.6
ATXN10	119.4	134.4	115.4	124.6	114.8	100.1	114.5	121.8
ATXN2	720.2	706.2	614.6	707.8	692	565.3	861	864.6
ATXN2L	148.3	140.7	126.7	136.7	132	121.6	165.9	152.7
ATXN2L	130.2	124.4	117.3	143.3	129.8	101.8	140.8	132.6
ATXN3	258.3	340.4	230.7	327.2	321.8	160.2	341	371
ATXN3	122.1	124	123.9	142.7	124.4	108.6	131.7	129.7
ATXN7	119.3	111.6	114	119.9	109.3	95	115.8	105.3
ATXN7L2	173.6	203.4	146.3	171	145.4	120.4	180.5	151.9
ATXN7L4	122.1	128	115.6	123.1	115.4	93	120	115.8
ATXN8OS	110.9	111	113.2	114.1	108.4	96.6	108.2	109.8
AUH	170	184.9	176.2	177	168.6	149.9	153.7	159.7
AUP1	173.6	156.7	162.3	172.7	146.2	168.1	152.5	162.9
AURKA	158.5	163.3	161.9	144.1	140.5	134.2	160.2	147.3
AURKA	139.5	140.2	136.7	148.1	137	118.3	136.1	149.1
AURKAIP1	1771.2	2014.5	1571.5	1854.6	1406.5	1125.3	1635	1707
AURKB	117.9	125	110.2	126.4	119.3	103.5	133.6	124.8
AURKC	101.1	115.2	108.9	123.5	103.5	89.1	115.9	110.2
AURKC	114.1	116.1	122.8	119.3	100.8	93.9	121.1	116.1
AURKC	117.8	122.7	121.2	126.3	108.7	104	119.5	113.3
AUTS2	398.1	477.4	613.3	496.5	431.2	536.1	438.5	438.9
AVEN	129.8	146.5	130.9	139.7	138.4	110.1	143.2	131
AVIL	129.4	128.8	128.4	137.7	111.7	107.8	134	154.8
AVP	121.5	123.8	117.1	135.6	121.4	105.7	124.8	117.6

AVPI1	282.5	324.7	223	398.6	324.1	287.5	438.5	386
AVPR1A	109.2	113	119.8	111.5	108	96	122.8	119.1
AVPR1B	124.4	135.3	141.6	125.7	119	107.5	121.4	129.3
AVPR2	109.2	122	113.8	117.2	108.7	104.4	116.3	115.1
AXIIR	1200.8	1092.8	1317.1	928.1	613.9	1039.6	463.5	370.9
AXIN1	541.3	579.4	430.6	594.7	520.7	428.6	812.2	842
AXIN1	122	135.4	122.2	132.2	123.4	105.9	139.8	140.2
AXIN2	408.6	477.4	277	343.6	358.7	231.5	333.3	314.5
AXL	116.6	118.4	123.8	116.2	107.8	102.7	119.6	115.5
AXL	116.8	120.8	121.5	130.4	113.4	99.8	113.2	112.9
AXUD1	480.8	596.3	352.3	639.6	462.9	419.7	753.4	844.4
AYP1	134.7	126.5	127.1	125.3	112.1	106.7	129.8	124.6
AYTL1	115.1	131	118	125.4	116.1	102.6	119.9	119.2
AYTL2	321.8	336.1	270.9	349.1	235.6	193.4	279.9	288
AZGP1	127.9	127.9	127.6	135.4	115.8	105.5	128.6	123.7
AZI1	216.9	256	233.8	189.6	158.7	167.8	195.8	169.6
AZI1	113	122.1	121.3	116	116.3	106.6	114	122.2
AZI2	153.6	154	162.3	157.8	157.5	157.2	158.3	216.5
AZIN1	732.1	907.5	822.3	767.6	720.4	664.4	887.8	1252.6
AZIN1	131.7	127.6	130.9	139.5	128	105.2	128.4	126
AZIN1	157.8	164.1	196.4	151.3	127.1	142.6	180.5	163.6
AZU1	116.7	114.5	117.3	117.5	123.8	93.8	121.2	127.5
B2M	4277.4	3789.2	4235.2	5882.2	3217.4	2669.3	5529	3373.1
B3GALNT1	109.8	130.6	120.9	123.1	113.3	104.6	120.6	116.9
B3GALNT2	114.6	115.4	118.4	126	115.9	105.8	124.3	114.1
B3GALT1	125.9	123.2	120.9	131.7	117.2	108.4	125.1	128.9
B3GALT2	135	118.2	141.2	136.4	118.2	106.8	138	127.6
B3GALT3	126.5	112.5	123.9	127.3	119.9	97	115.7	130.5
B3GALT3	104.2	111.3	119.2	117.1	101.6	92.1	111.9	119.4
B3GALT3	103.4	132.4	136.7	137.3	117.5	107.7	126.7	126.1
B3GALT4	237.9	200.9	214.2	230	175.1	177.2	216.8	186.2
B3GALT5	118.6	120	117.2	117.3	108.2	106.2	119.8	114.6
B3GALT5	113.5	116.9	115	119.5	120.7	114.5	122.9	121.7
B3GALT5	115.4	110.7	104.2	116.6	108.4	97.6	112.8	120.9
B3GALT6	247.7	294.1	216.3	279.1	225.5	183.5	285.4	208
B3GALT7	115	111.4	110.3	120.2	119.6	111.7	130.9	121.2
B3GAT1	126.7	123.2	125.8	126.5	115.9	104.3	126.3	142
B3GAT1	124.9	121	122.6	127.8	120.1	117.9	139.6	130.3
B3GAT1	113	129.3	115.1	126.5	113.1	98.6	127.3	117.2
B3GAT2	114.5	117.8	111.3	114.5	101.5	101.1	108.1	112.2
B3GAT3	174.2	190.4	212.9	166.8	155.8	152.7	158.8	140.3
B3GNT1	142.4	154.4	178.9	144.5	143.2	112.3	141.2	142.3
B3GNT3	125	120.9	127.7	135.5	112.9	109.1	150.1	125.7
B3GNT4	117.9	116	128.3	131.2	112.1	97.1	116.2	115.7
B3GNT5	121.7	129.8	135.2	118.2	106.3	107.2	137.6	141.6
B3GNT6	154.6	157.6	152	145.7	131.5	125.6	132.7	115.7
B3GN-T6	111.7	122.1	115.9	119	107.3	98	110.4	120
B3GNT7	116.6	121.5	116.6	122.8	116.4	97.2	108.7	112.6
B3GNTL1	119.9	116.5	117.4	129.9	101.5	110.3	111.2	112
B3GTL	194.9	184.9	180.8	194.5	197.2	144.4	186	195.8
B4GALNT2	111.9	113.9	118.2	118.7	104.8	96.8	120.2	112.7

B4GALNT3	119.6	111.9	124.8	109.7	104.1	97.4	119.7	114.8
B4GALNT4	111.3	100.3	109.8	109.5	107.8	95.1	112	103.5
B4GALT1	140.2	205.9	145.1	153.5	160.1	133.9	168.2	158.6
B4GALT2	108.4	135.4	106.3	126	111.2	109	116.4	116.7
B4GALT2	112.1	116.7	137	135	122.6	106.6	124.8	129.6
B4GALT2	115.3	138.9	133.5	142.9	129.7	114.6	136.3	134
B4GALT3	547.9	672.5	425.3	549.4	453.8	401.1	637.6	618.6
B4GALT4	210.2	190.2	183.5	192.7	161.2	172.8	227.1	213.5
B4GALT4	129.4	153.6	163.5	146.4	137	128.4	158.7	150.6
B4GALT5	336.3	362.8	273.9	353.8	247.1	182	426.8	292.4
B4GALT6	116.5	113.7	116.8	112.9	121.4	104.2	132.4	121.1
B4GALT7	283.5	268.5	319.5	286.1	213.4	208.6	228.4	213
BA16L21.2.	154.8	141.4	162.8	174.7	146.8	118.5	148.6	173.9
BA9F11.1	111.4	114	110.7	109.5	99.4	94.3	114.5	118.6
BAALC	114.9	112.7	113.4	121.9	104.4	105	123.1	120.8
BAALC	117.6	113.3	131.7	116.2	118.4	104.1	123.2	116.2
BAAT	114.2	109.9	108.9	116.4	109.2	102.2	117.7	108.7
BACE1	114	117.7	123.8	115.4	105.3	93.2	116.4	116
BACE1	120.6	122.8	128	137.4	128.1	108.3	124.3	124.4
BACE2	111.1	121.8	114.1	127	116.1	105.8	128.3	110.1
BACE2	115.1	114.3	112	116	117.9	105.4	123.9	118.4
BACH1	121.6	122.2	116.7	126.1	113.7	99.7	131.5	129.6
BACH1	122.3	118.9	116.4	119.9	113.2	98.2	108.9	116
BACH1	108.9	126.3	116.5	129.2	123.3	102.4	122.7	113.7
BACH2	394	389.6	275.6	340.8	322.9	251.3	394.9	493.5
BAD	124.2	122.5	132.3	129.7	110.1	111.8	120	116
BAD	185.9	194.9	178.2	182.1	154.2	156.2	172.8	145.3
BAD	113.7	120.4	119.5	123.9	109.6	100.7	111.7	122.2
BAG1	128.8	133	126.9	118.8	115.7	101.9	138.9	131.8
BAG3	367.7	437	360.7	425.4	375.9	299.9	870.7	1815.6
BAG4	117.6	126.8	135.4	135.7	111.7	109	127.1	138.5
BAG5	812.4	690.9	796.9	861.4	630.7	652.9	897.3	828.2
BAG5	110.1	113.9	108	109.7	112.6	95.4	116	101.6
BAG5	115.1	115.1	110.4	123.9	107.1	95.6	120.7	113.2
BAG5	162.7	177.3	166.7	207.3	152	139.9	197	192.7
BAGE	117.8	112.9	115.6	116.2	104	102.8	127.1	129.8
BAGE2	116.4	120.6	122.4	125.2	116.9	103.6	131.4	126.1
BAGE3	115.8	121.6	130.8	125	119.5	105.6	130.2	139
BAGE4	113	118.7	115.1	113.5	111.3	101.5	116.3	115.9
BAGE5	137.8	141.5	122.3	151	160.1	106.5	163.1	173.1
BAHD1	123.5	113	133.7	126.2	118.9	107.1	117.3	117.6
BAI1	115.7	111.8	120.9	115.9	106.3	100.4	110.6	105.8
BAI1	110.9	114.7	115.5	110.1	98.7	108.5	119.5	125.7
BAI1	113.1	109.2	116.2	115.9	99.9	97.4	117.3	114.2
BAI2	127.5	119	116.8	119.1	117.4	94.4	112.2	110.3
BAI3	104	102.1	110.2	110.4	108.9	100.5	117.5	114.9
BAIAP2	122.4	133.2	127.2	134.9	121.4	113.4	148.7	130.8
BAIAP2	108.7	116.5	119.9	123.7	104.8	99	123.6	119.3
BAIAP2	104.4	105.8	106.6	128.6	102.1	102	116.7	118.6
BAIAP2	108.3	111.7	117.8	117.8	102.6	97.6	123.2	120.5
BAIAP2L1	107.7	117.5	108.2	106.1	103.4	97.7	109.7	122.8

BAIAP2L2	151.8	159.8	178.2	203.6	167.2	145.8	170	189.8
BAIAP3	110.3	110	113.9	111.8	105.6	96.3	119.3	120.8
BAK1	133.2	162.2	147.4	145.9	131.6	117.9	131.2	143.6
BAMBI	113.1	107.1	99.6	121	104.6	97.4	126.8	122.7
BANF1	603.3	604	697.9	658.7	560.9	527.4	628.2	741.2
BANK1	418.6	340.7	301.6	532.7	325.5	227.2	358.9	274.5
BANP	154.1	150.4	135.7	155.3	124.9	121.8	184.3	179.1
BAP1	169.7	193	160.2	194.9	170	141.9	195.3	205.7
BAPX1	111.7	120.6	119.9	117.9	103.7	99.7	125.4	113.4
BARD1	203.9	172.4	204.4	197.6	184.2	141	219.5	212.6
BARHL1	118.8	120.8	115.7	118.5	102.2	103.1	123.4	114.4
BARHL2	122.3	127.6	132	124.7	121.3	108.5	117.8	119.4
BARX1	117.3	112	119.4	116.1	110.9	99.5	127.1	110
BARX2	110.6	109.6	123.6	119.3	106.9	107.5	121.1	115.7
BASP1	404.4	364.3	454.7	496.5	312.1	615.6	482.2	278.1
BAT1	1184.5	1101.6	1105.7	1071.4	826.5	1038.2	1379.7	1059.3
BAT1	111.5	122.2	120.1	117.2	115.3	104.8	113.8	122.2
BAT1	133.9	126.1	130	142.3	114.7	110.6	124.6	115.4
BAT2	169.3	195.4	143.4	172.7	175.2	146.5	213.3	185.7
BAT2	361.5	386.1	294.2	389.4	228	284.3	555.6	399.7
BAT2D1	716.5	823.8	635.9	855.8	807.7	522.9	844.6	992.4
BAT3	175.1	188.7	156.9	177.4	136.9	122	177.3	164.2
BAT3	156.6	162.4	187	170.4	138.7	158.7	175.1	154.5
BAT4	115.7	123.5	121.9	127.4	108.2	122.5	122.1	117
BAT5	324.2	292.4	335.1	318.2	269	285	302.1	271.4
BATF	189.1	200.7	163.1	175.4	152.7	122.7	155.3	154.9
BATF2	126.4	113.5	127.6	112.8	110.6	99.7	113.1	118.3
BAX	757.3	899.3	480.1	704.7	820.9	479.9	692.8	883
BAX	117.9	119.8	114.5	122.1	110	96.3	128.3	126.7
BAX	129.2	139.8	130.3	143.1	138.9	112.3	195.1	185.8
BAZ1A	782	737.8	695.5	881.5	674.3	520.2	1086.3	882.1
BAZ1A	242.5	203.3	187.4	230.4	182.3	143	247	302.2
BAZ1B	400.5	392.1	285.7	359.1	406.3	267.4	423	372.3
BAZ2A	133.4	118.6	127.3	126.4	116	107.9	133.3	121.4
BAZ2B	267.6	263.3	255	246.8	209	210.6	213.6	219.7
BBC3	108.2	116.1	124.6	127.4	113.7	109.9	119.2	130.2
BBOX1	107.5	128	121.6	124.6	110.4	105.7	119.9	118
BBS1	160.5	165.7	189.7	158.4	136.4	126.6	153.6	136.9
BBS12	117.1	116.4	125.6	125.8	116.5	113.9	125.6	117.1
BBS2	276.2	278.2	379.8	264.5	264	229.6	216.2	173
BBS4	160.6	162.8	231	182.1	138.3	158.2	156	142.2
BBS5	112.1	117.7	112.3	119.6	107.4	97.6	126.7	119.7
BBS5	117.3	114.6	117.4	113.6	104.4	98.3	106.1	113.8
BBS5	118.4	114.4	110.4	114.5	103.3	95.4	115.6	108.9
BBS7	120.5	121	117.6	127.4	110.4	108.9	113.8	109.2
BBS7	175.1	190.8	173.1	169.3	176.2	152.8	222.1	227.2
BBS7	107.8	117.4	111.9	118.4	102.9	101.5	110.2	114.9
BBS9	102.9	121.1	116.5	121.7	106.8	100.9	122.6	120.8
BBX	549.9	625.7	440.7	544.3	488.2	355.3	538.6	705
BC002942	399.7	381.1	497.9	443.2	282.9	346.6	519.2	413.9
BC37295_3	120	117.9	117.5	128.6	102.5	95.2	126.8	112.9

BCAM	111.9	121.3	120	120.5	108.7	103.9	118.4	124.7
BCAM	108.3	109.7	111.2	115.1	101.6	100.3	114.6	110.3
BCAM	113.2	121.4	113.2	123.6	118.1	99.5	123.4	113.5
BCAN	131.1	131.7	134.4	152.2	140.1	121.6	139.4	137.2
BCAN	106.8	121.7	107.2	117.3	107.3	89.6	107.9	114.5
BCAN	120.6	115.9	116.6	120.8	109.6	100.8	127.5	115.9
BCAP29	185	188.8	223.1	201	164.5	193.2	178	181.7
BCAP29	115.4	115.9	117.3	118.1	101.6	99.9	118.5	123
BCAP29	156.2	145.8	186.2	200.7	154.8	177.9	181.6	178.3
BCAP29	121.5	113.8	116.4	119.4	110.9	95.4	115.7	112
BCAP29	134.4	144.4	180.5	148.1	136.4	131.7	149.5	149.2
BCAP31	1248.4	1367.8	1207.1	1591.5	1120.5	1162	2305.9	1540
BCAR1	115.6	117.5	114.8	116.6	106.4	94.9	112.5	113.1
BCAR3	137	135.1	128.7	153.9	125.3	106.5	131.3	126.2
BCAS1	116	109.8	110.4	127.4	105.5	97.6	127.1	117.1
BCAS2	119.6	122.7	133.8	129.8	115.2	100.3	117.8	125.1
BCAS3	115.8	114.3	108.3	116.2	102.3	94.4	123	115.5
BCAS4	378.3	406.3	422.5	441.2	329.5	274.5	305.9	236.4
BCAS4	480.1	574.4	453.6	481.8	343.2	306.2	298.7	275.9
BCAS4	121.2	131.4	136.5	129.3	120.4	102.7	135.3	141.5
BCAT1	133.7	132.4	137.6	125.4	122.2	111.4	135.8	135.8
BCAT2	157.7	171.4	184.2	162.7	140.9	136.5	162.2	151.1
BCCIP	450.4	556.4	549	431.6	417.7	342.9	455.1	420.2
BCCIP	184	164.7	235.5	197.2	158	173.5	153.8	176.9
BCCIP	168.9	148.4	201.8	181.8	160.6	137.6	169.9	193
BCCIP	120.5	119.8	134.3	120.3	121.3	102.2	130.5	120
BCDIN3	919.8	1015.3	631.4	960	833.8	598.2	1297.9	1347.5
BCDO2	104.4	109.6	112.1	111	104	96.9	112.9	111
BCHE	110	118.1	121.8	116	109.1	97.5	110.8	118.5
BCKDHA	351.8	394.5	430.6	371.8	304.4	344.2	268.4	274.8
BCKDHB	128.8	123.5	128.2	136.6	117.2	109	120	116.7
BCKDHB	113.3	122.1	116.5	128.9	110.4	107.1	128.3	125
BCKDK	793.9	630.6	1051.8	865.4	397.5	608.3	601.4	416.2
BCL10	123	132.9	134.9	127.6	124.1	123	142.5	130.2
BCL11A	147.9	136.8	142.1	157	127.1	107.1	144.9	117.1
BCL11A	127.9	135.3	117.2	133.9	116.6	101.6	132.5	114.6
BCL11A	155.8	143.4	153.6	160.8	126.1	114.7	123.5	128.9
BCL11B	1596.1	1841.9	1361.8	1525.8	1494.7	1205.5	1050.4	1157.1
BCL11B	109.8	110.6	109	117.4	107.5	98.1	108.3	112.4
BCL2	108.2	115.2	113.4	114.3	101.3	92.9	111.6	108.1
BCL2	117.6	128.4	123.1	121.6	113.9	100.9	116.3	121.9
BCL2	461.6	457.9	393.3	452.1	340.7	349.8	383.3	329.1
BCL2A1	112.6	138.2	125.3	128.3	119.2	119.1	133.5	123.2
BCL2L1	392.4	390.4	392.1	367.2	312.7	335.8	383.2	315
BCL2L1	117.6	116.2	119.4	116.9	113	93.5	130.6	121.4
BCL2L10	105.2	115.4	113.6	122.2	109.9	102.1	129.4	114.4
BCL2L11	113.5	111.2	115.5	118.6	106.6	96.1	121.5	117.2
BCL2L11	108.2	116.8	112.4	114.8	108.4	101.1	112.7	115.8
BCL2L11	119.3	117.8	114.8	124.8	113	104.8	130.4	111.1
BCL2L12	121.7	124.2	134.9	135.3	118.6	118	132.7	126
BCL2L12	226.7	258.4	242.3	259.6	205.5	207.5	252.9	249.7

BCL2L12	213.4	216.7	220.6	192.7	190.1	185.6	210.7	233.3
BCL2L13	598.6	608.6	636.3	607.1	681.4	556.8	703	881.4
BCL2L14	122.8	127.1	123.3	120	112.1	109.1	123.4	123.6
BCL2L14	111.7	119.4	124.4	121.1	103.6	102.8	124.7	114.8
BCL2L14	119.8	121.3	130.1	116.7	116.3	106.6	122.3	124.8
BCL2L14	110.3	110.4	111.8	113.1	108.3	95.7	114.7	107.8
BCL2L2	146.8	130.4	176.9	159.7	136.3	138.1	147.2	135.2
BCL3	235.9	220.8	192.6	303.9	200.2	239.2	315.5	214.8
BCL6	113	111.9	116.9	121.4	107.2	96.5	118.5	117.1
BCL6	495.5	379.8	375.5	581.4	425.9	320.2	757	620.6
BCL6B	103.9	119.7	109	118.1	108.3	95.8	120	113.2
BCL7A	110.4	113.7	120.1	119.7	117	105.7	116.6	123.3
BCL7A	121	125.3	112.8	118.6	104.3	101.9	128.7	121.7
BCL7A	123.6	120.2	119.3	128.3	119.8	100.9	118.3	121.3
BCL7B	190.3	193.4	159.8	193.1	147.1	147	197.1	173.6
BCL7B	232.4	241	237.1	270.2	217.6	202.8	302.4	267
BCL7C	239.9	290.3	397.3	254.9	217.1	278.6	246.6	213.7
BCL9	150.2	150.5	151.4	157.4	153.2	144.7	155	162.9
BCL9L	170.3	160.7	159.1	163.2	134.8	138	159.6	143.9
BCLAF1	932.8	1027.6	894.6	1107.9	753	699.2	914.6	842.7
BCLAF1	343	284.5	270.5	264.6	277.7	220.3	210.4	220.8
BCMO1	112	117.3	123	111.9	112.4	103.6	127.5	111.5
BCNP1	133.7	123.7	146.4	133.9	111.1	110.7	130.5	117.8
BCOR	318.9	305.4	348.6	294.6	247.4	301.3	258.3	208.1
BCOR	122	126	131.7	124.6	107.2	106.4	125.8	120.9
BCOR	140.1	152.5	170.5	158	126.9	120.5	137.8	120.8
BCORL1	122.3	126.6	125.5	135.7	114.7	115.3	141.3	145.6
BCORL2	129	136.9	134.7	131	124.6	109.1	129.3	125.7
BCR	120.9	183.2	125.6	135.2	164.6	102.9	129.3	155.1
BCR	118.4	118.2	119.1	114.4	116.6	101.2	131.3	107.4
BCR	126	122.4	120.3	129.7	109.4	104.9	129.2	119
BCS1L	177.4	174.9	185.8	162	145.4	140.9	171.4	159.5
BDH	119.5	123	127.5	122.6	111	109.1	126	118.9
BDH	110.4	116.6	114.3	127.8	114.4	103.9	113	107.3
BDH	136.1	145.9	127.6	135.6	115.3	113.3	130	132.2
BDH2	118.1	117.6	131.1	119.7	109.2	102.3	123.2	109.2
BDKRB1	113.4	121.2	118.1	114	115.8	102	132.9	119.4
BDKRB2	119.4	110	133.4	114.9	96.6	97.7	108.3	110.1
BDNF	107.4	115.6	120	117.5	120.1	98.2	121.7	110.4
BDNF	112.7	117.3	120.4	118.2	108.9	104.8	116.3	117
BDNF	136.5	133.1	173.6	130.1	129.8	111.7	128	128.8
BDNF	121.5	123.2	126	142.5	115.4	107.2	115.5	132.3
BDNF	117.7	106.7	116.6	111.7	107.5	108.8	110.7	110.4
BDNF	125.3	132	143.5	134	117.4	122.4	124.3	119.8
BDNF	100.4	110.7	112.8	118.6	109.1	99.5	118.7	112.3
BDP1	140.5	124.4	131	137.9	126.1	106.9	137.6	131.7
BECN1	185.4	200.2	193.2	176.7	189.2	177.3	204.8	181.1
BET1	151.1	177.3	162.5	164.7	147.6	126.2	147.2	168.4
BET1L	155.8	152.3	148.1	144.8	143.9	120	142.9	136.9
BEX1	121.2	131.1	121.6	120	109.6	103.8	113.9	124
BEX2	457.3	556.7	540.7	472.4	426	374.6	369	388.3

BEXL1	242.4	265.8	285.2	251.7	212.6	272.9	175.9	187.9
BEXL1	545.7	668.4	688	575.5	368.9	484.8	430	478.8
BFAR	290	260.2	270.4	306.9	248.1	227	296.2	406.3
BFSP1	117.3	109.1	123.2	119.8	105.5	95.3	117.7	111
BFSP2	115.7	119	116.6	118.9	121.4	105.2	119.6	111.9
BGLAP	121.6	132.2	125.6	133.4	120.2	103.1	131.7	119.4
BGN	119.3	112.8	126.7	112.1	110.8	103.8	119.1	118.2
BHLHB2	602.8	638.6	485.1	641.1	434.2	344.9	659.8	544.9
BHLHB3	158.2	151.4	129.3	178.3	134.3	113.7	120.5	115.5
BHLHB4	110.7	122.8	120.7	123.8	112.5	102.7	117.5	105.3
BHLHB5	101.3	116.2	114.6	114.2	96.8	101.9	113.7	113.3
BHLHB8	118.6	118.3	111.5	120	117.4	105.4	118.8	121.3
BHLHB9	123.4	131.4	128.1	137	123.5	108.3	131.2	130.3
BHMT	110.2	113	113.5	113.6	103.4	95.2	120.4	103.5
BHMT2	112.6	111	115.1	111	102.6	98	117.1	109.6
BICC1	114	113	121	111.8	102.2	99	110.5	115.3
BICD1	117.2	119.1	117.9	121.4	119.3	104.4	123	116.5
BICD1	123.4	118	117.7	111.8	114.8	102.2	122.3	118.9
BICD2	110.9	112.8	112.7	122.2	113.4	104.9	116	125.2
BICD2	841.7	892.7	857.2	876.1	571.9	531.7	772.5	844.9
BID	205.6	248.1	226.1	260.2	221.2	238.9	220.3	178
BID	192.9	182.6	180.6	197.2	162	136.5	184.6	172
BID	109.6	100.5	103.6	109.9	96.5	89.1	102.6	108.8
BIK	112.1	120.5	109.4	118.8	112.3	110.9	120.5	115.6
BIN1	562.4	799.6	731.7	767.3	598.5	674.4	606.5	585.4
BIN1	1193.4	1344.3	1340.7	1325.8	1083.6	1245.5	1252.3	989.6
BIN2	138	128.7	141.7	135	130.2	110.6	135.6	134.3
BIN3	129.4	129.4	145.5	128.8	127.5	118.2	131.9	136.7
BIRC1	112.4	115.3	115.6	119.5	109	100.2	120.1	111.8
BIRC1	110.7	116.3	121.3	118.7	109.8	93.5	118.4	114.5
BIRC2	1207	1064.2	1385.4	1012.8	1043.1	956.5	1129.8	1192
BIRC3	1655	2057.3	1268.3	2047.3	1685.9	1029.1	1954.5	1705.4
BIRC3	1155.4	1481.4	901.3	1253.1	1262.3	652	1510.1	1292.3
BIRC3	325.5	388.4	217.9	327.9	364.3	123.8	378.1	369.8
BIRC4	126	120.9	124.9	127.9	127.4	115.5	120.2	128.1
BIRC5	109.6	116.1	119.4	115.1	108.1	93	116	119.1
BIRC5	107.5	120	113.8	111.2	109.5	98.4	111.3	114.4
BIRC6	168.4	177.6	168.1	176.1	172.2	131.2	194.5	172
BIRC7	116.2	121.8	114.1	118.9	102.9	97.3	116.9	116.8
BIRC7	113.1	120.5	121.4	121.1	120.5	95.7	113.2	122.4
BIRC8	114.6	113.4	114.1	120.6	107.8	100.3	124.2	120
BIVM	152	161.7	166.3	149.5	163.6	147	134.4	139.4
BLCAP	248	250.7	286.9	230.9	195.1	214.6	213.3	211.6
BLES03	139.9	156.9	151.6	144.7	131.5	131.8	133.5	138.7
BLK	177.3	182.9	201.3	189	121.7	168.4	160.9	125
BLM	113.8	118.5	133.1	120.1	102	113.1	117.9	122
BLMH	168	216.2	175.2	188.3	165	142	179	182.3
BLNK	122.4	117.5	115.2	124.3	109.5	102.3	116.3	112.6
BLOC1S1	211.1	237	250.8	227.6	203.4	202.9	191.1	197
BLOC1S2	119.4	108.2	119.6	124.6	111.2	103.5	120.4	110.3
BLOC1S2	124.5	124.3	132.9	133.3	126.5	109.2	140	144.6

BLOC1S3	105.9	108.9	114.8	113	103.9	101.3	122.6	116.3
BLR1	456.6	303.5	354.3	407.1	206.9	277.7	291.9	251.1
BLR1	180	166.3	167	180.8	140.9	139.2	174.9	148.3
BLR1	119.1	122.1	120.5	116.4	105.8	100.9	125.7	112.8
BLVRA	161.6	130.3	228.6	163.9	154.7	153.5	148.2	137.7
BLVRB	142.3	117.1	185.3	142.8	117.7	163.1	154.5	131.9
BLZF1	226.6	187.7	183.1	229	202.2	169	247.2	235.3
BM88	121.6	108.4	120.6	127.8	114.3	99.1	119.1	117.4
BMF	148.2	142.9	186.2	165.4	121.8	148.6	152.3	131.4
BMF	102.8	109.6	117.6	115.9	100.2	99.1	120.3	105.4
BMF	116.2	117.2	137.3	125.1	106.5	94.8	133.5	119.7
BMF	135.2	136.3	150.5	146.7	120.2	123.1	138.4	128.5
BMP1	105.2	111.5	113	98.7	110.6	98.1	115.9	112.6
BMP1	117.3	123.4	122.5	121.6	106.9	101.1	128.1	117.7
BMP1	126.9	126.9	126.7	134.3	123.3	111.1	143.9	144.9
BMP10	108.6	106.2	117.2	126.1	113.2	102.5	116.8	110.8
BMP15	111.9	115	124	113.2	108.1	93.3	114.9	112.8
BMP2	114.1	119.8	114	119.4	109.4	106.9	118.7	112.8
BMP2K	133	145.4	130.9	133.5	124.8	106.6	123	124.6
BMP2K	132.7	131.4	126.7	139.3	105.9	107.9	122.2	121.7
BMP2K	123.3	129.1	138.1	120.4	122.4	103.4	118.6	137.7
BMP3	115.9	114.8	112.2	131.9	109.6	97.9	124.3	125.8
BMP4	121	121.6	117	122.9	118.5	105.8	115	115.2
BMP4	113.7	111.8	116.7	119	106.2	99.9	121.1	113.2
BMP4	121.2	119.8	128.5	116.1	106.7	100	120.5	118.8
BMP5	111.4	106.3	115.2	123.9	111.4	101.8	117.4	124.9
BMP6	131.2	151.4	126	128.2	127.1	123.4	135.5	133.8
BMP7	117.9	118.3	124.1	116.4	117.4	103.6	115.8	116.8
BMP8A	109.2	112.4	118.4	119	100.1	90.3	115.2	108.1
BMP8B	125.6	105.9	129.4	138.7	122.4	104.6	127.5	127.1
BMPER	110.1	128.6	119	125.2	110.7	100.2	118.8	118.5
BMPR1A	113.1	116.1	122.3	119.6	105.6	99.9	119.7	117.9
BMPR1B	108.1	119.2	109.7	113.3	103.1	104	106.9	109.2
BMPR2	171.8	168.8	173.1	195.8	162.6	130.1	231.2	174.6
BMS1L	405.6	459	334	453.1	366.6	259.9	363.4	337.2
BMX	119.9	112.8	120.5	116.5	103.1	100.3	112.9	112.3
BMX	118.1	119.6	121.3	119.1	110	104.5	117.8	120.9
BNC1	135.6	115.2	116.2	123.7	111.8	104.7	116.2	118.3
BNC1	114.8	116.2	114.7	116.8	107.9	96.7	114.1	104
BNC2	120.6	121.2	150.5	135.3	107.5	112.6	124.1	132.9
BNIP1	138.6	131.8	128.3	128	113.4	101.5	119.1	132.4
BNIP1	139.6	149.2	143.3	158.5	127.7	115.4	126.2	128.7
BNIP2	610.4	758.1	597	782.5	667.4	646.7	910.3	1009.9
BNIP3	766.1	723.2	547.4	720.7	537.1	431	678.8	671.6
BNIP3L	147.7	153.5	158.1	179.3	132.5	127.8	160.5	193.6
BNIPL	121.4	122.1	124.5	139.1	119.5	102.3	134.2	126
BNIPL	132.8	154.3	153	162.4	139.3	115.4	165.7	180.1
BNIPL	126.1	122	120.9	118.5	122.8	95.2	120	120.5
BOC	105.5	106.4	103.1	113.8	101.5	91.8	111.8	111.3
BOK	128.6	124.5	132.7	137.2	121.2	114.9	142.3	135.4
BOLA1	111.6	119.5	133.8	139.1	111.5	105	127	115.1

BOLA2	791	1027.7	924.4	869.6	820.1	611.7	625	651.7
BOLA2	281.6	295.4	274.7	193.1	213.9	184.5	197.2	220.1
BOLA3	308.4	380.2	559.1	329.7	357.7	379.6	301.5	338.9
BOLA3	314.4	379.6	597.9	353.8	368.5	462	335.4	356.1
BOLA3	128.1	119.1	129.6	126.5	117.5	102.8	123.8	115.1
BOLL	103.2	105.9	110.6	112.8	108.2	86.3	111.3	106.5
BOLL	121.8	116.6	124.8	121.3	112	103.5	118	115
BOLL	119.5	121.7	116.8	115.4	106.8	102.9	116.2	111.7
BOP1	446.4	467.1	344.8	417.2	338.6	186.1	371.9	331.5
BP75	156.5	147.7	147.7	153.8	161.9	125.4	144.1	132.8
BPGM	176.4	160	206.1	164.3	151.3	172.8	170	158.3
BPGM	113.7	122.3	121.5	121.3	116.9	107.4	117	122
BPHL	184.9	242.9	229	235.2	189.1	181.6	195.2	187
BPI	105.1	128.1	115.6	113.1	115.3	99.6	113.4	130.1
BPIL1	110.7	103.6	110.1	120.1	106.9	99.2	115.1	113.5
BPIL2	118.9	114.3	123	119.5	107.3	97	127.9	116.8
BPIL3	110.4	122.8	123.1	121.2	102.9	106.2	115.4	114.1
BPNT1	142.1	137	143.5	151.8	134.4	113.2	130.8	141.1
BPY2	116	112.3	107.9	116.3	104.9	94.4	111.9	116.7
BPY2B	110.8	109.3	113.4	107	103.1	99	125.1	117.2
BPY2IP1	250.3	263.1	255.8	283.6	184.1	231.7	361.2	357.7
BRAF	126.2	124.5	116.8	137.4	123.1	113.4	130.6	128.6
BRAP	119.3	115.5	124.2	130.2	113.1	114.8	121	122.1
BRCA1	111.7	116.1	126.7	128.5	109.6	98.3	117.4	118.7
BRCA1	122.4	116.1	118.2	105.7	111.4	108.9	127.4	115.7
BRCA1	123	120	118.6	121	117.6	96.8	113.9	116.8
BRCA2	107.9	102.9	110.1	113.3	107.6	113.4	120.8	124.1
BRCC2	111.6	105.8	133.5	122	111.2	95.4	125.7	115.8
BRD1	206.5	186.5	160.4	195.9	169.9	157	192	216.9
BRD2	2144	2693.8	1438.4	2699.5	2408.8	1413.2	3377.5	3829
BRD3	481.2	532.6	464.6	608.3	476.7	400.9	643.1	583.8
BRD4	119.8	124.7	123.2	129.4	113.6	92.1	143	134.3
BRD4	125.2	118.2	109.3	131.6	123.6	98.3	147.3	141.7
BRD7	447.6	443.4	338.5	387.8	287.3	273.6	380.8	318.4
BRD8	260.9	317.8	263.8	338.1	229.7	212.8	321.5	287.6
BRD8	106.8	110.1	112.7	110.6	108.3	99.1	122.5	121.8
BRD9	550.3	616.6	597.7	612.3	439.6	473.7	657.8	652.4
BRD9	115.1	113.2	118.6	120.2	105.7	108	123.8	112.9
BRDG1	136.6	132.3	148.4	130.5	111	117.5	131.7	123.4
BRDT	113.5	109.1	114.8	121	105.5	95.7	116.6	110.6
BRDT	118.7	117.3	109.9	114.1	107.9	98.3	114.4	121.1
BRDT	112.2	108.9	105.8	108.9	104.1	90.8	108.5	112.6
BRE	211.6	244.9	278.2	247.6	201.7	227.8	218	204.2
BRE	209.8	195.7	226.4	196.7	172.2	186.7	220.3	210.7
BRE	116.3	126.8	125.1	115.8	121.9	101.2	129.4	118.6
BRF1	112.4	114.9	112.9	119.3	114.2	101	114.1	125.6
BRF1	142.3	126.4	141.6	143.1	121.7	117.1	135.6	143
BRF1	126.1	119.1	125.8	117.7	106.5	115.5	124.4	119.8
BRF2	265.8	321.2	328.8	318.7	273.8	270.8	345.5	397.3
BRI3	861.8	676.1	1023.2	889.5	480.6	940.1	1047.2	824.8
BRI3BP	111.8	123.9	122.4	120.8	109.8	107.1	121.8	121.4

BRI3BP	138	131.4	154.4	129.1	117.2	112.4	127.4	121.7
BRI3BP	131.6	117.8	138.6	114.4	127.8	106.6	129.7	131.4
BRIP1	114.1	117.2	134.8	122.7	109.3	105.9	127.8	117.4
BRMS1	1579	1629.8	1746.2	1549.2	1321.6	1102.2	1234.7	962.2
BRMS1	197.5	207.3	219.6	234.8	198.5	216.8	199.3	174.6
BRMS1L	112	114.7	117.3	109.7	107.1	106.9	120.6	122.4
BRP44	363.2	358.8	408.9	402.5	307.4	323.3	356.5	419.4
BRP44L	603.9	577.2	739.2	619.1	546.4	572.5	607.9	560.1
BRPF1	201.6	183.2	176.7	249.6	143.7	170.4	234.1	253.9
BRPF1	1864.4	2378.1	1588.2	2119.3	1680.6	1367	2345.4	2369.3
BRPF3	326.5	351.6	256.3	392.6	265.9	240.3	471.4	458.2
BRRN1	113.6	127.2	141.6	129.3	118.3	115.7	136.8	115.8
BRS3	114.6	112.5	124	111.9	122.7	105.2	121.7	119.6
BRSK1	332.1	202	581.2	510.8	223.8	1011.6	430.8	205
BRSK2	118.9	127.1	136.7	116.3	113.4	108.5	119.4	126.4
BRUNOL4	128.7	118.1	122.5	123.2	110.1	97.6	111	118.4
BRUNOL4	117.8	118.1	118.9	118.8	118.5	104.2	123.6	114.7
BRUNOL5	121.7	117.3	112.2	125.3	120.8	99.6	116.1	117.3
BRUNOL6	128.2	124	136.9	132.8	126.1	114.6	132.2	127.6
BRWD1	114.4	108	110.3	115.3	112	92.9	118.9	107.5
BRWD1	117.5	121.5	141.6	137.9	113.8	114.7	129.9	121.6
BRWD1	168.5	184.2	211.8	197.2	175.9	187.3	176.2	167.8
BRWD1	157.1	136.8	144.5	160.3	142.8	116.4	171.8	161.2
BRWD2	155.8	126.5	162.4	167.5	125.2	138.7	142.4	130.7
BRWD3	119.8	121.6	133.4	132.8	111.7	109.3	125.3	124.2
BSCL2	261.9	306.3	272.5	316.4	257.1	212.5	320.3	361.8
BSDC1	457.5	363.8	371.7	447.9	332.6	316.9	481.8	462.3
BSG	424.4	725.1	515.5	614.2	458.9	421.8	712.2	656.2
BSG	109.5	111.9	119.6	116.2	108.7	100.8	121.8	111.8
BSN	115	115.8	125.4	122	108.3	101.8	118.9	108.7
BSND	115	133.3	124.2	123.7	109.5	104.8	133.7	111.8
BST1	112.6	119.2	120.7	113.8	103.7	103.4	119.9	112.8
BST2	372.3	313.7	455.4	387	241.6	335.6	454.9	359.3
BTAF1	625.1	475.2	755.4	564	519.8	434.9	636	527.4
BTBD1	156.4	158.3	175.8	199.7	171	145.5	159.6	182.5
BTBD1	125.8	129.2	137.8	155.4	134.3	132.3	121.6	115.4
BTBD10	143.5	196.6	197.6	178.6	172.8	125.4	169.4	168.3
BTBD11	109.2	116.5	116.8	108.6	112	96.3	108	112.3
BTBD11	122.7	111.7	118	122.4	108.5	101.3	117.8	114.5
BTBD11	110	118	118.3	109.3	108.3	95.5	123.1	100.3
BTBD11	422.3	533	231.2	379.8	323.1	171.8	296.9	274.2
BTBD12	167.5	172.4	171.5	192.4	149.5	131	196.7	180
BTBD14A	143.6	143.7	140.1	151.1	128.2	105.3	143.8	143.1
BTBD14B	125.8	117.3	135.3	114.1	114.3	101.3	134.2	130
BTBD3	111.1	115.6	119.5	111.4	110.4	104.3	114.2	124
BTBD3	128.8	139.3	130.2	133.9	119.3	118.7	132.2	129.6
BTBD4	128.1	122	124.8	144.8	110.3	114.1	136.6	118.6
BTBD5	408.1	389.9	262.3	387.1	428.6	215.8	431.5	467.6
BTBD6	441.4	468.7	369.3	392.7	428.9	288.7	439.9	447.5
BTBD7	174.9	206.4	220.3	240.2	205.6	148.8	229.4	274.1
BTBD7	113.5	115	115.6	115.3	108.5	107.5	122	112.1

BTBD7	152.8	160.5	159	172.5	138	128.7	180.5	190.7
BTBD8	123	115.7	118.4	118.4	112.4	105.2	126.4	117
BTBD9	126.6	135.7	123.4	134.8	105.2	107.9	126.4	127.9
BTC	116.3	106.3	119.4	126.6	107.8	89.1	107.9	110.1
BTD	137.5	152.7	146.6	139.1	134.6	117.4	147.7	138.9
BTF3	4394.7	4167	5879.5	5679.7	3133.8	4706.1	4463.4	4778.5
BTF3	5558.2	4915.2	5907.8	6415.4	4673.5	5712.8	7379.7	4999.6
BTF3	155.4	158.1	182.3	176.4	136.4	151.9	154.1	160.8
BTF3L4	149.1	166.4	194.7	164.2	150.5	151.2	133.6	154.6
BTG1	3531.3	3402.1	3683.5	3761.8	2981.2	3512.5	3316.3	3732
BTG2	694.9	734.1	740.7	769.1	443.3	816	505.2	543.7
BTG3	230.7	176.8	166.4	226	148.4	175.1	232	225.7
BTG4	112.1	106.5	109.5	114.5	117	104.4	118.3	112.8
BTK	199.8	143.7	282.9	216.7	136.3	201.8	166.5	138.6
BTLA	201	198.7	188.7	180.6	147.3	132.9	154.8	140.2
BTN1A1	103.1	104.9	107.8	112.1	103.4	96	106.8	110.5
BTN2A1	121.2	126.5	127.2	131.3	109.9	106.3	136.9	129.5
BTN2A1	477.7	487.5	403.2	458.7	362.1	381.1	455.5	464.7
BTN2A2	126.7	140.8	141.7	133	113.2	111.4	131.1	136.3
BTN2A2	108.3	111.5	113.8	123.3	113.2	93.7	109.4	113.4
BTN2A3	112.3	111.1	116.9	118.4	104.3	97.9	119.9	112
BTN3A1	117	126.3	116.3	112	105.7	97.9	106.3	112.6
BTN3A1	319.8	247	370.4	272.7	178.2	235.3	189.2	171.6
BTN3A2	559	673.7	657.9	585.7	320.6	463	275.7	354.2
BTN3A3	276.3	246.5	244.8	258.9	188	197.9	178.9	155
BTNL2	128.2	135.9	117.5	126.9	117.1	100.8	122.6	125
BTNL3	113.6	113.7	120.8	114	105.8	103.4	113.7	118.2
BTNL3	113.4	105.8	113.2	125.7	100.8	99.5	118.4	117.9
BTNL3	113.9	118.3	119.1	121.1	112.6	101.2	120.2	114.1
BTNL8	107.6	108.9	117	105.8	108.4	105.8	115.3	109.8
BTNL9	114.6	118.6	125.7	112.5	113.3	101.4	129.5	120
BTRC	121.5	137.7	128.8	129	117.5	103.2	125.3	136.7
BTRC	117.9	116.4	115.9	119	114.8	98.6	117	115.9
BUB1	111.8	113.6	127.4	113.9	100.6	96.8	115.3	108.1
BUB1B	130	116.3	125.6	125.5	121.4	106.7	120.1	123.9
BUB3	2630.3	2796.8	2334.9	2748.8	1943.6	1724.8	2542.3	2770.9
BUB3	126.6	133.8	123.2	126.9	122.4	102.4	115	121.5
BUB3	197.9	201.3	162.9	284.1	180.2	144.1	254.1	178.3
BVES	114.3	114.3	114.1	119.9	108.5	108.1	118.7	116.4
BVES	116.8	113.6	110.5	115.3	105.4	99.1	121.3	106.8
BVES	115.6	110	121.1	113.9	120.7	95.5	118.2	119.8
BXDC1	403.2	453.5	453.1	437.1	496.9	312.4	494	477.4
BXDC2	192.4	208.8	191.6	234.1	179.3	149	198.7	223.6
BXDC5	269.6	254.5	242.3	236.3	219.3	192.7	249.4	283.5
BYSL	148.5	157.9	162.1	145.5	128.8	113.3	145.6	152.3
BZRAP1	180.8	159.7	189.4	166.8	136.5	145.4	165.3	155.8
BZRPL1	109.1	112.4	109.5	112.9	103.2	95.2	112.6	109.6
BZW1	128.3	116.5	133.7	125.7	113.7	109.5	129.4	126.5
BZW2	277.5	295.3	303.7	268.3	283.1	248.6	371.1	321.9
C10ORF10	106	111.1	120.3	124.2	120.4	94.9	123.8	117.3
C10ORF10	392.1	358.1	534.7	448.9	381.7	271	370.5	437.1

C10ORF107	117.9	118.6	129.7	122.2	107	100.2	119.8	123.7
C10ORF11	113	113.8	135.4	117.3	106.6	103.5	110.6	108.6
C10ORF111	114.6	122.2	121.2	124	104.8	99	129.4	126.9
C10ORF113	109.3	117.8	126.9	121	113.4	101.9	125.3	120.8
C10ORF114	106.2	120.2	121.1	111.5	108.6	101.1	119.4	114.9
C10ORF116	121.1	120.4	131.5	132.4	112.5	100.1	129.4	125.4
C10ORF118	123.3	125.8	132.9	138	113.8	107.4	130.9	137
C10ORF119	164.9	148.5	156	153.6	135.7	118.7	167.1	156.7
C10ORF12	115.5	122.9	114.2	125.6	112.7	102.1	136.7	119.9
C10ORF120	107.8	110.9	114.4	114.7	112.4	92.2	117.7	108.2
C10ORF125	138.1	140.4	195.1	142.5	114.3	125.2	129.9	124.2
C10ORF129	113	107.9	114	127.3	104.5	104.2	115.3	126.4
C10ORF13	122.9	115.6	125.2	127.5	112.5	105.5	123.6	114.2
C10ORF130	116	112.5	117.8	115.1	103.5	92.4	123.9	105.5
C10ORF132	127	119.6	131.5	131.3	110.2	99	114.4	116.2
C10ORF137	184.3	220	213.6	251.3	161.5	182.6	178.3	183.3
C10ORF22	349	351.8	259.6	369.7	250.5	251.8	299.7	238
C10ORF25	115.9	113.2	115.9	125.1	109.2	103.7	120.3	118.5
C10ORF26	229.4	227	252	245.4	222.7	218	237.5	241.6
C10ORF27	120.4	122.1	120.1	118.8	112.2	99.1	111.1	119.2
C10ORF28	138	151.8	137.9	152.6	144.4	135	170.5	184
C10ORF30	112.2	118.7	113.9	114.7	102.6	103	114.1	114.8
C10ORF32	284.3	343.8	280.6	294.8	315.6	246.8	305.7	296.1
C10ORF33	137.6	145.5	126.2	132	125.8	112.3	121.5	136.2
C10ORF35	192.2	193.7	174.4	198.2	162.1	129.9	175.1	187.1
C10ORF37	117.7	121.7	114.4	119	108.6	103	116.3	116.2
C10ORF38	169.7	167.2	173.8	179.9	158	175.6	144.6	147.7
C10ORF4	127.8	128.7	142	129	112.7	101.9	123.1	139.6
C10ORF4	114.2	117.4	114	121.5	115.2	103	124.4	120.7
C10ORF4	114.1	125	124	123.2	111.1	112.8	137.4	126.3
C10ORF4	115.4	116.4	116.5	118.8	106.3	99.9	118.5	114.5
C10ORF4	121.6	117.4	109.4	118.3	105.3	93.9	117.2	112.2
C10ORF42	164.9	140.7	162.2	178.8	140.2	129.4	167	164.5
C10ORF46	119.4	118.8	125.2	116.2	112.2	104.8	115	113.6
C10ORF47	116.9	117.7	127.3	119.4	107.1	110.2	119.1	110.3
C10ORF49	110	128.8	127.2	106.6	115.5	102.8	114.8	123.5
C10ORF53	109.5	117.6	123.1	120.1	112.5	106.3	109.5	112
C10ORF53	119.9	115	113.4	117.8	110.3	105	107	113.2
C10ORF53	117.1	114.3	124.2	115.9	110.9	100.6	129.8	123.3
C10ORF54	410.6	441.3	628.9	348.8	301.6	285	209.2	238.5
C10ORF55	122.2	110.5	121.2	122	110.2	102.9	123.2	112.8
C10ORF56	136.7	143.8	158.5	148.5	121.4	125.3	142.7	141.1
C10ORF57	144	144.3	184	152	130.4	117.1	131.1	126.7
C10ORF58	728	965.4	1151.6	872.9	1070.2	611.1	1659.2	919.2
C10ORF59	98.4	112.2	105.8	119.3	99.1	98.1	113.7	114.4
C10ORF59	143.5	145.9	143.2	145.2	122.1	116	132.3	139.5
C10ORF59	129.4	137.8	140.1	141.5	116.6	114.1	126.2	129.3
C10ORF6	327.9	316.2	323.4	318.4	319.1	200.6	318.8	324.8
C10ORF61	152.1	129.8	189.7	157.8	134.7	140.8	133.3	127.4
C10ORF61	112.2	110.3	115.1	114.2	107.5	99.6	119.1	114.5
C10ORF61	132.5	139.4	137	120.2	116.8	115.3	132.5	118.6

C10ORF62	108.3	106.7	110.7	125.1	103.9	98.3	119.5	106.3
C10ORF63	109.8	118.4	116.2	115.2	110.2	99.2	117	109.2
C10ORF65	121.6	129	122.5	121.1	109.2	108.2	111.2	112.6
C10ORF67	117.3	114	117.4	120.3	106.9	95.2	114.1	111.4
C10ORF68	116.6	112.2	113.8	118	112.5	96.6	111.4	113.2
C10ORF7	402.7	456.5	408.1	356.5	316.2	358.5	343.2	315.7
C10ORF71	104.3	104.1	105.4	112.5	104.1	98.3	107.2	109.7
C10ORF72	112.1	118.8	106.2	117	96.3	91.9	120.3	109.4
C10ORF72	117.8	121.5	128.1	124.1	110.1	105.1	135.2	123.6
C10ORF72	118.3	114.8	121.9	123.8	114.8	103	119.6	122.2
C10ORF76	240.4	229.7	228.6	254	227.6	276.9	261.9	210.1
C10ORF77	125.9	142.8	141.9	143.8	117	101.9	120.2	128.1
C10ORF78	121.5	123.8	134.3	134.3	110.5	97.6	127.5	119.5
C10ORF79	119	119.8	122.4	113.7	105.6	105.4	113.7	117.8
C10ORF80	118.8	119.9	115.5	122.4	116.2	107.1	125.5	111.4
C10ORF81	115.5	115.5	113.4	119.1	110.1	102.2	118.9	110.6
C10ORF82	121.8	110.8	117.2	123.4	106.6	110.2	125.7	128
C10ORF83	117.9	130.6	120	121.1	124.4	104.1	115.2	115.6
C10ORF84	126.5	140.6	131.8	125.4	112.9	111.2	127	138.7
C10ORF85	126.4	114.4	122.9	124.4	110.6	97	122.8	114.2
C10ORF86	615.6	647.2	812.7	500.8	465.7	463.2	436.1	435.1
C10ORF87	112.6	121.2	126.8	122.3	106.2	99.3	113.7	111.4
C10ORF88	141.5	139.9	140.1	138.8	119.8	130.6	130.4	147
C10ORF89	133.2	128.1	133.4	135.8	118.8	105.9	137.8	127.4
C10ORF9	164.2	168.2	155.1	161.8	153.8	174	157.9	178.4
C10ORF9	110.4	119.7	107.3	109.8	109	96.6	118.4	107.2
C10ORF90	127.3	115.6	107.9	112.5	109.8	102	114.8	106.6
C10ORF91	117.6	124.3	107.9	115.5	105.7	94.5	122.4	106.9
C10ORF92	125	130	115.9	128.5	119.9	102	122.6	122.1
C10ORF93	118	109.6	106.3	110.1	109.5	102.7	124.3	107.8
C10ORF93	110.2	106.1	106.8	111.4	110.9	98.3	109.3	108.4
C10ORF95	116.1	112.1	111.1	116.2	103.6	104.9	119.5	117.7
C10ORF96	111.3	113.7	117.1	114.3	109.1	99.4	115.5	113.9
C10ORF97	200.9	195.9	203.1	215.7	199.1	159.3	193.9	196.6
C10ORF99	125.2	119.3	113.4	113.9	111.5	98.1	118.3	119.1
C11ORF1	221.5	298.7	380.2	280.3	252.1	377.7	251.7	313
C11ORF10	1643.8	1996.4	2414.9	1606.9	1749.3	1821.8	1910.8	1805.2
C11ORF11	154.1	160	131.1	167	122.3	118.2	146.1	125.1
C11ORF16	120.5	121.6	125.3	110.9	116.1	103.7	120.8	114.2
C11ORF17	122.1	113.6	127.3	121.8	116.8	108.8	119	113.2
C11ORF17	185.8	194.7	232.9	175.4	157.7	174.6	156.3	163.9
C11ORF17	150.3	151.2	180.5	167.2	124.5	123.1	133.7	142.7
C11ORF17	116.9	120.1	116.6	125	110.9	103.9	121.5	115.3
C11ORF17	241.9	252.6	364.4	233.1	238.2	283.4	201.3	210.5
C11ORF2	2398.5	2731.9	1558	2430.8	1724.1	1149.7	2490.4	2534.7
C11ORF24	142.7	138.4	158.4	145.9	151.7	129.6	138.2	135.2
C11ORF30	122.5	144.6	125.7	134.2	121.8	98.8	118.8	124.6
C11ORF31	143.6	149.5	152.1	150	140.1	117	150.3	145.8
C11ORF35	123.6	133.5	122.7	134.3	111.3	111.1	129.4	122.5
C11ORF36	109.5	107.3	108	110.3	102.4	96.7	115.7	110.6
C11ORF38	141.9	137	140.7	151	139.7	114	158.2	154.6

C11ORF40	120.6	118.5	119.6	122.1	109.4	102	116.4	119
C11ORF42	113.8	119.7	121.2	121.7	109.4	96.3	111.3	119.2
C11ORF44	113.7	109.4	122	121.6	111.8	104.7	129.7	114
C11ORF45	123.5	124.1	118.9	124.8	119.6	108	142.1	124.4
C11ORF46	548.8	607.3	524.9	467.7	485.9	311.4	482.5	650.1
C11ORF47	128.5	127.1	139	147.9	127.2	106.7	129.8	140.1
C11ORF48	279	328.2	289.7	301.2	266.9	301.5	298.2	302.8
C11ORF49	119.3	120.5	106.9	117.7	110.5	98.6	116	118.7
C11ORF49	119.3	123.7	124.2	115	110.5	99.7	128.6	115.5
C11ORF49	127.3	125.8	139.1	127	106.2	104.2	129	125.5
C11ORF49	119.4	114.4	117.4	115.4	109.6	102.2	126.5	114.3
C11ORF51	134	145.7	136.3	139.9	114.1	129.5	133.7	125.2
C11ORF52	115.9	122.9	112	114	105.3	95.4	116.6	114
C11ORF53	121.7	111.4	112.4	118.3	105.6	100.3	120.9	111.9
C11ORF54	285.7	299	332.7	391.4	318.1	295.1	363.9	343.2
C11ORF55	126.2	116	125.3	127	114.5	105.6	125.9	125.8
C11ORF56	213	236.5	230.1	253.4	185.7	212.7	216	212.9
C11ORF61	117.2	145.3	139	146.1	122.9	105.9	143	131.6
C11ORF63	120.7	123.8	118.9	115.4	106	101.5	128.5	117.8
C11ORF63	114.9	129.4	121	113.4	108	105.1	120.1	119.6
C11ORF63	187.1	171.6	233.9	184.3	193.6	138.2	218	240.6
C11ORF65	103.9	116.8	103.8	116	103.5	92.4	110.6	109.8
C11ORF70	116.3	111.1	110.7	120.3	110.1	96.7	112.3	120.3
C11ORF71	124.8	120	148	129.6	124.5	117.3	144.8	115.5
C11ORF76	104.3	119.8	108.5	124.4	103.9	96.2	114.3	109.5
C11ORF77	132.1	121.1	129.8	129	124.6	104.3	139.4	145
C11ORF9	117.9	118.5	115.3	114.7	112.5	102	114.2	104.6
C12ORF10	402.6	454.7	473.7	404.3	323.9	315.9	271.1	306.5
C12ORF11	117.8	113.8	127.9	115.9	112.9	107.3	120.3	115.7
C12ORF12	123.5	119.9	125.5	125.2	114.3	102.1	120.8	113
C12ORF23	162.1	154.3	137.2	169.7	135.8	113.5	146.1	169
C12ORF24	242	240.2	191.8	218	198.5	114.3	182	162.5
C12ORF25	108.8	122.1	108.6	124.8	104.7	100	122.4	127
C12ORF26	306	397.5	316.9	313.8	305.3	217.4	297.6	322.4
C12ORF28	111.3	121.4	119.5	115.4	112.6	93.3	118.1	123.2
C12ORF29	216.3	285.5	246	270.5	254	187.5	267.8	255.2
C12ORF30	129	139	140.5	142.8	121	116.4	124.5	128.1
C12ORF31	200.2	203.5	187.1	198.3	187.3	147.1	181.5	200.8
C12ORF32	188.6	178.7	229.3	195.2	170.2	176.3	197	176.3
C12ORF34	112.1	123.9	117.4	129.2	114.8	106.4	122.6	123.7
C12ORF35	228.5	214.3	216.8	340.5	188.9	203.1	239	188.6
C12ORF36	119.3	114.5	112.8	115	108.1	98	124.2	106.3
C12ORF38	102.6	119.7	112.4	114	107	96.5	115.8	109.6
C12ORF39	107.6	118.9	115.7	122.1	111.8	96.7	125.3	110.5
C12ORF4	136.3	154.4	158.9	142.1	142.6	121.6	157.7	145.9
C12ORF40	110.7	111.6	118.7	123.1	106.8	97.8	105.3	111.1
C12ORF40	117.6	114	110.7	117.6	106.7	104.3	112	108.7
C12ORF41	689.2	911.3	645.6	737	574.7	620.5	755.3	944.9
C12ORF42	106.8	116	106.4	116.3	106.6	94.4	121.3	112.3
C12ORF43	200.1	191.8	258.5	221.2	162.3	168.9	203.5	195.7
C12ORF44	487.5	526	409.2	521.8	359.9	297.5	601.1	525.9

C12ORF45	145.2	158.2	176.9	143.5	137.4	131.4	148.5	147.6
C12ORF46	256.1	223.8	319.3	251.4	271	212.3	247.8	267.7
C12ORF47	459.9	476.1	588.4	374.6	347.4	371.5	469.6	495.3
C12ORF48	132.1	138	139.9	134.4	113.3	109.4	122.9	112.6
C12ORF49	155.5	171.5	145.4	175	172.5	130.7	161.8	194.4
C12ORF5	157.6	139.7	160.6	165	138.9	121.2	155.8	155.2
C12ORF50	115.6	116	110.7	125.3	112.7	99.7	119.6	119.1
C12ORF52	184.7	190	178.4	180.6	158.4	132.4	161.8	159.5
C12ORF53	116.6	120.7	107.4	116.2	110.4	97.1	130.9	109.9
C12ORF54	119	125.9	122.8	126.9	109.4	99.5	123.1	112.6
C12ORF57	1365.1	1719.1	1880.9	1606.6	1235.2	1439.5	1297.2	1626.8
C12ORF58	127.9	129.4	147.1	134.8	123.4	107.4	124	127.2
C12ORF59	108.1	111.3	114.8	114.1	101.5	97.6	114.4	116.5
C12ORF60	113.7	114.5	119.2	127.9	114.8	90.2	132.5	116.7
C12ORF61	106	111.2	116.5	121	119.7	101.5	122	125.7
C12ORF62	160.5	184	176.3	158.2	167	127.5	138.7	164.4
C12ORF64	129	111.6	116.5	113.1	114.3	92.6	113	113.8
C13ORF1	126.1	121.2	137.4	134.3	119.5	104.4	124.2	129.8
C13ORF10	228.5	287	224	259	246.9	220.7	277.4	286.3
C13ORF16	120.9	113.5	122.4	121.2	107.7	92.7	123	133.9
C13ORF18	144.5	137	133.7	183.1	132.2	130.6	180.1	146.1
C13ORF21	123	112.8	119.8	129	118.1	99.4	115.5	119.7
C13ORF23	281.4	367.1	291.8	321.4	278.2	242.7	269.8	298.7
C13ORF23	255.7	307.3	262.7	285.3	283.2	208.7	218.4	220.1
C13ORF23	111.9	112.9	107.9	119.9	101.9	103	114.4	108.4
C13ORF24	136.9	154.5	152.3	160.6	130.7	133.1	148	145.6
C13ORF26	113.7	109.8	100.8	119.8	111.7	95.8	106.6	110.3
C13ORF3	113.7	129.5	120	116.2	118.9	102	117.4	103.4
C13ORF7	166.6	177.8	197.1	151.8	179	133.7	153.6	175.4
C13ORF8	437.9	512.6	380.8	453.8	387.2	334.8	439	300.3
C14ORF1	126.4	129.4	139.8	136.5	126.5	112.4	134.9	116.7
C14ORF10(	275	241.6	283.3	273	211	306.7	182.3	182.5
C14ORF10:	115.1	112.1	125.5	117.4	115.7	103.4	115.8	118.3
C14ORF10:	365.1	452.1	410	450.5	521.7	341.8	436.1	432.4
C14ORF10:	214.9	198.6	175.2	197.1	158.5	141.8	195.5	190.3
C14ORF10:	146.3	149.7	156.5	157.1	142.5	129.5	163.5	174.2
C14ORF10	156.5	149	135.6	146	154.1	118.3	140	149
C14ORF10!	119.5	130.3	116.6	128.6	109.4	102.7	124.1	117.7
C14ORF10(	454.8	361.7	493.2	422.8	306.3	270.4	340.1	345.5
C14ORF10!	220.8	225	276.9	332.8	287.2	200.4	302.7	223.3
C14ORF11	112.7	115.3	119.1	117.7	116.8	97.1	118.9	112.9
C14ORF11:	267.6	359.1	457.6	385.7	328.4	352.7	273.3	354.6
C14ORF11!	112.6	105.5	109	114.8	107.3	97.5	112.1	116.2
C14ORF11!	139.8	126	123.3	128.2	118.9	104.3	130.8	125.6
C14ORF11!	133.1	128.3	125	128.2	126.7	109.7	132.5	138.3
C14ORF11!	136	138.8	157.6	140.4	123.6	106.2	138.1	143.7
C14ORF12(	240.6	238.3	223.6	239.3	266.2	197.4	226.6	248
C14ORF12:	126.5	125.6	135.9	131.2	122.3	108	120	133.6
C14ORF12:	138.4	155.1	157.4	146.3	135.1	128.1	145.2	131.3
C14ORF12	168.7	138.8	148.2	161.2	136	111.5	142.7	135.7
C14ORF12(	114.4	114.9	123.1	120.8	115.7	99.3	114.4	108.6

C14ORF12(	162.3	171.9	200.3	191.6	157.9	159.3	201.9	203.7
C14ORF13(	150.1	157.7	148.3	147.5	149.6	133.5	140.7	158.5
C14ORF13(	219.3	193.1	197.8	250.5	177	188.5	312.1	278.8
C14ORF13(	110.7	117.7	122.4	124.9	111.9	109.4	120.7	112.3
C14ORF13:	266.6	313	335.1	287.1	244.5	223.4	234.2	218.8
C14ORF13:	142.6	160.8	169.1	156.8	134.5	140	149.7	139.1
C14ORF13!	460.4	463.6	455	433.7	442.4	303.3	405.1	342.3
C14ORF13{	112.9	106.3	114.8	109.4	105.9	95.5	110.9	107.9
C14ORF13{	240.6	216.6	233.4	237.6	256.3	205	259.6	277.3
C14ORF13{	392.7	429.7	352.9	467.5	370.7	285.8	446.1	467.1
C14ORF13(	135.9	144.9	140.3	149.2	124.1	110.9	138.7	123.2
C14ORF14(	105.8	117.2	116.8	107.9	104.7	94.9	113.4	116.9
C14ORF14(	124.8	123.1	110	114.1	108.1	96.8	120.3	123.4
C14ORF14:	157.7	180.6	178.6	178.1	136.4	121.5	175.2	221.1
C14ORF14:	116.1	114.8	114.2	115.5	110.9	109.6	117	110.1
C14ORF14!	139.9	141	108.7	125.2	111.8	102.8	147.8	131.2
C14ORF14:	186.7	172.6	166.3	192.6	166.8	145.7	170.2	151.1
C14ORF14{	120.6	130.4	114.2	110.8	101.8	98.1	116.1	108.3
C14ORF14(	130.4	123.8	132.8	130.9	107.3	108.1	127.9	123.2
C14ORF15(	105	117.8	113	116.7	108.3	95.5	113.3	109
C14ORF15(	127.1	130.8	132.9	137.1	119.3	115.2	131.4	127.9
C14ORF15(	130.1	131.5	134	130.7	117.6	114.8	130.4	124.2
C14ORF15:	134.8	150	141.3	152	131.7	133	179.9	167.5
C14ORF15:	112.6	108.7	114	116.1	110.2	103.9	120.8	114.9
C14ORF15:	1912.6	1438.7	2008.9	1594.6	1976.7	909.1	2123.7	2355.9
C14ORF15!	115.6	110	110.6	118.2	106.2	98.9	114.4	113.8
C14ORF15(	1339.2	1446	1873.4	1413.9	1062.1	1201.2	977.3	1029.6
C14ORF15(	331	347.3	316.2	363.2	341.9	251.5	314.4	242.2
C14ORF16:	131.5	127.5	139.6	133.6	127	113.8	134.8	129.9
C14ORF16:	115.6	113.1	121.7	126.7	111.1	108.5	125.5	117.4
C14ORF16(	240.5	290.4	338.8	265.6	286.8	259.5	278.9	237.8
C14ORF16(	112.9	114.8	113.1	121.5	103.8	106.1	122.7	113.6
C14ORF16(	179.3	218.5	196.5	261.1	199.2	145.8	208.4	197.8
C14ORF17:	134.6	162.9	138.5	153.7	129.9	120.5	138.9	141.9
C14ORF17:	1169.6	1127.4	1033.8	966.8	828.2	731.5	863.7	841.5
C14ORF17:	196.2	206.8	178	193.2	153.3	153	172.6	167.5
C14ORF19	141.1	146.1	143.7	159.9	125.4	130.1	140.9	150.7
C14ORF2	305.4	332.8	410.2	325.5	257.2	274.3	302	291.8
C14ORF21	130.3	122.9	130	129.4	117	109.1	129.6	120.6
C14ORF24	2865.4	2670.6	2945.7	3588.1	2262	1173.1	2977.1	3035.9
C14ORF24	132.3	118.9	131.7	125.9	115.9	119.4	132.5	126.8
C14ORF24	134	124.9	126.5	151.2	116.9	115.3	134.1	127.5
C14ORF28	197.5	181.7	225.4	200.7	205.2	176.1	219.1	228.5
C14ORF29	110	116	117.2	115.3	110.7	96.4	115.4	130
C14ORF29	109.3	120.7	112	116.2	100.5	98.9	113.5	106.1
C14ORF32	391.3	464.6	371.2	488.1	511.6	335.2	506.4	563.1
C14ORF37	115.9	117.2	115.2	121.5	107.5	97.1	119.1	121.1
C14ORF39	119.1	116.1	111.6	119.5	111.6	93	115.7	107.7
C14ORF4	306.2	224.7	325.5	335.3	204.3	273.5	330.1	239.4
C14ORF43	111.2	104.1	122.1	113.2	105.2	96.1	105.1	103.8
C14ORF43	180.2	198.9	186.9	196.5	173.2	129.6	152.5	131.5

C14ORF43	256.3	387.2	272.2	348.5	244.9	220	202.9	176.4
C14ORF44	116.7	117.6	115.9	126.9	110.4	105.6	108.4	112.9
C14ORF45	115.8	116.7	123.2	113	102.5	102.7	119.2	123.3
C14ORF46	137.2	127.1	146.6	136.6	130.9	116.8	141.9	134.7
C14ORF48	122.2	124	129.7	129.1	113.1	103.8	121.6	116.6
C14ORF49	118.3	121.9	136.6	123	117	101.3	114.8	122.7
C14ORF50	109.1	114.5	108.9	118	105.8	102.4	110.9	110.5
C14ORF54	114.5	119.6	113.9	121.2	109.1	100.9	117.4	117.2
C14ORF58	162.7	133.3	276.9	190.4	113	198.7	142.9	120
C14ORF68	120.9	119.9	119.7	114.8	110.6	94.9	120.9	118.7
C14ORF73	113.9	116.3	114.9	113.7	104.6	102.7	112.3	113.4
C14ORF73	112.9	113.7	117.2	114.7	112.2	103.6	121.8	124.4
C14ORF79	128.3	123.4	128.8	127.6	123.8	107.2	121.1	124
C14ORF8	113.7	110.2	110.6	116.9	103.1	100.8	114.4	111.4
C14ORF80	144.7	166	162.8	146.3	148.7	132	142.1	156.6
C14ORF85	1701.2	1576.2	1514	1449.2	1186.9	936.4	1693.7	1306.5
C14ORF92	143.5	130.4	150.1	143.1	131.1	125.8	155.7	162.4
C14ORF93	297.5	304.7	278.4	219.6	225	190.4	253.2	221.8
C14ORF94	150.3	158.8	208.2	185.8	128.3	149.8	158.6	140.6
C15ORF15	695.5	1348.8	1002.3	927.5	899.3	678.3	930.1	1710.9
C15ORF17	248.4	273.5	266.3	230.2	262.2	198.5	209.6	192.8
C15ORF2	119.1	128.1	126.5	119.1	120.3	105.3	121.3	124.8
C15ORF21	117.3	114.5	121.7	126.4	107.1	100.8	123.1	115
C15ORF21	115.3	121.4	123.9	129.3	109.3	99.7	122.2	123.2
C15ORF21	112.7	117.1	112.2	108.9	111.3	105.3	119.1	110.6
C15ORF23	116.2	141.8	123.5	125.5	118.2	104.5	113.9	119.6
C15ORF24	788.5	928.4	904.2	886	749.2	821.7	803.6	746.4
C15ORF26	106.2	123.9	135.2	112.9	114.7	101.1	124.9	111.9
C15ORF27	111.7	110.5	117.4	122.7	105.8	100.9	107.9	112
C15ORF29	115.7	113	138.1	128.7	111.9	109.9	132.5	114.8
C15ORF32	113.8	111.5	126.6	111.4	111.1	99.4	117.4	120.8
C15ORF33	114.3	107.9	125.9	114.3	103.9	98.9	114.5	114.8
C15ORF37	118.2	116.9	111.7	116	110.3	104.7	122.9	112
C15ORF38	129.2	107.7	147.5	120.9	113	105.2	117.7	115.1
C15ORF39	163.2	169.1	147.7	154.6	134.9	141.3	151.1	164.8
C15ORF40	154.8	166.4	163.8	164.5	158.7	148.4	176.4	162.8
C15ORF41	140.3	111.3	123.5	137.1	138.9	111	130.6	119.8
C15ORF42	114.1	114.7	121.9	119.6	109.1	105.6	120.1	114.4
C15ORF43	116.1	112.6	111.4	117.7	106.3	93.6	116.7	109.9
C15ORF44	105.1	130.3	122.1	122.8	123.6	97.6	112.7	129.8
C15ORF44	134.4	155.2	160.3	150.4	150	132.5	165.9	151.4
C15ORF48	386	324.8	395.7	487.4	252.2	888.6	400.9	212
C15ORF48	170.8	142	129.7	183.7	122.9	180.9	186	130.6
C15ORF48	115.9	121.6	135.5	131.2	107.1	112.4	125.9	124.8
C15ORF5	127.5	117.1	130.3	128.9	119.6	103.9	129.7	137.1
C16ORF24	254.8	286.1	215.9	215.6	170.8	153.9	169.2	145.7
C16ORF25	116.5	117.4	112.6	114.2	108.1	102.9	126.2	108.5
C16ORF25	110.3	108.4	118	117.7	98	101.8	118.8	113.8
C16ORF28	147.6	144.7	171.1	166.7	147.7	151.3	193.5	173.2
C16ORF3	112.1	116.5	117.6	117.3	113.2	100.6	118	118.6
C16ORF30	518.8	511	381.1	523.8	346.6	323.1	369.5	338.2

C16ORF33	304	385.7	377.8	346.4	294.9	260.5	286.8	318.4
C16ORF34	136.2	134.2	157.8	130.2	126.4	119.7	148.9	157.4
C16ORF35	110.1	117.3	122	119.2	111.9	96.9	108.4	120.5
C16ORF35	330.7	378.5	257.3	386.2	286.1	190.6	382.7	274.1
C16ORF44	266	322.4	236.7	286.6	222.7	205.6	291.3	314.2
C16ORF45	116.6	118.5	117.1	116.8	120.9	108.3	125.4	109.4
C16ORF46	118.7	114.5	122.3	125.8	111	100.7	129.5	127.6
C16ORF48	138	171.2	161.1	149.2	129	130.8	147	137
C16ORF5	131.5	135.3	126.6	133.3	114.1	111.8	138.8	150.8
C16ORF50	119.9	129.9	121	125.7	117.3	111.2	125.3	117.8
C16ORF53	180.3	176.2	152	178.3	223.8	163	209.3	151.9
C16ORF54	116.4	124.2	120.4	135	116.5	118.6	124.2	127.1
C16ORF55	120.6	115.9	120	126.1	119.8	106.1	122.2	119.4
C16ORF56	391	357.2	419.8	407.7	279.6	361.2	329.5	335.5
C16ORF57	138.3	143.7	152.6	183.1	136.4	125.2	166.2	170.2
C16ORF58	479.5	546.4	590.9	492	425.4	402.9	411.8	382
C16ORF60	156.2	136	160.8	166.6	127	139.4	166.3	153.5
C16ORF61	979	1104.2	1166.2	1049.5	882.7	900.6	1082.6	1313.3
C16ORF63	616.9	641.7	568.3	753.2	459.6	576.9	576.7	473.8
C16ORF69	114.3	121.4	124.3	121.9	117.5	105.8	122.2	120
C16ORF7	302.6	255.7	296.8	260.2	167.9	188.9	242.1	272.2
C16ORF78	112.4	105.7	113.1	116.7	99.4	95.6	106.6	104.6
C16ORF80	252.3	353.7	261.1	347.3	255.3	218.2	270.3	314.7
C16ORF9	264	264.2	268.9	239	189.7	240.4	235.1	191.8
C17ORF25	225	284.9	330.8	294.7	249.8	233.6	262.6	227.1
C17ORF27	129.4	123	117.4	125.2	124.7	111.7	133	136.3
C17ORF28	110.7	118.2	111.5	113.1	104.4	101.9	125.4	125.1
C17ORF32	169.1	164.7	165.5	180.3	152.5	143.4	184.9	172.7
C17ORF37	134.4	135.4	142.5	127.3	120.4	121.7	134.1	128.8
C17ORF38	108.6	115.2	113.1	115.2	101.3	97.7	110.9	104.3
C17ORF39	174.1	138.2	141.7	144.4	127.3	122.8	133.1	133.1
C17ORF40	326	281.4	349.1	330.7	254.1	215	326.9	351.5
C17ORF42	108.5	119.7	125.3	122.9	106.4	98.9	116.6	117.8
C17ORF45	2634.2	3004.6	3186.6	3086.1	2211.2	2661.9	2990.9	4202.3
C17ORF46	113.2	126.2	121.9	125.6	114.2	103.6	128.4	120.2
C17ORF47	119.7	117.3	129.6	137.7	114	117.4	127.1	121.7
C17ORF47	124.1	125	146.6	132	116.6	114.8	131.8	124.5
C17ORF48	292.5	434.6	363.5	418.3	314.6	242.6	382.1	342.1
C17ORF49	551	552.8	549.6	491.8	345.6	366	497.2	399.3
C17ORF50	111.5	123.9	115.4	122.9	117.1	100.3	138	119
C17ORF53	123.9	139.2	128.4	126.8	119.1	110.3	120.1	118.6
C17ORF54	121.1	121	120.1	117.5	112.6	98.8	123.8	126.9
C17ORF55	110.9	109.4	123.7	114.5	113.2	86.8	120	109.6
C17ORF56	135.2	165	158.7	141.8	124.4	135	147.5	117.8
C17ORF57	114.9	117	120.4	122.3	108.4	101	112	117.5
C17ORF58	155.4	163.6	228.9	153	144.1	154.8	156.2	160.2
C17ORF58	154	142.4	185.5	149.4	124.2	123.7	127.6	128
C17ORF58	119	118.6	115.8	115.2	112.4	108.3	129.9	115
C17ORF59	154.7	205.1	155.2	170.3	142	138.4	152.1	139.6
C17ORF61	464.2	524.7	922.3	472.9	426	461.2	371.4	373.5
C17ORF62	367.7	356.2	482	344.2	262.5	337	253.2	224.8

C17ORF63	221.5	205.4	193.5	228	200.9	162.6	216	230
C17ORF64	124.8	129.1	120.2	122.6	115.4	112.3	131.3	117.5
C17ORF65	129.5	132.4	156.2	124.1	136.2	121.6	124	121
C17ORF66	117.8	117.9	122	123.4	109.7	106.2	116.1	119.1
C17ORF68	329.8	409.8	357.8	359.8	273.4	229.8	304.8	264.6
C17ORF68	217	220.4	184.6	201.2	162.4	141.4	189.2	164.8
C17ORF69	138.1	142.9	128.5	142.9	138.8	106.9	136.2	131.9
C17ORF70	568.4	670.5	603.2	602.1	388.7	329.3	450.7	407.9
C17ORF71	120.8	128.1	119.1	136.3	122.3	111.4	125.4	132.6
C17ORF73	122.9	123.4	126.9	148	118.2	106.6	138.2	135
C17ORF74	124.1	116.1	136.7	139.6	116.5	98.8	117.4	130.2
C17ORF75	142.4	137.9	143	157	152.9	110.2	129	150.3
C17ORF76	120.8	123.4	116.1	118.9	108.4	104.5	128	125.5
C17ORF77	107	98.8	108	112.7	94.3	99.9	113.4	110.1
C17ORF78	110.7	130.4	110.6	111.3	97.3	92.7	110	111.7
C17ORF79	144.8	135.9	129.1	138	125.5	116.6	144.9	126.2
C17ORF80	139.4	151.3	151	150.7	128.4	120.5	140.5	142.9
C17ORF81	126	134.7	129.3	135.2	127.5	110.5	143.6	154.9
C17ORF81	182.9	189.2	175	211.6	162.3	139.3	154.1	144.7
C17ORF81	178.7	184.9	172.6	183.7	158.2	129.5	185.3	163.4
C17ORF81	115	132.6	130.3	117.8	110.3	107.1	128.4	117
C17ORF83	103.5	109.6	115.8	113.1	105.4	97.7	114.6	111.5
C17ORF85	188.4	175.9	213	218.8	170.2	187.6	223.2	286.5
C18ORF1	122.5	108.5	134.7	129.9	110.4	135.5	120.5	112
C18ORF10	144.4	129.8	161.9	145.5	117.7	124.9	139.7	126.4
C18ORF14	104.1	121.6	116.3	115.3	108	99.6	113.2	115.7
C18ORF17	456.3	499.1	340.3	393.8	371.1	278.2	361.6	306.8
C18ORF19	144.9	143.1	141.1	141.9	135.7	115.8	131.3	147.2
C18ORF21	183.7	228.1	199.2	203.2	187.7	179.2	177.8	184.8
C18ORF22	132.4	144.9	141.8	144	132.3	108.7	143.6	131.2
C18ORF24	117.1	118.6	123.9	118	123.2	102.1	125.7	124.1
C18ORF25	695.6	619.1	605.5	754.7	700.4	533.5	783.1	756.1
C18ORF25	111.8	112	115.3	113.2	108.1	104.7	123	118.7
C18ORF26	117.6	115	124.9	128.7	113.8	104.9	119.2	113.6
C18ORF34	110.8	118.5	124.2	118.9	111.9	97.4	122.3	110.4
C18ORF37	213.3	203.6	186.4	227.4	212.2	149.1	209.3	187
C18ORF45	121.5	123.4	119.5	125.1	124	98.2	131.1	120.6
C18ORF51	112.3	113.3	119.9	112.1	107.3	104	122.5	116.5
C18ORF51	125.9	133.2	142.9	127.6	116	93.4	123.8	130.6
C18ORF54	123.6	131.4	138.1	135.7	126	112.9	129.5	131.2
C18ORF55	403.5	402.1	575.6	522.6	429.2	369.4	384.1	359.3
C18ORF56	115.9	117.2	119.7	109.4	115	97.2	108.7	131
C18ORF8	432.4	433	362.1	575.3	387.9	364.6	568.2	581.1
C19ORF10	272.4	272.5	405.9	280.1	253	301.9	325.9	340.9
C19ORF12	112.6	113.4	115	116.8	111.6	100.4	121.9	107.1
C19ORF12	320.3	418.1	243.4	312.5	342.1	169.3	291.6	373.8
C19ORF12	396.2	494.2	309.1	449.3	304.2	228.3	423.6	362.7
C19ORF12	118.3	107.2	131.2	138	117.8	100.7	123.3	118.9
C19ORF15	114.5	118	126.3	119.6	108.7	99.7	116.7	113.1
C19ORF18	118.1	108.1	133.1	119.5	114	99.8	122	120.7
C19ORF19	112.8	113.3	127.5	118.5	120	102.4	121.6	109

C19ORF2	252	239.8	248.9	226	232.4	149.5	265.2	228.8
C19ORF2	1045.6	1535.5	1235.2	1381.8	1308.7	998.6	1627.4	1684.1
C19ORF20	125.2	122.1	127.9	120.3	126.8	106.8	117.4	119.9
C19ORF21	119.3	125	125.9	134.5	115.6	105.6	120.9	126.5
C19ORF22	911.7	1008.8	921.8	988	717	970.7	1054.4	1075.4
C19ORF23	120.5	131.9	120.3	128.5	122.5	104.3	151.3	127.4
C19ORF24	225.9	225.9	184.6	226.2	169.7	142.4	190	191.2
C19ORF25	261.6	228.7	230.9	227.2	203.1	210.2	237.6	230.6
C19ORF26	117.5	135.9	137.9	134.8	133.3	117.3	154.9	142.7
C19ORF28	130.1	120.6	134.2	126.6	120.5	108.1	122.3	122.4
C19ORF28	158.8	176.7	146.9	153.7	128	119.2	153.8	133.8
C19ORF29	157.2	145.9	155.1	152.5	132.6	128.4	165.7	157.6
C19ORF29	125.1	117.8	125.5	131.9	126.3	112.1	136.3	133.6
C19ORF30	117.3	120.1	124.8	128.2	111.6	97.5	129.7	126.2
C19ORF31	12977.1	12563.5	13928.4	14179.7	11491.1	7590.2	11646.7	14013.1
C19ORF33	112.8	111.2	109.2	119.7	112.1	95	119.4	118.6
C19ORF34	110	118.2	117.6	117.7	112.5	87.6	121.8	110.9
C19ORF35	113.7	109.2	129.7	130.6	106.7	110	118.8	112.7
C19ORF36	119	123.4	129.3	128.2	123.5	106.5	120.6	112.7
C19ORF36	106.3	117.3	103	115.9	109.3	95.9	116.9	113.3
C19ORF37	755.7	835.6	850.5	660.8	737.5	627.2	491.7	476.8
C19ORF4	114.9	111.3	129.2	117.1	117.5	102.6	117.9	115.8
C19ORF40	125.7	122.4	123.7	120.2	114	110.7	136.7	121.9
C19ORF48	133.1	138.4	125.7	150.1	125.1	104	143.6	130.7
C19ORF55	115.4	113.5	120.7	122	105.5	97.2	119.4	113.2
C19ORF6	160.2	182.3	151.5	163.8	151.4	134.7	144.2	140
C19ORF6	380.6	387.4	345.4	400.2	264	290.4	335.1	367.7
C19ORF7	460.6	591.1	436	628.9	511.6	476.9	740.3	760.1
C1D	109	118.3	131.2	133.1	108.2	108.1	131	132.8
C1D	114.8	108.9	112.6	114.8	106.3	95.8	117.7	104.6
C1GALT1	197.6	225	196.6	229	229.7	128.6	264.4	298.9
C1GALT1C1	116.9	107.9	122.2	120.5	106.6	95.1	121.8	113.1
C1GALT1C1	154.7	143.2	159.3	151.6	146.7	120.8	162.3	143.3
C1ORF100	117.9	113.6	114	120.5	106.8	104.5	119.1	112.9
C1ORF101	127.9	130.3	126	130.6	125.6	111.3	136.1	145.5
C1ORF102	113.1	114.4	121.7	123.7	111	101.5	128	127.9
C1ORF102	118.2	120.6	134.4	123.9	110.8	100.6	125.3	120.5
C1ORF102	108	112.1	116.4	127.8	106.2	99.5	130.4	118.2
C1ORF103	111.5	116.8	118.5	108	102.5	102.4	121.4	111.7
C1ORF103	138.4	122.6	128.9	141.9	159	108	151.2	165.8
C1ORF104	123.3	131.2	115	133.3	116.9	100.9	126.1	127.7
C1ORF105	106	117.1	115.6	121.2	108.7	94.4	119.3	108.2
C1ORF106	110.2	119.5	127	112.1	115.1	104.3	113.2	114.2
C1ORF107	187.4	192.7	164.5	189.2	183.5	108.7	183.9	194.6
C1ORF108	593.9	600.9	465.1	640.1	452.1	312.4	685.3	660.9
C1ORF108	122.2	119.3	133.5	137.3	120.3	108.1	125	120
C1ORF108	271.5	309.5	322.8	304.6	307.6	214.7	302.3	356.2
C1ORF109	238.5	224	228.5	233.5	218.2	186.2	220.1	213.6
C1ORF110	122.3	114.9	114.4	120.9	107.5	100.7	122.1	119.9
C1ORF111	113.6	114.6	121.3	118.6	101.6	94.1	123.3	112.5
C1ORF112	143.4	139.4	145.8	147.5	128	122.8	133.5	126

C1ORF113	119.4	123.1	138.6	142	107.4	105.8	127	117.2
C1ORF114	108.7	116.9	127.4	123.9	104.6	99	119.5	121
C1ORF115	123.1	140.9	124.7	131.6	138.5	108.4	120.3	119.4
C1ORF116	114	122.1	120.2	118.3	105	108.9	112.8	114
C1ORF117	111.7	114.3	104.4	116.1	112.5	95	117.1	102
C1ORF118	116.2	119.7	119.3	115.5	119.1	99.7	114.3	123.4
C1ORF119	353.3	376.3	418.9	370.3	336	308.3	394.5	416.2
C1ORF121	185.8	216.1	214.8	235.5	209	161.3	201.8	230.1
C1ORF122	265.1	239.7	218.8	230.3	160.4	193.6	215.1	183.4
C1ORF123	483.1	418.5	497.9	393.5	318.3	317.1	293.1	273.4
C1ORF124	160.5	181.6	151.8	176.6	164.1	132	153.5	151
C1ORF124	119	123.4	125.2	136.1	119.7	108.1	124.7	119
C1ORF124	114.6	127	115.8	119	109.2	105.1	114.9	110.8
C1ORF125	126.5	115.8	113.6	122.3	118.4	101.8	110.2	125.4
C1ORF125	116.4	118.1	115.7	117.2	105	96.4	110.8	111.8
C1ORF127	131.5	119.8	144.9	130	110.1	105.4	129.2	133.2
C1ORF128	1189.3	1164.5	1212.8	1323.6	1190.5	1022.3	1605.8	1583.2
C1ORF129	110.6	113.4	123.3	122.2	106.3	94.2	109.5	113.4
C1ORF131	411.5	393.4	607.3	437	371.4	463.9	400.7	297.2
C1ORF135	108.5	111	113.3	115.9	105.1	98.3	119.1	121.3
C1ORF14	119.6	107.1	115.5	111.8	101.4	99.6	114.6	109.7
C1ORF140	112.1	121.2	118.5	124.4	106.6	102.2	112	117.2
C1ORF141	108.4	114.7	110.6	115.5	103.3	101.7	123.3	107.4
C1ORF142	149.7	140.6	134.5	160.4	121.3	116.8	125.9	159.4
C1ORF144	355.5	414.7	476.4	413.9	356.5	328.1	442.9	365.7
C1ORF146	112.4	115.1	116	116.1	106.3	93.4	108.1	110.1
C1ORF149	175.4	222.5	178.4	183.1	175.1	144.8	173.5	168
C1ORF150	118.4	120.4	123.2	128.1	126.1	102.6	125.1	122.8
C1ORF151	110.4	115	113.8	121.2	112.2	98.2	123	116.8
C1ORF152	123	122.3	116	123.2	99.3	103	125.4	106.4
C1ORF155	179.8	190.3	161.4	201.4	174.7	138.2	199.2	215.8
C1ORF156	116.2	127.9	123.7	140.7	114.2	105.8	122.9	119.5
C1ORF157	120.5	114.2	127.4	126.4	108	99.2	115.2	118.3
C1ORF158	116.9	110.6	128.3	122.1	110	113	125.6	112
C1ORF159	146.2	148.3	151.1	146.8	122.1	128.5	158.4	144.6
C1ORF160	157.5	174.2	210.5	201.2	176.1	164.8	164.3	201.1
C1ORF162	1004.9	1117.1	2980.3	927.6	810.9	956.8	519.7	639.3
C1ORF163	135.2	144.7	121.8	125.4	109.9	109.1	133.3	134.6
C1ORF164	422.8	471.8	435	457.8	377	327.7	415.9	406.6
C1ORF165	127.4	128.4	128	129.6	122.5	102.5	136.7	132.3
C1ORF166	162.4	155.6	149.3	162.7	146.1	134.4	155.8	172.2
C1ORF168	116	120.3	113.1	109.9	109.4	95	122.8	117.5
C1ORF171	179.8	152.6	166.7	164.7	161.2	127.2	144.6	168
C1ORF172	117.9	127.7	119.7	121.2	115	100.6	113.8	107.1
C1ORF173	114.9	114.3	112.7	113.2	111.9	97.8	121.7	115.4
C1ORF175	125.5	109.7	136.7	122	103.7	98.6	120.1	121.4
C1ORF176	650.8	577.3	617.4	451.7	581.2	357	660.2	561.8
C1ORF177	121.4	114.2	114.4	118.1	113.4	100.5	118.2	123.3
C1ORF178	116	111.2	117.4	118.7	109.3	99.6	124.7	107.9
C1ORF178	120.4	110.8	120	118.8	109.3	94.1	111.5	121.3
C1ORF179	115.4	123.4	117.7	116.2	108	99.4	113.7	116.5

C1ORF181	293.4	389.5	347	380.2	297.3	212.9	284.1	322.7
C1ORF182	109.8	114	112.3	110.6	109.4	104	123.1	115.1
C1ORF183	117.2	116.1	121.4	113.6	110.9	98.2	125.3	116.7
C1ORF183	112.8	114.8	125.9	121.5	111.8	103.5	116	116.7
C1ORF183	113.5	108.4	122.4	125.1	111	101.3	135.2	121.3
C1ORF186	115.4	118.9	119	117.1	102.2	98.1	126.4	111
C1ORF187	120.8	119.1	125.1	119.4	109.4	107.5	112.4	113.4
C1ORF188	117.4	111.2	117.6	114.5	109.4	97	114.2	110.5
C1ORF189	123.6	114.2	113.3	116	108.2	98.8	118	116.3
C1ORF19	382.1	424.7	481.5	400.9	397.7	317.2	335.8	380.3
C1ORF190	117.3	122.9	116.7	115.9	103.2	104.2	120.3	117.1
C1ORF192	119.1	115.8	119.7	125.2	119.4	112	129.3	126.8
C1ORF198	128.5	140.3	131	150.8	134.7	113.4	151.5	161.1
C1ORF2	262.8	352.2	207.1	276.6	232.3	171.5	223.5	207.4
C1ORF2	111.5	113.9	111	119.6	103.4	101.7	123.7	110.8
C1ORF2	112.9	135.6	118.4	123	108	92.8	117.9	119.1
C1ORF201	116.4	121.8	120.6	114	108.6	109.5	128.3	126.9
C1ORF21	157.2	150.1	171.3	177.5	152.1	154.7	202.1	177.2
C1ORF210	115.4	106.8	122.3	118	108.7	93.5	118	113.1
C1ORF24	388.1	433.5	342.3	370.3	330.1	201.3	315.7	322.9
C1ORF25	120.9	126.4	123.7	123.5	115.7	118.5	125.1	123.7
C1ORF26	142.2	125.7	129.5	135.4	128.8	121.6	132.4	128.1
C1ORF27	127	118.5	120.1	120.8	114.8	98.3	129.7	116.3
C1ORF31	189.6	148	157.7	186.7	134.2	145.5	156.2	149.4
C1ORF32	100.1	112	112	117.1	115.4	101.3	122.4	115.3
C1ORF33	157.5	168.9	152.2	153.9	162.4	126.3	155.2	144.3
C1ORF34	122.7	115.4	116.1	112.7	109.4	99.5	115.5	116.7
C1ORF35	229	262.8	213.4	246.1	225.8	187.4	249.3	244.8
C1ORF36	121.7	122.9	130.9	127.1	113.2	104.1	125.2	132.8
C1ORF37	813.8	967.8	1043	976.4	720.7	692.5	893.6	856.3
C1ORF38	128.1	139	125.7	134.7	116.9	104.6	134.7	132.7
C1ORF41	306.1	358.3	414.1	352.2	297.9	340.2	286.4	399.1
C1ORF42	117.6	113.7	112	118.7	111.5	98.2	118.6	108.1
C1ORF43	253.6	304.7	355.4	333.7	293	297.8	446	368.3
C1ORF43	135.6	150	169.2	169.9	134.4	141.3	146	164.2
C1ORF45	114.8	112.4	109.2	113.8	97.3	89.8	122.6	110.9
C1ORF48	862.9	925	1179.4	853.4	844.2	731	704.9	681.6
C1ORF49	117	118.8	124.4	117.6	118.3	97.8	130.5	114
C1ORF50	389.2	429	397.7	397.9	277.1	282.6	346.9	340.3
C1ORF51	111.5	106.5	117.1	114.5	108.6	98.5	119.6	122.5
C1ORF52	660.6	705.1	608.9	689.8	646.8	417.3	693.1	693.6
C1ORF53	124.3	119.7	132	119.5	116.4	105.1	118.9	121.1
C1ORF54	216.6	225.8	352.1	228.5	205.8	197.3	218.6	278.8
C1ORF55	268.2	292	277	310.2	297.9	226.7	345.6	438.1
C1ORF56	159.3	152.4	147	152	179.8	118.7	160.3	176
C1ORF57	162.2	197.3	242.1	168.6	186.5	156.4	162.1	151.1
C1ORF58	99.5	111.6	121.9	112.4	113.5	101.5	113.1	115.1
C1ORF59	692.2	795.9	776.5	707.8	597.7	581.1	617.9	656.2
C1ORF60	235.2	247.4	202.2	268.2	250.1	216.1	265.3	219.6
C1ORF61	108.5	115.9	117.7	122.3	107.4	97.9	108.7	110.7
C1ORF62	112	115.7	125.9	117	106.7	103.8	117.5	111.8

C1ORF63	214	192.1	210.9	186.7	170.3	242	193.8	194.5
C1ORF64	103	115.8	112.8	116.2	100.1	97.8	119	114.6
C1ORF65	117.2	115.5	121.2	110.2	103.9	93.6	119.5	111.2
C1ORF66	265.5	266.8	248.6	247.4	219.5	218.6	271.6	247.9
C1ORF69	141.4	154.3	134.9	162.3	138.3	136.9	155.8	148
C1ORF71	1251.7	1511	941.4	1438.7	1233.1	600.4	2204.4	2142
C1ORF73	131.7	130.5	137	138.6	127.1	104.3	131.6	129.9
C1ORF74	133.7	131.4	136.8	137.1	127.6	124.6	135.8	132.8
C1ORF75	116.1	122.4	118.1	124.5	114.7	105.2	117.3	109.5
C1ORF76	133.6	132.1	134.5	133.9	116.9	110.6	126.9	131.2
C1ORF77	284.2	383.1	315.1	417.3	312.5	278.2	412.8	489
C1ORF78	147.3	143.1	143.7	176.4	135.1	132.3	187.7	148.6
C1ORF82	251.8	255.2	269.2	289.3	276.7	168.6	305.7	316.1
C1ORF83	141.8	143.2	175.6	149.1	128.2	135.1	148.2	148.4
C1ORF84	122.2	113.8	113.4	123.7	114.3	111.9	124.8	117.4
C1ORF84	116.2	119.6	122.3	122.8	107.3	102.4	114.8	127.8
C1ORF84	130.2	133.3	121.8	125.6	116.7	107.6	125.8	120.8
C1ORF85	372.2	246.9	372.6	325.8	202.4	192.2	241.6	219.1
C1ORF86	229.1	256.3	276.5	197.9	163	215.1	202.7	196.4
C1ORF87	110	113.6	121.4	120.1	118.3	105.5	121.9	117.1
C1ORF88	131.3	125.3	134	144.2	115.1	116.1	133.3	125.7
C1ORF89	121.1	126	136.8	132.8	114.7	107	123.4	118.5
C1ORF9	112.2	109.4	110.3	109.2	105.3	93.3	111.1	109.2
C1ORF9	106.5	114.4	116.1	119.9	102.6	98.5	121.5	108.7
C1ORF9	126.7	122.1	130.9	132.1	116	111.3	128.4	143.6
C1ORF90	127.3	121.6	124.5	132.8	115.9	116.3	120.6	120.5
C1ORF91	107.5	104	110.7	122.4	100.5	102.5	121.7	112.6
C1ORF92	107.1	118.3	114.7	125.6	105.2	98.5	138.5	113
C1ORF94	113.1	119.7	115.9	125	112.2	109.1	111.5	112.6
C1ORF95	114	123.2	109.5	119	111.4	108	113.6	109.4
C1ORF96	124.5	123.7	112.9	106.9	107	102.9	122.9	113
C1ORF97	136.1	117	162.7	127.9	121.8	118	138.8	136.6
C1ORF99	121.7	115.4	116.8	113.9	105.9	106	114	114.1
C1QA	118.2	112.3	116	125.4	108.4	101.5	108	115.7
C1QB	122.5	123.1	144.2	131.3	107	103.9	143.7	123.7
C1QBP	723.1	971.9	731.2	923.2	702.1	497.2	788	740.7
C1QC	113.8	110.1	154.2	115.7	109.8	111.5	119.6	112.7
C1QDC1	189.7	177.5	186.7	203.2	173.4	168.2	189	163.7
C1QDC1	179.2	169.5	224.6	210.8	195.3	168.7	179.1	176
C1QDC2	114.3	123.8	121.5	119.7	111.6	103.1	126.7	120.5
C1QL1	115	116.3	117.3	116.7	114.3	102.3	119.8	108.8
C1QL2	121.6	115.7	116.9	117.1	108.5	93.3	114.2	116.1
C1QL3	116.7	120.6	119.1	132.1	112	101.5	117.6	119.5
C1QL4	115.7	106.4	112.3	118.7	111.8	96.4	111.8	114.5
C1QR1	127.9	128.7	184.7	125.5	116.8	104.1	126.4	118.3
C1QTNF1	129	125	132.2	131.7	114.9	110.4	129.2	121.1
C1QTNF1	110.5	113.6	116.9	112	106.1	99.2	109.8	113.5
C1QTNF1	120.6	116.4	115.8	113.5	108.3	92.4	125.5	118.8
C1QTNF2	116.9	118.4	123.3	110.9	109.1	107	125	119.6
C1QTNF3	112.6	116.5	117.9	118.5	108.7	97.9	117.1	108.8
C1QTNF3	109.5	119.7	113.2	119.9	108.7	102.7	113.4	113

C1QTNF4	117.4	117.1	116.2	120.3	109.6	97.8	115.8	114.7
C1QTNF5	113.2	119.3	132.4	126.7	121.4	105.3	117.1	117.8
C1QTNF6	115.9	122.3	119.4	121.2	109.2	95.6	116.6	117.6
C1QTNF6	132.3	140.9	194.5	155.7	135.2	142.3	133.4	136.6
C1QTNF6	108.4	105	119	114.7	108.3	91	124.5	113.1
C1QTNF7	120.4	119.1	114.5	117.1	106.9	100	112	113.2
C1QTNF8	121.1	110.9	122.2	124.6	111.9	107.4	116.5	125.1
C1R	117.8	120.2	118.8	125.2	103.9	107.2	116.5	120.8
C1RL	126.2	140.3	138.2	132.2	135.3	122.6	117.9	117.8
C1S	111.5	116.9	114.6	113.7	105.2	105.8	109.3	117.9
C1S	119.4	110.2	120.1	119.7	105.4	100.7	124	118.2
C2	121.3	116.5	126.9	122.3	120.7	103.1	123.3	117.4
C20ORF10	115.5	112.6	115	113.8	106.6	97.7	111.1	113
C20ORF10(	152	180.1	181.5	181.8	155.6	169.2	213.9	227.2
C20ORF10:	111.8	113.7	114.8	114.3	105.8	95.9	121.4	104.2
C20ORF10:	113.7	113.1	108	114.5	105.1	107	113.1	117.2
C20ORF10(	114.9	124.5	120.8	129.4	120.3	105.5	124.3	141.9
C20ORF10:	136.3	141.4	143.8	137.1	124.1	106.4	156.9	157.5
C20ORF10{	164.6	195.9	203.6	184.3	148.3	179.6	152.6	139.1
C20ORF11	802.3	967.2	727	1000.1	810.5	671.7	1011.3	1085.6
C20ORF11:	493.2	590.6	566.5	627.9	499.8	679.6	981.9	1444
C20ORF11:	107.1	112.7	129.2	128.7	115.9	104.9	123.2	113.9
C20ORF11·	112.3	108.9	126	119.8	111.4	107.6	120	117.7
C20ORF11(	375.7	399.2	389.7	363.4	277.3	285.7	446	370.4
C20ORF11:	128.1	131.4	136.7	151.3	118	122.2	180.6	177.2
C20ORF11:	106.5	113	118.9	122.2	106.7	105.6	113.8	114.5
C20ORF11{	112.8	113.1	138	118.7	107.9	101.2	120.4	126.1
C20ORF12	125.5	121.8	125.9	122.3	114.6	95	115.8	118.7
C20ORF12:	324.7	389.8	263.8	333.3	313.5	243	337.8	265.1
C20ORF12:	167.1	185.4	169.1	205.2	184.1	132.9	189.6	178.9
C20ORF12{	114.9	111.5	129.8	120.7	109.7	103.4	112.3	124
C20ORF12!	115.9	112.5	127.1	129	115.9	98.8	122.4	135.7
C20ORF13:	118.6	118.3	115	117.7	114.9	98.7	114.8	113.4
C20ORF13:	114.1	119.8	119.7	123.9	107.7	102.4	126.9	120.2
C20ORF13:	108.7	110.2	117.9	109.7	102	98.2	117.8	115.1
C20ORF13:	121.8	127.1	126.6	111.7	111.1	105.2	114	114.6
C20ORF13:	110	117.1	122.8	114.2	108.9	99.9	119.4	120.7
C20ORF13·	120.8	129.8	125.4	142.1	116.6	107.3	129.6	120.1
C20ORF13!	116.1	121.3	111.7	115.5	107.3	101.1	122.1	124.4
C20ORF14	124.7	116.8	145.8	130.6	114.6	114.1	127.9	115.7
C20ORF14:	109.6	110.1	111.2	119.1	102.8	99.3	115.5	111.8
C20ORF14:	110.9	120.1	118.1	128.7	110.3	104.1	119.7	110.7
C20ORF14·	106.5	107.2	122.4	117	106.9	89.4	119.1	114.7
C20ORF14!	144	137	137.1	149.5	134.8	120.9	140	139.8
C20ORF15:	116.9	124.7	136.3	124.3	105.7	109.1	117.9	119.5
C20ORF15:	116.8	115.2	120.9	127.8	102.1	107.6	118.7	109.8
C20ORF15!	376.3	422.1	453.9	381.8	361.5	274.1	370.6	363.2
C20ORF16(	116.7	121.6	118	124.9	110	101.6	120	118
C20ORF16:	118	104.5	118	118.4	112.4	109.7	122.7	113.6
C20ORF16:	117.3	128.7	129.3	120.3	121.7	102.1	115.9	123.7
C20ORF16!	101.9	109.3	120.9	117.2	106.8	99.8	107.6	111.5

C20ORF16(	112	124.4	111.6	109.8	106.9	104.5	116.7	115.4
C20ORF17:	135.4	141.6	160.3	141.7	134.7	119.5	146.6	142.1
C20ORF174	156.3	145.2	111.9	136.3	130.9	107.1	151.2	135.5
C20ORF174	334.1	353.8	240.7	425.1	280.8	194.2	279.5	302.7
C20ORF17	623.6	704.9	851.4	649.5	647.5	501.5	390.9	348.4
C20ORF17!	116.6	115.2	113.3	112.4	104.9	96	112.8	119
C20ORF17!	108.2	114.1	111.3	124.2	113.7	95.9	114.8	106.5
C20ORF18!	128.2	116.7	123	123.9	112.2	103.2	119.5	121.6
C20ORF18(	109.2	106.5	109.4	120.1	109.7	96.3	107.5	114.4
C20ORF19	122.5	120.9	131.8	141.2	118.1	102	129.6	139.1
C20ORF19:	172.9	203.2	174.9	221.7	204.8	148.2	206	220.6
C20ORF19!	113.2	125.7	122.7	126	115.5	104.7	132.9	120.1
C20ORF20	948.1	1090.8	690.4	996.2	786	535.5	956.1	845.4
C20ORF22	126.8	123.3	164.6	135.5	120.2	129.6	146.6	140.5
C20ORF23	132.1	139.1	136	123.2	139.8	123.9	133.2	141.1
C20ORF24	2728.4	2880.2	2817.1	3600.2	2451.8	2515.7	4706	3380.9
C20ORF24	3584.6	3025.1	3473.7	4759.9	2974.5	3689.5	5177.2	4183.1
C20ORF24	930.2	717.2	908.8	1229.6	691.9	928.2	1299	1160.8
C20ORF26	116.7	120	110.5	120.9	114.7	103.5	126.8	115.8
C20ORF27	194.2	200.5	244.7	204.6	164.8	146.9	161.8	150.4
C20ORF28	121.6	120.5	134.1	138.8	117.4	106.2	119.1	122.3
C20ORF29	161.9	173.4	146.1	164.5	142	136.8	132.5	139.9
C20ORF3	394.2	409	506.5	379.5	304.9	368.5	377.8	346.5
C20ORF30	853.5	689.6	997	733.2	702	715.8	739.8	693.8
C20ORF30	1103.9	1375.8	1282.2	1175.5	1026.4	1424.5	1170.3	1065.5
C20ORF31	166	169.7	192.2	170.2	160	151.9	177.5	166.7
C20ORF32	122.2	119.4	116.5	112.5	105.1	97.3	116.5	107.7
C20ORF35	250.1	287.1	319.5	295.1	216.5	266.9	262.8	248.4
C20ORF39	110.5	121.3	115.5	115.4	104.8	102.2	125.6	111.9
C20ORF4	353.9	402.6	355.5	387.3	297.2	332.6	397.6	329.3
C20ORF42	108.4	113.6	110.9	113.2	104.7	90.9	110.2	119.4
C20ORF43	1330.9	1417.1	1379.4	1529.2	1078	1294.6	1309.5	886
C20ORF44	144.4	159.7	152.6	164.6	134.4	134	155.3	158.1
C20ORF44	131.2	123	132.7	135.6	126.2	104.7	126.3	127.1
C20ORF44	308.8	354.4	312.1	279.4	280.8	204.2	277	336.6
C20ORF45	255.9	275.7	239.4	293.7	298.8	199.3	306.1	291
C20ORF46	120.5	110.5	130	116.5	119.1	106.8	121.1	116.8
C20ORF52	824.8	856.3	1202.7	877.9	878.6	921.5	715.4	859.1
C20ORF54	111.9	114.2	118.9	113.5	105.7	103.8	122.3	106.5
C20ORF55	214.4	231	166	195.7	146	186.3	227.4	159.5
C20ORF55	126.6	111.2	121.4	118.5	110.9	105.4	116.1	118
C20ORF55	366.5	369.7	274.3	386.3	280.5	281.4	406.5	242.2
C20ORF58	105.9	111.3	114	117.1	110.6	101.4	118.2	116.3
C20ORF59	117.2	119.6	125.6	129.1	110.5	107.5	121.3	119.2
C20ORF6	109.6	119.3	114	116.1	103.4	96.8	116.1	109.6
C20ORF67	145.7	169.4	138.2	170.8	137.5	121.9	156.9	168.7
C20ORF7	121.6	120.2	124.4	126	122.5	88.5	120.8	124
C20ORF7	208.3	202.3	266.7	236.1	186.7	189.9	225	241.7
C20ORF7	143.5	135.7	156.9	150.8	147.6	132.3	157.1	142.5
C20ORF70	116.2	119.2	117.9	117	119.9	97.8	114.8	117.3
C20ORF71	120.6	115.6	114.5	122.3	104.8	95.6	117.2	127.4

C20ORF71	109.6	122.5	122.6	120.1	110.8	98.6	127.4	115.7
C20ORF71	111.1	110.4	112.3	115.4	99.9	103.7	119.3	114.5
C20ORF72	372.2	298	423.4	303.3	294	224.3	269.7	261.5
C20ORF74	112.2	119.2	116.4	114.7	104.5	102.3	121.9	112.5
C20ORF74	114.2	108.3	126.8	117	118.6	118	126.9	123.5
C20ORF75	111.1	119.9	115.5	117	115.1	97.3	124.4	115.6
C20ORF77	118.7	112.4	112.5	111.3	111.3	100.7	120	109.2
C20ORF79	112.6	113.2	112.9	116	106.6	94.3	121.4	110.8
C20ORF85	119.5	120.9	119.1	122.7	104.9	100.6	115.6	118.6
C20ORF86	120.1	126.4	127.6	131.7	107.9	112.2	135.4	122.8
C20ORF94	150.1	136.5	131.4	152.3	151.8	105.7	142.5	154.9
C20ORF96	111.7	118.5	112.7	117.3	110.4	97	124.6	113.3
C20ORF98	141.4	114.2	124.1	127.9	116.5	102.8	128	121.7
C21ORF10(	109.3	115.9	114.3	127.4	114.3	106.2	119.9	121.3
C21ORF11!	127.1	121.1	133.8	126.7	119.1	112.1	126.2	129.1
C21ORF12:	108.9	119.5	115.7	110.3	108	96.4	114.5	116.2
C21ORF12.	144.4	148.2	139.7	134.6	137.7	111.3	137.5	134.4
C21ORF12!	118.4	113	111.2	110	102.2	95.8	119.4	118.6
C21ORF13	109.8	115.3	122.9	113.7	106.3	102.9	115.2	116.9
C21ORF2	553.5	576.4	514.7	529.7	367.9	306.3	536.4	464
C21ORF24	4234.5	4080.8	3545.5	3338	3853	2895.6	4730.8	4758
C21ORF25	116.2	131.1	135	123	115.9	117.2	119.5	125.2
C21ORF25	166.8	167	203.1	158.5	135.4	156.7	143.4	146.5
C21ORF29	107	113.6	114.6	123	110	93.7	115.3	126.1
C21ORF33	188.7	191.4	258	164.3	209.5	169.6	149.6	151.1
C21ORF33	617.1	581	713.3	571.4	433.1	544.1	439.4	372.2
C21ORF33	157.8	187.9	160.9	151.9	140.9	134.4	146.3	141.1
C21ORF34	125.6	117	120.4	130	114.6	114.8	124.9	116.9
C21ORF34	110.2	116.2	125	123.9	108.9	98.8	132.8	123.5
C21ORF34	116.5	100.3	108.2	120.1	111.9	99.2	112.2	113.4
C21ORF41	117.3	115	112.5	130.6	110.3	107	121.3	113.6
C21ORF45	125.7	119.5	121.7	123.6	111.6	102.3	123.4	113.4
C21ORF49	120.7	111.9	130.1	116.5	107.3	101.2	124.2	109.7
C21ORF51	221.2	264.6	323.1	255.8	266.3	220.2	216.8	234
C21ORF51	155.5	154.3	166.4	157.8	151.4	124.1	138.1	153.5
C21ORF55	123	112.6	125.7	125.8	110.3	103.4	125.7	114.4
C21ORF55	123.2	119.9	123	118.3	117.5	105	124	124.4
C21ORF55	116.9	123.2	127.1	117.1	112.8	95.7	109	123.8
C21ORF56	125.9	130.5	135.1	128.8	112.1	119.6	127.9	123.6
C21ORF57	245.1	258.9	249.3	254.9	216.7	210.2	277.6	295.4
C21ORF57	165.8	150.5	169.8	187.4	152.8	140.2	155	168.7
C21ORF57	124.8	126	124.8	122.6	111	98.7	122.6	123
C21ORF58	116.7	111.3	114.4	117.2	105.7	90.9	112.9	113.7
C21ORF58	107.7	116.8	108.9	121.6	115.1	103.8	121.2	116.6
C21ORF58	116.3	114	112.6	116.8	116	103.9	118.5	109.6
C21ORF59	103.7	117	111.7	117.3	103	93.1	109.8	108.9
C21ORF59	279.8	342.9	282.8	243.6	275.7	213.9	223.3	224.9
C21ORF6	122.9	129.6	137.3	142.8	127.8	109.9	120.9	128.6
C21ORF62	120.1	124	113.6	122.1	112.4	101.1	117.6	122.6
C21ORF63	129.4	137.6	136.3	148.2	111	105.1	128.9	128.8
C21ORF66	131.7	119.8	132.2	137	118.4	106.1	122.2	125.1

C21ORF66	130.9	128.2	164.2	143.4	126.9	124.8	133.7	132.2
C21ORF66	132	120.8	149	142.5	122.2	99.2	146.8	125.7
C21ORF66	429.3	432.2	387.6	458.4	425.6	255.9	429.5	460.7
C21ORF67	121.4	133.6	129.5	144.2	122.6	111.3	123.2	125.5
C21ORF69	108.5	116.4	117.8	116	116.2	101.6	126.1	103.7
C21ORF7	211.3	210.2	207.8	348.8	240.1	234.3	294.5	213.3
C21ORF70	146.1	151.7	143.3	143	126.2	121.6	137.8	132.2
C21ORF87	113.1	121.7	119.8	125.3	124.1	112.9	122.7	120.3
C21ORF87	119.8	122.6	125.3	119.7	109.4	105.2	120.2	123.2
C21ORF88	116.7	125.4	120	122	109.4	103.3	129.3	119.6
C21ORF90	118.1	112.8	124.7	127.8	115	96.3	125.1	116.9
C21ORF91	190.7	196.7	173	168.1	169.8	132.2	172.5	200.3
C21ORF94	109.5	114.4	118.5	117.4	109.1	100.6	112.4	122.3
C21ORF99	112.1	120.2	121.5	127.7	102.7	100.6	121.1	109
C22ORF13	564.9	551.1	438.7	605.7	431.1	400	800.1	728.4
C22ORF15	126.1	129.5	125.6	120.9	112.6	108.7	128.1	111.1
C22ORF16	365.3	395.1	523.1	370	290.4	373.6	346.9	299
C22ORF18	109.9	116.8	118.3	123.4	109.4	102.2	118.6	127.7
C22ORF23	117.9	121.3	116.8	132.3	117.7	103.9	130.5	122
C22ORF25	195.6	183.7	230.3	177.4	162	168.9	184.7	160.3
C22ORF5	217.5	229.1	182.9	221.4	184.2	157.5	242.1	221.7
C22ORF8	251.7	141.4	302.3	222.6	122.1	188.4	215.9	127.8
C22ORF9	116.7	115.2	121.7	122.4	112.2	96.8	113.4	116.1
C22ORF9	126.1	139.3	161	146.6	128.4	128.3	150.7	123
C22ORF9	112.5	108.5	110.6	112.5	110.3	96.8	111.4	110.9
C2ORF10	117.6	121	117.2	119.6	112.8	103.8	122.8	117.3
C2ORF11	108.2	115.4	119.3	113.1	110.8	96	115.9	116
C2ORF12	119.3	115.2	121.9	121	108.1	105	127.8	117.5
C2ORF13	105.9	107.4	117.5	122.1	107.3	102	109.1	112.6
C2ORF16	117.7	110.1	114.2	112.9	102.6	97.3	125.2	110.7
C2ORF17	296.8	335	229.6	403.8	255.8	233.5	359.8	331
C2ORF18	145	132.6	171	164.2	125.3	162.1	154.9	137.1
C2ORF21	121.2	116.6	128.2	129.3	110.1	107	122	118.9
C2ORF24	197.1	217.9	206.4	237.5	193.3	214.5	308.9	254.9
C2ORF25	936.3	838.2	851.8	817.5	762	843.7	855.8	1053.2
C2ORF26	228.7	166.6	354.5	247.5	133	319.3	289.1	147.9
C2ORF27	114.5	111.7	107.2	108.7	107	97.9	113.7	113.9
C2ORF28	978.1	1045.5	1595.4	1044	942.6	994.5	938	816.2
C2ORF28	482.1	496.1	498.1	453.5	391.3	366	493.5	401.9
C2ORF29	654.8	825.7	821.5	759.7	697.5	526.6	625.7	602.4
C2ORF3	133.9	126.5	127.5	133.7	109.5	106.2	118.5	118.6
C2ORF30	337.4	417.8	412.7	435.4	361.1	413.2	363.8	380.1
C2ORF32	117.6	114.1	121.5	117	105.4	99.9	120.3	108.9
C2ORF33	132.7	129.4	155.7	174	127.3	122.9	155	143.4
C2ORF34	133.4	149.5	145.3	155.9	132	121.9	135.8	121.2
C2ORF4	187	209	239.6	213.7	169.2	152.7	203.3	214.7
C2ORF52	115.5	120.6	110.4	117.8	114.4	103.4	117.5	117.2
C2ORF7	138.9	113.6	118.2	121.3	108.5	114.3	123.2	119
C3	119.7	117.4	128.2	117.9	113.1	100.7	114.5	115.5
C3AR1	201.3	127.4	202.2	193.5	117.8	166.4	152.9	129.5
C3ORF1	134.1	131.2	138.3	131.8	132.1	115.1	118.5	125.6

C3ORF10	405.6	356.3	454	446.2	332.3	366.2	427.7	452
C3ORF14	154.5	132.1	213	163.9	124.9	158.1	143	124.2
C3ORF15	124.4	108.6	124.4	135.8	112.1	110.3	117.9	118.9
C3ORF17	210.3	205.7	208.1	185.6	153.3	202.5	181.5	166
C3ORF17	111.4	114.5	117.6	123.5	98.3	108.8	113.6	119.8
C3ORF17	147.1	173.7	164.7	153.6	152	125.6	143.2	163.7
C3ORF18	123	128.2	125	133.6	112	107.8	124.7	120.5
C3ORF19	602.6	601.6	629.6	745.7	565.4	492.5	575.6	639.5
C3ORF20	116.3	117.9	114.3	126.2	116.3	102.8	119.9	113.1
C3ORF21	143.2	149.5	182.2	146.2	139.2	129.6	136.4	135.2
C3ORF22	119.4	116.1	117.9	117.9	117.8	104.1	125.2	119.8
C3ORF23	129.9	129	143.4	126.1	124	106.6	126.9	122.3
C3ORF23	114.9	109	111.8	116.3	106	94.7	108.7	112.9
C3ORF23	111.4	108.4	114.5	109.9	105.5	108.3	118.7	109.8
C3ORF23	122.4	126.1	133.4	139.4	120.5	110.7	135.1	132
C3ORF24	111.4	109.4	114.1	109.2	109.4	94.5	122.2	111.7
C3ORF25	114.6	120.8	120.1	122.2	109.1	100.9	125.2	113.9
C3ORF26	254.7	302.8	245.7	273.8	234.4	167.4	226.7	249.8
C3ORF27	124.7	115.9	130.4	117.5	111.7	109.5	122.1	123.2
C3ORF28	264.4	279.1	376.6	271.3	216.1	257	202.9	211.7
C3ORF29	579.6	597.3	564.8	700.3	444.6	489.9	739.1	781
C3ORF29	144.5	196.9	169	187	194.5	155.5	216.3	190.7
C3ORF30	110.6	116.1	123.2	118.9	107.6	100.7	114	119.3
C3ORF31	272.9	283.1	367.8	251.8	268.9	243.3	216.2	198.2
C3ORF32	114.1	117	116.8	131.1	114.2	104.7	128.6	113.5
C3ORF33	115.3	117.5	127.1	115.8	105.5	98.4	127.5	115.6
C3ORF34	148.2	184.6	162.9	186.6	157.9	134.1	178.9	159.2
C3ORF34	2959.5	2310.5	3429.8	2616.9	2795.5	1860	2959.6	3204.2
C3ORF35	114.9	127.1	127.6	118	116.2	104.4	119.4	126.3
C3ORF35	118.1	117.2	131.4	121	108.8	101.7	125.6	127.4
C3ORF36	112.2	113.6	114.8	115	110.4	101.5	122.6	116.3
C3ORF37	399.3	458.8	346.4	361.5	326.9	269.4	416.6	399.8
C3ORF37	109.2	111.6	122.7	122.5	115.3	97.5	118.9	121.4
C3ORF38	295.4	256.5	318.8	280.8	202.3	253.7	239.3	232.8
C3ORF39	156.2	161.6	147.5	147.2	140.2	117.9	159.9	156.7
C3ORF40	171.5	179.7	166.3	206.8	180.8	152.5	214.1	191.2
C3ORF42	118.5	132.6	115.8	137.5	119.9	111.2	134.7	129.9
C3ORF42	125.6	113.6	125.4	125.4	114.6	112.4	122.4	126.2
C3ORF45	111.2	121.4	109.2	120.6	108.9	97.6	118.1	117.6
C3ORF48	117.8	115.5	115.5	115.2	112.1	97.8	117.1	114.7
C3ORF52	107.1	121.4	116.3	121.8	109.7	93.9	126.9	115.7
C3ORF54	168.1	179.3	163.5	167.4	142	127	138.7	140
C3ORF56	114.7	114.3	116.4	117.2	102.1	106.2	115.9	123.1
C3ORF57	117.6	109	115.6	119	106.2	97.5	117.6	122.5
C3ORF58	175	195.8	191.4	162.2	174.5	136.7	163.9	168.1
C3ORF59	149.2	147.5	166.4	145.6	137.3	154.1	133.5	136.3
C3ORF60	117.2	105.7	121.2	118.6	110.4	103	116.4	116.6
C3ORF60	121.5	127.2	131	138	115	106	120.4	127
C3ORF60	103.4	116	122.3	132	110.6	103.3	116.3	116.6
C3ORF60	111.9	121.2	127.2	112.1	115.9	106.9	119.5	109.7
C3ORF60	1168.1	1352	1152.4	1230	776	839.3	736.6	540.9

C3ORF62	157.7	159.6	176.5	189.2	165.6	137.7	195.7	207.4
C3ORF63	184.4	169.3	208.8	173.9	184.2	157.5	191.7	151.8
C3ORF64	163.6	152.4	165.3	174.9	172.4	126.1	152.3	159
C3ORF65	116.4	115	117.5	114.7	109.4	101.4	115.7	121.1
C3ORF9	264.5	265	224.3	218.4	211.2	159.8	195.7	187.8
C3ORF9	1109.3	1300.2	962.3	1064.7	1094.8	498.5	874.5	685
C4A	115.3	129.9	142.2	133.1	121.2	110.8	136.2	126.9
C4BPA	109.2	118.9	115.8	130.1	106.1	101.7	119.4	114.9
C4BPB	118.4	120.6	126	126.7	117	107.4	117.3	115.5
C4BPB	115.2	120.6	114.4	123.3	112	102.4	122.6	110.8
C4ORF13	113.2	116.4	117.8	115.5	110	97.3	119.3	120.4
C4ORF13	136.6	138.4	171.9	147	134.2	134.7	128.8	119.3
C4ORF14	456.6	565.1	442.7	517.6	330.1	389.6	371.4	443.6
C4ORF15	126.9	115.2	119.1	114.2	126.2	104.6	132.7	121.5
C4ORF16	133.7	128.7	129.1	131	121.8	107.2	128.4	142.8
C4ORF17	103.4	113.1	108.2	110.1	110.3	93	120.5	106.4
C4ORF18	116.9	112.6	159.9	126.7	111.4	146.2	120.3	115.4
C4ORF18	153.8	117.8	208.3	184.4	124.1	194.9	146.3	116.8
C4ORF18	111.6	108.6	115.5	123.2	102.1	94.5	117.4	107.2
C4ORF28	106.4	109.4	105.9	104.4	108.8	97.5	105.8	110.9
C4ORF32	114	139.5	128.2	121.7	118.8	107.7	126.6	122.1
C4ORF34	281.6	318.9	390.3	304.7	260.2	177.4	308.7	287.5
C4ORF36	117.4	114.3	106.8	117.5	116.5	97.9	114.1	112.1
C4ORF6	109.9	120.6	115.7	114.8	109.1	99.9	106.4	111.3
C4ORF7	112	119.3	119.3	126.8	99.1	101.9	118.3	111.6
C4ORF8	191.3	203.4	161.8	186.7	218.4	169.8	222.6	224
C4ORF9	235.9	230.5	232.4	222.8	223.5	169.9	228.4	187.1
C5	115.8	127.9	153.2	145.9	133.5	106.7	131.5	129.8
C5AR1	334.7	282.7	460	745.2	334	1265.8	1004	325.9
C5ORF13	126.3	148.7	159.2	144.5	126.9	107.3	121.4	127.8
C5ORF14	129.3	129.9	123	126.2	129	109.1	128.3	127
C5ORF15	588.8	580.1	653.4	637	432.3	462.3	510.3	492.7
C5ORF16	163.1	170.6	130.3	172	149.7	123.5	185.7	183.5
C5ORF20	122.1	118.9	143.8	118.5	111.1	102.7	120.5	120.9
C5ORF21	306.4	326.3	496.8	319.7	291.2	363.9	356.4	334.9
C5ORF28	1435.6	1424	1249.2	1340.3	1326.8	615.8	1416.2	1689.3
C5ORF3	122.9	136.9	136.6	135.3	124.3	116.6	142.9	154.2
C5ORF34	124.1	128.1	125.2	135.4	113.8	102.9	124.2	120.7
C5ORF36	119.6	108.8	113.6	115.7	106.7	94.1	121.3	115.3
C5ORF37	188.9	194.5	208.8	195.7	180	165.3	198.2	176.5
C5ORF4	107.5	110.3	120.6	123.6	110.8	101.3	120.2	122.2
C5ORF4	116.3	104.3	104.6	119.8	111.7	95.8	121.2	119.9
C5ORF4	120.6	124.9	120.7	123.9	113.8	100.5	113	120.3
C5ORF5	459.8	609	514.5	567.3	537.3	376.2	578.2	645.8
C6	109	113.5	116.5	126.9	106.1	94.7	123.6	108
C6ORF1	119.7	119.7	129.6	122.4	108.7	102.2	117	119.2
C6ORF1	132.8	120.7	142.4	132.5	111.5	113.5	116.2	127.5
C6ORF1	127.8	118.3	118.3	120.2	110.9	96.5	126.6	116.1
C6ORF10	117.3	108.5	113.9	113.3	109.9	97.2	125.9	109.8
C6ORF105	163.6	131.8	171.3	158.3	118.4	130	131.3	113.4
C6ORF106	128.4	149.6	136.6	192.6	157.8	105.1	182.8	196.8

C6ORF106	188	239.2	179.2	242.5	164.9	183.9	315.8	285.8
C6ORF107	111.9	113.1	124.3	122.5	111.7	100.2	115.6	128.2
C6ORF108	120.1	116.3	120.8	124.5	118.5	103.2	115.4	113.6
C6ORF108	438.5	459.8	898.1	406.2	238.1	648.8	231	141.4
C6ORF111	769.9	572.6	1044.8	681.1	644.5	509.3	467.1	525.3
C6ORF113	171.9	180.1	205	203	175.2	156	189.7	203.6
C6ORF114	113.1	119.7	124.1	122.7	111.7	103.7	116.2	117.7
C6ORF117	123.1	113.2	125	120.2	110.5	99.1	117.8	120.2
C6ORF118	115.5	119.8	116.3	125.7	106.8	101.2	122.8	124.5
C6ORF120	158.4	169.4	181.9	156.3	169	123.1	155.3	150.8
C6ORF122	109	119.9	106.1	112.8	109.3	96.1	112.2	105.9
C6ORF125	295.2	381.2	310.1	353	261.9	218.8	255.5	212.1
C6ORF128	136.4	125.5	119.6	127.2	111	107.4	126.1	124.7
C6ORF129	220.7	278.2	329.4	230	196.4	303	209.3	210.5
C6ORF130	352	409.3	457	448.8	445.1	418.8	523.3	594.9
C6ORF134	117.1	120.4	116.8	127.5	111	99.3	124.8	122.4
C6ORF134	119.2	120	131.6	124.2	111.1	101.2	128.1	123
C6ORF134	109	105.5	110.1	123.6	109.5	98.7	115.2	114.8
C6ORF136	189.8	181.4	205.9	197.6	218.7	139.9	195.9	178.9
C6ORF139	137.4	129.3	145.2	135.2	115.6	113.2	139.1	133.3
C6ORF142	125.8	119	117.8	118.7	114.9	94.7	122.8	117.5
C6ORF143	109.8	119.5	114.5	116.8	106.7	101.8	112.4	118.3
C6ORF145	117	108	118.5	122	114.5	109.9	115.9	113.7
C6ORF146	111.6	121.5	113.5	106.7	105.2	99.6	112.3	110.2
C6ORF148	114.2	122.4	127.9	123	120.1	106.1	123.3	122.1
C6ORF149	153.3	165.1	139.8	146.1	133.6	121.7	154.8	148.5
C6ORF15	109.6	119	114.2	114.9	102.6	92	124.6	112.7
C6ORF150	123.9	121.9	129.4	128.3	112	111	121.9	132.3
C6ORF151	138.2	151.9	133.4	137.8	125.5	114.4	140	142.9
C6ORF152	113.5	118.8	118.8	119.6	105.2	101.9	112.7	109.6
C6ORF153	339.9	323.5	333.5	328.4	276.8	214.5	247.6	335.1
C6ORF154	111.1	115.6	120.2	113.4	110.3	101.4	108	122
C6ORF157	119.9	118.2	122.9	121.8	106.2	102.7	126.1	127.5
C6ORF159	115.2	117.8	123.5	128.2	107.3	103.3	128.8	130.3
C6ORF162	128.5	120	136.4	132.1	123	114.4	127.8	136.6
C6ORF162	134.6	130.5	142.1	128	126.7	115.6	141.8	125.2
C6ORF165	109.6	116	113.1	121.9	109.4	96.7	115.3	125
C6ORF165	119.6	127.5	116.7	116.7	106	102.1	124.7	124.8
C6ORF165	114.8	128.9	117.5	120.9	109.2	94.3	111	126.1
C6ORF166	479.3	492.5	477.2	517.9	540.9	651.9	710.1	825
C6ORF167	110.5	111.7	132.1	126.6	115.3	107.3	114.4	125.9
C6ORF168	121	118.5	114.5	116.9	109.7	97.6	116.7	107.5
C6ORF170	261.7	280.2	281.9	262.6	301.8	168.8	260.2	289.4
C6ORF173	138	115.9	141.6	133.2	127.9	111.2	126.6	133.9
C6ORF174	107.8	112	117	116.3	107.5	96.8	121.4	109.1
C6ORF182	106.6	119.8	120.7	122.2	109	92.9	117.9	118.6
C6ORF188	110	114	115	116.6	110.7	97.5	120.6	114
C6ORF190	173.6	214.5	176.8	196.6	199.2	149.2	270.2	442
C6ORF191	122	113.4	123.1	118.4	110.4	97.2	123.9	112.2
C6ORF192	261.2	263.5	246.9	270.2	236.5	202.6	171.9	166.6
C6ORF194	123.4	115.6	126.6	120.6	108.1	108	123.1	125.6

C6ORF195	111.6	126.1	104	114.1	105.6	98.2	113.8	118
C6ORF199	115.5	127.9	113.6	116	112.1	107.2	117.2	119.2
C6ORF201	116.6	110.7	121.7	122.2	112.7	106.9	119.5	114.4
C6ORF203	134.9	121.2	141.4	130.4	111.8	119.2	115	121.6
C6ORF204	188.6	159.9	141	175.1	159.5	111.9	148.6	167.2
C6ORF204	156.1	145.5	143.8	165	151.3	138.2	144.3	141.2
C6ORF205	122.9	133.9	125.5	129.2	118.4	105	119.5	126.7
C6ORF205	114.2	121.2	125.7	117.1	116.8	94.9	116.7	113.5
C6ORF206	112.5	108.2	113.3	119.7	100	92.7	98.4	106.1
C6ORF208	117.9	123.8	112.7	116.7	113.8	95.1	124.1	118.6
C6ORF21	115.1	109.7	118.4	115.9	115	97.1	121.4	112.7
C6ORF211	151	137.5	191.9	154	129.6	127.8	173.6	153.3
C6ORF213	113	118.2	115.8	118.1	106.1	103	114.1	114.4
C6ORF25	130.1	119.2	125.3	139.2	121.9	106	114.3	122.8
C6ORF25	112.4	121.6	125.6	128.7	114.7	100.2	118.2	115.4
C6ORF25	116.2	122	137.2	118.6	124.6	103.9	127.4	118.6
C6ORF25	113.8	115	113.5	115.3	106.4	92.6	112.9	116
C6ORF26	112.9	119.8	122.1	119.2	103	96.8	117.4	119.3
C6ORF27	124	121.3	129.5	120.3	114.2	105.6	126.9	110.9
C6ORF32	1147.9	1286.3	984.3	1201.2	1109.9	755.7	858.4	655.9
C6ORF35	117.6	125.7	129.1	106.7	110.2	101.7	125.1	119
C6ORF47	270.1	228.5	217.7	257.9	204.8	165.2	226.5	220.2
C6ORF48	114.6	110.4	112.9	110.6	111.1	97.6	122.7	112
C6ORF48	3086.5	3149.2	4104.9	3139.4	2460.6	3334.4	3071.9	3400.1
C6ORF49	606.6	624.4	855.6	640	567.9	534.2	439.7	615.1
C6ORF51	122.7	121.7	118	126	106.1	97.1	114	114
C6ORF55	181.2	186.5	189.4	187.8	149.6	161.3	187.5	191
C6ORF57	126.1	125.6	141.8	129.2	122.5	126.5	114.6	120.1
C6ORF59	117.6	120.6	124	122	119.3	107.8	117.3	126.3
C6ORF60	152	155.6	142.8	144.6	147.4	124.3	209.8	225.8
C6ORF62	116.3	114.3	123.1	120.4	112	106.3	124.1	120.7
C6ORF64	184.5	186.8	210	195.5	164.2	140.7	206.8	214.8
C6ORF65	111.2	114.7	117.3	115.5	103.3	95.3	119.1	113
C6ORF66	136.8	144.3	138.5	146.5	136.1	111.2	131.9	156.4
C6ORF69	395.1	445.1	584.4	495.3	366.3	313	450.1	564.7
C6ORF70	171.4	201.3	181.4	206.5	193.6	158.1	190.7	185.6
C6ORF71	116.4	107.4	111.2	109.5	98.7	88.6	112.9	107.9
C6ORF72	124.6	112.4	132.9	135.7	136.1	104.7	129.7	132.4
C6ORF75	116.9	115.4	127.1	118.4	106.6	106	119.2	114.6
C6ORF75	120.5	116.1	123.1	115	111	95.8	121.1	112.1
C6ORF75	244.7	240.3	260.2	226.4	246.2	192.3	191.4	177.9
C6ORF78	114.9	112.2	127.2	114.7	116	92.8	108.9	111.4
C6ORF79	198.7	184.4	179.2	220.2	175.1	152	204	217.8
C6ORF81	118.7	124.3	116.8	122.7	112.9	102.3	128.7	123.5
C6ORF84	133.4	124.8	133.1	129.3	124.4	120.9	131.7	121.3
C6ORF85	168.9	178.1	134.7	182.7	174.5	121	186.2	210.2
C6ORF89	123.7	128.8	139.4	145.5	128.8	124	145.6	133.7
C6ORF96	127.9	132.3	151.7	144.4	124.2	109.9	140.9	117.3
C7	108.5	111.8	115.1	121.1	106.1	101.7	113.7	112.7
C7ORF10	115.2	122.8	121.4	121.6	128.5	105.2	128.7	124.9
C7ORF11	153.1	155.3	161.1	165.9	128.2	127.3	170.4	131.8

C7ORF16	114.2	113.6	116.8	117.9	109.9	109.9	120.5	113
C7ORF20	181.3	177.3	217.6	197.8	175.9	182.5	217.4	213.3
C7ORF20	137.5	133.1	134.5	133.8	123.8	115.6	134.9	143.2
C7ORF21	584.3	556.4	473.5	518.7	377.6	449.6	645.1	674.4
C7ORF24	191.7	185.4	205	170.9	159	146.3	194.7	154.7
C7ORF25	156.2	171.3	158.9	165.7	144.2	126.9	177.3	162.3
C7ORF26	461.9	528.8	412	487.5	331	398.9	475.3	417.8
C7ORF27	289.2	290.5	291.7	362.9	271.7	245.8	206	258.5
C7ORF28A	116	118	103.3	117.1	110.1	101.2	115	118.8
C7ORF28B	352.2	451	324.2	351.1	373.3	262.3	299.6	536.6
C7ORF29	134.9	129.8	134.1	144	131.2	112.4	123.9	135.8
C7ORF30	795.3	889.1	905.1	762	727.5	767.5	588.3	618.6
C7ORF31	114.6	120	116.1	119.1	110.5	101	124.7	121.9
C7ORF33	114.5	109.7	123.8	108	104.4	92.4	115.2	112.9
C7ORF34	121	117.5	123	122	103.1	98.3	122.6	116.2
C7ORF36	158.7	167.2	155.8	160.3	144.3	127	127.8	135.5
C7ORF44	355.6	432.4	502.8	342.8	321.8	338.5	270.7	262
C7ORF9	116.9	113.2	120.2	116.5	101.5	101	116.7	107.6
C8A	117.6	106.7	114.7	107.5	110.1	97.3	100.4	113.9
C8B	121.9	122.2	114	121.3	112.7	93.9	117	98.3
C8G	122.5	114.3	118.3	121.5	110	105.9	127.7	127.3
C8ORF1	154	162.4	144.1	155	149.7	125.5	171.7	149.8
C8ORF13	113.6	119.2	123.5	120.6	116.3	107.6	120.4	128.9
C8ORF15	127.5	120.5	125.4	125.6	109.7	97	123.3	127.5
C8ORF22	106.4	112.3	112.5	117.1	119.3	109.7	116.6	119.5
C8ORF30A	100.1	115.9	106.2	109.7	111.5	102.7	110.7	109.6
C8ORF31	120.1	116.6	111.4	118.7	112.7	94	119.6	108.3
C8ORF32	147.4	130.8	125.7	138.8	124.8	110.1	138.5	137.6
C8ORF33	208.7	217.1	236.4	258.7	188.6	172.1	205	239.6
C8ORF34	131.8	134.9	140.2	133.5	128.6	110.9	133.8	124.6
C8ORF35	391.8	458.7	366.6	413.8	500.4	307.1	386.5	472.4
C8ORF36	184.5	200.7	209.7	204	177.5	162	176.7	211.7
C8ORF37	1552.9	1703.7	1997.1	1940.9	2161.8	1104.9	2205.8	2123.9
C8ORF38	145.7	154	140.6	147.1	134.1	124.4	142.7	140.9
C8ORF4	109.3	110.1	114.5	112.5	109.5	93.1	118	113.4
C8ORF40	252.3	257.2	429.5	270.1	278	308.5	230.5	260.4
C8ORF41	204.2	154.4	160.3	198.8	154.2	154.7	160.8	176.1
C8ORF42	108.3	115.2	101.8	110.1	99.6	90.2	110.3	108.5
C8ORF44	118.7	120.3	137	140.7	129.1	101.8	124.4	115.9
C8ORF45	939.9	613.2	794.1	726.3	621.4	414.1	621.2	801.9
C8ORF46	116.2	124.2	108.7	119.4	109.4	94.7	107.5	118.8
C8ORF47	114.9	110.7	128	119.8	114.8	99.8	113.4	117.8
C8ORF48	120.6	119.3	125.9	127	114.1	106	124.2	118.2
C8ORF49	108.8	115.4	122.4	118	114	96.9	114.2	113.5
C8ORF52	158.2	178	173.7	156.6	136.2	138.2	160.3	184.9
C8ORF53	127.5	141.5	130.3	133.9	128.2	108.4	135.1	174.3
C8ORF55	361.8	437.6	363.3	387.7	296.6	194.2	210.3	214
C8ORF58	141.2	128.6	131.4	142.5	121.3	129.3	160.8	134.7
C8ORF61	148.5	138.3	135.5	149.7	135.5	130.2	150.6	157.7
C8ORF70	132.1	134.2	140.7	143.4	125.9	109.9	132.6	121.5
C8ORF72	120.4	116.9	120.2	120.4	105.2	99.9	121.5	115.6

C8ORF74	110.1	112.3	117.3	121.7	107.4	95.3	113.5	113.1
C8ORF76	621.9	653.2	679.2	678.1	610.7	475.8	748.2	699.8
C8ORFK32	116.9	120.7	114.2	126.1	110.7	104.4	120.7	118.9
C8ORFK36	111.2	106.7	120.8	119.1	109.6	93.4	110.5	103.5
C8ORFK36	114.1	106.3	109	112.1	106.9	95.4	115.5	110.9
C9	108.5	116.1	110.3	114.1	110	97.9	118.7	124.6
C9ORF100	108.6	116.2	115.6	112.1	101.7	99.1	112.7	105.4
C9ORF100	112.4	114.9	122.5	118.3	117.7	103.5	116.9	122.5
C9ORF102	181.7	187	162.1	186	178.1	132.2	214.9	199.9
C9ORF103	165.9	183.9	283.4	188.8	182.4	238.8	184.8	227.5
C9ORF106	113.4	121.4	122.2	124	109.3	102.4	117	120.8
C9ORF10O	144.5	160.7	159.1	176.9	161.4	161.8	164	138.9
C9ORF11	117.1	104.6	111.5	112.2	104.3	104.2	112.4	110.4
C9ORF111	276.4	262.7	432.3	258.6	228.8	270.3	234.8	218.2
C9ORF112	118.3	120.5	132.8	127.7	119	117.9	124.5	124.7
C9ORF114	679.2	757.1	454.8	733.3	541.2	328.2	568.7	516.4
C9ORF116	116.1	117.6	119.3	119.9	103.4	110.7	112.7	118.3
C9ORF116	116.8	118.1	118.1	119	107.6	95	121	111.2
C9ORF117	111.2	110.1	119	113.1	104.1	106.3	111.1	114.5
C9ORF121	113.3	106.1	120.4	118.5	107	92.6	113.5	110.6
C9ORF123	203.9	197.4	247.4	225.4	187.7	183.4	200.8	175.5
C9ORF125	119.2	119	115.7	119.1	107.1	97.2	117.5	111.1
C9ORF126	113.6	111.9	111	115.7	105.8	93.6	111.9	106.8
C9ORF127	133.1	149.6	147.5	140.1	128	138.9	139.4	179.5
C9ORF127	119	110.9	174.4	119	111.1	119.4	130.9	124.5
C9ORF128	108.4	113.7	104.7	115	109.1	93.6	117	112.8
C9ORF132	201.3	212.7	218	229.1	179.6	282.9	226.6	217.1
C9ORF138	123.8	108.7	113	115.2	98.7	95.1	120.5	110.4
C9ORF140	107.7	120.7	121.8	114.6	104.9	105.4	121.5	122.6
C9ORF142	948	1249.7	1819.4	1056	768.1	1236.3	695.6	681.8
C9ORF150	117.4	114.6	115	111.1	104.9	97.1	114.8	105.1
C9ORF152	110.4	109	101.9	117.6	106.6	100	122.4	118.4
C9ORF153	115.1	114.2	123.1	118.1	103.9	99.1	123.3	116.5
C9ORF156	452.4	477.3	492.7	495.2	400.1	400.9	409.1	434
C9ORF157	117.2	114.7	126.6	113.2	111.2	99.9	114.8	116.3
C9ORF16	129.7	151.9	155.8	160.7	115.1	116.9	135.2	135.8
C9ORF166	117.6	119.5	120.1	113.8	116.6	104.5	125.1	122.1
C9ORF18	108.3	110.5	103.7	114.2	103.7	98.5	113.4	108.5
C9ORF19	941.7	952.7	1751.7	893.8	667.1	917.5	557.9	448.8
C9ORF21	138.1	144	148.1	153.1	120.3	115.4	161	136.1
C9ORF23	135.9	165.4	167.1	140.4	137.3	119.3	129.4	150.8
C9ORF23	131.7	124.3	128.6	142.1	125.4	111	133	126
C9ORF23	229	236.1	255.7	254.6	184.1	234	250.2	220
C9ORF24	121.6	129.1	120.5	125.1	117.4	97.3	120.2	137.1
C9ORF24	119.1	118.8	124.2	126.6	111.8	93.3	114.4	119.2
C9ORF24	113.5	113.8	128.8	136.1	109.9	97.8	112.4	111.1
C9ORF25	137.6	141.2	152	128.7	119.3	102.1	129.4	139
C9ORF28	167.2	187.1	180.8	180.7	148.4	166.5	170	145.1
C9ORF3	109.5	111.5	116.1	115.1	106.6	97.7	116.6	114.2
C9ORF30	142.6	151	144	152.9	148.5	118.3	167.2	137.1
C9ORF32	162.3	150.5	190.2	201.8	170.6	153.7	146.7	164.9

C9ORF36	113.4	121.9	110.5	125.4	118.6	96.8	113.6	111.5
C9ORF37	130.3	155.1	160.9	147.1	120.1	121.9	137.7	123.7
C9ORF39	123.8	111	116.5	118.2	105.1	106.5	117.6	125.7
C9ORF4	107.5	112.9	122.9	119.8	108.9	103.4	121.4	109.6
C9ORF40	156.1	153.9	149.9	166.8	149.7	128.5	187.5	180.1
C9ORF41	117.6	125.8	119.2	126.9	112.9	94.3	125.6	118.5
C9ORF43	108.1	115.8	124.6	107.9	116.6	103.2	129.4	121.9
C9ORF45	266.9	230.7	277.5	254.4	210.3	189.6	232.1	239.1
C9ORF46	381.3	358.1	656	387.2	288	426.9	263.7	201.7
C9ORF47	115.1	106.8	105.9	128.9	114.5	107.6	117.2	118.7
C9ORF48	119.4	132.4	118.2	119.5	119.8	99	116.3	110.3
C9ORF5	308.8	408.8	272.6	281.5	408.4	260	477.9	421.2
C9ORF50	110.4	115.7	122.6	118.4	117.6	97.7	111.3	126.7
C9ORF52	107.6	121.8	124.9	120.9	112.8	98.2	116.4	115.4
C9ORF58	126.4	118.3	116.3	127.8	114.9	101.4	113.7	112.6
C9ORF58	114.1	123.2	128.5	129.3	115.2	101.5	115.7	117.2
C9ORF6	156.1	177.6	209	179.2	174.3	169.1	159.6	149.8
C9ORF61	108.3	116.4	108.6	111.1	108.7	98.4	117.7	109.9
C9ORF64	119.9	126.8	126.4	126.1	124.5	118.5	135.1	128.7
C9ORF66	123.2	133.8	137.9	127.8	131	107.7	124.4	112.2
C9ORF68	109.6	119.4	109.7	118.5	98.8	99.9	113.4	106.8
C9ORF7	129.5	132.9	129.4	134.2	120	110.9	150.7	146.8
C9ORF71	111	115.8	112.2	113.6	102.1	102.1	118.7	117.6
C9ORF72	138.5	133.5	150	158.1	119.6	122.5	149.6	128.8
C9ORF72	113.1	111.6	122.5	115	108.1	103.5	120	114.2
C9ORF72	115.7	115	125.2	125.9	116.6	112.9	118.5	119
C9ORF74	482.5	562.8	620.3	511.6	420.7	532.1	433.5	375.2
C9ORF75	140.6	143.3	137.1	139.9	114.9	126.1	144.9	142.5
C9ORF77	125.8	137.9	154.9	142.9	132.6	118.5	141.2	130.9
C9ORF77	123.7	118.4	130.1	134.4	103.5	103.1	122.9	113
C9ORF77	192.6	233.9	188.8	186	147.9	153	191.8	253.4
C9ORF78	1871.7	2097.2	1921.1	1834.3	1745	1686.4	2003.4	2058.4
C9ORF79	117.7	124.8	114.7	115.6	109.4	97.1	131.3	118.2
C9ORF80	2830	3059.5	2637.8	3263.3	3010.4	1454.6	3091	4264.1
C9ORF82	147.9	171.8	171.9	186.9	148.3	135.4	148	152.8
C9ORF84	120.5	122.8	117.9	123.1	115.2	97.2	119.3	124
C9ORF84	115.4	114.8	118.6	119.3	102	100.7	127.6	113.5
C9ORF85	124.6	129.6	122.7	131.4	128.9	109	121.5	123.3
C9ORF85	122.2	127.3	126.9	125.3	113.2	115.4	126.1	106.8
C9ORF86	201.3	228	213.6	233.8	186.4	151.4	235.9	201.3
C9ORF88	757.7	1017.4	651.1	1293	736.1	747.1	892.9	439.8
C9ORF89	354.9	346	378.7	369.9	268.4	385	509.8	501.4
C9ORF9	118.1	116.5	117.3	125.4	109	101.3	113.5	113.8
C9ORF90	248.6	253.7	210.8	274.4	265	205.5	351.4	352.9
C9ORF91	171.8	177.5	156.6	161.2	150.9	119.8	141.2	153.8
C9ORF93	112.3	119.5	121.5	117.6	117.4	95.8	120.7	124.9
C9ORF95	173.8	193.8	212.4	198	196.2	180.8	200.9	164.1
C9ORF96	106.8	109.9	111.5	121.4	104.8	96	115.4	116.4
C9ORF97	128.7	151.4	135.2	136.1	135.6	105.3	137.9	128.5
C9ORF98	135.3	125.5	127.9	126.7	115.5	94.9	148.4	110.9
CA1	123.2	118.1	123.5	119.2	107.8	104.5	120.6	113.3

CA10	117.5	123.3	114.8	119.8	106.3	105.9	112.7	121.5
CA11	163.8	174.3	135.2	157.8	142.6	129.7	182.1	163.4
CA12	110.8	115.3	116.7	117.2	110.5	94.8	116.6	124.9
CA12	107.1	110.4	112.6	112.3	102.6	91.7	110.8	104.7
CA13	116	130.3	121.5	116.4	111.4	107	127	120.8
CA14	108.5	111.4	120.5	119	103.6	93.7	114.6	114.2
CA2	188.5	265	381.9	213.6	181.4	220.1	182.7	224.5
CA3	109	119.7	112.7	108.8	108.4	95.2	120.2	103.4
CA4	112.6	128.7	124.8	118.8	110	97.7	136.6	127.2
CA5A	112.8	105.5	107.6	106.9	100.4	97.1	115.1	113.3
CA5B	227.7	289	268	279.1	228.7	206.1	210.8	206.6
CA6	122.8	138	139.1	139.6	110.5	108.4	125	129.6
CA7	120.5	109.3	108.7	117.1	111	100.3	116.7	119.2
CA7	106.5	116.1	111.6	124.2	102.1	99.5	117.5	112.4
CA8	108.9	111.8	115.5	122.7	110.1	101.4	106.6	109.5
CA9	125	114.7	138.3	125.5	113	102.3	127.2	117.2
CAB39	1631.9	1816.7	1555.3	1739.2	1625.3	1552.7	1837.4	1922.3
CAB39L	123.3	127.2	136.6	132.1	127	115.5	167.5	148.6
CAB39L	107.7	118.7	107.9	129.9	117.1	96.5	123.1	110.9
CAB39L	121.6	122.2	122.6	130.4	113.2	97.5	117.4	116.2
CABC1	440.6	491.9	356.5	534.4	451.1	431.1	552	497.5
CABIN1	193.6	241.4	205.1	195.5	155.2	212	157.9	140.5
CABLES1	139.7	136.2	136.5	147.1	119.6	106.7	141.5	132
CABLES2	133.2	135.2	140.9	134.4	117.4	120.7	143	144.3
CABP1	130.4	110.6	121.8	123.1	109.8	93.5	120.5	122.2
CABP1	105.7	113.8	115.4	115.8	105	102.1	113.2	115.3
CABP1	118.7	117	112.7	124.5	107	99.1	118.2	107.7
CABP2	114.4	116.7	118.8	121.8	111.9	101	118	117.7
CABP2	115.8	131.2	113.7	120.6	104.6	98.5	121.7	119.7
CABP2	120.9	107.4	133.5	119.5	109.8	104.7	124.4	116.1
CABP4	118.5	119.5	123.9	127.1	117.1	94.1	130.6	124
CABP5	116.6	111.2	111.6	111.6	101.1	101.6	123.1	115.2
CABP7	124.5	126.7	132.6	123.7	123.5	114.9	132.3	123.3
CABYR	106.7	100.6	123.6	112.8	117.4	99.3	110.2	107.9
CABYR	120.8	123.7	125.4	136	135	107.1	189.2	245
CACHD1	125.5	135.6	117.6	124.2	114.1	99.7	123.9	128.1
CACNA1A	110.6	109.5	116.1	116	109.6	104.3	111.1	110.8
CACNA1A	119.5	113.8	128.8	129.4	111.6	107.2	132.1	115.1
CACNA1B	131.8	127.9	129.5	128.7	123.9	112.1	127.6	135.7
CACNA1C	123.5	122.4	126.9	116.4	117.5	113.9	133.5	108.1
CACNA1D	119	120.6	121.6	136.9	117.3	103.4	132.2	118.9
CACNA1E	118.3	115.7	118.5	124.2	107.8	94.9	126.5	110.8
CACNA1F	111.8	125	125.9	114	104.7	97.4	121.2	107.7
CACNA1G	106.8	112.7	110.6	104.5	105.2	97.5	106	112.3
CACNA1G	123.2	116.5	112.7	122.1	108.6	98	127.6	108.1
CACNA1G	113.8	112.6	109.2	116.5	105.3	92.8	114.2	112.7
CACNA1G	111.4	105.7	112.1	108.9	111.2	95.6	112.9	113.9
CACNA1H	148	137.1	145.2	166.8	125.9	123.4	146.4	142.2
CACNA1H	120.3	118.4	122.9	118.1	106.8	99	111.6	111.8
CACNA1I	114.1	113.8	118.2	121.7	101.8	101.6	106.7	113.7
CACNA1I	452.1	367.7	395.8	463.5	311.4	294.5	331.4	307.3

CACNA1S	113.4	114.6	110.1	120.2	100.8	96.3	119.3	110.9
CACNA2D1	114.7	122.2	123.5	125.8	109.8	97.3	114.4	113.6
CACNA2D2	126.9	150.8	137.2	131	138.9	104.8	135.6	130.5
CACNA2D2	111.6	112.7	117.2	117	109.3	103.7	115.3	115.6
CACNA2D3	117.9	109.2	128.2	121.8	119.5	101.4	117.8	121.6
CACNA2D4	136.3	117.4	125.6	121.2	105.9	106.9	117	120.1
CACNA2D4	130.1	147.4	125.3	153.4	117.9	121.1	120.1	128.8
CACNB1	120.5	122.4	103.1	127.3	112.1	98.7	118	112.7
CACNB1	114.8	113.5	120.2	119.4	107	100.5	121	118.2
CACNB1	112.2	120	113.3	109.5	106.3	94.7	110.1	113.8
CACNB1	117.2	120.7	111.8	123.8	109.1	98	118.7	114.3
CACNB2	109.4	115.1	116.3	130	111.8	92.3	107.9	113.8
CACNB2	118.9	122	114.6	118.2	107.3	98.3	123.3	123.9
CACNB2	119.7	115.9	126	117	103.2	93.5	113.4	118.3
CACNB2	120.9	115.4	120.5	123.9	102.2	95.8	113	113.9
CACNB2	116.3	109.3	116.9	121.9	97.5	96.6	106.3	112.9
CACNB3	188.2	179	150.2	180.8	187	131.6	266.9	255.8
CACNB4	121.9	127.2	128.1	121.1	118.2	98.9	114.6	117
CACNB4	101.9	106.7	114.2	113.7	106.2	94.1	113.5	122.1
CACNB4	105.8	108.7	111.8	122	100.3	95.1	115.2	112.8
CACNG1	112.3	109.9	109.9	124.2	100.3	102.4	118.2	112.2
CACNG2	114.7	116.9	115.9	108	101.5	99.1	117.8	115.5
CACNG3	110.4	110.4	111.1	114.9	102.5	96.8	117.9	106.2
CACNG4	111.6	108.9	112.6	111.8	103.3	90.7	109.3	105.4
CACNG5	117.4	120.7	119.7	122.2	114.5	94.6	125.9	111.2
CACNG5	121.7	123.4	122.9	109.1	115.2	97.3	112	112.9
CACNG5	120	105.7	119.4	122.7	113.4	97.1	121.4	114.6
CACNG6	109.2	116.5	124.3	122.1	116.1	101.1	117.4	110.2
CACNG6	121.4	115.1	127.1	117.2	110.7	93.8	111.2	120.7
CACNG6	110.2	114.5	132.9	125.5	113.4	106.7	121	118.2
CACNG7	115.8	112.5	115.6	115.3	112.4	95.4	109.2	109.5
CACNG8	130.1	121.4	128	132.4	127.8	101	114.3	134.2
CACYBP	322.6	297.9	415	420.1	380.1	350.3	657	1016.2
CACYBP	168.5	183.1	178.7	192.4	166	139.6	201.5	211.4
CACYBP	118.1	106.6	109.7	123.9	109.5	107.9	114.8	120.7
CAD	150.6	175.7	148.2	162.3	150.5	124.7	163.4	175.2
CADPS	120.8	109.2	120.3	124	108	97.9	115.3	115
CADPS	119.7	123.8	123.4	128.4	124.8	104.5	127.1	123.7
CADPS	105.2	116.4	117.9	118.5	105.7	94.3	114.7	118.1
CADPS2	104.7	107.8	107.5	109.6	104.8	100.8	106.8	105.9
CADPS2	111.3	121.7	133.4	119.4	122.8	94.1	117.6	113.6
CAGE1	125.9	150	129.2	131.3	121.6	108.6	139	141.6
CALB1	115.2	112.9	110.8	114.3	115	97.3	113	117.8
CALB2	132.1	136.8	138.2	138.2	126.8	115.1	115	139.1
CALB2	114.5	115	114.4	108.4	112.3	98.9	122.2	113.2
CALCA	112.8	112	111.6	120.8	111.3	107.1	122	117.6
CALCA	118.7	112.5	127.8	129.1	114.3	105.4	120.5	130.9
CALCA	108.8	123.5	118.7	122.1	102.7	97.5	117.4	111.7
CALCB	118.8	116.4	119.9	116.1	110	98.5	117.4	118.2
CALCOCO1	205.3	200.9	177.6	197.2	148.5	145.9	185.1	149.8
CALCOCO2	157.7	146.2	163.6	170	149.1	158.2	149.4	156.5

CALCR	111.7	117	120.4	119.9	111.2	106.5	128.3	110.3
CALCRL	105.9	104.9	108.8	109.4	105.8	98.1	119.9	114.1
CALD1	106.9	106.3	120.3	125.1	110	100.8	121.8	114.9
CALM1	2345	2920.2	2444.1	2426.9	2053.9	2341.3	2237	2609.8
CALM2	2572.5	2168.8	4323.1	2497.2	1922.4	2455.4	1905.7	2167.2
CALM3	1661.2	2245.9	1636.9	1687.8	1602.4	1357.4	1244.8	1523.8
CALML3	110.3	116.6	110.2	115.1	115.8	101.5	120.2	111.5
CALML4	112.9	121.4	107.1	119	111.6	97.6	110.6	123.9
CALML4	128.8	131.6	125.7	126.8	115.5	103.8	128.4	117.7
CALML4	405.2	656.4	659.7	616.7	418	333	462.7	506.7
CALML4	136.2	138.6	192.3	151.6	134.6	140.2	138.4	136.4
CALML5	118.8	114.5	115.6	129.5	119	111.9	119.7	116
CALML6	112	115.2	109.3	122.3	113.2	101.1	120	113.5
CALN1	127.9	122.4	118.9	132.3	113.4	105.3	128.3	122
CALN1	139.5	146.4	150.5	156.8	124.8	118.3	142.9	152.9
CALN1	125.9	115.2	116.3	114.7	108.5	100.7	119.3	112.5
CALR	268.1	209.2	299	260.2	221.7	389.6	255.7	336.5
CALR3	110.3	115.1	110.5	117.2	108.3	96.6	114.9	116.7
CALU	194	159.7	154	188.1	143.7	136	228.7	167
CAMK1	227.1	167.1	550.9	236.1	142.3	455.9	180.9	119.1
CAMK1D	152.8	157.4	192.7	175.8	146	119	161.9	133.9
CAMK1D	107.2	108.4	114.2	111.4	100.5	93.3	111.2	115.3
CAMK1D	123.1	112.3	112.1	108.1	116.7	107.4	126.8	108.7
CAMK1G	109.3	117.3	119.3	117.4	103.5	106.7	122	117.8
CAMK2A	119.5	114.6	115.9	114.6	104	104.8	116.9	115.4
CAMK2B	114	110.4	115.8	109.3	103.4	90.8	115	112.9
CAMK2B	105	105.5	108.7	117.7	106.8	98.8	116.8	109.6
CAMK2B	117.3	112.3	123.1	116.1	112.5	100.5	130.7	116.1
CAMK2B	124.5	120.2	132	131.8	116.6	103.1	120.8	127.3
CAMK2D	133.3	128.1	120.5	139.5	113.4	108.2	130.1	123.6
CAMK2D	119.9	122	117.7	109.4	113.9	94.4	129.1	117.8
CAMK2D	112.4	113.4	113.5	123.1	111.1	99.6	120.3	115.9
CAMK2D	113.4	114.2	117	119.1	109.7	100.4	116.2	110.3
CAMK2G	235.1	241.3	232.9	232.5	203.5	151.3	181.2	171.9
CAMK2G	156	160.3	143.9	168.1	128.2	112.5	136.1	131.7
CAMK2N1	205.3	194.1	201.4	204.6	197.1	184.9	205.7	191.8
CAMK2N2	120.4	122.3	117	111.4	103.5	100.9	124	114.6
CAMK4	105.7	112.4	112.4	115.8	103.7	97.3	124.4	113.2
CAMKK1	130.5	119	131.8	122	114.1	100	126.9	120
CAMKK1	117.3	107.7	117.2	119.3	106.7	99.7	124.3	114.3
CAMKK2	122	115	131.9	125	117.4	108.5	123.2	120.5
CAMKK2	122.9	116.7	132.9	133.4	112.5	103.3	120.9	129.9
CAMKV	116.3	111.9	119.1	115.7	105.7	118.2	116.9	107.6
CAMP	147.7	201.3	168.9	150.2	180.1	140.9	138.5	180.8
CAMSAP1	221	252.3	219	284	208.8	192.7	275.8	265.4
CAMSAP1L	188.2	213.5	198.5	239.3	197.9	170.2	230	240.9
CAMTA1	124.2	130.1	120.4	134.6	125.1	103.6	138.9	136.2
CAMTA2	120.5	118.8	123.1	128.2	103.8	98.2	121.8	117.4
CAND1	146.2	164.4	123.5	177.1	153.1	131.3	148.4	169.7
CAND2	106.3	123.7	119.8	109	107.5	94.3	119.3	107.7
CAND2	151.7	139.8	145.2	144.2	135.3	118.3	145.9	121.1

CANT1	303.3	306.4	250	361.8	250.7	307.2	425.5	396.1
CANX	702	797.5	1089.7	931.4	705.7	1001.8	1050.3	1095.3
CAP1	3445.8	3444.7	4499.3	3420.7	2254.6	3271.7	4129.9	3508.3
CAP2	110.7	117.4	108.6	113.8	107	91.3	124.2	110.2
CAPG	125.3	119	155.6	132.2	120.4	114.2	138	132.5
CAPN1	511.2	521.7	684.1	493.4	343.4	432.1	559.3	370.6
CAPN10	137	155.4	152	131.5	125.1	124.8	142.8	140.8
CAPN10	119.6	121.2	120.6	126.1	125.7	92	121.7	116.3
CAPN10	120.1	128.2	122	121	113.5	106.6	121.2	121.6
CAPN10	111.2	120.1	112.2	119.3	113.5	105.4	121.8	117.8
CAPN11	125.4	112.2	131.9	124.9	111.3	103.7	119.7	112.7
CAPN12	209.4	222.5	184.4	197.8	175.1	133	161.7	142.6
CAPN13	114.3	123.9	120	123.8	121.7	111.3	113.4	112.2
CAPN2	137	138.5	138	132.3	128.2	112.3	127.8	129.9
CAPN3	110.9	114.6	122.7	114	106.8	107	114.1	118.8
CAPN3	110.8	111.8	111.7	121.6	112.3	97.1	107.4	123.3
CAPN3	154.7	144.3	170.6	168.5	144.4	138.6	161.2	163.3
CAPN3	126.1	130.6	157.7	131.3	118	117	134.3	145.3
CAPN5	141.1	155.4	129.2	134.6	135.2	97.6	142.3	130.8
CAPN7	135.3	132	130.7	138.9	123.7	115.1	133.1	127.1
CAPN9	121.5	106.4	120.3	120.6	116.1	94.9	116.5	121
CAPN9	117.1	116.5	114.9	117.3	109.5	99.3	111.9	118.4
CAPNS1	693.3	767.7	882.2	727	550.4	800.5	834	719.2
CAPNS1	227.7	225.4	224.4	236.1	230.8	232.2	253.2	240.7
CAPNS2	116.2	117.7	126.5	121.4	104.1	101.7	122.2	113.8
CAPS	126.9	115.5	125.7	123.4	99.6	101.2	129.1	117.7
CAPS	170.2	207	206.6	190.9	196.2	160.3	164.3	186.3
CAPS2	108.3	112.9	113	123	113.7	103	117.7	126.1
CAPSL	124.2	110.4	122.5	131.9	112.1	102.1	128	116.3
CAPSL	114.8	115.6	126.6	113.2	103.5	101	127	117.2
CAPSL	115.7	133.4	119	121.7	114.5	100.1	122.6	116.8
CAPZA2	1046.7	885.9	1298.5	1164.9	914.1	1131.5	1326.6	1090.2
CAPZA3	103.8	117.3	109.4	113.4	99.3	95.2	109.2	109.6
CAPZB	164.7	202.2	202.3	181.1	169.6	188.9	169	176.2
CARD10	126.3	121.8	118	133.3	119.4	103.2	120.3	122.8
CARD11	420.7	470.9	302.5	446.3	356.8	259.5	322.7	302.8
CARD12	112.3	120.7	145	119.1	109.5	147.3	120.7	109
CARD14	117.2	113.6	126.1	123.1	100.2	97.4	120.5	119.1
CARD14	120.2	110.3	111.9	113.9	108.1	91.9	104.3	114
CARD14	122	121.8	114.5	119.6	113.9	97.5	115.5	123.3
CARD15	183.9	138.3	162.4	157.6	110.5	137.6	126.8	118.3
CARD6	122.6	123.6	118.2	119.2	122	104.3	120.8	119.5
CARD8	677.7	685.8	561.3	635.8	740.4	440.9	578.2	560.6
CARD9	171.2	156.5	553.7	164.8	125.8	171.7	154.3	152.4
CARF	385.1	339.7	359.6	372.7	328	206.5	350	415.9
CARHSP1	411	471.7	420.6	502.4	394	347.8	489.1	521.4
CARKL	200.5	225.3	269.3	216.3	177.3	180.8	186.3	169.6
CARM1	543.3	702	607.8	561.5	438.7	403.8	558.9	718.6
CARS	126.5	137.8	125.8	128.7	124.6	107.5	118.7	144.5
CARS	285.2	483.4	259.4	419.6	458.4	267.8	369	498.4
CART	112.2	123.5	118	124.2	115.4	102.7	127.3	118.5

CART1	105.7	107.6	110.3	111.9	103.4	95.4	109.9	111.1
CASC1	116.4	123	124.1	114.3	113.6	107.1	121.1	121.8
CASC2	127.6	118	137.1	133.1	116.4	102	136.8	117.4
CASC2	124.7	127.1	133	122.7	117.1	101.4	123.5	118
CASC3	295.5	321	246	365.4	229.2	251.1	373.2	316.2
CASC4	108	115	117.2	121	112	101.2	110.4	111.1
CASC4	215.8	201.8	275.8	230.6	182.5	246.1	206.2	158.8
CASC5	106.9	115.3	112.4	119.4	109.2	91.8	116.7	110.6
CASC5	114.5	123.6	114.9	118.7	103.7	103.2	118.4	123.1
CASD1	138.9	131.5	148.8	134.4	144.2	108.9	128.9	122.4
CASK	175.3	206.7	191.2	187.4	195.6	168.1	219.9	192
CASKIN1	112.4	111.1	120	119.9	114.4	89.1	116	110.1
CASKIN2	122.8	114.2	135.8	124	123.6	106.7	120.6	112
CASP1	332.8	315.6	931.7	259.2	251.5	429.9	174.1	229.2
CASP1	115.6	107	118.3	112.3	108.1	92.2	111.3	107.1
CASP1	232.2	229.8	535.6	205.2	160.6	254.4	162.5	151.6
CASP10	123.1	121.6	118.2	133.8	114.9	97.4	133.2	127.8
CASP10	116.7	119.2	122.2	125.1	110.5	102	123.4	118.8
CASP12	115.5	111.7	118.4	125.3	109.7	97.5	111.7	121.2
CASP14	109	115.5	128.6	116.5	113	96.7	116	118.9
CASP2	123.3	123.4	119.7	138.9	112.6	99.8	123.3	119.1
CASP2	1982	2260.7	1601.5	2272.4	2001.8	1331.4	2042.2	1597.4
CASP2	270.1	296.4	218.4	289.6	224.7	203.6	280.6	264.1
CASP3	266.8	350	256.9	353.6	281.9	233.4	343.5	376
CASP3	129.1	130.2	120.4	145	113.2	110.9	123.6	129.1
CASP4	113	114.1	121.1	110.9	107.2	97.1	118.9	117.2
CASP4	241.7	305.8	345.4	295	235.2	331.1	259.5	304.7
CASP4	116.3	114.3	119.2	115.4	116.6	96.8	117.2	111.1
CASP4	128.3	126.9	139.6	134.3	123	109.4	133.3	138.6
CASP5	118.7	124.2	120.3	114.2	107.5	104.7	125	118.1
CASP6	197.3	187	219.5	208.4	206.1	154.5	170.8	225
CASP6	139.4	136	127.3	133	122.3	106.3	129.6	124
CASP6	143.7	140.1	147.3	149.4	132.1	114.8	154.2	139.7
CASP7	151.9	144.3	142	133.8	129.9	128.5	144.4	125.8
CASP7	112.3	110.2	119.6	127.7	117.2	96.1	122.8	121
CASP7	112.2	106.7	115.9	118.3	104.5	99.4	109.1	106.5
CASP8	133.2	125.2	124.6	146	131.4	102.6	126.6	154.1
CASP8	123.1	116.2	135.5	134.2	131.4	110.7	129.3	124.3
CASP8	132.2	134.6	151	154.8	137.8	114.4	125.4	143.2
CASP8	124.5	133	147.4	138.6	134.3	108.8	133.2	132.2
CASP8AP2	112.2	117.4	114.4	115.5	106.2	101.5	105.2	112
CASP9	130.4	114.6	135.3	132.5	105	115.7	132.2	114.4
CASP9	167.9	178.4	156.6	203.7	167	158.1	216	195.6
CASQ1	126.5	121.9	123.8	124.5	112.1	107.1	117	112.4
CASQ2	114.7	117	110.9	120.2	111.1	93.1	115.3	110.3
CASR	118.6	122.2	112.5	126.8	105.5	100.9	121.8	128.5
CAST	199.6	173.2	172	196.6	191.2	164.2	187.7	162.5
CAST	111.9	114.6	118.9	120.5	106.9	96	117.5	109.8
CAST	107.6	112.5	113.5	108.8	104.7	91.7	112.7	104.3
CAST	229.4	275.6	215.5	214.1	223.8	201.4	243.4	196.7
CAST1	118.1	108.3	112.7	115.1	106.2	97.8	113	115.8

CASZ1	240.1	252.6	194.4	261.1	224.6	154.4	319.9	287.8
CASZ1	115.1	112.7	125.3	114	107.3	103.8	114.3	117.7
CASZ1	117.9	118.3	120.6	114	113.6	103.3	129.5	138.4
CAT	200.5	176.4	225.1	206.9	154.2	185	150.8	155.3
CATSPER1	113.1	124.1	141.4	120.4	106.6	100.6	121.7	114
CATSPER2	120.6	117.5	121	120.6	112.6	100.5	122.4	110.7
CATSPER3	129.2	123	118.1	121.7	115.5	104.2	108.6	111.5
CATSPER4	109.4	103.2	114.4	111.8	104.3	96.5	114.7	103.7
CAV1	113.1	111.2	115.4	109.8	103	94.9	119.7	116.5
CAV2	125.4	112.1	118	118.8	111.8	96.7	128.3	122.3
CAV2	116.6	121.3	133.1	139.4	116.9	109	132.6	123.4
CAV3	106.6	116.2	121	109.9	110.4	104.8	120.5	113.8
CAV3	114	130.4	129.9	122.2	110.1	111.8	133.5	129.9
CBARA1	176	167.9	186.7	185.3	151.4	158.3	183.9	203.6
CBFA2T2	171.6	167.4	151.9	153	147.9	114.8	159.4	175
CBFA2T2	112.6	126.1	122.3	116.5	121.5	110.6	114.8	124.4
CBFA2T2	129.4	115.9	145.4	126.9	113.6	112.2	140.6	131.6
CBFA2T3	123.4	119.9	116.5	125.4	106.1	104.8	127.8	115.9
CBFA2T3	128.5	128.3	146.7	149.5	131.6	117.9	145.5	130.6
CBFA2T3	113.4	135.8	121.4	111.3	116.8	102.6	132.3	124.2
CBFB	287.9	299.5	297.8	373.8	359.3	345.8	335.6	376.9
CBFB	109.6	112.7	103.6	110.9	111.4	97.3	114.4	113.3
CBL	158.7	151.5	141.8	173.2	179.2	155.1	198.9	204.3
CBLB	861.3	792.4	610.3	1166.5	1043.8	546.4	1482.3	1844.7
CBLC	114	116.4	109.8	115.1	103.4	89.3	117.8	110.9
CBLL1	501.1	557.7	455.9	481	528.9	310.7	542.8	611.7
CBLN1	113.7	104.2	121.2	121.5	116.4	108.3	115.4	111.4
CBLN2	123.8	115.7	130.1	119.4	108.6	104.8	121.9	120.8
CBLN3	151.7	150.3	194.7	193.1	128.6	155.6	145.5	136
CBLN4	120.4	122.1	117.8	121.7	108.2	96.6	116	119.6
CBR1	164.1	136.9	147.4	161.3	132	134.2	149.8	141.4
CBR3	179.8	239.2	169.5	189.8	187.2	148.9	166.5	202.5
CBR4	194.9	201.1	328.7	234	203.9	201.5	180.8	186.8
CBS	125.1	118.9	128.7	129.1	113.7	106.1	135	123.4
CBWD1	108.3	119.6	116	119.8	110.2	96.7	115.4	113.7
CBWD2	121.7	112.3	123.3	113.3	108.7	96	117.8	110.1
CBWD5	175.3	186.7	205	177.3	168.4	132.6	152.1	162.2
CBX1	131.9	143.5	138.8	146.5	136	129.5	168.4	132.2
CBX2	128.6	115.9	123.2	124.9	117.3	98.2	121.8	135.8
CBX2	105.9	118.3	111.6	116	106.1	93.6	116.3	111.1
CBX2	116.3	128.9	120.3	129.2	104.6	105.1	114	115.5
CBX3	147.9	152.7	147.1	170.5	144.5	114.7	177.1	188.4
CBX3	500.3	546.6	585.5	585	502.9	419.1	426.2	440.8
CBX3	106	106.4	115.4	115.5	110.6	101.2	116.5	118.3
CBX4	193.3	201.8	193	194.7	152.6	192.9	162.6	175.3
CBX5	190.8	206	171.6	209.8	213.5	126.6	225	235.7
CBX6	453.5	540.1	385.6	570.9	361.6	256.1	577.9	508.1
CBX7	646.3	686.1	673.1	540.1	428.7	650	391.8	368.6
CBX8	124.1	112.9	125.5	126.4	118.8	113.5	129.7	120
CBY1	116.9	107.4	125.5	120.3	108.1	100.8	124.8	118.3
CC2D1A	137.5	150.8	129.7	147.8	114.3	105.8	145.2	142

CC2D1B	214.2	235.4	228.1	262.5	219.8	191.5	226.4	219.1
CCBE1	264.7	245.8	302.4	303.9	323.5	165.7	336.1	310
CCBL1	178.2	175.7	166.7	161.7	144.7	128.8	141.1	138.1
CCBL2	251.3	325.7	248.3	302.3	261.5	188	247.6	180.8
CCBL2	121.1	115.3	130.1	139.3	112.7	113.3	126.5	133.8
CCBP2	116	112.8	122.2	123.6	112.2	98	119.9	111.1
CCDC104	111.7	121.7	111.1	121.7	107.7	108.7	117.3	127.1
CCDC108	113	110.9	115.7	121.4	108.2	99.4	114.8	117
CCDC108	113.8	118.4	117.4	116	106	97.4	116.4	113.4
CCDC11	111.8	121.9	115.7	112	106.2	102.8	119.6	115.3
CCDC110	127.9	119.6	117.4	117	118.1	99.7	112	121
CCDC117	121.5	128.2	120.2	126.4	122.5	105	134.7	134
CCDC12	711.1	698.5	680.9	676.8	575.2	462.7	658	739.3
CCDC121	113.2	125.2	121.9	115.4	116.4	109.7	111	109.4
CCDC127	171	171.8	162.7	180.7	173.5	143.1	176.5	235.5
CCDC129	116.8	106.1	119.5	125.9	106.5	99.1	111.7	106
CCDC13	108.4	111.2	118.4	125.1	101.2	91.5	111.5	115.1
CCDC14	195.4	233.5	304.4	236.1	205.4	199.9	180.8	193.7
CCDC16	554.9	330.5	428.3	521.5	461.7	415.5	460.1	476.4
CCDC17	140.1	128.4	137.9	133.6	124.1	103.2	140.2	122.3
CCDC18	127.6	123.2	133.3	118.6	104.5	102.2	122	125.8
CCDC21	171	166.5	139.7	164.5	136.2	126.5	152.7	145.9
CCDC22	134.1	131.4	150	135.5	113.8	121.3	127.3	138.7
CCDC23	496.7	447.6	704.2	450.6	386.7	451	520.7	445.1
CCDC24	125.7	135	106.8	124.2	129.9	103.6	140.1	130.2
CCDC25	133.7	167.6	167.7	168.2	157.8	120.2	139.7	150.1
CCDC25	509.5	545.4	484.3	484	418.9	381.1	403.3	348.7
CCDC27	120.7	129.3	123.6	128.5	117.9	103.7	128.6	122
CCDC28A	405.3	534.3	520.8	482.9	447.7	502	393	432.2
CCDC28B	146.8	142.4	127.9	140.9	126.7	99.2	130.5	130.6
CCDC3	107.7	116.7	109.5	118.5	106.7	89.3	114.3	106.1
CCDC32	129.4	159.3	152.7	142.3	133.2	139.8	144.1	157.4
CCDC32	745.4	588.3	653.2	734.2	445.8	582.5	670.6	624.5
CCDC33	109.5	117.2	122.9	122.8	110	102.1	127.5	112.7
CCDC34	112.5	102.4	116.1	112.9	105.8	97.8	114	104.6
CCDC34	146.1	144.3	147.8	121	123.3	108.5	126.4	124.5
CCDC34	196	299.3	276	243.8	203.9	202.2	192.3	163.7
CCDC36	125.4	127	126.9	137.2	123.9	102.8	125.7	123.1
CCDC37	115.7	127	115.2	125.4	108.3	99.6	118.4	113.1
CCDC38	118.7	123.9	119.2	127.7	114.1	100.8	119.4	110.1
CCDC4	112.3	119.9	114.8	120.6	104.2	91.1	117.4	114.5
CCDC4	119.5	147.4	115.1	132.8	126.9	105.9	139.8	150.1
CCDC41	115.1	121.2	115.5	126.5	111	98.9	124.5	114
CCDC41	136.7	143.3	157.4	141	148.6	121.9	143.4	149.5
CCDC42	103.5	108.4	108.1	118.1	97.5	90.6	106.2	110.3
CCDC43	128.4	124.9	150.5	138.4	129.3	117.3	127.1	130.4
CCDC44	273.8	328.7	325.5	292.1	237	240.9	247.8	239.3
CCDC45	269	272.1	382.6	266.5	286.7	224.1	318	369
CCDC46	123.9	130	121.6	116.7	110	105.4	126.8	120.2
CCDC46	106.8	116	118.7	134.2	112.1	99.7	120.9	109.5
CCDC47	202.4	184.3	201	216.2	172	160.3	207.9	214.3

CCDC48	102.2	116.4	112.1	115.7	104.5	97.6	107.7	115.4
CCDC49	298	309.2	302.1	336.9	267.6	196.5	350.8	385.9
CCDC5	159.3	154.3	169.8	161.1	146.5	133.8	150.6	143
CCDC50	394.8	331.2	365.6	425	263.4	288.4	387.7	295.1
CCDC50	108.6	112.8	118.5	118	108.7	96	119.5	110.7
CCDC51	128.9	132.5	125.5	138.8	125	111.3	130.7	127.6
CCDC52	122.2	111.9	116.3	118.9	105.9	96.2	116.4	113
CCDC53	507.7	710.8	693.8	546.5	488.2	552.1	512.5	549.8
CCDC54	103.4	118.1	108.4	112.1	104.1	101.4	115	107.8
CCDC55	124.1	134.2	135.4	153	128.5	140.1	151.1	130.4
CCDC56	667.5	714.5	726.7	720.6	453.4	445.2	522.2	463.6
CCDC57	125.6	122.5	135.4	127.7	111.3	115.1	124.3	128.6
CCDC58	162.9	181	161.9	167.1	227.8	119.8	153.6	184.1
CCDC59	1144	1359.4	1225.3	1238.3	974.5	1167.2	1172.4	1298.6
CCDC6	466.6	484.7	451.6	536.2	475.6	417.9	415.4	357.4
CCDC60	127.2	117.9	115.8	114.3	108.4	99.6	118	114.5
CCDC62	127	118.4	137.5	137	123.7	115.8	132.1	125.5
CCDC62	111	117.6	117.9	129.3	108.1	102.8	132.8	122.1
CCDC63	122.9	122.6	112.8	121	110.8	89.6	113.2	125
CCDC64	116.2	106.2	110.7	115.9	107.7	95.8	114.7	110.9
CCDC65	123.4	131.8	133.1	124.6	116.8	98.2	127.9	130.7
CCDC66	160.1	231.1	199.7	193.8	176.8	139.3	185.4	228.2
CCDC67	108.9	112.2	105.7	118.6	108.6	103.8	107.7	125.3
CCDC68	112.7	108.6	110.8	115	105.2	99.8	113	113.5
CCDC69	140.1	163.8	146.8	158.6	150	140.2	142.4	147.3
CCDC7	118.1	125.9	124.1	123.8	109.8	101.6	120.8	122.1
CCDC7	115.2	110.9	116.4	115.6	105.7	90.9	115.9	115.9
CCDC7	116.4	126.6	124.1	121.2	113.9	98.5	120.2	121.3
CCDC70	117.3	112.9	130	137.9	110.7	114.9	118.9	117.1
CCDC71	195.8	222.6	170.4	209.5	146	164	164.2	179.3
CCDC72	891.9	1350.7	1563.4	1079.6	1185.3	1295.8	1068.4	1029.5
CCDC74A	110.3	121.6	117.9	124.2	101.8	100.8	113.9	115.7
CCDC74B	118	113.3	121.8	127	111.8	106.2	126.6	125.4
CCDC75	122.1	122.5	130.6	126.2	111.3	104.1	124.4	116.6
CCDC76	220.2	205.1	276.8	229.2	252.5	236.9	211.6	222.4
CCDC77	247.5	254.7	240.8	285.7	255	219.1	276.2	245.5
CCDC8	114.8	119.6	126.1	120	113.4	109.6	115.1	118.6
CCDC85A	111.3	115.3	108.7	128.4	102.4	99.8	111	111.6
CCDC9	111.5	121.2	121.3	137	110.1	98.7	131.8	119.1
CCDC90B	733.4	792.3	1039.8	868.5	706.8	834.5	816.7	700
CCDC91	149.7	138.1	146.6	137.2	129	107.7	134.2	152.5
CCHCR1	117.6	118.7	129.9	110.9	105.6	103.4	122.4	113.6
CCIN	114.3	110	116.9	118.7	106.1	98.2	114.7	114.5
CCK	109.3	115.5	117	114.5	102.8	104	134.2	117.6
CCKAR	110.2	107.4	117.4	118.2	102	96.2	119.4	112.3
CCKBR	114.6	125.7	125.7	121.3	106	99.9	127.1	105.9
CCL1	118	126	110.8	115.7	107.7	92.2	116.8	105.4
CCL11	110.5	108.3	108.2	106.9	108.5	97.9	113.9	121.4
CCL13	131.2	118	132	134.4	111.5	110.4	121	122.4
CCL15	105.6	113.9	113.7	103.8	102.4	108.9	102.1	110.2
CCL15	124	117.5	117	110.5	119.3	95.6	118.4	113.5

CCL16	120.7	104.1	112.4	108.7	106.1	100.9	119.8	112.5
CCL17	113.6	117.5	114.5	121.2	107.3	94.6	120.8	117.3
CCL18	118.5	117.1	122.2	128.7	116.1	109.7	111.9	121.8
CCL19	120.9	124	125.1	128.5	120.3	114.3	130.2	132.5
CCL2	1469.9	461.8	525.3	2256.1	870.9	782	1927.9	760.4
CCL20	134.9	157.8	160.5	137	121.6	114	129.7	146.2
CCL21	115.6	113.9	115.4	118.1	113.5	99.7	115.2	124.1
CCL22	178.7	156.3	176.5	192.7	121	147.7	163.7	131.3
CCL23	106.9	107.1	112.9	117.2	109.2	99.5	109.9	107.1
CCL23	120.7	123.2	137	131.2	115.2	105.7	125.7	122.7
CCL24	1699	1715.9	161.2	2434.2	1291.4	217.4	1529.2	331.7
CCL25	117.5	113.6	119.9	111.4	107.4	99	118.6	108.4
CCL25	115.8	108.5	116.3	120.7	106.9	99.7	108.9	111.5
CCL26	109.5	107.4	112.6	121.7	107.8	95.8	118.7	108.4
CCL27	110.2	114.8	115.1	112.6	112.4	93	108.4	106.2
CCL28	123	114.4	128.4	110.6	115.2	99.7	114.9	119.3
CCL3	434.2	354.6	240	358	312.2	143.1	315.9	224.7
CCL3L1	184	163.4	132.4	188.1	135.5	119.3	181.1	134.2
CCL3L3	592.9	386.3	358	466.4	360.9	179.4	515.8	300.6
CCL4L1	360.6	179.7	324.9	330	135	237.9	260.3	147.6
CCL5	6190.4	8102.2	11607.2	7444.3	6098.8	8078.9	7226.3	6645.8
CCL7	122.4	124.7	116	128.5	124.8	105.1	131.9	127.5
CCL8	808.9	134	240.7	565	125.9	131.9	147.7	119.7
CCM2	142	128.8	133.8	123.1	108.4	107.4	129.2	129
CCM2	340.3	442.4	435.5	429.6	401.1	351.3	277.4	348.8
CCM2	1823.3	2065.2	1866.7	2124.1	1539.6	1328.5	1931.1	1824.2
CCNA1	122.5	126.4	118.5	125.8	114.2	109.2	124.7	127.4
CCNA2	113.9	118.8	128	120.8	112.6	105.3	144.3	133.9
CCNB1	120	112.8	127.2	115.8	100.5	107.5	109.1	121.8
CCNB1IP1	290.2	265.3	333.4	282.4	265.4	210.4	364.8	347.6
CCNB1IP1	115.9	127.1	125.4	134.9	105.2	105.4	126.8	125.4
CCNB1IP1	117.8	128.1	118.2	124.6	109.9	95.9	119.8	122
CCNB1IP1	206.3	220.1	197.5	192.7	154.6	155.1	175.7	183.4
CCNB2	113.4	117.3	108.7	118.4	108	97.1	114	124.8
CCNB3	108.5	114.6	112.1	126.9	118	102.7	120.9	124.6
CCNB3	111.6	115.1	123.3	117.4	106.5	101.8	124.5	113.5
CCNB3	111.7	126	115.9	123.3	103.6	98.1	119.4	109.5
CCNC	149.2	138.8	209	152.4	140.6	151.4	136.8	150.6
CCNC	177.6	199.9	271.4	214.4	189.4	232.6	190.9	229.6
CCNC	159.7	139.7	141.2	136.9	140.6	134.7	159	166.5
CCND1	122.6	122.2	125.3	117	112.2	105.6	124.8	119.7
CCND2	1675.3	1809.7	1240.1	1340.7	1239.7	837.2	1005.8	955.2
CCND3	3863.1	4076.9	2980.3	3921.7	2877.7	2320.4	3568.9	3403.6
CCNDBP1	127.6	128.9	134.8	140.8	121.6	120.2	119.5	134.1
CCNDBP1	125.5	116.4	124.4	119.8	123.5	114.9	121.5	114.6
CCNDBP1	2236.9	2290.4	2418.6	2328.2	2030.5	2179.5	1828.6	1993.3
CCNE1	155.4	171.8	136.6	174.7	172.4	117.1	205.8	244
CCNE1	116.3	114.6	111.2	121.2	104.2	104.1	117	116.9
CCNE2	105	110.3	123.6	122.1	105.3	102.1	122.6	115.4
CCNE2	125.2	120.4	116.4	134.6	117.9	108.9	139.8	134.2
CCNF	133.2	157.7	129.5	154.1	130.8	125	153.4	136.5

CCNG1	421.8	569.5	315.8	347.2	378.7	251.1	465.8	429.7
CCNG1	125.2	119.1	126.3	118.6	108.2	97.2	123	124.8
CCNG2	207.7	214.6	150.2	207.1	217.9	146.5	181.3	203.5
CCNH	180.2	199.6	221	219.7	185.1	174.8	220.2	192.2
CCNI	7967.2	8175	7218.9	8877.5	5794.7	5310.2	8220.7	8752.8
CCNJ	118.4	114.2	106	117.9	119.8	90	111.1	117.5
CCNK	343.3	464.8	463.3	438.8	432.9	474.9	649.2	680.6
CCNL1	561.5	503.9	664.9	506.5	489.9	414.1	477	700.6
CCNL2	115.3	130.7	126.6	135.6	113	109.5	126.7	129
CCNT1	143.4	145.8	141.1	150.9	149.5	117.1	161.9	164.5
CCNT2	123.6	127.2	118.6	132.5	120.2	104	136	133.5
CCNT2	243.3	228.3	263.1	252	248.9	235.4	209.4	298.2
CCNT2	109.3	120.2	127.8	131	102	104.4	125	120.1
CCNY	731.2	844.3	800.2	969.7	810.3	752.6	960.9	963.5
CCNYL1	141.2	133.4	152.5	152.5	143.3	127.7	144.5	162.8
CCPG1	120.9	123.6	123.1	123.6	110.8	110.4	120.6	111.1
CCPG1	225.1	244.4	287.7	287.8	203.9	274.4	204.3	160.1
CCR1	352	259	315.2	537.9	348	503.7	562.9	250.9
CCR10	116.1	122.2	120.8	125.1	105	98.4	128.2	115.5
CCR2	121	113.4	114.1	114.4	98.8	95.4	119.1	118.5
CCR2	109.7	112.2	133.1	124.6	105	97.3	121	118.9
CCR3	114.7	125.9	127.6	119.9	112.7	103.8	121.8	119.5
CCR3	113.3	109.9	123.7	116.2	114.8	101.2	111.4	115.3
CCR4	116.2	132.7	110.9	125.6	118.2	96.9	118.7	127.3
CCR5	119.2	124.3	125.6	127.8	105.5	113.3	116.7	107.4
CCR6	6999	7110.2	8072.7	6791.5	8664.2	3596.9	7051.7	8948.7
CCR6	118.9	114.2	117.9	118.7	112.7	97.1	117.5	114.5
CCR7	4151.7	5058.5	3788.4	4220.7	3498.5	2989.1	3449.2	3402.7
CCR8	123.3	108.9	115.2	115.7	105.5	97.8	120.7	114.1
CCR9	128.4	137.3	130.3	130.8	119	112.7	126.4	121.1
CCR9	118.7	109.8	114.1	119.7	109.5	104	115.7	107.9
CCRK	178.9	171.2	175.7	174.4	152	125.8	158.1	143.1
CCRK	127.5	125	135.1	139.4	120.8	111.1	133.5	125.7
CCRL1	118.3	108.1	119.9	109.9	104	106	121.3	114.2
CCRL1	114.4	120.1	119.6	123.5	110.9	104.5	128.1	116
CCRL1	110	112.4	115.5	121.5	105.7	94.1	112.6	107.5
CCRL2	117.3	121.3	114.3	125.4	111.8	93.2	113.7	114.8
CCRN4L	125.6	123.9	119.9	126.6	112.3	98.4	131.4	132.4
CCS	296.4	260.8	357.3	245.8	248.4	254.7	192.9	175.4
CCS	198.5	219.9	231.8	196.6	153.4	157.7	172.2	147.8
CCT2	814.7	969	623.8	932.7	751.7	559.5	934.4	1361.6
CCT3	136.1	143.4	145.5	147.1	136.2	124.7	133.8	163.5
CCT3	510.5	507.9	480	581.1	479.2	386	630.4	791.7
CCT4	184.4	178.7	180.3	208.1	144.1	133.8	223.5	242.9
CCT5	128.6	125.6	126.5	130.8	122.6	105.6	152.5	160.2
CCT6A	234.3	249.3	271.4	262.6	210.2	168.6	264.2	319.3
CCT6A	245.2	256.8	228.1	272.5	242.7	205.9	385.8	484.9
CCT6AP1	406.8	436.4	402.9	447	363.9	270.2	482.6	450
CCT6B	120.3	120.2	135.8	137	126.3	110.7	141.5	134.5
CCT7	1315.6	1318	1210.8	1532.2	1101	1021	1676.1	1864.8
CCT7	240.7	244	244.4	247.5	201.2	207.6	292.8	311.1

CCT7	376.9	425.3	412.2	434.7	370.2	312.4	390.3	415.6
CCT8	1317.6	1213.2	1371.8	1377.3	1227.5	1123.4	1235.3	1445.1
CD109	121.5	116.4	120.9	127	108.5	100.9	120.1	120.5
CD14	6172.2	1645.4	9051.8	5702.7	1114.4	9046.3	3525.2	540.9
CD14	953.1	291.9	1727.1	1010.6	231.7	1710	540.3	161.5
CD151	142.1	130.1	183.8	160.6	115.3	132.3	135	117.9
CD151	1197.3	753.4	1447.6	1230.8	530	834.5	981.3	572.9
CD151	129.5	118.8	120.7	130.3	112.8	118	128.4	127.7
CD160	131.4	108.7	132.5	127	110.5	99.3	127.5	124.5
CD163	213.2	125.8	920.8	166.7	109.7	397.8	150	118.4
CD163	142.3	117.6	275.2	132.9	103	181	128.4	120.6
CD164	153.8	147.6	157.5	180.6	145	135.5	187.7	166.7
CD164L2	111.4	106.3	114.4	112.7	101.3	95.3	111.8	114.6
CD177	112.5	117.1	122.7	131.8	110.1	98	119.9	123.1
CD180	116.6	120.9	114.7	126.6	113.5	105.8	118.7	121.6
CD19	435.5	322.9	553.5	406.7	193.1	337.4	227.4	168.3
CD1A	104.7	109.3	111.1	112.1	99.8	96.5	118.6	106.4
CD1B	108.8	110.2	126.2	114.4	106.5	94.1	116.6	114.8
CD1C	115.7	109.5	143.6	113.6	128.8	93.9	120	109
CD1D	118.4	118.3	135.7	126.7	114.6	108.6	108.2	113.6
CD1E	121.8	118.7	123.2	122.2	111.9	96.4	112.4	111.2
CD1E	117.7	121.7	133.1	121.1	111.2	112.2	124.9	116.1
CD2	2085.1	2414.2	2772.5	2033.5	1740.8	2386.3	1482.9	1322.6
CD200	142.3	138.7	147.2	142.1	119.7	107.8	116	128.2
CD200	115.7	115.4	110.1	127.6	104.9	100.3	117.8	111.7
CD200R1	121.2	121.6	119.6	117.3	107.8	102.9	113.7	118.1
CD200R2	107.7	116.8	109.1	111.7	103.7	98.3	109.8	114.1
CD207	123.7	115.7	116.7	125.8	111.9	107.8	106.6	111.6
CD209	142.1	120.4	129.7	144.3	112.9	103.6	129.6	116.5
CD22	132.3	124.6	130.7	126.2	114.8	112.2	125.3	116.5
CD226	186.2	201.7	155.8	167.1	191.7	147.8	164.4	173
CD24	295	286.2	402	250.4	217.6	265.9	179.6	159.5
CD244	145.3	170.4	148.2	186	142.2	121.6	180.5	163.6
CD247	6262.7	6139.8	5727.6	6285.6	4963.1	4986.5	5402.1	5373.2
CD247	5601.5	5146.5	4437.3	5020.5	4261.8	3525.7	4233.3	4289.7
CD248	159.6	248.5	149.2	161	188.2	121.2	148.3	158.6
CD274	114.1	110.6	107.2	124.5	113.6	98.8	122.9	110.9
CD276	138.5	121.3	146.7	142.6	116.8	108.9	134.9	127
CD276	112.9	108.7	104.7	108.6	116.7	98	116.8	113.4
CD2AP	129.8	129.8	141.8	141.2	121.2	108.8	131.5	155
CD2BP2	782.7	798.7	729.2	790.6	588.9	626	658.5	588.6
CD300A	285	271.6	272.7	235.4	220.5	157.2	268.6	221.5
CD300C	128.9	116.1	176.4	125.7	109.6	117.7	128.7	123.3
CD300E	116.1	118	125.6	109.9	113	94.4	118.3	123.9
CD300LB	116.9	118.7	124.3	121.4	113.4	94.1	133.6	115.2
CD300LF	121.4	113.4	144	117.9	98	103.9	114.5	114.3
CD300LG	122.4	124.7	139.7	134.8	105.2	104.8	123.7	127.6
CD302	107	105.3	125.9	112.7	113	98.5	110.3	113.7
CD320	253.5	283.3	184.3	256.5	217.6	143	194.4	163.7
CD33	130.2	116.5	208.2	130.4	104.5	97.4	121.5	115.2
CD33L3	107	115.5	115.1	122.2	118	98.7	107.7	110.5

CD34	142.1	132.7	137.8	128.3	119.2	112.9	123.8	129.7
CD34	114.8	108.4	119.8	111.7	102.9	99.7	114.8	116.5
CD34	119.2	116.3	116.6	119.5	114.8	107.8	124.8	122.9
CD34	106.9	114	116.7	117.4	106.8	103.2	112.9	117.9
CD36	314.7	116.3	1258.2	239.3	113.4	456.5	177.2	114.3
CD36	289.6	126	1594.5	288.9	121.3	672.5	168.8	115.7
CD36	113.2	115.7	112.3	113.8	103.5	103.5	115.9	110
CD37	433.6	431.3	399.9	411.7	346	336.3	347.4	330.3
CD37	562.3	543.2	532.3	505.2	456.1	373.9	604.3	488.2
CD38	113.4	115.4	109.3	108.2	95	87.2	116.9	109.8
CD3D	2763.7	3893.8	3730.1	3044.5	2881.3	2548.6	1952.8	2300.2
CD3D	1989.1	2725.5	2563.1	2010.7	1833.1	1539.5	1615.3	1423.2
CD3E	575.7	633.9	418.1	487.7	379.6	336.3	526.1	545.5
CD3EAP	128	108.6	120	124.5	119.2	99.3	121.4	112.4
CD3G	264.4	485.4	322.9	359.8	465.5	196.5	245.6	326.2
CD4	196.6	186.3	238.7	180.6	128.8	156	144.3	132.8
CD40	121.9	113.4	130.2	124.3	110.6	103.6	122.6	112.1
CD40	157.8	125.1	148	147.7	122.3	113.4	138.5	118.9
CD40LG	129.4	140.2	153.9	149.2	137.7	137.1	144	138.2
CD44	162.4	162.1	162.6	149.4	185.7	132.4	157	139.2
CD44	694.8	590.2	696.8	673	594.9	528.1	764.5	461
CD44	8339.5	7570	6287.4	7460	6440.7	6047.4	8794.8	6865.5
CD46	636.8	650.7	764.3	544.5	465	507.8	388.6	361.3
CD47	1193.9	1049.1	1134.6	1216.6	1107	882.3	762	724.3
CD47	161.7	183.4	185.7	170.9	139.3	151.1	152.8	152
CD48	815.9	852.7	778.2	1031.4	688.2	744.9	883.8	800.6
CD5	410.9	495.4	358.2	448	332.3	350.5	490.2	438.9
CD52	2363.6	3605.9	2968.2	2547	3175.7	2125.9	2000.2	1685.8
CD53	338.8	361.4	348.7	355.9	234.2	286.3	332.2	379.7
CD53	364.8	407.5	391.1	476.4	292.5	360.9	449.5	463.5
CD55	350.9	487.3	255.4	365.4	346.4	262.7	553.2	837.2
CD58	260.9	275.3	390.1	239.1	258.5	276.4	262.9	197.2
CD59	150.1	144.4	135	167.4	166.5	117.3	246	279.3
CD59	111.2	115.4	126.4	127.3	109.3	100.6	124.2	135.4
CD5L	123.8	110.9	120.1	129.3	119.8	101.5	115.8	121
CD6	1945.5	1922.5	2571	2088.5	1492.2	2135.2	1423.7	1473.6
CD63	264.2	258.2	217.6	302.9	244.2	212.5	307	227.7
CD68	909.7	523	1132.9	824.7	340	712.7	988.9	532.6
CD68	269.8	190.7	279.3	270.3	159.1	219.8	275.4	161.5
CD69	416.2	394.2	436.2	384.4	356.2	432.4	649.1	1385.7
CD7	736.8	1023.5	674.8	721.4	536	496.5	539.7	510.5
CD72	136.5	131.3	135.2	134.6	115.7	108.9	120.8	128.9
CD74	1407	1243.3	3368.8	1432.3	417.6	1535.4	1155	906.9
CD74	143.6	133.9	212	148.7	109.1	148	123.6	114.5
CD74	571.2	477.8	1520	486.6	315.3	945.8	471.4	334.1
CD79A	250.7	182.3	230.8	270.3	174.9	219.4	228.6	185.1
CD79A	1554.5	1183.9	1343.7	1769.3	767.7	1578.5	1299.5	858.7
CD79A	112.4	124.4	130.4	141.8	123.5	107.5	134.7	132.2
CD79B	1447.9	1229.9	1350	1589.6	837.5	837	823.1	459.8
CD79B	485.9	441.4	506.5	668	386	403	389.9	260.3
CD79B	325.3	343.2	296.8	348.4	242.3	214.8	179.6	165.4

CD80	131.6	120.8	123	123.3	110.8	105.6	123.1	122.1
CD81	4930.9	5360.4	7127.4	4949.1	3450.2	3681	3923.3	3688.5
CD82	282.3	292.3	284.4	249.5	259.7	251	260.9	190.4
CD82	138	143.2	155.3	156.5	130.3	120.4	134.9	127.6
CD83	613.3	443.7	504	544.8	350	417.4	727.2	441.9
CD83	371.8	333	326	443.8	316.1	295.4	443	353.8
CD84	142.2	136.8	150.5	160.3	143.4	121.5	124.6	152.4
CD86	121.8	117.6	236.7	125.1	110	148.3	122.9	125.5
CD86	201.7	176.4	407.9	171.3	134.7	371.8	134.5	137.8
CD86	115.5	123.6	118.4	113.2	112.4	102.5	110.4	118.4
CD8A	730.8	1308.1	865.1	1088.5	755.7	1007.5	636.5	548.7
CD8A	256.3	298	399.3	257.5	261.6	271.3	174.4	154.3
CD8A	1352.7	2142.7	1512.7	1210.6	1020.8	1155.4	739.5	618.6
CD8B	184.4	301.8	196.9	181.9	233.8	181.6	146.6	161.8
CD8B1	151.3	192.5	161.4	156.8	170.4	140.2	158.5	133.8
CD8B1	106.8	117.2	116.6	120.8	108.9	94	113.4	114.9
CD8B1	113.6	110.7	111.4	114.4	115.2	95.8	112.5	114.1
CD9	149.5	130.7	155	150	144.9	138	127.2	125
CD96	575.6	996.2	1027.1	645.4	912.8	812.6	669	844.6
CD96	604.5	1115.6	1032.7	719	1065.7	689.1	876.9	1173.1
CD97	180.7	199.4	169.8	170.4	148.2	136.8	146.2	159.1
CD97	1703.8	1283.6	1460.5	1093.2	740.8	922	698.1	773.3
CD99	206.2	216.6	212.2	211.7	160.9	196.9	215.4	166.3
CD99L2	132.3	148.7	165.7	146.4	135.7	144.8	151.7	143.8
CD99L2	595.8	678.3	830.4	608.6	420.7	601.7	603.9	338.9
CDA	158.9	193.6	133.3	191.5	156.8	105.5	163.6	127.8
CDADC1	118.1	121.4	120.8	133.4	110.9	98	123.1	116.8
CDAN1	200.6	175.5	171.4	223	189.1	162.3	237.4	173.3
CDAN1	3412.1	3075.9	3817.2	2914.4	2503.9	1383.2	2887.5	3570.4
CDC14A	131.1	136	135.5	127	117.6	109.5	136.6	131.9
CDC14A	120.4	112.8	111.4	127.8	117.8	103.7	123.8	109.8
CDC14A	111.1	113.6	106.9	115.4	106.4	94.5	114.9	116.5
CDC14A	172.5	150	147.2	162.2	159.4	110.4	147.1	153.6
CDC14B	137.6	126.5	139.6	125.8	119.3	107.7	121.9	124.5
CDC14B	156	160.9	148.4	162.9	144.7	132.1	147.9	134.9
CDC14B	289.4	228.2	147.7	364.9	217.5	125.1	303.1	258.4
CDC16	801.2	572.7	779	665.8	607	545.1	680	572.1
CDC16	571.8	582.5	612.1	568.7	369.4	377.4	579.3	464.7
CDC16	1133.1	988.6	1291.5	1167.5	807.7	894.1	1139.4	808.9
CDC2	107.4	116.8	118.2	114.5	114.7	97	124.5	119.2
CDC2	113.9	118.5	122.9	123.4	106.9	98.7	117.8	121
CDC20	117.1	116.7	110.8	117	112.6	101.4	130.9	116
CDC23	295.9	323.2	332.5	359.7	293.3	272.7	271.6	287.6
CDC25A	110	109.4	106.8	119.7	106.8	92.3	118.6	117.5
CDC25A	111.4	113.5	121.8	119.5	115.1	95	116.3	108.7
CDC25B	3164.9	3607.9	2086.2	3132.2	2647.8	1759.3	2413.1	1761.6
CDC25C	119.7	125.9	123.7	126.8	113.4	106	127.1	133
CDC25C	118.6	116.2	120	124.7	108.9	100.1	120.7	119.7
CDC25C	109.9	118.9	118.3	106.5	106.5	99.8	118.7	117.1
CDC26	1026.5	1254.7	720.7	1222.2	873.5	470.9	952	1174.9
CDC27	125.1	117.3	132.2	125.8	110.2	103.6	116.8	125.8

CDC2L1	107.5	104.6	112.6	111.5	103	100.2	110.4	112.1
CDC2L2	135.9	128.1	130.3	149.3	123.9	118.7	158.6	141.1
CDC2L5	275.9	310.4	253	276.4	228.7	231.6	285.2	279
CDC2L5	283	324	259.6	343.5	246	223.5	339.2	291.7
CDC2L6	381.2	407.2	340.4	436	329.5	313.4	420.2	463.1
CDC34	164.8	187.7	169.5	203.7	176.5	137.9	179.3	235.6
CDC37	5265.9	5367.9	5532.8	5850.7	4073.4	3690.2	4886.9	4237.1
CDC37L1	123.8	112.2	127.4	127.3	109.1	108.4	136.5	127.6
CDC40	505.8	598.9	543.7	555.3	385.3	426.1	410.8	435.4
CDC42	128.4	120.9	132.8	126.8	117.7	112.5	128	119
CDC42	104.7	110.5	105.7	118.2	104.7	91.6	118.4	116
CDC42	139.7	144.2	146.4	165.4	153.5	134.5	190.8	176.7
CDC42	333.3	237.3	324.3	325.3	283.6	307.7	525.1	315.7
CDC42BPA	109.3	124.4	125.9	129	115.4	106.7	122.9	110
CDC42BPA	105.2	102.9	112.3	112.8	104.5	97.4	117.6	110.8
CDC42BPB	125.8	125.1	134.1	133.7	116.2	123.2	125.5	126.2
CDC42BPG	117.2	119.4	106.9	106.8	101.9	91.4	103.8	109.8
CDC42EP1	120.5	121.9	127.7	130.8	112	106.3	121	125
CDC42EP1	123.8	120.1	115.4	128	115.8	101.2	115.2	123.8
CDC42EP2	128.7	126.6	116.5	127.3	113	120.4	132.7	116.8
CDC42EP3	132.6	138.9	127.3	140.6	154.3	124.5	133.1	130.3
CDC42EP4	207.1	178.7	219.3	225.7	188.5	193.4	213.8	210.3
CDC42EP5	110.5	119.8	112.7	114.6	110.4	96.7	121.9	119.7
CDC42SE1	364.5	389.2	347.6	386	325.6	312.8	395.5	326.8
CDC42SE1	162.7	181.5	179.1	175.9	156.7	150.5	156.3	165.2
CDC42SE2	189.6	195.6	150.4	186	191.5	121.5	173.1	203.6
CDC42SE2	1225	2138.6	990.6	1364.4	1641.8	1076.2	1703.1	1886.4
CDC45L	108	114.4	108.2	122.2	111.4	96.1	119.5	116.7
CDC5L	232.9	229.4	187.5	233.2	174.2	208.5	270.2	200.3
CDC6	111.4	125.2	113	121.3	102.4	107.7	125.5	120.9
CDC7	129.5	145.3	138.7	146.5	133.9	99.4	138.1	141.4
CDC73	116.2	124.6	122.8	118.5	107.1	104	115.1	125.9
CDC91L1	148.9	164.7	157.4	159.6	140.9	121.9	145.6	139.4
CDCA1	108.2	118.2	111.9	110.4	106.6	98.7	118.4	112
CDCA2	114.4	119.7	125.2	117.9	113.9	100.3	121.6	125.8
CDCA3	115.3	116.5	112.3	123.4	112	105.8	119.2	121.2
CDCA4	115.7	113	114.8	116.9	100.7	107.1	125.3	116.2
CDCA4	172.5	214.2	207.1	191.6	180.9	156.9	185.2	172.1
CDCA4	149.9	155.5	141.1	167.1	150.1	124	144.7	149
CDCA5	122.6	131.1	130.5	134.1	113.6	107.4	159.4	138.1
CDCA7	164	157.8	154.9	155.4	122.7	118.4	124.2	123.9
CDCA7	107.7	112.4	107.8	113.2	107.9	99.1	117.8	111.8
CDCA7L	140.4	152.1	150.7	133.6	117.1	117.4	122.3	129.8
CDCA8	116.5	122.7	128	112.7	119.8	105.6	133.5	127.2
CDCP1	121.9	114.1	123	119.7	118.9	99.4	121.1	119.7
CDCP1	119.1	125.1	124.4	105.3	108.8	98.9	123.5	120.5
CDCP1	122.5	113.5	135.8	133.9	108.9	105.5	121.3	119.7
CDCP2	113	116.8	111.5	121.6	104.8	101.7	106.4	116.9
CDGAP	115.6	118.1	124	126.3	113.7	105.9	123.8	109
CDH1	114.9	116.9	116.5	115.6	102.9	94.9	120.1	111.8
CDH10	118.2	110.8	115.4	125	101.3	102.6	118.8	112

CDH11	115.4	126.5	116.1	130.3	110.7	100	133.1	117.9
CDH12	109.5	113.5	108.8	108.4	106.5	98.4	115.1	108.4
CDH12P	118.5	118.5	122.5	116.3	106.9	100.8	121.6	117.4
CDH13	114.7	111.8	116.9	123.9	108.7	100.1	112.4	110.6
CDH15	119.3	109.2	115.3	115.2	103.7	98.3	121.7	114.8
CDH16	111.5	121.3	120	124.6	108.3	104	124.1	117.1
CDH17	114.8	118.5	122.4	122.8	107.3	98.6	120.2	113.9
CDH18	108.7	111.8	116.3	117.1	100.5	94.5	114.2	111.8
CDH19	122.2	113.8	118.3	122	111.7	98.3	110.7	118.4
CDH2	131.8	141.2	130.7	139.7	127.9	102.9	125.1	124.6
CDH20	114.9	119.1	117.8	127.3	117.5	106.5	126.1	120.5
CDH22	121.6	122.7	113.6	115.3	106.2	94.4	126.2	116.6
CDH23	119.6	106.1	123.4	128.3	111.4	96.3	139.6	122.9
CDH23	108.8	115.8	117.1	112.7	108.8	99	113.5	116.3
CDH23	276.8	293.6	233.2	226	188	147.4	156.3	194.4
CDH24	117.5	109.5	112.7	123.1	110.6	99.7	129.9	106.8
CDH24	115.9	116.4	113.7	130.6	117.2	109.6	126.3	123.6
CDH24	106.5	107.4	120.5	113.9	116.1	98.9	113.6	109.1
CDH26	111.2	122.1	121.8	117.7	106.8	102	118.5	119.6
CDH26	119.9	108.5	126	122.7	124	103.6	118	121.1
CDH3	118.3	117	122.3	124.7	105.7	97.6	117.5	122.6
CDH4	121.2	111.1	116.6	101.5	107.7	93.6	114.2	118.6
CDH5	113.4	115.8	118.5	114.6	109.9	100.1	118.6	120
CDH6	112.8	114.5	115.6	115.7	108.6	96.4	117.7	119.5
CDH7	122.5	122	121.5	125.9	108.1	104.8	115.5	116.3
CDH7	122	112.3	116.2	121.9	107.1	103.5	116	116.8
CDH7	124.7	124.7	122.5	120.2	113.8	104.4	122.9	118
CDH8	120.9	108.1	117.4	106.8	101.7	98.3	107.9	114.9
CDH9	127.1	130	134.9	127.3	123.4	101.8	118.7	114.7
CDIPT	141.1	160.8	134.1	153	122.4	124.6	152.8	148.1
CDK10	754	869.3	695.7	741.4	611.3	346.2	531	462.3
CDK10	122.8	121.1	125.4	120.6	109.4	106.5	117.5	125.8
CDK2	122.1	144.1	136.3	158	127.3	111.3	144.7	145.5
CDK2	232	223.8	233	272.9	242.3	198.8	287.6	324.2
CDK2AP2	166.7	163.2	182.9	172.6	145.5	163	149.3	161.1
CDK3	114.8	129.9	120.5	123.6	113.9	101.3	129	131.9
CDK4	229.2	297.4	205.6	277.5	246.8	188.7	261.8	301.8
CDK5	137.4	125	137.7	121.6	110.3	103.2	127.8	110.5
CDK5R1	118	123.2	131.9	118.9	117.7	120	123.4	119.8
CDK5R2	112.5	114.2	119	123.1	112.6	105.2	120.8	119.9
CDK5RAP1	332.5	315.7	318.2	297	229.6	234.1	207.2	223.2
CDK5RAP1	115.9	106.1	122.9	123.7	115.4	103.3	124.8	117.8
CDK5RAP2	198.1	200.3	150.7	313.8	264.6	142.1	391.2	303.7
CDK5RAP2	367.4	643.7	197.3	707.9	678.7	259.6	1203	1012.3
CDK5RAP2	119.6	121.1	120.1	130.1	111.5	96.9	118.1	122.2
CDK5RAP3	1052.8	1107.6	1213.2	935.3	899.9	841.2	780.3	735.6
CDK5RAP3	227.2	266.8	234.3	201.8	225.1	179.4	217.7	213.3
CDK5RAP3	1197.9	1286.2	1362.8	936.2	726.6	774.5	704.7	811.1
CDK6	160.7	167.2	158	174.9	160.2	134.7	187.6	203.4
CDK7	173.1	177.6	194	181.1	156	144.2	171.1	209.4
CDK8	115	130.3	133.5	129	127.2	107.9	126.4	121.2

CDK9	282.9	267.9	258.3	291.9	261.8	230.3	274.4	204
CDKAL1	126.7	135.9	129.6	124.1	121.4	104.5	124.8	132.1
CDKL1	122.3	124.2	124.7	118.2	117.2	98.6	111.8	119.1
CDKL2	105.1	111.3	112	114.8	106	88.5	117.2	110.6
CDKL3	120.8	136.8	128.4	128.9	122.3	119.7	160.2	178.1
CDKL4	115.4	109.3	119.8	116.1	114.1	101.6	125.5	119.1
CDKL5	114.4	116.2	114.1	117.4	117.4	100.3	112.5	112.8
CDKL5	113.2	115.3	112.6	107.4	105.6	102.7	109.3	106
CDKL5	115.1	128	108.3	121.3	108.7	97.7	117.9	113.6
CDKN1A	591.4	588	402.4	1234.6	844.7	738.2	1425.4	1294.4
CDKN1A	121.2	121.4	119.7	133.7	116.9	109.6	167.6	139.6
CDKN1B	1307	1146.1	1061	1190.7	830.1	1071.8	1059.8	1011
CDKN1C	120.6	115.7	117.6	124.3	110.8	103.9	119.3	124.2
CDKN2A	122.1	120.5	117.9	129.9	120.9	102.2	125.6	111.6
CDKN2A	114.5	107.6	113.5	121.6	112.5	103.6	123.3	113.5
CDKN2A	106.8	110.7	122.5	124	107.3	110.4	121.3	118.2
CDKN2B	186.6	152.1	139.3	286.1	145.3	167.1	271.8	145.9
CDKN2B	116.2	112.2	115.4	124.1	112.5	95.7	113.9	122.6
CDKN2C	142	147.9	178.4	161.2	145.5	150.7	143.1	146.6
CDKN2C	125	108.1	116.4	110.2	112.4	101.4	117.2	106.2
CDKN2C	119.7	116	130.1	137.4	120.2	96	116.3	127.4
CDKN2D	121.9	125.5	128.1	121.7	119.5	94.8	123.5	121.1
CDKN2D	373.1	383	265.7	362.7	274.6	213.8	347.9	358.5
CDKN3	124.5	110.8	129.7	140.4	120.3	107.3	120.5	123.5
CDO1	114.9	117.2	119.4	117.4	99.1	101.6	125.6	112.2
CDON	115.4	110	119.7	112.1	118.1	93	110.6	116.2
CDON	105	121.9	118.8	116.8	120.5	108.2	119.7	116.5
CDR1	114.1	109.7	105.6	116.5	108.2	96.7	113.7	106.7
CDR2	535.7	481.4	340	522.8	513.5	254.6	387.9	487.6
CDR2L	112.9	117.7	118.4	119.6	103.9	100.4	120.1	120.3
CDRT1	105.3	110.9	111.3	107.6	99.4	95.4	118	113.2
CDRT15P	128.9	115.4	114.5	116.8	109	99.4	118.1	119.4
CDS1	116	137.4	115.5	133.3	119.7	92.1	130.2	127.1
CDS2	281.7	262.8	221.8	283.8	247.8	234.6	376.6	350.6
CDSN	124.6	109.4	122.2	123.5	119.1	114.4	121.2	125.3
CDT1	112.3	121.4	113.9	128.3	117.3	92.6	119.3	107.9
CDV3	535.9	705.9	639.5	650.2	544.2	465.5	805.7	880.5
CDV3	847.2	697.2	559.5	681.8	507	520.2	847.7	974.4
CDX1	111.1	112.1	115.8	121.4	109.8	94.3	117.7	120.3
CDX2	114.6	118.2	114.7	106.5	102.3	93.8	115.3	111.9
CDX4	116.3	119.5	118.3	118.8	109.3	98.4	121	110.7
CDY1	106.7	111.5	112	127	107	98.3	112.6	117.2
CDY1	107.3	108.3	119.4	118.2	103.3	93.7	115.5	115.5
CDY1B	122.1	116.2	126.5	130.7	106.7	101.3	114.2	121.5
CDY2A	115.4	108.5	104.6	113.6	108.6	96.4	108.6	111.3
CDY2B	109.2	116.5	117.3	117.2	100.5	96.1	114.3	110.6
CDYL	132.6	146.5	133.1	139.5	138.5	118.6	145.1	130.4
CDYL	119.4	107.1	111.8	120.9	105.2	100.5	111.5	120.1
CDYL2	113	115.8	120.3	123.9	110.9	101.5	125.5	120.1
CEACAM1	118.8	124.1	126.3	139.8	125.2	112.4	143.7	134
CEACAM1	123.1	110.4	115	128.3	117.2	98.3	109.6	123.5

CEACAM1	129.2	121	126	123.3	129.2	104.2	124.8	117
CEACAM19	117	121.2	121.7	124.5	116.9	101.6	122.9	122.2
CEACAM20	117.6	117.8	131.5	108.1	106.6	97.1	113.2	118.7
CEACAM21	126.7	133	118.9	132.6	121.3	113	123.8	133.8
CEACAM3	111.3	116.4	112.3	107.2	109.5	101.4	124.1	116.1
CEACAM4	118.1	121.6	128	123.8	109.2	103.8	126.5	125.9
CEACAM5	117.5	121.8	116.8	118.8	109.6	101.9	117.2	113.9
CEACAM6	115.8	130.7	120.6	119	134.3	102.9	123.5	148.1
CEACAM7	109.9	116.3	115.9	122.5	105.1	99.3	117.5	113.1
CEACAM8	113.2	161.7	130.3	127.1	141.4	109.5	119.9	141.8
CEBPA	274.3	199.4	661.6	302.8	132.4	356.6	236.8	146
CEBPB	3618.8	2651.1	1527.3	4097.2	2451.4	2176.1	3508.8	1366.3
CEBPD	537.2	600.5	630.3	656.2	513.4	537.9	548.6	677.4
CEBPE	117.7	124.3	113.5	125.1	109.2	109.7	121.6	119.9
CEBPG	224.3	319.7	242.9	303.6	295.9	251.7	306.2	436
CEBPZ	902.6	1338.2	993	1302.8	1000.8	753.1	1047.1	1303.1
CECR1	123.9	123.9	131.3	118	117.8	101.2	118.3	103.8
CECR1	1100.8	1166.4	1676.7	1009	808.2	1134.6	750.2	687.7
CECR1	125.1	133.7	128.1	122.7	121	100.5	130.9	125.6
CECR5	139.5	130.2	129.2	154.9	125.1	122.5	140.2	124.7
CECR5	478.1	563.9	427.4	487.3	371.3	210.4	388.5	514.8
CECR6	133.2	144.3	125.1	155.6	126.9	100	156.9	121.5
CEECAM1	113.3	104.9	114.7	110.7	102.4	100.7	119.8	121.8
CEI	115.7	116.1	118.8	126.1	116.9	100.4	118.6	116.9
CEL	141.4	128.1	133	134.3	119.5	105.6	141.7	143.3
CELSR1	123.7	120.4	132.1	130.2	118.7	107.4	125.7	122.5
CELSR2	114.6	118.9	122	115.5	108	98.1	116.6	111.8
CELSR3	153.6	181.1	143.2	168.9	157.6	110.5	168.6	180.1
CEMP1	120.3	133.1	137.4	138.8	122.2	111.8	119.6	117.9
CENPA	121.3	121	125	119.3	118.8	104.5	129.6	120.7
CENPA	115.5	130.8	118.5	118.9	107.2	103.6	132.8	128
CENPB	936.9	1464.9	1049.6	1065.6	955.4	836.9	856.6	825.3
CENPC1	127.8	110.5	120.6	125.5	111.1	109.5	114.5	126.6
CENPE	121.9	117.2	117.4	117	106.6	101	111.9	114.9
CENPF	114.8	113.6	116	122.2	108.1	107.9	117.5	115
CENPH	122.4	121.7	121.2	126.8	106.2	107.3	122.6	117.4
CENPI	109.9	113.4	113.1	112	105.6	101.3	121.3	108.6
CENPJ	131.7	128.9	144	135.2	116.5	113.5	130.9	127.8
CENPM	123.9	130.1	127.8	128.4	114.9	106	118.1	123.2
CENPM	107.1	112.3	112.4	118	105.5	101	103.1	120.5
CENPO	111.1	120	118.8	123.4	111	97.7	120.4	125.1
CENTA1	810.4	698.5	1462.4	823.8	397.1	693	741.9	378
CENTA2	328.8	158.3	833.7	307.3	146.6	695.6	191.2	130.7
CENTB1	909.2	892.1	670.7	697.6	468.1	466.2	622	446.7
CENTB2	176.2	181.2	169.2	184.5	164.7	145.7	199.6	213.6
CENTB5	121.5	120.3	128.7	119.7	112.2	98	129.9	108.1
CENTD1	183	181	135.9	182.3	171.8	123.2	188.7	228
CENTD1	109.8	107.6	120.1	114.8	107.5	102.1	121.9	114.7
CENTD2	131.4	126.4	125.9	121.1	115.5	116.8	120.7	113.4
CENTD2	207.3	201.6	191	224.9	161.1	170.2	221.3	177.3
CENTD3	329.4	365.7	272.3	518.9	317.6	245.9	494	291.1

CENTG1	116.9	118.1	114.6	128.8	104.8	95.2	116.8	116
CENTG2	110.1	120.6	125.5	115.4	121.3	105.2	116.2	109.5
CENTG2	125.2	139.7	140.8	134.5	134.5	103.8	129.3	132.2
CENTG2	107.5	108.4	116	116.6	112.4	100.3	114.3	102.3
CENTG3	113.7	110.4	116.4	111.1	109.3	102.5	117.8	116.1
CENTG3	158.8	141.4	138.8	150.7	122.8	112.9	149.4	127.8
CENTG3	213.4	186.4	193.7	216	143.7	185.8	203.3	173.9
CEP1	228.9	195.8	263.1	249.1	211.5	221.4	223.7	204.7
CEP135	431.6	446.4	387.5	420.1	382	267.7	331.7	349.6
CEP152	126	118.4	138.6	133.1	124.1	121.4	130.7	116.7
CEP164	164	154.3	156.2	165.8	150.4	146.1	161.5	143.5
CEP170	128.6	132.7	124.4	135.7	119.2	96.9	123.7	125.7
CEP170	104.7	119.5	104.3	113.7	105.3	96.8	110.6	120.6
CEP192	245.2	254.5	217	258.2	289.9	195.5	217.9	207.4
CEP250	122.8	128	116.2	122.6	111.7	103.5	120.5	120.1
CEP250	121.1	120	132.7	121.2	113.7	108.4	132.9	132.9
CEP27	363.8	354	345.3	292.7	347.2	172.3	345.8	351.1
CEP290	162.2	158.3	157.4	176.6	173.7	133.5	178.5	177.7
CEP55	126.4	130.2	126.1	125.6	116.1	119.3	115.9	124.5
CEP57	173.3	178.9	161.4	173.1	141.4	137.5	169.7	167.4
CEP63	316.7	353.9	371.4	382.4	436.5	321.3	382.8	309.3
CEP63	120.2	122.1	126.5	118.8	107.3	102.1	126.3	115.7
CEP63	113.4	112.7	115.6	111.7	110.3	101.8	131.2	113.3
CEP63	117.4	128.9	140.7	125.2	113.8	119.6	125.7	128.7
CEP68	157.8	153.9	150.3	164.9	143.3	142.6	148.2	144.6
CEP70	119.3	122.8	117.2	122.8	120.5	101.9	123	129.6
CEP72	120.7	128.9	112.3	128.9	110.5	105.3	127.1	123.4
CEP76	144.1	116.5	131.9	114.8	121.1	109.6	132	122.4
CEPT1	126.1	111.5	118.5	118.2	110.2	103.7	124	118.7
CEPT1	146.3	142.9	145.6	159.5	147.2	122.9	157.5	147.5
CEPT1	230.5	238.5	286.4	265.2	230.9	162.3	258.8	241.9
CER1	105	108	113.5	110.4	115.4	92.8	111.9	110.7
CERK	860	1149.8	1140.8	991.2	990.8	978.3	907.7	976.8
CERK	801.2	736	771.4	770.3	542.5	523.8	729.9	796.1
CERK	122.3	118.4	133.2	141.6	125.9	105.9	139	119.9
CERKL	123.1	146.5	130.8	168.9	114	101.4	115.9	131.1
CERKL	106.8	112.2	121.4	121.9	103.3	95.8	106.2	122.2
CERKL	117.4	109.7	117.8	116.3	114.2	102.1	114.9	112.7
CERKL	111.4	114.6	109.9	122	103.6	92.3	110.4	118
CES1	272.1	158.5	145.5	417.2	150.6	139.6	443.2	140.9
CES2	292.2	314.1	423.4	333.4	246.8	300.2	300.1	245.9
CES2	130.2	121.4	136.8	149.5	136.4	121.3	131.7	142
CES3	116.4	117	120.8	114.4	101.4	99.3	112.6	112.8
CES4	140.5	111	115.2	157.1	118.6	104.2	137.5	117.4
CES7	109.2	117.2	108.9	127.1	102.2	94.1	119.8	119.9
CESK1	112.7	111.5	106.5	105.7	98	94.6	110.1	105
CETN1	117.1	109.2	122.5	116.6	107.5	98	109.1	115.7
CETN2	339.1	513.6	673.4	348.5	298.8	454.6	264.2	224.6
CETN3	111.3	130.4	117.3	108.3	104.1	110.2	122.9	109.1
CETP	121.8	116.9	132.3	130.5	119.1	110	122.6	123.7
CFB	124.6	112.8	118.8	118.4	106.7	92.3	120	115.9

CFC1	111	115.7	114.6	118.3	106.9	100.4	112.9	115.8
CFD	143.4	114.8	453.9	140.1	107.6	258.4	132.4	117.4
CFDP1	139.2	134.1	142.6	132.6	145.5	106.9	145.4	135.3
CFH	110.8	108.9	113.9	116.9	105.1	96.3	114.1	113.8
CFH	130	125.1	132.7	133.6	115.5	110	128.9	128.7
CFH	114.3	110.3	114.6	123.8	109.1	103	126.1	124.5
CFHR1	118	118.7	138.6	114.1	109.5	115.7	120.2	114.9
CFHR2	121.9	141.6	116.2	126.5	107.7	103.1	125.5	122.4
CFHR3	107.1	115.8	124	110.9	106.4	98.6	123.5	119.2
CFHR4	110.7	111	112.5	119.8	107.4	97.1	128.8	107.9
CFHR5	112.6	113.9	118.3	125.9	106.3	109	128.7	116.5
CFI	116.6	125.5	123.9	130.6	120.1	107.2	128.4	115
CFL1	1682.4	1541.8	1939.6	1959.2	1099	1690.2	1658.1	1797.7
CFL2	119.6	109.4	112.2	125.5	107.7	101	124.3	113.6
CFL2	112.7	107	106.5	121.7	107	99.8	111.7	115.7
CFLAR	425.6	430.4	409.9	517.6	411.7	304.3	455.3	417.5
CFTR	119.1	131.4	131.7	140.9	124.4	115.8	129.1	121.6
CG018	145.9	148.6	158.5	144.1	138.7	143.6	137.2	146
CG018	115.2	110.5	118.3	130.4	107.9	108.4	121.6	116
CG018	158	162.8	170	151.9	130.8	118.4	154	155.5
CGA	124.7	120.3	128.1	138.1	107.9	111.1	119.6	116.2
CGB	115	117.2	123.3	116	110.4	96.5	121.2	118.1
CGB1	119.4	124	132.5	121.3	106.2	112.8	118.3	114.5
CGB2	111.8	104.2	110.4	117.4	99.1	93.2	117.8	112.5
CGB5	114.8	115.2	115.7	119	108.3	99.4	111.8	113.4
CGB7	112.3	110.6	115.1	121.7	109.9	100.9	117.1	109.1
CGGBP1	734.6	712	598.8	844.9	581.8	481.5	881.3	794.2
CGGBP1	111.2	110.7	121.2	123.7	111.9	104.9	137.1	128.8
CGGBP1	1360.6	1551.1	1444.7	1549	1400.2	1123.7	1669.6	1900.2
CGI-09	120.1	124.8	123.1	119.9	112.4	112.7	114.3	126.3
CGI-115	228.6	276.4	240.8	229.2	224	156.2	203.4	229.7
CGI-38	116.5	114.7	113.3	125.2	110.9	92.4	114.8	131.4
CGI-38	118.4	114.7	112.5	115.3	114	95.2	114.2	112.4
CGI-69	1136	971.1	1019.8	1054.2	685.1	580.3	863.7	694
CGI-96	298.6	192.7	343.4	314.2	153.8	231.6	270.4	219
CGI-96	117.6	125.3	136.9	131.4	109.6	117.6	129.6	115.4
CGN	144.9	138.3	119	138.6	121.2	99.5	130.5	133.1
CGNL1	122	112.7	113.4	112.1	111.3	99.2	114.8	113.5
CGREF1	122.5	120	138.7	112	116.6	96.3	129.2	110.5
CGRRF1	232.2	242.5	252.7	225.5	204.7	183	203.6	203.9
CH25H	121.6	107.8	116	130	111.1	95.5	114.9	127.3
CHAC1	114.6	109.2	116.1	114.9	111.6	106.9	111.8	122.8
CHAC2	121.5	117.4	110	115	104	98.7	113.9	117.4
CHAD	109.9	112.3	120.6	117.1	105.6	95.9	115.6	104.5
CHAF1A	115.1	126.3	119.3	113.5	107.6	104.7	118.4	117.9
CHAF1B	132.3	134.3	125.3	132.5	114.1	108.5	155.1	139.9
CHAT	121.3	111.1	122.3	129.4	114.1	100.2	128.1	117.2
CHAT	121.7	114.8	115.1	116	106.4	97.2	130.5	118.8
CHAT	109.1	115.7	106.1	118.9	109.2	95.2	121.4	117.9
CHAT	106	121.4	110.1	121.9	98.5	95.8	115.6	113.6
CHCHD1	148.9	142.2	151.4	161.2	121	116.7	128.9	134.2

CHCHD2	1369.9	2595.5	1977.4	1517	1988	1271	1356.2	2713.2
CHCHD3	142.3	188.3	194.5	182.2	153	146.3	153.7	164.3
CHCHD4	328.1	315	270.6	329.5	257.1	200.2	230.1	227.6
CHCHD5	216.6	202.5	267.4	231.2	197.3	211.8	214.8	185.3
CHCHD6	127.8	145.4	124.7	147.8	118.3	111.3	134.6	131.6
CHCHD7	137.5	128.2	171.9	141.1	123.1	119.9	138.1	127.7
CHCHD7	257.2	321.1	339.9	261.5	242.5	256.7	249	231.2
CHCHD8	149	150.5	156.8	141.4	141	117.4	145.2	152.4
CHD1	555.5	385.2	416	472.9	299.7	288.7	528.6	704.3
CHD1L	156.2	142.6	147	139.7	142.9	121.2	160.6	147.6
CHD2	145.4	150.9	177.6	145.2	143.1	116.5	151.4	149.8
CHD3	130.7	130.9	125.7	143.6	127.3	107	120.6	136.9
CHD3	136.5	136.3	129.9	138.6	128.3	110.6	137.3	123.1
CHD4	1045.2	1132.5	644.9	1010.2	803.6	621	853.9	708.3
CHD5	117.2	105.4	112.1	118.5	103.1	102.6	121.2	121
CHD6	155.8	165.4	163.5	148.1	140.8	129.4	155.1	153
CHD7	301	368.4	440.8	387.6	378	334.5	536.5	599.8
CHD8	650.6	614.1	457.5	749.4	575	394.3	883.8	908.5
CHD9	562	588.6	535.8	646.7	489.2	383.4	710.8	705.3
CHDH	114.5	107	104.9	108.1	99.6	95.4	117.8	107.9
CHEK1	114.7	121	119.2	115.9	115.3	101.1	136	129.2
CHEK2	127	128.1	126.6	123.6	117.3	103.1	115.5	128.9
CHEK2	115.8	117.2	117.6	114.1	107.5	99.3	110.7	114.2
CHERP	149.6	149.5	130.5	158.3	132.6	117.6	154.1	162.8
CHES1	407.6	474.5	425.6	418.7	310.4	479.9	412.1	355
CHFR	704.3	652.3	736.6	706.5	581.4	525.6	740.8	551.2
CHGA	112.6	115.5	123.5	108.9	101.4	98	126.6	116.2
CHGB	116.5	113.6	119.2	120.6	108.4	95.6	128.2	111.1
CHGN	135.6	141.7	153.3	167.4	117.2	125.5	151.8	136.2
CHI3L1	112.9	112.6	115.1	120.7	111.1	93.6	126.2	113.3
CHI3L2	136.7	141.6	163.8	139.5	120.1	129	140.6	120.4
CHI3L2	116.5	117.5	123.4	125.2	107	104.4	111.9	110.9
CHIA	116.1	116.4	115.2	112	106.9	100.4	117.8	117.1
CHIA	114	118.7	116.3	123.5	114.4	100.8	124.1	115.7
CHIC1	115.5	120	112.5	130.9	109	100.5	124.9	129.8
CHIC1	115.3	115.7	124.9	122.9	110.1	107.5	119	116.7
CHIC2	822.7	933.6	1058.4	1301.3	953.6	1007.1	1383.6	1208.6
CHID1	121.1	122.2	115.2	110.4	108.5	101.3	126	108.4
CHKA	171.6	175	157.8	181.7	166.7	118.6	202.2	213.7
CHKA	113.1	122.9	113.2	134.6	115.6	105.1	121.7	128.2
CHKB	735.2	756.9	768.6	665.2	513	472.7	525.5	511.3
CHKB	123.9	115.5	117.3	120	112.4	100.1	115.6	104.9
CHL1	115.2	122	115.9	125	104.3	95.4	114.6	120.3
CHM	120.5	122.2	121.8	127.3	113	96	107	118.9
CHML	137.5	137.7	137	141.4	130.6	119.1	146.9	138
CHMP1B	560.6	588.4	583.5	723.7	548.8	635.5	783.5	893.6
CHMP2A	116.3	124.8	120.3	119	121.5	101.7	124.9	126.6
CHMP2A	148.7	137.1	180.3	178.8	142.5	170.1	147.9	159.1
CHMP2A	519.9	505.7	887.4	500.6	386.7	628.7	491	582.9
CHMP2B	271.6	244.1	226.4	268.3	217.5	207.9	262	318.2
CHMP4A	292	328.4	392.3	358.3	262.2	296.5	279.2	271.5

CHMP4B	366.3	426.1	454.9	467.5	394.6	377	381.3	428
CHMP4C	109.1	139.5	143.5	123.1	99.2	100	128.9	115.8
CHMP5	219.2	181.5	258.5	236.6	204.7	216.7	196.1	192.3
CHMP6	171.1	204.4	189.7	166.3	135.9	148.7	148.9	133.2
CHMP6	113.6	118.9	123.7	123.8	109	113.5	103.7	105
CHMP7	150.4	161.7	186.8	189.9	153.4	146.4	202.6	184.3
CHN1	150.5	165	144.6	142.6	142.6	115.3	139.5	148.5
CHN1	116.1	111.9	106.5	115.7	108.7	104.9	112.5	115.9
CHN2	121.5	127.7	143.7	125.9	117	101.1	114.1	109.4
CHN2	136.4	128.1	148.9	129.9	123.1	106.4	121.6	122.1
CHN2	120.3	116.4	109.1	112.7	100.6	104.1	111	106.3
CHODL	128.9	127.1	127.1	134.9	101.1	106.2	137.6	123.6
CHORDC1	112.3	123.6	123.8	144.1	103.1	103.6	113.4	112.4
CHP	147.8	154.5	147.6	165	128.2	145.2	174.2	163.4
CHPF	133.8	132.2	117.1	128.5	110.1	111	142.4	138.2
CHPT1	157.4	138.3	188.3	183.8	163.1	124.2	177.4	178.6
CHR415SYT	111.2	108.9	119.7	119.6	108.6	100.2	120.1	114.5
CHRAC1	151.2	166.5	151.8	184.6	149.9	129.2	164.7	168
CHRD	107.1	117.9	111.8	130.8	113.5	103.5	119.9	114.1
CHRDL1	103.9	105.9	103.9	112.9	105.8	95.6	113.7	107.7
CHRDL2	116.7	112	118.4	117	112.7	100	116.4	112.4
CHRFAM7A	106.2	120.2	110.1	115.3	104.5	93	111.5	111.3
CHRFAM7A	110.8	120.7	119.4	115.3	108.7	97	123.2	109.7
CHRFAM7A	110.4	113.9	111.3	112.8	109.8	103.4	109	113.9
CHRM1	122.6	115.4	116.7	119	111.2	102.6	123.5	120.7
CHRM2	110.5	114.1	109.7	111.4	112.4	95.2	114.6	115.3
CHRM2	115.8	112.4	123.8	113.4	102.4	99.1	112.8	121.9
CHRM4	108.7	116.4	122.3	116.8	108.5	103.3	117.7	103.8
CHRM5	111.7	118.1	118.6	120.8	107.7	107.9	119.8	117.5
CHRNA1	107.3	111.8	118.5	116.8	110.8	103.8	118.9	115.4
CHRNA1	115.6	114.6	113.6	127.2	104.9	98.8	115.6	106.8
CHRNA1	109.2	113.1	111.1	112.8	102.9	97.8	112.7	111.8
CHRNA10	116.5	114.4	124.7	133.3	109.2	96.7	130.8	122.6
CHRNA2	118	116.2	122.3	125.6	117.9	101.2	132.3	125.5
CHRNA3	121.6	119.5	120.7	125.4	106	104.1	116.6	118.6
CHRNA4	122.3	119.3	120.3	117.4	111.8	113.3	118.5	122.1
CHRNA5	1423.9	989.2	1578.8	1141.4	1221.9	1015.6	1521.8	1461.3
CHRNA6	128.4	126.6	122.5	134.6	115.7	107.1	131.7	119.9
CHRNA7	117	116.4	130.3	135.1	111.2	92.5	119.7	111.7
CHRNA9	119.9	113.8	117.7	124.5	111.2	95.6	108.6	120.2
CHRNB1	172.6	181.8	191.7	186.6	159.7	168.4	175.3	156.2
CHRNB2	107.8	107.6	120.3	116.4	101	92.5	108.8	109.2
CHRNB3	137	123.2	133.1	112	115.5	108.6	124.9	116.9
CHRND	110.9	115.1	116.1	123.8	113.8	102.4	119.6	117.6
CHRNE	116	116.5	116.4	126	110.4	102.7	123.9	111.6
CHRNG	115.7	123.8	120.2	115.4	108.9	104.8	120.7	121.7
CHST1	110.4	123.6	116.6	123.9	108.6	100.2	108.7	116.5
CHST10	130	116.7	118.8	138.4	119.1	101	133.9	124.1
CHST11	114.8	114.9	124.8	128.2	118.3	106.6	129.8	117.2
CHST12	451.9	384.6	403.6	431.9	333.8	232.8	406.9	279.2
CHST13	147.6	115.3	194.4	131	111.3	117.3	118.3	117.4

CHST2	136.2	112.7	135.6	131.5	131	135.3	135.5	133
CHST3	126.8	127.8	119.5	126.1	114.8	97.1	117.9	127.3
CHST4	111.9	109	112.8	109.4	104.4	99.2	115.3	116
CHST5	118.9	114.7	111	112.6	108.4	98.5	128.3	111.9
CHST5	112.1	108.4	117.8	114.2	112.4	94.6	103.9	112.6
CHST6	111	109	119.9	113.1	113.2	95	112	112.9
CHST7	256	242.4	208	236.7	183.2	164.7	230	193.6
CHST8	119.9	117.7	131	115.8	105.6	102.8	116.8	125.1
CHST9	113.3	114.4	114.6	112.9	99.9	100.3	121.5	113.2
CHSY1	436.8	390.6	414.8	483.8	445.2	419	472.3	450.3
CHTF18	174.9	149.5	147.6	157.6	132	119.1	158.1	147.8
CHUK	231.1	240.7	258.7	316.6	254.5	284.2	352.6	263.8
CHURC1	1005.4	504.4	1180.9	1049.4	519.2	957.2	884.7	625.9
CHX10	120	119.8	130.6	122.5	118.4	97.9	120.7	118
CIAO1	638.6	738.8	699.9	626.5	566.1	510.2	551.9	597
CIAPIN1	164.8	141.4	140.4	169.3	142	141.1	177.8	171.6
CIAS1	139	125.9	184.6	130.2	111.3	119.2	122.8	121.2
CIAS1	118.6	122.8	128.8	116.7	118	107.9	124	121.6
CIB1	3207.1	3492.2	4724.3	3278.8	1948.8	3027.3	2087.3	1750.1
CIB2	116.9	116.2	129.4	122.5	118.2	101	123.8	119.1
CIB3	116.5	119.9	117.5	122.3	120.4	106.6	111.7	121.2
CIB4	107.7	108.8	109.6	112.6	105.8	97.1	114.2	100.9
CIC	197.3	232.7	198.8	238.4	163.4	171.9	228.8	268.1
CICE	120.2	117.4	126.1	119	111.7	102.5	127.8	125.3
CICK0721Q	107.1	117.2	124.1	116	108.8	99.4	117.2	117.9
CIDEA	125.3	119.3	121.1	125.4	106.7	107.4	132.4	120
CIDEA	114.5	126.3	126.8	125.8	105.4	101	124.2	119.6
CIDEB	151.9	153.5	172.5	127.4	138.6	113.6	121.9	122
CIDEC	105.1	109.7	113.6	116.8	103.6	96.9	118.6	117.1
CIITA	142.2	118.3	146.9	136.8	118.4	108.2	113	121.1
CILP	117.2	112.7	107.8	121.3	112	94.6	125.8	118.4
CILP2	112.5	111.3	130.3	134.8	110.9	110.8	122	117.3
CINP	114.9	119.6	128.4	123.3	112.1	106.5	119.7	117.2
CIP29	694.2	719.5	922.2	830.1	741.8	838	786.4	744.2
CIR	157	158.4	138	173.8	151.6	132.3	140.4	152.2
CIRBP	771.4	783.6	1255.1	825.2	770.4	1007.3	763.8	735.5
CIRH1A	375	494	376.7	442.6	379.2	230.5	455.7	467.2
CISH	121.9	114.4	137.2	124.3	114.5	113.1	121.4	119.7
CIT	112.7	112.8	108.2	113.9	112.7	96.6	117.5	118.6
CITED1	112.3	122.1	108.4	124	116.2	92.8	115	109
CITED2	328.4	283.5	285.5	238.2	267.6	253.4	301	309
CITED4	145	155.4	160.4	156.5	127.7	134	138.7	122.9
CIZ1	124	128.5	120.1	127.7	124.3	100.1	131.6	131.8
CKAP1	362.1	318	359.3	300.7	263	227.8	284.2	267.6
CKAP2	171.6	177.1	208.5	185.1	147.9	153.4	161.7	174.1
CKAP4	211.7	295.9	249.3	261.9	204.2	286.8	280	225.3
CKAP5	368.1	445	332.3	398.8	363.7	313	348.1	308.8
CKAP5	122.4	117.4	136.1	127.8	122.4	101.8	125.8	112.3
CKB	119.7	112.1	128.8	128.5	105.4	99.4	126.9	117.7
CKLF	1070.1	1227.7	1622.6	1274.2	1117	997.2	845.7	765.6
CKLF	477.7	557.9	575.3	510.7	427	357.7	363.3	381.5

CKLF	116.7	120.4	122.6	113.7	118.8	98.2	118	115.3
CKLFSF4	126.1	111.7	114.3	108.4	107.7	100.1	114.9	114.7
CKM	111.6	113.6	117.3	117.3	106.8	94.2	115	111.4
CKMT1A	112.9	118.5	122.3	118.6	110.7	102.3	115.7	120.4
CKMT1B	116.6	110.9	110.9	110.2	107.7	90.7	112.5	114.8
CKMT2	124.1	121.7	120.7	113.2	106.3	109.6	130.4	126
CKS1B	223.8	183.4	178.3	189.9	154.6	132.9	201	169.8
CKS2	279.7	303.2	314.2	389.9	298	212.9	588.5	569.1
CLASP1	331.5	368.5	323.5	330.9	265.3	259	388.4	399.5
CLASP2	149.3	162.1	197.4	194.5	173.9	188.8	185.3	162.3
CLC	132.7	141.8	618.4	145	147.2	411.1	126.5	144.3
CLCA1	112.2	123	113.4	109.2	104.5	92.6	117.4	120
CLCA2	105.9	107.9	105.8	110.8	107	88.7	109.9	111.7
CLCA3	115.3	119.3	110.3	116.1	112.7	97.2	122.6	118
CLCA4	121.8	118.3	125.3	122.9	109.8	106.9	120.5	108.6
CLCC1	118.8	112.3	112.4	120.7	106.4	106.6	124.8	122.8
CLCC1	120	106.3	127.4	123.3	109.2	105.5	117.2	122.2
CLCF1	196.4	219.7	203.8	211	175.6	201.5	226.6	188.7
CLCN1	110.7	112.6	114.4	120.1	110.3	100.9	120.8	115.2
CLCN2	131.6	122	118.6	113.7	110.7	91.2	126.6	115.4
CLCN3	111.9	125.4	121	114.8	107	98.6	111.6	112
CLCN3	184.4	220.9	210.9	222.1	217.5	177.5	232.7	260.4
CLCN4	108.5	113.1	117.6	117.9	110.8	94.2	111.7	109.2
CLCN5	106.6	114	116.1	115.6	110.2	103.8	119.5	114.3
CLCN6	120.1	120.1	123.8	118.5	114.3	108.1	127.7	140.8
CLCN6	180.2	179.4	152.4	176.2	160.7	128.8	169.6	164
CLCN6	206.7	200.9	202.1	215.3	174.6	161.2	202.9	186.1
CLCN7	2572.8	3034	1962.5	3638.7	1802.2	1468.4	3523.4	2417.6
CLCNKA	124.4	112	114.8	118.3	110.8	95.7	119.9	115.2
CLCNKA	111.3	119.4	121.9	127.1	110.9	103.1	125.5	116.8
CLCNKB	117.8	125.9	114.5	115.1	107.2	103.2	121.3	122.7
CLDN1	117.6	116.4	112.5	126.4	117.7	96	115.4	118.1
CLDN10	114.9	112.6	114.7	116.3	110.6	100.9	113.1	113.8
CLDN10	110	106.8	113	105	105.8	93.2	108.4	112.1
CLDN10	112	115.1	108.8	120.9	113.5	95.9	116.2	108.5
CLDN12	127.8	125.5	118.2	131.4	118.3	100.2	122.3	125.1
CLDN14	216	206.3	181.1	203.5	166.6	147.7	202.9	193.1
CLDN14	109.4	103.7	102	104.1	108.2	88	111.2	106.3
CLDN14	112.4	110	112.9	115.7	109.6	89.5	118.6	105.5
CLDN15	118.2	133.8	109.2	128.8	122.9	100.4	131.4	139.7
CLDN15	422.6	555.6	479.6	595.3	494.9	328.3	554.4	768.9
CLDN15	137	143.9	134.9	145	129.7	96.2	142.3	163.7
CLDN16	105.4	113.3	113.5	118.1	107.8	96	113.2	113.6
CLDN17	114.8	115.4	117.8	126	103.3	96.8	121	118.6
CLDN18	121.5	110.6	123	121	107.6	97.1	118.3	121.9
CLDN18	130.4	130.2	117.9	131.1	118	108.5	126.4	137.6
CLDN18	112.4	122.9	118.9	107.7	112.7	105.4	118.1	118.5
CLDN18	125.5	118.1	120.6	115.7	113.4	100.6	117.2	117.8
CLDN19	109.5	116	121.1	115.1	109.6	106.1	118.1	109.1
CLDN2	111.4	111	101.1	114.2	103.2	95.3	111.1	114.8
CLDN20	125.5	125.9	129.9	123.9	109.1	105.5	131	116.6

CLDN23	159.1	148.9	154.8	177.7	124.9	118	145.5	131.7
CLDN3	111.3	132.7	123.6	122.5	118	102.5	119.1	122.4
CLDN4	101.8	114.7	113.9	127.5	106.8	99	120.9	108
CLDN5	113.1	121.1	112.2	118.8	118.8	99.9	119.4	110.8
CLDN6	117.3	112.8	113.7	118.1	116.2	99.9	125.3	115.5
CLDN7	113.4	127.1	116.9	124.3	112.5	98.4	115.5	115.4
CLDN8	107.3	105.3	115.5	109.6	102	95.3	119.8	103.5
CLDN9	120.3	132.5	125	121.9	115.5	109.5	131.9	133.7
CLDND1	629.7	591.3	662.5	769.5	473.6	552.5	736.3	671.9
CLDND1	747	662.2	805.9	832.6	793.3	743.1	760	888.8
CLDND2	235.3	222	241.1	282.1	226.7	188.9	303.2	335.6
CLEC10A	115.6	109.4	170.4	113.2	105.6	99.5	124.1	105.9
CLEC10A	105.3	112.1	167.5	108.9	100.9	104.4	121	112.5
CLEC10A	133.4	105.1	276	120.9	106.2	99.3	120.3	110.6
CLEC11A	124.1	137.1	139.9	134.6	122.4	113.8	126.6	122.2
CLEC12A	199.3	151.2	188.2	167.7	125.2	112.6	156.5	125.8
CLEC12A	207.6	161.7	187.4	165.7	129.5	114	187.3	149
CLEC12A	132.8	142.5	128.5	135.5	117.5	110.2	119.7	117.9
CLEC12B	113.3	116.8	122.2	121.8	108.1	104.9	114.8	114.9
CLEC14A	118.6	108.1	112.8	113.7	99.6	93.3	112.8	124.3
CLEC1A	110.1	110	126	116.6	102.6	103.9	111.7	118
CLEC1B	107.2	111	119.8	115.3	114.4	99.8	127.2	119.2
CLEC2B	277.9	166	331.8	324.4	160	336.3	365.5	224
CLEC2D	134.1	132.6	215.8	155.4	119.6	128.5	155.4	141.3
CLEC2D	497.2	519.7	560.7	634.1	669.5	382	570.3	518.8
CLEC3A	118.7	108.5	114.5	120.4	100.1	110.7	119.1	107.1
CLEC3B	111.4	121.9	130.4	119.1	106.8	96.3	122.3	114
CLEC4A	275	142.8	472	242.7	129.1	249	170.4	135.9
CLEC4C	121.8	121.2	121.5	126.8	107.4	100.5	122.9	118
CLEC4C	109.5	123.3	110.5	120.3	108.6	100.1	123.2	114.9
CLEC4D	119	119.2	111.6	131.3	110	102.6	140.4	122.3
CLEC4E	127	110.8	113.3	120.4	103.1	101.8	118.1	118
CLEC4F	109.6	117.6	117.2	123	106.4	95.3	114.6	116
CLEC4G	114	115.4	114	125.7	107.2	98.7	115.4	113.1
CLEC4M	109.1	112.8	114.4	106.1	111.3	98.2	114	114
CLEC5A	352.1	321.5	555.2	710.8	352.1	1144.5	543.9	200.2
CLEC6A	114.2	111.2	111.1	112	108.5	96.1	111.4	121.5
CLEC7A	137.6	137.7	167.6	152.9	115.1	157	139.8	116
CLEC7A	114.8	107.6	121.7	130	109.4	114.2	115.6	112.8
CLEC7A	145.2	134.8	184.4	179.2	127.9	164.9	157.3	126.5
CLEC9A	115.1	114.9	119.4	121.8	108.3	105	117.1	107.3
CLGN	112	117.6	117.5	117.6	109.9	95.3	109	111.5
CLIC1	777	526.2	1261.1	703.4	524.6	1053	878.3	458.2
CLIC2	125.3	117.5	130.9	126.4	118.3	103.1	120.6	137.9
CLIC3	244.4	256.1	439.6	266.4	211.8	362.7	209.2	196.6
CLIC4	235.3	195.7	225.4	235	171.9	220.9	192.2	165.7
CLIC5	136.5	138.3	164	136.2	114.3	116.3	133	126.3
CLIC6	124.8	125.5	125.4	121.1	123.2	107	132.3	134.5
CLIP1	375.9	322.4	358.4	509.8	302.2	445.5	457.7	437.7
CLIP2	149	142.7	114.1	148.3	126.1	125.6	182.7	158.3
CLIP2	151.8	135.3	151.9	190	129.9	144	208.6	140.5

CLIPR-59	142.1	221.9	127.2	149.1	207.3	106.1	143.1	236.5
CLK1	243.7	375.5	325.3	313.4	304.7	293.8	445.8	497.1
CLK1	137.4	138.7	153.9	145.2	147.2	126	149.6	179.1
CLK2	178.8	172.3	162.1	169.5	144.1	166.2	166.7	135.2
CLK2	372.4	377.3	374.5	319.4	245.1	300.6	299.2	269.8
CLK2P	109.2	113.6	124.1	122.4	111.4	93.2	118.9	109.2
CLK3	182.9	196.8	182.7	217.3	189.7	145.7	178.7	199
CLK3	542.1	615.6	467.4	608.6	572.9	377.3	541.2	659.8
CLK3	129.1	118.5	138.3	128.3	126.9	110.2	132.9	121.1
CLK4	296.9	292.3	323.4	345.8	305.8	229.1	268.4	299.5
CLLU1	108	113.9	112.1	107.4	102.2	87.7	113.7	123
CLLU1OS	142	117.6	118.8	149.4	106	101	126.2	108.5
CLMN	117.1	118.6	115.8	117.7	104.9	104.6	119.2	112.6
CLN3	293.5	342.6	244.9	267.4	193.8	211.7	276.9	208.1
CLN3	256.6	244.5	215.8	231.5	192.3	176.1	237.7	185.5
CLN5	197.8	192.5	240.1	203	179.8	177.2	209.6	236.8
CLN6	131.1	126.1	157.6	119	114.7	117.3	139.6	126.3
CLN8	117	139.9	143.9	128.7	156.3	101.7	157.3	156.7
CLNS1A	716	759.8	919.9	663	683.3	496.6	511.2	557.6
CLOCK	130.3	129.6	140.5	130.2	116.6	106	129.8	120.8
CLPB	114.1	118.2	117.6	116.6	107.1	96.4	119.4	117.5
CLPP	242.8	265.7	222.3	249.8	181	176	240.6	187.5
CLPS	110.2	111.5	113.8	110	98.3	89.2	112.7	108.3
CLPTM1	484.4	426.8	462.9	481.9	339.7	455.4	645.5	487.4
CLPX	228.3	263	201.8	210.1	232.1	200.5	227.8	231.7
CLRN3	111.6	109.8	114.5	115.9	99.3	98.3	122.5	108.9
CLSPN	114.1	117	141.7	120	107.6	108.3	134.3	119.8
CLSTN1	883.3	891.7	753.3	912.8	564.1	680.8	914.1	600.4
CLSTN1	122.8	118.1	117	130.1	114.1	101.5	117.9	109.5
CLSTN2	118.8	134.7	117.3	125.2	111.7	104.3	129.2	119.3
CLSTN3	125.4	118.7	118.5	121.9	112	97.6	134.1	117.6
CLTA	570.8	575.8	657.7	610	454.9	527.1	554.9	604.1
CLTA	115.8	123.9	116.4	113.1	105.3	99.4	120.1	112.1
CLTA	396.3	392	429.7	412.3	324.1	363.4	432.8	431.9
CLTB	377.6	321.7	473.4	348.5	307.4	409.1	335.5	364.2
CLTB	112.7	113.4	124.8	113.1	106.8	102.9	114.2	103.5
CLTC	123.2	131.6	135.2	139.1	130.2	116	182.3	157.3
CLTCL1	109.1	95.9	113.1	112.9	111.2	105.1	117.8	116.4
CLTCL1	114.2	119.1	135.5	130	107.6	105.4	130.3	124.8
CLU	110.2	105.8	105.7	112.2	102.6	101.5	106.6	108
CLU	109.3	105.4	102.8	111.7	96.6	99.7	114.2	110.9
CLU	122.3	106.9	113.5	115	105.8	97.1	113.2	115
CLUAP1	135.2	155.6	162.3	148.4	135.4	111.1	122.1	130
CLUAP1	15529.8	11579.9	12817.1	11469.8	13201.3	7646.4	12266.4	13790
CLUAP1	158.7	195.3	168.4	158.1	160	146.8	131.9	125.6
CLUL1	107.7	111.6	112.9	115.2	103.5	95.9	113.3	120.7
CLUL1	125.3	138.2	135.3	139.6	121.3	102.7	125.1	124.5
CLUL1	115.1	121.7	138.2	120.2	113.8	99.5	126.7	118.2
CLYBL	161.5	191.9	205.3	169.5	176.5	169.4	146.4	173.1
CLYBL	122.9	138.5	148.9	134.5	131.2	139	132.7	131.5
CMA1	118.8	115.3	130.8	112.7	103	96.8	118.3	110.8

CMAH	132.2	137.3	153.6	165	134.7	118.9	132.1	132.3
CMAS	439.7	425.6	425.2	477.5	387.5	350.2	494.4	422
CMIP	556.2	428.5	776.3	536.5	457.7	809.7	514	520.7
CMIP	115.8	109.1	113.7	121.2	101.3	98.5	128.9	119.6
CMIP	116.9	116.5	129.4	119.5	113.9	105.9	118.7	115.1
CMKLR1	128.7	120	142.7	127	108.1	124.1	126.4	113.2
CMKOR1	187.4	181.2	240.2	176.5	156.2	153.1	144.8	137.4
CML2	111.3	119.2	119.5	112.2	109.2	103.5	131.7	117.6
CMPK	701.9	724.3	638.1	686	551.3	632.2	530.9	659.7
CMTM1	117.3	111.2	113	124.2	114.9	93.2	117.1	124
CMTM1	114.7	110	118.1	116.9	110.4	103.4	122.5	121
CMTM1	112.3	128.9	128.7	125.6	127	105.2	122.6	110.5
CMTM2	109.3	110.6	119	110.3	107.1	99.6	113.6	110.6
CMTM3	98.8	107.2	123.6	128.4	112	104.5	122.2	119
CMTM3	744.3	911.5	754.2	766.6	564.6	664.9	809.8	799.1
CMTM3	122.7	129.1	128.6	128.5	118.9	112	130.3	121.2
CMTM3	254.2	296	279.4	258.8	226.3	240.6	285.3	260.8
CMTM4	127.3	112.3	146.6	125.8	125.5	116.7	131	134.1
CMTM4	117.3	118.7	129.6	120.7	115.1	107.3	115	121.4
CMTM5	118.4	115.6	121.7	122.5	120.3	103.7	123.2	116.8
CMTM7	1551.4	1681.8	2518	1571.2	1175.3	1351.6	1077.1	1036.6
CMTM7	192.1	164.5	220.1	181.4	176.5	153.8	152.8	162.5
CMTM7	197.2	173.1	201.1	203.3	162.7	167	188.1	164.8
CMTM8	992.9	357	822.6	1057.2	317	635.6	757.1	283.1
CMYA1	108.4	107.9	118.6	119.8	109.7	96.1	115.3	106.8
CMYA3	112	107.1	122.1	119.7	99.1	92.2	109.9	106.8
CMYA3	115.2	104.6	116.5	106.4	107.3	98.6	120.5	111.8
CMYA5	125	129.4	127.9	127.5	111.8	107.2	132.6	118.1
CNAP1	242.6	273.4	241.1	246.3	196.1	172	231.9	213.6
CNBD1	107.3	112.2	108.4	114.5	107.9	95.5	119.9	109.7
CNBP	1060.6	1079.4	1279.3	1285.1	957.9	1200.9	1290.1	1012.6
CNDP1	112.4	115.6	120	119	113.2	98.5	125.9	112.7
CNDP2	1322	1269.8	1948.6	1406.4	1016.4	1109.2	1239.4	1229.9
CNFN	139.1	133	125	140.9	115.2	112.8	132.6	117.9
CNGA1	105.2	110	118	114.6	106.4	96.7	118.3	113.5
CNGA2	110.5	114.3	118.8	115.2	106.8	102.5	109.6	112.1
CNGA3	120.8	118.9	119.4	115	109.5	95.8	122.9	110.6
CNGA3	114.3	115.1	100.3	116	107.3	95.9	114.4	110
CNGA3	124	125.2	122.7	119.7	110.5	102.7	122	118.2
CNGA4	113.4	110.4	124.3	120.7	103.6	105.1	117.3	118
CNGB1	129.9	133.1	135.5	131.3	127.8	112.6	140.1	129.8
CNGB3	111.3	119.7	127.8	113.1	102.8	103.3	117.5	113.8
CNIH	401.7	463.5	450.2	374.8	303.9	354.3	348.8	377.6
CNIH	257	291.7	352.3	302.9	205.3	267.7	256.8	249.8
CNIH2	110	113	108	113.4	108.1	94.4	104	109.6
CNIH2	115.3	113.2	114.6	118.4	107.9	104.4	118.7	109
CNIH3	120.9	116.7	117.5	123	104.2	105.1	115.8	122.3
CNIH4	248.4	398.5	416	357.3	283.9	376.2	322.1	341.4
CNKSR1	117.2	124	126.2	124.3	120.9	95.5	126.2	114.8
CNKSR2	120.8	118.1	119.3	124.3	105.2	101	126.1	114.4
CNKSR3	117	114.2	121.7	129.3	105.9	100.1	120.3	119.6

CNN1	114.3	108.5	119.6	112	107.6	98	111.3	104.1
CNN2	137.7	122.8	137.4	128.3	118.3	109.5	119.4	124.3
CNN2	1087.1	1047	986.1	880.5	661.6	650.3	542.5	464.9
CNN3	215.9	278.7	200.8	247.2	217.7	138.9	244.1	230.6
CNNM1	130.8	128.2	129	138.3	138.7	106.9	131.8	129.8
CNNM2	114.2	110.4	124.1	106.8	107.9	94	124.5	115.8
CNNM2	119.8	132.7	129.4	132.7	114.8	105.5	140.9	147
CNNM2	130.5	126.2	137.8	132.3	114.7	106.2	125.9	122
CNNM3	265.7	233.7	255.3	266.3	206.6	183.9	257.7	218.5
CNNM4	143.8	129.5	128.3	132.5	122.5	112.3	134.2	129.5
CNO	319.9	297.8	359.3	344.6	240.9	227.6	280.7	270.1
CNOT1	115.2	116.7	117.1	122.9	114.6	99.2	120.4	113.4
CNOT1	1532.5	1586.1	1404.4	1573.8	1433	1342.9	1912.1	2115.1
CNOT10	191	192.9	228	216.4	161.6	173.4	191.4	185.8
CNOT2	558.1	579.4	529	549.5	366.2	466.1	536.7	496.5
CNOT3	146.4	168.7	142.5	151	143.8	122	208.8	169.7
CNOT4	281.1	287.4	294.6	323.3	304.6	253.2	423.5	466.3
CNOT4	115.8	120.5	120.8	130.7	108	104.6	136.5	129.3
CNOT4	125.3	127.1	118	122	114.7	107	151.9	134.7
CNOT6	454.7	373.3	434.8	519.6	343	396.1	546.9	463
CNOT6L	218.3	228.5	207.4	233.2	236.7	178.6	258.3	270.2
CNOT7	239.8	264.9	279	327.5	203.5	243.7	256	262.7
CNOT7	636.6	676.6	514.5	583.4	492.1	381.6	461.2	483.6
CNOT7	111.7	113.2	129	122.6	100	111.2	111.4	110.2
CNOT8	337.6	384.7	337.7	307.5	321.5	229.3	370	336.8
CNP	110.5	128.1	114	128.4	107.2	102.3	135.5	118.1
CNR1	120.6	123.7	124.3	123.9	117.7	112	128.7	123.4
CNR2	108.2	118.4	112.4	120.5	112.6	93.4	112.1	114.9
CNTD	118.1	114.8	113	119.2	114.1	91.5	115	125.3
CNTF	113	117.1	116	117.6	118.5	103.8	116.8	117.2
CNTFR	118.1	115.9	125.9	118.4	110.7	103.6	120.7	119.9
CNTFR	119.8	124.5	120.2	121.7	110	97.4	126.2	116.5
CNTFR	114.6	109.7	105	119.8	100.6	94.2	115.8	116.7
CNTN1	111	112.7	119.4	109.8	109.2	94	117	110.2
CNTN1	106.1	118.6	121.5	119.2	103.9	97.5	109.7	119.3
CNTN2	114.7	116.5	110.1	115.4	104.9	100.4	110.9	108.3
CNTN3	114	117.9	117.1	116.8	107.5	96.5	115.5	115.6
CNTN4	116.9	130.3	141.5	130.9	117.9	110	132.5	117.1
CNTN4	108.3	111.9	105.9	121.5	107.3	92.8	115.1	109.3
CNTN4	119.5	109.8	114.9	134.1	115.1	103	125.4	117.1
CNTN5	105.5	109.1	112.9	116.4	107.2	92.5	120.2	110.4
CNTN5	119.1	118.9	121.5	123.8	107.1	107.5	117.9	113.5
CNTN6	112.6	117.3	110.3	109.6	113.1	103.8	124.6	117.6
CNTNAP1	115.5	113.7	125.7	125.4	117.6	107.3	119.6	113
CNTNAP2	400.7	332.9	189.8	299.2	177.5	131.7	174.1	141.1
CNTNAP3	121.5	117	122.8	125.6	110.4	99.9	121	115.5
CNTNAP4	116	121.4	117.2	119.6	114.5	96.7	115.9	110.4
CNTNAP4	111.7	112.6	108.9	115.8	104.3	106.1	122.3	110.2
CNTNAP5	110.4	116.4	119.7	118.5	110.9	103	111.8	122.9
CNTNAP5	113.5	110.2	111.2	114.7	105.8	93.6	115.6	112.8
CNTROB	138.8	122.7	131.2	137.8	110.1	111.2	134.1	117.5

COASY	325.6	371.8	457.9	362.7	307.1	243.3	284	279.4
COASY	133.8	143.5	148	150.4	131.3	141.6	153.4	126.3
COBL	118	106	113.1	118.5	117.7	98.5	113.4	118.2
COBLL1	415.5	346.9	234.3	487.9	256.6	208.5	364	252.1
COBRA1	137.7	142.8	143.9	137.3	124.9	110.3	128.6	125.1
COBRA1	311.1	328.1	308	349.2	251.1	264.2	374.9	309.3
COBRA1	537.4	483.9	453.8	532.6	349.6	423.2	580.2	462.1
COCH	114.5	114.4	112.5	120.2	114.4	97.2	122.5	112.3
COG1	169.9	172	199.3	171.2	155	138.4	163.9	168.2
COG2	306.1	313.9	257.8	313.1	223.1	197.2	313.1	225
COG3	173.8	190.8	211.8	215.3	197	168.5	164.7	229.1
COG4	134.2	153.9	128.2	147.1	122.4	116.5	146	139
COG5	122.9	115.1	139.9	124.6	113.4	108.9	126.4	127
COG5	140.3	137.3	148.3	184.1	146.2	118.2	167.1	137.3
COG6	217.6	256.7	199.6	223.3	215	161.7	225	192
COG7	122.7	128.7	136.2	144.5	118.2	111.1	123.6	126
COG8	128.1	129.3	133.9	135.6	121.2	116.4	124.3	122.4
COIL	271	348.4	278.7	355.4	251.2	270.5	284.1	327.3
COL10A1	116.6	118.1	118.8	117.7	108.2	100.9	123.1	114.8
COL11A1	112.2	111.3	105.6	126.3	97.3	90.4	116.3	113.9
COL11A1	114	119.5	120.5	113.3	109.7	96.1	117.3	113.2
COL11A1	118.5	117.6	112.1	118.1	106.5	104.8	118.8	113.9
COL11A1	114.2	118.2	120.4	109.8	105.5	95.9	115.2	123
COL11A2	123.6	117	121.8	118.3	109.9	105.2	118.8	119.2
COL11A2	120.9	118	126.1	121.9	118.7	106.4	117.9	121.4
COL12A1	124.9	121.2	121.1	119.6	118.6	100.4	125.6	125.4
COL12A1	125.7	120.7	125.9	128.8	121.9	109.6	120	120.4
COL13A1	125.4	118.2	109.1	120	106.3	96.4	117.9	118.1
COL13A1	112.2	114.2	129.6	117.8	104.2	99.5	117.8	118.5
COL15A1	107.6	113.8	111	112.8	102.8	93.1	111	103.7
COL16A1	117	122	122	118.2	111.8	97.9	123.3	121.2
COL17A1	103	123.1	116.7	119.1	104.9	99.4	115.7	119.9
COL17A1	117.5	125.1	124.8	109.3	118.4	101.6	123.8	115.8
COL18A1	121	151.5	132.5	131.2	128.1	105.4	117.4	132.5
COL18A1	115	125.6	127.8	119.8	118	104.6	126.9	122.5
COL19A1	114.4	117.7	114	111.8	112.1	97.9	121.8	118.3
COL1A1	110.9	115.5	113.1	120.1	116.2	96	111.2	117.3
COL1A2	114.8	114.7	119.5	119.1	116.3	99.4	121.9	114.1
COL20A1	125.2	114.9	126.5	118.3	112.1	97.6	125.5	122.7
COL21A1	108.7	111.7	118.9	111.7	101.3	93	111.9	109.5
COL22A1	117.5	115	119.8	128.8	112.7	103.6	122.3	120.3
COL23A1	118.1	126.1	159.1	132.2	125.3	136.4	131.9	125.2
COL24A1	122.6	111.2	109.9	123.3	111.1	105.4	119.5	112.3
COL25A1	121.1	116.7	129.8	121.4	116.2	101.4	131.3	115.2
COL25A1	125.9	119	116	113.4	108.9	94.4	117.2	110.6
COL25A1	108.8	108.4	109	106.5	109.9	97.3	121.1	104.6
COL27A1	120.5	122	123.9	113.6	100.4	95.1	120.8	116.4
COL2A1	122.1	124.5	132.1	118.1	117.9	112	124	128.8
COL2A1	112.8	103.8	120	115.5	106.4	111.1	116.4	114.8
COL3A1	137.2	129.2	123.9	123.8	111.2	97.8	131.6	112.8
COL4A1	122.9	126.5	133.2	131.9	121	105.1	127.5	116

COL4A2	126.5	130	123.9	142.3	120.6	109	128.7	116.4
COL4A3	110.3	112.2	113.5	113	111.5	98.6	124	107.2
COL4A3	122.2	128.2	125.8	122.3	122.2	101.9	125.1	125.9
COL4A3BP	118.3	130.3	120.7	135.2	115.2	99	134.8	157.5
COL4A3BP	384.6	394.2	327.1	374.9	368.7	235.2	528.1	503.4
COL4A4	117.2	128.7	116.1	116.8	112	103.6	119.8	113.3
COL4A5	105.1	112.2	108.8	118.3	109.6	99.8	114	108.1
COL4A5	102	110.9	111.8	118.9	99.8	98	111.8	102.3
COL4A6	110.3	108.2	111	110.4	103.1	95.1	107.5	109.8
COL4A6	107	117.3	118.6	111.5	120.5	100.6	118.7	111
COL4A6	112.3	113.2	114.2	123	108.4	101.4	116.2	121.1
COL5A1	128	119.9	136.7	125.8	114.9	94.6	116.6	130.1
COL5A2	119.7	110.9	117.3	124.4	117.3	104.2	136.2	115.9
COL5A3	111	115.8	122.8	121.9	111	102	116.6	123
COL6A1	116.1	122.7	125	114.7	113	96.8	131.5	121.8
COL6A2	111.5	126.1	119.8	113.6	109	116.9	126.9	109.5
COL6A2	116	126.5	118	123.4	107.4	103.8	118.2	118.4
COL6A2	126.7	121.4	121	124.8	112	106.9	128.4	112.2
COL6A3	120.9	129.1	111.4	117.9	110.8	94.7	105.6	110.6
COL6A3	128.9	147.4	122.6	126.1	131.8	101.1	125.8	132.5
COL7A1	140.4	125.4	128.4	143.3	124.8	111.6	153.1	145.4
COL8A1	125	108.3	117.5	124.4	105	96.2	113.4	122.7
COL8A1	107.9	109	113.9	112.5	105.6	93.8	112.5	116.3
COL8A1	110.8	110.1	105.7	121.5	102.5	97.4	124.7	116.5
COL8A2	128.8	126.4	126.4	129	107.1	99.2	124.6	122.7
COL9A1	109.8	121.3	117	118	118.3	97.5	118.6	122.3
COL9A1	111.5	133.5	114.2	119.1	109.3	99	128	126.1
COL9A1	125.5	122.9	142.2	131.8	133	104.8	119.1	125.1
COL9A1	115.6	110.1	108.3	107.5	110.7	99	121.2	113.2
COL9A2	120.9	124.6	129.3	130.1	104	103.2	125.7	114
COL9A3	115.9	118.2	119.1	119.7	109	111.1	123.9	114.7
COLEC10	120.9	107.4	118.8	116.1	104.3	111.3	124.7	107
COLEC11	111.6	114	114	120.5	106.2	100.2	116.6	109.5
COLEC11	108.3	110.6	110.9	111.4	107.6	95.3	108.8	112.8
COLEC11	135.7	133.1	124.5	127.7	125.4	129.9	134.1	131
COLEC12	118	129.7	124.7	109.6	103.9	111.2	119.8	117.2
COLEC12	107.3	111.5	116.8	121.9	104.6	92.1	121.8	114.8
COLQ	135.9	137	153.5	144.6	120.9	113.6	135.9	147.4
COLQ	113.6	111.1	112.1	121.4	104.6	102.5	122.6	111
COLQ	133	130.7	118.5	135	133.2	105.8	132.8	124.2
COLQ	119.6	116.3	120.2	129.1	121.4	107.3	122.2	119.8
COMMD1	302	334.9	498.6	325.5	256.2	293	250.5	235.5
COMMD10	208.1	194.3	350.2	227.6	177.1	205	152.5	140.2
COMMD2	253.8	268.9	315.2	274.7	192.5	226.5	201.9	208.9
COMMD3	1134.1	1134.1	1569.5	864.5	896.8	928.4	650.3	646.8
COMMD4	331.9	314.1	333.4	266.4	227.7	251.8	230.1	193.3
COMMD5	298.3	298.8	306.7	317.7	300.7	265.9	264.5	316.6
COMMD5	127.5	135.9	133.9	137.6	129.3	111.7	128.9	134.9
COMMD6	1953.2	2240.5	3789.5	2318.5	2499.1	2507.3	1978.6	2411
COMMD6	109.7	116.4	110.9	116.7	105.3	99.7	113.8	111
COMMD7	649.2	585.3	1183.6	630.8	464.3	784.6	437.5	490.2

COMMD8	132.6	124.5	148.1	138.9	115.1	125.1	122.6	125.4
COMMD9	215.5	248.3	255.6	235.6	189.9	199.8	194.6	231.3
COMP	115.7	109.7	114.1	116.9	112	106	117.2	109.7
COMT	130.4	128.1	137.6	127.1	121	121.3	124.8	123
COMT	811.4	676.2	1011.5	857.3	545.6	677.4	712.6	707.6
COMTD1	149.7	169.1	161.1	158.6	139.2	145.3	146.5	147.3
COP1	126.4	124.3	157.5	128.2	132.5	127	124.3	143
COP1	123.3	122.7	132.2	121.4	111.6	103.3	117.8	115.6
COPA	949.3	972	1073	1003.1	1243.6	819.6	1112	1095.5
COPB	575.3	702.9	560.3	817.7	540.8	576.8	815.8	1077
COPB2	444.3	531.8	443.9	527.9	393.4	421.1	488.4	432
COPE	325.9	368.3	415.2	428.9	307.2	412.3	343.3	332.4
COPE	382.9	424.3	549.7	463.2	333.2	371.1	413.7	360.4
COPG	175.3	181	193.6	185.9	164.3	178	177.4	160
COPG2	140.3	137.4	141.2	130.9	114.7	109.1	136.6	129.7
COPS2	108.5	107.9	110.5	117.7	115.2	95	110.4	111.1
COPS3	633.8	774.2	667.2	726.7	695	633.3	776.4	741.8
COPS4	218.9	289.2	258.8	238.9	210.3	217.8	228	257.8
COPS5	912	1041	1096.7	1005.6	879.3	1025.7	951	1114.1
COPS6	315.4	320.9	320.1	300.2	227.3	239.3	252	212.3
COPS7A	562.1	493.8	704.8	525.9	384.2	535.3	433.5	368.7
COPS7B	388.3	386.3	355.1	395.3	282.6	247.2	325.2	279.9
COPS8	112.3	100.8	110.4	114.8	105.6	101.5	115.7	121.4
COPS8	147.3	126.8	139.3	151.9	130.8	129.9	128.6	142.3
COPZ1	387.9	428.2	527	504.1	358	452.5	439.9	497.2
COPZ2	126.2	114.8	119.4	129	116.1	99	120.8	118.9
COQ10A	294.5	332.7	220.4	319.7	218.5	174.6	234.2	187.5
COQ10B	831	1101.7	702.8	867.9	874.7	568.8	1098.1	1236.5
COQ2	210.2	216.5	262.7	250.8	168.9	216.7	200.9	150
COQ3	144.3	126	136.1	138	137.1	126.7	166.4	159.8
COQ5	359.6	479.9	540	498.4	419.9	387.6	412.3	430.3
COQ6	137.6	137.7	134.5	138.7	120	109.7	126.4	120.1
COQ6	292.8	299	292.6	268.6	188.5	235	223.1	259.8
COQ7	126	135.2	145.5	134.3	125.8	116.6	131.9	131
COQ9	304.8	296.5	369.7	315.7	273.3	281.4	255.5	212
CORIN	108.1	108.9	119.6	116.4	103.8	94.5	110.6	114.6
CORO1A	1605.3	1409.9	1458.6	1321.6	899.2	1116.5	997.4	1155.6
CORO1B	138	134.5	160.2	128.9	117.3	127.3	123.7	112.4
CORO1B	393.1	266.9	633.2	383	205.4	450.1	234.1	174.5
CORO1C	186.5	156	171.4	194.5	149.3	163.7	205.5	164.4
CORO2A	247	264.5	274.7	234.2	246.1	191.4	213.9	213.1
CORO2A	131.6	128.5	120.3	131.3	120.2	104.5	123.6	131
CORO2A	113.7	121.8	138	137.4	120.8	110.3	124.8	116.6
CORO2B	106.3	118.5	125	120.9	114.9	102.8	114.3	121.1
CORO6	112.4	115.8	108.2	115.1	111.9	98.1	120.9	118.9
CORO7	254.5	244.1	252.4	249.8	215.7	217.1	316.7	274
CORT	121	113.2	107.4	109.2	102.7	95.9	116.3	116.9
COTL1	118.3	129.8	131.6	156.5	121.5	112.6	146	131.6
COVA1	199.1	194.6	178.6	172.9	163.5	156.8	176	166.2
COVA1	117.2	113.6	110.2	120	108.3	101.2	113.6	114.6
COVA1	129.3	126.1	131.9	126.1	117.6	102.8	124.2	127.7

COX10	246.7	290	271.6	272.5	240.1	207.7	219.2	240.6
COX11	130.1	148.3	159.7	146.7	134	120.1	140.1	139.3
COX11P	114.9	122.1	105.2	126	104.1	95	123.2	105.9
COX15	104.2	111.2	111.7	105.6	96.3	97.5	115.5	107.1
COX15	142	160	195.1	149.9	141.3	140.8	171.3	147.3
COX17	1554.3	1515.4	1690.7	1832.4	1571.8	1532.8	1932.7	1600.4
COX4I1	6084.6	8038.6	7865.8	7477.7	6243.4	5482.4	6955.1	8150.6
COX4I2	116	122.2	114.3	117.9	105.1	98.6	119.8	114.9
COX4NB	139.8	138.6	163.3	154.9	159.8	131.3	147.7	154
COX5A	975.7	1173.2	1543.3	1293.5	966.4	1280.9	1165.4	1263.9
COX5B	1195.4	1418.5	1624.4	1363	1139.2	1188.9	964.6	862.7
COX6A1	473.1	558.6	508.6	522.3	449.8	413.4	581.3	635.7
COX6A2	113.3	105.2	112.6	114.7	107.1	89.3	109.1	107.5
COX6B1	2422.7	2433.5	3784.1	2624	2468.9	2669.3	2412.8	2394.5
COX6B2	116.9	112.9	119.9	121.3	115.5	100.9	121.9	113.3
COX6C	1872.6	2055.8	2413.5	1607.5	1680.6	1932.6	1742.9	1994.4
COX7A1	120.3	117.3	115.6	123.7	114.4	110.1	118.4	121.6
COX7A2	2644.8	2846.1	4355.7	3188.5	2503.4	2644.9	2692.6	2909.4
COX7B	399.1	565	580.2	496.1	538.3	480.3	413.3	513.4
COX7B2	118.8	107.9	122.3	113	110.1	96.7	118.3	121.1
COX7C	5955	7020.7	8042.5	7221	7185.1	7271.4	6391.9	6082.4
COX8A	1898.7	1837.6	2510	2053.8	1371.5	1843.9	1726.3	1628.2
COX8C	114.2	113.1	113.1	124.4	112.9	101.4	119.6	109
CP	108.1	117.7	115.3	118.2	108.1	96.1	117.2	115.1
CP110	312.2	275.4	252.9	327.9	249.7	190.9	252.2	251.7
CPA1	117.7	104.3	120.7	114.4	101.9	98.7	117.8	125.8
CPA2	118	108.1	123.8	122.4	102.1	95.5	105.1	123.8
CPA3	118.3	120.3	144	118.7	113.8	125.1	120	113.2
CPA4	113.5	125.7	117.5	116.8	105.7	106.5	117.5	117.4
CPA5	122	132.7	116.9	118.8	111.7	103	120.4	122.3
CPA6	104.4	107.1	111.7	111.7	107.9	98.5	121.4	106.4
CPAMD8	105.2	121.6	117.5	125.2	116.4	101.2	114.6	117.9
CPB1	116.5	113.9	137.7	111.3	116.5	99.4	130.2	110
CPB2	130.7	116	124.2	120.3	109.7	103.4	117.1	119.7
CPB2	120.5	108.6	107	113.5	105.7	93.2	114.1	110.6
CPB2	109.8	109.2	113.4	113.8	107.7	95.7	116.4	114.7
CPD	258.6	304.6	213.3	337.5	248.8	202	333.5	366.3
CPE	106.7	120.9	110.9	122.6	113.5	105.9	126.1	110.3
CPEB1	124.4	114.5	123.1	130.9	120.7	101.8	126.8	118.2
CPEB1	112.1	121	109.1	118.7	110.6	105.9	120.9	117.3
CPEB1	112.1	120.8	115.7	121.5	113.3	92.7	116.1	120.3
CPEB2	112.8	111.6	131.1	128.9	125.9	117.2	124.7	132.8
CPEB2	170.4	157.7	170.5	171.3	163.3	171.9	160.3	149.1
CPEB3	127.2	121.4	120	132.1	110.1	101.7	121	122.1
CPEB4	142.9	161.6	131.2	180.6	174.7	106.4	176.9	227
CPLX1	113	122.1	114.1	114.2	113.5	103	118.4	116.6
CPLX2	123.4	115.3	132.2	120.7	116.6	100.3	110	127.3
CPLX2	108.1	107	112.2	111.1	106.5	96.7	111	118.2
CPLX3	121.3	118.1	116.2	120.7	109.6	100.3	121.3	125.1
CPM	112.8	107.3	125.3	124.4	107	100.9	124.4	130.6
CPM	112.1	113.5	111.5	124.3	111.6	95.3	118.8	106.6

CPN1	109.9	119.6	106.9	116.2	106.8	88.6	111.4	116.3
CPN2	121.4	113.9	121	123.7	106.9	97.5	118.3	121.8
CPN2	117	117.1	115.5	126.1	119	104.4	117.6	121.8
CPNE1	114.3	122.4	120.5	127.7	118	105.1	118.1	120.7
CPNE1	415.9	474.1	330.5	482.9	352.2	326.5	497	417.3
CPNE1	450.6	415.6	357.9	505.8	471	347.7	483.9	408.3
CPNE2	123	119.6	119.9	117.2	114.9	111.5	128.9	120.4
CPNE3	870.7	758	777.1	782	687.7	632.4	792.4	963.5
CPNE4	122.5	119.9	115.1	120.1	111.4	109.8	118.7	120.4
CPNE5	122.7	127.7	114.9	130.2	114.5	104.4	120.9	121.4
CPNE6	108.3	122.2	121.6	115	104.4	96.8	124.3	115.6
CPNE7	113.6	102.6	116.9	111.9	104.6	103.3	110	110.5
CPNE8	163.5	139.4	151.8	149.7	124.8	124.1	151.6	133.6
CPNE9	138.4	118.5	127.8	122.9	106	110.2	131.5	133.7
CPO	121.2	120.1	115.6	119.7	102.2	99.2	122.4	112.6
CPS1	113.6	115.6	116.8	118.9	102.3	96.3	114.4	111.1
CPSF1	336.9	300.1	266.7	430.5	230.3	206.5	259.2	233.9
CPSF2	193.1	225.2	210	225.5	183	176.3	271.4	234.5
CPSF3	251.4	251.3	296	232.4	268.7	220.3	246.8	242.3
CPSF3L	179.9	168	197.2	186.9	146.4	132.8	175.5	184.9
CPSF3L	138.7	158.1	154.4	158.8	133.4	128.3	154.5	138.6
CPSF4	816.2	893.6	797.9	1063.4	765.8	695.2	1179.2	946.3
CPSF4	119.7	119.4	132.1	122.8	110.3	101.4	114.3	126.3
CPSF6	149.9	125.1	131.6	114.6	120.9	99.7	142.1	131.5
CPT1A	112.7	116.6	113.2	131.1	112.7	105.7	113.6	119
CPT1A	113.7	110.5	111	107.4	108	99.2	114.2	122.1
CPT1A	124.4	128.8	134.7	132.4	119.5	116.3	130.9	121.9
CPT1B	150.1	146.5	197.4	151.5	138.9	144.1	177.8	172.3
CPT1B	118.6	136.1	125.7	122	116	105.9	130	123.4
CPT1C	132.5	123.4	126.9	129.3	116.1	99.7	127.5	122.6
CPT2	227.2	210.9	334.8	218.6	226.6	296.5	187.8	186.6
CPVL	171	124	471.3	150.8	112.7	196.5	132.9	120.9
CPVL	145.4	111.9	273.8	126.8	110.6	120.4	134.1	119.1
CPXCR1	122.6	114.8	128.6	128.2	120.9	103	123.9	118.8
CPXM	114	113.7	120.6	109.2	107.4	102.2	123.8	114.7
CPXM2	114.4	117.5	132.1	117.5	121.1	101.3	128.1	118.3
CPZ	117.8	113.6	114.4	121.8	106.5	101.6	121.1	112.2
CPZ	109.2	123	117.5	119.5	103.6	96.7	121.7	113.6
CPZ	113.6	106.4	112.6	109.7	108.7	91.6	119.6	112.9
CR1	119.9	121.9	122.5	113.7	112.9	95.9	115	120.1
CR1	113.6	115.6	116.7	116.7	106.2	104.3	117.3	118
CR1	110.2	112.3	116.7	118.7	115.7	105.9	126.3	113.1
CR2	111.4	117.2	124.2	113.3	105	96.4	116.9	112.6
CR2	130.3	131.6	126.9	135.3	123.3	116	124.6	128.9
CRABP1	112.8	125.4	127.1	117.6	110	93.3	126.4	120.3
CRABP2	127.2	123.3	120.1	116.4	111	101.7	128.6	120.9
CRADD	191	203.1	203.2	225	207.1	179.7	250.5	200.8
CRAMP1L	228.4	235.5	167.1	184.1	181.9	177.9	196.5	204.8
CRAT	127.4	115.2	121.4	127	111.3	105.3	131.5	132.8
CRAT	104.7	117.3	112.5	108.4	103.1	96	113.8	112.3
CRB1	115.2	107.2	117.2	109.8	113.2	104.5	124.2	117.5

CRB2	118.8	113.6	116.1	124.1	111.6	99.8	117.1	114.4
CRB3	119.3	109.8	110.2	133.2	106	88.1	117.3	110.7
CRB3	127.7	137.7	126.1	121.2	118.5	108.4	128.7	121.6
CRB3	119.6	122.1	131.7	143.3	132.6	114.5	135.8	142.7
CRBN	545.2	800.2	708.3	667.8	686	503.3	700	556.8
CREB1	1066.1	850.4	1156.9	970.2	1113.9	859.9	1136.3	1137.2
CREB3	133.7	141.8	138.6	132.6	128.9	122.2	133.4	125.7
CREB3L1	119.9	114.5	118.3	130.3	106.9	101.7	120.2	112.2
CREB3L2	303.8	289.2	293.1	291.9	295.2	202.2	292.4	246.4
CREB3L3	129.5	114.6	120.3	112.8	106	96.9	120.4	117.6
CREB3L4	118	114.6	125.7	113.2	107.1	98.7	124.4	119.4
CREB5	112.3	108.7	118.5	122.5	111.1	97	119	118.5
CREB5	111.3	117.2	112.7	126.4	103.5	102.3	122.4	115.1
CREB5	113.7	111.1	118.6	111.7	102	102.4	120.2	103
CREB5	116.5	113.7	149	127.7	110.7	101.1	126.2	116.6
CREB5	117.3	112.1	119.7	126.9	106.1	99.7	108.9	109.9
CREBBP	137.5	129.1	112.2	145.7	122.8	103	139.4	139.8
CREBBP	684.4	852.4	687.7	915.2	854.8	602.9	1009.2	1091.8
CREBL1	123.8	134.7	129.7	130.6	126.1	108.8	129	137.1
CREBL2	124.5	118	131.8	117.9	117.5	111.8	124	128.2
CREG1	549.1	370.9	634.4	1013.9	416.8	726.4	995	455
CREG2	103.6	117.3	123.2	127.8	114.7	99.8	126.5	121.9
CRELD1	118.7	118.7	116.4	121.2	111.4	111.9	131.1	115.6
CRELD1	117.9	112.2	128.6	113.5	119.6	106.5	111.9	112.5
CRELD1	353.2	368.5	448.8	305.5	227.4	315.8	229.5	231.4
CRELD1	107.5	118.6	118.9	114.5	107.1	97.5	114.1	112.9
CRELD2	358.6	372.4	681.5	388.9	356.4	331.2	362.2	411.4
CREM	128.1	123.6	118.9	120.3	128.3	98.6	133.7	124.5
CRH	113.8	124.9	139.3	122.8	107.6	104	119.3	111.6
CRHBP	119.7	115.4	117.9	123	112.5	94.5	119.6	115.7
CRHR1	158.8	152.8	141	149.4	132.8	108.3	138.5	146.2
CRHR1	122	118.6	114.1	115.1	110	100.8	128.3	130.6
CRHR2	104.7	104.6	104.5	114.9	104.2	96.2	106	108.9
CRI1	135	131.1	133.7	124.6	111.7	121.3	126.5	112.4
CRI2	180.2	214.8	181.7	222.7	187.5	149.4	169.6	169.6
CRIM1	119.4	113.2	109.8	131	118.5	98	119.1	110.9
CRIP1	456.6	509.6	539.5	347.7	333.9	397.7	260	244.9
CRIP2	124.9	121	131	125.8	103.9	114.6	117.2	104.5
CRIP3	110.4	119.5	127.6	116.6	115	104.1	128.6	128.3
CRIPAK	199.3	180.6	193.9	211.2	166.8	161.6	187.8	199.7
CRIPT	293.8	225.7	299.9	275.8	219.2	248.7	269.6	203.9
CRISP1	118	109.3	122.3	109.3	113.5	102.9	111.5	117.7
CRISP1	122.4	117.4	123.2	114.8	105.8	93.8	118.7	118.5
CRISP2	126.6	120.4	117.4	119.3	117.9	103.6	113.7	112.5
CRISP3	112.3	117.5	117	116.6	103.4	92	110.5	111
CRISPLD1	115.2	119	116.7	114.9	111.2	96.6	121.5	114.2
CRISPLD2	114.8	113.9	101.8	119.4	111.9	98.5	114.8	115.5
CRK	224.1	261.8	279.7	315.1	243.2	206.4	297.8	343.5
CRK	131.5	139.6	140.5	144.1	126.6	136.8	171.8	140.9
CRKL	1337.9	1623.8	1136.4	1653.3	1396.8	938.4	1651.7	1768.8
CRKRS	216.4	240.6	174.5	262.1	200.3	150.6	227.4	243

CRLF1	115.1	112	118.2	112.3	114	96.2	125.8	114.2
CRLF2	124.8	119.9	118.2	126.2	108.6	94.7	118.2	117.6
CRLF3	870.5	973.4	887.8	861.3	947.7	771.1	809.7	996.1
CRMP1	136.6	120.8	122	116.7	116.9	101.5	116.6	121.7
CRMP1	121.9	131.9	129.1	128.6	114.4	109.4	131.5	127
CRMP1	115	115.3	110.5	116.7	104.2	98.3	114.5	116.7
CRNKL1	166.2	162.3	142.9	169.5	132.7	140.6	189.2	191.2
CRNN	127.6	142	123.3	129.2	120.9	95.9	130.8	145.5
CROCC	138.8	124.2	144	134.3	128.1	111.7	144.9	122.5
CROP	197.9	203.1	253.5	204.3	171	193.5	179.8	176.4
CROP	627.5	947.2	988.6	797.6	661.2	539.9	999.3	728.3
CROT	157.5	182.3	148	167.5	157.4	126.3	152.3	147
CRP	111.5	116.8	110.9	116.5	110.8	95.8	112.7	114.1
CRR9	512.4	543.7	406	550	412.3	382.2	526.8	420.8
CRSP2	117.4	121.8	119.5	116.7	107.6	103.4	122.2	109.9
CRSP3	262.6	329	276.4	335.7	250.5	228.7	301.3	345.2
CRSP3	119.8	119.3	132.5	113.9	122.5	103.1	121	116.2
CRSP6	117.4	128.3	128.7	120.5	118.6	115.7	131.8	132.5
CRSP7	143.7	154.4	145.6	152.1	139.2	125.1	154.4	163.9
CRSP9	122.8	128.2	135.1	122.7	111.7	111.4	138.1	116.5
CRTAC1	111.7	118	117.7	118.9	110	100.7	107.6	108.7
CRTAM	115.1	113.2	122.9	112.6	107.1	108.4	122.3	107.3
CRTAP	185.8	189.3	230.9	186	173.1	174.7	181	159.3
CRTAP	139.3	132.8	168.3	151.7	129.7	126.4	158.8	125
CRTC1	130.5	122.9	112	129	114.7	101.6	137.5	123.9
CRTC2	146.3	166.1	164.4	157.7	141.6	163.8	146.5	161
CRX	124.1	116.3	124.3	119	107.3	101.4	119.7	114.6
CRY1	307.7	340.1	227.2	351.7	332.3	257	503	446.4
CRY2	171.2	173.3	168.9	171	140	149.3	155.5	153.9
CRYAA	112	109.5	112.1	124.1	104.8	97.1	117	118.1
CRYAB	122.6	117.5	125	122.6	107.3	103.5	115	112.1
CRYBA1	111	119	117.4	122	107.1	95.1	127.2	112.5
CRYBA2	114.2	113	111.3	109.3	102.3	96.4	119.8	104.1
CRYBA2	114	119.1	109.9	124.4	113.4	99.1	114.3	112.2
CRYBA4	121.6	120	116.9	120.7	112.8	99.4	118.2	117.6
CRYBB1	116.4	127.6	116.2	107.7	106.3	104.7	129.3	114.7
CRYBB2	139.2	143	140.6	140	121.4	113.7	150	159.7
CRYBB3	111.8	113.7	120	118.3	106.7	97.6	122.5	110.7
CRYGA	125.2	125.2	130	138.4	122.4	113.4	133.2	133.4
CRYGB	116.3	119.5	116.6	125.1	119	109.9	117.9	124.2
CRYGC	112.9	112.4	102	106.7	110.3	92.3	110.5	107.8
CRYGD	109.6	113.2	110.2	109.8	106.3	93.9	111.3	110.3
CRYGN	116.2	122	126.2	138.4	119.3	102.4	122.4	131.5
CRYGS	181	185.9	193.5	202	160.9	158.7	188.4	202.8
CRYL1	139.9	143.7	201.6	147.7	126.5	129.9	125.9	122.7
CRYM	114.9	127.2	133.4	129.3	132.5	117.2	121	124.8
CRYM	117.8	122.5	114	122	108.7	95.1	124.9	123.7
CRYM	108.4	118.3	114.6	123.7	114.1	101.4	117.4	117.5
CRYZ	305.6	461	256.4	274.4	368.6	181.7	215	378.7
CRYZL1	230.1	239.6	322.7	252.7	266.2	239.9	190.4	210.1
CS	113.1	113.3	115	118.6	108.1	101.2	119.4	116.2

CS	901	1095.7	1037.8	1015.7	762.4	1054.1	1204.3	974.9
CS	206.2	181.8	191.7	214.7	161.1	152.6	170.5	189
CSAD	176.4	181.5	302.4	218.3	198.4	165	172.5	169.4
CSAG1	118.8	124.3	119.5	110	112.1	100.2	125.4	119.1
CSAG1	116	117.1	108.3	121.7	111.3	107.8	118.2	121.3
CSAG2	113.9	116.7	106.9	122.7	111	106.2	115.8	117.7
CSAG2	122	116.2	125.2	120.8	113.4	104.8	110.4	119.6
CSAG2	110	112.7	107.4	108.2	108.3	96.2	121.3	113.1
CSAG3A	113.1	109.3	104.4	112.5	105.8	100.3	122.7	124.2
CSDA	344.6	297.9	462.5	328.5	256.5	335.9	263	271.4
CSDC2	124.2	121.3	108.2	111.6	105.5	93.5	120.3	106.6
CSDE1	152.1	141.5	168.3	163.4	150.6	154	140.5	154.9
CSDE1	133.9	139	150.4	152.1	143.5	118	150	134.9
CSE1L	357.3	406.4	377.8	425.8	298.6	324.7	437.9	504.1
CSE1L	472.8	481.8	497.5	559.5	438.2	411.8	619.7	676
CSEN	133.6	122.3	135.4	112.2	121.2	105	130.9	124.1
CSF1	112.3	122.5	119.4	120.4	111.2	101	119.2	112.9
CSF1	108.6	111.9	111.2	122.9	105.3	92.9	112.8	119.1
CSF1	109.1	105.8	108.1	119.4	102.6	96.2	117.7	108.8
CSF1R	1139	762.2	2989.8	1439.6	577.1	2551.7	812.6	292.2
CSF2	120.9	113.4	119.8	117.9	105.7	99.1	121.1	113.2
CSF2RA	263.5	184.8	374.9	546.1	205.6	538.7	404.1	216.3
CSF2RA	1014.9	850.8	1015.8	1592.8	869.9	1864.5	1638.1	1000
CSF2RA	316.9	244.3	474.9	637.5	323.8	898.8	611.4	315.3
CSF2RB	117.2	110.9	122.1	114.4	108.7	102.2	133.9	113.8
CSF3	116.8	113	112.8	120.1	111.8	99.1	128.2	116.9
CSF3	115.4	118	121.9	117.1	111.5	100	126.4	120.6
CSF3	115.5	119.5	139	118.8	131.7	106.5	127.4	131
CSF3R	121.5	114.2	129.6	114.3	101.8	93.8	123.9	116.8
CSF3R	122.8	116.2	260.7	120.1	105	123.2	117.9	114.1
CSGLCA-T	192.2	193.9	177.2	195	153.7	180.3	187.3	184.6
CSH1	117.5	118.2	124.6	118	100.2	95.3	119.3	121.5
CSH1	109.2	106.7	129.1	114.4	115.8	100	116.6	115.6
CSH2	117.1	116.2	122.2	131.4	116.9	100.8	133.6	121.7
CSH2	112.6	112.2	112.9	116.1	107.7	102.4	121.8	118.3
CSHL1	116	116.9	132.8	119.2	123.2	100.4	120.3	132.8
CSHL1	122.4	124.1	116.9	129.5	113	102.2	118.7	125.1
CSK	1180.6	1443.3	1263.4	1226.5	859.2	1099.5	1000	949.4
CSMD1	114.3	109.6	109.1	105.9	104.7	94.4	114.8	105.4
CSMD2	117.6	119.4	139	122.4	115	110.5	119.5	116.5
CSMD3	108.6	101.8	112.4	115.3	104.6	101.9	112.7	112
CSMD3	104.9	107	121	117.6	107.4	93	114.6	121.4
CSMD3	108.7	109.2	114.6	115.5	104.3	102.5	116	105.9
CSN1S1	121.5	119.3	131.7	116.6	102.6	100	117.7	112.5
CSN1S1	124.9	130.3	121.8	128.6	110.8	105.9	118.4	119.3
CSN2	112.2	116	112.9	111	108.5	96.8	110.1	112.8
CSN3	116.3	115.5	106.4	111.8	105.3	101.2	115.9	109.5
CSNK1A1	131.2	127.8	133	147.1	126.4	108.8	138.5	151.5
CSNK1A1	119.2	119.7	122.5	132.8	113.4	108.8	137.5	126
CSNK1A1	238.5	231.8	249.1	294	256.9	219	286.5	283.4
CSNK1A1L	109.2	121.2	119.1	119.5	111	98.1	120.1	116.4

CSNK1D	443.9	394.8	412.5	521.4	354.7	360.6	594.8	547.7
CSNK1D	219.7	206.7	272	270.8	200.8	253.7	291.2	230.6
CSNK1E	523.8	525.4	431.8	466.8	436.6	351.8	492.9	638.6
CSNK1E	115.7	120.1	121.2	126.8	113.9	105.1	126.8	122.6
CSNK1E	145.7	147	159.5	174.6	147.9	133.5	177.1	166.4
CSNK1G1	152.7	190.7	193.4	204.4	176.4	151.2	186.6	195.1
CSNK1G1	118.3	116.9	123.7	108.3	109.6	101	121.6	113.8
CSNK1G2	256.7	260.2	228.5	274.3	234.7	210.2	356.5	243.4
CSNK1G3	107.5	103.3	119.5	115.7	102.6	97.7	119.2	117.4
CSNK1G3	193.4	219.4	226.2	206.9	217.1	163.7	176.9	187.9
CSNK1G3	136.4	153.3	150.1	161.2	149.1	113.5	131.1	133
CSNK2A1	172.2	173	162.8	180.3	170	141.3	169.1	189.1
CSNK2A1	203	165.6	163.1	217	182.8	174	204.6	190.7
CSNK2A1	123.3	128.7	120.9	136	113.6	95.2	147.4	132.6
CSNK2A1P	144.2	152.6	117	141	126.8	112.5	153.2	143.2
CSNK2A2	382.7	457.8	349.4	457.1	329.5	359.5	531.8	515.2
CSNK2B	1108	1058.8	1093	1081.2	762.3	1104.2	1030.1	950.9
CSPG2	129.7	120.4	146.4	133	112.1	167.2	122.5	119.9
CSPG3	126.4	128	137.7	136.1	119.2	112.7	124.6	132.7
CSPG4	125	116.3	126.1	131.7	117.2	102.6	134.9	127.9
CSPG4LYP2	118.2	120.2	121.5	115.4	102.3	96.6	116.8	116.7
CSPG5	113.8	105.8	113	113.8	111.3	96.3	122.7	116.9
CSPP1	215.8	193.3	185.3	196.6	197.8	148.4	228.5	257.4
CSPP1	115.2	122.8	132	138.7	124.3	111.3	126.3	117.3
CSRP1	228.2	225.2	339	204.9	168.9	195.8	176.4	178.5
CSRP2	107.4	110.8	127	131.2	115.2	98.9	124.7	124.4
CSRP2BP	129.4	131.1	132.4	132.1	121.1	103.3	135	127.6
CSRP2BP	224.2	259.5	251.7	217.6	204.5	173.3	190.1	161.2
CSRP2BP	119.2	117.6	117.7	119.1	111.4	104.5	119.9	121.6
CSRP3	101.9	105.4	110.8	113.5	106	96.8	109.2	108.3
CSS3	116.3	124.6	118.6	115.2	115.6	96.6	126.2	117.6
CST1	105	112.5	117	124.4	108	100.1	124.1	121.2
CST11	105.3	117.2	109.4	109.7	98.5	89.3	124	110.5
CST11	112.5	111.6	121.4	112.1	97.6	97.3	119.5	108.8
CST2	108.5	117.2	121.4	121.9	131.7	100.7	112.7	122.2
CST3	533.2	211.9	3323.6	460.6	160.5	1824.1	351.9	225.3
CST4	117.6	118.2	109.1	113.9	110.1	105.6	118.9	126.5
CST5	113.6	118.5	125	123.9	111.7	107.7	107	113.5
CST6	118.6	108.2	280.6	121.5	110.4	120.5	109.9	114.1
CST6	127.8	119.8	224.5	121.4	109.9	126	132.3	116.9
CST7	620.4	929.4	477.4	758.4	621.9	399.9	787.3	870.5
CST8	111.7	117.1	116.4	116.6	111.6	91.9	123.9	112.7
CST9	120.1	130	120.8	127.1	114.9	102.8	130.7	122.9
CST9L	115	117.6	117.5	113.1	100.1	99.2	116	113.8
CSTA	130.4	124.3	122.5	116.4	105.1	102.2	124	116.1
CSTB	3028.2	1844.2	2248.4	3205.9	1765.4	2152.5	2967	1746.4
CSTF1	123.1	126.5	139.9	130.1	124.9	109.3	128	132.7
CSTF1	123.6	125.8	128	129	106.4	102.4	115.3	124.5
CSTF1	118.1	128.9	115.6	119.2	117.4	114.2	115.5	112.5
CSTF1	116	123.4	129.1	124.5	112.3	99.6	113	122.8
CSTF2	219.7	260	224.5	225.9	214.2	196.3	223.8	191.1

CSTF2T	146.8	159.7	160.9	155.5	126.4	128.7	153.3	144.5
CSTF3	243.2	222.6	363.7	252.1	255.8	243.9	199.9	190.8
CSTF3	184.2	191.1	180	164.9	184.3	136.2	141.8	145.2
CSTF3	249.5	255.9	290	298.5	268.4	241.2	399.1	492.4
CSTF3	168.5	156.3	183.4	168.3	162.1	150.1	145.8	142
CSTL1	107.1	119.2	117.7	118.8	109.6	97.7	117.6	110.9
CT45-1	108.5	116.5	112.7	111.6	109.7	109.5	113.5	110.5
CT45-4	114	122.8	127.4	113.9	110.2	103.2	116.4	109.7
CT45-5	112	109.1	119.4	114.9	105.5	96.8	118.4	108.2
CT45-5	114.5	122.4	117.2	124.7	109.6	97.5	114.3	123.5
CT45-5	121	111.7	112.8	125.1	116.9	95.8	121.8	110.3
CT47.1	110.3	111.5	120.2	116	99.7	92.2	114.2	114.4
CT47.6	115.8	112.2	103.8	127	110.8	107.1	125.5	132.4
CT47.7	114.3	115	124.3	119.6	109.2	98.3	114.1	114.1
CTAG1A	121.8	113.4	133.3	121.2	107.4	114.6	130.4	131.6
CTAG1B	112.5	115.8	122.3	108.6	102.3	99.5	113.8	111.6
CTAG2	124.3	114.4	120.1	109.4	106.7	98.7	116	125.6
CTAG2	117.9	110.3	122.5	120.6	107.8	98.3	119	115
CTAGE1	120.5	110.9	117.3	119.2	105.5	97.7	123.2	113.3
CTAGE5	119.9	117	120.2	119.8	114	107.9	116.2	113.9
CTAGE5	111.9	115.4	114.4	119.2	103.4	98.3	119.1	114.4
CTAGE5	111.8	113.3	115.1	113.9	101.9	93.5	117.4	110.7
CTAGE5	130.9	129.6	145.6	143.1	137.1	107.1	133.1	124.7
CTBP1	130.9	118	122.8	122.3	111.8	106.4	127	115.9
CTBP1	121.5	141.1	119.6	132.6	119.7	112.8	130.1	119.8
CTBP1	265.3	321.3	214.9	240.1	277.3	203.4	231.2	220.6
CTBP2	110.7	123.2	120.2	118.2	111.3	106	116.5	123
CTBP2	112.9	117.3	112.6	113.9	109.8	101.9	113.4	109.2
CTBS	230.5	209.4	258.3	292.2	251.3	191.8	237.7	260.4
CTCF	316.1	374.7	272.9	320.5	308.2	230.4	320.7	275.8
CTCFL	109.7	111.8	112.1	125.4	97.6	108.8	112.7	114.6
CTDP1	141.5	158.6	153.3	154.6	135.1	112.1	149.4	142.7
CTDP1	124	124.5	114	122.9	114.9	100	123.8	121.1
CTDP1	151.1	153.3	135.6	141.5	123.7	109.4	141.2	139
CTDSP1	254.9	396.3	330.7	287.5	268.6	301.2	233.5	206.7
CTDSP1	147.7	146.9	140.9	147.1	138.3	116.3	135.3	122
CTDSP2	1423.1	1592.2	1175.4	1519.4	1106.2	1002.7	1483.1	1441.6
CTDSPL	133.4	119.6	130.2	133.2	110.4	105.3	141.3	122.5
CTDSPL2	128.8	135.7	128	156.5	117.9	110.1	144.3	142
CTF1	122.6	113	121.2	121.5	111.8	95.5	120.8	113.7
CTF8	112.5	122.4	126.1	128.4	127.4	118.1	138.4	130.2
CTGF	124.4	121.9	121.1	127	120.2	112.1	119.2	132.6
CTGLF1	123.3	123.4	118.9	125.9	113.9	99.1	124.7	125.4
CTGLF3	697.3	624.9	765.7	656.7	559.7	476.1	735	606.2
CTGLF4	131.9	143.6	149.3	152.3	153.6	134.5	155	160.2
CTH	126.1	122.4	133.6	128.7	119.6	110.2	125.7	133.6
CTH	114.7	124.7	119.3	120.2	116	104	124.8	116
CTHRC1	108.6	113.2	119.5	111.7	101.1	98.7	114.1	111.4
CTLA4	130.7	130	129.7	141.6	134.4	112.3	131.7	141.1
CTLA4	128.6	113.7	132.1	120.2	120.6	110.2	117.8	123.1
CTLA4	157.9	149.4	146.8	190.1	134.5	133	172.3	191.6

CTNNA1	329.1	254.4	559.8	499.3	282.4	467	447.3	296.6
CTNNA2	111.1	119.1	110.2	119.8	107.3	95.4	116.3	117.3
CTNNA3	110.7	115.9	121.3	121.6	115.3	94.9	134.4	117
CTNNAL1	140	134.1	132.7	179.4	126.7	133.3	216.7	237.7
CTNNB1	168.5	161.2	192	176.1	169.9	162.9	164.9	151.5
CTNNB1	110.9	125.3	120.2	115.8	115.9	103.2	111.2	122
CTNNBIP1	109.2	114.5	111.8	109.1	104.8	101.2	117.1	117
CTNNBIP1	111.4	118.2	114.6	115.9	101	103.1	122.9	116.3
CTNNBL1	294.8	300.5	336.2	379.1	269.9	199.5	299.6	316.7
CTNND1	113.7	115.7	119.5	121.9	108.5	95.4	106.7	113.9
CTNND1	100.8	107.8	116.9	120.3	94.6	92.4	115.7	107.9
CTNND2	121.7	120.1	124.1	128.7	113.1	99.8	122.9	120
CTNS	109.9	102.9	118.3	120	100	96.7	115.9	115.5
CTNS	280.1	296	317.4	317.2	197.8	173.9	308	286.3
CTNS	130.5	134.3	135.9	127	123.3	106.7	129.7	120.3
CTPS	225.4	220.9	155.4	258.2	193.7	149.5	302.7	310.9
CTPS2	104.6	112.6	117.4	108.8	102.9	96.6	113.5	107.4
CTPS2	203.6	195	203.9	209.1	190.3	171.3	155.3	163.1
CTPS2	120.6	118.9	139	137.3	121.7	109.2	130.1	120.9
CTPS2	114.8	114.2	116.1	123.4	113.4	107.6	119	118.8
CTR9	132.5	139.2	133.9	131.7	121.3	119.2	136.7	136.2
CTRB1	110.1	105.8	113.9	115.6	104.5	94.8	112.7	107.1
CTRB1	119.7	118.5	131.5	113.3	120.2	101.3	114.4	114.8
CTRB2	124.9	127.4	130.8	129.7	115.2	106.9	123.8	122.7
CTRC	139.6	145	131.3	158.6	140.6	117.6	159.6	141.9
CTRL	177.2	158.5	160.4	190.9	159.1	122.5	242.2	220.2
CTSB	1435.1	1068.8	3152.3	2597.4	1109.2	3077.1	2329.9	870.9
CTSC	120.6	118.4	119.8	115.1	106.4	103.1	117.7	111.2
CTSC	180	173.3	241.7	171.5	157.2	159.9	191.4	159
CTSC	357.5	296.6	494.4	348.6	233.7	295.1	281.7	242.1
CTSD	311.2	243.1	322.8	362.9	205.8	353.9	406.8	218.8
CTSE	123.7	117	137.1	121.5	110.9	103.3	129.5	118.5
CTSE	117.8	121.9	115.6	127.8	112.3	102.5	122.4	111.5
CTSE	121.6	122.3	130.7	126.8	118.5	113.4	126.5	117.8
CTSF	199.8	195.5	177.4	162.5	128	129.4	138.3	143
CTSG	113	146.4	122.3	116.3	124.1	100.2	115.5	149.7
CTSH	832.7	445.7	1208.6	476.7	253.5	399.2	431.1	268.1
CTSH	114.2	123.2	141.5	136.1	113.7	124.2	136.7	129.5
CTSK	134.7	121.1	148.3	140	117	125	151.7	144.5
CTSL	135.9	134.5	189.5	193.5	171.2	162.7	264.8	213.5
CTSL1	575.3	541.3	1743.5	1654.5	1544.6	1442.9	4064.5	2397.5
CTSL1	496.8	494.1	996.9	1392.7	892.5	1389.5	3653.1	1964
CTSL2	125.6	122.4	123.8	118.4	110.9	104.6	128.4	117.9
CTSO	215.5	275.7	385.7	256.5	192.4	253	178.4	145.8
CTSS	280.7	170.3	285.8	229.7	156.1	187	184.8	157.3
CTSW	244.1	380.3	197	218.9	236.5	165.7	211.1	215.9
CTSZ	498.7	277.7	599.6	488.7	236.7	598.7	466.9	287.7
CTTN	116	112.1	113.1	120.3	112.6	105.9	129.2	115.3
CTTN	123.6	130	125.6	123.5	108.9	103.3	118.7	130.5
CTTN	107.3	114.1	108.6	110.1	101.6	100	117.2	111.3
CTTNBP2	120.4	111.4	119.4	117.2	104.4	106.3	120.1	122

CTTNBP2N	125.1	117.6	123.6	115.8	108.5	98.2	122.6	112.5
CTXN1	108.2	110	121.1	116.1	121.4	92.5	130.1	103.7
CTXN3	118.1	115.5	110.1	117.7	100.9	99.4	114.3	106.7
CUBN	129.1	114.2	130.3	131.4	113	110.6	129.5	125.9
CUEDC1	154.2	138.4	327.5	232.1	152	202.6	246.6	168.7
CUEDC2	239.8	300.4	324.7	254.8	213.5	213.8	217	236.1
CUGBP1	134	117.9	121.5	124.1	137.6	108.7	130.3	138.6
CUGBP1	132.8	139.5	134.9	136.3	128.3	115.6	129	117.8
CUGBP2	112	117.3	126.9	125.3	114	112.8	116.9	123.1
CUGBP2	257.9	261.7	276.7	259.5	287.9	270.6	247.9	300.7
CUGBP2	211.5	182.4	201	200.4	163	150.5	195.2	198.6
CUL1	215.8	190.8	199.5	197.6	173.1	173.2	214.5	251.3
CUL2	355.2	347	376.6	367.9	314.9	277.7	316.8	336.2
CUL3	107.1	120.5	125.1	118.3	116.8	103.6	117.3	145.1
CUL4A	114	119.9	130	121.6	118.2	106.2	124.1	124.2
CUL4A	427.5	378.5	437.6	450.7	356.2	280.6	532.1	595.9
CUL4B	133.7	155.5	146.7	149.3	153.7	122.4	146.3	170.8
CUL4B	139.4	141.2	143.6	149.7	141.2	133.9	131.1	134.6
CUL5	432.7	412.6	439.7	349.6	345.7	291.5	304	337.4
CUL7	114.9	123.4	130.4	115	110	108	134.8	123.8
CUTA	1123.8	1728.9	1095.9	1225.1	1270.3	869.8	1011.9	1340
CUTA	434.9	481.4	536	549.9	407.6	340.2	379.3	457.4
CUTA	116.5	121	118.7	129.6	111.6	109.4	126.2	117.5
CUTC	280.4	372.6	366.9	367.3	326.7	273.7	308.6	300.6
CUTL1	109.5	126.5	120.8	122.6	109.5	97.4	122.1	123.6
CUTL1	286.5	345.2	245.9	346.9	319.5	221.4	361.2	385.8
CUTL1	118.4	115.7	110.5	120.8	100.3	100.4	111.3	112.6
CUTL2	115	105	116.8	112.3	97.4	96.1	114.1	111.2
CUZD1	143	132.1	145.4	144.6	128.9	113.6	146.2	119.2
CWF19L1	835.3	696.3	848.9	1060.1	547.2	1195.2	784.8	529.4
CWF19L2	281.9	330.7	248.9	313.2	261.1	216.6	227.9	286.8
CX36	121.5	116.5	110.9	118.5	115.4	99	115.3	111.7
CX3CL1	107.1	115	115.3	114.4	111.5	97.5	113.9	109.6
CX3CR1	1081.9	1056.9	504.5	1019.6	722.6	383.9	911.2	801.1
CX40.1	105.4	114.7	115.9	116.1	99.1	94.8	123.9	119.4
CX62	115.8	118.3	111.3	114.6	108.6	111.7	107.6	112.7
CXADR	116.3	117.6	108.2	98.8	105.1	101.2	111.7	110.3
CXCL1	117.5	124.3	123.2	127.3	105.2	124.1	118	121.9
CXCL10	2676	194	1021.5	665.2	118.6	126.5	119.1	116
CXCL11	118.9	115	118.7	116.1	104	93.2	109.5	103.9
CXCL12	114.1	115.2	127	119.6	113.6	97.2	117.5	116.4
CXCL12	115.2	115.4	126.3	115.8	110.7	96.1	123	112.1
CXCL13	114.3	114.4	122.2	127.8	108.7	94.3	127.3	111
CXCL14	118.2	114.9	119.8	129.1	121.6	111.8	129.4	125.1
CXCL16	975.4	492.7	807	1880.5	420.7	2699.7	1331.2	394.8
CXCL2	133	141.3	123.7	157.1	142.4	284.2	205.1	146.4
CXCL3	122.4	134.2	124.3	142.8	121.3	110.2	134.1	120.5
CXCL5	180.5	269.2	126.4	294.8	207	182.2	198.9	135.6
CXCL6	111.7	117.5	113.3	121.7	105.9	150.1	119.5	110.6
CXCL9	309	127.2	187.3	120.1	116.3	111.8	116.6	113.9
CXCR3	201.5	252.4	278.2	179.2	170.6	178.7	147.5	151.5

CXCR4	329.6	381.3	452.9	377.7	408.7	355.3	333.6	332.9
CXCR4	111	110.8	126.5	137	98.4	98.9	109.4	112
CXCR4	1278.5	1607.9	1796.3	1553.9	1732.1	1454.9	1009.8	1273
CXCR6	203.8	280.4	234.2	259.5	251.3	237.2	289	331.9
CXCR7	121.5	133	134.6	128.2	110.4	104.8	124.9	111.7
CXORF1	111	114.3	106.9	111.3	102.2	93	114.7	105.9
CXORF12	193.6	241.6	213.3	208.3	189.4	167.4	184.4	229.7
CXORF15	114.5	113.7	120.6	122.3	114.2	99.9	120.4	114.8
CXORF2	120.2	130	118.4	116.4	114.7	105.8	126.4	122
CXORF20	111.3	121.4	115.5	112.7	116.5	101.4	121.9	125.6
CXORF21	120.8	109.5	125.3	121.6	109.3	106	118.9	112.8
CXORF22	112.7	113.4	115.5	120.4	108.1	104.1	120.7	111.2
CXORF23	119.9	115.5	132.3	123.2	112.8	107.7	114.4	112.6
CXORF26	321.2	377.8	339.9	334.5	432.6	262.6	349	381.9
CXORF33	129.7	127.9	132.4	136	119.1	101.3	121.2	120
CXORF34	109.1	113.4	108.6	117.5	104.8	101.7	103.6	116.7
CXORF36	133.7	121.3	135.3	134.7	130.3	121.7	130.7	141
CXORF38	261.5	238.9	323.7	266.9	220.3	201.2	220.7	198.6
CXORF39	167.4	180.5	156.6	204.4	154.2	143.5	177.8	177.9
CXORF40A	286.3	276.3	295.9	292.6	225	224	294.7	278.2
CXORF40B	212	249.9	251.3	252.2	214.5	181.8	224.3	194.1
CXORF41	121.3	105.1	110.3	117.6	106.9	98.9	115.9	110.8
CXORF43	130.5	115.1	118.9	114.3	116.5	104.3	131.6	123.2
CXORF44	152.1	152.1	175.9	142.6	131.8	127.9	155.2	143
CXORF45	166.5	146.3	198	184.3	160	159.4	164.2	171.9
CXORF48	114.4	110.4	114.4	115.3	102.5	100.2	116.7	105.1
CXORF48	107.7	113.2	112.4	110.2	103.7	99.4	114.5	108.6
CXORF53	187.6	205.5	189.2	203.3	217	167.2	182.4	206.8
CXORF55	114.6	127.5	112.6	113.8	110.4	98	122.6	113.4
CXORF56	127.7	127.3	126.2	136.7	116.7	113.1	125.4	121.2
CXORF6	133.9	138.4	120	136.6	113.5	101.7	119.2	126.9
CXORF9	541.5	512.8	611.8	455	378.1	442	447.3	422.7
CXX1	333	290.6	371	361.7	192.2	289.1	245.8	185
CXXC1	1065.1	1265.8	973.5	1162.8	768.7	736.1	1277.4	930.2
CXXC4	127.2	126.8	141.4	141.5	107.9	111.6	129	125.1
CXXC5	452.6	466.2	400.6	479.4	303.7	355.3	407.3	254.6
CXXC6	119.1	111.2	129.1	122.6	104.9	98.8	121.7	120.8
CXYORF3	1890.1	1927.4	2015.2	2439.1	1601.5	1228.5	2351.2	2750
CYB561	169.2	164.2	152.9	179.2	146.5	124.2	143.8	133.5
CYB561	190.4	231.7	178.1	190.9	163.8	134.6	144.4	150
CYB561	116.3	125.5	117	124.7	103.8	104.4	122.3	118.1
CYB561D2	271.5	302.1	346.1	292.7	206.9	247.5	229.2	180.2
CYB5A	252	262.9	296.9	262.4	256	181	219.1	237.6
CYB5A	152.2	191.9	148.9	167	155.9	107.8	138	136.7
CYB5D1	155.8	158.1	179.1	158.2	157.7	143.3	150.9	143.9
CYB5D2	213.8	198.9	167	199.8	193.2	171.3	220.8	212.6
CYB5-M	942.2	868.5	953.2	1004.2	850.5	817.9	1024.3	837.1
CYB5R1	331.3	265.5	310.7	350.9	230.7	299	306.6	296.1
CYB5R2	126.3	112.7	124.2	114.1	114.7	110.4	133.1	121
CYB5R3	143.3	135.3	148.9	159.5	126.8	144.6	165.6	159.3
CYB5R3	1290.5	1341.8	1699.3	1730.3	1058.9	1613.7	1413	1497.9

CYB5R4	226.4	254.9	301.4	344.7	305.5	308.2	352	215.1
CYBA	2993.2	2748.5	4405.5	3112.1	1716.1	2929.9	2243.8	1541.4
CYBASC3	642.6	568.6	464.6	622.1	340.2	446.1	498	286.8
CYBB	417	225.4	474.5	493.7	202.3	304.4	296.8	216.1
CYBRD1	134.6	142.3	186.6	150	129.5	134.1	144.3	135.2
CYC1	728.5	748.1	901.9	831.7	528.2	527.9	656.9	519.3
CYCS	195.4	223.9	197.8	233.3	186.9	154.3	219.2	225
CYCSL1	156.5	157.6	164.9	154.9	136	109.4	147.6	164
CYFIP1	122.5	126.3	124	122.7	114.7	107.8	130	117.9
CYFIP1	248.5	186.5	380.4	202.3	183.3	257.8	216.8	166.1
CYFIP1	120.1	115.2	121.4	125.1	125.9	105.4	112.4	124.2
CYFIP2	126.1	124.5	125.7	124.4	113.8	100.2	117.5	122.9
CYFIP2	436	518.9	417.7	501.7	399.8	443.5	562.2	673.7
CYFIP2	1456.8	1721.3	1041.4	1226.5	1081.4	605.2	1358.3	1722.6
CYGB	122.2	131.3	126.1	127.3	109.4	101.7	117	126.2
CYHR1	117.5	124.7	132.4	138.9	106.7	112.3	127	118.2
CYLC1	113.5	112.6	117.8	114.1	110.7	101.7	113.5	108.6
CYLC2	121.7	112.5	115.8	132.5	114.8	94.4	122	123.4
CYLD	123.5	124.7	129.6	129.7	116.7	98.6	122	131.8
CYLD	596.4	630.5	471.8	623.2	580.8	302.2	661.8	836.3
CYLN2	117	116.1	123.2	127	117.3	100.1	128.6	120.1
CYORF14	121.6	121.1	121.6	130.1	113.9	104.7	118.8	114.9
CYORF15A	276.9	344.7	335.2	274.8	295.4	271.5	249.8	276.7
CYORF15B	155.6	156.2	148.3	146.5	138.4	114.4	136.6	155.4
CYP11A1	119.8	124.9	132.5	140.3	117	117.3	128.5	115.4
CYP11B1	107.2	107.5	119.2	114.7	104.5	99.3	112.3	119.8
CYP11B2	123	121.2	130	117.8	120	102.6	124.8	122.6
CYP17A1	112.3	123	120.5	114	109.5	101.8	115.8	114.9
CYP19A1	107	111	113.4	116.3	113.6	102.7	116.4	113.4
CYP19A1	110.1	101.7	110.6	115.8	107.6	97.9	115.5	110.6
CYP19A1	127	130	132.5	143.1	119.5	128.1	120.9	120.4
CYP1A1	123.1	117.3	129.5	123.5	110.7	99.5	120.5	114.1
CYP1A2	115.5	111.7	113.8	111	101.6	95.5	115.2	116.4
CYP1B1	1399.8	1532.8	1318.8	2912.8	1012.7	1798.2	3090	576.9
CYP20A1	272.6	292.1	293.7	308.6	269	220.8	312.9	389.8
CYP20A1	111.6	108.6	119.6	118.1	109.1	102.6	121.9	117
CYP21A2	116.9	117.3	122	124.7	106.6	100.7	126.5	130.8
CYP24A1	107.6	112.5	118.7	120.8	109.8	97.5	116.1	106.5
CYP26A1	103.1	109.3	112.3	116	106.9	104.8	118	107.1
CYP26A1	115.3	111.3	112.6	125.2	107.6	108.4	120.9	121.6
CYP26A1	114.2	115.2	114.6	117.4	109.4	98	115.7	110
CYP26B1	120.2	120.3	133.1	127.5	107.7	109.3	111.2	129.1
CYP26C1	119.8	124.4	126.2	117.7	112.3	96.7	119.8	116.2
CYP27A1	2211.9	1469	234.9	2818.8	936.7	958.2	1957.9	532.1
CYP27B1	138.6	134.6	122.7	114.4	118.6	110.8	135.1	134.6
CYP2A13	112.2	120.3	120.3	124.3	103.3	105.4	117.8	117.6
CYP2A6	104.7	109.5	112.5	109	109.9	98.3	122.3	116.8
CYP2A7	116.4	116.4	117.3	120.6	108.3	98.7	129.5	121.7
CYP2B6	116.1	114.5	126.4	119.8	109.2	99.8	120.9	107.6
CYP2B7P1	106.4	111.2	114.7	121.1	106.1	97.9	118.6	114.1
CYP2C18	107.1	110.9	115.2	130.7	109.5	94.1	113.5	112.3

CYP2C19	113.1	116.6	112	107.9	104	95.7	121.6	108.7
CYP2C8	123.6	122.5	123.6	127.7	115.2	108.3	124.4	114.3
CYP2C9	113.9	102.3	115.2	119.8	104.4	95.3	115	116.1
CYP2D6	120.2	116.1	124.8	109.4	107.8	98.9	112	117.3
CYP2D7P1	124.7	123.4	120.8	131.8	122.2	113.2	128.3	135
CYP2E1	137.9	127.8	136.8	146.6	123.6	103.2	137.3	135.3
CYP2F1	112.3	114	133.9	127.8	116.2	107.4	115.7	113.5
CYP2J2	124.9	136.5	121.8	142.1	119.9	104.3	134.2	117.3
CYP2R1	197.6	196.7	235	215.1	193.5	174.6	240.2	233.4
CYP2S1	107.4	113.9	122.1	122.4	112.7	98.7	111.6	106.4
CYP2S1	129.3	118.9	176.8	138.9	109.7	126	130.3	118.2
CYP2U1	126.4	115.6	130.4	119	115.9	106.1	115.1	119.6
CYP2W1	126.4	119.4	115.3	112.2	112.7	109	120.9	121.5
CYP39A1	107.7	107.2	111.3	113.5	108	94.8	113.7	107.3
CYP3A4	108.1	114.3	123.3	112.5	107.1	95.2	114	108
CYP3A43	116.2	115.9	110.4	119.4	113.8	103.7	121.7	109.2
CYP3A43	107.9	110.7	111.1	115.9	103.7	94.3	116.9	121
CYP3A5	106.4	117.8	123.4	115.1	106.2	100	113.6	111.8
CYP3A7	109	114.7	131.6	121.2	107.6	109.4	120.8	120.2
CYP46A1	110.2	114.3	109.1	121.6	111.5	101.1	122.1	115.7
CYP4A11	104	114.2	114.8	123.3	109.6	91.2	124	113.2
CYP4A22	107.3	110.2	107.1	119	105.8	101.4	106	112.7
CYP4B1	117.3	107.6	117.6	123.7	109	97.2	122.1	113
CYP4F11	119.5	114.7	117.8	127	115.8	106.2	125.8	126.5
CYP4F12	121.2	120.3	119.2	120.1	106.5	100.9	113.4	105.3
CYP4F2	106	120.6	121.1	111.5	109.5	98.5	107.9	117.3
CYP4F3	113.7	115.4	112.1	116.9	105.4	101.2	111.1	106.6
CYP4F8	117	122.9	122.5	122.7	115.2	109.6	120.8	121.2
CYP4V2	275.1	310.3	364.9	301	264	208.7	200.6	210.9
CYP4X1	113.2	110.3	110.1	120.6	105.1	105.2	119	117.8
CYP4Z1	112.7	110.1	107.3	121.1	111.5	96	127	111.7
CYP4Z1	104.5	112	127.8	116.3	112.1	94.2	109.6	112.6
CYP4Z1	113.5	115.2	106.9	118.8	101	101.1	117.7	117.9
CYP4Z2P	111.8	108.8	114.7	120.2	107.1	103	115.6	117.1
CYP51A1	151.4	154.7	146.6	152.4	124.3	128.5	165.3	151.5
CYP7A1	124.4	125.4	127.5	111.7	110.4	101.5	114.1	126.2
CYP7B1	111.9	112.7	120.1	123.8	115.2	112.1	120.6	112.3
CYP8B1	116.5	109.6	130.3	126.3	118.6	103.1	120.5	107.1
CYR61	112.2	116.1	116.9	116.9	106.7	98.6	107.2	111.9
CYS1	117.1	113.3	119.4	120.6	110.8	105.5	120.2	130.5
CYS1	109.8	111.7	117.4	120.6	107.1	94.8	102.4	108.4
CYSLTR1	223.7	225.5	323.7	209.1	192.9	144	174.9	196.4
CYSLTR2	115.6	101.7	130.5	118.5	115.9	105.8	130.9	117.7
CYTL1	112.4	117	122.4	114.6	115.9	105.5	119.6	114.2
CYYR1	109.2	113.7	114	125.5	101.5	102.1	109.5	107.1
D15WSU75	243.2	304	228.3	297.4	229.5	244.7	279	288.5
D21S2056E	127.6	120.5	129.5	132.4	118	100.9	113.8	125.9
D2HGDH	580.2	504.7	839	566.5	409.6	380	351	298.8
D4S234E	378.9	370.1	352.9	414.6	364.2	291.9	349.9	325.6
D4ST1	182.5	183.3	146	163.8	157	144.8	171.3	156.8
DAAM1	150.1	147.8	133.8	148.8	114.4	130.7	144.8	149.8

DAAM2	123.4	120.3	119.4	123.1	116	102.7	126.2	118.4
DAB1	114.1	108.7	117.3	111.2	99	94.5	117.5	108.5
DAB2	218.5	177.8	318.6	230.7	133.9	204.2	208.7	161
DAB2IP	115.8	125.7	111.6	129.8	110.1	104.4	118.1	117.4
DAB2IP	110.8	114.7	117.6	122.4	100.4	97.9	114.4	107.9
DAB2IP	113	108.3	117.9	112.2	103.5	95.2	114.4	108.3
DACH1	110.5	116.1	110.4	118.2	108.7	98.8	121.4	104.3
DACH1	116.6	119.4	119.9	116.8	100.4	93.2	114.9	120.6
DACH1	114.5	115.5	126.2	113.9	103.3	103.1	116.6	111.1
DACT1	132.4	117.7	132.4	120.9	113	108.3	128.5	112.9
DACT1	129.1	130.9	127	124.4	109.6	108.2	131.7	125.7
DACT1	120	124.2	119.8	123.1	110.9	98.7	126.7	117.9
DACT2	124.8	120.8	133.1	124.8	118.2	97.7	119.3	111.1
DAD1	1565	1859.6	1942.1	1903.4	1376	1889.3	2111.8	2565.4
DAG1	144.6	144.8	151.3	146.8	135.3	128.5	149.2	136.6
DAK	112.8	120.7	124.4	118	119.5	120.3	112	118.1
DALRD3	117.5	124	135	140.1	121.2	114.1	118.2	120.3
DALRD3	104.3	106.7	114.7	115.1	101.8	100	125	113.1
DAND5	112.7	118.3	115.9	120	113.9	104.6	112.9	117.1
DAO	112.9	111.3	115.7	115.5	100.9	100.1	109.7	111.1
DAOA	118.8	119.7	119.6	120.1	110.9	102.4	108	118.9
DAP	161.1	179.4	172.3	154.3	134.1	150.9	206.2	151.5
DAP3	458.4	474.6	615	489.6	382.4	483.4	564.6	598.4
DAPK1	144.6	137.6	164.9	133.9	105.4	134.2	147.8	124.5
DAPK2	129	117.9	138.4	123.7	112.7	113.6	123.4	106.7
DAPK3	176.7	155.5	157.5	170.8	160.3	133.6	179.1	148
DAPP1	1278.6	1473	1917.5	1426.8	1625.3	981.5	1606.4	1648
DARC	112.1	125	137.3	139.9	115.3	112.5	119.8	123
DARS	1561.1	2129.7	1975.9	1567.2	1539.7	1277.4	1329.5	1431.2
DARS2	169.8	163.2	151.4	154.5	142.3	122.4	151.2	172.7
DAXX	205.1	220.8	190.6	228.6	184	181.9	206.8	231.4
DAZ1	117.8	113.3	112.2	115.1	110.2	98.2	126.1	110.6
DAZ2	117.2	118.3	122.9	114.4	111.3	93.2	125.8	111.3
DAZ2	119.2	122.6	119	127	113.2	100.5	119.7	121.3
DAZ4	117.7	121.9	115.2	110.8	103.4	99.5	115.5	114
DAZAP1	115	123.7	126.7	124.9	116.6	110	120.8	123.3
DAZAP1	262	250.3	315.7	263.3	253.5	261.2	340.7	276.7
DAZAP2	4585.4	5137	6160.4	5154.4	4615.2	5272.8	5491.2	4831.4
DAZL	117.1	122.2	117.5	132.6	114.2	100.8	116.5	112.8
DBC1	111.3	109.1	114.3	110.9	105.7	102.2	113.6	114.5
DBF4	120.2	117.9	120.5	120	105.3	104.4	117.2	121
DBF4B	121.1	115.1	127.3	122.6	119.1	96.3	122.5	119.6
DBF4B	112.5	116	110.5	117.8	101.5	101.6	117.7	108.7
DBH	106.9	109.4	120.5	131.9	107.5	115.8	120.3	113.5
DBI	1323.5	1320.1	1474.5	1334.1	1047.3	971.9	1091.3	948.1
DBI	214.8	242.6	213.8	193.4	190.9	137.7	229.9	186.9
DBN1	151.9	205.2	140.6	167.7	149.1	113.6	156.9	155.9
DBN1	117	118.6	114.2	119.7	104.5	106.2	121.6	125.5
DBNDD1	124.1	136.3	126.5	136.8	120.2	114.7	131.1	141.2
DBNDD2	112.9	112	114.3	117.6	111.4	90.5	117.5	115.2
DBNL	1040.3	1232.6	1411.2	1104.1	711.7	730.7	622.7	512

DBNL	242.1	236.6	252.2	200.1	187.5	167.6	180.3	142.8
DBP	130	145.3	151.9	145.1	123.7	125.9	128.6	125.7
DBR1	160.8	172.6	158.8	182.4	132.3	133.8	178.5	150.9
DBT	301.6	370.1	452.7	358.5	309.3	273.4	327	325.1
DBX1	116.9	124.1	127.4	125.3	115.4	111.9	118.3	111.3
DBX2	120	124.1	114	115.7	104.3	101.6	117.8	117.6
DC2	165.8	147.7	239.9	176.2	203.3	167.3	170.2	209.7
DCAKD	379.6	402.9	425.3	427.8	342.8	301.5	394.4	348.9
DCAL1	203	199.5	245.3	221.7	149.1	148.9	158.4	147.1
DCAMKL1	131.3	116.7	129.4	125	112.9	125.8	124.6	114.9
DCAMKL2	121.3	109.6	110.5	117.3	115.3	100.8	116.1	113.9
DCAMKL2	112.8	113.5	112	120.2	107.6	97.1	114.1	111.8
DCBLD1	111	123	129	122	116.1	107.5	121.7	130.9
DCBLD2	146.9	169	137.2	175.3	132.8	111.7	144.3	158.7
DCC	113.8	116.1	113.7	121.5	109.4	95.9	129.6	120.5
DCC1	117.9	117.8	126.6	127.2	117	104.3	114.8	119.2
DCD	115.9	110.3	114.3	114.2	105.8	97.7	118.6	109.3
DCDC1	107.1	114.9	118.1	117.3	108	101.3	114.6	117.3
DCDC2	102.9	112.7	123.1	113.3	106	96.1	117.9	119.7
DCDC5	160.2	224.4	210.1	192.3	183.5	154	179.5	219.4
DCHS1	133.1	114.8	129.4	137.5	122.3	108.8	121.5	125.4
DCHS2	113	122.4	113.5	109.9	105.4	101.6	115.6	118.2
DCI	128.6	144.6	132.6	144	124.1	114.2	126.5	131.9
DCK	148.5	141	151	162.1	151.3	129.8	162.2	145.7
DCLRE1A	132.4	142.6	137.3	140.4	125.2	124.3	142.5	137
DCLRE1B	111.9	117.7	116.2	121.8	117	99.7	122.8	120.3
DCLRE1C	159.4	186.6	175.3	180.9	153.1	150.3	166	153.2
DCLRE1C	124.1	130.3	127.1	119.6	101	104.3	115.5	117.1
DCLRE1C	239	226.1	236.9	240.1	275.6	145.8	214.9	226.6
DCN	118.4	114	115.2	111.1	105.6	98.4	111.2	113.7
DCN	116.5	124.7	120.9	118.6	106.5	91.4	122.8	126.5
DCN	118.3	119.7	118.9	115.6	109.1	96.1	119.9	116.4
DCP1A	203.1	210	178.3	199.8	210.1	182.8	270.5	303.9
DCP1B	125.6	137.8	138.5	129.9	119.9	101.5	136.1	126.6
DCP2	499.5	649.5	492.2	549.3	562.8	481	646.5	854.8
DCPS	133.1	150.9	154.1	155.7	131.3	123.8	118.8	143
DCST1	119.2	125.7	130.1	120	114.7	104.4	124.4	114.7
DCST2	112.4	112.4	110.2	127	107.5	100.4	117.7	110.9
DCT	117.3	116.7	118.7	119	107.9	104	114.2	114.5
DCTD	121	144.1	132	133.5	110.4	118.7	142	134
DCTD	377.7	497.2	395.5	420.5	335.7	382.3	389	392.5
DCTN1	412.1	350.8	282.3	334.7	264	330.1	347	363.3
DCTN1	115.1	117.2	129.2	123.5	114.9	97.3	121.7	129.3
DCTN1	156.2	154.2	157.5	168.5	135.5	146.6	172.4	168
DCTN2	1252.7	1202	1106.4	1297.7	935.9	1075.5	1414	1035.7
DCTN3	107.7	115.2	117.4	126.9	116.1	102.1	109.5	118.5
DCTN3	515.4	520.2	707.7	524.9	498	510.9	348.9	386.2
DCTN4	187.7	187.3	192.6	183.6	176.1	184.9	199.7	179.2
DCTN5	398.9	462.4	411.7	495.1	347.4	351.8	523.4	462.3
DCTN6	121.9	118.1	118.5	121.4	112.1	104.2	129.9	126.3
DC-UBP	181.1	166.7	140.1	200.4	152.3	123.2	195.3	164.3

DCUN1D1	107.4	111.1	108	126.1	109.7	102.9	114.3	111.6
DCUN1D2	125.7	137.7	114.7	150.5	120.7	119.7	141.7	131.6
DCUN1D3	225.3	246.5	159	260.1	185.4	149.1	255.3	269
DCUN1D4	129.9	135.8	143.5	146.6	116.7	107.6	148.7	124.4
DCUN1D4	380.3	382	364.2	402.1	329.9	264.2	383.2	443.3
DCUN1D5	286.1	321.2	329	320.4	257.7	270.4	268.1	315.2
DCX	126.5	115.3	129.9	119.6	105.7	102.3	107.8	121.7
DCX	115.1	113.9	113.5	115.9	111.2	100.5	118.3	112.9
DCXR	254.4	251.6	289.6	261	187.8	190.9	243.8	227.4
DDA1	575.5	562.3	538.1	609.2	376.9	545.7	700.2	626
DDAH1	111.8	118.3	121.3	116.7	105.3	98	117.6	109.4
DDAH2	114.5	126.1	138.3	137.3	123.4	135.8	131	136.2
DDB1	1785.1	2033.6	1460.9	2070.5	1458.5	1372.5	2312.5	2152.7
DDB1	136	137.8	129.1	128.4	140.1	101.7	131.5	132
DDB2	114.4	117.1	134.1	122.1	118.2	101.1	126.3	127.1
DDC	114.6	110.7	104.9	120.1	100.9	100.1	116.2	108.6
DDEF1	206.4	177.1	142.5	225.7	218.7	146.7	198.8	213.4
DDEF2	119	120.3	114	127.7	111.5	107.2	121	121.5
DDEFL1	135.9	136.6	138.1	131.6	118.8	112.8	135.5	125.8
DDHD1	122.8	136.3	127.4	122.8	111.2	104.6	130.6	116.2
DDHD2	127.9	129.9	136.7	131.2	109.5	112.9	124.6	125.8
DDI1	130.2	124.9	134.3	135.5	115.3	100.8	119.3	122.6
DDI2	112.1	123	119.8	113	102.6	92.4	126.6	121
DDIT3	195	230	227.9	257.7	249.7	189.4	289.9	508.1
DDIT4	173.8	228.4	246.9	200.1	173.6	250	196.6	257.2
DDIT4L	114.5	125.5	122.9	118	112.3	106.1	121.2	125.4
DDN	115.3	120.7	109.1	115.1	100.4	98.9	116.8	114.5
DDO	108.9	121.3	116.3	121.9	104.8	95.6	120	117.6
DDO	120.7	113	129.5	131.1	109	109.3	121.3	119.9
DDOST	451.5	369.4	576.7	465.9	401.1	414.7	374.3	366.4
DDR1	139.5	155.9	131.5	149.4	124	113.8	146.6	155.7
DDR1	124.4	126	142.6	133	118.3	110.7	123.7	126.5
DDR1	114	115.5	125.5	125.6	115.8	100	126.3	116.7
DDR2	112.9	121	115.3	116.8	110	98.6	113.6	105.2
DDR2	103.7	112.9	113.7	102.4	106.8	99.9	120.1	124.8
DDT	759.9	1114	1240.6	875.8	599.8	739.4	903.9	1028.9
DDX1	949.5	758.2	947	871.8	832.1	828.2	825.1	724.8
DDX10	282.5	307.4	224.2	247.9	279.5	189.5	249.1	253.4
DDX11	121.9	114.9	121	122.6	107.6	109.7	127.4	120.4
DDX11	152.4	157.3	163.3	150.7	121.8	134.1	153.1	131.8
DDX11	107.8	118.5	128.6	122.9	118.4	108.9	116.7	121.9
DDX17	793	1004.3	815.5	570	814.3	865.3	1048.8	1009
DDX17	162.3	162.1	149	159.4	157.7	123.8	158.2	134.2
DDX17	349.3	335.8	244.5	345.2	352.2	172.6	321.5	287.9
DDX18	952.3	1176.4	736.3	938	752.2	546.5	781.5	639.9
DDX19A	281.8	344	260.2	337.2	256.7	227.8	259	219.2
DDX19B	334.7	406	385.4	349	311	290.3	255	273.9
DDX20	128.5	134.1	115.9	127.6	123.9	112.6	125.2	120.6
DDX21	974.7	791.9	599.2	947.9	725.6	426.3	1003.2	1037.9
DDX23	363.5	381.7	405.1	386.1	309.6	362.8	394.8	348.1
DDX24	510.1	668.5	496.8	591.4	523.8	437.1	519.4	516

DDX25	114.5	112	110.3	128.1	106	100.9	117.1	116.3
DDX26	644.2	747.4	589	658	492.4	422.7	710.6	785.4
DDX26B	137.1	129.4	138.3	145.8	125.4	118.1	147.9	125.8
DDX27	202.3	191.6	190	231.6	190.7	127.8	196.4	197
DDX28	144.2	178.1	143.7	176.1	150.9	150	183.2	150.6
DDX31	204.1	190.7	181.7	180.1	169.8	134.6	184.2	166.4
DDX31	111.3	119.7	110.6	114.8	114.5	100.7	124.7	103.9
DDX31	126.1	122.9	126	129.1	113.5	102.3	127.8	124.6
DDX39	1821.9	1611.9	1113.5	1303.2	1714	916.8	1842.4	2090.1
DDX3X	116.8	119	119.9	127.6	100.2	100.1	124.2	113.6
DDX3X	1098.6	1090.4	952.9	1392.9	976.8	734.6	1454.7	1313.3
DDX3Y	139	126.2	130	141.8	131.1	108.4	119.4	149.2
DDX4	110.4	111.6	117.2	117.3	112.5	101.6	121.6	114.3
DDX41	180.7	182.6	174.8	191.1	164.9	152.4	208	170.2
DDX42	120.4	117.2	116.5	132.9	109.7	94.6	121.8	123.4
DDX42	563.2	467.6	508.1	560.7	486.2	392.1	456.6	356.2
DDX43	119.2	117.2	122.4	133.8	114.9	106.9	131.6	125.3
DDX46	313.8	388.5	383	396.1	349.4	252.8	312.2	309
DDX47	244.3	276.6	305.6	279.1	250.3	237.3	240.9	273.5
DDX47	615.2	694.8	608.7	579.8	495	377.2	580	662.8
DDX48	892.6	851.2	917.2	1044.9	734.7	815.3	1065.4	1029.7
DDX49	125.6	126.3	133.4	130.9	112.7	107.1	130.6	119.6
DDX5	3789.3	4310.5	4978	4005.4	4155.5	4486	4117.4	5353.2
DDX50	207.4	215.2	203.6	210.5	189.7	146	210.1	215.4
DDX51	2013.2	1870.5	2128.4	2074	2668.2	1188	2537.6	2214.6
DDX52	123.7	111.9	113.3	128.3	116.9	103.6	122.9	128.1
DDX53	112.2	123.8	122.7	124.9	113.4	102.7	120.1	123.5
DDX54	180.2	206	152	199.2	172.2	133.8	223.9	227.8
DDX55	362.4	332.6	348.4	399	239.4	275.2	313.8	268.8
DDX56	547.5	522.9	456.8	515.4	414.8	349.7	520	475.3
DDX58	153.4	176.7	156.5	163	157.3	128.5	145.1	139.2
DDX59	303	302.7	347.1	306.5	311	373.6	387.6	285.5
DDX59	119	114	117.2	121.4	106.2	103.1	111.2	114.8
DDX59	149.8	122.2	152.6	144.8	124.4	141.2	143.5	149.8
DDX6	130	129.5	132.9	140.8	125	99.5	137.9	130.5
DEADC1	120.1	113.3	115.8	112.9	110.8	103.1	116.4	119.7
DEAF1	124.4	141.9	135.6	127	112.1	105.1	138.4	126.8
DEAF1	119.1	115.3	125.2	127	122.9	104.7	116.5	117.8
Dec-01	119.9	109.2	118.1	120	106.1	98.4	123.5	121.5
DECR1	270.2	270	297.1	250.2	283.2	227.7	264.1	277
DECR2	138.2	158.7	171.6	152.9	127.3	118.8	152.2	150.2
DEDD	385.6	419.3	349	432.7	337.1	330.8	583.6	559.7
DEDD	120.7	133.5	131.8	127.4	124.3	109.2	129.7	133.2
DEDD	160.7	181.8	172.8	177.9	154.2	144.2	210.4	182.7
DEDD2	573.2	514.3	481.1	546.4	468.1	316.8	863.4	1188.1
DEF6	281.1	321.6	431.5	298.2	284.9	261.9	255.7	316.5
DEFA1	268.2	1438	646	420.2	1256.3	436.6	271.8	1450.5
DEFA3	185.4	779.7	434.3	229.6	685.5	268.9	187.8	840.3
DEFA4	121.8	212.4	122.6	122.8	166.3	104.2	118.9	221.2
DEFA5	111.3	115.9	131.4	125.9	108	99.4	118.5	116.2
DEFA6	114.2	105.6	116.1	111.5	105.7	102.9	113.8	113.3

DEFB1	107.7	106.5	115.1	111.6	110.7	97.6	116.4	119.4
DEFB103A	117.4	109.9	108	115.5	103.9	97.6	119.3	112.2
DEFB104B	111.8	113.2	111.5	112.1	105.7	97.5	115.1	116.8
DEFB105A	110.7	109.1	121.4	116.3	112	108.6	117.6	107.5
DEFB106A	109.7	112	104.7	113.7	102.6	99.8	113.6	107.5
DEFB106B	119.9	115.9	117	118.9	113.7	93.8	115.7	141.3
DEFB107A	122	123.1	120.1	129.1	113.7	105	123.7	120.9
DEFB108B	121.2	111.6	121.1	122.9	103.8	107.8	125.4	119.2
DEFB109	103	118.3	112.6	123.6	100.9	97.5	109.5	116.4
DEFB110	111.2	114.2	116.6	121.1	101.8	95.9	115	111.1
DEFB111	110.3	122.1	114.3	110.1	99.8	101.7	114.2	116.9
DEFB112	108.3	104	120.4	116.5	104.6	96.5	109.9	112.1
DEFB114	116.4	117.4	123.7	124.6	101.1	110.4	120.6	112.2
DEFB118	114.9	118	122.1	121.9	104.5	105.1	119.2	115.3
DEFB119	137.8	128.9	124.5	135.2	114.1	108	135.7	134.9
DEFB119	111.6	110.9	111.6	113.3	105.5	102.6	115.6	110.1
DEFB121	109.7	117.2	106.4	116.3	99.5	99.5	108.1	112.9
DEFB122	116.5	113.3	114.4	121.4	107.8	99.5	124.5	114.1
DEFB123	120	116.3	121.7	127	111.2	102.7	115.9	118
DEFB124	113	109.5	126.3	122	113	103.9	124.9	116.9
DEFB125	122.8	121.4	115.4	118.3	111.6	100.7	123.2	122.6
DEFB126	106.6	113.8	114.3	112	112.1	97.1	117	115.2
DEFB127	112.3	120.8	122.4	108.2	103.1	99	111.6	111.4
DEFB128	113.3	118.9	117.3	127.1	120.1	103	124.1	115.9
DEFB129	98.7	113.1	115.9	113.7	99.4	99.2	116.8	103.3
DEFB131	134.1	136	139.6	134.2	118.7	103	139.8	129.8
DEFB134	112.5	109.4	118	113.5	105.7	91.3	115.8	110.3
DEFB136	106.6	104.5	112.9	106.8	102.1	95.4	120.7	105.4
DEFB137	116.1	114.6	111.3	124.5	117.5	100.7	129.9	113
DEFB32	113.3	115.7	115.2	113.7	114.2	100.3	123.2	114.4
DEFB4	116.7	110.6	121.8	113.9	110.8	103.4	109.9	117.1
DEGS1	882.8	885.3	1050.4	854.7	617.6	769	987	1156.1
DEGS1	256.9	306	329.4	285.2	294.9	374.5	281.8	367.3
DEGS2	109.9	111.2	108.3	114.3	105.1	99.8	111	109.4
DEK	1291.5	1058.4	986	1219.2	1104.9	882.4	1223.6	1402.9
DENND1A	122.1	117.3	146.6	122	119.3	121.7	139.7	137.8
DENND1A	126.3	126.9	129.8	128.6	112.6	109.8	124.9	114
DENND1A	123.4	131.3	121.9	119.7	107.7	103.3	119.4	124.2
DENND1B	114.2	115.9	108.1	115	106.2	103.9	105.5	108.1
DENND1C	146.8	134.1	141.1	153	132.4	128.1	139.8	132
DENND2A	119.4	107.3	128.6	120.9	114	94.9	118.8	120.6
DENND2C	117.2	118.8	128	129.5	107.9	103.6	110.4	115.8
DENND2D	643.9	815.9	838.3	664.1	642.2	584.4	549.2	466.1
DENND3	137.3	126.7	145.8	135.3	114.5	131.8	144.3	128.8
DENND4A	146	158.7	141.6	153	141.8	135.9	140.2	141.9
DENND4C	246.7	284.1	203.5	258.3	232.1	194.6	277.8	293.7
DENR	310.3	258.1	255.3	263.1	261	131.7	283.7	317.6
DEPDC1	120	120.6	115.5	117.2	108.5	96.5	125.5	120.8
DEPDC1B	113.7	109	121	110.9	101.4	103.2	119	113.6
DEPDC2	104.1	121.7	119.4	118.1	113.7	107.5	115.9	114.1
DEPDC2	123.2	112.9	111.2	111.4	111.1	101.5	115.2	109.1

DEPDC4	111.5	117	117.3	119.2	104.1	99.3	129.2	120.7
DEPDC5	170.8	162.4	177.7	173.5	146.8	140.7	173.8	169.7
DEPDC5	117	116.8	124.9	120.2	107.3	101.9	124.3	110.7
DEPDC5	106.9	107.4	114.1	114.2	109.1	97.1	114.4	111
DEPDC6	112.3	140.1	129.2	125.6	116.4	103.4	110.8	125
DEPDC7	120.1	116.3	122.7	120.3	113.7	102.6	116.2	109.9
DEPDC7	114.4	111.7	111.3	122.1	111.1	100.2	110.9	106.8
DERA	451.9	504.6	712.2	499.3	381.5	428.3	379.8	457.3
DERL1	613.5	713.1	482.7	679.1	470.3	421.1	675.5	681.2
DERL2	202	254.6	207.6	244.3	177.9	193	250.4	281.8
DERL3	117.1	125	124.8	111.5	109.7	102.2	131.7	106.1
DERL3	116.2	119.9	126.4	115.8	99.3	102.6	118.2	120.1
DERPC	114.7	120.9	130.9	127.8	112.3	104.6	111.1	122.7
DERPC	124.9	132.3	128.3	141.4	124.7	112.5	132.3	136.4
DES	111	134.7	116.2	117.5	109.3	104.5	121.8	115.3
DET1	165.8	149	165.5	157.6	145.1	129.1	145.4	133.5
DEXI	525.2	612.7	441.9	579.1	465.9	384.8	537.7	607.3
DFFA	317.7	278.3	315.2	358.9	296.5	167	303.3	310.2
DFFA	122.8	130.4	126.9	134	120.8	106.3	118.9	122.4
DFFB	108.6	116.4	120	115.7	111.4	108.2	124.1	114.6
DFFB	216.7	218.5	212	210.5	177	162.9	164.8	190.2
DFNA5	126	132.8	118	136.7	127.1	132.7	148.4	161.6
DFNB31	135.7	123.6	122.9	133.8	123.1	107.4	127.6	120.1
DFNB59	122.1	114.6	126.9	122.1	122.8	105.8	125.8	116.2
DGAT1	201.2	248.1	205.3	231.9	176.9	150	190.6	172.5
DGAT2	120.1	127.3	135	131.7	130.6	117.3	125.7	125.1
DGAT2L3	118.2	123.9	118.8	124.8	106.2	106.4	120.9	119.4
DGAT2L4	109.9	115	107	113.2	100.9	97.3	115.1	110.6
DGAT2L6	106	114.4	111.1	110.9	99.7	100.6	111.1	110.2
DGCR13	141.2	141.5	124	139.4	126	119.5	158.6	164.6
DGCR14	139.5	153.9	145	146.7	139.6	118.1	134.6	131.9
DGCR2	181.9	231.4	173.7	213.1	187.5	174.9	244.3	209.4
DGCR5	110.2	115	112.8	119	106.4	105.9	120.1	115.1
DGCR6	138.1	149.5	151.1	139.5	126.9	114.6	119.4	137.9
DGCR8	253	242.1	197.6	248.6	200.4	227.9	281.1	204.6
DGKA	131.7	132.1	139.1	130.4	141.7	109.6	128.3	123
DGKA	116.7	109	119.5	122	115.4	104.7	114.5	125.9
DGKA	355.2	340.1	289	312.3	287.6	295.9	284.2	269.8
DGKA	125.2	126.9	121.4	125.4	109.1	113.8	122.1	117.8
DGKA	460.4	571.9	480.6	545.7	391.4	397.4	384.6	412.2
DGKB	119.7	116.7	107.1	117.7	106	85.3	127.8	114.5
DGKB	119.3	117.7	118.8	126.2	116.3	104.8	119	113.7
DGKB	110.9	114.1	114	114.3	107.7	100.7	118.7	114
DGKD	131.6	137	159.7	134.3	128.3	107.2	140.8	129.7
DGKD	125.5	124.7	124.6	139.7	123.5	102.7	124.8	132.1
DGKD	106.7	113	117.2	121.8	112.3	100.1	109.4	108.1
DGKE	129.4	138.7	136.2	150.3	126.1	107.2	134.2	112.8
DGKG	117.1	121.3	118.1	120.3	107.7	98.3	113.5	104.9
DGKH	109	106	115.8	113.6	102	96.9	115.5	110.4
DGKH	128.4	115.4	135.6	129.5	119.3	115.5	134.8	130.2
DGKI	112.6	119.6	127.1	116.3	105.5	103.8	120	123.3

DGKK	112.8	120	125.3	122.2	106.8	102.3	107.5	114
DGKQ	464.1	581.4	454.2	522.1	368.9	397	522.7	416.3
DGKZ	121.1	112.2	114.3	127.7	110.3	103.6	118.1	115.2
DGKZ	110.1	116.6	131.7	119	113	106.2	113.4	120.2
DGKZ	105.1	115.8	129	114.5	113.3	97.4	123.2	108.4
DGUOK	973.6	1117.1	998.1	1028.6	855.8	744.1	826.2	1178
DGUOK	990	1205.6	1158.2	903.3	981.9	893.6	996.8	1196.3
DGUOK	173.3	204.8	202.9	197.7	189.7	182.7	210.6	244.3
DHCR24	127.5	125.3	120.3	119.1	110	104.3	126.3	115.9
DHCR7	163.3	179.9	152.1	179.4	144	128.3	168.7	157
DHDDS	202.4	205.6	182.6	192.9	160.2	166.1	205.3	194.5
DHDDS	130.3	130.2	131.8	140.1	127.9	126.5	138.6	134.9
DHDH	127.2	128.5	125.6	124	129.4	110.9	134.8	167.2
DHFR	112.2	122.4	113.1	121.8	106	100	117.5	109.2
DHFRL1	151.7	145.8	149.8	142.8	120.4	121.4	143.9	122
DHH	131.1	124.6	132.9	128.1	115.7	122.1	132	123.8
DHODH	123.8	123.6	118.4	111.2	105.4	97.8	121.5	105.6
DHODH	116	122.2	111.5	122.5	112.1	100.8	117.9	112.9
DHPS	353.7	394.6	400.6	334.3	272.7	339.7	281.5	252.2
DHPS	359.9	415.7	372.5	379.2	264.9	289.4	332.6	233.3
DHPS	495.6	417.8	402	446.5	290.3	311.8	391.9	317
DHRS1	475.2	550	432.5	372.5	301.8	240.7	226.4	184.6
DHRS10	128.1	120.2	116.7	118.4	111.9	99.9	120.3	116.2
DHRS2	123.8	119.1	142.5	130.8	113.1	107.2	122.5	113.4
DHRS2	110.9	113.9	116.8	113.8	110	92.9	116.2	118.2
DHRS3	271.5	258.4	288.7	290.2	247.7	213.3	289.7	253.9
DHRS4	637.7	481.4	903.8	514.3	399.3	533.3	474	355.3
DHRS4L2	146.5	144.9	168.7	151.9	147.9	152	149.2	129.4
DHRS7	462.3	524.5	630.4	521	567.7	668.3	504.3	578.5
DHRS7B	146.6	129.7	152.6	132.3	121.1	129.9	146.2	131.6
DHRS8	211.3	258.1	350.9	259.8	237.3	254.8	204.4	235.2
DHRS9	238.9	155.2	188.9	684.5	249.1	1227.7	827.4	342.5
DHRS9	111.4	116.5	121.7	147.2	111.3	148.8	157.5	135.8
DHRS9	109.5	105.2	110.8	119.9	109.3	100	117.6	111.9
DHRS9	125.3	122	130.1	224.9	130.4	419.3	245.5	164.9
DHRSX	122.8	125.7	133.7	127.3	119.1	107.2	132.5	114.4
DHTKD1	129.2	125.7	137.4	142.3	130.9	110.3	142.8	125.5
DHX15	1394.4	1667.1	1380	1651.9	1636.5	1055.5	2042.1	2197.1
DHX16	635.9	662.7	522.8	617.8	453.8	460.3	535.9	482.5
DHX29	334.8	368.1	424.3	365	274.2	365.2	407	396.3
DHX30	409.5	514.2	369.1	416.5	369.4	371.2	550.3	456.3
DHX30	110.4	108.1	115.5	115.4	101.7	93.4	117.2	116.6
DHX30	215.2	207.1	175.8	196.7	153	154.1	206.5	208.3
DHX32	191.6	211.8	188.7	169.8	181.5	142.6	175.7	160.9
DHX33	197	218.3	202.7	212.4	217.6	177.2	200.6	193.2
DHX34	183.2	175.8	200.7	206.1	139.7	141.8	223.7	203
DHX35	170.1	174.5	179.6	152.8	140.4	146.6	151.6	146.6
DHX36	193.1	279	193.8	230.1	226.7	154.5	217.8	206.9
DHX37	325.9	424.3	269.4	323.9	240.8	182.3	270.7	256.7
DHX38	175.9	198.3	183.7	159.3	150.5	141.2	235.1	212.1
DHX40	304.6	297.9	231.4	277.7	253.5	239.2	300.6	412.1

DHX40	141	171.1	155.4	153.6	154.7	146.2	144.6	157.4
DHX57	121.8	124	127.9	114.2	110.6	92.7	130.7	115.1
DHX57	109.1	112.6	114.1	125.8	108.4	98.6	117.7	111.5
DHX8	147.8	155.5	147.1	153.5	138.2	132	142.4	157.6
DHX9	234.2	226.3	244.7	252	236.4	190.3	265.3	252.7
DIABLO	782	973.8	930.4	1050.7	773	930.2	976.9	1025.3
DIABLO	175.6	206.2	185.6	187.1	191.4	176	214.5	207.6
DIABLO	137.8	141	158.8	136.5	143.3	123.6	142.1	151.3
DIAPH1	213.6	214.6	179.2	186.8	200.5	188.7	253.2	250.3
DIAPH1	718.3	814.4	657	789.5	634.4	602.9	947.4	1110.1
DIAPH2	148.1	154.8	139.3	179.3	142.7	135.3	152.7	164.3
DIAPH3	117.6	124.9	130	119	118.3	100.7	119.7	125.8
DIAPH3	112.5	120.5	119.5	118.9	109.8	98.7	121.4	110.9
DIAPH3	109.5	112.7	121.4	118.3	113.4	105.6	124.5	108.6
DICER1	107.6	110.1	106.8	104	107.2	96.9	116.9	114.8
DICER1	121	118.7	127.1	130.1	112.3	103.7	116	126.8
DICER1	165.3	179.6	144.7	168.5	141.1	147.2	140.7	130.9
DIDO1	153.6	137.4	144.7	161.7	136.8	122.7	142.2	135.9
DIDO1	269.7	248.8	217.2	220.4	186.5	168.3	238.7	168.6
DIDO1	126.4	129.6	118.4	138.4	123	115	124.9	128
DIO1	117.3	111.4	120.4	116.4	107.8	94.6	128.4	112.7
DIO1	113.1	120.4	109.7	110.7	109.4	99.5	116.2	116.8
DIO1	109.1	101.9	114.1	105.4	108.5	102.2	119.7	115.3
DIO2	115.2	112.3	114.9	125.8	111.9	103.7	116.7	118.3
DIO2	137.5	114.8	116.1	120.5	105.2	90.5	115.7	111.7
DIO3	105.2	111.4	111.8	124	103.3	94.7	111.2	110.5
DIP	187.1	165.3	228.3	179.8	138.2	165	160	135.4
DIP13B	353.8	404.7	483.8	374	337.1	495.1	428.4	339
DIP2A	125.7	115	123.2	114.7	108.3	108.8	120.3	117.9
DIP2A	115.2	115.4	112.2	118.4	116	97.1	113.3	111.3
DIP2A	146	155.2	154.3	169.8	147.1	124.9	167.8	197.5
DIP2A	134.6	149.2	164.7	144.4	133.5	120.1	152.6	156.9
DIP2A	175.4	219.2	185.2	185.1	225.1	141.7	167	194.9
DIP2B	647.9	694.9	564.9	631.1	618.6	494.7	676.2	819.9
DIP2C	132.5	135.8	138.8	134.2	120.2	115.1	133.8	125
DIPA	118.6	119.7	113.5	123.4	113.4	114.1	124.3	122.8
DIRAS1	104.1	120.2	128.6	123.8	101.4	96.6	128.5	105
DIRAS2	112.2	114.4	115.3	117.3	109.6	102.8	114.7	115.9
DIRAS3	120.8	121.8	119.5	120.1	119.2	106.1	116.3	123
DIRC1	115.8	118.8	110.3	115.1	105.9	98.4	114.4	113.6
DIRC2	554.2	420.4	371.2	1003.2	450.4	730.4	789	280.2
DISC1	121.9	110.8	115.8	128.3	112.4	97.4	119.8	117
DISC1	112	113.2	110.6	125.6	112.8	106.6	124.8	115.8
DISC1	113.6	118.7	120.8	125.7	113	101.2	120.6	117.7
DISC1	126.4	124.9	108.8	120.2	106.6	94.5	123.2	106.5
DISP1	140.3	131	150.1	154.9	122.1	117.7	128	137.1
DISP2	119.1	112.7	109.1	124.3	107.7	102.4	120.6	122.1
DIXDC1	134.2	132	137.9	128.3	124.7	107	116.1	115.1
DIXDC1	103.6	116.4	110.8	117	103.2	99.4	121.4	109
DIXDC1	126.3	121.2	135.7	124	109.6	108	124.8	116.7
DJ122O8.2	422.6	472.2	510.8	395.5	429.2	308.7	323.9	304.7

DJ222E13.2	151	149.8	139.1	150	133.9	106.8	157.8	146.1
DKC1	975.4	1138.3	893.9	1094	805.2	646.3	1112.3	893.4
DKFZP434A	126.6	110	113.6	110.5	103.1	97	117.6	109.1
DKFZP434A	165.9	137.9	138.4	145.9	135	121.6	139.8	133.8
DKFZP434E	319.8	322.5	264	313.3	242.5	232.9	332.8	355.7
DKFZP434E	99.2	115	111.6	112.4	102.4	101.7	111.2	110.6
DKFZP434I	113.8	117	123.2	124.6	108.7	97.5	122	112.9
DKFZP434K	225.7	249.7	177.9	238.6	192.2	146.9	246.5	244.1
DKFZP434C	117.8	113.8	125.5	115.7	112.2	103.8	120.8	120
DKFZP434C	127.1	122.8	123.3	139.3	125	124	116.9	127.8
DKFZP547C	129.1	154.1	142.6	134.9	127.2	129.2	124.4	127.9
DKFZP547C	145.2	143.2	148.6	155.5	132.8	136.3	157.7	130.5
DKFZP547H	117	115.5	112.2	122.7	108.1	102.2	117	113.8
DKFZP564E	146.9	156.8	170.9	157.7	130.7	142.2	151.2	131.8
DKFZP564J	239.1	223.2	279.1	266.9	167.2	213.3	342.2	227.8
DKFZP564J	128.6	117.8	127.6	120.9	115.2	103.9	130.9	116.4
DKFZP564J	115.5	111.2	121.7	127.3	106.9	101.1	113.9	115.3
DKFZP564K	1069.4	798.8	936.4	935.2	925	527.7	998.6	1150.3
DKFZP564M	118.9	121.6	112.3	118.6	115.2	101.7	121.6	108.5
DKFZP564C	162.4	168.9	178	179.5	177.6	139.4	156	185.5
DKFZP564C	119.4	121.8	140.2	124.9	119.1	110.7	126	110.3
DKFZP566M	120.5	119.4	124.8	119.2	127.1	108.8	121.5	119
DKFZP586D	186.2	207.3	173.9	185.7	158.9	133	137.5	154
DKFZP586D	123.1	110.3	119.2	118.5	113.4	99.4	117.2	118.7
DKFZP586H	100.4	117.1	120.4	120.2	108.8	99.7	115.7	108.4
DKFZP586H	102.1	117.9	118.2	125.7	101.9	96.3	117.8	110.8
DKFZP586I	184.8	177.5	197.8	166.9	201.7	145.1	208.5	281.2
DKFZP586F	120	112.9	121.4	112.5	116.6	96.4	114.7	117.7
DKFZP666C	104.9	106.9	110.6	120.3	105.6	102.3	117	108.1
DKFZP667C	114.2	116.4	116.3	119.4	110	98	116.9	111
DKFZP686A	117	119.6	122.4	134.6	113	99.5	123.2	103.2
DKFZP686A	114.2	147.7	126.8	137.2	113.1	109.4	123	126.5
DKFZP686D	110.1	112.9	118.8	117.9	105.9	98.3	112.7	111.8
DKFZP686E	119.9	121.2	135.8	130.6	119.2	102.7	130.7	127.7
DKFZP686I	121.7	120.3	139.1	127.6	109.8	117.5	116.6	114.6
DKFZP686I	133.5	126	127.8	123.5	128.1	114.6	131.9	127.3
DKFZP686K	388.5	382.5	340.2	421.3	359.4	182.7	413.2	378.1
DKFZP686L	174.3	177.5	155.3	175.1	145.3	146.7	201.4	210
DKFZP761E	114	117	116.4	117.1	113.2	92.6	120.1	117.3
DKFZP761E	137.5	129.2	122.8	148.4	118.9	108.4	149.1	138.3
DKFZP761H	112.6	122.5	125.2	126.2	118.5	104.1	121.8	116
DKFZP761I	249.8	237	196.9	255.9	187.7	159.7	227	209.5
DKFZP762E	120.5	119.3	121.2	131.2	113	100.2	122.8	119.9
DKFZP762I	444	331.8	382.9	474.4	431.4	228.3	420.5	476
DKFZP779E	109.5	121.1	126.7	126.9	109.1	113.6	118.7	120.5
DKFZP779C	112.4	118.6	113.7	120.6	111.9	93.4	126.6	113.1
DKFZP779C	118.2	121.3	119.3	121.4	108.9	101.9	119.1	124.8
DKFZP781I	124.3	127.4	118.6	118.1	111.9	107.7	116.9	128
DKK1	108.9	111.9	115.1	116.2	103.7	99.3	108.1	114
DKK2	104.6	110.2	108.3	111.9	102	97.8	102.8	109.9
DKK3	114.6	119.8	123.4	128.2	114.1	106.6	116.8	126.4

DKK3	124.6	157.6	209.9	120.3	148	144.6	126	142.7
DKK3	108.9	117.1	117.8	112.5	101.7	102.4	116.2	112.4
DKK3	133.2	189.6	246.2	155.2	165.3	198.9	140.7	146.6
DKK4	115.9	118	126.6	129.2	110.7	100.9	126.2	119.6
DKKL1	117	120.3	127	131.9	111.5	102.1	124.3	114.6
DLAT	203.1	214.5	232.3	260.4	198.4	164.2	187.3	217.5
DLC1	114.9	122.7	126.2	125.3	105.9	103.9	126.5	122
DLC1	108.9	118.6	130.8	127.8	119.7	116.1	129.3	127
DLD	181.3	225.3	193.6	217.9	165.9	162.4	191.8	213.7
DLEC1	129.4	118.7	129.1	125.8	109.6	105	122.1	115.2
DLEC1	111.1	118.9	131.4	122	105.9	106.7	117.7	115.7
DLEU1	121.7	129.3	131.9	117.5	118	100.5	140.1	125.1
DLEU1	215	261.4	164.5	213.8	165.9	139.3	178.4	182.2
DLEU2L	113.2	108.2	108.6	124.5	104.6	99.7	123.8	107.8
DLEU7	113.2	117.7	118.3	114.2	114	108.9	117.9	109.8
DLG1	111.7	115.9	121.3	129.7	109.3	111	123.8	112.7
DLG2	109	113.8	113.1	123.5	106.7	99.1	114.5	113.7
DLG3	121.7	125.1	127.7	125.2	104.4	110	137.4	116.3
DLG3	119.3	112.1	119.5	114.2	107.9	96	110.3	119.8
DLG3	122.3	114.8	119.4	117.9	104	97.1	116.5	115.2
DLG4	136.2	135	127.2	144.2	117.7	105.7	132.2	127.1
DLG5	115.8	114.3	128.2	129.5	106.1	106.9	110.9	115.1
DLG7	112.5	122.9	125.1	138.2	112.1	108.4	123.2	118.5
DLGAP1	101.7	111.1	108	110	101	96	114.2	107.6
DLGAP1	126.8	130.4	122.2	121.7	104.6	99.8	128.6	119.3
DLGAP2	123.1	122.6	125.7	130.4	116.3	111.8	121.6	121.1
DLGAP3	121.7	113.2	121.8	118	116	106.5	123.7	118.2
DLGAP4	110.1	117	115.7	123.2	102.8	94.8	111.4	113
DLGAP4	116.1	111.9	111.7	116.3	108.6	101.3	113.8	114
DLGAP4	173.6	170.9	182.1	176.9	155.7	166.7	189.5	164.2
DLK1	108.4	114	119.9	117.2	104.8	97.8	111.8	110
DLK1	106.8	113.2	108.1	122.6	105.5	105.2	113.4	115.4
DLL1	124.1	126.1	134.1	138.5	128.8	104.5	118.9	127.4
DLL3	112.8	103.2	114.2	107.7	102	99.3	113.3	115.5
DLL3	110.1	122.5	118.6	123	111.3	102.5	119.4	117.7
DLL4	127	115.4	117.9	125.9	107.7	106.7	116.5	120
DLNB14	358.4	370.5	332.6	368.7	430	254.3	310.9	316.4
DLST	266.1	349.7	296.7	318.9	242.8	202	353.7	405.8
DLX1	120.6	125.9	127.8	118.8	107.4	103.3	125.5	131.2
DLX1	127.6	119.9	128.4	130.3	117.5	103.5	128.6	125.3
DLX2	127	112.8	116.1	115.3	108.4	98.1	113.7	110.6
DLX3	119.3	118.7	130.7	125.3	120.8	97.2	119.5	121.7
DLX4	118.3	115.6	118.1	105.8	102.4	100.7	122	113.8
DLX4	124.2	112.7	121.5	114.9	114.6	101.2	117.3	112
DLX4	104.4	116.3	117.9	121.8	101.9	103.2	107.9	115.5
DLX5	107.4	108.9	113.8	119.8	106.8	94.9	122.8	115.3
DLX6	116.3	110.1	112	121.4	105.5	95.5	120.1	112.1
DMAP1	505.4	479.1	400.6	446.8	330.7	316.8	377.2	403
DMAP1	304.8	341.7	351.5	322.7	241.4	224.7	260.1	283.6
DMBT1	114.5	112.8	124.2	116.6	112.7	101.9	118.2	112.3
DMBX1	118.5	131.9	116.8	124.2	110.5	98.2	121.5	119.2

DMC1	1680.1	1444	1627.2	1545.8	1302	954.6	1919	2153.3
DMD	119.9	113.8	123.6	113.2	108.2	95.2	120.5	121.6
DMD	107.1	122.7	107.2	110.3	111.7	99	112	110.9
DMD	141.9	157.3	158.9	177.9	149.2	134.7	154.6	141
DMD	114	116	122.3	111.5	110.7	103.1	114.7	121.5
DMD	102.4	108.9	109	115.3	105.7	96.9	112.8	116.4
DMD	113.5	120.4	114.2	121.4	102.9	98.5	124.5	115.3
DMD	126.1	121.8	125	132	110.4	112.9	133.9	125.4
DMD	113.9	125.9	123.6	129	116.6	98.6	115.3	113.4
DMGDH	116.4	120.2	115.6	122.2	108.9	111	119.8	115.7
DMN	172.9	146.3	119.8	159.7	145.4	121.9	181	152
DMN	116.3	117.4	116.4	118.3	109.8	100.1	123.9	107.1
DMP1	115.9	119.8	106	112.3	104.8	96.2	128.9	113.8
DMPK	109.6	119.3	112.3	119.3	109.8	98.2	113	112.3
DMPK	123	118	119.5	118.3	115.1	99	119.1	111.1
DMRT1	109.9	117.2	114.9	119.8	107.3	101.9	123	114.7
DMRT2	116.6	113.2	108.2	118.7	107.8	92.9	117.5	108.6
DMRT2	125.1	120.9	110.9	126	112.1	96.8	120.9	114.7
DMRT3	104.2	108.9	104.5	112.1	103.3	91.2	117.9	112.4
DMRTA1	113.2	116.1	117.9	116.9	111.5	103.4	129.4	120.6
DMRTB1	113.4	116.4	112.7	112.7	107.6	97.1	116.7	105.2
DMRTC2	118.9	114.3	120.1	110.5	107.6	97.5	120.3	127.5
DMTF1	458.6	451.9	422.8	452.4	440.4	322	433.2	500.5
DMWD	131.7	138.7	133.4	137.6	141.3	123.6	141.4	125.6
DMXL1	148.4	131	126.5	141.9	114.6	104.5	135.2	125.1
DMXL2	144.9	121.4	162.7	146.1	121.8	138.8	127.9	115.8
DNA2L	130.8	131.2	126	138.7	120.9	108.2	131.1	122.4
DNA2L	133.4	148.4	135.4	135.2	119.6	102.1	141	129.8
DNAH1	235.2	196.1	254.9	252.6	179.5	172.9	202.5	224.9
DNAH10	117.5	114.7	115.4	119.9	103.9	101.9	116.1	116.2
DNAH11	116.4	115.2	109.5	115.3	110.9	95.4	117.8	112.5
DNAH17	110.7	113.3	114.4	110.6	102	95.5	115.8	125.7
DNAH3	109.6	107.7	120.4	120.6	103.4	104.5	124.4	118.9
DNAH5	119.5	116.7	118.9	123.8	117.7	100.1	120	115.1
DNAH7	122.6	129.1	135.9	122.6	111.5	101.3	124.6	123.9
DNAH8	113.3	116.5	116.2	121.7	116.9	103.5	113.3	113.8
DNAH9	114.3	121.7	112.9	107	102.6	103.3	113.1	110.9
DNAH9	110.2	107.3	122.8	122.6	110.5	102	119	117.2
DNAH9	114.7	111.3	116.4	122	109	99.2	124.3	117.5
DNAHL1	110.9	112.3	126.1	112.5	108.4	103.3	119.4	112.7
DNAI1	109.9	121.4	113.1	113.5	104.5	93.6	117.9	116.1
DNAI2	135	155	130	141.3	120.9	115.5	142.8	146.2
DNAJA1	2311.9	2176.5	1887.2	3099.4	1747.2	1673.7	4599.6	7632.9
DNAJA2	636.5	828	565.7	885.6	663.7	551	939.6	818.2
DNAJA3	922.1	927	788.2	943.7	683.1	496.5	701	767.9
DNAJA4	267.4	269.2	263.5	289.5	304.5	243.2	711	1340.1
DNAJA5	133.8	132.4	135.4	141.4	125.7	109.8	131	128.5
DNAJA5	117.6	118.5	116	120.7	109.4	101	119.8	127.7
DNAJB1	579.2	664	486.9	639.4	583.5	510.9	1814.8	3471.5
DNAJB11	768.7	716.2	1098.6	898.7	672.4	995.3	848.2	896.9
DNAJB12	130.6	134.3	123.5	138.3	136.9	103.9	141	159.1

DNAJB12	160.6	174	171.8	172.9	146	118.1	193.4	161.4
DNAJB12	132	133.3	151.7	131.7	121.2	116.7	140	136.2
DNAJB13	114.4	119	124	121.9	111	105.5	127	126.6
DNAJB14	119.6	125.3	134.9	146.9	121.2	104.2	117.7	125.2
DNAJB14	311.5	248.6	247.2	339.9	283.8	196.9	287.7	316.6
DNAJB14	116.5	121.5	114.8	117	104.4	98.5	111.9	121.5
DNAJB2	122.2	129.7	125.3	136.7	116.7	109.6	129	124.5
DNAJB2	747.7	783.5	855.6	844.1	598.5	722.7	854.7	800
DNAJB4	129.7	122.7	128.2	136.5	121.4	113.8	169.7	175.7
DNAJB5	123.6	125.7	118.2	120.1	109.8	98.8	112.8	119.1
DNAJB5	126.2	133	126	130.8	116.8	95.6	137.3	122.7
DNAJB6	407.3	334.1	664.5	462.6	433.9	526.7	728.2	1106.1
DNAJB6	1102	983.1	974.4	1435.7	987.9	1172.6	3217.1	4181
DNAJB7	115.5	119.8	117.4	118	115.1	104.5	110.5	120.3
DNAJB8	134.4	128.8	132.2	127.2	116.1	114.5	115.1	130.9
DNAJB9	364.1	351.6	375.8	396.7	345.4	377.1	534.6	744.6
DNAJC1	119.8	124.7	127.4	126.4	118.3	106	116.1	122.5
DNAJC10	296.9	238.8	335.4	285.5	219.3	209	249.2	222.7
DNAJC11	119.5	121.1	131.9	114	119.1	110.2	126.2	124.5
DNAJC12	113.8	118.4	114.1	130	106.1	95.7	123.5	106.8
DNAJC12	110.8	114.6	110.4	114.9	99.6	92.5	117	108.3
DNAJC12	111.9	116.2	123.1	121.3	114.3	105.3	121.3	118.5
DNAJC13	190.7	217.8	168.3	225.7	163.8	146.2	216.1	184.7
DNAJC14	210.9	208.4	214.2	226.5	182.1	198.7	245.2	180.3
DNAJC15	174.4	200.6	257.5	199.6	158.5	176.2	197.5	205.5
DNAJC16	113.4	112.4	125.4	126.3	114.4	107.2	113.8	112
DNAJC17	151.4	155.2	151	158.4	131.7	129.3	142.8	140.6
DNAJC18	133.8	148	141	128.7	118.9	115.6	152.1	123.1
DNAJC19	156.7	152.3	175.3	186.4	143.8	131.9	153.7	133.2
DNAJC3	121.5	134.6	126	139.6	113.2	118.5	136.6	120.4
DNAJC4	122.4	127.3	124.1	122.7	113.3	113.9	125.2	114
DNAJC5	212.2	172.3	199.7	251	168.9	201.7	284.6	222.7
DNAJC5B	100.7	114.4	107.1	104.6	107.7	98.8	118.1	103.5
DNAJC5G	115.6	112.6	115.2	111	100	92.6	115.8	116.2
DNAJC6	117.1	111.5	114.3	113.7	106.1	93.3	116.4	115.7
DNAJC7	233.6	244.1	200.1	279.2	202.3	206	313.7	305.3
DNAJC8	2746.1	3258.2	2887.1	3336.5	2673	1976.9	3305.3	4235.3
DNAJC9	548.2	688.6	462.4	652.7	496.1	355	597.3	611.8
DNAL1	121	115.5	112.7	125.3	116.3	104	115.9	111
DNAL4	223.6	258.6	314.7	239.4	227	283	249.1	256.3
DNALI1	121.6	111.9	120.6	125.5	114.5	105.6	116.2	125.9
DNAPTP6	138.2	141.8	137.7	128.2	142.4	124.2	147.6	151.2
DNASE1	121.9	124.5	128.3	115.7	111.1	101.3	124.1	120.2
DNASE1L1	207.4	209	266.1	211.7	166.7	226.4	170.4	139.1
DNASE1L1	115.1	112.6	119.8	113.4	109.4	93.5	116.9	119.6
DNASE1L1	123.8	124.1	140.3	134.2	105.7	116	118.6	115.3
DNASE1L1	118.8	121.6	113.2	123.9	108.8	100.3	125.2	118.2
DNASE1L2	118.3	118.9	120.9	128.9	109.8	96.8	125.3	125.6
DNASE1L3	124.7	123.6	121.8	122.6	121.3	102	111.2	116.9
DNASE2	517.7	508.5	968.1	498.3	440.6	446.4	372	316.6
DNASE2B	111	115.1	117	126.9	110.3	100.8	120.8	120.2

DNASE2B	110.1	111.5	112.3	115.8	106.9	94.3	116.6	109.6
DNASE2B	112	117	114.1	114.2	105.4	95.4	112.4	117.2
DNCL2A	212.6	229.9	280.5	227.6	219.1	325.3	323.7	305.5
DNCL2A	156.9	173.4	213.9	185.6	168	173.7	160	180.8
DND1	115.4	120.4	117.6	129.6	113	97.9	118.1	113.6
DNER	117.9	113.1	121.8	114.9	106.5	99.5	124.2	119.2
DNHD1	171.2	201.2	150.5	179	129.4	125.1	149.9	151.6
DNHD2	112.4	114.8	128.4	120.8	105.4	100	118.2	111.7
DNHD3	110	114.9	116.3	116.9	105.5	99	123.7	118.4
DNM1	117.1	112.1	112.2	118	100.3	97.9	122.4	109.8
DNM1L	325.4	347.2	386.2	412.1	374.8	250.3	412.6	536.9
DNM1L	135.6	117.3	135	145.3	132.1	121.3	143	141.4
DNM2	167.5	189.1	218.6	175.1	141.6	162.4	207.6	175.1
DNM2	240.1	212.2	255.3	252.4	180.5	181.1	244.8	215.8
DNM3	115.4	117.6	123	120.5	111.5	100.5	119.1	119.5
DNMBP	115.3	119.7	117.1	127.7	114.3	97.1	129.1	120.1
DNMT1	2539.1	2465.2	2638.8	2136.3	1613.6	1669.8	1981.7	1641.8
DNMT2	124.5	127.9	133.3	125.4	112.8	103.3	118.7	119.9
DNMT3A	121.3	153.9	122.1	138.4	120.5	111.7	144.2	128.2
DNMT3A	120	126.8	134.9	136.6	108.5	111.3	120.6	132.8
DNMT3A	122.7	127	130.2	117.8	118	96.7	133.4	116.2
DNMT3B	128.7	129	129	124.9	119.6	97.1	126.3	129
DNMT3B	106.1	105.5	111.5	110.2	101.6	96.1	109.6	117.4
DNMT3B	116.2	115.6	115.3	106.3	103.1	100.1	118.9	115.3
DNMT3L	119.7	115.3	121.7	112.3	111	97.9	122.9	112.1
DNMT3L	120.1	124.3	116.7	129.1	114.1	98.7	125	121.5
DNPEP	138.7	139.2	158.5	152.4	127.4	127.6	153.6	139.5
DNTT	131.1	120.4	115.9	125.6	129.8	102.3	124.8	117.5
DNTT	119.5	115.2	116.4	116.8	108.7	106.9	130.6	115.6
DNTTIP1	163.9	164	196.9	162.2	163.3	157.3	174.6	160.2
DNTTIP2	124.3	126.3	113.7	125.3	116.3	115.4	153	159.8
DOC1	121.3	121.1	131.2	134.5	113.8	105.3	131.9	107.6
DOC1	127	121.4	125.9	136.3	118.7	109.7	134.8	110.2
DOC1	116.9	115.2	122.3	118.8	105.5	96.4	125.9	117.6
DOC2A	118.7	127.2	117.6	124.1	112.9	99.2	120.5	116.8
DOC2B	121.1	115.7	112.3	119.7	114.9	97.1	123.3	113.6
DOCK1	123.7	122.9	131.9	122	109.5	106.6	119.6	120
DOCK10	1253.9	1165.9	1307.6	1051	1096.9	874.5	1050.7	1004.1
DOCK11	745.1	614.5	483.5	740.8	832.6	534.9	848.8	1043.9
DOCK2	1053.8	1078	1091.4	958.2	754.3	657.7	659.6	656.4
DOCK3	114.5	110.3	109.6	119.3	111.9	92.7	109	115
DOCK4	123.6	116.6	122.5	125.1	124.1	106.1	121.3	114.1
DOCK5	114.1	117.8	138	128.3	107.5	116.5	129.2	111.7
DOCK6	112	109.1	115.6	115.8	106.6	97.3	121.6	109.6
DOCK7	102.1	107.9	122.1	114.8	111	96.5	107	121.3
DOCK8	699.4	653.1	760.5	737.9	531.1	548.8	582.1	497
DOCK9	163	169	161.4	177	148.8	143.2	147.2	157.6
DOK1	122.4	128.5	137.3	140.1	109	104.5	136	114.1
DOK2	111.7	114.7	119.2	111.6	103.3	96.5	115.9	102.3
DOK2	137	123	158.3	140.6	116.4	126.1	138.9	138.2
DOK3	115.7	114.6	130.7	117.8	106.2	112.8	122.5	110.6

DOK4	114.9	123.3	117.8	116	120.1	100.6	121.2	121.8
DOK5	113.8	115	112.7	113.8	108.5	105.1	113.5	117.6
DOK6	113.6	117.2	117.4	127.3	106.3	95.7	115.6	108.1
DOLPP1	170.1	193.2	172.7	165.2	145.4	138.7	158.6	168.1
DOM3Z	237.7	260.5	213.4	227.1	207	173.8	205.8	177.5
DONSON	164.8	142	169.7	170.3	155.4	144.3	169	224.7
DONSON	116.7	118.9	116.6	123.5	114.1	104.3	121.7	125.5
DOPEY1	142.7	128.9	139.7	144.8	129	110.7	144.1	134.4
DOPEY2	330	254.4	343.5	315.7	311.4	207	349.6	392.3
DOT1L	116.5	123.4	126.9	127.6	121.6	107.8	122.8	124.1
DP58	117.5	111.3	123.7	119.7	113.9	95.6	119.8	113.6
DPAGT1	123.5	135.6	135.8	126.9	113.8	116.2	119.9	127.2
DPAGT1	286.6	320.4	321.3	255.2	236.4	195.6	254	211.6
DPCR1	118.7	121.1	121.3	109.8	116.8	106	118.6	116.1
DPEP1	116.4	118.6	121.1	120.4	112.3	101.1	113.1	112.5
DPEP2	606.5	517.4	692.7	523.7	313.4	410.1	291	312.7
DPEP3	163.3	146.5	138.6	161.1	142.1	122.2	155	163.6
DPF1	114.8	125	118.7	124.8	110.4	114.9	132.9	127
DPF2	425.3	408	436	490.5	305.3	333.2	480.7	389.9
DPF3	118.1	124.9	118.7	130	110.8	108.4	120.2	126.7
DPH1	120.5	120	122.9	128.6	117.3	103.5	127.9	122.7
DPH2	119.7	138.3	123.3	132.6	117.5	99.3	130.9	135.4
DPH2	113.7	128.3	121.4	116.3	111	98.3	127.6	120.5
DPH2	352	343.7	294.8	335.4	260.6	205.8	356.8	308.6
DPH3	169.2	199.4	219.4	208.5	156.6	163.5	169.4	178.5
DPH3	143.8	140.6	133.3	146.4	110	135.6	128.1	148.9
DPH5	295.2	379.3	453	331	348.3	313.7	266.2	353.6
DPH5	233	231.4	314.3	249.8	197.7	211.3	233	233.2
DPM1	565.8	493.4	758.3	643.6	490.8	506.3	569.3	633.2
DPM2	150.8	142.4	151.9	146.6	140.3	124.3	149.6	157.1
DPM3	190.6	189	251.2	176.5	174.2	237.2	152.4	180.2
DPM3	307.3	323.4	438.1	305.3	290.5	358.3	221.9	201.9
DPP10	122.9	111.1	122.1	117.4	109.2	95.7	117.7	118.1
DPP10	131.6	113.4	118	123.3	108	98.2	111.5	121.4
DPP3	136.2	139.5	139	135.7	124	114.3	151.3	133.9
DPP3	135	122.4	132.7	121.8	113.8	99.4	123.7	120.7
DPP3	163.1	168.8	181.4	171.9	154.9	142.6	156	168.1
DPP4	131.1	146.2	157.6	148.4	141.4	141.9	144.5	133.8
DPP6	115.9	132	127.1	137.6	122.4	114.1	118.1	114.8
DPP6	107.6	106.1	100.5	121.3	106.4	96.9	120.3	108.9
DPP7	176.7	172.5	225.6	181.7	158.3	164.6	185.5	179.2
DPP7	294.8	328.7	331.3	310.1	242.4	189	267.8	291.8
DPP8	178.6	193.7	156.5	187.5	140.7	134.4	170.9	165
DPP8	254.7	191.2	253.5	264.8	239.5	200.2	255.7	257.1
DPP8	112.6	115.5	135.3	128.8	114.2	109.3	115.7	128.8
DPP9	349.8	369.1	318.9	397.5	263.4	262.5	373.8	371.1
DPPA2	113.7	116.5	134.4	125.2	111.8	97.6	121.7	116.5
DPPA3	115.8	122.1	121.7	116.2	106	100.4	122.5	117.3
DPPA4	110	114.7	117.2	120.4	112.5	101.5	120.3	113.3
DPPA5	110.9	110.6	121.2	109.6	112.3	96.2	119.3	108.2
DPRX	120.9	118.3	113.6	107.7	106.3	94.4	114.8	112.7

DPRXP4	118.1	108.6	126.5	116	110.8	102.7	127.9	118.4
DPT	120.4	124.5	135.2	123.2	110.8	114.1	133.5	123.8
DPY19L2	105.8	113.7	116.9	116.6	106.6	96	119.3	107.9
DPY19L3	113.1	107.5	129.8	123.7	105.5	102.6	120.3	111.2
DPY19L4	117.1	143.4	128.6	131.9	124.5	119.9	143.5	126.7
DPYD	198.6	175.7	225.8	196.9	187.2	171.1	191.2	145.6
DPYS	111.9	113.6	118.9	119.8	101.6	101.8	110.5	111.2
DPYSL2	758	668.6	1169.8	568.6	400.6	296	357.6	268.4
DPYSL3	172.1	341.7	119	227.7	336.1	107.7	282.4	259
DPYSL4	120.5	174.5	116.6	117	145.6	94.7	129.2	202.5
DPYSL5	113.8	121.5	120	121	117.6	103.4	127.6	119.9
DQX1	123.1	111	119.9	113.3	105.6	104.9	123.2	117.5
DR1	211.6	209.9	229	240.4	216	178.6	268	227.6
DRAP1	855.4	1019.2	1058.2	979.9	765.7	1121.6	1190.2	997.6
DRB1	173.2	203.3	202.7	196	173.2	143.6	145.2	137.6
DRD1	120	126.9	112.5	112.2	106.9	106.7	122.2	121.7
DRD1IP	116.2	138.4	151	144.9	121.8	110.5	127.5	123.4
DRD2	113.4	117.3	116.2	127.3	106.9	93.5	116.8	112.1
DRD2	116.3	115.9	114.8	110	112.1	106.5	122.7	124.2
DRD2	130.2	109.6	142.8	124.5	116.9	108.7	117.5	115.3
DRD3	112.1	116.7	116.1	122.9	115.9	103.4	121.5	117.3
DRD3	119.1	114.6	116.6	123	110.7	104.5	107.1	119.8
DRD3	102.2	107.2	120	112.1	112.3	99.2	105.9	118.5
DRD4	119	109.2	129.1	131.1	118.3	112.4	130.4	157.5
DRD5	113.3	113.6	117.7	116.9	108	99.7	117.5	114
DREV1	142.5	162.4	159.5	175.7	166.3	149.4	162.3	174.1
DRG1	355.6	344.7	387.1	362	346	313.7	307.7	329.2
DRG2	196.4	184.6	195.7	204	159.6	157.8	185.6	181.7
DRP2	103.6	105.4	115.5	116.1	110.1	92.4	113.5	111.7
DSC1	145.1	179.8	142.8	148.8	179.5	110.3	128.1	142.3
DSC2	138.2	119.2	140.5	121	109.2	101.8	123.8	115.5
DSC2	120.4	122.5	114.1	117.2	104.4	108.2	118.9	117.1
DSC3	105.2	112.7	112.3	114	103.3	97.1	116.1	110.4
DSCAM	135.2	156.4	140	137.8	134.3	125.7	156	139.5
DSCAM	123	116.6	141.6	123.1	117.1	105.2	136.1	127.9
DSCAM	122.4	122.9	129.8	129.3	112.2	109.4	124.4	116.7
DSCAML1	111.9	115	113.1	109.5	110.7	104.1	105.6	116.7
DSCR1	120.6	123.7	113	132	112.4	102	128.6	112.6
DSCR1	108.8	116.1	116.4	118.8	113	91.6	114.1	113.4
DSCR1	172	208	187.7	179.7	170.3	152	160.7	155
DSCR10	113.7	126.7	126.4	121.9	107.8	103.9	120.6	115.6
DSCR1L1	110.7	120.6	111.4	116.9	104.8	101.1	119.9	112.3
DSCR1L2	158.2	137.9	139.9	148.5	136.5	120.3	140.9	143.1
DSCR2	196	210.7	221.9	209.5	195.9	167	220.2	217.2
DSCR2	186.3	200.3	202.2	204.5	184	167.3	194.9	212.8
DSCR3	432.9	329.6	348.4	417.7	412.8	349.2	500.9	428.4
DSCR4	117.7	124.8	124.6	128.6	119.7	120.7	132.3	124.1
DSCR5	161	199.4	175.3	177.7	198.8	145.5	160.4	191.8
DSCR5	317.9	439.9	492.8	346.1	429.9	333.4	321	442.5
DSCR5	119.9	122.2	120.1	120.2	110	105.7	126.1	113
DSCR6	111.1	118	126.4	131.3	113.3	100	124	124.1

DSCR8	109.8	120.9	113.9	117.9	112.5	97.5	117.6	112.6
DSCR8	112.8	121.9	127.7	126.5	113.5	99.6	121.4	115.1
DSCR8	113.1	108.4	114	111.3	107	96.6	112	112.5
DSCR9	111.3	119.2	121.1	118.6	105.4	95.6	109	108.8
DSEL	127.6	130.4	135.3	142.9	108.2	109.3	128.9	150.1
DSG1	121.4	123.2	132.8	120.1	122.3	105.2	121.9	120.8
DSG2	110.2	120.9	116.6	123.8	103.9	95	108.4	105.5
DSG3	112.5	114.7	120.8	111.2	117.3	96	120.5	117
DSG4	120	117.8	113.9	124.8	112.6	104.5	121.9	115.8
DSP	117.9	118.1	114.9	114.9	107.3	102.4	122.7	113.3
DSPG3	106.4	119.1	112.9	109.4	109	95.3	113.8	104.5
DSPP	112	114.4	120.3	114	107.2	101.8	130.2	111.7
DST	116.9	125.7	116	135.6	113.9	101.1	122.3	130.2
DST	112.6	113.4	112.7	121.1	111	101.3	120.2	113.9
DST	122	119.1	118.2	122.2	106	98.5	116.9	109.1
DST	116.7	110.5	116.9	112	107.4	98.6	117.6	118.3
DST	106.5	113.4	118.5	110.2	109.2	97.6	121	122.5
DSTN	318.6	369.4	361.6	276.8	268	311.9	262.4	293.4
DSTN	128	130.3	121.5	133.4	118.8	111.4	119.5	117.9
DSTN	239.8	303.7	292.5	248.8	294.1	258.7	262.5	253.2
DSU	119.6	120.4	120.3	128.6	110.2	94	120.9	117.6
DTL	107.6	107.9	109.4	112.7	108.9	103.7	117.1	117.9
DTNA	120	111.9	112.4	112.2	115.4	94.9	114.8	115.6
DTNA	112.8	123.4	121.7	122.4	112.3	108.8	130.4	116
DTNA	110.7	119.3	113.8	127.5	105.6	92.6	111.6	116.5
DTNB	109.3	122	117.8	125	116.2	99.4	119.1	112.4
DTNBP1	128.7	130.1	113.1	127.4	115	96.3	132.6	134.3
DTNBP1	170	178.9	162.3	163.2	151.6	129.1	183.5	165.6
DTNBP1	127.1	135.4	125.5	131.9	120.7	100.1	122	123.6
DTWD1	118.6	113.3	121.3	119	111.4	106.6	114.8	117.3
DTWD2	549	386.7	493.9	609.1	658.9	293.4	637.8	736.7
DTX1	113.4	117.2	126.6	133.4	118.9	110.5	127.8	127.2
DTX2	495.6	478.1	473.2	539.8	324	341.6	444.8	377.4
DTX3	142.9	175.2	145.2	144.4	118.3	115.5	164.2	142.2
DTX3L	145.3	142.9	160.4	153.1	118.5	114	134.5	138.9
DTYMK	122.4	121.4	114.5	121.8	108.8	103.5	116.9	115
DUB3	113.3	119.6	114.4	121.8	111.4	99.2	114	118
DULLARD	204	185.8	162.1	193.9	149.7	153.4	207.6	208.4
DUOX1	122.5	115	123.8	122	107.3	97.6	114.4	122.6
DUOX1	136.3	130.1	114.6	124.6	117.6	109.9	127.7	127.7
DUOX1	113.7	110.8	110.8	112.4	103	98.5	107.2	111.6
DUOX2	110.4	114.7	114.1	109.1	109.1	98.8	119.7	109
DUPD1	119	120.2	116	124.3	100.5	95.7	112.1	114.1
DUS1L	161.6	166.8	162.2	140	145.1	126.1	151.4	140.7
DUS2L	159.5	180	214.5	167.6	173.1	129.7	165.3	146.8
DUS3L	260	342.6	271.7	307.8	239.5	198.2	274.2	261.9
DUS4L	152	153.6	201.4	159.6	137.2	139.9	155.2	153.1
DUSP1	365.2	305.3	362.8	598.6	422.7	619.9	968.1	1131
DUSP10	117.5	119.3	128	127.9	116.6	107.2	117.8	123.6
DUSP10	115.9	104.4	114.6	124.7	113.1	110.1	115.7	107.8
DUSP10	162.4	133.4	164.7	153.7	128.9	134.1	183.6	221.7

DUSP11	128.8	144.5	151.6	143.8	130.5	114.9	140.6	146.2
DUSP12	497.3	636.8	672.7	576.5	417.2	431.3	440.1	525.9
DUSP13	111	116.9	114	123	113	103.9	115.4	112.7
DUSP13	115.5	105.2	113.2	122.3	110	92.5	114.1	116.3
DUSP13	120.4	125.7	117.6	126.8	110.7	105.4	119.5	118.5
DUSP13	114.7	115.6	122.7	124.4	113.4	105.9	121.2	108.2
DUSP14	215.9	219.2	180.4	239.8	185.6	143.3	226.2	236.6
DUSP15	113.1	118	115.5	119.2	113.8	102.3	113.5	116.2
DUSP15	114.9	106	113.4	119.5	99.2	94.7	105.9	110.4
DUSP15	114.4	119.3	127.7	121.5	113.3	108.1	115.3	117.9
DUSP16	153.1	146.4	145.5	145.1	147.4	116.8	186.4	166.1
DUSP18	199	205.2	194.2	242	179.9	192.8	193.5	172.5
DUSP19	2017.9	1873.1	1919.3	1497.5	2274.2	1529	2025.1	2385
DUSP2	217.2	210.5	160.8	243.3	153.9	146.5	238.3	208.5
DUSP21	118.5	121.8	114.4	113.4	108.3	108.5	127.3	113.2
DUSP22	129.9	133.3	189	135.2	114.4	140.4	128.4	141.4
DUSP22	304.5	377.2	414.8	336	306.2	306.5	312.3	367.6
DUSP23	426.4	530	790.6	415.7	331.4	325.4	379.9	309.4
DUSP26	116.5	130	126.5	126.2	107.8	101.4	122.3	126.2
DUSP27	118.4	129.7	131.3	127.8	120.3	109.7	128.6	132.2
DUSP27	115.9	123.1	124.4	123.5	121.7	103.4	127.9	117
DUSP3	375.6	286.6	573	367.9	233.5	373.8	399.9	451
DUSP4	111.8	108.3	105.7	108.8	98.9	92.5	111.8	116.8
DUSP4	114.6	110.5	116.8	127.3	105.8	94.7	121.1	119.2
DUSP5	263.4	186.2	184.4	276.3	189.2	211.7	400.6	330
DUSP5P	119.4	121.3	121.2	120.7	115.4	101.4	127.3	111.9
DUSP6	217.1	154.9	281.4	195.7	176.6	246.3	207.9	146.7
DUSP6	149.1	133.1	186.7	187.2	138.6	216.8	162.5	125.5
DUSP7	120.5	113.6	125.5	125.6	106.6	104.7	113.8	123.5
DUSP8	142.6	155.6	148.2	158.7	134.4	113.3	180.9	210.5
DUSP9	110.1	128.8	118.8	123.7	105.7	102.8	126.2	117.6
DUT	121.8	131	154	141.8	117.8	113.7	124.1	133
DUT	137.7	140.8	124.8	142.8	124.8	125.3	124.5	137.3
DUT	145.5	139.6	160.6	164.2	139.1	130.8	134.9	158.4
DUX1	113.2	108.8	122	121.9	112.5	100.6	119	108.4
DUX2	116.7	122.2	128.8	120.8	116.1	105.9	119.4	130.7
DUX3	114.7	123.4	120.9	118	106.5	97.1	127.9	113.2
DUX4	112.1	110.5	117.5	119.2	111	102.9	115.4	120.5
DUX4C	114.2	123.6	116.1	109.3	114.3	102.3	115.6	115.7
DUX5	115.6	109.7	110.7	128.1	109.7	116.4	120.6	118.2
DUXA	104.9	112.3	117.4	120.2	108.3	102.7	106.6	112
DUXAP3	677.1	630.8	682.8	658	923.2	518.2	650.1	688.7
DVL1	120.4	124.1	114.3	127.7	112.1	104.8	122.9	126.4
DVL2	219.4	239.9	208.9	225.2	187.7	175.9	208.8	233.4
DVL3	203.9	231.7	267.4	224.1	219.6	222.6	226.3	261.8
DXS9879E	548.1	691.8	659.3	572.5	398.4	409.7	475.6	553.7
DYDC1	112.9	125.4	115.6	116.6	107	100	119.9	115.3
DYM	400.7	443.2	403	411.9	359.5	349.1	385.5	344.1
DYNC1H1	310.9	280.7	193.7	314.2	225.6	300.2	297	295.2
DYNC1I1	106.5	111.5	115.7	115.5	110.1	92	107.3	112.6
DYNC1I2	485.9	482.2	480	432.1	372.7	434.9	341.1	338.4

DYNC1LI1	120	129.4	138.6	132.1	125	109.6	143.4	150.8
DYNC1LI2	936.8	1029.8	989.2	1083.2	1034.7	771	938.2	970.9
DYNC2H1	116.2	135.2	132.3	121.2	121.5	109.2	132.7	146.3
DYNC2LI1	115.5	108.8	110.1	114.9	106.7	100.2	114.4	109.8
DYNC2LI1	132.3	142.2	161.2	136.6	125.1	112.5	118.4	113.4
DYNC2LI1	115.3	103.7	114.7	114	107.9	100.9	117.7	104.7
DYNLL1	1306.8	1014.3	1498.7	1331.3	873.6	1304.7	1097.8	963.2
DYNLL2	192.7	237.9	218.2	209.7	213.6	157.8	184.5	213.2
DYNLRB1	435.8	427.3	502	389.1	364.1	430	433.3	460.8
DYNLRB2	135.2	139.8	138.3	145.9	125.4	118.1	139.6	135
DYNLT1	448.1	411	473.9	471.5	405.5	534	423.8	371.1
DYNLT3	130.7	119	123.3	117.9	107	95.5	120.5	112.5
DYRK1A	322.7	411.8	346	394.6	283.6	314.1	345.8	458.8
DYRK1A	113.7	125.7	124.2	127.1	115.3	100.5	133.5	115.2
DYRK1A	411.7	511.1	336.9	432.9	422.6	384	501.7	539.2
DYRK1A	114.4	122.5	117.5	126.4	112	111.3	120.9	123.1
DYRK1B	136	136.9	124.9	145.3	132.5	118.9	149.5	140.5
DYRK1B	140.6	139.7	129.6	139.2	116.3	115.2	138.5	125.7
DYRK2	947	890.8	876.7	1004.9	839.5	443.6	800.8	733.5
DYRK2	119.2	121.3	126.5	136.1	113.9	103.3	135.2	115.5
DYRK3	118.6	118.1	122.5	116.7	104.7	103.2	116.7	108.5
DYRK3	122.5	115.6	106.3	118	103.6	94.1	114.8	113.6
DYRK3	102	108.1	110	107.9	99.9	90.1	115.2	108.7
DYRK4	182.9	194.4	227.4	168.7	158.4	147	157.1	131.8
DYSF	130	117.2	127.7	134	111.1	104.8	130.3	117.3
DYSFIP1	121.5	119.1	130	125.9	106.9	103.1	124.6	120.1
DYX1C1	108.1	109.6	119.1	116.1	106.3	103.8	131.9	111.9
DYX1C1	115.9	115	120.6	118.7	110.6	102.9	119.5	112.4
DZIP1	115.7	115.4	117.7	121.7	112.6	98.5	112.7	114
DZIP1	111.1	114.2	110.1	119.2	106.5	93.8	117.8	113.3
DZIP1L	121.8	124.2	133.2	122.5	111.5	104.1	123.2	120.6
DZIP3	115.3	116.9	114.5	116.8	107.1	107.3	121.1	124.8
E2F1	114	116.6	124.2	123.9	108.2	100.8	128.1	117.7
E2F2	143.6	149.2	146.1	144.8	111.6	105	133.9	132.9
E2F3	1237.9	1297.5	1043.6	1377.1	1085.1	906	1454.3	1198.1
E2F4	451.9	483.6	448.8	469.7	342	345.8	427.5	383.9
E2F5	250.1	224.5	251.8	267.1	202.5	191.9	247.4	170
E2F6	121.1	120.7	127.8	133.9	121.7	106.7	131.1	121
E2F6	263.2	284	207.4	330	279.5	226.2	475.8	584.1
E2F7	124.2	114.5	120.2	118.1	103.1	98.3	119.4	112
E2F8	116	113.4	117.1	120.3	113	105.4	109	109.6
E4F1	550.5	681.3	632.8	660.5	589.3	376.3	555.2	459.3
EAF1	142.7	155.2	140	140.1	131.8	131.8	164.3	127.9
EAF2	149.5	136.9	159.9	150.1	124	108.8	152.9	154.8
EBAG9	603.4	722	752.3	816.3	743.2	558	604	752.7
EBAG9	133.2	133.8	119	134.3	128	104.2	134.7	124.1
EBAG9	120	128.7	117.8	122.3	116.2	105.9	117.2	128.4
EBF	129.2	141.8	144.7	150.1	124.7	114	139.7	129.8
EBF2	110.9	111	111.9	115.4	100.6	97.7	108.7	117.7
EBF3	113.7	119.8	109.9	125.6	105.6	100	117.4	119.2
EBI2	359.4	527.4	532.2	637.3	368.6	505.6	603.6	587.6

EBI3	124.4	125.5	119.3	139.9	118.1	104.9	120.2	121.4
EBNA1BP2	435.3	441.6	559.4	467.7	430.3	393.4	400.8	355.9
EBP	193.4	217.1	200.1	180.8	196	161.6	181.2	178.3
ECAT1	117.6	105.8	112	111	112.9	98.3	109.4	107.7
ECAT11	123	107.5	141.7	120.6	131.8	114.1	126.7	124
ECD	160.4	154.5	141	159.8	143.6	154.1	172.8	139.5
ECE1	117.6	127.1	117.5	116.2	104.6	104	130	124
ECE2	130.6	130.8	135.6	124.8	125.7	115.4	122.8	119
ECE2	115.3	116	110.9	113.5	106.8	94.6	114.5	118.2
ECE2	123.6	111.1	114.3	116.4	114.2	102.4	121.9	123
ECEL1	115.6	116.3	116.2	111.4	111.8	98.7	116.8	107.6
ECGF1	1611	405.7	4170	1416.9	342.8	2314	661.3	243.9
ECH1	995.9	1301.6	1095.5	1038.6	894.3	819.2	650.1	657.9
ECHDC1	178.9	177	168.5	214.3	174.7	133.2	157.7	146
ECHDC2	366.9	505.4	775.5	455.5	378.2	424.1	341.8	320.6
ECHDC3	134.4	109.8	137.6	132.4	106	110.3	122.2	111.7
ECHS1	289.6	337.7	382.4	314	230.3	242.3	260.6	226.3
ECM1	120.8	114.1	118.6	126.8	109.4	103.9	118.9	126.8
ECM1	120.8	132.6	143.9	137	131.3	106.8	122.8	125.4
ECM2	108.6	110	113.9	122.9	111.9	101.9	120.6	119.4
ECOP	191.3	192.8	229.1	207.6	176	210.3	155.8	133
ECRG4	125.3	125.3	133.5	123.6	130.8	118	128.3	125
ECSIT	123.5	134.9	135.4	128.1	108.5	102.4	128.3	116.1
EDA	121.8	142.2	119.9	126.2	114.3	100.9	124.1	118.2
EDA	115	111.4	117.5	112.7	107.6	99.4	116.2	119.3
EDA	104.5	123.9	116	115.2	105.8	96.6	118.9	120.4
EDA	120.9	115.9	118.5	122.1	108.6	101.8	123.4	117.7
EDA	105.6	115.2	114.4	122.2	107.9	99.7	119.5	122.9
EDA2R	114.1	106.5	115.8	116.9	106.9	95.5	117.1	110
EDAR	155.9	159	152.5	163.9	140.7	137.2	156.5	143.9
EDARADD	116.4	113.6	119	113.4	106.6	99.7	112.4	121.3
EDARADD	133.9	159.3	136.6	151.2	135.4	109.3	127.3	143.2
EDARADD	121.9	119.8	124.7	114	103.3	103.5	123.6	111.9
EDD1	222.1	173.2	172.8	208.1	199.1	175.6	242	343.6
EDEM1	178.5	214.3	175.7	189.4	199.5	162.4	180.3	240.9
EDEM3	201.6	217.3	208.1	259.1	172.9	175.8	225.9	244
EDF1	314.6	318.3	347.9	302.7	246.3	250.7	206.5	310.5
EDF1	1672.5	2169.2	2329.8	2068.8	1810.2	2547.5	1655.3	1775.9
EDG1	443.2	560.3	483	660.8	554.2	713.1	521.5	571
EDG2	153.1	140.7	156.2	161.7	123.5	219.6	142.3	110.6
EDG2	113.6	114.7	129.1	119.8	107.9	99.6	114.9	114.6
EDG3	113.4	124	117.5	137.7	132.5	104	244.1	134.6
EDG4	238.2	228.9	307.4	266	191.7	269.7	239.4	301.4
EDG5	116.7	119.5	122.1	120	109	100.6	128.2	110.6
EDG6	953.8	1171.4	724.4	1013.8	680.3	607.3	839.9	716
EDG7	115.6	122.8	121.3	123.2	110.4	94.4	120.9	116.2
EDG8	388.9	455.3	260.9	416	387.6	210.1	330.8	261.4
EDIL3	113.8	112.2	130.3	123.9	109.7	111.7	118.6	122
EDN1	118.7	138.8	146.3	132.3	123.7	109.6	129.7	120.7
EDN2	107	114	108	111.5	110.4	92.9	121.8	104.8
EDN3	117.6	121.8	117.6	121.7	110.5	100.2	118.1	125.2

EDN3	112.2	112.7	120.3	116.2	112.2	97.5	111.8	120.1
EDNRA	108.3	110.3	107.3	116	109	98.1	111.9	107
EDNRB	124	130.3	145.3	141.3	114.2	248.3	138.7	136.4
EDNRB	112.9	109.5	115.7	119.2	110.2	108.6	128	112.8
EDNRB	107.4	115.2	119.1	112.2	115	94.7	113.4	114.7
EEA1	126.6	125.5	125.1	121.7	118.8	95.6	125.7	129.4
EED	120.2	121.8	124.2	125.2	123.5	112.6	133.7	128.3
EED	179.9	206.7	215.9	214.3	156.6	175.8	205.1	260.2
EED	125.7	133.3	118.6	131.6	129.1	125.2	144.2	132.6
EEF1A1	33711	35629.4	34130	35477.3	31777.4	30100.2	39463.4	35595.2
EEF1A1	18359.5	25334.6	23556.3	25917.9	21927	21987.3	23654	24050.1
EEF1A2	124.6	126.3	123.4	122.9	126	103.5	133	132.1
EEF1B2	135.8	130	183.5	150.2	130.2	121.6	133.3	140.2
EEF1B2	3690.6	4049	4483.3	4488.2	6322.5	3544.7	4761.8	5118.4
EEF1B2	323.4	322.8	376.2	341	310.1	281.1	371.1	372.2
EEF1B2	7249.7	7637.4	8457	8002.3	6359.1	6432.9	7907.5	9257.7
EEF1D	1646.2	1540.9	1615	1874.2	1660.3	1281.1	1449.9	1644.9
EEF1D	120.9	112.6	118.2	117.1	98	95.5	114	117.3
EEF1E1	115.7	111.6	118.1	122.7	101.9	91.2	118.2	113.8
EEF1G	4889.4	5293	4569.2	4579.4	5166.1	4339.6	4729.5	5546
EEF2	5154.7	5440.3	3464.7	4703.2	4307.7	3748	5813.5	5607.3
EEF2K	209.9	250.1	227.1	191.1	186.6	129.4	182.5	177.2
EEFSEC	114.7	123.8	125.5	129.5	113.4	105.8	118	133.8
EFCAB1	112.3	115.5	129.2	121.4	104.1	93.2	121	122.4
EFCAB2	117.3	110.3	118.3	122.6	102.1	99.2	107.7	105.2
EFCAB3	113.5	123.5	128.5	109.3	117.4	97.2	126.6	119.2
EFCBP1	124.1	123.7	118.9	114.4	119.1	101	137.4	129.7
EFCBP2	115.7	117.4	115.3	118.2	115.9	96.7	128.6	115.3
EFEMP1	116.1	109.6	127.9	127.2	108.7	110.9	120.8	115.7
EFEMP1	123.4	110	109.5	125	105.9	97.7	120	117.5
EFEMP1	124.7	113	114.2	122.2	112.5	98.5	115.9	114.6
EFEMP2	144.6	135.1	145.1	140.5	120.8	119.9	120.9	135
EFHA1	723.9	649.6	643.1	603.9	583.6	492.6	651.6	601.5
EFHA2	139.5	120	125.8	120.1	111.7	108.6	126.6	121.4
EFHB	115.1	109.5	130.3	109	107.8	99.1	121.2	137.5
EFHC1	124.8	119.4	133.6	121.5	112.8	109.5	126.2	116.5
EFHC2	133.3	116.1	131.4	125	101.2	102.7	121.2	120.7
EFHD1	136.6	125.3	132.5	138.5	131.3	117.3	127.4	128.4
EFHD2	1223.9	1330.8	1563.5	1589.2	837.8	1184	1332.8	945.7
EFNA1	116.7	114.7	122.3	121.9	109	107	114.4	125.8
EFNA1	158.8	188.3	143.2	164.1	152.7	115.6	148.4	145.6
EFNA2	113.9	111.4	116.7	110.8	110	96.5	118.8	105.4
EFNA3	116.4	122.5	114.9	115.9	105.9	95.6	124.8	120.3
EFNA4	130	134.5	139.5	136.2	119.2	119.2	127.9	127.3
EFNA4	219	206.4	192.4	216.3	182.3	154.7	183.3	198.1
EFNA5	126.6	118.2	120.1	117	116.6	103.1	117.7	113.2
EFNB1	117.2	118.2	117.3	128.8	113	102.9	121.5	128.5
EFNB2	139	116.3	130.9	125.5	123.2	105.1	153.7	163.1
EFNB3	108.1	114.8	126.8	109.7	110.9	94	117.1	112
EFS	125.3	114.8	119.6	117.9	114	100.5	115.7	117
EFS	113.6	123.6	114.4	125.8	115	99.6	128.5	116.4

EFTUD1	161.6	223.7	168.6	170.7	155.7	128.9	184.1	164.7
EFTUD1	186.4	175.8	185.5	200.4	167.3	165.6	148.3	173.2
EFTUD1	111.2	117.3	112.8	123.3	113.2	102.9	126.1	108.4
EFTUD2	408.9	435.6	466.5	498.4	373.9	390	451.4	338.4
EGF	121.9	115	111.6	116.7	111	105.5	122.5	116.4
EGFL5	110.9	111.8	116	115.7	111.3	95.3	117.2	108.6
EGFL6	121	119.8	116.8	121.5	112.4	103.8	122.3	121.5
EGFL7	118.5	123.8	129.1	121.6	112.1	96.6	122.5	116.2
EGFL7	116.3	115.3	116.1	111.6	107.1	100	124.6	108.7
EGFL8	120.5	110.6	131	132.1	123.3	105.1	120.3	105.1
EGFL9	111.4	108.9	127	125.5	109.4	104.9	121	108.6
EGFL9	128.5	125	115.2	120.6	112.8	101.1	127.8	127.8
EGFL9	112.9	116.7	120.3	129.6	106.9	97.5	114	118.3
EGFLAM	126.7	118	135.4	113.5	119.4	105.2	123.4	118.4
EGFR	109	110	109	115	105.8	94.3	108.7	116.3
EGFR	108.9	118.4	113.5	109.4	107.8	103.1	123.1	117.5
EGFR	115.8	114.4	120.2	126.2	100.7	105.9	111	107.7
EGFR	126.3	131.3	131.1	128.9	114.6	101.7	124.5	120.8
EGLN1	129.1	139.1	128.4	133.3	122	113	125.9	122.9
EGLN2	111.3	114.2	122.4	112.4	123.6	101.9	121.8	119.2
EGLN2	1944.9	2025.9	1656.4	2069.7	1735.9	1171.2	1889.7	2082.6
EGLN3	120.1	107.9	119.1	117.1	105.1	94.7	121.8	115.9
EGR1	142.6	132.4	132.9	132.2	127.4	104.5	138.2	165.6
EGR2	1003.6	1168.8	233.3	1475.5	857.3	561.4	1441.2	488.8
EGR3	128.4	116.2	111.6	131.1	111.1	100.3	127.5	117.9
EGR3	118.9	137.9	132.1	126.3	120.2	118.2	133.7	130.1
EGR4	113.8	112.9	131.7	117.6	105.2	100.2	109.3	111.6
EHBP1	197.1	204.8	181.2	213.2	189.1	149.3	243.5	212.9
EHD1	1573.3	1957.9	1668.9	1928	1361.2	1173.4	1288.3	1188.3
EHD2	112.5	123	127.4	119.7	110.1	98.5	129.9	127.8
EHD3	122.6	127.2	129.4	124.7	115.4	118	137.2	128.6
EHD4	274.8	256.7	284.7	288.6	179.4	213.7	226.4	192.1
EHF	117.2	117.9	116.5	119.7	117.1	100.9	122	118.1
EHHADH	103.1	116	116.2	112.3	99.2	101.7	113.3	119
EHMT1	118	111.7	121.7	122.2	115.9	108.6	121.7	110.8
EHMT2	133.5	137.4	124.3	133.8	119.9	102.5	129	124.2
EHMT2	108.3	121.6	126.5	117	118.6	112.2	123.7	109.8
EI24	333.5	359.1	389.8	386.1	359.7	322.2	317.6	379
EI24	173.4	149.9	166.6	156.9	135.6	155	159.2	157.1
EID2B	1387.2	1069.9	1587.8	1229.6	1316.7	871.5	1194.1	1307.3
EID3	119.7	127.1	138.8	136.6	122.9	104.5	133.2	148.4
EIF1	1924.5	1552.3	1510.6	1792	1854.5	1545	3108	5063.7
EIF1AX	126.8	121.5	120.7	127.1	114.9	105.1	129.8	123.4
EIF1AX	255.2	301.2	330.6	348.2	254.1	258.8	315.2	307.6
EIF1AX	202.1	190.2	184.4	163.3	136.7	139.6	191	203.8
EIF1AY	154.6	171.8	178.9	196.3	150	154.2	194.6	196.8
EIF1B	390.9	486.1	371.9	437.4	390.5	411.1	435.7	612
EIF2A	598.1	554.8	579.8	623	554.4	532.2	608.1	518.6
EIF2AK1	900.2	1048.7	906.8	1075.4	811.9	798.3	778.6	741.7
EIF2AK2	586.5	399.4	281.2	470	372.3	220	393.9	332.7
EIF2AK4	241.2	236.3	224.5	312	240.4	165.4	253.3	271

EIF2B1	310.4	350.2	406.1	308.6	261	326.4	269	257.6
EIF2B2	298.2	358.2	259	331.6	308.4	287.6	255.5	284.9
EIF2B4	823.4	981.9	880.5	1045.1	819.7	674.8	1024	805.5
EIF2B4	691.9	842.3	696.9	829.6	630.4	601.9	699.6	715.1
EIF2B4	116.1	119.3	121	123.2	125.8	106.1	125.7	129.7
EIF2B5	252.2	345.7	236.9	279.3	238.2	207.1	287.7	246.7
EIF2C1	227.1	195.9	183.7	211.4	180	176.5	169.6	151.7
EIF2C2	510.5	517.3	324.1	602.4	633.6	298.2	942.6	970.1
EIF2C3	146.6	131.9	123.3	142	138.3	126.2	162.1	159.3
EIF2C3	137.3	115.7	123.1	123.6	115.3	99.6	129.5	132.2
EIF2C4	143.3	138.6	137.8	134.2	130.8	129.8	135.8	131
EIF2S1	177.1	143.1	150.8	167.9	148.3	130.2	191.1	173.7
EIF2S2	146.4	131.6	127	144.3	136.2	114.4	160.3	161.5
EIF2S3	715.6	904.9	1049.3	830	670.2	927.3	814.4	817.7
EIF3S1	209.1	223.9	195.6	213.2	175.3	193.1	239.9	252.6
EIF3S10	383	397.3	339.5	403.1	354.9	267.9	434.3	572
EIF3S12	739.4	800	1370.9	1259.5	753.9	1120.3	1309.3	1063.6
EIF3S2	595.9	747.7	598	736	514.3	368.5	720.1	726.2
EIF3S3	1284.4	1289.4	1347	1500.8	1037.3	1152.3	1259.2	1898.5
EIF3S4	1416.3	1318.4	1254.6	1160.5	1169.1	1219	1058	1192.8
EIF3S5	2420.8	2573.3	2617.1	2448.8	2196.3	2522.3	2112	2675.3
EIF3S6	4970.4	5153.1	7748.2	6335.9	5433.1	7364.3	5823.7	6595.1
EIF3S6IP	1861.2	1796.5	1727.5	1512.3	1317.1	1603.8	1859.1	1854.2
EIF3S7	1621	1751.8	1977.3	2013.1	1130.4	1602.5	1792.1	1878.3
EIF3S8	111.9	108.2	116.9	107.2	101.9	97.2	120.3	120.6
EIF3S9	2479	2591.9	2154.4	2615.1	1708.8	1315.3	2870	2633.2
EIF3S9	208.8	192.3	187.9	209.4	174.9	162.1	200.4	182.4
EIF3S9	589.9	574.7	520.8	618.5	390.5	449.8	781.1	717.2
EIF4A1	5779.1	5247.8	5579.4	6725.9	5163.5	5345.7	7878.2	7955.4
EIF4A2	4994.6	4362.4	4609.4	4459.8	3059.3	3431	5279.9	4583.7
EIF4B	3512.6	4256.3	3475.9	3820.4	2959.2	2950.4	3703.1	4441.5
EIF4E	127.1	135.7	132.1	131.1	130.1	107.3	140.1	126.6
EIF4E2	219.9	199.8	235.1	222.2	159.1	215.9	197.6	183.4
EIF4E3	502.4	448.5	480	578.6	375.2	352.3	356.1	330.6
EIF4EBP1	135.1	110.4	139.7	111.9	112.2	108.7	119.6	121.2
EIF4EBP2	294.7	365.8	352.2	402.7	326.6	323.6	304.3	345.9
EIF4EBP3	114	109.4	115.6	118.5	113.7	97.6	118.1	117.1
EIF4ENIF1	189.3	211.3	189.6	246.4	196	176.6	221	228
EIF4G1	262.3	294.3	245.3	279.2	260.1	197.7	285.7	328.7
EIF4G1	122.4	106.8	123.4	113.9	114	93.5	121.4	109.2
EIF4G1	762.7	775.1	502.2	827.7	694	461.3	929.5	937.7
EIF4G1	125.9	107.8	122.1	121	107.7	98.7	116.6	115.3
EIF4G2	1573.5	1656.3	1451.5	1630.2	1215.6	2054.5	1893.5	2991.7
EIF4G2	176	179	197.4	188.6	180.6	199.1	198.4	193.9
EIF4G2	2864.8	2914.4	2861.3	3020.2	2632.3	2871.9	4104.6	3880.9
EIF4G3	335	285.1	244.7	321.5	229.3	185.7	265.9	232
EIF4H	723.6	856	944.2	1051.1	641.8	830.4	946.2	1155.9
EIF5	136.2	141.8	143.8	154.8	124.7	104.9	145.5	153.9
EIF5	490	514.1	546.3	635.4	513.1	370.8	664.1	795.3
EIF5	127.4	114.2	115.2	131.9	112.1	100.2	118.1	122.6
EIF5A	546	559	518.5	581.8	414.1	520.2	542.2	530.5

EIF5A2	123.7	125.5	123.2	116.6	124.7	103.5	130.8	139
EIF5B	221.3	259.8	248.5	248.3	206.7	166.3	234.5	261.3
ELA1	172.8	169.6	212.2	176.7	170.4	159.8	193.8	190.2
ELA2	123.6	165.1	131.6	134.8	150.5	115.2	122.4	165.6
ELA2A	100.6	104.2	115.7	110.8	104	101.7	103.8	106.9
ELA2B	125.5	118.6	128.5	135.4	133.6	111.1	122.6	118.3
ELA3A	106.1	107.8	117	112.3	103.8	94.9	112.4	119.6
ELA3B	109.4	112	122.9	114.9	107.8	97.7	120.8	127.5
ELAC1	121.1	131	120.7	132.9	122.1	122.4	121.5	122.9
ELAC2	193	183.3	175.3	179	154.1	151.4	195.1	155.8
ELAVL1	152	149.6	131.9	148.7	129.3	114.9	151.7	141
ELAVL2	113.8	117.3	106.9	126.7	111.1	104.3	120.5	111.8
ELAVL3	114.5	129.2	124.6	128.6	120	108.5	135.6	118.3
ELAVL3	115.1	119.6	130.5	123.2	109.5	106.4	128.9	122
ELAVL4	115.2	109.5	112.4	113.8	107.7	101.1	113.3	112.5
ELF1	753.7	1282.8	952.7	1392.3	941.4	748.1	1039.4	2063.5
ELF2	137.3	121.7	127.9	127.9	125.5	102.2	127.4	117.4
ELF2	288.1	330.7	455.1	344.7	293.8	356.3	290.5	243.1
ELF2	151.3	174.6	155.2	178.7	203.7	116	165.1	152
ELF3	115.4	106.6	104.2	117.3	107.1	95.5	113.5	107.6
ELF4	323.5	293.6	293.7	342.6	227.4	277.1	362.8	266.6
ELF5	105	114.2	106.7	108.5	108.1	94.4	118.4	110.8
ELF5	110.2	105.3	110.7	128.3	101.8	98.2	117.1	114.7
ELK1	231	216.5	200.6	237	181.2	202	205.5	260
ELK3	120.6	123.4	126.5	123.5	113.3	99.5	117.4	109.7
ELK4	141.2	132.3	120.6	137.7	123.9	105.7	143.8	138.5
ELK4	111.8	117.8	108.3	117.7	109.7	95.4	125.4	112.6
ELK4	135.4	140	126	137.4	123.7	108.4	144.4	136.1
ELL	190.5	228.1	186.4	223.7	162.7	161.1	204	223.3
ELL3	157.9	129.8	165.1	142.4	124.8	129.2	137.5	125.4
ELLS1	230	214.3	253	286.5	217.6	218.6	335.2	435.5
ELMO1	134.1	120.6	123.9	128.9	119.8	109.6	121.9	124.6
ELMO1	335.5	365.6	295.4	429.7	306.7	333	544.9	388.7
ELMO1	120.7	111.5	132.6	119.4	110.3	105.3	123.8	115.2
ELMO1	135	130.7	120.7	124	121.8	121.7	140	131.2
ELMO2	124.4	126.4	130.8	142.7	115.6	108.9	138.6	138.7
ELMO2	492.9	444.2	335.3	467.1	340.7	323.7	541	474.3
ELMO2	122.9	109.4	115.6	122.7	108.4	100.5	123.1	114.3
ELMO3	135.8	145.3	159.5	154	134.9	114.9	159	157.9
ELMOD1	118.5	119.5	123.4	119.1	112.3	97.8	120.5	109.2
ELMOD2	141.2	141.7	149.5	160.7	135.9	145.3	154.9	132.1
ELN	112.1	119.4	124.2	117.9	117.9	110.9	131.9	132.2
ELN	113.2	120.5	118.9	107.1	108.4	104.7	121.6	113.4
ELOF1	169	143.7	163	166.5	140.1	127.5	132.7	149.3
ELOVL1	155.4	172.2	193.9	172.6	143.1	154.5	179.6	157
ELOVL2	113.8	109.9	118.6	125.3	108.4	97.9	114.5	113.4
ELOVL3	111.2	115.3	122.3	117.6	106.4	98.2	107.8	111
ELOVL4	195.6	211.6	190.5	165.2	183.4	149.7	180.7	191.2
ELOVL5	659	523.8	623.1	654.1	603.8	532.3	739.8	589.4
ELOVL6	134	139.4	131.1	149.6	124	108.5	160.5	140.4
ELOVL7	110.8	115.6	110.1	110.6	108	102.2	116.6	112.4

ELP3	359	413.4	344.9	387.8	316.3	250.9	374.7	332.2
ELP4	147.1	136.2	163.5	141.2	115.5	125.6	140.1	131.5
ELSPBP1	105.7	111.1	105.8	118.3	108.3	95.9	118.6	112.3
EMCN	118.7	113	115.3	115.2	108.9	99.2	117.9	120
EMD	621.1	662.6	731.9	680	538.1	639.4	681.5	731.1
EME1	120.6	117	137	136.7	119.1	105.3	124.5	129.4
EME2	119.1	123.9	111.5	118.6	105.1	102.3	111.9	110.4
EMG1	376.1	547.2	372.6	414.2	387.8	323.6	377.7	463.9
EMID1	116.8	119.3	107.9	121.7	111.9	97.5	121.9	118.5
EMILIN1	115.7	127.4	116.7	124.3	112.4	101.1	124.7	115.1
EMILIN2	390.4	284.5	682.1	406.8	207.3	498.1	350.2	235.2
EMILIN3	116.9	109.6	110.9	117.7	106	96.1	111.1	114.8
EML1	116.3	118	115.2	119.3	118.6	104.3	120.3	117.8
EML1	118.7	128.9	115.5	120.2	109.6	98.7	121.4	108.7
EML1	121.4	119.2	119.4	113.5	111.1	99	119.2	116.3
EML3	1094.2	1256.1	2009.1	1171.1	939.9	1690.1	1082.3	1109.3
EML4	630.2	568.5	464.5	569	554.9	501.8	539.7	734.7
EML5	103.1	110.2	115.8	118.9	118	95.7	121	115.1
EMP1	787.3	950.9	246.8	1227.6	892.6	628.4	2113.6	794.2
EMP2	130.3	138.4	126.8	140.1	118.7	121.5	137.8	130.8
EMP3	2659.1	2859.9	2511.9	3324.2	2403.8	2386.7	3056	2749.3
EMR1	141.7	116	116.8	137.9	108.1	106.8	146.5	120.7
EMR2	252.6	201.7	338.4	313	160.4	303.6	228.2	169.9
EMR3	104.8	114.4	119.3	123.3	109.8	107.5	119.8	111.9
EMR3	120.2	126.7	119	121.9	117.9	103.1	124.7	110.5
EMR4	108.9	120.8	130.5	119.6	116.9	100.2	128	120.1
EMR4	113.3	122.7	116.8	109.8	114.5	99.2	113.7	112.6
EMX1	110.5	123.3	116	106.7	110.4	94.6	110.8	106
EMX1	116.7	121.6	122.1	130.2	110.1	96.3	120.3	115.9
EMX2	124.5	118	116.5	121	103.5	102.8	121.3	123.4
EN1	118.3	111.3	116.4	115	105.4	95.4	119.9	112.4
EN2	116.8	105.6	136.2	134.1	114.7	106.4	122.9	119.9
ENAH	114.7	111.6	112.3	114	105.1	97.7	116.6	108.4
ENAH	107.4	113.6	110.4	119.4	108.5	102.6	114.3	111.8
ENAH	115.6	107.1	119	124.3	98	101.1	114.8	126.5
ENAM	103.6	113.2	115.1	117.3	109.2	102.7	118	112.5
ENC1	129	132.5	137.4	134.3	127.7	106.3	137.4	129.4
ENDOG	195.2	153.2	167.7	155.3	127.1	131.6	150.9	146.2
ENDOGL1	115.3	118.7	122.5	134.5	118.8	101.4	160.5	120.4
ENG	293.6	187.5	404.2	325.7	160.2	368.3	331.2	148.1
ENO1	3598.7	3670.8	3566.5	3713.3	2336.6	3377.6	4233.8	3342.7
ENO2	285.2	266.6	347.5	301.2	219.7	289.7	281.6	222.9
ENO3	188.2	161.2	229.6	240.5	194.8	140	197	191.8
ENO3	123.8	120.7	135	129.3	117.9	121.9	125.8	122.8
ENOSF1	157.4	181.9	201.2	169	146.1	133.6	138.1	174.3
ENPEP	117.8	109	119	113.1	114.8	96.7	108.6	112.7
ENPP1	125.1	125.9	119.4	132.5	127.3	103.3	117.9	125.2
ENPP2	110.2	106.7	114.3	127.9	113.3	98.5	130.8	110.9
ENPP2	108.2	118.6	122.4	113	105.4	109.7	130.8	123.2
ENPP3	117.1	121.9	114.4	109.9	105.6	93.3	106.6	115.3
ENPP4	221.4	240.5	219.1	233.8	212.2	181.4	195	182.2

ENPP5	119.1	139.3	119.3	125.2	115.8	102.9	136.1	126.1
ENPP6	117.9	102.9	111.9	113.1	112	93.3	119.2	115.1
ENPP7	123.2	127.3	124.1	119.6	118.8	112.2	128.3	125.6
ENSA	121.9	121.4	114.7	117.7	112.8	103.9	128.1	120.4
ENSA	193.5	207.1	218.7	202.8	158	144.6	177.8	173.3
ENTH	629.5	879.2	778.5	762.8	652	598.9	724.3	814.7
ENTPD1	128.2	145.6	170.3	131	129	116.1	136.6	128.1
ENTPD2	115.1	115	111	119.2	102.2	97.3	110.5	110.5
ENTPD2	117.8	114.7	122.9	113.5	105.1	97.3	115.4	117.9
ENTPD3	123.5	116.8	120	119.1	107.3	105.5	113.5	116.4
ENTPD4	307.7	360.9	342.4	316	335.4	188.8	303.6	382
ENTPD5	112.6	122.6	118.4	128.4	111.9	104.1	123.3	112.4
ENTPD6	266.6	276.7	322.4	270.1	220	246.6	266.7	292.9
ENTPD7	120.5	125.2	121.7	126.7	114.7	115.2	122.8	120.8
ENTPD8	102.8	117.7	119.8	115.1	113.3	102.2	111.6	112.4
ENTPD8	126.9	125.4	126.2	133.1	118.4	121.3	121.1	128.7
ENTPD8	109.8	117.1	111.1	117	104.7	101.8	114.5	119.7
ENY2	672.7	601.1	767.6	643.4	536.4	888.7	628.5	548.6
EOMES	835.7	939.7	1226.4	788.7	544.6	828.8	578.2	507.9
EP300	244.5	248.8	181.2	285.2	235.1	221.3	331.4	375.9
EP400	351.7	395.5	347.7	431.7	412.2	333.3	395.2	432.8
EP400NL	112.5	115.9	114.7	116.3	107.1	101.8	116.1	117.4
EPAG	110.9	122.7	111.8	117	103.1	97.1	115.6	111.4
EPAS1	121	127.2	123.1	140.3	120.9	123.5	150.2	151.2
EPB41	123.5	131.3	138.7	137.9	113.7	127	141.3	142.4
EPB41	119.5	118.5	114	111.6	114.8	106	121.3	117.7
EPB41	141.7	161.9	158.4	156.2	156.2	137.7	147.8	192
EPB41L1	122.7	107.8	110.4	114.3	106.9	98.3	119.7	111.6
EPB41L1	122	124.9	122.9	128.5	116.9	109.4	128.4	108.1
EPB41L2	116.3	122.5	121	121.4	104.1	99.4	120.8	121.8
EPB41L3	341.8	151.3	1118.7	338.1	140.6	850.2	199.4	132.8
EPB41L4B	119.3	116.6	126.7	128.4	119.3	104.4	114.1	124
EPB41L4B	118.8	108.9	129.6	126.9	113.8	101.6	121.8	112.3
EPB41L5	120.1	117.2	120.9	121.3	117.9	101.6	126.4	116.9
EPB42	115	117.8	117	127	108.9	94.5	122.3	113.6
EPB49	122.9	124.3	114.7	118.9	116.6	108.4	120.6	119.9
EPC1	263.3	287.3	211.4	338.2	303.8	143.2	367.5	381
EPC2	135.7	152	132.7	146.8	120.5	109.7	138.9	137.9
EPDR1	121.5	124.2	121.3	128.9	119.1	112.4	130.3	139.6
EPGN	124.8	118	144.9	142.9	125.1	111.4	120.2	126.3
EPHA1	280.2	347.5	417.8	283.1	187.3	268.1	189.1	180.7
EPHA10	128.2	125.8	133.6	135.6	128	108	134.3	131
EPHA10	112.2	122.9	116.8	125	110.6	98.7	119.7	116.1
EPHA2	114.7	121.3	127.6	120	115.5	95.6	123.8	111.7
EPHA3	114.4	109.2	115.1	125.7	106.5	94.6	120.9	118.3
EPHA3	117.1	116.2	112	115.5	106.4	94	120.3	107.9
EPHA3	116.8	116.1	122.1	125.3	108.4	110.9	125.3	112.5
EPHA4	221.4	229.3	233.1	216.7	183.4	167.8	181.8	173.1
EPHA5	119.9	132.8	133.3	126.7	112.6	113.4	129.3	112
EPHA5	107.3	108.5	108.6	117.8	105.3	95	111.4	117.2
EPHA5	119.3	108.6	123.8	125.8	114.7	111.2	118.6	133.5

EPHA6	117.8	107	134.2	128	112.4	95.8	112.9	119.9
EPHA6	127.3	144.9	122.4	125	113.2	106.3	117.3	128.8
EPHA7	114.8	109.9	113.8	110.7	110.7	99.9	115.6	116.1
EPHA8	110.4	128.3	138.3	116.8	124.9	108.1	130.4	122.9
EPHA8	111.7	107.6	112.4	117.8	112.8	96	115.9	121.7
EPHB1	118.4	114.9	118.9	119.2	109.8	101	118	112.9
EPHB2	130.2	132.3	150	130.5	117.4	123.8	121.6	135.1
EPHB2	109.5	115.7	124.1	120	108.5	103.7	114.6	114.1
EPHB3	128.8	131.7	125.1	126.7	122.3	101	129.8	127.9
EPHB4	119.3	126	121.1	126.5	108.1	106.4	120.8	128.3
EPHB6	206.2	215.2	214.6	253.7	153.9	165.7	200.4	152.7
EPHX1	130.4	121.1	131.4	140.4	123	112.5	140.6	141.4
EPHX2	153.9	144.8	148	162.2	119.4	115.7	127.7	122
EPIM	125	119.6	132.2	127.9	127.7	107.6	117.5	131.2
EPM2A	124.2	128.8	128.5	117.3	119.8	114.5	113.3	137
EPM2A	108	100.9	107.9	111.5	109.2	97.1	110.7	114.6
EPM2AIP1	259.7	288.7	350.6	271.2	230.6	257.1	247.1	230
EPN1	950.5	915.9	910.9	1096.5	642.6	778.2	897.9	826.4
EPN2	119.4	113.8	118	117.3	115.6	108.9	136.8	110.4
EPN2	108.2	113.3	117.9	115.6	104.4	93.9	118.2	101.9
EPN2	102.6	123.3	115.9	122	111.5	96.6	123.4	115.1
EPN3	124.2	118.1	130.8	122.8	112	101.5	108.7	117
EPO	111.3	117.8	106.7	108.6	109.2	104.6	118.4	115.1
EPOR	113.5	109.9	118.8	117.8	107.6	108.1	121.1	110.9
EPPB9	109	112	117.7	119.9	111.5	107.4	117.1	118.6
EPR1	115.3	112.7	123.1	111.4	104.1	96.5	109.5	125.2
EPRS	795.2	925.9	821	934.5	723.2	745.9	753.8	911.4
EPS15	197.5	208.1	236.7	213.4	168.4	181.6	183.9	211.4
EPS15L1	144.6	135.7	130.8	138.6	132.2	112	140.8	139.9
EPS8L1	117.2	108.8	116.3	112.8	107.7	95.1	115.1	117.9
EPS8L1	122.6	118.1	123	121.6	111.7	97.5	121	109.1
EPS8L1	114.7	116.1	118.9	121.2	108.8	100	120.5	115
EPS8L1	123.3	110.9	122.2	133.1	110.8	105.7	120.9	117.1
EPS8L2	117	124.3	130.5	124.3	113.6	106.5	114.8	118.3
EPS8L3	110.8	118.1	121.5	116.7	109.7	99.9	122.2	115.2
EPS8L3	121.3	122.3	131.2	130.6	120.6	108.2	124.4	117
EPSTI1	764.4	361.7	838.9	671.3	276	501.5	419.9	222.9
EPX	125.2	119.2	128.8	132.4	124.5	101.2	118	119.6
ERAF	119.3	109.3	132.7	125.9	114.4	115.6	131.8	122.8
ERAL1	255.5	255.5	366.8	299.3	212.6	262.9	244.2	266
ERAS	105.9	108.9	113.6	118.3	107.7	102.7	120.7	121.5
ERBB2	118.1	119.5	116.2	108.3	108.6	99.5	125.1	109.8
ERBB2	117.5	118.5	125.8	127.6	115.4	105.7	117.4	107.1
ERBB2	118.4	133.7	132.5	130.7	117.5	104	128.1	126.2
ERBB2IP	119.2	121.9	128.6	133.2	122.7	116	130.5	124.8
ERBB2IP	124.6	119.9	125.9	127.3	111.6	102.3	127.4	115.2
ERBB3	116.2	124.1	134.5	130.2	123.9	107.2	130.6	129.3
ERBB3	110.2	112	120.6	118.5	101.8	97.3	119.4	118.9
ERBB3	134.6	116.9	134	121.1	119.7	113.7	118	110
ERBB4	129.9	133.4	133	136.1	129.5	105.9	133.2	145
ERC1	128	123.2	140.8	124.2	119	112.6	121.1	126.9

ERCC1	239.9	263.3	390.6	254.7	216.6	215.1	191.8	164.4
ERCC1	232.8	274.2	360.3	261.3	194.8	187.3	213.7	174.4
ERCC1	110.8	120.5	121.5	121.4	101.8	96.1	130.7	110.7
ERCC2	131.6	125.4	142.5	143.8	117.8	115.6	128.2	129.7
ERCC3	242.6	279.3	230.7	259.3	210.7	184.6	255.2	230
ERCC4	116.4	115.4	122.2	129.7	120.1	102.2	124.2	118.3
ERCC5	599.5	767	731.5	699.8	676.1	614.6	663	683.2
ERCC6	119.4	131.7	123.5	119.8	105.9	109.1	117.5	120.4
ERCC8	115.9	121.4	122.8	116.1	109.1	98.4	111.7	121.7
ERCC8	107.9	118.9	133	120.3	112.1	103.5	123.9	116.8
ERCC8	116.1	119.1	118.4	109.1	101.7	96.1	116.8	114.3
ERCC8	119.3	113.8	120	136	102.3	98.2	122.6	114.3
EREG	126.1	144.9	123.6	191.8	148.4	120.1	178.7	124.7
ERF	146.2	156	149.4	144.8	128	140.7	155.3	202.8
ERG	113.6	116.7	111.5	124.2	109.1	94.8	116.6	119.6
ERG	116.2	116.7	125.9	129.9	119.1	101.3	117	112.8
ERG	120	117.4	130	126.3	108	101.9	135.8	125.1
ERGIC1	181.4	230.1	217.8	195.5	198.4	177	159.8	153.2
ERGIC1	117.9	125.4	125.5	123.7	117.4	116.3	122.5	110.2
ERGIC1	135.8	135.1	129.5	130.9	128.3	112	134.6	131.5
ERGIC2	203.5	195.2	175.8	218.3	168.9	174.5	245.5	201.4
ERGIC3	283.4	287	234.8	298.9	209	217.1	222.7	236.5
ERGIC3	999.7	1303	1030.8	1189.5	1004.4	878.3	1178.9	1271.1
ERH	421.3	526.5	519.9	507.1	452.5	397.7	509.7	564.8
ERICH1	605.1	467.5	565	634.1	415.4	458.7	537.9	384.8
ERLIN2	114.1	126.2	126.9	124.3	125.5	105.5	123.6	127.4
ERMAP	118.5	104.9	106.7	112.4	108.6	102.7	134.7	119.2
ERMAP	137.3	168.2	150	147.7	152.9	114.6	139.7	172.8
ERN1	236.2	233.9	254.9	244.1	212.3	175.7	229.2	246.8
ERN1	123.7	110.8	113.9	119	101.8	102	105	106.8
ERN2	111.5	122.9	129.2	118.5	114.1	99.9	123.8	118.6
ERO1L	185.7	204.6	178.5	191.3	179.4	164.4	197.7	177.8
ERO1LB	134.6	123	130.2	132.1	120.1	119.2	127.1	122.5
ERP29	387.7	469.9	505.6	399.3	365.6	361.8	294.1	360.3
ERP29	3163.8	3871.7	3448.9	3373.1	2842.8	2628.5	2841.4	2882.4
ERRFI1	156.1	173.4	165.8	165.2	145.2	128.9	199.7	259
ERV3	114.4	118.5	128.7	119.2	117.3	114.5	112.2	123.2
ERVWE1	109.7	112.5	113.9	115.9	110.5	103	119	116
ESAM	124.2	121.7	123.6	120	109.9	99.5	134.4	119.7
ESCO1	140.1	142	129	149.8	122.6	112.5	139.7	157.5
ESCO2	110.7	107.4	115.5	112	101.9	98.5	124.9	108.6
ESD	152.3	149.7	148.8	160.2	141.8	129.6	149.1	142.2
ESM1	118.5	123.7	118.3	113.4	109.6	106.1	114.4	114.4
ESPL1	119.3	122.4	125.9	143.5	129.2	109.4	127.4	122.2
ESPN	119.2	112.4	117.3	120.4	104	98.7	119.4	117.5
ESPNL	110.3	117.7	114.3	119.6	100	100.6	112.5	115.8
ESR1	107	112.8	111.7	110.6	109	100.1	119.2	111.8
ESR2	130	121.1	128.9	124.9	121.5	109.1	133.5	123.5
ESR2	121.7	117.9	122.9	124.3	106.9	101.2	119	124.4
ESRRA	168.7	191.7	189.9	186.4	138.1	137.9	204.3	198.5
ESRRB	119.3	134.4	122.1	120.1	115.2	105.7	118.3	112.8

ESRRG	125.2	131.3	125.4	140.2	118.6	107.4	114.1	118.3
ESRRG	115.6	110.6	114	112.8	108.4	98.2	118.8	115.2
ESRRG	100.4	105.8	106.9	109.4	101.6	89.3	107.1	107.5
ESSPL	107.2	113.2	109.1	108.1	104.4	93.1	111.6	112.2
ESTRRA	150.4	164.2	150.2	155.1	155.1	146	145	157.5
ESX1	109.1	114	131.1	123.4	105.8	93	123.4	117.2
ET	377.3	402.9	380.7	381.5	343.4	277.4	509	533.9
ETAA16	125.5	124.2	128.5	140.3	115.9	112.9	134.9	115
ETF1	424.7	482.7	390	506.2	418.3	373	622.8	711
ETFA	573.8	647.3	742.3	560.4	512.3	506.8	372.9	388.4
ETFB	109.9	112.1	105.4	117	107.8	97.1	113.5	108.8
ETFB	221.2	209.5	387.3	245.7	232.6	284.5	191.8	190.4
ETFB	317.8	345.5	561.6	368.2	298.3	419.8	314.3	334.6
ETFDH	230.1	230.2	250.1	246.3	223.7	206.5	244.2	291.7
ETHE1	119.9	117.4	115.4	118.1	113.8	111.3	115.9	123
ETNK1	112.3	124.9	121.8	135.9	119.3	116.4	128.6	134.3
ETNK1	148.2	150	162.1	170.4	146.8	156.7	166.1	179.5
ETNK1	203.8	167.2	154.2	215.3	164.3	170.6	221.6	192.5
ETNK2	119.9	120	114.1	131.4	99.1	95.8	116.8	116.6
ETS1	2742.1	2816.2	2616.6	2296.3	1852.3	2508.4	2002.7	2401.4
ETS2	302.6	249.7	230.9	482.3	256.3	370	558.6	260.4
ETV1	108.3	110.6	109	110.4	104.8	97.7	114.1	104.6
ETV2	121	124.3	140	139.1	114.6	114.7	138.7	123.7
ETV3	135	148.1	140.9	142.2	132.3	109.2	154.3	145
ETV4	118.6	123.8	117.6	113.5	108.7	103.5	111.7	106.3
ETV4	110.3	117.7	111.4	108.3	110	96.5	114	109.8
ETV5	102.5	113.1	175.5	120.7	108.8	111.1	117.6	107.2
ETV5	122.2	137.6	135.9	120.8	109.5	100.1	122.5	116.3
ETV6	218.4	160.4	252.4	194.8	153.1	177.5	152.6	150.6
EVA1	119.8	122.2	126.5	117.8	114.5	104.7	117	118.7
EVA1	177.7	229.2	154.2	186.8	165.5	126	161	168
EVA1	104.9	104.3	112.1	113.8	98.2	96	110.7	114.5
EVC	123.9	113.1	124.8	121.7	114.2	96.1	117	116.9
EVC	128.5	118.3	125.6	118.6	109.9	103.5	135.4	119.9
EVC2	113.2	109.6	120	118.5	111	103.8	124.7	118.5
EVI1	112.4	114.2	111.8	119.4	101.6	89.4	114.5	113.3
EVI2A	218.5	204	194.5	239.1	178.9	168.4	231.8	196.6
EVI2A	143.4	131.4	133.9	152.6	125.6	110.6	144.1	178.1
EVI2B	105.5	118.9	119.5	122.8	109.6	103.2	124.3	108.8
EVI5L	179.6	201.9	193.1	201.5	189.2	174.3	203.3	187.1
EVL	4456	4927.4	5378.5	4555.9	3681.6	3409.4	3052.3	2970.4
EVPL	110.1	119	113.8	116.2	114.2	97.8	115.5	115.7
EVX1	121.6	130	124.5	136.2	111.4	114.4	133.4	123.7
EVX2	121.6	116.1	111.3	118.6	110.9	104.6	116.4	122.9
EWSR1	467	458.3	491.2	602.1	473	557.1	714.9	1050.8
EWSR1	133.4	134.5	146.6	134.1	119.4	121.4	132.1	122.9
EWSR1	684.7	691.5	822.5	845.6	824	867	1074.9	1276.4
EXDL1	109	116.7	116.5	120	109.2	97.4	122.5	119.7
EXDL2	164.5	178.6	182.2	165.5	162.5	133.4	150.4	136.7
EXO1	120.1	125	120.5	127.7	111.3	94.4	119.2	116.3
EXO1	113.1	116.4	119.3	120.3	103.6	99.2	115.3	115.6

EXO1	114.3	115.4	133.5	121.4	114.9	101.3	119.7	117.4
EXOC1	123.5	125	136.7	137.8	126.4	106.4	144.9	121.7
EXOC1	140.4	121.1	144.8	142.1	133.6	131.1	134.9	133.8
EXOC1	285.8	272.6	303.7	274.4	268.5	230.4	274.8	255.4
EXOC2	211.3	208.2	186.5	208.5	182.6	162.2	177.8	206.2
EXOC3L	112.8	124.8	115.7	111	119.8	97.7	119.3	115.6
EXOC4	144.1	127.9	145.5	127.1	131.2	110.8	126.5	141
EXOC6	115.5	125.1	119.9	133.1	116.4	101.7	121.7	119.5
EXOC6	163.1	190.8	161.4	206.7	158.9	137.7	156.9	160.5
EXOC6	122.8	124.1	141.1	122.2	104.6	100.2	122.2	120
EXOC7	630	697.3	530.6	800.8	561	484.6	899.4	875.9
EXOC7	148.1	130	121.8	129.1	135.9	111.7	124.8	145.9
EXOC8	124.3	120.8	127.2	149.5	118.8	112.6	131.5	132.8
EXOSC1	666.1	642.8	699.3	593.2	681.7	610.3	554.2	550.7
EXOSC10	336	382	404.6	371.8	335.5	364.4	389.7	402
EXOSC10	364.9	393.2	359.6	516.4	319	334.7	446.6	402.2
EXOSC10	321.8	326.6	340.1	361.1	335.5	286.1	292.9	288.1
EXOSC2	222.6	223	233.5	231.5	230.2	161.3	234.1	201.6
EXOSC3	295.8	333.4	308.6	320.5	235	212.8	261.6	249.5
EXOSC3	307.6	404.5	290	302.9	251.3	255.8	274.4	269.1
EXOSC4	171.6	169.3	168	179.3	149	147.3	178.7	143.6
EXOSC5	189.6	187.1	191.9	192.1	184.6	145.9	190.3	166.8
EXOSC6	497.2	546.5	369.8	459.7	380.1	282.8	495	579.2
EXOSC7	254	249.5	229.9	252.4	207.3	179.5	190	173.1
EXOSC8	391.4	435.8	434.7	433.3	406.2	410.9	426.7	439.6
EXOSC9	149.9	154.4	181.5	165.4	150.7	148.1	174.5	152
EXOSC9	162.6	196.9	229.2	195.3	162.4	177.9	176.4	189.9
EXPH5	124.7	134.1	118.5	136.3	114.9	104.4	132.6	141.5
EXT1	137.1	149.1	130.4	144.9	136.9	112.9	130.9	127.7
EXT2	110.8	119.4	118	118.4	106	100.2	115	116.9
EXT2	193.9	226.2	183.5	208.3	214.5	152.2	201.4	232.3
EXT2	168.8	156	151.8	154.5	149.1	120.8	166	158.4
EXTL1	119.4	120.6	118.7	119.4	115.2	108.4	124.5	125.9
EXTL2	114.5	121.1	117.6	132.9	119.7	104.7	135.1	125.7
EXTL2	153.4	169.4	140.1	176.8	141.4	108.1	161.4	154.7
EXTL3	202.5	209.4	176.3	187.8	151	166.2	217.6	181.1
EYA1	108.1	119.7	130.9	116.9	109.6	110.3	114.3	117.7
EYA1	109	110.8	110	112.5	109.2	92.7	118.1	110.8
EYA1	112.6	120.8	96.9	115.9	104.9	92.9	115.8	120.2
EYA2	113.2	113.4	113.8	122.5	103.4	97.9	141.3	116.8
EYA2	108.9	112.2	114.6	111.7	116.2	98.4	122.9	112.8
EYA2	111.1	119.9	120.6	113.1	113.7	100.7	120.4	122.7
EYA2	113.7	112.1	123.2	112.4	100.8	92.5	114.9	117.4
EYA2	108.7	117.1	123.9	120.1	110	101.1	122.5	111.5
EYA3	115.6	117.9	129.6	117.3	107.1	113.9	120.6	129.1
EYA3	115.3	123.4	116.4	115.8	105.3	102.7	108.8	121.7
EYA4	123.8	135.2	127.5	114.7	113.8	103.8	117.5	125.9
EYA4	114.4	118.7	118.3	126.2	113.2	107.4	120.3	125.6
EYA4	113.3	108.1	119.8	118.7	109.8	99.5	111.2	115.6
EZH1	152.6	131.5	149.1	130.4	136	110.7	146.2	144.8
EZH2	124.7	127.3	133	125.9	123.1	100	131	128.9

EZH2	115.5	121.8	123.4	137.6	113.5	108.8	126.9	129.4
EZH2	132.1	136	131.3	125.1	123.9	109	133.5	146.5
F10	107.6	110.6	110.5	113.9	111.2	95.2	111.2	111.5
F11	121.3	120.2	127.1	129.2	115.8	107.6	128.3	122.8
F11	116.8	116.6	108.9	110.8	103.3	91.2	110.1	124.1
F11R	111.7	119.3	118.2	114.8	117.2	99.1	114.7	106.6
F11R	117.7	111.7	122.5	118.6	103.7	97.7	111.8	116.9
F11R	153.7	162.4	201.1	160.9	144.1	145.4	168.9	166.8
F12	109.7	125.9	121.7	121.8	106.1	99.6	123.4	114.1
F13A1	112.3	113.9	124.2	109.3	111.1	97.3	125.3	111.7
F13B	112	107.6	118.9	122.7	107.9	101.5	110.9	109
F25965	136.2	133.5	146.8	128.7	126.4	120.1	150.9	139.2
F2R	15130	16512.2	13360.9	16107.4	14327.4	8948.1	16997.9	17229.2
F2RL1	147.6	146.2	153	141.8	126.3	137.4	132.7	136.2
F2RL2	118.9	117.5	116.7	114	110.7	103.7	116.7	103.6
F2RL3	122.3	124.6	121.6	113.9	113.3	102.4	116.4	114.9
F3	117.7	120.3	115.7	121.7	110.7	97.9	129.1	118.3
F5	128.7	135.3	127.1	130.9	137.7	114.6	131.1	127.3
F7	103.6	108.4	105.8	113.8	106	90.9	111.6	107.9
F7	119.5	122.2	120.9	122.2	121.7	106.9	125.7	128.7
F7	120	118.3	115.6	108.9	104.6	105.3	122.6	133.6
F8	115.1	115.3	118	116.9	107	102.1	121	109.5
F8	109.3	114.2	114.3	122.4	107.9	95.6	113.9	119.3
F8	124.6	120.6	118.9	115.5	114	99.4	131.5	114.3
F8A1	188.2	237.1	199	202.5	171.2	195.5	199.2	166.6
F8A3	124.3	123	115.1	132.2	108.5	104.4	137.7	123.5
F9	129.6	118.6	123.8	114.7	113.2	103.5	122.7	116
FA2H	109.2	126.3	129.3	113.9	113.9	103.2	117.3	116.7
FAAH	119.8	134.7	130.5	161.4	118	111.4	124	118.8
FABP1	112.9	109.4	122.1	119.4	108.7	93	117.7	112.7
FABP2	113.4	123.9	133.2	127.1	113.6	95.9	119	113.5
FABP3	110.1	108.2	112.1	111.4	98.7	95.5	110.3	125.8
FABP4	119.7	130.4	134.3	120.2	114.9	107.7	121.7	116.7
FABP5	114.3	120	121.6	124.1	111.8	111.7	121.6	115.6
FABP5L3	115.4	116.4	122.5	119.7	113.9	96.8	120.9	153.5
FABP6	109.7	111.9	114.1	115.6	101.1	100.6	112.6	115.6
FABP6	113.3	110.4	118.3	105.9	112.5	100	114.7	109.5
FABP7	121.4	119.2	113.6	119.8	109.7	106.8	122.6	123
FADD	445.9	487.5	462	434.8	325.7	295.6	353.8	343.2
FADS1	163	129.6	151.9	140.3	119.9	119.5	121.6	121.4
FADS2	118.7	125.3	120.3	123.1	110	102.3	121.5	111
FADS3	100.9	129.7	113.1	113.8	111.7	100.2	123.8	108.8
FADS6	114.4	120.6	136.2	141.1	126.7	103	123.2	128.1
FAF1	180	217	170.7	187.3	162.6	136.2	169.6	171.6
FAF1	142.3	145.7	162.4	153.6	144.7	113.6	134	125.5
FAH	127.3	109.5	161.2	126.9	118.7	112.4	132.7	123.5
FAHD1	173.9	176.7	174.6	172	138.5	147.5	139.5	148.1
FAHD1	126	142.1	135.6	142.2	124.9	113.7	127.7	132.7
FAHD1	112.8	123	118.4	116.5	105.5	96.3	113.5	117
FAHD2A	117.2	122.7	138.2	139.2	121.3	109.1	132.9	127.4
FAHD2B	173.2	165.5	163.5	150.6	132.4	137.1	125.6	135.9

FAIM	117	117.1	130.7	121.9	117.7	107.6	117.7	124.7
FAIM	110.7	106.9	110.8	121.4	108.1	98.6	119.5	103.9
FAIM	124.1	124.1	125.8	118.9	117.5	105.9	128.5	118.7
FAIM	125.5	118.9	114.9	112.5	105.6	102.6	110.8	120.7
FAIM	143.3	142.8	160.2	158.3	145.3	108.1	140.5	132.5
FAIM2	117.5	112.1	118.7	117	108	93.3	120.5	111.6
FAIM3	5734.7	5089.9	3374.9	4935	4164.3	3810.8	5848.2	6397.5
FALZ	115.7	111.7	113	122.2	112.6	94.8	112.6	121.9
FALZ	145.3	135.8	131	148.7	141.4	111.7	140.4	134.3
FALZ	119.9	120.3	115.4	130.4	126.9	109.5	128.1	125.3
FAM100A	211.4	189.9	177.9	193.3	135.8	145.9	260.9	219
FAM100B	243	232.5	170.5	223	185.8	183.2	334.9	379.6
FAM101A	117.8	113.3	113.4	124.9	112.1	98.6	109.6	121.6
FAM101B	192.3	167.8	164.8	180	157.7	123.2	171.3	204.4
FAM102A	511.6	798.6	747.2	509.1	588	531.4	496.5	512.2
FAM102A	125.7	154.4	135.8	135.4	128.7	117.1	130.8	144.7
FAM102B	124.6	130.6	165.7	149.8	132.9	132.4	128.2	136.8
FAM103A1	257.8	245.6	314.1	294.5	230.2	240.4	300.6	284.1
FAM104A	687.9	742.3	748	893.9	601.2	538.2	882.4	918.8
FAM105A	146.1	126.7	164	142.2	123.4	119.4	160.6	125.4
FAM105B	287.2	318.5	291	340.6	271.3	242.6	352.5	423.3
FAM107A	128.9	125.3	132.8	121.9	123.5	112.5	125.2	119.8
FAM107B	1651.5	1814	1180.1	1689.3	1257.1	1034.6	1746.3	2096.3
FAM108A1	97.4	115.5	112.4	105.9	99.4	97.2	108	109
FAM108A2	286	226.5	294.3	256	177.8	220	180.9	190.1
FAM108A2	124.6	138.2	128.5	122.6	126.3	120.8	142.8	115.6
FAM109A	136.4	140.4	129.2	131.9	118	108.6	141.8	129.1
FAM109B	113.1	116.4	113.1	120.4	111.8	102.1	127.5	115.5
FAM10A4	110.4	99.2	112.2	114.2	106.4	104.1	123.5	118.9
FAM10A6	122.6	110.2	109.6	117	113.2	94.3	115.9	117.5
FAM10A7	139.1	130.8	134.8	143.7	135.9	115.7	156.5	148.9
FAM110C	116.3	118.8	120.4	117.5	115.7	104.3	117.5	117
FAM111A	439.7	502.8	417.7	409.9	284.4	303	328.1	367.1
FAM111A	302.4	296.3	346.9	297.7	230.5	202.9	210.1	228.5
FAM111B	125.4	116.1	126.9	126.4	109	103.5	118.7	123.2
FAM112A	119.1	125.8	118.9	129.2	107.6	100.6	118.1	115.2
FAM112A	116.9	107.5	111.5	123.2	110	95.7	113.4	113.3
FAM112B	123.4	121.9	131.9	143.6	131.2	105.5	132.8	132.4
FAM113A	148.9	167.7	166.1	164.1	141.4	156.1	125.8	135.3
FAM113B	1070.4	1579.1	921.4	1130.8	775.2	721.8	636.3	698.7
FAM119A	1538.7	1633.9	1532.8	1384.4	1390.2	957.9	1595.5	1970
FAM120A	1079.3	910.1	1111.9	1109.8	921.3	814.3	999.6	939.1
FAM120C	118.6	101.6	116	114.1	110.1	96.5	114.4	106
FAM122A	119.5	135.9	127.8	151.1	113.3	109.1	120	123.1
FAM122B	305.6	345.8	375.8	352.6	301.6	268.5	298	284.1
FAM122C	112.8	116	118.2	112.5	112.1	96.1	110	117.8
FAM123A	113.3	102.1	112.7	117.1	108.6	100.9	121	107.6
FAM125A	255.9	213.1	305.4	254.3	193.2	198.7	196.8	184.4
FAM125B	125.9	121.3	119.2	110.3	103.7	96.7	118	119.6
FAM125B	113.7	107.5	104.6	111.8	106.7	94.7	113.1	112
FAM126A	111.9	107.2	109	128.5	107.1	94.4	115.4	108.5

FAM126B	108.1	109.8	108.3	110.7	107.1	94.3	123.5	115.6
FAM127B	113.2	111.2	119.2	121.8	118.4	104.1	114.3	108.8
FAM127C	115.1	105.6	117.6	117.3	108.2	95.2	107.7	113
FAM129B	128.2	114.4	118.2	126.5	117.6	102.9	124.7	123.2
FAM129B	176.9	179.8	161.6	214.5	157	159.6	213.9	155.8
FAM12A	121.7	143.9	132.3	141.2	115.9	107.8	140.4	129.8
FAM12B	121.9	118.2	112	115.6	105.5	102.9	129.8	106.5
FAM130A1	208.8	195	184.1	218.6	196	164.8	272.4	211.7
FAM130A2	117.4	119.4	109.2	123.4	111.1	101.1	114.9	112.1
FAM13A1	112.2	116.8	120.7	120.7	106.4	101.9	116.9	114.9
FAM13A1	113.5	109.7	117.8	123.6	111.9	99.9	119.4	122
FAM13A1	115.3	108.5	115.1	113.4	113.7	100.1	115.5	115.7
FAM13C1	111.7	116	115.9	115.7	112.4	98.7	116.2	112
FAM13C1	105.4	105.5	107.1	116.7	104.5	94.8	110.8	111.6
FAM13C1	114.1	113.9	122.1	118.6	107.3	100.6	119.1	108.7
FAM14A	160.9	161.2	167	185.3	136.6	170.6	154.9	141.4
FAM14B	130.7	122.2	132.3	128.4	112.9	100.1	125.1	114.1
FAM18B	153.5	146.8	196.6	170.2	160.6	140.5	146.2	162.9
FAM18B	350.3	361.5	332.2	355.2	343	314	343.2	421.8
FAM18B2	129.6	114.3	112.8	122.6	110.7	104.7	119	107.9
FAM19A1	126.8	127.3	117.7	124	114.3	96.5	114.7	117.9
FAM19A2	126.6	125	112.3	116	102.3	102.7	128.2	118.7
FAM19A3	110.1	115	116.2	114.7	101.9	98.6	112.2	109.3
FAM19A3	123.4	120.5	126.6	123.9	110.5	108.9	121.5	120.9
FAM19A3	113.7	113.3	114.9	117.7	95.5	93.7	108.6	100.2
FAM19A4	127.6	115.4	123.8	134.4	117.3	104.2	118.9	121.1
FAM19A4	118.8	114.3	115.7	125.1	107.4	103.3	119.1	112.1
FAM19A4	114.2	114.9	121.8	112.8	107.7	101.4	120.7	121.6
FAM19A5	113.9	111.3	122.7	118	110.1	107.3	113.4	117.6
FAM20A	118.9	115.4	113.1	130.1	104.3	105.2	113.6	116.3
FAM20B	270.4	254.6	267.3	278.8	220.9	247.8	266.6	284.3
FAM20C	109.9	116.4	114.1	116.5	105.2	99.8	117.1	111.4
FAM20C	269	183.6	252.1	287.7	138.3	241.6	253.5	151.4
FAM20C	125.4	129	131.1	139.5	122.1	112.2	131	129.3
FAM21C	131.6	136.3	131.8	126.4	117.7	120.7	155.5	145.8
FAM22G	113	123.6	127	118.7	110.4	98.1	118.5	117.1
FAM24A	114	116.9	112.8	126.6	109.1	99.5	110.2	122.1
FAM24B	146.5	146.8	162.1	145.3	135.1	127.1	128.7	155.7
FAM26B	153	174.4	158.6	149.1	148.1	129.2	144.6	143.1
FAM26C	160.8	165.3	164.4	163.8	127.4	127.6	168.1	141.5
FAM27L	123.2	124.2	111.9	112.5	115.3	98.9	110.1	108.9
FAM29A	120.3	113.7	125.1	112.7	108.1	97.8	116.9	114.7
FAM29A	133	107.2	125.9	118.5	107.8	111.1	107.8	115.8
FAM33A	140.7	158.8	172.9	148.9	137.8	120.8	159.5	133.6
FAM35A	130.7	143.6	130	168.3	123.5	129.7	150.4	152.4
FAM36A	146.5	155.4	152.5	158.7	159.2	135.4	150.5	187.7
FAM38A	391.2	365.8	302	407	344.8	253.9	395.4	406.1
FAM38B	111.4	124.2	131.4	127.9	111.9	105.1	124.6	123.7
FAM3A	552.3	625	550.3	521.1	414.8	565	526.5	493.2
FAM3B	114	124.4	125	118.2	111	96.1	110.6	111.7
FAM3B	112	107.1	123.8	108.4	100.6	96.2	125.2	114.8

FAM3C	141.3	155.8	138	157.3	141.7	136.7	133.5	139.8
FAM3C	151.3	149.9	151.8	156.2	138.6	143.1	136.3	134.4
FAM3D	118.1	121.6	118.7	119.4	115.1	101.5	110.8	125.6
FAM40A	185.1	199.5	168.8	192.6	184.9	144.7	206.6	164.3
FAM40B	303	247.3	293.8	289.2	312.2	186.9	280.8	296.5
FAM43A	325.3	328	392.2	272.4	260.1	232	202.7	221.5
FAM43B	110.9	113.7	111.5	111.8	107.2	99.3	114.5	112.6
FAM44A	114.9	109.5	110.8	110.2	112.7	97.4	109.2	116.6
FAM44B	267.5	328.4	339.4	323.7	292.3	242.3	263.7	252.9
FAM45A	204	223.7	306.5	240.6	214.7	189	180.8	207
FAM45B	110.3	127.7	110.6	126.2	110.4	97.5	118.1	115
FAM46A	491	421.7	601.2	500	326.6	327.5	440.3	467.3
FAM46B	111.9	113.1	115.1	117.1	104.8	100.4	113.7	116.4
FAM46C	524.8	416.2	494.7	576.8	427.5	494.8	564.3	554.5
FAM46D	115.1	108	112.8	115.1	106.9	97.6	104.9	109.5
FAM47A	119.7	116.3	123.9	109.1	107.5	102.1	105.9	119.8
FAM47B	123.1	114.1	119.3	123.2	106.7	103.6	119.3	117.1
FAM47C	112.9	114.9	125.4	119.5	124.7	108.3	115.9	118.3
FAM48A	216.6	243.7	224.6	242.8	197.9	166.1	215.9	211.8
FAM48A	117.8	102.3	113.4	121.2	102.2	98.8	126.7	122.1
FAM49A	129.5	132.6	117.1	118.2	107.8	111.4	116.7	115.6
FAM49B	210.8	167.9	256.9	195.7	184.8	265.5	243.3	267.6
FAM50A	1634.1	1502.1	1585	1773	1046.1	1192	1646.8	1466.2
FAM50B	162.4	232.9	186.8	186.5	227.9	177.1	186.2	168.4
FAM51A1	127.2	136.5	131.4	127.9	115.9	102.5	119.2	121.1
FAM53A	128	133.9	127.8	133.4	119.7	109.8	129.4	135.2
FAM53B	127.6	122.2	140.8	136.3	124.4	121.8	127.5	131.6
FAM53C	467	527	354.4	490	411.8	273.6	751.7	1055.1
FAM54A	116.1	119.1	112.4	115.7	111.7	104.9	123	114.4
FAM55A	111.2	107.1	110.5	102.6	106.9	97	116	112
FAM55B	122.3	117.6	113.2	114.2	106	98.9	126.5	113.1
FAM55C	139.8	156.1	142.3	136.5	153.5	113.2	134.9	145.5
FAM55D	117.7	114.8	115.5	116.9	109.8	96.4	133.4	110.9
FAM55D	116.4	105.1	113.5	121.4	103.5	93	116.2	112.7
FAM55D	118.5	113.7	124	118.3	115.4	98.6	113.5	106.3
FAM57A	130.5	139.6	127.9	142.3	112.9	115.5	127.3	131.4
FAM58A	310.4	346.8	320.7	361.2	282	259.3	308	265
FAM59A	113	120.7	119.8	123	109.9	101.2	115.6	119.6
FAM5B	118.5	110	116.5	122.9	110.9	100.1	117	124.4
FAM5C	119.7	122.1	132.2	126.7	114.7	106.3	128	117.4
FAM62A	426.3	906.6	602.2	678.2	487.8	422.6	518.3	522
FAM62B	2154.9	2973.4	1825.6	2240.8	2325.2	1465.1	2506.8	2795.9
FAM62C	112.6	122.1	116.6	122.7	112.2	106.2	119.2	117.1
FAM63A	404.6	302.6	394.8	324.4	343.2	327.4	391	307.2
FAM63B	123.2	119.1	129.7	120.9	115	107.7	122.7	124.9
FAM64A	110.5	113.4	112.4	112.6	107	98.1	120.9	122.3
FAM65A	458.3	471.6	474	629.8	342.6	331.2	491.2	552.6
FAM69A	141.4	135.3	155.9	154.6	140.1	115.5	143.6	128.3
FAM69B	137.9	128.5	140.9	148.1	117.9	115.3	130.8	113
FAM69B	117.3	117	120.7	111.8	109.9	102.2	110.8	121.1
FAM70A	113.4	116.9	117.5	119.1	104.3	101.2	131	114.6

FAM70B	119.7	116.3	127	123.2	103.6	99.4	119.9	118.8
FAM71A	126.3	116.2	117.2	99.4	101.7	99.7	108.6	116.8
FAM71C	109.9	110.8	125.1	124.4	104.8	97.5	115.2	119.7
FAM72A	117.5	122.9	119.6	119.9	120.4	99.5	128.5	119.5
FAM73A	116.4	115.8	120.9	115.4	114.3	99.6	126.1	110.1
FAM73B	176.6	170.2	155.7	167.4	144.7	151.7	177.2	178.7
FAM76A	160.8	155.3	136	154	129	119.3	147.8	135
FAM76B	211.9	227.4	250.8	235.8	177.2	183.7	217.9	219.3
FAM77C	112.8	108.5	110	119	103.3	94.3	116	117.4
FAM77D	120.2	114.6	123.6	133.9	126.3	104	119.6	121.6
FAM78A	143.2	155.3	157.7	156	134.7	129.5	143.9	137.7
FAM78B	126.9	126.4	116.4	126	118.2	106.2	127.5	136.4
FAM79A	968.3	1228	807.5	1185.5	1028.3	818.9	1518.1	1698.8
FAM79B	115	119.6	124.6	112.3	116.6	104.5	117.6	110.6
FAM7A2	120.8	105.8	119.7	114.3	107.7	103.8	122.2	116.7
FAM80A	121.2	125.5	116.4	116.9	96.9	98.4	125.8	120.7
FAM80B	131.7	134.7	131.5	133.5	136.7	118.5	131.5	152
FAM81A	116.3	105.8	122.8	125.9	109.2	104.7	123.6	110.6
FAM81B	123.3	114.2	123.6	120.8	109.1	115.1	129.3	132.8
FAM82A	126.9	114.4	125.1	117.8	102.7	100.1	117.3	124.2
FAM82C	220.1	238.4	259.6	225.7	196.3	229.4	205.8	208.8
FAM83A	114	107.1	109.4	113.5	106.4	111.3	124	116.7
FAM83A	110.5	114.2	114.6	109.7	96.8	96.1	105.1	99.4
FAM83A	109.5	104.8	111.2	123.3	106.1	101.6	111.1	107.7
FAM83E	119.4	114.2	124.6	118.4	104.8	102	119.4	119
FAM83F	125.1	116.6	124.3	120.7	119.4	99.5	133.8	121.8
FAM83G	112	115.2	119.7	114.6	100.4	101	123.3	117.4
FAM84A	119.4	116.1	114	116.6	105	102.8	114.5	116.1
FAM84B	377.9	392.4	274.3	382.1	405.8	240.1	431.2	457.8
FAM86A	131.7	134.5	132.5	126.8	124.6	118	135.8	141.1
FAM86A	113.6	126.3	126	129.2	123.5	104.8	130.9	129.1
FAM89A	153.6	163.4	161.1	167.3	152.1	119.6	171.8	195.4
FAM89A	331.4	368.6	281.4	441.1	320.7	196.7	476.7	462.7
FAM89B	495.8	621.8	497.2	473.9	305.7	341.5	412.9	405.1
FAM8A1	186.9	174.1	144	187.9	153.7	159.5	200.7	186.2
FAM90A1	109.6	114.6	118.1	131.9	111.9	103.3	115.2	121.7
FAM91A1	142.3	147.3	144.3	178.5	136	134.8	166.2	140.4
FAM92B	120.1	113.1	113.1	114.6	113.1	94.4	117.1	125.5
FAM96A	969.4	1017.2	1866.2	967.9	762.8	1018.3	792.1	771.4
FAM96A	153.7	171.5	232.8	197.8	147.9	160.9	179.2	171.1
FAM96B	365.6	418.5	437.2	455.9	324.5	371.1	314.2	293.5
FAM98A	223.4	241.1	255.7	286.5	194.4	190.8	256.4	209.8
FAM98B	127.2	120.9	132	132	119.1	118	130.6	116.1
FAM98B	121	109.7	117.5	116	112.2	103.3	116.2	117.6
FAM98C	137.7	143	170.9	163.1	152.2	130.4	151.6	150.5
FAM9A	117.7	122.7	108.8	113.2	111.1	92.9	112.4	114.1
FAM9B	110.1	112.3	119.1	123.3	114.4	98.3	129.7	109
FAM9C	115.1	111.3	110.9	118.1	115.9	96.2	120.8	126.2
FANCA	114	118	111.2	131.7	118.7	103.4	121.3	118.2
FANCA	117.3	126.2	126	125.4	116.2	113.3	123.9	128.2
FANCA	116.9	125.4	116.4	114.9	110.8	102.9	114.7	121.7

FANCB	124.3	131.6	135.3	133.3	127.6	101.3	124.9	124.3
FANCB	126.6	129.8	121.8	132.6	119.1	108.6	127	121
FANCC	127.6	119.1	121.3	126.7	116.2	98.6	123.9	123.7
FANCD2	138.7	159.6	194.8	155.7	148.5	167.5	156.6	134.1
FANCD2	117.4	114	110.8	115	119.6	90.8	116.3	111
FANCD2	121	113	120.2	119.6	108.7	100	125	115.5
FANCE	208.3	221.2	185	203.4	221	130.1	200	202
FANCF	112.7	123.8	119.1	126.3	118.9	102.9	126.8	117.4
FANCG	157.6	177.7	167.5	168.2	150.1	128.6	156.1	151.9
FANCL	117.4	116.6	112.3	116.8	103.8	103.8	107.4	123.2
FANCM	119.6	117.9	131.8	137.5	118.4	107.4	123.3	123.3
FANK1	124.3	120.1	125.4	128.3	109.9	102.3	130.8	119.8
FAP	106	115.2	116.9	110.7	113.5	96.6	108.8	115.3
FARP1	121.6	112.8	120.3	116.3	117.8	100.5	115.1	114.9
FARP1	112.3	119.8	117.3	110.7	106.1	100.5	119.4	112
FARP1	111.2	111.4	110.9	113.5	110.2	100.8	116.3	117
FARP2	134.5	138	144.3	127.7	122.9	108.1	125.7	130
FARS2	214.4	227.8	263.2	234.9	205.5	206.6	183.4	177.6
FARSB	120.8	113.9	115.9	119.7	118.2	108.9	117.9	123.1
FARSLA	154.1	181.5	178.7	184.3	124.4	126.2	153.3	164.1
FAS	123.6	115.1	132.6	134.2	108.8	100.7	127	113.1
FAS	128.7	119.3	136	133.4	111.8	109.6	134	129.1
FASLG	186.6	156.9	146.8	148.3	132.6	124.6	136.7	123.4
FASN	242.5	288.8	258.5	328.5	216.9	208.6	288.1	320.4
FASTK	443.3	495.7	496.5	472.3	349.2	275	331.1	406.6
FASTK	149.4	171.6	144.3	157.6	153.5	128.7	145.9	137
FASTK	168.7	184.8	192.1	168.6	147.8	170.7	190.4	168.6
FASTKD2	126.1	133.2	134	129.7	125.7	122.6	123.7	117
FASTKD3	152.4	176.2	176.2	188.2	151.5	139.5	154.9	162.6
FAT	117.3	124	118.3	126	115.4	95.1	113.6	114.2
FAT2	115.3	109.2	107.6	116	102.9	94	109.2	103.5
FAT4	114.5	132.1	122.3	123.8	118.1	107.9	115.1	120
FATE1	118.8	114.9	120.6	116	109.8	107.7	124	118.3
FAU	7001	9333.9	10174.2	8051.3	8026.6	7298.1	7291.6	8715.2
FBF1	117	122.5	121	122.7	119.6	102	129	117.9
FBL	956.5	1078.6	1014.9	997	817.2	787.2	931.5	1169.6
FBLIM1	111.5	111.5	105.9	113.2	108.4	101.9	125	110.2
FBLIM1	116.9	124.1	124.4	126.2	102.7	99.7	119.3	120.2
FBLN1	124.7	121.7	113.9	121.9	108	101.7	128	113.1
FBLN1	111.5	121.3	127.9	117.8	118.8	96.5	126.3	114.5
FBLN1	118.6	120.9	118	124.6	114.5	100.6	109.5	113.7
FBLN1	116.7	110.8	115	121.5	119.3	97.3	120.8	119.2
FBLN1	119	112.5	113.5	122	109.3	103	122.1	120.9
FBLN2	110.8	112.5	104.4	110.6	105.2	94.3	111.9	110.1
FBLN2	125.6	123.7	123.5	121.5	99.2	108.2	126.4	115.3
FBLN2	128.7	140.5	139.1	133.2	129.6	108.9	130.6	128.5
FBLN5	145.9	161.4	209.7	164.6	145.1	166.9	163.2	163.9
FBN1	108.9	121	125.7	129.5	115.4	104.3	133	136.5
FBN2	132.5	123.3	154.1	144.9	112.4	113.8	119.5	127.1
FBN3	113	131.3	118.5	116.7	108.6	97.6	117.8	113.3
FBP1	351.2	342.6	235.1	293.5	189.3	126.6	255	179.9

FBP2	110.2	108.3	107.8	121.5	107.7	100.5	113.6	114.3
FBS1	145.7	160.3	144	154.9	146.8	125.2	198.7	170
FBXL10	106.3	112.3	112.7	120.2	115.2	103.1	120	115.7
FBXL10	1206.9	1259.2	1298.5	1074.3	942.2	915.6	1147.9	1056
FBXL10	120.5	119.5	129.6	142.3	112.8	100.4	135.4	128
FBXL12	135.4	132.3	144.5	140.3	121.1	117.6	132.9	135.8
FBXL13	98.6	115.4	115.6	122.8	112.8	98.8	121.7	112.3
FBXL14	118.5	129.3	124.7	126.3	113.6	106.4	128.1	125.1
FBXL15	303	345.1	271.4	286.9	235.8	203.8	222	216.4
FBXL16	178	165.6	155.4	160.5	118.6	129.6	129.4	126.6
FBXL17	121.8	135.3	145.6	125.5	120.8	117.4	125.1	123.3
FBXL18	111.3	115.7	113.6	116.3	110.5	99.5	118.3	118.1
FBXL2	127	116.7	121.8	118.2	109.4	94.5	114.8	108.2
FBXL20	148.7	154.1	145.4	157	134.5	135.8	175.9	155.4
FBXL21	120.7	123	130.5	111.6	109.5	102.4	112.9	115.8
FBXL22	116	108.1	118.7	112.1	109	95.3	115	107.1
FBXL3	117.1	112.2	124.9	107.2	114.1	103.8	116.1	112.2
FBXL4	106.8	111.6	121.3	130.2	106.1	94	120.7	112.2
FBXL5	101.8	115	119.2	109.9	110.7	97.9	116.5	115.2
FBXL5	126.2	126.1	136.7	120.5	129.9	108.7	135	128.3
FBXL5	318.8	363.5	344.8	440.7	313.4	330	336.8	294.6
FBXL6	164.3	172.9	182.8	181.3	154.3	141.1	185.1	168.5
FBXL7	131.4	122.6	138.2	134.4	116.1	107.4	128.2	129.1
FBXL8	120.8	115	128.2	123.8	118.7	100.3	123.6	115.8
FBXO10	125.4	120.8	119.6	119.7	116.6	98.7	119.6	110.1
FBXO10	134.5	118.3	133.4	124.3	109.5	105.9	142.4	129.1
FBXO11	134.5	122.5	128.6	127.8	113.3	103.2	127.6	128.6
FBXO11	622.7	675	565.5	728.3	497	507.6	960.5	826.9
FBXO11	117.1	118.3	119.7	128.8	114	98.2	124.5	118.6
FBXO11	137.7	133.2	140.4	145.4	141.7	113.4	126.8	142
FBXO15	123.7	128.2	139.5	140.2	120.5	118	135.2	125.9
FBXO16	110	107.4	118.2	114.1	100.6	100.8	124.5	114.1
FBXO17	114.7	107.3	112.6	118	106.3	99.9	109.3	106.7
FBXO17	121.5	114.9	117.2	122.7	112.9	104.6	118.4	121.8
FBXO17	124.5	128.1	128.7	116.2	109.6	108.2	115.9	119.4
FBXO18	112.7	115.1	120.8	117.2	113.8	101.2	124.6	121.1
FBXO18	396.3	435.5	440	414.9	329.1	282.4	420.4	396.7
FBXO2	167.1	156.4	134	146.7	128.7	108.2	155.5	138.6
FBXO21	166.1	145.7	152.3	170.1	143.8	139.5	155.3	161.1
FBXO21	427.3	502.5	485.7	500.6	478.6	363.2	480.1	506.2
FBXO22	131.7	154.2	162.3	142.1	129.4	115.1	123.6	125.2
FBXO22	113.3	113.3	121.7	111	102	95.5	115.9	107.6
FBXO22	131.7	136.8	137.9	138	119.3	107.4	130.5	131.4
FBXO22OS	115	111.9	113.4	118.1	107.9	97	119.2	118.3
FBXO24	103.9	110	110.2	112.9	109	95.9	113.1	107.5
FBXO24	111.5	111	112.2	118.5	106.5	91.4	111.5	110.4
FBXO24	110.6	112.1	111.1	112	104.8	100.9	117.7	115.9
FBXO25	117.1	126.7	109.1	116.5	112.4	96	114.1	115.3
FBXO27	118	115.1	117.3	117.3	107.2	93.4	123.2	107.8
FBXO28	271.5	279.8	261.1	294.7	236.8	226.5	330.1	290.6
FBXO3	134.5	121.4	133.5	148.4	126.5	103.2	120.1	127.3

FBXO3	163	155.8	186.2	173.2	170.5	144.5	140.2	169.8
FBXO3	138.8	145.1	152	154.2	136.8	110.5	137	137.6
FBXO30	186.3	173.7	184.4	198.5	187.3	190.5	244.9	278.5
FBXO31	437.8	543.2	387.2	507.4	352.6	320.8	596.9	527.4
FBXO32	141.2	146.9	161.5	148.3	134.1	123.3	144.5	163.1
FBXO32	117.8	108.9	113.1	117.6	112.1	102.2	121	123.8
FBXO32	341.6	351.3	318.2	297.8	263.1	224.3	279.7	375.1
FBXO33	769.8	901.2	796.9	817.7	814.9	716.1	741.5	923.4
FBXO34	317.3	330.1	309.2	302	266.8	240.9	327.7	330.8
FBXO36	110.5	116.7	123.6	115.8	108.8	94.8	118	128.6
FBXO38	125.9	129.5	120.5	130.7	125.8	109.1	128.2	120.7
FBXO38	199.9	175.7	173.3	151.9	156.9	137.9	184	176.1
FBXO39	111.3	123	119.8	119.5	103.6	94	115.4	113.2
FBXO4	134	164.4	180.7	155.8	127.9	131.7	153.5	138.2
FBXO4	118.3	120.8	120.7	118.9	110.8	106.3	122.1	123.4
FBXO40	125.5	116.4	116.9	120.7	117.2	97.7	117.4	123
FBXO42	134.8	139.5	147	130.4	132.2	123.1	141.3	137.7
FBXO43	107.6	117.8	131.5	120.7	118.9	110.4	121.3	117
FBXO43	114.2	119.5	122.5	113.7	106.9	104.9	121.8	119.6
FBXO44	125	131.7	151.6	132.3	119.2	119.9	128.2	115.5
FBXO44	114.2	119.5	132.5	121.4	118	109	121.2	119.2
FBXO44	107.8	102.3	118.5	115	110.3	98.8	111.5	108.4
FBXO46	152.5	150.3	131.6	154.4	126.3	129.2	149.9	137.5
FBXO47	119.4	112.8	109.4	117.5	98.5	90.8	112.1	114.1
FBXO5	134.5	124.7	146.5	141.3	125.7	102.8	143.7	129
FBXO6	192.7	182.8	184.3	187.6	136.5	127.9	138.3	142
FBXO7	149.9	160.4	200.1	190.3	142.3	155.9	160.9	171.6
FBXO7	130.2	124	130.4	132	131.7	110.6	135	122
FBXO7	351.3	389.1	333.9	336.4	314.3	272.8	437.6	454
FBXO8	177	213.2	201.9	218	191.4	193.3	192.4	228.2
FBXO9	112.5	106.6	115.7	105.1	110.5	90.2	114	108.5
FBXO9	126.9	130.3	128.7	141.1	123.9	95.9	132	126
FBXO9	111.7	126.4	110.3	115.1	109.3	93.5	117.6	114.5
FBXO9	115.9	115.3	122.2	135.8	112.2	106.2	123.1	116.2
FBXW10	119.2	115.2	118.2	128.2	103.8	104	117.4	115.1
FBXW11	112.7	117.8	125.6	113.9	107.1	102.3	112.1	121.9
FBXW11	118.3	120.8	122.9	114.4	113.8	96.8	111.2	121.1
FBXW11	461.9	444.9	469.2	524.2	343.7	408.8	524.6	491.6
FBXW12	112.3	117.4	112.9	118.8	115.6	98.6	107.3	115
FBXW2	210.7	210.2	157.8	196.4	194.9	152.4	201	185.7
FBXW4	183	191.6	186.3	186.9	172	166.4	218.4	201
FBXW5	266.8	338.5	291.4	289.4	235.1	229.4	321.3	245.3
FBXW7	216.2	223.2	198.1	249.8	218.4	113.1	241.4	288.4
FBXW7	109.6	130.8	122.9	127.3	115.8	115.9	126.9	118.5
FBXW7	295.8	251.3	264.1	300.7	249.6	247.9	277.3	291.7
FBXW7	110.9	122.6	117.6	121.8	103.7	104.5	116.1	103.2
FBXW8	111.1	113.4	112	121.8	107.1	100.5	120.8	117.5
FBXW8	116.5	112	120.3	115.8	103.7	95.1	118.7	119.7
FBXW8	173.1	159.3	189	182.7	151.7	150.8	174.1	173.1
FBXW9	139.2	141.5	144.9	131.3	133.1	119.9	123	139.5
FCAMR	112.2	117.8	111.8	122.8	111.6	101	126.6	112.4

FCAR	449.4	361.2	489	399.9	395.7	328.7	651.7	471.4
FCAR	138.1	157.8	197.2	170.1	185.7	180.3	164.4	177.3
FCAR	128.8	136.1	125.8	171.5	143.7	211.9	149.4	118.1
FCER1G	1274.1	586.8	2731.1	1166.3	486.1	1686.9	736.8	435.3
FCER2	201.5	136.8	146.5	200.6	124.5	119.2	181.2	132.3
FCF1	127.9	124.4	132.5	136.4	121.1	109.3	144.7	143.5
FCGBP	148.6	142.2	131.4	135.3	126	110.4	141.8	157.8
FCGBP	854.8	1597	525.6	828.4	998	273.4	759.5	1022.1
FCGR1A	158.5	120.3	147.8	136	115.8	111.9	129.2	117.3
FCGR1B	272.6	118	312	179.6	114.1	140.4	116.8	118.7
FCGR1B	167.8	128.5	220.5	140.8	115.7	110.7	129.8	123.1
FCGR2A	125.8	126.7	180.6	129.4	109.3	155.6	158.7	128.2
FCGR2A	133.8	119.6	162.8	128.8	115.6	124.1	127.8	123.7
FCGR2B	121.6	129.8	178.8	134.1	123	127.3	125.9	124.1
FCGR2C	110.1	111.2	112.1	109.8	99.8	94.9	111	108.5
FCGR3A	111.1	112.2	119.6	114.5	100.9	101.8	108.1	106.2
FCGR3B	133.3	129.1	135.9	136.5	114.4	115.1	127.8	125.8
FCGRT	352.1	274.6	1188.4	370	204.9	650.8	240.5	210.3
FCHO1	225.9	239.9	219.5	221.8	168.4	162	214.7	193.6
FCHO2	118.2	123.4	118.3	125	116.1	108.4	137.7	120.8
FCHSD1	117.7	121.1	118.7	116.2	114.1	110.4	118	111
FCHSD2	301.3	231.4	229.1	274.4	210.1	192	209.7	202.6
FCMD	3652.3	2753.9	3117	4215.2	2732.5	2008.9	3293.4	2527.1
FCMD	113.2	110.9	125.1	112.5	110.1	93	129.4	118.5
FCN1	550.3	170.7	5839.6	562.1	170.8	2336.1	358.5	192.4
FCN2	116	113.2	114.5	122	117.3	93.9	115.7	119.9
FCN2	125.1	121.7	147.1	132.4	116.1	110.6	134.9	114.2
FCN3	115.2	109.2	118.2	115.3	95.5	108.4	117.4	125.1
FCN3	126.2	109.7	124.8	120.5	111.1	108.4	122.7	125.5
FCRL1	111.7	113.6	113	113.6	107.2	101.6	121.4	116
FCRL2	106.2	113.3	106.6	112.4	102.2	88.6	107.1	108.4
FCRL2	187.2	157	196.1	191.6	136.1	147.4	143.7	118.3
FCRL2	114.8	115.1	116.8	123.4	115.5	95.8	124.3	116.3
FCRL3	162	140.9	127.7	158.1	118.4	115	124.4	122.6
FCRL3	224.5	174.2	165.6	213.4	145.7	136.2	163.3	135.4
FCRL4	115.7	105.3	111.2	115.8	104.6	102.1	124.7	106.6
FCRL5	111.9	119.6	119.7	128.8	120.7	103.1	126.2	125.6
FCRL6	111.2	118.1	128.8	117.2	102.2	98.8	113.6	110.3
FCRLM1	288.5	216.2	304.2	242.2	162.6	165.8	158	130
FCRLM2	134.6	129.7	155	130.8	126.2	114.1	119.4	120.4
FDFT1	1246.7	1547.3	1145.8	1212.8	887.1	796.3	1214.2	1433.3
FDX1	118.5	118.3	127.5	128	125.4	105.9	118.6	130.9
FDXR	124.1	106.1	111.8	121	110.3	99.1	119.1	118.5
FDXR	118.8	125.1	126.7	129.5	104.6	106.4	122.8	121.4
FECH	110.2	118.8	116	122	110.3	99.2	123.5	120.5
FECH	157.5	169.9	149.1	161.8	170.6	135.5	160.1	197.9
FEM1A	399.9	420.8	367	456.8	403.4	316.7	442.9	378.2
FEM1B	135.3	134.9	138.3	130.3	130.5	112.4	145.5	123.6
FEM1C	244.6	276.6	249	240.5	251.6	236.5	297.8	393
FEN1	303.4	334.7	244.3	310.5	212.4	225.4	286.7	265.4
FER	123.3	112.6	119.5	131.8	115.6	102.4	123.5	117.9

FER1L3	291.6	221.7	508.1	240.7	159.8	160.8	214.7	146
FER1L3	123.6	134.3	179.3	131.1	111.8	110	136.5	115.9
FER1L4	118.4	123.2	112.9	126.3	113.1	92.3	131.2	123.2
FERD3L	118.8	110.7	131.3	127.7	104.9	105.7	113.2	118.2
FES	148.3	132.7	256.7	126.9	114.3	121.6	125	115.7
FETUB	117.9	110.6	117.9	115.9	102.3	94.8	121.2	110
FEV	112.5	119.2	120.1	124	108.2	97.3	122.1	121.5
FEZ1	123	124.2	116.4	109.4	118.6	106.4	132.2	125.6
FEZ1	159.1	169.6	168.9	155.1	164.6	124.8	155.6	147.8
FEZ1	241.4	247.3	139.9	189.2	176.3	112	176.2	156.9
FEZ2	307	295	387.6	309.8	300.2	329.9	349.4	327.6
FEZ2	442.9	534.8	540	478.9	489	523.8	542.5	536.5
FFAR1	125.8	119.7	118.8	126	116.5	109	125.8	110.5
FFAR2	133	116.5	131	114.8	112.4	99	110.7	114.9
FFAR3	112.2	119	120.5	116.1	117.4	97.3	123.9	112.7
FGA	117.1	118.7	116.4	111.4	109.3	113.9	121.8	119.1
FGA	172.9	170.9	174.7	172.1	163.2	134.7	163.2	153.9
FGA	105.6	106.2	110.5	125.8	110	98.1	113.2	120.2
FGB	119.9	126.8	129.3	124	116.3	101.2	125	114.6
FGD1	112.3	115	116.7	118.2	107.6	105	109.8	107.8
FGD2	115	122.4	127	127.7	110.4	105.8	115.9	116.4
FGD3	2285.3	2432.9	2358.3	2240.9	1800.8	1666	2863.2	2435.7
FGD4	106.7	118.7	137.8	117	102.7	98.2	118.7	115.6
FGD5	113.7	106.8	115.6	116	105.1	101.5	103.5	114.1
FGD6	120.9	111.5	114	117.4	110.8	105	124.3	111.2
FGF1	125.1	99.5	112.3	117.6	97.7	96.6	112.5	121.5
FGF1	122	112.3	122.3	130.8	119.7	113.3	113.8	119.2
FGF10	101.8	118	107.8	111.8	105.9	100.2	113.4	109.5
FGF11	108.4	116.6	114.8	117.4	111.6	104.9	124.8	128.7
FGF12	121.9	120.1	111.3	133.5	101.8	97.2	112.8	116.6
FGF12	115	117.8	122.3	116.4	109.9	97.8	117.3	123.9
FGF12	109.7	118.9	109.6	121.4	106	104	118.5	111.4
FGF13	115.2	110.8	114.6	121.4	114.9	100	110.7	113.8
FGF13	105.7	109.5	116.2	113.4	105.1	95.8	126.9	104
FGF13	108.4	112.6	113.4	113.3	108.1	92.6	115.1	112.7
FGF14	118.8	114.7	124.9	119.9	111.8	98.5	125.5	119.7
FGF14	104.6	109.5	126	110.3	113.4	95.3	119.3	107.2
FGF16	115.3	115.2	112.1	116.6	115.2	94.8	125.8	111.3
FGF17	121.8	115.3	119.6	111.6	106.2	100.5	117.9	119
FGF18	150.8	153.1	153	135.4	137.6	127	137.7	158.9
FGF18	108.7	113.4	110	109	107.8	94.8	132.6	111.7
FGF19	116.4	118.6	122.1	129.6	118.5	107.5	125.1	117.6
FGF2	125.5	140.5	135.5	135.2	118	119.4	129.5	123.6
FGF20	113.8	113.3	116.6	113.6	99.8	99.2	107.2	105.8
FGF21	118.3	115.5	114.9	126.1	109.1	103.2	116.5	116.3
FGF22	106.9	112.5	123.9	114.2	102.3	97.1	112.8	107.1
FGF23	106.2	110.9	112.8	116	105.9	87.4	123.7	108.1
FGF3	115.7	121.5	131.9	117.3	106.6	100.9	114.6	122.7
FGF4	117.9	115.6	130.8	124.1	112.1	115.6	121.6	117.7
FGF5	105.2	115.2	116.5	118.6	117.3	99.8	121.9	115.7
FGF5	114.4	120.2	124.6	125.5	121.1	102.9	122.9	120.7

FGF6	108.5	116.9	122.2	119.5	111	99.9	123.5	117.2
FGF7	111.9	105.9	122	109.7	113.3	99.8	115	106.5
FGF8	118.6	116	117	118.6	113.7	104.5	115.8	119.5
FGF8	107.6	117.2	109.2	115.8	97.4	95.1	117.1	114.6
FGF9	162	208.8	174.6	172.3	174.1	145.3	185.9	170.3
FGFBP1	108.1	110.4	108	118.4	103.5	96.4	112.4	111.2
FGFR1	115.9	111.7	116.7	120.9	109.3	103.9	123.2	109.1
FGFR1	113.2	119.8	116.7	123.1	108.1	102.1	117	111.1
FGFR1OP	123.2	128.4	128.4	135.5	129.5	115.6	142.5	129.4
FGFR1OP2	172.5	157.6	174.3	160.8	159.9	149.7	161.9	189.2
FGFR2	105.5	125.6	120	114.3	105.7	103.4	116.8	110.3
FGFR3	112.1	125.8	128.8	130.9	110	117.7	133	120.4
FGFR3	107.3	126.9	112.3	119.1	103.3	102	124.9	122.7
FGFR4	117.2	121.5	119.4	125.2	111.1	101.8	125.1	125.8
FGFR4	105.5	113.6	123.7	115.6	105.2	99.6	116.3	108.7
FGFR4	114.2	119	123.2	116	114.4	99.7	122.5	118.9
FGFRL1	169.9	172.5	192.3	183.3	150	203.8	241.5	234.5
FGFRL1	122.5	123	130.8	119	117.3	116	136.3	128.6
FGG	109.3	107.7	115.8	112	102	93.6	119	111.8
FGG	111.2	115.6	110.8	115	104.7	96.7	121	107.9
FGG	110	106.1	116.9	122.4	108.6	101.2	104.2	120.7
FGL1	114.4	115.3	114.3	110.4	105.2	98.5	121.2	110.4
FGL1	117.7	117.3	114.3	117.4	100.7	97.5	114.8	110.2
FGL2	442.5	249.3	3363.3	319	143.1	999.6	152.8	118.5
FGR	323.5	274.6	259.1	339.7	231.6	192.6	261.8	233.3
FGR	120.2	115.6	131	128.9	118.9	104	124.7	125.6
FGR	675.3	588.3	700.6	702.8	500.4	325	746.4	440.1
FH	362.4	384.2	429.6	350.5	228.3	322.8	299.5	312.2
FHIT	168.9	152.2	187.7	175.8	129.9	142.7	162.5	134.4
FHL1	146.3	148.5	139.9	159.2	141.4	133.9	151.6	136
FHL2	125.1	116.3	114.7	129.9	97.3	102.7	124.7	108.9
FHL2	113.5	128.3	119.8	121.2	107.1	101.1	126.7	113.7
FHL2	117.1	120.9	122.8	120.7	105.9	105.2	120.1	116.6
FHL2	115.1	118.6	111.1	121.2	108.9	96.9	121	111.2
FHL3	214.1	225.3	242.1	240.8	157.6	228.4	195.5	187.1
FHL5	113.5	118.3	119.7	123.8	111.1	95	122.4	114.5
FHOD1	312.7	308	427.9	346.8	233.4	308.3	319.6	248.1
FHOD3	112.6	110.6	113.3	118.7	109.4	104.5	116	125.1
FIBCD1	121.7	131.7	139.1	126.2	104.5	95.8	125.2	128.9
FIBP	431.1	453.2	520.6	360.2	269	260.2	319.1	251.8
FIBP	145.3	137	139.6	136.2	135.7	105.2	134	132.7
FIGF	116.8	118.6	128.3	118.9	108.8	103	129.6	120.1
FIGLA	117.4	118.5	119.2	118.7	107	103.9	119.4	112.3
FIGN	110.3	120.7	114.1	118.9	118.3	98.8	112.2	122
FIGNL1	135.9	125.3	147.4	128.3	132.6	120.6	146.6	128.3
FIGNL1	132.8	136.5	152.9	151.9	132.1	122.9	133.1	135.3
FILIP1	116	122.8	120.7	128.6	110.1	100.3	121.2	111.4
FILIP1L	138.9	117.2	115.8	131.1	114.6	104.8	126.5	121
FIP1L1	119.4	114.1	118.1	115.8	102.6	97.7	124.6	112.4
FIS1	223.6	254.1	288.4	246.5	210.5	235.5	204.6	180.2
FJX1	119.4	112.1	122.4	123.4	106.2	93	124.1	124.1

FKBP10	121.8	125.9	124.2	128.5	121.2	106	122.7	135.3
FKBP11	776.9	895.9	610.3	720.5	518.5	455.6	651	646.6
FKBP14	1362.3	1046.5	1258.1	1125.1	1117.8	658.7	1386.9	1101.1
FKBP1A	144.2	143.7	143.4	148.5	142.9	130	152.5	136.9
FKBP1A	160.1	222.2	187.5	194.5	187.6	114.8	175.9	165.6
FKBP1A	472.9	620.5	485.1	450.6	419.4	324.2	478.7	389.9
FKBP1B	116.5	129	121.6	116.4	113.5	107.1	118.8	112.7
FKBP2	231.4	216.6	230.4	279.2	199	221	255.9	239.9
FKBP2	128.6	124.6	130.6	132.9	129.4	134	134.4	122.3
FKBP2	128.2	117.2	130.6	122.2	123.6	103.7	127.8	121.4
FKBP3	182.5	166.7	224.1	204.7	158.4	181.3	178.8	240.8
FKBP4	236	211.1	174.4	237.9	198.4	161.2	513.5	612.3
FKBP5	350.7	416.1	290.4	314.5	369.2	218.9	370	510.6
FKBP6	114.4	115.9	119.2	112.7	100.9	98.4	116.1	109.2
FKBP7	113.6	114.1	109.7	117	106.4	105.5	116.3	115.9
FKBP8	121.8	125.2	138.2	137.8	114.2	114.3	125	131.5
FKBP9	104.8	109.5	121.8	116.5	102	90.1	114.3	110.2
FKBP9L	110.8	106.1	117.1	116.6	120	100	118.5	109.4
FKBPL	123.8	129.1	126.4	133.1	122.7	102.4	120.9	126.9
FKHL18	116.6	116.9	105.3	113.1	102.8	91.2	110.9	108.8
FKRP	184.3	203.6	193.3	203.8	176.1	135	238.7	226.1
FKRP	141.7	134.1	158.9	138.4	128.9	112	137.7	129.5
FKSG14	196.2	333.5	170.5	154.9	236.9	122.9	145	231.9
FKSG2	115.9	124.5	121.4	124.3	104.7	98.8	121.6	112.9
FKSG24	118.6	127.4	124.8	122.7	121.4	102.3	120.3	127.1
FKSG30	113.5	108.3	110.5	115.5	107.1	97.1	118.8	119
FKSG44	111.8	114.8	112.3	111.9	109.7	96.6	122.8	113.5
FLAD1	133.5	125.7	128.7	130.1	117.3	104.8	140.2	123.3
FLAD1	260.3	244.5	289.2	275.1	207.6	197.4	267	276.6
FLCN	122.3	133.1	120.7	150.6	131.2	105.4	144	154.8
FLCN	127.3	111.7	131.2	139.2	110.3	109.2	127	130.4
FLCN	117	123.9	117.2	129.8	117.5	102.8	132.6	122.8
FLG	111.1	120.2	110.1	115.7	103.9	95.8	122.9	110.1
FLG2	117.8	111	117.5	124.7	114.4	105.6	120.5	120.4
FLI1	114.6	126.2	124.8	136	107.3	109.4	124.9	117.4
FLII	224.3	280.5	259.5	239.2	193.3	250.2	250.6	269.2
FLJ00060	116	124.5	121.9	122.7	114.8	99	115.4	119.5
FLJ10006	318.1	336.2	362.8	419.8	303.9	256.5	374.5	358
FLJ10081	486.7	466.2	386.6	432.6	378.6	389.2	508.6	608.4
FLJ10099	226.1	270.7	206.9	265.1	244.2	282.4	270.8	256.5
FLJ10120	189.4	184.3	204.2	173.7	160.3	144.7	155.1	158.9
FLJ10120	120	127.1	130.3	121.4	113.1	107.8	128.9	128.6
FLJ10154	2470.3	2235.4	3211.1	2028.6	1776.4	1488.1	1854.6	1642.7
FLJ10159	113.1	116	125.3	123.8	108.5	96	120.8	110.6
FLJ10178	192.5	197.5	206.4	200.1	178.7	165.6	203.3	196.3
FLJ10213	160.5	145.7	149.9	154.3	163.7	130	161.7	192.8
FLJ10241	441.2	462.5	411.7	531.7	367.2	384.5	475.9	440.1
FLJ10260	127.6	135.9	145	146.6	122.9	103.6	122.2	115.7
FLJ10292	112.7	122.7	128.8	117.8	109.3	100.8	124.1	125
FLJ10324	115.3	108.4	114.9	116.4	105.3	94.2	119.4	114.3
FLJ10357	108.3	110.7	139.6	129.1	114.8	108.1	126	114.2

FLJ10374	138.5	145.1	157.2	163.7	141.7	123.4	125.2	149.5
FLJ10379	206.6	241.4	211.9	247.2	232.7	185.4	236.5	194.3
FLJ10490	120.8	122.5	112.4	136.3	108.2	109.8	110.1	125.9
FLJ10661	110.4	126.7	133.2	130.1	121.2	102.1	120.1	120.5
FLJ10726	132.2	152.1	137.7	150.4	134.1	118	150.1	180.4
FLJ10769	299.8	285	309.8	351.4	250	259.3	333.3	241.6
FLJ10781	125.4	117.7	114.4	113.7	119.3	103.3	120.7	116.5
FLJ10786	101.1	110.5	109.9	113.5	103.1	97.9	106.7	113.4
FLJ10815	121	115.6	121.2	127.1	113.9	107.6	125.2	119.9
FLJ10847	122	118.7	109.6	123.1	111.4	100.1	121.3	112.8
FLJ10916	125.4	123.6	130.1	127.3	114	108	130.9	130.2
FLJ10922	137.5	142.6	147.7	148.5	119.6	120.4	138.2	124.2
FLJ10925	212.2	199.8	194	190.8	157.8	145.4	182.3	167.9
FLJ10945	108.9	121.2	117.8	107	114.7	106	114.6	119.4
FLJ10986	155.4	140.2	173.8	146.5	128.7	138	133.2	120.6
FLJ10996	116.3	110	117	124.8	119.7	101.3	149.4	122.8
FLJ11000	280.4	271.9	271.3	340.8	281.7	274.4	342.2	310.1
FLJ11017	121.5	120.4	123.5	122.5	111.9	102.3	114.9	110.2
FLJ11151	121.4	120.8	123.9	124.5	106.2	102.6	124.5	119.7
FLJ11155	109.3	110.5	124.8	115.6	102.4	101.6	116.7	108.2
FLJ11171	258.3	214.1	257.4	235.1	202.2	210.7	207	228.8
FLJ11184	121.4	119.2	123.6	125.8	108	106.1	113.3	114.9
FLJ11193	118.9	125.3	138	122.4	116.1	107.2	128.2	125.6
FLJ11200	193	184.8	194.2	175.9	162	145.1	164.2	217.3
FLJ11259	286.3	158.5	220.9	256.8	155.6	153.7	202.2	134.1
FLJ11286	309.9	342.7	322.6	291.3	252.9	249.6	274	290.7
FLJ11305	1221.4	1065.2	1427.2	1240.7	999.5	946.3	1186.2	964.2
FLJ11331	114.5	124	118.1	117.3	107	92.7	121.5	118.3
FLJ11712	188.1	188.3	225.2	178.4	150.6	130.9	170.1	166.3
FLJ11783	129.1	127.7	133	149.2	130.4	101.9	130.9	122.5
FLJ11795	158.5	157.1	141.1	150.3	150.5	132.6	152	147.5
FLJ11806	119.7	111.7	120.1	118.8	116.5	101.5	123.1	117.4
FLJ11806	120.7	117.4	125.2	137.9	117.5	110.8	119.7	127.9
FLJ11806	115.6	117.3	122.6	119.9	112.7	93.6	103.8	114.6
FLJ11806	136.6	131.8	122.1	127.7	122.5	113.3	125.4	137.8
FLJ12056	113.9	118.6	125.6	121.5	110.9	107	117.4	114.2
FLJ12118	126	136.3	134	124.1	124.7	102.9	124.8	130.5
FLJ12331	113.2	128.7	120.2	122.2	113.2	93.9	111.4	111.2
FLJ12505	115.1	114.8	124.4	118.7	101.8	99.1	124.8	111.7
FLJ12529	111.6	113.1	115.5	117.3	110	104.3	131.7	124.6
FLJ12571	128.9	124.3	125.6	125.3	112	97.5	118.9	123.5
FLJ12681	125.4	118.4	135.1	137.7	122.7	110.8	122.3	124.4
FLJ12684	106.4	112.1	112.1	115.6	103.8	93.3	114.5	114.5
FLJ12716	161.9	161.6	185.9	159.9	143.9	144.1	136.3	147.7
FLJ12716	195.1	199	190.6	217.2	178.4	185.2	174	186.9
FLJ12788	384.3	393.4	409.5	356	280.4	279.8	268.6	207.9
FLJ12886	134.6	142	146.6	160.6	135.5	131.6	180.1	153.6
FLJ12949	133	152.3	132.9	142.6	136	128.5	127.5	136
FLJ12949	283	269.7	272.2	287.6	257.6	203.1	218.3	203.8
FLJ12998	127.2	135.4	134.8	142	116	123.8	143.2	133
FLJ13096	127	130.2	135.2	136.5	121.5	106.8	134.4	136

FLJ13149	353.8	502.7	357.4	443.4	325.5	268.1	445.4	467.2
FLJ13231	111.7	111.4	108	105	105.8	100.8	118.4	115
FLJ13236	120	120.4	120.4	114.6	105.8	96.4	122.2	124.3
FLJ13265	126.4	122.1	117.1	115.3	112	105.2	133.4	128.6
FLJ13273	126.4	125.7	132.4	135.6	125.4	105.3	126.2	122.2
FLJ13273	187.5	250.4	203.8	249.6	220.8	170.4	208.1	261
FLJ13305	129.9	124.6	128.4	125.8	116.9	101.8	119.9	125
FLJ13391	102.9	111.7	122.5	119.3	109.3	99.6	115.7	113
FLJ13576	138.2	145.8	146.2	151.4	139	125.1	134.6	121.9
FLJ13611	119.5	116.9	120.8	118.3	107	99	123.2	114.4
FLJ13614	116.6	103.4	112.3	118.2	98	100.8	113.7	109.1
FLJ13639	126.3	126.9	136.3	131.8	118.7	111.3	128.8	142.3
FLJ13639	132.6	139.9	151.7	133.1	119.2	117.1	139.8	125.7
FLJ13639	131.4	137.4	138.9	153.9	127.5	113.2	129.8	129.9
FLJ13841	113.9	115.8	123.5	117.8	103.6	104.6	122.2	111.9
FLJ13909	115	126.3	129.6	125.7	102.5	102	122.1	116
FLJ13910	129.8	127	123	135.1	127.3	128.4	145.7	138.4
FLJ13912	142.1	166.5	143.8	149.4	148.5	111.5	162.8	169
FLJ13946	113.6	109.5	130.1	121.3	116.1	101.1	118.3	119.7
FLJ13984	116.1	108.7	118	121.7	102.4	104.4	120.4	119.8
FLJ14054	119.1	113.1	118.7	117.6	110.2	95.7	117.4	114.5
FLJ14107	125.1	115.5	132.3	140.7	104.4	111.7	125.7	123.6
FLJ14154	141.1	138.2	145.3	161.2	118.5	119.4	173.1	164.6
FLJ14166	106.6	113.1	114.4	116.4	113	94	113.6	115.9
FLJ14213	300.3	248.9	355.3	295.6	208	270.6	229.2	187.6
FLJ14346	121.7	118.3	132.2	128.5	114.3	110.1	125.2	123.2
FLJ14397	113.2	116.6	118.3	114.2	113.1	102.8	114.6	115.1
FLJ14466	317.4	376.9	253.7	309.8	235	215.5	270.1	241
FLJ14640	125.9	125.4	119.2	124.3	109.7	113	122.7	118.4
FLJ14668	303.3	389	372.6	292.1	273.1	214.1	224.6	210.1
FLJ14768	179.8	171.6	144.2	180	146.6	116.5	192.5	187.6
FLJ14803	322.1	309.4	348.9	337.6	246.1	273	284.1	231.9
FLJ14816	112.8	113.3	107.7	111.9	100.9	99.8	113.9	108.7
FLJ14834	112.9	107.8	103.8	113	102.2	91.2	108.1	116.1
FLJ16008	119.5	139.7	121.8	125.5	116.2	103.4	123.6	123.8
FLJ16124	118.1	107	118.2	115	108.4	91.2	114.3	113.4
FLJ16165	115	111.8	120.5	117	122.4	98.8	112.4	108.8
FLJ16231	122.8	113.4	139.7	130.5	122.5	116.4	137.2	121
FLJ16237	119.5	108	113.6	129.8	114	99.6	118.4	115
FLJ16331	110	107.9	108.6	120.3	111.1	106.1	119.8	109.1
FLJ16478	112	115.2	108.3	118.9	99.1	95.2	115.3	117.6
FLJ16542	129.5	132.9	145.9	131.7	127.9	122.7	134.9	135.8
FLJ16636	113.6	112.1	138.8	122.6	105.9	105.4	126.4	117.5
FLJ16641	115.6	114	124.6	111.2	113.5	97.5	118.8	111.4
FLJ16734	131.6	136.2	141.1	128.5	115	112.9	125.5	126.3
FLJ16793	121.1	130.1	126.4	125.5	115.9	106.8	119.5	119.8
FLJ20032	110.8	121.5	119.6	116.9	104.6	99.3	118	118.4
FLJ20035	202.2	192.5	248.3	206.2	204.1	179.8	171.4	163.2
FLJ20054	302.2	271.5	393.4	372.4	282.8	349	303.1	402.5
FLJ20097	171.3	176.1	160.4	175.5	164.3	142	191	197.6
FLJ20097	137.7	118	127.2	119.7	115.4	105.2	127.9	116.6

FLJ20097	112.3	120.4	112.9	118	114.1	98.1	113.2	103.5
FLJ20105	123.1	115.5	113	115.6	111.8	100.4	118.2	118.6
FLJ20105	111.7	114.2	119.1	121.9	107.1	97	117.7	112.2
FLJ20125	144.9	159.6	171.5	156.5	152.6	145.5	159.2	143.2
FLJ20130	126.1	133.1	136.8	138.9	118.2	107.9	112	119.8
FLJ20152	155.2	182.8	175.3	195.9	161.4	150.7	166.7	187.1
FLJ20152	188.8	216.5	231.9	198.1	190.6	219.2	180.2	185.6
FLJ20152	447.2	554.7	485.2	494.5	348.5	324.1	348.3	472.8
FLJ20160	330.1	312.9	221.5	362.2	254.6	227.5	279.9	258.2
FLJ20184	108.9	101.9	119.7	109.4	107.9	101.6	116.6	112.9
FLJ20186	148.4	164.5	136.3	159.2	124.1	121.5	142.5	138.8
FLJ20186	132.6	148.5	154.5	154.2	122.3	141.5	152.6	150.7
FLJ20186	308.2	309.7	415.2	338.5	233.1	290.2	274.9	261.2
FLJ20232	326.8	406.9	319.2	366.3	307.5	268	336.8	309.4
FLJ20245	135.5	139.8	151.9	125.8	123	144.1	144.5	127
FLJ20254	143.6	150	175	172.1	139.7	134.7	150.8	150.2
FLJ20272	196	196.4	191.3	226.3	175.8	155.2	172.1	171.1
FLJ20273	179.2	187.2	173.8	209.1	135.3	216.5	214.8	137.5
FLJ20280	232.7	267.2	295.8	235.1	225.7	202.2	205.8	184.6
FLJ20294	130	125.2	127.3	120.5	119.2	108.1	124.6	115.7
FLJ20297	121.2	140.2	154	146.3	120.5	108.5	148.4	133
FLJ20298	122.2	109.2	132.3	126.8	113.9	100.2	118.3	118.9
FLJ20298	117.7	117.9	110.3	114.8	111.5	96.1	111.7	110.7
FLJ20309	122.1	127.6	114.1	120	109.7	96.5	128.4	136.7
FLJ20323	204	205.3	224.4	196	174.6	183.6	201.3	184.9
FLJ20364	154.3	142.3	148.9	166.9	146.5	127.8	158.3	157.8
FLJ20366	113.1	126.1	124.4	117.4	113.6	95.7	118.8	115.2
FLJ20397	107	106.5	116.8	112.9	108.5	95.6	111.8	116.9
FLJ20422	131.6	121.1	117.7	128.7	114.8	107.1	120.8	117.2
FLJ20433	120.2	122.2	136	124.2	112.3	102.7	118.9	111.5
FLJ20489	131	122.9	144.4	127.7	110.5	197.7	135.1	140.8
FLJ20512	684.3	869.9	784.5	755.9	567.4	478.7	549.2	387.9
FLJ20516	120.8	118.4	131.4	123.1	116	99.4	122.3	119
FLJ20534	167.1	205.8	217	201.1	168.6	158.6	164.2	131
FLJ20551	223	235	205.6	218.2	206.4	159.7	240.7	221
FLJ20558	237.2	225.5	220.4	240.2	226.5	203.3	277.9	249.2
FLJ20581	124.5	131.8	136.6	126	115.7	101	122.5	117.8
FLJ20582	208.4	239.9	196.6	195.7	228.9	187.3	225.6	236.5
FLJ20625	504.5	574.7	708	563.1	483.6	559.2	533.3	453.3
FLJ20628	144.5	152.6	144.4	144.7	136.6	118.9	134.7	133.8
FLJ20643	222.7	225.3	175.7	221.3	200.3	171.2	177.9	163.7
FLJ20647	1653.1	1634.8	1940	2188.2	1620.6	1438.5	1649.7	2012.9
FLJ20674	117.6	110	119	117.5	111.5	107.3	126.6	116.9
FLJ20674	120.8	135.2	116.2	129.1	106.1	113.7	121.3	112.7
FLJ20699	205.1	226.1	210.9	243.4	145.6	149.9	244	197.9
FLJ20701	240.7	190.3	486.6	199.9	138.9	261	164.1	125.1
FLJ20718	146.4	136.2	160.1	129.9	135.5	121.4	144.8	130.3
FLJ20718	119.5	129.6	120.9	132.1	121.4	100.7	139.5	135.4
FLJ20758	134.1	137.3	117	134.7	110.5	112.6	134.4	119.8
FLJ20850	134.7	149	146.8	133	116.6	104.7	134.4	119.4
FLJ20920	113.2	106.3	113.2	123.7	108.4	101.9	112.8	108.7

FLJ21062	123.2	116.6	118.2	124.8	114.1	105.8	112.3	111
FLJ21075	108.4	107.8	111.4	127.2	102.4	96.3	114	119.2
FLJ21103	126.4	130.7	123.6	124.5	110.7	104.5	126.9	133.9
FLJ21106	205.8	216.6	189.7	213.6	198.7	131.8	200.2	165.3
FLJ21125	223.3	264.5	274.4	214.8	195.3	224.7	157.3	153.3
FLJ21127	152.7	127.6	137.1	134.8	114.9	116.8	129.1	116.1
FLJ21159	120.8	120.6	119.7	112.7	117.5	98.4	122.1	120.5
FLJ21511	112.3	112.2	115.2	108.5	106.2	91.4	120.6	125.6
FLJ21687	127.5	136.6	139.3	124.1	113.7	102	128.9	124.5
FLJ21742	129.2	132.2	133	129.6	121.1	108.5	127.1	130
FLJ21749	317.4	283.9	416	299.6	249.6	276	244.4	243.2
FLJ21816	142.9	158.9	143	144.1	135.2	126.8	156.2	152.1
FLJ21820	148.4	142.9	163.3	152.5	143	137.3	143.1	127.1
FLJ21827	134.8	134.8	151	132.6	122.4	126.2	142.7	129.9
FLJ21839	159.7	163.7	139.8	154.7	116.2	118.5	128.7	127.3
FLJ21839	144.1	139.2	124.1	169.7	136.1	115	158.5	133.5
FLJ21865	313.3	271	328.2	275.4	180.4	248	226	216.8
FLJ21901	153.7	182.3	166.9	162.9	146.5	135	172.1	168
FLJ21908	120	114.6	118.1	114.5	102.5	95.7	113	107.7
FLJ21945	220.2	226.3	208.6	230.6	205.3	179.2	208.4	177
FLJ21963	120.5	117.7	122.6	127.4	115.2	105.8	121	120.9
FLJ21986	127.1	116	318.3	125.4	111.6	102.1	119.6	120
FLJ22104	137	157.6	140.8	140.6	142	122.1	151.1	151.9
FLJ22167	125.5	123	113.3	124.8	114	100.1	122.2	121.1
FLJ22184	125.3	109.2	116.1	120.8	101.6	97.5	114.7	118.6
FLJ22184	110.3	119.8	127	121	109.9	94.3	113.8	118.6
FLJ22222	127.8	127.1	134.5	134	119.2	110.5	126.7	126.6
FLJ22222	133.4	128.6	157.6	143.3	126	140	126.3	146.1
FLJ22222	120.5	126.8	131.3	132.8	114	102.7	130.8	134.5
FLJ22318	142.9	171.7	129.3	159.9	119.8	116.5	135.9	123.7
FLJ22349	122	129.8	125	131.7	114	112.1	120.5	116
FLJ22374	127.8	134.9	128.3	120.6	115.1	104.6	125	115.2
FLJ22386	134.2	120.5	133.5	128.1	110.6	104.5	125.4	130.2
FLJ22471	642.3	606.3	557.9	660.8	560	422.1	632.4	650.5
FLJ22531	137.8	186.4	141.9	144.7	141.1	115.5	145.3	139.4
FLJ22555	304.1	324.1	338.1	304.4	324.1	272.3	320.1	309.5
FLJ22624	165.2	155.4	163.7	193	153.4	135.9	174.2	182.3
FLJ22639	128.7	128.6	128.2	134.7	143.2	118	123.6	148.7
FLJ22662	333.3	199.5	760.4	272.2	170.2	512.8	246.3	155.9
FLJ22671	108.8	114.4	106.6	112.6	110.6	100.6	122.9	111.6
FLJ22688	126.6	131.1	132.7	133	128.1	109	124.1	125
FLJ22709	307.7	320.7	371.1	283.8	226.8	205.9	243.9	172.5
FLJ22746	118.1	108.6	117.8	119.6	109.3	105	115.2	106.3
FLJ22795	148.7	154.4	139.9	143.4	124.4	105.7	136.5	133.6
FLJ23049	123.4	109.3	119.1	125.3	128.7	102	128	114.2
FLJ23191	105.6	110.1	104.4	110.6	105.9	95.6	117.1	114.6
FLJ23356	118	133	117.5	121.9	121.8	98.6	120.2	117.4
FLJ23436	126.1	110.8	124.6	124.1	114.2	97	111.7	118.3
FLJ23447	125.1	111.8	116.2	126.3	115.2	98	110.3	111.9
FLJ23514	110.9	111.8	119.6	118.5	113.3	102.6	121.7	114.5
FLJ23518	119.4	121.4	116.9	115.9	113.8	97.7	121.1	112.4

FLJ23577	110.6	107.3	117.1	114.9	110.3	91.2	113.2	110.5
FLJ23577	120.2	129.5	145	135.1	112.2	112.4	130.4	125.5
FLJ23577	109.8	114.4	116.7	111.1	111.2	105.7	112.2	109.4
FLJ23584	157.3	170.7	162	168.6	148.2	140.6	170.6	167.7
FLJ23588	113.7	112.8	108.9	110.1	102.2	95.7	113.2	105.5
FLJ23588	121	115.8	110.4	117.1	115.1	101.4	119.2	114.9
FLJ23588	120.2	108.7	117.3	126.2	116.3	109.5	117.5	124.6
FLJ23657	118	119.1	111.7	110.8	105.5	100.3	113.7	111.9
FLJ23834	138	146.8	141	131.9	113.5	101.8	120.9	118.6
FLJ23861	124.1	110.6	129.1	120.4	111.7	106.6	129	121.7
FLJ23878	112.2	114.9	111.3	121.6	110.9	102.6	118.9	110.2
FLJ25006	122.8	120	150	121.2	119.5	109	102.8	126.8
FLJ25067	188	205.7	175.5	174.1	155.7	146.6	173.5	146.4
FLJ25084	132.1	128.1	124.5	131	117.5	106.2	138.7	137.4
FLJ25102	122.7	114.1	116	120.7	115.2	102.3	123.8	120.6
FLJ25143	119.5	116.8	120.7	126.4	114.3	106.1	126.9	138.1
FLJ25222	339.3	316.3	330.4	320.2	192.7	302.5	375.7	238.4
FLJ25328	112.5	109.3	114.2	130.3	110.4	99.7	109	116.6
FLJ25369	103.5	101.6	113.9	114.6	109	90.5	109.9	112.2
FLJ25371	111	122.2	117.6	122.4	105.1	98.5	119.3	113.1
FLJ25410	134.9	131.4	153.2	141.1	123.6	115.2	128.1	135.5
FLJ25416	115.4	142.1	120.8	126.2	115.2	101.3	147.8	131.8
FLJ25421	115.3	132.9	130.4	128.7	119	104.8	128.1	118.2
FLJ25439	122.3	123.2	120.1	122.1	108.9	100.4	121.1	120.9
FLJ25444	117.9	106.6	110.7	118.2	104.5	103.6	121.7	110.1
FLJ25476	202.1	239.3	305.3	218.3	181.1	206.1	192.8	174.1
FLJ25477	123.6	121.8	119.8	131.7	123.4	97.4	113.2	109.1
FLJ25660	121.5	111.1	118.2	115.9	107	99.1	127.1	112.7
FLJ25680	117.5	113.8	116.4	118.3	116.2	99.7	124.3	110.4
FLJ25770	107	109.9	107.4	133.7	105.9	99.6	110.8	116.5
FLJ25773	113.8	111.9	115.9	111	101.6	93.7	108.8	113.2
FLJ25791	108.4	136.6	133.1	130.7	128.9	109.4	141.7	139.6
FLJ25801	116.1	121.5	123	125.4	104.8	105.6	116.4	120.5
FLJ25976	106.5	107.4	108.8	114.2	102.7	89.8	107.9	114.4
FLJ25996	105.7	119.6	116.2	118.4	108.6	105.8	112.4	114.4
FLJ26175	143.2	147.1	144.8	147.9	139.1	120.6	141.6	137.4
FLJ26443	117.7	119.9	112.6	119.7	107.9	103	127.1	107.7
FLJ26850	136.8	121	140.2	125.6	128.2	109.1	124.2	118.7
FLJ27465	120.7	114.2	119.3	123.9	114.2	102	122.2	116.1
FLJ27465	115.2	107.4	116	136.8	119	103.5	127.9	116.6
FLJ27505	104.6	107.9	110.3	111.9	102	95.3	117.1	120.5
FLJ27523	105.7	121.6	124.3	132.2	116	107.8	129.1	130.4
FLJ30046	217.4	237.5	195.4	252.6	255	191.9	229.1	235.9
FLJ30046	126	132.6	109.9	129.9	124.8	106	118.8	133.5
FLJ30058	123.8	117.6	118.1	124.3	116.4	114.2	126.1	125.9
FLJ30277	112	110.8	117.1	114.1	113.6	94.4	126.5	129.6
FLJ30294	107.7	111	112.8	123.5	113.8	101.3	112.3	114.4
FLJ30313	120.2	112.4	113.5	123.4	113	99	118.1	116.4
FLJ30596	114.9	125.1	119	127	119.9	117	135.3	118.7
FLJ30655	115.5	120.7	131.1	124.9	116.5	106.6	115.7	119.4
FLJ30707	104.9	106.3	105.3	121.7	109.8	99.9	107.5	102.3

FLJ30834	118.5	116.9	123.7	134.9	113.7	106.6	116.1	118.2
FLJ30851	112.7	114.6	107.9	111.8	102.7	97.1	115.5	112.5
FLJ30934	115.8	117.4	114.9	120.3	106.9	99.1	117.4	119.8
FLJ30990	111.4	105.5	112.9	113.4	106.8	97.1	117.6	103.6
FLJ31196	119.9	114.2	128.5	115.9	104.6	109.1	120.6	122.1
FLJ31204	145	133.4	137.8	160.4	119.7	108.8	132.3	130.8
FLJ31401	113	114.1	109.5	114	111.5	98.2	116.2	109.4
FLJ31413	378.7	378.8	323.4	653	414.9	506.4	804.9	615.2
FLJ31438	122	117.1	131.3	108.3	128.3	100.8	117.7	111.8
FLJ31568	119.9	112.8	123.5	121.4	104.2	98.4	110.6	110.9
FLJ31659	116.2	121.7	120.9	112.8	104.8	96.7	120.2	118
FLJ31846	115.4	121.1	125	125.1	108.8	96.7	126.3	111.2
FLJ32011	121	124.4	122.5	134.5	105.3	105.4	122.9	120.6
FLJ32063	109.9	106.4	117.4	117.5	103.2	95.6	113.2	111.6
FLJ32110	115.7	115.9	113.3	119.2	106.2	99.3	115.1	113.2
FLJ32130	132.5	126.6	130	143.9	136	107	126.1	114.6
FLJ32206	114.7	112.9	119.8	122.6	105.2	97.1	124.5	130
FLJ32312	121.6	118.3	131.6	128.4	111.7	104.3	129.7	120.2
FLJ32447	138.9	138.9	147.3	134.9	124.3	110.5	152.3	134.6
FLJ32549	124.5	128.4	131	129.8	115.2	97	127.4	125.5
FLJ32569	109.3	123.1	114.4	122.9	112.5	101.8	117.5	109.9
FLJ32569	113.4	119.4	119.6	120.2	106	96.8	115	110.7
FLJ32658	123.7	128.2	122.9	119.2	111.3	103.7	114.9	118
FLJ32679	120	120.2	147.4	135.9	116.3	118	109.3	116.6
FLJ32745	128.4	129.9	136.7	135.4	128.3	105.7	137.1	125.3
FLJ32771	114	126.4	117.2	116.6	113.1	103.1	125.4	119.1
FLJ32784	122.7	129.1	114.3	121.4	114.8	98.3	125.3	113
FLJ32786	110.8	110	113.6	116.7	102.4	93.2	112	111.6
FLJ32867	110.7	102.8	111.1	113.9	112.2	94.4	111.5	114.5
FLJ32871	118.1	118	119.6	118.5	110.1	95.5	116.4	117.6
FLJ32894	123.5	124.3	116.8	117	110.8	101	135.6	112.9
FLJ32926	118.8	124.4	114.9	122.7	105	97.6	111.5	111.5
FLJ33360	113.6	118.9	120.5	112.1	120	103.2	115.4	114.9
FLJ33590	148.6	144.2	145.1	137.8	126.2	115	137.1	122.8
FLJ33641	316.4	157.7	2197.8	274.1	131.8	859	152.6	126
FLJ33718	114	114.2	135.5	125.8	114.7	107.9	117.8	130.4
FLJ33790	123.8	130	116.1	128.2	128.2	109.3	127.9	128.1
FLJ33860	116.5	118.1	117.8	120	108	98.1	119.7	118.5
FLJ34306	110.2	107.3	111.7	119.2	102.8	101.8	109.7	107.6
FLJ34503	117.8	117.9	129.7	124.1	116.3	102.7	120.3	119.3
FLJ34931	118.6	115.8	118.2	126.9	116.1	116.2	127.7	109.6
FLJ34969	260.3	241	270.7	303.3	242.2	258.2	278.3	246.2
FLJ35119	153.3	138.9	155.7	141.4	132.9	109.6	144.4	149
FLJ35220	117.1	120.4	116.1	117.6	108.6	95.9	111.6	122.1
FLJ35409	118.5	114.3	116.3	113.9	107.5	96.8	112.5	112.9
FLJ35695	116.4	107.4	117.3	117.7	103.6	101.6	117.3	125.5
FLJ35725	129.6	128.7	124.3	127.6	111.8	110.8	131.3	129.9
FLJ35725	116.7	119.6	115.5	121.8	105.9	104.4	127	127.9
FLJ35767	118.1	113	117.6	113.7	108.6	100.6	130.2	121.7
FLJ35773	110	113.3	119.1	114.1	107.5	94.3	116.6	112.7
FLJ35776	116.5	120.2	114.8	121.6	111.6	88.2	120.6	115.6

FLJ35784	115.4	118.4	118.2	125.8	113.9	103	116.6	115.9
FLJ35816	118.6	110	115.9	123.1	113	97.9	108.6	120.4
FLJ35834	118	124.5	117.6	123.5	105.8	99.9	120.4	112.9
FLJ35848	108.6	110.1	118.5	109.9	106.8	100.5	114.2	111.2
FLJ35880	107.8	116.8	126.3	119.2	113.9	99.3	119.9	113.6
FLJ35894	111	110.9	119.1	117.8	105.6	104.5	113.1	109.9
FLJ36004	109.9	113	120.5	108.6	106	100	111.4	108.9
FLJ36031	109.2	110.1	112.2	111.8	109.4	100.3	118.2	110.3
FLJ36046	115.3	109.2	109.4	116.8	114.7	101.8	123.6	111.3
FLJ36070	124.9	115.5	122	136.8	113	96	121.6	122.4
FLJ36090	128.7	126.3	133.8	136.4	129.2	131	129.3	124.8
FLJ36166	103.2	113.1	116.3	113.4	111.6	100.4	117	115.3
FLJ36180	118.5	115.1	125.7	137.6	114.7	107.3	132.7	121.9
FLJ36208	116.7	126.1	119.8	131.5	116	99.6	120.7	119.2
FLJ36268	114.4	112.1	109.6	109.6	105.6	94.6	116.4	114.9
FLJ36445	116	120	119.5	122	107.2	103.7	120.1	118.3
FLJ36492	118.3	115.6	118.9	123.6	111.7	91.2	122.8	116.9
FLJ36492	114.7	116.3	118.6	116.6	107.3	94.6	114.7	115.6
FLJ36748	126.4	122.3	120.5	128	115.4	99.7	134.4	120.6
FLJ36779	111.9	125.8	113.8	120.6	110.6	97.2	121.8	113.4
FLJ36874	619	668.6	649.9	730.8	599.7	532.5	752.8	842.8
FLJ36888	135.4	118.6	126.9	135.4	112	106.2	138	127.8
FLJ36980	114.1	128	124	132.9	113.2	99.4	119.6	114.6
FLJ37078	122.2	119.5	111.8	128.4	111.3	102.3	113.4	124.5
FLJ37266	114.3	117.1	114.4	115.8	114.3	101.3	113.9	116.3
FLJ37357	124.9	123.2	121.5	127.9	109.4	96.5	124.7	123.6
FLJ37440	159.1	165	233.1	180.6	134.7	267	145.8	136.9
FLJ37464	137.8	134.8	158.3	130.9	114.3	114.3	124	124.1
FLJ37538	116	109.9	113.5	115.3	107.2	102.8	112.8	129.5
FLJ37562	166.8	163.9	164.5	173.3	153.3	124.2	184.6	161.1
FLJ37927	110.2	113.8	116.4	122.6	113.4	105.6	123.6	119.5
FLJ37953	118	123.3	110	123	115	95.7	128.7	116.6
FLJ37970	156.3	164.7	151.4	147.1	155.7	112.8	154.5	155.8
FLJ38020	112	120.2	128.6	125.4	112.2	101.9	116.8	121.3
FLJ38159	124	127	149.1	132.8	122.6	116.7	122.9	121.7
FLJ38288	130.8	136	139.7	131.7	122.2	112.4	133.1	127.7
FLJ38377	115.3	113.8	100.6	115.3	105.9	95.8	118.9	112.1
FLJ38451	323.5	373.1	301.3	312.8	250	242.6	260	236.7
FLJ38482	254.7	245.6	319.6	316.8	239.8	227.8	270.4	311.3
FLJ38596	111.4	110.4	123.8	111.8	109.9	89.7	110.5	127
FLJ38663	173.7	203	201.4	212.4	204	186.8	186.8	183.8
FLJ38723	117.4	116	115.1	119.3	106.2	104.5	120.8	113.1
FLJ38725	131.5	122.5	148.9	155.9	117.5	127.2	141.3	127.6
FLJ38964	127.3	131.9	135.5	123.4	121.7	109.4	141.3	201.5
FLJ38973	117.2	125.7	132.2	126.1	114.2	100.4	121.9	116
FLJ38984	138.2	152.1	135.1	152.7	143.6	123.2	176.5	172.4
FLJ38991	119.3	117.2	117.5	115.5	105.8	101	120.3	119.7
FLJ38991	136.3	124.8	136.3	125	120.4	112.7	130.9	113.8
FLJ39061	112.8	117	114	121.7	109.9	98.9	113.2	117.9
FLJ39155	113.2	115.1	121.3	114.5	110.9	95.8	116.2	123.7
FLJ39155	111.5	114	119.9	114.1	107.7	97.5	121.1	117.1

FLJ39155	107.9	115.4	107.6	109.9	110.7	101.2	112.1	115.9
FLJ39237	114	116.5	121.4	112.9	112.1	96.3	117.8	109.7
FLJ39378	136.8	133.2	155.4	153.3	141.6	124.1	154	145.9
FLJ39501	116.1	121.5	122.2	126.7	106.9	107.6	128.1	120.5
FLJ39502	112.1	120.7	113.5	119	112.4	98.9	108.8	108.2
FLJ39531	113.7	119.6	122.5	122.9	111.1	97.8	112	126.1
FLJ39575	135.7	130.2	138.5	157	142.8	117.1	199.1	192.6
FLJ39599	124.3	117.8	127.6	123.4	121.8	118	123.1	127.3
FLJ39660	110.2	113.2	115.1	115.9	113.4	95.2	116.3	117
FLJ39743	106.9	108.6	108.8	113.7	98.8	95	112.7	114.9
FLJ39779	119.4	124.1	134.4	124.2	114.8	105.2	121.1	121.9
FLJ39822	138.2	120.3	130.1	127.2	113.4	110.4	113.8	122.6
FLJ39827	197.7	218.7	183.3	187.8	170.9	139.3	155.5	150.5
FLJ40113	115.6	112.6	111.3	116.1	102.1	107.4	113.4	114.9
FLJ40125	119.3	128.4	127.4	123.6	115.7	102.8	126.1	124.3
FLJ40142	224	233.5	250.3	201.3	187.2	183	159.7	133.2
FLJ40172	107.6	105.5	112.6	120.8	102.4	93.8	111.6	102.7
FLJ40235	125.5	117.5	121.7	120	107.9	98.2	126.5	119.3
FLJ40243	109.5	108.3	111.9	117.7	102.3	99.6	109.5	108.5
FLJ40244	104.7	115.2	108.7	113.4	102.2	95.3	106.5	118.2
FLJ40288	114.8	124.4	141.6	121.6	122.3	102.8	121.9	128
FLJ40296	118.4	120	117.1	129.8	98.7	99.7	121.2	112.8
FLJ40298	100.8	109.6	109.1	113.5	108.2	96.7	119.6	113.3
FLJ40365	117.1	112.1	115.7	112	107.9	102.9	120.3	111.6
FLJ40411	125.3	118.4	116.2	121.6	112.8	108.5	114.2	115.4
FLJ40448	118.3	127.4	127.2	138.1	127.5	116.3	139.2	135
FLJ40453	111.8	108.4	122.1	131.3	108.9	98.3	121.7	114
FLJ40504	113.8	122.4	118	120.1	116.2	95.1	117.4	112.3
FLJ40629	117.7	110.3	117.4	116.9	114.2	98.7	116.8	111.3
FLJ40722	117.1	122.8	123.3	144.7	122.1	115.3	127.8	107.4
FLJ40852	125.9	123.6	143.7	132.8	117.3	101.5	131.4	126.8
FLJ40869	105.6	116	116.8	105.5	105.8	98.5	109.2	117.3
FLJ40919	111.6	117	116.7	114.4	109.9	103.2	112.8	121
FLJ41046	116.2	121.8	118.1	112.8	110	102.4	115.9	115.4
FLJ41131	129.6	135	147.5	125.1	127.6	118.8	132.4	146.2
FLJ41170	124.3	122.5	119.9	125.3	109.8	98.9	128.6	110.2
FLJ41423	242.1	233.9	230.1	236.4	258.7	151.4	219.9	266.4
FLJ41603	110.6	119.8	114.2	118.3	112.9	104.7	121	124.3
FLJ41649	116.2	119.7	111.3	122.8	115.6	99.6	119.3	119.9
FLJ41733	116	113.3	133.4	124.2	114.9	107.5	130	130.9
FLJ41821	104	109.9	117.3	108.5	102.8	95.4	115	115.6
FLJ41841	112.3	115.7	112.8	113.9	99.7	92.7	126.5	119.5
FLJ41993	118.3	117.1	113	120.2	103.8	100.6	117	121.7
FLJ42102	110.7	106.5	116	117.7	108.4	97.1	115.6	114.9
FLJ42117	120.8	118.9	118.7	130.5	110.7	110.4	126.3	116.3
FLJ42133	110.8	112.9	105.1	112.9	109.6	101.6	118.8	108.1
FLJ42177	115.8	116.6	124	124.4	117.3	106.5	114.8	116.1
FLJ42258	119.8	121.4	113	122.4	106.7	97.3	114	107.8
FLJ42280	117	111.5	120.1	119.2	108.1	104.9	116.4	119.4
FLJ42289	110.3	113.5	128.3	126.1	108.9	102.7	125.2	113.1
FLJ42461	109	106.3	112	108.9	100.5	90.5	112	109.6

FLJ42486	114.1	120	116.1	123.8	107.1	97.7	115.1	115.7
FLJ42953	109.2	121.5	115.3	108.2	102.2	100.5	111	112.7
FLJ42957	119.4	108.1	119.8	126.6	112.9	102.2	124.3	113
FLJ42986	112.4	115.3	114.4	114.6	111.9	97.5	118	107.6
FLJ43093	116.7	114.7	121.2	118.6	102.4	97.6	116.9	107.9
FLJ43276	105.5	113.2	114.4	116.4	112.7	98.4	129.8	114.4
FLJ43339	127.6	125.3	145.5	121.3	118.1	115.3	125.5	119.7
FLJ43374	117.3	117.3	114.6	114.1	107.1	93.4	120.7	108
FLJ43505	112.1	120	122.1	120.5	104	102.9	113.8	108.9
FLJ43692	161.5	178.2	136.2	181.2	168	120.3	180.7	199.6
FLJ43752	116.4	116.8	123	125.1	121.6	106.6	124	121.7
FLJ43763	114.7	103.1	108	109.7	119.9	95.6	117.2	111.7
FLJ43806	113.6	112.8	112.7	117.2	102.8	93.4	118.1	110.7
FLJ43855	113.3	116.9	112.4	115.1	104.9	95.5	109.2	111.6
FLJ43855	122.1	123.4	130.2	121.9	115.7	115.4	130.6	133.3
FLJ43860	115.6	117.9	117	114.3	106.8	95.5	118.8	112.3
FLJ43860	111.3	121.5	106.5	113.1	104.1	100.5	114.9	113.1
FLJ43980	111.3	121.2	109.7	112.3	114.2	95.2	126.9	119
FLJ43987	123.6	113.5	121.1	115	112.4	97.7	123.2	125.3
FLJ43987	110	122.7	127.8	122.2	118.3	104.4	123.3	116.9
FLJ44048	115.9	111.1	121.2	121.3	117.9	107.4	121	117.5
FLJ44060	115.5	113.9	107.9	118.5	103.6	100.3	121.9	114.1
FLJ44076	115.6	112.3	112.6	113.8	107.3	102.3	114.8	112.2
FLJ44112	116.1	109.9	110.2	112.8	110.2	101.4	112.9	118.5
FLJ44124	1276.6	1052.4	1021.7	1003.2	903.3	646.4	1568.7	1321.2
FLJ44216	206.3	172.9	173.7	224.5	164.7	172	240.6	216.7
FLJ44216	115.4	123.8	114.4	123.5	108.9	97.3	118.9	117.9
FLJ44290	117.9	118.7	118.5	111.9	113.7	101.8	117	113.5
FLJ44379	112.4	114.2	119.5	126.8	116.9	97.9	114.4	124.2
FLJ44635	117.9	128.9	119.6	127.2	106.2	121.4	131.6	119.1
FLJ44635	114.5	116.1	115.5	116.6	104.3	99.3	110.3	115.3
FLJ44653	122.6	127.2	131.6	119.4	114.9	104.1	125	119.2
FLJ44691	118.6	118.6	119.2	118.5	111.2	101.8	115	118.9
FLJ44815	114.7	107	110.3	112.1	100.8	94.5	111.5	110.3
FLJ44894	109.7	111.8	129.3	115.8	108.4	98	137.7	119.1
FLJ44955	107.8	113.9	130.3	124.2	117	104.4	115.7	110.2
FLJ44968	112.4	113.3	116.9	126.4	121.3	100.2	123.4	129.9
FLJ45032	136.9	121.7	130	139.6	114.9	115.5	134.7	126.1
FLJ45079	110.5	110.4	116.3	122.6	112.1	94.4	133	116.6
FLJ45121	112	124.7	114.6	124.4	115.7	92.8	127.9	112.4
FLJ45139	111.2	111.6	109.5	117.6	103.5	98.6	113.8	110.9
FLJ45224	118.7	116.7	121.9	125.8	118.4	104.9	125.8	115.5
FLJ45256	127.7	121.5	124	119.6	121.3	99.6	137.5	118.2
FLJ45337	131.9	136.7	136.2	148.2	130.6	118.4	139.2	145.1
FLJ45422	127.1	116.8	130.3	124.6	116.6	105	117.7	129.6
FLJ45445	119.5	119.5	134.6	122.8	115.4	95.5	129.4	126.5
FLJ45455	114.2	116.2	115.1	119.9	102.9	101.8	121.9	116.8
FLJ45530	110.4	124.5	107.3	108.5	103.4	91.8	113.4	115
FLJ45537	122.7	124.7	127.3	117.8	105.5	102.5	121.5	106.7
FLJ45557	127.9	127.1	133.4	132.8	116.9	109.1	128.9	114.3
FLJ45645	116	115.3	116.5	121.5	110	114.1	125.3	116.9

FLJ45717	125	108.3	115.1	120.5	109.4	92.9	109.4	113.3
FLJ45721	108.4	113.7	124	119.7	110.8	99.6	118.7	117.6
FLJ45803	109.5	121	129.9	118.2	108.6	103.3	118.7	108.6
FLJ45831	113.9	113.9	122.8	120.6	110.1	95.5	115.9	122.4
FLJ45850	115.8	126.5	123.1	132.5	106.6	93.5	122.5	118.4
FLJ45909	193.2	184.7	207.4	179.3	176.6	154.2	185.9	190.8
FLJ45910	113.4	109.8	119	117.6	111.1	96.7	114	109.7
FLJ45964	107.2	108.6	111.3	120.8	109.6	95.8	111.7	109.1
FLJ45966	118.9	121	111.8	110.5	107.6	104.7	110.3	112.8
FLJ45974	121.5	118.6	117.1	119.4	109.3	106.7	125.7	122
FLJ45983	121.9	120.3	118.9	135	110.7	111	123.3	114.2
FLJ46010	107.7	108.7	118.6	110.7	113.8	98.6	115.5	121
FLJ46072	126.6	125.2	135.9	135.5	130.2	112.6	130.9	118.3
FLJ46082	111.9	107.1	111.9	115.5	110.2	93.2	117.7	110.2
FLJ46109	112.6	120.1	118.4	109.2	100.7	98.8	120.5	120.8
FLJ46109	188.7	169.4	165.4	169.6	153.7	126.3	164.8	173.2
FLJ46210	119.7	115.6	118.5	123.1	106.1	99.5	120.6	115.3
FLJ46247	116.6	109.6	115.1	127.4	101.6	97.4	113.7	126.8
FLJ46247	111.6	119.9	121.7	126.7	111	95	120.9	110.8
FLJ46247	107.5	117.3	116	117.8	103.8	103	115.4	124.3
FLJ46257	110.5	109.7	119.3	105.8	94.9	100.4	123.1	114.3
FLJ46266	114.9	110.4	107.4	110.3	106.7	93.3	116.3	111.8
FLJ46309	1397.8	1106.2	1249.2	814.2	1051	898.3	1143.2	1234.3
FLJ46321	120.8	119.1	119.9	116.8	105.9	99.7	114.3	116.4
FLJ46347	106.2	113.5	109.3	113.2	104.2	102.1	109.3	114.7
FLJ46385	121.7	120.8	113.4	108.7	116.1	97.9	118.1	115.8
FLJ46481	123	124.7	141.2	133.1	105.2	110.2	125	128.1
FLJ46536	110.3	112.3	110.7	116.5	109.3	101.6	116.6	109.2
FLJ46688	123.1	120.5	126.6	131.4	116.4	104.1	129	126.3
FLJ46831	118.6	124.2	114.2	120	111.9	92.9	116.5	118.6
FLJ46836	134	139	146.3	141.6	126.4	126.6	132.4	136.2
FLJ46838	113.3	113.9	120.4	125.5	113.8	109.7	137.4	120.4
FLJ90013	131.2	156.8	166.1	158	153.2	130.7	131.6	148.4
FLJ90166	125.2	139.8	119.8	179.5	151.2	99.3	210.1	150.1
FLJ90396	195.2	194	244.9	251.5	176.4	170.8	220.6	239.5
FLJ90575	138.9	111.9	125.1	130.1	118.9	106.7	129.2	120.1
FLJ90586	108.7	113.9	106.1	113.6	106.4	89.4	105	115.1
FLJ90650	114.7	119.5	112.9	112.2	106.6	99.3	120.8	117.2
FLJ90652	212.9	247.1	225.5	245.8	177.7	216.2	247.2	193.4
FLJ90709	137.5	168.8	146.9	157.5	157.1	136.8	141.6	151.4
FLJ90757	121.5	124.1	119.1	121.2	116	110	128.2	122.5
FLJ90805	121.2	121.1	121.3	126.6	119.7	104.9	131	127.9
FLJ90834	122.9	116.2	113.8	122.7	110.1	94.3	123.1	119.4
FLNA	124	121.1	144.6	128.3	116.6	114.3	146.7	116.1
FLNB	350.2	390.3	209.9	331.1	340.7	222.4	271.9	332.6
FLNC	117.3	113.4	113.7	118.7	115.2	101.3	122	108.6
FLOT1	323.7	256.1	276.5	281.9	190.8	225.2	247.4	210.6
FLOT2	2282.6	2924.5	2354.1	2160.6	1767.8	1478.1	1740.6	1476.7
FLRT1	107.9	114.1	105.6	115.3	105.7	94.2	115.5	114.6
FLRT2	128.2	114.2	105.4	127.6	108	107.4	119.7	111.9
FLRT3	122.4	118.6	117.2	123.6	112	96.2	118.3	112

FLRT3	115.7	112.1	119.5	122.6	104.5	98	123.2	117.4
FLT1	116.2	111.1	118.2	121.8	108.6	96.6	119.9	119.4
FLT3	112.3	119	120.4	135.8	102.1	99.6	118.2	120.5
FLT3LG	273.5	318	392.3	265.7	262.1	267.9	310.2	291.2
FLT4	111.8	111.3	115.4	115	110.7	100.7	110.9	109.6
FLT4	113.8	114.4	123.6	113.1	107.2	101.9	116	114.5
FLVCR	140	135.6	158.6	158.4	134.3	121.8	135.4	134.9
FLYWCH1	118.8	121.1	121.6	135.2	114.4	107.8	118.3	115
FLYWCH1	119.8	119.9	135.1	125	119.6	115.2	134	155.3
FMN2	114.8	101.6	112.2	116.1	105.1	96.7	121.4	111.7
FMNL1	156.3	141.9	142.8	141.5	125.6	129.3	144.9	134.7
FMNL2	115	115.3	125.9	117.9	105.9	100	110.5	119.9
FMNL3	162.1	158.4	239.2	166.1	145.9	193.3	154.1	132.9
FMNL3	117.6	121.3	103.7	115.5	108.4	108.6	109.1	114.5
FMNL3	124.6	128	137.6	131.5	112.4	122.2	123	116.8
FMO1	117.7	110	112.7	121.3	105.5	105.9	126	117.2
FMO2	111.3	116.5	121.3	119.9	112.5	95.4	109.5	113.7
FMO3	108	114.3	111.4	110.9	104.6	91.3	118.7	115.7
FMO3	106.6	120.8	115	120.2	102.9	97.7	119.3	118.2
FMO4	151.5	117.7	148.2	142.6	125.8	114.8	130.2	127.3
FMO5	121.4	123.6	125.3	112.1	111.1	107.7	116	121.1
FMO6	136	155.1	159.6	130.7	131.9	125.7	139.7	149
FMO6P	112.1	122.8	130.9	117.1	118	101	118.2	118.6
FMOD	117	118.3	116.6	128.5	110.3	100.3	119.4	122.4
FMR1	111.7	118.5	120.8	111.5	110.8	98	122.9	112.4
FMR1NB	118.4	121.4	123.6	127.4	112.2	100.9	128.3	112.9
FN1	118.3	121.7	133.7	127	99.7	109.3	126.7	115.9
FN1	109	111.3	112.2	114.4	108.9	100.6	117.3	114.8
FN1	119	115.5	121.6	123.3	114.1	111.5	115.8	124.1
FN3K	114.9	115.4	124.4	129.1	107.1	98	116.3	120.5
FN3KRP	323.4	315.2	317.1	302.9	221	260.1	241.9	254.8
FN5	145.8	143.7	200.7	145.2	123.7	113.8	129.6	123.8
FNBP1	2816.3	3639.9	2944.1	3120.9	2553.5	2343	3083.7	3057.4
FNBP1L	107.7	105.4	117.7	109.9	105.4	96.8	117.9	113.4
FNBP1L	117.2	116.7	136.9	118.3	117.5	94.2	118.4	117.6
FNBP4	2358.7	2312.8	1767.2	2482.9	2444.5	1459.2	2412.4	2587.5
FNDC1	106.8	112.5	121.5	126.7	116.3	104.5	118	113.9
FNDC3A	173.4	155.3	158.2	175.7	145.1	190.3	172.9	190.7
FNDC3A	115.8	128	112.8	126	111	102.8	118.7	116.7
FNDC3A	183	174.4	187.6	208.9	199.3	227.4	192.3	189.2
FNDC4	117.5	126	124	126.4	110.9	95.6	123.1	113.9
FNDC5	113.1	111.2	114.8	119	109.3	103.5	119.4	111.7
FNDC6	117.9	119.9	114.8	121.5	119	104.7	137.5	149.3
FNDC7	114.9	112.6	114.3	119.3	111.6	95.4	116.4	112.7
FNDC8	115.2	120.1	121.7	124.8	113.5	104.4	123.3	117.1
FNTA	218.9	273.6	348.3	282	253.2	239.3	215.5	235.6
FNTA	124.1	115.2	142.1	126.4	110	109.1	119.9	115.6
FNTA	987.2	1230.6	1281.1	1112.7	1030.2	974.6	1241.1	1401.7
FNTB	151.3	160.3	156.6	152.7	133.5	115.2	147.9	146.9
FOLH1	112.5	113	114.4	121.8	107.4	97.3	109.4	112.5
FOLH1	135	136.4	129.8	147.4	122.1	117.9	128.7	132

FOLR1	115.2	126.5	119	118.3	110.6	101.4	115.5	119.8
FOLR1	113.1	113.6	120	118.9	106.2	101	111.7	113.3
FOLR1	105	120.5	112.3	117.7	120.8	104.5	120.3	112.3
FOLR1	113.3	115.3	109.2	120.8	99.5	98.8	110.1	116.9
FOLR2	114	111.3	116.1	116	112.1	107	121.5	114.6
FOLR3	132.2	114.4	154.8	123.8	117.2	106	120.3	123.3
FOS	204.9	189.7	401.2	345.1	188	394.2	353.9	206
FOSB	126.5	128.8	122.6	120.9	132.4	106.3	158.4	228.9
FOSL1	107.7	116.8	116.2	124.7	116.9	104.3	148.2	123.6
FOSL2	122.5	116.7	125.4	119.5	113.8	104.8	127.1	106
FOXA1	104.8	113.4	112.7	102.6	105.5	95	109.9	117.9
FOXA2	110.7	109.4	116.8	122	112.7	98.5	117.9	124.9
FOXA2	105.4	105.9	108.4	113.6	105.1	100.9	111.4	113
FOXA2	118.8	114.3	115.6	120.2	109.1	102	127.4	119.8
FOXA3	112.1	118.5	111.7	114.8	105.1	95.2	112.6	117
FOXB1	115.2	126.9	128.9	129.6	121.5	103.1	123.7	114.5
FOXC1	114	118.5	124	111.9	108.7	103.3	113.8	116.1
FOXC2	123.9	125.3	125.8	129	121.3	119.6	129.2	125.6
FOXD1	115.3	110.8	128.9	124.4	101.7	96.9	121.8	108.6
FOXD2	129	145.9	138.1	137.6	126.5	104.5	138.1	147.2
FOXD3	113.3	114	118.3	118	107.4	94.1	113.1	114.7
FOXD4	119.7	133.5	131.8	142.2	125	112.1	169.1	198.6
FOXD4L1	123.8	121.5	130.3	132.6	106.6	95.4	134.3	166.7
FOXD4L2	111.6	112.2	107.9	109.8	107.8	92.9	120.5	110.3
FOXD4L3	122.1	133.2	149.6	137.3	118.3	110.3	132.4	120.7
FOXE1	122.1	115.5	111.6	116.8	105.6	92.8	122.5	119.4
FOXE3	120	111.7	118.3	118.6	101.3	102.4	129.4	115.8
FOXE3	122.1	123.2	126.2	111.1	121.5	105.7	119.5	127.4
FOXF1	118.7	114	119.7	128.5	116.7	109.1	108.8	119.7
FOXF2	110.7	110.8	108.8	118	112.2	97.1	108.1	117.7
FOXG1B	118.1	124.5	132.4	140.8	113.4	109.8	122.5	110.8
FOXH1	119.7	118.4	124.9	128.8	116.2	98.4	125	120.3
FOXI1	114.3	117.1	128.8	117	115.4	101.7	125.6	116.8
FOXI1	131.5	135	119.4	126.5	115.1	94.4	124	117.7
FOXJ1	133.1	123.1	140.8	124.3	119.2	107.4	112.5	129.5
FOXJ2	663.6	778.5	792.6	748.4	679.2	574	829.9	990.8
FOXJ3	1205	1324.5	1006.2	1326.2	1215.7	720	1431.3	1692.3
FOXK1	434.5	482.5	449.2	464.8	506.4	430.1	512.6	506.8
FOXK2	110.1	108.5	114.5	106.4	102.6	96	115.6	108.9
FOXK2	131.2	128.2	136.7	125.2	142.1	100.5	139.9	149.9
FOXL1	115.8	119	115.9	115.6	110.4	92.1	116.1	110.6
FOXL2	106.3	111	116.6	111.5	102.8	96.9	114.9	103.1
FOXL2	111.8	118	132.8	116.5	112.8	101.3	125.6	126.3
FOXM1	114.1	114.5	129.2	120.7	107.4	89.8	106.1	117.4
FOXM1	113.6	119.8	125.2	120.7	114.8	97.5	120.6	115.9
FOXN1	114	114.6	111.9	106.3	103.8	95.7	114	115.7
FOXN4	104.9	117	114.7	118.6	105.4	95.2	117.8	110.2
FOXO1A	706	937.4	561.3	725.7	619.2	466.8	578.6	625.5
FOXO3A	110.7	107.2	116.2	119.7	104.4	101.8	117.7	117.3
FOXO3A	652.1	754.6	572.6	724.9	626.5	400.7	897.1	751.9
FOXP1	125.6	121.8	127.3	146.8	129.3	118.8	139.3	127.6

FOXP1	130.8	138.8	136.8	140.5	119	117.8	128.8	147.8
FOXP1	135.3	134.6	125.6	142	124.6	109	155.7	143.5
FOXP2	113.5	109	109	112.2	113.9	93	110.6	106
FOXP2	117.3	111.7	112.9	108.1	112	99.2	119.6	114.2
FOXP3	124.8	121.5	120.1	120.2	119.6	102.8	118.8	115.8
FOXP4	123.4	134.2	129.9	131.4	105.1	107.8	133.9	131.5
FOXP4	113.7	116.3	125.2	117.4	112.3	94.9	126.1	113.9
FOXQ1	111.3	121.4	117.4	118.5	107.4	100.2	130.2	119.7
FOXR1	112.2	120.9	111	122.3	110.3	99	117.9	107.4
FOXR1	125.4	108.1	113.3	121	102.5	96.2	117.8	116
FOXR2	113.8	107.9	116.1	110.6	110.3	97	111	118.8
FOXRED1	176.6	192.4	204.7	176	142.5	148.2	167.6	155.2
FOXRED2	145	138.8	173	137.8	117.9	105.2	141.5	128.2
FPGS	122.2	113.3	120.5	132	111.4	97	121.7	114.4
FPGS	133.2	140.7	141.7	126.8	120.8	121.8	126.4	137.2
FPGS	111.2	116.7	121.4	125	102.5	111.8	121	115.3
FPGT	112.5	111.3	116.5	123.8	110.1	95.4	119.7	114.5
FPR1	128.7	113.2	250.4	120.8	105.5	124.4	121.5	107.7
FPRL1	109.5	116.8	118.2	119.7	106.2	101.1	125.4	116.4
FPRL1	116.7	111.7	115.5	114.3	106.4	98.3	112.4	113.9
FPRL2	230	116.3	974.7	270.8	108.6	812.9	159.5	124.7
FRAG1	151	149.8	158.7	149.9	137.5	124.4	149.2	156.8
FRAP1	383.4	389.7	337	377.1	352	300.9	448.7	418.9
FRAS1	112.4	112.9	112.6	115.8	105.7	97.5	109.3	111.2
FRAT1	149.3	145.4	152.4	146.8	143	115.2	140.6	126.3
FRAT1	142.6	133.1	129.3	143	126.6	108.8	135.4	130
FRAT2	578.2	704.2	555.5	594	499.3	392.4	494.6	509
FREM1	113.2	113	124.4	118.7	113	105.9	114	115.3
FREM2	111.7	108.9	116.4	115.1	111.2	108.2	123	121.5
FREQ	121.8	108.4	120.9	116.1	108.2	99.2	126	116.5
FRG1	233.7	279.2	210.8	344.3	278.5	361.7	258.5	189.5
FRG2	116.5	108.5	117.6	108.9	105.2	100.2	111.8	110.7
FRK	104.2	107.8	106	117.7	112.6	104.6	117.8	117.2
FRMD1	113	119	121.6	118.6	101.5	98.5	126.7	109.4
FRMD4A	117.7	119.9	135.3	125.1	130.8	105.3	139.1	121.2
FRMD5	110.3	116.8	122.2	116.1	111.9	99.2	117.4	105.7
FRMD6	117.4	121.1	126.1	118.4	115	105.7	149.9	142.9
FRMD6	133.2	148.6	130.3	171.9	147.4	129.8	216	241.3
FRMD6	121.4	122.3	123.7	125.2	115.8	108.1	114.3	121.2
FRMPD1	116.4	115.8	120.8	121.1	111.1	96.9	113.1	114.9
FRMPD2	115.9	121.3	117.5	114.9	107.7	105	122.2	112.8
FRMPD2	109.1	115.4	125.2	117	109.2	99.1	117.5	118.9
FRMPD2	110.4	115.5	123.8	113.6	118.2	100.7	129.8	116.7
FRMPD2	120.4	112.3	109.7	110.5	108.3	102.1	113	108.7
FRMPD2	108.3	117	108.2	125.4	114.2	106.8	129.4	116.7
FRMPD2	105.1	120.1	120.7	119.3	114.1	102.9	112.9	121.7
FRMPD4	121.4	125.7	115.3	119.5	105	90.6	118.6	129.1
FRRS1	110.1	123.7	120.7	120.3	112	108.8	116.7	116.4
FRS2	116.3	123.4	119.3	120.2	109.3	98	124.7	110.1
FRS2	123	122.4	124.7	125.1	108.1	101.1	117.7	123.5
FRS2	106.5	120.1	107.3	117.1	103.2	98.7	112.1	122.4

FRS3	150.6	159.5	170.1	149.2	143	128.1	130.2	132.5
FRY	117.3	126.5	124.3	140.2	117.3	104.9	128.7	119.1
FRYL	125	118.3	126.9	129.4	118.8	101.6	134.1	114.7
FRZB	118.3	114.7	116.5	130.9	119.5	106.7	117.9	123.9
FSCN1	190.3	181.6	178.4	196	146.5	126	157.5	131.4
FSCN2	112.7	123.9	114.5	120.2	104.8	100.6	113.6	109.1
FSCN2	111.4	117	116.5	110.5	108.4	99.8	114.4	115.2
FSCN3	128.3	116.9	120.2	123.3	111	98.2	123.4	115.6
FSD1	116.5	117.9	118.5	119.5	108	100.7	118.2	124.8
FSD1	119.6	109.2	121.5	121.4	110	97.9	118.2	128
FSD1	130.7	150.5	131.3	134.3	125.4	102.7	128	121.2
FSD1CL	111.3	113.9	112.7	104.1	112.2	99.1	109.7	109.8
FSD1NL	118.3	130.1	122.8	135.2	116	101	123.9	121.4
FSD2	121.2	114	110.7	116	102.7	100	115.5	119.8
FSHB	111.7	112.1	111	112.8	106.8	93.9	126.8	117.9
FSHB	120.8	119.5	148.6	133.9	119.8	121	122.9	120.6
FSHR	111.2	108.3	111.8	119.3	108.8	99.5	118.8	112.1
FSHR	110	110.6	111.3	118.2	108.6	103	114.5	117.9
FSIP1	125.4	113	113.8	125.3	115.7	103.3	122.4	119.2
FSIP2	115.6	123.7	115.8	121.2	110.1	97	118.2	116.5
FST	132.1	109.2	123.2	118.4	108	109.4	120.3	111.5
FST	188.6	176.3	180.6	212.5	144.4	182	177	168.3
FSTL1	110.8	107.3	122.2	120.4	103.7	95.2	111.2	104.7
FSTL3	131.7	145.6	118.2	132.5	117.1	107.4	128	119.6
FSTL4	104	117.8	108.4	111	104.5	97.4	115.9	115.1
FSTL5	110.2	115.6	114.4	109.7	109.7	93.5	120.6	106.7
FTCD	115	116.5	115.1	116	111.1	97.5	119.8	120.9
FTCD	116.2	112.1	117.9	114.6	107.9	101.2	112.8	110.2
FTHL11	645.4	399.8	412.6	583.6	246.5	559.3	521.2	520
FTHL12	1714.4	1051.5	1492.2	2427.6	1307.5	3102.8	2269.7	1466.3
FTHL17	117.3	121.6	116.7	134.5	111.2	100.2	125.8	106.8
FTHL2	168	126.4	153.8	199.9	142	173.6	186.9	145.9
FTHL3	656	452.6	627.5	719.2	351.5	924.8	1064.7	599.3
FTHL7	7378.1	6379.4	9770.1	13617.5	8113.7	15280.1	11363.6	9856.5
FTHL8	113.5	114.9	121.8	116.7	110.9	106.1	121.9	117.5
FTL	16636.9	16042.4	24481.1	24112.6	22591.5	27883.5	33145.9	31583.6
FTMT	112.9	114.3	115.6	117.6	108.6	101.6	115.9	111.3
FTO	132.5	153.7	128.5	145	132.1	117.3	126.5	136.2
FTS	163.2	217.6	154.6	182	200.3	146.8	178.5	153.7
FTSJ1	242.5	282	238.2	255.6	201	235.6	210.2	274.1
FTSJ1	118.1	116.2	126.6	119.6	104.2	94.5	119.4	108.2
FTSJ2	151.2	150.1	152.4	151.6	140.5	122.1	137.7	137.5
FTSJ3	129.4	143.9	138.2	151.6	131.7	122.9	133.4	156.5
FUBP1	138.1	144.1	132.4	144.4	135.1	125.6	140.8	154.9
FUBP3	532.8	578.8	502.5	621.3	556.5	469.2	571.3	600.4
FUCA1	226.1	283	403	246.4	278.1	347.6	204.3	217.9
FUCA2	108.8	107.2	108.8	114.3	99.7	91	106.9	117.7
FUK	258.8	264.5	220.9	230.1	188.7	168.8	193.9	206.1
FUNDC1	147.6	150.9	152.4	161.4	128	137	138.6	142.7
FUNDC2	112	118.6	128.2	127.2	125.4	105.5	120.9	117.7
FURIN	141.4	138.8	173.6	146.1	124	129.7	160.3	152.5

FUS	129	130.9	127.7	139.3	124.5	108.3	138.8	138.2
FUSIP1	111.8	110.2	114.9	110.5	107.3	101.3	116.7	111.8
FUSIP1	210.1	198.6	231.7	196.2	214.3	152.4	189.2	194.3
FUT1	114.4	120.6	122.7	126.5	114.7	103.9	125.4	121.5
FUT10	108.8	111.3	107.3	114.5	103.4	98	106	119.2
FUT11	128.8	125.7	126.8	135.9	121.6	102	132.7	128.6
FUT2	117.9	107.3	104.3	115.2	102	100.7	119.1	114.7
FUT3	116.5	109.3	117.2	120.8	106.1	93.2	129.6	111.4
FUT4	181.3	188.1	173.8	195.7	201.5	163.2	205.7	219.1
FUT5	121.6	125.9	121.8	122.8	111.7	101	120	116.4
FUT6	132.8	142	150	155.1	144.7	115.2	149.6	135.3
FUT6	111.1	124.6	123.2	120.4	112.3	103.6	116.1	112.8
FUT7	114.2	113.3	116.6	126.6	104.6	100.3	106.4	120.4
FUT8	138.7	147	146.1	135.5	122.4	127.9	133.2	138.8
FUT8	105	115.7	113.9	114	102.5	95.8	115.5	108.9
FUT9	120	112.9	106.6	120.2	110.7	99.9	112.7	108.8
FVT1	268.2	268.5	218.7	243.2	201.3	164.8	302.5	308.4
FXC1	170.6	163.7	151.6	179.6	161.9	138.2	148.9	150.7
FXN	128.4	126.7	125.9	136.9	116	107.4	127.9	121.9
FXN	117.8	123.9	123.5	124.5	111.8	105.7	128	109.2
FXR1	336.6	394.3	386.5	422.9	416.9	319.8	595.8	767.2
FXR1	119.7	117.5	117.9	124.4	116.5	104.4	129.5	136.7
FXR1	215.3	228.9	229.8	222.8	225.5	215.9	269.5	320
FXR2	229	235	210	274.7	188.8	181.3	303.9	260.7
FXYD1	129.2	122.3	121.8	120.3	111.9	112.6	121.1	126.3
FXYD1	113.7	120	121.5	121.3	112.3	104.8	127.1	113.7
FXYD1	137	122.8	124.6	144.8	122.4	117.9	119.4	127.8
FXYD2	120.8	123.7	114.5	125.1	115.3	102.1	127.2	123.5
FXYD2	112.7	120.4	107.5	121.3	103.6	97.5	115.9	107.6
FXYD2	121.5	116.5	108.2	119.9	113.9	98.1	119.4	123.3
FXYD2	110.7	121	115.1	125	104.5	101.1	127.2	114.8
FXYD3	117.1	113.1	121.6	126.1	107.1	99.6	109.8	112.5
FXYD3	114.3	130.9	127.6	123.9	107.5	102.2	115.5	115.1
FXYD3	117.5	115	119.8	123	112.5	90.4	124	116.8
FXYD4	118.6	122.1	130.9	120.7	105.2	111.7	116.7	113.4
FXYD5	855.1	984	980.9	909.3	792.8	1125.9	803.1	1026.7
FXYD5	135.4	151.4	137.4	155.7	126.5	126.9	143.9	152.1
FXYD5	3607.4	3975.4	4221.9	4304.8	3505.1	3483	3241.9	3682.9
FXYD6	110.7	116.6	108.7	119.3	109.1	89.6	120.5	118.7
FXYD7	123.2	129.5	130.7	128.3	112.7	112.1	122.1	119.2
FYB	127	120.3	124.9	136.1	114.3	99.5	120.8	140.7
FYB	2611.1	2475.3	2556.5	2087.5	1929.8	2019	1906.7	1674.4
FYCO1	251.6	278.4	291	329.1	282.7	294.7	314.9	243.6
FYN	706.4	912.1	532.9	756.4	511.5	456.6	714.4	746.4
FYN	399.2	448.6	338.4	448.8	421	329.7	416.4	426
FYN	342.4	345	390.5	366.2	368.6	321.6	377.1	443.5
FYN	487.7	426.8	443.2	490.6	435.7	345.2	364.4	369.4
FYTTD1	141.3	155.9	168	186.4	157.6	119.8	194.1	192.6
FYTTD1	258.3	264.7	222.5	284.3	227.5	245.1	273.2	235.4
FYTTD1	125.5	133	140.3	138.9	128.2	106.6	127.4	124.5
FZD1	118.5	119	118.2	125.5	112	100	120.8	124.4

FZD10	116.5	106.6	120	119.5	104.7	105.3	115	117
FZD2	124.7	113.7	183.5	123.9	106.9	110.4	112.9	110.8
FZD3	112.4	126.2	119.2	115.5	109.2	103.7	125.3	125
FZD4	115.5	118.3	117.5	132.8	112.6	100.2	126	115.2
FZD5	112.1	117.6	123.5	120.9	108	106.7	114.1	119.4
FZD6	127.4	132.6	129.9	122.5	110.7	98.5	124.3	125.4
FZD7	148.8	161.3	167.4	174.1	163.6	123	145.6	170.1
FZD8	119.1	120.6	123.6	116.4	109.4	109.8	125	119.7
FZD9	127.2	131.1	123.3	128.8	118.3	106.8	147.1	148.1
FZR1	132.2	137.9	121.6	142.5	123.3	115.1	131.5	137.2
G0S2	116.1	112.5	117.5	120.4	103.9	94.3	110	112.5
G10	367.8	467.3	546	516.1	571.6	700.4	739.4	671.4
G1P3	981.6	733.3	936.7	1132.1	547.3	513.8	830.6	505.1
G3BP1	297.5	268.4	251	333.9	236.2	230.8	290.6	247.5
G3BP1	136.8	137.5	133.3	139	129.2	109.1	135.6	139.1
G3BP2	124	123.8	123.2	124.8	113.4	95.4	119.4	123.2
G3BP2	715.1	785.5	714.9	713.7	708.6	619.3	861.3	1015.7
G6PC	114.7	121.7	111.3	113.8	116.5	94.7	125.3	116.5
G6PC2	118.8	122.1	117.1	127.5	110.6	91.5	121.3	124.2
G6PC2	126.9	118.7	111.5	122.5	107	98.2	129.3	117.6
G6PC2	104	112.8	119.6	110.9	104.8	104.3	118.2	111.1
G6PC3	161.7	156.6	137.5	129.4	116.1	114.8	131.5	126.3
G6PD	375.2	381.3	234.7	559.6	249.1	390.1	613.5	319.3
G6PD	945.6	1009.6	668.8	1665.7	640	1275.7	1730.3	800.2
G6PD	117.6	133.6	120.4	121.7	112.2	101.6	119.2	107.9
GAA	151.4	124.7	196	140.5	114	144.5	145.5	130.4
GAA	126.4	129.5	179.8	130	117.8	114.2	133.3	114.9
GAB1	106.6	111.6	107.2	114.3	97.7	95.8	115.6	110.5
GAB1	108	120.6	115.9	126.7	99.6	97.6	113.6	112.7
GAB1	124.3	116.2	137.1	112.2	109.2	99.2	132.7	118.7
GAB2	147.7	156.2	166.7	179.5	138.5	184.9	189.9	132.8
GAB2	120	105.9	105.3	114.5	98.1	102.6	124.6	110.4
GAB2	107.9	108.3	113.1	117.4	109.7	103.9	116.9	113.1
GAB3	138.9	135.7	164.7	146.9	124.7	126.8	140.5	128.7
GABARAP	152.1	151.7	141.8	166.4	140.5	141	172.6	146.6
GABARAPL	477.5	592.8	566.3	936.1	886	1000.8	1326.3	1925.3
GABARAPL	153.7	169.5	158.3	180.4	151.1	160.3	159.5	165.7
GABBR1	123.3	115.5	130.1	138.4	112.5	104.5	127.7	120.3
GABBR1	117.6	115.8	112.7	112.9	104.6	100.7	128.7	105.2
GABBR1	145.3	133	142.4	148.1	142.5	141.1	138.4	139.9
GABBR2	115.4	113.2	117.3	130.7	110	101.3	119.6	118.8
GABPA	119.3	107.5	113.7	118.1	110	99.5	112	110.7
GABPB2	107.8	113.5	113.5	110.8	110.5	100.6	120.5	116
GABPB2	147	143.5	161.4	159.3	139.2	135.2	155	171.3
GABRA1	114.4	113.4	111.5	115.6	107.9	94.2	112.5	112.6
GABRA2	119.4	120.3	117.4	130.6	110.1	92.5	122.1	110.1
GABRA3	114.6	120.7	118.5	127.5	105.2	103.3	110.8	121.8
GABRA4	108.7	113.9	123.5	118.7	112.2	98.1	119	110.8
GABRA5	116.6	108.1	116.6	114.6	107	95.8	120.8	122
GABRA6	120.5	117.8	130.2	127	117.4	108.4	120.8	118
GABRB1	109.9	110.7	108.7	105.8	106.8	98	116.5	110.5

GABRB2	119.8	113.1	118.5	113.2	103.7	98.8	126.4	113.6
GABRB2	115.1	118.5	117.1	126	110.8	97.4	124.2	118.1
GABRB3	112.1	112.4	115.2	116.9	105.5	99.6	113.9	116.6
GABRB3	116.6	122.5	114.5	133.9	119.1	112.7	123.5	109.9
GABRB3	114.8	115.2	120.8	123.6	110.7	96.8	124.6	114
GABRB3	118.5	121.6	119.5	122.6	107.5	102.1	119.9	106.3
GABRD	117	119.6	126.1	126.4	110.3	101.7	120	109.3
GABRE	128.2	118.3	122.8	132.1	117.5	99.3	121.8	124.3
GABRE	114.5	114.6	112	120.9	104.8	99.1	109.7	112.9
GABRE	129.8	131.2	126.8	132.1	117.1	108.5	136.6	129.2
GABRG1	114.1	111.6	122.2	110.9	110.7	98	124.4	111.7
GABRG2	113.5	119.2	112.7	128.2	109.2	92.8	118.5	112.3
GABRG2	111.9	120.6	108.6	121.6	115.6	96.3	117.7	121.7
GABRG3	119.8	117.2	131.9	115	106.5	98.5	121.7	118.1
GABRQ	104.1	104	109.5	120	98.6	92.9	106.6	111.7
GABRR1	120.5	112.1	112.7	117.3	105.1	98	111.8	122.2
GABRR2	113	125	124.3	124.3	107.4	106.5	122.5	121
GAD1	103.1	102.5	113.3	114.8	106.2	91.6	116	107.8
GAD1	116.1	115.4	120.6	116.1	110.3	102.6	121.3	109.1
GAD1	113	112	117.2	111.3	101.4	95.5	114.8	106.8
GAD2	112.4	121.7	123.8	119.4	107.8	103.9	120.9	121.5
GADD45A	161.8	164.5	164.4	172.2	152	136.5	168	214.6
GADD45B	141.3	137.4	160.9	152.1	144.7	128.3	135.6	143.5
GADD45G	123	125.3	124.5	126.3	117.4	109.1	139.7	144.8
GADD45GI	110.5	118.3	111.7	123.8	111.7	99.9	118.6	109
GADL1	99.9	111.3	118.1	111	104.9	100.1	112.6	111.1
GAGE1	119	109.5	117.2	117.3	110.2	97.5	121	112.1
GAGE1	113.8	109.9	105.4	122.4	113.7	105.6	107.4	118.6
GAGE1	112.7	110.5	114.1	121.4	119.1	99.7	123	112.9
GAGE2	106.5	119.5	117.2	116.7	112.5	96.8	118.9	109.1
GAGE4	107.4	121.2	120.4	123.2	115.4	104	122.9	118.1
GAGE5	115.5	121.8	112.7	113.7	108.9	95.3	118.3	111.9
GAGE6	126.8	126.1	136.4	122.8	106.6	107.6	126.4	123.5
GAGE7	112	116.9	118	123.3	106.4	110	127.5	119
GAGE7B	111.2	113.4	110.1	116.8	112.3	100.2	128.9	113.7
GAGE8	118.5	117.2	126.6	116	99.7	101.6	109.9	118.1
GAK	1554.3	1688.5	1310.3	1744.6	1325.8	1095.6	1597.3	1477.8
GAL	109.9	121.2	111.4	118.7	116.7	100.7	120.3	111.9
GAL3ST1	119.1	125.2	131.1	123.4	120.4	109.7	119.5	119.3
GAL3ST2	119.2	125.4	124	129.1	112.3	112.5	121.4	127.6
GAL3ST3	121.7	118.7	117.7	123	111.3	96.7	119.5	115.6
GAL3ST4	135.8	156.5	166.9	138.7	132.4	130.2	142	130.2
GALC	138.5	134.2	162.7	132.4	120.6	110.1	138.8	124.6
GALC	281.7	282.9	474.1	300.8	263.5	305.5	238.4	217.3
GALE	105.8	116.5	117.6	117	108.1	99	117.7	112.4
GALE	133.1	124	146.2	138.6	114.9	140	141.2	144.4
GALE	117.9	116.5	119.6	121.5	108.9	100.1	117.9	106.3
GALGT	121.4	124.7	123.6	121.5	118.3	102.8	119.3	114.6
GALIG	120.5	123.5	118.1	124.5	118.9	99.1	128.9	116.5
GALK1	461.8	380.5	707	344.9	238.9	264.7	211.3	198.8
GALK2	127.4	113.4	160.6	133.3	126.4	118	137.5	145.5

GALK2	178.1	181.2	154.5	168.3	165	114.4	142.3	163.8
GALK2	117.4	117.8	120.5	121.6	106.8	105.2	122.5	114.2
GALM	228.6	311.7	297.5	270.8	255.9	226.3	187	195.6
GALNAC4S-	222.7	193.2	263.6	274.1	176.8	235.8	282.4	166.5
GALNACT-2	139.2	137.6	145	149.8	150.8	122.2	139.2	129.2
GALNS	133.2	114.9	123.6	138.9	110.8	116.3	129.8	132
GALNT1	114.2	121.7	121.8	119	108.8	100.5	120	118.4
GALNT10	126	109.4	124.6	125.5	111.3	101.9	125.5	119.8
GALNT10	140	168.8	199.5	157.7	153.5	157.4	162.3	145.2
GALNT10	115.4	120.3	122.4	123.1	110.1	95.3	114.3	111.2
GALNT11	226.6	314	226	246.7	222.3	188	197.2	249.1
GALNT12	121.4	123.4	130.1	125.1	117.2	110.7	118.7	119.4
GALNT13	116.3	118.5	117.4	121.4	112.3	97.6	122.5	117.8
GALNT14	123.3	121.2	120.6	130.5	116.1	110.5	119.9	123.3
GALNT17	108.8	118.8	121.8	120.4	105.3	97.4	127.5	125.7
GALNT2	122.4	134.1	129.8	123.4	122.3	107.9	129.2	122.5
GALNT3	208.2	183.2	224.2	186	199.5	143.7	223	165.3
GALNT4	149.4	141.8	168	157.1	153.7	144.4	166.2	149.7
GALNT5	106.6	106.5	116.4	105.8	101.8	95.7	109.3	107.1
GALNT6	119.8	116.6	124.1	122.8	111.1	109.4	125.4	121.3
GALNT7	110.5	109.5	114.5	115.4	108.5	98.4	111.1	108.4
GALNT8	119.1	122.2	121.9	120.5	115.4	106.8	119.9	120.9
GALNT9	118.4	121.3	124.3	128.1	115.6	103.1	120.2	112.4
GALNT9	124.3	109.6	119.3	115.1	100.8	106.7	116.6	127.6
GALNTL1	115.4	110.3	110.8	122	104	99.5	121	116.7
GALNTL2	120	119.1	124.2	122.4	111.8	97.5	118	113.6
GALNTL4	119.2	112.4	122.6	122.9	110.7	100.6	109.9	115.9
GALNTL5	111	108.5	111.8	113.2	111.3	95.6	110.2	110.6
GALP	118.1	116.1	129.7	106.5	102.7	106	124.5	110.4
GALR1	114.4	107.2	121	115.1	105.6	95.5	122.8	115.6
GALR2	112	117.1	119.2	123	104.8	93.4	116.2	112.9
GALR3	121.2	114.7	122.1	114.3	110.4	101.1	117.3	112.4
GALT	325.5	390.4	336.7	300.1	232.6	170.1	183.8	189.7
GALT	210.5	198.5	217.7	194.5	172.4	128.1	158.7	145.1
GAMT	216.9	239.8	224.9	247.6	176.6	143.7	179.9	152
GAMT	126.3	128.1	142.4	145.5	117.6	124.6	119.5	125.6
GAMT	148.5	144	148.9	149	138.6	123.6	130.4	139.5
GAN	116.9	117.2	118.8	124.9	104	100.8	121.3	118.5
GANAB	189.9	183.9	199.2	244.4	189.1	187.1	203.7	211.4
GANC	112.8	122.6	116.4	120.4	113.4	100.6	122.7	120.3
GAP43	107.7	116.5	117.3	118.2	113	98.2	120.8	117.3
GAPDH	678.4	774.7	902.2	763.7	660	646.9	910	852.5
GAPDH	253.5	244.5	310.1	337	217	223.2	293	239.9
GAPDH	1472.4	1718.5	1842.7	2312	1477.2	1707.6	2492	2669.9
GAPDHS	114.4	112.4	129	129.4	114.8	107.6	120.9	121.4
GAPVD1	366.4	391	405.6	432.1	426.8	349.3	382.1	439
GARNL1	397.2	336.1	319.7	385.7	260.4	253.2	448.6	519.5
GARNL3	102.4	109.4	109.4	108.9	110.1	93.8	114.7	109.1
GARNL4	145.8	145.4	173.9	193.2	136	116.1	141.1	134.3
GARS	593.2	714.4	565.5	674.9	638.7	543	770.6	923
GART	490.8	477.2	425.2	483.9	412.1	318.5	597.5	576.1

GART	134.3	149.9	154.2	145.4	151.7	122.9	150.7	142.5
GART	120.4	117.2	119.7	111.3	112.7	97.1	122.7	110.5
GAS1	119	120.9	114.9	121.1	106.2	95.6	114	116.7
GAS2	119.9	110.4	128.9	120.8	110.8	102.3	115.8	109.5
GAS2	113.3	118.2	109.8	116.8	111.4	95.9	108.6	107.8
GAS2L1	120.7	116.9	129.1	129.2	111.4	101.5	127.6	121.7
GAS2L1	120.6	117.1	132.3	120.8	107.1	98.6	124.6	106.6
GAS2L1	111.4	114.7	128.6	120.3	114.5	104.2	125	112
GAS2L2	114.5	124	136.5	121.8	117.3	112.2	119.1	123
GAS2L3	156.2	138.9	152.8	139.8	143.3	109.2	165.2	141.7
GAS6	133	135.6	145.6	131.1	127.4	122.7	119.6	116.6
GAS6	126.2	127.7	143.1	130.9	112	114.2	119.2	110
GAS6	119.7	119.3	124.9	116	117.6	94.6	114.9	121
GAS7	112.9	115.9	114.7	121.8	114.2	98.6	118.4	112.1
GAS7	158.2	135.6	265	158.7	122.9	192.5	149.9	131.8
GAS7	116.1	110.5	115	115.7	110.9	96.9	113.3	111.1
GAS7	116.9	109.9	121.9	122.9	111.5	99.7	124	118.6
GAS8	131.5	123	128.3	129.4	112.2	102.7	118.3	127.2
GAST	121.5	119.7	123.5	118.4	115.8	102.3	126.3	120.9
GATA1	118.9	120.8	112.3	117	109.2	101.8	118.9	116.6
GATA2	123.7	111.5	123.9	125	114.2	102.8	124.1	121.8
GATA3	167.2	164.7	178.3	143.6	152.5	138.4	144.2	143
GATA4	118.7	113.1	119.8	121.8	114.9	98.2	119.3	109.7
GATA5	117.6	109.1	116.1	113.1	107.2	95.6	115.9	122.1
GATA6	112.7	117.9	110.7	118.1	104.5	97.2	121.3	112
GATAD1	129.7	139.2	172.8	142.6	139	139	140	132.7
GATAD2A	693.9	862.4	447.2	796	646.9	529.1	1107	961.1
GATAD2B	271.1	276.5	207.2	281	215.8	186.4	304.9	336.3
GATM	113.3	117.2	125.3	121.1	107.9	95.3	119.9	105.6
GATS	334.5	289.4	301.6	343.2	299.4	260.6	409.1	303.4
GBA	349.3	337.1	386.3	335.6	228.4	208	288.4	248.8
GBA2	171.6	192.2	160.8	186.5	166.5	156.1	177.4	189.1
GBA3	114.2	109.3	109.4	113.4	111.6	92	118.1	110.9
GBAS	255.2	284	311.4	269	298.8	237	233.4	246.1
GBE1	179.5	197.9	158.7	207.4	170.1	151.6	210.3	177
GBF1	183.7	170.2	155.7	158.2	148.6	123.8	182.7	162.5
GBGT1	159.8	136.1	182.8	159.4	120.7	128.3	138.4	118.1
GBL	189	185.7	187.6	181.4	151.1	156.8	172.1	154.9
GBP1	504.9	214.7	413	355.4	195.4	251.4	221.1	168
GBP2	1937.7	1781.4	2045.3	1977.1	1146.9	1359.5	1675.1	1125.7
GBP3	122.3	138.6	132.5	128.6	110.4	122.2	127.5	134.2
GBP4	621.7	557.6	414.9	485.6	363	326.9	338	312.6
GBP5	512.2	313.2	402.3	335.6	186.2	362.5	219	171.6
GBP6	121.8	117.9	130.7	132.8	126.8	102.2	125.7	123.2
GBP7	104.8	99.8	106.5	116.4	108.8	93.3	125.5	106.1
GBX2	109.5	116.5	121.2	125.3	108.3	100.8	127.1	112.2
GC	114	119.5	121.2	122.8	107.5	97	113	108.7
GCA	347.3	298.8	467.8	334.2	217.6	344.1	256.5	308.1
GCAT	119.3	122.9	125.5	121.4	116.9	107	121.7	117.7
GCC1	304.3	227.7	274.1	294	184.1	203.7	347.8	355.7
GCC2	126.6	124.1	119.8	124.3	121	101.2	119.8	109.4

GCC2	119.6	117.7	114	111.4	108.9	95.4	120.8	108
GCC2	124.9	130.1	139.8	142	134.3	114	125.7	133.3
GCDH	133	143.9	124.3	138.4	117.8	116.1	138.7	125.1
GCDH	204	198.4	177.8	197.1	166.3	131.3	154.8	152.2
GCET2	117.4	116.6	125.2	121.7	113.8	100.7	120	117.2
GCET2	126.5	124	135.6	132.1	125.6	108.1	133.8	124.2
GCG	112.6	120.9	117.4	121.2	112.5	104.4	120.2	130.3
GCGR	112	125.4	115.2	131.9	110.7	100.3	127.2	113.5
GCH1	186.6	164.2	158.9	183.4	155.5	131.3	153.5	164.2
GCH1	121.7	115.8	125.6	117.1	116	99.6	121.8	119.5
GCH1	118	123.3	134.5	131.8	136.3	105.6	122.5	111.8
GCHFR	285	337.2	453.3	291.1	239	302	197.1	193.4
GCK	114.5	119.8	134.5	140.5	108.9	99.3	117.2	116.7
GCK	115	122.1	118.6	120.4	108.9	96.7	119.8	108.9
GCK	112.4	114	115.5	120.9	103.3	99.1	116.5	114.1
GCKR	119	122.1	115.2	129.5	103.8	98.7	114.3	121.7
GCLC	123.7	127.1	124.4	147.3	119.5	111.1	140.8	117.5
GCLM	132.3	136.8	138	167	171.5	140.5	320.4	466.5
GCM1	108.1	113.6	120.4	121.2	114.9	107.1	124.8	137.6
GCM2	119.8	116.7	116.3	124.9	110.8	99	131	114
GCN1L1	221	248.4	206.4	179.6	164.3	173	252.8	183.3
GCN5L2	477.8	484.6	492.1	463.8	334.8	264.4	290.4	294.8
GCNT1	115.9	116.8	128.4	119.6	107.8	110.2	122	122.5
GCNT2	109.1	113.9	109.9	118	104.3	94.9	114.4	111.9
GCNT2	108.8	117.1	117.5	117.2	113.4	100.2	113.5	120.9
GCNT2	137.8	114.5	144.1	134.7	119.5	110	131.2	115.6
GCNT2	116.1	115.5	117.2	123.6	111.3	93.8	120.4	114.4
GCNT3	114.8	107	110.1	119.2	109.7	101.5	115.6	110
GCOM1	117.9	118.2	114.9	116.6	117.6	97.4	117.8	119.2
GCOM1	115.4	115.5	126	109.1	109.5	96.3	119.3	116.5
GCOM1	119	117.1	125.3	119	107.7	98.6	114.2	117.6
GCOM1	117	119.5	111.2	114.7	111.3	97.4	114.1	109.7
GCS1	158.7	162.6	177.6	162.8	141.5	120.9	169.2	141.3
GCSH	129.7	129.9	139.9	144.1	126.6	123.9	135.9	123.8
GDA	119.4	121.6	113.8	112.2	102.3	100.4	114.4	111
GDAP1	112.2	114.9	123.4	119.8	108.4	99.3	123	115
GDAP1	117.9	116.8	116.5	125.2	108.9	103.2	118.6	111.3
GDAP1L1	117.5	115.4	121.1	115	116	97.9	112.3	109
GDAP2	161.5	179.8	140.2	178.8	148.9	128.2	196.4	173.8
GDDR	114.9	118.2	106.7	116.6	115.1	101.4	110.7	121.7
GDF1	123	119.6	123.4	129.5	114.5	105.2	125.6	115.8
GDF10	119	114.7	118.8	117.3	104.3	102.5	123.2	119.1
GDF11	120.2	129.3	124.5	137.5	115.8	123.2	126.2	126.8
GDF15	120.8	123.9	115	135.5	118	95	186.4	136.3
GDF2	104.7	114.7	104.1	109.1	106.2	93.7	121.1	102.3
GDF3	117.6	122.2	115.8	112.9	103.8	100.3	111.1	110.8
GDF5	114.9	104.3	110.5	113.4	109.2	102.9	114.9	118.6
GDF6	110.7	115.1	115.2	117.5	104.8	96.4	119.1	118
GDF7	111.9	115.7	112	120.1	109.6	100.7	115.4	114.7
GDF8	114.1	110.9	112.5	115.1	103.5	104	122.3	113.8
GDF9	125.6	120.5	119.4	117.4	114.8	101.4	119.5	112.1

GDI1	277.3	296.2	283.8	314.6	218.5	328.5	375.6	314.1
GDI2	525.5	523.6	602.1	481.3	339.3	373.1	493	443.1
GDNF	113.4	101.1	122.5	118.2	109.1	98.6	118.8	114
GDNF	111.3	111.4	109.6	115.2	110.3	108.8	110.2	112
GDNF	119.9	129.3	131.6	129	118.8	104.5	130.2	127
GDPD1	196.8	199.4	210.7	163.5	170.2	141.6	214.9	166.8
GDPD2	113.4	120.7	130.4	121	113.3	101.9	117.5	113.8
GDPD3	171.4	201.5	224.4	189.9	176.7	194.4	179.3	170.6
GDPD3	106.7	107.9	113	117.3	113.9	99.7	125.4	117.5
GDPD4	111.4	135	133.5	125.2	120.5	115.9	135.8	126.5
GDPD5	162.2	192.6	145.8	163.3	152.3	139.3	158	157
GEFT	117.5	123.6	126.1	132	113.7	101.1	120.3	123.1
GEFT	115.8	123.9	108.5	116.9	106.8	91.7	104.6	112.1
GEFT	123.3	123.2	128.1	124.8	107.2	101.8	123	116.7
GEM	113	118.4	123.5	127.8	109.7	97.9	127.2	118.6
GEM	117.5	118	126.1	116.9	112.1	91.2	118.2	111.8
GEMIN4	585.5	722.7	465.5	634	439.5	298.2	530.7	576.9
GEMIN5	135.9	151.9	148.6	146.4	132.8	138.6	134.6	132.3
GEMIN6	226.2	239.3	232.5	234.5	217.4	185.3	223.6	208
GEMIN7	115.9	121.5	118.9	128.9	106.4	105.3	128.6	123.6
GEMIN7	105.7	128.2	116	121.2	114.8	103.3	126	112.8
GEMIN8	120.3	130.5	138.2	140.4	124.3	116.6	125.4	132.9
GENX-3414	113.8	113.3	123.4	122.7	121.2	102.1	123.9	129.3
GFAP	114.5	117.9	128.1	120.6	111.3	104.9	115.7	122.4
GFER	120.3	115	126.8	139.1	118.7	97.2	116.1	139.3
GFI1	260.9	283.8	210	284.8	186.3	149.1	326.2	231.5
GFI1B	113	116.5	118.9	115.2	113.5	96	122.6	112.1
GFM1	300.7	362.2	295.9	251.1	269.6	241.6	269.8	330.8
GFM2	110.4	111.1	124.4	114.1	110.8	95.9	116	108.3
GFOD1	205.2	184.6	173.3	214.6	168.1	145.3	186	184.6
GFPT1	216	215.9	195.1	270.5	189	187.3	300	268.5
GFPT2	121.1	126.9	116.1	125.1	117.4	105.6	120	124.6
GFRA1	119	124.7	119.6	130.4	110.8	108.8	130.2	117.9
GFRA1	121.9	114.6	118.5	114.1	97.5	96.4	118.5	114
GFRA1	124.4	123.6	125	128.3	122.4	106.8	117.9	113.2
GFRA2	151	154.1	150.4	147.8	137.2	116	138.9	150.5
GFRA3	114.2	113.8	120.1	121.9	106.7	95.2	120.9	123.4
GFRA4	114.6	117	115.6	118.3	109.9	99.3	123.1	110
GFRA4	146.2	130.3	140.2	137.7	126	108.8	138.1	142.8
GGA1	117.3	124.6	137.5	128	121.2	101.2	117.6	120.6
GGA1	9785.2	12288.7	11422.6	9251.6	10005.9	8814.1	10416.6	12034.4
GGA1	129.2	128.9	118.4	135.7	122.3	108.9	118.4	130.1
GGA1	140.4	117.2	121.4	138.5	129.6	105.5	133.9	120
GGA2	351.7	334.8	485.1	358.6	289.2	348.2	316.5	274.8
GGA3	212	229.5	253	280.3	238.4	170.5	261.4	242.6
GGA3	151.2	173	179.7	149.5	139.8	139.5	185.1	150.7
GGA3	117.7	125.6	131.9	122.5	122.4	122.3	129.6	120.3
GGCX	154.2	132.5	142	147.9	121.3	115.1	138.6	130.1
GGH	176.3	141.1	127.9	167.2	128.2	107.6	162.4	128.9
GGN	116.8	109.4	111.1	123.7	108.8	100.5	123.2	112.3
GGPS1	115.7	131.2	115.4	142.1	120.3	104.7	120.8	132

GGPS1	474.2	370	418.5	495.7	367.1	473.4	513.6	475.3
GGT1	119.7	116.3	115.7	111.2	101.1	95.6	117.5	115.1
GGT1	115.4	116.5	124.3	124.3	109.1	104.2	123	117.3
GGT3	113.5	118.3	128.7	134.3	109.9	101.4	124.4	115.9
GGT6	117	117.2	120.7	124.9	106.4	104.7	121	116.9
GGTA1	116.2	125.1	127.1	118.3	108.8	102.7	119.2	114.6
GGTL3	121	135.3	134.5	130	129.7	100.3	132.5	137.9
GGTL4	106.3	116.5	106.3	119.3	106.8	98.8	110.6	107.7
GGTLA1	116.5	125.9	123	125.9	117.9	105.9	124.4	124
GGTLA4	118.8	114.3	122.9	118.7	110.6	101.1	129.8	121.4
GGTLA4	116.8	124.3	124.4	124.6	108.1	105	132.6	117.2
GGTLA4	120.7	120.5	122.2	125.3	108.8	105.5	117.9	125.7
GH1	120.3	117.9	131.3	119.6	108.1	97.5	113.5	110.8
GH1	110.2	115	119.6	116	105.8	100	119.1	114.8
GH2	115.2	122.8	116.8	118.6	107	104.6	111.5	122.8
GH2	115	110.6	114.1	123.8	112.4	97.9	117.5	116.1
GH2	117.3	113.8	117.3	118.7	108.8	99.9	116.1	123.6
GHITM	2167.4	2577.8	2328.9	3035.6	2146.1	2432.6	2924.9	3221.4
GHR	104	109.3	109.5	113	105.2	86.6	116.6	111.7
GHRH	119.2	117.8	115.2	111.3	113.1	108	114	114.9
GHRHR	116.6	122.2	118.3	118.4	98.7	97.9	118.1	107.2
GHRHR	110.7	105.3	107	115.2	112.4	89.5	123.5	122.2
GHRHR	113.7	115.2	110.6	120	110.7	104.8	121.2	105.7
GHRL	106.3	112.9	116.8	119.7	103.4	96.7	127.8	117.6
GHSR	121.9	113.8	125	115.6	110.1	92.8	117.6	98.9
GHSR	132.2	115.6	118.1	117.2	107.2	95	114.8	121.6
GIF	114.5	107.2	119.6	128.7	106.4	98.8	115	106.7
GIMAP1	244.5	261.9	238.7	231.1	247.6	171.5	204.1	189.4
GIMAP2	833.7	703.9	525.6	844.3	679.6	445.7	581.5	626.1
GIMAP4	2935.4	3830	2792.2	3398.5	2764.3	2305.5	2598.4	2162.8
GIMAP5	2794.2	3920.1	2037.8	2898.5	2290.2	1639	2078.6	1657.6
GIMAP6	119.3	113.8	130.5	140.9	112.1	108.9	128	110.6
GIMAP6	1121	1407.2	683	981.5	874.5	472.2	664.2	762
GIMAP7	185.8	198.8	189.8	190.7	160.9	153.6	197.6	177
GIMAP8	606.8	913.3	603.4	611.6	583.8	504.3	582.3	637.5
GIOT-1	106.2	105.5	122.9	112.4	105.1	91.9	114.4	110.7
GIP	116.9	113	126.9	122.9	103.8	99.9	114.7	110.6
GIPC1	116.4	123.8	135	127.4	111	105.1	117.3	116.2
GIPC1	344.9	417	388.7	310.5	253.2	239.2	294.8	237.5
GIPC2	113.4	118	126.1	125.2	106.4	96.9	120.3	106
GIPC3	116.5	112.7	114.2	120	109.6	93.7	113.7	106.6
GIPR	109	110.8	109	108.3	104	92.2	116.2	106.4
GIT1	153	129.2	137.2	144.2	123.2	116.6	163.9	153.7
GIT2	165	196.2	256.7	184.2	202	166.1	165	167.8
GIT2	131.2	140.4	155.5	139	141.5	123	134	125
GIYD1	138.7	150.7	146.6	152.4	128.6	125.5	145.5	130.5
GIYD1	128.2	109.5	139.5	119.3	114.8	101.6	122.5	111.3
GIYD2	112.3	112.8	114.9	111.7	102.6	97.1	106.4	114
GJA1	116.9	115.1	108.2	108.4	107.4	103.2	113.4	117.9
GJA10	116.6	117.5	120.4	150.3	118.6	89.5	131	126.6
GJA12	155	184.3	125.8	168.6	154.6	113	186	219.2

GJA3	106.5	117.2	115.2	118.4	109.8	114.1	117.7	119.4
GJA4	112.5	113.4	110.6	121.5	107.5	95.5	120.9	112.8
GJA5	115.6	115.5	115.8	120.4	101.1	105.5	114.1	114.6
GJA5	126	122.9	131.9	120	117.9	100.5	116.2	119.7
GJA7	115.5	121.2	135.1	123.2	120.8	107.6	126.6	122.2
GJA7	4614.4	4948.8	4265.9	4687.9	3523.3	2716	4824.5	4656
GJA8	120	112.9	113.6	121.6	111.7	102.6	114.6	109.9
GJB1	116.1	122.1	125.7	118.1	109.4	110.3	132.5	119.1
GJB2	127.3	131.9	112.2	123.8	113.2	105.3	128.3	122.8
GJB3	113.8	126.6	127.5	129.6	115.1	112.8	132.4	117.3
GJB3	111	111.6	116.2	120	109.1	101.8	119.3	111.8
GJB3	114.2	112.3	109.7	117	103.5	95.5	113.7	111.2
GJB4	108.6	113.5	127.2	112.2	112.3	94.5	120.7	126.3
GJB5	117.1	123.7	115.2	123.5	114	100.4	113.9	115.9
GJB6	123.6	124.1	120.4	115.1	112.9	110.6	114.7	112.8
GJB7	113.7	117.5	113.3	110.2	106.3	107.8	112.9	121.6
GJC1	119.7	109.5	132.3	116.3	106.4	101.2	121.4	115.6
GJE1	110	107.1	125.8	119.1	107	104.9	110.3	113.5
GK	358.6	276.8	203.2	481.8	293	372	474.2	226.4
GK2	164.2	162.6	169.2	169.2	139.6	137.5	181.6	167.4
GKAP1	120.3	132.1	131.6	123.4	117.4	103.9	133.7	129.1
GKN1	116.9	127.9	120	122.8	107.1	98.8	121.7	118.7
GLA	1819.9	1918.8	800.8	3115.3	1534.2	1111.7	3805.9	2345.2
GLB1	526.5	518	713	583.4	437.8	641.1	448.4	374.8
GLB1	123.9	120.2	120.1	118.3	107.2	100.5	126.6	113.1
GLB1	501.5	456.8	689.9	499.9	383.3	570.4	497.7	310.8
GLB1L	122.2	118.2	124	121.9	115.8	104.2	109.6	106.9
GLCCI1	155.1	168.4	188.3	159.4	147.1	152.9	154.7	140
GLCE	137.2	138	149.5	129.5	130.5	115.1	133.4	116.2
GLDC	113.3	119.2	116.8	112.3	101.2	93.5	108.7	113.3
GLDN	104.1	107.3	112.1	118.8	109.4	98.3	118.8	121.4
GLE1L	435.8	494.1	433.8	483.9	339.2	463.3	412.6	403.1
GLE1L	135.2	130.2	140.2	140.9	127.6	113	125.2	142.8
GLG1	723.1	797.2	904.2	827.9	726.5	642.8	762.4	792.2
GLI1	127.2	119.5	121.8	126.3	109.6	105.6	132.2	115
GLI2	108.2	113.3	108	116.1	107.9	94.5	112.9	113
GLI3	107.2	121.5	111	112.4	104.5	96.3	117.6	113.8
GLI4	117.2	123.5	126	129.9	118.8	103.7	119.5	117.3
GLIPR1	294.2	349.4	620.7	321.7	306.4	402.1	288	239.3
GLIS1	108.6	120.1	111.6	114.1	103.9	94.4	118	110.6
GLIS2	122.2	122.6	115.4	118.8	103.2	96.9	115.9	111.9
GLIS3	113.3	115.3	110.8	113.4	104.6	96.3	115.6	108.2
GLIS3	125.2	109.7	115.7	107.9	113.4	102.5	113.5	108
GLIS3	122.6	113.4	119.6	147.3	131.3	170.7	166.2	130.3
GLMN	119.2	113.6	123.1	123.2	112.2	102.5	118.7	117.9
GLMN	245.2	248.2	217.9	232.6	215.4	165.7	262.5	256.3
GLO1	725.3	935.6	1026.3	838.2	644	944.4	684.9	747.3
GLOXD1	106	120.9	108.4	113.4	108	98.9	119.7	128.5
GLP1R	113.1	112.3	118.1	116.5	105.9	93.6	115.4	113.9
GLP2R	117.8	112.1	114.4	116.3	102.2	93.2	118.8	114.4
GLRA1	114.5	104.8	117.3	113.5	94	96.2	115.5	112.6

GLRA2	117.8	114.6	115.9	112.9	113.3	94.3	125.6	111.7
GLRA3	117.3	115.3	120.1	117.3	111.5	100.3	126.7	117.8
GLRA3	106.6	117.5	109.6	115.6	114.1	100.3	121.2	111.4
GLRB	103.3	113.6	112.4	116.1	107.5	103.6	110.4	112.9
GLRX	362.2	376.5	470	664.1	442.7	432.8	774.7	513.1
GLRX2	147.9	153.8	166.7	173.4	136.6	131.6	146	135.2
GLRX2	120.6	122.6	130.7	138.7	113.2	96.4	125.4	120.9
GLRX2	156.7	175.5	166.6	205.4	151.3	157	150.1	164.7
GLRX5	1224.6	1422.1	1290.4	1362.1	1021	798.2	876	756.6
GLS	246.5	259.1	244	327.8	220.4	192.8	286.8	323
GLS2	116.4	114.3	117.6	113.8	105.2	100.2	113.5	111.1
GLS2	114.3	112	105	123.5	110	93	122.3	115.5
GLT1D1	122	127.4	135.3	119.1	117	106.6	117.9	115.1
GLT25D1	614.7	611.2	754.9	625.3	486.5	425.2	662.5	510.1
GLT25D2	145	129	140.7	149	125.5	113	141.2	122.4
GLT6D1	106.3	116.7	115.1	122	108.5	107.9	125.4	111.8
GLT8D1	137.8	140.1	156	132.4	123.9	116	123.2	138.1
GLT8D1	113.4	108.9	115.4	112.8	98.2	98.5	120.3	112.1
GLT8D1	123.8	122.7	119	123.7	108.6	107.8	135.7	128.4
GLT8D1	113.1	115.5	123.5	110.2	111.7	107.6	132.1	115.1
GLT8D2	118.2	130.7	118.9	120.6	114.3	97.8	111.7	119.7
GLTP	1150.6	1263.7	1300.1	1374.1	1090.9	1045.2	1315.4	1345.8
GLTSCR1	193.9	236	179.2	279.9	230.7	170.8	275.3	333.5
GLTSCR2	8410	11385.9	8497.7	9147.1	8475.3	8761.9	11116.9	12111.4
GLUD1	542.8	504.2	578.9	611.1	357.1	585.4	485.3	507
GLUD2	128.5	119.6	121.8	138.9	122.2	109.8	128.4	136
GLUL	122.8	117.6	127	128.5	125.3	102.9	118.9	123.4
GLUL	117.7	111.6	111.3	111	106.3	94	106.1	113.9
GLUL	116.1	115.4	141.9	120.3	112.3	123.9	124.9	113.3
GLULD1	113.1	108.4	110.4	112.1	117.5	97.4	114.6	113.6
GLYAT	106.3	112.8	112.3	114.2	100.8	94.9	116.9	113.7
GLYAT	110.4	123.9	117.6	114	112.3	98.7	117.2	120.5
GLYAT	109.4	125.9	124.8	130.7	111.6	108.6	136.5	116.4
GLYATL1	119.6	116.3	128.4	115.7	104.5	92.2	125.8	116.3
GLYATL2	120.6	111.1	108.4	112.6	102.3	96.2	121.5	114.5
GLYCAM1	106.5	111.4	132.5	118.6	104.5	104.5	119.7	116.1
GLYCTK	163.4	172.7	199.2	163.8	132.4	124.7	138	134.6
GM2A	122.8	119.6	121.7	123.3	118.1	100.2	133.8	127.3
GM632	146.5	143.2	151.3	152	136.5	107.3	134.8	115.4
GMCL1	374.8	430.1	291.3	421.1	406.4	202.1	409.1	370.4
GMCL1L	108	113.5	102.7	112.7	108.6	95	117.1	116.7
GMDS	277.4	260.6	238.7	251.6	245	173.7	207.1	195.3
GMEB1	151.5	170.3	141.6	166.2	152.7	117.9	178.2	165.6
GMEB1	159.5	164.9	149.6	166	126.3	132.6	170.3	155.5
GMEB2	416.3	427.5	383.6	471.7	377.7	363.3	491.5	459.2
GMFB	734.1	674.6	862.8	805.9	566.3	767.9	757.2	848.7
GMFG	739.6	594.9	1016.2	691.6	508.9	688.4	447	521.5
GMIP	305.9	281.1	367.3	348.4	209.3	303.4	310.2	242.8
GML	125.9	110.6	124.1	129.7	119.7	98.5	121.6	120
GMNN	122.3	147.7	137.5	163.5	127.8	118.3	163	136.9
GMPPA	261.2	248.2	347.3	271.7	191.6	276.3	248.6	250

GMPPA	124.8	128.8	115.3	120	120.3	108.2	118.8	122.4
GMPPB	185.6	186.4	241.3	218.4	146.1	177.5	160.5	164
GMPPB	137.6	128.2	159.2	127	121.5	119.1	130.9	129.5
GMPPB	127.4	111.7	121.3	123.3	113	100.5	114.8	124.5
GMPR	127.4	109.6	127.4	120.6	113.2	100	115.6	130.2
GMPR2	198.7	189.5	205.1	182.9	165.1	151.4	159.7	178.8
GMPR2	128.5	120.1	140.8	139.3	103.2	107.1	126.6	108.4
GMPR2	426.2	453.7	508.7	438	340.5	337	308.1	371.5
GNA11	140.6	156.1	124.6	140.4	130.9	107.7	133.9	138.1
GNA12	249.5	201	264.5	269.3	171.3	233	264.7	193.4
GNA13	263.6	319.8	233.3	240.6	253.7	292.2	341.3	330.4
GNA14	111.5	115.6	111.1	118.4	107.1	97.8	120.4	109.4
GNA15	259	184.1	506.1	279.3	183.6	377	352.8	184
GNAI1	109.7	119.2	124	130.2	108.8	103.6	124.4	124.7
GNAI2	835.7	824.9	1214.8	952.2	688.3	1035.7	681.7	749
GNAI3	242.6	193.4	177.9	250.6	178.2	170.4	264.9	214.7
GNAL	123.1	119.7	123	127.2	117.2	100.7	118.6	124.9
GNAL	109.1	122	111.1	119.9	109.8	104.2	120.6	117.2
GNAO1	125.8	114.6	126.2	122.8	114.6	103.5	128.4	119.2
GNAO1	117.5	131.8	120.6	120.3	114.7	108	127.4	116.1
GNAO1	114.7	120.2	110.8	125.7	108	95.3	121.5	102
GNAQ	134.4	146.1	151.5	142.1	140.6	126.3	149.6	131.2
GNAS	119.7	124.9	120.9	120.7	118.2	97.5	129.3	121.7
GNAS	110.7	108.4	118.7	111.9	118.9	98	122.4	112.4
GNAS	5901.9	5211.8	6154	5408.8	3814.3	4692	6215.6	6751.3
GNAT1	115.8	108	116.3	127.1	114.2	100.4	132	114.9
GNAT1	123.2	117	123	129.5	114.1	103.5	118.2	111.6
GNAT2	113.5	113.1	134.5	124	111.8	104.1	121.9	115.9
GNAZ	121.5	118.9	137.2	125.1	107.8	108.7	123.7	122.7
GNB1	2085.4	2343.6	1992.1	2316.5	1762.6	2230	2787.4	2640.4
GNB1L	145.7	131.8	156.6	173	133.7	147.5	163.1	145.3
GNB2	146.2	148.9	155	171.2	121.9	130.4	153.1	158.1
GNB2L1	10784.2	11283.6	9726.8	9883	10386.9	8673.1	8664.3	12742.9
GNB3	114.8	117.5	129	114.9	115.2	102.6	115.8	116
GNB4	141.3	118.2	178	143.3	116.3	119.1	126.6	128.8
GNB5	233.1	233.1	200.4	221.5	186.5	159.7	180.6	191.2
GNB5	112	129.1	116.6	112.8	109.5	98.9	119.7	115.8
GNB5	108.7	110.6	113.7	115.5	102.8	95.9	114.5	107.6
GNE	219.7	196	196.4	258.9	182.5	190.4	216	220.5
GNG10	155.5	168.1	203.4	207.3	170.7	192.3	146	169.8
GNG11	129.4	139.8	169.2	153.2	118.8	119.9	135.4	140
GNG12	115.6	123	118.7	119.5	108.3	96.3	108.9	119.4
GNG13	107.4	109.9	116.3	109.6	99.5	100.9	113.6	104.9
GNG2	190.3	224.6	188.8	231.6	171.5	177.8	221.1	211.8
GNG3	126.4	113	135.9	124.2	104.7	103.4	119.2	125.1
GNG4	113.8	115.2	114.8	118.4	108.4	98.1	119.1	108.7
GNG5	191.8	215.2	188.8	222.1	199.6	221.5	221.3	185.7
GNG7	181.1	164.2	173.7	186.8	144.5	148.7	183.1	185.6
GNG8	134.9	123.8	127.4	143.2	119.9	110.2	124.6	128.4
GNGT1	119.3	112.7	125.6	120.5	111	105.8	126.7	121.1
GNGT2	157.9	141.7	164.4	149.9	120.7	114.9	142.6	121.2

GNL1	365	390.1	311.2	422.7	312.7	230.8	407.3	404.4
GNL2	397.7	409	479.3	447.9	350.7	304.4	442.5	455.7
GNL3	210	240.2	269.9	285.2	203.9	216.4	213.8	315.7
GNL3	393.9	403.8	439.9	437.9	359.6	319.4	387.2	429.2
GNL3	117.8	116.7	121.2	121.3	119.4	87.1	115	116.8
GNL3L	325.1	420	413.9	363.6	381.3	213.4	372.4	459.9
GNLY	146	151.1	143.4	158.9	130.6	114.9	129.3	124.5
GNLY	6558.5	3918.9	4487.1	6395.8	2475.7	3349.1	5889.6	2535.3
GNLY	2164.3	1365.1	1405.7	2018.5	899.1	1277.2	1766.4	1015.7
GNMT	125	111.9	122.9	126	118	98.7	119.4	122
GNPAT	337.9	349.8	392.2	359.2	309.6	283.7	323.5	376.2
GNPDA1	569	764.5	678.6	735	697.9	474	946	1378
GNPDA2	117.1	124	119.4	116.3	105.7	107.7	110.7	113.8
GNPNAT1	124.6	121	112.3	117.6	108.3	94.6	116.3	125.6
GNPTAB	113.8	115.2	113.5	118.7	107.3	106.7	118.6	127.4
GNPTG	587.8	519.1	461	467.5	420.7	296	433.6	418.9
GNRH1	124.6	147.3	158.7	162.2	140.8	123.5	179.7	200.5
GNRH2	114	105.9	112.2	117.6	108.7	94.9	117.9	108.2
GNRH2	113.1	120.9	118	111.8	100.7	101	120.1	120.1
GNRHR	104.5	113.5	109	117.2	112.7	94.8	118.4	112
GNRHR2	116.1	124.3	127.4	119.1	112.9	106.9	121.6	131.9
GNS	2479	1538.6	2662.6	2178.9	1227.7	1644.2	2177.8	1656.6
GOLGA	146.9	145	167.1	136.2	121.3	125.5	151.8	126.9
GOLGA1	146.8	149.7	164.5	153.2	144.8	140	153.4	144.5
GOLGA2	167.1	147.3	149.2	166.9	158.8	129.7	159.9	175.4
GOLGA2L1	121.9	123.6	116.9	125.6	105.3	117.6	124.9	125
GOLGA2LY	125.3	118.9	121.5	117.1	107.9	98.2	116.1	111.3
GOLGA3	502	590	411.3	562.4	586.4	393.9	537.7	601.1
GOLGA4	142.8	122.6	131.7	139.4	132.3	114.6	131.8	130.9
GOLGA6	120.1	111.9	112.2	138.9	104.8	93.9	117.9	113.8
GOLGA7	1743.1	1945.5	1898.2	1975.8	1487.7	1408.4	1908.3	1627.8
GOLGA7	115.3	121.5	139.7	128.2	110.6	99.7	122.4	136.3
GOLGA7	124.6	133.1	127.6	153.5	124.9	122.1	152.4	125.7
GOLGA8A	161.4	181.4	286.1	204	176.3	124.7	180.2	159.4
GOLGA8B	1617.2	1491.3	2293.3	1381.2	1688.2	1315.7	1365.5	1282.5
GOLGA8E	113.8	117.4	110.7	120.4	103.4	98.5	117.7	111.1
GOLGA8F	118	114.5	108.5	110	101	100.4	119	114.7
GOLGA8G	108	118.9	115.7	111.3	104.9	100.9	123	116.9
GOLGB1	173.1	146.4	146.4	127.4	148	116.2	173.4	192
GOLPH2	115.8	109	110.2	113.8	111.4	95.7	112.6	126.2
GOLPH2	114.8	110.8	122.5	127.9	126.6	105.7	118.1	118.2
GOLPH3	431.3	644.7	470.8	477.4	450.7	423.4	634.4	585.8
GOLPH3L	211	217.1	178.1	201	184.3	126.7	194.4	186.5
GOLPH4	150.6	136.6	147.8	149.4	129.1	116.6	142.4	133.8
GOLT1A	128.8	109.2	123.8	113.6	104.8	99.9	106.9	113.5
GOLT1B	129.4	144.4	142.1	149.8	143.6	144.3	133.4	133.7
GON4	114.7	120.5	117.3	121.6	111.3	93.1	118.7	116.7
GOPC	166.8	165.6	183.3	155.2	146.8	138	152.6	149.3
GORASP1	202.6	184.1	235.1	185.2	152.3	208	151.3	122
GORASP2	400.8	430	306	426.8	368.6	261	464.4	460.8
GOSR1	125	138.7	155.1	136.2	136	123.3	147.7	135.2

GOSR1	119	110.3	130.7	124.7	107.8	99	122.9	127.1
GOSR2	124.4	123.4	131.7	137.5	120.4	106.6	147.3	133.3
GOSR2	156.5	143.1	159.1	155.7	145.7	136.8	135.7	146.6
GOSR2	124.4	124.9	121	121.8	114.9	109.8	122.3	119.4
GOT1	293.8	318.4	279.4	328.8	241	254.4	241.6	339.3
GOT1L1	115.2	119.7	119.1	119.5	104.1	97.2	117.5	110.5
GOT2	540.9	673.7	402.7	524.8	436.2	347.6	505.9	476.7
GP1BA	131	137.3	139	133.3	122	124.6	130.7	130
GP2	124.3	117.4	131	128	120.1	103.5	126.3	119.1
GP6	121.7	129.4	117.8	113.3	111.5	103.2	128.7	117.4
GP9	103.6	113	108	116.3	115.8	97.4	108.1	116.7
GPA33	159.8	178.6	156.7	177.2	137.8	124.9	147.5	134.5
GPAA1	259.5	261	308	245.8	169.3	231.7	222.3	183.1
GPAM	122.8	129.8	133.4	133.3	128.8	120.3	131.3	119.5
GPATC1	170.2	171.3	170.5	175.3	147.3	144.2	151.6	144.7
GPATC2	119.2	120.9	147.1	135.9	120.1	130.1	119.7	120.1
GPATC3	185.3	163.2	190.7	185.3	145.5	151.3	200.8	193.5
GPATCH4	113.2	123.6	118.7	119.6	109.2	97	126.3	116.8
GPATCH4	234.7	295.1	219.8	230.7	230.9	164.8	204.1	234.5
GPATCH4	119.7	160.8	110.4	124.8	154.6	95.6	118.2	159.4
GPBAR1	126.7	124.7	143	125.4	116.4	97.8	125.5	125.3
GPBAR1	130.3	123.1	164.3	126.8	105.5	111.8	132.4	127.2
GPBP1	173.3	147.9	155.5	187.9	213.9	175.1	194.5	217
GPBP1L1	168.7	177.8	139.7	157.8	147.1	130.7	180.8	169.4
GPC1	111.8	110.4	113.7	118.6	112.4	95.3	117.5	119.6
GPC2	151.2	154.2	220	160.7	140.9	146.6	128.7	132.9
GPC3	115.4	112.9	117.1	107.2	104.6	93.1	110.8	120.4
GPC4	149.6	193.3	124.1	138.6	152	97.5	136	121.8
GPC5	119.1	130	127.3	127	111.7	100.6	128.1	117.9
GPC6	123.9	133.5	131.9	131.9	131.2	123.1	139.9	137.6
GPD1	113.5	109.2	109.9	114.5	108.8	93.8	115.4	100.5
GPD1L	359.6	191.5	258.9	320.3	190	199.8	225.9	200.7
GPD2	104	109.8	122	113.1	105.9	98.1	114.1	115.6
GPHA2	110.1	127.6	123.1	133.6	117.5	106.9	132.7	111
GPHB5	132.7	131.6	125	118.8	116.1	101.2	118.6	120
GPHN	137.8	136.9	135.5	142	123.8	108	141.6	127.8
GPHN	113.4	120	120	122.2	116	96.6	121.8	112.4
GPI	413.2	438.8	324	444.5	328	347	351.6	327.5
GPIAP1	127	131.8	128.6	126.8	137.2	107.1	131.3	122.5
GPIAP1	179.9	198.4	195.5	210.3	196	162.3	190	210
GPIAP1	159	176.7	183.4	176.9	172.9	172.5	149.2	162.7
GPKOW	138	131.9	143.7	155	144.4	144.1	131.1	168
GPLD1	122	116.5	124.9	113.7	108.6	95.6	115.9	115.4
GPLD1	108.8	112.4	119.5	120.1	103.1	95	109.8	115.9
GPM6A	113.6	122.1	114.6	134.2	114	104.9	125.4	113.8
GPM6A	115.6	121.2	118.3	120.8	108.6	93	117.4	117.1
GPM6A	128.7	128.5	128.9	127	128.1	109.1	141.5	133.1
GPM6B	120.3	120.6	119.6	120	109.6	100.6	114.9	111.6
GPM6B	114.8	118	110	107.3	109.7	94.1	119.5	111.9
GPM6B	121.3	119.6	117.4	136	118.8	99.3	117.8	119.6
GPNMB	469.8	167.7	154.4	313.6	152.2	110.5	320	157.2

GPNMB	878.6	269.9	262.9	586.9	214.2	163.2	461.5	188.5
GPR1	425.1	357.4	464.6	331.6	479.1	296.7	371.4	321.1
GPR101	110.6	119.8	118.8	126	105.2	112.1	115.2	109.6
GPR103	118.3	114.3	106.2	115.4	107.9	96.2	122.5	109.2
GPR107	113.7	114.3	125.9	132	113.8	104	122.7	119.5
GPR108	173.3	196.6	199	177.2	155.1	183.2	212.9	177.4
GPR109A	111	111.2	124.9	113.9	102.1	103.3	124.1	111.3
GPR109B	118.9	114.3	108.3	117.7	106.5	102.8	111.2	107.2
GPR110	112.8	110.9	108	121.5	111.5	98.8	110.7	113.4
GPR110	110.9	116.3	122.3	119	110.3	100.8	117.5	115.8
GPR110	112.5	114.9	117.9	112.1	107.7	103.4	115.8	112.1
GPR111	113.2	116.9	129.4	113.4	109	99.4	116.2	111.5
GPR112	101.4	106.5	112.9	104.6	102	93.5	116.5	113
GPR113	120.7	129	127.9	129.4	117.6	100	114.8	128.3
GPR114	255.3	274	218.6	245.7	203.5	147.2	227.3	180.5
GPR115	110.8	110.8	116.3	111.4	108.2	97	115.2	118.5
GPR116	112.7	116.4	116.1	119	108.9	92.7	118.1	116.7
GPR119	136.4	135	147.8	142.8	125.7	117.6	133	128.8
GPR12	104.8	108.2	113.7	119.3	104.5	101.2	114.5	107.6
GPR120	110	116	126.2	119.5	111.1	105	117.1	119.7
GPR123	125.3	111.1	107.4	123.1	109.7	102.9	120.7	115.8
GPR124	110	125.2	119.7	120.5	108.6	102.9	117.4	124.2
GPR125	125.6	134.9	130.9	128.8	113	101.5	123.5	125.2
GPR126	103.7	112.9	113.3	114.1	100.5	107.4	109.8	108.6
GPR128	124.6	113.6	127.6	117	107.8	98.6	113.9	123.6
GPR132	247	265.1	221.4	236	171.6	160.9	206.5	180.7
GPR133	110.8	115.6	116.9	119.1	103.9	101.2	124.2	116.9
GPR135	120.2	114.5	116.8	119.7	112.4	103.3	123.5	118.4
GPR137	786.5	933.5	493.8	856.2	662.4	500.5	1033.2	1185.6
GPR137B	175.6	173.2	223.1	204.6	176.7	201.3	188.3	157.4
GPR139	118.3	117.4	115.1	128.8	104	98.5	124.2	115.3
GPR141	115.6	111.7	117	122.6	108.3	103.4	117.6	109.7
GPR142	112	110.7	119.6	121	114.8	105.9	121.3	114.1
GPR143	127.9	121.1	143.5	117.7	113.9	121.1	120.7	121.9
GPR146	132.7	128.9	135	129.3	114.4	110.5	126.6	118.6
GPR148	113.8	117.6	122.9	123.2	113.5	105.5	124.5	107.9
GPR149	108	115.3	117.2	114.4	100.9	94.8	115	122.2
GPR149	124.3	121	130.3	124.8	112.1	107	114.3	121.7
GPR15	124.8	128.5	122.1	127.5	114	103	120.3	121.7
GPR150	111.4	117.5	129.3	111.6	106.9	96.3	124.6	115.1
GPR151	110.2	113.3	124.5	113.2	102.4	98.9	121.9	124.5
GPR152	115.2	113.2	115.3	124.9	101.8	104.4	120.7	117.6
GPR153	121.7	111.9	118.1	119.5	115.3	99.7	111.6	120.9
GPR154	111.1	118.2	116.1	122.6	105.6	98.2	128.6	114.7
GPR154	110.4	114.6	112	113.3	105.9	92.4	113.9	106.5
GPR154	161.8	189.7	221	150.2	176.6	148.7	153.7	177.9
GPR155	127	122.5	125.3	124.3	126.8	98.4	125.6	121.6
GPR155	140.5	131.4	122.7	138.9	125.1	100.3	124.4	134.2
GPR156	118	129.1	141.6	126.6	132.8	116	129.2	125.7
GPR157	107.9	109.6	112.7	124.6	106.1	96.7	118.9	112.6
GPR158	113.2	126.7	112.2	117.3	119.2	102.9	125.9	119

GPR160	136	210.5	154.2	151.3	190.5	122.6	136.1	181.8
GPR161	126.6	136.1	125.2	133	118.2	112.2	118.8	120.9
GPR161	112.9	114.7	120.3	119.1	99.5	97.8	110	110.6
GPR162	246.6	167	476.5	338.3	162.5	482.7	287.5	139.2
GPR162	118.2	105.9	127.2	120.7	104.5	102.7	124.2	115.6
GPR17	113.9	122	127.3	122.4	107.2	101.1	124.5	113.6
GPR171	118.4	119.4	120.7	112	107.2	104.2	120.8	121.6
GPR172A	377.8	417.9	299.2	436.5	265.1	295.4	508.5	422.7
GPR172B	112.7	119.1	113.4	127.3	109.3	104.5	123.5	118
GPR173	116.5	113.3	120.9	128.6	114.3	103.9	124	119.2
GPR173	127.4	125	116.3	110.6	108.1	106.9	129.3	117.6
GPR174	117.6	134.9	125.1	111.1	107	94.6	125.7	120.1
GPR175	177.2	190.8	180.7	184.7	145.7	180.2	208.9	180.8
GPR176	111	120.8	130.4	134.7	112	99.1	129.6	114.3
GPR177	109.2	103.1	104.3	114	108.4	94.4	115.1	110.3
GPR177	124.5	134.1	116.3	126.1	106.7	106.6	122	120
GPR177	114.1	115.2	115.7	130.8	123.2	103.8	137.6	121.2
GPR179	115	121.6	113.8	123	106.9	104.8	119.6	116.3
GPR18	374	513.3	358.5	420.3	361.2	300.7	338.9	383.5
GPR19	116.8	107.5	118.5	121.4	104.2	97.9	118.6	116.6
GPR20	119.6	116.8	125.8	117.9	106.1	101.7	121.2	114.4
GPR21	113.3	112.6	132.5	124.5	110	104.6	121.4	121.1
GPR22	119.1	117.5	112.5	133.7	106.5	105.5	121	114
GPR23	121.3	121.6	116.9	113.8	112.5	100.9	121	114.1
GPR24	114.6	119.5	118	122.6	111.2	108	119	108.2
GPR25	119.8	117.7	119	131.3	112.1	109.2	113.4	114.9
GPR26	128.8	125.6	128.3	130.2	106.8	104.1	118.9	117.4
GPR27	135	142.8	142.2	136	120.9	120.6	142	141.7
GPR3	110.2	117.7	115.9	110.1	112	102.6	125.4	125.9
GPR30	132.1	128	125.7	157.7	121.7	106.7	134.9	118.6
GPR30	138.2	148.7	132	148.3	114.9	102.3	141.4	134.2
GPR30	111	130.9	121.6	113.3	110.2	101.5	126.2	117.3
GPR31	109.8	116.1	124.4	123.6	110.6	99.9	116.2	117.4
GPR32	118	114.2	115.5	113.5	108.5	100.7	136.1	116.4
GPR34	115.1	122.7	115.3	120.4	116.1	97.3	128.5	113
GPR34	114.9	130.7	208.6	122.7	115	105.8	123.2	112.8
GPR34	115.7	114.3	118.8	131.6	103	103.8	125.5	110.8
GPR35	122.7	136.3	133.9	133.4	122.7	117.2	132	129
GPR37	112.7	110	111.8	117.4	101.7	103	116.3	113.4
GPR37L1	112.6	106.2	111.6	113.9	103.2	98.6	107.3	111.2
GPR39	122.8	115.1	118.2	115.4	108.2	102.3	127.6	112.5
GPR4	110.8	106.8	108.6	102.5	104.3	94.3	111.2	107.2
GPR42	108.8	108.3	121.8	114.1	111.8	97.5	116.2	112.9
GPR44	112.8	116.2	114.7	117.5	103.2	102	113.9	115.9
GPR45	117.3	118.3	109.2	112	106.9	96.7	111.6	111.4
GPR50	119.2	113.1	119.4	110.5	106.9	104.2	119.2	113
GPR52	110.3	108	107.4	115.9	106.7	97.6	111.6	111.3
GPR55	123.7	124.8	139.9	125.9	132.2	112.2	122	125.7
GPR56	117.2	128.4	112.1	115.8	108.4	104.9	119.3	121.4
GPR56	1037.9	614.1	828	1006.8	441.9	657.4	927.2	380.1
GPR56	1075.1	563.1	891.2	1196.3	380	646.3	832.9	400.8

GPR6	107.8	113.5	113	114.8	117.5	94.7	110	103.8
GPR61	121.1	118.6	127.9	125.7	112.8	100.8	122.8	113.5
GPR62	107.6	112.4	116.4	119.8	107.8	98.7	121.2	116
GPR63	116.5	114.5	111.5	113.2	112.2	95.3	118	123.4
GPR64	122.5	125.6	122	127.2	128.2	101.8	119.4	120.3
GPR64	112.6	112.5	124.9	114.6	105.4	110	120.9	113.1
GPR65	320.9	370.6	397.1	343	236.4	304.2	347.7	359.6
GPR68	207.3	257.4	223.9	262.8	194.4	183.2	181.2	143
GPR75	113.7	119	118.1	123.4	108.9	103.4	123.9	113.1
GPR77	119.6	106.5	116	118.6	110.3	100.8	117.7	103.5
GPR78	129.7	124.6	124.8	122.1	111.7	107.2	122.4	131.6
GPR81	125.2	110.8	114.6	108.3	109.2	101.1	125.4	110
GPR82	106.3	107.8	151.3	111.9	108.5	93.5	107.8	111.6
GPR83	108.4	115	117.3	121.2	111.2	104.3	122.8	132.2
GPR84	124.6	137.6	170.8	121.3	117.1	104.4	127	116.5
GPR85	124.2	119.3	126.5	121.1	109.3	98.6	121.8	115.6
GPR87	114.8	108.6	113.8	114.6	102.6	98.2	120.9	108
GPR88	114.4	120.6	106.9	118.5	112.8	107.1	124	118.8
GPR89A	137.4	138.3	162.6	146.7	152.2	124	140.9	147.1
GPR92	372.8	464.4	371.4	358.2	341.1	237.2	272.9	257.2
GPR97	118.1	121.7	118.6	123.3	111.8	102.7	117.3	125.1
GPR97	119.9	117.2	121.7	123.8	111.1	105.8	113.2	116.3
GPR98	114.2	109.3	116.8	121.4	107.2	106.2	121.6	109.6
GPRASP1	116.4	107.6	123.5	126	111.2	99.8	122.3	119.2
GPRASP2	132.6	156.7	143.5	143.4	139.1	111.7	139.5	147.1
GPRASP2	131.8	114.8	143.1	131.1	121.4	118.4	144.5	120.7
GPRC5A	110.3	121.3	113	121.2	115.3	101.5	124.7	114.8
GPRC5B	123.1	124.5	122.8	127.9	131.2	112.4	125.9	122.3
GPRC5C	121.1	118.5	113	123.9	107.4	103.3	115.3	112.4
GPRC5C	118.7	113.2	109.4	121.2	112.1	101.6	123.7	122.8
GPRC5C	107.4	112.7	112.6	113.1	121.3	101.4	113.2	116.2
GPRC5D	116.3	123.1	123.1	125.7	115	103.5	127.9	117.5
GPRC6A	118.2	116.5	119.1	129.7	110.1	96	116.6	124.3
GPS1	123.5	127.4	140.8	131.8	123	104.7	123.2	125.2
GPS1	559.3	626.9	572.7	628.4	388.4	361.5	423.5	424
GPS1	134.8	150.1	144	146.9	123.1	113.1	141.1	129.9
GPS2	345.3	325.9	267.3	338.5	250.5	267.2	329.2	323.7
GPSM1	111.3	117.2	119.7	120.4	116.9	110.8	114.7	113.5
GPSM2	119.4	127.1	127.7	135.8	124.9	97.5	117.3	126.5
GPSM3	484.5	528.9	335.6	484.2	330.4	488	402.6	603.7
GPSN2	1050.7	1271.6	1353.8	1269.8	798.2	943.8	973	856
GPT	121	130.8	123.2	124.3	111.3	102.1	119.8	121.9
GPT2	166.3	172.7	141.1	177.4	133.5	110.8	151.8	146.1
GPX1	1099.7	845	3213.4	847.6	430	1116.9	408.4	332.1
GPX1	115.7	111.3	141.6	114.9	102.6	104.8	124.8	116
GPX2	117.7	117.8	114.2	114	112.5	100.4	114.9	121.5
GPX3	126.1	129.6	142.2	131.9	113.4	117.8	123.8	120.4
GPX4	565.5	540.9	793.3	783.9	632.1	668.4	836.5	486.8
GPX4	4039.9	3506.3	3973.2	4122.8	2950.4	4126.1	4190.5	3203.2
GPX5	115.9	123.3	107.5	121.8	109.8	106.6	108.7	109.4
GPX5	109.2	111.4	110.3	109.1	100.2	94.3	115.6	115.4

GPX6	118.3	129.2	124.7	131.7	111	106.1	120.9	112.1
GPX7	208.7	200.9	266.5	222.7	186.7	186.3	178.8	192.4
GRAMD1A	479.9	765.1	480.1	487.6	402.9	324.8	404.9	474.2
GRAMD1C	113.6	118.9	121.4	125.1	109	101	113.4	117
GRAMD2	111.4	111.7	117	120.9	101.2	105.4	121.3	116.4
GRAMD3	124.7	144.3	145.9	136.9	130.6	117.4	123.3	139.3
GRAP	134.2	125.7	140	127.2	124	116.5	126.1	128.5
GRAP	1057.4	1215.8	1498.9	1139.8	1017.7	1231.2	813.9	1104.5
GRAP2	136.5	140.4	125.2	132.8	120.3	115.2	128.8	155.4
GRASP	130.2	130.6	125	141.5	120.4	111.6	145.2	130.6
GRB10	123.3	110.8	111.4	125	109.7	101.2	124.1	129.9
GRB10	128.6	125.4	134.5	126.9	117.6	112.4	131.7	142.4
GRB10	116.3	122.7	124.8	120.5	110	102.2	126.4	112.1
GRB10	113.5	113.5	121	121.4	107.2	108.3	128.4	121.5
GRB14	104.9	113.1	115.1	127.6	105.5	104.9	120.9	114.7
GRB14	117.7	127.4	134.6	127.5	119.9	125.7	122.9	131.2
GRB2	286.4	267.1	344.2	240.5	175.5	303	190.5	206.6
GRB2	403.3	391.5	648.4	516.3	315	341.6	347.5	309.8
GRB7	118	115	115.5	117.5	110.9	105.1	104.7	111.9
GRB7	111.1	106.8	122.3	117.6	100.6	97.8	120.5	105.4
GRB7	121.9	126	116.3	122.9	111.4	100.1	124.6	128.3
GREB1	109.9	105.3	116.3	116.8	102.3	98.5	115.9	118.1
GREB1	116.1	128.9	127.1	120.7	110.5	102.3	125.5	119
GREB1	119.3	119.1	122.3	125.6	107.9	100.1	116.2	117.4
GREB1	114.2	117.6	117.5	119.8	117	105.6	129.3	111.5
GREM1	116.4	117.3	114.4	115.5	110.2	105.6	119.8	121
GREM2	116.4	128.1	113.6	124.1	106.8	100.3	117.6	113.3
GRHL1	116.3	111.8	121.6	125.6	115.1	110.4	111.7	117.8
GRHL1	121.4	118.8	126.6	120.5	109.5	101.1	123.5	117.3
GRHL1	111.7	123.3	112	107	105.6	98.8	109.7	107.3
GRHL2	112.5	114.9	115.1	125.6	101.3	95.4	117.1	121.5
GRHL3	106.3	112.8	119.8	107.9	111.7	101.7	126.2	115.4
GRHL3	113.3	112.6	110.6	111.1	110.1	88.3	113.3	119.4
GRHL3	111.8	122.6	127.9	128.8	113.5	110.8	121.9	115.4
GRHPR	128.7	178.8	152.6	158.1	152.4	149.3	140.1	154.1
GRIA1	105.8	113.4	111.1	100.5	95.5	98.6	108.4	105.2
GRIA2	118.9	111.1	108.2	123.3	117.7	99.6	118.9	118.8
GRIA3	120.7	115	122.9	120.7	112.8	100.9	131.2	118.9
GRIA3	112.6	115.5	117.8	119.7	114	93.9	114.1	126.4
GRIA3	124.3	120.8	126.9	131.6	118.8	108.1	135.9	125.9
GRIA4	115.8	116	127.6	118.2	112.5	97.9	108	121.7
GRIA4	107.7	106.8	110	116.3	101.6	93.3	110.7	111.3
GRID1	120.7	122.2	126.3	124.5	115.8	104.4	117.8	111.5
GRID2	106.6	111.7	119	107.2	108.8	97.9	107.9	108.7
GRIK1	118.5	115	124.6	121	119.1	110	122.3	124.3
GRIK1	124	118.7	113	120	112.1	91.3	117.6	103.3
GRIK1	109	110.7	111.6	111.7	103.7	94.7	117.3	114.3
GRIK2	111.5	110.4	113.1	110.9	106.7	97.9	118.5	106.5
GRIK2	118.8	109.2	111.9	107.9	107.4	99.2	117.4	112
GRIK3	110	114.4	114.6	126.2	104.1	97.6	121.7	108.9
GRIK4	111.7	115.9	129.3	122.1	108.5	97.9	116.4	110.8

GRIK5	120.3	118	121.1	123.1	114.4	108.3	123.4	119.2
GRIN1	108.3	114	117.1	114.8	104.9	96.6	112.7	111.4
GRIN1	113.4	117.2	112.9	119	107.8	102.4	115.1	118.1
GRIN1	173.1	144.6	177.6	156.2	151.8	138.6	173.3	155.5
GRIN2A	120.3	130.5	137.9	124.4	113.6	103.5	121.2	117.6
GRIN2B	115.3	112.8	132.5	132.2	112	107.7	123.7	121.9
GRIN2C	110.5	106.4	109.1	104.8	103.8	99.8	117.2	113.4
GRIN2D	105.9	112	110.1	111.8	97.1	100.3	110	115.6
GRIN3A	115.8	112.5	117.9	113.3	105.1	101	108.8	109.3
GRIN3B	116	129.8	131.6	120.9	115.1	94.5	131	121.8
GRINA	260.1	221.5	209.2	250.7	149.2	162.6	226.5	193.4
GRINA	489	200.3	385	369	164.9	355	293.8	184.4
GRINL1A	111.5	111.7	112.8	117	105.8	97.4	113.3	106.1
GRINL1A	141.6	141.7	145	138.4	121.2	135.2	157.4	167.7
GRINL1A	113.9	117.4	126.5	119.7	119.8	108.4	118.8	125.3
GRIP1	112.2	103.6	109.8	114.9	102.2	93.4	113.5	112.6
GRIP2	118.6	120	122	109.8	105.2	98.6	114.7	116.6
GRIPAP1	186.2	222.1	196.8	218.9	169	176	190.8	158.5
GRIPAP1	135.4	140	144.3	133.2	142.2	116	141	143.4
GRIPAP1	1842.1	1177.1	1589.8	1518.6	1541.3	1200.1	2077.3	1872.2
GRK1	113.7	113.6	107.3	118.6	110.7	89.9	111.4	111.7
GRK4	113.3	116.9	117.4	117.1	103.1	96.7	115.1	108.9
GRK4	113.7	120.7	118.2	111	116.5	103.8	123.2	133.4
GRK4	117.3	108.3	117.2	115.1	117.7	98	115.4	135.2
GRK5	180.5	210.7	166.6	181.8	164.5	149.1	199.9	175.8
GRK6	154.2	144.2	142.2	154.6	138.3	138.8	146.4	143.7
GRK6	166.8	177.7	137.8	177.6	134.5	149.3	162	148.2
GRK7	114.7	103.1	118.2	107.4	109.7	100.8	112.2	111.9
GRLF1	121.5	129.6	116.3	119.8	108.9	99.7	125.6	122.6
GRM1	115.6	125.4	115.9	119.6	109.4	101.4	117.3	109.9
GRM2	131.9	123.8	162.6	128.8	115.4	121.2	137.2	137
GRM3	114.8	108.5	117.7	124.3	109.1	95.9	113.5	116.4
GRM4	121.8	117.5	114.9	126.8	106.9	99.8	118.5	111.1
GRM5	111.1	114.7	119.2	128.9	106.6	105.3	126.7	130.6
GRM6	111.2	115.4	120.8	116	109.5	95.4	114.9	114
GRM7	102.8	111.9	106.1	116.8	112.2	101.6	118.2	111.2
GRM7	113.4	118.1	118.2	111.9	104.5	97.4	117.3	116.2
GRM7	113.2	115.2	108.1	123.4	102.6	99.5	114.5	114.6
GRM8	118.2	117.3	115.1	123.4	112.4	101.2	123.1	119.8
GRN	495.2	241.4	600.9	408.9	182	438.9	298.2	216
GRN	394.6	248.6	545.3	328.5	185.3	304.6	301.5	211.1
GRP	122.7	126.5	123.6	115.1	117.2	98.8	127.1	108.9
GRP	117.7	118.1	111.7	116.6	113.8	96.6	113.2	123.1
GRPEL1	238.4	255.7	258.6	252	229.5	206.8	256	294.5
GRPEL2	645.4	859.2	662	798.1	664.7	569	747.9	843.6
GRPR	119.8	110	122	122.8	112.8	107.7	118.6	115.6
GRRP1	120.3	116.3	118.5	108.5	111	102.6	116.2	111
GRSF1	428.1	488.4	452.2	511.2	385.4	316.1	477.9	560.6
GRTP1	117.4	116.2	110.9	110.9	106.8	96	120.9	114
GRWD1	288.1	337	275.3	316.7	251	217.2	273.4	321.6
GSC	118.1	129.9	136.8	127.1	118.9	105.5	133.5	131.6

GSCL	115.5	111.7	115.7	111.6	110.7	100.7	117.4	111.3
GSDM1	133.9	146.2	134.5	137	122.7	106.1	124.9	128.5
GSDMDC1	596.7	616.1	522.3	514.3	390.5	330.5	447.7	378
GSDML	245.8	304	267.6	229.3	281.5	200.3	246	223.2
GSDML	161.5	183.7	156.7	170.3	191.2	127.7	186.4	165.2
GSDML	340.8	411.3	336.9	352.1	408.6	218.7	314.8	339.6
GSG1	117.7	111.5	116.3	131.7	104.7	94.9	124.2	114.1
GSG1	111.5	110.5	115.7	109.5	110.9	97.3	114	108.6
GSG1	118.1	120.5	117.6	127	106.2	99.8	126.3	122.4
GSG2	113.6	123	121.1	129	113.9	96.8	121.9	114.8
GSH1	104.6	111.1	122.9	118	112.4	89.4	112	111
GSH2	113.9	112.1	110.6	119.7	110.2	98.7	118.3	114.3
GSK3A	116.2	132.2	128.8	133.1	131.1	123.1	128.7	135
GSK3B	291.7	308	270.3	336.2	226.9	200.5	323.5	292.5
GSN	143.2	129.7	184.7	150	109.5	132	134.9	112.1
GSN	114.6	124.7	105.6	115.6	113.7	94.4	115.4	111.8
GSPT1	1464.1	1366	1197.9	1656	1313.7	1001.9	1836.1	1852.7
GSPT2	273.7	325.9	288.1	289.7	237.3	224	242	214.3
GSR	117.5	122.9	130.3	127.3	128.7	116.5	127.8	124.4
GSS	248.9	207.4	242.7	254.6	197.3	228.1	253.3	238.7
GSTA1	114.7	111.7	115.5	113.9	107.8	93.7	110.6	111.7
GSTA2	124	113.1	117.3	129.3	120	100	121.6	131.3
GSTA3	119.5	112	123.4	115.7	106.8	96	122.3	123.8
GSTA4	105.3	118.8	117.6	119.6	109.3	101.1	123.3	113.5
GSTA5	125.9	120.1	118.5	122.5	109.3	99.7	124.6	111.6
GSTCD	121.8	118.8	125.2	118	105.5	93.8	114.7	110.4
GSTK1	1897.2	2127.8	2227.8	1873.4	1083.6	1133.8	763.5	904.5
GSTM1	148	139.5	166.3	161.1	124.4	158	148.8	125
GSTM1	881.3	586.8	1341.5	789.8	323.8	815	439.2	272.2
GSTM1L	113.1	112.2	112.8	121.2	103.9	98.3	113.6	112
GSTM2	861.2	462.6	1297.2	783.8	296.2	862.2	433.1	242.8
GSTM3	124.9	157.4	124.3	123.7	140.8	100.7	134.7	198
GSTM4	114.7	126	129.1	125.5	112.1	104.1	119	116.1
GSTM4	131.2	145.6	124.9	132.5	133.7	120.8	119	133.4
GSTM5	104.3	123.5	114.5	124	110.5	103.7	119.7	131.6
GSTO1	787.2	848.8	1416.2	829.2	619.6	977	913.2	692.1
GSTO2	119.2	124.7	126.8	122.7	116.1	110.3	132.5	124
GSTP1	1206.9	1439.9	1892.9	1948.9	1321.9	1715.8	1920.7	1951.4
GSTT1	226.3	182.6	210	208.9	140.8	154.8	145.2	124.9
GSTT2	107.9	127	124.8	122.4	107.2	99.4	125	119.1
GSTT2	121	118.8	120.1	116.7	107.7	98.2	107.4	105.6
GSTZ1	115.4	126.3	134.5	126.3	122.6	102.7	128.2	119
GSTZ1	139.9	121.9	178.8	144.5	119.7	115.4	136.6	132.2
GSTZ1	122.2	111.7	123	124.5	109.6	101.9	116.2	125.2
GTDC1	117.7	127.5	134.3	132.2	114.1	112.5	128.3	121.5
GTDC1	143.5	125.7	145.1	147.9	117.5	114.5	131.4	130.4
GTDC1	118.7	128.9	116.1	129.4	112.3	100.6	116	121.8
GTF2A1	112.2	123.4	129.1	120.8	107.8	105.1	126.2	120.1
GTF2A1	119.5	119.9	109.1	129.4	106.1	98.5	118.8	122.6
GTF2A1	120.6	107	128.1	130	125.8	109.7	124	123.3
GTF2A2	358.2	343.3	526.6	517.2	445.5	399	430	452.5

GTF2B	393.9	398.2	412.7	422.2	347.5	317.7	529.2	511.8
GTF2E1	229.2	275	286.1	297	227.6	248.2	275	251
GTF2E2	713.4	687.1	841.9	799.9	575.8	671.4	801.3	743.8
GTF2F1	362.3	381.7	410	417.4	332	340.3	393.2	386.5
GTF2F2	624.2	500.1	502.6	669.6	314	405.1	460.6	357.5
GTF2H1	140.2	143.2	153.4	158.2	122.6	120	171.1	160.6
GTF2H2	116.4	114.4	119.9	125.5	110.5	100.9	123.4	112.9
GTF2H3	145.4	143.9	202.9	161.4	145	135.2	149.2	161.7
GTF2H4	285.5	287	274.6	296.5	234.4	180	230.5	211.4
GTF2H5	324.6	443.2	594.8	335.1	389	366.9	320.2	282.7
GTF2I	143.4	160	148.6	147.6	143.5	117.5	144.1	144.8
GTF2I	124.2	125.8	127.6	120.6	123.6	106.3	127	129.5
GTF2IRD1	113	121.6	118.6	131.1	116.9	100	128	126.9
GTF2IRD1	124.9	117	117.1	131.1	113.5	105.3	120.2	122.5
GTF2IRD2B	116.9	111.1	118.4	119.5	113.2	103.5	118.6	111.5
GTF2IRD2P	109.4	114.7	118.7	119.5	112.7	99.1	120.2	121.8
GTF3C1	159.2	151.7	129.5	169.2	141.7	126	166	151.1
GTF3C2	466.2	546	624.1	582.8	413.6	450.6	438.7	402.3
GTF3C2	175.4	152.4	143.2	143	128.3	127.1	149.5	132.4
GTF3C3	205.8	250.2	243.5	207	212.5	214.8	229	298.5
GTF3C4	115.4	116.8	128.3	123.3	116.8	105.7	109.2	116.1
GTF3C5	248.9	306.8	291.8	322.8	218.6	222.8	266.8	244.6
GTPBP1	123.7	124	122.8	133.1	107	107.5	125	125.1
GTPBP10	119.8	131	126.7	111.6	123.5	107.8	114.8	107.6
GTPBP2	121.3	133.5	122.7	128.3	120.8	109.9	128.2	126.3
GTPBP3	133.9	142.3	154.1	144.8	123.7	111.7	129.2	134
GTPBP3	106.7	114.2	121	124.5	115.2	93.6	119.5	112.4
GTPBP3	539.4	615.6	635.4	537.6	464.7	391.8	429.5	320.3
GTPBP4	583.1	539.6	466.6	780.6	423.6	341.4	610.6	628.2
GTPBP5	119.3	117.3	124.5	127.4	118.3	96.4	127.9	120.4
GTPBP6	938.4	1380.3	983.8	1228.4	1230.2	625	1216.3	1337.5
GTPBP8	336.3	329.1	377.1	359.9	336.8	225.9	267.5	337.4
GTPBP8	181	199.2	182.5	194	189.7	143.7	168.9	221.5
GTPBP8	174.4	169.9	175.1	174.9	146.4	143.1	155.6	188.9
GTPBP9	111	113.9	112	117.7	107.6	94.8	113.3	110.8
GTSE1	125.8	124	128.7	115.7	106	116.2	128.6	124.8
GUCA1A	117.3	125.1	136.2	131.4	126.8	109.4	123.7	125.5
GUCA1B	148.5	136.6	162.8	150.5	135.4	124.8	139.7	145.7
GUCA1C	140.6	135.4	133	135	120.6	105.8	132.7	124.1
GUCA2A	128.2	123.9	119.7	125.2	118.3	94.4	122.9	121.7
GUCA2B	115.9	113.6	121.4	121.8	118.9	107.7	111.1	109.4
GUCY1A2	116	116.9	122.7	124.7	112.6	100.5	117.9	120.4
GUCY1A3	108.7	109.7	116.3	115.5	103.5	96.6	107.1	106.5
GUCY1B3	120.2	120.4	124.5	124.9	123.9	102.3	117.3	113.4
GUCY2C	106.9	103.9	117.2	104	110.1	102.8	113.5	114.5
GUCY2D	118.9	116.9	122.8	120.1	109.3	96.8	119.5	125.3
GUCY2F	114.1	116.3	113.6	128.9	112.4	106.2	118.1	111.5
GUF1	131.5	135.2	135.9	136.6	128	119.9	130.2	130
GUK1	516.8	661.3	559.5	594.5	359.2	545.1	403.2	410.9
GULP1	112.2	111.4	119.2	112	111.4	98.3	123.3	124.1
GUP1	113.9	126	115.3	117.2	113.2	103.1	121.7	118.8

GUSB	399.7	383.7	402.7	404.3	316.1	292.3	316.3	281.1
GUSBL2	137.7	160.7	149.3	142.8	155.1	113.4	141.4	145.2
GYG1	350.5	438.6	536	416.9	368.8	387.8	395.7	448.3
GYG2	109.6	117.4	104.8	112.7	103.5	97.4	113.9	112.8
GYG2	112.2	121.3	114.3	110.7	102.4	99.6	122.5	110.3
GYG2	113.5	111.9	107.4	110.5	109.1	95.7	115.7	112.8
GYLTL1B	107.4	109.6	112.9	111.1	106.8	95.6	116.6	115.9
GYPA	111.4	108.4	114.1	115	105.7	96.1	113.6	111.3
GYPB	107.9	114.7	112.8	118.1	109.8	97.8	115.8	116
GYPC	907.1	997.4	591.4	833.4	668.6	582	850.1	686.9
GYPC	942.3	835.4	729.5	840.9	849.1	530.4	785.1	790.5
GYPE	111.3	111.7	124.8	119.9	112.6	98.8	122	116.6
GYPE	112.4	124.5	112.8	124.9	113.3	105.9	118.8	125.3
GYPE	110.8	110	122.7	116.1	98.7	97.4	110.3	99.5
GYS1	143.4	137.4	162.4	152.9	127.9	121.9	133.5	129.1
GYS2	116.6	117.4	114.7	114.4	106.6	91.9	121	122
GZMA	744.7	818	751.3	921.8	693.1	516.5	649.7	658.9
GZMB	1113	887.3	682.6	933.9	575.2	516.6	752.5	612.9
GZMH	505.3	422.5	645.5	470.4	297.4	442.8	375.1	264.9
GZMK	1177.8	1530.4	1200.1	1129.3	1110.7	869.1	1091.1	1182.9
GZMM	297.1	263.7	248.3	295	176.4	182	189.9	186.4
H19	111.5	104	109.4	112.6	102.5	97.9	118.3	112.2
H1F0	221.2	139.9	231.6	168.1	118.3	157.9	132.3	121.3
H1FOO	117	123.4	110.8	119.6	106	94.4	113.8	116.3
H1FX	162.3	176.1	201.8	152.1	131.7	163.1	138.8	133.3
H1T2	117.2	109.5	118.3	114.2	105.2	96.4	110.1	114.9
H2AFB1	121.4	125.3	120.9	125.7	115	100.7	124.1	115.8
H2AFB2	121	116.1	123.2	117.8	116.9	109	116.1	126.6
H2AFB3	109	115.9	105.2	114.9	102.1	94.8	111.7	118.1
H2AFJ	112.4	112.5	119.4	113.5	120.5	103.5	125.9	121.3
H2AFJ	148.7	142.3	184.7	150.8	131.1	164.7	146.4	161
H2AFV	130.2	144.4	135.2	139.2	136.6	106.8	151.6	148.1
H2AFX	122.3	120.3	137.6	130.7	125.9	110.6	139.7	129.2
H2AFY	107.9	104.9	108.8	110.8	99	92.3	122.7	119.1
H2AFY	228.6	187.7	569.3	199.7	166.4	214.2	144.5	132.2
H2AFY	2697.8	2105.4	4482.4	3254.2	2191	2450.4	3238.6	2594.7
H2AFY2	195.8	231.7	331.6	203.4	169.6	259.6	136.3	150.6
H2AFZ	3777.6	3928.7	2917.5	3606.8	2870.6	2646.8	3852.6	4030.7
H2BFS	265.7	262.2	203.1	279.4	237.3	193.6	240.9	246.1
H2BFWT	102.3	114	112.7	111.8	107.7	108.2	118.8	105.2
H2BFXP	113.5	116.4	118.5	110.5	106.8	99.5	121.1	114.4
H3F3A	7536	8844.9	11675.1	8748.1	8067.9	9761.4	8755.6	8285.7
H3F3B	462	492.9	507.7	500.2	366.9	464.1	816.3	1221.8
H6PD	110.4	115.2	120.1	117.9	103.2	95.5	112.1	123.6
HAAO	118.9	113.2	110.9	123.1	108.4	100.4	129.2	120.7
HABP2	105	110.3	116.8	113.9	100	106	115.5	115.5
HABP4	174.6	219.6	213.5	177.8	164.8	174.5	161.9	203.2
HACE1	127.8	133.4	148.4	136.6	117.5	119.4	127.6	121.3
HADH2	155.2	157	168.5	159.6	135.7	152.9	140.3	147.5
HADH2	176.1	174.3	199.3	181.1	154.9	150.4	174.5	200.6
HADHA	195	231.9	222	212.2	170.2	164.4	250	206.4

HADHB	167.2	138	152.2	158.7	139.4	135.3	141.1	152.8
HADHSC	369.7	365.5	442.8	378.3	358.7	304.2	289.2	296.7
HAGH	184.6	144.9	148.5	144.9	129.8	135.2	129.6	130.4
HAGH	763.3	603.4	750.2	807.1	414.7	482.7	445.1	321.5
HAGHL	149.2	188	165.7	155.7	173.9	123.2	148.6	145.2
HAGHL	127.8	120.4	129.8	123.1	112.4	107.6	119.9	119
HAGHL	167.3	204.2	173.6	168.3	163.8	141.6	155.9	150.9
HAK	129.5	114.3	130.3	126.5	110.2	108.2	121.7	114
HAL	124.2	117.5	123.4	125	111.5	99.4	127.3	113.3
HAMP	125.8	134.9	118.2	164.7	134.5	122.8	159	132.9
HAND1	115.1	107.5	122.4	119.4	110	102	125.5	116.7
HAND2	117.6	113.7	114.6	123.9	112	101.1	133.6	123.3
HAO1	107.8	114.6	122.8	124.7	106	101	119.2	125.3
HAO2	118.7	123.4	124.7	125.4	125.6	111.8	121.7	115.7
HAO2	110.1	111.9	120.9	111	110.6	98.7	114.1	124.6
HAP1	113.9	108.3	113.9	126.6	104.9	99.1	118	114.4
HAP1	116.1	107.6	111.9	117.7	114.2	108.8	112.5	106.6
HAP1	112.3	121.5	114.2	125.1	113.6	96.1	132.4	113
HAPLN1	104	103.8	108.7	121.5	99.2	98.7	121.8	115.8
HAPLN2	126.5	126.4	131.1	123.3	113.5	104.5	121.6	123.6
HAPLN3	179.8	186.4	204	165.7	134.4	167.2	145.1	157.3
HAPLN4	123.7	115.6	118.5	114	123.9	105.4	117.5	118.4
HARS	479.6	672.8	446.9	645	487.9	362.6	685.6	614.9
HARS2	173	172.6	199	176.2	153.2	143.6	164.5	139.1
HARSL	644.1	901.3	782.8	844.7	709.9	757.4	646.1	721.5
HAS1	119.8	120.8	122.9	118.3	107.7	97.6	122.4	114.6
HAS2	107.8	108	115.2	118.5	110.8	96.8	113.2	105.2
HAS2	102.6	113.8	110	107.7	97.3	98.9	109.5	107.9
HAS3	114.2	113.1	115.2	119.8	97.9	98.4	117.5	118.7
HAS3	114	113.5	117.4	123.1	110.9	101.3	136.1	118.4
HAT1	207.6	190.9	235.4	238.6	150.8	210.6	252.4	186.1
HAT1	142.3	135.2	145.8	136	122	124.6	128.7	147.4
HAVCR1	116.1	118.8	109.9	109.1	110.3	96	114.4	112.2
HAVCR2	439.4	314.1	702.5	589.5	330.2	514.1	932.9	537.3
HAX1	306.3	299.2	286.9	351.2	283.2	260.2	237	273
HAX1	154.5	138.4	165	149	123.9	135.3	122.3	137.2
HAX1	145.1	174.7	174.1	155.6	132.5	129	147.6	148.5
HB-1	112.1	105.2	109.8	113.4	106.5	95.7	113.9	104.2
HBA2	784.1	289.4	3584.2	1278.4	234.6	2562.6	1055.4	270.4
HBB	1838.7	536.9	8474.4	3134.4	539.1	6996.4	2676	650.6
HBD	129.4	121.3	162.9	150.3	124.8	149.2	143.7	137.5
HBE1	114.7	116.3	116.4	106.6	106	100.1	122.4	109.3
HBEGF	137.4	133.1	140.2	127.5	119	131.8	140.4	153.9
HBG1	139.2	117.1	221.3	153	112.9	173.4	134.9	121.7
HBG2	133.4	121.6	274.3	167.7	110.1	235.4	142.8	121.2
HBM	114.7	116.1	125.3	118.6	108	100.7	116.5	108.6
HBP1	429	431.7	377.5	491	404.3	316.8	515.6	679.5
HBQ1	121	119.1	128.7	118.9	121.1	112	126.8	130.1
HBS1L	116.1	109.9	117.7	122.2	104.4	96.4	114.8	119.4
HBXAP	129.8	118	128.2	127	120	106.8	136.1	125
HBXIP	1455.8	1677.1	1714.8	1411.8	1393.5	1614.6	1342.6	1504.1

HBZ	112.9	101.9	111.6	116.2	103.7	94.1	103.4	108.3
HCA112	150.5	104.4	196.4	138.5	107.2	112.9	129.3	120.1
HCAP-D3	152.1	142.6	139.1	146.4	134.1	116.3	133.4	144.8
HCAP-G	116.4	113.3	137.7	114.4	115.4	106.7	131.3	115.3
HCAP-H2	141.1	166.5	139.4	149.9	134	128.4	146.8	151.7
HCAP-H2	117.7	116.4	118.8	116.9	107.8	114.3	117.5	120.1
HCAP-H2	122.4	123.5	137	123.3	114.4	105.3	123.8	126.4
HCCA2	233.1	248	180.2	246.9	172.4	181.9	291	312.6
HCCS	143.8	133.8	144.2	151.2	122.2	116.9	142.1	159
HCFC1	685	766	529.7	805.9	647.1	541.2	1145.1	1443.2
HCFC1R1	141.6	150.9	136.7	164.2	132.8	105.9	143.1	135.7
HCFC1R1	391.8	326.6	315.2	370.1	248.7	240.3	325.5	264.3
HCFC2	167.4	155.2	176.3	178.6	158.7	130.8	157.5	163.9
HCG27	117.7	119.7	147.6	127.6	118.7	112.8	113.1	122.9
HCG2P7	1606.9	1441.3	1246.5	1175.9	1530.6	1647	1843.4	1265.5
HCG3	123.6	122.9	132	118.1	103	105	122.3	111.8
HCG4	119.2	103.6	109.3	108.1	100	98.8	116.2	108.9
HCG9	118.1	118.8	113.2	120.5	104.6	103.9	124.2	123.1
HCK	503.4	207.7	506.7	322.9	145.6	428.6	208.4	146.5
HCLS1	1570.4	2674.3	2679.4	2552.1	1779.1	2560.9	2080.2	1950.5
HCN1	112.3	112.7	111.1	116.4	106.7	111	113.3	111.8
HCN2	110	115.3	113.3	111.7	105.5	97.8	113.9	114
HCN3	140.4	139.3	137.2	135	124.3	114.1	144.2	117.7
HCN4	109.3	124.7	116.8	127.5	116	103	129.2	124.4
HCP1	114.2	121.5	124.4	110.6	103.9	98.8	111	113.5
HCP5	3092.1	3820.9	3256	2688.1	2095.6	1799.9	1741.8	1467.3
HCRT	124.7	125.5	122.3	130	125.6	104.9	125.9	132.3
HCRTR1	114.9	124.3	109.3	121.1	107.6	112.7	122.7	113.8
HCRTR2	121.7	125.8	129.7	133.7	111.6	98.7	134.9	129.3
HCST	1934.4	2575.5	3501.2	1840.5	1429.6	2166.2	1202.8	1152.7
HCST	258.1	281.2	296.6	254.5	206.7	209.8	189.2	209.8
HCTSL-S	106.4	104	120.8	121	110.8	101.7	114.4	122.9
HDAC1	1469	1986.7	1621.5	2056.4	1626.2	1292.9	1622	1970.8
HDAC10	129.2	119.2	133.3	126.7	112.8	102.7	119.8	123.1
HDAC11	118	120.5	140.5	133.6	115	107.7	125.8	130
HDAC2	410.6	407.4	317.7	449.2	478.8	383.1	462.2	456.6
HDAC3	263	355.3	395.4	270.1	287.5	297.8	336.6	371.1
HDAC4	218.9	238.2	160.7	194.9	173.6	137.6	198.6	191.5
HDAC5	123	123.5	136.1	123.8	108.4	115.8	122.6	128.2
HDAC5	127.1	132.1	136.6	135.1	121.3	110.9	124.6	143.1
HDAC6	241.8	216.4	234.2	250.5	180.8	189.3	188	235
HDAC7A	119.1	123.5	121.4	125.9	110.1	104	119.5	119.8
HDAC7A	131.5	129.1	136.4	132.2	119.3	117.6	140.2	123.6
HDAC8	126.7	122.6	128.3	121.4	113.2	108.4	119.1	118.6
HDAC9	133.1	138.9	131.7	123.5	116.2	104.4	143.5	123.6
HDAC9	114.8	105.4	114.7	113.1	108.4	94.7	114.2	118.1
HDAC9	116.5	110.5	115	110.5	111	102.6	119.2	119.2
HDAC9	105	117.2	114.8	119.3	105.3	96.9	122.3	110.9
HDAC9	113.9	111.7	119.4	124.4	109.5	99	119.3	117.3
HDC	122.3	133.5	173.6	136.8	122.9	136.6	115.4	113.4
HDCMA18I	245.6	287.4	282.6	284.9	319	261.7	275.7	277.1

HDDC2	674.7	705.5	662.9	735.5	572.2	657.8	634.2	716.3
HDDC3	213.2	212.1	238.1	219	172.5	177.1	159.4	160.6
HDGF	355.6	396.2	338.5	390.9	270.6	304.5	361.1	357.8
HDGF2	177	179.1	189	175.3	154.4	152.1	180.9	184.3
HDGF2	175.5	165.9	149.7	189.8	161.1	156.4	162.2	158.1
HDGFL1	105.3	112.9	112.5	128.1	110.7	93.8	119.9	117.1
HDGFRP3	121.1	124.1	122.4	127.9	119	112.4	129.1	118.2
HDHD1A	155.8	163.2	173.1	156.7	139.7	124.6	143.9	141.6
HDHD2	148.6	163.7	184.4	141.8	134.3	112.3	135.2	142.3
HDHD3	219	216.1	191.2	192.6	171.6	134.3	176.3	193.1
HDLBP	118.3	126.8	127.4	141.1	114.7	106.8	121.4	142.3
HDLBP	118.9	113.2	117.4	121.5	105.7	90.5	112	118.6
HDLBP	114.4	131.6	131.3	127.2	121.7	107.6	124.7	129.7
HEAB	247.9	287.1	242.3	281.7	221.8	191.8	254.7	313.4
HEATR1	330.6	476.8	363.8	430.6	338.4	162.8	292.7	305.3
HEATR5B	327	363.5	300.8	392	373	253.7	322.5	334
HEBP1	187.4	202.1	308.4	346.2	227.6	354.6	436.6	241.9
HEBP2	284.9	393.8	323.6	349.3	291.5	228.5	281.9	263.7
HECA	595.2	732.9	437.4	590.2	609.9	417.1	697.8	961.1
HECTD1	870.4	921.4	780.3	960	978.6	688.4	1027.5	892.9
HECTD2	114.6	120.1	122.2	124.1	123.3	107.8	120.1	119.6
HECTD2	110.2	114	116.6	116.8	105.7	103.5	111.1	110.6
HECTD2	149.5	124.6	123.3	133.1	115.1	115.2	126.8	129.9
HECTD3	212.2	192	195.8	204.8	151.4	149	148.4	155.8
HECW1	107.4	105.3	126.6	117.3	107	94.4	120.9	112.7
HECW2	115.6	119.9	133.9	114.5	111.5	95.3	131	111.5
HEL308	287.5	273.1	273.5	288.8	272.5	231.4	278.5	276.7
HELB	128.2	142.3	124.3	128.6	127.6	110.7	136.8	150.5
HELLS	118.5	124.9	122.6	133.3	111.4	94.4	128.7	115.6
HELT	111.1	116.7	115.1	117.2	110.5	101.9	121.4	120
HELZ	217.1	227.7	176.8	242.1	210.7	133.7	249.5	326.8
HEM1	182.6	199	190.2	187.1	147.9	162.3	167.5	133.6
HEMGN	115.4	118.7	118.2	126	110.4	106.5	128.2	122.5
HEMGN	112	111.2	128.7	126	111.5	94.9	127.4	110.5
HEMGN	122.8	131.9	116.4	121.7	129.6	103.2	125.8	120
HEMK1	133.4	128.5	150.8	132	121.8	120.4	127.7	127.2
HEPACAM	117.6	118	128.2	110.8	108.9	104.4	118.7	109.8
HEPH	115.8	119.7	107.9	112.5	101.5	98.6	115.8	102
HEPH	108.8	111.3	115.9	112.8	100.5	98.2	109.1	116
HERC1	389	362.4	381.4	436.5	372.4	312.6	445.1	489.3
HERC2	198.7	240.5	247	220.5	188.6	162.1	233.7	224.2
HERC2P2	118.8	119.9	136.9	124.3	118.5	102.1	131.7	118
HERC2P4	112.3	108.1	113.3	125.3	109.3	96	115.6	113.1
HERC3	169.4	157.2	172.7	155.3	165.3	129.6	143.7	145.6
HERC4	141.7	138	150.2	150.5	122.9	123.8	153	157.3
HERC4	131.1	134.8	125.3	130	123.3	107.2	121.4	135.2
HERC4	363.4	331.2	361.6	394.9	441.5	316.3	463.3	500.1
HERC5	716.9	588.6	371.7	719.3	487.5	316.5	504.8	560.3
HERC6	306.1	312	266.1	322.6	325.1	233.4	316	248.4
HERPUD1	357.7	336.2	399.4	401	374.5	294.9	428.3	620.2
HERPUD2	170.1	148.6	155.5	171.9	147.3	185.5	174.8	161.4

HERV-FRD	112.7	117.5	116.3	121.6	113.7	102	115.6	118.2
HES1	105.4	112.5	113.4	109.1	110.6	99.3	115.9	113.3
HES2	115.7	119.3	134.4	124.3	113.8	105.2	132.9	120.8
HES4	119.3	116.4	118.9	113.8	105.2	95.9	122	126.5
HES6	147.3	153.5	212.1	181.2	130.8	145.6	187.6	150.3
HES7	108.2	113.4	106	109.2	95.5	92.1	109	108.8
HESX1	113	114.1	115.5	115.5	104.9	95.7	114.7	112
HEXA	131.3	120.1	122.4	132.7	115.2	108.4	141.6	128.1
HEXB	1612.3	1194.6	2503.2	1814.9	1057.6	1773.8	1984.1	1190
HEXDC	131.4	145.4	134.9	129.3	105.6	129.3	133.6	116.8
HEXIM1	113.6	123.1	117.1	138.9	105.1	102.1	130.4	116.1
HEXIM2	124.7	125.7	120.1	118.5	116.4	93.4	126.3	126.5
HEY1	118.4	119.6	118.1	123.3	110.3	101.6	125.6	122.8
HEY1	118	118.7	119.8	117	112.8	106.9	122.2	117.1
HEY2	122.8	127	128	128.5	118.3	101	120.9	138.3
HEYL	116.9	108	109.5	118.4	106.5	98.7	116.6	118.5
HFE	113.9	117	118.2	123.2	105.4	103.4	116.1	109.7
HFE	109.7	117.6	122.7	118.1	111.5	103.7	122	112
HFE	118.8	117.9	112.2	135.1	116.3	106.4	121.3	113.8
HFE2	118.3	108.4	124	125	111.5	103.7	126.9	127.4
HFE2	121.8	120.4	120.2	121.8	117.4	102.9	129.7	117.4
HFE2	104.4	109.1	107.2	110.6	102.8	89.7	107.7	112.3
HGD	117.3	124	123	117.4	112.1	100.3	118.1	126.3
HGF	118.1	118.8	129.6	130.3	120	108.8	126.5	115
HGF	109.5	124.9	116	116.5	113.7	99.9	126.7	111.4
HGF	113.6	118.8	116.9	121.5	104.9	100.5	124.6	117
HGFAC	114	110.6	108.2	116	110.8	101.9	117.9	121.7
HGS	1375.7	1256.4	1148	1702.1	980.1	960.6	1538.1	1416.2
HHAT	105.5	114.2	117.2	124.8	113.9	105.5	114.4	112.2
HHEX	133.5	132	173.4	147.9	114.6	124.6	135.6	138.6
HHIP	116.9	120.8	117.3	112.4	108.4	100.2	116.6	110.2
HHLA1	116.8	117.6	116.2	115.6	104.3	91.5	115.1	118.7
HHLA2	111.1	111	121.3	126.6	110.3	97.9	110.6	123
HHLA3	123	122.5	113.3	112.5	120	108.8	121.9	109.1
HHLA3	118	114.4	116.6	119	115.3	113.1	116.5	111.2
HHLA3	114.8	116.6	123.2	120.4	110.2	103.5	111.8	109.4
HIAT1	147.7	129.5	143.1	159.2	123.1	119.5	185.2	126.7
HIATL1	322.8	422	287.9	394.5	399.4	296.8	374.3	422.5
HIBADH	304.1	330	299.8	346.5	283.3	274.7	269.6	236.1
HIBCH	203.4	219.1	252.4	232.3	170.1	161.3	169.3	164.8
HIC1	122.9	120.9	122.3	130.1	119.1	99	123.4	115.9
HIC2	227	266.8	250.1	269.4	250	226.1	316.2	287.3
HIF1A	330.6	352.7	286.9	319	281	257	429.8	416.1
HIF1A	187.6	148.9	195.2	242.8	180.5	158.5	192.8	227.5
HIF1AN	148.7	164.4	141.7	151.5	138.1	129.3	161.7	177.2
HIF3A	114.8	112	123.2	119.2	106.2	96.3	117.8	115.3
HIF3A	115.8	121.7	131.7	140.8	116.5	103.8	125.4	123.1
HIF3A	119.2	119.3	109.3	118.8	103.9	102	124.6	110.9
HIF3A	115.3	115.2	110.5	117.2	109.4	99.9	113.1	111.8
HIF3A	122.6	115	111.9	113.4	106.4	103.7	120.1	125.6
HIG2	125.8	127.3	130.4	133.5	125.6	115.9	124.9	140

HIGD1A	479	498	503.6	569.9	361.7	379.4	695.8	805.2
HIGD1B	114.9	125.5	117.6	120.9	118.9	96.5	118.3	115.3
HIGD2A	865	822.8	783.9	895.9	541.9	636.9	664.6	613.3
HIGD2BP	112.5	109.3	115.1	115.8	112.1	101.7	111.2	109.8
HIGD2BP	110.8	110.5	122.5	119.7	109.7	98.6	117.1	111.6
HILS1	111.1	122.6	120	121.3	111.5	104.5	116.8	124.2
HINT1	4478.1	4734.7	5664.4	5400.3	5459	4852.1	5432.5	6201.9
HINT2	185.4	239.5	276.5	211	219.1	183.5	190.4	170.8
HINT3	123	115.8	114.4	129.6	107.6	90.3	112.4	109.5
HIP1	134.5	121.8	143.7	119.5	119.7	149.9	127.7	115.9
HIP1R	151.6	154	148.2	146.4	149.6	137.2	160	151.2
HIP1R	134.6	118.5	143.5	120.5	124.4	124.9	138.4	128.3
HIP1R	114.2	112	125	120	108.8	103.9	126	119.3
HIP2	110.3	108.3	111.2	118.8	108	100.4	117.8	119.5
HIPK1	116.7	114.5	111.4	107.4	112.3	98.3	112.5	99.8
HIPK1	119.5	111.8	121.7	121.8	106.8	98.9	113.1	115.7
HIPK1	112.4	116.4	115.4	118.2	105	98.4	118	109.6
HIPK1	114.9	113.1	111.2	108.9	101.7	96.7	119.6	100.3
HIPK2	172.6	156.2	151.6	196.1	171.6	142	199.2	206.5
HIPK3	122.8	114.8	122.2	130	111.6	104.7	125.9	124.3
HIPK3	124.8	122.2	133.5	119.3	110.2	112.9	141.9	113.2
HIPK4	119.9	114.5	117.4	122.1	107.3	98.4	118.4	112.1
HIRA	128.8	123.3	139	131.2	130.2	106.3	128.3	119.1
HIRIP3	194.8	243.8	209.6	236.6	183.4	139.7	216.6	203.9
HISPPD1	107.9	108	108.9	127	106.6	93.9	115.9	110.4
HISPPD2A	182.4	189.7	195.3	173	159	147.1	156.1	153.6
HISPPD2A	110.9	108.9	110	111.8	106.2	89.9	106.9	107.6
HISPPD2A	110.3	114	120.4	122.5	116.1	98.8	112.9	116.9
HIST1H1A	112.1	115.5	115	118.9	114.5	102.9	114.1	117
HIST1H1B	116.6	116.2	112.5	117.3	112.1	96.7	113.4	120
HIST1H1C	127.9	129.5	152.9	146.1	118.6	107	124.8	126.3
HIST1H1D	116.3	121.7	120.9	121.3	111.7	100.8	121.7	111.7
HIST1H1E	109	117.8	119.3	116.9	113	94.3	118.5	116
HIST1H1T	113.4	110.2	119.9	126.8	110.1	99.1	116.1	117.3
HIST1H2AA	114.8	114.9	111.6	122.8	112.1	96.5	121.8	119.9
HIST1H2AB	107.6	113.3	124.1	118.9	105.5	104.8	110.4	123.1
HIST1H2AC	166.1	183.2	132.5	204.2	170.4	144.7	173.7	151.2
HIST1H2AD	100	107.1	109.1	117.4	107.9	96	113.8	109.3
HIST1H2AE	106.1	116.2	119.6	118.2	107.5	105.8	117.1	113.4
HIST1H2AG	105.9	106.2	105.2	115.3	113	94.7	116.3	112.8
HIST1H2AH	101	117.1	108.4	110.9	106.7	97.4	118.4	111.8
HIST1H2AI	118.7	120.7	112.8	121.9	104.9	103.1	118.3	124.2
HIST1H2AJ	117.7	119.4	120.7	118	108.2	95.1	120.8	114.5
HIST1H2AK	119.6	107.6	110.7	117.9	105.8	105.5	122.5	106
HIST1H2AM	110.8	109.5	120.4	118.3	116.8	99.5	129.3	123.5
HIST1H2BA	103.5	118.7	114	110.8	111.4	90.7	117.6	101.1
HIST1H2BB	111.5	110.6	119.7	112.8	110.1	96.7	110.1	113.8
HIST1H2BC	116.7	109.5	122.4	118.5	110.2	95.5	126.4	115.7
HIST1H2BD	209.6	271.7	148.5	250.6	253.7	143.5	274.9	298.5
HIST1H2BD	151.4	165.5	145	166.5	155.5	128.6	153	158.3
HIST1H2BD	105.3	116.8	107.2	108.9	100.2	98.7	112.7	104.5

HIST1H2BE	114.8	124.8	114.2	117.4	116.6	98.2	118.3	116
HIST1H2BF	118.9	117	110.9	122.1	102.7	106.3	118.4	120
HIST1H2BG	122.9	122.4	116.3	126.9	121.2	103.4	130.5	142.9
HIST1H2BH	109.6	121.4	119.7	108.4	115.9	100.3	124	125.7
HIST1H2BI	101.8	105.6	115.7	118.1	99.1	94	117	118.7
HIST1H2BJ	240.3	243.4	304.6	262	257.6	223.3	251.9	291.6
HIST1H2BK	2712.8	1986	1508.3	2513	1150.3	1223.3	2480.3	2381.4
HIST1H2BL	108	127.5	110.4	112.5	105	93.3	116.6	112.1
HIST1H2BN	116.4	117.5	122.8	111.9	108.9	104.3	124.7	113.2
HIST1H2BO	117.3	126.7	116.2	125.2	101	108.6	125.6	123.7
HIST1H3A	125.2	115.3	118.6	133.5	107.1	103.6	129.6	121.4
HIST1H3B	120.4	110.2	119.8	112.9	106.5	100.9	110.1	119.2
HIST1H3C	110.2	114.1	116	105.2	106.9	93.8	117.4	118.4
HIST1H3D	136.5	137.3	135.5	123.8	115.2	107.1	137.4	128
HIST1H3F	116.3	131.8	137.7	130.5	108.1	110.7	129.6	118.6
HIST1H3G	117.4	123.2	122.5	129.4	114.3	104.5	119.6	121.5
HIST1H3H	124	117.1	130.6	131	109.5	111.9	122.2	111.6
HIST1H3I	114.9	119.7	119	125.4	116.7	99.6	123.4	116.6
HIST1H3J	114.4	120.7	112.5	123.5	111.7	99.1	120.7	126.1
HIST1H4A	131.1	111	129.1	114.5	112.4	110	124.2	119.6
HIST1H4B	118.8	120.7	120	128.6	110.1	100.7	121.4	112.2
HIST1H4C	2605.4	3179.4	2898	2671.4	2171.5	1170.3	1611.5	2150.7
HIST1H4D	107.8	110.4	106.3	111.2	110.4	91.7	117.3	110.3
HIST1H4E	117.9	117.4	123.4	115.2	109.6	107.6	114.9	121.7
HIST1H4F	118.5	114.4	109	112.3	106.2	93.8	116.2	121.2
HIST1H4G	113.8	119.1	120.1	132.8	109.9	110.2	117	109
HIST1H4H	158.2	142.6	132	172.4	140.2	113.2	161.4	175
HIST1H4I	108.4	118.6	125.2	117.4	116.9	102.7	118.3	117.9
HIST1H4J	117.8	120.1	116.7	122.9	111.4	105	113.2	119.9
HIST1H4L	106.8	110.8	123.9	116	110.3	100.7	118.2	116.2
HIST2H2AA	252.5	218.1	266.3	295.6	164.6	190.2	270.9	270.8
HIST2H2AB	113.2	115.1	123.4	121	113.5	93.1	126.2	120.5
HIST2H2AC	202.9	188.6	220.2	190	152	173.9	214.2	217
HIST2H2BE	131.3	133.8	145.8	149.4	117.5	119.7	136.8	151.5
HIST2H2BF	117.2	125.4	125.6	132.9	113.3	102	127	128.3
HIST2H3C	113.8	112.5	116	108.9	108.4	96.4	111.5	108.7
HIST2H4A	113.8	123.9	114.6	128.6	119.1	99.3	122.5	122
HIST3H2A	142.4	132.1	190.4	162.8	151.1	132.6	148.3	153.8
HIST3H2BB	113.9	121.5	119.6	116	105.3	102.4	115.7	116.3
HIST3H3	119.1	120.7	116.9	122.5	108.5	103.3	120	117
HIST4H4	109.8	105.1	115.3	122.6	106.5	93	123.5	109.2
HIVEP1	147.2	143	143.8	148.9	136.3	120.2	169.3	174.6
HIVEP2	134	148.3	142.5	162.8	144.8	126.5	159.3	140.7
HIVEP3	113.2	119.1	112.8	107.1	112.9	97.4	127.7	117.5
HK1	1918.2	1921.8	2527.6	1740.5	1368.1	1795.5	1783.5	1798.8
HK1	119.5	122.5	125.9	129.4	118	103.3	124.7	117.5
HK1	109.6	129.1	116.9	125.2	109.4	92	115.4	110.9
HK1	120.4	114.5	114.2	115.4	102.5	97.1	107.8	107
HK2	191.9	163.9	175.7	176.4	144.3	121.5	171.9	167.5
HK3	109.5	147.2	198.9	137.2	117.2	115.9	126.9	114.7
HKDC1	114.8	130.7	136.2	131	118.3	113.6	118	118.3

HKR1	141.5	138.5	136.6	133.3	123.1	117.4	139	153.3
HKR2	120.5	138.2	129.2	118.1	109.4	104	127.2	112.6
HKR3	366	344.9	435.6	366.9	228	327.2	380.2	308
HLA-A	19966.2	18095.9	24113.6	21647.4	15175.4	21955.8	18614.1	14532.8
HLA-C	103.4	110	118.1	117.6	102.7	102.7	119.9	110
HLA-DMA	1358.9	889.6	3591.1	1180.5	457.8	1144	615	401.2
HLA-DMB	1686.1	1048.9	4855.3	1459.1	471.9	1568.3	721.7	349.9
HLA-DOA	268.1	232.8	349.5	220.4	184.9	232.6	168.1	153
HLA-DOB	197.3	197.8	216	181.9	140.9	134.3	132.5	147.9
HLA-DPA1	1596.9	1032.4	7556.7	1464.7	503	4149.3	678.7	418
HLA-DPB1	155.1	131	320.8	145.1	122.9	209.8	127.5	120.9
HLA-DPB2	105.7	107.5	109.3	114.4	110.8	104	116	122.1
HLA-DQA1	647.5	290.3	2461	554.6	230.4	1470	287.6	211.4
HLA-DQA2	111.3	116.9	118.4	117.6	109.4	94.8	116.1	114.6
HLA-DQB1	168.3	119.3	300.6	167.9	108	161.7	137	116.9
HLA-DQB2	114.8	113.2	119.8	114.6	109	97.1	109.3	114.3
HLA-DRA	1970.7	1277.4	7885	2132.9	1051.9	7414.9	1330.2	912.8
HLA-DRB1	126	192.5	125.2	122.7	169.2	104.8	121	160.5
HLA-DRB3	178.5	150.2	503.6	169.2	144.4	332.4	150.1	136.9
HLA-DRB4	157.2	116.9	258.7	163.2	116.5	218.7	148	115.8
HLA-DRB5	113.2	123.2	118.1	113.9	112.3	99.2	109.6	113.8
HLA-DRB6	247.5	177.2	519.6	252.3	138.5	517.4	236.3	195.4
HLA-E	784.5	755.2	1164.1	774.1	636	666.2	399.2	595
HLA-F	1536	2254.9	1971	1975.3	1504.3	1245	1385.3	1460.9
HLA-G	330.7	377.9	302.1	257.1	216.9	288.1	251.2	249.9
HLA-H	4853.1	6723.1	5825.4	5089.9	3524.2	4413.7	3258.5	3720
HLCS	108.4	119.5	106.5	112	107.8	102.9	116.5	123.4
HLF	116.8	133.3	119	137.9	121.6	109	128.1	130.1
HLRC1	117.5	126.7	144	133.3	132.6	120.1	122.7	135.1
HLX1	145.1	159.9	164.8	170.6	152.2	154.2	235.2	168
HLXB9	118.9	120.5	122.8	122.2	113	100.1	115.2	119.3
HM13	114.8	113.8	115.1	115.1	107	97.9	120.4	104
HM13	258.5	207.9	252.3	264.7	210.7	229.4	253.6	208.5
HM13	160.8	145.9	141.4	168	160.9	110	162.4	140.4
HMBOX1	132.5	142.7	127	154.7	132.1	135.4	153.8	186
HMBS	123.5	122.3	126.4	125.7	114.5	105.5	121.2	123.8
HMBS	117.2	139.7	135.5	147.7	128.6	111.5	138.4	123.8
HMBS	139.3	187.6	178.8	165.3	143.6	126.8	137.7	146.8
HMCN1	119	106.3	111.6	126.4	103.6	100.5	121.1	115
HMFN0839	348.7	447.8	223.7	683.3	429.6	377.1	657.5	427.2
HMG1L1	167.6	158	192.1	182.4	165.6	128.9	152	165.7
HMG20A	137	143.5	128.4	130.8	129	135.7	136.3	141.4
HMG20B	474	549.3	306.6	525.6	371	318	409.6	417.1
HMG2L1	119.8	141	143	135.5	118.4	115.8	123	120.6
HMG2L1	104.7	127.7	122.9	134.3	109.5	111.5	122.3	112.3
HMG4L	109.2	114.8	108.5	120.2	102.1	95.4	114.6	104.6
HMGA1	120.2	136.6	117.7	127.2	122	112.4	135.1	136
HMGA1	120.7	120.2	121.2	115.7	103.4	100	114.6	110.5
HMGA1	119	125.8	122.5	129.3	106.4	106.3	126.7	116.8
HMGA1	414.5	498.4	343.6	612.6	371.2	345.1	707.7	416.3
HMGA2	124.3	114.8	117.5	131.4	118	93.9	129.3	109.1

HMGA2	124.4	118.4	117.1	127.4	112.7	93.9	119.4	114.1
HMGB1	205.5	171.4	179.1	184.3	137.2	149.3	169.4	167.6
HMGB2	360.8	334.7	346.6	333.6	279	284.5	318.6	407.6
HMGB3	131.3	123	131.4	123.9	117.9	107.2	121.6	127.8
HMGB4	113.7	118.3	119.9	124.2	111.8	96.1	124.6	115.2
HMGB4	111.8	114.6	129.1	113.1	105.2	100.6	115.3	115.2
HMGCL	137.7	138.3	159.6	151.8	134.3	137.1	149.7	139
HMGCLL1	112.4	113.6	117	112.9	108	96.7	111.2	110.1
HMGCLL1	116.3	115.9	115.6	114.1	110.1	93.9	106.4	126.7
HMGCLL1	101.1	105.8	106.2	106.2	107.9	99.2	109.2	108.9
HMGCR	336.1	385.8	297.1	409.4	283.3	284.6	381.1	297.1
HMGCS1	319.4	320.5	279.7	273.4	253.5	211.3	369.7	328.7
HMGCS2	117.2	127.1	112.7	122.3	112.2	110.5	115.5	120.2
HMGN1	3189.3	2461.6	3265.7	3406	2479.2	2955.8	3336.1	2930.5
HMGN2	296.1	252.6	370.7	249.1	232.8	449.2	229.6	287.4
HMGN3	110.7	109	115.4	129	106.6	102.7	119.9	123.2
HMGN4	1122.2	969	1091.4	907.8	776.3	690.7	990.8	980.5
HMHA1	494.7	552.9	348.6	439.3	355.2	271.2	308.1	387.1
HMMR	122.7	112.5	119.9	117.1	109	104.9	127.4	118.3
HMMR	121.5	118.2	112.7	124.7	109.7	102.5	114.8	118.8
HMOX1	193	170.7	316.1	550.6	669.8	2186.7	4348.5	5093.7
HMOX2	116.9	126.6	118.7	134.4	121.5	111	113	109.3
HMP19	122.4	111.6	119.4	118.7	115.1	112	123.4	118.9
HMX1	111.9	124.3	115.4	120	108.8	95.4	123.4	125.2
HMX2	115.9	107.8	116.8	111.8	110	98.5	120.1	114.5
HN1	133.7	146	160.3	139.8	129.7	120	129.7	151.1
HNF4A	119.8	116.7	113.3	121.4	105.6	99.9	125.2	120.7
HNF4A	119.2	120	118.5	122.8	110.2	98.5	125.3	122.4
HNF4G	113.7	108.2	112.3	115.9	107.9	97	125.9	111.8
HNMT	207.4	116.6	282.3	163.5	120.2	180.6	134	115.5
HNMT	214.1	133.5	345.8	187.9	113.3	243.6	157.7	117.1
HNMT	136.7	114.4	186.8	124.6	112.2	124.4	121	118.6
HNMT	113.2	117	115.3	110.2	107.6	95.5	109.9	111.6
HNRNPG-T	109.2	115	123.7	121.5	116.5	103.4	112.6	113.5
HNRPA0	701	632.4	612.5	779.8	645.8	584.4	836.2	1069.8
HNRPA1	124.8	140.7	125.6	136.9	121.3	119.5	135.3	140.8
HNRPA1	129.9	137.3	143.1	132	133.1	120.5	122.3	132.2
HNRPA1L-2	224	234.4	262.3	252.8	275.2	199.6	271.9	332
HNRPA2B1	2134.6	1972.3	2115.5	2232.3	2015.3	2082.8	2606.9	2955.9
HNRPA3	138.5	136.9	134.7	138.9	137.1	121.2	135.2	148.2
HNRPA3P1	133.4	115.7	134.3	132.6	119	116.8	131.5	121.4
HNRPAB	212.2	241.7	239.3	227.9	209.9	196.9	194.6	193.6
HNRPAB	388.3	464.2	419.5	428.6	382.2	342.2	403.6	530.4
HNRPAB	2033.6	2351.1	1945.9	2415.5	1529.3	1769.7	2654.4	2643
HNRPC	153.7	157.1	150	189.9	172.6	135.5	199.1	221.7
HNRPC	408.3	402.9	493.6	535.7	377.7	394.8	554.6	688.7
HNRPCL1	109.4	115.8	115.3	117.1	110.1	100.6	125.1	110.5
HNRPD	2654.5	2676	2538	2370	2391.9	2469.2	3016.3	2839.4
HNRPDL	296.5	293.8	285.5	339.3	281.5	224	377.6	429
HNRPDL	758.6	822.4	850.7	714.8	673.2	458.7	489	620.4
HNRPF	132.3	138.4	135.9	146.1	138.9	126.2	131.6	139.4

HNRPH1	118.6	129.4	128.4	123.8	117.4	106.5	136	120.5
HNRPH2	163.7	156	197	173.2	148	141.8	176.6	200.2
HNRPH3	267.8	271.4	275.1	289.3	345.4	261.9	286.4	474.2
HNRPH3	122.8	111.8	134.5	127.3	118.3	103.6	118	125
HNRPK	1642.3	1805.9	2052	1940.7	1648.9	1489.1	1697.2	2219.8
HNRPL	355	299.4	246.9	359	333.7	275.3	409.2	474.9
HNRPLL	123.8	151.7	150.6	148	129.6	114.4	150.7	145.4
HNRPM	118.6	123.4	119.6	128	113.7	101.1	134	120.3
HNRPM	1112.4	1356.3	1331.8	1393.2	1170.2	1052.3	1757.5	1916.4
HNRPR	869	1098.7	904.1	1128.1	922.7	788.7	1082.2	1078.5
HNRPU	698.6	497.6	606.1	478.9	577.5	396.4	621.9	565.9
HNRPU	260.9	326.3	228.3	251	279.9	189	289.3	348.7
HNRPUL1	688.8	657.5	771.4	739.3	481.7	517.7	633.1	708.6
HNRPUL1	1545.5	1834.3	1396	1696.2	1507	1305	1552	1552.9
HNRPUL2	320.5	443.5	298.9	424.6	391	263.3	535.5	399.6
HNT	115.1	115.6	109.2	109.3	104.9	94.9	111.9	119
HNT	121.4	108.7	118.8	118.9	103.3	104.9	114.1	127.6
HNT	120	110.6	118.6	113.1	108.6	93.7	125.3	115.7
HOMER1	114.8	119	112.4	132.5	119.7	102.2	125.6	123.4
HOMER2	109.4	110.2	120.1	118.9	107.5	97.3	109.3	111
HOMER2	122.7	110.5	130.5	128.2	111.7	120.4	110.7	112.2
HOMER3	167.4	152.2	169.6	224.5	136.8	153.5	186.1	124.1
HOM-TES-1	114.9	121.6	125.2	124.6	121.5	99.8	110.2	117.2
HOM-TES-1	134.1	128.7	137.6	141.5	128.1	111.3	119	116.2
HOM-TES-1	686	521.4	725.9	509.7	375.7	419.6	403.8	342.8
HOOK1	195.8	209.3	191	200.4	208.1	136.2	229.9	177.5
HOOK2	172.8	149.1	148.9	190.9	145	116.2	181.7	157.2
HOOK3	126.1	129.7	127.8	133	113.5	120.7	140.6	136.4
HOP	110.2	111.9	129.1	116.9	106.5	100.1	122.6	116
HOP	125.1	120.1	146.2	122	114.1	108.7	132.1	122.9
HOP	839.3	709.1	652.1	800.4	500.4	449.9	570.1	446.3
HORMAD1	113.5	109.9	118.2	111.7	107.8	97.2	118.5	106.9
HORMAD2	110.1	119.4	115.3	113.3	106.9	100.2	132.2	119
HOXA1	109.9	114.2	116.1	120.1	100.3	103.4	122.6	116
HOXA1	122	124.6	128.8	133	115.7	114.3	115.9	134
HOXA10	115.5	118.2	115	114.3	109.6	103.8	120.4	115.7
HOXA10	113.2	111	118.5	112.5	109.6	99.8	108.8	115.1
HOXA10	106.2	112.1	121.5	110.6	105.1	91.9	120.6	106.2
HOXA11	107.6	108.7	111.2	109.6	105.5	99	119.1	116.5
HOXA13	124	111.4	121.8	115.9	109.4	92.9	120.7	110.3
HOXA2	116.9	107.8	119.8	112.2	111	99.6	121.2	107.4
HOXA3	111.6	111.4	121	113.2	108.6	103.1	114.1	117
HOXA3	115.6	111.6	120.2	110.2	109.7	102.6	116.9	117.8
HOXA3	112.2	111.4	112.9	125.5	104	100.3	109.9	108.6
HOXA3	109	109.6	113	115.5	107.5	97.6	114.7	107.9
HOXA4	114.7	114.3	119.9	116.8	121.6	98.9	119.1	115.7
HOXA5	124.8	135.5	124.5	126.6	109.7	109.4	131.4	116.1
HOXA6	124.6	112.9	120.4	127.8	125	104.9	137.9	120.6
HOXA7	114.6	110	116	120.3	107.6	91.1	128	109.3
HOXA9	120.3	120.7	125.2	132.1	103.8	106.4	135.5	117.2
HOXA9	116.8	119.1	113.3	121.4	108.7	104.7	119.9	112.3

HOXB1	123.8	123.2	142	136.1	113.8	115.2	139.9	137.7
HOXB13	120.3	109.7	107.4	113.6	107.4	91.3	108.5	107.5
HOXB2	314.5	202.8	309.3	259.3	156.1	189.6	207.4	150.6
HOXB3	123.3	126.1	122.7	113.8	113.9	111.6	120.4	112.4
HOXB4	117	132.1	125.1	128.1	114	106.7	124.4	118.1
HOXB5	118.7	122.3	130.2	123	111.7	108.8	124.6	122.9
HOXB6	170.6	148.3	147.1	150.3	132.4	129.9	135.3	141.9
HOXB6	132.6	124.1	127.2	140.4	117.5	98	144.1	125.5
HOXB7	108	124	124.7	119.7	114.4	99.7	117.4	117.2
HOXB8	102.5	113.3	117.6	116.4	100	91.5	107.3	110.6
HOXB9	127.1	120.2	119	120.8	106.1	98.6	121.2	111.5
HOXC10	120.5	119.6	121.5	126.6	105.1	99.2	130.2	124.9
HOXC11	117	136	139.7	128.4	124.5	120.9	131.6	134.6
HOXC12	103.3	108.4	119	127.3	113.1	86.8	114.3	113
HOXC13	118.8	127.3	118.5	131.5	111.7	113.5	120	122.9
HOXC4	125.5	118.7	117.7	111.5	102.3	101.7	120.3	113.6
HOXC4	163.7	207.4	178.5	181.1	181.3	123.3	160.4	159.6
HOXC5	117.2	117.3	121.8	125.8	109.4	99.2	120.4	119.4
HOXC6	113.2	118.1	118.6	123.9	106.7	99.1	122.6	109.9
HOXC6	124.6	123.4	113.5	114.1	117.9	105.2	127.1	125.4
HOXC6	121.8	115.6	120.6	119.8	115.1	105.2	120.5	124.9
HOXC8	125.3	111	115.3	120.3	111.1	99.6	126.9	122.5
HOXC9	109.6	115.4	119.3	121.6	104.9	104.6	119.1	126.1
HOXD1	105.9	110.4	116.4	121.1	107	96	119.5	124.4
HOXD10	105.3	116	111.2	114.2	105.6	101.4	116.6	106.1
HOXD11	128	123.3	139	137.4	119.5	108.8	110.4	118.5
HOXD12	132.1	124.8	122.4	124.3	113.1	117.5	124.6	120.1
HOXD13	113.8	129.1	130.1	139.8	114.7	112.9	143.9	133.9
HOXD3	132.3	137.4	129.6	138.1	127.4	100.7	121.8	140.5
HOXD4	126.1	117.2	113.7	127.3	115.1	108.4	118.7	128.1
HOXD8	117.3	115	114.2	118.6	115.7	109	125.3	115.2
HOXD9	117.8	116.7	123	120.3	106.5	101.3	110.8	119.6
HP	119.1	116.4	123.6	113.2	113	103.8	126.4	112.9
HP1BP3	148.3	128.3	152.9	127.2	127.4	124.3	164.1	139.6
HPCA	106.9	115.8	112	114.2	102.3	102.7	116.6	117.1
HPCAL1	123.2	123.3	110.8	119.4	114.8	103.4	126.5	99.2
HPCAL1	964.9	1535.2	1044.5	1560.2	1018.6	1349.1	1780.3	1165.8
HPCAL1	123.1	122.4	131.3	126.9	116.5	114.6	129.1	121.3
HPCAL4	140.5	163.7	156	176.7	143.2	123.7	143.4	154.2
HPD	118.7	127.9	121.9	131.6	109.5	99.1	127.7	120.7
HPGD	117.9	137.2	131	129.7	116.8	115.3	122.9	138.6
H-PLK	107.7	115.6	122.5	115.1	104.9	92.6	111.8	110.6
HPN	113.2	126	111.9	118.2	116.5	97.5	122.1	108.1
HPN	117	117.3	123.5	113.4	116.9	104.9	129.7	116.1
HPN	114.1	114.6	118.1	120.1	114.3	93.5	120.9	112.5
HPR	107.5	114.5	120.6	121.1	103.4	98.1	118.3	109.5
HPRT1	412.8	375	571	509.3	347.6	459.5	412.1	595.2
HPS1	129.2	128.7	134.8	133.2	119.6	111.2	134.4	132.1
HPS1	155.7	138.2	134.2	139.8	145.2	118.8	156.6	135.2
HPS1	121.8	130.6	115.5	133.6	120.7	103.7	122.6	143.7
HPS3	164.9	154.7	220.6	147.3	149.7	183.6	138.9	134.5

HPS4	127.1	105.7	115.7	112.7	105.3	92.3	118.1	120.3
HPS4	134.6	117.2	138.6	131	116.3	108.6	124.1	128.5
HPS4	149.2	141.9	156.2	140.5	146.9	134.8	143.6	149.8
HPS4	120.1	116.1	131	115.4	119.1	108	126.2	125.3
HPS5	182.8	188.6	201.2	213.5	195.3	180.8	247.4	270.9
HPS5	114.6	126.2	113.1	107.8	101.8	98.1	121.6	109.1
HPS6	724.8	778.3	778.9	722.9	500	532.3	592.1	561.3
HPSE	132.5	137.5	143.6	130.1	116.3	124.4	122.7	128.1
HPSE2	112.1	121.1	118.7	108	108.7	98.9	118	110.4
HPX	117.5	105	114.2	115.6	106.8	95.9	116.4	118.2
HR	104.6	108	108.2	107.9	102.5	91.5	107.6	107.8
HR	117.3	113.2	119.3	115.8	108.3	96	118.9	111.6
HRAS	170.1	183.8	228.4	209.5	165.2	154.8	215.6	194.7
HRAS	114.1	112.8	124.2	129.2	108.5	104.4	117.4	121.8
HRASLS	124.6	117.6	125.3	126	113.8	99.1	120.2	117.4
HRASLS2	121.5	123.4	132.5	135.2	111.2	105.8	116.8	120.3
HRASLS3	165.2	198.3	271.5	187.3	142.8	177.9	154.2	154.5
HRASLS5	108.1	111.9	111.2	112.8	106	92.8	115.9	115.6
HRB	100.7	106.3	121.3	111.4	110.9	99	113.8	113.2
HRB2	152.1	138	148.4	149.5	130.1	131.5	151	151.1
HRBL	127.1	124.7	127.7	131.1	110.4	98.7	133.1	125
HRC	109.5	108.9	123.1	119.9	101.1	97.9	118.2	100.8
HRG	106.2	109.7	110.9	123.4	98.9	93	110.3	114.2
HRH1	126	115.4	123.6	124.6	111.3	99.3	118.3	114.9
HRH2	117.5	114.3	116.3	111.1	109.7	101.9	117.9	122.7
HRH3	119.2	105.2	116.3	125.5	113	100.3	118.3	111.9
HRH4	114	107.6	117.4	116.4	106.3	93.2	113.8	108.1
HRIHFB212	126.4	125.5	144.7	139.3	132.5	112.3	128.4	126.5
HRIHFB212	115.2	116.4	119.1	118	114.1	103.7	122.1	112.7
HRIHFB212	187.8	201.3	277.4	173.7	164.6	218.9	178.2	156.5
HRK	102.3	111.5	114.3	117.6	106.4	103.6	112.1	116.5
HRNR	109	116	123.9	118.6	109.3	100.7	110.3	124.9
HRSP12	109.7	139.2	125.5	130.7	129.7	109.1	129.2	115.3
HS1BP3	184.8	157.1	172.5	186.5	137.7	150.3	169.2	159.7
HS2ST1	126	134.3	123.1	127.8	129.3	106.9	124.9	127.3
HS3ST1	122.5	104.8	255.8	126	114	297.2	111.4	107.5
HS3ST2	120.6	113.1	120.3	121	114	105.1	121.8	117.2
HS3ST3A1	110.6	112.2	116	111.1	104	101.5	108.9	109.4
HS3ST3B1	127.8	124	121	124.5	111.1	103.1	126.7	119.6
HS3ST4	115.3	106.3	133	120.9	116.5	98	115.8	117.4
HS3ST5	121.7	122.3	118.9	127.8	106.5	98.3	137.6	117.4
HS3ST6	108.4	112.9	115.1	105.9	108.4	100.3	114	106.1
HS6ST1	127.4	129.8	127.9	125.8	115.1	105.5	133.9	126.3
HS6ST2	103.4	107.9	109.4	112	107.9	93.2	115.5	110.3
HS6ST2	120.4	119	138.4	123.4	116.9	100.2	124.9	121.3
HS6ST2	100	118.4	111.2	113.3	105.6	100.1	117.8	110.3
HS6ST3	111.4	113.3	121.1	120.7	101.3	97.2	119.9	119.2
HS747E2A	116.1	115.5	118.8	120.5	105.5	95.6	128	125.9
HSA9761	487.9	574.4	456.9	533.1	438.3	256.9	506.8	555.1
HSBP1	715.6	666.6	737	740.5	499.7	570.3	705.1	539.3
HSCARG	339.1	356.1	351.9	302.7	312.2	252.8	227.2	243.7

HSCB	291.4	236.5	264	252	235.5	203.6	262.4	405
HSD11B1	122.7	117.6	122.6	124.1	106.5	98.9	115.3	110.1
HSD11B1	116	117.9	112.5	117.5	109	100.3	121.4	113.1
HSD11B1	121.5	111	112.2	116.1	108.3	97.7	114.2	122.2
HSD11B1L	135.6	143.8	134.6	135.4	124.6	111.4	117.9	123.9
HSD11B2	110.3	113	115	107.3	103.7	98	121.6	110.7
HSD17B1	113.2	117.9	120.7	122.7	111.2	101.4	127.5	116.1
HSD17B12	531.5	456.5	781	556.6	393.9	533.2	716.2	584.1
HSD17B13	113.7	123	119.8	118.5	109.3	99.7	116.3	114.5
HSD17B2	103.6	115.9	112	114.7	103.5	88.8	115.7	110.5
HSD17B3	117.6	116.4	120.7	118.1	106.8	95	113	109.1
HSD17B4	319.3	343.4	446.3	369.9	269.4	305	232.7	239.3
HSD17B6	131.5	111.3	104.1	115.1	122.1	94.3	121	122.7
HSD17B8	149.2	162.9	243.8	149	132.4	180.5	128.5	132.5
HSD3B1	110.1	111.7	115.1	112.3	110.9	93.5	118.5	111.7
HSD3B2	112.5	107.7	112.9	128	105.5	102.1	122.7	107.6
HSD3B7	137.3	130.5	118.1	148.8	113.3	105	131	116.9
HSDL1	223	230.4	203	215.3	221.3	151	225	205.5
HSDL2	146.9	152.8	170.2	136.9	133.3	133.2	145.9	136.9
HSF1	121.2	125.2	119.4	133.3	106.4	112.3	119.9	125.6
HSF2	167.6	263.1	207.3	219.7	225.2	194.4	268.3	276.6
HSF2BP	121.7	119.1	121.8	121.4	117.4	104.6	117.3	120.2
HSF4	107	116.6	118.2	110	113.5	92.9	111.9	113.5
HSF4	112.3	116.9	115.5	121.7	117.4	104.2	114.7	116.2
HSF5	147.7	126.1	151.7	143.4	125.5	106.1	149.1	133.7
HSFX1	111.8	121.4	117.4	111	102.7	95	120.3	118.9
HSFY1	120.3	116.6	123.2	122.7	113.5	98.2	122.6	117.4
HSFY1	103.2	114.9	115.9	117.2	102	96	122.5	118.5
HSFY2	111.2	112.6	106.2	111.5	103.2	99.6	120.2	112.3
HSFY2	134.6	116.7	128.8	122.6	118.9	103.3	117.1	118.9
HSH2D	225.2	229.5	209.3	236.6	178.4	165	177	178
HSMPP8	444.9	392.5	498.5	420.9	394.8	423.5	345.1	291.5
HSN2	119.7	119.2	123.5	130.3	129.6	100.7	123.3	125.9
HSP90AA1	121.4	119.7	121.9	127.7	121.9	110.8	154.1	148.3
HSP90AA1	4161.2	3963.2	3674.6	4697.8	3601.6	2942	8350.2	9592.9
HSP90AB1	987	957.7	837.3	1183.9	965.4	846.8	3179.1	5019.3
HSP90AB2I	119.6	128.7	128.2	121.7	114.1	104.6	124.9	127.2
HSP90AB4I	112.5	118.5	113.9	120.4	107.3	97.4	116.4	116.9
HSP90B1	1721.3	1829.3	2564.3	2112.1	1888.3	2059.3	2037.8	2050.2
HSP90B3P	112.5	118.2	117	119.9	117.2	99.7	125.4	111
HSPA12A	119.8	109	113.3	124.7	108.8	102.4	133	122.6
HSPA12B	126.3	114.7	129.8	122.3	112.8	105.4	126.5	124.5
HSPA14	200	225.9	253	210.1	186.7	197.5	211.8	275
HSPA1A	1058.8	563.6	1153.1	945.7	598.7	1012.5	3699.3	7544.3
HSPA1B	505.5	385	491	548.2	498.4	451.3	3322.1	6990.1
HSPA1L	250.4	252.7	231.9	255.7	229.7	206	276.3	451.5
HSPA2	118	129.9	108.8	125.5	110	96.9	140.2	168.8
HSPA4	135.9	153.1	137.8	184.7	122.7	115.6	160.3	144
HSPA4	128.1	124.1	141.7	142.2	128.4	105.5	135.6	138.9
HSPA4	393.9	501.7	466.7	586	357.8	295.5	634	639.8
HSPA4L	130.2	106	121.7	120.3	114.7	97.9	121.9	117.5

HSPA5	225.3	286.9	466.3	266.8	273.7	216.6	332.4	390.2
HSPA8	3250.5	3555.7	3653.1	3909.5	2415	2486.9	5223.3	7210.7
HSPA8	2976.7	2482.6	2198.2	2418.5	2325.8	2675	4041.5	5021.7
HSPA9B	398.1	408.1	368	446.5	322.7	312.5	504	579.3
HSPB1	529	503.7	411.9	570.4	308.3	286.3	676.1	1161.5
HSPB2	108.5	114.5	111.2	120.6	109.6	105.8	112.6	115.3
HSPB3	125.5	113.4	116	115	114.4	100.7	121	117.1
HSPB6	114.4	110.8	122.7	113.6	103.6	98.7	123.5	120.1
HSPB7	111.6	115.2	117.2	109.2	100.9	101.3	115.8	111.9
HSPB8	109.3	106.5	110.9	120.6	105.7	95.9	113	119.7
HSPB9	122.7	121.9	114.8	116.1	110.9	102.2	120.6	113
HSPBAP1	238.8	228.5	204.3	229.5	209	166.3	222.6	208.6
HSPBP1	121.6	114.5	124.8	121.5	119.7	97	119.1	109.4
HSPBP1	129.7	108.8	114.9	125	107.8	102.7	117.6	117.6
HSPC023	849.8	854.7	826.9	740	646.7	582.4	664.6	660.9
HSPC049	167.8	169.1	191.7	181.3	142.9	210.1	205	158.4
HSPC065	118.6	113.4	118.3	124.9	104.2	95.4	114.6	116
HSPC072	110.6	115.6	120.2	115.2	110.4	96.7	127.4	117.6
HSPC105	118.1	130	115.1	114.4	108.6	93.8	121.2	112.8
HSPC111	166.3	156.6	158.3	197.4	135.9	124.8	174.9	164.1
HSPC117	314.2	374.6	333.5	323.5	253.5	227.2	278	220
HSPC142	160.9	214.3	226.6	218.6	181.3	193.6	168.3	151.2
HSPC142	609.8	774.3	876.9	653.9	505.8	591	517.2	395
HSPC148	492.5	416.9	501.1	474.3	345.3	346.3	414.9	470.7
HSPC152	900.7	826.7	960.8	928.3	965.2	710.5	737	1003.5
HSPC159	109.3	113.4	116.5	123.6	105.9	96.7	115.5	115
HSPC171	267.2	247.8	311.3	311.1	229.6	255.6	245.4	221.9
HSPC176	576.7	478.2	894.5	555.5	433.5	432.4	489.4	354.7
HSPC196	254.5	234.7	228.5	267.6	173.5	218.9	225.6	186.7
HSPC268	585.9	452	552.8	430.9	526.9	433.9	572.4	585.3
HSPD1	1111.1	850.3	881.3	1014.6	898.8	608.2	1692.3	1583
HSPD1	117.8	118.1	126.2	126.8	111.1	99.9	120.6	119.3
HSPD1	121	126.5	133.2	140.5	114.3	105	122.2	125.9
HSPE1	363.6	321.8	337.6	349.2	280.9	236.5	384	628.9
HSPG2	118.1	117	113.5	124.5	112.3	98	118.2	115.8
HSPH1	723.1	538.2	591.9	658.8	602.7	458.9	2086.8	4413.7
HSU79303	235	227	209	232.6	205.7	148.6	192.8	162.9
HSZFP36	228.1	264.7	230.8	238.1	222.3	153.4	202	212.1
HSZFP36	182.4	188.9	149.5	174.6	149.2	125.5	145.2	143.2
HTATIP	123.1	119.6	125.9	132.8	114.1	107.4	131.4	128.4
HTATIP	137.6	136.7	147.7	154.7	125.8	129.4	135.9	135
HTATIP2	371.4	419.8	563	647.7	451.6	823.3	523.9	575
HTATSF1	126.8	123	113.6	115.4	105.3	95.1	116.1	113.7
HTF9C	132.4	134.5	131.4	139.7	106.6	113.5	136	132.6
HTF9C	112.6	116.6	110.1	121.6	108.4	99.3	121.6	114
HTF9C	131.6	131.2	121.5	136.1	113.4	106	133.9	127.6
HTLF	603.5	644.4	570	705.2	571	476.2	479.9	424.5
HTN1	105.2	118.3	112.9	118.1	109.5	94.3	113.2	116
HTN3	114.9	121.2	122.1	123.7	113.7	107.3	124.2	120.1
HTR1A	110.9	120.7	125	122.1	115.3	107	122.4	114.6
HTR1B	113.1	106.4	108	111.5	104.9	96.7	118	113.7

HTR1D	116.5	124.8	124.1	128.9	119.1	112	127.4	114.6
HTR1E	128.5	122.4	132.3	141.3	122.5	117.9	145.1	117.6
HTR1F	106.4	109.9	109.7	113	104.6	97.4	116.1	111.8
HTR2A	266.7	319.4	329.1	291.1	271	279.3	374.7	367.6
HTR2B	113.9	122.2	118.8	110.4	108.9	101	112.9	111.7
HTR2C	115.3	107.8	117.1	124.3	112	96.2	119.9	114.3
HTR3A	121.1	121.3	117.3	132.7	121.6	99.3	123.4	125.1
HTR3A	119.7	112.9	122.4	117.8	113.1	99	115.2	120.3
HTR3A	123.2	120.2	112.7	120.3	116.4	101.3	118.8	116.5
HTR3B	113	117.4	110.9	111.2	114.9	95.1	118.8	115.7
HTR3C	121.3	118.1	109.2	114.3	102.3	101.5	109	121.7
HTR3D	110.2	115.1	116.2	118.7	109.7	101.6	118.3	114.1
HTR3E	113.3	116.7	104	115.6	109	93.8	116.9	109.2
HTR4	115	114.1	121.6	119	106.4	97.3	114.3	117.4
HTR4	113.8	116.7	116.1	126.1	108.1	90.7	129.1	112.8
HTR4	114.3	121.9	114.4	121.9	111.6	100.1	126.2	113.7
HTR4	116.2	116.8	129.3	133.9	106.9	104.6	124.2	114.2
HTR5A	117.2	117.1	117.3	127.2	105	98	111.4	106.9
HTR6	128.4	132.7	134.5	129.8	128.5	108.6	130.5	127.7
HTR7	125.7	117.3	118.8	118.5	111.1	107.8	125.7	122.5
HTR7	116.8	123.4	137.1	122.3	112.6	106.9	122.6	131.8
HTR7P	117.2	131.6	121	132.9	118.4	114.2	117.6	123.2
HTRA1	161.4	179.6	241.8	198.1	158.2	224.6	197.1	141.3
HTRA2	169.1	172.4	183.8	204.1	188	163	197.7	204.6
HTRA2	192.6	184.5	175	201.5	168.4	156.5	250.2	188.1
HTRA3	133.3	123.2	120	122	107.8	102.9	119.2	114.8
HTRA4	136.1	133.2	124.1	139.4	113	100.1	129.5	121.6
HUNK	123.4	111.4	114.9	107.2	100.5	85.3	126.1	110.6
HUS1	113.4	123.9	107.7	121	103.7	92.8	118.7	109.5
HUS1B	129.5	138	132.8	134.7	114.9	108.6	133.5	136.4
HUWE1	134.8	134.9	135.1	142.5	134	116.4	159.1	146.3
HYAL1	119.6	116.3	110.2	118.5	113.5	97.1	119.6	118.1
HYAL1	115.7	114.8	120.3	115.8	106.1	99.8	108.6	115
HYAL1	118.7	110.7	118.7	118.4	101	100.7	119.3	117.7
HYAL1	105.2	123.4	124.6	133.1	106.1	101.1	120.1	115.1
HYAL2	122.7	120.2	119.7	130.5	113.6	105.1	118.3	123.5
HYAL2	136.8	132.4	138.1	136.5	118	105.7	125.5	132.3
HYAL2	120.1	127.5	121	115.2	112.5	98.2	117.9	113.9
HYAL3	111.7	129.5	117	125	121.4	101	129.4	127.9
HYAL4	119.1	117.8	121.6	118.5	106.9	100.3	118.9	111.6
HYDIN	111.1	113.7	107.5	114.1	109	95.6	113.2	110.4
HYDIN	111	115	119.2	113.5	101.3	97.1	114	113.3
HYDIN	118.1	111.8	108	116	106	101.7	121.7	115.7
HYDIN	112.6	116.6	122	116	103.5	100	114	107.4
HYI	130.5	121.1	132.9	138.4	118.6	103.5	123.1	122.7
HYLS1	256.7	303.5	231.5	278.5	224.7	171	260.9	257.4
HYOU1	185.3	164.9	181.4	160.5	151.2	158.8	183.5	176.7
HYPB	598.2	689.7	536.2	726.9	741.3	500.3	783.9	741.9
HYPE	176.9	169.7	195.1	156.3	170.8	147.3	181	185.8
HYPK	2436.9	2000	2029.4	2029.8	2094.4	1248.9	2210.4	2214.9
IAH1	369.3	428	504	438.8	377.8	397.7	347.1	237.6

IAPP	114.8	124.3	123.2	111.7	106.2	100.2	127.2	116.6
IARS	266.6	337.6	267.6	330.5	340.3	278.4	318.1	320.8
IARS	107.6	108.4	105.9	117.4	100.1	94.9	117.8	108.5
IARS2	425.6	614.1	486.4	453.2	449.3	433.3	520.8	619.3
IBRDC1	122.2	114	124.4	111.2	110.8	103.4	109.2	111.3
IBRDC2	148.8	123.5	157.2	133.3	112.2	109.4	117.8	111
IBRDC3	154.5	135.8	160.7	150	130.1	169.3	144.8	141.2
IBSP	123.6	126.3	128.7	116.3	127	111.4	131.1	129
IBTK	252.5	265.8	308.2	327.4	229.1	286	267.3	271.4
ICA1	114.6	125.7	130.3	114.8	111.2	98	113.2	109.3
ICA1	125.5	124.7	141.4	124.5	114.7	114.5	121.7	119.1
ICA1	116.9	128.7	138.6	133.3	120.3	111	131.1	138.8
ICA1L	117.3	116.3	115.5	119	104.9	105.8	117.6	119.8
ICA1L	113.3	111.7	117.4	130.1	108.1	96.8	118.3	115.8
ICAM1	121.3	138.3	157	153.1	133.4	119.7	143.8	133.8
ICAM2	1499.6	1884.7	1283.1	1491.9	1186.7	855	1251.1	1104
ICAM3	1632.1	1941	1946.3	1762.7	1443.3	1541.6	1682	1868.4
ICAM4	111.4	114.5	115.3	115.4	99	101	105.3	110.5
ICAM4	124.4	124.9	127.7	128.3	120.8	111.7	113.7	121.4
ICAM4	119.9	115.2	111.2	133.1	104.1	97.1	113.8	112.8
ICAM5	117.4	122.8	127.8	118.9	109.5	106.3	121.9	114.4
ICEBERG	104.8	123.1	107.3	111.2	104.6	98.8	110.3	103.5
ICK	112.9	137.7	122.2	120.5	113.6	105.2	124.9	129.7
ICK	114.6	120.4	117.8	110.1	105.1	94.3	117.2	116.7
ICMT	215.3	209.5	193.5	207.6	155.5	159.5	180.2	152.6
ICOS	545.4	677.3	350.8	668.7	631.8	390.7	1280	1390.5
ICOSLG	118	121.7	138	133.1	111.7	113.7	123.3	110.9
ICT1	143.9	159.4	190.3	158.4	155.7	145.7	145.5	172
ID1	119.8	115	109.9	116.9	107.6	96	122.8	116.6
ID1	116.7	114.9	125.8	123.9	105.4	109.6	114.7	123
ID2	479.7	527.6	626.3	456.7	405.8	337.8	416.7	399.7
ID3	410.2	344.4	567.8	352.7	296.3	340.1	270.3	253.8
ID4	104.4	119.1	112.5	120.8	110	101.4	119.4	118.1
IDE	167.5	139.3	167.2	155.3	148.9	147.3	160.9	136.5
IDH1	300.2	246.1	407.4	262.8	206.7	273.9	210.2	182.4
IDH2	213.3	240.1	551.4	202.7	158	284	164.8	166.7
IDH3A	186.9	180.7	169.3	201.9	167.4	138.6	202.3	171.5
IDH3B	889.9	955.6	972.5	949	721.2	739.2	813.3	807.9
IDH3B	120.5	121.4	120.3	127.6	109.4	111.4	125.4	116.8
IDH3B	641.1	643.8	585.8	552	495.3	459.4	457.2	398.1
IDH3G	129.2	119.4	146.8	145.2	115.4	113.9	143.9	142.2
IDH3G	401.8	455.6	494.9	380	262.7	343.7	266.1	234.6
IDI1	157.2	164.1	204.7	162.5	164.6	153.4	184	184.3
IDI2	108.5	111.7	111.6	121.2	104	91.9	115.6	110.4
IDS	410.7	467.5	497	620	498.2	644.5	626.1	668.8
IDS	146.7	132.3	202.6	156.6	131.2	150.1	145.7	166.6
IDS	291.8	308.3	403.6	385.5	268.2	371.8	295.5	300.4
IDUA	236.9	240.7	245.8	226.5	171.1	172.7	231.7	166.4
IER2	128.3	161.3	172	135.8	139.2	138.1	151.2	142.8
IER3	546.6	990.4	453.1	899.3	586	295.1	945.9	398.9
IER3IP1	157.9	177.1	204.9	179.6	150.7	134.8	162.1	166.6

IER5	264.7	313.3	226.1	315.4	241.5	221.2	423.9	369.6
IER5L	133.1	130.9	161.7	131.9	116.3	120.3	142.1	134
IFI16	501.8	440	466.5	542.4	439.2	326.8	441.1	559.5
IFI27	111	115.8	116.6	116.5	108.7	95.7	115.2	115
IFI30	398.9	179.8	935.2	436.9	199.7	461	487.6	222.6
IFI35	373.7	384.7	470.7	337.7	233.7	292.4	205.9	181.9
IFI44	306	328.8	387.2	344	305.9	244.7	284.2	404.1
IFI44L	230.2	163.3	162.7	238.9	141	123.1	148.3	137.3
IFI6	198.1	155.5	193.7	205.6	166.6	131.4	147.2	153.9
IFIH1	369.9	240.9	292.3	370.9	261	225.2	284.1	226.5
IFIT1	173.7	145.6	148.3	182.1	131.2	116.9	142.2	125.4
IFIT1L	117.4	115	106.9	115.9	105.2	103.3	121.5	111
IFIT2	319.4	281.8	291.2	291.4	229.9	248.4	223.9	202.4
IFIT3	153.1	133.5	151.2	143.2	125.7	119.5	136.5	127.4
IFIT3	295.2	174.7	208.9	248.3	131.4	126.1	174.4	135.8
IFIT3	116.5	114.9	122.4	114.7	112.6	93.7	115.7	99.9
IFIT5	130.8	137.4	140.5	135.2	119.6	129.1	129.6	120.5
IFITM1	2233.8	2661.8	1484	1852	1081.2	859.8	803.5	594.7
IFITM2	684.3	916	619.6	639	526.6	377.7	393.1	410.2
IFITM3	146	189.3	176.7	172.4	145.9	109.6	128.8	146.9
IFITM4P	118.7	107.3	120.2	120.6	104.7	101.2	123.7	110.1
IFITM5	107.5	103.2	110.2	119.2	97.9	105.6	122.2	108.8
IFNA1	117	111.4	115.4	116.4	104.7	108.5	113.6	114.5
IFNA10	128.7	121.7	146	131.1	120.1	112.7	124.1	115.6
IFNA13	113.8	108.5	117.3	116.5	106.4	97.5	119	119.9
IFNA14	126	121.6	143.1	124.6	117.1	104.5	126	127.3
IFNA16	111.4	113.7	119.3	117	110.2	99.7	118.1	106.8
IFNA17	109.5	111.9	118	112.7	108.4	102.3	113.9	114.2
IFNA2	109.4	115.5	118.1	113.2	107.9	88.7	120.4	114.3
IFNA21	124.8	124.2	129.9	128.2	117.8	105.9	124	125.8
IFNA4	109.2	109	123.1	115.3	105	97.3	112.6	107.6
IFNA5	117.4	121.2	121.1	111.2	118.4	99	127.8	123.8
IFNA6	111.5	110.6	111.2	106.1	108.1	90.3	109.2	112
IFNA7	125.2	132.9	138.2	132.1	113.5	107.8	112.7	120.8
IFNA8	137.5	154.1	131	129.6	129.9	113.4	134.1	146.4
IFNAR1	280.7	319.8	288.3	292.4	235.3	202	250.2	252.5
IFNAR2	259.4	222.9	215.2	239.7	188.5	171	223.5	206.8
IFNAR2	188.3	235.3	270.5	189.1	175.1	205.9	154.2	170.5
IFNAR2	111.1	105.1	118.3	119	110.6	110.9	119.8	109.8
IFNB1	107.1	113.8	116.6	110.7	105.5	98.1	105.7	106.7
IFNE1	117.3	116.3	111	116.8	111.4	104.4	114.4	117.9
IFNG	142.9	121.2	155.5	133	119.2	107.7	125.2	125.2
IFNGR1	871.2	705.5	2369.1	925.3	505.4	1484.6	685.6	576.7
IFNGR2	950.9	638.3	1913.9	663.6	489.6	682.7	529.5	418.6
IFNK	113.2	105.4	126.8	130.5	109.1	92.5	119.4	109.4
IFNW1	111.4	114.6	109.8	114.6	103.3	94.9	117.1	116.6
IFRD1	143.3	169.4	144.2	145.7	138.9	127.9	163	180.3
IFRD1	104.8	112.9	117.3	123.9	111.7	107.2	122.4	115.7
IFRD2	160.1	161.3	132.9	155.6	132.6	108	161	145.9
IFRG15	117.6	120.6	121.1	125.2	108.1	96.2	121.8	117.1
IFT122	113	105.2	110.8	111.1	103.2	98.9	120.1	107.7

IFT122	121.2	121.3	123	122.1	115.9	106.8	122.4	123.9
IFT122	150.3	169.6	162.7	150.3	131.9	124.4	171.2	141.6
IFT140	114.8	119.2	121.8	124.3	108.2	99.5	120.6	119.7
IFT172	129.6	125.1	126.7	134.4	120.4	104.3	121.4	120.2
IFT52	120.5	148.8	141.2	128.5	122.5	103.1	123.2	128.9
IFT57	149.9	144.2	168.2	152.5	143.1	127.5	151.1	166.1
IFT74	138.2	145.1	163.8	143.8	137.9	120.4	134.9	141.5
IFT80	113.7	121.3	116	117.7	106.2	101	114.5	120.9
IFT81	108.5	116.9	115.3	113.6	105	101.8	124.2	113
IFT81	117.2	118.7	121.8	114.2	113.6	99.9	113.4	109.5
IFT81	120.6	126.9	123.4	120.3	114.1	111.2	117.6	116.9
IFT88	124.2	118.9	124.9	130.2	113.5	99.1	123.2	124.2
IGBP1	365.9	375.7	302.1	274.9	312.7	246.3	325.1	321.7
IGF1	114.5	113.4	117.4	124.4	108	95.8	119.2	116.2
IGF1R	115.4	126.7	124.9	125.8	117.5	102.4	127.8	122.3
IGF2	109.4	112.3	108.4	114	104.5	103.1	114.2	110.1
IGF2	109.4	118.5	116.9	116.2	107.8	95.9	124.8	121.4
IGF2AS	114.3	114.6	122.6	121.1	110.8	96.7	116.5	127.8
IGF2BP1	112.2	131.5	125.7	121.9	119.6	113.8	116.6	128.9
IGF2BP2	141.3	147.2	150.4	167.6	133.9	162.6	153.1	135.1
IGF2BP3	109.7	121.7	120.2	126	114	105.1	133.1	125
IGF2R	850.5	754.7	572.4	1072.4	928.6	429.8	1082.3	1169.9
IGFALS	124.4	122.8	104.7	117.9	102.2	101.4	123.7	107.6
IGFBP1	116.7	114.3	126.5	113.1	108	104.2	120.9	119.2
IGFBP2	111.8	111.1	128.1	124.4	112.4	96.6	114	116.5
IGFBP3	126.6	122.6	130.7	127.6	109.5	107.9	113.9	115.6
IGFBP3	125.3	124	123	131.7	122.8	105.1	119.8	123.8
IGFBP4	130.7	123.7	138.7	145.1	118.7	108.9	145.6	129.9
IGFBP5	105.3	120.2	114	125.1	107	106.8	113.3	121.1
IGFBP6	119.1	121.3	108.9	117.3	112.6	103.3	118.8	117.6
IGFBP7	128.5	134.7	144.1	142.1	114.1	106.2	130.9	125
IGFBPL1	136.6	134.8	142.8	132.4	131.2	114.3	122.7	128
IGFL1	120.5	113.3	119.3	118.6	113.4	101.4	117.4	107.9
IGFL2	110.3	109.8	106.2	110.6	103.6	96.3	112.4	106.3
IGFL3	114.1	123.7	120.7	113.1	120.9	96.2	124.8	120.9
IGFL4	115.2	112.4	120.1	116.9	110.1	103.7	115.5	106.1
IGHMBP2	134.3	144.3	155.1	127.3	136.1	122.6	139.8	157
IGJ	141.7	142.6	137.7	135.8	129.9	124.3	118.1	131.4
IGLL1	624.1	783.1	494.8	496.8	403.3	487.8	499.8	391.5
IGLL1	102.4	118.7	129.9	116.9	113.1	102	107.5	119.6
IGSF1	115.6	118.5	116.8	123.5	111.9	98.5	119.5	113
IGSF1	114.9	124.7	124.1	128.1	104	102.8	113.6	115.3
IGSF1	115.7	124	130.6	129.4	118.8	96.7	126.5	115.5
IGSF10	106.8	108.9	105.3	116.3	105	97.1	115.7	117.6
IGSF11	115.3	108.2	112	116.9	104.6	103.4	113.2	112.6
IGSF11	116.6	118.2	125.4	125.3	113.3	102.5	119.8	122.1
IGSF11	120.7	117.1	126.3	123.6	117.5	104.6	120.6	115.9
IGSF2	113	127	116.5	131.7	112.8	104.1	123.5	124.5
IGSF21	104.9	117.3	116.8	115.9	104.9	99.8	116.7	115.7
IGSF22	108	105.7	115.9	115.4	110.7	102.4	118.3	119.3
IGSF3	114.9	106.7	110.9	113.8	98.1	101.8	124	108.6

IGSF3	114.8	120.5	122.8	116	103.5	98.4	125.4	114.4
IGSF4	141.9	162	118.6	141.5	138	110.7	141	135.8
IGSF4B	117.6	114.4	114.5	114.4	112.7	103.3	118.5	118.3
IGSF4C	103.5	112.2	107.2	114.8	101.9	94.5	111	108.3
IGSF4D	109.8	110.7	109.5	112.9	105.6	92.4	112.1	105.6
IGSF6	224.7	169.7	230.7	142.3	121.7	105.6	154	123.7
IGSF8	126.7	140	134.4	119.4	124.5	108.1	123.8	133.1
IGSF9	109.7	111	122.1	117	109.8	92.9	135.4	119.4
IHH	120.4	101.1	115	118	104.7	97.2	107.2	114.5
IHPK1	119.8	123.3	125.7	122.9	116	96.1	133.6	117.9
IHPK1	324.5	318.8	288.6	346.4	253.7	270.4	466.1	586.4
IHPK1	352.5	410.7	350.1	422.5	323.1	358.2	568	701.6
IHPK2	144	154	160	152.6	138.8	141.8	159.4	173.7
IHPK2	131.8	121.7	143.1	147.8	124.2	132.5	139.8	148.2
IHPK3	133.8	118.5	138.1	128.5	130.7	106.2	121.9	133.1
IIP45	146.7	141.4	157	160.9	124.5	119.4	151.3	138.8
IIP45	113.1	105	124.3	113.6	113.9	106.6	120.8	112.8
IK	423.8	395.5	488	442.4	371.3	448.4	397.7	465.6
IKBKAP	130.8	127.4	132.5	128.6	119.1	102.5	135.1	126.1
IKBKB	144	147.5	147.3	149.6	132	124.1	145.6	124.9
IKBKE	244.7	301	231.9	248.1	178.2	180	174.1	157.4
IKBKG	505.2	573.7	644.4	749	553.6	595.9	753.2	631.7
IKIP	122.1	126.1	138	131.5	119.1	104.7	121.2	119.4
IKIP	110.6	127	122.5	115.2	105.7	107.5	123.1	122
IKIP	115.4	120.1	109.1	115.9	103.6	97.1	111.5	114
IKZF3	135.9	131.6	131.4	132.7	120.1	95.8	118.9	114.6
IL10	833.9	665.2	910.9	800.4	1035.8	607.9	789.8	899.6
IL10RA	397.3	484.3	414.8	511	315.7	408.1	381.5	342.7
IL10RB	662.4	796.4	1317	724.9	564.1	974.4	635.5	623
IL11	107.8	110.9	116.4	113.8	107.9	93.4	112.9	114.4
IL11RA	177.9	173.3	187.4	174.2	127.5	129.2	136	117.7
IL11RA	1135.9	1082.6	1318.3	874.8	643.5	687.7	481.5	375.9
IL11RA	114.9	109.3	113.8	125.4	108	99.2	115.2	118.6
IL12A	122.7	126.9	123.9	129.8	119.4	106.6	129.1	122.9
IL12B	106.5	106.9	111.2	107.5	101.7	94.7	115.5	115.6
IL12RB1	121.8	126.4	118.4	112.3	116.8	97.7	114.6	118.3
IL12RB1	185.8	170.1	162.3	161	154.9	126.5	137.8	137.5
IL12RB1	156.5	177.2	159.5	175	154.2	120.1	146.3	150.4
IL12RB2	127.5	131.7	128.1	122.4	113.1	110.2	137	133.2
IL13	100.5	102.8	110.5	111.9	97.5	95.8	123.1	104.7
IL13RA1	410	193	858.8	405.6	169	645.2	268.9	147
IL13RA2	108.8	107.6	128.9	130.1	105.9	106.7	121	108.7
IL15	202.6	186.5	224.8	226	145.5	172	167.2	147
IL15	125.5	134.1	147.3	134.1	132.1	111.3	124.5	122.1
IL15	124.1	123.2	135.7	120.3	107.2	102.4	115.9	114.9
IL15RA	124.2	111.8	110.8	128.4	106.7	98.2	134.1	121.8
IL15RA	116.5	119.2	114	127.9	113	110.5	120.7	121
IL15RA	120.3	104.5	111	117.2	107.3	95.6	117.3	116.4
IL16	159.1	195.3	206.6	185.4	190.5	179.1	172.7	172.8
IL16	192.5	196.8	247.3	214.3	188.9	247.3	189.6	158.4
IL17	111.2	115.8	122.5	113.5	104.8	96.2	116.2	103.3

IL17B	120.2	116.7	111	116.4	108.8	102.3	122.1	114.7
IL17C	119.5	121.5	111.7	117.9	118.8	117.8	118.1	115.1
IL17D	126.7	121.6	134.8	139	114.3	104	129.4	137.2
IL17E	131.9	124.9	129	127.3	110.6	108.9	119.5	128.7
IL17F	116.5	104.5	105.7	105.9	103.6	92.3	118.3	106.6
IL17R	210.9	206.8	187.6	219.5	160.7	159.4	221.7	194.6
IL17RB	101.9	120.3	119.9	114.4	110.5	97.5	111.1	110.1
IL17RC	110.8	110.4	114	123.4	106.3	105.5	121.3	114
IL17RC	118.5	122	128.3	123	113	104.5	118	119
IL17RC	130.3	132.8	124.7	130.3	119.7	106.5	120.2	127.1
IL17RD	102.4	109.4	120.9	107.5	103.3	94	115.4	115.2
IL17RD	818.9	661.1	712.8	969.5	809.3	339.6	848.6	763.2
IL17RD	111.4	108.6	113.5	110.7	109.6	98.6	112.7	118.2
IL17RE	127.4	121	131.1	133.6	105.4	96.8	119.1	130.8
IL17RE	119.3	119.5	124.2	122	115.4	105.8	117.2	107.5
IL17RE	115.3	112.6	118.3	121.2	104.5	101.1	117.8	118
IL18	4119.5	4051.1	3940.8	4049.9	4059.8	1733	3612.1	4989.1
IL18BP	108.1	104.5	113.2	114.5	102.5	95.8	112	109.6
IL18BP	365.8	219.7	1156.1	460.8	175.3	941.2	265.6	210.5
IL18R1	218	216.9	161.8	206	186.3	123.2	215.2	196.4
IL18RAP	856.9	710	643.8	843.5	542.5	422.9	733.4	588.6
IL19	121.9	110.5	116.3	116.1	106.6	93.8	124.5	116.9
IL19	118.2	119.4	122.3	127.1	108.7	104	121	122.2
IL1A	117.6	123.6	123.6	127	114	106.8	131.5	112.1
IL1B	352.8	703.4	213	594	514.1	197.4	473.1	199.2
IL1F10	116.5	103.4	105	118.8	111.7	89.2	112.2	109.6
IL1F10	116.1	113.9	122.4	114.7	111.1	99.1	120.7	111.1
IL1F5	119.2	108.3	112.3	111.8	114.7	93.8	119.2	112.3
IL1F5	108.3	105.3	112.9	101.4	113	96	118.3	113
IL1F6	113.7	114	112.6	116	105.4	93.9	109.8	113.5
IL1F7	117.9	112.7	106.2	108.3	111	100.9	118.2	106.3
IL1F7	135.7	118.2	128.9	124.5	123.2	104.4	124.2	113.9
IL1F7	118	117.5	108	113.3	111.8	102.1	115.9	120.5
IL1F8	115	118.6	124.4	119.4	110.6	100.6	117.4	127.7
IL1F8	115.7	121.7	125.8	133	108.9	99.8	113.3	110.8
IL1F8	119.7	118.5	121.2	130.3	122.2	101.2	125.4	122.4
IL1F9	112.3	107.4	108.5	113.4	105.3	93.9	115.5	109.1
IL1R1	99.9	109.8	120.8	134.5	115.7	97.4	122.9	114
IL1R2	132.3	120.8	123.3	148.3	115	110.6	124.8	116.5
IL1R2	201.5	151.2	182.8	373.6	146.3	205.2	240.7	135.5
IL1R2	111.9	110.7	116.6	115	98.3	99.9	110.1	111.1
IL1RAP	111.6	114	115	120.5	110.6	99.1	116.6	111.2
IL1RAP	121.8	118.1	121.1	115.1	111.7	100	117.2	126.8
IL1RAP	121	116	139.1	131.8	124.9	111.1	117.3	130.2
IL1RAPL1	117.6	109.2	108.3	109.6	106.4	93.9	114.1	110.4
IL1RAPL2	112.9	108.4	110.5	117.9	105.7	104.4	102	110.8
IL1RL1	111.9	113.4	107.3	116.9	106.4	102.8	112.9	110.7
IL1RL1	112.5	119.5	117.7	115.6	115.9	108.5	119.7	128.3
IL1RL1	113.3	118.9	111	123	109.7	95.9	113.5	116.6
IL1RL2	112	109.3	117.6	119.5	111.8	102.4	122	120.4
IL1RN	116.6	119.6	129.4	122.2	105.7	94.6	125.9	120.8

IL1RN	210.2	198	132.3	293.7	225	105.9	250.9	131.6
IL1RN	285.9	404.2	142.4	453.9	302.2	126	682.3	162.7
IL2	112	118.7	113.2	110.6	100.2	91.3	120.9	106.8
IL20RA	116.8	110.6	115	126.3	116	105.5	111.2	124.6
IL21	108.9	122.5	113.9	115.1	101.3	97.1	123.2	112.6
IL21R	161.7	176.7	164.4	160.7	144.7	121	166.4	160.5
IL21R	122.1	115.5	118	126.7	101.3	96.2	113.5	121.3
IL21R	116.8	107.8	124	127.9	117.5	99.4	117.1	127
IL21R	129.2	116.2	108.5	117.2	105	98.4	109.9	113.5
IL22	107.8	116.7	106.9	115.7	106.3	91.3	115.9	116.2
IL22RA1	104.2	123.7	119.7	123.5	126.8	98.6	115.9	122.8
IL22RA2	123.1	117.4	142.5	138.1	123.6	105.2	122.4	133.8
IL22RA2	112.5	110.7	125.3	111.9	109.3	100.5	121.3	116.1
IL23A	163.4	204.4	148.4	168.4	157.8	122.8	133.5	144.5
IL23R	117.8	125	107.6	124	115.3	101.4	122.1	136.7
IL24	112.1	119.5	111.4	120.2	104.6	105.6	114.5	108.9
IL24	139.3	190.2	140.7	204.9	153.9	122.1	147.6	168.1
IL24	112.7	120.4	122.9	128.1	112.7	107.9	117.2	114.7
IL25	118.1	117.8	111.5	117	108.1	99.4	112.5	115.8
IL25	114.2	129.4	135.9	132.3	111.8	105.1	126.2	119.1
IL26	111.4	118	112.1	113.4	109.7	97.6	117.5	110.3
IL27	114.4	104.8	112.4	110.9	106	99.7	115.8	108.3
IL27RA	626.4	854.6	501.1	683.5	568	606.1	743.4	548.2
IL28A	114	116.1	117.1	118.7	106.1	99.6	112.6	119.3
IL28B	115.4	117.7	134	136	111.6	112.3	137.2	117.8
IL28RA	170.4	142.7	145.1	153.8	132.1	121.8	132.1	136
IL29	118.4	120	121.3	112.5	110.9	101.5	116.6	111.8
IL2RA	114.1	120	128.8	126.8	125.7	108.1	121.7	118.5
IL2RB	1105.7	1162.6	1130.4	1111.8	660.4	683.8	603.5	547.9
IL2RG	183.4	187.9	152.4	184.3	145.5	121.3	173.4	199.5
IL3	106.2	122.8	113.8	110.5	119	95.1	123.9	107.4
IL31	117	110.9	111.3	112.2	97.2	97.8	104.9	113.7
IL31RA	114.1	116.8	118.8	117	109.6	102.4	120.6	114.2
IL32	324.2	425.6	237.2	342.1	349.1	221.2	253.2	253.1
IL32	415.4	570.1	339.7	452.2	367.3	256.9	319.7	298.6
IL33	127	113.7	119.8	114.9	108.8	93.2	129	110.7
IL3RA	143.7	116.3	134.9	127.4	121.3	123.2	134.1	124.7
IL4	104.1	117.6	115.6	118.8	112.6	99.6	125.6	117.4
IL4	123.3	115.5	123	113.1	110.9	110.8	119.1	109.1
IL4I1	159.1	123.3	142.7	148.2	115.1	103.4	128.6	116.8
IL4I1	121.2	116.2	109.9	116.5	108.3	96.3	123.6	117.2
IL4R	116.9	123.5	118.3	126.3	112.7	101	118.8	118.9
IL4R	201.1	189.3	225.3	207.5	160	154	187.2	182
IL5	108.6	106	114.6	120.4	107.4	98.6	120.7	109.4
IL5RA	127.5	138.9	131.9	132.9	118.6	112.8	129.7	128
IL5RA	119.9	110.1	113.5	115.4	108.6	96.4	113.4	116.5
IL6	118.5	119	124.3	132.5	116.1	106.4	128.5	138.5
IL6R	129.9	129.9	150	137.4	117.5	116.7	141.4	126.6
IL6R	209.5	184.9	227.4	232.2	214	145.2	220.2	179.3
IL6ST	119	119.1	113.7	133	112	102.2	128.7	121.4
IL6ST	116.1	126.6	118.7	120.4	124.3	99	127	151.9

IL7	108.5	111.8	120.7	122.8	112.7	95.5	111.4	110.5
IL7R	6358.2	7962	4474.6	6637.6	6323.9	3287.6	7339.1	8564
IL8	7966.8	13707.5	918.8	20570.9	20408.5	18566.6	32605.4	16844.7
IL8RA	115.8	131.2	134.1	139.4	111.1	114.9	118	119.7
IL8RB	127.4	131.2	131.6	135.9	116	111.4	125	128.5
IL9	125	119.3	124.3	119.7	104.8	99.6	118.1	112.8
IL9R	118.1	110.6	128.2	121.2	112.1	101.9	111.1	110.8
ILDR1	109.7	118.4	108.7	114.3	107.5	97.2	109.2	116.3
ILF2	840.6	1156.6	887.6	912.2	743.7	771.2	1293.5	1324.9
ILF3	760.6	934.8	466.5	805.8	510.7	497.3	888.9	1069.9
ILF3	123.8	130.6	141.9	127.5	117.4	112.9	121.9	120.1
ILF3	219.1	223.3	221.1	217.9	237.8	210.1	216.7	213.8
ILK	117.3	114.6	119.3	126.3	117.7	98.8	116.9	119.1
ILK	1351.3	1199	1447.9	1139.5	765	1047.8	1142.4	965.1
ILK	150.9	149.8	165.8	166.7	133.4	124.4	138.4	139.8
ILKAP	202.5	229	202.8	196.1	178.9	164.8	264.2	220.3
ILKAP	110.5	111.9	124.3	118.3	104.1	97.5	116.8	125
ILVBL	217.7	226.7	229.5	250.7	173.7	203.1	242.4	226.6
ILVBL	426.4	480.2	411	427	343.5	272.6	408.3	423.1
IMAA	115.9	110	116.6	134.2	112	101	128.9	121.9
IMAA	18335.4	16543.4	15888.8	16588.5	16329.6	12371.5	17292.8	18157.3
IMMP2L	125.4	130.8	153	145.2	127.7	134	140.9	134.5
IMMT	287.4	323.9	345.1	309	207.1	304.3	322.9	268.5
IMP3	1455.1	1629.7	1528.8	1456.3	1073.3	1051.8	820.4	739.2
IMP4	876.2	913.7	946.4	864.8	621.3	499.8	668	595.8
IMP5	118.7	124.1	130.2	123.1	118.4	106	123.6	122.7
IMPA1	238.2	206.7	232.5	217	165.8	180.5	246.4	220.6
IMPA2	178.5	150.3	352.1	188.6	146.3	303.4	222.3	194.4
IMPACT	111.5	116.1	117.4	122.3	107.1	114.9	117.6	107.7
IMPAD1	142.4	161.5	149.1	145.8	124.8	117.3	149.9	150.5
IMPDH1	249.5	239.7	346.1	214.3	167.5	205	191	162.5
IMPDH1	131.5	120.3	127.1	123.9	110.9	118.2	123.9	125.3
IMPDH1	120.5	111.5	137.2	140.3	117.1	102.6	124.1	119.9
IMPDH2	505.4	688.7	369.4	524.4	523.6	397.5	610.6	540.6
IMPG1	111.6	108.9	111.4	118.3	98.9	95.5	117.1	113.9
IMPG2	120.4	136.5	131.2	135.2	125.2	104.4	124	134.9
INA	115.6	115.1	112.6	113.6	114.4	95.9	119.6	116.8
INADL	111.5	117	126.5	122.3	107.4	111.3	114.8	124.8
INADL	115.7	123.8	113.4	123.1	105.2	95.2	112.7	126.1
INCA	117.7	113.3	121.9	115	110.6	98.5	118.8	109.9
INCA1	111.6	108.1	115.7	118.8	97.1	97.4	113.5	119.2
INCENP	135.3	136.4	128.8	139.2	121.3	106.2	143.6	131.8
INDO	136.9	120.4	131.9	147.2	119.8	108.3	117.8	113.7
ING1	110	113.8	117.9	119.6	107.9	95.3	110.1	113.2
ING1	236.4	226.7	237.6	219.5	200.1	176.6	217.7	220.6
ING1	123.3	128	132.8	126.3	139.4	108.4	129.1	126.3
ING1	113.6	120.5	120.1	112.6	107.4	97.7	127.3	126.9
ING2	179.5	202.9	211.1	199.6	163	198.7	204.5	190.4
ING3	174.2	165.9	191.2	163.8	155.7	128.5	166.5	197.4
ING3	123.8	118.6	130.3	121.5	117.5	101.8	119.7	117.9
ING3	570	659.3	548	654.1	551.7	490.8	686.5	801.7

ING4	176.4	172.3	171.6	191.7	157	146.8	182.3	172.3
ING5	119.9	119.1	121	125.3	119.3	96.8	113.4	122.6
ING5	117.4	109	121	119.6	117.1	105.8	123	115.9
INGX	117.5	123.8	121	116.9	112.8	95.9	113.6	119.9
INHA	119	118.8	114.7	125.2	116.7	105.1	115.8	120.1
INHBA	116.7	111.8	117.7	112.7	104.9	99.3	123	115.4
INHBB	118	117.3	117	119.9	111.4	103.3	113.6	122.7
INHBC	113.4	127	140.7	118.2	102.8	105.8	118.7	111.9
INHBE	127.3	116.8	131	131.8	117.6	112.4	132.2	123.1
INMT	113.5	110.9	109.6	113.7	108.7	99.6	114.4	116.9
INOC1	108.9	130.2	115.7	130.5	107.9	102.9	128.4	115.2
INOC1	202	192	184.5	223.6	193.5	155.1	207.3	200.3
INPP1	216	213.2	222.7	222.5	147.5	173.7	172	157.3
INPP4A	111.5	107.6	115.3	110.3	111.2	96.1	120.6	114.1
INPP4A	149.4	144.6	141.1	145.7	128.8	119.8	155.4	143.2
INPP4A	128.3	119.3	129.7	135.1	116	107.1	128.9	116.6
INPP4B	185.7	183.1	135.4	187.1	166.1	133.6	186.4	173.8
INPP5A	164.3	166.6	176.6	156.9	147.3	178.2	155.5	168.3
INPP5B	160.7	166.4	187.7	177.7	149.1	144.1	164.9	162.8
INPP5D	318.2	281.2	351.1	370	210.1	298.9	257.4	282.6
INPP5E	159.7	164.2	155.4	186.9	151.5	149.3	216.6	184.8
INPP5F	103.1	114.1	123.9	128.8	112.5	92.3	118.7	113.8
INPP5F	117.5	109.4	116.2	125.8	116.4	103.6	115.8	116.7
INPP5F	142.9	152.1	154.3	161.9	137.6	137.9	159.1	161.1
INPPL1	574.8	558.3	562	621.6	479	378.5	651	652.1
INS	109.3	113.1	108.2	118.3	98.8	93.3	115.2	117.2
INSC	113.2	112.6	116.4	118.8	108.3	103.2	121	120.1
INSIG1	133.8	143.5	186.1	166.4	136.1	209.8	140.9	129.1
INSIG2	334.4	396.5	322.5	393.9	299.4	270.6	311	307.1
INS-IGF2	114.7	114.7	115.7	118.3	114.8	101	115.5	118.5
INS-IGF2	114.2	116.8	118.7	120.3	109.1	99.7	116.3	116.7
INSL3	143.4	132.9	126.1	149	119.8	109.2	147.8	143.3
INSL4	116.9	123.8	120.8	117.8	116	105.3	118.1	127.5
INSL5	121.9	107	113.4	119.3	109.3	101.8	115.2	118.2
INSL6	110.9	118.7	122.8	114.6	109.3	102.2	125.9	110.1
INSM1	120.2	124.2	134.4	135.8	115.8	115.4	128.9	122.5
INSM2	108.8	111.4	111.8	112.3	105.1	103.2	129.3	112
INSR	126.7	136.9	163	137.3	122.1	112.3	126.4	147
INSRR	111	120.4	115	122.1	112	102.6	119.2	125.6
INT1	183.2	185.2	178.7	188.3	144.2	139.8	149.6	173.2
INT4	299.9	274.2	247.2	279.8	232.3	248.2	286.9	234.1
INTS1	217	335.6	243.2	299.1	208.3	165.3	254.5	239.6
INTS6	111	121.5	127.3	116.7	123.8	105.6	118.3	123.1
INTU	107.4	110.2	120.5	119.4	111.7	109	122	119
INVS	121.6	127.3	116.3	132.5	103.9	96.8	120.1	112.6
INVS	168.1	156.3	169.9	192.2	148.2	129.2	175.6	170.7
INVS	113	125.7	114.5	129.7	110.5	100.9	128.4	117.1
IPF1	111.5	117.6	107.6	117.2	107.6	94.1	128.7	108.3
IPMK	131.1	131.2	127.2	145.3	128.6	101.3	129.4	119
IPO11	265.2	307.4	253.3	273.3	218.8	208.9	255.1	252.5
IPO13	174.3	166.1	156.1	143.3	148.2	127.9	150.5	156.9

IPO4	167.5	172	146.7	157.8	136.1	118.7	163.1	138.6
IPO7	123.7	137.3	120.6	150.6	141.4	107.4	143.7	133.1
IPO8	223.4	217.2	198.7	224.6	198.9	194.4	217.2	223.3
IPO9	145.8	184.5	172.5	192.6	157.5	161.6	193.9	169
IPP	134.9	129.6	153.1	144	121	120.2	138.9	140
IPPK	122.9	120.9	132.2	129	115.9	94	124.6	121.1
IQCA	123.6	119.5	123.3	121	116.4	102.7	118.5	115.5
IQCB1	116.6	124.7	123.2	122.2	106.9	106.9	114.8	111.1
IQCB1	152.7	170.5	162.8	186.8	160.7	168.5	196.6	201.6
IQCC	134.2	119.9	122.3	124.9	125.3	102.3	119.8	119.1
IQCD	116.8	111.1	113.3	109.3	99.6	96.3	120.1	112.6
IQCE	111	118.1	114.3	119	110.1	101.4	118.4	123.6
IQCF1	124.1	113	112.9	111.7	107.1	95.9	117.7	116.3
IQCF2	120.3	122.5	125.8	125	117.8	97	121.1	111.2
IQCG	131	136.3	126.3	134.5	119.8	111.4	130	118.1
IQCH	105.6	106.8	112.6	112.8	106.3	92.8	118.6	115.9
IQCH	117.3	113.2	112.6	114.2	99.9	96.1	110.7	117.3
IQCK	126.8	136.7	138.2	136.4	122.7	110.6	122.6	127.6
IQGAP1	264.7	306	379.6	312.6	195.1	321.1	406.5	275
IQGAP2	247.1	222	196	223.5	156.9	162	175.7	164.5
IQGAP3	122.1	120.5	133.7	122.4	127.7	106.5	119.9	124.1
IQSEC1	339.6	298.6	280.9	400.6	252.2	329.4	420.7	277.3
IQSEC2	116.8	122.8	123.3	122.5	108.4	100.1	122.6	108.4
IQSEC3	104.8	113.5	121.4	119.2	117.9	104.6	125.1	115.4
IQWD1	396.6	381.5	339.4	381.2	383.4	348.9	472	533.3
IQWD1	132.3	142.5	132.3	135.3	113.6	102.1	127.7	129.7
IQWD1	122.8	124.5	122.3	127.3	117	110.6	132.6	143.7
IRAK1	339.5	327.2	269.1	328.8	269.2	252.9	267.5	247.1
IRAK1BP1	111.4	116.1	119	109.7	110.9	94.7	113.6	111.7
IRAK2	379.9	404.5	217.5	475.5	345.4	223.7	550	395.1
IRAK3	182.3	150.5	218.2	160.6	129.4	160.5	172	146.6
IRAK4	157.1	151.8	218.4	174.3	146.6	190.3	171.8	161.1
IREB2	220.5	258.4	197.7	233.6	210.3	191.5	267.9	198
IRF1	2062	1182.4	1216.8	1338.8	743.7	828.9	958	683
IRF2	117.7	127.2	124.7	128.1	106.4	107.5	121.3	130.4
IRF2BP1	166.6	165.8	144.4	159.9	149.4	134.1	170.2	149.2
IRF2BP2	306.4	307.6	388.5	244.4	254.1	227.8	252.2	281.5
IRF2BP2	174.3	189.9	166.2	197.6	213.4	130.8	212.3	178.8
IRF3	343.2	353.6	441.4	303.4	250.8	303.9	267.1	273.2
IRF4	117.2	115.5	122.5	122.7	111.1	108.4	136	136.4
IRF5	115.4	126.1	129.5	126.8	115.7	102.3	119.2	118.3
IRF6	110.7	114.9	115.5	119.4	104.3	98.1	116.5	114.2
IRF7	202.6	190.7	185.5	195	175.4	174.1	193	158.8
IRF7	122.5	112	115.6	116.5	109.2	99.7	119.7	117.8
IRF7	754.6	601.4	618.8	656.3	338.3	338.8	608.3	401.2
IRF8	921.8	486	765.4	804.6	337.1	411.3	627.3	394.3
IRGC	124.2	112.4	122.5	114.1	108.9	104.2	119.9	127.3
IRGQ	115.2	106.2	106.8	101	107.1	92.9	110.8	106.3
IRS1	117.7	121.5	113.9	131.5	115.3	102.6	121.7	120.6
IRS2	217.8	184	188.2	197.2	237.4	180.1	279.5	375.5
IRS4	120.4	122	122.9	128.6	117	102	121.6	124.2

IRX1	127.2	129.4	144.2	134.5	121.8	112	125	127.3
IRX2	108.5	118.9	120.4	117.5	115.9	110.1	119.1	115.5
IRX3	108.1	118.2	109	126.1	107.6	103	109.2	109.6
IRX4	114.6	117.1	122.9	121.3	119	96.6	125.2	113.7
IRX5	123.1	114.1	121.7	121	107.6	101.4	118	110.4
IRX6	123.8	117.5	120.5	126.2	99.4	103.1	117	114.9
IRXL1	111.6	119.9	119.6	109.6	106.5	95.3	122.1	108.9
ISCA1	192.3	169.4	176.4	203.8	183.9	160.6	210.2	199.5
ISCA2	168	183.3	201	205.5	180.7	150.8	191.5	197.1
ISCU	1945.4	1805.2	2049.9	2091.5	1459.2	1542.3	1789.9	1645.6
ISG15	247	222.7	214.4	292.9	187.7	182.5	204	158.4
ISG20	1608.1	2077.1	1499.8	1616.3	1258.7	988.5	1125	1423.4
ISG20L1	278.9	231.2	208	315.8	236	169.3	385.2	306.4
ISG20L2	339.6	380.7	402.6	460.8	352.7	384.9	505	470.5
ISGF3G	710.6	616.5	762	792.8	556.7	548.4	724.4	583.3
ISL2	121.7	125.3	129.7	144.1	127	105.5	131.8	130.2
ISLR	102.3	114	115.2	110.6	103.1	105.7	111.3	105
ISLR	111.7	121.8	119.1	119.4	108.1	102.2	116.6	122.3
ISLR	115.1	117.8	124.5	123.4	113.4	94.9	125.9	121.1
ISLR2	110	117	115.7	119.2	110.5	98.3	114.9	115.5
ISOC1	144	189.5	190.8	172.8	166.1	163.8	180.7	175.9
ISOC2	127.1	131.9	156.8	132.2	113.8	123.3	148.6	133
ISYNA1	143.5	134.9	135.3	154.3	124.8	115.7	141.5	141.5
ITCH	261.9	311.5	283	299.3	305.9	212.4	280.4	262.4
ITFG1	112.9	121.6	111.3	129.1	119	98.5	116.7	118.2
ITGA1	128.4	110.7	128.7	125.9	109.6	102.6	124.4	114.3
ITGA10	158.9	148.3	131.9	157.4	149.4	119.4	190.9	187
ITGA11	116.1	113.3	112.7	112.8	108.1	102.2	116.9	110.8
ITGA11	122.8	125.8	127.2	111.8	108.8	110.8	129.4	110.5
ITGA2	112.9	113.4	108.2	122.5	118.2	96.6	123.5	110.3
ITGA2B	115.6	108.9	120.4	122.8	104.8	102.9	117.4	138
ITGA3	114.1	116.7	121.3	117.4	114.3	101.8	116.3	112.1
ITGA3	166.7	160.9	171.7	195.8	165.4	152.1	195.5	219.5
ITGA4	249.1	228.3	253.7	285.7	236.5	173.1	198	186.5
ITGA5	342.8	327.9	430.1	367.4	234.7	283.8	421.7	400
ITGA6	161	159.3	148.6	185	136.9	114.5	176.4	162.4
ITGA6	113.7	128.4	111.7	122	102.9	98.3	119.7	112.5
ITGA7	112.9	123.1	112.9	117.4	105.6	104.6	109.4	121
ITGA8	105.9	112.2	106.5	113.3	105	90	109.6	108.9
ITGAD	106.2	107.9	120.8	113.4	116.1	104	115	118.9
ITGAE	220.8	266.4	301.5	280.8	259.1	257	225.6	217.4
ITGAL	514.4	676.8	666.4	764.5	441.5	525.9	460.6	376.6
ITGAM	157.5	153.8	149.8	141.7	139.3	110.1	124	128.8
ITGAV	151.8	164.4	297.5	171	158.4	213	180.6	195.7
ITGAX	340.5	245.6	226.8	384.2	234.6	334.1	392.6	180.9
ITGAX	113.7	117.6	118	122.1	113.5	111.5	121.8	119.2
ITGB1	294	309.7	465	342.6	282.4	388	260.2	289.1
ITGB1	1259.3	1494.1	1707.6	1780.5	1101.1	1797.4	1562.6	1362.7
ITGB1	763.4	772.2	1103.5	936.6	675.8	881.7	676.3	617.8
ITGB1	108.1	109	118.3	128.3	109.8	102.7	112.6	108.7
ITGB1	111.9	113.9	122.2	122.8	118	97.6	125.1	109.6

ITGB1	114.6	104.2	120.8	117.1	101.6	97.9	112.4	115.8
ITGB1BP1	290.2	170	223.6	277.6	172.9	195.2	334.8	251
ITGB1BP1	179.8	330.9	166.3	204.4	192.9	180.3	196.1	272.4
ITGB1BP2	118.1	117.6	120.5	122.3	106.4	106.3	117.2	126.6
ITGB1BP3	122.8	114.1	120.9	114.9	115.1	106	130.3	118.9
ITGB1BP3	114.7	113.1	119.6	116.4	109	96.1	118.4	113.5
ITGB2	7940.1	7781.8	8946.7	6789.6	4752.2	4685.8	4735.6	3047.8
ITGB3	116.8	112.1	113.6	110.7	106.6	89.3	112.4	111.7
ITGB3BP	130.8	148.6	160.9	153.3	145.6	128.1	138.2	141.2
ITGB4	115.4	103.6	110.1	108.7	110.7	97.5	119.9	108.6
ITGB4	102.5	112.5	110.6	114.1	109.2	92.7	122	111
ITGB4	113	111.9	124.6	115.3	99	98.1	119.6	114.2
ITGB4	118.5	112.4	123.6	127.4	112.8	114.2	127.8	122.3
ITGB4BP	539.2	583.1	448.5	590.8	429.7	459.7	662.1	501.1
ITGB4BP	114.6	117.9	111.6	137.1	122.4	108	123.7	136.2
ITGB4BP	827.5	821.9	788.9	1091.7	654.2	728.8	1186	874.8
ITGB5	112.8	119.2	166	121.3	112.7	123.1	117.4	124.6
ITGB5	138.6	135.6	393.3	143.3	122.3	193.4	147.4	128
ITGB6	111.2	118.3	114.8	115.9	104.7	96.4	115.9	118.9
ITGB7	1136.5	1218.4	1019.9	1029.7	781.5	1032.9	785.1	609.3
ITGB8	117.6	118.2	117.4	117.4	111.9	106	120.2	121.3
ITGBL1	121	117.8	116.1	114.6	111.4	113	122.4	122.3
ITIH1	121.2	126	127.6	116.1	105.9	101.6	120.5	116.3
ITIH2	110.6	123.8	121.7	118	110.1	100.1	113	116.4
ITIH3	121.8	115.8	117.8	112	122.5	107.6	123.1	132.8
ITIH4	130.7	132.1	113.8	129.7	117.7	108.3	123.3	115.1
ITIH5	115.7	115.7	112.7	116.9	98.7	95.3	117.4	112.8
ITIH5	10169	10355.9	10729.6	10341.9	11561.8	4907.5	9722.5	10021.2
ITIH5	114.4	120.7	119.6	112.7	105	96.7	119.5	117.5
ITIH5	111.3	117	119.8	119.7	110.1	102.5	118.5	120.6
ITIH5L	107.6	119.4	117.1	120.6	110.6	93	111.1	115.4
ITK	2040.3	2618.1	1579.7	2540.9	2142.8	1180.6	2171.7	2323.7
ITLN1	116.4	120.5	117.8	117.3	111.2	97.5	122.4	123.5
ITLN2	112.3	118.2	108.1	113.3	102.6	93.7	108.9	117.7
ITM2A	170.1	161.9	150.2	181.9	145.7	137.6	163.5	156
ITM2B	107.5	129.3	125.4	125.9	112.7	118.2	125.2	125.6
ITM2C	353.6	492.9	441.9	376.8	259.8	332.1	243	279.2
ITM2C	489.3	617.3	604.7	449.6	418.3	413.1	347	424.3
ITPA	162.2	158.8	185.7	200	167.8	132.6	159.4	191.4
ITPA	254.1	274.3	257	295.1	218.7	199	241.8	262.7
ITPK1	169.1	187.5	155.6	174.2	142.5	132.7	211.6	178
ITPKA	125.7	146.5	125.7	118	114.1	109.1	125	127.5
ITPKB	426.7	536.9	375.2	463.2	386.6	323	429.5	384.4
ITPKC	130.2	139.8	131.5	122.1	119.8	112.9	137.2	130.1
ITPR1	278.6	284	281.2	293.7	257.9	244.7	279.8	218.7
ITPR2	146	156.2	171.8	152.7	154.6	143.8	144.5	161.4
ITPR3	1226.1	1185.6	1248.1	1430.9	1005.1	833.1	1170.9	1240
ITR	181.5	162.9	191.9	209.6	154.8	142.3	152.3	146.9
ITSN1	109.6	121.9	121.2	115.1	103.1	100.3	118.2	118.1
ITSN1	111.6	110.7	118	116.9	102.9	100.5	111.7	112.4
ITSN1	147.6	143.5	136.1	161.4	125.2	110.1	147.1	155.2

ITSN2	119.1	117.2	139.4	109	112	108.9	132	130.6
ITSN2	109.4	117	119.3	113.9	113	101.5	117.9	116.4
IVD	154.1	149.1	149.3	143.5	122.9	110.3	131.1	124
IVL	118.5	116	124.4	126.5	110.4	105	125.4	128.2
IVNS1ABP	362.7	361.8	475.8	462.3	393.8	334.5	397.5	403.3
IVNS1ABP	296.1	402.4	307.6	369.6	328	259.3	323.5	269.4
IVNS1ABP	130.5	122.6	145.1	130.5	121	104.6	125.8	129.6
IXL	290.5	246.3	294.6	296.7	216.6	217	266.4	215.9
IZUMO1	111.5	117.3	119.7	118.5	100.4	94	115.9	112.5
JAG1	113.7	114.3	120.5	116.3	105.9	110.7	126.5	118.5
JAG2	129.6	127.5	130	136.4	115.6	102.6	143.8	147.1
JAG2	126.2	118.9	115.6	119.7	121.3	101.6	114.9	123.6
JAG2	111.2	122.4	124.6	130.1	115.3	99.7	125	122.4
JAK1	736.2	793.3	970.5	824.5	798.9	741.2	629.4	775.4
JAK2	160.9	151	209.3	153.5	139.8	150.4	144.7	139.5
JAK3	115.2	120.6	122.4	125.2	103.3	98	122.3	108.2
JAKMIP1	123.3	124.2	126.9	112.7	109.7	99.8	116.2	110.5
JAKMIP2	165.6	149.6	131	148.8	136.4	103.4	130.9	136.8
JAM2	115.9	117.1	116.8	118.4	109.3	98.7	114.2	110
JAM3	254.4	318.2	229.3	234.8	270.9	162.5	204.6	243.3
JARID1A	95.5	113.8	110	116	99.4	94.3	111.5	98.9
JARID1B	274.9	298.1	280.5	294.3	252.6	227.6	265.5	331.3
JARID2	341.6	310.9	334.6	378.8	348.8	363.4	332.5	293.5
JAZF1	173.4	216.1	245.2	177.7	138.8	177	164.4	160.8
JDP2	120.5	130.7	154	143.1	127.4	115.9	151.9	122.8
JM11	165.2	171.3	155.8	173.7	147.3	122.8	191.7	189.1
JMJD1A	138.2	182.3	157.3	193.6	176.7	155.8	182.4	235.4
JMJD1B	197.6	221	167.1	216.5	184	133.3	176	209.9
JMJD1C	459	449.2	471.7	510.5	400.6	502	441.2	468.3
JMJD1C	334	340	405.1	379.7	334.1	302.3	418.7	373.9
JMJD2A	122.8	114.6	125.3	124	118	103.9	137.9	117.2
JMJD2B	114.4	116.6	128.1	119.4	110.5	114.8	119.3	112.3
JMJD2C	123.2	121.5	128.2	128.2	124.9	107.5	131.4	117.5
JMJD2D	114.1	107.1	118.1	134.1	107.8	96.5	124	118.4
JMJD3	119.1	144.3	127.1	128.3	123.6	111.1	147.6	130.9
JMJD4	151.6	150.7	143	153.9	128.1	114.5	159.1	139.1
JMJD5	116.1	120.5	122.8	119.4	111.2	102.1	121.7	117.1
JMY	126.8	139.6	124.1	127.6	131.1	114.1	155	155.3
JOSD1	183.3	182.1	148.2	165.3	183.8	128.3	247.6	234.6
JOSD2	133.7	128.9	137.1	136.1	121.4	113.5	131.7	123.4
JOSD3	138.2	128	140.4	141	115.3	117.2	141.6	121.4
JOSD3	176.7	152.1	187.4	205.4	173.8	141.1	211.8	176.1
JPH1	106.9	120.5	114.8	127.2	104.9	93.5	116.3	112.9
JPH2	118.6	134.9	129.3	133.9	116	106.9	128.6	119
JPH2	125.1	124.9	125.2	128	117.9	103.7	128.4	128.6
JPH2	114.7	105.1	118.1	112.3	104.4	103.3	115.4	109.5
JPH3	109.2	128.6	140.7	123.3	112.1	101.4	120.2	120
JPH4	118.2	113.2	122.4	128.8	115.3	112.2	125.2	123.2
JRK	117.9	117	112.6	123.7	113.5	99.8	119.8	107.9
JRKL	118.3	117.6	121.1	127.8	118.1	107.2	131.7	124.8
JSRP1	133	128.9	127.4	133.1	119.2	103.1	126.4	125.9

JTB	4781.7	4554.1	5168.5	4858.5	4500.8	4229.4	5609.6	3978.9
JTV1	301.7	334	370.9	335	248.3	214.1	284.2	283.6
JUB	119.9	119	140	130.2	114.1	106.6	123.2	119.3
JUB	109.2	109.8	111.7	115.3	102.8	92.5	112.4	119.9
JUB	110.9	106.4	121.2	120	109.8	95.8	115.2	118.3
JUN	337.1	334.4	626.8	425.1	303.1	535	496.6	548.4
JUNB	169	175.9	163	189.1	155.2	138	200.6	172
JUND	4207	4874.5	3596.1	4749.2	3460.2	3993.9	6094.1	4671.4
JUP	112.7	112	121.1	130.2	119.4	97.3	118.9	121.9
JUP	127.9	118.4	117	133.3	109.4	102.5	116.5	116.4
JUP	165.4	153.1	149.6	140.8	123.6	109	133.7	115.6
K5B	114.8	115.1	116.1	123.1	109.9	109.4	116.3	116
K6HF	114.3	109.5	117.2	121.5	114.2	105.8	114.5	115.3
K6IRS2	413.2	915	294.8	397.1	800.9	234.3	299	546.2
K6IRS3	210.6	270.4	163.3	206	191.7	124.8	154.2	161.6
K6IRS4	113.3	120.4	113.5	110	117.8	97.3	108.3	114.7
KA21	112	109.7	120.1	114.9	106.1	98.5	114	111.1
KAAG1	114.1	114.8	116.7	113.5	111.8	96.9	116.2	116.9
KAL1	112.4	116.4	114.8	120	105.1	95.4	121.9	104.2
K-ALPHA-1	3126.5	3104.7	5690.3	3051.8	2278.6	2927.6	3122	3739.2
KALRN	132.5	126.1	132.9	135.8	123.8	107.3	120.3	117.4
KALRN	116.5	113.8	110.1	106.6	107.5	99.3	118.3	109.9
KALRN	127	113.5	107.7	111.4	100.9	100.1	112.5	111.6
KARCA1	118.9	120.7	122	118.5	115.4	105.7	118.3	110.9
KARCA1	100.3	122	117	116.6	111.3	98.3	115.7	118.3
KARS	292.3	324.6	330	338.4	262	318.8	256.4	273.4
KATNA1	279.4	373.1	356.7	425.4	358.7	312.4	422.8	446.8
KATNAL1	132.5	129.4	122.3	137.2	122.5	108	126	118.9
KATNAL1	118.9	129	124.4	133.1	134.2	114.6	145.9	145.4
KATNAL1	113.5	116	117.1	119.1	109.3	92.8	113.8	121.7
KATNAL2	110.1	125.4	127.6	119.6	110.7	103.9	119.9	123.3
KATNAL2	122.7	116.9	127.9	123.7	106.6	107.5	118.5	112.2
KATNB1	240.5	253.5	239.8	255.6	197.1	193.2	249.2	223.3
KAZALD1	106.1	113.8	115.6	121.9	109.6	98.9	127.2	114.7
KBTBD1	114.2	121.5	138.9	138.9	120.9	108	125.3	129.6
KBTBD10	105.7	110.1	117.3	118.4	117.1	98.4	126.1	116.2
KBTBD11	337.8	411	323.6	350.2	298.3	263.3	323.4	310.9
KBTBD2	550.4	618.8	498.1	577.9	476.6	446.1	757.9	963.9
KBTBD3	125	122.2	120.5	122.9	115.7	107.2	117.6	121.1
KBTBD3	134	132.1	161.4	139	128.9	114.6	151.7	151.7
KBTBD4	112.2	115.2	137.8	121.9	109.1	97.2	116.8	112.6
KBTBD4	138.5	135.1	132.8	134	120.5	110.5	129.9	137.6
KBTBD5	109.8	105.6	123	118.9	97.5	94.4	110.5	109.7
KBTBD6	117.1	130.5	135.1	128.6	114.2	105.1	133.2	120.2
KBTBD7	156	181.9	156.7	174.4	152.5	128.7	152.7	141.6
KBTBD8	194.9	185.6	175.3	189.3	178	142.8	199.5	224.8
KCMF1	122.5	113.3	119.5	134.4	117.7	110.6	132.8	142.2
KCMF1	114	111.1	123	125.1	115.1	107.9	128.1	136.7
KCMF1	129.2	142	140.4	141.2	153.6	112.1	157.3	177
KCNA1	108.9	117.3	117.5	117.2	109.8	97.4	128.8	122.5
KCNA10	104.4	108.6	106	110.2	103.5	96.3	109	106.6

KCNA2	113.8	108	115.3	111.4	108.6	96.7	113.5	115.3
KCNA3	126.9	141.9	123.9	132.2	124.1	97.9	136.6	141.9
KCNA4	113	109.3	118.7	112.3	110.7	94.5	115.1	120.1
KCNA5	117.7	120.6	121.5	119	107	95.8	113.2	115
KCNA6	111	125.1	121.3	127.3	126.3	110	123	129.7
KCNA7	121.8	130.1	125.1	129.3	111.8	104.4	118.4	113.2
KCNAB1	126.5	118.4	126.8	130.7	118.3	96.2	123.1	129.9
KCNAB1	119.1	117	121	118.4	105.1	98.8	124.7	122
KCNAB1	119.7	117.6	122.5	111.6	105.1	99.7	121.5	115
KCNAB1	112	114.8	121.8	112.9	107.3	99.3	111.4	120.4
KCNAB2	122.3	125.7	132.6	133.4	121	108.3	143.3	113.8
KCNAB2	119.3	119.3	112.2	130.1	107.8	105.1	126.8	123
KCNAB3	122.3	126.6	117	117.4	114.1	103.5	120.6	112.6
KCNB1	114.2	125.1	123	122.2	114.5	108.2	127.9	120.6
KCNB2	114.2	119	109.7	117.4	109.5	104.1	111.1	117.2
KCNC1	114.1	115.2	115.9	116.5	110.3	105.6	120.1	110.4
KCNC2	104.3	104.1	104.8	106.5	102.6	92.9	111.7	102.7
KCNC2	114.6	106.7	117.6	112.5	109.6	91.4	112.2	121.6
KCNC3	117.5	111.9	115.3	116.3	105.9	97.3	112.3	118.4
KCNC4	114	117.3	112.1	124.7	112.6	101.3	110.9	119.3
KCNC4	119.2	105.8	127.5	114.1	116.9	97.6	112.5	116.4
KCNC4	106.2	109.2	109.3	118.1	105.1	97.3	112	112.1
KCNC4	120.1	114	118.4	125.1	111.8	96.9	112.3	115.2
KCND1	119.8	118.3	122.8	121.6	109	104.6	123.2	124.1
KCND2	113.4	113.7	111.7	117.1	109.1	101.5	105.4	115
KCND3	128.5	119.5	111.9	126.7	103.4	102.4	122.4	119.3
KCND3	127.5	124.7	121.6	125.4	119.9	113.3	120.8	116.9
KCND3	114.4	117	116.5	114.6	106.3	99.2	116.1	112.7
KCNE1	107.5	120.5	109	110.1	99.3	90.5	113.9	110.3
KCNE1L	124.5	118.1	119.8	126.7	116.7	109.7	122.1	118.9
KCNE2	113.9	112.9	117.1	122.2	117.3	99.9	117.9	115.5
KCNE3	116.9	111.3	109.4	114.1	103.5	103.2	122.2	108.8
KCNE4	111.1	106.5	114.8	109	110.2	94.7	117.2	114.5
KCNF1	102.6	114.1	112.4	119.4	106.8	98.5	117.6	111.4
KCNG1	185	155.7	170.5	182	121.6	115.9	147.5	121.1
KCNG1	112.4	114.3	125	125.4	108.9	102.5	111.7	117.4
KCNG1	113	114.4	116.1	119.2	107.8	96.5	113.4	112.2
KCNG2	126.3	128	127.7	134.6	133.8	111.6	153	149.4
KCNG3	109.2	110.7	118.4	109.5	101.6	94.4	109.4	106.7
KCNG3	109.1	109.2	108.7	109.3	112.8	94.1	121.8	111
KCNG4	107.7	111.5	108.6	120	110.1	93.8	118	111.6
KCNG4	111.7	117.6	109.5	114.5	104.6	96.6	107.4	109.2
KCNH1	121.3	104.9	116.9	121	110.8	101	118	107.5
KCNH1	111.2	111.7	114.3	111.2	103.8	97.6	115	104
KCNH2	112.3	107.6	112.2	116.4	106.7	104.3	117.6	117.3
KCNH2	120.5	122.7	118.5	127.3	116.3	95.4	121.4	118.8
KCNH2	127.2	132.4	128.8	142.5	126.7	99.5	126.9	126.4
KCNH2	108.9	118.7	119.2	124	109.6	100.9	106.1	115
KCNH3	160.5	134	150.2	145.9	114.5	118.7	151.6	115.7
KCNH4	125.4	116.6	126.7	119.2	107.1	102.4	123.8	117.9
KCNH5	110.5	119.5	110.3	117.9	104.5	98.5	114.8	120.1

KCNH5	120.8	116.1	115.4	113	110.5	94	116	115.2
KCNH6	122.3	109.5	110	113.2	111.1	94.5	113	105.2
KCNH6	1914.4	2488.8	2921.5	2926.9	2264	1122.9	3284.8	2044.8
KCNH7	123.4	110	115.8	124.5	109.7	99.5	115.5	115.1
KCNH7	111.4	116.7	123.1	123.9	118.5	106.6	115	117.7
KCNH7	112.7	117.1	126.1	126.6	110.2	100.1	112.1	122.9
KCNH8	123.6	123.5	126.9	119.8	107	102.9	123.4	133.1
KCNIP1	121.8	113.3	116.8	118.9	109.5	108.3	120.1	118.4
KCNIP1	109.5	107.7	117.2	111.4	100	97.5	117.4	109
KCNIP1	107.6	119.1	113.9	115.4	107.3	93.4	120.8	108.6
KCNIP2	119.9	119.5	122.8	119.5	116.5	99.9	122.8	115
KCNIP2	120.1	112.3	124.9	117.4	104.8	106.5	112.8	120.7
KCNIP3	117.8	118.6	116.4	122.1	113.7	105.7	119	113.3
KCNIP3	130.6	122.1	124.1	114.7	118.2	106.8	118.6	117.4
KCNIP4	113.8	109.6	120.8	117.7	119	97.2	116	108.5
KCNIP4	121	119.9	125.7	113	120	98.8	117.5	113.8
KCNIP4	118.7	109	113.9	121.7	106.4	98	122	118.4
KCNIP4	107.6	101.7	116	122.3	113.5	103.4	112.3	108.3
KCNIP4	110.7	116.1	117.3	118.2	103.5	93.5	135	112.6
KCNIP4	114	116.2	120.6	105.1	113.5	97.5	114.9	116.2
KCNJ1	114.6	108.8	117.4	117.1	104.5	94.9	125.3	110.8
KCNJ1	111.5	118.7	117.4	122.4	109.1	100.7	116.4	121.8
KCNJ1	103.9	109.6	117.1	115.7	111.8	96.8	125.1	110.5
KCNJ10	109.5	115.7	112.9	114	111.3	93.6	116.2	119
KCNJ11	120.1	111.5	108.2	112.5	104	98.2	103.5	109.6
KCNJ12	113.8	111	109.4	115.7	109.4	95.3	111.4	104.3
KCNJ13	116.1	117.1	125.8	122.4	111.7	100.3	124.4	115.9
KCNJ14	112.9	115.3	116.6	127.8	111.8	96.8	115.4	108.4
KCNJ14	113.5	124.1	116.4	122.1	106.8	102.3	119.9	122.8
KCNJ14	119.9	132.5	130.6	132.3	119.4	106	129.3	128.6
KCNJ15	136.3	137.4	128.8	156.4	128.9	105.8	163.4	132.6
KCNJ15	114.2	115.8	120.3	122.3	120.5	102.1	129.4	119.4
KCNJ15	107	117.3	128.5	123.3	111.6	107.1	135.3	122.3
KCNJ16	117.6	113.1	120.7	123.7	116.8	94.7	125.7	116.2
KCNJ2	117.7	124.6	132.5	132.1	108.4	110.8	116.1	109.2
KCNJ3	121.5	113.7	106.6	124.8	103.6	102.1	116.6	120
KCNJ4	113.9	127.1	116.8	115.9	108	100.4	121.2	117
KCNJ4	108	116.6	123.8	120	114.9	99.9	120.2	110.4
KCNJ4	114.7	117.7	113.5	112.6	108.4	99.5	121.6	113.7
KCNJ5	119.9	119.7	127.1	125.6	114.5	102.1	111.9	119.3
KCNJ6	115.3	119	121.3	111.8	108.7	103.5	112.3	114.8
KCNJ8	115	124.8	124.5	128.8	111.8	105.7	129.7	120.9
KCNJ9	108.9	113.3	107.7	121	114.1	97.2	122.4	113.7
KCNK1	115	116.4	116.1	110.9	107.2	105.8	120.5	126.4
KCNK10	115.1	114.6	119.1	116.8	107.3	108.7	128.1	112.2
KCNK10	117.2	127.6	129.4	129.3	106.8	112	126.4	119.6
KCNK10	110.1	114.6	116.5	115.6	102	105.6	116.4	106.4
KCNK10	112.5	112.8	109	115.1	108.9	99.8	114.9	112.6
KCNK12	117.8	128.8	168.3	129.8	121.9	113.9	119	121.8
KCNK13	105.7	115.8	143.1	128.7	99.3	112.1	131.7	116.3
KCNK15	125.1	125.9	121.4	126.3	117.7	115.2	120.2	131.3

KCNK16	113.1	107.9	118.7	115.8	115	103.4	124.8	112.5
KCNK17	124.5	112.7	117	117.4	106	110	113.4	102.6
KCNK18	110.8	125.9	118.8	120.7	104	104.4	120.2	117.1
KCNK2	118.8	121.2	118	118.1	110.5	95.4	111.3	114.1
KCNK2	110.4	118.5	109.3	113.8	103.4	102.5	122.7	113.3
KCNK2	106.3	113.4	111.8	103.9	106.4	92.1	114.7	107.8
KCNK2	116.6	113.4	124.9	118.5	110.2	102.9	118.5	122.2
KCNK3	116.9	125.9	119.3	124.7	111.9	106.3	117.7	117.5
KCNK4	114	113.7	113.7	113.3	115.4	93.7	113.4	120.1
KCNK4	115.5	110.4	124.8	123.9	114.4	101.2	119.1	110
KCNK5	127.1	120.1	122.7	125.6	113.8	108.2	119.5	119.6
KCNK6	128.5	128.9	137.4	133.6	122.3	110	130.8	130
KCNK7	126.6	129.5	126.3	132.2	113.9	102.7	128.1	127
KCNK7	109.1	125.9	113.9	119.5	108	98	119.3	121.5
KCNK9	110.7	120.1	125.1	119.1	112	99.8	127	124.8
KCNMA1	109.9	114	111.3	115.6	112.7	105.7	108.2	115
KCNMA1	114.7	126.3	120.7	133.6	116.4	121.2	130.4	119.4
KCNMA1	110.7	114	107.3	110.1	101	99.4	114.4	114.7
KCNMB1	140.8	112.2	226.4	122.7	110.9	102.1	120.6	116.2
KCNMB2	108.6	131.5	122.8	120.9	118.6	96.9	119.7	118.9
KCNMB2	117.6	112.4	110.1	108.6	104.8	96.6	121.5	109.8
KCNMB2	110.6	125	111.3	114.1	114.1	100.6	118.3	108.8
KCNMB3	110.6	115.8	111.9	108.6	107	98.9	114.5	106.5
KCNMB3	127	115.8	125	123.2	110.3	104.3	132.7	113.7
KCNMB3	145	123.1	127.1	135.4	136.1	114.8	127.9	122.8
KCNMB4	127.3	127.5	143.2	132.8	126.1	114.4	124.2	129.2
KCNN1	114.4	123.2	123.5	125.1	112.6	99.4	118.9	124.7
KCNN2	112.8	122.5	124.9	111	103	103.1	118	125.9
KCNN2	114	121.1	122.3	119	104.3	103.6	118.2	111.9
KCNN3	113	109.6	121.4	121.1	105.3	92.2	119.6	109.2
KCNN3	116.4	107.2	127.5	117.7	106.6	106.6	119.2	116.2
KCNN4	142.7	136	138.8	140.1	123.4	115.5	135.5	123
KCNQ1	119.4	113.2	111.3	129.5	108.4	98.8	117.9	120.8
KCNQ1	123.7	142.6	142	141.1	126.4	106	150.7	145.5
KCNQ1	114.5	124.9	111.3	116.6	109.6	97.8	110	112.1
KCNQ1DN	102.9	118.5	110.9	122.4	105.9	90	113.9	119.8
KCNQ2	104.9	111.7	116.9	125.4	104.5	94.9	123.9	112.7
KCNQ2	117	130.7	105.6	123.5	118.2	97	129.9	118.4
KCNQ2	123.2	115.3	108.1	122.2	107.9	105.5	118.9	121.9
KCNQ3	140.5	133.2	127.3	130.7	108.3	105.6	121.9	121.1
KCNQ4	115.6	119.5	114.2	118.7	105.5	96.7	114.1	115.1
KCNQ4	108.2	108.1	118.4	118.7	91.1	90.1	116.8	109.4
KCNQ4	120.6	131.1	118	122.5	112.5	107.1	116.2	122
KCNQ5	115.1	111.5	118.7	108.1	109.3	97.3	109.2	114.5
KCNRG	106	97.2	118.1	131.5	109.3	97.2	110.4	96.1
KCNRG	141.5	116.3	127.9	132.6	118.6	106.8	137.5	127.5
KCNS1	117.5	119.5	122.8	132.4	114.1	102.9	121.3	116.6
KCNS2	116.5	117	115.8	110.6	108.8	95.8	108.2	112.8
KCNS3	106.2	108.4	107.1	114.9	106	94.6	119.9	121.6
KCNT1	104.7	112.1	108.6	124.6	103.4	101.9	113.9	116.2
KCNT2	112.3	109	115	122.1	107.3	100.6	115.9	114.3

KCNV1	105.5	113.6	111.1	119.7	103.6	97.6	120.6	122.2
KCR1	122.3	117.3	119.2	122.8	112.8	102.7	141.4	114.2
KCTD1	121.4	117.3	119.3	134.1	115.1	107.1	128.5	119.9
KCTD10	203.1	160.5	201.8	198.9	147.4	181.3	183.6	157.6
KCTD11	129	127.3	148.2	149.2	121.2	127	133.2	126.1
KCTD12	117.5	112.2	118.1	119.5	105.5	106.6	116.6	113.8
KCTD13	261.5	264.9	229.1	300	233.1	232.3	316.5	256.4
KCTD14	114	115.2	117.8	115.8	111.4	100.1	128.3	115.6
KCTD15	123.6	120.1	127	124.4	100.4	100.7	119.9	113.9
KCTD16	111.5	116.7	121.5	123.8	109.9	93	118.8	112.4
KCTD17	119.7	113.2	117.1	114.8	99.5	106.2	121	120.7
KCTD18	160.7	161.8	172.5	159.3	141.8	135.8	146.9	156.7
KCTD2	117.4	112.9	120.5	132.2	107.9	101.5	129.9	119
KCTD2	189.6	193.3	181.1	209	177.1	174	222.1	174.8
KCTD3	129.8	127.1	127.8	128.3	115.7	99.7	113.9	123.6
KCTD4	113.5	114.6	116.4	115.4	104	95.7	128.3	113.5
KCTD5	482.2	476	585.2	588	406.7	514.8	732.4	715.6
KCTD6	464.4	460.4	412.2	450.4	314.2	446.4	459.2	467.5
KCTD7	127.1	119.8	132.4	134.9	127.9	116.7	131.4	125.3
KCTD8	117.2	115.7	118.9	110.7	107.6	100.4	112.6	119.1
KCTD9	189.1	197.6	241.3	230.4	218.5	202.2	246.9	252.7
KDELC1	101	118.1	126.2	117	105.6	100.7	115.5	109.4
KDELC2	143.2	152.5	162.2	145.3	130.4	127.9	173.5	155.7
KDELR1	282.3	267.8	388.9	299.1	265.7	362.5	276.6	278.5
KDELR2	204.2	198.3	252.6	196	179.6	197.4	193.6	216.3
KDELR3	129.1	115.7	118.3	134.6	113.3	112.2	116.5	115
KDELR3	112.6	111.1	122.1	117.3	114.7	96.6	115.7	122.2
KDELR3	106.5	110.1	113.2	115.1	101	97.8	110.2	117.5
KDR	113.6	117	119.7	117.9	105.3	103.2	114.1	121.9
KEAP1	218.3	222.9	162.2	254.4	172.5	222.1	292.8	304.5
KEAP1	116.8	107.5	117.4	123.2	106.6	98.5	112.4	109.7
KEAP1	170.4	206.6	199.9	265.1	270.2	221.4	348.1	327
KEL	120.2	115.7	115.4	121.5	112	102.3	143	118.3
KENAE	125.9	121.1	117.2	122.8	111.4	103.8	119.4	124.1
KERA	117.3	118.8	116.2	109.5	112.3	98.1	118.2	121.6
KGFLP1	97.5	111.3	106.5	112.1	111.2	92.4	115	112.9
KHDRBS1	1253.3	1211.2	1335.9	1317.7	1119.2	1077.3	1770.8	1562.2
KHDRBS2	119.3	113.9	113.7	132.1	106.3	107	113.8	109.4
KHDRBS3	109.3	107.7	110.4	119	116	102.9	123.5	113
KHK	121.6	127.6	118.4	134.1	121.9	105.6	113.4	110.1
KHK	119.3	120.9	121.9	119	126.7	104.3	114.6	120.8
KHK	119.5	123.3	119.2	130.5	117.3	104.4	120.1	119.1
KHSRP	299.7	287.4	235.7	292.4	234.4	186.5	311.2	293.9
KIAA0020	338.6	354.5	300.3	355.4	325.3	258.8	287	348.5
KIAA0082	123.2	115.5	127.4	122.9	116.9	119.2	123.3	117.5
KIAA0090	183.3	193.6	179.9	204.4	184.5	163.4	205.9	168.7
KIAA0100	116.4	125.5	120.2	121	114	100.1	118.6	122.8
KIAA0101	111.9	118.3	115.6	120.2	106.5	99.9	119.3	111.7
KIAA0101	118.2	111.5	127	132.4	113.5	104.8	117.5	110
KIAA0101	8255	6325.4	6670.4	6497.9	7611.1	5080.5	7815.9	8400
KIAA0103	135.7	131.8	131.5	137.7	113.5	115	133.9	121.8

KIAA0133	138.3	147.7	146.5	147.7	125.1	123.6	168.7	143.8
KIAA0141	284.3	327.6	325.1	285.5	207.5	223.3	269.6	223.6
KIAA0143	911.6	819	702.6	929	713.2	796	829.5	645.9
KIAA0152	291.3	354.3	445.6	350	303.6	297.2	325.1	292.4
KIAA0157	311.8	347	359.1	368.2	259.1	271.8	334.9	295.7
KIAA0174	1360.6	1502.5	1332.9	1614.3	1147.5	1005	1504.6	2033.9
KIAA0179	158.8	179.4	162.6	185.2	158	139.2	134.6	134.4
KIAA0182	285	248.9	249.2	232.9	224.5	187	232.8	203.7
KIAA0195	202.4	198	167.8	189.1	182.4	131.8	183.4	169.9
KIAA0196	444.9	433.6	472.6	458.5	391.8	394	382.8	364.6
KIAA0232	138.8	140.9	130.7	150.9	148.8	116.3	144.7	163.4
KIAA0240	649.2	676.3	446.2	744.3	625.9	455	871.2	837.9
KIAA0241	126.6	137.1	126.4	150	124.4	116	145.7	135.1
KIAA0247	293.5	254.1	270.3	301.4	213.8	297.7	248.9	216.4
KIAA0251	127.5	130.9	152.7	138.4	117.7	109.7	132.5	141.5
KIAA0256	343.7	257.2	302.3	315.7	252.2	224.8	300.4	272.9
KIAA0258	117.6	118.5	124.6	118.2	108.9	102.1	114.3	114.5
KIAA0261	230.6	257.5	247.4	243.2	251.6	199	290.9	280.5
KIAA0265	125.7	125.1	118.4	123.8	115.4	104.5	126.1	116.3
KIAA0274	178.5	176.1	188.3	193.1	162.8	145.6	170.1	181.4
KIAA0280	117.6	119.6	135.8	116.3	107.7	100.8	130	119.9
KIAA0284	113.5	110.6	116.3	116.9	99.1	95.9	127.2	115.1
KIAA0286	174	182.1	169.1	243.8	168.2	182.7	220.4	241.3
KIAA0310	569	539.2	536.3	676.9	569.5	605.8	788.2	785.1
KIAA0319	117.4	122.1	118.3	125.2	109.2	109	125.5	111.1
KIAA0319L	125.4	157.1	136.8	151	133.3	115.8	144.9	145.3
KIAA0319L	179.5	174.9	177.9	161.7	144.7	148.3	166.2	149.3
KIAA0319L	126.9	118.7	120.1	121.7	114.9	105.5	114.8	122.7
KIAA0323	309.1	332.2	269.9	373.4	239.6	332.8	384.7	481.2
KIAA0329	110.6	112.3	114.3	119.6	110.8	99.9	113.7	116
KIAA0350	423.6	384.6	307.2	378.8	356.6	226.8	407.4	367.3
KIAA0355	621.7	633.6	641	692	748.6	480.4	729.2	796.1
KIAA0367	108.8	111.8	120.4	121	108.8	100.9	110.3	115.2
KIAA0368	142.4	128.7	145.1	127.5	124.9	116.3	155.1	162.5
KIAA0372	323.4	303	258.3	306.7	281.9	239.5	302.2	318.5
KIAA0376	862.2	1143.9	691.8	1096.4	856.5	514.5	1017.1	856.8
KIAA0391	382.8	483.2	332.8	401.6	364.1	321	326.5	303.7
KIAA0404	247.6	284.8	213	288.5	190.3	193.3	317.8	361.7
KIAA0406	127.9	139.2	134.1	142.9	115	114.4	123.5	128.3
KIAA0408	140.4	161.9	161.1	148.6	136.1	118.6	153.4	157.4
KIAA0409	129.9	167.6	153.3	133	142.4	125.5	144.3	137.1
KIAA0415	145.9	145	147.5	153.9	119.9	115.7	181	161.9
KIAA0423	167.5	157.6	156.8	158.4	135.5	123.2	153.6	156.8
KIAA0427	138.4	129.7	136	138.7	121.3	128.1	161.5	135.7
KIAA0446	232	231.2	245.7	285.9	219.5	183.3	214.4	244.8
KIAA0460	152.3	139.3	127.3	138.9	134.4	126.9	160.5	136.5
KIAA0467	119.4	114.1	112.8	121	105	102.7	116.8	112.1
KIAA0494	446.8	529.7	451.1	525.4	359.9	407.9	370.3	343.9
KIAA0495	144.4	143.9	131.3	130.2	130	139.6	131.8	135.4
KIAA0513	218.3	227.7	287	170	155.1	194.7	180.7	169.3
KIAA0514	109.5	118.3	102.5	108.2	106.4	97.2	115.5	113

KIAA0523	118	123.8	123	112.3	111.6	110.5	124.8	121.9
KIAA0528	349.3	356.9	330.9	361.7	267.1	231.9	228	216.1
KIAA0553	124.3	125.3	132.1	130.7	130.3	101.9	140.8	130.6
KIAA0556	153.9	163	155.7	137.9	139.9	122.7	141.4	144.2
KIAA0562	162.5	157.3	143.7	158.7	160.2	127.5	197.2	177.2
KIAA0564	133.6	127	129.7	134.5	119	114.3	117.7	119.9
KIAA0564	129.2	148.8	129	139.7	131.1	110.2	129.3	127.7
KIAA0586	141.1	130.6	155.3	160.4	136.3	119.8	132	140.2
KIAA0644	114.5	121.7	116.8	119.5	103.4	103.2	116.9	111
KIAA0649	123.4	120.1	117.3	123.6	106.8	103.6	130.6	118.7
KIAA0652	115.5	122.3	128.3	118	117.5	97.8	128.4	117.9
KIAA0664	172	175.7	154.3	170.3	153.7	132.5	199.3	190.5
KIAA0672	121.9	116.3	113.2	106.4	99.4	101.5	110.5	108.7
KIAA0683	194.9	155.9	161.2	166	143.4	126.5	159.5	180.1
KIAA0690	143.9	169.7	161.4	150	143.5	118.4	136.4	177.1
KIAA0701	125	122.8	119.3	118.3	109.6	111.3	133.3	127.1
KIAA0703	111.2	114.8	115.1	116.9	116.8	95.6	125	114.9
KIAA0738	1702.9	1915.4	1822.3	1642.6	2160.8	1240.3	1677.3	1746
KIAA0746	346.5	316.2	293.9	323.6	297.3	293.3	389.7	415.7
KIAA0753	117.4	127.4	148.2	128.3	113.2	111.7	134.6	119.5
KIAA0773	122.4	129.2	127.5	127	121	113.8	126.3	125
KIAA0773	113.5	130.7	123.5	124.2	111.4	104	123.3	116.1
KIAA0773	108.2	104.3	113.8	110.4	100	103.5	110.8	121.1
KIAA0774	118.7	111.7	113.7	119.8	109.9	95.4	118.5	115.8
KIAA0774	117.4	114.7	108.7	116	110.1	95.6	117.7	111.9
KIAA0774	109	102.2	114.3	119.5	106.7	97.2	117.6	108.7
KIAA0776	131.7	130.8	134.8	144.2	123	112.1	129.2	122.9
KIAA0789	106.9	125.7	125.1	123.8	113.8	99.8	133.7	118.6
KIAA0802	105.8	112.2	114.2	111.8	107.1	92.5	120.1	112.8
KIAA0828	160.2	169.5	175.3	176.9	148.6	140.3	192	163.2
KIAA0831	215.7	189.3	233.6	207.4	170.3	175.9	203.8	241.9
KIAA0853	115	104	118.4	115.7	109.2	97	108.2	112.2
KIAA0859	199.3	233.2	228	215.5	196.2	184.6	226.4	231.8
KIAA0859	461.2	544.7	505.3	585.7	383.9	353	469	529.5
KIAA0892	258.2	288.4	296.5	317.5	275.9	295.7	359.7	320.7
KIAA0895	125.1	114.2	126.1	128.2	103.4	97.6	119.4	113.2
KIAA0907	395.1	397	372.3	465	309.2	191.7	437.5	475
KIAA0913	481.1	473.9	426.2	576.3	385.6	426.5	657.4	537.4
KIAA0922	248.1	223.4	243.2	212	226.7	204.5	207.3	196.5
KIAA0961	119	126.5	127.1	121	136	107.4	121.9	121.6
KIAA0963	155.7	158.8	149.1	153.3	120.5	122.6	132.5	121.4
KIAA0980	106.7	106.4	116.4	114.1	117.8	98.5	126.9	126.5
KIAA0999	326.8	295.4	230.5	311.7	291.9	216.6	360.7	478.6
KIAA1005	120.3	119.2	118.3	120.3	110.3	97	117.4	109.8
KIAA1008	119.7	120.2	131	136.5	131.2	106.6	141.5	122.8
KIAA1012	788.1	971.3	740.6	984.6	774.7	538.9	921.1	870.9
KIAA1024	122.3	118.1	116.6	127.9	112.6	97.6	116.6	131.6
KIAA1026	120.7	119.5	133.6	128.7	114.3	95.8	120.4	115.4
KIAA1026	118.2	120.6	112.6	114.6	114.5	94.4	122.1	110.2
KIAA1026	133.2	121.5	119.7	123.3	104.7	93.7	116.1	113.6
KIAA1026	123.7	128.6	121	126.7	108.5	97.3	114.6	117.6

KIAA1026	111.3	105.9	122.8	118.1	118.1	102.6	116.6	118.7
KIAA1033	136.8	126.8	139.9	133.9	123.5	113.1	159.8	139.6
KIAA1128	471.2	753.1	540.7	488.4	608.2	460.1	578.1	708.3
KIAA1143	105.8	116.2	109.7	118	101	99.7	119.9	109
KIAA1147	789.4	1103	1284.9	1094.3	1162.3	1192.6	937	891.9
KIAA1160	175.9	190.1	219.4	196	177.6	158.2	167.7	193.8
KIAA1161	124	110.3	123.4	120.1	125.3	105.4	126.2	122.6
KIAA1166	126.1	123.8	140.2	136.3	122.4	106.9	132.7	128.5
KIAA1171	108.8	112.3	113.9	120.8	110.8	95.1	112.3	115.5
KIAA1189	117.7	106.5	115.3	119.9	106.8	99.3	114.8	111.3
KIAA1189	115.5	116.1	113.2	121.1	102.3	88.8	117	109.8
KIAA1189	123.1	123.1	120.4	124.4	103.3	102.4	128.6	114.8
KIAA1191	374.4	428.2	498.1	426.5	419.3	413.9	437.9	391.2
KIAA1191	361.2	359	304.6	360.4	295	236.3	321.1	354.4
KIAA1193	126.1	123.1	131	134.4	123.6	114.9	136.9	130
KIAA1199	106.1	115.5	115.5	118.3	112.6	103.5	117.5	116.6
KIAA1212	148.5	129.5	125.8	133	125.3	111	134.5	140.2
KIAA1217	118.3	114.5	115	113.1	107.8	99.5	114.6	107.3
KIAA1219	212.7	224.6	208.7	229.6	188.4	160.1	246.9	252.2
KIAA1244	128.4	108.3	130.4	121.9	111.2	93.4	123	126.6
KIAA1267	837.7	675.3	741.2	782.9	729.6	538.7	837.2	1087.9
KIAA1274	125	116.6	133.1	131.9	107.7	115.5	123.9	130.4
KIAA1279	127.6	126.5	123.2	129.1	116.3	105.7	132.4	140.2
KIAA1285	104.6	112.7	114	131.8	106	107.9	125	120.1
KIAA1287	188.6	151.5	171.5	164.8	166.3	150.4	176	210.9
KIAA1324	128.5	145.3	131.5	137.3	124	108	142	137
KIAA1324L	160.4	150.9	152.6	149.9	145.8	130.6	138.3	151.8
KIAA1328	120.2	129.9	139.2	123.8	111.5	102.9	121.6	122.9
KIAA1333	123.4	123.9	135.4	132.9	133.4	113.1	132.3	142.5
KIAA1344	124.2	120.1	120.7	120.5	119.6	110.2	138.4	120.5
KIAA1345	107.2	118.7	119.9	110.5	97.8	92.5	123.1	113.4
KIAA1370	243.8	251.3	491.4	331	255.2	300.8	292.9	343.3
KIAA1377	111	117.8	112.6	123.1	105.7	97.4	108.8	111.7
KIAA1407	130.6	134.1	140.3	131	121.2	108.7	117.3	130.1
KIAA1409	107.8	118.3	114.1	114.4	103.2	106.1	125.6	114.7
KIAA1411	145.2	140.7	133.6	171.9	139.1	112.5	156.5	129.2
KIAA1429	149.6	142.8	148.3	141.1	124.9	119.8	128.3	128.6
KIAA1432	121.4	118	115.8	114.5	107.1	98.9	125.5	113.1
KIAA1434	172	197.9	235	191.9	198.7	173.4	213.5	223.1
KIAA1443	120.7	119.1	123.5	120.3	105.7	100	125.5	124.1
KIAA1446	123.8	131	127	127	119.2	109.3	123.7	125.9
KIAA1456	110.5	116.6	116.6	119.4	109.6	107.4	119.3	112.4
KIAA1467	129.2	128.8	123.4	134.4	121	110	120	114.8
KIAA1468	140.8	156.1	179.8	166	149.1	133.6	138.8	162.4
KIAA1505	134.6	139.9	157.6	155.4	158.2	127.3	135.2	149.8
KIAA1522	118.8	119.4	115.8	123.8	113.3	101.5	125.9	118.2
KIAA1524	114.7	115.8	118.2	123.6	115.7	105.4	113.9	110.6
KIAA1529	110.3	112.9	113.6	121.6	115.9	98	127.4	113.3
KIAA1530	277.8	216.2	290.6	281.5	252.6	216.1	283.5	258.5
KIAA1530	118.8	115.3	127.9	126.9	120.4	100.4	129	122.1
KIAA1539	196.7	178.4	219.6	208.5	147.7	223.7	253.7	188.7

KIAA1542	121.3	115.8	123.6	114.9	107.9	100.8	133.8	127.5
KIAA1543	115.8	120.2	123.5	114.2	113.2	101.2	124	126.3
KIAA1543	106.8	118.5	108.1	117.2	115.6	97.5	121	114.9
KIAA1553	117.3	155.1	150	131.9	113.1	104.6	122.1	127.9
KIAA1553	111.7	123.6	124.6	123.9	121.8	104.3	128.4	140.4
KIAA1559	120.9	130.6	135	124.6	115.6	106.8	120.9	129.3
KIAA1576	126.2	119.8	124.9	122.7	118	104.1	124.8	131.1
KIAA1586	129.6	125.6	113.1	136.8	109.4	105	115.7	114.3
KIAA1598	279.5	186.6	366.8	275.1	146.7	332.8	205.9	131.9
KIAA1600	398.3	326	389.3	423	323.6	390.9	388.3	440.8
KIAA1604	119.1	127	121.8	124.4	116.2	115.3	124.2	147.5
KIAA1609	123.2	117.6	120.8	126.3	103.7	105.6	128.4	111.7
KIAA1618	233.5	242.7	221.8	333	242.3	183.2	283.2	288.5
KIAA1622	111.6	115.3	116	121.7	116	104.8	122.4	115
KIAA1622	105.5	113.5	103.7	118.6	106.1	91.9	112.8	118.6
KIAA1622	112.7	113	112.3	126.7	108.6	99.5	116.6	121.1
KIAA1627	123.9	110.5	121.3	142.6	119.2	111.1	121.4	132.6
KIAA1632	131.7	121.3	140.4	130.9	118	107.5	140.6	137.5
KIAA1641	127.8	128.5	128.9	129.2	119.4	109.2	130.1	121
KIAA1683	138.5	135.3	139.3	139.4	136.7	103.4	156.7	227.4
KIAA1688	121.2	114	115.7	122.1	104.4	97.8	118.8	114
KIAA1698	188.7	239.4	163.4	200	187.4	156	202.4	210.6
KIAA1704	121.7	118	123.7	115.7	103.3	92.5	120.2	114.3
KIAA1706	137.7	122	157	153.4	119.5	152.9	156.9	131.4
KIAA1712	399.8	384.7	484.7	449	306.6	349.9	387.8	319
KIAA1715	267.8	283.5	262.4	293.6	222.6	218.2	236.6	236.3
KIAA1727	111.8	115.8	120.9	119.7	111.2	96.8	121.2	112.4
KIAA1729	393	332.4	380.7	397.3	392.1	323.6	349.3	370
KIAA1737	263.1	235.8	234.2	246.1	190.3	220.2	228.2	198.7
KIAA1751	433.5	442	492.7	694.1	503.1	331.6	531.4	495.7
KIAA1754	308	357.1	248.3	338.7	234.7	236.3	305.6	294.2
KIAA1754L	128.7	134.6	123.5	139.2	131.5	104.9	148	138.4
KIAA1754L	119.3	119.6	123.8	130.3	120.4	106.7	141.1	129.2
KIAA1754L	116.5	116	112.9	119.4	112.5	97.4	115.6	114.2
KIAA1755	115.8	113.5	132.9	135.1	115.4	116.3	128	127.2
KIAA1772	120.6	115.4	113	112.9	106.6	100.1	118.7	114.5
KIAA1787	174	181.1	153.2	169	164.6	129.6	198.1	176.3
KIAA1794	126.6	139.4	119.7	130.4	120.6	106.8	126.2	118.1
KIAA1797	123.8	135.2	119.2	136.6	127	106.2	119.1	141.2
KIAA1799	114.8	110.4	122.4	118.8	105.9	97.5	121.8	116.8
KIAA1804	119.5	109.2	122.2	125.9	111.9	106.1	125.8	132.2
KIAA1815	143.6	129.5	147.5	173.3	153.2	139	143.2	138.8
KIAA1822	107.3	112.6	111.3	116.3	111.2	100	117	110.7
KIAA1822L	134.2	127.5	136.5	134.6	115.2	109.5	125	125.9
KIAA1826	298.4	294.5	287.1	286.2	255.8	214.6	252.2	304
KIAA1838	525	661.4	686.9	531.5	536.7	533.7	495	488.2
KIAA1840	606.1	551.8	548	550.2	370.3	445.3	439.6	479.5
KIAA1841	111.6	101.6	111.8	113.6	112.5	91.3	113.1	118.1
KIAA1853	111.9	114.8	128.1	124	109.6	103.5	120.5	129
KIAA1862	149.2	155.1	143.4	141	132.4	113.3	157.4	127.1
KIAA1875	154.9	149.5	155.7	151.4	124.1	115.2	141	145

KIAA1893	111.9	115.9	115.3	112.3	106.8	104	124.8	117
KIAA1900	106.7	127.4	127.3	120.6	112.7	97.1	117.2	113.4
KIAA1904	125.9	116.9	129.8	114.7	121	114.2	118.9	126.5
KIAA1909	100.3	112.5	118.9	114.1	107.2	94.5	105.6	116.5
KIAA1912	120.9	103.9	114.7	110.5	105.3	108.2	113.7	124.1
KIAA1913	115.8	132.5	120.3	121.8	114	103.4	121.8	119.5
KIAA1914	164.7	150.9	136.5	140.7	122.3	109.7	138	125.3
KIAA1919	111.8	116	121.2	124.5	117.6	105.4	110.1	104
KIAA1920	118.6	118.3	125	119.7	106.2	101.1	119.1	116.1
KIAA1924	110.9	116.5	118.9	129.2	117.2	114.5	113.8	112.2
KIAA1944	110	124.8	114	110.7	107.1	90.8	127	118.2
KIAA1946	117	129.9	121	129.5	109.9	102.6	116.4	123.1
KIAA1958	122.7	134.7	130.3	134.6	120.7	102.5	143.6	134.3
KIAA1961	109.4	115.1	115.2	119.4	109.7	96.5	112.3	117.7
KIAA1961	322.3	354.3	289.6	416.8	318.6	228.1	353.2	433.2
KIAA1967	183.7	197	161	196	163.3	150.6	184.4	208.6
KIAA1967	116.3	117.1	115.3	122.9	118.4	97.9	116.6	132.5
KIAA1967	120.6	124.4	123.5	130.7	116	105.7	126.2	121.4
KIAA1970	150.9	145	167.2	150.2	145	126	155.5	136.9
KIAA1984	114.8	122.8	119.4	119.2	106.2	106.5	123.1	117.8
KIAA2010	118.8	107	118	113.7	103.2	94.1	117.4	112.9
KIAA2010	434.7	504.5	410.7	513.5	584.3	397.2	558	673.7
KIAA2013	268.5	256.8	227.4	255.7	189.7	173.2	229.1	214.3
KIAA2018	97.8	119.8	111.6	119.5	109.3	106.8	109.5	127
KIAA2022	111.6	118.3	124.7	118.8	111.9	102.8	113.7	118.1
KIAA2026	252.8	304.8	254.3	300	320.1	239.8	355	306.8
KIDINS220	393.4	445.2	406.9	447.6	390.5	451.3	449.5	506.7
KIF11	117.3	115.8	111.8	112.7	117.2	98.1	127.8	121.6
KIF12	109.9	112.2	112.3	115.2	108.6	97.8	115.7	107.8
KIF13A	115.7	128	120	116.6	109.4	106.4	120.4	120.6
KIF13B	230.2	236.4	260.3	272.4	200.5	205.6	302.4	305.4
KIF14	135.3	131	119.8	123.9	120.2	103.5	128.7	116.7
KIF15	113.4	107.2	117	119.5	106.2	96.7	112.3	116.6
KIF17	115.3	110.5	116.9	122.9	106.6	100.9	117.2	120
KIF18A	117	124.7	116	115.7	104.1	92.9	122.7	114.9
KIF19	116.3	119.7	128.4	124.7	123	109.1	124.5	111.8
KIF1A	111.8	118	115.8	120.4	108.7	91.7	110.3	112.1
KIF1B	125.1	117.8	121.9	123.7	116	106.5	136	117.7
KIF1B	123.2	129	129	140.2	123.9	111.6	159.9	143
KIF1B	424.2	408.7	327.7	544.5	402.2	307.5	567	359.8
KIF1C	131.1	123.4	134.7	153.2	109.6	117.4	132.9	123.7
KIF2	532.8	612.5	290.4	625.2	510.9	334.5	657.1	536.2
KIF20A	109.9	116.4	113.1	110.1	100.4	95.3	115.4	110.2
KIF21A	137.7	124.8	146.9	167.6	135.2	104.5	143.1	157.7
KIF21B	114.8	109.9	114.7	115	109.8	94.7	113.1	110.5
KIF21B	162.3	133.8	167.2	144.8	134.3	133.5	173.9	164
KIF23	111.9	118.6	109.9	120.9	112.3	98.5	119	111.7
KIF23	117	121.8	115.6	120.9	114.7	103.3	123.6	118.3
KIF25	119.6	110.3	110.8	115.2	102.2	88.4	106.8	103.9
KIF25	128.1	117.6	129.1	129.7	111.3	106.3	124.8	117.3
KIF26B	106.5	110.3	114.4	105.8	117	94.2	111.5	107.3

KIF27	127	119.2	130	130.2	106.2	99.1	123.4	135.1
KIF27	116	116.4	117.4	115	121.7	96.5	120.3	115.7
KIF2B	108.8	119.8	116	126.5	110.8	93.9	115.5	119.3
KIF2C	118.7	112.4	121.9	122.1	113.8	101.7	129.8	118.4
KIF3A	116.4	102.8	107.3	123.8	114.2	95.3	113	111.1
KIF3B	167	176.9	150.9	170.7	171	166.6	160	151.4
KIF3C	119.6	131.9	127.2	129.7	119.4	110.2	119.7	126.4
KIF4A	125.7	119.8	126.1	120.3	109.4	96.2	115.8	118.4
KIF5A	114.9	119	113.3	117.8	112.1	101.6	107.9	110
KIF5B	176	172.5	190	188.7	160.4	160.7	186.2	175.9
KIF5C	218.9	215.3	159.9	206.3	170.4	151.3	223.5	198.8
KIF6	130.1	132.4	132.1	135	121.7	109.4	125.9	139.1
KIF7	116.6	127.1	119.3	111.9	105.4	106	124.8	123.1
KIF9	112.4	119.7	122.5	114.1	108.2	98.5	113	117.8
KIFAP3	248.8	273.2	237.6	235.8	257.2	214	228.6	244.8
KIFC1	119.2	111.8	122.9	124.2	114.3	98.9	122.9	118.3
KIFC2	186.1	157.5	154.4	171.5	168	161.9	195.9	190.9
KIFC3	109	124.9	126.8	121.1	108.2	109.9	122.9	124.2
KIN	117.1	115.3	114.7	124.9	109	103.6	111.3	115.1
KIR2DL1	130.8	238.8	140.1	132.6	202.1	112.3	137	177.5
KIR2DL3	119.3	131	133.6	129.9	118	119.1	122	124.6
KIR2DL3	256.1	297.1	264.5	274.7	263.9	310.7	228	251.5
KIR2DL4	107.1	122.7	117.8	124.2	117.3	110.3	123.9	117.8
KIR2DL5A	121.9	128.8	133.2	127.9	122.7	109.4	111.1	128.5
KIR2DL5A	117.7	110.6	123.7	124.3	109	106.4	110.8	119.3
KIR2DL5A	119.6	116.7	119.2	113	102.7	104.3	110.9	111.5
KIR2DL5B	117.4	113.1	124.8	123.1	122.3	102.7	111.8	125.7
KIR2DS1	128.6	108.7	114.6	134.6	110.6	96.5	123.8	129
KIR2DS3	111.1	117.4	118.9	116	107.1	99.2	119.8	126.1
KIR2DS4	122.6	118.7	114	122.4	119.5	110.1	118.8	117.1
KIR2DS5	133.1	128.2	122.1	133.7	119.3	107.1	120.6	142.5
KIR3DL1	144.6	173.5	163.7	146.2	127.6	136.5	141.5	145.1
KIR3DL2	125	128.8	129.6	129.3	128.4	115.9	124.7	131.7
KIR3DL3	127.1	132.5	125.7	136.9	117.9	109.9	122	124.9
KIR3DP1	119.4	125.3	128.4	131.3	120.4	98.9	121	118.9
KIRREL	113.7	113	120.3	113.2	115.4	105.4	113.2	109.2
KIRREL2	120.4	115.5	125.4	121	114.7	104.4	114.3	109.2
KIRREL2	113.5	118.8	113.4	126.2	108.8	100	127.7	111.5
KIRREL2	113.5	116.3	112.3	122.4	102.1	97.1	111.6	114.8
KIRREL3	118.4	127.5	122.7	113.2	110.1	100	113.8	116.3
KISS1	117.2	123	111.3	126.1	104.4	99.6	121.9	129.6
KISS1R	110.5	119.6	128.3	128.1	112.9	106.8	112.7	112.4
KIT	171.3	155.5	176.5	152.2	138	117.9	134.2	128.4
KITLG	108.4	115.6	117.4	125	112.1	156	127.1	113.5
KITLG	112.2	115.4	113.8	118.6	106.9	96.7	120.2	115
KL	106	121.2	106.9	113	101.7	98.2	105.1	107.1
KLB	115.2	123.4	115.3	125.9	116.9	102.5	116.3	113.6
KLC2	117	123.7	121.6	117	106.5	107.5	117.5	128.7
KLC2	126.6	116.4	121.6	120.2	109.2	105.3	117.6	130
KLC3	114.5	109	121.5	116.1	104	95.3	130.2	115.6
KLC3	117.4	117.1	132.2	118	113.8	94.3	115.9	122

KLC4	122.1	111.1	128.9	124.5	109	95.6	121.3	117
KLC4	113.8	124.7	113.1	111.1	106.4	101.9	121.6	104.5
KLC4	115.5	129.6	124.8	125.7	122.2	97.4	120.9	121
KLF1	116.1	112.2	127.9	128.8	105.9	100.2	117.3	112.5
KLF10	121.5	112.4	129	129.4	113.2	105.8	131.3	122
KLF10	121.3	127.3	136.9	132.9	124.1	107.5	132.8	120.8
KLF10	109.8	110.2	130	112.5	109.2	106.1	130.3	125.7
KLF11	131.9	109.7	131.9	127.8	113.6	100.5	123.8	123.4
KLF11	211.6	206.1	284.3	223.9	186.5	206.3	241.2	307.1
KLF12	196.4	192.6	169.8	180.2	175.5	174.8	207.3	190.8
KLF12	129.3	127.4	129.1	133.2	121.6	107.9	126.1	135.4
KLF13	1033.5	1286.2	1220.6	1439.8	922.7	1143.4	994.5	1122.4
KLF14	107.2	111.2	112.2	114.7	110.6	104.9	128.7	118.2
KLF15	117.5	133.7	118.6	125.2	129.8	114	121.5	124.2
KLF16	119.7	114	112.4	108.3	107.6	94.9	116.4	119.6
KLF17	117.8	128.6	119.3	115.7	106.4	94.2	110.3	125.5
KLF2	3374.4	3309.7	2927.9	3112.9	3289.8	2735.6	3759.9	3348.7
KLF3	120.9	140.7	129.3	140.7	122.8	107.1	121.9	124.2
KLF4	131.7	126.4	132.5	122.4	115.3	116.4	141.2	142.8
KLF5	117.7	115.5	111.8	117.1	111.5	96.2	118.7	119.8
KLF6	413.3	330.8	411.7	516.2	436.8	454.8	493.6	528.2
KLF6	1383.5	1259	1503.6	1583	955.3	1026	1420.9	1611.2
KLF7	122.8	124.2	131	132.1	118.9	101.5	121.5	115.8
KLF8	115.9	136.8	126.8	127.6	116.2	116.8	131.1	118.1
KLF9	531.9	559.9	777.6	538.9	393	769.4	382.8	438.2
KLHDC1	111.8	115.4	110.8	114	103.7	107.9	112.1	111.2
KLHDC2	353	357.8	471.4	396.7	463.4	349.7	321.2	448.4
KLHDC3	457.1	587.2	562.8	476.9	431.1	540.7	438.5	474.9
KLHDC4	231	185.2	246.4	230.9	156.5	196.4	225.2	182.8
KLHDC5	176.9	195	228.8	224.7	195	182.3	166	191.5
KLHDC6	119.8	109.6	123.8	116.6	103.9	96.3	113.1	115.4
KLHDC7A	123	127.4	118.8	137.2	109.7	105.7	121.9	129.4
KLHDC7B	105.8	119.5	122.7	116.5	113.6	101.1	127.1	122.8
KLHDC8A	114.5	119.1	132.7	139.5	118.2	104.7	128.1	146.4
KLHDC8B	171.7	192	155.8	175.1	153.8	121.2	168.4	167.6
KLHL1	118.3	123.2	112.6	122.8	117	98.1	122.4	107.3
KLHL10	107.3	111.6	118.1	115.4	107	100.2	120.2	112.6
KLHL11	110.4	119.4	121.6	117.1	107.4	95.3	119.9	128.6
KLHL12	404.9	412.2	442.9	475	329.1	429	388.6	373.1
KLHL13	109.8	125.3	113.1	121.4	114.1	101.7	116.4	111.7
KLHL14	123.8	118.5	115.2	120.4	111.2	101.1	112.4	113.4
KLHL15	103.7	118.3	119.3	112.9	108.4	103.3	114.2	116.6
KLHL17	118	114.3	117	123.4	110.8	98	119.6	119.8
KLHL17	157.3	174.8	147.5	162.7	124.2	119.3	167.3	137.3
KLHL18	121.2	138.8	141.9	141.8	124.8	119.5	138.9	121.8
KLHL2	144.4	168.1	153.8	178	142.7	134.9	182.5	163.6
KLHL20	178.3	153.6	161.3	193	179.7	150.6	151.6	176.6
KLHL21	184.1	218.2	160.3	261.1	200.3	177.6	395.3	446.3
KLHL22	648	681.8	560	709.7	523.3	397.4	459.7	489.4
KLHL23	116.2	120	126.3	117	100.3	100	124.3	116.2
KLHL25	112.3	119.7	116.7	135.3	112.3	111	126.9	119.3

KLHL26	130.7	137.6	127.1	139.4	119.4	108.7	149	136.1
KLHL3	280.5	280.2	330	315.1	298.8	215.7	226.1	228.1
KLHL4	107	110.5	109.8	117.4	106.3	94.3	107.5	112.2
KLHL4	109.2	107.2	111.7	111.3	102.6	100.8	112	108.5
KLHL5	122.3	124.1	140.3	129.5	112.4	102.5	120.3	119.1
KLHL5	415.4	429.9	491.4	530.9	338.9	397.5	500.5	363.7
KLHL5	230.3	167.6	193.1	209	148.2	151.9	217.9	157
KLHL7	171.2	240.9	211.2	244.4	183.3	170.4	223.8	199.5
KLHL7	110.2	118.1	113.4	120.1	104	95.8	118	117.9
KLHL8	365.6	431.6	371.6	418.9	322.4	228.7	337.9	313.4
KLHL9	247.1	252.4	341.9	277.2	221.9	287	223.5	193.3
KLK1	115.8	127.5	117.2	122.8	109.8	97.5	131.6	111.5
KLK10	104.8	115	111.9	113.6	111.4	96	122.1	98.6
KLK10	121.6	127.8	131.2	128.7	115.4	112.5	131.9	129.9
KLK11	124.7	119.1	119.2	126.6	110.4	105.4	120.3	121.5
KLK11	120.6	119.9	122.1	116.2	110.9	105.7	115.6	112.3
KLK12	116.5	123.2	123.8	132.6	115.8	101.1	126.1	112.5
KLK12	115.2	128.5	123.9	120.2	117.8	105.1	121.4	116.9
KLK13	106.3	112.4	114.7	109.8	102.8	91.6	115.2	117.4
KLK14	118.9	118.1	125.3	119.7	112.7	99.6	122.6	113.5
KLK15	112.8	127.1	117.5	122.8	108	101.2	120.3	118.6
KLK15	110.4	108.2	109.5	123.2	103.5	95.2	120.9	104.6
KLK15	115.2	109.1	119.1	120.1	111.6	102.1	110.3	117.9
KLK2	110.3	120.3	112.6	113.4	106.5	98.5	120	112.6
KLK2	118.8	121	129.3	124.3	109.9	106.1	123.8	116.9
KLK3	115.5	114.7	116.4	115.5	102.8	101.9	116.6	122.5
KLK3	110.3	110.9	123	114.6	106.6	93.3	107.3	116.7
KLK3	118.6	126.7	125.3	116.7	121.1	104.9	123.2	123.5
KLK4	117.8	111.3	114.8	115.9	108.8	100.7	119.6	115.5
KLK5	108.8	114.2	110.1	112	105.2	98.2	108.1	108
KLK5	106.6	119.2	128.8	117.1	108	104.5	118.9	111.5
KLK5	118.6	105	114.4	116.8	106.7	95.3	107.5	121.4
KLK6	119.7	121.5	116.4	108	103.9	99	113.4	115.3
KLK6	114.3	118	118.9	127.4	105.7	100.7	113.9	123.6
KLK7	123	117.4	120.2	122.3	110.9	99.9	125.2	122.3
KLK7	124	140.1	126.6	130.7	125.2	108.6	138.5	117.5
KLK8	119.4	119.3	114.8	108.9	105.1	109.2	126	119.4
KLK8	115.6	112.7	112.3	126.6	109.1	101.3	116.9	113.1
KLK9	117.8	107.6	106.1	109.9	103.4	92.9	106.7	112.2
KLKB1	142.9	128.3	136.8	141	121.7	115.3	126.6	140.9
KLKBL4	105.9	105.3	111	109.6	101.5	99.9	113.9	107.3
KLKP1	116.7	107.1	112.8	115.3	103.2	91.5	124.8	107.4
KLP1	118.1	120.7	154	137.2	122.1	112.8	143.1	122.4
KLRA1	124	122.1	132.1	133.1	119.3	106.9	118.7	123.2
KLRB1	4737.6	4856.9	4945.5	5662.6	4192	4153.3	5639.7	5533
KLRC1	157.1	189.6	161.3	163.2	159.6	127.3	141.2	168.5
KLRC2	165.7	178.5	184	200.5	133.9	147.6	159.7	148
KLRC3	110.9	111.3	107.2	108.5	108.7	105.5	115	114.2
KLRC3	239.8	315.9	321.1	226.8	212.3	200.6	220.5	240.3
KLRC3	111.9	126.4	119.4	118.6	102.9	100.8	111.9	122.8
KLRC4	132.4	122.4	126.2	126.4	121.3	104.4	129.2	121.2

KLRD1	313.3	341.4	297.6	343.2	313.9	181.2	234.2	273.1
KLRD1	114	123.8	121.2	120.6	110.3	110	114.8	109.8
KLRF1	507.4	378.6	572.5	408.6	289.9	350	334	392.2
KLRG1	116.1	112.7	115.4	111.8	113.1	98.9	117	117.6
KLRG2	110.9	124.4	124.7	121.6	116.1	101.1	120.8	118.3
KLRK1	128.3	139.7	137.6	139.4	134.8	111.1	125.5	135.5
KMO	130.9	116.1	144.7	138.1	115.8	110.7	121.8	121.2
KNDC1	108.7	109.2	117	117.8	112	105.6	125.6	124.3
KNDC1	124.1	113.1	119.5	117.7	109.5	101.7	118	112.1
KNG1	113.1	112.1	119.7	118.8	103.3	98.8	112.1	119.1
KNS2	123	141.5	147.7	145.9	124.4	108.5	139.6	129.8
KNS2	174.6	172.7	165.4	192.8	169.4	161.5	191.7	176.8
KNTC1	195.9	198	200.7	204.5	192.3	171.8	201.7	199.1
KNTC2	126.5	137.7	139.2	139.5	127.6	112.4	144.4	176.6
KPNA2	137.1	135.5	136.9	143	120.1	118.3	130.1	131.3
KPNA3	244	285.8	259.4	308.7	282.5	214.6	328.9	319.7
KPNA4	513.1	605.9	509.9	664.3	583.8	444	924.3	1094.2
KPNA5	153.4	137.9	141.2	157.9	136.3	116.6	137	172.7
KPNA6	459.8	583.2	451.9	531	502	380.5	667.3	586.7
KPNB1	1832.3	1755.1	1877.8	2064.7	1678.6	2632	2831.1	1980.1
KPTN	118.2	115.6	118.8	108.2	107.3	97.3	122.3	120.9
KRAS	244.7	243	274.9	240.6	256.8	275.7	290.8	319.2
KRAS	111.5	113.9	119.2	117.7	108.7	95.6	118.1	117.2
KRCC1	161.7	183.8	227.5	192.7	203.1	165.2	192.2	212.8
KREMEN1	109.9	112.5	114.8	124.2	119.7	96.6	112	122.7
KREMEN1	136.1	128.7	137.2	139.8	130.5	122.1	127.3	139.4
KREMEN2	107.9	117.3	117	121.4	110.8	97.2	117.3	123.1
KREMEN2	112.2	117.7	116.1	122.9	118.9	100	108.4	114.8
KRIT1	119.4	110.3	117	116.9	113.4	98.6	120.8	113.1
KRT1	105.8	127.4	116.6	120.6	126.7	96.5	115.4	131.2
KRT10	633.1	728.5	606.3	683.1	610.7	447	590.9	566.8
KRT12	103.4	111.2	106.8	113	106.4	95.3	108.9	109.5
KRT13	119.8	112	122.1	131.9	108.2	101	123.1	121.7
KRT14	111.2	105.6	112.4	113.8	102.7	94.7	110	110.6
KRT15	117.5	115.8	118.4	112.6	116.4	102	105.7	106.5
KRT16	109.1	110.1	111.5	120.2	113	103.4	115.3	117.6
KRT17	116.3	132	126.3	120	113.1	105.7	124.2	117
KRT18	120.3	114.4	116.5	121.2	109.3	105.6	115.6	115.6
KRT18	130.2	120.2	134.3	133.2	118.9	112.4	130.6	118.7
KRT19	112.2	110	113.5	111.2	110.1	93.9	111.6	107.4
KRT1B	110.5	108.3	111.8	123.8	105	101.5	116.3	122.7
KRT20	114.8	113.7	114.5	116.3	114.3	96.4	117.3	121.4
KRT23	122.1	118.1	125.7	135.2	123.1	103.7	123.9	118
KRT24	118.4	112.5	123.4	123.7	115.4	104.8	122	120.2
KRT25A	105	104.4	117	118.3	107.1	101.5	111.8	104.5
KRT25C	110.6	116.3	121.1	116.2	103.1	102.2	127.7	116.1
KRT25D	111.5	115.6	108.2	108.2	107.2	107.8	117.1	111.3
KRT26	117.1	119.3	114.2	120.4	114	101.4	117.4	113.8
KRT2A	130.9	145.1	119	146	119.3	111.8	114	118.9
KRT2B	116.4	113.8	115.5	118.4	105.7	98.8	112.3	113.2
KRT3	116.2	121.1	111.6	119.3	106.9	92.5	127.4	110.4

KRT33A	115.7	118	116.2	121.7	115.6	96.6	129.4	118.3
KRT39	116.4	124.7	125	117.6	113.1	100.9	113.1	121.4
KRT4	115.2	107	119.8	124	105.6	113.8	112.5	117
KRT40	107.5	113.5	109.7	118.1	104	95.9	117.2	99.7
KRT5	109	115.3	116.4	121.2	107.9	96.5	116.6	109.2
KRT6A	117.9	118.2	121.1	116.8	106.5	94.2	112.3	114.9
KRT6B	105.1	118.2	117.1	120.7	113.3	101.3	119.9	110.4
KRT6E	113.4	109.2	116.7	121.1	114.2	95.8	118	107.7
KRT6IRS	115.3	118	122.8	121.1	106.9	106.3	122.5	107.5
KRT6L	119.7	113.7	110.5	107.2	105.4	96	111.3	109.8
KRT7	108.3	123.9	119	124.3	108.2	97.6	120.5	101.8
KRT8	114.6	115.5	115.4	118.5	103.7	107.4	123.8	112
KRT86	118.5	124.7	179.9	133.3	112.7	122.9	115.2	117.7
KRT9	132.9	128.4	133.1	121	118.5	105	152.4	124.5
KRTAP10-1	109.6	115.3	129.8	122.3	106.7	99.6	125.4	110.4
KRTAP10-1	135	120.7	138.1	126.3	119.6	96.5	117	127.9
KRTAP10-1	130.1	110.2	124	122	105.2	99.4	120.3	118.5
KRTAP10-1	106	114.1	110.9	113.3	104.6	94.5	115.1	110.7
KRTAP10-2	114.8	115.5	118.3	122.7	117.2	110.8	120.6	126.2
KRTAP10-2	108.1	109.8	105.8	111.2	117.6	94.5	101.4	112.8
KRTAP10-3	113.3	117.1	120.2	133.8	101.1	95.9	116.6	111.7
KRTAP10-4	110.5	125.4	123.3	127.2	112.4	97.9	122.2	115.1
KRTAP10-5	116.6	128.6	116.2	132	118.8	124.2	121.8	122.9
KRTAP10-7	105	118.4	108	115.5	100.7	97.9	113.4	117.9
KRTAP10-8	111.6	114.6	120.7	117.8	104.2	97.1	128	108.1
KRTAP10-9	112.9	116.4	122.7	115.1	114.2	96.6	119.6	121.5
KRTAP1-1	109.9	111.1	114.2	120	108.7	101.2	118.4	118.4
KRTAP11-1	109.5	116.6	117.4	119.6	109.4	108	120.1	118.5
KRTAP12-1	118.6	122.3	115.4	120.7	110.6	99.3	119.4	119
KRTAP12-2	117.7	115.4	117	117	104.2	94.5	128.6	117.1
KRTAP12-3	112.1	117	115.9	124.3	112.7	101.2	110.1	114.3
KRTAP12-4	111.5	124.8	119.2	120.3	117.5	105.8	120.4	130.3
KRTAP1-3	109.1	120.7	120.8	120.9	105.4	96.7	118	121.4
KRTAP13-1	110.1	109.7	106	121.2	109.4	103.6	119.7	107.9
KRTAP13-2	103.6	117.7	114.4	111.4	115	101.4	124.7	114.8
KRTAP13-3	118.2	115.2	115.4	113.4	106.9	96.4	119.6	121.9
KRTAP13-4	107.9	114.4	119.9	114.7	107	101.3	121.8	112.8
KRTAP15-1	106.9	98.6	116.8	131.1	103.2	99.5	126.8	107.2
KRTAP17-1	118.2	110.2	122.7	123.5	114.8	101.8	123.3	110.9
KRTAP19-1	111.4	108.5	112	110	106.4	97.1	113.3	113.9
KRTAP19-2	116.6	112.5	112.7	116.5	99.2	102.6	117.6	111.3
KRTAP19-3	103.6	114.7	116.4	113.6	104.7	96.8	115.5	104.3
KRTAP19-4	115	108.8	122	128.7	104.2	99.5	120.3	119.6
KRTAP19-5	129.4	122.4	129.3	115.5	112.5	108.1	131.7	114.3
KRTAP19-6	114.2	134	122.7	139.1	124.8	100	124.6	122.1
KRTAP19-7	113.7	127.2	107.5	133.6	118.8	112.7	120.8	120.5
KRTAP20-1	109.3	107.9	103.9	116.2	98.7	97.2	107.7	111.2
KRTAP20-2	111.6	130	118.9	130.5	118	112.8	115.7	130.7
KRTAP21-1	120.2	107.4	117.4	113	109.4	99.5	112.2	112.2
KRTAP21-2	117	111.4	115.3	118.5	113.9	94.7	120.5	118.2
KRTAP22-1	115.1	130.9	117.4	121.5	110.1	103.2	118.8	119.3

KRTAP23-1	110	123.3	112.6	120.1	106.8	95.3	125.2	111.1
KRTAP26-1	127.2	121.1	126	125.5	122.5	115	125	120.5
KRTAP27-1	116.6	117.3	121.1	114.5	121.1	100.1	119.3	116.8
KRTAP3-1	110.1	105.3	116.5	111.1	104.3	96.7	109.8	108.1
KRTAP3-2	111.8	114.9	110.9	113.7	108.9	102.9	120.7	112.7
KRTAP4-10	117.6	112.3	120.3	114.7	104.2	101.9	115.6	117.9
KRTAP4-12	117.7	115.5	128.1	119.8	114.8	107	123	118.1
KRTAP4-14	114.3	111.7	115.8	121.8	111.3	100	121.8	115.6
KRTAP4-2	104.8	106.6	111	111.3	100.8	96	105.4	107.9
KRTAP4-4	106.8	117	122.4	129.3	110.3	101.7	111.2	120.7
KRTAP4-5	108	112.3	114.8	110.7	100.8	99	116.2	106.7
KRTAP4-7	118.1	122.5	122.9	132.4	116	95.7	116.5	132.7
KRTAP5-1	106.3	115.5	121.3	120.7	102.1	94.2	111.6	111.1
KRTAP5-10	113.4	111.5	113.4	122.7	110.8	98.8	117.1	121
KRTAP5-11	121.7	118	125.2	122.1	112.6	101.3	115.7	114.3
KRTAP5-2	117.7	114	113.1	111.6	102.6	99.4	116.6	115
KRTAP5-3	113.9	112.9	117.9	119.5	110.8	102.1	130.1	119.9
KRTAP5-4	115.4	109.6	113.7	119.7	106	99.2	118.9	117.1
KRTAP5-5	114.5	109	111.1	113.9	105.3	102.4	107.1	107.8
KRTAP5-6	107.3	111	106	111.2	98.8	90.9	103.6	115
KRTAP5-7	114.9	120.1	109.6	117.6	109.9	92	118	115.7
KRTAP5-8	107.5	118.2	111.6	114.9	110.8	99.7	108.2	118.2
KRTAP5-9	112.6	117	130.2	123.6	113.2	103	121.4	118.2
KRTAP6-1	119.4	118.3	111.2	122.5	110.9	95.9	120.2	113.4
KRTAP6-2	122.9	112.8	120.7	118.6	107.1	94.5	120.7	116.6
KRTAP6-3	115.2	113.3	116.8	110.1	109.5	102.5	127.9	110.8
KRTAP8-1	113.6	113.7	119.4	116.7	109.5	94.1	111.8	105.2
KRTAP9-3	107.7	115.9	116.6	110.8	100.3	93.6	124.5	108
KRTAP9-4	116.5	116.6	129.7	115	113.7	98.3	124.8	112.1
KRTCAP2	1244	1556.4	1411.3	1258.9	1158	880.3	1342.2	1201
KRTCAP3	123.8	129.1	125.1	120.1	115.2	107	120.2	123.9
KRTHA2	123.7	132.3	118.9	124.5	105.7	100.1	129.1	120.1
KRTHA3B	115.5	112.6	112.1	116.3	106.3	102.2	114.1	119.4
KRTHA4	110.3	109.2	115.3	112.9	118.1	100.6	119	112.5
KRTHA5	113	113.9	111.2	113.7	105.4	100.7	109.9	108.8
KRTHA6	115	123.5	120.6	137.9	109.5	98.4	117.2	114.4
KRTHA7	124	118.4	117.4	125.9	110.2	114.5	129.1	112.3
KRTHA8	106.6	117.8	109.1	109	107.8	95.1	110.2	107.4
KRTHB1	112.1	120.8	129.7	122.3	119.3	121.2	122.9	120.4
KRTHB2	111	125.2	120.4	116.7	109.9	97.7	108.7	126.6
KRTHB3	123.4	123.4	121.6	125.7	116.9	102	117.5	120.5
KRTHB4	111	107	116	122.1	115.7	98.2	120.5	114.6
KRTHB5	114.2	118	108.4	110.8	105.7	96.3	117	125
KSP37	1256	1024.9	2116	1629.1	916.9	1728	1275.8	678.9
KSR1	126.6	112.7	119.7	131.5	104.9	94.7	133.9	117.5
KSR2	106.1	112.4	117.7	113.3	107.6	98.9	121.6	122.7
KTI12	175.3	197.2	172	187.7	164.9	139.6	164.4	163.7
KTN1	296.2	298.7	253.6	247.3	308.3	246.4	309	402.2
KTN1	176.3	170.6	167.7	210.8	187.5	151.8	249.5	223.4
KUA	288	276.2	216.1	315.7	208.3	195.2	279.5	236.7
KUA-UEV	126.7	123.5	135.3	142.9	122.1	101.7	130.6	122.2

KY	126.5	118.8	139.9	119.3	122.7	116.8	124.1	131.9
KYNU	2504.4	2300.2	572.2	3376.5	1414.2	1741.6	2609.6	895.5
KYNU	1204.2	878.2	249.9	1425.2	645.7	731.7	1107.6	376.7
L1CAM	124.9	123.4	118.2	124.7	109.5	104.9	128	122.1
L1CAM	130.1	128	132.2	144	125.5	106.7	130.2	127.2
L3MBTL	113.3	112	115.1	128.2	100	96.1	117.9	108.5
L3MBTL	114.7	115.1	125.6	117.8	117.7	103.2	119.8	121.7
L3MBTL	117.3	117.4	116.6	120	110.3	101.6	120	119.9
L3MBTL	118.2	129.2	124.5	128.6	114.5	117.3	119.7	124.5
L3MBTL2	125.5	128.5	111.8	118.4	110.9	113.4	118.5	105.5
L3MBTL2	430.5	460.3	424.2	409.8	303.7	329.5	343	300.5
L3MBTL3	193.8	197.8	200.9	184.4	193.9	173.5	204.1	216.2
L3MBTL3	118	120	126.3	100.5	104.1	100.9	120	131.7
L3MBTL3	114.4	123.9	108.6	124.6	114.6	95.2	122.7	113.9
L3MBTL4	111	115.2	117.2	114.3	104.8	105.2	110.6	115.8
LACE1	116	114.5	119	110.2	111.3	99.7	122.3	124
LACRT	115.5	108.6	116.8	119.5	105.5	99.5	126.3	121
LACTB	159.5	144.7	180.7	174.2	147.4	148.2	165	154.3
LACTB	938.8	636.8	770.5	991.2	453.2	805.2	664	570
LACTB	161.2	141.5	154.1	153.9	139.1	118.7	160	148.8
LACTB2	180.6	171.9	190.4	183.5	174.5	137.6	179.3	196.9
LAD1	120.4	113.3	118.9	130.4	118	113.3	116	114.5
LAD1	121.7	114.7	116.7	122.9	115.4	100.4	122.7	119.5
LAG3	263.9	295.9	192.5	250.8	174.9	131.4	203	170
LAIR1	140.8	132.6	194.1	141.7	130.7	113	125.3	121.3
LAIR1	18779.5	16429.2	16965.8	19019.9	16050.6	14684.6	18487.5	18259.9
LAIR2	126	120	143	128.3	119.6	109.8	130.2	115.4
LAIR2	141.1	133.4	147.7	137.8	109.8	116.3	123.2	124.4
LALBA	112.6	120.8	116.7	114.5	108.9	97.8	122.7	112.4
LAMA1	113.4	117.1	110.7	121.1	101.9	98	108.6	111.4
LAMA1	113.7	113.7	107.9	113.7	109.3	95.3	120.2	108.7
LAMA2	118.5	122.8	111.3	119.3	111.3	97.8	130.2	122.7
LAMA2	108.7	111	115.6	117.4	110.6	96.4	121.9	109.8
LAMA3	109.9	116.2	113.8	106.6	112.7	96.7	115.5	106.4
LAMA3	112.6	109.1	111.1	111.1	113.4	92.7	113.1	112.3
LAMA3	117.2	119.3	125.1	130.6	106.1	102.4	116.5	120.4
LAMA3	114	118.9	115.8	112.6	103.4	94	114.2	109
LAMA4	128.1	108.7	115.5	122.9	115.8	98	124.4	116.8
LAMA5	177.6	153.9	164.7	173.1	152.6	130.6	204.7	181.2
LAMB1	106.3	111.7	115.1	118.7	100.4	99.2	117.6	112.6
LAMB2	112	111.3	126	121.3	105.5	100	125.2	111.8
LAMB3	127.1	119.3	118.2	119.9	106.6	103.5	131.6	124.6
LAMB3	116.6	111.8	118.8	118.3	108	99.8	116	116.6
LAMB3	113.2	122.6	117.5	115.1	108.3	100.7	120	110.2
LAMB4	115.3	124.1	117.1	113.2	105.8	100.8	119.6	118.8
LAMC1	134.3	123.5	152.6	164.3	121.2	156.5	182.5	124.7
LAMC2	119.2	120.9	116.9	119.5	114.2	102	129.3	111.7
LAMC2	104.2	110.3	113.6	118.1	109.4	92.2	119.5	110.9
LAMC2	113	116.8	114.1	114.3	110.7	94	110.2	112.3
LAMC3	110.3	121.3	130.5	118	102.8	117.2	129.8	117.7
LAMP1	1310.5	1576.5	1128.5	1433.2	1334	1066.4	1541.9	1847.4

LAMP2	900.2	870.6	907.6	994.6	720.1	866.6	1058.2	1012.8
LAMP2	248.6	231.1	276.7	263.5	189	255.1	253.9	176.7
LAMP2	240.7	237.4	291.7	330	201.1	291	352.7	221.4
LAMP3	156.7	152.5	154.8	162.4	132.7	126.9	159.1	133
LANCL1	294.1	304.9	356.6	288.3	285.3	250.9	222.8	191.6
LANCL2	139.8	151.7	146.9	138.1	141.2	123.6	166.7	137.4
LANCL3	115.4	117.2	121.5	112.7	112.8	97.7	110.8	118.4
LAP3	406.1	200.3	467.5	360.6	174.3	403.6	285.1	194
LAPTM4A	148.6	149.3	143.7	152.6	143.8	140.5	163.4	185.9
LAPTM4B	137.6	158.5	158.7	166	149.8	146.1	170.5	171.6
LAPTM5	1307.4	1619.3	2081.8	1655.3	1430.6	2196.7	1317.4	1531.3
LARGE	171.9	165.8	198.2	171.5	143	161.2	157.4	160.1
LARGE	111.7	120.3	112	103	108	103.9	112.8	114.8
LARGE	125.8	127.9	122.7	124	108.5	102.4	120.7	114.7
LARP1	116	120.4	115.8	126.8	108.4	98.8	122.5	112.4
LARP1	294.5	262.4	204.5	276.9	206	198.1	263.8	285.9
LARP2	113.4	116.3	111	122	120.3	101.9	144.8	126
LARP2	128.6	127.5	134.9	120.5	127.1	112.7	130	156.4
LARP2	117.4	128.5	124.4	124	109	106.5	119.3	116.4
LARP2	215.6	208.5	205.5	243.1	208.4	195.7	298.4	391.2
LARP4	140.6	129.9	147.7	144.5	121.9	113	133.5	131.6
LARP4	210.7	207.5	208.7	232.5	215.2	149.3	271	356.4
LARP5	536.4	498	431.7	548.7	461.2	424.7	668.2	626.5
LARP6	116.6	117.8	116.2	121.2	106.1	101.2	120.3	118
LARP6	114.4	107.3	112.2	118.3	107.3	114.6	114.8	117.8
LARP7	580.7	547.4	595.9	626.4	550.2	480.9	571.6	584.1
LARS	254	239	253.2	236.5	235.7	205.4	240.3	204.9
LARS2	147.6	139.1	137.1	156.6	125.9	113.1	148.1	148.2
LAS1L	125.1	139.5	139.9	153.5	146.4	122.5	140.1	137.8
LASP1	3055.1	2520.4	3057.5	3305.1	2083.1	2212.9	3319.2	2073
LASS1	124.1	115.7	118.9	112.2	117.2	98.1	118.8	115.3
LASS1	110.3	113.8	119.2	114.2	106	98	113.6	115.6
LASS1	114.8	115.6	116.3	117.3	105.5	99.9	114.6	120.2
LASS2	251	279.4	221.1	257.5	190.9	210.4	263.2	296.3
LASS2	361.9	335.7	299.3	375.5	258.6	237.2	430.3	414.6
LASS3	106.2	118.4	119	107.8	103.1	96.5	107.5	115
LASS4	116	115.9	125.5	132.3	119.8	103.5	129.7	134.6
LASS5	238.5	213.6	166.9	231.2	190.6	157.9	255.6	255.7
LASS6	585	506.6	527.7	567.7	585.5	467.4	707.4	942.9
LAT	1039.2	1110.2	863.8	986.5	688.1	678.6	724.6	726.4
LAT	576.9	775.4	480	527.8	491.6	411.9	427.2	385.4
LAT	111.7	108.4	115	111.2	105.6	95.9	108.8	111.9
LAT	127.7	133.3	120.4	123.5	119.9	106.8	126	112.1
LAT1-3TM	292	246.9	383.8	326.9	235.2	310.8	372.9	364.1
LAT1-3TM	114	115.3	109.6	108.1	113.5	101.8	123.8	119.1
LAT1-3TM	112.8	116.5	127.3	125.1	115.8	99.8	123	114.8
LAT2	185.3	156.3	411.3	197.5	124.2	246.1	190	157.8
LAT2	181.9	147	384.4	201	126.9	234.9	164.1	147.3
LAT2	118.4	122.4	140.8	120.4	105.3	103.5	129.1	110.4
LATS1	117.6	138.5	127	131.9	115.3	114.1	136.4	122.8
LATS2	131.4	139.6	136.6	133.4	127.4	103.7	137.6	148.2

LAX1	296.6	272.1	366.1	299.4	285.7	308.1	278.9	345.8
LAYN	123.7	121.2	118.3	117.1	119.3	102.6	116.6	115.4
LBH	136.8	145.2	130.6	131.1	139.2	98.9	127.9	135.3
LBH	721.7	962	552.7	760.5	500.3	516.6	583.3	394.6
LBP	124.5	123.9	132.9	128	116	111.3	124	125.3
LBR	133.4	141.8	133.4	159.4	137.2	117.4	160.1	164.2
LBR	238.5	258.3	222.8	321	342.6	204.3	436	584.2
LBR	112.8	111.7	108.5	117.2	113.8	107.7	118.7	134.4
LBX1	105	114.1	119.1	117.2	108.2	101.6	117.5	115.2
LBX2	108.6	120.1	113.8	114.1	102.4	98.8	122.2	120
LBXCOR1	111.5	118	118	121.7	113.1	103.1	129.7	111.7
LCAP	116.6	133.1	116.4	127.3	113.1	104.2	132.1	123.8
LCAT	128.6	110.2	123.7	129.8	113	105.6	132.7	121.7
LCE1A	117.2	110.2	117	119.5	105.7	96.7	113.4	108
LCE1B	112.7	116.5	116.7	124.5	109.6	108.4	124.4	121.5
LCE1D	110.7	118.9	110.9	115.1	105.3	98.5	117.5	112.6
LCE1E	121.1	118.8	123.8	128.6	117.7	104.5	115.6	114.5
LCE1E	111.3	116.3	119.4	127.7	98.3	101.6	110.8	110.6
LCE1F	117.1	129.3	124.3	128.8	113.4	101.8	115	121.3
LCE2A	115.4	109.6	105.6	116.7	98.8	95.7	120.2	112.4
LCE2B	111.8	113.1	123.4	119.3	112.2	101	112.9	113.7
LCE2C	120.6	118.9	116.3	114.8	107.1	99.2	115.5	115.3
LCE2D	115.1	113.6	115.9	109.1	101.3	91.7	117.4	114.8
LCE3A	114.7	116.2	117.6	132.6	110.6	105.4	105.2	114.4
LCE3B	106.9	115.6	115.1	116.4	101.5	106.8	113.8	110.9
LCE3C	104.9	111.6	119.5	117.5	109.5	97.8	115.9	109
LCE3D	120.9	106.2	125.1	131.7	113.9	104.7	124.7	119.3
LCE3E	115.9	111.7	112.5	110.8	105.8	95.6	124	112.9
LCE5A	116.8	114.6	117.5	114.7	107.5	102.9	114.9	113.2
LCK	209	205.1	177.3	198.3	166.1	146.2	157.2	176.6
LCK	164.5	217.8	149.9	164.4	179	141.4	153.8	139.6
LCK	128.9	139.2	116.8	135.4	115.4	96.4	136.5	130.5
LCMT1	494.5	560.4	516	515.4	354.6	304.9	407	319.9
LCMT1	347.4	336.9	360.3	324.8	267.9	261.2	251.9	227.4
LCMT1	241.8	271	268.2	280.6	234.7	195.4	203.9	185.4
LCMT2	261.5	314.7	235.7	271	252.3	198.7	254	217.1
LCN1	105.2	105.5	114.5	117.6	109.3	102	129.8	114.3
LCN10	123.1	119.4	123	112.3	109.3	96.7	116.3	113.4
LCN12	110.9	101.1	118	115.9	110.5	99.5	112.1	107
LCN2	124.6	160.1	150.7	134.9	172.1	124.6	132.4	156.6
LCN6	107.1	119.2	111	105.2	107.2	95.5	115.6	114.7
LCN8	115.6	108.8	116.3	109.6	103	100.5	114.5	107.8
LCN9	120.1	111.2	117.1	113.6	114.8	101.1	122.2	107.6
LCOR	432.3	331.1	280.5	334.8	297.9	231.1	456.4	481.6
LCORL	110.7	104.3	106.8	111.9	102.3	99.9	113.9	115.6
LCP1	5626.2	4585.2	6203.3	5512.9	3438.1	5022.8	6141.9	5592.9
LCP2	181.2	142	162.6	146.5	139.6	120.6	137.4	144.4
LCT	114.7	119.5	117.2	109	107.9	102.4	116.7	122
LCTL	117.9	119.1	132.8	130	126.6	111.1	119.9	126.3
LDB1	121.2	124.7	133.6	135.1	128.4	106.5	131.9	118.7
LDB2	127.8	155.5	159.8	142.2	134.7	118.4	119.5	131.1

LDB3	119	133.7	127.4	124.3	112.8	100.3	128.9	125.7
LDB3	112.7	115.2	119.1	116.6	103	96.9	117.2	101.9
LDB3	103.4	115.1	114.2	114.3	102.6	96.5	113.8	108
LDHA	1778.7	1852.1	2001.1	1861.7	1425.9	1775.3	1853.7	1611.4
LDHAL6A	120.8	128.6	132.7	142.1	118.2	95.8	119.6	121
LDHAL6B	110.7	113.6	118.4	120.4	117.2	95.8	113.9	120.2
LDHB	1043.6	1175.8	778.1	1453.4	828.8	798.3	798.3	959.1
LDHC	121.8	132.7	121.2	130.9	115.5	96.5	127.7	129.8
LDHC	119	113.8	106.1	119.6	103.4	94.9	114	111.7
LDHD	113.7	113.6	107.3	114.8	114.1	101	114.6	109.5
LDHD	111.4	114	112.5	114.9	98.5	107	111.4	111.2
LDLR	506.2	480.3	377.6	401.8	260.4	234.8	327.6	300.8
LDLRAD1	105.9	107.2	116.7	117.8	114.4	98	119.6	114.4
LDLRAD2	121	115.7	111	116.7	108.5	100.4	109.8	114.5
LDLRAD3	106.3	117.3	112.3	117.4	100.4	91.8	103.8	114.1
LDLRAP1	316.6	325.1	370.1	281.2	236.5	265.3	230.1	239.4
LDOC1	140.5	173.7	148.6	131.5	144.2	128.2	132.3	137.6
LDOC1L	240.9	320.4	246.4	250.9	245.1	183.3	230.9	235.8
LEAP2	136.1	157.4	141.6	148	140.8	109.7	144.3	151.9
LECT1	123	132.9	124	128.5	114.1	101.7	122.1	113.1
LECT2	106.9	116.4	101.5	110.2	100.5	91.9	108.7	107.3
LEF1	2029.2	2199.8	1187.7	1716.5	1689.8	1161.9	1617.1	1905.4
LEFTY1	120.2	116.7	118.1	127.9	106.8	100.4	121.7	116.7
LEFTY2	111.8	114.3	118.6	125.4	106.7	94.8	121.9	120.9
LELP1	117	119.5	115.5	123.5	105.4	100.1	116.4	116.3
LEMD1	114.8	115.8	115	132.4	115.7	100.5	122.3	108.4
LEMD2	289.8	325.5	275.2	285.6	234.4	236.4	300.5	305.2
LEMD3	428.7	592.1	527.5	660.3	553.9	457.8	751.1	847.7
LENEP	106.6	106.7	111.6	113.6	106.6	95.2	117.4	111.9
LENG1	120.8	115.7	123.2	130.3	110.8	118	140.9	129
LENG4	231.7	254.8	250.2	272.6	193.8	314.7	304.9	274.2
LENG8	139.1	139.7	152.8	135.1	121.9	118.1	129.7	113.7
LENG9	105.4	117.7	120.3	115.2	106.7	94.6	120.1	117.6
LEO1	122.6	119.4	122.6	113.3	113.3	98.5	125.1	121.4
LEP	213.4	171.7	195.1	196.8	220.8	136.6	227	207.3
LEPR	121.5	122	127.6	112	109.2	100.2	107.5	123.5
LEPR	114.3	127	116.2	115.6	114.2	116.2	119.4	113.1
LEPR	110.1	109.8	113	116.9	102.8	99.5	111.3	121.7
LEPRE1	199.2	169.4	174	176.1	155.4	143.8	180	176.8
LEPREL1	124.2	124.6	131.5	121.5	107.3	116.2	115.7	113.1
LEPREL2	104.3	116.3	115.9	111.2	103.5	105.6	113.3	109.6
LEPROT	308.9	419.4	373.8	433.9	369.8	207.5	311.3	395.8
LEPROTL1	1063.2	1522.9	944.9	1788.3	1292.6	711.5	1337.2	1614.5
LETM1	131.8	133.6	121.8	128.7	109.4	108	139.5	143.2
LETM2	116.4	118.4	123.2	118.6	107.3	103.8	121.4	112.9
LETMD1	149.2	141.2	154.5	148.6	127	116.8	137.4	130.4
LETMD1	377.4	403.4	432.5	396	312.9	336.5	373.9	333.8
LFNG	117.4	111.4	126.2	115.7	114.7	103.3	133.1	112.8
LFNG	129.1	138.6	124	126.1	118.6	99.9	135.6	114.3
LFNG	1054.9	1274.4	1272.4	1064	657.6	906.6	660.4	434.8
LGALS1	661.3	586.3	2162.8	672	365.5	1648.1	797.3	448.7

LGALS12	113.2	117.5	120.9	115.3	112.5	104	115.7	112.9
LGALS13	108.9	117.6	115.1	118.8	117.1	107.5	119.9	122.6
LGALS14	130	118.7	139.2	126.7	126.6	115.6	135.7	112.8
LGALS14	106.7	113.7	117.2	114.7	112.8	95.1	124.6	113.5
LGALS2	122.4	118.8	130.5	135.2	115.9	111.8	128.9	118.2
LGALS3	398.1	326.7	422.8	389.5	216.9	255.8	343.3	277.4
LGALS3BP	160.8	168.7	121.6	146.5	139.9	118.6	141.9	132.4
LGALS4	118.9	127.4	122.2	137.9	114.5	105.7	114.8	121.4
LGALS7	124	127.9	128.4	121	119.5	99.5	132.5	114.2
LGALS8	121.1	108.5	127	109.2	107.7	103.7	129.5	116.3
LGALS8	172.9	241.6	255	191.7	175.6	175.4	201.6	198.4
LGALS8	118.4	113	121.4	127.4	116.8	107.5	114.6	127.1
LGALS8	285.9	205.8	289.4	276.1	223.7	226.9	242.8	208.9
LGALS9	161.9	127.9	164	142.5	115.1	126.7	126.2	125.2
LGALS9	136.2	127.6	124.5	131.2	109.7	108.1	133.9	120.8
LGI1	115	117.3	115.1	121.9	104.3	98.6	118.1	108.4
LGI2	134.2	120.8	119.5	136.9	121.3	119.1	128.8	136.5
LGI3	111	105.9	119	117.7	108.7	93.2	102.3	123.6
LGI4	120.2	121.6	129	119.3	124.8	111.6	135.9	118.5
LGICZ1	124.9	115	118.3	120.7	114.4	107.5	117.8	122.4
LGMN	209.6	131.2	306.5	227.6	130.9	257	194.8	138.6
LGMN	120	109.3	124.4	125	111.5	105.9	126.9	119.1
LGMN	253.6	151.7	540.5	345.5	130.6	450	234.2	176.1
LGP1	187.5	193.8	189.5	173.5	144.1	134	163.1	170.9
LGP2	151.9	167.6	175.8	176.9	135.1	137.1	138.6	152.2
LGR4	115.8	107	108.7	124.4	102.5	95.5	113.3	103.4
LGR5	115.7	116.5	116.6	119.1	103.5	100.6	118.7	121
LGR6	111.5	122	125.6	118.6	119.8	97.9	120	119
LGR6	126.6	128.4	125.5	136.8	118.7	108.6	125	113.4
LGR6	116.2	116.3	124	123.2	117.5	104.1	117.7	123.3
LGR6	112.4	110.8	118.8	121.6	108.3	97.9	117	108.5
LGR7	115.6	108.6	108.2	114.5	108.8	100.7	108	115.9
LGR8	107.9	107	110.2	118.9	103.9	95.8	114.6	108.1
LGTN	255.1	284.2	296.1	298.8	199.3	243.6	247.1	254.3
LHB	108.3	109.9	109.3	112.9	101.8	99.8	119.3	107.5
LHCGR	120.6	112.3	118.3	115.9	110.6	108.9	107.4	114.4
LHFP	118.9	112.8	114.5	113	107.9	100.5	113.6	114.8
LHFPL1	135.9	141.2	161.1	151.2	118.4	132.1	139.4	137.7
LHFPL2	649.5	240.3	382.5	872	461.3	857.8	1135.9	469.2
LHFPL3	122.5	123.5	130.8	118.7	116.6	110.3	121.2	128.8
LHFPL5	112.5	109.7	118	119	106.9	92.5	116	109.8
LHPP	255	212.2	279.8	252.9	164.6	168.7	180.9	171.6
LHX1	129.9	124	121.6	129.6	119.6	101.8	123.9	116.8
LHX2	123.3	127.4	114.5	121.6	116.6	104.1	112.3	115.8
LHX3	117.4	121	115.7	114.4	104.1	93.5	119.2	124.6
LHX3	120.4	112.7	116.4	113.8	108.3	106.9	119.9	113.1
LHX3	109.8	109.9	117.7	120.4	110.1	97.1	119.1	113.4
LHX3	109	103.8	116.7	120.1	104	97.8	115.7	115.5
LHX3	108.9	118	112.3	119.5	112.3	99.7	111.5	119.5
LHX4	109.4	117.7	119.7	120	111.7	103.7	118.4	113.9
LHX5	108.1	112.4	105.9	112.8	102.9	106.8	110.7	108.2

LHX6	132.6	135.7	136.3	142.8	134.3	119.9	149.9	146.2
LHX6	111.8	119	111.6	122.3	109.9	103.4	118.9	109.9
LHX8	118.6	104.5	110.2	111.8	103.2	101.8	124.2	109.6
LHX9	110	105.9	108.1	114.8	103.8	89.4	107	107.3
LHX9	118.8	112.7	113.2	116.4	108.7	99.5	116.8	114.3
LIAS	140	136.1	148.3	136.8	121.1	123.8	142.9	123.6
LIAS	261.3	229.1	343.5	270	225.3	226.9	218.8	246.7
LIF	116.2	104.9	118.3	121.5	109	96	115.2	120.5
LIFR	111.2	116.1	113.7	115.3	112.2	98.9	123.7	118.3
LIG1	220.2	260.4	315.8	229.6	176.4	231.7	209.5	199.8
LIG3	112.8	115.5	115.8	113.8	107.9	95	112	116.3
LIG3	125.8	136.7	118.6	136.3	120.5	107.8	129.6	119.6
LIG3	164.9	177.5	162.1	169	129.4	125.6	154.8	151.4
LIG4	144.9	132.6	137.2	146.6	126.8	115.9	141.7	136.9
LIG4	120.4	124.3	115.5	119.3	106.5	99.8	125.6	108.7
LILRA1	117.5	116.5	125.7	119	110.9	101.3	118.2	120.4
LILRA2	175.5	146.5	190.3	174.4	127.7	104.7	159.8	120.5
LILRA3	132.8	121.7	312.2	128.9	111.2	173.9	135.8	120.1
LILRA3	109.9	115	131.5	121.2	102.8	109.3	116.4	113.4
LILRA4	123.4	133.6	131.5	128.4	116.6	113.4	141.2	125.3
LILRA5	127.8	124.2	118.9	118.5	113.4	98.6	120.7	108.4
LILRA5	156.1	108.5	237.6	130.9	106.8	114.9	119.4	106.3
LILRA5	115.7	118	121.4	120.4	106.7	97.7	107.5	116.7
LILRA6	104.4	136.8	113.9	121.4	107.1	102.9	113.8	111.5
LILRB1	142.2	126.2	146.1	144.5	112.6	111.8	129	118.3
LILRB1	2830.1	2505.8	2638.1	3018.9	2213.7	1675.8	2737.5	2450.4
LILRB2	435.3	157.3	921.7	377.8	155.8	674	301.2	141.8
LILRB3	662.5	302.3	626.4	624.5	277.9	446.9	570.5	292.7
LILRB3	10747.6	8518.5	10418.1	11176.1	10394.3	5980.7	10777.5	11076.8
LILRB4	151.5	120.2	189.8	170	124.8	169.7	138.5	122.7
LILRB5	110.4	113.8	125.8	118.4	99.9	101.5	117.1	111.6
LILRB5	123.7	122	122.9	135.4	117.8	101.5	125	126.5
LIM2	111	121	124.6	121.9	106.5	103.1	106.8	105.4
LIMA1	152.3	175.9	157	155.1	144.6	111	147	143.6
LIMD1	108.2	108.6	109.9	106.3	102.7	93.8	117.4	109.1
LIMD2	115.7	122.7	124.5	131.1	108.3	102.7	137.1	129.3
LIME1	797.7	803.4	1384.1	904.6	756	946.4	677.2	604.1
LIMK1	108.4	120.3	118.6	117.5	109.4	98	126.8	120.8
LIMK1	120	125	117.2	117.2	110.9	101.7	118.1	122.9
LIMK2	127.2	121.6	129.7	127.2	121	98.5	119.6	121.3
LIMK2	111.1	115.7	120.2	121.5	109.5	101.5	115.6	119
LIMK2	132.6	130	128.5	127.1	109.9	100.6	122.8	117.3
LIMK2	114.1	120.6	104.1	115.2	108.1	96.3	119	106.8
LIMS1	252.4	224.9	255.3	334.4	211.1	191.4	318.4	223.1
LIMS1	118.5	122	141.5	125.1	120.3	102.1	118.3	126.5
LIMS2	119.9	124.6	117.3	124.2	114.5	112.3	113.9	116.6
LIMS3	103.1	116.9	123.7	132.9	106.5	104.5	135.7	112.4
LIN10	154.8	156.3	151.2	167.7	146.7	136.9	189.8	147.3
LIN28	112.3	118.1	117.4	112.6	108.3	96.6	120.1	112
LIN28B	124.8	128	114.7	122.2	113.9	102.9	119.5	127.4
LIN7A	114.1	118.9	120.8	117.3	117.4	94.1	121.9	121.8

LIN7B	163.8	162.1	176.3	189.2	130.8	135.5	151.3	134.1
LIN7C	159	133.5	138.6	155.7	132.1	126.5	159.5	134.4
LIN9	131.6	139.8	124.8	147.4	127.3	116.7	134.8	134.2
LINCR	115.6	124.2	128.3	123.4	107.7	99.8	122	117.2
LINGO4	121.8	122.9	125.1	121.7	109.5	102.7	111.1	112.8
LINS1	119.6	139.1	133	124.5	128.4	114.5	140.6	133
LINS1	123	122.6	121.6	137.3	118.5	105.2	115.8	112
LIPA	771.2	728.8	1339.6	690	603.4	712.6	598.6	484.4
LIPC	115.8	116.6	118	118.3	112.5	97.5	107.8	110.4
LIPE	120.2	145.7	129	123.2	122.5	110.8	126.4	133.3
LIPF	116.5	115.8	120.9	117.6	108.7	105.5	119.9	110.7
LIPG	116	107.5	110.8	110.5	108.6	97.1	115.2	120.3
LIPH	115.6	116.8	113.2	111	110.8	108.6	115.2	112.4
LIPI	117.3	117.6	109.6	119.3	107	101.7	109.1	115.3
LIPK	135	120.3	129.7	122.3	115.8	104.2	130.9	133.7
LIPL1	125.6	129.5	136.1	129.4	112.3	108.8	121.3	118.2
LIPT1	227.2	245.9	268.2	238.9	205	238.9	192.3	215
LIPT1	126.1	125.5	129.8	126.3	116.6	111.7	130.2	129.8
LITAF	2291.3	2598.9	1935.7	2438.2	1939.4	2306.8	2401.1	2710.2
LIX1	114	110.2	123.7	116.1	107	98	118.2	113.2
LIX1L	200.2	208.2	192.3	195.8	175.8	162.1	152.9	154.3
LKAP	1588.7	1614.6	1400	2040.5	1811.3	1749	2251.3	1679.3
LLGL1	298	368.7	247.4	353.1	333.9	217.6	334.8	304.3
LLGL2	115.4	125.2	121	111.4	118.9	104.9	118	126.1
LMAN1	118	133.6	133.8	127.4	112.3	102	117.9	128.4
LMAN1L	122.8	135.1	119.4	134.2	124.4	108.1	124.5	134.2
LMAN2	130.1	144.4	157	137.7	136.1	128.6	132.9	119.5
LMAN2L	148.7	142	141.6	149.6	129	140.4	148.5	157.1
LMBR1	236.9	228.7	234.7	292.5	215.4	221	281.2	299
LMBR1L	115.7	125.5	117.6	136	122	105.7	138.8	146.7
LMBRD1	555	568.5	613	654.9	509.5	465.6	483.6	597.8
LMBRD2	121.7	114.2	127.5	130.4	106.4	103.2	114.6	134
LMCD1	113.8	121.4	120.9	117.6	118.7	104.1	120.1	116.2
LMLN	133.9	113.5	131.2	126.2	109.9	109.7	122.3	122.1
LMNA	168.4	147.5	314.2	207.4	156.3	312.7	228.2	170.9
LMNA	316.1	216.7	818.1	435.4	235.8	529	404.5	232.7
LMNB1	156.2	127.9	127	143.1	132.8	131.2	119.9	114.5
LMNB2	213.1	239.3	203.8	224.2	231.6	182.4	326.5	250.1
LMO1	111.2	116.1	132	106.7	110.3	98.6	123.5	118.3
LMO2	160.6	127.8	241.2	150.5	116.7	125.7	117.5	137.8
LMO3	115.1	117.3	112.4	123.3	104.9	102.3	123.4	120.9
LMO3	110.6	120.1	118	125.6	114.7	101.2	120.3	115.8
LMO3	110.4	116.9	111.9	109.5	105	98.4	111.7	109.2
LMO4	616.9	569.2	565.5	546.7	470	411.7	652.9	460.1
LMO6	124.3	113.8	119.7	127.2	105.5	99.7	115.6	113
LMO7	113.4	122.3	124.1	134.2	122.5	111.9	123.4	137.7
LMOD1	125.8	121.3	114.4	125	113.3	94.1	113.3	116.1
LMOD3	584.9	408	529.6	460.2	459.5	281.3	568.7	508
LMTK2	125.1	134.7	129.9	120.9	123.3	100.8	131.4	118
LMTK3	185.4	173.5	207.8	218	162.6	160.9	192.5	149.5
LMX1A	109.1	106	115.6	109	107.1	96.9	115.5	105.7

LMX1A	109	119.3	117.1	113.2	99.9	91.6	108.3	107.1
LMX1A	118.7	114.6	109.2	122.1	101.7	95.4	116.5	110.2
LMX1A	113.5	116.4	118.5	115.4	110.2	97.3	114.7	108
LMX1B	120.7	114	123.5	115.1	115.9	99.7	120.9	118.7
LNK	809.6	743.3	787.3	833.1	558	698.5	1089.3	747.9
LNPEP	115.8	115.2	119.3	114.2	109.3	107.1	115.8	118.9
LNPEP	186	182.8	181.4	176	178.6	159.1	204.6	186.5
LNX1	117.5	122.9	121.5	117.4	129.3	100.4	115.5	120
LNX2	151.5	163.2	148.7	165.5	142.4	147	142.3	176.9
LOC11270[illegible]	117.9	121.1	127.5	137.9	120.2	101.8	143.9	121.4
LOC11271[illegible]	124.8	120.3	132.6	121.8	124	101.2	121	116.7
LOC11286[illegible]	147.8	171.2	159.8	184	151.8	136.4	165.2	147.4
LOC11286[illegible]	363.6	374.1	260.2	410.6	279.2	202.5	372.6	298.5
LOC11317[illegible]	150.4	183.6	142.2	170.8	127.3	125.5	187.3	148.7
LOC11338[illegible]	247.2	396.8	261.3	252	281.6	212.8	213	284.7
LOC11344[illegible]	165.2	199	190.1	221.8	171.4	163.8	207.6	186.3
LOC11498[illegible]	148.9	141.9	161.4	156.9	137.5	141.8	139.9	136.2
LOC11509[illegible]	133.9	144.5	121	143.3	122.1	109.4	131.4	133.9
LOC11614[illegible]	158.8	163.4	142.8	142.4	145.6	113.2	140.9	138.1
LOC11623[illegible]	140.4	158	150.8	156	125.8	134.2	142.5	121.5
LOC11971[illegible]	130.8	133.6	145.6	130.4	122.9	126	122.8	127.6
LOC12037[illegible]	106	115.6	117.6	118.7	106.5	101.3	113.7	116.6
LOC12225[illegible]	115.9	114.5	106.5	123.2	111.4	98.2	115.6	115.7
LOC12368[illegible]	188.3	158.6	166.1	173.8	152.5	132.3	199.3	194.5
LOC12387[illegible]	107.7	111.6	110.8	125.3	109.7	98.3	117.9	115.5
LOC12422[illegible]	106.6	105.3	112.8	110	104.4	95.6	116.2	111.7
LOC12444[illegible]	376.6	448.8	542.4	434.6	327.1	347.2	348.4	288.9
LOC12451[illegible]	154.4	170.3	185.1	174.4	152.1	132.3	144.7	122.1
LOC12451[illegible]	189.3	182.7	176.2	182.7	160.1	130.1	150.1	149.4
LOC12451[illegible]	143.1	170.5	180.4	157.7	130.3	133.2	146.4	123.8
LOC12475[illegible]	122.4	114.9	128.6	129.8	114	101.9	122.5	112.3
LOC12484[illegible]	117.6	109.9	117.4	122	115.4	107.1	110.1	113.8
LOC12589[illegible]	323.7	369.2	342	316.7	244.7	261.1	249.2	233.7
LOC12607[illegible]	121.9	108.6	120.3	119.5	104.1	93.3	118.9	118.8
LOC12614[illegible]	114.2	111.5	119.1	114.2	107.9	97.7	108.8	115.6
LOC12620[illegible]	662.4	739.6	596.6	762.7	624.5	483.4	899	867.6
LOC12624[illegible]	108.9	114.7	125.7	123.9	110.8	107.6	121.1	108.7
LOC12629[illegible]	117.7	115	103.5	117.9	104.2	109.1	127.3	105.5
LOC12653[illegible]	118.7	120.8	126.9	115.3	110.7	99.4	117.6	116.2
LOC12815[illegible]	116.1	108.4	127.5	109.3	112.9	107.1	123.6	113.1
LOC12843[illegible]	132.1	141.3	150.9	141.4	118.9	109.7	133.3	122.7
LOC12897[illegible]	148	159.9	159.4	146.9	136.9	134.9	139.7	130.2
LOC12913[illegible]	191.4	211.8	166.1	181.1	170.6	141.6	192.1	227.4
LOC12928[illegible]	183.8	185.5	232.3	192.4	182	169.7	207.4	175.2
LOC12953[illegible]	171.8	141.5	147.5	152	144.7	127.5	147.5	147.3
LOC12953[illegible]	498.7	576.9	635.4	613.7	610.8	373.6	522.6	619.9
LOC12960[illegible]	132.7	117.2	129.5	122.3	106.9	97.1	115.5	115.1
LOC13007[illegible]	172.7	183.1	142.2	174.3	170.5	135.6	162.4	222.8
LOC13035[illegible]	128.1	149.5	151.9	142.8	125.2	132.3	145.9	140.3
LOC13057[illegible]	117	113.5	118.8	120.8	110	104.2	130.2	119.5
LOC13094[illegible]	111.8	116.1	116.6	113.9	101.7	95.4	121.1	116.1

LOC130951	117.7	122.7	117.5	135.1	107.7	115.6	131.3	126.3
LOC131149	114.7	118.6	121.9	107.6	106.2	100	117	118.8
LOC132203	110.6	113.8	114.5	108.8	105.3	94.6	118.6	116.8
LOC132241	129.3	118.9	110	125.9	115.7	106.7	119.3	133.7
LOC132321	125.6	124.4	132.3	119.1	126.8	118.3	132.3	131.9
LOC133308	119	115.5	120.3	128	105.8	111.6	114.4	115.8
LOC133619	212.3	199.8	218.2	227.7	224.6	200.8	170.2	198.3
LOC134145	117.5	117.4	115.5	117.2	108.5	97.7	113	123.2
LOC134147	113.7	121.1	118.3	114.7	106.5	95.7	115.7	105.9
LOC134997	1285.7	991	1452.1	1305.2	1135.9	850.1	1182.6	1310.1
LOC136242	114.3	113.7	113.9	114.7	108	96.3	113.4	119.7
LOC136263	117.6	116.5	129.9	117.7	106.8	108.1	127.8	124.6
LOC136306	106	109.4	113.3	112.6	110.1	93.4	110.8	111
LOC138046	107.1	107.5	114.2	112.7	99.9	95.3	121.8	109.8
LOC138255	114.3	112.7	126.1	121.5	121.4	102.3	106.1	123
LOC139886	247.4	235.3	231.9	221	157.7	138	176.1	159.2
LOC143188	112.8	119.9	128.4	126.5	102.4	99.8	120.6	122
LOC143543	172.4	177.2	159.9	167.2	153.6	160.5	175.3	174.7
LOC143941	110	122.1	122.1	113	110.7	98.7	119.9	109.8
LOC144097	141.3	122.6	144.5	133.7	111.7	115	126.3	132.1
LOC144233	296.5	335.3	347	323.9	363.4	274.2	316.7	336.3
LOC144363	475	411.6	676.4	440.1	373.1	413.3	347.6	285.3
LOC144404	112.1	119.4	109.6	116.7	110.4	98	112.1	103.6
LOC144404	109.4	109.5	116.5	120.6	107.3	98.4	113	107.4
LOC144501	113.3	108.4	110.4	109.1	101.7	101.5	107.4	113.2
LOC144501	106.5	109.9	128	117	112.8	103.9	120.3	113.8
LOC144983	122	117.9	124.9	125.6	126.6	110.8	127.4	124.1
LOC144983	108.4	110.6	116	108.8	105.8	95.6	114.5	112.7
LOC146167	113.7	107	112.5	118	109.4	99.9	112.5	107.8
LOC146325	113.6	120.3	111.8	108.1	108.8	93.2	112.3	102
LOC146562	111.1	108.8	112	120.1	103.3	96.7	108.5	113.1
LOC146713	119.4	111.3	109.4	108.2	103.6	104.1	110.7	118.9
LOC146909	116.3	118.9	110.9	117.9	106.6	94.4	115.5	109.7
LOC146909	107.1	113.1	115.5	112.5	100.9	97.1	113.8	111.3
LOC147808	157.8	133.2	161.5	171	140.4	116.4	146.2	140.4
LOC148898	119.2	129.5	123.2	132.5	112.5	114.6	114.9	122.5
LOC149643	132.9	117.1	113.3	119.8	106.9	106.3	120.3	113
LOC149950	120.7	110.1	123.3	111.6	108.9	101.2	126.5	115.6
LOC150084	103.7	119.8	121.1	126.9	106.1	103.7	117	113.5
LOC150223	152	149.8	173	152.4	137.9	140.2	160.6	157
LOC150297	229.6	125.2	127.5	293.6	113.6	104.4	209.5	111.5
LOC150383	113.8	113.6	121.1	126.3	119.8	100.5	114.7	126
LOC150383	130.5	135	145.6	152.1	125.7	128.4	142.2	133.9
LOC150383	119.3	120.8	124	120.6	106.2	110.4	120.2	119.6
LOC150763	122.7	122.5	131.9	130.8	123.1	108.5	125.9	131.1
LOC150786	114.9	117.2	121.5	110.4	110.7	106.4	112	109.6
LOC151121	117.6	119.9	120.1	120.6	109	97.6	112.9	111.1
LOC152195	116	111.9	119	113.2	105.8	97.1	114	118.3
LOC152485	221.7	250	236.7	245	193.6	182.7	240.9	209.4
LOC152573	109.4	110.4	109	119.8	111.7	97.1	112.5	118.8
LOC152667	113.9	103.1	114.8	122.9	108.4	92.1	120.8	113.9

LOC153222	768.9	800.2	573.6	886.8	810.7	584.1	1239.5	1579
LOC153328	114.8	125.9	130.5	128.7	121.8	109.1	127.7	122.7
LOC153364	169.4	187.1	164.2	170.7	172.3	130.5	136.4	150.9
LOC154907	111.6	110	113.1	104.8	99	92.4	113.9	106.8
LOC155100	113	112	119.2	111.7	109.4	103.8	115.3	105.9
LOC158160	149	180	174.8	175	138.2	126.7	185.7	191.4
LOC158160	121.3	120.4	133.1	116.3	110.2	101	122.6	117.8
LOC158160	139.8	144.2	154.5	139.4	124.3	112.6	142.1	132.9
LOC158381	119	120.2	119.9	122.9	119.5	107.9	126.3	117.8
LOC158830	136.4	147.9	141.4	139.1	123.4	117.4	139.8	130.2
LOC161247	143.5	137	142.4	148.4	139.4	114.3	124.1	136.7
LOC161931	117.1	115.7	118.3	120.4	115.5	98.3	122.5	117.1
LOC162073	111.8	116.1	117.7	123.3	103.1	108.2	114.1	120.6
LOC162427	138.5	131.8	141	159	122.8	115.4	154.7	137.8
LOC163131	113	118.6	120.7	119.1	108.2	98.8	123.1	114.7
LOC164380	117.3	118.2	109.2	116.3	106.2	100.8	112.4	101.5
LOC164714	117.6	119.9	111.1	118.2	109.3	104.1	117.1	114.7
LOC165186	118.9	107.6	125.8	136.1	113.1	110.6	125.6	116.6
LOC168474	120.4	117.1	124.8	115.4	105.4	96.5	119.2	122.6
LOC168850	589.5	580.3	605.5	622.3	517.1	481.4	649.9	607.5
LOC196264	116	127.5	118.2	124.9	114.3	116.3	129.2	123.5
LOC196394	133	128.7	121.9	141.3	125.2	104	129.4	135.6
LOC196463	144.5	138.7	174.2	156.1	112.6	154.3	145.7	122.2
LOC196541	110.3	102.9	121.1	120.9	106.4	102.5	113.8	103.1
LOC196913	122.6	119.1	126.2	117.2	105.7	104	122.4	110.1
LOC197322	122.7	122.9	113.8	125.7	120.6	104.2	126.1	124.7
LOC197336	141.2	138	123.9	132.6	129.4	109.9	136.4	134.1
LOC198437	110	108	110	104.5	106.9	94.4	112.5	110.5
LOC200312	120.4	111.2	119.4	113.3	102.6	95.5	114.8	107.8
LOC200420	130.3	127.4	123.6	127.9	119.6	102	121.3	122.1
LOC200810	140.5	130.4	123.5	126.5	106.6	119.1	132.7	124.1
LOC201164	129.4	149.1	141.7	137.7	113.7	102.4	119.2	120.4
LOC201175	114.5	120.3	123.9	121.8	122.4	112.2	121.5	119.8
LOC201181	124.6	128.8	136	141.9	103.1	110.2	114.7	113.2
LOC201229	119.4	120.6	115.7	117.8	111.4	112.3	122.5	112.3
LOC201229	117.9	130	122.1	114.9	109.4	105.1	125.7	124.1
LOC201725	118.1	127.4	129.7	127.1	119.7	104.3	124.3	131.9
LOC202134	235.8	231.9	233.6	212.1	220	140.9	217.9	193.9
LOC202459	120.9	118.7	125.6	120.1	114	101.1	125	120.6
LOC203076	109.2	114.2	117.3	120.5	107.4	101.7	122	118.5
LOC203413	111	114.8	115.5	115	102.2	100.3	121.4	113.5
LOC203547	646.3	759.2	598.4	722.8	555.4	426.2	652.2	558.7
LOC205251	129.8	138.3	146.9	131.3	137.2	120.8	129.7	118.6
LOC219854	247.5	260.7	392	224	185.5	208.6	217.7	171
LOC220115	111.5	117.4	108.8	110	108.6	90.6	113.8	111.2
LOC220115	123.3	103.9	109.7	118.5	101.3	98.6	106.1	106.8
LOC220429	115.9	119.8	122.4	122	111.5	92.3	117.9	109.9
LOC221091	112	107.3	100.6	113.2	100.6	95.5	101.6	109.8
LOC221091	119.1	110.4	120.1	114.7	109.4	99.8	116	126
LOC221143	151.8	171.5	175.9	167.4	146.2	159.3	159	155.7
LOC221711	116	121	117.4	116.5	106.9	97.1	119.7	117.4

LOC22195[illegible]	294	375.8	458.7	310.8	251.8	220.3	295	328.6
LOC22296[illegible]	113.9	124.8	115.5	127	108.8	97.6	131.1	122.3
LOC22296[illegible]	127.4	120.9	122.1	115	115	101.2	124.9	118.3
LOC25301[illegible]	115.2	120.5	126.9	127	115.6	98.5	126.2	118.4
LOC25301[illegible]	116.1	116	113.2	116.4	107.1	93.9	123.9	121.5
LOC25301[illegible]	107.3	112.7	107.6	111.5	104.9	105	105.7	109.4
LOC25537[illegible]	117.1	116.3	117.2	119.6	115.7	100.4	119.6	109.6
LOC25735[illegible]	112.9	109.5	119.9	120.7	105.7	102.6	114.4	117.4
LOC26033[illegible]	110.1	101	112	115.5	105.4	102.8	120.6	112.9
LOC26034[illegible]	115.8	117.7	126.6	121.3	105.9	98	121.9	115
LOC28312[illegible]	111.2	119.4	113.6	112.3	116.7	106.6	107.7	113.7
LOC28312[illegible]	114.5	104.9	111	112.5	99.3	94.3	114.5	110.5
LOC28313[illegible]	119.7	115	130.1	119.3	115.6	104	127.1	112
LOC28315[illegible]	112.2	118.3	114.9	109.9	110.5	102.5	110.7	110.8
LOC28315[illegible]	107.4	108.1	114.8	116.9	105.3	98.7	110.6	107.6
LOC28317[illegible]	131.6	133.8	118.9	131.1	133.3	106.8	119.4	129.1
LOC28321[illegible]	291.5	234.5	322.4	303.3	232.3	214.5	234.4	357.2
LOC28334[illegible]	475.9	559.1	470.8	487.3	437.7	468.9	537	576.9
LOC28337[illegible]	128.5	124.9	139.9	123.7	117.2	113	139.3	110.7
LOC28339[illegible]	111.5	111.3	108.8	117	108	102.2	117.6	112.1
LOC28351[illegible]	120	125.9	153.4	127.6	107.1	109.3	119.2	122.2
LOC28353[illegible]	291.3	257.3	299.9	232.8	215.4	189	179.9	189
LOC28355[illegible]	110.8	119.1	113.7	119.3	107.5	100.9	108.4	118.5
LOC28367[illegible]	115.8	127.8	118.3	113.3	113.2	96.1	108.2	120.4
LOC28375[illegible]	103.9	106.4	107.1	117	106	91.7	109.2	108.2
LOC28387[illegible]	167.2	165.4	155.5	149.9	130.4	122.1	144.3	129.7
LOC28387[illegible]	132.2	131.3	142.3	134.9	122.9	130.9	141.1	126.5
LOC28395[illegible]	234.9	231.1	208.6	260.1	218.2	205.8	251.1	234.4
LOC28400[illegible]	114.6	113.9	114.6	113.2	110.5	101.9	125.8	106.8
LOC28419[illegible]	116.7	137.8	126.7	130.6	117.4	115.6	124.1	122
LOC28427[illegible]	114.9	114.1	115.4	119.9	100.2	96.9	100.5	111
LOC28432[illegible]	109.6	116.2	118.5	120.1	113.6	101.7	112.7	116.2
LOC28436[illegible]	99.8	110.7	111.4	122.7	109.5	99	113.2	121.3
LOC28436[illegible]	124.7	113.1	124.5	125.6	109.8	102	122.1	110.3
LOC28437[illegible]	118.3	111	115.1	121.5	102	95	127.2	114.8
LOC28440[illegible]	105.5	115.6	114.9	112.5	110.7	100	114.8	105.9
LOC28443[illegible]	114.4	118.7	112	108.1	112	103.5	117	114.4
LOC28444[illegible]	109.3	115.4	126.1	123.4	113.4	97.8	130.1	116.4
LOC28473[illegible]	116.9	118.6	125.2	113.6	120.1	98.9	114.3	122.3
LOC28475[illegible]	125.8	116.9	118.6	114.2	114.6	101	114.4	117.4
LOC28475[illegible]	126.1	117.6	127.6	112.1	109.8	103.5	117.1	117.8
LOC28491[illegible]	107.6	118.7	139.4	128.2	112.3	105.5	117.5	119.3
LOC28494[illegible]	113.2	108.6	117.6	117.8	107.6	102.1	121.6	113.1
LOC28501[illegible]	120.2	113.6	122.2	134.5	108.7	101.5	119.9	111.5
LOC28503[illegible]	119.4	120.3	117.9	126.6	119	102.2	118.1	124.3
LOC28507[illegible]	315.7	433.7	469.3	325.6	347.9	315.7	429.9	433.2
LOC28519[illegible]	274.2	278.1	309.1	314.2	270.9	232.5	291.7	258.2
LOC28535[illegible]	115.5	129.2	133.3	129.5	110.6	104.4	121.7	122.3
LOC28538[illegible]	120	127	122.8	119.5	111.2	105.5	135.6	122.7
LOC28542[illegible]	130.6	131.2	144.9	122	110.4	111.5	126.3	138.6
LOC28545[illegible]	131.6	137.6	128.7	128.8	123.2	113.2	133.1	128.4

LOC28549	121.3	112.2	115.8	123.1	109.5	100.1	122.3	119.3
LOC28551	139.7	148.3	153.7	153.5	133.9	120.1	151.1	160.3
LOC28552	109.9	111.6	112.6	125.6	111.1	100.1	115.8	115.3
LOC28563	401.9	365.9	420.9	443.9	356.5	344.1	406.2	416.2
LOC28573	126.7	106.3	111.6	123.1	105	96.4	122.9	108.6
LOC28590	123.8	114.8	131.3	133.1	121.2	115.4	131.2	134.3
LOC28598	170.8	176.5	194.5	163.3	159.3	138.6	155.4	166.4
LOC28598	111.4	121.6	110.5	116.2	108.7	101.5	114.3	112.7
LOC28598	156.3	141	191.2	164.4	152.3	119.3	160.2	145.1
LOC28607	118.4	106	120.4	114.9	104.3	92.9	106.7	111.6
LOC28618	127	112.6	118.8	120.2	109.3	107.9	127.4	108.2
LOC28652	110.1	113.3	108.5	117.9	108.1	93.6	117.6	108.8
LOC33832	121.5	112.3	119	122.4	108	93.3	113.6	117.7
LOC33832	108.5	111.8	111.8	112.5	110.9	99.8	117.9	110.3
LOC33879	308.1	285.7	352.1	291.3	247.3	215	229.4	235.6
LOC33912	1659.4	1804.6	1514.9	1811.9	1174.5	901.7	1099.3	945.6
LOC33922	607.8	1020.9	852.3	643.2	570.1	421.9	563	429.6
LOC33922	147.6	149.4	167.2	170.6	141.6	122.9	133.3	131.1
LOC33924	117.3	116.1	125.1	114.5	114.9	100.8	116.2	122.9
LOC33934	431.5	417.9	353.2	488.6	393	341.9	447.6	408.1
LOC33945	112.9	121.1	111.5	124.2	104.4	93.8	111.3	108.9
LOC33974	135.6	130.8	139.7	137.6	123	118.6	125.6	139.6
LOC33997	109.7	117.5	122	118.6	108.3	105	114.7	119.8
LOC34006	106.7	116.2	117.5	112.3	111	95.4	119.7	107.4
LOC34015	113.9	118.2	126.3	138.1	115.1	96.7	123.7	124
LOC34026	111.2	104.6	116	118.9	103.8	105.2	120.4	113.3
LOC34052	116.3	110.9	133	125.9	116.4	106.4	117.6	123.3
LOC34052	124.5	124.7	118.3	130.3	124.8	109.5	109.9	121.8
LOC34052	116	115.8	111.1	116.3	111	94.1	112	113.6
LOC34052	122.4	119.5	124.3	126.8	117.9	97.5	121.6	109.8
LOC34057	112.1	120.6	116	118.7	103.4	100.6	121.2	108.1
LOC34060	107.9	114.2	113.9	111.9	113.3	95.7	117	126.4
LOC34084	138.7	115.5	135	130.2	108.9	109.8	133.5	117
LOC34289	114.4	117.3	121.7	123.9	113.7	99.7	117.9	116.6
LOC34306	121.8	109.5	128.8	117.1	113.8	103.3	112.8	110.3
LOC34352	120.3	120.2	116.6	132	108.1	101.5	115.1	116.5
LOC34440	128.2	135.3	147.3	128.3	126.3	118.7	132.7	119.2
LOC34440	114.9	119.8	142	119.4	124.7	109.3	121.2	124.6
LOC34465	121.4	114.1	128.5	114.3	99.6	99.7	122.2	118.2
LOC34522	112.7	117.5	115.9	119.9	109	102.6	113.2	123.2
LOC34522	112.8	121.5	114.7	119.5	106.2	100.2	122.2	121.8
LOC34615	109	107.6	113.4	113.1	100.1	93.1	109.3	109.9
LOC34665	112.3	122.9	116.2	118.2	102.8	102.1	108	110.7
LOC34667	116.8	114	123.6	111.7	105.6	102.4	107.9	119.6
LOC34727	133.3	131.9	135.4	124.6	129.5	115	143.9	130
LOC34748	111.8	106.5	114.1	114.1	114.1	99.8	115	111.8
LOC34817	116	113.7	125	124.4	108.1	97.8	127	119.7
LOC34818	117.9	121.8	119	122.2	110.6	99	131.2	112.4
LOC34818	137.7	126.7	135.4	134.9	117.3	100.8	118.2	132.3
LOC34818	108.8	125.8	139.8	121.7	121.8	107.3	120.1	125.9
LOC34826	207	205	201.8	203.4	172	150.1	159.1	149.6

LOC348645	138.5	121.1	129.9	142.1	130.7	102.6	137.5	118.4
LOC348840	126.6	121.9	118.7	121	117.6	116	145.3	126.9
LOC349114	141.5	119.7	165	133	116.1	134.4	144.4	122.5
LOC349136	136.2	121	142.5	132.4	116.8	111.4	132	120.6
LOC349236	193.8	218.4	183	203.7	173.3	168.7	206.1	232.2
LOC349338	176.7	173.1	199	183.1	147.2	179.5	178.3	198.9
LOC352909	112.6	121.1	115.2	119.4	104.2	101.6	115.7	109.6
LOC374395	981	966.7	1459.4	1027.1	648.3	971.1	686.3	561.4
LOC374491	113.7	115	114	121.3	105.8	96	111.3	119.8
LOC374491	116.7	119.8	116	131.7	118.5	93.6	120.5	132.8
LOC374920	118.7	121.4	127.5	122.3	113.2	109.4	124.4	126.7
LOC375323	108.4	115.1	113.5	119.3	110.6	97.6	131.9	109.8
LOC375449	121.3	118.8	120.3	111.9	110.6	105.3	112.5	118.8
LOC375748	118.3	117.6	112.4	115.9	120.6	102.5	118.5	119
LOC387601	122	120	116.2	119.1	107.3	97.8	124	109.3
LOC387646	113.8	115.2	110.8	121.1	102.1	102.6	113.1	111.3
LOC387680	127	135.3	125.7	131.6	128.9	109.1	135.5	142.5
LOC387755	120.5	119.2	128.3	128.4	105.8	98.8	126.2	112.1
LOC387758	117.3	115.4	123	120	117.9	104.3	120.2	119
LOC387775	119.7	124.1	118.3	115.2	117.9	100	119.4	125.8
LOC387775	115.5	111.8	119.7	119.6	110.4	99.4	117.1	109.3
LOC387778	119.2	109.1	109.3	122.9	108.7	101	120.8	120.1
LOC387856	113.6	113.4	120.4	117.1	110.3	99.4	118.2	118.8
LOC387856	114.4	126.1	118.8	123	116.5	96	122.7	114
LOC387856	110.4	112.3	112.5	107.7	108.7	105.4	110.6	105.6
LOC387911	123.6	135.2	141.1	129.6	111.9	109.6	122.6	116.7
LOC387921	110.4	116.8	117.9	116.4	117	106	125	119.7
LOC387921	184.2	200.3	272.1	228.8	218.4	195.6	142.9	154.4
LOC388135	127.7	118.6	121.7	121.3	108.4	105.5	118.6	128.7
LOC388199	115.9	108	109.4	108.2	109.9	99	115.8	112.4
LOC388284	127.2	123.8	150.2	128	123.8	115.1	126.5	124
LOC388323	105.2	118.8	108.9	117.1	101.5	88.5	113.1	110.6
LOC388335	138.4	152.3	140.1	154.7	150.7	100.6	129.5	158.7
LOC388389	120.1	111.3	110.1	118.5	115.5	97.6	119.5	116.7
LOC388407	117.2	115.9	116.6	121.5	112.2	102	114.3	123.1
LOC388419	108.9	100.7	118	117.5	111.3	100.4	109.6	103.6
LOC388503	122	114.6	121.6	118.9	117.7	102	131.1	117.1
LOC388524	12133.2	14031.2	14375.1	13645.8	8461.5	9216.9	13018.7	12107.6
LOC388595	117.2	113.3	118.4	114	103	99.6	119.7	103.9
LOC388610	113.1	113.3	118.8	117.7	109.8	98.9	117.3	116.6
LOC388692	282.6	319.8	266.8	288.8	271.5	207.6	304.1	361.8
LOC388882	109.6	116.3	112.6	116	109.3	96.6	111.6	120.2
LOC388886	111.6	120.7	120.8	115.5	104.9	96.6	109.8	117.2
LOC388910	111.8	119.2	121.1	124.9	105.9	92.1	109.7	108.3
LOC388915	107.4	104.4	110	115.8	106.8	93.4	109.7	106.9
LOC388931	110.9	111.9	115.6	125.8	103.5	94.5	122.6	119.1
LOC388955	111.7	116.9	116.4	121.8	106.3	101.3	117.4	106.7
LOC388965	121.1	115.7	115	117.7	109.3	106.7	116.5	114.2
LOC389031	125.2	129.1	122.5	123.8	117.4	97.2	123.5	117.7
LOC389072	112.7	115.7	116.8	119.6	100.2	97.7	119.5	115.8
LOC389118	127.1	114.2	125.9	118.6	106.3	102	123.8	119.9

LOC389151	108.3	119.4	117.1	117.5	102.9	93.4	118.5	115.6
LOC389257	110.8	120.3	111.9	114.3	109.4	96.1	132.4	110.3
LOC389257	123.6	116.2	114	119.9	120.5	98.3	118.3	119.4
LOC389286	206.6	236.9	222	264.1	202.9	115.8	173.5	215
LOC389293	104	115	111.2	110.2	108.7	95.2	109.5	113.1
LOC389429	105.7	106.7	117	108.9	102.9	101.5	112.9	114.1
LOC389432	113.2	108.6	123.4	120.4	101.1	102.2	115.5	107.7
LOC389458	113.6	116.7	119.7	124	108	98.4	119.9	112
LOC389517	108.6	112.3	111.8	119.1	100.1	92.6	111.5	107.7
LOC389541	390	352.9	395.4	348.5	341.8	309.3	321.1	286.6
LOC389549	113.7	113.5	118.8	116.9	106.9	96.7	121.1	109.7
LOC389607	106.4	114.7	114.5	113.9	107.2	98.3	121.1	112.3
LOC389813	109.4	110.1	119	110.2	112.7	97.6	114.8	117.7
LOC389816	130.6	122.3	120.7	132.2	110	112	117.5	114.3
LOC389816	129.7	114.7	120.1	110.4	109.2	107.4	117.5	110.9
LOC389827	116	116.5	113.1	114.6	107.9	102.3	124.2	117.6
LOC389827	119.3	107.8	105.8	118.1	105.4	100.2	115	118.4
LOC389832	116.8	125.5	122	122.9	115.4	108	115.1	119.1
LOC389834	120.6	133.5	115.5	110.4	141.2	101.3	108.4	131.3
LOC389936	104.4	115	111.1	117.8	106.6	98.7	117.7	117.1
LOC390110	112	109	118.5	115.8	103	97.1	119.4	119
LOC390243	107.5	108.5	103.1	113.4	107.3	92.1	116.9	117.9
LOC390243	111.4	112.3	116.9	117.3	97	97.1	116.1	105.9
LOC390637	117.8	118	122.5	119.7	110.1	103.4	113.3	124.5
LOC390667	116.8	131.4	151.6	137.5	121.7	109.4	131.1	128.9
LOC390688	119.2	126.4	124	122.8	124.5	96.3	128	117.5
LOC390748	113.8	113.1	120.6	113.7	98.7	101.9	118.4	119.7
LOC390748	122.9	122.8	118.4	120.9	113.8	104.5	121.3	125.6
LOC390927	118.5	121.8	113.4	116.4	107	98.9	121	114.2
LOC390999	119.2	115.1	116	116	107.7	103	117.1	119.3
LOC391356	132	178.7	146.1	147.4	138.8	123.5	161.4	144.4
LOC392196	106.3	111.1	109.8	103.3	104.4	94.3	100.1	105.1
LOC392395	111.1	125	112.2	122.2	114.1	96.9	114.5	113.4
LOC392465	111.2	127.4	128.5	134.4	135.3	114	131.4	128.5
LOC399706	115.8	115.3	108.2	117	100.5	99.8	118.1	118.1
LOC399744	118.1	131.2	122.6	139.2	114.5	111.7	143.5	130.3
LOC399818	119.9	122.1	114.6	132.6	109.6	104.2	121	126.6
LOC399898	105.9	105.6	116.2	115.7	106.4	97.5	114.2	113.3
LOC399900	5719.9	5388.7	5602.7	4990.5	6452.1	4509	7401.9	6639.1
LOC399947	113.4	113.1	110.4	120.9	104.9	98.4	116.1	114.9
LOC400120	122.5	140.8	123.4	136.9	119.5	115.6	131.2	135
LOC400258	115.2	115.4	116.5	119.9	107.9	101.9	124.8	118.5
LOC400451	115.3	130.4	121.6	123.1	109.3	112.1	125.5	124.7
LOC400464	170.4	153.4	205.2	180.1	138.5	142	158.4	139.4
LOC400464	140.1	146.1	156	141.2	164.7	106.7	151.8	143.6
LOC400499	110.9	108.1	117	122.5	109.6	98.5	120.7	127.2
LOC400506	118.2	117.3	130.1	136.2	117.1	91.6	121.9	125.6
LOC400566	112.9	116.7	130.1	118.3	112.1	106.3	115.6	121.7
LOC400566	108.4	119.1	127.4	124.2	115.3	114.2	131.2	115.1
LOC400657	363.7	375.3	321	349	269	309.6	318.6	364.7
LOC400707	115.3	109.3	109.8	108.8	110.1	99.1	108.7	117.6

LOC400891	120	115.4	112.6	119.9	110.4	96.9	121.6	113.3
LOC400924	144.7	129.7	133.3	132.8	120.2	117.4	148.7	129.4
LOC400965	119.9	111.4	117	120.9	111.6	98.6	117.4	113.3
LOC400986	114.2	106.9	110.3	119.8	102.8	93.8	120.5	114.8
LOC400986	149.7	152.7	160.5	147	162.7	116.6	147.3	150.3
LOC401010	118.1	113.8	110.1	123.6	116.3	110.7	124.4	115
LOC401019	4186.2	3121.2	4664.3	3317.3	2312.3	2880	3467	3879.7
LOC401052	143.6	135.2	163.9	137.4	120.1	131.8	154.6	127
LOC401072	107.4	117.1	113.1	117.7	105.6	103.3	123.2	114.8
LOC401137	117.8	115.9	126.2	126.2	116.5	99.2	118.9	119.1
LOC401152	448.7	430.2	499.9	478.5	329.9	334.3	393.2	334.7
LOC401233	154.8	131.5	188.9	181.3	131.1	151.5	147.9	139.5
LOC401252	106.7	104.9	116.8	114.6	114.8	103	117.2	115.2
LOC401286	108.8	107.2	114.2	111.5	106.6	107	115.1	113.9
LOC401296	112	125.1	120.4	118.8	113.6	101.8	117.9	114.5
LOC401357	197.5	150.8	180.4	225.5	150.2	176	183.9	149.7
LOC401387	111.2	109	129.9	121.6	112.3	107.7	121.3	113.7
LOC401398	112.2	115.2	109.3	112.7	106.7	93.5	121.9	115.6
LOC401410	116.4	124	125.1	124.6	103.7	94.9	115.9	106.6
LOC401431	165.6	172.8	159	154.5	151.9	119.1	161.6	153.3
LOC401498	118.7	120.6	110.8	108.1	108.3	98.6	119.9	118.1
LOC401551	118.5	116.6	116.5	129.9	118.7	101.2	120.5	127.9
LOC401565	112.9	123.7	116.7	113	116.5	100.5	117.2	111.5
LOC401589	116.6	121.4	126.5	115.8	115.4	98.9	121	120.2
LOC401898	121.1	124.2	114.6	125.4	116.9	100.5	117.6	118.8
LOC402055	134.1	126.1	139.8	137	125.6	110.2	123.7	123.3
LOC402057	169.8	140.8	174.7	168.8	127.3	169.9	177.9	143.5
LOC402110	121.9	120.9	121.4	122.2	109.4	98.3	113.3	121.8
LOC402117	118.8	111.8	121.7	118.3	109	95.3	120.6	113.3
LOC402176	137.8	144.9	145.3	132.8	148.7	112.2	149.4	185
LOC402573	112.2	120.7	118.8	125	116.8	105.3	129.2	131.3
LOC402682	133.1	139.8	128.9	137	115.9	100.8	127.1	127.9
LOC405753	123.1	107.6	129.9	125.4	118.8	103.1	119.6	120.1
LOC407835	260.6	292.1	302.8	288.8	221.4	255.1	325.2	232.4
LOC432369	6106.5	7472.7	9392.8	6488.4	6337.4	6675.5	7446.9	7464.9
LOC439985	111.6	113.7	110.5	120.8	105.4	99.3	125.1	117.1
LOC440053	109.3	109.4	111.4	105.2	100.3	97	117.1	118.8
LOC440087	110.2	118.6	118	123.9	105.3	97.9	108	118.2
LOC440093	125.5	110.2	112.2	109.7	108.7	98.5	123.9	120.5
LOC440145	150.2	148	144.7	150.2	141.1	142	158.5	130.3
LOC440145	123.2	136.9	137.3	147.1	133.2	117.1	135.2	143.1
LOC440157	141.2	133.7	139.1	138.3	134.7	109.2	132.3	137.2
LOC440248	115.9	120.1	122.8	127.3	105.7	103.5	114.9	122
LOC440295	117.1	114.6	112.8	115.4	114.1	103	118.3	125.5
LOC440313	111.4	117.9	123.6	130.8	102.5	101.2	120.3	114.2
LOC440348	341.4	366.5	328.7	294	335	255.2	418.1	470.5
LOC440350	129.9	129.5	127.4	126	121.2	101.5	129.3	125.1
LOC440354	131.1	127.5	134.4	143.2	124.1	104.2	134.3	145.4
LOC440396	111.3	118.9	122.3	124.5	105	100.6	114.8	126.3
LOC440508	119	115	114.7	118.2	103.8	109.6	116.3	120
LOC440508	105.9	112.3	109.8	115.6	112.1	101.4	111.9	111.4

LOC440558	106.4	104.6	112.5	112	98.6	95.7	114.5	114.4
LOC440570	104.9	105.6	111	123.8	107.3	96.2	109.8	112.9
LOC440686	129.5	118.5	125.7	124.7	118.2	100.1	125.3	135.4
LOC440742	115.1	119.6	118	117.6	117.2	102.4	118.6	121
LOC440799	112.8	102.9	125	124.6	105	98.5	115.6	113.2
LOC440836	114.6	112.5	130.9	122.3	104.7	99.1	120	111.4
LOC440925	108.8	112.6	122.2	115.4	103.8	101.2	116.6	112
LOC440926	216.7	220.4	249.3	253.6	194.8	268.7	236.2	232
LOC440944	119.6	119.6	116.1	119.8	109.4	102.8	121.7	115.2
LOC441046	111.9	117	118.8	128.8	114.8	99.8	117.1	117.2
LOC441070	112.7	138.2	121.7	132.3	117.3	111.2	125.1	132
LOC441108	122.4	119.1	133.2	131.9	111.5	95.3	124.8	127.2
LOC441120	122.2	114.8	121.8	123	112	101	114.9	114.7
LOC441150	154.6	162.4	200.5	180.1	139.8	132.5	144.7	129.1
LOC441161	119.7	119.3	117.1	122.6	113.3	100.8	121.8	122.9
LOC441168	156.8	132.3	175.4	138.8	130.5	123.6	127.8	125.5
LOC441177	103.8	108.2	110	113.1	105.1	97.3	107.5	109.4
LOC441178	108.8	115.6	120.6	121.7	110.6	100.6	111.5	110.6
LOC441208	110.8	120.5	115.9	124	105.2	104.3	113.8	119.9
LOC441233	114.9	112.1	114.1	114.1	104.5	105.1	117.7	115.9
LOC441242	127.1	133.5	115.7	121	114.7	96.6	119.2	118
LOC441244	122.8	123.6	141.6	122.4	114.8	108.8	134.9	133
LOC441251	119.6	116	127.4	126.7	113.4	107.2	123.6	110.2
LOC441257	106	116.9	114.6	116.5	107.8	102.3	113.4	113.6
LOC441268	232.7	198.2	153.2	184.9	156.9	126.3	154.5	140.1
LOC441282	109.3	114.9	116.7	107.9	110.2	101.8	107.5	119.8
LOC441294	118.2	126.7	125.5	134.5	122.5	109.9	125.4	120.4
LOC441376	105.8	106.3	110.1	116	107.5	93.5	115	104.4
LOC441426	116	109.9	123.1	117.9	111.9	97.9	112.6	103.6
LOC441476	118.1	113.7	112.8	118.2	104.7	108.4	123	116.2
LOC441581	112.1	119.2	119.4	120.1	115.7	97	118	122.7
LOC441581	112.5	123.7	116.8	120	108.7	97.8	122.6	113.4
LOC441601	122.3	116.4	119	109.7	103.1	105	114.6	117.3
LOC441734	111.3	110.7	110.2	109.5	104.4	92.7	111.6	115.7
LOC441743	188.1	188.1	162.2	204	159.6	146.1	162.1	168.9
LOC441931	119.4	108.6	128.3	117.9	107.5	99.6	117.2	115.1
LOC441956	118.1	115.8	117.2	119.6	99.1	98.4	120.1	117.3
LOC441956	118	113.4	111.8	117.6	104.6	97	116.5	111.8
LOC442213	118.1	124.4	122.4	128.8	110.9	103.6	135.8	126.4
LOC442245	115.1	122.1	135.2	128.6	113.3	98.1	118.3	120.6
LOC442424	111.4	110.9	111.4	120.7	100.2	94.4	123.1	115.3
LOC442425	113.6	109.6	122.2	129	108.3	113.9	123.3	128.6
LOC442578	933.8	764.6	790.6	793.1	898.6	449	548.8	582.3
LOC442582	144.1	126.7	135.5	146.4	122.9	117.7	134.4	132.8
LOC442715	115.9	111.3	117.3	114.7	109.4	107.2	122.3	120.2
LOC492303	107.2	112.9	117.5	115.7	105.9	97.6	113.2	116.1
LOC492311	238.6	199.1	253	249.9	204.3	188.2	202.2	156.1
LOC493829	141.3	119.5	120	117.6	110.4	102.8	115.7	127.8
LOC493856	133.3	155.9	155.2	148.9	133.5	130.6	135.1	148.7
LOC493860	117.9	111.8	104.4	114.2	103.9	96	119.1	102
LOC493869	108.2	106.1	111.6	112	111	100.9	120.2	109.9

LOC497190	113.8	114.6	128.3	117.9	109.3	96.7	124.6	117
LOC497661	137.1	164.4	182	144.3	130.2	144.5	146	152.5
LOC51035	1566.1	1935.2	1538	1683.8	1053.1	1192.7	1706	1444.6
LOC51057	118.6	112.4	125.2	121	123.7	103.7	120.7	119.1
LOC51057	112.7	127.4	121.5	112	117.7	106.7	123.5	118.3
LOC51136	172.1	131.3	191	170.7	131.9	147.5	144	135.8
LOC51149	117.8	124.7	127.5	127.9	124.2	104.7	125.3	128
LOC51149	107.7	109.9	117.3	106.5	105.6	100.4	121.4	116.6
LOC51233	115.9	116.4	110.7	117.4	109.6	97.7	118.2	107.2
LOC51252	120	122.9	121.4	129	103.7	99.4	122.4	117.4
LOC51255	524.5	658.2	791.3	632.3	422	695.3	544.7	577.4
LOC51333	127.6	124.1	132	133	125.4	101.8	134.2	137.3
LOC51334	131.6	123.2	128.8	129.7	120.6	110.8	132.7	118.1
LOC54103	133.4	116.6	145.9	148.3	118.3	116.3	136.3	128
LOC541469	128.8	121.1	138.5	130.3	111.6	107.9	117.8	115.3
LOC541473	117.3	123.1	116.2	128.6	108.6	108.7	124.7	111.1
LOC550631	131.6	140.3	124.7	116.6	113.2	95.1	124.8	115.6
LOC552891	111.9	112.6	113.8	120.2	106.6	97.7	110.7	127.9
LOC554226	113.2	112.8	110.9	107	110	95.3	118	124.1
LOC554235	118.1	125.1	113.7	123.8	109.9	107	118.8	114.8
LOC554251	122.7	118.9	121.6	128.8	127.3	105.8	116.3	108.7
LOC55565	193.8	170.4	177.8	170.8	134	125	134.8	132.3
LOC55908	129.5	126.4	125.5	124.5	108.4	99.3	130.7	120.7
LOC56901	121	107.8	109.4	126.3	116.1	100.4	119.5	113.8
LOC56902	145.2	141.7	140.7	140.3	130.9	122.3	148.4	140.2
LOC56964	108.9	115.2	110.5	104.6	111.4	99.8	117.2	111
LOC57149	370.2	417.5	561.4	485.8	402.1	480.8	559.2	726.8
LOC57228	126	134.8	124.1	142.3	136.2	117.6	140.8	138
LOC57228	190.6	221.1	172	219.4	187	135.3	271.4	272.1
LOC57228	111.7	111.9	124	116.4	107	104.3	113.2	119.8
LOC57228	118.8	117.3	120.6	125.7	109.3	96.5	122.2	116.1
LOC594834	112.3	117.5	108.8	112.9	103.3	95	110	113.5
LOC594834	115	108.2	123.2	129.3	127.8	95.2	113.2	120.7
LOC594834	116.6	115.9	126.6	125.4	107.5	103.2	113.4	122.1
LOC595101	124.3	130.5	128.2	123.6	120.8	107.1	129.7	127
LOC606495	113.9	116	122.2	131.8	114.4	102.6	122.5	114.5
LOC606724	834.4	1013	804.8	812.6	531.3	704.5	700.8	716.8
LOC613037	2102.4	1700.7	1352.4	1872.8	1615.1	725.3	2608.9	2204.7
LOC613212	121.6	119.7	109.2	113.5	106.3	99.9	114.9	114.4
LOC619207	112.4	119.6	121.2	117.5	108.4	103.5	125.5	115.7
LOC619208	117.7	123.1	127.6	117.5	111.9	102.7	123.1	118.4
LOC63920	116.5	137.7	140.9	135.6	119.3	112.5	123.7	125.7
LOC63928	109	111.6	123.6	125.2	107.1	104.4	126.1	119.7
LOC641522	121.2	121.6	123.7	122.4	115.5	98.1	127.5	123.4
LOC641847	113.6	114.7	111.5	123.9	110	94.4	117.6	117.3
LOC641949	131.5	124.6	127.8	125.7	112.7	105.2	116.9	129.9
LOC642090	333.5	431.6	333.9	435.2	378.8	287.5	495	479.4
LOC642181	104.1	107.6	108	107.3	105.1	94.7	118	105.2
LOC642201	114.1	111.8	118.2	115.8	110.8	98.2	113.3	111
LOC642265	115.9	113.1	110.9	113.1	110.9	91.5	105.9	109.1
LOC642293	112.5	108.3	106.4	108.2	107.4	91.3	112.7	110.7

LOC642316	110.4	112.3	114.4	121.9	110.9	96.8	116.2	111.8
LOC642323	125.8	138.1	148.7	154.8	136.8	110.7	143	136.3
LOC642782	118.6	116.6	120.8	122.9	108.9	101.1	116.6	108.1
LOC642944	115.6	110.2	114.8	112.8	112.4	99.9	117.8	121.8
LOC642947	4886.9	5226.2	4816.2	6273	5431.8	2616.7	4997.7	6101.6
LOC642968	128.6	120.4	117.2	124.9	117.2	102	124.1	116.5
LOC642980	113.4	125.3	121.2	124.9	104.4	100	121.7	122.8
LOC642980	120.5	127.9	118.1	122.6	115.7	106.3	123.5	120.6
LOC643057	22298	19164.3	18661.8	20836.3	14684.6	18465.6	21352.9	23919.7
LOC643165	126.6	125.4	112.5	130.5	106.5	100	132.2	123.9
LOC643196	118	108.3	119.8	120.4	109.1	103.3	123.6	121.3
LOC643226	115.4	111.7	117.7	112.7	108.4	100.4	118.7	103.7
LOC643226	104	107.6	105.3	112.6	107.1	102.1	118.1	113.5
LOC643253	112.8	122.1	112	118	110.5	91.3	122.6	108.5
LOC643298	124.7	120.8	121.8	127.8	121.6	104.5	131.9	132.9
LOC643366	117.8	131.8	114.2	115.3	117.7	114.5	134.6	127.7
LOC643394	115.3	119.4	118.2	121.5	108.6	100.9	113.8	113.4
LOC643414	115.4	117.3	110.1	118.7	106.2	96.2	113.9	106.6
LOC643425	114.9	111.4	116.3	116.8	113.5	101.5	109.9	113.4
LOC643516	827.2	4082	983.9	830.2	2973.3	687.4	805	3891.3
LOC643542	110.6	114	118.8	118.2	111.5	100.8	117.4	115.4
LOC643591	177.5	157.3	169.1	193.3	189.6	118.9	156	156.9
LOC643668	1136.3	907.5	1485.9	1265.7	1052.6	1210.4	1438	1497
LOC643736	116.4	116.8	115.8	121.3	110	102.6	124.6	120.9
LOC643812	128.2	120.8	125	117.1	123.3	109.6	121.2	122.6
LOC643842	123.9	117.2	122.2	131.1	113.6	100.6	111.2	120.8
LOC643853	112.4	119.1	111	118.5	116	94.5	117.9	119.9
LOC643853	116.3	118.6	120.1	118.7	113	106.2	125.5	120.8
LOC643866	117	118.4	117.6	120	107.4	97.5	117.3	119
LOC643905	111.9	115.2	107.2	109.6	95.8	96.8	115.3	107
LOC643923	113.3	121.9	120	118.8	106.1	98.8	112.2	111.5
LOC643923	120.4	113.4	110	121	110.2	100	125.8	113.6
LOC643940	382	374.1	486.5	470.2	366.1	394.7	473.2	568.5
LOC643995	158.4	134.1	143.7	136.3	129.9	112.5	139.2	148.9
LOC644076	108	118.6	119.9	115.3	103.7	94.3	116.3	109.1
LOC644096	383.6	429.3	405.4	447.8	341.2	307.5	356.5	291.8
LOC644168	113.7	116.2	114.1	122.4	108	103	109.9	110.9
LOC644376	111.9	112.1	120.3	119.8	112.7	104	111.8	120.5
LOC644398	114.1	125.7	117.6	132.2	104.3	106.9	116.2	116.6
LOC644407	114.9	119.8	124	117.1	116.4	97.2	131.3	116.2
LOC644529	120.7	118.1	124.5	117	110.4	100.1	121.6	128.5
LOC644613	114.7	127	113.9	121.8	102.4	91.9	127.3	121.1
LOC644615	159.7	130.7	130.8	132.3	108.8	110.4	123.6	119.5
LOC644685	116.2	115.3	123.7	119	115.5	105.7	133	131.9
LOC644784	119.8	118.3	119.4	123.9	103	103.9	127.3	115.7
LOC644792	115.5	119.2	123.2	118.2	123.5	95.6	127.1	115.1
LOC644890	114.6	115.8	121	124.7	110.5	110.2	127.9	112.6
LOC644943	116.8	117.3	105.6	114.5	114.1	92.7	116.3	117.2
LOC644997	114.7	123.6	112	118.4	103	96.3	122.2	113.5
LOC644997	118	117.2	138.8	121.6	119.8	110.7	116.9	127.3
LOC645039	120.1	116.7	117.2	123.6	108.1	92.7	123.3	111.3

LOC645437	117.4	114.7	135	117.6	109.4	111.2	124.4	116.4
LOC645562	113.7	115	118.4	121.6	108.8	94.3	115.2	118
LOC645849	115.3	107.4	114.2	118.8	109.6	99.9	119.9	114.7
LOC645922	103.5	112.5	110.3	113.7	103.1	86.3	121	106.8
LOC645974	116.3	111.8	112.9	126.7	111	100.5	119.8	110.5
LOC645974	109.3	107.5	120.4	117.5	103	101	114.9	114.9
LOC646182	109.3	115.7	113.7	114.3	109.7	93.6	112.9	110
LOC646200	4535.9	4612.1	5617.3	4743.8	4800.5	3958.9	4616.8	5937.3
LOC646480	104.2	105.2	112	113.5	99.3	95.3	116.4	107.8
LOC646600	105.3	114.7	108.3	113.5	107.6	99.3	112.1	108.8
LOC646962	113.2	107.1	111.9	108.6	104.5	98.1	124.4	106.8
LOC647043	113.9	108.7	115.1	115.2	110.2	100.4	112.8	112.8
LOC647141	119.4	112.7	119.1	118.7	109.3	107.8	123.4	114
LOC647649	101.5	111.1	109.5	103.8	105.8	94	104.2	116.9
LOC647649	108.9	117.2	120.1	122.9	110.3	94.3	121.9	112.5
LOC647760	116.7	128	121.7	119.1	117.4	100.6	118.3	120
LOC647881	115.3	124.8	130.5	128.1	105	106.8	124.8	123.9
LOC648148	111.5	108.5	109.6	110.2	106.9	90.8	114	105.3
LOC648293	470.5	531.2	567.3	494.5	487.9	428.6	502.7	698.3
LOC648303	113.3	128.3	130	126.8	119.7	101.7	121	117.3
LOC648367	121	121.4	112.2	127.5	110.1	95.9	115.2	118.7
LOC648832	111.9	111	118.7	122.7	118	101.8	123.1	111.4
LOC649019	113.8	123.4	106.8	117.6	107.9	101	117.1	116.1
LOC649159	110	112.6	113.1	116.9	104.8	95	106.1	105.4
LOC649159	130.8	122.2	127.6	119.8	116.6	99.2	117.8	125.9
LOC649230	111.5	112.2	123.5	121.5	105.3	105.3	122	111.9
LOC649377	123	124.9	136.2	133.1	109.5	105.6	124.3	118.4
LOC649387	121.4	133	128.8	130.8	118.6	114	139.4	133.3
LOC649387	114.4	108.1	120.9	115.8	116.7	93.6	110.6	118.3
LOC649431	110.4	124.2	122.7	114.1	105	97	116.5	131.6
LOC649446	113.8	108.2	122	116.7	115.3	95.6	113.6	113.3
LOC649516	119.5	128.2	118.2	120.5	104.4	105.2	121.7	121.1
LOC649555	115.9	115.8	124.1	134.6	115.4	105.7	128.4	113.9
LOC649588	119	109.7	112.8	119.4	109.7	98.6	121.7	121.7
LOC649708	122.4	127.3	121.1	124.9	115	110.9	119.6	120.7
LOC649853	1318.5	1222.5	2217.8	1721.1	1255.1	1608.9	1256.1	666.2
LOC649946	1227.6	1216.5	1396.4	1256.3	728	817.2	1130.9	1085.1
LOC650000	108.1	112.4	111.4	116.6	114.8	98.7	113.8	106.5
LOC650040	113.2	116.2	131.8	136.7	109.9	105.6	114.5	116
LOC650137	112.8	119.4	119.8	127.7	106.5	103.7	132.2	121.4
LOC650137	104.1	109.7	108.6	124.5	110.1	99.7	125.1	111.8
LOC650239	116	116.4	113.7	121	109.8	104.8	113.2	119.5
LOC650254	121.8	113.9	120.4	125.7	106.9	100.9	114.2	110.9
LOC650293	109.2	110.9	108.9	118	109.7	94	122	117.8
LOC650586	124.3	128.6	131.3	118	126.8	105	136.8	143.1
LOC650645	121.1	115.6	126	121	111.2	104.5	122.8	123.8
LOC650695	108.2	112.4	105.8	107.4	109.3	93	123.6	110
LOC650798	128.2	115.6	117.9	113.1	111.8	109.3	115.6	124.9
LOC651076	147.3	145.5	148.4	148.7	144	112.3	148.5	129.4
LOC651137	106.9	101.8	116.4	109.4	109.2	91.9	119.2	112.2
LOC651321	117.8	130.3	127.4	120.3	116	98.7	116.7	117.9

LOC651423	188.9	217.7	201.7	215.9	170.8	153.5	197.2	203.6
LOC651503	123.9	123.2	116.1	122.5	111.7	109	120.8	115.8
LOC652020	125.3	107.5	118.7	116.4	118.8	103.4	118.3	111.5
LOC652236	116.4	118	116.9	117.9	114.3	98.6	121.5	125.8
LOC652461	118.5	126	133.1	126	119.9	120	133.1	129.8
LOC652480	108	123.3	115.1	115.4	105.9	107.5	129.2	118.6
LOC652705	114.1	114.7	118.2	120.1	106.8	101.2	129.2	109.1
LOC652744	112.7	117.6	114.6	112.8	105.7	101.9	117.4	116.1
LOC652870	119.1	111.7	120	109	109.8	96.6	117.5	107.8
LOC652888	114.4	124.2	122.4	128.1	104.7	94.3	118.4	121.1
LOC652924	124.2	126.8	133.9	139.7	124.7	110.4	126.4	120.9
LOC652968	137.3	159.5	150.7	151.2	149.8	133.6	141	151.6
LOC653033	113.3	121.7	117.5	113.8	106.3	104	110.7	113.8
LOC653056	118.3	121.8	125.4	128.6	112.9	104.2	118.1	118.1
LOC653062	109.4	104.4	118.3	123	108.6	95.2	119.6	121.8
LOC653084	118.7	125.3	115	120.2	111.6	102.3	113.9	113.6
LOC653098	125.7	124.6	137.5	141.8	107.3	116.5	123.8	125.1
LOC653101	121.7	119.5	137.7	139.1	116.3	117.5	137.1	122.6
LOC653122	107.8	108.9	117.2	112.7	103.1	101.4	109.7	112.9
LOC653131	116.2	117	122.8	117.7	107	98.4	119	124.6
LOC653133	152.8	145.2	155.3	180	136.4	161.3	160.6	139.9
LOC653142	113.4	106.1	111.9	111.8	105.8	93	101.7	116.9
LOC653150	133	143.1	136.3	127.8	131.7	121.8	145.6	130.4
LOC653152	124.7	125.9	141.4	148.7	118.9	103.9	132.1	136
LOC653170	114	113.4	122.5	122.4	104.6	101.4	115.6	120.7
LOC653179	125.4	138.6	121.7	119.4	120.6	103.5	131.6	125.8
LOC653216	108.9	116.1	123.5	123.3	110.1	103	116.1	110.1
LOC653216	120.5	121.6	127.1	114.3	119.5	101.8	120.4	115.2
LOC653216	108.1	116.5	118.7	113.2	106	100	117.3	107.5
LOC653281	125.5	121	109.9	120.1	111.3	103.1	128	119.9
LOC653282	116.5	115.6	120.8	120.8	109.1	100.9	119.4	115.3
LOC653285	120.1	117.9	123.9	121.4	112.8	106.1	122.7	118.4
LOC653314	1580.3	2224.7	1637.4	1959.8	1825.7	974.3	1762.7	2387.8
LOC653316	121.1	113.2	108.1	118.9	99.3	91.5	111.2	109.8
LOC653319	110.8	117.6	122.5	132.7	112.2	98.7	123.4	119.7
LOC653328	3273.7	2998.7	3525.2	3499.9	2477.3	2904.1	4047.9	4335.4
LOC653375	127.5	131.6	127.4	127.3	127.2	122.4	128	123.1
LOC653386	118.7	131.8	112.6	122.9	117.3	103.4	127.8	119.8
LOC653417	124.2	123.4	132.6	116.2	117.6	115.9	129.3	118.1
LOC653419	114.4	116.6	134.3	123.2	105.9	113.5	113.5	115.8
LOC653423	115.8	114.1	127.9	112.8	114.6	105.2	125.9	122.2
LOC653466	121.6	115.2	115	116.5	107.4	96.7	122.2	117.7
LOC653484	138.3	126.6	126.4	143.5	119.3	119	126.8	121.8
LOC653490	115.1	121.6	119.6	119.9	118.9	105.1	125.3	121.1
LOC653499	119.4	113.8	111.8	122	109.3	99.8	120.4	119.4
LOC653502	115	123.4	122	131.3	113.2	106.9	134.5	119.4
LOC653517	117.4	107.7	116.3	105.5	106.6	107	110.6	122.9
LOC653542	145.9	141.3	196.5	153.5	158.7	149.3	158	150.6
LOC653546	112.5	112.7	114.6	117.9	112	96.9	112.6	105.8
LOC653558	115	116.9	117	125.6	108.9	101.5	115.2	108.3
LOC653566	212.4	185.6	341.4	257.4	256.3	283.9	259.8	219.3

LOC653568	105.7	115.3	116.2	112.8	106.6	98.5	114.3	110
LOC653600	167.6	767.9	328.3	214.5	579.3	274.6	179.4	770.4
LOC653602	113.4	121.5	119.1	117.4	110.4	107.2	114.4	115.1
LOC653613	106.5	108.2	108	115.4	110.1	92.1	114.5	112.1
LOC653614	116.3	112.6	114.3	116.4	106.5	101.3	121.3	116.6
LOC653620	112.6	112.9	114.5	125.8	107.8	100	120.7	111.7
LOC653623	108.5	114	119.3	113.3	106.4	98.1	130.2	118.3
LOC653626	111.4	113.5	114.2	120.6	108.1	107.8	119.2	112.5
LOC653655	109.5	120.6	139.5	123.2	104.2	103.1	123.8	129.9
LOC653685	123.5	118.4	126.4	125.2	109.7	100.4	127.7	110.4
LOC653686	103.8	112	110.8	116.2	98.4	94.2	115.3	104.6
LOC653693	109.7	114.9	121.6	122.1	107.3	100.8	122.2	114.3
LOC653697	109.5	123.1	118.2	114	107	95.1	112.6	105.3
LOC653703	112.7	121.3	116.9	119.2	104	98.2	119.3	114.5
LOC653705	114.4	109.5	124	111.2	112.5	99.1	117.5	115.7
LOC653709	123.6	128.9	138.3	131.2	117.7	117.8	138	144.7
LOC653716	104.9	108	116.5	102.9	115.7	93.5	111.8	109.5
LOC653723	117.3	117.3	122.6	128.7	110.4	105.9	118.3	123.4
LOC653742	133.2	121.1	124.4	135.6	127.7	110.5	124.4	122.6
LOC653744	118.9	119.3	119	113.7	104.1	102.1	118.5	112.7
LOC653745	167.9	174.2	148.6	164.2	146.6	109.3	158.5	148.3
LOC653746	104.5	113	118	112.4	98.9	95.9	112.7	116.6
LOC653749	114.6	117.2	116.8	112.2	106.6	105.9	126.4	116.1
LOC653757	150.7	147.8	145.2	148.9	129	130.8	136	128.4
LOC653759	109	114	105.6	115.5	108.6	91.9	112.7	108.2
LOC653762	117	123.5	127.7	119.7	115.8	102.3	127.5	122.5
LOC653767	112.8	121.3	116.2	113	104.5	102.9	127.3	115.5
LOC653771	117.1	109.5	107	120.3	114.4	99.3	121.3	105
LOC653792	104.1	110	116.1	107.3	102.3	91.7	111.3	112
LOC653795	116.3	110.3	120.8	115.1	104.4	97.9	120.1	111.5
LOC653797	118	120	124.2	122.3	110.5	96.8	114.8	114.8
LOC653803	124.6	120.5	126.2	118.7	113.5	104.4	118	124.3
LOC653813	112.7	108.8	110.1	122.7	105.2	104.7	112.1	109.3
LOC653814	111.9	112.2	119.5	119.7	107.2	94.4	119.2	113.4
LOC653827	112	105.3	103.8	120.7	97.7	96.7	109.7	114.7
LOC653866	126.2	110.8	120	121.4	103.1	98.2	115.7	110.5
LOC653906	110.5	119.8	115	117.7	104.7	95.6	124	106.6
LOC653950	118.5	114.6	113.8	122.2	114	107.5	107.8	118.9
LOC653980	115.5	106.4	112.6	117.3	116.5	99.4	111.6	117.7
LOC653980	118.6	120	128.9	139.8	117.2	98.3	132	124.1
LOC654017	173	226.1	231.4	221.6	199.2	170.3	211.8	185.9
LOC654052	135.9	151	128.9	157.5	138.2	110.8	159.4	142.3
LOC654084	118.7	114.8	117.5	123.2	107.4	105.9	117.5	122.8
LOC654119	112.9	112.9	107	115.2	108.8	96.3	117.4	109.1
LOC654123	151.1	152.7	142.8	153.7	142.3	129.3	157.4	129.6
LOC654123	117.6	120.3	114.7	117.1	107.9	102.9	116.4	121.1
LOC654129	115.6	122	117.5	123.7	109.8	100.8	121.3	118
LOC654146	109.3	117.7	114.8	119.5	107.6	101	123	124.1
LOC654151	111.9	125	122.1	116	102.3	95.1	121.6	109.9
LOC654182	117.1	111.9	125	110.9	107.3	96.9	116.8	125.6
LOC654229	113.7	121	115.5	131	120.5	100.9	110.2	113.7

LOC654346	111.8	108.8	132.5	132.2	112.6	103.4	133.8	119.5
LOC723972	131.6	123.5	137.8	142.8	128.4	118.9	124.9	128.5
LOC727936	107.5	110.6	116.4	120.7	114.3	99.6	126.3	117.4
LOC728137	120.6	115.9	136.1	136.9	115.3	112.2	118.6	124.4
LOC728215	111.4	113.8	106.8	117.8	108.2	102.2	113	116.8
LOC728269	118.2	121.9	127	128.5	116.3	99.5	119.1	117.9
LOC728489	491.3	587.3	426	548.5	398.5	270.3	481.7	477.3
LOC728621	110.3	107	114.3	114.3	108.2	102.1	105.5	123.8
LOC728635	123.7	106	119.1	124.2	110.8	99.6	140.2	118.3
LOC728643	239.9	219.1	259.9	292.9	234.9	202.4	297.9	305.8
LOC728758	173	176.4	197.8	183.2	165.4	124.9	190.1	180.4
LOC729164	120.4	125.6	113.8	115.2	104.4	93.6	124.3	114.8
LOC729440	111.2	126	135.6	123.4	128.8	101.8	132.3	133
LOC729603	4482.3	2693.3	4274.4	3829	4821.2	2175.5	3516.2	4310.4
LOC81558	113.9	140	126.1	133.7	135.7	106.6	128.1	121
LOC81691	135.5	153.1	144.6	151.8	142.5	123.8	148.5	146.4
LOC84661	767.5	1017.5	1176.7	898.9	886.6	885.8	820.4	785.8
LOC89944	122.8	128.6	121	123.1	108.4	98.1	124.7	113.6
LOC90167	105.5	115.9	123.6	111.3	107.3	99.1	117.1	112.2
LOC90321	113.4	121.6	143.9	134.3	136.3	112.7	130.1	131.5
LOC90355	139.5	127.3	143	141.1	122.2	108.3	160.9	138
LOC90379	113	112.8	121.1	117.3	103.4	93.7	112.4	110
LOC90379	188.4	241.1	200.3	206.9	169.8	165.5	272.5	212.8
LOC90580	232	265.7	202.6	282.8	205.8	185.8	274.7	218
LOC90586	2052.3	1544.7	1900.8	1454.3	1644	1041.3	1830	2215.3
LOC90639	336.2	356.9	380.4	370.9	383.5	278	407.9	502.3
LOC90693	132.9	153.4	133.2	141.2	140.8	118.4	148.7	132.9
LOC90826	149.1	144.4	159.1	154.4	135.3	135.7	158.7	145.1
LOC90835	136.4	140.2	174.7	142.3	117.1	109.3	136	144.3
LOC90925	180.1	156.2	282.5	168.8	127.1	270.4	134.6	119.6
LOC91137	266.3	279.4	290.4	307.6	256.6	188.5	267.6	242.9
LOC91353	128.7	137.2	122.7	138.2	112.8	111.9	143.6	133.4
LOC91431	124.1	133.8	138.1	142.5	124	104.9	129.9	133.2
LOC91461	121.8	118.6	117.2	118.9	110.4	100.7	115.9	121.5
LOC91561	25979.7	28269.2	23119.3	26371.9	21446.3	21199.6	26520.5	28801.2
LOC91614	111.6	123.7	121.5	118.1	106.9	100.2	121.2	126.3
LOC91689	185.1	167.5	208.2	186.1	157.6	169.5	166.9	159.7
LOC92017	155.2	135.8	138.7	145.9	141.2	109.9	136.1	137.6
LOC92196	114.3	137.9	117.7	123.9	115.1	104.9	123.2	120.7
LOC92270	124.1	123.8	143.8	135.2	120.6	120.3	150.3	128.5
LOC92345	128.7	129.6	137	135.6	129	103.6	123.5	135.8
LOC92689	110	111	111.5	110.9	107.7	96	116.2	117.1
LOC92691	130.5	124.4	140.5	129	116.3	112.4	124.7	118.9
LOC93081	807.2	726.7	687.7	889.9	710.9	645.1	686.7	887.4
LOC93349	121.7	131.4	120.1	137.7	109.8	103.1	134.2	115.7
LOC93622	245.5	379.2	330.5	309.3	229.6	270.8	243.4	306.9
LOH11CR2A	121.5	138.8	131.5	131.3	123.3	116.6	125.5	113.8
LOH11CR2A	127.1	114.6	139.7	137.9	120.2	101.5	135.3	127.7
LOH12CR1	124.7	117.8	110.7	120.2	112.7	102.6	127.9	124.1
LOH3CR2A	107.5	110.1	113.7	114.9	106.5	102.7	111.9	111.7
LONP2	133.3	138.4	140.3	150	134.9	107.5	135.4	133.5

LONRF1	138.8	148.6	139	161.6	128	126.6	138.2	165.2
LONRF2	110.7	112.2	111.7	123.4	106.2	94.1	116.1	128.2
LONRF3	150.6	164.8	140	134	149.2	111.1	173.4	153.9
LONRF3	111.9	112.1	110.1	113.4	112.4	109.1	115.4	126.9
LOR	126.8	113.1	106.7	112.3	95.8	103.5	114.9	112.2
LOX	110.6	109.9	121.1	105.9	109.2	101.9	119.9	119.3
LOXHD1	124.6	129.4	124.4	135.1	121.3	100.4	137.4	122.9
LOXL1	108.4	117	113.3	114	110	99.4	109.8	111.8
LOXL1	106	107	117.3	114.4	104.6	99.2	114.1	113.7
LOXL2	107	115.4	115.4	113.9	108.3	94.8	103.2	111.8
LOXL3	119.6	117.7	144.4	123	108.7	100.4	112.7	118.8
LOXL4	109.6	107.9	117.3	117.8	113	91.4	115.6	109.5
LPA	122.8	109.5	123.1	116.8	111.4	94.6	133.6	115.7
LPA	106.8	109.6	115.6	117.2	100.9	95.2	114.9	116.2
LPAL2	121.8	112.5	129.6	133.5	109.3	101.5	121	113.4
LPAL2	122.1	113.4	123.8	115.1	110	98.7	118.3	110.3
LPAL2	112.1	119.5	118.6	112.8	111.3	98.4	116.8	106
LPGAT1	117.7	118.7	130.9	117.7	104.5	98.5	122.1	119.3
LPHN1	241	264.4	206.8	251.2	205.3	191.8	274.9	287.2
LPHN1	115.6	123.9	122.7	119.6	104.8	103.9	111.7	121.7
LPHN2	124.2	125	134.5	129.1	116.4	106.6	126.5	116.3
LPHN3	128.7	112.4	126.6	116.5	115.5	101.6	114.2	122.8
LPIN1	1257.6	1256.6	1340.2	1274.2	1308	1204.6	1251.4	1632.3
LPIN2	141	126.2	126.3	141.7	134.1	109.2	148.4	143.8
LPIN3	117.5	119.4	110.4	120.2	111.3	99.5	116	106.3
LPL	135.7	148	124.4	125.4	111.2	98.6	130.3	112.1
LPO	111.6	115.7	109.8	115.5	108.1	100.8	115.2	116.3
LPP	265.6	274.9	252.6	266.7	294.2	178.4	233.9	243.3
LPPR2	118.3	123.8	143	129.5	109.2	119	129.2	123.8
LPPR4	113.9	104.2	125.1	114	105.5	97.3	122.1	127.7
LPXN	2734.8	2571.4	3278.5	2935.9	2489.5	3180.1	3679.9	2993.3
LRAP	3554.8	3262.7	3989.9	2853.7	3417	2415.2	3758.6	3647.1
LRAT	111.5	118.2	117.6	110.9	112.1	95.5	117.5	114.6
LRBA	353.8	394	339.5	370.5	337.1	286.5	451.6	377.2
LRCH1	112	106.4	115.1	110.6	112.1	93.9	122.5	107.5
LRCH2	124	121.8	127.9	122.4	108.9	104.2	112.9	110
LRCH3	171.2	160.8	170	166.9	162.7	127.6	171.9	188.1
LRCH4	669.1	689.1	519.7	696.2	465.6	273.6	507.1	468.4
LRDD	197.3	202.3	210.6	195.9	186.7	156.2	201	191.5
LRDD	166.3	168.2	165.1	165.6	141.5	152.4	182.5	177.3
LRDD	112	113.4	113.4	117.4	106.8	102.6	117.6	112.7
LRFN2	108.4	105.2	112.7	118.4	103.6	97.7	116.7	110
LRFN3	339.2	443.7	216	304.8	257.4	174	245.1	198.6
LRFN4	116.1	133.3	129.2	128.1	115.5	113	115.2	117
LRFN5	110.7	110.5	113.9	118.2	107.9	96.9	114.9	108.2
LRG1	111.9	118.4	136.5	137.7	122.4	95.2	121.7	124.9
LRIG1	514.6	515.2	449	539.2	480.5	395.1	640.3	563.5
LRIG2	142.3	173.7	165	153.6	141.7	124	162.5	146.8
LRIG3	115.7	111.4	111.8	113.6	103.3	94.8	115	114.6
LRMP	265.3	293.6	321.7	333.1	231.3	248.5	263.5	239.5
LRP1	112.1	107.7	120	118.6	103.9	107.1	116.6	116.3

LRP10	189.9	179.9	186.3	224.8	172.3	177.2	206	170
LRP11	111.8	116.3	111.8	125.7	110.1	103.1	127.3	113.6
LRP12	116.5	116.3	114.2	127.3	123.4	96.2	117.7	124.1
LRP16	118.2	121.2	121.8	127.1	118.2	101	124.2	117.2
LRP1B	109.1	119.1	119.2	124.1	109.4	105	122.1	112.7
LRP2	119.8	110.8	108.2	115.9	99.3	95.5	114.9	112.3
LRP2BP	118.1	120.8	117.2	115.8	107.2	105.8	119.1	117.6
LRP3	133.8	158	140.3	149.4	115.6	107.5	133.1	115.8
LRP4	121.2	110.5	112.5	117.6	110.1	93.7	113	118.8
LRP5	104.9	112.1	119	111.3	102.6	99.8	125	125.1
LRP5L	208.8	258.4	271.9	237.2	219	156.9	217	246
LRP6	123.1	138.9	135.6	136.3	116.2	114.1	132.4	133.3
LRP8	121.2	115.7	126.7	125.9	108.1	101	119.8	116.4
LRP8	122.1	124	143.1	146.2	131.6	152.4	140	141.5
LRPAP1	220.9	264.6	235.5	233.8	208.9	210.7	206.8	191.3
LRPPRC	242.6	266.9	208.2	261.7	240.3	190	223.3	185.8
LRRC1	121.5	117.9	118.6	120.7	117.7	103.7	115.7	110.4
LRRC1	186.8	172.9	147.7	187.9	163.9	125.2	178.3	159.2
LRRC10	102.3	117.9	118	121.4	104.9	98.8	125	111.9
LRRC14	180.8	142.4	174.5	161.4	135.3	137.5	155.6	134.1
LRRC15	123.9	122.1	125.1	136	122.3	102.5	133.7	133.9
LRRC16	120.3	132.8	122.7	125	119.1	106.6	117.8	118.3
LRRC17	111.5	117.4	114.5	117.5	109.3	108.8	110.3	104.8
LRRC17	116.5	121.6	109.9	114.8	108	99.1	122.1	112.9
LRRC17	110	107.9	108.4	109.9	110.3	98.5	111.8	114.5
LRRC18	121.2	112.9	117.5	127.2	122.6	102.4	115.5	111.1
LRRC19	117.6	117.4	129.5	126	110.7	97.5	117.6	114.9
LRRC2	109.9	113.4	118.3	115.5	119	103	119.4	116.3
LRRC20	111.3	106.6	113.8	124.9	100.8	91.4	101.3	104.2
LRRC20	158.9	173.5	144.8	163.7	145.8	105.2	129.9	122.1
LRRC20	112.1	115.2	125.2	122.1	103.3	107.9	120.2	109.9
LRRC21	124.1	118.9	118.9	121.4	104.1	98.2	115.5	110
LRRC22	112.7	109.8	110.7	112.1	104.2	91.2	112.9	115.2
LRRC23	111.7	123.1	133.6	125.4	115.9	112.3	131.6	135.4
LRRC23	120.2	116.8	119.6	118.5	110.4	103	126.7	120.6
LRRC24	111.8	111.7	120.8	123.4	108.7	99.2	112.8	120
LRRC25	195	124.1	292.3	201	107.6	218.2	169.3	109.2
LRRC27	118.7	121.4	117.1	114.7	115.1	103.4	122.4	121.3
LRRC28	157.2	139.8	167	155.8	122.9	117.5	148.4	139.2
LRRC29	112.9	123.7	112.5	115.8	106.3	103.1	118.7	111.7
LRRC29	127.5	138.3	148.4	135.9	124.8	117.6	126.6	123.2
LRRC3	118.3	120.3	137.6	126	97.7	100.4	120.3	114.8
LRRC31	115.6	116.3	122.9	115	112.9	103.7	113.5	121.2
LRRC32	118.6	120.5	109	115.7	111.4	95.1	116.2	118.2
LRRC33	220.7	204.1	285.8	208.3	169.9	165.5	206.8	150.8
LRRC34	104.6	108.1	134.3	114.8	110.1	97.6	115.3	109
LRRC35	121.6	124.3	121.9	119.1	113.3	120.6	119.6	122
LRRC36	111.4	115.2	130.1	122.8	110.5	100.3	124.6	110.3
LRRC37A2	125.9	121.5	109	107.1	111.4	96.1	118.9	112.6
LRRC37B	433.5	454.4	395.7	436.1	478.5	295.4	448.4	542.4
LRRC39	121.9	110.6	113.5	116.9	113.7	96	112.4	122.2

LRRC3B	109.1	121.8	106.3	120.4	108.7	90.3	111	114.7
LRRC4	116.4	116.5	129	126.2	112.2	93.7	121.2	120.1
LRRC40	127.1	119.4	120.9	129.4	122.9	109.8	125	143.4
LRRC41	135.9	153.8	148.6	150.1	141	99.6	144.5	170.6
LRRC42	286	285.9	265.9	331.6	271.1	238.4	451.7	498.3
LRRC43	110.9	109.3	132.9	113.4	110.5	95.3	109.6	109.3
LRRC45	158.3	151	157.4	142.2	124.5	111.4	140.9	118.5
LRRC45	118.8	115.1	113.6	110.9	111.5	101.9	119	119.3
LRRC46	119.5	113.6	131.6	130.5	111.2	111.5	127.1	114.8
LRRC47	266.1	236.6	230.5	214.1	184.7	188	235.1	241.5
LRRC48	107.5	109.4	117.2	110.3	110.6	98.8	116.9	109.5
LRRC48	107.4	112.6	117.1	120.1	107.9	99.9	131.2	115.5
LRRC49	104.9	114.1	118.5	119.6	116	99.4	121.8	113.2
LRRC4B	127.4	109.7	118.3	114.2	107.6	97.1	126.3	118.2
LRRC4C	121.6	111.9	114.6	120.5	111.3	109.7	111.4	114.3
LRRC50	121.6	129.7	122.5	120	109	103.1	116.9	111
LRRC51	121.8	112.1	118	129.7	113.9	105.4	119.9	120.2
LRRC52	115.5	113.2	120.9	110.8	107.1	91.9	112.6	118.7
LRRC54	114.3	114.9	107	121.4	104.1	93.7	110	108.5
LRRC55	112.7	112.1	116.9	117.7	103.2	100.3	120.9	112.9
LRRC56	131.1	125	125.7	139.7	113	103.8	130.6	119.2
LRRC57	131.4	134.1	148.2	139.7	131.3	121.3	142.2	124.6
LRRC59	124.3	120.6	127.1	112.5	117	107.3	129.3	118.3
LRRC6	123.6	113.6	115.7	131.7	111.2	107.7	125.6	118.5
LRRC61	116.3	129.6	117.3	114.7	111.9	100.3	130.7	111.3
LRRC7	110.1	111.3	114	111	106.7	91.7	117.1	106.2
LRRC8A	168.5	174.8	144.4	176.2	147	131.3	187.8	204.8
LRRC8B	115.4	119.7	118.5	121.4	113.7	100.8	114.2	120.4
LRRC8C	148.4	221.1	166.6	167.2	207.3	137.2	203.3	246
LRRC8D	152.9	184.7	159.5	185	187.8	136.6	189.9	219.5
LRRC8E	118.9	127.5	127	117	105.4	111.9	126.3	132.6
LRRCC1	110.9	111.1	119.7	121.3	106.2	92	109.9	113.7
LRRCC1	145.4	162.6	148.9	168.3	186.4	118.2	145.6	159.6
LRRCC1	115.4	104	118.8	110.6	113.5	93.5	113.6	117.4
LRRFIP1	1116.2	938	1324.6	1189	1110.4	1168	1362.5	1104.4
LRRFIP2	103.9	111.6	112.8	117.7	105.2	91.2	107.7	112.9
LRRFIP2	253.5	274.9	244.1	326	262.6	247.9	418.8	313.2
LRRFIP2	171.5	211	169.6	211.9	169.7	169.7	202.9	179.5
LRRIQ1	102.6	111.3	104.3	115.2	107.3	96.2	117.1	112.8
LRRIQ2	113.2	104.4	108.7	112.6	101.4	94.9	112.4	107.9
LRRK1	114.3	120.9	120	128.7	110.6	108.1	125.1	111.2
LRRK2	114.7	121.5	121.4	125.1	103	96.1	124.6	111.4
LRRK2	132.8	126.7	133.1	141.9	112.9	112.2	124.3	120.7
LRRN1	113.7	126.6	119.8	126.8	114.6	98.3	114.7	118.5
LRRN3	743.7	1031.4	353.8	640.5	628.1	311.6	557.6	886.8
LRRN5	114.4	112.7	118.8	128.7	103.9	91.4	119.7	114.7
LRRN5	121.8	117.2	105.9	119.1	107.2	106.1	116.6	109.9
LRRN5	112.1	119.5	106.8	127.5	106.5	97.9	117.2	118.4
LRRN6A	114.5	121.9	116	122.1	105.6	103.5	116.5	113.3
LRRN6C	124.7	120.4	124.3	139	112.2	103.2	134.9	128.1
LRRTM1	113.9	120	105.8	123.5	106.4	96.4	116.2	113.6

LRRTM2	118.3	115.1	115.2	130	114.5	99.5	124.9	113.4
LRRTM3	108.1	118.1	105.5	112.5	99.6	102.1	122.1	113.6
LRRTM4	115	109.9	115.5	119.4	112.4	103.9	123	126.8
LRSAM1	110.8	117.9	118.8	124.8	111.2	98.9	116.7	106
LRSAM1	293.9	249	291.3	265.5	226.1	200.5	240.8	234.8
LRSAM1	116.8	119.5	118.8	126.3	109.8	106	122.7	112.4
LRTM1	108.8	114.6	129.3	121.2	110.4	102.4	130.8	124.2
LRTM2	124.2	131.8	122.7	129.5	117.5	110.9	120.5	123
LSAMP	113.2	128.2	123.8	118	122.2	113.9	125.7	121.3
LSDP5	159.7	137.4	158.2	157	148.5	132.6	158	171.4
LSG1	150.7	150.2	162.5	165.5	142.9	113.5	147	148.4
LSM1	1539.5	1762.4	1889.9	1820.2	1761.9	1464.9	2050.3	2171.9
LSM10	268.2	236.8	294.9	287	242.8	210	233.4	208.2
LSM11	115.1	119.4	124.1	138.5	114.5	102.5	130.1	116.7
LSM12	157.8	155.9	155.7	176	159.7	132.1	164.4	154.3
LSM14A	1332.4	1550	1177.9	1439	1372.2	1051.7	1648	1663.1
LSM14B	122.2	127.3	132.1	138.7	118.7	105.5	125	132.5
LSM16	169.5	186	181.7	178.9	151.2	141.7	165.2	158.2
LSM2	450.2	507.7	609.9	329.9	472.5	534.5	372.9	442
LSM3	472.2	624	803.4	588.2	576.6	649.3	578.3	560.2
LSM4	316.2	474.3	572.3	475.3	320.8	316.2	392.4	375.1
LSM5	166.2	151.7	196.8	170.4	150.5	129.9	146.8	168.5
LSM6	135.2	133.2	134.6	123.1	123.6	118	119	124.1
LSM7	692	723.2	862.5	730.4	543.2	720.9	563.5	557.2
LSM8	164.9	183.6	173	174.1	163.5	158.6	162.5	167.6
LSMD1	140.9	169.9	190.6	163	135.9	122.7	134.5	133.7
LSP1	160.1	207.1	162.7	184.4	134.8	154.7	153.3	152.8
LSP1	117	110.9	117.8	116.5	110.8	98.8	120.8	120.5
LSP1	203.9	196.2	219.3	189.7	160.6	250.8	170.6	164.5
LSP1	122.5	102.2	113	119.6	108.8	103.1	116.5	107.3
LSR	125.2	132.5	125.2	123.5	111.2	108	131.3	121.6
LSR	124.4	113.3	123.4	126	115.4	114.5	117.2	124.9
LSR	121.2	131	145.2	136.8	113.1	121.6	121.7	122.4
LSS	130.9	117.6	119.2	116.8	113.4	100.5	121.9	121.1
LSS	120.3	116.8	113.9	123.3	112.7	99.3	122.9	117.1
LSS	259.7	252.1	241.5	266.3	179.8	166.5	280.4	222.6
LST1	277	185.9	1154.4	265.3	159	377.1	190.8	150.8
LST1	111.1	110.2	112.1	117.8	107	97	115.9	116.7
LST1	163.7	138.7	284.6	166.6	120.2	156.5	152	139.9
LST1	196.4	158.1	320	174.9	120.5	134.7	147.8	141.2
LST-3TM12	119.2	118.5	119.8	123.7	111	101.8	129.4	118.4
LTA	355.9	405.1	301.7	385.5	338.1	234.4	326.7	410.1
LTA4H	1621.8	750.7	1573.7	1050.8	557.7	961.6	566.1	525.7
LTB	5773.7	7226.9	3602.3	5926	5234.3	2967	5716.2	4579.3
LTB	2003	2259.3	818.7	1928.2	1782.9	1034	1714.6	1710.8
LTB4DH	173	180	125.4	290.1	227	145.4	373.6	245.2
LTB4R	124.1	141.5	164.4	130.8	102.4	107.9	124.1	117.3
LTB4R2	113.4	113	116.1	119.8	100.5	101.3	118.1	117
LTBP1	118.2	117.7	112.4	114.8	101.5	104.5	111.9	109.7
LTBP1	109.1	119.8	120	121.5	112.7	98.5	111.5	116.9
LTBP3	128.2	117.9	123.2	113	109.7	95.8	121.7	116.7

LTBP3	115.7	107.3	111.2	118.5	116.8	95.3	109.2	118.6
LTBP3	362.1	322.6	336.8	332.2	249.6	266.6	249.4	218.8
LTBP4	124.8	110.3	124.6	122.1	108.9	105.1	123.7	110.1
LTBP4	143.2	138.2	156.5	142.1	121	133.6	149.1	148.4
LTBR	234	145.7	297.4	243.8	126.8	282.6	202	159.5
LTC4S	111.8	116.6	136.1	125.7	108.6	100.2	120.6	113.4
LTF	128.1	125.8	131.1	136.5	120.2	107.5	144.7	116.6
LTK	137.9	117.4	144.1	129.1	117.8	104.4	113.7	138.2
LTK	119.7	117.1	110.7	117.8	109.3	110.6	123	124.3
LTV1	242.3	202.1	211.9	208.6	168	161.8	169.1	159.6
LUC7L	122.3	121.8	119.3	134.4	119.9	107.7	127.4	122.9
LUC7L	362.8	419.8	429.5	346	309.4	298	302.1	322.2
LUC7L2	145.8	145.6	165.3	154.2	142.9	141	188.4	159.8
LUM	110	118.5	114.6	117	110.9	97.8	118.6	112.3
LUZP1	331.4	444.1	318.7	357.4	300.9	329.2	488.9	617
LUZP2	119.3	120.5	120.8	119.6	105.8	103.2	115.5	116.3
LUZP4	111.8	122.3	113	116.8	104.7	97.2	121.5	118.2
LXN	175	215.2	233.7	208.6	180.8	144.3	167.7	193.3
LY6D	115.7	120.8	121.8	117.9	97.2	107.4	121	114.3
LY6E	423.9	447.5	368.7	457	264.9	294.3	322.3	271.6
LY6G5B	111.9	124.8	126.3	116.5	113.9	102.7	121.2	121.1
LY6G5C	139.3	149.1	147	146.9	128.7	111.6	127.3	147.6
LY6G5C	119.8	133.2	132.3	116.8	115.9	107.5	127.1	116.7
LY6G5C	116.5	121.8	116.9	115	106.3	94.6	119	115.5
LY6G6C	124.7	113.6	141.9	130.6	114.5	111.9	120.8	122.8
LY6G6D	116.4	118.5	121.9	119.7	106.5	95.9	117.5	124.8
LY6H	144.3	153.8	167.2	132.2	129.7	123.1	123.9	125.8
LY6H	106.1	113.7	133.2	122.3	115	101	117.7	118.7
LY6K	113.2	104	116.2	118	110.7	103.3	114.3	115.4
LY75	126.3	114.8	119.7	129.9	113.4	92.1	122	112.1
LY86	214.6	162.4	374.1	183.9	142.5	161.6	154.9	143.4
LY9	347	386	281.9	336.9	265.9	201	288.3	309.8
LY9	276.4	364.1	253	320.7	316.6	229.8	365.4	445.7
LY9	126.9	121.8	127.4	134.3	124.1	106	119.3	119.6
LY96	726.3	454.3	892.9	721.5	433.3	739.6	705.1	477.3
LYAR	679.8	847	885.5	740.8	650.6	502.3	967.9	901.9
LYCAT	115.1	114.6	112.6	118.6	115.8	109.6	114.9	111.2
LYCAT	206.2	440.3	210.3	199.5	252.3	167.1	171.6	215.3
LYG2	107.7	105.9	113.6	125.9	106.4	100.2	114.3	119.9
LYK5	129.8	116.4	121.5	119.8	107.1	104.3	109.4	103.9
LYK5	339.2	352.5	373.4	423.4	276	340.3	319.5	318.2
LYK5	134.9	149.3	148.1	142	112.3	115.3	144	134.8
LYL1	496.3	324.1	711	397	235.3	350.3	263	211.9
LYN	2061.9	1114.6	2360.8	1867.6	682.5	1578.6	1604.1	602.7
LYNX1	113.8	130	107.5	121.4	110.5	95.1	121.8	123.4
LYNX1	102.6	122.3	126.9	118.5	105.1	97.7	112.2	110.6
LYNX1	116.5	122.9	118.3	122	114.9	100.2	123.4	121.3
LYPD1	124.1	112.4	114.7	128.6	109	101.3	130.1	118.3
LYPD1	123	125.2	128.6	133	112.9	105.6	125.4	133.2
LYPD2	124.1	118.6	129.8	117.9	110.1	111.6	124.6	119.2
LYPD3	128.1	122.3	124.8	130.9	117.1	106.1	127.9	124.4

LYPD4	111.5	100.1	111.1	112.2	112.5	97.9	123.3	114.9
LYPD5	109	112.4	117.8	112.4	108.5	98.6	121.4	117.6
LYPD5	116.9	115.5	125.3	125.6	100.1	97.6	129.3	120.5
LYPD5	106.9	106.1	108.7	117.8	109.6	98.9	118.4	108.7
LYPLA1	112.6	118	109.8	114.2	109.7	100.6	114	110.5
LYPLA2	150.9	164	163.3	153.3	141.6	124.5	177	151.5
LYPLA2P1	124.1	119.6	129.5	121.1	113.6	112.2	111.9	124.9
LYPLA3	129.9	136.5	137	149.1	112.9	132.8	140.8	128.6
LYPLAL1	214	228.4	307.7	243.6	229.3	262.3	217.8	223.9
LYSMD1	117.7	114.3	114.7	129.2	106.2	96.6	118.6	126.6
LYSMD2	1349.1	1315.5	1451	1356.3	1159.1	864.7	926.8	779.3
LYSMD3	124	118.5	123.8	130	124.6	108.1	132.2	127.9
LYSMD3	109.1	109.2	119.4	118.2	98.6	97.8	124.6	111.4
LYSMD4	204.9	208.1	268	209.3	207.1	200.9	221.4	193.4
LYST	265.1	196.8	152.1	240.5	212.8	147.9	276.1	177.1
LYST	109.5	120.1	119.8	122.9	104.1	100	119.6	113.1
LYST	112.8	116.8	114.6	121.7	109	99.7	117.5	119.7
LYZ	371.2	287	483.3	395.8	161.7	283.2	213.9	180.4
LYZL1	121	123.3	111.1	114.6	106.6	101.1	108.8	111.2
LYZL2	116.6	120.4	113.5	110.4	107.6	101.4	120.8	113.7
LYZL4	114	110.9	109.1	117.6	99.3	92.8	115.8	115.2
LYZL6	137.3	124.6	122.4	134.9	121	103.4	120.2	115.9
LZIC	134.3	147.6	146.6	154.5	132.8	122.8	137	142.9
LZTFL1	157.1	153.6	157.9	163.8	142.7	129	141.9	152.4
LZTR1	735.2	850.4	665.2	760.4	553.3	431.2	747.9	749.2
LZTR2	122.1	120.9	124.1	114.6	110.8	96.3	127.5	116.1
LZTS1	116.4	114.2	111.3	123.3	105	94.6	116.3	112.9
LZTS2	136.7	119.9	126.3	138.4	117.3	106.1	122.1	131.8
M160	115.2	111.3	192.4	121.5	106.3	150	114.9	105.2
M6PR	551.5	549.5	677.2	550.4	532.1	430.1	524.4	445.9
M6PRBP1	578.1	495.7	714.7	630.9	482	847.1	708.2	498.5
MAB21L1	122.7	117.3	124.3	132.8	113.4	104.8	118.8	118.4
MAB21L2	103.8	117.3	115.9	120.6	102.2	102.6	111	108.1
MAB21L2	127	118.3	115.4	121.5	111.7	104.4	109.1	125.9
MACF1	128.7	127.5	132.6	126.8	121.8	105.7	132.7	151.9
MACF1	117	117.9	114.6	117.5	111.3	100.4	114.8	105.7
MACF1	380	386.5	311.6	346.8	439	246.6	337.3	327.1
MAD1L1	296	247.5	283	254.5	191.3	219	245.5	202.2
MAD2L1	119.1	121.5	123.7	111	104.9	109.4	127.7	110.3
MAD2L1BP	112.4	111.5	113.4	121.6	106.1	105.6	112.6	124.9
MAD2L1BP	187.3	190	186.2	216.3	165.9	142.4	165	174.6
MAD2L1BP	336.1	380.4	292.2	327.3	312.9	248	309	534.1
MAD2L2	604.9	650.9	597	633.9	413.4	497.1	595.2	472.8
MADCAM1	114.7	118.4	118.3	119.3	107.4	92.6	118.9	112.9
MADCAM1	110.5	118.8	124.2	116.6	112.9	97	118.1	111.3
MADD	110.9	108	112.4	109.7	99.1	93.6	111.9	115.1
MADD	298.3	276.7	206.9	255.8	229.9	178.9	264.4	251.9
MADD	236.1	277.3	207	237.6	172.8	162.4	210.3	252.8
MAEA	292.6	275.8	385.2	304.9	322.3	332	316.7	333.6
MAEA	141.1	148.4	154.6	157.6	147.1	142.4	140.7	140.8
MAEL	106.4	113.6	117.6	119.2	106.1	96.4	111.3	115

MAF	131.7	118.2	135.3	123.9	114.3	109	119	120.9
MAF	294.1	275.3	318.5	281.9	207.8	196.1	195.2	194
MAF1	212.8	237.9	200	218.8	155.9	183	209.7	184.2
MAFA	120.5	133.7	125.2	140.5	121.4	107.5	130.2	138.9
MAFB	682.9	253.4	1900.1	1110.9	364.6	2252.8	1081.2	394.2
MAFF	108	115.9	116.4	112.6	107	102.6	115.9	116.1
MAFF	249.1	239.1	158.6	212.9	191.7	161.8	175.4	198.4
MAFF	116.6	126.9	124.1	120.2	115.2	112.2	122.6	134
MAFG	155.6	172.2	147.7	203.3	155	116.8	278.8	269.5
MAFG	109.5	114.8	126.2	127.6	102.7	104.2	127.2	125.8
MAFK	123.3	123	135.3	141.6	108.7	108.8	132	123.6
MAG	108.8	119.1	120.8	126	102.3	104.3	112.5	116.2
MAG	119.2	119.3	106.8	115.4	109.6	101.4	115.7	113.2
MAGEA1	120.4	121.1	112.9	124.9	110.6	102.9	112.8	116.4
MAGEA10	110.3	114.9	117.4	117.4	102.2	99.5	109.8	108
MAGEA10	108.1	112.3	117.8	114.6	103.2	101.9	111.8	111.7
MAGEA11	118.2	119.6	123.1	123.8	119.3	95.2	116.4	113.7
MAGEA11	113.5	114.1	115.9	118.4	102.7	102	121.5	111.2
MAGEA11	128	117.3	114.8	125.8	112.7	100.4	123.3	125.4
MAGEA11	109.1	118.9	121.1	112.1	112.8	106.7	126.6	116
MAGEA12	121.9	112.4	118.4	123.8	105.9	96.2	131.7	112.3
MAGEA2	112	112.4	109.5	116.3	100.4	95	117.8	107.4
MAGEA2	111.7	107.2	111.3	112.4	105.6	95.4	116.7	120.2
MAGEA2B	113	116.4	116.6	118	98.5	96.8	103.9	111.2
MAGEA3	105.6	109.5	119.8	122.1	106.1	99.6	114.7	106
MAGEA4	112.6	106.7	113.8	110.5	100.9	97.9	126.5	118.9
MAGEA5	119.2	119.1	137	120.8	119.5	101	123	129.7
MAGEA6	127.7	129.6	129.4	126.1	112	109.9	127.8	130.9
MAGEA8	114.3	117.9	105	113.3	102.6	89.1	106.9	115
MAGEB1	107.9	108.9	110.2	113	102.9	94.1	111.5	112.5
MAGEB1	109.7	105.1	108.5	116.7	109.5	100.1	127.3	118.1
MAGEB10	101.7	112	119.1	121.4	104.7	98.4	107.8	114.4
MAGEB18	113	112.6	115.5	122.4	112.6	98.7	117	119.2
MAGEB2	113.8	119.4	123.9	130.2	111.2	95.9	121.2	115.1
MAGEB3	118.1	119.6	130.4	123.2	115.4	105.8	122.5	123.7
MAGEB4	110.7	115.9	122.7	111.8	103.9	98.2	119	114.3
MAGEB6	143.6	108	125.4	122.7	106.9	99.7	124.6	112
MAGEC1	110.3	115.4	113.4	108.2	109.8	99.1	119	114.7
MAGEC1	106.4	108.8	115.6	110.3	104.6	94.8	112.9	101.3
MAGEC2	119.2	117.5	118.3	116.7	103.5	98.6	120.7	118.1
MAGEC3	112.1	112.9	114.9	114.3	112.2	86.7	121.1	105.8
MAGEC3	117.7	118.3	120.4	118	102.6	104	117.8	111.9
MAGEC3	104.6	98.3	116.2	117.2	106.8	95.5	114.9	114
MAGED1	510.1	576.4	439	568.4	443.8	354.1	406	362.7
MAGED1	214.9	221.5	186	202.6	170.7	151.9	168.1	173.1
MAGED1	117.5	113.4	121.2	116.5	115.3	98.6	120.8	121.3
MAGED1	123.6	113	118.2	123.9	104.1	96.4	117.7	118.2
MAGED2	127.3	124.9	127.1	139.9	130.1	110.2	125.9	131.8
MAGED2	128	123.2	125.7	130.2	119.9	125.7	139.6	121.6
MAGED2	131.9	133.3	150.3	140.6	116.5	119.1	124.1	133.5
MAGED2	126.1	126.3	134.4	128.5	134.1	117.2	138.5	126

MAGED4	118.8	126.6	142.2	143	122.9	115.6	134.8	127.8
MAGEE1	366.9	342	291.8	298.4	251.6	209.2	236.5	257.6
MAGEE2	125	113	116.8	112.9	104.1	94.9	122.7	109.2
MAGEF1	120	115.3	112	126.3	113.7	99.9	124.9	114
MAGEH1	154	144	154.2	146.2	140.2	125.1	128.1	146.5
MAGI1	108.1	122.5	107.7	111.1	101.5	103	124.7	125.6
MAGI1	102.6	113.2	112.9	111	112.7	105.1	116.3	108.4
MAGI1	115.6	124.9	134.6	122.8	111	113.1	118.8	122.7
MAGI2	117.9	114.7	123.8	118.5	109.1	100.1	112.6	107.5
MAGI3	105.4	114	111.7	115.5	108.5	93.9	106.9	114.8
MAGI3	108.2	118.9	120.1	117.7	110.2	99.6	115.4	113.1
MAGMAS	253.2	274.8	316.1	251.4	220.6	257.4	210.7	209.3
MAGOH	189	205.3	189	192	185.9	183.7	194.8	198.9
MAK	113.8	109.8	121.5	119.5	110.3	102.3	122	116.9
MAK10	112.9	113.2	117.2	118.8	116.8	100.7	114.3	112.7
MAL	1106.9	1286.7	999.5	1218	1032.1	815.4	646.9	708.3
MAL	447.9	581.5	451.6	487.7	368.9	390.2	307.6	380.5
MAL2	117.2	110.7	116.2	115.5	115	102.2	124.2	115.2
MALAT1	110.1	114.1	118.5	134.4	105.1	98	109.9	116.6
MALL	109.8	115.4	109	112.7	113.9	96.5	115.8	113
MALT1	256	229.9	216	290.8	206.2	177.9	243.2	265
MALT1	113.4	114.8	117.9	120.7	108.9	98.8	121.8	114.1
MAMDC1	112.5	111.5	120	111.4	108.5	100.4	121.6	117.6
MAMDC2	107	115.3	109.2	109	115.1	102	109.2	104.5
MAMDC4	120.2	123.6	122.5	121.4	106.7	103.1	125.4	113.6
MAML1	114.4	114.1	123.5	117.1	111.8	96.7	122.2	125.7
MAML1	161.5	172.5	163.9	176.1	158.8	131.1	176.5	176.8
MAML2	131.3	127.2	130.8	135	126.7	108.7	127.4	136.2
MAML3	114.7	106.5	109.2	116.7	104.7	100.3	125.8	116.6
MAN1A1	149	144.8	140.2	160.5	138.4	121.7	140	160.3
MAN1A2	117.1	115.1	114.2	121.2	122.8	95.2	122.1	115.8
MAN1B1	423.3	467.1	462	466.4	330.9	320.8	362.8	377.3
MAN1C1	243	327.9	310.6	244.1	222.1	258	176.3	166.7
MAN2A1	109.5	121.5	113.2	124.6	102.7	101.9	112.4	106.8
MAN2A2	192.6	216.6	213.6	185.8	172.1	167.1	180	176.1
MAN2B1	197.6	200.5	344.2	227.6	157.7	238.5	169.1	158.3
MAN2B2	174	190.2	171.9	167	152	166.5	196.3	149.1
MAN2C1	301.7	307.4	296.1	260.3	206.9	251.3	215.2	240.1
MANBA	234.9	167.3	252.2	209	168.3	190.4	159.6	166.3
MANBAL	346.5	322	427.7	452	292.7	329	360.2	270.4
MANBAL	112.2	108.9	115.1	112.4	104.4	100.6	113.2	106.3
MANBAL	130.9	136.2	151.6	143.6	117.1	118.7	139.4	129.9
MANEA	122.4	106	122.7	114.7	102.9	98.2	111.3	114.4
MANEA	135.7	124.8	152.7	146.9	124	112.1	126.6	125.7
MANEAL	109.9	115.1	117.7	116.2	107.7	100.2	114.9	119.4
MANEAL	113.2	124.5	116.1	121.7	106.4	99	110.7	113.4
MANEAL	124.8	126.3	113.1	117.9	108.6	103.3	118.9	103
MANSC1	111.9	118.6	106.9	116.8	115.5	107.7	123.2	117.8
MAOA	109.7	121.6	120.7	113.8	104.8	101.8	123.5	110.3
MAOB	107.9	109.8	122.2	128.6	110.3	104.3	109.2	118.4
MAP1A	140.1	131.2	137.9	146	130.2	117.9	151.6	134

MAP1B	110.8	113.7	111.6	118.4	109.1	101.1	113.6	112.4
MAP1B	158.6	146	142.4	151.6	145.3	119.4	141.2	150.2
MAP1B	119.8	118.8	114.2	110.6	112	97.4	122.4	112.3
MAP1D	114	124	127.2	131.9	119.3	106.4	127.1	131.2
MAP1LC3A	100.8	117.8	111.8	114.4	107	93.1	113.1	108.5
MAP1LC3A	116.9	104.5	120.1	114.4	105.1	94.5	125.2	121.9
MAP1LC3A	212.1	234.2	195.8	233.1	204.9	191.6	364.9	364.8
MAP1LC3B	467.5	450.4	505.3	554.2	467.9	473.8	696.6	916.1
MAP1LC3C	129.6	115.5	121.5	128	108.8	94.7	115.7	118.5
MAP2	109.8	116.2	119	119.8	113.4	100.7	112.9	112.8
MAP2	116.9	113.6	116.6	119.9	106.9	97.1	121.5	114.9
MAP2	110.6	118.2	109.5	113.2	113.8	100.6	121.9	105.3
MAP2	117.5	118.1	125.1	124.2	109.1	103.6	115.2	110.1
MAP2K1	459.6	451.8	427.5	456.4	345.7	421.5	489.2	418.6
MAP2K1IP1	533.7	554.2	617.5	549.6	474.9	478.2	648.4	682.1
MAP2K3	110.9	108	113.7	121.3	108.5	96.6	123.7	112.1
MAP2K3	117	124.7	123.1	140.2	117.6	111.2	129.6	126.5
MAP2K3	117.9	122.2	121.1	113.8	114.1	99	128.7	114.6
MAP2K3	122.2	114.7	124.1	140.9	120.4	100.3	123.9	113.5
MAP2K4	257.1	252.4	222.9	254.6	203.1	205.6	290.4	256.7
MAP2K5	150.5	152.8	173	162.2	135.9	149.4	140.9	137.5
MAP2K5	133.7	134.5	130.6	140.1	118.5	117.9	132.7	129.1
MAP2K6	121.8	116.1	114.6	119.4	108.5	94.4	110.3	110.1
MAP2K7	138.8	136.2	142.8	132.6	130.3	110	150.1	137.6
MAP3K10	125.6	116.1	136.4	130.1	112	108.1	118	120.7
MAP3K11	282.2	267.9	281.4	273	197	217.4	353.4	261.5
MAP3K12	129.9	149.1	133.1	142.7	135.3	107.6	127.7	126.1
MAP3K13	110.1	111.8	117.1	120	109.1	96.7	118.9	120.7
MAP3K14	168.1	159.8	160.2	184.1	124.2	165.8	191.3	151.4
MAP3K15	119.6	129	129.1	128.5	123.9	100.9	136.4	126
MAP3K2	133.9	207.4	139.3	142.3	192.6	119.5	158	214
MAP3K3	117.6	115	124.5	116.3	109.4	98.8	111.9	113.4
MAP3K4	122.2	120	128.2	119.1	113.3	106.7	129.9	118.1
MAP3K4	383.6	453.8	360.3	428.7	484.9	290.5	366.7	357.6
MAP3K6	123.8	131	128.8	137.9	123	106	126	120.2
MAP3K7	139.8	143.8	142	135.7	120.4	123.4	140.6	140.6
MAP3K7	472.4	439	459.4	407.7	376.8	353.5	469.4	437
MAP3K7IP1	115.7	122.5	129.5	114.6	113	102	121.4	122.2
MAP3K7IP1	157.9	180.5	166.4	170.9	144.9	152.4	165.1	148.8
MAP3K7IP1	123.4	115.8	124	127.8	114.7	104.7	120.5	115.5
MAP3K7IP2	146.1	145.9	150.9	166.7	131	120.2	143.4	142.4
MAP3K8	284.6	188.9	280.2	390.4	215.4	303.5	336.6	244.2
MAP3K9	131.7	113.1	118.6	118.8	112.4	95.2	109.9	127.1
MAP4	132.1	143.2	133	136.4	124.5	109.1	156.8	160.7
MAP4	115.2	120.8	116.4	109.4	111.7	96.4	115.4	106
MAP4	122.3	110.1	115.4	116.2	103.2	98.9	122.8	113.1
MAP4K1	536.4	741.5	531.9	634.3	449.7	429.1	368	485.2
MAP4K1	388.1	450.9	398.2	433.9	331.8	438.1	353.1	326
MAP4K2	2360.2	2251	2020	2128.3	1500.4	1388	1540.5	1358.8
MAP4K3	142.1	139.3	146.5	137	131.7	111.1	144.1	124.3
MAP4K4	105.5	122.4	119.7	118.5	107.6	97.3	117.1	107.5

MAP4K4	184.4	225.1	215	224.2	209.7	165.9	221.7	249.8
MAP4K5	110.8	131.2	153.3	140.5	132.1	115.1	131.8	134
MAP4K5	165.5	199.5	213.7	194.4	170.4	153.6	181.9	193.9
MAP6	106.4	108.8	110.9	116.9	104.6	95.1	109.2	112.5
MAP6	105.1	109.3	114.7	111.8	100.1	91.3	114.2	108.9
MAP6	112.3	115.4	121.7	125.8	105.8	102.9	122.5	111.6
MAP6D1	156.5	165.5	161.8	177.6	129.8	120.7	158.6	168.8
MAP7	120.7	130.5	125.4	128.6	116	110.2	124.2	134.5
MAP7D2	123.5	107.5	108.9	113.3	100.9	96.2	109	100.2
MAPBPIP	327.8	297.8	436.1	286.5	273.4	305.6	247.4	179
MAPK1	144.6	127	139.3	152.2	130.1	126.1	146.5	143.1
MAPK1	429.9	540.4	539.7	544.5	536.6	601.9	549.8	594.6
MAPK1	256.8	280.5	311	348.1	303.5	312.8	323.5	279.6
MAPK10	115.7	112.2	112.2	128.5	110.1	104.5	116.1	116.8
MAPK10	117.2	119.8	120.3	120.6	110.2	100.3	120.9	118.5
MAPK10	108.7	118.7	120.1	123.5	114.2	99.3	128.9	115.4
MAPK10	113.5	113.3	120	121.7	105.9	97.4	116	110.9
MAPK10	115.1	108.8	114	117	111.1	102.2	123.2	116.6
MAPK11	121.7	119.8	116.3	113.2	111.6	99.3	115.2	110.7
MAPK11	112.5	135.1	119.6	117.6	108.5	108.1	122.6	113.1
MAPK12	122.3	116.1	119.9	112	108.3	103.4	117.5	116.4
MAPK13	245	266	272.2	237.3	215.2	199.3	203.6	192.6
MAPK14	115.6	117.3	124.6	124.2	109.7	106.6	118.5	124.8
MAPK14	123.6	129.9	127.6	130.7	125.6	113	123.7	120.1
MAPK14	124.4	138.6	119.3	123.2	117	102.4	118	131.2
MAPK15	111	115.8	118.8	118.2	113.1	94.6	118.5	126
MAPK3	491.2	434.8	581.8	441.6	376.1	455.5	487	346.3
MAPK3	230.4	216.9	332	248.5	187	345	244.2	207.4
MAPK4	108.4	104.9	116.7	122.2	103.8	101.8	120.6	111.8
MAPK6	408.4	388.9	400.1	485.8	433.5	502.7	611.1	623.4
MAPK7	128.2	128	117.1	135.1	112.5	104.9	136.8	117.6
MAPK7	184.8	166.5	164	201.9	148.3	156.7	216.6	183.3
MAPK7	116.7	119.2	121	116.7	111.1	104.1	121.4	121.2
MAPK8	115.5	110.9	111.8	115.2	104.6	107.2	121.2	122.7
MAPK8	104.2	107.6	108.1	120.1	108.4	98.5	110.7	107.8
MAPK8IP1	113.7	108.7	127.5	120.7	102.3	96.2	113.2	120.9
MAPK8IP2	117.4	123.6	153.5	124.8	128.3	104.7	131.1	128.4
MAPK8IP2	111.6	118.2	112.7	113.2	108.5	96.7	117.5	110.8
MAPK8IP2	113.3	116.2	113.3	121.1	110.4	101.7	115.2	112.5
MAPK8IP2	118.2	111	118.6	114.5	110.9	99	125.2	124.4
MAPK8IP3	121.8	119.8	122	119	108.6	93.7	126.5	117.9
MAPK8IP3	683.2	627.6	676.4	629.4	376.2	507.6	813.4	708
MAPK9	300.2	358.7	350.5	334.5	270.7	268.8	237.9	241
MAPKAP1	116.8	127.7	124.4	123.7	123.4	121.3	149.5	145.1
MAPKAP1	177.2	170.5	207.8	174	169.4	176.8	157.6	168.7
MAPKAPK2	121	114	121.7	114.2	111.7	113.4	114.1	121
MAPKAPK2	165.2	158.9	155	159.2	122.9	125.4	186.6	162.8
MAPKAPK3	494.9	453.9	791.9	518.6	289.1	423.3	321	309.5
MAPKAPK5	186.1	188.8	178.6	228.9	172.9	149.2	181	221.8
MAPKBP1	127.7	138.9	127.5	131.5	126.1	110	139.1	130.5
MAPRE1	742.9	816.1	993.3	789.9	640.5	1003	1035.9	1314.1

MAPRE2	466.6	606	596.9	541.7	445.2	401.6	536.2	522.6
MAPRE3	141.4	140.8	149.8	134.2	133.6	121.5	146.4	149.3
MAPT	111.8	120	117.6	113.7	115.1	107.9	118.7	121.6
MAPT	118.3	118.2	116.1	115.1	108.5	101.7	119.3	117.3
MAPT	115.7	115.4	112.3	120.7	106.7	89.1	108.4	122.1
Mar-01	116	122.5	138.1	135.5	112	114.8	137.1	121.4
Mar-02	140.4	149.1	182.2	152.1	124.9	150	134.9	142.4
Mar-02	121.1	114.2	145	134.1	116.7	120.5	130.8	115.7
Mar-02	118.3	121.7	123.6	128.2	110.2	103.2	118.3	117.6
Mar-02	116	123.6	105.8	120.2	102	104.2	126.4	113.9
Mar-03	142.8	135	141.8	134.2	135.5	111.1	135.1	136.2
Mar-03	297.6	312.5	271.8	312.5	249.3	188.9	296	296.5
Mar-03	277.9	315.1	233.8	264.3	230.6	183.7	298.9	257.4
Mar-04	117.8	107.4	113.1	121.8	108.5	96.9	115.5	114.8
Mar-05	122.1	127.3	147.7	137.7	126.8	118	121.5	125.6
Mar-06	272.9	282	241.4	316.3	332.9	233.3	410.9	381.4
Mar-07	1461	1477.9	1284.4	1611.2	1232.1	953.9	1634.2	2023.5
Mar-08	117.4	107.2	115.5	115.4	108.1	91.8	113	107.6
Mar-08	130.8	134.1	126.6	128.9	113.8	102.8	140.9	136.9
Mar-08	106.9	112.9	115.6	108.8	113.9	96.4	111.3	109.1
Mar-09	126.2	113.7	108.6	140	105.4	94.4	132.3	127.3
MARCKS	728.2	440.3	2081.8	514.5	276.2	632.4	356.7	210.7
MARCKSL1	539.9	506.5	503.7	521.8	401.6	508.2	642.4	735.2
MARCO	123.2	119	127.5	133.1	108.4	106.8	119.9	117.7
MARK1	124.1	118.4	112.6	125.9	110.1	91.2	126.7	118.5
MARK2	122.4	127.9	124.5	126.3	114.3	99.2	130.3	130.5
MARK2	117.8	119.3	120.3	122.8	109.4	104.2	122.5	121.7
MARK2	132.3	136.8	133.6	147.9	136.2	133.8	178.1	167.2
MARK3	161.4	146.5	149.7	163.1	152	146.7	177.2	156.3
MARK4	124.5	121.3	132.9	131.3	132.3	126.8	138.5	128.5
MARS	218	202.6	187.9	221.5	195.9	182.2	211.9	248
MARS2	118.5	125.8	130.9	138.1	118.7	107	113.4	120.6
MARVELD2	125.7	122.1	112.9	120.1	113.9	99.1	139.7	141.2
MARVELD2	116.8	118	108.7	121.3	106	91.8	116.3	114.5
MARVELD2	104.5	108	102.2	109.9	102.3	87.8	110.8	106.2
MARVELD3	111.8	112.4	109.8	122.7	107.2	96.5	119.5	112.5
MARVELD3	112.4	117.5	112.7	118.5	109.6	93.9	121	124
MARVELD3	104.6	113.7	108.4	112.5	107.3	98.9	99.2	107.7
MAS1	124.7	113.2	117.8	114.8	110.9	98.5	108.9	112.7
MAS1L	118.7	116.9	114.9	123.5	110.2	111.3	119.6	120.4
MASA	390.1	471.8	385.4	465.4	461.1	317.4	429	488.4
MASK	480.8	513.2	528.4	630.3	431.5	512.9	637.9	688.8
MASK-BP3	135.2	124	119.1	129.6	127.7	115.2	132.1	132.4
MASP1	115.7	121.2	125	114.6	109.9	107.9	109.5	113.8
MASP1	133.8	125.8	111	116.6	118.2	97.1	113.5	122
MASP1	113.2	117.1	123.1	109.6	118.2	108.3	109.3	134.3
MASP1	117.9	111	117	122.1	109.3	93.5	124.5	119
MASP2	120.2	111.6	109.5	123.5	111.2	99.7	118.8	123.3
MASP2	106.3	108.5	107.7	116.6	106.1	95	116.1	114.4
MASP2	113.3	119.4	122.9	124.2	100.4	105.1	119.7	114.2
MASS1	117.2	113.5	113.4	114	112.7	96.6	114.8	111

MAST1	114.4	115.1	122.1	114.4	108.7	85.8	115.4	107.2
MAST2	119.1	124.9	144.2	137.3	126.5	108.1	118.7	136.9
MASTL	136.3	119	118.2	139.6	127	107.5	130.5	126.5
MAT1A	114.4	118.3	119.1	115.5	106.5	96.6	121.2	120.6
MAT2A	2071.1	2253.4	1454.5	2204.3	1701.2	978	2650.9	2867.8
MAT2B	2081.5	2326.9	2027	1756.5	1605.3	1977.2	2519.2	1865.9
MAT2B	141.8	161.8	170.1	161.5	134.6	138.6	135.1	139.4
MAT2B	117.4	118.8	131.9	115.4	109.9	103.4	113.6	117.5
MATK	131.7	127.6	124.5	115.1	115	106.9	117.5	131.9
MATK	519.4	561.5	503.9	567	364.3	305.1	391.1	368.2
MATK	138.5	143.9	157.7	142.3	140.5	116	125	133.1
MATN1	106.2	109.3	114.2	108	105.6	97.5	109.9	111.7
MATN2	117.1	111.3	112.4	115.7	108.7	96.5	113.5	112.3
MATN2	114.8	116.8	106.8	118.4	104	102.7	112.4	115.5
MATN3	116.5	121.3	131.4	123.9	109.6	94.1	126.8	118.6
MATN4	105	112.7	103	118.4	101.9	97.2	116.9	104.9
MATN4	123.4	122.6	117.8	117.8	104	103.1	117.3	110.2
MATN4	111.9	119.4	113.5	121.1	101.2	104	109.2	110.8
MATR3	118.4	114.8	132	128.3	114.5	99.4	124.6	126.5
MATR3	119	119.1	131.6	128.2	126.8	108.4	121.5	120.5
MATR3	706.1	586.7	755.2	794.7	900.1	547.4	986.2	1257.8
MATR3	200.5	219.7	266.4	224.2	254.7	201.5	233.9	226.8
MAWBP	109.7	109.3	110.3	117	104.6	99.9	112.6	104.6
MAWBP	115.5	112.1	122.9	110.3	105.9	97.9	118.4	113.7
MAWBP	116.1	106.7	110.1	109.3	110.1	90.9	113.9	114.5
MAX	145.4	170.5	157.6	184	136.2	124.4	190.9	182
MAX	133.2	139.6	140	143.7	116.1	109.7	126.1	137.5
MAX	133.9	140.9	133.5	142.2	141	119.8	143	154.6
MAX	125.9	134.1	129.5	137.5	125.8	105.2	122.3	133.1
MAX	120.8	114.5	117.1	126.2	105.2	99.8	119.5	120.2
MAZ	115.3	128.5	136.2	126	117.5	107.9	136.9	115.2
MAZ	116.6	111.3	117.1	114.9	106	91.1	116.9	123.6
MAZ	147.1	145.7	125.7	160.1	129.8	122.2	151	126.7
MB	122.8	120	113.4	122.6	109.3	106.2	118.9	127
MB	126.4	113.7	109.5	119	110.5	100.4	123.7	118
MB	114.2	121	133.3	132	107.5	98.6	122.3	114.8
MB	109.2	108.2	112.1	121.5	102	99.6	127.8	111.6
MBD1	174.8	179.9	173.4	211.9	163.4	156.8	221	206
MBD1	201	204.4	184.6	208.3	202.5	160.3	236.6	215.6
MBD2	111.4	108.8	116.6	117.6	104.4	99.6	122.3	110.9
MBD2	113.8	108.1	111.4	114.4	103.4	95.2	121.6	110.6
MBD2	118.9	118.2	128.3	125.1	113.7	94.1	111.3	121.2
MBD3	195.7	208.4	190.4	216.7	170.7	149.6	228.9	207.7
MBD3L1	109.2	112.9	110.2	116.6	111.5	97.9	115.3	107.6
MBD3L2	122.4	106.3	114.4	116.7	110.7	100.5	122.5	120.9
MBD4	1360.7	1323.6	1495.1	1488.6	1418.4	836.1	1571	1580.1
MBD5	115.6	125.3	114.8	117.7	115	108.3	125.3	117.2
MBD6	469.6	407.9	468.9	455.6	362.5	271	433.4	391.2
MBIP	158.2	188.2	189.3	189.1	155.5	176.2	142.7	160.4
MBL2	116.1	126	105.6	116.8	105.7	102.1	118.4	115.8
MBNL1	371.5	530.9	344	327.6	347.7	267.5	371.1	513.8

MBNL1	683.9	711.7	692	804.4	848.4	388.1	747	848.8
MBNL1	116.9	116.1	115.4	125.1	103.1	103.1	125.1	110.4
MBNL2	111.6	106.4	105.4	121.3	101.9	101.4	118.9	114
MBNL2	333.8	374.4	365.1	397.6	268.1	234.1	271.8	403.4
MBNL2	182.5	187.5	202.7	236.6	174.7	167	187.3	211
MBNL3	114	125.7	111.1	126.7	105.2	107.9	115	124
MBNL3	122	118.2	114.4	117.8	110.1	95.1	116.6	110.1
MBOAT1	116.2	124.5	114.4	123	107.5	113	116.4	119
MBOAT2	139.5	132.2	140.4	138.9	121.9	103.2	143.3	146.9
MBP	490.2	397	546.9	630.2	341.4	340.2	552.7	447.5
MBP	2509.6	2504.9	2092.5	2628.8	2097.4	1869.8	3026.3	2486.6
MBP	361.9	312.4	451.5	451.9	314.9	304.3	362.2	380.5
MBTD1	132.3	139.3	123.6	135	142.9	106.4	125.1	145.8
MBTPS1	647.1	607.7	537.3	605.7	462.7	510	622.9	593.7
MBTPS1	169.7	151.8	158.1	175.4	135.2	138.1	174.6	163.7
MBTPS2	117.2	112.2	129.5	120.2	116.8	102.8	120.4	120.3
MC1R	141.7	132.6	150	134.8	128.6	119.3	197.2	237.4
MC2R	115.8	117.9	118.7	118.7	109.2	100.4	115.7	114.1
MC3R	114.5	119.3	110.7	118.8	100.7	95.8	108.3	109.8
MC4R	117.8	126.1	115.2	124.1	112	108.1	120.5	123.2
MC5R	120.1	113.5	116.4	123.4	113.2	99.6	119.9	116
MCAM	114.4	120.1	118.5	130.4	109.9	94.1	117	118.6
MCART1	123.3	119.8	121	123.3	133.8	108.9	128.6	130.7
MCART2	117.3	117	107.6	106.2	110.6	97.6	120.6	112.9
MCART6	122.4	130	165.3	125	114.3	134.4	120.6	115.7
MCC	121.6	116.7	121	126.3	112.6	96.6	121.6	127.9
MCCC1	260.8	285.9	337.4	272.2	254.5	192.9	220.2	222.7
MCCC2	129.6	132.5	125	127.6	120.2	114	128.6	122.9
MCCD1	117.8	120.2	123.7	114.5	119.6	106.4	122.9	130.3
MCEE	345.2	273.9	451.8	315.4	304.6	243.7	247.2	250.9
MCEMP1	403.8	428.3	200.9	462.5	261.5	132	368	192.1
MCF2	116.4	122.6	124	124.4	107.6	106.5	127.7	122.9
MCF2L	107.6	110.6	117.8	115.5	108.9	100.7	120.9	115.1
MCF2L2	116.1	122.3	118.7	113.9	108.5	107.6	122.8	117.8
MCFD2	132.4	143.9	146.4	149.9	127.3	115.4	157.2	137.5
MCHR2	115.6	114.5	111.4	125.1	108.9	101.5	119.5	112
MCHR2	128.1	116.5	126.5	138.6	119.4	113.4	124.3	134.4
MCL1	279.6	280.5	300.1	300.3	267.2	202.7	217.7	260.5
MCL1	161	164.3	139.3	185.2	146	141.2	139.2	156.9
MCM10	104.3	104.7	105.2	114.1	107.9	97.7	108.8	116.9
MCM10	113.4	105.8	113.1	114.1	107.5	99.4	119.7	124.8
MCM2	141	134.6	141.2	144.9	131.5	119.5	146.7	133.6
MCM3	1367.2	1583.1	1054.7	1370.7	1100.1	801.7	983.2	932.2
MCM3AP	414.5	376.8	369.6	410.9	326.8	263.4	358.5	400
MCM3APA	139.1	139.1	130.9	137.3	153.9	100.9	153.7	150.9
MCM4	118.8	118.4	116.5	122	121.7	102.3	122.2	127.5
MCM4	144.6	137.7	143	151.2	133.1	112.1	145.6	137.5
MCM5	228.3	264.7	299.6	301.7	197	252.2	472.9	413.1
MCM6	694.5	1004.9	586.6	677.5	751.3	478.8	404.5	507.1
MCM7	124	133.2	118.6	119.8	117.8	103.6	123.1	117.2
MCM7	830.1	1140.1	727.4	812.8	723.2	604.5	891.2	1076.7

MCM7	117.4	131.9	125.1	150.9	112.2	113.4	136.3	131.2
MCM8	118	131	137.9	136.5	121.8	104.6	131.6	122.4
MCM8	113.5	123.7	121.1	127.4	119.1	101.6	120.8	110.7
MCM8	1177.7	796.2	726.9	855.4	867.3	622.5	1167.7	1309.1
MCMDC1	122.2	110.8	128.2	121.4	115.8	102.6	123.5	121.1
MCOLN1	329.3	258	365.9	328.8	205.9	291.2	396.1	244.2
MCOLN2	237.5	241.5	253.3	237.2	191.8	213.5	184.2	189.5
MCOLN3	124.1	116.3	109.7	119.8	106.9	94.6	113.2	114.4
MCPH1	155.9	144.2	151.1	159.1	140.5	133.4	147.2	146.7
MCRS1	653.1	723.7	659.6	731.5	564.3	513.7	600	533.6
MCRS1	159	146.9	184.1	162.4	154.3	141.9	163.8	142.3
MCTP1	107.8	110.7	113.3	116.7	108.3	101.6	115.1	112.8
MCTP1	139.8	110.2	139.2	110.7	119.2	102.1	127.3	114.4
MCTP1	112	114.8	116.9	116.6	110.7	94.8	119.5	111.5
MCTP2	121.8	120.8	132.2	126	123.5	97.9	126.4	113.9
MCTS1	149.7	157.1	159.3	157.3	123.2	144.6	167.8	160.9
MDAC1	109.8	113.2	120.2	114.7	103.7	96.4	110.7	116.3
MDC1	263.5	256.3	255.1	237.8	219.3	219.3	247.6	232.1
MDFI	121.6	137.8	131.4	128.1	114.4	107	146.2	134.2
MDFIC	162.6	149.2	200.7	190.1	161.6	151.9	162	144.1
MDGA1	113.8	108.4	122.5	123.9	106.2	100.4	122.9	116.6
MDH1	1565.3	1483.9	1762.8	1626.1	1037.2	1134.1	1271.9	1110.9
MDH1B	112.8	107.2	116.5	118.6	107.8	87.5	114.2	112.1
MDH2	2474	2779.2	2838.5	2344.1	1782.6	1774.6	1637.1	1353.3
MDK	104.4	112.8	111	111.9	107.2	92	114.5	112.6
MDK	117.5	114.8	120	119.7	106.9	105.2	124.5	116.4
MDK	114.4	124	119.8	121.7	108.6	103.1	120.3	120.8
MDK	131.2	116.7	133.1	130.5	112.3	104.4	127.5	129.8
MDK	112.7	118.9	118.8	123.4	108.5	94.6	114.3	109.4
MDM1	109.7	110	111.9	117.3	110.7	99.6	114.8	110.8
MDM1	128.5	119.4	130.3	122	114.4	107.5	120.5	120.3
MDM1	128.7	136.6	134.2	125.7	121.1	97.3	120.5	134.3
MDM2	118	112.1	123.1	124.8	107.1	99.7	126.9	123.9
MDM2	122.5	112.9	116.8	122	106.5	101.9	113.3	115.8
MDM4	126.8	120.4	127.1	134.7	128.4	98.5	141.7	125.9
MDN1	134.8	137.8	121.3	131.1	124	105.1	139.1	131.8
MDP-1	133.5	132.8	153.2	144.7	138.8	121.7	133.7	125.6
MDS028	507.4	525.3	527.2	449.4	378.3	348.5	368.6	276.4
MDS032	140.5	179.8	131.7	153.1	129.7	123.7	153.7	139.1
MDS1	118.8	111.4	123.2	119.8	109.5	102.4	112.5	109.7
ME1	289.3	250.6	180.6	486.6	300.2	418.9	566.9	271
ME2	247.6	235	313.4	278.3	231.8	226.8	283	272.9
ME3	117.3	119	117.7	121.7	113.2	103.2	120.4	118.5
ME3	111.7	124.7	128.3	123.4	113.4	101.2	130.5	120.2
ME3	114	118.4	105.5	115.2	106.3	102.1	109.4	115.1
MEA1	209.1	199.6	203.4	207.6	193.6	157.2	201.3	220.2
MECP2	154.5	143.8	121.3	152.5	133.2	117.8	143.2	155
MECR	120.6	131.6	107.6	119.4	115.6	98.4	130	109.9
MECR	121	136.2	123.2	131.4	123.2	111.6	124.4	138.3
MED12	194.2	190.3	159	180.6	175.4	137.2	199.1	183.6
MED12L	112.7	109.1	111.9	121.8	110.6	95.7	114.9	114

MED18	106.9	120.9	108.4	116.7	112.2	91.1	106.4	119.8
MED19	275.5	277.2	272.9	281.7	263.4	213.7	299.5	260.4
MED25	192.7	174.5	194.9	187.8	138.9	176.5	161.2	137.8
MED28	413.9	424.5	435.7	524.2	360.5	417.8	503.1	382.7
MED31	104	113	119	124.8	106.8	98.8	121.9	102.6
MED4	308.4	565.4	339	391.8	529.6	347.8	429.4	576.4
MED6	722.3	838.6	842	921.3	799.9	783.6	1038.9	977.7
MED8	175.2	170.6	130.9	146.5	132.7	128.7	149.9	157.3
MED8	155.7	183.5	167.5	159.1	154.6	143	166.6	149.3
MED8	150.3	139.2	157.3	176.9	139.1	140.3	157.1	136
MED9	119.9	140	122.2	136.2	122.8	111.3	131.3	126.8
MEF2A	153.4	136.8	144.2	138.1	130.7	117.1	161.8	156.4
MEF2B	114	122.8	123.2	125.9	121.5	102.2	126	121
MEF2C	166.4	152.6	196.8	181.1	121	123.6	156.8	128.5
MEF2D	491.2	543.7	492.3	570.8	536	398.6	649.6	871.3
MEFV	115	113.5	122.5	117.4	111.9	96.4	116.9	116.3
MEG3	116.2	114.3	124.1	122.3	113.1	106.8	119.6	123.1
MEG3	116.5	110.9	116.8	115.7	107.1	96.9	113.1	109.4
MEGF10	110.5	104.3	108.9	117.2	102.8	93.9	119.2	108
MEGF11	110.2	112.3	126.4	120.2	115.7	97.6	115	103.9
MEGF8	107.9	114	110.7	123	102.5	103.4	116.7	119.5
MEGF9	129.8	123.6	139.6	146	110.8	122.4	134.1	119.8
MEI1	147.9	160.8	147.3	175.8	137.6	126	144.9	152.4
MEIG1	113.7	107.9	106.6	115	110	93.9	116.9	112
MEIS2	118	119.5	120.2	123.8	110.1	100.4	119.4	124.3
MEIS2	110.4	116.6	117.2	110.9	104.8	99.9	119.4	114.3
MEIS2	145.3	115.9	129.2	120	111.2	102.6	111.1	121.3
MEIS2	112.9	110.7	124.5	122.8	112.3	105.5	114.7	119.3
MEIS3	114.2	109.7	122.1	118.2	115.1	104.1	115.3	117.2
MEIS3P1	122.3	125.2	119.5	122.1	120	109.4	128.2	125.1
MELK	109.9	107.4	113.5	120.4	99.9	100	116	117.6
MEN1	133.3	133.3	120.5	140.2	117.6	109.1	145.9	131.3
MEN1	116.4	120.3	108.8	118.1	110.1	97.2	126.4	110.4
MEN1	129.3	117.7	118	127.2	122.1	105.2	129.8	126.2
MEN1	182.1	182	153.9	185.1	153.5	141.8	187.1	159.1
MEOX1	118.9	135.2	129.3	125.5	111.2	99.3	109.2	110.3
MEOX1	116	118	111.5	114.9	100.4	101.8	107.9	108.9
MEOX1	110	123.8	124.3	122	113.6	110.6	130.1	121.6
MEOX2	109.8	116.1	118.6	122.6	101.6	102.6	118	113.2
MEP1A	128.9	119.2	128.8	136.9	108.7	115.8	133.3	123.2
MEP1B	117.4	117.6	116.1	119.2	109.5	98.1	113.7	112.4
MEPE	115.9	115.3	109.2	125	111.2	97.5	132	113.7
MERTK	133.2	117.7	287.7	147.3	123.8	306	141.7	130.7
MESDC1	129	137	124.3	147.4	137.8	142.8	181.9	143.7
MESDC2	116.2	118.5	122.8	119.6	115.4	96.8	115.8	109.2
MESP1	119.5	122.2	121.2	121.2	114.2	99.6	118.2	113
MEST	114.2	114	101.5	110.1	104.8	92.6	108	102.4
MEST	109.8	118.1	114.5	116.7	110.1	104.3	119.9	110.1
MEST	107	109	114	112.9	104.3	102.2	110.8	104.6
MET	111	117.5	118.8	110.4	122.2	110.8	122.3	114.6
METAP1	811.7	833.6	701.5	791.2	745.5	502.9	750.2	815.4

METAP2	759.9	910.3	697.3	961.3	769.9	651	1123	1043.3
METRN	125.2	139.7	117.6	131	114.9	117.3	133.5	120.1
METRNL	159.1	146.2	158.2	171.8	149.1	161	178.9	165.3
METRNL	109	113.5	120.4	121.9	105.4	108.5	122.1	123.9
METRNL	113.2	118.8	140.2	137.1	117.7	124.1	155.3	134.2
METT10D	136.4	140	137.5	157.3	146.2	120	164.5	144.5
METT11D1	112.7	111	107.5	114.7	106.4	100.6	116.3	110.4
METT11D1	161.7	159.5	162.2	172.8	138.6	126.5	150.9	138
METT5D1	129.9	123	151.3	155	131	111.9	130.4	128.1
METTL1	149.2	164.2	135.6	152.4	135.7	115.2	152.1	135.3
METTL1	111.7	120.4	129.2	115.1	130.1	103.4	128	117
METTL2A	131.3	142.2	145.2	154.3	141.3	111.4	178.9	158.4
METTL2A	113.6	116.1	119.8	117.9	113.8	103.5	119.3	120.1
METTL2B	117.4	114.1	126.2	118.2	109.1	92	131.4	114.4
METTL3	357.3	456.7	458.5	434.4	403	327.4	336.9	298.8
METTL4	203.2	203.9	190.4	209.4	196.9	157.2	245.8	243.7
METTL5	384.6	519.7	590.2	445.7	376.1	440.1	354.3	411.1
METTL6	112.8	129.4	125.6	116.8	112.2	92	119.8	104.3
METTL7A	145.4	161.7	190.9	160.3	148.7	130.9	135.4	133
METTL7B	143.4	160.6	112.7	135.4	138.1	101	146.5	114.6
METTL9	111.8	130.8	119.5	122.6	117.8	101.3	121	129.7
MFAP1	185.2	171.2	193.6	177.8	174.6	172.5	199.4	211.2
MFAP2	123.8	110.6	117.9	119.7	109.1	110.7	132.2	113.2
MFAP2	111.4	104.6	109.4	118.6	105	104.4	118.6	110.2
MFAP2	115.2	121.2	124.2	118.2	107.7	102.4	120.7	111.9
MFAP3	159.8	170.2	154.9	160.2	149.7	132.4	166.8	159
MFAP3L	118.8	111.6	125.5	111.7	114.2	102.5	120.1	109
MFAP3L	114.6	110.5	116.4	123.6	110.9	96.3	123.4	114.3
MFAP3L	119.1	131.2	122	107.8	102.8	99.8	111.7	115.5
MFAP4	112.2	110.7	112.5	120.1	114.8	103.4	118.3	122.7
MFAP5	116.7	114	130.5	117.1	106.9	104.5	127.9	115.6
MFGE8	560.1	602.3	646.1	528.9	522.9	318.9	342.2	465.7
MFHAS1	149.6	167.1	144	163	143.4	117.2	152.7	174.4
MFI2	111.1	129.2	123.7	124.7	118.7	106.3	122.4	122.2
MFI2	112.6	117.6	116.4	121.1	109	96.5	120.9	124.8
MFI2	114.4	117.6	121.6	120.5	108.4	94.8	117.2	116.2
MFN1	115.3	112.8	113.8	117.1	100.5	101.4	123.3	109.5
MFN1	125	140.7	135	141.7	129.2	106.2	128.6	130.1
MFN2	172.3	224.3	191.8	218.3	171.1	155.7	236.6	211.3
MFNG	896.5	1029.2	1033.9	858.6	776.9	870.1	781	695.6
MFRP	118.4	111.2	111.9	115.2	111.2	99.1	115.7	115.1
MFSD1	582.9	561.7	970.5	547.2	411.5	693.8	404.6	558.8
MFSD2	117.7	112.7	116.5	126.2	110	103.6	124.7	115.8
MFSD3	263.8	316.8	282.6	244	188.7	142.6	167	160.4
MFSD4	120.5	113.2	115.8	122.1	102.9	101.9	120.2	106
MFSD5	352.4	358.6	280.5	362.2	264.8	260.3	312.1	304.6
MFSD7	118.4	125.5	127.4	123.8	110.5	95.7	121.2	118.3
MFSD8	256.8	311.3	372.2	337.7	278.7	245.7	220.9	216
MGA	167.6	167.8	150.3	157.6	169.5	120.4	189.5	170.8
MGAM	124.4	115.5	120.4	113.3	114.9	109.6	123.5	112.9
MGAM	110.4	115	130.1	119.2	109.9	102.8	113.9	115.9

MGAT1	934.1	843.8	737.1	1003.5	485.2	582.2	1286.2	1056.9
MGAT2	412	416.4	388	438.3	368.6	350.9	477.9	385.7
MGAT2	111.7	125	127.6	120.4	107.7	102	112.4	121.9
MGAT3	101.9	112.5	110.5	112.2	105.2	99.1	125.6	112.6
MGAT4A	398.5	467.8	368.6	450.8	439.7	241.1	493.7	499.9
MGAT4B	110.6	114.3	124.6	131.1	123.8	114.3	134.9	123.1
MGAT4B	114	106	113.8	124.6	107	98.2	121.2	111.1
MGAT4B	164.7	188.8	154.8	191.7	167.6	149.9	184.8	192.1
MGAT4C	111.8	110.5	112.2	120.4	115.6	97.3	112.9	115.4
MGAT5	129	124.8	121.5	117	111.8	101.3	125.3	119.7
MGAT5B	120.9	120.2	115.4	117	108.2	99.4	113.3	117.3
MGAT5B	109.7	115.4	111.9	111	103.7	94.5	114.5	110.3
MGC10334	159.6	161.9	157.4	173.8	135.5	128.7	160.1	153.6
MGC10433	354	372.2	354.6	377.9	226.6	383.4	360.7	368.9
MGC10471	2082.1	1806.1	2827.1	2159.1	1629.5	1834.6	1874.4	1596.2
MGC10911	136.8	161	145.1	157.5	134.6	136.8	132.6	139.6
MGC10992	291.1	350.6	260.8	252.7	236.7	179.7	209.8	267.4
MGC10993	169.2	206.5	178.9	176.3	173.7	138.1	164.2	166.8
MGC10997	1009.7	1055.2	916.9	956.8	863	523.3	998	1179.9
MGC11102	126.1	129.6	133.9	139.7	113.7	118.3	123.6	123.7
MGC11257	325.3	326.4	790.3	304.5	257.3	363.6	219.9	254.2
MGC11271	117.9	113.1	121.6	118.5	104.6	97.4	118.4	112.4
MGC11332	124.7	125.4	122.2	119.7	113.9	101.7	132	125.8
MGC11335	166.1	172.3	155.4	175.1	160.2	122.8	169.1	193.6
MGC11929	128	126.3	144.4	129.7	119.1	104.3	126.6	128.6
MGC12965	136.2	142.5	147.4	155.7	142.6	115.3	135.8	148.4
MGC12966	129	111.7	127.5	125.3	110.1	112.9	116.5	125.4
MGC12966	425.1	414.7	486	455.2	385.7	287.3	381.6	407.8
MGC12981	326.4	360.8	390.1	385.6	335.3	370.9	242.6	239.3
MGC13017	6429.5	4451.9	5267.3	5824.7	4988.7	3341.9	6211.2	6026.1
MGC13024	122.9	128.2	134.9	131.5	119	110.9	139.7	119.6
MGC13034	114.7	115.5	131.2	127.7	122	97.4	119.4	113.3
MGC13057	101.3	116.5	115	113.9	107.1	104.9	108.6	109.3
MGC13057	109.9	115.8	122.3	120.1	106.5	106.2	117.1	106.2
MGC13096	222.2	233.9	174.4	239.8	182.5	134.2	192.8	187.6
MGC13114	260	296.6	246.6	229.2	210.5	194.5	206.6	202.4
MGC13114	137	138.4	129.4	129.2	124	110.4	149.7	132.7
MGC13125	148	166.8	152.4	153.3	142.1	123.1	143.7	152
MGC13138	117.9	122.2	137.4	129.4	122.2	110.7	129.5	116.9
MGC13170	127.6	112.5	119.5	120.1	115.4	104.6	112.9	118.6
MGC13170	180.1	236.6	171.9	217.9	184.9	154.8	230.9	211.7
MGC13379	313.9	373.7	316.7	319.8	282.6	202.8	250	207.6
MGC14141	157.2	139.8	169	152.3	118.2	133.1	148.9	144.5
MGC14327	738.4	731.1	733.1	680.8	613.1	689.7	631.9	648.9
MGC14376	248.7	254.7	219.1	397	313.2	419.2	667.5	678.5
MGC15416	141.7	151.7	145.5	151.6	141.2	119.6	132.4	128.3
MGC15476	122.9	140.4	137.1	148.4	126.5	114.9	135.1	154.7
MGC15523	151	157.6	167.6	156.3	137.8	127.6	149.4	159.6
MGC15523	115.3	120.7	120.6	115.9	101	103.5	115.5	116
MGC15523	139.8	141.6	137.3	148	120.5	124.4	140	129.3
MGC15619	161.6	166.7	223.5	171	144.5	144.7	141.6	147.1

MGC15619	190.3	164.7	265.2	185.1	142.5	147.4	155.9	141.3
MGC15763	191.8	228.1	306.3	222.5	221.9	226.4	181.4	184.5
MGC15875	339	326.6	299.3	317.6	219.5	221.7	283.7	274.8
MGC15875	135.9	147.4	143.3	139.2	119.7	121	138.4	130.5
MGC15912	190	177.8	200.5	175.9	192.8	156.5	193.6	207.2
MGC16028	168.7	178.3	202.7	175.9	183	181.4	143.8	153.8
MGC16169	136.8	152.2	159.3	155.4	124	137.5	136.1	125.1
MGC16186	124	120.3	131.6	131	116.1	113.5	126.7	125.3
MGC16291	105.9	106.6	116.7	119.8	101.6	91.9	113.9	106.8
MGC16372	116.4	112.6	117.7	119.4	106.9	99.4	116.9	114.8
MGC16703	8003.9	7050.1	7398.1	7805.7	7961.1	4184.5	8480.4	8986.9
MGC16824	122.4	120.5	135.8	133.2	117.6	112.1	129.6	121.9
MGC16943	115	117.5	128.2	120.4	116.4	107.1	118.3	113.1
MGC17330	1926.2	2238.4	2520	1803.3	1380.5	1912.2	1298.4	1194
MGC17624	125.7	143.2	119.4	163.6	125	105.7	126.5	128.7
MGC17839	110.3	119	114.6	117.2	104.3	99.2	128.7	102.4
MGC18079	165.6	232.1	307.7	222.8	245	249.1	258.3	218.9
MGC18216	118.1	130.3	152	129.3	138.1	139.8	137.9	154.4
MGC19604	167.7	169.9	181.3	165.7	146.4	139.2	137.4	149.3
MGC19764	180.1	216.3	195.5	190.7	220.7	132.1	171.4	169.1
MGC20255	246.2	279.4	274	298.6	206.5	238.6	325.6	279.9
MGC20470	112.2	112.5	124	115.9	106	102.3	129.1	116.3
MGC20983	114.8	113.5	110.6	117.8	106.7	96.8	122.2	117.4
MGC21644	118.2	114.3	114.3	119.8	108.5	96.9	118.4	113.5
MGC21675	120.5	124.6	115.8	127.4	105.9	100.8	128.1	116.9
MGC21830	179.7	154	168.2	171.8	159.2	147	170	158.7
MGC22001	106.7	117.3	110.8	112.2	95	95.6	115.1	109.7
MGC22793	356.6	473.6	494.6	341.8	355.5	461.8	331.5	260.6
MGC23280	136.7	127	158.6	138.4	136.3	108.4	141.1	125
MGC23909	155.2	147	186	148.8	151.1	128.2	136.8	135.6
MGC23985	119	109.6	116.7	111.1	110.5	98.4	114.7	114
MGC24039	130.1	135.9	133.7	143.2	126	106	123.4	124.7
MGC2408	136.2	158.1	135.1	147.1	122.1	120.1	133.2	140.1
MGC24381	148.1	169.4	157.9	157	145.8	132.3	148.5	151.3
MGC2463	1055.2	1098.2	1002.2	971	649.7	844.7	545.6	430.8
MGC24665	150.4	168.5	149.7	147.2	133.7	109.4	148.3	136.9
MGC2474	122.7	112.4	127.5	121.2	108.8	105.4	122.1	114.7
MGC24975	116.7	135.3	128.7	134.3	110.2	109.3	126.2	123.6
MGC2654	208.7	213.6	224.6	209.8	187.4	182.6	219.1	212.4
MGC26647	119.3	120.5	126.1	122.4	113	102.6	134.8	115.8
MGC26718	126.1	109.9	125.9	134.6	122	103.5	117.9	120.6
MGC26733	115.5	117.4	114.9	121.2	116.3	101.7	116.2	127.8
MGC26816	109.3	111.5	114.2	108.1	102.2	97.5	120.3	116.9
MGC26856	112.6	120	114.2	116.5	111.1	97.1	113.7	116.3
MGC26885	149.5	149.7	163	170	164	117.6	171.2	160.1
MGC26963	117.3	116	129.5	114.5	105.6	100.9	116.2	117.7
MGC27016	105.6	104.5	105.3	116.6	100.7	92.6	104.1	104.4
MGC27121	112	115.7	115.6	112.9	110.8	96.1	115.2	112.2
MGC2747	163.2	164	182.7	167.5	160.5	138.6	128.3	139.8
MGC2749	174.5	181.9	208.9	192.5	181.3	182.6	195	193.7
MGC2752	131.6	131.3	141.1	137.4	130.2	110.4	129.9	134.4

MGC2803	239.3	264.9	261.4	297.2	312.2	206.3	401.2	316.8
MGC29506	131.9	127.5	143.2	161	123	111.2	124	119.8
MGC29671	121.7	121.6	147.3	127.5	117.5	108.9	129.2	115.7
MGC29891	3873.1	2876.8	3652.9	3457	3559.7	1535.7	4235.2	4434.3
MGC3101	112.2	111	114.3	123.7	111.5	94.3	122	125.7
MGC3121	796.6	1053.2	726.9	914.2	732.8	566.6	867	793.8
MGC3123	119.6	115.7	126.5	119.7	107.6	109.8	130.4	129.3
MGC3196	193	197.6	210.9	198.2	168	151.2	164.7	157.6
MGC3196	164.2	169.1	162.7	155	140	114.6	140.9	136.9
MGC31967	158	161.4	165.2	149.5	118.9	122.8	134.8	129.4
MGC3207	559.1	549.2	621.1	524.2	466.7	416.8	265.1	272.8
MGC3207	130.1	115.8	134.7	130.3	114.4	121.1	121.2	116.2
MGC3207	228.9	228.3	250.3	209	175.2	140.7	171	149.3
MGC3207	736.4	1019.9	1111.2	845.6	757.3	561	479.2	424.2
MGC3265	121.9	111	125.9	123.4	107.7	100.3	121	110.9
MGC33212	117.3	127	136.1	128.3	131.9	107.4	139.7	145.9
MGC33212	252.4	320.8	301.5	318.1	319.9	301	322.7	457.2
MGC33214	124.7	125.9	133.4	124.4	115.1	124.6	128.1	137.3
MGC33407	118.7	130.7	128.9	115.4	104.9	103.7	119.3	112.6
MGC33486	106.4	113.6	113.4	117	110	97.9	110.8	107.8
MGC33530	110.2	109.3	118.9	116.9	112.8	104.8	121.5	115.9
MGC33556	218.9	337.9	259.2	236.4	170.5	162.3	154	164.4
MGC33584	155.4	158.2	138	158	120.8	137.8	146.4	141.5
MGC33600	112.2	106.7	108.9	113.5	103.9	93.1	116.6	114.1
MGC33648	163.8	215.3	155.9	184.2	169.7	143.1	190.5	333.8
MGC33657	124.7	124.6	117.5	125.7	113.2	104.1	123.1	122.5
MGC33692	156.8	152.4	195.6	150	125.9	118.7	132.4	129.4
MGC33846	115.2	107.1	116.9	113.1	108	95.2	122	114.5
MGC33887	125.6	122.4	124	126.3	118	100.7	118.2	122.4
MGC33894	109.6	114.5	108	120.1	111.4	93.3	110	108.2
MGC34646	110.3	118.9	112.7	125.9	107.2	102.1	124	116
MGC34647	112.5	119.9	119.1	123.7	108.1	98.4	112.3	124.9
MGC34725	116.2	116.9	113.5	117.2	106.9	101.4	114.8	109.2
MGC34732	114.4	119.4	117.9	114	113.7	100	117.7	121.9
MGC34761	118.5	120.7	120.4	128.4	113.9	105.4	136.2	121.3
MGC34774	103.9	114	104.1	108.3	107	98.8	105.2	100.6
MGC34821	113.1	123	121.2	121.5	110	100.1	118.3	110.7
MGC34824	124.1	123	135.2	136.8	115.4	102.6	132.7	123.7
MGC34830	116.8	125.6	117.4	121.8	112.9	98.2	114.7	119.8
MGC35043	114.1	123.3	117.7	128.3	113.1	110.8	126	128.1
MGC35154	118	115.3	121	111.8	114.6	105.7	119.7	110.2
MGC35206	118.2	122.3	125.4	125.1	119.5	110.4	124.3	109.4
MGC35212	117.6	115	123.1	125.7	102.7	97	114	119.3
MGC35295	110.8	114.4	119.1	115.9	113.5	95.4	121	116.6
MGC35308	120.5	116.6	126.8	123.4	113.4	103.9	119.6	121.2
MGC35361	132.6	125.4	122.7	132.6	113.5	108.7	121.2	121
MGC35402	111.7	120.6	112.1	115.9	115.8	96.7	113.6	118
MGC35440	108.8	128.9	132.1	123.6	112.9	104.3	126.1	117.3
MGC39497	118.4	113.4	113.8	121	108.8	104.8	124.8	117
MGC39581	117.9	118.8	123.1	121	110.3	102.7	123.4	115.5
MGC39606	122.5	118.1	117.8	121.8	111.7	101.4	119.9	106.7

MGC39633	146.2	161	205.4	159.6	132.9	138.1	134.7	125.8
MGC39662	107.1	114	111.4	109.4	100.6	104.2	118.5	105.3
MGC39681	125.5	105.1	115.5	121.4	106.3	99.4	116.3	115.2
MGC39715	120.4	121.6	119.2	113.3	106.8	106.2	131.6	119.9
MGC39900	140.7	142.8	152.4	133.9	124	118.6	133.6	132.7
MGC39900	113.1	121	129	130.8	118.4	110	119.2	127.1
MGC39900	128.8	134.7	144.6	127	129.8	120.7	128.8	126.5
MGC40069	152.6	152.7	157.9	148.4	130.8	116.4	135.8	125.1
MGC40222	110.6	114.4	115.4	119.4	106.1	99	106.9	115.4
MGC40405	258.4	305.6	253.9	290.2	227.7	236.5	279	354.4
MGC40405	268	277.1	267.6	281.7	228.9	176.3	254.5	258.1
MGC40499	125.3	138.7	140.6	127.7	124.5	111.2	144.6	127.5
MGC40499	142.1	149.4	135.5	145.7	125.5	120.8	120.3	123
MGC40579	180.7	149.5	168.4	203.6	162.6	119	163.6	141.7
MGC4093	178.8	194.9	192.4	210.6	176	145.4	182	184
MGC4172	138.3	127.4	168.5	141.3	118.8	135.5	131.7	143.6
MGC41945	118.3	126.6	130	138.5	118.9	106.2	135.2	130.5
MGC41945	132.3	113.8	112.8	122.5	109.6	103.1	112.9	114.1
MGC42090	114.7	113.3	108.7	109.9	103.6	96.3	116.7	110.4
MGC42105	111.9	111	113.2	111.1	103.6	98	121	107.1
MGC42174	161.8	150	165.7	165.7	149.1	141.6	160.8	145
MGC42367	193.8	150.8	177.2	161.3	148.7	128.9	201.6	187.2
MGC4266	116.5	116.4	125.7	118.5	115.5	97.7	119.4	106.6
MGC4268	149.9	165.3	152.5	164.7	152.1	128.7	160.6	205.7
MGC4399	120.7	120.9	120.6	122.5	106.6	96.3	125.3	123.9
MGC44328	117.5	109.3	116.9	123.8	109.4	102.3	130	114.9
MGC44505	120.8	116.7	129.8	121.8	116.2	103.3	129.7	117.2
MGC45438	116.9	130.7	118.8	121.6	120.1	108.9	122.7	118.3
MGC45491	105.4	120.5	120.5	127.4	110.3	98.6	115.6	107.1
MGC4562	346.4	457	462.9	414.6	501.6	317.2	300	371.7
MGC45800	111.5	116.7	112.4	111.1	108.7	99.3	132.2	114.4
MGC45840	139	137.1	147.6	145.1	131	122	147.2	139.7
MGC4618	272.2	291.2	390.6	255.2	197.1	202.8	194.9	183.9
MGC46496	115.4	107.7	122.7	116.5	103	99.4	126.9	114.3
MGC4655	111.6	115.5	116.8	125.7	114	103.4	130.8	112.2
MGC4677	105.4	113.8	115.8	114.4	102.4	104.4	113.9	115.2
MGC4677	519.9	672.9	677.2	719.4	573.4	668.1	980.2	1009.2
MGC4728	239.5	222.4	253.3	235.5	223.2	222.8	260.5	221.3
MGC4825	145.7	145.6	135.4	164.7	146.9	125.5	162.9	150.1
MGC48595	108.4	109.2	122.2	118.5	107.7	97.7	116.2	112.6
MGC48628	117.6	118.5	114.9	128.8	111.6	101	116.9	109.4
MGC48915	111.9	103.3	112.2	116.7	111.7	95.3	109.3	114.6
MGC50273	120.6	106.3	119.9	113.7	97.8	95.9	112.4	114.5
MGC50372	114.4	116.1	111.9	115.3	105.5	93.8	116.8	116.5
MGC50559	121.4	115.5	111.4	123.9	109.3	108.3	122.3	109.2
MGC50721	110.2	112.4	119.2	120.2	106.6	100.2	121	122.1
MGC50722	120.6	121.1	122.2	123.5	112.6	103.5	127.4	122
MGC50811	114.5	112.1	116.5	116.4	105.2	92.6	108.3	115.8
MGC51025	110.2	112.9	115	116.6	110.9	97.9	109.7	111.9
MGC5139	112.9	117.7	107.1	116.3	113.7	102.2	116.1	115.8
MGC52057	116.1	116.5	113.8	125.3	113.9	104.5	113.2	113.7

MGC5211C	315.9	313.6	408.8	266.7	261.4	224.4	222.3	211.2
MGC5242	133.6	165.8	138.7	148.9	124.1	118.6	132.4	120.7
MGC52498	259.1	268.9	274.8	238.5	180.7	243.3	200.4	185.1
MGC5509	161.9	157.3	156.8	155.8	148	125.1	158.8	167.5
MGC57359	104.5	115.4	114.1	120.6	110.7	99.7	116.4	110
MGC57359	121.9	113.2	128.2	115.7	107	109.3	111.6	108.9
MGC59937	113.2	111.8	116.8	120	105.6	98.7	120.4	122.2
MGC61571	155.9	160.6	162.7	174.5	142.8	133.3	135.6	146.4
MGC61598	116.1	110.4	117.3	116	102.6	89.6	117	106.8
MGC6210C	110.1	120.5	124	118.7	125.1	102.1	114.2	122.2
MGC7036	272	278.6	213.3	273.2	260	205.3	221.3	246
MGC70857	180	159.6	168.8	184.3	133.6	142.8	160.1	143.7
MGC70863	112.3	118.4	120.3	127.9	105.3	102.1	118.2	114.4
MGC70863	118.3	133.9	120.7	120.9	117	108.2	133.8	124.1
MGC7087C	107.9	114	109.6	115.8	104.3	100.2	119.1	108.8
MGC70924	115.7	121.8	127.5	135.2	112.4	93.2	134.1	117
MGC71993	1108.3	1562.8	1293.7	1543.5	921.7	1169	1521.5	1264.3
MGC72075	123.9	119.2	128.1	129.7	110.3	113.4	127.8	122.4
MGC7208C	126.4	121.8	132.5	127.5	114.5	111	127.4	124.2
MGC7536C	117.9	112.4	111.4	118.7	105.9	99	110.6	112.2
MGC87631	114.7	125.9	124.5	135.3	109.9	98	115.7	120.4
MGC88374	107.1	109.9	118.7	116.7	103.7	96.1	112.8	109.6
MGC88387	124.2	128.6	123.2	129.4	123.1	107.9	124.2	116.1
MGC9712	101.2	123.9	120.4	111.1	102.2	96.5	108.2	109.4
MGC9850	229.8	244.1	226.9	237.1	220.7	234.8	252.1	239.9
MGC99813	117.9	126.6	124.2	125.5	117.5	112.2	121.5	127.7
MGEA5	1435.7	1418.8	1320.2	1602.7	1480.5	1396.6	1992.5	2715.2
MGLL	129	126	115.6	128.3	122.8	105.6	126.6	108.8
MGLL	118.1	118.3	114.4	125	99.4	99.9	125	116.8
MGLL	147.5	143.5	151	154.2	117	148.1	136	114.6
MGMT	186.8	194.2	244.5	215.7	175.2	180.6	163.6	176.7
MGP	115.7	106	119.5	114	106.4	108.8	126	134.8
MGRN1	222.6	209.7	200.6	210.1	153.7	162.6	230.2	185.5
MGST1	121.6	112	116.5	116.5	104.5	97.1	114.7	116.7
MGST1	144.9	176.8	148.9	227.3	187.6	174.6	199.9	157.7
MGST1	112.8	115	127	127.3	116.1	105	124.5	113.5
MGST1	194	320.2	223.2	441.7	371.3	376.7	433.1	337.6
MGST2	167.9	144.6	332.5	147.9	129.6	153.7	140.2	118.6
MGST3	792.1	742.5	1248.9	761.8	634.2	821.7	561.1	439.8
MIA	117.3	108.2	133.5	120.4	124.9	108.7	121.1	122
MIA2	106.5	116.7	106.5	110.3	108.3	101.6	113.8	106
MIA3	128.6	128.7	136	125.4	120.8	109.4	123.6	127
MIB1	135.5	133.6	132.7	144.5	127.6	119.8	140.8	128.9
MIB2	284.4	332.3	256.4	302.9	267.1	224.6	332.8	292.6
MIB2	233.6	275	192.2	275.5	195.5	144.1	290.9	276.8
MICA	129.3	117	129.7	135.5	135.2	118.5	133.2	122
MICAL1	237.8	198	284	220.8	171.7	210.3	217.6	194.2
MICAL2	111.9	114.5	116.4	129.3	114.6	105	121.5	117.7
MICALCL	122.8	116	112.8	118.9	115.8	99.6	121.9	121.4
MICAL-L1	175	171.5	154.4	188.6	130.7	140.5	196.7	176.2
MICAL-L2	122.8	116.1	120.5	123.6	111.2	101.1	119.8	122.2

MICAL-L2	116.3	111.9	119.9	119.8	108.3	100.9	120	119.4
MICB	323.1	347.1	274.3	317.2	210.3	249.1	321.1	275.3
MID1	130.2	149.4	135.4	136.9	136.5	122.8	132.7	139.5
MID1	109	110.5	114.8	115.1	108.6	99.6	114.6	110.9
MID1	118.5	102.8	115.5	117.3	104.9	101.4	117.7	110.7
MID1IP1	206.9	253.7	191.2	218.1	165.9	199.5	185.4	199.5
MID2	124.8	126.1	121.8	122.1	125.4	105.3	121.5	133.7
MID2	118	126.5	120.1	116.8	114.3	98.8	120.4	120.2
MID2	129.9	147.3	133.1	157.7	138.1	110	140.4	136.9
MIDN	250.9	196.7	304.4	252.4	229.2	249.3	333.3	309.9
MIER1	113.2	122.7	120.6	117.4	109	100.7	115.4	114.3
MIER1	301.6	350	288.2	316.8	303.3	239.3	422	484.8
MIF	819.6	878.2	815.7	837.5	743.4	576.6	504.1	774
MIF4GD	174.9	185	200.1	160	144.7	147	167.7	148.1
MIMITIN	226.1	234.4	257.5	231	241.1	214.2	278.7	326.5
MINA	134	136.9	122.3	151.6	134.2	105.6	122.8	125.5
MINA	122	116.6	117.2	121.1	108.1	104.4	116.5	118.6
MINK1	122.7	115.3	124.2	121.9	110.6	94.8	110.2	126.6
MINK1	124.7	128.2	119	131	119	98.3	132.7	129.8
MINPP1	127.6	143	134.2	143.1	122.7	113.3	124.5	124.5
MIOX	115.9	113.3	121	125.5	105.2	96.6	112.4	110.8
MIP	108.6	104.4	106.6	118.6	106.1	90.5	118.8	112.5
MIPEP	125.8	140.7	143.4	129	119.4	117.9	120.6	121.3
MIPOL1	111.6	116.4	119.5	118.8	105.3	91.3	113.7	118
MIR16	197.2	197.4	237	207.1	165.2	179.6	199.1	180.7
MIS12	732.5	636.4	657.9	769.4	577.9	501.8	862.4	729.9
MIST	118.5	119.8	126.9	125.5	110.4	97.5	128	121.3
MITF	155.9	127.3	134	161.6	122.9	123	145.7	132.5
MITF	135.3	108.4	119.3	117.4	109.2	99.1	117	121.4
MITF	110.5	129.3	116.1	144.2	109.3	111.3	118.9	127.6
MIXL1	110.1	108.1	119.4	119.4	107.6	95.9	121.2	114.9
MIZF	125	116.4	115.9	122.1	117	104.2	115.7	114.4
MIZF	252.1	273.5	206	242.2	232.2	178.5	255.3	266.9
MIZF	201.6	276.2	175.6	213.4	189.1	151	230.2	241.9
MKI67	111.7	112.9	120.5	125.9	110.2	105.4	111.5	120.4
MKI67IP	487.5	435.3	390.5	431	398.6	327.4	432.7	471.5
MKKS	138.5	122	146.1	121.2	111.9	120.3	127.5	114.2
MKKS	335.9	508.5	332.6	417.6	333.2	285.8	404.1	422.2
MKKS	111	108.8	131.5	115.5	119	108.3	128.2	107.7
MKL1	220.5	271.5	214.1	257.4	183	204.3	244	277.2
MKL2	140.8	147.5	144.5	161.7	140	126.3	120.6	122.4
MKLN1	560.1	556.1	574.6	717	645.1	411.3	745.8	751.4
MKNK1	117	115	114.3	119.2	106.5	96.9	119.6	113.7
MKNK1	470.1	400.7	659.3	572.7	334.9	674	490.1	383
MKNK2	790.1	720.4	704.2	688.7	616.4	448.9	808.7	543.4
MKNK2	214	198.9	190.7	207.3	159.1	150.6	210.2	162.9
MKRN1	1659	2040.9	2070.9	1899.1	1377	1481.4	2050.6	1726.8
MKRN2	372	400.1	407.3	444.3	337.3	374.5	422.6	370.9
MKRN3	107.3	124.8	126.9	133.4	126.5	103.6	126.3	113.2
MKS1	117	120.8	124.9	118.4	125.3	99.2	113.4	118.6
MLANA	109.4	108	101.8	109.1	109	95.2	123.7	111

MLC1	114.7	121	108.1	124.9	108.5	95.9	120.5	116.4
MLC1	125.3	122.7	123.2	114.5	118.2	106.1	122.7	114.8
MLC1	120.9	117.2	121.2	127	110.5	108.5	130.7	109.8
MLC1SA	261.5	273.4	173	228.8	182.2	141.8	193.5	176.6
MLCK	116.1	116.4	121.2	123.5	106.4	93.9	124.8	117.4
MLF1	106.6	117.7	118.6	114.9	102.1	94.8	113.8	120.1
MLF1IP	118.2	114.2	113.7	125.6	120.5	111.9	121.9	123.9
MLF2	297.3	313.6	304.1	322.4	191.1	301.6	360.3	314.6
MLH1	153.7	138.9	191.2	165.2	150.1	165	177.8	153
MLH3	161.8	152.2	153.8	158	124.7	109.2	156.7	126.6
MLH3	123.7	121.4	122	134.9	120	108.8	117.7	116.3
MLKL	192.5	203.2	212.4	188.8	140.4	136.5	147.7	161.6
MLKL	122.2	127.3	141.4	127.1	114.8	115.1	116.1	115.6
MLL	177.9	196.5	143	157.3	149.6	133.6	209.6	188
MLL2	128.5	134	136.7	135.8	126.8	113.3	125.4	117.2
MLL3	135.9	119.8	143.1	141.3	148.7	103.8	147	148.6
MLL3	147.9	135.7	125.1	154.5	152.6	119.6	147.7	152.5
MLL3	122.8	114.8	122.9	127.7	114.6	93.5	119.8	117.2
MLL3	190.2	192.5	194.9	230.4	209.9	157.5	234.9	254.1
MLL4	280.7	237.7	191.3	247.5	208.5	184.1	332	299.9
MLL5	161	155.1	137.9	178	159.4	134.8	176	196.6
MLL5	146.8	133	128.6	136.2	119	107.6	134	130
MLL5	390.9	417.8	408.9	482.7	410.4	253	348.1	439.4
MLLT1	110.9	103.8	111.4	109.4	107.8	95.5	115.8	113.1
MLLT10	113	115.7	132.4	120.4	101.9	112.8	125.2	117.5
MLLT10	287.2	270.7	227.4	276.3	308.1	210.1	224	207.2
MLLT11	232.9	270.4	210.1	254.1	237.7	189.6	299.4	433
MLLT3	106.1	121.1	118.5	116.8	107.8	96.9	118.9	124
MLLT6	780.5	790.1	726	680.4	622.9	417	530.5	494.7
MLLT7	209.5	205.2	180.9	196.5	196.6	197.7	199.3	207.7
MLN	112.8	108.2	122.3	114.1	113.7	103	123.1	111.3
MLN	111.9	117.5	128.4	128.3	110.4	106.8	128.1	130.4
MLNR	114.2	109.4	116.1	115	108.9	98.4	117.5	110
MLPH	114.3	121.8	121.3	113.8	103.5	98.8	122.9	115.9
MLSTD1	131.5	129.7	155.1	137.3	119.6	113.5	144.4	113.8
MLSTD2	222.3	229.2	268.2	264.9	218.1	210.3	250.7	273.7
MLX	115.4	118.1	116.7	127.6	101.3	92.8	114.2	102.4
MLX	191.4	193.2	160.8	160	148.7	121.4	146.5	146.2
MLXIP	105.1	112.7	113.8	124.5	112.2	92.8	120.7	114.2
MLXIPL	116	113.9	118.5	115.8	109.4	93.1	118.1	110.7
MLYCD	151.5	147.6	155.4	171.4	143.4	112.8	147.9	132.4
MLZE	116.6	123.8	116	120.3	104.2	97.1	109.6	118.7
MMAA	119.1	120	126.1	116.9	117	98.9	124.8	112
MMAB	150.5	135.5	155.6	146.9	132.4	119.5	137	137.1
MMACHC	129.1	134.8	135.7	140.2	129.7	111.2	135.2	140.2
MMD	188.5	164.1	268.6	185	178	174.9	186.4	212.5
MMD2	103.5	118.2	114.9	112.2	102.1	92.9	112.4	108.6
MME	121.1	113.6	103.9	131.9	109.4	104.4	111.4	125.4
MME	108.4	114.2	99.5	106.7	105.8	97.1	115.8	105.2
MME	111.6	113	116.3	125.5	113.7	100.1	110.9	115.8
MME	110	112.4	110.1	115.6	102.4	107.5	124.6	106.4

MMEL1	115.6	120.2	107.9	128.1	103.2	99.5	110.5	106.6
MMP1	107.5	110.8	111.8	119.7	107.9	96.4	119.4	114
MMP10	116.4	112.5	111.1	112	104.2	100.2	118.3	111.2
MMP11	127.4	106.2	115.2	115.3	119	108.2	116.7	114.2
MMP12	110.4	113	114.9	118.4	103	97.3	116.2	112.3
MMP13	112.1	111.2	113.7	108.7	102.7	98.5	103.1	107.5
MMP14	111.3	118.2	128.3	121.4	103.6	94	114.6	114.5
MMP15	131.1	133.7	148.2	124.5	120	100.1	135.8	133.8
MMP16	110.3	112.8	110.1	108.4	110.1	87.6	113.5	110.7
MMP16	110.3	107.4	105.2	114.3	102.6	102.2	114.4	111.9
MMP16	127.9	115.2	122.3	125.3	113.4	111.4	122.7	129.2
MMP17	122.7	116.2	124.3	107.1	114.9	97.7	121.1	137.3
MMP19	114	114	109.2	116.3	104.9	98.1	122.5	122.1
MMP19	112.6	113.9	122.3	119.2	111	106.3	119.7	115.4
MMP2	119.6	136.9	129	125.4	112	102.6	126.7	122
MMP20	119.7	112	119.4	117.2	108.1	99.5	122.6	116.5
MMP21	111.9	122.7	109.3	116.5	109.1	95.8	118.8	111.3
MMP23A	124.8	132.9	133.5	123.8	116.9	111.1	122.1	118.2
MMP23B	196.9	207.2	169.1	176.7	155.1	134.5	170.1	151.8
MMP24	119.3	121.1	120.2	126.8	114	104.6	129.5	111.6
MMP25	105.8	115.4	111.6	114.5	105.6	91.2	107.2	113.9
MMP25	131.2	131	138.6	133.2	123.5	105.3	127	118.4
MMP26	108.5	113.4	111.2	124.3	108.9	97.2	110.4	108.4
MMP27	115.6	110.8	120.1	119.4	105.6	97.3	115	110.4
MMP28	115.2	112.4	117.8	123.6	115.7	98.5	120.9	114.6
MMP28	215.5	264.8	284.1	211.5	172.1	179.8	144.4	143.1
MMP3	111.6	115.9	117.3	129.7	115.8	96.1	114.2	128.7
MMP7	113.3	149.7	148.7	133.8	125.3	122.7	127.7	118.7
MMP8	103.8	106.9	115.8	113.6	102	98.5	122.2	113.5
MMP9	1122.2	546.9	1536.7	1443.2	485.5	1762.1	1153.8	246.9
MMRN1	103.7	120.3	112.6	119.1	107.3	99.9	123.7	106.8
MMRN2	122.9	143.6	132.5	134.9	124.1	109.7	130.3	112.8
MMS19L	682.5	616.6	601.3	702.1	550.5	434	670.4	656.7
MN1	116	115.3	157.8	127.1	106.3	131.3	117.2	129.8
MNAB	158.2	163.3	158.9	160.2	137.8	129.7	169.2	208.7
MNAT1	116	114.1	121.8	118.5	113.2	110	116.1	114.9
MND1	113.1	105.8	114.8	120.6	111.8	90.7	118.5	113.7
MNDA	142.2	149.8	386	126	118.2	134.9	126.6	127.1
MNS1	110.9	113.5	111.9	120.1	99.7	104.4	115.2	122.6
MNT	261.1	272.3	231.4	271.1	262.1	246.5	328.4	315.1
MOAP1	425.6	514.9	451.8	618.9	495.8	487.2	542.3	662.1
MOBK1B	121.5	116.2	115.9	116.6	118.9	90	113	113.9
MOBKL1A	112.2	112	132.5	151.8	120.4	113.8	129.8	128.7
MOBKL2A	293.7	273.7	312.9	335.9	204.4	249.9	257.8	223.8
MOBKL2B	114.6	115.6	117.6	120.7	110.1	98.3	117.4	113.7
MOBKL2C	114.7	118.6	129.1	120.6	106.2	112.8	119.4	115.3
MOBKL2C	490.5	419.6	366.3	542.7	375.5	426.7	595.7	435.9
MOBKL2C	104.1	110.1	114.3	116.8	106.4	94.3	116.5	111.7
MOBP	105.2	113.5	116	118.7	99.7	93.8	118	117.4
MOBP	118.7	114.7	119.1	112.4	112	99.9	118	121.1
MOBP	126.7	110.8	136.1	120.8	110.8	101.7	121.6	109.8

MOBP	111.1	115.9	112.9	114.8	114.7	100.1	117.9	113.6
MOCOS	120	116.5	118.6	135.2	118.5	111.9	119	126
MOCS1	136.4	146.2	151.5	149.4	116.3	104.2	140.3	132
MOCS1	116.9	128	111.7	123.4	114.4	104.3	117.6	114.2
MOCS1	131	136.8	120.5	143.7	108.7	110.7	120.7	125.7
MOCS2	115.4	120.9	118	115.2	109.3	98	118.3	123.1
MOCS2	125.5	125.2	117.4	126.1	110.4	105.3	121.2	125.7
MOCS2	145.5	139.7	170.8	159.1	155.3	135.3	145.9	155.1
MOCS3	120.3	116.7	126.6	124.2	115.8	102.1	133.7	121.7
MOG	110	114.5	115.5	109.6	109.3	95.3	109.6	116.3
MOGAT1	116.2	118.7	112.9	116.2	115.4	103.1	122.5	122.6
MOGAT2	118.2	107.1	117.7	120.6	108.9	108	109.7	111
MOGAT3	107	113.8	108.9	113.9	99.6	93.3	113.1	104.2
MON1A	112.2	130.3	116.1	124.9	112.3	107.9	128.9	115.2
MON1B	205.7	199.6	214	200.5	187.2	180.9	252.1	229.9
MON2	395.2	404.8	349.9	465.9	386.2	288.7	368.4	374.8
MORC1	116.4	125.5	113.3	125.6	108.4	103.3	131.4	111.2
MORC2	191.9	189.3	151	194.8	178.8	139.8	179.5	169.4
MORC3	173.4	182.3	262.3	209.7	207	204.2	176.8	198.8
MORC4	105.1	127.6	117	118.7	113.4	99.5	124.4	123.3
MORF4	131.4	118.8	118.4	129.1	110.8	98.1	121	121.2
MORF4L1	800.4	588.4	787.3	751.5	523	843.8	774.2	705.8
MORF4L1	112.3	103.5	105	113	104.5	91.4	116.6	109.1
MORG1	131.1	143.8	141.4	139.9	131	110.3	145.1	160.9
MORN1	121.5	118.1	124.4	118.6	119.9	99.7	130.5	125.5
MORN3	132.1	131.5	128.1	127.7	111.2	106	125.3	129.2
MOS	105.9	108	127.1	133.2	111.6	100.9	116.2	106.6
MOSC1	109.8	109.2	106.1	121	96.4	93.1	113	119
MOSC2	114.2	117	116.8	129.1	108.3	105.8	119.9	117.7
MOSPD2	119.5	114	124.1	118.4	121.3	104.4	127.4	133.1
MOSPD3	143.3	154.6	152.4	134.6	126	119.6	148.2	146.3
MOSPD3	136.4	130.2	130.2	131.6	121	116.8	131.1	128.5
MOV10	200.6	220.1	220.3	200.9	149.6	173.3	197.8	161.3
MOV10L1	117.8	115.8	121.6	133	111	107.4	123.7	119.2
MOXD1	115.4	121.3	122.1	131.4	112.1	99.3	130.3	114.9
MOXD1	110.8	112.9	113.6	123.1	109.3	96.2	116.9	115.8
MPDU1	147.8	152.7	178.4	146.8	127.2	123.2	145.5	133
MPDZ	111.2	117.8	115.5	116.1	106.8	101.4	121	124.5
MPFL	119.5	115.2	129.6	119.5	102.2	97.4	117.1	116.3
MPG	105.4	113.3	109.5	109.9	106.5	100.2	113.2	119.5
MPG	126.6	116.3	117.5	128	111.6	98.4	122.5	112.7
MPG	118.5	118.8	115	120.2	102.9	102.2	108.1	103.5
MPHOSPH1	212	227.8	200	253.5	188.9	166.6	198.2	174.8
MPHOSPH1	119.3	113.9	113.5	117.4	113.9	94.4	110.1	129.4
MPHOSPH(	145.3	124.3	141.9	139.7	119.9	123.3	127.1	136.2
MPI	135.7	154.9	142.5	151.1	116	110.3	147	122.8
MPL	128.8	114.9	139.2	128.7	113.5	105.9	125.8	119.9
MPN2	109.2	111	122.9	116.3	114	97.1	116.8	112.8
MPND	125.3	133.1	129.2	116.4	114.1	105.8	122.4	125.4
MPO	123.2	191.2	133.2	138.5	139	96.3	121.7	148.7
MPP1	315.3	159.3	314.2	503.6	194.3	313.4	392.4	203.2

MPP2	116.2	118.9	125.7	109	108.2	93.1	125.1	116
MPP3	115.1	116.2	114.6	121.7	111.4	104.2	114.6	118.6
MPP4	101.3	110	104.9	114	99.7	92.1	116.9	106.1
MPP5	165.2	185.1	184.9	171.1	138.2	134.4	149.1	156.5
MPP6	185.7	178.1	163.8	167.4	147.5	135.2	169.4	184.5
MPP7	117.4	127	118.9	124.5	117.8	98.7	123.8	116.8
MPPE1	385	442	569	485.4	354.7	303.2	301.3	386.6
MPPED1	114.1	119.8	116.5	119.3	119.7	101.4	126.4	109.9
MPPED2	112	122.7	112	111.7	108.9	97	108.7	117.8
MPST	123.8	123.9	134.8	131.3	111.5	128.9	127.9	131.4
MPST	127.8	129.4	135.5	136.5	115.3	101.4	129.9	147.4
MPST	129.8	125.5	125.2	123.2	114.9	104.6	129.6	132.9
MPV17	274.5	286.5	314.4	281.3	239.5	243.5	211.3	243.7
MPZ	123.7	121.7	128.8	120.6	109.5	112.8	115.2	108.1
MPZL1	121.5	108.2	121.1	116.8	106.2	94.6	122.1	110.7
MPZL1	132.3	136	126	132.1	120	99.8	140.7	119.2
MR1	175	147.1	198.7	182.7	146.3	146.1	160.3	158.1
MRAP	111	115.8	108.2	110.5	100.8	95.2	116.3	114.5
MRAP	108.5	119.2	118.7	119.9	102.5	100.2	122.8	110
MRAS	120.1	122.5	116.7	134	107.2	96.3	126.5	115.2
MRC1	113.1	116.4	113.8	111.3	112.7	92.1	107.3	113.1
MRC1L1	119.1	116.9	117.2	118.8	103.1	106.7	117.2	118.6
MRC2	106.7	118.7	121.5	112.9	109.2	91.9	112.6	114.5
MRCL3	3707.6	3494.3	3610.3	2998.2	3394.8	4280.2	3770.9	3072.4
MRE11A	110.9	133.5	130.5	119.9	117.2	110.3	121.7	124
MRE11A	131.1	138.3	134	138.7	129.4	110.4	130.6	130.9
MRE11A	152.2	146	153	143.7	125.6	121.5	135.3	137.7
MRFAP1	1152.4	1163.1	1232	1197.1	1010	908.2	982.3	975.2
MRFAP1L1	1568.7	2060.4	1475.8	1572.5	1301.9	1178.4	1376.1	1597.3
MRFAP1L1	184.3	189.7	167.4	222.6	218.5	145	191.3	209
MRFAP1L1	125.9	132.2	143.7	133.5	114.3	107.1	123.9	130.6
MRGPRD	113.3	114.5	122.3	118.6	109.8	99.3	121.3	125.2
MRGPRE	123	112.7	136.9	128.7	118.9	108.6	130.2	116.4
MRGPRF	108.4	109	111.7	118.1	105.3	94.8	121.5	108.5
MRGPRX1	106.9	109.6	118.6	109.8	107.4	100.4	113	117.9
MRGPRX2	110.1	114.7	112.5	116.7	96.4	103.6	103.6	110.6
MRGPRX3	124.4	117.1	111.6	117.1	105.8	104.6	126.3	119.2
MRGPRX4	121.9	106.4	103.3	117.3	97.4	95.8	122.1	115.5
M-RIP	116	120.6	125.4	118.8	109.3	98.6	122	115.6
M-RIP	299.3	314.7	214	298.9	309.1	199.8	241.9	264.3
MRLC2	3129.7	3082.4	4311.6	3308	3068.3	3627	3644.9	4100.4
MRM1	112.2	120.5	122.9	112.2	105.9	103.2	112.9	108.5
MRO	104.1	118	120.4	111	102.3	93.4	109.4	113.9
MRP63	220.1	217.5	222.9	226.2	183.3	163.8	207.1	170
MRPL1	172.2	156.2	147.5	181.8	165.6	111.3	135.5	139.1
MRPL10	133.3	144.1	140.8	165.6	140.1	141.6	124.7	136.4
MRPL10	115.5	124.6	136	132.2	108.8	104.6	127.6	120.5
MRPL11	193	179	196.3	199.5	168.8	147.1	192.5	182
MRPL11	110.6	114.6	131.1	129.1	118.3	100.9	121.8	125
MRPL11	429	417.2	477	385.1	348.7	318.6	330.6	362.8
MRPL12	214.3	230.7	204	222.5	193.6	163.4	220.4	161.5

MRPL13	184.1	184.7	199.7	196.1	197.9	175.8	206.1	248.9
MRPL14	418.5	598.6	531.7	598.7	559.3	418.7	574.1	562.3
MRPL15	239.5	247.3	260.8	247	198.4	176.6	199.8	175.1
MRPL16	130.8	140.7	152.3	149.3	136.1	115.8	150.6	130
MRPL17	314.1	324	246.2	307.9	285.1	162.3	235.2	187.2
MRPL18	432	419.4	536.2	408.9	415.2	397.1	630.7	862.2
MRPL19	117.6	123.6	121.9	132.6	119	108.2	121.7	119.2
MRPL2	177.2	182	161.6	179.2	145	135.9	158.3	156.3
MRPL20	227.4	283	297.3	248.3	258.2	256.8	204.8	247.5
MRPL21	223.1	319.1	297.8	241.4	234.2	214.9	183.1	202.9
MRPL21	316.4	318.8	485	391.2	306.1	343.5	271.4	289.8
MRPL22	250.2	254.9	281.6	264.6	269.2	181.9	248.4	280.9
MRPL22	441	424	477.2	498.8	388.9	365.7	466.4	581
MRPL23	357.7	411.1	587.3	435.8	358.7	370	385.6	344.3
MRPL24	172.5	139.8	165.5	166.7	148.8	159.3	155.6	162.5
MRPL24	414.9	439.4	632	494.9	494.6	446.1	428.7	398.5
MRPL27	216.9	231.5	270.4	242.3	192.8	155.1	222.4	217.4
MRPL27	111	112.5	121.2	120.4	116.2	98.1	114.6	118.2
MRPL27	120.5	138.2	147.8	144.3	125.4	120.8	133.3	145.6
MRPL28	120.2	124.9	145.1	147.2	124.6	112.3	147.2	146.8
MRPL3	123.9	114.8	120.5	118.1	111.6	102.6	118.7	114.6
MRPL30	123.7	121	138	119.4	113.8	109.6	131.8	126.1
MRPL30	138.3	132.5	169.4	133.2	138.9	134.2	139	123.2
MRPL32	282.1	225.1	335.9	333.5	266.7	202.6	270.7	303.1
MRPL33	337.8	381.7	369.6	351.2	346.1	294.2	314	385.6
MRPL33	1527.4	1947.9	2235.7	1539.1	1731.5	1474.2	1682.3	1698.7
MRPL34	398	426	372.5	416	337.4	312.1	319.3	252
MRPL35	294.7	317.3	432.1	303.3	254.2	293.3	211.1	231.4
MRPL35	139.3	147	159.6	141.4	133.5	118.7	134.2	152.6
MRPL35	145.9	144.6	184.1	137.8	142.4	138.7	137.5	132
MRPL36	507.3	493.7	397.4	486.8	517.7	304.2	504.1	567.5
MRPL37	508.3	626.3	606.3	617.1	423.7	400.9	449.8	428.1
MRPL38	166.3	159.7	141.8	170.5	139.4	122.4	166	163.5
MRPL39	120.3	122.3	114.7	119	114.9	105.4	121.3	124.6
MRPL39	320	320	364.4	347	327.6	283.4	298.4	284.2
MRPL4	110.7	118.1	115.4	119.1	109.9	98.3	121.3	121
MRPL4	132.5	138.4	126.1	133.4	122.7	120.4	122	131
MRPL4	122.4	133.2	137.9	133.1	121.2	105.7	152.3	125.6
MRPL40	263.8	246.3	282.9	221	223	198.6	179.4	196.6
MRPL41	398	429.6	653	488	320.1	369.9	333.6	289.2
MRPL42	131.7	140	132.6	140.9	109.2	107.5	138.4	122.3
MRPL42P5	115.5	122.1	117.3	120.9	113.4	98.7	124.5	112.6
MRPL43	567.6	506.6	674	650.4	403.8	462.3	429.8	351.1
MRPL43	140.7	150.5	143.8	137	125.2	112.6	137.7	134.3
MRPL43	143.8	217.6	218.4	159.4	170.8	185.8	147.7	158
MRPL44	109.1	117	116.7	123.2	117.6	109.5	128	117.7
MRPL45	225	234.1	211.4	225.1	203.6	204	220.4	305
MRPL45	181.9	171.1	216.7	244.6	163.5	182.9	191	174.8
MRPL46	290.3	327.8	303.4	355.8	285.9	200.7	211.5	244.5
MRPL47	123.8	142.8	139.4	143.2	128.5	121.4	127.4	141.6
MRPL47	161.9	170	165.9	181.9	158.7	124.6	181.3	192.2

MRPL48	216.2	250.9	191.9	222.6	179.7	154.6	190.2	190.2
MRPL49	227.7	255.8	233.7	246.6	202.5	204.4	238.6	254.7
MRPL50	349.2	432	359.1	328.8	351.5	266.5	438.9	412.8
MRPL51	656.2	696.6	683.1	817.9	658	644	558.4	720.8
MRPL52	215.3	228.9	264.6	222.9	216.8	163.4	260.3	262.9
MRPL52	157.2	158.9	152.4	172.7	167.1	151.1	181.1	223.8
MRPL53	213.9	200.6	242.9	214.4	175.6	194.4	195.9	198.6
MRPL54	275.1	408.5	377	323.2	311.1	227.1	283.8	201.8
MRPL55	316.8	226.1	339.2	245.7	234.7	257.6	297.2	273.3
MRPL55	246.8	213.8	280.1	224.6	174.7	174	184.9	181
MRPL55	296.9	391.1	315.5	329.8	235.1	269.6	221.2	209.3
MRPL9	192.6	197.9	199	181.4	178.9	149.4	180.2	190.7
MRPS10	550.9	620.6	569.8	551.1	526.5	360.5	503.4	494
MRPS11	453.5	519.9	523.5	512.4	374.7	390.9	389.8	388.7
MRPS11	259.5	263.6	275.5	245.6	227.5	218.2	240.3	222.8
MRPS12	448.7	559.3	448.9	465.6	405.7	307.3	429	418.6
MRPS12	119.2	120.4	129.6	129	111.5	100.3	121.7	124.3
MRPS12	229.5	254.3	212.5	223.6	182.2	183.1	216.4	198.4
MRPS12	140	149.3	139.2	144.9	141.4	110.2	141.3	134.9
MRPS14	127.3	135.8	133.7	136.4	121.8	109.6	136.2	131.9
MRPS15	340.7	391.7	475.5	373.6	302.3	324.3	357.1	341.7
MRPS16	163.2	237.7	212.6	211.2	204.8	182.2	236.8	252.5
MRPS17	185.7	200	233	205.9	196.8	186.1	190.9	184.4
MRPS18A	158.2	192.9	166.8	180.3	159.2	153	173.6	192.5
MRPS18B	334.1	363.2	384.3	436.2	378.6	372.1	405.3	397.4
MRPS18C	124	116.1	121.5	121	115	100.5	119	119.6
MRPS2	220.6	241.3	201.2	247	180.2	164.6	218.8	184
MRPS21	497.7	445.4	629.4	539.7	308.2	452.6	299	298.2
MRPS21	349	321.3	520.8	312.2	277.1	299.2	249.9	226.4
MRPS22	577.9	526.8	591.8	596.5	573.1	533.6	631.7	575.8
MRPS23	168.8	177.7	171.7	186	158.6	133.1	170.1	170.6
MRPS24	676.1	846.6	706.5	736.8	671.8	507.9	540.1	479.7
MRPS26	222.5	216.2	195.1	236.1	178.6	126	193	153
MRPS27	219.8	307.3	277.2	303.1	245.3	248.1	294.7	282.3
MRPS28	198.9	186.6	242.3	193.9	152.7	147.1	167.2	186.7
MRPS30	542.8	671.2	542.5	565	505.7	418.3	607.3	535.9
MRPS31	524	575.8	596.9	573.3	427.6	408.6	448.8	535.8
MRPS33	432	493.1	538.7	483.4	453.7	381.3	394.2	467
MRPS33	148.2	153.6	151.2	161.3	158.3	141.2	158.4	161.3
MRPS34	147.4	157.2	138.5	149.9	146.6	115.2	127.5	129.2
MRPS35	181.3	199.7	196.5	176	168.9	158.8	198.9	176.5
MRPS36	168.1	183.4	168	163	151.9	149.9	183.1	199.6
MRPS5	277.4	292.9	306.9	344.6	272.4	219	288.5	337.8
MRPS6	6675	5828.5	3068.1	8515.4	6256.4	3339.6	10515.4	7335.2
MRPS7	234.1	270.4	312.2	239.3	201.1	161.2	181.6	188.3
MRPS9	399.7	472.7	431.7	428.4	373.4	382.3	371	405.7
MRRF	243.6	209.4	238.5	263.3	219.5	178.3	228.7	228.4
MRRF	246.4	217.6	266.2	225.4	208.4	182.2	240.4	214.2
MRRF	120.5	118	123.7	129.4	108.4	102.7	120.7	126.6
MRS2L	142	139.8	166.9	166.1	133.9	135.5	144	127.1
MRVI1	114.7	104.7	114.9	126.4	105	102.7	117.1	112.9

MRVI1	112.4	119.5	120.8	120.7	110	99	111.6	119.7
MS4A1	137.9	141.1	146.8	154.2	146.8	119.9	156	140.4
MS4A1	110	110.9	119.7	115.3	102.9	90.7	111.2	114.1
MS4A1	148.4	138.2	163.6	153.8	129.5	114.8	109.2	123
MS4A10	111.5	113.9	109	117.5	112.2	93.2	113	106.7
MS4A12	115.5	126.8	126.5	109.9	106	102.7	117.8	119.9
MS4A13	115.1	115.7	126.6	120	114.6	100.8	121.1	116
MS4A2	122.2	113.8	136.6	118.1	113.4	104.3	126.2	120.8
MS4A3	112.4	122.3	120.6	128.1	116.7	103.1	119.6	118.9
MS4A3	112.7	117.3	112	108.1	105.8	97.7	125.6	121.2
MS4A3	116.2	156.5	174.3	130	146.8	119	121.3	150.5
MS4A4A	96.7	105.9	119.1	120.5	113.2	95.6	117.9	114.5
MS4A4A	114.7	120.5	121	118.7	110.6	94.2	113.2	124.8
MS4A4A	119.4	113.8	122.8	113.5	111	98.8	120.8	118.9
MS4A5	113.1	108.4	108.4	120.5	102.2	97.5	130.7	113
MS4A6A	123.4	117.6	371.9	120.6	111	107.2	123.7	123.9
MS4A6A	120.2	129.4	254.6	119.9	118.8	110.6	131.9	110.8
MS4A6A	121.6	114.1	243.5	120.6	112.4	102.4	119.4	115.8
MS4A6E	117.6	116.1	120.8	120.1	103.9	105.6	124.1	110.2
MS4A7	465.2	157.6	1492.8	366.7	118.8	890.1	292.4	130.5
MS4A8B	110.2	121.8	115.4	119.6	112.7	97.9	125.2	118.5
MSC	211.6	330.9	134	218.4	172.2	195.8	224.5	150.2
MSH2	121.7	115.5	118.4	116.9	113.3	97.8	125.2	125.8
MSH3	8031.9	7270.5	6743.2	7601.4	7378.9	3449.1	5725.2	8450.2
MSH4	127.2	121.6	123.8	119	113.9	102.7	135.1	116.9
MSH5	126.4	129.4	135.8	133.1	124.2	102	122.8	125.1
MSH5	136.4	119.3	121.3	116.2	110.5	98.2	111.1	108.7
MSH5	118.9	112.8	119.7	124.5	112.6	98.4	118.7	125.5
MSH5	117.2	116.7	124.9	123.3	109.7	96.6	124.3	115.1
MSH5	120.1	115.4	114.2	131.2	113.1	102.5	124.7	128.6
MSH6	615.3	816.8	527.1	765.7	582.7	357.2	603.7	849.3
MSI1	109.6	123.4	120.3	122.6	112.7	99.1	117.7	111.8
MSI2	112.8	116.6	114.1	118.6	107.7	98.4	121	115.4
MSI2	132.1	137.6	129.7	148.1	121.3	99.5	143.5	140.5
MSI2	137.4	164.1	149.9	157.9	144.3	126	170.8	181.4
MSL2L1	123.7	117.7	133.2	118.5	115.4	99.2	124.2	112.3
MSL3L1	461.7	381.3	314.4	466.1	514.7	271.1	473.3	595.3
MSL3L1	121.7	110.3	112.3	120	111.9	94.7	122.2	111.9
MSL3L1	174.9	204.3	180.2	198	162.2	156.1	161.2	184.3
MSL3L1	202	201.6	233.5	186.7	178.1	178.4	178.1	171.9
MSLN	104.5	106.6	102.9	106.7	108.1	100.5	108.3	109.1
MSMB	113.5	116.7	118.2	127	110.2	97.7	119.1	110.3
MSMB	107.6	111.3	108	110.5	100.6	88.8	110.5	114.1
MSMB	114.2	118.4	113.1	114.9	108.4	101.1	126.6	113.5
MSN	1808.6	2119.3	1774.1	1800.1	1602.7	1868.4	2559.1	3096.5
MSR1	108.7	94.2	119.9	116.8	104.2	114.2	114.7	105.5
MSR1	122.4	118.4	135.6	133.2	119	110.4	128.1	127
MSR1	111.2	109.9	125.7	110.5	104.7	103	117.7	109.6
MSRA	226.1	234	251.8	247.5	192.7	186.8	211.1	183.4
MSRB2	214.1	220.6	364	225.4	203	192.7	167.7	191.8
MSRB3	107.4	117.8	120.7	120.5	95.4	96.1	121.8	109.4

MSRB3	115.3	118	113.2	105.1	100.9	96.8	110.7	114.3
MSRB3	112.8	113.4	109.6	121.4	113.8	95.7	113.4	124.5
MST150	134.5	129.8	129.2	131.4	127	111.5	136.1	138.9
MST1R	121.8	115.2	122.3	124.8	114.1	96.5	114.7	120.4
MST4	324	336.3	264.6	344.3	351.2	296.4	348.4	475.8
MSTP9	121.6	126.2	123.9	140.2	110.1	113.5	129.8	117
MSX1	108.1	104.5	107.6	110	101.6	96	110.7	119.4
MSX2	117.5	112.2	117.1	124.8	116	111.1	130.2	124.6
MSX2P	118.1	124.7	117.7	110.5	112.6	96.9	119.2	115.6
MT	119.2	131.8	125.4	142.7	127.2	108.9	119.5	122.1
MT	114.3	118.7	135.9	123.8	116.6	104.8	118.5	125.1
MT1A	400.9	582.3	455.2	392.7	311	257	1014.5	556.1
MT1B	114.6	117.4	107.4	116.9	102.2	98.1	119.3	117
MT1E	135.3	136.9	132.6	145.8	125.3	110.3	183.3	155.3
MT1F	325.4	493.4	309.9	371.1	354.6	201	598	354.7
MT1G	120.4	149.6	129.6	175.2	182	100	510.8	319.1
MT1H	129	153.4	122.7	172.7	123.4	98.3	286.1	178.2
MT1M	120.3	119.4	122.6	134.5	114.5	93.3	146.3	125.1
MT1P2	116.4	109.7	114.9	115.4	105.2	100	116.7	123.1
MT1X	339.1	531.8	392.4	324.9	352.4	213.9	552.9	447.9
MT2A	362.9	357.7	363.5	411.5	304.2	217.1	928.5	493.2
MT3	110.5	112.5	121	123.5	111.1	98	119.6	111.6
MT4	109.8	110.6	103.7	112.7	105.8	100.4	111.6	102.8
MTA1	275.8	296.9	247.3	268.1	223.3	207	309	261
MTA2	219.4	249.4	225.7	258.1	230.2	253	299.8	239.2
MTA3	126.4	130.3	131.4	130	126.8	108	130.7	127.2
MTAC2D1	241.8	354.3	263.1	337.7	306.7	191.9	192.8	243.6
MTAP	221.1	260	196.7	278.6	209.3	165.8	233.3	169.7
MTBP	125.6	136.9	128.6	125.7	127.3	109.1	131.8	124.4
MTCH1	2658.2	2951.9	3426.8	2731.1	2307.4	2678.7	2793.1	3207
MTCH2	107.7	120.2	129.4	134.9	117.5	107.7	122.2	126.9
MTCP1	410.1	442.2	551.5	451.5	404.4	493.9	371.6	433.3
MTDH	248.4	224.8	270	232.6	224	215	274.4	298.3
MTE	125.2	131.8	131.2	126	115.4	109.2	140.2	126.6
MTERF	111.9	126.4	111	121.6	110.8	100.8	119.7	115.4
MTERFD1	226.5	223	265.2	237.7	216.4	189.9	227.5	251.2
MTERFD2	134.4	119.5	132.6	135.6	126.7	112.5	134.2	130.9
MTERFD3	111.7	108.8	110.9	118.6	104.9	100.4	125.6	116.1
MTERFD3	117.8	122.7	116.8	119.6	113.4	101.6	120.2	122.4
MTF1	327.7	301.9	270.1	391.7	253.9	246.3	387.7	368.6
MTF2	174.8	188.4	171.9	227.9	206.2	146.8	199	244.4
MTFMT	111.5	117.5	113.9	114.9	104.4	96.6	114.9	109.9
MTFR1	176.1	170.7	174.1	187.4	181.6	140.2	193.5	169
MTG1	158.1	171.8	143.4	154.6	129.7	119.5	136.2	135.8
MTHFD1	121.5	118.4	123.7	127.8	116	103.1	132.6	116.8
MTHFD1L	152.6	162.5	137.6	182.6	139.5	117.1	170.6	149.7
MTHFD2	163.9	152.7	142.9	186.9	136.6	177.2	200.8	240.2
MTHFD2	116.3	111.8	106.4	113.1	111.5	88.7	121.8	109.2
MTHFD2	359.4	291.9	368.6	370.3	275.4	392.1	392.6	587.8
MTHFD2L	138.6	157.3	163.2	164.1	176.6	151.1	244.7	191.9
MTHFR	243.8	212.7	235.9	243.1	159.6	169.6	241.6	243.7

MTHFS	325	325.1	336.1	393.6	275.9	259.7	342.8	288.5
MTIF2	268.5	290.9	402.2	292.4	307.1	253.9	241	243.7
MTIF2	112.1	115.4	119.7	119.7	107.2	98.9	122.6	112.7
MTIF3	286.1	219.6	301.8	259.6	214.6	253.7	243.2	226.2
MTL5	119.3	123.1	117.1	126.4	100.5	105.2	118.3	110.5
MTL5	116.1	114.6	113.4	122.5	115.9	97.7	127	115.8
MTM1	180.3	210	183	218.6	187.2	168.1	193	191.6
MTMR1	106.3	112.3	116.4	117	102.2	99.2	128.4	111.5
MTMR11	127.9	120.7	117	129.3	113.8	108.1	124.1	122.8
MTMR12	138.4	133.6	132.6	142.2	133	112.8	145.5	166.3
MTMR14	146	130.6	152	158.8	129.1	123.1	149.2	164
MTMR15	118.6	134	158	157.1	137.7	128.1	139.5	134.2
MTMR2	113.1	120.2	116.9	116.3	104	102.7	118.6	122.7
MTMR2	125.9	126.5	129.5	128.2	124	109.7	128.3	130.1
MTMR3	473.6	562.9	394.9	602.8	417.5	389.5	644.8	675
MTMR3	107.7	109.9	112.2	120.2	104.4	102.5	117.5	119
MTMR3	139.2	128.2	142	148	141.3	119.7	141.5	155
MTMR4	242.4	238.7	269.6	274.2	229	312	242.7	304.6
MTMR6	258	266.3	237.5	358.7	258.1	208.2	338.5	373
MTMR7	110.4	115.1	112	115.1	110.7	93.9	114.9	115.8
MTMR8	122.6	115	118.3	124.2	119	102.1	120.3	115.1
MTMR9	381.7	343	408.2	398.8	340.3	309.9	439.3	381
MTNR1A	114.4	117.9	112.9	111.3	110.3	107.3	115.9	115.4
MTNR1B	110.7	115	117.6	118.8	108	94.3	109.8	115.3
MTO1	120	131.3	118	128.4	113.7	102	113.4	122.3
MTO1	437	536.1	512.2	510.1	361.4	405.7	418.1	367.1
MTP18	148	160.2	149.5	145.2	130.8	115.9	150.9	155.4
MTP18	127	132.5	114.9	140.9	118.8	94.7	131.2	120.9
MTP18	245.5	243.8	250.8	245.4	244.9	176.8	230.6	240.1
MTPN	1418.8	1312.9	2032.5	1568.8	1432	1472.4	2094.4	1715.4
MTR	140	157.9	160.4	162	169.1	139.4	168.5	156.2
MTRF1	141.5	139.4	136.9	147.1	136.4	109.6	141.9	140.4
MTRF1L	326.2	258.6	306.1	272.4	234.6	203.6	273.7	273.7
MTRR	118.9	123.4	126.7	119.5	115	99.6	122.5	114.3
MTRR	122.5	122.4	127.5	139.1	115.6	109.3	132.6	131.7
MTRR	379.8	546.2	320.2	395.3	501.7	220.7	375.1	506.2
MTSS1	525.3	464.9	539.8	567.7	349	383.6	543.2	328.2
MTTP	109.6	110.4	100.1	124.6	109.1	101.5	113.7	114.6
MTUS1	107.9	110.4	107	116.4	103.2	98.6	118.5	105.7
MTUS1	121.8	112.2	121.8	124.2	115.8	103.8	123.1	117.2
MTUS1	116	119.2	112.4	124.1	113.5	105.4	120.8	118.6
MTUS1	105.4	115.5	112.7	108	115.1	100.1	111.1	110.8
MTUS1	129.1	136	133.3	138.4	131	104	127.7	131.9
MTX1	138.3	148.1	163.7	150.7	146	134.8	148.8	139.5
MTX1	186.4	204.3	207.9	204	189.2	151.1	192.1	195.5
MTX2	200.2	257.8	229	202.8	223.8	215.9	233.1	229.7
MTX2	117.3	105.4	117.7	118.9	107.6	94.4	116.2	115.2
MTX3	217.4	214.3	253	248.6	192	183.6	167.5	166.8
MUC1	113.9	116.9	120.9	127.5	114	101.2	120.2	123.7
MUC1	113.7	124.2	128.3	108	112.5	101.7	119.2	114.1
MUC1	124.7	124.6	130.3	143.2	130.9	109.6	138.5	126.6

MUC13	109.1	114.9	124.7	121.5	106.3	99.1	114.4	118.7
MUC15	118.1	106.6	116.1	138.1	114.3	97.3	115.4	119.7
MUC16	116.4	121	115.8	115.3	113	105.3	127.7	116.9
MUC17	113.1	112.5	122.1	130.6	115.9	110.6	119.8	110.3
MUC2	124.7	115.2	124.7	131.1	122.2	106.4	136.4	126.7
MUC20	154	137.3	136.5	151.8	120	116.2	154	134.8
MUC4	112.1	124.5	131.6	138.4	114.6	102.2	113.9	119
MUC4	117	116.5	117.1	121.9	107.3	99.2	110.8	120.7
MUC4	105.7	116.5	110.4	118	105	92.9	105.8	111.6
MUC4	117.6	115.7	135.4	123	113.2	102.6	121.1	124.5
MUC7	110.3	113.6	111.3	112.8	103.9	101	114.1	112.5
MUCDHL	109.1	114.6	112	113.9	102.5	104.6	118.3	109.7
MUCDHL	114.9	105.7	126.2	118	108.2	99.2	125.3	119.7
MULK	205.5	222.7	227.9	228.3	211.7	151.2	207.8	199.6
MUM1	323.8	411	364.5	361.1	282.3	314.4	528.1	634.5
MUM1L1	110.3	119.7	108.4	112.9	108.6	100.9	115.2	111.1
MUS81	271.7	310	291	260.9	202.2	192.4	231.9	247.3
MUSK	135	129.3	136.9	141.4	126.4	112	139.3	130.4
MUSTN1	118.5	135.4	129.8	121.4	118.4	108.8	113.2	126.5
MUT	114.1	111.4	112.2	119.3	117.2	99.7	123.7	111.9
MUTED	336.3	347.4	293.5	332.9	233.9	196.4	247	242.7
MUTYH	133.3	144.7	124	123.6	125.9	104	135.2	123.3
MUTYH	322.3	314.9	236.5	307.2	230.2	209.2	256.7	259.6
MUTYH	118.9	131.1	120.8	140.5	115.4	109.1	116.8	118.2
MVD	174.4	154.1	155.9	158.3	129.9	138.4	170.3	176.4
MVK	128.7	138.4	130.4	146.4	121.5	100.1	134.7	124.3
MVP	198.9	182.9	196.3	242.7	142	184	174.9	143.4
MVP	472.5	544.7	470.6	423.9	298	309.4	407.5	331.3
MX1	1585.7	1318	832.5	1442.7	910.6	611.3	858.1	794.4
MX2	307.7	309.3	317.4	293.2	268	233.6	250.7	229.7
MXD1	230.5	206.7	207.3	240	201.7	268.9	360.5	429.8
MXD3	138.5	140.6	129.5	144	115.6	106.4	133.8	140.6
MXD4	1549.8	1481	1867.9	1574	1025.8	1616.6	1503.8	1537.6
MXI1	121.8	116.8	118.6	124.9	107.1	99.4	118.5	118.9
MXI1	105.7	109.5	116	112.9	109.9	97	112.7	116.7
MXI1	125.3	129.9	137.8	145.8	133.1	101.5	134.9	130.2
MXI1	107.1	116.9	122.5	125.7	108.3	98	123.9	115
MXRA5	115	127.1	134.3	114.2	135.9	97.6	130.1	123.1
MXRA7	120.5	118.1	129.6	137.9	121	111.7	125.7	115.9
MXRA7	162.8	156.6	140.3	191.5	140.2	132.6	147.9	139
MXRA7	129.6	130.3	123.2	126.1	111.7	101.3	114.7	112.4
MXRA8	109.4	113.7	127	114	106.1	101.2	108.1	116.3
MYADM	113.4	124.3	120.5	133.3	97.9	99.2	117.9	117.4
MYADM	127.7	134.7	134.4	113.7	107.7	102.4	127.3	129.2
MYADM	563.8	585.1	604.5	478	470.6	348.3	424.3	563.4
MYADML	116.2	116.3	111.4	122.2	111	109.6	131.7	113.5
MYB	199.9	225.9	182.1	203.5	211.5	136.7	216.4	209.7
MYBBP1A	132.4	121.4	146.4	125.2	113	112.7	134.7	134.9
MYBL2	133.7	116.6	124.5	131.8	121	99.7	119.1	125
MYBPC1	108.4	111.4	116.4	105.4	104.6	95.6	112.3	112.9
MYBPC1	123.6	106	117.9	119.4	104.7	105.5	122.1	107.9

MYBPC1	128.6	120.9	127.6	119.1	116.9	103.8	132.1	118.7
MYBPC1	116.9	121.5	119.4	124.2	107.9	98.9	123.4	120.5
MYBPC2	106.2	106.1	121.4	118.4	105.8	99.6	120.3	116.4
MYBPC3	123.3	116.7	135.2	120.8	105.5	114.8	130.5	109.7
MYBPH	136.8	112.4	118.3	123.8	110	97.2	127.8	111.5
MYBPHL	103.6	104.3	110.3	116	115.4	94.5	111.4	108.9
MYC	336.3	381.6	222.6	288.1	256.3	228	293.5	326.5
MYCBP	116.8	122.6	136	122.1	118.5	120.7	119.6	119.4
MYCBP2	855.7	1044.7	736.9	1126	920	594.7	1060.4	991.6
MYCBPAP	120.4	120.6	120.9	120.7	106.2	100.7	119.2	124.5
MYCL1	109.5	115.3	116.3	119.4	121.9	99.5	116.7	115.7
MYCL1	126.9	125.5	134.8	136.4	115.2	106.1	131.3	122.7
MYCL1	117.8	125.5	139.4	134.9	120.6	105.5	140.1	129.8
MYCN	115.2	120.6	133.2	125	120.4	112.4	114.8	124.3
MYCT1	129	124.5	122.3	123.9	113.7	97.4	124.4	126.4
MYD88	391.7	356.3	401.7	286.2	236.6	279.4	285.7	219.7
MYEF2	123.4	119.3	131.8	120.9	117.8	109.4	137.6	129.2
MYEOV	120.7	119.3	113.4	112	99.6	97.3	113.9	118.6
MYEOV2	116.5	120.9	128.7	139.2	117.8	113.5	126.7	119.9
MYF5	109.4	112	120.1	109.1	112.2	97	117	103.7
MYF6	107.4	105.3	124.8	113.9	111.6	96.1	110.6	115.2
MYH1	112	111.5	116.9	117.9	110.3	97.9	114.2	115
MYH10	133.5	146.1	123.4	151.4	120.8	95.8	144.8	134
MYH11	128.6	122.6	117.1	112.5	111.7	104.9	123	121.2
MYH11	108.3	110.5	107.8	108.8	109.6	93	120.8	108.9
MYH13	115.7	116.1	137.6	121.3	105.2	99.5	113.5	117
MYH14	115.4	107.6	115.2	121.5	105.9	100	124.3	115.5
MYH14	106.2	113.7	109.7	127.4	111.3	94.3	111.5	101.7
MYH16	111.6	116.5	124.9	118.3	116.6	100.1	120.8	115
MYH2	110.3	119.8	116.7	113.5	97.2	96.4	119.9	114.5
MYH3	166	155.5	149.4	174.4	166.2	121.5	176.1	180.8
MYH4	152.7	128.1	158.9	138.4	120.7	114.3	141.2	140.5
MYH6	116.8	110.9	115.9	127.5	117.2	111.2	118.9	127.5
MYH7	120.2	112.4	114.6	122.5	108.4	103.6	115.4	113.4
MYH7B	113.7	115	116.8	120.8	108.2	96.8	122.6	116.3
MYH8	110.2	110.7	113.1	116.5	113.5	96.4	117.3	119.9
MYH9	3114.2	3461.5	3493.3	3654.1	3338	3308.9	3043.2	3181.1
MYL1	116.9	112.3	114.1	124.4	104.8	104.6	123.3	121.6
MYL1	113.9	115	117.9	114.2	103.1	99.2	114.7	109.8
MYL2	107.4	113.3	112.3	120.4	100.4	103.8	113.4	104.5
MYL4	111.4	121	105.8	114.2	107.4	95.2	113.8	118
MYL4	116.7	109	115.5	119.9	106.5	100.4	118.9	118.4
MYL5	160.9	166.8	187.9	160.5	144.6	170.8	162.2	147.9
MYL6	5852.2	6320.6	8385.9	6414.1	4909.7	7303.1	5807.1	5462.5
MYL6	5666.2	6319.1	8103.8	6142	5320.3	6605.7	5746	5535.5
MYL7	120.1	120.8	111.9	139.6	115.2	102.6	126.8	120.2
MYL9	110.9	128.4	117.7	115.4	120.7	107.7	125.8	126.2
MYL9	113.3	123.5	115.5	110.8	110.3	99.2	111.8	108.5
MYLC2PL	110.9	120.3	115.4	109.7	109.6	93.3	112.5	105.1
MYLIP	419.7	306.7	384.2	447.5	349.5	261.4	506.5	523.3
MYLK	117.5	133.1	112	115.2	107.6	100.6	110.9	118.9

MYLK	107.6	105.8	107.9	130.6	115.5	96.8	109.9	110.1
MYLK2	106.9	120.6	109.1	113.1	103	94.1	106.9	113.7
MYLPF	119.3	116.7	114.9	120	118.8	108.4	110.3	127
MYNN	111.2	127.1	120.1	114.4	100.6	95.5	110.7	114.8
MYO10	122.9	119	117.4	124.2	105.6	110.3	124.8	122.8
MYO15A	115.6	108.4	116.4	121.8	112.3	110.8	109.4	115.9
MYO18A	134.6	163.9	174.6	149.4	120.3	126.4	141.5	129.2
MYO18A	113.9	125.6	113.9	122.2	108.1	96.3	111.3	118.5
MYO18B	111.2	111.4	115.6	125.7	108.4	98.6	115.3	113.2
MYO1A	116.2	114.1	117.5	121.3	116.5	100.4	120.3	122.6
MYO1B	103	114	110.2	123.8	116.2	99.4	119.4	115.4
MYO1C	117.9	119.1	119.3	115.7	115.1	106.8	117.4	120.5
MYO1C	119.2	109	110.8	117	102.6	88.7	113.2	112.6
MYO1C	136.8	122.8	148.3	138.7	117.6	127.4	149.5	146.4
MYO1D	139.6	147	127.1	132.1	118.6	120.1	139.1	119.5
MYO1E	119.9	120.6	122.9	123.3	114.5	110	120.7	125.9
MYO1F	278.3	271.8	262.8	278.2	170.3	299.6	306.2	176.9
MYO1G	307.3	285.1	366.3	276	216.7	286.3	242.8	194.1
MYO3A	117.2	113.4	102.2	112.3	108.2	96.3	117.6	115.7
MYO3B	176.7	189.4	159.9	180.2	176.9	142.1	214.8	170.9
MYO5A	185.8	192.3	201.6	204.7	175.7	228.4	240.9	210.9
MYO5B	113.7	112.8	120.1	110.2	112.9	101.6	121.1	122.1
MYO5C	118.4	123.6	119.2	115.7	115.9	105.2	112.2	129.4
MYO6	117	113.4	126.6	130.5	109.3	102.5	125.6	116.3
MYO7A	120.5	115.5	125	126.5	107.4	99.8	114.5	114.3
MYO7B	121.5	127.3	121.4	114.2	101.6	97.7	116.6	119.8
MYO9A	258.4	254.6	201	286.6	269.9	193.2	410.6	371.8
MYO9B	344.9	311.2	283.8	318.7	280.4	304.8	467.8	352
MYOC	102.4	125.2	118.1	124.1	111.7	102.8	112.2	113
MYOCD	113.7	109	119.7	122.8	113.5	103.3	112.9	110.8
MYOD1	115.7	119.1	115.7	127.5	109.1	98.3	114.7	122.6
MYOG	117.9	118.1	126.9	125.9	110.6	104.9	116.2	115.1
MYOHD1	117.6	106.7	109.8	110.1	113.4	89.9	105.4	120.3
MYOHD1	115.6	118.5	126.1	136.3	106.4	101.6	121.5	125.8
MYOHD1	123.5	126.2	127.2	135.2	114.5	112.3	149.8	135
MYOM1	140.1	144.6	172.2	153.8	144.9	129	162	151.3
MYOM2	208	184.4	186.7	229.9	167.6	144.2	262.3	276.3
MYOM3	117.2	119.6	118.8	119.3	110.8	103.8	109.9	112.5
MYOT	117.2	119.4	110.7	120.9	108.8	108.9	114.3	122.9
MYOZ1	117.7	112.8	112.9	119.2	109.6	102.1	117.4	114.7
MYOZ2	113.8	128.6	116.2	116.5	107.9	102.7	123.6	118.9
MYOZ3	118.3	117.4	130.1	119.9	109.1	95.2	116.1	114
MYPN	115.9	110.3	119.4	118.6	103.9	98.7	117.1	115.9
MYR8	109	124.4	113.5	122.5	109.8	100.8	112.9	123.2
MYRIP	108.7	114.9	111.9	115.7	106.5	97.1	117.6	114.2
MYST1	242.4	272.5	207.2	223.2	211.9	187	227.5	194.7
MYST2	131.9	135.9	138.1	143	117.6	113	127.5	136.1
MYST3	1017.3	1050.1	1060.5	1137.3	874.9	1114.8	1659.3	1575
MYST4	155.2	137.6	136.5	161.8	144.4	106.3	151.6	157.2
MYT1	116.5	130.4	113.4	119.3	121.3	102.5	117	129.4
MYT1L	115.2	118.1	125.5	118.6	102.9	98	117.5	105.9

N4BP1	221	271.2	211.9	248.3	213.8	226	281.3	296.5
N4BP2	556.3	347.4	413.5	389.9	595.4	230.8	444.2	474.1
N4BP3	118.4	115.1	130.9	129.5	117	104.9	126.2	113.5
N6AMT1	183.1	213.3	183.4	187.9	143.8	133.1	154.9	141.1
NAALAD2	119.5	123.5	141.9	127	116.7	106.2	130.7	134.9
NAALADL1	148.9	155.3	159.4	152	123.8	133.9	137.2	135.9
NAALADL2	115.1	108.1	111.2	112.9	114.5	102.4	116.6	111.1
NAB1	188.4	169.8	206.8	187.2	176.3	207.6	224.4	190.6
NAB2	130.7	123.6	114.9	131.1	122.6	118.1	142.5	119.9
NACA	3813.1	4319.6	3622.7	3738.3	3167.5	2772.7	3128.4	4344.2
NACAL	117.1	106.4	109.9	115.4	103.5	102.7	113.4	111
NACAP1	243.1	162.4	246.5	211.9	222.9	172.2	289.6	238.3
NADSYN1	182.9	184.7	217.6	213.9	145.7	203.2	164.5	179
NAG	152.7	145.6	162.2	151.4	129.5	123	154.8	141.1
NAG18	7462.5	7742.8	5650.4	5572	7248.8	5690.8	7881.9	7301.7
NAG6	189.4	268.1	157	199	174.2	117.2	156.9	172.2
NAG8	172	160.6	150.8	190.3	219.5	128.4	204.1	200.8
NAGA	117.5	123.3	142.9	122.2	109.4	115.1	117.1	119.4
NAGK	841.9	526.5	980.8	644.9	414.2	360.8	509.9	528.8
NAGLU	145.2	126.4	169.1	149.1	141.3	112.3	148	138.7
NAGPA	355.1	353.6	369.3	386.8	240.3	266.2	382.7	276.9
NAGS	115.5	114	134.5	121.1	111.9	107.1	119.6	125
NAIP	110.6	127.8	121.4	119	111.2	98.9	122.3	122.2
NAIP	112.5	111.7	127.3	122.1	118.9	102	120.2	106.9
NALP1	133.4	130.7	124.5	121.1	114.7	106.3	121.4	132
NALP10	118.1	108.7	123.3	119.4	105.7	103.8	121.8	115.1
NALP11	111	108.2	118.3	113.9	110.8	98.9	113.2	109.9
NALP12	115.1	126.7	135.2	121.6	107.5	114.9	119	114.5
NALP12	116.3	111.2	182	137	110.4	124.3	125	122.5
NALP12	104.6	109.8	117.2	118.6	115	96.8	123.1	118.2
NALP13	110.2	117.4	112.7	110.2	105.6	101.1	117.8	119.8
NALP14	113.2	115.1	114.7	120.8	110.4	100.5	106.3	109
NALP2	140.3	117.8	118.6	142.8	104.4	108.2	133	109.3
NALP4	114.9	117.7	124.2	120.6	103.4	99.9	128.3	118.9
NALP5	105	122.7	112.5	109.8	114.7	102.4	113.7	108
NALP6	119.4	120.6	115.9	118.1	111.8	97.6	119.2	110.2
NALP7	119	116	127.1	119.7	118.3	98.5	128.8	113.7
NALP7	126.5	118.5	115.9	127.4	105.1	105.9	117.8	111.4
NALP7	113.7	111.5	107.1	111.5	101.6	99	111.4	120.8
NALP9	109.9	115.9	112.4	112.9	114.9	98.6	113.6	119.2
NANOG	110.9	116.6	114.4	119.5	100.7	96.2	116.9	110.9
NANOS1	125.3	145.4	134.9	137.5	122.5	107.7	138.3	137.3
NANOS1	101.8	104.6	117.6	100.3	93.9	92.4	110.1	101.4
NANOS1	114.9	111.2	116	114.6	105.7	95.9	109	109.2
NANOS2	126.8	120.2	124.7	124.8	114.8	100.1	120.8	112.2
NANP	113.7	98.8	113.7	122.6	105.3	98.8	118.6	107.3
NANS	148.2	131.4	131.6	143.2	120.8	122.3	131.8	147.7
NAP1L1	298.9	271.5	199.3	401.9	225.3	147.2	249	245.1
NAP1L1	118.3	126.8	136.2	127.1	108.6	99.4	122	116.5
NAP1L2	120.9	119.5	116.4	112	101.8	96.6	114.6	117.2
NAP1L3	123.9	105.9	121.3	120.7	114.7	111	115.2	107.3

NAP1L4	1185.3	1376.6	1264.4	1516.1	1312.8	1312.7	2307.1	2197.6
NAP1L5	136.8	168.6	128	155.4	147.1	119.3	153.4	177.5
NAP5	109.6	109.5	112.6	116.3	106.2	91.6	111.4	113.4
NAP5	126.1	115.7	131.9	119.5	117.2	101.6	109.3	117.7
NAP5	109.7	109	116.4	100.3	99.9	101.7	112.6	114.6
NAPA	122	126.9	131.2	120.4	124.5	112.3	124.3	126.9
NAPB	285.7	208.8	300.9	252	245.3	204.3	248	186.2
NAPE-PLD	151.4	150.2	131.1	145.8	135	118.9	129.2	122.6
NAPG	130.9	131.5	135.7	135.1	113.2	106.9	149.2	124.4
NAPRT1	194.3	245.9	395.1	252.1	222.9	212.3	188.4	228.6
NAPSA	121	132.3	140.4	130.2	118.3	104.8	118.4	124.9
NAPSB	190.3	168.7	279	189.2	146.7	197.5	145.9	156.8
NARF	355.2	439.1	416.5	438.3	334.9	266.7	435.9	378.8
NARFL	141.6	185.3	164.1	171.4	144.6	149.4	173	167.2
NARG1	139	145.2	153.4	154.8	134.2	118.4	152.9	162
NARG1L	217.4	222.5	197.9	210.7	207.8	169.5	209.4	297.6
NARG1L	167	189.3	160	194.1	195.7	143.3	205	243.9
NARG1L	122.2	115.1	128	124.1	120.1	106.5	113.5	118.1
NARG2	132	123.6	124.9	124.9	127.5	120.5	133.9	135
NARG2	121.8	129.1	126.8	128.1	123.9	109	123.3	126.6
NARG2	129.6	129.2	131.3	133.8	116.6	112.8	130.7	122.9
NARS	897.3	1154.8	775.6	798.7	507	433.4	749.9	939.4
NARS2	165.6	168.7	179.6	190.4	133.8	136.6	162.9	156.5
NASP	116.6	120.1	121.2	132.1	117.7	96.7	118.8	129.7
NASP	203.4	206.1	199.7	205.7	201.1	148	208.7	207.2
NAT1	121.7	113.7	123	123.1	107.4	93.3	118.4	116.9
NAT1	165.4	149.6	154.4	154.9	139.2	147.9	142.9	134.4
NAT10	457.3	498.2	323.7	457.4	413.9	293.5	451.9	387.3
NAT11	120.6	135.5	133.3	138.5	123.7	104	116.8	118.7
NAT12	129.7	123.3	143.1	148	134.1	107.7	155.8	150.7
NAT13	134.4	152.8	157.1	162.5	143	138.8	176	150.5
NAT2	108.7	117.7	120.2	116.2	107.8	105.1	119.3	108
NAT5	1306.7	1280.4	1525.1	1350.8	960.6	1065.4	1212.6	1106.3
NAT5	114.3	118.2	112.4	126.5	109.8	96.6	115	120.1
NAT5	606.9	635.8	754.5	571.1	568.1	543.4	478.3	500.3
NAT6	200.6	199.7	191.8	203.5	140.7	181.1	158.4	140.5
NAT8	110.7	105.9	117.4	108.4	102	96.4	111.1	119.5
NAT9	332.4	317.4	273.3	351.6	246.1	231.8	289.9	367.5
NAV1	118.4	112.9	124.4	121.4	108.9	96.4	133.5	116.5
NAV2	118.9	109.4	118.4	130.6	105.8	99.3	120.6	121.1
NAV2	121.7	120.4	147.4	126.7	118.5	107.8	127.6	116.4
NAV3	117.1	123.3	118.9	127.1	126.3	95.8	133.1	130.8
NBEA	112.8	95.1	105	107.5	101.4	91.3	88	96.9
NBEAL1	120	114.6	110.5	108.6	101.7	93.8	119.1	103.9
NBL1	242.5	193.2	314.6	279.3	181.5	376.4	353	259.5
NBL1	159.7	154.9	184.4	190.1	127.1	168.8	177.5	178
NBLA1038[illegible]	153.8	144	156.4	152	139.6	112.7	161.2	144.1
NBN	109.2	116.7	122.5	119	112.6	102.9	124	121.5
NBN	134.8	130.1	138	127.2	125.1	107.6	121.9	123.1
NBPF1	157.8	156.6	188.2	167.2	156.7	109.6	154.3	132.9
NBPF1	109.5	124.1	125.2	132.9	117	102.3	135.1	126.6

NBPF10	4055	4800.4	3664.8	4552.6	6509	1729.1	5101.3	5907.9
NBPF11	929.2	765.1	731.8	895.9	771.1	598	805.5	788.2
NBPF14	210.4	224.8	215.6	227.5	275.3	158.1	257.5	243.2
NBPF15	107.5	101.3	113.1	117.2	105.7	91.2	114.6	107.5
NBPF20	7123.2	7089	4425	8389.5	9599.6	2895.6	10482.6	10340.5
NBPF3	349.4	318.9	286.8	349.5	362	306.4	309.7	355
NBPF3	116.6	124.1	123.8	118.1	113.6	97.9	111.8	110.1
NBPF7	109.6	126.7	119.5	118.8	115.5	95.7	123.4	112.8
NBPF9	136.5	121.9	134.6	146.7	125.3	103.4	121.7	127.5
NBR1	120.9	125.6	125.4	131.9	123.4	102.1	124.2	122.7
NBR1	122.6	112.5	115.6	116.6	106	104.6	114.8	111.9
NBR2	114.2	112.5	115.3	112.8	108.3	105.5	110.7	115.3
NCALD	256.9	301	230.2	251.1	233.7	164.5	205.4	184
NCALD	246.1	261.4	197.4	204.3	197.5	163.8	176.2	178.6
NCALD	122.8	107.1	106.6	115.1	102.2	99.6	111.7	113.2
NCAM1	102.8	108	122.2	133.1	109.4	100.9	112.2	111.4
NCAM1	109	109.7	108.4	111.8	102.6	93.8	106	107.8
NCAM1	160.7	138.4	146.1	162.7	121.8	118.9	138.7	132.5
NCAM2	112.4	117.8	120.8	119.5	113.5	100.8	117	118.6
NCAPG2	126.8	140.6	132.2	132.1	127.4	107.4	131.3	139
NCBP1	202.6	225.5	216.1	200.1	169.5	196.7	201.5	202.4
NCBP2	258.3	344.7	323.9	340.4	275.3	267.4	298.4	327.6
NCBP2	133.3	129.9	132	118.9	116.6	99.4	125.3	116.2
NCBP2	154.2	149.9	180.7	161.9	142	127.6	146	139.7
NCDN	124.3	141.2	128.5	139.7	122.2	116.8	142.3	132.6
NCDN	121.9	132.4	140.4	137.2	123.5	105.3	119.3	125.7
NCDN	148.4	159.5	131	159.7	138.8	126.1	149	144.4
NCF1	526.2	229.8	861.4	427.7	162.4	827.5	185.5	139.3
NCF1B	134.5	116.9	137.7	137.4	112.9	126.4	130.3	121.9
NCF1C	233	170.8	271.3	193.8	147.6	212.6	159	122.2
NCF2	172.6	163.8	154.3	208.1	150.9	153.6	212.8	140.7
NCF4	144.4	127.4	178.6	132	122.1	140	127.3	118.4
NCF4	152.1	129.3	170.6	153.9	112.1	136.7	135.1	126.3
NCF4	235.5	179.3	673.7	212.4	140.8	361.3	156.9	137.5
NCK1	584.9	619.2	576.5	726.7	603.5	477.3	604.5	661
NCK2	128.4	132.5	125.8	117.8	119.4	111	134.4	123.6
NCKAP1	120.9	113.9	119.8	118.7	112.9	98	111.2	121.3
NCKAP1	120.9	121.7	111.6	123	107.4	100.3	126.4	125.5
NCKIPSD	125.7	128.6	122.9	122.7	117.9	107.6	123.3	113
NCKIPSD	155.9	171	151.9	159	146.8	116.8	143.4	129
NCL	1288.4	1095.6	978.4	1044.6	804.4	773.6	1037.7	1282.1
NCOA1	419	475.4	445.2	532.2	438.2	325.2	625.9	663.2
NCOA1	149.1	135.5	137.7	165.3	126.4	135	171.3	170
NCOA1	135.7	122.2	120.6	119	119.9	110.5	154.5	138.3
NCOA2	107.6	117.8	119.5	128.3	103.3	102.1	116.1	117.6
NCOA3	118.3	116.9	125.5	124.3	115	113.6	126.2	129.2
NCOA3	758	599.1	786.4	962.4	737.7	809	1089.7	1057.4
NCOA4	5722.6	5377.4	5917.2	5955.7	3782.5	4656.5	4884.5	4108.8
NCOA5	478	470.2	352.5	469.2	328	328.1	560.6	491.3
NCOA6	617.8	526.2	392.5	629.1	575.7	380.2	744.3	764.3
NCOA6IP	154.7	147.9	149.5	140.2	134.5	116.1	165.7	145.1

NCOA7	615.5	721.9	454.7	603.3	634.8	378.4	714.2	912.5
NCOR1	184.3	184.1	171.7	178.1	167.9	145.4	175.9	176.8
NCOR2	244.6	222.8	248.3	268.2	218	230.2	317.4	288.8
NCOR2	635.2	692.2	797.7	708.8	485.9	556.2	948.6	655.1
NCOR2	112.9	118.9	116.6	114.2	108	104.2	121.7	119
NCR1	137.5	128.8	137.4	138.3	117.3	114.3	143.2	108.5
NCR2	105.1	111.6	123.7	115.5	109.4	98.2	121.6	113.1
NCR3	271.5	289.7	160.5	293.6	214.5	126.6	246.6	163.2
NCSTN	660.8	693.3	860.6	722.1	619.6	883.3	647.9	609.9
NDE1	465.5	474.4	483.2	653.8	450	275.9	430.7	439.4
NDEL1	1217.1	1479.3	1193.2	1731.8	1388	1233.2	1949.9	2144.8
NDEL1	161.5	193	161.7	178.3	153.8	144.6	171.4	221.3
NDFIP1	183.3	207.3	211.5	196.7	196.4	168.2	226.4	233.2
NDFIP2	186.5	181.9	164.3	196.3	168.4	121.6	212.5	217.7
NDN	111.8	113.3	130.2	124.1	100.3	98.9	124.7	113.3
NDNL2	129.2	125.4	131.9	140	127	118.4	141.1	132.3
NDOR1	107.4	124.2	124.5	130.8	113.9	114.5	123.6	121.7
NDP	111.2	120.2	125.3	130	119.4	102.7	126.3	115.4
NDRG1	204.1	193.9	192.7	202.6	162.1	172.1	189.6	237.6
NDRG2	120.2	122	148.7	124.3	132.2	113.1	121.4	115.7
NDRG2	153.3	176.4	169	159.1	146.1	146.1	158.1	139
NDRG3	222.4	228.9	217	207	177.2	184.4	157	192.7
NDRG3	162	175.5	176	173.7	176.6	149.5	173.6	157.2
NDRG4	127.8	124.3	121.6	124.9	118.2	113.2	127.1	130.4
NDRG4	111.1	112.1	120.7	123.6	118.5	100.7	116.5	123.2
NDRG4	134.7	122.6	134.4	131.1	108.7	108.1	135.7	121.5
NDST1	114.8	124.8	126.6	137.1	115.4	111.9	129.8	125.4
NDST2	490.5	463	430.5	448	353.9	406.3	412.8	397.9
NDST3	117.8	117.7	111.1	116.7	106.4	96	122.1	114.2
NDST4	110.7	117.7	111.1	121.8	101	98.4	109.6	121.2
NDUFA1	3653	5004.7	5595.5	4583.1	4681.2	3669.2	4333.7	3907.5
NDUFA10	148.9	134.4	163	138.9	129.9	134.5	130	143.4
NDUFA11	645.8	666.1	1078.6	637.8	555.5	602.2	457.4	412.2
NDUFA12	1027.1	1406.4	1104	1152.5	1329.3	811.5	1002.1	1424.6
NDUFA13	563.3	580.6	588.6	618.9	530.4	672.1	428.4	555.4
NDUFA3	1094.4	1549.3	1562.6	1039.5	1423	1336.4	800.1	796.2
NDUFA4	1874.5	2057.1	2605.2	1779.8	1287.9	2024.8	1844.3	1925.6
NDUFA5	116.5	118	116.1	124.1	110.9	105.4	114.9	114.3
NDUFA7	185.9	208.8	276.5	218.8	194.4	197.7	174.5	179.2
NDUFA8	629.6	717.4	706	564.8	553.5	514.7	586.6	620.5
NDUFA9	280.1	260.6	339.7	294.8	257.4	233	244.5	229
NDUFAB1	713.6	714.3	859.9	732.9	766.2	643.6	673.7	647.1
NDUFAF1	164.1	157.6	194.1	183.4	157.2	160.7	188.8	164.6
NDUFB1	124.3	133.1	133.3	128.7	122.2	105	125.2	126.9
NDUFB10	371	339.6	269.5	262	245.4	195	294.3	194.1
NDUFB11	923.4	1202.1	1019.6	1099.6	807	807.3	956.6	1111.3
NDUFB2	1362	1395.4	1356	1272.1	1192.2	931	1000.2	1330.9
NDUFB3	778.8	726	1085.8	885.2	764.6	896.3	862.5	1000.7
NDUFB4	111.5	112	122.4	111.6	100.8	98.6	117.3	110.7
NDUFB5	1149.1	1291.5	2117.8	1452.2	1238.7	1225.1	1455.7	1540.5
NDUFB6	223.6	316.2	323.7	239.9	244.5	216.7	217	240.5

NDUFB7	542.6	715.7	735.1	576.1	449.2	414.1	395.2	357.5
NDUFB8	2818.3	2958.6	3285	3013.7	2492	2421	2444.8	2411.8
NDUFC1	225.2	235	293.9	230.2	213.4	309.2	211	230.9
NDUFC2	122.5	123.7	125.6	128	108.8	100.7	125.8	115
NDUFS1	149.4	170	134.8	134.4	138.8	116.4	167.9	155.4
NDUFS2	109.8	125.8	86.3	126.7	125.8	111.3	135.6	120.4
NDUFS3	579.2	700.2	482.6	544.6	523.9	382.2	450	445.4
NDUFS4	393.6	383.8	721.5	454	360	571.9	310.3	330.8
NDUFS5	1214.6	1389.5	2259.3	1440.8	1239.2	1628.6	1286.1	1069.5
NDUFS6	111.1	116.3	118.5	116.4	109.8	95.4	122.2	116.9
NDUFS7	716.9	814.3	976.4	717.4	510.2	506.5	519.5	435.6
NDUFS8	768.1	1073	917.9	816	621.3	627.5	654.2	624.2
NDUFV1	658.5	669.8	815	587.6	438.8	459.7	488.2	419.3
NDUFV2	765.7	696.3	857.9	860.2	659.1	745.4	868.2	663.6
NDUFV3	108.6	115.1	118.4	116.3	111.6	100.4	118.2	119.3
NDUFV3	132.4	132.8	143	132.2	129	111	130.9	132.3
NEB	124.5	117.4	122.7	127.9	116.6	100	120.7	117.7
NEBL	113.4	116.3	123.4	114.9	116.5	113	125.6	117.7
NEBL	110.1	109.3	109.9	119.2	101.6	98.6	114	109.3
NEBL	117.1	127.8	127.4	124.4	120	103.5	115.7	122.7
NECAP1	553.1	452.7	561.3	463.2	388.7	463.9	474.7	421.6
NECAP2	814.8	1049.1	925.7	1225.2	830.6	752.4	1210	1108.2
NEDD1	120	111.8	129.8	126.5	111.3	104.8	125.2	120.6
NEDD4	117.7	116.2	121.3	119.8	117.2	93.9	118.8	123.3
NEDD4	113.7	111.8	110.3	117	105.3	107.2	117.1	112.3
NEDD4	133	129.9	117.7	123	125.5	105	128.1	134.5
NEDD4L	121.2	119	127.1	126.6	113.9	98.9	126.3	131.5
NEDD8	3915.7	4279.1	5004.8	4635	3265.7	3459.8	3674.9	3834.5
NEDD9	128.3	118.9	121.3	123.1	121.5	95.6	125.4	114.8
NEDD9	149	167.9	147.2	161.5	137.3	124.6	138.6	142.1
NEDD9	125.4	147.1	141.2	140	127.6	122.6	135.7	138.3
NEFH	129.7	137.2	129.4	125.4	116.3	116.7	125.5	126.6
NEFL	120.5	120.2	114.6	119.7	107.2	99.1	118.9	115.5
NEFM	110	115.3	101.6	124.9	106.1	98.4	113.4	104.9
NEGR1	110.6	113.2	116	121.3	108.1	102.6	113.2	113.3
NEIL1	118.3	131.8	136.6	126.7	124.3	111.4	122.3	137.2
NEIL2	131	127.9	150.7	148.7	115.2	119	143.6	133.6
NEIL3	105.3	116.1	117.4	117.3	115.2	106	118.1	116.9
NEK1	200.4	208.7	178.7	207.6	176.3	153.7	212.5	211.7
NEK10	118.2	133.2	129	139	120.9	101.7	126.6	122.5
NEK10	128.4	113	124	121	122.7	104.6	119.3	118
NEK11	114.5	121.1	120.2	119.8	113.6	103	115.9	117.1
NEK11	122.4	125.6	123	116.5	119	104.9	120.5	126
NEK2	114.6	122.9	117.1	108.6	116.6	98.2	123	117.7
NEK3	109.7	114.7	123.2	119.4	106.5	99.9	117.8	119.6
NEK3	121.3	133.9	122.7	114.8	114	108	121.8	118.3
NEK3	117.3	124.6	121.3	130.5	119.2	99.2	114.6	121.8
NEK4	121.7	115.4	129.4	119.1	115	104.8	126.9	120.2
NEK5	109.4	113.2	116.6	110.8	102.4	97.6	115.2	117.3
NEK6	151.6	155.9	182.5	153.9	129.6	133.7	171.5	136
NEK7	121.9	114.9	124.7	115.9	114.4	102.1	129.2	103.3

NEK8	155.3	152.1	156.9	152.7	148	118.5	161.1	146.7
NEK9	118.6	108.4	117.4	119.2	107.3	103.6	117.6	114.5
NELF	142.9	147.7	169.2	171.9	141.3	140.2	170.2	181.4
NELL1	107.7	115.5	129.4	118.7	112.6	99.7	118.8	119
NELL2	1810	2246.7	1280.9	2058.6	1823.8	1190.7	2056.9	2502.2
NENF	539.4	656.7	599.6	779.9	463.9	422.3	754.6	528.3
NEO1	125.7	149.8	142	169.2	124.4	117.2	170	154
NES	111	117.8	119.9	121.1	110	94.1	115.2	118.5
NET1	276.4	317.4	265	368.2	253.6	263.6	304.3	322.5
NET1	129.4	128.7	122	130.3	113.5	105.3	127.3	116.7
NET1	120.7	133.8	124.7	134.8	130.4	106.9	127.7	120.3
NETO1	111.1	116.2	116.7	122.5	106.3	93.5	120.4	112.6
NETO1	110.7	112.8	117.8	121.3	109.5	98.6	122.2	113
NETO1	120	102	114.1	112.8	99.5	99.2	119.1	114.9
NETO1	119.3	116.6	118.7	103.4	109	100.5	123.9	112
NETO2	145	144	191.1	183.3	137.4	150.3	208.8	179.5
NEU1	244.2	236.6	233.3	287.3	226.5	221.3	320.8	315.6
NEU2	112.1	115	109.2	117.3	107.1	97.4	116.8	111.9
NEU3	118.6	120	120.4	136	116.3	100.9	120.1	124.2
NEU4	108	110.6	103.9	107.3	105.9	98.7	111	112.8
NEURL	121.8	132.9	131.2	128.7	114	108.4	127.7	112.5
NEURL2	128.1	130.4	129.1	141.2	117.3	108	130.8	127.7
NEUROD1	121.4	116	119	114.6	111.6	101.7	113.8	116.6
NEUROD2	104.7	121.8	100.1	120.4	100.6	102.4	117.9	108.8
NEUROD4	117.2	115.3	113.7	116.8	106.2	95	113.9	114.7
NEUROD6	122.7	125.5	119	122.1	103.5	99.7	122.5	116
NEUROG1	123.9	111.2	117.7	117.3	105.9	104.5	114.4	119.4
NEUROG2	104.2	116.8	121.2	116.7	107.3	112.3	116.4	120.9
NEUROG3	123.2	119.8	120.7	117.2	116.6	106.4	112	122.2
NEXN	135.9	136.9	130.9	130.4	115.2	105.2	121.5	132
NF1	127.3	117	120	122.9	123.3	110.5	130.6	121.4
NF2	109.2	113.8	122	123.3	106	98	117.7	121.2
NF2	110.6	121.6	118.5	119.2	104.2	99.2	115.3	111.3
NF2	126.6	122.8	126.9	124.1	114.2	108.5	118.5	119
NFAM1	131.4	119.9	143.3	144.2	124.5	122.2	124.6	141.5
NFASC	112.1	117.7	117.7	124.3	100.5	99.8	120.4	109.9
NFAT5	121.2	119	116.2	136.2	123.8	101.4	130.3	128.2
NFAT5	154.6	129.1	136.8	144.7	133.9	107.6	155.2	165.4
NFAT5	109.6	120.5	114.6	110.9	107.2	97.7	120.9	112.8
NFATC1	162.4	157.6	138	167.7	129	133.9	192.2	186.6
NFATC1	128.7	131.6	122.6	122.1	119.3	109.8	137.2	123
NFATC1	117.8	123.2	124.4	116	109.8	106.1	118.7	113.6
NFATC1	116.4	110.6	118.9	119.3	107.6	100.7	118.3	107.2
NFATC2	118.1	114.5	117.2	112	100.5	84.7	119	110.6
NFATC2	108	117.8	113.6	120.4	104.3	99.9	115.5	117.9
NFATC2IP	136	152.3	154	153.9	136.5	135.2	151.2	142.8
NFATC2IP	119	129.6	128	116.9	115.6	99.6	119.7	122.5
NFATC3	227.7	265.3	227.3	255.7	261	173.3	259.6	249.2
NFATC3	131.2	117.5	119	126.3	121.8	102.8	119	115
NFATC3	246.3	229.2	173.7	210.8	214.7	146.1	230.4	240.9
NFATC3	154.3	170.2	148.8	197.9	157	128.3	167.8	164.7

NFATC3	147	133.3	117.5	137.6	121.9	112	132.4	145.2
NFATC4	125.4	122.5	115.6	123.9	106.6	103	119.8	113.5
NFE2	134.9	137.2	147.5	159.6	120.8	127.4	132.4	116.1
NFE2L1	325.3	289.7	281.9	310.4	225.7	319.9	399.8	389.4
NFE2L2	751.3	611.8	546.3	746.4	592.7	686.6	788	955.7
NFE2L3	184.3	150.9	198.6	155.6	126.3	116.4	138.9	121.1
NFIA	116.8	127.8	117.7	126.1	115.5	94.1	113.3	115.8
NFIB	122.7	120.5	129.8	126.1	109.2	103.8	123.3	120.8
NFIC	121.8	113.1	126.2	118.8	106.3	100.7	117.4	121.1
NFIC	112.8	119.2	114.6	114.8	105.2	95.9	122.4	115.3
NFIC	130.5	123.1	119.9	126	109.6	95.9	121.4	117.7
NFIL3	125.9	123	134.4	131.5	118.4	112.4	130.5	140.4
NFIX	167.6	165.9	140.9	151.6	124.7	121.4	145.8	132
NFKB1	2512.5	2374.3	2001.8	2329.6	2203.2	1484.7	2628.5	2440.4
NFKB2	141.1	180.5	125.1	156.3	135.5	130.5	209.9	186.2
NFKB2	147.5	171.9	133.5	154.6	140.1	124.4	189.9	196.3
NFKB2	120.2	112.7	110.8	116.4	102.3	95	126.5	124.7
NFKBIB	133.4	136.1	116.8	156.6	110.7	122	132.7	142.2
NFKBIB	115.4	120.9	130.5	129.1	113.6	115.7	115.4	134.9
NFKBIB	156.4	147.7	129.7	135.9	140.2	117.4	149.6	149.3
NFKBIE	232.8	226.9	223	273.2	170.7	182.4	222.7	216.5
NFKBIL1	117.3	122.9	115.5	123.6	110.2	105.8	121.8	115.1
NFKBIL2	117.2	111.9	113	130.1	109.1	95.3	119.9	111.1
NFKBIZ	122.2	129.5	122.1	123.2	106.4	110.7	125.4	127.7
NFKBIZ	677.9	672.7	745.7	615.1	616.4	438.9	991.2	1056.5
NFRKB	136.4	141.3	127.9	141.7	137.8	122.7	150.9	156.1
NFS1	121.4	132.6	136.8	126	117.6	104.7	121.7	122.2
NFS1	618.4	614.7	1141.2	567.6	545.1	600	450.3	426.3
NFU1	342.2	472.4	396.9	402	312	352.2	323	360.2
NFX1	150.9	136.5	148.4	158.6	142.3	125.2	176.2	177.4
NFX1	171.7	176.6	181.8	209.7	156	131.3	185.6	186.1
NFX1	183	167.7	159.4	175	160.3	118.4	191.8	192.7
NFX1	307.1	331.6	310.9	310.9	331.4	297.5	332	396.8
NFXL1	265.9	231.1	364.5	250.6	181.9	246.5	218.8	216.7
NFYA	145.1	140.9	160.8	159.7	134	138.5	136.7	151.9
NFYA	120.7	110.7	121.1	122.4	110.6	100.9	117.3	111.6
NFYB	120.8	129	135.5	133.2	122.1	109.2	134	138.5
NFYC	210.5	239.8	207.4	225.8	184.3	207.6	227.8	209.2
NGB	120.4	124.6	117.9	121.5	109.1	101.8	131.7	120
NGDN	151.5	134.8	152.8	147.3	141.8	128.5	139.9	137
NGDN	495.1	511.2	438	594.5	497.1	468.1	583.2	605.6
NGEF	116.1	118.9	113.3	106.6	99.4	97.7	121.6	111
NGFB	124.2	116	121	129.6	123.1	108.5	129.9	124.4
NGFR	115.9	110.1	119.9	126.1	121.4	117.1	117.1	125.6
NGFRAP1	165.9	164.7	162.7	153.3	156	120.7	140.1	129
NGFRAP1	155.6	159.9	170.8	159.2	141.2	119	136.2	126.6
NGFRAP1	429.5	489.7	367.2	494.8	280.6	277.6	250.8	232.4
NGFRAP1L1	143.9	160.6	147.1	147.9	142.2	124.2	151.7	174.7
NGLY1	244.5	235.6	232.6	272.3	195.6	175.7	283.7	268.4
NGRN	1367.1	1373.9	1390.8	1413.7	1197.3	1123	1472.9	1570.7
NGRN	199.5	182.4	263.2	213.9	200.4	181	218.3	199.2

NHLH1	113.6	110.6	116.1	123.4	103.9	101.8	113.3	120.9
NHLH2	114.9	115.9	130.1	129.2	113.9	106.6	118.5	116.6
NHLRC1	114.1	110.6	113.5	125.1	113.7	105.3	109.5	114.6
NHLRC2	155.3	165.9	139.1	147.8	125.6	126.3	143.8	133.7
NHN1	185.4	158.2	173.5	177.1	138.5	150.1	182.3	175.2
NHP2L1	798.1	860	629.1	875.5	624.1	645.4	790.8	942
NHP2L1	336.9	307.8	377.1	336.9	356.8	289.5	341.3	382.6
NHP2L1	135.2	133.6	166.8	147.2	152.9	131.9	145.2	155.6
NHS	116.9	117.9	116.3	110.9	112	93.9	112.6	117.9
NIBP	140.7	147.1	140.6	141.7	125.9	131	143.3	145.2
NICN1	345.9	339.2	304.7	281.8	251.9	189.4	198.2	214.6
NID1	110.7	114.1	113.1	119.4	110.1	94.5	119.2	116.7
NID2	121.5	118.3	125.2	129.6	113.6	99.4	120.5	123.1
NIF3L1	473.8	613.6	702.7	532.2	448.9	539	397.3	369.7
NIF3L1BP1	692.8	721.3	979.4	730.6	690.2	744.6	658	964.4
NIFIE14	663.2	752.9	876.3	741.4	559.8	552.8	497.8	382
NIFUN	273.9	259.9	230.8	293.6	248.8	203.3	255.3	264.9
NIN	153.5	160.8	174.6	167	162.6	128.2	159.3	140.1
NIN	166.2	165	166.8	176.5	160.1	154.2	175.3	158.6
NIN	116.6	106.4	114	124.5	108.9	99.9	113.7	119
NIN	697.2	815.2	689.9	830	797.1	495.7	639.9	680.6
NINJ1	1205.7	973.3	870.5	1247.7	642.1	1153.9	797.9	453.8
NINJ2	230.4	258.2	216.9	244.5	198	165.5	254.9	265.2
NIP	114.9	112.4	110.8	123.6	111.6	104	117.5	117.5
NIP30	143.9	168.2	167.9	169.4	142.7	137.9	151.3	131.8
NIP7	351	493	399.1	444.6	408.4	336.6	438.9	485.7
NIPA1	196	188.6	172.1	194.8	170.6	137.2	182.7	193.3
NIPA2	118.9	130.9	120.6	121.3	108.9	109.7	124	117.3
NIPA2	696.8	658.2	545.5	716	632.3	566.4	902.8	1069.8
NIPBL	114.5	108	111.1	117.8	101.2	104	117.7	115.6
NIPBL	148.8	158.6	152	148.4	157.3	136.9	162.4	177.5
NIPBL	531.7	496.9	470.1	574.7	618	370.3	764.2	921
NIPSNAP1	174.4	150.5	183.1	160	152.7	147	134.1	126.3
NIPSNAP3/	112	112	110.7	110.7	102.6	93	115.7	105.1
NIPSNAP3E	127.6	115.1	136.5	122.4	116.2	109.1	130.4	126.1
NISCH	750.4	569.9	927.5	827.5	409.3	875.8	656.2	577.1
NIT1	132.5	127.2	144.8	146.5	116.4	120	123.6	125.6
NIT2	186.2	188.6	203.4	194.7	178.2	145.4	178.4	170
NKAP	127.1	120.1	111.5	138.6	106.6	105.5	122.7	133.4
NKD1	118.7	117.4	145.1	122.8	117.6	115.2	122.9	122.3
NKD2	108.6	118	110.7	106.4	104.7	100.2	105.7	113.3
NKG7	2374.2	3275.9	1733.7	2443.2	2076.1	1446.7	2518.8	2502.9
NKIRAS1	155.4	177.9	162.2	193.3	172.3	140.9	159.6	216.3
NKIRAS2	107.1	124	119.8	125.9	107.4	102.1	121	114.4
NKIRAS2	114.3	114.5	116.1	118.4	115.6	104.4	121.7	117.9
NKIRAS2	147.1	134.5	140.5	156	121.7	131.8	119	150.2
NKPD1	114.2	125.7	109.2	127.2	110	97.3	123.7	106.8
NKRF	199.1	231.6	201.1	188.2	163.2	149.9	191.6	231.3
NKTR	213.7	210.2	230.1	204.2	253.2	185.8	192	222.4
NKTR	1360.4	1180.3	1424.8	1402.6	1038.2	777.1	1063.7	1047
NKX2-2	115.8	111.3	118.7	113.2	109.3	92	113.1	101

NKX2-3	111.9	122.7	112.9	125.5	106.8	100.9	113.1	119
NKX2-5	109	109.7	114.7	109.9	111.5	98.8	109.2	105.3
NKX2-8	118.1	136.8	133.8	123.7	103.7	112.8	123.5	115.7
NKX3-1	116.3	130.6	136.6	132.1	137.3	96.8	135.4	130.5
NKX6-1	117.1	100.4	115.5	118.6	117.9	105.4	110.3	113.2
NKX6-2	118.4	114.1	104.7	118.7	104.5	97.6	104.4	114
NKX6-3	128.8	122.3	154.3	129.3	129.1	119.3	122.7	124.1
NLE1	120.3	125.8	119	125	115.5	110.8	129.8	114.4
NLE1	131.7	133.8	116.8	124.8	118	100.2	134.2	126.7
NLF1	108.1	115.9	111.3	106.2	109.4	95.8	116.7	112
NLF2	114.7	104.8	119.5	126.3	106.8	94.4	120.3	105.5
NLF2	115.6	117.7	123.8	120.2	114.4	113.6	119.6	125.4
NLGN1	111.5	110.9	111.8	123.3	107	92.9	121	112.4
NLGN2	147.3	148.9	131.4	145.6	121	110.4	140.6	151
NLGN3	120.3	117.2	121	117.1	108.7	97.7	120.3	118.9
NLGN4X	119.8	115.7	115.3	106.4	103	100	115.4	125.2
NLGN4X	110.3	111.3	117	122.4	112.8	94.6	115.7	118.9
NLGN4Y	107.3	106.9	112	113	108.9	93.5	108.5	112.1
NLK	141.5	143.8	141.8	127.1	124.7	115.3	128.9	123.3
NLN	117.7	118.1	126.5	134.7	124.5	110.6	133.6	129.1
NLRP1	145.6	163.2	177	146	140.3	126.1	140	137
NLRP3	128	132.5	163.4	151.9	121.2	141.5	131.2	121.9
NLRP8	2479.7	1692.4	2034	1849.6	2325.2	946.7	1727.3	2168.5
NMB	144.4	164.1	132.1	140.3	136.7	116.2	145	145.3
NMBR	111.4	114.8	116.1	112.7	109.7	96.1	117.2	117.2
NMD3	252.6	179.3	216	253.4	187.3	154.6	204.4	185.3
NME1	430.3	443.6	523.4	472.5	342.4	237.8	418.1	348.4
NME1	110.5	121.4	136.9	124.6	110.2	98.7	112	111.5
NME1	122.5	129.8	129.3	124.4	119.1	96.4	122.9	127.6
NME1-NMI	119.8	129.3	118.1	117.8	109.5	111.5	125.4	119.8
NME2	125.7	116.9	115.9	119	110	97.5	118	106
NME2	131.1	158.4	139.2	161.3	150.5	110.4	145.8	157.6
NME2	121.6	115.6	120.8	124.8	113.2	101.2	129.6	121.1
NME2	149.7	133.5	171.5	146.7	126.4	105.8	143	155.8
NME2P1	124.8	111.4	120.8	111.8	111.3	102.7	120.1	117.6
NME3	613.5	635.1	777.2	532.8	413.5	453.7	319.4	284.3
NME4	291.1	293.7	297.2	284.1	229.4	271.1	281.5	207.3
NME5	109.7	110.6	115.9	116.2	114.9	97.4	113.4	114.5
NME6	182	187.7	180.1	204.2	167.8	129.5	180	180.3
NME7	116.9	124.5	132.6	139	108.1	101.4	124.6	124.8
NME7	163.1	156	157.6	159.8	138.8	134.5	136.5	171
NMI	385.7	276.1	348.6	372.3	308.5	318.1	344.9	318.2
NMNAT1	122.2	114.2	123.2	122.9	106.6	106.2	115.9	118.2
NMNAT2	113.2	111.5	120.7	118.3	117.1	103	116.9	118.1
NMNAT2	106.2	116	124.8	123	105.2	97.9	110.8	114
NMNAT2	110.5	120.5	114.8	120.4	104	97.3	113.8	110.3
NMNAT3	128.5	148.7	153.2	150.8	121.6	119	148.6	129.1
NMS	124	111.8	111.6	115.6	111.3	94.5	111.8	115.3
NMT1	163.7	191.4	181.2	200.6	187.5	146.8	218.1	262.1
NMT2	184.5	193.2	161.5	211.1	162.9	165.3	162	162.6
NMU	106.5	112.8	106.5	106.4	100.9	91.8	113.2	104.6

NMUR1	131.1	120.5	122.9	126.2	111.8	97.9	121	109.9
NMUR2	116	113.4	113.4	112.9	113.5	102.5	123.4	112.8
NNAT	119.9	120.8	114.4	118.6	108	99.8	118.1	119.2
NNAT	121.3	117.7	124.7	119.7	119.6	106.6	132.9	118.6
NNMT	134.1	132.6	142.3	151.8	134.2	139	162.3	142.7
NNT	105.7	116.6	103.4	114.4	102.6	98.6	117.2	122.9
NNT	218.6	277.2	248.3	258.2	224.4	190.8	160.8	219.6
NNT	177.8	158.4	176	198	171.8	139	148.3	154
NOB1P	152.2	154.1	134.5	161.7	122.1	131	155.6	161.6
NOBOX	115.3	116.5	113	111.7	109.4	92.5	112.6	106.8
NOBOX	118.9	131.2	119.6	127.8	114.6	106.3	121.3	119.8
NOC2L	110	113.6	108	119.9	114.3	106.5	118.7	128.4
NOC3L	287.9	383.9	308.5	320.7	323.2	196.3	262	253.7
NOC4L	118.9	132.7	120.2	129.7	104.6	100.1	116.7	112.8
NOC4L	144.8	149.9	151.4	153.7	130.3	119.7	144.7	161.9
NOD1	151.3	148.6	174.6	153.1	129.2	127.7	144.9	133.4
NOD27	205.2	226.6	222.4	195.3	199.7	199	161.6	159.2
NOD3	122.1	121.4	125.2	127.4	113.6	103.9	120.7	125.7
NOD9	159.4	164.4	176.1	157.2	141.5	136.4	162.4	163.8
NOD9	129	123.5	118.1	135.3	114	113.4	134	125.2
NODAL	107.7	118.6	113.2	115.3	114	93.8	121.7	110.3
NOG	148.1	175.9	150.2	153.5	140.6	129.9	138.8	122.2
NOL1	925.1	1163.7	726.9	1083.5	771.6	472.3	1060.4	876.1
NOL1	115.3	116.5	120.7	115	119.1	106	116.3	125.1
NOL1	148.8	133.7	142.8	151.9	134.6	131.2	145.7	145.4
NOL10	125.2	121.3	123.8	138.4	121.4	109.3	130.4	125.4
NOL11	340.7	341.4	341.7	364.4	336.7	308.4	386.2	374
NOL12	281.3	239.1	351.9	253.9	183.5	228.1	232.8	213
NOL3	123.7	111.3	116.2	127.8	116.3	113.6	132.5	124.1
NOL4	120.8	126.6	118.9	125.4	115	97.8	117.3	120.1
NOL5A	663	633	478.8	508.2	438.7	192	448.3	574
NOL6	273	376.7	341.6	351.8	306.5	192.4	325.5	327.8
NOL6	123.2	119.6	112.7	122.7	113.6	109.7	123.2	111.5
NOL7	1761.6	1888.2	1600.8	1834.2	1199.6	1157.3	1490	1436.8
NOL8	244.7	235.2	256.9	230.4	186.1	239.3	180.5	241.9
NOL9	142	141.7	128.4	140.4	129	103.3	141.4	121.4
NOLA1	177.3	174.4	156.3	184	173.3	132.8	161.7	193.3
NOLA1	592.7	810.9	509.3	637.9	674.3	436.4	697.9	839.7
NOLA1	161.2	159.8	165.2	181.1	153.8	138.7	174.6	160.2
NOLA2	180.3	207.8	190.9	208.4	181.1	176.2	214.3	185.5
NOLA2	171.2	188.3	170.7	167.6	148.5	147.9	168.1	156.1
NOLA3	1645.3	1926.8	1965.2	1937.1	1801.9	1665.9	1758.8	2277.6
NOLC1	174.7	171.3	168.3	164.4	147.2	125.4	190.7	172.7
NOM1	116.8	118.6	116.5	121.4	110.8	96.7	120.5	117.2
NOMO1	137.2	161.7	124.3	172.6	152.8	144.7	152.4	159.6
NOMO2	123	122.1	113.3	117.9	107.2	100	127.3	117.2
NOMO3	128.1	117.8	130.1	132.4	112.1	103.9	123.6	122.9
NONO	1476.8	1123	1429.6	1511.6	1237.6	1486.4	1431.2	1753.5
NOP5/NOP	194.1	235.5	214	159.5	190.8	137.1	174.2	196.6
NOPE	119.3	122.7	120.6	117.1	116.9	103.5	115.4	123.4
NOS1	118.2	110.5	110.9	119.1	104.6	93.6	117.9	115.8

NOS1AP	107.4	120.5	106	123.1	107.7	96.7	116.6	112.5
NOS2A	109.1	116.8	116.6	116.7	110.3	105.2	109.9	114.2
NOS2A	110.5	124.5	116.3	110.6	103.5	95.3	126.7	119.6
NOS3	117.3	115.8	118.7	123.1	100.3	96.6	117.1	117.7
NOSIP	994.1	1282.2	975.4	1052.5	867.7	624.8	984.3	1026
NOSTRIN	118.3	116.4	109.4	117	108.5	92.2	120	125.5
NOTCH1	557.8	498	719.3	525.9	491.8	341	702.8	1033.2
NOTCH2	187.8	166.6	178.6	202.4	162.8	153.4	196	175.5
NOTCH2NL	122	114.6	121.1	123.6	112.5	99.8	114.8	110.9
NOTCH3	153.4	176.5	113.6	240.8	164.6	191.8	222.2	159.1
NOTCH4	114.6	113.9	126.8	118.9	100.7	114.2	136.5	118.6
NOTUM	110.2	130.4	127.4	121.8	113.8	104.1	129.5	108.9
NOV	109.7	110.4	109.6	119.5	104.7	96.3	118	113
NOVA1	114.1	114.1	124.4	115.9	105.8	98.2	115.5	112
NOVA1	108.5	107.9	118.5	115.2	105.6	93.9	115.6	108.5
NOVA2	116.8	127.5	121.6	124.9	106.9	102.8	121.6	117
NOX1	117.5	126.3	119.1	121	104.3	105.2	128.1	119.4
NOX1	106.9	117.6	114.3	106.8	104.5	98.4	110.6	104.7
NOX1	113.2	114.9	110.4	122.3	110.7	101.4	119.2	112.3
NOX3	107.3	114.2	117.3	124.6	111.8	97.4	115	112
NOX4	114.7	120.2	118	129.7	110.1	101	128.6	120.5
NOX5	110.7	106.4	107.5	108.9	108.7	106.1	111.7	109.3
NOXA1	129.1	119.2	171.2	149.8	118.1	132.5	120.2	112.9
NOXO1	116.5	128.2	128.8	134.6	115.4	111.1	128.3	135.4
NOXO1	113.5	125.2	115.1	117.1	111.6	100.1	121.4	124.5
NOXO1	129.3	126	129.2	129.6	115.3	111.5	116.6	132.8
NP	363.1	501.8	482.6	404.9	438	344.7	360.6	439.8
N-PAC	188.2	251.9	278.2	237.4	240.3	240.9	408.1	280.2
NPAL1	119.9	112.7	121.3	119.7	103.6	95.3	122.3	121
NPAL2	113.8	107.6	112.8	118.6	100.5	94.9	114.9	110.6
NPAL3	319.1	322.6	237.7	290.7	324.9	199.6	322.8	308.1
NPAS1	120.9	121.7	118	121	111.4	100.2	124.4	115.5
NPAS2	120.9	117.4	121.1	140.3	113.1	103.8	134.6	122.8
NPAS3	115.3	109.6	117.5	119.8	112.7	96.5	122.8	108.3
NPAS3	114.3	110.5	111.6	112.5	113.4	105.5	116.8	116.2
NPAS3	108.8	121.7	114.3	114.9	102.7	100	116.5	115.2
NPAS4	115.4	117.4	119.5	116.4	102.5	94.3	115.5	106.4
NPAT	173.8	242.8	180.6	223.4	212.1	176.9	269.7	241.7
NPB	116.1	123	120.2	121.1	110.6	99.3	123.6	121.1
NPBWR1	113.9	115.1	111	121.6	104.4	99.3	113	117
NPBWR2	117.6	116.2	119.4	118.3	112.7	98.1	128.9	123.5
NPC1	449.5	512.4	315.8	492.3	513.1	311.8	759.1	956.3
NPC1L1	118.3	120.6	112.8	112.2	107	100.6	130.7	134.4
NPC2	5912.7	2297.2	11749.2	6750.8	2525.6	8237.2	5930.5	2934.2
NPDC1	114.7	108.3	111.7	117.9	110.5	100.4	107.4	115
NPEPL1	349.4	358.9	427.8	315.1	254.5	266.9	224.8	193
NPEPPS	282.2	322.2	263.4	379.4	276.8	239.5	272.6	295.2
NPFF	121.7	112.8	125.2	129.9	111.5	97.6	119.9	123.7
NPFFR1	120.4	117	114.8	118.1	105.8	96.1	115.3	119.4
NPFFR2	113.1	114.2	124.9	120.8	112.5	97.5	124.9	119.1
NPFFR2	118.6	127.7	118.3	132.4	120.5	115.9	118.2	122.3

NPHP1	119.6	110.8	119.5	119.5	113.3	101.5	114.2	110.3
NPHP1	111.2	108.1	122.8	129.5	106	100.9	115.7	114.8
NPHP1	106.4	116.5	116.4	119.7	105.5	93.2	118.5	116.4
NPHP3	264.2	288.1	391.8	321.2	339.1	248.2	302.8	372.9
NPHP4	136.4	147.3	126.8	131	124.5	114.9	139.7	146.9
NPHS1	104.9	110	110.1	111.9	103.1	98.3	122.1	105.9
NPHS2	108.5	123.5	126.2	116	116.9	104	114.4	115.4
NPIP	286.8	264.8	226.2	300.4	248	173.6	300.2	331.3
NPL	425.5	184	665.5	675.3	225.9	921.1	615	266.6
NPLOC4	164.5	189.6	165.4	192.5	155.3	178.5	226.6	225.8
NPM1	113.8	114.6	108.1	123.4	112.2	98.8	110.2	116.9
NPM2	116	117.2	116.5	121	109.1	98.2	116.7	115.5
NPM3	262.1	271.4	220.4	237.5	243	181.3	234.9	224.5
NPNT	111.3	101.8	105.8	114.6	100	95.8	109	109.2
NPPA	113.4	106	119.7	115.2	101	99.6	108.1	121.8
NPPB	111.3	112.7	115.9	111.6	107.6	96.9	114.8	115.5
NPPC	115.9	116.8	123.4	129.1	111.9	100.6	119.7	127.2
NPR1	131.8	110.6	130.1	131.4	114.5	107.3	120.9	122.5
NPR2	155	168.5	155.1	161.9	134.7	123	154.3	161.1
NPR3	117.9	108.2	115.8	111.5	104.8	95.5	117.1	110
NPTN	1772.9	1608.9	2014.9	2079.1	1251	1937.6	1881.4	1390.5
NPTX1	106.3	101.3	113	119.3	99.1	101.8	114.6	122.2
NPTX1	127.1	114.3	120.9	123.8	112.4	106.3	128	119.3
NPTX2	111.5	111.1	113.9	119.5	103.4	94	116.2	112.3
NPTXR	121.5	129.3	108.5	131.7	113.5	98.3	127.9	116
NPY	108.4	114.6	111.6	118	99.5	97.5	118	114.4
NPY2R	119.3	115.5	117.9	117.4	112.8	96.6	119.8	113.8
NPY5R	123.4	105.5	115.2	126.3	121.7	111.2	117.4	114.4
NPY6R	121.7	112.3	117.8	120.4	106	95.1	115.8	115.6
NQO1	123	114.3	126.5	126.4	115.6	104.9	129.9	116.5
NQO1	217.3	229.7	188.8	588.8	401.3	619.7	957.4	841.8
NQO2	142.5	121.6	171.5	152.9	128.1	138.3	158.7	207.2
NR0B1	121.2	111.9	116.7	122.3	96.4	100.9	110.6	112.6
NR0B2	105.8	116.1	111.8	114.4	104.1	95.1	109.5	114.4
NR1D1	121.2	114.6	119.8	112.7	105.7	101	119.7	118.2
NR1D2	178.6	177.9	153.7	163.7	187.3	119.7	165.5	226.2
NR1H2	218.6	240.3	229.4	253.1	191.6	198.2	259.3	264.2
NR1H3	142.8	146.6	136.4	150.9	118.3	111.7	155	137.2
NR1H4	114.8	126.5	110	112.4	106.5	99.9	113.4	112.9
NR1I2	110.5	106.3	121.4	110.4	109.1	98.8	128.5	106
NR1I2	128.8	123.3	117.4	140.4	105.7	116	114.8	134.2
NR1I3	111.6	114.7	120	131.1	110.2	99	112.6	120.2
NR1I3	108.1	112.4	105.8	116.3	107.7	100.6	117.9	111.6
NR2C1	151.7	158.5	157.3	145.2	132.4	109.7	126	139.1
NR2C1	142.9	133.5	139.2	149.4	122.9	115.8	136.8	131.7
NR2C1	174	167	165.3	169.4	161.1	121	129.9	137.5
NR2C2	164.8	165.3	141.4	203.9	166.2	142.4	234.6	208.8
NR2E1	118	112.9	120.7	117	109.7	103.2	116.2	114
NR2E3	120	113.7	123	123.7	109	99.8	122.2	119.6
NR2E3	106.5	114.9	116.3	112.7	105.1	95.2	112.5	116.9
NR2E3	116.8	116.2	120.9	113.4	109.3	92.9	121.5	110.7

NR2F1	104.6	105.4	112	113.7	104.1	89.4	120.5	106.4
NR2F2	112	117.3	120.6	130.4	107.9	103.3	115.8	119.5
NR2F6	130.1	155.2	134.1	138.4	121.1	107.7	122.5	122.9
NR3C1	152.5	164.9	148.7	161.2	145.5	124.2	178.6	177.1
NR3C1	129.7	123.4	135.2	115.9	124.7	106.9	122.4	116.7
NR3C1	115.2	129.6	145.4	125.6	121.6	103.5	123.2	121.7
NR3C2	193.4	220	206	184.3	175.9	170.3	162.9	180.9
NR4A1	113	109.7	124.1	116.8	106.8	100.2	125.2	116.4
NR4A1	113.4	119.2	118.1	106.6	110.2	101.2	125.7	113.2
NR4A1	121.4	120.4	110.5	108.6	100.6	99.6	102.9	104.7
NR4A2	115.8	122.3	120	139.3	118.2	98.5	145.8	179.6
NR4A3	117.9	114.1	117.6	110.6	110.1	100.1	107.8	113.2
NR4A3	112	121.9	117.6	118.9	107.8	102.5	117.3	114.4
NR4A3	112.7	105.1	109.2	106.1	99.6	100.4	113.4	107.3
NR4A3	114.5	116.8	111.1	119.9	108.7	100.9	121.7	117.1
NR4A3	114.8	116.9	115.2	116.4	107	95.4	118.7	117.4
NR5A1	111.1	109.8	112.5	105.7	102.1	100.5	115.3	112.4
NR5A2	110.8	107.6	117.5	119.6	109.2	95.9	110.9	112.4
NR5A2	119.7	107.7	110.2	100.7	108.1	99.8	124.8	104.8
NR5A2	121.7	122.1	125.6	131.4	120.7	112.4	124.3	124.1
NR6A1	126	114.9	118.3	113.3	98.7	103.6	115.9	119.4
NR6A1	113.2	114.9	118.6	115.4	112.3	91.6	111.2	116.3
NRAP	107.7	116.5	112.6	113.1	101.4	95.2	117	103.1
NRAP	110.2	122.6	113.1	119.8	108.9	99.6	110.6	116.5
NRAS	195.5	242.4	200.8	234.7	194.1	187	252.6	232.6
NRBP1	160.6	143.8	154.1	169.3	144.2	129.3	179.1	171.4
NRBP2	238.9	228.8	319.1	250.5	196.3	269.9	201.4	214
NRCAM	121.6	122.3	132.8	119.6	115.2	106.2	112.4	126.3
NRCAM	114.3	130.4	136.7	116.3	107.6	114.4	120.1	123.4
NRD1	1057.7	874.3	823.7	1151	757.8	803.2	1134.2	1056.6
NRF1	125	120.9	119.4	108.6	109	98.7	122.7	125
NRF1	130.3	131.8	121.5	132.2	124.5	109.2	133.4	129.5
NRF1	115	114.8	115.2	118.4	119.3	101.2	117.5	119.6
NRG1	102.7	115.6	103.3	116.7	106.6	94.1	115.7	109.9
NRG1	118.6	114.2	154.9	119.2	99.1	106.1	125	110.3
NRG1	126.6	121.4	114.2	114.2	108	99.7	117.8	118.4
NRG1	117.7	122.4	115.4	124.3	113.1	110.9	123.2	113.9
NRG2	121.7	114	124.5	119.8	118.3	116.6	124.9	117.3
NRG2	111.9	112.5	110.4	133.9	104.4	89.7	115.7	115
NRG3	113.8	113.2	115.3	117	108.7	99.4	112	119.8
NRG4	115.6	110.9	112.9	113.1	96.6	95.8	114.8	112.3
NRGN	125.2	113.9	133.8	150.4	113.3	101.5	124.1	128.1
NRIP1	254.9	305.3	317	316.6	308.5	288.1	299.2	267.8
NRIP2	113.2	126.3	136.4	126.2	128	108.4	133.1	137.9
NRIP3	132.7	164.9	124.5	221.5	192.7	122.9	520.1	359.2
NRK	129.3	121	141.1	130.2	131.7	117.1	137.4	129.5
NRL	106	114.9	118.6	112.5	110.3	100.9	107.1	111.1
NRM	148	121.8	134.1	141.6	116.3	102.8	126.1	126.5
NRN1	104.5	108.1	122.9	113.1	105	96.3	120.7	110.9
NRP1	115.2	113.7	124.7	134	116	103.7	130.8	127.7
NRP1	154.8	147.4	161.7	227	205	129.6	319.5	219.2

NRP2	117.3	111.4	117.8	126.8	112.2	103.6	116.3	116.1
NRP2	107.6	104.4	110.8	115.5	101.8	94.4	118.6	112.7
NRP2	110.3	117.1	113.9	119.2	111	99.7	120.9	112.6
NRTN	108.3	119.1	112.2	124.7	118.6	98.6	122.5	109.6
NRXN1	119.5	121.4	122.7	116.9	112.6	96.8	116.1	116
NRXN1	111.8	113.1	116	113	104.6	100.7	119	115.3
NRXN1	109.9	107.9	118.5	121.9	109.1	101.7	116.8	118.6
NRXN2	111.8	104.5	110.9	120.9	107.4	99.7	112.3	114
NRXN2	122.5	121.5	123	119.7	117	94.6	136.8	118.1
NRXN3	103.7	111.4	110.7	117.4	107.3	88.7	113	104.3
NRXN3	106.4	110.8	116.9	109.7	107.8	92	119.8	109.5
NRXN3	122.2	128.6	129.8	119.6	108.7	101.1	129.4	111.5
NS4ATP2	162.4	211.8	172.3	179.4	167.3	195	183	174.2
NSBP1	108.1	111.3	110.9	113.2	103.5	97.2	112.1	108.1
NSD1	127.6	121.2	124.6	144.4	112.5	109.3	122.9	129.1
NSD1	148.3	134.8	130.5	143.7	158.8	112.6	126.9	117.6
NSDHL	168.6	200.5	211.3	192.6	161	169.7	170.2	177
NSF	115.1	123.7	120.9	103.1	108	101	118.2	116
NSF	176.3	176	195	170.6	160	189.9	170.3	157.8
NSF	805.9	762.7	877.7	844.3	652.6	662.9	619.2	539.1
NSFL1C	161.5	195.2	226.3	203.3	157.4	196.8	208.3	210.6
NSFL1C	223.8	225.2	288.2	220.5	211.9	181.4	251.9	253.5
NSL1	115.1	120.6	132.4	115.7	105.8	97.8	118.7	119.8
NSL1	121.6	124.4	140.2	127	111.2	97.4	122	122.5
NSMAF	776	818.1	730.7	937.8	651.7	684.2	1171	727.2
NSMCE1	282.4	228.5	278.3	250.8	256.8	214.3	204.1	244.6
NSUN2	490.3	586.4	515.3	655.8	541.6	445.4	702.4	601.2
NSUN3	141.6	157.7	155.6	156.8	144.2	121	176.3	155.1
NSUN4	222.8	236.7	265	233.2	213.4	204.8	216.4	219.7
NSUN5	285	291.4	305.4	254.3	200.2	247.1	228.8	179.7
NSUN5	211.9	200.7	175	182.1	158.1	132.3	170.8	148.9
NSUN5	184.9	161.6	209.2	184.2	164.8	149.1	164.1	159.3
NSUN5B	306.8	267.6	345.8	304.4	229.9	213.5	191.2	188.4
NSUN5C	135	137.7	135.4	131.1	131.4	114.2	137.9	129.4
NSUN5C	110.1	121.6	148.7	138.8	116.1	113.8	121.6	123.1
NSUN6	121.2	122.7	148.6	121.8	131.7	120.4	132.4	121.4
NSUN7	124.5	119.5	127.9	130.1	109	110.7	120.1	125.5
NT5C	597	615	653.9	609.9	452.2	315.5	355.2	369.5
NT5C1A	106.4	108.9	127.5	112.5	107.2	95.9	114.4	114.2
NT5C1B	115.2	106.1	111.1	122	107.7	97	118.8	111.6
NT5C1B	112.9	119.1	119.9	117.9	111.9	95.5	108.5	112.6
NT5C2	333	381.7	337.5	393	283.7	352.2	605.9	449.5
NT5C3	631.6	671.3	702.3	807.3	730	444	641.1	1080.5
NT5C3L	162.6	171.8	155.5	158.8	155	132.9	194.6	185.5
NT5DC1	116.8	121.8	118.3	126.9	112.7	115.5	120.8	116.3
NT5DC2	161	142.1	192.7	189.5	131.3	184.3	146.1	129.5
NT5DC3	123.8	110.6	125.7	122.1	107.1	99.6	119.9	112.7
NT5DC3	157.9	161.6	144.5	147.5	136.6	120.3	154.5	144.3
NT5E	159.1	163.3	133.3	145.3	130.5	119.2	134.2	140.6
NT5M	110.2	124.4	116.4	115.3	111.2	102.6	112.9	117.2
NTAN1	383.8	348.3	355.1	427.6	305.3	344.3	470.5	352.7

NTE	741.2	706.7	895	831.1	448.4	762.8	840.2	595.1
NTF3	114.2	136.9	117.4	115	109	98.9	121.2	117.9
NTF5	113.9	116.5	126.1	118.2	112.9	92.6	129.4	119.5
NTHL1	161.9	164.9	133.4	161.2	144.8	131.6	131.2	132.4
NTN1	118.7	123.3	118.4	125.5	109.7	98.7	120.8	123.3
NTN2L	109.6	109.7	120.2	117.6	108.1	102.5	116.9	104
NTN4	115.5	113.9	130.7	124.2	115.7	105.5	126.6	116.5
NTNG1	100.7	114.4	107.2	107.8	109.2	94	114.7	108.5
NTNG2	116.4	118	124	123.3	111.6	104.9	121.7	117.6
NTRK1	120.6	117.5	127.2	117.8	112.1	104.2	122.1	117.7
NTRK1	126.9	118.6	126.7	128.5	106.8	102.9	119	131.1
NTRK1	127.2	118.6	122.9	129.4	116.3	101.4	123.2	112.9
NTRK2	117.1	108.3	118	121.2	110.8	99.8	106.4	114.6
NTRK2	117	121.5	119.9	126.2	112.5	102.4	111.9	116.4
NTRK2	114.6	112.3	116.5	114.1	103.5	94.1	113.2	109.1
NTRK3	120.6	120.1	118.4	120.7	118.5	100.6	116	121.4
NTRK3	115	116.9	122.3	114.8	115.5	97.5	134.6	117.4
NTS	111.5	109	117.7	115.4	107.1	99.1	109.2	110
NTSR1	124.7	122.6	117.5	117.7	109.4	102.8	118.4	112.4
NTSR2	114.3	118.6	119.7	125.5	102.7	100.1	118.6	125.3
NTSR2	114.7	118.8	121.4	121.1	111.1	101.1	119.4	121.2
NUAK1	119.9	128.6	112.2	116.7	105.8	99.6	119.3	124.6
NUAK2	190.7	194.8	185	207.5	158.7	178.8	159.5	171.7
NUB1	114.8	119.8	119.3	114.8	107.7	103.9	116.7	120.5
NUBP1	242.2	276.4	250.6	260.9	183.1	211.2	212.3	228.6
NUBP2	136	151.3	148.8	157.9	123.4	123.9	146.9	142.2
NUBPL	726.2	531.2	715.8	748.8	689.4	419.1	639.5	751.6
NUCB1	2348.1	2517	3257.7	2794.3	1768.8	3256.1	2403.5	2406.5
NUCB2	328.5	379.1	304.2	358.1	368.4	278.3	332.4	376.7
NUCKS1	126.2	123.6	135	135.9	124.1	113.1	153.3	146
NUDC	1069.2	1141.5	926.1	1000.9	738	551.7	970.2	1203.5
NUDCD1	120.4	120.5	126.2	138.3	113.2	99.4	123	124.2
NUDCD2	142.7	144.2	146.5	151.2	131.2	118.2	137.9	134.7
NUDCD3	259.2	285.9	228.1	227.3	176.8	180.5	254.2	212.2
NUDT1	120.9	114.7	115.3	120.9	117	103.2	109.7	116.8
NUDT1	303.4	299.9	397.6	244.8	246.7	187.7	209.2	202.1
NUDT10	115.7	114.5	116.4	113.5	106.9	102	124.5	123.8
NUDT11	126.2	117.5	128	130.6	115.5	112	127.9	136.5
NUDT12	107.4	112.8	116.5	119.8	105.5	99.2	119	109.5
NUDT13	124.2	118.1	130.6	129.6	125.2	101.5	135	124.9
NUDT14	277.7	315	304.3	309.7	201	218.3	280.5	230.1
NUDT15	126.1	126.8	125.9	132.9	107.3	103.9	122	118.5
NUDT16	128.8	121.1	146.6	122.3	117.3	106.6	126.9	124.3
NUDT16L1	334.6	406	353.6	356.6	328.9	293	292.5	342.9
NUDT17	115.2	116.2	119.2	118	120.8	108.2	130.3	111.2
NUDT18	248.6	216.5	241.8	200.7	140.3	167.2	161.5	140.8
NUDT2	144	143.3	179.6	150.6	149.1	164.2	160.6	159.9
NUDT2	213.6	201.6	286.7	221.8	182.4	222.4	225.8	211.1
NUDT21	116.1	125.9	144.7	126.3	116.4	110.7	113.1	121.4
NUDT22	169	173.2	212.9	191.3	172.5	153.8	152.7	160.2
NUDT3	268.4	283.6	349.5	314.2	265.7	266.2	315.2	302.1

NUDT4	117.2	107.8	110	111.5	110.2	97.7	117.5	124.6
NUDT4P1	127.4	117.2	112.9	129.3	102.7	104.7	124.9	130.9
NUDT5	358.1	297.9	310.2	327.7	257.9	233	241.1	261.9
NUDT6	112	117.1	125.9	110.6	114.3	96.9	113.3	117.6
NUDT6	121.2	114.9	117.8	119.1	101.7	94.6	117.9	107.2
NUDT6	117.8	117.5	120.8	123.7	105.1	100.2	120.5	121.7
NUDT8	121.6	123.5	118.2	114.9	107.1	100.9	109.2	112.2
NUDT9	129	125.3	125.9	133.1	112.8	102.5	130.2	132.8
NUDT9	317	371.1	340.6	367.5	313	280.4	331.3	344.9
NUDT9	126.7	116.3	120	121.8	113	101.2	118.7	117.9
NUDT9P1	110.1	114.5	117.5	119.3	104.4	104.8	123.7	135.2
NUF2	109.7	115.3	133.7	122.5	114	102.3	119	123.2
NUF2	113.1	110.4	108.3	109.6	107.6	95.7	124.4	115.8
NUFIP1	120.5	132.9	134.5	134.3	117.9	108.6	109.3	120.5
NUFIP2	437.4	524.5	269.3	392.5	294.8	282.3	524.4	628.5
NULP1	1058.7	1075.7	931.8	1067	977.2	734.5	1330	1206.4
NUMA1	261.8	276	253.8	245.7	228.5	240.5	312.5	287.6
NUMB	602	628.3	519.2	839.6	552.5	666.8	1135.8	641.3
NUMBL	125.4	134.4	139.8	139.8	130.9	108.8	132	125.5
NUP107	192.9	240.4	245.2	195	208.4	208.1	213.1	223.9
NUP133	305.1	304.7	335.8	301.1	269.1	283.4	288	279.1
NUP153	364.3	420.9	281.7	437.1	334.6	330.7	506.6	627.6
NUP155	115	123.1	113.3	122.1	107.4	101.8	125.5	111.4
NUP155	239.3	300.7	290.9	274.8	223.7	242.4	279.2	249
NUP160	633.4	587.9	465.3	651.6	502.5	372.6	688.5	686.6
NUP188	283.6	220.9	201.1	264.5	197.4	166.4	265.6	254.6
NUP205	478.2	431.4	328.7	474.4	412.4	393.4	482.9	495.5
NUP210	198.2	203.6	206.2	169.1	178.9	152.9	223.8	169.5
NUP210L	115	114.7	112	120.5	111.9	99.3	106	127.6
NUP214	387.9	322.6	680.9	401.2	247.9	462.2	393	294.6
NUP35	131.6	162.1	166.4	160.2	137.8	124.6	141.3	136
NUP35	126.4	127.3	134	127.1	131.1	98.7	136.1	128.3
NUP37	337	306.1	501.1	291.1	263.1	305.5	251	232.1
NUP43	137.6	117.9	160.4	126.7	122.7	114.1	123.7	112.3
NUP50	174.3	170	203.4	210	142.8	160	184.3	158.7
NUP50	114.1	119.6	107.9	115.1	106.4	102.3	113.9	122.5
NUP50	118.2	119.1	117.9	114.8	114.9	98.7	124.5	120.4
NUP54	485.2	551.6	526.2	475.8	511.5	469.3	441.8	541.2
NUP62	160	155.8	131.9	154.9	129.7	132.1	172.3	152.2
NUP62	1731.5	1560.7	1894.6	1560.9	1236.3	1409.6	1518.4	1528
NUP85	447.5	382.5	448.3	440.6	336.7	297.7	361.2	328.7
NUP88	620.7	679.2	632	638.9	520.1	469.4	720.2	719.8
NUP93	229.9	289.9	258.5	252.3	256.2	205	211.1	203.8
NUP98	120.5	128.4	119.2	122.9	125.7	106.3	127.5	132.7
NUP98	144.7	164.1	140.5	155.6	141.5	119.4	166.6	158.7
NUP98	122.6	117.9	125.1	133.4	128.6	104.9	135.3	117.3
NUPL1	106.5	114.8	124.7	112.8	107.3	92.9	124.4	121.3
NUPL1	118	127.4	136.4	131.8	125.4	103.6	139.6	131.8
NUPL1	143.7	139.1	146	150.4	137.7	117.9	147.5	166.6
NUPL2	341.5	261.1	280	275.8	210.1	174	243.6	217.7
NUPR1	133.4	121.5	119.3	136.4	115.1	105.9	121.7	121

NUS1	135	132.3	135	137.1	123	112.3	137.3	147.1
NUSAP1	126.8	134	170.2	147	134.9	126.3	158.2	167.7
NUSAP1	114.6	127.3	135.9	115.1	116.5	106.1	130.1	121.4
NUT	112.4	115.3	111.8	114.5	102	98.7	122.1	113.9
NUTF2	118.3	122.6	120.3	127.2	113.3	109.9	121.5	124.8
NVL	115.1	122.3	112.8	118.9	107	95.8	122.3	113.6
NVL	172.6	216.1	167.7	190.5	158.4	130.2	167.3	178.9
NXF1	769.4	746.6	631.6	790.3	710.3	793.4	958.7	1400.3
NXF1	761.7	673.2	663.7	782.1	586.8	423.2	958.9	1309
NXF2	126.3	128.4	118.4	130.4	110.1	101.8	123.2	113.5
NXF2	115.1	114.4	110.3	113.4	105.3	93.1	118.9	110.3
NXF3	110.4	125.6	128.4	129	110.8	104.3	125.4	119.1
NXF4	120.1	115.3	122.9	114.7	108.4	109.4	119.6	106.4
NXF5	104.9	120.5	111.8	121.1	107.8	101.9	120.5	114.7
NXN	120	123.1	117.3	119.2	114	100.5	123.3	119.3
NXPH1	128.7	114	120.6	122	112.2	99.4	112.6	119.8
NXPH2	104.1	103	115.3	113.8	102.2	103.6	114.2	109.3
NXPH3	115	115.6	116.3	106.5	115.7	96.5	116.5	117.3
NXPH4	111.9	115.8	133.9	117.2	105.3	115.4	115.9	115.1
NXPH4	105.6	104.3	119	113.9	102.2	95.2	113.4	120.3
NXT1	584	695.4	534.1	673.2	587.5	342.9	680.9	624.2
NXT2	144.3	137.4	168.4	153.1	120.4	132.1	146.9	158
NYD-SP14	117	125	114.9	120.3	106	95.1	116.6	112.7
NYD-SP18	110	105.5	113.1	117.8	103.4	100.3	110.6	111
NYD-SP21	146.3	116.4	208.1	140.9	109.7	147.6	155.4	118.9
NYD-SP21	133.3	119.9	152.1	116.6	120.6	136.1	132.9	118.4
NYD-SP26	117.8	113.6	117.7	116.1	109.8	97.6	109.9	117.2
NY-REN-7	110.2	118.7	115.8	116.6	112.2	101.1	120.9	113.4
NY-SAR-48	132.6	137.5	124.4	131.8	118.7	118.8	135	122.7
NY-SAR-48	153.1	146.6	147.7	160.8	134.5	117.2	164.4	162
NYX	111.8	109.1	120.4	112.6	108.2	100.6	115.5	114.6
OACT2	109	117.9	121.1	118.5	111	102.2	120.1	115.5
OACT5	173.6	138.3	153.3	136.2	130.9	125.4	147.8	147
OAF	169.2	139.9	147.3	167.7	135.5	127.2	175.2	133.3
OAS1	114.8	119.2	118.1	118.2	107.3	97.9	120.4	107.4
OAS1	156.4	129.5	140.6	139.9	116.2	111.4	131.8	124.1
OAS1	176.8	132.9	148.1	174.8	121.8	114.4	139.5	122.6
OAS1	138.7	143.7	137.8	139.3	126.4	116	123.1	131.7
OAS2	167.6	147.4	146.4	154.3	144.7	131	140.1	126
OAS2	137.9	149.3	143.3	138.3	132.2	118.7	123.3	135.4
OAS2	122.8	116	115.7	123.8	107.4	106.1	118.1	108.1
OAS2	1151	1226.8	785.2	1252.2	713.6	565.7	749.6	621.7
OAS3	107.2	108.5	112.2	115.9	104.1	108.5	114.3	108.9
OASL	125.7	117.7	142.1	136.8	107.5	105.8	114.3	109.4
OASL	226.7	179.5	255	215.8	134.8	223.7	169.2	149.9
OAT	364	319.5	303.2	310.1	275.8	272.8	334.9	347.8
OATL1	122.8	111.2	133.7	124.2	117.2	104.2	112.3	122.8
OATL1	134.7	155.1	130	141.1	126	121.9	174.3	170.5
OAZ1	15474.9	16280.3	19165.5	18429.7	13848	17365.7	18675.1	18730
OAZ3	136.9	131.5	127.6	121.9	122.4	107.1	134.2	126.1
OBFC1	282	312.7	320.1	299.2	225.2	219.6	228.8	189.4

OBFC2A	286.5	305.4	290	348.9	247.2	246	353.7	414.5
OBFC2B	197	251.6	197	194.7	173.9	143.2	240.7	207.7
OBP2A	116.3	117	117.9	124.8	118.4	103.9	120.2	126.3
OBSCN	140	140.9	118.9	131.1	119.2	101.5	150.1	130.3
OC90	111.7	119.8	135.1	121.8	104.1	95.9	120.7	105.5
OCA2	118.7	115.4	114.2	113.1	113.3	104.6	122	116
OCIAD1	1669.3	1207.3	1513.2	2171.6	2037.1	995.3	1870.5	1974.9
OCIAD1	894.6	1072.2	897.7	909.5	925.3	994.4	729.3	988.4
OCIAD2	402.6	419.8	574.9	431.1	321.7	564.1	291.3	285.8
OCIAD2	1377	1863.6	4176.8	1683.8	1805.5	3171.2	1546.4	1697
OCLM	117.3	124.9	124.6	127.4	110.8	102.8	126.4	119.7
OCLN	111.1	115.1	114.1	110	109.5	94	109.7	108.3
OCLN	117.3	119.2	108	121.6	108.5	98.1	118.5	120.2
OCM	113.2	114.4	109.6	121.5	103.6	102.4	116	114
OCRL	123.5	124.6	136.7	127.7	116.1	110.5	131.7	122.4
OCRL	137.7	127.1	125.4	125.9	109.5	108.1	115	116.7
ODC1	267	247.4	300.3	286.6	197	241.6	427.7	518.5
ODF1	123	121.3	119.4	113.4	109.8	99.8	111.6	113
ODF2	131.4	135.2	140.4	126.5	130.5	120.7	133.8	132.9
ODF2	108.2	119.2	122.5	122.5	107.3	102.8	124.8	121.3
ODF2	180.7	203.4	179.8	178.5	166	172.1	208.6	210.2
ODF2L	138.4	141	154.1	145.6	140.1	116.1	125.6	138.5
ODF2L	119.8	108.4	113.9	116.9	100.2	96.4	121	114.6
ODF3L1	128.5	118.6	124.9	130.6	113.8	107.2	125.8	124.4
ODF4	124.4	115.5	118.6	126.3	110.8	108.6	122.1	117.7
ODZ1	108.8	108.9	109.2	115.7	100.3	93.8	111.4	109.9
ODZ2	125.2	120.4	118.5	130.2	119.6	107.8	117.8	118.9
ODZ3	120.6	116.3	116.6	135.4	116.3	102.4	111	119.2
OFCC1	112.4	106.9	115.7	112	105.7	98.5	114.3	117.3
OFD1	191.6	192.2	197	218.1	199.1	220.2	205.5	213.7
OGDH	136.4	132.1	131.7	130.7	119.6	110.3	127.7	130.8
OGDH	125.9	102.4	117.4	123.7	117.7	99.4	110.6	117.4
OGDH	229.4	280.6	205.9	219.7	189.7	160.9	267.2	222.6
OGDHL	112.9	113	119.9	118.1	103.7	95.6	119.4	108.1
OGFOD1	104.7	108.7	113.2	113.1	105.7	99.3	120.9	115.8
OGFOD1	184.7	204.5	184.5	184.9	163.5	173.8	188.3	193.9
OGFOD1	267.3	338.6	376.5	339.5	351.1	309.5	340	319.3
OGFOD2	123.5	121.3	121.6	133.6	122.1	109.6	126.7	125.8
OGFR	399.8	430.4	344.3	449.2	272.9	310.4	431.7	361.1
OGFRL1	128.2	119.6	176.2	126.9	126.4	134.6	120.7	123.6
OGG1	130	125.9	126.2	129.8	114.1	104.8	132.2	116.3
OGG1	174.7	152.6	158.4	156.3	141.3	130.7	142.4	134.7
OGN	107.2	112.8	123.7	130.1	111.2	107.7	124.4	118.3
OGN	121	111	118.5	113.2	108.3	104.7	108.7	114.4
OGN	113.9	107.3	107.1	113.4	103.7	92.7	121.1	119.2
OGT	1579.1	1970.1	2388.1	1646	1710.1	1329.8	1583.7	1445.8
OGT	533.8	645.9	1015.6	539.6	683.9	510	407.7	361
OIP5	137.5	126.1	132.9	142.1	107.1	94.1	128.2	123.1
OIT3	125.4	107.7	126.5	123.1	116.4	93.5	118.4	120.1
OLFM1	118.3	124.1	121	121.2	108.7	100.5	116.2	115.5
OLFM1	115.6	117.9	199.4	115	105.1	108.9	119.9	117.8

OLFM1	119.8	115.5	135.6	115.5	116.2	103	124.7	125
OLFM2	117.1	118	124.1	124.6	119	95.9	117.2	125
OLFM3	116.1	111.8	109.8	105.9	104.6	91.5	105.4	97.8
OLFM4	127.1	167.3	137.7	140.2	140.3	119.3	126.8	138.6
OLFML1	115	107.3	117.6	114.5	111.3	101.4	121.4	113.3
OLFML2A	115.6	115.8	118.9	110.1	111.6	104.4	115.9	115.8
OLFML2B	116	121.4	140.2	122.3	108.7	122.7	117.6	112.8
OLFML3	125.5	118.9	134	125	110.7	104.9	111.5	113.7
OLIG2	118.7	116.2	117.4	123.1	110.7	95.3	120.6	114.9
OLIG3	110.6	120.9	124.1	116.9	116.2	107.9	125.4	120.3
OLR1	120.5	131.7	834.8	130	128	337	125.1	132.4
OMA1	129.7	129.4	148.1	135.8	124.6	109.2	125.4	121.5
OMD	105.5	126.3	121.8	116.9	110.9	105.2	116.3	114.7
OMG	106.8	121.4	124.3	114.8	121.4	98.7	134.2	136.7
OMP	105.8	106.1	117.4	106.4	97.2	92	115	114.5
ONECUT1	112.8	109.7	112.9	118.3	98.9	95.4	112.6	112
ONECUT2	118.1	128.5	123.1	118.8	113.4	102.4	127.8	124.7
ONECUT3	112.6	113.5	117	117.8	112.2	104.2	119.4	115.9
OPA1	181.7	187	179	183.4	180.7	161.6	189.7	186.2
OPA1	103.7	113.9	119.3	119.3	102.7	96.1	120.8	117.5
OPA3	122.6	113	120	127.3	109.1	115.8	123.5	113.7
OPA3	191.9	178.9	176.3	194	168.7	129.2	201.9	173.8
OPCML	113.8	108.4	119.4	111.6	108.2	93.2	114	107.2
OPCML	117	118.6	121.4	126.9	116.2	102.8	117.6	115.8
OPHN1	115.1	111.3	126.5	118.9	118.5	97.6	112.6	113.1
OPLAH	114.3	110.8	118.5	123.2	100.1	97.8	101.7	104
OPLAH	139.5	123.7	156	131.9	110	102.7	128.6	104.8
OPN1LW	121.2	112.7	111.5	116.9	104.4	100	124.5	122.7
OPN1MW	118.9	121.4	118	131	115.1	99.5	118	113.9
OPN1SW	121.4	119.1	125.6	127.7	114.3	107.2	118.2	124.8
OPN3	201.1	191.8	255.4	208.2	177.7	175.3	182.4	153.6
OPN3	129.5	129.2	132.5	128.5	110.6	105.9	115.3	117.4
OPN3	118.5	121.2	128.2	131	112.6	99.6	123.3	121.9
OPN4	114.5	109	125.7	112.2	105.2	98.9	120.5	112.2
OPN4	109.6	111.8	112.7	122.2	106.8	99.3	117.7	114.3
OPN5	110.9	117.6	123.2	111.7	109.9	97	123.9	109.6
OPN5	122	111.1	122.6	123.4	117.1	100	112	119.6
OPRD1	125.6	115.2	115	122.8	112.1	99.9	126.2	114.5
OPRK1	119.3	123.7	116.5	120.1	118	96.9	114.3	120
OPRL1	135.7	123.5	149.7	137.9	122	119	130.9	124
OPRL1	114.1	117.4	131.8	119.1	111.7	105.5	124.8	122.1
OPRL1	111.9	109.9	104.4	108.1	107.9	95.9	113.2	109.4
OPRM1	114.1	119	126.4	120.3	109.8	102.5	126.1	122.5
OPRM1	116.9	109.4	113.6	117.8	108	96.1	107.9	111.2
OPRM1	118.1	113.3	113.1	132.8	110.6	99.6	129.1	122.7
OPRM1	108.3	112.1	119.6	112.9	99.1	97.7	108.7	112.1
OPRM1	104.9	110.2	110.3	111	99.9	96.5	111.1	108.2
OPRM1	119.8	113.7	130	115.9	114.9	106.9	127.6	112.7
OPRS1	130.8	148	145.7	138.9	117.3	120.3	119.2	126.3
OPRS1	208.8	188.7	170.8	173.4	144.3	160.1	157.2	143.1
OPTC	120.1	123.5	116.5	113	105.2	100.1	122.5	108.4

OPTN	515.3	674	445.2	562.6	631.7	448.6	790.2	858.5
OR10A2	110.1	115.7	118.6	115.9	105.1	99.8	129.4	115.1
OR10A3	113.7	113.1	128.7	118.1	112.2	102	123	114.4
OR10A4	114.6	113.4	129.3	111.8	108.1	101.6	122.8	107.9
OR10A5	105.2	113	104.7	114.8	113.5	101	103.4	112.3
OR10A6	116.5	118.5	110.3	114.3	114.4	98.7	113.3	122.6
OR10A7	116.4	108.2	121.5	122	110.7	108.5	130.1	115.8
OR10AD1	113.7	117.9	119.2	112.2	109.1	101.5	109.3	115.2
OR10AG1	113.9	111.9	120.3	112.6	109	99	121.9	108.6
OR10C1	125.7	109.1	114.9	118	103.1	95.1	113.7	116.4
OR10G2	118.5	109.2	116.9	122.3	102.8	107.3	120.4	121.9
OR10G3	116.7	117.6	116	112.7	108.7	104.3	116.9	120.7
OR10G4	111.7	112.1	119	121.7	107.4	104.4	117.2	118.4
OR10G7	114.3	121.1	123	119.4	111	100.8	121.7	112.5
OR10G8	111.1	112.5	123.1	112	109.8	98.7	115.8	128.8
OR10G9	108.1	120.8	120.8	120	106	98.8	114.8	113.6
OR10H1	112.2	114.6	115	112.3	107.7	95.1	117.5	109.5
OR10H2	118.1	105.6	114.8	114.4	118.5	94.6	108.3	105.7
OR10H3	108.5	122.7	114.8	115.8	106.7	97.6	111.6	116.4
OR10H4	109.9	110.6	116.1	113.6	112.2	95	117	113.9
OR10H5	110.6	118.5	109.8	121.3	106.7	93.8	117.7	109.9
OR10J1	121	121.6	126.1	127.5	113.7	109.8	124.1	122.3
OR10J3	109.1	111.8	120.6	113.3	109.5	95.6	122.8	111.9
OR10J5	110.4	119.6	123	120.7	116.2	100.6	114.9	123.7
OR10K1	118.8	119.5	113.6	117.2	109.5	97.5	110.7	114.2
OR10K2	106.8	112	118.9	121.8	106.7	95.3	109.2	112.3
OR10P1	116.2	113.5	107.7	111.7	109.5	103.3	112.1	109
OR10Q1	118.1	117.5	116.6	122.2	104	101.3	118.9	116.2
OR10R2	111.6	113.5	116.6	114.1	116.8	99.2	121.1	112.1
OR10S1	120.3	120.8	122.8	127.4	115.9	106.5	119.7	122.1
OR10T2	119.2	111.1	116.1	116.8	113.4	103.3	123	111.3
OR10V1	119.5	120.9	122.8	119.5	108.1	104.7	119.4	114.9
OR10W1	110.4	113.5	108.5	112.5	113.3	92.9	124.8	106.5
OR10X1	115.7	109.5	126.9	121.3	111.8	96.5	119.9	107.8
OR10Z1	115.9	112.9	109	117.6	112	94.2	112.1	114.5
OR11A1	107.7	112.9	113	117	108.3	99.5	113.2	115.9
OR11G2	120.2	112.9	113.7	119.1	98	92.9	111.7	108.8
OR11H1	110.9	119.3	113.8	120.5	106.5	113.4	124.7	116.4
OR11H12	114.6	127.4	118	115.3	102.9	113.9	124.9	127.2
OR11H12	139	109.9	134.6	138.4	109.2	107.3	129.8	119
OR11H4	116	114.1	116.7	122.2	111.3	101.7	126.1	117.5
OR11H6	113	116.6	117	126.3	106.8	93.7	112.8	104.3
OR11L1	136.9	123.2	133.1	140.5	117.9	114.5	124.2	133.7
OR12D2	125.7	128.2	130.5	131.3	115.4	109.4	116.3	122.4
OR12D3	119	114.9	115.8	114	97.6	93.7	112.5	117.2
OR13A1	120.9	116.1	108.7	122.2	115	102	118.3	112
OR13C2	116.7	100.7	110.4	115.4	108	95.9	113.2	111
OR13C3	119	118.6	113.5	110.9	114.2	98.4	121.7	114.2
OR13C4	118	103.8	111.6	125	114.8	95.6	122	110
OR13C5	113	122.3	124.3	124	105.7	99.5	124.5	112.2
OR13C8	111.9	116	114.8	117.5	104.6	102.2	121.2	115.8

OR13C9	114.2	125.7	115.3	120.9	110.7	99.7	124.3	118.7
OR13D1	112.5	116.4	104	104.8	104.6	93.5	125.6	105.7
OR13F1	102.7	116.9	111.4	114.3	97.2	96.5	110.6	105.8
OR13G1	116.2	112.9	117.3	127.6	105.7	98.7	114.4	110.1
OR13H1	106.5	118.2	121.2	112.1	99.7	94.3	121.5	107.8
OR13J1	112.7	126.7	112.1	111.5	109.6	94.4	115.6	114.7
OR1A1	112.9	123.8	113.6	112.5	117.4	96.3	117.8	119.1
OR1A2	116.2	126.7	121.6	123.7	111.7	101.6	132.3	128.7
OR1B1	113.8	117.7	106.9	118.4	108.4	93.1	112.6	110
OR1C1	114.6	119.7	123.2	115.8	115.1	109	106.2	122.2
OR1D2	119.6	118.5	118.2	117.9	111.1	110	122.1	119.2
OR1D4	116.4	118.7	118.7	121.3	105.8	107.7	121.8	114.4
OR1E1	115.1	114.8	112.3	114.5	108	98.1	123.4	123.5
OR1E2	106.1	112.1	116.6	130.4	104.1	95.3	117.6	104.9
OR1F1	110.4	113	117.9	118.8	104.8	102	114.7	112.6
OR1F2	105.6	113.6	106.4	105.6	103.4	102.7	115.6	114.6
OR1G1	108	112	116.2	113	114.3	101.8	110.3	113.8
OR1I1	105.5	112.6	124.5	121.7	109.9	107.1	115.2	112.8
OR1J1	118.5	117	135.5	127.3	112.6	96.4	122.1	125.4
OR1J2	114.2	120	112.8	127.7	110.1	101.1	129.5	120.3
OR1J4	122	116.7	127.5	129.6	115.8	97.8	118.6	122.2
OR1K1	111.8	119.9	113.9	110.1	109.4	92.9	117.3	122.6
OR1L1	123.6	109	119.8	119.8	105.7	108.5	105.2	115.7
OR1L3	110.5	108.8	110.3	120	96.8	96.2	109.8	110.9
OR1L4	115.8	111.2	125.8	119.3	102.2	94.6	126.3	119.1
OR1L6	110.1	113.6	121.2	120.3	115.2	101.7	116.3	114.8
OR1L8	117.4	120.1	114.4	117.4	113.2	106	122.6	117.4
OR1M1	114.9	117.7	113.2	108.7	106	100	120.1	113.8
OR1N1	111.5	121.4	114.6	113.7	107.1	100.3	120	115.8
OR1N2	120.2	115.7	115.4	130	109.6	106.2	125.9	118.9
OR1Q1	101.9	105.7	110.3	102.4	106.7	97	111.7	106.9
OR1S1	104.4	116.4	115.1	110.6	105.7	92.2	119.1	114.4
OR1S2	113.6	117.7	119.7	118.7	112.7	99.3	119.4	120.3
OR2A1	133.9	134.5	135	138.1	124.6	101.7	133	129.2
OR2A12	112.1	111.3	128.6	112.2	104.2	99.1	111.3	110.7
OR2A14	122.7	113.2	111	122.2	115.5	106	129.3	110
OR2A2	123.7	126.5	119.2	116.4	114.5	97.2	114.9	129
OR2A20P	118	113.4	114.8	124.3	114.4	100.4	113.4	112.7
OR2A25	112.7	119.7	112.7	117.2	108.8	95.4	125	118.4
OR2A42	123.4	120.7	117.8	127.4	115.4	100.8	125.4	121.4
OR2A5	115.2	127.3	125.6	125.9	111.5	103.1	124.1	113.5
OR2A7	123.3	122.8	122.4	123.9	109	104.1	126.1	113.8
OR2A9P	189.3	182.6	168.5	174.8	169.2	147.2	168.1	198.7
OR2AE1	112.2	120.9	108.9	116	108.6	101.5	131.8	113.4
OR2AG1	115.8	119.4	119.8	124.2	109.1	103.7	120	116.5
OR2AG2	123.6	120.9	113.7	121.2	106.1	95	117.9	126
OR2AK2	113.8	124.4	113.4	124	127.4	108.2	115	114.9
OR2AT4	118.2	115.3	116	116.2	109	105.2	118.4	117.5
OR2B11	109.3	110.6	111.3	111	105.7	100.9	120.3	123
OR2B2	111.1	120.8	113.7	112.2	113.5	99.6	129	113.2
OR2B3	111.4	118.6	114.9	116.3	105.8	95.9	113.7	106.6

OR2B6	114	117.4	120.2	108.7	107.5	100.9	109.8	109.8
OR2C1	116.3	117.4	124.6	125.9	111.6	95.7	115.6	127.2
OR2C3	112.6	103.9	112.3	116.9	104.7	99.4	109.6	112
OR2D2	117.4	111	111.2	116	103.1	100.7	114.4	113.6
OR2D3	116.6	120.4	114.3	117.4	108.1	105.2	112.4	108.6
OR2F1	105.7	111.4	111.1	127.4	103.3	100.4	119.3	107.7
OR2F2	117.6	117.6	115.8	125.6	103.3	98.9	114.6	115.3
OR2G2	113.2	118	112.2	106.5	97.7	97.5	116.7	109.5
OR2G3	115.1	110.1	124.4	126.5	118	103.1	119.6	113.7
OR2G6	109.1	112.6	103.4	106	102.3	96.1	110.1	106
OR2H1	116.5	109.3	110.9	116.6	106.5	106.5	116.6	110.9
OR2H2	126.6	124.6	131	136.1	129.4	112.7	137.9	132.9
OR2J2	112.6	106.7	109.6	114.8	99.3	97	118.9	117.1
OR2J3	123	112.3	118.8	114.1	113	95.6	119.1	112.6
OR2K2	111.4	110.8	98.6	117.2	107.1	95.6	115	107.9
OR2L13	113.6	117	113.5	114.4	106.5	94.5	110.8	116.4
OR2L1P	108.4	105.2	116.1	111.4	95	98	118.4	107.8
OR2L2	111.2	112.3	105.7	111.8	102.8	91.9	110.1	109.2
OR2L3	119.7	117.3	127.8	127.7	114.7	117.8	122.1	124.7
OR2L8	126.6	124.3	121.2	130.2	116.7	100.3	124.4	116.4
OR2M1P	118.8	111.1	130.2	113.1	110.5	94.1	115.6	108
OR2M2	112.4	123.8	114.7	115.2	107.3	101.1	119.3	119.7
OR2M3	116.5	123.1	117.8	120.4	104.4	100.6	122.4	115.5
OR2M4	112.1	109.4	116	123.5	107.6	94.3	117.4	111.1
OR2M5	116.8	120.3	120.7	123.4	108.7	98.7	117	123.6
OR2M7	110.8	112.8	114.6	111.8	105.7	93.1	116.1	112.5
OR2S2	113.1	123	121.6	117.2	110.8	90.5	124.6	116.7
OR2T1	134.6	120.1	130.8	143	124.4	114.6	122.8	126.8
OR2T10	117.4	120.9	124	120.8	102.9	101	125.5	119.5
OR2T11	112.9	114.5	121.8	129.4	118.3	110.8	126	125.7
OR2T12	120.9	111.9	115.3	113.6	105.7	97	114.1	114
OR2T27	104.4	123.2	112	110.1	108.1	100	106.8	107.7
OR2T3	118.5	105	123.7	110.9	110	92.2	130.4	108.6
OR2T33	123.4	117.7	119.8	132.5	113.2	104.3	119	110.7
OR2T35	114	111.5	115	114.9	115.4	98.6	114.6	119.3
OR2T4	111.6	110.8	116.8	120.5	104.2	95	108.7	115.6
OR2T5	116.5	111.6	122.7	120.9	108.1	96.5	114.9	114
OR2T6	122.7	121.9	116.9	126.5	113	100.2	123.5	117.5
OR2T8	116	115.1	130.3	120.3	103.1	105.1	126.3	114.1
OR2V2	115.6	119	115.9	111.7	115.7	100.3	110.5	115.2
OR2W1	107.9	111.3	111.9	125.6	105.6	94.9	113.9	111.8
OR2W3	118.2	124.1	109.6	120.7	107.3	96.6	110.3	115
OR2W5	127.7	119.6	124.9	129.9	120.8	102.8	126.3	122.4
OR2Y1	107.6	107	111.8	112.8	101.1	100	121.5	114.9
OR2Z1	114.8	117.2	121.3	116	107.3	101.8	121.3	115
OR3A1	119.1	113.4	122.1	116.7	113	109.1	119.2	114.6
OR3A2	114.3	119.6	117.6	120.3	117.5	108.6	129.4	114.7
OR3A3	115.1	107.5	109.4	108.9	105.7	88.3	116.8	108.9
OR3A4	118	118.4	119.2	130.5	120.6	109.3	131.5	129.8
OR4A15	122.8	112.7	126	126.3	115.5	106.5	126.2	123.3
OR4A16	111.6	118.5	114.9	117.9	112.2	93.3	119.5	111.3

OR4A47	110.9	121.1	115.1	113.7	106.7	98.8	120.7	118.8
OR4A5	102.8	105	108.6	112.7	109.7	93.5	116.4	111.5
OR4B1	118.5	113.9	125.6	122.8	112	98.3	122.8	120.1
OR4C11	116.2	116.5	115.2	118.1	110.5	98.9	119.2	110.8
OR4C12	111.8	113.5	115.2	118.5	115	100.2	125.4	110.9
OR4C13	111.5	124.3	112.7	121.9	107	101.6	115.8	117.1
OR4C15	109.8	120.5	119.7	118	115.6	99.8	114.2	123.9
OR4C16	125.5	120	124.1	120.2	111.8	105.9	114.8	122.5
OR4C3	114.5	114.2	113.4	114.2	100.2	99.5	119.4	114
OR4C45	130.3	121.7	118.1	123.3	110.7	100.3	122	122.7
OR4C46	116.5	115.9	112.1	120.2	100.4	100.2	126.5	106
OR4C6	113.7	114.4	124.9	108.1	112.8	97.9	121.4	110.5
OR4D1	101.1	112.9	110.4	117.6	111.2	110.3	125.2	118.5
OR4D10	110.1	111.8	110	118.6	106.8	103.4	117.2	121.5
OR4D11	120.2	104.8	112.2	121.4	112.9	105.6	131.4	122.8
OR4D2	113.7	116.1	118.4	124.7	108.3	96.6	117.9	117.2
OR4D5	118.7	119.1	114.7	124.4	118.9	113.7	120	115.7
OR4D6	110.2	122.6	119.7	114.1	106.6	108.7	120.1	115.5
OR4D9	113.4	119.4	105.1	114.9	100.9	89.5	115	113.4
OR4E2	110.9	121.1	110.6	118	110.5	95.9	117.2	115.9
OR4F15	112.8	118.7	107.9	121.6	110.8	102	124	103.4
OR4F21	111.4	119.9	118.3	120.9	120	101.7	121.1	114
OR4F3	108.5	122.4	119.5	118.4	108.2	99.5	111.5	114.9
OR4F4	121.8	123.8	126.5	126.1	120.7	106.3	124.9	116.8
OR4F5	116.3	119	116.4	113.8	108.7	101.2	129.9	120.5
OR4F6	115.7	112.7	116.6	117.5	106.7	100.9	115.6	109.1
OR4K1	120	119.8	117.5	116.9	107.9	98.8	129.2	110.7
OR4K13	107.5	104.8	111.2	121.7	103.6	104.3	122.9	111.5
OR4K14	109.2	118.6	117.7	118.3	102.9	99.1	115.8	113
OR4K15	116.1	115	116.4	113.4	108.6	103.6	124	118.7
OR4K17	108.9	118	126.8	110.6	117	102	122.7	109.1
OR4K2	109.5	111.2	122.7	115	104.9	102.7	115.6	117.2
OR4K5	113.7	119.3	117.8	117.3	109.2	103.1	111.9	112.9
OR4L1	111.8	116	114	119.1	109.6	102	119.9	116.2
OR4M1	115.1	109	113.7	126.2	102.6	94.1	120	119.2
OR4M2	109.6	110.6	114.1	115.9	109.4	105.7	124.5	122
OR4N2	118.6	111.1	116.6	128	115.1	102	119	123.1
OR4N4	117.3	116	115.9	114.2	101.4	100.1	114.5	118.3
OR4N5	119.8	114.1	122.9	112	106	93.4	125.5	111.1
OR4P4	109.2	113.1	116	121.5	107.5	97.4	113.1	113.5
OR4Q3	114.2	107.6	110.5	114.9	103	97.1	114.4	107.8
OR4S1	120	121.2	112.8	135.4	105.2	105.7	126.4	118.3
OR4S2	112.8	117.3	117.1	120.8	112.2	102.2	111.4	117.3
OR4X1	120.1	119.3	113.6	125.7	107.6	101.4	117.6	119.8
OR4X2	108.2	112.1	108.8	121.9	102.7	92.8	113.3	107.1
OR51A2	111.1	112.7	115.3	117.1	101.6	102.3	113.8	126
OR51A4	102.2	111.5	114.7	110.7	103.5	97.8	121	116
OR51A7	116.7	110.9	117.7	121	100.2	102.6	127.1	117.2
OR51B2	121.7	110.3	110.6	112.5	114	102.9	122.5	118.2
OR51B4	118	112.7	115.2	112.5	103	95.8	121.1	113.8
OR51B5	116.3	118.9	119.1	118.7	111.6	97.3	119.9	108.6

OR51B6	115	120	123.4	122.2	112.1	100.4	119.5	115.6
OR51D1	118.4	124.5	122.6	115.3	110.6	109.3	118.1	112.4
OR51E1	112.3	114.3	113.5	118.3	107.2	96.4	105.5	104.9
OR51E2	130.5	122.8	123.9	120.8	115.1	100.4	116.3	111.4
OR51F1	111.6	111.3	104.9	105.3	103.6	97.9	114	112.7
OR51F2	116.8	110.1	117.3	112.6	105.8	96.5	114.2	115.4
OR51G1	118.3	116.2	121.9	115.6	113.6	101.7	115.2	119.1
OR51G2	114	120	118.8	116.2	112.3	102.5	117.5	120.2
OR51I1	112.7	110	115.8	112.5	101.9	95.8	114.4	114.9
OR51I2	108.4	115.1	114.8	117.6	105.4	99.1	117.2	115.7
OR51L1	104.8	111.7	114.7	119.4	105.4	98.9	110.8	109.5
OR51M1	117.3	110.6	116.5	120.5	110.5	99.2	116.4	118.8
OR51Q1	115.8	114.8	124.1	118	101	107	115	117.2
OR51S1	116.5	107.9	109.6	121.7	103.4	102.6	119.8	115.9
OR51T1	124.5	122	127.7	115	115.3	105.6	130.8	135.4
OR51V1	108.2	109.4	109.3	116.8	98.4	92	104.6	108.7
OR52A1	105.9	108	116.1	121.9	106	97.4	115.5	123.3
OR52A4	117.8	110.7	130.1	118.5	108.3	96.3	109.6	111.6
OR52A5	118.5	126.5	124.6	123.2	110.1	101.5	126.9	121.8
OR52B2	119.8	107.8	122.7	115.1	109.3	100.6	113.9	113.1
OR52B4	113.3	109.4	118.3	117.2	113.4	97.6	117.8	108.1
OR52B6	116	123.5	122.5	114.9	109.3	105	121.7	121
OR52D1	114.3	115.1	131.9	119.3	109.7	106.1	120.8	118.6
OR52E2	117.5	112	112.4	112.8	100.9	96	120.1	111.5
OR52E4	108.7	122	114	115.4	118.4	113.8	126.7	114.5
OR52E6	120.3	116.8	116.2	118.6	114.2	97.1	118.5	117.9
OR52E8	114.6	113.7	109	111.4	109	95.5	114.4	112
OR52H1	112.6	111.9	115.5	121.1	109.6	100	116.6	113.2
OR52I1	111.3	114.3	109.4	111.5	110.2	99.8	117.2	110.3
OR52I2	116	116.2	119.7	127.8	106.2	103.3	120	103.5
OR52J3	110.1	122.2	117.4	117	110.4	100.8	118.3	121.5
OR52K1	122.3	127.3	117.6	133.3	117.8	110.7	129.8	120.5
OR52K2	116.7	116.5	115.4	115.8	107.4	101.2	118.5	127.6
OR52L1	114.7	115.7	109.7	120	109.1	96.2	122.2	110.9
OR52M1	109.9	121.8	114.6	120.7	111.8	105.4	123.8	111.7
OR52N1	124.3	126.6	118.7	116.4	113.8	97.8	121.7	113.3
OR52N2	130.4	118.2	133.3	125.2	118.9	106.6	130.2	121.6
OR52N4	111.2	115.2	109.5	110.8	104.8	101	109.4	111.9
OR52N5	119	116.6	130.4	118.2	115.5	113.3	118.2	117.7
OR52R1	117.3	109.4	125.9	122.2	108.1	105.5	129.5	116.7
OR52W1	115	111	124.1	111.3	108.8	96.7	114.6	119.6
OR56A1	111.4	122.6	114.3	111.4	104.1	98.6	119.7	113
OR56A3	115.6	116.6	108.3	112	104.3	92.2	113.2	112.4
OR56A4	113.4	113	114.2	109.6	102.9	94.4	104.6	110.1
OR56B1	117.5	121.5	128.2	126.3	124.2	101.7	121.4	132.1
OR56B4	116.4	121.6	130.9	120.9	106	105.6	117.2	120.6
OR5A1	109.3	126.1	117.1	110.4	114.4	97.1	116.4	107.8
OR5A2	115.5	113.7	118.1	116	103.9	94.2	120.5	108.8
OR5AC2	112.9	109.6	126	111.7	104.6	96.5	114.7	114.9
OR5AK2	114.1	117.6	118.6	118	104.9	99.2	110.2	104
OR5AN1	116.4	113.4	109.7	116.8	107.3	102.2	112.3	112.2

OR5AP2	114	127.1	119.3	122.4	105.2	103.2	111.5	122.4
OR5AR1	124	109.8	119.4	123.6	104.3	103.2	122.9	116
OR5AS1	121.1	110.8	111.7	115.9	114.3	100.6	117.9	116.5
OR5AT1	119.7	109.5	127.8	117.1	111.4	98.7	118.1	113.7
OR5AU1	109.2	124.1	116.8	122.3	110	96.6	115.2	114.7
OR5AY1	116.7	107.7	114.4	115.1	104.1	96.5	107.3	111.9
OR5B12	120.8	113.7	122.7	121.5	107.8	101.7	115.9	114.5
OR5B17	106.5	122.3	114	111.8	97.9	100.3	118.7	121.1
OR5B2	118.4	121.9	123.4	124.2	109.1	98.9	116.9	123.9
OR5B21	110.8	113.2	117.7	119.5	121.1	103	125.1	115.3
OR5B3	116	118.6	118.4	121.6	107.8	107.4	105.7	110.5
OR5BF1	119.1	120.7	121.5	118.5	108.3	101.1	114.1	109.3
OR5BU1	111.8	115	111.7	123.7	114.8	97.7	117.2	114.2
OR5C1	114.3	113.9	119.6	114.6	108.7	96.4	125.8	113.9
OR5D13	112.1	108.6	114.6	111.6	108.3	96.2	120	105.3
OR5D14	111	124.9	118.7	124	103.4	105.5	124.1	111.4
OR5D16	110.4	120.9	117.9	116.8	119.3	107.5	126.9	124.5
OR5D18	107.6	112.9	111.4	114.5	103.4	92.3	104.2	110.5
OR5F1	116.8	115.6	120.7	115	104.3	105.2	111	114
OR5H1	117	114.3	113.1	116.9	112.6	107.8	112.8	120.6
OR5H14	105.8	115.7	115.7	101.8	101	91.2	114.4	104.5
OR5H15	110.9	115.4	116.6	116.1	98.3	103.2	121.5	112
OR5H2	109.7	109.5	112.6	113.3	102.3	91.9	113.3	108
OR5H6	115	120.7	116.5	112.2	115.5	90.5	117.9	117.1
OR5I1	118.6	115.6	113.1	115.7	101.9	95.6	117.5	115.2
OR5J2	111.8	114.7	120.2	114.3	109.4	104.2	113.4	109.8
OR5K1	109.2	107.2	111.2	114.8	108.5	101.5	119.8	120.5
OR5K2	112.9	122.2	131.8	120.5	108.7	106.5	113.7	113.7
OR5K3	110.8	114.7	112.6	112.5	105.1	102.6	107.6	107.6
OR5K4	111.7	125.8	128.6	129.6	108.9	104.2	111.4	120.2
OR5L1	121.3	122.4	119.6	123.8	111.6	99.6	113.4	122.9
OR5L2	118.5	115.9	122.2	115.9	107.3	92.2	118.3	115
OR5M10	111.4	117.8	119.8	121.1	113.4	103.8	118.5	109.5
OR5M11	110.4	114.8	108.6	119.5	105.3	99	112.5	114.4
OR5M3	115.4	108.4	121.4	123.6	111.6	101.5	112.2	118.2
OR5M8	122.5	118.3	115.5	117.6	105.4	97.2	122.9	120.9
OR5M9	118.8	119.9	112.5	121	107	102.8	125.3	110.1
OR5P2	104.8	107.6	110.2	107.8	102.3	93.3	110.7	111.6
OR5P3	121.4	120.4	118.5	118.3	116.6	101.1	125.6	109.7
OR5R1	116.8	118.3	120	115.3	116.1	100.4	128.7	119.5
OR5T1	123.8	118.7	118.9	111.9	113.9	99.1	117.9	116.6
OR5T2	107.6	109	106.5	120.1	105.7	88.9	110.3	102.3
OR5T3	113.1	113.7	110.2	125.7	105.2	96	124.3	116.6
OR5U1	115.7	114.8	114.7	108.9	117.3	96.5	122.9	112.5
OR5V1	114.5	128	112	121.2	109.9	99	108.8	118.3
OR5W2	115.9	126.5	123.4	114.6	108.1	103.6	114.9	113.6
OR6A2	125.5	114.9	131.7	120.6	106	101.2	115.4	117.8
OR6B1	115.6	118.2	117.3	117.9	105.9	90.8	112.4	118.3
OR6B2	113.5	123	120.4	127.8	112.2	105.1	115.8	122.2
OR6B3	115.1	112.8	123.6	121.4	105.2	103.7	114.5	116.4
OR6C1	112.4	119.5	125.3	118.3	110.5	101.4	117.5	118

OR6C2	108.2	114	115.9	109	106.9	98.4	120.2	111.7
OR6C3	113.7	114.4	112.6	129.2	106.3	98.5	110.4	107.7
OR6C4	117.8	113.3	120.9	117.5	102.1	101	115.7	112.2
OR6C6	107	119.5	104.8	114.7	102.8	98.8	112	111.5
OR6C65	113.9	113.9	111.2	113.6	100.5	95	114.5	114.7
OR6C68	117.6	113.4	112.3	123.4	102.1	96.6	105.1	111.1
OR6C70	104	119.3	104.8	101.7	111.7	98	114.3	112.7
OR6C74	112.3	110.1	121.8	107.5	105	92.4	109.6	109.9
OR6C75	111.9	115.1	116.7	111.6	104.8	93	108	114.4
OR6C76	102.4	110.1	105.2	119.8	117.8	99.1	113.6	105.7
OR6F1	115.6	117.9	127.9	115.8	112	98.5	127.4	123.1
OR6K2	115.7	127.6	126.1	104.3	110.3	95.8	131.2	114.4
OR6K3	119.4	120	118.5	111	113.9	101.1	117.1	112.9
OR6K6	120	104	120.9	114.9	100.3	94.7	117.3	112.7
OR6M1	128.1	109.7	114.2	122	113.7	101.5	124.7	113.3
OR6N1	117.2	113.4	118.5	121.9	111.8	101.5	118.4	125
OR6N2	114.9	107.8	124.6	123.1	124.1	108.8	124.6	124.6
OR6Q1	117.8	115.6	111.4	122.4	108.3	93.9	114.8	120.3
OR6S1	106.9	109.4	115.4	118.8	100	106.9	112.8	113.3
OR6T1	107.8	107.2	112.2	111.6	106.2	95.4	111.7	104
OR6V1	121.1	126.9	132.8	118.5	115.1	107.8	120.4	119.4
OR6W1P	115.9	131.2	133.3	115.4	112.5	105.4	123.6	124.4
OR6X1	120.4	112.2	121.8	122.1	99.4	98.8	125.3	115.9
OR6Y1	108.5	116.6	110.1	123.3	114.9	98.8	120.9	113.5
OR7A10	114.6	117.6	117.2	120.3	104.4	98.8	119.7	117.3
OR7A17	124.2	123.5	142.9	125.2	127	107.3	127.1	121.2
OR7A5	114.7	115.9	116	116.1	100.6	101.7	117	113.2
OR7C1	112.8	118.7	120.1	115	107.3	95.9	118.1	118.8
OR7C2	117.5	119	118.4	116.3	110.5	105.3	116.8	109.5
OR7D2	143.5	119.3	138.2	132.8	108.6	106.7	130.4	111.7
OR7D4	111.9	108.1	111.6	116.5	104.1	102.6	116.9	109.2
OR7E156P	128.5	134.7	139.5	140.5	120.1	106.6	131.4	132.9
OR7E24	117.3	119.7	128.6	121.2	106.4	100.2	122.1	118.9
OR7E37P	113.5	114.6	109.6	116	106.2	99.2	112.5	115.2
OR7E91P	117.8	113.3	111.1	115.5	107.7	96.7	123.7	129.4
OR7G1	113.7	125.1	124.7	124.1	117.9	105.4	108.1	117.8
OR7G2	121.7	115.8	128.3	129.4	124.1	128.1	121.2	127.2
OR7G3	119	122.7	130.6	124.5	113.1	106.6	130.3	119.8
OR8A1	114.1	113.8	112.8	115.7	114.9	104.4	118.8	115
OR8B12	130.3	121.4	137.2	128.1	109.1	105.6	124.6	121.5
OR8B2	114.4	117.3	118.1	122.4	119.1	102	123.9	121.5
OR8B3	111.2	115.4	113.4	118.2	111.1	99	120.1	112.8
OR8B4	113.5	114.6	124	120.5	120.7	98.4	125.4	110.8
OR8B8	108.5	119	113.4	121	108	103.7	113.5	111.5
OR8D1	121.8	119.5	113.6	124	111.2	99.6	125.5	113.3
OR8D2	118.1	110.5	113.8	121.4	109.2	101.2	126.4	115
OR8D4	121.9	109	118.9	120.2	111.5	92.4	115.8	110.5
OR8G1	116.6	122.1	124.3	119.8	116.8	105.9	113.9	119.8
OR8G2	114	126.8	130.4	121	105.2	108.6	121.4	118.7
OR8G5	116	117.6	119.7	114.4	106.6	97.7	115.6	109
OR8H1	113.7	111.5	113.1	125.5	111.4	97	126.6	124

OR8H2	113.4	108.8	109.9	114.9	98.2	93.9	114.6	115.5
OR8H3	115	117.7	141.1	118.7	113.1	110.3	120	118
OR8I2	120	107.3	105.2	110.3	98.2	100.3	107	107.9
OR8J1	112.4	114.6	113.1	108.7	103.9	97	117.2	113.5
OR8J3	110.5	120.1	114.9	119.3	100.8	99.8	114.3	113.5
OR8K1	107	104	112	113.4	108.5	101.4	111	114.3
OR8K3	117.1	118.4	137.2	117.7	113.3	107.3	113.6	118.5
OR8K5	117.9	119.8	116.7	123.5	113.6	94.2	115	114.1
OR8S1	112	106.7	117.2	122.6	110.7	99.8	110.3	112.6
OR8U1	122.1	111.5	117.4	122.4	105.7	101.9	115.6	117.4
OR8U8	113.4	114.4	110.4	116.8	103.5	93.3	114.6	109.2
OR8U9	102.5	114	111.2	112.4	109.9	91	105.4	112.6
OR9A2	113.5	107.6	121.6	118	105.1	96.9	124.7	118.2
OR9A4	113.2	113.8	143.1	120.2	105.7	99.7	123.7	111.4
OR9G1	112	122.5	122.1	112	111	99.6	125.6	119.1
OR9G4	109.9	114.4	121.4	123	104.8	101	113.4	107
OR9G9	112.6	113.2	115.6	119	108.7	91.4	110.7	114.8
OR9I1	115	124.2	113.4	120.3	112.8	105.7	115.3	122.7
OR9K2	118.4	107.4	111.3	117	102.7	97	118.1	112.8
OR9Q1	107.9	110.9	107.8	111.9	97.6	97.1	104.5	113.3
OR9Q2	111.7	115.3	119.4	112.3	107.6	100.8	115.2	116.2
ORAOV1	315.8	339.9	319.5	315.5	332.2	186.9	322.4	346.5
ORC1L	108.5	117.8	120.2	125.1	119.5	99.2	128.4	125.1
ORC2L	342.9	430.4	336.3	365.6	327.1	237.4	359.9	400.6
ORC3L	192.8	172.8	221.2	200.8	167.6	221.6	184.9	183.4
ORC4L	133.3	130.9	123	147.5	128.8	117.2	144.7	137.2
ORC5L	273.1	321.6	285.6	319.5	286.5	236	283.7	278.4
ORC5L	111.9	121.8	112.5	122	118.4	95.8	127.7	126.2
ORC5L	122.1	114.1	124.7	123.6	105.6	100.8	110.2	117.4
ORC6L	8893.3	8168.7	7169.8	6445.5	7790.6	4695.2	7198.3	8495.9
ORF1-FL49	182.2	158.5	140.6	173.9	130.2	139.7	183.3	225.3
ORM1	115.5	129.4	124.1	125.3	115.2	104	117.2	112.7
ORM2	117.6	122.6	118.7	119.5	113.3	94	116.5	123
ORMDL1	136.1	123.6	124.9	133.2	123.5	113.6	127.1	119.8
ORMDL2	138.1	119.5	130.8	125	116.6	108.7	135.9	129.8
ORMDL3	179.3	204.6	154.5	186.2	187.1	155.1	167.7	157.3
OS9	134.8	141.6	170.3	146.1	129.5	151.6	158.4	158.5
OSAP	112.4	119.9	118.3	119.9	116.3	106	123	115.8
OSBP	764.1	875.3	725.8	962.2	690.1	661.3	946.7	1296.7
OSBP2	112.2	106.7	114.9	116.4	109.9	100.7	120.1	115.3
OSBPL10	193.1	170.9	152.6	204.7	157.7	127.2	169.4	140.5
OSBPL11	147	153.9	165.4	163.1	136.9	136.8	159.4	151.4
OSBPL1A	121.3	126.3	121.3	124.5	109.8	107.5	126.7	116.3
OSBPL1A	124.2	119.1	126.8	118.5	123.9	103.3	118.7	117
OSBPL1A	132.5	125.6	147.1	129	115.5	110.3	129.5	124.8
OSBPL2	130.8	121.1	114.8	130.2	113.1	103.6	120.1	134.1
OSBPL2	226.5	249.2	188.2	204.5	189.4	158.2	228.7	196.6
OSBPL3	134	126.1	121	128	113.4	100.1	132	121.9
OSBPL3	134.5	157.7	163.1	164.1	185	120.2	184.4	179.2
OSBPL5	220.6	219.7	217.4	242	166.9	166.6	222.4	182.2
OSBPL5	254.9	248.5	243.2	267	168.6	170.8	217.3	185.3

OSBPL6	134.5	142.2	144.3	136.3	134.6	117	141	154.5
OSBPL6	120.7	116.2	109.5	115.5	108	95.9	114.1	115.7
OSBPL7	337.3	255.7	289.1	264.9	206.5	237.1	235.6	198.1
OSBPL7	130.9	128	122	125	113.7	107.6	134.8	121.8
OSBPL8	272.7	254.6	253.3	379.5	205.2	168	290.2	344.5
OSBPL8	222.7	316.4	221.6	195.8	261.9	204.5	248	322.2
OSBPL9	105.4	113.4	109.2	122	112.8	94.6	128.9	121.5
OSBPL9	396.7	459.2	447	491.8	471.1	344.7	388.1	433.8
OSCAR	131.4	134.3	191.6	132.6	107.8	150.9	126.4	118.2
OSCAR	215	164	319.7	169.8	128.4	178.8	145.3	135.2
OSGEP	160	167.1	173.9	155.6	159.1	115.3	135.3	138.1
OSGEPL1	169.5	162.9	196.8	177.9	167.2	158.7	142.9	123.9
OSGIN1	119.1	117.6	121.3	128.8	110.7	108.9	139.8	137.3
OSGIN1	116.5	109	115.3	112.6	109.5	100	120.4	113.3
OSM	115.2	121.6	123.5	125.1	116.6	108.7	132.9	126.4
OSMR	112	116.8	107.2	105.7	111.9	96.6	113.4	113.4
OSR1	111.6	108.6	112	108.4	109	93.3	112.4	100.5
OSR2	118.8	122.9	127.2	117.8	111.6	95.4	131.1	115.5
OSR2	115.3	112.9	117.5	116.7	108.3	109.4	128.1	117.6
OSRF	161	152.8	156.5	156.8	156.8	155	173.6	189.8
OSTALPHA	108	124.7	118.4	110.1	109.5	100.7	107.1	111.9
OSTBETA	116.3	120.1	115.1	116.6	102.9	95.6	119.9	115.7
OSTF1	933.6	918.3	1457.9	1002.5	869.8	1002.3	921.7	531
OSTM1	178.3	189.5	198.8	226.6	150.1	164.5	200.1	178.5
OSTN	112.2	108.9	109.4	117	106	102.3	113.4	114.3
OTC	106.7	109.3	115.7	112.9	115.3	105.5	118.9	107.5
OTEX	117.2	120.2	101.6	122.3	109.3	98.7	117.4	112.2
OTOA	110.7	111.1	110.2	122.7	104.8	94.4	118	115.6
OTOA	118.2	116.8	115.4	126.4	105.4	97	117.9	120
OTOA	118.9	121	117.4	112.2	112.2	100.9	118.1	121.1
OTOF	106.7	111.8	114.6	112.1	108.7	95.3	107.8	119.7
OTOF	121.9	121.2	129	138.6	103.6	104.1	119.1	125.4
OTOF	113.3	103.9	120.6	124.4	110	95	114.7	112.6
OTOP1	113.1	111.5	122.2	122.5	109.5	101.5	116.1	119.8
OTOP2	112.4	107.7	113.4	110	113.2	98.5	112.2	122.8
OTOP3	118.4	116.5	119.9	120	109.1	98.9	111.5	105.9
OTOR	118.5	109.7	124.8	115.7	96.4	90	128.8	109.4
OTOS	107.5	112	114.2	112.3	101.6	93.9	110.9	111.7
OTP	128	119	125.5	144.3	119.4	111.8	117	107.6
OTUB1	118.9	120.3	118.4	123	115.3	110	122.8	116.2
OTUB1	137.4	130.7	128.5	136.5	118.2	121.9	143.4	140.4
OTUB1	161.7	170.7	188.8	172.2	144.8	135.8	172.3	178.4
OTUB2	125.9	118	124	125.9	111.9	101.7	120.6	119.9
OTUD4	258.6	219.5	240.4	279	209.3	198.2	226.7	225.4
OTUD4	112	120.1	122.9	114.7	105.3	101	116.4	110.9
OTUD4	113.3	122.1	129.9	112.4	112.5	100.6	129.8	127.2
OTUD5	129.4	128.4	136.5	126.9	121.3	119.7	152.4	157.6
OTUD6A	106	116	122.5	111.6	99.3	101.7	120.6	120.3
OTUD6B	177.4	169.5	198	154.6	143.2	127.6	159.2	142.4
OTUD7	110.7	109.2	112.9	119.6	110.2	103.1	108	112.8
OTUD7A	122.1	123	122.5	133.7	108.8	109.1	115.4	112.7

OTX1	109.6	117.1	105.4	124.5	107.2	98.2	113	114
OTX2	120.8	116.2	116.5	114.2	107.8	93.7	121.5	120.4
OTX2	120.9	118.8	115.4	120.5	110.1	98.1	118.3	113.3
OTX2	123.2	117.4	124.2	117.1	115.9	100.9	122.3	116.3
OVCA2	128.9	128.2	118.8	134	118.9	104.6	131.7	132.2
OVCH1	113.5	121.9	123	104.2	110.9	101.3	117.9	105.9
OVCH2	117.7	116.2	110.6	114.7	115	105.9	123.2	118
OVGP1	167.2	192.5	200.3	194.2	185.1	147.7	191.6	218.1
OVOL1	111.2	114.5	110.8	109.2	108.5	96.8	104.8	109
OVOL2	122.3	118.4	125.1	117.7	102.2	97.2	118.4	112.7
OVOS2	111	111.4	111.6	117.7	104	95.5	120.5	102.2
OVOS2	131	124.5	125.9	129.6	124.2	104.5	126.3	123.6
OXA1L	484.6	589.6	485.8	503.9	397.2	377.7	352.3	343.7
OXCT1	130.2	154.6	154.3	147.8	129.2	124.7	158.1	159.3
OXCT2	115.8	129.6	121.4	120.3	107	104.5	119.6	123.7
OXER1	120.4	122.3	121.3	124	102.6	103.6	110.1	112.3
OXGR1	111	115.2	108.4	114.3	107.4	93.1	117.1	115.6
OXR1	835.1	725.2	807.8	835.7	570.6	585	715.4	666.9
OXSM	128.6	125.8	127.1	128.1	115.8	99.2	124.4	124.4
OXSR1	617.1	609	497.6	713.9	651.6	490	787.1	756.8
OXT	129	124.6	120.6	123.1	101.3	104.9	119.9	119.5
OXTR	131.5	136.1	126.8	128.3	123.1	113.8	129.4	125.9
P11	120.4	118.7	118.8	118.7	106	109.1	124.3	112.7
P15RS	365.7	438.8	340.5	423.4	343.5	343.4	346.1	301.3
P18SRP	112.6	116	113.7	115.7	120.6	95	123.1	113.9
P2RX1	137.9	119.5	144.4	135.9	112.6	110	129.4	117.2
P2RX2	114.1	120.4	113.6	119.9	106.6	97.7	119.1	112.6
P2RX2	108	110.2	112.4	113.3	106.1	94.7	109.3	107.8
P2RX2	121.4	112.8	116.5	118	116.5	97.6	119	127.2
P2RX2	122.1	130.1	140.7	133	127.3	113.8	133.9	117.6
P2RX3	115	120.4	123.4	125.5	106.3	98.4	113.3	114.2
P2RX3	111.8	102.3	107.6	110	107.8	100.9	118.7	110.8
P2RX4	359.6	241.3	342.7	312.2	197.6	277.7	293	193.1
P2RX5	127.6	140.2	126.8	129.4	121.4	106.9	129	113.9
P2RX5	113.4	113.2	119.1	125.2	107.7	100.5	107.6	122.1
P2RX7	301.3	153.9	216.8	214.4	156.2	176.1	209.1	155.6
P2RXL1	113.3	118.5	124.4	114.1	98.3	101.8	114.3	116
P2RY1	119.3	115.3	113.9	118.1	105.5	99.1	114.3	121.6
P2RY10	468.9	543.8	448.3	607.6	420.1	343.7	530.4	664.7
P2RY10	151.1	160.6	195.2	170.4	184.6	161.2	201.7	191.8
P2RY11	218.1	206.2	323.8	229.2	161.8	224.1	208.8	193.7
P2RY13	115.7	122.4	131.6	123.4	110	94.7	116.9	119.3
P2RY13	130	124.5	120.9	125.8	107.9	101.2	122.3	113.7
P2RY13	104.9	108.4	108.8	108	98.5	88.9	110.6	108.1
P2RY14	110.3	110.1	113.2	121.6	103.1	96.6	110.1	116.1
P2RY14	111.7	113.7	116.3	119.5	105.5	94.2	105.7	111.6
P2RY14	111.1	121.1	109.9	116.5	104.8	102.7	112.3	110.6
P2RY2	116	111.9	120.6	126.2	106	105.1	114.4	120.1
P2RY2	120.2	120.8	116.4	130	115.7	105.8	125.1	118.7
P2RY2	116	115.6	131.9	113.8	104.7	103.3	109.1	115.5
P2RY2	115.5	100.4	107.9	122.4	114.2	104.8	122.7	114.5

P2RY4	125.3	111.8	111.3	123.4	108.2	107.7	112.1	108
P2RY5	356.8	376.4	893.9	465.1	296.4	784.1	312.1	279
P2RY6	121.6	116.9	124.8	124.4	112.9	100.6	123.8	119.3
P2RY6	133.1	114	178.8	118.9	102	129	121.8	121.1
P2RY6	118.1	115.2	137.9	135.4	107.4	111.9	131.4	115.6
P2RY8	1763.3	2439.1	1057.3	1778.6	1304.5	830.2	1409.4	966.4
P4HA1	111.1	112	112.7	110.1	108.7	102.5	115.1	107.2
P4HA1	152.2	163.6	141.8	155.3	127	128.2	142.9	200.8
P4HA2	109.6	113.9	112.1	114.3	105.4	100	112.5	117.4
P4HA2	109.8	112.2	115.1	119.9	104.5	102.6	120.3	110
P4HA3	104.4	110.7	127.5	114	105.6	108.4	117	118.5
P518	122.4	119.3	118.3	123.9	112.8	104.5	127.5	117.5
P53AIP1	110.1	113.8	122.9	110.8	103.1	99.7	116.8	107.6
P8	124.2	117.5	133.7	123.4	108	103.7	128	124.4
PA2G4	336	300.4	277.6	314.2	284.7	297.3	377.2	336.2
PA2G4P4	114.4	115	129.5	134.8	113.1	113	122.9	118.8
PABPC1	363.4	324.5	481	440.2	462.6	648.3	744.4	360.3
PABPC3	108.5	117	113.2	112.8	112.5	97.6	117.8	109.1
PABPC4	1132	1109.7	954.4	1386.5	1050.9	1143	1479.7	1274.6
PABPC5	96.1	109.8	119	121.3	95.5	94.3	110.1	113.9
PABPC5	120.5	105	116.4	117.7	110.5	101.1	121.7	116
PABPC5	113.3	110.6	116.5	124.2	103.5	101.4	122.6	115.5
PABPN1	551	615.6	511.6	594.8	519.7	407.7	666.7	595.1
PACRG	116.7	115.9	121.8	121.3	121.9	96.8	111.3	120.3
PACRG	108.8	113.4	123.3	122.5	114.8	102.6	119	109
PACRG	119.5	107.7	118.7	111.6	102.2	99	118.6	116
PACRG	109.1	112.3	114.6	112.8	104	101.2	111.2	112.8
PACS1	284.5	338	248.6	285.8	212.2	241.8	304.7	278
PACS2	165.5	150.5	145.2	144.6	123.3	124.1	156.1	129.6
PACSIN1	146.3	170.4	162.5	146.4	149.1	117.2	143.2	137.6
PACSIN2	749.7	676.4	659.3	735.9	492.7	544.1	635.3	617.1
PACSIN3	118.9	121.3	125.3	127.1	112.7	102.9	130.7	123.3
PADI1	112.5	118.5	127.8	114.6	115.8	93	120.3	124.5
PADI2	118.4	117.3	147.6	142.5	118.2	108.9	130.2	139.1
PADI3	107.8	111.7	117.4	124	110.7	96.4	115.5	117.2
PADI4	139.5	171.3	182.5	153.2	162.5	131.7	128.8	136.9
PADI6	125.2	114.9	111.9	115.5	112.5	115.2	124.8	131.9
PAEP	113.4	116	117.1	115.1	103.5	96.8	116.6	113.3
PAEP	120.3	119.8	114.4	116.3	110.8	105.6	110.9	108.6
PAF1	316.5	373.3	220.4	273.3	238.4	224.4	267.9	290.6
PAFAH1B1	375.1	421.9	340.3	378.2	315.5	377.1	550.5	604.8
PAFAH1B2	117.7	116	115.8	120.7	103.9	109.5	117.9	113.7
PAFAH1B3	467.7	554.4	305.6	388.6	345.1	197.2	311.3	313.2
PAFAH2	233.5	256.5	271.5	249	222.4	272.1	229.2	234.7
PAG1	510.6	635.7	414.3	541.7	573.1	310.5	472.6	614.1
PAGE1	110.2	108.2	118	108.3	113.3	90.1	118.6	114.4
PAGE2	116.8	121	120.1	119	112.4	100.9	115.3	119.7
PAGE2B	114.3	108.6	113.9	117.2	110.4	110.6	120.8	112.7
PAGE3	115.6	115.9	114.5	126	99.6	98.8	115.6	125.3
PAGE4	112.6	113.4	120.1	112.9	104.2	104.8	112.5	105.8
PAGE5	111.4	113.8	119.2	116.8	108.2	101.2	115	120.3

PAGE5	117.8	107.1	118.4	116.7	98.3	97.3	115.7	131.1
PAH	104.4	115.1	121.1	116.4	104.9	98.3	115.2	113.4
PAICS	358.1	402.2	298	433.6	423.1	268.1	513.8	463.3
PAICS	112.8	114.5	112.2	116.5	105.3	102.8	111.7	115.9
PAICS	444.1	559.5	399.1	548	478.7	334.4	511.5	531.2
PAIP1	132	134.7	142.4	136.9	130.4	120.8	137.1	145.6
PAIP1	120.9	123.8	119.4	113.7	105.9	98.5	119.4	120.3
PAIP2	1487.7	1422.7	2066.9	1794.9	1535.3	1679.4	1906.3	2222.7
PAIP2	113.2	114.2	107.7	114.5	108.5	94.6	119.8	110.2
PAIP2	129	129.2	136	138.1	126.1	110.1	130.8	126.7
PAK1	139.7	126	198.4	142.4	122.1	176.7	150.5	138.3
PAK1IP1	120.1	126.5	125.7	126.8	125	105.7	139.9	130.6
PAK2	356.2	343.1	298.8	312.7	267.1	221.5	412.4	380.6
PAK2	303.7	287.4	239.5	366	328	157.3	375.3	395.8
PAK3	117.6	123.4	121.8	114.3	108.1	99.6	115.2	118.8
PAK4	115.1	117.6	116.1	118	109.2	96.3	124.2	132.1
PAK4	119	118.3	108.3	110.4	107.8	101.6	137.7	114.4
PAK4	141.8	136.2	131.1	170.1	114	110.5	140.5	148.4
PAK6	111.4	114.7	110	124.3	100.1	110.2	114.4	124.6
PAK7	112	109.5	109.8	111.9	110.3	99	114.3	119.6
PAK7	116.7	111.4	112.3	117.1	112.3	96.6	116.5	111.2
PALLD	145.3	133.6	143.3	147.5	121.5	119.3	135.2	127.6
PALM	108	102.5	103	120.4	102.8	91.7	107.9	109.3
PALM	124	119.7	121.1	110.4	115.2	102.1	124.9	124.8
PALM	118.6	115.1	131.7	120.5	115.4	106	120.6	117.2
PALM2	110.7	110.4	126.1	114.2	105.5	108.8	128.9	116
PALM2	112.2	123	113	127.8	109.6	98.7	113.4	110.7
PALM2-AK/	113.1	121.1	111.2	124.5	106.8	96.6	116.4	109.1
PALM2-AK/	113.6	113.2	128	115.5	111.3	94	114.9	106.9
PALMD	116.5	126.7	116.8	126.4	122.3	104.5	131.5	123.6
PAM	378.4	404	409.7	416	397	332	391.4	447.8
PAMCI	119.4	110.9	123.1	121.3	108.1	103.4	131.7	115.1
PAN3	739	778.4	708.6	885.9	797.8	606.6	1063.7	920.5
PANK1	120.2	116	114.1	126.5	114.2	97.8	121.9	111.6
PANK1	119.7	148	115.4	132.8	121	98.7	126.9	126.2
PANK2	506	636.8	757.6	623.6	443.7	516.4	661	638.6
PANK2	338.8	337.6	404	321.5	293.7	318.9	343	458.9
PANK2	105.8	115.6	119.8	115	107.6	95	117.3	113.4
PANK3	118.8	121.9	126.8	159.5	112.3	107	126	129
PANK4	252.4	256.7	224.6	248	185.7	175.2	240.8	223.7
PANX1	121.9	130.9	132.6	163.3	119.2	103.3	129.6	124.7
PANX2	112.3	122.6	117.1	127.7	123.9	128.2	147.4	130
PANX3	119.1	119.5	111.2	126.6	119.5	105.6	123.8	120.1
PAOX	135.1	141.5	141.4	135.6	120.7	115.5	124.6	120.9
PAOX	215.5	209.7	224.4	184.6	157.4	139.4	155.9	147
PAOX	115	121.3	148.8	131.6	115.3	117.9	135.2	125.3
PAP2	106.5	115.8	117.9	114.9	107.4	101.8	109.9	114.8
PAP2D	103.6	103	107.5	112.4	104.4	98.4	117.4	108.3
PAPD1	289.9	323.9	339.3	323.2	300.3	211.9	265.1	260.7
PAPD4	554.4	553.5	648.7	642.4	504.3	601.3	585.9	507
PAPD5	199.3	239.4	212.2	227.2	239	136.6	242.5	284.4

PAPLN	113.1	115.1	112.6	124	104.2	101.8	112.5	111.8
PAPOLA	1109.7	1213.3	1100	1223.4	791.2	841.7	1757.9	1569.8
PAPOLB	115.6	109.7	110.9	122.7	111.9	93.4	113.6	111.4
PAPOLG	142.2	126.5	135.3	142.4	137	112.7	143.9	139.4
PAPPA	106.3	109	109	113.8	102.8	92.8	118.5	117.8
PAPPA2	110.5	115.2	115.3	110.6	109.2	99.1	119.7	116.5
PAPPA2	113.6	117.4	120.8	119.1	105.6	93.1	119.7	112.7
PAPSS1	220.8	179	198	237.6	151	153.7	179.1	153.6
PAPSS2	116.5	116	110.2	118.4	114	103.8	111.6	122.3
PAQR3	181.2	179.7	162.3	186.1	162.9	130.2	182.8	160.4
PAQR4	134	124.5	191.2	135.8	114.9	130.2	137.9	116.9
PAQR5	104.4	117.6	115.2	118.5	108.4	98.5	121.5	109.7
PAQR6	125.6	114.4	150.1	136.4	117.6	112.3	121.3	120.3
PAQR6	114.6	118.5	120.6	111.9	114.7	94.8	120.2	111.8
PAQR6	119.6	118.3	117.9	126.6	108.1	96.1	116.6	112
PAQR7	142.8	140.5	122	139	108.7	103.6	140.8	121.8
PAQR8	291.5	270.3	304.2	308.2	270.3	177.8	256	205.4
PAQR9	116.1	117.5	114	116.9	104.8	101.4	119.5	117
PARC	139.3	154.5	154.1	162.1	131.3	126.6	141.7	146.5
PARD3	117.8	117.4	123.8	127.4	117.8	103.7	122.9	113.7
PARD3B	116.1	122.8	120.7	121.3	121.3	105.8	129.1	121
PARD6A	180	181	144.6	171.5	164.6	125.6	146.3	152.2
PARD6A	181.6	168.2	147	166.4	156.2	121.4	147.8	146.3
PARD6B	119.7	110.3	120.2	116.3	109.2	102	125.4	120.2
PARD6G	112.8	114.3	120.7	121.3	109	99.2	109.8	115.4
PARD6G	113.4	108.7	110.8	119.8	104.9	99.2	118	117.2
PARG	137.2	110	136.8	122.5	115.3	106.4	123.9	114.9
PARK2	111.8	110.5	117.4	113.5	102.1	98.2	129.6	119.1
PARK2	112.3	127.4	112.8	119.3	111.6	95.8	122.1	114.5
PARK7	879.1	919.9	1076.7	1093	820.7	625.5	825.6	964
PARL	480.9	522.4	854.3	484.4	488.3	521.7	471.2	395.6
PARL	202.1	222.8	265.6	224.5	205.3	227.3	180.4	175.6
PARN	141	146.2	170.2	175.6	153.4	149.7	142.6	163.9
PARP1	845.9	970.5	895.4	870.6	715.1	639	947.6	905
PARP10	141.4	130.8	140.9	146.1	114.4	123.4	140.6	126.3
PARP10	237.1	250.6	211.1	241.9	167.5	155.3	190.2	180.7
PARP11	160.4	170.6	155	164.2	152.7	148.9	142.2	162.5
PARP12	456.7	448.8	383.9	400.3	281.7	272.9	291.9	269.4
PARP14	291.8	249	219.7	302.7	200.7	205.1	254.2	211.3
PARP15	143.8	134.2	147.6	146.9	133.8	122.3	137.1	136.6
PARP16	139.1	152.1	145.9	152.7	123.6	117.1	142.4	125.3
PARP2	163.5	170.3	174.7	189.1	155.7	175	167.3	159.6
PARP2	117.5	127.6	136.8	152.5	123	116.7	151.4	138.9
PARP3	116.7	112.2	127.7	132.2	108.1	97.6	117.7	131.1
PARP3	184.9	210.1	252.4	217.1	171.6	189.2	176.9	182.3
PARP3	139.8	149.7	144	131	113.7	115	132.9	119.1
PARP4	698.4	853.2	756	810.1	670.1	523.4	609.5	633.1
PARP6	137.9	129.3	123.7	139.8	126.9	126.8	129.1	133.2
PARP6	161.2	175.7	159.4	200.4	146.1	131.2	188.8	218.3
PARP6	126.8	125.2	130.7	119.9	107.6	107.1	124.6	131
PARP8	225.8	260.2	245.4	292	324.2	243.3	309.9	361.5

PARP9	150.2	141.4	163.8	143	117.9	107.1	156.4	138.6
PARS2	116	114.7	107.4	122.1	115.9	108.3	114.7	118.5
PART1	113.6	123.9	117.7	125.3	110.7	107.5	116.2	121
PARVA	117.7	115.3	118.4	108.9	103.4	97.4	113.2	117.4
PARVB	150	137.1	162.1	153.2	127.2	118.4	144.6	126.5
PARVB	131.3	140.2	136.6	143.9	129.3	104.2	136.1	148.7
PARVB	117.6	116.1	118.7	129.2	104.8	111.4	124.1	122.1
PARVG	929.1	810.9	1162	846.2	453.7	592.2	712.4	437.8
PASD1	114.4	101.4	108	121.8	111	98.7	118.9	107.1
PASK	1214.9	2013.6	1206	1227.4	1669.7	981.8	1147.2	1723.1
PATE	119.8	119.4	117.8	118.5	105	101	113.2	119
PATZ1	120.9	128.5	139.4	137.7	124.8	111.3	128.1	121
PAX1	120.4	121.5	117.4	137.2	119.2	106.5	123.2	121.3
PAX2	122.3	124.2	120.4	123.2	106.5	94.9	121.7	118.2
PAX2	110.8	120.2	116.6	115.6	110.3	93	120.4	118.6
PAX2	119.6	114	121	114.8	113.2	97.7	122.2	114.1
PAX3	117	117.7	115.5	114.6	109	97.9	120.6	106.3
PAX3	117	124.5	127.7	122.8	115.4	103.9	137.8	126.4
PAX3	111.5	109.3	117.6	121.4	110	105.5	122.7	112.4
PAX3	108.1	114.6	114.6	113.5	107.3	103.9	115.6	107.1
PAX4	115.9	115.5	138.5	123.8	116.2	105.6	120	119.9
PAX5	112.2	113.6	108	116.3	106.3	104.6	119.5	113.1
PAX6	109	115.6	119.4	115.8	110.9	92.2	119.8	110
PAX6	131.3	123.7	119	122.9	126.8	95.6	122.1	123.9
PAX7	195.3	213.6	189	223.2	177.1	185.4	212	227
PAX8	115.7	118.7	129.9	135.4	115.1	108.9	124.4	112.6
PAX8	118.4	108	123.1	128.7	113.2	103	120.5	117.1
PAX8	110	120.9	108.6	121.6	111.7	97	118.2	118.7
PAX9	121.8	123.7	119.9	116.2	106.4	107.1	127.8	116.5
PAXIP1	254.6	251.5	253.8	247.6	203.7	189.2	265.6	249.8
PB1	126.9	126.4	136.1	126.5	110.7	105.7	128.6	124.2
PB1	129.1	132.7	132.8	139.2	132.6	106.4	130	132.6
PB1	156.1	152.3	148.8	153.3	131.2	127.7	152.1	140.3
PBEF1	159.9	163.5	150.6	228.6	182	217.1	187.2	177
PBEF1	175.3	146.8	132.2	213.7	181	264	232.7	179.4
PBK	112.9	113.5	115.2	115.9	95.7	100.3	120.7	118.2
PBOV1	117.9	123.3	114.5	117.4	110.1	101.5	119.6	119.3
PBX1	118.9	123.5	114.5	120.2	113.4	99.4	121.4	111.1
PBX2	320.1	363.2	290.4	366.8	301.6	213.9	357.1	343
PBX3	306.3	285.7	489	308.8	300.8	334.3	309.8	309.9
PBX4	193.6	218.4	217.1	193.9	208.7	184	207.9	173.3
PBXIP1	338.5	324	287.3	347	230.3	208.4	344.3	287.4
PC	111.2	113.4	110.7	111.8	114.2	104	122.6	111.8
PC	126.9	118.4	109.4	134.1	111.3	108.6	142.3	123.3
PC	132.8	117.6	134.2	135.2	122	127.1	128.6	123.8
PC-3	127.3	127	132.5	126.9	114.6	116.9	122.9	130.8
PCAF	126.2	114.3	125.4	119.7	113.1	106.8	121.6	116.4
PCBD1	133.5	139.7	153.4	132	131.3	130.1	138.1	148.8
PCBD1	158.7	167.4	211.4	162.9	165	142.6	166.2	180.8
PCBD2	137.5	143.6	141.1	138.2	133.2	122	134.6	134.5
PCBP1	3276.1	3961.8	3194.9	4082.3	3305.3	2953.2	4045.7	3421.4

PCBP2	2915.5	3046.6	2765	3052.1	2922.3	2457.3	3173.9	3211.9
PCBP2	1284.6	1121	744.9	966.3	713.4	983.7	1318.6	1514.7
PCBP3	113	114.9	110.8	117.1	110.3	93.2	118	105.1
PCBP4	139.8	157.3	158	156.7	143.5	151.9	150.3	152.4
PCBP4	119.2	116.3	121.4	120.1	112.9	103.7	117.1	116
PCBP4	111.2	118.6	134.6	116.7	111.9	122.8	127.4	112
PCBP4	188.2	140.2	170.2	183	133.6	129.9	175.9	163.5
PCCA	155.1	139.5	140.7	150.3	122.5	118.8	135.1	136.9
PCCB	322.8	313.5	392.5	355.9	242.2	216	253.9	252.4
PCDH1	113.9	114.9	128.1	129.9	104.3	100.1	119.7	113.9
PCDH1	120.4	128.2	120	120.3	118.3	98.7	121.8	114.4
PCDH10	109.3	114.1	111.1	116	104.9	104.8	114.3	113.5
PCDH10	117.1	120.2	106.9	135.3	108.1	99.1	123.3	117.5
PCDH10	113.7	114.4	112.5	120.4	109.6	98.8	120.3	119
PCDH11X	120.5	120	124.7	120.2	109.7	97.9	119.8	114.1
PCDH11X	109.4	123.9	108.9	115.2	109.8	99.9	119.7	118.3
PCDH11X	120.8	113.3	123.1	119.7	114.2	101.8	125.1	112.7
PCDH11Y	105.3	109.3	112.4	107.4	104.4	97.8	114.1	105
PCDH11Y	113.5	108.5	119.4	116.7	109.3	102.4	111.6	115.5
PCDH11Y	115.6	113.1	112.7	123.3	109.3	104.1	124	116.7
PCDH12	118.6	131.9	132.3	128.1	118.8	113.9	111.3	126.1
PCDH15	105.9	114.8	120.8	112.7	106.6	94.6	115	114.8
PCDH17	106.6	121.5	119.9	127.4	100.3	98.8	124.1	104.3
PCDH18	124.8	125.1	121.8	134.1	119.4	106.9	127.3	138.3
PCDH19	121.8	126	114.4	126.6	109.5	95.3	116.3	114.9
PCDH20	105.9	115.2	111.4	116.7	106.7	95	115.1	115.8
PCDH21	113.7	120.2	122.6	128.9	117.8	106.8	118.1	114.9
PCDH7	113	117.7	120.7	113.8	112.4	98.4	128.7	115.3
PCDH7	114.4	109.7	117.5	114.4	103.3	94.4	118.7	116.9
PCDH7	117.6	130.2	135.6	115.6	105.5	104.3	119.1	139.3
PCDH7	110.6	105.6	113.4	114	105.2	93.8	117.3	122.4
PCDH8	111.7	106.1	113	121.6	109.5	91.1	117.9	105.3
PCDH8	112.7	113.9	105.6	117.6	103.8	98.4	116	111.2
PCDH9	111.8	108.7	116.3	114.4	107.4	100.1	112.4	112.3
PCDH9	111.1	117.9	116.9	111.7	116.2	103	116.9	109.5
PCDH9	121.5	134.9	128.2	118.8	120.5	112.7	128	121.4
PCDHA1	116.4	124.7	131	127.5	111.7	100.9	117.7	114.1
PCDHA1	120.5	118.7	120.3	123.1	108	100.5	116	113.2
PCDHA1	111.1	114.3	112.4	120.8	108.5	100.8	116.9	109.9
PCDHA10	110.8	109.4	117.7	118.9	107.3	98.7	121.8	113.1
PCDHA10	111	122.1	111.5	117.3	114.3	96.3	124.7	107.9
PCDHA11	116.6	120.8	117.7	122.8	119.2	102.8	115.5	123.6
PCDHA12	121.1	115.9	120.2	114.4	107.9	100.3	120.9	120.5
PCDHA12	109	119.1	115.9	120.3	114.3	100.7	124.8	120.3
PCDHA13	116.1	111	107.5	106.1	103.5	91.5	102.7	116.3
PCDHA2	116	119.4	112.8	112.9	110.9	98.1	114.6	123.3
PCDHA3	118.2	117.1	125.6	115.6	106.6	95.4	124.9	115
PCDHA3	107.8	111.6	115.5	107.2	108.1	95.1	111.8	109.8
PCDHA3	118	125.5	113	120.2	107.7	91.7	108.8	118.5
PCDHA4	119	113.8	125.8	119.8	127.2	102.7	125.7	133.9
PCDHA5	125.2	123.4	122.7	109	119.6	107.3	126.3	112.9

PCDHA6	110.8	113.4	115.7	119.8	104.1	102.9	117.1	114.6
PCDHA6	112.5	108.7	112.6	108.5	107.8	94.1	125	105
PCDHA7	117.2	119.3	125.3	130	106.4	100.5	123.2	117.4
PCDHA7	130.8	125.4	127.3	138.8	122.7	111.2	123.4	122.8
PCDHA7	104.3	112	122.3	120.7	110.4	97.9	119.6	122.5
PCDHA8	109.6	117	112.7	120	104.6	95	124.1	107.8
PCDHA9	113.8	115.4	121.5	114.2	103	96.4	115.8	114.7
PCDHA9	112.6	108.9	108.9	111.8	111.6	97.3	112.1	111.2
PCDHAC1	109.5	110.4	118.4	116.8	111.1	100.2	121.6	117.3
PCDHAC1	104.5	107	116.1	115.7	111	99.6	120.8	111
PCDHAC2	123.7	132.6	122.4	128.2	115.1	103	131.1	130.9
PCDHAC2	117.8	115.1	113.6	118.8	112.8	98.1	116	108.1
PCDHAC2	98.3	112	112.8	110.4	102.6	95.4	111.4	105.3
PCDHB1	101.3	111.4	110.6	113.1	115.1	96.2	113.4	107.9
PCDHB10	114.4	109.6	113.5	115.3	105.1	97.6	112.9	119.3
PCDHB11	113.3	115.2	107.3	114.3	109.1	100.2	113.8	109.7
PCDHB12	108	112	109.1	115	103.2	95.1	116.7	110.4
PCDHB13	104.6	113.8	113.7	120.2	106.4	94.1	114.4	119.6
PCDHB14	125.2	124.6	129.4	136.2	104	103.7	122.8	116.7
PCDHB15	113.5	122.7	112.4	119.2	107.4	100.5	115	108.9
PCDHB16	106	122.5	118.2	119.6	111.6	89.4	115.4	113.8
PCDHB17	113.4	112.8	125.2	119.3	105.6	104.1	119.4	116.4
PCDHB18	107	112.5	119.9	110.2	105.6	96.8	101.9	108.7
PCDHB2	109.3	113.7	118.7	117.9	109.5	105.9	114.8	110.6
PCDHB3	112.6	113.3	108.6	111.5	116.3	101.1	122.8	110.2
PCDHB4	110.7	112.8	118.6	118.9	108.4	104.2	113.5	115.9
PCDHB5	108.1	113.2	108.6	122.7	102.2	97.7	115.1	106
PCDHB6	108.1	104.1	114.1	110.7	103.9	83.9	112.9	104.2
PCDHB7	109.6	105.1	112.9	116.4	111.9	100.1	109.9	107.7
PCDHB8	108.5	110.6	119.7	123.1	113.5	99.6	117.6	108.2
PCDHB9	206.7	198.7	247	213.8	248.8	154.7	260.2	301.5
PCDHGA1	121.8	115.2	129.9	121.5	113.9	98.9	110.1	116.8
PCDHGA1	122.7	113.1	119.5	118.6	113.8	94.3	117.4	111.1
PCDHGA1	113.2	116.9	129.6	110.9	104.4	91.8	115.6	114.6
PCDHGA10	121	116.9	124.7	124.8	109.2	95	125.5	118
PCDHGA11	114.5	116.7	110.1	113.9	105.8	100	116.8	107.3
PCDHGA11	118.4	115.4	116.5	115.2	108.1	98.9	113.8	115.4
PCDHGA12	117.4	124.4	111.1	112.5	109.6	94.4	121.5	115.6
PCDHGA2	107.2	112	112	117.3	102.5	100.2	109.3	113.5
PCDHGA3	112.1	114.4	109.6	112.7	111.1	98.6	107.7	114.3
PCDHGA3	114.6	117.9	120.6	122.6	102.7	99.1	125.3	114.3
PCDHGA4	113.4	114.3	111	110.4	106.3	94	114.7	103.5
PCDHGA4	113.1	109.9	111.7	119.9	98.6	93.5	109.6	107.2
PCDHGA5	115.2	114.2	112.8	114.5	113.1	100.6	114.3	112.3
PCDHGA5	115.3	122.5	117.5	117.3	103.9	105.6	119	103.9
PCDHGA6	123.8	109.8	118.2	120.3	119.4	104.2	120.5	114.2
PCDHGA7	104.8	113.1	119	112.6	106.2	95.2	122.6	112.2
PCDHGA7	105.4	107.6	116.5	116	99.2	94	112.3	108.5
PCDHGA8	114.2	116.8	118.7	122.2	107.8	102.9	114.2	118.1
PCDHGA9	115.3	129.8	123.9	129.6	115.5	104	132.7	104.2
PCDHGA9	126.1	118.8	123.8	123.5	112.2	96.6	124.7	119.8

PCDHGB1	115.4	119.6	114	112.6	104.4	101.8	121.4	117.4
PCDHGB2	119	123	103.4	118.9	109	98.1	117.5	106
PCDHGB3	119.9	116.6	114.6	122.1	108.1	101.2	121.5	122.4
PCDHGB4	111.2	109.5	130.9	121.1	117.6	109.5	119.1	121.6
PCDHGB5	121.6	113.3	122.5	117.3	111	97.8	113.9	113.1
PCDHGB6	107.6	116.3	109.6	117	103.6	99.7	112.3	118.2
PCDHGB6	145.2	153.4	150.2	149.4	131	119.1	138.4	139.9
PCDHGB7	112.3	107.6	115.7	118.4	104.8	100.7	115.4	116.8
PCDHGB7	109	112.2	113.6	115.6	94.9	94.6	112.5	107.3
PCDHGC3	116.5	110.8	118.9	117.9	109.7	99.4	121.2	120.1
PCDHGC3	114.5	119.6	116.6	116	106.9	103.3	119.2	115.8
PCDHGC3	119.7	120.6	116.3	123	122.3	103.9	134.4	124.2
PCDHGC4	109.5	120.3	119.7	119.9	108.5	94.7	122.8	117
PCDHGC4	105.9	110.8	114	111.6	110	95.6	113.4	110.9
PCDHGC5	111.3	109.5	112.7	120.1	99.8	100.5	113.3	113.7
PCDHGC5	110.2	110	120.1	117.6	111.4	100.8	117.4	122.6
PCF11	242	243.6	257.5	235.5	306.2	238.1	348.1	535.1
PCGF1	155.8	177.1	194	178.8	136.6	146	187.9	173.5
PCGF2	117.6	106	109.8	120.7	100.4	93.7	121.1	108.3
PCGF3	127.3	121.5	131.2	121.3	114.9	104.8	129.5	122.3
PCGF4	626.8	712.9	528.7	645.1	660.6	512.7	555.1	660.5
PCGF5	135.9	132.6	133.2	146.8	130	113.7	127.9	132.1
PCGF6	165.3	145.9	181.4	152.1	142.9	126.7	151.3	169.2
PCGF6	127	122.6	139.7	133.2	122.1	108.9	122.1	114.4
PCID1	348.9	310	534.2	374.2	358.8	283.8	301.6	398.5
PCK1	117.2	109.8	115.5	120.4	106.7	102.5	112.9	113.3
PCK2	116.2	125.7	124.4	120.6	106.2	115.2	103.7	113.4
PCK2	263.5	199.6	265	219.4	177.7	303.3	225.9	238.1
PCK2	112.1	116.1	116.2	131.2	109.8	97.1	112.6	114.2
PCLKC	126.4	128.4	120.1	133	107.9	111.9	132.8	131.8
PCM1	471.1	364.7	530.7	554.7	399.6	414.8	496.6	455
PCMT1	1620.2	1689.8	1416.4	1607	1271.9	1132.7	1692.4	1524.7
PCMTD1	310.4	248	268	345.2	333.3	266.5	302.9	304
PCMTD2	632.7	878	673.5	754.7	852.3	503.2	574.6	609.7
PCNA	121.8	128.8	129	127.6	122.9	107.8	115.6	116.3
PCNA	115.1	125.6	126.4	124.4	121.2	109.4	125.1	131.9
PCNA	284.2	308.1	304.7	406.2	275.5	287.2	395.9	376.7
PCNP	1414.3	1626.8	1940.2	1917.5	1669.9	1719.6	2201.6	2454.9
PCNT2	368.8	478.8	357	441	403.8	287	551.8	595.5
PCNX	596.3	628.6	405.6	729.2	577.3	387	848.9	757
PCNXL2	134.2	129	143.3	143.8	122.9	114.9	133.9	126.6
PCNXL2	122	131.2	127.4	115.9	123.9	104.3	117.7	125.2
PCNXL2	132	140.2	140.1	142.4	127.6	115.6	134	131.2
PCOLCE	125.4	130.8	125.7	141.8	109.4	108.1	123.7	118.3
PCOLCE2	115	127.5	121.7	117.3	115.8	98.2	116.7	120.9
PCOLN3	319.8	289.2	279.4	355.3	214.1	300.8	436.1	415.2
PCP2	116.2	113.9	132.8	128.7	118.4	100.7	119.4	121.1
PCP4	127	127.3	132.7	121.5	117.6	96.1	116.9	131.6
PCQAP	156.7	147.1	139.4	142.5	141.2	132	140.3	152.6
PCQAP	143.8	141.7	149.8	152.6	140.8	112.8	149	148.2
PCSK1	114	113.8	124.6	122.1	111.6	99.5	121.4	110.4

PCSK1N	118.9	117.6	121.7	133.7	102.9	99.2	119.2	114.2
PCSK2	120.7	108.7	125	128.1	113.4	97.4	119.9	112.1
PCSK4	118.2	109.9	123.8	125.7	104.3	105.9	125.1	127.9
PCSK6	118.7	110.1	105	114.9	100.9	96	113.9	106
PCSK6	111	108.1	117.9	121	115	102.5	114.4	111.4
PCSK6	113.2	115.3	116	114.3	112	92.8	119.2	115.2
PCSK6	111	117.3	108.1	112.1	108.2	100.7	118.1	103.8
PCSK6	112.1	110.1	117.9	119.8	107.7	95.6	126.6	119.5
PCSK7	370	398.1	332.8	326.5	305.2	204.8	327.2	299.6
PCSK9	110.4	121.5	122.2	131.2	116.6	98.7	126.6	129.7
PCTK1	112	119.3	111.5	116.2	109.8	101.3	116.1	112
PCTK1	111.9	114.9	114	112.2	106.3	94.4	114.6	116.6
PCTK2	130.1	131.1	131.3	137.9	121.6	118	123.6	133.1
PCTK3	134.4	125.7	118.6	136.5	117.7	94.3	132.1	122.1
PCTK3	107.2	120.4	124	123.2	109	101.8	120.1	115.9
PCTK3	117	106.6	120.6	114	117.8	104.4	121	121.7
PCTK3	107.2	117	119.5	110.8	98.6	101.2	114.4	109
PCTP	119.4	123	138.8	131.2	117	107.4	133.8	124.5
PCYOX1	326.1	268.4	380.2	347.1	184.3	245.1	255.4	228.7
PCYT1A	112.9	122.5	130.6	121.6	105.7	99.5	132.9	119.2
PCYT1B	128	118.6	118	125.4	108.8	99	115.7	105.7
PCYT2	125.1	124.8	129.4	134.6	127.5	103.3	129.2	119.1
PDAP1	113.2	135.9	121.7	129	123.7	127.2	131	124.8
PDC	111.8	124	125.3	124.2	110.4	104.1	109.6	109.9
PDC	115.3	103.6	109.5	108.2	102.6	97.5	112.6	107.6
PDCD1	128.7	133.6	123.7	127.9	119.4	108.2	135.8	137.1
PDCD10	153.7	164.5	163.5	166.6	149.7	129.4	153	142
PDCD10	117.8	113.7	119.5	109.3	108.4	101.2	119.3	115.4
PDCD10	372	473.7	422.2	447.7	368.9	369.1	448	473.8
PDCD10	151.2	135.2	185.5	169.2	141.2	129.9	163.6	162.3
PDCD1LG2	114.2	107.1	106.3	109.7	104.5	100	121.8	100.7
PDCD2	330.6	423.7	331.5	326.9	270.7	250.2	305.9	219.5
PDCD2	626.7	758.9	620.2	704.9	508.9	442.8	696.4	732.8
PDCD4	2513.7	2777.2	2761.3	2637.1	2198.6	2174	3437.4	3232.5
PDCD5	237.4	282.6	294.8	311.1	272.6	221.7	282.2	289.1
PDCD6	810.3	1050.5	901.2	933.4	716.5	750.8	742.1	809.9
PDCD6IP	118.6	115.4	107.4	122.1	108.3	99.8	116.9	114.8
PDCD7	4260	4285.2	3396.8	4386.6	3360.2	3089.5	5076.5	3613
PDCD8	141.3	127.5	146.1	135	123.2	112	124.2	129.9
PDCD8	157.2	169.4	173.6	188.1	149.6	141.3	167.8	164.1
PDCD8	117.7	121.6	129.4	115.5	118.6	101.9	123.6	130.6
PDCL	148.1	160.8	137.3	151.2	141.6	110.7	150.2	142.8
PDCL2	126.3	109.7	121.3	114.9	108.4	96.7	123.7	113.7
PDCL3	238.9	286.9	211.3	234.1	212.4	145.9	243.1	231.4
PDDC1	122.7	118.1	114.4	111.6	99.2	100	117.8	113
PDDC1	181.5	210.1	200.6	198.3	173.6	154	204.4	176.2
PDE10A	113.7	119	119.9	112.8	117.4	100.9	119.5	110.7
PDE11A	112.7	117.6	118.6	121.7	106.2	100.8	110.7	115.1
PDE11A	111.6	116.2	114.5	114.2	112.6	99.4	123.2	117.9
PDE11A	114.1	112.2	113	123	109.2	103.2	116.9	122
PDE1A	98.1	111.5	109.8	119.2	106.8	95.1	110.2	117.1

PDE1A	113.8	113.3	119.6	114.4	109.8	96.5	113.5	112.7
PDE1A	131.5	118.2	115.2	115.8	111.3	104.3	117.9	119.1
PDE1B	131.4	118	123.4	125.1	115	116.5	133.2	122.2
PDE1C	113.4	113.3	107.1	118.5	110.7	91.7	119.4	115.3
PDE2A	122.7	103.8	116.7	123.3	108	96.1	113.3	110.3
PDE3A	104.2	118.7	111.3	116.3	109.4	105.7	116.3	118.7
PDE3B	711.2	1139.7	561	722.6	828.2	548.1	726.1	863.4
PDE4A	121.1	124.2	124.3	129	111.4	101.1	117	119.5
PDE4B	526	483	519	538.9	468.5	491.8	430.1	385.2
PDE4B	169.1	176.7	190.2	171.5	167.5	164.9	157.5	142.1
PDE4B	116.6	113.6	113.5	123	111.3	94.4	115.2	113.1
PDE4B	124.6	119.3	124.2	113.9	105.2	103.9	129.7	111.1
PDE4C	116.1	110.8	113.1	114.2	118.5	104	125.2	110.9
PDE4D	220.4	255.4	217.7	261.5	237.3	177.7	225.6	250.6
PDE4DIP	122.8	123.3	127.3	126	112.3	104.8	125.8	111.9
PDE4DIP	108.6	107.1	109.3	116.2	99.7	96.5	110.1	108.8
PDE4DIP	115.5	105	111.7	120.4	107.6	98.3	117.5	108.1
PDE5A	113.3	114.6	110.9	112.7	103.2	100.7	112.4	112
PDE5A	136.9	135.8	130	146.2	118.2	108.1	136.8	139.3
PDE5A	115.9	108.9	124.3	117.3	108.3	94.3	121.3	104.9
PDE5A	117.4	113.6	116.3	123	113.5	100.8	125.4	112.4
PDE5A	108.7	106	112.2	131.3	109.5	99.7	117.2	113.5
PDE6A	115	108.8	111	112.1	109.9	95.4	114.8	116.3
PDE6B	119.5	122.9	133.8	119.5	110.7	103.3	112.2	118.6
PDE6C	113.7	112.8	118.2	132.6	106.9	98.3	111.4	118
PDE6D	134.3	155.8	139.7	162.6	133.3	117.2	152.6	149.6
PDE6G	128.4	135	127	131.9	106.4	108.1	129.3	129.1
PDE6H	115.4	121.9	118.9	120.2	121	109.7	123.5	118.7
PDE7A	183.9	171.4	183.1	189.3	202.7	149.2	146.8	160.7
PDE7A	509.7	398.5	411	462.8	553.7	263.4	365.4	358.6
PDE7A	114.6	113.9	124.4	130.2	112.9	99.2	131.6	109.8
PDE7B	126.8	118.3	134.2	136.7	120.3	100.5	124.6	119.1
PDE8A	138.7	126.9	124.5	144.3	138.3	125.1	130.3	147.6
PDE8A	117.1	114.2	130.1	133.2	120.2	106.6	118.2	116.3
PDE8A	118.1	120.4	118.6	117.5	104.2	98.5	119.3	108.9
PDE8B	118.3	122.2	118.9	129.9	120.2	107.6	125.9	123.5
PDE8B	111.9	115	110.3	114.3	97.5	96.5	113	110.3
PDE9A	116.3	108.5	108.3	117	110.6	103.7	120.1	116.6
PDE9A	121.8	180.5	162.7	138.1	151.2	125.6	128.9	154.3
PDE9A	115.8	124.6	119.7	126.4	110.7	107.9	134.6	121.3
PDE9A	119.5	108.8	118.2	127.6	106.8	108.7	127.9	115.5
PDF	109.8	118.8	117.8	118.1	108.1	101.4	115.4	108.2
PDGFA	126.8	110	124.7	107.2	113.3	102.7	121.6	122.4
PDGFA	116.5	115.6	123	112.6	108.7	105.2	114.3	116.2
PDGFB	121.6	121	120.9	119.4	118	104.6	119.3	121.7
PDGFB	130.2	116.8	128.2	122.1	112.4	115.1	126.8	116.4
PDGFB	125.1	128.4	122.1	126.3	111.3	101.4	124	125
PDGFC	130	115	133.8	139.1	110.8	104.7	128.1	113.7
PDGFD	127.1	122.9	122.1	128.8	105.6	100.8	121.4	112.4
PDGFD	127.2	133.3	130.5	147.5	112.8	106.7	132.6	119.1
PDGFRA	129.5	126.7	120.2	107.1	137.3	99.8	134.1	121

PDGFRB	125.1	159.1	133	126.8	138	103	139	122.1
PDGFRL	113.4	117.5	108.1	113.7	113.3	91	118.1	114.1
PDHA1	180.4	191.1	204	221.7	185.5	178.2	190.9	221.2
PDHA2	106.4	112.3	112.1	106.6	100.4	93.7	114	104.4
PDHB	1729.5	1744.2	1765.9	1818.4	1341.3	1092.9	1270.8	1095.2
PDHX	209.2	220.4	226.9	221.9	181	161.9	209.6	196.5
PDIA2	112.7	104.2	113.1	116.6	115.9	95.1	112.1	114.2
PDIA3	128.8	126.4	130.9	132.3	123.4	113.4	126.9	123.9
PDIA3P	130.8	141.1	141.8	132.7	126.7	130.5	152.1	146.8
PDIA4	120.3	136.4	124.2	130.5	109.6	113.8	132.7	125.3
PDIA5	120.1	136.5	137.9	123.7	119	109.7	122.7	122.5
PDIA6	163.7	164.2	233.7	199.6	168.5	174.6	174.9	188.4
PDIK1L	233.5	268.1	223.6	233.2	219.9	189.3	209.8	186.2
PDILT	112.5	108	113.7	116.5	113.3	100.8	126.6	119.6
PDK1	128.6	129.9	134.7	153	126.7	118.2	130	136.6
PDK2	134.8	117.2	128	124.5	118.1	120.8	128	122.4
PDK3	137.1	146.6	160.1	147.9	116	120	136.4	135.5
PDK4	126.5	115	309.3	141.3	118	195.6	126	111.6
PDLIM1	158.9	145.7	142.7	155	123.7	126.3	131.3	128.3
PDLIM2	115.9	109	120.9	128.1	119.3	98.6	120.1	118.1
PDLIM2	108.3	113.8	120	125.6	115.2	97.1	110.7	115.6
PDLIM3	122.3	110.2	104.2	123	102.7	100.4	127.7	108.7
PDLIM4	109.4	117.4	113.4	115.8	104.4	96.6	109.6	107.6
PDLIM5	130.1	125.4	120.4	132.2	117	106.8	128.4	126.7
PDLIM5	121.1	108.8	113.3	127.7	115.7	115.5	124.7	124
PDLIM5	118	116.3	112.4	128.7	114.6	101.2	118.5	126.2
PDLIM7	113.1	114.7	119.7	120.6	114.2	104.9	114.1	103.6
PDLIM7	136.8	137.3	145.6	137.6	129.3	128.1	141.5	139.6
PDLIM7	162.7	143.7	210.4	196.5	137.2	182.6	144.1	133
PDP2	137.2	125.2	140.3	145.6	122.4	114.9	137.4	140.4
PDPK1	117.4	127.6	123.5	120.4	108.6	101.9	127.1	116.7
PDPK1	194.3	211.7	202.9	243	225.1	156.2	222.4	223.9
PDPK1	761.7	686.5	538.1	818.9	746.9	571.4	848.9	874.8
PDPN	113.2	121.4	124.8	124.2	110.2	114.4	117.9	113.4
PDPN	116.9	115.2	116	117.9	99.6	106.8	115.8	122
PDPR	275.1	184.6	245.5	241.3	164.2	200.9	281.4	194.3
PDPR	479.5	352.8	454.7	489.2	326.9	351.7	649.6	348.7
PDPR	121.6	118.2	126.1	121.9	107.3	98.6	117.4	114.2
PDRG1	199	235	230.9	261	196.4	218.7	248.4	267.5
PDSS1	141.3	142.6	142.7	139.3	146.9	127.1	147.5	148.9
PDSS2	416.6	457.1	382.9	373.6	438.6	300.8	364.2	403.9
PDXK	610.2	707.3	723.9	765.2	562.8	506.1	829.8	768
PDXP	390.7	426.8	332.5	378.7	245.2	183.9	245.2	209.5
PDYN	124.4	118.4	126.3	120.9	108.8	104.7	120	111.1
PDZD11	112.8	109.8	114.3	121.9	110.6	95.6	124.3	116.1
PDZD3	110.5	112.9	109.6	112.9	103	87.5	112.6	107.1
PDZD4	119.1	119.3	131.1	132.8	112.1	105.9	119.4	116.8
PDZD7	108.3	114	117	110.8	103.6	98.7	117.5	106
PDZD8	161.3	171.9	149.4	157.6	137.3	132.6	159.7	172.6
PDZK1	107.6	115.7	124.6	121.5	115.9	103.8	121.9	111.9
PDZK1IP1	109	111.2	111.2	119.6	101.5	101	119.5	104

PDZK3	118.7	116	106.9	115	105.5	103.1	115.1	111.8
PDZK3	105.5	112.8	115.7	114.4	106	94.9	111.6	107.6
PDZK5B	123.7	108.7	119	118.9	109.8	94.5	107.8	108.2
PDZK5B	111	106.8	110.2	103.4	117.2	93.7	118	113.4
PDZK5B	113.9	111.4	118.4	120	113.8	101.5	116.9	121.7
PDZRN3	121.1	117.4	126	125.4	105.4	103	111.7	117.2
PDZRN3	110.4	113	115.3	111.1	106	96	118.7	112.8
PDZRN3	109.5	126.1	127.8	122.7	112.2	94.4	118.8	117.6
PDZRN4	117.5	112.3	121.6	118.6	107.9	99.7	117.4	110.3
PEA15	694.9	572	1492	717.2	475.2	1082.2	803.1	571.1
PEAR1	127.7	133.4	116.4	128.8	105.8	87.4	117.5	113.8
PEBP1	217.3	228.5	207.7	195.6	216.1	224.5	165.6	194.2
PEBP4	119.6	114	124.7	123.9	116.8	101.4	118.7	111.9
PECAM1	201.1	237.8	314.4	198.6	175.2	211.4	154.7	177
PECI	191.5	272.5	292.8	285.6	248.3	236.9	233.6	184.2
PECI	258.1	235.3	314.5	300.1	218.3	240.8	191	213.1
PECR	182.7	182.1	188.4	173.5	164.6	136.4	129.7	140.5
PEF1	248.4	246.1	237.1	234.3	196.2	226.4	289.2	384.5
PEG10	111.8	123.4	118.9	132.9	117.6	109.8	129	121.5
PEG3	122.5	116.1	117.7	117.7	102.2	102.4	118.7	124
PELI1	204.2	211.5	234	278.8	237.9	236.9	377.4	315.2
PELI2	187.1	203.9	183.3	218.4	205.1	199.2	247.4	292.3
PELI3	121.2	116.8	111.4	120.8	110.9	106.1	113.2	112.5
PELO	485.7	596.3	510.5	611.4	415.7	598.9	783.4	652.5
PELP1	208.8	229.1	160.9	170.7	167.4	145.2	170.6	169
PEMT	197.1	209	257.3	195.6	161.3	154.2	197.2	163.3
PEMT	113.1	114.2	115.4	115	101.1	93.6	108.7	110.7
PEMT	114.1	122.2	111.9	119.6	115.2	105.9	122.7	116.2
PEMT	126.1	129	149.1	125.6	118.4	100.2	133.2	134.6
PENK	118.2	107.2	112	115.1	107	95.3	115.7	111.1
PEO1	162.5	130.1	140.3	144	132.9	117.7	145.2	129.9
PEPD	558.5	591	592.2	584.5	423.8	374.8	532	433.4
PER1	131.6	130.3	126	126.5	116.3	118.2	125.7	136.7
PER2	119.2	109.1	112.4	117.7	104.7	92.5	102.3	107.1
PER2	204.5	239.1	201.5	228.8	195.6	167.9	220.4	217.7
PERF15	114.6	115.7	113.9	123.6	110	101.8	115.4	112.1
PERLD1	262.1	272.9	296.1	254.4	219.3	182.7	200.9	217.1
PERP	105.8	117.9	113.5	120.8	100	100	107.9	116.8
PERQ1	134	132.2	138.4	139.6	128.5	106.7	139.8	152.8
PES1	118.9	138.7	138.1	134.5	122.3	106.8	123.8	115.8
PET112L	147.2	155.4	186.8	159.5	147.9	119	150.1	156.1
PEX1	178.3	187.7	181.6	175.7	146.8	149.2	185.3	171.7
PEX10	116.3	124.6	140.6	139.6	126.6	103.2	129.9	120.9
PEX10	111.7	116.9	113.1	124.1	108.3	108.2	120.2	118.8
PEX11A	124.4	117.2	129.3	132	127.7	117.9	128.9	138.7
PEX11B	547.8	517.3	579.6	504.5	497.4	500.3	409.8	386.8
PEX11G	116	121.2	127.4	129.4	112	98.9	126.6	112
PEX12	107.1	132.3	125.5	133	111.9	101	117.7	111.2
PEX13	107	110.7	119.7	113.5	105.3	106.8	108.8	109.2
PEX14	137.5	168.6	150.5	177.3	137.6	138.4	160.6	154.5
PEX16	450.6	422.5	427.9	430.6	335.3	263.3	427.1	358.2

PEX16	119.2	124.4	124.3	129.1	126.3	112.3	131.3	125.7
PEX16	492.1	581.7	466.4	480.6	393.8	399	454.1	386.3
PEX19	237.2	281.5	251.5	241.1	240.5	189.7	235.6	268.9
PEX26	113.3	112.9	122.5	120.9	108.9	101.8	111.3	122.3
PEX3	122.4	122.7	122.8	123.8	116.9	95.7	114.9	118.1
PEX5	445.7	399.2	372.7	464.4	400.7	295.6	494.1	398.9
PEX5L	115.6	113.8	119.2	119.6	114.9	98.1	119.2	118.2
PEX6	140.8	148.3	135.3	163.6	136.1	123.6	150.1	154.3
PEX7	144.1	154.2	166.8	147.6	123.5	127.5	136.5	128.5
PF4	110.2	108.2	107.4	110.9	107.6	93.5	115.5	111.4
PF4V1	120.8	125.8	123.3	121.2	125.6	105.6	122.4	126.4
PFAAP5	314.7	261.7	392.8	335.8	266.9	282.4	273.6	226.6
PFAS	182	178.1	160.4	185.5	168.4	124.5	152.9	138.8
PFC	121.1	141.6	186.4	140.7	117	120.1	126.9	120.5
PFDN1	457	539.8	541.3	494	346.2	446.9	496.6	476.7
PFDN2	128.2	127.7	122.4	140.2	117	112.1	116.6	121.1
PFDN4	118.6	133.4	125.9	123.9	110.6	99.8	133.1	132.1
PFDN5	1768.4	2567.1	3189	2358.7	2280.9	2948	2517.7	2984.6
PFDN5	1301.3	1828.4	1892.6	2535.7	1993.9	1620.5	1942.3	1433.2
PFDN6	157.2	171.9	178.3	195.2	179.9	152.4	160.6	170.4
PFKFB1	120.5	121	117.6	117.1	121.8	106.3	115.4	120
PFKFB2	113.7	139.1	112.8	119	106.8	104.3	125.5	121.9
PFKFB2	122.6	128.4	110	122.3	107.2	100.4	116.3	112.3
PFKFB2	121.1	120.2	123	122.1	109.2	105.7	135.7	120.1
PFKFB3	627.3	668.4	947.1	989.1	565.2	1026.6	1235	551
PFKFB4	188.1	204.4	200.6	222.5	160	148.8	238.5	192.6
PFKL	113	115.1	114.2	122.5	105.3	93.3	118.6	121.9
PFKL	234.1	205.2	279.5	248.3	152.6	243.6	204.4	176.8
PFKM	158.9	153.3	133.9	161.6	138.6	111.7	145.1	127
PFKP	256.7	245	242.8	277.5	200.7	282.6	331.6	258
PFN1	6967	8885.6	8455.3	7031.8	6552.7	8019	7852.3	5900.9
PFN2	113.1	109.1	120.1	123.6	111.8	99.3	118.5	114.3
PFN2	123.4	124.4	134.1	124.6	125.5	108	149	126.7
PFN3	115.5	113.2	122.3	121.8	106.8	105	126.8	121.3
PFN4	113.1	117.6	116.8	118	110.4	102.6	118.2	108.2
PFS2	129.1	111	130.4	125.6	118.6	103.9	129.7	130.6
PFTK1	134.3	134.5	133.9	133	105.2	105.5	125.6	125.1
PGA3	126.1	114.9	126.8	108.9	110.7	111.6	117.3	113.5
PGA4	114.1	117.9	130.3	114	111.4	103.4	121.2	106.8
PGA5	117.5	109.9	118.5	110.4	104.6	96.3	122	118.6
PGAM1	465.6	489.4	553.6	535.1	392.8	422.2	418.1	479.5
PGAM2	114.8	126.6	122.3	123.6	113.9	104.2	121.5	125.2
PGAM4	122.2	121.6	113.8	111.6	112.5	100.5	118.3	114.8
PGAM5	106.1	114.7	115.3	113.9	105.6	102.1	113.6	113.4
PGAP1	116.3	111.4	111.3	121.3	115.6	105	130.1	120.6
PGBD1	123.4	132.6	129.8	133.4	129.8	109.6	130.8	124.8
PGBD2	195	178.6	182.9	201.2	202.4	132.4	188.5	197
PGBD2	122.6	120.8	122.2	109.8	109.1	97.2	130.2	123.9
PGBD3	119.1	130.8	116.2	127.1	113.8	107.5	124.1	116.6
PGBD4	123.4	123.1	123.3	120.3	117.7	103	124.6	125.9
PGBD5	116.1	118.6	127.5	121.9	112.7	102.9	122.6	117.1

PGC	118.5	106.6	116.3	123	96.5	96.5	107	111.2
PGCP	281.2	197	374.1	307.6	148.8	307.7	224.8	148.7
PGD	1304.4	870.3	908.9	2380	1052.2	2194.6	2181.9	910.6
PGDS	115.6	106.9	111.7	117	107.8	97	117.9	110.5
PGEA1	162.4	196	221.9	171.1	178.7	188.4	171.8	150.3
PGEA1	116.6	115.7	116.1	120.7	109.9	111.6	118.7	111.4
PGF	111.6	111.3	115.4	111.3	101.5	95.6	115.5	112.5
PGGT1B	123.5	133.1	136.1	141.8	136.1	115.1	137.9	137.4
PGK1	355.8	294.3	432.1	409.9	347.9	314.7	466.5	550.6
PGK2	108.7	112.2	110.4	121.2	106.7	100.5	115.5	116.3
PGLS	473.9	462.6	548.1	520	344.6	380.4	495.6	399.4
PGLYRP1	115.7	138.2	125.6	125.2	130.7	104.8	123.5	137.1
PGLYRP2	117.1	117	130.6	114.2	110.9	105.8	114.5	115.4
PGLYRP3	112.9	111.2	114	118.1	107.9	101.9	122.1	115.8
PGLYRP4	120.1	119	122.6	125.3	117.5	102.8	124.1	122.8
PGM1	449.4	652.4	407	543.2	397.6	297.1	450.9	442.2
PGM2	133.7	142.9	168.3	147.5	143.3	117.1	150	142.1
PGM2L1	278.8	249	233.8	243.5	252	211.2	297.1	325.6
PGM3	141	133.6	145.9	130.5	118.9	109.5	137.1	126.8
PGM5	111.4	104.7	105	101.8	110.2	99	115.9	116.5
PGM5	110.6	113.5	112.1	114.4	105.7	94.4	107.6	121.7
PGM5	108.9	113.3	107.7	118	102.5	99.5	112	105.4
PGM5P2	120.6	117.1	108.5	118.5	110.8	97.5	118.9	111.7
PGM5P2	112.3	102.3	125.4	121.4	109.4	105.9	115.9	115.3
PGPEP1	115.8	110	122.8	118.8	116.4	100.8	114.2	107.8
PGR	109	117	115.2	119.7	107.5	96.1	123.3	115.6
PGRMC1	325.5	316.9	391.6	306.3	284.9	359.6	328.6	326.5
PGRMC2	878.7	965.8	892.1	895.5	833.3	750.4	1004.8	937.8
PGS1	354.7	345.8	332.8	339.8	249	255.6	265.5	248.8
PH-4	132.5	136.3	137.1	123.8	119.2	118.2	135.1	121
PH-4	140.7	156.5	154.3	161.1	141	129.8	133.3	129.8
PH-4	213.2	210.7	274.9	194.5	176.6	181.5	185.7	179.3
PHACS	125	132.8	139	121.5	123.8	104.5	126.8	120.8
PHACTR1	129.1	129.4	125.8	135.3	119.5	104.4	173	166.4
PHACTR2	353.6	381	284.6	395.4	391	193.8	294	315.4
PHACTR3	112.3	113.9	120.7	121.9	115	101.7	115.9	116.3
PHACTR3	114.8	118.6	126.7	116.6	104.9	97.6	112.7	113.3
PHACTR3	119.1	107.1	115	112.2	102	100.2	117.2	112.4
PHACTR4	603	780.5	607.8	721.1	673.3	445	552	585.6
PHACTR4	126.6	125.4	122.9	129.4	110.6	102.8	123.9	127.7
PHACTR4	116.3	105.7	116.2	116.8	108	100.2	122.8	114.3
PHB	337	372.2	282.2	393.1	270.3	203.6	327.4	316.7
PHB2	196	246.3	215.8	215.7	171.3	212.2	215.2	260.3
PHC1	190.5	155.3	168.6	147.8	146.8	124.4	155.3	155.6
PHC2	112.8	114.8	120.4	121	112.3	96.5	125.6	119.6
PHC2	142.5	139.4	157.4	148.4	143.5	128.7	173	165.9
PHC2	124.9	126.2	126.1	125.2	112.8	105.6	121.2	122.2
PHC3	160.3	162.1	146.6	168.8	170.5	132.3	195.3	182.6
PHCA	178	169.9	220.3	194.5	156.1	149.4	252.3	219.2
PHEX	106.9	113.6	120.1	118.1	106.7	109.1	123.2	108.7
PHF1	136.9	126.6	120	125.9	130.9	122.2	144.5	132.2

PHF1	222.9	223.1	167.9	220.9	164.1	167.1	258.4	209.6
PHF1	303.5	272.1	248.2	278	213.3	300.4	328.1	294.6
PHF10	187.2	167.2	207	194.2	181.1	199.2	206.3	213.2
PHF11	1154.7	1289.3	1177.6	1211.8	1197.2	974.9	808.4	1157.6
PHF11	1260.2	1505.9	1953.9	1711	1339	1170.7	1354.6	1211.4
PHF11	121.7	130.2	129.3	141.4	119	116.3	119.4	118.9
PHF12	119.5	114.7	129.3	112.5	118.8	107.9	122.6	107
PHF12	222.4	187.1	180.6	179.3	163.9	134.4	188.7	170
PHF12	112.8	125.2	111	113.4	109.3	93.8	112.3	114.5
PHF13	338.6	432.8	276.1	448.5	386.1	316.5	627.3	711.9
PHF14	121.3	123.6	128.3	121.3	115.1	100.8	117.9	129.8
PHF14	126.5	117.8	126.3	127.5	112.9	110.9	122.5	109.1
PHF14	162.9	160.5	145.9	136.3	138.2	130.9	138.5	141
PHF15	860.3	1125.5	543.4	837.7	769.7	556.8	878.8	797
PHF16	135.8	143.7	147.2	134.1	133	147.5	167.9	157.3
PHF16	121.6	121.9	143.1	127	107.8	108.5	133.6	118.9
PHF17	120.2	127	130.5	124	125.1	106.4	123	128.9
PHF17	237	275.4	265	246.7	208.6	222.2	210.3	213.2
PHF17	144.2	120.6	148	137.6	122.6	125.1	138.2	140.6
PHF19	212.2	224.6	182.7	192.2	209.9	165	196.5	195.8
PHF19	190.4	189.2	163.5	188.6	165	129	184.5	188.7
PHF19	167.3	165	152.4	153.1	152	115.2	174.5	155.8
PHF2	124.7	123.4	125.3	125.9	117.2	108.4	114.1	119.1
PHF2	258.1	308.1	290	262.7	237.4	279.5	306.6	338
PHF20	166.2	197.5	192.7	218.8	192.1	162.4	211.2	204.3
PHF20L1	123	122.8	130.6	154.9	141.7	118.2	134.3	129.8
PHF20L1	442.7	452	390.1	482.4	362.8	352.7	466.7	471.6
PHF20L1	127.7	119.6	127.8	129.1	111.2	108.2	126.6	111.4
PHF20L1	129.3	119.7	114.9	130.2	117.4	109.2	128.6	139.1
PHF20L1	162.8	157.7	178.8	184.6	158.2	141.3	172.8	178.9
PHF21A	864.7	852.4	700.5	881.5	706.6	541.4	1004.8	1025
PHF21B	116.4	116.6	119.8	120.5	106.1	104.9	113.8	123.5
PHF22	307	421.3	443.3	403.4	320.6	342.3	429.7	403
PHF23	179.4	153	134.4	164.8	178	121.5	168.5	160.7
PHF3	156.4	149.1	130.5	146.6	145.8	124	151.4	144.6
PHF5A	440.5	447	618.4	453.2	430.7	363.5	430.4	443.5
PHF6	117.5	124.7	122.4	114.6	105.5	97	126.8	114.3
PHF6	112.2	111.2	104.7	110.9	103.5	99	112.5	116.6
PHF7	116.8	121.8	121.6	127.4	114.3	105.3	119	120.5
PHF7	111.9	120.3	120.6	115.3	110.1	96.1	113.7	108.2
PHF7	115.4	115	120.8	125.6	110.7	102.1	124.5	129.7
PHF8	110.9	108.1	120.6	123.3	108.6	106.5	119.2	115.3
PHGDH	225.7	315	167.3	281.7	272.8	178.2	192.9	292.3
PHGDHL1	251.2	279.1	285.5	292	269.8	205.3	268	263.5
PHIP	321.8	422.1	309	328.3	332.1	296.2	432.7	439.1
PHKA1	144.1	129.5	123.7	123.7	127.8	103.1	122.2	121.9
PHKA2	339.7	331.3	319.2	376.6	315.6	239.4	412.1	425.7
PHKB	166.6	150.7	196.5	177.4	145.9	166.6	159.3	169.1
PHKB	122.3	123.3	154.3	148.6	120.2	123.6	131.9	129.7
PHKB	322.8	332.1	428.2	365.3	292.5	353.5	312	312.5
PHKG1	133.7	128.1	140.6	133.2	124.7	98.3	126.3	126.8

PHKG2	139.5	148.5	161.5	163.1	131.6	122.4	154.5	150.1
PHLDA1	142.5	161.9	165.1	184.6	208	354.7	237.6	198.3
PHLDA2	117.8	122.6	129.5	125	114.4	101.3	121.6	121.4
PHLDA3	116.9	114.6	117.9	114.7	109.1	100.9	112.3	110.2
PHLDB1	127.9	135.7	132.7	132.4	119.5	104.6	130.8	126.1
PHLDB2	122.8	125.5	130.2	134.4	114.7	108.7	125.4	118.5
PHLDB3	126.9	120.3	114.3	114.7	109.2	106.5	121.3	115.9
PHLPP	129.6	123.2	116.1	121.8	113.7	109.9	124.1	121.5
PHLPPL	151.9	150.6	150.3	145.7	155.6	135.5	206.7	209.6
PHOSPHO1	109.6	114.3	113.8	121.8	105.9	93.5	117.6	118.1
PHOSPHO2	104.8	112	105.6	114.9	103.9	97.3	117	104.8
PHOX2A	115.9	114.1	119	112	106.9	98.4	119.5	115.7
PHOX2B	114.8	111.4	118.2	122.3	112.8	93.2	108.4	111.1
PHPT1	219.8	220	300.1	207	186	190.3	227.2	184.7
PHTF1	148.3	121.5	166.8	152.3	161.1	160.5	177.7	230.8
PHTF2	114.5	114.4	121.6	132.9	114.2	107.1	126.5	122.5
PHYH	121.3	116.9	115	118	107.3	110	129	121.2
PHYH	112.4	118.9	142.9	133.1	119.6	112.4	122.9	127.5
PHYH	161.1	162	211.9	164.7	155	167	164.7	167.2
PHYH2	393	401.4	526.1	516.8	356	474.7	549.2	680.5
PHYHD1	114.1	109.6	111.6	116.9	99.5	92.7	104	112
PHYHIP	120.1	119.4	139.9	119.5	107.8	97.1	115	125.6
PHYHIPL	107.2	109.8	110.7	106.4	98.5	94.4	106.7	114.4
PI15	135	133	117	123.9	115.7	116.3	141.3	114.7
PI16	118.7	145.1	130.8	126.7	116.5	108.2	113.9	119.5
PI3	111.4	122.6	122	119.3	109.9	92.6	110	114.8
PI4K2B	135.3	129.2	127.7	132.5	138	104	124.8	128.2
PI4KII	167.1	181.7	161.1	228.6	153.7	144.9	259.2	176.4
PIAS1	404.3	295.8	370.6	329	292.5	227	355.9	374.9
PIAS2	108.9	116.5	121.1	108.4	99.7	93.3	109.8	108.5
PIAS2	162.8	143.1	196.3	149.3	147.5	160.5	165.1	165.9
PIAS2	117.2	124.9	124.5	122	118.4	111.3	126	116.1
PIAS3	131.9	144.5	145.2	138.5	121.1	120.2	133.6	142.8
PIAS4	694.4	814.6	554.4	809.3	766.9	420.6	893.4	910.8
PIB5PA	115.5	112.3	120.7	121.6	106	100.6	123	110.8
PIB5PA	108.6	117.5	109.2	111.5	115	99.7	116.2	112.5
PICALM	125.4	116.9	137.5	130.1	122.1	106.1	128.7	136.8
PICALM	515.1	286.9	564.2	513	331	493.4	513.5	531.7
PICK1	209.8	268.5	216.4	191.4	183.1	154.3	254.1	218.4
PIF1	113.5	118.8	118.5	120.8	106.1	102.2	132.9	111.9
PIG38	113.4	122.2	123.8	124.3	112.1	115.5	126	124.8
PIGA	366.9	328.2	402.5	392.3	346.8	293.8	369.3	423.2
PIGA	114.4	109.5	124.4	129.6	109.7	109.7	115	106
PIGA	122.4	120.7	144.5	131.7	118.8	113.6	127.8	115.7
PIGB	193.2	198	247.4	205.3	194.7	187.5	184.4	186.6
PIGC	535.7	567.7	819.6	508.3	400.2	612.9	386.2	304.5
PIGC	195.9	200.6	251	217	183.7	186.7	155.7	144.8
PIGF	243.3	239.6	357.2	243.4	185.6	271	215.2	164.6
PIGF	117.6	130.7	137.6	133.3	118.1	107	124.8	124.5
PIGG	135.5	130.1	162.4	150.9	138.9	118.5	133.9	169.9
PIGH	238.6	181.4	224.6	203.3	175.4	136.3	161	203.7

PIGK	162.2	186.3	173	176.5	144.3	149.3	151.1	148.3
PIGL	116	117.2	125	123.6	110.8	109.1	132.1	125.5
PIGM	144.7	143.4	148.7	186.6	151	121	142.5	144.3
PIGN	117.7	122.1	119.4	123.4	109.4	99	114.2	120.1
PIGN	159.5	157.3	210.5	182.1	143.6	127	162.3	143.5
PIGN	201.5	202.7	238	197.6	199.9	179.7	192.6	203
PIGO	142.4	129.2	139.3	144.8	118.9	122.9	133.7	129.1
PIGO	172.6	196.8	206	183	166.1	174.4	181.3	158.9
PIGO	115.4	122.2	135.5	132.8	115.1	103.8	133.2	127.2
PIGQ	188.1	189.1	193.7	205	157.9	158.6	153.3	184.2
PIGR	121	119.2	111.8	121.2	107.9	99.5	119.2	113
PIGS	205.5	221.3	243.2	204.2	182.2	210.6	210.5	194.1
PIGT	230.4	249.6	280.3	258.3	178.6	196.8	232.3	184
PIGV	248.8	277.1	253.6	248.4	231.2	199.7	228.4	244.9
PIGW	123.5	129.7	135.8	135.2	111	101.5	122.7	111.6
PIGX	152	136.3	143.4	151.4	139.8	122.5	151.3	161.8
PIGY	729.9	974.2	937.9	748.1	697.8	826.9	756.1	706.4
PIGZ	116.4	120.6	124.1	116.9	101.7	98.5	125.3	108.9
PIK3AP1	192.9	145.4	185.8	172.4	124.9	149.6	207.3	147.8
PIK3C2A	124.5	115.7	124	142.7	121.5	107.8	134.8	127.6
PIK3C2B	135.1	131.8	121.1	141.2	135.4	120.4	137.1	125.2
PIK3C2G	111.5	108.9	111.9	107.7	107.8	96.5	110.6	99.8
PIK3C3	125.3	123.6	128.7	129.8	107.5	110.3	120.4	127.8
PIK3CA	145.7	148.2	140.7	159.3	155.3	125.7	179.2	147.8
PIK3CB	130.3	115.7	143.3	130.5	132.9	114.8	137	113.8
PIK3CD	476.9	597.6	467.1	570.9	531.7	379.2	543.2	541.1
PIK3CG	169.4	181.2	198.3	191.1	150.4	141.4	166.7	158.8
PIK3R1	510.7	706.9	310.5	565.1	548.3	250.8	487.7	560.8
PIK3R1	112	101.3	112.3	112.2	111.1	97.4	125.9	109.7
PIK3R1	117.5	125.5	129.6	142.4	121.2	106.8	129.3	120.1
PIK3R1	112.1	107.9	120	118.5	105.8	107.4	119	114.3
PIK3R1	157.4	135.2	137.3	149.9	147.6	114.7	149.1	143.6
PIK3R2	592.8	638.3	507	541	424.3	345.7	328.6	277.8
PIK3R3	123.8	122.9	125.6	130.4	112.9	112.9	137.2	140.1
PIK3R4	131.5	116.3	130.2	130.7	128.3	124.1	123.6	122.4
PIK3R5	143.1	130.6	159.8	143	117	136.7	138.8	148.9
PIK4CA	1299.8	1179.3	856.8	1414.5	840	592.8	1192.7	1524.8
PIK4CB	247.6	304.6	246.9	283.8	275.2	203.1	322.4	301.3
PILRA	417.8	226.5	626.7	469.7	190.7	580.1	472.8	223.5
PILRA	206.7	140	288.7	206.8	131.2	236.4	205.6	151.3
PILRA	280.6	152	354.1	284.6	141.7	342.9	273.7	147.9
PILRB	130.7	123.3	127.7	136.8	116.9	103.6	126.2	120
PILRB	297.3	340.7	356	327.7	275.8	223.1	309.4	333.2
PILRB	126.9	118.8	118.3	115.9	104.3	103	124.9	120.3
PILRB	120	118.4	119.9	143.8	110.7	93.4	114.3	121.6
PIM1	684	745.2	624.4	655.8	552.4	584.7	650.7	562.2
PIM2	669.8	948.8	421.6	651.4	513.4	415.7	679	492.1
PIM3	218.8	231.6	169.3	264.5	203.4	160.6	278.5	200.4
PIM3	105.5	111.7	114.6	121.1	101.6	99.7	128	111.4
PIN1	847.2	788.2	1200.5	754.1	701.4	721.6	722.1	639.1
PINK1	155.8	172.2	156	188.5	163.3	141.1	174.6	192.9

PINX1	143	139.1	139.1	140.3	125.6	110	140	132.2
PIP	121.8	100.6	113	114.5	113.6	102	112.3	106
PIP3-E	293.5	226.2	227.2	259.5	245	185.8	265.7	309
PIP5K1A	122.3	130.8	129.8	127.6	115.7	111.6	126.6	124.1
PIP5K1B	116.2	115.3	113.4	116.4	105.7	97.6	130.5	124.1
PIP5K1C	327.2	289.7	274.4	318.5	223	273	343.4	276.9
PIP5K2A	153.3	188.7	168.8	166.9	167.1	143	193	194.6
PIP5K2B	135.9	161.2	132.4	143.4	128.5	107.6	128	131.8
PIP5K2B	115.4	118.5	121.7	138.6	117.9	101.2	130.1	127.1
PIP5K2C	130.9	131.8	134.8	141.3	137.6	131.6	128.9	137.4
PIP5K3	125.3	115.8	116.5	127	112.7	100.8	121.2	111.4
PIP5K3	111.7	111.4	106.2	108.5	110.4	99.2	111.1	109.9
PIP5K3	110.4	124.7	114.7	124	113.3	97.3	134.6	112.2
PIP5KL1	116.4	117	111.5	116.4	111.2	97.1	117.4	112.3
PIPOX	111.9	117.8	116	108.1	104.8	100	120.7	115.3
PIR	134.8	116.4	139.5	155.7	134.4	152.3	154	136.1
PIR	135.3	120.9	131.2	149.3	132.5	161.6	162.7	134.2
PISD	196.2	195.3	177.2	214.8	190	178.2	211.5	187.9
PITPNA	134.8	151.3	130.6	150.6	123.3	120.7	156.2	154.5
PITPNB	232.5	269.8	206.6	260.7	207.5	190.3	262.8	309.5
PITPNC1	340.7	483.7	378	471.8	363	401.9	373.4	425.4
PITPNC1	153.9	201.4	139.5	166.1	158.2	136.8	171.5	172.6
PITPNM1	1135.4	1303.5	984.6	1174	661.5	889.7	1187.9	1197.8
PITPNM2	151.6	141.3	188.6	168.4	132	109	131.1	130.6
PITPNM3	111.4	108.6	119.1	112.3	112.9	101.4	115	113.2
PITRM1	202.6	205.7	200.7	256.9	203.3	174.8	245.6	236.3
PITX1	108.1	105.6	112.3	120.4	107.5	97.4	108.6	110.3
PITX2	117.3	123.2	123.7	126.7	103.2	97.4	121.2	117.9
PITX2	133.7	128.4	132.3	128.2	124.1	109.7	124.6	123.9
PITX2	113.7	116.6	118.1	112.5	104.1	103.4	115.7	120
PITX3	110.1	109.4	113.8	107.4	107.7	95.7	121	110.4
PIWIL1	113	111.7	105.6	119	111	100.4	116.6	114.8
PIWIL2	116.3	115.9	131.9	119.2	104.8	99.3	132.8	121.7
PIWIL2	114.3	102.9	116.5	107.2	104.1	91.7	115.1	113.2
PIWIL3	125.8	107.4	123	117.9	104.9	102.9	119.2	118.4
PIWIL4	113.3	123.9	118	128.3	108.6	102.2	133.5	134.5
PJA1	125.5	116.7	114.9	132.1	114	114.6	127.3	111.1
PJA1	241.4	275.6	245.6	272	238.9	204.7	228.3	245.9
PJA1	109	114.5	130.2	123.5	105.4	102.6	107.6	120.2
PJA2	1117.6	949.8	1163.8	990.6	715.3	971	1103.7	964
PJCG6	113.4	125.1	112	116	111.8	103.2	127	114
PKD1	174.3	185.5	162.3	217.1	175.5	174.4	253.9	209.1
PKD1	140.5	142.2	153.2	160.7	131.5	129.3	149.3	150.7
PKD1L1	116.4	130.8	119.2	131.3	110.9	108.3	127.6	123.6
PKD1L2	115.5	104.7	119.2	114.3	107.6	92.8	134.5	110.1
PKD1L2	117	124.9	116.2	122	105.1	100.1	121.7	122.8
PKD1L2	117.5	102.8	118.4	118.2	103	97.8	110.7	125.2
PKD1L2	119.5	113.4	113.1	112	106.8	89.5	127.5	109.4
PKD1L3	124.3	113.1	127.6	130.5	112.4	101.2	114.5	123.7
PKD2	155.3	160	172.7	138.5	144.3	137.3	164.6	152.5
PKD2L1	115.4	114.7	118.3	107.7	111.9	105	120.6	111.4

PKD2L2	112.8	109.4	108.7	117.6	109.4	94.6	113.3	109.3
PKDREJ	116.2	114	112.6	112	106.6	104.3	116.3	119.3
PKHD1	116.7	113.1	116.7	129.2	107.2	100	120.8	115.2
PKHD1	124.7	114.1	121.5	118.8	102.4	103.2	120.2	116.4
PKHD1	127.2	121.1	142.2	124.5	118.6	119	124.7	131.5
PKHD1L1	116.3	128.2	123.9	125.8	115.9	107	115.4	117.8
PKIA	378.5	402.2	487	342.4	395.8	308.6	343.2	316.8
PKIA	118.9	120.1	113.7	119.4	105.5	104.5	128.1	122.1
PKIA	166.2	166.1	189.6	163.4	168.3	105.6	126	141.5
PKIB	117.4	125.2	115.8	118.1	105.5	98.7	109.7	114.7
PKIB	120.1	121.4	142.9	123.4	109.3	117	119.3	116.9
PKIG	106.1	126.8	122.5	128.9	108.4	110.2	108.2	129
PKIG	132.5	119.6	125.1	134.2	122.4	113	122.7	126.8
PKIG	115.2	113.7	132.8	115	117.3	110.7	125.7	128.5
PKLR	122.1	120	118	119.5	104	101.7	123	116.2
PKLR	105.9	119.8	121.3	120.4	110.2	96.1	114.1	116.9
PKLR	116.9	117	121.8	119.8	115.2	105.9	120	107.9
PKLR	121.8	118.7	118.4	117.8	108.2	93.2	127.4	116.4
PKM2	1117	1179.2	935.5	1357.8	523.7	804.5	1379.1	1018.6
PKM2	207.9	209	168.6	223.3	151.1	169.1	260.2	193.1
PKM2	201.5	154.1	139.9	187.1	141.6	154.5	207.1	166.1
PKMYT1	115.8	117.9	117.7	109.9	119.2	101.9	118	112.2
PKN1	106.7	111.6	124.8	114.1	106.9	105.3	113.5	111.7
PKN1	131.6	131.9	123	139.8	113.9	106.6	128.8	138.2
PKN1	155.9	148.6	137.7	152.4	119.9	118.8	166.6	144.9
PKN1	134.2	121.6	140.4	138.5	134.9	122.5	134.1	120
PKN2	133.7	128.6	118	142.1	127.6	106.5	129.8	125
PKN3	119.5	120.8	122.7	117	121.8	95.9	113.2	133.8
PKNOX1	167.2	164.3	160.8	156.1	139.2	126.2	169.6	207.4
PKNOX1	131.6	123.7	131.2	131.9	113.9	104.3	131.7	121
PKNOX2	118.8	109	117.5	115.2	103.2	97.4	113.9	110.6
PKP1	121	109.5	112.9	127.6	103.8	103.3	120.8	118.2
PKP1	116.4	109.8	123.1	126.2	108.6	101.7	121.1	110
PKP2	113.2	111.7	119.3	115.6	117.1	94.9	112.1	115.4
PKP3	124.3	127.4	129.5	123.4	103	100.7	112.8	111.8
PKP4	115.6	110.3	129.5	122.5	110.9	98.1	120.5	109.9
PKP4	164.3	161.5	202.8	176	165.1	200	190.7	185.6
PLA1A	115.9	119.8	119.4	111.5	107.7	100.6	117.7	114.2
PLA2G10	114.3	123.4	123	115	112.7	103.8	116.1	119.3
PLA2G12A	121	112.2	119.7	122.3	113	102.2	118	113.4
PLA2G12B	116.8	122.8	121.2	120.1	107	107.6	120.8	115
PLA2G1B	112.9	113.6	117	118.1	101.3	100.8	116.4	117.4
PLA2G2A	110.1	123.3	112.9	121.7	105.6	102.5	124.5	116.3
PLA2G2D	201.9	158.7	222.6	188.5	218	134.8	224.6	190.3
PLA2G2E	112.4	110.1	124.5	122.9	108.2	97.1	120.1	120.2
PLA2G2F	108.2	121.1	114.1	114.3	109.7	99.3	114.1	121.1
PLA2G3	114.3	111.5	125.7	122.1	109.8	105.3	119.3	113.3
PLA2G4A	112.9	113	132	123.2	107.3	138	120.9	113.7
PLA2G4B	954.3	870.1	1033.3	866.9	589	682.8	695.5	530.8
PLA2G4C	151.9	151.3	139.1	149.9	133.3	126.2	145.7	172.3
PLA2G4D	109.3	116.6	129.8	116.7	117.6	101.3	113.7	116.3

PLA2G4E	112.8	118	118.6	110.7	112.3	96.1	115.3	120.5
PLA2G4F	112.8	104.8	113.5	108.9	103.6	95.4	119.1	117.1
PLA2G5	106.6	114.9	113.6	120.9	102	99.2	109.5	108.5
PLA2G6	133.7	130.5	134.1	151.3	127	121.4	131.9	134.4
PLA2G6	114.5	112.4	120.1	112.4	104.8	104	119	114.7
PLA2G7	1407.2	612.6	1342.7	2255.2	788.2	2784.9	1670	529.6
PLA2R1	114.3	120.4	115.1	124.9	110.8	95.3	125	120.4
PLA2R1	113.6	106.2	120	114.7	108.8	93.9	111.9	107.6
PLAA	131.2	134	136.5	126	118.8	106.4	117.3	123.4
PLAA	121.1	115.5	111	115.7	119.3	101.3	119.6	118
PLAA	126.7	132.4	131.1	138.3	125.1	115.4	140.6	135.5
PLAC1	114.2	113	108.1	117.6	104.8	97.5	113	120.4
PLAC2	130.2	120.3	129.4	133.6	109.9	115.4	132.5	133.8
PLAC4	118.9	117.2	122.7	126.1	117	91.7	116	115.6
PLAC8	924.4	711.6	711.8	820.8	720.5	706.8	915.1	814.7
PLAC8L1	134.2	128	115.8	144.9	124.8	101.9	155.8	139.9
PLAC9	113	115.9	117.1	118.1	106.3	95.8	119.5	116.9
PLAG1	118.7	119.4	130.4	117.7	111.3	100.5	128.1	118.7
PLAG1	148.9	160	148.3	154.7	138.5	131.1	140.8	142.8
PLAGL1	135	134	131.7	140.6	115.2	105.4	133.3	129.6
PLAGL1	123.5	109.5	118.4	117.8	103.6	102.4	124.2	127.2
PLAGL2	261.1	248.1	174.5	264.3	210.7	188.1	328.3	379.5
PLAT	112.4	104.7	106.7	119.3	107.4	98.8	115.3	109.2
PLAU	130.4	120.9	172.7	126.6	107.7	109.3	116.2	116.2
PLAUR	154.2	134.8	146.9	181	155.7	159.5	236.2	143
PLAUR	148	141.2	164.7	208.9	147	177.2	249.1	160.2
PLAUR	226.9	193.6	261.7	436.1	338	430.8	613.1	318.6
PLB1	116.4	119.1	122.9	117.8	110	100.7	111.7	121.1
PLCB1	142.3	143.7	138.1	161.2	123.3	142.7	155.9	133.2
PLCB1	111.7	108.7	125.1	115.6	108.2	102.5	116.2	110.4
PLCB2	276.8	254.6	284	222.1	200.1	237.2	187.6	171.6
PLCB3	124.3	122.2	119.8	121.7	118.3	100.1	127.2	114.6
PLCB4	127.7	122.2	130.9	130.3	117.7	93.6	131	126.9
PLCB4	123.2	116.2	124.6	118.1	108.3	100.1	120.1	114.1
PLCD1	169.5	198.2	147.9	161.5	159.8	147.5	153.6	146.8
PLCD3	120.2	108.9	116.7	131.2	113.9	112.1	120.6	113.9
PLCD4	111.7	114.8	110.3	109.5	119.1	97.2	117.9	116.9
PLCE1	120.5	118.7	123.8	133.7	102.9	105.6	118.4	119.8
PLCG1	169.9	175.6	153.9	173.6	181.5	138.4	144.6	148.4
PLCG1	274.7	254	227.2	259.8	239.6	214.2	212.5	213.2
PLCG2	643.6	475	608	771.8	329.6	524.3	495.2	319.8
PLCH1	121.7	106.3	123.4	116.3	104.7	100.1	116.8	105.6
PLCH2	259.8	181.4	330.4	225.9	152.4	189.7	174.4	162
PLCL1	132	129.8	132.1	127.7	127.8	112.5	132.1	147
PLCL2	789	1073.5	586.6	874.5	765.5	475.9	798.7	575.6
PLCXD1	213.3	188	268.5	223.9	172.9	157.3	229.1	243.7
PLCXD2	109	115.7	121.2	120.7	111.2	96.9	114.5	119.7
PLCXD3	104.9	117.1	113.3	114.8	113.6	98	115.7	107.2
PLCZ1	109.5	114.7	116	114.2	105.6	100.9	126.1	113.8
PLD1	119.4	124.1	130.6	136.3	121.7	108.2	117.4	129.1
PLD2	133.9	123.2	141.1	141.9	109.3	119.9	143.6	128.9

PLD3	121.3	120.2	127.3	124.2	107	107.9	125.6	120
PLD3	1416.6	898.4	992	1289.2	641.1	641.6	920.8	529.8
PLD3	117.6	124	117.2	122.2	104.6	100.5	125.9	105.7
PLD4	121.2	123.4	118.8	131.7	115.9	99.6	135.2	131.1
PLD5	107.3	112.7	116.7	120	105.9	100.5	110.4	114.2
PLDN	617.4	653.3	686.7	590.1	512.9	593.1	728.9	687.8
PLEC1	191	165.7	239.1	200.7	145.9	203	164.1	145.7
PLEC1	111.4	121	121.2	124.1	113.7	99.7	119.7	109.6
PLEC1	124.7	113.5	108.1	126.5	102.5	95.9	121	111.7
PLEC1	117.7	116.7	121.6	121.7	112.9	102	122.5	120.1
PLEC1	115.5	113.1	113.2	119	107	95.5	121.9	113
PLEC1	110.2	124	117.1	115.8	107.2	94.9	121.5	125
PLEC1	121.6	113.1	115.1	130	117.8	102.2	110.2	112.7
PLEC1	110.8	116.5	125.5	130.1	119.5	91.1	113.5	114.2
PLEK	822.3	513.6	665.7	859.9	568	492.2	1084.5	885.2
PLEK2	124.6	128.5	132	127.2	115.1	120.1	123.7	124
PLEKHA1	825.4	718.4	762.8	1032.1	968.5	434.4	1171.8	1005.7
PLEKHA1	738.8	1004.8	577.9	756.9	692.8	542.5	855.7	757.6
PLEKHA3	165.2	154.2	164.5	169.9	155.7	131	183.8	169.2
PLEKHA4	130.3	122.4	122	115.9	109.1	95.9	117.9	134.1
PLEKHA5	133.5	132.9	116.4	129.3	113.9	108.2	131.3	122.2
PLEKHA6	112.2	115.6	124.5	120.9	110.4	95	123.4	120.1
PLEKHA7	114.6	123.9	124	125.6	113.3	101.3	115.8	122.6
PLEKHA8	120.8	109.2	117.1	132.9	107.1	107.8	113.7	122.2
PLEKHA9	183	208.4	188.6	192.1	138.9	145.3	167.5	158.9
PLEKHB1	248.9	244.1	205.3	230.5	168.6	184.4	183.5	155.3
PLEKHB2	195.8	197.2	242.1	244.3	169.1	173	273	282.1
PLEKHB2	253.2	204.5	346.3	285.9	240.9	221.6	295.2	310.9
PLEKHB2	116.1	112.6	121.2	119.2	110.9	108	122.1	116.8
PLEKHC1	114.8	122.5	115.8	112.2	116.6	105.1	136.1	145.2
PLEKHF1	768.5	882.7	711.1	953.4	642.8	602	1090.3	1176.3
PLEKHF2	120	133.4	135.1	120.8	126.3	123.3	141	128.4
PLEKHG1	125.8	116.8	120.3	124.5	111.6	110.3	129.3	120
PLEKHG2	119.9	134.2	124.2	130.7	113.5	99.7	118.2	114.2
PLEKHG3	226.5	218.9	168.1	218.4	166.6	149.8	176.5	179.9
PLEKHG4	170.6	162.5	157.4	182.9	141.3	128.3	145.5	122.6
PLEKHG5	116.5	120.7	112.6	116.7	105	96.2	113.2	110.9
PLEKHG5	123.2	111.7	122.1	125.5	110.5	117.7	126.4	126.8
PLEKHG5	107.6	113.9	108.9	120.4	106.6	104.8	114.8	115.4
PLEKHG5	115.1	119.1	116.8	118.2	109.9	98.2	112.9	121.4
PLEKHG5	122.7	119	123.3	118.1	117.3	95.8	120.7	129.7
PLEKHG6	122.2	115.6	112.7	112.6	110.1	105.2	113.7	110.6
PLEKHH1	113.6	110.5	115.7	115.1	106.1	99.8	124.4	120.9
PLEKHH2	117.5	118.6	119.4	120.8	117.6	96.9	116.7	125.4
PLEKHH3	119	121.1	130.8	119.3	116	101.3	125.5	120.7
PLEKHJ1	153.5	163.1	158.7	158.5	138.8	130.7	138.5	122.8
PLEKHM1	201.9	202.9	196	210.5	164.2	191.1	252.4	228.1
PLEKHN1	127.6	127.8	126.7	129	124.7	112.6	130.6	129.9
PLEKHO1	377.7	398.4	386.9	384.7	296.6	394.9	374.2	294.4
PLEKHQ1	195.7	166.6	230.7	255	159.8	265.9	287.3	204.2
PLG	118.2	117.2	113.5	119.3	109.4	101.3	116.4	112.6

PLGLB1	195.3	183.5	189.7	203.8	206.2	126.4	199.6	233.6
PLIN	116.7	111.8	131.2	116.6	120	104.9	124	115.9
PLK1	127.5	136	138.9	146.1	121.3	117.2	143	143.2
PLK2	124	118.4	123.5	113.9	118.1	106.4	121.3	114.8
PLK3	126.8	141.2	153.9	159.9	139.4	125.9	136.8	140.7
PLK4	138.3	133.7	134.4	128.8	113.5	113.9	131.3	128.7
PLN	117.8	122.5	130.6	118.1	115.5	103.8	129.9	120.8
PLOD1	241.5	174.2	382.9	296.4	139.2	188.9	256.1	157.2
PLOD2	115.7	120.3	115.9	117.4	121.7	97.9	108.5	108.1
PLOD2	110.2	117.9	114	122.2	106.5	99.1	119.7	117.8
PLOD2	122.4	120.3	129.9	129.6	118.6	115.5	126.4	121.6
PLOD3	380.1	332	437.4	378.3	259.4	361.5	432.8	343.4
PLP1	111.7	117.2	113.9	113.2	100.6	96.9	113.6	117.7
PLP1	112.5	107.9	115.6	113.6	111.1	99.4	117	122.8
PLP1	121.4	124.5	120.8	130.6	119.3	103.8	122.1	127.3
PLP2	131.3	134.7	150.3	124.2	125.3	128.1	139.5	123.7
PLRG1	388.5	479.9	501.1	437.8	399.7	379.9	385.5	381.6
PLS1	150.1	158.5	133.5	151.9	136.5	111.3	156.3	152.1
PLS3	116.8	109.2	110.5	114.4	109.6	102.5	113.4	102.8
PLSCR1	124.6	119.2	124.1	118.9	108.8	108	117.4	121.4
PLSCR2	117.4	117.5	115.4	124.6	106.1	103.2	115.7	118.4
PLSCR3	2782	3167	1910.5	2759.1	2432	1705.1	2035.5	2007.2
PLSCR4	119.2	133.7	115.6	123.4	102.9	99.9	112	128.5
PLTP	118.4	109.6	128.2	112.9	111.4	103.2	119.2	116.3
PLTP	115.4	117.1	119.1	125.4	124.2	103.4	124.6	115.6
PLUNC	109.1	115.6	106.7	114.8	105.3	97.5	113.1	108.3
PLVAP	110.4	109.8	126.9	112.5	107.3	101	111.9	109.1
PLXDC1	118.9	125.6	124.5	125.7	112.5	98.4	120.2	129.2
PLXDC2	169	137.7	246.7	127	110.3	187.5	127.7	117.2
PLXNA1	140.4	142	138.2	146.1	149.3	122.2	161.1	162.9
PLXNA2	116.3	126.6	125.6	144.3	118.5	113.6	136.3	116.4
PLXNA3	164.8	150.2	139.2	148.3	143.1	117.3	164.3	139.3
PLXNA4B	107.6	107.8	114.1	111.3	104.9	101.3	126.4	108.6
PLXNB1	113.1	130	117.5	132.3	110.3	96	122.3	109.1
PLXNB3	124.5	110.8	115.5	114.2	109.4	96.5	120.1	113.4
PLXNC1	125.1	127.6	143.6	131.8	120.4	101.8	115	114.1
PLXND1	107.5	129	146.3	150.2	124.4	111.7	135.1	123.8
PMAIP1	158.2	150.2	136	155.7	151.4	134.5	293	363.1
PMCH	119.6	117.7	112.5	120.1	113.5	105.4	115.7	122
PMCHL1	112.8	123.3	133.4	126.5	112.5	111.3	125.8	126.1
PMFBP1	138	129.9	144.1	131.1	114.3	107.7	125	112.6
PML	120.5	122.4	128.8	124.6	113	108	126.3	109.9
PML	113.1	115	115.4	119.4	102.5	98.7	126.8	110.3
PML	127.6	121	127.9	133.8	103.5	104	129.5	126
PML	129.9	122.4	123.5	118.5	111.9	102.3	122	116.3
PML	116.6	106	119.2	123.5	110.1	103.4	118.4	117.7
PMM1	334.3	348.8	373.8	380.2	284.8	350.8	381.8	402.7
PMM2	159	164.5	169.9	154.5	150.4	137.2	166.1	172.3
PMP2	117	118.7	110	117.4	108.7	95	117.3	118.6
PMP22	108.9	119.5	117.8	117.2	109.5	94	116.6	114.8
PMP22	120.7	119.3	121.6	122.3	108.5	106.9	125.3	110.2

PMP22	129.3	115.2	314.9	137.6	110.4	148.1	132.9	111.4
PMP22CD	112.9	116.4	123.9	149.8	102.8	103.8	117.3	118.3
PMPCA	339	394.3	322.4	319.8	227.4	250.9	325.8	279.8
PMPCB	495.7	620.1	730	459	367.5	554.7	383.7	531.3
PMS1	148.7	146.5	156	143.6	138.7	110.3	135.2	136.4
PMS2	130.9	139.1	128.7	140.8	132.8	107.4	129	129.8
PMS2	117.7	119.9	123.2	123.9	117.1	100.3	120.3	119.1
PMS2CL	121.6	124	137.7	142.2	117.7	101.8	131	128
PMS2CL	109.8	113.3	116.7	111.4	110	99.9	105.7	123.1
PMS2L2	117.2	115.8	119.3	117.4	110.4	100.4	123.2	120.3
PMS2L3	142.1	151.5	160.5	149	125.7	128.6	138.1	120.5
PMS2L5	147.5	124.5	152.8	132	128	123	144.3	148.9
PMVK	140	160.7	174.9	167.5	150.8	127.1	156.3	156
PNCK	117.1	124	118.3	129.8	119.4	100.4	126.3	128
PNKD	205.9	171.1	171.5	300.1	189.1	384.8	346.1	295.2
PNKD	142.1	125.9	140.5	135.7	118.4	110.1	133.3	133.1
PNKD	148.3	146.4	166	148.1	137.1	114.7	120.8	131.9
PNKP	300.5	339.7	297.9	323.8	235.5	240.3	261.1	239.3
PNLDC1	121.5	101.6	110.7	115.6	112.9	101.4	120.9	124.6
PNLIP	117.1	124.3	119.4	119.1	109.5	97.6	124	120
PNLIPRP1	113	115.7	113.6	121.3	105.7	99.7	112.5	111.6
PNLIPRP2	107.6	105.5	109.4	111.8	108.3	91.1	106.6	111.5
PNLIPRP3	103.7	109.9	111.9	107.3	98.9	95.1	113.1	112.5
PNMA1	151.7	193.9	178.7	172.4	157.4	142.7	170.1	153.2
PNMA2	125.2	119.8	124.7	128	117	103.6	123.4	131
PNMA3	195.5	215.1	182.6	190.3	178.5	127.2	150.6	146.5
PNMA5	106.9	118.3	114.5	114.3	113	99.5	117.4	112.5
PNMA6A	116.6	116.2	129.7	116.2	112.5	104.2	118.7	121.4
PNMT	104	110.3	110.9	112.6	99.2	96.3	114	113.3
PNN	659.8	653.5	833.4	557.1	557.6	553.3	725.6	585.7
PNOC	116.5	113.9	113.4	114.1	108.6	106.9	115.9	113.7
PNPLA1	113.5	112.8	118.9	114.1	102.4	98	122.2	114.1
PNPLA1	110.3	120.9	120.4	125.5	115.2	104.7	127.2	106.1
PNPLA2	219.6	286	218.8	258	167.7	193.8	278.2	289.8
PNPLA4	110.1	124	113.6	110	112.4	103.2	115.2	109.5
PNPLA5	111.1	111.9	111.7	116.3	102.7	99.7	110.5	103.4
PNPLA8	160.7	171.8	158.3	155.2	164.7	126.1	152.9	157.3
PNPO	325.9	298.9	342	306.3	211.7	222.5	218.8	221.4
PNPT1	3843.1	4010.9	2845.2	3966.7	3725.7	1287.5	4623.6	4535
PNRC1	129.2	138.8	140.8	129.9	117.5	107.1	116.8	133.2
PNRC2	776.5	887.6	760.3	924.8	803.2	773.9	890.1	950.3
PODN	119.1	126	125.6	129.8	118	99.2	117.4	117.7
PODXL	115.7	115.5	120.8	130.3	114.8	98.3	122.9	126.7
PODXL	116.1	108.1	108.3	123.3	103.4	101.2	118	109.9
PODXL	125.4	118.5	122	127.9	110.9	98.8	122.2	130.9
PODXL2	127.3	124.6	136.1	125.9	114.3	99	116.2	123.5
POF1B	117	121	123.7	134	110.7	110.6	127.7	129.2
POFUT1	590.7	856.3	823.9	763.2	890.4	559	1024.2	885.4
POFUT1	119.5	112.2	110.5	114.2	119.3	96.1	110.7	117.3
POFUT1	119.9	111.2	116.4	113.4	108.2	94.6	113.8	110.7
POFUT2	246.7	254.6	273.2	250.6	197.3	228.7	289.9	265.4

POFUT2	127.5	116.7	131.8	155	119.1	115.2	131.7	125.3
POFUT2	225.3	241.6	239.7	240.5	206.5	216.5	287.7	222
POGK	899.4	1050.6	744.8	964.1	760.3	621	1054.4	1049.9
POGZ	153.3	148.5	147.4	160.4	147.1	112.4	143.2	149.1
POGZ	129.1	122.6	135.9	133.5	119.9	107.6	134.3	128.6
POGZ	122.7	120.3	115.9	126.4	124.4	106	114.4	117
POL3S	153.1	148	127.9	148.4	131.6	106.2	145.1	134.7
POLA1	114.5	127.4	123.5	124	122.2	97.1	118.6	135.9
POLA2	193.6	202.8	207.5	207.7	164.5	157.6	165.5	150.3
POLB	296.1	326.4	422.6	322.8	298.8	269.6	258.3	331.3
POLD1	170.3	177.4	158.5	163.1	138.9	132.9	177.9	148.3
POLD3	110.5	121.5	126.8	125.2	115	104	115.5	125.4
POLD4	149.9	172.1	144.8	145.1	122.3	129.2	134	134
POLDIP2	156	158.8	147.5	174.2	147.4	130.9	159.4	164.6
POLDIP3	322.5	352.6	350.1	410.7	279.4	393.9	445.9	466.3
POLDIP3	119.3	127.6	122.8	131	114.5	101.7	129.7	117.4
POLE	158.4	159.6	150.5	161	145.3	137.4	195.3	200.1
POLE2	128.9	133.1	127.5	144.6	117.3	96.6	128.9	125
POLE3	1328.9	1443.4	1534.3	1610.3	1232.5	987.3	1563.1	1512
POLE4	1005.1	1255.7	1058.1	1164.5	970.9	716.4	1092.1	1106.5
POLG	299.2	333.3	300.5	421.8	288.7	265	301.7	340.3
POLG2	111.1	120.8	131.5	134.9	125.1	128.8	144	123.8
POLH	130.6	124.2	118	127.4	110.7	104.2	116.7	115.8
POLI	113.2	113.5	120.7	116.8	108.1	104.5	113	113.5
POLK	118.8	119.3	115	114.1	100.1	97.8	122.5	120.7
POLL	127.1	127.3	138.6	128.5	115.8	118.9	131	133.2
POLM	196.8	204.3	238.9	207.2	162.7	215.8	228.2	192.3
POLN	119.5	121.7	123.8	132.7	117.5	99.6	130.3	130.4
POLQ	116.2	106.3	127.4	121.3	116.2	104.6	123.4	115.1
POLR1A	116.3	119.6	110.7	128.7	115.2	103.4	128	124.5
POLR1B	125.7	126.3	117	118.4	118.2	103.6	130.3	109.8
POLR1C	311.5	386.3	257.3	336.3	303.9	204.6	351.1	324.3
POLR1C	291.1	285.9	286.2	283.9	286.8	202.2	282.8	242.1
POLR1D	1944.1	2337	2316.3	1867.1	1950.4	1675.2	1945	2431.1
POLR1E	239.7	254.4	300.2	263.7	209	212	271.6	284.2
POLR2A	841.9	809.3	605.9	899.3	753.2	619.9	1311.1	1468.2
POLR2B	277.7	284.1	292.1	277.8	273.8	298.9	344.5	244.6
POLR2C	142.2	150.4	159.2	159.5	136.3	143.6	168.6	144.8
POLR2C	587.1	568.4	549.9	641.4	430.1	421.2	691.6	672.2
POLR2D	206.5	207.4	178.7	205	190.3	167.9	238	221
POLR2F	680.3	812.6	748.7	775.5	545.5	611.3	541.5	515.4
POLR2G	503.3	600	493.9	585.7	483.1	610.1	424.1	481.8
POLR2H	284.3	382	260.1	352.5	264.2	279	310.5	263
POLR2I	308.6	318.1	385.1	370.5	265.2	289.8	254.6	221.8
POLR2J	159.4	180.4	148.9	193	136	143.2	168.9	153.5
POLR2J2	112.8	117.4	122.8	120.7	112.2	101.2	119.4	127.8
POLR2J2	122.2	127.8	127.4	118.7	132.4	106.7	130.2	139.9
POLR2J3	277.4	214.9	287	286.6	191.2	150.4	169.4	206.7
POLR2K	126.5	135.9	130.6	127.2	112.8	117.7	135.5	142.3
POLR2L	171.5	183.7	202.6	219.6	146.4	163.7	200.2	175.1
POLR3A	220.6	224	219.9	243.4	213.5	191	246.5	235.4

POLR3B	218.6	272	224.6	261.6	215.8	161.1	209.3	184.1
POLR3C	407.2	501.5	574.8	468	329.5	395	489	414.2
POLR3D	136.4	133.8	129.2	132	119.8	110.5	127.5	125
POLR3F	126.3	135	124.7	136.8	119.7	120.6	128.5	131
POLR3G	117	115.8	118.3	115.2	108.5	103.3	105	119.7
POLR3GL	583.8	610.4	806	737.6	580.3	687.3	526.1	546.7
POLR3H	288.4	352.5	283.4	318.4	258.5	202.5	319.5	313.8
POLR3H	120.8	111.6	123.2	124.4	106.6	96.2	123.5	120.7
POLR3H	107.1	114.2	104.4	108.8	101.8	99.5	132.9	112.4
POLR3K	212.6	273.1	247.4	237.4	209.4	200.3	220.5	202.1
POLRMT	364.4	364.3	309.5	325	256.8	236.3	306.1	269.4
POLS	292.1	314	273.9	342.4	317.9	249.5	338.7	337.2
POM121	110.6	113.7	108.8	118.6	115.4	100.9	125.9	123
POMC	140.4	138.7	142.8	144.1	120.5	115.3	137.3	127
POMC	107.7	117.7	110.2	114.4	117.1	105.4	116.2	124.9
POMGNT1	184.4	194.7	212.6	213.8	163.8	142.9	185.2	186.7
POMP	106.9	114.2	119.4	116.1	105.4	95.7	110.7	107.9
POMT1	126.2	126.7	132.4	126.7	118.4	106	121.2	118.9
POMT1	226.9	205.2	198.1	197.7	151.8	150.4	175.9	172
POMT2	162.2	145.3	146.4	173.6	123.5	122.1	163.3	158.7
POMZP3	123.4	129.2	120	128.3	115.7	107.1	125.3	123.9
PON1	110.8	112	112.9	122.6	108.4	90.5	116.9	114.8
PON2	150.9	142.8	235	158.8	144.9	109.5	155.4	137.3
PON3	102	110.9	116.2	113	114.5	93.9	123.8	111.9
POP1	138.8	134.2	142.6	143.4	125	121.4	130.9	136.6
POP4	134.7	146.8	147.1	147.2	131.4	133.8	152.9	146.3
POP5	213.5	219.8	221.3	222.4	188.4	142	180.6	148.7
POP7	175.1	188.5	198.5	172.8	175.6	151.4	178.7	191.1
POPDC2	112.1	118.7	120.9	118.5	108.6	103.6	121.9	119
POPDC3	112.1	119.4	126.3	130.4	113.9	101.9	122.4	108.1
POR	173.4	196.4	150.7	189.2	148.1	195.5	255.7	186.6
PORCN	134.2	144.1	143.7	135.8	121.1	102.8	124.6	117.1
PORCN	127.9	122.7	141	136.9	125.4	110	137.6	134.4
POSTN	109.3	114.2	119.8	119.9	103.5	103.4	118.2	125.2
POT1	116.1	119.1	122.8	130.1	106.5	101.5	109.2	125.6
POT1	126	137.1	140.2	140.3	120.8	117.7	126.6	148.2
POT1	115.7	113.7	118.8	114.5	100.6	105.8	118.2	106.2
POTE15	120.5	121	126.1	130.9	107	104.2	116.6	119.1
POTE2	109.9	116	102.4	108.6	108.3	90.4	115.1	105.5
POTE8	111.9	121.1	116.7	111.7	102.3	101.2	111	123.5
POU1F1	110.2	113.7	105.9	115.1	102	103.5	110	111.9
POU2AF1	133.2	145.3	141.5	161.4	122.8	119.2	133.1	121.3
POU2F1	131.7	146.9	142.7	140.9	140.9	137.3	164.1	146.2
POU2F2	124.3	141.5	111.6	118.1	119.8	96.3	129.4	117.7
POU2F3	118.6	128	129.4	124.5	108.7	100.7	127.9	127.2
POU3F1	109.2	116.3	117.8	120.6	105.4	96	111.9	110.4
POU3F2	121.4	117.2	126.9	115.6	107.5	102	124.8	111.1
POU3F3	127.8	115.8	115.8	124.5	110.7	108.3	119.1	108.1
POU3F4	128.9	116.9	123.7	111.1	103.5	96.2	125.3	109.2
POU4F1	117.2	112.4	108.5	119.6	102.2	96.5	121.5	117.9
POU4F2	104.3	115.9	116.1	117.6	107.1	98.5	119.7	112.5

POU4F3	115.7	114.4	117.4	108.9	111	99.8	116.3	118
POU5F1	102.5	116.1	106.8	118.4	105.8	93.6	115.3	114.2
POU5F1P1	115	125.1	125.7	113.7	111	103.1	107.3	117.2
POU6F1	127.3	134.3	144	134.3	120.8	109.3	118.6	123.7
POU6F2	112.7	111.3	111.9	109.1	104.9	94.8	107.1	108.4
PP2447	1502.7	2263.7	2742.1	1841.2	1333.3	1505.6	1084.4	982.2
PP8961	114.9	120.4	115.7	120.7	116.2	104.7	120.2	116.7
PPA1	1488.8	1458.4	1376.8	1745.6	1197.4	1194.1	1681.2	1580.9
PPA2	293.7	246.5	327.1	291.1	205.5	316.1	257.8	205.9
PPA2	2538.1	1916.2	1633.3	2107.1	2040.5	1042.3	2207.1	2198.4
PPA2	112.4	113.7	118.7	121.7	101.4	100	124.7	117.7
PPAN	228.3	190.1	186.3	191.4	187.8	139.2	187.8	155.1
PPAN-P2RY	114.7	120.2	108.6	126.4	112.3	105.5	128	116
PPAP2A	112.5	126.5	117	120.9	114	102.5	127.2	112.9
PPAP2A	121.1	120.2	126	141.4	121.7	104.3	131.8	124.2
PPAP2A	151	149.9	129.6	149.8	140.8	110.4	163.1	154.2
PPAP2B	129.8	125.5	136.9	113.7	120.7	99.4	113	127.4
PPAP2B	112.9	115.8	110.1	113.3	120.5	96.9	127.5	118.5
PPAP2C	131.9	113.7	125.5	132.4	120.3	106.9	134.8	125
PPAP2C	118.8	118.9	121.5	124	110	106.5	127	122
PPAP2C	113.2	120	118.4	122	111.7	97.9	124.8	130
PPAPDC1A	113.1	112.6	123.5	122.8	100.8	102.8	116.7	117.7
PPAPDC1B	145.7	145.2	159.8	141.1	125.5	104.2	154	145.6
PPAPDC2	169	164.7	221.8	209.3	159.8	179.9	150.8	138.6
PPAPDC3	121.2	105.9	123.4	113.2	110.1	94.9	115.5	111.5
PPARA	110.4	119.4	116.1	120.4	116.7	107	121.5	121
PPARA	120.2	119.6	115.5	119.5	112.3	94.9	120.2	121.8
PPARBP	184.9	187.5	159.1	194.9	143.8	137.5	182.5	174.2
PPARD	120.1	118.7	118.2	122.3	118.3	98	125.3	116.6
PPARD	169.8	173.2	155.7	187.9	138.2	139.6	163.8	149.9
PPARG	114.3	117.3	118.2	114.4	109.5	99.9	121.9	112.8
PPARG	117	145	147.2	116.5	122.7	113.4	128.9	119.4
PPARG	116.5	121.3	117.8	128.9	114.2	96.2	124.4	118.2
PPARG	122.1	118.9	121.7	131.7	113.1	97.3	119.9	131.4
PPARGC1A	111.9	129.1	123.8	114	120.6	98.4	122.9	111.5
PPARGC1B	116.1	131.8	120	122.3	124.2	96.6	121.2	117.3
PPAT	155	158.1	147	151.7	143.2	122.8	147.7	155.9
PPBP	126.5	116.3	140.5	118.4	108.7	114.4	123.2	113.9
PPCDC	117.1	120.8	121	130.6	110.6	99.6	118.5	124.1
PPCS	334.7	398.7	571.7	378.3	300.1	468.9	255.5	221.3
PPCS	465	542.5	763.8	477.7	444.4	465.6	400.5	327.4
PPEF1	112.4	121.2	127.1	128	103.7	100.8	118.8	117.6
PPEF1	114.4	114.3	121.6	123.8	110	97.5	116.9	117.2
PPEF2	113.3	110.6	111.6	120.3	104.5	95.5	122.3	117
PPFIA1	385	300.3	421.5	483.2	393	373.6	594	531.8
PPFIA1	141.8	140.8	154.6	155.9	147.3	113.7	151.1	137.8
PPFIA1	153.9	154.8	180.6	165.4	144.5	146.9	154.1	168.6
PPFIA2	117.6	121.5	119.5	126.1	111.5	103.5	121.7	118
PPFIA3	109.5	115.8	112.1	116.7	111.2	93.5	112.3	104.3
PPFIA4	120.8	121.4	120.1	120.5	108.5	95.1	111.9	118.7
PPFIBP1	111.7	112.1	113.2	117.2	106.5	98.1	115.4	108.7

PPFIBP1	117.3	113.5	113	119	106.6	98.3	117.1	113.1
PPFIBP1	109.1	117.7	118.6	121.1	111	102.7	113.4	120.3
PPFIBP2	242.3	259.5	279	277.9	213.4	176.2	259.2	259.3
PPGB	245.7	197.3	234.6	231.5	157.4	189.6	194.2	168.1
PPHLN1	386.8	327.1	419.8	382.3	418.8	315	336.7	308.5
PPHLN1	124.4	121.9	136.4	133.9	123.8	112.6	124	132.6
PPHLN1	139.8	127.8	135.7	139.3	123.1	107.2	134.4	136.5
PPHLN1	417.6	380.7	509.1	443	407.2	404.3	385.6	405
PPIA	131.1	124.1	131.5	133.7	110	115.3	131.8	136.3
PPIB	137.8	147	139.7	131.4	115.1	118.4	137.6	135.2
PPIC	115.8	102.4	99.1	117	107.9	105.6	109	115.5
PPID	118.6	121.4	118.6	123.4	110.6	94.5	121.9	122
PPIE	218.6	231.9	263	220.1	205.5	222.4	185.4	193.5
PPIE	127.1	113.2	126.4	125.4	121.6	110.8	128.4	117.8
PPIF	128.8	145.3	128.4	142.2	116.8	114.4	174.6	136
PPIG	128.3	122.1	134.2	125.8	120.8	112.9	121	118.4
PPIH	234.6	223.3	267.7	201.8	183.6	202.6	172.4	156.9
PPIL1	176.2	184.7	180.7	186.8	167.8	130.9	174.6	194.5
PPIL2	118.4	110.3	125.7	122.8	108.6	102.6	123.5	121.9
PPIL2	126.1	124.2	117.3	123.6	116.1	104.7	134.1	118.8
PPIL2	113	117.6	111	122.2	111.1	94.9	125.2	107.3
PPIL3	124.5	151.9	144	137.9	120.4	107.9	134.7	126.5
PPIL3	102	113.8	120.7	112.1	111.3	99.9	118.8	119.2
PPIL3	729.5	701.7	942.1	847.6	682.9	597	543.4	551.5
PPIL3	130.8	132.8	154.3	136.9	127.2	103.4	118.8	127.4
PPIL4	117	121.4	122.5	119.1	118.6	104.4	112.5	108.5
PPIL5	289.3	242.8	284.6	304.1	225	263.1	358	291.4
PPIL5	109.3	112.2	109.5	111.4	115.1	97.6	111.8	110.3
PPIL5	113.8	117.6	115.3	116	107.3	102.1	127.5	117.4
PPIL6	106.2	102.1	112.6	122.8	111.5	101.1	120	118.8
PPL	110.4	113.7	117.4	120	97.6	95.7	114.5	107.9
PPM1A	114.2	110.4	113.4	115.6	107	100.6	114.7	106.1
PPM1A	169.8	177.1	179.2	198.4	190.2	137.5	192.7	184.3
PPM1A	121.8	126.9	134	127.7	122.7	108.4	123.1	119.6
PPM1A	125.1	127	134.5	140.5	134.2	112.3	127	139.1
PPM1B	107.7	119.9	128.8	119.9	114.6	107.4	117.6	121.6
PPM1B	1121.6	980.2	970.8	1039.9	857	713.6	1173.6	1022.8
PPM1B	183	148.4	147.5	156.2	174.1	108.5	169.3	174.5
PPM1B	138.2	132.2	154.5	152.6	131.2	116.3	126.7	129.6
PPM1B	124.4	119.5	121.6	124.5	117.1	104.7	117.8	123.9
PPM1D	231	265	178.3	281.5	172	174.9	275.6	342.3
PPM1E	126.1	118.1	130.2	124	116.6	94.9	123.3	120.2
PPM1F	512	514	587.8	555.5	390.5	325.8	371.9	328.4
PPM1G	111.6	115.7	121.3	119.1	111	101.4	117.2	110.9
PPM1G	1241	1416.7	1057.3	1392.7	1138.9	754.2	1321.5	1044.7
PPM1J	115.6	116.8	124.8	116.6	105.7	96.7	117.6	119.4
PPM1K	231.1	186.9	196	198.2	198.1	131.7	202.6	201.4
PPM1L	116.4	114.4	122.3	116.1	113.8	99.1	122.8	120.6
PPM1M	945.1	1123.8	1273.5	1033.7	596.7	816.7	616.6	505.7
PPM2C	152.7	144.6	149.8	141.2	131.4	139.6	168.5	145.3
PPME1	145.9	168.6	158.6	172.1	139.7	124.6	155.3	149.5

PPOX	212.7	166.5	247.2	164.3	148.1	160.3	132.9	126.9
PPP1CA	219.8	240.6	226.3	257.5	187.1	202.3	221.9	208
PPP1CB	411.7	485.8	459.8	431.8	445.8	335.2	488.6	624.4
PPP1CC	1651.5	2046.6	1556.9	1600.9	928.6	819.2	1346.4	1638.1
PPP1R10	168.7	153.1	143.8	169.6	172.8	142.6	226.4	262.1
PPP1R11	1241.5	1399.8	1381.4	1329.9	1061	1323.9	1291.3	1357.9
PPP1R12A	216	211.5	157.2	239.6	215.6	174.8	281	219.3
PPP1R12B	135	125.4	134.7	131.7	126.4	109.4	132.3	143
PPP1R12C	170.8	161.5	155.5	148.4	130.6	118.4	139.2	144.2
PPP1R13B	188.8	211.4	206.5	199.4	171.3	168.8	226.6	248
PPP1R13L	139.1	120.7	125.7	135.8	110.3	108.3	119.4	116.7
PPP1R14A	136.7	148.5	145	139.7	127.5	110.2	130.8	133.4
PPP1R14B	451.7	597.8	992.9	445.9	445.5	843.1	391	338
PPP1R14C	112.1	119.9	121.4	115.9	112.7	105.4	117.8	118.4
PPP1R14D	113.6	130.3	120.1	127.6	114.6	109.7	130.5	116.6
PPP1R15A	682.6	836.1	556.5	1246.6	1004.2	781.1	1926.7	2536.3
PPP1R15B	135.7	144.1	135.9	134.4	114.5	104.1	138.8	125.8
PPP1R16A	135.6	123.3	133.6	139.3	119.1	108.9	119.5	119
PPP1R16B	329.9	451.5	435.6	453.1	348.1	405.7	470.2	479.6
PPP1R1A	120.9	109.3	118.5	115.1	103.9	101.8	121.3	114.3
PPP1R1B	112.5	117.7	128.8	110.2	108.1	102.4	119.9	126.8
PPP1R1B	110.4	110.2	112.8	112.1	107.4	104.2	122.4	116.2
PPP1R1B	122.2	124.5	132.6	138.6	117.7	116.2	131	136.6
PPP1R1C	113.9	115.7	114.4	118	104	106	113	122.3
PPP1R2	304.7	339.3	381.7	322.3	293	378.8	484.7	620.7
PPP1R2P3	102.4	112	109.6	112.3	109.4	101.2	105	107.9
PPP1R2P9	116	113.5	114.6	114.7	106.6	100.1	123.3	112.2
PPP1R3A	113.7	109	122.1	119.6	115.9	106.5	120.8	114.9
PPP1R3B	104.5	118.3	113.3	118.3	103.3	99.2	116.5	122.6
PPP1R3C	117.3	108.4	104.4	121.3	103.7	97.2	116	111.7
PPP1R3D	164.3	152.3	145.2	156.5	158.7	128.2	147.2	135.9
PPP1R3F	236.8	252.3	181.4	246.6	201.5	146.4	272.4	291.8
PPP1R7	216.5	243.2	285.1	278	247.2	313.6	270	226.7
PPP1R8	180	220.4	210.3	205.2	215.8	173.6	195.2	191.8
PPP1R8	124.8	154	142.2	141.1	125.3	108	140.1	150.4
PPP1R8	107.8	116.2	119	119.4	108.8	104.8	112.1	110.7
PPP1R9A	117.1	121.7	110.8	116.6	106.8	105.9	111.9	109.8
PPP1R9B	124.8	128	125.5	121.4	100	100.3	120.5	112
PPP2CA	584.8	648.4	648.8	756.3	528.1	560.1	821.9	957.1
PPP2CB	118.6	121.7	128.4	132.8	112.3	101.6	134.9	125
PPP2CB	149.7	170.3	160	175.4	144.6	136	207	208
PPP2R1A	432.8	508.1	558.5	641.7	466.8	511.1	853	664.6
PPP2R1B	116.9	118.9	119	131.8	113.5	103.6	115.2	122.6
PPP2R1B	116.8	115.3	125.5	119.4	115.2	99.2	120.3	126.9
PPP2R1B	118	131	123.2	140.3	124.4	113.6	126	128.5
PPP2R2A	728.1	774.9	557.1	885.5	658.2	550.9	1219.3	1332
PPP2R2B	205.4	209.6	188.3	172.2	148.1	138.2	197.9	196
PPP2R2B	111.8	118.4	124.9	135.7	115.1	106.3	124.6	115.6
PPP2R2B	113.9	114.6	112	111.9	114.9	96.6	116.2	112.7
PPP2R2B	121.4	118.9	119.5	113.8	110.3	101.3	117.3	122.6
PPP2R2B	371.3	318.6	344.8	376.3	294.5	233	304.3	268

PPP2R2B	112	106.8	118.2	115.7	105.2	97.4	120.2	121.8
PPP2R2C	125.4	118.6	122.6	120.9	106	106.7	116.3	110.5
PPP2R2C	119.8	127.6	121	114.7	106.5	98.3	117.9	119.2
PPP2R2C	115.4	115.7	121.3	123.8	105.2	98.6	123	120.1
PPP2R2C	115	108.6	126.1	123.9	106.7	95.9	123.4	120.9
PPP2R2D	125.6	127.3	151.4	146.1	134.6	123.1	152.5	134.5
PPP2R2D	119.2	126.9	126.5	134	119.8	99.7	117.1	125.7
PPP2R2D	311.1	397.6	318	393.9	360.3	269.5	422.6	423.6
PPP2R3A	109.1	120.5	120	126.4	111.4	104.9	121.3	120.5
PPP2R3A	115.1	121.3	119.7	119.4	114.1	101.7	120.3	113.9
PPP2R3A	116.3	118.8	138.7	120	110.5	116.7	126.6	125.7
PPP2R3B	121.7	138.7	114.7	126.3	117	107.9	123.9	123.4
PPP2R3B	139.4	137.8	131.6	132.4	126.3	110.8	140.3	128.6
PPP2R3B	127.4	126.1	128.9	117.3	118.8	113.6	117	115.9
PPP2R3C	433.9	493.7	495.5	431.7	413	304.4	460.6	480
PPP2R4	108.2	122.6	120.1	116.9	107.5	95.1	127.7	118.3
PPP2R4	116.6	121.5	127.4	140.9	113.2	100.2	121.3	131.7
PPP2R4	109.9	123.8	116.8	122.5	118.3	102.1	124.1	118.5
PPP2R4	240.2	299.9	383.1	360.2	245.8	280.5	276.6	286.3
PPP2R5A	318.2	371.4	386.4	372.1	319	336	386.2	409.1
PPP2R5B	178.1	201.1	156.2	179.6	149.7	126.2	174.5	160.7
PPP2R5C	979.6	965.7	985.5	1105.5	1022.3	1062.5	1120.5	1122.8
PPP2R5C	122.7	129	127.2	133.2	122.8	116.9	133.8	139.9
PPP2R5C	135.1	128.8	145.2	148.2	125	117.1	136.9	131
PPP2R5D	119	118.5	119.3	129.6	119.7	109.9	131.1	115.9
PPP2R5D	127.6	150.9	139.1	138.3	117.3	122.1	145.1	148.7
PPP2R5D	194.4	179.4	203.5	216.4	166.3	177.8	199.1	196.7
PPP2R5E	867.6	893.7	790.4	865.7	737.3	479.2	1037.2	1164.5
PPP3CA	144.3	135.3	138	134.7	120.7	126.7	122.3	135.2
PPP3CB	871.2	854	783.8	891.8	716.6	743.3	868.9	799.4
PPP3CC	297.8	344.1	254.5	327.7	349.9	214.8	302.9	316.8
PPP3R1	500.5	666.4	419.9	687.2	644.3	409.3	636.8	687.3
PPP3R2	117.4	126.1	115.1	112	116.8	90.7	125.3	109
PPP4R1	254.7	308	216.7	241.5	201.1	244.1	293.6	370.6
PPP4R1	163.9	196.9	172.8	227.4	178.6	158	275.4	265.6
PPP4R1L	119.5	116.5	122.6	123	115.5	95.6	121.7	114.7
PPP4R2	115.8	107.3	115.4	116.2	105.4	98.7	119.9	113.4
PPP5C	119.4	129.2	136.1	137.5	129.4	102.3	121.7	113.6
PPP6C	1051.6	969.2	957.3	1201.9	905.6	880.2	1086.5	1244.1
PPRC1	337.7	361	235.5	401.2	278.7	181.4	422.8	450.8
PPT1	1401	1159.5	1824.9	1581	963	1178.5	1366.6	748.1
PPT2	115.6	108.3	114.3	115	116.2	99.9	116.8	111
PPT2	138.1	129.9	117.7	139.5	114.4	102.2	122.9	123.5
PPWD1	293.6	319.7	383.1	372	465.3	302.2	286.9	385.2
PPY	117.9	113.4	112.8	112.8	98.6	96	114	107.8
PPY2	109	110.6	128.8	114.2	111.6	110.3	116.2	125.5
PPYR1	115	111.9	116.9	118.2	110.1	96.2	108.7	110.1
PQBP1	133.4	139	141.2	138.6	113.3	114.6	119.3	147.1
PQBP1	339.2	284.2	330.8	345.3	288.2	217.2	327.2	280.8
PQBP1	137.2	143.3	128.8	137.6	116.5	104.7	131.8	122.8
PQLC1	732.5	776.4	918.9	939.3	562.4	733.3	888.4	803.3

PQLC2	114.2	112.7	115.6	120.4	109.8	98.9	123.4	121
PQLC2	114.5	116.4	112	112.1	104.9	97.5	113.4	117.3
PQLC3	455.9	381.3	915.4	481.5	342.1	521.3	334.3	294.5
PRAC	122.7	123.9	114.5	114.9	106.5	101.8	127.1	108.5
PRAF2	144.5	135	132.3	129.9	118.4	104.5	125.8	127.7
PRAM1	125.2	134.9	167.5	120.8	113.3	105.9	118.6	116.8
PRAME	108.4	117.3	125.5	129.4	115.5	105.4	111.4	112.3
PRAME	135.3	159.5	128.5	142.5	138.3	119.1	148.5	139.2
PRAME	119.5	118.1	120.2	117.3	108.7	103.5	117.3	126.1
PRAMEF1	113.6	118.5	115.6	126.2	109.5	93.9	112.5	114.2
PRAMEF10	115.5	123.7	118.7	119.8	118.8	103.7	127.9	116.1
PRAMEF10	101.5	108.9	109.2	106.7	100.2	96.8	109.6	102.1
PRAMEF10	106.3	119.6	109.7	115.6	98	96	113	110.9
PRAMEF16	108.5	121	113.6	117.5	107.2	94.8	120.5	113
PRAMEF4	117	112.9	122	124.8	113.2	103.8	122.5	106.8
PRAMEF5	142.5	160.2	142.8	146.8	146.7	126.8	148	159.8
PRAMEF6	116.9	109.3	112.7	107	108.2	102.5	117.2	114.8
PRAMEF7	115.6	108	121.1	123.1	117	97.7	126.9	115.9
PRAMEF8	120.7	118.6	125.3	125	108.9	100.2	126.5	116.9
PRAMEF9	117	121.6	134.4	119.5	105.2	104.1	115.7	117.1
PRAP1	121.1	109.8	123.2	117.2	108.7	101.8	126.8	115.5
PRB1	105.8	112.7	116.6	107.9	108.1	96.9	110.8	111.5
PRB1	118	116.9	112.2	119.7	102.3	101.4	114.5	119.2
PRB2	116.2	120.5	119.6	119.1	102.2	95.2	113.8	115.2
PRB3	115.9	125.1	117.5	119	108.1	97	121	119
PRB4	111.3	114.9	112.1	115.8	112.4	89.9	116.2	115.7
PRC1	138.7	142.8	161.9	143.4	130.9	135.9	142.8	148.5
PRCC	134.6	136.6	124.9	139	131.1	115.5	127.3	130.1
PRCC	178.9	182.6	187.5	187.6	159	145.8	213.5	184
PRCP	1109.9	1200.7	1464.2	1085.2	693.5	760.9	890	684.5
PRCP	364.5	290	415.2	271.7	212.5	199.9	326.9	258.6
PRDM1	118.4	134.3	116.5	128.6	117.6	103.8	123.7	125.6
PRDM1	141.2	134.4	128.8	130.4	120.1	112.8	131.1	107.2
PRDM1	119.2	122.2	124.3	127.4	110.6	96.4	121.8	113.7
PRDM1	675	405.3	612.4	551.9	352.4	406.9	554.8	538.5
PRDM10	277.6	284.5	219.1	292.3	331.7	185.4	379.4	372.5
PRDM11	115.7	108.7	105.9	110.4	104.1	98.3	120	114.8
PRDM12	113.7	113.7	113.6	116.5	103.4	95.3	115.9	119.2
PRDM13	112.1	128.6	125.6	116.1	114.4	101	119.7	123
PRDM14	111	112.6	115.7	116.7	100.9	100.1	114.5	113.2
PRDM15	122.9	117.8	120.1	122.8	102	102.3	120.3	115
PRDM16	123.2	119.7	113.5	117.9	113.3	100.9	116.2	121.2
PRDM16	114.9	108.7	122.2	120	107.1	91.5	120.2	117.7
PRDM16	118.2	117.1	113.9	125	108	99.3	133	114.3
PRDM2	146	152.7	138.8	142.4	125.7	116	138	132.6
PRDM2	115.8	125.9	108.4	110.1	110.1	95.4	125.7	110.8
PRDM4	288.8	259.9	222.4	284.1	265.4	211.7	310.7	367.7
PRDM5	112.7	115	121.4	124.3	105.7	101.5	120.9	120.1
PRDM7	112.4	132.4	108.3	114.5	108.8	99.8	118.1	125.8
PRDM8	263.7	287.6	234.8	256.4	222.9	185.5	208.2	256.2
PRDM9	123.6	120.1	120.9	123.6	114.6	110.7	122.5	120.6

PRDX1	2819.1	1914.1	2981.6	4059.7	2420.4	3987.7	4418.9	3773.5
PRDX1	1203.5	790.4	1107.6	1340.7	835.3	1486.9	1477.7	1742.9
PRDX2	112.8	109.5	114.8	113.2	105.4	98.8	116.6	110.3
PRDX2	293.2	303.7	229.4	271	268.4	181.3	221.4	202.7
PRDX3	607.6	692.6	607.2	692	486.1	461.8	561.4	651.8
PRDX4	250.2	165.8	311.6	222.4	217.7	184.3	232	223.9
PRDX5	580	621.6	689.7	598.5	496.3	439.1	452.2	470.2
PRDX5	1875.2	1752.1	2092.7	1605.4	1277.3	1166.6	1320.1	1299.6
PRDX5	1628.4	1701	1989.6	1657.5	1492.4	1231	1332.4	1316.1
PRDX6	942.8	1103.4	876.3	1118.1	888.8	793.8	1297.7	1545.1
PREB	239.1	214.2	209.9	208.7	162.3	184.3	242.6	209
PRED57	107.8	112.7	113.1	118.9	94.9	105.9	118	119
PREI3	239.2	243.7	233.6	248	236.5	194.8	258.5	262.6
PREI3	146.2	155.6	153.5	171.4	131.5	124.2	145.4	159.5
PREI3	114.7	116.9	124	117.3	100.4	99.6	113.9	105.7
PRELID1	417.7	454.8	530.2	481	294.9	349.7	393.8	349.1
PRELID2	117.1	113	119.3	114.2	117.2	94.1	113	112
PRELP	117.8	123.5	124.6	118.8	111.3	103.1	116.7	119.8
PREP	161.3	151.6	155	162.9	137.2	164.2	142.1	143.6
PREPL	191.2	204.9	198.5	177.5	179.4	156.6	158.6	164.3
PREX1	153.1	159.5	188.1	164.9	136.3	143.3	139.9	138.6
PRF1	816.2	815.3	445.3	769.3	472.6	404.4	1034.6	704.6
PRG2	114.3	109.3	117.8	117.2	112.2	89.7	126.8	110.4
PRG2	128.1	116.2	136.8	124.4	120.8	102.5	125.1	113.2
PRG3	115.7	115.1	114.8	127.5	108.9	103.5	111.6	118
PRG-3	118	124	129.2	115.3	116.1	106.1	130.8	123.6
PRG-3	110.6	108.4	117.8	112.6	105.7	99.2	118.1	117.5
PRG-3	110.9	103.5	108.6	113.1	110.9	98.7	105.4	112.8
PRH1	110.9	106.6	125.7	122.2	108	108	126	120
PRH2	117.5	113.5	110	119.3	106	104.8	114.9	115.3
PRIC285	517.2	348.4	372.8	548.5	298.2	336	343.4	274.7
PRICKLE1	173.9	152.7	153.3	173.1	141.1	117	171.2	133.6
PRICKLE2	107.3	116.6	119.5	116.9	101	106.2	114.6	113.8
PRIM1	241.7	269.4	220.3	254.8	205.9	182.4	241.7	210.6
PRIM2A	114.3	119	118.4	124.4	111.8	103.9	129.7	119.3
PRIMA1	112.6	114.4	116.8	111.4	109.1	96	119.2	124.7
PRKAA1	228.6	249.4	218	244.3	215.4	162.2	227.6	253.5
PRKAA1	370.7	433.9	330.1	422.1	368.3	292.8	419.4	418.8
PRKAA2	114.1	113.6	113.6	109.7	105.4	103.7	118.9	109.4
PRKAB1	168.5	192.9	181.2	169.5	159.4	144.6	173.2	167.9
PRKAB2	147	163.6	188.1	166.9	162.7	141	136.6	148.8
PRKACA	126.1	118.1	131.3	129.9	121.4	101	127.2	122.3
PRKACA	118.7	132.1	119.4	127.9	108.9	100.8	117.8	118.3
PRKACB	130.6	115.4	127.4	123.3	109.4	101.7	119.3	124.6
PRKACB	126.3	142.2	154.6	138.2	146.8	129.8	133.7	141
PRKACB	121.4	122.3	131.8	124.6	113.1	106.2	116	119.9
PRKACB	115.4	121.1	125.9	118.8	101.4	100.9	127.3	119.1
PRKACG	118.4	122.7	130.4	127.7	107.6	99.7	115	118.6
PRKAG1	147.7	171.9	206	174.2	148.3	157.2	174.6	155.7
PRKAG1	224.6	234.6	276.4	227	234.7	233.7	236.8	264.9
PRKAG2	302.7	317.4	279	327.6	275.4	272.1	342.2	282

PRKAG2	142.6	145.5	150.9	150	142.9	119.8	155.5	144.2
PRKAG2	118.3	110.3	117.2	122.3	103.5	101.4	122.4	118.3
PRKAG3	113.3	124.9	120.4	123.8	110.6	99	118.5	113.4
PRKAR1A	112.3	112.1	118.1	117.8	98.3	98.8	113.5	113.3
PRKAR1A	944.5	468.8	839.1	754.4	456	630.1	778.6	593.2
PRKAR1B	134.4	129.5	123.3	140.4	136.6	112.9	126.3	120.7
PRKAR2A	125.5	132.5	124.1	134.3	134.8	97.9	121.6	120.4
PRKAR2B	108.5	118.5	117.7	118.9	110.5	95.7	123.3	126.4
PRKCA	429.9	586.7	351.5	543.7	395.9	266.1	519.4	595.3
PRKCABP	122	147.4	131.6	144.6	113.3	99.7	117.2	139.7
PRKCB1	153.4	143.1	139.9	187.7	151.6	118.1	163.3	162.9
PRKCB1	192.1	187.1	168.2	232.3	257.7	155.8	350.5	272
PRKCB1	1037.7	1114	765	1857.2	1856.3	752.3	2243.3	2200.3
PRKCBP1	193.7	190.5	191.1	245.4	194.1	149.4	172.1	159.9
PRKCBP1	124.1	122.9	133.6	125.5	110.4	108.5	117.9	117.4
PRKCD	570.3	449.1	837.8	564.2	321.3	449.3	620.3	483.7
PRKCD	116.7	114.8	127.2	119.8	119.3	108.9	121.8	116.8
PRKCDBP	114	129.1	134.1	131	105.6	106.3	113.8	115.8
PRKCE	133.6	141.3	125.7	166.1	113.1	113.3	113.3	136.3
PRKCG	115.1	113.4	117	124.1	115.3	98.2	110	118.4
PRKCH	1595.3	1887	1151.4	1728.5	1597.5	1042.1	2329.5	2452.3
PRKCI	145.8	146.2	148.6	147	133.2	116.1	142.3	133.2
PRKCQ	466.7	578	416.6	518.8	511.1	367.4	498.1	417
PRKCSH	299.4	349.9	345.2	298.3	240.9	296.1	286.7	295.2
PRKCSH	121.3	131.2	134.4	125	106.6	110.6	125	121.1
PRKCZ	122.1	126.2	129.5	122.3	119.3	102.9	121.1	127.4
PRKCZ	110.2	108.8	122	118.9	113.9	93.6	115.6	116.6
PRKCZ	171.6	187.4	152.8	188.4	181.6	138.9	196.6	195.8
PRKCZ	105.4	114.7	114.3	117.1	114.5	97	123.2	114.1
PRKD1	104.2	119.7	106.3	118.9	114.7	96.3	116.3	111
PRKD2	171.5	163.7	139.5	147.3	143	155.2	165.5	192
PRKD2	131.8	146.2	123.3	119.8	112.9	111.8	135	131.9
PRKD3	120.5	127.7	137	126.3	122.7	114.5	129.4	132.6
PRKDC	220.4	237.1	172.5	196.5	209.3	137	200.3	219
PRKDC	129.8	131.4	117.9	127.6	114	107.5	124.2	118
PRKDC	122.7	128.6	122.2	120.4	117.1	100.8	122.4	123.6
PRKG1	117.2	118.6	116	113.1	104.5	97.1	118.3	110.6
PRKG2	109.6	120.9	111.1	116.2	101.1	95	106.1	112.6
PRKRIP1	251	245.5	297.8	245	206.3	185.5	248.4	230.1
PRKRIR	1174.2	1305.4	1268.4	1405	1035.9	1053.3	1350.2	1138.5
PRKX	251.5	230.4	229.2	217.4	201.9	175.4	273.9	237.9
PRKY	233.8	215.4	183.4	193.7	210.5	127.6	206.1	182.7
PRL	105	124.1	111.1	117.6	106.2	94.4	128.1	115.7
PRLH	119.2	117.4	120.8	115.8	107.6	103.2	116.5	117.4
PRLHR	105.3	109.1	112.8	120.2	96.9	96.6	114.1	108.4
PRLR	104.8	112	114.2	105.7	110.9	94	114	103.4
PRM1	115.1	111.8	120.2	113.8	102.6	96.8	126.3	113.9
PRM2	108.2	105.5	112.7	115.1	106	88.8	119	114.1
PRM3	107.1	122	119.9	115.1	105	100.1	121	107.8
PRM3	120	111.8	128.6	131.7	113	101.5	114.7	123.3
PRMT1	130.9	126.2	123.5	128.4	119.3	98.6	125.7	116.7

PRMT1	411.7	424.2	329.3	390.3	349.3	240.2	323.6	280.1
PRMT1	896.5	934.9	784.5	897.8	681.2	611.9	803.6	729.9
PRMT2	330.8	524.4	529.1	394.6	426.6	376	346.3	389.3
PRMT2	143.1	188.7	254.4	143.3	169.7	152.3	141.1	182
PRMT2	503.8	708.5	603.2	409.9	523.4	444.8	380.9	563.8
PRMT5	171	176	146.9	181.9	152.3	135.1	198.6	188.4
PRMT5	175.8	160.9	185.7	156.8	161.4	141.7	204.2	161.8
PRMT6	177.2	231.8	198.8	179.6	218.3	143.2	139.9	160.5
PRMT7	138.2	146.3	141.7	131.7	127.2	111.5	136.1	131
PRMT8	127.9	112.8	128.2	121.3	106.5	106.2	119.9	124.1
PRND	110.5	110.4	129.4	111.2	107.4	93.3	119.7	117.6
PRNP	346.3	370.9	401.3	445.3	324	360.1	462.1	595.7
PRNP	1659.5	1876.1	1480.1	1949.2	1637.3	1705.3	2625.8	2935.1
PRNPIP	127.7	115	123.6	121.5	107.2	115.6	118.7	118.6
PRNT	124.5	108.8	118.9	111.4	111.1	97.5	113.9	115.2
PRO0149	426.4	407.5	397.2	524.2	337.6	348.3	723.5	643
PRO0611	106.3	119.3	122.8	113	115.1	105	123.2	114.5
PRO0628	138.7	133	143.9	173.3	136.1	103.8	185.4	187.1
PRO1853	200.3	139	167.8	161.6	146.4	127.9	170.6	193
PRO1853	121.4	126.1	136.3	137.9	130.5	112.2	124.4	125.1
PROC	116.2	114.5	147.3	113.5	110.3	110.6	110.8	117.4
PROCA1	115.1	121.5	107.7	121.4	111.4	106.8	113.4	118.5
PROCR	115.9	127.2	127	126.4	112.6	108.5	118	120.7
PRODH	108.7	111.6	115	124.6	102.4	96.1	114.8	112
PRODH2	128.1	129.1	123	131.8	109.7	110.5	116	116.3
PROK1	128	125.4	127.5	118.3	114.1	100.7	121.5	117.3
PROK2	197.2	142.4	212.2	223.8	151.4	184.3	248.9	168
PROKR1	114.9	114.4	116.3	131.8	97.5	94.4	121.3	109.3
PROKR2	109.7	113	106.5	121.9	100.2	100.7	120.1	116.4
PROL1	103.4	118.8	103.7	117.1	100.5	100.2	114.6	108.9
PROM1	113.9	108.9	124	127.6	109.9	103.1	118	118.3
PROM2	116.5	113.7	116.7	130.6	106.9	109.6	116.1	112.1
PROP1	119	109.4	115.3	117.5	118.2	102.5	115.1	108.5
PROS1	112.9	114.8	107.3	120.7	108.7	100.1	124.3	110.1
PROSAPIP1	141.3	143.4	161.7	147.5	139.1	138.1	151.9	147.7
PROSC	673	682.5	818.7	734.4	669	690.6	706.2	724.4
PROX1	109.5	120.5	113.6	116.3	106.4	105.9	120.1	121
PROZ	111.5	112.3	112.4	113.1	100.4	93.8	116.1	107.3
PRP2	109.3	115.7	114.2	116.9	108.5	94.3	121.2	117.2
PRPF18	170.4	168.9	167.9	178.9	161.6	141.9	176.4	157.1
PRPF19	149.7	195.9	178.1	214.2	158	123.9	186.6	170.8
PRPF3	387.5	314.5	360.8	391.6	302.2	314.4	388.5	299.9
PRPF31	331	268.6	403.9	342.9	261	337.8	379.3	220.7
PRPF38A	190.8	206.2	208.4	209.4	201.1	168.4	224.7	212.5
PRPF38A	113.8	124.4	127.3	134.1	112.3	102.1	126.6	118.7
PRPF38B	192.4	222.1	244.7	247.5	216.2	164.7	232	226.8
PRPF39	127.3	130.8	117.8	127.6	112.1	107.1	120.8	120.8
PRPF4	364.7	441.1	406.9	399.2	459.3	400.2	491.4	479
PRPF40B	114.3	115.4	109.1	116.2	106.9	102	115.4	108.8
PRPF40B	111.4	113.9	115.6	111.9	103.4	95.9	114.5	109
PRPF4B	136	136.7	148.5	130.3	130.6	127.3	149	156.6

PRPF4B	106.4	104.9	114	111.7	106.7	98.1	114.9	108.2
PRPF4B	338.3	300.9	313.2	375.7	330.1	239.4	290.5	272
PRPF8	597.9	811.3	598.1	908.4	519.9	530.4	948.2	832.3
PRPH	114.6	118.8	122.5	122.3	115.2	114.1	129.4	112
PRPS1	360.5	463.5	450.3	487.4	358.2	344	353.9	422.9
PRPS1L1	115.2	119.6	126	115	103.5	97.3	119.1	113.8
PRPS2	177.4	167.4	170.2	161	157.7	123.9	147.9	152.3
PRPS2	178	191.3	194.2	197.6	172.7	162.2	190	184
PRPSAP1	751.2	636.6	536.2	645.5	611.1	431.3	603.5	464.5
PRPSAP2	337.1	310.4	327	341.8	279.2	241.6	358.8	329.2
PRR11	118.1	122.9	126.9	111.6	117	99.8	128.8	124.6
PRR13	4530.4	4788.9	6304.4	4747.4	4133.8	5524.4	4773.6	4201.3
PRR15	134.2	122.9	127.6	133.5	119.2	114	130.8	122.3
PRR3	131.9	125	138.6	143	114.6	108.2	124.7	121.8
PRR3	119.2	148.2	121.3	125.7	122.5	110.7	127.3	128
PRR4	118.3	113.8	121	121.7	108	103.8	117.9	117.2
PRR5	111.2	113.4	114.2	121	108.1	100.5	114.5	121.7
PRR5	311.9	402.9	288.1	393.2	280.7	249.7	392.5	437.2
PRR5	175.1	241.4	179.2	208.9	208.2	173.2	255.2	258.8
PRR6	196.3	250.4	220.7	199.9	183.1	168.1	219.7	226.2
PRR7	130.9	137.3	128.6	139.6	113.3	99.9	137.9	166.4
PRR8	126.5	126	124.7	123.9	110.7	107.7	122.8	118.3
PRRG1	122.8	107.7	118.4	122.5	107.7	97.1	121.3	117.7
PRRG2	121.5	127.9	114.6	127	119	90.1	126.4	126.8
PRRG3	109	109	109.9	122.4	108.9	97.3	113.5	120.5
PRRG4	139.7	155	148.9	131.1	138.2	122.7	175.4	150.3
PRRT1	124.8	118.8	119.6	126.3	102.6	100.1	116.5	111.9
PRRT2	137.7	145.7	150.8	141.3	122.7	115.6	135	124.1
PRRT3	125.7	128.2	145	150	145.3	122.2	148.1	129.1
PRRX1	113.5	117.3	119.9	122.4	110.9	105.1	125	110.2
PRRX1	112.9	117.6	129.2	126.1	103.3	96.3	113.9	107.8
PRRX2	118.2	110.1	118.4	124.4	108.5	103.9	121.1	108.3
PRSS1	117	102.7	126.6	118.8	107.3	91.8	113	112.8
PRSS12	110.1	115.2	120.5	122.6	111.5	97.7	115.2	109.5
PRSS15	351.8	462.8	301.6	396.7	249.4	276.5	400.2	377.2
PRSS16	116.3	118.7	119.7	114.7	110.5	91.5	113.3	111.5
PRSS2	119.7	119	114.3	122.8	117	109.8	124.1	105.7
PRSS21	119.9	127.1	143.9	128.4	114.3	115	126.1	125.2
PRSS21	112.5	111.3	114	116.8	106.3	98.9	117.7	114.6
PRSS22	116.6	118.4	127.3	124.1	108.6	104.4	128.9	125.5
PRSS23	135.1	124.4	127.1	135.1	115.7	114.7	135.1	124.3
PRSS27	113.6	114.4	125.8	110.6	106.7	103.8	115.5	123.4
PRSS3	118.8	114.9	116.5	116.4	109.4	99.6	112.3	115.7
PRSS33	110.1	115.2	119.2	112	115.7	93.7	110.9	115.8
PRSS35	134.9	160.3	158.8	145.5	149.9	130	141.2	143
PRSS7	131.1	139.8	134.8	137.7	127.1	122.1	131.5	139.7
PRSS8	112.1	125.4	128.9	128.2	113.4	107.9	125.3	117.2
PRSSL1	116	115.6	126.7	133.5	113.4	106.9	112.6	122.1
PRTFDC1	119.1	122.1	118.9	127.2	121.3	96.5	110.4	113.8
PRTG	113.7	125	116.8	118.1	111	101.1	119.5	114.4
PRTG	120	110.3	114.7	113.1	105.9	99	118.3	109

PRTN3	119.9	116.8	122.1	132.8	113.9	105.1	120.3	118.3
PRUNE	189.2	248	181.2	220	202.6	167.7	220.5	218.9
PRUNE2	124.8	123	124.9	131.1	120.6	99.8	122.4	115.6
PRX	121.8	125.1	123.3	126.5	114.3	108.5	111.9	132.3
PRX	112.4	123.8	123.3	126	116.8	111.2	116.8	116.5
PRX	113.4	118.9	116.2	119	108.7	105.7	120	122.5
PRY	113.6	122	115.5	121.8	108.2	105.7	116.2	113.6
PSAP	1983.3	1471	3207.3	1803.7	1087.1	2277.4	2216.4	1574.6
PSAP	2231.1	1674.7	3374.1	2465.7	1509.6	2535.1	2192.5	1710.6
PSARL	504.7	542.8	865.3	577	486.1	616.6	552	450.7
PSAT1	153.7	183.4	145.5	190.1	180.5	136.9	182.1	270.1
PSAT1	106.2	114.5	108.3	117	100.5	97.2	119.4	114
PSCA	117.1	130.2	118.9	114.4	107.5	102.5	118.2	114.5
PSCD1	310.9	294.6	227.1	338.7	248.7	195.2	381.7	406.5
PSCD1	3450.8	3122.3	2446.8	3669.5	2566.5	2068	4592.7	4251.1
PSCD2	132.5	137.6	144.9	130.3	125.2	130	166.4	156.1
PSCD2	125.8	126.5	145.5	135	114.6	112.5	135.4	136.9
PSCD3	140.6	157.3	138.1	151.8	136.3	127.3	168.3	162.3
PSCD4	1010	1007	1394.7	1193	719.9	1159.3	880.5	407.5
PSCDBP	127.8	141.2	131	147.6	130.4	136.9	152.4	145.9
PSD	114	117	114	116.7	113.9	94.5	110.5	119
PSD2	112.5	119	126.8	131	101.6	104	123.3	116.2
PSD3	120.8	125.9	123	126.8	111.1	99.1	125.4	116.7
PSD3	142.3	132.2	140	140.9	127.4	118	130.1	125.8
PSD3	121.1	119	128.8	124.4	121	104.1	118.8	126.1
PSD4	378.1	419.6	464.8	367.1	257	339.4	283.6	226.1
PSEN1	123.5	117.1	124.5	121.1	108.8	102.3	120.6	127.2
PSEN1	110.6	114.4	120.4	123.9	103.5	122	115.4	112.9
PSEN2	141.8	126.1	143.8	149.3	112.8	116.4	128	134.9
PSEN2	157.8	154.4	164.9	151.9	118.8	125	136.2	134.4
PSENEN	142.8	138.4	163.6	153.7	125.7	124.1	144.3	128
PSF1	118.7	120	142.9	120.7	125	95	133.1	128.2
PSG1	115.1	113	117.6	113.1	101.3	94.3	112.2	112.8
PSG11	120.9	111.8	118.7	123.6	105.7	96.4	118.3	108.3
PSG2	105.3	109.6	110.8	113.8	104	98.3	114	115.4
PSG3	115.8	118.4	126	121.8	110.2	105.9	122.8	113.6
PSG4	111.1	117.6	112.9	120.4	110.1	92.6	115.7	112
PSG4	118.3	121.9	124	138	118.7	97.1	127.8	132
PSG4	115.5	118.4	108.8	123.4	104.7	97.1	112.1	106.9
PSG5	122	108.9	106.4	118.6	110.3	95.6	114.6	106.7
PSG6	112.9	108.8	113.5	116.1	112.6	98.4	120.8	108.6
PSG6	122	114.1	118.9	127.4	119.5	99.1	112.6	113.7
PSG6	110.7	110.4	112.3	120.5	111.5	97.3	117.7	109.1
PSG7	114.9	111.5	113.6	125	106.3	98.3	122	107.4
PSG8	105.6	127.3	125.1	122.4	104	102.9	124	109.7
PSG9	109.9	119.5	114.8	124.5	106.1	94.4	119.9	124.7
PSIP1	111.1	116.7	109	112.8	120	106.7	105.9	109.7
PSIP1	396.9	462.3	468	398.4	447.1	413.5	350.6	244.8
PSITPTE22	105	110.6	108.3	110.5	103	93.1	117.2	107.3
PSKH1	110.6	122.7	127.3	119.8	109.7	98.5	126.1	119
PSKH2	111.6	111.2	119.6	109.2	98.1	106.3	111.2	106.3

PSMA1	108.1	137.1	120.1	119.3	124.4	99.9	123.8	125.8
PSMA1	196.2	195.9	207.1	230.2	227.5	187.3	266.6	323.4
PSMA1	925.2	945.3	1408.2	1522.4	1258	1286.9	1814.5	1677.6
PSMA2	292.6	228	228.4	262	194.1	160.1	271.3	301
PSMA3	360.5	424.6	364.8	425.1	371.5	608.3	511.3	716.1
PSMA3	131.6	148.3	169.1	176	146.3	171.3	200.9	229.1
PSMA4	300.6	339.3	471	452.7	402	412.9	458.4	403.7
PSMA5	1272.4	1209.7	1451.2	1343.1	926.1	1375.4	1000.6	1188.5
PSMA6	3371.7	3256.7	4014.2	3524.3	2632.7	3809.5	4052.2	3585.5
PSMA8	111.1	134	119.9	124.8	105.9	101.8	124	106.6
PSMAL	118.3	116.4	113.8	116.2	104.6	96.5	120.5	106.3
PSMB1	1577.3	1518.1	1568.7	1834.4	1652.1	1832.4	1522.6	1865.1
PSMB10	1783.4	1853.4	2147.7	1623.3	1176.5	1372.9	1050.5	712.2
PSMB2	392	420.2	484.4	426	344.5	475.1	475.3	451.1
PSMB3	1484.7	1529.3	1612.9	1542.1	1065.6	1373.3	1373	1160.1
PSMB4	1055.8	966.1	1122	1283.7	784.6	1057.9	954.2	1025.4
PSMB5	273.1	260.6	273.1	319.7	255	238.9	358	345.7
PSMB6	1333.5	1424.5	1458.7	1676.7	1323.9	1372.8	1877.8	2198.4
PSMB7	1036	1091	1141.2	1178.3	794.2	996.3	1143.5	1312.6
PSMB8	296.5	219.9	317	237.9	188.6	180.6	169.2	155.7
PSMB8	145.1	162.7	186	166	143.2	125.7	155.3	135.6
PSMB8	135.3	137.5	117.7	142.1	139.5	110.1	126.2	130.3
PSMB8	1264.9	1371.5	1326.7	1162	728.5	840.1	712.1	687.9
PSMB9	350.5	441.7	621.5	327.4	290.1	307	179.2	183.7
PSMB9	134.1	151	148	146.3	139.7	120.2	128.4	123.8
PSMC1	1743.7	1709.4	2177.8	1954	1481.1	1833.7	2390.1	2572.1
PSMC2	814.9	815	1079.5	1006.6	779.2	879.1	1036.5	1372.8
PSMC3	295.6	274.7	335	343	320.6	245.6	400.5	379.7
PSMC3IP	116.2	117.2	110	114.9	109.7	93.3	125.8	121.5
PSMC3IP	124.1	116.4	127	123.5	112.5	105.9	121.3	123.7
PSMC4	282.7	299.9	361.3	382.3	258.2	327.7	330.1	331.6
PSMC4	197.4	218.3	244.2	200.3	185.3	185.9	210.2	198.6
PSMC5	432.2	463.1	353.5	667.6	388.4	465.7	357.6	547.7
PSMC6	126.7	129.1	132.4	136.9	117.1	116	141	133.5
PSMD1	348.8	373.4	445.2	420.7	365.5	520.3	612.9	577.2
PSMD10	437.1	532.1	544.3	564.3	498.2	479.9	543.9	546.2
PSMD10	121.2	131.7	111.8	121.4	114.5	106.3	123.5	108.3
PSMD10	155.5	190.1	196.5	199.7	188.6	189.6	180.9	191.1
PSMD11	136.2	146.5	146.3	165	134.5	121.8	163.1	148.9
PSMD12	381.7	399.4	384.7	408.3	302.8	340.2	420.1	611.8
PSMD13	131	125.2	121.9	120.9	116	108.3	113.6	122.5
PSMD13	158.8	131.8	153.7	147.6	149.7	134.1	162.1	154.7
PSMD14	195.3	262	297.9	244.7	234.5	246.6	330.7	434.6
PSMD2	290.8	352	286.5	338.3	268.2	256.2	374.8	413.1
PSMD3	129.1	133.6	137	144.7	130.7	107.1	138.1	148.6
PSMD4	108.1	116.7	104.4	101.5	103.5	98.3	143.4	131.3
PSMD4	431.4	504.4	591.4	548.8	402.4	459.4	453.2	590
PSMD4	804.4	878.6	962.7	962.2	666	742.3	948	946.7
PSMD5	149.9	158.8	133.9	160.2	136	128.7	148.2	152.6
PSMD6	1103.7	994.1	794.6	1028	886.1	784.3	1160.3	1292.4
PSMD7	536.9	723.1	694.1	736	677	750.8	950.9	870.9

PSMD8	241.7	321.3	290.7	325.5	257	230.4	338.3	366.3
PSMD9	139	131.6	132.6	138.6	120.1	115.1	153.8	133.5
PSME1	3243.9	2871.2	3693.8	3245.3	1671	3055.9	1926.2	1895.7
PSME1	141.7	138.2	127.3	140.7	133.3	117.3	145.5	143.3
PSME2	709.7	514.5	975.2	600.8	452.7	591.1	522.9	414.5
PSME3	334.4	344.6	309.3	327.5	295.1	284	299	321.2
PSME3	264	290.5	279.7	307.1	236.8	281	313.9	305.3
PSME4	485.2	550.8	335.8	500.7	449	331.2	575.5	494.6
PSMF1	108.3	107.6	112	124.7	112.9	103.9	119.1	119.6
PSMF1	597.3	702.3	900.8	800.4	520.2	581.4	566	644.4
PSORS1C1	125.2	135.4	132	130.4	119	111.3	131.8	128.6
PSORS1C2	109.6	118.9	120.1	115.6	112.7	102.1	122.8	119.9
PSPC1	134.8	142.9	137.4	156.7	128.3	127.8	146.1	160.4
PSPH	122.5	118.8	114.2	112.6	117	94.5	116.3	117.9
PSPN	105.4	114.7	108.6	123.6	108.6	102.3	119.8	110.6
PSRC1	120.6	122.7	123.6	122.4	105.9	101.7	118.1	115
PSRC1	115.5	104.4	115.2	127.1	116.6	102.2	115.3	119.2
PSRC1	118.3	118.7	128.1	128.9	123.4	92.6	125.4	122.2
PSRC2	186.4	193.7	191.8	154.9	156.9	137.8	155.2	145.5
PSTPIP1	240.2	267.8	209.6	206.1	180.6	188.2	179.2	165.6
PSTPIP2	153	130.7	182.9	142.9	113.7	121.4	123	114.6
PTAFR	133.2	118.5	176.7	152	123.6	178.7	150.7	119.7
PTBP1	2488.3	2907	2123.1	3170.5	2353.9	2214.2	4218.7	3594.8
PTBP1	4733.7	5310.9	3509.7	4898.1	4641.9	3269	7140	6377.8
PTBP2	189.1	196.3	189.3	228.2	209.5	160.5	237.7	243.6
PTCD1	306	373.1	339.7	329.4	261.1	224.6	297.8	352.3
PTCD2	136.8	133	148.4	140.3	136.5	124.6	129	124.7
PTCH	137.7	131.7	130.7	135	131.9	107.8	133.6	122.6
PTCH2	121.4	119	117.9	121.2	111.2	102.1	119.9	118.4
PTCHD1	113.3	108.3	105.3	116.4	106.9	91.7	117.8	113
PTCHD3	115.6	129.4	116.7	128.3	111.4	95	115.2	118.6
PTCRA	112.1	108.1	120.7	108.7	106.1	99	112.2	106
PTD004	123.3	116.8	118.9	117.4	113.1	98.2	126.6	117.7
PTD004	108.1	112.1	116.3	116	111.3	102.2	115.5	113.8
PTD008	692.5	808.7	757.4	719	513.7	609.7	761.4	759.9
PTD015	140.2	143	174.8	148.2	142	162.8	129	141.6
PTDSR	123.2	115.7	119.4	115.2	110.4	100	116.7	119.9
PTDSR	148.7	146.8	141.5	156.1	133.8	130.4	170.3	211.3
PTDSS1	941.4	1264.6	771.3	1143.4	891.8	704.9	1076.7	1122
PTDSS2	136.7	132.2	152.1	142.8	119.7	124.9	138.6	142.9
PTEN	448.2	440.4	363.8	468.8	367.6	324.2	459.3	449.9
PTER	112.4	116.2	116.7	121.5	111	102.9	116.6	116.1
PTER	117.4	118.6	115.9	121	105.3	107.2	119	123.2
PTER	187.4	179.8	186.1	199.1	179.4	126.4	200.3	184.6
PTF1A	113.8	107	109.8	123.6	106.8	102.4	113.2	105.9
PTGDR	169.2	164.9	160	165	141	119	141	131.6
PTGDS	114.6	118.3	114.8	113.4	111	107.2	124.4	119.7
PTGER1	117.2	111.2	117.4	113.4	98	101	113.1	100.7
PTGER2	481.7	329.6	426.1	384.1	217.7	208.1	302.7	244.2
PTGER3	109.9	119.5	115.2	121.6	104.8	93.1	108.5	109.4
PTGER3	114.7	122.1	120.8	116.9	111	110	114.7	114.6

PTGER3	114	117.9	111	110.6	107.8	99.7	122.7	111.7
PTGER3	107.4	109.7	112.8	115.2	106.3	94.4	104.1	105.4
PTGER3	118.1	117	119.8	120.5	112.1	111.3	123	116
PTGER3	114.7	111.9	112.8	119.1	105.8	96.3	120.3	122.3
PTGER4	327	333.6	566.8	367.2	345.3	315	438.5	450.6
PTGES	109.2	115.4	119	119.2	106.1	92.3	117.5	106.6
PTGES2	292.3	270.7	286.1	215.2	219.4	184.8	189.2	201.9
PTGES2	346.2	444.9	478.8	430.6	270.7	273.4	288.4	260.2
PTGES3	652.4	738.7	891.5	764.7	678.6	722.4	1198.7	1663.4
PTGFR	116.9	117.5	124.6	122.5	111.6	99.2	124	115.5
PTGFR	113.7	117	129.9	132.1	125.1	110.9	124.9	129.8
PTGFRN	122.7	127.5	121.4	122.8	112	102.4	107.3	118.4
PTGIR	119.1	118.5	111.2	118.1	109	103.4	113.1	116.3
PTGIS	108.1	116.5	112.5	116.9	102.2	95.7	113.6	108.4
PTGS1	119.9	120.4	160.5	114.8	104.9	103.9	112	114.4
PTGS1	113.9	127.6	139.6	127.3	117.8	107.6	121.7	121.3
PTGS2	149.6	143.1	182.1	165.6	126.9	134	151.6	130.7
PTH	119.1	110.1	113.7	123	112.2	104.6	114.7	113.1
PTHLH	115.7	119.7	104.6	116.8	105.4	102.2	110.6	117.4
PTHLH	119.5	119.1	120.4	125.7	113	100.6	110.4	109.6
PTHR1	136.8	130.9	127.1	139.3	127.5	100.1	130.9	129.7
PTHR2	112.4	122	114.3	121.8	112.4	103.7	119.8	115.7
PTK2	203.1	214.4	310	211.9	207.8	292.4	290.8	252.1
PTK2	110.4	118.1	110.1	117.6	100.5	98.4	120.6	110.4
PTK2B	122.7	114.8	117.3	123.1	127.2	114.7	123.6	116.4
PTK2B	138.4	125.2	145.3	135.6	119.9	106.7	138.6	131.4
PTK2B	120.8	117.9	122.6	124.7	127.6	102.3	124.5	119.3
PTK6	120.5	132.1	133.9	128.7	122.7	111.3	140.7	139
PTK7	114.8	120	119.8	125.3	107	97.2	120.4	119.5
PTK7	113.6	116.7	125.3	124.5	109.7	107	125.3	123.5
PTK9	118.6	114.9	120	119.9	108	102.4	129.5	124.8
PTK9	121.9	121.3	123	118.4	125	109.2	116.8	112.8
PTK9L	658	791.6	571.2	781.6	482.5	551.9	707.7	487
PTMA	512.1	343	766.6	452.4	310.9	788.8	507.6	410.8
PTMS	131.6	138.9	152.4	166	122.2	128.3	150.7	135.7
PTN	132.6	166.3	170.5	158.9	141	131.5	147.7	153.3
PTOV1	672.1	669.1	638.1	750.4	495.2	600.4	740.4	756.5
PTP4A1	114	122.3	123.8	115.7	113.3	101.2	124.2	123.8
PTP4A2	3026.9	3918.9	2872.1	3311.3	2667.5	2407.6	4415	4331.6
PTP4A3	115.8	114.2	129.4	123.5	114.9	110.5	119.3	113.4
PTP4A3	119.3	123.3	125.1	126.3	111.9	106.2	130.6	121.3
PTPDC1	111.3	112	121.3	113.5	106.9	98.7	112.7	110.6
PTPDC1	147.1	145.2	142.5	147	127.9	126.3	151.4	137.4
PTPDC1	110.5	112.1	111.5	115.4	114.5	96.2	117.9	111.3
PTPLA	128.9	127.5	132.1	140	124	109.1	138.5	123.7
PTPLAD1	359.3	375.1	317.3	419.7	335.9	218.8	284.5	300.9
PTPLAD2	308	308.6	314.5	318.3	305.3	197.4	320.3	347.5
PTPLB	847.4	934.5	899.1	1009.6	699.5	676.9	995.4	934.7
PTPN1	766.4	652.2	504.2	740.2	485.8	411.3	732	631.2
PTPN11	464.9	435.3	393.1	430.8	422.3	443.3	474.6	525.3
PTPN12	584.7	420	389.5	637.6	400.2	434.1	549.9	348.4

PTPN13	107.2	111.2	119.7	124.2	115.3	95.7	104.7	113.1
PTPN14	111.1	108.6	112.3	111.1	104.2	99	113.7	122.1
PTPN18	110.5	124.6	119.6	114.5	106.8	98.8	131.2	118.6
PTPN2	115.5	111.5	113.7	109.5	109.3	96.6	122.4	115.8
PTPN2	250.2	254.1	255.1	255.9	215.5	195	208.3	214.7
PTPN20A	102.5	115.3	111.1	108.6	102	92.7	115	117.6
PTPN20B	105.5	112.8	107.6	107.1	103.1	96.4	110.4	109.3
PTPN21	114.1	112.8	113.8	120.9	101.5	98.9	112.7	108.3
PTPN22	143	147.2	145.1	155.8	134.5	114.3	132.6	136.9
PTPN22	122.8	118.4	112.9	119.7	103.9	94.3	116.2	108.6
PTPN22	281.1	330.8	267.1	269.4	236	198.1	307.4	355.2
PTPN23	156.5	156.1	141.8	171.8	127.5	125.2	183.5	146.1
PTPN3	116.1	117.5	111	121.7	109.3	92.2	118.9	115.6
PTPN4	610.7	607.2	630.9	733.4	502.1	448.5	637.6	565.7
PTPN5	126.2	116.1	141.7	120.2	116.1	138.4	119.8	123.3
PTPN5	134.7	133	130.9	130.9	135.8	107.6	128.3	123
PTPN6	120.1	125.7	126.8	120	115	104.4	118.5	121.4
PTPN6	694.6	680.5	742.9	810.2	329.2	620.6	493.7	262.3
PTPN7	111	118.6	123.7	119.2	109.6	98.7	130.2	117.5
PTPN7	261.8	244.4	214.1	251.4	178.5	196.1	292	272.5
PTPN9	133.9	118.3	129.3	138.8	128.8	122.2	136.4	140
PTPN9	113.2	124.6	123.2	127.3	117	102.1	123.3	111.3
PTPNS1	563.6	349.2	426.6	797.2	316.8	775	607.6	274.5
PTPNS1	281.9	183.6	212.4	311.5	154.5	281	285.1	153.5
PTPRA	124.8	128.1	121.1	127.1	109.6	100.2	125.6	117.1
PTPRA	231.4	200.5	213.3	223.9	192.6	155	198.6	182.8
PTPRA	190.9	181.9	219.7	190.6	165	162.9	169.3	181
PTPRA	144.5	148.8	155.6	161	143.8	111.7	143	132.9
PTPRB	109.8	117.9	114.4	110	109.7	96.6	119.3	109.1
PTPRC	106.3	109.2	107.1	112.6	103.4	96.2	105.9	107.7
PTPRC	162.8	150.9	153.1	162	152.8	109.1	141.8	160.5
PTPRC	189	183.9	192.6	197	197	128.1	172.1	168.7
PTPRC	125.4	135.6	122.3	143.7	136.7	109	134.1	135
PTPRCAP	702.3	996	554.6	700	462.7	390.4	649	514.9
PTPRD	112.8	116	122.6	112.4	111.3	111.9	119.6	127.8
PTPRD	106.1	117.1	119.7	113.2	108.7	99.7	123.1	122.5
PTPRE	1575	1241.3	698.5	1971.1	1100.4	1101.2	2093.8	1119
PTPRE	110.2	100.8	108.8	118.4	114.3	96.8	115.1	108.1
PTPRE	138	150	130.7	139.2	117.4	112.1	152.5	131
PTPRF	109.5	122	136	125.4	110.3	98.3	123.5	117.7
PTPRF	105.1	111.7	114	118.6	102.9	98.5	117.6	113.8
PTPRG	139	125	138.7	128.3	131.2	116	125.6	125.6
PTPRH	120	127.8	129.3	120.8	116.1	113.4	124.3	117.1
PTPRK	122.4	143	125.8	118.5	126.3	137.7	137.5	156.5
PTPRM	152.8	192.5	132.9	146.9	166.9	112.9	149.9	149.9
PTPRN	118.6	106.6	113.4	120.2	107.8	101.5	113.8	116.3
PTPRN2	121	118.8	121.1	134.9	121.9	103.3	128.7	111.7
PTPRO	150.8	145.3	259.8	164.9	116	148.2	139.8	133.6
PTPRO	146.5	131.1	242.8	149.2	120.3	142.5	128.6	118.7
PTPRR	118.1	110.7	115.8	123.4	113.2	107.2	121.3	122.2
PTPRR	124.8	129.9	123.6	141.1	120.1	114	126	133.6

PTPRS	119.4	122.6	120.8	116.4	101.8	100.8	116.7	117.2
PTPRS	121.2	112.9	121.9	116	107.6	101.9	115.4	113.3
PTPRT	113.3	115.9	114.8	110.2	106.6	103.5	121.9	116.4
PTPRT	112.7	120.3	119.4	114.4	94.7	105.9	114.1	112.1
PTPRT	115.8	112	114.3	111.1	104.7	92.7	121.8	121.5
PTPRU	117.1	123.7	127.6	125.9	118.2	103.6	123.2	113.9
PTPRU	112.6	107.7	123.1	129.7	117.6	109.2	130.3	123.6
PTPRZ1	115.3	121.3	123.6	117.1	103.2	99.2	124.4	115.4
PTRF	120	128.7	138.4	137.4	110.7	110	127.4	118.8
PTRH1	152.3	167.5	167.9	148.1	125.8	128.8	144.6	127.7
PTRH2	398.1	445.9	388.7	422.5	327.2	311.8	415.3	386.3
PTRH2	118.9	120.4	125.7	117.8	123.5	103.9	126.8	129.9
PTS	189.7	254.9	233.5	245.7	199.9	216.2	304.5	281
PTTG1	148.9	194.9	201	159.7	135.8	141.5	192.1	200.8
PTTG1IP	869	769	1258.6	1114.6	652.8	883.8	859.6	754.9
PTTG2	126.4	152.4	149.6	130.6	131.1	107	132.4	126.8
PTTG3	125.3	150.1	159.9	141.3	128.2	120	154	141.6
PTX3	125	109.8	113.9	119.3	106.5	100.6	116.7	117.6
PUM1	1176.8	1221.3	958.3	1345.2	1203.1	788.1	1767.2	1774.5
PUM1	848.4	995	750.1	1006.1	969.6	583.4	941.6	1015.4
PUM2	855.3	970.3	726.7	1047	1050	722.1	1091.4	1244.9
PUNC	114.4	119.1	114.2	115.4	105.8	96.1	114.3	104.8
PURA	253.3	336.6	272.7	281	293.2	269	251.8	242
PURB	729.7	757.5	650.8	919.8	858.4	691.4	1121.5	1092.8
PURG	140.5	147.8	138.4	139.9	143	114.9	151.8	137.4
PURG	114.2	110	110.3	121.1	104.9	101.9	106.1	116.7
PUS1	681.6	600.8	580.3	601.1	519.9	346.1	580.9	423.6
PUS1	121.1	117.2	129.3	120.3	111.1	101.7	125.8	123.4
PUS1	149.6	207.5	159.1	176.7	162.9	144.7	182.3	156.5
PUS3	160.8	165.7	163.7	164.1	145.3	143.2	146.2	157.7
PUS7	205.2	192.4	182	215.6	175	120	242.8	203.9
PUS7L	137.3	147.2	159.8	140.9	130	127.2	136.9	140
PUSL1	132.8	136.1	121.6	154.4	137.6	123.6	137.9	126.8
PVALB	104.9	111.4	119.8	112.4	109.6	97.8	120.5	117.5
PVR	120.1	115.5	130.9	128.4	110.7	101.9	125.2	122.3
PVRL1	115.4	113.4	116.4	120.1	104	93.7	110.3	117.2
PVRL1	116.2	118.3	115.3	121.1	109	101.4	108.8	123.8
PVRL1	121.1	113.4	116.5	123.6	116.1	96.4	121.6	122
PVRL1	107.7	110.8	112.7	105.7	101.2	99.8	124.7	105
PVRL2	126.7	119.2	131.4	116.1	107.1	119.9	135.7	124.2
PVRL2	134.7	128.5	156	158.1	112.7	167.3	158.8	129.5
PVRL2	112.8	112	113.6	118.6	102.7	110	132.2	121.3
PVRL3	133.8	156.6	142.9	153.6	141.3	112.2	140.7	146.7
PVRL4	118.3	113.6	118	114.7	109.2	106.3	112.6	116.6
PWP1	452.2	494	389.1	467.3	373.1	382.4	418.1	418.8
PWP2H	124.5	119.7	125.6	127.8	122.9	119.3	132.5	117.3
PWWP2	199.8	193.3	171.3	203.9	171.1	143.3	210.3	202.8
PWWP2	112.2	138.3	136.3	141.3	124.9	106.3	137.5	134.1
PXDN	123.3	105	129.3	124.9	126.1	108.1	119.6	118.3
PXDNL	107	113.3	109.5	115.8	110.4	96.5	107.9	114.1
PXK	135.4	138.5	142.4	148.3	125.8	135.9	148.3	134.3

PXMP2	198.8	224.4	177.1	204.8	166.9	155.9	169.3	168.7
PXMP2	135.5	143	136.3	134.2	132.9	110.1	131.1	126.8
PXMP3	269.2	317.9	407.2	277.1	237	281.6	227.7	249.6
PXMP4	122.4	124.4	137.5	127.3	118	109.3	124	122.2
PXMP4	127.5	134.1	139.9	131.3	124.5	113.3	123.6	117.4
PXN	138.4	138.2	120.6	141.7	134.7	104.7	126.2	148.3
PXT1	112.4	106.5	115.5	118.5	106.9	93.6	113.1	110.9
PYCARD	122	132.4	146.3	123.5	125	107.6	119.4	123.1
PYCARD	608.7	695	1326.3	518.9	429.6	434.1	287.5	235.7
PYCR1	108.8	120.9	109.9	113.4	103.9	102.2	124.6	121.1
PYCR2	408.4	535.4	355.9	391.9	459.2	314.4	359.7	361.7
PYCRL	109.2	120.1	120.9	126.8	108.1	101.4	115.9	124.3
PYDC1	113.8	111.8	118.3	116.1	109.4	91.5	112.7	114
PYGB	219	192.5	211.5	193.5	142.8	144.6	195	172.9
PYGL	134.1	122.4	153.5	120.1	107.6	113.2	120.9	120.6
PYGM	114.4	117.3	120.7	123.6	110.6	103.2	128.7	126.5
PYGO1	112.8	119.3	120.3	109.9	116.9	97.4	120.5	117
PYGO2	148.7	157.9	138.4	157.6	139.4	127.1	171.6	135.6
PYHIN1	182.7	220	162.3	194.2	207.2	180.2	199.4	239.5
PYHIN1	544.2	583.9	486.9	651.8	535.8	449	597.7	566.3
PYHIN1	657.6	650	496.6	689.7	598	370.2	656.1	593.5
PYY	103.9	112.9	111.9	112.7	104.7	95.4	111.5	120.3
PYY2	104.1	107.7	111.8	112.2	104.5	97.8	112.1	100.1
PZP	116.4	116	118.1	115.9	105.7	98.3	117.9	125.7
QARS	1688	1593.2	1694	1344.2	1177.9	1070.7	1233.7	1343.9
QDPR	239.6	273	333.7	277.6	206.5	229.2	220.2	192.9
QIL1	622.7	647.8	733.6	515.8	446.9	436.8	376.9	397.7
QKI	126.8	119.3	134.6	123.5	116.2	106.2	118	127.7
QKI	138.8	117.3	131	127.4	121.8	109.6	127.5	129.8
QKI	122.5	129.4	133.8	124.4	124.3	107.9	132.2	130.5
QP-C	1060.3	1309	1929	1188.4	1017.5	1138.6	824.2	642.2
QPCT	127.8	132.4	143.2	141.1	126.7	123.8	139.7	137.9
QPCTL	119	135.7	124	125.8	106.7	98.8	121.9	118.5
QPRT	122.4	119.2	114.8	118.7	119.2	96.5	131.4	123.2
QRICH1	128.9	107	126.1	125	114.9	99.2	127.7	118.2
QRICH1	115.4	115.8	119.9	111	104.9	100.7	113.5	122.3
QRICH1	857.5	1048.4	918.6	1115.5	996.2	570.7	1145.8	1105.3
QRICH2	110.4	105.8	109.7	111	107.5	95.9	126.2	119
QRSL1	126.6	139	128.9	125.6	122.6	104.9	132.6	125.2
QSCN6	141.9	125.8	159.8	130.6	123	147.9	135.9	131.4
QSCN6	123.4	116.8	124.7	120.6	105.9	100.9	113.9	112.9
QSCN6L1	219.4	198.5	189.3	204.2	167.6	135.5	219.8	163.4
QSER1	128.2	122.3	135.9	132.1	113.4	101.9	123.5	123.5
QTRT1	127.9	148.3	141.3	134.6	122.1	101.3	135.4	120.1
QTRTD1	130.8	123	133.5	137.8	118.4	120.2	138.8	127.5
R3HDM1	145.5	138.4	127.1	143	137.6	115.4	146.2	130.7
R3HDM2	140.9	134	130.2	139.6	106.4	115.9	138.6	132.5
R3HDML	121.5	112.2	111.2	116.8	108.1	97.9	115.1	112.8
RAB10	1578.4	1461.1	1449.3	2036.2	1464.9	1582.3	3095.2	2089.3
RAB11A	608	690.3	690.9	750.4	642.7	558.9	727.3	726.7
RAB11B	112.6	117.7	113.9	122.4	108.6	98.1	119.4	117.7

RAB11FIP1	109.5	112.2	120.9	109.4	111.2	103.8	128.5	124.1
RAB11FIP1	121.6	122.4	125.4	126.1	118	107.3	126.6	119.2
RAB11FIP1	494.2	410.9	601.6	494.5	377.5	382.4	417.9	370.8
RAB11FIP2	325.5	324.7	318.6	348.2	316	262	271.4	266.5
RAB11FIP3	381.1	344.8	303.6	354.7	347.8	238.2	404.2	404.4
RAB11FIP4	138.1	158.4	171.7	159.6	142.9	140.4	139.3	141.5
RAB11FIP5	253.5	287.4	192.5	251.3	202.8	130.6	249.3	254.2
RAB12	119.2	116.9	132.8	116	116.5	102.6	120.2	121.7
RAB13	116.5	132.1	124.3	132.5	108.8	106.1	117.7	111.4
RAB14	113.7	129.4	121.8	116	104.4	99.7	125	118.2
RAB15	136.7	113.5	119.7	129.5	109.2	99.8	119.2	117.3
RAB17	112.9	108.6	108.2	109.3	93.8	101.2	112.7	110.1
RAB18	142.6	143.8	143.1	181.3	135	134.3	157.9	147.4
RAB19B	122.8	116.5	115.5	122.3	114	99.8	108.5	115.9
RAB1A	191	167.5	204.1	211	162	167.7	228.2	219.8
RAB1B	144	161.7	128.4	145.4	127.5	121.7	151.3	128
RAB2	182.1	178.2	211.2	213.7	192.8	199.6	264.1	253.3
RAB20	136.9	119.3	127	134.8	115.5	100.6	130.8	121.6
RAB21	222.5	258.1	227.1	373.5	264.3	234	336.4	383.5
RAB22A	591.7	657	496.1	664.3	576.4	444.6	614.1	602.9
RAB23	108.2	119.3	108.7	113.8	106.5	95.6	111.4	107
RAB23	113.2	123.5	112.3	126.9	115.2	99.2	117.3	111.4
RAB23	240.2	287.9	155.7	261.5	228.2	139.2	251.6	268.3
RAB24	159.6	149.3	163.1	148.7	126.5	116.7	139.1	123.6
RAB24	437.4	418.3	517.1	345.7	245.8	303.2	263.6	258.8
RAB24	201.3	216.3	186.3	190.6	136.9	133.1	186	141.9
RAB24	136.3	132.1	140.3	134	122	108.1	125.7	129.4
RAB25	117.9	117.2	121.9	124.8	114.8	100.5	119.6	127.2
RAB26	117.8	116.2	118.6	120.3	107.7	94.6	118.6	121.7
RAB27A	161.9	182.3	213	192.6	158.3	183.1	193.2	173.8
RAB27A	115.6	110	122.2	114.1	109.9	101	116.2	110
RAB27A	109.4	115.9	120.9	110.2	104.9	89	112	108
RAB27B	119	117.8	125.3	124.8	101.3	96.9	120.8	123
RAB28	158.4	162.1	157.5	140.8	150.7	134.4	145.2	133.1
RAB28	163.2	153.6	158.9	165.5	161.6	136.2	140.8	158.9
RAB2B	815.8	896.1	871.2	883.6	856.5	653.2	1144.2	1015.3
RAB30	132.3	140.1	143	144.6	122.7	115.6	147.6	134.6
RAB31	564	353.3	2224.4	592.5	250.9	1281.7	380.4	223.5
RAB32	276	157.8	681	304.8	167.7	512.1	336.2	196.5
RAB33A	161.1	169.1	286.9	157.9	150.4	221.6	128.7	137.6
RAB33B	196.5	194.7	221.6	212.9	173.7	147.2	205.4	168.4
RAB34	140.4	122.5	136.5	144.8	100.8	123.6	132.3	120.1
RAB35	336.9	370.2	335	423.3	298.3	291.8	420.6	477.2
RAB36	118.6	116.6	123.7	113.2	123.3	95.5	118.8	123.4
RAB37	207	234.8	223.5	212.9	155.9	158.6	139.1	127.9
RAB37	154.5	164.7	149.2	141.5	128.5	119.7	134.5	126.2
RAB37	130.5	125.9	117	132.6	115	100.7	122.7	113.7
RAB38	116.4	176.4	129.1	127	119.5	106.8	126.5	122.3
RAB39	113.3	112.4	118	109.6	113.4	100.2	120	115
RAB39B	112.3	119.6	117.8	123.1	119	102.3	114.7	112.4
RAB3A	114.3	125.8	121.4	131.9	114.9	102.1	125	118.7

RAB3B	115.5	115.2	119.6	137.4	119.1	98.4	114.7	119.4
RAB3C	113.1	118.8	132.9	110.9	113.1	100.6	123.6	116.7
RAB3D	120.9	106.7	115.6	130.9	118.1	102.3	131.9	122.2
RAB3GAP1	843.3	888.4	871.9	1000.6	711.4	830	1013.1	960.4
RAB3IL1	119	115.8	134.1	119.9	113.2	99.2	116.7	116.2
RAB3IP	254.8	247.4	304.9	290.3	254	221.6	264.1	235.2
RAB3IP	382.5	390.4	339.2	496.2	375.8	266.9	585.5	412.3
RAB40A	110.5	107.4	111.9	114.7	106.1	90	107.3	106.3
RAB40B	148.9	135.7	170.5	141.3	138.7	147	146.9	141.8
RAB40C	708.8	677.7	582.6	672.7	500	434.9	643.2	536.7
RAB41	119.4	116.1	125.1	115.9	114.3	101.1	123	109.3
RAB42	116.1	122.9	125.5	130.2	112.1	101.3	123.8	119.2
RAB43	489.2	524.4	310.5	461.2	297.8	285	553	577.8
RAB4A	146.9	158.1	165.7	159	135.3	142.7	132.4	129
RAB4B	193.7	186.2	229.5	213.3	163.3	180.8	183.7	170.1
RAB5A	268	285.5	315.5	281.2	278.3	292.7	340.3	334.5
RAB5B	1058.2	1110.3	1106.4	1132.5	960.6	961.6	1192.4	1053.1
RAB5C	319.8	250.8	340.4	326.5	203	271.3	434.2	266.7
RAB5C	165.5	187.3	182.4	180.3	186.1	192.9	180.4	178.3
RAB6A	130.3	135.6	139.3	160.1	128	117.7	153.7	145.4
RAB6B	125.9	121.1	138.4	124.6	114.5	100.1	125.5	126.4
RAB6C	115.1	117.6	118.5	113.8	102.5	99.6	117.5	118.7
RAB6IP1	584.3	673.7	589.3	670.9	636.4	474.5	881.8	1277.6
RAB6IP2	116.1	113.8	125.5	121.1	112.3	99.3	120.5	115.7
RAB7	1326	1500.3	1606.3	1727.4	1517.5	1420.7	2342.9	2498.8
RAB7B	172.8	159.2	198.1	245.9	178.3	271.2	315.3	172.8
RAB7L1	526.3	534.9	414.1	555.5	467.2	278.8	460.7	388.5
RAB8A	601.6	720.5	753.2	729.2	578.6	657.5	801.9	770.1
RAB8B	551.4	685.4	559.6	667.6	745.8	560.9	1177.8	1463.3
RAB9A	543.2	552.4	533.4	736.7	487.1	485.9	625.4	628.4
RAB9B	114.4	119.6	123.5	123.2	126.2	97.3	122.2	128.2
RABAC1	579.7	499.7	602	617	400.5	444.1	472	377.5
RABEP1	447.4	619.5	479.3	545.4	493.3	360.7	496.1	616.8
RABEP2	112	122.7	112.2	127.9	105.9	99.7	124.5	107.1
RABEP2	119.6	117.2	117.9	126	101.5	93.6	119.6	105.6
RABEPK	282.5	322.1	296.6	313	239.1	260.4	283.5	275.2
RABGAP1	546.4	655.4	527.4	628.8	449.2	402.8	774.6	622.1
RABGEF1	127.9	128.3	122.7	174.9	138.5	113.8	141.4	167.4
RABGGTA	114.5	122.5	120.5	119.9	113.5	93.9	114.2	117.6
RABGGTA	145.4	117.5	130.8	131.5	114.3	104.1	121	113.1
RABGGTB	246.5	255.6	250.9	324.7	252	219.5	283.6	261.5
RABIF	120.1	118	144.7	138.2	106.4	104.6	139.2	122.9
RABL2A	113.8	123.3	135.1	126	118.5	118.5	132.7	128
RABL2A	188.1	181.3	214.4	143.8	144.6	141	141.5	123.1
RABL2B	253.7	280.6	272.3	215.9	235.6	164.2	151.2	164.8
RABL3	185.8	178.7	183.2	210.3	171.2	162.2	155.9	149.6
RABL4	150	157.8	170.8	162.4	124.8	146.6	157.3	160.3
RABL5	125.4	116.4	132.4	117.9	110.8	102	121.2	115.7
RAC1	921.6	1116.1	1473.1	978.7	788.2	1056.1	925.9	913.9
RAC1	115.4	114.4	123.8	117.5	115.1	104	108.6	107.6
RAC1	106.7	110.6	116.1	110.1	102.5	93.2	112.9	111.5

RAC2	2458.2	2886.7	2220.9	2209.9	2609.6	1752.3	1938.1	1445.3
RAC3	125	131.6	123.7	125.2	120.9	106.6	129.5	115.1
RACGAP1	153.1	140.5	157.7	164.9	153.4	135.8	164.9	167.9
RAD1	133.4	131.8	163.4	147.7	124.4	126.7	151.8	156.8
RAD1	108.9	112.1	114	116.9	105.8	96.8	120	116.9
RAD17	118.8	113.1	111.6	115.9	115.6	103.8	115.5	114.1
RAD17	148.7	146.5	156.7	151.9	151.9	120.8	132.8	145.9
RAD17	116.8	121	123.5	121.4	116	104.3	112.4	118.3
RAD18	115.4	110.2	113.8	110.3	100.2	93.8	122.7	117.6
RAD21	596.6	566.8	646.2	544	551.1	338.9	733	695.2
RAD23A	456.4	407.9	338.1	416.8	296.3	370.7	431.7	599.9
RAD23B	264.5	299.4	267.1	320	346.6	246	368.4	377.6
RAD50	171.6	218.9	157.1	201.9	181	127.6	195.6	187
RAD50	114.6	120.6	119.9	117.1	109.1	103.9	131.8	118.1
RAD51	162.3	163.6	152.1	165.4	143	130.6	146.5	154.2
RAD51	123.4	123.5	129.6	140.8	115.1	106.8	130.5	124.5
RAD51	119.7	111.1	111.3	117.2	106.4	97.3	115.5	110.1
RAD51AP1	130.8	122	135.6	119.3	119.8	101.4	136	127.5
RAD51C	201.5	234.3	196.8	226.4	195.4	163.5	182	177.3
RAD51C	109.5	110.8	117	110.3	110.2	93.7	118.6	112.3
RAD51C	204.5	218.3	252.1	207.7	179.8	166.9	161.4	149.2
RAD51L1	125.4	125.2	109.8	118.2	106.9	97.7	107.3	110.2
RAD51L1	120.1	122.9	120	121.7	108.5	106.1	126.9	126
RAD51L1	104.8	112.3	124.5	129.3	111.5	99.9	127.5	113.3
RAD51L3	125.1	132.2	120.1	117.6	115	105.2	116.3	118.6
RAD51L3	118.6	112.1	121.9	118	104.7	92.9	121.4	115.3
RAD51L3	124.8	119.1	121.2	116.8	119.5	103.9	122.3	123.8
RAD52	111.7	116.3	115.5	114.9	107.1	93.2	121.1	116.8
RAD52B	125.8	129.1	133.1	125.9	124.8	108.4	132.5	111.5
RAD54B	112.1	108.6	110.4	117	98.1	97.2	114.1	116.1
RAD54B	127.5	132.9	135.1	128.6	122.2	109.1	128.1	133.2
RAD54L	117.4	125.8	116.6	126.8	114.5	94.7	125.1	116.3
RAD9A	233.7	238.5	254.9	248.3	189.9	228.3	321.9	267.3
RAD9B	113.4	117.5	123.2	117	115.1	95.4	123.6	121.8
RAE1	122.7	131.3	132.4	140	121.6	125.4	133.8	135.6
RAE1	107.6	107.6	119.1	105.9	104.7	97.1	110.9	111.8
RAE1	409.5	431.8	446.6	466.6	386.4	362.7	497.7	528.9
RAET1E	119	113.3	115.3	115.6	106.4	104.3	116.6	113.6
RAET1G	117	115.2	109	111.3	106.2	102.5	104	110
RAET1L	123	122.4	108.7	119.3	106.7	100.3	122.4	112.2
RAF1	298.3	348.9	255	299.2	225.9	211.1	309.6	314.5
RAFTLIN	540.6	484.4	607.6	486.2	553.9	560.4	457.8	366.9
RAG1	113	118.4	119.7	123.3	102.2	96	126.8	116.5
RAG1AP1	123.6	131.4	137.8	132.3	126.2	118	124.5	132.8
RAG2	107.7	122.6	111.9	111.2	107.9	96.2	112	114.9
RAGE	150.9	162.7	156.9	144.3	139.6	130.1	151.1	131
RAI1	179.5	244.2	186.2	194.3	165.3	152.3	207.2	223.6
RAI14	119.5	135.7	118.3	136.4	131.6	109.5	137.2	130
RAI16	141.9	132.6	140.6	142.3	125.8	117.3	130.9	138.3
RAI17	445.2	410.8	576.2	639.2	390.9	489.9	630.5	334.1
RALA	1146.3	1002.4	847.7	1122	786.3	792.9	1099.6	876.3

RALB	289.3	313.3	577.6	354.8	270.2	457.6	304.6	277.4
RALBP1	222.3	201.3	207.9	247.1	210.5	197.9	261.3	239.4
RALGDS	116.5	102.3	112.3	110	110.6	91	115.8	106.8
RALGDS	1741.3	1983.3	2022.1	1767.2	1287.4	1506.5	1703	1647.5
RALGPS1	153.4	161	135.8	152	144.1	110.4	133	150.1
RALGPS2	122	122.5	128.8	123	111.9	106.1	126.4	114.4
RALGPS2	114.6	119.7	122.2	142.1	107.4	106.3	124.2	124.4
RALGPS2	124.4	113.6	116.7	113.1	105.9	100.3	119.6	110.2
RALY	279.3	354.7	270.7	382.9	311	295	356.6	381
RALY	683.1	852.8	666.3	806.5	539.9	683.3	994.6	1007.4
RAMP1	109.8	121.3	116.7	122.1	118	105.5	118.4	131.9
RAMP2	128.2	129.5	133.6	137.6	117.2	110.1	133.5	134.1
RAMP3	122.9	117.5	116.1	128.6	116.2	101.4	128.1	120.2
RAN	198.9	213.7	191.5	241.6	228.1	193.5	268.8	337.6
RANBP1	264.4	255.9	353.5	251.5	262.4	248.3	285.4	294.6
RANBP10	197.3	215.9	189.8	231.8	169.1	186.4	220.1	205
RANBP17	114.7	119.3	115	119	109.4	98.8	118	100.8
RANBP2	110.4	118.7	130.3	125.1	120.1	105.5	121.2	123.6
RANBP3	236.9	298.7	273.1	301.1	238.8	273.1	339	277.3
RANBP3	168.1	152.9	161.6	158.7	140.8	128.3	189.2	173.1
RANBP3L	109	116	106.5	128.5	103.4	97.8	110.7	110.8
RANBP5	129.1	131.1	138.3	138.4	135.6	112.3	137.9	159.2
RANBP6	125.7	121.9	134.1	118.5	113.9	104.5	121.9	114.5
RANBP9	119.5	135.2	125.9	133.9	111.4	100.4	129.1	142.2
RANGAP1	755.4	775.5	559.3	660.3	561.5	416.1	902.6	826
RANGNRF	129.7	125.6	117.2	119.6	118.1	102.9	127.9	127.9
RAP1A	129.1	142.1	142.5	135.3	125.5	116.7	131.1	149.7
RAP1A	135.7	131.3	128.1	137.1	144.5	107.4	131.8	150.8
RAP1B	123	144.8	134.6	140.2	120.6	107.7	135.8	129.1
RAP1B	279.8	297.5	301.8	266.7	223.3	280.1	368.2	341.2
RAP1GA1	116.8	117.8	118.8	133.2	106.7	95.9	124.7	122.5
RAP1GDS1	187.8	144.3	216.9	159.4	136.9	153.9	165	147.8
RAP2A	353.2	308.7	486.6	350.7	325.7	374.3	470.1	509.1
RAP2B	119.2	124.7	122	121.6	115	108.1	128.9	116.4
RAP2C	188	180.9	196.7	177.5	168.1	164.3	178.8	182.2
RAP80	806.3	933.9	611.7	891.7	823.3	411.2	1046.3	1121.4
RAPGEF1	114.5	125.2	121.5	125.4	116.2	109.9	126.6	121.9
RAPGEF1	1265	1238	1075.8	1428.4	838.3	1054.9	1530.9	1108.3
RAPGEF1	119.2	118.7	111.3	136	130.3	103.8	134.2	117.6
RAPGEF3	119.2	114.6	113.8	114.9	107.1	95.8	115.9	117.3
RAPGEF4	133.9	126.5	128	128.3	120.5	105.1	122.7	119.2
RAPGEF6	438.8	672	485.4	569.3	709.9	679.8	669.8	660.9
RAPGEFL1	149.2	133.4	128.7	131.6	125	108.7	129.3	129.4
RAPH1	115.7	105.3	124.6	119.3	103.7	97.2	107.9	105.6
RAPH1	117.4	114.4	126.6	120.3	110.9	96.7	120.2	113.1
RAPH1	117.3	115.2	115.5	114.5	106.6	98.4	123.6	113.2
RAPH1	119.2	115.1	123.7	113.4	108	95.1	130.8	123.1
RAPSN	136.4	151.5	127.1	144.4	130.7	107.9	155.4	131.4
RAPSN	121.3	121.4	119.2	121.2	114.5	105.9	125.8	124.6
RAPTOR	114.8	115.7	118.9	112.9	104.9	98	109.4	113.5
RARA	127.1	128.1	149.8	125.8	123.9	110.6	126.7	120.1

RARA	503.9	642.1	780.8	606.2	444.1	600.2	721.8	582.2
RARA	108.4	116	112.8	114.5	111	100.3	112.3	114.9
RARB	114.8	131.5	127.8	135.9	115.1	102.5	132.5	121.3
RARB	113.8	114.3	105.5	124.8	111.3	87.3	120.1	104.4
RARB	119.4	115.7	113.1	117.2	103.6	97.7	114.7	114.7
RARG	111.4	121.4	116.8	126	110.7	96.3	115.4	116.9
RARRES1	110	112.4	107.2	108.9	100.6	87.6	116.4	100.9
RARRES1	102.9	109.5	123.1	116.6	109.4	101.6	113.2	108.6
RARRES1	110.4	116	114.3	123.7	106.6	100.1	126.1	114.8
RARRES2	116.6	115.9	118.7	117.4	104.8	97	113.5	120.6
RARRES3	1600.8	1681.9	2407.9	1694.1	1372.8	2101	1080.6	1169.1
RARS	699.1	705	664.4	738.3	490.1	495	608.3	612.3
RARSL	274.1	312.1	360.1	313	299.8	267.4	336.2	268.2
RASA1	111.8	110.4	120.8	125.2	115.8	97.9	129	110.9
RASA1	458.3	539.5	595.3	536.4	428.1	442.4	510.3	596.4
RASA1	108.7	121.2	111.2	112.9	108.7	93.7	119.5	115.6
RASA2	187.4	209.3	189.5	233.2	220.3	131.3	252	247.9
RASA3	195.8	224.1	204.8	218.3	178.8	174.9	186	186.8
RASA4	119.6	112	118.6	112.4	106.7	102.1	115.5	117.5
RASAL1	128	144.2	176.9	161.5	125.4	180	137.6	128.6
RASAL2	125.8	121.2	132.4	134.4	115	112.9	114	126.6
RASAL2	103.4	117.1	111.9	110.7	104.6	103.9	107	112.2
RASAL2	106.8	112.6	108	119.6	104.4	103.8	111.2	113.5
RASD1	123.9	118.2	117.2	121.4	117.3	104.3	120.3	129.3
RASD2	112.2	109.3	119.5	121.7	104.4	100.4	117.6	127.2
RASEF	110.1	129.8	119.2	123.6	114.6	104.3	122.7	112.2
RASGEF1A	104.8	105.6	107.2	109.3	102.4	93.8	108.2	110.6
RASGEF1B	123.6	124.2	118.2	114.7	103.4	95.3	109.7	117.3
RASGEF1C	114.4	123.8	134.2	118.2	112.2	101.4	121.7	122.4
RASGEF1C	113.8	116.1	104.5	119.6	98.8	90.7	108.6	109.5
RASGEF1C	117.8	126	112.7	114.6	109	106.7	115	113.7
RASGRF1	117.2	119.9	121.3	122.6	108.1	104.3	115.1	117.7
RASGRF1	120.3	117.4	122.7	119.4	109.5	107.6	127.7	124
RASGRF2	120.5	127	117.4	119.4	114	91.2	124.1	113
RASGRP1	445.6	514.8	391.3	553.1	353.1	346	402	563.5
RASGRP2	1505.1	1327.6	1314.3	1585.9	1050.6	1110.4	1090.2	1113.1
RASGRP2	621.1	649.1	525.8	627.6	497.2	402	502.4	553.2
RASGRP2	144.6	162.9	140.4	149.4	156.5	112.2	133.4	131.6
RASGRP3	325.7	228.9	216	384.7	192.2	140.5	308.7	179.6
RASGRP4	115	119.3	113.8	119.8	106	101.6	120.8	110.8
RASGRP4	113.4	121.9	121.5	118.2	117.9	98.5	118	114.5
RASGRP4	106.8	112.4	115.1	107.4	110.5	90.4	112	111.9
RASIP1	116.8	122.2	113.7	120.9	107.8	101	115.3	112.9
RASL10B	118.6	114.7	123.5	117.5	109.7	97	118	105.8
RASL11A	114.4	122.2	121.3	118.8	112.8	102.1	121.7	115.6
RASL11B	111.4	109.1	113	110.1	110.9	102.4	123.2	115.5
RASL12	117.5	114.7	116.2	121.5	113.3	98.7	114.3	112.2
RASSF1	355.7	339.5	302	395.9	245.2	224.2	336.7	309.1
RASSF1	494.5	467.5	488.3	464.8	379.2	308.9	424.9	433.4
RASSF1	116.9	116.5	116.8	121.6	110.5	102.7	117.8	113
RASSF1	149.5	142.2	141.5	132.5	148	121.2	184.5	180.5

RASSF2	266	345.8	434.8	273.4	279.3	414.6	285.9	292.3
RASSF2	118.9	107.8	128.6	127.3	115.1	104.1	131.5	121.2
RASSF2	541.8	766.9	964	829	667.5	1065.9	748.1	815.4
RASSF3	112.2	113.4	102.5	107.1	104.1	102.8	122.6	116.5
RASSF4	125.5	118.1	161.4	122.3	110	101.4	115	116.2
RASSF4	116.2	129.9	124.5	121.6	114.8	103.5	119	115.2
RASSF5	144.9	140.1	146.9	151.8	129.2	133.5	131.6	153.4
RASSF5	131.7	136.5	115	122.6	119.1	97.5	116.2	117.4
RASSF5	1385.6	1912.8	1317.3	1640.4	1297.3	1219.3	1170.2	1208.2
RASSF6	144.3	154.3	175.3	151.5	152.2	131.2	179	159.3
RASSF6	109.3	111.4	108	115.3	105.9	97.7	114.9	103.8
RASSF6	112.2	123.2	117.6	127.2	113.3	106.7	114	117.7
RASSF7	547.7	698.2	856.3	674.9	530.9	630.2	670	636.9
RASSF8	123.3	118.3	124.4	116.9	108	103.7	117	117.1
RAVER1	256	298.7	191	313.7	206.8	236.8	304.7	275.5
RAVER2	119.6	117	124.8	115.1	118.1	108.3	126.3	135.3
RAX	120.3	121.5	117.7	109.4	115.4	103.9	117.7	107.1
RAXL1	305.8	225.6	215.7	255.4	268.2	142.5	246.7	248.7
RAXLX	120.2	122.5	115.7	110.2	108.2	95.7	112.3	119.9
RB1	162.3	143.5	174	175.9	144.2	155.7	178.1	164
RB1CC1	386	479.7	330.3	507.1	371.8	345.9	508.4	572
RBAF600	494.6	487.1	377.4	587.4	551.2	380.2	885.5	858
RBAK	148.1	136.5	135.6	134.4	116.2	102.9	134.9	128.6
RBBP4	131.6	138.5	132.4	142.6	117.9	112.7	138.1	141.6
RBBP5	168.7	188.7	161.3	184.7	183.7	177.2	193.6	189.2
RBBP6	102.2	110.2	114.5	109.6	107	94.3	114.4	112.7
RBBP6	125.2	123.6	124.1	129.1	121.4	115.3	120.6	133.8
RBBP6	116.1	113.5	117.4	119.9	108.6	96.4	118.7	110
RBBP7	211.3	154.7	162.6	172.9	154.8	148.9	191.4	176.7
RBBP8	113	110.8	123.3	116.2	110.1	100.5	125.5	122.3
RBBP8	130.7	116.6	120.8	132.2	115.6	106.1	117.5	121
RBBP8	187.1	197	174.3	225.5	213.3	208	288.3	302.9
RBBP9	112.1	132	131.6	128.7	108.7	109.5	125.3	121.8
RBBP9	247.5	256.7	241.5	220.2	204.8	181.7	208.8	197.4
RBCK1	193.6	171.2	188.6	187.9	157.9	143.5	172.5	207.9
RBCK1	200.7	205.5	213.9	201.4	166.6	161.7	200.2	195.5
RBED1	160.1	145.6	165.6	153.6	130.7	125.7	142.2	141.9
RBJ	185.5	193.6	225.7	204.4	192.7	188	178	214
RBKS	139.6	143.4	140.4	137.8	122.5	121.6	133.3	134.7
RBL1	115.3	117.1	109.3	110.9	101.9	104.7	118.8	108
RBL1	128.7	134.4	138.3	152.4	130.6	118.6	138.1	131.7
RBL2	1515.9	1650	1457.3	1685.8	1292.4	967.5	1100.2	1034.5
RBM10	163.6	185.7	171.7	198.2	154.8	139.6	191.3	272.1
RBM10	1279.9	1188.6	1264.1	1367.3	1118.9	1116.1	1700.5	1878.2
RBM10	118.8	117.8	126.5	132.8	112.5	93.5	124.5	122.1
RBM11	125.4	127.3	130.6	133.7	127.5	107	147.7	130.8
RBM12	158.9	162.5	154	151.9	141.1	124.7	153	187
RBM12	270.5	286.5	291.8	267.1	264.7	281.4	381.8	446.5
RBM12B	178.6	141.5	204.3	188	196	118.9	168.9	232
RBM13	195.8	202.6	195.1	224	184.5	157.8	185.5	196.9
RBM14	694.8	587.2	592.2	544.6	475.3	434.3	601.7	805.3

RBM15	413.7	418.7	357.2	445.8	462.1	291.1	403.1	396.2
RBM15B	133.1	156.9	129.6	136.2	134.8	121.8	166	134.7
RBM17	544	730	695.3	641.7	578.2	564.8	583.7	486.2
RBM18	198	159.2	192.6	181.6	159.7	149.3	186.7	161.4
RBM19	121.9	114.3	134.8	128	115.2	104.9	122.8	127.7
RBM21	158.4	154.7	152	159	135.6	135.1	157.2	142
RBM22	2097.8	2259.5	1858.9	2121.5	1896.7	1560.3	3297.4	3282.2
RBM23	412.2	447.9	386.1	430.5	345.5	320.3	664.4	573
RBM23	1032.6	1134	780.4	1129.6	764.8	804.1	1611.4	1592.7
RBM24	107.5	118.8	110.3	118.6	107.4	97.3	109.8	107
RBM25	462.7	367.5	318.1	432.4	356.5	225	401	375.2
RBM28	171.6	168.3	155.5	176	146.7	131.3	182.8	168.4
RBM3	115.5	117	138.3	129.6	110.5	105	120.9	126.3
RBM3	149.7	162.9	161	185.7	145.3	129.7	152.3	156.2
RBM33	716.1	746.1	707.1	679.4	676.8	500.6	695.1	683.5
RBM34	206.9	194.5	243.5	240.2	194.4	228.7	261.6	224.6
RBM35B	131.6	141.4	135	140.2	124.3	93.9	124	143.8
RBM38	448	307.4	416.2	377	252.3	404	470.4	502.2
RBM39	424.1	468.3	357.6	500.6	590.3	302.8	666.8	720.1
RBM4	219.2	209.9	208.8	234.9	231.3	174.6	227.6	216.9
RBM4	517.7	586	500.5	447.9	513.6	453.4	564.1	624.6
RBM4	155.9	198.9	191.2	191	185.3	151.8	196.1	208.1
RBM41	109.3	107.4	122.8	123.6	106.9	92.8	115	107.8
RBM4B	223.1	235.4	214	228	184	169.2	193.7	196.8
RBM5	1350.9	1268.8	1430	1328	1261.2	1227.4	1607.6	1523.4
RBM6	334.4	400.5	338.6	439.8	376.2	306	350.4	340.5
RBM7	174.4	207.2	157.3	190.4	166.6	146.6	187.5	221.7
RBM8A	133.2	137	115.5	133.2	116.2	110.3	140.6	126
RBM9	122.9	127.6	130.8	131.1	123.3	101.4	147.5	157.3
RBM9	113.7	110	117.5	126.7	115.4	100.4	121.3	117.2
RBMS1	362.6	373.2	417.6	384.3	361.9	254.9	305.2	347.4
RBMS1	116.6	117.6	108.6	116.7	106	99	125.1	115.9
RBMS2	116.3	113.7	120	116.2	106	102.6	125	112.4
RBMS3	110.1	109.5	111.4	111.1	106.9	92.8	124.4	110.8
RBMS3	114.9	120.8	119.8	118.6	113.3	102.6	130.7	118.7
RBMS3	111.5	114.5	118.6	136.5	111.3	98.3	119.7	112
RBMX	347.6	360.2	422.6	369.1	382.2	315.7	386.9	398.9
RBMX2	132.1	139	141.3	137.2	144.6	162.7	140.3	153.4
RBMXL1	105.6	107	114.5	117	107.6	110.6	124.4	128.6
RBMY1A1	113.6	111.8	120.8	114.1	108.8	94.7	111.8	112.9
RBMY1A1	105.8	122	106.8	111.6	110.6	97	115.3	116.2
RBMY1A3P	111.1	116.8	118.2	117.4	101.9	106.8	116.7	117.1
RBMY1B	114.9	112.5	112.6	119.5	103.8	97.2	112.2	117
RBMY1E	112.7	120.4	117.4	124.4	106.1	104.3	117.1	126.2
RBMY1F	123.8	106	118.3	116.2	110.9	97.2	120.3	114.9
RBMY1J	122.2	104.5	119.3	114.2	107.3	98.7	121.2	118.1
RBMY2EP	116.2	115.2	129	124.8	105.3	93.7	105.7	119.9
RBMY2FP	119.7	117	113.3	114.6	120	100.6	119.9	119.1
RBP1	113.8	120	132.2	110.3	109	101.5	119.6	115.7
RBP2	111.8	113.5	108.4	114.4	105.7	98.3	115.1	112.9
RBP3	112	118.3	115.6	118	110.1	103.3	104.2	115.8

RBP4	113.2	115.9	117.8	114	106.5	100.9	121.6	111.3
RBP5	112.9	121.4	120.8	117.3	106.7	98.7	118.1	114.1
RBP7	111.1	117.9	179	115	109.4	111.7	131.4	119.1
RBPJ	106.5	116.7	110.2	119	109.9	95.3	121.7	113.7
RBPMS	114.5	118.2	120.5	120	110	101.6	119.5	119.2
RBPMS	110.1	105.2	118.3	115.3	113	101.9	121.5	109.8
RBPMS	126.1	111.5	116.6	123.9	103.5	95.9	116	112.7
RBPMS2	123	163.4	120.2	132.3	135.9	110.3	130.8	179.6
RBPSUH	109	113.3	130.2	116.1	105.6	101	120.1	127.2
RBPSUH	326.1	353.8	453.4	375.1	316	306.6	313.4	332.6
RBPSUHL	110.8	111.3	122.1	119.8	121.9	112	115.4	117.9
RBX1	713.5	662.1	798.2	715.9	627.7	496.1	846.2	870
RC3H1	116.9	116.6	118	118.7	117	94.4	138.4	115.6
RC74	267.3	324.8	286.3	338.5	261.9	205.3	311.5	286
RCBTB1	136.4	128.5	141.6	149.6	125.8	108	133.4	121.3
RCBTB2	566.1	389.4	833.6	818.6	480.9	1029	626.3	338.1
RCC1	122.8	124.5	123.9	121.4	105.2	95.8	120.4	125.4
RCC2	947	1103.5	778.8	1012.9	896.3	593.6	1027.4	953.9
RCCD1	112.3	126.6	122.9	116.7	111.4	97.5	115	120.3
RCCD1	110.6	114.9	117.6	118.5	107.2	105.9	116.6	113.3
RCCD1	128.3	130.9	152.4	127.5	121.7	105.5	119.3	138.9
RCD-8	684.4	740	537.2	679.1	530.8	415.9	814.1	760.5
RCE1	270.2	326.5	282.5	291.6	193.6	218.8	327.8	274.2
RCE1	143.5	145.7	133.2	143.2	126.3	106	140.8	140.7
RCHY1	131	138.1	159.7	150.5	134	128.1	150	147.7
RCHY1	118.5	123.3	127.4	129	115.2	105.8	121.8	122.9
RCL1	171.5	145.8	150	166.9	150.2	132.2	167	161.5
RCN1	108.7	115.8	107.2	117.1	115.4	103.7	103.4	106.5
RCN2	117.6	121.2	120.5	119.7	114.8	99.2	117.3	110.9
RCN3	128.3	129.8	136.6	126.2	104.6	111.4	122.4	116
RCOR1	110.9	107.7	105.3	114.7	105.2	95.7	116.5	115.2
RCOR2	109.4	104.9	106.4	125.4	100.5	103.2	115.6	110.4
RCOR3	447	472.6	443.3	443.3	409.5	331.1	353.9	384
RCP9	883.8	734.5	612.8	742.8	785.1	368.4	613.7	687.1
RCP9	112.8	117.6	114.3	123.6	111.5	97.9	114.5	120
RCP9	106	102.9	104.2	111	110.2	89.7	114.3	114
RCSD1	333.6	289.2	281.7	310.7	242.9	256.3	214.2	202
RCV1	124.6	120.9	136.8	116.8	108.8	118.3	118.4	117.6
RDBP	195.6	170.5	197	189.4	162.3	146.4	178.2	164.2
RDH10	185.6	191.1	155.8	241.4	189.1	140.7	276.4	284
RDH11	238.7	224.7	232.2	234.1	181.8	202.8	196.1	221.8
RDH12	105.7	114.6	112.3	118.8	105.9	105.8	124.3	110.7
RDH13	146.6	176.5	173.7	158.6	154.1	129.3	140.1	150.9
RDH14	1603.6	1680.9	1137.6	1473.2	1178.2	810.4	1547.3	1516.8
RDH16	126.3	128	121.7	129.5	117.4	98.5	119.5	125.1
RDH5	197.1	232.7	198.6	175.7	187.9	143.1	185.8	206.6
RDH8	116.9	114.1	124.4	110.8	110.3	97.2	116.1	117
RDHE2	113.2	119.6	125.4	111.4	108.8	105.4	116.7	114.3
RDM1	119.5	121.1	120.6	107.7	109.3	98.8	109	104.2
RDS	122.2	128.7	112.8	114.3	125.8	105.9	115.3	115.9
REC8L1	268	238.4	275.6	310.3	214.7	187.3	284.4	264

REC8L1	160.6	149.1	140.3	152.5	143.4	107.2	164	158.6
RECK	189	216.9	213.2	224.4	155.1	140.6	177.6	190.5
RECQL	288.8	223.6	254.3	254.5	204.1	178.7	247.9	281.2
RECQL	342.4	377.1	392.4	429.2	340.2	277.7	364.2	348.7
RECQL4	109.3	115.9	115.1	119	112.2	102.2	127.1	112.4
RECQL5	112.3	120.3	120.9	120.9	114.3	101.1	121.9	115
RECQL5	143.6	141.9	134.6	154.5	131.4	112.8	149.8	137.7
RECQL5	117.5	140.5	118.3	121.5	118.3	104.7	123.6	126.2
REEP1	115.3	117.1	111.5	109.3	101.9	100	110.8	105.7
REEP2	114.8	117.2	110.5	114.1	116.9	93.9	114.6	107.7
REEP3	115.6	118.2	121.2	122.9	118.2	101.4	116.6	120.5
REEP4	150.4	164.2	167.9	155	129.6	125.4	141.7	122.2
REEP5	640.4	755.4	821.6	690.1	610.2	732.5	623.4	644
REEP6	132.4	134.8	140.8	138.8	110.7	106.7	124.4	131.6
REG1A	118.6	116.2	117.7	111.8	95.6	97.5	111.1	109.8
REG1B	111.9	118	112.6	119.4	102	100.4	127.9	119
REG3A	114.5	121.6	126.1	142.2	114.2	101	121.8	122.9
REG3A	112.3	120	125	114.8	111.2	99.5	125.5	109.3
REG3G	106.2	113.9	111.5	109	108.3	101.7	112.9	107.6
REG3G	111.2	122	121.8	124.3	110.9	100.4	126.6	116.3
REG4	126.8	137.6	118.9	131.6	152.7	103.5	129.8	154.4
REL	135.3	132.6	129	127.7	127.2	125.2	133.6	142.5
RELA	169.5	186.6	150.8	191.8	148.9	127.6	182.6	158.7
RELB	229.4	257.8	166.1	252.5	210.5	171.7	411.6	399.3
RELN	129.2	117.5	129.3	121.2	113.9	94.8	120.2	121.5
RELN	117	115.5	123.3	126.2	118.1	107.7	121	120.3
REM1	112.5	115.9	107.1	117.6	106.8	88.3	122.9	110.6
REN	113	124.3	118.5	119.4	116.7	103	119.2	122.9
RENBP	285.4	215.4	391	258.7	185.2	242.5	239.4	169.6
RENT1	207.7	263.6	172.2	228.6	194.1	157.5	263.7	300.5
REP15	108.2	110.5	109.9	100	105.6	90.7	116.5	118.3
REPIN1	110.3	118.2	121.8	129.1	117.5	103	122.6	112.2
REPIN1	116.6	125.9	109.3	122.5	115.6	96.5	127.7	126.5
REPIN1	660.8	630.7	598.8	491.4	358.7	312.4	349.3	338.1
REPS1	125.3	133.6	130.3	136.5	137.8	112.6	142.5	149.9
REPS2	115.7	122.2	125.6	135.6	116.6	98.5	124.6	120.4
REPS2	120.8	115.4	124.5	124.7	109.7	100.8	121.5	117.4
REPS2	121.5	120.3	123	118.7	106.8	101.9	116.4	114
REPS2	124.7	115	200.4	128.5	107.5	110	130.2	120.2
REPS2	118.8	122	113.5	111.6	108.5	98.4	122.9	120.4
RER1	145	135.4	131.8	128.7	123.6	117	131.2	135.6
RERE	322.4	349.2	330.8	378.9	385.4	252.9	375.3	353.5
RERE	121	111.8	127.9	118.6	112.9	101.4	122.8	115.4
RERE	185.3	192.4	180	182.8	177.4	144.2	189	204.4
RERG	117.7	114.1	117.7	109	101.5	96.2	120.3	107.3
RESP18	125.1	109.2	117.6	124.3	113.1	103.1	129.2	118.8
REST	123.6	126.2	120.3	128.8	119.9	105.1	136.5	131.9
RET	122.3	118.3	119.4	124.6	113.8	102	117	121.3
RET	119.7	113.6	119	127.7	109.8	105.1	113.6	107.8
RET	128	105.7	123.1	121.8	108.6	102.3	116	114.2
RETN	135	105.7	118	119.1	107.1	97.4	115.5	117.8

RETNLB	112.5	117	121.1	123.1	107.6	100.9	118.7	114.6
RETSAT	152.8	155.6	159.3	166.3	142.8	133.1	166	149.6
REV1	253.5	290.2	278.5	299.1	202.9	205.2	254.8	304.3
REV1L	273.9	379.9	323.9	322	318.2	249.1	326.3	442.5
REV3L	119.4	134.8	126.3	126.5	122.2	110.8	129.1	122.2
REXO1	169.5	165.3	159.6	174.7	156.6	127.8	181.5	178.1
REXO1L1	113.1	115	122.7	129.1	105.7	102	117.8	113
REXO2	969.2	910.1	718.8	1039.7	927.2	588.4	966.7	1179.2
REXO4	251.8	320	207.3	271	230.3	168.7	256.3	245
RFC1	293.2	349.1	253	310.1	258.9	223.6	235.6	246.5
RFC2	118.4	136.8	121.3	121	112.7	99.3	134	125.2
RFC2	122.9	133	130.7	132.3	129.7	124.7	125.4	131
RFC3	118.8	117.7	127.6	123.7	111.4	107.9	123	113.9
RFC3	127.9	130.6	133.8	118.5	111.1	109.1	116.6	113.4
RFC3	113	122.8	110.8	115.9	107.6	106.8	123	116.1
RFC4	225.6	227.8	235.3	167.8	193.3	193.2	179.2	182.3
RFC4	207.6	229.8	265.5	263	189.2	199.3	172.7	174.9
RFC5	216.9	225.5	228.2	219.9	174.9	161.3	171.2	167.7
RFC5	112.2	113.3	114.2	119.3	111.6	91.4	112.3	107.2
RFESD	143.6	161.8	167.9	155.8	126.3	125.9	148	146
RFFL	114.8	121.8	109	115.6	107.4	96.5	111.1	113.1
RFFL	111.1	113.5	112.6	124.6	109.6	108.4	121.7	114.5
RFK	122.7	117.6	131	125	107.1	104.9	129.6	116.8
RFNG	121.6	115.2	123.5	129.6	119.1	101.1	122.9	113.7
RFNG	399.2	480.8	450.7	456.2	303.9	436.1	405.6	360.3
RFNG	113	121.8	131	120.6	111.9	103.2	119.4	117.3
RFP	170.2	171.5	166.8	170.3	130.6	134.6	139	148.2
RFP2	116.1	129.5	126	130.5	115.5	101.7	125.3	117.1
RFPL1S	109.2	118.5	130.4	120	106.5	100.4	116.2	116.8
RFPL3S	127.4	135.7	121.7	116.7	110.9	108.7	122.1	129.4
RFPL3S	119.3	122.5	135.1	128.8	112.6	101.1	124.9	122.3
RFPL4B	112	110.9	110	111.1	106.2	91.4	110	109.6
RFT1	113.8	121.4	112.9	120.4	110.3	109.8	120.5	118.4
RFWD2	561.9	598.4	699.1	651.2	558.4	563.3	594.2	536.3
RFWD2	114.7	114.8	125.7	121.9	116	99.4	113.8	118.1
RFWD2	532.4	569.1	708.4	664.1	457.7	476.5	672.8	593.7
RFWD3	245.8	259.2	260	249.7	240.3	185.9	249.9	233.3
RFX1	228.4	229	203.6	213.7	197.5	183.5	230.7	183.9
RFX2	121.5	117.6	120	117.4	119.7	108.7	117.1	127.3
RFX2	117.6	113.5	112.3	122.4	107.1	104.9	122	113.8
RFX3	115.7	115	131.9	123	110.8	102.8	127.9	115.2
RFX3	121.8	132.2	134.2	136.6	119	117.1	138.1	129.7
RFX3	110.2	115.8	125.8	120.2	106.8	106.8	126.1	114.3
RFX4	104.6	113.3	112.1	108.3	106.7	106.2	109.5	118.3
RFX4	120.4	131.9	129	122	109.3	103.2	119.9	120.7
RFX4	117.5	112.2	111.4	124.4	110.2	95.4	114.7	117
RFX4	121.8	111.5	110	122.2	112	99.4	114.1	115.5
RFX5	123.2	126.8	126.6	119.3	113.1	104.9	115.5	116.1
RFX5	748.8	647.3	621.7	706.1	421.2	421.3	512	445.5
RFXANK	173.3	159	192.5	174.9	152.1	141.4	157.9	151.1
RFXANK	129.3	141	128.3	134.5	120.2	108.3	124.8	129.9

RFXANK	360.4	407.8	394.6	375.2	255.5	284.3	356.7	280.2
RFXAP	133.1	111.5	127.6	121.9	106.2	96.6	121.5	117.6
RFXDC1	112.7	113.2	115	117.2	107.4	100.1	116.7	115.1
RFXDC2	152.3	155.2	143.2	149.3	136.5	130.9	158.6	139.4
RG9MTD1	208	217	195.5	227.7	181.5	180.6	218.7	237.2
RG9MTD2	130.7	116.2	132.3	125	120.1	113.3	141.9	128.3
RG9MTD3	148.7	145.8	165.4	188.7	152.6	120.7	156.5	157.6
RGAG1	120.8	112.5	129.8	120.5	112.4	101.2	111.3	123.5
RGAG4	111.7	120.8	121.7	126	104.8	106.1	113.6	114.2
RGAG4	136.4	119.6	140.7	129.7	110.8	120.6	125.7	118.6
RGC32	3908.8	5331.1	2974.4	5274.3	5284.8	3567.1	5536.3	3724.8
RGL1	689.9	240	1088.6	855.4	191.5	1280.2	526.5	180.4
RGL2	169.6	162.7	163.9	161.2	137.1	126.6	143.3	159.5
RGL3	117.5	130	133.5	117.4	114.6	118.1	138.5	122.9
RGMA	179.7	151.5	145.9	250	119.4	118	200.3	122
RGMB	114.2	120	122.7	122.1	109.8	100.4	117.4	117.7
RGMB	141.2	185.6	142.1	156.4	151	135.1	134.8	180.2
RGMB	126.2	119.8	116.1	122.2	108.5	99.5	124.1	116
RGN	121.9	109.4	114.5	124.2	115.7	109.8	124.1	133.6
RGN	118.1	114.7	122.2	126.3	109.8	100.9	114.1	112.9
RGNEF	130.1	127.1	125.2	116.4	116.3	113	120.7	117.6
RGNEF	109	114.6	110.1	109.4	105.6	96.2	113.7	105.6
RGPD1	161.8	248.8	236.5	171.5	216.8	229.9	169.8	222.7
RGPD2	122.7	121.3	130	119.7	114.6	104.5	123	122.3
RGPD5	116.8	112.7	122.4	114	106.9	101.8	111.6	114.7
RGPD5	110.8	110.1	114.4	112.5	102.9	101.1	113.5	107.7
RGPD5	120.2	121.8	117.2	122.4	118.6	93.3	121.3	113
RGPD7	127.2	119	117	121.9	120.7	94.4	125.2	113.8
RGR	107.4	115.9	111	123.7	104.5	102.7	116.2	117.4
RGR	124.8	126.1	133.2	137.6	115.3	107.7	138.9	134.1
RGR	134.1	169.7	203.5	133.5	149.7	169.5	131.3	145.9
RGS1	187.7	241.8	171.5	206.2	196.2	158.5	335.3	530.4
RGS10	332.1	378.5	668.6	368.7	332.2	457.7	284.3	300.9
RGS10	581.2	517.3	988.4	479.5	639.2	633.1	434.5	655.7
RGS10	118.6	114.3	122.4	116.4	109.2	98.6	117.4	132.4
RGS11	120.5	121.4	127.9	124.2	105.9	106.9	118.4	111
RGS11	117.1	113.2	123.5	117.7	117.1	100.2	123	116.4
RGS12	120.5	116.5	120.8	118.7	111.4	101.6	117	117.3
RGS12	182.5	159.7	348.5	172.9	133.8	151	211.9	153.1
RGS12	111.9	106.1	110.1	118.2	105.1	102.8	117.1	114.4
RGS13	120	112.6	117.8	115.7	103	98.2	120.4	116.2
RGS14	152.6	146.8	144.9	154.7	140.1	132.2	145.1	134.5
RGS16	115.6	119.4	117	116.6	104.6	102.7	121.9	107.8
RGS17	122.1	121.5	122.8	123.5	115.9	94.4	122.2	123.9
RGS18	140.7	195	270.2	149.2	160.3	185.2	153.5	148.8
RGS19	464.2	416.8	698	467.5	247.1	443	337.8	328.5
RGS2	574.7	602.1	597.4	606.6	528.1	752.5	755.3	1014.4
RGS20	108.9	111.9	115.3	118.2	108.4	97.4	115.6	108.6
RGS20	109.3	112.3	110.6	115.6	101.6	96.9	119.1	107.9
RGS22	112.2	116.3	107.6	118.8	111.7	100.2	113.1	112
RGS3	118.9	116.5	117	117.2	105.2	103	112.5	106.9

RGS3	113.8	113.6	109	115.8	111.6	106.9	117.8	109
RGS3	120.4	107.8	119.1	114.1	105.4	99.3	120.3	134.8
RGS3	102.3	108.4	104.3	119.1	101.6	102	115.2	103.8
RGS3	113.6	117.6	115.1	120.4	125.2	102.6	122.1	113.4
RGS3	126.2	137.5	126.7	126.1	113.6	103.4	131.2	115.6
RGS4	117.1	117.5	114.2	119.8	111.7	99.5	126.8	122.8
RGS5	111.9	114.2	120.4	110	105.9	99.3	132.2	125.7
RGS6	119.8	111.9	122	117.1	115.9	104.7	119.3	119
RGS7	112.1	113.5	113.6	108.9	103.6	94.2	124.6	107
RGS8	118	111.2	126.2	129.7	111.9	103.9	120.4	122.6
RGS9	112.9	116.9	128	129.5	113.4	106.8	117.8	124.1
RGS9	110.3	112.6	115.1	115.8	101.3	92.5	120.1	116.9
RGS9BP	112.5	115.7	121	117	104.7	101.4	115.9	109
RHAG	107.9	117.3	114.1	110.2	105.9	100.9	117.7	110.1
RHBDD1	165.8	151.5	192.9	173.4	131.6	159.8	189.3	148.5
RHBDD2	1378	1299.6	758.3	1545.3	514.7	1222.2	1231.9	718.7
RHBDD2	1069.4	1305.6	841.6	1439.1	593.9	1073.4	1180.9	672.2
RHBDD3	126.8	138.4	160.4	142.2	141.9	117.5	148.9	153.6
RHBDF1	118.9	123.8	126.2	132.6	111.8	104.9	122.3	141.1
RHBDF2	511.4	447.2	466.1	491.8	420.7	313.3	445.7	426.4
RHBDF2	131.3	127.9	143.8	132	111.9	108.1	137.4	124.8
RHBDF2	421.4	395.2	381.4	450.7	314.1	265.4	382.7	430.9
RHBDF2	115.2	105.2	106.3	115	106.9	96	116.9	108.2
RHBDL1	112.3	120.6	115.1	133.3	111.8	102.2	127.3	122.4
RHBDL2	143.8	129	139	137.8	119.6	111.2	135.7	129.2
RHBDL3	121	117.5	114.2	119.6	108.7	104.4	117.7	111.6
RHBG	104.8	110.5	110.9	117.1	110.2	101.4	116	119.5
RHCE	120	120.6	116.8	124.6	107.4	101.9	118.2	114.1
RHCE	117.5	114.7	126	115	110.5	101.9	118.2	124.7
RHCG	110.6	120.2	117.3	116.2	104.1	95.4	121.1	119.1
RHD	116.1	112.9	107.3	110	110.3	98.7	118.1	111.4
RHEB	588.3	514	483.9	719.1	551.8	506.6	973.2	953
RHEBL1	128.7	115.1	128	124.3	117.3	104.6	128.6	127.5
RHO	118.7	114.6	122.9	117.7	110.5	88.3	116.3	126.4
RHOA	1135.3	1075.1	1770.6	1119.6	1384.1	1746.1	1916.9	1826.2
RHOB	115.5	114.9	114	124.3	108.6	98.4	131.1	112.9
RHOBTB1	104.8	103.3	108.3	111.1	100	100.3	110.8	118.3
RHOBTB1	106.8	106.5	112.3	115.3	96.6	87.9	102.9	103.5
RHOBTB1	115.1	98.9	112.3	110.7	102.2	90	112.5	109.4
RHOBTB1	124.3	117.9	121.6	118.5	113.1	101	125.3	120.7
RHOBTB2	122.3	124.2	131.5	112.5	105.4	114.3	121.9	122.3
RHOBTB3	163.2	150.4	181.4	182.8	163.5	136	193.7	154.2
RHOC	220.6	226	215.6	236.8	232.5	211.7	257.6	246.2
RHOC	142.2	155.9	152.1	172.3	166.6	128.8	174	168.5
RHOD	117.1	117.8	117.2	124.2	109.7	100.7	114.8	117
RHOF	124.8	136.4	123.5	128.9	124.8	111.8	118.6	125.9
RHOG	1489.5	1433.5	1727.5	1512	845.4	1520.5	1777.4	1521
RHOH	121.1	124.3	121.8	133	113.1	99.4	121.6	117.3
RHOJ	119.2	123	111.8	124.3	108.3	103	114.8	119.9
RHOQ	1129.5	872.7	958.4	1030.3	759.1	489.8	1161.7	812
RHOT1	207.2	233.3	301.6	243.8	238.4	191.8	179.4	167.6

RHOT1	345.2	363.2	441.8	327.4	327.3	259.6	275.7	251.8
RHOT1	118.2	129.3	118	134.3	132.1	129.5	126.5	129.9
RHOT2	570.2	539.3	479.7	499.1	308	394	563.9	438.3
RHOV	128.6	111.9	118.2	113.3	108.8	102.7	116.9	105.7
RHPN1	125.8	136.8	133.6	142.2	131.2	110.4	134.4	137.4
RHPN2	140.3	139.8	137.1	145.9	120.2	115.9	151.9	146.9
RIBC1	130.6	133	155.3	126.7	130.4	124.7	134.5	138.9
RIBC1	113.5	116.8	109	125.4	110.1	102.1	119.8	112.3
RIBC2	112.8	117.3	121	118.1	109	103.1	118.6	126.2
RIC3	119.7	117.3	119.9	118	106	96	113.6	112.2
RIC8A	569	669	543.6	576.5	459.9	520.7	666.1	593
RIC8B	123.9	114.2	129.6	139.3	119.6	103	125.6	135.7
RICS	158.5	141.8	136.1	154	150.1	117.1	172	176.8
RICTOR	116.5	124.2	121.2	118.5	103.1	112.9	113.7	114.2
RIF1	187.9	182.3	202.5	217.6	201.4	169.5	240.4	198
RILP	138.5	134	152.7	159.7	119.1	127.8	144.3	130.8
RIMBP2	115.4	117.4	109.8	111.5	112	97.7	117.3	111.3
RIMS1	112.3	113.6	115.8	113.1	106.7	101.5	113.6	109.9
RIMS2	118.1	117.9	121	122.2	115	104.9	117.5	120.6
RIMS3	166.7	157.9	150.4	165	136	134	168.4	153.7
RIMS4	118.4	111	122.4	117.1	116.1	103.5	132.1	123.1
RIN1	115.2	138.2	133.4	131.6	110.4	107.1	110.3	120.9
RIN2	234.7	133.3	373	252.2	118.1	294.6	234.3	114.9
RIN3	152	141.4	139.2	150.3	144.2	118.5	148.9	132.9
RING1	1408.4	1131.2	975.9	1355	1193.1	849.2	1217.6	1091.9
RINT-1	141	157.5	144	143.6	154.1	121.5	153.7	152.7
RIOK1	286.9	310.9	241.9	312.5	273.6	175.7	331.2	341.8
RIOK1	112.2	109.2	114.5	106.6	109	100.3	113.3	109
RIOK1	128.8	135.9	137.3	144.6	135	108.9	143.7	146.6
RIOK2	260.1	293.1	303.1	233.2	255.6	233.5	278.1	253.9
RIOK3	228.8	208	329.9	296.1	203.7	209.4	298	273.5
RIOK3	486.5	429.6	638.2	533.6	491.3	463.4	544.3	646.7
RIOK3	120.1	116.5	123.1	118.5	117.6	100.4	114.4	116.5
RIP	815.4	892	926.5	813.1	660.8	565.6	637.1	710.3
RIPK1	285	348.7	274.6	349.3	279.8	227.7	370	417.2
RIPK2	341.6	281.2	258.9	307.5	234.1	196.9	231.7	215.3
RIPK3	167.5	186.7	217.1	181.7	125.6	156.3	180.3	160.6
RIPK4	124.3	141.7	145	143.9	120	112.7	142.8	134.5
RIPK5	161.8	167.8	162.8	167.8	146.8	133.4	171.1	139.5
RIPK5	296.9	256.9	296.9	288.6	263.5	216.2	236.2	250.7
RIS1	248.1	242.6	212.6	384.1	200.9	480.6	357.5	165.9
RIT1	180.7	174.8	190.1	304.7	216.8	246.6	398.1	295.7
RIT2	112.1	115.1	108.4	114.4	99.5	94.4	117.6	113.5
RKHD1	121.8	121.5	125.6	114.3	108.4	102.8	125.1	108.1
RKHD2	416.3	480.9	433.2	496.8	497.8	386.4	543.4	557.9
RKHD3	111	116	127.7	124.3	122	110.1	117.4	126.4
RLBP1	109.5	117.2	110.2	121.2	114.7	100.1	123.2	120.4
RLF	134.5	140.5	154.7	150.7	138.7	116.9	137.3	157
RLN1	113.5	130.2	136.5	121.1	113.4	108.9	121.3	125.8
RLN2	120	127	122.2	116.5	113.1	104.6	113.8	122.1
RLN2	127	125.7	144	129.9	131.9	111.8	137.4	136.4

RLN3	107.5	112.6	117.4	118.2	109.5	98.2	121	119.6
RLN3R1	118.8	115.5	110.7	118.2	108	103.3	121.8	117.5
RLN3R2	116.5	120.7	123.8	116.4	105.5	100.2	123.2	115.8
RLTPR	166.6	196.2	170.4	199.2	155	163.3	166.4	172.4
RMI1	206.2	181.2	184.3	200.1	148.6	155.6	215.8	227.2
RN7SK	810	498.5	402.7	797.2	623.6	289.7	1494.7	1264.8
RN7SL1	9516.3	9389.8	8669.7	10940.9	9267.6	7216.8	9524.5	11018
RNASE1	126.6	119.2	417.2	120.7	106.7	324.5	115.9	112.6
RNASE1	136.5	120.4	1081.7	141.7	113.9	789.2	136.4	124.6
RNASE10	146.2	134.5	133.4	134.9	121	109.1	135.2	128.3
RNASE11	119.1	118.8	118	120.6	108.8	98.2	119.9	109.6
RNASE12	120.7	113.3	109.3	114.2	105.8	100.9	118.7	118
RNASE13	110	110.8	120.1	113.9	109.9	98.2	115.7	105.4
RNASE2	124	135.5	146.4	124.1	130.1	109.9	121.7	132.1
RNASE3	114.2	138.5	108	114.7	121.3	102.8	118.5	133.9
RNASE4	113.4	112.9	116.7	116.2	110.9	96.3	122.5	116.1
RNASE4	121.7	113	136.4	115.9	105.4	115.8	121.9	130.4
RNASE4	133.4	123.9	201.2	134.3	116.5	173.6	128.6	123.8
RNASE4	118.6	122.8	157.4	130.3	109.5	142.5	118.8	129.4
RNASE6	150.5	141.2	505.7	143.1	122.2	395	126.3	116.7
RNASE7	109.3	114.5	119.7	118.3	106.7	92.8	127.7	113.8
RNASE8	126.6	112.3	111.2	109.1	101.8	100.3	127	112
RNASE9	106.7	113.4	109.2	112.7	104.3	100.6	109.2	108.4
RNASEH1	490.4	460.2	428.1	507.9	384.6	329.4	438.7	439.3
RNASEH2A	139.4	150.1	141.5	147.3	140	109.7	140.7	132.9
RNASEL	250.4	240.6	307.9	278.4	222.2	210.5	209.9	199.5
RNASEN	115.9	118.1	125.7	119.5	107.6	104	122.1	116.8
RNASET2	2307.8	2939.9	2392.9	2119.3	2133.6	1199.2	993.9	1508.8
RND1	138.7	123.5	120.2	118.6	117	97.6	128.4	135.7
RND2	120.7	125.9	125.7	120.7	111.1	99.9	121.8	136.8
RND3	107.7	112.1	106.9	113.9	104.9	102.5	104.6	111.1
RNF10	168.7	183.2	189.4	194	171	154.6	191.3	189.1
RNF11	127	126.8	127.9	122	116.1	110.4	120.4	141.2
RNF111	118.4	114.2	125.4	136.4	114.1	107.4	122.7	108.9
RNF113A	216.8	255.9	248	198.5	223.5	180	209	245.3
RNF113B	112	127.8	118.7	127.7	118.6	99.5	110.4	123.1
RNF12	111.2	116.6	117	113	117.1	101.1	121.9	110.1
RNF12	124.7	115.5	115.6	124.9	105.4	96	120.1	115.5
RNF12	117.1	114.9	117.7	131.2	114.1	103.3	115.3	110.4
RNF121	157.2	146.3	148.7	173.6	139.4	142.2	146.5	157.1
RNF121	294.9	309.8	288.9	333.3	218.2	228.4	296	281.4
RNF122	126.3	138.1	135.7	135	131.2	112.1	130.1	132.1
RNF123	189.5	197.4	174.4	186	164.4	164.9	217.4	215.2
RNF125	175.4	224.1	219.5	188.6	218.6	143.4	170.9	185.6
RNF126	371.9	353	333.7	351.3	257.6	212.1	345.2	254
RNF126	168	164.9	188.7	198.7	138	138.4	161.7	168.1
RNF126P1	124.6	125.4	125.1	127.2	122	106.1	127.9	129.4
RNF126P1	116.7	125.3	114.2	121.1	107.7	103.4	115.4	116.5
RNF128	106	119.5	127	123.8	113.1	102.8	121.9	114.4
RNF128	128.1	122.9	131	130.9	114.6	108.8	131.7	134.3
RNF128	115.5	132.4	138.6	140.3	141	110.4	133.6	136.3

RNF13	347.1	275.2	279.4	409	246.4	345.4	449.5	284.5
RNF13	118.1	107.4	114.8	129.8	117.4	109.8	117.7	122.6
RNF130	293.2	243.7	358.1	260	161.3	249.9	227.7	184.6
RNF133	122.5	114.2	122.9	120.7	112.8	98.9	121.2	114.8
RNF135	204.7	160.5	315.3	187.9	139.5	329.6	173.1	161.8
RNF135	131.8	128.8	194.3	138.3	110.9	149.9	134.6	128.2
RNF135	117.2	126.8	142.8	122.2	100.5	122.2	114.2	123.6
RNF138	165.5	193.3	209.7	206	170.6	160.6	192.8	299.7
RNF139	119.1	123.1	124.8	122.6	134.2	118.7	118.5	139.1
RNF14	122.1	122.3	127.5	122.5	105.9	105.2	120	112.8
RNF14	243	218.2	324.2	304.3	248.1	235.7	358.6	389.7
RNF14	209.2	186.9	271	252.3	179.8	193.4	250.3	260.8
RNF141	127.6	140.4	151.2	131.6	130.3	113.6	138.1	115.5
RNF144	264.8	322.8	301.2	285	244	233.1	249.1	223.1
RNF146	200.1	222.9	216.7	220.7	210.4	197.7	218.1	206.9
RNF148	117.2	116.5	118.6	121	109.8	100.8	118.4	119.4
RNF149	142.4	153.9	157	180.7	146.7	130.6	174.4	213.6
RNF150	118.9	111	114.3	126.5	112.3	99.5	114.6	116.3
RNF151	110.3	124.4	109.8	113.9	104.7	94.4	116.2	109.1
RNF151	114.5	115.1	107.7	111.4	111.8	100.1	110.4	102.9
RNF152	117.4	114.6	111.3	119.2	110.9	98	115.6	121.5
RNF157	156.7	174.7	154.5	185.7	166.3	122.9	168.4	175.9
RNF165	133	128	126.5	136.6	111.2	107	127.1	131.5
RNF166	139.3	141.9	138	143.4	120.8	112.8	138.4	139.1
RNF167	167.9	224.5	154.7	191.9	176.6	164.3	177.1	256
RNF168	119.4	123.4	123.5	114.6	114.3	100.3	118.3	119.8
RNF17	114.4	105.8	116.6	110.6	98.6	100.7	120.4	115.7
RNF17	112.8	110	109.7	106.6	107.3	97.2	126.2	110.4
RNF170	152.6	140.4	187.1	158.8	148.1	130.7	173.2	161.5
RNF175	152.2	157.6	151.2	183.7	151.6	116.1	171.2	138.8
RNF180	109.6	120.4	112.7	117.4	109.7	102.7	126.9	109.4
RNF183	141.8	115.7	125.1	134.3	117.3	97.4	124.4	136.6
RNF185	144.1	150.1	148.2	154.9	129.8	118.5	165.6	192.4
RNF186	122.3	112.5	127.6	128.5	113.6	105.9	107.7	115.7
RNF19	164.6	154.3	167.8	168.8	149	108.9	153.6	140
RNF19	116.5	116.3	124.3	122.6	112.4	99.5	115.9	113.9
RNF19	406.5	409.9	474.1	421.6	366.9	396.4	332.9	377.2
RNF190	114	124.3	109.7	117.2	121.2	99.5	116.4	124.7
RNF2	119.5	112.7	115.6	125	114.2	103.8	130.9	110.2
RNF20	312.5	319.5	385.6	352.6	287	315.3	313.2	301.1
RNF24	184.9	192	190.9	255	191.2	222.8	318.1	204.2
RNF25	109.5	129.3	134.2	143.7	116.5	109.2	132.8	128.7
RNF26	145.4	153	159.3	131.7	117.3	132.5	155.8	145.4
RNF31	246.4	216.1	218.6	269.2	211.2	173.3	237	258
RNF32	123.9	105.2	113.2	117.3	105.8	96.8	119.8	114
RNF34	975.1	894.3	964.7	1036	826.4	884.3	951.9	1071.6
RNF34	119.2	128.5	126.1	129.9	117.7	112.7	121.1	129
RNF34	107	111.9	118.8	117.8	118	98.6	113	113.6
RNF36	153.6	144.6	133.4	161	132.6	120.7	156.9	159
RNF36	122.8	134.3	132.2	130.9	121	102.8	130.2	133.9
RNF38	120.6	127.6	169.7	132	128.5	112.1	152	135

RNF38	133.2	141.7	146.1	161.8	138.7	130.8	156.2	139.9
RNF38	668.8	713	545.2	700.8	588.8	503.5	727.8	757
RNF39	111.2	119.5	120.8	118.7	112.8	97.1	115.2	119.8
RNF39	122.8	109.4	116.2	126.7	107.5	99.8	122.5	118.4
RNF4	190.6	197.1	183.9	248.1	151.8	160	222.8	186.9
RNF40	203.6	262.2	200.1	244.5	176.1	188.8	293.3	205.5
RNF41	151.8	142.8	146.3	179	132.1	127.2	153.6	160.9
RNF41	116.4	116.4	114.9	126.8	113	105.2	128.3	120.5
RNF43	120.5	119.1	116.8	116.1	120	103.2	121.1	121.1
RNF44	471.1	502.4	378.3	476.4	373.7	296.9	442.2	494.4
RNF5	178.9	164.7	186.6	202.6	139.3	166.6	183.8	182.2
RNF5P1	456.5	501.3	447.4	466.4	407	330.5	410.5	353.9
RNF5P1	170.7	180.1	195.3	182.9	155.7	153.2	169.2	163.8
RNF6	134.3	134.1	131.9	133.8	117.5	110.4	127.5	126
RNF6	108.3	113.6	112.1	121.4	109.8	107	112.8	120
RNF6	117.4	112.9	118.5	121.4	110.1	106.5	129.4	118.8
RNF7	109.9	118.6	126.5	126.2	107.6	107.7	112.4	112.6
RNF8	119.7	114.3	122.8	134.1	110.7	104.1	118.1	113.5
RNF8	128.6	134.4	152.4	145.1	129.4	117.8	133.7	127.9
RNGTT	316.5	425.2	385.6	342.9	351.7	280.4	355.2	361.4
RNH1	399.2	348.9	431.5	378.3	263.4	322.2	422.5	305.5
RNH1	153.9	149.5	180.9	170.3	135.8	172.1	198.6	169.9
RNMT	297	406.1	326.2	362.1	304.4	298.8	406	362.1
RNMTL1	245.3	292.4	287.7	268.2	230.7	197.7	244.1	235.2
RNPC1	191	218.4	248.3	235.2	190.1	246.7	292.9	264.6
RNPC2	117.2	115.9	107.8	121.8	113.9	100.1	118.5	117.9
RNPC3	140.2	151.7	208.2	157.3	140.9	144.4	140	164.3
RNPEP	146.2	149.6	170.3	150.6	142	127.7	134.3	136.3
RNPEPL1	169.5	179.3	153.5	178.3	123	123.7	164.7	145.2
RNPS1	494.9	453.8	456.5	643.2	376.1	346.8	680.3	534
RNPS1	134.2	130.9	138	133.5	115.8	113.8	138.9	136.7
RNU2	118.5	121	111.3	126.9	111.6	107.5	114	113.3
RNU3IP2	120	118.4	138.3	130.4	119.5	110.1	132.9	120.7
RNUT1	297.1	340.5	263.8	275.3	284.9	239.8	349.8	317.1
RNUXA	269.4	205.7	239.3	215.2	199.6	162.8	202.9	233.9
ROBO1	120.4	116.3	112.8	116.8	104.5	96	117.3	107.7
ROBO1	111.7	121.1	109.8	117.2	107.1	92.7	113.3	110.5
ROBO1	132	122.9	116.8	120.2	112.3	91.1	123.4	125.4
ROBO2	110.8	108.9	117.5	111	104.1	103.2	116	111.3
ROBO3	129.6	121.6	127	127.4	114.2	107.2	131.7	124.9
ROBO4	113.9	114.7	116.5	120.5	110.8	96	124.8	116.3
ROCK1	200.5	198.5	153.7	230.1	184.3	136.4	266.9	276
ROCK2	6157.2	7347.3	6171	6996.4	6190	3706.8	6402.9	7041.6
ROD1	1156.4	961.9	1116.6	1043.3	810.3	799.1	1289.8	850.6
ROM1	135.9	119	153.4	132.2	121.1	117.4	117.6	131.1
ROPN1	117.8	114	120.6	119	116.1	107.1	134.7	127.1
ROPN1B	109.1	119.9	117	118.7	103.5	102.8	113.8	109.9
ROPN1B	126.9	113	128.1	115.6	116.9	100.1	125.1	117.6
ROPN1L	122.7	110.5	118.8	120.2	111.7	98	130.8	130.9
ROR1	107.4	114.8	126.4	126	117.3	103.4	121.5	106.3
ROR2	107.9	114	112.7	123.7	119.4	101.1	114.9	117.4

RORA	286.7	308.7	184.3	300.8	330	146.9	318.4	400.6
RORA	131.6	137.6	120.2	133.8	140.2	118.2	128.6	152.8
RORA	127.2	122.3	121.3	123.6	110	98.5	132.6	118.5
RORA	123	123.6	118.9	115.1	111.4	104.4	125.2	117.2
RORA	114.9	114.4	124.3	116.9	98.2	99.9	118.8	110.7
RORA	117.9	116.3	115.3	124.1	112.1	93.6	120	111.3
RORB	125.3	130.9	125.6	125.9	115.1	99.2	120.1	109.1
RORC	128.6	126.1	114.3	120.6	106.4	92.4	122.4	120.4
RORC	110.8	107.8	121.8	118.6	108	96.4	125.6	109.6
RORC	123.8	142.5	125.8	135.2	125.3	107.6	127.3	137.5
RORC	116.5	114	123.7	126.2	121.6	102.6	112.8	114.6
ROS1	113	118.7	116.5	123.2	108.5	105.5	120.1	112.8
RP1	119.1	124.5	113.4	115.8	108.5	94.2	117.1	113
RP1-112K5	287	244.3	241.6	302.9	242.6	256.3	253.4	315.9
RP11-19J3.	130.2	127.5	117.9	122.9	108.6	100.6	122.1	118.6
RP11-450P	127.7	121.5	138.8	123.9	115.7	120	112.2	111.5
RP11-529I1	114.1	112.7	114.8	115.1	108.9	95.9	110.4	115.4
RP11-564C	120	132.3	131.3	117	112.3	107.3	121.4	128.7
RP13-11B7	117.8	126.8	125.1	125	126.1	103.7	125.2	127.8
RP13-15M1	108.8	109.7	114.2	120.3	105.2	98.9	118.4	110.4
RP13-15M1	113.4	114	113.6	115.8	108.4	94.5	118	118.1
RP1-32F7.2	114.1	113.1	122.7	114.6	104.4	100.1	127.1	121
RP1L1	126	116.3	117.5	119.8	108.1	100.3	120.4	111.9
RP2	209.9	184.4	208.6	219.9	169.2	244.1	210.5	170.5
RP9	289.3	328.8	266.5	295.6	261.1	240	278.3	259.7
RPA1	686.4	377.3	693	750.7	309.7	584.1	574.9	398.5
RPA2	1191.1	1559	1298	1384.5	1443.6	1347.1	1466.1	1315.3
RPA3	533.1	574.7	653.7	727.5	554.8	573.4	621.9	616.7
RPA4	116.7	122.9	114.7	119.8	109.7	96.4	127.7	135.3
RPAIN	1214.8	1289.7	1386.8	1340.3	1307.6	1118.4	1045.8	1126.8
RPAP1	131	128.7	129.2	127.1	118.3	114	146.5	129
RPE	120.2	123.7	126.7	119.9	114.3	100.6	128.4	132.6
RPE	160.1	166.5	160.5	179.8	134.9	119.6	156.7	128.4
RPE	111.6	117.1	118.8	120.3	110.7	96.9	125.4	120.8
RPE65	116.1	115.5	123.6	119.3	108.5	105.8	111.7	111.7
RPESP	123.4	119.9	122.9	141.6	114.7	95.3	128.7	123.2
RPGR	110.2	114.7	118.1	126.2	105.6	99	112.2	113
RPGR	121.1	137.9	143.2	124.3	142.9	107.2	129.9	118.5
RPGR	117.4	116.7	122.1	127.3	120.8	105.3	119.8	118.6
RPGRIP1	111.7	126.3	114.6	115.8	104.8	104.1	116	116
RPH3A	116.8	120.9	123.4	118.8	113.3	103.7	120.3	114.2
RPH3AL	120.6	127.4	139.8	121.1	108.1	115.1	117.3	108.7
RPIA	263.5	270.5	246.3	233.7	229.1	156	204.1	244.6
RPIB9	109.4	114.8	121.5	125	110	103	121.4	121.1
RPIP8	101.1	110.8	108.6	107.9	105	96.4	115.2	104.8
RPL10	113.2	113.8	120.8	126.3	124.8	103.5	125.1	119.6
RPL10A	5888.1	7847.6	8409.7	6527.7	7105.1	7440.6	6800.4	7183.5
RPL10L	135.5	133	138.3	143.2	123.1	117.6	140.5	132.5
RPL11	9011.5	10494.3	9311.4	10195.6	9397.7	7169.5	9525.6	9886.2
RPL12	8535	13109.8	12450.3	10124.1	11383.4	11933.7	7948.3	13189.7
RPL13	133.9	132.8	128.5	127	113.7	95.2	125.1	125.2

RPL13	556.1	534.2	554.5	663.5	413.3	384.9	580	787
RPL13A	1705.5	1951.1	1274.1	1601.4	1505.3	1039.8	1435.4	2008.7
RPL14	452.7	510.8	369.2	414.3	268.1	388.3	365.6	451.9
RPL14	361.1	925.8	315.4	319.5	547.3	252.6	339.7	901.3
RPL17	9203.9	10124.9	12234.7	10176.4	8625	7827.4	11408.3	10406
RPL17	1477.7	1626.2	2440.4	1869	1623.5	1601.6	1474.5	1723.6
RPL18	9265.2	11768.8	11476	10886.5	10978.8	10798.2	10075	14415
RPL18A	13006.2	15182.9	17043.9	17629.8	12787.9	14203.4	15301	18065.8
RPL19	10735.4	13136.5	12945.5	13731.2	12025.9	11431.1	14098.2	13577
RPL21	3623.8	4537.9	3984.8	3173.4	3891.9	3005.2	3538.5	5123.4
RPL22	1866.7	2334.5	2167.7	2084.8	2187.4	1891.4	1939.5	2224.2
RPL23	1177.2	1138	1467.2	1466.9	918.4	1163.5	999.7	1049.6
RPL23A	258.8	265.6	239.8	219.3	201	274.7	212.6	271.9
RPL23AP13	136.6	140.7	149.6	141.3	118.1	126.8	144.5	138.6
RPL23AP7	185	201.7	225.3	195.1	198.4	147.8	209.2	207.5
RPL24	8847.1	10028	13078.5	10340.4	10704.1	8950.7	9643.3	10620.7
RPL26	2534.3	3174.6	3256.8	3080.3	2768.9	2467.4	2856	3760.6
RPL26L1	258.4	257.2	333.5	230.5	230.7	262.7	250	244.8
RPL27	12612	12826.5	15214	12218.8	12121.4	10891.8	12985.3	14460.3
RPL27A	5679.9	5962.3	7997.9	5218.4	5365.3	6203.7	5870.2	5065
RPL28	185.5	202.4	174.6	240.3	170.3	172.9	254	239.8
RPL29	127.6	141.3	129.1	161.6	150	121.4	164.8	173.6
RPL29P2	132.8	136.6	130	140.3	123.8	107	131.3	123.3
RPL3	4393.2	4739.9	4613.6	5268.2	4348.1	4770.8	4921.1	6359.9
RPL3	6682.9	6234.7	5574.2	7767	5175.4	7359	6955.1	6935.9
RPL30	10540.8	12413.6	12590.2	12070.3	11653.2	9566.8	11482.2	13522.7
RPL31	1688.9	2104.9	1589.4	2088.2	1942.2	2255.9	1692.9	2502.9
RPL32	180.7	215.7	223.1	208	192.6	154.5	205.5	234.5
RPL32P3	130.6	129	125.7	143.1	122.8	106.3	133.2	129.6
RPL34	511.2	622.3	446.7	536.6	501.9	320.8	533.5	583.6
RPL34	187.9	265.4	225.9	251.6	184.4	161.5	212.5	203.2
RPL35	3582.8	4858.1	3761	4110.3	4053.7	3319.4	3848.6	6198.5
RPL35A	16875.6	16667.2	20213.2	17451.7	18931.1	16397.6	17509.5	18399.2
RPL36	1867.3	2282.8	3735.4	2715.2	2521.2	2990.1	2181.3	2755.6
RPL36	141.4	150.1	135.8	149.6	137.9	112.2	151.9	153.1
RPL36A	143.9	125.5	129.1	134.3	114.1	112.7	117.6	128.5
RPL36AL	2290.8	2886.2	2938.4	2765.5	2408.7	2700.3	3813.2	2513.9
RPL37	133.5	126.8	140.4	151.5	128.4	110.3	155	120.9
RPL37A	2619.9	3491.5	3026.4	2817.3	2893.9	2401.6	3622.4	3913.3
RPL38	16422.9	20716.9	25618.9	21970.4	20737.9	21390.3	25028.8	22274.4
RPL38	13614.9	17085.8	14799.9	13043	9712.4	12503.6	13817.5	16239.2
RPL39	15512	17762	18064.4	17288.2	15096.8	15211.2	15974.5	17173.5
RPL39L	206.4	174.5	198.5	184.1	157.8	141.4	175	172.5
RPL3L	108	107.8	109.8	114.6	108	95.6	111	117.1
RPL4	3674.5	3679.1	3804.2	4195.9	2764.3	2623.2	3831.3	4359.1
RPL41	127.5	136.8	137.3	147.5	120.1	114.8	155.8	153.3
RPL41	20227.7	21684.5	23926.4	19178.4	19452.2	19407.5	21973.4	21240.1
RPL5	4199	3984.7	4115.1	4945.6	3629	3371.4	4366.6	4933
RPL6	205.9	189.9	174.3	193.2	188.4	129.7	180.5	190.1
RPL7	496.3	379.4	438.4	492.9	438.6	432.8	448.1	540.1
RPL7A	187	169.2	185.7	236.6	202.2	174.9	198	206.9

RPL7L1	3052.6	2879.4	3251	3493.2	3225.9	1657.6	3417.5	3546.6
RPL8	156.9	163.9	173.4	156.6	162.2	137.2	151.5	143.6
RPL8	8904.8	10838.2	11319.5	9871.6	9110.3	9167.4	9390.7	11765.6
RPL9	270.1	317.2	380.1	351	310.5	278.5	369.3	385.4
RPL9	1710	1816.2	1781.6	1316	1258.5	1713.3	2085	1879.1
RPLP0	112.6	111.1	111.7	114.4	101.8	95.4	120.8	118.4
RPLP0	822.7	831.3	632.9	861	742.6	544.9	745.2	880.3
RPLP0P2	117.5	117.7	126.9	120.8	111.5	103.2	129.6	122.8
RPLP1	9209.9	10531.6	8432.2	7899.4	10277.2	6493.2	8585.6	8845.2
RPLP1	4507.4	3390.8	3683	5000.6	3950.9	2933.3	4372.4	5349.6
RPLP2	10110.6	12236.1	15300.8	12738.4	13300.5	11290.5	14083.7	15081.7
RPN1	1305.8	1173.8	1278.4	1482.5	993.5	1041.3	1384.4	1240
RPN2	208	206.2	280	242.6	168.1	210.3	229.1	260.5
RPP14	116.9	120.5	110.1	131.8	114.9	108	112.1	113
RPP21	1165.5	1365.9	1083.2	1376.2	1098.9	712.6	1214.9	1342.1
RPP25	121.7	117.3	116.2	118.9	112.9	103.5	119.3	121.7
RPP30	105.6	116.7	109	123.2	108.2	98.1	112.8	110.9
RPP38	168.8	223	183.7	171.2	189.5	138.8	160.4	179.9
RPP38	112.7	123.6	126.5	126	107.6	107.1	119.3	119.4
RPP40	293	357.5	271.2	300	321.5	175.7	275.9	289.3
RPRC1	2271	2539	1907.1	2988.2	2139	1933.3	2840.1	2577.6
RPRM	113.7	127	129	125.6	110	103.2	119.6	108.2
RPRML	115.3	115.2	115.7	113.7	108.1	103.3	111.1	113
RPS10	14359.7	13598.8	15826.3	13166.8	14118	13598.9	15200.1	16129
RPS11	11697	13080.8	12607.3	12550.6	9917.8	9069.2	13484.3	11457.5
RPS12	19243.8	20139.1	20836.8	20620.6	19065.9	16888.1	19816.2	22602.4
RPS13	3517.6	5443.1	6309.7	4813.6	4970.5	4453.8	4594	5712.6
RPS14	9075.8	9571.2	10377.8	9664.8	10227.6	7681.5	7469.8	10683.9
RPS14	4429.2	5519.7	5259.9	4567	4991.6	4170.7	4321.7	6054.8
RPS15	3107.8	4402.1	4383.2	5309.9	3722.4	4418	3330.9	4581.6
RPS15A	2098.9	1941.1	2538.1	2028	1823.6	1589.3	1635.9	2316.9
RPS15A	14582.2	16828.5	17348.4	17284	13845.7	11195.3	15753.7	16389.1
RPS15A	1391	1545.2	1814.5	1927.8	1438.1	1529.1	1346.1	1911.5
RPS16	12874.6	14783.2	14462.3	11330	11272.7	9953.5	11527	11687.2
RPS17	11412.5	11649.4	12363.3	11563.7	10127.3	11246.5	13358.6	11809.4
RPS19	4421.6	5262	3969.9	3903.4	3109.4	4171.4	5843.2	5224
RPS19BP1	300.6	328.5	281.5	309.3	300.7	265.7	284.1	299.6
RPS2	6115.1	7598.1	7478.3	5940.2	6383.5	5192.3	5533.4	7616.1
RPS20	6100.4	6373.6	8686.3	8794.2	5901.7	5748.8	6014.6	6558.3
RPS21	345.7	391	489.6	404.8	377	333.3	378.7	426.1
RPS23	463.3	2158	383.7	518.2	1914.7	368	523.4	2511.2
RPS24	182.6	182.1	218.7	194.4	191	179.9	192.5	248.2
RPS24	1332.5	1481.2	1944.8	1461.3	1535.3	1752.3	2176.9	2117.3
RPS25	25748.8	26790.5	32115.2	30199.1	27069.5	22958.4	27197.6	30203.8
RPS26	654.9	2519	723.7	640.3	2248.2	841.3	635	1850.8
RPS26L	396.7	1346.4	358.2	427.3	830.9	440.8	390.1	1474.5
RPS26L1	112.3	123.2	119.8	121.2	108.7	108	114.4	112.9
RPS27	30628.2	32712.2	31420	36033.3	29543.9	24684.5	34847.4	33196.8
RPS27A	21738.7	18185.5	23149	20061.2	20600	16037.6	22282.3	25626.2
RPS27L	113	115.3	112.2	115.6	115.7	101.2	123.6	112.9
RPS28	2303.2	1582.5	2901.1	1803.4	2369.4	2156.6	2156.2	2680.7

RPS29	6306.9	7747.5	7246.7	6897.2	6821.9	6721.3	7123.5	7607
RPS29	894.3	786.9	842.8	914.7	574.3	579	900.3	786.6
RPS29	22711.6	28200.5	27390.2	29628.2	25830.3	18227.7	26900.7	29749
RPS29	14808.1	14367.4	19110.4	16385.4	11978.2	13733.2	17403.5	18476.2
RPS3	11901.8	17652.9	20120.8	14955.1	15216.2	15783.4	15515.8	18365.8
RPS3A	2619.7	5030.2	5626.3	4692.2	3273.7	4376.3	4237.9	5725.5
RPS4X	4037.5	5830.6	5366.9	4316	4197.4	3230.9	4440.7	5215.7
RPS4Y1	1442.8	1840.2	1710.8	1710.6	1237.9	1702.3	1606.8	1745
RPS4Y2	227.3	238.8	229.8	245	194.4	175.9	217.2	220.3
RPS5	6009.5	5011.3	5480.9	5813.8	4577.6	4653.3	7111.1	7033.9
RPS6	4949.2	3705.9	4469.2	5139.3	3260.4	2778.6	4034.1	5664.1
RPS6KA1	113.7	109	116.4	121.1	106.4	91.9	110.9	114
RPS6KA1	388.2	486.6	277.5	661	314.2	254.2	554.9	309.4
RPS6KA2	116.8	109.1	123.4	122.8	104.9	102.3	113.5	111.3
RPS6KA2	214.7	195.2	209.1	252.2	167.2	187.2	216.9	169.9
RPS6KA2	114.4	127	111.4	121.2	111.4	104	116.7	111.1
RPS6KA3	270.6	280.6	214.6	290.2	255.2	170.5	255.1	275.5
RPS6KA3	119	124.8	120.8	124.1	117.2	113.3	127	135.2
RPS6KA3	144.5	135.2	146.9	144.7	131.4	113.3	141.2	151.9
RPS6KA4	159.8	151.2	169.7	150.7	134.2	137.1	147	142
RPS6KA5	375.9	405.5	278.3	407.1	358.4	240.9	354.2	343.4
RPS6KA5	118.3	112.1	116.3	117.8	106.9	92.1	112.4	112.2
RPS6KA6	115.1	129.2	106.1	114.1	105.1	98.9	122.2	115.5
RPS6KB1	616.4	649.5	547	805.2	557.5	404.1	644.6	681.1
RPS6KB2	875.7	999.7	977	954.9	769.8	524.1	880.1	765.4
RPS6KC1	136.5	135.6	159.9	151.5	144.7	144.9	139.8	144.2
RPS6KL1	119.9	130.2	130.6	131.8	115.2	102.1	128.1	125.2
RPS7	125.7	118.2	153.5	142.4	141.6	129.1	208.2	301.6
RPS9	4238	4207.1	4513.8	4729	4447.4	3592.5	4282	5001.7
RPS9	5259.7	7144.9	6010.4	6186.8	5473.6	4889.7	5964.2	6362.1
RPSA	13510.9	16259.4	16014	17221.1	14352.7	13576.5	15172.8	17956.8
RPSA	134.8	129	132.8	137.9	120.3	117.9	134.2	153.5
RPUSD1	229	266.4	214.9	272.7	191.7	170.7	263.8	229.6
RPUSD2	462.1	470.7	424.4	484.3	326.1	285.9	333.9	294.2
RPUSD3	312.3	342	398.4	354.9	236.5	273.3	283	255
RPUSD4	775	938	752.3	815.6	604.3	491.5	826.3	790.1
RQCD1	205.6	210.2	257.8	210.5	188.8	196.3	246.7	203.9
RRAD	110.1	112.9	114.9	114.1	107.1	99.8	114.5	110.4
RRAGA	1372	1352.7	1162.9	1325.2	800.2	967.9	1283.5	769.8
RRAGB	118.5	123.2	156.4	159.2	117.4	111.9	135.4	139.9
RRAGB	112.7	108.6	112.3	116.5	104.9	92.7	107.6	104.1
RRAGB	221.9	251.8	291.9	250.9	230.2	188.3	225.7	244
RRAGC	303.8	305.8	249.1	353.1	321.7	226.6	470.8	543.3
RRAGD	187.6	162	185.7	215.9	175.8	173.8	298.2	268.5
RRAS	201	170.1	367	170	134	250.8	180.1	155.4
RRAS2	218.2	222.4	202.3	245.8	220.5	186.7	267.5	238.4
RRBP1	137.5	129.5	130.7	132.2	121.1	106.8	133.6	137.8
RRBP1	341	298.8	342.9	349.6	293.7	299.4	376.1	438.8
RRBP1	138.3	138.3	153.5	139.6	133.9	100.9	129.2	150.7
RREB1	121.6	113.1	111.3	120.8	117.1	108	117.2	127.8
RREB1	120	119.7	131.2	131.3	108	108.8	127.4	120.8

RREB1	111.3	110.4	111	116.7	106.3	99.1	119.9	119.6
RREB1	119.8	120	119.8	117.7	108.1	100.4	126.1	124.6
RRH	108.9	118.7	110.5	114.9	98.9	93.3	118	107.7
RRM1	704.1	846.6	922.7	808.6	622.1	743.4	748.7	720.3
RRM2	113.9	112.8	120.2	128.7	109.5	105.9	130.4	112.9
RRM2B	134	147.2	154.3	152.4	130.6	148.1	139.9	156.1
RRN3	140.7	161.6	164.9	185.2	146.7	140.7	156.6	157.9
RRP22	115	122.4	127.5	123.3	109.5	97.4	131.7	120.5
RRP22	119.3	120.3	130.4	123.9	113.9	102.7	113.7	117
RRS1	252.7	317.9	187.2	223.8	199.3	164.5	186.9	192.2
RS1	111.9	111.2	109.8	124.7	107.3	103.5	113.7	119.3
RSAD1	138.1	146.5	151.2	157.1	126	123.1	139.7	150.7
RSAD2	124.3	120.5	122.8	133.6	111.5	101	118.7	124.9
RSAFD1	196.4	185.2	154.3	176.2	169.8	136	175.8	178.8
RSBN1	801.2	929	671.7	902.9	919	558.4	672.8	734.1
RSBN1L	205.9	271.7	208.5	197.1	201.1	168.2	212.5	237.5
RSC1A1	140.2	144.5	139.7	137	123.7	106.5	140.4	128.1
RSHL1	107.6	116.8	110.3	109.7	108.8	101.4	115.6	114.9
RSHL2	125.2	135.5	152.1	123.1	122.9	111.1	126.2	121.3
RSHL3	106.5	120	114.4	114.5	116.7	93.1	125.8	121.3
RSL1D1	560	677.8	653.8	641.6	592.2	497.6	651.7	692.5
RSN	104.8	117.6	119.5	121	129.3	104.4	135.3	127.4
RSNL2	210	220.6	238.7	217.2	248.5	177.5	214.4	218.2
RSPO1	111.5	127.6	110.9	116.6	110.8	96.6	114.5	110.2
RSPO2	134.8	135.4	160.9	137.1	144.4	115.8	157	150.7
RSPO3	124.7	124.6	118.7	112.8	113.1	101	130.5	129.5
RSPO4	121.9	120.2	123.9	120.7	107	97.4	114.6	121.8
RSPO4	120.6	111.4	122.5	114.8	111.7	95.2	111.1	116
RSPRY1	634.8	606	501.9	683.1	601.4	335.4	846.9	873.1
RSRC1	181.2	158.1	168	182.3	167.3	149.8	156.4	148.8
RSRC2	236.7	308.7	293	318.1	343.6	294.2	347.5	507.3
RSU1	438.8	461.4	408.9	361.5	349.1	261.4	281	275.6
RSU1	126.2	124	131.3	132.7	123.2	106.5	130.5	126.3
RTBDN	113.2	105.4	112.3	123.5	108.9	102.9	116.8	114.5
RTBDN	120.7	115.5	115.6	120.6	106	99.5	113.6	117.4
RTBDN	108.5	106.4	116	119.2	104.5	93	111.1	114.3
RTCD1	137.8	151.8	173.4	181.7	156.1	141.7	159.9	173.2
RTDR1	111	117.2	107.8	106.8	108.2	98.3	125.4	102.9
RTEL1	116.9	119.2	131.3	113.7	121	101.8	130.9	130.5
RTEL1	115.8	111.1	134.5	133.4	111.5	102.6	131.8	118.4
RTF1	125.7	124.9	134.4	129.8	119.3	117.1	142.8	176.8
RTKN	118.1	125.5	123.2	136.1	118.2	103.2	131.9	120.3
RTKN	124.6	135.4	128.7	125.1	131.1	106.9	132	119.8
RTKN	120.2	128.6	126.3	119	115.1	113.3	115.7	121.5
RTKN	120.5	115.4	109.8	113.9	103.8	101.8	112.6	118.4
RTN1	108	114.5	112.6	118.1	102.6	97.8	115	112.3
RTN1	121.3	118.8	121.1	123.4	106.1	103.1	108.9	117
RTN1	211.9	144.4	452.9	174.3	110.8	173.2	129.7	121.1
RTN2	129.4	113.5	132	125.4	123	106.7	119.9	124.8
RTN2	261	289.2	320.5	299.2	252	232.9	269.4	242.4
RTN2	120.3	114.6	134.6	153.1	112.9	96.7	115.9	118.1

RTN3	167.5	166.7	162.9	178.2	172.8	139.7	183.2	172
RTN3	120	115.2	122.4	135.3	104	95.4	122.5	119.5
RTN3	108	121.5	110	111	109.3	96.2	120.6	117.8
RTN3	402.3	373.8	327.8	398.1	338	285.4	585.5	510.4
RTN4	377.8	344.8	523	443.4	325.4	554.4	412.8	502.3
RTN4	110.2	108.7	107.4	109.3	106.8	99.3	108.6	109
RTN4	1884.7	1307.8	2433.7	2028.8	1340.1	2358.9	2378.7	2355.4
RTN4	119.6	112.1	128.6	126	109.8	101.3	123.8	114.1
RTN4IP1	131.8	125.4	154.5	142.6	120.6	114.9	132.1	128.8
RTN4R	124	129.1	125.2	122.7	119.2	100	112.3	117.1
RTN4RL1	127.7	119.7	118.2	121.6	114.4	93.9	132	124.9
RTN4RL2	114.2	118.1	114.4	121.3	108.4	96.6	113.2	109.8
RTP1	119.2	119.2	121.8	120.7	112.9	103	116.6	112
RTP2	119.5	109.7	107.1	122.7	106.3	96.7	129.1	109.3
RTP3	113	111.6	113.9	129	106.8	90.1	117.4	107.3
RTP4	129.3	141.5	149.2	127.5	121.5	125.1	138.9	119.6
RUFY1	126	116.2	114.2	112.9	107.4	93.2	111.4	117.5
RUFY1	157.1	172.3	172.2	166.4	126.6	136.6	162.8	149.5
RUFY2	122.2	126.4	131.2	132	115.5	110.2	129.6	123.7
RUFY2	131.7	142.6	141.1	137.6	117.2	109.5	137.2	130.3
RUFY2	116.9	109.5	120	113.7	107.1	98.9	112.3	117.2
RUFY3	139.4	126.1	146.9	137.7	134.4	129.2	145.7	151.9
RUFY3	126.3	137.8	144.2	131.8	132.9	104.6	142.8	156
RUFY3	102.8	119.7	110.1	120.2	104.9	102.4	113.1	117.1
RUNDC1	380.1	351.4	341.4	370.1	266.7	277.2	252.3	193.4
RUNDC2A	122.8	120.9	136.2	140.7	117.1	108.9	132.4	123
RUNX1	117.7	126	126.4	113.6	112.4	96.4	113.8	114.8
RUNX1	134.1	114	114.5	131.2	127.1	106	124.5	126.8
RUNX1	246.9	236.8	229.7	303.7	224.6	197.5	219.8	177.7
RUNX1T1	116.7	121.1	123.2	114.2	110.7	103.1	121.3	107.4
RUNX1T1	118.8	117.5	119.5	129.9	105.2	103.4	125.1	113
RUNX1T1	114	112.8	107.4	124.3	104.5	97.4	122.1	113.7
RUNX1T1	113.9	131.1	134.9	120.8	118.5	101.5	127.6	126.7
RUNX1T1	114.7	109.7	114	116.9	107.5	99.2	123.4	115
RUNX2	120.6	132.3	133.8	143.6	113.4	106.7	128.9	139.2
RUNX2	138	158.3	126.3	127.6	127.1	105.5	121.3	123.6
RUNX2	116.4	119.1	107.6	118.3	108.3	96.9	113.5	112.5
RUNX3	2118.9	2638.1	2130.8	2300.8	1906.7	1632.2	2659.1	2225
RUNX3	117.3	116.7	125.4	126.6	114.1	102.2	116	128.1
RUSC1	480	501	515.2	514.2	384.7	417.3	548.6	538.5
RUSC2	130.2	109	124.2	121.1	109.4	108.3	132.2	127.4
RUTBC1	1832	2021.8	2453.6	1903.5	1726.1	1694.3	1892.1	2144.4
RUTBC2	112.5	114.3	117.6	109.2	105.7	98	117.9	108.8
RUTBC3	162.2	165.7	148.2	155.3	123.7	135.2	172.4	149
RUVBL1	125.5	165.9	143.9	148.3	120.1	123.1	136.9	129.5
RUVBL2	172.3	184.4	192	178.8	148.4	143.8	162.8	170.9
RWDD1	116.2	118.8	120.8	132.6	115.1	101.8	122.3	140.8
RWDD1	1209	1303.8	1515.5	1248.5	1194.3	1025.3	1298	1256.8
RWDD2	182.2	231.6	269.7	231	163.2	198.6	177.1	180.5
RWDD4A	303.9	259	334.2	324.6	242.5	260.8	298.3	313.3
RXRA	474.5	409.8	1100.3	702.5	411.8	1245.5	771.8	380

RXRB	230.4	222.5	219.3	229	227.8	173.9	237.3	271.6
RXRG	122.2	117.7	115	112.2	103.7	96.7	122.4	116.2
RXRG	111.2	106	111.6	112.3	103.8	100.1	110.3	108.5
RXRG	117.7	115.8	116.9	115.8	107.7	102.7	122.7	116.5
RY1	435.8	422.2	427.7	424.4	330.9	343.4	358.1	351.9
RYBP	438.5	609.4	432.6	534.5	613.3	232.2	730	1148.5
RYK	157.1	166.6	148.3	162.4	155.5	124.5	157.2	143.9
RYK	218	224.6	205.2	212	214.9	173.2	207.2	165.5
RYK	409.1	486.9	411.6	517.9	486.2	322.5	393.8	328.6
RYR1	111.5	113.5	121.8	116.7	114.3	90.5	117.9	115.8
RYR1	121.5	132.2	126.4	124.7	108.7	105.1	121.3	126.9
RYR2	114.5	115.8	119.9	120.9	114.8	98.8	121.7	106.4
RYR3	114.9	114.8	117.1	126.3	100.6	95.1	137.7	118
S100A1	119.4	109.7	120.4	118.9	112.4	106.1	136.8	122.2
S100A10	2424.8	2735.8	4716.8	3011.1	2054	3769.5	2733.6	2353.4
S100A11	381.3	314.3	597	432.1	269.2	633.8	454.9	332.4
S100A12	164.6	135.8	278.2	157.9	114.8	235	152.1	124.2
S100A13	108.3	113.8	114.3	125	105.1	97.3	116.9	114.6
S100A13	110.3	113.9	117.5	117.3	114.1	95.6	110.3	110.4
S100A13	118.8	125.7	126.4	126.9	111.5	106	112.5	118.9
S100A13	135.3	123.1	141.4	139.7	131.9	119.8	148.8	134.9
S100A13	119.5	114.8	115	116.6	113.1	100.2	127.5	127
S100A13	125	117.7	119.1	124.1	116.1	105.6	121	136.8
S100A14	116.9	112.2	124.6	124.7	108.7	106.1	123.7	119.1
S100A16	126.1	113.3	126.7	127.5	102.8	100.6	117.5	117.5
S100A2	130.1	133	135.4	137.4	111.3	115.1	132.9	134.5
S100A3	109.1	117.9	114.7	127.8	109.5	107.8	117.3	114.9
S100A4	6111.4	4610.3	9610.7	5224.5	2831.7	4677.9	3045	2015.2
S100A4	6872.2	5369	12823.5	6471.6	3768	6373.1	3477.6	1688
S100A5	120.4	124.3	138.2	129.4	120.8	104.1	119.7	121.5
S100A6	1490.1	1062.9	1973.4	1220.2	677.8	1211.5	943.1	546.3
S100A7	119.6	119.8	114.4	111.8	107.7	92.9	112.9	105.7
S100A7L1	117.2	116.4	112.3	108.4	106.1	98.4	116.9	114.7
S100A7L2	121	101.7	115.3	113.5	112.8	106.1	115.2	125.4
S100A8	667	472.9	2702.1	790.9	363.6	1740.9	465.1	451
S100A9	482.2	317.2	2817.4	749.9	446	1656.7	595.7	379.6
S100B	109.6	115.6	118.1	116.2	109.5	96.6	110.9	108.7
S100G	112.2	110.6	120	125.9	117.7	102.2	110.9	118.2
S100P	116.1	129.2	131.5	123.9	131.7	117.7	125.3	146
S100PBP	132.9	123.6	137.2	139.7	114.3	109.1	128.1	120.6
S100PBP	159.6	136.9	183.2	160.1	135.6	107.2	147.7	141.9
S100Z	109.3	110.7	110.7	113.8	108.9	88.5	113.4	114.5
SAA1	119.2	116.5	124.9	119.9	119.4	106.9	119.2	129.3
SAA1	116.9	120.8	131.8	121.5	115.6	105	119.1	117.8
SAA1	120.6	112.4	125.9	126.6	117.7	98	127.4	131.2
SAA2	111.9	115.7	121.6	129.1	108.5	105.6	122	109.7
SAA4	113.5	111.5	122.9	111.9	102	94.9	105.1	111.1
SAAL1	191.7	213.3	192.5	198.7	229.6	159.2	192.6	179.9
SAC	117.8	128.8	122.2	126.6	111.6	104.4	109.8	108.2
SACM1L	527	380.6	525.2	678.3	391.8	387.4	548.1	593.9
SACS	167.5	143.2	163.1	162.7	145.1	144	163.4	174.1

SAE1	1444	1414.8	1358.2	1635.6	1345.4	1204.9	2106.1	2087.8
SAFB	536.9	578.8	403.7	587.6	482.9	285.9	640.2	715.5
SAFB2	404.3	459.6	372.1	414.2	346.8	390.9	487.5	422.4
SAG	111.3	115.6	116.4	108.4	117	104.6	119.4	113.1
SAGE1	108.5	112.1	106.9	111.7	102.2	96.6	106.6	110.4
SALF	121.2	112.7	110	121.2	101.4	100.6	117.8	116.3
SALL1	114	116.1	118.4	127.8	112.9	100.8	113.5	109.4
SALL2	169.7	198.7	150.1	168.6	153.9	144.8	156.4	148.1
SALL3	155.2	173.7	165.4	162.6	155.3	146.8	158.2	160.1
SALL4	119.1	112.3	112.4	125	111.2	103.1	123.3	111.4
SAMD1	129.3	133.8	131.4	139.5	116.7	111.3	150.8	145.9
SAMD10	124.7	121.3	122.6	141.2	117.9	114.9	116.7	126
SAMD11	129.1	134.1	127.2	131.5	121.4	105.9	133	121.7
SAMD12	112.6	110.2	124.6	115.7	109.7	105.7	124.2	127.9
SAMD13	116.6	117.8	114.9	117.5	106.2	93.9	116.9	111.9
SAMD14	127.8	130.1	141.1	129.1	104.4	105.6	121.4	123.6
SAMD3	756.7	821.1	617.8	773	710.9	402.2	622.7	584.4
SAMD3	170.3	174	162.5	188.5	173.4	138.1	171	165.7
SAMD3	146.5	176.8	166.5	165.3	182.9	128.6	166.4	157.9
SAMD4A	110.5	105.1	105.4	113.9	102.2	96.2	115.2	100.7
SAMD4B	176.5	158.4	154.5	178.3	148.9	154.2	240.5	233.3
SAMD7	124.3	116.6	122	133.9	110.6	96.6	132.6	120.1
SAMD8	117.5	114.2	118.7	107.4	107.2	97.3	116.2	111.9
SAMD9	576	582.8	569.6	579	404.6	398.7	441.1	472.6
SAMD9L	213.1	170.6	287.8	234.2	146.7	246.3	192.9	151.2
SAMHD1	113.4	118.6	126.2	140.6	115	110.4	131.8	132.6
SAMM50	604.1	704.6	640.4	609.1	516.1	459.6	486.6	472.9
SAMSN1	121.9	126.7	145.8	143.3	113.2	124.1	124.9	136.4
SAP130	231	267.8	257.9	275.8	238.8	233.1	313.2	351.5
SAP18	207.5	249.3	243.1	255.3	205.2	221.3	302.3	302.1
SAP30	193.4	170.4	260	197.4	156.1	144.3	181.8	210.7
SAP30BP	145.4	169.5	153.8	160.4	133.1	122.9	178.9	156.8
SAPS1	226.5	260.8	216.6	287	217.7	166.6	274.2	271.1
SAPS2	119	114.6	127.1	113.6	126.5	100.5	104.3	114.6
SAPS3	453.1	511.9	369.9	470.3	306.1	301.8	735.7	687.9
SAR1A	177.1	164.3	168.9	203.3	165.2	161.6	206.6	214
SAR1B	122.2	116	110.9	122.7	114.1	105.2	127	138.4
SAR1B	428.4	363.5	442.7	517.6	350.9	398.9	460.1	430.8
SAR1B	173.9	154	187.4	173	151.3	174.7	170.9	187.9
SARDH	118.9	115.8	135	115.6	112.6	109.8	112.9	116.7
SARM1	182.8	176.6	146.3	182.9	148.2	123.6	179.4	146.7
SARS	145.4	202.4	160.4	158.9	128.5	141.5	152.8	154.1
SARS2	192.7	168.3	201.6	190.2	158.8	164.6	172	144.5
SART1	130.4	126.3	124.8	141.3	125.2	101.7	132.3	126.2
SART2	111.8	110.9	114.3	115.6	100.5	94	105.7	109.1
SART2	111.8	116.6	124.2	114.3	110.5	104.5	120.4	112.5
SART2	525.9	390.6	1150.6	571.1	232.3	711.9	406.4	268.9
SART3	147.8	156.3	189.6	156.4	137.7	138.1	151.9	154
SAS10	113.4	112	114.4	112	105.2	100.8	114.8	111
SASH1	142.6	119.7	158.7	137.7	119.9	110.8	139.8	131
SASS6	138	151.4	133.6	146	128.8	115	141.6	135.4

SAS-ZFAT	115.4	117.7	114.5	126.8	114	96.6	107.5	120
SAT	1227.2	888.8	1719.2	1587	866.7	2669.6	1782.9	942.7
SAT2	246.2	226.2	271.3	305.6	190.4	256.4	302.4	300.1
SATB1	144.3	138.9	125.7	150.8	134.4	104.4	140.1	153.8
SATB2	114	119.7	118.9	124.1	108.1	109	118.2	117.1
SATL1	112.3	128.2	126.3	132.6	113.1	100.5	116.6	115.8
SAV1	142.5	143.8	143.6	135.8	123.7	129.9	147.4	134.3
SBDS	330.6	348.1	380.4	344.8	307.1	244.4	444.9	448.6
SBDSP	154.2	134.2	152.1	139.3	140.9	120.6	166.6	173.3
SBEM	111	118.6	119.8	116.5	103.8	105.2	119.6	115
SBF1	745.7	784.9	693.5	722	495.4	561.9	648.3	649
SBF2	116.8	116.1	127.4	116.8	109.8	102.5	117.5	118.5
SBK1	110.3	111.2	121.8	121.5	99.2	95.8	125	102
SBK1	769.2	850.5	513.2	609.9	703.9	356.3	451	492.4
SBNO1	111.4	111.2	115.5	127.6	118.6	95.5	120.4	122.4
SBP1	115.4	132.1	135.7	133.1	123	110.1	125.9	131.7
SBSN	107.5	110.4	111.4	113.3	107.9	95.6	112.4	115.8
SC4MOL	136.1	154.7	132.5	146.2	137.7	117.2	134.2	134.2
SC4MOL	244.5	264	245.2	231.8	206.5	141.9	229.5	201.3
SC4MOL	110.8	122.9	121.6	123.9	114	99.2	118.6	115.6
SC5DL	286.6	291.9	239.7	310.8	222.7	184.6	307.7	288.1
SC65	124.6	141.2	138.6	144.9	120.6	108.9	134.6	119.1
SCAMP1	799	706.4	732.3	716.1	521.9	574.8	763.1	536.8
SCAMP1	134.2	134.2	148.6	140	124.8	108	129.7	131
SCAMP2	189.9	165	182.4	183.2	148.8	125.7	179.5	182.6
SCAMP3	134.2	129.3	141	128.3	129.2	110.2	148.3	127.7
SCAMP3	670.9	632.7	628.4	675.6	496.1	478.3	623.9	614.6
SCAMP4	121.7	127.5	134.8	125.1	116.9	110.9	126.8	107.2
SCAMP5	118.8	117.1	122.5	126.5	115.9	107.3	119.2	114.5
SCAND1	224.9	277.8	267.5	278	226.7	217.8	250.4	254
SCAND1	146.9	136.8	146.4	159.6	128.4	129.3	149.9	158.9
SCAND1	424.8	464.9	452.6	422.9	400.9	381.2	445	404.8
SCAP	331.1	437.9	352.9	386	357.1	239.9	352.2	366.1
SCAP1	402	437.7	459.4	533.4	441.3	399.2	381.4	308.2
SCARA3	124.5	117.2	117.7	119.7	112.5	101.5	111.4	119.6
SCARA3	110	115.8	117.4	118.5	103.8	92.6	113.5	110.7
SCARA3	110.8	112.8	106.9	114.8	107.7	96.7	109.7	113.8
SCARA5	111.3	113	108.5	114	99.5	91.9	117.4	113.3
SCARB1	116.4	109.6	123.4	112.7	110.6	96.4	124.1	119.2
SCARB2	688.5	478.7	1262.5	881.5	417.9	1011.4	910	528.1
SCARF1	112.8	116.6	114.3	118	103.4	95	116.1	111
SCARF1	115.3	128.7	124.7	125.2	118.6	108.8	119.7	120.2
SCARF2	113.4	116.3	114.4	131.9	115.8	102.5	137.4	111.6
SCARF2	120.2	104	121.3	120.1	113.9	100.6	115.9	107.8
SCARNA15	117.8	109.4	109.4	108.7	104.2	94.3	110.6	112.3
SCC-112	322	275.2	222.1	270.6	298.6	280.1	297.1	331.4
SCCPDH	114.9	124.4	119.6	120.4	107.1	100.1	122.4	118.9
SCD	203.9	194.7	233.8	164.6	157.2	126.8	181.8	185
SCD5	140.4	121.2	125.2	131.3	118	102.1	139.5	131.6
SCD5	114.6	112.7	118.4	117.3	100.2	91.9	119.1	109.4
SCD5	119.6	128.2	133.2	135.5	128.6	113.7	122.4	122.5

SCDR10	130.6	147.5	126.3	130	121.7	109.2	136.1	126.6
SCEL	115	128.8	115	118	111.5	110.9	113.2	111.1
SCFD1	678.9	614.5	656.8	720.2	470.3	478.9	721.1	623.5
SCFD1	123.9	122.3	116.5	113.6	115.6	103.9	118	120.3
SCFD2	128.1	113.3	128.7	128.3	118.8	107.8	128.2	119.4
SCG2	117.2	115.2	118.7	117.2	113.2	98.1	118.7	111.3
SCG3	112.2	116.4	121.8	117.8	109.5	104	117.8	108.3
SCG5	118.6	118	112.8	124.7	116.3	101.5	123.4	116.2
SCGB1A1	114.2	122.3	122.6	111.6	110.9	107	116.2	120.4
SCGB1C1	114.6	110.6	118.9	111.6	108.5	98.9	120	109
SCGB1D1	116.2	113.9	119.7	123.3	109.7	99.6	122.5	113.3
SCGB1D2	119.8	115.1	111.3	117.6	114.5	95.9	128	113.3
SCGB1D4	109.6	109.8	120.7	113.7	103.8	102.6	110.1	121.6
SCGB2A1	116.6	119.4	116.4	121	110.6	96.1	118.2	113.6
SCGB2A2	115.8	121.4	126	128.6	107.1	101.2	119.7	116.6
SCGB3A1	160.7	120.4	223.2	184.2	109.1	182.3	136.1	114.5
SCGB3A2	116	122.4	120.6	138.4	112.2	95.8	118.2	116.6
SCGN	119	114.2	109.7	112.2	115.4	95.8	118.4	110.2
SCHIP1	118.3	129.8	116.9	127.8	113.6	106.2	127.8	122.7
SCIN	115.3	115.1	109.7	114.3	113	94	113.7	119.7
SCLY	144.4	145.8	141.9	142.5	128.8	118.1	156.1	150.6
SCMH1	119.3	104.4	104.2	117.2	105.9	101.8	110.2	116.3
SCMH1	165.8	163.7	147.4	155.5	134.7	136.1	180.7	158.9
SCMH1	125.3	118.2	114.8	116.4	114.5	100.2	125.1	118.9
SCMH1	120.4	116.5	131.6	128.1	122.6	110.6	127.5	122.6
SCML1	230.3	122.4	329	222.9	123.6	273.9	254.3	146.9
SCML1	177.9	148.7	216.6	177.4	141.1	164.7	181.7	201.9
SCML1	107.3	108.8	120.8	114.7	107.6	96.6	115.2	111.3
SCML2	140.6	128.9	136.1	149.2	126.1	117.6	149.5	142
SCML4	144.7	140.3	149.3	135.6	123.6	115.6	121.2	117.8
SCN10A	114	118.2	117.5	123	109.1	103.4	122.9	122.5
SCN11A	112.5	108.4	117.5	126.3	102.9	94.3	118.2	110.2
SCN1A	107.2	117.2	107.5	130.4	103.1	100.2	120.9	122.5
SCN1B	107.8	112.3	113.4	113.3	115.2	100	123	118.2
SCN1B	120.7	113.3	107.6	119.9	111.2	100.7	114.9	113
SCN2A2	110	109.3	118.8	122.5	103.6	96.3	116.6	115.5
SCN2A2	110.2	109.5	117.3	133.3	114	98.3	121.4	119.4
SCN2A2	118.1	117.7	117.7	126.2	103	100.5	115.6	119.6
SCN2B	115.2	119.4	108.4	115.9	116.9	107.2	119.8	117.3
SCN3A	114.5	111.8	116	111.5	102.5	97.4	111.3	117.6
SCN3A	161.5	159.5	164.6	176.7	124.8	127.2	157.4	144
SCN3A	123.5	121.7	124.7	121.7	117.7	101.8	124.9	125.2
SCN3B	110	110.5	113.2	114.5	107.6	101.5	114.3	113
SCN3B	115.5	110.9	104.6	116.5	102.4	97.7	122.7	104.6
SCN4A	128.3	114.5	121.2	118.8	111.9	100.6	108.5	114.1
SCN4B	123.5	127.7	124	127	118.6	105.6	125.4	125.3
SCN5A	105.7	112.3	115.8	115.4	109.3	98.9	107	105.7
SCN5A	111.5	121	116.9	120.4	105	103.2	117.5	114.8
SCN7A	115	122.2	118.5	122.6	111.8	101.3	114.4	114.3
SCN8A	115.4	117.2	109	120.3	109.3	93	112.7	110.8
SCN9A	110.7	118.1	121	116.3	106.1	97.8	121	122

SCNM1	219.6	215.9	243.4	234.8	207.7	183.1	235.3	234.6
SCNM1	232.5	249.5	322.4	264.6	228.6	197.8	222.4	251.5
SCNN1A	132.2	129.6	136.6	137.7	122.8	112.5	125.8	121.7
SCNN1B	115.1	118.4	112.2	130.6	111.9	101.8	120.7	116
SCNN1D	146.2	141.2	159.4	162.9	130.6	121.5	142.4	142.2
SCNN1G	115.4	110.7	122.6	124.6	101.8	98.7	115.7	115.9
SCO1	292.1	331.4	381.6	333.8	249.8	255.6	326.3	238.9
SCO2	326.7	340.6	562.3	322.6	242.7	275.1	283.6	250
SCOC	278.6	236.9	256.4	294.4	204.6	210.7	237.4	217.1
SCOTIN	926.3	694.4	1204.8	869.6	679.8	1127.7	825.8	841.4
SCP2	121.1	128.7	118.4	118.1	113.2	104.9	126.3	111.4
SCP2	116.8	130.3	123.2	126	116.5	101.6	120.3	116.6
SCPEP1	283.8	246	446.4	310.8	214.1	253.3	264.4	253.7
SCRG1	115.1	105.8	114.6	112.9	107.4	97.8	111.2	113
SCRIB	120.6	129.2	123.1	133	118.7	105.7	130.7	126
SCRIB	300.1	302.7	222.2	282.7	232.9	178.9	238	319.5
SCRIB	109.1	115.9	118.5	126	116.6	98.8	120.9	113.4
SCRL	107.8	115.3	106.6	119.8	104.9	99.7	111.4	113.9
SCRN1	189.7	196.5	192	202.9	174.7	153.5	212	182.1
SCRN2	117.7	130	132.9	129.7	117.4	113.6	123.4	131.2
SCRN3	127.9	119.4	129	130.1	110.1	101.6	121.8	112.1
SCRT1	120.5	108	108.9	119.1	107.4	97.2	114	117
SCRT2	116.5	123.3	121.4	122.4	110.7	102.8	120.2	115.8
SCT	127.1	116.1	119.9	131.2	121.6	104.6	125.2	123.1
SCTR	119.3	131.2	142	135.2	118.9	104.1	122.7	131.3
SCUBE1	111.1	123	118	119.9	118.9	102.6	110.2	120.2
SCUBE2	110.8	110.8	112.7	114.8	107.5	95.6	127.1	117.7
SCUBE3	112.9	110.2	117.4	117.7	111.8	99.1	114.6	122.6
SCYE1	115.6	114.2	113.2	111.6	106.6	95.1	120.8	109.5
SCYL1	183.5	177.4	168.1	182.4	158.9	178.3	200.4	180.8
SCYL1	538	335.8	431.8	561.9	338.3	358.7	490.9	491
SCYL1BP1	143.8	148.2	158.5	155.9	139.5	110.5	127.8	143.8
SCYL2	389	443	404.2	523.1	345.6	421.1	576.4	632.5
SCYL3	169.8	196.5	183.2	186.2	176.8	142.2	177.5	229
SCYL3	148	179.8	144.2	193.4	151.7	121.8	159.3	174.2
SCYL3	118.5	120.3	121	111.1	112.9	106.4	125.5	116.7
SDAD1	871.1	956.3	837	831.9	783.4	742.3	932.9	678.8
SDC1	126.8	115.8	122.3	133.8	113.1	101.3	119.1	125.5
SDC1	116.4	126.2	121.2	120.2	123.7	101	130.4	119.3
SDC1	113.6	115.8	117.9	122.3	106.3	91.9	111.7	111.8
SDC2	113.7	123.3	129.7	136.9	114.1	142.2	137.5	119.2
SDC3	124.7	115.2	122.4	128.6	105.4	103.2	110.1	118.1
SDC3	116.7	107.5	109.7	116.9	109	95.8	112.8	118.9
SDC4	107	108.4	120.4	119	107.4	113.3	118.7	114.1
SDCBP	1496.1	912.9	814.5	1273.5	932.2	1453.6	1403.3	1143
SDCBP2	115.5	122.5	120.1	127.2	111.8	105.8	137	129.5
SDCBP2	102.1	113.6	114.6	120.1	110.1	91.4	110	119.1
SDCBP2	112.4	120	122.4	123.2	107.9	102.1	136	125.2
SDCBP2	107.5	111.3	123	120.8	109.9	101.3	117.3	114.2
SDCCAG1	283.6	250.3	286.5	255.1	240.9	182.8	242.2	210.3
SDCCAG10	245.4	263.7	277.8	231.2	210.6	209.7	212.5	239.4

SDCCAG3	228.2	261.5	178.1	196.1	201.3	150.6	180.8	166.1
SDCCAG3	129.8	131.9	128.9	132	119.6	108.8	135.9	142.3
SDCCAG3	503.9	539.1	402.7	519.2	358.8	306.7	414.3	359.9
SDCCAG33	331.5	428	290.4	342.3	336.6	253.8	277.5	312.1
SDCCAG8	129.7	116.2	146.5	126.6	120.9	117.6	148.5	129.1
SDF2	586.2	584.5	539.1	635	537.4	538.8	634.8	787.6
SDF2L1	660	846.2	1268.7	720	534	713.8	666.4	718.4
SDF4	404.8	541.9	433.1	453.2	368.8	458.7	492.3	573.6
SDF4	115.5	122	114.2	117.8	105.3	100.6	122	112.6
SDF4	202.6	287.3	228.8	244.5	219.2	212.9	260.7	211.4
SDFR1	241.3	221	294.6	283.9	244.8	415.7	276.3	230.7
SDFR1	131.5	110.5	116.3	122.2	108.5	97.6	128.2	126
SDHA	417.3	374.6	425.1	511.7	382.2	364.4	356.8	380.5
SDHAP3	142.1	127.8	125.8	170.3	124.5	101.7	125.8	145.3
SDHB	1158	1099.2	1068.5	1110.3	730.5	799.8	1210.6	1062.2
SDHC	156.2	177.2	184.4	169	175.3	166.5	196.5	204.9
SDHD	288.8	345.2	384.4	282.1	246.2	330.5	382.6	341.6
SDK1	117.9	110.9	109.3	120.6	109.8	96	117.7	117.3
SDK1	118	116.2	117.6	117.8	108.2	102.4	114	118.2
SDK2	129.6	120.8	129.8	133.8	123	107.6	132.1	122.5
SDPR	125.8	135	145.4	123.7	119.2	116.3	138.6	134.5
SDR-O	110.8	119.8	120.1	125.3	109.6	110.2	118.3	106.8
SDS	112.5	114.6	127.1	120	105.3	104.2	117.9	121.7
SDSL	122.1	115.6	134.4	128.9	108.9	101.8	113.9	115.1
SEBOX	111.9	122.7	108	114.9	105.2	96.7	111.1	111.7
SEC10L1	142.1	145.3	150.5	133.6	142.5	118.3	140.6	137.6
SEC11L1	1064.7	1074.7	1554.5	1173	1062	1222.7	1234.8	1598.7
SEC11L3	175.5	173.6	253.3	206.7	179.3	196.9	171.7	227.8
SEC14L1	152	166.9	145.4	187.4	132.4	149.7	195.5	154.7
SEC14L1	209	181.8	149	211.1	153.1	157.5	196.4	171.1
SEC14L2	117.6	122.2	113.2	117.8	111.8	98.6	116.9	108.6
SEC14L3	111.8	119.8	120.6	120.8	109.6	90.6	110.6	108.5
SEC14L4	123.2	126.7	129.6	123.2	115.2	105.3	113.2	128.4
SEC22C	382.7	424.2	397.3	414.7	352.2	420	328.4	321.4
SEC22C	185.7	181.9	199.2	201.4	171.1	170	182.2	173
SEC22L1	314.2	374.5	333.7	412	314.2	259.7	375.2	338
SEC22L2	219.6	241.6	215.9	264.9	227.5	235.7	250.6	270.3
SEC22L3	134.8	139.4	150.2	144.1	135.1	118.2	162.5	132.1
SEC23A	103.9	115.6	114.1	123	110.3	103.9	116.5	113.4
SEC23B	267.6	196.1	246.8	277.9	177.7	230.6	301.8	251.4
SEC23B	366	390.8	373.2	378.9	293.9	371	442	505.5
SEC23IP	190.2	189.5	224	225.7	200.6	186.5	227.6	185.1
SEC24A	123	134.9	135.2	143	143	110.9	154.2	163.4
SEC24B	342.9	246.2	216.6	258.9	254.3	197.8	261.1	332.1
SEC24B	112.7	114	117	116.6	108.6	100.2	107.2	109.3
SEC24B	148.4	154.1	141.9	181.3	148.4	142.7	165.2	164.2
SEC24B	134.6	139.1	135.8	142.4	145.2	123.1	133.4	148.8
SEC24C	1281.8	1351.3	1106.8	1552.8	856.6	970.1	1591.6	1483.5
SEC24C	142.7	139.5	137.5	151.1	120.4	122.1	134	129.2
SEC24D	162.2	139.5	155.2	166.6	133.2	163.7	156.9	178.3
SEC31L1	119.6	124.9	121.3	129.5	117.6	111.2	133.4	128.3

SEC31L1	461.1	549.2	508.3	435.4	324.6	272.4	529	647.2
SEC31L2	119.5	107	119.2	110.1	109	98.3	113.8	106.3
SEC31L2	134.4	117.9	114.3	126.6	109.9	114.5	128.9	121.6
SEC61A1	1029.2	1154.6	891.8	1201.7	854.4	877.3	1469.8	1336.9
SEC61A2	126.6	128	123.5	137.2	125.4	106.1	143.5	135.8
SEC61B	860.7	822.8	988.5	799.4	830.1	1003.6	1040	1038.4
SEC61G	1374.1	1496.4	2480.1	1374.1	1588.2	1880.2	1527.9	1595.9
SEC61G	1416.6	1780.1	2067.7	1749.7	1290	1778	1350.9	1940
SEC63	113.9	116.3	116.5	119	111.2	98.6	112.6	121.4
SEC6L1	319.1	314	342	355.7	251.3	319.6	294.7	215.7
SEC8L1	117.2	121.1	141.1	140.7	112.9	112.8	127.9	132.1
SECISBP2	244.1	262.3	264.1	272.8	260.2	213.9	263.9	236.1
SECTM1	118.5	126.2	124.3	121.1	116.2	106.1	120.8	126
SEDLP	143.9	166.5	140.7	148.4	165.2	163.7	139.9	170
SEH1L	170.9	191.1	148.2	159.4	148.6	127.2	178.4	155.3
SEH1L	134.3	141.8	146	159.5	130.4	116.4	137.5	153.1
SEH1L	159.7	154.4	174.7	174	160.1	147.4	178	181.2
SEL1L	130.3	140.6	146.9	132.1	129.4	123.5	154.6	148.9
SELE	124.4	118.4	125.2	137.6	116.6	94.1	128.5	114
SELENBP1	111.2	116.8	116.6	119.7	105.4	115.4	115.4	112.5
SELI	164.8	139.2	140.1	173.4	138.2	119.2	166.8	168.7
SELK	336.7	390.7	363.7	464.4	289.8	301.2	492.6	656.3
SELL	889.7	1333.6	866.9	1273.9	981.6	1018	1433.9	1388.3
SELM	173	153.1	221.4	186.1	136.9	196	167.8	120.8
SELO	250.3	235.8	251.9	221.1	180	184.2	225.9	230.6
SELP	112.9	108.7	111.3	118.1	103.8	99.2	109.5	110.9
SELPLG	222.5	266.8	202.1	276.6	259.4	133.7	200.8	280.4
SELS	119.7	112.2	126.5	132.6	122.4	110.3	124.3	128.9
SELS	895.5	1067.8	927.1	1239.1	776.1	1075	1228.7	1228.6
SELS	356.1	347	313.4	412.2	324.8	292.7	394.4	413.5
SELT	110.2	111.7	119.9	122.9	109.2	104.5	126.1	115.6
SELV	117.8	124.1	116.3	108.6	102.8	97.8	117.8	112.5
SEMA3A	117.3	114.8	127.7	124.7	114.3	101.5	130.6	120.2
SEMA3B	119.5	129.6	117.5	132.4	109.5	103.6	141.8	122.4
SEMA3B	120.2	122.7	115.8	117.1	118.9	106.2	128.1	122.5
SEMA3C	122.5	120.7	107.9	112.7	109.7	102.6	110.4	122.4
SEMA3D	115	105.6	112.6	110.1	106.3	98.7	116	112.5
SEMA3E	756.1	657.5	864.8	633.5	609.2	565.4	838.1	846.3
SEMA3F	115.7	118.8	115.1	119.2	110.4	98.3	123.3	109.2
SEMA3G	124.5	131.3	126.7	121.5	112.4	108	117.6	118.3
SEMA4A	149	133	178.9	142.4	119.6	139.7	131.8	122.4
SEMA4B	223.1	206.8	192.3	201.6	165.3	171.9	190.6	170.1
SEMA4B	112.6	109.8	116.7	112.6	105.1	93	117.7	117.6
SEMA4C	124.8	128.8	129.1	126.1	117	109.1	117.4	115
SEMA4D	1949.3	2429.5	1448.7	1882.5	1482.7	1297.3	1622	1719
SEMA4F	172	190.3	172.5	199.4	150.1	145.3	209.3	180
SEMA4G	111.5	109.5	118.7	112.9	112.4	99.1	112.3	125
SEMA5A	110.5	117.6	114.7	114.2	109.8	96.3	111.1	114.3
SEMA5B	111.2	116.9	116.2	121.6	110.5	96.7	117.2	117.6
SEMA6B	135	131	168	140.7	123.9	206.7	168.7	128.4
SEMA6B	116.4	113.2	115.1	124.2	109.2	100.3	116.8	117.8

SEMA6B	114	107	111	118.8	111.9	101.6	118.7	121
SEMA6C	120	120.2	134.6	127.2	120.8	107.1	126.4	130
SEMA6D	114.7	113.8	110	124	118.6	101.3	130	112.9
SEMA6D	111.6	104.7	115.9	117.1	103.7	97.1	117.6	109
SEMA6D	114	124.1	128.2	127.2	122.8	110.4	123.5	128
SEMA7A	115.8	116.3	118.7	124.2	113.9	102.6	120.9	121.3
SEMG1	129	114.4	117.2	123.8	107.5	102.5	125.1	115.2
SEMG1	130.2	128	125.5	138.5	119.3	96.9	123.7	115.6
SEMG2	113.3	108.8	112	113.3	105.8	96.2	110.5	107.7
SENP2	167.9	183.9	153.9	171.4	139.7	139.2	205.8	185
SENP3	120.8	125.2	130.3	126.6	116.4	106.3	118.1	130.7
SENP5	122.7	122.2	130.9	131.3	128	112.2	140.5	133.3
SENP6	120.6	123.4	125.4	135.3	110.1	95.9	128.6	131.8
SENP7	123.6	133.9	153.1	149.2	139.9	119.9	124.3	128
SENP7	312	373.4	381.5	293.6	361.4	273	294.3	327.3
SENP8	115.2	112.3	117.2	117.2	108.1	109.2	110.2	113.6
Sep-15	1480.1	1493.8	1681	1912.8	1496.9	1522.4	1943.4	1816.5
SEPHS1	268.2	263.7	291.3	255.7	241.9	217.3	262.4	331.8
SEPHS2	168.4	163.4	170.6	147.6	138	125.6	155.1	148.8
SEPN1	109.5	114.3	117.6	115.8	105.6	100.1	113.4	109.3
SEPN1	143.7	136.6	153.5	162.2	136.3	121.7	140	139.7
SEPN1	254	293.8	294.8	224.3	176.7	308.4	236.7	191.7
SEPP1	116.8	115.2	109.6	116	104.7	98.1	122.8	114.9
Sep-01	129.9	131.1	129.7	132.1	110.3	109.4	116.6	124.6
Sep-10	115.4	111	113.3	116.3	103.6	108.8	111.7	117.3
Sep-10	108.6	114.3	121.9	117.8	97.1	92.5	109.3	107.7
Sep-11	209.5	217.2	189	228.6	155.6	151.1	173.6	186.9
Sep-02	2190.2	2236	2669.5	2723.5	2087.2	1908.9	2561.2	2757.9
Sep-02	1672.5	1825.7	2224.9	1938.4	1948.8	2270.6	2134.8	2838.5
Sep-02	119.3	114.2	135.7	130.5	115.5	101.5	134.3	121.8
Sep-02	119.9	120	131.1	140.8	121.7	99.7	110.7	118.2
Sep-03	108.8	110.9	115.7	113.4	104.9	100.7	117.6	110.7
Sep-03	120.5	113.5	133.6	122.9	111.9	91.9	129.9	117.7
Sep-04	124.3	123.5	116.3	127.6	106.7	95.5	120.3	119.8
Sep-04	122.9	119.6	124.1	127.2	113.1	106.2	120.6	116.5
Sep-04	115.3	116.6	123.7	124.3	111.2	110.5	115.2	113.9
Sep-04	107.3	115.7	112.5	102	105.4	107.6	120.6	118.1
Sep-05	115.6	125.7	122.6	121.3	108.4	101.1	121.3	117.3
Sep-06	123	143.3	119.1	129.8	118.6	106.8	127.2	123.1
Sep-06	281.4	312.2	282.4	298.6	256.1	212.1	212.5	204.6
Sep-06	308.4	352.6	388.4	283.5	270.4	179.1	221.6	208.2
Sep-07	151.7	161.5	142.8	153	153.3	139.7	178.5	156
Sep-09	7908.1	9663.5	7706.2	7832.4	6294.9	6584.3	5733.9	4988.2
SEPW1	286	287.6	367.7	254.5	190.5	242.8	208.9	232.1
SEPX1	497.1	466.5	578.8	570.5	337.3	662.7	546.6	432.4
SERAC1	107.8	117.9	123.2	121.2	112.6	100.8	135.2	117.6
SERF1A	116.7	114.9	114.4	128.3	116.4	93	130.5	126.9
SERF1A	115.8	129.8	128.6	133.3	115.4	101.7	123.5	115.7
SERF1B	109	109.4	110.2	116.9	109.4	98	114.8	115.3
SERF2	1467.2	1505.3	1990.9	1538.2	1365.9	1695.4	1099.4	1198.9
SERGEF	214.7	260.7	247.4	254.7	186.7	193.7	216.1	197.4

SERHL	120.4	118.3	120.4	124.1	120.4	104.9	116.2	125
SERHL2	112.7	111.1	114.6	117.8	112.2	98.4	114.6	113
SERINC1	222.7	326.6	235.6	282.4	239.3	355.7	329.4	399.8
SERINC2	113.5	115.1	121.5	110.4	104.8	96.3	116.6	116.8
SERINC3	116.2	132.1	121	132.2	119.5	100.8	114	148.2
SERINC3	123.9	126.6	130.2	145.3	126.8	116.6	129.1	140.2
SERINC3	906.2	1178	803.8	1246.3	1032.2	607	1171.8	1417.2
SERINC4	125.6	116.6	131.7	109	121.1	109.2	119.3	114.9
SERINC5	134	140.9	129.8	141.4	141.8	110.8	133	139.5
SERP1	140.6	153.6	172.1	167.6	146.5	159.8	161.6	244.8
SERPINA1	162.4	118.2	485.7	153.6	108.9	267.4	155.8	118
SERPINA1	106.5	114.2	115.1	108.1	100.7	96.9	111.5	104.3
SERPINA1	160.8	116.4	530.6	164.1	110	269.6	143.3	121.5
SERPINA1	100.7	116.5	122.4	120.7	107.4	90.2	116.2	116.7
SERPINA10	113.5	115.7	114.9	115.5	114	99.8	116.2	116.1
SERPINA11	117.2	127.6	117	132.7	117.2	105.8	122.1	128.8
SERPINA11	113.9	117.7	107.9	116.5	109.2	104	117.1	117.6
SERPINA12	111.7	119.7	109.2	117.4	114.5	97.5	116	116.6
SERPINA13	113.3	116.6	118	115.9	108.2	97.3	115.4	114.2
SERPINA3	103.8	112.5	124.5	112.1	103.6	88.7	114.6	107.9
SERPINA4	114.8	121.7	112	128.7	117.8	105	136.5	130.1
SERPINA5	113.4	111.3	117.9	121.3	108.6	101.5	118.8	115.9
SERPINA6	117.9	119.2	123.9	114.3	113.3	98	118.9	116.2
SERPINA7	123.5	117.1	121.2	120.5	107.5	99.9	124.6	122.1
SERPINA9	118.3	116.4	118.1	118.4	110.8	97.9	113.2	116.3
SERPINA9	107.1	116.7	113.8	122.7	104	97.2	117.8	112.1
SERPINB1	324.6	292.2	363.2	312	227.4	268.7	235.4	199.8
SERPINB10	122.3	122.1	125.6	118.6	114.5	103.6	125.8	110.7
SERPINB11	123.1	116.1	115.3	116.1	109.1	103.7	119.2	115.2
SERPINB12	103	101.9	114.9	103.9	98	97.1	108.8	104.8
SERPINB13	124.8	114.1	119.1	123.7	112.7	102.6	112.4	124.1
SERPINB2	251.9	324.6	124.1	528.1	529.6	738.7	558.9	419.8
SERPINB3	120.4	119.7	122.6	122.3	114.9	102.3	118.8	109.2
SERPINB4	112.3	116	107.9	118.8	108.8	97.2	108.9	117.2
SERPINB5	111	122.2	124.2	125.7	116.6	104.2	129.2	119.4
SERPINB6	541	460.5	730.9	652.9	351.1	420.2	436.8	349.6
SERPINB7	113.1	109.1	105.4	122.8	99.8	104.3	129.2	112.7
SERPINB7	112.9	117.7	119.7	124.2	117.3	97.2	120.1	125.6
SERPINB8	192.7	193.2	230.9	208.7	162.9	197.5	248.4	162.7
SERPINB8	166.9	189.6	231.8	227.8	163.6	187.4	245.8	163.5
SERPINB9	114.1	119.2	116.5	116.5	108.7	104.2	109.9	116.2
SERPINC1	110.7	108.9	123.1	118.1	105.1	96	115.1	119.2
SERPIND1	109.4	124	111.2	121.1	103.7	106.1	120.4	115.8
SERPINE1	113.2	121	108.5	142.5	133.3	140.8	138.2	117
SERPINE2	240.8	356.4	239.9	285.6	296.2	189	388.4	449.3
SERPINF1	117.4	122.3	117.1	116.3	101.1	102	120.7	114
SERPINF2	113.6	124.8	115.2	115	104.9	103.7	119.9	117.7
SERPING1	126.2	121	124.8	126.7	127.1	106.6	120.3	123.7
SERPING1	116.5	118.9	125	130.3	122.4	103.6	132.3	129.2
SERPINH1	139.1	137.4	124.5	138.7	116.6	121.4	131.8	180.4
SERPINI1	124.1	112.1	118.4	121.6	107.1	113.2	121.4	122.8

SERPINI2	127.5	115.7	126.9	113.3	107	100.2	111	117.2
SERTAD1	314.9	348.8	249.1	380.3	274	272.3	632.8	577.1
SERTAD2	904.7	956.1	948.1	983	1142.4	633.1	897.4	1033.3
SERTAD3	170.1	178.6	194	207.6	167.6	208.1	243.1	245.6
SERTAD3	115.7	115.1	118.9	124.6	110.6	97	121.3	109.9
SERTAD3	127.2	121.7	146.3	138.3	118.2	121	139	133.1
SERTAD4	116.6	126.1	124.6	120.2	120.7	99.5	124.1	114.6
SESN1	316.1	481.1	347.6	329.9	298.6	339.8	368.2	343.9
SESN2	130.1	118.7	125.1	123.4	117.1	100.4	128.7	120.5
SESN3	161.4	179.9	133.9	197.4	162.5	121.4	169.7	170.4
SESTD1	178.7	169.4	191	207.7	148.3	204.1	200.6	178.5
SET	591.1	696.8	584.6	786.7	538.9	646.6	876.3	744.6
SET7	116.5	106.2	108.4	118.1	114.6	96.2	114.3	114.2
SETBP1	158.6	146.1	142.8	159.7	132.6	117.1	136.8	129.3
SETD1A	403.8	439.5	309.6	431	245.3	266.6	499.9	457.5
SETD3	113.2	134.1	129.1	129.9	127.1	111.8	126.6	124.9
SETD3	124.1	122.6	113.2	125.1	105.7	107.7	116.5	128.6
SETD3	969.1	1156.3	1087.4	1295	916.5	884	1424.8	1270.8
SETD4	136.2	150.5	146.1	133.8	136.2	128.6	136.2	122.7
SETD6	116.8	123.6	117.8	126.1	117.5	101.9	122.4	125.5
SETD8	149.2	153.8	172.3	167.5	143.1	142.6	190.6	183.6
SETDB1	226.1	229.2	234.6	248.1	178.9	189.4	224.6	211.6
SETDB2	184.9	217.7	224.9	212.6	162.8	158.8	213.9	178.9
SETMAR	236.3	194.6	188.7	209.5	192	148.8	197.9	193.5
SETX	154.8	150.7	158	176.2	188.4	127	169.3	186.6
SEZ6	117.1	121.5	122.6	120.3	106.6	101.3	115.5	126
SEZ6L	113.2	108.9	110.2	104.8	101.6	92.7	121.8	109
SEZ6L2	118.7	124.7	112.5	130.8	107.2	110.8	120.8	114.2
SEZ6L2	112.6	115.4	122.9	106.4	110.6	105.7	106.3	113.2
SEZ6L2	106	114.1	110.2	110.6	106.5	101.2	119.5	114.8
SF1	128.3	121.7	128.4	126.2	124.7	99.6	127.4	125.5
SF1	182.6	180.2	134	207.4	210.3	107.2	195.6	194.3
SF1	145.2	154.6	152.7	153.2	123.5	117.6	141.5	132.2
SF3A1	113.5	117.2	119.6	109.8	109.1	103.3	117.9	114.9
SF3A1	241.2	373.8	233.7	233.6	246.8	220.8	227.8	299.3
SF3A2	638.8	741.7	715.8	627.3	492.8	721.2	826.6	872.2
SF3A3	499.2	639.2	691.6	609.1	455.7	428.2	624.2	687.9
SF3B1	119.6	125.6	136.2	136.1	133.6	121.3	127.7	122.1
SF3B1	1371.5	1421.4	1464.5	1393.9	1252.5	1177.9	1658.3	1588.6
SF3B1	225.5	207.1	235.8	217.9	221.7	136.4	222.5	272.4
SF3B14	161.4	228.9	225.6	226.5	207.9	251.5	195.2	229.5
SF3B2	589.8	669.1	368	477.6	409.7	359.7	541.4	479.3
SF3B3	370	414	341.7	443.3	379.1	303.8	489.1	475.4
SF3B4	663.6	880.9	610.6	818.6	718.1	773.5	976.9	1073
SF3B5	787.9	891.7	940.5	867.3	826.3	813.6	644.5	632.3
SF4	348	301.9	285.7	268.3	218.4	225.2	321.9	255.8
SFI1	112.4	129.1	127.9	124.8	116	100.6	121	125.8
SFI1	156.8	163.1	129	145.8	133	111.9	149.2	141.8
SFI1	130.1	139.8	135.4	126.6	117.4	113.2	113.6	126.6
SFMBT1	151.3	175.7	155.1	183.2	201.7	137.2	224.5	234
SFMBT1	191	196.7	191.9	216.9	196.3	168.2	259.6	286.5

SFMBT1	114	117.6	116.4	113.3	109.3	101.7	112.9	115.5
SFMBT2	142.8	141.4	123.5	148.8	140	106.3	140.4	136.4
SFN	112.6	105.6	120.9	122.2	108.5	94.5	121.5	111.6
SFPQ	138.7	135.8	156.2	154.3	138.8	123.2	164.7	159.8
SFRP1	109.7	98.5	112.7	110.3	110.8	92.7	125.6	112.3
SFRP2	118.5	114.8	114.2	115.5	104	97.3	122	110.3
SFRP4	116.1	112.8	126.9	116.6	110.1	105.7	115.6	117.5
SFRP5	108.1	118.6	112.4	118.5	127	93.9	117.2	113
SFRS1	494.6	567.9	465.2	737.1	545.8	497.5	508.8	397.6
SFRS10	791.2	817.2	1017.9	953.6	773.5	853.1	979.1	1368.2
SFRS11	193.6	208.3	200	207	212.5	184.3	175.2	294.7
SFRS12	188.6	181.8	166.1	182.2	147.6	109.9	194.4	191.9
SFRS12	244.5	261.6	209.6	311.2	277	155.9	333.9	317.1
SFRS12	128.4	126.4	134.6	140.1	116.2	98.5	127.1	120.6
SFRS14	693.5	593.5	519.4	549.9	425.4	348	511.3	466.5
SFRS14	314.2	282.8	266.4	260.5	197.1	193.8	223.7	259.4
SFRS14	160.6	161.8	174.6	171	133.6	122.4	164.3	152.4
SFRS15	255	240.3	224.6	314.6	248	183.3	377.3	414
SFRS16	149.4	172	152.5	145.6	143.2	113.6	179	146.1
SFRS2	3406.6	3190.3	3307.5	3514.3	3052.8	2571.9	3272.8	3986.3
SFRS2IP	287.9	303.4	300	279.5	258.1	224.6	268.4	286.5
SFRS3	115.7	112.6	116.8	124.1	115.2	102	123.6	125.5
SFRS4	1453.4	1907.2	1212.2	1901.7	1614.1	1271.4	1499.6	1429.4
SFRS5	3135.6	2567.6	3155.8	2458.2	1866.2	1605.2	1860.3	1674.9
SFRS5	3107.6	3011	3262.9	2877.9	2267.7	1935.8	1944.9	1903.5
SFRS6	2861	2798.3	2423.9	2985.6	2331.8	1889	2971.4	3321
SFRS7	425.7	374.2	552.6	421.7	411.9	393.8	349.7	470.8
SFRS8	131.6	149.8	126.1	163.4	138	134.4	156.1	154.6
SFRS9	1472.9	1459.4	1713.4	1521.8	1285.8	1302.5	1532.6	1423.7
SFT2D1	1245.7	1012.2	2142.9	1515.6	812.6	1697.3	1064.1	610.9
SFT2D2	128.9	123.6	124.1	125.4	116.4	105.1	114.5	123.6
SFT2D3	129	156.8	143.3	141.8	131.5	121.7	119.5	142.7
SFTPA1	111.9	112.3	121.6	119.9	105.7	102.4	105.3	106.1
SFTPA2	113.1	120	106.7	118.3	110.3	98.3	119.2	107.1
SFTPB	117.6	104.8	118.6	115.7	108.2	96	113.5	119.3
SFTPB	115.5	120.7	107.1	115.3	119.1	96.9	130.8	113.4
SFTPC	118.2	115.6	120.3	110.2	105.3	97.5	114.5	113.5
SFTPD	110.2	105.3	125.6	121.1	104.7	98.3	112.4	119.6
SFTPG	111.6	114.6	111.8	124.6	106.9	96.8	124.5	123
SFXN1	178	175.5	151.6	169.5	170	129.4	165.2	153.4
SFXN2	126.5	120.4	115.9	131.3	117.9	101.2	116.2	115.4
SFXN3	123.1	120.6	115.9	121.6	109.1	97.9	127.4	118
SFXN4	183.3	209.6	220.5	176.5	180.7	162	186.6	192.3
SFXN4	461.1	420.6	435.7	376.4	236.5	223.8	299.7	259.8
SFXN4	148.4	128.6	135.9	148.5	130.4	121	142.8	126.1
SFXN5	141.7	153.6	135.2	139.2	125.9	124.4	169.4	159.8
SGCA	125.2	130.5	128.3	119.1	107.3	100.7	127.2	116.7
SGCB	122.6	124	116.2	140.4	111.1	104.3	124.9	116.4
SGCD	116.5	112	120.2	115.8	105.2	94.4	119.2	118.6
SGCD	107.8	113.7	120.9	115.8	107.4	101.8	114.4	116.8
SGCD	107.2	123	114.3	122.1	108	96.8	122.7	114.5

SGCE	153.4	132.5	145.6	142.6	137.2	123.9	147.1	130.1
SGCG	117.1	120.5	112.5	123	109.6	99.9	112.5	118.6
SGCZ	117.8	112.1	113.7	121.1	109.5	99.3	119.3	114.2
SGEF	112.7	108.8	115.1	112.6	105.9	101.1	116.4	117.2
SGIP1	109.9	107.5	111.7	122.6	109.3	98.5	115.1	114.4
SGK	1278	1154.7	750.6	1369.1	915.6	587.9	2265.8	1660.3
SGK2	111.4	109.6	108.4	117.8	99.3	94.5	110.1	108.7
SGK2	113.6	111	99	117.3	103.2	99.4	121.3	103
SGK2	118.1	123.3	125.4	128	123	108.2	136.5	120.9
SGK3	118.8	113.1	121.4	123.6	109.6	96.8	116.7	128.2
SGK3	331.4	310	453.1	347	280.5	279.1	368.7	275.3
SGK3	251.1	218.2	334.1	297.8	196.7	171.6	287.1	226.4
SGOL1	119.8	111.2	111.5	121.5	105.5	95.2	120.4	114
SGOL2	118.3	117.8	107.4	123.2	111.6	108.9	111.8	117.4
SGPL1	176.4	175.8	189.5	175.2	137.5	156.7	169.9	175.4
SGPP1	116	107.5	127.4	113.3	110.8	99.8	119.4	115.2
SGPP2	115.8	115.4	119.9	138.6	116.7	119.3	122.3	126.2
SGPP2	111.8	118	119.6	119	111.8	95.5	118.9	119.7
SGSH	721.5	541.9	527.2	656.4	362.5	503.9	654.3	379.7
SGTA	130	130.7	109.9	126.8	115.8	110.2	124.4	125.5
SGTB	115.2	123.3	127.9	119.7	109.3	108.2	117.4	125.1
SH2B1	213.2	198.4	191.7	205	159.7	175.9	192	184.7
SH2D1A	370.1	529.9	366.8	361	452.6	342.1	378.6	579.2
SH2D1B	313.3	283.2	469.3	356.3	236.7	374.1	227.1	211.7
SH2D2A	129	116.1	122.1	133.1	122.1	101.9	128.2	137.1
SH2D3A	124.4	144.1	135.4	138.6	127.4	106	144.7	118.2
SH2D3C	413.9	401.4	349.6	357.2	240	299.9	296	229.7
SH2D3C	125.3	149.7	136.5	128.6	128.8	108.2	139.1	136.1
SH2D3C	113.2	122.5	138.6	129.4	118.8	106	114.7	129
SH2D4A	111.6	113.9	119.4	115.8	111	104.4	123.8	113.1
SH2D4B	114.8	112.7	120.8	116	113	107.4	117.4	107.3
SH3BGR	125.1	127.6	132.9	144.5	108.4	99.6	124.9	125.2
SH3BGR	123.5	117.4	118.7	127.4	119	102	126.9	125.7
SH3BGRL	113.5	115.3	113.5	112.9	106.8	100.5	109.9	117
SH3BGRL2	135.3	164.5	126.5	135.4	161.8	108.6	156.8	179.3
SH3BGRL3	1681	1563.2	2164.2	1671.3	1104.7	1677.7	1574.1	1096.8
SH3BP1	301	268.4	246.5	275.7	170.4	224.1	234.8	185.5
SH3BP2	114.6	118.6	125.3	116.4	113.1	105.2	115.9	117.9
SH3BP4	135.6	119.4	129.3	132.8	112.6	134.4	134.7	115.3
SH3BP5	116.7	111.8	113	119.8	113.2	94	111.5	117.3
SH3BP5	109.4	115.7	115.2	115.1	109.1	92.9	121.6	115.8
SH3BP5L	243	214.8	232.5	263.6	188.5	222.1	264.4	252.9
SH3D19	108.3	118.8	104	112.6	101.7	91.1	108.7	107
SH3GL1	125.2	151.5	162.5	153.8	125.9	119.2	147.7	137.2
SH3GL2	116.7	124.9	114.7	123.4	101.7	102.4	119.7	119.4
SH3GL3	115.8	114.1	115.2	121.7	110.3	96.7	120.9	106.4
SH3GLB1	559	587.3	521.1	722.8	650	567.8	781.6	662.6
SH3GLB2	930.4	1006.5	1068.4	968.6	497.3	725.3	526.9	453.2
SH3KBP1	1551.4	1596.1	2034	1669.8	1689.7	1284.7	1543.2	1587.4
SH3KBP1	203.4	199.9	192.1	233.3	200.9	129.5	178.5	177.9
SH3KBP1	1713.6	1980.8	1455.9	1852.3	1413.6	1206.2	2043.5	1847.5

SH3MD2	106.4	119.4	117.8	124.5	109.7	105	119.2	116.9
SH3PX3	127.3	118.8	116.8	118.3	113.9	100.8	122.9	122.3
SH3PXD2A	390.9	319.2	324.4	361.4	257.4	254.3	304.4	209.2
SH3PXD2B	123.7	115.4	120.7	124.6	117	109.8	121.3	118.7
SH3RF2	118.7	140	117	123.8	112.3	100	120.8	116.1
SH3TC1	146.1	155.6	155.8	167.3	143.7	134.2	172.2	153.9
SH3TC2	119.4	113.3	113.2	119.3	109	104.3	120.6	124.2
SH3YL1	180.6	174.9	183.9	193.4	148.9	125.8	162	170
SHANK1	119.4	114.7	113.9	113.7	113.5	92.7	110.7	115.2
SHANK2	115.2	105.1	129	116.3	107.5	97.7	121.2	109.8
SHANK2	108.6	111.8	112.3	113.4	114.2	96.8	121	121
SHANK3	112.7	119.1	120.2	118.6	111.8	99.3	126.9	114.5
SHARPIN	143.2	142.6	144.1	141.3	123.5	124.4	129.8	128.8
SHB	130.9	138	135.6	143.6	116.4	116.2	136.8	135.4
SHBG	107.2	110.7	106.5	116.7	101.9	98.6	120.6	116.5
SHC1	117.5	113.3	119.8	122.8	110.5	107.2	122.3	108.4
SHC1	115.5	118.2	121.4	115.7	103.7	100.7	121.8	118
SHC1	624.1	468.7	499.6	516.2	418	543.3	556.3	802.4
SHC3	121.7	110	128.2	136.3	110.7	107.2	118.4	129.4
SHC4	114.2	116.6	128.6	111.5	107.9	103.8	124.7	110.8
SHCBP1	4118.8	3107.9	3342.9	3949.4	3354	1834.2	3669.3	3614.8
SHD	109.2	119.9	116.1	129.2	106.2	99.8	126.6	119.1
SHE	124.7	119.8	122.8	120.6	114.7	100.5	114.2	116.4
SHF	119.7	119.9	127.6	121	115.4	98.6	121.1	130.3
SHFM1	1390	1566.5	2269.9	1839	1662.4	1952.2	1389.7	1596.7
SHH	125.2	112.6	125.9	120.1	110.8	101.7	115.7	126.1
SHKBP1	230.2	187.3	188.9	200.9	143	173.7	213.2	162.5
SHMT1	197.9	203.6	169.5	189	154.9	129.5	171.7	139.9
SHMT1	134	135.1	119.1	137.4	126	113.4	127.3	133.5
SHMT1	122.2	118.7	126.6	123.3	111.8	99.3	116.6	118.3
SHMT2	930.9	930.8	718.5	835.8	568.8	565	589.5	493.5
SHOC2	143.8	151.8	149	163.7	155.8	130.5	180	177.2
SHOX	111.4	107.1	110.4	116.3	106	104.4	109	114.1
SHOX	114.2	110.4	119	120.1	108.5	103.7	113.1	115.4
SHOX2	142.1	133.4	134.5	129.8	117	106.4	132.7	140.8
SHOX2	106.5	122.9	117.5	120.9	108.5	101.3	118.7	120
SHPRH	121.5	123.6	118.3	129.1	115.2	103.2	126.2	116.5
SHPRH	132.8	144.4	134.8	147.2	150.2	107.5	141.6	120.9
SHQ1	143.4	134.6	164.5	172.8	148.5	125.8	135.1	134
SHRM	113	124.4	113.2	122.8	102.1	103.1	121.1	112
SHROOM4	1124.9	883.1	1218.4	871.8	980.6	764	1339.6	1177.8
SI	112.6	117.8	116	127	113.9	96.7	121.5	109.7
SIAE	124.4	125.7	114.5	119.8	107.6	101.9	118.8	118.7
SIAH1	114.6	110.8	116	115.3	110.3	107.1	116.6	116.1
SIAH1	114.3	116	115	117.3	109.2	97.6	106.8	109.3
SIAH1	303.1	332.1	259.3	319.5	290.2	216.4	385.6	427.4
SIAH2	163.7	148.7	153.7	167.3	145.4	143.8	167.2	153.2
SIAHBP1	1473.8	1783.3	1188.1	1627.3	1074	865.3	1656.6	1655.5
SIDT1	177.8	150.9	206.1	167.4	151	171.9	153.2	153.8
SIDT2	273.7	286.9	415.6	319.2	239.9	294	268.5	259.4
SIGIRR	144.7	151.6	153.5	138	141.7	114.3	131.9	153.8

SIGIRR	162.2	167.7	182.7	165.1	155.6	125.7	140.3	144.4
SIGLEC10	107.3	114	142	111	99.7	105.2	117.1	106.1
SIGLEC11	112.8	112.4	112.8	111.1	104.6	91.8	115.1	117.4
SIGLEC12	114.7	132.7	125	125.4	123	110.5	133.5	126.7
SIGLEC12	113.9	115	124.5	121.3	102	101.3	119.8	121.3
SIGLEC12	120.3	114.9	114.9	115.5	107	107.1	119.9	105.8
SIGLEC5	111.7	119.9	125.3	115.3	114.2	97.9	119.3	110.1
SIGLEC6	114.8	114.7	119	115.4	111.7	95.4	121.9	113.6
SIGLEC7	119.5	130.4	135.4	130.8	116.9	104.2	124.5	122.7
SIGLEC7	116.5	118.7	129.9	122.1	111.3	99.4	118.3	108.6
SIGLEC7	115.7	116.6	126	125.6	117.2	102.3	126	118.3
SIGLEC8	116.9	109.9	106.8	106.4	109	100.4	110.6	113.6
SIGLEC9	148.2	128.5	158.8	154.3	119.7	136.4	151.4	116.4
SIGLECP16	123.2	118	206.8	119.2	105	108.2	111.4	112.8
SIGLECP3	117.8	120.1	118.1	112.6	110	102.9	126.1	113.9
SIGLECP3	121	126	109.4	132.6	114.4	103.5	129.3	114.9
SIKE	121.9	123	126.6	127.9	117.5	106	153.6	134.7
SIL1	136.6	109.1	152.1	137.7	105.2	119.3	134.6	121.6
SIL1	117.5	114.7	130.9	120.2	116.8	99.3	111.3	108.8
SIL1	195.4	172.9	302.4	196	166.1	189.7	176.1	193.3
SILV	113.5	120.5	108.1	113.6	105.4	98.6	119.5	113
SIM1	116.1	117.5	121.3	118.9	104.2	95.5	114	113.9
SIM2	114.5	110.3	114.7	110.1	110.2	97.1	113.7	110
SIM2	120.7	111.8	118.4	122.8	108.9	112.8	118.5	116.2
SIN3A	581.1	678.4	524.8	675.9	592.9	520.5	787.4	776.4
SIN3B	136.4	140.1	147	142.3	128	135	152.9	140.9
SIP1	208.2	218.1	259	247.6	219.9	191.5	213.8	208.3
SIPA1	146.1	148.3	159.4	166.9	126	118.2	135	140.8
SIPA1	654.7	813.4	760.3	679.7	498.3	567.6	453.6	476.8
SIPA1L1	130.1	128.8	120.9	134.4	105.5	96.2	125.3	114.2
SIPA1L2	212.1	193.2	190.8	269.8	160.7	194.8	303.4	217
SIPA1L3	123.7	125.2	139.9	144.6	113.6	130.9	141.2	119.6
SIRPB1	119	113.4	143	121.2	100.9	117	114.8	117.6
SIRPB2	120.5	134	130.4	132.3	117.4	113.3	118	124.7
SIRPD	116.7	107.7	122.8	128.6	122.8	104.3	120.6	120.8
SIRPG	200.5	227.3	234.9	241.7	160.5	233.8	204.5	204.8
SIRPG	145	160.7	153.5	145.8	139.1	146.2	137.1	139.9
SIRT1	318.2	347.7	291.5	423.9	336.1	292.4	493.6	447.6
SIRT2	134.8	143.3	156.5	129.8	123.4	119.9	133.8	138
SIRT2	117.8	119.4	126.8	126.5	116.6	96.4	120.1	122.1
SIRT3	117.9	115.6	127.7	125.8	120.9	96.7	125.9	118.1
SIRT3	120.1	129.9	134.9	111.8	119.3	111.4	130.9	118.6
SIRT4	178.8	174.8	158.4	176.8	157.3	122.7	158.5	169.9
SIRT5	136.8	139.8	147.8	157.6	126.8	122.3	132.5	147.1
SIRT5	123	130.2	129.1	135.1	126.6	109.3	146.6	130.4
SIRT5	166.7	185.9	205.9	195.2	172.7	177.6	189.2	205.3
SIRT6	120.2	124.2	128.6	121.5	119	105	135.8	120.7
SIT1	140.2	135.4	123.9	128.8	113.2	109.4	123.5	119.2
SIVA	549.7	603.1	484.7	493.9	410.8	397.2	458.3	349.1
SIVA	285.9	314.3	368.9	289.1	250.7	296.5	269.7	222.4
SIX1	113.3	112.6	118.8	111.5	107	98.7	115.7	119.9

SIX2	101.6	114.3	115.8	108.7	97.4	94.8	112	109.6
SIX3	119.7	117.1	110.3	120.3	110.7	97.1	119.1	118.2
SIX4	121.5	116.2	121.7	123.5	111.3	100.6	112.2	119.5
SIX5	112.6	120.1	119.2	117.3	111.5	106.4	117.3	118.5
SIX6	115.2	121.3	126.4	121.7	117.6	100.4	117.8	118.3
SKAP1	190.3	218.7	269.2	275.3	230.1	210.2	218	206.2
SKAP2	167	143.9	180.2	147.9	137.3	139.1	167.2	139.2
SKI	144.4	157.5	152.7	164.1	155.8	133.2	143.3	149
SKIL	111.1	117.8	109.3	112	106	98.7	119.6	108.8
SKIP	124.1	135.5	116.1	119.1	132.6	104.7	135.8	125.1
SKIP	111.2	116.9	120.8	111.6	104.2	102.2	113.5	117.8
SKIV2L	547.1	566.4	475.9	563	392.5	312.9	505	341.5
SKIV2L2	252.3	259	255.9	287.2	214	205	257.2	247.1
SKP1A	420.2	440.7	472.4	490.3	366.5	446.7	410.3	475.4
SKP1A	112.6	111.8	116.7	110.7	108.6	99.3	117.1	118.7
SKP2	249.3	329.9	358.2	291	264	267.2	217.3	200.9
SKP2	118.2	135	142.3	129.2	112.7	117.1	124.9	124.6
SKP2	128.2	115.7	121.1	116.8	104.5	109.6	123.2	124.5
SLA	136.2	135.1	161.4	141.9	131.6	109.4	130.1	116.8
SLA	212.1	253.9	269.6	249.4	206.8	205.9	231	215.2
SLA/LP	137.6	137.6	139.6	136.2	127.9	122.2	124.9	112.9
SLA/LP	116.3	122	124.9	119.7	113.4	102.6	123.6	120
SLA2	119.2	122.1	122.8	133.3	110.9	96.2	127.3	120.5
SLA2	223.8	247.1	189.9	226.3	166.2	195.4	196	172.9
SLAMF1	303.9	452.7	244.9	360.7	355.3	196.7	523.1	569.8
SLAMF6	1497.1	1658.9	1266.9	1546.8	1030.9	803.7	1133.6	898.8
SLAMF7	151.8	133.8	138.9	148.1	124.7	112.3	153.3	152.7
SLAMF8	223.2	152	235.9	247.1	139.3	208.6	215	148.5
SLAMF9	127.2	114.4	117.1	120.3	112.3	98.8	108.9	110.7
SLBP	193.7	208.9	247.1	250.7	224.4	191.5	274.4	318.1
SLC10A1	117.8	111.2	117.3	116.5	104.6	96.2	110.3	112.9
SLC10A2	114.6	112.5	119.1	124.5	114.2	110.2	120.2	108.9
SLC10A3	278.7	268.9	231.4	283.9	202.4	236.9	263.7	280.3
SLC10A4	107.7	108.8	109.1	118.4	109.8	93.9	111.2	109.6
SLC10A5	119.6	110.3	121.7	113.2	108.7	104.9	124.9	111
SLC10A6	113.8	109.8	112.2	117.4	106.9	97.4	109.4	106.7
SLC10A7	113.5	103.3	122.4	117	104.9	94.1	111.4	110.7
SLC11A1	203.6	148.4	430	253.8	151.8	276.7	240.5	126.7
SLC11A1	108.1	115	112.2	100.9	97.6	97.9	119.5	103.3
SLC11A2	372.5	283.2	359.4	314.8	269.9	246.5	297.9	229.3
SLC12A1	114	121.8	125	113	106.5	93.2	111.1	119.8
SLC12A2	152.2	151.8	125.7	157.5	136.1	116.5	168	174.1
SLC12A3	111.7	113.2	113	114.1	104.5	92.5	113.1	103.5
SLC12A4	113.7	136.5	129.7	129.6	121.8	104.5	118.9	126.6
SLC12A5	114.1	123.5	110.2	116.6	112	96.9	120.7	121
SLC12A6	147.9	128.5	138.9	144.8	113.3	114.1	144.1	141
SLC12A6	110.7	106.3	117.8	105.1	105.9	100.1	109.2	110.5
SLC12A7	106.8	109.6	113.2	112.3	112.4	101.8	118.3	111.1
SLC12A8	134.3	140.8	112.6	163.5	125.4	110.2	195.8	166.7
SLC12A9	748.4	831.1	670	702.4	526.1	404.5	479	437.7
SLC13A1	109.6	111.4	120.2	116.8	108.4	98	127.3	114.9

SLC13A2	114.4	117	119.7	120.7	111.3	104.7	127.1	120.4
SLC13A3	157.6	171.2	185.2	175.4	179.3	141.8	173.8	175.8
SLC13A3	109	115.1	115.1	122	120.2	97.9	118.6	120.1
SLC13A3	116.6	109.8	115.6	110.3	101	97.4	126.8	107.8
SLC13A4	107.8	115.9	117.9	111.7	106.5	105.9	126.5	124.6
SLC13A5	111.8	109.9	123.8	130.1	115.8	99.8	130.5	125.9
SLC14A1	117.8	117.9	120.5	118.8	120.8	102	118.4	111.4
SLC14A2	119.4	112.7	116.3	113.6	118	101.4	116.2	126.4
SLC15A1	114.7	116.5	124.4	125.9	113.6	98.7	120.1	113.9
SLC15A2	119.6	124.1	119.3	120.6	117.2	100.6	127.2	121.2
SLC15A3	623.3	337.9	657.9	509.9	240.5	380.2	485.9	259.2
SLC15A4	409	316.7	529	431.9	341.1	345.2	482.8	342.2
SLC16A1	110.8	116.9	127.3	132.6	109.9	98.6	112.9	109.7
SLC16A10	204.7	222.5	201.7	218.9	250.6	204.5	224.4	263.2
SLC16A11	122.7	114.1	124.9	112.6	113.5	97.6	121	122
SLC16A12	3360.6	2950.4	3107	3757	4236.3	1693.4	3990.7	4026
SLC16A13	115.3	121.3	118.3	135.3	111.9	112.3	111.5	125.5
SLC16A14	109.8	116.2	121.1	117.9	104.7	97.2	107.7	114.2
SLC16A2	127.1	119.2	127	121.5	107.2	96.9	126.6	120.6
SLC16A3	637.9	559.1	1012.4	858.7	328.3	1123.8	550	265.7
SLC16A4	111.8	135.3	130.5	135.7	111.4	103	127.6	114.8
SLC16A5	191.1	174.1	248	172	149.8	144.8	152.9	146.7
SLC16A6	145.9	185.3	163.5	247.2	202.1	261.6	231.1	140.6
SLC16A7	119.3	121.6	121	131.1	117.7	103.3	134.8	125.3
SLC16A8	108.9	117.1	121.5	124.5	108.7	100.8	122.5	118.8
SLC16A9	109.6	125.3	117	116.2	112.7	104.1	116.8	119
SLC17A1	127.2	104.8	133.2	123.6	111.8	105.3	121.7	112.6
SLC17A2	116.6	117.7	115.1	129.7	111	100.5	122.8	115.9
SLC17A3	119.2	115.6	124.1	136.9	106.4	111.6	131.4	129.9
SLC17A4	103.2	112.7	113	115.2	108.1	97.7	125.5	110.2
SLC17A5	106	104	115.9	113.6	111.3	95.8	119.2	120.3
SLC17A6	117.4	118.5	114.5	113.5	112.2	102.6	116	112.4
SLC17A7	118.5	120.1	123.3	121.4	112.1	105.1	120.3	124.1
SLC17A8	110.5	114.3	108.5	112.4	108.6	103.7	110.7	116
SLC18A1	129.2	120.2	123.9	121.5	111.9	106.3	121.5	111.7
SLC18A2	116	111.6	111.5	111.6	106.3	106.2	110.8	113.1
SLC18A3	102.5	115	114.7	121.1	105.7	96.4	109.6	104.3
SLC19A1	119.7	126.4	135.6	124.7	109.1	105	122.8	118.6
SLC19A2	111	116.3	121.5	122.1	104.1	101.9	109.7	113.6
SLC19A3	111.5	110.8	121.1	127	112	95.4	122	119.1
SLC1A1	110.8	108.8	116.5	113.9	103.2	97.6	116.2	118.8
SLC1A2	122.7	130.8	134.7	138.1	125.2	121.2	116	118
SLC1A3	183.4	171.8	128.7	196.1	156.7	102.3	226.1	159.9
SLC1A4	123.9	132.5	135.1	131.7	121.1	111.9	142.4	126.9
SLC1A5	165.5	139.5	168.6	142.9	121	172.7	145	182.6
SLC1A6	108.7	108.6	106.1	113.1	110	96.2	110.5	110
SLC1A7	129	125.2	135.8	124.6	126.2	106.2	119.2	124.2
SLC20A1	1054.4	1152.8	788	1200.4	955.6	564.7	1245.2	1162.3
SLC20A2	137.1	154.5	158.8	149.8	140.6	131.3	173.2	129.5
SLC22A1	120.9	124	129.5	141.9	113.2	107	117.3	128.8
SLC22A1	123	122.6	115.1	119.5	115.9	101.5	124	124.4

SLC22A10	114.7	111.9	108.5	114.9	101.2	93.2	126.1	115.7
SLC22A11	119.1	113.6	112.8	134.1	102.4	98.1	120.2	115
SLC22A12	113.4	117.2	119.4	119.3	110.8	92.5	121.1	116.7
SLC22A12	113.6	114.3	121.2	132.5	107.1	94.8	115.8	119.1
SLC22A12	110.2	116.4	120.6	111.8	102.5	97.9	122.1	115.3
SLC22A13	128.1	115	113.7	120	108.7	109	114.3	112.2
SLC22A14	120.4	120.6	113.8	117.5	113.4	101.9	115.8	122.4
SLC22A15	121.4	116.3	116.2	115.9	106.6	99.2	111.2	107.4
SLC22A16	107	116.9	109	113.8	112.5	93	115.3	114
SLC22A17	127.7	110.5	121.9	121.1	107.9	105.6	119.8	107.4
SLC22A17	214.6	181.6	228.9	208.7	149.4	169	173.7	158.8
SLC22A18	109.7	115.5	132.8	117.4	109.2	104.9	116.1	118
SLC22A18	141.4	139.3	177	146.8	124.1	122.1	133.3	138.2
SLC22A18A	125	124.1	142.4	128.4	115.9	115.4	131.4	127.9
SLC22A2	114	114.8	115.1	122.2	114.9	103.5	126.3	115.2
SLC22A2	118.1	108.1	120.1	110.5	108	106.9	105.7	107.2
SLC22A3	109	116	118.1	118.5	107.3	97.2	111.3	111.5
SLC22A4	116.6	106.4	130.3	121.6	115.1	113.7	116.9	114.6
SLC22A5	131.9	142.6	130.6	138.5	127.1	117	135.9	134.6
SLC22A6	114	122	115	125.8	107.7	100.1	114.8	123.7
SLC22A6	111.8	120.6	117.9	112.4	104.3	100.4	114.9	109.3
SLC22A7	111.7	107.2	120.8	110.7	108.3	99.7	126.5	115.3
SLC22A7	109.4	115.6	106.6	110.3	102.8	97.5	108.1	106.5
SLC22A8	121.9	113.2	113.9	119.3	111.6	105.4	107.7	114.7
SLC22A9	103.3	109.4	115.8	114.6	110.6	100.2	112.3	116.9
SLC23A1	128.4	116.9	128.6	112.8	107	122.6	121.4	119
SLC23A1	123.6	125.6	131.1	128.3	118.8	109.1	128.9	141.7
SLC23A2	122.4	115.7	114.7	115.3	113.6	111	117.2	114.9
SLC23A2	174	153.2	200.7	249.9	158.2	273.5	253.8	185.6
SLC23A2	115.4	111.9	120.2	122	112.7	96.4	115	113.5
SLC23A3	131.1	124.6	144.5	146.3	117.1	103.6	132	131.2
SLC24A1	120.3	120.3	129.8	128.1	111.3	107.6	125	117.9
SLC24A2	116.1	108.5	114.7	123.2	106.5	96.8	115.4	107.5
SLC24A3	108.2	121.1	130.6	123.4	117.4	106.5	135.5	125.6
SLC24A4	114.5	101.9	115.4	113.3	106.3	105.8	117	110.5
SLC24A4	114.6	119	124.4	119.8	110	106.4	131.8	115.6
SLC24A4	123.1	123.5	121.2	134.5	114.9	107.8	119.7	122.4
SLC24A5	113	113.6	118.6	124.5	109.8	105	124.4	115.7
SLC24A6	511.7	444.2	496.2	628.1	454.9	433.6	547.6	689.2
SLC25A1	269.4	315.5	255.2	266.4	210.5	210.6	289.4	243.8
SLC25A10	128.5	132.2	128.1	122.2	116.3	97.2	117.1	123
SLC25A11	148.2	141.5	132.7	156.9	131.1	123.2	134.6	138.8
SLC25A12	174.2	161.8	162	163.9	142.1	134.9	147.8	148.5
SLC25A13	173.2	143.3	151.3	166.4	133.6	121.3	160.6	152.3
SLC25A14	155	142.9	147.8	162	132.1	117.3	129.8	134.9
SLC25A14	131.7	152.6	146	152.1	121.5	109.8	122.5	120
SLC25A15	128.2	135.5	125.1	126.9	116.4	103	126.1	126.3
SLC25A16	122.2	118.8	132.3	128.2	112.1	97.6	116.5	108.7
SLC25A17	133.6	143.3	151.5	157.6	135.6	123.7	136.4	154.3
SLC25A18	112	116.4	108.3	120.7	101.6	96.3	113.6	117
SLC25A19	213.6	204.9	310.8	212.5	149	218.1	140.6	187.9

SLC25A2	109.5	114.9	115.1	115.1	105.8	95.5	113.9	121.4
SLC25A20	129	132.8	147.2	125.7	115.8	126.3	124.4	124.9
SLC25A21	107.1	111.2	110.1	124.6	110.4	95.2	116.5	109.3
SLC25A22	223.6	238.2	184.7	219.9	198.1	136.7	193.6	206
SLC25A23	399.7	427.1	350.2	384.9	243	257.1	256.4	201.4
SLC25A24	122.9	114	121.2	119.2	110	99.7	114	122.7
SLC25A24	114.5	123.7	126.2	132	113	101.9	123.9	120.9
SLC25A24	130.8	116.5	146.1	134.4	125.3	126.5	134.4	136.7
SLC25A25	341.4	365.3	329.1	368.7	277.5	266.5	319.4	310.9
SLC25A25	117.7	119.6	116.9	121.3	107	109.4	114.4	121.4
SLC25A25	116.7	123.1	114.1	124.9	110.8	105.3	125.9	125.1
SLC25A25	132.8	131.8	123.1	125.2	122.7	105.3	129.4	118
SLC25A26	190.1	165.7	171.7	190.6	171.7	163.1	168.2	170.8
SLC25A26	126.2	144.4	133.5	136.9	133.9	124.1	140.1	129.9
SLC25A26	133.1	134	143.7	129.2	121.9	109.1	126.7	130
SLC25A27	121.3	109.6	121.6	122.3	113.9	103.7	124	118.8
SLC25A28	499.5	495.3	635.3	467.8	407.3	437.4	449.4	459.2
SLC25A29	124.5	125.2	119.8	137.8	113.9	99.8	124.5	135.3
SLC25A29	135.9	130.3	142.2	129.8	131.1	111.4	133.4	153.5
SLC25A3	1589.2	1908.4	1907	1470.4	1516.9	1497.3	1674.2	1581
SLC25A3	116.4	130.1	120.8	111	129.1	111.1	129	125.4
SLC25A30	120.5	118.2	117.9	120.8	115.9	108	118.1	124.9
SLC25A31	121	116.4	124	111.6	107.4	101.2	113.6	116.1
SLC25A32	119.8	115.2	127.3	119.4	113.4	104.7	109.3	117.5
SLC25A34	122.9	120.8	126.7	129.3	114.7	103.4	139.3	127.9
SLC25A35	115.8	117.7	131.3	121.7	110.8	103.8	126	116.3
SLC25A36	244	215.4	203.8	270.4	217	142.5	236.1	240.6
SLC25A37	161.9	183.5	180	169.7	148.3	145.3	166.6	154.2
SLC25A4	379.5	461	256.6	374	290.3	197.5	326.4	270
SLC25A40	165.3	169.4	205.2	166.7	139.3	135.2	155.2	139.9
SLC25A42	752	811.9	648.5	680.9	441.8	483.9	491.6	457.2
SLC25A43	218	198.4	255.7	184.3	155	139	156	142.2
SLC25A45	115.4	118.6	124.1	117.2	112.9	106.2	123.5	119.5
SLC25A5	3591	4106.7	3326.7	3813.9	2807.3	2532.3	4252.2	4511.9
SLC26A1	114.8	122.6	120	124.5	117.8	100.6	125.6	126.7
SLC26A1	122.4	126	116.2	116.9	105.5	106.5	128.4	121.7
SLC26A1	105.9	119.9	119.6	115.7	111.8	100.1	108.3	111.8
SLC26A10	118.6	111.1	117.4	115.8	112.5	106.1	126.5	119.6
SLC26A11	113.1	116.5	122.2	115.2	106.5	103.1	114.4	113.6
SLC26A11	244.4	265.9	155.3	267.6	196.6	149.8	313.9	291.2
SLC26A2	121.8	116.7	132	122.3	122.2	120.4	123.2	128.2
SLC26A3	109.4	117.9	118.6	111.3	110.1	95.4	114.2	114.5
SLC26A4	113	113.9	121.5	122.1	109.2	107.6	109.3	113.6
SLC26A5	115.4	119.6	114.4	109.8	113.9	103.9	110.3	109.2
SLC26A5	122.3	127.3	119.7	120.7	104.9	104.7	129	116.5
SLC26A5	123.7	123.6	115.5	115	112.1	105.2	117.7	110.8
SLC26A6	220.5	210.1	237.7	238.1	180	157.3	177.2	174.1
SLC26A6	119.6	107.3	115	114.6	97.2	88.2	114.7	103.4
SLC26A6	119	121.7	124.6	112.8	111	97.1	115.3	109.7
SLC26A7	111.8	114.6	118.5	116.3	105.1	102.5	116.7	114
SLC26A7	117	121.2	122.8	112.2	109	104	124.4	115.9

SLC26A8	108.5	117.3	128.5	133.2	111	100.7	126.8	116.4
SLC26A8	109	115.5	107.7	109.5	111.7	93.8	117.1	113.2
SLC26A9	102.7	117.2	116.4	123.1	110.2	104	107.5	117.5
SLC26A9	112	126.9	123.5	110.2	104.7	105.5	136.9	124.5
SLC26A9	114.5	108.7	115.8	113.8	104.5	100.5	116.5	112
SLC27A1	170.9	173.1	215.3	165.1	140.9	158.8	166	142.2
SLC27A2	113.5	124.7	128.4	118	120.4	108.1	122.6	118.1
SLC27A3	222.6	231.2	261	194.5	173.2	145.6	147.4	154.5
SLC27A4	120.6	113.6	140.8	151.4	113.3	106.7	128.3	121.6
SLC27A5	146.6	127.7	139.6	133.4	114.9	119.3	125	135.2
SLC27A6	116.6	123.1	119.3	114.5	104.7	103.9	113.8	121.6
SLC27A6	117	130.7	119.1	114.6	112.5	104.5	107.1	125.8
SLC28A1	109.5	118.4	113.6	121.5	102.1	96.3	110.7	101.6
SLC28A1	132.7	135.2	134	127.6	129.6	117.5	132.8	126
SLC28A1	112.6	119.6	126.1	120.8	110.1	105.8	119.6	110.7
SLC28A3	115.1	106.6	119.5	120.6	106.5	101.6	125.9	101.9
SLC29A1	120.6	114.6	130.5	119.1	124.3	109.6	125.1	121.3
SLC29A1	118.3	123.3	120.4	119.2	108.4	102.6	123.4	115.9
SLC29A1	112.3	124.1	123.9	123.6	104.8	106.6	116.8	126.3
SLC29A2	147.6	143.2	155.8	159.4	140.1	128.4	143.9	136.8
SLC29A3	145.3	137.5	138.5	126.3	126	118.2	144	137.3
SLC29A4	116.1	115.4	114.5	116.5	110.8	96.8	122.8	121.1
SLC2A1	290.9	320.1	267.8	298.3	240.5	186.6	285.6	311.1
SLC2A10	112.6	110.4	115	115	105.8	96	114.4	109.4
SLC2A11	115.8	115.2	130.6	119.2	116.4	102.3	114.4	109
SLC2A11	129.9	120.9	145.6	124.8	117.7	111	130.8	113.8
SLC2A11	137.6	132.5	130	139.4	128.8	115.2	137	136.5
SLC2A12	109.2	109.6	118.7	119.3	102.7	101	122.6	111.4
SLC2A13	108.9	118.1	104.4	124.3	105.7	101.2	118.2	107.1
SLC2A14	131.3	131.1	118.6	146.8	130.7	108.4	122.8	131.5
SLC2A2	110.4	117.2	110.5	115.4	109.8	101.2	115.5	113.3
SLC2A3	1626.7	1834.2	1210.5	1922.6	1608	1257.6	2280.4	2227.9
SLC2A4	115.7	115.6	125.4	129	115.9	105.7	119.1	124
SLC2A4RG	147.9	161	167.6	175.7	133	125.9	164	164.4
SLC2A5	198.2	197.4	200.9	191.8	144.7	155.7	153	136.2
SLC2A6	339.3	285.1	225.7	256.6	157.2	143	155.8	144.6
SLC2A7	105.6	107.7	114.7	110.5	100.6	105.6	109.8	110.2
SLC2A8	164.4	156.1	209.9	178.5	143.4	167.9	160.5	172.5
SLC2A9	112.7	119.6	116.2	133.3	119.3	116	133.5	129.5
SLC2A9	122.9	106.4	112.4	135.3	116.8	98.4	121.1	122.8
SLC2A9	117.1	122.9	138.7	119.2	107.5	103.1	123.9	112.7
SLC30A1	161.2	153.6	151.8	183.9	177.9	183.2	286	197.1
SLC30A10	112.2	124.7	119.8	117	105.6	99.7	119	114.3
SLC30A2	113.4	119.3	112.4	116.2	110.9	95.1	123.4	119
SLC30A2	116.7	120.4	131	112.3	114.3	106.6	124.3	122.3
SLC30A3	119.9	130.3	128.1	130.6	112.7	104.8	130.8	125
SLC30A4	114.1	120.9	120	122	112	98.3	122.6	114
SLC30A5	269.6	335.9	287.7	317	250.7	200.7	276.4	257.3
SLC30A5	138.8	138.6	131	133.9	113.4	116.4	132.3	134.2
SLC30A5	118.3	123.6	123.8	117.4	110.3	106.9	126.2	118.9
SLC30A6	118.7	128.3	130	130	110.7	103.5	123.6	110.7

SLC30A7	379.3	330.8	361.5	391.9	434.7	306.1	471	506.8
SLC30A8	116.6	104.3	105.3	123.6	102.5	105.5	113.8	115.3
SLC30A9	260.6	250	288.4	237	217.3	204.2	225.4	220.8
SLC31A2	484.6	344	448.6	650.5	365.2	571.4	689.6	392.3
SLC32A1	108.6	104.3	108.3	113.8	103.6	88.6	114.4	111.5
SLC33A1	139.1	134	144.7	155.6	133.1	117.1	128.1	129.4
SLC34A1	107	108.8	119.6	115.6	106	99.7	119.3	111
SLC34A2	109	110.4	109.5	110.9	107.8	95.5	114.2	111.9
SLC34A3	116.6	127.7	121.1	126.3	115.8	95.4	121.7	119.5
SLC35A1	555.4	647.1	693.2	740.9	526.2	518.1	532.6	398
SLC35A2	248.7	271.2	236.1	283.5	214.4	254.9	284.9	324.3
SLC35A2	118.2	117.8	122.8	112	107	100.1	123.7	104.4
SLC35A2	122	123.5	129.3	126.5	105.3	110.8	135.7	121.6
SLC35A2	116.1	127.2	122	110.1	106.1	105.7	128.5	121.8
SLC35A3	195.2	206.7	216.8	189.3	190.9	165.7	193.3	180
SLC35A4	275.3	295.9	274.3	339	179.1	307.6	339.6	306.4
SLC35A5	392.7	357.5	523.6	478.3	328.6	363	380.3	285.2
SLC35B1	534.8	489.8	777.3	529.7	392	511.8	487.7	560.2
SLC35B2	396.8	421.7	336.5	423.4	383	256.2	406.8	323.8
SLC35B3	297.8	326	311.5	340.6	283	258.4	414.8	477.9
SLC35B4	136.8	129.2	143.3	144.3	133.5	119.3	134.8	143.7
SLC35C1	168	192.4	169.9	195.3	138.8	138.2	196.1	178.9
SLC35C2	180.2	183.9	190.4	193.9	152.3	165	177.9	149
SLC35C2	267	280.9	261.4	238	191.2	192.3	241.9	289.9
SLC35C2	112.2	109.9	111	112.5	114.7	94.5	118.4	112.5
SLC35D1	110	122.3	116.6	124.8	109.8	100.3	122.9	113.2
SLC35D2	203.5	215.2	231.4	189.5	168.3	173.2	170	203.2
SLC35D3	120.9	113.2	120.6	111.6	109.2	101.2	110.3	111.6
SLC35E1	783.4	531	681	700.7	740	420.1	697.4	727.1
SLC35E2	126.9	132.2	123.5	156	133.6	107.3	144.8	130.8
SLC35E3	356.6	315.2	326.9	373	291.2	225.6	252.1	211.8
SLC35E4	118.4	114.9	125.3	134.8	105.2	105.2	117.3	115.6
SLC35F1	115.5	115.5	117.3	126.3	107.4	98.6	117.6	111.4
SLC35F2	153.4	150.4	163.3	159.7	136.9	128.1	149.5	149.6
SLC35F3	112	123.5	114.6	118.9	106.9	101.2	116.7	109.6
SLC35F3	118.4	113.3	123.4	116.4	117.5	102.4	118.9	114.3
SLC35F4	119.4	112.4	121.2	117.1	114.8	98.2	116.7	125.8
SLC35F5	140	134.9	138.9	145.7	122.1	122.1	142	150.3
SLC36A1	130.2	123.2	137.9	127.4	115.5	121	120.8	120.8
SLC36A2	110.3	112.7	104.9	107.6	106.8	94	110.6	117.1
SLC36A3	114.7	116.6	112.7	118	107.6	105.5	119.9	116.2
SLC36A4	310.4	297.6	299.4	352.9	293.7	232.5	380.4	337.2
SLC37A1	133.3	126.4	147.3	138.5	122.5	123.2	137.8	139.5
SLC37A2	118.7	112.8	118.5	119.3	106.6	97.1	117.7	115.2
SLC37A3	196	188.3	218.6	187.4	179.2	151.4	207.8	198.2
SLC37A3	121	121	117.4	120.8	111.5	108.2	122.4	116.7
SLC37A4	254.4	262.9	261.9	227.2	198.4	174.9	227.9	232.6
SLC38A1	122.5	125.4	118.6	126.1	115.8	104.4	134.5	127.2
SLC38A1	828.2	997.7	653.5	1117.6	1527.2	401.6	1419.4	1836.8
SLC38A2	1381.5	1218.8	574.7	1452.3	1011.6	717.3	1967.4	1853.4
SLC38A3	114.7	122.3	119	106.7	99.9	95.8	117.7	127.1

SLC38A5	132.5	137.9	148.8	130.2	124	119.9	132.2	138.8
SLC38A6	210.4	137.5	214.1	238	130.6	245.4	248.7	164.1
SLC39A1	487.9	467.8	474.4	498.3	277.7	338.9	378.5	345.4
SLC39A10	145	173.4	136.3	161	168.6	128.9	164.7	166.5
SLC39A11	136.8	130.6	198.5	144.9	120.9	139.1	135.7	120.2
SLC39A12	116.1	112.6	117.5	112	109.4	101.7	126.7	112.4
SLC39A13	127.3	130.6	125.8	136.1	121.2	117.9	128.5	137
SLC39A14	132.8	137.2	124.8	141.6	130.9	118.3	162.9	149.2
SLC39A2	122.1	124.2	121	129.2	108.4	102.2	123.3	114.4
SLC39A3	134.3	129.7	145.4	143.1	130.8	105.4	143.6	129.9
SLC39A3	342.7	376.6	418.3	364.8	274	278.9	259.9	225.2
SLC39A3	127.4	146.1	132.7	133	116.9	123.3	136.5	135.8
SLC39A4	113.2	108.6	104.1	112.9	107.8	94.2	111.9	110.1
SLC39A4	113.9	123.4	117.7	133.9	126.2	119.9	132.3	116.5
SLC39A5	123.9	115.4	122.2	117.1	111.2	99.4	115.9	113.5
SLC39A6	148.9	172.2	154	158.5	176.6	132.1	173.5	207.7
SLC39A7	118	126.6	138.2	136.4	114.1	120.5	136.7	156.7
SLC39A7	120.4	135.7	123.7	138.8	116.7	114.2	126.5	134.5
SLC39A8	166.8	122.2	178.3	158	116.1	172.8	171.7	123.1
SLC39A9	121.5	127.4	125.9	126	123.4	116.1	124.5	112.9
SLC3A1	113.1	117.4	117.3	111.2	105.1	99	114.9	109.3
SLC3A2	214.8	258.4	203.3	305.5	283.9	328.6	350.7	488
SLC3A2	131	140.2	132.8	141.1	120.6	125.1	137.5	149.7
SLC3A2	358.5	371.8	261.1	414.9	309.1	474.8	859.1	808
SLC40A1	308.6	276.7	298	262.6	203.9	239.9	259.3	195.1
SLC41A1	151.6	152	141.1	144.7	138.8	121.4	164.2	172.4
SLC41A2	118.3	135.6	109.7	133.8	120.2	109	149.9	114.4
SLC41A3	380.5	417.2	375	400.1	331.7	271.3	440.5	476
SLC41A3	113.2	114.1	118	112.6	104.9	93.9	118.5	117.8
SLC41A3	195	195.1	202.8	251.9	194.7	164.5	255.3	215.8
SLC43A1	102.4	105.3	122.8	124.7	109.8	99.4	120.5	104.7
SLC43A2	236.4	152.1	347.1	247.4	129.5	276.3	193.8	145.2
SLC43A3	242.2	197.2	180.8	181.4	147.7	133.5	237	154.9
SLC43A3	115.7	119.9	120.7	134.6	102	100.3	115.6	122
SLC43A3	133	121.1	128.6	140.8	120.5	108.2	133.1	121.7
SLC43A3	111.7	118	120.4	129.4	110.5	104.3	121.8	117.3
SLC44A1	1125	1000.4	568	1961.5	1143.8	539.5	1886.5	635.5
SLC44A1	671.7	520.2	254.9	1032.3	410	414.9	835.3	304.4
SLC44A2	864.2	1020.5	926.9	865.3	710.9	637.4	923.3	1112.7
SLC44A3	119.3	119.9	121.6	125.5	114.8	104.7	127.6	117.1
SLC44A4	110.6	115.9	119.4	121.6	117.4	98.5	120	111.5
SLC44A4	4178.9	2891.5	2961.8	3021.1	3727.4	1593.9	3633.3	2737.1
SLC44A4	112.7	112.1	109.4	106.6	107.8	97.3	119.3	114.3
SLC44A5	111.1	116.8	118	118	114.2	102	112.4	111.8
SLC45A1	120.6	123	127.1	133.5	124.4	111.6	132.9	134.7
SLC45A2	123	124.9	133.4	134.9	116.1	104.7	119.2	124.9
SLC45A2	113.3	114	112.5	112.3	109.3	97.8	114.1	111.2
SLC45A2	114.6	125.3	129.9	123.6	118.2	104.1	130	118.3
SLC45A3	120.9	120.5	131.1	124.9	114.3	112	122.3	135.9
SLC45A4	159.7	167.8	158.1	163.3	137.4	134.5	163.4	157.2
SLC4A1	124	124.3	129.7	126	108.8	108.8	119.1	119.7

SLC4A10	115.2	121.4	124.3	120.8	112	101.2	116.7	115.1
SLC4A11	121.2	124.3	120	126.3	110.7	104.8	128.2	121.1
SLC4A1AP	134.2	120.6	143.8	136.2	127.6	114.4	137.6	128.2
SLC4A2	143.8	161.3	128	155.5	130	115.2	176.3	143.8
SLC4A3	119.6	124.4	121.9	132.5	110.5	104.8	112.9	121
SLC4A3	124.5	120.4	133	119.8	120.9	111.8	125	127.7
SLC4A4	114.9	118.6	123.6	129.3	108.1	100.9	122.9	116.3
SLC4A5	451.5	367.8	407.6	438.2	391.1	194.2	417.7	415.9
SLC4A5	112.1	107.3	126.2	118.6	111.1	98.1	116.9	114.1
SLC4A5	116.1	123.1	118.8	111.8	109.2	99.7	120.8	111.8
SLC4A7	592.2	478.8	613.9	617.2	416.8	454.6	558	488.5
SLC4A8	110.7	114.7	120.8	132.4	109.6	101.7	123.9	116.3
SLC4A8	121.3	104.5	109.3	112.2	108.9	92.2	127	113.1
SLC4A9	121.3	109.2	110	120.3	105.1	97.3	108.8	109.7
SLC5A1	109.5	106.5	114.1	115.5	102.7	96.1	125	111.1
SLC5A10	115.3	112.1	111.3	113.2	103.7	91.4	114.1	113.8
SLC5A10	109.9	118.5	112.7	114.6	108.4	95.5	115.9	111.1
SLC5A10	107.1	113.1	110.8	118.9	110.1	94	108.6	114.3
SLC5A10	113.1	130.4	129	136.4	105.5	107.4	132.1	123
SLC5A11	128	119.3	115.4	121.3	111.1	109.5	117.2	119.2
SLC5A12	118.5	109.5	106.6	112.5	103.6	96.2	120.2	107.5
SLC5A12	117.7	120	111.9	120.3	112.2	94	117	109.8
SLC5A12	113.4	110.2	115.3	109.3	110.4	97.3	109.6	108.4
SLC5A2	109.6	103.8	102.4	115.8	104.8	97.6	115.5	101.4
SLC5A3	242.2	192	118.3	311.6	196.6	114.5	355.6	314.8
SLC5A4	111.5	111.7	116.7	117.7	106.5	99.9	114.9	112.8
SLC5A5	110.7	118.3	112.7	124.8	105.7	101.7	121.7	123.2
SLC5A6	230.9	247.8	227.4	280.1	191.5	189	269.7	242
SLC5A7	114.4	114.6	111.6	119.4	105.7	94.1	108.2	110.1
SLC5A8	205.2	182.3	184.3	185.9	176.3	133.9	191.3	200.2
SLC5A9	118.3	122.6	109.6	124.7	106.6	98.5	113.7	112.5
SLC6A1	111.5	111.1	119	116.5	110.9	93.5	120.4	120.1
SLC6A11	114	116.9	113.3	118.2	109	98.5	128.6	122.6
SLC6A12	140.3	138.9	142.2	131.4	120.7	109.5	133.6	131.2
SLC6A13	120	132.5	126.3	122.1	119.7	100.9	135.6	128.2
SLC6A14	109.4	112.7	110.4	120.1	103.6	90.3	114.9	110
SLC6A15	122.9	121	131.7	114.1	111.4	99.3	116.7	127.5
SLC6A15	116.6	112.8	116.6	121.1	113.6	97	114.2	109.6
SLC6A15	114.2	109.2	110.7	113.1	110.6	95.8	116.7	117.3
SLC6A16	182.3	161.1	178.5	180.4	177.2	122.1	171.5	166
SLC6A17	111.5	113.2	113.2	127.2	116	100.8	124.4	108.6
SLC6A18	110.6	112.5	120.3	113	114.3	101.5	123.6	110.6
SLC6A19	120.8	108.8	128.9	112.4	114.2	95.4	105.3	120.5
SLC6A2	109.1	113.8	117.2	123.5	101.1	93.5	114.7	113.4
SLC6A20	109.7	120.6	120.3	127.2	112.6	101.5	118.3	119.4
SLC6A20	109	117.6	124.7	124.5	109.3	99.5	118.9	113.7
SLC6A20	109.8	115.7	117.8	118.3	105.3	98.6	121.3	113.3
SLC6A3	114.6	100.3	112.6	122	107.7	101	120.3	118.7
SLC6A4	112.6	113.9	120	115.4	104.6	97.6	120.8	115.9
SLC6A5	110.2	105.9	106.4	116.1	110.6	96	110.9	98.3
SLC6A6	140.2	152.8	127.5	184.7	131.5	141.5	217.8	139.4

SLC6A7	119.3	110.7	131.8	117.6	123.4	105.1	123.7	115.8
SLC6A8	125	121.4	118.4	132	107.5	109.1	133.6	128.1
SLC6A9	114.9	116.7	112.2	122.7	113.1	99.8	124.6	112.8
SLC6A9	111.5	120.4	119.8	118.4	108.5	106.4	118.4	111.8
SLC6A9	122	113.2	115	126.7	107.5	105.5	116.3	124.7
SLC7A1	249.4	270.9	190.7	282.6	218.1	235	287.9	364.8
SLC7A10	108.1	112.8	114.1	119.2	112.9	91.4	123.1	115.9
SLC7A11	237.2	266.9	130	460.9	251.3	392	433.8	200.3
SLC7A13	113.2	118	112.6	120.7	103.7	101.9	122.5	115.6
SLC7A14	117.3	114.1	129.1	120.5	110.5	107.5	122	120.4
SLC7A2	124.7	126.6	123.1	120.7	112.5	98	119.2	119.5
SLC7A2	103.8	110.2	106.3	116.7	113.4	101.1	112.8	109.7
SLC7A2	99.2	109.9	111.9	115.1	105.5	93.8	110.2	111.2
SLC7A3	115.1	121.2	123.1	116.5	104	99.2	129.8	122.6
SLC7A3	113.9	123.2	118.1	119.5	108	98.4	123	114
SLC7A4	111.5	108.8	109.4	120.8	99.8	99.7	111.5	116.1
SLC7A5	249.4	238.1	224.2	299.8	193.4	337.2	446.9	546.8
SLC7A6	218	282.9	264.7	289.8	222.4	205	202	249.1
SLC7A6	223.8	257.5	222.7	191	243.4	169.7	181.7	225.5
SLC7A6OS	157.6	132.2	149.8	167.6	132.7	110.3	135.2	156.4
SLC7A7	247.1	128.2	954.3	162.8	121.8	159.8	132.7	117.6
SLC7A8	110.8	118.8	119.7	134.1	121.2	111	112	120.7
SLC7A8	116.5	111	115.1	124.1	111.7	104.4	108.7	113.4
SLC7A8	116.6	135.2	165.6	129.1	112.6	184.2	130.4	119.5
SLC7A9	115.8	133	125.6	127.9	122.8	107.9	133.9	121.7
SLC8A1	112.6	109.2	118.4	116.5	123.8	98.4	117.8	119.8
SLC8A2	112.6	115.8	117.8	135.3	104.9	97.9	119.5	110.4
SLC8A3	105.5	119.8	114.3	121.1	104.7	100	114.3	108.3
SLC8A3	122.2	115.4	116.2	120.9	109.2	101.9	120.1	113.2
SLC8A3	111.6	119.8	117.6	118.8	106.8	103.2	125	112.7
SLC9A1	388.1	408.4	332.2	370.7	281.5	206.3	339.3	272.9
SLC9A10	113.5	116	115.1	112.5	111.5	96.3	119.2	117.5
SLC9A11	116.3	109.7	112.6	121.5	101.6	101.3	118.1	107.2
SLC9A2	112.3	124.7	125.2	134.6	117.6	107.6	120.5	115.6
SLC9A3	124.7	124.6	124.2	128.2	118.4	105.6	138.4	124.9
SLC9A3R1	721	847.8	755.8	745.1	576.2	487.7	587.2	565
SLC9A3R2	127.3	112.5	121	118.7	105.7	99.2	114.1	106.1
SLC9A4	136.6	142.5	165.1	158.9	118.1	114.4	128.2	145.7
SLC9A5	111.2	119.5	116.4	117.5	103.9	98.5	120.4	122
SLC9A6	123	108.4	129.1	118.2	119.3	97.4	115.2	120.3
SLC9A6	106.5	111.6	118	113.6	107.8	99.6	121.7	114.1
SLC9A6	272.8	307.6	250.2	285.3	274.8	286.3	263.7	262.5
SLC9A7	120	120.8	112.8	125	107	99.7	129.3	124.5
SLC9A8	156.7	155.4	155	156.7	148.5	120.3	168.8	173.5
SLC9A9	128.1	127.6	161.8	139.6	121.7	120.8	144.2	124
SLCO1A2	108.5	110.7	112.9	107.1	102.5	92.9	112.8	115.9
SLCO1A2	110.7	113.6	114.3	123.6	106.4	100.1	121.4	124.6
SLCO1A2	112.1	111.6	108.1	117.1	106.8	94.6	110.6	117.9
SLCO1A2	110	127.5	128.1	113.8	106	94.9	122.2	127
SLCO1A2	113.7	117.9	117	122.5	116.3	106.6	120.2	117.9
SLCO1B1	114.6	116.5	114.3	116.7	104.1	98	111.1	106.4

SLCO1B3	109.1	115.8	106.6	115.2	106.7	99.7	121	107.1
SLCO1C1	106.7	113.6	118.7	121.2	113.3	98.5	109.3	105.2
SLCO2A1	120.2	119.4	113.8	120	110.9	108.1	123.1	111.3
SLCO2B1	132.2	121.3	410.2	139.4	111	143.1	139.2	120.5
SLCO3A1	130.8	137.2	133.3	129.9	130	108.7	136.6	129.3
SLCO3A1	123.9	118.7	121.1	131	115.8	134.2	147.1	132.1
SLCO4A1	132.6	149.4	136.4	166.4	141.9	128.2	181.4	171.8
SLCO4C1	120.8	116.7	124.5	134.6	114.9	102.4	120.9	116.4
SLCO5A1	109	113.4	116.8	121.5	105.2	104.8	118.8	117.7
SLCO6A1	111	120.1	122.7	117	111.1	97.7	119	114.7
SLD5	129.7	119.4	131.1	133.7	116.2	102.5	126.2	121.7
SLFN11	204.9	167.4	206.7	194.1	159.7	136	198.1	186.7
SLFN13	160.2	133.5	158.8	151.6	141.2	124	137.2	119.4
SLIC1	119.6	130.7	125.8	123.4	111.6	108.1	123.4	123.6
SLIC1	123.1	110.6	130.8	114.5	126.9	97.7	125.8	126.5
SLIT1	112.7	117.1	114.6	113.5	104.8	97.1	122.8	118.7
SLIT2	114.3	112.2	122.4	111.8	108.9	94.4	113	110
SLIT3	105.6	110.7	109.7	111.3	106.2	94	111.5	120.2
SLITL2	120.4	104.3	116.1	118.3	115.7	108.6	136.9	127.5
SLITRK1	116.1	112.6	123.8	124	110.9	101	123	109.7
SLITRK2	115.2	119.6	122.4	114.1	114	98.6	111.9	116.6
SLITRK3	118.8	116.5	122.2	120	111.3	101.8	115.5	118.5
SLITRK4	121.9	117.5	121.8	121.1	112.5	96.8	107.9	114.9
SLITRK5	108	117.6	115.9	118	105.1	99.9	118.2	103.8
SLITRK6	116.5	119.5	119.4	118.3	106.3	104.1	121.5	111.5
SLK	277	256.1	234.3	262.7	238.2	212.7	212.7	260.5
SLMAP	429.6	430.8	364	394.2	414.2	282.6	522.5	615.6
SLMO1	121.8	118.1	127.6	112.7	108.5	106.1	115.1	120.3
SLN	120.3	115.1	122.7	112.5	112.1	96.5	119.4	117.6
SLPI	118.4	112.6	121.3	122.3	115	98.6	111.5	123.1
SLTM	867.8	774.4	717.4	1100.6	977.5	637.2	922.4	1338.4
SLTM	505.2	415.4	436.1	540	389.2	363.3	425.5	761.2
SLTM	193.6	185.6	198.3	199.9	199.5	143.7	231.6	282.5
SLU7	297.2	277.3	308	307.7	248.2	369.2	416.1	306.4
SLURP1	110.2	121.8	114.4	120.6	104.6	101.4	126.2	112.8
SMA4	149.9	200.3	152.1	151	157.3	123	134.3	154.7
SMA5	112.4	111.4	113.5	119.9	113.8	93.8	119.1	108.9
SMAD1	119.2	114.5	108.1	135.5	107.6	98.2	111.9	109.7
SMAD1	112.3	115.3	125.1	112.6	109.2	106.8	121.9	114
SMAD2	140.9	138.2	130.5	154.1	154.3	137.1	167.6	143.8
SMAD2	116.8	129	123.4	122.3	124	107	126.6	109.7
SMAD3	440.9	432.1	434.9	481.3	413.6	321.4	504.6	513.9
SMAD4	414.3	594	441	514.9	490.1	413.7	554.4	545.3
SMAD5	118.7	118	118.9	123.5	104.3	94.9	122	119.8
SMAD5	117.6	121.8	118.4	127	116.9	105.3	123.3	118
SMAD5	253.9	275.7	289.7	276.7	219.6	168.8	199.7	213.6
SMAD6	108.2	121	139.9	114.9	111.6	94.5	120	118.1
SMAD7	151.9	156.8	257	166.8	138.2	148.4	188.6	166.1
SMAD9	119	116.9	125.8	115.3	113	101.6	121.3	122.3
SMAF1	116.3	115.4	116.7	124.1	105.1	95.2	130.6	122.9
SMAP	136.8	135.1	120	139.4	111.5	113.3	136.8	125

SMAP1	340.4	371.3	342.2	379.8	318.4	326.3	389.7	368.1
SMAP1	125.1	137.4	129.1	171.9	115.2	117.9	130.8	136.8
SMAP1L	1837.7	2360.8	1834.3	2322.1	1702.5	1529.8	1835.9	2032.8
SMARCA1	108.9	109.4	117.4	121.5	104.9	101.1	119.3	119.6
SMARCA2	114.3	109.5	125.9	115.5	113.7	99.2	125.4	121.1
SMARCA2	990.1	1050.8	885.3	947.4	749	858.5	720	713.5
SMARCA3	126.2	137.6	126.1	128.5	119.6	108.3	119.2	125.1
SMARCA3	242.1	248	230.4	296.3	244.6	156.9	205.5	195.4
SMARCA4	374.4	451.9	312.1	441.4	385.1	252.2	463.3	574.1
SMARCA5	168.4	144.6	166.5	181.1	147.9	140.9	183.5	147.3
SMARCAD1	158.3	168.4	178.5	151.8	143.3	130.2	143.3	140.1
SMARCAL1	264.6	255.7	201	234.5	217.8	192.1	227.4	217.9
SMARCB1	182.2	170.3	181.3	175.1	135.5	137.2	190	160.6
SMARCB1	211	219.2	262.6	278.1	184.9	222.4	194.1	177.2
SMARCC1	451.3	502.8	367.1	442.4	474.4	337	477.1	537.9
SMARCC2	118.6	105.4	122	118.3	108.2	104.5	115.9	118.3
SMARCC2	595.9	585.1	499.4	617.2	528	494.8	754.8	566.7
SMARCC2	152.5	168	145.4	172.4	149.9	130.8	173.6	184.4
SMARCD1	111.8	118.9	122.8	118.5	108.9	105	118	112.5
SMARCD1	552.4	580.7	434.3	585	437.2	424.7	644.7	577.3
SMARCD2	219.7	237.9	216.1	254.6	234.2	200.5	261.6	231
SMARCD3	116	126	123.1	123.2	109.3	108.7	115	114.2
SMARCD3	113.9	110.4	122.4	129.6	101.4	100.3	123.7	107.4
SMARCD3	131.3	137.1	159.9	131.8	147.2	159.2	118.4	149.4
SMARCE1	200.1	203.3	193.1	188.2	178.9	161.5	196.5	214
SMBP	178.8	194	202.6	245.7	176.9	194.9	240.6	234.7
SMC1L1	143.4	168.9	143.8	154.3	138	118	139	144.8
SMC1L2	114.9	116.4	116.7	123.4	115	99.8	126.7	123.4
SMC2	115.6	112.6	125.2	121.4	103	103.9	126.7	113.7
SMC2	183.5	197	189.8	177.7	166	138	165.2	154.9
SMC2L1	135.6	142	146.2	146.8	123.2	120.6	131.8	125
SMC3	122.2	128.7	136.8	129.5	124.7	117.3	125.5	122.5
SMC4	297.8	346.3	275	308.6	309.8	233.1	415.9	440.1
SMC4	121.9	116.9	126.7	138.1	110.3	108.6	132.4	125.5
SMC4L1	126.1	108	115	114.5	107.4	94.9	122.2	122.1
SMC5L1	117.6	115.5	124	118.5	105.7	100.4	119.5	103.9
SMC6L1	165.5	151	165.6	172.3	132.3	114.1	156.3	141.9
SMCP	120.5	122.9	131.4	128.9	108.6	108.6	112.3	120.2
SMCR7	117.5	122.8	114	123.2	101.2	97.8	117.1	121.1
SMCR7	119.5	125.4	123.8	128.2	114.5	109.8	113.8	119.1
SMCR8	114.5	114.5	112.7	123.8	105.1	92.8	114	109
SMCX	118.3	121.3	114.6	120.4	109.9	109.4	123.1	122.4
SMCY	265.4	291.7	277.1	275.9	234.9	211	248.1	234.6
SMEK2	367.1	388.7	334.3	480.2	348.9	320.3	472.5	426.3
SMEK3P	115.8	102.2	118.3	111.3	105.4	98.5	110.3	113.1
SMG1	567.8	551.2	583.3	680.6	677	451	628.1	919
SMG5	194.7	227.3	224.7	236.5	162.1	150.4	198.6	196.9
SMG6	118.5	134.4	125.4	135.7	117.6	115.4	147.6	141.1
SMG7	168.9	152.4	152.2	167.1	134.3	128.1	150.2	138.5
SMG7	124.2	115.9	119.2	125.3	117.1	100.5	134.4	119.8
SMG7	285.9	278	231	257.4	244.9	200.6	300.2	290

SMN1	120.3	118.5	128.2	119.2	107.8	101.9	119.5	122.3
SMN2	135.9	133.4	131.5	134.4	119	113.3	144.3	125.1
SMNDC1	440.9	450.4	466.3	520.4	351.5	385.6	617.6	493.1
SMO	122.6	128.9	124.6	123.2	111.1	108.6	133.2	111.8
SMOC1	105.2	103.3	112.1	119	115	108	115.1	117.7
SMOC1	119.4	121.1	124.9	125.1	113.2	96.5	111.3	118.4
SMOC2	115.4	115.2	119.3	109.2	105.2	99.9	116.8	109.3
SMOX	115.9	127.9	129.3	133	120.3	110.3	143.3	123.6
SMOX	115.3	115.8	117.4	131.5	109.7	101.4	122.9	112
SMOX	115.5	116.4	111	110.6	111.5	108.1	111.9	116.8
SMPD1	118.4	133.2	152.3	145.4	127.9	117.6	128.6	122.5
SMPD1	131.7	136.8	123.7	129.9	126	119.2	131.1	136.3
SMPD2	137	165.5	139.7	157.2	130.6	124.6	155.9	134.6
SMPD3	119.2	124.9	128.3	124.7	105.4	107.1	115.7	122
SMPDL3A	123	107.7	119.6	115.7	106.4	100	109.1	110.7
SMPDL3B	120.7	114.7	118.4	115.5	106.6	100.5	113	114.8
SMPDL3B	114.9	107.7	121.1	115.5	103.7	101	118.3	117.6
SMPDL3B	121.1	114.2	118.9	117.1	115.4	104.6	124.5	118.6
SMPX	105.7	109.6	111.6	112.8	109.6	92.3	108.8	113.9
SMR3A	108.9	115.8	121.1	110.3	105.3	95.9	119.5	119.5
SMR3B	125.5	115.3	111.8	121.8	110.3	101	119.9	116.9
SMS	221	207.6	320.1	216.1	221.6	238.5	200.3	170
SMTN	115.4	114.5	118.4	114.8	103.5	107.5	125.6	109
SMTN	117.4	118.6	121.8	121.7	104	101.7	117.3	122.1
SMTN	139	133.8	125.7	149.1	121.8	115.4	160.1	138
SMU1	135	158.1	166.8	158.3	149.1	125.6	140.8	137
SMUG1	231.2	201.4	231.1	225.9	193.1	178.9	180.1	164.3
SMURF1	115.4	114.7	118.9	128.7	105.3	105.7	129.2	121.4
SMURF1	112.6	113.1	116.5	122.1	105.4	99.2	109.6	113.4
SMURF2	130	137.3	141.7	131.2	123	103.8	125.3	138.2
SMYD1	111.3	115.9	109.6	111	105.9	99.8	110.4	105.9
SMYD2	169.7	191.2	213.2	177.6	154.8	131.3	153.7	138.3
SMYD3	155.1	164.1	158.9	155.2	140.5	142.5	148.1	144.5
SMYD4	188.3	170.7	240.2	200.9	195.9	210.6	175.5	186.4
SMYD5	123.1	127.3	135.8	121.2	117.5	99.5	125.5	122.1
SN	115.6	113.8	111.6	124.8	110.8	103	106.7	111
SNAG1	127.6	120.9	122.7	128	112.7	104.9	120.5	129.8
SNAI1	103.5	115.8	102.3	112.5	98.3	90.9	116.3	111.7
SNAI2	113.1	107.3	105.2	102.2	106.4	94.3	107.3	110.4
SNAI3	141.9	159.2	132.7	138.8	125.7	111.7	145.7	131.6
SNAP23	144.4	140.8	182.4	151.6	133.5	160.2	135.2	144.7
SNAP23	216.6	245.7	231	256.4	187.1	200.4	210.8	222.8
SNAP25	126	124	128.3	120.1	112.5	111	124.6	125.4
SNAP25	117.6	119.5	120.6	123.9	104.1	97.5	121.7	112.2
SNAP29	179.6	181.7	192	202.3	182.9	148.6	175.4	201.8
SNAP91	106.9	109.6	117.2	116.2	98.8	96.9	117.8	112.3
SNAPAP	145.7	126.5	129.2	142.6	124.5	120.6	135.7	130.2
SNAPC1	193.9	182.9	208.7	205.1	244.3	142.3	210.4	227.1
SNAPC2	198.8	217.5	225.8	243.6	160.2	188	221.4	213.3
SNAPC3	106	110	125.6	123.6	108.9	101.1	121.7	115.6
SNAPC4	463.4	546.1	391.5	449.6	376.2	288.4	473.8	428

SNAPC5	143.6	136.7	146.9	140	122.4	119	141.4	136.1
SNCA	254.5	168.9	237.1	229	148.3	169.1	307.4	160.1
SNCA	121.3	119.7	131.9	138.5	111.2	102.6	151	116.9
SNCAIP	109.8	110.4	110.7	116.3	103.3	94.8	119.1	113.5
SNCB	112.3	106.1	118	111.8	104	99.6	102.6	108.9
SNCB	110.9	111.1	112.5	117.9	108.9	97.5	118.5	100.9
SNCG	105	118.8	122.2	124.3	111.3	94.2	126.2	118
SND1	391.8	382.2	386.1	465.9	302.9	330.2	423.6	402.5
SNF1LK	123.8	118.9	122.8	139.3	119.2	110.8	153.7	160.7
SNF1LK2	124.7	119.8	112.8	127.8	112.1	99.4	125.4	117.6
SNF8	152.1	175.3	164.9	163.8	156.1	136.4	161.1	154.6
SNFT	136.2	130.1	128.3	128.8	129.4	110.3	131.9	158.3
SNHG10	117.4	122.4	126.3	122.2	113	98.4	116.5	115.9
SNHG3	107.8	114.9	124.3	126.7	113.9	104.5	124.8	109.8
SNHG3-RC(	112	124	120.2	116.7	105.5	99.3	103.3	110.9
SNHG5	1325	2831.9	1332.9	1421.8	2209.5	969.5	1215.3	2499.9
SNHG8	116.9	125.6	126	118.9	112	98.4	129.3	109.1
SNIP	114.7	121.2	102.8	106.7	105.3	91.4	110.4	108.5
SNIP1	214.9	231.2	201.5	224.5	213	171.9	265.4	265.3
SNN	138.3	143	144.8	147.1	125.4	111.6	133.1	190.1
SNORA32	174.9	153.6	177.9	173.6	129.8	134.8	157	148
SNORD107	109.1	115.3	112	119.9	106.8	96.7	114	120.6
SNORD116	118.5	116.2	111.7	124.2	112.5	100.7	118.4	113.7
SNORD116	111.8	109.7	115.6	125.1	100.9	92.4	120	109.2
SNORD36A	149.2	151.5	148.8	148.9	144.3	123.9	128.1	125.4
SNORD36C	154.6	190.4	148.9	168.1	164.5	134.8	172	154.2
SNORD48	132.5	139.6	119.2	127.5	128.8	106.6	123.6	133.6
SNORD56	267.8	275.6	189.9	290.3	278.4	159	345.5	384.8
SNPH	144.4	148.7	141.3	145.1	130.6	131.2	119.4	128.8
SNRK	1458.4	1728.1	1114.7	1585.4	1270.9	812.9	1597.4	1993.5
SNRP70	207.7	192.3	195.5	206.6	188.5	188.3	198.6	175.4
SNRP70	1892.9	1453.2	2033.4	1809.1	1109.2	1320.7	1492.3	1479.3
SNRP70	114	115.1	110.6	110.8	101.1	97	118	117.2
SNRPA	196.5	208.8	270.7	196	170.1	191.3	164.7	170.8
SNRPA1	928.7	1004.5	1161.7	1025	800.7	749.5	873.2	893.8
SNRPB	775.9	750.6	618.8	738.8	569.9	586.8	755.8	917.4
SNRPB	1214.2	1404.5	1167.6	1366.5	1160.8	1202	1983.3	1991.6
SNRPB2	1112.5	982.7	1121.3	1008.8	889.9	986.9	935.5	1155.7
SNRPB2	566.7	686.5	664.4	654	589	469.8	514.7	572.4
SNRPC	288.1	251.8	260.4	272.3	210.7	266.1	209.4	217.1
SNRPD1	122	116.9	142.5	128.8	128.6	109.2	126.2	123.7
SNRPD2	133.5	124.5	126	131.5	113.7	101	129.7	120.7
SNRPD2	5899.7	6705.8	7235.8	6571.1	5724.7	4925.1	5872.6	6580.4
SNRPD3	221.1	175.7	225.1	207.1	171.1	137.1	173.3	185.6
SNRPE	107.5	105.9	116.5	112.4	105.7	91.4	110.7	115.1
SNRPF	1044.1	1219.3	1357.5	1192.1	1077.4	869.7	1020.9	1195.1
SNRPG	2767.2	2867.7	3455.6	2889	2715	2890.5	3052.5	3106.8
SNRPN	806.3	971.2	777.5	798.3	581.7	564	551.9	586.4
SNRPN	121.4	110.7	120.9	117	106.6	106.4	119.8	115.3
SNTA1	175.1	157.4	163.8	159.3	134.6	139.2	170.1	161.9
SNTB1	204.6	208.2	271.8	234.4	201.9	175.1	179.6	179.5

SNTB2	116.6	129.3	122.3	131.3	123.2	106.6	133.8	127.2
SNTB2	302.6	357.2	321.1	345.2	299.2	199.1	344.6	326.5
SNTG1	118.1	110	110	119.2	108.7	97.7	105.2	108.8
SNTG2	112.9	120.3	122.1	118.9	111.5	97.5	119.6	116.5
SNUPN	234.7	267.1	227.7	217.6	237	180.5	256	244.6
SNURF	629.1	839.4	747.9	714.4	629.9	547.3	368.9	456.2
SNURF	120.7	129.7	135.7	130.3	119.6	109.7	121.2	118.4
SNW1	133.4	117.8	155.9	159.6	140	111.2	144.2	132.8
SNX1	172.9	182.2	189.1	187	175.2	178	153.7	179.5
SNX10	350.5	211.6	338.6	246.2	162.9	176.8	173.3	179.8
SNX11	332.1	326.5	375.9	324.4	232.3	296.3	324	322.6
SNX11	119.2	113	116.2	122.7	110	95.2	128.4	114.8
SNX12	132.9	130.4	151.3	132.4	125.2	108.7	129	133.1
SNX13	136.7	138.3	140.5	148.1	140.2	123.8	152.5	143.7
SNX14	109	115.5	107.8	105.7	101.6	98.9	106	113.7
SNX14	284	300.5	290.6	306.9	260.1	197.8	305.7	257.9
SNX15	128.7	124.3	117.2	134.3	115.1	107	131.3	122.7
SNX15	119.3	119.5	118.5	131.5	114.4	101.1	121.5	129.8
SNX16	178	208	158.6	201.8	203.3	123.2	204.4	258.5
SNX16	110.4	106.6	114.4	120.2	104.2	91.3	113.6	117.6
SNX17	885	916.3	946.4	957.8	591.3	669.7	621.4	613.8
SNX19	205.7	182.8	172.7	214.7	155	148.5	174	173.1
SNX2	695	628.4	875.2	630.5	467.8	581.8	817	607.2
SNX21	113.3	104	113.8	117.3	104.1	95.9	114.3	111.1
SNX22	107.2	109.7	117.8	117.9	112	102	102.8	116.3
SNX24	112.4	120.9	124.3	125.1	111.5	112.4	125.4	112.4
SNX25	217.2	231.7	289.3	276.6	198.3	198.1	225.1	227.9
SNX26	163	164	212.8	204.1	163.4	156.8	165.3	158.5
SNX27	754.4	672.8	578.4	745.8	592.9	411.3	567.3	548.5
SNX3	1348.6	1378.9	2015.3	1593.7	1120.5	1324.2	1576.8	1981.3
SNX4	237	241	252.4	256.9	263.5	199	253.6	266.7
SNX5	140.7	136.8	154.3	131.7	127.6	121.6	140.1	129.4
SNX5	203.8	225.4	194.3	208.4	202.5	189	182.5	173.1
SNX6	326.2	424.2	265.7	476.8	315.6	252	515.6	528.9
SNX6	126	119.4	132.1	129.4	115.2	109.1	135	127.9
SNX7	106.6	113.1	109.2	111.9	101.1	95.4	115	105.6
SNX7	102.7	112.1	114.8	116.5	111.1	98.1	119.9	115.7
SNX7	106.2	111.3	124.3	112.9	107	102.2	114.2	104.1
SNX8	146.2	135.7	173.3	165.8	115	148.5	186.5	138.6
SNX9	108.7	107.8	115.7	112	105.8	100.7	120.1	110.3
SOAT1	156.1	129.4	144.7	143.3	129.7	123.5	161.5	139.6
SOAT2	113.1	111.2	114.3	113.3	96.7	92.3	114.8	115.6
SOCS1	144	141.3	139.7	138.5	118.9	122.5	134	131.8
SOCS2	143.1	170.9	145.6	155.4	145.2	109.2	136.1	161.6
SOCS3	109.5	111.8	113.2	119	106	94.4	114.1	109.4
SOCS4	127.5	124.6	132.9	145.9	128.4	108.2	148.8	138.4
SOCS4	273.6	275.6	235.3	307.7	217.9	230.6	276.9	252.1
SOCS5	112.2	114.3	116.6	121.3	105.4	99.2	118.6	116.3
SOCS5	111.4	111.2	113.6	121.7	112.6	99.1	119.9	114.7
SOCS5	128.5	117.6	124.1	136.6	122.2	111.7	131.3	125.9
SOCS6	116	115	120.7	129	120.1	102.6	119.5	120.1

SOCS7	119.4	118.3	125.6	119.1	107.7	98.6	125.8	118
SOCS7	119.2	116.9	125.2	138.2	117.2	105.1	125.8	112.8
SOD1	1640.4	1591.9	1338.7	1495.5	1588.7	1516.8	2854.2	4594.3
SOD2	460.9	363.1	220.3	390.6	209	206.9	235	227.3
SOD2	3213.5	2851.5	1481	2658.7	1083.6	1481.7	1975.5	1442
SOD2	123.5	126	114.6	120.5	104.3	94.6	118.8	105.3
SOD3	117.1	113.5	117.2	119.8	110.6	92.6	114	112.5
SOHLH2	109.8	114.7	114.7	113.5	113.7	96.8	117.8	116.9
SOLH	122.3	137.4	140	142.5	127.5	107.6	136.3	138.3
SON	251.2	267.9	242.5	252.2	261.5	222.3	264.8	270.2
SON	141.8	139.4	168	147.4	127.9	117.2	150	145.4
SON	181.3	193.8	170.1	212.4	196.3	147.1	210.1	257.3
SON	1126.7	1364.7	1172.2	1521	1646.5	1203.5	1980.9	2255.3
SORBS1	122.8	110.5	126.3	123.8	107.7	95.7	124.3	126.5
SORBS1	125.1	123.4	127.3	124.5	112.9	93.9	126.1	122.5
SORBS1	123	114.1	115	126.2	100.8	93.6	113.6	105
SORBS1	114	107	133.5	121.7	125.9	90.5	112	110.1
SORBS1	116.3	116.4	112.2	121.7	109.1	94.5	118.4	106.8
SORBS2	113.5	120.1	119.9	130.6	111.2	106.5	112.8	114.1
SORBS2	110.4	113.9	111.9	106.4	106	98.3	111.7	119.2
SORBS2	112	114.4	117	118.9	117.3	100.4	120.1	109
SORBS3	108	122.4	119.4	121.5	107.5	102.6	117	116.8
SORBS3	127.4	126.1	119.4	122	115.8	105.2	123.3	114.6
SORBS3	206.4	205.6	177.3	195.8	160.5	136.7	184.3	166.8
SORCS1	115.2	118.6	115.8	122.3	104.2	94.7	120.2	115.7
SORCS1	124	117.8	127.2	115.7	113.2	108.1	121.1	136.1
SORCS1	112.5	120.4	116.8	118.6	107.8	95.9	116.2	113.5
SORCS2	111.3	107.7	117.6	110.9	104.1	97.8	116.7	100.4
SORCS3	107.3	112	112.1	118.6	106.9	103.4	112	107.3
SORD	114.8	115.7	110.9	120.1	105.2	97.8	121.7	112.5
SORD	129	116.7	129.6	115.4	117.1	102.6	115.5	118.4
SORL1	540.1	678	505.1	697.7	559.3	432.9	473.8	510.4
SORT1	147.7	139.4	167.6	141.2	119.5	114.4	140.4	118.2
SOS1	155	160.5	142.9	159.8	135.8	128.9	136.8	167.4
SOS2	131.5	121.9	138.1	129.8	124.9	119.6	128.1	133.8
SOST	117.3	115.4	113.7	118	103.5	101.1	123.2	111.3
SOSTDC1	112.1	116.1	122.2	126.7	108.8	100.1	133.7	117.7
SOX1	106.6	111	120.3	122.4	115	97.4	112.3	117.6
SOX10	113.8	108.6	119.6	113.3	116.5	109.2	120.5	119.4
SOX11	113.5	111.3	119.3	113.8	119.9	98.4	122.9	122.3
SOX12	120.7	132.5	133.9	131.1	120.4	104.7	127.7	131.1
SOX13	133.3	126.9	129.4	136.4	136.8	105.4	129	132.7
SOX14	124.7	121.2	131	126.2	114.5	108.9	119.2	116
SOX15	113.6	116.8	117.9	117.8	109	95.3	129.5	124.5
SOX17	103.7	117.3	110.6	112	109.1	99.3	117.1	110.6
SOX18	106.7	118.5	124.1	112.1	110.6	98.4	121.8	119.4
SOX2	115	109.5	107.4	111.6	100.4	96.8	113.5	110
SOX21	111.2	112.2	118.5	105.9	109.7	107.2	109.5	110.8
SOX3	123.9	120.4	115.5	125	111.2	98.7	108.5	107.8
SOX30	109.5	116.6	127.9	117.1	109.9	99.8	125.3	117.2
SOX30	122.1	115.8	130.9	119.8	106.8	106.3	126.5	119.5

SOX4	126.8	126.6	133.5	141.6	127.7	116.7	120.9	129.3
SOX5	113.9	115.9	108	114.5	110.7	91.7	110.7	109.4
SOX5	120.2	109.1	121.4	120	107.3	88.6	124.8	110.1
SOX5	120.2	118.3	113.3	113	107	93.4	117.5	113.3
SOX6	115.9	116.9	115.2	118.8	105.9	91.6	113	112
SOX6	102.1	112.2	114.7	114	99.1	93.9	117.7	112.6
SOX6	105.6	110.6	113.7	119.6	108.2	91.5	109.2	107.6
SOX6	105.9	107.9	112.3	109.6	104.1	96.5	114.7	108.5
SOX7	116.3	110.1	117.7	116.3	115.7	97.2	116.7	114.4
SOX8	324.3	384.2	191.1	283.5	272.5	141.8	284.5	253.4
SOX9	116.6	108.6	109.4	116.5	106.1	94.4	121.4	112
SP1	192.8	229.2	225.6	241.6	184.2	161	280.2	263.3
SP100	196.7	198.9	186.7	234	164.4	151.8	226.7	184.9
SP100	142.6	150.2	147	164.4	139.4	128.9	168.7	188.4
SP100	180	142.2	171.8	208.1	162.7	142.2	188.3	209.8
SP110	751.6	713.6	807.9	767.2	603.5	552.8	605.3	697.8
SP110	682.1	549.7	548.4	658	498	482.2	421.2	527.7
SP110	182.2	172.2	167.4	163.3	157.4	113.8	155.1	140.5
SP140	144.3	139	154	147.3	126	120.3	137.3	137.6
SP140	212.7	190.1	247	229	182.3	182.9	184.6	189.4
SP140	178.2	197	185.1	183.7	179.5	147.2	184.3	183.9
SP2	287.3	268.9	268	330.7	264	242.7	354.1	334.8
SP3	209.9	159.6	199.1	185.1	194.6	134.3	235.7	215.3
SP3	113.6	118.6	116.1	130.3	110.7	105.4	125.4	122.4
SP3	123.2	110.7	110.3	123.1	104.1	100.7	115.7	116.8
SP4	210.4	201.7	204.9	209.7	175.6	160.2	249.7	207.6
SP5	118.5	124.2	124.5	129.2	107.7	109.4	121.9	117.5
SP6	115.8	114.6	115.8	120	103.6	105.8	117.3	118.1
SP7	111.3	125	124.8	123	107.4	103.3	123.6	125.7
SP8	112.7	117	116.4	120.8	110.1	97.4	114.9	116.2
SP8	118.6	108.5	121.9	108.4	99.4	93.6	119	115.9
SPA17	109.3	120.9	133	121.7	116.2	110.4	116.5	121.6
SPACA1	121.1	112	116.1	121.4	106.2	102.8	118.7	118.2
SPACA3	122.6	125.4	124.8	123.6	118.9	104.5	123.1	120.9
SPACA4	114.2	112.3	119.4	115.5	111.7	98.5	110.2	114.8
SPACA5	122.8	123.1	109.6	121.8	109	99.7	113.6	117.7
SPACA5B	114	113.4	113.1	122.4	101.8	96.8	112.2	114.7
SPAG1	117.6	126.6	114.7	123.3	110.8	102.3	132.1	120.1
SPAG1	115	119.3	108.6	111.7	111.7	96.6	119.4	115.2
SPAG1	119.4	108	124.3	116.9	113.5	99.4	124.9	121.3
SPAG11	109.1	116.8	112	107.9	103	96.3	116.7	108.7
SPAG11A	120.7	124.6	121.9	122.6	106.8	102.4	122	125
SPAG16	113.7	109.4	113.1	119.5	105.9	100.4	118	108.1
SPAG16	125.4	139.7	150.1	123.1	120.4	120.1	121.7	127.7
SPAG16	116.6	119.3	113	118.3	107.2	99.9	131.1	124.6
SPAG17	119.1	125.6	117	122.3	111.9	96.9	117.7	113
SPAG4	126.1	111.1	126.9	119.2	115.4	105.6	125	123.2
SPAG4L	116.2	120.3	121.3	136.6	106.5	107	117.5	121.7
SPAG5	118.7	136.1	118.4	115.1	121.3	102.8	119	119.4
SPAG6	114.3	119.3	122.7	117.7	105	98.1	121.6	123.1
SPAG6	114.4	111.2	115.4	123.4	104.1	103.7	123.1	116.2

SPAG7	217.9	225.1	202.1	215.5	173.1	174.9	227	202.6
SPAG8	125.6	114.1	110	103.3	96.2	101.4	105.2	101
SPAG8	101.8	104.2	112.5	115.1	101.6	98.4	115.3	109.6
SPAG9	150.7	169.1	168.1	196.5	160.5	145.6	246.2	204.8
SPAG9	132.2	126.6	138.4	132.7	126.1	113.8	138.7	132.4
SPAG9	292	359.1	281.4	575	402.2	302.1	1014.5	713.1
SPAM1	120.3	121.4	119.4	122.8	114.4	96.8	122	127.6
SPAM1	117.7	113.2	115.4	118	107.9	95.9	119.3	113.3
SPANXA1	113.4	119.1	112.1	107.5	101.6	93.7	110.4	116.8
SPANXB1	108.3	114.2	115.4	117.6	105.6	94.7	107.8	109.9
SPANXC	109.8	111.3	111	108	102.1	98.7	114.5	106
SPANXE	117.3	109	122.1	120.4	102.9	98.6	115.5	105.8
SPANX-N1	107.6	118	114.9	117.3	106.8	96.6	116.9	118.9
SPANX-N2	108.6	115.9	120.2	103.1	106.3	94.4	114.7	113.7
SPANX-N3	121.5	112.1	123.6	113.8	112.6	107	112.3	123.3
SPANX-N4	118.8	108.8	118.4	111.7	105.9	90.4	114.6	111.6
SPANX-N5	111	106.3	106	111	105.9	93.7	116	109
SPARC	108.5	105.7	119.4	113.7	108.3	97.4	115.3	111.8
SPARCL1	114.7	105.8	118.7	114.2	110.1	99.6	112.5	110.2
SPAST	148.6	144.1	159.5	137.5	143.5	142.3	170.2	162.1
SPAST	119.7	116.7	117.6	126.8	110.5	100.7	114.7	115
SPATA1	109.7	117.8	113.2	117.5	106.7	99.6	113.1	112.2
SPATA12	117.7	120.1	124.5	128.1	106.4	108.4	114.3	114
SPATA13	226.1	215.1	249	218.3	165.1	136.2	211	221.4
SPATA16	113.6	113.8	114.3	112.9	118.8	98.1	110.8	109.7
SPATA18	116.5	114.6	114.4	113.7	107.4	101.8	118.4	114.1
SPATA19	119.2	120.5	119.3	116.5	116.3	99.4	97.2	118.4
SPATA2	143.8	142.8	134.4	153.4	133.6	123.9	160.4	160.8
SPATA20	185.2	238.4	183.2	203.9	169.5	134.8	152.3	161.1
SPATA21	110.8	111.5	115	121.2	109.1	101	123	109.4
SPATA22	109.9	111.4	119.5	113.9	103	99.2	112.2	115.2
SPATA3	112.8	111.3	118.6	122.3	102.4	102.5	117	114.6
SPATA4	114.4	121.7	113.8	116.2	106.3	99.6	107.3	114.3
SPATA5	103.3	114.6	112.3	119.6	108.1	100.4	109.6	116.9
SPATA5L1	239.7	261.6	293.5	298.7	259.6	242.9	245.9	237.2
SPATA6	117.7	119.2	121.5	116.2	115.7	98.8	117.4	110.2
SPATA7	124.2	148.4	121	136.7	112	113.8	113.4	108.9
SPATA7	116.7	122.3	123.3	120.4	105.3	104.4	122.6	118
SPATA7	99.4	119.5	117.2	124.7	112.8	103.9	116.2	123
SPATA8	117.4	114	118.1	118.2	110.1	99.9	131.3	112.8
SPATA9	115	116.2	108.6	124	105	108.9	119.7	120.5
SPATC1	124.2	115.8	124.1	112.7	102.1	102.5	124.5	119.1
SPATC1	112.5	111.7	121	116.8	111.3	101.4	116.1	117.6
SPATS1	107.5	106.4	106.4	108	106.6	99	121.9	108.4
SPATS2	129.6	120	125.8	146.4	115.8	112.1	150.1	137.6
SPC24	141.2	128.4	137.4	135.1	120.9	118.3	120.1	138.4
SPC25	119	114.1	118.3	110.5	110.5	100	123.3	108.9
SPCS1	2200.7	2326.7	2419.8	2050.2	1836	2183.7	1471.5	1556.1
SPCS2	111.9	119.7	122.3	115.2	109	107.4	110.3	120.8
SPCS3	144.9	148.5	180.3	168.8	136.1	153	144.4	136.7
SPDEF	118	113	108.5	109.3	110	92.5	113.9	107.7

SPDY1	117.6	120.3	125.3	120.4	109.6	100.4	120.5	124.3
SPDY1	119.2	116.5	117.4	122.3	109	94.4	120.2	133.8
SPDY1	111.6	107.6	107.3	112.5	102.1	95.5	106.4	106.1
SPECC1	116	113.6	125.9	127.4	120.8	103.9	114.2	109.7
SPECC1	112.3	116.9	121.7	116.1	105.1	95.3	111.3	119.7
SPEN	665.4	747	558.5	740.7	679.2	565.9	862.2	817.9
SPERT	112.4	106.9	117.7	137.8	104.6	96.6	113.4	127.9
SPESP1	119	115.6	120	119.7	116.2	102.5	132.4	124.2
SPFH1	129	122.8	128.1	122.3	123	104.6	130.3	129.8
SPFH2	116.8	128.3	124.4	119.1	110.7	97.1	121.4	107.3
SPFH2	164	157.6	193.2	166.4	140.7	133.8	152.5	145.1
SPG20	149.5	132.8	142	148.3	146	111.1	185.1	177.4
SPG21	129.1	131.1	154.8	138	123.9	127.4	126.3	121.5
SPG21	256.6	257.2	418.9	260.6	265.2	256.2	223	176
SPG3A	129.2	141.4	124.1	133.2	132.9	113.1	118.4	138.8
SPG7	156	156.6	154.9	147.5	141.4	114	142.3	142.7
SPG7	127.2	119.1	115.3	119.3	120.2	108.2	125.9	114.5
SPG7	1143.7	1140	1015.1	1181.2	919.5	554.8	896.4	869.3
SPHAR	119.5	118.4	129.7	134.8	113.1	105.9	135.5	125.4
SPHK1	231.5	273	145.2	318.3	178	184.3	382.6	224.3
SPHK1	114.1	108.4	126.8	108.7	102.6	104.2	113.4	112.1
SPHK2	621.9	592.2	530	661.9	491.2	413.5	806.3	820.4
SPI1	164.6	144.2	217.2	201.2	125.6	231.3	170.1	133.1
SPI1	317.5	240.5	425.6	422.6	190.5	484.6	334.3	149.3
SPIB	144.3	133.3	147.2	140.6	109.1	121.6	124.8	110.2
SPIC	113.5	110.2	124.3	115.1	97.8	94.1	112.5	115.7
SPIN	163.8	197.3	175.5	217.7	183.2	115.6	192.9	197.9
SPIN1	442.6	369.6	406.7	454.7	325.3	267.9	399	377
SPIN2	116	119.4	113.8	117.8	107.2	95.4	117.2	119.7
SPIN-2	128.9	140.4	130.9	139.1	119	116.8	133.7	126.4
SPIN2B	109.7	119.3	126	122.9	121.4	111.9	118.4	105.2
SPINK1	326.8	419.5	115.4	532.4	537.2	94.7	724.6	751.1
SPINK2	132.5	117.2	124.7	141.4	115.6	112.9	120.5	118.7
SPINK4	112.7	109.5	108.7	112.6	104.1	92.9	112.9	109.5
SPINK5	117.8	114.8	109.4	121.1	108	96.6	121	118.3
SPINK5L2	109.4	114.3	114.4	111	106.7	100.9	116.1	119.9
SPINK6	107.8	122.6	119.4	128.2	107.7	101.7	121.2	117
SPINK7	116	113.4	116.4	113.1	109.7	99.9	115	112.5
SPINK9	128	117	125.1	132.7	111.7	105.7	120.1	120.2
SPINLW1	113.2	119.1	116.3	113.7	104.6	101.8	115.6	113.9
SPINLW1	127.6	127.9	129.2	129.9	126.5	104.3	129.9	125.4
SPINLW1	113.6	115.4	119.9	124.3	111.5	106	116.8	115.4
SPINT1	116	113.7	126.3	124.3	115	101.1	125.1	110.3
SPINT1	114.9	118.9	114.5	122.7	114.3	95.6	116.1	107.6
SPINT2	291.2	263.5	288.6	277.3	188.9	214	207.7	194.9
SPINT4	108.7	120.3	128.3	119.2	110.6	102.5	116.5	122.6
SPIRE1	198	151.8	189.6	224.9	144.1	191.7	336.7	198.5
SPIRE2	129.8	131.6	125.7	137.1	119.1	107.2	124.4	129.1
SPN	627.3	657.1	908.6	674	641.8	480	401	360.5
SPN	118.7	113.5	123.9	117	113.2	107	130.1	125.3
SPN	109.9	139.3	135.3	127.4	114.4	103.2	123.8	135.1

SPO11	114.6	112.9	113.8	116.1	106.3	98.7	122.2	112
SPO11	117.6	130.3	139.7	137.9	122.6	106.6	127.3	124.6
SPOCD1	122.7	122.1	133.1	123.7	110.2	112.5	119.6	125.1
SPOCK	114.6	115.2	117.9	119	113.3	103.8	117.9	127.3
SPOCK2	4363.6	5250	6564.8	4527.4	3347.7	5269.4	3667.6	3682.1
SPOCK3	110.7	117.8	121.8	103.6	107.1	96.3	111.1	112.5
SPON1	309.6	396.9	392.2	287.5	290.7	248.3	237.7	247.1
SPON2	125.1	117.2	137.1	126.9	112.1	109.6	119.5	116.9
SPOP	1183.4	1378.2	1052.5	1313.9	1245.9	740.1	1477.1	1674.5
SPOP	118	122.3	127.6	120.9	104.3	111.8	136.9	121.2
SPP1	141	223	126.1	214.7	230.8	101.7	224.7	170.7
SPP1	138.8	188.7	116.1	179.1	172.7	97.5	182.3	144.9
SPP2	109.9	118	116.3	116	112.3	97.6	128.9	113.7
SPPL2A	622.7	557.9	829.7	711.8	334.3	633.3	562.7	381.9
SPPL2B	122.4	123.5	121.5	127.9	113.1	104.3	124.8	111.9
SPPL2B	110.8	109.9	113.3	109.1	99.4	99.6	115	111.1
SPPL2B	136.2	125.2	183.1	131.8	113.4	118.9	135.7	122.2
SPPL3	120.4	116	124.1	126.3	114.5	110.2	120.1	120.7
SPR	120.2	128.8	116.4	134	106.7	109.6	116.8	107.4
SPRED1	245.3	153.4	585.6	211	136.1	431.8	163.8	128.8
SPRED2	121.2	120.7	130.8	128.2	107.7	112.9	129.1	116.5
SPRED3	116.5	134.8	135.6	134	116.2	105.1	126.9	110.5
SPRED3	112.5	123.9	118.9	124.4	114.4	102.4	116.2	121.1
SPRED3	105	116.3	115.7	123.8	118	94.1	108.6	113.5
SPRN	111.8	117.5	116.2	126	110.8	103.1	134.9	114.9
SPRR1A	122.5	126	130.4	146.9	122.2	130	144.3	116.8
SPRR1B	128.1	137.8	122.8	124.7	115.5	112.1	123.6	121
SPRR2A	113.3	120.3	125	114.6	105	90.8	114.4	113.1
SPRR2B	114	113.8	126	122.3	110.3	104	129.2	111.2
SPRR2C	109.1	123.4	113.8	121.3	104.7	95.3	114	116
SPRR2D	106.9	110.9	120.7	112	102	96.2	109.4	112.9
SPRR2E	106.1	107.6	107.1	112.3	106.5	91	112.4	100.4
SPRR2F	107	110.6	111.4	106.1	100.4	94.9	108.3	108
SPRR2G	110.3	126.9	113.6	119.2	116.3	112.7	127.3	117.1
SPRR3	122.7	123.4	132.7	116.3	111.4	104.7	127	116.6
SPRR4	115.1	114.6	125.3	124.2	106.8	105.7	114.6	118
SPRY1	104.9	115.7	116.4	117.4	106.8	95.7	105.8	111.9
SPRY1	165	151.5	186.6	162.6	125.4	124	135.9	142.7
SPRY2	119.8	130.1	122	135.1	116.7	98.8	119.2	116.2
SPRY3	124.4	112.2	126.8	115.6	115.4	100.9	125.4	121.8
SPRY4	109.6	112.7	110.2	125.2	107.6	102.3	109	103.8
SPRYD3	261.1	358.7	300.3	265.3	239.8	188.4	184.6	221.4
SPSB1	126.8	125.2	133.7	152.5	128.6	125	154.4	137.5
SPSB2	173	131.3	170.2	146	137.5	134.1	160.1	157
SPSB3	1831.8	2294.2	2031.3	2189.1	1778.2	1178	1888.7	2397
SPSB4	111.3	118.8	126.3	125.5	119.8	107.3	127.6	114.8
SPTA1	114.7	125.6	117.7	132.8	108.6	105.5	129.2	123.2
SPTAN1	630.1	727.4	452.4	638.7	507.3	367.8	624.2	498.5
SPTB	104.5	122.5	128.3	125.4	107.9	104.9	117.2	115.9
SPTB	118.5	116.8	107.5	117.9	106.8	109.6	113.2	115.9
SPTBN1	128.6	117.1	116.2	124.6	105	100.3	120.5	121.7

SPTBN1	121.6	126.5	124.8	118.5	104.5	106.7	118.9	119.9
SPTBN1	168.4	185.5	169.6	186.4	198.4	135.8	160.4	166.9
SPTBN2	120.3	121.6	117	123.7	114.5	105.5	125.6	121.6
SPTBN4	112.1	115.2	125.3	122.5	108.6	103.9	123.4	116.3
SPTBN4	114.2	111.3	128.8	120.7	112.5	103.8	121.2	122
SPTBN5	145.6	119.6	133	129.8	107.1	105.5	118.5	124.7
SPTLC1	2265.1	1673.4	1876.4	1810.7	1369.4	1407.7	1819.7	2401.2
SPTLC1	135.9	131.6	141.1	141.2	142.6	124.6	137.5	132
SPTLC1	140.3	147.5	147.7	160.9	139.1	155	150.6	151.7
SPTLC2	166.5	169.6	174.9	176.4	164	137.4	172.4	147.7
SPTLC3	113.4	122	115.4	121.2	110.6	96.8	122.1	114
SPTY2D1	177.5	205.4	205.2	209.3	189.9	177.6	219.6	249.6
SPZ1	118.6	119.8	119.4	124.9	101.5	106.1	120.3	124.4
SQLE	339.9	318	304.9	285	247.8	195.6	302.4	247
SQRDL	243.7	217.2	320.7	291.5	205.9	250.2	285.9	203.3
SQSTM1	665.7	1112	410.8	1309.3	989.6	890	2046.1	3659.3
SR140	317.9	304.6	280	353.3	320.9	245.3	295.6	338.4
SRA1	181.9	158.4	168.4	189.4	134.9	165	178.6	146.9
SR-A1	120.4	118.7	122.6	127.7	112.1	100.5	125.2	124
SRC	113.3	120.3	116.8	110.2	116.2	99.7	118.7	113
SRC	114.6	121.3	128.8	119.8	107.1	103.1	126.9	119.7
SRC	433.6	345.8	330.2	487.2	260	502.2	525.5	227.2
SRCAP	134	137	136.3	134.4	122.9	112.9	129.9	123.2
SRCRB4D	115.6	121.8	121.3	110.7	99.6	96	116.7	116.4
SRD5A1	110.8	130.1	125.6	130.1	121.4	106.3	131.3	124.1
SRD5A2	128.2	114.3	125.5	117.1	120	107.8	126.3	116.1
SRD5A2L	113.4	114.9	131.1	125.1	105.1	104.7	118.5	105.9
SRD5A2L2	114.1	108	109.8	103	106.4	99.5	113.7	112.3
SREBF1	139.9	149.3	150.6	137.7	127.6	117.7	161.7	161.3
SREBF1	123.7	120.9	135.4	122.6	107.1	115.1	120.1	106.2
SREBF1	134.3	139.3	127.1	123.2	128.5	116.8	138.5	147.7
SREBF2	105.1	124.2	119.3	117	127.1	109.5	122.9	119.2
SRF	459.9	480.3	433.1	531.7	399.2	404.6	424.2	489.9
SRFBP1	183.3	203.4	186.4	167.1	169	113.4	179.4	166.7
SRGAP1	111.5	110	121.1	119.4	127.1	98	121.3	103.7
SRGAP2	139.1	124.7	151.8	128.1	126.2	112.6	127.8	132.5
SRGAP2	112	116.3	112.2	107	100.9	94.4	110.4	106.7
SRGAP2	123.9	128.2	125	128.1	121.2	107	123.8	128.6
SRGAP2	117.9	117.3	109.5	116	115.9	95.9	121.3	117.9
SRGAP3	129.7	117.8	135	126.8	111	105.5	123.4	118.6
SRGAP3	130.1	129.1	147.7	129	129.5	107.3	135.9	127.5
SRGN	1450.8	1410.6	2334.9	2148.5	2185.7	2750.8	2378.5	3390.4
SRI	109.9	108.5	117.7	109	108.9	96.1	119.7	109.3
SRI	154.5	161.5	158.8	172.6	145	131.5	133.1	133.8
SRI	183	203.3	215.2	210.2	163.4	177.8	193.4	188.8
SRM	280.2	261.8	254.1	232.9	161.7	164.6	221.6	216.8
SRMS	113.7	117.1	128.8	117.6	108.7	103.5	130	111.8
SRP14	1649.6	1584.4	1669	1719.6	1976.4	1325.3	1497.2	1813.7
SRP14P1	515.7	469.6	441.6	516.8	381.1	473.1	421.1	722.4
SRP19	281	316.9	386.2	318.2	318.5	278.3	265.4	322.8
SRP46	594.6	650.4	554.5	632.6	477.1	453.8	419.5	464.1

SRP54	626.7	539	710.5	923.8	573.3	675.6	783.6	840
SRP68	553.8	508.4	398.1	514.9	427.3	474.5	466.2	564
SRP72	213.8	181.6	215.8	227	158.2	159.8	189.1	192.5
SRP9	1772.2	1684.1	2055.6	1455	1504.8	1561.7	1791.1	1970
SRPK1	495	384.7	343.6	433.9	441.4	290	435	382.1
SRPK2	161.4	199.7	199.7	180.1	186	144.5	159.4	168.9
SRPK2	130	135.8	149.9	136.9	130.2	111.9	137.7	131.9
SRPK2	137.8	125.2	129.2	143.4	134.4	107.3	130.3	132.2
SRPR	276	324.4	289.8	361.5	246.9	270	340.8	294.5
SRPRB	540.5	721.3	572.6	548.5	564.5	347.3	614.4	602.1
SRPX	110.4	110.1	114.2	115.9	109.4	99.4	117.7	115
SRPX2	110.5	109.9	114.1	121.9	104.2	98.3	111.6	116
SRR	123.9	127.9	131.3	122.3	120.7	110.7	142	140
SRRM1	867.3	1006.3	956.4	1087.3	1091.5	719.4	1075.8	983.5
SRRM2	2453.8	2455.2	2498.1	2516.1	2152	1813.3	2552.4	2407.4
SRRP35	106.7	117.9	113.5	120.7	115.6	101.7	123.2	120.4
SRXN1	653.4	599.8	254.1	841.7	501	710.7	1439.7	1149
SRY	111.1	111.6	117	120.1	107.3	102.2	118.7	113
SS18	130.6	143.2	144.2	140.3	121.9	104.4	134.8	128.4
SS18	110.3	116.8	126.9	126.4	109.1	100.6	121.7	120.2
SS18L1	153.7	162.5	190.1	165.4	175.5	154.5	168.9	155.6
SS18L1	114.1	109.7	134.3	115	108.4	111.2	136.1	114.1
SS18L2	3099.8	3569.4	3129.7	3514.4	2702.9	2345.1	2982.1	2689.6
SSB	128.4	134.8	116.9	121.8	107.9	101.9	121.3	117.2
SSBP1	607.7	724.5	577.9	727.3	493.7	703	658.6	657.4
SSBP2	242.7	268.9	266.7	258	213.4	239.1	220.7	215.8
SSBP3	152.3	135.9	142.8	146	129.3	111.6	152.8	129.5
SSBP3	138.2	149.4	134.6	137.9	109.9	116.1	147.8	121.5
SSBP4	201.4	194.4	178.6	165.5	126	140.7	133.6	122.7
SSFA2	129	124	115.2	135.9	107.9	108.8	125.8	128.1
SSH1	174.5	185.6	164.1	208.1	172.5	127.7	239.3	184.9
SSH2	316.5	282.7	263.8	358.3	293.2	329	340.8	236.6
SSH3	125	141.1	166.4	143.4	138.3	133.1	141	144
SSH3	108	118	115.7	118.5	107.3	92.6	115.1	108
SSNA1	287.1	370	326.9	321.3	293.5	262.6	326	319.4
SSPN	123.5	125.3	120.6	129.3	111.7	100	134.3	115.4
SSR1	780.9	966	832.3	857.2	755.7	615.3	653.5	759.4
SSR2	626.9	717.8	828.6	740.5	640.9	625.9	547.5	716.1
SSR3	132.7	124.8	139.5	128	114	112.7	130.2	113.7
SSR4	1216.3	1401.5	1534.4	1358.2	1109.4	1167.7	1123.8	1288.9
SSRP1	152.6	155.3	130.7	153.4	146.4	110.4	150.2	144.1
SSSCA1	118.4	135.8	126.4	138.5	111	114.5	141.7	132.1
SST	103.8	116.3	109	112.9	103.2	96.8	114.3	106.5
SSTR1	102.3	115.3	124.2	112.1	112	104.8	116.9	118.9
SSTR2	315.7	288.2	294.6	279.5	201.7	183.5	317.8	293.5
SSTR3	113.4	125.7	118.2	124.8	115.7	98.7	122.5	119.5
SSTR4	111.5	117	111.7	116.1	105	99.1	109.9	117.8
SSTR5	109.6	116.2	116.8	121	110.6	91.9	114.9	108
SSU72	3424.2	3433.4	2661.8	3621.8	2430.7	2460.4	4027.5	3138.8
SSX1	115.8	115	112.1	119	105.3	100.8	112.8	111.1
SSX2	115.3	114	114.1	114.5	116.7	105.6	117.8	110.3

SSX2	113.6	117.2	123.7	118.4	112.1	99	121.7	118.5
SSX2IP	116.6	121.3	130.6	119.4	118.3	99.3	128.1	115.8
SSX3	113.4	120.5	116.2	124.4	108.8	100.6	117.3	110.2
SSX3	121.3	120.3	129.7	118.2	113.9	112.5	134	115.6
SSX3	113	123.1	122.5	111.5	109.4	96.9	111.1	106.5
SSX4	115.4	115.5	108.2	110.5	105.5	106.8	120.2	119.3
SSX4	121.3	120.7	118.2	113.4	109.8	100	115.6	120.5
SSX4B	107.1	110.7	115.5	122	106.9	102.8	123.1	121.3
SSX4B	116.9	119.6	121.2	125.2	110.3	98.9	118.6	111.8
SSX5	124.9	118.6	120.9	131.7	110.9	102.7	117.2	129.2
SSX5	113.8	113.7	110.5	113.9	102	106.5	117.7	117.1
SSX6	114.5	114.3	122.5	120.5	108.5	100.1	116.3	112.8
SSX7	112.6	110.3	121	113.5	107.2	97.3	118.9	111.7
SSX9	109.5	117.8	114.8	120.8	110	101.9	117.7	121.3
SSX9	112.5	122.3	110.3	112.8	101.8	92.8	111.8	108.7
ST13	307.3	347.6	306.4	482.3	272.3	277.6	545.4	703.8
ST14	122.2	125.4	131.8	122.6	114.7	105.4	130.5	117.1
ST18	111	110	115	112.2	112.3	91	127.2	108
ST3GAL1	782.4	887.9	488.7	888.5	592.3	456.9	799.5	861.1
ST3GAL1	136.1	124.1	136.7	146.1	137.3	112.9	127.9	124.2
ST3GAL2	162.6	165.8	160	177.3	145.4	114.9	149	144.6
ST3GAL3	117.5	125.2	119.1	125.9	117.7	105.2	120.8	113.9
ST3GAL3	123.7	125.1	133.7	126.4	115.9	117.5	130.7	117.5
ST3GAL4	123.4	122.2	120.5	129.3	114.6	107	129.2	117
ST3GAL5	159.2	152.3	193.7	181.7	151.3	151.6	148.7	129.1
ST3GAL5	316.5	263.5	367	306	252	249.9	220.6	212
ST3GAL6	145.8	136.7	175.7	171	141.3	140.2	238.1	156.8
ST5	118.7	111.1	125.1	122	102.7	105	115.9	119.7
ST5	124.1	110.9	121.4	125.6	108.5	106.4	122.9	122.4
ST5	104.5	117.8	121.1	111.4	106.2	99	115.2	106.2
ST5	113.7	116.6	114.3	118	104.9	100.6	121.8	121.2
ST6GAL1	123.6	129	124.8	134.6	117.8	108.8	122	119.1
ST6GAL1	768.9	738.8	738.7	837.7	718.4	701.4	597.8	745.4
ST6GAL1	124.8	120.6	114.7	132.3	121.5	100.7	141.5	121.4
ST6GAL2	113.8	117.3	114.9	127.5	106.9	100.8	128.4	117.4
ST6GALNA(	114.1	117.6	121.2	117.2	102.7	97.4	114.2	108.4
ST6GALNA(	117	128.5	120.5	120.7	110.8	106.2	128.7	125.1
ST6GALNA(	120.6	125.2	122.8	131	112.9	105.1	123	112.1
ST6GALNA(	159.9	197.2	178.2	188.3	171.7	153.7	206.3	144.5
ST6GALNA(	108.4	107.3	120.4	113.3	110.9	99.7	120.1	111.6
ST6GALNA(	145.4	163.6	161.2	169	135.8	130.4	159	149.3
ST6GALNA(	108.2	122	122.6	124.6	108.5	105.7	117.5	122.9
ST6GALNA(	325.5	439.3	438.6	331.1	325	302.1	417	371.1
ST7	118.2	136.2	145.5	139.5	115.8	103.4	121.6	120.6
ST7	110	123.6	128.4	127.4	118.1	111.4	133	121.5
ST7	123.2	127.4	130.5	135	116.4	117.6	123.8	128.5
ST7L	102.1	108.9	122.2	109.5	108.9	94	115.9	117.7
ST7L	105.5	110.8	133.2	116.5	114.5	101.1	111	112.2
ST7L	108.5	112.9	120.4	114.8	107.5	97	120.7	114.3
ST8SIA1	113.3	117.9	114.3	121.5	101.4	100.1	128.1	111.8
ST8SIA2	110.4	116.6	113.1	118.6	111.9	100.6	117.7	118.1

ST8SIA3	109.3	124	121.4	122.1	108	101.5	116.5	113.5
ST8SIA4	129	116.1	130.1	120.2	111.2	134.5	124.2	120.2
ST8SIA4	110.8	109.6	113.4	113.6	109.6	97.8	123.8	109.4
ST8SIA4	166.1	168.1	234.3	166.9	137	206	143.8	141.3
ST8SIA5	115	109	117.4	111.1	103.1	98.2	116.7	115.5
ST8SIA6	119.3	115.9	116.5	117.2	113.2	99.2	119.3	119
STAB1	195.1	147.4	612.5	177.3	116.2	327.1	154.4	126.9
STAB2	116	104.8	109.5	106.8	101.9	98.4	114.4	104.2
STAC	111.4	115.6	134.7	120.6	114.4	91.9	114.7	116.9
STAC2	120.5	121.6	118.5	123.5	121.7	105.9	117.6	119.5
STAC3	115.9	119.5	127.2	129.9	111.8	102.4	110.7	132.4
STAG1	368.1	353.2	278	390.4	278	241.6	388.8	406
STAG2	125.1	125.7	116.6	131.2	119	105.8	131	140.7
STAG2	432.4	500.8	391.7	552.7	346.9	318.8	511	593.6
STAG2	116.2	127.8	132.8	129.9	133.7	113.7	150.5	128.1
STAG3	118.3	122.2	129.5	120.5	111.9	109.2	123.6	124
STAM	239.8	264.8	235.2	236.5	225.6	188	263.6	247.3
STAM2	132.3	124.7	145.3	132.5	117.6	110	130.7	127.8
STAMBP	391	502	500.8	441.7	317.6	358.1	400.2	405.9
STAMBP	112.1	108.7	114.7	115.5	114.5	94	104.6	113.2
STAMBP	232.8	247.1	270.4	223.7	210	227.8	184.3	192.2
STAMBPL1	266.7	359.2	429.6	274.5	292.8	330.9	225	197.5
STAP2	118.6	119.2	126.1	119.2	105.7	95.7	121.7	112.7
STAP2	115.2	109.8	113.5	120.6	117.1	104.9	116.3	114.4
STAP2	123	124.5	139.1	129.8	119.8	100.8	121.7	125.1
STAP2	109.2	119	114.6	122.4	112.9	103.4	128.2	119.1
STAR	121.5	112.4	110.1	113.4	108.7	95.6	123.6	118.5
STAR	109.9	124.3	119.8	115.4	108.9	101.4	119.6	112
STAR	125.4	121.5	127.4	123.2	125.5	107.1	129.4	129.7
STARD10	225.3	210.2	163.1	220.8	177.4	141.7	217.9	180.2
STARD13	106.7	113.8	99.4	124.2	101.8	99.5	118.6	113.7
STARD13	112.1	120.6	127.1	122.1	108.2	98	119	106
STARD13	118.4	120.7	119.5	118.2	113.2	99.7	117.8	118.9
STARD13	125.7	132.6	125.4	124.7	123.4	105.1	132.8	133.2
STARD3	176	156.5	176.6	181.9	131.8	158.4	150.9	163.9
STARD3NL	214.8	217.3	215.3	255.8	199.8	202.9	233.6	210.2
STARD4	112.4	103.9	115.1	115	105.6	96.6	128	118.1
STARD5	114.6	122.9	124.5	118.5	120.1	125.3	119.3	109.6
STARD5	165.1	159.7	156.1	168.8	144.1	129.5	156.8	140.3
STARD6	109.6	115.1	106.2	128.8	101.2	97.7	108.7	103.5
STARD7	1620	1718.2	1275.4	1818.6	1359.2	1330.7	1728	1131.3
STARD7	486.3	474.4	431.5	567.6	372.1	392.3	457.4	365.7
STARD7	109.5	117.4	127.2	115.1	101.7	100.1	120.4	111.7
STARD8	116.2	119.9	144	136.8	123	103.2	114.8	126.3
STAT1	540.9	221.7	697.9	424.4	185.8	496.1	195.9	122.4
STAT1	2155.5	897.1	3241.8	1663.5	556.4	2001.9	880.8	326.4
STAT1	2931.8	1590.5	3305.7	2536.4	787.4	1872.2	1330.1	614.3
STAT2	1938.7	1469.6	1541.5	1735.3	1121.4	1019.8	1283.2	970.3
STAT3	493.8	421.3	354.3	413.5	424.1	412.9	550.6	431.9
STAT4	1397.7	1409.2	1554	1378.3	1420.8	1217.9	1194	1283.6
STAT5A	632	704.5	543.9	704.3	484	500.4	555	451.7

STAT5B	557.2	671.6	388.1	569.9	594.1	374.4	531.3	503
STAT6	482.9	564.4	426.5	487.8	459.5	303.1	437.6	561.8
STATH	108.9	126.2	126.6	121.1	114.4	100.5	118.2	122.2
STATIP1	278.7	260.5	287.2	306.5	209.5	217.4	275.8	248.4
STAU	116.8	120.6	124.5	126.2	114.8	99.9	129.7	122.7
STAU	292.7	329.4	305.4	369	325.8	318.6	364.8	421.4
STAU1	1472.3	1737.5	1312.9	1756.1	1640.9	1491	2311.9	1937.8
STAU2	159.7	160	164.8	174.5	155.2	150.2	163.5	146.9
STC1	111.8	124.2	120.7	121	117.6	100.4	118.1	111.4
STC2	113	105.5	120.1	120.2	109.7	97.2	118	111.9
STCH	184.8	214.8	183.8	219.4	173.1	147.4	251.7	281.4
STEAP1	109.7	110	117.8	114.5	105.8	93	117.4	114.2
STEAP2	109.5	106.2	106.3	110.1	100	96.2	109.8	114
STEAP2	104.3	107.8	105.2	118.5	104.8	94.7	102.7	112.6
STEAP2	118	114	118.3	126.3	109	107.1	120	110.4
STEAP3	112	105.5	119.1	122	103.9	95.3	126.5	109.1
STEAP3	117.1	107.2	112.9	118	117.6	96.5	118.6	117.8
STEAP3	102	101.9	124.2	102.4	108.7	92.7	108.5	117.1
STEAP4	119.9	117.1	111.3	126.9	107	95.7	115.2	109.9
STGC3	118.8	119.9	117	108	100.9	98.2	112.1	109.5
STH	113.7	122.3	119.4	116.9	112.3	99.4	121	127.9
STIL	160.9	169.9	152.4	164.8	138.9	120.1	167.7	188.3
STIM1	193.5	175.7	172.3	167.1	156	154.8	168.5	177
STIM2	153.8	146.8	135.9	148.5	129.5	119.4	146.2	154.8
STIP1	370.7	370.4	276.9	448.9	388.7	315	1040.1	1343.6
STK10	421.6	501.6	317	416.6	329.4	271	516.8	431.4
STK11	128.4	155.7	130.1	150.2	151.4	118.3	138.4	156.2
STK11IP	258.5	288.9	256.4	252.7	188.3	184	301.9	286.8
STK16	123.5	110.9	121.8	117.7	116	104.5	119.5	111.5
STK16	125	119.4	155.3	126	117.4	120.2	116.9	121
STK16	118.8	122.3	124.8	119.6	113.7	104.2	131.6	128.8
STK17A	112.6	128.8	131.8	125.6	122.1	107.5	132.8	134.8
STK17B	115.8	116.8	129.1	126.1	109.1	105.7	115.1	120.5
STK19	287.1	315.2	221.4	263.3	264.3	215.8	278.4	308.6
STK19	197.3	233.3	227.9	245.9	187.3	225.3	264.2	227.1
STK23	111	125.5	114.5	123	104.2	98	119	108.8
STK24	108.6	114.8	121.9	121.2	113.7	97.1	126.8	118.8
STK24	108.5	131.8	125.6	123.5	110.4	100.6	118.3	112
STK24	2267.7	2643.5	2480.9	2582.7	2109	1975	2110.2	1974.5
STK25	318.7	364.9	351.7	355.9	271.2	285.7	300.1	234.1
STK3	209.7	201.9	222.8	247.5	188.2	195.6	366.6	259.3
STK31	123	117.5	111.7	120.1	116.4	105.3	113.9	115.7
STK31	115.1	117.1	118.6	119.4	106.5	97.9	122.5	126.2
STK31	117.4	114.3	115.7	121	108.9	98.7	109	125.4
STK32A	120.1	109.7	113.2	122.3	106.9	100.8	115.4	116.7
STK32B	118.3	105.8	113.6	114.4	107.6	92.1	116.1	115.3
STK32C	124.9	126.8	134.1	128.1	124.3	108	119.2	122.5
STK33	119.1	121.3	125.3	123.7	114.4	103.2	114.6	110.3
STK35	142.4	142	135.5	137.5	126	134.1	155.4	148.5
STK36	335.6	331.5	312.1	295.1	219.7	174.1	224.3	219.5
STK38	2121.1	1994.4	1059.7	2211.6	1920.3	1009	1760	1604.5

STK38L	131	124.5	124.1	143.3	118	109.7	131.2	123.5
STK39	483.9	562.2	514.3	536.5	471.8	436.1	420.9	395.5
STK4	2156.7	2734.6	2115.6	2774	2533.5	3018.2	3342.3	3313
STK40	794.6	796.4	658.8	1057.3	531.9	1004.5	1445.8	1229.5
STK6P	120.8	120.1	114.8	134.2	124.4	106.9	130.1	136.3
STMN1	117.7	120.6	110.4	103.5	111.9	97	112.5	113.2
STMN1	271.3	273.9	226.7	253.9	216.3	197.4	334.1	325.5
STMN1	129.5	139.6	139.7	144.2	144.1	126.3	142.8	173
STMN1	117.4	124.5	122.4	140.2	111.3	106.8	127.9	128.7
STMN2	126.2	118.6	116.5	132.7	113	102.7	118.5	118.3
STMN4	120.3	112	117.8	115	104.8	94.8	119.1	115.2
STOM	397.1	464.6	464.6	482.7	373.3	329	422.7	569.7
STOM	153.6	165	197.9	152.8	138.7	130.2	149.7	156.9
STOML1	120.2	136.4	122.7	131.9	115.4	108.7	115.8	127.1
STOML2	474.9	587.7	469.8	560.3	463.7	368.5	464.7	408.1
STOML3	114.7	114.4	125.4	114.5	115.7	102.4	111.6	111.8
STON1	112.4	113	128.9	116.3	107.6	99.7	114.6	118
STON2	121.9	109.9	125.9	123.2	106.7	94.9	118	112.7
STOX1	119.1	126.4	126.8	123.1	110.6	105.4	117.2	122.4
STOX2	112.1	120.9	111.8	118	110.3	103.4	130.4	117.8
STRA13	405.5	444	510	498.9	325.6	342.7	418.5	385.4
STRA6	109.1	114.6	110.3	113.7	102.4	98.5	111.5	112.9
STRAP	226.4	234.2	254.4	218.2	223	207.5	239.3	323.8
STRBP	196.3	249.6	281.5	219.8	219.4	206	229.5	227.8
STRC	126.6	143.9	161.1	134.1	148.1	113.5	133.1	150.4
STRN	127.3	126	132.7	133.4	114	103.5	138.2	133.7
STRN3	347.4	365	307.2	439.6	316.2	198	403.2	456.7
STRN4	154.4	167.6	149.7	163.6	142.7	141.4	181.5	184.3
STRN4	121.6	120	120.2	119.8	100.4	103.7	122.2	124.3
STS	121.2	111.3	123.6	117.5	105.2	97.3	118.4	116.6
STS-1	323.1	388.3	333.3	329	321.1	286.2	257	266
STT3A	309.3	301.2	331.6	331.3	298.8	267.3	306.7	296.4
STT3B	160.1	191.1	187.3	210.8	173.5	168.8	181.8	167.1
STUB1	279.9	303.3	333	300.2	300	280.4	321	282.2
STX10	508.9	566	610.5	472.1	388.4	400.9	385.3	321.5
STX11	390.6	227.7	310	376.4	203.3	319.3	288.7	237.2
STX12	162.8	169.5	175.9	176.3	154.2	154.6	188.9	182.3
STX16	112.5	112.2	114	127.6	106.9	105.4	110.6	120.9
STX16	1041	946	1393.3	1045.2	925.2	676.3	684.5	737.4
STX17	120.3	127.6	116.9	118.7	113	108.5	119.7	122.7
STX18	121.1	141	134.2	108.8	116.7	109.9	122	124.4
STX19	113.1	113.4	117.2	121.5	106.6	96.5	118.9	123.1
STX1A	123.8	148.7	124.1	130.9	116.1	109.6	129.9	151.2
STX1B2	120.2	111.5	119.2	118.4	111.2	102.3	126.7	112.7
STX2	355.4	367.6	282.7	367.6	273.3	208.3	324.6	270.7
STX2	386.7	396.9	335.5	359.5	354.8	247	347.5	344.4
STX3A	183.3	165	167.2	184.2	157	139.7	255.5	222.5
STX4A	520.7	530.1	444.5	564.4	440	433.4	579.1	489.2
STX5A	857.8	1040.8	899.2	1042.3	821.8	1025.2	1289.3	1184.9
STX5A	112.8	115.5	117.4	129	107.6	105.7	127.4	118.3
STX5A	232.5	212.1	273.4	271.2	201.5	263.8	404	335.4

STX6	419	386.2	476.6	409.5	280.8	314.7	420.2	306
STX7	121.5	107.7	120.8	117.3	109.5	102	119.2	128
STX8	151.6	183.8	239.1	161.4	155.2	144.9	147.4	141.8
STXBP1	125.8	119.2	123.3	129.5	106.3	98.6	122.5	128.8
STXBP1	120.5	125.2	122	150	122.7	116.7	134.3	122.9
STXBP1	127.4	123.9	123.8	109.9	115.4	98.5	120.9	127.5
STXBP2	474.5	401.2	453.2	404.4	267.3	476.2	380.8	308.6
STXBP3	253.3	269.1	251.9	272.2	234.7	204.8	257.3	290.8
STXBP5	257	267.9	253.5	283.7	232	225.6	350.2	390.7
STXBP6	106.2	107.2	124	120.7	99.3	98.1	107.8	108.5
STYK1	111.7	105.2	116.8	117.7	101.8	95.2	118.6	113.7
STYX	108.1	113.2	120.5	113.3	108.5	96.1	114.9	109.5
STYXL1	228.1	194.7	296.5	227.8	196	267.2	209.1	181.1
SUB1	322.6	347.1	338	297.9	265.1	255.9	331.5	415.9
SUCLA2	303.3	274.8	311.5	299.1	226.1	220.1	261.5	178.1
SUCLG1	444.1	421.6	375.4	483.3	394.7	294.9	389.9	395.2
SUCLG2	174	160.6	201.7	222.9	152.6	147.4	143.5	128.2
SUDS3	118.7	128.1	115.8	119.2	121.2	107	122.7	117.1
SUFU	107.7	113.6	113.8	111.2	108	91.7	110.1	117.3
SUGT1	230.4	218.9	196.5	212	226	185.3	215.9	225.8
SUHW1	116.9	113.2	109.5	109.3	109.7	98	111.5	104.2
SUHW2	119	114.2	124.8	122.2	108.7	115.8	117.4	111.3
SUHW3	166.4	167.2	152	197.4	172.8	137	187.2	219
SUHW4	145.4	155.3	158.2	150.7	125.8	126.5	136.5	121.9
SUHW4	134.1	128.8	145.8	142.2	110.4	122.3	132.3	126
SUHW4	128.4	114.8	125.8	116.7	115.2	105	117.8	123.2
SULF1	110.4	124.2	114.4	127.1	106.5	102	114	114.3
SULF2	119.6	117.4	138	134.4	125.6	112.3	128.9	120.6
SULF2	114.5	122	112.4	111.7	113.6	96.8	121.6	107.4
SULT1A1	133.3	134.8	127.1	123.9	117.7	111.8	130.5	132
SULT1A1	118.3	109	115.2	121.7	104.6	97.8	114.4	114.5
SULT1A1	1438	1433	1179.9	1330	1012.7	765.4	1829.6	1196.4
SULT1A1	115.8	115	124.5	129.2	115	105.8	112.9	119.6
SULT1A2	118.8	107.7	118.9	120.2	107.6	98.8	128.3	117.9
SULT1A2	138.9	146.1	174.8	156.8	135.8	126.8	158.8	173.3
SULT1A3	128.9	127.3	130.4	134.7	116.4	108.1	124.1	121.7
SULT1A4	242.4	274.7	264.4	242.4	221.1	203.3	255.4	256.6
SULT1C1	114.3	111.9	118.3	121.6	104	96.4	110.4	115.8
SULT1C1	108.1	104.6	108.7	118.9	103.8	92.8	105.1	106.3
SULT1C2	112.1	115	122.8	108.5	107.2	91.7	118.5	116.5
SULT1C3	111.5	114.2	115	116.1	105.1	96.7	111	109.2
SULT1E1	116.7	124.9	119.1	127	118.4	101.3	118.4	126.7
SULT2A1	114.9	113.5	112.7	114	107.9	96.2	115.5	109.3
SULT2B1	109.4	115.6	113.7	114	107.4	104	124.3	118.5
SULT2B1	145	149.6	153.4	160.1	126.6	138.1	167.3	153.5
SULT2B1	111.5	113.4	113.1	120.9	106.4	99.1	115.7	112.9
SULT4A1	116	117.8	119.5	117.9	106.7	103	110.4	109.4
SULT6B1	126.4	128.9	121.5	125.6	119.4	104.4	130.6	120.6
SUMF1	218.9	169.1	219.6	211	143.9	164	166.7	184.7
SUMF2	141.7	135.7	148.3	142.4	142.3	126.8	126.1	131.1
SUMF2	924.9	929.9	1311.2	1057.1	983.1	892.9	836.5	839.8

SUMO1	117.5	124.1	130	124.6	115.5	130.6	127.1	135.2
SUMO1	117.3	118.5	114.7	121.7	114.8	103.5	123.7	113.7
SUMO1P1	122.4	113	123.3	118.2	114.2	98.2	112	113.5
SUMO1P3	113.8	103	116.4	106.9	111.3	98.1	118.2	111.5
SUMO2	293.5	330.6	406.8	290.7	286.1	417.1	364	394.4
SUMO2	1107.1	1077.1	987.9	1107.5	1083.9	690.3	895.5	928.3
SUMO3	1491.7	1605.3	2110	1533	1144.7	1498.9	1222.7	1149.8
SUMO4	116	120	120.4	116.2	116.2	96.5	121.7	100.9
SUNC1	126.7	114.7	118.9	129.6	115.7	109	128.9	122.5
SUNC1	114.2	117.3	120.2	130.5	114.8	96.5	111.8	114.2
SUOX	157	144.3	206.9	158.3	133.7	152.6	170.2	197.5
SUOX	119	111.5	110.9	117.7	115.1	103.8	123.9	113.4
SUOX	114.3	115.7	132	124.7	101.4	101.7	115.9	132.9
SUPT16H	591.4	669.7	542.3	687.4	596.4	499.2	693.7	657.1
SUPT3H	116.1	115	130.7	118.9	114.2	114.9	123	120.7
SUPT3H	148.2	135.5	148.5	132.5	107.1	150.8	124.7	118.5
SUPT4H1	338.3	385.2	456.4	390.2	308.2	381.7	426.5	416.6
SUPT5H	472.7	461.4	450.5	514.1	388.7	350.1	554	556
SUPT6H	125.1	134.5	130.8	142.7	135.2	118.6	158.1	140.2
SUPT7L	120	131	133.8	117.9	123.4	102	115.7	117.5
SUPV3L1	179.7	210.8	179	191.8	176.6	137.5	185.4	170.6
SURB7	122.9	124.6	132.9	133.3	121	105.6	123.4	122.9
SURF1	249.5	240.6	304.7	211.5	200.2	219	208.9	155.5
SURF2	133	130	143.1	139.1	128.2	103.2	129.6	135.3
SURF4	428.4	452.8	451.3	450.7	344.8	458.2	558.4	555.4
SURF5	140.3	155.8	149.5	156.8	134.6	134.8	142.1	134.6
SURF5	401.3	479.2	453.5	502.1	374	310.8	435.4	369.8
SURF6	329.6	426.9	309.1	349.5	339.8	286.3	371.3	323.1
SUSD1	205.4	179.1	273	255.3	153.2	219.5	250.2	186.9
SUSD2	131.6	122.1	132.9	130.3	110.5	105.8	131.7	120.3
SUSD3	461.1	506.4	565.5	422.1	388.9	462.6	321.2	344
SUSD4	116.8	114.3	105.8	113.4	106.9	90.2	111.5	106.1
SUSD4	121.7	101.4	128.8	114.4	112.6	96.6	108.7	122.4
SUV39H1	281	310.5	278.3	306.5	221.3	195.7	240.9	256.6
SUV39H2	137.9	146.6	131.5	147.4	125.4	112.6	141.2	133.4
SUV420H1	126.4	118	116.8	136.6	130.4	110.3	140.1	134.9
SUV420H1	170.2	166.4	178.5	167.1	160.9	136.6	148.2	159.4
SUV420H1	129.9	167.3	154	145.4	137.6	151.2	163.7	145.2
SUV420H2	112.9	118.9	122.2	136.3	114.9	104.4	126	109.4
SUZ12	2059.5	2312.1	1983.1	2552.6	2078.2	1542.8	2213.2	2679.9
SV2A	131.8	142	140.4	132.2	118.3	99.1	122.2	121.3
SV2B	112.7	125.8	119.7	130.9	110.9	110	121.2	111.5
SV2C	114.1	110.9	123.8	113.9	108.3	98	118.8	116
SVEP1	109.9	116.1	119.7	119.7	107.9	103.8	118.1	119.4
SVIL	115.5	115.7	109	118.3	103.4	98.1	116.1	115.3
SVIL	139.5	135.3	137.3	143.8	130.7	106	137.9	137.2
SVIL	354.6	353.6	250.7	356	245.1	221.2	260.9	275.3
SVOP	118.6	115.6	109.8	123.3	107.5	101.8	114.9	114.3
SWAP70	481.8	315.2	547.8	557.1	309.1	675.9	514	386.2
SYAP1	187.9	183.7	173.5	205.8	160.9	141.8	181.1	194.7
SYBL1	462.7	455.7	582.5	558.1	449.5	501.2	553.6	440.6

SYCE1	111.1	116.6	113.7	129.5	112.4	100.4	123.9	115
SYCE1	107.2	114.6	111.2	121.6	102.7	101.7	120.2	114
SYCN	111.5	115.3	119.8	113.9	114.5	99.6	117.3	116.3
SYCP1	121	113.6	111.7	115.3	104.8	93.4	112.5	109
SYCP2	122.8	119.7	121.9	132.8	116.2	102.8	122.8	116.7
SYCP3	116.7	114.8	121.7	115	110.6	103	125.9	119.7
SYDE1	118.2	115.9	127.1	107.9	105.2	99.3	116.7	108.3
SYF2	508.3	561.9	544.1	612.6	510.3	472.2	633.5	714.3
SYF2	117.2	114.3	117.2	111.9	107.1	99.1	117.9	106.7
SYF2	1215.8	1341.2	1300.4	1570.4	1204.8	1083.1	1238.4	1730.8
SYK	358.7	300.2	551.5	380.6	230.7	331.1	272.5	220.4
SYMPK	143.5	152.3	156.7	144.6	128.7	118.4	143	139.9
SYN1	116.3	106.5	113.7	117.8	109	96.5	112.9	106.7
SYN1	128.8	139.7	120.7	133.9	127.6	103	134	137.3
SYN1	107.9	116.6	113.3	119.4	105.6	98.7	115.1	116.5
SYN2	110	122.3	132.8	117	115.3	102.9	118.9	114.2
SYN2	108.9	109.5	115.2	117.8	109.4	99.7	119.2	111.7
SYN3	116.9	116.8	129.2	114	107.4	97.5	112.6	116.7
SYN3	124	111	112.8	134.4	114.7	98.4	125.3	121.7
SYNC1	119.2	114.8	125.3	120.9	111.7	102.2	126	122.7
SYNCRIP	280.4	346.2	290.7	333.6	335.5	237.4	309.3	335.7
SYNE1	112.2	119.6	120.2	125.6	113	106.1	118.9	119.5
SYNE1	119.9	111.5	115.1	119.1	108.9	94.5	116.5	115.3
SYNE1	128	129.4	136	119.6	110.2	108	126.7	130.8
SYNE1	124.9	119.1	118.7	115.2	109.9	103.9	123.3	117.2
SYNE2	129	130.2	144.6	164.6	133.5	126.5	153.3	139.2
SYNE2	167.3	211.1	145	171.7	190.4	149.9	201.9	185.5
SYNE2	114.8	126.5	119.2	120.9	108.5	104.3	126.7	136.7
SYNE2	109.3	116.2	118.9	116.3	119.1	94.6	121.3	107.1
SYNGAP1	117	115.4	120	115.3	113.1	99.5	123.7	109.6
SYNGR1	112.5	116.8	123	123.1	109.4	103.9	127.5	117.5
SYNGR1	124.9	118.8	133	119.6	118.1	98.7	116	118.3
SYNGR1	118.5	118.4	119.9	129.9	125.1	105.2	120.2	122.8
SYNGR1	141.9	146.5	152.7	147.3	125.8	110.3	130.8	118.5
SYNGR2	104.3	110	113.7	115.3	108.9	100.2	113	115.2
SYNGR3	108.2	108.2	111.4	105.4	111.5	101.6	113.4	108.6
SYNGR4	114.5	108	119.8	123.6	118.5	97	119.7	114.9
SYNJ1	220	198	191.1	247.4	212.5	179	286.6	238.9
SYNJ1	121.8	110.1	128.1	126.8	107.2	108.3	126	114.2
SYNJ1	102.2	107.7	118.4	116.9	103.9	97.2	101.7	114.3
SYNJ2	159.8	178.3	147.2	175.8	164.2	125.6	187.8	214.3
SYNJ2BP	272.4	284.2	348	255.4	231.8	184.6	217.7	213.8
SYNPO	124.3	116.7	117.2	125.6	111.7	101.4	131.8	121.1
SYNPO2	115.7	118.9	113.9	116.9	107.5	99.6	128	119.2
SYNPO2	117.4	121.9	127.2	119.2	114.7	101.5	119.6	117.7
SYNPO2L	110.3	109.7	118.9	113.2	112.8	104.7	117.6	103
SYNPR	113.8	108.1	116.2	115.8	107	96.9	107.2	111.7
SYP	116.3	123.1	125.4	130.9	110.3	101.3	109.7	119.7
SYPL1	791.7	650.4	829.9	741.5	798.5	659.5	763.2	833.9
SYPL1	174	166.1	173	173.4	151.2	144.4	160	157.6
SYPL1	117.1	114.7	120.2	120.8	115.5	102.5	114.7	116.8

SYPL2	117.5	118.5	114.2	119.4	104.6	101.3	108.2	112.3
SYT1	113.4	109.6	113.4	115.5	109.9	94.6	119.9	112.2
SYT10	109.9	113.5	113.2	114.1	108.3	98.6	124.4	119.5
SYT11	571.9	670	692.6	769.6	598.6	350.8	585.6	542.1
SYT12	121.9	107.1	119.1	118.1	113.8	94.1	125.8	117.6
SYT13	113.9	115.7	113.3	111.6	106.3	92	110.8	113.7
SYT14	110	120	111.9	112.3	100.5	95.9	116.6	115.9
SYT15	146.4	152.8	154.2	141	143.4	128.8	142.1	157.8
SYT15	129.2	131.1	125.1	139.5	119.7	99.9	119.4	119.5
SYT16	122.6	111.8	133.2	122.2	108.3	108.5	135.5	135.5
SYT17	118.8	128.8	117.6	125.1	113.6	101.8	125.7	112.2
SYT2	109.9	111.4	112.6	116.5	107.2	97.9	111.2	105.3
SYT3	113.4	126.8	143.3	142.3	124.4	105.8	137.9	131.3
SYT4	122.5	119.2	115.7	124.1	106.5	102.4	118.6	112.6
SYT5	127.5	110.6	126.6	118.8	105.3	97.1	115.8	121.3
SYT6	115.2	109.3	123.7	121.2	105.8	100	122.2	108.7
SYT7	117.1	129.6	122.5	145.7	113.5	109.3	123.6	111.5
SYT7	141.3	131.1	125	130.5	115.3	112.4	131.6	123.6
SYT8	111	115	113.5	111.4	107.6	101.1	116.5	116.5
SYT9	108	115.5	104.2	119.7	104.2	102.3	119.7	133.9
SYTL1	252.2	202.2	226.5	186.2	147	157.4	172.2	122.1
SYTL2	121.8	116.6	123	119.6	114.4	99.9	137.5	127.8
SYTL2	131.1	141.9	123	121.7	109.2	107.3	122.9	116.4
SYTL2	119.1	109.6	118.3	124.9	105.8	101	117.3	120.3
SYTL2	604.1	529.7	412.5	808	466.6	323.2	916.4	864
SYTL3	215.1	213.5	151.6	193.4	171.5	141.6	188.8	197.7
SYTL4	113.5	107.4	106.2	120.6	107.3	99.8	114.7	119.8
SYTL5	125.3	115.2	127.3	132.7	103.9	104.1	118.6	122.3
SYVN1	294.3	257.5	224.8	354.4	239.2	224.8	284.7	275.2
SYVN1	129.6	120.7	126.3	127.6	119	109.2	136.7	134.7
T	110.8	107.6	103.2	106	103.8	95.4	112.8	112.1
T2R55	109.7	114.8	122	119.7	109.7	100.9	126.6	115
TAAR1	122.5	127.5	126.2	121.9	117.6	103	125	118.1
TAAR2	112.1	111.1	128.2	119.1	111.8	99.4	112.3	121
TAAR5	117.7	119.7	120.5	118.3	117.6	99.6	119	114.7
TAAR8	128.4	131.8	123.6	130.3	111.1	96.8	116.7	125.6
TAAR9	116	111.6	121.9	115.1	108	104	118.1	110.5
TAB3	119.3	111.1	111.7	116.2	105.4	98.7	130.6	112.9
TAB3	111.9	119.6	113.1	121.7	109.7	98	120	117.5
TAC1	119.9	116.4	119.5	114.8	108.7	92.5	110.1	110.4
TAC3	111.9	112.1	120.4	119.6	107.3	97.4	123.2	111.3
TAC3	119.1	123.7	125.9	133.2	105.8	105.9	128.6	136.3
TAC3	105.2	109.2	114.2	115.1	107.6	93.1	116.1	114.8
TAC4	102.1	116.4	119.2	118.8	107.8	100.9	115.3	113.4
TAC4	115.8	115.5	117.4	115.4	104.8	97.8	123.8	117.1
TACC1	1770	1476.9	1838.5	1696.1	1286.1	1596	1904.6	1617.6
TACC2	115.2	114.3	113.6	110.7	102.4	95.8	109.7	114
TACC2	113.9	107.9	112	121.2	106.3	93.4	113.9	112.6
TACC2	123.2	122.8	117.3	119.2	109.1	113.6	115.6	109.7
TACC3	178.2	166.4	188.3	162.8	127.2	161.7	173.6	174
TACR1	113.8	125.1	116.1	122.6	102.7	99.3	119.9	115.3

TACR1	116.2	119.2	123.8	117.6	111.1	103.2	129.7	124.6
TACR1	116.8	118.5	118.4	119.6	115	98.6	113	120.9
TACR2	115.2	111.8	105.8	113.2	99.8	101.7	122.2	112.2
TACR3	119.7	118.3	135.4	132.7	120.5	104.8	132.7	125.3
TACSTD1	118.2	131.8	137.4	130.9	115.6	107.9	126.4	123.6
TACSTD2	113.6	131.3	119.6	132.9	112.3	108.1	124.6	111.9
TADA1L	186.6	209.4	179.5	215.2	179.9	135.7	216.5	226.7
TADA2L	117.1	120.9	123.5	117.4	111.1	96.9	115.4	112.7
TADA2L	135.7	120.9	126.6	115.8	119.3	110.2	125.9	112.4
TADA2L	110.3	110.5	118.8	115.1	106.2	102.8	111.7	106.8
TADA3L	149.1	143.8	167.9	169.1	146.7	126.5	151.1	165.8
TADA3L	168.8	153.4	145.5	157.6	138.1	131.6	164.8	141.2
TADA3L	105	117.9	116.7	117	113.2	104.8	124.1	116.6
TAF1	134.5	128.1	121.4	148.7	136.1	117.8	127.7	148.9
TAF1	130.6	131.8	127.5	139.8	117.8	112.6	144.6	133.8
TAF10	478.1	670.2	601.2	500.2	430.1	471.2	448.4	402.1
TAF11	118.4	113.4	116.6	121.3	108.5	102.5	118	124.9
TAF13	106.2	122.7	109.3	124.7	112.6	102.9	122.8	117.3
TAF15	2188.3	2281.1	1151.3	2284.5	2167.4	1296.8	2308	2236.7
TAF15	200.1	216	165.8	228	233.5	146	187.3	206.4
TAF1A	133.2	118	122.4	134	113.5	107	130.4	122.6
TAF1A	113.4	109.1	118.7	118.1	107.6	99.1	117.5	111.4
TAF1B	159.3	175.7	152.1	145.4	151.3	122.7	156.6	163.8
TAF1C	111.8	105.2	123.1	123.8	116.6	100.5	119.2	113.3
TAF1C	174.9	153.1	193	167.7	118.5	143.4	167.1	143.1
TAF1C	909.1	958	853.8	896.2	663.6	536.3	761.2	589.6
TAF1L	189.1	192.1	210.7	210.3	184.3	145.2	166.3	183.7
TAF2	259.1	246.3	241.2	277.9	239	192.7	252.4	281.2
TAF4	484.8	512.7	688.3	621.1	586.5	530.7	612	579.3
TAF5	225	231.6	196.7	195.9	178.8	159.8	174	169.3
TAF5L	117.1	115.6	112.5	120.4	112.6	97	112.9	109.2
TAF5L	150.2	135.5	111.9	148	138.5	106.1	145.8	168.1
TAF6	128	111	123.8	126.8	128.2	109.3	132.8	123.1
TAF6	126	133.1	122.8	125.5	110.8	108.8	133.7	129.6
TAF6L	372.6	424.6	351	447.1	287.2	230.8	493.2	476
TAF7	219.5	164.7	257.9	220.8	216.1	225.9	215.2	326.2
TAF7L	110	113.3	108.6	116.2	107	99	117.3	112.6
TAF9	142.3	136	140.2	145	139.3	123.9	140.4	145.1
TAF9	130.6	130.6	144.9	139.3	139.6	118.4	139.4	150.5
TAF9B	108.2	115.9	119.4	119	115.8	96.7	118.6	110.9
TAGAP	177.2	166.1	152	171.2	161.4	113.8	167.1	159.4
TAGAP	192.7	190.1	183.7	201.6	189.1	137.6	182.5	199.2
TAGAP	187.1	157.7	185.8	180.1	152.7	125.6	159	165.1
TAGLN	135.1	134.6	167.8	148.2	141.5	139.6	169.2	209.7
TAGLN	128.3	123.2	137.6	159.7	113.8	115.4	125.2	136.8
TAGLN	117.6	117.8	104.7	117.9	106.8	95.2	120.9	111.1
TAGLN2	733.7	1033.4	773.3	801.1	551.8	689	586.4	452.9
TAGLN3	118.4	121.7	108.3	115.4	106.3	97.8	120.5	114.3
TAGLN3	117.8	118.5	112	124.5	110.3	96.6	117.5	117.8
TAL1	132.4	120.3	128.8	124.5	116.4	116.8	133.4	128.2
TAL2	114	114.3	113.9	119.1	99.9	100.2	115.5	105.2

TALDO1	3548.8	2418.3	2904.1	5197.7	1932.4	5188.6	3603.6	2455.3
TANC1	128.3	125.6	117.3	132.1	114.7	107.2	136.4	112.4
TA-NFKBH	130.1	131.2	118	139.5	123.7	121	156	132.1
TANK	144.6	141.6	155.2	176.2	141.4	154.3	144.9	154
TANK	115.7	110.7	122	110.5	103.6	96.7	118.3	118.2
TAOK1	195.3	205.7	158.8	241.5	181.3	121	240.6	258.7
TAOK2	187.1	193.5	167.1	168.8	139.1	120.3	194.5	185.6
TAOK2	134.2	106.9	121.6	128.4	116.4	107	116.5	131.7
TAOK2	115.5	107.5	119.9	127.4	113.6	98.8	120.2	114.3
TAOK3	111.1	106	115.6	121.3	109.6	93.5	115.6	113.4
TAP1	3046.2	2627.3	2233.2	2962.5	1570.4	1655.3	1545.1	1103.3
TAP2	217.3	210.2	204.8	211.1	169.2	146.2	185.7	147.1
TAP2	339.7	392.3	416.2	375.7	350.7	274.7	297	253.7
TAPBP	1593	1453.6	1326.2	1383.5	719.9	1029	1064.2	797.3
TAPBP	126.7	136.7	133.8	131.6	129.5	106	131.3	125.1
TAPBP	119.8	122.7	136.8	136	121.1	110.1	118.3	117.4
TAPBPL	170.4	175.9	202.4	162	157.8	141.4	167.6	128.3
TA-PP2C	291.3	269.7	349.3	347.8	243.5	226.3	290	308.4
TARBP1	159.1	163.2	167.4	151.2	131.6	135.2	141.5	126.8
TARBP2	143.2	158.2	160	158.6	144.4	123.1	151.9	142.7
TARBP2	160.3	156	194.5	169.5	151.6	138	156.4	166.2
TARDBP	149.9	137.9	150.1	174.6	131.3	132.6	148.2	139
TARP	168.4	202.8	174.8	190.5	175.2	159.5	165.2	180.8
TARS	279	317.5	272.8	295.2	255.8	255.8	369.3	592.6
TARSL1	125.1	141.3	134.5	133	118.7	114	134.7	122.1
TAS1R1	119.5	109.3	114.8	116.7	106.4	94.7	118	109.4
TAS1R1	110.1	112.2	114.5	115.9	106.6	104.2	106.4	114.2
TAS1R2	120.6	112.4	129.8	120.6	114.8	97.8	119.2	123.2
TAS1R3	117.5	106.8	109.9	114.1	108.3	104.2	117.5	111.9
TAS2R1	107	111.2	110	109.2	106.3	94.3	111.7	121.9
TAS2R10	118.4	115.6	110.3	118	110.9	96.1	122.2	115
TAS2R13	111.9	106.4	117.5	113.5	109.5	101.4	114.7	112.9
TAS2R14	110.2	127.2	121.7	125.5	107.3	106.3	114.3	122.8
TAS2R16	114.2	110.9	124	122.1	115.5	102.5	114.2	118.1
TAS2R3	121.4	121.3	121	116.8	109.8	105.7	117.4	119.4
TAS2R38	114.2	113.5	130.5	127.6	113.6	103.2	118.7	112.3
TAS2R39	119.6	113.9	115	114	104.5	100.9	114.7	116.3
TAS2R4	113.9	112.4	114.8	123.2	116.6	96	121.4	114.7
TAS2R40	105.3	106.1	119.1	121.3	113.2	97.1	116.6	112.1
TAS2R43	118.5	125.1	126.8	127.8	114.3	97.6	123.8	120
TAS2R44	120.7	112.4	125.3	116.4	116.4	100.3	118.4	112.5
TAS2R45	113.1	119.7	119.5	128.6	113.1	94.3	110.1	108.7
TAS2R46	117.2	112.3	105.9	116.6	114	101.5	123.6	122.9
TAS2R48	108.7	114.9	117.3	116.9	105.2	95	124.1	108.3
TAS2R49	120.9	117.6	120.5	121.5	113	101	118.3	111.5
TAS2R5	116.9	117.6	110.3	125.3	112.2	97.6	114.4	118.8
TAS2R50	114.3	116.1	114.1	118.2	105.2	96.5	117.2	116.1
TAS2R60	120	122.6	119.8	123	115.2	112.4	113.8	121.8
TAS2R7	114.9	114.6	127.3	127.9	107	98.8	120.3	116.1
TAS2R8	116.1	111.6	119.5	127.3	110.8	94	126.6	121.8
TAS2R9	118.1	113	126	113	109.7	98.7	119.2	119.2

TASP1	136.5	144.1	148.9	139.4	137.4	115.5	142.4	134.8
TAT	119.6	113.2	117.1	117.2	112.7	96.9	111.6	112.9
TATDN1	164.4	145.6	187.7	179.3	136.3	170.6	158	165.1
TATDN2	207.1	198.8	172.8	210.8	172.2	168.2	208.7	204.9
TATDN3	164.3	156.2	251.9	175.8	144.3	164.1	179.8	158
TATDN3	150.3	144.9	262.9	167.1	145.6	160.9	137.7	148.4
TAX1BP1	1002.5	1164.5	907.2	1148.5	1031.3	948.5	1309.7	1351.6
TAX1BP1	1036.5	1070.1	881	1327.8	1101	1181.4	1252.3	1721.8
TAX1BP3	257.1	244.6	384.4	237.5	177.6	171.6	173.6	150
TAZ	151.6	183.9	181.6	171.7	159.8	142	173	160
TAZ	124.2	122.5	135.2	116	126.5	107.7	118.3	114.4
TAZ	198.3	193.4	176.3	204.9	171.8	156.5	189.7	205.4
TBC1D1	113.7	114.6	127.2	127.6	119.5	102.6	134.2	118
TBC1D10A	164.7	150.9	165.4	154.5	155.8	148.4	140.1	156.4
TBC1D10B	149.4	166.6	156.7	156.7	152.5	133.1	188.1	169.1
TBC1D10C	2561.9	2920.5	2573.4	2665.8	1805.4	2211.2	2483.8	2681
TBC1D13	281.3	195.1	234.2	257.2	161.8	226.3	256.9	182.5
TBC1D14	530.6	589.8	629.9	649.1	540.2	552.4	684.9	708.7
TBC1D15	510.4	517.2	359.7	596	611.7	357.6	886.9	1304.5
TBC1D16	127.2	136.2	157.3	138.2	134.6	122.1	135.9	144.9
TBC1D17	125.2	146.7	130.8	133.4	116.1	111.1	153.4	159.6
TBC1D19	128.6	128.4	137.6	139.8	124.2	113.2	127.8	130
TBC1D2	277	200.7	171.4	347.5	225.3	143.4	551.3	363
TBC1D20	195.5	209.5	191.7	212.7	195.2	181	247.4	256.4
TBC1D21	107.8	107.1	128	122	106.1	108.4	122.4	122.6
TBC1D22A	125.9	113.7	121.9	122.1	104	102.1	123.1	112.2
TBC1D22B	178.6	170.4	138	157.3	144.5	122.4	186.9	182.8
TBC1D23	155.1	177	159.8	182.6	152.5	130.4	216.7	180.4
TBC1D24	126	154.4	123.1	131.2	114.8	105.9	129.4	135.7
TBC1D2B	603.1	695	652.2	505.6	472.8	567.3	456.2	348.4
TBC1D3	117	110.3	109.7	130.4	114.4	91.4	112.7	110.7
TBC1D3B	148.4	149.2	154.9	162.6	132.4	121.9	143.8	149.4
TBC1D3C	109.9	115.7	116.5	129.3	107.7	99.4	116.1	117.5
TBC1D3G	138.4	146.5	138.7	165.8	124	122.4	137.2	144.8
TBC1D4	289.6	335.9	309.4	341.9	336.3	323.1	383.3	368.5
TBC1D5	121	121.6	137.9	129.9	116	104	137.6	120.8
TBC1D7	133.6	196.5	154.7	132.7	164.2	135.5	218.5	206.2
TBC1D8B	114.2	105.7	112	112.8	105.4	98.8	127.1	115.9
TBC1D9	184.5	172.6	230.1	177.4	132.1	125	131	145
TBC1D9B	161.7	155.7	147.6	143.5	122.3	116.5	151.4	171.3
TBC1D9B	138.1	148.9	146.2	153.2	130.7	130.4	156.1	131
TBCA	2242.3	1945.9	2489.8	2480.3	2225.4	2611.4	2848.9	2678.7
TBCC	360.6	434.4	302.8	375.7	296.5	279.8	264.9	306.8
TBCCD1	116	121.9	127.8	117.7	109	99.3	117.8	116.1
TBCD	144.9	131.1	185.9	142	124.4	141.7	139.4	133
TBCD	443.4	531.4	900.5	469.1	341.4	679	394.3	365.6
TBCE	238.7	232.7	262.5	249.1	217.5	202.9	202.3	293.7
TBCE	132.4	122.5	131.3	130.2	121.7	103.2	137	132.7
TBK1	395.2	490.1	374.2	617.4	475.3	433.6	711.6	610.2
TBKBP1	121.8	117.6	107.3	124.5	108.4	102	124.5	118.1
TBL1X	435.9	446.9	435.7	558.7	464.7	385.4	671	638.4

TBL1XR1	128.7	136.5	138.9	139.8	121.4	116.6	141.5	145.1
TBL1Y	113.4	118.5	110.9	116.7	111	106.5	117.2	121.1
TBL1Y	112.8	115.6	112.2	118.9	110.2	94.4	123.5	113.4
TBL2	138	147.5	159.6	163.6	148.5	135.6	132.6	153.7
TBL3	136.1	137.2	139	138.6	121.7	106.8	141.3	119.4
TBN	115.1	117.4	120.6	122.3	113.3	106.7	123.4	131.9
TBP	319.5	280.8	297.9	311.8	286	235	287.8	306.9
TBPIP	113.8	119.7	111	120.8	110.7	98.6	119.9	119.8
TBPL1	392.6	481	501.1	417.2	403.9	285.2	424.8	459.1
TBPL2	112.6	123	124.4	116.5	111.3	103	123.8	106.1
TBR1	106.1	108.1	116.4	116.2	111.9	101.5	111.6	104.3
TBRG1	107.3	113.1	122.5	119.3	116.7	112.6	119.3	129.1
TBRG4	264.1	255.5	200.2	286.8	206.5	213.2	282.6	240.7
TBRG4	272.4	381.6	353.5	378.1	266.9	298.1	418.5	341.7
TBRG4	118	123.8	118.8	117.9	112.3	97.5	121.8	117.4
TBX1	121.1	114.3	114.3	106.3	114.3	106.6	119.1	114.6
TBX1	118	110.4	107	116.1	105	102.6	114.3	111.4
TBX1	119.1	109.3	115.1	112.8	107.2	98	109.1	100.9
TBX1	115.1	115	111.2	119.6	98.9	104.6	110.1	114.9
TBX10	101.5	114.7	123.6	124	105.3	97.4	111.6	112.2
TBX15	117	121.6	132.6	128.4	117.2	103	121.8	117.8
TBX18	118.7	122.1	134.9	123.5	114.7	108.4	118.7	116.4
TBX18	115.1	111.6	116.9	124.2	109.2	100.3	116.8	120.7
TBX19	138.4	143.2	138.8	137.7	120.4	115.6	134.7	123.6
TBX2	112.1	117.8	121.3	121.6	109.7	100.2	123.1	122.4
TBX20	113.5	114.5	119.1	119.1	114.1	99.8	115.9	114.5
TBX20	117.2	121.1	117.7	120.9	110.1	103.1	116.3	114.3
TBX21	130.7	130.6	132.4	143.1	114.5	103.8	126	132.4
TBX22	118	102.9	115.7	115.8	107.3	93.8	118.2	120.3
TBX3	111.9	131	121.9	122.5	116.5	95.5	120.6	117.3
TBX3	119.2	123.5	121.8	122.2	116.1	97.8	121.7	122.9
TBX4	109.9	111.7	117.3	115.3	101.9	99.1	124.1	113.9
TBX5	114.2	108.4	110.4	107.4	113.1	99.3	112.9	106.5
TBX5	111.8	114.5	115.3	117.5	104.8	99.2	116.8	116
TBX5	116	110.6	110.8	123.1	104.6	94.4	120.9	117.6
TBX6	122.9	111.4	134.4	122.5	117.8	99.2	121.3	120.2
TBX6	114	125.5	120.3	128.6	103.7	99.3	115.4	116.8
TBX6	121.8	112.8	114.7	123.5	118.9	101	120	115.8
TBXA2R	121.5	122.7	123.2	124	114.9	95.5	110.1	113.3
TBXA2R	116.2	121.8	109	127.9	112.4	104.3	113.2	112.9
TBXA2R	116.6	120.7	131.8	128	114.9	111.3	124.8	143.2
TBXAS1	402.8	260.1	925	558.6	261.7	716.5	392	249.5
TCAP	109.7	119.7	122.4	112.8	115.3	115.7	122.7	122.5
TCBA1	111.8	116.5	123.3	107.3	106.1	94.3	122.4	109.4
TCEA1	282.2	311.7	309	410.4	303	288.4	262.3	260.6
TCEA1	118.1	117.2	121.1	118.1	107.4	109.5	117.4	120.3
TCEA1	125.5	133.2	134.3	136.3	119	118.3	125.5	119.7
TCEA2	199.5	183.9	231.8	194	150.9	166.7	157.6	154.4
TCEA2	112.9	122.9	120.9	113.9	111.4	103.7	115.4	119
TCEA2	124.5	119.9	129.5	132.5	113.1	117.1	121.7	116.9
TCEA3	185.5	236	185.2	175.3	161.8	131.3	138.1	153.6

TCEAL1	143.4	131.6	127.4	152.1	136.4	116	127	145.6
TCEAL1	126.5	122.8	120.8	130	114.9	103.5	126	115.2
TCEAL2	148.1	137.5	150	163.3	124.1	118.7	142.9	127.8
TCEAL3	267.7	315.9	258	250.4	216.9	210.6	243	215.9
TCEAL3	508.8	540.3	489.8	576.9	450.9	386	401.8	401.6
TCEAL4	125	124.5	124	118.5	114.3	102	122	118.3
TCEAL4	135.9	154.5	168.7	139.3	130	116.4	129.5	148.1
TCEAL4	530.1	679.7	558.2	489.8	541.4	371.3	401.7	553.7
TCEAL5	116.6	126.3	121.5	128.1	118.4	111	136.6	123.4
TCEAL6	105.5	119.8	111.8	108.8	106.5	102.1	118.8	109.2
TCEAL7	116.3	110.8	112.7	107.1	100.9	97.3	116.4	108.9
TCEAL8	342.2	324.1	384.4	400.1	292.1	232.1	220.2	247.4
TCEAL8	209.4	216.4	212.8	220.4	215	159.8	171.6	175.6
TCEAL8	359.6	414.8	307.1	351	265.4	216.4	279.9	279.3
TCEB1	496.8	517.3	681	685.3	650.3	578.3	984.8	918.9
TCEB2	126.3	137.7	143.2	133.6	135.9	128.4	148.8	191.6
TCEB2	1429.2	1652.7	1818	1678.2	1319.1	1594.5	1402.7	1515.8
TCEB3	208.6	219.4	175.2	213.3	185	161.5	238.8	206.2
TCEB3B	107.5	109.1	116.8	101.6	103.2	93.2	114.1	111.7
TCEB3C	111.3	111.4	111.8	107	104.5	97.6	121.5	114.6
TCERG1	157.9	160.2	177.6	165.1	174.3	146.3	193.2	179.8
TCERG1	116.7	125.8	111.6	121	107.4	99.7	123.9	115.8
TCERG1	164.7	162	140.3	187.7	179.8	136.5	200.1	178
TCERG1L	119.6	110.9	113.8	119.8	113.4	101.7	110.3	113.1
TCF1	106.6	118.7	99.4	126.3	99.5	106.4	120.1	116.6
TCF12	262	291.2	319	331.3	303.7	221.1	320.5	363.8
TCF12	107.4	120.3	110.7	116.4	110.7	96.7	116	115.9
TCF15	128.6	120.5	125.1	126.3	113.7	108.9	133.9	115.4
TCF19	112.5	114.6	105.8	119.8	120.9	99.5	116	120.8
TCF19	112.2	126	116.5	125.5	118.7	106.3	116.3	124.8
TCF2	115.6	109.8	117.1	112.6	118.4	99.8	129.3	102
TCF2	109.7	120.3	119.2	119.6	107.6	96.2	122.5	113.2
TCF2	109.9	117.5	109.8	116.8	111.3	104.1	118.2	116.4
TCF20	142.1	137.6	149.4	145	125.4	116.8	149.8	147.4
TCF20	131.7	118.8	122.5	126.2	110.9	109	131.5	118.5
TCF20	527.4	522.6	350.7	486.1	561.6	312.5	566.4	651.8
TCF21	114.3	114.9	119.4	123.8	111.5	100.4	122.8	115.4
TCF21	113.9	106.4	113.4	111.6	102.9	92.2	116.1	107.1
TCF21	109.7	120.5	114.3	120.9	106.5	109.3	125.8	111.5
TCF23	114.4	114.1	108.1	110.8	113.1	105.7	129.6	105.5
TCF3	185.3	190.2	154.3	233.3	186.5	151.4	218.3	251
TCF4	181.5	139	139	143.2	134.4	107.5	157.9	129.7
TCF7	122.1	142.2	121	136.2	129.1	114.2	126	136.8
TCF7	148.4	158.5	127.5	160.4	148.1	115	153	163.6
TCF7	114.8	111.3	114.1	122.4	122.1	101.5	126.1	117.4
TCF7	115.2	113.6	122.7	121.7	111.5	90.3	114.9	114.8
TCF7	120.1	121.1	120.7	122.8	116.3	103.9	120.1	117.1
TCF7	111.6	110	107.3	117.7	100.6	100.2	114	117.5
TCF7L1	109.1	120.8	116.3	114.6	110.1	105.3	116.2	118.1
TCF7L2	117	118.6	112.6	125.3	109.4	102.6	123.1	117.8
TCF8	123.7	128.3	115	116.8	109.6	99.9	120.3	122.5

TCFL5	264.6	260.4	261.7	319.8	239.7	225.2	250.5	277.5
TCHHL1	115.3	112.5	111.7	112.8	106.7	93.3	114.4	105.6
TCHP	147.1	141.9	148.9	141.8	126.7	132.7	136.3	125
TCIRG1	703.4	561.1	825.3	622.4	326.1	553.5	460	353.6
TCIRG1	115.2	117.5	116.4	128.6	104.9	99.7	121.7	117.7
TCIRG1	126.4	117.9	137.1	130.7	111.3	118.9	120.5	108.2
TCL1A	191	145.7	176.1	186.8	124	147.4	152	117.1
TCL1B	131	125.1	138.9	154.8	128	117.8	127.6	134.2
TCL1B	105.4	112.6	108.4	108.2	99	93.4	111.7	101.5
TCL6	109.1	113.7	118.1	119.9	95.5	105.8	102	116.5
TCL6	118.4	110.2	108.1	111.3	109.4	99.8	112.8	99.9
TCL6	112.8	120.9	120.5	125.6	111.1	97.3	122.7	109.5
TCN1	115.6	131.4	126.6	121.7	127.5	110	118.3	134
TCN2	113.7	114.3	119.1	114.9	107	93.6	111.8	115.3
TCOF1	106.6	115.6	111.7	111.8	110.8	97.3	121.1	123.6
TCOF1	114.5	119.9	124.5	112	113.4	104.1	119.3	115.2
TCOF1	108.1	110.3	124.7	120.7	114.8	97.2	111.8	117.1
TCOF1	120.4	118.8	119.6	120.5	118.6	105.6	123.9	117.9
TCP1	153	163.7	163.8	173.4	144.6	141.4	148.9	262
TCP1	121.6	133.9	132.6	135.6	115.6	114.8	136.5	167.3
TCP10	114.5	117.9	103.5	113.9	104.6	90.5	116.3	114.4
TCP10L	116.1	121.7	121	122	107.8	106	130.8	122.1
TCP11	112.6	122.4	110.4	116.8	109.2	104.2	112.9	116.7
TCP11L1	113.9	109.6	108.1	114	114.6	102	111.6	108.5
TCP11L2	126.9	125.5	129	127	124	114.7	147.8	136
TCTA	159.9	166.3	197.6	201.5	151.2	141.7	159.9	188.3
TCTEX1D1	121.5	120.5	117.3	123	115.5	113.3	124	137
TDGF1	116.8	106.5	111.2	117.9	104.1	98.2	114.2	111
TDGF3	113.6	116.3	118.1	118.5	110.8	96.4	124.6	113.7
TDH	114.3	105.8	117	119.7	113.3	100.3	115.6	108.3
TDO2	114.5	113.9	111.7	117.4	104.3	105.1	113.4	113.9
TDP1	411.8	369.7	530.1	393.1	406.5	353.6	449.1	514.4
TDP1	135.9	135.1	130	117	125.5	112.5	137.9	129.3
TDP1	158.3	193.7	159	183.7	152.9	127.4	172.6	164
TDRD1	425.3	333.4	461.8	383.5	347.7	307.3	417.7	447.8
TDRD10	116.7	116.8	110.3	111.8	113.9	100.6	122.7	113.4
TDRD3	124	133.5	151.5	134.3	119.6	128	141.7	131.8
TDRD4	112.9	119.9	115.8	114.3	103.8	97.5	114.3	115
TDRD5	113.7	114.7	113.9	118.6	105.7	90.9	114.5	116.5
TDRD6	120.9	133.4	129.1	148.3	125.8	109.9	133.5	141.9
TDRD7	410.8	394.2	365.3	430	319.6	300.4	385.9	346.9
TDRD9	115.7	133.1	125.5	125.5	111	105.5	128.9	116.4
TDRKH	125.8	124.6	126.3	125.2	125.2	106.6	126.1	132.1
TEAD1	117.5	120.5	120.7	121.6	103.8	104.6	122.5	120.5
TEAD2	174	182.5	172	200.7	142.5	125.7	159.9	144
TEAD3	117.3	118.6	125.1	137.4	119.6	112.3	136.6	120.3
TEAD4	120.6	114.4	137.2	122	113	112.9	122.9	130.4
TEAD4	109.6	115.9	116.7	118.7	103.4	93.8	119.3	115.4
TEAD4	118.3	116.8	120.4	119.9	117.6	109.2	119.3	110.3
TEC	124.1	123.6	120.4	123.1	116.9	104	125.1	124.8
TECTA	117.5	130.1	119.6	132.2	111.6	104.4	128	110.5

TECTB	128.7	124.4	109.8	110.2	111.4	108.4	118.4	122.3
TEDDM1	120.1	113.8	116.8	114.8	112.5	106.5	114.8	113.6
TEF	162.9	156.5	181.2	168	156.3	145.3	170.7	182.6
TEGT	2808.5	3283.6	3929	3666.1	2280.6	3396	4062.8	4428.2
TEK	111.2	116.1	127.5	113.9	112.2	110.2	124.3	123.7
TEKT1	129.1	120.6	131	141.6	117.5	104.7	130.3	123.2
TEKT2	121.8	122.2	118.1	125.8	119.4	107.1	122.5	117.3
TEKT3	118.8	108.7	120.5	110.7	110	100.3	114.9	117
TEKT4	104.6	119.7	105.8	116.3	105.6	85.6	106.2	106.1
TENC1	120.5	113.7	115.6	127.8	109.1	96.1	112.5	123
TENC1	114.4	115.2	118.2	118.4	122.4	101.4	113.2	104.2
TENC1	111	119.8	113.8	119.4	111.4	96.5	124.3	126.9
TENC1	109	109.6	109.8	121.3	102.2	98	119.3	115.9
TENR	107.4	113.3	116.5	116.8	110.5	102.1	114.3	121.9
TEP1	126.4	122.7	132.5	123.6	110.4	104.9	116.6	115
TEPP	112	114.3	124.8	116.4	112	100.5	112.8	115.2
TEPP	114	128.6	121.3	134.5	113.2	108.7	132	117.2
TEPP	107.4	119.9	114.8	117.1	108.4	101.3	126.6	117.2
TERF1	128.3	135.5	134.9	147.2	128.4	113.1	159.2	173
TERF1	109.3	110.5	112.9	113	106.3	93.6	106.9	107.1
TERF1	120	120.9	120.5	127.3	113.8	109	117.9	114.7
TERF2	241.9	256.2	222.3	240.2	226.2	218.4	292.5	295.1
TERF2IP	491.2	565.1	451.6	595	431.7	440.5	625.4	688.4
TERT	118.4	108.6	112.9	119.8	114.2	99.4	122.6	110.3
TERT	119	115	120.4	123.2	108.5	108.6	125.9	113.4
TES	148.7	165.9	150.7	143.6	126.3	130.8	172.4	171.9
TES	136.3	154.4	136.3	151.1	146.2	126	153.6	154.9
TESC	127.4	124.1	141.1	132.5	130.2	115.9	119.6	125.8
TESK1	386.5	371.2	300.7	419.2	304.2	315.8	563.5	506.3
TESK2	166.4	165.5	143.9	163.4	148.4	138.9	185.9	198.9
TESSP2	111.7	124	126.8	137.4	118.4	106.5	131.6	120.4
TESSP5	108.3	104.6	115.3	118.6	106.5	100.8	112.8	107.8
TETRAN	1814.5	1839.2	1394.4	2058.1	1648.7	984	1469.8	1238
TEX10	272.1	264.8	238.8	278.9	246.1	198.5	377	393
TEX101	105	107.4	113.9	114.2	101.2	88.9	111.8	113.7
TEX11	114.3	123.7	125	120.3	107.5	98.1	122.2	116.1
TEX11	106.2	118.3	113.9	120	111.1	97.3	119.9	130.8
TEX11	111.5	106.1	105.3	112.4	106.1	98	114.2	105.6
TEX12	118.2	123.3	118.1	108.2	99.5	104.4	115.4	112.9
TEX13A	108.6	117.3	129.9	124.5	112	105.5	129.2	123.4
TEX13B	120.3	116.5	116.8	117.5	118.2	93.1	129.5	120.7
TEX14	106.3	117.6	110.9	119.4	110.1	96.5	129	110.1
TEX14	104.9	109.1	119	110.8	108	88.1	121.1	106.7
TEX15	110.6	114.8	116.2	120.2	104.7	103	105	109.2
TEX2	258.5	273.5	254.1	258.1	236.1	192.4	293.1	271.2
TEX261	165.8	154.5	169.9	166	151.7	117.1	161.7	159
TEX264	158	207.3	159	185.1	167.3	153.7	159.5	180.4
TEX9	114.4	112.2	115.6	115	107.1	103.3	112.6	113.8
TF	117.6	113.1	113.6	120.4	111.8	101.8	125.6	114.3
TFAM	376.3	322.7	346.1	373.5	276.2	277.4	361.4	364.9
TFAP2A	158.1	160.6	149.1	175.1	152.2	130.1	192.8	165.7

TFAP2A	122.5	109.5	100.2	115.4	100.6	98	107.4	111.2
TFAP2A	117.5	122.1	123.3	113.3	117.8	105.2	130.7	117
TFAP2A	116.6	115	122	124.9	102.7	98.5	117.4	115.5
TFAP2B	116.5	114.4	114.9	113.1	102.5	94.3	120.6	107.9
TFAP2C	114.4	114.1	112.1	110.8	108	97.7	115	109.1
TFAP2D	113.1	114.6	118.9	112.6	104.9	95.4	121.5	115.9
TFAP2E	122.4	118.7	120.9	123.7	116.2	102	131.8	114.1
TFAP4	113.3	119.4	126.3	127.3	114.1	100.2	126.9	121.4
TFB1M	207.5	226.3	268.6	259.9	248.7	185.1	198	212.7
TFB2M	249.6	272.4	245.5	275.3	218.6	174.5	217.1	307.4
TFCP2	324.3	317.1	277.6	372.6	294.5	295.2	380.2	246.7
TFCP2L1	113.4	120	117	110.4	108.9	93.6	115.1	117.3
TFDP1	312	406.4	457.3	365.5	276.9	330.8	273.3	242
TFDP2	125.5	131.6	118.4	118.3	117.8	99.9	118.6	115.8
TFDP3	111.1	116.8	117.5	120.1	106.8	98	117.1	118.4
TFE3	150.2	155.4	158.9	193.9	138.4	159.8	233.8	192
TFEB	116.5	119.7	112.4	119.6	112.9	107.8	119.8	113
TFEC	120.6	114.2	121	123	110.1	102.8	119.5	115.8
TFEC	120.7	116.1	115.8	125.2	102.9	101.5	115.8	118.9
TFF1	110.2	114	121.1	126.4	121	110.4	114.1	114.8
TFF2	120.8	115.8	118.5	108.4	115	105.6	128.2	111.9
TFF3	112.9	121	113.5	122.6	112.2	100.6	120.4	119
TFG	124.5	119.8	131	123.4	118.9	105.2	121.8	124.1
TFG	696.2	161.7	890	833.5	157.2	717.3	729.1	188.5
TFIP11	280.6	251.5	247.6	339.7	238.9	239.4	344.5	297.1
TFIP11	233.6	244.2	219.6	243.1	181.1	208.1	279.3	237.4
TFIP11	144.8	137.5	149.1	169.4	144.9	111.9	162.1	161.4
TFPI	116.8	114.5	110.5	125.3	142.6	115.7	133.3	125
TFPI	111.4	110.9	103.3	111	107.5	104.6	113.7	111
TFPI	116.9	122.7	119.9	147.4	148.1	146.2	154.3	141.9
TFPI2	113.8	120.7	122.3	116.8	107.9	97.6	114.7	108.7
TFPT	213.8	258	205	208.3	198.9	164.9	205.9	253.1
TFR2	114.3	110.9	122.6	119.4	113.8	103.1	114	115.8
TFRC	714.5	717.3	579.4	815.2	531.3	312.8	906.4	913.7
TG	112.6	116.4	116.6	110.1	105.3	107	116.5	125.6
TGDS	240.7	228.6	254.9	238.1	217.7	210.6	205.7	231.6
TGFA	127.4	139.1	161.8	138	110.8	121.7	132.8	122.9
TGFB1	106.7	102.9	112.9	119.1	102.9	97.9	108.4	107.8
TGFB1I1	122.3	111.1	117.9	122.4	111.4	102.5	122.8	122.6
TGFB1I1	117.1	115.4	117.9	117.1	102.1	102.7	117.8	118.5
TGFB2	108.8	115.5	126.9	122.7	105.5	104.2	119.4	109.4
TGFB3	129.6	130	148	124.1	130.1	114.4	131.8	130
TGFBI	1344.3	195.8	9080.4	2097.2	396.5	10356.9	2465.7	414.8
TGFBR1	121	113.2	110.3	118.3	101.5	107.8	122.5	120.8
TGFBR2	134	122.9	145.4	132.2	127.9	112.3	138.5	136.4
TGFBR2	1534.2	1339.1	1710.3	1460.2	1201.7	1584.6	1816.9	1547
TGFBR3	881.4	1132.3	562.4	1135.3	868.3	516.2	1625.8	1437.3
TGFBRAP1	214.6	236.6	186.5	219.5	181	147.9	233.7	232
TGIF	117.2	118.8	117.9	125.6	111.9	103.2	114.3	117.3
TGIF	111.2	120.5	129	118.8	107.3	94.6	130	119.9
TGIF	111.7	110	122.4	120	108.4	108.8	123.7	115.5

TGIF1	114.6	113.2	112.7	108.6	102.7	96.6	113.4	116.8
TGIF1	296.8	416.1	436.8	368.2	334.7	390.5	362.4	404.4
TGIF1	116.2	129.1	124.3	123.9	114.3	94.8	125.5	115.8
TGIF2	147.4	192.1	148.3	165.3	163.3	132	180.4	186.9
TGM1	120.5	118.6	112.4	124.5	108.6	100.2	119	111.5
TGM2	116.7	127.2	127.5	125.9	123.1	104.6	122.8	124.7
TGM2	117.2	120.2	110.8	116.8	107.4	101.6	118.2	120.7
TGM2	119.8	128.7	121	130.2	117.5	106.1	130.4	118.7
TGM3	112.5	107.2	119.3	120	108.2	105.3	117.6	117.2
TGM4	109.1	119.6	115.2	114.4	115.4	98.5	111.8	115.8
TGM5	114.9	117.2	144.2	117.9	108.7	110.4	120.9	117.6
TGM5	113.6	108.2	126.7	118.5	100.5	108.3	118.4	110.8
TGM5	113.8	113	115	130.7	111.8	104.8	114	107.1
TGM6	107.4	110.5	124.5	111.6	102.3	103.2	113.1	117.6
TGM7	124.8	113.3	119.1	122.1	108.8	103.4	111.2	123.8
TGOLN2	489.4	511	670.3	584.6	550.5	578	611.3	469.6
TH	114.1	122.1	122	117.1	112.5	97.6	120.5	106.8
TH	109.9	116.4	124.5	110.5	109.2	102.7	124.4	122.4
TH1L	573.6	451.5	391.6	500	427.7	336.4	707.5	416.4
THADA	188	179.9	171.5	184.6	152.1	148.8	174.9	166.7
THADA	110.2	111.4	107.4	118.3	109.1	93.7	109.9	106.1
THAP1	149.9	149.4	170.3	174.4	156.2	152.6	164.8	149.3
THAP1	127.3	150	145.2	146.3	146.3	144.4	154.5	174.3
THAP10	112.7	125.9	119.9	129.1	103.2	92.8	123.1	117.9
THAP11	1415.3	1358.5	1027	1228.5	1087.8	875	1186.8	1003.6
THAP2	117	122.4	119.8	121.8	114.7	99.5	119.1	115
THAP3	121	115.8	128.2	119.8	111.6	107.8	120.3	130.5
THAP4	125.3	124.3	117.9	126.1	114.7	112.8	123.9	121.1
THAP5	117.2	120.7	131.1	125.1	107.1	105.5	132.4	119.1
THAP6	130.3	149.5	139.7	145.3	140.5	126	138.4	140.5
THAP7	139.3	136.9	136.3	123.4	123.3	103.7	131.9	118.1
THAP7	204.7	209.4	218.1	203.2	177.2	161.6	184.1	149.9
THAP7	121.9	113.6	121.8	117.8	117.2	95.1	127	116.8
THAP8	115.4	115.7	120.8	123.6	103.9	111.5	122.5	115.9
THAP9	123	126.4	121.2	122.2	120.7	97.7	125.3	121.2
THBD	108.9	117.7	127.3	113	107.8	100.9	121.6	111
THBS1	186.8	134.3	605	341.9	165.4	1841.4	184.9	126.1
THBS2	113.4	114	126.1	123.3	112.2	101.3	130.1	117.1
THBS3	119.2	121.3	127	131.8	127.2	109.3	122.2	132.7
THBS4	109.4	115	123	118.5	106	94.9	119.1	117.5
THEDC1	124.9	120.8	113.5	121	127.6	107.9	121.7	123.7
THEG	122.5	129.1	127.1	128.3	124.4	104.6	136.1	118.6
THEG	122.2	125.5	121.7	124.4	110.3	100.9	131.9	119
THEM2	302.5	255.5	324.4	411.4	287.4	377.4	292.3	240.4
THEM4	132.7	124.6	141.2	157.2	115.4	125.7	129.1	119.9
THEM5	108.5	109	115.8	112.5	112	92.6	122	108.2
THEX1	112.8	124.9	120.9	115.4	109.5	100.2	116.8	115.6
THG1L	150.2	142.1	138.1	145.9	125.1	108.9	132.9	131.7
THNSL1	182.1	165.9	181.3	170.1	159.5	139.1	158.4	131.8
THOC1	214.8	235.5	238	247.1	231.4	158.8	228.4	197.8
THOC2	295.5	240.3	272.3	329.1	255.2	186.9	354.2	292.2

THOC2	140.6	126.1	182.8	169.4	140	128.1	148.5	147
THOC3	184.6	181.1	230.6	205.9	200.3	190.6	174.1	158
THOC3	296	248.8	263	272.9	216.5	196.8	214.3	183.9
THOC4	139.4	136.7	150.2	149.3	142.8	138.2	133.8	132.4
THOC5	216.6	261.6	251.9	244.6	185.9	184.7	258.1	220.4
THOC5	250.1	262.9	274.4	247.7	201.4	182.9	230.4	203.7
THOP1	231.4	243.9	200.8	221.1	175.2	165.6	254.1	203
THPO	114.2	111.5	113.1	112.2	105.5	106.2	118.2	121.6
THRA	115	133.6	126.6	122.3	126	106.4	138.4	127.9
THRA	120.1	132.4	124	131.4	116.7	102	126.7	119.5
THRA	108.1	119.3	126.4	119.5	113.9	102.8	118.1	113.3
THRAP1	117.6	125.1	127.9	129.5	120.2	99.3	124.1	121.8
THRAP2	145.2	139.3	154.3	163.5	163.2	130.5	157.7	176.2
THRAP3	111.2	117.8	125.6	137.2	119.6	109.2	120.4	136.8
THRAP4	162.6	225.8	162.9	189.3	148.7	131.5	164.3	168.7
THRAP4	233.6	264.1	235.5	272.4	185.2	181.2	235.5	226.6
THRAP5	287.5	326	246.2	321.7	249	246.9	331.8	328.8
THRAP5	424.8	422.4	409.6	484.7	304.4	366.4	459	446.6
THRAP5	158	162.6	165.6	188.1	138.4	157.8	175.4	157
THRAP6	428.6	516.4	546.2	477.6	475.5	423.9	475.6	604.4
THRB	108.3	114.2	132.1	128.6	114.7	108.4	124.7	132
THRSP	105.4	111.1	108.1	104.1	104.8	95.3	116.1	113.8
THSD1	103.9	112.6	114.5	123.3	105.3	93.6	116.3	117.8
THSD1	112.3	115.8	113.6	108.2	117.3	102.6	118.2	121
THSD1	108.4	117.7	118.4	110.4	107	99.4	118.8	107.4
THSD1P	131.2	128.9	135.8	131.9	109.2	107.2	121	117.8
THSD1P	115.4	114.1	111	107.5	104.7	101.9	119.4	118.4
THSD3	106.5	114.3	129.3	112	113.5	120.6	120	116.2
THSD3	115.8	115	124.4	116.4	113.9	104	114.5	114.8
THSD3	106.1	106.7	113	115.4	105.8	104.3	123.6	111.5
THSD4	121.9	111.7	118.4	120.6	103.5	94.3	125.8	116.1
THSD7B	105.9	120.2	118.4	119	104.7	103.1	117.9	108.1
THTPA	115.5	127.1	109.2	122.1	108.5	95.4	124	129.6
THUMPD1	402.5	375.7	329.5	351	338.4	299.8	312.2	351.6
THUMPD2	130.5	141	159.4	121.4	128.9	108.2	128.6	136
THUMPD3	119.5	134.4	127.2	130.5	123.1	116.5	125.9	131.8
THY1	110.6	105.2	122	117	100.6	105.6	115.2	113.9
THYN1	535.3	494.8	517.3	516.8	496.4	374.4	306.2	310.7
THYN1	164.9	164.4	169	183.8	154.6	128.7	129.4	134.5
THYN1	339.2	391.1	325.7	242.2	320.3	240.1	203.9	187.1
TIA1	577.6	593.7	561.6	602	546.2	375.1	521.6	527.2
TIAF1	457.1	398.6	484.5	405.1	280	428	293.2	276.1
TIAL1	274.8	312.3	361.7	315.1	248	189.9	222	270.6
TIAL1	526.3	537.8	623.5	513.7	433.6	561	677.5	537
TIAL1	201.8	170	184.8	208.7	168.8	144.8	189.9	213.7
TIAM1	163.9	168.8	165.3	175.1	148	113.6	130.2	147.4
TIAM2	113.5	118.9	118.6	121.6	109.4	96.4	113.2	111.9
TIAM2	113.7	112.6	118.8	111.5	107.5	97.9	121.7	110.9
TICAM1	126.6	135.9	127.4	142.6	116.3	112.7	146.4	157
TICAM1	482.1	486.7	371.2	611.7	390.5	436	732.3	728.2
TICAM2	237.3	163	344.7	206.6	157.2	209.2	230.6	255.5

TIE1	118.6	113.7	115.3	120	111.8	97.4	124.8	116.9
TIFA	135.7	125.9	131.9	129.3	121.8	112	128.1	127
TIGD1	111.5	112.1	113.3	117.6	109.3	99.2	124.7	118.9
TIGD2	154.1	161.9	177.7	164	163.2	150.9	163.3	158.5
TIGD3	136.4	135.2	127.9	118.7	129.8	107.6	128.8	138.6
TIGD4	109.5	101.8	107.7	114.2	104.4	92.8	107.1	107.5
TIGD5	233.6	299.9	180.4	247.1	243.7	158.2	247	239.3
TIGD6	124.8	121.9	126.2	127.8	116.9	98.7	124.8	118.6
TIGD7	150.9	161.4	179.1	174.2	157.9	128.8	209.4	165.6
TIMD4	122.5	131	122.7	137.8	120	102.1	135.5	135.1
TIMELESS	249.1	241.2	192.5	247.5	198.6	147.9	196.1	177.4
TIMM10	303.5	516.2	376.6	306.3	427	244.8	345.3	477
TIMM13	112.9	119.8	133.7	121.6	111.6	114.2	140.4	125.8
TIMM17A	123.1	126.1	134.1	129.3	117.4	114.7	129.4	122.8
TIMM17B	138.2	159.6	180.8	157	137.4	132.2	154.4	145.3
TIMM22	324.8	400	340.4	393.3	284.8	234.3	330.9	274.1
TIMM23	194.5	288.8	232.2	265	207	186.9	189.8	229.4
TIMM23	638.5	456.4	515.7	521.2	413.5	352.4	488.6	470.3
TIMM23	303	312.2	390.6	349.1	258.6	307.6	304.8	289.1
TIMM44	148.3	146.4	151.4	159.2	141	136.4	197.7	221.5
TIMM50	115.2	109.4	124.9	114.7	108.7	94.7	113.8	119.7
TIMM8A	113.2	132.2	121.7	138.4	116.2	100.3	134.2	125
TIMM8B	132.7	132.7	139.7	126.9	132.2	122.3	128.4	123.7
TIMM9	171	155.1	184.8	147.9	144.6	161.6	160.7	176.3
TIMP1	1949.9	2995	2305.7	2039.3	1836.1	1733.3	1283.1	1177.3
TIMP2	417.4	259.2	623.3	383.5	193.8	330	295.6	202.6
TIMP2	168.9	121.8	152.4	140.5	124	129.4	141.5	125.1
TIMP3	119.9	116.5	114.1	116.1	103.7	99.9	118.7	115
TIMP4	114.8	105	112.3	115.5	114.9	100.6	111.7	115
TINAG	118.1	122	132	140.2	109.4	109.1	123.1	116.6
TINAGL1	124	111.1	124	123.9	102.7	95.5	123.2	114.4
TINF2	763.9	952.8	764.8	774	607.3	498	668.6	695.7
TINP1	2163.9	2099.5	2440.7	2666.4	2057.7	2091.5	2251.1	2225.1
TIP39	108.3	119	122.4	115.6	113.2	102.4	124.4	125.2
TIPARP	436.4	649.3	880.6	887.2	593.7	1098	831.2	986.3
TIPRL	253	265.9	248	320.4	237.2	232.4	249.7	260.4
TIPRL	175.1	182.8	224.9	197.6	171	170.1	215.5	206.1
TIRAP	117.6	136.9	129.5	133.2	114	102.8	119.6	117.8
TIRAP	116.1	115.2	119.2	119.6	108.5	102.6	118.7	118.5
TIRAP	122	116.4	126	116.3	116.6	107.9	133.4	118.4
TITF1	101.3	110.6	107.4	113.6	111.5	94.5	112	115.7
TITF1	108.9	107.8	119.5	115.6	108.7	98.2	116.1	116.2
TJAP1	585.1	629.1	588.6	622.7	515.9	433.2	684.1	780.2
TJP1	113.6	105.4	109.9	106.9	100.3	90.8	109.3	117
TJP1	111.5	120.4	130	109.9	117	101.9	119.4	117.3
TJP2	123	118	122.6	116.7	105.5	98	121	119.2
TJP2	116	109.6	117.9	121.7	114.3	98.8	110.2	125
TJP3	222.2	237	169.1	232.2	172.3	149.2	187.4	186
TK1	116	119.7	125.9	121.5	113.6	99.7	126.9	124.6
TK2	130.8	121.1	129.5	122	114.8	113.8	119.1	114.2
TKT	1072.2	891.1	1167.6	1484.1	772.1	1475.5	1691.3	913.7

TKTL1	138.6	158.7	124.3	143.9	133.5	109.4	149.9	147.5
TKTL2	115.8	108.9	107.7	123	117	103.2	113.5	103.8
TLCD1	115.9	136.3	121.4	119.9	114.4	110.8	118.5	114.9
TLE1	127.9	130.4	141.8	137.1	119.7	112.8	137.2	127.1
TLE2	200.3	235.2	163.9	201	173.5	139.4	216.5	232.6
TLE3	126.8	126.3	139	139.1	120	114.7	141.1	143.8
TLE4	643.6	678.2	511	749.2	647	559.7	844.4	977.1
TLE6	107.7	118.3	116.1	118.9	113	106.2	115.4	110.7
TLK1	403.8	426.8	307.4	417.9	342.1	354.1	451.1	355.5
TLK2	215.6	253.7	228.1	239.8	243.1	212.1	229.5	268.2
TLL1	109.1	102.6	111.5	111.1	106.7	95.2	105.9	111.9
TLL2	107.9	117.2	110.5	114.1	98.2	100.8	110.4	114.1
TLN1	433	389	464.6	451.6	335.2	384.1	507.8	443.4
TLN2	113.6	121	116.6	121	107.6	109.9	133.9	116.2
TLOC1	221.8	271.7	238.1	244.7	223	200.8	216.9	242.2
TLR1	179.8	153.7	218.6	175.5	118.4	180.7	171.1	157.6
TLR10	123.1	109.3	119.8	119.9	106.2	96.2	122.9	111.9
TLR10	156.8	147.3	157.1	147.2	123.5	144.9	146.2	152.4
TLR2	116	117.9	125.4	116.7	109.2	97.1	121.6	113.3
TLR3	112.1	116.3	106.4	106.9	113.4	97.7	121.9	108.4
TLR4	136.7	110.3	177.2	127.3	103.2	143.4	136.5	118.7
TLR5	123.6	126.8	171.5	124.6	106.1	93	115	114.2
TLR6	124.9	123.5	126.5	133.7	107.7	108.3	121.5	125.5
TLR7	188.7	145.6	358.3	156.4	135.4	130.2	135.7	125.7
TLR8	168.6	123.7	274.2	150	110.3	122.5	134.8	111.2
TLR8	107	117.8	118	119.2	104.7	96.3	115.8	117.5
TLR8	220.3	130.6	440.5	188.4	113.2	145.5	153.9	112.3
TLR9	120.3	121.5	125.1	116	106	110.9	140.4	118.5
TLX1	116	118.2	124.4	132.1	116.8	106	125.2	115.1
TLX2	114.4	112	126.2	120.2	116.9	100.3	122.3	120.5
TLX2	108.8	112.6	127.6	131.2	108.3	102.1	120.7	120.2
TLX2	110.5	105.9	107.1	106.6	103.6	93.3	110.9	107
TLX3	110.3	127	130.2	124.8	110.2	108	121.2	120.6
TM2D1	547.7	566.2	782.8	644.3	462	426.2	673.6	747.2
TM2D2	170.9	163.1	183.3	174.4	150.3	153.3	152	177.6
TM2D3	135.8	127.7	140.6	149.1	143.5	117.1	140.8	146.5
TM2D3	574.7	587.1	630.5	773.2	631.3	664.9	712.9	694.6
TM4SF1	117.7	120.6	119	128.8	148.3	102.5	145.4	137.2
TM4SF11	134.7	138.1	149.8	136.2	129.3	115.7	129.3	123.6
TM4SF18	113.7	111.2	106.1	118.6	106.5	95.8	121.8	112.7
TM4SF19	165.2	151.8	111.3	211.9	175.4	107.9	296.1	189
TM4SF19	191.8	175.9	116.5	316.2	219.4	119.7	379.6	210.8
TM4SF20	117.2	103.3	115.1	114.3	107.5	95.2	111.7	107.5
TM4SF4	111.8	112.6	117.8	116.3	100.8	96.8	109.5	115.4
TM4SF5	107.2	108.2	105.5	106.9	102.2	97.7	104.6	109
TM6SF1	130.2	133.5	309.5	129.4	119.5	204.3	137.7	123.3
TM6SF2	110.1	119	113	115.3	104	97.6	114.3	111.5
TM6SF2	112.7	117	116.6	119.6	118.1	96.5	117.4	121.2
TM6SF2	119	117.8	110.6	116.7	113.3	95.8	110.1	118.7
TM7SF2	182	191.1	197.6	188.6	164.7	153.4	185.3	165.1
TM7SF3	164	159	173.8	158.7	145.5	125.4	133.2	136.6

TM7SF4	181.1	222.6	119.6	253.2	181.9	137.2	217.2	167.9
TM9SF1	187.7	173.8	174.2	192.5	153.5	163.9	170.6	143.6
TM9SF1	130.9	145.5	143.2	147.4	120.2	106.1	138.8	119.9
TM9SF1	133.7	134.4	129.8	138.8	124.1	114.9	135.9	125.5
TM9SF2	692.1	538.3	954.6	847.1	617.3	679.9	807.5	661.3
TM9SF4	209.4	225.8	196.3	201.2	180.2	185.5	239	255.2
TMBIM1	263.8	275.6	201.9	297.7	246	220	429.9	454.8
TMBIM4	2506.7	2259.1	4630.1	2741.3	2329.1	2878.5	2361.7	1737.8
TMC2	118	116.8	124.3	129.6	113.7	96.9	121.6	122.1
TMC4	130.4	113.5	122.9	115.4	107.8	102.9	104.3	111.3
TMC5	111.6	118.3	132	124.5	108.5	106	121.2	126.9
TMC6	537.3	463.6	406	429.5	314.6	289.2	434.1	366.5
TMC7	112.4	116.6	121.8	122.2	117.6	107.2	119.8	124.8
TMC8	131	125.7	113.5	133.5	123.3	98.3	125	121.7
TMCC1	115.6	114.3	113.3	116.3	109.2	105.9	120.7	119.2
TMCC1	127.9	125.1	130.5	125.8	118	100	126	121
TMCC2	120.5	116.1	114.3	124	114.6	97.1	120.5	116.7
TMCC3	130.5	122.9	116.1	132.5	123.9	109.8	123	127.5
TMCO1	871	846.9	1065.9	979.3	700.2	975.6	911.3	797.1
TMCO2	115.1	114.8	128.9	126.2	125.6	112.3	144.1	126.3
TMCO3	244.3	240.6	208.9	336.1	230.9	246.1	318.2	288.8
TMCO4	144	144	137.5	144.9	136.3	121.9	138.9	139.2
TMCO5	106.8	125.4	131.8	139.8	136.1	121.1	141	118.9
TMCO6	137.3	138.3	136.7	131.8	114.9	115.5	128.7	127.6
TMDCII	112.7	120.8	120.1	121.4	114.9	98.6	125.3	113.5
TMED1	182.2	166	175.5	162.9	136.9	158.1	203.6	174.3
TMED10	367.3	444.2	583.8	425.7	347.8	482.1	331.1	347.7
TMED10P	850.2	822.8	1407.5	890.8	554	888.7	670.2	750.7
TMED2	250.9	219.2	264.8	224.5	244.7	227.8	288.5	352.3
TMED3	918.8	881.2	980.2	835.8	593.4	532.5	698.6	671.9
TMED4	772.1	704.6	568.6	695.5	710.5	363.3	510.7	489.8
TMED5	611.7	642.5	582.9	776.2	620.1	628.3	848.5	843
TMED6	128.9	113.3	128.7	130.2	115.3	105.8	139.1	140.9
TMED7	441.1	483.8	518.5	505	403.8	472.9	416.6	489.2
TMED8	118.8	121.1	113.9	122.1	116.4	96.4	121.8	124.3
TMED9	587	578.4	819.7	850.6	468.7	659.3	638.3	816.4
TMEFF1	122.5	112.2	113.8	123.7	115.1	100.6	114.9	113.6
TMEFF2	114.6	107.7	110.3	106.6	105.1	93	109.2	117.6
TMEM1	225.9	192.6	183.9	244.1	188.6	171.2	197.1	190
TMEM1	112.4	115.3	118	124	108.4	96.8	119.5	124.4
TMEM1	135.6	140.9	147.4	131.4	118.6	109.8	135.7	121.8
TMEM10	111.1	109.7	120.5	109.1	104.9	99.1	111.3	124.3
TMEM10	112.5	106.2	117.8	119.9	113.2	95.9	113.3	121.9
TMEM100	122.4	116.4	121.5	130.7	109.7	100.8	133.9	118.6
TMEM101	450.6	473.5	509	423.1	291.7	377.3	322.3	235
TMEM102	106.8	114.7	117.5	123	109.7	92.8	118.9	120
TMEM103	282	267.5	270.1	277.1	220.5	203.2	298	254.4
TMEM103	137.4	148.8	144.2	135.2	148.5	109.9	126.3	123.7
TMEM104	115	122.7	114.2	115.1	108.6	110.9	124.8	121.2
TMEM105	121.3	123	130.4	136.3	123.2	101.2	125.9	123.6
TMEM106I	150.9	189.4	152.3	157.8	162.3	116	159.3	158

TMEM106(	203.3	177.1	223.5	197.3	243.3	175.2	205.9	188.8
TMEM107	111.3	137.6	125.3	111.5	123.2	101.8	118.1	122.4
TMEM108	114.9	118.2	125.5	122.2	110	99.4	118.7	118.7
TMEM109	308.4	342	397.8	335.7	209.3	280.4	278.1	273.3
TMEM11	122.2	131.5	129.1	134.3	129	111.4	136.6	127.9
TMEM110	121.2	120.4	114.2	121.3	113.4	92.7	131.8	110.1
TMEM113	859	1165.7	898.3	1472.1	1151.7	743.7	1441.1	1408.2
TMEM115	343.6	365	319.4	346.5	276.9	285.8	367.3	362
TMEM116	223.4	363.6	231.8	264.4	300.3	189	251	403.6
TMEM117	116.8	131.8	107.9	127.9	130.7	100	102.3	112.2
TMEM118	168.2	166.5	161.6	182.5	157.1	131.1	159.3	136.9
TMEM119	119.9	132.6	116.1	125.2	119.5	102.1	130.7	124.4
TMEM121	107.1	109.9	114.1	119.4	102.7	101.2	119.1	110.8
TMEM122	119.9	119.6	112.4	126.8	113.7	95	116.6	116.3
TMEM123	1901.6	2317.8	2411	1971	1943.5	1937.5	1632.1	1802.6
TMEM125	110.2	111.6	115.7	115	101.2	98.8	110.1	119.2
TMEM126/	578.3	617.8	828.6	607.1	568.3	544.9	509.4	581.8
TMEM126I	1131.8	993	1044.9	1226.9	912.6	573.7	1299.2	1400.1
TMEM127	145.6	164.5	164	170.5	154.7	157.4	185.4	151.3
TMEM128	173	144.2	198.6	153.6	122.8	157.2	151.8	128.8
TMEM129	130.6	125.4	144.4	149.1	121.3	99.8	121.7	115.7
TMEM130	118.8	133.7	117.7	121.3	110.4	101.3	130.8	125.8
TMEM132/	116.1	114.9	132.2	133.4	107.7	102.7	127.6	109.3
TMEM134	316.2	315.2	389.3	276.4	229.8	270.7	248.5	206.9
TMEM14A	188.7	220.3	239.9	216.9	220.2	186.4	150.5	132.1
TMEM14B	461.3	443.4	801	375.9	366.7	553.7	410.9	518.9
TMEM14C	784.3	745.5	1419.4	735.2	554	732.6	553.4	498
TMEM15	196.7	191	197.3	204.9	171.5	194.2	201.1	158.9
TMEM150	328.4	275.1	239.1	224.9	219.9	176	182.8	152.1
TMEM151	127.4	120.1	123.8	122.6	109.7	93.8	103.9	116.3
TMEM151	113	116.8	122.4	114	107.3	98.5	119.3	120.4
TMEM154	221.2	215.9	213.8	194.9	193.1	140.8	165.9	163.5
TMEM156	215.3	162.7	239.9	250.9	193.3	188.5	332.1	223.1
TMEM157	114.5	125.4	123.1	123.9	121.9	106.7	129	125.5
TMEM159	120.1	133.1	120	139.4	121.1	109.1	123.1	127.2
TMEM164	120.7	117.9	125.4	118.9	113.1	104.5	124	111.6
TMEM16A	119.7	107.4	117.9	114.2	102.2	92.8	116.9	109.6
TMEM16B	118.5	116.7	103.8	119.8	103.7	96.4	121.8	113.6
TMEM16C	110.1	113.3	114.2	111.8	104.1	95.1	114.9	105.6
TMEM16D	113.7	125.3	116.7	115.5	112	96.6	114.6	119.2
TMEM16E	118.3	112.3	122.4	117.1	112.5	113.2	126	116.5
TMEM16F	210.7	198	217.7	215.5	176.4	211.9	200.6	226.9
TMEM16G	107.4	117.9	108.2	114.7	102	89.1	113.8	112.7
TMEM16G	121.7	117.6	123.2	118.1	122.7	105.8	132.7	132.2
TMEM16G	108.2	111.3	116.3	113.9	106.6	91.4	113.4	110.5
TMEM16H	150	139.6	156.6	161.1	130	118.3	142.9	137.5
TMEM16J	119.9	135.1	121.3	127.2	112.7	108.3	127.6	117.4
TMEM16J	119.2	129.9	132.2	115.6	118.1	108.3	118.3	113.1
TMEM16K	141.5	140	134.8	134.2	112.7	107	148.5	132.9
TMEM17	3597.8	3277.9	3871	3832.8	5005	2732.4	3949.1	3502
TMEM170	182.1	187.1	158.3	223.4	181.1	175.4	208.1	219.2

TMEM173	360	535.4	740.8	436.3	291.1	412.8	266.8	199.5
TMEM176I	119.9	129.6	137.5	127.6	105.2	114.6	129.4	117.2
TMEM178	116.4	108.1	112.8	122.8	105.8	100	115.5	121
TMEM18	160.9	172	192.7	194.8	136.7	151.6	153.9	147
TMEM183I	846.4	863.4	904.6	1062	786.3	694.9	760.5	821.4
TMEM185/	192	224.7	204.7	205.2	144.5	185.7	220.8	185.4
TMEM185I	169.7	178.8	153.2	173	139	139.8	175.8	167.9
TMEM186	121.2	136.4	135.7	128.4	125.5	113.9	132.1	127.6
TMEM19	127.9	132.8	123.5	120.6	127.5	95.3	124.2	119.6
TMEM2	290.7	344.3	238	407	302.7	241.1	372.6	366
TMEM20	124	124.1	128.3	128	117.9	96.8	116.5	121.4
TMEM22	117.2	115	117.7	120.2	104.9	104.3	123.5	119.2
TMEM23	123	125.3	141.8	136.2	115.4	114.5	118.6	124
TMEM24	142.2	131	132.7	135.2	112.5	119.6	134.2	129.6
TMEM25	128.2	135	119.9	121.7	109.7	108.3	125.2	116
TMEM26	115.1	122	118.5	128.9	107.8	100.4	121.6	117.8
TMEM27	113.9	122.2	119.7	119.5	113.5	101.6	118.4	116.6
TMEM28	115.1	117.5	114.8	125	104.7	105.1	121.6	128.2
TMEM29	176.6	186.2	241.3	183.7	141.5	147.7	147.6	150.9
TMEM30A	255.1	265.4	234.6	216.4	217.2	199.3	269.6	272.4
TMEM30B	118.1	126.9	135.2	138.5	122.8	118.3	159.1	168.7
TMEM31	118.3	118.8	134.6	131	115.8	99.1	120.9	121.2
TMEM32	1296.8	1377.3	1031.2	1365.9	848.4	1173.1	1463.2	1367.4
TMEM34	135.5	127.2	136.6	139.8	125.5	113.8	133.5	122
TMEM35	123	115.8	119.5	115	109.3	95.6	121.6	116.1
TMEM37	118.5	114.7	115.8	122.6	118.4	104.9	116.6	113.7
TMEM38A	107.1	111.4	113.1	122.8	113.3	94.8	116	116.6
TMEM38B	121.7	127.1	121	125.7	112.7	100.4	116.1	121
TMEM39A	143.5	157.5	161.4	161.9	147.4	140.2	164.2	170.1
TMEM39B	307.5	288	284.7	295.4	243.6	221.8	240.3	254.8
TMEM4	286	238.4	229.8	343.5	252.5	221.1	222.3	203
TMEM40	119.8	118.9	115.7	122.8	108.9	99.3	114.2	111
TMEM41A	158.1	171.3	163.2	163.7	154.5	135.1	154.4	166.8
TMEM41B	526.9	614.3	634.9	520.8	481.2	517.5	544.9	465.2
TMEM42	280.2	279.5	391.5	252.7	235	264.6	208.7	210.7
TMEM43	544.1	568.6	677.7	582.4	513.9	577.2	581.5	506.3
TMEM44	139.5	130	123.7	135	109.5	112.6	136.1	138
TMEM44	114.5	108.4	125.7	144.5	128.8	97.7	118.8	114.5
TMEM45A	112.6	110.3	118.3	123.2	98.8	105.3	112.1	114.7
TMEM45B	143.8	152.8	156.8	161.9	136.3	117	169.3	140.4
TMEM46	125.6	134.6	118.8	119.9	118.3	102.2	115.1	126.9
TMEM47	113	117.6	120.1	118.3	112.1	102	127.2	113.5
TMEM48	147.1	162.6	137.2	148.5	131.4	120.1	145.9	129.1
TMEM49	615.3	463.9	731.1	736.7	436.2	976.1	744.1	530.7
TMEM5	426.6	503.1	547.9	467.6	419.8	380.2	482.6	561.2
TMEM50A	187.6	186.6	195.3	184.9	186.6	156	231.4	231.9
TMEM50B	532.7	556.6	704	468.3	409.3	434.4	398.4	375.2
TMEM51	284.5	160.8	920.5	300.8	159.5	595.6	256.6	189.8
TMEM52	121.1	113.3	107.6	123.8	106.9	103.2	120.7	116.9
TMEM53	113.5	127.6	121.2	121.5	124	105.9	125.3	118.2
TMEM54	114.3	110.4	108.2	111.4	99.6	95.3	104.8	113.6

TMEM55A	220.5	266.7	242.8	228.8	190.3	153.9	180.4	159.1
TMEM55B	404.3	388.8	371.8	422.4	409.3	352.9	447.2	454.6
TMEM56	111.7	114	121.9	120.1	104	103.9	117.7	116.1
TMEM57	145.3	168.5	158.9	155.6	152.6	144.1	200.8	189.5
TMEM58	113	131.1	125.1	122.9	116.7	109.5	129	127
TMEM59	1268.6	1386.9	1519	1581.9	1233.7	1321.2	1505.9	1272.6
TMEM60	794.8	775	813	729.3	570.8	522.2	640.6	686.3
TMEM61	112.4	121.3	117.5	134.8	107.7	101.5	124.1	118.3
TMEM62	240.4	249.5	258.1	232.7	189.3	197.5	219.3	209.4
TMEM63A	287.1	293.2	193.8	272.9	176.3	145.6	233.4	163.4
TMEM63B	113.3	123.6	117.3	125	108.9	99.7	124.5	123
TMEM63C	128.5	102.4	114.6	119.5	109	101.4	115.6	105.4
TMEM64	145.7	165.1	128.9	165.4	139.8	120.9	175.2	168.6
TMEM65	119	117.8	105.8	122.4	113.8	100.8	120.2	121.1
TMEM66	7024.2	7742.1	5624.2	6311.3	5926.9	5978.7	7919.9	8566.8
TMEM67	117.1	113.8	110.7	123	109.1	97.1	121.2	111.4
TMEM68	122.5	117.2	132	129.9	111.7	109.4	122.6	111.8
TMEM69	299	287.4	334.9	324.6	261.6	242.8	229.5	246.5
TMEM70	129.6	138.8	148.2	149.3	115	136.7	151.7	140.9
TMEM70	182.4	195.2	182.5	193.9	160.2	131	176.9	164.7
TMEM71	426.7	558.1	495.3	507	524.8	318.2	393.6	373.6
TMEM74	115.7	115.2	101.5	108.6	101.6	97.5	119.6	117
TMEM75	107.5	116.4	108.2	125.3	104.5	100	111.6	112.1
TMEM77	188.6	185.4	270.6	217.4	193.1	226	184.5	182.4
TMEM79	337.3	333.2	308.2	369.9	340.4	245.8	323.6	321.9
TMEM8	216.6	322.8	214.9	228.9	203.9	189.2	189.4	227.9
TMEM80	199.3	184.5	192.8	162.5	148.2	153.4	142.7	157.3
TMEM80	173.3	151.6	174.1	174.1	131.2	123.5	144	141.3
TMEM80	125.3	113.2	136.8	119.6	117.9	102.6	114.3	115.3
TMEM81	114.3	117.2	134.9	135.2	133.9	103.7	126.7	137.9
TMEM83	114.5	113.3	111.8	122.1	115.5	97.9	114	109.3
TMEM85	779.4	962.2	991.6	811.8	690	813.7	729.2	665.4
TMEM86A	120.4	117.8	115.9	129.7	112.2	118.4	134.1	116.2
TMEM86B	210.4	218.3	225.7	177	167.2	185.1	190.5	183.8
TMEM87A	1481.4	1444.8	1669.9	1623.3	1675.1	1288.3	2113.9	2135.7
TMEM87B	124	118.4	131.3	126.9	108.6	105.3	135.4	117.3
TMEM88	115.9	113.7	118.2	118.8	113.4	105.7	120.6	117
TMEM89	120	119.3	122.6	118	109.3	108.1	121.4	133
TMEM9	158.4	148.4	151.7	159.4	130.7	133.4	138.3	149.6
TMEM91	157.8	190.4	191.2	156.9	149.2	124.8	138.7	127.4
TMEM92	109.4	109.8	111.6	110.6	107.4	92	111.6	111.5
TMEM93	296.6	308.1	307	340.4	249.4	192.1	272.4	248.8
TMEM93	139.8	132.1	149.1	145.7	131.4	121	156.3	142.9
TMEM93	511	555.7	469.8	492.7	394	447.1	474.6	506
TMEM95	114.6	115	106.2	122.6	107.5	95.3	117.5	108.1
TMEM97	201.6	226.3	196.4	191.5	161.5	129.1	172.7	177.3
TMEM98	114	125.1	120.2	121.4	118.5	98.6	112.7	109.6
TMEM98	113.5	132.5	114.9	122.3	108.4	103.8	117.9	117.4
TMEM99	219.1	241	223.7	261	248	223.7	298.1	311.8
TMEM9B	665.2	674.3	876.4	665.2	530.6	540.3	606.9	541.2
TMEPAI	164.3	137.5	354.4	163.8	131.2	284.4	158	153.4

TMEPAI	109.9	109.7	116	116	99.7	93.7	107.1	114.9
TMEPAI	141.1	135.7	236	139.7	129.8	200.9	143.8	137.9
TMEPAI	114.6	114.9	129.8	114.2	107.8	89.9	121.2	110.6
TMF1	203.5	216.3	239.2	174.8	200.8	153.6	204.8	181.5
TMIE	125.4	120.2	121.2	136.5	113.3	114.5	115.5	116.5
TMIGD	114.1	130.6	121.3	125.9	115.8	111.6	122.7	127.1
TMIGD2	117.3	117.1	121.2	113.9	110.6	97	119.7	110.7
TMLHE	121.9	119.5	144.5	139.7	118.1	107.1	135.5	118
TMLHE	120	125.2	116.8	121.3	107.4	104.7	124.2	121.2
TMOD1	117.1	129.8	113.2	136.1	120.1	106.1	132.2	133.2
TMOD2	110.8	115.5	109.2	115.2	113.9	99.6	119.3	113
TMOD3	139.3	148.9	141.1	158.5	129	119.7	163.8	137.3
TMOD4	123.8	128.3	138.8	127	115.2	115.6	135.1	120.8
TMPIT	147.1	139.9	151.8	140.5	117	140.2	140.1	121.2
TMPO	118.2	123.1	130.5	124.6	117.8	95.5	117.5	118.2
TMPO	132.3	129.1	128.6	130.8	124	114.7	124	125.3
TMPO	116.9	113.1	112.8	121	109.7	92	110.9	113.8
TMPRSS11	120.4	110.4	119.3	122.2	112.2	96.7	119.5	110.9
TMPRSS11	110.1	113.9	118.7	117	110.9	102.5	114.4	115.6
TMPRSS11	117.8	108.5	118.5	123.4	110.9	102.8	129.4	114.8
TMPRSS12	111.1	115.8	116.2	116.8	105.9	96.2	119.1	112
TMPRSS13	118.3	124.6	125.1	115.3	105.1	94	131.3	114.3
TMPRSS2	117.3	114.3	119.1	115	122.4	99.2	121.3	119
TMPRSS3	122	115.8	120.3	124	112.2	106.3	114.9	113.6
TMPRSS3	114.6	100.9	110.4	124.1	112.6	95.2	109.9	118.5
TMPRSS3	126	130.4	127.4	126.4	117.9	104.6	131	124.8
TMPRSS3	128.2	119.1	125.9	126.4	123.1	110.1	116	120.7
TMPRSS3	125.8	124.9	134.1	136.6	115.7	102.4	124.8	113.3
TMPRSS4	108	120.3	116.9	119	111.8	98.7	120	119
TMPRSS4	120	120.7	121.6	125.6	113.5	100.1	124.8	123.6
TMPRSS5	119	112.6	111.1	116.1	103.2	93.9	116.6	113.4
TMPRSS6	116.8	115.2	118.1	123.8	111.1	106.2	123	116.2
TMPRSS7	112.5	105.6	115.2	117.5	105.5	114.6	110.4	109.4
TMPRSS7	121.3	113.5	110.5	106.6	103.5	95.1	111.2	107.4
TMPRSS9	112.7	116.4	118.7	118.3	104.3	101.8	112.4	103.1
TMSB10	6720.5	6894.7	6895.2	7085.1	5630.7	7175.3	6413	5162.6
TMSB4X	131.7	140.9	131.8	147.8	124.7	116.8	132.7	119.2
TMSB4Y	123.9	135.4	131.4	130.9	123.5	117	122.6	125.3
TMSL8	109.8	113	112.8	121	107.9	97.1	115.8	112
TMTC2	115.6	118.9	121.8	113.6	118.9	105	126.5	121.2
TMTC3	120.9	133.3	130.6	152	127.6	104	130.1	125.3
TMTC4	126.9	124.3	120	121.2	123.4	104.7	114.8	120.8
TMTC4	123.7	109.5	119.4	121.9	114.4	103.6	124.2	120.6
TMTC4	127.9	141.6	128.7	136.9	125.2	112.8	146.5	123.8
TMUB2	213	200.5	192.6	213.6	176.1	183.6	211.4	204
TMUB2	282.7	289.6	278.8	298.4	247.1	192.6	305	288.2
TNC	149.7	120.2	136.1	124.6	124.6	114.8	134	117.6
TNF	352.4	256.4	299.6	404.2	251	325	670.6	452.9
TNFAIP1	172	158.2	183	174.4	151.8	136.5	163.3	155
TNFAIP2	210.7	137.5	171	172.9	116.8	143.8	186	131.1
TNFAIP3	857.4	748.2	478.8	730.7	559.2	502.2	732.4	955.7

TNFAIP6	119	121.7	126.1	120.7	124.5	104.9	116.5	127.7
TNFAIP8	450.5	493.6	510.5	432.3	509.7	382.2	328.3	345.4
TNFAIP8	120.9	126.7	130	126.5	114.4	103	116.9	129.9
TNFAIP8L1	214.3	234.6	269.7	247.6	246.6	184	239.4	234
TNFAIP8L2	198.3	185.7	184.6	218	148.9	137.3	160.4	158.9
TNFAIP8L3	115.4	121.6	121.8	119.6	116	110.4	123.5	123
TNFRSF10A	272.1	264.9	219.2	323.6	252.9	174.6	380.9	401.7
TNFRSF10B	115.5	113.7	126.9	122.9	107.7	104.8	123.5	127.1
TNFRSF10B	118.3	110.1	120.2	112.4	107.9	101.3	116.6	118.7
TNFRSF10B	258.2	254	303.6	269.5	210.8	196	357.8	302.2
TNFRSF10C	137.6	115.4	141	134.5	114.2	110.2	142	131.5
TNFRSF10D	116	122.7	142.9	138.5	135	106.5	142.1	161.7
TNFRSF11A	120.7	109.8	113.2	114.8	105	95.9	109.7	110.6
TNFRSF11B	115.5	117.4	125.4	107.8	108.5	99.7	126.6	119.4
TNFRSF12A	115.2	113	117.3	125.7	119.4	115.9	130.1	114.4
TNFRSF13B	160.7	163.4	155.5	166.3	138.8	137.3	143.1	135.5
TNFRSF13C	141.9	128.8	137.2	143.6	111.3	113.6	125.4	117.5
TNFRSF14	4181.4	3413.9	3858.2	3918.9	2771.8	2221	2915.1	2748.8
TNFRSF17	134.4	139.2	117	143.2	121.6	107.4	122.5	122
TNFRSF18	140.4	135.3	125.1	141	114.4	111.1	132.4	124.5
TNFRSF18	113.9	121	113.9	116.8	101.6	107.5	111.5	109.2
TNFRSF19	112.2	118.9	131.4	111.4	108.8	110.2	133.7	131.2
TNFRSF19	132.2	126.7	127.1	125.3	111.2	103.2	123.6	120.5
TNFRSF19	112.7	122.6	115.2	112.4	105.9	89.8	120.6	115
TNFRSF19L	113.2	113.4	106.2	110.4	112.9	94.2	113.4	102.8
TNFRSF19L	128.8	130.2	122.4	124.5	109.1	114.7	125.2	127.3
TNFRSF19L	119.6	111.9	122.4	118.1	104.6	97.2	123.1	113.4
TNFRSF1A	568.1	583.3	693.9	618.2	410.7	613.7	679.4	573
TNFRSF1B	179.8	187.5	206.3	225.2	178.7	166.5	199.3	196.9
TNFRSF21	119.2	120.7	122.8	130.4	112.1	105.7	120.9	122.4
TNFRSF25	166.2	165.8	226	151.7	183.1	172.6	161.1	164.4
TNFRSF25	1410.1	2145.1	2476.6	1515.3	1471.7	1994	1461.6	1400.2
TNFRSF4	276.8	254.2	160.7	254.8	140	128	171.5	119.7
TNFRSF6B	114.3	111.1	123.2	117.1	105.6	102.2	124.7	112.1
TNFRSF6B	130.9	138.8	123	132.3	124.3	100.5	121.5	128.2
TNFRSF7	221.3	257.1	221.6	211.4	215.3	244.1	188.3	163.6
TNFRSF8	113.2	117.8	112.9	117.6	116.6	95.9	118.8	115.6
TNFRSF8	124.3	124	140.9	128.8	110.1	104.1	116.4	116.6
TNFRSF9	131.1	117.7	123	132.2	117.7	110.9	133.7	117.6
TNFSF10	292.3	302.3	442.8	248.7	238.7	215.6	196.3	191.9
TNFSF11	114	117.8	124.7	126.5	113.8	109.9	121.8	117.2
TNFSF11	117	116.2	105.8	122.5	112.6	93.6	122.2	111.3
TNFSF11	114.1	116.3	117.2	114.6	112	97.5	117.2	111.4
TNFSF12	281.5	250.7	362.5	249.2	183.9	233	167.9	154.7
TNFSF13	117.3	122	120.6	111.4	119.2	105	107.7	106.5
TNFSF13	119	121	123.2	116.3	100.6	102.6	121.1	123.1
TNFSF13B	205.1	212.2	342.7	223.4	179.3	174.1	159.5	150.1
TNFSF14	266.7	375.8	231.9	353.1	374.4	171.7	404.9	363.6
TNFSF14	132.7	160.1	122.6	158.2	174.1	100.8	152.3	161.3
TNFSF14	375.2	401.1	307.8	421.2	361	232.9	454.6	457.3
TNFSF15	252.5	253.8	270.1	241.6	255	184.7	290.7	262.8

TNFSF18	111.8	122.2	120.6	123.7	109.2	106	126.5	117.6
TNFSF4	123.4	115.2	148	130.6	116.9	117.6	126	136.5
TNFSF5IP1	967.7	1091.7	1264.7	1267.1	1015.7	1076.1	979.2	976.3
TNFSF7	130.5	125.7	129.4	136.3	115.5	112	126.6	119.3
TNFSF8	128.7	140.4	121.6	129.8	119.2	117.9	128.7	126.7
TNFSF9	122.3	119.2	122.7	129.5	127.1	115	141.9	121.6
TNIK	153.2	196.5	170.6	159.4	166.7	127.7	152.2	152.7
TNIP1	281.2	352.5	196.8	294.2	198.7	292.6	366.9	304.7
TNIP2	144.3	138.1	125.7	163.6	131.1	132.4	137.3	147
TNIP3	129.3	123.9	116.5	129.9	124.1	103.2	119.9	125.4
TNK1	114.8	117.1	117.8	113	103.5	96	114.3	99.5
TNK2	700.6	740.2	815.4	748.8	556	486.1	547.8	520.2
TNK2	122.4	123.8	129.9	119.4	115.7	121	123.6	120.6
TNK2	122.3	117	111.3	116.1	116.9	102.9	115.6	121.1
TNKS	122.7	125.8	124.6	126.8	110.5	103.7	120.4	113.4
TNKS1BP1	122.5	122.9	115.6	127	111.4	110.1	135.1	118.9
TNKS2	122.9	125.9	140.9	123	110.7	117.3	133.6	118.6
TNMD	119.8	114.2	115.6	133.7	116.7	101.8	125.8	121.5
TNN	119.7	115.2	116.9	118.4	111.2	101.3	124	115.2
TNNC1	126.9	110.6	130.1	131.2	108.9	102	123.8	119.3
TNNC2	106.5	111.6	110.4	113.9	110.2	105.6	119	104.6
TNNI1	108.3	111.2	115.5	118.8	108.2	97.9	112.9	108.5
TNNI2	115.5	120	125.7	115.1	105.6	95.7	123	113.8
TNNI3	131.8	116.8	117.5	140.1	113.1	105.1	118.5	120.7
TNNI3K	112.6	116.4	120.8	113	112.4	109.9	121.6	116.2
TNNT1	111.2	119.8	113.3	104.5	103.9	99.5	120.5	113.4
TNNT2	118.8	117.2	132.6	129.1	119.4	114.5	128.6	113.5
TNNT2	115.6	130.1	126.7	123.3	110.4	117	113.9	121.8
TNNT3	117.5	118.4	125.1	129.2	117.6	110.1	124.9	119.8
TNNT3	116.9	110	122.2	118.3	106.4	96.9	117.8	109
TNP1	110.7	117.3	116.2	111.4	106.5	97.4	113.9	120.8
TNP2	112.6	118.4	112	116.8	111.2	100.5	125.2	114.2
TNPO1	111.5	125.2	133	121.7	124.6	107	130.8	135.7
TNPO1	115.2	116.4	113.3	118.2	112.5	105.6	118.8	119
TNPO1	386.8	458.4	271.8	561.1	379.4	227.8	611.6	510
TNPO2	524.8	613.7	626.1	670.2	551.9	493.1	699.6	563.7
TNPO3	387.4	385.2	335.5	441.8	374.8	262.6	457.7	495.1
TNR	124.5	109.9	125.4	111.8	100.2	103	114.8	118
TNRC15	262.5	282.4	197.6	256.2	215.3	204	308.2	328.7
TNRC4	116.8	120.8	118.4	122.3	107	96.1	113.8	111.1
TNRC5	226.4	224.4	190.8	219.4	215.2	167.3	203.4	177.6
TNRC5	230.4	240.9	224.1	203.3	197	164.8	169.4	193.8
TNRC6A	117.1	127.9	124.5	126.8	117.9	105.9	137.9	130.3
TNRC6A	319.3	310.1	282.3	296.2	298	266.7	364.7	288.4
TNRC6A	115.7	124.9	126	122.6	115.3	101.9	123.5	117.1
TNRC6B	537.9	541.3	460.3	628.4	597.2	369.5	506.8	775.2
TNRC6B	109.7	115.4	134.8	133.1	114.7	105.9	116.2	109.8
TNRC6B	118.1	111.8	110	121	106.9	101.8	116	108
TNRC6C	118.9	113.4	108.9	114.7	107.5	95.2	114.5	111.1
TNRC9	113.6	114.1	119	123.6	110.6	99.8	114.3	118.4
TNS1	118.9	115	133.4	129.1	107.5	117.8	137.2	113.8

TNS3	291	163	451.5	214.3	140.9	206.5	173.7	137.8
TNS4	106.6	114.6	115.9	118.6	104.5	92	117.8	114.8
TNT	118.2	111.3	112.9	122.2	108.5	95	112.3	111.6
TNXA	110.5	116.1	111.4	118.6	103.7	95.1	114.1	114.8
TNXB	126.7	118.3	108.2	111.8	110.1	116.7	120.3	113.9
TNXB	119.4	119.2	112.2	112.6	107.1	101.2	119.9	109.8
TOB1	374.6	499.8	243.2	398.6	373.1	194.9	394.9	404.2
TOB2	142.3	146.7	129.2	133.3	145.4	105.1	146	143.6
TOE1	126.5	135.3	123.6	129.6	131	123.3	129.6	148.8
TOLLIP	201.3	233.9	181.5	208.9	176.3	194.4	257.5	258.4
TOM1	356.3	311.7	334	320.5	272.9	340.6	468.4	417.6
TOM1L1	105.1	114	107.2	125.4	99.3	93.6	108.4	115.5
TOM1L2	121.5	118.7	121.7	133.8	118.3	107.1	133.8	118.4
TOM1L2	126.6	124.7	118.5	123.5	112.2	102.6	133	129.3
TOM1L2	130	125.1	128.8	131.5	120.8	115.1	128.6	125.6
TOMM20	451	531.9	484.3	518.5	506.3	403.1	427.2	394
TOMM22	240	307.6	235.7	253.5	255.1	206.2	245.4	240.6
TOMM34	249.3	236.1	219.4	277.5	247.3	169.9	266.6	302.9
TOMM40	762.8	880.1	625.3	815.2	611.5	431.1	686.4	675.9
TOMM40L	146.2	139.3	144.7	150.4	125.3	115.2	138.3	127.5
TOMM7	4649	5471.5	7321.4	4356.2	4617	6248.9	4529.9	5751.8
TOMM70A	641	629.7	635.6	615.5	508.8	509	573.1	575.5
TOP1	126.4	134.3	129.5	124.3	114.9	101.7	134.3	137.1
TOP1MT	280.4	300.5	247	299.2	221.3	187.3	268.5	268.6
TOP1MT	155	150.1	133.9	127.2	137.6	107.4	140.8	132.3
TOP1MT	131	140.5	128.2	126.3	119.2	106.3	119.5	111.3
TOP1P1	110.7	133.1	131	113.7	109.7	103.5	126.3	122.7
TOP1P2	175.1	174.1	186.5	184.3	159.1	176.1	225.3	253
TOP2A	132.7	123.7	130.1	116.6	115.8	110.2	124.7	118.3
TOP2B	1018.3	1218	1152.2	1105.9	1309.5	1124.7	1483	1191.3
TOP3A	251.4	268.9	239.5	256.4	235.9	145.2	309.4	401.9
TOP3B	290.9	362.9	302	342	238.2	250.9	298.4	297.5
TOPBP1	195.3	223.8	184.9	214.4	210.4	178.3	205.5	220.5
TOPORS	127.7	136.5	121.2	132.5	121.7	114.9	148.1	135.7
TOR1A	168.2	171.5	178.2	210.7	140.2	150.4	208.8	176.6
TOR1AIP1	488.6	606.6	575	631.1	347	465.3	458.9	496.9
TOR1AIP2	145.7	146.1	132.9	155	123.3	129	160.6	179.8
TOR1B	186.3	236.2	196.9	244.7	226	197	236.7	312.1
TOR2A	127.4	115.7	120.1	118.5	111.8	113.9	127.4	131.1
TOR3A	244.7	230.5	231.5	228.3	220.2	154.9	224.3	193.1
TOX	130.5	136.6	149.7	120.5	117.2	107.5	129.8	118.8
TP53	132.9	133.6	119.1	130.1	147.6	108.8	134.5	129.4
TP53BP1	247.1	237.9	205.4	270.4	201.2	172.5	215.2	251.5
TP53BP2	327.9	329.1	271.8	368.4	299.7	251.8	450.4	409.9
TP53BP2	122.4	122.1	124.2	137.6	118.3	113	117.9	119.8
TP53I11	118.9	115.4	115.3	128.8	111.3	98.5	122.9	116
TP53I11	125.3	128.3	125.5	129.8	115.8	97.8	120.5	121.9
TP53I13	237.2	257.1	365.1	262.5	204.5	255.7	238.1	192.9
TP53I3	115	110	120.6	119	114.1	92.8	117.8	111.4
TP53I3	127.1	115.5	110.2	113.1	105.2	99.9	114.5	115.3
TP53INP1	165.8	153.8	162.1	154.6	122.9	134.5	137.1	145.7

TP53INP2	178.6	169.3	171.3	210.9	170.1	200.6	281.2	256.9
TP53RK	386	424.4	328.3	461	360.3	284.3	357.2	328
TP53TG3	122.1	118.3	120.8	123.9	113.5	101.7	124.1	128
TP53TG3	124.6	116	127.6	115.4	120.9	108.2	133.1	115.4
TP73	123	118.6	121	132	105	103.5	123.7	105.6
TP73L	118.2	108	121.4	117.1	103.2	96.3	111.8	104.4
TPARL	148.6	122.4	135.5	142.1	134	133.5	149.9	135.3
TPCN1	114.4	117.7	142.8	121.1	114	102.4	115.9	122.6
TPCN2	156.8	156.2	156.5	176.7	135.5	123.4	146.6	144
TPD52	117.7	110.9	121.2	116.3	107.2	96.6	128.2	108.5
TPD52L1	115.1	121.3	121.9	105.5	117.4	97	126.7	123.3
TPD52L1	118.1	126.8	125.4	112.1	120.7	104.1	121.4	124.8
TPD52L1	112.4	113.3	109.3	117.6	110.1	95.3	110.1	107.8
TPD52L2	184.7	185	196	244.9	167.6	210.6	200.9	173.5
TPD52L2	914.9	829.1	846.3	1066.4	594.1	795	1240.6	849.6
TPD52L3	124.5	119	118.2	119.2	113.5	100.1	115.2	122.9
TPD52L3	119.9	117.3	118.3	122.1	107.6	105.3	120.5	107.2
TPD52L3	114.1	119.4	118.6	119.5	111.4	107.8	122	121.2
TPH1	128	113.9	118.1	122.8	113.2	100.6	123.3	128.7
TPH2	119.3	115.4	117.7	114.3	114.6	102.2	127.6	108.8
TPI1	1655	2200.4	1953.2	1925.7	1756.7	1640.9	2229.2	2027.1
TPK1	202.8	242.5	294.5	192.6	218.8	228.2	192.4	181.6
TPK1	113.2	130	119.5	120.7	120.6	104.4	121.2	122.9
TPM1	140.1	127.5	151.9	140.3	114.9	110.4	126.8	114.9
TPM1	117.9	120.5	134.6	128.2	120.4	104.2	124.3	111.1
TPM1	131	136.2	171.1	141.7	118.5	119.4	131.9	117.4
TPM2	446.3	611.4	502.5	327.6	381.9	278.6	293.3	365.2
TPM2	114.5	125.1	121.3	124.9	103.4	97.9	134.9	115.7
TPM2	227.9	323.6	251.1	232.2	237.3	197.4	199.9	201.2
TPM3	317.4	306.1	338.6	300.1	327	263.1	333.2	385.9
TPM3	109.4	121.1	126.6	130	121.7	104.2	120.2	118.9
TPM3	126.1	118.7	143.6	140.2	115.9	109.6	130.3	124.2
TPM4	123.3	121.8	126.6	115.4	118.5	123.4	122.5	120.5
TPMT	297.2	290.9	391.5	347.9	282.9	263.7	328	315.5
TPO	103.2	109.3	109.2	111.5	111.4	101.3	111.7	118.4
TPO	127.3	114.8	114.5	121.4	109.1	98.5	115.4	125
TPP1	759.3	676.6	847.2	744.4	545.7	673.3	669.5	683.3
TPP2	134.2	147.6	161.5	150.6	152.8	123.7	136.1	150.9
TPPP	121.1	120.5	121.1	123.7	116.5	99.4	123.3	111.8
TPR	726.7	683.1	618.2	785.9	494.5	577.4	858.1	718.8
TPRKB	132.1	122.7	122.9	129.2	119.1	112.2	114.7	123.4
TPRX1	117.4	104.4	118.1	116.8	107.7	97	117.3	116
TPRXL	114.1	116.3	115.4	110.5	105	98.2	110.7	116.5
TPSAB1	116.9	120.3	118	121.3	105.8	95.1	112.4	116.8
TPSD1	107.4	123.2	121.1	116.1	109.7	100	115.1	121.6
TPSG1	118.9	109.9	111.6	116.7	119.5	104.9	123.4	114.7
TPST1	143.2	144.9	120.9	163	135.8	111	164.3	145.5
TPST2	800.3	996.2	862.4	833.3	639.4	569.8	937.4	864.1
TPT1	31362	33809.7	33891.9	34564.6	32779.3	26575.3	39105.9	36593.7
TPTE	107.8	113.4	108.6	113.8	106.7	95.5	117.1	115.9
TPTE2	118.8	108.4	110	119.4	114.3	104.2	114.1	122.4

TPTE2	115.3	120.8	121.9	129.5	109.5	101.4	113.1	114
TPTE2	110.8	114.8	112.9	120.7	104.6	93.8	122.8	113.6
TPX2	118.1	111.2	122.2	126.8	111	101.3	128.8	115.6
TR2IT1	110	110.5	126.7	118.6	104.3	92.1	110.8	106.2
TRA16	161.7	199.4	159.8	199.4	140.2	125.1	176.4	155
TRA1P2	178.6	181.2	223.5	188.3	179.9	207.6	156.8	191.7
TRA2A	240.6	240.7	209.1	250.1	261	162.3	218.9	262.6
TRADD	176.4	196.5	178.8	173.7	163.2	151.7	157	140.7
TRADD	262.7	236.2	229.9	234.9	200.7	188.2	218.7	191.7
TRAF1	119.5	123.8	113.8	127.6	111.4	108.2	125.3	111.3
TRAF2	164.8	158.5	147.1	184	126.8	135.3	190.9	165.9
TRAF3	166.8	166.3	141.8	172.9	149.3	126.9	200	168.2
TRAF3IP1	123.6	122.4	130.6	137.1	121.1	113.4	127.2	124.4
TRAF3IP2	109.6	110.6	120.1	118.4	115.7	92.7	111.3	105.8
TRAF3IP2	114.4	130.7	122.5	114.2	110.6	103.6	127	129.2
TRAF3IP2	184.1	276.2	210.1	219.3	194.4	159.1	190.7	200.3
TRAF3IP3	947.9	1299.8	1266.1	946.4	872.9	836	571.9	570.7
TRAF4	113.2	114.9	128.9	122.2	114.8	98.3	133.7	121.8
TRAF4	106.2	129.6	120	130.4	106.3	103.9	109.3	115.2
TRAF5	123.2	114.2	117	123.6	110.2	97.5	116.1	116.3
TRAF5	137.1	132.9	132.4	126.1	118.8	122	124.3	132.3
TRAF5	112.8	112.8	128.9	128.4	119.2	102	121	118.7
TRAF5	112.8	114.1	125	116.3	114.8	101.5	122.6	116.8
TRAF5	274.4	293.1	383.3	290.1	244.5	221.9	192.2	219.9
TRAF6	112.6	130.5	117.8	114.1	114.4	112	116.5	107.3
TRAF6	177.8	157.9	162.1	187.6	146.9	147.1	191.7	199.1
TRAF6	180	215.3	174.4	203.1	152.8	153.1	188.3	189.4
TRAF7	133.1	120	124.9	139.2	111.3	113.3	128.1	128.8
TRAF7	116.5	118.7	126.3	115.8	105.7	101.5	115.6	115.2
TRAFD1	238.6	203.4	234.8	196.2	151.4	198.6	214.1	211.9
TRAIP	128.8	120.6	129.8	136	120.4	117.9	138.8	128.4
TRAK1	177	189.8	183.4	189.8	140.4	169.6	178	158.6
TRAK1	254.6	240.7	255.8	242.6	195	208	220.1	198.7
TRAK1	152.7	142.4	181.7	148.8	131.1	122.8	156.6	152.3
TRAK2	325.2	334.6	304.4	375.4	313.1	277.7	342.5	324.1
TRAM1	2505.5	2878.7	3547.8	3104.9	2768.6	3066.8	3143.5	3776
TRAM1L1	118.3	108.6	119.5	122.8	114.1	100.9	115.9	119.6
TRAM2	164.8	180.4	140.9	146.6	163.3	126.7	147.7	145.1
TRAP1	183	194.4	182.7	188.6	163.1	130.2	182.4	218.6
TRAPPC1	401.1	530.4	504.7	397.2	347.6	421.6	342.5	273.9
TRAPPC2	134.7	124.6	138.5	123.8	116.5	102.1	124.9	107.4
TRAPPC2	332.7	405.3	432.8	346.3	345.4	364.9	288.7	304
TRAPPC2	109.5	117.8	126.4	127.9	109.4	101.4	124.4	122.5
TRAPPC3	499.4	477.1	530	553.1	484.8	454.1	594.3	654.8
TRAPPC4	524.5	569.9	620.7	614	529.8	527.7	566	570.5
TRAPPC5	345.6	302.8	403.3	358.3	323.9	253.5	367.2	320.9
TRAPPC6A	176.6	181.7	195.1	174.2	139.8	152.4	141.8	152.1
TRAPPC6B	155.6	160.9	149.7	136.8	129.5	119.8	136.3	148
TRAPPC6B	126.7	115.7	114	125.1	109.1	109.9	140.3	113
TRAPPC6B	200.6	240	185.2	184.8	156.2	135.4	187	234.6
TRAT1	116.8	116.3	121	129.7	106.7	106.9	124.9	116.8

TRDMT1	138.5	161.1	157.4	151.1	124.5	106	123.5	132.1
TRDN	111.1	123	129.1	123.5	115.9	107.9	125.2	121.1
TREH	110.3	126.7	113.7	125.3	108.3	107.4	117.6	112
TREM1	119.1	125.2	123.8	128	119.3	98.2	122.5	113.9
TREM2	113	117.3	111.3	114.1	104.6	99.4	117.8	113.5
TREML1	109.6	123.4	117.6	113	112.3	102.3	124.2	121.1
TREML2	128.8	133.6	135.8	129.9	128.9	100.5	128.5	132.2
TREML2P	110.7	108.5	114.2	113.6	103.2	95.2	123.3	118.6
TREML4	122.1	116.4	103.6	113.2	112.9	105.9	119.3	107.7
TRERF1	157.4	159.8	154.8	155.6	139.8	116.7	143.2	124.9
TRERF1	116.4	113.3	109.8	113.2	111.5	94.2	119.9	114.6
TREX1	104.9	123.6	124	122.7	115.6	102.5	116.4	132.7
TREX1	176.6	197.2	195	190.5	149.7	170.2	183.1	169.9
TREX1	143.2	170.2	160	169.4	146.3	137.2	173.9	152.4
TREX1	121.9	114.3	118.7	119.7	111.4	106.8	119.8	115
TREX2	120.3	118.4	124.4	117.5	107.6	97.4	121.7	104.5
TRFP	315.4	333.3	352	405.2	338	297.4	408.6	485.7
TRG20	441.5	534.2	459.5	621.9	456.4	431.5	660.6	805.1
TRH	110.9	116.7	112.6	112	101.5	94	109.8	112.9
TRHDE	114.9	112.4	109.9	122.9	103.9	96	115.7	110.3
TRHR	121.2	114.6	119	116.9	102.3	106.8	122.5	124.3
TRIAD3	116.3	113.3	122.3	110.6	111.1	101.5	123.1	109.7
TRIAD3	611.1	577.2	597	653.7	617.8	437.8	572.8	651.8
TRIAP1	246.2	229.9	233	239.1	183.6	182.3	182.6	197.9
TRIB1	347.4	316.6	291.7	483.9	300.5	447.9	517.9	270.6
TRIB2	410.1	406	262.8	332.9	322.5	155.1	277.6	214.8
TRIB3	148.7	149.7	146.6	141.7	138.6	119.1	146.6	152.9
TRIM10	112.8	119.2	109.4	120	99.9	89.8	122.3	116.4
TRIM10	108.2	115	123.5	121.1	109.7	102	126.1	111.4
TRIM10	117.3	120.8	117.9	123.2	106.3	94.5	120.5	117
TRIM11	139	166.7	141.8	144	135.9	141.4	170.2	156.4
TRIM13	352.8	351.8	385.1	344.5	451.7	246.2	379.7	466.5
TRIM13	188.7	187.7	173.4	194.4	189.6	140.6	165	158
TRIM13	219.9	223.9	225.5	253	258.8	196.8	307.4	293.2
TRIM14	116.3	112.2	117.6	121.8	118.3	99	120.7	110
TRIM14	124	122	124.4	128.5	121	105.7	127.8	134.8
TRIM14	126.7	120.5	141.5	128.2	111.4	100	122	116.9
TRIM14	111.3	116.1	118.3	125.2	115.4	101.9	122.3	108.1
TRIM15	110.3	120.5	125.1	120.1	103.7	97.9	112.5	119.3
TRIM15	109.7	110.1	107.4	113.5	100.8	92.2	109.4	107.2
TRIM15	113.9	119.3	125.4	121.9	109.6	96	116	112.7
TRIM16	109.9	109.2	112.3	111.9	97.4	97.4	119.6	106
TRIM16L	115.3	111	115.2	117.6	107.1	98.7	115.3	123.2
TRIM17	120.5	105.2	120.4	124.7	116.1	102.9	123.8	118.6
TRIM17	119.9	122.7	122.8	117.1	107.9	104	116.3	117.2
TRIM2	121.7	141	114	127.4	128.3	103	120.7	130.3
TRIM21	227.5	216.1	282.3	259	174.2	245.3	224.3	216.4
TRIM22	547.2	556.6	574.1	557.9	488.7	439.4	484.5	463.6
TRIM23	168.7	192	190.8	210.5	199.5	146.4	183.6	219.5
TRIM23	112.5	119.1	121	120.8	111.4	97.8	124	124.6
TRIM24	127.4	126.7	123.9	148.5	118.4	100.8	128.4	124.7

TRIM24	117.8	110.3	116.5	112.8	101.5	105.1	109.6	104.1
TRIM24	251.2	221.9	243.2	291.6	241.7	175.7	306.7	328.4
TRIM25	258.6	280.6	225.1	348.7	255	285.1	316.7	213.3
TRIM26	247.5	252.7	259.2	262.5	191.6	220.1	261.2	265.7
TRIM28	295.5	346.2	243.9	320.9	214.7	226.4	426.6	336.7
TRIM29	106.6	111.7	115.4	107.3	107.7	99.3	103.7	103.7
TRIM29	122.3	127.8	124.2	122.4	115.2	102.7	115	131.9
TRIM3	142.2	152.4	148.2	140.6	124	130.2	138.3	143.9
TRIM3	104.6	106.7	115.8	112.9	114.1	97.2	116.3	115.4
TRIM31	116.5	110.6	139.2	115.9	110.7	93.6	121.7	113.7
TRIM31	115.1	118.6	120.2	123.4	114.7	109	124.8	119.7
TRIM32	199.1	204.2	173.8	204.7	199.6	137.3	156.1	190.5
TRIM33	220.1	219.1	188.7	242.6	233.7	212.4	318.5	280.5
TRIM33	619.4	648.2	710.5	691	706.4	543.5	778.1	774.1
TRIM33	103.9	113.2	115.5	116.3	107.6	89.7	126.5	105.6
TRIM34	115.4	118.5	114	118.4	106.6	96	127.5	114.9
TRIM34	114.9	116.3	119.5	115.1	104.4	95.4	109.9	116.2
TRIM34	115.5	111.7	114.3	122.7	103.4	91.7	114.5	110.6
TRIM34	119.3	131.9	124.2	126.3	111	104.3	126.6	120.7
TRIM35	133.6	129.1	132.6	120.8	125.8	110.5	128.6	118.2
TRIM36	120.6	107.5	126.7	129	115.8	105.1	127.6	125.7
TRIM36	106.4	109.6	132.1	117.3	111.6	101.3	115.3	110.7
TRIM36	127.2	112.4	117.2	116.4	108.3	102.6	117.3	112.1
TRIM36	112.6	113.2	120.4	120.1	111	95.4	118.3	121.4
TRIM37	127.6	123.6	126.4	124.1	117.8	117.6	122.9	134.2
TRIM37	143.7	170.1	156.3	165.9	156.1	132.7	147	145.4
TRIM38	256.3	230.6	286.6	243.8	232.5	185.9	270.4	285.3
TRIM39	107.5	115.8	122.2	121.5	108.4	105.6	115.5	113.8
TRIM39	317.9	390.5	347.7	312.7	271.1	237.7	341.7	350.6
TRIM39	152.2	172.6	132.5	149.1	119.2	126.5	136.9	147.4
TRIM4	1016.6	1541.7	982.4	1121.4	966.9	801.7	1074.3	1041.7
TRIM4	129.5	119.4	129.9	124.9	105.2	108.7	121.4	118.8
TRIM40	117.8	115.7	130.1	123.4	116.4	103.6	118.7	129
TRIM41	187	197	195.9	203.3	179.9	187.6	254.3	183
TRIM41	143.2	136	129.8	144.8	112.4	111.2	146.4	126.7
TRIM42	117.3	106.3	114.8	118.9	111.6	107.5	120.4	121.1
TRIM43	113	107.3	124	126.2	109.2	108	113	111.5
TRIM44	871.9	1089.3	707.1	888.9	893.4	610.8	895	976.2
TRIM45	117.1	124.4	118.8	128.2	113.1	117.2	124.5	120.4
TRIM46	136.4	140.9	123.3	144.3	121.4	101.2	125.1	117
TRIM47	125.6	132.3	117	136.6	117.4	107.5	118.1	124.5
TRIM48	111.6	108.3	112.3	111.6	109.7	103.9	114.5	112.3
TRIM49	141.7	133.7	138.8	140.7	127.5	125.8	155.1	148.6
TRIM5	265.2	346.1	255.7	285.8	271	178.3	210.2	254.7
TRIM5	123.1	124.8	129.7	128.5	115.7	105.1	117.8	118
TRIM5	166.7	139.9	152.1	174.4	143.5	129.8	159.6	139.2
TRIM50A	113.6	112.8	114.8	112	111.1	97.9	114.8	114
TRIM51	115.7	115	135.5	125.6	115.6	109.3	116.7	114.3
TRIM52	400.8	400.4	464.4	324	335.7	211.5	329.4	349.5
TRIM54	127.8	130.5	132.4	137.1	124.4	113.7	132.6	121.9
TRIM54	113.2	117.2	120.9	121.5	110.9	94.8	113.8	117.5

TRIM54	111.7	119.3	119.7	121.3	106.4	105.3	124.6	116.9
TRIM55	113.8	117.9	118.4	125.2	112.8	109	117.3	111.2
TRIM55	106.4	112.7	122.7	137.8	106.9	97.4	121.8	113.6
TRIM55	119.6	116.2	110.5	117.5	109.3	109.1	119.5	123.2
TRIM56	236.2	252.7	183	277.9	201.2	187.5	239.8	205.6
TRIM58	118.6	119.8	114.8	119.2	122.5	100	116.1	114.6
TRIM59	106.9	120.4	114	115.4	107.1	105.5	111.1	113.2
TRIM6	108.9	110.3	110.3	116.5	113.8	95.1	113.6	119.2
TRIM6	115.3	114.4	120.2	128.5	115.6	103.8	126.2	122.7
TRIM60	112.6	105.7	115.8	116	110.3	97.7	117.4	115.1
TRIM62	130.1	130.7	165.3	136	115.3	105.4	115.2	122.8
TRIM63	113.4	118.1	128.9	114.8	108.4	105.4	129.4	126.5
TRIM67	120.5	124	118.8	112.9	119.7	104.1	126.5	121.9
TRIM68	230.9	267.4	278.3	277.5	223.2	231.4	252.4	257.6
TRIM69	123.9	128.3	128.9	131.6	121.5	114.1	134.4	126.3
TRIM6-TRII	110.7	110.6	114.6	118.7	104.9	96.6	120.2	107.3
TRIM7	116.9	119.7	119.3	120	110.9	102.2	114.9	124.7
TRIM7	117.6	123.4	119	128.4	115.1	113.8	119	121.4
TRIM7	114.6	129.1	125.9	115.5	111.9	100.9	118.8	117.9
TRIM71	112.5	116.1	111.3	126.5	106.8	102.5	125.1	113.9
TRIM74	126.3	121.8	138.3	129.2	124.3	113.1	124.4	123.1
TRIM8	682.7	614.1	660.8	651	467.3	543.1	544.1	410.6
TRIM9	118.8	121	125.1	130.6	107.4	107.6	126.3	105.1
TRIM9	121.2	120.7	126.1	129.3	117.1	103.3	127.3	133.2
TRIM9	140.1	129	131.9	130.6	125.3	115.1	137	122.8
TRIO	123.3	121.8	126.1	134.5	121.1	101.4	131.2	136.7
TRIOBP	113.2	115.2	120.4	112.9	110.6	102.5	118.7	113.9
TRIP10	126.3	124.4	118.9	129.3	112	102.7	121.8	133
TRIP11	192.3	212.5	193.6	202.6	193.9	160.9	220.3	265.1
TRIP12	327.5	331.1	298.9	482.4	320.8	325.8	552.4	560.4
TRIP13	115.1	116	119.9	122.9	107.6	93.5	134.1	122.8
TRIP4	240.2	298.9	290.7	251.9	226	234.4	282.2	247.8
TRIP6	171.2	169.2	183.8	187.5	156.7	166.4	183.9	154.6
TRIT1	189.2	176	218.5	182	157.8	128.5	147.4	133.3
TRMT1	318	335.5	352.6	317.1	244.6	205.9	283.8	229
TRMT12	164.6	163.9	177	146.9	162.8	153.1	159.3	158.6
TRMT5	477.9	547.1	729.3	411.3	374.5	556.7	418.1	608.7
TRMU	133.4	128	152	136.5	126.7	106.6	128.5	137.8
TRNT1	107.6	113.8	125.4	112.3	108.5	110.4	111.7	111.7
TRNT1	284.2	327.2	220.3	250.6	266.6	174.8	214	208
TRNT1	114.5	118.1	121.3	123.5	116.2	108.5	129.5	117
TRO	147.2	139.4	141.2	150.3	117.2	107.7	139.9	115.8
TRO	117.6	120.6	117.9	120.8	102.8	90.6	111.2	115
TRO	131.4	123.1	136.3	141.3	110.2	108.4	134.3	126.3
TROAP	122.6	123.3	121.5	120.3	108	98	123.3	120.4
TROVE2	401.8	514.1	455.9	566.6	528.8	511.2	519	582.7
TROVE2	117	129.4	123.6	137.7	117.4	107.8	125.9	127.1
TROVE2	159.4	182.6	177.8	183.1	182.4	180.8	166.9	169.9
TROVE2	795.9	970.8	707	965.1	787.3	603.5	1103.2	999.9
TRPA1	114	121.1	118.3	122.6	108.8	94	117.8	112.5
TRPC1	120.2	126.1	139.5	122.3	122.4	109	142.9	144

TRPC2	114.8	116.8	121.6	125.2	113.3	96.8	114.2	111
TRPC3	110.2	108.8	107.5	120.7	106	98	117.4	111.5
TRPC4	116	120.5	120.3	127.3	109.4	95.1	132.4	121.4
TRPC4AP	481.1	330.8	556	538	279.3	363.6	611	393.8
TRPC4AP	575	512.4	545.8	632.3	505.9	530.5	836	703.2
TRPC5	123.7	134.6	135.9	142.1	126.4	128	140.2	128.7
TRPC6	120	122.5	114.1	124.4	108.2	99	111.7	107.5
TRPC7	122.3	112.9	106	112.8	104.4	97.3	120	117.1
TRPM1	113.6	110.3	115	114.6	105.5	102.6	110	112.7
TRPM2	121.7	111.9	136	126.8	117	108.2	111.6	120
TRPM2	114.9	113.3	129.7	116.8	104	102.2	112.9	116.5
TRPM3	121.5	124.6	112.9	125.1	116.2	97.5	122.7	114
TRPM3	110.8	110.4	118.4	120.9	99.4	101	111.6	110.7
TRPM4	110.2	105.4	108.6	126.5	101.8	99.1	108.5	113.5
TRPM5	108	112.3	118.8	116.9	110.3	95.3	128.4	108.9
TRPM6	109.5	111.1	111.6	129.2	109.2	98.8	122.5	110.6
TRPM8	107.5	108.5	114.2	113.5	103.1	102.4	119	115.5
TRPS1	131.1	126.8	139.9	141.3	128.9	116.3	130.8	136.8
TRPT1	107.4	112.9	110.3	113.2	107.3	101.5	120.7	106.3
TRPT1	840.5	783.3	812.6	826.6	792.1	562.3	798.3	610
TRPV1	122	128.8	123.1	127.4	116.6	107.6	132.6	120.8
TRPV1	127.7	123.7	119.8	114.3	115.4	112.7	134.4	114
TRPV1	114.2	107.2	110.9	129.7	113.1	95.4	117.2	117.7
TRPV1	119.5	121.4	106.5	114.8	109.8	91.4	115.8	120.8
TRPV1	112	111.3	119.8	120.5	100.9	103.9	116.3	119.3
TRPV2	379.1	326.5	357.4	398.3	244.9	288.1	265.8	232.1
TRPV3	114.6	111.1	119.6	120.8	117.8	104.3	122.7	124.5
TRPV4	110.8	117	109.6	120.6	103.6	94.9	109.7	111.9
TRPV4	105.6	117.8	120.1	110.3	103.7	96.4	111.6	110.1
TRPV5	108.6	112.2	131.5	125.1	114.7	93.2	112.2	119.1
TRPV6	113.8	119.1	108.1	118	105.5	97.5	118.6	110.3
TRPV6	105.7	108.2	121.1	121.3	111.6	100.7	110.8	109
TRPV6	117.1	108.4	105.4	122.9	116.1	100.5	120.3	119.4
TRRAP	571.5	385.7	461.1	576.8	650.7	313.7	802.6	608.8
TRSPAP1	125	137.7	157.7	147.8	125.5	138	142.4	148.5
TRSPAP1	125.6	119.8	130.1	121.9	110.8	101.8	118.2	117.3
TRUB1	114.2	121	118.4	130.6	107.9	101.3	117.4	123.5
TRUB2	124.5	131.9	135.3	136	129.9	102.8	127.9	129
TRY1	124.6	99	115.8	115.9	107.3	104.7	126.2	118.4
TRY6	123	118.1	118.3	128.6	114.3	94.1	123	123.2
TSC1	117.9	129.9	129.5	115.1	110.8	96.5	128.2	123
TSC1	216.5	181.7	200.1	232.3	200.9	182.7	245	386.2
TSC1	119.1	113.1	122.4	130.2	120.9	98.8	130.7	117.9
TSC2	141.7	153.9	150.7	167.9	144.1	117.7	162.8	157.1
TSC2	109.1	118.3	115.2	113	102.7	94.3	109.5	106.9
TSC22D1	127.2	160.4	139.8	140.4	138.4	125.3	150.5	134.9
TSC22D1	116.1	139.2	129.9	141.1	117.2	101.3	113.1	118.1
TSC22D1	110.5	107.8	121.7	128	115.3	101.1	120.5	117.4
TSC22D2	244.7	335	268.8	311	267.1	243.8	449.4	377.9
TSC22D3	140.2	136.5	136.9	138.1	133.2	120.6	121.6	148
TSC22D3	426.3	588	508.7	515.9	547.7	409.7	456.5	650

TSC22D3	406.2	344.9	363.2	406.1	356	354.6	432.5	480.9
TSC22D3	123	120.7	118.9	120.5	102.5	96.4	122.4	127.4
TSC22D3	115.5	105.5	121.1	115	111.8	95.7	112.5	99.9
TSC22D4	164.3	173.8	151.8	132.6	131.5	139.5	189.2	156.5
TSCOT	125.2	141.6	141.4	144	129.2	127	135.4	140.7
TSEN2	194.5	230	206.8	202.9	208.6	161.6	179.2	161.8
TSEN34	399	547.2	742.6	616.5	489.6	527.8	683.8	643.4
TSEN34	369.2	363.3	445.5	441.5	311.4	451.6	465.2	383.5
TSEN54	274.6	275.1	189.3	282.9	208.5	156.4	243.5	245.4
TSFM	158.6	141.5	140.9	159.3	148.9	118.8	144	130.5
TSG101	627.4	713.1	650.4	687.5	594.2	683.7	768	670.7
TSGA10	121.4	126.1	113.4	123.6	119.2	108.1	133.2	134
TSGA10	137	141.8	134.4	149	139.6	109.2	130.7	122.7
TSGA10	110.7	116.1	123.6	111	106.6	96.4	112.8	114.6
TSGA10IP	121.6	111.6	118.4	123.4	108.9	93.3	115.8	116.8
TSGA13	126.3	123.3	119.5	122.4	114	99	116	115.9
TSGA14	146.7	167.7	180.8	153.2	150.7	143.2	136.7	155.1
TSGA2	107.6	116.1	120.5	121.3	106	98.1	107.9	115.4
TSHB	108.5	114.2	117.9	124.5	110.2	96.9	113.4	109.9
TSHR	118.8	106.3	117.9	125	114.2	97.1	116.7	112.4
TSHR	116.3	117.9	117.7	135.9	120.5	105.2	124.5	119.7
TSHR	119.3	113.9	123.6	112.9	111.2	95.7	119.8	115.1
TSKS	110.3	120.1	131.2	116	109.1	99.7	122.4	114.6
TSLP	116.7	122.5	118.6	126.1	107.8	100.3	125.3	124.2
TSLP	111.5	115.7	117	118.6	100.6	103.4	122.9	116.2
TSN	128.6	110.9	132.2	126.8	118.5	101.3	134.5	130.7
TSNARE1	107.8	116.5	114.6	115.9	105.6	105.3	117.7	128.8
TSNAX	144.1	143.4	137.9	133.9	140.1	119.8	133.6	122.5
TSNAXIP1	128.1	114.2	134.1	118.4	118.9	97.8	132.3	119.6
TSP50	111.4	132.6	121	116.1	105.3	101.4	121.5	104.4
TSPAN1	118.3	132.3	134.2	122.3	121.4	104.6	116.2	124.3
TSPAN10	126.1	117.3	125	121.4	110.8	100.2	116.7	122.4
TSPAN11	111.2	110.7	114.5	113	105.9	100.3	113.9	119.9
TSPAN11	110.6	110.7	118.3	116.9	109.1	94	111.8	109.9
TSPAN12	114.5	110.1	117.8	121.4	111.9	96	116.2	107.7
TSPAN13	140.8	137.4	136.2	139.5	121.4	112.3	146.6	124.8
TSPAN14	137.3	141.1	161.2	146.6	130.6	145.2	138.1	145.9
TSPAN15	117.7	113.1	126.7	125.4	113.6	96.8	115.9	126.3
TSPAN16	118.5	116.8	121.3	119.6	109.3	100.2	124.2	124.4
TSPAN17	137.3	162.1	133.3	141.5	128.3	126.5	123.9	137
TSPAN17	836.5	1141.6	712.5	911.3	602.5	344.5	640.7	556.3
TSPAN18	111.6	118	125.2	125.1	107	98.5	108	105
TSPAN18	135.4	144.9	136	147	130.5	120.8	129.5	127.8
TSPAN18	134.7	120.3	130	133.4	120.8	110.6	127.3	125.6
TSPAN2	116.3	117.4	122.7	118.4	104.3	98.6	123.4	118.6
TSPAN3	237.3	255.8	233.5	290.5	265	161.9	327	282.8
TSPAN3	405.1	475.3	359.5	447.1	396.2	307.7	321.1	422.4
TSPAN31	356.3	432	438.3	352.9	302.6	304.3	332.7	334.4
TSPAN32	901.4	980.3	884.5	614.1	469.9	428	251.2	249.1
TSPAN32	286.1	280.9	243.8	199.2	157.8	172.2	141.6	138.4
TSPAN32	156.9	144.2	138.5	143.5	143.9	99.3	123.8	118.9

TSPAN33	192.4	243.2	204.9	195.1	154.6	152.9	160.6	144.3
TSPAN4	129.8	130.3	142.9	143.2	117.9	120.8	136	123.1
TSPAN4	109.3	110.8	121.6	117.3	110.5	97.7	122	106.4
TSPAN4	120.3	121.3	126.7	130	111.5	101.6	117.9	118.8
TSPAN4	109.8	106.9	132.3	122.2	107.1	103.8	123.3	127.1
TSPAN5	157	171.6	147.5	166.9	141.7	111.9	140.8	147.2
TSPAN6	126.6	131	132.1	135.1	111.2	110.5	125.1	121
TSPAN7	116.4	113.3	115.9	116.6	120.5	102.5	122.7	113.4
TSPAN8	121.1	114.2	121.3	125.5	114.3	101.4	124.7	106.7
TSPAN9	145.3	155.5	155.9	184.6	146.2	132.9	178.8	180.9
TSPO	439.9	308.1	756.2	389.7	306.3	385.1	291.2	234.9
TSPO	183.3	190.7	244.5	205	150.8	183.5	172.5	167
TSPO	3489.2	2934.4	5411.3	2785.2	1845.8	2389.5	2024.9	1600.8
TSPY1	104.8	106.1	109	115.2	100.6	91	109.5	102.3
TSPY2	117.1	120	111.6	119	107.5	100.6	114.7	112
TSPYL1	434.8	404.5	422	418.9	379.8	373	454.9	413.4
TSPYL2	121.1	114.1	110.8	120.3	105.7	105.2	123.8	133.9
TSPYL2	184.3	178.4	161.8	176.4	181.9	159.5	229.5	221.3
TSPYL2	127.5	117.3	116	131.6	113.9	107.3	127.7	130.9
TSPYL2	349.8	351.7	270.3	368.1	341.4	259.9	649.7	516.6
TSPYL3	108	117.9	116.9	120.4	117.2	97.7	114.7	120.8
TSPYL4	118.7	127.6	139.3	122.9	117.7	113.2	119.7	131.2
TSPYL5	133.8	132.8	144.4	120.9	104.5	116.1	132.3	114.5
TSPYL6	124.1	111.1	123.5	120.8	117.9	96.7	134.9	121.1
TSR1	116.3	119.9	135	130.8	115.6	100.8	133.7	119
TSRC1	116.9	119	139.5	116.9	103.9	104.4	111.4	113.8
TSRC1	102.2	108.7	118.1	117.2	103.2	97.9	120.1	114.9
TSRC1	112.9	123.5	121.3	118.1	105.5	97.5	115.5	109
TSSC1	269.9	283.5	307.5	272.7	246.4	214	241.8	227.1
TSSC4	434.7	467.3	371.3	497.8	360.3	290.8	494.7	515.2
TSSK1	127.6	124.1	118.6	114.6	113.2	98.7	128.1	126.6
TSSK2	117.2	112.3	113.7	114.5	105.5	93.8	116.9	118.8
TSSK3	117.4	117.2	126.1	119.2	113.5	105.3	136.7	125.3
TSSK4	109.6	112.2	119.5	119.7	121.1	94.9	112.1	113.6
TST	263.2	200.1	351	245.5	161.9	201.3	168.7	165.6
TSTA3	141.6	132.9	135.6	138.9	116.9	112.5	149.8	141.5
TTBK1	112.4	110.7	116.8	113.4	102.4	93.3	125.5	116.5
TTBK2	119.4	118.3	109.1	118.5	108.3	99.3	124.6	108.4
TTC1	235.9	254.8	238.5	260	211.9	244.4	262.2	230
TTC10	121.6	119.2	125.1	119.7	112.5	103.8	117.9	110.4
TTC10	122.7	136.6	142	132.7	119.5	120.7	147.2	123.3
TTC12	130.2	124.6	144.1	127.4	118.8	112	125	118.7
TTC13	417.6	444.9	384.4	418.3	437.5	247.7	367.8	290.9
TTC13	113.9	126.1	114.8	140.5	109.2	106.2	133.8	130.3
TTC13	192.6	209	215.8	189	168.9	145.9	177	188
TTC14	412.2	428.2	498.9	415.4	457.8	278.7	305.4	281.1
TTC14	334.2	322.8	399.1	347.3	296.3	251.7	222.3	188.4
TTC15	423.5	474.4	486	388.2	386.4	307.4	426.4	311.6
TTC16	142.4	133.4	133.4	162.3	134.4	126.7	142.2	137.1
TTC17	226.7	204.1	271.6	252	219.4	184.1	225.9	239
TTC18	114.7	118.4	122.3	125.5	119.8	102.2	119.2	133.5

TTC19	411.9	496.2	485.6	439.7	441	371.1	400.3	311
TTC21A	115.2	112.4	123.8	116.5	116.7	111.7	135.8	125.8
TTC21B	110.2	111.2	111.2	119.3	103.8	98.2	109.8	109
TTC22	107.7	103.6	108.1	114.6	109.7	97.6	109.3	110.2
TTC23	122.4	126.7	129.4	118.4	111	106.5	130.3	125.9
TTC23	115.2	115.7	121.5	120.6	108.3	101.3	119.9	123.2
TTC25	111.5	110.1	117.3	126.3	110.6	96.5	114.5	117.1
TTC3	1447.3	1344.8	1435.6	1111.3	1051.6	905.3	1062.2	1166.4
TTC3	116.8	116.1	130.7	118	110.3	103.3	112.4	117.2
TTC32	199.2	200.8	268	191.2	233.9	195.5	257.1	305.7
TTC4	549.6	648.6	541.4	575.1	606.2	402.4	727.1	497.1
TTC5	140.8	168	170.2	167.9	159.3	132.1	134.8	128.8
TTC6	104	105.7	115.4	110.9	103.4	97.1	123.8	109.8
TTC7A	120	111	125.2	132.5	108.4	110.6	106.1	114.1
TTC7B	118.2	115.7	119.7	121.2	111.4	98.9	122.2	115.7
TTC8	126.7	121.8	122.7	119.1	115.8	97.1	116.5	124.5
TTC8	112.3	123.2	130.1	111.8	117.6	101.2	124.8	112.8
TTC9B	119.3	118.1	112	133.4	109.6	101.2	110.2	112.8
TTC9C	183	174.8	158.7	165.5	169.9	130.7	154.9	138.2
TTF1	110.8	122.6	121	118.9	99.9	95.9	111.3	120.8
TTF2	216.7	215.8	232.7	251.9	198.3	183.8	211.6	194.1
TTK	122.5	121	112.2	130.6	113.1	113.2	127.4	118.8
TTL	184.9	193.5	169.9	234.5	176	176.5	198.5	191.3
TTLL1	112.5	116.3	122.4	113.4	111.5	96	112.6	109.6
TTLL1	116.3	117.9	126.4	127.6	108.6	104.1	113.9	115.9
TTLL1	115	120.4	133.6	137.2	131.6	102.7	128	125.8
TTLL10	114.7	116.1	114.7	116	103.7	99.1	119.4	116
TTLL12	183	194.8	180.9	212.9	164.1	146.6	208.6	188.1
TTLL13	120.4	122.1	121.6	121.2	108.6	99.9	124	112.9
TTLL2	111.1	110.9	104.8	112.2	99.6	91.7	118.3	114.1
TTLL3	206.5	194.6	249	209.1	169.5	178.5	202.9	177
TTLL3	122.1	111.7	132	121.8	113.7	99.9	124.2	108.1
TTLL4	160.5	137.2	128.4	157	129.4	121.6	160	127.2
TTLL5	141.9	151	146.4	137.4	116.6	114	136.8	128.5
TTLL6	116.1	114.2	111.3	116.1	106.6	98.5	119	108.8
TTLL7	113	112.2	113.1	124.1	118.3	105.2	123.6	117.1
TTLL9	115.3	124.3	122.1	126.7	108.9	113.4	123.3	121.8
TTMA	112.5	112.2	108.7	117.9	101	95.9	119.4	112
TTMB	116.5	120.1	122.3	124.6	117.9	105.9	113	115.1
TTN	120.8	127.6	117.6	111.8	106.8	103.5	120.1	112.4
TTN	108.2	112.6	106.4	118.2	113.4	100.6	117.3	112.6
TTN	109.3	108.5	122.7	118.6	106	101.8	112.9	122
TTN	115.2	111.4	120.2	109.4	107	93.2	117	109.4
TTPA	116.3	107.9	120.1	115.3	105.4	97.3	117.5	104.5
TTR	114.6	118	121.1	122.2	114.2	96.6	129.6	121.5
TTRAP	141.3	150	140	177.5	147.9	119.5	207.2	167.4
TTTY1	104.9	112	115.5	117.1	104.1	100.9	114.5	109.9
TTTY10	118.1	122.4	134	139.9	114.6	108.3	137	129.6
TTTY11	135.6	117.7	120.8	124.2	113.7	103.7	121.6	115.4
TTTY12	107.6	114.6	118.4	112.2	100.6	97.3	116.5	106.6
TTTY13	115.1	112	109.9	106.7	111.3	95.3	112.2	113

TTTY14	116.1	108.7	114.3	111.7	115.9	106	117.1	113.8
TTTY17A	113	113.3	118.4	126.3	106.2	94.1	115.3	102.3
TTTY19	132.8	129.2	128.2	137.7	120.5	104	118.9	123.2
TTTY2	117	111.9	115	112.5	110.2	98.1	122.3	113.4
TTTY21	112.5	121.1	121.9	114.9	108	101	125.9	114.5
TTTY5	106.1	110.1	113.8	120.2	104.6	96.5	121.2	106.1
TTTY6	111.4	115.7	124.3	118.5	106.1	98.6	110.2	112.8
TTTY6B	109.1	109.3	113.1	121.7	111.6	97.2	120.2	110.7
TTTY7	116.7	116.1	113.5	128.9	106.5	93.4	121	115.3
TTTY8	125	124.1	119.2	125.2	108.1	104.7	132.6	117.6
TTTY9B	113.9	108.8	109.8	115.9	111.6	102.7	115.1	119.3
TTYH1	110.5	116.3	116.4	123.7	111.4	91.3	126	116.2
TTYH1	121.4	114.5	115.5	119.1	111.7	102.9	114.6	114.3
TTYH2	136.6	127.9	122.8	130.6	125.9	105.3	131.6	127.7
TTYH2	109.7	122.8	119.8	119.3	109.7	94.6	118.1	115.3
TTYH2	111.8	117.2	108.7	111.5	99.8	100.4	117.3	109.1
TTYH3	188.3	166	235	225.2	157.8	192.2	229.5	159.2
TUB	116	115.1	114.1	115.5	110.7	89.8	115.2	111.5
TUB	119.2	118.7	113.1	118.5	106.9	104.8	116.2	111.4
TUB	118.6	114.3	117.4	118.9	106.9	90.4	126.3	120.8
TUBA1	428.3	459.6	333.1	492.3	286.3	285	372.3	360
TUBA1A	1053.9	908.2	1952	1261.3	734.9	1096.4	1442.3	1312.9
TUBA2	114.5	120.7	110.8	112.9	107.3	102.6	112	110.4
TUBA3D	122.4	110.4	121.4	118.9	108.9	99.7	119.6	112.9
TUBA4	110.5	105.8	113.8	109.8	103.1	91.8	121.7	109.8
TUBA6	884.2	893	2040.7	973	735.3	1178.4	1000.2	1292.8
TUBA8	113.3	110	113.2	115.7	106.3	99.9	114.2	124.4
TUBAL3	113.9	107	112.3	113	99.3	98.7	118.4	119.2
TUBB	126.8	121.6	134.5	123	116.4	111.3	128.2	132.9
TUBB	1181.9	1277.5	1383.7	1129.8	819.8	795.8	854.9	670.3
TUBB2A	114	116.8	124.8	134.5	115.7	102.5	137.6	123.2
TUBB2B	120.4	115.4	127.9	121.3	118.7	106.7	122.6	118.7
TUBB2C	252.8	286.3	271.8	261.3	206.5	210.6	332.2	259.4
TUBB3	107.9	113.8	120.5	122.2	107.7	97.2	121.5	124
TUBB4	139.5	146.1	136.8	140.7	119.8	111.3	126.2	123.7
TUBB4Q	125.3	127.9	132.6	132.2	115.2	108.4	132.4	139.7
TUBB6	122.1	125.9	125.6	137.6	119.1	114.1	135.4	120.7
TUBB6	152.8	138	172.8	175.5	135.9	130.7	166.7	134.5
TUBB8	114.5	106.9	114.5	106.5	113.6	95.2	114.1	115.2
TUBD1	154.5	154.1	179.2	155.2	128.6	122.6	139	132
TUBE1	132.4	126.8	136.9	137.1	123.2	117.7	121.5	131.1
TUBG1	120	117.8	125.3	128.6	118.8	109.7	123.7	118.2
TUBG1	224.4	244.4	219.7	230.7	192	170.8	181.4	190
TUBG1	183.5	169.1	190.6	172.9	145.9	161.6	165.2	150.3
TUBG2	183.9	229.6	184.4	184.4	208	140.2	163.4	193.8
TUBGCP2	329.1	324.1	344.2	341.4	221.3	281.4	276.7	224.2
TUBGCP3	118.8	119	115.2	123.5	107	96.6	133.1	119.3
TUBGCP5	111.3	100	124.4	126.6	106.8	113.7	116	105.3
TUBGCP6	132.1	137.9	123	134	121.2	112.6	123.9	125.1
TUBGCP6	243.7	224.2	228.5	220	179.3	149.9	192.3	180.6
TUBGCP6	118.5	124.3	120.2	119.8	114.8	96.1	116.9	112.3

TUFM	352.5	414.5	421.1	410.9	277.9	336.2	453.5	401.6
TUFT1	124.9	128.1	145.9	136.4	135.1	120.7	149.2	193.4
TULP1	103.4	114.2	112.5	110.5	104.8	95.9	109.2	109.4
TULP2	106.6	114.2	112.6	114	105.9	92.3	122.9	118.5
TULP3	136.4	132.6	119.7	135.9	121.2	112.3	137.3	145.1
TULP4	147.2	154.6	169	158.5	172.2	133.2	161.9	171.2
TULP4	229	236.3	229.8	221.3	241.4	194.1	238	260
TULP4	112.4	114.4	114	116.6	112.5	100.6	122.1	126.3
TUSC1	111.6	114.4	113.6	126	109.7	104.9	117.9	119.2
TUSC2	173.4	184.4	155.2	172.1	151	148.5	165.8	160.1
TUSC3	122.8	112.1	118.6	115.1	106.2	93	113.4	111.4
TUSC3	110	116.1	110.1	116.3	102.3	90	115.3	119.7
TUSC4	918.8	984	879.2	911.7	695	482.6	618.3	613.3
TUSC5	114	122.3	121.1	120.4	110.8	108	126.5	121
TWIST1	138.3	132	127.4	132.6	124.3	106.7	113.1	128.6
TWISTNB	116.5	127.4	130.9	128.1	123.9	110.6	121.1	121.8
TWSG1	263.3	279.8	234.1	331	246.5	224.2	362.3	438.7
TXK	181.3	207.7	229.2	198.1	171.2	170.6	218.9	224.4
TXLNA	608.2	629.2	478.2	659.6	450.4	321.5	704.8	488.1
TXLNB	112.5	109	124.6	115.7	107.9	95.8	121.9	115.7
TXN	1033.2	839.9	758.5	1790.5	1465.8	1989.4	2247.2	2260
TXN	2782.3	2559	2199.1	6171.1	4727.9	5223.9	7803.7	6723.9
TXN2	138.7	133.8	138.3	164.2	117.7	118.4	131.6	122.6
TXNDC	1253.6	1258.4	972.7	1269.1	822.9	679.3	1187.4	838.9
TXNDC10	201.4	221.1	263.5	243.2	239.6	183	213	236
TXNDC11	148.7	189.3	146.9	178.9	145.5	133.3	173.3	183.4
TXNDC12	1257.1	1371.6	1820.9	1652.7	1129.8	1715.7	1681	1422.4
TXNDC13	331.3	268.9	312.2	331.8	250.2	182.1	273.3	247.1
TXNDC14	380.8	540.7	633.2	511.4	355.4	437.6	525.2	410.6
TXNDC3	129.4	133.7	124.2	127.5	116.3	116.1	126.6	116.4
TXNDC4	111.7	120.5	121.7	125.1	106.7	99	114.7	123.5
TXNDC5	111.3	103.7	115	119.4	105.5	95.5	110.4	118.2
TXNDC5	120	124	151.1	135.6	119.7	136.1	133.6	126.4
TXNDC5	113.1	118.4	122.6	126.6	114.9	95.3	111	111.2
TXNDC6	118.5	121.5	122.4	119.8	122.3	100.1	125.2	113
TXNDC8	116.3	108.6	119.4	114.4	96	98.9	122.6	108
TXNDC9	199.6	224.4	193.2	233.1	204.1	201.5	216.4	214.6
TXNIP	364.1	476	491.9	409.8	335.8	344.6	369.1	470.2
TXNL1	133.3	141.3	120.2	133.2	132.5	127.3	147	165.9
TXNL2	310.7	320.8	349	374.7	297.3	283.9	352.7	407.3
TXNL4A	134.6	145.4	134.1	133.9	147.2	115.8	130.7	130.6
TXNL4B	115.4	124	142.5	134.5	119.9	105.1	127.5	127.8
TXNL5	750.8	762.9	1342.7	782.4	530.6	644.7	433.6	486.5
TXNL6	118.8	131.1	133.1	131.7	113	98.6	119.8	128.9
TXNRD1	830.3	957	377.6	2000.3	1286.2	1474.1	2496.8	2374.6
TXNRD1	252	332.1	215	519.8	444.2	678.2	684.4	619.1
TXNRD2	163.4	169.4	195.3	162.6	135.1	145.8	150	149.7
TYK2	1140.6	1163.6	1188.6	1040.6	1055.8	742.8	1155.2	903.3
TYMS	121.8	133.4	134.3	131	121	104.4	128.3	133
TYR	105.4	120.6	123.3	121.9	110.5	95	116.6	123.8
TYRO3	124.5	113.9	118.8	121.5	115.2	109	118.9	127.9

TYRO3P	117.6	114.6	114.2	120.2	106.2	103	122.8	105.3
TYROBP	3354.9	1756.9	8752.4	3817.6	1212	7015.1	2377.4	978.1
TYRP1	113	108.6	106.2	110.4	106.3	94.2	123	108.2
TYSND1	407.1	462.5	442.1	504.5	323.6	291	321.4	281.6
TYSND1	130.7	144.4	121.8	138.3	120.9	109.6	121	123.3
TYSND1	142.3	144.6	154.6	153.4	141.4	137.6	125.9	149.2
U1SNRNPB	269.2	268.6	291.2	332.5	293.5	263.7	255.2	233.5
U1SNRNPB	140.4	138.1	143.6	139.4	118.7	112.4	129.5	127.8
U2AF1	228.5	288.5	196.6	288.5	270.3	214.7	256.7	292.9
U2AF1	146	169.5	154.5	158.4	153.3	156.4	141.6	165.1
U2AF1L2	377.3	284.9	421.4	385.7	262.6	274.6	328.9	288.3
U2AF1L3	245.7	268.6	206	214.2	154.5	180.4	187.6	191.2
U2AF1L3	167.4	165.6	191.7	162.4	138.2	125.6	144.3	134.5
U2AF1L4	664.3	572.4	1019.7	606.7	307.1	685.4	360.2	289.6
U2AF2	984.8	1107.7	707.7	1128.3	1001.8	631.9	1310.6	1198.2
U2AF2	134.7	123.8	128.1	135.4	115.9	110.9	121.2	127.8
UACA	110.5	107.1	119.1	111.4	108	97.2	113.2	113.8
UACA	120.8	118.2	128.2	125.6	112.6	102.9	121.7	116.5
UACA	123.1	109.4	124.2	114	113.6	111.8	115.8	119.9
UAP1	144.2	131.9	145.2	147.1	130.6	122.1	150.6	162.4
UAP1L1	157.5	148.5	154.9	185.4	132.1	133.1	181.7	185
UBA2	111.3	114.6	118.7	124.2	104.3	96.7	114.4	112.1
UBA52	19254.1	23825.6	23873.2	22438.9	19535.8	18816	22758	24742.5
UBA52	203.2	258.2	212.5	250.3	188.4	167.8	277.7	295.4
UBADC1	567.9	635.6	767.5	577.5	444.8	481.9	365.9	424.6
UBAP1	373.9	470.9	447.6	583.9	377.1	415.9	844.4	685.7
UBAP2	127.1	128.5	126	125.3	111.5	110.5	149.5	159.3
UBAP2L	235.5	216	207.9	245.9	212	205.5	309.9	219.8
UBASH3A	304.4	333.7	258.7	351.2	257.4	209.2	234.7	213
UBASH3A	126.6	110	122.8	119.1	116.6	98.3	112.9	109.6
UBB	8048.4	8492.3	7822.9	9316.7	7061.2	6436.2	13017.1	19339.6
UBC	12690.7	12491.3	13528.6	13364.1	12618.2	11762.5	18527.1	21957.8
UBC	10407.9	11352.8	12408.5	13431.9	12302.2	13190.6	18429.7	22553.9
UBC	119.4	139.8	113.1	121.1	113.2	105	143.8	199.3
UBD	124.5	121	116.7	126	107.3	97	109.7	108.6
UBE1	403.8	469.8	317.6	439.6	283	290.1	523.5	507.2
UBE1	118.8	104	120.8	113.5	112.1	104.3	119.8	114.2
UBE1C	182	186	236.2	213.7	190.9	200.1	171	198.7
UBE1C	130.8	134.2	152.8	151.7	117	114.3	144.9	133.2
UBE1DC1	147.1	144.1	160.1	168.5	154.6	132.4	149	146.1
UBE1DC1	113.2	109.6	123.2	117.2	114.3	102.6	114.3	105.6
UBE1DC1	130.2	125.7	130.1	123.9	123.8	116.9	117.7	124.7
UBE1L	506.6	585.2	641.4	522.3	339.1	318.1	290.4	314.3
UBE1L2	188.1	182.7	195.3	227.1	162.8	166.3	246.8	262.1
UBE2A	114	116.7	131.8	118.7	109.8	112.5	122.5	139.5
UBE2A	506.3	489.1	653	602.9	611.6	720.5	868.9	1046.5
UBE2B	139.2	134.5	139.6	150.9	140.7	105.1	147.1	189.2
UBE2C	114.3	107.5	120.2	122.6	108.3	102.6	123	118.7
UBE2C	111.9	112.8	101.6	113.2	103	102.4	118.3	109.1
UBE2C	116.8	123.5	131.3	123.9	107.2	102	127.5	120.6
UBE2C	108.3	114.2	111.9	105.4	101.5	95.3	106.6	113.4

UBE2D1	119.7	118.7	130.2	117.1	113.4	101.1	118.4	119.3
UBE2D2	188	207.5	182.7	213.4	157.3	147.1	185.8	247.3
UBE2D2	143.8	172.2	160.6	174.6	149.9	132.4	146.6	145.1
UBE2D2	159.2	139.2	132.9	145.8	132.3	123.9	144.9	159
UBE2D3	107.5	109.7	103.3	109.7	112.3	93	121.7	117.6
UBE2D3	1348.8	1598.6	1377.6	1510.7	1327.5	1231.1	2098	2727.6
UBE2D3	2211.8	2254.6	2262.8	2418.4	2375.6	2247.9	2674.3	3467.9
UBE2D3	105.2	124.5	117	118.8	104.3	93.8	121.5	111.4
UBE2D4	203.4	188.2	179	226.7	178.6	160.4	183.8	176.4
UBE2E1	956.3	917.8	1182.4	1135.4	703.3	1221.3	709.2	781.2
UBE2E1	136	134.6	141	148	121.6	118.4	130.9	129.3
UBE2E2	253.6	201.8	304.1	247.7	157.1	207.3	188.7	142.9
UBE2E3	155.7	162	167	157.9	132.7	129	165.6	134.5
UBE2E3	513.8	577	456.5	636.8	551	615.4	508.4	580
UBE2F	766.3	828.7	931.2	848.9	617.6	680	578.2	582.9
UBE2G1	213.5	269.7	242.5	287.8	278.8	208.9	304.3	346.5
UBE2G1	238.7	258.2	277.7	281.5	247.7	225.8	296.9	291.6
UBE2G2	431.8	481.7	390.3	379.6	359.9	341.2	423.3	349.8
UBE2G2	117.5	115.9	109.9	122.3	105.6	95.2	119.9	117.1
UBE2H	125.3	155.3	129	135.6	133.1	116.3	146.2	154.6
UBE2H	171.1	171.7	178.9	216.5	159.6	129.5	182.1	226.3
UBE2I	1063.4	1123	1041.6	1081.9	860.4	913.9	882.3	1137.6
UBE2I	106	113.8	120.6	112.1	103.6	96.2	122.8	110.1
UBE2I	123.4	122	121.6	125.4	120.7	99.2	118	113.9
UBE2I	122	106.2	133.1	120.6	110.2	106.7	125.2	127
UBE2J1	393.6	388.2	353.7	480.5	362.8	337.9	490	460.3
UBE2J2	132.7	122.3	123.9	133.6	120	106	146.2	127.9
UBE2J2	139.3	161.6	138.1	157.3	147.1	118	172.7	147.7
UBE2J2	110.3	111.9	115.9	108.3	104.9	103.3	105	112.3
UBE2L3	249.2	310.1	241.5	315.1	258.4	251.2	334.7	419.6
UBE2L3	124.4	119.3	136.8	129.9	118.1	110.7	130.4	127.5
UBE2L6	113.4	132.1	120.2	111.5	109.3	95.6	121.2	114.2
UBE2L6	1619.5	1350.1	1928	1692.5	767.4	1237.3	1264.9	1155.6
UBE2L6	312.4	261.9	393.3	316.2	240.7	307.6	209.4	216.9
UBE2M	690.4	831	674.8	840.9	574.2	540.9	894.8	648.9
UBE2MP1	113.4	115.4	130.3	115	107.1	101.2	119.8	116.3
UBE2N	458.8	599.8	596.7	542.6	402.2	446.7	571.8	694.9
UBE2NL	121.1	106.9	124.8	121.9	107.1	96	131.7	115.8
UBE2O	259.3	237.8	179.9	230.3	178.7	152.8	216.1	242.2
UBE2Q1	266.7	364.6	229.9	301.9	283.2	196.4	336	326.1
UBE2Q2	507.1	540.7	517.4	523.9	554.8	490.7	524.4	667.8
UBE2R2	126.8	118.7	136.2	135.8	117.2	115.1	119.1	122.1
UBE2S	119.3	120.1	110.7	123.8	107.8	104.1	108.2	113.9
UBE2T	125.8	144.2	131.4	149.9	141.6	107.8	124.9	137.9
UBE2U	120.2	121	133.4	122.2	130.8	103.7	123.6	126.7
UBE2V1	121.5	120.9	118.4	123.9	128.3	111.2	136	135.8
UBE2V1	134	124.8	131.1	121.7	122.8	105	121.9	131.7
UBE2V1	115.8	123.4	129	113.1	104.2	106.7	121.9	107.4
UBE2V2	112.6	120.3	108	112.5	104.9	98.3	124.3	112.1
UBE2W	117.4	117.3	125.9	117.5	101.8	95.7	119.6	117.2
UBE2Z	195.9	188.7	166.8	223.1	195.3	133	231	208

UBE2Z	308.9	287.3	246	386.1	373.2	286.2	421.1	370.3
UBE2Z	130.5	129.6	125.4	126.3	114.1	105.1	123.4	123.9
UBE3A	551.1	715.9	562.5	662.2	498.7	471.3	677.1	807
UBE3A	343.8	429.5	410.9	457.8	311.7	318.4	396.6	443
UBE3A	128.8	114.8	135.8	130.2	113.3	108.9	126.4	118.8
UBE3B	120.9	132.4	147	140.6	112.3	119.5	124.7	131
UBE3C	564.9	769.1	420.2	616.3	573.5	315.3	644.8	626.8
UBE4A	233.3	246.4	294.3	344.7	242.9	250.6	254.5	271.1
UBE4B	175.4	164.5	159.3	155.8	162.3	146.1	180.1	201.1
UBIAD1	141.2	210.6	165.4	192.1	174.5	151.5	153.5	190
UBL3	403.2	544.7	575.8	592.9	445.6	435.3	452.7	556.3
UBL4A	311	340.4	323.4	350.2	245.1	213.6	286.1	245.2
UBL4B	115	112.3	110.1	122.5	107.7	98.9	120.7	112.9
UBL5	123.4	142.6	141	132.8	126.4	106.5	137	139.6
UBL5	315.6	323.7	316.5	410	325.3	265.8	286.3	270.3
UBL7	152.2	147.3	156.6	157.1	156.2	119.3	154.4	142.5
UBL7	189.8	208.5	204.5	211.6	156.5	158.3	165.6	167.5
UBLCP1	370.3	395.1	334.6	384.3	346.6	238.3	290.1	318.3
UBN1	126.3	121	128.4	147.7	127.3	109	131.7	133.5
UBN1	867.4	808.7	622.7	1012.4	696.2	570	1038.3	1217.5
UBOX5	115.8	120.5	130.3	117	118.1	108.4	128.5	127.1
UBOX5	119.3	109.4	135.7	133.3	112.4	101.8	124.7	120.2
UBP1	584	708.4	674.1	646.3	551.1	537.7	691.5	627
UBPH	131.7	122.5	126.7	135.7	118.2	117	145.7	123
UBQLN1	236.3	243.2	248.8	294.1	236.4	233.3	360.7	360.3
UBQLN1	624.2	620.7	815.9	818.7	636.1	741.8	818.2	1008.9
UBQLN2	776	639.1	758.5	688.4	506.6	684.2	682.9	774.7
UBQLN3	121.9	125.5	140.1	130.1	129.4	117.6	131.4	130.7
UBQLN4	470.4	489.4	372.8	612.6	363.9	309.7	598.2	475.5
UBR1	142.3	148.3	139.7	148.8	141	116.4	171.7	142.2
UBR2	233.7	186.6	236.5	276.7	264.1	215.9	315.2	288.5
UBTD1	189.5	201.1	164.3	213.1	155.3	140.8	267.7	196.4
UBTF	145.4	159.8	181.3	151.5	152.9	168.8	184.4	158.1
UBTF	137	126.6	124.2	142.6	108.9	108.8	126.5	130.3
UBTF	148.1	173.8	134.4	173.1	166.3	126.8	143.5	148.3
UBTF	114.4	120	115.9	112.6	115.7	100.6	130.1	118
UBXD1	302.3	348.1	302.8	344.7	241.5	372.2	348.2	260.9
UBXD2	721.4	744.7	738.3	968.1	681.6	809.1	918.6	866
UBXD3	137.1	126.9	141.7	139.8	132	111.1	151.1	126.7
UBXD4	300.6	266.9	252.9	327.6	261.8	203.7	324.6	310.1
UBXD5	103.5	107.1	109.9	104.6	112.2	102.3	106	108.7
UBXD5	124.5	117	115.7	116.4	111.2	98.8	119.7	118.4
UBXD5	119.3	123.4	127	120.3	110.9	111.8	119.6	114.4
UBXD5	274.9	252.1	283.3	212.3	177	196.4	179.1	201.2
UBXD5	114.4	124.4	126.5	126.7	110	114.9	118.2	113
UBXD6	117	112.3	126.6	121.6	110.6	96.2	134.6	123.2
UBXD8	668.9	642.8	669.8	826.2	689.8	609.9	912	809.5
UCHL1	121.2	140.2	116.4	116.7	105.3	107.3	109.7	116.9
UCHL3	220.1	262.2	301.4	235.3	211.4	178.3	222.1	195.5
UCHL5	201.9	236.8	253.5	266.3	215	184.5	194.5	197.1
UCHL5IP	337.8	383.3	372	369.2	285.5	257.4	310.6	320.5

UCK1	121.1	120.1	123.3	126.1	117.2	104.1	126.4	124.2
UCK2	124	118.1	152	148.4	122.9	108.5	142	145
UCKL1	640.6	593.2	549.1	673.3	467	423.3	505.8	352.9
UCN	135.1	116.2	125.3	133.8	118.5	110	124.5	128.9
UCN2	119.4	116.8	127.8	119.2	112.1	95.4	127.8	125.3
UCN3	120.7	122.5	116	114	109.3	100.4	118	113
UCP2	816.3	775	842.8	669.1	432.4	487.9	415.6	345.2
UCP3	119.8	124.7	122.2	117.1	107.6	104.5	117.7	121.3
UCP3	114.6	117.3	113.8	123.5	114.5	99.6	139	120.1
UCP3	132	127.6	121.6	134.5	124.1	108.6	125.6	122.2
UCRC	224.1	222.7	228.5	226.9	209.1	171.4	216.9	245.4
UCRC	297	348.1	341.3	295.6	251.2	255.6	243.1	258.2
UCRC	313.8	299.2	321.5	305.7	240.7	204.2	263.9	236
UEV3	131.9	121.6	144.8	129.6	121.8	114	123.8	119.1
UEVLD	114.4	127.2	122.4	130.4	110.7	112.5	118.2	124.9
UFC1	966	1026.8	962.6	1028.6	943.6	665.7	1214.8	1270.8
UFD1L	129.2	129.4	123.3	142.6	129.4	108.5	143	131.4
UFD1L	126.3	121.7	131.1	135.4	116.9	106.6	125	122.6
UFM1	185.3	152.5	207.2	157.1	177.2	137.5	190.7	174.4
UGCG	146.9	151.2	181	212.7	180	132.6	248.2	235.6
UGCGL1	101.8	113.6	113.2	111.6	105.3	98.7	114.9	105.5
UGCGL1	140.8	147.6	129.9	160.1	131.2	109.6	144.5	149.3
UGCGL1	208.4	242.4	196.5	275.2	228.1	137.8	229	243.8
UGCGL2	125.5	123.8	133.2	132.7	116.2	106.5	117.2	120.4
UGDH	233.4	329.2	209.4	341.5	263.1	141.5	365.6	355.1
UGP2	355.7	346.5	443.7	424.6	310.9	352.2	326.2	313.8
UGP2	127.4	122	121.7	122.4	114.7	96.2	119.8	115.4
UGP2	589.5	717.8	1050.3	826.2	681	706	550.3	581.2
UGT1A1	123.4	117.5	120.6	113.6	106.1	101	118.8	128
UGT1A10	102.2	109.9	105.4	111.9	111.8	96.7	113.8	107.3
UGT1A3	109	100.7	109.9	110.4	97.1	94	111.6	110.1
UGT1A4	113.9	120.4	121.7	123.1	115	109.5	125.2	119.8
UGT1A5	113.1	120	118.6	111.1	112.8	98.2	114.3	121.3
UGT1A6	110.1	108.8	124.4	120.9	109.1	101.4	112.7	115.1
UGT1A6	113.2	112.8	116	118.4	103.1	99.4	112.8	111.7
UGT1A6	117.1	104.1	113.4	123.2	106.9	104.3	118.3	110.1
UGT1A7	110.9	122.6	115.3	115.1	103.2	98.4	113.5	114.2
UGT1A8	111.4	114.3	110	112.2	98.9	89.7	115.4	107.7
UGT1A9	112.2	112.8	113	127.5	111.5	97.8	125.3	112.2
UGT2A1	120.5	105.1	112.9	124.4	105.1	96	104.9	114.7
UGT2A3	103.9	114.1	112.7	119.1	101.1	96.6	112	113.5
UGT2B10	112	107	107	114.1	100.1	93.7	112	114.1
UGT2B11	127.6	121.2	119.6	118.6	98.9	100.6	126.8	117.2
UGT2B15	121.2	122.2	110.6	115.6	118	103.4	119	115
UGT2B17	119.7	114.3	114.3	122.7	105.8	98.4	115.3	112.5
UGT2B17	112.4	113.5	120	121.9	110.5	102.7	113.4	115.2
UGT2B28	119.1	118.9	110.7	120.9	108.7	105.5	119.8	125.2
UGT2B4	117.4	119.7	113.6	128.2	107.5	102.7	122.3	111.8
UGT2B7	114.2	110.7	116.9	119.7	111.9	98.7	126.2	113.2
UGT2B7	114.2	113.8	110.8	127.1	109.8	97	113.3	113.4
UGT3A1	123.3	106.6	110.9	117	102.7	107.6	111.6	110.9

UGT3A2	113.5	113	116.8	114.2	101.3	100.8	109.3	107.7
UGT8	124.1	107.1	123	117	103.6	97.9	110.8	106.4
UHMK1	254.7	281	179.8	303.9	216.7	121.1	244	252.2
UHRF1	115.4	110	112.2	109.5	104.6	91.1	114.5	116.6
UHRF2	147.7	159.6	203.3	151.4	156.9	135.2	166.2	150.5
UIP1	124.9	128.6	121.8	119	107.3	103.9	120.3	110.3
ULBP1	123.9	129.2	121.4	126.6	116.8	99.2	124.4	141.9
ULBP3	121.6	117.8	137.4	122.2	112.5	97.7	126.8	125.7
ULK1	853.1	993.7	778.4	1205.1	994.2	668.7	1382.9	1751.8
ULK1	815.2	842.1	739.3	901.6	721.6	574.8	1275.1	1518
ULK1	113.1	109.4	109.6	121.3	104	90.3	110.5	110.5
ULK2	119.6	120.9	136.1	126.1	111.8	106.9	112.1	119.8
ULK3	128.7	142.2	143.4	151.5	124.1	111.3	153.1	144.3
UMOD	110.4	103.4	112.4	116.6	106.8	98.7	108.7	107.3
UMOD	120.2	122.4	125.6	126	125.9	107.7	120.2	115.5
UMODL1	112.9	115.9	125.2	119.3	111.8	99.8	118.5	118.4
UMODL1	108.1	130.8	119.8	111.8	115.4	100.3	116.8	117.2
UMPS	110.6	126.9	128.3	122.5	117.7	98.8	128.9	115.9
UNC119	124.6	130.6	137.4	138.3	134.7	106.1	124.5	124.4
UNC119	175.9	161.1	168.4	167.3	130	117.8	136.1	159.8
UNC13A	118.8	114.9	112.2	117.6	109.3	103.9	114.8	116.4
UNC13A	131.1	121.8	126.1	120.8	112.5	110.3	129.7	127.3
UNC13A	127.4	121	137.1	124	117.1	110.5	124.9	120.3
UNC13B	117.3	115.4	118.5	119.7	108.5	100.8	130.7	114
UNC13C	109.3	117.8	111.3	111.5	109.3	99.6	108.1	117.2
UNC13D	191.7	198.7	196.5	220.1	156.3	135.9	169.9	167.9
UNC45A	417.9	462.6	435	503.2	339	367.5	487.8	494.3
UNC45A	128.4	138.9	152	136.9	126.6	112.2	129.6	135.9
UNC45A	383.8	480.1	352.7	456.7	332.2	323	520.1	386.4
UNC45A	119	110.9	121.1	118.3	108.5	104.5	129.4	117.9
UNC45B	111.1	117.1	114	113.5	107.7	104.4	114.5	123.6
UNC50	797.2	940.5	1361.2	978.3	597.5	942.8	923.7	753.2
UNC5A	114.2	109.4	113.2	117.7	112.5	98.9	112.5	113.9
UNC5B	109	114.2	120	112	110.3	106.2	120.8	124.6
UNC5C	123	125.3	124.2	117.9	121	102.5	127.7	123.3
UNC5CL	116.2	112.2	121.6	127.3	106	94.7	113.7	108.8
UNC5D	111.3	112	110.9	106.2	104.3	94.5	113.3	107.8
UNC84A	283.8	312.6	361.5	371.4	307.3	324	301.6	337.7
UNC84B	6711	6610.2	5142.7	6954.7	5967.2	3983.9	5558.4	5420.2
UNC93A	111.8	119.1	124.4	123.1	112.3	103.7	119.3	122.8
UNC93B1	261.2	176.1	325.2	226.5	157.8	205.3	177.3	151.4
UNCX4.1	116.7	120.4	109.8	127.5	112.1	101	129.8	119.3
UNG	119.2	109.3	126.1	119.4	116.6	97	117.4	123
UNG	238.1	256.6	199.6	245.9	213.2	201.3	201.4	230.7
UNG	119.8	116.9	133.2	124	109	109.4	112.9	129
UNG2	116.4	128.6	130	120.7	131.9	108	126.6	125.9
UNK	107.4	117.1	116.6	112.3	113.3	92.6	117.3	106.9
UNKL	129.4	124.8	126.3	130.4	110.9	111.2	126.9	118.9
UNQ1940	112	122.6	115.7	124.3	108	100.7	126.6	110.2
UNQ2446	114.8	113.5	121.3	115.9	116.9	94.8	122.5	118.2
UNQ2541	101.9	114.4	121.8	117	116.9	99.2	117.8	113.3

UNQ3033	115.5	127.6	122.1	115.5	105.5	94.9	122.3	120.5
UNQ3045	118.4	113.6	104.1	112.8	111	96.2	109.7	114.4
UNQ467	113.7	116	114.4	118.5	107.5	98.5	127.4	115
UNQ470	117.1	123.5	112	118	102.7	94.6	115.6	113.6
UNQ473	115.3	116.6	121.8	114.6	107.6	96.3	124.7	117.7
UNQ501	445.9	445	776.5	403.5	342.3	432.8	295.3	285.4
UNQ5783	129.6	135.8	133.1	130.3	114.5	98.8	118.7	118.5
UNQ5810	112.6	108.7	116	123.5	111	98.7	119	118
UNQ6411	119.8	115.2	113.5	113.1	111	101.4	120.7	118.1
UNQ689	112.1	111.4	111.6	114.1	108.5	94.7	113.2	107.5
UNQ739	115.6	112.6	118.4	122.8	114.2	106.9	119.5	116.6
UNQ830	111.7	116.2	115.2	122.4	107.5	103.9	122.3	114.6
UNQ846	131.3	135.7	149.6	149	140.2	119	148	147.6
UNQ9217	122.8	117.6	122.4	130.3	115	104.6	120.9	117.7
UNQ9356	119	117.8	119.3	119.7	108.5	95.9	116.8	122
UNQ9391	116	111.2	131.1	145.3	112.9	105.7	117.5	121.7
UNQ9433	105.3	113.3	125.8	117.6	114.7	101.2	117.6	117.4
UNQ9438	117.1	120.1	110.7	117.2	99	103.9	112.8	115.6
UPB1	117.8	118.5	117.1	123.5	124.2	102.7	123	131.7
UPF2	1030.3	1086.9	872.6	1298	963.1	818.7	1519.2	1633.7
UPF2	123.9	122.5	119.6	120.8	125.5	102.7	143.5	138.8
UPF2	443.2	453.1	422.6	440.6	508.3	290	444.1	465.7
UPF3A	471	470.9	486	633.4	467.5	328.1	518.6	416.8
UPF3A	392.1	378.1	315.7	450.4	314.8	254.9	332.9	291
UPF3B	265.4	337	263.8	331.5	283	234.8	394.8	329.3
UPF3B	287.6	344.3	321.8	406.9	254.6	279.3	388.8	359.2
UPK1A	121.1	132.4	124.3	119.5	114.6	101.2	135.5	123
UPK1B	105.3	115.2	119.3	125.6	117.2	100.8	121.9	117
UPK2	108	114.9	114	120.9	118.3	99	124.7	126.3
UPK3A	114.5	127.8	126.1	117.1	125.6	111.3	110.9	124
UPK3B	113.7	119.6	111.5	121	103.1	95.6	113	117.8
UPK3B	108.5	115	119	115.2	110.6	103	119	117.2
UPK3B	128.8	120.2	130.5	126.6	115	112	142.5	132.5
UPP1	126.6	114.2	133.1	129.3	114.2	103.3	125.6	125.4
UPP1	116.7	121.6	128.3	127.8	122	109.1	122.4	118.3
UPP1	458.3	506.7	617.8	483.6	432.6	451.8	616.4	633.6
UPP2	110.6	116.4	120.2	131.9	113.1	107.8	119.5	109.9
UPRT	255.8	216.1	219.6	227.4	266.9	224.4	248.5	281.5
UQCR	162	165.6	165	150.3	130.8	141.8	193.7	154.9
UQCRB	125.9	137.4	135.3	139.8	113	111.3	129.6	141.6
UQCRC1	293.1	359.1	428.7	374.9	240.4	352.2	334.1	236.7
UQCRC2	228.7	231.4	205.4	221.4	178.2	211.4	222.2	216.5
UQCRFS1	1445.2	1744.6	1722.8	1818.3	1364.8	1290.5	1585.6	1605.2
UQCRH	2723.2	3365	3403.4	3574.9	2850.9	2797.1	3667.5	3397.6
URB	128.4	124.7	118.3	118.5	119.4	106.1	119.8	121.3
URB	112.4	119.7	103.8	118.4	107.3	103.9	121.2	116.7
URG4	202.2	234.6	181.2	217.5	208.6	188.1	269.1	251.4
URG4	221.1	252.4	192.7	239.7	192.3	165.6	242.6	245.5
UROC1	106.7	115.2	117	121.8	107.5	96.5	116.1	117.7
UROD	335.7	373.1	472.6	363.6	282.7	316.7	301.1	241.7
UROS	442.9	554	523	401.5	332.6	315.8	285	332.3

URP2	1212.7	1023.9	1051.8	1527.1	777.8	1125.3	1595.5	987.8
URP2	221.6	203.3	194.8	257.1	191.7	221.6	287.4	197.3
USF1	143.2	132	145.9	143.6	131.9	109.6	131.4	131
USF1	176.6	215.3	170.5	204	178.4	141.4	179.6	164.2
USF2	130.3	120.6	127	127.7	116.7	112.1	132.9	127.9
USF2	446.1	458.8	337.7	448.9	305.5	299.9	424.7	350.7
USF2	241.7	314.4	229.1	327.2	177.5	193.5	271	212.5
USH1C	118.3	120	123.1	114.8	115.4	98.4	117	117.1
USH1C	108.5	112.8	118.8	128.7	107.7	107.4	130.3	123
USH1G	123.2	123	131.2	113	123.8	106.2	132.4	133.6
USH2A	113.4	113.4	122.9	120.9	109	99.1	122.6	120.7
USH2A	107.4	110.4	108.3	111.7	109.2	95.7	113.9	116.4
USH2A	107.5	118.5	122.8	109.8	111.6	95	110.3	112.6
USH3A	128.6	126.2	129.1	118.1	117.9	114.3	123.6	125.1
USH3A	111.4	108.4	112.1	107.5	111.4	96.1	116.8	113.3
USHBP1	116.1	116	115.6	120	112.9	97.4	128.1	119.3
USMG5	174.8	119.6	209.9	171.8	116.6	154.4	177.4	122
USP1	106.2	114.5	111.9	117.2	106.5	93.2	114.8	120
USP1	368.8	327.2	287.8	260.3	244	250.8	334.9	376.5
USP1	174.8	215	205.2	197.5	188	136.6	154.7	158.4
USP10	168.7	230.3	182.9	218.2	199.1	154.1	165.9	219.8
USP11	212	232.5	164.7	227.4	166.1	159.4	208.7	221.9
USP12	111.8	113	123.3	120.1	108.9	109.2	119.4	109.1
USP13	121.7	128.4	144.8	143	117.8	109.5	155.5	126.4
USP14	235	337.3	301.3	350.1	270.7	235.3	391.6	508.1
USP14	162	180.1	167.7	185.9	189.8	159.9	179.8	191.8
USP15	180.1	187.5	174.6	196.5	171.1	164.1	181.7	228
USP16	449.1	455.8	530.2	526.9	476.8	467.3	523.4	525.6
USP16	121.9	112.4	114.2	117.2	105.3	106	115.1	127.4
USP19	125.3	130.6	126.1	130.7	123.4	103.2	161.5	119.6
USP2	107.7	109.6	113.7	115.4	103	94.2	113.4	107.9
USP2	119.4	114.9	117.6	121.8	112	100.8	119.4	115.8
USP2	114.1	114.6	122.6	119.7	113.5	98.1	119.2	112
USP20	98.4	101.3	104.2	110.4	98.6	97.6	110.3	108.2
USP20	126	128.8	117.3	140	116.6	98.1	139.4	120.1
USP21	173.5	189.2	171.9	212.7	162.8	155.1	182.6	171.7
USP21	117.9	128.5	121.6	104.9	110	87.2	122.7	120.1
USP21	205.6	214.6	175	216.1	196.2	177.1	207.1	192.9
USP25	136	134.9	145.8	126.3	121.9	110	134.1	128.7
USP26	121.1	121.5	124.3	132.2	117.2	108	127.7	118.7
USP28	118.9	114.5	120.9	123.8	110	100.6	116.8	120.5
USP29	113.2	114.1	117.4	110.3	111.2	96.1	117.8	106.2
USP3	1146.7	1040.2	1172	1291.6	971.3	984	1079	783.8
USP30	119.9	121	125.2	121.6	118.2	103.9	122.6	128.8
USP31	108.4	113.8	116.8	121.5	109.3	98.1	122.5	125.2
USP32	121	119.2	112.8	111.6	115	100.6	121.4	105.7
USP32	127.9	131.1	118.7	132.6	143.2	118.5	133	140.1
USP33	143	143.8	133.5	137.3	132.1	116.3	135.1	134.9
USP33	188	222.3	214.4	260.3	199.6	151.9	235.8	205.8
USP34	349.2	462.8	301.6	406.2	399.9	259.1	407	461.9
USP37	131	151.1	137.5	149.7	145.6	121.7	160	145.7

USP38	491.3	508.7	360.5	495.5	385.5	362.2	494.9	591.7
USP39	427.8	410.5	408.1	456.4	327.9	335.7	454.3	465
USP4	130	121.8	131.5	122.1	128.2	104.7	134	134.1
USP4	299.8	281.1	302.8	318.3	291.5	266.7	327.1	237.4
USP40	117.6	124.2	117.6	126.1	113.4	100.6	120.9	123.9
USP42	140.1	134.9	147.8	155.5	133.5	113	139.7	145.6
USP42	545.1	514.7	443.7	652.9	587.9	506.4	639.8	706.6
USP43	103.8	112.9	110.8	117.9	110.8	97.3	116	122.1
USP44	124.3	123.3	111.5	118.8	118.2	99.9	131.8	137
USP44	123.4	119.5	121.2	122.8	120.7	106.9	131.1	117.1
USP45	118	131.1	115.6	121.9	129	102.5	132.2	135.8
USP46	117	120.8	133.7	122.2	110.1	95.8	108	110.9
USP47	132.7	166	145.7	163.7	144.5	143	163.6	150.7
USP48	178.6	190.5	159.6	182.9	185.3	131.1	165.9	177.8
USP48	145.8	133.1	140.5	147.3	115.6	124.5	137	136
USP48	192.1	157.5	158	192.3	144.8	136	145.9	147.5
USP49	1473.6	844.6	1046.7	1374.9	1061	511.8	1160.9	1165.5
USP5	555.4	732	664.5	711.7	422.1	571	672	630.2
USP50	113.4	115	115	108.4	107.5	91.6	113.5	105
USP51	112.3	118.2	109.7	117.3	106.1	97.5	126.5	121.8
USP52	211.7	242.8	228.3	235.9	207.4	192.2	205.2	215
USP53	120.5	125.2	129.2	139.5	120	109	125	120.6
USP54	118	117.8	116.1	112.9	107.7	101.9	115.9	114.2
USP6	143.1	129.3	118	137.2	120.6	107	127.4	144.8
USP6NL	174.3	155.3	167.5	195.8	141.3	140.1	159.7	146.4
USP6NL	113.3	113.3	116	116.5	101.1	91.1	107.9	111.8
USP7	307.1	341.8	268.3	325.4	293.6	241.3	354.9	405
USP8	199.1	238.1	261	244.5	178.8	213.9	194.1	273
USP9X	122.5	131.1	119.9	120.8	115.6	107.5	115.2	130.3
USP9X	287.8	307.1	250.4	300.8	357.3	230.4	384.9	425.1
USP9Y	120.9	128.8	136.4	134	129.3	102.5	133	137.3
USPL1	203.5	187.7	217.4	171.3	184	171.1	206.1	204.8
UST	146.7	136	136.2	214.9	124.5	210.2	161.2	126.3
UTF1	115.4	109	113.1	112.8	112.6	97.8	124.8	117.3
UTP11L	227.8	234.6	262.4	269.7	247.2	254.2	293.7	273.2
UTP14A	272.6	294.1	286.8	267.4	284.7	247.8	323	265.6
UTP14C	257.1	239.5	298.1	290.1	280.8	227.8	234.3	339.6
UTP15	139.9	145.3	135.2	139.9	133.3	106.5	138.3	139.6
UTP20	115.9	113.7	124	113.7	112.8	100.4	124.1	117.1
UTRN	129.7	124.2	130.9	119.9	126.5	105.2	132.7	129.6
UTS2	112	114.5	119.7	116.2	117	97.4	121.3	118.7
UTS2	112.7	118.3	122.6	127.4	112.2	106.2	128.5	111.9
UTS2	124.2	114.8	120.6	112.6	112.5	91.5	114.9	115.7
UTS2D	122	108.9	115.5	120.1	118.3	107.3	132.4	120
UTS2R	119.6	111.4	116.5	111.1	103	104.9	123.9	119.2
UTX	161.5	163.8	154.6	166	168	160.7	182.3	198.2
UTY	124.4	125.7	136	133.6	117.3	110.4	126.3	124.2
UTY	144.7	152.5	148.3	157.1	130.6	110.8	161.9	177.4
UTY	117.5	113.3	117.1	114.2	102.2	103.5	117.1	116.7
UVRAG	148.7	159.1	178.1	162.7	151.7	133.7	158.8	148.1
UXS1	132.5	146.9	169	158.4	157.4	116.3	144.9	137.7

UXT	1565.3	2178.4	2081.8	1895.1	1670.4	1884.5	1751.6	1936.9
UXT	111.9	126.5	120.1	123.6	116.9	102.7	144.2	132.2
VAC14	125.5	119.1	131.9	131.7	123.8	99.7	129.5	130.3
VAC14	169.9	171.3	146.6	176.9	123.5	130.5	184	150.4
VAMP1	116.4	124	115.4	125.5	115.1	107.8	115.4	116
VAMP1	111.2	107.9	111.2	127.4	103.1	101.6	116.9	115.5
VAMP1	658.4	614.2	893.8	670.9	538.2	452.9	389.7	399.3
VAMP2	233.9	314.2	228.8	287.2	208.8	222.6	300.2	260.3
VAMP3	291.4	286.7	445.3	324.3	241.2	335.7	369.7	281.4
VAMP4	137.9	133.5	146.9	127.4	123.9	126.8	132.6	134.5
VAMP4	147.4	148.8	155.6	142.1	131.8	123.3	130.1	131.1
VAMP5	547.7	656.8	1126.9	520.4	411.1	673.6	397.4	434.9
VAMP8	1379.8	1980.3	2330.2	1252.1	1425.3	1068.5	991.4	1346.9
VANGL1	113	124.7	119.1	117.3	112	103.3	122.8	126.2
VANGL2	112	120.8	111.5	110.7	110.8	100.1	122.4	115.4
VAPA	119	115.1	126.9	137.2	122.5	107.4	130	129.1
VAPA	121.5	116	124.4	126.1	114.1	101.8	128.6	123
VAPA	138.1	124.1	146.3	126.7	124.4	113.5	141.6	144.2
VAPB	108.3	121.6	121.4	116.6	99.7	94	118.2	114.5
VARS	120.5	120.6	119.9	120.3	111.6	108.2	126.3	114.2
VARS	233.7	193.9	184.4	191.1	140	120.4	193.3	180.1
VARSL	245.4	330.9	280	352.5	279	199.7	328.6	348.8
VASH1	154.2	167.3	285	133.4	122.6	148	129.5	123.8
VASP	140.8	134.4	155.8	139.8	115.2	110.1	151.4	116.7
VASP	111.6	116.3	116.5	122.7	106.3	104.1	119.9	103.5
VAT1	185.3	171	148.1	162.2	137.9	130.4	186	157.8
VAV1	208	194.5	165.1	204.3	147.6	153.5	187.5	173.5
VAV2	118.7	106.6	119.4	112	103.8	98.7	109.8	125.6
VAV3	124	110.1	120.1	118.2	117.8	98.1	120.8	110.6
VAV3	140.3	142.2	162.2	171	145.5	166.4	185.3	154.9
VAV3	115.9	221.6	125.8	120	230.8	99.6	128.4	282.6
VAX1	115	118.5	115.3	122	106.9	100	124.5	119.8
VAX2	118.4	116.2	119.4	114.1	103.8	103.7	130	111.9
VBP1	791.2	926.9	758.2	776.9	706.2	790.8	1072.6	1060.4
VCAM1	123	120.7	117.6	123.8	114.1	104.5	114.1	121.9
VCL	433.9	449.9	532.9	410.6	299	240.6	354.9	330.4
VCL	123.1	120.9	130	138.3	122.9	109.9	129.8	119.5
VCL	106.6	114.5	107.5	109.3	105.2	89.8	105.3	110.5
VCP	375.5	451.8	363.9	520.7	336	347.7	676.8	766.3
VCPIP1	307.4	309.1	271.1	348.2	284.5	247.9	360.3	418.6
VCX	118.5	111.6	124.4	122.4	118.5	106.5	125.9	112
VCX2	113.8	122.9	111.9	108.9	107.6	100.3	116.2	110.5
VCX2	111.8	112.6	115.7	109.1	113.9	98	114.7	101.7
VCX3A	108.3	120.4	115.7	116.3	106.6	96.3	113	110.5
VCX3A	116.2	119.3	116.9	109.4	119.5	92.8	120.4	124.4
VCX-C	116	109.8	114.8	127.3	109.2	92.9	102.6	110.6
VCY	111.7	120.6	115.8	113.6	110.6	97.8	114.5	114.9
VCY1B	111	104.3	109	108.1	97.8	91.9	113.6	114.5
VDAC1	971.6	951.8	1070.4	1164.2	1011	1171.9	1256.4	1069.9
VDAC2	100	116.2	124.3	113.5	110	102.5	124.6	110.1
VDAC3	1615.5	2297.7	2563.8	2484.1	1673.9	1880.2	1892.8	1742.9

VDP	360.7	356.7	425.6	510.9	389.4	330	574.5	493.4
VDR	106.9	109.5	105.1	120.5	107	98.7	109.8	101.5
VDR	121.7	116.9	140.5	142.5	110.6	109.6	123.1	129.4
VEGF	115.8	114.3	116.1	122.9	101.3	97	126.3	111.7
VEGF	113.7	114.1	121.8	120	104	94.6	121.6	110.9
VEGFA	126.3	132.4	130.4	141.5	120.9	113.4	151.4	141.7
VEGFB	1697.7	1488.7	1490.2	2252.9	1645.1	1112.1	1535	1636
VEGFB	113.5	110	107.8	121.4	111	101.4	118.1	118.1
VEGFB	173.8	179.1	173.5	204.6	165	156.2	200.2	169.5
VEGFC	106.8	109.6	112.8	117.7	104.4	93.5	106.9	114.8
VENTX	135.6	130.9	140.8	137.7	109.1	119.2	122.8	123.5
VENTXP7	115.2	120.5	119.9	118.5	103.2	93	118.6	112.6
VEPH1	115.9	115.5	115.5	117.1	109	99.7	127.8	113.7
VEZT	126.2	127.6	117.7	118.5	110.2	101.2	126.6	108.2
VGCNL1	113.9	111.7	129.6	124	115.6	95.7	122.3	113.3
VGF	111	109.6	126.7	131.7	109.1	100.8	120.4	122.5
VGLL1	119.6	111	121.7	115.9	115	101.6	113.7	120.9
VGLL2	117.4	122.6	121	116.8	112.8	108.5	113.2	116.2
VGLL2	126.9	125.6	129.8	125.9	111.5	114.8	124.5	121.8
VGLL3	112.8	125.4	115.1	124.3	107.4	111.1	115.2	118.8
VGLL4	352	335.3	330.8	338.4	289.6	244.2	330.3	375.4
VHL	181.8	223.7	196.9	228.2	182.7	147.2	200.9	197.9
VHL	660.5	664.2	689.6	654.8	721.2	533.7	651.1	577.6
VHL	136.8	133.1	138.5	150	124.6	120	139.5	140.9
VHLL	111.3	114	117.4	113.9	107.3	99.8	111.1	108.3
VIL1	111.3	106.4	125.5	102.7	110.6	99	114.6	119.8
VIL2	2386.2	2713.7	1742.1	3441.8	2782.4	1943.9	5546.2	4753.1
VILL	141.7	147	150.7	155.8	129.5	126.1	136.7	124.1
VIM	1313.8	671.7	1201.4	815.4	502	961.7	687.8	557.5
VIP	116.7	125.3	128.1	117	105.8	95.9	115.9	115.1
VIP	116.2	120.3	115.9	116.6	114.4	96.7	120.7	109.8
VIPR1	749.5	940	803	596.5	618.3	451.5	511	503.3
VIPR2	135.4	135.5	134.6	135.5	114.1	113.5	119.4	119
VISA	123.2	129.5	128.8	124	109.9	110.7	129.9	118.1
VIT	110.5	116	111.2	124.2	107.2	96.3	115.2	112.3
VKORC1	1138.2	1066.9	1165.7	1191.6	727.7	813	1035.9	833.4
VKORC1	155.3	174.3	169	169.5	131.9	131.3	129.5	131
VKORC1L1	168.7	188.5	191.9	176.4	152	133.1	195.3	195.1
VLDLR	110.5	120.9	122.5	117.2	111	103.4	124.4	119.9
VLDLR	106.4	102.2	107.5	111.6	108.6	96.3	110.9	104.9
VMAC	119.1	119.8	127.8	130.6	121.8	118.4	122	127
VMD2	140.6	126.1	194.5	137.1	117.5	127.5	124.1	114.6
VMD2L1	116	119.5	121.2	132.1	114.8	100.6	104.2	113.9
VMD2L2	113.3	128.2	128.8	123.4	109.6	105.7	123	122.4
VMD2L3	119.8	121.4	114.5	114.5	110.2	93.5	113.4	125.2
VMD2L3	125.1	116.5	114.6	120	107.7	96.1	120.5	120.6
VMO1	148.7	119.6	431	236.5	117.9	743	161.9	124.7
VMP	117.3	115.4	116.7	114.6	106.5	91.7	114.1	108.7
VN1R1	106.9	115.4	119.5	121	104.2	106.6	117.3	113.3
VN1R2	118.3	116.9	120.2	126.3	122.8	98.8	123.9	113.6
VN1R4	111.9	117.8	119.6	116.5	110	99.6	113.9	117.2

VN1R5	119.2	108.6	107.8	118.5	100.1	98.3	129.2	116.8
VNN1	128.3	120.7	119.2	128.6	116.2	117.3	118.4	120.2
VNN2	300.6	389.5	395.4	309.7	289.9	354	260.6	297.6
VNN2	112.7	113.7	123.5	106.7	107.4	104.3	110.5	115.8
VNN2	135.8	129.2	147	153.7	133.5	134.4	122.3	127.5
VNN3	111.7	116.8	113.8	120.4	110.6	100.6	111.8	122
VNN3	110.9	121.9	118.3	117.9	104.1	94.1	124.1	113.3
VNN3	108.5	115.4	119.7	127.8	110.8	103.7	121.7	116.1
VPRBP	150	150.7	140.6	180.2	142.9	111.1	162.1	144.3
VPREB1	108.7	111	110.4	126.1	105.1	94.3	117.1	115.8
VPREB3	145	154.2	231.6	130.2	111.2	130.7	112.3	118.5
VPS11	158.2	160	151.3	160.6	146.1	139.5	162.8	143.9
VPS13A	112.4	122.2	115.3	112.3	112.6	97.4	110.8	107.5
VPS13A	169	178.1	176.1	193.1	195	125	156.7	175.9
VPS13A	115	115.9	119.9	119.7	111.3	96.5	120.9	116.3
VPS13A	160.7	188.1	172.6	179.7	185.5	133.5	174.5	182.9
VPS13B	116.3	117.6	113.2	117.4	104.6	106	122	114.6
VPS13B	133.1	126.7	138.4	134.5	123.3	96.8	132.8	132.6
VPS13B	108.6	118.4	114.1	110.5	103.6	97.5	120.9	119.2
VPS13B	123.7	124.2	116.8	118.6	114.2	98.2	127.6	124
VPS13B	117.5	113.7	119.4	121.5	109.5	104.7	124	112.4
VPS13B	124.2	143.2	139.1	140.5	126.2	108.8	147.7	132.3
VPS13C	121.4	119	133.2	129.3	125.8	102.5	131	120.2
VPS13C	175.7	159.5	131	187.6	151.2	107.1	182.9	175.9
VPS13D	120.7	114.3	119.2	118.4	106.4	100.2	119.5	114.7
VPS13D	112.6	116.3	123.9	125.6	114.8	98.4	119.5	120.7
VPS13D	126	130.7	136	128	113.3	112.4	125.6	126.1
VPS16	138.1	123.8	137.6	139.2	114.3	118.4	128.6	128.8
VPS16	531.9	526.6	534.2	506.9	329	322.7	411	327.2
VPS16	114.3	118.9	111.5	120.8	118.5	110	118.8	122.8
VPS18	199.4	210.5	185	207.3	140.8	153.3	217.5	240
VPS24	177	213.1	213.6	200.8	150.8	218.3	165.4	172.1
VPS24	116.7	112.9	124.1	119.4	105.1	104.4	114.6	115.2
VPS25	157.9	155.5	169.3	162	141.2	146.7	146	160.2
VPS26	152.7	157.4	157.2	181.4	146.5	132.5	186.6	208.5
VPS26A	641.3	653.8	602.6	807.4	557.8	661.4	1039.3	1142.9
VPS26B	214.7	223.9	233.1	195.8	175.5	155.8	174.2	160.2
VPS28	567.9	546.9	639	601.5	435.5	532.1	561.8	560.9
VPS28	295.7	285.1	294.7	272.7	206.7	213.4	266.1	225.1
VPS29	943.2	965	1518.1	1069.5	793.8	1022.4	781.2	932.5
VPS33A	168.7	171.4	172.1	166.6	149.5	154.3	173.2	148.8
VPS33B	213.9	206.9	234.1	233.8	189.5	206.6	214.6	199.3
VPS35	615.8	610.9	731.6	631.5	584.5	492.5	493.4	593
VPS36	309.9	295.5	390.7	302.4	262.8	214.5	227	282
VPS37A	262.3	244	243	264.2	213.2	200.4	284.9	272.8
VPS37B	236.6	239.5	226.3	258.7	215.3	269.2	410.3	520.8
VPS37C	1162.7	812.9	755	1453.1	731.3	757.9	1885.5	826.8
VPS37D	142.5	139.1	146.3	146.5	134.3	119.4	148.3	145.3
VPS39	175.6	185.3	174.8	185.3	156.5	161.6	184.4	177.8
VPS41	135.6	131.1	127	151.2	122.2	103	138.6	119.8
VPS41	344.4	292.2	272.3	328.9	353.3	185.9	281.3	324.3

VPS41	672	605.4	712.2	724.3	501.6	493.2	631.5	497.4
VPS41	124.5	115.9	122.5	131.9	115.1	112.5	118	115.6
VPS45A	228.3	240.3	272.9	239.1	219.6	202.7	184.6	188.1
VPS4A	184	187.4	181.8	196.7	173.9	207.5	219.2	200.7
VPS4B	959.7	844.7	698	947.6	813.4	704	1078.8	1174
VPS52	240.1	272	260.6	264.3	191.4	208.4	203.4	216.8
VPS53	115.5	115.3	116.2	121.4	111.8	99.8	128	118.7
VPS54	117.6	132.4	130.7	134	128.7	116	130.2	126.4
VPS54	154.9	142.9	147.7	146	135.9	129.5	156.4	147.4
VPS72	172.6	203.6	188.4	190.6	174.2	192.2	173.3	169.4
VPS8	250.2	322	285.2	312.3	223.8	189.6	312.7	330.9
VPS8	114.6	114.5	125	117	118.5	108.1	125.6	119.9
VRK1	148	140.4	139.2	148.6	150	114.1	149.5	164.8
VRK2	136.4	137.6	198.1	154.2	127	162.2	150.5	122.3
VRK3	162.7	166.9	158.8	181.8	177.3	166.9	184.6	209.5
VRK3	148.3	156.6	168.2	153.8	144.3	125.5	140.5	153.5
VRK3	403.8	467.3	419.1	406.4	409.2	264.6	438.8	554.7
VSIG1	121	120.2	108.3	129.8	127.8	102.2	127.7	109.6
VSIG2	126	122.7	124.8	137.8	111.4	101.1	121.4	126.2
VSIG4	113.4	121.3	170.3	122.8	121.5	118.3	125.4	114.8
VSIG8	118	121.1	119.7	118.9	111.8	98.8	131.4	114
VSIG9	191	153	158.9	204.4	142.7	138.2	154.7	123.9
VSNL1	112.3	112.8	112.3	116.1	108.6	97.5	112.7	119.3
VSX1	108.2	104.8	108.2	109.8	99.1	99.2	112	106.4
VSX1	111.7	116.9	127.6	118.1	110.1	102.1	122.3	118.9
VSX1	114.9	106.4	117.5	116.4	105.2	96.6	109.2	108
VTCN1	111.4	117	114.2	114.7	113.4	97.8	115.1	113.6
VTI1B	158.1	147.9	153.8	188.7	141.8	148.4	150.6	148.3
VTN	111.5	115.8	115.8	117.6	106.2	95.4	108.9	107.8
VWA1	109.3	106.8	121.6	125.4	115.7	99.3	122.4	111.7
VWA1	124.2	120.7	125.4	130.3	117.8	102	126	122.6
VWA2	111.8	113.4	106.9	116.1	110.2	97.9	105.7	110.1
VWCE	111.5	119.7	129.3	119.1	110.7	96	127	119.8
VWF	122.9	125.9	128.9	136.8	115.5	126	138.8	116.8
WAC	670	963.2	724.8	942.6	736.9	578.1	977.8	1122.4
WAC	127.2	147.3	123.7	138.5	127	106.6	129.8	133.3
WAC	2054.5	827.4	1465.5	1597.1	943.3	1118.6	2276.8	1144.1
WARS	830.1	256.5	422.9	716.7	183.5	383.8	467.6	201.1
WARS	1003.2	345.7	482	848.4	200.1	531.9	593.4	253
WARS2	117.2	113.3	122.7	129.6	116.7	101.6	128.5	115.3
WAS	1705.3	1692.6	2702.4	1862.5	1067.9	2087.1	1295.3	1086.2
WASF1	113	115.4	119.3	114.7	105.7	98.3	122.8	116.9
WASF1	121.3	117.9	121.1	118.4	110	101.8	120.6	111.9
WASF2	260.3	336.6	251.1	322.5	247.5	269.9	476.5	349.6
WASF3	111.9	114.3	122.1	115.3	112.6	97	118.6	122.5
WASL	279.2	311.9	244.8	311.8	278.6	223.7	367.8	295.7
WASPIP	658.9	829.9	1053	983.2	882.1	979.4	900.2	886.5
WBP1	146.7	131.7	171.5	154.8	143.5	146.7	138.9	133.2
WBP11	807.2	997.8	766	955.7	791.2	857.8	1036.3	1113.9
WBP2	3081.6	2888.8	3058	3604.1	1912.3	4281.3	4251.7	3967.1
WBP4	228.7	255.9	232.9	242.1	207.1	241.4	273.6	297.9

WBP5	113.8	122.1	128	120.6	112.5	103.6	123.1	115.5
WBSCR14	121.1	114.8	121.2	123.8	108	102.4	121.5	115.6
WBSCR16	114.7	118.3	126.1	142.1	112	106.8	120.3	120
WBSCR17	126.5	120.7	118.9	131.2	106.1	94.5	120.5	124.2
WBSCR18	214.3	201.6	216.7	208.2	185.1	154.6	185	174.9
WBSCR19	114.9	109.4	108.1	118.9	104.7	96.5	117.8	108.3
WBSCR19	114.2	117.7	116.7	115.8	111.9	95.7	118.5	112.1
WBSCR22	368	454.7	399.1	389.3	356.1	423.6	399.3	501.5
WBSCR23	109.6	111.2	108.3	109.4	106.4	91.4	111.8	113.2
WBSCR27	110	117.2	113.8	112	112.1	99.2	109.9	118.7
WBSCR28	117.7	117.1	119.1	117.7	118.7	101.7	113.7	124.1
WDFY1	599.5	536.3	679.1	515.7	357.2	545.4	409.6	307.2
WDFY2	164.6	165.1	197.9	172	196.6	144.7	210.5	165.9
WDFY3	117.7	118.2	116.6	117.4	112.6	102.8	120.4	109.4
WDFY3	122.9	122.5	124.1	124.6	115	106.7	128.5	110.2
WDFY3	114.4	103.4	112.2	107.5	108.1	91.9	118.3	111.6
WDHD1	117	121.1	119.1	126.9	115.1	100.2	115.4	122.2
WDHD1	111.8	113.7	105.5	124.5	110.7	97	115.2	114.5
WDHD1	110.5	117.8	120.8	122.9	110	95.4	123	118.9
WDR1	181.8	184.5	193.7	204.4	159.2	172.2	195.6	147
WDR1	1950.1	1878.7	2625.3	2402.8	1759.1	2106.8	2246.3	1870.4
WDR12	156.4	175.4	178.1	154.1	175	129.6	168.8	161.8
WDR13	174.8	244.2	215.6	215	167.8	164.7	178.3	191
WDR16	104	113.4	115.6	113.4	102.3	96.1	120.7	110.1
WDR16	113.1	131.3	136.4	127.8	124.5	103.5	141.2	133.4
WDR17	112	111.5	115.6	112.3	109.2	95.7	112.3	118.1
WDR17	127.7	117.7	121.8	122.8	104.4	105.6	124.9	122.4
WDR18	425.7	540.5	519.4	482.7	376	358.3	372.3	310.7
WDR19	363.5	368.1	345.4	411	318.3	252.7	438.1	353.9
WDR20	387.1	365.7	320.9	431.9	341.7	306.9	540.7	676
WDR20	136.5	163	158.2	150.2	147.7	159.3	159.3	181.9
WDR20	131.3	127.1	125.3	132.6	127.7	121	134.9	140.7
WDR21A	182.2	199.3	146.8	180.2	176.3	122.6	157	153.5
WDR21C	119.6	109.6	118.8	122.5	106.9	99.3	128.8	115
WDR22	300.2	274.9	301.9	292.3	279	207.7	269.7	281.6
WDR23	124.7	127.1	130.8	137.9	120.4	107.1	111.8	125
WDR23	289.7	283.7	305.7	299.9	231.7	201	233.5	266.5
WDR24	139.1	128.3	134.1	158.2	123.8	109.2	135.8	120.6
WDR25	130.4	133.4	118.6	127.6	122.4	107.6	124.2	122.3
WDR26	198	235.8	233.7	216.3	197.2	191.8	354.8	293.7
WDR27	120.4	119.9	141.2	132.5	122.1	116.5	122.9	119.3
WDR3	116.2	115.8	109.8	122.6	111.3	106	130.4	123.1
WDR31	118.4	124.7	132.7	124.1	109.6	101.8	115.2	108.7
WDR33	210.2	195	203	196.2	248.2	146.6	177.1	147.8
WDR33	516.4	703.9	589.6	667.6	582.8	456.8	436.2	472.1
WDR33	142.9	142.3	136.2	136	124.8	116	131.6	147.3
WDR33	126	133	121.1	135.7	117.3	98.2	123.4	129.9
WDR34	117.1	124.1	136.4	127.5	107.6	108.9	121.6	131.2
WDR35	117	115.7	121.4	128.3	105.8	105.8	125.2	117.9
WDR36	232.8	280.6	202.5	226.3	225.6	168.3	205.5	214.5
WDR37	463.2	505.6	468.4	544.5	406.2	406.2	488.5	481.5

WDR4	289.8	295.9	269.8	319.1	280.8	192.7	247.6	191.2
WDR4	122	125.1	131.7	123	112.6	108.1	113.9	120.5
WDR4	122.1	134.9	122.7	130.1	125.6	109.2	138.4	122.2
WDR40A	189.7	221.2	200.4	199.3	186.9	157.8	198.3	228.4
WDR40B	113.9	117.6	116	131	115.4	100.7	123.5	110.1
WDR41	118.4	132.1	125.3	126.3	123.2	100.9	136.5	136.8
WDR42B	103.9	106.5	115.5	112.6	105.3	97	106.2	105
WDR44	138.3	127.6	133.8	133.2	119.6	113.6	126.7	128.3
WDR45	330.7	380.6	252.9	380.1	246.4	238.5	397.5	356.6
WDR45	118.9	125.2	124.6	120.8	104.5	109.5	129.1	111.1
WDR45	115.5	123	125	123.4	113.8	107.3	125.8	114.6
WDR45L	213.1	219.1	281.2	234	198.2	208.6	200.8	225.2
WDR46	391.7	365.6	291.2	363.6	303.8	220.2	367.2	307.4
WDR47	107.8	130.7	116.2	115.7	120.7	93.2	120.4	132.1
WDR48	280.1	276.8	256.5	315.4	227.1	254.2	360.6	349.6
WDR49	110.1	113.6	116.4	111	103.3	95.7	107.6	108.8
WDR5	113.6	110.8	119.3	126.9	114.5	104.5	114.1	119.3
WDR5	238.1	236.1	229.9	212	198.8	199.3	298.1	256.8
WDR50	198.5	181.9	220.3	203.2	162.6	160.7	180.7	204.1
WDR51A	153.3	167.1	140.9	163.1	136.8	119.2	137.5	133.1
WDR51B	132.8	151.1	151.8	168.8	125.9	138.5	148.1	157.8
WDR52	120.1	128.9	139.1	132.8	123.4	102.8	125.4	119.4
WDR53	170.1	164.4	211.8	176.3	155.9	137.3	171.9	184.8
WDR54	770.7	904.3	820.4	790.3	463.1	471	451.7	347.7
WDR55	157	172.5	145.1	186.2	160.8	133.5	177.6	170.1
WDR57	322.2	375.3	357.3	399.1	398.9	295	377.6	366.8
WDR58	173.4	180.3	192.3	187.3	157.8	131.5	154.3	137.2
WDR59	511.4	594	481.3	589	478.5	446.6	636.5	643.4
WDR5B	118.9	133.2	123.2	136.7	113.5	117.5	132.9	119.9
WDR6	1823.8	1906	1569.5	2001	1139	1128	1930.1	1544.4
WDR60	162.3	213.1	173.4	176.1	147.9	122.1	166.6	152.2
WDR61	451.3	464.3	606.1	432.6	417.4	439.7	323.2	330.5
WDR62	123	114.2	118.2	113.9	107.6	95.8	115.1	119.9
WDR63	117.5	110.6	121.2	122.6	110.5	94.1	114.1	120.8
WDR64	108.5	111	113.8	102.7	101.7	107.5	113.2	117.8
WDR65	118.8	114.7	121.2	115.6	112.6	105.7	128	118.3
WDR66	119.4	127.3	117.8	119.9	114.5	104.3	107.8	120.4
WDR67	215.1	212.8	235.6	187.9	186.9	185.8	197.4	183.4
WDR68	712.5	868.9	751.9	822	718	744.2	778.9	614.7
WDR69	109.6	126.2	105.1	116.5	113.9	98	109.9	109.9
WDR7	126.9	112.6	122.4	122.4	124.8	104.2	123.7	118.4
WDR7	227.7	242.7	212.4	245.3	206.2	177	204.3	183.8
WDR70	193.2	222.1	239.3	205	207.3	206.1	224.9	177.9
WDR71	243.2	199.4	280.4	251.9	193.4	223.6	243.3	227.3
WDR72	118.9	119.4	119	125.6	108.4	102.8	113.8	114.9
WDR73	193.5	204.5	218.4	229.1	186.2	147	173.4	154
WDR74	425.6	460.7	323.4	442.1	296.2	214.1	347.5	362.9
WDR74	246.9	268.9	250.9	227.8	310.9	180.8	264.7	236.1
WDR74	165.4	158.1	163.6	188.1	163.7	146.5	170.9	163.2
WDR75	606.2	778.7	663.2	580.5	639.1	394	625.8	524.5
WDR77	179.1	192.1	154.8	163.1	161.4	139.3	155.7	139.4

WDR78	113.2	112.5	120.3	111.3	102.7	96.7	121	114.5
WDR78	114.5	118.3	119.4	125.8	113	109.7	115.7	120.3
WDR78	114.2	117.9	107.1	110.3	109.5	99.4	123.3	121.7
WDR79	177.1	188.4	171.4	177.7	158.4	142.2	155.5	151.3
WDR8	472.3	541	430.3	424.4	367.5	338.2	433.9	421
WDR81	157.9	174.8	134.5	160.5	131	160.3	121	149.4
WDRPUH	119.5	112.6	119.6	117	112.2	92.5	112.8	119.8
WDSOF1	119.6	122.5	122.5	115.2	119.8	110	125.5	124.7
WDSUB1	252.7	253.4	269.4	295.5	256	202.8	247.3	243.1
WDTC1	120.6	118	120.4	115.5	107.3	113	124.2	124.6
WEE1	130	131.7	125.3	140.2	121.4	112	147.2	129.9
WFDC1	111.6	116.1	117.8	119.6	110.4	110.6	115.7	111.9
WFDC10A	108.1	117	117.4	114.5	105	105	112.8	106.9
WFDC10B	111	119.2	118.7	121.6	104.4	102.9	116.8	106.4
WFDC10B	123.2	119.2	117.8	127.1	116.2	99.8	114.1	118
WFDC11	110.9	118.2	113.2	118.3	102.5	107.7	119.2	124.5
WFDC12	105.7	121.3	120.3	125.2	108.1	94.8	109.5	111.6
WFDC13	116.9	117.3	128.3	106.6	112.1	106.1	124.8	113.4
WFDC2	126.2	111.6	133.2	117	112	110.5	122	119
WFDC2	113.2	121.5	116.1	120.7	110.1	102.8	118.4	114.6
WFDC3	114.1	114.6	122.6	121.9	110.3	107.6	125.4	121.9
WFDC3	118	117.7	123.3	115.9	108	99.8	119.1	116.4
WFDC3	108.5	127.1	121.1	124.3	109.1	108	119.4	115.5
WFDC5	113.6	106.1	111.5	116.5	105	91.5	122.9	104.3
WFDC6	120.8	123.2	124.9	124	114.6	110.2	121	116.1
WFDC8	125.2	118.6	115.4	118.5	113.7	106.1	119.3	119.4
WFDC8	113.4	129.1	131.5	129	116.2	114.6	135.8	130
WFDC9	112.9	112.8	128	117.6	109	106.3	114.6	114.5
WFIKKN1	113.5	117.2	108.3	120.5	112.8	100.3	115.3	115.8
WFIKKN2	111.2	114.8	117.5	119	112.5	100.6	125.2	109.3
WFS1	156.2	185.5	153.4	164.3	147	120.9	165.1	150.1
WHDC1	305.2	271	222.4	298.3	272.2	139.4	367.8	366.2
WHDC1	163.5	150.5	149.9	168.4	151.8	124.7	174.5	153.5
WHSC1	115.9	116.3	117.8	120.6	107.7	103.7	120	110.7
WHSC1	117.3	111	122.7	120.5	107.4	113.5	128.8	123.8
WHSC1	107.7	123.2	138.5	118.6	106.3	103.1	119.2	129.8
WHSC1	107.9	115.6	112.2	123.7	107.4	95.6	119.6	113.9
WHSC1	118.3	121.4	146.6	132.3	118.2	111.2	134	125.1
WHSC1L1	102.6	108.1	114	120.7	115.4	92.2	114.7	111.4
WHSC1L1	140.1	125.3	129.3	140.1	123.2	103.3	128.2	123.8
WHSC1L1	153.9	167.4	137.2	165	132.2	126.6	164.8	180.7
WHSC2	136.3	137.8	149.3	151.9	129.3	117.3	140	144.7
WIBG	152	174.7	157.5	162	148.7	145.1	172.5	157.7
WIF1	116.7	116.4	107.4	113.3	109.1	97.6	111.7	109
WIPF1	122.4	114.3	115.1	119.3	102.5	100.7	116.2	110.6
WIPF1	238.4	183.8	220.3	270.1	263.2	220	270.9	222.8
WIPF3	107.3	110.7	120.5	113.8	105.8	101.3	111.5	111.5
WIPI1	141.4	137.2	121	131.3	121.8	130.3	139.3	141.7
WIPI2	197.2	285.4	187.6	241.5	243.3	186.3	283.1	309.3
WIPI2	113	120.1	120.5	124.9	109.7	101.3	117.1	109.5
WIRE	129.3	136.3	126.5	126.4	120.7	114.4	134.2	129.7

WISP1	116.9	114.4	113.1	114.3	111.9	99.8	108.8	110
WISP1	114.5	115.3	109.6	116.4	105.1	94.3	121.2	110.4
WISP2	111.4	124.1	118	116.9	107.7	95.8	112.6	114
WISP3	114.3	118.7	120.6	124.1	114.6	103.2	123.2	110.9
WISP3	110.8	124.2	115.4	112.9	111.4	99.8	113.5	114.3
WISP3	113	121.3	129.4	124.6	126.7	101.7	116.8	116.2
WIT-1	104	108.4	104.6	114.9	107	94.3	109.5	107.9
WNK1	161.1	162.3	157.1	178.3	173.9	144.2	196.3	191.1
WNK2	116.3	115.7	126.4	121.1	111.4	98	119.5	118.6
WNK3	123.8	130.4	125.4	118.6	111	108.8	121	118.6
WNK3	120.7	114.5	113.4	117.8	113.7	99.8	128	116.1
WNK4	108.3	113.3	121.1	118.2	106.3	97.6	114.4	122.2
WNT1	133.2	123.7	114.8	121.7	110.4	97.7	114	119.7
WNT10A	119.7	122.6	122.6	129.7	105.5	107.9	121.9	117.2
WNT10B	121.7	119.4	127.3	121	104.2	107.2	119	107.5
WNT11	116.7	113.9	115.6	111.7	112.8	99.5	116.4	108.3
WNT16	126.1	131.9	127.1	130.2	114.1	106.8	127.8	127.5
WNT16	122.8	117.6	120.1	112.2	110.1	100.1	109.3	118.4
WNT2	118.9	125.3	130.3	119	119.6	105.3	127.8	120.1
WNT2B	123.7	126.8	136.6	132.3	118.2	112.9	122	124.7
WNT2B	115.9	116.6	118.1	128.4	112.5	97.6	114.5	123.6
WNT3	105.6	110.2	122.6	112.8	115	103.6	110.7	115.4
WNT3A	116.1	116.2	115.4	118.9	106.7	98.8	113.6	111.7
WNT4	123.1	117	113.3	111.1	108.5	99.1	121.4	116.4
WNT5A	105.8	108.5	108.8	116.4	100.6	99.1	122.6	115.4
WNT5B	122.6	134.1	122.6	136.2	117.7	113.6	139.9	124.8
WNT5B	109.4	111.9	114.5	113.3	108	92.6	118.2	111.8
WNT6	122.1	123.1	120.5	115.3	108.6	102.7	124.9	134.5
WNT7A	157	144.8	140.3	163.8	123.6	119.8	137.7	138.4
WNT7B	114.6	124.5	114.4	120.8	111.7	103.9	114.5	121.5
WNT8A	111.7	113.1	116.3	113.7	106.3	99.2	123.4	112.7
WNT8A	121.4	120.8	118.2	111.7	106.8	101.4	118.4	114.2
WNT8B	121.9	114.6	117.8	121.9	106.3	105.4	121.6	120.6
WNT9A	118.7	109.3	111.2	124.8	111.7	93	120.1	110.7
WNT9B	111.5	107	129.8	119.6	112.4	106	127.9	108.8
WRB	181.5	160.1	176.8	200.3	188.7	149	181.9	177.7
WRN	206.2	197.9	243.8	209	219.6	151.7	220.5	269.2
WRNIP1	149.6	172.4	167.7	176.9	160.2	135.9	158.2	167.7
WRNIP1	1221.4	1294.6	965.3	1428.2	1296.7	826.8	1351.7	1429.2
WSB1	311.5	275.1	319.2	292.1	284.2	252.2	336.5	291.5
WSB1	453.3	475.9	393.7	369.3	517.5	356.8	445	539
WSB2	141.9	148.5	152.6	143.7	140.6	146.5	155.6	143.4
WT1	106.8	111.8	106.8	118.1	100.6	97.8	123.1	117.8
WT1	125.3	137.2	130.5	127.8	116	108.5	120.8	126.6
WTAP	877.8	956.3	825.8	1056.7	799.2	443.2	1003.8	1186.2
WTAP	119.9	114.9	117.2	116.9	106.8	104.3	124.4	113.9
WTAP	126	129.6	123.3	137.8	124.8	109.5	124.6	136.6
WTIP	112.8	117.4	109.2	119.9	104.2	103.7	117.8	109.4
WTIP	121.7	112.3	113	136.2	112.1	103.5	126	121.2
WWC1	123.2	111.8	114.3	119.2	106.5	102.6	114.9	114.2
WWC2	112.6	112.3	111.1	119.1	107.1	96.1	119.7	113

WWC3	246.8	247.7	252.6	258.3	254.2	213.6	226.7	226.9
WWOX	111.2	120.3	117.4	111	112.4	96	112.2	115.3
WWOX	120.7	126.9	118.9	124.2	118.2	105.1	121.2	132.7
WWOX	120.9	123.2	116.3	118.1	105.4	103.6	112.7	115.8
WWOX	120.1	127.5	114.7	115.7	115.1	101.7	119.6	105.4
WWP1	118.2	117.4	121	115.8	111.7	105.4	138	115.2
WWP2	107.3	113.3	107.1	123.2	107.2	94.5	121.3	108.5
WWP2	120.6	112.6	130.4	136.1	118.3	106.6	117.2	121
WWP2	183.7	177	195.3	182.7	160.7	155.3	227.4	204.3
WWTR1	102.9	110.2	113.3	116.7	102.7	93.7	110.8	116
XAB1	628.4	687.4	614.3	583.2	494.8	501.7	665.4	781.7
XAB2	478.3	472.6	506.9	494	298.6	355.1	547.7	441.1
XAF1	256.4	214.7	307.7	253.4	209.3	177	197.7	141.8
XAF1	170.4	145.7	164.7	151.3	127.8	126.6	144.9	128.1
XAGE1	119.6	112	120.5	114.2	109.1	98.4	122.7	123.7
XAGE1	105.1	108.5	116.9	117.4	111.8	93.3	109.6	113.6
XAGE2	114	113	119.1	117	110.8	96.9	115.5	114.9
XAGE3	121.5	116.4	127.2	116.1	108.6	114.3	112.6	116.1
XAGE3	112	124.4	108.9	116.5	101.2	93.4	107.2	113.8
XAGE5	111.4	116.8	121.7	117.3	102.9	94.7	116.2	109.4
XBP1	815.6	1044.5	794.3	927.3	825.8	885	1035.4	1559.7
XBP1	1350.6	1445.2	1181.7	1210.2	1482.7	1470.9	1524.5	1946.9
XCL1	126	130.2	130.7	127	126.3	101.7	120.8	119.7
XCR1	108.3	115.1	113.2	119.7	102.6	95.6	123.1	115.5
XCR1	114.2	105.5	117.6	111.8	113.2	99.4	110.4	112.6
XCR1	121.4	120.5	119.3	113.8	111.3	108.9	110.6	121.3
XDH	108.2	119.2	129.9	124.2	109.6	92	119.2	119.1
XG	107.2	113.8	111.6	110.2	103.1	95.7	111.6	109.5
XK	124.9	116.5	116.8	118.1	101	101.4	120.9	113.5
XKR3	111.4	113.6	115.6	114	117.9	111.7	119.1	128.9
XKR4	105.5	118	118.9	110.8	98.7	96.7	106.5	113.2
XKR5	110.6	117.2	110.2	117.8	108.5	99	123.8	108.9
XKR6	108.4	124.9	109.7	112.3	104.7	91.7	116.5	114.1
XKR6	122.4	116.6	144.3	121.7	113.4	95.2	132	119.5
XKR6	158.1	151	157	164	138.6	117.1	151.4	148.7
XKR7	119.1	111	111.3	112.4	103.2	97.3	113.2	117.3
XKR8	180	204.1	177.3	199.5	182.7	162.2	180.9	171.2
XKR9	107.7	114.9	115	110.1	96.4	100.1	112	117.9
XKRX	122.5	117.1	118.9	121.9	111.7	97.8	112.8	112.7
XKRY	119.3	116.5	111.1	111	104	88	108.4	109.1
XLF	116.7	106.5	115	124.3	108.9	100.8	118.1	110.7
XLKD1	105.8	118.6	116.2	122.1	109	94.6	124.1	117.4
XPA	121.8	109.1	115.9	128.4	119.1	103.7	130.5	116.5
XPC	948	832.6	939.5	787.7	748.3	864.1	820.9	719.8
XPNPEP1	578.5	606.3	679.6	625.9	451.7	441.9	512.1	506
XPNPEP2	113.1	117.5	116.2	117.8	105.1	106.8	128.8	116.7
XPNPEP3	3704.6	4007	3612.7	3993.3	3168.5	3188.2	3803.3	4439.2
XPO1	676.7	757.6	708.3	682	524.6	394.9	764.9	893.7
XPO4	183.9	214.7	186.5	191.7	172.3	145	185.9	154.9
XPO5	150.3	164.4	142.1	173.1	149.8	122.3	157.8	161.7
XPO6	430.2	475.3	419.2	473.4	380.3	279	546.8	657.9

XPO7	239.7	243.1	199.2	240.6	200.4	167.5	244	252.8
XPOT	149.8	144.7	164.4	166	139.2	120.2	138.7	137.2
XPR1	336.5	333.2	276.5	362.7	303.9	244.2	432.4	352.8
XRCC1	207.8	180.7	254.1	184.3	148.4	163.5	149.3	133.4
XRCC2	937.8	722.1	930	764.3	805.5	497.9	887.9	868.7
XRCC3	123.2	143.2	130.4	136.4	121.5	106.4	138.9	138.2
XRCC4	111.7	109.9	117.2	116.5	114.1	94.8	117.7	116
XRCC5	152	194.1	177.9	198.2	145.3	136.4	169.7	173.6
XRCC6	962.3	1161.1	901.4	1133.9	932.3	794.2	1180	1258.9
XRN1	117.3	121	129	132.4	117.2	105.9	127.4	108.3
XRN1	564.1	524.6	488.5	544.3	506.1	403.4	547.7	553
XRN2	107.4	170.8	109.8	116.2	167.8	102.9	116.5	182.5
XTP3TPA	449.6	536.3	434.1	408.2	333.9	279	364.6	333
XTP7	121.2	117.3	125.7	119.7	110.1	101.1	124.4	113.9
XYLT1	107.9	117.2	115.6	120	114.5	104.8	118.9	118.9
XYLT2	355.3	366.1	291.1	313.4	295.4	227.3	318.9	254.9
YAF2	112.6	124.6	117.2	135.5	116	106.8	127.8	135.6
YAF2	114.6	115.3	130.4	124.1	106.7	100.4	124.8	113.7
YAF2	185	187.6	179.7	159	165.8	127.3	184.7	195.6
YAP1	106.9	113.2	116.3	116.6	101.3	98.8	114.6	107.6
YARS	473.5	625.4	709.1	698.8	588.9	649.7	731.8	872.8
YARS2	237.3	220	227.9	235	208.1	183.3	189.8	201.1
YBX1	630.4	688.4	423.9	516.5	438.8	498	627	1037.2
YBX2	111.6	118.1	107.4	129.4	102.4	104.2	120.8	113.7
YEATS2	222.7	224.6	195.9	258.8	229.5	197.8	238.2	226.3
YEATS4	329.9	325.1	292.2	289.6	253.5	234.2	319.3	229.3
YES1	147.1	168	145.5	147.9	137	131.3	174	255.6
YIF1A	178.2	165.1	250.2	186.1	157.7	156.9	172.1	166.4
YIF1B	109.1	112.3	118.7	118.1	111.5	93	110.4	112
YIF1B	149.2	164.1	176.8	166.8	123.5	122.9	138.8	135.2
YIF1B	176.2	153	167.9	165.1	135.8	125	152.7	133.8
YIPF1	260.2	240	400	258.3	193.4	244.6	272.2	249.8
YIPF2	129.4	126	128.8	127.4	116	113.6	129.3	130
YIPF3	407.6	463.7	415.1	443.7	293.8	384.7	368.5	401.9
YIPF4	110.3	124.3	120	120.6	112.3	102	109.7	116.5
YIPF5	162.2	163.8	201.2	159.1	147.1	141.8	195.7	179.8
YIPF5	105.9	107.5	117.5	117.6	108.4	96.3	118.6	113
YIPF6	300.9	317.4	309.9	395.7	280.1	277.3	337.3	344.6
YKT6	130.8	133.2	135.5	121.6	130.3	113.5	134.6	125.1
YME1L1	444.2	402.6	436.4	425.3	435	357.7	429.7	420.6
YOD1	109.5	109.6	114.2	111.3	109.8	97.2	114.4	106.5
YPEL1	184.6	239	210.6	239.1	196.2	153.1	187.9	171.3
YPEL2	290.2	282.9	328.4	271	284.7	358.1	265.9	316.9
YPEL3	870.9	1053.8	976.5	801.5	536	799.3	784.1	691.6
YPEL4	137.1	128.3	125.2	128.3	104.7	103.5	111.6	109.7
YPEL5	928.7	994.8	997.6	1017.5	960.5	1145.1	1591.5	1905.8
YRDC	315.8	357.2	321.7	348.4	244.6	248.6	392.8	412
YSK4	102.7	109.6	116.6	116.2	115.6	101.1	108.7	108.7
YSK4	113.5	120.4	111.8	113.9	117.8	97.1	122	115.6
YSK4	118.4	108.2	110.5	121.1	99.5	98.2	117.9	118.2
YTHDC1	120.7	117.7	133.4	127.9	119.9	109.4	122.7	126.3

YTHDC1	162.3	150.5	171.1	179.2	179.8	124	188.2	282.6
YTHDC1	2104.6	2273.9	2101.8	2588.2	2170.9	1918.1	3308.1	3064.3
YTHDC2	119.6	136	141.5	137	140.4	133.2	141.6	129.4
YTHDF1	306.4	340.1	299.7	364.3	361.2	296.2	420.6	430.2
YTHDF2	358.1	476.6	359.4	441.4	388.5	299	470.1	542.7
YTHDF3	155	144.5	179.9	178.8	160.5	147.2	190.3	189.7
YWHAB	3427.6	3401.4	4097.1	3201.6	3129.8	3973.8	3894.5	3308.1
YWHAB	240.7	269.5	203.3	204	198.9	167.6	211.6	217.8
YWHAE	165.2	153.6	149.8	172	134.3	181.4	156.5	157
YWHAG	278.9	260.9	247.9	322.6	240.4	271.9	335.2	421.6
YWHAH	1413	1756.6	2515.8	1564.8	1119.5	1192.3	1480.3	1709.4
YWHAQ	2442.1	2915.2	2568.9	2396.7	2503.8	2023	2618.6	3310.9
YWHAZ	3559.8	3973	4150.7	4490.7	3552	4752.9	4825.8	5827.4
YWHAZ	122.3	141.9	131.8	124.4	121.2	112.1	133.2	118.1
YY1	500	500.4	444.1	583.1	365.5	330.8	777.6	815.3
YY1AP1	516.9	649.5	531	647.2	570.1	453.5	666.5	789.5
YY2	111.1	117.1	120.5	126.7	105.7	95.1	116.2	117
ZA20D2	1864.6	1858.6	1867.3	2738.6	2154.2	2258.4	2978.9	3428.5
ZADH1	244.3	190.2	247.6	234.4	237.7	170.2	207.4	226
ZADH2	239.7	280.3	266.9	227.6	255.5	154.3	223.2	225.1
ZAK	111.8	117.5	134.1	121.6	111	101.2	125.4	113.4
ZAK	143.1	134.6	148.9	129.2	120.7	121	132.6	122.9
ZAN	114.2	116.6	112.9	114.2	113.2	100.8	117.1	116.9
ZAN	123.8	137.8	127	123.6	118.8	109.6	128.5	130.5
ZAN	118.6	124	125.2	127.7	115.8	105.6	121	124.6
ZAP70	120.2	120.7	124.9	126	115.1	105.4	137.9	128.7
ZAP70	643.3	720.8	731.5	667.6	488.5	686.7	719.1	473
ZAP70	117.6	128.9	115.7	117.3	125.9	102.3	126.7	121.6
ZAR1	108.5	119.5	121.3	107.9	100.7	98.9	107	111.4
ZBED1	490	466.7	636.1	464.2	348.2	507.5	496.9	550.5
ZBED2	112	116.8	119.7	111.8	117.6	94.4	117	119.8
ZBED3	114.8	114.5	136.9	116	118.3	111.3	135.9	108.7
ZBED4	221.1	245	208.3	220	200.3	183.2	245.4	309.7
ZBED5	466.6	258.6	337.7	390.4	278.1	260.5	277.3	282.8
ZBP1	288	254.7	421.4	251.1	159.2	220.6	208.7	178.6
ZBTB1	125.7	130.2	119.9	122.3	111.7	99.2	122.7	127.1
ZBTB10	111.8	126.9	127.9	128.6	108.9	104.7	125	125.6
ZBTB11	150.1	169.4	172	164.7	130.5	125.3	178	171.8
ZBTB12	114.5	104.6	116.9	107.2	107	96	115	115.3
ZBTB16	127.8	143.5	126.6	147.1	121.2	98.8	144.7	133.6
ZBTB16	178	175	150.5	178.1	147	129.6	163.4	139.7
ZBTB16	112.9	115.7	114.2	112.6	113.7	104.2	112.8	120.1
ZBTB17	311.1	332.7	260	352.7	269.9	228.1	412.5	319.5
ZBTB2	132.6	134.5	136.7	139.6	129.4	104.3	144.5	135.3
ZBTB20	201.2	179.5	202.1	185.7	171.7	161.1	186.7	199.3
ZBTB24	324.1	302.1	221.6	302.3	291.8	186.9	280	244.2
ZBTB25	208.2	256.3	225.7	205.4	220.9	160.7	189.5	187.4
ZBTB26	119.1	123.3	118.4	123.9	105.9	92.7	118.9	123.3
ZBTB3	127.8	133.5	150.4	133.6	131	123.9	126.1	135.7
ZBTB32	124.2	125.7	125.1	125.3	111.4	100.7	122.4	118
ZBTB33	905.1	944.8	879.9	1060.5	646.8	642	931	913.4

ZBTB37	110.3	109.1	109.3	120.5	103.9	102	119.7	113.1
ZBTB38	118.2	124.6	144.3	141.2	129.2	116.9	129	123.5
ZBTB39	123	129.2	123.9	130.9	117	110.9	127.8	129.5
ZBTB4	1047.1	923.6	999	981.8	1193.4	739.8	928.9	877.5
ZBTB40	245.7	239.8	201.1	250.5	174.1	132.1	223.5	263
ZBTB41	118.8	127.3	114.9	115.9	117.1	98.2	113.5	126.9
ZBTB44	134.5	159.7	155.2	147.5	128.1	120.8	145.2	154.6
ZBTB5	172.7	183.6	173.5	197.4	177.8	139.7	203.5	202.4
ZBTB7A	124.8	126	133.2	135.7	125	111.6	133.7	131.5
ZBTB7B	125.8	128.4	126.4	132.6	117.1	108	140.1	129.5
ZBTB8	116.7	111.1	124.4	121.5	110.8	96.5	116.6	115.6
ZBTB8	109	110.5	111.4	114.5	104.2	102.3	121	107.7
ZBTB8OS	126.6	126.8	128.2	142.9	121.3	110.4	122.6	131.9
ZBTB9	193.5	217.9	207.4	216.9	162	179.5	219.3	205.7
ZC3H10	179.6	202.1	192.8	244.9	150.9	164.4	176.7	170.1
ZC3H11A	123.6	108.6	119.9	124	114.4	93.7	116.5	124.2
ZC3H12A	324.1	355.7	256.9	320.6	274.9	239.2	414.8	369.8
ZC3H12B	128.1	133	149.3	128.3	117.2	111.8	115.6	124.4
ZC3H14	243.8	234.2	198	262.7	257.2	180.2	223.7	204.2
ZC3H15	237	215.1	230.6	272.6	235.4	206	256.7	240.9
ZC3H3	443.9	443.2	478.8	473.9	325.4	450.1	417.2	349
ZC3H6	139.9	137.4	137.2	137.5	141.3	117.1	157.9	142.2
ZC3H7A	120.3	115.3	128.9	135.1	125.4	101.3	128.1	139.2
ZC3H7B	114.5	117.1	118.6	120.4	102.5	100.4	117.4	120.8
ZC3H8	129.4	127.9	139.4	134.8	117.9	121.2	129.4	128
ZC3HAV1	166.6	204.1	150.6	197.5	175.2	159.8	196.8	222.9
ZC3HAV1	169.7	146.6	139.7	178.1	203	134.3	182	205.9
ZC3HAV1	983.6	1004.2	858.1	1214.2	1485.2	850.8	2486.2	3804.2
ZC3HAV1L	114.6	116.3	113.2	113.4	109	100.1	122.1	109.8
ZC3HC1	473.3	507.8	498.3	534	439.6	372.5	547.4	465.3
ZCCHC10	118.6	116.8	115.1	117.3	106.1	97.6	118.7	117.1
ZCCHC11	290.5	292.3	333.3	311.1	297.6	274	329	364.3
ZCCHC11	196.7	204.2	193.7	214.5	178.6	143.1	185.8	196.2
ZCCHC12	119	120.9	125.2	124.7	113.4	102.4	125.6	129.7
ZCCHC13	113.2	118.8	111	107.9	107.1	107	108.7	117.8
ZCCHC14	232.1	258.2	210.6	224.3	222	204.2	251.2	214.6
ZCCHC16	111.6	113.5	106.1	119.7	107.2	95.8	120.7	106
ZCCHC17	336.3	331.9	311.3	329.3	272.8	318.2	336.3	297.3
ZCCHC2	112.7	117.4	111.4	110.2	116.3	93.4	123.1	112.9
ZCCHC3	237.2	220.7	174.5	225.8	179.1	164.8	236.9	234.5
ZCCHC5	117	119.8	114.9	116.9	104.7	107.5	120.7	113.2
ZCCHC6	139.4	165.7	131.1	164.9	169.9	123	183.3	167.5
ZCCHC7	425.1	402.4	418.4	435.2	359.9	324.6	394.2	351
ZCCHC8	234.4	223.4	222.2	289	237.7	221.3	230.9	256.3
ZCCHC9	318.2	408.7	469	447.3	337.5	375.6	252.7	315.4
ZCD1	669.4	811.6	806.6	707.2	708.5	471.8	613	516.6
ZCRB1	133.1	137.5	143.6	141.2	137.1	130.5	146.8	154.2
ZCSL2	153.3	160.3	165.4	177.5	141.2	162.1	181.9	154.9
ZCSL3	196	226.6	225.2	209	201.1	225.6	195.3	175
ZCWPW1	159.2	165.8	167.9	193.6	177.9	140.4	214.4	233
ZCWPW2	109	123.9	117.8	120.3	106.4	104.4	120	122.9

ZD52F10	121.7	117.3	117.9	114.1	112.3	100.7	110.1	112.3
ZD52F10	108.2	121.1	118.6	107.7	109.7	93.9	115.7	112.5
ZD52F10	113.4	125.7	120	127.2	111.9	102	119.6	116.2
ZDHHC1	116.9	107.9	120.3	115.4	105.9	90.4	115.7	113.4
ZDHHC11	162	173.7	127.7	165	143.9	111.6	176.9	179.3
ZDHHC11	127.5	122.9	129.5	120	118.8	110.9	130.7	111.2
ZDHHC12	160.5	180.8	186.3	166.9	129.1	169.8	176.2	155.9
ZDHHC13	169.1	177.8	184.7	194.6	170.7	146.4	170.4	165.1
ZDHHC13	127.9	149	119.6	130.3	132.7	105.9	128.5	119.5
ZDHHC13	105.2	108.8	116.8	116.8	114.6	106.2	112.5	121.8
ZDHHC14	169.5	180	140.8	188.5	159.1	120.9	216.5	196.9
ZDHHC15	120.7	114.8	111.3	107.4	108.7	96.9	114.6	116.2
ZDHHC16	221.3	224.2	188.1	195.2	174.9	166.6	178.7	200.4
ZDHHC16	122	117.6	124.4	123.4	113	98.7	123.1	125.4
ZDHHC16	782.3	764.5	555.6	755.8	464	391.2	609.6	558.8
ZDHHC17	231	260.3	237.4	268	241.9	224.5	266.2	267.8
ZDHHC18	183	197.9	162.9	176	180.7	156.7	185.4	181.8
ZDHHC19	117.5	111.9	115.4	120	103.9	92	117	114.7
ZDHHC2	128.7	136	137.8	146.8	138.8	113.5	144.2	160.7
ZDHHC20	120.6	125.2	125.7	131.7	112.9	97.8	135.7	125.8
ZDHHC21	118.4	118.8	117.5	110.2	113.2	94.3	112.8	114.7
ZDHHC22	124.4	139.5	149.4	133.3	116.1	104.7	138.4	122
ZDHHC23	115.1	118.4	125.1	115.9	128.2	109.6	127.9	120.9
ZDHHC24	134.3	137.1	146.8	133	117.3	117	128.2	129.1
ZDHHC3	147.8	154.1	159.1	166.4	132.1	126.8	141.5	148.1
ZDHHC4	109.8	122.1	128.4	114.5	112.4	109.8	118.2	113.5
ZDHHC5	305.2	304.5	324.7	451.4	289.5	255.1	404.1	427.9
ZDHHC6	537.3	540.4	755.3	629.7	484.5	477.6	471	486.7
ZDHHC7	522.7	578.8	865.3	527.6	396.9	564.2	553.5	416.8
ZDHHC8	1269.1	1369.9	991	1120.5	803.1	721.5	861.4	744.3
ZDHHC9	185.4	187.9	182.8	187.3	164.6	118.6	195.8	184.8
ZDHHC9	115.9	111.2	111	114.3	109.7	95.5	111.5	114.3
ZDHHC9	114.5	115	107.5	119	109	95.7	112.9	111.7
ZF	155.1	188	190.4	184	152.7	155.2	162.2	181.9
ZFAND1	174.8	164.4	186.2	169.2	159.8	132	170.3	168.5
ZFAND2A	243.6	262.2	215.5	285.6	287.1	180.2	493.5	1389.5
ZFAND2B	508.1	493.6	389.4	547.6	376.5	454.8	507.4	456.2
ZFAND3	179.9	173.8	170.7	184.1	160.3	170.6	200.9	209.9
ZFAND6	151	166.1	177.2	185.3	152.6	163.1	177.8	170.9
ZFHX1B	316.8	203.6	336.6	295.4	216.8	280.4	250.4	183.4
ZFHX4	117.3	113.7	115.1	111.2	103.5	92.2	115.1	115.9
ZFP1	146.7	131.6	114.9	116.1	116	108.7	136.4	126.9
ZFP106	413.6	381.9	484	410.7	341	313.3	346.2	324
ZFP161	364.3	384.1	372.9	388.8	301.9	275.1	240.5	271.6
ZFP2	116.2	123.2	122.8	123.7	110	112.2	130.4	140.3
ZFP260	167.3	232.9	223.5	212.3	172.4	169.6	186.2	208.4
ZFP28	116.9	120.7	118.7	131.4	110.6	103.4	124.7	117
ZFP3	171.9	162.4	222.6	188.1	160.2	146.5	136.6	141.4
ZFP36	839.4	981	1128.3	783.4	633.6	812.3	786.9	795.6
ZFP36L1	876.6	614.9	998.4	897	722.1	1017.8	801.7	556.3
ZFP36L2	453.6	415.5	455.4	389.9	402.7	311	280.9	307.7

ZFP37	177.1	193.7	128.7	163.7	170.9	124.4	158	172.8
ZFP41	110.3	124.5	120.7	125.4	107.8	103.9	131.2	115.1
ZFP41	117.8	122.7	124.4	122.3	112.1	104.7	112.9	118.6
ZFP42	130.2	125	122.9	128.9	113.6	108.1	125.1	120.4
ZFP64	112.1	117.2	123.4	121.1	113.9	93.8	112.5	115.4
ZFP64	114.5	121.6	124.6	117.3	106.2	97.2	122.6	123.6
ZFP90	321.6	317.1	354.9	333	253.5	260.2	265.5	236.9
ZFP91	1012.9	974.1	820.4	829.3	986.7	709	1014.3	1056.5
ZFP91	135.6	162.7	133.9	158.1	139	107.5	168.3	172.6
ZFP95	109.9	120.3	115.8	124.6	108	101.5	122.6	113.6
ZFP95	110.5	105.6	120.6	111.1	101.6	98.8	116.2	110.7
ZFP95	131.5	133.1	134.2	145.6	119.5	115	158.9	181.3
ZFPL1	131.2	125	147.5	143.8	110.2	116.2	134.9	139.8
ZFPM1	253.6	240.5	228.3	244.2	174.6	194.4	235.4	219.9
ZFPM2	114.3	111.7	118.9	117.4	102.8	101.6	118.4	116
ZFR	220	157.7	232.2	389.8	381.8	272	339	378.5
ZFX	146.1	142.7	135	157.8	142.6	114.7	158.4	164.9
ZFY	157.9	160.6	155.2	190.7	194	145.6	179.8	184.3
ZFYVE1	111.2	121.3	117.5	132.2	107.9	98	115.8	116.8
ZFYVE1	119.4	119.5	113	126.9	112.6	112.2	121.5	117.8
ZFYVE16	165.2	144	156.2	173.4	158.5	146.5	195.3	176.5
ZFYVE19	292.9	366.9	370.8	277.8	256.2	231.6	293.4	324.7
ZFYVE20	577.1	506.6	548.7	553.9	463.3	403.8	484.8	450.3
ZFYVE21	306.9	267.4	364.5	272.3	266.7	321.5	361.1	311.2
ZFYVE26	435.2	398.9	394.1	442.7	380.8	301.1	452.5	494
ZFYVE27	124.6	134.4	125.6	129.9	109.8	106.9	132.8	139.3
ZFYVE27	164.2	165.4	148.4	179.3	132.4	144.6	173.9	170.9
ZFYVE28	111.6	112.3	117.4	118.6	108.8	107.9	122.6	122.3
ZFYVE9	112.1	119.6	119.9	129.6	106.6	94.5	125.8	117.6
ZFYVE9	114.9	117.9	111	114.6	110.2	100.1	114.7	111.3
ZFYVE9	114	110.2	113.9	110.3	117.3	98.6	126.1	114.3
ZFYVE9	112	117	125.5	122.6	116.3	101.2	122.2	112.5
ZGPAT	142.6	134	149.5	153	144.4	124.5	130.5	132
ZGPAT	117.2	120.2	128.5	120.9	108.1	110.7	121.3	117.6
ZGPAT	537.1	662.3	586.7	636	435.1	512.1	474.1	403.9
ZGPAT	117.2	125	129.2	125.4	118.3	108.5	137.2	124.7
ZHX1	135	132.3	141.8	154.2	127.5	123.8	150	140.3
ZHX1	111.8	119.8	128.6	121.9	110.4	103.7	124.7	117.6
ZHX1	119.5	128.7	127.1	129.3	117.3	98.1	115.5	130.8
ZHX2	291.8	411.8	220.5	273.9	420	182.7	293.3	446.1
ZHX3	153	159.9	150.6	154	142.7	117.1	149.3	134.6
ZIC1	113.6	115.8	107.9	112.4	114.8	95	126.9	110.4
ZIC2	112.4	108.8	121.1	119.5	111.8	100.3	119.9	118.1
ZIC3	111	106.7	107.9	114.4	99	100.5	117.4	107.9
ZIC4	105.8	108.8	108.1	111.2	101.3	96.8	115.3	117.4
ZIC5	117.4	116.3	114.4	123.2	109.1	104.4	121.3	106.2
ZIK1	152.7	184.6	156.5	164.4	156.2	134	151.3	157.2
ZIM2	116.4	109.2	117.9	117.1	114	101.2	109	116.3
ZIM3	119	125.6	117.2	130.4	112.1	102.2	119.3	127.4
ZKSCAN1	122.4	134.3	151.8	122.8	129.4	104.3	123.8	132.8
ZMAT1	123.3	111.7	115.3	119.1	111.4	96.8	111.1	113.6

ZMAT1	106.9	117.6	119	117.7	109	97.9	119.5	111.5
ZMAT1	109.8	117	104.3	110.6	101.7	91.6	113.3	106.8
ZMAT2	491.9	584.9	571	658.5	527	526	654.8	645.8
ZMAT3	1421.5	1277	1330.5	1386.5	1313.2	1163	1528.9	1726.9
ZMAT5	106	108.6	117.8	117.1	105.9	100.4	112.1	108.3
ZMAT5	151.2	155.2	174.4	151.9	127.9	134.1	146.1	146.7
ZMIZ2	129.3	147.2	144.5	135.3	125.5	110.3	150.4	128.9
ZMPSTE24	723.7	828.2	850.5	945.7	567.6	572.9	1020.7	953.7
ZMYM1	217.4	216.7	213.5	196.1	193.9	146.1	206.8	208.5
ZMYM3	115.7	115.2	119.8	113.4	104.1	99.9	121.6	111.6
ZMYM3	138.5	131.5	159.9	142.1	116.6	120.4	134.2	125.1
ZMYM3	118	119.1	116.1	119	105.2	98.5	111.8	111.5
ZMYM4	204	242.2	230.3	260.4	208.5	209.2	265.3	277.9
ZMYM5	150.5	153.8	160	177.1	148.6	118.6	164.2	159.5
ZMYM5	147	153.6	167.1	180.9	161.8	119.1	158.4	179.9
ZMYM6	110.7	122.6	120	120	107	99.4	112.6	113
ZMYND10	120.1	112.4	116.1	118.1	112.7	103.7	123.3	114.7
ZMYND11	117.6	114.3	131.9	117.4	110.7	105.8	120.9	114.3
ZMYND11	122.9	115.1	114.9	117.9	112.4	100.9	129.7	121.4
ZMYND12	112.8	106.4	116.5	112.6	103.5	92.6	120.3	106.3
ZMYND15	164.2	126.1	207.8	166.3	117	299.4	118.7	114.5
ZMYND17	119.2	115.6	121.5	111.1	106.7	106.1	122.3	115.1
ZMYND19	302.2	327.9	232.3	272.5	253.1	175.4	326.8	283.3
ZMYND8	172	174.5	195.3	161	158.8	139.2	152.5	139.3
ZNF10	151.6	161	155.4	164.7	149.3	133.4	166	165.4
ZNF100	114.9	124.1	119.7	122.7	112.2	97.5	122.5	116.2
ZNF101	253.8	305.2	323.6	274.2	287.2	198.7	288.7	272.3
ZNF114	124.9	123	116.7	120	109.8	96.5	128.1	117.7
ZNF117	110.3	103.4	115.7	116.6	104.1	96.3	117.2	112.1
ZNF11B	508.7	678.6	489.1	503.7	422	403.7	456	386
ZNF12	165.4	153.4	156.5	157.3	137.6	119.3	158.5	154
ZNF121	107.1	107.5	114.9	113.5	110	92.1	112	113.5
ZNF124	121.8	122	123.2	118.5	107.6	94.5	124.1	115.2
ZNF131	118	128.7	113.9	123.8	117.9	97.8	131.3	114.1
ZNF132	113.4	125.4	119.1	124.2	106.7	111.6	128	135.9
ZNF133	157	157	144.7	156.8	133.1	114.2	139.1	146
ZNF134	138	169.6	143.1	137.6	131.3	132.3	155.5	140.1
ZNF135	128.7	136.3	131.8	128.6	129.2	107.8	119.2	129.3
ZNF136	133.1	142.5	144.3	151.5	139.9	112.6	143.5	125.6
ZNF136	158.4	154.2	175.6	158.6	148.4	125.4	143.2	160.4
ZNF137	128.6	132.1	147.3	140.1	129.3	112.9	141.2	136.4
ZNF14	119.2	119.5	111.6	113.7	104.5	109.1	128.9	117.9
ZNF140	133	131.3	163.7	154.3	136.3	120.2	142.5	148.4
ZNF141	109.9	114.3	128.4	121.3	105.3	100.1	125.2	137.5
ZNF142	172.9	188.8	189.9	194.7	149.8	165.4	208.8	206
ZNF143	347.2	341.8	327.5	383.8	332.3	342.8	428.4	502.2
ZNF146	444.5	393.5	355.7	544.8	366.8	322.2	483.1	501.8
ZNF148	1091.5	1136.2	823.1	1189.5	911.5	656.6	1370.8	1311
ZNF155	126.2	114.5	129.4	113.1	97.9	99.7	133.3	119.7
ZNF155	117.9	123.7	123.9	126.5	116.4	105.6	125.1	121.4
ZNF155	113.2	116.2	121.7	119.2	111.1	108.2	132.1	118.7

ZNF157	109.4	112	120.8	124.4	113.2	100.8	127.3	109.3
ZNF16	145.2	149.5	133.8	146.5	157.1	121.9	155.7	163.4
ZNF16	116	107.7	116.1	107.1	99.5	98.5	126.8	122.5
ZNF16	123.2	129.3	121.2	124.4	127.4	113.3	132.9	126.9
ZNF160	177.3	161.6	155.8	180.3	165.5	127.9	150.8	176.7
ZNF160	114.7	125.6	119.1	115.7	116.9	101.6	120.4	122.6
ZNF160	218	204.9	211.9	197.1	229.1	135.1	165.2	245.9
ZNF161	1245	1566.4	1018.5	1241.4	1123.7	866	1159.7	1218.3
ZNF165	126.9	128.6	125.1	136.5	122.4	108.1	141.8	156
ZNF167	128.7	125.5	133.7	127.8	110.5	108	124.7	116.1
ZNF167	115.8	114.9	116.6	120.1	106.1	97.8	112	117.9
ZNF167	113.4	118.4	112.3	113.5	110.2	104.9	112.6	120.5
ZNF169	128.9	150.8	141.9	143	132	117.9	145.4	136.3
ZNF17	256.8	298.5	286.2	286.7	243.1	210.1	238.5	236.7
ZNF174	137.7	139.3	127.5	131.7	114.6	123.8	129.9	142.4
ZNF174	119.3	143.2	132	133.7	118.2	108.2	139.1	121.5
ZNF174	132	125.8	126.6	126.3	117.9	111.8	119.7	123.5
ZNF175	152.1	142.2	145.7	149.5	139.8	125.9	150.5	140.8
ZNF177	127.5	115.7	125.5	129.1	121.3	113.8	142	141.7
ZNF179	122.3	138.8	120.3	142.5	120.8	112.2	134.3	140
ZNF18	174.4	221.8	251.6	223.1	179.9	215	207.3	208.8
ZNF181	124.4	137.4	127.1	132.9	120.9	114.2	127.5	128
ZNF182	253.5	291.9	237.9	256.4	270.5	183.7	221.3	236.1
ZNF184	125.1	119.6	118.6	119.1	130.2	109.7	122.5	121.2
ZNF187	111.9	108.4	116.3	119.6	116.2	102.5	125.8	109.9
ZNF187	152.9	151.9	182.2	163.1	150.5	138.4	133.9	152
ZNF189	226.7	238.1	227.5	237.4	250.6	217	260.9	254.7
ZNF189	103.1	110.7	108.2	114.2	109.5	94.4	120.6	119.6
ZNF19	112.3	120.1	113.5	126.2	101.9	95.9	110.4	111.2
ZNF192	120.8	125.6	121.4	137	116.9	103.4	121.4	114.7
ZNF193	144.6	137.3	156.4	165.4	128.6	131.7	135.6	125.4
ZNF195	223.5	217.1	208.9	227	218.9	158.8	240.4	249.4
ZNF197	129.9	142.4	128.1	138.1	115.9	119.4	129.7	130.2
ZNF197	123.8	119.3	114.6	115.3	109.9	98.6	117.4	113.4
ZNF197	135.3	128.4	145.4	144.7	129.8	127.8	132.6	130.8
ZNF2	146	134.8	144.2	137.4	139.4	129.2	145.1	128.1
ZNF2	104	110.4	111.5	109.2	104.1	100.3	129.4	105.5
ZNF20	186.1	208.1	172.2	208.6	172.4	137.2	182	178.3
ZNF200	104.9	105.8	116.5	122.3	108.5	102	115.9	112
ZNF200	149.4	153.6	160.9	144	139.9	128.8	127.4	130
ZNF200	217.9	237.1	196.7	223	203.7	171.5	236.7	232.9
ZNF202	130.5	148.9	127.6	147.7	142.6	115	160.3	151.6
ZNF204	135.9	135.6	135.3	136.7	106	107	120.3	115.8
ZNF205	115.2	112.8	113.8	120.8	108.4	97.9	119.6	119.6
ZNF205	122.2	120.9	121.9	118.7	111.9	102.1	117.9	111.2
ZNF206	120.6	113	118.1	119.9	106.1	96.7	116.9	115.6
ZNF207	497.9	560.6	510.6	582.8	528.6	389.6	538.8	483.5
ZNF207	637.5	737.1	616	860.6	596.5	591.4	843.5	672.2
ZNF208	109	108.4	113.7	115.4	111.6	93.4	117.1	113
ZNF21	133.4	131.9	126.1	133.9	118.4	103.8	134.2	127.1
ZNF211	265.8	291	235.7	286	265.1	217	242.6	223.1

ZNF212	294.7	316.7	288.6	333.5	260.6	233.5	359.6	342.1
ZNF213	159.8	186.9	187.4	197.4	149	151.6	177.1	148.7
ZNF214	114.5	125.5	114.5	115.7	105.5	93.3	113	120
ZNF215	123.8	110.3	113.7	117.5	112	102.1	120	116.6
ZNF217	313.3	449.9	425.5	451.3	399.5	314.2	278.1	348.5
ZNF218	275.4	212.5	246.6	255.7	166.8	244	291.7	207.9
ZNF219	228.8	212.4	149.3	216.1	164.1	136.8	201.4	157.3
ZNF22	496.4	624.7	633.9	560.2	572.7	391.7	524.3	527.4
ZNF221	108	119.3	115.7	118.7	106.3	99.6	120.3	116.2
ZNF222	129.2	119.8	122.9	124.5	110.7	104.8	127.3	111.5
ZNF223	357.6	431.8	436.7	426.5	392.5	202.7	513.5	420.6
ZNF224	162.6	175.7	168	185.6	145.3	147.7	164.3	152.8
ZNF225	156.1	162.4	144.6	145.5	155.7	132.1	151.9	140.5
ZNF226	130.7	149.7	138.8	132.2	124.6	107	121	128.2
ZNF226	122.1	131.8	146.7	134	110.2	109.3	126.6	132.1
ZNF227	143.6	162.8	128.4	145.3	126.9	125.2	149.5	151.5
ZNF228	150.4	117.9	119.2	142.7	117.7	106.2	137.5	125.9
ZNF229	113.1	113.7	113.5	114.4	101.4	98.2	117	112.6
ZNF23	140.7	124.6	159.1	133.2	128.7	110.9	127.4	127.2
ZNF230	128.5	131.3	131.4	143.4	132.1	121.4	130.7	126.4
ZNF232	173.6	166	171.4	162.7	171.8	137.2	156.6	154.8
ZNF233	113.9	106.6	113.9	120.8	98.1	94.2	110.4	113.5
ZNF234	249.4	192.1	194.6	247	187.6	141.6	219.3	236.2
ZNF235	192.7	221.8	187.6	209	192.4	161	204.4	175.7
ZNF236	136.2	135.9	124.5	148.5	126.4	111.4	146.2	133.4
ZNF238	123.8	117	118.1	136.2	113.3	107.4	126.8	122.8
ZNF238	109.7	115.8	116.6	116.8	110	101.8	111.1	111.1
ZNF238	123.8	126.9	118.3	122.6	120.5	98.9	122.3	124.1
ZNF239	160.3	158.1	177	172.7	148.5	124.2	157.9	134.7
ZNF24	142.2	156.6	148	172.8	156.4	136.9	153.4	166.5
ZNF248	137.6	135.4	138.4	152.2	132.9	126.3	136.8	122.6
ZNF25	199.3	215.8	235.1	232.7	198.7	188	183.3	201.6
ZNF250	151.4	138.6	141.6	152.7	153.2	123.6	148.1	146
ZNF253	106.7	116.2	118.2	111.7	113.5	103.4	123.6	111.5
ZNF254	132.1	136.6	116.6	125.4	130.7	108.8	130.9	127.7
ZNF256	166.7	197.9	183.7	190.2	195	141	188.7	228.6
ZNF257	113.4	111.7	116.1	122.1	106.7	99.3	122.1	109.1
ZNF259	155.9	180.3	168.9	162.6	136.4	148.6	153.8	154.1
ZNF26	187.1	210	198.6	210.2	187.3	150.5	195.2	171.2
ZNF263	246	294.5	231.9	226.2	240.8	224.4	297.2	290
ZNF264	230.8	237.7	272.3	235.9	253.9	216.2	265.1	287.4
ZNF265	164	174.6	176.8	182.9	171.6	136.4	174.6	194.3
ZNF265	582.1	404.3	654.4	435.8	469.6	375.4	408.2	452.2
ZNF266	247.1	280.3	252.8	324.7	288.3	213	235.3	334.7
ZNF266	180.1	192.4	182.4	193.1	185.3	169.3	181.2	292.1
ZNF266	121.1	110.8	122.3	131.9	111.4	103.6	113.9	117.3
ZNF267	115.8	112.7	129	124.5	107.9	95	113	117.1
ZNF268	120.9	106.4	118.8	118.7	115.7	100.7	115.7	117.6
ZNF268	107.6	112.3	108.5	120.3	106	90.4	111	119.5
ZNF271	158.7	189.4	192.6	203.7	167.9	166.8	234	258.4
ZNF273	124.4	121.1	116.1	121.6	104.8	99.1	125.5	115.5

ZNF273	107.4	116.4	110.4	116.2	114.2	101.4	119.6	113.2
ZNF274	465.7	408.4	537.8	457.3	438.6	454.5	471.8	515.5
ZNF274	116.3	123.3	123.4	132.5	112.9	107.8	132.1	118.5
ZNF274	198.9	192	205.8	178.4	156.4	169.4	175.3	197.1
ZNF275	403.6	395.2	367.2	404.8	313.8	248.9	461.8	462.2
ZNF275	122.3	115	121.5	119	111.5	98.6	117.9	113.9
ZNF276	121.2	153.9	150.1	146.8	124.7	134	153.8	144.4
ZNF277	442.2	484.5	563.9	477.1	410.9	421.9	403.7	430.2
ZNF278	117.8	121.8	123.2	126.2	110.5	114.5	118.2	106.9
ZNF278	110.6	113.6	107.4	114.8	106.4	99.4	115.8	116.1
ZNF28	125.7	123.9	122.6	124	110.8	100.7	124.2	111.6
ZNF284	104.8	122.2	114.9	114.6	102.3	97.4	104.4	114.8
ZNF285A	141.2	132.2	145.3	143	133	125.8	121.9	115.8
ZNF286	118.6	132	132.8	134.5	133.7	107.3	133.1	128.9
ZNF287	109.6	119.6	124.2	119	108.7	107.1	108.3	118.1
ZNF289	205.1	245.1	253.5	199.9	159.5	196	223.7	193.8
ZNF291	157.6	159.1	151.7	182.2	137.3	130.4	181.3	191.6
ZNF294	121	131.8	135.1	146.1	116.2	117.5	122.4	131.4
ZNF295	207.8	205.2	194	242.7	215.6	167.7	348.7	412
ZNF297B	205.9	181.9	174.5	201.6	193.2	130.9	242.9	291.7
ZNF3	136.5	143.2	149	136	144.3	115.6	141.1	134.6
ZNF3	131.1	132.5	142	154.3	129.8	123.4	144.1	135.2
ZNF3	147.2	142.4	147.4	137.3	123.9	116.4	140.1	130.5
ZNF3	116.2	111.3	112.6	112	106.3	96.9	111.3	117.6
ZNF30	223.1	236.4	228.7	221.2	188.4	187	170.1	178.1
ZNF300	105.3	105.8	116.3	116.3	107.2	103.3	124.9	115.2
ZNF302	107.9	114.9	118.9	120.6	124.2	100.4	120.3	115.5
ZNF304	127	133.1	137	135.8	123.3	125	129.8	132.7
ZNF306	128.2	139.2	154.3	136.3	125.3	112.5	136.2	121.8
ZNF307	175.3	183.6	190	185.6	155.1	128.9	159.6	144.3
ZNF31	115.4	125.9	119.1	127.9	114.4	103	121.4	121.9
ZNF311	103.7	105.8	103.7	113.3	114.7	95.4	119.7	112.2
ZNF312	121.4	115.1	120.7	131.3	116	100.8	113.6	123.3
ZNF313	550.9	512.7	780.3	726	530.6	495.8	694.1	851.3
ZNF317	187.3	240.8	190.7	213.6	153.7	157.5	195.9	217.6
ZNF318	171.9	167.7	146.3	183.8	133.3	119.7	165.2	161.6
ZNF319	278	274.1	266.1	310.2	253.5	220	345.7	282.1
ZNF32	117.7	114.8	126.7	123.3	118.8	107.4	129.6	116.2
ZNF32	232.5	218.6	200.1	200.6	193	189.1	223.1	199.8
ZNF32	151.4	153	173.5	155.2	153.7	131	141.9	129.2
ZNF320	139.8	137.2	131.7	137.4	123.6	104.2	126.4	137.2
ZNF322A	124.4	124.3	143.5	120.6	129.2	113.2	139.1	132.5
ZNF322B	127.4	109.4	117.7	118.1	126.4	105.7	125.1	115
ZNF323	126.6	118.7	127.2	124.5	115.2	107.3	118.6	115.8
ZNF323	104.4	109.9	106	111.6	97	94.2	115	111.7
ZNF324	252.7	264.6	253.2	288.3	195.7	220.5	301.6	326.8
ZNF326	115.6	124	125.6	129	119.3	106.6	137.8	138.7
ZNF326	116.7	126.8	121.1	115.1	110.9	103.9	131.8	119.2
ZNF326	134.3	133.8	141.1	143.4	140.3	108.6	155.5	167.3
ZNF329	232.4	256	276.8	235.7	257.8	287.3	315.7	314.4
ZNF330	180	163.6	235.8	169.5	164.7	156.7	172.6	196.9

ZNF331	143	150.7	173.2	149.9	130	158.1	154.8	137.9
ZNF331	116.5	113.5	95.5	111.3	109.4	99.5	110.3	108.3
ZNF331	134.5	150.4	140.3	167.4	129.8	148.5	145.5	153.5
ZNF333	155.4	143.7	145.3	163.7	123.3	128.1	129.4	136.7
ZNF334	119.2	104.4	119.4	116.5	110.3	99.4	117.9	112
ZNF334	111	118.9	113.6	126.3	108.7	103.7	119.9	114.7
ZNF335	142.2	135.8	128.3	140.4	122.5	101.7	156.2	139.7
ZNF336	122.6	126.8	126.2	130	118.8	101.6	123.9	136.4
ZNF337	322.1	335.6	335.6	272.2	274	310.1	327.6	300.5
ZNF33A	191.9	163.1	174.9	195.9	168.9	147.2	152.6	169.8
ZNF33A	146.8	143.4	163.6	146.9	145.2	120.5	141.8	133.4
ZNF34	146.3	159.4	150.5	152.7	126.7	122	149.8	120.2
ZNF341	172.4	154.1	138.7	171.4	145.6	143.2	175.4	143.2
ZNF342	221.6	212.1	213.1	211	168.5	170	201	186.3
ZNF343	127.6	118.5	125.8	117.5	117	113.6	120.9	115.2
ZNF345	114.2	124.8	121.8	126.2	107.9	104.6	121.4	124.1
ZNF346	144.5	159	134.8	138.9	127.6	111.5	150.2	136.4
ZNF347	119.9	116.6	123.7	122.4	118.7	104.5	124	126.4
ZNF35	148.6	163.7	150.5	162.3	134	127.5	161.2	146.1
ZNF350	152.9	158.8	184.7	160.1	152	153.8	155.5	159
ZNF354A	150.4	170.6	142.2	152.5	136.2	120.3	163.5	169.8
ZNF354B	127.7	120.9	121.5	118.5	111.3	99.2	123.8	117.5
ZNF354C	112	109.7	120.4	113.5	104.8	91.9	123.5	108.5
ZNF358	245.6	242.9	208.5	247.9	183.5	149.4	203.4	223.1
ZNF364	556.7	585.2	481	656	504.4	321.5	664.2	686.1
ZNF365	113.4	112.7	114.1	125.8	106.1	97.9	113.6	118.9
ZNF365	119	113	114.5	129.4	107.7	103.6	114.8	102.9
ZNF365	109	119.4	115	118.7	117.6	105.1	117	113.4
ZNF365	117.5	132.5	120.5	129.2	121.1	105.6	125.6	125
ZNF366	120.8	133.7	134.7	128.5	128.1	109.4	130.9	123.7
ZNF367	112.6	105.9	113.6	121.6	118.7	94.8	119.3	110.9
ZNF37A	107.1	118.3	110.1	116.9	104.2	95.6	108.7	98.1
ZNF37A	134.1	117.4	128.2	130.3	113.6	111.9	123.2	117.6
ZNF37A	138.7	146.1	129.9	165.4	130.1	112.6	137.6	140.6
ZNF382	112.9	115	112.1	127.7	98.6	102	117.1	113.7
ZNF383	165.1	163.4	146.8	145.2	162.9	124.3	163.6	147.3
ZNF384	117.7	121.4	122.9	114.6	109.5	101.2	130.8	129
ZNF384	177.3	186.2	176.4	194.4	156.7	144.4	179.4	182
ZNF385	141.3	146.9	146.5	197.9	127.5	139.2	222.3	145.3
ZNF394	1000.6	933.7	1057	807.4	1000.5	619.8	1101.5	1229
ZNF395	347.3	436.5	404.5	369.6	300.2	259.7	371.9	331.2
ZNF396	116.7	116.2	121.2	119.5	113.9	104.3	122	112.9
ZNF397	127.9	136.4	147.5	137.3	127	110.1	132.1	126.1
ZNF398	116.3	127.3	126.1	122.9	118.6	105.3	127.3	119.6
ZNF398	211.9	212	154.9	181	173.5	148.4	207.7	176.7
ZNF403	806.8	934.4	880.9	920	859.6	721.4	874.3	835.2
ZNF404	129.7	123	126.7	120.5	109.2	109.4	129.2	112.4
ZNF406	124.7	117.9	114.9	120.4	111.1	98.9	120	124.7
ZNF406	109	108.9	128.2	118.4	122.5	111	121.9	115.5
ZNF407	112.4	107.7	121.2	112	106.2	96.1	110.9	110
ZNF408	132.4	149.3	129.6	136.9	117.5	126.5	141.9	140.2

ZNF41	111.8	120.1	117	127.5	116.9	102.9	121.4	117
ZNF41	119.2	119.6	119.1	120.9	114.5	110.1	118.7	118.7
ZNF41	120.2	124.8	126.3	116.8	105.5	104.9	123.5	119.7
ZNF410	249	302.1	307.1	324.8	246.8	296.6	345.7	427.8
ZNF414	127.1	125	126.5	121.4	115.9	97.8	127.2	111.4
ZNF415	124.6	125.4	133.3	141.5	136.8	116.4	132.7	127.9
ZNF416	139.4	146.6	144.1	154.9	130.5	122.1	143.2	153.3
ZNF417	119.9	127.3	124	128.5	122.5	108.8	127.8	126.3
ZNF418	119	141.2	127.6	123.5	120.4	102.3	121.9	122.8
ZNF419	225.5	240.5	257.7	238.5	211	184.1	196.7	208.4
ZNF42	525.3	501	524	457	362.1	260.7	407.2	342.1
ZNF42	102.7	118	124.7	116.6	118.6	110.7	123.7	121.5
ZNF420	129.3	128	125.5	125	116.1	99.3	136.9	131.5
ZNF423	118.5	125.1	115.6	113.9	104.3	104.1	121.6	114.9
ZNF425	130.4	128.7	122.7	145.9	129.4	101	152	135.1
ZNF426	146	171.7	190.8	166.1	136.9	146.5	161.6	192.9
ZNF429	114.9	119.7	128	121.7	104.8	102.2	114.2	115.7
ZNF43	115.2	116.8	125.6	117.3	105.5	96.9	117.1	116.5
ZNF430	2315.2	1585.2	1794.6	1685.2	1600.8	1235.1	1614.1	2336.2
ZNF431	139.9	128.7	151.5	135	128	117.9	129.9	129.6
ZNF432	131.6	156.1	162.1	156	122.4	135.4	146.8	154.5
ZNF433	115.5	122.2	122.1	126.7	108.7	98.4	128.8	125.1
ZNF434	190.8	242.1	232	233.2	221.9	176.3	235.1	258.8
ZNF435	264.8	277	245.4	307.7	260.9	228.5	228.7	206.7
ZNF436	118.5	109.3	118.6	116.3	113.2	99.3	125.7	119.1
ZNF436	116.6	111.9	114.3	122.1	107.2	94.1	118.9	117.5
ZNF436	115.6	129.6	148.2	120.6	122.9	111.3	119.5	133.1
ZNF438	154.1	139.9	155.4	169.9	139.3	144.4	200.3	179.1
ZNF439	229.3	235.2	195.9	230.3	232.9	170.5	184.7	210.4
ZNF44	115.4	115.4	121.2	121.2	103.7	98.4	117.8	110.3
ZNF440L	135.9	159.5	162.8	156	153.3	125.7	150.5	164.1
ZNF441	117.9	112.1	123.5	122.8	114	100	115.9	117.9
ZNF442	133.8	130.9	132.9	128.6	121.8	110.5	128.4	135.1
ZNF443	185.2	218.3	181.7	194.2	166.8	154.9	186.6	180.9
ZNF444	178.8	205.2	196.1	180.8	163.3	161.4	179.9	163.2
ZNF445	118.9	120.2	121.2	128.6	106.8	93.4	107.5	114.7
ZNF446	163.9	183.5	145.5	158	136.7	146.5	153	157.8
ZNF447	352.9	392.5	694.6	422.2	403.6	523.9	356.9	412.6
ZNF449	115	121.3	115.6	129.3	120.7	107.3	125.3	113.7
ZNF45	186.2	173.6	199.2	184.9	191.5	153.8	179.2	190.8
ZNF451	114.3	117.3	119.7	127.2	106.6	99.8	122.1	118.9
ZNF451	320.2	279.6	327	312	296.9	311.7	355.6	283
ZNF451	134.6	142.7	135.4	136.9	137.7	126.3	135.2	125.1
ZNF452	115.8	111.1	113.9	115.7	105.8	99.6	113.7	116.4
ZNF454	124.8	123.5	126.9	118.2	116.1	111.3	130.1	124.8
ZNF460	119.6	128.7	138.6	127.4	122.2	109.5	131.7	118.5
ZNF462	121.1	119.3	106.6	127.9	112.9	99.9	118.7	115.4
ZNF467	164.7	164.4	208.8	194.2	138.8	146	169.4	163.9
ZNF468	109	107.7	118.8	117.3	102.8	94.9	121.7	103.2
ZNF471	118.9	116.6	116.5	119.8	120.5	95.1	121.6	133.4
ZNF473	155.6	156.4	164.4	158.5	132.5	135.2	181.9	173.5

ZNF473	120.1	121.9	122.6	118.8	109.3	101.1	110.9	116.9
ZNF474	112.9	110.7	113.8	118.1	115.3	96.4	120.5	108.2
ZNF480	157.3	157.9	197.9	158.7	165.7	167.1	171.7	169.6
ZNF482	130.3	133	155.8	150.7	124.1	120.3	146.4	135.4
ZNF483	111.4	113.4	133.5	120.8	112.4	104.3	118.7	116.5
ZNF483	376.2	325.5	414.1	337.3	316.3	241.2	427.5	336.5
ZNF483	129.1	131.5	123.1	127.2	126.2	107.4	135	132.8
ZNF484	159.9	163.9	158.8	170.1	157.3	131.5	175.4	171.7
ZNF485	114.9	115.9	122.5	126.9	116.4	107.7	118.4	119.4
ZNF490	121.6	122.6	118.8	115.6	118.5	109.4	123.6	116.6
ZNF491	111.5	106.8	108	120.7	110.9	93.1	115.6	121.6
ZNF493	141.5	140	135.3	128	132.6	110.8	132.3	124.2
ZNF493	128.2	124.9	124.1	127.6	111.4	113.4	135.2	127.1
ZNF496	111.8	111.8	115.2	122.2	114.1	99.1	116.4	112.4
ZNF497	110.4	120	121.9	135.2	114.2	101	108.8	115.1
ZNF498	127.3	134.5	139.4	139.3	135.4	118.6	146.1	133.8
ZNF499	166.4	141.9	154	174.1	144.3	137.3	157.9	132.4
ZNF500	211.3	227.3	189.5	234.2	173.1	177.4	179.3	186.6
ZNF501	104.3	117.9	118	112.3	113.3	107.6	108.9	102.4
ZNF502	123.2	130.1	124	125	116	114	128.2	130.9
ZNF503	118.3	120.5	142.8	113.7	105.3	102.8	118.8	112.2
ZNF507	124.9	121.7	126.7	127.2	116.7	104.3	118	122.8
ZNF508	222.9	239.3	215	238	236.2	173.7	273.5	317.5
ZNF509	183.2	202.5	159.8	194.8	181.7	140.4	241.4	219.5
ZNF510	116.7	121.9	123.2	118.7	109	104.9	109.8	122.5
ZNF511	259	304.6	258.8	270.1	216.3	199.1	200.5	221.6
ZNF512	551.9	594.6	595.5	516	574.7	450	405.4	395
ZNF513	138.9	138	151.5	145.7	134.5	116.2	146.5	127.2
ZNF514	182.3	179.6	213.1	195.6	149.7	134.5	194.1	180.7
ZNF517	107.1	110	111.4	120.5	117.6	99.7	115.2	115.9
ZNF518	162.1	160.9	171	179.7	168.6	139.9	153.4	159.3
ZNF519	111	115.6	116.6	116.4	106.7	101.7	118.8	118.4
ZNF519	120.8	125.7	122.6	125.2	109.5	104.6	119.8	113.3
ZNF521	117.6	119.3	111.6	113.5	109.2	97.2	118.3	114.8
ZNF524	451.1	415.7	422.2	412.9	305.4	334.8	396.1	356.5
ZNF526	152.8	135.1	144.8	143.1	138	118.2	141.1	142.9
ZNF528	108.2	107.6	116.8	118.5	106.2	93.5	114.9	115.8
ZNF529	250.3	321.8	336.5	308	269.9	264.3	309	255
ZNF530	118.6	124.9	117.4	120.6	121.7	100.8	119.7	117.7
ZNF532	121.3	106.4	143.6	140.5	121.6	102.8	134.2	132.4
ZNF533	107.3	109.9	113.2	113	107.2	98.5	112.4	112.6
ZNF536	113.2	120.5	107.4	116.3	106.5	98.6	117.7	120.2
ZNF537	132.4	124.2	166.8	150.1	125.2	137.3	146.8	132.9
ZNF539	116.3	118.7	127.8	131.4	112.9	105.7	125.7	123.2
ZNF540	139.2	150.5	146.5	151.3	126.8	107.1	126.5	124.7
ZNF542	116.3	115.5	121.4	120.8	106.9	94.4	116.8	124.8
ZNF543	139.4	163.8	140.7	155.4	136.6	128.7	140.3	157.1
ZNF544	113.5	108.8	120.6	128.7	117.7	102	116.7	112.3
ZNF545	183.6	193.8	164.6	222.5	170.9	155.2	190.7	176.3
ZNF546	109.5	109.6	111.8	114	108.7	101.3	113.4	113
ZNF547	113.5	119.6	112.6	124.6	113.9	106.6	116.4	117.2

ZNF548	257.9	248.2	277.1	263.5	240	202.9	263.8	256.5
ZNF549	5995.6	4764.5	5774.1	4859.1	6031	3568.6	5647	5238.7
ZNF550	118.2	124.1	123.7	122.6	107	106.9	126.7	114.9
ZNF551	147.1	156.7	134.5	159.3	130	115	136.9	143.3
ZNF552	119.4	129.8	122.7	122.4	113.7	98.2	114.8	117
ZNF553	108.4	110.7	121.4	122.8	108.1	101.7	130.6	122.8
ZNF554	116.3	116.6	119.2	117.5	105.4	93.2	122.4	124.5
ZNF555	121.6	117.8	121.8	123.8	113.9	111	119.1	117.4
ZNF556	104.3	111.9	116.3	115.3	106.7	109.2	119.8	127.5
ZNF557	118.1	131.2	109.3	130.5	111.3	102.4	122.5	117.7
ZNF557	239.4	159.4	208.1	196.8	174	136	178.2	231.2
ZNF558	147.2	146.8	146.5	155	126.7	125	128.2	132.5
ZNF559	194.1	185.8	233.6	247	193.9	199.4	257.1	259.6
ZNF560	109.2	116.3	118	120.7	109.3	100.5	119.9	122.3
ZNF561	122.6	126	118.2	119.8	104	106.1	129.6	120
ZNF562	195.9	208.5	206	216.3	216.7	179.7	260.5	245.3
ZNF563	115.6	129.5	121.1	137.3	125.5	111.5	116.8	127.8
ZNF564	315.5	306.2	296	297.9	275.7	224.2	279.1	229.5
ZNF565	140.7	133.6	136.2	157.5	127.2	117.7	170.4	152.4
ZNF566	136.1	160.2	123.2	129.2	132.4	106.3	125.2	145.4
ZNF567	123.4	121.2	115.5	121.1	120.5	95.9	114.7	133.3
ZNF568	126.4	128.6	119.2	126	118.4	96.8	116.3	113.7
ZNF569	113.9	113.2	123.7	123.2	102.3	102.5	115.9	123.8
ZNF570	125.1	127.5	116.1	125.4	110.2	119.3	127.5	131.2
ZNF571	148.7	150.9	160.6	172.6	116	123.2	143	145.9
ZNF572	111.9	117.8	118.6	128.5	103.9	95.9	121.3	110.9
ZNF573	232.5	281.2	282.4	281.4	295.6	170.7	232.7	228.7
ZNF574	140.4	138.8	121.5	136.9	126.1	110.9	136.5	135.2
ZNF575	116.4	119.6	123.5	128.8	114	108.8	119.9	109.9
ZNF576	162.2	155.4	149.8	166.2	136.8	122	149.2	144.2
ZNF577	386.4	254.2	272.8	260.9	319.1	255.9	437.2	443.8
ZNF579	117.8	116.2	126	123.8	117.3	102.4	130.3	131.8
ZNF580	117.5	126.5	147.4	125.4	109.4	106.2	138.3	123.6
ZNF580	171.3	157	182.3	177	146.1	140.3	169.4	157.4
ZNF580	122.2	121.6	129.5	128.2	116.2	117.4	116.8	114.8
ZNF581	825.4	770.2	515.9	769.7	436.8	385.5	763.1	591.6
ZNF582	133.7	123.1	127.7	120.3	117.1	109.2	112.9	126.8
ZNF584	116.2	118	119	124.7	122.8	99	120.1	120.8
ZNF585A	153.6	209	262.7	212.5	225.4	183.7	182.6	193.2
ZNF585A	162.2	165.1	173.7	184.6	163.2	130.2	164.1	169
ZNF585B	113.3	121.4	128.2	126.7	130.3	100.6	127.1	125.7
ZNF586	365.6	516.3	437.5	423.5	343.6	256	423.8	406.4
ZNF586	475.2	620.6	547.1	532.9	381.7	346.6	461.7	552.9
ZNF586	121	115.9	121.6	126	115.7	111.3	117.6	131.5
ZNF587	107	112.9	116.3	114.5	106.1	103.9	117.6	109.8
ZNF588	105.1	112.9	107.8	109.4	99.6	102.3	107.6	104.4
ZNF589	248.8	274.9	304.9	233.2	208.5	185.7	189.8	150.5
ZNF592	114.4	118.2	119	128.3	109.5	102.4	122.3	113.9
ZNF593	176	183.6	176.9	185.7	157.9	133.1	168	157.6
ZNF595	118	105.8	104.1	104	103.8	95.5	115.2	112.7
ZNF596	109.5	110.6	118.2	123.5	113.1	104.5	121.2	106.2

ZNF596	110.9	117.5	113.5	108.7	109.7	93	117.1	120
ZNF597	136.7	121.1	128.7	144.6	123.5	115.5	124	121
ZNF598	428	442.1	274.5	456.5	326.3	291.9	369	369.1
ZNF599	117.6	120.5	119.9	123.9	122.3	106	125.1	131.7
ZNF599	118.5	115	124.9	115.4	111.4	103.5	121.2	123.6
ZNF6	117.1	126	120.6	117	106.5	106.2	108.5	117.1
ZNF600	131.5	137.8	143.9	138.5	122.5	112.8	140.8	129.1
ZNF606	125.4	128.8	121.9	125.9	109.1	101.2	125.3	119.3
ZNF607	115.4	123.7	107.3	120.5	107.3	92.9	114.2	109.3
ZNF608	108.7	119.6	111.6	114	102.6	100.8	112.5	119.1
ZNF609	161.4	166.7	156.2	176.9	153.4	145.1	196.3	186.6
ZNF610	103	105.7	109.1	115.6	104.9	91.6	109	109.3
ZNF611	128.8	116.6	143.2	135.5	111.4	110.6	125.5	129
ZNF613	222	264.1	228.8	242.5	215.6	196.6	212	236.6
ZNF613	117.7	120.4	119.5	122.8	110.8	99.3	115.6	114.9
ZNF614	171.8	204.7	211.3	184.8	167.8	150.6	188	185.7
ZNF615	210.4	264.8	228.4	230.8	238.7	200.7	239.9	313.1
ZNF616	146	160.7	160.8	173.5	149	135.4	174.1	187
ZNF618	142.2	128.9	128.8	123	118.8	111.9	137.7	128.3
ZNF619	113.7	110.7	113.4	111.9	98.4	94.4	110.7	112.2
ZNF620	121.5	112.4	119.2	126.2	108.5	100.4	123.3	118
ZNF621	214.4	234.5	221.4	260.2	198	172.2	214.7	238.3
ZNF622	361.7	383.7	319.7	435.7	323.7	277	419.6	479.5
ZNF623	115.7	111.9	110	113.6	103.4	98.2	126.7	118.5
ZNF624	117.4	137.4	138.8	139	119	103.8	127.7	124.3
ZNF625	115.6	115.6	118.7	123.2	113.7	96.1	126.5	109
ZNF626	133.5	137.9	171.5	157.6	140.1	129.1	137.4	151.7
ZNF626	139.3	123.7	156.8	148.9	135.4	111.7	132.8	129
ZNF626	131.7	132.9	134.7	134.3	120.5	113	134.7	137.8
ZNF627	279.3	263.8	287.3	273.7	218.9	241.2	248.2	249.1
ZNF628	121.5	133.7	122.3	121.1	116.9	104.9	131.3	121.9
ZNF629	125	116.5	124.9	119.2	110.9	98.7	122.3	122.1
ZNF629	123.8	123	124.1	123.9	91.9	90.8	121.8	115.4
ZNF630	135.4	117.3	138.5	131.5	112.4	106	131.7	130.1
ZNF630	124.2	127.5	138.1	126.2	118.2	114.5	134.7	139.7
ZNF639	114.2	98	113.1	108.3	102.8	86.6	123.7	107.5
ZNF641	237.8	219.7	224.8	214.3	166.7	139	152.8	196.7
ZNF642	122.9	141.3	116.2	123.7	117.9	109.9	118.6	115.5
ZNF643	124.1	128.8	125	126.7	118.8	103.4	127.5	126.3
ZNF644	111.9	118.8	111.5	121.9	103.5	98.7	121	127
ZNF644	258.6	263	266.1	314.2	250.8	226.4	336.3	299.8
ZNF644	136.2	145.3	143.4	143.9	140.3	111.8	164.9	180.4
ZNF645	114.7	118.9	114.7	114.4	104.4	96.4	125.7	106.5
ZNF646	138.6	123.4	137.4	127.6	114.2	106.8	137.6	135.1
ZNF648	124.8	118.6	123.6	120.9	109.2	103.3	121.8	108.8
ZNF649	120.2	137.6	134.8	126.1	127.2	111.1	134.3	124
ZNF650	223.2	249.7	265.3	250.2	232.8	168.9	227.1	191.5
ZNF651	117	108.4	121.9	112.3	103.1	109.2	115.4	106.8
ZNF652	1071.9	995.2	825.6	1031.8	810.6	456.2	967	1144.4
ZNF653	340.9	313.4	249.4	340.8	270.4	221.7	284.1	320.2
ZNF654	123	137.6	141.5	149.5	128.5	113	138	147.8

ZNF654	159.2	177.9	174.4	230.1	178.1	129.5	234.4	236.1
ZNF654	164.6	177	155.5	198.7	169.6	129.9	180.6	256
ZNF654	131	136.8	133.7	142.4	136.1	106.5	130.6	136.3
ZNF655	129.1	108.6	125.9	129.5	123.5	109	128.9	133.3
ZNF655	127.5	121.9	141.9	130.1	125.8	107	116.7	119.6
ZNF655	110.4	123.2	142.6	124.8	110.5	109.9	128.5	119.2
ZNF655	178.6	176	189.9	185.8	165.2	126.5	167.6	172.8
ZNF658	118.3	130.7	119.8	115.7	113	102.4	108.9	117.1
ZNF658B	108.2	120.7	114.3	113.4	110.2	93.2	116.1	107.5
ZNF659	120.5	113.9	123.1	127.4	114.9	100.9	124.4	120.6
ZNF660	152.2	137.4	154.9	162.5	159.7	133.7	144.9	162.2
ZNF662	125.1	132.5	110.6	124.7	112.2	94.5	131.2	120.5
ZNF664	137.3	129.1	142.4	147.1	128.6	107.9	142.9	126.2
ZNF665	114.9	110.5	120.6	122.8	116.7	112.8	114.5	119.2
ZNF667	119.2	118.3	121.5	124.9	111	103	122.5	123.4
ZNF668	245.2	230.8	172.4	257.9	198	157.1	260.5	272.6
ZNF669	255.8	214.1	244.3	248.3	272.4	149.2	242.2	251.5
ZNF670	118.7	112.1	112.3	112.4	118.2	96.8	117.8	116
ZNF671	257.9	291.7	304.6	278.4	236.4	232.7	254.3	270.2
ZNF672	383	403.5	337.3	461.3	321.8	278.3	537.4	498.1
ZNF673	121.6	129.1	115.6	121.9	113.1	97.9	113.9	131.7
ZNF675	114.5	119.4	109.6	122.1	113.5	101.2	113.5	115.9
ZNF677	114.5	121.5	113.3	119.2	110.1	101.7	122.2	118.9
ZNF678	104	105.6	97.5	111.5	105.6	96.7	109.9	111.6
ZNF680	148.4	161	140.7	155.7	131.5	121.2	136.7	130.3
ZNF681	125.5	121.1	124.3	116.2	127.9	122.9	129.2	123.9
ZNF682	127.6	109.7	121.8	120.9	110.6	102	121.2	115.4
ZNF682	130.4	137.5	131.2	137	130.2	106.1	127.1	131.7
ZNF682	1191.3	660.9	748.4	709.6	860.7	398.7	1000.3	1127.4
ZNF683	315.6	182.2	545.7	320.9	175	353.4	189.2	158.9
ZNF684	161.2	153.3	156.8	164	164.3	119.8	184.2	163
ZNF687	139.8	127.4	134.1	134.3	113.7	117.2	138.2	119.9
ZNF688	158.5	165.3	168.2	181	136.9	130	165.7	151.9
ZNF688	122.9	116.4	111	127.7	112	108.5	120.7	113.6
ZNF689	511.1	524.3	432.1	503.9	393.8	264.8	453.2	495.4
ZNF69	757.3	811.6	1002	795.3	936.6	487.4	835.2	839.5
ZNF690	122.5	121	129.6	135.7	123.5	109.6	130.9	118.4
ZNF691	132.7	125.8	116.7	136.2	116.1	110.1	118.5	120.1
ZNF692	283.6	293.6	248.7	251.1	199	192.1	257.6	208.7
ZNF694	165.8	175.2	171.2	165.6	149.2	134.7	162.8	168.6
ZNF695	107.2	113	122.6	113.3	112.2	99.5	125.4	115.5
ZNF696	169	176	154.6	164.9	157.9	134.2	145.7	154.5
ZNF699	127.2	115.5	122.9	130.1	108.2	108.8	128	118
ZNF7	220.1	232.9	269.9	251.6	206.2	214	233.1	203.6
ZNF70	108.1	109.5	112.6	120.4	100	98.6	102.2	110.6
ZNF700	409.9	407.6	407.5	439.4	394.9	286.2	384.6	382.3
ZNF701	129.1	133.4	142.6	138.1	125.7	129	124.1	130.6
ZNF702	121.4	142.5	119.7	124.8	131.8	105	138.3	136.3
ZNF703	118.7	119.5	125.1	122.4	109	102.4	120.4	127.7
ZNF703	117.5	110.3	121.5	121.3	107.6	96.9	120	117.3
ZNF704	117.3	117.4	124.8	118.4	112.1	104.7	113.1	124.5

ZNF706	141.2	151.1	146.5	150.6	144.5	130.3	150.5	158.6
ZNF706	112.4	113.7	116.7	117	110.7	98	117.5	110.2
ZNF706	150	142.2	167.5	177	149.1	122.9	171.6	169.6
ZNF707	162.5	166	142.6	182.6	143.3	126.3	178.5	167.4
ZNF708	125.3	136.1	136.7	128.3	111.8	120.9	131.4	120.8
ZNF709	109	115.8	123	125.7	112.4	98.7	124.8	116.7
ZNF71	111	116.3	124.3	129.4	118.3	94.1	116.7	114.5
ZNF710	108.7	119.4	122.5	116.5	105	97.7	110.2	109.9
ZNF713	111.9	120.4	119.1	116.6	103.5	98.7	119.7	118.3
ZNF714	119.3	121.8	118.3	126.6	106.4	105	125.6	123.8
ZNF718	127.6	120.5	130.2	131.6	122	108.8	124.9	124.3
ZNF721	315.2	322.6	330.9	338.7	388.7	268.6	340.7	412.8
ZNF74	109.7	116.3	109.2	114.8	113.5	97.9	113.8	110.8
ZNF74	130.8	123.7	129.7	126.2	110.8	100.5	127.1	115
ZNF74	154.3	173.4	144.8	177.4	146.3	114.2	149.8	150
ZNF740	134.2	135.9	132.1	125.1	118.1	105.8	141	123.5
ZNF749	110.2	122.7	117.1	114.4	100.8	95.4	112	115
ZNF75	131.8	194.7	122.1	128.2	168.9	103.4	126.1	189.5
ZNF75A	154.2	145.6	141.9	139.5	129.4	112.8	157.4	155.6
ZNF76	179.5	171.1	163.9	164.3	143.1	130.8	169.8	166.7
ZNF765	107.9	106.6	108.2	111.5	106.1	90.1	105.9	107.2
ZNF767	204.3	239.6	211.3	231	193.7	171.8	212.4	193.6
ZNF77	133.8	134.1	140	139.1	133.8	118.2	137.4	140.9
ZNF781	120.7	121.8	119.2	123.7	111.5	96.9	115.9	119.4
ZNF79	125.2	144.8	131.2	149.3	120.2	108.7	132.5	117.5
ZNF8	122.8	121.9	134.8	124	114.3	109.5	122.1	132.9
ZNF80	148	153.6	146.4	147.4	135.4	114.5	134.2	141.6
ZNF805	147.8	141.4	145.6	161	132.3	125.5	135.1	158.4
ZNF808	116.2	120.9	113.9	117.9	115.1	101.9	121	114.8
ZNF81	118.5	121.3	111.3	129.3	113.4	99.7	110	113.7
ZNF83	176.1	238.5	228.3	200.8	201	156.4	164.3	207.5
ZNF85	131.3	123.8	123.5	114.8	106	111.8	113.7	111.2
ZNF91	230.1	224.6	245.4	226.3	234.8	245.1	222.4	190.3
ZNF92	125.2	118.7	129.9	124.7	110.2	98.7	124.2	118
ZNF92	121.1	111.9	115.2	127.5	110.1	108.9	113.3	110.6
ZNF93	161.5	170.6	198.5	153.1	137.2	133.5	178.1	166.9
ZNF93	128.3	128.6	115.9	114.2	105.5	105.5	132.4	116.8
ZNF96	117.9	124	126.1	127	112.1	112.5	135.4	121
ZNF99	127.5	108.9	115.2	113.2	106.5	97.6	118.5	119.6
ZNFN1A1	583.7	851.4	512	674.7	545	306.5	541.6	678.5
ZNFN1A2	134.1	120.6	125.8	127.7	113.3	110.9	136.3	130.2
ZNFN1A2	123.4	126.8	137.1	124.9	115.4	96.7	113.3	117.6
ZNFN1A3	137.8	140.7	138	140	136.1	113.3	130.8	134.6
ZNFN1A4	129.2	139.5	132.6	134.1	122.8	117.8	143.8	131.5
ZNFN1A5	198.4	211.2	208.2	210.8	188.1	177.7	234.3	261.4
ZNFX1	427	396.9	382.6	448.4	380.4	333.2	510.3	611.5
ZNHIT1	415.7	439.4	511.1	445.6	342.3	386.6	393.1	341
ZNHIT2	130	141.1	136.6	154.7	127.4	115.2	132	129.3
ZNHIT3	1478.3	1736.4	1588.5	1482.7	1474	1239.2	1258.5	1790
ZNHIT3	110.8	122.2	126.1	116.6	110.3	96	118.1	110.1
ZNHIT3	215.2	254.7	282	244.4	191.8	194	176	201.1

ZNHIT4	180.8	174.6	149.6	175.7	152	136.8	176.4	157.8
ZNRD1	218.1	252	206.3	213.7	203.5	152.9	188.5	242.6
ZNRD1	327.5	418.6	332.8	367.7	321.4	240.2	316.4	356.9
ZNRD1	239.4	299.6	301.5	293.8	227.2	242.5	301.5	254.7
ZNRF1	116	124.7	130.8	130.3	118.2	108.4	127.8	120.1
ZNRF2	110.4	123.2	125.7	129.8	112.8	104.2	129.2	118.3
ZNRF4	119	116.3	118.5	113.8	100.6	98	129.9	105
ZP1	110.9	120.1	117.8	121.8	103.6	97.1	117.3	115
ZP2	120	118.7	115.1	127.4	109.3	114.8	120	112.6
ZP3	111.6	116.9	125.4	119.8	105.8	102.7	120.9	116.6
ZP4	102.6	109.7	113.4	112.7	96.6	95.7	111.2	102.6
ZPBP	116	116.2	114.2	107.3	105.9	100.3	115.1	111.7
ZPBP2	117.1	106.7	118.3	122	107.1	103.9	105.8	115.5
ZPBP2	109.3	122.7	115.5	114	106.1	102.2	115.7	115.6
ZPBP2	113.8	109.2	113.8	124.6	107.4	97	117.9	111.6
ZPLD1	113.4	111.3	117.9	116.5	98.6	98.1	112.5	110.9
ZRANB1	170.5	329	152.2	217.5	287.9	127.2	259.5	471.9
ZRANB3	110.7	116	113.6	114.8	113	95.3	116.6	116.6
ZRF1	118.4	111.7	126.7	128	107.5	100.7	124.4	124
ZSCAN1	115.8	129.8	120.9	119.5	112.1	109.1	119.8	126.7
ZSCAN2	126.8	128.9	125.1	125.6	115.3	109.6	134.1	129.7
ZSCAN2	192.1	188.8	199.4	172.4	142.7	150.8	160.3	160.3
ZSCAN2	115.3	130.1	128.4	121.3	112.8	97.6	116.9	116.5
ZSCAN2	120.5	111.3	113.3	123.3	111.8	94.7	132.4	107.1
ZSCAN4	118.5	103	116.7	132.6	114.6	100.9	119.2	107.8
ZSCAN5	141.6	143.8	133.4	122.5	121	111.4	136.9	153.1
ZSWIM1	233.9	278.2	270.7	259.5	231.5	214.9	204.7	225.2
ZSWIM2	111.1	108.9	114.7	118.7	105.4	88.8	119	111.4
ZSWIM3	119.7	123.4	117.9	121.9	108.5	95.3	126.5	128.8
ZSWIM3	161.6	179.8	153.8	178.7	163	134.9	164.8	161.7
ZSWIM3	114.9	134.4	111.9	127.4	115	101	125	122
ZSWIM3	117.8	120.3	120.7	130	116.3	115.4	113	117.5
ZSWIM4	192.4	207.3	229.1	240.3	208.3	208.7	332.1	239.6
ZW10	271.6	310.7	220.2	297.3	294.8	194	316.5	290.4
ZWILCH	111.5	110.8	122.1	114.2	109.4	96.9	108.3	105.8
ZWILCH	115.4	117.5	118.8	125.6	115.7	95.3	125	124.8
ZWILCH	201.9	241.2	249.8	222.8	200.5	193.1	234.6	263.9
ZWINT	113.2	122.3	111	121.7	109.9	104.8	113	110.9
ZWINT	119.6	110.5	121.6	115.4	107	97.5	103.7	110.6
ZWINT	123.4	118.2	121.1	135.1	123.5	112	132.4	115.3
ZXDA	125	123.3	128.7	139.4	116.9	109.6	127.1	124.9
ZXDA	123	120.1	117.5	125.7	102.1	98.1	117.8	112.6
ZXDB	135.7	147.2	122.5	146.6	131.3	125.1	154.1	156.3
ZXDC	134.3	129.2	132.8	126.9	122.7	98	143.9	141.4
ZXDC	191	170.1	180.4	194.4	177.5	135	195.6	207.4
ZYG11A	104	106.7	110.4	114.7	106.2	98.2	107.8	106.2
ZYG11B	689	721.5	538.6	641.3	601.9	398.6	508.3	481.1
ZYG11BL	142.7	137.4	145.3	165.4	136.5	126.7	163.7	146.8
ZYX	382.8	329.9	518.9	439.2	220.2	320.2	408.3	232.1
ZYX	836	835.3	1309.7	960.4	600.7	1059.8	884.4	517.4
ZZEF1	265.7	276.2	257.9	310	271.4	240.7	316.9	269.8

ZZZ3	346.3	421.6	324.5	518.7	338.4	243.8	460.1	569.8

AVG_Signa	AVG_Signa	AVG_Signa	AVG_Signa	AVG_Signa	AVG_Signa	AVG_Signa	AVG_Signa	AVG_Signa
114.3	120.9	120.3	109	101.2	123.4	112	139.8	110
147.5	182.5	164.7	161.1	187.1	197.2	163.7	260.1	226.5
94	109.9	104.9	98.9	89	102	99.2	106.6	100.8
105	121	109.2	111.4	95.8	114.6	101.7	121.9	125.1
106	99.9	93.2	91.2	93.1	104	92.3	99.4	109.2
98.9	107.2	95.8	97.5	98.2	109.2	105.2	107.7	103
109.6	111.4	106.1	107.9	97.6	109.9	96.3	107.7	107.9
101.3	111.9	109.4	108	101	115.3	104.8	113	119.1
111.1	110.3	102.3	122.8	101.6	111.7	110.2	107.7	110.5
101.3	104.3	94.7	95	93.6	97.6	100.8	101.9	103.4
104.3	104.6	95.8	98	89.5	114.8	98.6	110.5	106.5
102	100.5	95.3	98.8	94.6	107.9	95.2	108.4	103
100.9	108.8	106.8	99	101	109.1	109	113.5	112
108.3	114.3	105.5	105.5	103.4	106.1	102.4	107.4	117.9
104.5	116	102.6	92.3	94.5	107.2	100.8	101	108.4
104.9	105.9	103.9	97.1	98.6	109	106.4	102.7	119.7
105.4	110.8	100.7	101.2	97.3	109.5	101	102.5	116.7
100.4	100.7	100.6	97.8	97.5	102.2	97.6	107	102.7
97.3	105.4	106.2	101	96.1	102.7	105.8	113.7	106.1
110.8	125.2	119.4	118.2	103.1	118	108.4	120.7	117.2
137.5	175.7	139.5	154.9	145.1	156	134.4	210.2	159.2
104.3	108	102.4	95.1	93.2	106.9	97.2	109.6	100.5
107.3	150.9	151.1	178.5	151.7	155.1	166.5	224.7	134.3
105.6	108.6	103.5	101.3	93.8	106.3	103.6	109	103.1
106	109.4	98.6	98.5	92.8	107.5	99.6	114.3	111.3
103.6	108.9	97.5	98.7	95.7	106.8	101	105.7	112
107.7	109.1	107.1	97.7	97.2	110.8	105.8	117.3	107.6
106.9	106.9	96.7	94.2	104.6	116.7	103.4	111.1	114.2
163.9	147.7	145.7	153.2	139.1	178.3	179.6	258.4	181.2
102.7	112.4	112.8	103.9	91.5	110.2	103.8	105	101.8
914.2	1626.1	1631.9	983.3	1702.4	2097.6	1441.3	3278.6	2185.6
146.8	204.5	181.5	175.8	158.3	206.9	208.7	255.1	182.1
127.4	134.3	132.1	123.1	127.7	150.6	128.6	200.2	148.1
145.6	186.1	187.7	188.2	154.8	168.5	172.2	209.7	150.8
264.6	262.1	234.6	272.2	232.5	240.1	244	467.7	311.1
100.5	108.9	106	105.9	104.9	110.5	107.4	119.8	119
361	373.4	366	349.6	332.4	403	359.4	599.7	377.1
112.5	119.9	108.6	120	105	120.3	118.9	110	120.8
105.7	107	102.8	100.1	97.6	111.1	108.9	98.1	104.8
105.1	108	103	98.1	107.9	101.8	106.7	110.5	109
111.1	116	114.8	111.2	103.4	115.3	119.7	126.7	117.2
123.7	115.2	115.3	103.2	99.5	119.6	110	106.6	106.3
114.6	139	121.6	125	111	136.5	128.8	139.6	114.9
329.2	231	184.2	294	308.9	246	554.3	474	248
119.8	109.2	121.4	109.4	104.7	112.6	102.5	116.6	114.4
117.5	114.5	112.3	101.8	101.2	107.6	106.9	126.9	115.2
103.9	104.9	94.5	101.4	98	119.9	111.1	118.6	105.4
112.6	115.5	98.5	107.6	94.9	108.1	100.4	128.3	112.1
99	108.2	101.5	102.3	87.2	106	107	113.6	104.2
105.7	108.4	100.3	99	91	107	99.7	109	108.8

102	115.6	116.8	110.7	107.6	112.8	112.1	106.5	108.6
108.8	124	113.2	113.7	103.5	109.4	116.4	116.7	107.9
137.4	136	132.3	113.4	117.7	140.1	127.8	143.2	145.7
110.5	129.3	112.8	120.1	106.7	133.8	121.6	133.5	129.1
103.5	108.3	109.6	99.7	105.8	108.6	99.5	115	111.1
106	119.8	112.8	107.9	98.5	111.4	107	115	113
105.8	104.6	96.5	99.8	91.1	102.4	103.7	104.7	107.3
106.7	118.1	107	98.5	109.2	122.4	116.5	123.3	122.8
103	115.3	106.8	99.8	99.7	110.2	96.4	106.4	105.5
145.1	311.3	353.8	353.6	231.2	252.9	271.4	220.5	178.7
107.6	112.7	97.4	87.9	93.5	105.3	100.8	106.4	108.4
105.4	111	102.9	103.3	96.9	108	101.8	112.7	107.8
109.9	119	119.1	99.1	98.6	112.1	106	112.5	109.9
105.9	106.5	113.1	108.9	99.2	109.9	106.2	126.2	104.4
374.8	187.1	236.5	199.6	187.6	253.7	190.8	244.1	296.9
145.5	183.5	181.8	189.8	169.4	192.8	175.3	225.4	187.1
101.4	109.3	109.6	92.8	99.8	107	101.5	105.7	117.2
111.4	104.4	113.8	96.4	100.6	102.6	104.5	103.5	108.2
102.3	108	107.4	96.3	94	111.1	107.6	108.7	102.5
175.8	145.3	140	124.3	155.7	280.5	177.5	635	334.2
155.8	263.4	160.5	179.2	196.9	184.4	137.3	323.8	202.3
106.6	113.7	104	107.7	97.4	109.2	103.6	108.7	111.1
100.9	108.6	99.1	102.5	100.1	104.7	100.5	108.5	106.8
110.5	119	110.6	108.3	115.9	122.1	114.5	117.8	111.3
118.5	118.6	116.4	122.4	112.1	125.8	130.4	125.1	127.2
114.2	106.5	101.8	97.6	102.5	107.1	102.5	111.9	113.3
114.4	125.7	115.3	107.7	128.3	120.6	134.6	151.4	141
130.3	134.4	141.6	152.5	131.2	133.1	140	144.1	124.9
101.5	101.3	102	96	98.2	106	101	101.6	107.6
109.9	105.9	102.9	106.5	100.2	109.9	105	108.7	112.6
109.7	106.9	98.6	94.2	101.6	120.4	108	108.1	114.5
111.8	113.1	105.6	103.6	93.6	115.3	111.7	127.1	117
107.4	103.4	107.8	119.9	103.9	117.5	112.7	124.2	116.8
108.6	120.5	101.7	99.7	100.8	109.7	106.4	117.5	116.8
116	130.7	114.3	104.2	108.4	113.8	117.3	118.9	110.9
150.8	276.6	226.9	227.3	235.9	202.3	588.2	234.6	120.2
126.1	131.3	117.5	138.3	112	128	132.9	133.4	106.4
102.7	104	97.6	101.3	92.5	105.6	101.6	104.2	110.4
149	234.5	221.4	217	235.3	220.1	264.9	314.4	190.9
126.2	190.3	196.1	222.7	168.5	184.7	223.3	184.1	166.2
109.8	111.6	101.1	106.7	97.9	110.4	102.1	115.3	109.1
108.8	105.7	103.1	130	105.7	115.9	112.5	117.6	126.5
101.8	111.4	107.9	92.8	86.8	105.6	99.1	96.3	98.9
111.1	113.9	100	103.9	99.9	105.3	100.7	109.6	105.1
105.2	108.8	112.2	100.9	97.5	118.4	109.6	122.6	117.9
102.8	103	100.5	103	94.7	105.4	101.2	106.6	105.1
109.6	106.8	99.1	99.2	97.1	99.9	101.4	103.2	94
110.8	116.3	106.7	120.1	102	114.6	104.7	114.5	112
115.2	123.2	110.5	120.8	105.9	116.2	124.2	138.6	111.8
103.7	107.1	102.6	98.1	98.1	104.5	101.1	110.6	102
103.7	112.8	110.3	100.8	110.9	120.1	108.4	117.3	111.8

111.2	132.4	113.3	95.1	103.9	106.4	111	115.4	113.3
228.8	358.2	369.1	331.8	371	311.4	315.2	416.5	368.5
190.6	389.3	368	297.6	342.7	345.9	300.8	504.3	310.1
296	363.8	419.9	297.4	312.1	388.6	403.7	719.8	471.7
658.6	885.9	1019.7	649.2	865.5	1071	799.8	1471.8	935
126.5	121.8	112.4	112.2	103	121.9	124.4	114.4	120.7
122.2	154.9	142.2	126.9	135.6	169	160.4	211.1	196.8
116	130.9	151.1	134.9	119.4	142.7	137.3	167.7	144.5
113.5	125.9	152.4	117.6	114.2	130.9	130.6	190.4	143.5
120.9	139.7	140.9	114.6	113.7	139.6	114.8	144.5	136.9
109.6	106.5	108	102.2	99	109	102.7	102.6	100.3
105.2	113.2	100.2	101.9	99.4	107.1	100.1	111.5	95.1
115.8	110.9	111.5	110.6	106	126.7	119.9	131.4	110.2
113.9	131.4	108.7	121.5	104.4	127	130.4	119.9	110.9
110.8	115.7	98.8	107.1	97.6	115	109.8	112.9	111.2
106.1	104.6	116.2	100.6	91	104.5	97.5	113.7	103.7
107.7	107	103.3	98.3	94.9	106.1	101.5	109.7	116.4
116.4	129.5	106.6	110.5	100.4	107.9	104.9	129.7	115.2
104.1	108	105	92.4	89.7	115.2	98.2	111.3	111.9
112.8	110.2	105.6	114.7	95	108	104	105	111.1
110.6	138.4	120.8	138.5	121.1	120.5	139.8	143.1	129
116.6	116.1	110.4	121.3	107	122	123.2	143.4	126.7
105.2	116.3	99.4	100.3	102.1	111.6	110	108.5	106.2
129.8	128.6	111.1	130.1	106.8	132.5	132.2	145	130.2
115.2	149.7	125.9	167.1	117.2	137	161.3	157.5	145
139.2	215.9	142	343.6	154.8	149.5	257.5	184.1	151.5
112.2	105.8	99.1	105.9	99.6	109.7	108.3	105	112.5
104.8	166	174.4	176.2	154	165.1	169.7	137.6	120.4
147.2	180.4	183	178.9	161.9	164.9	164.2	147.1	121
111	126.5	119.8	108.7	109.6	125.7	133.6	154.9	124.5
96.6	104.9	104.2	99.9	93.4	102.9	95.5	107.6	104.5
110.9	114	110.8	120.7	115.8	121.8	108.1	125.9	113.4
492.5	253.6	261.7	282.3	233.5	312.6	379.4	896.4	1060.1
176.2	129.9	118.1	134.5	127	155.4	116.3	241.4	228.5
139.5	148.5	115.8	123.2	130	133.4	125.8	302.2	153
130	152.5	134	150.9	130.4	156.5	146.7	175.5	132.4
121.7	137.5	128	122.2	108	132	105.8	118.7	109.8
164.1	250.8	200.6	264.3	246.1	226.9	248.4	342.7	197.9
115.6	107.7	99.3	108.7	98.4	100.7	105.7	124.9	115.4
108.4	107.3	101.7	95.4	95.2	104.7	95.6	107.2	115.5
269.4	235	248.8	220.9	201.7	198.9	226.1	307.2	296.6
107.8	113	108.5	114.6	96.5	115.8	108.9	109.7	113.1
123.4	147.8	143.6	127.5	124	146.3	140.7	185.7	151.2
113	112.5	106.3	106.7	92.5	115.2	98.8	126.8	127.5
124.8	186.8	151.9	196.1	145.7	143.4	207.2	171.3	149.4
110.5	105	104.9	110.8	106.2	122.4	106	114.8	118.1
187.8	215.2	185.4	203.4	178.5	213.2	227.5	205.7	174.4
111.7	115.6	111.1	96.5	105.2	117.8	107.9	123	124.5
105.2	108.5	96	99.8	101.7	102.2	99.8	107	112.4
109.8	111	106.4	103.1	97.7	120.2	104.9	125.9	121.7
109.4	113.1	101.4	104.7	91.6	113.8	103.4	120.1	114.5

109.5	106	104.2	93.3	97.1	107.6	113.2	104	120.5
2539.7	1866.7	2347.4	2216.6	2367.3	3460.9	2810.7	4185.9	2714.9
207.7	203.7	192.2	165.6	171.9	247.4	145.2	342.3	244.6
107.4	114.5	105.7	100	105.5	108.4	104.5	116	112.1
671.5	669.1	613.5	450.7	583.1	864.6	750.6	1686.2	1080.7
110.7	113.4	127.5	122.4	122.7	115.8	123.9	122.5	114.4
120.5	113.6	107.8	102.1	96.8	118.6	112.5	113.9	127.3
107.6	110.1	108.5	95.2	101.8	116.6	102.3	104.4	112.6
106	118.5	104.7	99	91.6	113.6	104.6	106.5	108.7
465.3	655.1	481.7	595.1	609.3	561.1	632.7	1328.1	496.4
113	114.6	103.3	103.5	97.4	116.9	112.2	127.7	132
98.4	106.5	99.2	101.7	95.9	110	104.8	112.3	110.7
109	109.6	108.4	92.9	93.7	108.8	101.9	103.4	107.5
118.9	136.2	119.2	121.3	107.7	124.8	125.4	151.5	156.1
383.9	442.7	365.2	406.3	342.4	413.2	431.9	525.2	317.3
187.6	193.5	198.2	150	184.1	211.5	203.7	234.7	186
105.5	109.9	100.7	100.5	96.6	100	104	114.6	108.9
220.5	364.1	398.6	349.6	302.6	362.3	328.1	505	288.2
132.5	143.9	136.2	166.2	134.5	147.1	130.8	172.9	166.9
157.8	173.5	162.5	182	163.4	183.2	175.9	187.4	174.3
137.2	145.5	130.3	129.9	118.6	131.8	132.4	144	128.1
105.6	103.5	104.2	96	99.1	106.7	103.6	116.8	106.9
123.2	171.2	194.2	167.1	170.4	177.1	174.2	169.4	138.9
123	126.8	119.6	137	115	133.8	128.5	144.3	118.6
205.7	267	201.4	239.7	172.1	189.9	232.1	230.1	226.4
194.2	300.1	281.4	261.9	293.2	311.7	276.9	398.1	250.5
146.8	256.4	352.5	203.4	225.3	310.1	248.8	332.5	212.2
103.1	105.3	102.1	104.1	96.8	111.8	101.8	105.5	105.5
496	682.1	643.9	659.5	680.1	686.7	623.7	1001.5	682.8
108.1	117.7	113.5	123.8	110.4	120.5	128	137.7	121.2
113.6	114	120.8	111.7	101.2	118.1	120	132.9	120.1
105.2	116.7	107.8	117.1	97.7	104.1	122.9	119.6	114.6
602.6	1091.7	1101	1341.1	951.5	1155.7	1795	2378.7	1501.1
202.5	347.4	377.9	347.6	328	338.2	657.6	567.6	421.8
233.7	329.3	269.2	356.5	263.1	288.2	340.4	526.9	356.7
107.7	104.7	112.3	107.8	99.3	116.3	100.6	112.2	114.3
293.5	426.2	284.9	300.3	295	288.6	259.4	484.7	389.4
116.9	108.2	120.2	114.5	107.3	118.7	110.5	110.4	112
121.8	118.5	115	122	106	114.7	112.6	115.9	126.7
107.9	106.6	102.2	102.4	93.8	103.1	100	96.7	112.3
101.3	108.5	102.4	104.9	94.4	110.3	97.9	104.1	105.6
118.9	138.1	129.2	132.5	128.5	140.2	135.6	148	138.3
104.8	108.3	105.5	96	94.6	103.6	99	126	111.5
104.6	107.7	107.1	104.4	99.3	109.9	106.4	110.5	106.6
103.4	99.8	102.6	95.9	90.4	107.2	100.5	112.4	110.6
107.7	107.2	105.6	100.6	101.6	107.9	99	115.1	111
99.8	106.5	105.2	102.2	92	102.5	98	112	107.8
113.2	121.6	112.7	121.9	102.6	117	112.1	138.6	118.3
105.3	109.2	105.7	102.3	100.4	108.1	107	114.3	98.9
108.1	110.4	106	110.5	100.5	118.8	106.9	116.8	118.4
100	104.5	101	92.5	94.2	100.3	99.1	98.3	108.7

105.1	106.3	100.7	97.8	94	106.2	103.7	105.9	110.2
104.5	103.6	102.3	100.6	97.4	107	101.9	115.7	109.3
232.2	181.2	186.7	179.8	181.4	261.3	213.6	347.1	222.7
103.2	109.1	104.2	101.8	93.5	105.7	107.3	118.4	120.4
112	112.4	102.5	101	100.4	108.1	105.9	113.4	104.6
111.1	105.4	103	90.8	93.1	110.1	106.4	110.2	107.5
103.7	112.7	98.8	109.3	96	110.2	100.7	107.4	109.6
102.5	101.5	103.3	103	91.7	106.8	88.2	108.2	112.2
111.1	118.1	108.1	104.6	97	105.7	100	111.8	112.5
108.4	113.6	104.5	105.8	98.4	107.2	99.8	116.2	110.7
102.7	99.2	98.1	108.3	92.8	105.2	99	97.6	103.2
96.3	104.4	103.9	89.5	93.3	103.8	97.5	110.8	102
338.8	407.6	364.8	313	391.7	394.7	394.5	596.9	422.3
542.9	767.3	617	469.2	513.3	663.1	524.3	761.8	600.7
122.7	123.9	124.3	124.2	118.5	136.3	119.6	148.9	127.3
160.3	320.2	315.4	366.6	317.9	247.6	403.6	371	227.6
145.2	300.1	269.2	312.7	266.7	267.1	346.6	307.6	211.4
142.8	147.4	148.9	141.3	142.5	133	146.4	207.5	160.9
102.6	104.2	106.5	106.1	97	111.1	103.9	113.8	103.7
108.5	118	110.4	109.3	106.7	120.2	114.3	123.8	124
103.3	140.4	101.6	146.8	107.1	120	153.5	119.3	106.7
116.3	110.5	106.1	102.2	99.9	111.8	110.5	108	119
106.3	115.2	105.1	102.5	98.1	107.1	101.2	116.3	117.6
93.5	105.9	104.1	101.6	96.4	106.1	110.4	111.7	120.2
112.3	124.4	107.6	112.2	109.1	117	103	119.4	132.5
95.1	104.1	96.9	97.3	98.9	107.1	91	101.8	107.1
105.5	106.5	97.3	107.4	95	105.6	99.8	105.9	108.9
103.8	108.1	106.8	97	96	109.4	94.9	105.5	116.7
345.4	151.1	156.9	157.4	141.1	168	185.3	386.9	413.2
105.5	115.7	109.1	106.3	101.5	118.3	108.9	118.2	108.9
225.2	128.9	113.9	103.1	103.2	158.8	121.4	225.8	242.9
109.9	108.3	106.4	110.7	94.8	111.6	107.2	105.9	101.5
106.6	124.8	114.7	111.8	108.4	113.4	124	124.1	111.7
142	135.7	116.9	127.9	112.6	127.1	131	140.2	116.6
202.6	203.7	154.1	174.8	176	185.9	207.6	262.6	181.2
119.7	126.4	113.7	114.3	111.3	125.3	119.2	148	134.3
104.8	106.9	102.5	97.7	90.4	106.4	103.9	112.5	108.4
113.4	111.1	98.9	86.2	93.9	105.9	100.9	105.4	97.7
104.5	107.6	96.6	97.8	94.5	103.7	103.5	106.8	105.3
129.6	151.3	140.2	165.6	127.3	147.7	159.5	182.5	158.2
100.8	108.7	101.2	88.9	97.6	108.5	98.1	95.9	111.8
485.5	520.1	476.8	395.5	374.7	459.4	516.1	736.7	607
714.3	944.3	945.4	755.8	761.2	940.7	816.3	1365.8	1111.4
114.2	134.9	144.7	149.2	135.5	140	142.8	204	145.7
135.6	884.7	771.1	708.4	1317.6	867.5	1030.6	1478.6	270.9
104.8	127.8	129	105.9	103.7	119.1	114.6	121.8	116.4
136.5	146.2	157.1	132.7	118.4	154.1	132.6	132.5	128.6
102.4	110.3	100.7	107.3	85.2	99	109.4	106.2	104.7
153	161	143.5	125.6	135.3	155.3	149.7	175.2	152.8
102.6	104.7	105	113.9	105.5	103.4	103.5	100.8	114.7
109.4	116.8	105.3	105.4	103.5	117.3	105.7	118.7	120

111.4	115.2	102.3	104.9	95.3	106.9	100.7	103.3	109.3
145	137.3	128	137.9	124.8	127.8	120.9	200.9	161.8
116.1	117	106.3	106.5	102.1	119.9	110.9	111.4	113.5
108.7	105.6	102.3	106.8	100	109.2	102.5	114	110.2
100.4	106.2	105.1	98.6	97.2	105.3	104	111.6	114.9
111	113.2	100.9	102.9	99.7	104.9	106.7	105.8	106.2
158.8	147.3	125.9	139.9	121.3	158.6	142.3	210.3	176
249.2	293.4	318.4	270.6	279.5	286.8	364.9	523.1	300.8
112.9	112.5	109.9	120.2	105	116.3	110.1	125.5	121.2
129.4	143.2	127.4	131.7	112.8	147.1	139.1	133.5	155.1
108.6	111.6	110.4	120.3	103.2	115.8	109.2	120.8	116.3
134.5	146	129.5	157.2	131.5	128.9	126.1	159.5	143.2
293.8	301	222.7	317.3	263.9	249.6	350.6	377.2	306.7
152.3	183.9	159.1	157.3	174.2	169.2	162.1	217.3	175.7
109.9	106	103.8	102	94.6	104.4	109	107.9	109.3
107.1	103	98	106	99	107	98	101.4	97.9
103.7	102.8	100.6	105.4	94.5	112	100.4	118	111.7
378.8	596.3	527.7	514.2	620	496.2	507.2	625.7	535.5
103.1	112.2	104.5	97	93.8	101.4	99.1	101.6	108.5
99.7	113.2	107.9	111.3	100	109.8	97.1	106.7	107.8
112.8	103.8	104.6	95.2	95.8	115.1	106.9	109.2	112.4
102.3	101.6	97.1	94	92.1	109.8	96.8	101.1	102.8
102	106.8	99.3	95.3	90.7	108.8	103.3	113.4	104.2
99.5	107.4	93.1	97	99.1	106.3	99.6	103	106.5
105.6	114.7	107.4	108.4	100.4	118.8	104.3	117.6	107.4
105.3	108.7	94.2	112	108.5	106.9	106.9	109.9	113.3
156.9	123.9	125.2	139.1	113.6	130.4	132	122	118.2
151.2	273.9	326.5	182.3	242	242.6	224.3	276	211.8
106.5	107	100	109.8	100.5	116.4	98.4	107	101.6
109.7	111.3	108.5	104.5	90.1	109.6	106.3	107	116.7
229.3	388.5	298.4	452.4	373.2	359.2	445.1	385.6	217.1
325.3	473.2	430	581.5	417	467.7	598.8	519.7	262.1
118	111.3	102.7	99.6	97.1	104.4	98.1	118.9	120.8
206.8	299.1	172.2	280.9	209.3	156.1	230.5	206.8	216.9
1819	4310.8	3692.6	7287.6	3388	4435.9	6724	5222.8	4581.3
1186.2	2908.1	2333.6	3973.4	2551.6	2379.1	4312.9	3574	2917.7
109.3	109.4	104.8	102.4	98.2	110.1	102.2	114.1	101.9
110	112.1	97.7	101.1	104.7	113.7	108.4	114.4	109.5
3549.4	8121.9	6708.5	8226.7	7113	8069.9	8628.7	8968.6	6679.2
101.4	104.7	97.8	108.4	100.4	104.7	106.6	124.2	104.7
347.9	617	603.1	445.7	546	570.3	532.7	683.2	481.8
140.3	232.7	211.5	212.7	166.5	229.6	250.2	375	273.4
107.6	107.9	105.6	106.6	96.7	109.4	102.8	104.4	105.1
101.9	111.9	103.5	104	89.5	100.1	100.8	121.8	102.6
108.4	108.1	111.9	97.8	92.8	108.6	99.2	115.4	101.3
103.4	105.9	118.5	115.5	105.3	114	103	112.3	106.3
656.2	921.6	1005.3	733.9	857.7	975.3	569.2	840.2	547
117.4	109	110	109.3	93.5	118	106.4	129	125.9
107.4	108.8	100.5	102.9	101.9	104.3	103.8	120.2	113.5
276.3	258.8	281.4	273.6	335.7	291.1	266.2	450.4	244.6
390.3	432.8	326.8	339.3	304.3	342.9	358.3	633.5	511.1

537.6	407.9	363.5	457.9	386.5	458.1	409.6	769.6	501.5
165	313.7	334.8	317.8	219.2	236.8	309.8	280.9	165.5
2252.8	2226	2109.2	2810	2178.8	2015.6	2453.8	3715.1	3232
573.7	434	353.4	483.5	336	460.1	419.1	677.1	608.2
107.3	121.4	107.2	106.8	105.4	113.4	106.5	114.1	107.1
110	112.6	101.3	104.2	96.2	107.7	109.8	104.1	105.3
111.4	113.2	111.3	105.1	115.9	113.6	103.8	120.3	118.4
146.5	173	173.6	203.5	176.6	197.4	186.2	266.4	168.2
328.3	457.7	363.4	544.7	349.8	363.2	448.9	664.3	550.8
113.5	174.3	138.4	124.3	131.2	143.6	136.9	163	142.3
116.3	107.5	111.2	111.2	106.5	111.8	114	125	124.3
292.7	434.3	413.9	294.1	423.3	435.8	313.2	839.7	319.6
108.8	113.1	106.5	98.9	103	113.4	104.5	125	121.7
113.6	124.9	103.4	111.6	110.8	132.4	106.7	140.1	130.8
171.7	255.4	197.6	169.8	179.5	235.2	223.2	260.7	210.8
109	111.7	98.1	111.2	97.8	112.6	103.5	116	114.5
104.6	107.7	103	99.7	94.7	111.4	98.7	122.2	108.4
153.5	170.7	159	168.3	168.9	183.5	172.2	202.6	173.5
109.9	103.8	105.9	109.6	101.1	105.9	102.6	103.5	107.6
101.5	109	98.9	100.6	94.9	106.1	105.4	98.6	106.5
113.6	130.5	147.5	144.7	116.1	138	155.3	134.2	137.6
124.7	130.9	125.6	115.5	104.4	123	119.2	139.5	119.9
101.7	106.5	99.8	101.1	93.6	106.9	100.4	98.9	103.3
126.8	121.6	114	128.9	109.1	125.8	117.4	135.8	129.4
172	259.4	227.4	237.8	226.1	225.1	261.3	376.2	299.3
110.5	112.8	107	107.1	105.4	113	111.9	134.3	129.1
162.7	178.6	174.7	146.8	157.4	172.5	151.7	326.8	259.3
106.2	115.1	105.2	110.9	100.4	121.4	104.6	104.8	105.8
273.7	396.3	384.4	580.6	288.3	329	466.8	286.5	248.8
109.1	125.7	111.6	107.6	108.4	134	113	136.7	127.5
106.6	131.2	108.4	118.8	99.2	118.5	106.4	121.5	116.2
114.3	100	103.4	101.8	101.3	114.1	105.4	124.6	115.7
103.7	112.7	102.2	110.1	94.3	113.1	112.8	114.1	114.4
109.6	116.6	114.3	113.2	102.5	113.1	108	124	104.9
100	108.1	107.1	100.7	98.2	112.2	105.1	104.9	100.8
105.8	112.8	114.6	121.3	107.9	122.4	127.2	133.6	115.2
272.9	311	243.4	550.3	293.9	324.4	374.7	558.8	437.2
104.7	106.3	104.9	104.1	94.9	104.4	105.4	119.7	110.6
197.6	155.7	135.9	130.4	138.3	148.7	185.5	260.2	267.1
106.2	106.7	105.2	103.9	92	106.2	98.2	103.4	107.2
107.2	117.8	106.6	114.7	103.5	121.1	114.9	122.2	110.7
104.7	114.9	99	101.3	91.7	112.5	96.7	113.7	109.6
157.1	267.5	258.3	259.6	204.4	212.9	263.9	172.2	147.4
110.8	116.3	109.7	104.4	98.1	110.8	104	116.3	122.8
114.8	117.3	100.9	98.8	100.9	103.6	104	103.5	106.3
110.6	114.8	109.3	106.1	93.8	113	110.2	125	109.4
103.6	114.6	107.5	90.2	104.9	114.5	100.4	119.2	107.5
109.4	104.4	100.8	108.7	93.3	106.8	98.3	105.1	104.2
130	113.2	107.7	118.4	99	111	103.6	127.7	116.9
109.9	108.5	121.2	100.8	98.7	116.4	104.6	113.4	109.4
111.7	117.9	99	110.4	98.8	120.4	108.9	105.6	118

109.8	115.6	107.9	106.3	103.1	117.7	111.9	118.5	110.3
109.7	110.8	105.8	119.6	100.7	105.4	119.8	116	112.4
117	106.3	102.9	101	103	116.5	111.2	118.1	112.5
109	109.9	95.2	96.2	95.5	107.7	103.7	104.5	103.1
107.9	105.2	104.9	99.4	100	108.4	105.6	109.8	113.8
115.4	104.8	98.8	108.2	88.9	117.9	108.3	109.7	101.8
116.4	115.7	106.4	110.3	101.1	115.3	109.6	116.3	126.8
103.9	114.4	109.6	105.2	100.5	117.5	107.3	119.7	116.1
111.4	101.2	100.2	95.3	94.5	104.3	102.9	102.1	110.9
110.6	113.3	106	103.6	96.7	113	102.1	118.2	114.3
135.3	167.2	161.4	179.2	128.2	148.3	152.8	148.3	134.4
146.3	200.3	237.5	215.4	174	177.5	188.4	190.5	180
125.5	120.1	105.9	125.6	112.5	124	131	160.3	133.2
141.1	490.3	219.8	405.5	257.9	221.5	291.8	295.6	296.5
106.1	111.7	106.5	112.7	99.1	114.2	115.4	117	106.1
111.1	111.3	111.3	115.8	106.2	110.5	111	110.6	105.9
99.9	108.4	101.5	94	93.6	110.1	105.2	113.5	105.9
113.5	122	106.7	120.6	105.6	119.6	106.7	125.6	117.2
102.6	103	101.9	93.3	101	102.9	102.4	99.7	99.9
110.9	123	109.6	119.5	100.1	124.3	111.3	116	119.2
105.9	119.1	102.2	101.5	95.1	104.3	102.3	101.1	113.7
105	106.7	104.1	122.5	101.3	117.5	99	112.9	111.6
116	109.6	100.1	103.7	98.1	112.5	101.2	112.4	111.2
102.9	107.5	100.6	107.8	101.8	102.9	106.8	114.3	105.8
108.6	103.9	102.1	102	95.6	114	103.8	104.7	119.6
107.5	104.5	99.9	111.7	97.8	118.1	107	115.4	108.5
106	104.3	102	106.5	93.9	106	96.2	109.6	108.3
125	138.7	128.6	115.2	110.1	122.5	124.2	132.2	132.4
107.7	104	109.7	90.5	94.2	106.5	102.1	111.1	111.4
98.1	109.1	102.4	99.9	96.1	105.7	96	114.6	101.9
105.2	112.5	102	108.2	100.2	102.9	101.2	112.5	111.3
110.1	109	98.1	102.4	89.7	108.1	94.9	102.1	102.7
110.3	107.3	104.9	99.9	98.8	106.8	110.2	110.3	107.2
102.6	102.2	109.3	102.7	100.7	113.7	114.5	104.6	102.6
108.3	101.1	98.2	99.6	102.7	106.2	98.4	100.8	108.5
104.1	116.1	123.1	104	109.4	114.7	113	116	118.2
113.8	109.4	110.6	107.9	95.4	109	102.3	113.1	110.1
101.4	92.2	96.7	113.6	97.4	106.4	101.6	104.6	103
105	101.2	96.3	102.7	88.2	106.8	91.6	112.8	96.3
105.1	105.7	105.7	101.6	93.4	113.5	102.4	111.3	120.9
108.8	103.1	101	110	98.2	111.3	98.1	103.9	107.8
112.7	112.6	103.6	108	96.8	109.9	101	118.6	103
107.8	106	95.8	95.8	98.3	102.5	99.1	106.5	109
113.3	105.1	113.1	104.2	99.5	109.9	101.4	105.6	103.9
113.3	105.8	105.4	109.5	100.6	120.5	111.4	114.4	97.8
113.3	124.7	112.8	117.7	109.7	114.3	116.3	118.8	110.1
485.7	911.5	716.5	596.5	584.7	739.4	498.7	1345.4	704.1
109.3	115.1	107	105.3	101	106.5	101.7	123.9	111.5
279.8	560.3	506.3	437.2	500.9	457.3	532.1	576.5	520.8
119.7	111.5	101.7	104.5	100	118.7	114.2	123.5	114.2
137.4	187.4	173.9	169.7	159.4	184.8	183.9	185.4	143.5

110.2	112.8	103.9	113.1	95	110	103.6	125.1	108.6
283.8	421.2	444.5	424.8	439.2	458.8	463.5	436.2	274.8
107.5	104.6	116.1	115	109.4	124	109.9	120.2	116
173.9	240.6	254.2	195.5	246.9	233	227.7	278.8	187.5
113.1	114.8	108.2	107.4	105.1	113.4	116.8	115.7	117
117.4	162.3	144.8	157.5	125.7	147.9	128.3	150.1	117.3
243.2	248.1	294.5	295.4	225.1	270.4	302.8	267.3	211.6
115.8	131.6	121.1	111.8	109.8	119.3	105.9	129.4	110.6
117.2	157.6	134.3	129.3	121.9	145.4	134.5	142.7	131.4
107	118	106.8	101.5	94.7	104.8	103.1	116.3	109.3
114.3	122.1	105.7	109.6	101.8	113.4	107.5	125.9	121.3
205.9	428.1	441.5	318.1	359.8	387.8	349.5	517.9	253.1
134	113.2	108.2	110.3	105.7	122.7	122	136.2	122.5
107.1	113	98.6	109.8	92.2	104.8	103.8	107.2	107.6
105.9	115	105.1	103.8	94.2	108.8	98.4	110.4	101.9
114.9	111.5	106.6	101.7	95.2	105.3	94.9	118.5	112.8
162.6	188.3	184.6	190.6	161.7	170.6	216.8	248	206.6
109.4	117.1	100	89.2	96.7	113.4	99.4	118.9	110.3
113	132.2	126.8	125.6	135.5	125.7	136.2	142.1	127.3
106.9	108.3	107.3	113.4	95	109.8	101.6	110.4	112.3
119.1	109.1	103.8	98.6	102.2	123.4	101.3	106.8	104.8
119.6	124.8	109.4	113	107	121.1	110.5	130.1	118.8
1415.5	1711.6	1677.8	1685.3	1776.5	1646.8	1556.8	2541	1719.5
99.6	112.1	104.8	97.9	97.3	103.2	97.1	113.3	102.3
108.9	113.4	105.2	110.1	94.7	106.8	106.4	108.8	109.1
106.2	108.2	104.6	94.4	102.2	110.7	114.1	123	111.3
105.4	98.3	99	103.6	93.5	111.3	94.9	112.8	109.6
423.4	554	613.6	589.4	561.3	560.9	316.7	875.2	908.4
672.2	768.9	889.7	775.4	585.5	1072.2	841.6	1598.2	1241.1
399.8	544.7	545.8	460	534.9	649	316.8	865.9	798.1
1044	934.1	828.5	455	933.6	1457.2	805.2	2483.2	1136.4
105.8	103.8	97.4	99	93	108.2	100.4	102.6	106
107.6	102.9	99.7	99.4	95.7	104.6	96.8	102	100.5
109.3	111.2	101.9	98.9	91.1	105.3	99.8	103.8	103.6
109.7	105.5	102.3	87.9	89.5	104.3	98.5	99.2	105.5
112.1	117	110	119.5	116	118.6	107.5	117.4	123.5
111.2	117.9	102.6	105.7	98.3	111.9	108.6	115.3	125.3
105.4	116.2	104.1	96.5	94.6	101.2	102.5	112	102.9
129.5	160	146.7	164.7	128.5	130.5	171.4	136.5	149.3
98.9	114.1	109.2	108	95.4	106.5	106.8	114.2	117.7
118.6	125.1	108.3	111.9	103.4	122.8	120	124.3	120.4
106.3	106.1	103.7	101.7	93.9	107.5	103.2	119.7	117.8
289.3	243.2	236.6	223.3	246.7	231.4	295.1	520.6	275.1
356.3	466.8	382	309.8	363.8	428.9	407.6	633.1	468.4
107	121.6	109.1	112.3	102.9	116.7	109.9	125.1	108.2
108.5	113	108.4	101.8	94.7	111	110.6	100	113.5
121	144	128.3	140.9	118.7	134.9	118.5	136.3	135.8
2548.4	180.2	152.1	158.6	216.9	270	335.9	917.5	713.8
134.3	137.9	123.6	139.6	124.2	169	142.7	182.7	178.9
104	105.8	109.9	101.6	94.8	107.2	105.1	100.6	111
140	178.6	179	187.4	134.1	184.8	156.5	252.3	186.1

119.6	129.6	138.8	116.9	121.1	144.9	114.8	144.9	131.2
106.6	109.8	103.8	109.5	98.4	103.8	97.1	103.7	107.2
111	110.6	109.4	109.1	97.6	108.5	108	108.6	111.9
136.5	262.2	291.4	182.9	241.3	269.2	209.1	235.4	174.9
112.5	124.8	103	115.7	112.1	111.4	117.1	144.9	103.1
102.8	110.2	91.6	308	92.8	98.3	148.2	107.6	105.2
116.8	121	120.1	221.6	111.9	129.3	176.5	151.3	124.3
231.4	306.5	291.6	305.4	287.4	249.1	355.6	422.4	387.1
103.4	100.4	102.8	101.4	94.9	107.4	99.7	112.6	112.6
120.1	134	110.6	116.9	110.2	116.2	116.5	146.8	112.9
109.2	107	108.4	103.5	93.8	107.5	99	113.8	103.7
112.5	106.5	106.6	104.5	104.9	113.8	102.5	115.9	120.2
115.1	103.6	106.8	116.3	99.9	106.1	108.7	114.4	115.8
200.4	213.6	182.3	177.4	199.9	203.7	207.9	375.6	237.3
102.8	98.5	102.1	91.4	92.6	107.1	96.4	108.6	118.5
103.1	101.9	97.3	94	93	105.1	101.3	98.1	114.6
110.3	100.7	108	92.3	105.1	108.9	97.3	119.2	106.9
110.1	129.3	102.8	107.6	107	117.6	117.6	124.7	119.9
114.2	111.1	101.4	98.8	98	116.8	100.6	115.6	110.1
113.8	113.5	107.3	100.5	107.8	115.6	107.6	117.7	121
112.2	107.5	112.9	105.1	102.4	119.3	105.5	119.2	109.7
113.3	107.3	97.9	104.8	101.5	101.9	112	127.6	112
131	116.9	113.5	121.9	107.8	122.2	112.6	123.1	119.3
111.1	114.7	111.2	105.9	106.2	120.5	108.2	121.5	113.6
108.1	106.2	98	103.5	101	109	101.3	107.5	111.7
161.9	361.3	367.8	290	360.9	380.3	248.7	540	327
99.7	107	100.8	105.8	101.4	112.8	111.4	117.4	119
97.3	104.4	97.6	89.5	86.5	107.2	92.6	103.8	102.3
113	105.7	107.3	104.4	100.2	109.2	103	109.9	99
212.8	195.3	194.1	164.4	185.9	249.8	227.6	536.3	491.8
419.3	272.1	208.8	211.1	323.5	251	310.3	1081.6	1240.5
388.2	977	918.6	693.6	694.7	862.6	781.9	1042.9	657.5
662.7	771	809.8	590.5	741.1	833.7	910.3	1045.6	730.3
102.6	109.5	98.4	102.2	100	110.8	103.6	118.3	109.3
119.4	109.6	114.2	112.7	92	116	120.6	123.5	121.3
115.7	103.4	107.7	92.7	95	106.2	104.1	115.1	107.9
126.8	133.4	135.4	124.8	123.2	137.9	138.2	162	123.8
131.3	130.3	126.5	132.9	129.8	140	132.2	176.7	148.4
1208	1544.7	2041.1	1751.7	1446.1	1948.3	1217.1	2085.8	859.3
314.1	523.8	552.9	441.7	553.5	419.2	459.3	822.1	479.4
102.5	113.3	101.8	102	99.8	113.2	98.4	119.1	114.6
107.1	107.6	105.6	95.4	89.5	106.7	102.2	106.6	102.8
115	121	113	117.1	109.8	121.2	129.7	124.5	117.3
111.8	118.9	98	100.7	100.7	109.2	107.5	115.4	108.5
118.8	118.4	109.2	113.9	105.4	114.6	121.3	116.3	128.9
109.8	116.1	109.5	111	114.6	122	102.3	121.2	119.5
107.6	105.5	112.1	97.5	107.7	106	94.3	115.2	102.4
100.6	104.9	100.3	93.6	92.4	114.4	101.4	102.5	113.2
109.1	124.8	129.6	115.7	112.6	119.5	127.5	185.7	167.3
110.3	118	104.1	116.7	98.5	115	105.3	116.3	108.2
121.5	138.4	129	132.5	107.9	118.9	123.8	116	111.3

109.5	118.7	105.9	107.5	99.6	104	101.5	98.8	105.7
215.1	167.9	174	147.1	173.6	186.1	155.5	321.5	243.4
277.1	326.4	324.7	214.4	285.3	320.9	266.8	404.9	270
122.8	134.6	125.8	115.7	102.3	123.3	109.5	127.1	121.2
104.6	110.2	108.3	103.7	96.3	109.6	99.2	115.9	111.9
110.2	106.7	105.4	94.6	92.2	108.4	100.7	104.6	110.1
272.7	372.7	350.2	250.8	290.3	332.2	325.9	494.9	366.1
97.5	104.3	99.1	96.9	83.9	106.1	95.9	100.9	104.2
113.4	118	107.4	142.8	105.2	111.5	116.2	105	103.2
104.4	114.4	107.1	108.9	99.9	118	107.5	116.2	111.2
159	142.2	136.8	130.4	130.9	148.2	133	164.9	129.6
115.4	122.6	112.9	114.5	111.4	114.8	107.7	185	154.7
199.9	188.7	191.6	172.7	193.5	200.8	196.2	323.5	255.5
111.8	215.7	183.3	206.5	175.4	169.3	170.5	178.4	155
100.1	103.7	107.5	103.1	91.4	100.6	96.9	101.7	106.2
102.9	110.4	98.3	98.4	92.7	111.5	100.1	109.9	100.1
106.2	99.5	97.7	104.2	93.2	100.4	99.9	118.2	123.2
107.1	105.6	111.4	93.1	94.2	112.1	105	108.8	108.1
107.2	107.7	99.1	96.4	89.6	102.1	92.6	110.2	106.2
110.7	106.9	109.1	107.6	104.5	112.2	113.4	115.9	105.2
119.4	121.9	109.7	112.9	101.2	117.2	127.6	132.5	130
224.6	270.3	313.9	279.6	300.9	362.5	327	389.4	295.2
110.4	118.3	97.3	109.1	95.1	113.8	112.4	113.7	107.1
110	116.1	98.6	102.5	101.8	106.3	102.7	101.2	112.9
177.3	293.6	314.6	282.3	284.9	276.7	265.9	305.1	197.5
108.1	108.9	109	104	96.9	108.4	99.8	102.2	111.7
106.5	109.5	100.4	105.9	98.5	107.6	101.7	110.4	105.8
111.8	133.3	134.3	118.3	117.7	129.6	125.9	138.1	131.6
119.1	176.6	169.4	146.2	132.6	167.9	146.2	156.9	129.8
101	105.2	102.6	95	91.1	107.1	101.2	107.3	108
100	103.9	98.4	99.8	94.5	108.5	98.7	98.8	103.5
128.6	140.4	138.4	143.4	133.1	142.8	142.8	153.8	120.2
111.7	138.3	108.8	129.4	119.8	122.4	145.3	138	123.4
117.1	120.9	103.1	134.4	114.5	116.9	125.2	129.3	125
116.8	130.9	115.8	127.2	109.4	129.7	133.2	135.8	129.3
116.4	109.8	102.9	100.2	101.6	112.3	102	109.5	111.9
116.8	137.5	118.7	121.5	109.3	121.5	124.7	135.7	118.5
153	167.7	158.7	157.3	143.5	145.1	177.6	160.5	139.6
130.9	206.8	192.6	138.9	189.4	180	142.5	195	122.9
106.6	108.7	113.5	114.7	97	118.3	98.6	113	110.3
110.3	137.4	130.3	124.8	124.5	118.4	120.7	136.1	120.2
165.1	243.8	179.8	208.2	199.4	201.3	207.9	354.6	249.8
113.8	109.4	111.6	119.4	118.4	129.8	111.8	117.9	109.8
109.1	107.7	97.9	97.2	95.4	105.1	109.1	106.4	111.7
104.7	112.9	106.1	101.5	94.5	114.4	107.4	103	105.3
104.1	111	107.4	116.2	98.1	106.4	101.1	98.5	105.6
111.4	98.6	107.8	101	93.5	108.9	103.4	105.8	111
104.9	106.4	94.3	99.5	91.1	107.2	95.2	99.1	99.3
112.2	126.2	113.5	143.6	117.1	127.9	121.7	153.8	122.8
100.5	104.6	99.5	94	93	106.2	96.7	107.2	110.6
105.9	112	92.6	99.3	97	97.9	90.8	117.4	108.2

113	118.7	113.5	116.2	104.5	117.3	110.6	114.5	115.8
112.4	133.6	119.8	143.3	112.8	115.8	124	122.7	117.9
108.3	130.2	112.7	126.3	110.5	115.2	118.9	117.2	102.3
105.2	108.5	102.8	105.4	94.1	108.5	106.2	112.4	110.5
107.7	111.5	108.5	109.4	94.6	114.1	104.2	124.8	116.8
103	108.6	99.9	103.8	93.2	106.8	101.9	114	108
104.3	106.3	99.9	104.8	94.9	115.9	105.9	104.1	105.9
240.7	245.4	233.8	191.8	207.1	255.4	219.2	307.9	236.8
238	241.6	257.1	183.7	256.1	228.6	237.3	357.8	305.7
281.5	570.6	587.2	545.5	509.7	494.3	556.9	680.2	392.3
282.2	462.6	347.2	387.8	492.2	566.1	356.7	759.3	535
124.1	116.7	128.4	102.6	115.1	130.7	112.8	120	117.4
130.5	133	109.8	119.2	135.1	126.4	113.3	163.4	130.7
317.2	602.5	489.5	628.7	713.1	434.3	738.2	822.5	583.8
123.1	109	111.3	108.9	97.1	109.7	113.2	126.7	123.3
112.4	108	110.8	114.7	94.5	110.3	108.4	113	115.5
116.4	117.7	106.4	112.5	106.1	114.3	127.5	133.1	126.5
101.6	117.6	105.3	108	99.7	108	110.8	121.1	106.8
938.5	325.7	227.9	285.4	225.7	260.3	265.1	927.2	1277.3
125.6	159	135.6	188.4	128.6	132.2	140.6	182.7	187.7
111.5	108.9	96.5	109	94.3	101.1	110.8	112.3	118.8
112.3	108.3	102.4	106.8	93.5	99.2	104.3	119.3	102.4
259.9	328.4	129.8	1447.6	242.8	147.3	1069.7	225.7	227.3
631	806.7	343.8	3472.1	565.7	330.1	2050.8	392.9	267.7
102.4	110	98.3	94	89.8	109.3	96.5	117.1	108.7
110.2	113.6	111.6	107.1	107.6	120.5	112.4	106.7	110.5
197.2	290.8	267.8	253.9	225.7	274.4	303.6	393.1	247.4
117.4	114.4	111.3	108.7	99.2	120	111.1	113.6	113.1
134	138.8	119.1	135.9	103.6	130.1	115.7	142.5	142.8
101.8	108	106.9	110.8	98.7	111	105.8	113.4	103.3
120.9	117.6	114.1	113.7	101	114.9	105.4	116.4	125.3
426.5	835.3	908.2	652	708.7	939.7	694.3	1078	679.4
122.6	103.9	105.1	108	99	116.3	115.1	108	122.2
107.1	109.2	106.5	95.8	100.4	108.1	109.2	118.4	118.8
108.1	108	110.4	108	102.1	106.1	107.5	120.8	122.2
3198.1	3239.4	2532.2	3422.3	2392.3	3529.6	2041.2	5915.6	7133
101.9	102.3	105.4	108.6	96.4	108.8	104.9	112.2	116.9
102.4	110.3	102.2	115.4	100.3	108.3	98.1	104.4	112.9
105.8	120.4	109.7	100.3	107.7	121.5	107.3	123.4	116.7
213.4	260.7	216.9	242.6	169.9	212.2	229.3	211.2	140.1
119.1	139.3	135.1	120.8	108	137.6	125.3	169.1	127.3
135.6	156.7	127.9	125.8	131.2	151.1	131.5	143.1	121.8
155.9	387.5	299.8	279.8	309.1	335.3	306.8	388.5	200.9
237.3	403.7	492.3	368.4	382.1	508.2	391.7	387.6	381.5
115	125.9	145.5	104.1	115.4	129	108.6	134	122.3
110.2	94.2	99.5	106.1	89.6	105.1	103	106.4	112.9
115.4	113.5	105.6	107.4	101	109.6	99.5	110.4	105.9
108	112.1	106	103.4	100.5	101.3	107.4	106	110.2
104	142.1	147.4	113	134.3	140.9	111.6	109.4	107.1
112.2	107.5	99.5	93.4	96.7	107.2	106.1	104.5	107
127.4	144.5	128.4	118.5	130.6	119.8	114.8	144.3	122.4

121.3	126.8	122.7	115.8	113.4	128.9	116	158.9	154.8
104	104.9	100.2	102.2	95.2	105.2	101.3	104.9	99.6
189.5	300.2	307.2	334.9	234.6	279.5	336	353.1	242.1
109.1	130.8	125.7	113.2	123.5	130.9	109.2	128.8	119.6
110.4	108.2	101.3	103.3	94.6	110.1	106.4	112.7	108.7
115.6	113.5	109.3	116.5	102.7	121.9	100.1	113.2	108.1
112.9	117	108.2	110.3	98.9	109.3	114.1	123.6	108.9
202.6	181.3	165.1	157.7	184.5	171.3	161.2	229.8	203.9
785	843.8	837.4	783.7	805	1019	1012.1	1431.5	1019.5
102	96.9	94	97.6	97.2	99.9	101	109.5	115.1
100	110.6	100.3	92.4	93.4	110.1	100.7	110.7	109
100.1	109.9	100.8	97.3	95.2	101.6	104.8	112.6	106.8
105.7	103.8	99.3	105.7	102.2	103.4	103.9	108.5	106.7
103	112.6	109.2	106.1	111.7	113.1	117	118.4	113.8
104.5	104.6	101.4	96.5	101.2	105.7	100.1	110.2	108.4
104.8	104.7	110.7	102.8	94	106.3	103.6	110.7	109.8
107.6	117.5	104	111.8	105.2	117.2	112	126.9	110.4
101	110	102	96.4	100.5	119.1	101	113.6	105.3
100.5	104.1	96.6	98.6	88.8	104.4	102.3	97	101
95.9	95.4	100.5	85.8	89.7	101.7	106.8	104.5	109
111.7	118.6	119.1	108.9	110.1	115.9	103.6	125.5	118.1
136.1	275.1	271.7	328.6	218.5	266.6	262.4	188.8	176.1
106.9	114.5	110.8	100	97.9	122.7	115	114.6	115.9
322.4	336	391.7	314.4	340.1	353.6	364.7	521.9	368.1
195.4	227.1	183	175	191.7	206.5	171.1	341.7	302.7
106.8	116.3	96.2	101.9	94.2	109.3	97.4	114.6	113.2
111.4	115.1	104.6	106.8	98.2	103	114.8	111.3	107.4
404.1	947.7	880.5	729.3	730.3	714	752.9	1047.8	664
319.4	829.6	644.5	1297.1	690.1	580.5	1078.1	873.4	507.1
103.1	108.1	102.3	99.4	95	110.9	106.3	107.5	110.2
240.4	497.5	496.1	804.2	467.4	421.1	761.2	906.1	483.2
393.2	1314.6	1692	635.6	1181.7	1324	681.9	1901.5	674.3
103.5	103.7	98.7	97.9	94.5	108.7	101.5	109.2	108.2
105.9	109.3	99	95.2	95.6	109.3	93.3	107.3	97.1
113.6	101.9	104.9	102.9	104.6	116.4	109.5	127.5	109
417.3	190.9	217.8	164.6	200.9	316	314.6	758.7	315.6
106.8	111.8	107.8	103.5	108.1	117.1	103.7	117.9	114.7
113.9	104.6	114.8	106.4	110	113.7	111.5	138.6	114.8
105.3	110.8	95.7	103.7	96.5	108.9	102.1	102.8	101.2
3246.8	3501.3	4145.4	3086.9	2830.3	3039.7	3242.2	7705.4	8308.3
182.7	486.9	420	703.4	380.4	358.1	599.3	330.1	227.4
118.2	152.1	136.1	160.2	125.8	132	159.3	140.3	127.4
597.1	591.5	535.5	667.3	614.4	681.1	861.8	914.6	582.3
430.1	404.9	394.9	491.8	318	493.2	541.4	734.9	459.5
119.8	118.7	111.4	103.2	112.7	123.5	105.9	130.6	113.7
117.8	108.3	99.5	88	92	101.6	103.8	115.4	116.2
101.8	110.2	105.2	100.9	99.5	108.3	105.2	109.1	109
111.9	108.1	104.4	96.6	95.3	106	105.9	123.2	116.1
110.2	110.5	102.2	108.1	99.8	119	113	107.6	110.7
118.1	117.5	110.7	111.5	101.8	107.5	112.1	121.8	131.3
322.7	764.4	786.3	522.5	612.5	956.7	649.2	1095.7	530.1

118.1	118.6	119.7	111.1	110.1	111.4	111	128.3	112.7
131.5	210.6	219.1	222.8	170.4	226.6	248.7	213	164.8
103.7	119.6	106.9	100.9	96.6	102.2	100.3	111.1	110.7
121.3	113.8	107	110.6	90	113.9	106.7	108.7	120.5
114.5	129.2	122.6	108.7	105.1	122.7	118.4	119.1	129.2
112.5	115	103.5	114.6	102.3	119.2	120	109.9	117.1
107.6	109.1	109.7	105.8	104.6	109	104.6	111.4	112.3
136	113	102.7	107.7	100.9	114	118.6	110.2	113.8
115.8	114.4	109	100.2	100.7	103.7	102.4	115.8	121.1
108.2	104	91.7	89.9	93.2	106	100	100.7	102.2
140.2	149.6	125.6	156.4	130.2	123.7	140.4	154.5	116.7
130.5	186.7	187.7	161.3	159.9	176.7	170.3	154.7	152.2
120.3	160.5	129.5	118.7	118.8	129.9	135	144.6	114.4
109.6	463.2	249.2	424	304	179.5	249.7	333.3	191.9
117.3	121.4	105.1	106.2	104.5	121.5	110.1	127.3	118
122.6	115	112.5	108	104.8	109.9	107.3	112.4	125.6
105.1	113.3	103.7	106.8	97.9	106.8	106.9	107.5	97.6
108.4	109.7	111.1	100.9	104.8	113.4	105.1	111.6	110
113.6	111.2	99.5	96.4	101.6	110.9	98.2	108.2	116.1
128.6	110.3	110.7	105.4	101.1	123.5	111.3	120	110.6
121.5	173.8	121.4	692.1	106.9	119.5	182.3	104.3	115.7
109	103.5	100.9	104.9	96.8	114.5	102.4	100.4	108.5
100.3	111	104.9	94.2	96.7	115.8	111.4	115.1	108.5
134.9	236.4	177.6	196.7	226.9	171.3	272.8	232.1	151.5
102.8	100.7	102.9	109.4	95.5	110.4	95.5	105.8	113.6
113	125.3	116.1	130.8	109.1	106.4	128.2	113.5	108
117.5	110.1	106	107.5	105.2	112	107.2	111.4	118.5
100.8	102.8	110.8	108.2	91.7	97.3	101.3	102.2	106.5
109.3	106.3	98.2	96.8	90.2	105.3	102.3	107.7	103.7
104.2	108.3	105	96.2	90.5	98.6	100.5	97.2	103.7
111.9	115.5	115.5	109.4	105.9	115.9	105.7	121.1	113.6
109.6	112.4	98.6	101.3	96	110.5	113.2	111.9	124.8
116.5	146.1	152.6	147.5	138.5	149.3	150.7	142.7	120.6
111.7	118.6	106.8	111.2	94.7	111.1	110.8	106.5	107.8
110.5	128.4	115.9	120	103.7	118.1	104.6	116.4	113.6
105.6	105.2	103.1	98.1	94.7	119.9	105.3	111.3	130.8
102.7	103.4	91.6	99.6	97.6	106.4	99.6	98.5	105.7
1128.7	2209.7	2001.7	2000.3	2066.4	2107.5	1773.9	2900.6	1682.3
117.2	118.6	110.2	100.8	98.9	118.4	105.9	119.8	114.9
1213.6	1293.8	1065.4	1061.3	1240.6	1573.2	1315	2955.7	2880
106.8	121.4	101.6	107.1	97.2	113.3	104	109.3	103.4
712	961.9	887	865.2	1040.8	906.5	1034.5	2189.4	1906.7
105.6	103.4	112.9	95.2	100.2	118.6	104.9	114.8	120.1
324	592.6	485.9	476.2	432.2	530.6	496.6	595.7	373.2
110.2	111.5	102.7	98.1	89.7	109.6	105.9	109.1	107.5
97.1	111.9	111.1	90.7	95	111.9	99.3	99	103.6
108	133.1	122	123	116.9	132.4	110	122.7	120.3
103.5	106.6	99.8	110.2	92.4	104.2	99.5	114.8	106.6
107.8	116.5	96.4	101.5	95.7	120.8	98.1	117.5	113.5
108.7	116.6	115	108.5	110.2	115.5	114.7	123.8	117.6
122.1	130.7	121.2	126.8	100.9	126.4	111.9	146.8	133.8

120	118.4	110.1	110.5	94.7	117.4	112	132.3	126.5
118.9	108.4	105.1	98.2	93.8	111.1	108.3	120.6	105.3
139.6	238.7	265.3	232.2	187.4	220.5	243.5	285.2	228.8
211.5	332.4	234.4	356.3	255	280.6	332.1	473.5	343
178.3	335.9	293.6	244.1	232.5	243.8	267.3	293.1	205
220	246.7	194.8	223.7	202.5	229.6	217.3	244.5	178.5
104.8	116	96.3	97.7	96.8	104.8	103.1	103.1	111.6
240.2	433.3	343.4	349.4	295.1	295.5	338.5	301.3	206.1
165	150.4	170.1	184.8	150.9	180.5	192.8	210.8	183
103.9	106.6	102.7	104.9	98.5	114.1	108.1	114.6	124.3
160.3	189.9	182.3	160.4	174.8	194.5	189.2	222.7	207.3
105.6	110	104.6	112.5	99.7	112	113.5	125	116.5
171.1	214	172.3	166.9	188	197.8	186.2	256.9	222.6
114.8	177.2	187.4	153.5	144.9	165.5	164.8	202.3	155.3
157.2	307.3	233.9	222.3	227.5	253.5	248.6	261.4	231.3
114.6	130.8	128.2	120.7	118.2	125.3	129.3	148.5	134.5
1323.3	2426.3	1950	1944.7	2066.5	2148.7	2023.3	3280.1	2402.6
224.2	233.7	203.4	200.6	179	231.2	215.3	321	287
183.8	184.3	181.8	173.2	177.4	185.1	223	290.7	226.5
124.6	174.3	189	156.4	164.7	181.8	127.8	194.3	170.2
101.9	107	100.3	94.7	91.6	102.2	99.9	111.1	110.3
104.4	105.2	94.6	106.3	99	108.5	98.2	112.5	109
105.1	108.5	111.3	99.2	93.4	107.5	106.8	114.1	107.4
133.5	132.8	135.4	132.9	132.7	146	132.6	144	131.5
102.9	111.5	105.4	107.7	92	102.5	109.8	112.1	103.6
103.2	104.2	101.8	105.2	100.7	105.3	105.6	103	118.5
106.8	104.3	103.4	109.3	96	109.3	106.1	129.9	135.2
110.1	114.6	116.1	104.7	96.5	114.7	112.4	115.7	114.2
109.6	105.4	110.7	109.2	104.7	109.9	101.5	116.7	107.7
105.5	116	103.1	99.7	91.4	114.2	105.3	117.7	125.1
110.9	106.6	103	104	101.7	107.2	104.5	101.4	104.7
114.1	106	107	111.9	103.4	114.9	116.2	110.1	125.6
144.4	564.7	325.8	1263.3	338.5	220.8	410.2	173.6	161.8
106.7	109.2	98.6	104.7	97.6	122.3	99.5	111.4	103.9
443.7	1290	1353.1	1242	874.1	1313.7	1478.8	1168.4	928.8
103.8	115.3	106.7	92.8	98.9	113.4	100.7	118.8	117.9
114.2	109.8	106.6	103.1	103.9	104	105.2	130.5	105.2
132.5	165.7	146.4	148.3	155.5	147.9	155.6	156.5	133
103.3	100.5	97.2	103.3	89.5	113.1	93.6	105.7	110.6
111.9	103.7	98.6	95.5	93.2	110.5	102.1	113	102.4
1370.2	1415.5	1283.2	843.5	1164.1	1203.1	732	2226.2	2996.1
112.4	110.3	109.3	106.5	103.1	118.8	110.7	125.3	120.3
190.9	179.2	173.7	166.1	142.5	201.2	164.4	364.2	310
103.5	104.1	97.8	102	101.1	106.7	108.9	108.7	107.9
108.7	111.6	93.8	129.2	94.2	118	105.7	104.5	107.2
108.3	104.1	105.2	93.6	91.8	100.6	102.8	99.7	109.6
107	98.4	99.7	90.8	93.8	98.5	104.1	105.5	105.4
103.7	107	102.4	101.5	104.5	118.6	98.2	104.5	106.5
519.7	848.7	1032.4	768.1	818.4	874.6	735.5	1228.7	660.2
103.8	107	101.9	100.4	94.8	108.9	103.3	122.6	108.7
107	116	111	108.2	102.9	106.2	108.8	131.2	106.8

108.4	114.9	103.1	100.7	90.9	112.2	103.7	113.9	112.9
146.5	187.6	152.1	164.5	141.1	167.9	179	198.1	165
107.3	129	130.8	123	106.8	141.5	124.3	125.6	129.8
101.6	106.3	99.7	110.2	98.7	104.6	103.7	106.5	102.5
113.1	112.3	113.1	109.1	107.5	119.8	102.6	113.5	116.3
104.1	105.2	99.5	94.1	89.7	101.7	101.7	103.9	107.4
110.8	107.2	102.1	103.2	102.9	114.5	110.6	110.3	111
110.8	116.8	108	105	100.5	118.5	103.4	120.1	114
112	110.8	100.9	97.3	103.1	103.7	103.2	107.9	115.9
114.9	116.1	106.5	115.1	101.5	121.6	111.8	122.6	133.2
105.6	116.8	124	105.7	111.7	123.8	115	129.5	119.5
108.8	103.3	109.2	112.4	102.2	104.6	95.6	112.1	115.1
106.3	102.4	100	99.6	94.4	114.8	101.8	104.7	103.7
109.5	113.5	101.8	101.7	100.6	117.6	111.1	127.1	111.5
566.8	734.2	692.8	744.5	572.9	751.1	625.9	1305.2	918.8
221.7	327.3	222.2	337.4	209.4	299.7	177	352	333.1
106.7	113.5	112.5	113.7	106.6	113	106.2	121.9	115.2
108.3	199.4	163.8	180.2	132.7	142.3	145.7	169.4	118.8
104.9	112	103.9	93.4	90.9	100.7	99.3	103.3	103.1
119.4	116.4	110.3	113.4	103.3	119.1	117.2	122.5	113.6
266.1	254.9	288.6	222.3	246.5	328.5	236.7	363.3	343.5
111.7	119.7	115.9	114.3	112.9	113.7	119.5	147.4	132.1
123.7	117.6	104.2	97.8	96.6	107.7	104.6	113.3	112.4
113.1	105.6	103.5	96.8	99.5	108.4	106.3	103.3	117.8
291	842.9	814	1067.7	551.8	732.2	1018.2	709.9	458.7
115.9	110.4	126.3	103.5	111.9	123.5	114	150.1	169.3
109.7	112.6	109.8	112.6	102.1	121.6	106.2	125.2	117
104.1	101.5	112.4	102.3	108.7	116.4	109.5	130.2	116.7
107.6	118.4	107.3	106.8	95.7	107.7	112.4	102.6	113.4
102	107.7	112.8	109.2	91.5	109.1	103.9	113.1	105.2
132.1	139.7	135.9	141.3	123.9	129.7	155	173	138.6
113.1	127.4	119	124.6	110.2	126.1	129.3	166.1	126.1
102.6	106	104.2	95.4	99.4	111.4	100.9	105.6	106.7
116.1	123.6	123.9	119.3	108.2	114.8	120.8	143	140.7
115.3	111.7	104.3	109.2	94.7	107.6	111.6	111.1	120.4
104.1	112.9	97.6	101.2	97.9	107	102	104	115.5
106.4	109.1	109.4	105.1	103.6	108.6	98.7	112.6	113.8
104.4	107.4	104.1	106.1	97.3	106.6	100.7	102.8	103.5
109.1	99.9	96.5	102.7	102.3	104.2	103.1	103.1	103
111.1	125.5	122.5	114.1	108.3	120	125.2	123.7	112.3
104.7	105.2	96.8	102.5	98.4	104.9	99.9	104.1	116.7
110	112.5	100.7	99.1	97.3	109.4	99.7	99.9	112.3
107.4	110	105.3	98.5	95.2	104.7	104.4	112.3	109.3
114.2	115.9	105.3	109.7	97.4	118.4	113.9	129.1	113.5
109.8	128.3	121.1	106.7	100.1	107.8	104.5	109.6	106
130	169.6	152.5	182.9	137.9	140.2	168	141.5	119.7
103.2	103.8	94.9	103.1	93	108	97.5	107.5	114.2
116	160	155.7	200.4	137.9	148.3	159.2	186.7	177.9
104.1	100	100.7	96.4	101	103.6	106.5	107.5	101.6
116.1	121.1	115	118	92.8	106.6	110.7	117.3	123.4
96.4	105	103.3	108	98.5	107.2	103.7	102.4	107.1

148.7	129.8	130	145.1	110.2	139.7	149.9	165.7	145.7
105.1	117.7	98.2	108.5	100.7	110.6	101.5	103.9	105.5
101.5	106.7	106.2	103.4	98.7	114.6	113.7	118.7	112.2
749.3	912.2	809.3	796.4	767.5	936.9	930.1	1161.3	939.6
222.8	384.5	395.8	302.5	367.6	398.6	341.3	384.3	265.2
155.7	236.2	189.1	224.5	192.3	203.5	200.2	335.7	288.4
170.8	244.1	233.8	241.2	219.1	253.9	294.9	316.7	222.1
110.5	108.3	102.1	96.2	97.3	100.6	108.7	103.5	111.4
175.5	226.3	165.9	204.3	192.9	230.9	242.5	373.8	289.8
608.4	1015.3	844.9	706.8	617.2	676.9	661.2	1111.8	721.3
106.4	108.5	102	98.8	98.9	108.9	98	104	102.8
118.8	118.2	114.5	112.9	101	109.3	122.7	112.4	117.3
187.1	314	380.1	263	287	346.3	298.5	392	261.4
338.4	735.8	678.7	676.1	604.1	708	640.8	996.3	645.3
126.8	146.7	125.6	123.9	121.4	133.3	137.1	167.5	140.4
111.5	122.1	107	202.8	109.8	131.6	182.1	140.3	128
114.9	146.7	136.9	146.3	141.5	149.7	145.3	146.1	129.8
585.8	710.7	634.9	536.5	531.5	661.9	629.3	851.9	742.5
107.8	118.9	107.2	105.6	102.8	116	114.7	118.6	116.3
113.4	111.3	99	100.9	95.4	108.2	106.1	109.2	112.9
110.2	114.5	101.6	106.4	98.5	110.1	107.5	107.6	104.4
105.7	107.5	98	96.3	95.3	109.1	106.6	108.2	102
112.9	110	107.3	102.5	104.1	108	105.2	113.1	112.5
101.9	108.8	102.6	101.1	92.9	108.8	102.7	112.8	113.5
110.5	113.3	103.6	101.7	100.5	115.8	94.3	115.5	110.5
116.9	119	99.5	108.5	102.2	131.7	101.6	121.9	104.1
115.2	119.1	111.5	105.6	99.9	116.6	108	111.9	120.4
108.8	106.6	97.3	93.7	87	97.6	109.6	102.1	105.6
107.8	119.4	110.7	113.3	102.4	112.3	112.5	115.6	114.8
107.6	103.1	111.1	101.5	101	113.1	105.3	117.1	118
107.5	110.5	111.6	104.7	97.6	107.7	102.8	115.2	113.5
114.2	117.3	118	114.8	105.8	112.6	108.7	129.6	105.6
109	100.5	96.1	91.9	94.6	103.1	102.5	105.8	110.7
110.6	108.8	98.3	98.7	99.7	106.3	96.8	108.2	104.4
116.6	120.6	110.4	104.5	99.2	125	112.2	127.2	120.3
124.6	122.1	120.3	121.1	120.1	110.7	139.7	144.2	129.5
107.6	121.3	112.7	117.3	104.4	115.3	102.3	107.3	100.5
119.4	114.4	110.9	106.4	93.7	114.3	106.1	118.3	115.2
169.7	150.2	153.8	128.5	169.4	151.1	130	316.6	190.4
109.1	115.5	106.3	99.4	88.5	110	102	106.6	109.6
158	186.2	187.6	154.7	180.9	194.5	205.7	260.6	173.8
117.8	134.7	132.5	118.1	139.4	133.6	135.3	160.6	144
100.1	110.2	109.3	105.5	105.1	114.6	109.7	116.4	115.6
121.6	122.4	108.9	116.9	95.2	124.6	96.8	105.1	104.3
106.6	119.8	110	107.1	100.4	118.9	111.9	137.9	117.6
106.9	102.9	104.3	102.9	93.5	100.3	100.5	112.8	116.6
116.4	107.5	115.4	117.8	91.8	107.5	104.9	118.7	104.6
147.7	213.6	238.3	185.9	186.2	225.5	198.6	183.6	164.2
109.1	118.2	105	101.7	109.1	106	101.4	104.9	103.7
101.1	103.8	102.2	94.1	92.7	107.6	101.3	108.1	110.5
128.3	150.5	145.3	159.9	134.8	157.7	158.4	238.8	195.4

371.9	391	385.9	388.3	348.6	404.5	399.1	457.1	355.9
101.8	106.1	105.9	102.4	94.6	101.5	100.6	110.6	113
142.5	208.9	202.5	180.3	185	194.6	185.7	282	206.5
504.1	587.3	605.2	315.1	465	494.1	418.1	1579.7	977.8
425.1	500.6	391.1	464.4	546.8	446.4	582.3	867.9	640.1
326.5	541.6	403.9	505.2	408.9	469.3	554.1	791.4	510.6
114.2	110.9	109.2	99.4	95	121.2	102.7	128.1	110.6
162.5	189.9	154.8	145.9	153.7	176.4	156.9	216.5	216.3
113.4	106.8	108.3	103.2	94.9	110.3	96	112.5	108.8
102.3	108.7	99.8	101.1	99.2	107.7	105.1	104.5	109.8
105.1	109.9	99.6	98.4	97.6	108.6	96.9	108.6	98.5
151.9	201.7	178.5	175.4	217.4	177.2	201	259.2	125.5
108.4	140.2	123.8	108.6	119.8	128.4	127.6	138.9	131.2
110.4	128.4	120.4	120.3	107.4	121.1	108.8	124.7	105
110	115	118.5	113.7	101.2	122.6	122.8	130.8	119.6
115	112.9	121.9	109	102.9	121.8	104.8	127.1	130.2
162.6	171.7	159.8	167.9	150.3	170.1	176.3	281.1	191.6
224.4	242	272.9	189.8	238.4	283.4	273.3	391.8	250.5
105.9	124.4	109	105.8	112	120	108.6	136.2	126.5
105.2	108.5	97.9	97.8	100.6	106.9	100.1	97.5	105
113.6	123	135	116.9	128.7	159.5	122.4	136.4	140.3
98.1	101.1	99.4	96.2	88.6	101.6	89	104.8	99
106.9	116.3	98.6	109.6	101.3	109.9	119	115.4	115.1
97.9	107.5	100.4	99.1	95.4	98.7	105.4	115.3	112.5
102	103.9	104.3	101.6	95.9	104.7	104.2	110.3	104.1
105.8	101.8	93.4	99.2	95.7	105.7	97.2	110	102
105.6	130.1	108.8	130.6	95.1	111	110.2	115.1	109.5
118.4	106.6	109.9	114.9	101.9	120.4	116.5	132.6	125
102.1	111.2	104.4	101.4	95.6	107.3	95.5	106.4	105.8
101.1	115.6	107	98	98.6	103.3	103.1	99.7	116.5
129.9	142.9	138	117.7	106	130.3	124	139.7	124.6
122.7	132.5	115.7	112.3	114.6	146	122.3	147.4	130.8
131.2	145.2	173.3	148	133.2	171.8	153.8	225.6	208.3
113.8	110	109.8	98	98	112.6	108.3	107.8	107.3
105.3	99.7	92.2	96.4	98.1	108.7	91.1	107.3	103.8
99.1	108.9	100.7	94.2	94.3	109.8	107.1	100.1	99.6
104.7	114.1	104.8	100.9	93.5	107.6	109.1	110.4	103
102	113.3	111.7	130.5	111.3	122.9	109.2	133.5	115.4
171.1	265.2	170.3	409.1	233.4	219.9	325.2	431.7	255.3
112.5	127.4	106.4	106.3	106.4	127.7	113.2	119.1	124.2
112.3	119.6	108.8	113.8	98.1	107.9	112.9	111.7	113.5
108.1	120.8	103.2	103.1	97.5	129.4	105.4	119.5	111.2
108.2	111.8	100.2	99.6	96.2	106.5	104.7	108	103.5
126.5	166.3	157.3	122.5	140.1	143.6	107.6	142.7	140.5
205.8	166.7	154.5	132	147.1	176.1	131.3	217.7	225.2
109.5	118.1	110.2	106.6	106.2	111.8	111.7	117.6	118.2
147.8	170.6	166.1	140.8	157.9	179.5	159.8	232.8	243
109.3	115.2	105.5	120.3	101.2	124.1	110.2	117.9	117.5
112.5	108	104.6	104.1	105.2	119.2	114.9	127	109.2
109.3	106	107.8	95.1	94.1	115.1	99.5	110.4	107.6
107.6	105.8	101.5	106.8	98.2	105.8	99.7	117.9	107.7

147.5	144.3	146.1	148.2	112.7	150.4	120	193.7	221.2
104.5	111.4	102	96.9	96.7	115.5	98.2	119.8	111.5
141.3	241	246.1	202.4	218.5	189.3	223.3	241.2	193.6
112.6	108.9	110.7	102	105.3	111.4	113.2	117.1	109.9
291	526.1	459.5	374.7	400.8	415.6	443.2	551.5	436.9
103.3	106.9	99.3	97.6	95.6	100.7	95.8	97.7	105.1
112	109.5	95.6	93.9	93.6	98.6	101.2	105.6	99.4
105.1	111	104	106.8	100.9	108.3	99.7	110.3	115.6
113.7	119.8	111.6	122.3	97.6	116.7	121.3	128.9	116.3
118.8	129.5	128.2	131.2	119.9	133.9	108.1	140.5	126.3
102.2	110.5	105	91.8	91.3	104	99.1	103.9	95.5
143.6	154.7	139.1	121.5	126.4	148	123.7	164.8	133.4
114.7	112	103.7	111.7	101.6	106.1	116	120.5	125.6
105.9	112.7	103.9	98	98	110.1	109.1	110.6	111
127	138.5	141.1	152.7	137.2	151.6	156.5	198.6	138.5
154.7	205.4	198.6	185.3	177.2	200.5	195.9	251.6	161.1
109.5	118.4	101.5	108.9	95.5	106	114.2	108.6	102.4
105.2	97	93.7	88	93.7	102.4	107.8	106.6	99.2
105	110.4	104.5	100	98.4	106.2	96	117	108.9
115.7	125.8	123.7	117.3	115.7	140.3	124.9	141.4	122.3
105.3	102.1	100.3	93.8	91.5	98.7	107.3	95.8	96.8
115.3	105.5	96.9	97.1	98.3	105.9	101.8	109.2	104.3
172.2	292.7	308.5	270.5	253.8	234.6	306.1	349.7	237.6
111.7	110.9	101	100.6	101.8	112.2	102	116	114.5
107.1	109.3	107.1	105.1	105.9	124.7	117.2	123.8	107.9
113.1	116.5	107.2	116.1	95.2	115.5	95.4	112.5	115.3
107.2	111.6	109.6	109.6	106.8	112.9	121.9	104.7	110.4
3017.5	3811.9	3871	3305	2899.3	3119.7	3784.8	5362.1	4024.1
109.9	106.5	103.9	94.3	92	110.6	118	110.3	111.2
105.4	105.5	97	100.1	96.4	109.2	97.9	107.4	113.1
109.9	110.4	90.1	128.8	103.8	110.1	102.6	106.5	105.7
227.8	194.6	159	415.4	195.5	159.2	418.4	184.3	118.5
119.1	123.7	114.4	107.4	103.3	115.6	108.6	126.4	128.2
125.9	118.8	110.7	139.1	112.7	142.2	141.2	146	135.7
109.1	116.5	103.6	105.9	95.2	107	105	109.9	108.6
199	259.4	276.1	331.2	222.3	218.2	231.7	265.9	197.9
109.3	109.1	108.7	100.9	103.6	113.1	110.1	107.5	100.8
4280.5	2350.3	2416	1920.2	1915.7	2918.9	1854.8	5544.5	6108.7
109	105.1	105.2	100	99.3	104.8	100.1	108.7	106.7
173.3	173.7	189	182.5	216.4	191.4	174.4	259	182.7
353.7	506.2	509.5	485.5	511.9	604.2	357.1	624.9	438
121	126.6	109.3	107.9	106.8	122.6	110.6	133.3	112.4
107.9	106.6	97.4	102.1	94.2	104.3	99	120.2	100.9
110.9	107.3	98.3	98.5	95.7	105.6	101.4	106.1	104.5
105.1	105.3	102	103.5	92.4	109	100.9	107.2	107
526	661.7	353.7	528.8	442	435.5	468.9	612.1	510.9
183	204.9	165.2	207.8	174.7	170.5	197.1	253.3	173.1
2478.9	2994.5	1808.1	3835.6	3262.8	2621.6	3617.6	6398.5	4234
675.5	571.2	456.8	1319.6	582.3	410.4	1122.5	1211.8	716.4
107.9	114	100.2	106.5	99.6	105.9	102.1	114.6	103.5
108.5	124.8	114.3	115.9	115.1	115.3	121.5	130	122.7

1626.1	1728.6	1467	1448.9	2302.9	2818.8	2259.3	4680.2	1271.4
106.8	131.3	138.7	131.1	116.4	135.4	124.3	149.2	145.3
116.1	181.5	182.1	144.3	124	186.8	139	161.5	197.4
109.2	110.7	98.1	96.6	100.4	112.8	103.3	115.4	108.6
1000.8	1031.5	790.9	856.1	764.5	912.2	1060.6	2265.9	2089.4
109.4	114.5	111.8	103.4	103.8	111.6	109	120.9	119.5
112.6	105.6	97.8	99.6	102.3	112.6	104.7	108.4	97.7
112.9	106.8	102.5	94.6	88.4	112	103.1	98.2	100.4
143.1	187	184.1	190.4	158.1	138.3	193.3	206.1	185.1
112.9	106.2	114.8	106.2	100.2	116.7	109.2	123.8	120.2
106.6	108.1	100.3	102.7	99.7	108.5	96.4	116.9	115.1
105.8	111.2	105	96.8	93	106.3	96.6	105.8	111
107.8	110.3	102.8	135.5	110.2	122.9	121.9	121.5	104.3
101.9	103.9	105.9	100.2	93.2	110.4	104.2	114.1	106.9
164	265.5	284.7	223.8	236.8	234.5	274	335.5	223.8
102.4	109.3	101.9	101.5	93.9	106.3	105.4	109	99.7
122.4	110.3	113.7	125.5	102.6	114.2	129.6	127.6	112.9
197.3	255.2	257.4	350.1	228.3	217.8	295.1	305	220.9
238.2	255.6	217.5	209.1	247.1	266.8	229.5	349.9	462.3
101.6	107.5	96.3	96.8	90.6	102.5	102.3	105.7	102.2
334.4	551.8	657.4	449.1	464.7	557.7	455.8	613.8	390.8
117.3	118.5	126.6	109.8	118.6	136.5	108.6	127.3	134.1
122.5	120.8	119.7	113.2	106.6	127.5	106.8	138.1	126.9
289	662.1	536.8	580.1	526.8	494.8	569.2	763	494.4
108.7	113.3	104.2	109.2	100.5	118.3	97.5	116.5	116.2
118.5	163	134.7	156.3	154	166.9	148.6	210.2	150.4
126.1	177.9	159.6	147.5	161.5	160.6	162	217.5	173.6
581.1	740.1	598.5	1296.6	662.7	538.8	838.2	1179.1	728.6
105.4	102	107	89.7	97.9	103.2	101.1	105.2	104.7
108.5	124.4	116.8	118	103.2	128	113.8	131.7	122.1
108.6	103.1	97.5	98.8	92.2	105	99.8	107.1	100.8
107.1	107.4	107.3	103.6	93.9	106.1	114.5	108	103.1
147.1	150.4	161	172.8	139.5	161.3	170.8	170.3	140.3
130.3	130.5	148.8	143.8	142.4	165.3	171.8	191.8	169.8
125.2	141.5	144.1	141.3	121.7	167.1	144.3	178.7	179.6
233.5	285.3	349	367.4	309.3	358	394.4	494.9	271.5
254.3	244.2	265.6	364.7	288.7	339.1	430.9	515.5	296.2
352.3	580.3	500.2	763.7	438.8	502.1	663.1	877.4	614.6
2020	3700.9	2612.2	5268.3	2954.6	3034.3	4906.9	3931.1	2586.9
495.3	803.6	560.9	646.8	666.7	587.8	760.3	794.5	545.4
102.8	109.7	99.8	99.1	100.4	108.8	100	110.1	111.5
99.3	108.3	102.1	105.6	91.8	102.2	103.2	107.4	110.5
228.3	299.4	286.3	271.2	298.4	327.9	327.3	448.1	248.7
212.8	317	249.3	238.2	277.7	272.8	307.2	418.2	292.3
248.6	266.4	228.8	249.3	281.8	288.5	279.5	324.1	354.6
112.6	105.6	106.7	97.9	99.7	109.7	102.8	104.9	106.4
282	294.3	279.9	317.6	282.8	303.5	393	447.6	430.4
245	316	276.9	249.2	251.2	285.7	333.1	334.9	235.4
208.3	221.8	195.7	198.1	188.8	212.4	274.6	266	272
125.2	111.2	114.2	120.2	107.7	124.6	121.2	142.5	118.5
453.8	432.3	420.9	356.3	387.9	459.7	518.6	941.8	793.2

179.7	230	211.9	158.8	185	208	242.7	279.5	215.5
116.1	112.4	102.9	108.7	94	106.6	108.2	111.4	106.3
123	117.8	125.6	116.2	112.4	121.7	114.7	131.8	123
246.5	280.7	246.8	266.4	195.5	248	341.3	384.5	332.6
116.4	117.6	102.3	116.2	103.8	118.3	115.3	128.1	111.7
107.5	110.1	97.5	102.4	100.5	103.2	104.9	116.9	105.2
110.3	112.9	125.1	113.8	109.6	130.6	116.7	125.5	120
103.7	119.2	110.4	103.9	106.2	110.2	114.2	112.4	112.1
110.1	110.3	103.4	105.4	92.2	108	98.7	99.8	103.2
108.9	116.7	116	125.1	106.4	113.6	112.1	112.8	116
194.5	290.1	428.3	271.4	246.4	378.1	318.5	395.6	296.9
105.8	118.8	95.6	124.1	100.7	124.9	115.2	119.9	111
109.1	111.6	105.3	104.7	96.3	105.4	104	111.5	102.7
101.2	103.4	110.5	96.7	100.1	102.3	100.2	110.2	113.2
310.1	581.8	519	706.2	556.6	483.4	515	614	401.5
95.5	106.7	97.7	93.2	91.8	104	102.8	96.2	105
245.5	580.4	503.3	420.4	435.1	431.8	491.2	433.3	229
162.3	279.4	291.8	242.1	233.6	227.2	302.2	258.3	161.8
104.4	106.3	99.4	97.7	100.1	112.2	101	104.6	111.5
102.2	106.5	97.7	97.8	92.7	108	97	112.9	107.3
107.6	110.1	101.9	105.6	99.8	110	101.2	110.4	107.6
134.6	132.8	115	122.4	110	135	122	124.6	123.5
142	237.1	357.5	263.3	257.5	273	380	378.2	213.1
235.2	816.8	811.8	643.1	548.8	652.6	548.7	669.7	358.9
368.6	1463.5	1471.5	1245.9	1016.2	1079	1101.3	1049.8	519.1
166	205.9	195.7	215.8	184	212.2	215.2	289.9	158.9
369.7	605.7	579.9	535.4	540.1	530.8	597.9	724.2	480.4
136.4	165.8	123.9	156.2	127	141.4	138.6	186.5	159.3
159.6	274.8	189.4	302.5	193.8	182.6	298.9	231.5	148.6
267.7	367.1	297.5	366.4	314.2	281.6	350.3	672.2	568.4
112.3	111.3	101.4	94	99.2	111.5	100.4	108.8	115.5
151.3	188.7	148.5	131.6	137	157.1	162.9	189	161.9
103.2	106.6	102.5	105.1	96.8	114	102.6	112.3	118.6
134.4	171.2	182.6	159	140.1	178.2	139.1	205.6	160.7
113.5	115.4	104.7	112.2	102.8	109.8	108.9	118.7	100.1
109.4	117.9	103.4	95.8	99	114.1	108.6	111.1	106.8
103.6	109.3	96.3	98.2	100.8	99.6	100.3	104.5	105.8
106.9	111.8	101.5	95.5	92.7	106.5	106.8	105.6	105
103.8	108.8	102.6	92.6	99.4	112.3	110.3	120.2	112.5
205.9	542.7	624.6	371	530.1	535.4	503.7	663.3	400.7
105.2	110.7	101	103.6	102.9	112	108.7	111	110.5
105.3	102.2	101.8	105	95.9	103.6	95.2	106.5	104.8
98	106.9	104.5	96.6	92	106.6	95.7	113.3	103.4
98.3	114.4	112	110	98.4	113.4	105.3	124.6	118.6
134.2	155.8	171.9	179.6	176.7	164.8	199.2	176.1	142.2
109	105.8	96.9	94.4	96.3	111.5	104.8	108.3	107.5
106.9	104.5	99.8	100.4	93.1	102.7	102.5	105.8	107.7
108.5	139.2	103.2	110.5	97.8	110.1	101	112.2	105.6
104.4	106.3	107.2	94	100.8	102.3	104.2	111.4	107.8
116.3	130.8	146.6	150.7	110.7	128.4	138.7	128.6	130.6
106.3	116.1	110.9	112.2	97.9	113	100.1	112.5	115.9

134.3	201	151.8	172.3	141.3	144.1	194.8	204.9	185.1
158.9	284.1	168.5	197.9	178.7	158.7	163	219.9	179.5
122.3	148.3	120.3	123	117.2	127.6	112.6	134.6	130.6
235.1	446.1	320.9	328.5	315.1	248.2	263.5	351.3	322.8
103.1	115.6	103.7	104.8	103.2	101.2	101	108.9	113.6
100.5	135	118.8	122.7	104.3	116.2	111.6	111.5	113.2
109.1	109.9	115.5	98.9	106	104.6	117.9	103.6	110.2
107.9	111.9	97	104.7	96.7	106.1	103.9	111.6	107.1
113.1	99.8	99.8	105.1	97.6	109.4	107.4	105.4	105.2
107	101.3	101.2	97.4	90.4	100.5	99.6	109.1	116.9
127.1	179.6	234.8	103.9	131.6	206.3	105.8	144.6	132
112.5	105.2	103.5	97.2	95.2	110.4	99.9	110.9	117.9
108.9	107.3	100.6	99.4	95.3	104.4	100.5	106.7	105.1
115.4	108.9	110.3	115.9	104.6	116.6	103.4	121.1	111.1
108.9	108.5	96.8	98.8	97.5	107.7	101.4	109.6	106.2
113.4	115.5	103.3	103.1	107.2	116.7	106.1	120.4	117.3
109.6	99.3	101	102.9	101.8	113.6	106.4	110.1	117.6
159.9	130.9	128.7	136.6	115.8	128.3	162.1	218.8	262.6
103	104.3	98.9	95.9	92.7	102.6	103.3	102	99.5
910.3	1408.3	1412.4	1024.7	1080.5	1091.7	1035.5	1269.5	1001.4
106.4	113.3	104.3	113.8	100	112.7	109.9	106.9	113.4
106.1	112.1	116.7	103.2	103.9	108.9	110.9	115.2	109.2
106.2	112.2	104.7	108.1	94.3	101.2	105.9	112.9	107.8
105.5	108.5	105.1	105.8	97.8	110.6	100.1	106.1	112.2
117	135.8	117.3	116.5	103.5	118.9	104.9	123.7	140.6
106.8	115.2	111.2	104.7	108.3	107.7	107.5	114.6	105.9
120.3	112.7	113.6	116	100.8	108.6	110.6	107.7	110.1
222.8	275.4	231.7	203.8	201.3	200.8	203	212.6	168.7
104.5	116.3	113	110.6	105.9	106.5	105.3	118.6	123.3
150.4	168.1	147.9	155.8	132.4	151.9	150.6	176.5	158.7
101.4	109.6	101.6	99.8	93.5	104.1	118.3	126.5	110.3
325.1	449	495.3	455.3	320.3	485.3	487.2	602	486.2
271.4	384.7	357.9	369.2	303.9	337.6	368.8	445.2	359.5
415.3	400.8	418.4	369	274.2	361.2	360.6	487.3	381.8
131.3	155.1	149.3	168.8	128.4	154.3	143.2	193.3	154.5
158	151.7	140.4	138.2	135.4	147.6	160	180.1	157.5
199	220	184.6	155.5	167.9	235.5	172.8	286.7	229.1
440.9	716.1	830.4	960	714.3	724.2	817	667.4	450.8
194.4	348.3	307.9	336.9	244.7	304.8	362.4	312.3	229.4
113.6	124.8	109.6	98.1	96	115.6	104.2	112.1	109.2
195.1	234.9	195.6	192.6	191.9	213.9	210.6	296.7	259.2
103.1	102.2	98.6	93.7	99.6	107.5	95.7	104.7	105.9
155.5	141.2	148.6	153.2	136.4	149.9	158.2	183.5	165.4
98.5	114.2	104	91.1	97.2	105.8	102.5	109.4	104.2
110.3	111.4	107.4	105.5	98	114.2	113.1	105.8	110.8
110.1	131.9	113.8	116.8	103.7	119.3	112	112.6	116.8
104.2	109.5	107.8	99.5	96.8	107.9	107.2	112.5	98.4
103.1	104.7	99.8	97.3	91.9	100.8	92.3	100	106.8
101.6	123.4	115.2	104.7	104.4	112.4	103.4	123.8	118.4
104.1	110.8	102.6	98.5	95.9	107.6	110.7	104.5	113.9
102.7	115.2	103	97.4	96	106.4	110.7	107.7	108.6

111.6	131.2	117	133.2	118.2	124.5	109.1	131.1	112.2
113.1	116.8	111.4	98.9	101.2	114	103.2	109.7	115.2
108.1	106.4	98.8	102.4	92.9	102.9	98.9	107.5	103
110.6	108.9	108.2	98.9	95.1	107.8	100.5	103.1	105.6
112.7	118	107.6	109.4	102.8	106.3	109.7	128.8	109.2
105	112.6	95.8	98.4	100.4	108.2	102.4	112.5	110
101.6	112.7	104.4	106.5	100.7	114.1	109.1	116.6	117.7
115.5	110.5	102	96.8	93.8	102.9	96.2	110.8	108
131.9	167.4	191	99.4	133	149.1	103.9	144.5	113.4
114.3	118.4	119.3	107	104.3	117.3	107	121.8	114.5
112.9	108.6	106.3	111.7	99.6	123.7	110.4	115.7	104.2
103.7	104.4	96.7	99	92.3	104.3	96.9	104.6	103.8
112.4	106.9	107.7	107.8	96	109.4	102.2	103.6	105.8
263.5	553.9	563.9	425.8	453.7	497.9	448	530	303.8
111.6	110.2	105	110.5	104.3	115.3	112.3	132.2	127
613.9	579.4	583	466.8	555.9	665.5	710.4	1150.6	984
163.6	195.9	195	201.7	193	241.1	241.1	328.2	184.5
108	105.1	96.6	96.3	98.2	110.7	110.1	103.9	109.5
148.9	141.5	132.4	122.3	116.4	137.3	152.4	191.8	171.3
1131.4	971.4	835	840.7	1003.6	1085.4	1300	1953.4	1525.9
122	118.2	110.1	123.8	101.3	115.3	105.8	124.6	115.4
136	243.9	200.6	269	218.9	238.5	265.3	267	191.4
197.6	196.6	141.9	151.7	149.6	197.8	161.2	339.5	402.8
747	553.4	538.7	640.1	647.7	562.2	477.9	1630.9	1602.2
439.6	444.6	392.9	536.9	384.3	483.7	450.3	639.4	476.7
109.7	112.4	106.1	117.2	106.7	120.1	102	119	110.1
105.2	112.3	115.7	104.3	95	107.8	111.3	105.8	106.3
182.3	220.8	260.1	207.3	222.8	282.7	233.9	329.4	250.1
106	112.9	105.4	101.5	98.9	107.4	104.4	108.2	111.3
134.3	140	139.2	149.7	139.2	153	140.6	228.1	168.5
118.7	117.7	113.2	102.3	108.9	130.6	105	164.2	120.4
123.5	197.2	165.5	164.3	155.4	172.9	191	258.5	176.4
110.3	108.1	101.4	101.5	99.3	112	99.7	106.8	113.3
110	124.4	116.1	122	106.8	118.3	100.6	121.7	111.5
112.4	143.4	134.6	132.5	124.5	146.8	133.8	149	125.7
104.5	112	106.2	116.3	94.5	112.5	99.3	113.8	121.3
134.4	153.1	126.8	106.4	140.7	156	119.3	452.6	220
113.8	109.3	102.1	108.5	94.3	101.8	105.6	114.2	116.5
108.2	115.5	105.8	101.6	91.8	104.9	97.2	107.8	107.7
109.1	109.6	104.4	107	95.2	109.4	104.6	104.4	103.8
237.6	339.3	335.2	337.4	312.5	373.2	353.6	356	284.8
118.7	134.2	129.4	135.9	119	123.5	146	186	140.1
124.3	138.6	120.2	123.6	105.8	152.3	120.5	167.1	133.5
105.3	106.5	102.3	101.6	94	107.7	100.7	106.2	102.6
118	117.1	106.4	107.1	102.3	116.4	106.5	119.3	119.3
118.5	137	125.2	106.8	110.5	121.3	118	161.1	125.5
809	1280.9	1457.9	1015.9	1065.2	1315.8	889.9	1380.6	1139.6
593.1	860.7	701.8	682	706.5	602.4	742.1	882.3	662.2
143.4	146.3	140.7	141.2	123.1	130.2	134.3	189.1	149.1
107.1	116.8	99.7	97.9	98.1	109.9	105.2	111.7	113.4
104.5	118.5	111.3	123.7	107.7	109.1	132.7	127.7	121.6

178.7	208.5	197.4	211.8	178.4	201.2	232.6	262.1	212.7
107.1	108.5	106.3	106.7	95.5	106.3	104.8	112.5	117.9
240.6	224	224.2	254.9	248.2	237	298.1	427.3	337
102.4	130.3	110.7	129.1	109.5	120.3	121.8	137.3	116
107.6	127.1	105.8	113.6	102.5	112.9	102.8	109.4	112.1
109.4	122.4	113.3	115	103.9	117.1	112.7	107.6	115.3
120.6	117.8	104.1	105.9	107.8	112.3	110.5	125.1	116.5
125.5	164.2	129.9	143.1	137.5	148.9	126.2	127.8	140.4
104.5	105.4	107.2	109.2	101.2	115.5	97.7	114	109.5
320.5	565.1	675.3	555.4	679.1	659.1	519.8	1222	631.8
111.7	113	105.7	102.6	98.5	115.2	99.8	117.4	113.5
110.7	114.1	103	111.4	101.5	113.9	110.4	119.9	127.1
104.6	109.6	103.6	102.5	105	109.2	106.2	125.7	115.3
101.9	101.2	95.1	92	93.1	108	99.1	100	106
113.2	105.1	101.2	107.4	102.9	116	105.2	111.2	115.7
106.1	100.3	100.6	115.2	95.4	103.1	99.3	105.7	104.7
109.5	108.6	95.1	97.2	95.7	107.1	108.9	104.6	111.3
328	717.6	711.6	507.7	592.1	738.1	608.9	973.6	630.1
760.3	1131.6	892.4	870.4	727.9	933.7	936.6	1295	972.4
179.6	332.3	275.3	227.7	221.9	317	243.5	334.2	268.5
369.7	649.3	732.6	649	508.8	420.2	541.8	405	246.8
105.9	114.7	110.6	112.4	104.6	116	106	121	123.4
102.6	104.3	107.2	99.7	92	104.1	107.3	113.5	109.3
106.1	102.9	99.7	104.9	95.6	115	103.4	106.2	112
102.9	104.6	94.5	94.4	91	97.4	97	107.9	108.1
101.8	104.3	91.9	105.3	102.4	107.3	104.6	106.4	108.4
104.6	100.9	97.6	93.8	97.6	106.7	102.4	100.2	105.1
101.2	109.4	103.8	113.1	104.8	108.6	92.9	106.4	112.1
108.8	105.2	103	105	90.6	104.5	98.6	106	108.6
610.8	1734.1	1292.8	1471.7	1201.5	1258.6	1014.3	1566.1	898.9
155	351.5	400.2	345.2	332.3	328.2	347.2	376.9	256
349.2	392.8	372	365.2	331.1	463.5	402.3	749	491.8
5515.1	8684.6	8973.9	9931	7100.6	9239.5	9757.3	9877.3	6579
100.8	111.5	104.8	86.9	100.9	98.5	100.5	108.5	113.9
161.3	182.4	147.2	157	154.3	177.5	202.4	197.9	168.5
107.8	109.4	103.6	101.8	103.6	108	101.1	129.2	112.1
119.3	135.6	133.5	118.8	114.3	130.6	134.6	140.1	131.2
127.8	156.2	192.1	133.9	142	163.3	139.7	138.5	115.3
121.3	119.5	112.8	126.1	106	124.4	110.9	125.3	109
114.7	116.5	112.3	107.3	105	130.3	113.8	131.6	116.5
107.1	118.2	108.5	113.8	106.6	110.3	108.9	108.3	106.3
101.2	103	96.5	101.5	97	112.4	103.3	99.8	101.3
111.4	121	118.4	115.7	110.5	115.8	115.9	113.9	127
107.5	112.7	119	124.6	114.2	120.5	109.2	115.6	115.7
105.6	115.1	103.1	110.2	96.2	115	101.8	114	110.4
105.1	108.9	104.8	103.2	99.4	104.6	104.2	116.9	115.4
112.7	108.6	117	107	107.7	112.7	105.8	125.5	121.2
104.8	113.3	98.7	96.5	87.7	113.2	103.3	115.6	110.6
906.1	2640.6	2799.1	2298.8	2234.8	2448.5	2147.8	2325.7	1031.9
155	158	150.2	158.1	149.4	189.1	187.4	245.9	219.4
755.4	1202.6	1126.4	1232.1	915.2	1147.6	1153.9	2041.6	928.5

1183.9	1561.6	1413.2	1552	1342.2	1498.5	1316.3	2074.8	1514.2
98.1	108.7	98.7	100.1	96.2	107.1	103.3	106.6	105.8
115.3	118.2	99.9	112.8	106.1	105.2	106.4	108.9	109.2
123.1	118.2	116.2	109	111.9	122.4	110	129.1	116.4
788.9	1450.8	1795.5	1604.7	1274.7	1423.9	1643.4	1092	804.6
445	508.4	528.1	424.8	437	453.1	524	878.5	508
104.7	106.6	99.3	99.4	90	107.7	112.5	117	107
109.1	111.4	106.5	106.2	93.4	110.4	118.3	124.3	126.5
110.6	111.4	100.5	103.5	102.5	106.8	104.2	105.3	105
110.2	107.4	101	105.3	99.1	114.5	101.6	114.1	116.4
515.1	712.3	676	644.7	765.5	773.5	679.3	1080.3	695.5
109.5	106.3	107.2	90.9	93.4	100.5	102.2	104.6	103.2
104.9	110.4	105.9	104.6	91.2	103.1	104.3	120.3	110.1
137.8	154.2	116.5	130.1	129.5	146.4	141.6	196.7	153
231	293.3	233.2	218.4	291.4	248.8	270.7	394	228.6
103.9	115.4	107.6	102.8	99.9	112.1	107.1	115.8	108.7
137.4	154.7	134.9	136.7	138.5	143.5	133.9	197.5	159.6
136.8	140.6	136.9	126.6	116.1	144.6	116.9	187.6	154.5
194.5	188.2	145.2	152.7	160.9	158.9	171.7	285.1	219.5
120.4	134.7	135.5	109.1	115.4	128.7	131.1	174.1	173.3
591.4	516.1	530.1	474.5	478	415.5	194.7	836.3	660.9
194	200.4	179.5	153.3	176.9	192	124.2	262.6	238.9
127.5	111.3	112.5	97.1	104.3	116.1	112.6	116.1	119.4
103.2	106.5	91.3	94.9	99.8	114.5	97.5	101	109.6
135.3	137.3	134.5	140.2	126.9	131.7	134.4	132.4	125.7
323.1	483.9	459.8	348.8	418	476.2	370.6	679.9	559.8
200.3	171.6	165.2	153.7	184.1	175.9	152.8	382.8	305.2
834.6	870.7	886.6	553.4	661.6	972.9	761.6	1084.4	816.5
193.6	256.6	252.1	281.2	223.7	282.4	275.2	328.3	246.6
105.3	111.4	102.1	101	100.5	109.4	104.3	109.5	110.1
106.5	113.8	100.6	106.4	98	108.3	104.7	105.1	102.7
102.8	101.4	98.4	105.7	94.7	109.6	104.7	103.5	96.6
108.5	110.5	100.3	115.1	115.4	109.8	106.2	112.1	127.8
108.1	111.5	99.2	99.3	95.8	116.3	101.2	110.2	113.4
106	102.8	106.2	97.5	92.2	106.8	99.5	107.6	112.1
114.1	123.6	119.2	109.8	112.4	130.4	103.4	145.6	121.6
583.6	638.3	638.9	528.5	498.7	518.9	381.3	1416.6	1230.6
321.8	675.1	612	627.2	507	592.9	566.2	694.8	316.9
348.8	436.1	534.1	510	530.9	409.6	518.1	638.2	462.3
111	109.3	108.8	118.3	107.3	121.2	117.5	135	113.3
111.5	137.6	131.3	103	119	126.3	117.1	150.7	131.5
122.7	149.2	145.9	107.7	117.3	137.5	108	144.5	127.2
116.2	130	120	126.5	110.6	129.5	105.5	132	115.8
106.5	112.9	107.7	99.8	94	113.7	101.6	108.8	113.9
127.8	136.3	133.3	150.2	135.9	131.2	158.4	147.9	140.7
113.3	105.8	106.6	98.1	98	117.2	108.4	115.1	121.8
113.4	139	128.5	137.9	120.2	130.1	120	142.3	135.1
719.9	1159	1049.2	1317.7	832.6	1020.6	842.7	1732.4	1162.5
232.6	454.1	290.8	369.3	269.5	324.3	281.5	524	326.7
126.5	129.2	117.6	106.5	101.4	137.2	114.9	154.4	140.7
101.5	115.8	96.6	96.2	99	104	92.1	108.4	110.6

107.6	109.9	99.8	98.6	103.6	103.1	101.6	119.9	107
101.3	103	100.5	96.2	90	110	98.1	96.9	106.8
126.3	120	111.8	137.8	110.9	116.3	117.4	142.4	164.3
138.5	168.5	162.7	151.4	145.7	176.4	171.3	234.8	166.4
128.7	149	126.5	134.5	124.3	119.3	140.9	159.1	136.3
112.2	125	122.2	115.5	114.9	136.8	121.7	139.5	123.9
133.4	194.1	157.4	164.2	169.3	144.8	174.2	157.9	146.7
618.9	1139.2	1509.3	1894.9	868.2	1672.2	1397.6	1896.7	1055.9
105.9	102.5	96.9	100.4	95.4	111.9	103.8	106	106.9
107.2	112.3	103.1	106.5	94.4	104.7	103.3	111.8	111.4
344.7	395.1	322.1	345.1	345.6	364.7	322.9	669.2	518.6
110.5	112.2	102.3	104.9	97.9	116.1	105.4	112	112.9
142.3	191.1	177	161.7	163.9	189.6	156.9	285.8	206.4
257.5	408.2	358.4	398.5	357.7	408.4	365.1	519.1	360.3
103.5	111.3	101.2	109.1	94.1	115.2	108	112.6	104.2
108	103.9	98.3	101.6	100.2	105.9	99	108.7	109.5
121.3	112.5	107	119.3	109.5	120.5	112.8	129.2	106.7
119.5	134.5	122.4	127	115.7	132.4	125.6	150.7	132.8
168.1	260.2	302.9	255	231.9	261.1	282.2	250.8	216.6
110.9	115.5	112.2	101.4	99.8	114.5	105.7	112.3	114.2
158.2	197.5	165.5	131.3	177.4	167.4	177.6	214.5	181.2
116.9	107.8	111.3	101.5	105.8	118.1	114.1	123	122.2
111.2	115.1	107.2	105	96.3	105.4	110.9	122.9	117.8
108.9	116.6	97.4	111.7	102.1	109.2	104.6	129.2	110
107.9	106.7	103.2	111.1	95.9	109.9	96.5	114.6	102.4
189.1	214	172.1	183.5	167.7	163.2	168.7	221.3	192.8
184.6	202.9	181.3	203.5	150.1	180.9	200.9	295.3	323.3
106.8	115.5	103.6	93.6	93.1	111.8	103.6	118.9	118
118.3	133.4	133.1	137.8	113.6	124.7	121.9	150.5	158.7
176.4	212.4	173.8	162.4	155.3	192.4	161.8	272.9	252.3
154.1	192.4	138.5	162.3	131.8	149.6	129.8	190.8	200.2
1133.1	1041.4	831.6	818.2	896.6	1116.1	1179.2	1448.8	1345.3
116.1	112.6	95	103.2	98.1	103.5	113	111.2	114.8
124.3	123.3	109	134.4	105.4	122.5	125	124.7	131.2
116.7	117.5	99.6	96	97.2	103.5	105	105.9	103.9
105.7	112.2	99.9	100.8	97.1	103.3	103.9	112.2	111.3
115.7	116.8	111.8	127.8	110.8	116.7	114.4	120.7	121.6
477.2	445	441.8	392	413.8	544.2	470.4	847.7	620.7
103	114	98.1	104.6	100.4	107	104.3	115.3	109.2
122.5	131.5	123.3	116.5	102.8	116.4	139.8	119.7	126.5
254.9	317.9	338.1	269.4	284.4	297.3	370.8	434.8	328
209.3	324.4	269.2	398.1	300	301.4	414.7	457.8	361.3
108.1	106.8	103.2	95	101.7	110.2	99.3	107.7	101.7
2977.7	2959.6	2497.7	3359.7	2955.8	2743	3267.6	4937	3705.7
116.8	111.3	109.4	117.1	103.6	116.9	111.9	137.7	121.1
2557	3268.6	2544.8	3124.4	2125.9	2312.4	3191.7	3435.6	3217.9
108.6	109.7	104.4	101.8	92.1	106.4	111.8	105.8	115.7
564.1	566.4	431.4	605.3	511.1	510.4	814.5	950.1	914.2
404.7	323.1	327.8	400.1	264.6	323	422.1	659.8	511.1
1093	1123.2	776.7	1214.1	1030	840.7	1108.5	1965.7	1129.6
341.4	485	395.2	311.1	361.8	420.3	374	707	753

109.6	117	109.9	107.2	102.1	119.4	106.1	119.6	111.1
105.8	115.3	100.9	98.5	96.6	107	102.1	106.2	103.6
152.5	183.6	240.3	185.7	130.2	176.5	176.3	292.9	222.3
101.1	101.6	102.7	99.3	97.9	110	96.4	104.5	110.6
96	97.3	104.9	98.6	94.2	104.3	104.8	110.9	106.1
106.6	107.8	105.9	97.5	94.2	116	96.8	107.4	108.9
110.1	102.1	99.4	100.1	97.1	102	96.8	102.6	106.5
108.6	110.1	99.9	100.7	100.6	112.9	99.9	106.4	117.2
119	186.6	137.7	210.1	141.3	147.6	187.2	205.2	136.4
110.8	160	133.7	164.9	125	138.8	168.6	161	118.6
114.7	111.3	108.7	102.5	102	124.3	107.2	121.5	116.8
124.2	138.8	135.4	135	146.3	147.2	145.7	181.1	153.4
110.6	133.9	117.1	110.3	109.9	120.7	121.5	125.1	103.2
110.4	130	108.1	111.2	108	120.2	100.3	108.6	104
128.2	131.2	119.7	114.3	108.2	134	113.9	164.3	154.6
334.5	278.7	245.5	233.6	249.8	235	279.5	452.6	237.5
189.9	143.2	140	138	124.4	166.4	154	278.2	431.7
130.3	115.2	108.5	126.8	105.6	111.3	120.9	145.5	126.1
122.7	143.9	137.8	136.2	116.5	146.5	131.1	146.7	126.3
1194.2	1681.8	1580.5	1113.9	1406.5	1559.3	1363.7	3020.2	2179.7
98.9	108.1	104.9	101	94.8	107.9	106.5	120.5	111
115.7	138.9	147.2	150.8	140.6	154.7	140.1	148.9	118.9
108.1	109.2	99.4	100.1	101.2	107.4	99	102.3	98.8
124.7	156.9	126	162.7	141.4	132.9	148	172.5	138.9
116.2	110.3	117	119	104.1	112.7	103.6	126.1	107.1
117.4	104.8	108.1	119.5	104.1	113	107	107	110.2
115.5	118.2	106.6	114.8	100.4	113.8	105.7	121	120
124.5	156.6	161.7	190.4	126.2	139.3	180.1	136.6	130.5
112.1	102.1	103.8	99.5	95.2	113.1	104.1	112.1	104.3
97	100.8	103.4	92.7	96.4	106.3	101.5	108.7	98.9
120.1	111.8	111.5	114.9	94.7	107.3	106.6	123	108.4
103.9	110.1	101.9	105.5	98.9	109.8	112.7	96.3	110.7
103.1	105.1	101.3	101	95.4	106.4	106	113.3	104.2
116.7	110	95.5	98.6	99.9	105.2	110.8	109.2	111.5
108.2	145.1	141.2	115.1	122.2	133.7	124.9	154.4	127
116.3	116.1	106.7	112	104.6	116.3	107.9	124.6	112.1
103	108.8	104	102.1	97	112.3	100.6	115.1	123.1
108.4	104.6	109.6	105.9	93.8	103.3	103.4	110.5	107.1
109.1	107.9	98.8	96.1	90.9	104.1	101	102.2	104
100.3	108.7	103.6	89.9	88.2	105	105	112.2	111.1
97.5	104.1	95.6	99.1	90.3	107	95	108.7	97.2
104.4	110.2	103.4	99.5	96.3	110.6	95	106.2	112.5
99.1	95.9	101.3	96.9	98.3	104	106.2	102.6	113.4
110	109.6	96.7	99.6	92.2	101	108.5	105.9	110.3
118.7	111.6	129.3	112.9	115.8	132	116.6	122.6	122.8
110.2	118.4	109	120.5	100.2	111.2	114	119.4	107
142.5	186.3	189.9	167.7	175.8	181.4	242	272.8	180.9
106.5	105.4	106.1	101	89.4	103.5	91.1	105.7	113.5
108.6	113.7	107.5	100.9	107	113.1	103	118.1	111.3
109.3	106.7	104.5	96	99.3	106.1	102.6	110.3	112.2
108.5	105.4	107.3	101.6	93.9	102.4	108.8	105.4	100.5

128.6	159.4	124.3	177.4	142	139.8	163.8	181.5	180.1
107.2	112.9	102.9	114.1	103.5	114.3	103.8	106.2	121.5
106	108.1	101.7	104.8	96.7	104.1	101.7	106.7	104.7
104.3	108.2	100.7	101.1	95.5	105.8	99.9	115	108.3
99.2	105.9	104.3	100.7	90.3	105.3	100.2	97.2	106
139.2	204.4	187.2	265.6	153.8	183.8	210.6	206.7	185.3
158.9	178	201	284.5	183.6	203	259	214.6	188.6
134	180.9	126.3	224.5	149.7	169.6	181	155.6	129.6
105.5	106.8	97.1	102.4	96.9	109.1	99.4	103	106.7
163.5	192.1	188.5	156.8	184.5	203.9	211.6	312.1	203.3
102.1	113.6	100.7	105.3	96.2	111.8	108.5	119.2	111.1
113.1	112.8	99.7	99.4	94.1	108.9	104.9	119.3	107.8
123.5	108	114.5	112.1	94.9	116	103.6	114.8	100.4
118.5	118.6	112.1	109.5	108.8	120.4	114.2	135.9	123.8
105.4	104.2	104.5	99.9	95	105.8	99.7	108.5	104.2
109.6	191.7	142.4	191.2	151.9	129.7	151	143.3	115.2
105.1	104.5	106.2	96.7	97.5	106.9	103	113.6	108.8
102	106.7	101.1	92.7	95.5	106.3	97.4	104.5	97.6
104.9	109.3	102.2	98.7	98.1	104.4	107.1	112.7	112.3
107.5	106.4	97.8	90.6	99.7	102	102.6	105.9	107.3
117.1	118.7	106.4	106.5	103.3	116.6	108.1	115.9	114.9
153.7	147.2	132.8	149.1	132.3	157.5	133.3	178.3	181.5
133.5	150.3	138.7	122.5	125	141.9	128.5	172.7	169.1
105.5	109.1	104	104	100.8	117.9	100.9	107.1	120.5
100.6	105.6	106.5	94.1	107.1	116.1	103.9	97.8	103.2
105.7	107	103	95.3	99.2	111.2	98.3	112	105.7
108.2	105.7	97	111.7	100.9	112.5	105	109.6	101.9
99.1	113.5	111.6	102.1	95.8	102.4	101.4	127.1	102.3
171.2	151.9	170.5	143.6	138.1	162.1	172.1	218.8	193.7
160.3	156.1	136.4	169.6	156.9	165.5	160.1	220.9	175.5
112.2	107.5	105.9	112.8	97.4	114.4	99.3	127.9	103.5
107.9	115.2	101.4	100.4	99.1	116.7	102.8	118.8	120.4
104.4	113.4	114.1	116.5	97.2	125.9	115.2	154.4	134.2
147.6	192.6	241.1	226.1	152.9	174.7	145.3	329.2	181.9
105.5	108	102.7	102.7	90.9	108.2	101.4	111.4	106.8
99.3	103.3	103.2	92.9	97.1	105.7	104.7	108.6	103.8
283.1	381.5	304.6	282	258.9	312.6	291.6	400.2	247.7
424.5	583.8	578.2	635.7	484.9	628.6	531.2	720.6	537.2
108.5	110	113.3	115.1	112.4	119.3	111.9	129	128.1
131.5	145.2	114.6	127.4	118.7	124	130.6	140.3	133.4
114.5	110.3	108.8	114.5	99.7	112	105.8	112.6	108.3
118.9	117	127.1	116.2	126.5	134.5	123.1	165.4	148.9
128.4	161.6	149.8	163.6	131.3	146.5	161.3	184.3	149.3
111.1	104.9	101.6	104	101.5	106.8	102.2	106.9	107.8
140.9	406.1	184.5	249	288.4	198.6	260.8	309.6	161.2
105.4	105.5	106.3	105.9	91.4	106.6	99.1	114.1	110.3
110.9	109.6	102.4	110.8	98.2	109.8	107.1	112.5	113.9
725.5	410	358.4	393.1	401.8	641.1	537.4	1362.2	1855.4
111.9	109.3	103.4	107.7	101.7	106.2	109.8	116.3	120.7
117.7	133.5	108.3	194.9	112.8	114.6	135.7	120.7	128.5
97.3	103.1	100.1	97.5	92.5	105.6	99.3	115.7	112.4

108.3	109.3	106.7	200.7	94.9	115.9	119.8	115.6	106.7
110.7	109.1	100.9	127	94.2	105.5	107.6	119.7	117.4
113.9	119.9	112.2	114.4	95.4	113.2	108.2	118.6	116.6
167.4	389	321.9	357.6	348.4	330.5	346.6	368.6	241
103.3	114	102.6	98.1	96.5	105.2	103.8	100.2	103.4
107.5	111.8	103.1	100.5	99.1	113.8	96.5	116.4	104.8
110.2	115.9	105.6	100.3	100.5	115.2	104.6	127	103
118.4	122.5	122.5	121.9	110.4	115.2	104.7	155.4	128.2
105.7	109.1	103.1	98.5	97.5	103.6	100.8	106	103.5
156.9	130.3	136.9	131.2	142.7	148.7	162.8	207.8	195.3
106.1	106.7	102	113.1	96.7	107	105.5	109.8	100.7
453.5	569.1	598.2	374.1	471.7	656.7	608.6	957.6	644.5
138.2	132.5	144.3	110.1	119.6	131.1	121.7	161.1	122.5
737.6	1062.5	1115.8	760.7	1064.3	1250.8	873.6	1314	738.7
107.4	109.9	102.7	96.6	106.1	106.9	106.4	98.9	107.3
122.7	120.4	111.6	126.6	108	124.9	121.1	183.5	124.1
124.9	134.1	108.5	101.8	133.2	131.8	114.5	284.2	146.1
115.6	109.1	106.1	106.5	95.7	105.9	108.3	121.4	110.4
108.3	107.3	107.3	103.2	108.2	106.9	105.2	123.2	118.7
103.8	110.4	104.7	103.3	94.9	116.9	100.2	107.8	114.3
112.6	132.7	121.4	109	116.6	126.6	118.2	138.4	120.5
113.6	104.8	99.6	102.9	98.7	118.4	107.3	115.4	116.5
104.9	111.2	105.6	104.5	97.3	98.9	106.6	108.4	103.8
106.7	110.7	100.9	105.2	93.7	105.5	100.2	116.5	113.3
426	634.9	615.1	431.1	450.9	510.5	472.6	868.2	587.9
103.8	128.1	107	137.6	116.4	108.3	130.6	143.6	114.5
108.3	114.5	101.2	103.9	100.9	101.8	99.9	109	109
105.1	105.1	98.9	106.4	98.1	110.4	104.9	110.7	108.3
126	147.4	165.1	198.2	150.3	163.2	151.4	224.8	158.4
105.9	111.5	103.6	105.3	100.3	122.1	109.6	118.1	123.1
99	106.9	100.3	95.1	98	107	100.7	112.4	109.9
106.1	108	101.1	102.1	98.2	117.3	106.7	104.5	99.4
108.4	110.4	115	100.4	99.1	111.8	105.4	114.5	112.4
107.2	110.5	100.7	104.4	94.8	109.9	103.3	117.5	110
109.4	113.4	104.2	107.5	89.4	107.2	104.7	96.6	104.6
182.4	219	256.8	198.5	183	231.9	248.7	263.3	171.6
375	361.3	340.4	433	459	411.8	292.4	719.1	581.1
112.3	100.4	102.3	100	94.9	106.8	97.8	98.6	105.3
116.9	134	119.6	121	124.2	132.3	125	158.9	165.9
120	133.4	121.1	124.5	121.8	127.7	141.9	149.9	142.9
127	124.5	134.9	107.9	115.3	132.8	135.7	172.5	120.3
115	121.5	114.7	95	97	114.8	109.9	118	120.7
111.2	110.5	102.8	96.6	99.3	100.8	102.2	116.9	109
120.1	119.6	116.3	116.3	111.1	129.5	119.4	152.6	138.8
111.8	121.4	110.5	156.1	110.7	112.4	120.6	120.5	108.4
114.1	112.9	104.6	102.6	98.8	112.9	105.4	118.1	114.5
109.2	103.6	106.4	107.4	102.9	104.8	103.6	126.3	115.2
96.8	107.8	103.8	94.2	101.9	108	103	118.5	118
120.7	121.1	110.9	103.3	108.6	125.9	110.7	142.5	126.3
129.8	144.7	135.7	125.9	104.4	115.7	128.1	202.8	150.2
126.6	130.2	122.2	116.5	108.8	137.6	125.1	171.1	152.1

736.2	196.1	215.3	131.4	267.2	270.9	155.4	1053.6	902
114.7	107.1	102.4	103.1	99.9	115.7	97.6	122.6	116.8
101.8	110.6	105.5	100.6	99.3	107.8	102.4	107.8	114.3
4186.4	3842.1	2837.4	2918.2	3229.1	3596.3	2473.4	8994.7	6807.8
119.9	119.2	129.5	114	125	121.9	114.9	169.1	187
228.7	192.5	201.2	149.3	175.5	207.9	138	485	418.8
243.6	266.4	178.4	258.5	184.9	197.7	253.5	416.3	346.7
193.3	321.5	329	273.2	267.1	320.9	283.9	323.2	242.6
115.4	105.9	105	100.7	96.6	112.4	99.1	112	111.2
110.1	116.7	119.1	110.5	102	117	102.9	118.7	123.3
131.3	180.6	220.2	154.5	168	216.3	175.9	182.3	158.9
117.7	117.6	117.5	99.5	107.3	123	119	119	108.4
701.9	798.6	715.8	706.8	662.6	741.9	546.6	1076.3	826.6
286.6	328.7	349	240.2	315.5	381.3	333.2	589.6	352.1
310.5	371.7	436.8	322.2	452	497.8	446.4	622.6	390.6
127.3	160.7	143.5	158	149.3	159.5	161.6	202.9	144.3
123.1	117.1	113.7	116.2	106.6	125	114.2	124.8	138.8
170	157.1	155.4	135.1	146.9	159.4	152	249.1	215.3
96.3	107.8	108.7	109.1	97	106.7	99.6	110.5	110.6
104.5	111.2	106.7	95.6	93.7	107.9	96.4	106	105.9
258.7	287.3	234.3	251	242.7	279.5	229.8	418.8	304.5
105.2	110	95.7	93	93.8	104.2	99.5	99.6	100.8
111.5	110.8	110.3	111	100.2	114.7	103.4	117.7	110.4
143.2	134	132.5	129.4	117.9	147.5	142.5	168.6	174.6
131.5	228.7	147.8	257.3	189.9	172.9	268.7	263.7	156.8
107.2	100.9	99	91.6	87.8	100.5	99.7	101	101.5
186.2	234.5	211.9	200	230.9	247.2	264.7	309.9	225.8
187.5	207	228.2	225.8	189.4	231.8	264.7	304.2	198.3
107.4	107.2	108.3	104.5	102.5	114.4	108.9	123.7	121.2
695.4	553.1	803.1	416.2	556	720.9	585	1054.6	682.7
294	883.9	750.7	696.1	711.7	803.9	668.9	871.7	548.2
111.4	110	100.2	108	99.8	113.7	114.8	119.1	107.7
1149.1	1479.9	1652.3	1128.4	1186.4	1565.5	1198.8	2116.1	1255.1
102.6	100.9	101.7	99.4	93.3	103.4	97.9	110	109.7
106.3	104.3	104.5	105.4	101.2	114.2	104.3	117.3	111
206.1	213.1	207.6	202.5	193.9	213.9	200	321	166.3
105.5	108.6	105.9	97.3	99.4	111.2	97.9	111.2	101
111.3	109.9	103.9	112.2	94.8	111.2	103.6	101.2	117.3
107.4	105.9	100.1	106.9	92.3	101.6	106.1	108.2	108.2
103.6	102.3	111.7	100.8	97.8	107.9	99.6	116.8	111.8
296	564.3	395.6	636.7	482.2	332.2	568.8	488.3	375.8
108.5	123.4	113.6	103.6	109.2	126.2	105.7	133.7	122.9
112.8	115.8	110.4	120.4	99.7	115.5	106.1	114	106.2
101.6	106	102.9	98.6	87.5	106.6	104.6	105.2	103
105.5	104.2	99.8	96.7	87.6	105.1	100.3	103.1	103.8
263.3	285	250.5	276.5	200.5	223.2	257.5	450.1	308.6
117.1	98.7	115	117.5	100.9	108.6	110	123.7	111.9
189.3	181.2	193	189.1	176.9	174.8	178.7	245.2	174
111	115.1	113.1	114.4	102.7	114.2	111.2	118.6	107.9
172.5	271	267.5	206.4	227.9	255.8	265.8	379.3	198.1
124.7	139.1	131.9	130.8	140.8	144.8	128.3	148.4	139.8

103.1	109.8	96	102.7	93.6	104.1	99.1	109.4	103.5
106.6	103	105	97.4	98.1	107.7	100.4	106.1	109.9
606.1	981.3	931.3	522.2	955.4	1006.7	863.9	1480.2	876.7
812.7	1249.4	1211.2	1161.9	1213	1316.1	898.6	2495	1105.7
246.1	243.4	261.3	232.3	297.5	317.1	241.9	695.8	342.4
100.1	99.3	99.4	95	91	103.2	99.3	106.1	101.3
116.9	117.6	105.2	116	111.7	115.8	106.5	119	113.5
106.5	103.1	104	103.1	98.8	106.1	103.9	103.9	105.1
111.4	112.2	110.4	108.6	97.2	108.8	107.8	114.1	118.7
126.4	131.5	125.2	178.8	126.1	122.7	134.7	147.8	146.7
177.9	229.6	138.7	236.8	149.4	153.2	240.4	203	250.8
136.6	144.7	112.5	193.1	125.1	120.2	153.2	150.6	187.7
126.5	159.9	139.7	138.3	140	162.6	128.9	168.7	163.3
543	789.8	480.1	754.5	785.1	966.2	631.3	1603.3	1094.6
191.2	181.3	164.2	150.2	225	181.6	154	549	266.8
386.9	598.9	334.5	445.2	419.3	352.7	341.6	1092.7	698.6
101.1	115.6	103.6	103.1	104.2	109.6	107.5	118.5	104.1
106.2	105.4	102.2	93.2	90.2	107.9	97.4	104	113.1
239.1	201.7	159.9	156	181.8	199.9	195.2	263.8	292.8
374.3	289.2	280	341.2	261.2	346.6	372.3	369.6	399.1
328.6	213.6	222	220	258.3	254.7	290.1	313.7	284.2
118.8	143.3	125.6	119.3	115.7	119.1	118.3	135	133.1
128.1	131	131.6	129.4	128.8	144.5	135.9	155.7	127.6
118	126.7	112.1	144.4	105.4	135.2	126.3	134.3	133.9
109.2	111.9	110.6	129.4	96.6	120.5	107.3	119.2	124.1
99.9	116.3	106.2	113	95.6	107.4	104.3	109.7	103.2
104.2	107.3	103.4	98.5	94.8	110.3	100.7	117.2	113.7
100.2	109.8	100.4	100.8	95.4	108.2	97.5	107.1	110.1
101.3	112.8	107.8	102	93.3	109	102.8	127.8	111.9
114.7	114.6	118.3	109.4	102.7	115.3	98.1	105.2	107.2
102.7	107.5	107.7	93.4	97.8	103.4	101.8	100.4	107.9
115	124.4	114.9	108.5	117.1	119.5	113.2	123.7	125.4
233.4	304.4	303.7	325.2	358.6	350.8	386.4	372.7	404.8
190.4	156.5	148.9	148.8	142.9	169	149.8	231.9	223.1
127.5	124	116.8	97.3	109.2	123.1	113	145.9	152
247.9	221.3	176.7	199.8	188.1	230	190.1	325.3	359.6
95.9	104.3	101.5	94.8	101.4	101.8	99.9	98.2	107.3
109.8	109.2	108	97	101.5	110.2	101.7	117.5	110.3
111.1	106.8	115.6	98.6	103	109.6	108.7	109.7	114.2
1955.7	4169.4	3762	4284.9	3009.9	3463.3	3893.7	4235.9	2395.2
1950.5	3912.6	3629.1	3371.8	2854.4	3337.9	3447.6	3812.7	2364.3
3184.3	4421.9	5688.5	5019.4	3592.6	5045.8	4508.8	7206	5490.9
330.4	624.5	568.6	525.5	521.9	545.1	357.2	848.7	605
146.3	173.6	187.1	160.2	182.2	228.2	217.7	231.4	164.3
412.5	937.5	976.9	1014.4	804	914.8	935.3	819.3	456.7
187.2	249.4	250.9	301.4	192.8	247	304.7	425.9	309.9
888.1	1452.8	1557.3	1422.7	1127.3	1012.6	1363	2213	1462.1
113.6	124.7	106.7	110.2	104	128.2	118.5	119.5	119.4
418	711.2	704.2	689.5	659.2	669	772.5	773.7	488.1
112	107.8	106.4	99.4	99	104.7	106.3	104.1	104.8
112.1	113	104.2	104.1	97.4	111.4	109.6	130.8	113.7

1181.4	1270.4	1106.7	1777.3	1205.1	1320.3	1532.8	1576.1	1396.8
107	120.3	119.9	122.5	119.8	135.6	121	135.9	119.2
1130.8	1602.1	1322.8	1797.8	1361.8	1207	1790.5	2027.7	1471.9
1226.5	2134.2	1761.6	2331.3	1703.2	1548.5	2305.6	1986	1637
1805.2	2273.7	2197.4	2714	2286.1	2125	2831.5	2671.4	2404.4
250	308.2	254.8	269.8	257	287.5	274	332.3	295.7
186.7	248.8	253.8	203.2	162.6	217.1	284	261.7	286.5
1378.9	1740.5	1665.6	1868.9	1667.3	1892.1	1866.1	2508.2	1962.5
1587.1	2077.5	1832.6	1961	1800.1	2082.2	1960.6	2985.8	2214.1
412.6	615.2	502.1	721.8	517.3	644.4	753.3	806.4	642.8
600.1	629.2	725.1	907.6	700.9	826.4	1032.7	1122.9	856.3
214.4	152.9	149	201.7	131.7	174.3	188.4	335.1	265.2
1980.2	1712.3	1358.5	2045.9	1203.8	1652.8	1779.1	2400.4	2202.8
132	188.9	155.8	158.4	120.2	158.9	131.8	217.8	170.9
120	111.9	112.5	124.5	96.9	111.4	103.3	122.1	119.3
1165.2	2647.4	1965	2138.4	2756.8	2526	2670.3	4785.9	1584.3
1088.2	1003.6	1016.1	1977.6	1043.9	778	1837.2	1901.8	1078.8
541.1	528.4	241.6	477	426.3	293.7	526.6	697.3	360
168.3	195.8	229.8	161.7	199.6	212.1	173.1	300.2	194.7
105	113.4	105.6	120.4	97.7	106	99.2	104.4	106.9
108.1	107.1	106.9	104	99.9	109.9	104.7	113.1	110.1
105.1	101.6	97.3	108.9	99.6	109.9	105.5	125.5	109.6
108.2	102.2	108.5	104	93.8	94.1	98.1	113.3	117.5
1820	2006.2	1542.7	1698.3	1808.6	1641.6	2151.1	2822.5	1530.3
153.7	135.3	133.7	156.1	151.1	147.2	151.1	210.5	147.4
299.4	290.8	218.4	311.6	246.8	258.7	304.8	482.1	348.6
421.9	537.3	415.8	521.5	538.3	464.7	594.8	878.8	471.1
99.8	102.3	105	105.1	94.5	106	104.2	108.1	114.1
2434.5	2269.9	2159.1	2557.5	2042.7	1914.9	2551.8	4319.9	3120.2
166	1223	1524.7	933.3	824.9	1025	754.3	797.2	326.3
1007	1270.3	971.6	1249.8	1285.5	979.7	1289.7	2343.4	1357.6
105	113.7	101	100.2	92.3	111.3	97.6	112.7	118.1
2465.5	2455.5	1538.3	2486.3	2223.6	1593.7	3055.3	3724.6	1602
186.6	197.4	167.5	193.7	201.3	177.7	194.4	323.1	194.5
112.7	119.2	100.8	111.3	103.6	119.3	106	140.5	128.2
107.4	106.7	100.2	104	99.1	106.6	108.2	109.5	109.9
104.7	106.5	97.4	104.5	89.2	99.4	99	104.8	101
438.6	644.9	498.6	641	569.1	586.7	668.6	1396.6	648
1184.1	1513.5	1195	1285.7	1451.9	1409.9	1302.2	2568.8	1277.5
860.2	1187.8	988.1	940.3	1036	1121.1	1194	1815.3	1213.5
179.8	156.7	151.9	141.5	143.2	172.2	167.7	170.4	166.2
841.5	1222.7	1054.5	1179	1107.5	1123.8	1139.4	2343	1011.7
1267.4	1478.3	1227.2	1504.5	1084.2	1441.1	1483.7	3110.2	2133.8
117.7	126.8	112.3	106.3	107.4	127.5	110.8	133.1	130.2
104.6	103.3	110.9	105.3	99.8	112	98.2	108.4	101.4
103.5	104	104.3	94.1	93.3	105.1	93.8	102.8	107
108.7	109.3	103.8	108.8	92.9	113.3	99.9	116.1	112.4
188.7	199.1	217.8	155.1	195.6	242	200.3	471.9	258
138.3	133.4	128.6	124.6	113.9	123.7	139.6	143.2	142.5
114.4	114.5	112.8	106.1	103.6	117.2	105.7	129.7	116.6
119.8	115.9	106.6	103.3	102.2	111.8	103.5	112.9	111.7

112.3	115.6	102.4	103.9	109.9	118.2	106.6	118.5	122.3
114.8	113.7	104.6	107.8	106.9	105.5	107.6	130.6	117.9
103.5	111	106.8	95.9	97.9	111	105.9	115.5	115.9
107.7	125.5	110	118.1	108.8	103.1	106.8	111.4	114
149	247.1	305.4	203.7	200.1	217.8	236.7	322.8	227.2
111	105.4	103.7	99.4	97.6	113.2	100.3	113.7	114.5
147.6	150.5	170.4	292.2	147.4	156.2	190.2	155.6	153.8
184	259.4	167.6	203.7	199.6	171.6	180.8	169.5	167.4
110.2	119.1	136.7	113.1	109.9	121.6	126.3	147.6	112
160.9	195	195.2	208.1	201.9	199.5	213.5	212.9	188
146.2	188.8	163.3	179.9	170.8	183.5	195.9	195.9	190.7
114.1	124.1	111.9	127.6	115.8	134.8	128.5	138.7	126.5
200.3	314.7	234.3	266.5	266.3	249.3	251.1	444.4	259.6
102.4	111.7	107.1	91.5	88.9	100.8	103.1	109.3	102.2
116.3	125.9	130.9	129.8	118.4	139.1	115.4	130.5	129
110.3	116.1	111.4	110.7	106.5	114.9	113	117.9	113.1
150.9	224	219.8	266.3	190	234.7	259.1	301.5	237.2
127.8	174.9	182.5	162.2	158.9	146.8	179.3	228.6	146.7
109.1	104.1	103.6	106.6	93.5	103.6	100.2	112.4	98.9
102.9	112	99.4	103.1	101.3	106.3	100.9	110.3	111.6
117.5	139.3	124.6	121.8	109.6	134.9	110	135.3	136.8
115.7	119.1	104.9	117.7	99.5	108.1	113.6	113.3	109.6
107.2	101.3	95.8	108.3	89.7	106.3	99.9	105.8	112.8
105.3	108	109.1	97.9	101.7	113.6	104.8	116.3	103.3
107	115.7	108.5	114.8	98.7	114.9	103.9	121.4	110.7
99.9	104.6	102.4	104.3	96	99.9	101.6	110.6	96.6
117.1	110.3	99.2	102	100.9	107.4	100.6	112.2	106
196.2	215.9	208.1	259.1	227.5	212.2	221.8	442.6	267.4
105.8	110.1	105.4	93.6	101.8	122.1	100.6	106	107.5
736.5	642	676.6	483.9	469.7	595.5	469.5	876	634.2
127.4	118.7	122.1	113.3	119.1	135.1	130.1	170.6	140.2
110.8	113.8	120.3	113.2	98.8	128.9	100.5	172.4	164.1
247.7	230.2	256.1	195.2	209.5	283.6	181.6	477.5	359.2
121	124.3	119.8	110	107.3	129.6	114.2	136.3	133.2
98.1	104.2	102.3	104.2	95.6	102.9	96	106.3	100.1
124.5	141.6	165.2	123.1	143.4	155.9	122.8	211.8	172.9
109.1	110.3	107.6	122.9	119	119.4	106.3	110.6	111.8
107.4	100.7	101	99.5	100.4	102.9	99.2	105.5	112.7
117.1	197.5	172.3	156.9	142	180.9	170.6	180.4	149.6
156.9	159	162.9	182.9	123.6	168	171.3	300.3	192.3
137	135.3	121.1	143.1	123.1	125.6	146.3	154	142
112.4	121.6	110.2	120.4	129.3	122.9	133.5	140	136.5
973.6	1644.2	1645.6	1528.5	1571.9	1866.2	1583.5	2465.6	1649
108.1	114.5	110.9	108.7	98.1	118.1	103.8	126.5	117.1
101.5	99.7	99.2	98.3	96.9	106	103.1	109.1	103.1
96.5	104.7	108.8	99.9	92.6	102.7	99.2	111.1	99.7
107.1	106	96.9	106.4	100.6	113.4	111.8	111.7	109.4
430.6	464.3	403.9	495.2	284.8	448.4	465.6	474.1	274.6
102.8	125.4	125	127.3	121.1	138	120.1	155.1	152.1
138.1	123.4	114.8	101.7	110.1	120.6	115	134.4	140.7
106	112.4	102.6	105.5	94.8	107.8	107.8	115.7	105.7

569.9	352.9	303.8	210.7	409	495.2	332.4	656.9	293.3
109.5	109.7	98.7	92.5	95.9	108.6	99.4	115.6	112.5
105.8	110.5	107.3	122.9	98.9	103.2	104.6	101.7	104.3
112.8	105.1	103.9	98.7	97.4	117.8	102.9	119	123.5
286.8	1210.1	1228.3	1198.5	847.5	786.5	851.2	536.8	392
540.3	505.4	580	387.3	510.7	599.1	452.8	787.1	524.5
116.3	130.1	120.1	121.1	106.5	107.7	108.9	134.4	113.5
221.1	375.2	339.4	205.5	254.2	315.3	238.5	319	233
115.4	107.4	92.8	115.7	92	104.1	107.6	99.9	102.5
101.7	112	104.7	117	97.3	101.3	101	102.5	109
635.5	332.6	354.7	289	374.5	433.4	358.1	956.1	648.4
117.7	122.2	113.7	103.8	104.2	114.5	109.8	132.9	118.4
110.3	109.2	115.2	104.4	100.8	117.2	100	116.3	114.6
193.1	256.3	247.8	252.5	227.3	306.6	251.1	319.3	207.4
122.3	122.3	103.2	123.2	106	124.1	109.9	126.7	118.9
145.2	200.7	175.7	180	167.2	203.3	235.8	231.5	179.4
110.7	108.6	112.7	110.3	98.7	119.5	103.5	114.1	108.8
153.4	159	169.3	136.3	136.2	133.5	136.8	254.7	215.4
1199.7	683.2	577.9	648.5	608.4	500.4	645	1187	1415
134	112.9	100.6	114.3	108.2	125	114.8	131.9	133.9
184.9	166.2	130.7	183.6	130.4	109.3	143.5	274.1	167.8
100.4	97.1	112.2	101	95.8	104.6	109	108.9	118.9
4211.8	4418.4	4757.6	4181.8	3261.9	4149.1	5478.8	8102.7	5664.2
109.5	113.2	113.4	107.8	97.3	106.3	97.4	112.8	112.8
104	115.7	105.6	100.2	104	117.5	103.2	121.7	116.4
112.2	118.1	105.2	110.5	106.9	117.6	112.4	125.3	115.6
120.1	124.7	128.7	108.7	104.4	125.2	107.7	158.7	126.2
114.8	112.8	109.7	106.5	92.4	115.5	102.5	101.7	111.5
105.4	104.7	100.8	102.3	90	111.4	105.7	108.8	117.2
104.4	120.3	112.3	115.1	98.4	115.3	111.8	129	106.6
149	183.5	193.5	186.9	167.7	181.1	157.1	229.7	155.1
107.7	108.5	102.6	101.7	101.8	103.3	101.8	104.2	109.3
103.6	116.3	105.4	107.2	93	121.9	114.2	134.6	108.9
107.7	105.3	103	102	92.1	102.9	105.2	99.9	115.9
144.2	236.6	233	173.5	236.3	209.8	192.4	274.4	174.1
113.6	119.3	115	103.1	109.3	115.7	113.5	128.6	113.7
117.9	117	108.9	100.2	109.9	130.1	104.7	110	119.2
117.6	124.9	109.3	113	103.4	125.9	109.6	136.8	136.4
116.2	117.8	111.1	107.5	103.7	114.7	109.3	118.2	121.6
104.2	106.6	101.2	96.3	90.1	108.5	97	108.4	110.8
124.1	157.5	178.7	153.7	153.2	171.2	168	160.4	135.8
131.7	136	134.6	136.2	123.4	138.1	140.5	173.2	164.4
107	109.8	105.2	113.4	103.3	117.5	112.7	110.1	104.1
106.9	120.3	102	103	105.9	111.9	101.7	110.2	111.2
108	117.6	103.4	119.5	105	104	101	114.1	106.7
107.7	142.6	146.8	118	122	134.5	120.1	108.4	109.7
101.2	108.6	108	103	102.8	105.4	108.2	100.9	94.5
110.6	106	100.6	105	94.6	107.5	100.7	108.2	109.1
98.7	113.2	105	108.9	94.1	120.1	105.5	113.7	116.6
173.2	178.3	194.9	149.1	146.4	183.2	147.4	195.1	165.9
103.5	103.4	100.1	103.7	108.2	111	97.7	112.4	102.2

106.2	112.9	103.7	101.4	96.6	97.6	101	100.6	110.7
106.1	102.3	105.5	91.8	90.8	101.7	99.8	101.1	97.6
140.7	140	145.8	125.9	143.4	165.9	141.9	179.8	159.5
108.5	113.5	104	99.4	94.6	111.9	102.8	113.8	111.8
107.6	116.9	109.5	114.8	103.9	117.2	107.9	126.6	113.6
123.3	108.6	106.9	106.9	116.6	120.7	111.6	117.4	110.3
453.5	586.7	429	390.4	458.1	491	518.8	950.5	537.3
223.2	195.8	154.6	196.4	149.8	170.6	172.9	391.7	256.3
158	127.4	133.6	138.9	141.2	134.5	128.9	188.3	169.4
238.6	341	258.5	280	347.9	278.4	281.3	586.4	357.5
112.6	107.1	107.3	104.7	98.4	103.4	112.2	110	99.9
175.5	302.3	272.4	254.7	242.6	279.8	280.7	288.9	214.1
136.1	164.5	139.5	139.4	128.1	157.7	139.8	202.9	166.8
107	107.3	106.9	97.4	98.8	109.2	98.6	114.4	107
103.7	118.9	108	102.6	97.2	114.1	109.4	116.8	115.2
113.3	111	100.3	112.9	96.2	111.3	102.5	114	112.9
105.1	110.5	99	100.2	93.3	107.1	103.1	100.4	105.9
109.8	113.8	105.9	109.7	98	109.1	95.6	104.8	102.2
113.1	115.9	106.9	121.2	105.5	128.4	109.3	115.6	97.9
112	112.7	107.3	108.6	98.7	118.6	98.6	119.4	105.1
110.1	102.8	97.1	102.9	94.7	104.9	102.1	111.1	106.3
110.2	117.9	105.2	96.7	96.5	112.9	104.6	144.2	142.7
109.9	108.6	107.9	100.5	98.5	104.2	112.4	112.8	110.2
105.6	104.7	99.9	100.7	93.6	104.9	103.7	116.8	113.4
271.8	271.3	292.5	266.6	245.5	301.3	276.9	564.4	402.1
114.7	114.9	115.2	100.9	96.8	116.4	105.2	104.5	111.5
122.4	191.3	167.4	158.6	147.9	173.9	156.7	168.9	148.7
114.4	107.4	105.1	105.4	97.4	103.1	98.7	109.3	107
113.3	117	112.2	110.9	111.3	126.1	118.4	138.4	117.7
2240.9	361.2	342.5	288.7	331.4	449.7	280.9	1757.6	2705.3
105.7	114.8	109.3	102.1	96.7	111.4	110.6	145.8	114.6
572.7	752.3	679.4	671	757.8	743	762.1	1022.2	763.9
98.6	109.3	96.8	92.6	96.1	107.8	98.2	101.8	110.3
108	112.2	99.9	103.4	101.3	97.7	103.2	114.1	106.9
174.2	165	178.4	157.4	137.4	155.7	172.3	193.6	154.6
118	119	106.3	110.4	100.7	112.4	102.7	116	110
117	106.6	104.8	97.2	101.4	103.1	99.2	114.7	117.8
114.4	112.7	107.6	114.7	97.1	108.5	98.5	121.2	114.3
110.2	102.8	108.9	96.9	97.7	107.3	98.4	109.7	105.8
135.2	123.8	111.7	114.6	123.8	130.7	103	142.7	134.8
109	120.2	104	111.2	105.1	124.4	105.4	116.1	114.8
110.1	112.6	99.1	87.5	101.8	107.6	112.5	106.9	104.7
102.5	115.2	98.3	93	95.3	110.9	99	104.3	110.1
110	106.3	92.6	97.5	96.6	105.5	108.7	104.3	113.6
112.5	103.7	99.1	105.8	104.5	104.3	99.6	106	97
100.4	102.9	101.1	90.5	91	105.4	97.4	102	101.8
118.8	130.1	120.1	118.8	108.3	128.2	121.6	145.5	124.7
104.7	109.1	102	95.4	92.1	107.2	113.5	136.2	116.6
108.4	108.3	104.5	103	98.1	107.7	97.6	106.3	102.5
107	105.5	102.1	104.6	100.1	105.1	102	104.5	113
112.8	102	101.1	108	89.2	107.7	102.5	99.2	110.7

187.7	151.3	154.3	138.6	141.8	161.7	173.2	194.2	196.5
105	110.5	105.3	98.2	96.4	106.1	100.7	102.6	103.5
119.9	159.7	129.3	147	125.3	129.1	119.7	159.7	140.3
115.3	104.4	94.3	91.4	93.3	105	113.5	149.4	129
582	665.1	716.4	530.1	575.1	679.3	615.8	738.5	901.6
171.4	565.6	360	356.7	400.9	258.7	234.6	206.2	197.4
156.4	152.5	122.9	115.7	114.3	141	118.6	194.7	160.3
167.3	137.5	147.9	159.7	140.4	184.6	151.8	231.5	192.7
107.5	98.2	99.1	106.3	91.7	113.3	104.4	102.3	106.8
174.7	168.1	164.1	169.8	157.2	183.9	148.6	204.4	174.7
109.2	104.6	100.3	97	97.5	108	108.9	109.8	104.9
108.6	122	107.9	105.8	95.5	105.7	112.5	125.5	105.7
106.3	108.4	104.6	103.9	98.6	111.5	105.5	106.3	116.2
108.3	101.4	100.1	103.1	94.7	110.3	107.2	106.5	112.7
487.4	583.2	432.2	519	559.5	474.2	394.4	1031.8	385.2
722.7	1017.7	1164.7	740.6	894.4	965.5	923.1	1275	911.2
107.6	110.9	107.6	108.9	95.6	113.4	98.9	117	105.8
117.2	106.7	99.7	103.3	102.1	120.5	117.5	111.3	108.9
136.5	171.2	155.3	131.3	167	177.2	152.2	218.2	157.2
242.6	247.1	246.9	237.6	265.7	306.7	288.6	409.7	251.5
683.7	829.9	697.5	566.5	777.9	742	670.7	1231.1	909.9
147.3	148.4	147.7	161.7	137.1	162.5	139	200.5	138.6
136.2	143.2	140.3	155.4	138.6	149.3	170.3	154.6	133.1
112.5	130.1	105.1	102.3	103.7	120.1	101.5	144.3	119.5
265.5	262.6	245.5	269.6	250.4	294.9	294.4	341.6	296
128.3	196.9	202.8	205.8	161.7	169.1	191.6	188.2	224.9
100	120.6	107.7	109.7	109.4	108	110	112.5	112.2
452.8	870.2	920.1	458.7	825.2	1018.3	685.7	1241.5	739.2
102.2	104	108.3	100.2	99.6	105.6	99.4	113.8	109.1
133.8	136.8	124.2	110.7	114.5	139.8	126.9	173.9	165
687.5	625.5	539.8	565.9	466.4	510.4	525.7	916.9	795.3
184.7	158.9	156.1	156.2	147.6	188.4	132.2	259.2	232.6
298.8	344.5	300.8	244.5	323.2	357.5	276.2	412.1	368.5
107	127.4	118.9	99.9	107.9	122.8	110.7	119.3	120.3
185.1	212.8	191.6	225	208.3	208.4	217.3	211.9	197.7
106.3	114.8	102.9	120.3	101.9	115	102.9	120.7	115.1
107.2	107.6	108.3	109.2	104.4	113.1	108.1	115.3	114.6
121.9	143.8	140.9	120.9	135.9	142.3	147.6	136.9	133.6
111.1	113.9	108.2	113.3	96.9	112.3	112.8	115.8	117.3
133.4	274.9	252	238.4	230.7	202.5	262.8	227.9	160.1
150.5	150.9	147.5	170.6	142.7	149.2	160.7	155.4	153.3
105	113.2	99	95.2	100.5	113.2	102.1	107.4	105.8
100.9	108.4	103.5	103.3	96.2	110.6	101.9	101.4	107.8
104.4	98.8	98.9	101.1	92.7	102.6	100	105.3	110.2
112.1	111.6	106.2	107.7	103.3	114.9	112.4	121.9	109
156.4	174.2	172.9	143	159.5	172.5	156.6	206.6	194.2
103	107.9	101.7	100.7	94.5	96.1	103.2	110.8	109.6
100.9	121.6	102.5	93	99.7	103.5	96.3	123.7	101.7
399.6	490.8	388.4	515.6	452.7	444.5	245.4	767	473
371.1	310.9	334.6	336.7	331.6	340.8	382.3	474.9	285.8
113.7	111.4	101.7	97.3	100.5	115.1	105.2	104.8	105

109.9	124.6	109.8	106.7	99.8	115.5	100.5	111.9	103.9
99.6	107.3	101	99.7	92.6	105.5	99.8	103.3	103.3
105.9	113.6	109	95.9	94.1	105.3	104.7	113.4	116
119.8	124.1	110	133.8	104.1	141	117.1	130.1	128.9
105.9	101.7	100.9	94.5	96.9	109	98.1	103.9	100.4
109	97.1	108.6	99.4	102.6	108.1	107.8	99.2	104.1
168.9	160.9	144.4	155.7	146.3	156.3	158.3	192	144.6
108	112.8	100.3	100.6	97.4	100.4	102.9	105.8	106.2
167.2	165.7	137.2	184.8	136.6	150.5	145.7	183.1	152.4
103.4	101.4	101.7	90.6	97.8	109.3	105.1	109.9	105.4
149.3	134.6	121.4	127.1	116.3	136.4	146.1	121.9	125.6
1246.4	1224.5	1206.2	1104.8	1735.9	1713.3	1481.6	3024.1	1760.7
105.9	99.5	106.7	107.3	93.6	110.9	98.6	109.8	104.5
116.7	127.2	126.4	109.7	114.8	132.2	111.1	141.3	124.7
109.6	108.4	101.8	102.5	98	106.7	108.8	112.3	102.5
117.7	122.1	106.5	111.7	107.9	115.7	105	122.3	120.6
106	111	105.9	95.9	103.7	104.2	105.4	104.3	109.4
163.1	368.9	376.1	319.1	337.9	346.2	300	236.6	182
191.1	425.3	457	346.5	350.9	417.5	370.8	306.4	187.9
120.3	125.9	116.3	110.4	115	116.1	108.2	149.2	142.1
119.6	135.1	126.6	134.4	124.5	137.3	113.5	171.8	115
127.7	170.5	153.8	160.1	135.1	170.2	183.2	162.8	144.6
318.4	624.8	406	345.5	429.5	429.4	471.8	825	583.3
180.8	233.5	211.3	165.7	182.9	164.3	195.1	327.4	251.6
187.7	191.5	182.5	167.2	160.4	182.8	185.3	229	210
113.3	120.6	120.4	115.5	108.4	119.3	101.8	127.4	110
952.3	867.4	801.8	614.9	708.7	863.1	634.7	1609.1	1132.6
96	115	95.3	87.3	89.5	97.6	103.3	96.5	96.4
108.8	108.3	102.7	99.9	92.9	106.2	98.5	112.2	109.3
188.8	392.1	355.3	360.2	317.8	396.2	415.8	449.8	295.6
115	113.3	117.6	122.5	109.2	108.9	117.8	131.3	127.6
112.4	113	107.5	108.6	95.9	116.9	114.6	122.9	115.2
345.2	797	650.7	835.4	684	649	956.5	925.4	379.6
137.6	138.7	123.3	111.6	108.7	131.3	134.6	161.2	171.5
112.6	150.7	124	119.6	112.5	114.5	111.3	128.9	113.6
105.9	122.6	95.9	108.2	98.8	112.7	107.5	107.2	104
121.2	157.3	124.5	122.4	116.1	127.8	119.3	121.6	113.5
403.9	1468.5	1370	1130.3	1181.1	1205.7	1168.6	1138.7	634.5
104.2	106.1	103.3	94.5	95.9	105.8	100	107.7	106.4
106.8	106.7	95.6	96	99.9	102.2	107.9	103.5	107.2
111.7	109.4	104.3	115.3	100.5	117.2	109.2	114.5	111.9
245.1	567.4	471.7	398.9	458.1	323.1	349.5	301.1	245.8
117.8	127.8	116.4	110.6	102.3	117.4	106.2	138.2	134
225.5	383.7	381.9	311.6	305.9	310.1	345.7	374.1	275.1
111.1	106.2	108.5	109.1	94.8	110.6	110.7	111.9	111.7
116.8	109.4	99.7	103.5	96.3	105.9	97.2	101.3	112.9
107	110.6	101.5	99.2	95.7	102.8	98.3	110.5	109
104.5	104.9	98.4	98.3	93.4	109.1	97.9	115.2	104.4
110.7	112.7	107.8	103.6	100.7	112.9	105.1	114.1	116
120.5	124.5	115.3	114.2	107.8	126.2	127.3	124.7	135.2
204.2	265.5	226.8	232	219.9	260	259	403.5	366.8

176.8	212.9	199	187.7	201	223.5	195.2	330.9	307.9
699.1	743.6	580	447.4	606.5	604	580.7	916.4	699.4
103.3	113.4	96.8	104.6	103.5	111.8	92.2	108.9	111.9
103.2	119.2	105.2	100.1	101.5	111.7	102.3	106.5	106.2
109.3	108.7	108.5	101.6	99.3	109.5	105	118.5	114.2
105.5	104.1	97.9	92	86.6	99	101.5	102.9	108.5
121.2	132.9	129.1	133.8	129.2	123.1	125.7	175.1	139.3
187.2	179.3	183.8	153.5	172.2	171	202.6	227.2	167
102.9	110.3	94.7	96.6	95	111.7	100.1	110.4	106
571	466.8	488.6	365.2	322.2	346.5	413.9	902.5	692.3
107.8	108.6	97.1	99.7	88.6	98.8	105.9	120.7	99.6
112	104.9	106.8	102.6	97.9	119.7	103.4	115	101.1
103.4	118.2	110.8	113.1	98.9	113.3	104.5	112.1	110.9
109.8	116	104.6	108.2	98.8	110	108.6	110.1	105.5
146	151.7	172.1	131.5	122.3	160.7	150.2	257.3	200.1
252.1	262.7	214.6	209.7	180.4	240.4	200.9	319	247.2
175.5	256.9	244.7	237.9	245.2	294.6	259.9	290.5	263.5
160.4	139.3	127.2	132.8	126.4	149.1	157.1	160.9	149.3
120.3	148.2	143.2	154.2	137	148.2	153.1	172.5	129.5
545	1073.9	964.8	781.3	662.9	849.5	864.3	1291	763.3
190	266.5	325.8	266.9	234.4	223.2	223.7	416.3	260.3
103.4	105.1	98.9	99	92.5	104.2	107.3	98.7	101.6
108.8	129	116.3	120.3	102.2	119.8	113.1	115.1	116.4
204.5	282.7	264.5	245.7	211.9	220.2	281	301.6	233.6
106.7	114.5	107.7	115.5	99.3	115.5	100.9	114	114.9
121	116.6	126.1	130.7	118.1	127.3	116.3	134.1	124.3
121.2	111	111.5	101.6	109.6	131.1	112.4	127.6	122.8
119.2	129.4	119.7	103.8	101.9	116.8	116.4	127.3	117.7
110	123.4	146.9	107.3	107.8	157.5	116.6	135.7	116.7
110.2	106.8	113.9	101.9	101.2	113.6	102.5	125.1	114.5
112.7	108.6	123.4	108.6	106	108.7	107.7	126.4	108.8
132.9	151.1	174.7	184.5	148.8	159.3	169.3	227.6	130.2
112.3	103.1	111.8	105.2	113.2	129.6	110.3	111.7	112.7
106.5	102.1	105.3	96.6	101.8	105.6	101.9	105.5	109.7
109.6	132.7	145.3	116.1	118.3	128.1	119.9	131.6	116.6
108.9	113.3	105.7	116	97.9	104.3	101.7	110.8	107.8
108.2	113.5	107.7	95.7	97.1	117.9	109.1	114.8	124.1
113.2	113.8	101.2	103.1	97.3	101.8	106.8	105	109
100.2	110.9	98.2	101.6	99.7	108.5	106.8	107.7	105.6
109.8	108.1	101	109.5	103.4	114	108.9	120.3	120.2
115.7	132.2	118.1	138.9	98.1	119.8	106.5	123.4	120.3
115.3	106.5	104.7	106.5	95.6	113.5	112.3	117.4	118.9
110.8	103.9	94.7	99.2	92.4	107.1	107.2	109.3	106
107.6	108.9	115.1	110.5	112	128.1	112.5	122.7	126.3
109.1	107.1	96.9	96.8	95.7	105.1	102.6	107.1	113.8
115	107.7	109.4	114	97.7	116.6	104.1	122	122.9
183	206.3	177.6	194.5	140	226	177.2	324.6	298.2
159.2	160.4	141.2	147.5	141.2	137.8	184.6	230.9	194.2
119.2	126	126.5	132	129.1	131.3	133.6	137.8	113.2
109.3	109.7	114.2	111.4	99.2	114.9	109.6	118	108.2
298	446.6	523.8	422.8	394.4	412	442.1	516	543

224.7	275.1	218.8	320.2	249.7	182.6	195.7	331.9	256.6
424.1	654.7	456.7	636.8	403.3	420.2	554	768.6	543.7
223	262.3	243.4	195.8	214.5	269.1	216.3	412.5	287.7
102.7	103.5	103.8	97.4	90.4	111.4	102	120	112.6
116.4	115.1	101	107.9	99.9	110.9	107.1	116.7	120
104.4	104.4	115.4	113.6	94.3	122.8	118.3	125.3	123.7
110.4	110.5	104.7	96.4	98.4	110	108.8	107.3	116.8
448.6	570.5	513.5	371.7	564.5	590.8	393.1	890	533.9
107.2	211.2	169	106.8	144.1	116	166.7	129.5	116.3
114	108.5	100.8	103.2	98.6	112.1	109.3	99.3	111
107.4	101.8	98.1	97.8	95.9	103.8	96.9	105.8	110.6
111.2	115.4	101.1	98.7	100.8	105.1	105.3	104.6	104.4
113.3	125.2	111.9	124.5	104.1	116.7	107.8	133.9	131.7
106.5	106.1	99.7	101.1	89.6	106.9	101.5	105.3	106.9
101.5	107.4	101.9	91.9	96.6	101	104.2	107.7	108.4
108.7	103.7	103.1	97	92.2	109.7	103.2	109.7	107.1
108.8	111.2	108.4	95.7	99.1	104.6	100.8	112.7	118.6
114.2	110.4	95.4	102.1	101.6	108.1	105.9	120.3	113.2
98.6	108.6	100.7	102	95.3	115.4	103.5	105.2	109.1
433.7	713.6	693.5	738.9	682.2	738.2	491.9	1011.3	592
164.2	234.6	203.2	227	193.4	180.9	259.7	256.5	189.9
139.2	181.9	152.9	164.3	151.1	168	181.7	221.8	167
103.2	97.5	90.7	92.5	91.5	104.7	94.1	103.9	104.2
104.6	104.6	112.4	100.7	91.9	110	111.6	108.8	104.9
481.7	668.7	576.8	587.7	538.2	745.6	637.2	739.9	553.4
749.4	993	1170	1108.4	967	1281.9	1116.7	1267.6	968.8
114.2	127.3	122.3	113.1	117.6	118.2	120.7	144.7	141.1
126.9	120.9	123.9	116.1	105.2	132.6	109.9	146.7	131.8
107.2	115.7	96.7	103.3	96	108.1	100.2	111.2	113
110.2	101.2	103.6	107.8	99.9	106.8	100.9	111.2	112
884.4	884.2	892.7	964.4	697.6	890.2	834.2	1434.3	679.2
893	1315.5	1701.7	1118.8	1165.6	1013	1033.8	1865.5	1241.5
445.8	1009.4	1574	726.7	1048.9	950.1	922.5	1177.7	614.5
176.5	269	278.3	154.1	193.4	215.9	122.8	263.6	114.7
123.2	124.9	114.2	118.2	117.7	126.7	107.8	132.3	122.9
110.4	106.7	110.9	101.7	102.1	114	108.8	111.7	111.1
109.2	110.8	98	105.4	93.5	102.6	101.9	103.7	113.9
138.5	135.6	136.6	138.1	131.1	157.4	155.2	177.5	143.5
99.5	112.9	97.1	103.7	90	102.7	101.4	98.9	110.7
103.9	115.1	104.4	100.4	96.5	110.4	96.6	110.7	108.9
110.5	105.2	108.7	95	94.5	107.5	99.2	103.4	114.9
128.4	133.9	136.6	150.7	124.2	165.3	129.8	139.2	130.2
161.3	205.4	200.5	209.3	196.1	209	224.8	261.2	186.9
121.1	124.6	139.5	139.5	128.5	134.2	128.2	145.1	128.8
128.9	172.4	142	191.9	164.4	131.1	165.1	141.3	127.6
107	119.7	108.8	107.6	109.8	124.5	121.9	115.6	126.8
157	172.7	163.5	148.2	156.7	176.4	143	256.7	180.4
106.3	109.4	99.7	97.4	92.8	104.5	104	108.5	108
209.8	249.6	235.5	230.7	240.9	244.2	263.5	406.5	290.5
108.8	111	103.6	97.2	95.7	110.4	101.4	120.3	127.2
155.6	130.8	117.5	100.4	109	124.7	114.2	215.3	183.3

103.5	106.2	101	94.9	92.9	108.4	98.7	106.4	106.2
184.2	284.1	255.9	239.7	270	247	284.5	284.3	150.9
129.5	143.1	137.8	149.2	131.3	148.6	131	165.1	137.2
101.6	118	110.2	108.6	95.7	111.4	105	113.6	105.6
147.4	161.3	127.1	177.5	141.9	138.9	199.6	166.6	140.7
152.9	130.7	123.5	209.1	119.1	138.3	163.2	175.6	185
201.4	177.2	182.9	144.7	149.2	187.2	159.9	232.7	314.6
112.9	110.2	105.4	98.6	98.3	114.9	107.7	118.8	120.1
121.6	139.1	125.9	170.3	111.7	122.2	165.2	139.3	115.6
104.3	99.7	106.6	96.7	101.7	102.7	98.9	107.3	113.1
113.8	107.7	100.6	109.2	100.2	112.7	108	118.2	102.7
110.5	130	126.3	140.7	132.9	120	147.4	133.1	117.2
101.1	107.5	101.2	95.4	98.4	101.1	105.2	115.5	108.9
105.8	109.7	109.6	105.7	96.1	111.2	109.2	119.1	107.5
116.5	118.3	126.6	123.4	107.4	122.9	123.6	120.6	120.8
102.3	108.6	96.3	98.2	97.3	106.2	106.7	99	114.2
110.6	112.3	98.6	108.8	96.8	105.1	107.7	110.6	111.6
107.8	109.6	108.1	101.5	94.9	110.8	97.6	104.5	101.4
111.5	135.7	123.7	114.4	121.5	118.9	124.5	141.4	119.2
110.7	123.1	129.6	117.1	108.7	126.4	115	125.3	127.1
109.5	117	106.3	124.3	101.8	119.6	106.1	113.9	124.3
110.3	105.9	105.1	106.8	91	121.7	106.5	116	95.6
106.9	103.9	107.9	95.9	98.3	117.3	104.1	107.4	109.1
103.9	98.2	104.5	103.9	100.6	102.7	100	108.1	104.1
115.1	100.5	96.6	106.9	95	106.6	117.1	110.9	115.4
111.3	115.9	110.7	105.5	96.9	111.5	103.6	103.9	108.8
108.3	111.5	121.4	115	110.4	117.8	117.8	126.3	107.5
117.1	110.6	104.9	110.9	97.9	115.3	104.4	112.7	110.7
103.8	98.4	95.8	99.1	92.7	106.9	101.6	107.2	106.8
121.4	114.8	113.9	104.2	102.6	119.3	115.3	118.9	125.4
105.1	115.4	108.6	97.5	104.6	104	108.1	117	106.9
109.4	113.5	101.6	95	99.3	104.5	100.2	113.3	110.2
101.8	107	102.7	91.8	93.4	101.8	97.2	102.8	98.1
144.8	135.3	150.8	145	141.8	167.2	164	166.5	142.7
234.5	437.7	405.1	314.5	361.2	430.6	331	501.2	331.5
108.8	113.9	106.6	102.2	93.9	109.1	99	105.5	108.2
103.9	106.1	100.7	99.6	92.8	107.9	107.7	116.2	117.3
112	107	111.3	105.8	104.2	115	107.2	116.9	109.5
106.4	106.4	98.7	104.3	92.7	102.9	106.2	100.8	109
119.1	116.3	109.6	112.1	104.4	117.9	112	121.1	120.3
110.5	114.9	108.5	102.5	103.2	112.9	112.5	133	137.3
121.4	126.4	137.5	121.5	127.1	150.3	126.5	150.1	182.4
664.6	755.7	665.1	566.1	576.4	827	591.3	1200.4	866.4
393.7	583.5	648	497.5	582.2	642.6	484.4	1108.3	702.5
147.3	129	120.7	136.3	109.2	136.1	121.4	167.6	144.1
114.1	116.8	113.5	115	114.8	116.7	109.2	127.4	112.3
151.3	143.1	145.8	133.6	135.2	153	141	167.7	153.7
97.7	99.6	110	95.4	93.4	105.7	97.5	111.7	99.9
108.5	106.8	103.5	93.5	86.9	97.8	96.3	97.9	98.7
119.4	136.6	108.4	137.2	112.5	118.5	126.1	155.2	127.3
112.8	105.8	108.5	110.4	100.8	114.6	105.5	125.8	119.5

359.2	1125.1	942.2	662.6	854.1	946.2	742.4	1170.7	795.3
129.6	231.5	239.9	199.4	205.2	208	216.2	277.4	210.3
333.7	323.1	322.4	419.1	279	352.9	397.7	330.1	293.8
384.2	378.2	398.5	412.8	294	377.9	478.8	340.8	327
121.5	121.1	106.5	114.7	110.5	113.3	113.4	113.7	125.5
97.4	104.6	93.8	96.5	85.7	99.6	95.5	103.8	108.7
98.8	109.6	99.3	99.7	94.2	109.4	104.4	121.2	107.8
109.3	109.7	106	100.2	97	114.3	107.1	106.6	104
161.8	373.2	467.7	316	336.4	425.8	327.8	577.5	275.5
123.1	155.5	132.1	151	132.6	155.5	141.7	175.5	158.9
164.3	210	153.4	161.7	148	166.4	173.2	215.5	179.7
119.1	109.1	111.9	106.7	95.7	111.8	103.5	108.9	108.6
152	217.6	196.2	219.8	184.5	205.6	193.4	179.4	147
103.2	104.9	111.5	105.6	95.8	116.3	106.1	108.8	118.6
107.7	110.5	104.3	103	99.2	106.7	99.6	106.3	101.8
109.9	114.3	102.1	93.1	95.9	110.6	103.4	113.7	105.9
108.7	104.5	102.1	98.3	94.6	107.8	107.1	109.2	106.5
118.1	133.6	132.3	118.5	111.4	128.9	113.9	140.9	134
107.6	103.8	99.9	103.1	93.4	104.5	102.6	100.8	108.8
107.8	101.1	100.3	96.4	91.2	100	108.4	104.2	106.7
238.7	219.8	206.7	226.2	208.5	228.1	239.1	323	211.1
115.7	113.3	122.6	106.4	103.5	129.4	109.1	146.6	123.4
108	113.9	108.9	106	99.6	116.4	105	124.4	118.3
109.9	112.5	100.6	100.8	101.1	109.5	112.1	115.5	107.6
113.9	108.5	99.7	105.6	101.2	110	101	111.9	98.4
111.7	114.9	103.7	108.4	94.5	119.9	108.3	116.2	111.7
98	107	105.7	97	92.3	115.7	95.2	113.4	103.8
112.1	123.4	112.2	106.4	113.6	112.6	99.9	107.4	103.1
171.2	168.5	184.2	159	165.4	192.9	188.1	240.6	192.6
2279.6	1778.6	1798.6	1316.4	2028.3	2605	1906.4	3866.2	3496.1
299.9	368.3	439.1	427	445.5	495.2	342.9	534.7	293.2
116.2	112.5	110	102.9	104.6	116.5	106.8	132.9	125.7
117	115.4	108.2	105.3	109.9	111.7	108.4	116.2	114.8
248.3	357.5	320.7	269.8	277.7	286.1	277.5	452.2	263.7
216.1	259.3	252	214.8	173.9	231.2	266.1	550.3	354
110.9	107.9	106.3	89.3	94.9	110.9	96.4	116.1	109.1
433.1	562.7	496	476.3	477.8	588.6	582.7	898.2	689.4
106.9	108.6	112.1	107.8	100.6	111.9	106.3	128.7	116.4
121.9	127	111.5	107.9	108.5	110.9	112	120	114.5
103.8	100.6	102.8	92.9	95.1	109.5	100.3	108.9	107.8
102.4	106.9	101.8	96.4	94	102.6	94.7	97.3	99.4
99.2	98.2	95.9	97.9	92.5	105.8	95.6	102.5	100.6
244.6	239.6	193.6	212.4	209	213.4	241.2	276.4	245.3
184.3	207.7	175.5	167.2	158.9	185.1	193.7	248.2	211.9
108.5	109.7	108.4	107.1	98.6	110.8	100.6	115.6	119.3
113.5	103	97.7	99.1	95.8	105.4	102.3	112.3	107.4
116.7	118.1	112.5	106.2	120.8	121.8	118.1	149.1	123.5
104.4	114.5	98.2	107.7	94.3	113.6	108.3	118.7	113
316.4	267.2	252.5	235.7	269.7	345.3	305.5	638.9	672.5
671.4	866.2	625.3	1047.5	907.9	681.6	930.9	1182.8	703.8
107.7	110.3	113.6	111.5	96.6	112.9	116.8	108.4	119.6

112.1	118.3	115.5	132.8	104.2	124.2	126.2	117.1	125.4
119.9	120	116.1	131.3	110.2	114	113.3	122.5	120.3
106.8	124.1	109.9	103.8	98.9	116.7	114.8	123.7	107.3
722.6	1702.4	1398.5	1098.7	1594	1526.1	1383.1	1924.6	1369.9
154	233.2	191.9	188.5	185.8	233.4	218.5	298.2	197.1
101.5	106.3	106.5	100.2	101.2	110.1	109	110.4	109
381.6	289.5	288.1	346.3	250.6	341.6	332.1	573	495
586.9	663.6	554.3	650.5	496.8	645.5	678.6	1039.8	650.1
173.2	173.3	159	149.1	154.3	194.9	168.5	313.2	203.5
1642.3	1814.3	1681.4	1372.6	1547.8	1700.7	1378.5	2809.9	1755.1
353.2	306.4	267.8	209.8	253.2	297.7	267.8	723.5	398.9
111.3	107.6	105.9	108.1	105.1	114.1	109.7	117.5	113.1
110.8	106.2	110.8	103.6	96	116.5	115.3	113.2	104.5
381.7	399.5	191.9	520.4	319.1	173.9	658.6	331.8	179.6
115.7	113	115.4	105.1	100.5	119.6	99	119.5	119
112.6	110.2	98.5	103.5	102.2	105.4	104.5	118.4	111.3
105	107	105.8	102.6	96.8	101.8	104.3	101.7	105.6
106.6	105.4	99.6	103.2	94.1	116	108.3	109.3	116.3
122	130.5	118	109.1	105.8	113.4	117.5	131.8	132.9
98.1	96.7	95.3	99.9	92.9	104.4	92.8	114.8	105.7
121.6	114.1	115.8	113.9	112	121.9	99.7	122.5	125.6
187.1	168.7	177.3	172.3	150.7	178.9	204.8	206.7	195.7
130.2	137.2	115.7	122.6	133.3	119.5	135.4	168.9	136.5
108.7	146.5	126.1	159	117.9	111.1	141.8	155.4	136.8
121.3	111.8	102.2	101.4	105.2	116.9	106	148.8	118.4
350.9	281.6	278	310.1	259.4	277.2	265.8	396.3	341.8
418.1	339.3	335.6	309.8	288.7	343.2	344.8	1005.3	888.2
428.9	402.6	613.1	473	564.7	654	549.8	759.7	433.3
97.2	105.7	99.9	103	96.6	106.7	104.5	116.2	112.6
105	105.6	110	101.7	95.8	106.6	116.4	112.7	105.5
114	114.1	108.1	106.4	103.5	117.9	108.2	113.8	104.9
109.6	111.5	112.6	117.2	99.2	110.8	102.4	104.2	112.6
380.6	397	321.5	587.1	342.3	278.9	615.2	558.2	698.4
536	492.3	460.9	477.3	476.2	534.1	692.7	771.1	589.8
118.1	159.1	148.6	147.8	155.3	144.4	146.7	237	165.5
131.8	144.6	132.9	132.4	116.1	140.6	133.5	172.9	157.4
154.2	152.3	128.6	154.2	133.4	143.2	162.6	232.6	171
108.7	103	103.8	102.6	96.3	100.7	102	108.8	114.9
109.3	115.4	103.4	108	96.5	111.4	109.2	110.6	101
98.8	108.9	102.2	106.5	90.4	109.9	100.6	107.8	103.4
161	227.2	248.3	193	172.3	243.3	187.4	197.4	140.3
145.9	142.3	139.3	128.8	143.1	148.3	172.2	209	136.7
124.6	151.6	132.4	111.9	132.7	146.2	120.2	169.2	132.3
108.8	114.2	103.8	107.3	100	117	109.9	131.1	134.4
108.5	111.2	103.7	100	93.5	103.7	113.4	111.7	115.3
117.3	124.2	121.1	115.6	117.9	125	105.1	130.7	126.6
111.9	117.2	98	116.1	107.4	110.4	118.3	112.4	110.9
301.6	239.2	254.5	224.3	255	250.3	189.4	264.4	242.6
243.8	408.8	397	333.8	492.6	497	326.8	555.6	349.7
204.3	195.2	163.3	174.4	200.7	203.3	199.7	293.2	191.2
108.5	106	98.9	103.1	98.4	126.7	103.1	111.5	104.2

144.1	153.2	158.8	128.9	149.2	153.9	123.5	227.8	148.9
106.7	106.5	97	102.4	94.8	107.1	99.5	113.4	110.3
112.1	107.3	118.5	100.5	103	113	100.1	107.6	112.4
110.2	108.5	103.6	93.2	95.5	100.7	99	110.8	98.2
109.4	145.8	140.6	140.6	121.6	129.6	134.6	165.7	150.2
4096.3	4320.1	3572.2	4334.7	3636.2	3579.4	4237.3	7051.6	5876.6
5570.7	5699.4	4737.3	4588.9	4783.7	4207.9	4843.1	7054.4	6275.6
164.9	160.9	132.3	149.3	160.9	129.2	132.8	219.8	207.8
126.6	152.5	147.3	159.1	130.2	153.7	173	208.5	170.5
2327.6	4464.7	2922.6	3073.6	2677.1	2359.1	3144.2	6342.5	4161.1
336.4	654.8	634.3	566.2	545.3	544.4	720.1	597.9	371.5
217.6	158.6	181.1	158.6	156.8	147.4	164.5	276.4	299.4
104.7	110.3	107.2	107.1	93.4	110	101.3	114.4	117.8
132.8	177.1	145.3	246.8	167	129.5	235.8	153.3	136.4
103.6	184	147.6	157.1	137.1	136.9	132.5	153	125.8
100.4	103.4	98.9	97.6	89.4	100.5	96.8	113.8	103.4
115.5	120.1	108	102.9	100.2	123.4	105.5	135.1	120.9
324.6	426	368.6	472.3	307.2	280.1	305.8	652.7	494
124.8	120.5	127	114.2	111.5	119.1	109.2	129.9	111.7
97.3	111.3	100.7	98.6	91.8	104.9	91.8	111.4	102.2
100.9	107.6	107	94.1	93.1	104	95.5	113.8	107.3
104.8	115.6	107.9	96.8	99.4	102.4	103.6	113.5	103.4
145.2	247.1	220.8	260.1	187.2	184.2	254.8	183.8	156.2
202.1	482.3	429.4	541.6	226.2	315.9	527.1	335.4	195.7
135.7	209	164.7	244	171.6	161.9	208.3	168.9	142.9
113.3	120	109.3	100.7	102	121.9	105.2	119.7	130.7
110.4	109.1	99.4	93.4	95.8	104.4	98	110.7	110.9
101.9	110.3	98.6	105.7	89.8	108.7	103.6	116.4	108.1
105.4	104.5	101.4	107.8	99.3	108.5	100.1	115.8	118.8
97.2	106.1	92.9	102.2	94.8	103.5	101.9	94.5	103.6
110.7	119	109.8	108.1	100.5	117.6	105.6	123.2	113.2
109	121.2	107.8	108.6	97.3	126.3	120	128.4	115
109.2	109.1	102.3	100.9	97.9	109.3	101.2	101.6	117
112.2	108.4	109.6	108.2	96.6	107	99.1	99.2	114.7
110.2	113.4	101.6	100.3	103.4	109.1	108	110.1	107
1550.1	2596.5	2385.9	1968	2034.3	2482.7	2029	3124.3	2062.5
110.5	111.5	107.1	105.2	102.4	121	116.9	106.2	112.5
129.6	168	248.9	187.8	149.6	163.9	171.9	375.6	212
105.7	115.4	103.9	111.9	92.2	108.7	100.6	115.4	116.4
111.9	117.5	102.7	98.3	100.8	114.3	110.9	111.4	115.4
107.5	109.1	106.9	100.2	100.6	107.9	103.3	109.4	112.3
276.7	391.9	463.2	363.7	389.4	442.4	368.5	541.5	400.4
141.2	189.6	213.4	179	151.2	200.8	172	304.4	222
185.6	276.8	202.3	186.6	197.9	241.5	228.5	380.1	324.2
134.2	157.7	143.5	125.7	135.4	152	154.3	206.5	165.5
145.6	156	139.2	143.8	132.5	128	149.8	132.7	142.4
104.5	110.2	97.4	96.5	89.1	100.5	104.4	109.8	111.3
119.3	120.5	109.5	106.3	104.9	117.4	110.5	118.5	123.5
240.2	297.7	282.8	214.1	239	292.5	250.3	449.9	277.1
110.8	110.7	103.2	116.1	96.6	109.6	111	112.8	106.7
228	426	295.8	405.8	332.1	359.6	276	551.3	342.5

109.2	109.8	98.2	107.5	101.7	116.1	96.7	107.6	110
107.9	102.7	105.7	148.9	95.6	106.6	117.5	107.2	98.8
108.9	109.3	105.2	102.1	93.5	105.9	100.8	120.5	116.5
108.3	110.2	104	102.1	100.7	107.4	102.7	113.1	109.8
100.9	109.7	98.1	104.5	100.8	110.6	103.7	106.9	121.2
109.4	113.5	103.7	102.3	96.8	116.4	115	106.7	123.4
106.9	120.6	108.8	122.5	108.6	122.8	107.1	152.8	116.3
126.1	158.4	127.1	145.4	138.8	140.8	140	184.8	133.7
107.6	109	114.3	110.5	99.3	119.2	107.7	130.2	117.4
97.5	99.7	107.1	100.8	92	106	100	103.8	108.7
106.2	138.6	135.8	163.7	122	126.8	149.1	120.8	114.8
115.1	111.5	104.1	107.8	99.5	111.3	109.7	104.9	105.6
115	119.3	109.8	111.4	99.2	108.6	100.6	114.4	108.4
104.7	108.3	98.6	97.1	99.8	109.6	105.2	107.5	109.4
103.5	114.3	109.5	102.5	99.7	102.1	113.8	111.8	118.2
159.8	229	181.7	166.8	163.3	179.4	170.2	381.6	211.9
198.4	316.2	301.8	296	293.7	245.8	360.4	498.7	306.3
109	102.5	100.2	99	87.4	103.4	102.6	100.5	97
194.8	263.5	253.9	194.5	199.4	233.1	213.1	292.5	248.3
109.2	110.7	109.1	100.3	109	117.9	99.9	114.5	106.8
175.4	137.5	140.7	130.2	124.2	149.5	131	230.1	191.4
104.9	105.6	98.3	101.3	90.8	110.8	107.4	102.5	105.9
188.4	292.2	292.1	211.2	257.4	338.9	269.7	486.2	326.8
134.1	125.2	138	124.9	120.9	138.7	118.2	131.2	145.3
149.7	200	167.3	147.7	196.9	199	166.5	491.1	257.2
106.1	109.2	103.8	106.5	94.6	109.3	96	111.5	104.8
129.6	141.4	163.4	136.9	134.6	155.2	150.3	131.1	130.7
117.1	128.2	117.5	111.4	108.9	119.7	124.8	123	119.9
112	119.6	106.3	111.8	102	108.5	108.5	117	102.1
112.7	112.1	109.7	105.9	100.3	115	112.7	111.3	115.9
104.3	101.9	100.9	101.7	87.8	111.2	103.3	101.9	107.1
104.4	112.2	113.3	100.6	96.3	111.7	98.5	108	110.5
123.6	141.9	134.5	131.5	133.6	144.1	133.6	165.6	153
114.9	109.8	105	101.4	89.2	114	113.4	116.4	106.8
111.3	110.1	101	113.8	100.2	108.6	109.3	113.4	100.6
113.7	115	115.8	97.3	96.2	116.8	112.1	120.8	105.8
111.6	115.7	103.7	115.1	96.9	104.2	99.9	122.8	113.9
105.5	110.2	111.2	101.6	97.2	107.6	106	111.1	102.7
106.6	111.1	106.5	102.7	100.1	113.9	107.1	105.4	108.2
162	392	361.2	495.4	349.6	333.9	487.5	283.5	280.3
109.4	108.3	108.5	102.6	99.8	103.5	100.6	105.8	104.5
127.2	129	116.3	119.4	114.2	126.7	130.4	147.1	139.3
121	139.2	117.8	129.9	114.3	129.8	127.2	140.4	115.5
579.4	972.4	676.5	1455.8	915.7	709.1	1010.6	1776.5	1700.6
100.3	110.8	103.4	103.7	88.6	105.1	95.3	103.6	102.6
111.6	148.2	112.4	121.9	119.9	143.9	121.2	137.4	128.2
109.3	118.2	123.4	139	114.4	128.3	120.1	146.4	121.8
220	351.6	270.9	269.6	256.8	288.4	311	285.3	280.4
121	154.8	142.6	144.3	132.5	153.8	148.8	139.3	146.7
104.4	109.8	101.2	99	93.2	107.3	101.3	105.8	103.8
112.9	126.8	127.7	108.7	99.8	130.3	128.5	130.4	120.6

104.7	105.8	110.7	102.4	94.8	107.8	104.7	104.5	99.5
110.1	107.9	101	98.5	92.3	107.7	102	105.6	109.7
109.7	106.3	101.9	104.6	100.6	108.4	103.8	105.3	111.3
104	103.3	97.4	101.8	100.1	110.4	98.1	114.2	109.8
106.8	104.1	102.1	98	92.6	111.5	99.7	104	111.8
292.6	434.7	332.9	329.2	341.7	381.4	402.3	517	480.4
106.6	103.7	98.4	100.6	89.5	104.8	99.8	111.9	106.3
105.6	105.7	106.9	98.8	98.6	100.7	99.5	114.4	106.1
116.8	129.7	93.1	103.5	97	109.1	102.1	114.9	100
108.5	113.2	103.5	105.8	100.1	116	108.7	111.6	111.7
182.9	213	187.1	195.9	170.8	228	228.4	243.4	200
109.9	117.1	119.8	122	101.5	126.5	112.8	130	119.4
112.5	111.4	106.1	114.1	107.3	115.6	121.4	117.3	111.9
105.4	109.3	105.3	109.7	91.6	111.3	111.5	109.9	111
103.7	112.9	107.2	99.2	98.2	104.9	102.2	106	109.7
111.3	107.6	107.9	103.5	92.3	108.6	91.8	112	108.5
100	103.7	109.9	101.7	99.7	107	110.9	110	112.1
105.9	111.4	103.8	107.3	97.6	106.6	109.5	121.8	111
117.1	128	120.9	112.4	109.4	116	110.5	139.1	114.4
113.7	109.7	111.6	110.5	99.5	115.1	106.1	112.5	110.8
262.2	512.9	527.5	646.6	403.2	490.7	623.9	664.9	485.8
101.3	111.6	103.2	115.1	99.2	117.6	99	111.8	109.4
129.3	131.2	134.9	135.2	114.7	136	133.5	160.6	134
115.7	120.9	112.7	118.7	107.9	116.4	117.2	125.5	128.5
144.1	153.4	134.7	149.4	122.6	135.2	168.7	208.4	146.6
106.4	103.1	96.4	98.1	92.5	100.8	102.4	103.8	106.9
106.2	116.2	107.2	97.7	96.3	105.8	99.9	111.7	107
106.7	99.9	104.8	91.5	92.9	98.4	92	98.4	102.2
114.6	117.6	108	105.7	101.1	113.3	108.2	115.4	110.7
103.6	104.5	96.7	98.4	98.2	112.3	97	104.8	106.2
97.9	101	101.6	103.2	89.9	99.9	97.3	109.1	107
110.7	111.7	103.7	99.4	100.2	105.8	106.6	110.6	109
111.8	111.9	102.3	100.9	92.3	108.3	102.2	101.1	102
158.1	209.2	183.2	160.9	161.3	200.2	181.3	305.8	213.1
112.2	109.5	105	107.1	94.9	115.9	105.7	114	103.7
275	283.7	233.6	298.4	252.6	252.9	322.7	297.9	266.6
1385.8	1897.1	1640.4	1884.3	1565.4	1918.1	2432.5	2490.8	2462.2
108	132	132.1	103.9	147.6	143.6	140.5	183.6	114.1
110.2	107.2	99.9	112	95.7	118.5	102.9	127.6	112
108.7	115.9	106.8	111.9	98.7	117.7	101.3	109.9	126.8
118.7	196.6	178.3	228	155.4	185.9	234.6	210	172.4
126.4	171.4	131.5	172	129	140.9	153.4	134.7	132.3
100.3	108.3	100.5	107.6	93.7	114.5	100.7	109	105.4
150	274.4	213.4	275.9	182.7	224	251.2	280.6	194
1177.2	2242.4	2337.1	1258.9	2085.6	2098.7	1581	2483.9	1004.9
122.3	135.1	137.2	134.4	124.3	134.1	122.5	151.7	128.7
116.1	120.4	119.6	108.4	99.8	122	111.8	129.8	121.8
125.5	163.3	155	128.3	121.4	147.4	123.7	150.4	124
108.4	108.6	109.3	111.2	94.1	119.4	112.2	106.9	111.1
101.5	105.4	95.1	94	96.9	108.3	106.1	110.5	109
136.1	143.7	133.5	126.1	118.6	131.7	127.6	177.1	229.4

106.2	109.7	100	95.2	102.4	103.9	103.3	104.4	107.7
109.6	114.8	93.1	100.2	91.5	102.2	101.1	102	117.3
102.8	113.1	95.1	113.7	99.3	107.7	107.3	109	112.2
106.7	128.7	123.1	105.7	119.8	132.1	113.9	131.7	114.5
329.1	494.6	535.6	416.2	398.1	444.7	230.9	569.6	421.9
128.3	126.6	108.3	119.6	109.9	126.6	116.2	150.4	134.2
228.9	312.2	274.6	265	228.3	261	299.4	537	400.2
112	109.2	101.4	97.5	103.6	99.6	104.2	101	108.9
106.1	113.1	106.5	99.8	98.9	101.9	102.8	107.4	111.5
112.7	123.6	106.4	116	108.3	109.4	107.5	123.7	112.6
105.5	111.4	111.8	101.5	97.5	111.2	106	110.9	111.4
122.7	137.5	120.9	126.7	110.7	127.7	125.6	137.8	138.7
99.2	106.9	103.4	99	93.6	105.8	99.8	105.6	105.6
105.2	107.6	101.5	103.3	94.5	116.3	101.9	109.4	107.2
308.1	273.7	253	277.4	265	314.9	295.1	306.5	272.9
100.8	119.4	98.9	105.5	97.4	107	106	122.7	123.7
180.6	195.9	208.2	168	185.2	201	195.2	264.6	199.8
121.7	125.2	109.1	110.4	108.2	131.2	106.8	166.8	124
106.8	111.1	101.8	94.7	93.5	110.7	106.1	120.1	109.3
120.3	114.6	99.1	106.7	95.7	113.4	98.8	114.5	124
174.5	158.4	160.9	138.8	157.4	172.9	135.6	284.8	289.9
104.7	105.3	99.6	99.5	97.2	104.5	99.7	120.2	104
100.2	107.4	103	93.3	93	107.8	97.4	116.4	106.4
127.4	123.5	112.1	106.3	111.5	121.1	124.6	148.2	146.4
104.7	104.4	101.6	100.7	94.1	108.6	101.4	103.3	104.1
129.3	120.2	108.5	114.4	111.4	118.8	102.7	142.6	155.7
106.5	115.5	103.8	110.3	99.5	111.5	105.3	105.9	115.1
258.8	357.3	345	356.8	360.6	324.7	395.5	452.2	355.9
106.9	105.8	106.7	108.7	100.4	113	101.5	123	120
112.3	109.2	101.2	97.5	98.7	106.2	106.7	108.7	116.5
123.8	136.7	141.9	150.2	129.9	134.2	134.8	188.6	147.9
122.4	203	193.9	159.6	210.9	188.4	146.8	173.3	134.6
104.2	111	104.9	109.3	97.3	107.9	101.8	120	113.9
178.3	356.2	354.6	264.6	290.8	314.5	303.2	435.7	314.8
118.3	108.4	108	102.4	96.5	115.5	100.6	108.8	112.3
150.8	242.6	242.2	186.4	200.7	274.3	192.4	334.6	258.1
115.2	114	118.5	98.5	101.4	118.5	104.4	124.5	124.5
184.7	208.2	203.5	160.2	234.3	219.5	163	319	258.1
146.3	212.8	195.2	189.1	164.4	156.7	189.3	167.7	158
113.7	109.5	105.4	103	100.3	114	103	131	111.7
167.7	205.9	168.1	191.9	244.4	232.1	201	487.9	324
99.8	102.1	101.6	96.6	96.6	107.9	106.3	100.3	109.8
115.1	115.9	101.2	105.2	93.1	107.1	98.3	109.8	109.5
108.1	111.2	102.5	93	93.7	110.2	101.9	114.9	99.6
132.8	162.7	125.5	148.6	137.5	152.8	149.5	155	155.6
103.5	104.8	103.7	105.1	90.5	103.3	104.2	103.5	108.4
103	107.6	102.3	103.3	93.5	107.1	102.9	106.5	104
537	704.4	719.9	541	650.3	677.9	580	1153.8	781.5
111.7	108.9	105.6	98.3	94.6	108.3	100	105.5	103.5
129.9	171.2	173	174.4	178.5	158.9	160.2	243.5	176.3
371.9	473.7	487.4	307.6	411.9	535.1	401.4	639.1	462.6

128.3	143.7	146.5	134.4	121.4	130.9	136.1	174.8	156.2
244.1	262.8	264.8	224.8	214.9	257.1	217	273.6	214.4
374.2	407.4	428.2	508	354.8	374.8	354.5	1035.6	656.6
106.7	118.6	116.3	104.1	103.4	109.3	101.7	115.7	108.5
135.7	152.5	144.9	112.1	144.5	162.5	128.5	208.8	191
130.7	154.2	129.1	123.1	139.6	151.8	150.7	260.6	211.2
110.6	109.2	105.9	100.1	102.1	110.9	102.4	110.7	115.6
122.9	178.8	182.5	166.7	137.7	170.6	175	215	166.8
103.3	120.3	100.4	92.9	88.6	105.2	96.5	112.6	98.2
112.3	116.3	101.6	100.4	95.7	115.1	104.8	112.6	112.5
1168.2	1869.3	1609.4	1724.9	1083.7	1297.8	1555	1930.9	1607.2
112.6	114.9	115.8	120.1	102.1	121.8	106.4	127.2	119.8
108.5	105.4	103.4	93.4	98.2	109	104.8	105.6	106.4
110.5	106.4	101.2	108.3	92.6	108.2	100.7	109.5	120.2
107.5	112.6	108.6	97.4	90.1	109.4	102.1	108.6	107.4
131.3	148.1	152.9	188.4	129.8	123.5	158.9	178.1	156.2
102.5	119.8	99	98.3	102.7	108.6	113.6	112.3	112.3
107.2	114.3	102.6	112.6	91.9	114.5	114.5	125.4	113.2
186	217.4	262.5	229.1	167.3	212.2	186.9	315.4	259.8
118.1	106.7	99.5	100.6	100.9	105.2	107	112.4	108.9
128.2	166.7	135.9	113.1	130.3	129.5	124.4	160.9	117.4
99.8	118.2	107.7	112.1	100.8	112.4	106.5	111.3	112.2
184.3	300.5	310.4	267.8	244.2	274.8	266.9	376.8	324.9
178.9	282	264.1	236.1	229.2	272.3	249.7	305.4	253.3
107.5	106.2	101.1	102.9	92.3	104.6	106.4	106.8	115.7
123.7	131.4	122.5	126.8	132	125	130.5	161.1	130.4
103.9	101	91.1	100	91	104	101.1	101.6	112.5
108	111.5	99.2	96.9	104.7	114	106.7	112.1	114.1
135.2	168.6	162.9	137.7	144	181	165.8	239.9	230
203.8	432	428.8	373.7	402.8	335.5	351.4	525.7	277
110.2	118.2	108.9	120.3	111.4	120.4	101.6	127.2	118.9
148.5	211.4	203.3	231.3	211.5	225.5	254.5	330.4	269.9
107.3	121	105	104.8	98.4	120.7	108.4	124.1	117.3
328.3	430.7	416.1	343.5	315.8	453.7	268	768	799.8
133.6	168	170.8	159.4	145.5	187.4	158.8	266.1	219.5
132.5	144.9	144	115.8	126.2	148.5	135.7	156.4	147.9
112.4	148.9	129.9	129.7	120.6	128.3	140.1	155.1	135.8
101.7	113	107.8	100.7	99.4	111.3	110.1	110.2	110
227	373.6	330.3	397.3	298.9	297.9	335.8	423.1	309.8
206.3	240	262.8	234.9	201.2	208	158.3	358.5	259.7
110.4	108.7	102.7	106.1	107	112.4	118.5	116.7	106.3
282.1	311.7	341.5	300.7	298.9	387.1	420.1	559	508
102.1	103	102.5	96.1	92.1	101.8	103	104.3	99.7
110.7	112.6	113.7	101	102.3	116.4	111.4	142.2	124.4
124.1	121.4	109.4	104.3	111.9	123.3	131.4	187.2	186.5
111	124.8	121.2	131.3	97.5	130.5	108.3	109.8	111.5
252.8	267.7	258.2	249.5	221	273.1	259.5	492.8	390
117.4	127.8	121	109.5	116.5	132.8	113.9	131.7	121.4
123.7	158.7	119.9	122.7	135.7	133.4	133.7	195.2	132.4
111.4	169.2	130.6	123.6	132.9	151.2	120.6	193	131.3
106.5	118	101.4	97.4	88.9	112.4	100.9	111.2	115.1

178.4	167.9	158.4	153.4	152.9	149.2	155.4	224.5	182.1
123.6	135.3	137.4	131.2	114.7	136.4	129.8	159.3	175.9
241.9	192.7	201.7	177.2	161.2	196	228.3	337	260.3
100	110.9	102.6	105.3	96.6	105.5	106.9	110.4	102.4
173.5	272.2	303.8	230.3	227.4	267.2	256.4	284.7	182.4
148.7	159	134.5	151.5	109.7	128.9	140.2	163.1	150.7
275.1	408.3	360.8	345.3	349	398.5	350.4	503.6	270.3
104.1	102.6	102.2	97.9	91.8	101.5	99	98.3	114.2
250.7	247	227.1	178.3	234.8	242.7	231.8	362.4	302.9
342.7	457.2	327.3	298.9	329.5	433.9	338.1	643.3	444.2
115.8	128.6	111.8	131.9	112.2	130.7	120.1	124.3	120.1
109.4	112.9	97.6	113.6	98.4	111.6	94.2	134.1	119.8
104	108.4	97.2	97.8	88.9	107.7	97.6	98.1	104
191.4	166	160.7	154	175.3	168.5	142.3	259.2	248.8
110.2	114.9	103.7	107.4	97.8	113.3	107.4	113	108.3
105.8	124.6	114.8	108.1	102.6	113.6	118.9	125.2	112
135	172.8	139.6	173.4	144.2	151.3	162.3	137.9	144.1
105.1	99.8	106.5	95.7	93.3	102.5	101.8	97	107.8
110.6	118.3	114.4	113.2	99.3	116.1	106.1	127.8	123.9
103.6	109	104.3	105.7	91.2	107.9	107	114.5	115.1
102.6	118.8	115.2	116.4	113.5	118.8	112.9	125.2	112.3
111	124.3	110.9	109.4	101.6	117.8	111.2	119.4	120.3
129.6	137.4	132.8	126.7	132.4	146.5	134	192.4	152.8
105.7	105.8	101.2	100	96.6	108.6	98.7	114.5	109
1963.6	1339.7	1866	1205.1	1301.1	1559.7	1103.4	2192.2	2971.8
106.8	118.3	102.7	98.1	91.8	107.9	105.8	107.6	112.1
783.1	1620.9	1408.5	1441.2	1423	1382.3	1367.7	1916.2	1467.5
179.2	412.8	379.9	453.6	290.1	301.4	350.4	403.6	319
117.2	135.9	104.1	124.5	118.1	124.9	126.8	153.7	109.7
114.8	116.1	101.9	108.7	98.9	109.8	102.1	110.1	107.2
221.5	257.9	249.2	271.3	228.1	242.7	279.9	602.4	409.2
105.4	110.1	101.8	102	99.9	105.6	109.2	113.9	111.2
128.4	183.1	189.2	180	161	220	154	273.7	196.9
111	143.4	143.4	113.1	119.5	144.7	133	155.1	120
596.8	855.4	919.9	862.2	811.2	889.2	911.9	1115.7	555.9
143.9	152.3	163.4	167.9	146.8	153.9	176.5	185.6	140.5
142.8	121.7	122.2	118.2	106.1	124.5	138.9	144.4	149.6
303.7	309	260	309.6	240	286.9	351.6	379.6	317.7
110.4	117.7	106.7	101.5	106.2	114.8	122.8	135.8	116.8
2670.6	2455.5	1723.6	2291.8	2149.1	3068	1705.5	6226.7	6154
121.1	136.6	112.5	101.5	118	131.6	110.2	135.8	140.1
131.6	127.5	113.3	113.6	113.4	124	118.5	126.7	142.2
146.9	207.4	178.8	160.1	164.4	199.9	147.1	233.2	214.1
110.2	110.1	104.1	105.3	103.8	116.3	103.6	107.1	102.3
97.2	101.6	98.3	95.3	91.1	102.8	96.5	105.2	106.5
523.7	417.3	378.7	331.7	357.8	398	368.2	707.4	542.6
103.9	102.6	102.6	100	97.8	114.1	107.3	104.2	103.4
107.7	106.7	95.4	96.7	98.1	111.6	96.4	103.2	108
224.9	302.8	201.9	260	253.5	204.5	303.1	469.7	228.3
104.6	100	94.5	87.7	92.2	105	105.1	101.9	96
113	161	194.2	144.6	154.8	162.4	172	169	147.1

127.3	256.7	239	233.1	235.2	214.7	281.8	300.9	205.5
102.7	109.4	97.6	95.3	97.7	109.4	112.5	110.8	105.1
108.6	107.8	103	96.6	99.4	106.8	108.3	111.3	102.8
130.3	125.8	127	117.4	115	133.2	120.8	154.2	154.9
111.6	111.8	104.7	103.8	103.3	114.4	110.3	117	110.8
104.6	110.6	105.4	104.2	100.5	113.7	111.3	110.6	111.4
[truncated: 482,007 more chars]
